# Deciphering the Dynamics of Non-Covalent Interactions Affecting Thermal Stability of a Protein: Molecular Dynamics Study on Point Mutant of *Thermus thermophilus* Isopropylmalate Dehydrogenase

**DOI:** 10.1371/journal.pone.0144294

**Published:** 2015-12-11

**Authors:** Reetu Sharma, G. Narahari Sastry

**Affiliations:** Centre for Molecular Modeling, CSIR-Indian Institute of Chemical Technology, Tarnaka, Hyderabad, 500007, India; CNR, ITALY

## Abstract

*Thermus thermophilius* isopropylmalate dehydrogenase catalyzes oxidative decarboxylation and dehydrogenation of isopropylmalate. Substitution of leucine to alanine at position 172 enhances the thermal stability among the known point mutants. Exploring the dynamic properties of non-covalent interactions such as saltbridges, hydrogen bonds and hydrophobic interactions to explain thermal stability of a protein is interesting in its own right. In this study dynamic changes in the non-covalent interactions are studied to decipher the deterministic features of thermal stability of a protein considering a case study of a point mutant in *Thermus thermophilus* isopropylmalate dehydrogenase. A total of four molecular dynamic simulations of 0.2 μs were carried out on wild type and mutant’s functional dimers at 300 K and 337 K. Higher thermal stability of the mutant as compared to wild type is revealed by root mean square deviation, root mean square fluctuations and Cα-Cα distance with an increase in temperature from 300 K to 337 K. Most of the regions of wild type fluctuate higher than the corresponding regions of mutant with an increase in temperature. Cα-Cα distance analysis suggests that long distance networks are significantly affected in wild type as compared to the mutant. Short lived contacts are higher in wild type, while long lived contacts are lost at 337 K. The mutant forms less hydrogen bonds with water as compared to wild type at 337 K. In contrast to wild type, the mutant shows significant increase in unique saltbridges, hydrogen bonds and hydrophobic contacts at 337 K. The current study indicates that there is a strong inter-dependence of thermal stability on the way in which non-covalent interactions reorganize, and it is rewarding to explore this connection in single mutant studies.

## Introduction

*Thermus thermophilus* isopropylmalate dehydrogenase (IPMDH, EC 1.1.1.85) is involved in the leucine biosynthetic pathway, catalyzes oxidative decarboxylation and dehydrogenation of isopropylmalate (IPM). Thermostability is an extremely important phenomenon with potential industrial applications as several reactions have to be carried out at elevated temperature [1, 2]. Supramolecular assembly, protein structure and stability are the net result of how various non-covalent interactions (NCI) occur and manifest themselves. Some of NCI which contribute to thermal stability are an increase in hydrogen bonds (HBs) [3], networks of salt bridges [4], tighter packing [5], higher secondary structure content [6] and a highly hydrophobic core [7]. Combination of one or more of NCI contribute to protein’s thermal stability [8]. Apart from structural aspects, dynamic features of the NCI contributing to the stabilization of proteins are unclear and need investigation [9].

IPMDH has been one of the most widely studied proteins for understanding the factors affecting thermostability [10–16]. Numerous site-directed mutagenesis studies have been conducted to improve the thermal stability of the protein, especially at 172 position of the protein [10,15,17–19]. The thermophilic bacteria has optimum growth temperature of about 337 K [15]. Crystallographic structures of the enzyme from *T*. *thermophilus* have been determined for wile type (*wt*, PDB code: 1IPD) [20] and the mutant (*mut*, A172L, PDB code:1OSJ) [19] (S1 Fig). It is a 690 residue protein, a homodimer with two domains in each subunit. Residues from 1–99, 252–345 constitute domain 1 with both N and C termini of the subunit, remaining 100–251 residues form domain 2. Similarly, in second subunit, residues from 346–444, 597–690 and 445–596 form domain 1 and domain 2, respectively [20]. The hinge region connecting the two domains plays an important role in the thermal stability [21]. Alanine 172 is located at the hinge region, at C terminus of alpha helix. Introduction of leucine at position 172 improves the thermal stability of the enzyme [10,19].

Circular dichroism experiments suggest that the *mut* has maximum thermal stability among the known substitutions at the site and a melting temperature of 3^°^C higher than that of *wt* [10]. Crystallographic studies by Qu et al. [19] proposed that enhancing the local packing of the side chains and shifting of backbone of opposite domain contribute to the thermal stability of the protein. IPMDH undergoes rearrangement of domains in the *mut* due to interaction of side chains of residues 172 and 300 [19]. The *wt* and *mut* structures are highly similar as their structural alignment shows a root mean square deviation (RMSD) of less than 1 Å for Cα atoms of all chains by the use of Superpose [22]. Crystallographic structures, although valuable are static in nature, a limited conformation among possible ones, dynamics of NCI underlying the adaptation remains poorly understood. Moreover, conformations of the protein can be affected by different crystallization conditions and temperatures at which data is collected. Hence, detailed analysis of the adaptation mechanism regarding dynamic NCI to the thermal stability of a protein needs to be investigated.

Extending our ongoing efforts in understanding NCI such as *π*-*π*, hydrophobic interactions and HBs [23–26], this is the first classical method applied on IPMDH to gain an insight in analyzing the overall change in NCI with respect to a single mutation before the onset of denaturation. Considering the dynamic nature of biomolecules, the approach provides an advantage as the systems were treated at identical conditions, only modifying the temperature at 300 K/337 K in this case. Thus, only the effect of temperature on the system led to an overall change in main NCI in *mut* can be monitored and compared with that in *wt* at atomic level before denaturation. In the present work, an effort has been made to extract the dynamic changes in overall main NCI, viz., saltbridges, HBs and hydrophobic interactions to be quantitatively addressed as a deterministic feature at 300 K and 337 K. The study makes an attempt to bridge the gap between crystallographic static conformation and biochemical findings by observing the dynamic changes in the NCI at atomic level. The detailed analysis provides a new direction to improve the biocatalytic applications in industry.

## Materials and Methods

### Description of systems

The atomic coordinates of the *wt* (A172; PDB code: 1IPD) and *mut* (A172L; PDB code: 1OSJ) were downloaded from protein data bank (PDB). Dimer of *wt* was generated using symmetry equivalent molecules. Functional dimers of both systems were used for all simulations at the different temperatures.

### All atom molecular dynamics simulation

All atom molecular dynamics simulations were performed using Gromacs program, version 4.5.3 [27], with the optimized potential for liquid simulations (OPLS) force field [28]. The topologies of protein were generated by Gromacs package directly. The simulation system was solvated in a periodic cubic box with a distance of 1.0 nm between wall of box and the protein, filled with TIP4P water molecules. Appropriate sodium ions were added to neutralize the charge of *mut* and *wt* system. Temperature of the protein and solvent (water and counterion) were separately coupled to an external bath held at 300 K/337 K, using the (V-rescale) modified Berendsen thermostat with 0.1 ps relaxation time [29]. The pressure of the system was isotropically coupled to a barostat at 1 bar using the Parrinello-Rahman method [30]. Simulations were carried out using the isothermal-isobaric (NPT) ensemble with an isotropic pressure of 1 bar. The electrostatic interactions were treated using the Particle Mesh Ewald (PME) method [31] with a coulomb cutoff of 1.0 nm. The van der Waals interactions were treated using switch potential with a cutoff of 1.0 nm with the switch function applied from 0.9 nm. The bond lengths were constrained using the LINCS [32]. Following energy minimization, temperature and pressure equilibration was performed by applying the position restraints on the system. To remove possible unfavorable interactions between solute and solvent, 100 ps equilibration with position restraints were performed. All production simulations were run for 50 ns with a time step of 2 fs with the atomic coordinates and velocities were saved after 2 ps. The simulations were conducted till equilibrium structures were obtained for *wt* and *mut* at both temperatures. Simulations were analyzed in region, 10 ns to 40 ns, unless otherwise mentioned.

RMSD was calculated as a measure of the deviation between the respective Cα atom of the proteins with respect to initial structure’s Cα atom, average was taken over the Cα atoms. In case of root mean square fluctuations (RMSF), the average was taken over the time. Total solvent accessible surface area (SASA) of hydrophilic and hydrophobic residues was calculated at a time gap of 2 ps, and was averaged over the trajectory. Further, the solvent accessibility of each residue and each atom of the residue was calculated.

HBs were calculated using a maximum cutoff distance between the donor and acceptor as 0.35 nm and the donor-hydrogen-acceptor angle to be ≥ 120°. Average number of HBs as a function of time were calculated as the sum of the total number of HBs in each frame and then dividing it by the total number of frames. All unique interactions and their occurrence were calculated for the trajectory with a gap of 2 ps. The hydrogen bond formed between the same donor and acceptor but with different hydrogen atom was considered unique. Charged (arg, lys, asp, glu), polar (gln, asn, ser, thr, tyr, cys) and hydrophobic (ala, ile, leu, phe, val, pro, gly, met, trp) residues were considered for calculating HBs within them.

The salt-bridges (SBs) were calculated between the atoms of negatively charged residues asp (Oδ1, Oδ2), glu (Oε1, Oε2), and positively charged residues arg (Nε, Nη1, Nη2), lys (Nζ), within distance of 0.4 nm [33]. Time averaged saltbridges were calculated by dividing the total saltbridges at a frame to the total number of frames, where each frame was noted after every 2 ps of the trajectory. Terminal carboxylic or amide groups and histidine were ignored for saltbridge calculation as are deeply affected by local environment.

Stojanovic and Zaric reported that all C atoms within 0.39 nm interacts through hydrophobic contacts [34]. Unique hydrophobic contacts were calculated between all the hydrophobic atoms (C, Cα, Cβ, Cδ, Cδ1, Cδ2, Cε, Cε1, Cε2, Cε3, Cγ, Cγ1, Cγ2, Cζ, Cζ2, Cζ3, Cη2) within a distance of 0.4 nm, without redundancy. The results of MD simulations were finally visualized by Visual Molecular Dynamics 1.9.1 (VMD) [35] and PYMOL [36]. Figures were prepared using open source gnuplot and Microsoft office Excel 2007. Perl, awk and sed scripts were used for some calculations.

## Results and Discussion

Despite the high structural similarity between *wt* and *mut*, our objective is to decipher the changes in NCI that control them for thermal stability. To fulfill the aim, at first, structural properties of the systems, that is, RMSD, RMSF, SASA and long distance network analysis are compared among the systems at both temperatures to confirm that the systems are in correlation with the experimental observations. Then the time-averaged NCI are assessed for both systems at different temperatures. Unique NCI and the percentage of time their interaction existed are monitored for *wt* and *mut* at both temperatures. Changes in unique interactions are analyzed within *mut*, with an increase in temperature and are compared with that in *wt* at both temperatures. The details are mentioned in the materials and methods section.

### Stability of the structures

**Fig 1:**
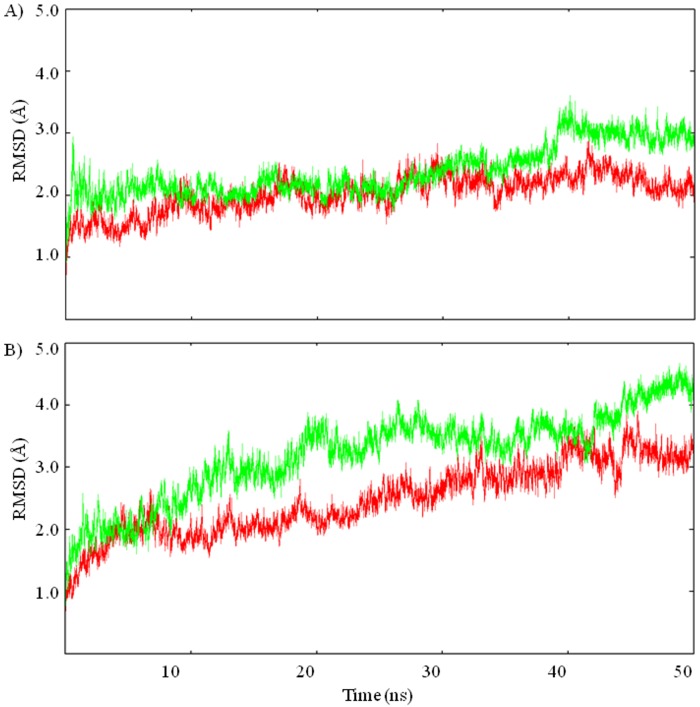
Stabilization of the structures. RMSD plot of Cα atoms of A) *mut* (PDB: 1OSJ, A172L) and B) *wt* (PDB: 1ipd, A172) at 300 K (red) and 337 K (green).

To gain an insight into the deviation in tertiary structure of the proteins at both temperatures, RMSD with respect to initial structure, suggests that the *mut* stabilizes much earlier than the *wt*, nearly at 2 ns (Fig 1). Average RMSD of Cα atoms of the *mut* at 337 K and 300 K is 2.3 ± 0.19 and 2.1 ± 0.25 Å, respectively and for the corresponding in *wt* is 3.5 ± 0.30 and 2.4 ± 0.57 Å (Fig 1 and Table 1). The RMSD of the *mut*, equilibrated and simulated at different temperatures nearly overlaps till 40 ns (Fig 1A) and had negligible variation between 300 K and 337 K (Fig 1). This suggests that the deviation in the three dimensional structure of the *mut* is less as compared to the *wt* with respect to initial structure with the shift in temperature.

**Table 1:** Average and standard deviation (SD) values of RMSD and SASA of *mut* and *wt* at 337 K and 300 K.

Systems	RMSD^a^	SASA^b^_pho_	SASA^c^_phil_	SASA^d^_total_
***Mut*, 300 K**	2.1 ± 0.25	158.36 ± 1.81	156.69 ± 1.56	315.05 ± 3.37
***Mut*, 337 K**	2.3 ± 0.19	155.76 ± 1.64	159.33 ± 1.45	315.09 ± 3.09
***Wt*, 300 K**	2.4 ± 0.57	157.60 ± 1.44	157.14 ± 1.27	314.75 ± 2.71
***Wt*, 337 K**	3.5 ± 0.30	158.42 ± 1.40	159.69 ± 2.05	± 3.45

^a^Average RMSD ± SD, units in Å.

Average SASA ± SD by ^b^hydrophobic residues, ^c^hydrophilic residues and ^d^total residues, units in nm^2^.

### Analysis of fluctuations

**Fig 2:**
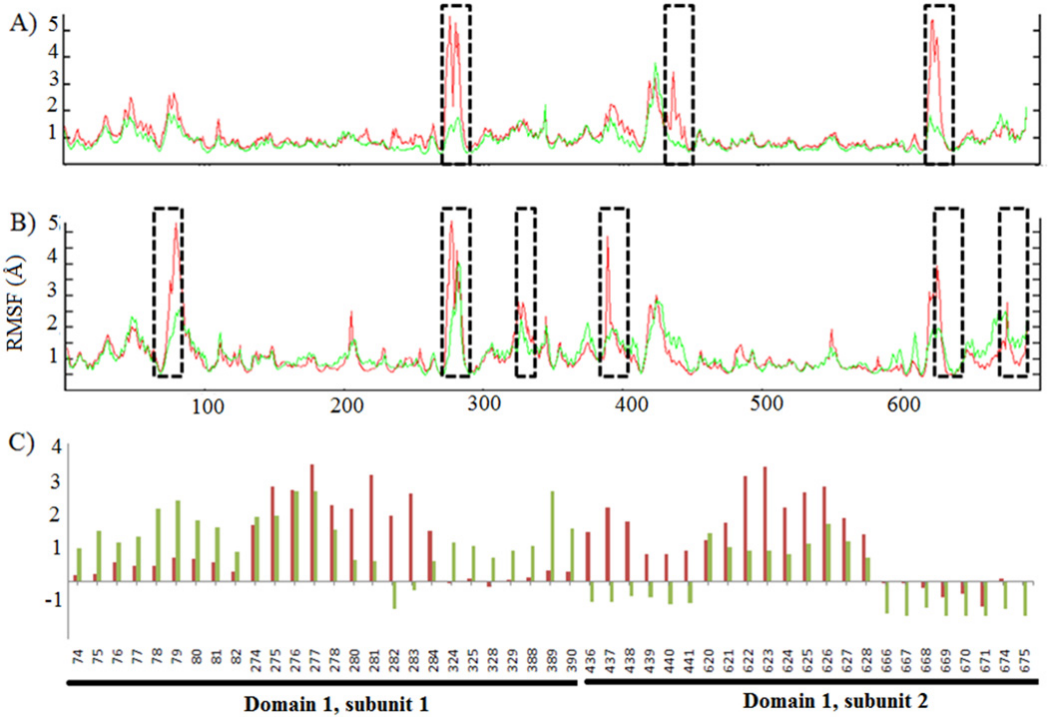
RMSF of all Cα atoms in A) *mut* and B) *wt* at 300 K (green) and 337 K (red) with reference to initial structure. Deviations more than 1 Å are highlighted with black dashed brackets. C) Deviated Cα atoms of *mut* and *wt* are shown with deviation (Å).

**Fig 3:**
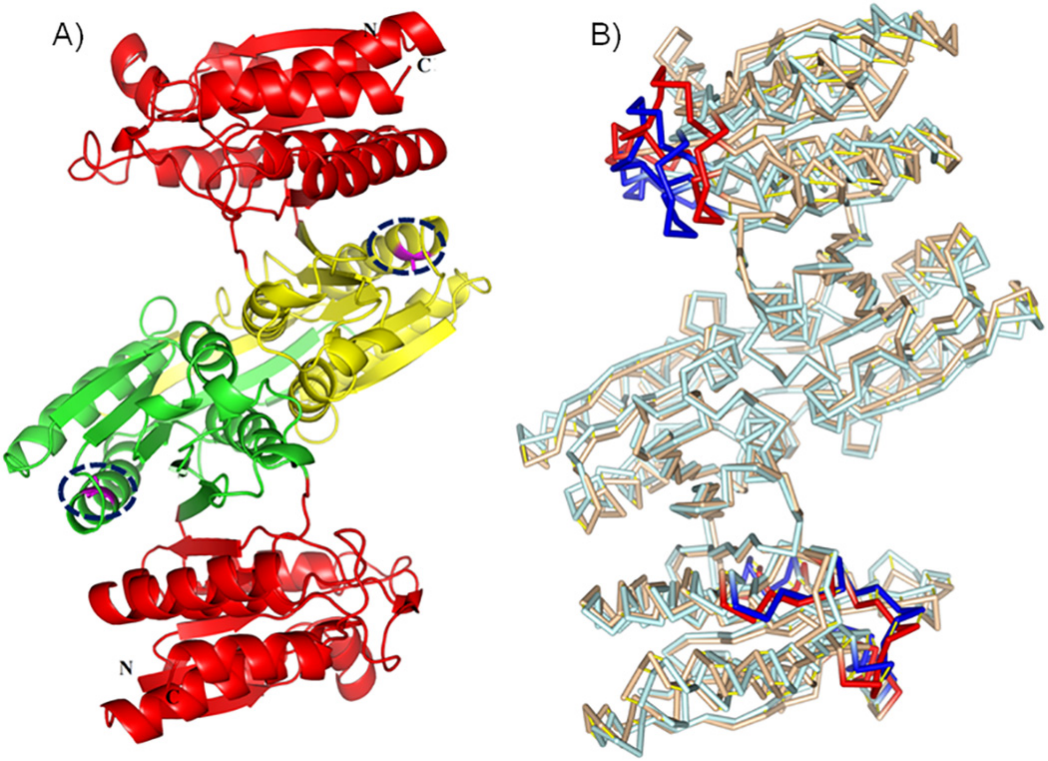
Three dimensional structure of IPMDH. A) Location of A172 is highlighted by blue dashed oval in domain 2 [19]. Domain 2 is highlighted as yellow and green in subunit 1 and 2 respectively. B) The regions fluctuated higher than 1 Å are emphasized in superposed *mut* (cyan) and *wt* (pink)’s structure as blue and red, respectively.

Thermostable proteins have lower fluctuated regions and are more compact [5]. Hence, in this section, fluctuations in the deviation of residues are monitored by RMSF of the Cα atoms of the *wt* and the *mut* (Fig 2). Regions fluctuated higher than 1 Å are highlighted in Figs 2C and 3. More fluctuations are observed in case of *wt* as compared to *mut* with an increase in temperature from 300 K to 337 K. Six regions (residues range: 74–82, 274–278, 324–329, 388–390, 620–627 and 666–675) fluctuated more as compared to three regions (residues range: 274–284, 436–441 and 620–628) in *mut* at 337 K. Two common regions (residues range: 274–278 and 620–628) fluctuate in *mut* as well as *wt*, corresponding to same loop regions in both subunits. Most of the fluctuations are observed in domain 1. However, the fluctuation in domain 2 is less as compared to domain 1 of both subunits. Still, fluctuations in domain 2 are higher in *wt* as compared to the *mut*. Loop deletion [37] or anchoring of loops [8] has been considered to increase thermal stability of the proteins, hence reducing the fluctuating regions has increased thermal stability of the enzyme. The mutation has enhanced thermal stability by reducing the fluctuations of the regions as compared to *wt* (Fig 3B).

### Distance network analysis

**Fig 4:**
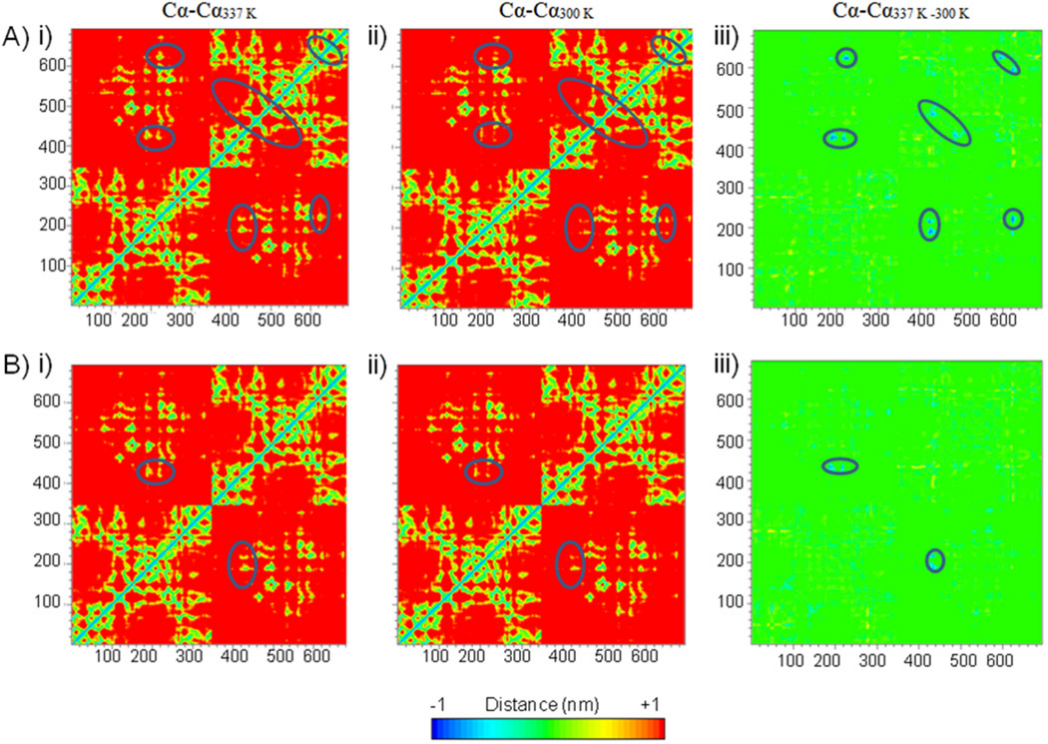
Long distance networks in A) *wt* and B) *mut*. A and B represents the Cα-Cα distance in *wt* and the *mut*, respectively. i, ii, iii represents the corresponding distances at 337 K (Cα-Cα_337K_), 300 K (Cα-Cα_300K_), difference in distances at 337 K and 300 K (Cα-Cα_337K-300K_), respectively. The regions showing extreme deviation (-1 nm) are marked with blue ovals and are represented in iii, as difference of i and ii. The temperature at which simulation is conducted is written in subscript. All residues, 1–690, are represented by their respective Cα atoms in x and y axis.

Cα-Cα distance (cut off 0.25 nm) is analyzed to observe the effect of the mutation on long distance network. The matrix defines the distance between each residue (Cα atom) to all 690 Cα atoms (Fig 4). The matrix is symmetrical about the diagonal representing the distance between a residue to itself. The distance between a residue to another is represented by the increase in amplitude of colors from blue to red. The matrix (690 X 690) is divided into four quadrants; the average distance within residues of subunit 1 is represented in square, lower left section, 345 X 345 corresponding to 345 rows to 345 columns, while for distance within subunit 2 is represented by the upper right quadrant. The upper left and lower right quadrant represents the Cα-Cα distance between the subunits (Fig 4). The regions showing extreme deviation in Cα-Cα distance are more in *wt* as compared to the *mut*.

In contrast to *mut*, *wt* shows delay in attaining the structural stability, highly fluctuated regions and higher perturbation in long distance network. These properties validate the models showing higher thermal stability for the *mut* as compared to the *wt* and are in agreement with the experimental observation that the *wt* has lower thermal stability than the *mut* [10]. Following it the dynamics of most influential NCI, viz., saltbridges, HBs and hydrophobic interactions are studied as a governing feature of monitoring thermal stability of a protein. The influence of NCI in a supramolecular assembly manifest in a cooperative or anticooperative fashion [38,39]. Thus, discussions on the individual NCI are given below.

### Saltbridges

**Fig 5:**
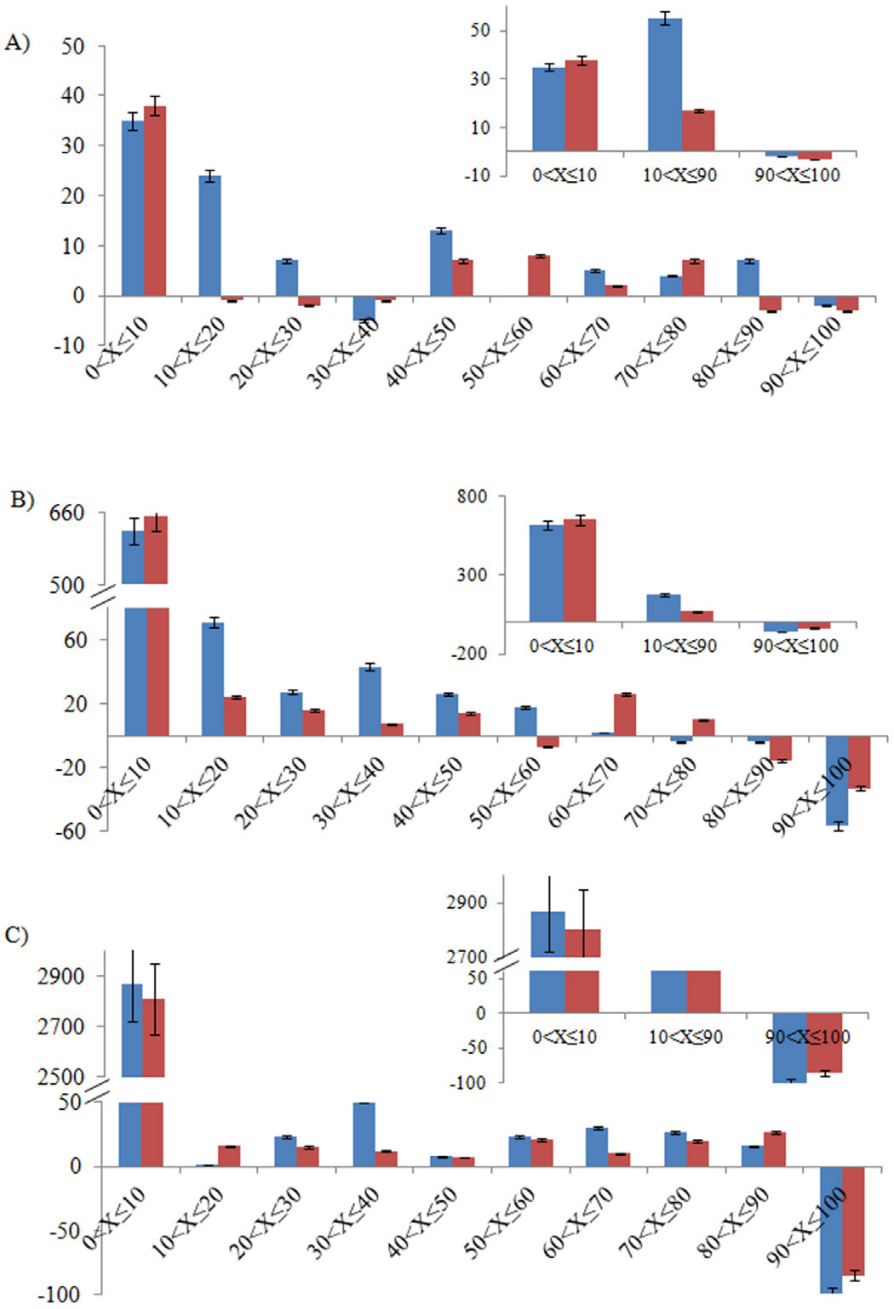
Difference in A) saltbridges, B) IP HBs and C) hydrophobic contacts (y-axis) based on percentage of the interaction existed (x-axis) at 337 K and 300 K in *mut* (blue) and *wt* (red) is represented by bar graphs. Insets in panel A, B and C represents short lived (0 < X ≤ 10%), substantially lived (10 < X ≤ 90%) and long lived (90 < X ≤ 100%) interactions. The criterion for saltbridge, IP HBs and hydrophobic contacts are mentioned in materials and methods. 5% of the series value is displayed as error bars.

Ionization properties of the participating groups, influenced by its environments can significantly affect saltbridge formation. Thermal fluctuations, their lifespan in solution and dynamic behavior has profound effect on saltbridge’s contribution to thermal stability of a protein [40]. In present section, number of time averaged and unique saltbridges with their percentage of existence over the trajectory are assessed at cutoff of 4 Å. The difference in time averaged saltbridges at 337 K and 300 K is nearly the same in the *mut* and *wt* as the number of saltbridges increases from 300 K to 337 K to the same extent. However, in contrast to *wt*, average saltbridges are more in the *mut* at both the temperatures. With an increase in temperature, the unique saltbridges also increase in *mut* to attain thermal stability at higher temperature in comparison to *wt* (Table 2). The similar trend is observed even after ignoring contacts having percentage of existence ≤ 5% (Table 2) and short lived contacts (percentage of existence ≤ 10%) (S9 Table). This indicates that the saltbridge distribution seems to play an important role for the *mut*’s thermal stability. A significant increase in the short lived saltbridges in *wt* as compared to *mut* suggests that most of the contacts are transient in nature at high temperature. In contrast to the *mut*, *wt* has higher short lived contacts. In addition, the decrease in long lived contacts (90 < X ≤ 100%) at 337 K indicates that the structure attains higher flexibility at higher temperature that lead to momentary contacts. Unique saltbridges with their percentage of existence in the systems at 300 K and 337 K are listed in S1 Table. The difference in substantially lived saltbridge contacts (10 < X ≤ 90%) of *mut* at 337 K and 300 K is nearly three times higher than that of *wt* (Fig 5A). This suggests that contribution from the substantially lived contacts can be significant for the higher stability of the *mut*.

**Table 2:** Difference in total unique NCI of *mut*/*wt* at 337 K and 300 K.

Interaction type	*mut*_337K-300K_	*wt*_337K-300K_	*mut*_337K-300K_ ^1^	*wt*_337K-300K_ ^1^
**Saltbridges** ^**a**^	76	52	38	29
**HBs**_**IP**_^**b**^	742	693	146	84
**HBs**_**mainch-sidech**_^**c**^	392	344	46	12
**HBs**_**sidech-sidech**_^**d**^	235	209	68	46
**HBs**_**mainch-mainch**_^**e**^	146	159	35	29
**HBs**_**pol-pol**_^**f**^	53	30	4	4
**HBs**_**char-char**_^**g**^	206	175	65	36
**Hydrophobic contacts** ^**h**^	2948	2850	84	69

^1^Excluding interactions existed for less than or equal to 5% of the time.

^a^Difference in total unique saltbridges of the *mut* or *wt* between 337 K and 300 K, cut off 0.4 nm.

Difference in total unique ^b^intra-protein, ^c^mainchain-sidechain, ^d^sidechain atoms, ^e^mainchain atoms, ^f^atoms of polar residues, ^g^charged residues HBs of the *mut* or *wt* between 337 K and 300 K with the criterion mentioned in materials and methods.

^h^Difference in total unique hydrophobic contacts of the *mut* or *wt* between 337 K and 300 K.

### HBs

**Fig 6:**
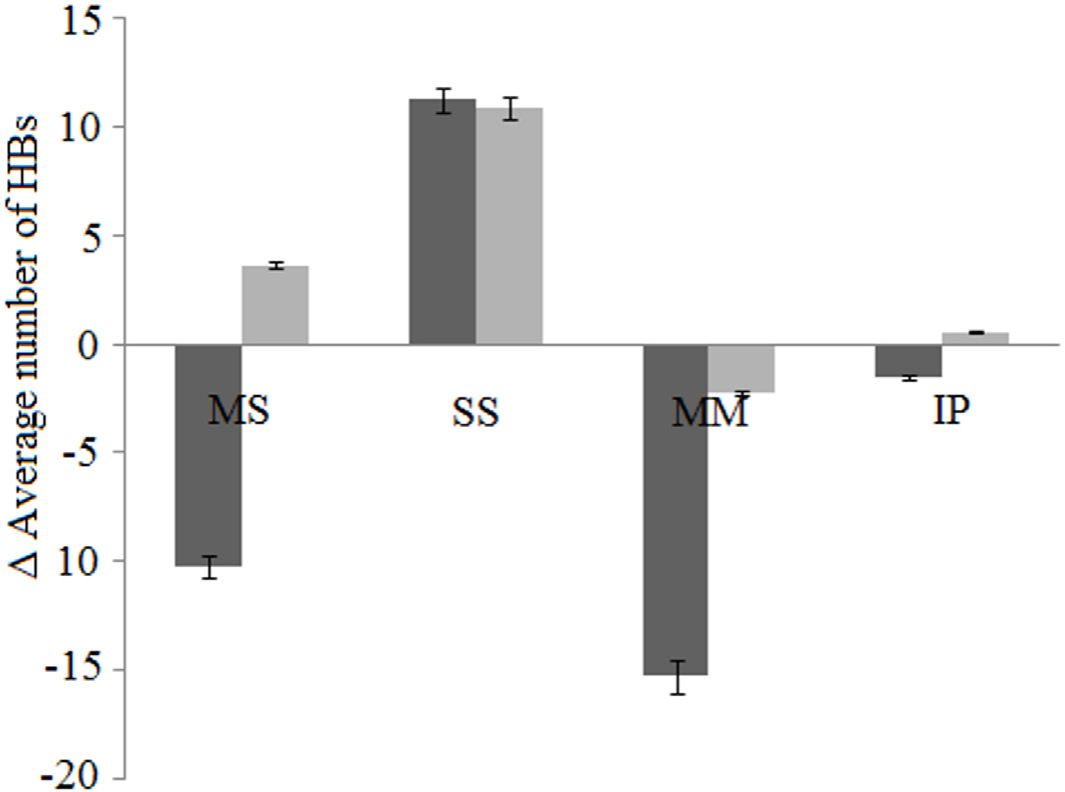
Difference in average HBs of *mut* (grey) and *wt* (black) at 337 K and 300 K. The change in average number of HBs formed between MS, SS, MM, and IP in case of *mut* and *wt* at 337 K and 300 K are highlighted. Error bars are displayed for the selected series with 5% of the value.

HBs are one of the important NCI governing the stability of a protein. Change in the intra-protein (IP) HBs depicts an increase in *mut* in contrast to *wt* at 337 K. The difference in time averaged IP HBs are positive in *mut* whereas is negative in *wt* (Fig 6). In contrast to *wt*, the difference in total unique IP HBs between 337 K and 300 K is higher in *mut* (Table 2). Even after ignoring short lived HBs, similar trend is observed (S9 Table). Considering the percentage of existence of these IP HBs in both cases, short lived contacts increase at 337 K suggesting that significant numbers of bonds break at high temperature. Long lived contact decreases at high temperature in both cases, possibly the contacts are unable to maintain for long time due to thermal vibrations. However, the decrease in long lived contacts is significantly higher in *wt* in comparison to the *mut*. This suggests that three dimensional structure of *wt* is not able to withstand high temperature as relatively higher stable and sustainable bonds are not able to maintain at 337 K (Fig 5B). The increase in substantially lived contacts in *mut* at 337 K is similar to that in saltbridges (Fig 5B). S2 Table lists the unique IP HBs in *wt* and *mut* at 300 K and 337 K.

Dissecting the contribution of IP HBs into components, HBs between mainchain to mainchain atoms (MM), mainchain to sidechain atoms (MS), sidechain to sidechain atoms (SS), polar to polar residue atoms (PP), hydrophobic to hydrophobic residue atoms (HH) are a few among several components.

HBs between MM are involved in maintaining the secondary structure of protein and are local in nature [41,42]. HBs within α-helix and β-sheets, responsible for stability of the secondary structures are between MM. Hence, analyzing their dynamic behavior can provide an indication that the secondary structure of the protein is changing with temperature. It has been observed that the number of time averaged HBs between MM decreases in both the cases at 337 K. However the decrease in *wt* is significantly higher (~ seven times) in comparison with the *mut* (Fig 6) suggests that even though *mut* is also affected with an increase in temperature but the change is much higher in *wt*. Similar to all the above cases, the substantially lived HBs formed between MM are more in *mut* (S2 Fig). In contrast to *wt*, difference in unique HBs formed between MM at 337 K and 300 K, even after ignoring interactions existed ≤ 5% of the duration or short lived interactions are higher in *mut*. This indicates that new yet moderately lived interactions increase to maintain the conformation at high temperature (Table 2, S3 and S9 Tables). This is supported by the fact that the short lived interactions between MM are less in *mut* as compared to the *wt*. The HBs formed between MM stays for relatively long time thus an increase in substantially lived contacts can be observed (S2 Fig, S3 Table).

Similar to MM, HBs between MS are also local in nature [42,43] and stabilizes mainly the start sites of α-helices [42,43]. On an average, HBs between MS of *wt* are highly fragile and nearly 10 bonds are broken at higher temperature while there is an increase of 3 bonds in *mut* (Fig 6). In contrast, time averaged HBs between MS decrease more than five times in case of *wt* with an increase in the temperature (Fig 6). Similar to MM, unique MS HBs are more in *mut* though short lived contacts are nearly two times less than that in *wt* (Table 2, S4 Table). More number of short lived (S2 Fig) and less unique interactions in *wt* in comparison to *mut* at 337 K (S2 Table) suggests that the share of short lived contacts in the overall unique interactions are higher in *wt*, fragile in nature, and fail to stabilize the contacts for long time. This advocates the instability of *wt* in comparison to *mut* at high temperature (S2 Fig).

The average HBs between SS increase in both cases to same degree with an increase in temperature at 337 K (Fig 6). In contrast to *wt*, there is a significant increase in unique interactions between SS in *mut* at 337 and 300 K (Table 2). Difference in short lived contacts formed between SS at 337 K and 300 K are higher in *wt* in comparison with *mut* whereas the long lived contacts decrease at 337 K (S2 Fig, S5 Table). In contrast to *wt*, substantially lived HBs formed between SS are higher in *mut* (S2 Fig).

Network of HBs formed by PP is responsible for stabilization of protein’s structure in solution [43,44]. Difference in unique HBs between PP at 337 K and 300 K is more in *mut* than *wt* (Table 2). The same trend is followed by HBs between charged residues (CC) (Table 2). The degree of increase in short and long lived contacts between CC is nearly the same, while the substantially lived contacts are more in the *mut* (S2 Fig). Difference in short lived HBs formed between HH shows that it is nearly 1.5 times more in *wt* as compared to *mut* and long lived contacts decreases at 337 K (S2 Fig).

Considering the effect of water on thermal stability of protein at higher temperature, surface water promotes both folding rate and stability of a protein [45]. Extensive network of HBs among waters in proteins hydration shell exists in thermophilic proteins [46]. There is a decrease in protein-water HBs at higher temperature in both *mut* and the *wt*, however, the decrease is significantly more in the *mut* as compared to *wt*. Similarly, HBs between mainchain atoms to water, sidechain residue atoms to water, polar residue atoms to water, charged residue atoms to water and hydrophobic residue atoms to water in *mut* shows considerably less interaction at higher temperature in comparison to *wt*. The least interaction between *mut*’s hydrophobic residue atoms with water is at 337 K, more than three-fold difference in comparison with *wt* where there is an increase of water-hydrophobic residue atoms interaction (Table 3). The substitution of alanine to leucine at position 172 may lead to the folding of protein such that the percentage of hydrophobic residues bury inside at higher temperature and minimal interaction with water contributing its stability.

**Table 3:** Difference in time averaged HBs of *mut*/*wt* with solvent (water) between 337 K and 300 K.

Interaction type	*mut*_337K-300K_	*wt*_337K-300K_
**Protein-water** ^**a**^	-27.85	-17.3
**Mainchain-water** ^**b**^	-23.87	-19.45
**Sidechain-water** ^**c**^	-53.00	-35.69
**Polar-water** ^**d**^	-7.19	-1.02
**Charge-water** ^**e**^	-47.91	-44.74
**Hydrophobic-water** ^**f**^	-21.66	9.63

Difference in average HBs formed between ^a^protein-water, ^b^mainchain-water, ^c^sidechain-water, ^d^polar residues-water, ^e^charged residues-water, ^f^hydrophobic residues-water between 337 K and 300 K.

### Hydrophobic contacts

Hydrophobic interactions at the protein surface contributes to protein stabilization [47]. Average and unique hydrophobic contacts at both temperatures are calculated as mentioned in materials and methods section. Percentage of time a unique contact exists is calculated based on the number of times the interaction existed out of the total number of frames considered. In contrast to *wt*, unique hydrophobic contacts exceeds in *mut* at both temperature. The difference in time averaged hydrophobic contacts remains unchanged in the *mut* however more than six hydrophobic bonds are broken in *wt* with shift in temperature. Further, the *mut* is stabilized with more unique hydrophobic contacts than *wt* at both temperatures (Table 2, S8 Table). Short lived unique hydrophobic contacts increase at 337 K in both *mut* and *wt* with an overlap of error bars with 5% of the value. In correlation with hydrogen and saltbridge contacts, the momentary hydrophobic contacts are more in *wt* at 337 K in comparison with *wt*. Similar to hydrogen and saltbridge contacts, difference in substantially lived hydrophobic contacts at 337 K and 300 K is higher in the *mut* in comparison to *wt* (Fig 5C). Unique hydrophobic contacts and their percentage of existence in *wt* and *mut* at 300 K and 337 K are listed in S8 Table.

SASA of whole protein, hydrophilic and hydrophobic residues provides an indication of folding state of a protein [9,48] (Table 1). Total SASA of *mut* remains the same but increases in *wt* at 337 K. Buried hydrophobic residues are more exposed to water in *wt* as indicated by increase in SASA of hydrophobic residues in *wt* while it decreases to the same extent in the *mut* at 337 K (Table 1). The result is further complemented by the fact that HBs between hydrophobic residue atoms and water in *mut* significantly lost at 337 K as it further gets buried in protein core (Table 3). Monera et al. [48] reported that buried hydrophobic residues of a protein get exposed to water during its unfolding. Hence, the increase in SASA of whole protein and of hydrophobic residues in *wt* at 337 K suggests that buried hydrophobic residues gets exposed to environment resulting in its instability and on the other hand, highly persistent hydrophobic interactions in *mut* thus provides additional capacity to withstand high temperature.

## Conclusions

The present molecular dynamics study examines and identifies key changes in NCI responsible for controlling thermal stability of *mut* and *wt* of IPMDH. The mutation effectively modifies the protein’s structural and flexibility properties. RMSD in quaternary structure (Cα atoms) is significantly higher in *wt* with respect to its initial structure and the protein fails to stabilize fast at both temperatures.

The SASA remains indifferent in *mut* while it increases in *wt* at 337 K. In contrast to *mut*, hydrophobic residues in *wt* are noticeably exposed to water at higher temperature. In contrast to *mut*, hydrophobic residues in *wt* are noticeably exposed to water at higher temperature. Further, the fluctuating regions (Cα atoms) are more in *wt* in comparison to *mut* at 337 K. Long distance networks (Cα-Cα) are significantly affected in *wt* as compared to *mut* at higher temperature. RMSD, RMSF, SASA, long range networks suggests the instability of *wt* in comparison with the *mut* at 337 K.

The dynamic features that demarcate the two systems permit us to conclude that thermal stability of the *mut* at 337 K is maintained to a greater extent as long and substantially lived saltbridge contacts increases. The increase in electrostatic and hydrophobic short lived contacts is less in *mut* than that of *wt* at 337 K. Less exposures of hydrophobic residues to solvent and more unique saltbridges, HBs and hydrophobic interaction in *mut* support its stability at high temperature.

The dynamics of the factors are analyzed as a feature to explain the thermal stability of *mut* to *wt*. The mutation produces complex distal effects that underlie the intimate interplay between the dynamic character of electrostatic and hydrophobic interactions, led to thermal stabilization of it. Accessing the causative factor for thermal stability is of prime industrial importance. Establishing a relationship between a mutation and structural transitions triggered by NCI can aid in optimal protein engineering.

## Supporting Information

S1 Fig Tertiary structures of A) *wt* (PDB id: 1IPD) [20] and B) *mut* (PDB id: 1OSJ) [19]. Subunit 1 and 2 are represented in green and blue, respectively.
(TIFF)

S2 Fig Difference in HBs between A) MM, B) MS, C) SS D) CC, E) HH (y-axis) based on percentage of time the interaction existed (x-axis) at 337 K and 300 K in *mut* (blue) and *wt* (red). (TIFF)

S1 Table Unique salt bridges and their percentage of existence in *wt* and *mut* at 300 K and 337 K. Drnona and Arnona represents donor and acceptor residue number followed by its abbreviation (3 Letter), respectively. D and A indicates interacting donor and acceptor atom, respectively. The color formatting indicates the percentage of time the interaction (precen) existed within 10% of the range, 0 < X ≤ 10%: grey background, 10 < X ≤ 20%: blue background, 20 < X ≤ 30%: yellow background, 30 < X ≤ 40%: green text, 40 < X ≤ 50%: red border, 50 < X ≤ 60%: red text, 60 < X ≤ 70%: pink background and black text, 70 < X ≤ 80%: green text and background, 80 < X ≤ 90%: orange background, 90 < X ≤ 100%: pink background and text.
(PDF)

S2 Table Unique IP HBs between donor and acceptor atom of *wt* and *mut* at 300 K and 337 K. The color formatting indicates the percentage of time interaction existed as in S1 Table.
(PDF)

S3 Table Percentage of time HBs existed between MM of the *wt* and *mut* at 300 K and 337 K. The color formatting pattern is as followed in S1 Table.
(PDF)

S4 Table Unique HBs between MS of *wt* and *mut* at 300 K and 337 K. The color formatting indicates the percentage of time interaction existed is as in S1 Table.
(PDF)

S5 Table Unique HBs between SS of *wt* and *mut* at 300 K and 337 K. The color formatting indicates the percentage of time interaction existed is as in S1 Table.
(PDF)

S6 Table Percentage existence of HBs between PP of *wt* and *mut* at 300 K and 337 K. The color formatting indicates the percentage of time interaction existed is as in S1 Table.
(PDF)

S7 Table Percentage of existence of unique HBs between CC of *wt* and *mut* at 300 K and 337 K. The color formatting indicates the percentage of time interaction existed is as in S1 Table.
(PDF)

S8 Table Unique intra hydrophobic contacts and the percentage of their existence in *wt* and *mut* at 300 K and 337 K. The color formatting indicates the percentage of time interaction existed is as in S1 Table.
(PDF)

S9 Table Difference of unique NCI in *mut* and *wt* at 337 K and 300 K after removing the interactions having percentage of existence less than or equal to 10%. (PDF)
