# Supplementary figures and images for: Deciphering the Dynamics of Non-Covalent Interactions Affecting Thermal Stability of a Protein: Molecular Dynamics Study on Point Mutant of Thermus thermophilus Isopropylmalate Dehydrogenase
Source: PLoS One. 2015 Dec 11;10(12):e0144294. doi: 10.1371/journal.pone.0144294 (PMC4689552; doi:10.1371/journal.pone.0144294)

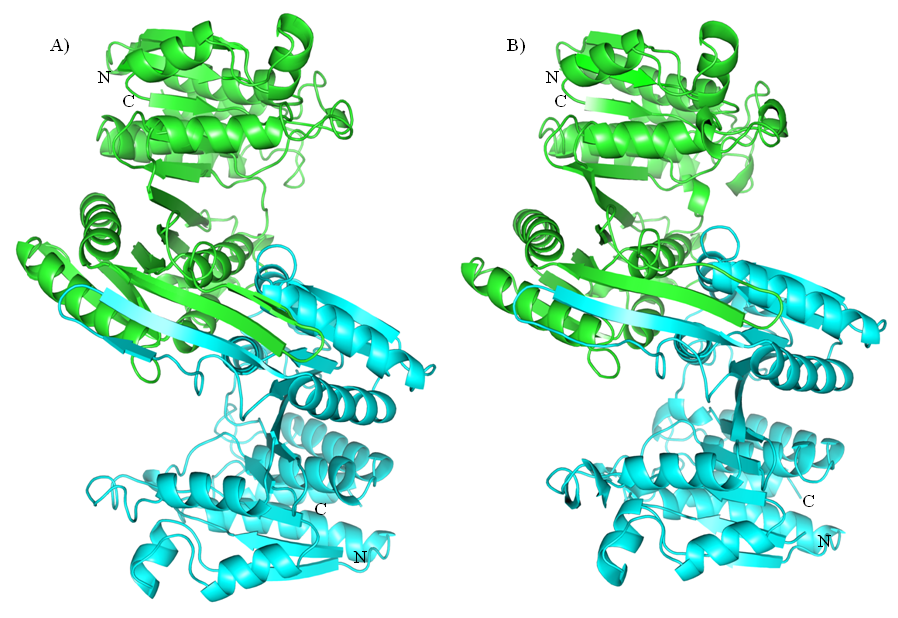

Supplement: S1 Fig — Subunit 1 and 2 are represented in green and blue, respectively. (TIFF) [file pone.0144294.s001.tiff]

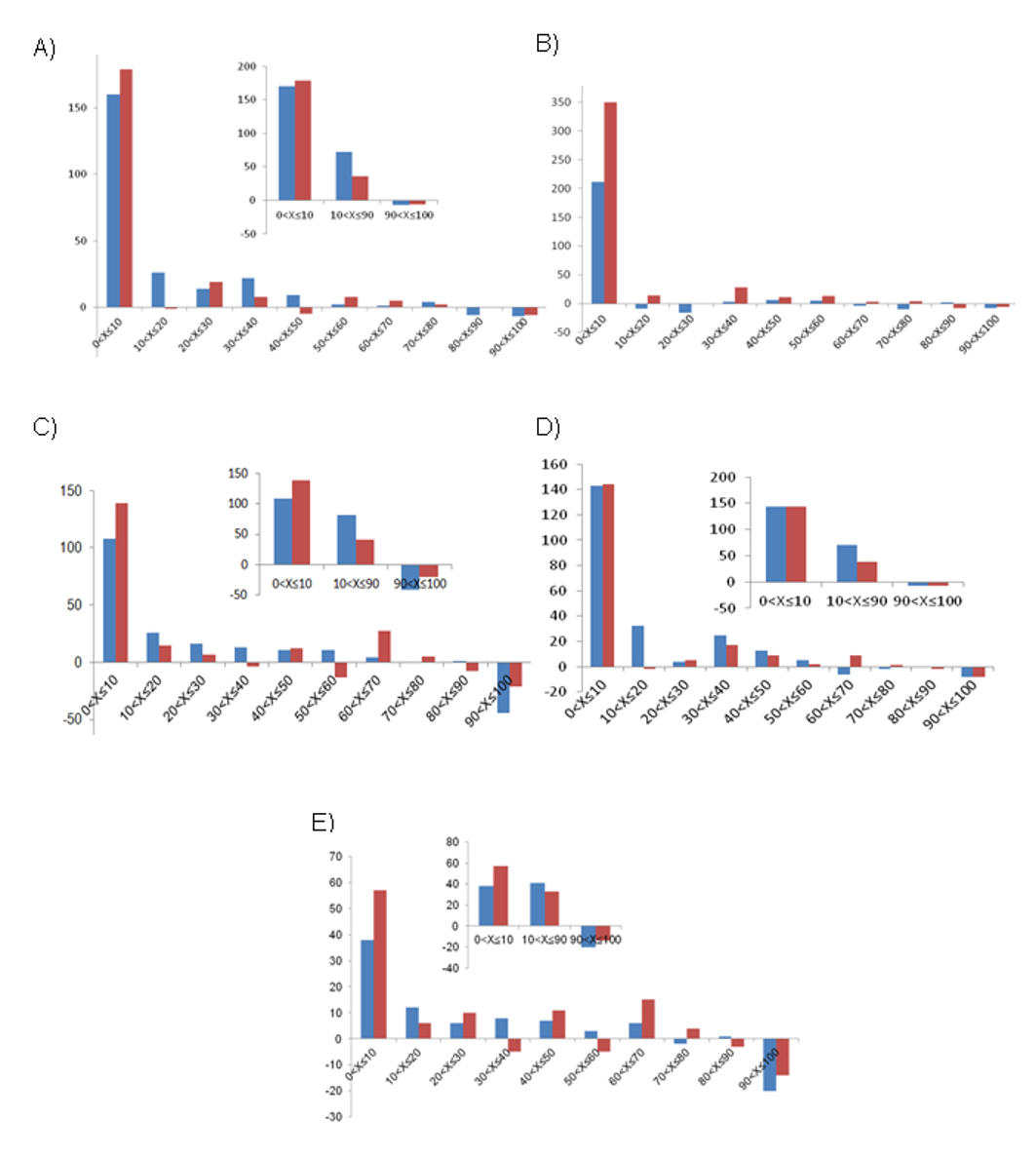

Supplement: S2 Fig — (TIFF) [file pone.0144294.s002.tiff]
