# Supplementary material for: Deciphering the Dynamics of Non-Covalent Interactions Affecting Thermal Stability of a Protein: Molecular Dynamics Study on Point Mutant of Thermus thermophilus Isopropylmalate Dehydrogenase
Source: PLoS One. 2015 Dec 11;10(12):e0144294. doi: 10.1371/journal.pone.0144294 (PMC4689552; doi:10.1371/journal.pone.0144294)
Supplement: S1 Table — Drnona and Arnona represents donor and acceptor residue number followed by its abbreviation (3 Letter), respectively. D and A indicates interacting donor and acceptor atom, respectively. The color formatting indicates the percentage of time the interaction (precen) existed within 10% of the range, 0 < X ≤ 10%: grey background, 10 < X ≤ 20%: blue background, 20 < X ≤ 30%: yellow background, 30 < X ≤ 40%: green text, 40 < X ≤ 50%: red border, 50 < X ≤ 60%: red text, 60 < X ≤ 70%: pink background and black text, 70 < X ≤ 80%: green text and background, 80 < X ≤ 90%: orange background, 90 < X ≤ 100%: pink background and text. (PDF) [file pone.0144294.s003.pdf]

S1 Table. Unique salt bridges and their percentage of existence in *wt* and *mut* at 300 K and 337 K.

1) *Wt* 300 K

Drnona	D	Arnona	A	percen
133GLU	OE2	94ARG	NH2	0.14
133GLU	OE1	94ARG	NH2	0.12
87GLU	OE2	94ARG	NH1	39.97
87GLU	OE1	94ARG	NH1	36.64
87GLU	OE1	94ARG	NH2	27.49
87GLU	OE2	94ARG	NH2	24.14
87GLU	OE2	94ARG	NH1	0.85
87GLU	OE1	94ARG	NH1	0.28
133GLU	OD2	82ARG	NH2	0.05
133GLU	OD1	82ARG	NH2	0.03
133GLU	OD2	76LYS	NZ	0.00
47ASP	OD1	76LYS	NZ	12.56
47ASP	OD2	76LYS	NZ	9.93
47ASP	OD2	76LYS	NZ	0.58
47ASP	OD1	76LYS	NZ	0.45
9ASP	OD2	76LYS	NZ	45.51
9ASP	OD1	76LYS	NZ	40.10
55GLU	OE2	59LYS	NZ	58.83
55GLU	OE1	59LYS	NZ	51.93
55GLU	OE1	59LYS	NZ	41.32
55GLU	OE2	59LYS	NZ	19.75
51GLU	OE2	58ARG	NH1	36.81
51GLU	OE1	58ARG	NH1	33.31
51GLU	OE2	58ARG	NH2	24.65
51GLU	OE1	58ARG	NH2	21.04
51GLU	OE1	58ARG	NH2	20.18
51GLU	OE2	58ARG	NH2	16.82
51GLU	OE2	58ARG	NH1	14.59
51GLU	OE1	58ARG	NH1	10.71
51GLU	OE2	58ARG	NE	1.85
51GLU	OE1	58ARG	NE	1.52
55GLU	OE2	58ARG	NH2	66.07
55GLU	OE1	58ARG	NH2	42.37
55GLU	OE2	58ARG	NE	17.48
55GLU	OE1	58ARG	NE	14.76
55GLU	OE2	58ARG	NH1	8.02
55GLU	OE1	58ARG	NH1	5.09
55GLU	OE2	58ARG	NH1	3.74
55GLU	OE1	58ARG	NH1	3.02
55GLU	OE1	58ARG	NH2	0.93

2) *Wt* 337 K

Drnona	D	Arnona	A	percen
9ASP	OD2	76LYS	NZ	76.79
87GLU	OE2	94ARG	NH2	29.08
87GLU	OE1	94ARG	NH2	27.90
87GLU	OE2	94ARG	NH1	40.52
87GLU	OE2	94ARG	NH1	0.00
87GLU	OE1	94ARG	NH1	54.60
51GLU	OE2	83LYS	NZ	0.55
51GLU	OE1	83LYS	NZ	0.36
78ASP	OD2	82ARG	NH2	1.75
78ASP	OD1	82ARG	NH2	3.13
193GLU	OE2	82ARG	NH2	46.79
193GLU	OE1	82ARG	NH2	44.63
190GLU	OE2	82ARG	NH2	53.91
190GLU	OE1	82ARG	NH2	41.03
78ASP	OD2	82ARG	NH1	0.85
78ASP	OD1	82ARG	NH1	0.37
78ASP	OD1	82ARG	NH1	0.00
193GLU	OE2	82ARG	NH1	9.94
193GLU	OE1	82ARG	NH1	8.80
190GLU	OE2	82ARG	NH1	54.00
190GLU	OE1	82ARG	NH1	60.07
78ASP	OD2	82ARG	NE	0.09
78ASP	OD1	82ARG	NE	0.50
193GLU	OE2	82ARG	NE	31.67
193GLU	OE1	82ARG	NE	35.50
9ASP	OD2	76LYS	NZ	59.82
9ASP	OD1	76LYS	NZ	71.23
9ASP	OD1	76LYS	NZ	58.51
47ASP	OD2	76LYS	NZ	0.39
47ASP	OD2	76LYS	NZ	0.26
47ASP	OD1	76LYS	NZ	0.36
47ASP	OD1	76LYS	NZ	0.30
55GLU	OE2	59LYS	NZ	34.10
55GLU	OE2	59LYS	NZ	29.15
55GLU	OE1	59LYS	NZ	46.39
55GLU	OE1	59LYS	NZ	42.60
55GLU	OE2	58ARG	NH2	52.08
55GLU	OE2	58ARG	NH2	49.45
55GLU	OE1	58ARG	NH2	38.99
55GLU	OE1	58ARG	NH2	33.40

3) *Mut* 300 K

Drnona	D	Arnona	A	percen
113GLU	OE2	114ARG	NH1	0.02
113GLU	OE1	114ARG	NH1	0.00
121GLU	OE1	124ARG	NH1	49.50
121GLU	OE2	124ARG	NH1	47.67
120GLU	OE2	124ARG	NH2	44.41
120GLU	OE1	124ARG	NH2	39.35
121GLU	OE2	124ARG	NH1	37.65
121GLU	OE1	124ARG	NH2	33.34
121GLU	OE2	124ARG	NH2	30.91
113GLU	OE1	124ARG	NE	30.11
121GLU	OE1	124ARG	NH1	29.28
120GLU	OE1	124ARG	NH2	27.21
113GLU	OE1	124ARG	NH2	22.91
113GLU	OE2	124ARG	NH2	19.98
120GLU	OE1	124ARG	NE	17.84
121GLU	OE1	124ARG	NH2	15.46
121GLU	OE2	124ARG	NH2	14.04
120GLU	OE2	124ARG	NH2	13.83
120GLU	OE2	124ARG	NH1	13.09
120GLU	OE2	124ARG	NE	13.03
120GLU	OE1	124ARG	NH1	11.34
113GLU	OE2	124ARG	NH2	8.84
113GLU	OE1	124ARG	NH2	6.07
120GLU	OE1	124ARG	NH1	5.98
113GLU	OE1	124ARG	NE	5.96
113GLU	OE2	124ARG	NE	5.08
120GLU	OE1	124ARG	NE	3.28
121GLU	OE1	124ARG	NE	3.10
113GLU	OE2	124ARG	NE	2.56
113GLU	OE1	124ARG	NH1	2.24
120GLU	OE2	124ARG	NH1	1.63
120GLU	OE2	124ARG	NE	1.54
121GLU	OE2	124ARG	NE	1.43
113GLU	OE2	124ARG	NH1	0.62
113GLU	OE1	124ARG	NH1	0.04
121GLU	OE2	124ARG	NE	0.01
121GLU	OE1	124ARG	NE	0.00
190GLU	OE1	144ARG	NH2	32.86
190GLU	OE2	144ARG	NH2	31.74
190GLU	OE2	144ARG	NE	29.73

4) *Mut* 337 K

Drnona	D	Arnona	A	percen
9ASP	OD1	76LYS	NZ	43.17
9ASP	OD2	76LYS	NZ	39.38
9ASP	OD1	76LYS	NZ	34.32
9ASP	OD2	76LYS	NZ	34.05
98ASP	OD1	164ARG	NH1	62.44
98ASP	OD2	164ARG	NH2	58.15
98ASP	OD1	164ARG	NH2	45.96
98ASP	OD2	164ARG	NH1	45.12
98ASP	OD2	264ARG	NH2	35.01
98ASP	OD1	264ARG	NE	33.48
98ASP	OD1	264ARG	NH1	31.02
98ASP	OD1	264ARG	NE	27.75
98ASP	OD2	264ARG	NH1	26.95
98ASP	OD1	264ARG	NH2	24.45
98ASP	OD2	264ARG	NE	23.69
98ASP	OD1	264ARG	NH1	23.16
98ASP	OD1	264ARG	NH2	19.91
98ASP	OD2	264ARG	NH1	7.88
98ASP	OD2	164ARG	NH2	3.86
98ASP	OD1	164ARG	NH1	2.64
98ASP	OD2	164ARG	NH1	2.12
98ASP	OD2	264ARG	NH2	1.20
98ASP	OD1	164ARG	NH2	1.08
98ASP	OD2	264ARG	NE	1.00
97GLN	OE1	264ARG	NH2	0.19
97GLN	OE1	264ARG	NE	0.00
97GLN	OE1	264ARG	NH1	0.00
87GLU	OE1	144ARG	NH1	0.09
87GLU	OE1	144ARG	NH2	0.02
87GLU	OE2	144ARG	NH1	0.01
87GLU	OE2	144ARG	NH2	0.00
78ASP	OD2	85ARG	NH1	27.58
78ASP	OD1	85ARG	NH1	23.70
78ASP	OD2	85ARG	NH2	11.48
78ASP	OD1	85ARG	NH2	9.73
78ASP	OD1	196ARG	NH1	0.06
78ASP	OD2	85ARG	NH2	0.04
78ASP	OD2	196ARG	NH1	0.04
78ASP	OD1	196ARG	NH2	0.01
78ASP	OD1	85ARG	NH1	0.00

55GLU	OE2	58ARG	NH2	0.89
133GLU	OE1	342ARG	NE1	0.19
133GLU	OE2	342ARG	NE	0.02
321GLU	OE1	342ARG	NH2	51.38
321GLU	OE2	342ARG	NH2	42.02
321GLU	OE1	342ARG	NH2	41.78
321GLU	OE1	342ARG	NH1	40.70
321GLU	OE2	342ARG	NH1	38.91
321GLU	OE1	342ARG	NH1	37.03
321GLU	OE2	342ARG	NH2	35.80
321GLU	OE2	342ARG	NH1	32.95
321GLU	OE1	342ARG	NE	4.87
321GLU	OE2	342ARG	NE	3.56
313ASP	OD1	317LYS	NZ	95.08
313ASP	OD1	317LYS	NZ	85.48
313ASP	OD2	317LYS	NZ	64.58
313ASP	OD2	317LYS	NZ	63.61
133GLU	OE2	310LYS	NZ	0.03
133GLU	OE1	310LYS	NZ	0.01
30GLU	OE1	310LYS	NZ	11.49
30GLU	OE2	310LYS	NZ	11.45
30GLU	OE2	310LYS	NZ	6.84
30GLU	OE1	310LYS	NZ	5.55
133GLU	OE2	309ARG	NH1	0.02
299GLU	OE1	309ARG	NH1	32.24
299GLU	OE2	309ARG	NH2	30.46
299GLU	OE1	309ARG	NH1	23.76
299GLU	OE2	309ARG	NH1	23.63
299GLU	OE2	309ARG	NH1	23.01
299GLU	OE2	309ARG	NH2	20.72
299GLU	OE1	309ARG	NH2	14.20
299GLU	OE1	309ARG	NH2	9.58
306GLU	OE2	309ARG	NH2	36.14
306GLU	OE2	309ARG	NH2	27.92
306GLU	OE1	309ARG	NH2	25.20
306GLU	OE1	309ARG	NH2	24.08
306GLU	OE2	309ARG	NE	20.22
306GLU	OE1	309ARG	NE	18.86
306GLU	OE1	309ARG	NE	18.47
306GLU	OE2	309ARG	NE	15.46
278ASP	OD2	282LYS	NZ	63.90
278ASP	OD2	282LYS	NZ	51.86
278ASP	OD1	282LYS	NZ	50.44

51GLU OE2	58ARG NH2	21.14
51GLU OE2	58ARG NH2	20.28
51GLU OE1	58ARG NH2	21.75
51GLU OE1	58ARG NH2	18.68
55GLU OE2	58ARG NH1	7.92
55GLU OE2	58ARG NH1	1.72
55GLU OE1	58ARG NH1	6.22
55GLU OE1	58ARG NH1	3.40
51GLU OE2	58ARG NH1	39.33
51GLU OE2	58ARG NH1	37.04
51GLU OE1	58ARG NH1	40.14
51GLU OE1	58ARG NH1	39.13
55GLU OE2	58ARG NE	14.10
55GLU OE2	58ARG NE	13.17
55GLU OE1	58ARG NE	11.25
55GLU OE1	58ARG NE	9.61
51GLU OE2	58ARG NE	0.26
51GLU OE2	58ARG NE	0.11
51GLU OE1	58ARG NE	0.23
51GLU OE1	58ARG NE	0.06
321GLU OE2	342ARG NH2	40.31
321GLU OE2	342ARG NH2	36.59
321GLU OE1	342ARG NH2	41.62
321GLU OE1	342ARG NH2	36.55
321GLU OE2	342ARG NH1	31.92
321GLU OE2	342ARG NH1	29.13
321GLU OE1	342ARG NH1	35.90
321GLU OE1	342ARG NH1	30.78
321GLU OE2	342ARG NE	0.01
313ASP OD2	317LYS NZ1	74.18
313ASP OD1	317LYS NZ1	76.38
313ASP OD2	317LYS NZ	76.15
313ASP OD1	317LYS NZ	71.90
30GLU OE2	310LYS NZ	8.88
30GLU OE2	310LYS NZ	2.36
30GLU OE1	310LYS NZ	8.31
30GLU OE1	310LYS NZ	3.68
306GLU OE2	310LYS NZ	1.16
306GLU OE2	310LYS NZ	0.54
306GLU OE1	310LYS NZ	0.66
306GLU OE1	310LYS NZ	0.19
306GLU OE2	309ARG NH2	46.22
306GLU OE2	309ARG NH2	42.47

142GLU	OE2	144ARG	NE	29.61
142GLU	OE1	144ARG	NH2	29.32
142GLU	OE1	144ARG	NE	28.06
142GLU	OE2	144ARG	NH2	26.16
190GLU	OE1	144ARG	NE	17.73
142GLU	OE2	144ARG	NH1	7.58
142GLU	OE1	144ARG	NH1	6.45
190GLU	OE1	144ARG	NH1	1.74
190GLU	OE2	144ARG	NH1	1.26
142GLU	OE2	144ARG	NH1	0.56
142GLU	OE1	144ARG	NH1	0.02
150GLU	OE2	156ARG	NH1	46.88
150GLU	OE2	156ARG	NH2	34.21
150GLU	OE1	156ARG	NH2	33.81
150GLU	OE1	156ARG	NH1	30.83
150GLU	OE1	156ARG	NE	23.12
150GLU	OE2	156ARG	NE	17.60
142GLU	OE2	156ARG	NH2	0.00
148GLU	OE2	159LYS	NZ	29.20
148GLU	OE1	159LYS	NZ	26.41
98ASP	OD2	164ARG	NH2	97.30
98ASP	OD1	164ARG	NH1	92.48
98ASP	OD2	164ARG	NH1	77.24
133GLU	OE2	164ARG	NH2	69.17
133GLU	OE2	164ARG	NE	55.34
133GLU	OE1	164ARG	NH2	48.85
133GLU	OE1	164ARG	NE	36.80
98ASP	OD1	164ARG	NH2	34.26
161GLU	OE1	164ARG	NH2	26.63
161GLU	OE2	164ARG	NH2	25.76
161GLU	OE1	164ARG	NH1	17.32
161GLU	OE2	164ARG	NH1	15.58
98ASP	OD1	164ARG	NH1	0.68
161GLU	OE2	164ARG	NH1	0.58
161GLU	OE1	164ARG	NH1	0.36
161GLU	OE1	164ARG	NE	0.05
98ASP	OD2	164ARG	NH1	0.04
161GLU	OE2	164ARG	NE	0.02
161GLU	OE2	164ARG	NE	0.00
163GLU	OE2	167ARG	NH2	65.12
163GLU	OE1	167ARG	NH2	61.87
163GLU	OE2	167ARG	NE	52.84
163GLU	OE2	167ARG	NH2	48.40

78ASP	OD1	85ARG	NH2	0.00
62GLU	OE2	264ARG	NH1	19.05
62GLU	OE1	264ARG	NH2	18.50
62GLU	OE2	264ARG	NH2	16.84
62GLU	OE1	264ARG	NH1	16.47
62GLU	OE2	264ARG	NE	0.02
55GLU	OE1	58ARG	NH2	49.16
55GLU	OE2	58ARG	NH2	41.29
55GLU	OE2	59LYS	NZ	40.19
55GLU	OE2	59LYS	NZ	39.31
55GLU	OE1	59LYS	NZ	37.60
55GLU	OE2	58ARG	NH1	33.62
55GLU	OE1	59LYS	NZ	32.41
55GLU	OE1	58ARG	NH1	31.49
55GLU	OE1	58ARG	NH2	18.01
55GLU	OE1	58ARG	NE	13.05
55GLU	OE2	58ARG	NH2	11.76
55GLU	OE2	58ARG	NE	10.61
55GLU	OE1	58ARG	NH1	5.62
55GLU	OE2	58ARG	NH1	5.00
55GLU	OE1	58ARG	NE	0.01
55GLU	OE2	58ARG	NE	0.00
51GLU	OE1	58ARG	NH2	48.69
51GLU	OE2	58ARG	NH2	42.60
51GLU	OE1	58ARG	NH1	39.36
51GLU	OE2	58ARG	NH1	38.64
51GLU	OE2	58ARG	NH2	22.03
51GLU	OE1	58ARG	NH2	21.53
51GLU	OE1	58ARG	NE	10.52
51GLU	OE2	58ARG	NE	9.34
51GLU	OE2	58ARG	NH1	8.86
51GLU	OE1	58ARG	NH1	7.96
51GLU	OE1	58ARG	NE	2.05
51GLU	OE2	58ARG	NE	1.88
51GLU	OE1	83LYS	NZ	1.39
51GLU	OE2	83LYS	NZ	1.27
51GLU	OE2	95LYS	NZ	0.96
51GLU	OE1	95LYS	NZ	0.75
47ASP	OD1	76LYS	NZ	17.54
47ASP	OD2	76LYS	NZ	13.16
47ASP	OD2	76LYS	NZ	1.09
47ASP	OD1	76LYS	NZ	1.00
326ASP	OD1	225ARG	NH2	79.32

278ASP	OD1	282LYS	NZ	47.59	306GLU	OE1	309ARG	NH2	44.74	163GLU	OE1	167ARG	NE	47.49	326ASP	OD2	225ARG	NH1	70.23
133GLU	OE2	264ARG	NH1	0.09	306GLU	OE1	309ARG	NH2	42.23	163GLU	OE1	167ARG	NE	44.97	326ASP	OD2	225ARG	NH2	62.72
133GLU	OE1	264ARG	NH2	0.06	299GLU	OE2	309ARG	NH2	14.70	163GLU	OE1	167ARG	NH2	42.45	326ASP	OD1	225ARG	NH1	45.93
133GLU	OE2	264ARG	NH2	0.05	299GLU	OE2	309ARG	NH2	4.08	163GLU	OE2	167ARG	NE	39.19	326ASP	OD2	225ARG	NE	0.04
133GLU	OE1	264ARG	NH2	0.04	299GLU	OE1	309ARG	NH2	8.13	163GLU	OE2	167ARG	NH1	9.08	321GLU	OE1	342ARG	NH2	49.37
133GLU	OE1	264ARG	NH2	0.02	299GLU	OE1	309ARG	NH2	5.85	163GLU	OE1	167ARG	NH1	8.06	321GLU	OE2	342ARG	NH2	48.81
133GLU	OE2	264ARG	NH1	0.00	306GLU	OE2	309ARG	NH1	12.91	163GLU	OE1	167ARG	NH1	2.49	321GLU	OE1	342ARG	NH1	37.87
133GLU	OE1	264ARG	NH1	0.00	306GLU	OE2	309ARG	NH1	11.29	163GLU	OE2	167ARG	NH1	0.28	321GLU	OE2	342ARG	NH1	35.84
161GLU	OE1	264ARG	NH1	23.61	306GLU	OE1	309ARG	NH1	13.38	171GLU	OE2	174ARG	NH1	32.07	321GLU	OE2	342ARG	NH2	34.67
161GLU	OE2	264ARG	NH1	7.72	306GLU	OE1	309ARG	NH1	12.74	171GLU	OE2	174ARG	NH1	27.28	321GLU	OE1	342ARG	NH2	33.67
161GLU	OE2	264ARG	NH2	1.04	299GLU	OE2	309ARG	NH1	12.28	171GLU	OE1	174ARG	NH1	22.52	321GLU	OE2	342ARG	NH1	31.93
161GLU	OE1	264ARG	NH2	0.69	299GLU	OE2	309ARG	NH1	6.08	171GLU	OE1	174ARG	NH1	15.63	321GLU	OE1	342ARG	NH1	31.07
62GLU	OE2	264ARG	NH2	0.71	299GLU	OE1	309ARG	NH1	16.44	171GLU	OE2	174ARG	NE	0.01	321GLU	OE2	342ARG	NE	0.49
98ASP	OD1	264ARG	NE	64.59	299GLU	OE1	309ARG	NH1	6.63	171GLU	OE2	175LYS	NZ	0.97	321GLU	OE1	342ARG	NE	0.12
98ASP	OD1	264ARG	NH2	62.96	306GLU	OE2	309ARG	NE	24.38	171GLU	OE1	175LYS	NZ	0.74	321GLU	OE2	342ARG	NE	0.04
98ASP	OD2	264ARG	NH2	54.84	306GLU	OE2	309ARG	NE	21.86	171GLU	OE1	175LYS	NZ	0.23	313ASP	OD1	317LYS	NZ	76.26
98ASP	OD2	264ARG	NE	44.37	306GLU	OE1	309ARG	NE	27.45	171GLU	OE2	175LYS	NZ	0.14	313ASP	OD2	317LYS	NZ	75.28
98ASP	OD1	264ARG	NE	33.03	306GLU	OE1	309ARG	NE	20.54	127ASP	OD1	176ARG	NH2	97.99	313ASP	OD2	317LYS	NZ	72.15
98ASP	OD2	264ARG	NE	29.79	299GLU	OE2	309ARG	NE	0.07	127ASP	OD1	176ARG	NH2	70.73	313ASP	OD1	317LYS	NZ	63.28
98ASP	OD2	264ARG	NH2	22.58	299GLU	OE2	309ARG	NE	0.01	127ASP	OD1	176ARG	NH1	60.31	30GLU	OE1	310LYS	NZ	14.72
98ASP	OD1	264ARG	NH2	19.06	299GLU	OE1	309ARG	NE	0.18	127ASP	OD1	176ARG	NH1	35.26	30GLU	OE2	310LYS	NZ	11.62
98ASP	OD1	264ARG	NH1	8.25	299GLU	OE1	309ARG	NE	0.09	127ASP	OD2	176ARG	NH1	34.83	30GLU	OE2	310LYS	NZ	6.34
98ASP	OD1	264ARG	NH1	6.26	78ASP	OD2	282LYS	NZ	0.93	127ASP	OD2	176ARG	NH2	33.18	30GLU	OE1	310LYS	NZ	5.69
98ASP	OD2	264ARG	NH1	3.48	78ASP	OD1	282LYS	NZ	1.57	127ASP	OD2	176ARG	NH2	19.42	306GLU	OE1	309ARG	NH2	42.93
98ASP	OD2	264ARG	NH1	0.37	278ASP	OD2	282LYS	NZ	66.75	127ASP	OD2	176ARG	NH1	11.08	306GLU	OE2	309ARG	NH2	42.92
17GLU	OE2	24ARG	NH2	35.32	278ASP	OD2	282LYS	NZ	44.09	127ASP	OD2	177ARG	NH2	5.64	306GLU	OE1	309ARG	NH1	21.10
17GLU	OE2	24ARG	NH2	28.89	278ASP	OD1	282LYS	NZ	65.47	127ASP	OD2	177ARG	NH2	1.69	306GLU	OE2	309ARG	NE	18.08
17GLU	OE1	24ARG	NH2	23.89	278ASP	OD1	282LYS	NZ	43.45	127ASP	OD2	177ARG	NH1	1.65	306GLU	OE1	309ARG	NE	16.99
17GLU	OE1	24ARG	NH2	22.14	98ASP	OD2	264ARG	NH2	59.20	127ASP	OD2	177ARG	NH1	0.18	306GLU	OE2	309ARG	NH1	16.80
17GLU	OE2	24ARG	NH1	21.63	98ASP	OD2	264ARG	NH2	25.60	127ASP	OD1	177ARG	NH2	0.00	306GLU	OE1	310LYS	NZ	2.02
17GLU	OE1	24ARG	NH1	20.11	98ASP	OD1	264ARG	NH2	61.37	127ASP	OD2	177ARG	NE	0.00	306GLU	OE2	310LYS	NZ	1.83
17GLU	OE1	24ARG	NH1	19.82	98ASP	OD1	264ARG	NH2	13.70	241ASP	OD1	185LYS	NZ	81.38	306GLU	OE2	310LYS	NZ	0.70
17GLU	OE2	24ARG	NH1	18.09	97GLN	OE1	264ARG	NH2	0.02	241ASP	OD2	185LYS	NZ	57.30	306GLU	OE1	310LYS	NZ	0.12
133GLU	OE1	229ARG	NH1	0.03	62GLU	OE2	264ARG	NH2	0.26	184ASP	OD1	196ARG	NE	98.72	299GLU	OE2	309ARG	NH1	62.00
133GLU	OE1	229ARG	NE	0.02	62GLU	OE1	264ARG	NH2	0.49	184ASP	OD1	196ARG	NH2	98.22	299GLU	OE1	309ARG	NH2	58.48
133GLU	OE1	229ARG	NE	0.02	161GLU	OE2	264ARG	NH2	3.06	184ASP	OD2	196ARG	NE	95.65	299GLU	OE1	309ARG	NH1	57.63
133GLU	OE2	229ARG	NE	0.01	161GLU	OE2	264ARG	NH2	0.30	184ASP	OD2	196ARG	NH2	95.13	299GLU	OE2	309ARG	NH2	55.24
212GLU	OE2	229ARG	NH1	98.77	161GLU	OE1	264ARG	NH2	1.35	184ASP	OD2	196ARG	NE	77.95	299GLU	OE2	309ARG	NH1	8.02
212GLU	OE1	229ARG	NH2	97.73	161GLU	OE1	264ARG	NH2	0.15	184ASP	OD1	196ARG	NE	62.29	299GLU	OE1	309ARG	NH1	7.91
212GLU	OE1	229ARG	NH1	66.02	98ASP	OD2	264ARG	NH1	12.37	184ASP	OD1	196ARG	NH2	48.47	299GLU	OE2	309ARG	NH2	6.62
212GLU	OE2	229ARG	NH2	50.23	98ASP	OD2	264ARG	NH1	10.02	184ASP	OD2	196ARG	NH2	45.81	299GLU	OE1	309ARG	NH2	4.78
212GLU	OE1	229ARG	NH1	50.21	98ASP	OD1	264ARG	NH1	6.81	193GLU	OE1	196ARG	NH1	0.01	299GLU	OE2	309ARG	NE	0.03
212GLU	OE1	229ARG	NH2	47.84	98ASP	OD1	264ARG	NH1	1.74	148GLU	OE2	197LYS	NZ	52.43	299GLU	OE1	309ARG	NE	0.01

212GLU	OE2	229ARG	NH1	46.71	97GLN OE1	264ARG NH1	0.04	148GLU	OE1	197LYS	NZ	51.87	278ASP	OD2	282LYS	NZ	69.68
212GLU	OE2	229ARG	NH2	46.27	62GLU OE2	264ARG NH1	0.40	193GLU	OE2	197LYS	NZ	2.84	278ASP	OD1	282LYS	NZ	69.11
214GLN	OE1	229ARG	NH2	1.94	62GLU OE1	264ARG NH1	0.20	193GLU	OE1	197LYS	NZ	2.55	278ASP	OD2	282LYS	NZ	58.89
214GLN	OE1	229ARG	NH1	0.77	161GLU OE2	264ARG NH1	4.51	193GLU	OE1	197LYS	NZ	0.69	278ASP	OD1	282LYS	NZ	51.67
214GLN	OE1	229ARG	NH2	0.58	161GLU OE2	264ARG NH1	0.07	193GLU	OE2	197LYS	NZ	0.04	245ASP	OD1	185LYS	NZ	10.31
214GLN	OE1	229ARG	NE	0.26	161GLU OE1	264ARG NH1	4.91	200GLU	OE1	204ARG	NE	88.68	245ASP	OD2	185LYS	NZ	0.28
326ASP	OD1	225ARG	NH2	2.26	161GLU OE1	264ARG NH1	0.02	200GLU	OE2	204ARG	NH2	88.56	241ASP	OD2	185LYS	NZ	66.59
326ASP	OD2	225ARG	NH2	2.24	133GLU OE1	264ARG NH1	0.07	200GLU	OE1	204ARG	NE	85.17	241ASP	OD1	185LYS	NZ	45.49
326ASP	OD1	225ARG	NH1	1.92	98ASP OD2	264ARG NE	55.98	200GLU	OE2	204ARG	NH2	82.58	214GLN	OE1	229ARG	NH2	1.34
326ASP	OD2	225ARG	NH1	1.27	98ASP OD2	264ARG NE	34.16	200GLU	OE2	204ARG	NE	68.16	214GLN	OE1	229ARG	NH1	0.51
133GLU	OE1	21LYS	NZ	0.07	98ASP OD1	264ARG NE	47.37	200GLU	OE2	204ARG	NE	61.98	214GLN	OE1	229ARG	NH2	0.21
133GLU	OE2	21LYS	NZ	0.00	98ASP OD1	264ARG NE	16.77	201GLU	OE2	204ARG	NH1	60.81	214GLN	OE1	229ARG	NE	0.04
133GLU	OE1	21LYS	NZ	0.00	17GLU OE2	24ARG NH2	48.44	201GLU	OE1	204ARG	NH1	55.83	214GLN	OE1	229ARG	NE	0.02
133GLU	OE2	21LYS	NZ	0.00	17GLU OE2	24ARG NH2	0.22	201GLU	OE1	204ARG	NH2	55.49	214GLN	OE1	229ARG	NH1	0.01
17GLU	OE2	21LYS	NZ	1.74	17GLU OE1	24ARG NH2	49.30	200GLU	OE1	204ARG	NH2	53.53	212GLU	OE1	229ARG	NH1	49.75
17GLU	OE2	21LYS	NZ	0.48	17GLU OE1	24ARG NH2	0.51	200GLU	OE1	204ARG	NH2	49.92	212GLU	OE2	229ARG	NH2	49.61
17GLU	OE1	21LYS	NZ	0.46	17GLU OE2	24ARG NH1	46.96	201GLU	OE2	204ARG	NH1	45.81	212GLU	OE2	229ARG	NH1	48.47
133GLU	OE1	204ARG	NH1	0.01	17GLU OE2	24ARG NH1	0.02	201GLU	OE1	204ARG	NH1	44.28	212GLU	OE1	229ARG	NH2	46.53
133GLU	OE2	204ARG	NH1	0.01	17GLU OE1	24ARG NH1	40.29	201GLU	OE1	204ARG	NH2	44.13	212GLU	OE1	229ARG	NH2	9.23
133GLU	OE1	204ARG	NE	0.01	17GLU OE1	24ARG NH1	0.30	201GLU	OE2	204ARG	NH2	37.25	212GLU	OE2	229ARG	NH2	9.10
133GLU	OE2	204ARG	NE	0.00	214GLN OE1	229ARG NH2	2.39	201GLU	OE2	204ARG	NH2	28.30	212GLU	OE2	229ARG	NH1	6.26
200GLU	OE2	204ARG	NH2	68.48	214GLN OE1	229ARG NH2	0.16	17GLU	OE2	21LYS	NZ	1.60	212GLU	OE1	229ARG	NH1	5.53
200GLU	OE1	204ARG	NE	63.46	212GLU OE2	229ARG NH2	80.51	17GLU	OE1	21LYS	NZ	1.00	212GLU	OE1	229ARG	NE	0.64
200GLU	OE2	204ARG	NE	50.08	212GLU OE2	229ARG NH2	22.48	17GLU	OE1	21LYS	NZ	0.78	212GLU	OE2	229ARG	NE	0.50
200GLU	OE1	204ARG	NH2	37.89	212GLU OE1	229ARG NH2	70.14	14GLU	OE1	21LYS	NZ	0.01	212GLU	OE1	229ARG	NE	0.44
200GLU	OE1	204ARG	NH2	15.54	212GLU OE1	229ARG NH2	27.39	17GLU	OE2	21LYS	NZ	0.01	212GLU	OE2	229ARG	NE	0.41
200GLU	OE1	204ARG	NE	14.93	214GLN OE1	229ARG NH1	1.32	14GLU	OE1	21LYS	NZ	0.01	201GLU	OE2	204ARG	NH1	51.59
200GLU	OE2	204ARG	NE	13.38	214GLN OE1	229ARG NH1	0.20	14GLU	OE2	21LYS	NZ	0.00	201GLU	OE1	204ARG	NH1	49.65
200GLU	OE2	204ARG	NH2	11.24	212GLU OE2	229ARG NH1	79.90	326ASP	OD1	225ARG	NH2	15.25	201GLU	OE2	204ARG	NH2	44.89
200GLU	OE1	204ARG	NH1	0.52	212GLU OE2	229ARG NH1	8.42	326ASP	OD2	225ARG	NH2	14.39	201GLU	OE1	204ARG	NH2	44.22
200GLU	OE2	204ARG	NH1	0.31	212GLU OE1	229ARG NH1	83.35	326ASP	OD2	225ARG	NH1	13.13	201GLU	OE2	204ARG	NH1	42.84
201GLU	OE2	204ARG	NH1	52.01	212GLU OE1	229ARG NH1	7.30	326ASP	OD1	225ARG	NH1	13.05	201GLU	OE1	204ARG	NH1	42.19
201GLU	OE1	204ARG	NH1	49.95	214GLN OE1	229ARG NE	0.18	212GLU	OE2	229ARG	NH2	86.18	201GLU	OE1	204ARG	NH2	29.48
201GLU	OE1	204ARG	NH1	41.38	212GLU OE2	229ARG NE	2.34	212GLU	OE1	229ARG	NH1	81.87	201GLU	OE2	204ARG	NH2	25.93
201GLU	OE2	204ARG	NH2	38.38	212GLU OE1	229ARG NE	3.14	212GLU	OE2	229ARG	NH1	77.53	201GLU	OE1	204ARG	NE	0.00
201GLU	OE2	204ARG	NH1	33.27	326ASP OD2	225ARG NH2	19.43	212GLU	OE1	229ARG	NH2	73.43	200GLU	OE2	204ARG	NE	74.31
201GLU	OE1	204ARG	NH2	30.79	326ASP OD1	225ARG NH2	15.04	212GLU	OE2	229ARG	NH2	1.68	200GLU	OE1	204ARG	NE	70.84
201GLU	OE2	204ARG	NH2	6.07	326ASP OD2	225ARG NH1	12.71	212GLU	OE1	229ARG	NH2	1.17	200GLU	OE1	204ARG	NH2	65.05
201GLU	OE1	204ARG	NH2	3.50	326ASP OD1	225ARG NH1	13.93	212GLU	OE2	229ARG	NE	0.15	200GLU	OE2	204ARG	NH2	64.25
201GLU	OE2	204ARG	NE	0.36	17GLU OE2	21LYS NZ	9.75	212GLU	OE1	229ARG	NE	0.07	200GLU	OE2	204ARG	NE	54.85
148GLU	OE2	197LYS	NZ	36.07	17GLU OE2	21LYS NZ	6.48	212GLU	OE2	229ARG	NH1	0.05	200GLU	OE1	204ARG	NH2	52.72
148GLU	OE1	197LYS	NZ	35.75	17GLU OE1	21LYS NZ	7.53	214GLN	OE1	229ARG	NH1	0.04	200GLU	OE1	204ARG	NE	52.41
193GLU	OE2	197LYS	NZ	4.51	17GLU OE1	21LYS NZ	4.72	214GLN	OE1	229ARG	NH2	0.02	200GLU	OE2	204ARG	NH2	47.85

193GLU	OE1	197LYS	NZ	3.63
193GLU	OE1	197LYS	NZ	3.46
193GLU	OE2	197LYS	NZ	3.00
133GLU	OE2	196ARG	NH1	0.08
133GLU	OE1	196ARG	NH1	0.04
184ASP	OD2	196ARG	NE	99.99
184ASP	OD1	196ARG	NH2	99.78
184ASP	OD1	196ARG	NH2	99.58
184ASP	OD2	196ARG	NH2	99.35
184ASP	OD2	196ARG	NE	96.04
184ASP	OD1	196ARG	NE	78.14
184ASP	OD1	196ARG	NE	61.70
184ASP	OD2	196ARG	NH2	40.66
241ASP	OD2	185LYS	NZ	86.69
241ASP	OD1	185LYS	NZ	72.47
127ASP	OD1	177ARG	NH2	24.92
127ASP	OD2	177ARG	NH1	14.26
127ASP	OD2	177ARG	NH2	11.48
127ASP	OD2	177ARG	NH2	11.14
127ASP	OD2	177ARG	NE	0.53
127ASP	OD1	177ARG	NE	0.44
133GLU	OD2	177ARG	NH1	0.14
127ASP	OD1	176ARG	NH2	96.91
127ASP	OD2	176ARG	NH1	66.16
127ASP	OD2	176ARG	NH2	49.27
127ASP	OD1	176ARG	NH1	41.76
127ASP	OD1	176ARG	NH1	38.11
127ASP	OD2	176ARG	NH2	32.13
127ASP	OD1	176ARG	NH2	21.72
127ASP	OD2	176ARG	NH1	21.24
171GLU	OE2	175LYS	NZ	3.41
171GLU	OE1	175LYS	NZ	3.25
171GLU	OE2	175LYS	NZ	1.64
171GLU	OE1	175LYS	NZ	1.50
171GLU	OE2	174ARG	NH2	85.84
171GLU	OE2	174ARG	NH2	81.40
171GLU	OE1	174ARG	NH2	81.06
171GLU	OE1	174ARG	NH2	76.50
171GLU	OE2	174ARG	NE	49.85
171GLU	OE2	174ARG	NE	44.49
171GLU	OE1	174ARG	NE	43.47
171GLU	OE1	174ARG	NE	37.72
163GLU	OE2	167ARG	NH2	53.13

14GLU	OE2	21LYS	NZ	0.01
14GLU	OE2	21LYS	NZ	0.00
14GLU	OE1	21LYS	NZ	0.03
14GLU	OE1	21LYS	NZ	0.02
201GLU	OE2	204ARG	NH2	44.85
201GLU	OE1	204ARG	NH2	39.12
201GLU	OE1	204ARG	NH2	0.03
200GLU	OE2	204ARG	NH2	78.46
200GLU	OE2	204ARG	NH2	29.53
200GLU	OE1	204ARG	NH2	75.63
200GLU	OE1	204ARG	NH2	31.66
201GLU	OE2	204ARG	NH1	4.02
201GLU	OE2	204ARG	NH1	0.33
201GLU	OE1	204ARG	NH1	3.98
201GLU	OE1	204ARG	NH1	0.80
200GLU	OE2	204ARG	NH1	13.19
200GLU	OE2	204ARG	NH1	0.01
200GLU	OE1	204ARG	NH1	7.81
200GLU	OE1	204ARG	NH1	0.26
201GLU	OE2	204ARG	NE	30.26
201GLU	OE1	204ARG	NE	32.83
200GLU	OE2	204ARG	NE	70.96
200GLU	OE2	204ARG	NE	27.82
200GLU	OE1	204ARG	NE	70.18
200GLU	OE1	204ARG	NE	22.26
193GLU	OE2	197LYS	NZ	4.92
193GLU	OE2	197LYS	NZ	4.72
193GLU	OE1	197LYS	NZ	5.54
193GLU	OE1	197LYS	NZ	5.52
148GLU	OE2	197LYS	NZ	45.71
148GLU	OE1	197LYS	NZ	46.55
184ASP	OD2	196ARG	NH2	99.44
184ASP	OD2	196ARG	NH2	98.53
184ASP	OD1	196ARG	NH2	99.41
184ASP	OD1	196ARG	NH2	97.82
193GLU	OE2	196ARG	NH1	0.02
193GLU	OE2	196ARG	NH1	0.01
193GLU	OE1	196ARG	NH1	0.01
193GLU	OE1	196ARG	NH1	0.00
184ASP	OD2	196ARG	NE	100.00
184ASP	OD2	196ARG	NE	56.28
184ASP	OD1	196ARG	NE	99.16
184ASP	OD1	196ARG	NE	60.19

214GLN	OE1	229ARG	NH1	0.00
17GLU	OE1	24ARG	NH2	56.75
17GLU	OE1	24ARG	NH1	54.65
17GLU	OE2	24ARG	NH1	51.89
17GLU	OE2	24ARG	NH2	49.29
17GLU	OE2	24ARG	NH1	34.92
17GLU	OE1	24ARG	NH2	34.18
17GLU	OE2	24ARG	NH2	31.27
17GLU	OE1	24ARG	NH1	29.41
98ASP	OD2	264ARG	NE	54.22
98ASP	OD2	264ARG	NH2	52.69
98ASP	OD1	264ARG	NH2	35.77
98ASP	OD1	264ARG	NE	28.40
98ASP	OD2	264ARG	NE	18.74
98ASP	OD2	264ARG	NH1	18.09
98ASP	OD2	264ARG	NH2	12.99
98ASP	OD2	264ARG	NH1	10.40
98ASP	OD1	264ARG	NH1	10.02
62GLU	OE1	264ARG	NH2	5.24
62GLU	OE2	264ARG	NH1	5.14
62GLU	OE1	264ARG	NH1	3.29
62GLU	OE2	264ARG	NH2	2.90
98ASP	OD1	264ARG	NE	2.31
98ASP	OD1	264ARG	NH2	1.47
98ASP	OD1	264ARG	NH1	0.88
97GLN	OE1	264ARG	NH2	0.37
97GLN	OE1	264ARG	NH1	0.00
278ASP	OD2	282LYS	NZ	40.30
278ASP	OD1	282LYS	NZ	39.70
278ASP	OD1	282LYS	NZ	3.54
278ASP	OD2	282LYS	NZ	3.38
299GLU	OE1	309ARG	NH2	75.00
299GLU	OE2	309ARG	NH2	69.45
299GLU	OE2	309ARG	NH1	65.96
306GLU	OE1	309ARG	NH2	63.97
306GLU	OE2	309ARG	NH2	59.79
299GLU	OE1	309ARG	NH1	51.73
306GLU	OE2	309ARG	NE	34.43
306GLU	OE1	309ARG	NE	31.06
306GLU	OE2	309ARG	NH1	10.58
306GLU	OE1	309ARG	NH1	5.38
299GLU	OE1	309ARG	NH1	5.18
299GLU	OE2	309ARG	NH1	4.96

200GLU	OE2	204ARG	NH1	4.57
200GLU	OE1	204ARG	NH1	4.43
200GLU	OE1	204ARG	NH1	0.01
200GLU	OE2	204ARG	NH1	0.00
193GLU	OE1	82ARG	NH2	41.86
193GLU	OE2	82ARG	NH2	39.09
193GLU	OE1	82ARG	NH1	32.15
193GLU	OE2	82ARG	NH1	31.23
193GLU	OE2	197LYS	NZ	14.34
193GLU	OE1	197LYS	NZ	14.04
193GLU	OE1	82ARG	NE	5.51
193GLU	OE2	82ARG	NE	4.72
193GLU	OE2	197LYS	NZ	1.40
193GLU	OE1	196ARG	NH1	0.34
193GLU	OE1	197LYS	NZ	0.29
193GLU	OE2	196ARG	NH1	0.18
193GLU	OE1	196ARG	NH1	0.00
193GLU	OE2	196ARG	NH1	0.00
190GLU	OE2	144ARG	NE	90.67
190GLU	OE1	144ARG	NE	90.22
190GLU	OE2	144ARG	NH2	79.94
190GLU	OE1	144ARG	NH2	60.51
190GLU	OE1	83LYS	NZ	1.28
190GLU	OE1	82ARG	NH2	1.00
190GLU	OE1	82ARG	NH1	0.80
190GLU	OE2	82ARG	NH1	0.33
190GLU	OE2	82ARG	NH2	0.26
190GLU	OE2	83LYS	NZ	0.04
190GLU	OE2	82ARG	NE	0.02
190GLU	OE1	82ARG	NE	0.01
184ASP	OD2	196ARG	NE	100.00
184ASP	OD1	196ARG	NH2	99.08
184ASP	OD2	196ARG	NH2	98.23
184ASP	OD1	196ARG	NH2	92.71
184ASP	OD2	196ARG	NE	88.78
184ASP	OD2	196ARG	NH2	71.55
184ASP	OD1	196ARG	NE	70.87
184ASP	OD1	196ARG	NE	61.33
17GLU	OE2	24ARG	NH2	63.03
17GLU	OE1	24ARG	NH1	62.61
17GLU	OE1	24ARG	NH2	60.95
17GLU	OE2	24ARG	NH1	54.34
17GLU	OE2	24ARG	NH2	50.03

163GLU	OE1	167ARG	NH1	49.08
163GLU	OE1	167ARG	NH2	49.02
163GLU	OE2	167ARG	NH1	40.14
163GLU	OE1	167ARG	NH2	37.84
163GLU	OE2	167ARG	NH2	33.11
163GLU	OE1	167ARG	NE	27.09
163GLU	OE2	167ARG	NE	25.83
163GLU	OE2	167ARG	NH1	14.60
163GLU	OE1	167ARG	NH1	13.96
163GLU	OE1	167ARG	NE	2.33
163GLU	OE2	167ARG	NE	1.49
133GLU	OE2	164ARG	NH1	1.46
133GLU	OE2	164ARG	NH2	0.20
133GLU	OE1	164ARG	NH2	0.04
133GLU	OE2	164ARG	NH1	0.04
133GLU	OE1	164ARG	NH1	0.02
133GLU	OE2	164ARG	NE	0.01
133GLU	OE1	164ARG	NE	0.00
161GLU	OE2	164ARG	NH2	47.49
161GLU	OE1	164ARG	NH2	30.71
161GLU	OE2	164ARG	NH1	22.01
161GLU	OE1	164ARG	NE	20.96
161GLU	OE1	164ARG	NH1	19.26
161GLU	OE2	164ARG	NE	16.64
161GLU	OE2	164ARG	NH1	10.49
161GLU	OE1	164ARG	NH1	7.82
161GLU	OE2	164ARG	NH2	0.21
98ASP	OD2	164ARG	NH1	64.13
98ASP	OD1	164ARG	NH1	52.65
98ASP	OD2	164ARG	NH2	48.96
98ASP	OD1	164ARG	NH2	48.41
98ASP	OD1	164ARG	NH1	20.30
98ASP	OD2	164ARG	NH2	19.87
98ASP	OD1	164ARG	NH2	16.11
98ASP	OD2	164ARG	NH1	5.18
148GLU	OE1	159LYS	NZ	32.39
148GLU	OE2	159LYS	NZ	24.87
142GLU	OE1	156ARG	NH2	1.05
142GLU	OE1	156ARG	NH1	0.92
142GLU	OE2	156ARG	NH2	0.83
142GLU	OE2	156ARG	NH1	0.34
150GLU	OE2	156ARG	NH2	78.24
150GLU	OE1	156ARG	NH2	76.20

241ASP	OD2	185LYS	NZ	45.96
241ASP	OD1	185LYS	NZ	67.88
127ASP	OD2	177ARG	NH2	8.74
127ASP	OD2	177ARG	NH2	7.84
127ASP	OD1	177ARG	NH2	36.56
127ASP	OD1	177ARG	NH2	3.06
127ASP	OD2	177ARG	NH1	18.09
127ASP	OD2	177ARG	NH1	1.58
127ASP	OD1	177ARG	NH1	11.37
127ASP	OD1	177ARG	NH1	2.79
127ASP	OD2	177ARG	NE	0.49
127ASP	OD1	177ARG	NE	2.57
127ASP	OD1	177ARG	NE	0.02
127ASP	OD2	176ARG	NH2	64.91
127ASP	OD2	176ARG	NH2	51.06
127ASP	OD1	176ARG	NH2	65.67
127ASP	OD1	176ARG	NH2	13.28
127ASP	OD2	176ARG	NH1	55.15
127ASP	OD2	176ARG	NH1	40.19
127ASP	OD1	176ARG	NH1	35.68
127ASP	OD1	176ARG	NH1	29.71
127ASP	OD2	176ARG	NE	0.00
171GLU	OE2	175LYS	NZ	1.27
171GLU	OE2	175LYS	NZ	0.92
171GLU	OE1	175LYS	NZ	1.72
171GLU	OE1	175LYS	NZ	1.27
171GLU	OE2	174ARG	NH2	69.37
171GLU	OE2	174ARG	NH2	53.73
171GLU	OE1	174ARG	NH2	68.53
171GLU	OE1	174ARG	NH2	55.58
171GLU	OE2	174ARG	NH1	25.48
171GLU	OE2	174ARG	NH1	18.48
171GLU	OE1	174ARG	NH1	23.08
171GLU	OE1	174ARG	NH1	15.73
171GLU	OE2	174ARG	NE	29.87
171GLU	OE2	174ARG	NE	28.67
171GLU	OE1	174ARG	NE	33.56
171GLU	OE1	174ARG	NE	25.10
163GLU	OE2	167ARG	NH2	55.55
163GLU	OE2	167ARG	NH2	49.46
163GLU	OE1	167ARG	NH2	56.62
163GLU	OE1	167ARG	NH2	53.30
163GLU	OE2	167ARG	NH1	9.09

299GLU	OE2	309ARG	NH2	4.50
299GLU	OE1	309ARG	NH2	1.78
30GLU	OE2	310LYS	NZ	8.64
30GLU	OE2	310LYS	NZ	5.65
30GLU	OE1	310LYS	NZ	4.66
30GLU	OE1	310LYS	NZ	2.62
306GLU	OE1	310LYS	NZ	0.83
306GLU	OE1	310LYS	NZ	0.24
306GLU	OE2	310LYS	NZ	0.13
306GLU	OE2	310LYS	NZ	0.12
313ASP	OD2	317LYS	NZ	93.07
313ASP	OD2	317LYS	NZ	82.68
313ASP	OD1	317LYS	NZ	75.18
313ASP	OD1	317LYS	NZ	64.47
321GLU	OE1	342ARG	NH2	45.65
321GLU	OE2	342ARG	NH2	43.89
321GLU	OE1	342ARG	NH2	42.79
321GLU	OE2	342ARG	NH2	40.42
321GLU	OE1	342ARG	NH1	37.88
321GLU	OE2	342ARG	NH1	31.22
321GLU	OE2	342ARG	NH1	30.13
321GLU	OE1	342ARG	NH1	25.11
321GLU	OE1	342ARG	NE	4.36
321GLU	OE2	342ARG	NE	1.18
321GLU	OE1	342ARG	NE1	1.17
321GLU	OE2	342ARG	NE1	0.90
55GLU	OE1	58ARG	NH2	39.51
55GLU	OE2	58ARG	NH2	35.81
51GLU	OE2	58ARG	NH2	35.23
51GLU	OE1	58ARG	NH1	28.54
51GLU	OE2	58ARG	NH1	26.97
51GLU	OE2	58ARG	NH2	23.30
51GLU	OE1	58ARG	NH2	22.96
51GLU	OE1	58ARG	NH2	19.42
55GLU	OE2	58ARG	NH2	15.58
55GLU	OE1	58ARG	NH2	14.90
55GLU	OE2	58ARG	NH1	13.25
55GLU	OE1	58ARG	NH1	11.78
55GLU	OE2	58ARG	NH1	10.22
55GLU	OE1	58ARG	NE	8.69
51GLU	OE2	58ARG	NH1	8.66
55GLU	OE2	58ARG	NE	8.20
55GLU	OE2	58ARG	NE	7.32

17GLU	OE1	24ARG	NH2	47.86
17GLU	OE1	24ARG	NH1	45.64
17GLU	OE2	24ARG	NH1	39.32
17GLU	OE1	21LYS	NZ	5.50
17GLU	OE2	21LYS	NZ	3.68
17GLU	OE1	21LYS	NZ	2.77
17GLU	OE2	21LYS	NZ	0.72
17GLU	OE2	24ARG	NE	0.00
171GLU	OE2	174ARG	NH1	21.38
171GLU	OE1	174ARG	NH1	20.50
171GLU	OE1	174ARG	NH1	18.74
171GLU	OE2	174ARG	NH1	16.60
171GLU	OE1	175LYS	NZ	1.46
171GLU	OE2	175LYS	NZ	1.27
171GLU	OE2	175LYS	NZ	1.18
171GLU	OE1	175LYS	NZ	1.17
171GLU	OE1	174ARG	NE	0.00
163GLU	OE2	167ARG	NE	63.68
163GLU	OE1	167ARG	NH2	54.80
163GLU	OE1	164ARG	NH2	50.77
163GLU	OE2	164ARG	NH2	47.94
163GLU	OE2	167ARG	NH2	46.96
163GLU	OE2	167ARG	NH2	45.63
163GLU	OE1	164ARG	NE	45.52
163GLU	OE1	167ARG	NE	44.35
163GLU	OE2	167ARG	NE	42.20
163GLU	OE1	167ARG	NE	28.31
163GLU	OE1	167ARG	NH2	22.90
163GLU	OE1	167ARG	NH1	8.66
163GLU	OE2	164ARG	NE	8.21
163GLU	OE2	167ARG	NH1	6.27
163GLU	OE2	167ARG	NH1	6.26
163GLU	OE1	167ARG	NH1	5.44
163GLU	OE1	164ARG	NH1	2.24
163GLU	OE2	164ARG	NH1	0.22
163GLU	OE2	264ARG	NH2	0.13
163GLU	OE1	264ARG	NH2	0.03
163GLU	OE2	264ARG	NH1	0.01
161GLU	OE2	164ARG	NH1	28.35
161GLU	OE2	164ARG	NH2	15.24
161GLU	OE1	164ARG	NH2	14.43
161GLU	OE1	164ARG	NH1	13.84
161GLU	OE2	164ARG	NH2	9.31

150GLU	OE1	156ARG	NE	56.45
150GLU	OE2	156ARG	NE	53.95
150GLU	OE2	156ARG	NH1	2.87
150GLU	OE1	156ARG	NH1	0.43
133GLU	OE2	144ARG	NH2	0.17
133GLU	OE2	144ARG	NE	0.02
133GLU	OE1	144ARG	NE	0.00
142GLU	OE1	144ARG	NH1	6.59
142GLU	OE2	144ARG	NH1	5.30
142GLU	OE2	144ARG	NH1	0.64
142GLU	OE1	144ARG	NH1	0.31
142GLU	OE1	144ARG	NH2	0.27
190GLU	OE1	144ARG	NH2	37.98
190GLU	OE2	144ARG	NH2	34.07
190GLU	OE2	144ARG	NE	27.59
190GLU	OE1	144ARG	NE	26.41
113GLU	OE2	124ARG	NH1	41.22
113GLU	OE1	124ARG	NH2	19.20
113GLU	OE2	124ARG	NE	15.00
113GLU	OE2	124ARG	NH2	8.69
113GLU	OE1	124ARG	NE	5.04
113GLU	OE2	124ARG	NH2	2.59
113GLU	OE2	124ARG	NH1	2.20
113GLU	OE1	124ARG	NH1	1.57
113GLU	OE2	124ARG	NE	1.21
113GLU	OE1	124ARG	NH1	0.48
120GLU	OE2	124ARG	NH1	36.59
120GLU	OE1	124ARG	NH1	36.06
120GLU	OE2	124ARG	NH2	30.54
120GLU	OE1	124ARG	NH2	29.40
120GLU	OE1	124ARG	NH2	16.48
120GLU	OE1	124ARG	NH1	8.84
120GLU	OE2	124ARG	NH2	7.37
120GLU	OE2	124ARG	NH1	7.08
120GLU	OE1	124ARG	NE	3.23
120GLU	OE2	124ARG	NE	0.94
121GLU	OE1	124ARG	NH2	60.85
121GLU	OE2	124ARG	NH2	52.92
121GLU	OE2	124ARG	NH2	44.94
121GLU	OE1	124ARG	NH2	41.56
121GLU	OE1	124ARG	NE	29.97
121GLU	OE2	124ARG	NE	29.53
121GLU	OE1	124ARG	NH1	25.60

163GLU	OE2	167ARG	NH1	4.00
163GLU	OE1	167ARG	NH1	7.16
163GLU	OE1	167ARG	NH1	2.80
163GLU	OE2	167ARG	NE	43.67
163GLU	OE2	167ARG	NE	43.62
163GLU	OE1	167ARG	NE	52.18
163GLU	OE1	167ARG	NE	43.54
98ASP	OD2	164ARG	NH2	65.86
98ASP	OD1	164ARG	NH2	22.97
161GLU	OE2	164ARG	NH2	47.02
161GLU	OE2	164ARG	NH2	7.96
161GLU	OE1	164ARG	NH2	49.20
161GLU	OE1	164ARG	NH2	6.29
133GLU	OE2	164ARG	NH2	0.22
133GLU	OE1	164ARG	NH2	0.09
98ASP	OD2	164ARG	NH1	58.34
98ASP	OD1	164ARG	NH1	64.88
161GLU	OE2	164ARG	NH1	63.03
161GLU	OE2	164ARG	NH1	24.17
161GLU	OE1	164ARG	NH1	59.08
161GLU	OE1	164ARG	NH1	26.25
133GLU	OE2	164ARG	NH1	0.88
133GLU	OE1	164ARG	NH1	0.38
163GLU	OE2	164ARG	NE	0.00
161GLU	OE2	164ARG	NE	0.04
161GLU	OE1	164ARG	NE	0.06
161GLU	OE1	164ARG	NE	0.00
148GLU	OE2	159LYS	NZ	22.39
148GLU	OE1	159LYS	NZ	20.17
150GLU	OE2	156ARG	NH2	61.20
150GLU	OE1	156ARG	NH2	64.28
142GLU	OE2	156ARG	NH2	0.00
142GLU	OE1	156ARG	NH2	0.06
142GLU	OE1	156ARG	NH2	0.01
150GLU	OE2	156ARG	NH1	3.39
150GLU	OE1	156ARG	NH1	1.95
142GLU	OE2	156ARG	NH1	0.05
142GLU	OE2	156ARG	NH1	0.01
142GLU	OE1	156ARG	NH1	0.26
142GLU	OE1	156ARG	NH1	0.06
150GLU	OE2	156ARG	NE	53.61
150GLU	OE1	156ARG	NE	58.27
142GLU	OE2	156ARG	NE	0.00

51GLU	OE1	58ARG	NH1	6.58
55GLU	OE1	58ARG	NH1	5.98
51GLU	OE1	58ARG	NE	4.66
55GLU	OE1	58ARG	NE	4.30
51GLU	OE2	58ARG	NE	3.74
51GLU	OE2	58ARG	NE	0.16
51GLU	OE1	58ARG	NE	0.04
55GLU	OE1	59LYS	NZ	41.89
55GLU	OE1	59LYS	NZ	38.81
55GLU	OE2	59LYS	NZ	38.63
55GLU	OE2	59LYS	NZ	31.48
9ASP	OD1	76LYS	NZ	70.95
9ASP	OD2	76LYS	NZ	59.64
9ASP	OD2	76LYS	NZ	2.60
47ASP	OD1	76LYS	NZ	1.97
47ASP	OD2	76LYS	NZ	1.73
9ASP	OD1	76LYS	NZ	1.58
47ASP	OD1	76LYS	NZ	0.79
47ASP	OD2	76LYS	NZ	0.69
78ASP	OD1	85ARG	NH1	3.34
78ASP	OD1	85ARG	NH2	1.93
78ASP	OD2	85ARG	NH1	0.86
78ASP	OD1	85ARG	NH1	0.67
78ASP	OD2	85ARG	NH1	0.54
78ASP	OD2	85ARG	NH2	0.38
78ASP	OD2	85ARG	NH2	0.18
78ASP	OD1	85ARG	NH2	0.13
78ASP	OD2	85ARG	NE	0.02
87GLU	OE2	94ARG	NH1	0.07
87GLU	OE2	94ARG	NH2	0.04
87GLU	OE2	94ARG	NH1	0.01
87GLU	OE1	94ARG	NH2	0.00

161GLU	OE1	164ARG	NE	4.16
161GLU	OE2	164ARG	NE	3.06
161GLU	OE2	264ARG	NH2	2.05
161GLU	OE1	264ARG	NH2	1.90
161GLU	OE2	164ARG	NH1	1.22
161GLU	OE2	264ARG	NH1	1.08
161GLU	OE1	264ARG	NH1	0.57
161GLU	OE1	164ARG	NH1	0.48
161GLU	OE1	164ARG	NH2	0.03
161GLU	OE2	264ARG	NH2	0.00
161GLU	OE1	164ARG	NE	0.00
150GLU	OE1	156ARG	NH2	78.04
150GLU	OE2	156ARG	NH2	75.85
150GLU	OE2	156ARG	NE	64.65
150GLU	OE1	156ARG	NE	61.86
150GLU	OE2	156ARG	NH1	0.16
14GLU	OE2	21LYS	NZ	0.05
14GLU	OE1	21LYS	NZ	0.05
14GLU	OE1	21LYS	NZ	0.01
14GLU	OE2	21LYS	NZ	0.00
148GLU	OE1	159LYS	NZ	27.36
148GLU	OE1	197LYS	NZ	22.88
148GLU	OE2	197LYS	NZ	22.51
148GLU	OE2	159LYS	NZ	19.24
142GLU	OE2	144ARG	NE	17.27
142GLU	OE2	144ARG	NH2	17.05
142GLU	OE1	144ARG	NE	16.58
142GLU	OE1	144ARG	NH2	14.29
142GLU	OE1	144ARG	NH1	2.65
142GLU	OE2	144ARG	NH1	1.30
142GLU	OE1	156ARG	NH1	0.56
142GLU	OE2	156ARG	NH1	0.29
142GLU	OE1	156ARG	NH1	0.21
142GLU	OE1	156ARG	NH2	0.06
142GLU	OE2	156ARG	NH1	0.04
142GLU	OE1	144ARG	NH1	0.03
142GLU	OE2	144ARG	NH1	0.03
142GLU	OE1	156ARG	NH2	0.02
133GLU	OE2	164ARG	NH2	27.51
133GLU	OE2	164ARG	NE	20.84
133GLU	OE1	164ARG	NH2	6.39
133GLU	OE1	164ARG	NE	0.22
133GLU	OE2	164ARG	NH1	0.02

121GLU	OE2	124ARG	NH1	21.97
121GLU	OE1	124ARG	NH1	10.94
121GLU	OE2	124ARG	NH1	10.42
121GLU	OE1	124ARG	NE	7.23
121GLU	OE2	124ARG	NE	6.80
133GLU	OE1	124ARG	NE	0.18
133GLU	OE2	124ARG	NE	0.12
133GLU	OE1	124ARG	NH2	0.04
120GLU	OE1	119LYS	NZ	0.58
120GLU	OE2	119LYS	NZ	0.46
133GLU	OE2	114ARG	NH1	0.08
133GLU	OE1	114ARG	NH1	0.06
133GLU	OE2	114ARG	NH1	0.00

142GLU	OE1	156ARG	NE	0.01
193GLU	OE2	144ARG	NH2	0.00
193GLU	OE1	144ARG	NH2	0.01
190GLU	OE2	144ARG	NH2	50.00
190GLU	OE1	144ARG	NH2	53.02
142GLU	OE2	144ARG	NH2	7.34
142GLU	OE1	144ARG	NH2	7.99
190GLU	OE2	144ARG	NH1	0.84
190GLU	OE1	144ARG	NH1	1.66
142GLU	OE2	144ARG	NH1	2.92
142GLU	OE2	144ARG	NH1	2.79
142GLU	OE1	144ARG	NH1	3.78
142GLU	OE1	144ARG	NH1	2.08
190GLU	OE2	144ARG	NE	40.54
190GLU	OE1	144ARG	NE	53.69
142GLU	OE2	144ARG	NE	6.50
142GLU	OE1	144ARG	NE	7.87
87GLU	OE2	132ARG	NH2	0.02
87GLU	OE1	132ARG	NH2	0.01
121GLU	OE2	124ARG	NH2	29.20
121GLU	OE2	124ARG	NH2	10.83
121GLU	OE1	124ARG	NH2	30.90
121GLU	OE1	124ARG	NH2	10.24
120GLU	OE2	124ARG	NH2	41.99
120GLU	OE2	124ARG	NH2	14.90
120GLU	OE1	124ARG	NH2	46.75
120GLU	OE1	124ARG	NH2	15.90
113GLU	OE2	124ARG	NH2	35.13
113GLU	OE2	124ARG	NH2	18.97
113GLU	OE1	124ARG	NH2	34.67
113GLU	OE1	124ARG	NH2	22.59
121GLU	OE2	124ARG	NH1	32.88
121GLU	OE2	124ARG	NH1	22.95
121GLU	OE1	124ARG	NH1	35.86
121GLU	OE1	124ARG	NH1	21.64
120GLU	OE2	124ARG	NH1	17.06
120GLU	OE2	124ARG	NH1	15.40
120GLU	OE1	124ARG	NH1	14.80
120GLU	OE1	124ARG	NH1	12.52
113GLU	OE2	124ARG	NH1	1.53
113GLU	OE2	124ARG	NH1	0.12
113GLU	OE1	124ARG	NH1	1.15
113GLU	OE1	124ARG	NH1	0.06

127ASP	OD2	176ARG	NH2	83.23
127ASP	OD1	176ARG	NH2	82.81
127ASP	OD1	176ARG	NH2	52.32
127ASP	OD1	176ARG	NH1	40.34
127ASP	OD2	177ARG	NH2	37.30
127ASP	OD2	176ARG	NH1	35.27
127ASP	OD2	176ARG	NH2	31.43
127ASP	OD2	176ARG	NH1	30.21
127ASP	OD1	176ARG	NH1	27.41
127ASP	OD2	177ARG	NH1	20.17
127ASP	OD1	177ARG	NH2	14.85
127ASP	OD2	177ARG	NH2	4.24
127ASP	OD1	177ARG	NH1	3.82
127ASP	OD1	177ARG	NH2	2.48
127ASP	OD2	177ARG	NH1	1.17
127ASP	OD1	177ARG	NH1	0.18
127ASP	OD2	177ARG	NE	0.03
127ASP	OD1	177ARG	NE	0.02
127ASP	OD2	309ARG	NH2	0.00
127ASP	OD1	177ARG	NE	0.00
121GLU	OE2	124ARG	NH2	51.42
121GLU	OE1	124ARG	NH2	48.54
121GLU	OE2	124ARG	NH2	39.71
121GLU	OE1	124ARG	NH2	35.29
121GLU	OE1	124ARG	NE	27.96
121GLU	OE2	124ARG	NH1	26.30
121GLU	OE1	124ARG	NH1	25.83
121GLU	OE2	124ARG	NE	23.69
121GLU	OE1	124ARG	NE	17.96
121GLU	OE2	124ARG	NH1	17.95
121GLU	OE1	124ARG	NH1	16.09
121GLU	OE2	124ARG	NE	15.56
120GLU	OE1	124ARG	NH2	26.09
120GLU	OE2	124ARG	NH2	21.12
120GLU	OE1	119LYS	NZ	18.42
120GLU	OE1	124ARG	NH1	17.61
120GLU	OE2	124ARG	NH1	17.02
120GLU	OE2	124ARG	NH2	16.71
120GLU	OE1	124ARG	NH2	15.64
120GLU	OE1	124ARG	NH1	15.47
120GLU	OE2	119LYS	NZ	15.41
120GLU	OE2	124ARG	NH1	11.46
120GLU	OE1	124ARG	NE	0.18

121GLU OE2	124ARG NE	7.20
121GLU OE2	124ARG NE	0.01
121GLU OE1	124ARG NE	7.54
120GLU OE2	124ARG NE	7.71
120GLU OE2	124ARG NE	0.98
120GLU OE1	124ARG NE	8.58
120GLU OE1	124ARG NE	0.90
113GLU OE2	124ARG NE	23.72
113GLU OE2	124ARG NE	9.77
113GLU OE1	124ARG NE	28.10
113GLU OE1	124ARG NE	26.22
120GLU OE2	119LYS NZ	4.64
120GLU OE1	119LYS NZ	8.66
113GLU OE2	114ARG NH2	31.80
113GLU OE1	114ARG NH2	32.08
113GLU OE1	114ARG NH2	0.01
113GLU OE2	114ARG NH1	0.02
113GLU OE2	114ARG NH1	0.00
113GLU OE1	114ARG NH1	0.11
113GLU OE1	114ARG NH1	0.02
113GLU OE2	114ARG NE	32.07
113GLU OE1	114ARG NE	31.16

120GLU	OE2	124ARG	NE	0.07
120GLU	OE2	124ARG	NE	0.02
113GLU	OE2	124ARG	NH2	18.83
113GLU	OE1	114ARG	NE	18.65
113GLU	OE2	124ARG	NH1	18.36
113GLU	OE1	114ARG	NH2	18.30
113GLU	OE1	124ARG	NH2	17.96
113GLU	OE2	114ARG	NE	16.13
113GLU	OE1	124ARG	NE	15.06
113GLU	OE2	114ARG	NH2	14.63
113GLU	OE1	124ARG	NH1	13.46
113GLU	OE2	124ARG	NE	13.24
113GLU	OE1	124ARG	NH1	11.50
113GLU	OE1	124ARG	NH2	7.47
113GLU	OE2	124ARG	NH1	6.54
113GLU	OE1	124ARG	NE	6.20
113GLU	OE2	124ARG	NH2	3.16
113GLU	OE2	124ARG	NE	3.12
113GLU	OE1	114ARG	NH1	0.03
113GLU	OE1	114ARG	NH1	0.03
113GLU	OE2	114ARG	NH1	0.02
113GLU	OE2	114ARG	NH1	0.01
