# Supplementary material for: Deciphering the Dynamics of Non-Covalent Interactions Affecting Thermal Stability of a Protein: Molecular Dynamics Study on Point Mutant of Thermus thermophilus Isopropylmalate Dehydrogenase
Source: PLoS One. 2015 Dec 11;10(12):e0144294. doi: 10.1371/journal.pone.0144294 (PMC4689552; doi:10.1371/journal.pone.0144294)
Supplement: S2 Table — The color formatting indicates the percentage of time interaction existed as in S1 Table. (PDF) [file pone.0144294.s004.pdf]

S2 Table. Unique IP HBs between donor and acceptor atom of *wt* and *mut* at 300 K and 337 K.

1) *Wt* 300 K

Drnona	D	Arnona	A	percen
1MET	N	33GLY	O	88.96
1MET	N	65GLU	OE1	0.50
1MET	N	65GLU	OE2	0.02
345ALA	N	341LEU	O	1.80
345ALA	N	342ARG	O	7.17
345ALA	N	343HIS	O	6.62
344LEU	N	340VAL	O	29.19
344LEU	N	341LEU	O	17.19
344LEU	N	342ARG	O	0.19
343HIS	NE2	310LYS	O	0.01
343HIS	NE2	313ASP	OD1	15.50
343HIS	NE2	313ASP	OD2	0.01
343HIS	NE2	317LYS	O	0.26
343HIS	NE2	321GLU	OE1	9.17
343HIS	NE2	321GLU	OE2	32.62
343HIS	N	339THR	O	33.51
343HIS	N	340VAL	O	42.31
343HIS	N	341LEU	O	0.05
342ARG	NH2	321GLU	OE1	41.75
342ARG	NH2	321GLU	OE2	20.54
342ARG	NH2	338ALA	O	0.08
342ARG	NH2	343HIS	NE2	0.00
342ARG	NH2	345ALA	O1	0.00
342ARG	NH2	345ALA	O2	0.53
342ARG	NH1	321GLU	OE1	16.25
342ARG	NH1	321GLU	OE2	30.87
342ARG	NH1	338ALA	O	0.17
342ARG	NH1	343HIS	NE2	0.00
342ARG	NE	321GLU	OE1	3.64
342ARG	NE	321GLU	OE2	2.02
342ARG	NE	338ALA	O	0.92
342ARG	NE	342ARG	O	0.04
342ARG	NE	343HIS	NE2	0.00
342ARG	NE	345ALA	O1	0.41
342ARG	NE	345ALA	O2	0.01
342ARG	N	338ALA	O	27.41
342ARG	N	339THR	O	30.68
342ARG	N	340VAL	O	0.38
341LEU	N	337THR	O	96.30
341LEU	N	338ALA	O	0.28

2) *Wt* 337 K

Drnona	D	Arnona	A	percen
1MET	N	32LEU	O	0.05
1MET	N	33GLY	O	65.50
1MET	N	65GLU	OE1	9.34
1MET	N	65GLU	OE2	8.34
345ALA	N	341LEU	O	5.80
345ALA	N	342ARG	O	4.94
345ALA	N	343HIS	O	15.23
345ALA	N	345ALA	O2	0.00
344LEU	N	340VAL	O	29.93
344LEU	N	341LEU	O	27.91
344LEU	N	342ARG	O	0.08
343HIS	NE2	310LYS	O	0.01
343HIS	NE2	313ASP	OD1	39.48
343HIS	NE2	313ASP	OD2	26.72
343HIS	NE2	317LYS	O	0.14
343HIS	NE2	321GLU	OE1	0.85
343HIS	NE2	321GLU	OE2	1.54
343HIS	N	339THR	O	10.62
343HIS	N	340VAL	O	60.19
343HIS	N	341LEU	O	0.27
342ARG	NH2	317LYS	O	0.02
342ARG	NH2	321GLU	OE1	25.86
342ARG	NH2	321GLU	OE2	27.66
342ARG	NH2	338ALA	O	0.00
342ARG	NH2	341LEU	O	0.00
342ARG	NH2	343HIS	ND1	0.02
342ARG	NH2	343HIS	NE2	0.01
342ARG	NH2	345ALA	O1	0.26
342ARG	NH2	345ALA	O2	0.53
342ARG	NH1	321GLU	OE1	21.38
342ARG	NH1	321GLU	OE2	18.69
342ARG	NH1	338ALA	O	0.11
342ARG	NH1	341LEU	O	0.01
342ARG	NH1	342ARG	O	0.11
342ARG	NH1	345ALA	O1	0.04
342ARG	NH1	345ALA	O2	0.09
342ARG	NE	338ALA	O	0.17
342ARG	NE	339THR	O	0.18
342ARG	NE	341LEU	O	0.01
342ARG	NE	342ARG	O	0.18

3) *Mut* 300 K

Drnona	D	Arnona	A	percen
1MET	N	1MET	O	0.00
1MET	N	33GLY	O	57.77
1MET	N	65GLU	OE1	15.32
1MET	N	65GLU	OE2	24.08
345ALA	N	341LEU	O	4.20
345ALA	N	342ARG	O	13.72
345ALA	N	343HIS	O	11.17
344LEU	N	340VAL	O	36.51
344LEU	N	341LEU	O	17.30
344LEU	N	342ARG	O	0.17
343HIS	NE2	313ASP	OD2	29.42
343HIS	NE2	317LYS	O	0.61
343HIS	NE2	321GLU	OE1	5.81
343HIS	NE2	321GLU	OE2	9.63
343HIS	N	339THR	O	34.66
343HIS	N	340VAL	O	39.86
343HIS	N	341LEU	O	0.11
342ARG	NH2	321GLU	OE1	30.01
342ARG	NH2	321GLU	OE2	24.83
342ARG	NH2	338ALA	O	0.02
342ARG	NH2	343HIS	NE2	0.06
342ARG	NH2	345ALA	O2	0.00
342ARG	NH1	321GLU	OE1	14.63
342ARG	NH1	321GLU	OE2	22.60
342ARG	NH1	338ALA	O	0.18
342ARG	NH1	339THR	O	0.00
342ARG	NH1	342ARG	O	0.05
342ARG	NE	321GLU	OE1	3.84
342ARG	NE	321GLU	OE2	0.07
342ARG	NE	338ALA	O	0.36
342ARG	NE	342ARG	O	0.25
342ARG	N	338ALA	O	29.89
342ARG	N	339THR	O	27.81
342ARG	N	340VAL	O	0.68
341LEU	N	337THR	O	94.42
341LEU	N	338ALA	O	1.04
340VAL	N	336PHE	O	74.22
340VAL	N	337THR	O	6.15
340VAL	N	338ALA	O	0.01
339THR	OG1	321GLU	OE1	0.01

4) *Mut* 337 K

Drnona	D	Arnona	A	percen
1MET	N	1MET	O	0.00
1MET	N	32LEU	O	0.01
1MET	N	33GLY	O	66.65
1MET	N	65GLU	OE1	7.49
1MET	N	65GLU	OE2	6.86
345ALA	N	341LEU	O	3.92
345ALA	N	342ARG	O	5.63
345ALA	N	343HIS	O	10.81
345ALA	N	345ALA	O2	0.01
344LEU	N	340VAL	O	33.15
344LEU	N	341LEU	O	19.68
344LEU	N	342ARG	O	0.14
343HIS	NE2	313ASP	OD1	11.68
343HIS	NE2	313ASP	OD2	1.62
343HIS	NE2	317LYS	O	0.72
343HIS	NE2	321GLU	OE1	10.14
343HIS	NE2	321GLU	OE2	10.36
343HIS	N	339THR	O	27.94
343HIS	N	340VAL	O	48.59
343HIS	N	341LEU	O	0.16
342ARG	NH2	321GLU	OE1	22.94
342ARG	NH2	321GLU	OE2	23.57
342ARG	NH2	338ALA	O	0.04
342ARG	NH2	343HIS	ND1	0.01
342ARG	NH2	343HIS	NE2	0.04
342ARG	NH2	345ALA	O1	0.27
342ARG	NH2	345ALA	O2	0.86
342ARG	NH1	321GLU	OE1	20.61
342ARG	NH1	321GLU	OE2	21.35
342ARG	NH1	338ALA	O	0.39
342ARG	NH1	339THR	O	0.00
342ARG	NH1	342ARG	O	0.16
342ARG	NH1	343HIS	ND1	0.02
342ARG	NH1	343HIS	NE2	0.00
342ARG	NH1	345ALA	O1	0.52
342ARG	NH1	345ALA	O2	0.62
342ARG	NE	321GLU	OE1	0.01
342ARG	NE	321GLU	OE2	0.38
342ARG	NE	338ALA	O	0.82
342ARG	NE	339THR	O	0.01

340VAL	N	336PHE	O	66.23
340VAL	N	337THR	O	6.13
339THR	OG1	321GLU	OE1	6.39
339THR	OG1	321GLU	OE2	2.37
339THR	OG1	335ALA	O	62.11
339THR	OG1	336PHE	O	3.65
339THR	OG1	339THR	O	0.10
339THR	OG1	342ARG	NE	0.02
339THR	OG1	342ARG	NH1	0.18
339THR	OG1	342ARG	NH2	1.00
339THR	OG1	343HIS	NE2	0.00
339THR	N	335ALA	O	37.76
339THR	N	336PHE	O	22.74
339THR	N	337THR	O	0.22
338ALA	N	334GLU	O	66.57
338ALA	N	335ALA	O	4.57
337THR	OG1	18ALA	O	0.02
337THR	OG1	333THR	O	75.24
337THR	OG1	334GLU	OE1	0.02
337THR	OG1	334GLU	O	2.78
337THR	OG1	338ALA	N	0.01
337THR	N	332GLY	O	0.36
337THR	N	333THR	O	24.22
337THR	N	334GLU	O	10.75
337THR	N	335ALA	O	2.39
336PHE	N	332GLY	O	92.68
336PHE	N	333THR	O	0.32
336PHE	N	334GLU	O	0.31
335ALA	N	332GLY	O	7.89
335ALA	N	333THR	O	0.02
335ALA	N	337THR	OG1	0.01
334GLU	N	332GLY	O	0.70
333THR	OG1	14GLU	OE1	0.90
333THR	OG1	14GLU	OE2	0.50
333THR	OG1	283GLY	O	2.91
333THR	OG1	285ALA	O	1.01
333THR	OG1	332GLY	O	0.00
333THR	OG1	334GLU	N	0.00
333THR	OG1	334GLU	OE1	0.04
333THR	OG1	334GLU	OE2	0.08
333THR	N	283GLY	O	10.66
333THR	N	285ALA	O	3.62
332GLY	N	283GLY	O	0.03

342ARG	NE	345ALA	O2	0.04
342ARG	N	338ALA	O	33.79
342ARG	N	339THR	O	13.64
342ARG	N	340VAL	O	0.90
341LEU	N	337THR	O	95.95
341LEU	N	338ALA	O	0.42
340VAL	N	336PHE	O	31.81
340VAL	N	337THR	O	21.14
340VAL	N	338ALA	O	0.00
339THR	OG1	314ALA	O	0.00
339THR	OG1	321GLU	OE1	0.02
339THR	OG1	335ALA	O	73.70
339THR	OG1	336PHE	O	2.40
339THR	OG1	339THR	O	0.06
339THR	OG1	340VAL	N	0.00
339THR	OG1	342ARG	NH2	0.02
339THR	OG1	343HIS	NE2	0.00
339THR	N	335ALA	O	61.34
339THR	N	336PHE	O	13.14
339THR	N	337THR	O	0.02
338ALA	N	334GLU	O	75.65
338ALA	N	335ALA	O	2.15
338ALA	N	336PHE	O	0.01
337THR	OG1	18ALA	O	0.01
337THR	OG1	333THR	O	79.68
337THR	OG1	334GLU	OE2	0.02
337THR	OG1	334GLU	O	1.58
337THR	OG1	338ALA	N	0.04
337THR	N	332GLY	O	0.01
337THR	N	333THR	O	39.56
337THR	N	334GLU	O	23.99
337THR	N	335ALA	O	6.55
336PHE	N	332GLY	O	84.83
336PHE	N	333THR	O	2.52
336PHE	N	334GLU	O	0.23
335ALA	N	332GLY	O	11.34
335ALA	N	334GLU	OE1	0.07
334GLU	N	332GLY	O	0.05
334GLU	N	334GLU	OE1	0.48
334GLU	N	334GLU	OE2	0.50
333THR	OG1	14GLU	OE1	2.50
333THR	OG1	14GLU	OE2	1.94
333THR	OG1	280ALA	O	2.12

339THR	OG1	321GLU	OE2	0.02
339THR	OG1	335ALA	O	55.99
339THR	OG1	336PHE	O	1.80
339THR	OG1	338ALA	O	0.00
339THR	OG1	340VAL	N	0.00
339THR	OG1	342ARG	NH1	0.00
339THR	OG1	342ARG	NH2	0.03
339THR	N	335ALA	O	41.07
339THR	N	336PHE	O	20.31
339THR	N	337THR	O	0.00
338ALA	N	334GLU	O	63.42
338ALA	N	335ALA	O	5.29
338ALA	N	336PHE	O	0.04
337THR	OG1	18ALA	O	0.00
337THR	OG1	333THR	O	52.79
337THR	OG1	334GLU	OE1	0.00
337THR	OG1	334GLU	OE2	0.13
337THR	OG1	334GLU	O	1.93
337THR	OG1	337THR	O	0.01
337THR	OG1	338ALA	N	0.06
337THR	N	333THR	O	80.44
337THR	N	334GLU	O	1.64
336PHE	N	332GLY	O	84.32
336PHE	N	333THR	O	2.65
336PHE	N	334GLU	O	0.00
335ALA	N	332GLY	O	9.26
333THR	OG1	14GLU	OE1	0.71
333THR	OG1	14GLU	OE2	0.19
333THR	OG1	283GLY	O	0.19
333THR	OG1	285ALA	O	69.71
333THR	OG1	334GLU	N	0.01
333THR	N	283GLY	O	45.71
333THR	N	285ALA	O	0.04
331ALA	N	282LYS	O	0.01
331ALA	N	283GLY	O	0.02
331ALA	N	323PRO	O	59.85
331ALA	N	329GLY	O	0.01
330SER	OG	329GLY	O	0.36
330SER	OG	330SER	O	0.34
330SER	N	323PRO	O	2.43
329GLY	N	324PRO	O	28.34
329GLY	N	325PRO	O	0.25
328GLY	N	324PRO	O	64.22

342ARG	NE	341LEU	O	0.01
342ARG	NE	342ARG	O	0.08
342ARG	N	338ALA	O	24.27
342ARG	N	339THR	O	31.00
342ARG	N	340VAL	O	0.78
342ARG	N	344LEU	N	0.00
341LEU	N	337THR	O	91.93
341LEU	N	338ALA	O	0.58
340VAL	N	336PHE	O	75.72
340VAL	N	337THR	O	6.21
340VAL	N	338ALA	O	0.00
339THR	OG1	321GLU	OE1	0.16
339THR	OG1	321GLU	OE2	0.11
339THR	OG1	335ALA	O	63.67
339THR	OG1	336PHE	O	4.80
339THR	OG1	339THR	O	0.01
339THR	OG1	340VAL	N	0.00
339THR	OG1	342ARG	NH1	0.04
339THR	OG1	342ARG	NH2	0.07
339THR	OG1	343HIS	NE2	0.00
339THR	N	335ALA	O	35.50
339THR	N	336PHE	O	25.87
339THR	N	337THR	O	0.06
338ALA	N	334GLU	O	58.77
338ALA	N	335ALA	O	3.98
338ALA	N	336PHE	O	0.02
337THR	OG1	18ALA	O	0.04
337THR	OG1	332GLY	O	0.00
337THR	OG1	333THR	O	67.29
337THR	OG1	334GLU	OE2	0.00
337THR	OG1	334GLU	O	1.14
337THR	OG1	338ALA	N	0.05
337THR	N	332GLY	O	0.11
337THR	N	333THR	O	54.74
337THR	N	334GLU	O	2.12
337THR	N	335ALA	O	0.59
336PHE	N	332GLY	O	90.14
336PHE	N	333THR	O	1.84
336PHE	N	334GLU	O	0.05
335ALA	N	332GLY	O	8.54
335ALA	N	333THR	O	0.10
335ALA	N	337THR	OG1	0.02
334GLU	N	283GLY	O	0.01

332GLY	N	330SER	O	5.21
332GLY	N	335ALA	O	0.03
332GLY	N	336PHE	N	0.00
331ALA	N	323PRO	O	0.15
331ALA	N	329GLY	O	0.18
330SER	OG	329GLY	O	0.08
330SER	OG	330SER	O	0.10
330SER	OG	331ALA	N	0.01
330SER	N	323PRO	O	0.03
329GLY	N	283GLY	O	0.08
329GLY	N	324PRO	O	0.14
329GLY	N	325PRO	O	1.18
328GLY	N	282LYS	O	13.06
328GLY	N	324PRO	O	8.19
328GLY	N	325PRO	O	6.51
328GLY	N	326ASP	O	2.45
327LEU	N	324PRO	O	16.98
327LEU	N	326ASP	OD1	2.28
327LEU	N	326ASP	OD2	4.36
327LEU	N	328GLY	O	0.16
326ASP	N	282LYS	O	0.07
326ASP	N	324PRO	O	0.13
326ASP	N	326ASP	OD1	0.02
326ASP	N	326ASP	OD2	0.02
326ASP	N	328GLY	N	0.00
326ASP	N	328GLY	O	52.76
322THR	OG1	318ALA	O	5.84
322THR	OG1	321GLU	O	0.03
322THR	OG1	322THR	O	3.82
322THR	OG1	323PRO	N	0.00
322THR	OG1	323PRO	O	0.36
322THR	OG1	330SER	OG	0.06
322THR	N	318ALA	O	86.94
322THR	N	319LEU	O	0.08
321GLU	N	317LYS	O	14.57
321GLU	N	318ALA	O	48.95
321GLU	N	319LEU	O	0.16
320LEU	N	316ALA	O	31.72
320LEU	N	317LYS	O	20.20
320LEU	N	318ALA	O	0.02
319LEU	N	315VAL	O	77.64
319LEU	N	316ALA	O	5.78
318ALA	N	314ALA	O	54.72

333THR	OG1	281GLY	O	0.01
333THR	OG1	283GLY	O	0.99
333THR	OG1	285ALA	O	18.38
333THR	OG1	332GLY	O	0.00
333THR	OG1	334GLU	N	0.05
333THR	OG1	334GLU	OE1	0.72
333THR	OG1	334GLU	OE2	0.84
333THR	N	280ALA	O	0.00
333THR	N	281GLY	O	0.13
333THR	N	282LYS	O	0.02
333THR	N	283GLY	O	26.11
333THR	N	285ALA	O	1.29
332GLY	N	283GLY	O	0.00
332GLY	N	334GLU	OE1	0.00
332GLY	N	334GLU	OE2	0.03
332GLY	N	335ALA	O	0.18
331ALA	N	283GLY	O	0.04
331ALA	N	323PRO	O	20.94
331ALA	N	329GLY	O	0.10
330SER	OG	328GLY	O	0.00
330SER	OG	329GLY	O	0.98
330SER	OG	330SER	O	0.46
330SER	OG	331ALA	N	0.01
330SER	N	323PRO	O	1.91
330SER	N	328GLY	O	0.00
329GLY	N	278ASP	OD1	0.88
329GLY	N	278ASP	OD2	0.02
329GLY	N	323PRO	O	0.00
329GLY	N	324PRO	O	12.62
329GLY	N	325PRO	O	7.06
329GLY	N	327LEU	O	0.02
328GLY	N	324PRO	O	46.49
328GLY	N	325PRO	O	22.54
328GLY	N	326ASP	O	0.06
327LEU	N	324PRO	O	70.54
327LEU	N	325PRO	O	0.85
326ASP	N	254LEU	O	2.63
326ASP	N	284ILE	O	0.41
326ASP	N	324PRO	O	0.46
326ASP	N	326ASP	OD1	0.60
326ASP	N	326ASP	OD2	0.66
326ASP	N	328GLY	N	0.00
326ASP	N	329GLY	O	0.01

328GLY	N	325PRO	O	21.06
327LEU	N	324PRO	O	73.83
327LEU	N	325PRO	O	0.00
326ASP	N	284ILE	O	3.28
326ASP	N	324PRO	O	0.24
326ASP	N	326ASP	OD1	0.01
326ASP	N	326ASP	OD2	0.02
326ASP	N	328GLY	N	0.01
322THR	OG1	318ALA	O	19.39
322THR	OG1	322THR	O	11.79
322THR	OG1	330SER	O	0.19
322THR	OG1	335ALA	O	0.00
322THR	OG1	339THR	OG1	0.06
322THR	N	318ALA	O	97.64
322THR	N	319LEU	O	0.00
321GLU	N	317LYS	O	43.39
321GLU	N	318ALA	O	24.80
321GLU	N	319LEU	O	0.19
320LEU	N	316ALA	O	23.69
320LEU	N	317LYS	O	36.72
320LEU	N	318ALA	O	0.01
319LEU	N	315VAL	O	80.60
319LEU	N	316ALA	O	3.33
318ALA	N	314ALA	O	58.53
318ALA	N	315VAL	O	8.07
318ALA	N	316ALA	O	0.00
317LYS	NZ	313ASP	OD1	36.91
317LYS	NZ	313ASP	OD2	69.11
317LYS	NZ	313ASP	O	0.16
317LYS	NZ	343HIS	ND1	0.05
317LYS	NZ	343HIS	NE2	0.01
317LYS	NZ	343HIS	O	0.00
317LYS	N	313ASP	O	73.28
317LYS	N	314ALA	O	6.80
317LYS	N	315VAL	O	0.01
316ALA	N	312GLU	O	38.63
316ALA	N	313ASP	O	4.78
315VAL	N	311VAL	O	87.35
315VAL	N	312GLU	O	0.62
314ALA	N	310LYS	O	80.16
314ALA	N	311VAL	O	2.31
313ASP	N	309ARG	O	65.40
313ASP	N	310LYS	O	6.52

334GLU	N	332GLY	O	1.87
334GLU	N	334GLU	OE1	0.05
334GLU	N	334GLU	OE2	0.02
333THR	OG1	14GLU	OE1	7.76
333THR	OG1	14GLU	OE2	4.65
333THR	OG1	14GLU	O	0.00
333THR	OG1	281GLY	O	3.50
333THR	OG1	282LYS	O	0.00
333THR	OG1	283GLY	O	1.96
333THR	OG1	285ALA	O	13.20
333THR	OG1	332GLY	O	0.07
333THR	OG1	334GLU	N	0.03
333THR	OG1	334GLU	OE1	0.15
333THR	OG1	334GLU	OE2	0.28
333THR	N	281GLY	O	5.79
333THR	N	282LYS	O	0.00
333THR	N	283GLY	O	9.37
333THR	N	285ALA	O	3.97
332GLY	N	286ASN	OD1	0.00
332GLY	N	330SER	O	0.18
332GLY	N	335ALA	O	0.02
332GLY	N	336PHE	N	0.00
331ALA	N	282LYS	O	0.01
331ALA	N	283GLY	O	3.08
331ALA	N	284ILE	O	0.01
331ALA	N	323PRO	O	32.31
331ALA	N	329GLY	O	0.34
330SER	OG	278ASP	OD1	0.00
330SER	OG	278ASP	OD2	0.01
330SER	OG	283GLY	O	0.72
330SER	OG	329GLY	O	0.88
330SER	OG	330SER	O	0.61
330SER	OG	331ALA	N	0.00
330SER	N	323PRO	O	4.42
329GLY	N	323PRO	O	0.39
329GLY	N	324PRO	O	36.99
329GLY	N	325PRO	O	0.72
329GLY	N	327LEU	O	0.01
328GLY	N	324PRO	O	27.71
328GLY	N	325PRO	O	13.65
328GLY	N	326ASP	O	0.27
327LEU	N	324PRO	O	51.45
327LEU	N	325PRO	O	14.63

318ALA	N	315VAL	O	7.97
317LYS	NZ	313ASP	OD1	77.89
317LYS	NZ	313ASP	OD2	25.69
317LYS	NZ	313ASP	O	0.18
317LYS	NZ	343HIS	ND1	0.01
317LYS	NZ	343HIS	NE2	0.01
317LYS	NZ	343HIS	O	0.38
317LYS	NZ	345ALA	O2	0.03
317LYS	N	313ASP	O	77.30
317LYS	N	314ALA	O	6.59
317LYS	N	315VAL	O	0.02
316ALA	N	312GLU	O	27.51
316ALA	N	313ASP	O	9.28
315VAL	N	311VAL	O	92.39
315VAL	N	312GLU	O	0.42
314ALA	N	310LYS	O	77.49
314ALA	N	311VAL	O	2.73
313ASP	N	309ARG	O	53.59
313ASP	N	310LYS	O	10.00
313ASP	N	311VAL	O	0.00
312GLU	N	308ALA	O	80.87
312GLU	N	309ARG	O	0.73
311VAL	N	307LEU	O	55.34
311VAL	N	308ALA	O	13.82
310LYS	NZ	30GLU	OE1	8.20
310LYS	NZ	30GLU	OE2	8.76
310LYS	NZ	306GLU	OE2	0.03
310LYS	NZ	306GLU	O	0.01
310LYS	NZ	313ASP	OD1	0.10
310LYS	NZ	344LEU	O	2.12
310LYS	NZ	345ALA	O1	25.53
310LYS	NZ	345ALA	O2	21.79
310LYS	N	306GLU	O	27.71
310LYS	N	307LEU	O	20.19
310LYS	N	308ALA	O	0.00
309ARG	NH2	175LYS	O	17.21
309ARG	NH2	299GLU	OE1	0.84
309ARG	NH2	299GLU	OE2	29.92
309ARG	NH2	306GLU	OE1	16.40
309ARG	NH2	306GLU	OE2	23.28
309ARG	NH2	312GLU	OE1	0.52
309ARG	NH2	312GLU	OE2	9.40
309ARG	NH1	299GLU	OE1	31.53

322THR	OG1	318ALA	O	10.88
322THR	OG1	321GLU	O	0.14
322THR	OG1	322THR	O	5.55
322THR	OG1	323PRO	N	0.00
322THR	OG1	323PRO	O	0.20
322THR	OG1	330SER	OG	0.01
322THR	OG1	331ALA	O	0.00
322THR	OG1	335ALA	O	0.00
322THR	OG1	339THR	OG1	0.02
322THR	N	318ALA	O	78.96
322THR	N	319LEU	O	0.07
321GLU	N	317LYS	O	29.37
321GLU	N	318ALA	O	36.72
321GLU	N	319LEU	O	0.17
320LEU	N	316ALA	O	25.72
320LEU	N	317LYS	O	26.53
320LEU	N	318ALA	O	0.07
319LEU	N	315VAL	O	84.14
319LEU	N	316ALA	O	3.16
318ALA	N	314ALA	O	45.71
318ALA	N	315VAL	O	16.45
317LYS	NZ	313ASP	OD1	54.19
317LYS	NZ	313ASP	OD2	50.61
317LYS	NZ	313ASP	O	0.54
317LYS	NZ	321GLU	OE1	0.00
317LYS	NZ	321GLU	OE2	0.03
317LYS	NZ	342ARG	O	0.03
317LYS	NZ	343HIS	ND1	0.19
317LYS	NZ	343HIS	NE2	0.01
317LYS	NZ	343HIS	O	0.00
317LYS	N	313ASP	O	79.08
317LYS	N	314ALA	O	4.70
317LYS	N	315VAL	O	0.02
316ALA	N	312GLU	O	69.81
316ALA	N	313ASP	O	2.55
315VAL	N	311VAL	O	84.72
315VAL	N	312GLU	O	2.35
314ALA	N	310LYS	O	77.07
314ALA	N	311VAL	O	2.83
313ASP	N	309ARG	O	73.29
313ASP	N	310LYS	O	3.90
312GLU	N	308ALA	O	78.70
312GLU	N	309ARG	O	1.52

312GLU	N	308ALA	O	81.88
312GLU	N	309ARG	O	1.03
311VAL	N	307LEU	O	71.47
311VAL	N	308ALA	O	5.79
310LYS	NZ	30GLU	OE1	1.39
310LYS	NZ	30GLU	OE2	4.26
310LYS	NZ	306GLU	OE1	0.65
310LYS	NZ	306GLU	OE2	0.10
310LYS	NZ	306GLU	O	0.04
310LYS	NZ	313ASP	OD2	0.00
310LYS	NZ	343HIS	O	0.12
310LYS	NZ	344LEU	O	2.12
310LYS	NZ	345ALA	O1	13.17
310LYS	NZ	345ALA	O2	13.43
310LYS	N	306GLU	O	44.68
310LYS	N	307LEU	O	13.10
309ARG	NH2	299GLU	OE1	0.37
309ARG	NH2	299GLU	OE2	4.03
309ARG	NH2	306GLU	OE1	51.46
309ARG	NH2	306GLU	OE2	40.73
309ARG	NH1	299GLU	OE1	4.83
309ARG	NH1	299GLU	OE2	2.28
309ARG	NH1	306GLU	OE1	0.52
309ARG	NH1	306GLU	OE2	10.43
309ARG	NH1	312GLU	OE1	0.03
309ARG	NH1	312GLU	OE2	0.42
309ARG	NH1	313ASP	OD2	0.00
309ARG	NE	305VAL	O	0.01
309ARG	NE	306GLU	OE1	19.43
309ARG	NE	306GLU	OE2	23.94
309ARG	N	305VAL	O	75.12
309ARG	N	306GLU	O	3.56
308ALA	N	304LEU	O	79.11
308ALA	N	305VAL	O	2.75
307LEU	N	304LEU	O	18.76
306GLU	N	304LEU	O	0.00
306GLU	N	306GLU	OE2	0.00
305VAL	N	303GLY	O	58.77
304LEU	N	298LEU	O	75.65
304LEU	N	302PHE	O	0.04
303GLY	N	298LEU	O	34.35
303GLY	N	299GLU	O	7.91
303GLY	N	300HIS	O	0.06

326ASP	N	324PRO	O	0.04
326ASP	N	328GLY	O	0.37
326ASP	N	329GLY	O	0.06
322THR	OG1	318ALA	O	14.61
322THR	OG1	321GLU	OE1	0.05
322THR	OG1	321GLU	O	0.33
322THR	OG1	322THR	O	15.72
322THR	OG1	323PRO	O	0.23
322THR	OG1	330SER	O	0.01
322THR	OG1	339THR	OG1	0.03
322THR	N	318ALA	O	94.24
322THR	N	319LEU	O	0.06
321GLU	N	317LYS	O	19.61
321GLU	N	318ALA	O	41.78
321GLU	N	319LEU	O	0.66
320LEU	N	316ALA	O	32.23
320LEU	N	317LYS	O	21.46
319LEU	N	315VAL	O	85.63
319LEU	N	316ALA	O	2.63
318ALA	N	314ALA	O	56.48
318ALA	N	315VAL	O	11.38
318ALA	N	316ALA	O	0.00
317LYS	NZ	313ASP	OD1	53.18
317LYS	NZ	313ASP	OD2	47.01
317LYS	NZ	313ASP	O	0.34
317LYS	NZ	321GLU	OE1	0.26
317LYS	NZ	321GLU	OE2	1.11
317LYS	NZ	343HIS	ND1	0.10
317LYS	NZ	343HIS	NE2	0.01
317LYS	NZ	343HIS	O	0.17
317LYS	N	313ASP	O	77.91
317LYS	N	314ALA	O	5.01
317LYS	N	315VAL	O	0.05
316ALA	N	312GLU	O	81.71
316ALA	N	313ASP	O	1.32
315VAL	N	311VAL	O	86.32
315VAL	N	312GLU	O	2.26
314ALA	N	310LYS	O	75.96
314ALA	N	311VAL	O	2.72
313ASP	N	309ARG	O	80.06
313ASP	N	310LYS	O	2.28
313ASP	N	311VAL	O	0.00
312GLU	N	308ALA	O	76.62

309ARG	NH1	299GLU	OE2	2.71
309ARG	NH1	306GLU	OE2	0.02
309ARG	NH1	312GLU	OE1	23.02
309ARG	NH1	312GLU	OE2	17.71
309ARG	NE	306GLU	OE1	14.28
309ARG	NE	306GLU	OE2	8.38
309ARG	N	305VAL	O	81.17
309ARG	N	306GLU	O	3.54
308ALA	N	304LEU	O	64.89
308ALA	N	305VAL	O	9.17
307LEU	N	304LEU	O	28.10
307LEU	N	305VAL	O	0.00
307LEU	N	306GLU	OE1	0.01
307LEU	N	306GLU	OE2	0.02
306GLU	N	306GLU	OE1	14.44
306GLU	N	306GLU	OE2	9.94
305VAL	N	303GLY	O	37.83
305VAL	N	306GLU	OE1	0.08
305VAL	N	306GLU	OE2	0.06
304LEU	N	298LEU	O	77.20
304LEU	N	299GLU	O	0.00
304LEU	N	302PHE	O	0.01
303GLY	N	298LEU	O	53.65
303GLY	N	299GLU	O	0.86
303GLY	N	300HIS	O	0.06
302PHE	N	297MET	O	68.24
302PHE	N	298LEU	O	1.53
302PHE	N	299GLU	O	0.01
302PHE	N	300HIS	O	0.03
301ALA	N	296MET	O	0.01
301ALA	N	297MET	O	61.29
301ALA	N	298LEU	O	0.00
301ALA	N	299GLU	O	0.02
301ALA	N	300HIS	ND1	0.01
300HIS	NE2	299GLU	OE2	0.07
300HIS	N	296MET	O	94.25
300HIS	N	297MET	O	0.67
299GLU	N	295ALA	O	86.91
299GLU	N	296MET	O	1.39
298LEU	N	294ALA	O	47.46
298LEU	N	295ALA	O	13.04
297MET	N	293SER	O	72.95
297MET	N	294ALA	O	1.31

312GLU	N	310LYS	O	0.00
311VAL	N	307LEU	O	66.66
311VAL	N	308ALA	O	7.52
310LYS	NZ	30GLU	OE1	5.85
310LYS	NZ	30GLU	OE2	6.33
310LYS	NZ	306GLU	OE1	0.50
310LYS	NZ	306GLU	OE2	0.95
310LYS	NZ	306GLU	O	0.09
310LYS	NZ	313ASP	OD1	0.02
310LYS	NZ	313ASP	OD2	0.02
310LYS	NZ	343HIS	ND1	0.07
310LYS	NZ	343HIS	NE2	0.00
310LYS	NZ	343HIS	O	0.37
310LYS	NZ	344LEU	O	0.95
310LYS	NZ	345ALA	O1	8.26
310LYS	NZ	345ALA	O2	8.85
310LYS	N	306GLU	O	37.26
310LYS	N	307LEU	O	16.00
309ARG	NH2	175LYS	O	6.16
309ARG	NH2	299GLU	OE1	2.55
309ARG	NH2	299GLU	OE2	13.46
309ARG	NH2	306GLU	OE1	31.42
309ARG	NH2	306GLU	OE2	30.98
309ARG	NH1	299GLU	OE1	14.82
309ARG	NH1	299GLU	OE2	4.24
309ARG	NH1	305VAL	O	0.01
309ARG	NH1	306GLU	OE1	10.12
309ARG	NH1	306GLU	OE2	7.27
309ARG	NH1	312GLU	OE1	0.89
309ARG	NH1	312GLU	OE2	1.17
309ARG	NH1	313ASP	OD1	0.02
309ARG	NH1	313ASP	OD2	0.01
309ARG	NE	299GLU	OE1	0.06
309ARG	NE	305VAL	O	0.02
309ARG	NE	306GLU	OE1	13.24
309ARG	NE	306GLU	OE2	15.41
309ARG	N	305VAL	O	76.89
309ARG	N	306GLU	O	3.66
308ALA	N	304LEU	O	69.13
308ALA	N	305VAL	O	5.50
307LEU	N	304LEU	O	24.04
307LEU	N	305VAL	O	0.01
306GLU	N	306GLU	OE1	2.08

302PHE	N	297MET	O	57.29
302PHE	N	298LEU	O	1.23
302PHE	N	299GLU	O	0.26
302PHE	N	300HIS	O	0.06
301ALA	N	297MET	O	68.17
301ALA	N	299GLU	O	0.03
300HIS	N	296MET	O	96.08
300HIS	N	297MET	O	0.44
299GLU	N	295ALA	O	88.29
299GLU	N	296MET	O	1.16
298LEU	N	294ALA	O	82.04
298LEU	N	295ALA	O	1.71
297MET	N	293SER	O	75.30
297MET	N	294ALA	O	1.85
296MET	N	292LEU	O	87.60
296MET	N	293SER	O	0.70
295ALA	N	291ILE	O	76.00
295ALA	N	292LEU	O	1.87
294ALA	N	290ALA	O	70.29
294ALA	N	291ILE	O	7.91
293SER	OG	259SER	O	0.02
293SER	OG	270GLU	O	0.01
293SER	OG	289ALA	O	98.24
293SER	OG	293SER	O	0.01
293SER	N	289ALA	O	36.69
293SER	N	290ALA	O	9.62
292LEU	N	288THR	O	98.10
292LEU	N	289ALA	O	0.01
291ILE	N	287PRO	O	85.57
291ILE	N	288THR	O	2.00
290ALA	N	273HIS	NE2	0.02
290ALA	N	287PRO	O	59.09
289ALA	N	255GLY	O	0.28
289ALA	N	273HIS	NE2	0.04
288THR	OG1	256LEU	O	0.02
288THR	OG1	286ASN	OD1	86.10
288THR	OG1	289ALA	N	0.02
288THR	N	286ASN	OD1	62.41
288THR	N	286ASN	O	0.06
288THR	N	288THR	OG1	0.00
286ASN	ND2	255GLY	O	0.03
286ASN	ND2	288THR	OG1	7.37
286ASN	ND2	326ASP	OD1	22.97

312GLU	N	309ARG	O	1.37
312GLU	N	310LYS	O	0.00
311VAL	N	307LEU	O	58.25
311VAL	N	308ALA	O	10.98
310LYS	NZ	29ALA	O	0.00
310LYS	NZ	30GLU	OE1	11.72
310LYS	NZ	30GLU	OE2	8.60
310LYS	NZ	306GLU	OE1	0.04
310LYS	NZ	306GLU	OE2	0.52
310LYS	NZ	306GLU	O	0.06
310LYS	NZ	313ASP	OD1	0.00
310LYS	NZ	313ASP	OD2	0.05
310LYS	NZ	343HIS	ND1	0.00
310LYS	NZ	343HIS	O	0.27
310LYS	NZ	344LEU	O	1.66
310LYS	NZ	345ALA	O1	20.48
310LYS	NZ	345ALA	O2	19.95
310LYS	N	306GLU	O	37.87
310LYS	N	307LEU	O	15.77
310LYS	N	308ALA	O	0.00
309ARG	NH2	299GLU	OE1	2.76
309ARG	NH2	299GLU	OE2	5.10
309ARG	NH2	306GLU	OE1	29.89
309ARG	NH2	306GLU	OE2	31.59
309ARG	NH2	312GLU	OE1	0.04
309ARG	NH2	312GLU	OE2	1.45
309ARG	NH2	313ASP	OD2	0.16
309ARG	NH1	299GLU	OE1	6.21
309ARG	NH1	299GLU	OE2	5.00
309ARG	NH1	305VAL	O	0.01
309ARG	NH1	306GLU	OE1	17.04
309ARG	NH1	306GLU	OE2	10.31
309ARG	NH1	309ARG	O	0.00
309ARG	NH1	312GLU	OE1	3.30
309ARG	NH1	312GLU	OE2	7.12
309ARG	NH1	313ASP	OD1	0.01
309ARG	NH1	313ASP	OD2	0.00
309ARG	NE	299GLU	OE2	0.01
309ARG	NE	305VAL	O	0.01
309ARG	NE	306GLU	OE1	10.68
309ARG	NE	306GLU	OE2	13.05
309ARG	NE	312GLU	OE1	0.52
309ARG	NE	312GLU	OE2	0.07

296MET	N	292LEU	O	84.14
296MET	N	293SER	O	1.69
295ALA	N	291ILE	O	39.37
295ALA	N	292LEU	O	12.82
294ALA	N	290ALA	O	89.63
294ALA	N	291ILE	O	1.53
293SER	OG	259SER	O	0.30
293SER	OG	270GLU	O	0.06
293SER	OG	289ALA	O	96.25
293SER	OG	293SER	O	0.01
293SER	N	289ALA	O	49.14
293SER	N	290ALA	O	7.48
292LEU	N	288THR	O	95.11
292LEU	N	289ALA	O	0.14
291ILE	N	287PRO	O	95.02
291ILE	N	288THR	O	0.20
290ALA	N	287PRO	O	23.43
289ALA	N	255GLY	O	17.94
289ALA	N	286ASN	OD1	0.12
288THR	OG1	255GLY	O	7.92
288THR	OG1	286ASN	OD1	86.26
288THR	OG1	289ALA	N	0.02
288THR	N	255GLY	O	0.00
288THR	N	286ASN	OD1	84.70
288THR	N	286ASN	O	0.01
286ASN	ND2	255GLY	O	0.27
286ASN	ND2	288THR	OG1	1.90
286ASN	ND2	325PRO	O	4.84
286ASN	ND2	326ASP	OD1	0.05
286ASN	ND2	331ALA	O	10.29
286ASN	N	284ILE	O	0.14
285ALA	N	283GLY	O	9.28
285ALA	N	333THR	OG1	0.12
284ILE	N	279ILE	O	0.01
284ILE	N	282LYS	O	0.84
284ILE	N	326ASP	O	15.75
284ILE	N	328GLY	O	0.26
284ILE	N	329GLY	O	0.02
283GLY	N	278ASP	O	0.64
283GLY	N	279ILE	O	12.50
283GLY	N	280ALA	O	0.14
283GLY	N	281GLY	O	3.86
283GLY	N	327LEU	O	0.02

306GLU	N	306GLU	OE2	0.51
305VAL	N	303GLY	O	48.43
305VAL	N	306GLU	OE1	0.05
305VAL	N	306GLU	OE2	0.01
304LEU	N	298LEU	O	72.44
304LEU	N	302PHE	O	0.02
303GLY	N	298LEU	O	50.27
303GLY	N	299GLU	O	3.78
303GLY	N	300HIS	O	0.03
302PHE	N	297MET	O	47.62
302PHE	N	298LEU	O	1.26
302PHE	N	299GLU	O	0.61
302PHE	N	300HIS	O	0.20
301ALA	N	296MET	O	0.01
301ALA	N	297MET	O	63.56
301ALA	N	299GLU	O	0.04
300HIS	NE2	168VAL	O	0.08
300HIS	NE2	172ALA	O	0.00
300HIS	NE2	261SER	O	0.00
300HIS	NE2	299GLU	OE2	0.03
300HIS	N	296MET	O	94.35
300HIS	N	297MET	O	0.87
299GLU	N	295ALA	O	75.30
299GLU	N	296MET	O	4.50
298LEU	N	294ALA	O	62.16
298LEU	N	295ALA	O	10.81
297MET	N	293SER	O	67.13
297MET	N	294ALA	O	2.90
296MET	N	292LEU	O	85.86
296MET	N	293SER	O	1.04
295ALA	N	291ILE	O	62.96
295ALA	N	292LEU	O	6.42
294ALA	N	290ALA	O	65.57
294ALA	N	291ILE	O	9.55
293SER	OG	259SER	O	0.68
293SER	OG	269PHE	O	0.00
293SER	OG	270GLU	O	0.21
293SER	OG	271PRO	N	0.01
293SER	OG	289ALA	O	94.11
293SER	OG	293SER	O	0.04
293SER	N	289ALA	O	44.26
293SER	N	290ALA	O	7.36
292LEU	N	288THR	O	96.74

286ASN	ND2	326ASP	OD2	11.58
286ASN	ND2	327LEU	N	0.14
286ASN	ND2	331ALA	O	0.03
286ASN	N	284ILE	O	2.18
286ASN	N	326ASP	OD1	0.03
286ASN	N	326ASP	OD2	0.38
285ALA	N	279ILE	O	9.39
285ALA	N	283GLY	O	1.02
285ALA	N	333THR	OG1	4.19
284ILE	N	279ILE	O	2.28
284ILE	N	282LYS	O	0.38
284ILE	N	333THR	OG1	0.00
283GLY	N	279ILE	O	1.62
283GLY	N	281GLY	O	0.00
282LYS	NZ	278ASP	OD1	21.77
282LYS	NZ	278ASP	OD2	22.26
282LYS	NZ	278ASP	O	19.03
282LYS	NZ	280ALA	O	0.14
282LYS	N	278ASP	O	0.02
282LYS	N	279ILE	O	19.73
282LYS	N	280ALA	O	2.02
281GLY	N	14GLU	OE2	0.02
281GLY	N	279ILE	O	0.08
280ALA	N	276ALA	O	5.94
280ALA	N	277PRO	O	60.23
280ALA	N	278ASP	O	0.06
279ILE	N	276ALA	O	10.36
279ILE	N	277PRO	O	0.14
279ILE	N	278ASP	OD1	0.02
278ASP	N	276ALA	O	0.25
278ASP	N	278ASP	OD1	0.11
278ASP	N	278ASP	OD2	0.02
276ALA	N	274GLY	O	4.34
275SER	OG	72VAL	O	6.35
275SER	OG	73GLY	O	2.72
275SER	OG	275SER	O	0.45
275SER	N	72VAL	O	0.00
273HIS	ND1	271PRO	O	0.12
273HIS	ND1	273HIS	O	0.46
273HIS	ND1	274GLY	N	0.04
273HIS	ND1	274GLY	O	0.66
273HIS	N	255GLY	O	0.00
273HIS	N	271PRO	O	9.24

309ARG	NE	313ASP	OD2	0.00
309ARG	N	305VAL	O	77.75
309ARG	N	306GLU	O	3.54
308ALA	N	304LEU	O	70.05
308ALA	N	305VAL	O	5.28
307LEU	N	304LEU	O	26.31
307LEU	N	305VAL	O	0.01
306GLU	N	306GLU	OE1	0.24
306GLU	N	306GLU	OE2	0.24
305VAL	N	303GLY	O	46.18
305VAL	N	306GLU	OE1	0.00
304LEU	N	298LEU	O	76.40
304LEU	N	302PHE	O	0.03
303GLY	N	298LEU	O	55.24
303GLY	N	299GLU	O	3.92
303GLY	N	300HIS	O	0.04
302PHE	N	297MET	O	41.17
302PHE	N	298LEU	O	1.96
302PHE	N	299GLU	O	3.72
302PHE	N	300HIS	O	0.31
301ALA	N	296MET	O	0.01
301ALA	N	297MET	O	61.92
301ALA	N	299GLU	O	0.15
301ALA	N	300HIS	ND1	0.00
300HIS	NE2	65GLU	O	0.06
300HIS	NE2	261SER	O	15.12
300HIS	N	296MET	O	94.56
300HIS	N	297MET	O	0.65
299GLU	N	295ALA	O	63.05
299GLU	N	296MET	O	10.53
298LEU	N	294ALA	O	55.64
298LEU	N	295ALA	O	13.19
297MET	N	293SER	O	69.89
297MET	N	294ALA	O	3.48
296MET	N	292LEU	O	81.53
296MET	N	293SER	O	1.86
295ALA	N	291ILE	O	65.47
295ALA	N	292LEU	O	4.52
295ALA	N	293SER	O	0.00
294ALA	N	290ALA	O	71.80
294ALA	N	291ILE	O	7.16
293SER	OG	259SER	O	1.12
293SER	OG	270GLU	O	0.24

283GLY	N	328GLY	N	0.00
282LYS	NZ	278ASP	OD1	34.76
282LYS	NZ	278ASP	OD2	41.61
282LYS	NZ	278ASP	O	2.98
282LYS	NZ	326ASP	OD1	3.27
282LYS	NZ	326ASP	OD2	0.16
282LYS	NZ	326ASP	O	1.10
282LYS	NZ	327LEU	O	0.62
282LYS	NZ	328GLY	O	0.00
282LYS	N	278ASP	O	27.60
282LYS	N	279ILE	O	29.72
282LYS	N	280ALA	O	0.15
282LYS	N	283GLY	O	0.05
281GLY	N	14GLU	OE2	0.00
281GLY	N	277PRO	O	2.17
281GLY	N	278ASP	O	51.85
281GLY	N	279ILE	O	0.86
281GLY	N	283GLY	O	0.03
280ALA	N	276ALA	O	28.85
280ALA	N	277PRO	O	25.30
279ILE	N	276ALA	O	55.95
279ILE	N	278ASP	OD1	0.01
279ILE	N	278ASP	OD2	0.01
278ASP	N	276ALA	O	0.57
278ASP	N	278ASP	OD1	0.04
278ASP	N	278ASP	OD2	0.02
278ASP	N	280ALA	O	0.03
276ALA	N	274GLY	O	0.53
275SER	OG	9ASP	OD2	0.02
275SER	OG	9ASP	O	61.55
275SER	OG	71SER	O	5.00
275SER	OG	72VAL	O	0.02
275SER	OG	73GLY	O	7.35
275SER	OG	274GLY	O	4.12
275SER	OG	275SER	O	0.07
275SER	N	72VAL	O	0.32
275SER	N	73GLY	O	2.13
273HIS	ND1	254LEU	O	0.02
273HIS	ND1	272VAL	O	0.00
273HIS	ND1	273HIS	O	0.64
273HIS	ND1	274GLY	N	0.05
273HIS	ND1	274GLY	O	82.15
273HIS	N	271PRO	O	10.49

292LEU	N	289ALA	O	0.09
291ILE	N	287PRO	O	72.18
291ILE	N	288THR	O	4.30
290ALA	N	273HIS	NE2	0.03
290ALA	N	287PRO	O	64.12
289ALA	N	255GLY	O	0.27
289ALA	N	286ASN	OD1	0.01
288THR	OG1	255GLY	O	0.05
288THR	OG1	256LEU	O	0.02
288THR	OG1	286ASN	OD1	94.83
288THR	OG1	289ALA	N	0.01
288THR	N	286ASN	OD1	75.46
288THR	N	286ASN	O	0.45
286ASN	ND2	255GLY	O	0.04
286ASN	ND2	283GLY	O	0.01
286ASN	ND2	288THR	OG1	0.90
286ASN	ND2	322THR	O	0.08
286ASN	ND2	323PRO	O	0.20
286ASN	ND2	326ASP	OD1	8.44
286ASN	ND2	326ASP	OD2	5.29
286ASN	ND2	327LEU	N	0.02
286ASN	ND2	331ALA	O	0.12
286ASN	N	273HIS	NE2	0.20
286ASN	N	284ILE	O	0.45
286ASN	N	326ASP	OD1	0.04
286ASN	N	326ASP	OD2	0.00
285ALA	N	279ILE	O	13.09
285ALA	N	283GLY	O	7.48
285ALA	N	331ALA	O	0.22
285ALA	N	333THR	OG1	2.88
284ILE	N	279ILE	O	52.61
284ILE	N	280ALA	O	0.03
284ILE	N	282LYS	O	0.43
283GLY	N	14GLU	OE2	0.02
283GLY	N	279ILE	O	35.65
283GLY	N	280ALA	O	2.14
283GLY	N	281GLY	O	0.00
283GLY	N	333THR	OG1	0.00
282LYS	NZ	277PRO	O	1.22
282LYS	NZ	278ASP	OD1	43.23
282LYS	NZ	278ASP	OD2	43.89
282LYS	NZ	278ASP	O	2.02
282LYS	NZ	280ALA	O	0.21

272VAL	N	257LEU	O	60.62
270GLU	N	259SER	O	98.81
269PHE	N	67VAL	O	96.42
268VAL	N	261SER	O	96.78
266THR	OG1	61VAL	O	75.03
266THR	OG1	62GLU	O	0.00
266THR	OG1	64ALA	O	0.62
266THR	OG1	97GLN	OE1	13.30
266THR	OG1	265GLY	O	0.01
266THR	N	97GLN	OE1	91.74
266THR	N	97GLN	O	0.19
265GLY	N	97GLN	O	23.35
265GLY	N	98ASP	O	0.11
265GLY	N	263GLY	O	1.49
264ARG	NH2	96SER	O	0.17
264ARG	NH2	98ASP	OD1	18.90
264ARG	NH2	98ASP	OD2	26.88
264ARG	NH1	96SER	O	0.00
264ARG	NH1	98ASP	OD1	5.94
264ARG	NH1	98ASP	OD2	15.68
264ARG	NH1	98ASP	O	0.02
264ARG	NE	98ASP	OD1	25.06
264ARG	NE	98ASP	OD2	47.58
264ARG	NE	98ASP	O	0.01
264ARG	N	97GLN	O	6.04
264ARG	N	98ASP	OD1	0.00
264ARG	N	98ASP	OD2	0.00
264ARG	N	98ASP	O	74.49
262LEU	N	101ALA	O	99.10
261SER	OG	101ALA	O	1.66
261SER	OG	102ASN	OD1	0.16
261SER	OG	260ALA	O	0.76
261SER	OG	268VAL	O	0.02
261SER	OG	270GLU	OE1	1.64
261SER	OG	270GLU	OE2	1.26
261SER	N	268VAL	O	97.68
260ALA	N	102ASN	OD1	0.01
260ALA	N	103LEU	O	94.82
259SER	OG	259SER	O	0.01
259SER	OG	270GLU	O	88.98
259SER	N	270GLU	O	10.32
259SER	N	293SER	OG	0.18
257LEU	N	254LEU	O	0.58

293SER	OG	271PRO	N	0.00
293SER	OG	289ALA	O	92.45
293SER	OG	293SER	O	0.04
293SER	N	289ALA	O	42.83
293SER	N	290ALA	O	10.00
292LEU	N	288THR	O	90.57
292LEU	N	289ALA	O	0.34
291ILE	N	287PRO	O	71.99
291ILE	N	288THR	O	3.61
290ALA	N	273HIS	NE2	0.02
290ALA	N	286ASN	OD1	0.00
290ALA	N	287PRO	O	41.04
289ALA	N	255GLY	O	15.41
289ALA	N	273HIS	NE2	0.01
289ALA	N	286ASN	OD1	7.01
289ALA	N	286ASN	O	0.05
288THR	OG1	255GLY	O	21.98
288THR	OG1	286ASN	OD1	66.52
288THR	OG1	286ASN	O	0.00
288THR	OG1	289ALA	N	0.03
288THR	N	286ASN	OD1	56.25
288THR	N	286ASN	O	2.33
288THR	N	288THR	OG1	0.01
286ASN	ND2	255GLY	O	2.11
286ASN	ND2	274GLY	O	0.01
286ASN	ND2	288THR	OG1	3.17
286ASN	ND2	322THR	O	0.50
286ASN	ND2	323PRO	O	0.01
286ASN	ND2	325PRO	O	2.52
286ASN	ND2	326ASP	OD1	0.56
286ASN	ND2	326ASP	OD2	0.06
286ASN	ND2	331ALA	O	20.17
286ASN	ND2	332GLY	O	0.61
286ASN	N	284ILE	O	0.06
285ALA	N	279ILE	O	16.58
285ALA	N	283GLY	O	1.76
285ALA	N	333THR	OG1	0.83
284ILE	N	279ILE	O	21.30
284ILE	N	282LYS	O	0.54
283GLY	N	14GLU	OE1	0.15
283GLY	N	14GLU	OE2	0.44
283GLY	N	279ILE	O	14.43
283GLY	N	280ALA	O	0.06

272VAL	N	257LEU	O	98.41
270GLU	N	259SER	O	94.04
269PHE	N	67VAL	O	96.26
268VAL	N	261SER	O	95.80
266THR	OG1	61VAL	O	86.48
266THR	OG1	62GLU	O	0.00
266THR	OG1	64ALA	O	0.53
266THR	OG1	97GLN	OE1	4.87
266THR	N	97GLN	OE1	91.37
266THR	N	97GLN	O	0.00
265GLY	N	97GLN	O	71.98
265GLY	N	263GLY	O	0.02
264ARG	NH2	96SER	O	7.64
264ARG	NH2	98ASP	OD1	5.71
264ARG	NH2	98ASP	OD2	10.41
264ARG	NH2	161GLU	OE1	0.02
264ARG	NH2	161GLU	OE2	0.00
264ARG	NH1	96SER	O	0.88
264ARG	NH1	98ASP	OD1	6.08
264ARG	NH1	98ASP	OD2	2.14
264ARG	NH1	264ARG	O	0.10
264ARG	NE	98ASP	OD1	32.15
264ARG	NE	98ASP	OD2	28.26
264ARG	NE	264ARG	O	0.00
264ARG	N	97GLN	O	21.99
264ARG	N	98ASP	O	55.04
262LEU	N	101ALA	O	90.12
261SER	OG	101ALA	O	4.18
261SER	OG	260ALA	O	0.63
261SER	OG	261SER	O	0.00
261SER	OG	262LEU	N	0.00
261SER	OG	262LEU	O	0.02
261SER	OG	268VAL	O	0.58
261SER	OG	270GLU	OE1	0.10
261SER	OG	270GLU	OE2	0.01
261SER	N	268VAL	O	92.16
260ALA	N	103LEU	O	94.45
259SER	OG	102ASN	OD1	0.19
259SER	OG	103LEU	O	17.89
259SER	OG	258PRO	O	3.63
259SER	OG	259SER	O	0.01
259SER	OG	260ALA	N	0.01
259SER	OG	260ALA	O	0.09

282LYS	NZ	281GLY	O	0.01
282LYS	NZ	282LYS	O	0.01
282LYS	NZ	329GLY	O	1.08
282LYS	NZ	330SER	OG	0.00
282LYS	N	278ASP	O	28.76
282LYS	N	279ILE	O	34.23
282LYS	N	280ALA	O	0.15
281GLY	N	14GLU	OE2	0.01
281GLY	N	276ALA	O	0.00
281GLY	N	277PRO	O	6.83
281GLY	N	278ASP	O	53.04
281GLY	N	279ILE	O	0.21
280ALA	N	276ALA	O	34.81
280ALA	N	277PRO	O	21.36
280ALA	N	278ASP	O	0.09
279ILE	N	276ALA	O	10.85
279ILE	N	277PRO	O	14.86
279ILE	N	278ASP	OD2	0.01
278ASP	N	10GLY	O	0.01
278ASP	N	276ALA	O	0.41
278ASP	N	278ASP	OD1	0.05
278ASP	N	278ASP	OD2	0.01
276ALA	N	274GLY	O	0.14
276ALA	N	325PRO	O	9.66
276ALA	N	326ASP	OD1	0.14
276ALA	N	326ASP	OD2	1.17
275SER	OG	9ASP	O	0.20
275SER	OG	72VAL	O	0.01
275SER	OG	73GLY	O	0.12
275SER	OG	273HIS	NE2	0.04
275SER	OG	273HIS	O	0.00
275SER	OG	274GLY	O	5.97
275SER	OG	275SER	O	0.60
275SER	OG	276ALA	N	0.03
275SER	OG	276ALA	O	2.37
275SER	OG	280ALA	N	0.00
275SER	OG	284ILE	O	0.04
275SER	OG	325PRO	O	0.01
275SER	OG	326ASP	OD1	0.04
275SER	N	72VAL	O	0.09
275SER	N	73GLY	O	1.93
275SER	N	273HIS	O	0.02
275SER	N	326ASP	OD1	6.42

257LEU	N	255GLY	O	0.06
257LEU	N	272VAL	O	0.02
256LEU	N	253SER	O	3.36
256LEU	N	254LEU	O	0.81
255GLY	N	253SER	O	0.11
255GLY	N	326ASP	OD1	0.00
255GLY	N	326ASP	OD2	0.04
254LEU	N	326ASP	OD1	0.02
254LEU	N	326ASP	OD2	1.75
254LEU	N	225ARG	NH1	0.01
253SER	OG	252GLY	O	0.08
253SER	OG	253SER	O	0.12
253SER	OG	326ASP	OD1	20.24
253SER	OG	326ASP	OD2	39.19
253SER	OG	326ASP	O	6.02
253SER	OG	327LEU	N	0.02
253SER	N	249VAL	O	0.10
253SER	N	251PRO	O	1.01
252GLY	N	248SER	O	0.00
252GLY	N	249VAL	O	85.44
252GLY	N	250LEU	O	0.01
250LEU	N	247ALA	O	46.49
249VAL	N	245ASP	O	0.05
249VAL	N	246LEU	O	19.07
249VAL	N	247ALA	O	0.05
248SER	OG	244SER	O	64.37
248SER	OG	245ASP	O	1.36
248SER	OG	248SER	O	0.55
248SER	OG	254LEU	O	16.26
248SER	N	244SER	O	8.15
248SER	N	245ASP	O	55.79
248SER	N	246LEU	O	0.07
247ALA	N	243LEU	O	71.63
247ALA	N	244SER	O	3.68
246LEU	N	242ILE	O	81.30
246LEU	N	243LEU	O	0.91
245ASP	N	241ASP	O	49.53
245ASP	N	242ILE	O	3.11
244SER	OG	240GLY	O	71.45
244SER	OG	241ASP	O	12.59
244SER	OG	245ASP	N	0.08
244SER	OG	245ASP	OD1	1.10
244SER	OG	245ASP	OD2	1.57

283GLY	N	281GLY	O	0.12
282LYS	NZ	278ASP	OD1	44.93
282LYS	NZ	278ASP	OD2	44.58
282LYS	NZ	278ASP	O	5.23
282LYS	NZ	280ALA	O	0.04
282LYS	NZ	330SER	O	0.02
282LYS	N	14GLU	OE1	0.04
282LYS	N	14GLU	OE2	0.12
282LYS	N	278ASP	O	4.10
282LYS	N	279ILE	O	36.60
282LYS	N	280ALA	O	2.14
281GLY	N	14GLU	OE1	1.72
281GLY	N	14GLU	OE2	2.54
281GLY	N	278ASP	O	2.85
281GLY	N	279ILE	O	0.16
281GLY	N	283GLY	N	0.00
281GLY	N	333THR	OG1	0.07
280ALA	N	276ALA	O	20.02
280ALA	N	277PRO	O	3.22
280ALA	N	278ASP	O	0.37
279ILE	N	276ALA	O	2.90
279ILE	N	277PRO	O	69.11
279ILE	N	280ALA	O	0.00
278ASP	N	276ALA	O	0.05
278ASP	N	278ASP	OD1	0.00
278ASP	N	278ASP	OD2	0.00
278ASP	N	280ALA	O	0.23
276ALA	N	274GLY	O	2.64
275SER	OG	9ASP	O	1.24
275SER	OG	71SER	O	0.03
275SER	OG	72VAL	O	0.78
275SER	OG	73GLY	O	1.13
275SER	OG	274GLY	O	0.40
275SER	OG	275SER	O	0.68
275SER	OG	276ALA	N	0.00
275SER	OG	276ALA	O	0.04
275SER	OG	326ASP	OD1	0.18
275SER	N	73GLY	O	0.00
274GLY	N	286ASN	ND2	0.02
273HIS	ND1	255GLY	O	0.06
273HIS	ND1	271PRO	O	37.50
273HIS	ND1	272VAL	O	0.31
273HIS	ND1	273HIS	O	0.40

259SER	OG	270GLU	OE1	0.02
259SER	OG	270GLU	OE2	0.00
259SER	OG	270GLU	O	67.17
259SER	N	270GLU	O	5.08
259SER	N	293SER	OG	7.24
257LEU	N	254LEU	O	0.28
257LEU	N	255GLY	O	0.00
256LEU	N	253SER	O	0.75
256LEU	N	254LEU	O	0.74
255GLY	N	253SER	O	11.60
255GLY	N	326ASP	OD1	0.16
255GLY	N	326ASP	OD2	0.02
254LEU	N	326ASP	OD1	5.05
254LEU	N	326ASP	OD2	4.04
253SER	OG	115LEU	O	0.00
253SER	OG	252GLY	O	0.01
253SER	OG	253SER	O	0.48
253SER	OG	325PRO	N	0.00
253SER	OG	325PRO	O	14.64
253SER	OG	326ASP	OD1	12.20
253SER	OG	326ASP	OD2	7.24
253SER	N	115LEU	O	0.01
253SER	N	248SER	O	0.00
253SER	N	251PRO	O	4.00
252GLY	N	248SER	O	1.11
252GLY	N	249VAL	O	13.76
252GLY	N	250LEU	O	0.00
250LEU	N	246LEU	O	0.44
250LEU	N	247ALA	O	34.36
249VAL	N	245ASP	O	2.81
249VAL	N	246LEU	O	36.96
249VAL	N	247ALA	O	0.01
248SER	OG	244SER	O	71.15
248SER	OG	245ASP	O	14.64
248SER	OG	247ALA	O	0.00
248SER	OG	248SER	O	0.05
248SER	N	244SER	O	21.58
248SER	N	245ASP	O	31.85
248SER	N	246LEU	O	0.07
247ALA	N	243LEU	O	73.88
247ALA	N	244SER	O	4.69
246LEU	N	242ILE	O	88.37
246LEU	N	243LEU	O	1.00

275SER	N	326ASP	OD2	17.63
274GLY	N	255GLY	O	6.57
273HIS	ND1	70GLY	O	0.08
273HIS	ND1	254LEU	O	0.02
273HIS	ND1	255GLY	O	1.32
273HIS	ND1	271PRO	O	0.17
273HIS	ND1	272VAL	O	0.49
273HIS	ND1	273HIS	O	1.62
273HIS	ND1	274GLY	N	0.15
273HIS	ND1	274GLY	O	3.47
273HIS	ND1	275SER	OG	0.05
273HIS	ND1	286ASN	O	1.54
273HIS	N	254LEU	O	0.00
273HIS	N	271PRO	O	2.62
273HIS	N	273HIS	ND1	0.05
272VAL	N	257LEU	O	47.91
272VAL	N	270GLU	O	0.02
270GLU	N	259SER	O	91.35
270GLU	N	268VAL	O	0.02
270GLU	N	293SER	OG	0.00
269PHE	N	67VAL	O	94.78
268VAL	N	261SER	O	93.06
266THR	OG1	61VAL	O	41.21
266THR	OG1	62GLU	OE1	7.42
266THR	OG1	62GLU	OE2	7.85
266THR	OG1	62GLU	O	0.02
266THR	OG1	64ALA	O	10.57
266THR	OG1	65GLU	O	0.76
266THR	OG1	97GLN	OE1	5.56
266THR	OG1	265GLY	O	0.01
266THR	OG1	266THR	O	0.38
266THR	OG1	267PRO	N	0.00
266THR	OG1	267PRO	O	1.31
266THR	N	61VAL	O	0.00
266THR	N	62GLU	OE1	7.34
266THR	N	62GLU	OE2	7.21
266THR	N	97GLN	OE1	27.61
266THR	N	97GLN	O	0.15
266THR	N	263GLY	O	0.04
266THR	N	264ARG	O	0.02
265GLY	N	97GLN	O	15.48
265GLY	N	98ASP	O	1.68
265GLY	N	263GLY	O	3.72

244SER	N	240GLY	O	78.30
244SER	N	241ASP	O	8.67
244SER	N	242ILE	O	0.00
243LEU	N	239PHE	O	10.13
243LEU	N	240GLY	O	4.28
242ILE	N	238ILE	O	9.88
242ILE	N	239PHE	O	3.64
242ILE	N	240GLY	O	0.00
242ILE	N	241ASP	OD1	0.01
242ILE	N	241ASP	OD2	0.02
241ASP	N	237ASN	O	91.89
241ASP	N	238ILE	O	1.45
240GLY	N	236GLY	O	2.99
240GLY	N	237ASN	O	69.02
240GLY	N	238ILE	O	0.37
239PHE	N	236GLY	O	0.22
237ASN	ND2	133GLU	OE2	0.00
237ASN	ND2	133GLU	O	0.20
237ASN	ND2	134LEU	O	36.37
237ASN	ND2	136GLY	O	89.43
237ASN	ND2	137GLY	O	2.18
237ASN	N	136GLY	O	11.94
235THR	OG1	183VAL	O	4.79
235THR	OG1	236GLY	N	0.00
235THR	OG1	236GLY	O	1.62
235THR	OG1	239PHE	O	5.43
235THR	OG1	240GLY	N	0.08
235THR	N	131VAL	O	63.96
234VAL	N	181VAL	O	90.94
233VAL	N	129LEU	O	91.47
232VAL	N	179HIS	O	9.60
232VAL	N	231ASP	OD2	0.00
231ASP	N	229ARG	O	0.00
230PHE	N	227PRO	O	38.63
230PHE	N	228ALA	O	1.96
229ARG	NH2	212GLU	OE1	0.98
229ARG	NH2	212GLU	OE2	1.46
229ARG	NH1	212GLU	OE2	0.04
229ARG	NH1	222HIS	ND1	0.02
229ARG	NH1	228ALA	O	0.06
229ARG	NH1	229ARG	O	0.02
229ARG	NE	212GLU	OE1	0.00
229ARG	NE	212GLU	OE2	0.00

273HIS	ND1	274GLY	N	0.05
273HIS	ND1	274GLY	O	4.52
273HIS	ND1	275SER	N	0.00
273HIS	ND1	275SER	OG	0.00
273HIS	ND1	286ASN	OD1	0.13
273HIS	ND1	286ASN	O	0.04
273HIS	N	271PRO	O	0.33
273HIS	N	273HIS	ND1	0.02
272VAL	N	257LEU	O	36.61
270GLU	N	259SER	O	80.76
270GLU	N	268VAL	O	0.39
269PHE	N	67VAL	O	96.53
268VAL	N	261SER	O	68.43
266THR	OG1	61VAL	O	36.32
266THR	OG1	62GLU	OE1	6.67
266THR	OG1	62GLU	OE2	5.52
266THR	OG1	62GLU	O	0.00
266THR	OG1	64ALA	O	7.61
266THR	OG1	65GLU	O	0.49
266THR	OG1	97GLN	OE1	7.88
266THR	OG1	266THR	O	3.16
266THR	OG1	267PRO	O	0.04
266THR	N	62GLU	OE1	7.13
266THR	N	62GLU	OE2	4.04
266THR	N	97GLN	OE1	29.15
266THR	N	263GLY	O	0.01
266THR	N	264ARG	O	0.07
265GLY	N	97GLN	O	28.55
265GLY	N	98ASP	O	0.08
265GLY	N	263GLY	O	0.68
264ARG	NH2	62GLU	OE1	13.56
264ARG	NH2	62GLU	OE2	9.01
264ARG	NH2	96SER	O	8.48
264ARG	NH2	97GLN	OE1	0.04
264ARG	NH2	97GLN	O	0.38
264ARG	NH2	98ASP	OD1	7.83
264ARG	NH2	98ASP	OD2	0.73
264ARG	NH2	163GLU	OE1	0.00
264ARG	NH2	163GLU	OE2	0.13
264ARG	NH2	266THR	OG1	0.00
264ARG	NH1	62GLU	OE1	9.29
264ARG	NH1	62GLU	OE2	13.99
264ARG	NH1	96SER	O	1.67

245ASP	N	241ASP	O	51.59
245ASP	N	242ILE	O	6.53
245ASP	N	243LEU	O	0.00
245ASP	N	245ASP	OD2	0.00
244SER	OG	240GLY	O	44.67
244SER	OG	241ASP	O	1.38
244SER	OG	245ASP	N	0.56
244SER	OG	245ASP	OD1	1.05
244SER	OG	245ASP	OD2	2.46
244SER	N	240GLY	O	54.93
244SER	N	241ASP	O	6.68
243LEU	N	239PHE	O	28.23
243LEU	N	240GLY	O	5.20
242ILE	N	238ILE	O	3.44
242ILE	N	239PHE	O	4.62
242ILE	N	241ASP	OD1	3.30
241ASP	N	237ASN	OD1	0.09
241ASP	N	237ASN	O	25.93
241ASP	N	238ILE	O	1.49
241ASP	N	241ASP	OD1	0.24
240GLY	N	236GLY	O	1.08
240GLY	N	237ASN	OD1	0.00
240GLY	N	237ASN	O	25.80
240GLY	N	238ILE	O	0.05
239PHE	N	236GLY	O	0.06
238ILE	N	236GLY	O	0.00
237ASN	ND2	133GLU	OE1	0.03
237ASN	ND2	133GLU	OE2	0.00
237ASN	ND2	133GLU	O	2.12
237ASN	ND2	134LEU	O	3.97
237ASN	ND2	136GLY	O	89.70
237ASN	ND2	137GLY	O	0.00
237ASN	ND2	236GLY	O	0.02
237ASN	N	136GLY	O	0.03
235THR	OG1	183VAL	O	10.70
235THR	OG1	236GLY	N	0.02
235THR	OG1	236GLY	O	0.40
235THR	OG1	239PHE	O	1.21
235THR	OG1	240GLY	N	0.00
235THR	N	131VAL	O	61.08
234VAL	N	181VAL	O	92.05
233VAL	N	129LEU	O	96.43
232VAL	N	179HIS	O	4.35

264ARG	NH2	98ASP	OD1	38.90
264ARG	NH2	98ASP	OD2	28.39
264ARG	NH2	98ASP	O	0.01
264ARG	NH2	99LEU	O	0.28
264ARG	NH2	161GLU	OE1	0.47
264ARG	NH2	161GLU	OE2	2.72
264ARG	NH1	98ASP	OD1	4.58
264ARG	NH1	98ASP	OD2	8.27
264ARG	NH1	98ASP	O	1.06
264ARG	NH1	99LEU	O	0.01
264ARG	NH1	133GLU	OE1	0.05
264ARG	NH1	161GLU	OE1	3.03
264ARG	NH1	161GLU	OE2	2.02
264ARG	NE	98ASP	OD1	34.29
264ARG	NE	98ASP	OD2	46.43
264ARG	NE	98ASP	O	0.25
264ARG	NE	99LEU	O	0.57
264ARG	N	97GLN	O	8.20
264ARG	N	98ASP	OD1	0.75
264ARG	N	98ASP	OD2	0.82
264ARG	N	98ASP	O	76.84
263GLY	N	265GLY	O	0.02
263GLY	N	266THR	O	0.21
262LEU	N	101ALA	O	96.36
262LEU	N	260ALA	O	0.01
261SER	OG	99LEU	O	0.00
261SER	OG	101ALA	O	3.07
261SER	OG	102ASN	OD1	0.49
261SER	OG	260ALA	O	2.03
261SER	OG	261SER	O	0.09
261SER	OG	262LEU	N	0.01
261SER	OG	268VAL	O	3.39
261SER	OG	270GLU	OE1	10.81
261SER	OG	270GLU	OE2	0.82
261SER	N	268VAL	O	92.78
260ALA	N	102ASN	OD1	0.04
260ALA	N	103LEU	O	93.38
259SER	OG	102ASN	OD1	0.02
259SER	OG	103LEU	O	11.70
259SER	OG	258PRO	O	0.56
259SER	OG	259SER	O	0.07
259SER	OG	260ALA	N	0.02
259SER	OG	260ALA	O	0.02

229ARG	NE	228ALA	O	0.10
229ARG	NE	229ARG	O	0.71
229ARG	N	226SER	O	28.81
229ARG	N	227PRO	O	10.60
228ALA	N	226SER	O	0.02
226SER	OG	222HIS	ND1	14.71
226SER	OG	222HIS	O	2.63
226SER	OG	226SER	O	7.68
226SER	OG	229ARG	NE	0.01
226SER	OG	229ARG	NH1	0.01
226SER	N	222HIS	O	97.84
226SER	N	223LEU	O	0.12
225ARG	NH2	222HIS	ND1	3.32
225ARG	NH2	115LEU	O	0.04
225ARG	NH2	248SER	OG	0.02
225ARG	NH2	252GLY	O	30.79
225ARG	NH2	253SER	OG	0.02
225ARG	NH2	326ASP	OD1	44.00
225ARG	NH2	326ASP	OD2	21.72
225ARG	NH2	326ASP	O	0.33
225ARG	NH1	222HIS	ND1	0.02
225ARG	NH1	252GLY	O	29.37
225ARG	NH1	326ASP	OD1	11.02
225ARG	NH1	326ASP	OD2	36.50
225ARG	NE	222HIS	ND1	0.16
225ARG	NE	226SER	OG	0.01
225ARG	NE	252GLY	O	0.14
225ARG	N	221MET	O	20.63
225ARG	N	222HIS	O	60.61
225ARG	N	223LEU	O	0.17
224VAL	N	220ALA	O	0.59
224VAL	N	221MET	O	66.27
224VAL	N	222HIS	O	0.02
223LEU	N	219MET	O	89.61
223LEU	N	220ALA	O	0.75
222HIS	NE2	214GLN	OE1	0.39
222HIS	NE2	226SER	OG	0.03
222HIS	NE2	226SER	O	0.01
222HIS	NE2	229ARG	NE	0.26
222HIS	NE2	229ARG	NH1	0.18
222HIS	NE2	229ARG	NH2	0.01
222HIS	NE2	229ARG	O	0.44
222HIS	N	218ALA	O	86.09

264ARG	NH1	97GLN	O	0.03
264ARG	NH1	98ASP	OD1	21.90
264ARG	NH1	98ASP	OD2	3.19
264ARG	NH1	98ASP	O	0.26
264ARG	NH1	264ARG	O	0.08
264ARG	NE	62GLU	OE2	0.00
264ARG	NE	96SER	O	3.93
264ARG	NE	97GLN	O	0.98
264ARG	NE	98ASP	OD1	26.71
264ARG	NE	98ASP	OD2	0.89
264ARG	NE	264ARG	O	0.29
264ARG	N	97GLN	O	18.75
264ARG	N	98ASP	OD1	0.00
264ARG	N	98ASP	O	41.24
263GLY	N	265GLY	O	0.04
263GLY	N	266THR	O	0.59
262LEU	N	101ALA	O	78.22
261SER	OG	101ALA	O	2.54
261SER	OG	102ASN	OD1	1.03
261SER	OG	259SER	O	0.00
261SER	OG	260ALA	O	0.87
261SER	OG	261SER	O	0.27
261SER	OG	262LEU	O	0.00
261SER	OG	268VAL	O	33.55
261SER	OG	270GLU	OE2	0.02
261SER	N	268VAL	O	60.91
260ALA	N	102ASN	OD1	0.00
260ALA	N	103LEU	O	90.39
259SER	OG	102ASN	OD1	0.20
259SER	OG	103LEU	O	11.34
259SER	OG	258PRO	O	7.06
259SER	OG	259SER	O	0.08
259SER	OG	260ALA	N	0.03
259SER	OG	260ALA	O	0.02
259SER	OG	270GLU	O	69.19
259SER	OG	271PRO	O	0.00
259SER	N	270GLU	O	4.43
259SER	N	293SER	OG	20.02
257LEU	N	253SER	O	0.00
257LEU	N	254LEU	O	21.02
257LEU	N	255GLY	O	1.47
257LEU	N	272VAL	O	0.11
256LEU	N	253SER	O	16.99

232VAL	N	231ASP	OD2	0.77
231ASP	N	229ARG	O	0.07
230PHE	N	227PRO	O	63.28
230PHE	N	228ALA	O	2.62
229ARG	NH2	212GLU	OE1	29.19
229ARG	NH2	212GLU	OE2	31.85
229ARG	NH2	214GLN	OE1	0.21
229ARG	NH2	229ARG	O	0.01
229ARG	NH1	179HIS	ND1	0.60
229ARG	NH1	212GLU	OE1	31.51
229ARG	NH1	212GLU	OE2	28.32
229ARG	NH1	214GLN	OE1	0.60
229ARG	NH1	228ALA	O	0.06
229ARG	NH1	229ARG	O	9.73
229ARG	NE	214GLN	OE1	0.02
229ARG	NE	229ARG	O	10.34
229ARG	NE	230PHE	N	0.00
229ARG	N	226SER	O	18.80
229ARG	N	227PRO	O	1.29
228ALA	N	226SER	O	0.07
226SER	OG	222HIS	ND1	0.77
226SER	OG	222HIS	O	5.39
226SER	OG	225ARG	O	1.50
226SER	OG	226SER	O	5.77
226SER	OG	228ALA	O	0.61
226SER	OG	229ARG	NE	0.01
226SER	N	222HIS	O	94.72
226SER	N	223LEU	O	0.55
225ARG	NH2	218ALA	O	0.01
225ARG	NH2	221MET	O	0.08
225ARG	NH2	222HIS	ND1	2.76
225ARG	NH2	115LEU	O	0.00
225ARG	NH2	252GLY	O	42.35
225ARG	NH2	326ASP	OD11	46.44
225ARG	NH2	326ASP	OD21	5.14
225ARG	NH2	326ASP	O101	1.77
225ARG	NH1	222HIS	ND1	0.78
225ARG	NH1	115LEU	O	0.14
225ARG	NH1	252GLY	O	0.61
225ARG	NH1	326ASP	OD11	6.28
225ARG	NH1	326ASP	OD21	40.90
225ARG	NH1	326ASP	O101	0.01
225ARG	NE	221MET	O	11.52

259SER	OG	270GLU	OE1	0.06
259SER	OG	270GLU	OE2	0.02
259SER	OG	270GLU	O	73.87
259SER	N	270GLU	O	8.20
259SER	N	293SER	OG	1.94
257LEU	N	253SER	O	2.15
257LEU	N	254LEU	O	1.32
257LEU	N	255GLY	O	0.30
257LEU	N	272VAL	O	10.38
256LEU	N	253SER	O	57.61
256LEU	N	254LEU	O	0.12
255GLY	N	253SER	O	1.01
254LEU	N	248SER	O	0.51
254LEU	N	252GLY	O	62.46
253SER	OG	106ALA	O	0.00
253SER	OG	107LYS	O	57.99
253SER	OG	248SER	O	1.12
253SER	OG	251PRO	O	0.02
253SER	OG	252GLY	O	0.06
253SER	OG	253SER	O	2.36
253SER	OG	254LEU	N	0.00
253SER	OG	256LEU	O	0.91
253SER	OG	326ASP	OD1	0.06
253SER	OG	326ASP	OD2	0.03
253SER	N	248SER	O	4.24
253SER	N	251PRO	O	0.39
252GLY	N	248SER	O	15.46
252GLY	N	249VAL	O	24.49
250LEU	N	246LEU	O	21.32
250LEU	N	247ALA	O	7.39
249VAL	N	245ASP	O	6.48
249VAL	N	246LEU	O	60.14
248SER	OG	105PRO	O	0.00
248SER	OG	244SER	O	30.46
248SER	OG	245ASP	OD1	0.23
248SER	OG	245ASP	OD2	0.08
248SER	OG	245ASP	O	43.87
248SER	OG	247ALA	O	0.00
248SER	OG	248SER	O	0.04
248SER	OG	249VAL	N	0.00
248SER	OG	253SER	O	0.20
248SER	OG	254LEU	N	0.00
248SER	OG	256LEU	O	0.16

222HIS	N	219MET	O	1.50
222HIS	N	220ALA	O	0.00
221MET	N	217ASP	O	14.16
221MET	N	218ALA	O	31.11
221MET	N	219MET	O	0.02
220ALA	N	216VAL	O	76.15
220ALA	N	217ASP	O	2.88
219MET	N	215TYR	O	93.74
219MET	N	216VAL	O	0.69
218ALA	N	215TYR	O	31.05
218ALA	N	216VAL	O	0.35
218ALA	N	217ASP	OD1	5.42
218ALA	N	217ASP	OD2	0.23
217ASP	N	217ASP	OD1	30.69
217ASP	N	217ASP	OD2	0.00
216VAL	N	184ASP	O	79.96
215TYR	OH	184ASP	OD1	0.00
215TYR	OH	187ASN	OD1	0.21
215TYR	OH	275SER	OG	0.01
215TYR	N	214GLN	OE1	9.31
214GLN	NE2	212GLU	OE1	2.63
214GLN	NE2	212GLU	OE2	4.12
214GLN	NE2	213HIS	O	9.85
214GLN	NE2	215TYR	N	0.00
214GLN	NE2	215TYR	O	2.67
214GLN	NE2	218ALA	O	0.05
214GLN	NE2	219MET	N	0.16
214GLN	NE2	222HIS	ND1	5.65
214GLN	NE2	222HIS	NE2	0.32
214GLN	NE2	229ARG	NE	0.01
214GLN	NE2	229ARG	NH2	0.01
214GLN	N	182SER	O	83.73
213HIS	NE2	196ARG	O	0.35
213HIS	NE2	200GLU	OE1	0.56
213HIS	NE2	200GLU	OE2	0.34
213HIS	NE2	215TYR	OH	1.68
213HIS	N	212GLU	OE1	9.49
213HIS	N	212GLU	OE2	10.40
213HIS	N	213HIS	ND1	0.07
212GLU	N	180VAL	O	72.08
210ALA	N	178LYS	O	94.72
209VAL	N	206TYR	O	64.97
209VAL	N	207PRO	O	0.12

256LEU	N	254LEU	O	12.67
255GLY	N	253SER	O	0.26
255GLY	N	286ASN	ND2	0.02
255GLY	N	326ASP	OD1	0.12
255GLY	N	326ASP	OD2	0.08
254LEU	N	252GLY	O	0.00
254LEU	N	326ASP	OD1	3.64
254LEU	N	326ASP	OD2	5.30
253SER	OG	252GLY	O	0.17
253SER	OG	254LEU	N	0.00
253SER	OG	325PRO	O	0.00
253SER	OG	326ASP	OD1	16.86
253SER	OG	326ASP	OD2	77.29
253SER	OG	326ASP	O	0.01
253SER	OG	225ARG	NH2	0.00
253SER	N	248SER	O	2.49
253SER	N	251PRO	O	0.12
252GLY	N	248SER	O	53.45
252GLY	N	249VAL	O	19.41
250LEU	N	246LEU	O	0.02
250LEU	N	247ALA	O	36.97
249VAL	N	245ASP	O	0.14
249VAL	N	246LEU	O	76.66
249VAL	N	247ALA	O	0.04
248SER	OG	244SER	O	29.76
248SER	OG	245ASP	OD1	0.07
248SER	OG	245ASP	O	50.02
248SER	OG	246LEU	O	0.02
248SER	OG	249VAL	N	0.00
248SER	OG	253SER	O	0.01
248SER	OG	254LEU	O	0.06
248SER	N	244SER	O	2.44
248SER	N	245ASP	O	60.75
248SER	N	246LEU	O	0.36
247ALA	N	243LEU	O	57.93
247ALA	N	244SER	O	1.84
246LEU	N	242ILE	O	82.52
246LEU	N	243LEU	O	2.78
245ASP	N	241ASP	O	24.22
245ASP	N	242ILE	O	31.32
245ASP	N	243LEU	O	0.00
244SER	OG	240GLY	O	57.06
244SER	OG	241ASP	O	11.52

225ARG	NE	222HIS	ND1	2.08
225ARG	NE	225ARG	O	0.52
225ARG	NE	252GLY	O	0.02
225ARG	N	221MET	O	34.97
225ARG	N	222HIS	O	37.37
225ARG	N	223LEU	O	0.92
224VAL	N	220ALA	O	7.60
224VAL	N	221MET	O	25.91
223LEU	N	219MET	O	83.26
223LEU	N	220ALA	O	1.96
222HIS	NE2	214GLN	OE1	0.15
222HIS	NE2	229ARG	NE	0.08
222HIS	NE2	229ARG	NH1	0.40
222HIS	NE2	229ARG	NH2	0.00
222HIS	N	218ALA	O	70.09
222HIS	N	219MET	O	9.92
222HIS	N	220ALA	O	0.00
221MET	N	217ASP	O	34.49
221MET	N	218ALA	O	11.44
221MET	N	219MET	O	0.00
220ALA	N	216VAL	O	89.18
220ALA	N	217ASP	O	1.00
219MET	N	215TYR	O	90.80
219MET	N	216VAL	O	1.19
218ALA	N	215TYR	O	24.30
218ALA	N	216VAL	O	0.01
218ALA	N	217ASP	OD1	3.01
218ALA	N	217ASP	OD2	1.58
217ASP	N	217ASP	OD1	17.66
217ASP	N	217ASP	OD2	22.19
216VAL	N	184ASP	O	96.88
215TYR	OH	186ALA	O	0.03
215TYR	OH	187ASN	OD1	0.10
215TYR	N	214GLN	OE1	0.20
214GLN	NE2	212GLU	OE1	31.47
214GLN	NE2	212GLU	OE2	29.35
214GLN	NE2	213HIS	O	1.14
214GLN	NE2	215TYR	O	0.04
214GLN	NE2	218ALA	O	0.01
214GLN	NE2	219MET	N	0.01
214GLN	NE2	222HIS	ND1	12.98
214GLN	NE2	222HIS	NE2	0.00
214GLN	NE2	229ARG	NE	0.10

248SER	N	244SER	O	8.10
248SER	N	245ASP	O	58.53
248SER	N	246LEU	O	0.68
247ALA	N	243LEU	O	80.96
247ALA	N	244SER	O	2.50
246LEU	N	242ILE	O	35.35
246LEU	N	243LEU	O	1.81
246LEU	N	244SER	O	0.02
245ASP	N	241ASP	O	8.79
245ASP	N	242ILE	O	4.32
244SER	OG	240GLY	O	74.69
244SER	OG	241ASP	OD2	0.00
244SER	OG	241ASP	O	11.38
244SER	OG	243LEU	O	0.01
244SER	OG	245ASP	N	0.03
244SER	OG	245ASP	OD1	0.28
244SER	OG	245ASP	OD2	0.36
244SER	N	240GLY	O	39.76
244SER	N	241ASP	O	25.20
244SER	N	242ILE	O	0.02
243LEU	N	239PHE	O	29.88
243LEU	N	240GLY	O	12.86
243LEU	N	241ASP	O	0.02
242ILE	N	238ILE	O	52.37
242ILE	N	239PHE	O	3.35
242ILE	N	240GLY	O	0.01
242ILE	N	241ASP	OD2	0.01
241ASP	N	236GLY	O	0.14
241ASP	N	237ASN	O	32.98
241ASP	N	238ILE	O	22.56
241ASP	N	239PHE	O	0.01
240GLY	N	236GLY	O	55.27
240GLY	N	237ASN	O	12.19
240GLY	N	238ILE	O	0.02
239PHE	N	236GLY	O	1.57
238ILE	N	236GLY	O	0.16
237ASN	ND2	133GLU	OE1	0.03
237ASN	ND2	133GLU	OE2	3.96
237ASN	ND2	133GLU	O	3.90
237ASN	ND2	134LEU	O	16.01
237ASN	ND2	135THR	O	0.00
237ASN	ND2	136GLY	O	79.87
237ASN	ND2	137GLY	O	0.04

209VAL	N	208ASP	OD1	0.10
209VAL	N	208ASP	OD2	0.08
208ASP	N	206TYR	O	0.34
208ASP	N	208ASP	OD1	18.73
208ASP	N	208ASP	OD2	21.77
206TYR	OH	167ARG	O	0.10
206TYR	OH	171GLU	OE1	67.16
206TYR	OH	171GLU	OE2	26.79
206TYR	N	202VAL	O	28.67
206TYR	N	203GLY	O	33.97
205GLY	N	201GLU	O	1.02
205GLY	N	202VAL	O	71.08
205GLY	N	203GLY	O	0.11
204ARG	NH2	197LYS	O	0.00
204ARG	NH2	200GLU	OE1	21.76
204ARG	NH2	200GLU	OE2	74.71
204ARG	NH2	201GLU	OE1	47.23
204ARG	NH2	201GLU	OE2	23.90
204ARG	NH1	201GLU	OE1	26.81
204ARG	NH1	201GLU	OE2	44.41
204ARG	NE	200GLU	OE1	72.86
204ARG	NE	200GLU	OE2	27.00
204ARG	NE	200GLU	O	0.02
204ARG	N	200GLU	O	44.39
204ARG	N	201GLU	O	22.38
204ARG	N	202VAL	O	0.00
203GLY	N	199VAL	O	58.85
203GLY	N	200GLU	O	6.58
202VAL	N	198THR	O	41.68
202VAL	N	199VAL	O	13.36
202VAL	N	200GLU	O	0.00
201GLU	N	197LYS	O	27.30
201GLU	N	198THR	O	23.26
200GLU	N	196ARG	O	28.37
200GLU	N	197LYS	O	7.19
200GLU	N	198THR	O	0.00
199VAL	N	195TRP	O	95.71
199VAL	N	196ARG	O	0.41
198THR	OG1	194PHE	O	93.95
198THR	OG1	195TRP	O	0.99
198THR	OG1	199VAL	N	0.00
198THR	N	194PHE	O	55.05
198THR	N	195TRP	O	24.99

244SER	OG	244SER	O	0.04
244SER	OG	245ASP	N	0.12
244SER	OG	245ASP	OD1	0.60
244SER	OG	245ASP	OD2	1.24
244SER	N	240GLY	O	64.39
244SER	N	241ASP	O	8.92
244SER	N	242ILE	O	0.01
243LEU	N	239PHE	O	42.72
243LEU	N	240GLY	O	1.56
242ILE	N	238ILE	O	13.33
242ILE	N	239PHE	O	7.79
242ILE	N	240GLY	O	0.01
241ASP	N	237ASN	OD1	0.01
241ASP	N	237ASN	O	61.94
241ASP	N	238ILE	O	4.24
240GLY	N	236GLY	O	36.20
240GLY	N	237ASN	O	28.24
240GLY	N	238ILE	O	0.02
239PHE	N	236GLY	O	26.40
238ILE	N	155GLU	OE2	0.13
238ILE	N	236GLY	O	0.00
237ASN	ND2	133GLU	OE1	0.13
237ASN	ND2	133GLU	O	23.83
237ASN	ND2	134LEU	O	4.18
237ASN	ND2	136GLY	O	55.16
237ASN	ND2	137GLY	O	0.01
237ASN	ND2	155GLU	OE2	0.00
237ASN	ND2	236GLY	O	0.04
237ASN	ND2	237ASN	O	0.01
237ASN	ND2	241ASP	OD1	0.10
237ASN	ND2	241ASP	OD2	0.30
237ASN	N	136GLY	O	0.04
237ASN	N	155GLU	OE2	0.00
237ASN	N	237ASN	OD1	0.02
237ASN	N	237ASN	ND2	0.02
235THR	OG1	183VAL	O	13.58
235THR	OG1	236GLY	N	0.09
235THR	OG1	236GLY	O	1.70
235THR	OG1	239PHE	O	0.40
235THR	OG1	240GLY	N	0.02
235THR	OG1	240GLY	O	0.01
235THR	N	131VAL	O	56.09
234VAL	N	181VAL	O	93.70

214GLN	NE2	229ARG	NH1	0.01
214GLN	NE2	229ARG	NH2	0.03
214GLN	N	182SER	O	92.25
214GLN	N	212GLU	O	0.03
213HIS	NE2	196ARG	O	0.02
213HIS	NE2	200GLU	OE1	4.14
213HIS	NE2	200GLU	OE2	4.19
213HIS	N	212GLU	OE1	1.48
213HIS	N	212GLU	OE2	1.73
212GLU	N	180VAL	O	71.91
210ALA	N	178LYS	O	96.86
209VAL	N	206TYR	O	52.50
209VAL	N	207PRO	O	0.42
209VAL	N	208ASP	OD1	0.01
208ASP	N	206TYR	O	0.74
208ASP	N	208ASP	OD1	2.94
208ASP	N	208ASP	OD2	1.57
206TYR	OH	167ARG	O	0.76
206TYR	OH	171GLU	OE1	50.37
206TYR	OH	171GLU	OE2	37.37
206TYR	N	202VAL	O	8.11
206TYR	N	203GLY	O	62.57
206TYR	N	204ARG	O	0.14
205GLY	N	201GLU	O	0.27
205GLY	N	202VAL	O	51.16
205GLY	N	203GLY	O	0.12
204ARG	NH2	197LYS	O	0.00
204ARG	NH2	200GLU	OE1	14.65
204ARG	NH2	200GLU	OE2	61.81
204ARG	NH2	200GLU	O	0.00
204ARG	NH2	201GLU	OE1	21.79
204ARG	NH2	201GLU	OE2	30.01
204ARG	NH1	200GLU	OE1	0.51
204ARG	NH1	200GLU	OE2	0.28
204ARG	NH1	200GLU	O	0.12
204ARG	NH1	201GLU	OE1	34.23
204ARG	NH1	201GLU	OE2	32.21
204ARG	NH1	201GLU	O	0.04
204ARG	NE	200GLU	OE1	56.73
204ARG	NE	200GLU	OE2	18.18
204ARG	NE	200GLU	O	0.09
204ARG	N	200GLU	O	35.85
204ARG	N	201GLU	O	19.69

237ASN	ND2	237ASN	O	0.02
237ASN	N	133GLU	OE1	0.00
237ASN	N	133GLU	O	0.04
237ASN	N	136GLY	O	0.63
237ASN	N	237ASN	ND2	0.01
236GLY	N	240GLY	N	0.00
235THR	OG1	183VAL	O	14.13
235THR	OG1	236GLY	N	0.05
235THR	OG1	236GLY	O	2.28
235THR	OG1	239PHE	O	1.98
235THR	OG1	240GLY	N	0.09
235THR	N	131VAL	O	84.04
234VAL	N	181VAL	O	97.69
233VAL	N	129LEU	O	95.80
233VAL	N	231ASP	O	0.00
232VAL	N	179HIS	O	29.11
232VAL	N	231ASP	OD1	0.17
232VAL	N	231ASP	OD2	0.10
231ASP	N	179HIS	ND1	0.00
231ASP	N	229ARG	O	0.10
230PHE	N	227PRO	O	57.20
230PHE	N	228ALA	O	2.09
229ARG	NH2	179HIS	ND1	0.02
229ARG	NH2	212GLU	OE1	22.34
229ARG	NH2	212GLU	OE2	15.87
229ARG	NH2	214GLN	OE1	1.64
229ARG	NH2	228ALA	O	0.00
229ARG	NH2	229ARG	O	0.01
229ARG	NH2	278ASP	OD1	0.02
229ARG	NH2	278ASP	OD2	0.56
229ARG	NH1	179HIS	ND1	0.01
229ARG	NH1	212GLU	OE1	3.80
229ARG	NH1	212GLU	OE2	5.80
229ARG	NH1	214GLN	OE1	0.66
229ARG	NH1	222HIS	ND1	0.42
229ARG	NH1	228ALA	O	0.21
229ARG	NH1	229ARG	O	0.36
229ARG	NH1	278ASP	OD1	0.13
229ARG	NH1	278ASP	OD2	0.78
229ARG	NE	179HIS	ND1	0.00
229ARG	NE	212GLU	OE1	1.23
229ARG	NE	212GLU	OE2	1.06
229ARG	NE	214GLN	OE1	0.07

198THR	N	196ARG	O	0.02
197LYS	NZ	193GLU	OE1	2.15
197LYS	NZ	193GLU	OE2	2.21
197LYS	NZ	193GLU	O	0.08
197LYS	NZ	147SER	O	0.26
197LYS	NZ	148GLU	OE1	26.38
197LYS	NZ	148GLU	OE2	23.78
197LYS	NZ	148GLU	O	0.27
197LYS	N	193GLU	O	46.74
197LYS	N	194PHE	O	12.61
197LYS	N	195TRP	O	0.02
196ARG	NH2	184ASP	OD1	94.25
196ARG	NH2	184ASP	OD2	5.32
196ARG	NH2	213HIS	NE2	0.00
196ARG	NH2	214GLN	O	0.00
196ARG	NH2	215TYR	OH	3.66
196ARG	NH1	193GLU	OE1	0.00
196ARG	NH1	215TYR	OH	0.01
196ARG	NE	184ASP	OD1	7.49
196ARG	NE	184ASP	OD2	93.26
196ARG	NE	213HIS	ND1	0.00
196ARG	N	192GLY	O	95.74
196ARG	N	193GLU	O	0.26
195TRP	NE1	183VAL	O	0.20
195TRP	NE1	234VAL	O	0.22
195TRP	N	191VAL	O	61.58
195TRP	N	192GLY	O	2.04
194PHE	N	190GLU	O	92.66
194PHE	N	191VAL	O	0.06
193GLU	N	189LEU	O	69.99
193GLU	N	190GLU	O	5.27
192GLY	N	189LEU	O	57.11
192GLY	N	190GLU	O	0.03
191VAL	N	153ASN	OD1	97.82
190GLU	N	190GLU	OE1	6.06
190GLU	N	190GLU	OE2	2.88
190GLU	N	153ASN	OD1	28.81
190GLU	N	153ASN	ND2	1.01
189LEU	N	185LYS	O	74.73
189LEU	N	186ALA	O	5.43
188VAL	N	185LYS	O	79.59
188VAL	N	186ALA	O	0.06
188VAL	N	187ASN	OD1	0.16

233VAL	N	129LEU	O	92.90
232VAL	N	179HIS	O	12.18
232VAL	N	231ASP	OD1	0.16
232VAL	N	231ASP	OD2	0.63
231ASP	N	179HIS	ND1	0.24
231ASP	N	179HIS	O	0.00
231ASP	N	229ARG	O	0.08
230PHE	N	227PRO	O	52.78
230PHE	N	228ALA	O	1.81
229ARG	NH2	179HIS	ND1	0.00
229ARG	NH2	212GLU	OE1	7.28
229ARG	NH2	212GLU	OE2	5.45
229ARG	NH2	214GLN	OE1	0.08
229ARG	NH2	222HIS	ND1	0.06
229ARG	NH2	228ALA	O	0.01
229ARG	NH2	229ARG	O	0.01
229ARG	NH1	179HIS	ND1	0.18
229ARG	NH1	212GLU	OE1	2.72
229ARG	NH1	212GLU	OE2	5.19
229ARG	NH1	214GLN	OE1	0.00
229ARG	NH1	222HIS	ND1	0.02
229ARG	NH1	228ALA	O	0.25
229ARG	NH1	229ARG	O	2.84
229ARG	NE	212GLU	OE1	0.22
229ARG	NE	212GLU	OE2	0.16
229ARG	NE	214GLN	OE1	0.01
229ARG	NE	222HIS	ND1	0.00
229ARG	NE	226SER	O	0.09
229ARG	NE	228ALA	O	0.06
229ARG	NE	229ARG	O	1.12
229ARG	N	226SER	O	27.92
229ARG	N	227PRO	O	5.02
228ALA	N	122ILE	O	0.02
228ALA	N	226SER	O	0.06
226SER	OG	222HIS	ND1	8.33
226SER	OG	222HIS	O	10.41
226SER	OG	225ARG	O	4.33
226SER	OG	226SER	O	5.09
226SER	OG	229ARG	NE	0.01
226SER	OG	229ARG	NH1	0.00
226SER	OG	229ARG	NH2	0.00
226SER	N	222HIS	O	88.10
226SER	N	223LEU	O	1.41

203GLY	N	199VAL	O	40.31
203GLY	N	200GLU	O	18.74
203GLY	N	201GLU	O	0.01
202VAL	N	198THR	O	64.70
202VAL	N	199VAL	O	6.57
201GLU	N	197LYS	O	51.49
201GLU	N	198THR	O	13.19
200GLU	N	196ARG	O	28.85
200GLU	N	197LYS	O	8.09
200GLU	N	198THR	O	0.00
199VAL	N	195TRP	O	97.45
199VAL	N	196ARG	O	0.24
198THR	OG1	162VAL	O	0.00
198THR	OG1	194PHE	O	91.17
198THR	OG1	195TRP	O	0.99
198THR	OG1	199VAL	N	0.03
198THR	N	194PHE	O	64.35
198THR	N	195TRP	O	15.51
197LYS	NZ	193GLU	OE1	2.94
197LYS	NZ	193GLU	OE2	2.23
197LYS	NZ	193GLU	O	0.18
197LYS	NZ	201GLU	OE1	0.02
197LYS	NZ	201GLU	OE2	0.28
197LYS	NZ	147SER	O	1.22
197LYS	NZ	148GLU	OE1	18.08
197LYS	NZ	148GLU	OE2	23.75
197LYS	NZ	148GLU	O	0.70
197LYS	N	193GLU	O	44.68
197LYS	N	194PHE	O	14.90
197LYS	N	195TRP	O	0.02
196ARG	NH2	184ASP	OD1	93.68
196ARG	NH2	184ASP	OD2	7.04
196ARG	NH2	213HIS	O	0.00
196ARG	NH2	214GLN	O	0.00
196ARG	NH2	215TYR	N	0.00
196ARG	NH2	215TYR	OH	0.36
196ARG	NH1	193GLU	OE2	0.06
196ARG	NH1	215TYR	OH	0.02
196ARG	NE	184ASP	OD1	13.45
196ARG	NE	184ASP	OD2	91.45
196ARG	N	192GLY	O	91.57
196ARG	N	193GLU	O	1.19
195TRP	NE1	183VAL	O	0.23

229ARG	NE	222HIS	ND1	0.01
229ARG	NE	228ALA	O	0.14
229ARG	NE	229ARG	O	1.44
229ARG	N	226SER	O	16.97
229ARG	N	227PRO	O	3.77
228ALA	N	226SER	O	0.08
226SER	OG	222HIS	ND1	1.46
226SER	OG	222HIS	O	5.07
226SER	OG	225ARG	O	2.58
226SER	OG	226SER	O	4.44
226SER	OG	228ALA	O	0.02
226SER	OG	229ARG	NE	0.00
226SER	N	222HIS	O	93.01
226SER	N	223LEU	O	1.36
225ARG	NH2	218ALA	O	0.01
225ARG	NH2	221MET	O	0.14
225ARG	NH2	222HIS	ND1	0.53
225ARG	NH2	115LEU	O	0.00
225ARG	NH2	252GLY	O	6.16
225ARG	NH2	253SER	OG	0.00
225ARG	NH2	278ASP	OD1	24.01
225ARG	NH2	278ASP	OD2	16.16
225ARG	NH2	278ASP	O	0.00
225ARG	NH2	326ASP	OD11	74.46
225ARG	NH2	326ASP	OD21	14.03
225ARG	NH2	326ASP	O101	0.24
225ARG	NH1	222HIS	ND1	0.00
225ARG	NH1	252GLY	O	6.30
225ARG	NH1	253SER	OG	0.01
225ARG	NH1	278ASP	OD2	0.03
225ARG	NH1	326ASP	OD11	9.33
225ARG	NH1	326ASP	OD21	70.50
225ARG	NH1	326ASP	O101	0.03
225ARG	NE	221MET	O	5.28
225ARG	NE	222HIS	ND1	0.10
225ARG	NE	252GLY	O	0.13
225ARG	NE	278ASP	OD1	0.72
225ARG	NE	278ASP	OD2	0.49
225ARG	N	221MET	O	8.49
225ARG	N	222HIS	O	54.35
225ARG	N	223LEU	O	3.25
224VAL	N	220ALA	O	13.01
224VAL	N	221MET	O	11.60

187ASN	ND2	215TYR	OH	0.04
187ASN	N	185LYS	O	0.10
187ASN	N	187ASN	OD1	2.91
187ASN	N	187ASN	ND2	0.24
186ALA	N	184ASP	OD1	29.62
186ALA	N	184ASP	OD2	46.68
185LYS	NZ	187ASN	OD1	0.32
185LYS	NZ	217ASP	OD1	73.22
185LYS	NZ	217ASP	OD2	2.73
185LYS	NZ	139TYR	OH	0.43
185LYS	NZ	241ASP	OD1	53.71
185LYS	NZ	241ASP	OD2	14.40
185LYS	N	184ASP	OD1	0.01
185LYS	N	184ASP	OD2	2.32
184ASP	N	182SER	O	0.06
184ASP	N	214GLN	O	82.16
183VAL	N	195TRP	NE1	0.00
183VAL	N	234VAL	O	68.55
182SER	OG	181VAL	O	0.00
182SER	OG	183VAL	N	0.01
182SER	OG	183VAL	O	15.28
182SER	OG	184ASP	OD1	0.77
182SER	OG	184ASP	OD2	1.78
182SER	OG	212GLU	O	0.74
182SER	OG	213HIS	ND1	3.23
182SER	OG	234VAL	O	3.09
182SER	N	212GLU	O	96.14
181VAL	N	232VAL	O	92.84
180VAL	N	178LYS	O	0.02
180VAL	N	179HIS	ND1	0.00
180VAL	N	210ALA	O	95.48
179HIS	NE2	211LEU	O	0.02
179HIS	NE2	212GLU	OE1	10.05
179HIS	NE2	212GLU	OE2	9.34
179HIS	NE2	229ARG	O	7.54
179HIS	N	177ARG	O	83.64
179HIS	N	231ASP	OD2	0.63
178LYS	NZ	174ARG	O	0.07
178LYS	NZ	176ARG	O	0.92
178LYS	NZ	208ASP	OD1	42.97
178LYS	NZ	208ASP	OD2	35.93
178LYS	NZ	208ASP	O	0.16
178LYS	N	173ALA	O	0.88

225ARG	NH2	222HIS	ND1	0.27
225ARG	NH2	115LEU	O	0.00
225ARG	NH2	252GLY	O	7.47
225ARG	NH2	253SER	OG	0.04
225ARG	NH2	326ASP	OD1	73.65
225ARG	NH2	326ASP	OD2	16.52
225ARG	NH2	326ASP	O	3.23
225ARG	NH1	222HIS	ND1	0.00
225ARG	NH1	115LEU	O	0.00
225ARG	NH1	252GLY	O	58.61
225ARG	NH1	253SER	OG	0.24
225ARG	NH1	326ASP	OD1	9.18
225ARG	NH1	326ASP	OD2	75.98
225ARG	NH1	326ASP	O	0.04
225ARG	NE	222HIS	ND1	0.38
225ARG	NE	226SER	OG	0.00
225ARG	NE	252GLY	O	0.19
225ARG	N	221MET	O	33.03
225ARG	N	222HIS	O	40.42
225ARG	N	223LEU	O	1.93
224VAL	N	220ALA	O	2.96
224VAL	N	221MET	O	33.84
224VAL	N	222HIS	O	0.02
223LEU	N	219MET	O	89.76
223LEU	N	220ALA	O	0.56
222HIS	NE2	214GLN	OE1	0.74
222HIS	NE2	226SER	OG	0.01
222HIS	NE2	229ARG	NE	0.75
222HIS	NE2	229ARG	NH1	0.65
222HIS	NE2	229ARG	NH2	0.20
222HIS	NE2	229ARG	O	0.04
222HIS	N	218ALA	O	73.87
222HIS	N	219MET	O	4.49
222HIS	N	220ALA	O	0.00
221MET	N	217ASP	O	13.05
221MET	N	218ALA	O	32.24
221MET	N	219MET	O	0.09
220ALA	N	216VAL	O	64.32
220ALA	N	217ASP	O	4.13
219MET	N	214GLN	OE1	0.00
219MET	N	215TYR	O	75.36
219MET	N	216VAL	O	12.87
219MET	N	217ASP	O	0.16

195TRP	NE1	184ASP	OD2	0.00
195TRP	NE1	234VAL	O	0.02
195TRP	N	191VAL	O	63.56
195TRP	N	192GLY	O	1.74
194PHE	N	190GLU	O	92.08
194PHE	N	191VAL	O	0.09
193GLU	N	189LEU	O	72.85
193GLU	N	190GLU	O	4.98
192GLY	N	189LEU	O	47.18
192GLY	N	190GLU	O	0.04
191VAL	N	153ASN	OD1	95.60
190GLU	N	190GLU	OE1	6.73
190GLU	N	190GLU	OE2	2.10
190GLU	N	144ARG	NH2	0.01
190GLU	N	153ASN	OD1	35.92
190GLU	N	153ASN	ND2	0.51
189LEU	N	185LYS	O	55.87
189LEU	N	186ALA	O	10.71
188VAL	N	185LYS	O	76.05
188VAL	N	186ALA	O	0.04
188VAL	N	187ASN	OD1	0.13
187ASN	ND2	215TYR	OH	0.02
187ASN	N	185LYS	O	0.26
187ASN	N	187ASN	OD1	2.68
187ASN	N	187ASN	ND2	0.50
186ALA	N	184ASP	OD1	19.57
186ALA	N	184ASP	OD2	31.30
185LYS	NZ	187ASN	OD1	0.18
185LYS	NZ	187ASN	ND2	0.01
185LYS	NZ	217ASP	OD1	49.25
185LYS	NZ	217ASP	OD2	49.61
185LYS	NZ	139TYR	OH	0.03
185LYS	NZ	241ASP	OD1	37.27
185LYS	NZ	241ASP	OD2	62.17
185LYS	N	184ASP	OD1	0.00
185LYS	N	184ASP	OD2	1.31
184ASP	N	182SER	O	0.17
184ASP	N	214GLN	O	97.11
184ASP	N	216VAL	N	0.00
183VAL	N	234VAL	O	44.77
182SER	OG	183VAL	N	0.02
182SER	OG	183VAL	O	1.30
182SER	OG	184ASP	OD1	0.42

223LEU	N	219MET	O	77.73
223LEU	N	220ALA	O	3.70
222HIS	NE2	214GLN	OE1	0.20
222HIS	NE2	226SER	OG	0.07
222HIS	NE2	226SER	O	0.00
222HIS	NE2	229ARG	NE	0.47
222HIS	NE2	229ARG	NH1	0.26
222HIS	NE2	229ARG	NH2	0.04
222HIS	NE2	229ARG	O	0.01
222HIS	NE2	278ASP	OD1	0.12
222HIS	N	218ALA	O	46.84
222HIS	N	219MET	O	18.43
222HIS	N	220ALA	O	0.02
221MET	N	217ASP	O	64.43
221MET	N	218ALA	O	2.41
220ALA	N	216VAL	O	89.49
220ALA	N	217ASP	O	0.77
219MET	N	215TYR	O	80.16
219MET	N	216VAL	O	3.64
218ALA	N	215TYR	O	19.71
218ALA	N	217ASP	OD1	4.34
218ALA	N	217ASP	OD2	1.09
217ASP	N	217ASP	OD1	30.59
217ASP	N	217ASP	OD2	7.10
216VAL	N	184ASP	O	97.36
215TYR	OH	186ALA	O	0.94
215TYR	OH	78ASP	OD1	9.15
215TYR	OH	78ASP	OD2	6.11
215TYR	N	214GLN	OE1	7.00
214GLN	NE2	212GLU	OE1	5.74
214GLN	NE2	212GLU	OE2	7.26
214GLN	NE2	212GLU	O	0.02
214GLN	NE2	213HIS	O	33.96
214GLN	NE2	215TYR	N	0.02
214GLN	NE2	215TYR	O	0.54
214GLN	NE2	218ALA	O	0.36
214GLN	NE2	219MET	N	0.16
214GLN	NE2	222HIS	ND1	6.06
214GLN	NE2	222HIS	NE2	0.03
214GLN	NE2	229ARG	NE	0.00
214GLN	NE2	229ARG	NH1	0.05
214GLN	NE2	229ARG	NH2	0.18
214GLN	N	182SER	O	69.14

178LYS	N	176ARG	O	0.12
178LYS	N	231ASP	OD2	0.46
177ARG	NH2	127ASP	OD2	3.60
177ARG	NH2	127ASP	O	1.15
177ARG	NH2	179HIS	ND1	0.06
177ARG	NH2	229ARG	O	0.02
177ARG	NH2	230PHE	O	0.46
177ARG	NH2	231ASP	OD1	36.49
177ARG	NH2	231ASP	OD2	2.64
177ARG	NH1	127ASP	OD2	1.17
177ARG	NH1	179HIS	ND1	0.06
177ARG	NH1	228ALA	O	0.04
177ARG	NH1	229ARG	O	24.94
177ARG	NH1	231ASP	OD1	0.00
177ARG	NH1	231ASP	OD2	18.64
177ARG	NE	179HIS	ND1	0.02
177ARG	NE	231ASP	OD1	0.34
177ARG	NE	231ASP	OD2	37.21
177ARG	N	173ALA	O	0.08
177ARG	N	177ARG	NE	1.22
177ARG	N	177ARG	NH2	0.08
177ARG	N	231ASP	OD1	3.14
177ARG	N	231ASP	OD2	6.82
176ARG	NH2	127ASP	OD1	61.18
176ARG	NH2	127ASP	OD2	0.91
176ARG	NH2	127ASP	O	0.01
176ARG	NH1	127ASP	OD1	35.15
176ARG	NH1	127ASP	OD2	14.10
176ARG	NH1	127ASP	O	45.69
176ARG	NH1	128VAL	O	0.00
176ARG	NH1	177ARG	NH2	0.00
176ARG	NH1	231ASP	OD1	69.40
176ARG	NH1	231ASP	OD2	0.00
176ARG	NH1	231ASP	O	0.10
176ARG	N	172LEU	O	11.26
176ARG	N	173ALA	O	58.39
176ARG	N	174ARG	O	0.18
175LYS	NZ	171GLU	OE1	0.19
175LYS	NZ	171GLU	OE2	0.02
175LYS	NZ	171GLU	O	0.00
175LYS	NZ	299GLU	OE1	52.68
175LYS	NZ	299GLU	OE2	39.88
175LYS	NZ	299GLU	O	0.28

218ALA	N	215TYR	O	29.46
218ALA	N	216VAL	O	0.07
218ALA	N	217ASP	OD1	3.43
218ALA	N	217ASP	OD2	0.63
218ALA	N	245ASP	OD1	0.16
218ALA	N	245ASP	OD2	13.67
217ASP	N	215TYR	O	0.12
217ASP	N	217ASP	OD1	15.31
217ASP	N	217ASP	OD2	3.23
216VAL	N	184ASP	O	91.63
215TYR	OH	186ALA	O	0.08
215TYR	OH	187ASN	OD1	21.11
215TYR	OH	217ASP	OD1	0.23
215TYR	OH	78ASP	OD1	2.26
215TYR	OH	78ASP	OD2	0.75
215TYR	OH	87GLU	O	0.00
215TYR	OH	275SER	OG	0.18
215TYR	N	213HIS	ND1	0.00
215TYR	N	214GLN	OE1	6.08
214GLN	NE2	182SER	O	0.01
214GLN	NE2	212GLU	OE1	6.93
214GLN	NE2	212GLU	OE2	9.68
214GLN	NE2	212GLU	O	0.06
214GLN	NE2	213HIS	O	9.58
214GLN	NE2	215TYR	N	0.01
214GLN	NE2	215TYR	O	0.10
214GLN	NE2	218ALA	O	0.08
214GLN	NE2	219MET	N	0.03
214GLN	NE2	222HIS	ND1	7.00
214GLN	NE2	222HIS	NE2	0.17
214GLN	NE2	229ARG	NH1	0.07
214GLN	NE2	229ARG	NH2	0.02
214GLN	N	182SER	O	81.04
214GLN	N	184ASP	OD1	0.02
214GLN	N	212GLU	O	0.19
213HIS	NE2	184ASP	OD1	5.08
213HIS	NE2	184ASP	OD2	27.29
213HIS	NE2	196ARG	O	0.43
213HIS	NE2	200GLU	OE1	0.61
213HIS	NE2	200GLU	OE2	0.37
213HIS	N	211LEU	O	0.08
213HIS	N	212GLU	OE1	5.78
213HIS	N	212GLU	OE2	5.84

182SER	OG	184ASP	OD2	1.39
182SER	OG	212GLU	O	0.01
182SER	OG	213HIS	ND1	4.39
182SER	OG	234VAL	O	0.02
182SER	N	212GLU	O	98.91
181VAL	N	232VAL	O	92.92
180VAL	N	179HIS	ND1	0.04
180VAL	N	210ALA	O	95.21
179HIS	NE2	211LEU	O	0.00
179HIS	NE2	212GLU	OE1	31.05
179HIS	NE2	212GLU	OE2	30.67
179HIS	NE2	214GLN	OE1	0.00
179HIS	NE2	229ARG	NH1	0.02
179HIS	NE2	229ARG	NH2	0.08
179HIS	NE2	229ARG	O	2.10
179HIS	N	231ASP	OD2	0.14
178LYS	NZ	174ARG	O	0.05
178LYS	NZ	176ARG	O	0.14
178LYS	NZ	208ASP	OD1	45.17
178LYS	NZ	208ASP	OD2	51.13
178LYS	NZ	208ASP	O	0.09
178LYS	N	173ALA	O	12.20
178LYS	N	176ARG	O	0.08
178LYS	N	231ASP	OD2	0.11
177ARG	NH2	125GLY	O	0.18
177ARG	NH2	127ASP	OD1	16.13
177ARG	NH2	127ASP	OD2	9.50
177ARG	NH2	127ASP	O	13.47
177ARG	NH2	228ALA	O	7.11
177ARG	NH2	229ARG	O	0.05
177ARG	NH2	230PHE	O	7.78
177ARG	NH2	231ASP	OD1	27.75
177ARG	NH2	231ASP	OD2	0.00
177ARG	NH1	125GLY	O	0.08
177ARG	NH1	127ASP	OD2	0.14
177ARG	NH1	228ALA	O	37.10
177ARG	NH1	229ARG	O	1.51
177ARG	NH1	230PHE	N	0.00
177ARG	NH1	230PHE	O	3.61
177ARG	NE	231ASP	OD1	18.99
177ARG	NE	231ASP	OD2	8.04
177ARG	N	231ASP	OD1	0.12
177ARG	N	231ASP	OD2	93.14

214GLN	N	184ASP	OD1	0.04
213HIS	NE2	200GLU	OE1	50.43
213HIS	NE2	200GLU	OE2	32.24
213HIS	N	212GLU	OE1	7.62
213HIS	N	212GLU	OE2	7.93
212GLU	N	180VAL	O	54.86
211LEU	N	209VAL	O	0.01
210ALA	N	178LYS	O	94.26
209VAL	N	206TYR	O	4.64
209VAL	N	207PRO	O	44.54
208ASP	N	206TYR	O	0.03
208ASP	N	208ASP	OD1	0.08
208ASP	N	208ASP	OD2	0.04
206TYR	OH	167ARG	O	0.65
206TYR	OH	170PHE	O	0.96
206TYR	OH	171GLU	OE1	22.50
206TYR	OH	171GLU	OE2	29.92
206TYR	OH	202VAL	O	0.02
206TYR	N	202VAL	O	27.35
206TYR	N	203GLY	O	26.27
206TYR	N	204ARG	O	0.07
205GLY	N	201GLU	O	0.68
205GLY	N	202VAL	O	43.18
205GLY	N	203GLY	O	0.18
204ARG	NH2	197LYS	O	0.12
204ARG	NH2	200GLU	OE1	23.92
204ARG	NH2	200GLU	OE2	11.57
204ARG	NH2	201GLU	OE1	25.62
204ARG	NH2	201GLU	OE2	33.83
204ARG	NH2	211LEU	O	0.26
204ARG	NH2	213HIS	NE2	0.19
204ARG	NH1	200GLU	OE1	5.92
204ARG	NH1	200GLU	OE2	10.18
204ARG	NH1	201GLU	OE1	2.69
204ARG	NH1	201GLU	OE2	2.72
204ARG	NH1	211LEU	O	6.77
204ARG	NH1	212GLU	OE1	0.02
204ARG	NH1	212GLU	OE2	0.03
204ARG	NE	200GLU	OE1	10.56
204ARG	NE	200GLU	OE2	23.30
204ARG	NE	200GLU	O	0.02
204ARG	NE	201GLU	OE1	25.25
204ARG	NE	201GLU	OE2	19.24

175LYS	NZ	300HIS	ND1	5.91
175LYS	N	171GLU	O	1.23
175LYS	N	172LEU	O	66.03
175LYS	N	173ALA	O	0.36
175LYS	N	176ARG	O	0.11
174ARG	NH2	205GLY	O	0.03
174ARG	NH2	208ASP	OD1	39.07
174ARG	NH2	208ASP	OD2	45.15
174ARG	NH1	171GLU	OE1	6.84
174ARG	NH1	171GLU	OE2	29.83
174ARG	NE	208ASP	OD1	46.51
174ARG	NE	208ASP	OD2	40.74
174ARG	N	170PHE	O	7.18
174ARG	N	171GLU	O	57.85
174ARG	N	172LEU	O	0.15
173ALA	N	169ALA	O	56.77
173ALA	N	170PHE	O	4.58
172LEU	N	168VAL	O	91.07
172LEU	N	169ALA	O	1.74
171GLU	N	167ARG	O	30.54
171GLU	N	168VAL	O	20.18
170PHE	N	166ALA	O	93.44
170PHE	N	167ARG	O	0.61
169ALA	N	165VAL	O	79.87
169ALA	N	166ALA	O	2.53
168VAL	N	164ARG	O	19.61
168VAL	N	165VAL	O	25.08
168VAL	N	166ALA	O	0.18
167ARG	NH2	163GLU	OE1	21.19
167ARG	NH2	163GLU	OE2	27.51
167ARG	NH2	171GLU	OE2	0.02
167ARG	NH2	201GLU	OE2	0.04
167ARG	NH2	201GLU	O	0.01
167ARG	NH1	163GLU	OE1	1.90
167ARG	NH1	163GLU	OE2	0.21
167ARG	NH1	206TYR	OH	1.12
167ARG	NE	163GLU	OE1	40.67
167ARG	NE	163GLU	OE2	43.71
167ARG	NE	163GLU	O	0.01
167ARG	NE	206TYR	OH	0.18
167ARG	N	163GLU	O	93.24
167ARG	N	164ARG	O	0.84
166ALA	N	162VAL	O	59.81

212GLU	N	180VAL	O	77.28
212GLU	N	210ALA	O	0.00
211LEU	N	209VAL	O	0.02
210ALA	N	178LYS	O	96.52
209VAL	N	206TYR	O	54.15
209VAL	N	207PRO	O	0.41
209VAL	N	208ASP	OD1	0.16
209VAL	N	208ASP	OD2	0.34
208ASP	N	206TYR	O	0.12
208ASP	N	208ASP	OD1	18.57
208ASP	N	208ASP	OD2	24.39
206TYR	OH	167ARG	O	0.38
206TYR	OH	171GLU	OE1	41.65
206TYR	OH	171GLU	OE2	50.63
206TYR	N	202VAL	O	19.43
206TYR	N	203GLY	O	41.21
206TYR	N	204ARG	O	0.04
205GLY	N	201GLU	O	0.78
205GLY	N	202VAL	O	62.40
205GLY	N	203GLY	O	0.19
204ARG	NH2	200GLU	OE1	48.77
204ARG	NH2	200GLU	OE2	44.88
204ARG	NH2	200GLU	O	0.00
204ARG	NH2	201GLU	OE1	34.84
204ARG	NH2	201GLU	OE2	34.16
204ARG	NH1	200GLU	OE1	0.00
204ARG	NH1	201GLU	OE1	29.50
204ARG	NH1	201GLU	OE2	31.97
204ARG	NE	200GLU	OE1	45.45
204ARG	NE	200GLU	OE2	50.84
204ARG	NE	200GLU	O	0.13
204ARG	N	200GLU	O	40.84
204ARG	N	201GLU	O	20.09
204ARG	N	202VAL	O	0.02
203GLY	N	199VAL	O	46.42
203GLY	N	200GLU	O	12.66
202VAL	N	198THR	O	70.89
202VAL	N	199VAL	O	4.56
201GLU	N	197LYS	O	53.82
201GLU	N	198THR	O	14.04
200GLU	N	196ARG	O	26.05
200GLU	N	197LYS	O	11.74
200GLU	N	198THR	O	0.02

176ARG	NH2	127ASP	OD1	7.05	204ARG	N	199VAL	O	0.04	166ALA	N	163GLU	O	3.67	199VAL	N	195TRP	O	93.78
176ARG	NH2	127ASP	OD2	17.65	204ARG	N	200GLU	O	36.06	165VAL	N	161GLU	O	2.56	199VAL	N	196ARG	O	0.22
176ARG	NH2	127ASP	O	0.00	204ARG	N	201GLU	O	24.15	165VAL	N	162VAL	O	8.74	199VAL	N	197LYS	O	0.00
176ARG	NH2	299GLU	OE1	0.01	204ARG	N	202VAL	O	0.12	165VAL	N	163GLU	O	0.03	198THR	OG1	194PHE	O	84.37
176ARG	NH2	299GLU	OE2	0.01	203GLY	N	199VAL	O	54.16	164ARG	NH2	133GLU	OE1	42.45	198THR	OG1	195TRP	O	0.03
176ARG	NH1	127ASP	OD1	22.99	203GLY	N	200GLU	O	13.90	164ARG	NH2	133GLU	OE2	63.97	198THR	OG1	198THR	O	0.00
176ARG	NH1	127ASP	OD2	47.43	202VAL	N	198THR	O	40.37	164ARG	NH2	135THR	O	0.00	198THR	N	194PHE	O	53.79
176ARG	NH1	127ASP	O	23.13	202VAL	N	199VAL	O	20.99	164ARG	NH2	161GLU	OE1	24.31	198THR	N	195TRP	O	19.22
176ARG	NH1	231ASP	OD1	94.57	202VAL	N	200GLU	O	0.12	164ARG	NH2	161GLU	OE2	23.66	198THR	N	196ARG	O	0.03
176ARG	NH1	231ASP	OD2	0.58	201GLU	N	197LYS	O	30.88	164ARG	NH1	98ASP	OD1	0.02	197LYS	NZ	193GLU	OE1	0.23
176ARG	NH1	231ASP	O	0.10	201GLU	N	198THR	O	25.19	164ARG	NH1	160PRO	O	0.01	197LYS	NZ	193GLU	OE2	1.30
176ARG	NE	299GLU	OE2	0.07	201GLU	N	199VAL	O	0.02	164ARG	NH1	161GLU	OE1	5.25	197LYS	NZ	197LYS	O	0.00
176ARG	N	172ALA	O	0.65	200GLU	N	196ARG	O	66.67	164ARG	NH1	161GLU	OE2	4.34	197LYS	NZ	200GLU	OE1	0.54
176ARG	N	173ALA	O	83.72	200GLU	N	197LYS	O	2.08	164ARG	NH1	264ARG	NH2	0.04	197LYS	NZ	200GLU	OE2	0.04
176ARG	N	174ARG	O	0.32	199VAL	N	195TRP	O	95.73	164ARG	NE	133GLU	OE1	33.27	197LYS	NZ	201GLU	OE1	3.95
175LYS	NZ	171GLU	OE1	1.81	199VAL	N	196ARG	O	1.09	164ARG	NE	133GLU	OE2	46.03	197LYS	NZ	201GLU	OE2	3.44
175LYS	NZ	171GLU	OE2	0.91	198THR	OG1	194PHE	O	95.71	164ARG	NE	161GLU	O	2.69	197LYS	NZ	147SER	O	0.85
175LYS	NZ	171GLU	O	3.74	198THR	OG1	195TRP	O	0.58	164ARG	N	160PRO	O	72.97	197LYS	NZ	148GLU	OE1	30.19
175LYS	NZ	299GLU	OE1	35.89	198THR	OG1	199VAL	N	0.00	164ARG	N	161GLU	O	4.58	197LYS	NZ	148GLU	OE2	30.16
175LYS	NZ	299GLU	OE2	37.74	198THR	N	194PHE	O	54.38	163GLU	N	159LYS	O	52.40	197LYS	NZ	148GLU	O	1.54
175LYS	NZ	299GLU	O	4.62	198THR	N	195TRP	O	24.69	163GLU	N	160PRO	O	5.76	197LYS	N	193GLU	O	50.49
175LYS	NZ	300HIS	ND1	4.91	198THR	N	196ARG	O	0.02	162VAL	N	158SER	O	36.24	197LYS	N	194PHE	O	14.32
175LYS	NZ	300HIS	O	0.01	197LYS	NZ	193GLU	OE1	4.82	162VAL	N	159LYS	O	14.96	196ARG	NH2	184ASP	OD1	71.17
175LYS	N	171GLU	O	1.14	197LYS	NZ	193GLU	OE2	3.80	162VAL	N	160PRO	O	0.01	196ARG	NH2	184ASP	OD2	31.86
175LYS	N	172ALA	O	63.35	197LYS	NZ	193GLU	O	0.15	161GLU	N	158SER	O	41.71	196ARG	NH2	213HIS	ND1	0.00
175LYS	N	173ALA	O	0.05	197LYS	NZ	201GLU	OE1	2.09	161GLU	N	159LYS	O	0.02	196ARG	NH2	213HIS	O	0.00
174ARG	NH2	171GLU	OE1	48.43	197LYS	NZ	201GLU	OE2	2.46	161GLU	N	161GLU	OE1	0.02	196ARG	NH2	214GLN	O	0.01
174ARG	NH2	171GLU	OE2	50.15	197LYS	NZ	147SER	O	0.31	159LYS	NZ	163GLU	OE1	5.83	196ARG	NH2	215TYR	OH	0.72
174ARG	NH2	208ASP	OD1	0.02	197LYS	NZ	148GLU	OE1	21.65	159LYS	NZ	163GLU	OE2	11.45	196ARG	NH2	78ASP	OD1	0.56
174ARG	NH2	208ASP	OD2	4.19	197LYS	NZ	148GLU	OE2	19.58	159LYS	NZ	197LYS	O	0.00	196ARG	NH2	78ASP	OD2	0.24
174ARG	NH1	205GLY	O	0.01	197LYS	NZ	148GLU	O	0.06	159LYS	NZ	198THR	OG1	3.91	196ARG	NH1	193GLU	OE1	0.22
174ARG	NH1	208ASP	OD1	4.77	197LYS	N	193GLU	O	73.29	159LYS	NZ	201GLU	OE1	8.44	196ARG	NH1	193GLU	OE2	0.07
174ARG	NH1	208ASP	OD2	6.21	197LYS	N	194PHE	O	4.22	159LYS	NZ	201GLU	OE2	7.10	196ARG	NH1	213HIS	ND1	0.00
174ARG	NE	171GLU	OE1	25.62	196ARG	NH2	184ASP	OD1	18.30	159LYS	NZ	148GLU	OE1	6.65	196ARG	NH1	215TYR	OH	0.45
174ARG	NE	171GLU	OE2	30.53	196ARG	NH2	184ASP	OD2	89.63	159LYS	NZ	148GLU	OE2	6.87	196ARG	NH1	78ASP	OD1	0.26
174ARG	NE	171GLU	O	0.20	196ARG	NH2	186ALA	O	0.35	159LYS	NZ	148GLU	O	1.27	196ARG	NH1	78ASP	OD2	0.43
174ARG	N	170PHE	O	37.38	196ARG	NH2	213HIS	O	0.02	159LYS	N	149ALA	O	86.70	196ARG	NH1	82ARG	NE	0.00
174ARG	N	171GLU	O	21.28	196ARG	NH2	214GLN	O	0.04	158SER	OG	158SER	O	0.01	196ARG	NH1	82ARG	NH1	0.00
173ALA	N	169ALA	O	76.91	196ARG	NH2	215TYR	N	0.02	158SER	OG	161GLU	OE1	24.41	196ARG	NE	184ASP	OD1	33.89
173ALA	N	170PHE	O	2.96	196ARG	NH2	215TYR	OH	0.10	158SER	OG	161GLU	OE2	24.10	196ARG	NE	184ASP	OD2	68.32
172ALA	N	168VAL	O	65.11	196ARG	NH2	78ASP	OD1	0.00	158SER	OG	150GLU	OE1	0.01	196ARG	N	192GLY	O	86.41
172ALA	N	169ALA	O	4.84	196ARG	NH1	193GLU	OE2	0.00	158SER	OG	150GLU	OE2	0.42	196ARG	N	193GLU	O	2.80
171GLU	N	167ARG	O	79.64	196ARG	NH1	215TYR	OH	0.91	158SER	N	156ARG	O	0.01	195TRP	NE1	183VAL	O	6.89

171GLU	N	168VAL	O	1.03
170PHE	N	166ALA	O	49.14
170PHE	N	167ARG	O	14.07
169ALA	N	165VAL	O	5.73
169ALA	N	166ALA	O	36.89
168VAL	N	164ARG	O	26.87
168VAL	N	165VAL	O	0.31
167ARG	NH2	163GLU	OE1	29.91
167ARG	NH2	163GLU	OE2	37.64
167ARG	NH1	163GLU	OE1	38.54
167ARG	NH1	163GLU	OE2	26.34
167ARG	NH1	163GLU	O	0.03
167ARG	NH1	206TYR	OH	0.35
167ARG	NE	163GLU	OE1	1.71
167ARG	NE	163GLU	OE2	0.82
167ARG	NE	206TYR	OH	1.26
167ARG	N	163GLU	O	82.00
167ARG	N	164ARG	O	4.66
166ALA	N	162VAL	O	45.41
166ALA	N	163GLU	O	4.03
165VAL	N	161GLU	O	4.63
165VAL	N	162VAL	O	7.82
164ARG	NH2	98ASP	OD1	43.88
164ARG	NH2	98ASP	OD2	37.47
164ARG	NH2	98ASP	O	0.52
164ARG	NH2	161GLU	OE1	0.04
164ARG	NH2	161GLU	OE2	0.11
164ARG	NH2	264ARG	N	0.02
164ARG	NH2	264ARG	NE	0.12
164ARG	NH2	264ARG	NH1	0.02
164ARG	NH2	264ARG	NH2	0.01
164ARG	NH1	98ASP	OD1	22.07
164ARG	NH1	98ASP	OD2	44.06
164ARG	NH1	133GLU	OE2	1.31
164ARG	NH1	161GLU	OE1	16.36
164ARG	NH1	161GLU	OE2	18.98
164ARG	NH1	264ARG	NH2	0.02
164ARG	NE	160PRO	O	0.00
164ARG	N	160PRO	O	23.63
164ARG	N	161GLU	O	37.88
163GLU	N	159LYS	O	47.07
163GLU	N	160PRO	O	1.10
162VAL	N	158SER	O	78.10

196ARG	NH1	78ASP	OD1	0.79
196ARG	NH1	78ASP	OD2	0.68
196ARG	NE	184ASP	OD1	96.82
196ARG	NE	184ASP	OD2	3.79
196ARG	N	192GLY	O	97.83
196ARG	N	193GLU	O	0.04
195TRP	NE1	183VAL	O	0.29
195TRP	NE1	234VAL	O	0.06
195TRP	N	191VAL	O	50.92
195TRP	N	192GLY	O	3.10
194PHE	N	190GLU	O	92.17
194PHE	N	191VAL	O	0.10
194PHE	N	192GLY	O	0.01
193GLU	N	189LEU	O	84.40
193GLU	N	190GLU	O	3.03
192GLY	N	189LEU	O	52.15
192GLY	N	190GLU	O	0.14
191VAL	N	153ASN	OD1	92.95
190GLU	N	190GLU	OE1	2.84
190GLU	N	190GLU	OE2	2.32
190GLU	N	153ASN	OD1	28.36
190GLU	N	153ASN	ND2	0.20
189LEU	N	185LYS	O	81.41
189LEU	N	186ALA	O	2.04
188VAL	N	185LYS	O	75.55
188VAL	N	186ALA	O	0.02
188VAL	N	187ASN	OD1	0.30
187ASN	ND2	185LYS	O	0.00
187ASN	ND2	186ALA	O	0.02
187ASN	ND2	215TYR	OH	1.22
187ASN	ND2	217ASP	OD1	0.05
187ASN	ND2	217ASP	OD2	0.11
187ASN	ND2	82ARG	NH1	0.02
187ASN	ND2	87GLU	OE1	0.00
187ASN	N	185LYS	O	0.96
187ASN	N	187ASN	OD1	4.82
187ASN	N	187ASN	ND2	0.02
186ALA	N	184ASP	OD1	2.36
186ALA	N	184ASP	OD2	97.30
185LYS	NZ	187ASN	OD1	1.75
185LYS	NZ	187ASN	ND2	0.08
185LYS	NZ	217ASP	OD1	69.20
185LYS	NZ	217ASP	OD2	15.72

158SER	N	161GLU	OE1	19.76
158SER	N	161GLU	OE2	18.63
157TYR	OH	133GLU	OE1	0.01
157TYR	OH	133GLU	OE2	0.04
157TYR	OH	136GLY	O	22.53
157TYR	OH	155GLU	OE1	3.36
157TYR	OH	155GLU	OE2	39.22
157TYR	OH	161GLU	O	0.03
157TYR	N	151ALA	O	97.22
156ARG	NH2	142GLU	O	29.95
156ARG	NH2	150GLU	OE1	2.78
156ARG	NH2	150GLU	OE2	4.22
156ARG	NH2	152TRP	NE1	0.04
156ARG	NH1	142GLU	O	4.89
156ARG	NH1	150GLU	OE1	28.23
156ARG	NH1	150GLU	OE2	61.30
156ARG	NH1	152TRP	NE1	0.00
156ARG	NE	142GLU	O	52.97
156ARG	NE	150GLU	OE1	5.92
156ARG	NE	150GLU	OE2	3.75
156ARG	N	154THR	O	0.03
156ARG	N	155GLU	OE1	6.39
155GLU	N	153ASN	O	97.14
154THR	OG1	143PRO	O	81.72
154THR	OG1	153ASN	O	0.16
154THR	OG1	154THR	OG1	1.88
154THR	N	141GLY	O	0.28
154THR	N	143PRO	O	3.47
153ASN	ND2	138ILE	O	3.03
153ASN	ND2	141GLY	O	0.00
153ASN	ND2	152TRP	O	0.02
153ASN	ND2	154THR	N	0.01
153ASN	ND2	154THR	O	0.00
153ASN	ND2	188VAL	O	0.50
153ASN	ND2	190GLU	N	0.01
153ASN	ND2	190GLU	OE1	40.21
153ASN	ND2	190GLU	OE2	45.05
153ASN	N	155GLU	O	97.69
152TRP	NE1	143PRO	O	0.03
152TRP	NE1	144ARG	O	0.24
152TRP	N	145GLY	O	99.44
151ALA	N	157TYR	O	95.25
150GLU	N	147SER	O	3.92

195TRP	NE1	234VAL	O	0.10
195TRP	N	191VAL	O	51.19
195TRP	N	192GLY	O	3.66
194PHE	N	190GLU	O	92.51
194PHE	N	191VAL	O	0.12
193GLU	N	189LEU	O	78.92
193GLU	N	190GLU	O	4.08
192GLY	N	189LEU	O	56.14
192GLY	N	190GLU	O	0.05
191VAL	N	153ASN	OD1	98.77
191VAL	N	153ASN	ND2	0.00
190GLU	N	190GLU	OE1	19.39
190GLU	N	190GLU	OE2	35.31
190GLU	N	153ASN	OD1	5.54
190GLU	N	153ASN	ND2	1.23
189LEU	N	185LYS	O	24.03
189LEU	N	186ALA	O	33.50
189LEU	N	187ASN	O	0.00
188VAL	N	185LYS	O	37.89
188VAL	N	186ALA	O	0.09
188VAL	N	187ASN	OD1	0.80
187ASN	ND2	215TYR	OH	4.21
187ASN	ND2	85ARG	NH2	0.03
187ASN	ND2	87GLU	OE1	0.06
187ASN	ND2	87GLU	OE2	5.48
187ASN	ND2	87GLU	O	34.35
187ASN	ND2	88THR	OG1	0.01
187ASN	ND2	92SER	OG	0.08
187ASN	N	185LYS	O	0.93
187ASN	N	187ASN	OD1	2.02
187ASN	N	187ASN	ND2	0.20
186ALA	N	184ASP	OD1	28.58
186ALA	N	184ASP	OD2	32.93
186ALA	N	187ASN	OD1	0.00
186ALA	N	196ARG	NH2	0.01
185LYS	NZ	187ASN	OD1	3.03
185LYS	NZ	187ASN	ND2	0.06
185LYS	NZ	215TYR	OH	9.45
185LYS	NZ	217ASP	OD1	48.54
185LYS	NZ	217ASP	OD2	25.65
185LYS	NZ	139TYR	OH	5.38
185LYS	NZ	241ASP	OD1	48.91
185LYS	NZ	241ASP	OD2	27.27

162VAL	N	159LYS	O	7.82
161GLU	N	158SER	O	58.66
161GLU	N	159LYS	O	0.08
161GLU	N	161GLU	OE2	0.04
159LYS	NZ	163GLU	OE1	15.57
159LYS	NZ	163GLU	OE2	12.60
159LYS	NZ	197LYS	O	0.00
159LYS	NZ	198THR	OG1	0.49
159LYS	NZ	201GLU	OE1	11.26
159LYS	NZ	201GLU	OE2	6.06
159LYS	NZ	148GLU	OE1	12.97
159LYS	NZ	148GLU	OE2	12.73
159LYS	NZ	148GLU	O	1.47
159LYS	N	149ALA	O	94.14
158SER	OG	156ARG	O	0.01
158SER	OG	157TYR	O	0.03
158SER	OG	158SER	O	0.08
158SER	OG	161GLU	OE1	0.47
158SER	OG	161GLU	OE2	0.20
158SER	OG	149ALA	O	2.19
158SER	OG	150GLU	OE1	11.52
158SER	OG	150GLU	OE2	9.79
158SER	N	161GLU	OE1	2.28
158SER	N	161GLU	OE2	0.58
157TYR	OH	136GLY	O	1.56
157TYR	OH	155GLU	OE1	0.01
157TYR	OH	155GLU	OE2	0.34
157TYR	OH	191VAL	O	0.01
157TYR	N	151ALA	O	95.64
156ARG	NH2	142GLU	O	31.86
156ARG	NH2	150GLU	OE1	0.66
156ARG	NH2	150GLU	OE2	0.38
156ARG	NH2	152TRP	NE1	0.02
156ARG	NH1	150GLU	OE1	56.90
156ARG	NH1	150GLU	OE2	39.56
156ARG	NE	142GLU	O	9.52
156ARG	NE	150GLU	OE1	0.06
156ARG	NE	150GLU	OE2	0.56
156ARG	N	155GLU	OE1	64.35
155GLU	N	153ASN	O	99.41
154THR	OG1	141GLY	O	0.00
154THR	OG1	143PRO	O	68.78
154THR	OG1	153ASN	O	0.44
185LYS	NZ	139TYR	OH	0.03
185LYS	NZ	241ASP	OD1	64.09
185LYS	NZ	241ASP	OD2	17.36
185LYS	N	184ASP	OD1	0.03
185LYS	N	184ASP	OD2	0.21
184ASP	N	182SER	O	0.00
184ASP	N	214GLN	O	95.64
183VAL	N	234VAL	O	83.39
182SER	OG	183VAL	O	0.02
182SER	OG	184ASP	OD1	4.09
182SER	OG	184ASP	OD2	0.34
182SER	OG	195TRP	NE1	0.00
182SER	OG	212GLU	O	0.00
182SER	OG	213HIS	ND1	0.26
182SER	OG	234VAL	O	0.00
182SER	N	212GLU	O	91.03
181VAL	N	232VAL	O	90.34
180VAL	N	178LYS	O	0.08
180VAL	N	179HIS	ND1	0.02
180VAL	N	210ALA	O	90.75
179HIS	NE2	211LEU	O	0.02
179HIS	NE2	212GLU	N	0.01
179HIS	NE2	212GLU	OE1	8.59
179HIS	NE2	212GLU	OE2	6.78
179HIS	NE2	229ARG	NE	0.18
179HIS	NE2	229ARG	NH1	0.12
179HIS	NE2	229ARG	NH2	0.58
179HIS	NE2	229ARG	O	13.14
179HIS	NE2	230PHE	N	0.00
179HIS	N	177ARG	O	0.05
179HIS	N	231ASP	OD1	4.66
179HIS	N	231ASP	OD2	6.81
178LYS	NZ	174ARG	O	0.07
178LYS	NZ	176ARG	O	0.06
178LYS	NZ	177ARG	O	0.01
178LYS	NZ	208ASP	OD1	47.95
178LYS	NZ	208ASP	OD2	53.82
178LYS	NZ	208ASP	O	0.25
178LYS	N	173ALA	O	15.28
178LYS	N	176ARG	O	0.16
178LYS	N	231ASP	OD1	6.91
178LYS	N	231ASP	OD2	6.78
177ARG	NH2	125GLY	O	4.02
149ALA	N	147SER	O	0.64
147SER	OG	146MET	O	0.14
147SER	OG	150GLU	O	70.71
147SER	N	150GLU	O	43.97
145GLY	N	152TRP	NE1	0.04
145GLY	N	152TRP	O	90.14
144ARG	NH2	139TYR	O	0.61
144ARG	NH2	187ASN	O	0.14
144ARG	NH2	188VAL	O	55.85
144ARG	NH2	190GLU	OE1	47.41
144ARG	NH2	190GLU	OE2	47.67
144ARG	NH1	138ILE	O	0.02
144ARG	NH1	139TYR	O	80.86
144ARG	NH1	141GLY	O	0.18
144ARG	NH1	142GLU	OE1	0.01
144ARG	NH1	142GLU	OE2	0.55
144ARG	NH1	188VAL	O	0.20
144ARG	NE	190GLU	OE1	50.77
144ARG	NE	190GLU	OE2	59.61
144ARG	N	142GLU	OE1	0.18
144ARG	N	142GLU	OE2	0.28
142GLU	N	142GLU	OE1	0.03
142GLU	N	142GLU	OE2	0.01
141GLY	N	137GLY	O	0.12
141GLY	N	138ILE	O	44.34
141GLY	N	139TYR	O	0.00
141GLY	N	144ARG	NH1	0.10
140PHE	N	137GLY	O	99.50
140PHE	N	138ILE	O	0.01
139TYR	OH	237ASN	O	0.06
139TYR	OH	238ILE	O	9.92
139TYR	OH	241ASP	OD1	54.49
139TYR	OH	241ASP	OD2	32.74
139TYR	N	137GLY	O	0.06
138ILE	N	155GLU	OE1	88.73
138ILE	N	155GLU	OE2	9.73
137GLY	N	135THR	O	0.00
137GLY	N	140PHE	O	0.00
137GLY	N	156ARG	O	0.25
136GLY	N	133GLU	OE1	42.02
136GLY	N	133GLU	OE2	29.13
136GLY	N	133GLU	O	0.02
135THR	OG1	99LEU	O	0.12
185LYS	N	184ASP	OD1	0.12
185LYS	N	184ASP	OD2	0.65
185LYS	N	192GLY	O	0.00
184ASP	N	182SER	O	0.01
184ASP	N	214GLN	O	83.17
184ASP	N	216VAL	N	0.00
183VAL	N	234VAL	O	54.46
182SER	OG	182SER	O	0.27
182SER	OG	183VAL	O	0.18
182SER	OG	184ASP	OD1	0.00
182SER	OG	184ASP	OD2	0.12
182SER	OG	212GLU	O	18.61
182SER	OG	213HIS	ND1	14.60
182SER	OG	213HIS	NE2	0.00
182SER	OG	234VAL	O	3.67
182SER	N	212GLU	O	94.19
181VAL	N	232VAL	O	92.51
180VAL	N	179HIS	ND1	0.02
180VAL	N	210ALA	O	88.85
179HIS	NE2	211LEU	O	0.01
179HIS	NE2	212GLU	OE1	14.52
179HIS	NE2	212GLU	OE2	10.68
179HIS	NE2	229ARG	NE	0.02
179HIS	NE2	229ARG	NH1	0.01
179HIS	NE2	229ARG	NH2	0.09
179HIS	NE2	229ARG	O	7.91
179HIS	N	177ARG	O	0.00
179HIS	N	231ASP	OD2	1.48
178LYS	NZ	174ARG	O	1.02
178LYS	NZ	176ARG	O	1.37
178LYS	NZ	177ARG	O	0.04
178LYS	NZ	208ASP	OD1	34.33
178LYS	NZ	208ASP	OD2	21.35
178LYS	NZ	208ASP	O	0.33
178LYS	N	173ALA	O	34.23
178LYS	N	176ARG	O	0.08
178LYS	N	231ASP	OD2	2.54
177ARG	NH2	125GLY	O	0.72
177ARG	NH2	127ASP	OD1	1.49
177ARG	NH2	127ASP	OD2	33.56
177ARG	NH2	127ASP	O	2.94
177ARG	NH2	228ALA	O	4.56
177ARG	NH2	229ARG	O	0.04

154THR	OG1	154THR	OG1	4.35
154THR	N	141GLY	O	0.48
154THR	N	143PRO	O	10.09
153ASN	ND2	138ILE	O	3.06
153ASN	ND2	141GLY	O	0.01
153ASN	ND2	143PRO	O	0.02
153ASN	ND2	152TRP	O	0.01
153ASN	ND2	154THR	N	0.02
153ASN	ND2	154THR	O	0.02
153ASN	ND2	190GLU	N	0.01
153ASN	ND2	190GLU	OE1	6.18
153ASN	ND2	190GLU	OE2	0.68
153ASN	N	155GLU	O	99.00
152TRP	NE1	143PRO	O	0.01
152TRP	NE1	144ARG	O	0.02
152TRP	N	145GLY	O	99.48
151ALA	N	157TYR	O	84.44
150GLU	N	147SER	O	3.41
149ALA	N	147SER	O	2.16
148GLU	N	148GLU	OE1	0.23
148GLU	N	148GLU	OE2	0.08
147SER	OG	146MET	O	0.08
147SER	OG	150GLU	O	73.77
147SER	N	150GLU	O	44.17
145GLY	N	152TRP	NE1	0.08
145GLY	N	152TRP	O	91.05
144ARG	NH2	139TYR	O	0.24
144ARG	NH2	187ASN	O	0.01
144ARG	NH2	188VAL	O	1.32
144ARG	NH2	190GLU	OE1	43.81
144ARG	NH2	190GLU	OE2	42.78
144ARG	NH1	138ILE	O	0.03
144ARG	NH1	139TYR	O	2.62
144ARG	NH1	141GLY	O	0.04
144ARG	NH1	142GLU	OE1	0.24
144ARG	NH1	142GLU	OE2	0.50
144ARG	NH1	187ASN	O	0.00
144ARG	NH1	188VAL	O	0.58
144ARG	NE	153ASN	ND2	0.01
144ARG	NE	190GLU	OE1	44.76
144ARG	NE	190GLU	OE2	37.12
144ARG	N	142GLU	OE1	0.76
144ARG	N	142GLU	OE2	0.93

177ARG	NH2	127ASP	OD1	29.73
177ARG	NH2	127ASP	OD2	5.62
177ARG	NH2	127ASP	O	10.93
177ARG	NH2	179HIS	ND1	0.06
177ARG	NH2	228ALA	O	2.86
177ARG	NH2	229ARG	O	1.15
177ARG	NH2	230PHE	N	0.00
177ARG	NH2	230PHE	O	9.92
177ARG	NH2	231ASP	OD1	24.49
177ARG	NH2	231ASP	OD2	13.60
177ARG	NH2	309ARG	NH2	0.00
177ARG	NH1	125GLY	O	0.81
177ARG	NH1	127ASP	OD1	5.05
177ARG	NH1	127ASP	OD2	0.70
177ARG	NH1	127ASP	O	0.36
177ARG	NH1	177ARG	O	0.26
177ARG	NH1	179HIS	ND1	0.00
177ARG	NH1	228ALA	O	22.88
177ARG	NH1	229ARG	O	1.16
177ARG	NH1	230PHE	N	0.01
177ARG	NH1	230PHE	O	4.70
177ARG	NH1	231ASP	OD1	1.95
177ARG	NH1	231ASP	OD2	0.95
177ARG	NE	127ASP	OD1	0.18
177ARG	NE	127ASP	OD2	0.04
177ARG	NE	230PHE	O	0.02
177ARG	NE	231ASP	OD1	10.68
177ARG	NE	231ASP	OD2	25.34
177ARG	N	173ALA	O	0.04
177ARG	N	177ARG	NE	0.06
177ARG	N	231ASP	OD1	36.55
177ARG	N	231ASP	OD2	36.09
176ARG	NH2	127ASP	OD1	2.55
176ARG	NH2	127ASP	OD2	27.12
176ARG	NH2	127ASP	O	0.06
176ARG	NH2	128VAL	O	0.01
176ARG	NH2	299GLU	OE1	0.00
176ARG	NH1	127ASP	OD1	12.37
176ARG	NH1	127ASP	OD2	36.36
176ARG	NH1	127ASP	O	41.11
176ARG	NH1	177ARG	NH2	0.00
176ARG	NH1	231ASP	OD1	50.88
176ARG	NH1	231ASP	OD2	42.51

135THR	OG1	100PHE	O	0.01
135THR	OG1	133GLU	OE1	61.20
135THR	OG1	133GLU	OE2	36.43
135THR	OG1	136GLY	N	0.02
135THR	OG1	164ARG	NH2	0.03
135THR	N	100PHE	O	0.03
135THR	N	133GLU	OE1	62.27
135THR	N	133GLU	OE2	36.61
134LEU	N	100PHE	O	73.87
134LEU	N	133GLU	OE1	10.01
134LEU	N	133GLU	OE2	1.26
133GLU	N	235THR	O	12.96
133GLU	N	236GLY	O	52.36
132ARG	NH2	102ASN	OD1	0.01
132ARG	NH2	133GLU	O	0.00
132ARG	NH2	237ASN	OD1	98.16
132ARG	NH2	237ASN	O	0.00
132ARG	NH2	241ASP	OD1	0.28
132ARG	NH2	241ASP	OD2	9.80
132ARG	NH2	245ASP	OD1	0.02
132ARG	NH2	245ASP	OD2	0.02
132ARG	NH1	102ASN	OD1	0.03
132ARG	NH1	241ASP	OD1	6.10
132ARG	NH1	241ASP	OD2	13.97
132ARG	NH1	244SER	OG	5.48
132ARG	NH1	245ASP	OD1	0.07
132ARG	NH1	245ASP	OD2	0.64
132ARG	NE	102ASN	OD1	0.08
132ARG	NE	133GLU	O	78.81
132ARG	NE	237ASN	OD1	4.01
132ARG	NE	237ASN	ND2	0.04
132ARG	NE	237ASN	O	0.20
132ARG	N	102ASN	O	97.55
131VAL	N	233VAL	O	97.16
130ILE	N	104ARG	O	96.57
129LEU	N	231ASP	O	85.23
128VAL	N	106ALA	O	97.92
126VAL	N	123ALA	O	4.99
126VAL	N	124ARG	O	0.02
125GLY	N	123ALA	O	0.02
124ARG	NH2	113GLU	OE1	1.88
124ARG	NH2	113GLU	OE2	5.08
124ARG	NH2	120GLU	OE1	24.58

177ARG	NH2	230PHE	O	1.34
177ARG	NH2	231ASP	OD1	31.22
177ARG	NH2	231ASP	OD2	25.37
177ARG	NH1	125GLY	O	0.44
177ARG	NH1	127ASP	OD1	0.03
177ARG	NH1	127ASP	OD2	9.13
177ARG	NH1	127ASP	O	1.79
177ARG	NH1	179HIS	ND1	0.06
177ARG	NH1	228ALA	O	15.24
177ARG	NH1	229ARG	O	1.11
177ARG	NH1	230PHE	O	0.51
177ARG	NH1	231ASP	OD1	13.15
177ARG	NH1	231ASP	OD2	0.03
177ARG	NE	231ASP	OD1	32.14
177ARG	NE	231ASP	OD2	25.53
177ARG	N	173ALA	O	0.11
177ARG	N	177ARG	NE	0.25
177ARG	N	231ASP	OD1	10.64
177ARG	N	231ASP	OD2	44.19
176ARG	NH2	127ASP	OD1	69.77
176ARG	NH2	127ASP	OD2	6.41
176ARG	NH2	127ASP	O	0.07
176ARG	NH2	128VAL	O	0.00
176ARG	NH1	127ASP	OD1	10.70
176ARG	NH1	127ASP	OD2	7.68
176ARG	NH1	127ASP	O	71.17
176ARG	NH1	177ARG	NH1	0.00
176ARG	NH1	177ARG	NH2	0.00
176ARG	NH1	231ASP	OD1	62.55
176ARG	NH1	231ASP	OD2	32.83
176ARG	NH1	231ASP	O	0.12
176ARG	N	172LEU	O	6.58
176ARG	N	173ALA	O	70.49
176ARG	N	174ARG	O	0.40
175LYS	NZ	171GLU	OE1	1.02
175LYS	NZ	171GLU	OE2	0.59
175LYS	NZ	171GLU	O	0.99
175LYS	NZ	174ARG	O	0.00
175LYS	NZ	175LYS	O	0.00
175LYS	NZ	299GLU	OE1	29.66
175LYS	NZ	299GLU	OE2	25.06
175LYS	NZ	299GLU	O	0.55
175LYS	NZ	300HIS	ND1	1.42

142GLU	N	140PHE	O	0.02
142GLU	N	142GLU	OE1	0.01
142GLU	N	154THR	O	0.01
141GLY	N	137GLY	O	0.05
141GLY	N	138ILE	O	40.42
141GLY	N	139TYR	O	0.15
141GLY	N	144ARG	NH1	0.00
140PHE	N	137GLY	O	95.74
140PHE	N	138ILE	O	0.02
139TYR	OH	237ASN	OD1	0.22
139TYR	OH	237ASN	ND2	0.10
139TYR	OH	237ASN	O	1.96
139TYR	OH	238ILE	N	0.00
139TYR	OH	238ILE	O	52.66
139TYR	OH	241ASP	OD1	0.66
139TYR	OH	241ASP	OD2	19.96
139TYR	N	137GLY	O	0.02
138ILE	N	155GLU	OE1	4.04
138ILE	N	155GLU	OE2	56.96
138ILE	N	237ASN	O	2.27
137GLY	N	155GLU	OE1	0.11
137GLY	N	155GLU	OE2	0.01
137GLY	N	156ARG	O	0.08
136GLY	N	133GLU	OE1	32.94
136GLY	N	133GLU	OE2	35.00
136GLY	N	134LEU	O	0.12
135THR	OG1	98ASP	OD2	0.00
135THR	OG1	99LEU	O	15.91
135THR	OG1	100PHE	O	0.06
135THR	OG1	133GLU	OE1	13.88
135THR	OG1	133GLU	OE2	34.07
135THR	OG1	136GLY	N	0.02
135THR	OG1	161GLU	OE1	0.02
135THR	OG1	161GLU	OE2	0.02
135THR	N	99LEU	O	0.01
135THR	N	100PHE	O	0.01
135THR	N	133GLU	OE1	38.45
135THR	N	133GLU	OE2	58.56
134LEU	N	100PHE	O	18.89
134LEU	N	133GLU	OE1	25.95
134LEU	N	133GLU	OE2	43.81
133GLU	N	235THR	O	0.23
133GLU	N	236GLY	O	82.30

176ARG	NH1	231ASP	O	0.10
176ARG	NE	299GLU	OE2	0.10
176ARG	N	172ALA	O	1.38
176ARG	N	173ALA	O	74.15
176ARG	N	174ARG	O	0.49
175LYS	NZ	171GLU	OE1	1.12
175LYS	NZ	171GLU	OE2	0.55
175LYS	NZ	171GLU	O	2.34
175LYS	NZ	299GLU	OE1	35.38
175LYS	NZ	299GLU	OE2	33.34
175LYS	NZ	299GLU	O	6.14
175LYS	NZ	300HIS	ND1	5.57
175LYS	NZ	300HIS	O	0.01
175LYS	NZ	303GLY	O	0.22
175LYS	N	171GLU	O	1.84
175LYS	N	172ALA	O	53.82
175LYS	N	173ALA	O	0.44
174ARG	NH2	171GLU	OE1	46.30
174ARG	NH2	171GLU	OE2	45.36
174ARG	NH2	206TYR	OH	0.90
174ARG	NH2	207PRO	O	0.18
174ARG	NH2	208ASP	OD2	0.25
174ARG	NH1	170PHE	O	0.02
174ARG	NH1	171GLU	OE1	9.96
174ARG	NH1	171GLU	OE2	13.90
174ARG	NH1	205GLY	O	0.00
174ARG	NH1	206TYR	OH	7.28
174ARG	NH1	207PRO	O	0.16
174ARG	NH1	208ASP	OD1	0.87
174ARG	NH1	208ASP	OD2	0.08
174ARG	NE	170PHE	O	0.02
174ARG	NE	171GLU	OE1	23.94
174ARG	NE	171GLU	OE2	19.29
174ARG	NE	171GLU	O	0.08
174ARG	NE	206TYR	OH	3.62
174ARG	NE	207PRO	O	0.06
174ARG	NE	208ASP	OD2	0.29
174ARG	N	170PHE	O	35.41
174ARG	N	171GLU	O	19.47
173ALA	N	169ALA	O	76.86
173ALA	N	170PHE	O	3.37
172ALA	N	168VAL	O	56.29
172ALA	N	169ALA	O	8.78

124ARG	NH2	120GLU	OE2	33.44
124ARG	NH2	120GLU	O	0.00
124ARG	NH2	121GLU	OE1	22.92
124ARG	NH2	121GLU	OE2	21.36
124ARG	NH1	113GLU	OE1	0.03
124ARG	NH1	120GLU	OE1	7.21
124ARG	NH1	120GLU	OE2	8.57
124ARG	NH1	121GLU	OE1	37.19
124ARG	NH1	121GLU	OE2	34.15
124ARG	NE	113GLU	OE1	4.81
124ARG	NE	113GLU	OE2	1.10
124ARG	NE	120GLU	OE1	14.88
124ARG	NE	120GLU	OE2	7.71
124ARG	NE	120GLU	O	0.02
124ARG	N	120GLU	O	19.82
124ARG	N	121GLU	O	7.65
124ARG	N	122ILE	O	0.68
123ALA	N	119LYS	O	76.20
123ALA	N	120GLU	O	6.85
122ILE	N	119LYS	O	73.47
122ILE	N	120GLU	O	0.01
121GLU	N	120GLU	OE2	0.00
121GLU	N	121GLU	OE1	2.53
121GLU	N	121GLU	OE2	8.81
119LYS	NZ	113GLU	O	7.57
119LYS	NZ	114ARG	O	22.27
119LYS	NZ	116SER	O	27.90
119LYS	NZ	118LEU	O	10.50
119LYS	NZ	120GLU	OE1	4.42
119LYS	NZ	120GLU	OE2	11.73
119LYS	N	117PRO	O	81.47
118LEU	N	116SER	O	0.00
116SER	OG	250LEU	O	96.51
116SER	N	112LEU	O	6.90
116SER	N	113GLU	O	45.20
116SER	N	114ARG	O	0.02
115LEU	N	112LEU	O	63.29
115LEU	N	113GLU	O	0.00
114ARG	NH2	111GLY	O	0.84
114ARG	NH2	120GLU	OE1	22.30
114ARG	NH2	120GLU	OE2	10.97
114ARG	NH1	111GLY	O	0.10
114ARG	NH1	120GLU	OE1	0.15

175LYS	NZ	300HIS	O	0.16
175LYS	N	171GLU	O	11.78
175LYS	N	172LEU	O	48.38
175LYS	N	173ALA	O	0.06
174ARG	NH2	205GLY	O	0.34
174ARG	NH2	208ASP	OD1	41.52
174ARG	NH2	208ASP	OD2	57.11
174ARG	NH1	171GLU	OE1	14.87
174ARG	NH1	171GLU	OE2	11.34
174ARG	NE	208ASP	OD1	58.33
174ARG	NE	208ASP	OD2	44.35
174ARG	N	170PHE	O	39.77
174ARG	N	171GLU	O	23.78
173ALA	N	169ALA	O	64.19
173ALA	N	170PHE	O	4.19
172LEU	N	168VAL	O	88.60
172LEU	N	169ALA	O	1.71
171GLU	N	167ARG	O	58.60
171GLU	N	168VAL	O	8.58
170PHE	N	166ALA	O	57.96
170PHE	N	167ARG	O	10.89
169ALA	N	165VAL	O	35.42
169ALA	N	166ALA	O	17.44
168VAL	N	164ARG	O	32.43
168VAL	N	165VAL	O	9.19
168VAL	N	166ALA	O	0.05
167ARG	NH2	163GLU	OE1	36.11
167ARG	NH2	163GLU	OE2	27.67
167ARG	NH2	171GLU	OE1	0.01
167ARG	NH2	201GLU	OE2	0.17
167ARG	NH2	201GLU	O	0.02
167ARG	NH2	202VAL	O	0.02
167ARG	NH2	206TYR	OH	0.01
167ARG	NH1	163GLU	OE1	7.46
167ARG	NH1	163GLU	OE2	5.49
167ARG	NH1	163GLU	O	0.03
167ARG	NH1	201GLU	O	0.00
167ARG	NH1	202VAL	O	0.02
167ARG	NH1	206TYR	OH	2.43
167ARG	NE	163GLU	OE1	33.69
167ARG	NE	163GLU	OE2	34.03
167ARG	NE	163GLU	O	0.02
167ARG	NE	171GLU	OE1	0.01

132ARG	NH2	102ASN	OD1	0.01	172ALA	N	170PHE	O	0.00	114ARG	NE	111GLY	O	0.01	167ARG	NE	206TYR	OH	0.41
132ARG	NH2	133GLU	O	1.56	171GLU	N	167ARG	O	68.43	114ARG	NE	120GLU	OE1	14.94	167ARG	N	163GLU	O	89.06
132ARG	NH2	237ASN	OD1	44.72	171GLU	N	168VAL	O	3.71	114ARG	NE	120GLU	OE2	25.80	167ARG	N	164ARG	O	2.34
132ARG	NH2	237ASN	ND2	0.15	171GLU	N	169ALA	O	0.00	114ARG	N	111GLY	O	0.20	166ALA	N	162VAL	O	45.35
132ARG	NH2	241ASP	OD1	8.61	170PHE	N	166ALA	O	90.17	114ARG	N	112LEU	O	0.00	166ALA	N	163GLU	O	7.70
132ARG	NH2	241ASP	OD2	9.44	170PHE	N	167ARG	O	1.64	114ARG	N	113GLU	OE2	0.01	165VAL	N	161GLU	O	4.64
132ARG	NH1	102ASN	OD1	0.08	169ALA	N	165VAL	O	29.64	113GLU	N	111GLY	O	0.08	165VAL	N	162VAL	O	9.43
132ARG	NH1	240GLY	O	0.00	169ALA	N	166ALA	O	18.74	113GLU	N	113GLU	OE1	13.11	165VAL	N	163GLU	O	0.02
132ARG	NH1	241ASP	OD1	4.24	168VAL	N	164ARG	O	40.43	113GLU	N	113GLU	OE2	76.81	164ARG	NH2	98ASP	OD1	18.97
132ARG	NH1	241ASP	OD2	2.63	168VAL	N	165VAL	O	3.66	112LEU	N	109PHE	O	88.90	164ARG	NH2	98ASP	OD2	50.27
132ARG	NH1	244SER	OG	48.21	167ARG	NH2	163GLU	OE1	35.55	112LEU	N	110PRO	O	0.01	164ARG	NH2	98ASP	O	0.57
132ARG	NE	102ASN	OD1	0.14	167ARG	NH2	163GLU	OE2	29.20	112LEU	N	113GLU	OE1	0.14	164ARG	NH2	99LEU	O	0.96
132ARG	NE	133GLU	O	35.23	167ARG	NH2	171GLU	OE2	1.60	112LEU	N	113GLU	OE2	0.38	164ARG	NH2	133GLU	OE1	5.44
132ARG	NE	237ASN	OD1	4.26	167ARG	NH2	202VAL	O	0.00	111GLY	N	113GLU	OE1	52.92	164ARG	NH2	133GLU	OE2	24.14
132ARG	NE	237ASN	ND2	0.01	167ARG	NH2	205GLY	O	0.00	111GLY	N	113GLU	OE2	54.40	164ARG	NH2	161GLU	OE1	9.32
132ARG	NE	237ASN	O	1.40	167ARG	NH2	206TYR	OH	0.03	109PHE	N	107LYS	O	0.02	164ARG	NH2	161GLU	OE2	8.20
132ARG	N	102ASN	O	92.81	167ARG	NH1	163GLU	OE1	5.59	108VAL	N	126VAL	O	76.11	164ARG	NH1	98ASP	OD1	51.67
131VAL	N	233VAL	O	96.17	167ARG	NH1	163GLU	OE2	7.71	107LYS	NZ	127ASP	OD1	1.58	164ARG	NH1	98ASP	OD2	20.18
130ILE	N	104ARG	O	88.37	167ARG	NH1	163GLU	O	0.00	107LYS	NZ	127ASP	OD2	2.26	164ARG	NH1	98ASP	O	0.50
130ILE	N	128VAL	O	0.00	167ARG	NH1	202VAL	O	0.10	107LYS	NZ	312GLU	OE1	44.73	164ARG	NH1	161GLU	OE1	0.16
129LEU	N	230PHE	O	0.01	167ARG	NH1	205GLY	O	0.07	107LYS	NZ	312GLU	OE2	55.74	164ARG	NH1	161GLU	OE2	0.48
129LEU	N	231ASP	O	90.87	167ARG	NH1	206TYR	OH	2.87	106ALA	N	128VAL	O	97.39	164ARG	NH1	264ARG	NE	0.01
128VAL	N	106ALA	O	93.20	167ARG	NE	163GLU	OE1	32.47	104ARG	NH2	102ASN	OD1	0.06	164ARG	NE	99LEU	O	0.00
127ASP	N	125GLY	O	0.02	167ARG	NE	163GLU	OE2	34.91	104ARG	NH2	132ARG	NH1	0.00	164ARG	NE	133GLU	OE2	18.20
127ASP	N	227PRO	O	0.00	167ARG	NE	163GLU	O	0.03	104ARG	NH2	244SER	OG	0.58	164ARG	NE	160PRO	O	0.11
126VAL	N	123ALA	O	21.66	167ARG	NE	167ARG	O	0.02	104ARG	NH2	245ASP	OD1	16.25	164ARG	NE	161GLU	OE1	0.88
126VAL	N	124ARG	O	0.03	167ARG	NE	171GLU	OE2	0.00	104ARG	NH2	245ASP	OD2	25.55	164ARG	NE	161GLU	OE2	0.75
125GLY	N	122ILE	O	20.54	167ARG	NE	206TYR	OH	1.76	104ARG	NH2	248SER	OG	3.26	164ARG	NE	161GLU	O	0.43
125GLY	N	123ALA	O	0.60	167ARG	N	163GLU	O	82.14	104ARG	NH2	270GLU	OE2	0.00	164ARG	N	160PRO	O	39.59
124ARG	NH2	113GLU	OE1	0.03	167ARG	N	164ARG	O	3.88	104ARG	NH1	244SER	OG	10.57	164ARG	N	161GLU	O	19.18
124ARG	NH2	113GLU	OE2	2.38	166ALA	N	162VAL	O	45.32	104ARG	NH1	244SER	O	0.71	163GLU	N	159LYS	O	36.73
124ARG	NH2	120GLU	OE1	17.17	166ALA	N	163GLU	O	11.25	104ARG	NH1	245ASP	OD1	8.26	163GLU	N	160PRO	O	10.61
124ARG	NH2	120GLU	OE2	20.28	165VAL	N	161GLU	O	15.01	104ARG	NH1	245ASP	OD2	8.83	163GLU	N	161GLU	O	0.00
124ARG	NH2	121GLU	OE1	49.21	165VAL	N	162VAL	O	8.36	104ARG	NH1	248SER	OG	50.73	162VAL	N	158SER	O	63.31
124ARG	NH2	121GLU	OE2	40.22	165VAL	N	163GLU	O	0.04	104ARG	NE	102ASN	OD1	16.37	162VAL	N	159LYS	O	10.49
124ARG	NH1	113GLU	OE1	0.06	164ARG	NH2	133GLU	OE1	0.02	104ARG	NE	244SER	OG	0.23	162VAL	N	160PRO	O	0.01
124ARG	NH1	113GLU	OE2	40.25	164ARG	NH2	133GLU	OE2	0.08	104ARG	N	130ILE	O	99.55	161GLU	N	158SER	O	36.87
124ARG	NH1	120GLU	OE1	28.25	164ARG	NH2	160PRO	O	0.00	103LEU	N	260ALA	O	99.54	161GLU	N	159LYS	O	0.01
124ARG	NH1	120GLU	OE2	27.95	164ARG	NH2	161GLU	OE1	32.77	102ASN	ND2	103LEU	N	0.01	161GLU	N	161GLU	OE1	0.08
124ARG	NH1	121GLU	OE1	7.30	164ARG	NH2	161GLU	OE2	27.55	102ASN	ND2	104ARG	NE	0.01	161GLU	N	161GLU	OE2	0.82
124ARG	NH1	121GLU	OE2	7.67	164ARG	NH1	133GLU	OE1	0.31	102ASN	ND2	132ARG	NE	0.01	159LYS	NZ	163GLU	OE1	2.80
124ARG	NE	113GLU	OE2	0.42	164ARG	NH1	133GLU	OE2	0.82	102ASN	ND2	259SER	OG	0.05	159LYS	NZ	163GLU	OE2	8.88
124ARG	NE	120GLU	OE1	3.12	164ARG	NH1	160PRO	O	0.01	102ASN	ND2	260ALA	O	2.46	159LYS	NZ	197LYS	O	0.01

124ARG	NE	120GLU	OE2	0.05
124ARG	NE	121GLU	OE1	18.58
124ARG	NE	121GLU	OE2	18.97
124ARG	N	120GLU	O	46.67
124ARG	N	121GLU	O	5.99
124ARG	N	122ILE	O	0.25
123ALA	N	119LYS	O	79.82
123ALA	N	120GLU	O	4.16
122ILE	N	119LYS	O	75.04
122ILE	N	120GLU	O	0.00
121GLU	N	121GLU	OE1	0.71
121GLU	N	121GLU	OE2	0.08
120GLU	N	120GLU	OE1	1.93
120GLU	N	120GLU	OE2	2.18
119LYS	NZ	113GLU	O	2.00
119LYS	NZ	114ARG	O	22.22
119LYS	NZ	116SER	O	26.90
119LYS	NZ	118LEU	O	9.32
119LYS	NZ	120GLU	OE1	9.28
119LYS	NZ	120GLU	OE2	4.16
119LYS	N	117PRO	O	82.98
116SER	OG	118LEU	O	0.02
116SER	OG	249VAL	O	3.82
116SER	OG	250LEU	O	91.97
116SER	OG	251PRO	O	0.00
116SER	N	112LEU	O	4.21
116SER	N	113GLU	O	49.89
116SER	N	114ARG	O	0.16
115LEU	N	112LEU	O	83.16
114ARG	NH2	111GLY	O	2.58
114ARG	NH2	120GLU	OE1	1.91
114ARG	NH2	120GLU	OE2	1.93
114ARG	NH2	319LEU	O	0.02
114ARG	NH2	327LEU	O	0.03
114ARG	NH1	111GLY	O	0.21
114ARG	NH1	113GLU	OE2	0.02
114ARG	NE	111GLY	O	1.36
114ARG	NE	120GLU	OE1	12.64
114ARG	NE	120GLU	OE2	12.53
114ARG	N	111GLY	O	0.06
114ARG	N	113GLU	OE1	0.02
113GLU	N	109PHE	O	0.01
113GLU	N	111GLY	O	0.00

164ARG	NH1	161GLU	OE1	42.43
164ARG	NH1	161GLU	OE2	49.74
164ARG	NH1	161GLU	O	0.01
164ARG	NH1	264ARG	NH1	0.02
164ARG	NE	160PRO	O	0.02
164ARG	NE	163GLU	OE2	0.00
164ARG	NE	167ARG	NH2	0.00
164ARG	N	160PRO	O	62.70
164ARG	N	161GLU	O	6.56
163GLU	N	159LYS	O	66.69
163GLU	N	160PRO	O	5.20
163GLU	N	161GLU	O	0.01
162VAL	N	158SER	O	32.47
162VAL	N	159LYS	O	19.96
161GLU	N	158SER	O	45.28
161GLU	N	159LYS	O	0.06
161GLU	N	161GLU	OE1	0.08
161GLU	N	161GLU	OE2	0.08
159LYS	NZ	163GLU	OE1	2.09
159LYS	NZ	163GLU	OE2	3.56
159LYS	NZ	197LYS	O	0.02
159LYS	NZ	198THR	OG1	5.67
159LYS	NZ	201GLU	OE1	11.80
159LYS	NZ	201GLU	OE2	12.26
159LYS	NZ	148GLU	OE1	21.60
159LYS	NZ	148GLU	OE2	22.98
159LYS	NZ	148GLU	O	3.07
159LYS	N	149ALA	O	91.22
158SER	OG	157TYR	O	0.00
158SER	OG	158SER	O	0.06
158SER	OG	159LYS	N	0.00
158SER	OG	161GLU	OE1	0.17
158SER	OG	161GLU	OE2	0.18
158SER	OG	149ALA	O	11.70
158SER	OG	150GLU	OE1	1.23
158SER	OG	150GLU	OE2	0.90
158SER	N	161GLU	OE1	0.36
158SER	N	161GLU	OE2	0.65
157TYR	OH	133GLU	OE1	14.07
157TYR	OH	133GLU	OE2	42.99
157TYR	OH	136GLY	O	3.19
157TYR	OH	155GLU	OE1	5.73
157TYR	OH	155GLU	OE2	13.29

102ASN	ND2	261SER	N	0.01
102ASN	ND2	261SER	OG	71.42
102ASN	ND2	270GLU	OE1	0.03
102ASN	ND2	270GLU	OE2	0.04
102ASN	N	100PHE	O	0.00
102ASN	N	132ARG	O	98.95
101ALA	N	262LEU	O	54.23
100PHE	N	98ASP	O	0.04
100PHE	N	262LEU	O	94.93
99LEU	N	94ARG	O	94.12
98ASP	N	93LEU	O	0.00
98ASP	N	94ARG	O	10.84
98ASP	N	95LYS	O	23.60
98ASP	N	96SER	O	0.01
97GLN	NE2	61VAL	O	0.04
97GLN	NE2	62GLU	OE1	45.28
97GLN	NE2	62GLU	OE2	39.59
97GLN	NE2	96SER	O	0.02
97GLN	NE2	266THR	OG1	0.00
97GLN	N	93LEU	O	72.13
97GLN	N	94ARG	O	1.04
96SER	OG	92SER	O	85.92
96SER	OG	93LEU	O	7.95
96SER	N	92SER	O	93.50
96SER	N	93LEU	O	1.73
96SER	N	94ARG	O	0.08
95LYS	NZ	91LEU	O	0.01
95LYS	NZ	135THR	O	0.24
95LYS	N	91LEU	O	70.74
95LYS	N	92SER	O	5.31
95LYS	N	93LEU	O	0.00
94ARG	NH2	87GLU	OE2	0.04
94ARG	NH2	261SER	OG	0.02
94ARG	NH2	270GLU	OE1	3.68
94ARG	NH2	270GLU	OE2	10.00
94ARG	NH1	87GLU	OE2	0.07
94ARG	NH1	134LEU	O	0.08
94ARG	NE	90LEU	O	0.00
94ARG	NE	261SER	OG	0.93
94ARG	NE	270GLU	OE1	0.00
94ARG	N	90LEU	O	92.64
94ARG	N	91LEU	O	0.50
93LEU	N	89GLY	O	81.05

159LYS	NZ	198THR	OG1	4.75
159LYS	NZ	201GLU	OE1	6.76
159LYS	NZ	201GLU	OE2	6.70
159LYS	NZ	148GLU	OE1	23.47
159LYS	NZ	148GLU	OE2	22.05
159LYS	NZ	148GLU	O	2.16
159LYS	N	149ALA	O	94.29
158SER	OG	157TYR	O	0.02
158SER	OG	158SER	O	0.06
158SER	OG	161GLU	OE1	16.24
158SER	OG	161GLU	OE2	13.16
158SER	OG	149ALA	O	1.34
158SER	OG	150GLU	OE1	2.24
158SER	OG	150GLU	OE2	1.48
158SER	N	161GLU	OE1	13.89
158SER	N	161GLU	OE2	9.57
157TYR	OH	133GLU	OE1	1.33
157TYR	OH	133GLU	OE2	0.10
157TYR	OH	136GLY	O	9.49
157TYR	OH	155GLU	OE1	0.46
157TYR	OH	155GLU	OE2	4.72
157TYR	N	151ALA	O	94.31
156ARG	NH2	142GLU	O	4.32
156ARG	NH2	158SER	OG	0.04
156ARG	NH2	161GLU	OE1	0.49
156ARG	NH2	161GLU	OE2	0.60
156ARG	NH2	150GLU	OE1	36.15
156ARG	NH2	150GLU	OE2	34.05
156ARG	NH2	152TRP	NE1	0.05
156ARG	NH1	142GLU	OE1	0.20
156ARG	NH1	142GLU	OE2	0.03
156ARG	NH1	142GLU	O	21.64
156ARG	NH1	156ARG	O	0.43
156ARG	NH1	158SER	OG	0.01
156ARG	NH1	161GLU	OE1	1.04
156ARG	NH1	161GLU	OE2	0.72
156ARG	NH1	150GLU	OE1	5.64
156ARG	NH1	150GLU	OE2	0.86
156ARG	NH1	152TRP	NE1	0.00
156ARG	NE	142GLU	O	2.16
156ARG	NE	150GLU	OE1	41.68
156ARG	NE	150GLU	OE2	45.91
156ARG	N	154THR	O	0.00

113GLU	N	113GLU	OE1	97.08
112LEU	N	109PHE	O	56.48
112LEU	N	110PRO	O	0.02
112LEU	N	113GLU	OE1	10.11
111GLY	N	113GLU	OE1	90.65
111GLY	N	113GLU	OE2	8.72
109PHE	N	107LYS	O	0.20
108VAL	N	126VAL	O	86.50
107LYS	NZ	127ASP	OD1	5.11
107LYS	NZ	127ASP	OD2	5.66
107LYS	NZ	309ARG	NH1	0.00
107LYS	NZ	312GLU	OE1	51.99
107LYS	NZ	312GLU	OE2	45.61
107LYS	N	105PRO	O	0.00
106ALA	N	128VAL	O	97.48
104ARG	NH2	102ASN	OD1	0.32
104ARG	NH2	241ASP	O	0.00
104ARG	NH2	245ASP	OD1	0.64
104ARG	NH2	245ASP	OD2	1.54
104ARG	NH2	259SER	OG	0.14
104ARG	NH1	244SER	O	1.19
104ARG	NH1	245ASP	OD1	13.61
104ARG	NH1	245ASP	OD2	6.92
104ARG	NH1	248SER	OG	19.60
104ARG	NE	102ASN	OD1	66.21
104ARG	NE	259SER	OG	2.17
104ARG	N	130ILE	O	96.74
103LEU	N	260ALA	O	99.62
102ASN	ND2	104ARG	NE	0.18
102ASN	ND2	104ARG	NH2	0.05
102ASN	ND2	259SER	OG	2.90
102ASN	ND2	260ALA	O	0.67
102ASN	ND2	261SER	N	0.01
102ASN	ND2	261SER	OG	56.01
102ASN	N	100PHE	O	0.00
102ASN	N	132ARG	O	98.90
101ALA	N	262LEU	O	34.25
100PHE	N	98ASP	O	0.47
100PHE	N	262LEU	O	80.34
99LEU	N	93LEU	O	0.01
99LEU	N	94ARG	O	93.50
98ASP	N	93LEU	O	0.07
98ASP	N	94ARG	O	3.38

157TYR	N	151ALA	O	88.04
156ARG	NH2	142GLU	OE1	0.01
156ARG	NH2	142GLU	O	3.00
156ARG	NH2	150GLU	OE1	50.16
156ARG	NH2	150GLU	OE2	38.03
156ARG	NH2	152TRP	NE1	0.02
156ARG	NH1	142GLU	OE1	0.05
156ARG	NH1	142GLU	O	30.49
156ARG	NH1	158SER	OG	0.00
156ARG	NH1	150GLU	OE1	2.50
156ARG	NH1	150GLU	OE2	4.75
156ARG	NH1	152TRP	NE1	0.00
156ARG	NE	142GLU	O	2.71
156ARG	NE	150GLU	OE1	38.39
156ARG	NE	150GLU	OE2	50.10
156ARG	N	137GLY	O	0.23
156ARG	N	140PHE	O	1.62
156ARG	N	155GLU	OE1	0.82
156ARG	N	155GLU	OE2	2.67
155GLU	N	153ASN	O	96.60
154THR	OG1	143PRO	N	0.01
154THR	OG1	143PRO	O	76.10
154THR	OG1	153ASN	O	1.38
154THR	OG1	153ASN	O	0.00
154THR	OG1	154THR	OG1	0.19
154THR	N	138ILE	O	0.06
154THR	N	141GLY	O	2.15
154THR	N	143PRO	O	2.40
153ASN	ND2	138ILE	O	11.65
153ASN	ND2	141GLY	O	0.21
153ASN	ND2	152TRP	O	0.10
153ASN	ND2	154THR	N	0.04
153ASN	ND2	154THR	O	0.03
153ASN	ND2	188VAL	O	0.01
153ASN	ND2	190GLU	N	0.00
153ASN	ND2	190GLU	OE1	15.57
153ASN	ND2	190GLU	OE2	18.26
153ASN	N	155GLU	O	97.29
152TRP	NE1	143PRO	O	0.04
152TRP	NE1	144ARG	O	0.27
152TRP	N	145GLY	O	98.78
151ALA	N	157TYR	O	93.28
150GLU	N	147SER	O	6.60

93LEU	N	90LEU	O	1.64
92SER	OG	88THR	O	35.93
92SER	OG	89GLY	O	1.08
92SER	OG	91LEU	O	0.00
92SER	OG	92SER	O	0.04
92SER	OG	93LEU	N	0.12
92SER	N	88THR	O	31.95
92SER	N	89GLY	O	5.85
92SER	N	90LEU	O	0.34
91LEU	N	87GLU	O	89.82
91LEU	N	88THR	O	0.64
90LEU	N	86PRO	O	47.50
90LEU	N	87GLU	O	21.09
89GLY	N	85ARG	O	0.00
89GLY	N	86PRO	O	92.94
89GLY	N	87GLU	O	0.12
88THR	OG1	51GLU	OE1	0.00
88THR	OG1	82ARG	O	1.01
88THR	OG1	83LYS	O	0.04
88THR	OG1	85ARG	O	85.67
88THR	OG1	86PRO	N	0.00
88THR	OG1	89GLY	N	0.01
88THR	N	82ARG	O	0.02
88THR	N	85ARG	O	88.26
87GLU	N	85ARG	O	0.52
87GLU	N	87GLU	OE1	27.84
87GLU	N	87GLU	OE2	8.79
85ARG	NH2	72VAL	O	0.02
85ARG	NH2	74GLY	O	0.00
85ARG	NH2	78ASP	OD1	1.00
85ARG	NH2	78ASP	OD2	0.33
85ARG	NH2	87GLU	OE1	32.57
85ARG	NH2	87GLU	OE2	65.04
85ARG	NH1	77TRP	O	15.62
85ARG	NH1	78ASP	OD1	2.29
85ARG	NH1	78ASP	OD2	0.13
85ARG	NH1	78ASP	O	7.88
85ARG	NH1	80LEU	O	7.11
85ARG	NE	87GLU	OE1	67.20
85ARG	NE	87GLU	OE2	23.78
85ARG	N	81PRO	O	3.93
85ARG	N	82ARG	O	53.83
85ARG	N	83LYS	O	0.01

156ARG	N	155GLU	OE1	40.48
155GLU	N	153ASN	O	97.56
154THR	OG1	143PRO	N	0.01
154THR	OG1	143PRO	O	81.40
154THR	OG1	153ASN	O	0.11
154THR	OG1	154THR	OG1	3.02
154THR	N	141GLY	O	0.22
154THR	N	143PRO	O	10.20
153ASN	ND2	138ILE	O	1.97
153ASN	ND2	152TRP	O	0.00
153ASN	ND2	190GLU	N	0.01
153ASN	ND2	190GLU	OE1	24.61
153ASN	ND2	190GLU	OE2	31.95
153ASN	N	155GLU	O	97.82
152TRP	NE1	143PRO	O	0.12
152TRP	NE1	144ARG	O	0.18
152TRP	N	145GLY	O	98.52
151ALA	N	157TYR	O	83.22
150GLU	N	147SER	O	4.40
149ALA	N	147SER	O	2.09
149ALA	N	148GLU	OE1	0.20
149ALA	N	148GLU	OE2	0.17
148GLU	N	148GLU	OE1	6.24
148GLU	N	148GLU	OE2	8.68
147SER	OG	146MET	O	0.05
147SER	OG	147SER	O	0.01
147SER	OG	148GLU	OE1	0.18
147SER	OG	148GLU	OE2	0.10
147SER	OG	150GLU	OE2	0.04
147SER	OG	150GLU	O	62.43
147SER	N	150GLU	O	46.47
145GLY	N	152TRP	NE1	0.21
145GLY	N	152TRP	O	88.11
144ARG	NH2	139TYR	O	1.08
144ARG	NH2	140PHE	O	0.01
144ARG	NH2	142GLU	OE1	7.54
144ARG	NH2	142GLU	OE2	11.50
144ARG	NH2	153ASN	ND2	0.00
144ARG	NH2	187ASN	O	0.00
144ARG	NH2	188VAL	O	24.88
144ARG	NH2	189LEU	N	0.00
144ARG	NH2	190GLU	OE1	33.57
144ARG	NH2	190GLU	OE2	32.76

98ASP	N	95LYS	O	24.56
97GLN	NE2	61VAL	O	0.05
97GLN	NE2	62GLU	OE1	34.44
97GLN	NE2	62GLU	OE2	46.69
97GLN	NE2	96SER	O	0.03
97GLN	N	93LEU	O	62.22
97GLN	N	94ARG	O	0.82
96SER	OG	92SER	O	83.31
96SER	OG	93LEU	O	9.59
96SER	N	92SER	O	86.52
96SER	N	93LEU	O	2.31
96SER	N	94ARG	O	0.12
95LYS	NZ	91LEU	O	0.04
95LYS	NZ	95LYS	O	0.01
95LYS	NZ	98ASP	OD1	0.06
95LYS	NZ	98ASP	OD2	0.67
95LYS	NZ	135THR	O	0.02
95LYS	N	91LEU	O	80.38
95LYS	N	92SER	O	3.76
95LYS	N	93LEU	O	0.00
94ARG	NH2	87GLU	OE1	20.08
94ARG	NH2	87GLU	OE2	11.36
94ARG	NH2	87GLU	O	0.01
94ARG	NH2	134LEU	O	0.00
94ARG	NH2	261SER	OG	0.63
94ARG	NH2	270GLU	OE1	0.20
94ARG	NH2	270GLU	OE2	0.52
94ARG	NH1	87GLU	OE1	27.35
94ARG	NH1	87GLU	OE2	34.65
94ARG	NH1	134LEU	O	0.78
94ARG	NE	90LEU	O	0.06
94ARG	NE	261SER	OG	0.04
94ARG	N	90LEU	O	78.36
94ARG	N	91LEU	O	2.78
93LEU	N	89GLY	O	53.93
93LEU	N	90LEU	O	10.49
92SER	OG	51GLU	OE1	0.10
92SER	OG	88THR	O	20.40
92SER	OG	89GLY	O	0.88
92SER	OG	92SER	O	0.02
92SER	OG	93LEU	N	0.03
92SER	N	88THR	O	14.13
92SER	N	89GLY	O	9.46

149ALA	N	147SER	O	1.80
149ALA	N	148GLU	OE1	0.04
149ALA	N	148GLU	OE2	0.07
148GLU	N	146MET	O	0.01
148GLU	N	148GLU	OE1	0.51
148GLU	N	148GLU	OE2	0.40
148GLU	N	150GLU	O	0.09
147SER	OG	146MET	O	0.35
147SER	OG	147SER	O	0.00
147SER	OG	148GLU	N	0.00
147SER	OG	148GLU	OE1	0.17
147SER	OG	148GLU	OE2	0.29
147SER	OG	150GLU	O	51.40
147SER	N	150GLU	O	52.66
145GLY	N	152TRP	NE1	0.09
145GLY	N	152TRP	O	88.36
145GLY	N	190GLU	OE2	0.00
144ARG	NH2	138ILE	O	0.00
144ARG	NH2	139TYR	O	1.04
144ARG	NH2	153ASN	OD1	0.01
144ARG	NH2	188VAL	O	16.91
144ARG	NH2	190GLU	OE1	33.50
144ARG	NH2	190GLU	OE2	35.71
144ARG	NH1	138ILE	O	0.16
144ARG	NH1	139TYR	O	27.82
144ARG	NH1	141GLY	O	0.20
144ARG	NH1	142GLU	OE1	1.88
144ARG	NH1	142GLU	OE2	2.57
144ARG	NH1	188VAL	O	0.29
144ARG	NE	139TYR	O	0.82
144ARG	NE	144ARG	O	0.01
144ARG	NE	153ASN	ND2	0.02
144ARG	NE	190GLU	OE1	43.51
144ARG	NE	190GLU	OE2	48.23
144ARG	N	142GLU	OE1	1.08
144ARG	N	142GLU	OE2	1.34
142GLU	N	140PHE	O	0.06
142GLU	N	142GLU	OE1	0.70
142GLU	N	142GLU	OE2	0.42
141GLY	N	137GLY	O	0.65
141GLY	N	138ILE	O	11.69
141GLY	N	139TYR	O	0.77
141GLY	N	144ARG	NH1	0.08

84ILE	N	81PRO	O	96.73
84ILE	N	82ARG	O	0.00
83LYS	NZ	193GLU	OE2	0.00
83LYS	N	81PRO	O	0.30
82ARG	NH2	80LEU	O	0.04
82ARG	NH2	87GLU	OE1	2.07
82ARG	NH2	87GLU	OE2	2.04
82ARG	NH2	186ALA	O	0.00
82ARG	NH2	187ASN	OD1	0.09
82ARG	NH2	187ASN	O	0.12
82ARG	NH2	193GLU	OE1	10.26
82ARG	NH2	193GLU	OE2	7.43
82ARG	NH2	215TYR	OH	2.58
82ARG	NH1	87GLU	OE1	5.52
82ARG	NH1	87GLU	OE2	7.98
82ARG	NH1	88THR	OG1	0.00
82ARG	NH1	139TYR	O	0.24
82ARG	NH1	186ALA	O	0.00
82ARG	NH1	187ASN	OD1	0.04
82ARG	NH1	187ASN	O	0.08
82ARG	NH1	193GLU	OE1	0.32
82ARG	NH1	193GLU	OE2	0.27
82ARG	NH1	215TYR	OH	0.18
82ARG	NE	80LEU	O	0.00
82ARG	NE	87GLU	OE1	5.42
82ARG	NE	87GLU	OE2	3.10
82ARG	NE	187ASN	OD1	0.14
82ARG	NE	187ASN	O	0.01
82ARG	NE	193GLU	OE1	0.05
82ARG	NE	193GLU	OE2	6.46
82ARG	N	82ARG	NE	0.00
80LEU	N	76LYS	O	1.66
80LEU	N	77TRP	O	47.63
80LEU	N	78ASP	O	1.06
79GLY	N	75PRO	O	0.00
79GLY	N	76LYS	O	18.24
79GLY	N	77TRP	O	0.01
79GLY	N	78ASP	OD1	0.00
79GLY	N	78ASP	OD2	0.04
78ASP	N	74GLY	O	27.07
78ASP	N	75PRO	O	16.19
78ASP	N	76LYS	O	0.05
78ASP	N	78ASP	OD2	0.39

144ARG	NH1	87GLU	OE1	0.02
144ARG	NH1	87GLU	OE2	0.00
144ARG	NH1	138ILE	O	0.06
144ARG	NH1	139TYR	O	47.42
144ARG	NH1	140PHE	O	0.01
144ARG	NH1	141GLY	O	1.22
144ARG	NH1	142GLU	OE1	2.38
144ARG	NH1	142GLU	OE2	0.91
144ARG	NH1	188VAL	O	2.29
144ARG	NH1	190GLU	OE1	1.74
144ARG	NH1	190GLU	OE2	0.51
144ARG	NE	141GLY	O	0.06
144ARG	NE	142GLU	OE1	13.48
144ARG	NE	142GLU	OE2	12.66
144ARG	NE	144ARG	O	0.00
144ARG	NE	153ASN	ND2	0.00
144ARG	NE	190GLU	OE1	35.27
144ARG	NE	190GLU	OE2	38.28
144ARG	N	142GLU	OE1	1.66
144ARG	N	142GLU	OE2	1.67
142GLU	N	142GLU	OE1	0.02
142GLU	N	142GLU	OE2	0.04
141GLY	N	137GLY	O	2.56
141GLY	N	138ILE	O	45.33
141GLY	N	139TYR	O	0.16
141GLY	N	144ARG	NH1	0.03
140PHE	N	137GLY	O	96.22
140PHE	N	138ILE	O	0.04
139TYR	OH	136GLY	O	0.01
139TYR	OH	237ASN	OD1	0.04
139TYR	OH	237ASN	O	2.30
139TYR	OH	238ILE	O	5.66
139TYR	OH	241ASP	OD1	36.15
139TYR	OH	241ASP	OD2	33.41
139TYR	N	137GLY	O	0.04
139TYR	N	237ASN	OD1	0.00
138ILE	N	155GLU	OE1	28.84
138ILE	N	155GLU	OE2	40.19
137GLY	N	155GLU	OE1	0.44
137GLY	N	155GLU	OE2	0.01
137GLY	N	156ARG	O	0.08
136GLY	N	133GLU	OE1	59.02
136GLY	N	133GLU	OE2	0.67

92SER	N	90LEU	O	0.00
91LEU	N	87GLU	O	73.05
91LEU	N	88THR	O	0.04
91LEU	N	89GLY	O	0.21
90LEU	N	86PRO	O	59.91
90LEU	N	87GLU	O	21.22
90LEU	N	88THR	O	0.00
89GLY	N	85SER	O	7.99
89GLY	N	86PRO	O	64.05
89GLY	N	87GLU	O	0.35
88THR	OG1	82ARG	O	32.65
88THR	OG1	83LYS	O	0.68
88THR	OG1	85SER	O	32.15
88THR	OG1	86PRO	O	0.03
88THR	OG1	89GLY	N	0.02
88THR	N	85SER	O	30.15
88THR	N	86PRO	O	8.32
87GLU	N	85SER	O	1.89
87GLU	N	87GLU	OE1	0.85
87GLU	N	87GLU	OE2	0.46
85SER	OG	77TRP	O	0.09
85SER	OG	80LEU	O	0.04
85SER	OG	81PRO	O	20.55
85SER	OG	82ARG	O	24.02
85SER	OG	84ILE	O	0.03
85SER	OG	85SER	O	0.04
85SER	OG	86PRO	N	0.00
85SER	OG	86PRO	O	0.02
85SER	OG	87GLU	OE1	0.03
85SER	OG	88THR	OG1	8.47
85SER	N	81PRO	O	1.40
85SER	N	82ARG	O	70.35
85SER	N	83LYS	O	0.06
85SER	N	88THR	OG1	2.60
84ILE	N	81PRO	O	94.92
84ILE	N	82ARG	O	0.00
83LYS	NZ	50GLY	O	0.06
83LYS	N	81PRO	O	4.12
82ARG	NH2	77TRP	O	0.01
82ARG	NH2	78ASP	OD1	0.03
82ARG	NH2	78ASP	OD2	0.01
82ARG	NH2	80LEU	O	0.69
82ARG	NH2	85SER	OG	0.14

141GLY	N	154THR	O	0.47
140PHE	N	136GLY	O	0.03
140PHE	N	137GLY	O	92.61
140PHE	N	138ILE	O	0.02
140PHE	N	154THR	O	0.25
139TYR	OH	134LEU	O	0.03
139TYR	OH	136GLY	O	0.41
139TYR	OH	237ASN	OD1	10.68
139TYR	OH	237ASN	ND2	0.35
139TYR	OH	237ASN	O	3.77
139TYR	OH	238ILE	O	0.54
139TYR	OH	241ASP	OD1	6.54
139TYR	OH	241ASP	OD2	16.48
139TYR	N	137GLY	O	18.04
139TYR	N	154THR	O	0.01
139TYR	N	237ASN	ND2	0.00
138ILE	N	155GLU	OE1	9.72
138ILE	N	155GLU	OE2	24.31
137GLY	N	135THR	O	0.30
137GLY	N	155GLU	OE1	0.86
137GLY	N	155GLU	OE2	5.54
137GLY	N	156ARG	O	8.97
137GLY	N	157TYR	OH	1.00
136GLY	N	133GLU	OE1	14.44
136GLY	N	133GLU	OE2	42.53
136GLY	N	133GLU	O	0.00
136GLY	N	134LEU	O	0.22
136GLY	N	139TYR	OH	0.12
136GLY	N	157TYR	OH	0.20
136GLY	N	237ASN	ND2	0.18
135THR	OG1	99LEU	O	1.22
135THR	OG1	100PHE	O	0.56
135THR	OG1	133GLU	OE1	57.03
135THR	OG1	133GLU	OE2	27.28
135THR	OG1	134LEU	O	0.04
135THR	OG1	135THR	O	0.24
135THR	OG1	136GLY	N	0.02
135THR	OG1	157TYR	OH	0.14
135THR	OG1	161GLU	OE1	0.01
135THR	N	100PHE	O	0.03
135THR	N	133GLU	OE1	61.99
135THR	N	133GLU	OE2	43.80
134LEU	N	100PHE	O	68.63

77TRP	NE1	43GLY	O	0.08
77TRP	NE1	47ASP	OD1	0.10
77TRP	NE1	47ASP	OD2	0.00
77TRP	NE1	84ILE	O	1.38
77TRP	N	74GLY	O	59.12
77TRP	N	75PRO	O	17.96
76LYS	NZ	9ASP	OD1	0.59
76LYS	NZ	9ASP	OD2	2.30
76LYS	NZ	47ASP	OD1	1.54
76LYS	NZ	47ASP	OD2	1.06
76LYS	NZ	77TRP	NE1	0.00
76LYS	N	74GLY	O	0.24
76LYS	N	78ASP	OD2	4.68
74GLY	N	275SER	OG	1.94
73GLY	N	9ASP	OD1	1.94
73GLY	N	9ASP	OD2	20.12
73GLY	N	9ASP	O	1.23
71SER	OG	9ASP	O	0.20
71SER	OG	70GLY	O	0.00
71SER	OG	71SER	O	0.13
71SER	OG	72VAL	N	0.00
71SER	OG	72VAL	O	0.02
71SER	OG	270GLU	OE2	0.03
71SER	OG	271PRO	O	0.04
71SER	OG	273HIS	NE2	0.02
71SER	OG	273HIS	O	4.88
71SER	OG	274GLY	N	0.02
71SER	OG	274GLY	O	11.45
71SER	OG	275SER	N	0.00
71SER	OG	275SER	OG	0.30
71SER	N	7PRO	O	2.90
70GLY	N	6LEU	O	10.15
69LEU	N	269PHE	O	96.87
68LEU	N	4ALA	O	99.39
67VAL	N	267PRO	O	88.22
66ALA	N	2LYS	O	9.32
65GLU	N	2LYS	O	98.36
65GLU	N	65GLU	OE2	0.01
64ALA	N	60GLY	O	25.13
64ALA	N	61VAL	O	11.90
64ALA	N	62GLU	O	4.22
63GLU	N	59LYS	O	12.61
63GLU	N	60GLY	O	30.73

136GLY	N	133GLU	O	0.04
136GLY	N	134LEU	O	0.30
136GLY	N	237ASN	ND2	0.00
135THR	OG1	99LEU	O	38.13
135THR	OG1	100PHE	O	0.41
135THR	OG1	133GLU	OE1	26.64
135THR	OG1	133GLU	OE2	1.48
135THR	OG1	135THR	O	0.02
135THR	OG1	136GLY	N	0.02
135THR	OG1	161GLU	OE1	0.02
135THR	OG1	161GLU	OE2	0.04
135THR	OG1	164ARG	NH2	0.02
135THR	N	99LEU	O	0.00
135THR	N	100PHE	O	0.00
135THR	N	133GLU	OE1	98.43
135THR	N	133GLU	OE2	1.08
134LEU	N	99LEU	O	0.06
134LEU	N	100PHE	O	9.99
134LEU	N	133GLU	OE1	80.79
134LEU	N	133GLU	OE2	0.04
133GLU	N	235THR	O	3.32
133GLU	N	236GLY	O	29.80
133GLU	N	237ASN	OD1	1.82
132ARG	NH2	102ASN	OD1	0.04
132ARG	NH2	133GLU	O	0.27
132ARG	NH2	139TYR	OH	0.02
132ARG	NH2	237ASN	OD1	26.15
132ARG	NH2	237ASN	O	0.06
132ARG	NH2	241ASP	OD1	6.32
132ARG	NH2	241ASP	OD2	3.18
132ARG	NH2	241ASP	O	0.02
132ARG	NH2	244SER	OG	5.93
132ARG	NH2	245ASP	OD1	0.02
132ARG	NH2	245ASP	OD2	0.48
132ARG	NH1	102ASN	OD1	0.04
132ARG	NH1	133GLU	O	32.05
132ARG	NH1	139TYR	OH	0.14
132ARG	NH1	237ASN	OD1	34.91
132ARG	NH1	237ASN	ND2	0.03
132ARG	NH1	237ASN	O	1.14
132ARG	NH1	240GLY	O	0.01
132ARG	NH1	241ASP	OD1	39.62
132ARG	NH1	241ASP	OD2	5.24

82ARG	NH2	87GLU	OE1	5.70
82ARG	NH2	87GLU	OE2	6.01
82ARG	NH2	186ALA	O	0.08
82ARG	NH2	187ASN	O	0.19
82ARG	NH2	190GLU	OE1	0.01
82ARG	NH2	193GLU	OE1	3.46
82ARG	NH2	193GLU	OE2	6.15
82ARG	NH2	215TYR	OH	0.02
82ARG	NH1	82ARG	O	0.00
82ARG	NH1	85SER	OG	0.18
82ARG	NH1	87GLU	OE1	3.95
82ARG	NH1	87GLU	OE2	5.65
82ARG	NH1	144ARG	NH2	0.00
82ARG	NH1	186ALA	O	0.01
82ARG	NH1	187ASN	O	0.14
82ARG	NH1	190GLU	OE2	0.02
82ARG	NH1	193GLU	OE1	6.89
82ARG	NH1	193GLU	OE2	2.82
82ARG	NE	87GLU	OE1	3.60
82ARG	NE	87GLU	OE2	0.85
82ARG	NE	88THR	OG1	0.00
80LEU	N	76LYS	O	0.20
80LEU	N	77TRP	O	36.61
80LEU	N	78ASP	O	0.14
79GLY	N	75PRO	O	0.37
79GLY	N	76LYS	O	3.01
79GLY	N	77TRP	O	0.06
79GLY	N	78ASP	OD1	0.03
79GLY	N	78ASP	OD2	0.01
78ASP	N	74GLY	O	22.84
78ASP	N	75PRO	O	31.54
78ASP	N	76LYS	O	0.24
77TRP	NE1	9ASP	OD1	51.62
77TRP	NE1	9ASP	OD2	34.82
77TRP	NE1	9ASP	O	0.00
77TRP	NE1	43GLY	O	0.01
77TRP	NE1	47ASP	OD1	0.32
77TRP	NE1	47ASP	OD2	0.21
77TRP	NE1	73GLY	O	0.00
77TRP	NE1	275SER	OG	0.00
77TRP	N	74GLY	O	74.58
77TRP	N	75PRO	O	0.04
76LYS	NZ	9ASP	OD1	18.84

134LEU	N	133GLU	OE1	11.26
134LEU	N	133GLU	OE2	3.61
133GLU	N	235THR	O	58.37
133GLU	N	236GLY	O	6.39
132ARG	NH2	133GLU	O	0.02
132ARG	NH2	139TYR	OH	0.22
132ARG	NH2	237ASN	OD1	82.40
132ARG	NH2	237ASN	ND2	0.07
132ARG	NH2	237ASN	O	1.16
132ARG	NH2	241ASP	OD1	5.21
132ARG	NH2	241ASP	OD2	11.49
132ARG	NH1	102ASN	OD1	2.01
132ARG	NH1	240GLY	O	0.08
132ARG	NH1	241ASP	OD1	8.49
132ARG	NH1	241ASP	OD2	14.16
132ARG	NH1	244SER	OG	9.06
132ARG	NE	102ASN	OD1	0.07
132ARG	NE	133GLU	O	26.49
132ARG	NE	236GLY	O	0.11
132ARG	NE	237ASN	OD1	22.22
132ARG	NE	237ASN	ND2	0.05
132ARG	NE	237ASN	O	14.42
132ARG	NE	241ASP	N	0.00
132ARG	N	102ASN	O	97.35
132ARG	N	130ILE	O	0.04
131VAL	N	233VAL	O	98.32
130ILE	N	104ARG	O	96.55
130ILE	N	128VAL	O	0.01
129LEU	N	231ASP	O	92.25
128VAL	N	106ALA	O	83.79
127ASP	N	125GLY	O	0.10
126VAL	N	122ILE	O	0.02
126VAL	N	123ALA	O	13.68
126VAL	N	124ARG	O	0.16
125GLY	N	121GLU	O	0.04
125GLY	N	122ILE	O	35.86
125GLY	N	123ALA	O	0.47
124ARG	NH2	113GLU	OE1	9.62
124ARG	NH2	113GLU	OE2	13.30
124ARG	NH2	120GLU	OE1	11.74
124ARG	NH2	120GLU	OE2	10.83
124ARG	NH2	121GLU	OE1	24.65
124ARG	NH2	121GLU	OE2	22.83

63GLU	N	61VAL	O	0.46
62GLU	N	58ARG	O	7.64
62GLU	N	59LYS	O	2.08
62GLU	N	60GLY	O	0.00
61VAL	N	57THR	O	91.97
61VAL	N	58ARG	O	0.21
60GLY	N	56PRO	O	5.57
60GLY	N	57THR	O	57.85
60GLY	N	58ARG	O	0.36
59LYS	NZ	55GLU	OE1	33.53
59LYS	NZ	55GLU	OE2	25.65
59LYS	NZ	55GLU	O	0.06
59LYS	NZ	63GLU	OE2	0.03
59LYS	N	55GLU	O	66.78
59LYS	N	56PRO	O	6.05
58ARG	NH2	51GLU	OE1	11.97
58ARG	NH2	51GLU	OE2	14.48
58ARG	NH2	53PHE	O	1.06
58ARG	NH2	55GLU	OE1	35.02
58ARG	NH2	55GLU	OE2	30.04
58ARG	NH2	92SER	OG	1.71
58ARG	NH1	51GLU	OE1	21.07
58ARG	NH1	51GLU	OE2	19.04
58ARG	NH1	53PHE	O	1.66
58ARG	NH1	55GLU	OE1	3.90
58ARG	NH1	55GLU	OE2	8.85
58ARG	NH1	92SER	OG	1.67
58ARG	NE	53PHE	O	21.34
58ARG	NE	55GLU	OE1	3.33
58ARG	NE	55GLU	OE2	2.25
58ARG	NE	92SER	OG	0.00
58ARG	N	54PRO	O	93.84
58ARG	N	55GLU	O	1.58
57THR	OG1	40PRO	O	98.32
57THR	N	54PRO	O	8.14
55GLU	N	51GLU	OE1	0.00
55GLU	N	55GLU	OE1	0.01
55GLU	N	55GLU	OE2	0.03
53PHE	N	51GLU	OE1	0.30
53PHE	N	51GLU	OE2	0.48
53PHE	N	51GLU	O	0.00
51GLU	N	45ALA	O	4.83
51GLU	N	49PHE	O	1.40

132ARG	NH1	241ASP	O	0.00
132ARG	NH1	244SER	OG	0.53
132ARG	NH1	245ASP	OD1	0.00
132ARG	NH1	245ASP	OD2	0.00
132ARG	NE	102ASN	OD1	0.26
132ARG	NE	133GLU	O	18.12
132ARG	NE	236GLY	O	0.01
132ARG	NE	237ASN	OD1	3.85
132ARG	NE	237ASN	ND2	0.04
132ARG	NE	237ASN	O	0.53
132ARG	NE	240GLY	O	0.43
132ARG	NE	244SER	OG	29.69
132ARG	N	102ASN	O	84.39
131VAL	N	233VAL	O	91.74
130ILE	N	104ARG	O	85.56
129LEU	N	231ASP	O	90.68
128VAL	N	106ALA	O	83.56
126VAL	N	108VAL	O	0.67
126VAL	N	123ALA	O	6.16
126VAL	N	124ARG	O	0.04
125GLY	N	108VAL	O	2.11
125GLY	N	123ALA	O	0.09
124ARG	NH2	113GLU	OE1	5.37
124ARG	NH2	113GLU	OE2	1.93
124ARG	NH2	120GLU	OE1	10.27
124ARG	NH2	120GLU	OE2	11.04
124ARG	NH2	120GLU	O	0.01
124ARG	NH2	121GLU	OE1	33.54
124ARG	NH2	121GLU	OE2	39.25
124ARG	NH1	113GLU	OE1	9.34
124ARG	NH1	113GLU	OE2	16.34
124ARG	NH1	120GLU	OE1	13.53
124ARG	NH1	120GLU	OE2	12.87
124ARG	NH1	120GLU	O	0.03
124ARG	NH1	121GLU	OE1	11.25
124ARG	NH1	121GLU	OE2	13.44
124ARG	NH1	124ARG	O	0.00
124ARG	NE	113GLU	OE1	4.35
124ARG	NE	113GLU	OE2	2.54
124ARG	NE	120GLU	O	0.07
124ARG	NE	121GLU	OE1	21.10
124ARG	NE	121GLU	OE2	14.38
124ARG	NE	121GLU	O	0.03

76LYS	NZ	9ASP	OD2	35.39
76LYS	NZ	43GLY	O	0.11
76LYS	NZ	47ASP	OD1	0.20
76LYS	NZ	47ASP	OD2	0.46
76LYS	NZ	77TRP	NE1	0.02
76LYS	N	74GLY	O	0.05
73GLY	N	9ASP	OD1	0.00
73GLY	N	9ASP	OD2	1.46
73GLY	N	9ASP	O	1.85
73GLY	N	77TRP	NE1	6.94
73GLY	N	275SER	OG	0.04
71SER	OG	9ASP	O	9.86
71SER	OG	71SER	O	2.63
71SER	OG	273HIS	ND1	0.03
71SER	OG	273HIS	O	0.00
71SER	OG	274GLY	O	9.89
71SER	OG	275SER	OG	49.10
71SER	N	7PRO	O	1.11
70GLY	N	6LEU	O	34.20
70GLY	N	7PRO	O	0.00
69LEU	N	67VAL	O	0.01
69LEU	N	269PHE	O	97.26
68LEU	N	4ALA	O	99.21
67VAL	N	267PRO	O	88.78
66ALA	N	2LYS	O	3.33
65GLU	N	2LYS	O	99.22
65GLU	N	65GLU	OE1	0.00
64ALA	N	60GLY	O	11.93
64ALA	N	61VAL	O	8.58
64ALA	N	62GLU	O	8.37
63GLU	N	59LYS	O	4.66
63GLU	N	60GLY	O	42.98
63GLU	N	61VAL	O	0.23
62GLU	N	58ARG	O	1.37
62GLU	N	59LYS	O	2.03
62GLU	N	60GLY	O	0.01
61VAL	N	57THR	O	85.98
61VAL	N	58ARG	O	0.06
60GLY	N	56PRO	O	2.38
60GLY	N	57THR	O	70.86
60GLY	N	58ARG	O	0.13
59LYS	NZ	55GLU	OE1	37.89
59LYS	NZ	55GLU	OE2	17.07

124ARG	NH1	113GLU	OE1	0.81
124ARG	NH1	113GLU	OE2	1.34
124ARG	NH1	120GLU	OE1	10.02
124ARG	NH1	120GLU	OE2	14.20
124ARG	NH1	120GLU	O	0.02
124ARG	NH1	121GLU	OE1	16.34
124ARG	NH1	121GLU	OE2	18.54
124ARG	NE	113GLU	OE1	23.59
124ARG	NE	113GLU	OE2	8.38
124ARG	NE	120GLU	OE1	0.66
124ARG	NE	120GLU	OE2	0.44
124ARG	NE	120GLU	O	0.02
124ARG	NE	121GLU	OE1	4.42
124ARG	NE	121GLU	OE2	3.88
124ARG	NE	124ARG	O	0.01
124ARG	N	120GLU	O	59.41
124ARG	N	121GLU	O	3.41
124ARG	N	122ILE	O	0.20
123ALA	N	119LYS	O	75.30
123ALA	N	120GLU	O	5.08
122ILE	N	119LYS	O	79.88
122ILE	N	120GLU	O	0.04
121GLU	N	119LYS	O	0.02
121GLU	N	120GLU	OE2	0.00
121GLU	N	121GLU	OE1	0.88
121GLU	N	121GLU	OE2	1.26
120GLU	N	120GLU	OE1	9.57
120GLU	N	120GLU	OE2	18.90
119LYS	NZ	121GLU	OE1	0.05
119LYS	NZ	121GLU	OE2	0.06
119LYS	NZ	113GLU	O	0.21
119LYS	NZ	114ARG	O	45.52
119LYS	NZ	115LEU	O	0.07
119LYS	NZ	116SER	O	32.95
119LYS	NZ	118LEU	O	0.70
119LYS	NZ	120GLU	OE1	0.14
119LYS	NZ	120GLU	OE2	0.21
119LYS	N	117PRO	O	84.36
116SER	OG	112LEU	O	0.02
116SER	OG	113GLU	O	0.03
116SER	OG	118LEU	O	0.12
116SER	OG	249VAL	O	10.22
116SER	OG	250LEU	O	80.57

51GLU	N	51GLU	OE1	0.01
50GLY	N	45ALA	O	76.92
50GLY	N	46ILE	O	3.42
50GLY	N	47ASP	O	0.02
49PHE	N	45ALA	O	81.07
49PHE	N	46ILE	O	0.10
49PHE	N	47ASP	O	0.04
48ALA	N	44ALA	O	77.52
48ALA	N	45ALA	O	3.39
48ALA	N	46ILE	O	0.65
47ASP	N	43GLY	O	58.73
47ASP	N	44ALA	O	10.01
47ASP	N	45ALA	O	0.00
46ILE	N	42GLY	O	34.51
46ILE	N	43GLY	O	18.76
45ALA	N	41PHE	O	0.08
45ALA	N	42GLY	O	25.45
45ALA	N	43GLY	O	0.01
44ALA	N	9ASP	OD1	9.20
44ALA	N	9ASP	OD2	2.04
44ALA	N	41PHE	O	0.07
44ALA	N	42GLY	O	0.00
43GLY	N	8GLY	O	0.01
43GLY	N	9ASP	OD1	7.48
43GLY	N	9ASP	OD2	28.01
42GLY	N	52PRO	O	80.30
39PHE	N	5VAL	O	97.15
39PHE	N	37GLU	O	0.01
38VAL	N	37GLU	OE1	6.37
38VAL	N	37GLU	OE2	6.37
37GLU	N	3VAL	O	42.21
36TYR	OH	17GLU	OE1	0.00
36TYR	OH	17GLU	OE2	0.00
35ALA	N	1MET	O	64.19
35ALA	N	33GLY	O	0.02
34LEU	N	27ASP	OD1	71.55
34LEU	N	27ASP	OD2	6.56
33GLY	N	27ASP	OD1	98.87
33GLY	N	27ASP	OD2	0.09
33GLY	N	27ASP	O	0.00
32LEU	N	26LEU	O	0.06
32LEU	N	27ASP	OD1	67.95
32LEU	N	27ASP	O	0.96

124ARG	N	120GLU	O	24.33
124ARG	N	121GLU	O	8.52
124ARG	N	122ILE	O	2.99
123ALA	N	119LYS	O	64.66
123ALA	N	120GLU	O	11.11
122ILE	N	119LYS	O	74.79
122ILE	N	120GLU	O	0.04
121GLU	N	119LYS	O	0.01
121GLU	N	120GLU	OE1	0.02
121GLU	N	120GLU	OE2	0.10
121GLU	N	121GLU	OE1	0.42
121GLU	N	121GLU	OE2	0.58
120GLU	N	120GLU	OE1	15.06
120GLU	N	120GLU	OE2	20.53
119LYS	NZ	121GLU	OE1	0.01
119LYS	NZ	121GLU	OE2	0.05
119LYS	NZ	113GLU	O	3.03
119LYS	NZ	114ARG	NH2	0.00
119LYS	NZ	114ARG	O	19.86
119LYS	NZ	115LEU	O	0.00
119LYS	NZ	116SER	O	33.59
119LYS	NZ	117PRO	O	0.01
119LYS	NZ	118LEU	O	19.11
119LYS	NZ	120GLU	OE1	15.64
119LYS	NZ	120GLU	OE2	18.37
119LYS	N	117PRO	O	87.93
118LEU	N	116SER	O	0.00
116SER	OG	113GLU	O	0.86
116SER	OG	118LEU	O	0.01
116SER	OG	249VAL	O	9.94
116SER	OG	250LEU	O	82.14
116SER	OG	251PRO	O	0.02
116SER	N	112LEU	O	19.50
116SER	N	113GLU	O	37.55
116SER	N	114ARG	O	0.28
115LEU	N	111GLY	O	2.32
115LEU	N	112LEU	O	59.85
115LEU	N	113GLU	O	0.02
114ARG	NH2	111GLY	O	1.51
114ARG	NH2	113GLU	OE1	12.34
114ARG	NH2	113GLU	OE2	6.16
114ARG	NH2	120GLU	OE1	10.01
114ARG	NH2	120GLU	OE2	9.32

59LYS	NZ	55GLU	O	0.02
59LYS	N	55GLU	O	54.47
59LYS	N	56PRO	O	11.26
58ARG	NH2	51GLU	OE1	14.16
58ARG	NH2	51GLU	OE2	8.20
58ARG	NH2	55GLU	OE1	35.45
58ARG	NH2	55GLU	OE2	57.39
58ARG	NH1	51GLU	OE1	21.43
58ARG	NH1	51GLU	OE2	28.99
58ARG	NH1	53PHE	O	2.24
58ARG	NH1	55GLU	OE1	2.79
58ARG	NH1	55GLU	OE2	6.67
58ARG	NH1	92SER	OG	10.03
58ARG	NE	55GLU	OE1	8.08
58ARG	NE	55GLU	OE2	7.71
58ARG	N	54PRO	O	94.22
58ARG	N	55GLU	O	1.05
57THR	OG1	40PRO	N	0.00
57THR	OG1	40PRO	O	97.35
57THR	N	54PRO	O	9.10
55GLU	N	51GLU	OE1	0.00
55GLU	N	51GLU	OE2	0.00
55GLU	N	55GLU	OE1	0.01
55GLU	N	55GLU	OE2	0.04
53PHE	N	51GLU	OE1	0.06
53PHE	N	51GLU	OE2	0.03
53PHE	N	51GLU	O	0.02
51GLU	N	45ALA	O	10.64
51GLU	N	49PHE	O	0.84
51GLU	N	51GLU	OE1	0.00
51GLU	N	51GLU	OE2	0.00
50GLY	N	45ALA	O	80.87
50GLY	N	46ILE	O	1.48
50GLY	N	47ASP	O	0.02
49PHE	N	44ALA	O	0.58
49PHE	N	45ALA	O	73.54
49PHE	N	46ILE	O	0.04
49PHE	N	47ASP	O	0.06
48ALA	N	43GLY	O	0.17
48ALA	N	44ALA	O	91.42
48ALA	N	45ALA	O	1.00
48ALA	N	46ILE	O	0.07
48ALA	N	47ASP	OD1	0.00

116SER	OG	251PRO	O	0.04
116SER	OG	252GLY	N	0.09
116SER	N	112LEU	O	1.84
116SER	N	113GLU	O	44.10
116SER	N	114ARG	O	0.66
115LEU	N	111GLY	O	0.66
115LEU	N	112LEU	O	65.63
115LEU	N	113GLU	O	0.09
114ARG	NH2	111GLY	O	5.65
114ARG	NH2	113GLU	OE1	19.00
114ARG	NH2	113GLU	OE2	15.86
114ARG	NH2	120GLU	OE1	4.13
114ARG	NH2	120GLU	OE2	2.27
114ARG	NH2	327LEU	O	1.08
114ARG	NH1	111GLY	O	1.79
114ARG	NH1	120GLU	OE1	0.92
114ARG	NH1	120GLU	OE2	0.06
114ARG	NH1	327LEU	O	0.20
114ARG	NE	111GLY	O	3.60
114ARG	NE	113GLU	OE1	18.19
114ARG	NE	113GLU	OE2	22.35
114ARG	NE	120GLU	OE1	5.24
114ARG	NE	120GLU	OE2	8.63
114ARG	N	111GLY	O	2.80
114ARG	N	112LEU	O	0.03
114ARG	N	113GLU	OE1	9.51
114ARG	N	113GLU	OE2	10.61
113GLU	N	109PHE	O	1.46
113GLU	N	111GLY	O	0.01
113GLU	N	113GLU	OE1	20.20
113GLU	N	113GLU	OE2	45.67
112LEU	N	109PHE	O	68.58
112LEU	N	110PRO	O	0.95
112LEU	N	113GLU	OE1	0.27
112LEU	N	113GLU	OE2	0.56
111GLY	N	109PHE	O	0.00
111GLY	N	113GLU	OE1	46.00
111GLY	N	113GLU	OE2	52.72
111GLY	N	114ARG	NE	0.04
111GLY	N	114ARG	NH2	0.31
108VAL	N	126VAL	O	49.34
107LYS	NZ	108VAL	O	0.10
107LYS	NZ	127ASP	OD1	2.69

31GLY	N	26LEU	O	91.94
31GLY	N	27ASP	O	0.74
30GLU	N	25ALA	O	0.00
30GLU	N	26LEU	O	67.50
30GLU	N	27ASP	O	0.14
30GLU	N	28GLU	O	0.06
29ALA	N	25ALA	O	84.52
29ALA	N	26LEU	O	3.16
29ALA	N	27ASP	O	0.18
28GLU	N	24ARG	O	45.94
28GLU	N	25ALA	O	17.15
28GLU	N	26LEU	O	0.02
27ASP	N	23LEU	O	83.91
27ASP	N	24ARG	O	1.39
26LEU	N	22VAL	O	83.46
26LEU	N	23LEU	O	2.94
25ALA	N	21LYS	O	49.71
25ALA	N	22VAL	O	10.45
24ARG	NH2	17GLU	OE1	42.21
24ARG	NH2	17GLU	OE2	34.23
24ARG	NH2	17GLU	O	0.02
24ARG	NH2	36TYR	OH	12.60
24ARG	NH1	17GLU	OE1	33.33
24ARG	NH1	17GLU	OE2	35.08
24ARG	NH1	36TYR	OH	58.63
24ARG	NE	20LEU	O	0.16
24ARG	NE	36TYR	OH	12.07
24ARG	N	20LEU	O	98.07
24ARG	N	21LYS	O	0.11
23LEU	N	19ALA	O	70.36
23LEU	N	20LEU	O	1.91
22VAL	N	18ALA	O	21.27
22VAL	N	19ALA	O	21.37
21LYS	NZ	17GLU	OE1	0.98
21LYS	NZ	17GLU	OE2	0.00
21LYS	NZ	334GLU	OE1	28.09
21LYS	NZ	334GLU	OE2	36.07
21LYS	NZ	334GLU	O	0.02
21LYS	NZ	337THR	OG1	10.57
21LYS	NZ	337THR	O	0.02
21LYS	N	17GLU	O	88.87
21LYS	N	18ALA	O	0.82
20LEU	N	16THR	O	92.57

114ARG	NH2	124ARG	NH2	0.00
114ARG	NH2	319LEU	O	0.00
114ARG	NH1	111GLY	O	0.12
114ARG	NH1	113GLU	OE2	0.02
114ARG	NH1	120GLU	OE1	0.16
114ARG	NH1	120GLU	OE2	0.08
114ARG	NE	111GLY	O	0.32
114ARG	NE	113GLU	OE1	10.54
114ARG	NE	113GLU	OE2	12.02
114ARG	NE	120GLU	OE1	16.92
114ARG	NE	120GLU	OE2	18.86
114ARG	N	111GLY	O	9.18
114ARG	N	112LEU	O	0.02
114ARG	N	113GLU	OE1	3.79
114ARG	N	113GLU	OE2	7.43
113GLU	N	109PHE	O	1.40
113GLU	N	111GLY	O	0.04
113GLU	N	113GLU	OE1	37.33
113GLU	N	113GLU	OE2	41.94
112LEU	N	109PHE	O	82.72
112LEU	N	110PRO	O	0.15
112LEU	N	113GLU	OE1	0.56
112LEU	N	113GLU	OE2	0.48
111GLY	N	113GLU	OE1	46.12
111GLY	N	113GLU	OE2	59.04
111GLY	N	114ARG	NH2	0.01
109PHE	N	107LYS	O	0.34
108VAL	N	126VAL	O	69.95
107LYS	NZ	127ASP	OD1	0.78
107LYS	NZ	127ASP	OD2	0.50
107LYS	NZ	299GLU	OE1	0.16
107LYS	NZ	299GLU	OE2	0.19
107LYS	NZ	309ARG	NH1	0.01
107LYS	NZ	312GLU	OE1	48.17
107LYS	NZ	312GLU	OE2	55.79
107LYS	N	105PRO	O	0.38
106ALA	N	104ARG	O	0.01
106ALA	N	128VAL	O	95.72
104ARG	NH2	102ASN	OD1	0.21
104ARG	NH2	244SER	OG	0.02
104ARG	NH2	245ASP	OD1	1.16
104ARG	NH2	245ASP	OD2	0.36
104ARG	NH2	259SER	OG	2.52

47ASP	N	43GLY	O	20.35
47ASP	N	44ALA	O	47.77
47ASP	N	45ALA	O	0.46
46ILE	N	42GLY	O	6.22
46ILE	N	43GLY	O	10.04
45ALA	N	41PHE	O	0.00
45ALA	N	42GLY	O	6.45
45ALA	N	43GLY	O	0.01
44ALA	N	9ASP	OD1	1.14
44ALA	N	9ASP	OD2	1.41
44ALA	N	41PHE	O	0.01
44ALA	N	42GLY	O	0.12
43GLY	N	8GLY	O	0.25
43GLY	N	9ASP	OD1	0.34
43GLY	N	9ASP	OD2	3.42
43GLY	N	41PHE	O	0.04
42GLY	N	52PRO	O	13.69
41PHE	N	39PHE	O	0.00
39PHE	N	5VAL	O	95.92
39PHE	N	37GLU	O	0.08
38VAL	N	37GLU	OE1	3.68
38VAL	N	37GLU	OE2	3.48
37GLU	N	3VAL	O	55.32
36TYR	N	34LEU	O	0.02
35ALA	N	1MET	O	51.95
35ALA	N	33GLY	O	0.24
34LEU	N	27ASP	OD1	27.29
34LEU	N	27ASP	OD2	56.27
33GLY	N	27ASP	OD1	33.00
33GLY	N	27ASP	OD2	62.98
32LEU	N	26LEU	O	0.01
32LEU	N	27ASP	OD1	26.17
32LEU	N	27ASP	OD2	40.20
32LEU	N	27ASP	O	0.03
31GLY	N	26LEU	O	93.92
31GLY	N	27ASP	O	0.02
30GLU	N	26LEU	O	63.89
30GLU	N	27ASP	O	0.09
30GLU	N	28GLU	O	0.04
30GLU	N	30GLU	OE2	0.02
29ALA	N	25ALA	O	85.50
29ALA	N	26LEU	O	2.80
29ALA	N	27ASP	O	0.11

107LYS	NZ	127ASP	OD2	1.29
107LYS	NZ	312GLU	OE1	50.20
107LYS	NZ	312GLU	OE2	53.43
107LYS	N	105PRO	O	0.09
107LYS	N	253SER	OG	0.00
106ALA	N	104ARG	O	0.02
106ALA	N	128VAL	O	96.17
104ARG	NH2	102ASN	OD1	2.17
104ARG	NH2	132ARG	NH1	0.01
104ARG	NH2	241ASP	OD1	0.09
104ARG	NH2	241ASP	OD2	0.25
104ARG	NH2	244SER	OG	0.13
104ARG	NH2	245ASP	OD1	8.63
104ARG	NH2	245ASP	OD2	12.19
104ARG	NH2	248SER	OG	0.25
104ARG	NH2	259SER	OG	0.36
104ARG	NH2	270GLU	OE1	0.00
104ARG	NH2	272VAL	O	0.11
104ARG	NH1	105PRO	O	0.00
104ARG	NH1	241ASP	OD1	0.59
104ARG	NH1	244SER	OG	3.07
104ARG	NH1	244SER	O	24.87
104ARG	NH1	245ASP	OD1	34.52
104ARG	NH1	245ASP	OD2	29.63
104ARG	NH1	248SER	OG	9.62
104ARG	NE	102ASN	OD1	12.18
104ARG	NE	244SER	OG	0.06
104ARG	NE	244SER	O	0.00
104ARG	NE	259SER	OG	1.88
104ARG	N	130ILE	O	97.14
103LEU	N	102ASN	OD1	0.01
103LEU	N	260ALA	O	99.08
102ASN	ND2	103LEU	N	0.02
102ASN	ND2	103LEU	O	0.00
102ASN	ND2	104ARG	NE	0.46
102ASN	ND2	104ARG	NH1	0.03
102ASN	ND2	104ARG	NH2	0.14
102ASN	ND2	132ARG	NH1	0.00
102ASN	ND2	259SER	OG	10.06
102ASN	ND2	260ALA	O	1.68
102ASN	ND2	261SER	N	0.02
102ASN	ND2	261SER	OG	58.01
102ASN	ND2	270GLU	OE1	0.03

20LEU	N	17GLU	O	0.79
19ALA	N	15VAL	O	44.13
19ALA	N	16THR	O	20.76
18ALA	N	14GLU	O	67.86
18ALA	N	15VAL	O	2.30
17GLU	N	13PRO	O	77.03
17GLU	N	14GLU	O	2.07
16THR	OG1	7PRO	O	2.65
16THR	OG1	12GLY	O	89.34
16THR	N	12GLY	O	81.47
16THR	N	13PRO	O	1.82
15VAL	N	11ILE	O	76.75
15VAL	N	12GLY	O	2.02
14GLU	N	11ILE	O	39.62
14GLU	N	14GLU	OE1	0.01
12GLY	N	9ASP	O	1.06
12GLY	N	71SER	OG	0.10
12GLY	N	71SER	O	0.00
11ILE	N	9ASP	O	0.07
11ILE	N	275SER	O	2.90
11ILE	N	276ALA	O	0.04
10GLY	N	9ASP	OD1	4.26
10GLY	N	9ASP	OD2	3.10
9ASP	N	71SER	O	93.56
8GLY	N	41PHE	O	1.93
6LEU	N	68LEU	O	99.65
5VAL	N	37GLU	O	95.45
4ALA	N	66ALA	O	96.98
3VAL	N	1MET	O	0.01
3VAL	N	35ALA	O	92.96
2LYS	NZ	36TYR	O	0.01
2LYS	NZ	37GLU	OE1	0.00
2LYS	NZ	37GLU	OE2	0.06
2LYS	NZ	63GLU	OE1	39.37
2LYS	NZ	63GLU	OE2	28.47
2LYS	NZ	63GLU	O	2.09
2LYS	NZ	65GLU	OE1	0.05
2LYS	NZ	65GLU	OE2	0.05
2LYS	N	65GLU	OE1	17.55
2LYS	N	65GLU	OE2	28.13
1MET	N	32LEU	O	0.00
1MET	N	33GLY	O	94.35
345ALA	N10	341LEU	O	0.90

104ARG	NH1	244SER	OG	0.18
104ARG	NH1	244SER	O	50.87
104ARG	NH1	245ASP	N	0.00
104ARG	NH1	245ASP	OD1	33.30
104ARG	NH1	245ASP	OD2	49.72
104ARG	NH1	248SER	OG	25.95
104ARG	NE	102ASN	OD1	7.46
104ARG	NE	244SER	OG	0.01
104ARG	NE	258PRO	O	0.98
104ARG	NE	259SER	OG	7.72
104ARG	N	102ASN	O	0.01
104ARG	N	130ILE	O	88.65
103LEU	N	101ALA	O	0.00
103LEU	N	102ASN	OD1	0.01
103LEU	N	260ALA	O	98.23
102ASN	ND2	102ASN	O	0.01
102ASN	ND2	103LEU	N	0.01
102ASN	ND2	104ARG	NE	0.15
102ASN	ND2	104ARG	NH2	0.13
102ASN	ND2	132ARG	NE	0.06
102ASN	ND2	132ARG	NH1	0.11
102ASN	ND2	132ARG	NH2	0.05
102ASN	ND2	259SER	OG	12.92
102ASN	ND2	260ALA	O	0.66
102ASN	ND2	261SER	N	0.01
102ASN	ND2	261SER	OG	35.92
102ASN	N	100PHE	O	0.01
102ASN	N	132ARG	O	98.23
102ASN	N	133GLU	O	0.00
101ALA	N	99LEU	O	0.01
101ALA	N	262LEU	O	39.59
100PHE	N	98ASP	O	0.33
100PHE	N	262LEU	O	70.88
99LEU	N	94ARG	O	87.68
99LEU	N	97GLN	O	0.00
98ASP	N	94ARG	O	20.14
98ASP	N	95LYS	O	14.56
98ASP	N	96SER	O	0.10
97GLN	NE2	58ARG	O	0.05
97GLN	NE2	61VAL	O	13.13
97GLN	NE2	62GLU	OE1	15.66
97GLN	NE2	62GLU	OE2	12.08
97GLN	NE2	93LEU	O	0.20

28GLU	N	24ARG	O	50.84
28GLU	N	25ALA	O	14.13
28GLU	N	26LEU	O	0.02
27ASP	N	23LEU	O	86.24
27ASP	N	24ARG	O	1.13
26LEU	N	22VAL	O	85.80
26LEU	N	23LEU	O	2.91
25ALA	N	21LYS	O	53.41
25ALA	N	22VAL	O	9.03
24ARG	NH2	17GLU	OE1	13.06
24ARG	NH2	17GLU	OE2	21.63
24ARG	NH2	17GLU	O	0.02
24ARG	NH2	27ASP	OD2	1.35
24ARG	NH2	34LEU	O	0.74
24ARG	NH2	36TYR	OH	16.89
24ARG	NH1	17GLU	OE1	15.97
24ARG	NH1	17GLU	OE2	11.94
24ARG	NH1	27ASP	OD1	0.02
24ARG	NH1	27ASP	OD2	0.04
24ARG	NH1	34LEU	O	0.21
24ARG	NH1	36TYR	OH	15.27
24ARG	NE	20LEU	O	0.60
24ARG	NE	24ARG	O	0.00
24ARG	NE	27ASP	OD2	0.00
24ARG	NE	36TYR	OH	35.38
24ARG	N	20LEU	O	96.07
24ARG	N	21LYS	O	0.30
23LEU	N	19ALA	O	78.52
23LEU	N	20LEU	O	1.45
22VAL	N	18ALA	O	30.49
22VAL	N	19ALA	O	15.85
21LYS	NZ	17GLU	OE1	0.25
21LYS	NZ	17GLU	OE2	0.29
21LYS	NZ	17GLU	O	0.02
21LYS	NZ	333THR	O	0.75
21LYS	NZ	334GLU	OE1	22.78
21LYS	NZ	334GLU	OE2	21.52
21LYS	NZ	334GLU	O	0.32
21LYS	NZ	337THR	OG1	13.51
21LYS	N	17GLU	O	65.38
21LYS	N	18ALA	O	8.02
20LEU	N	16THR	O	88.28
20LEU	N	17GLU	O	1.33
102ASN	ND2	270GLU	OE2	0.20
102ASN	N	132ARG	O	97.69
101ALA	N	262LEU	O	56.45
100PHE	N	98ASP	O	0.01
100PHE	N	262LEU	O	94.10
99LEU	N	94ARG	O	30.57
99LEU	N	97GLN	O	13.21
99LEU	N	98ASP	OD1	0.02
98ASP	N	62GLU	OE2	0.04
98ASP	N	93LEU	O	0.00
98ASP	N	94ARG	O	13.44
98ASP	N	95LYS	O	5.09
98ASP	N	96SER	O	0.59
98ASP	N	264ARG	O	0.29
97GLN	NE2	61VAL	O	3.57
97GLN	NE2	62GLU	OE1	22.80
97GLN	NE2	62GLU	OE2	19.06
97GLN	NE2	93LEU	O	0.17
97GLN	NE2	96SER	O	0.30
97GLN	NE2	97GLN	O	0.03
97GLN	NE2	264ARG	O	0.01
97GLN	NE2	266THR	N	0.03
97GLN	NE2	266THR	OG1	28.11
97GLN	NE2	266THR	O	17.55
97GLN	NE2	267PRO	O	0.05
97GLN	N	93LEU	O	86.34
97GLN	N	94ARG	O	1.08
97GLN	N	95LYS	O	0.01
96SER	OG	62GLU	OE1	0.15
96SER	OG	62GLU	OE2	0.02
96SER	OG	92SER	O	42.21
96SER	OG	93LEU	O	0.96
96SER	OG	95LYS	O	0.02
96SER	OG	96SER	O	0.14
96SER	OG	97GLN	OE1	0.27
96SER	N	92SER	O	60.80
96SER	N	93LEU	O	18.94
96SER	N	94ARG	O	0.05
96SER	N	97GLN	O	0.20
95LYS	NZ	91LEU	O	0.13
95LYS	NZ	95LYS	O	0.05
95LYS	NZ	98ASP	OD1	2.74
95LYS	NZ	98ASP	OD2	1.96
345ALA	N104	342ARG	O	10.66
345ALA	N104	343HIS	O	8.02
344LEU	N104	340VAL	O	45.87
344LEU	N104	341LEU	O	12.49
344LEU	N104	342ARG	O	0.08
343HIS	NE21	317LYS	O	0.92
343HIS	NE21	321GLU	OE1	5.45
343HIS	NE21	321GLU	OE2	12.02
343HIS	N104	339THR	O	44.97
343HIS	N104	340VAL	O	32.45
342ARG	NH2	321GLU	OE1	26.52
342ARG	NH2	321GLU	OE2	31.24
342ARG	NH2	338ALA	O	0.00
342ARG	NH2	343HIS	ND1	0.00
342ARG	NH2	343HIS	NE2	0.05
342ARG	NH1	321GLU	OE1	28.63
342ARG	NH1	321GLU	OE2	19.74
342ARG	NH1	338ALA	O	0.01
342ARG	NH1	342ARG	O	0.17
342ARG	NH1	343HIS	NE2	0.00
342ARG	NE10	321GLU	OE1	0.74
342ARG	NE10	321GLU	OE2	0.63
342ARG	NE10	338ALA	O	0.04
342ARG	NE10	342ARG	O	0.02
342ARG	N104	338ALA	O	26.37
342ARG	N104	339THR	O	31.42
342ARG	N104	340VAL	O	0.84
342ARG	N104	344LEU	O	0.00
341LEU	N103	337THR	O	97.78
341LEU	N103	338ALA	O	0.08
340VAL	N103	336PHE	O	89.83
340VAL	N103	337THR	O	1.81
339THR	OG1	321GLU	OE1	0.07
339THR	OG1	321GLU	OE2	0.10
339THR	OG1	335ALA	O	73.35
339THR	OG1	336PHE	O	1.50
339THR	OG1	338ALA	O	0.00
339THR	OG1	340VAL	N	0.02
339THR	OG1	342ARG	NH1	0.02
339THR	OG1	342ARG	NH2	0.03
339THR	N103	335ALA	O	45.75
339THR	N103	336PHE	O	19.77
339THR	N103	337THR	O	0.01
97GLN	NE2	96SER	O	0.10
97GLN	NE2	97GLN	O	0.06
97GLN	NE2	264ARG	O	0.08
97GLN	NE2	266THR	N	0.03
97GLN	NE2	266THR	OG1	10.97
97GLN	NE2	266THR	O	17.59
97GLN	NE2	267PRO	O	0.04
97GLN	N	93LEU	O	76.35
97GLN	N	94ARG	O	2.14
96SER	OG	51GLU	OE1	0.01
96SER	OG	92SER	O	52.50
96SER	OG	93LEU	O	6.70
96SER	OG	96SER	O	0.07
96SER	OG	97GLN	N	0.00
96SER	OG	97GLN	OE1	0.09
96SER	N	92SER	O	71.99
96SER	N	93LEU	O	8.06
96SER	N	94ARG	O	0.03
95LYS	NZ	91LEU	O	0.00
95LYS	NZ	95LYS	O	0.08
95LYS	NZ	98ASP	OD1	0.67
95LYS	NZ	98ASP	OD2	4.06
95LYS	NZ	135THR	OG1	1.37
95LYS	NZ	135THR	O	0.54
95LYS	NZ	140PHE	O	0.00
95LYS	NZ	161GLU	OE1	0.05
95LYS	NZ	161GLU	OE2	0.02
95LYS	N	91LEU	O	70.57
95LYS	N	92SER	O	5.38
95LYS	N	93LEU	O	0.01
94ARG	NH2	270GLU	OE1	33.81
94ARG	NH2	270GLU	OE2	49.63
94ARG	NH1	90LEU	O	0.01
94ARG	NH1	134LEU	O	5.84
94ARG	NE	90LEU	O	0.01
94ARG	NE	261SER	OG	0.00
94ARG	NE	270GLU	OE1	0.04
94ARG	NE	270GLU	OE2	0.16
94ARG	N	90LEU	O	91.97
94ARG	N	91LEU	O	0.76
93LEU	N	89GLY	O	71.31
93LEU	N	90LEU	O	3.84
92SER	OG	51GLU	OE1	0.01

19ALA	N	15VAL	O	65.19
19ALA	N	16THR	O	8.02
18ALA	N	14GLU	O	59.81
18ALA	N	15VAL	O	3.37
17GLU	N	13PRO	O	83.60
17GLU	N	14GLU	O	1.62
16THR	OG1	7PRO	O	1.77
16THR	OG1	12GLY	O	94.75
16THR	N	12GLY	O	78.68
16THR	N	13PRO	O	1.73
15VAL	N	11ILE	O	84.13
15VAL	N	12GLY	O	2.44
14GLU	N	11ILE	O	27.51
12GLY	N	9ASP	O	1.43
12GLY	N	71SER	OG	5.44
12GLY	N	275SER	OG	51.51
11ILE	N	9ASP	O	0.02
11ILE	N	71SER	OG	0.00
11ILE	N	275SER	OG	43.22
11ILE	N	275SER	O	33.79
10GLY	N	9ASP	OD1	0.29
10GLY	N	9ASP	OD2	0.03
9ASP	N	71SER	O	93.50
8GLY	N	41PHE	O	51.79
6LEU	N	68LEU	O	99.35
5VAL	N	37GLU	O	95.27
4ALA	N	66ALA	O	97.72
3VAL	N	35ALA	O	87.03
2LYS	NZ	37GLU	OE1	0.42
2LYS	NZ	37GLU	OE2	0.64
2LYS	NZ	63GLU	OE1	29.05
2LYS	NZ	63GLU	OE2	40.71
2LYS	NZ	63GLU	O	0.78
2LYS	N	65GLU	OE1	1.33
2LYS	N	65GLU	OE2	0.02
1MET	N	33GLY	O	90.94
1MET	N	65GLU	OE1	0.95
1MET	N	65GLU	OE2	0.02
345ALA	N1042	341LEU	O103	1.78
345ALA	N1042	342ARG	O103	11.86
345ALA	N1042	343HIS	O104	6.76
344LEU	N1040	340VAL	O103	32.65
344LEU	N1040	341LEU	O103	17.08

95LYS	N	91LEU	O	36.67
95LYS	N	92SER	O	26.21
95LYS	N	93LEU	O	5.14
95LYS	N	97GLN	O	0.01
94ARG	NH2	102ASN	OD1	0.08
94ARG	NH2	261SER	OG	0.96
94ARG	NH2	270GLU	OE1	47.51
94ARG	NH2	270GLU	OE2	40.80
94ARG	NH1	87GLU	OE2	0.00
94ARG	NH1	90LEU	O	0.01
94ARG	NH1	270GLU	OE1	0.04
94ARG	NH1	270GLU	OE2	1.29
94ARG	NE	261SER	OG	0.11
94ARG	NE	270GLU	OE1	0.05
94ARG	NE	270GLU	OE2	0.07
94ARG	N	90LEU	O	94.10
94ARG	N	91LEU	O	0.38
93LEU	N	89GLY	O	75.98
93LEU	N	90LEU	O	3.32
92SER	OG	51GLU	OE1	0.00
92SER	OG	51GLU	OE2	0.02
92SER	OG	88THR	O	23.17
92SER	OG	89GLY	O	1.37
92SER	OG	91LEU	O	0.00
92SER	OG	92SER	O	0.08
92SER	OG	93LEU	N	0.08
92SER	N	88THR	O	28.46
92SER	N	89GLY	O	8.45
92SER	N	90LEU	O	0.27
91LEU	N	87GLU	O	53.94
91LEU	N	88THR	O	3.85
90LEU	N	86PRO	O	20.68
90LEU	N	87GLU	O	41.49
89GLY	N	85SER	O	0.19
89GLY	N	86PRO	O	79.62
89GLY	N	87GLU	O	0.20
88THR	OG1	82ARG	O	2.25
88THR	OG1	83LYS	O	0.23
88THR	OG1	85SER	O	80.88
88THR	OG1	87GLU	OE1	0.05
88THR	OG1	87GLU	OE2	0.13
88THR	N	82ARG	O	0.06
88THR	N	85SER	O	78.74

338ALA	N103	334GLU	O	78.21
338ALA	N103	335ALA	O	1.73
337THR	OG1	18ALA	O	0.03
337THR	OG1	333THR	O	75.07
337THR	OG1	334GLU	OE1	0.01
337THR	OG1	334GLU	O	1.03
337THR	OG1	338ALA	N	0.03
337THR	N103	333THR	O	65.03
337THR	N103	334GLU	O	0.88
336PHE	N103	332GLY	O	92.93
336PHE	N103	333THR	O	1.01
336PHE	N103	334GLU	O	0.00
335ALA	N103	332GLY	O	2.72
335ALA	N103	337THR	OG1	0.00
334GLU	N102	283GLY	O	0.12
334GLU	N102	332GLY	O	0.01
334GLU	N102	334GLU	OE2	0.01
333THR	OG1	14GLU	OE1	0.40
333THR	OG1	14GLU	OE2	1.15
333THR	OG1	283GLY	O	6.97
333THR	OG1	285ALA	O	75.85
333THR	OG1	334GLU	N	0.00
333THR	OG1	334GLU	OE1	0.24
333THR	OG1	334GLU	OE2	0.06
333THR	N102	283GLY	O	66.07
333THR	N102	285ALA	O	1.08
332GLY	N102	285ALA	O	0.00
332GLY	N102	286ASN	OD1	0.02
331ALA	N102	286ASN	OD1	0.22
331ALA	N102	323PRO	O	18.66
331ALA	N102	329GLY	O	0.06
330SER	OG1	329GLY	O	1.22
330SER	OG1	330SER	O	0.47
330SER	OG1	331ALA	N	0.00
330SER	OG1	331ALA	O	0.00
330SER	N102	323PRO	O	7.96
329GLY	N102	324PRO	O	26.56
329GLY	N102	325PRO	O	0.19
329GLY	N102	327LEU	O	0.00
328GLY	N102	324PRO	O	64.43
328GLY	N102	325PRO	O	22.92
328GLY	N102	326ASP	O	0.01
327LEU	N102	324PRO	O	74.93

92SER	OG	51GLU	OE2	0.02
92SER	OG	88THR	O	36.07
92SER	OG	89GLY	O	0.12
92SER	OG	92SER	O	0.06
92SER	OG	93LEU	N	0.00
92SER	OG	96SER	OG	0.00
92SER	N	88THR	O	47.10
92SER	N	89GLY	O	3.03
92SER	N	90LEU	O	0.03
91LEU	N	87GLU	O	88.78
91LEU	N	88THR	O	0.18
90LEU	N	86PRO	O	38.17
90LEU	N	87GLU	O	27.84
90LEU	N	88THR	O	0.00
89GLY	N	85ARG	O	0.02
89GLY	N	86PRO	O	90.37
89GLY	N	87GLU	O	0.02
88THR	OG1	82ARG	O	13.90
88THR	OG1	83LYS	O	2.94
88THR	OG1	85ARG	O	1.40
88THR	OG1	88THR	O	0.80
88THR	N	82ARG	O	0.10
88THR	N	85ARG	O	84.85
87GLU	N	85ARG	O	0.06
87GLU	N	87GLU	OE1	0.16
87GLU	N	87GLU	OE2	0.28
85ARG	NH2	78ASP	OD2	0.04
85ARG	NH2	78ASP	O	0.00
85ARG	NH2	87GLU	OE1	53.78
85ARG	NH2	87GLU	OE2	45.75
85ARG	NH2	187ASN	O	45.89
85ARG	NH1	77TRP	O	0.26
85ARG	NH1	78ASP	O	0.10
85ARG	NH1	80LEU	O	90.43
85ARG	NH1	187ASN	O	0.38
85ARG	NE	87GLU	OE1	42.51
85ARG	NE	87GLU	OE2	52.91
85ARG	N	81PRO	O	1.77
85ARG	N	82ARG	O	74.62
85ARG	N	83LYS	O	0.01
84ILE	N	81PRO	O	96.58
84ILE	N	82ARG	O	0.02
83LYS	N	81PRO	O	0.02

344LEU	N104(	342ARG	O103	0.23
343HIS	NE21	317LYS	O100	1.02
343HIS	NE21	321GLU	OE11	10.05
343HIS	NE21	321GLU	OE21	15.16
343HIS	N103!	339THR	O103	50.47
343HIS	N103!	340VAL	O103	26.99
342ARG	NH21	321GLU	OE11	21.62
342ARG	NH21	321GLU	OE21	29.48
342ARG	NH21	343HIS	ND11	0.01
342ARG	NH21	343HIS	NE21	0.04
342ARG	NH11	321GLU	OE11	33.68
342ARG	NH11	321GLU	OE21	20.91
342ARG	NH11	338ALA	O103	0.00
342ARG	NH11	342ARG	O103	0.45
342ARG	NH11	343HIS	ND11	0.04
342ARG	NH11	345ALA	O110	0.12
342ARG	NE10	338ALA	O103	0.02
342ARG	NE10	342ARG	O103	0.32
342ARG	N103(	338ALA	O103	35.08
342ARG	N103(	339THR	O103	22.75
342ARG	N103(	340VAL	O103	0.20
341LEU	N103-	337THR	O103	97.99
341LEU	N103-	338ALA	O103	0.05
340VAL	N103:	336PHE	O102	88.15
340VAL	N103:	337THR	O103	2.42
339THR	OG11	321GLU	OE11	0.04
339THR	OG11	321GLU	OE21	0.02
339THR	OG11	335ALA	O102	78.57
339THR	OG11	336PHE	O102	0.03
339THR	OG11	339THR	O103	0.00
339THR	OG11	342ARG	NH11	0.02
339THR	OG11	342ARG	NH21	0.03
339THR	N103:	335ALA	O102	42.55
339THR	N103:	336PHE	O102	21.32
338ALA	N103(	334GLU	O102	85.92
338ALA	N103(	335ALA	O102	0.95
337THR	OG11	333THR	O102	93.98
337THR	OG11	334GLU	O102	0.00
337THR	N102!	333THR	O102	83.47
337THR	N102!	334GLU	O102	1.15
336PHE	N102(	332GLY	O102	90.48
336PHE	N102(	333THR	O102	0.60
335ALA	N102!	332GLY	O102	1.20

88THR	N	86PRO	O	0.02
88THR	N	87GLU	OE1	0.02
88THR	N	87GLU	OE2	0.11
87GLU	N	72VAL	O	0.22
87GLU	N	85SER	O	0.31
87GLU	N	87GLU	OE1	23.44
87GLU	N	87GLU	OE2	18.43
85SER	OG	72VAL	O	0.05
85SER	OG	77TRP	O	0.06
85SER	OG	80LEU	O	0.06
85SER	OG	81PRO	O	1.98
85SER	OG	82ARG	O	11.58
85SER	OG	84ILE	O	0.00
85SER	OG	85SER	O	0.02
85SER	OG	87GLU	OE1	24.97
85SER	OG	87GLU	OE2	17.72
85SER	OG	88THR	OG1	0.04
85SER	N	81PRO	O	3.42
85SER	N	82ARG	O	70.59
85SER	N	83LYS	O	0.12
85SER	N	88THR	OG1	0.27
84ILE	N	81PRO	O	56.05
84ILE	N	82ARG	O	1.26
83LYS	NZ	51GLU	OE1	0.18
83LYS	NZ	51GLU	OE2	0.36
83LYS	NZ	186ALA	O	0.92
83LYS	NZ	187ASN	O	0.02
83LYS	NZ	189LEU	O	0.13
83LYS	NZ	190GLU	OE1	0.08
83LYS	NZ	190GLU	OE2	1.45
83LYS	NZ	193GLU	OE1	10.90
83LYS	NZ	193GLU	OE2	4.51
83LYS	N	81PRO	O	1.72
83LYS	N	85SER	OG	0.00
83LYS	N	85SER	O	0.79
83LYS	N	88THR	OG1	1.57
82ARG	NH2	74GLY	O	0.00
82ARG	NH2	77TRP	O	0.70
82ARG	NH2	78ASP	OD1	2.45
82ARG	NH2	78ASP	OD2	1.41
82ARG	NH2	78ASP	O	0.36
82ARG	NH2	80LEU	O	0.00
82ARG	NH2	85SER	OG	1.10

327LEU	N102	325PRO	O	0.02
327LEU	N102	326ASP	OD1	0.00
326ASP	N101	284ILE	O	0.44
326ASP	N101	324PRO	O	0.03
326ASP	N101	326ASP	OD1	0.00
326ASP	N101	326ASP	OD2	0.00
322THR	OG1	318ALA	O	18.03
322THR	OG1	322THR	O	15.95
322THR	OG1	330SER	O	0.10
322THR	OG1	339THR	OG1	0.06
322THR	N101	318ALA	O	96.40
322THR	N101	319LEU	O	0.05
321GLU	N101	317LYS	O	27.67
321GLU	N101	318ALA	O	30.88
321GLU	N101	319LEU	O	0.50
320LEU	N101	316ALA	O	51.11
320LEU	N101	317LYS	O	12.86
319LEU	N100	315VAL	O	82.17
319LEU	N100	316ALA	O	3.94
318ALA	N100	314ALA	O	54.30
318ALA	N100	315VAL	O	9.31
317LYS	NZ10	313ASP	OD1	16.07
317LYS	NZ10	313ASP	OD2	85.53
317LYS	NZ10	313ASP	O	0.14
317LYS	NZ10	343HIS	ND1	0.02
317LYS	NZ10	343HIS	O	0.02
317LYS	N100	313ASP	O	87.66
317LYS	N100	314ALA	O	1.81
316ALA	N100	312GLU	O	84.05
316ALA	N100	313ASP	O	1.01
315VAL	N100	311VAL	O	92.58
315VAL	N100	312GLU	O	1.17
314ALA	N100	310LYS	O	78.46
314ALA	N100	311VAL	O	2.50
313ASP	N100	309ARG	O	77.40
313ASP	N100	310LYS	O	2.42
312GLU	N	308ALA	O	89.70
312GLU	N	309ARG	O	0.20
311VAL	N	307LEU	O	70.31
311VAL	N	308ALA	O	7.33
310LYS	NZ	30GLU	OE1	2.95
310LYS	NZ	30GLU	OE2	6.75
310LYS	NZ	306GLU	OE1	0.16

82ARG	NH2	87GLU	OE1	7.50
82ARG	NH2	87GLU	OE2	6.34
82ARG	NH2	139TYR	O	0.01
82ARG	NH2	187ASN	OD1	0.00
82ARG	NH2	187ASN	O	10.00
82ARG	NH2	188VAL	O	0.20
82ARG	NH2	190GLU	OE1	0.60
82ARG	NH2	190GLU	OE2	13.50
82ARG	NH1	87GLU	OE1	0.08
82ARG	NH1	139TYR	O	0.64
82ARG	NH1	187ASN	O	0.96
82ARG	NH1	188VAL	O	0.00
82ARG	NH1	190GLU	OE1	16.99
82ARG	NH1	190GLU	OE2	5.42
82ARG	NE	85ARG	NH2	0.04
82ARG	NE	87GLU	OE1	50.37
82ARG	NE	87GLU	OE2	42.07
82ARG	NE	187ASN	O	0.14
82ARG	N	85ARG	NH1	0.00
80LEU	N	77TRP	O	50.57
80LEU	N	78ASP	O	0.36
79GLY	N	75PRO	O	0.00
79GLY	N	76LYS	O	0.02
79GLY	N	77TRP	O	0.04
79GLY	N	78ASP	OD1	0.05
79GLY	N	78ASP	OD2	0.05
78ASP	N	74GLY	O	5.28
78ASP	N	75PRO	O	45.95
78ASP	N	76LYS	O	0.55
78ASP	N	78ASP	OD2	0.01
77TRP	NE1	43GLY	O	0.22
77TRP	NE1	46ILE	O	0.01
77TRP	NE1	47ASP	OD1	9.54
77TRP	NE1	47ASP	OD2	35.61
77TRP	NE1	84ILE	O	0.94
77TRP	N	74GLY	O	50.55
77TRP	N	75PRO	O	6.46
76LYS	NZ	9ASP	OD1	24.38
76LYS	NZ	9ASP	OD2	24.14
76LYS	NZ	47ASP	OD1	12.57
76LYS	NZ	47ASP	OD2	7.91
76LYS	NZ	75PRO	O	0.00
76LYS	NZ	76LYS	O	0.01

333THR	OG11	14GLU	OE1	0.00
333THR	OG11	283GLY	O	0.67
333THR	OG11	285ALA	O	90.50
333THR	N1022	283GLY	O	57.18
333THR	N1022	285ALA	O	0.06
331ALA	N1021	323PRO	O101	36.15
331ALA	N1021	329GLY	O101	0.01
330SER	OG10	329GLY	O101	1.00
330SER	OG10	330SER	O102	0.24
330SER	N1020	323PRO	O101	1.71
329GLY	N1019	324PRO	O101	38.61
329GLY	N1019	325PRO	O101	0.10
328GLY	N1018	324PRO	O101	51.19
328GLY	N1018	325PRO	O101	30.38
328GLY	N1018	326ASP	O101	0.02
327LEU	N1016	324PRO	O101	69.94
327LEU	N1016	325PRO	O101	0.08
326ASP	N1015	324PRO	O101	0.19
326ASP	N1015	326ASP	OD11	0.02
322THR	OG11	318ALA	O100	19.50
322THR	OG11	322THR	O101	17.26
322THR	OG11	339THR	OG11	0.01
322THR	N1009	318ALA	O100	98.46
322THR	N1009	319LEU	O100	0.02
321GLU	N1008	317LYS	O100	34.27
321GLU	N1008	318ALA	O100	24.27
321GLU	N1008	319LEU	O100	0.16
320LEU	N1006	316ALA	O100	48.20
320LEU	N1006	317LYS	O100	11.41
319LEU	N1004	315VAL	O100	86.69
319LEU	N1004	316ALA	O100	2.35
318ALA	N1003	314ALA	O	50.11
318ALA	N1003	315VAL	O100	10.38
317LYS	NZ100	313ASP	OD1	88.24
317LYS	NZ100	313ASP	OD2	15.34
317LYS	NZ100	313ASP	O	0.07
317LYS	NZ100	343HIS	O104	0.06
317LYS	N1001	313ASP	O	79.97
317LYS	N1001	314ALA	O	3.29
316ALA	N1000	312GLU	O	36.89
316ALA	N1000	313ASP	O	5.58
315VAL	N	311VAL	O	92.86
315VAL	N	312GLU	O	0.29

82ARG	NH2	87GLU	OE1	31.23
82ARG	NH2	87GLU	OE2	23.17
82ARG	NH2	217ASP	OD1	0.13
82ARG	NH2	217ASP	OD2	1.01
82ARG	NH1	77TRP	O	0.17
82ARG	NH1	78ASP	OD1	0.18
82ARG	NH1	78ASP	OD2	0.58
82ARG	NH1	78ASP	O	0.12
82ARG	NH1	80LEU	O	0.13
82ARG	NH1	85SER	OG	0.72
82ARG	NH1	87GLU	OE1	4.44
82ARG	NH1	87GLU	OE2	11.53
82ARG	NH1	187ASN	OD1	0.00
82ARG	NH1	187ASN	O	0.01
82ARG	NH1	215TYR	OH	1.26
82ARG	NH1	217ASP	OD1	3.02
82ARG	NH1	217ASP	OD2	6.85
82ARG	NE	77TRP	O	0.16
82ARG	NE	78ASP	OD1	0.27
82ARG	NE	78ASP	OD2	0.02
82ARG	NE	78ASP	O	0.16
82ARG	NE	85SER	OG	0.01
82ARG	NE	87GLU	OE1	20.74
82ARG	NE	87GLU	OE2	28.18
82ARG	N	80LEU	O	0.01
82ARG	N	215TYR	OH	3.90
80LEU	N	76LYS	O	7.51
80LEU	N	77TRP	O	29.75
80LEU	N	78ASP	O	1.38
79GLY	N	75PRO	O	0.06
79GLY	N	76LYS	O	6.18
79GLY	N	77TRP	O	0.06
79GLY	N	78ASP	OD1	0.04
79GLY	N	78ASP	OD2	0.04
79GLY	N	80LEU	O	0.06
79GLY	N	82ARG	NH1	0.04
79GLY	N	82ARG	NH2	0.00
78ASP	N	74GLY	O	3.21
78ASP	N	75PRO	O	22.18
78ASP	N	76LYS	O	4.50
78ASP	N	78ASP	OD1	0.01
78ASP	N	80LEU	O	0.02
77TRP	NE1	9ASP	OD1	7.72

310LYS	NZ	306GLU	OE2	0.10
310LYS	NZ	343HIS	O	0.14
310LYS	NZ	344LEU	O	0.48
310LYS	NZ	345ALA	O1	13.88
310LYS	NZ	345ALA	O21	22.27
310LYS	N	306GLU	O	27.98
310LYS	N	307LEU	O	20.91
309ARG	NH2	299GLU	OE1	52.61
309ARG	NH2	299GLU	OE2	31.95
309ARG	NH2	312GLU	OE1	86.09
309ARG	NH2	312GLU	OE2	12.64
309ARG	NH1	299GLU	OE1	35.92
309ARG	NH1	299GLU	OE2	56.90
309ARG	NH1	305VAL	O	0.03
309ARG	NE	312GLU	OE1	14.40
309ARG	NE	312GLU	OE2	87.01
309ARG	N	305VAL	O	72.28
309ARG	N	306GLU	O	4.18
308ALA	N	304LEU	O	61.52
308ALA	N	305VAL	O	9.08
307LEU	N	304LEU	O	25.86
307LEU	N	305VAL	O	0.01
307LEU	N	306GLU	OE2	0.01
306GLU	N	304LEU	O	0.00
306GLU	N	306GLU	OE1	10.01
306GLU	N	306GLU	OE2	6.03
305VAL	N	303GLY	O	38.72
305VAL	N	306GLU	OE1	0.07
305VAL	N	306GLU	OE2	0.04
304LEU	N	298LEU	O	77.58
304LEU	N	302PHE	O	0.00
303GLY	N	298LEU	O	58.35
303GLY	N	299GLU	O	0.48
303GLY	N	300HIS	O	0.03
302PHE	N	297MET	O	70.12
302PHE	N	298LEU	O	1.14
302PHE	N	299GLU	O	0.04
302PHE	N	300HIS	O	0.04
301ALA	N	296MET	O	0.00
301ALA	N	297MET	O	61.43
301ALA	N	298LEU	O	0.00
301ALA	N	299GLU	O	0.02
300HIS	NE2	299GLU	OE1	0.01

76LYS	NZ	77TRP	NE1	0.23
76LYS	N	74GLY	O	1.42
76LYS	N	78ASP	OD1	0.01
76LYS	N	78ASP	OD2	0.07
74GLY	N	72VAL	O	0.00
74GLY	N	275SER	OG	0.04
73GLY	N	9ASP	OD1	0.01
73GLY	N	9ASP	OD2	0.81
73GLY	N	9ASP	O	10.78
73GLY	N	42GLY	O	0.00
73GLY	N	71SER	O	0.07
73GLY	N	275SER	OG	0.07
72VAL	N	70GLY	O	0.01
71SER	OG	9ASP	O	0.46
71SER	OG	70GLY	O	0.09
71SER	OG	71SER	O	0.21
71SER	OG	72VAL	N	0.01
71SER	OG	72VAL	O	1.72
71SER	OG	270GLU	OE1	0.74
71SER	OG	270GLU	OE2	1.53
71SER	OG	271PRO	O	0.06
71SER	OG	273HIS	ND1	0.04
71SER	OG	273HIS	NE2	5.92
71SER	OG	273HIS	O	0.27
71SER	OG	274GLY	O	1.93
71SER	OG	275SER	OG	1.72
71SER	N	7PRO	O	3.09
71SER	N	273HIS	NE2	43.73
70GLY	N	6LEU	O	25.76
69LEU	N	67VAL	O	0.01
69LEU	N	269PHE	O	90.09
68LEU	N	4ALA	O	98.57
67VAL	N	267PRO	O	86.15
66ALA	N	2LYS	O	6.77
65GLU	N	2LYS	O	96.74
65GLU	N	65GLU	OE1	0.10
65GLU	N	65GLU	OE2	0.20
65GLU	N	66ALA	O	0.00
64ALA	N	60GLY	O	19.23
64ALA	N	61VAL	O	7.90
64ALA	N	62GLU	O	7.34
63GLU	N	59LYS	O	7.75
63GLU	N	60GLY	O	40.87

314ALA	N	310LYS	O	73.29
314ALA	N	311VAL	O	3.23
313ASP	N	309ARG	O	63.90
313ASP	N	310LYS	O	5.62
313ASP	N	311VAL	O	0.00
312GLU	N	308ALA	O	79.19
312GLU	N	309ARG	O	1.02
311VAL	N	307LEU	O	46.59
311VAL	N	308ALA	O	13.65
310LYS	NZ	29ALA	O	0.03
310LYS	NZ	30GLU	OE1	4.20
310LYS	NZ	30GLU	OE2	5.77
310LYS	NZ	306GLU	OE1	0.00
310LYS	NZ	306GLU	O	0.10
310LYS	NZ	313ASP	OD1	3.18
310LYS	NZ	343HIS	O104	0.08
310LYS	NZ	344LEU	O104	2.39
310LYS	NZ	345ALA	O110	23.96
310LYS	NZ	345ALA	O210	24.35
310LYS	N	306GLU	O	22.26
310LYS	N	307LEU	O	25.49
309ARG	NH2	175LYS	O	9.52
309ARG	NH2	299GLU	OE1	8.87
309ARG	NH2	299GLU	OE2	17.00
309ARG	NH2	306GLU	OE1	17.53
309ARG	NH2	306GLU	OE2	29.70
309ARG	NH1	299GLU	OE1	19.02
309ARG	NH1	299GLU	OE2	12.35
309ARG	NH1	312GLU	OE1	17.73
309ARG	NH1	312GLU	OE2	13.06
309ARG	NE	306GLU	OE1	13.44
309ARG	NE	306GLU	OE2	12.35
309ARG	N	305VAL	O	84.86
309ARG	N	306GLU	O	2.29
308ALA	N	304LEU	O	63.13
308ALA	N	305VAL	O	7.77
307LEU	N	304LEU	O	32.45
307LEU	N	305VAL	O	0.04
307LEU	N	306GLU	OE1	0.03
307LEU	N	306GLU	OE2	0.02
306GLU	N	306GLU	OE1	23.12
306GLU	N	306GLU	OE2	22.67
305VAL	N	303GLY	O	30.61

77TRP	NE1	9ASP	OD2	12.30
77TRP	NE1	9ASP	O	0.01
77TRP	NE1	47ASP	OD1	0.25
77TRP	NE1	47ASP	OD2	0.04
77TRP	NE1	72VAL	O	0.28
77TRP	NE1	73GLY	O	0.03
77TRP	NE1	74GLY	O	0.22
77TRP	NE1	80LEU	O	0.28
77TRP	NE1	81PRO	O	40.37
77TRP	NE1	82ARG	N	0.04
77TRP	NE1	84ILE	O	0.73
77TRP	NE1	85SER	OG	0.00
77TRP	N	73GLY	O	0.00
77TRP	N	74GLY	O	41.77
77TRP	N	75PRO	O	0.38
76LYS	NZ	9ASP	OD1	48.52
76LYS	NZ	9ASP	OD2	50.93
76LYS	NZ	9ASP	O	0.04
76LYS	NZ	43GLY	O	0.02
76LYS	NZ	47ASP	OD1	0.16
76LYS	NZ	47ASP	OD2	0.27
76LYS	NZ	73GLY	O	0.56
76LYS	NZ	74GLY	O	0.06
76LYS	NZ	77TRP	NE1	0.17
76LYS	NZ	275SER	O	1.02
76LYS	N	74GLY	O	4.28
76LYS	N	77TRP	NE1	0.11
74GLY	N	9ASP	OD2	0.01
74GLY	N	77TRP	NE1	0.14
74GLY	N	78ASP	OD2	0.02
74GLY	N	82ARG	NE	0.00
74GLY	N	82ARG	NH1	0.03
74GLY	N	82ARG	NH2	0.08
74GLY	N	87GLU	OE1	0.62
74GLY	N	87GLU	OE2	0.27
73GLY	N	9ASP	OD1	0.09
73GLY	N	9ASP	OD2	0.99
73GLY	N	9ASP	O	9.67
73GLY	N	71SER	O	0.00
72VAL	N	273HIS	O	0.78
71SER	OG	9ASP	O	29.23
71SER	OG	70GLY	O	0.02
71SER	OG	71SER	O	1.44

300HIS	N	296MET	O	94.86
300HIS	N	297MET	O	0.64
299GLU	N	295ALA	O	83.32
299GLU	N	296MET	O	1.66
298LEU	N	294ALA	O	56.78
298LEU	N	295ALA	O	11.53
297MET	N	293SER	O	71.71
297MET	N	294ALA	O	1.87
296MET	N	292LEU	O	86.00
296MET	N	293SER	O	0.92
295ALA	N	291ILE	O	53.85
295ALA	N	292LEU	O	7.04
294ALA	N	290ALA	O	79.22
294ALA	N	291ILE	O	4.34
293SER	OG	259SER	O	1.77
293SER	OG	270GLU	O	0.28
293SER	OG	289ALA	O	92.56
293SER	OG	290ALA	O	0.08
293SER	OG	293SER	O	0.03
293SER	N	289ALA	O	41.55
293SER	N	290ALA	O	10.24
292LEU	N	288THR	O	80.66
292LEU	N	289ALA	O	0.46
291ILE	N	287PRO	O	94.92
291ILE	N	288THR	O	0.58
290ALA	N	287PRO	O	27.19
289ALA	N	255GLY	O	0.17
288THR	OG1	255GLY	O	46.10
288THR	OG1	256LEU	O	0.02
288THR	OG1	286ASN	OD1	33.68
288THR	OG1	289ALA	N	0.04
288THR	N	286ASN	OD1	27.52
288THR	N	286ASN	O	2.52
288THR	N	288THR	OG1	0.00
286ASN	ND2	255GLY	O	2.45
286ASN	ND2	284ILE	O	0.12
286ASN	ND2	288THR	OG1	16.68
286ASN	ND2	322THR	O	0.25
286ASN	ND2	326ASP	OD1	10.26
286ASN	ND2	326ASP	OD2	2.00
286ASN	ND2	326ASP	O	0.00
286ASN	ND2	331ALA	N	0.00
286ASN	ND2	331ALA	O	0.94

63GLU	N	61VAL	O	0.70
62GLU	N	58ARG	O	3.06
62GLU	N	59LYS	O	4.30
62GLU	N	60GLY	O	0.15
61VAL	N	56PRO	O	0.00
61VAL	N	57THR	O	75.40
61VAL	N	58ARG	O	0.19
60GLY	N	56PRO	O	8.26
60GLY	N	57THR	O	58.71
60GLY	N	58ARG	O	0.91
59LYS	NZ	55GLU	OE1	30.14
59LYS	NZ	55GLU	OE2	31.59
59LYS	NZ	55GLU	O	0.21
59LYS	NZ	62GLU	OE1	0.25
59LYS	NZ	62GLU	OE2	0.40
59LYS	NZ	63GLU	OE1	0.11
59LYS	NZ	63GLU	OE2	0.16
59LYS	N	55GLU	O	45.42
59LYS	N	56PRO	O	18.19
59LYS	N	57THR	O	0.02
58ARG	NH2	51GLU	OE1	43.38
58ARG	NH2	51GLU	OE2	36.90
58ARG	NH2	53PHE	O	5.10
58ARG	NH2	55GLU	OE1	11.48
58ARG	NH2	55GLU	OE2	5.58
58ARG	NH2	92SER	OG	0.30
58ARG	NH2	96SER	OG	0.18
58ARG	NH1	51GLU	OE1	4.09
58ARG	NH1	51GLU	OE2	6.38
58ARG	NH1	53PHE	O	0.12
58ARG	NH1	55GLU	OE1	24.15
58ARG	NH1	55GLU	OE2	28.59
58ARG	NH1	92SER	OG	0.38
58ARG	NH1	96SER	OG	0.88
58ARG	NH1	97GLN	OE1	0.01
58ARG	NE	51GLU	OE1	2.58
58ARG	NE	51GLU	OE2	2.09
58ARG	NE	53PHE	O	19.04
58ARG	NE	96SER	OG	0.20
58ARG	N	54PRO	O	75.69
58ARG	N	55GLU	O	9.18
57THR	OG1	40PRO	O	86.22
57THR	OG1	54PRO	N	0.01

305VAL	N	306GLU	OE1	0.24
305VAL	N	306GLU	OE2	0.31
304LEU	N	298LEU	O	76.56
304LEU	N	299GLU	O	0.00
304LEU	N	302PHE	O	0.01
303GLY	N	298LEU	O	63.44
303GLY	N	299GLU	O	0.89
303GLY	N	300HIS	O	0.03
302PHE	N	297MET	O	52.77
302PHE	N	298LEU	O	2.64
302PHE	N	299GLU	O	0.86
302PHE	N	300HIS	O	0.18
301ALA	N	297MET	O	72.09
301ALA	N	298LEU	O	0.02
301ALA	N	299GLU	O	0.02
300HIS	NE2	65GLU	O	0.00
300HIS	NE2	261SER	O	0.01
300HIS	NE2	299GLU	OE2	0.02
300HIS	N	296MET	O	91.62
300HIS	N	297MET	O	1.21
299GLU	N	295ALA	O	77.62
299GLU	N	296MET	O	4.14
298LEU	N	294ALA	O	54.03
298LEU	N	295ALA	O	12.90
297MET	N	293SER	O	64.71
297MET	N	294ALA	O	2.88
296MET	N	292LEU	O	88.26
296MET	N	293SER	O	0.90
295ALA	N	291ILE	O	50.63
295ALA	N	292LEU	O	8.55
295ALA	N	293SER	O	0.00
294ALA	N	290ALA	O	74.91
294ALA	N	291ILE	O	8.98
293SER	OG	259SER	O	0.06
293SER	OG	270GLU	O	0.00
293SER	OG	289ALA	O	98.46
293SER	OG	293SER	O	0.00
293SER	N	289ALA	O	36.81
293SER	N	290ALA	O	11.89
292LEU	N	288THR	O	98.68
292LEU	N	289ALA	O	0.01
291ILE	N	287PRO	O	95.04
291ILE	N	288THR	O	0.52

71SER	OG	73GLY	O	0.05
71SER	OG	270GLU	OE1	0.05
71SER	OG	271PRO	O	0.03
71SER	OG	273HIS	NE2	0.23
71SER	OG	273HIS	O	5.04
71SER	OG	274GLY	N	0.06
71SER	OG	274GLY	O	0.14
71SER	OG	275SER	OG	0.16
71SER	N	7PRO	O	6.49
70GLY	N	6LEU	O	13.72
70GLY	N	7PRO	O	0.06
69LEU	N	269PHE	O	86.24
68LEU	N	4ALA	O	98.48
67VAL	N	267PRO	O	87.65
66ALA	N	2LYS	O	5.66
66ALA	N	64ALA	O	0.00
65GLU	N	2LYS	O	92.70
65GLU	N	65GLU	OE1	0.01
65GLU	N	65GLU	OE2	0.17
64ALA	N	60GLY	O	14.40
64ALA	N	61VAL	O	14.72
64ALA	N	62GLU	O	5.00
63GLU	N	59LYS	O	4.38
63GLU	N	60GLY	O	44.87
63GLU	N	61VAL	O	0.60
62GLU	N	58ARG	O	0.84
62GLU	N	59LYS	O	3.13
62GLU	N	60GLY	O	0.06
62GLU	N	62GLU	OE1	0.01
62GLU	N	62GLU	OE2	0.07
61VAL	N	57THR	O	76.32
61VAL	N	58ARG	O	0.06
61VAL	N	59LYS	O	0.00
61VAL	N	62GLU	OE2	0.01
60GLY	N	56PRO	O	4.60
60GLY	N	57THR	O	64.47
60GLY	N	58ARG	O	0.42
59LYS	NZ	55GLU	OE1	36.36
59LYS	NZ	55GLU	OE2	24.16
59LYS	NZ	55GLU	O	0.05
59LYS	NZ	59LYS	O	0.03
59LYS	NZ	62GLU	OE2	0.00
59LYS	NZ	63GLU	OE1	0.65

286ASN	N	284ILE	O	0.03
286ASN	N	286ASN	ND2	0.01
286ASN	N	326ASP	OD1	0.42
285ALA	N	14GLU	OE2	0.00
285ALA	N	279ILE	O	0.07
285ALA	N	283GLY	O	3.18
285ALA	N	333THR	OG1	6.78
284ILE	N	279ILE	O	1.07
284ILE	N	282LYS	O	0.34
283GLY	N	14GLU	OE1	0.09
283GLY	N	14GLU	OE2	1.52
283GLY	N	279ILE	O	0.41
283GLY	N	281GLY	O	0.42
282LYS	NZ	278ASP	OD1	2.48
282LYS	NZ	278ASP	OD2	1.84
282LYS	NZ	278ASP	O	1.35
282LYS	NZ	280ALA	O	0.47
282LYS	N	14GLU	OE1	0.08
282LYS	N	14GLU	OE2	2.07
282LYS	N	278ASP	O	0.00
282LYS	N	279ILE	O	38.93
282LYS	N	280ALA	O	0.86
281GLY	N	10GLY	O	0.02
281GLY	N	14GLU	OE1	1.41
281GLY	N	14GLU	OE2	5.30
281GLY	N	279ILE	O	0.25
280ALA	N	276ALA	O	0.05
280ALA	N	277PRO	O	4.59
280ALA	N	278ASP	OD1	0.60
280ALA	N	278ASP	OD2	0.68
280ALA	N	278ASP	O	3.56
279ILE	N	276ALA	O	0.51
279ILE	N	277PRO	O	18.78
279ILE	N	278ASP	OD2	0.00
278ASP	N	276ALA	O	0.73
278ASP	N	278ASP	OD2	0.00
276ALA	N	73GLY	O	0.01
276ALA	N	273HIS	O	0.00
276ALA	N	274GLY	O	1.82
275SER	OG	72VAL	O	0.18
275SER	OG	73GLY	O	0.83
275SER	OG	274GLY	O	0.60
275SER	OG	275SER	O	0.73

57THR	OG1	54PRO	O	2.73
57THR	OG1	58ARG	N	0.00
57THR	N	54PRO	O	15.49
57THR	N	57THR	OG1	0.00
55GLU	N	51GLU	OE1	0.03
55GLU	N	51GLU	OE2	0.04
55GLU	N	55GLU	OE1	0.25
55GLU	N	55GLU	OE2	0.28
55GLU	N	58ARG	NH2	0.00
53PHE	N	51GLU	OE1	0.13
53PHE	N	51GLU	OE2	0.05
53PHE	N	51GLU	O	0.00
51GLU	N	45ALA	O	20.88
51GLU	N	49PHE	O	1.08
50GLY	N	45ALA	O	63.70
50GLY	N	46ILE	O	6.39
50GLY	N	47ASP	O	0.00
49PHE	N	45ALA	O	84.07
49PHE	N	46ILE	O	0.18
49PHE	N	47ASP	O	0.00
48ALA	N	44ALA	O	86.68
48ALA	N	45ALA	O	1.42
48ALA	N	46ILE	O	0.04
47ASP	N	43GLY	O	57.01
47ASP	N	44ALA	O	11.78
47ASP	N	45ALA	O	0.00
46ILE	N	42GLY	O	1.51
46ILE	N	43GLY	O	18.55
46ILE	N	44ALA	O	0.00
45ALA	N	42GLY	O	0.86
45ALA	N	43GLY	O	0.01
44ALA	N	9ASP	OD1	0.37
44ALA	N	9ASP	OD2	0.25
44ALA	N	42GLY	O	0.01
43GLY	N	9ASP	OD1	1.09
43GLY	N	9ASP	OD2	2.36
42GLY	N	52PRO	O	2.62
41PHE	N	39PHE	O	0.12
39PHE	N	5VAL	O	94.87
39PHE	N	37GLU	O	0.12
38VAL	N	36TYR	O	0.01
38VAL	N	37GLU	OE1	4.59
38VAL	N	37GLU	OE2	5.10

290ALA	N	287PRO	O	53.29
288THR	OG1	286ASN	OD1	95.95
288THR	N	286ASN	OD1	68.87
288THR	N	286ASN	O	0.02
286ASN	ND2	288THR	OG1	0.38
286ASN	ND2	322THR	O101	0.08
286ASN	ND2	331ALA	O102	0.00
286ASN	N	284ILE	O	0.01
285ALA	N	283GLY	O	2.10
285ALA	N	331ALA	O102	0.00
285ALA	N	333THR	OG1	3.10
284ILE	N	279ILE	O	0.08
284ILE	N	282LYS	O	0.22
283GLY	N	279ILE	O	3.06
282LYS	NZ	278ASP	OD1	30.39
282LYS	NZ	278ASP	OD2	33.40
282LYS	NZ	278ASP	O	10.31
282LYS	NZ	280ALA	O	0.03
282LYS	N	278ASP	O	0.33
282LYS	N	279ILE	O	42.64
282LYS	N	280ALA	O	1.37
281GLY	N	10GLY	O	0.01
281GLY	N	14GLU	OE2	0.00
281GLY	N	277PRO	O	0.02
281GLY	N	278ASP	O	0.79
281GLY	N	279ILE	O	0.45
280ALA	N	276ALA	O	31.96
280ALA	N	277PRO	O	18.87
280ALA	N	278ASP	O	0.06
279ILE	N	276ALA	O	29.28
279ILE	N	277PRO	O	0.03
278ASP	N	276ALA	O	0.10
278ASP	N	278ASP	OD1	0.02
276ALA	N	274GLY	O	0.70
275SER	OG	9ASP	O	5.37
275SER	OG	71SER	O	0.02
275SER	OG	72VAL	O	0.21
275SER	OG	73GLY	O	87.29
275SER	OG	274GLY	O	0.01
275SER	OG	275SER	O	0.00
275SER	OG	276ALA	N	0.00
275SER	OG	276ALA	O	0.03
275SER	N	72VAL	O	0.19

59LYS	NZ	63GLU	OE2	0.04
59LYS	N	55GLU	O	48.91
59LYS	N	56PRO	O	15.74
59LYS	N	57THR	O	0.01
58ARG	NH2	51GLU	OE1	13.03
58ARG	NH2	51GLU	OE2	11.59
58ARG	NH2	53PHE	O	0.58
58ARG	NH2	55GLU	OE1	33.09
58ARG	NH2	55GLU	OE2	45.21
58ARG	NH2	92SER	OG	0.93
58ARG	NH2	96SER	OG	0.00
58ARG	NH1	51GLU	OE1	29.73
58ARG	NH1	51GLU	OE2	29.76
58ARG	NH1	53PHE	O	12.76
58ARG	NH1	55GLU	OE1	4.34
58ARG	NH1	55GLU	OE2	6.47
58ARG	NH1	92SER	OG	0.31
58ARG	NH1	96SER	OG	0.27
58ARG	NE	51GLU	OE1	0.02
58ARG	NE	51GLU	OE2	0.04
58ARG	NE	53PHE	O	7.10
58ARG	NE	55GLU	OE1	5.50
58ARG	NE	55GLU	OE2	5.98
58ARG	NE	58ARG	O	0.00
58ARG	N	54PRO	O	86.64
58ARG	N	55GLU	O	4.14
57THR	OG1	40PRO	O	97.00
57THR	OG1	54PRO	O	0.00
57THR	N	54PRO	O	13.39
55GLU	N	51GLU	OE1	0.02
55GLU	N	51GLU	OE2	0.02
55GLU	N	55GLU	OE1	0.05
55GLU	N	55GLU	OE2	0.00
53PHE	N	51GLU	OE1	0.44
53PHE	N	51GLU	OE2	0.30
53PHE	N	51GLU	O	0.04
51GLU	N	45ALA	O	4.98
51GLU	N	46ILE	O	0.06
51GLU	N	49PHE	O	1.40
51GLU	N	51GLU	OE1	0.04
51GLU	N	51GLU	OE2	0.02
50GLY	N	45ALA	O	63.60
50GLY	N	46ILE	O	6.40

275SER	OG	276ALA	N	0.00
275SER	OG	276ALA	O	0.02
275SER	N	273HIS	O	5.48
273HIS	ND1	271PRO	O	0.03
273HIS	ND1	272VAL	O	0.04
273HIS	ND1	273HIS	O	0.06
273HIS	ND1	274GLY	O	0.03
273HIS	ND1	275SER	O	0.01
273HIS	ND1	286ASN	O	0.00
273HIS	N	271PRO	O	0.26
273HIS	N	273HIS	ND1	0.01
272VAL	N	257LEU	O	37.17
270GLU	N	259SER	O	97.73
270GLU	N	293SER	OG	0.04
269PHE	N	67VAL	O	96.54
268VAL	N	261SER	O	97.16
266THR	OG1	61VAL	O	83.76
266THR	OG1	64ALA	O	0.52
266THR	OG1	97GLN	OE1	6.41
266THR	N	97GLN	OE1	91.84
265GLY	N	97GLN	O	68.25
265GLY	N	98ASP	O	0.03
265GLY	N	263GLY	O	0.01
264ARG	NH2	62GLU	OE1	5.06
264ARG	NH2	62GLU	OE2	0.65
264ARG	NH2	96SER	O	26.19
264ARG	NH2	98ASP	OD1	0.55
264ARG	NH2	98ASP	OD2	5.58
264ARG	NH1	62GLU	OE1	0.38
264ARG	NH1	62GLU	OE2	5.02
264ARG	NH1	96SER	O	2.32
264ARG	NH1	98ASP	OD1	0.73
264ARG	NH1	98ASP	OD2	8.90
264ARG	NH1	264ARG	O	0.28
264ARG	NE	97GLN	O	0.01
264ARG	NE	98ASP	OD1	2.28
264ARG	NE	98ASP	OD2	17.87
264ARG	NE	264ARG	O	0.06
264ARG	N	97GLN	O	8.27
264ARG	N	98ASP	O	79.76
262LEU	N	101ALA	O	97.62
261SER	OG	101ALA	O	1.58
261SER	OG	102ASN	OD1	0.01

37GLU	N	3VAL	O	55.96
37GLU	N	35ALA	O	0.09
36TYR	OH	23LEU	O	1.02
36TYR	OH	27ASP	OD1	4.08
36TYR	OH	27ASP	OD2	3.55
36TYR	OH	34LEU	O	0.01
36TYR	N	34LEU	O	0.09
35ALA	N	1MET	O	66.95
35ALA	N	33GLY	O	0.50
34LEU	N	27ASP	OD1	10.15
34LEU	N	27ASP	OD2	41.50
34LEU	N	32LEU	O	0.48
33GLY	N	27ASP	OD1	11.17
33GLY	N	27ASP	OD2	58.82
32LEU	N	26LEU	O	0.05
32LEU	N	27ASP	OD1	7.96
32LEU	N	27ASP	OD2	42.26
32LEU	N	27ASP	O	0.85
32LEU	N	30GLU	O	0.90
31GLY	N	26LEU	O	78.36
31GLY	N	27ASP	O	1.02
31GLY	N	28GLU	O	0.02
31GLY	N	30GLU	OE1	2.32
31GLY	N	30GLU	OE2	2.80
30GLU	N	25ALA	O	0.24
30GLU	N	26LEU	O	60.63
30GLU	N	27ASP	O	0.66
30GLU	N	28GLU	O	0.08
30GLU	N	30GLU	OE2	0.00
29ALA	N	25ALA	O	78.12
29ALA	N	26LEU	O	5.65
29ALA	N	27ASP	O	0.29
29ALA	N	30GLU	O	0.02
28GLU	N	24ARG	O	45.87
28GLU	N	25ALA	O	17.01
28GLU	N	26LEU	O	0.09
28GLU	N	30GLU	O	0.04
27ASP	N	23LEU	O	77.79
27ASP	N	24ARG	O	3.04
27ASP	N	25ALA	O	0.22
26LEU	N	22VAL	O	81.28
26LEU	N	23LEU	O	3.23
26LEU	N	24ARG	O	0.00

273HIS	ND1	255GLY	O	5.43
273HIS	ND1	271PRO	O	0.02
273HIS	ND1	273HIS	O	0.81
273HIS	ND1	274GLY	O	3.64
273HIS	ND1	286ASN	O	2.66
273HIS	N	255GLY	O	0.09
273HIS	N	271PRO	O	6.83
273HIS	N	273HIS	ND1	0.01
272VAL	N	257LEU	O	91.14
270GLU	N	259SER	O	91.20
270GLU	N	268VAL	O	0.01
269PHE	N	67VAL	O	97.48
268VAL	N	261SER	O	96.02
266THR	OG1	61VAL	O	31.23
266THR	OG1	62GLU	OE1	28.47
266THR	OG1	62GLU	OE2	32.47
266THR	OG1	64ALA	O	0.13
266THR	OG1	97GLN	OE1	7.14
266THR	OG1	265GLY	O	0.05
266THR	N	62GLU	OE1	25.24
266THR	N	62GLU	OE2	28.60
266THR	N	97GLN	OE1	37.62
266THR	N	97GLN	O	0.59
265GLY	N	97GLN	O	26.45
265GLY	N	98ASP	O	1.05
265GLY	N	263GLY	O	2.66
264ARG	NH2	62GLU	OE1	0.00
264ARG	NH2	62GLU	OE2	0.61
264ARG	NH2	96SER	O	1.28
264ARG	NH2	97GLN	OE1	0.03
264ARG	NH2	98ASP	OD1	32.17
264ARG	NH2	98ASP	OD2	34.57
264ARG	NH2	98ASP	O	0.00
264ARG	NH2	99LEU	O	0.25
264ARG	NH2	161GLU	OE1	0.14
264ARG	NH2	161GLU	OE2	0.94
264ARG	NH1	62GLU	OE2	0.02
264ARG	NH1	96SER	O	1.18
264ARG	NH1	98ASP	OD1	7.60
264ARG	NH1	98ASP	OD2	0.36
264ARG	NH1	161GLU	OE1	17.90
264ARG	NH1	161GLU	OE2	3.69
264ARG	NE	98ASP	OD1	51.64

50GLY	N	47ASP	O	0.02
49PHE	N	44ALA	O	0.05
49PHE	N	45ALA	O	79.46
49PHE	N	46ILE	O	0.28
49PHE	N	47ASP	O	0.03
48ALA	N	44ALA	O	81.20
48ALA	N	45ALA	O	3.08
48ALA	N	46ILE	O	0.42
47ASP	N	43GLY	O	36.07
47ASP	N	44ALA	O	17.19
47ASP	N	45ALA	O	0.03
46ILE	N	42GLY	O	6.75
46ILE	N	43GLY	O	30.67
45ALA	N	41PHE	O	0.01
45ALA	N	42GLY	O	5.22
45ALA	N	43GLY	O	0.04
44ALA	N	9ASP	OD1	0.20
44ALA	N	9ASP	OD2	1.10
44ALA	N	41PHE	O	0.00
43GLY	N	8GLY	O	0.90
43GLY	N	9ASP	OD1	1.13
43GLY	N	9ASP	OD2	2.40
43GLY	N	41PHE	O	0.16
42GLY	N	52PRO	O	47.28
39PHE	N	5VAL	O	92.92
39PHE	N	37GLU	O	0.13
38VAL	N	37GLU	OE1	5.22
38VAL	N	37GLU	OE2	5.10
37GLU	N	3VAL	O	56.22
37GLU	N	35ALA	O	0.04
36TYR	OH	17GLU	OE1	0.08
36TYR	OH	17GLU	OE2	0.12
36TYR	OH	23LEU	O	0.23
36TYR	OH	27ASP	OD1	0.21
36TYR	OH	27ASP	OD2	2.60
36TYR	N	34LEU	O	0.05
35ALA	N	1MET	O	62.51
35ALA	N	33GLY	O	0.10
34LEU	N	27ASP	OD1	37.08
34LEU	N	27ASP	OD2	29.03
34LEU	N	32LEU	O	0.05
33GLY	N	27ASP	OD1	45.66
33GLY	N	27ASP	OD2	37.90

261SER	OG	260ALA	O	0.49
261SER	OG	268VAL	O	0.05
261SER	N	268VAL	O	94.83
260ALA	N	102ASN	OD1	0.00
260ALA	N	103LEU	O	87.50
259SER	OG	259SER	O	0.04
259SER	OG	270GLU	O	90.02
259SER	OG	271PRO	O	0.00
259SER	N	270GLU	O	9.54
259SER	N	293SER	OG	2.71
257LEU	N	254LEU	O	10.35
257LEU	N	255GLY	O	0.18
257LEU	N	272VAL	O	0.06
256LEU	N	253SER	O	18.36
256LEU	N	254LEU	O	4.60
255GLY	N	253SER	O	0.09
254LEU	N	326ASP	OD1	0.00
253SER	OG	251PRO	O	0.00
253SER	OG	252GLY	O	0.70
253SER	OG	253SER	O	0.02
253SER	OG	326ASP	OD1	15.89
253SER	OG	326ASP	OD2	49.52
253SER	OG	253SER	O	0.03
253SER	N	248SER	O	1.29
253SER	N	251PRO	O	0.06
252GLY	N	248SER	O	65.30
252GLY	N	249VAL	O	2.44
250LEU	N	246LEU	O	0.00
250LEU	N	247ALA	O	52.20
249VAL	N	245ASP	O	0.06
249VAL	N	246LEU	O	70.19
248SER	OG	244SER	O	69.89
248SER	OG	245ASP	O	18.07
248SER	OG	247ALA	O	0.00
248SER	OG	248SER	O	0.04
248SER	OG	253SER	O	0.27
248SER	N	244SER	O	2.94
248SER	N	245ASP	O	54.31
248SER	N	246LEU	O	0.16
247ALA	N	243LEU	O	85.00
247ALA	N	244SER	O	1.02
246LEU	N	242ILE	O	91.60
246LEU	N	243LEU	O	0.72

25ALA	N	21LYS	O	43.49
25ALA	N	22VAL	O	11.61
24ARG	NH2	17GLU	OE1	35.53
24ARG	NH2	17GLU	OE2	44.37
24ARG	NH2	17GLU	O	0.00
24ARG	NH2	36TYR	OH	16.71
24ARG	NH1	17GLU	OE1	48.46
24ARG	NH1	17GLU	OE2	36.19
24ARG	NH1	17GLU	O	0.00
24ARG	NH1	36TYR	OH	12.10
24ARG	NE	20LEU	O	0.06
24ARG	NE	36TYR	OH	37.07
24ARG	N	20LEU	O	96.56
24ARG	N	21LYS	O	0.35
23LEU	N	19ALA	O	56.17
23LEU	N	20LEU	O	4.42
22VAL	N	18ALA	O	8.36
22VAL	N	19ALA	O	29.57
22VAL	N	20LEU	O	0.00
21LYS	NZ	17GLU	OE1	5.21
21LYS	NZ	17GLU	OE2	3.55
21LYS	NZ	17GLU	O	0.04
21LYS	NZ	333THR	O	7.20
21LYS	NZ	334GLU	OE1	24.81
21LYS	NZ	334GLU	OE2	34.65
21LYS	NZ	334GLU	O	0.03
21LYS	NZ	337THR	OG1	11.80
21LYS	N	17GLU	O	84.10
21LYS	N	18ALA	O	1.47
20LEU	N	16THR	O	87.67
20LEU	N	17GLU	O	1.75
19ALA	N	15VAL	O	45.37
19ALA	N	16THR	O	21.67
18ALA	N	14GLU	O	53.89
18ALA	N	15VAL	O	5.59
18ALA	N	16THR	O	0.01
17GLU	N	13PRO	O	76.18
17GLU	N	14GLU	O	2.90
16THR	OG1	6LEU	O	0.02
16THR	OG1	7PRO	O	3.89
16THR	OG1	12GLY	O	85.64
16THR	OG1	16THR	O	0.03
16THR	N	12GLY	O	74.87

264ARG	NE	98ASP	OD2	31.06
264ARG	NE	98ASP	O	0.42
264ARG	NE	99LEU	O	0.01
264ARG	NE	264ARG	O	0.03
264ARG	N	97GLN	O	9.52
264ARG	N	98ASP	OD2	0.15
264ARG	N	98ASP	O	79.58
262LEU	N	101ALA	O	97.57
261SER	OG	101ALA	O	0.09
261SER	OG	102ASN	OD1	0.06
261SER	OG	260ALA	O	0.55
261SER	OG	261SER	O	0.14
261SER	OG	268VAL	O	5.52
261SER	OG	270GLU	OE1	0.01
261SER	OG	270GLU	OE2	0.01
261SER	N	268VAL	O	93.86
260ALA	N	102ASN	OD1	0.01
260ALA	N	103LEU	O	94.86
259SER	OG	102ASN	OD1	0.01
259SER	OG	103LEU	O	2.91
259SER	OG	258PRO	O	0.02
259SER	OG	259SER	O	0.05
259SER	OG	260ALA	O	0.02
259SER	OG	270GLU	OE2	0.01
259SER	OG	270GLU	O	85.25
259SER	N	270GLU	O	5.01
259SER	N	293SER	OG	2.77
257LEU	N	253SER	O	0.88
257LEU	N	254LEU	O	7.20
257LEU	N	255GLY	O	2.28
257LEU	N	272VAL	O	2.52
256LEU	N	253SER	O	17.50
256LEU	N	254LEU	O	1.55
255GLY	N	253SER	O	0.16
255GLY	N	326ASP	OD1	0.01
254LEU	N	248SER	O	0.05
254LEU	N	252GLY	O	9.10
254LEU	N	326ASP	OD1	0.06
253SER	OG	107LYS	O	8.62
253SER	OG	251PRO	O	0.11
253SER	OG	252GLY	O	0.20
253SER	OG	253SER	O	0.08
253SER	OG	326ASP	OD1	50.63

32LEU	N	26LEU	O	0.06
32LEU	N	27ASP	OD1	32.68
32LEU	N	27ASP	OD2	26.97
32LEU	N	27ASP	O	0.08
32LEU	N	30GLU	OE1	0.00
32LEU	N	30GLU	O	0.33
31GLY	N	26LEU	O	87.56
31GLY	N	27ASP	O	0.12
31GLY	N	30GLU	OE1	0.83
31GLY	N	30GLU	OE2	0.66
30GLU	N	26LEU	O	60.09
30GLU	N	27ASP	O	0.50
30GLU	N	28GLU	O	0.09
30GLU	N	30GLU	OE2	0.00
29ALA	N	25ALA	O	79.19
29ALA	N	26LEU	O	5.06
29ALA	N	27ASP	O	0.21
28GLU	N	24ARG	O	46.59
28GLU	N	25ALA	O	17.52
28GLU	N	26LEU	O	0.04
27ASP	N	23LEU	O	81.01
27ASP	N	24ARG	O	2.10
26LEU	N	22VAL	O	78.81
26LEU	N	23LEU	O	4.40
25ALA	N	21LYS	O	45.93
25ALA	N	22VAL	O	11.74
24ARG	NH2	17GLU	OE1	34.92
24ARG	NH2	17GLU	OE2	28.44
24ARG	NH2	17GLU	O	0.03
24ARG	NH2	27ASP	OD2	2.92
24ARG	NH2	28GLU	OE1	0.90
24ARG	NH2	28GLU	OE2	1.83
24ARG	NH2	34LEU	O	0.00
24ARG	NH2	36TYR	OH	17.86
24ARG	NH1	17GLU	OE1	25.61
24ARG	NH1	17GLU	OE2	36.63
24ARG	NH1	17GLU	O	0.00
24ARG	NH1	24ARG	O	0.30
24ARG	NH1	28GLU	OE1	1.05
24ARG	NH1	28GLU	OE2	2.38
24ARG	NH1	34LEU	O	0.01
24ARG	NH1	36TYR	OH	13.46
24ARG	NE	20LEU	O	0.40

245ASP	N	241ASP	O	12.69
245ASP	N	242ILE	O	16.69
245ASP	N	245ASP	OD2	0.02
244SER	OG	240GLY	O	74.22
244SER	OG	241ASP	O	0.01
244SER	OG	245ASP	N	0.08
244SER	OG	245ASP	OD2	13.43
244SER	N	240GLY	O	62.86
244SER	N	241ASP	O	4.17
243LEU	N	239PHE	O	91.99
243LEU	N	240GLY	O	0.14
242ILE	N	238ILE	O	9.92
242ILE	N	239PHE	O	27.57
242ILE	N	240GLY	O	0.00
241ASP	N	237ASN	O	91.88
241ASP	N	238ILE	O	0.90
240GLY	N	236GLY	O	18.58
240GLY	N	237ASN	O	8.40
240GLY	N	238ILE	O	0.02
239PHE	N	236GLY	O	4.39
238ILE	N	155GLU	OE2	0.01
237ASN	ND2	133GLU	OE1	2.13
237ASN	ND2	133GLU	O	24.15
237ASN	ND2	134LEU	O	3.01
237ASN	ND2	136GLY	O	57.53
237ASN	N	133GLU	O	0.08
235THR	OG1	183VAL	O	7.17
235THR	OG1	236GLY	N	0.06
235THR	OG1	236GLY	O	2.31
235THR	OG1	239PHE	O	0.53
235THR	OG1	240GLY	N	0.01
235THR	N	131VAL	O	77.16
234VAL	N	181VAL	O	92.78
233VAL	N	129LEU	O	95.78
232VAL	N	179HIS	O	2.08
232VAL	N	231ASP	OD2	3.10
230PHE	N	227PRO	O	80.96
230PHE	N	228ALA	O	0.05
229ARG	NH2	212GLU	OE1	41.49
229ARG	NH2	212GLU	OE2	59.88
229ARG	NH2	229ARG	O	0.00
229ARG	NH1	212GLU	OE1	59.45
229ARG	NH1	212GLU	OE2	41.53

16THR	N	13PRO	O	3.22
15VAL	N	11ILE	O	52.30
15VAL	N	12GLY	O	5.92
14GLU	N	11ILE	O	44.35
14GLU	N	12GLY	O	0.01
14GLU	N	14GLU	OE1	0.00
14GLU	N	14GLU	OE2	0.00
12GLY	N	9ASP	O	0.54
12GLY	N	10GLY	O	0.00
12GLY	N	71SER	OG	2.10
12GLY	N	71SER	O	2.82
11ILE	N	9ASP	O	0.39
11ILE	N	71SER	OG	0.04
11ILE	N	71SER	O	0.04
11ILE	N	73GLY	O	0.28
11ILE	N	274GLY	O	0.02
11ILE	N	275SER	OG	0.02
11ILE	N	275SER	O	1.86
11ILE	N	276ALA	O	0.01
10GLY	N	9ASP	OD1	0.80
10GLY	N	9ASP	OD2	0.84
9ASP	N	71SER	O	77.46
8GLY	N	41PHE	O	25.61
6LEU	N	68LEU	O	98.56
5VAL	N	37GLU	O	93.07
4ALA	N	66ALA	O	93.09
3VAL	N	1MET	O	0.00
3VAL	N	35ALA	O	87.38
2LYS	NZ	36TYR	O	0.02
2LYS	NZ	37GLU	OE1	5.91
2LYS	NZ	37GLU	OE2	3.70
2LYS	NZ	63GLU	OE1	29.35
2LYS	NZ	63GLU	OE2	27.47
2LYS	NZ	63GLU	O	5.10
2LYS	NZ	65GLU	OE1	1.81
2LYS	NZ	65GLU	OE2	1.33
2LYS	N	65GLU	OE1	11.25
2LYS	N	65GLU	OE2	10.63
1MET	N	33GLY	O	78.25
1MET	N	65GLU	OE1	0.36
1MET	N	65GLU	OE2	2.66
345ALA	N104	341LEU	O	2.88
345ALA	N104	342ARG	O	4.66

253SER	OG	326ASP	OD21	12.14
253SER	OG	326ASP	O101	0.01
253SER	N	248SER	O	1.33
253SER	N	251PRO	O	0.62
252GLY	N	248SER	O	45.63
252GLY	N	249VAL	O	15.20
250LEU	N	246LEU	O	10.30
250LEU	N	247ALA	O	61.21
250LEU	N	248SER	O	0.01
249VAL	N	245ASP	O	1.78
249VAL	N	246LEU	O	31.90
249VAL	N	247ALA	O	0.06
248SER	OG	244SER	O	39.94
248SER	OG	245ASP	O	12.99
248SER	OG	248SER	O	0.06
248SER	OG	253SER	O	0.65
248SER	OG	254LEU	O	0.15
248SER	N	244SER	O	8.63
248SER	N	245ASP	O	57.46
248SER	N	246LEU	O	0.32
247ALA	N	243LEU	O	76.39
247ALA	N	244SER	O	2.76
246LEU	N	242ILE	O	92.80
246LEU	N	243LEU	O	0.98
245ASP	N	241ASP	O	12.44
245ASP	N	242ILE	O	26.21
245ASP	N	245ASP	OD2	0.01
244SER	OG	240GLY	O	89.65
244SER	OG	244SER	O	0.04
244SER	OG	245ASP	OD2	0.00
244SER	N	240GLY	O	77.97
244SER	N	241ASP	O	2.08
243LEU	N	239PHE	O	87.44
243LEU	N	240GLY	O	0.09
242ILE	N	238ILE	O	5.10
242ILE	N	239PHE	O	27.29
241ASP	N	237ASN	O	89.18
241ASP	N	238ILE	O	0.52
240GLY	N	236GLY	O	15.14
240GLY	N	237ASN	O	7.99
240GLY	N	238ILE	O	0.03
239PHE	N	236GLY	O	5.05
237ASN	ND2	133GLU	OE1	0.97

24ARG	NE	28GLU	OE1	0.61
24ARG	NE	28GLU	OE2	0.06
24ARG	NE	36TYR	OH	30.18
24ARG	N	20LEU	O	95.76
24ARG	N	21LYS	O	0.36
23LEU	N	19ALA	O	65.04
23LEU	N	20LEU	O	2.80
22VAL	N	18ALA	O	15.06
22VAL	N	19ALA	O	22.73
22VAL	N	20LEU	O	0.00
21LYS	NZ	17GLU	OE1	3.92
21LYS	NZ	17GLU	OE2	5.83
21LYS	NZ	17GLU	O	0.02
21LYS	NZ	333THR	O	0.01
21LYS	NZ	334GLU	OE1	18.86
21LYS	NZ	334GLU	OE2	21.58
21LYS	NZ	334GLU	O	0.01
21LYS	NZ	337THR	OG1	15.07
21LYS	N	17GLU	O	82.04
21LYS	N	18ALA	O	2.57
20LEU	N	16THR	O	82.42
20LEU	N	17GLU	O	2.96
19ALA	N	15VAL	O	48.65
19ALA	N	16THR	O	15.17
18ALA	N	14GLU	O	75.78
18ALA	N	15VAL	O	1.55
17GLU	N	13PRO	O	47.70
17GLU	N	14GLU	O	3.15
16THR	OG1	7PRO	O	0.73
16THR	OG1	12GLY	O	89.49
16THR	OG1	13PRO	O	0.28
16THR	OG1	17GLU	N	0.01
16THR	OG1	69LEU	O	0.00
16THR	N	12GLY	O	83.70
16THR	N	13PRO	O	1.03
15VAL	N	11ILE	O	86.61
15VAL	N	12GLY	O	3.58
14GLU	N	11ILE	O	49.94
14GLU	N	12GLY	O	0.02
14GLU	N	14GLU	OE1	0.01
12GLY	N	9ASP	O	0.43
12GLY	N	10GLY	O	0.00
12GLY	N	71SER	OG	28.10

229ARG	NH1	214GLN	OE1	0.02
229ARG	NE	229ARG	O	38.74
229ARG	NE	230PHE	N	0.00
229ARG	N	226SER	O	28.57
229ARG	N	227PRO	O	0.71
228ALA	N	226SER	O	0.02
226SER	OG	222HIS	ND1	7.65
226SER	OG	222HIS	O	1.01
226SER	OG	225ARG	O	3.45
226SER	OG	226SER	O	3.15
226SER	N	222HIS	ND1	0.01
226SER	N	222HIS	O	96.65
226SER	N	223LEU	O	0.26
225ARG	NH2	252GLY	O	30.21
225ARG	NH2	326ASP	OD1	10.53
225ARG	NH2	326ASP	OD2	9.14
225ARG	NH2	326ASP	O	1.15
225ARG	NH2	221MET	O	0.00
225ARG	NH2	222HIS	ND1	0.41
225ARG	NH1	252GLY	O	0.88
225ARG	NH1	326ASP	OD1	7.95
225ARG	NH1	326ASP	OD2	7.97
225ARG	NH1	326ASP	O	0.02
225ARG	NH1	221MET	O	0.56
225ARG	NH1	222HIS	ND1	8.11
225ARG	NE	252GLY	O	0.21
225ARG	NE	222HIS	ND1	0.03
225ARG	NE	226SER	OG	0.00
225ARG	N	221MET	O	1.09
225ARG	N	222HIS	O	70.30
225ARG	N	223LEU	O	3.06
224VAL	N	220ALA	O	3.18
224VAL	N	221MET	O	21.76
224VAL	N	222HIS	O	0.01
223LEU	N	219MET	O	66.06
223LEU	N	220ALA	O	9.05
222HIS	NE2	214GLN	OE1	0.32
222HIS	NE2	226SER	OG	0.05
222HIS	NE2	226SER	O	0.05
222HIS	N	218ALA	O	43.56
222HIS	N	219MET	O	18.78
222HIS	N	220ALA	O	0.00
221MET	N	217ASP	O	47.31

345ALA	N104	343HIS	O	16.62
345ALA	N104	345ALA	O1	0.00
345ALA	N104	345ALA	O2	0.00
344LEU	N104	340VAL	O	34.53
344LEU	N104	341LEU	O	23.68
344LEU	N104	342ARG	O	0.03
343HIS	NE21	310LYS	O	0.01
343HIS	NE21	313ASP	OD1	46.71
343HIS	NE21	313ASP	OD2	24.36
343HIS	NE21	317LYS	O	0.08
343HIS	NE21	321GLU	OE1	1.85
343HIS	NE21	321GLU	OE2	0.47
343HIS	N104	339THR	O	12.22
343HIS	N104	340VAL	O	58.31
343HIS	N104	341LEU	O	0.02
342ARG	NH21	317LYS	O	0.04
342ARG	NH21	321GLU	OE1	36.77
342ARG	NH21	321GLU	OE2	35.64
342ARG	NH21	338ALA	O	0.01
342ARG	NH21	343HIS	ND1	0.00
342ARG	NH21	343HIS	NE2	0.00
342ARG	NH21	345ALA	O1	0.06
342ARG	NH21	345ALA	O2	0.20
342ARG	NH11	321GLU	OE1	25.63
342ARG	NH11	321GLU	OE2	24.20
342ARG	NH11	338ALA	O	0.09
342ARG	NH11	339THR	O	0.01
342ARG	NH11	342ARG	O	0.06
342ARG	NH11	343HIS	ND1	0.00
342ARG	NH11	345ALA	O1	0.02
342ARG	NH11	345ALA	O2	0.00
342ARG	NE10	321GLU	OE2	0.00
342ARG	NE10	338ALA	O	0.15
342ARG	NE10	339THR	O	0.25
342ARG	NE10	342ARG	O	0.09
342ARG	NE10	343HIS	NE2	0.00
342ARG	NE10	345ALA	O2	0.00
342ARG	N104	338ALA	O	30.32
342ARG	N104	339THR	O	14.07
342ARG	N104	340VAL	O	1.29
341LEU	N103	337THR	O	97.45
341LEU	N103	338ALA	O	0.27
340VAL	N103	336PHE	O	21.66

237ASN	ND2	133GLU	OE2	2.09	12GLY	N	71SER	O	0.01	221MET	N	218ALA	O	5.34	340VAL	N103	337THR	O	22.87
237ASN	ND2	133GLU	O	0.23	12GLY	N	275SER	OG	0.00	220ALA	N	216VAL	O	90.38	340VAL	N103	338ALA	O	0.00
237ASN	ND2	134LEU	O	16.70	11ILE	N	9ASP	O	0.01	220ALA	N	217ASP	O	0.91	339THR	OG11	314ALA	O	0.00
237ASN	ND2	136GLY	O	90.14	11ILE	N	71SER	OG	1.53	219MET	N	215TYR	O	87.35	339THR	OG11	318ALA	O	0.00
237ASN	ND2	137GLY	O	0.01	11ILE	N	275SER	OG	7.97	219MET	N	216VAL	O	1.88	339THR	OG11	321GLU	OE1	0.04
237ASN	ND2	237ASN	O	0.06	11ILE	N	275SER	O	8.02	218ALA	N	215TYR	O	23.14	339THR	OG11	321GLU	OE2	0.11
237ASN	N	133GLU	O	0.00	10GLY	N	9ASP	OD1	0.44	218ALA	N	217ASP	OD1	0.02	339THR	OG11	335ALA	O	76.11
237ASN	N	237ASN	ND2	0.01	10GLY	N	9ASP	OD2	0.18	218ALA	N	217ASP	OD2	5.23	339THR	OG11	336PHE	O	0.28
235THR	OG1	183VAL	O	70.94	9ASP	N	71SER	OG	0.01	217ASP	N	217ASP	OD1	40.61	339THR	OG11	342ARG	NH1	0.02
235THR	OG1	236GLY	N	0.02	9ASP	N	71SER	O	88.48	216VAL	N	184ASP	O	98.40	339THR	N103	335ALA	O	64.82
235THR	OG1	236GLY	O	0.43	8GLY	N	41PHE	O	38.93	215TYR	OH	78ASP	OD1	1.78	339THR	N103	336PHE	O	8.79
235THR	OG1	239PHE	O	0.49	6LEU	N	4ALA	O	0.00	215TYR	OH	78ASP	OD2	3.16	339THR	N103	337THR	O	0.00
235THR	OG1	240GLY	N	0.02	6LEU	N	68LEU	O	98.08	215TYR	OH	78ASP	O	0.49	338ALA	N103	334GLU	O	72.45
235THR	N	131VAL	O	54.53	5VAL	N	37GLU	O	95.21	215TYR	OH	186ALA	O	0.01	338ALA	N103	335ALA	O	1.46
234VAL	N	181VAL	O	89.80	4ALA	N	66ALA	O	92.68	215TYR	OH	187ASN	OD1	0.01	337THR	OG11	18ALA	O	0.02
233VAL	N	129LEU	O	96.39	3VAL	N	1MET	O	0.02	215TYR	N	214GLN	OE1	0.30	337THR	OG11	333THR	O	85.12
233VAL	N	231ASP	O	0.00	3VAL	N	35ALA	O	87.75	214GLN	NE2	212GLU	OE1	57.08	337THR	OG11	334GLU	OE1	0.01
232VAL	N	179HIS	O	2.82	2LYS	NZ	35ALA	O	0.01	214GLN	NE2	212GLU	OE2	39.54	337THR	OG11	334GLU	OE2	0.07
232VAL	N	231ASP	OD2	3.38	2LYS	NZ	36TYR	O	0.02	214GLN	NE2	213HIS	O	0.08	337THR	OG11	334GLU	O	0.90
230PHE	N	227PRO	O	64.27	2LYS	NZ	37GLU	OE1	2.70	214GLN	NE2	218ALA	O	0.00	337THR	OG11	338ALA	N	0.02
230PHE	N	228ALA	O	0.04	2LYS	NZ	37GLU	OE2	5.87	214GLN	NE2	222HIS	NE2	0.00	337THR	N103	333THR	O	26.38
229ARG	NH2	212GLU	OE1	97.44	2LYS	NZ	63GLU	OE1	26.53	214GLN	NE2	229ARG	NH1	0.02	337THR	N103	334GLU	O	28.81
229ARG	NH2	212GLU	OE2	1.10	2LYS	NZ	63GLU	OE2	33.58	214GLN	N	182SER	O	93.26	337THR	N103	335ALA	O	10.39
229ARG	NH2	214GLN	OE1	0.26	2LYS	NZ	63GLU	O	8.74	213HIS	NE2	196ARG	O	1.08	336PHE	N103	332GLY	O	82.20
229ARG	NH2	229ARG	O	0.03	2LYS	NZ	65GLU	OE1	2.07	213HIS	NE2	200GLU	OE1	0.99	336PHE	N103	333THR	O	2.60
229ARG	NH1	212GLU	OE1	3.44	2LYS	NZ	65GLU	OE2	2.86	213HIS	NE2	200GLU	OE2	0.14	336PHE	N103	334GLU	O	0.28
229ARG	NH1	212GLU	OE2	97.65	2LYS	N	65GLU	OE1	11.09	212GLU	N	180VAL	O	64.41	335ALA	N103	332GLY	O	20.03
229ARG	NH1	229ARG	O	0.24	2LYS	N	65GLU	OE2	14.49	211LEU	N	209VAL	O	0.00	335ALA	N103	337THR	OG1	0.01
229ARG	NE	222HIS	ND1	0.30	1MET	N	1MET	O	0.00	210ALA	N	178LYS	O	95.32	334GLU	N102	283GLY	O	0.05
229ARG	NE	229ARG	O	38.96	1MET	N	32LEU	O	0.00	209VAL	N	206TYR	O	59.98	334GLU	N102	332GLY	O102	0.19
229ARG	N	226SER	O	29.60	1MET	N	33GLY	O	85.09	209VAL	N	207PRO	O	0.56	334GLU	N102	334GLU	OE11	0.94
229ARG	N	227PRO	O	1.32	1MET	N	65GLU	OE1	4.57	209VAL	N	208ASP	OD1	0.10	334GLU	N102	334GLU	OE21	0.49
228ALA	N	122ILE	O	0.01	1MET	N	65GLU	OE2	1.12	208ASP	N	206TYR	O	0.07	333THR	OG11	14GLU	OE1	0.83
228ALA	N	226SER	O	0.00	345ALA	N	341LEU	O	5.46	208ASP	N	208ASP	OD1	48.61	333THR	OG11	14GLU	OE2	1.07
226SER	OG	122ILE	O	0.22	345ALA	N	342ARG	O	3.53	206TYR	OH	167ARG	O	0.39	333THR	OG11	283GLY	O	11.78
226SER	OG	222HIS	ND1	0.58	345ALA	N	343HIS	O	22.34	206TYR	OH	171GLU	OE1	56.00	333THR	OG11	285ALA	O	1.04
226SER	OG	222HIS	O	1.15	344LEU	N	340VAL	O	32.53	206TYR	OH	171GLU	OE2	32.23	333THR	OG11	334GLU	N102	0.08
226SER	OG	225ARG	O	7.99	344LEU	N	341LEU	O	28.26	206TYR	N	202VAL	O	12.22	333THR	OG11	334GLU	OE11	1.28
226SER	OG	226SER	O	2.81	344LEU	N	342ARG	O	0.04	206TYR	N	203GLY	O	57.33	333THR	OG11	334GLU	OE21	0.76
226SER	N	222HIS	O	96.18	343HIS	NE2	310LYS	O	0.00	206TYR	N	204ARG	O	0.02	333THR	N102	283GLY	O	30.77
226SER	N	223LEU	O	0.51	343HIS	NE2	313ASP	OD1	43.24	205GLY	N	201GLU	O	0.39	333THR	N102	285ALA	O	2.05
225ARG	NH2	115LEU	O	0.25	343HIS	NE2	313ASP	OD2	29.83	205GLY	N	202VAL	O	69.18	333THR	N102	333THR	OG11	0.00
225ARG	NH2	248SER	O	0.02	343HIS	NE2	317LYS	O	0.01	205GLY	N	203GLY	O	0.07	332GLY	N102	283GLY	O	0.08

225ARG	NH2	252GLY	O	0.19
225ARG	NH2	326ASP	OD1	1.38
225ARG	NH2	326ASP	OD2	1.64
225ARG	NH2	218ALA	O	0.00
225ARG	NH2	221MET	O	0.09
225ARG	NH2	222HIS	ND1	13.48
225ARG	NH1	115LEU	O	3.87
225ARG	NH1	252GLY	O	0.05
225ARG	NH1	326ASP	OD1	1.58
225ARG	NH1	326ASP	OD2	0.34
225ARG	NH1	221MET	O	0.30
225ARG	NH1	222HIS	ND1	0.14
225ARG	NE	221MET	O	21.20
225ARG	NE	222HIS	ND1	9.24
225ARG	NE	225ARG	O	2.17
225ARG	NE	226SER	OG	0.00
225ARG	N	221MET	O	7.85
225ARG	N	222HIS	O	62.01
225ARG	N	223LEU	O	1.90
224VAL	N	220ALA	O	6.08
224VAL	N	221MET	O	16.48
223LEU	N	219MET	O	77.46
223LEU	N	220ALA	O	3.62
222HIS	NE2	214GLN	OE1	0.51
222HIS	NE2	226SER	OG	0.04
222HIS	NE2	229ARG	NE	0.07
222HIS	NE2	229ARG	NH1	0.10
222HIS	NE2	229ARG	NH2	0.01
222HIS	N	218ALA	O	43.55
222HIS	N	219MET	O	22.50
222HIS	N	220ALA	O	0.01
221MET	N	217ASP	O	53.76
221MET	N	218ALA	O	4.73
220ALA	N	216VAL	O	97.29
220ALA	N	217ASP	O	0.13
219MET	N	215TYR	O	85.72
219MET	N	216VAL	O	2.60
219MET	N	217ASP	O	0.00
218ALA	N	215TYR	O	29.75
218ALA	N	216VAL	O	0.01
218ALA	N	217ASP	OD1	0.60
218ALA	N	217ASP	OD2	1.32
217ASP	N	217ASP	OD1	12.57

343HIS	NE2	321GLU	OE1	0.10
343HIS	NE2	321GLU	OE2	0.18
343HIS	N	339THR	O	5.32
343HIS	N	340VAL	O	67.74
343HIS	N	341LEU	O	0.06
342ARG	NH2	317LYS	O	0.04
342ARG	NH2	321GLU	OE1	29.97
342ARG	NH2	321GLU	OE2	31.63
342ARG	NH2	338ALA	O	0.02
342ARG	NH2	345ALA	O1	0.02
342ARG	NH2	345ALA	O2	0.05
342ARG	NH1	321GLU	OE1	25.24
342ARG	NH1	321GLU	OE2	19.99
342ARG	NH1	338ALA	O	0.12
342ARG	NH1	342ARG	O	0.03
342ARG	NH1	343HIS	ND1	0.00
342ARG	NE	338ALA	O	0.10
342ARG	NE	339THR	O	0.48
342ARG	NE	342ARG	O	0.08
342ARG	N	338ALA	O	29.82
342ARG	N	339THR	O	14.47
342ARG	N	340VAL	O	0.82
342ARG	N	344LEU	N	0.00
341LEU	N	337THR	O	96.12
341LEU	N	338ALA	O	0.30
340VAL	N	336PHE	O	46.77
340VAL	N	337THR	O	17.59
340VAL	N	338ALA	O	0.00
339THR	OG1	314ALA	O	0.01
339THR	OG1	321GLU	OE1	0.01
339THR	OG1	335ALA	O	60.64
339THR	OG1	336PHE	O	10.17
339THR	OG1	339THR	O	0.00
339THR	OG1	340VAL	N	0.04
339THR	OG1	342ARG	NH1	0.01
339THR	OG1	342ARG	NH2	0.02
339THR	N	335ALA	O	39.57
339THR	N	336PHE	O	23.78
339THR	N	337THR	O	0.01
338ALA	N	334GLU	O	74.36
338ALA	N	335ALA	O	3.06
337THR	OG1	18ALA	O	0.06
337THR	OG1	332GLY	O	0.03

204ARG	NH2	200GLU	OE1	17.97
204ARG	NH2	200GLU	OE2	81.99
204ARG	NH2	201GLU	OE1	37.42
204ARG	NH2	201GLU	OE2	18.91
204ARG	NH1	201GLU	OE1	21.55
204ARG	NH1	201GLU	OE2	32.67
204ARG	NE	200GLU	OE1	80.56
204ARG	NE	200GLU	OE2	19.41
204ARG	NE	200GLU	O	0.05
204ARG	N	200GLU	O	35.88
204ARG	N	201GLU	O	24.72
204ARG	N	202VAL	O	0.00
203GLY	N	199VAL	O	50.91
203GLY	N	200GLU	O	10.52
202VAL	N	198THR	O	78.98
202VAL	N	199VAL	O	3.86
201GLU	N	197LYS	O	65.26
201GLU	N	198THR	O	8.34
200GLU	N	196ARG	O	35.91
200GLU	N	197LYS	O	7.76
199VAL	N	195TRP	O	96.95
199VAL	N	196ARG	O	0.12
198THR	OG1	194PHE	O	92.57
198THR	OG1	195TRP	O	0.01
198THR	N	194PHE	O	73.25
198THR	N	195TRP	O	10.30
197LYS	NZ	148GLU	OE1	35.87
197LYS	NZ	148GLU	OE2	33.84
197LYS	NZ	148GLU	O	0.08
197LYS	NZ	193GLU	OE1	0.65
197LYS	NZ	193GLU	OE2	0.04
197LYS	NZ	201GLU	OE1	16.00
197LYS	NZ	201GLU	OE2	10.32
197LYS	N	193GLU	O	64.22
197LYS	N	194PHE	O	8.36
196ARG	NH2	184ASP	OD1	10.26
196ARG	NH2	184ASP	OD2	87.53
196ARG	NH2	213HIS	O	0.02
196ARG	NH2	214GLN	O	0.01
196ARG	NH2	215TYR	OH	2.58
196ARG	NH1	215TYR	OH	0.20
196ARG	NE	184ASP	OD1	95.07
196ARG	NE	184ASP	OD2	10.54

332GLY	N102	285ALA	O	0.02
332GLY	N102	286ASN	OD1	0.44
332GLY	N102	330SER	O102	0.01
332GLY	N102	335ALA	O103	0.18
331ALA	N102	284ILE	O	0.02
331ALA	N102	286ASN	OD1	0.02
331ALA	N102	323PRO	O101	6.66
331ALA	N102	329GLY	O102	0.23
330SER	OG1(	284ILE	O	0.00
330SER	OG1(	328GLY	O102	0.00
330SER	OG1(	329GLY	O102	0.45
330SER	OG1(	330SER	O102	0.49
330SER	OG1(	331ALA	N102	0.00
330SER	OG1(	331ALA	O102	0.05
330SER	N102	323PRO	O101	1.69
330SER	N102	328GLY	O102	0.00
329GLY	N102	323PRO	O101	0.19
329GLY	N102	324PRO	O101	26.15
329GLY	N102	325PRO	O101	7.91
328GLY	N102	324PRO	O101	12.39
328GLY	N102	325PRO	O101	19.38
328GLY	N102	326ASP	O102	0.51
327LEU	N102	324PRO	O101	58.41
327LEU	N102	325PRO	O101	10.19
326ASP	N101	324PRO	O101	0.36
326ASP	N101	326ASP	OD11	0.02
326ASP	N101	328GLY	O102	0.17
326ASP	N101	329GLY	O102	0.06
322THR	OG1:	318ALA	O100	3.32
322THR	OG1:	321GLU	O101	0.05
322THR	OG1:	322THR	O101	4.70
322THR	OG1:	323PRO	N101	0.01
322THR	OG1:	323PRO	O101	0.44
322THR	OG1:	330SER	O102	0.22
322THR	OG1:	339THR	OG11	0.00
322THR	N101	318ALA	O100	67.11
322THR	N101	319LEU	O101	0.16
321GLU	N101	317LYS	O100	19.46
321GLU	N101	318ALA	O100	42.39
321GLU	N101	319LEU	O101	0.14
320LEU	N101	316ALA	O100	32.39
320LEU	N101	317LYS	O100	17.16
320LEU	N101	318ALA	O100	0.04

217ASP	N	217ASP	OD2	13.87
216VAL	N	184ASP	O	97.91
215TYR	OH	186ALA	O	0.27
215TYR	N	214GLN	OE1	0.53
214GLN	NE2	212GLU	OE1	18.51
214GLN	NE2	212GLU	OE2	61.67
214GLN	NE2	213HIS	O	6.74
214GLN	NE2	215TYR	N	0.00
214GLN	NE2	215TYR	O	0.02
214GLN	NE2	222HIS	ND1	0.12
214GLN	NE2	222HIS	NE2	0.01
214GLN	NE2	229ARG	NE	0.01
214GLN	NE2	229ARG	NH1	0.02
214GLN	NE2	229ARG	NH2	0.26
214GLN	N	182SER	O	78.21
214GLN	N	184ASP	OD2	0.06
213HIS	NE2	200GLU	OE1	41.63
213HIS	NE2	200GLU	OE2	44.39
212GLU	N	180VAL	O	56.49
210ALA	N	178LYS	O	97.06
209VAL	N	206TYR	O	9.50
209VAL	N	207PRO	O	41.71
208ASP	N	206TYR	O	0.08
208ASP	N	208ASP	OD1	0.02
208ASP	N	208ASP	OD2	0.02
206TYR	OH	167ARG	O	0.69
206TYR	OH	170PHE	O	0.89
206TYR	OH	171GLU	OE1	40.81
206TYR	OH	171GLU	OE2	23.64
206TYR	N	202VAL	O	39.90
206TYR	N	203GLY	O	25.83
206TYR	N	204ARG	O	0.03
205GLY	N	201GLU	O	0.60
205GLY	N	202VAL	O	63.75
205GLY	N	203GLY	O	0.20
204ARG	NH2	197LYS	O	0.00
204ARG	NH2	200GLU	OE1	11.29
204ARG	NH2	200GLU	OE2	5.13
204ARG	NH2	201GLU	OE1	1.87
204ARG	NH2	201GLU	OE2	4.95
204ARG	NH2	211LEU	O	0.14
204ARG	NH2	213HIS	NE2	0.01
204ARG	NH1	197LYS	O	0.00

337THR	OG1	333THR	O	59.55
337THR	OG1	334GLU	OE1	0.07
337THR	OG1	334GLU	OE2	0.16
337THR	OG1	334GLU	O	4.83
337THR	OG1	336PHE	O	0.04
337THR	OG1	338ALA	N	0.04
337THR	N	332GLY	O	0.08
337THR	N	333THR	O	31.12
337THR	N	334GLU	O	13.56
337THR	N	335ALA	O	0.40
337THR	N	337THR	OG1	0.00
336PHE	N	332GLY	O	74.87
336PHE	N	333THR	O	5.82
336PHE	N	334GLU	O	0.03
335ALA	N	332GLY	O	8.23
335ALA	N	333THR	O	0.01
335ALA	N	337THR	OG1	0.02
334GLU	N	332GLY	O	0.42
334GLU	N	334GLU	OE1	0.13
333THR	OG1	14GLU	OE1	2.06
333THR	OG1	14GLU	OE2	1.99
333THR	OG1	14GLU	O	0.00
333THR	OG1	281GLY	O	0.02
333THR	OG1	283GLY	O	0.36
333THR	OG1	285ALA	O	7.70
333THR	OG1	334GLU	N	0.05
333THR	OG1	334GLU	OE1	0.09
333THR	OG1	334GLU	OE2	0.09
333THR	N	283GLY	O	4.52
333THR	N	285ALA	O	0.07
332GLY	N	285ALA	O	0.01
332GLY	N	286ASN	OD1	0.03
332GLY	N	330SER	O	0.14
332GLY	N	335ALA	O	0.01
331ALA	N	323PRO	O	6.22
331ALA	N	329GLY	O	0.06
330SER	OG	323PRO	O	13.29
330SER	OG	329GLY	O	0.77
330SER	OG	330SER	O	0.41
330SER	N	323PRO	O	23.64
330SER	N	328GLY	O	0.01
329GLY	N	323PRO	O	0.02
329GLY	N	324PRO	O	14.95

196ARG	N	192GLY	O	96.53
196ARG	N	193GLU	O	0.68
195TRP	NE1	183VAL	O	0.03
195TRP	N	191VAL	O	60.37
195TRP	N	192GLY	O	1.62
194PHE	N	190GLU	O	96.16
194PHE	N	191VAL	O	0.05
193GLU	N	189LEU	O	84.75
193GLU	N	190GLU	O	2.01
192GLY	N	189LEU	O	48.37
192GLY	N	190GLU	O	0.01
191VAL	N	153ASN	OD1	97.87
190GLU	N	153ASN	OD1	10.36
190GLU	N	190GLU	OE1	20.94
190GLU	N	190GLU	OE2	21.50
189LEU	N	185LYS	O	24.04
189LEU	N	186ALA	O	30.13
188VAL	N	185LYS	O	53.84
188VAL	N	186ALA	O	0.05
188VAL	N	187ASN	OD1	0.03
187ASN	ND2	215TYR	OH	0.04
187ASN	ND2	217ASP	OD1	0.03
187ASN	N	185LYS	O	0.10
187ASN	N	187ASN	OD1	0.90
187ASN	N	187ASN	ND2	1.13
186ALA	N	184ASP	OD1	11.17
186ALA	N	184ASP	OD2	42.56
185LYS	NZ	241ASP	OD1	67.62
185LYS	NZ	241ASP	OD2	34.81
185LYS	NZ	187ASN	OD1	0.01
185LYS	NZ	217ASP	OD1	99.68
185LYS	NZ	217ASP	OD2	0.07
185LYS	N	184ASP	OD1	0.14
185LYS	N	184ASP	OD2	0.01
184ASP	N	182SER	O	0.04
184ASP	N	214GLN	O	95.95
183VAL	N	234VAL	O	72.53
182SER	OG	183VAL	O	0.03
182SER	OG	184ASP	OD1	0.09
182SER	OG	184ASP	OD2	0.44
182SER	OG	212GLU	O	0.01
182SER	OG	213HIS	ND1	0.23
182SER	OG	234VAL	O	0.00

319LEU	N100	315VAL	O100	84.30
319LEU	N100	316ALA	O100	3.74
318ALA	N100	314ALA	O100	41.11
318ALA	N100	315VAL	O100	18.27
318ALA	N100	316ALA	O100	0.02
317LYS	NZ1C	110PRO	O	0.14
317LYS	NZ1C	313ASP	OD1	46.53
317LYS	NZ1C	313ASP	OD2	53.54
317LYS	NZ1C	313ASP	O	0.08
317LYS	NZ1C	321GLU	OE	0.51
317LYS	NZ1C	321GLU	OE2	0.38
317LYS	NZ1C	343HIS	ND11	0.63
317LYS	NZ1C	343HIS	NE21	0.01
317LYS	N100	313ASP	O	86.77
317LYS	N100	314ALA	O	2.52
317LYS	N100	315VAL	O	0.00
316ALA	N100	312GLU	O	82.11
316ALA	N100	313ASP	O	1.38
315VAL	N100	311VAL	O	81.82
315VAL	N100	312GLU	O	3.80
314ALA	N100	310LYS	O	78.74
314ALA	N100	311VAL	O	1.97
313ASP	N100	309ARG	O	75.19
313ASP	N100	310LYS	O	2.69
312GLU	N	308ALA	O	84.11
312GLU	N	309ARG	O	0.62
311VAL	N	307LEU	O	73.46
311VAL	N	308ALA	O	5.99
310LYS	NZ	30GLU	OE1	3.90
310LYS	NZ	30GLU	OE2	4.12
310LYS	NZ	306GLU	OE1	1.49
310LYS	NZ	306GLU	OE2	1.16
310LYS	NZ	306GLU	O	0.12
310LYS	NZ	313ASP	OD1	0.01
310LYS	NZ	343HIS	ND1	0.01
310LYS	NZ	343HIS	NE2	0.00
310LYS	NZ	343HIS	O	0.12
310LYS	NZ	344LEU	O	0.56
310LYS	NZ	345ALA	O1	7.89
310LYS	NZ	345ALA	O2	9.80
310LYS	N	306GLU	O	28.44
310LYS	N	307LEU	O	23.26
309ARG	NH2	299GLU	OE1	35.86

204ARG	NH1	201GLU	OE1	36.62
204ARG	NH1	201GLU	OE2	27.00
204ARG	NH1	211LEU	O	1.01
204ARG	NE	200GLU	OE1	9.09
204ARG	NE	200GLU	OE2	11.21
204ARG	NE	200GLU	O	0.02
204ARG	NE	201GLU	OE1	0.01
204ARG	NE	201GLU	OE2	0.18
204ARG	NE	201GLU	O	0.01
204ARG	N	200GLU	O	25.16
204ARG	N	201GLU	O	36.99
204ARG	N	202VAL	O	0.01
203GLY	N	199VAL	O	47.87
203GLY	N	200GLU	O	15.66
202VAL	N	198THR	O	41.01
202VAL	N	199VAL	O	20.38
201GLU	N	197LYS	O	24.96
201GLU	N	198THR	O	27.50
200GLU	N	196ARG	O	37.75
200GLU	N	197LYS	O	4.50
199VAL	N	195TRP	O	98.47
199VAL	N	196ARG	O	0.26
198THR	OG1	194PHE	O	98.01
198THR	OG1	195TRP	O	0.00
198THR	N	194PHE	O	64.38
198THR	N	195TRP	O	17.71
197LYS	NZ	147SER	O	0.18
197LYS	NZ	148GLU	OE1	21.80
197LYS	NZ	148GLU	OE2	25.13
197LYS	NZ	148GLU	O	0.04
197LYS	NZ	193GLU	OE1	3.21
197LYS	NZ	193GLU	OE2	2.86
197LYS	NZ	193GLU	O	0.07
197LYS	NZ	201GLU	OE1	1.52
197LYS	NZ	201GLU	OE2	0.84
197LYS	N	193GLU	O	64.61
197LYS	N	194PHE	O	6.48
196ARG	NH2	184ASP	OD1	93.52
196ARG	NH2	184ASP	OD2	13.52
196ARG	NH2	213HIS	O	0.02
196ARG	NH2	214GLN	O	0.00
196ARG	NH2	215TYR	N	0.02
196ARG	NH2	215TYR	OH	0.02

329GLY	N	325PRO	O	0.44
328GLY	N	324PRO	O	74.39
328GLY	N	325PRO	O	12.50
328GLY	N	326ASP	O	0.01
327LEU	N	324PRO	O	65.79
327LEU	N	325PRO	O	0.01
327LEU	N	326ASP	OD1	0.01
326ASP	N	282LYS	O	0.08
326ASP	N	283GLY	O	0.01
326ASP	N	285ALA	O	0.01
326ASP	N	324PRO	O	0.42
326ASP	N	326ASP	OD1	0.07
326ASP	N	326ASP	OD2	0.04
322THR	OG	318ALA	O	17.93
322THR	OG	321GLU	O	0.08
322THR	OG	322THR	O	13.04
322THR	OG	323PRO	N	0.00
322THR	OG	323PRO	O	0.20
322THR	OG	329GLY	O	0.01
322THR	OG	330SER	OG	0.05
322THR	OG	330SER	O	1.07
322THR	OG	335ALA	O	0.00
322THR	OG	339THR	OG1	0.00
322THR	N	318ALA	O	92.18
322THR	N	319LEU	O	0.06
321GLU	N	317LYS	O	27.11
321GLU	N	318ALA	O	33.40
321GLU	N	319LEU	O	0.18
320LEU	N	316ALA	O	40.00
320LEU	N	317LYS	O	16.55
320LEU	N	318ALA	O	0.01
319LEU	N	315VAL	O	84.58
319LEU	N	316ALA	O	3.29
318ALA	N	314ALA	O	43.13
318ALA	N	315VAL	O	16.70
318ALA	N	316ALA	O	0.00
317LYS	NZ	313ASP	OD1	58.03
317LYS	NZ	313ASP	OD2	48.87
317LYS	NZ	313ASP	O	0.11
317LYS	NZ	343HIS	ND1	0.14
317LYS	NZ	343HIS	NE2	0.02
317LYS	N	313ASP	O	86.03
317LYS	N	314ALA	O	2.62

182SER	N	212GLU	O	98.94
181VAL	N	232VAL	O	87.88
180VAL	N	179HIS	ND1	0.07
180VAL	N	210ALA	O	90.65
179HIS	NE2	212GLU	N	0.00
179HIS	NE2	212GLU	OE1	37.13
179HIS	NE2	212GLU	OE2	52.17
179HIS	NE2	229ARG	NH1	0.07
179HIS	NE2	229ARG	NH2	0.08
179HIS	NE2	229ARG	O	0.16
179HIS	N	231ASP	OD2	0.01
178LYS	NZ	174ARG	O	1.84
178LYS	NZ	176ARG	O	1.42
178LYS	NZ	177ARG	O	0.03
178LYS	NZ	208ASP	OD2	18.85
178LYS	NZ	208ASP	O	0.19
178LYS	N	173ALA	O	26.29
178LYS	N	176ARG	O	0.12
178LYS	N	231ASP	OD2	0.07
177ARG	NH2	125GLY	O	0.03
177ARG	NH2	127ASP	OD2	1.49
177ARG	NH2	127ASP	O	0.97
177ARG	NH2	179HIS	ND1	0.06
177ARG	NH2	228ALA	O	26.93
177ARG	NH2	229ARG	O	0.29
177ARG	NH2	230PHE	O	0.74
177ARG	NH2	231ASP	OD1	27.97
177ARG	NH2	231ASP	OD2	0.34
177ARG	NH1	179HIS	ND1	0.07
177ARG	NH1	228ALA	O	33.25
177ARG	NH1	229ARG	O	10.67
177ARG	NH1	230PHE	N	0.00
177ARG	NH1	230PHE	O	0.48
177ARG	NE	231ASP	OD1	9.24
177ARG	NE	231ASP	OD2	21.14
177ARG	N	177ARG	NE	0.28
177ARG	N	231ASP	OD1	0.02
177ARG	N	231ASP	OD2	63.71
176ARG	NH2	127ASP	OD1	96.26
176ARG	NH2	127ASP	OD2	1.49
176ARG	NH2	127ASP	O	0.06
176ARG	NH1	127ASP	OD1	2.95
176ARG	NH1	127ASP	OD2	2.36

309ARG	NH2	299GLU	OE2	27.49
309ARG	NH2	312GLU	OE1	42.39
309ARG	NH2	312GLU	OE2	53.91
309ARG	NH1	299GLU	OE1	45.33
309ARG	NH1	299GLU	OE2	51.50
309ARG	NH1	305VAL	O	0.19
309ARG	NE	312GLU	OE1	56.03
309ARG	NE	312GLU	OE2	40.87
309ARG	N	305VAL	O	59.87
309ARG	N	306GLU	O	6.70
308ALA	N	304LEU	O	68.71
308ALA	N	305VAL	O	5.43
307LEU	N	304LEU	O	21.55
307LEU	N	306GLU	OE1	0.04
307LEU	N	306GLU	OE2	0.02
306GLU	N	306GLU	OE1	4.86
306GLU	N	306GLU	OE2	4.21
305VAL	N	303GLY	O	43.47
305VAL	N	306GLU	OE1	0.01
305VAL	N	306GLU	OE2	0.02
304LEU	N	298LEU	O	75.87
304LEU	N	302PHE	O	0.00
303GLY	N	298LEU	O	53.54
303GLY	N	299GLU	O	0.93
303GLY	N	300HIS	O	0.03
302PHE	N	297MET	O	68.33
302PHE	N	298LEU	O	1.12
302PHE	N	299GLU	O	0.10
302PHE	N	300HIS	O	0.14
301ALA	N	296MET	O	0.02
301ALA	N	297MET	O	60.91
301ALA	N	298LEU	O	0.00
301ALA	N	299GLU	O	0.02
300HIS	NE2	261SER	O	0.00
300HIS	N	296MET	O	93.43
300HIS	N	297MET	O	1.16
299GLU	N	295ALA	O	84.14
299GLU	N	296MET	O	1.76
298LEU	N	294ALA	O	73.25
298LEU	N	295ALA	O	4.82
297MET	N	293SER	O	69.40
297MET	N	294ALA	O	3.20
296MET	N	292LEU	O	81.69

196ARG	NH1	215TYR	OH	0.75
196ARG	NE	184ASP	OD1	1.41
196ARG	NE	184ASP	OD2	99.18
196ARG	N	192GLY	O	99.54
196ARG	N	193GLU	O	0.00
195TRP	NE1	183VAL	O	0.00
195TRP	N	191VAL	O	77.90
195TRP	N	192GLY	O	0.48
194PHE	N	190GLU	O	94.80
194PHE	N	191VAL	O	0.09
193GLU	N	189LEU	O	80.31
193GLU	N	190GLU	O	3.20
192GLY	N	189LEU	O	45.96
192GLY	N	190GLU	O	0.01
191VAL	N	153ASN	OD1	96.80
190GLU	N	153ASN	OD1	22.76
190GLU	N	190GLU	OE1	1.80
190GLU	N	190GLU	OE2	0.07
189LEU	N	185LYS	O	78.70
189LEU	N	186ALA	O	0.80
188VAL	N	185LYS	O	91.18
188VAL	N	187ASN	OD1	0.59
187ASN	ND2	185LYS	O	0.00
187ASN	ND2	215TYR	OH	0.09
187ASN	ND2	217ASP	OD1	0.03
187ASN	ND2	217ASP	OD2	0.01
187ASN	N	185LYS	O	2.34
187ASN	N	187ASN	OD1	5.27
187ASN	N	187ASN	ND2	0.01
186ALA	N	184ASP	OD1	99.92
185LYS	NZ	241ASP	OD1	17.78
185LYS	NZ	241ASP	OD2	78.77
185LYS	NZ	187ASN	OD1	0.03
185LYS	NZ	217ASP	OD1	46.47
185LYS	NZ	217ASP	OD2	52.33
185LYS	N	184ASP	OD1	0.16
184ASP	N	214GLN	O	98.99
183VAL	N	234VAL	O	89.67
182SER	N	212GLU	O	98.63
181VAL	N	232VAL	O	90.37
180VAL	N	179HIS	ND1	0.06
180VAL	N	210ALA	O	92.80
179HIS	NE2	211LEU	O	0.00

317LYS	N	315VAL	O	0.02
316ALA	N	312GLU	O	76.56
316ALA	N	313ASP	O	1.95
315VAL	N	311VAL	O	84.26
315VAL	N	312GLU	O	2.49
314ALA	N	310LYS	O	82.27
314ALA	N	311VAL	O	1.58
313ASP	N	309ARG	O	73.93
313ASP	N	310LYS	O	4.23
312GLU	N	308ALA	O	72.73
312GLU	N	309ARG	O	2.06
311VAL	N	307LEU	O	64.63
311VAL	N	308ALA	O	8.80
310LYS	NZ	30GLU	OE1	2.44
310LYS	NZ	30GLU	OE2	1.39
310LYS	NZ	306GLU	OE1	0.11
310LYS	NZ	306GLU	OE2	0.42
310LYS	NZ	306GLU	O	0.07
310LYS	NZ	343HIS	ND1	0.12
310LYS	NZ	343HIS	NE2	0.00
310LYS	NZ	343HIS	O	0.31
310LYS	NZ	344LEU	O	0.89
310LYS	NZ	345ALA	N	0.00
310LYS	NZ	345ALA	O1	7.56
310LYS	NZ	345ALA	O2	6.53
310LYS	N	306GLU	O	37.63
310LYS	N	307LEU	O	15.88
310LYS	N	308ALA	O	0.00
309ARG	NH2	175LYS	O	1.29
309ARG	NH2	299GLU	OE1	4.71
309ARG	NH2	299GLU	OE2	3.05
309ARG	NH2	305VAL	O	0.00
309ARG	NH2	306GLU	OE1	31.08
309ARG	NH2	306GLU	OE2	35.58
309ARG	NH2	313ASP	OD1	0.07
309ARG	NH1	175LYS	O	0.02
309ARG	NH1	299GLU	OE1	3.80
309ARG	NH1	299GLU	OE2	4.33
309ARG	NH1	305VAL	O	0.04
309ARG	NH1	306GLU	OE1	10.92
309ARG	NH1	306GLU	OE2	8.80
309ARG	NH1	312GLU	OE1	1.03
309ARG	NH1	312GLU	OE2	3.54

176ARG	NH1	127ASP	O	91.12
176ARG	NH1	231ASP	OD1	96.46
176ARG	NH1	231ASP	OD2	0.38
176ARG	NH1	231ASP	O	0.14
176ARG	N	172LEU	O	1.61
176ARG	N	173ALA	O	82.43
176ARG	N	174ARG	O	0.21
175LYS	NZ	171GLU	OE1	0.41
175LYS	NZ	171GLU	OE2	0.56
175LYS	NZ	171GLU	O	0.49
175LYS	NZ	299GLU	OE1	1.46
175LYS	NZ	299GLU	OE2	1.05
175LYS	NZ	299GLU	O	0.48
175LYS	NZ	300HIS	ND1	1.47
175LYS	N	171GLU	O	3.19
175LYS	N	172LEU	O	60.41
175LYS	N	173ALA	O	0.05
174ARG	NH2	205GLY	O	0.17
174ARG	NH2	208ASP	OD1	99.92
174ARG	NH2	208ASP	OD2	1.34
174ARG	NH1	171GLU	OE1	14.37
174ARG	NH1	171GLU	OE2	22.19
174ARG	NE	208ASP	OD1	4.22
174ARG	NE	208ASP	OD2	98.15
174ARG	N	170PHE	O	33.33
174ARG	N	171GLU	O	26.02
173ALA	N	169ALA	O	68.96
173ALA	N	170PHE	O	3.63
172LEU	N	168VAL	O	90.50
172LEU	N	169ALA	O	1.35
171GLU	N	167ARG	O	68.55
171GLU	N	168VAL	O	6.07
170PHE	N	166ALA	O	71.88
170PHE	N	167ARG	O	4.72
169ALA	N	165VAL	O	43.20
169ALA	N	166ALA	O	15.59
168VAL	N	164ARG	O	33.07
168VAL	N	165VAL	O	6.52
168VAL	N	166ALA	O	0.02
167ARG	NH2	163GLU	OE1	35.31
167ARG	NH2	163GLU	OE2	46.50
167ARG	NH2	171GLU	OE1	0.12
167ARG	NH2	206TYR	OH	0.04

296MET	N	293SER	O	1.53
295ALA	N	291ILE	O	71.67
295ALA	N	292LEU	O	2.88
294ALA	N	290ALA	O	69.25
294ALA	N	291ILE	O	7.06
293SER	OG	259SER	O	0.55
293SER	OG	270GLU	O	0.08
293SER	OG	271PRO	N	0.00
293SER	OG	289ALA	O	95.14
293SER	OG	293SER	O	0.04
293SER	N	289ALA	O	38.46
293SER	N	290ALA	O	9.63
292LEU	N	288THR	O	94.59
292LEU	N	289ALA	O	0.18
291ILE	N	287PRO	O	63.66
291ILE	N	288THR	O	3.56
290ALA	N	273HIS	NE2	0.01
290ALA	N	287PRO	O	66.78
289ALA	N	255GLY	O	51.33
289ALA	N	286ASN	OD1	0.61
288THR	OG1	255GLY	O	12.85
288THR	OG1	256LEU	O	0.01
288THR	OG1	286ASN	OD1	78.41
288THR	OG1	289ALA	N	0.06
288THR	N	255GLY	O	0.00
288THR	N	286ASN	OD1	66.33
288THR	N	286ASN	O	0.36
286ASN	ND2	255GLY	O	1.76
286ASN	ND2	284ILE	O	0.00
286ASN	ND2	288THR	OG1	3.10
286ASN	ND2	322THR	O	0.15
286ASN	ND2	323PRO	O	0.01
286ASN	ND2	325PRO	O	0.81
286ASN	ND2	326ASP	OD1	0.58
286ASN	ND2	326ASP	OD2	0.02
286ASN	ND2	331ALA	O	1.74
286ASN	ND2	332GLY	O	0.02
286ASN	N	284ILE	O	0.00
285ALA	N	279ILE	O	0.02
285ALA	N	283GLY	O	3.70
285ALA	N	331ALA	O	1.39
285ALA	N	333THR	OG1	0.12
284ILE	N	279ILE	O	16.33

179HIS	NE2	212GLU	OE1	62.23
179HIS	NE2	212GLU	OE2	34.13
179HIS	NE2	229ARG	NH1	0.02
179HIS	NE2	229ARG	NH2	0.09
179HIS	NE2	229ARG	O	0.12
179HIS	N	177ARG	O	0.01
179HIS	N	231ASP	OD2	1.15
178LYS	NZ	174ARG	O	0.01
178LYS	NZ	176ARG	O	0.02
178LYS	NZ	208ASP	OD1	83.10
178LYS	NZ	208ASP	OD2	18.15
178LYS	NZ	208ASP	O	0.02
178LYS	N	173ALA	O	19.52
178LYS	N	176ARG	O	0.06
178LYS	N	231ASP	OD2	1.13
177ARG	NH2	125GLY	O	2.84
177ARG	NH2	127ASP	OD2	6.03
177ARG	NH2	127ASP	O	3.08
177ARG	NH2	228ALA	O	19.64
177ARG	NH2	229ARG	O	0.63
177ARG	NH2	230PHE	O	2.38
177ARG	NH2	231ASP	OD1	10.70
177ARG	NH2	231ASP	OD2	0.01
177ARG	NH1	125GLY	O	0.04
177ARG	NH1	127ASP	OD2	13.16
177ARG	NH1	127ASP	O	0.10
177ARG	NH1	179HIS	ND1	0.01
177ARG	NH1	228ALA	O	25.09
177ARG	NH1	229ARG	O	8.06
177ARG	NH1	230PHE	N	0.00
177ARG	NH1	230PHE	O	0.24
177ARG	NH1	231ASP	OD1	12.92
177ARG	NH1	231ASP	OD2	0.03
177ARG	NE	230PHE	O	0.00
177ARG	NE	231ASP	OD1	6.76
177ARG	NE	231ASP	OD2	4.74
177ARG	N	177ARG	NE	0.00
177ARG	N	231ASP	OD1	0.28
177ARG	N	231ASP	OD2	71.54
176ARG	NH2	127ASP	OD1	92.64
176ARG	NH2	127ASP	OD2	1.10
176ARG	NH2	127ASP	O	0.02
176ARG	NH1	127ASP	OD1	3.96

309ARG	NH1	313ASP	OD1	0.17
309ARG	NH1	313ASP	OD2	0.05
309ARG	NE	299GLU	OE1	0.07
309ARG	NE	299GLU	OE2	0.04
309ARG	NE	305VAL	O	0.02
309ARG	NE	306GLU	OE1	19.17
309ARG	NE	306GLU	OE2	16.12
309ARG	NE	309ARG	O	0.00
309ARG	N	305VAL	O	75.63
309ARG	N	306GLU	O	3.62
308ALA	N	304LEU	O	70.98
308ALA	N	305VAL	O	5.00
307LEU	N	304LEU	O	24.19
307LEU	N	305VAL	O	0.01
306GLU	N	306GLU	OE1	1.63
306GLU	N	306GLU	OE2	1.44
305VAL	N	303GLY	O	39.48
305VAL	N	306GLU	OE1	0.02
305VAL	N	306GLU	OE2	0.00
304LEU	N	298LEU	O	73.23
304LEU	N	302PHE	O	0.01
303GLY	N	298LEU	O	58.39
303GLY	N	299GLU	O	1.15
303GLY	N	300HIS	O	0.08
302PHE	N	297MET	O	48.60
302PHE	N	298LEU	O	2.13
302PHE	N	299GLU	O	0.50
302PHE	N	300HIS	O	0.62
301ALA	N	296MET	O	0.03
301ALA	N	297MET	O	65.83
301ALA	N	299GLU	O	0.02
300HIS	NE2	65GLU	O	0.06
300HIS	NE2	261SER	O	0.15
300HIS	N	296MET	O	90.13
300HIS	N	297MET	O	1.82
299GLU	N	295ALA	O	76.55
299GLU	N	296MET	O	3.96
298LEU	N	294ALA	O	61.78
298LEU	N	295ALA	O	9.63
297MET	N	293SER	O	65.16
297MET	N	294ALA	O	3.79
296MET	N	292LEU	O	86.12
296MET	N	293SER	O	1.14

167ARG	NH1	163GLU	OE1	5.99
167ARG	NH1	163GLU	OE2	6.55
167ARG	NH1	163GLU	O	0.01
167ARG	NH1	171GLU	OE2	0.00
167ARG	NH1	206TYR	OH	0.15
167ARG	NE	163GLU	OE1	36.89
167ARG	NE	163GLU	OE2	27.57
167ARG	NE	163GLU	O	0.02
167ARG	NE	171GLU	OE1	0.02
167ARG	NE	206TYR	OH	0.98
167ARG	N	163GLU	O	91.94
167ARG	N	164ARG	O	1.21
166ALA	N	162VAL	O	57.74
166ALA	N	163GLU	O	3.89
165VAL	N	161GLU	O	7.20
165VAL	N	162VAL	O	9.78
165VAL	N	163GLU	O	0.01
164ARG	NH2	98ASP	OD1	6.19
164ARG	NH2	98ASP	OD2	93.95
164ARG	NH2	98ASP	O	0.08
164ARG	NH2	264ARG	NE	0.04
164ARG	NH2	264ARG	NH2	0.01
164ARG	NH1	98ASP	OD1	89.44
164ARG	NH1	98ASP	OD2	9.29
164ARG	NH1	98ASP	O	0.00
164ARG	NH1	161GLU	OE1	0.14
164ARG	NH1	161GLU	OE2	0.38
164ARG	N	160PRO	O	47.08
164ARG	N	161GLU	O	15.35
163GLU	N	159LYS	O	58.93
163GLU	N	160PRO	O	7.44
162VAL	N	158SER	O	62.72
162VAL	N	159LYS	O	9.87
162VAL	N	160PRO	O	0.00
161GLU	N	158SER	O	35.76
161GLU	N	161GLU	OE2	0.12
159LYS	NZ	148GLU	OE1	24.85
159LYS	NZ	148GLU	OE2	27.61
159LYS	NZ	148GLU	O	2.09
159LYS	NZ	163GLU	OE1	1.08
159LYS	NZ	163GLU	OE2	6.55
159LYS	NZ	198THR	OG1	3.43
159LYS	NZ	201GLU	OE1	12.86

284ILE	N	282LYS	O	0.76
283GLY	N	14GLU	OE1	4.56
283GLY	N	14GLU	OE2	26.60
283GLY	N	279ILE	O	15.89
283GLY	N	280ALA	O	0.17
283GLY	N	281GLY	O	0.03
282LYS	NZ	277PRO	O	0.09
282LYS	NZ	278ASP	OD1	31.65
282LYS	NZ	278ASP	OD2	36.39
282LYS	NZ	278ASP	O	15.33
282LYS	NZ	279ILE	O	0.06
282LYS	NZ	280ALA	O	0.60
282LYS	NZ	281GLY	O	0.00
282LYS	NZ	330SER	OG	0.00
282LYS	N	14GLU	OE1	4.24
282LYS	N	14GLU	OE2	25.31
282LYS	N	278ASP	O	9.37
282LYS	N	279ILE	O	26.09
282LYS	N	280ALA	O	0.39
281GLY	N	10GLY	O	0.01
281GLY	N	14GLU	OE1	13.23
281GLY	N	14GLU	OE2	33.30
281GLY	N	277PRO	O	0.24
281GLY	N	278ASP	O	7.57
281GLY	N	279ILE	O	0.32
281GLY	N	284ILE	O	0.10
280ALA	N	10GLY	O	30.04
280ALA	N	276ALA	O	23.10
280ALA	N	277PRO	O	2.11
280ALA	N	278ASP	O	0.56
279ILE	N	10GLY	O	4.99
279ILE	N	276ALA	O	4.17
279ILE	N	277PRO	O	43.65
278ASP	N	276ALA	O	0.42
278ASP	N	278ASP	OD1	0.00
278ASP	N	278ASP	OD2	0.01
278ASP	N	280ALA	O	0.22
276ALA	N	274GLY	O	2.48
276ALA	N	279ILE	O	0.00
275SER	OG	9ASP	O	0.02
275SER	OG	72VAL	O	0.72
275SER	OG	73GLY	O	11.33
275SER	OG	87GLU	OE1	0.66

176ARG	NH1	127ASP	OD2	3.00
176ARG	NH1	127ASP	O	88.95
176ARG	NH1	177ARG	NH1	0.00
176ARG	NH1	177ARG	NH2	0.02
176ARG	NH1	231ASP	OD1	92.68
176ARG	NH1	231ASP	OD2	1.88
176ARG	NH1	231ASP	O	0.06
176ARG	N	172ALA	O	1.84
176ARG	N	173ALA	O	83.99
176ARG	N	174ARG	O	0.20
175LYS	NZ	171GLU	OE1	1.14
175LYS	NZ	171GLU	OE2	0.94
175LYS	NZ	171GLU	O	1.48
175LYS	NZ	299GLU	OE1	23.69
175LYS	NZ	299GLU	OE2	36.19
175LYS	NZ	299GLU	O	7.84
175LYS	NZ	300HIS	ND1	3.59
175LYS	NZ	300HIS	O	0.01
175LYS	N	171GLU	O	5.34
175LYS	N	172ALA	O	50.84
175LYS	N	173ALA	O	0.04
174ARG	NH2	171GLU	OE1	49.71
174ARG	NH2	171GLU	OE2	54.91
174ARG	NH2	206TYR	OH	0.04
174ARG	NH1	206TYR	OH	0.05
174ARG	NH1	207PRO	O	0.00
174ARG	NH1	208ASP	OD1	0.30
174ARG	NH1	208ASP	OD2	0.00
174ARG	NE	171GLU	OE1	31.89
174ARG	NE	171GLU	OE2	36.43
174ARG	NE	171GLU	O	0.01
174ARG	NE	206TYR	OH	0.86
174ARG	N	170PHE	O	54.33
174ARG	N	171GLU	O	11.73
173ALA	N	169ALA	O	83.52
173ALA	N	170PHE	O	2.01
172ALA	N	168VAL	O	58.53
172ALA	N	169ALA	O	6.97
171GLU	N	167ARG	O	82.92
171GLU	N	168VAL	O	1.37
170PHE	N	166ALA	O	91.36
170PHE	N	167ARG	O	1.32
169ALA	N	165VAL	O	26.38

295ALA	N	291ILE	O	61.12
295ALA	N	292LEU	O	5.24
294ALA	N	290ALA	O	66.34
294ALA	N	291ILE	O	11.52
293SER	OG	259SER	O	0.04
293SER	OG	270GLU	O	0.04
293SER	OG	289ALA	O	94.39
293SER	OG	290ALA	O	2.14
293SER	OG	293SER	O	0.02
293SER	OG	294ALA	N	0.00
293SER	N	289ALA	O	30.27
293SER	N	290ALA	O	15.74
292LEU	N	288THR	O	93.25
292LEU	N	289ALA	O	0.18
291ILE	N	287PRO	O	90.78
291ILE	N	288THR	O	1.61
290ALA	N	273HIS	NE2	0.04
290ALA	N	287PRO	O	41.10
289ALA	N	255GLY	O	9.52
289ALA	N	273HIS	NE2	0.03
289ALA	N	286ASN	OD1	0.01
288THR	OG1	255GLY	O	15.58
288THR	OG1	256LEU	O	0.14
288THR	OG1	286ASN	OD1	71.20
288THR	OG1	286ASN	O	0.02
288THR	OG1	287PRO	O	0.12
288THR	OG1	289ALA	N	0.02
288THR	N	286ASN	OD1	64.29
288THR	N	286ASN	O	1.22
288THR	N	288THR	OG1	0.00
286ASN	ND2	254LEU	O	0.03
286ASN	ND2	255GLY	O	0.32
286ASN	ND2	273HIS	NE2	0.14
286ASN	ND2	288THR	OG1	13.27
286ASN	ND2	322THR	O	0.02
286ASN	ND2	326ASP	OD1	0.82
286ASN	ND2	326ASP	OD2	0.07
286ASN	ND2	331ALA	O	0.03
286ASN	ND2	332GLY	O	0.04
286ASN	N	273HIS	NE2	47.77
286ASN	N	283GLY	O	0.26
286ASN	N	284ILE	O	1.88
285ALA	N	279ILE	O	1.50

159LYS	NZ	201GLU	OE2	18.27
159LYS	N	149ALA	O	96.82
158SER	OG	149ALA	O	2.65
158SER	OG	150GLU	OE1	0.55
158SER	OG	150GLU	OE2	0.84
158SER	OG	158SER	O	0.01
158SER	OG	161GLU	OE1	0.73
158SER	OG	161GLU	OE2	7.63
158SER	N	161GLU	OE1	0.63
158SER	N	161GLU	OE2	6.08
157TYR	OH	133GLU	OE1	21.24
157TYR	OH	155GLU	OE2	0.03
157TYR	N	151ALA	O	96.22
156ARG	NH2	150GLU	OE1	18.92
156ARG	NH2	150GLU	OE2	23.46
156ARG	NH2	142GLU	O	9.24
156ARG	NH2	158SER	OG	0.01
156ARG	NH1	150GLU	OE1	25.21
156ARG	NH1	150GLU	OE2	40.97
156ARG	NH1	142GLU	O	6.66
156ARG	NE	150GLU	OE1	19.00
156ARG	NE	150GLU	OE2	12.33
156ARG	NE	142GLU	O	16.82
156ARG	N	137GLY	O	0.01
156ARG	N	154THR	O	0.03
156ARG	N	155GLU	OE1	51.34
155GLU	N	153ASN	O	98.72
154THR	OG1	143PRO	O	78.70
154THR	OG1	153ASN	O	0.23
154THR	N	141GLY	O	0.78
154THR	N	143PRO	O	10.67
153ASN	ND2	188VAL	O	0.00
153ASN	ND2	190GLU	OE1	12.33
153ASN	ND2	190GLU	OE2	5.66
153ASN	ND2	138ILE	O	2.68
153ASN	ND2	141GLY	O	0.04
153ASN	ND2	154THR	N	0.00
153ASN	N	155GLU	O	98.32
152TRP	NE1	143PRO	O	0.03
152TRP	NE1	144ARG	O	0.07
152TRP	N	145GLY	O	99.34
151ALA	N	157TYR	O	94.06
150GLU	N	147SER	O	9.24

275SER	OG	87GLU	OE2	1.10
275SER	OG	274GLY	O	2.54
275SER	OG	275SER	O	1.11
275SER	OG	276ALA	N	0.01
275SER	OG	276ALA	O	0.12
275SER	OG	284ILE	O	0.01
275SER	N	273HIS	O	0.02
274GLY	N	254LEU	O	0.14
274GLY	N	272VAL	O	0.05
273HIS	ND1	254LEU	O	0.64
273HIS	ND1	255GLY	O	0.01
273HIS	ND1	271PRO	O	8.02
273HIS	ND1	272VAL	O	0.03
273HIS	ND1	273HIS	O	0.09
273HIS	ND1	274GLY	N	0.38
273HIS	ND1	274GLY	O	4.89
273HIS	ND1	286ASN	OD1	0.01
273HIS	ND1	286ASN	ND2	0.00
273HIS	ND1	286ASN	O	1.36
273HIS	N	271PRO	O	0.78
273HIS	N	273HIS	ND1	0.01
272VAL	N	257LEU	O	77.77
270GLU	N	259SER	O	96.05
270GLU	N	268VAL	O	0.01
270GLU	N	293SER	OG	0.01
269PHE	N	67VAL	O	96.78
268VAL	N	261SER	O	93.68
266THR	OG1	61VAL	O	38.67
266THR	OG1	62GLU	OE1	0.19
266THR	OG1	62GLU	OE2	0.26
266THR	OG1	62GLU	O	0.01
266THR	OG1	64ALA	O	9.40
266THR	OG1	65GLU	O	3.46
266THR	OG1	97GLN	OE1	6.80
266THR	OG1	265GLY	O	0.03
266THR	OG1	266THR	O	2.75
266THR	OG1	267PRO	N	0.00
266THR	OG1	267PRO	O	3.56
266THR	N	62GLU	OE1	0.14
266THR	N	62GLU	OE2	0.13
266THR	N	97GLN	OE1	52.49
266THR	N	97GLN	O	0.18
266THR	N	263GLY	O	0.10

169ALA	N	166ALA	O	22.81
169ALA	N	167ARG	O	0.00
168VAL	N	164ARG	O	33.66
168VAL	N	165VAL	O	3.16
167ARG	NH2	163GLU	OE1	25.76
167ARG	NH2	163GLU	OE2	20.47
167ARG	NH2	201GLU	OE1	0.00
167ARG	NH2	201GLU	OE2	0.01
167ARG	NH2	201GLU	O	0.00
167ARG	NH2	206TYR	OH	0.11
167ARG	NH1	163GLU	OE1	12.25
167ARG	NH1	163GLU	OE2	12.72
167ARG	NH1	202VAL	O	0.05
167ARG	NH1	205GLY	O	0.01
167ARG	NH1	206TYR	OH	1.82
167ARG	NE	163GLU	OE1	19.32
167ARG	NE	163GLU	OE2	19.58
167ARG	NE	163GLU	O	0.12
167ARG	NE	171GLU	OE1	0.01
167ARG	NE	206TYR	OH	1.71
167ARG	N	163GLU	O	87.18
167ARG	N	164ARG	O	2.42
166ALA	N	162VAL	O	77.72
166ALA	N	163GLU	O	3.11
165VAL	N	161GLU	O	19.57
165VAL	N	162VAL	O	12.53
164ARG	NH2	98ASP	OD1	0.97
164ARG	NH2	98ASP	OD2	19.83
164ARG	NH2	133GLU	OE2	0.11
164ARG	NH2	160PRO	O	0.01
164ARG	NH2	161GLU	OE1	14.86
164ARG	NH2	161GLU	OE2	41.49
164ARG	NH1	98ASP	OD1	20.27
164ARG	NH1	98ASP	OD2	0.09
164ARG	NH1	161GLU	OE1	6.91
164ARG	NH1	161GLU	OE2	9.95
164ARG	NH1	264ARG	NE	0.01
164ARG	NH1	264ARG	NH2	0.01
164ARG	NE	160PRO	O	0.42
164ARG	NE	161GLU	OE1	18.29
164ARG	NE	161GLU	OE2	4.81
164ARG	N	160PRO	O	64.89
164ARG	N	161GLU	O	5.36

285ALA	N	283GLY	O	2.49
285ALA	N	331ALA	O	0.00
285ALA	N	333THR	OG1	0.32
284ILE	N	278ASP	OD1	0.02
284ILE	N	278ASP	OD2	0.00
284ILE	N	278ASP	O	0.01
284ILE	N	279ILE	O	23.96
284ILE	N	281GLY	O	0.00
284ILE	N	282LYS	O	0.68
283GLY	N	14GLU	OE2	0.18
283GLY	N	277PRO	O	0.56
283GLY	N	278ASP	OD2	0.00
283GLY	N	278ASP	O	0.88
283GLY	N	279ILE	O	25.26
283GLY	N	280ALA	O	0.34
283GLY	N	281GLY	O	2.04
282LYS	NZ	78ASP	OD1	1.37
282LYS	NZ	78ASP	OD2	0.79
282LYS	NZ	277PRO	O	0.20
282LYS	NZ	278ASP	OD1	32.29
282LYS	NZ	278ASP	OD2	31.55
282LYS	NZ	278ASP	O	2.90
282LYS	NZ	279ILE	O	0.00
282LYS	NZ	280ALA	O	0.04
282LYS	NZ	281GLY	O	0.02
282LYS	NZ	282LYS	O	0.15
282LYS	NZ	325PRO	O	0.01
282LYS	NZ	326ASP	OD1	20.97
282LYS	NZ	326ASP	O	0.32
282LYS	NZ	330SER	OG	0.01
282LYS	N	14GLU	OE2	0.22
282LYS	N	277PRO	O	12.94
282LYS	N	278ASP	O	16.80
282LYS	N	279ILE	O	18.34
282LYS	N	280ALA	O	0.67
282LYS	N	283GLY	O	0.00
282LYS	N	284ILE	O	0.40
281GLY	N	10GLY	O	0.00
281GLY	N	14GLU	OE1	0.11
281GLY	N	14GLU	OE2	0.69
281GLY	N	276ALA	O	0.00
281GLY	N	277PRO	O	7.76
281GLY	N	278ASP	O	14.20

150GLU	N	148GLU	O	0.02
149ALA	N	147SER	O	0.80
148GLU	N	148GLU	OE1	0.10
148GLU	N	148GLU	OE2	0.09
147SER	OG	146MET	O	0.27
147SER	OG	148GLU	OE2	0.00
147SER	OG	150GLU	OE1	0.02
147SER	OG	150GLU	O	58.02
147SER	N	150GLU	O	58.77
145GLY	N	152TRP	NE1	0.14
145GLY	N	152TRP	O	89.38
144ARG	NH2	188VAL	O	2.55
144ARG	NH2	190GLU	OE1	21.94
144ARG	NH2	190GLU	OE2	16.52
144ARG	NH2	139TYR	O	0.06
144ARG	NH2	140PHE	O	0.00
144ARG	NH2	142GLU	OE1	19.32
144ARG	NH2	142GLU	OE2	13.14
144ARG	NH1	188VAL	O	0.04
144ARG	NH1	190GLU	OE1	1.38
144ARG	NH1	190GLU	OE2	0.74
144ARG	NH1	139TYR	O	4.52
144ARG	NH1	140PHE	O	0.02
144ARG	NH1	142GLU	OE1	5.30
144ARG	NH1	142GLU	OE2	6.40
144ARG	NH1	144ARG	O	0.02
144ARG	NE	190GLU	OE1	8.64
144ARG	NE	190GLU	OE2	21.94
144ARG	NE	139TYR	O	0.00
144ARG	NE	141GLY	O	0.17
144ARG	NE	142GLU	OE1	20.32
144ARG	NE	142GLU	OE2	24.25
144ARG	NE	144ARG	O	0.01
144ARG	NE	153ASN	ND2	0.00
144ARG	N	142GLU	OE1	0.52
144ARG	N	142GLU	OE2	0.42
142GLU	N	140PHE	O	0.09
141GLY	N	137GLY	O	0.61
141GLY	N	138ILE	O	37.00
141GLY	N	139TYR	O	0.02
141GLY	N	144ARG	NH1	0.00
140PHE	N	137GLY	O	96.45
140PHE	N	138ILE	O	0.01

266THR	N	264ARG	O	0.05
265GLY	N	97GLN	OE1	9.36
265GLY	N	97GLN	O	20.68
265GLY	N	98ASP	OD1	0.03
265GLY	N	98ASP	OD2	0.11
265GLY	N	98ASP	O	0.03
265GLY	N	263GLY	O	1.97
264ARG	NH2	96SER	O	0.04
264ARG	NH2	98ASP	OD1	3.94
264ARG	NH2	98ASP	OD2	29.77
264ARG	NH2	99LEU	O	0.00
264ARG	NH2	161GLU	OE1	1.02
264ARG	NH2	161GLU	OE2	1.23
264ARG	NH1	96SER	O	0.00
264ARG	NH1	98ASP	OD1	26.04
264ARG	NH1	98ASP	OD2	23.90
264ARG	NH1	98ASP	O	0.58
264ARG	NH1	99LEU	O	0.72
264ARG	NH1	161GLU	OE1	0.20
264ARG	NH1	161GLU	OE2	0.92
264ARG	NE	97GLN	O	0.02
264ARG	NE	98ASP	OD1	32.49
264ARG	NE	98ASP	OD2	12.53
264ARG	NE	98ASP	O	0.01
264ARG	N	97GLN	OE1	0.02
264ARG	N	97GLN	O	21.02
264ARG	N	98ASP	OD1	0.22
264ARG	N	98ASP	OD2	1.91
264ARG	N	98ASP	O	56.65
263GLY	N	266THR	O	0.52
262LEU	N	101ALA	O	96.43
261SER	OG	101ALA	O	3.87
261SER	OG	102ASN	OD1	0.68
261SER	OG	260ALA	O	0.40
261SER	OG	261SER	O	0.03
261SER	OG	262LEU	N	0.02
261SER	OG	262LEU	O	0.01
261SER	OG	268VAL	O	1.81
261SER	OG	270GLU	OE2	0.01
261SER	N	268VAL	O	92.57
260ALA	N	102ASN	OD1	0.01
260ALA	N	103LEU	O	90.90
259SER	OG	102ASN	OD1	0.05

163GLU	N	159LYS	O	71.33
163GLU	N	160PRO	O	1.49
162VAL	N	158SER	O	46.81
162VAL	N	159LYS	O	19.94
161GLU	N	158SER	O	40.85
159LYS	NZ	148GLU	OE1	29.50
159LYS	NZ	148GLU	OE2	22.31
159LYS	NZ	148GLU	O	0.96
159LYS	NZ	163GLU	OE1	0.79
159LYS	NZ	163GLU	OE2	0.72
159LYS	NZ	198THR	OG1	5.18
159LYS	NZ	201GLU	OE1	12.20
159LYS	NZ	201GLU	OE2	11.32
159LYS	N	149ALA	O	95.89
158SER	OG	149ALA	O	1.38
158SER	OG	150GLU	OE1	0.04
158SER	OG	150GLU	OE2	0.03
158SER	OG	158SER	O	0.01
158SER	OG	161GLU	OE1	0.49
158SER	OG	161GLU	OE2	0.08
158SER	N	161GLU	OE1	1.01
158SER	N	161GLU	OE2	0.97
157TYR	OH	133GLU	OE1	40.23
157TYR	OH	133GLU	OE2	42.16
157TYR	OH	136GLY	O	8.51
157TYR	OH	155GLU	OE1	0.01
157TYR	N	151ALA	O	82.91
156ARG	NH2	150GLU	OE1	44.27
156ARG	NH2	150GLU	OE2	49.12
156ARG	NH2	140PHE	O	0.76
156ARG	NH2	142GLU	OE1	0.50
156ARG	NH2	142GLU	OE2	0.66
156ARG	NH2	142GLU	O	1.27
156ARG	NH1	150GLU	OE1	0.24
156ARG	NH1	150GLU	OE2	2.75
156ARG	NH1	142GLU	OE1	0.78
156ARG	NH1	142GLU	O	69.38
156ARG	NE	150GLU	OE1	47.02
156ARG	NE	150GLU	OE2	41.34
156ARG	NE	140PHE	O	0.05
156ARG	NE	142GLU	O	1.18
156ARG	N	137GLY	O	0.00
156ARG	N	155GLU	OE1	3.28

281GLY	N	279ILE	O	0.51
281GLY	N	283GLY	O	2.68
280ALA	N	276ALA	O	46.07
280ALA	N	277PRO	O	24.29
280ALA	N	278ASP	O	0.42
280ALA	N	283GLY	O	0.02
279ILE	N	276ALA	O	40.82
279ILE	N	277PRO	O	3.78
279ILE	N	278ASP	OD1	0.17
279ILE	N	280ALA	O	0.02
278ASP	N	276ALA	O	0.40
278ASP	N	278ASP	OD1	0.62
278ASP	N	278ASP	OD2	0.55
278ASP	N	280ALA	O	1.70
276ALA	N	274GLY	O	28.39
275SER	OG	217ASP	OD2	0.10
275SER	OG	9ASP	O	9.10
275SER	OG	71SER	O	0.30
275SER	OG	72VAL	O	0.20
275SER	OG	73GLY	O	1.44
275SER	OG	245ASP	OD1	1.46
275SER	OG	245ASP	OD2	0.70
275SER	OG	272VAL	O	0.53
275SER	OG	273HIS	O	0.02
275SER	OG	274GLY	O	0.60
275SER	OG	275SER	O	0.36
275SER	OG	276ALA	N	0.01
275SER	N	72VAL	O	4.04
275SER	N	73GLY	O	0.59
275SER	N	272VAL	O	0.07
275SER	N	273HIS	O	1.90
275SER	N	275SER	OG	0.01
274GLY	N	272VAL	O	0.14
274GLY	N	279ILE	O	15.20
273HIS	ND1	254LEU	O	2.90
273HIS	ND1	255GLY	O	2.04
273HIS	ND1	271PRO	O	0.00
273HIS	ND1	272VAL	O	2.82
273HIS	ND1	273HIS	O	3.23
273HIS	ND1	274GLY	N	0.33
273HIS	ND1	274GLY	O	3.20
273HIS	ND1	279ILE	O	8.22
273HIS	ND1	280ALA	O	0.00

139TYR	OH	237ASN	OD1	1.45
139TYR	OH	237ASN	ND2	0.02
139TYR	OH	237ASN	O	4.13
139TYR	OH	238ILE	O	68.43
139TYR	OH	241ASP	OD1	4.15
139TYR	OH	241ASP	OD2	11.59
139TYR	N	137GLY	O	0.02
138ILE	N	155GLU	OE1	19.06
138ILE	N	155GLU	OE2	64.03
137GLY	N	140PHE	O	0.00
137GLY	N	155GLU	OE1	0.69
137GLY	N	155GLU	OE2	0.00
137GLY	N	156ARG	O	2.44
136GLY	N	133GLU	OE1	52.73
136GLY	N	133GLU	OE2	11.24
136GLY	N	237ASN	ND2	0.01
135THR	OG1	99LEU	O	0.28
135THR	OG1	100PHE	O	0.01
135THR	OG1	133GLU	OE1	3.32
135THR	OG1	133GLU	OE2	83.85
135THR	OG1	136GLY	N	0.01
135THR	OG1	161GLU	OE1	0.06
135THR	OG1	161GLU	OE2	0.07
135THR	N	133GLU	OE1	13.83
135THR	N	133GLU	OE2	92.27
134LEU	N	100PHE	O	10.97
134LEU	N	133GLU	OE2	62.96
133GLU	N	235THR	O	11.98
133GLU	N	236GLY	O	63.26
132ARG	NH2	102ASN	OD1	0.18
132ARG	NH2	133GLU	O	0.65
132ARG	NH2	237ASN	OD1	43.19
132ARG	NH2	237ASN	ND2	0.10
132ARG	NH2	237ASN	O	0.01
132ARG	NH2	241ASP	OD1	1.49
132ARG	NH2	241ASP	OD2	15.30
132ARG	NH1	102ASN	OD1	0.26
132ARG	NH1	240GLY	O	1.04
132ARG	NH1	241ASP	OD1	0.40
132ARG	NH1	241ASP	OD2	0.46
132ARG	NH1	244SER	OG	60.10
132ARG	NH1	245ASP	OD2	0.01
132ARG	NE	102ASN	OD1	5.27

259SER	OG	103LEU	O	2.03
259SER	OG	258PRO	O	2.48
259SER	OG	259SER	O	0.04
259SER	OG	260ALA	N	0.01
259SER	OG	270GLU	O	83.16
259SER	OG	271PRO	O	0.00
259SER	N	270GLU	O	6.48
259SER	N	293SER	OG	1.81
257LEU	N	254LEU	O	1.37
257LEU	N	255GLY	O	0.26
257LEU	N	272VAL	O	1.77
256LEU	N	253SER	O	7.64
256LEU	N	254LEU	O	0.90
255GLY	N	253SER	O	0.81
255GLY	N	272VAL	O	39.97
255GLY	N	326ASP	OD1	0.01
254LEU	N	326ASP	OD1	0.28
254LEU	N	326ASP	OD2	1.32
253SER	OG	252GLY	O	0.05
253SER	OG	253SER	O	0.00
253SER	OG	254LEU	N	0.02
253SER	OG	254LEU	O	0.44
253SER	OG	326ASP	OD1	3.44
253SER	OG	326ASP	OD2	78.68
253SER	OG	326ASP	O	0.05
253SER	N	248SER	O	0.33
253SER	N	251PRO	O	0.14
252GLY	N	248SER	O	55.05
252GLY	N	249VAL	O	10.15
252GLY	N	250LEU	O	0.00
250LEU	N	246LEU	O	1.20
250LEU	N	247ALA	O	41.47
249VAL	N	245ASP	O	1.39
249VAL	N	246LEU	O	53.69
249VAL	N	247ALA	O	0.05
248SER	OG	244SER	O	77.98
248SER	OG	245ASP	OD1	0.01
248SER	OG	245ASP	OD2	0.01
248SER	OG	245ASP	O	0.89
248SER	OG	247ALA	O	0.01
248SER	OG	248SER	O	0.01
248SER	OG	253SER	O	0.05
248SER	OG	254LEU	O	0.01

156ARG	N	155GLU	OE2	1.50
155GLU	N	153ASN	O	96.39
154THR	OG1	143PRO	O	88.60
154THR	OG1	153ASN	O	0.19
154THR	N	138ILE	O	0.00
154THR	N	141GLY	O	12.49
154THR	N	143PRO	O	2.59
153ASN	ND2	188VAL	O	0.01
153ASN	ND2	190GLU	OE1	14.72
153ASN	ND2	190GLU	OE2	4.98
153ASN	ND2	138ILE	O	68.59
153ASN	ND2	141GLY	O	0.84
153ASN	ND2	152TRP	O	0.01
153ASN	ND2	154THR	N	0.08
153ASN	ND2	154THR	O	0.07
153ASN	N	155GLU	O	97.27
152TRP	NE1	144ARG	O	0.05
152TRP	N	145GLY	O	99.34
151ALA	N	157TYR	O	93.30
150GLU	N	147SER	O	8.89
150GLU	N	148GLU	O	0.01
149ALA	N	147SER	O	2.29
148GLU	N	146MET	O	0.01
148GLU	N	148GLU	OE1	0.17
148GLU	N	148GLU	OE2	0.16
148GLU	N	150GLU	O	0.08
147SER	OG	146MET	O	0.03
147SER	OG	147SER	O	0.02
147SER	OG	150GLU	N	0.00
147SER	OG	150GLU	OE1	0.09
147SER	OG	150GLU	O	65.72
147SER	N	150GLU	O	56.46
145GLY	N	152TRP	NE1	1.08
145GLY	N	152TRP	O	70.65
144ARG	NH2	188VAL	O	13.79
144ARG	NH2	190GLU	OE1	26.98
144ARG	NH2	190GLU	OE2	22.28
144ARG	NH2	139TYR	O	0.27
144ARG	NH2	142GLU	OE1	0.00
144ARG	NH1	188VAL	O	0.40
144ARG	NH1	138ILE	O	0.06
144ARG	NH1	139TYR	O	18.44
144ARG	NH1	140PHE	O	0.02

273HIS	N	255GLY	O	0.07
273HIS	N	271PRO	O	7.29
272VAL	N	255GLY	O	0.00
272VAL	N	257LEU	O	47.51
270GLU	N	259SER	O	98.27
270GLU	N	293SER	OG	0.01
269PHE	N	67VAL	O	95.50
268VAL	N	261SER	O	93.54
266THR	OG1	61VAL	O	73.29
266THR	OG1	62GLU	O	0.01
266THR	OG1	64ALA	O	1.01
266THR	OG1	97GLN	OE1	9.78
266THR	OG1	265GLY	O	0.02
266THR	OG1	266THR	O	0.50
266THR	OG1	267PRO	O	0.00
266THR	N	97GLN	OE1	83.72
266THR	N	97GLN	O	0.00
265GLY	N	97GLN	O	55.27
265GLY	N	98ASP	O	0.06
265GLY	N	263GLY	O	0.10
264ARG	NH2	62GLU	OE1	0.47
264ARG	NH2	62GLU	OE2	0.01
264ARG	NH2	96SER	O	3.16
264ARG	NH2	97GLN	O	0.00
264ARG	NH2	98ASP	OD1	5.56
264ARG	NH2	98ASP	OD2	10.98
264ARG	NH2	161GLU	OE1	0.05
264ARG	NH2	161GLU	OE2	0.14
264ARG	NH1	62GLU	OE2	0.40
264ARG	NH1	96SER	O	0.90
264ARG	NH1	97GLN	O	0.14
264ARG	NH1	98ASP	OD1	1.56
264ARG	NH1	98ASP	OD2	10.44
264ARG	NH1	161GLU	OE1	0.00
264ARG	NH1	161GLU	OE2	0.02
264ARG	NH1	264ARG	O	0.24
264ARG	NE	97GLN	O	0.01
264ARG	NE	98ASP	OD1	15.94
264ARG	NE	98ASP	OD2	32.29
264ARG	NE	264ARG	O	0.28
264ARG	N	97GLN	O	15.51
264ARG	N	98ASP	OD2	0.00
264ARG	N	98ASP	O	62.07

132ARG	NE	133GLU	O	15.28
132ARG	NE	237ASN	OD1	17.32
132ARG	NE	237ASN	ND2	0.04
132ARG	NE	237ASN	O	1.19
132ARG	N	102ASN	O	96.32
131VAL	N	233VAL	O	97.85
130ILE	N	104ARG	O	97.48
129LEU	N	231ASP	O	90.51
128VAL	N	106ALA	O	83.21
126VAL	N	123ALA	O	2.98
126VAL	N	124ARG	O	0.10
125GLY	N	123ALA	O	0.02
124ARG	NH2	113GLU	OE1	7.22
124ARG	NH2	113GLU	OE2	16.30
124ARG	NH2	120GLU	OE1	21.30
124ARG	NH2	120GLU	OE2	11.80
124ARG	NH2	121GLU	OE1	12.25
124ARG	NH2	121GLU	OE2	8.08
124ARG	NH1	113GLU	OE1	1.91
124ARG	NH1	113GLU	OE2	0.54
124ARG	NH1	120GLU	OE1	2.36
124ARG	NH1	120GLU	OE2	0.73
124ARG	NH1	121GLU	OE1	21.81
124ARG	NH1	121GLU	OE2	32.51
124ARG	NE	113GLU	OE1	27.51
124ARG	NE	113GLU	OE2	2.93
124ARG	NE	120GLU	OE1	3.10
124ARG	NE	120GLU	OE2	0.35
124ARG	NE	121GLU	OE1	2.73
124ARG	NE	121GLU	OE2	0.27
124ARG	N	120GLU	O	38.85
124ARG	N	121GLU	O	7.51
124ARG	N	122ILE	O	1.74
123ALA	N	119LYS	O	55.71
123ALA	N	120GLU	O	9.14
122ILE	N	119LYS	O	85.53
122ILE	N	120GLU	O	0.01
121GLU	N	119LYS	O	0.01
121GLU	N	120GLU	OE1	0.31
121GLU	N	120GLU	OE2	0.62
121GLU	N	121GLU	OE1	1.28
121GLU	N	121GLU	OE2	0.98
120GLU	N	120GLU	OE1	20.24

248SER	N	244SER	O	4.23
248SER	N	245ASP	O	63.00
248SER	N	246LEU	O	0.14
247ALA	N	243LEU	O	80.36
247ALA	N	244SER	O	1.86
246LEU	N	242ILE	O	87.10
246LEU	N	243LEU	O	0.77
245ASP	N	241ASP	O	19.74
245ASP	N	242ILE	O	19.93
245ASP	N	245ASP	OD1	0.02
244SER	OG	240GLY	O	87.35
244SER	OG	241ASP	O	0.53
244SER	OG	244SER	O	0.03
244SER	OG	245ASP	N	0.02
244SER	OG	245ASP	OD1	0.11
244SER	OG	245ASP	OD2	0.01
244SER	N	240GLY	O	57.11
244SER	N	241ASP	O	6.40
244SER	N	242ILE	O	0.01
243LEU	N	239PHE	O	74.71
243LEU	N	240GLY	O	0.16
242ILE	N	238ILE	O	63.21
242ILE	N	239PHE	O	4.08
241ASP	N	237ASN	O	58.98
241ASP	N	238ILE	O	11.42
240GLY	N	236GLY	O	68.24
240GLY	N	237ASN	O	11.57
239PHE	N	236GLY	O	2.40
238ILE	N	237ASN	OD1	0.01
237ASN	ND2	133GLU	OE1	0.83
237ASN	ND2	133GLU	OE2	0.02
237ASN	ND2	133GLU	O	0.00
237ASN	ND2	134LEU	O	14.52
237ASN	ND2	136GLY	O	87.02
237ASN	ND2	137GLY	O	0.05
235THR	OG1	183VAL	O	34.03
235THR	OG1	236GLY	N	0.25
235THR	OG1	236GLY	O	4.57
235THR	OG1	239PHE	O	1.08
235THR	OG1	240GLY	N	0.15
235THR	N	131VAL	O	63.25
234VAL	N	181VAL	O	89.97
233VAL	N	129LEU	O	90.16

144ARG	NH1	141GLY	O	7.56	262LEU	N	101ALA	O	92.32	120GLU	N	120GLU	OE2	11.56	232VAL	N	179HIS	O	4.82
144ARG	NH1	142GLU	OE1	5.80	261SER	OG	101ALA	O	7.41	119LYS	NZ	113GLU	O	0.13	232VAL	N	231ASP	OD1	2.69
144ARG	NH1	142GLU	OE2	4.47	261SER	OG	260ALA	O	0.32	119LYS	NZ	114ARG	O	54.89	232VAL	N	231ASP	OD2	0.01
144ARG	NE	190GLU	OE1	13.11	261SER	OG	261SER	O	0.05	119LYS	NZ	115LEU	O	0.01	231ASP	N	179HIS	ND1	0.12
144ARG	NE	190GLU	OE2	16.92	261SER	OG	268VAL	O	1.51	119LYS	NZ	116SER	O	28.00	231ASP	N	179HIS	O	0.00
144ARG	NE	139TYR	O	0.32	261SER	N	268VAL	O	92.42	119LYS	NZ	118LEU	O	0.25	231ASP	N	229ARG	O	0.07
144ARG	N	142GLU	OE1	1.63	260ALA	N	102ASN	OD1	0.00	119LYS	NZ	121GLU	OE1	0.00	230PHE	N	227PRO	O	62.64
144ARG	N	142GLU	OE2	1.28	260ALA	N	103LEU	O	93.41	119LYS	NZ	121GLU	OE2	0.06	230PHE	N	228ALA	O	0.33
142GLU	N	140PHE	O	0.40	259SER	OG	103LEU	O	0.04	119LYS	N	117PRO	O	84.54	229ARG	NH2	179HIS	ND1	0.01
142GLU	N	142GLU	OE1	0.01	259SER	OG	259SER	O	0.04	116SER	OG	118LEU	O	0.07	229ARG	NH2	212GLU	OE1	30.26
142GLU	N	142GLU	OE2	0.25	259SER	OG	270GLU	O	86.42	116SER	OG	249VAL	O	54.90	229ARG	NH2	212GLU	OE2	34.72
142GLU	N	156ARG	NE	0.06	259SER	OG	271PRO	O	0.02	116SER	OG	250LEU	O	35.54	229ARG	NH2	214GLN	OE1	0.94
142GLU	N	156ARG	NH1	0.06	259SER	N	270GLU	O	8.41	116SER	N	112LEU	O	8.73	229ARG	NH2	222HIS	ND1	0.12
142GLU	N	156ARG	NH2	0.30	259SER	N	293SER	OG	1.30	116SER	N	113GLU	O	49.35	229ARG	NH2	229ARG	O	0.00
141GLY	N	137GLY	O	2.63	257LEU	N	254LEU	O	0.88	116SER	N	114ARG	O	0.16	229ARG	NH1	212GLU	OE1	32.24
141GLY	N	138ILE	O	18.43	257LEU	N	255GLY	O	0.18	115LEU	N	111GLY	O	0.03	229ARG	NH1	212GLU	OE2	27.97
141GLY	N	139TYR	O	0.01	257LEU	N	272VAL	O	0.00	115LEU	N	112LEU	O	66.81	229ARG	NH1	214GLN	OE1	0.08
141GLY	N	144ARG	NH1	0.12	256LEU	N	253SER	O	1.81	115LEU	N	113GLU	O	0.02	229ARG	NH1	222HIS	ND1	1.02
140PHE	N	136GLY	O	0.02	256LEU	N	254LEU	O	0.20	114ARG	NH2	111GLY	O	3.78	229ARG	NH1	228ALA	O	0.04
140PHE	N	137GLY	O	96.84	255GLY	N	248SER	O	0.01	114ARG	NH2	120GLU	OE1	7.51	229ARG	NH1	229ARG	O	4.29
139TYR	OH	237ASN	O	3.96	255GLY	N	253SER	O	0.10	114ARG	NH2	120GLU	OE2	3.81	229ARG	NE	212GLU	OE1	0.32
139TYR	OH	238ILE	N	0.00	255GLY	N	326ASP	OD1	0.01	114ARG	NH2	319LEU	O	0.00	229ARG	NE	212GLU	OE2	0.15
139TYR	OH	238ILE	O	68.07	254LEU	N	326ASP	OD1	3.44	114ARG	NH2	320LEU	O	0.38	229ARG	NE	222HIS	ND1	0.15
139TYR	OH	241ASP	OD1	2.64	254LEU	N	326ASP	OD2	24.80	114ARG	NH2	327LEU	O	0.09	229ARG	NE	228ALA	O	0.02
139TYR	OH	241ASP	OD2	16.08	253SER	OG	252GLY	O	0.76	114ARG	NH1	111GLY	O	1.45	229ARG	NE	229ARG	O	17.68
139TYR	N	137GLY	O	0.01	253SER	OG	253SER	O	0.30	114ARG	NH1	120GLU	OE1	0.06	229ARG	N	226SER	O	29.88
138ILE	N	155GLU	OE1	66.37	253SER	OG	254LEU	N	0.03	114ARG	NH1	120GLU	OE2	0.00	229ARG	N	227PRO	O	2.57
138ILE	N	155GLU	OE2	15.27	253SER	OG	254LEU	O	0.21	114ARG	NH1	320LEU	O	0.39	228ALA	N	226SER	O	0.04
137GLY	N	155GLU	OE1	0.06	253SER	OG	326ASP	OD1	18.79	114ARG	NE	111GLY	O	3.94	226SER	OG	122ILE	O	0.00
137GLY	N	156ARG	O	6.56	253SER	OG	326ASP	OD2	65.23	114ARG	NE	120GLU	OE1	9.94	226SER	OG	222HIS	ND1	1.73
136GLY	N	133GLU	OE1	57.55	253SER	OG	326ASP	O	0.03	114ARG	NE	120GLU	OE2	18.32	226SER	OG	222HIS	O	10.72
136GLY	N	133GLU	OE2	34.36	253SER	OG	327LEU	N	0.00	114ARG	N	111GLY	O	7.21	226SER	OG	225ARG	O	3.99
136GLY	N	157TYR	OH	0.07	253SER	N	248SER	O	0.10	114ARG	N	112LEU	O	0.05	226SER	OG	226SER	O	5.22
136GLY	N	237ASN	ND2	0.02	253SER	N	249VAL	O	0.07	114ARG	N	113GLU	OE2	0.04	226SER	N	222HIS	O	88.30
135THR	OG1	99LEU	O	5.91	253SER	N	251PRO	O	0.54	113GLU	N	109PHE	O	0.00	226SER	N	223LEU	O	1.80
135THR	OG1	100PHE	O	0.01	252GLY	N	248SER	O	1.89	113GLU	N	111GLY	O	0.01	225ARG	NH2	252GLY	O	18.48
135THR	OG1	133GLU	OE1	46.89	252GLY	N	249VAL	O	85.41	113GLU	N	113GLU	OE1	18.60	225ARG	NH2	326ASP	OD1	70.28
135THR	OG1	133GLU	OE2	38.97	252GLY	N	250LEU	O	0.02	113GLU	N	113GLU	OE2	73.11	225ARG	NH2	326ASP	OD2	24.91
135THR	OG1	136GLY	N	0.00	250LEU	N	246LEU	O	0.08	112LEU	N	109PHE	O	81.74	225ARG	NH2	326ASP	O	2.44
135THR	N	100PHE	O	0.01	250LEU	N	247ALA	O	61.97	112LEU	N	110PRO	O	0.03	225ARG	NH2	222HIS	ND1	3.78
135THR	N	133GLU	OE1	65.17	250LEU	N	248SER	O	0.01	112LEU	N	113GLU	OE1	0.19	225ARG	NH1	252GLY	O	56.21
135THR	N	133GLU	OE2	40.85	249VAL	N	245ASP	O	0.03	112LEU	N	113GLU	OE2	1.21	225ARG	NH1	326ASP	OD1	11.16
134LEU	N	100PHE	O	73.60	249VAL	N	246LEU	O	10.53	111GLY	N	113GLU	OE1	37.64	225ARG	NH1	326ASP	OD2	61.61

134LEU	N	133GLU	OE1	10.38
134LEU	N	133GLU	OE2	2.92
133GLU	N	235THR	O	13.34
133GLU	N	236GLY	O	63.77
132ARG	NH2	133GLU	O	0.04
132ARG	NH2	134LEU	O	0.00
132ARG	NH2	237ASN	OD1	87.73
132ARG	NH2	237ASN	ND2	0.12
132ARG	NH2	237ASN	O	0.01
132ARG	NH2	241ASP	OD1	20.23
132ARG	NH2	241ASP	OD2	16.31
132ARG	NH1	102ASN	OD1	0.58
132ARG	NH1	241ASP	OD1	4.10
132ARG	NH1	241ASP	OD2	2.86
132ARG	NH1	244SER	OG	1.29
132ARG	NE	133GLU	O	57.51
132ARG	NE	236GLY	O	0.01
132ARG	NE	237ASN	OD1	20.34
132ARG	NE	237ASN	ND2	0.03
132ARG	NE	237ASN	O	1.07
132ARG	N	102ASN	O	93.11
132ARG	N	130ILE	O	0.00
131VAL	N	233VAL	O	96.62
130ILE	N	104ARG	O	96.89
129LEU	N	231ASP	O	89.36
128VAL	N	106ALA	O	89.81
126VAL	N	123ALA	O	18.23
126VAL	N	124ARG	O	0.07
124ARG	NH2	113GLU	OE1	17.66
124ARG	NH2	113GLU	OE2	0.70
124ARG	NH2	120GLU	OE1	14.00
124ARG	NH2	120GLU	OE2	3.86
124ARG	NH2	121GLU	OE1	28.66
124ARG	NH2	121GLU	OE2	36.72
124ARG	NH1	113GLU	OE1	1.17
124ARG	NH1	113GLU	OE2	2.08
124ARG	NH1	120GLU	OE1	4.20
124ARG	NH1	120GLU	OE2	5.34
124ARG	NH1	120GLU	O	0.01
124ARG	NH1	121GLU	OE1	20.97
124ARG	NH1	121GLU	OE2	12.90
124ARG	NE	113GLU	OE1	0.74
124ARG	NE	113GLU	OE2	14.74

249VAL	N	247ALA	O	0.02
248SER	OG	244SER	O	1.16
248SER	OG	245ASP	OD1	0.01
248SER	OG	245ASP	O	93.65
248SER	OG	249VAL	N	0.01
248SER	OG	254LEU	O	0.00
248SER	N	244SER	O	23.78
248SER	N	245ASP	O	35.55
248SER	N	246LEU	O	0.17
247ALA	N	243LEU	O	63.99
247ALA	N	244SER	O	6.46
246LEU	N	242ILE	O	89.22
246LEU	N	243LEU	O	0.71
246LEU	N	244SER	O	0.00
245ASP	N	241ASP	O	68.14
245ASP	N	242ILE	O	6.48
244SER	OG	104ARG	O	0.00
244SER	OG	240GLY	O	77.18
244SER	OG	244SER	O	0.02
244SER	OG	245ASP	OD2	0.01
244SER	N	240GLY	O	62.88
244SER	N	241ASP	O	6.64
243LEU	N	239PHE	O	57.45
243LEU	N	240GLY	O	0.44
242ILE	N	238ILE	O	45.63
242ILE	N	239PHE	O	5.83
241ASP	N	236GLY	O	0.00
241ASP	N	237ASN	OD1	0.00
241ASP	N	237ASN	O	25.99
241ASP	N	238ILE	O	26.78
241ASP	N	239PHE	O	0.01
240GLY	N	236GLY	O	64.08
240GLY	N	237ASN	O	4.03
240GLY	N	238ILE	O	0.01
239PHE	N	236GLY	O	6.13
238ILE	N	236GLY	O	0.02
237ASN	ND2	133GLU	OE1	1.82
237ASN	ND2	133GLU	OE2	50.11
237ASN	ND2	133GLU	O	13.20
237ASN	ND2	134LEU	O	0.72
237ASN	ND2	136GLY	O	13.72
237ASN	ND2	137GLY	O	0.09
237ASN	ND2	238ILE	N	0.00

111GLY	N	113GLU	OE2	64.34
109PHE	N	107LYS	O	0.04
108VAL	N	126VAL	O	72.44
107LYS	NZ	127ASP	OD1	0.37
107LYS	NZ	127ASP	OD2	0.65
107LYS	NZ	299GLU	OE1	0.02
107LYS	NZ	299GLU	OE2	0.01
107LYS	NZ	309ARG	NE	0.08
107LYS	NZ	309ARG	NH2	0.08
107LYS	NZ	312GLU	OE1	42.93
107LYS	NZ	312GLU	OE2	67.66
107LYS	N	105PRO	O	0.25
106ALA	N	104ARG	O	0.00
106ALA	N	128VAL	O	94.79
104ARG	NH2	244SER	O	0.23
104ARG	NH2	245ASP	OD1	13.14
104ARG	NH2	245ASP	OD2	5.37
104ARG	NH2	248SER	OG	0.56
104ARG	NH1	102ASN	OD1	42.66
104ARG	NH1	132ARG	NH2	0.02
104ARG	NH1	245ASP	OD1	0.34
104ARG	NH1	245ASP	OD2	0.39
104ARG	NE	244SER	OG	0.01
104ARG	N	130ILE	O	99.14
103LEU	N	101ALA	O	0.00
103LEU	N	260ALA	O	99.60
102ASN	ND2	132ARG	NE	0.00
102ASN	ND2	259SER	OG	0.11
102ASN	ND2	260ALA	O	0.38
102ASN	ND2	261SER	N	0.00
102ASN	ND2	261SER	OG	74.38
102ASN	N	100PHE	O	0.01
102ASN	N	132ARG	O	98.70
101ALA	N	262LEU	O	73.85
100PHE	N	98ASP	O	0.28
100PHE	N	262LEU	O	90.30
99LEU	N	94ARG	O	97.18
98ASP	N	94ARG	O	4.26
98ASP	N	95LYS	O	16.16
98ASP	N	96SER	O	0.00
97GLN	NE2	61VAL	O	0.02
97GLN	NE2	62GLU	OE1	44.91
97GLN	NE2	62GLU	OE2	42.78

225ARG	NH1	326ASP	O	0.12
225ARG	NH1	221MET	O	0.00
225ARG	NH1	222HIS	ND1	0.43
225ARG	NE	252GLY	O	0.08
225ARG	NE	221MET	O	0.02
225ARG	NE	222HIS	ND1	2.51
225ARG	NE	226SER	OG	0.01
225ARG	N	221MET	O	21.76
225ARG	N	222HIS	O	47.59
225ARG	N	223LEU	O	2.44
224VAL	N	220ALA	O	2.69
224VAL	N	221MET	O	25.26
224VAL	N	222HIS	O	0.01
223LEU	N	219MET	O	83.79
223LEU	N	220ALA	O	1.90
222HIS	NE2	214GLN	OE1	0.10
222HIS	NE2	226SER	OG	0.02
222HIS	NE2	229ARG	NE	0.57
222HIS	NE2	229ARG	NH1	0.34
222HIS	NE2	229ARG	NH2	0.08
222HIS	N	218ALA	O	63.31
222HIS	N	219MET	O	7.53
222HIS	N	220ALA	O	0.01
221MET	N	217ASP	O	32.37
221MET	N	218ALA	O	15.42
221MET	N	219MET	O	0.03
220ALA	N	216VAL	O	88.07
220ALA	N	217ASP	O	1.37
219MET	N	215TYR	O	83.69
219MET	N	216VAL	O	3.96
218ALA	N	215TYR	O	31.81
218ALA	N	217ASP	OD1	3.64
218ALA	N	217ASP	OD2	3.24
217ASP	N	217ASP	OD1	14.25
217ASP	N	217ASP	OD2	13.95
216VAL	N	184ASP	O	95.75
215TYR	OH	78ASP	OD1	0.98
215TYR	OH	78ASP	OD2	1.60
215TYR	OH	186ALA	O	0.14
215TYR	OH	187ASN	OD1	0.00
215TYR	N	214GLN	OE1	6.35
214GLN	NE2	212GLU	OE1	22.15
214GLN	NE2	212GLU	OE2	27.51

124ARG	NE	120GLU	OE1	0.08
124ARG	NE	120GLU	OE2	0.00
124ARG	NE	120GLU	O	0.01
124ARG	NE	121GLU	OE1	4.19
124ARG	NE	121GLU	OE2	4.09
124ARG	NE	121GLU	O	0.00
124ARG	N	120GLU	O	52.87
124ARG	N	121GLU	O	3.55
124ARG	N	122ILE	O	3.31
123ALA	N	119LYS	O	70.90
123ALA	N	120GLU	O	9.29
122ILE	N	119LYS	O	78.23
122ILE	N	120GLU	O	0.04
122ILE	N	121GLU	OE2	0.00
121GLU	N	119LYS	O	0.00
121GLU	N	121GLU	OE1	6.27
121GLU	N	121GLU	OE2	3.92
120GLU	N	120GLU	OE1	7.17
120GLU	N	120GLU	OE2	9.38
119LYS	NZ	113GLU	O	0.06
119LYS	NZ	114ARG	O	35.55
119LYS	NZ	116SER	O	27.35
119LYS	NZ	118LEU	O	0.24
119LYS	NZ	120GLU	OE1	0.31
119LYS	NZ	120GLU	OE2	0.27
119LYS	NZ	121GLU	OE2	0.01
119LYS	N	117PRO	O	88.22
116SER	OG	113GLU	O	1.48
116SER	OG	118LEU	O	0.01
116SER	OG	249VAL	O	44.79
116SER	OG	250LEU	N	0.00
116SER	OG	250LEU	O	47.37
116SER	OG	251PRO	O	0.00
116SER	N	112LEU	O	0.30
116SER	N	113GLU	O	71.35
116SER	N	114ARG	O	0.13
115LEU	N	112LEU	O	56.84
115LEU	N	113GLU	O	0.11
114ARG	NH2	111GLY	O	3.10
114ARG	NH2	120GLU	OE1	8.08
114ARG	NH2	120GLU	OE2	6.04
114ARG	NH2	320LEU	O100	0.01
114ARG	NH2	327LEU	O101	0.44

237ASN	N	133GLU	OE1	38.75
237ASN	N	136GLY	O	0.17
235THR	OG1	183VAL	O	31.41
235THR	OG1	236GLY	N	0.06
235THR	OG1	236GLY	O	0.60
235THR	OG1	239PHE	O	0.09
235THR	OG1	240GLY	N	0.06
235THR	N	131VAL	O	81.95
234VAL	N	181VAL	O	88.61
233VAL	N	129LEU	O	94.56
232VAL	N	179HIS	O	2.32
232VAL	N	231ASP	OD1	1.05
232VAL	N	231ASP	OD2	1.34
230PHE	N	227PRO	O	63.87
230PHE	N	228ALA	O	0.07
229ARG	NH2	212GLU	OE1	43.56
229ARG	NH2	212GLU	OE2	57.64
229ARG	NH2	214GLN	OE1	0.05
229ARG	NH2	229ARG	O	0.01
229ARG	NH1	212GLU	OE1	57.69
229ARG	NH1	212GLU	OE2	44.00
229ARG	NH1	214GLN	OE1	0.01
229ARG	NH1	229ARG	O	0.02
229ARG	NE	222HIS	ND1	0.62
229ARG	NE	229ARG	O	48.20
229ARG	N	226SER	O	42.39
229ARG	N	227PRO	O	1.76
228ALA	N	226SER	O	0.00
226SER	OG	122ILE	O	0.00
226SER	OG	222HIS	ND1	1.26
226SER	OG	222HIS	O	1.11
226SER	OG	225ARG	O	6.55
226SER	OG	226SER	O	5.69
226SER	OG	228ALA	O	0.00
226SER	N	222HIS	O	96.28
226SER	N	223LEU	O	0.47
225ARG	NH2	115LEU	O	0.04
225ARG	NH2	252GLY	O	0.44
225ARG	NH2	275SER	O	0.00
225ARG	NH2	326ASP	OD1	9.22
225ARG	NH2	326ASP	OD2	15.75
225ARG	NH2	326ASP	O	4.35
225ARG	NH2	327LEU	O	0.02

97GLN	NE2	93LEU	O	0.00
97GLN	NE2	96SER	O	0.06
97GLN	N	93LEU	O	72.39
97GLN	N	94ARG	O	0.34
96SER	OG	92SER	O	88.20
96SER	OG	93LEU	O	5.42
96SER	N	92SER	O	92.92
96SER	N	93LEU	O	1.53
95LYS	N	91LEU	O	78.01
95LYS	N	92SER	O	3.82
94ARG	NH2	87GLU	OE1	0.00
94ARG	NH2	134LEU	O	2.12
94ARG	NH2	135THR	O	0.10
94ARG	NH2	270GLU	OE1	5.18
94ARG	NH2	270GLU	OE2	0.27
94ARG	NH1	134LEU	O	0.46
94ARG	NH1	135THR	O	0.02
94ARG	NH1	261SER	OG	3.95
94ARG	NH1	270GLU	OE1	0.01
94ARG	NH1	270GLU	OE2	6.52
94ARG	NE	90LEU	O	0.43
94ARG	NE	94ARG	O	0.05
94ARG	NE	99LEU	O	0.01
94ARG	NE	134LEU	O	0.00
94ARG	N	90LEU	O	87.34
94ARG	N	91LEU	O	1.06
93LEU	N	89GLY	O	87.37
93LEU	N	90LEU	O	1.24
92SER	OG	88THR	O	37.97
92SER	OG	89GLY	O	0.46
92SER	OG	92SER	O	0.05
92SER	OG	93LEU	N	0.02
92SER	N	88THR	O	33.67
92SER	N	89GLY	O	7.11
92SER	N	90LEU	O	0.00
91LEU	N	87GLU	O	86.87
91LEU	N	88THR	O	0.60
90LEU	N	86PRO	O	65.87
90LEU	N	87GLU	O	10.93
89GLY	N	85ARG	O	0.56
89GLY	N	86PRO	O	84.47
89GLY	N	87GLU	O	0.18
88THR	OG1	42GLY	O	0.02

214GLN	NE2	213HIS	O	13.52
214GLN	NE2	215TYR	N	0.00
214GLN	NE2	215TYR	O	0.23
214GLN	NE2	218ALA	O	0.12
214GLN	NE2	219MET	N	0.07
214GLN	NE2	222HIS	ND1	4.25
214GLN	NE2	222HIS	NE2	0.01
214GLN	NE2	229ARG	NE	0.01
214GLN	NE2	229ARG	NH1	0.01
214GLN	NE2	229ARG	NH2	0.03
214GLN	N	182SER	O	72.93
214GLN	N	184ASP	OD2	0.02
213HIS	NE2	196ARG	O	0.83
213HIS	NE2	200GLU	OE1	10.82
213HIS	NE2	200GLU	OE2	15.12
213HIS	N	212GLU	OE1	1.71
213HIS	N	212GLU	OE2	1.90
212GLU	N	180VAL	O	66.39
211LEU	N	209VAL	O	0.00
210ALA	N	178LYS	O	96.34
209VAL	N	206TYR	O	53.06
209VAL	N	207PRO	O	0.55
209VAL	N	208ASP	OD1	0.49
208ASP	N	206TYR	O	0.16
208ASP	N	208ASP	OD1	44.03
206TYR	OH	167ARG	O	0.11
206TYR	OH	171GLU	OE1	39.83
206TYR	OH	171GLU	OE2	45.55
206TYR	N	202VAL	O	18.68
206TYR	N	203GLY	O	42.74
206TYR	N	204ARG	O	0.02
205GLY	N	201GLU	O	0.39
205GLY	N	202VAL	O	75.07
205GLY	N	203GLY	O	0.16
204ARG	NH2	197LYS	O	0.01
204ARG	NH2	200GLU	OE1	40.20
204ARG	NH2	200GLU	OE2	32.40
204ARG	NH2	201GLU	OE1	23.01
204ARG	NH2	201GLU	OE2	18.10
204ARG	NH2	203GLY	O	0.00
204ARG	NH1	200GLU	OE1	4.02
204ARG	NH1	200GLU	OE2	4.19
204ARG	NH1	200GLU	O	0.12

114ARG	NH2	328GLY	O101	0.00
114ARG	NH1	111GLY	O	4.40
114ARG	NH1	120GLU	OE1	0.01
114ARG	NH1	120GLU	OE2	0.01
114ARG	NH1	320LEU	O100	0.00
114ARG	NE	111GLY	O	1.54
114ARG	NE	120GLU	OE1	4.53
114ARG	NE	120GLU	OE2	14.01
114ARG	N	112LEU	O	0.01
114ARG	N	113GLU	OE1	0.01
113GLU	N	111GLY	O	0.01
113GLU	N	113GLU	OE1	88.82
113GLU	N	113GLU	OE2	3.68
112LEU	N	109PHE	O	92.32
112LEU	N	110PRO	O	0.01
112LEU	N	113GLU	OE1	0.11
112LEU	N	113GLU	OE2	0.02
111GLY	N	109PHE	O	0.01
111GLY	N	113GLU	OE1	29.52
111GLY	N	113GLU	OE2	62.95
109PHE	N	112LEU	O	0.04
108VAL	N	126VAL	O	33.85
107LYS	NZ	127ASP	OD1	11.08
107LYS	NZ	127ASP	OD2	1.02
107LYS	NZ	309ARG	NE	0.00
107LYS	NZ	309ARG	NH1	0.03
107LYS	NZ	312GLU	OE1	52.80
107LYS	NZ	312GLU	OE2	54.43
107LYS	N	105PRO	O	0.03
106ALA	N	128VAL	O	87.82
104ARG	NH2	102ASN	OD1	0.50
104ARG	NH2	132ARG	NH1	0.02
104ARG	NH2	245ASP	OD1	0.24
104ARG	NH2	245ASP	OD2	0.30
104ARG	NH2	259SER	OG	0.12
104ARG	NH2	270GLU	OE1	0.00
104ARG	NH2	270GLU	OE2	0.06
104ARG	NH1	244SER	O	9.83
104ARG	NH1	245ASP	OD1	25.49
104ARG	NH1	245ASP	OD2	31.27
104ARG	NH1	248SER	OG	12.12
104ARG	NE	102ASN	OD1	27.66
104ARG	NE	244SER	OG	0.01

225ARG	NH2	221MET	O	0.01
225ARG	NH2	222HIS	ND1	0.91
225ARG	NH2	226SER	OG	0.03
225ARG	NH1	252GLY	O	0.00
225ARG	NH1	326ASP	OD1	10.22
225ARG	NH1	326ASP	OD2	6.64
225ARG	NH1	326ASP	O	0.23
225ARG	NH1	221MET	O	0.34
225ARG	NH1	222HIS	ND1	0.44
225ARG	NE	221MET	O	0.22
225ARG	NE	222HIS	ND1	0.36
225ARG	NE	226SER	OG	0.09
225ARG	N	221MET	O	7.80
225ARG	N	222HIS	O	56.96
225ARG	N	223LEU	O	1.00
224VAL	N	220ALA	O	11.49
224VAL	N	221MET	O	13.26
224VAL	N	222HIS	O	0.02
223LEU	N	219MET	O	72.33
223LEU	N	220ALA	O	4.86
222HIS	NE2	214GLN	OE1	0.31
222HIS	NE2	225ARG	NH2	0.00
222HIS	NE2	226SER	OG	0.08
222HIS	NE2	226SER	O	0.01
222HIS	N	218ALA	O	44.09
222HIS	N	219MET	O	17.61
221MET	N	217ASP	O	54.34
221MET	N	218ALA	O	3.38
220ALA	N	216VAL	O	95.34
220ALA	N	217ASP	O	0.18
219MET	N	215TYR	O	81.14
219MET	N	216VAL	O	4.02
218ALA	N	215TYR	O	28.49
218ALA	N	216VAL	O	0.01
218ALA	N	217ASP	OD1	2.79
218ALA	N	217ASP	OD2	1.26
217ASP	N	217ASP	OD1	16.48
217ASP	N	217ASP	OD2	7.33
216VAL	N	184ASP	O	97.35
215TYR	OH	77TRP	O	0.12
215TYR	OH	186ALA	O	0.86
215TYR	N	214GLN	OE1	1.68
214GLN	NE2	78ASP	OD1	0.04

88THR	OG1	82ARG	O	15.42
88THR	OG1	83LYS	O	0.21
88THR	OG1	85ARG	O	64.69
88THR	N	85ARG	O	76.94
87GLU	N	85ARG	O	1.63
87GLU	N	87GLU	OE1	2.73
87GLU	N	87GLU	OE2	2.18
85ARG	NH2	77TRP	O	0.26
85ARG	NH2	78ASP	OD1	0.00
85ARG	NH2	78ASP	OD2	0.01
85ARG	NH2	78ASP	O	1.77
85ARG	NH2	80LEU	O	55.98
85ARG	NH1	78ASP	OD1	0.26
85ARG	NH1	78ASP	OD2	0.14
85ARG	NH1	78ASP	O	0.04
85ARG	NH1	87GLU	OE1	0.80
85ARG	NH1	87GLU	OE2	2.07
85ARG	NE	77TRP	O	22.00
85ARG	NE	80LEU	O	60.27
85ARG	NE	81PRO	O	0.08
85ARG	N	81PRO	O	0.20
85ARG	N	82ARG	O	75.50
85ARG	N	83LYS	O	0.04
85ARG	N	88THR	OG1	0.02
84ILE	N	81PRO	O	94.27
84ILE	N	82ARG	O	0.04
83LYS	N	81PRO	O	0.06
82ARG	NH2	187ASN	OD1	0.01
82ARG	NH2	187ASN	O	3.58
82ARG	NH2	87GLU	OE1	38.89
82ARG	NH2	87GLU	OE2	35.21
82ARG	NH1	187ASN	OD1	0.03
82ARG	NH1	187ASN	O	6.00
82ARG	NE	85ARG	NH1	0.00
82ARG	NE	87GLU	OE1	36.10
82ARG	NE	87GLU	OE2	42.91
82ARG	NE	88THR	OG1	0.00
80LEU	N	76LYS	O	0.27
80LEU	N	77TRP	O	64.59
80LEU	N	78ASP	O	0.44
80LEU	N	85ARG	NE	0.00
80LEU	N	85ARG	NH2	0.01
79GLY	N	75PRO	O	1.93

204ARG	NH1	201GLU	OE1	26.95
204ARG	NH1	201GLU	OE2	30.15
204ARG	NE	200GLU	OE1	32.45
204ARG	NE	200GLU	OE2	38.59
204ARG	NE	200GLU	O	0.07
204ARG	NE	203GLY	O	0.08
204ARG	N	200GLU	O	36.65
204ARG	N	201GLU	O	22.52
204ARG	N	202VAL	O	0.02
203GLY	N	199VAL	O	56.47
203GLY	N	200GLU	O	10.59
202VAL	N	198THR	O	35.24
202VAL	N	199VAL	O	16.76
202VAL	N	200GLU	O	0.00
201GLU	N	197LYS	O	28.23
201GLU	N	198THR	O	27.29
201GLU	N	199VAL	O	0.00
200GLU	N	196ARG	O	38.85
200GLU	N	197LYS	O	5.03
199VAL	N	195TRP	O	96.52
199VAL	N	196ARG	O	0.36
198THR	OG1	194PHE	O	96.44
198THR	OG1	195TRP	O	0.01
198THR	N	194PHE	O	52.80
198THR	N	195TRP	O	26.89
198THR	N	196ARG	O	0.01
197LYS	NZ	147SER	O	0.20
197LYS	NZ	148GLU	OE1	13.34
197LYS	NZ	148GLU	OE2	15.22
197LYS	NZ	148GLU	O	0.00
197LYS	NZ	193GLU	OE1	11.23
197LYS	NZ	193GLU	OE2	11.90
197LYS	NZ	193GLU	O	0.22
197LYS	NZ	197LYS	O	0.05
197LYS	NZ	200GLU	OE1	0.01
197LYS	NZ	200GLU	OE2	0.24
197LYS	NZ	201GLU	OE1	0.68
197LYS	NZ	201GLU	OE2	0.84
197LYS	N	193GLU	O	62.77
197LYS	N	194PHE	O	6.95
196ARG	NH2	184ASP	OD1	88.52
196ARG	NH2	184ASP	OD2	18.33
196ARG	NH2	213HIS	O	0.00

104ARG	NE	259SER	OG	0.91
104ARG	N	130ILE	O	95.64
103LEU	N	102ASN	OD1	0.00
103LEU	N	260ALA	O	99.18
102ASN	ND2	104ARG	NE	0.01
102ASN	ND2	104ARG	NH2	0.06
102ASN	ND2	259SER	OG	9.31
102ASN	ND2	260ALA	O	1.36
102ASN	ND2	261SER	N	0.01
102ASN	ND2	261SER	OG	44.49
102ASN	N	100PHE	O	0.01
102ASN	N	132ARG	O	98.49
101ALA	N	262LEU	O	46.94
100PHE	N	98ASP	O	0.34
100PHE	N	262LEU	O	80.34
99LEU	N	94ARG	O	58.40
99LEU	N	97GLN	O	8.96
98ASP	N	94ARG	O	3.25
98ASP	N	95LYS	O	13.33
98ASP	N	96SER	O	0.55
98ASP	N	264ARG	O	0.20
97GLN	NE2	61VAL	O	1.36
97GLN	NE2	62GLU	OE1	40.35
97GLN	NE2	62GLU	OE2	40.00
97GLN	NE2	93LEU	O	0.21
97GLN	NE2	96SER	O	0.04
97GLN	NE2	97GLN	O	0.00
97GLN	NE2	266THR	N	0.00
97GLN	NE2	266THR	O	24.78
97GLN	N	93LEU	O	85.81
97GLN	N	94ARG	O	0.27
97GLN	N	95LYS	O	0.00
96SER	OG	92SER	O	86.21
96SER	OG	93LEU	O	6.72
96SER	OG	97GLN	N	0.01
96SER	OG	97GLN	OE1	0.07
96SER	N	92SER	O	76.86
96SER	N	93LEU	O	12.78
96SER	N	94ARG	O	0.04
96SER	N	97GLN	O	0.20
95LYS	NZ	91LEU	O	0.03
95LYS	NZ	95LYS	O	0.02
95LYS	NZ	98ASP	OD1	0.03
214GLN	NE2	78ASP	OD2	0.02
214GLN	NE2	212GLU	OE1	66.53
214GLN	NE2	212GLU	OE2	24.19
214GLN	NE2	213HIS	O	0.92
214GLN	NE2	215TYR	N	0.01
214GLN	NE2	215TYR	O	0.02
214GLN	NE2	218ALA	O	0.01
214GLN	NE2	222HIS	ND1	0.04
214GLN	NE2	222HIS	NE2	0.00
214GLN	NE2	229ARG	NE	0.00
214GLN	NE2	229ARG	NH1	0.04
214GLN	NE2	229ARG	NH2	0.07
214GLN	N	182SER	O	77.16
213HIS	NE2	200GLU	OE1	47.27
213HIS	NE2	200GLU	OE2	49.33
212GLU	N	180VAL	O	47.76
210ALA	N	178LYS	O	85.85
209VAL	N	206TYR	O	2.89
209VAL	N	207PRO	O	36.49
208ASP	N	206TYR	O	0.03
208ASP	N	208ASP	OD1	0.00
206TYR	OH	167ARG	O	0.17
206TYR	OH	170PHE	O	1.97
206TYR	OH	171GLU	OE1	10.85
206TYR	OH	171GLU	OE2	13.58
206TYR	N	202VAL	O	6.38
206TYR	N	203GLY	O	37.19
206TYR	N	204ARG	O	0.25
205GLY	N	200GLU	O	0.00
205GLY	N	201GLU	O	0.36
205GLY	N	202VAL	O	10.12
205GLY	N	203GLY	O	0.18
204ARG	NH2	200GLU	OE1	41.19
204ARG	NH2	200GLU	OE2	45.63
204ARG	NH2	201GLU	OE1	0.02
204ARG	NH2	211LEU	O	1.18
204ARG	NH2	213HIS	NE2	0.21
204ARG	NH1	200GLU	OE1	0.17
204ARG	NH1	201GLU	OE1	0.69
204ARG	NH1	201GLU	OE2	0.24
204ARG	NH1	203GLY	O	0.01
204ARG	NH1	211LEU	O	22.18
204ARG	NE	200GLU	OE1	49.70
79GLY	N	76LYS	O	3.08
79GLY	N	77TRP	O	0.04
79GLY	N	78ASP	OD2	0.00
78ASP	N	74GLY	O	9.74
78ASP	N	75PRO	O	45.87
78ASP	N	76LYS	O	0.38
78ASP	N	78ASP	OD1	0.02
78ASP	N	78ASP	OD2	0.04
77TRP	NE1	9ASP	OD1	0.00
77TRP	NE1	42GLY	O	1.74
77TRP	NE1	43GLY	O	0.03
77TRP	NE1	47ASP	OD1	0.54
77TRP	NE1	47ASP	OD2	0.01
77TRP	NE1	84ILE	O	4.98
77TRP	N	73GLY	O	0.00
77TRP	N	74GLY	O	40.51
77TRP	N	75PRO	O	0.84
76LYS	NZ	9ASP	OD1	54.77
76LYS	NZ	9ASP	OD2	45.58
76LYS	NZ	47ASP	OD1	0.48
76LYS	NZ	47ASP	OD2	0.50
76LYS	NZ	73GLY	O	0.19
76LYS	NZ	77TRP	NE1	0.01
76LYS	NZ	278ASP	OD2	0.06
76LYS	N	74GLY	O	8.82
76LYS	N	78ASP	OD2	0.00
74GLY	N	274GLY	O	0.00
74GLY	N	275SER	O	2.82
73GLY	N	9ASP	OD2	2.01
73GLY	N	9ASP	O	11.68
71SER	OG	9ASP	O	0.37
71SER	OG	70GLY	O	0.12
71SER	OG	71SER	O	0.06
71SER	OG	73GLY	O	0.01
71SER	OG	270GLU	OE1	0.01
71SER	OG	271PRO	O	0.20
71SER	OG	273HIS	NE2	3.34
71SER	OG	273HIS	O	0.23
71SER	N	7PRO	O	21.11
70GLY	N	6LEU	O	33.47
70GLY	N	7PRO	O	0.01
69LEU	N	269PHE	O	95.30
68LEU	N	4ALA	O	99.18
196ARG	NH2	214GLN	N	0.00
196ARG	NH2	214GLN	O	0.02
196ARG	NH2	215TYR	N	0.00
196ARG	NH2	215TYR	OH	1.61
196ARG	NH1	78ASP	OD1	0.04
196ARG	NH1	78ASP	OD2	0.02
196ARG	NH1	215TYR	OH	1.63
196ARG	NE	184ASP	OD1	2.73
196ARG	NE	184ASP	OD2	98.54
196ARG	N	192GLY	O	97.05
196ARG	N	193GLU	O	0.07
195TRP	NE1	183VAL	O	0.01
195TRP	NE1	234VAL	O	0.01
195TRP	N	191VAL	O	61.27
195TRP	N	192GLY	O	2.53
194PHE	N	190GLU	O	92.34
194PHE	N	191VAL	O	0.08
193GLU	N	189LEU	O	77.74
193GLU	N	190GLU	O	3.98
192GLY	N	189LEU	O	52.66
192GLY	N	190GLU	O	0.03
191VAL	N	153ASN	OD1	92.63
190GLU	N	153ASN	OD1	20.84
190GLU	N	190GLU	OE1	11.19
190GLU	N	190GLU	OE2	17.26
189LEU	N	185LYS	O	67.37
189LEU	N	186ALA	O	1.65
188VAL	N	185LYS	O	83.09
188VAL	N	186ALA	O	0.03
188VAL	N	187ASN	OD1	0.57
187ASN	ND2	187ASN	O	0.00
187ASN	ND2	215TYR	OH	0.11
187ASN	ND2	217ASP	OD1	0.09
187ASN	ND2	217ASP	OD2	0.29
187ASN	N	185LYS	O	1.42
187ASN	N	187ASN	OD1	6.27
187ASN	N	187ASN	ND2	0.03
186ALA	N	184ASP	OD1	99.72
185LYS	NZ	241ASP	OD1	22.32
185LYS	NZ	241ASP	OD2	50.08
185LYS	NZ	241ASP	O	0.00
185LYS	NZ	245ASP	OD1	4.56
185LYS	NZ	245ASP	OD2	0.04

95LYS	NZ	98ASP	OD2	0.08
95LYS	N	91LEU	O	49.70
95LYS	N	92SER	O	16.22
95LYS	N	93LEU	O	4.14
94ARG	NH2	87GLU	OE1	0.11
94ARG	NH2	87GLU	OE2	0.04
94ARG	NH2	270GLU	OE1	40.58
94ARG	NH2	270GLU	OE2	42.92
94ARG	NH1	87GLU	OE1	0.21
94ARG	NH1	87GLU	OE2	0.80
94ARG	NH1	134LEU	O	0.00
94ARG	NE	90LEU	O	0.00
94ARG	NE	270GLU	OE1	0.36
94ARG	N	90LEU	O	95.24
94ARG	N	91LEU	O	0.26
93LEU	N	89GLY	O	81.41
93LEU	N	90LEU	O	2.75
92SER	OG	88THR	O	38.90
92SER	OG	89GLY	O	1.42
92SER	OG	92SER	O	0.04
92SER	OG	93LEU	N	0.17
92SER	N	88THR	O	30.52
92SER	N	89GLY	O	8.16
92SER	N	90LEU	O	0.25
91LEU	N	87GLU	O	91.18
91LEU	N	88THR	O	0.18
90LEU	N	86PRO	O	58.53
90LEU	N	87GLU	O	13.82
89GLY	N	85SER	O	2.06
89GLY	N	86PRO	O	83.06
89GLY	N	87GLU	O	0.10
88THR	OG1	82ARG	O	9.24
88THR	OG1	83LYS	O	0.07
88THR	OG1	85SER	O	82.38
88THR	OG1	87GLU	OE1	0.21
88THR	OG1	89GLY	N	0.00
88THR	N	82ARG	O	0.02
88THR	N	85SER	O	84.47
87GLU	N	85SER	O	0.02
87GLU	N	87GLU	OE1	1.77
87GLU	N	87GLU	OE2	0.09
85SER	OG	77TRP	O	0.04
85SER	OG	81PRO	O	7.16

204ARG	NE	200GLU	OE2	48.04
204ARG	NE	200GLU	O	0.01
204ARG	N	199VAL	O	2.01
204ARG	N	200GLU	O	66.12
204ARG	N	201GLU	O	2.60
204ARG	N	202VAL	O	0.01
203GLY	N	199VAL	O	49.32
203GLY	N	200GLU	O	12.95
203GLY	N	201GLU	O	0.01
203GLY	N	204ARG	O	0.02
202VAL	N	198THR	O	66.59
202VAL	N	199VAL	O	6.18
202VAL	N	200GLU	O	0.03
201GLU	N	197LYS	O	57.23
201GLU	N	198THR	O	11.26
201GLU	N	199VAL	O	0.07
200GLU	N	196ARG	O	76.50
200GLU	N	197LYS	O	1.54
199VAL	N	195TRP	O	97.09
199VAL	N	196ARG	O	0.60
198THR	OG1	194PHE	O	95.83
198THR	OG1	195TRP	O	0.01
198THR	N	194PHE	O	67.43
198THR	N	195TRP	O	14.71
198THR	N	196ARG	O	0.00
197LYS	NZ	147SER	O	0.02
197LYS	NZ	148GLU	OE1	30.12
197LYS	NZ	148GLU	OE2	30.16
197LYS	NZ	148GLU	O	0.35
197LYS	NZ	193GLU	OE1	4.92
197LYS	NZ	193GLU	OE2	3.98
197LYS	NZ	193GLU	O	0.01
197LYS	NZ	201GLU	OE1	7.82
197LYS	NZ	201GLU	OE2	7.48
197LYS	N	193GLU	O	83.73
197LYS	N	194PHE	O	1.91
196ARG	NH2	184ASP	OD1	90.88
196ARG	NH2	184ASP	OD2	15.65
196ARG	NH2	213HIS	O	0.02
196ARG	NH2	214GLN	N	0.01
196ARG	NH2	214GLN	O	0.04
196ARG	NH2	215TYR	N	0.02
196ARG	NH2	215TYR	OH	1.27

67VAL	N	267PRO	O	91.25
66ALA	N	2LYS	O	3.06
65GLU	N	2LYS	O	99.00
64ALA	N	60GLY	O	20.89
64ALA	N	61VAL	O	8.94
64ALA	N	62GLU	O	4.71
63GLU	N	59LYS	O	12.66
63GLU	N	60GLY	O	30.33
63GLU	N	61VAL	O	0.22
62GLU	N	58ARG	O	4.24
62GLU	N	59LYS	O	2.27
62GLU	N	60GLY	O	0.01
61VAL	N	57THR	O	86.74
61VAL	N	58ARG	O	0.22
60GLY	N	56PRO	O	5.54
60GLY	N	57THR	O	60.43
60GLY	N	58ARG	O	0.26
59LYS	NZ	55GLU	OE1	34.60
59LYS	NZ	55GLU	OE2	29.31
59LYS	NZ	55GLU	O	0.02
59LYS	NZ	62GLU	OE1	0.36
59LYS	NZ	62GLU	OE2	0.02
59LYS	NZ	63GLU	OE2	0.04
59LYS	N	55GLU	O	58.61
59LYS	N	56PRO	O	7.83
59LYS	N	57THR	O	0.00
58ARG	NH2	51GLU	OE1	12.69
58ARG	NH2	51GLU	OE2	24.24
58ARG	NH2	53PHE	O	2.60
58ARG	NH2	55GLU	OE1	11.26
58ARG	NH2	55GLU	OE2	7.06
58ARG	NH2	92SER	OG	7.94
58ARG	NH1	51GLU	OE1	5.00
58ARG	NH1	51GLU	OE2	4.92
58ARG	NH1	53PHE	O	0.12
58ARG	NH1	55GLU	OE1	10.62
58ARG	NH1	55GLU	OE2	10.02
58ARG	NH1	92SER	OG	3.82
58ARG	NE	51GLU	OE1	2.94
58ARG	NE	51GLU	OE2	1.26
58ARG	NE	53PHE	O	44.29
58ARG	NE	54PRO	O	0.01
58ARG	NE	55GLU	OE1	1.52

185LYS	NZ	187ASN	OD1	0.91
185LYS	NZ	217ASP	OD1	42.73
185LYS	NZ	217ASP	OD2	40.65
185LYS	N	184ASP	OD1	0.38
184ASP	N	214GLN	O	95.76
183VAL	N	234VAL	O	75.49
182SER	OG	183VAL	O	0.00
182SER	OG	184ASP	OD2	0.08
182SER	OG	213HIS	ND1	0.12
182SER	OG	234VAL	O	0.01
182SER	N	212GLU	O	97.40
181VAL	N	232VAL	O	88.07
180VAL	N	179HIS	ND1	0.08
180VAL	N	210ALA	O	89.86
179HIS	NE2	211LEU	O	0.01
179HIS	NE2	212GLU	N	0.00
179HIS	NE2	212GLU	OE1	31.96
179HIS	NE2	212GLU	OE2	25.09
179HIS	NE2	229ARG	NE	0.00
179HIS	NE2	229ARG	NH1	0.01
179HIS	NE2	229ARG	NH2	0.19
179HIS	NE2	229ARG	O	2.44
179HIS	N	177ARG	O	0.00
179HIS	N	231ASP	OD2	0.01
178LYS	NZ	174ARG	O	3.19
178LYS	NZ	176ARG	O	1.54
178LYS	NZ	208ASP	OD2	36.03
178LYS	NZ	208ASP	O	0.33
178LYS	N	173ALA	O	24.44
178LYS	N	176ARG	O	0.06
178LYS	N	231ASP	OD1	0.02
178LYS	N	231ASP	OD2	0.02
177ARG	NH2	125GLY	O	0.83
177ARG	NH2	127ASP	OD1	14.16
177ARG	NH2	127ASP	OD2	4.03
177ARG	NH2	127ASP	O	1.86
177ARG	NH2	179HIS	ND1	0.03
177ARG	NH2	228ALA	O	15.74
177ARG	NH2	229ARG	O	0.40
177ARG	NH2	230PHE	O	0.89
177ARG	NH2	231ASP	OD1	6.54
177ARG	NH2	231ASP	OD2	40.26
177ARG	NH1	125GLY	O	0.28

85SER	OG	82ARG	O	47.50
85SER	OG	84ILE	O	0.11
85SER	OG	85SER	O	0.06
85SER	OG	87GLU	OE1	0.02
85SER	OG	88THR	OG1	0.60
85SER	N	81PRO	O	1.10
85SER	N	82ARG	O	71.36
85SER	N	83LYS	O	0.00
85SER	N	88THR	OG1	3.44
84ILE	N	81PRO	O	71.61
84ILE	N	82ARG	O	0.02
83LYS	N	81PRO	O	1.36
82ARG	NH2	187ASN	O	0.16
82ARG	NH2	87GLU	OE1	50.35
82ARG	NH2	87GLU	OE2	40.33
82ARG	NH1	187ASN	OD1	0.00
82ARG	NH1	187ASN	O	3.18
82ARG	NH1	87GLU	OE1	0.07
82ARG	NH1	87GLU	OE2	0.03
82ARG	NE	87GLU	OE1	34.17
82ARG	NE	87GLU	OE2	44.02
80LEU	N	77TRP	O	25.67
80LEU	N	78ASP	O	0.95
79GLY	N	76LYS	O	0.00
79GLY	N	77TRP	O	0.03
79GLY	N	78ASP	OD1	0.08
79GLY	N	78ASP	OD2	0.10
78ASP	N	74GLY	O	0.68
78ASP	N	75PRO	O	39.34
78ASP	N	76LYS	O	1.46
78ASP	N	78ASP	OD1	0.01
77TRP	NE1	43GLY	O	0.01
77TRP	NE1	47ASP	OD1	0.57
77TRP	NE1	47ASP	OD2	0.28
77TRP	NE1	80LEU	O	0.00
77TRP	NE1	81PRO	O	23.31
77TRP	NE1	84ILE	O	26.24
77TRP	N	74GLY	O	7.21
77TRP	N	75PRO	O	0.09
76LYS	NZ	43GLY	O	0.12
76LYS	NZ	47ASP	OD1	10.69
76LYS	NZ	47ASP	OD2	8.17
76LYS	N	9ASP	OD1	0.32

196ARG	NH1	193GLU	OE2	0.02
196ARG	NH1	213HIS	O	0.00
196ARG	NH1	215TYR	OH	1.08
196ARG	NE	184ASP	OD1	1.95
196ARG	NE	184ASP	OD2	99.02
196ARG	N	192GLY	O	98.90
196ARG	N	193GLU	O	0.02
195TRP	NE1	183VAL	O	1.14
195TRP	NE1	234VAL	O	0.04
195TRP	N	191VAL	O	49.53
195TRP	N	192GLY	O	2.44
194PHE	N	190GLU	O	95.46
194PHE	N	191VAL	O	0.03
193GLU	N	189LEU	O	86.06
193GLU	N	190GLU	O	2.52
192GLY	N	189LEU	O	55.92
192GLY	N	190GLU	O	0.21
191VAL	N	153ASN	OD1	96.73
190GLU	N	153ASN	OD1	14.07
190GLU	N	190GLU	OE1	7.76
190GLU	N	190GLU	OE2	8.50
189LEU	N	185LYS	O	76.49
189LEU	N	186ALA	O	2.60
188VAL	N	185LYS	O	81.99
188VAL	N	186ALA	O	0.04
188VAL	N	187ASN	OD1	0.21
187ASN	ND2	187ASN	O	0.00
187ASN	ND2	215TYR	OH	0.09
187ASN	ND2	217ASP	OD1	0.00
187ASN	ND2	217ASP	OD2	0.01
187ASN	N	185LYS	O	2.50
187ASN	N	187ASN	OD1	4.55
187ASN	N	187ASN	ND2	0.01
187ASN	N	215TYR	OH	0.00
186ALA	N	184ASP	OD1	99.76
185LYS	NZ	241ASP	OD1	55.53
185LYS	NZ	241ASP	OD2	31.65
185LYS	NZ	187ASN	OD1	0.32
185LYS	NZ	187ASN	ND2	0.00
185LYS	NZ	217ASP	OD1	72.85
185LYS	NZ	217ASP	OD2	23.46
185LYS	N	184ASP	OD1	0.10
184ASP	N	214GLN	O	96.94

58ARG	NE	55GLU	OE2	7.66
58ARG	NE	92SER	OG	0.16
58ARG	N	54PRO	O	89.62
58ARG	N	55GLU	O	3.34
57THR	OG1	40PRO	O	97.65
57THR	N	54PRO	O	12.09
55GLU	N	55GLU	OE1	0.13
55GLU	N	55GLU	OE2	0.04
53PHE	N	51GLU	OE1	0.41
53PHE	N	51GLU	OE2	0.70
53PHE	N	51GLU	O	0.01
51GLU	N	45ALA	O	4.25
51GLU	N	49PHE	O	1.67
51GLU	N	51GLU	OE1	0.00
51GLU	N	51GLU	OE2	0.04
50GLY	N	45ALA	O	71.76
50GLY	N	46ILE	O	3.42
50GLY	N	47ASP	O	0.02
49PHE	N	44ALA	O	0.03
49PHE	N	45ALA	O	82.60
49PHE	N	46ILE	O	0.08
49PHE	N	47ASP	O	0.02
48ALA	N	44ALA	O	86.32
48ALA	N	45ALA	O	2.35
48ALA	N	46ILE	O	0.23
47ASP	N	43GLY	O	41.61
47ASP	N	44ALA	O	17.23
47ASP	N	45ALA	O	0.01
46ILE	N	42GLY	O	7.30
46ILE	N	43GLY	O	29.89
45ALA	N	41PHE	O	0.02
45ALA	N	42GLY	O	4.29
45ALA	N	43GLY	O	0.00
44ALA	N	9ASP	OD1	1.51
44ALA	N	9ASP	OD2	0.67
44ALA	N	42GLY	O	0.00
43GLY	N	8GLY	O	0.11
43GLY	N	9ASP	OD1	1.39
43GLY	N	9ASP	OD2	3.75
43GLY	N	41PHE	O	0.18
42GLY	N	52PRO	O	34.89
39PHE	N	5VAL	O	92.46
39PHE	N	37GLU	O	0.11

177ARG	NH1	127ASP	OD1	0.38
177ARG	NH1	127ASP	OD2	0.13
177ARG	NH1	179HIS	ND1	0.06
177ARG	NH1	228ALA	O	30.61
177ARG	NH1	229ARG	O	5.69
177ARG	NH1	230PHE	N	0.03
177ARG	NH1	230PHE	O	0.54
177ARG	NH1	231ASP	OD2	0.07
177ARG	NE	231ASP	OD1	32.08
177ARG	NE	231ASP	OD2	15.87
177ARG	N	173ALA	O	0.01
177ARG	N	177ARG	NE	0.25
177ARG	N	231ASP	OD1	58.95
177ARG	N	231ASP	OD2	4.53
176ARG	NH2	127ASP	OD1	17.31
176ARG	NH2	127ASP	OD2	73.56
176ARG	NH2	127ASP	O	0.04
176ARG	NH1	127ASP	OD1	6.00
176ARG	NH1	127ASP	OD2	5.39
176ARG	NH1	127ASP	O	79.36
176ARG	NH1	231ASP	OD1	8.25
176ARG	NH1	231ASP	OD2	87.40
176ARG	NH1	231ASP	O	0.11
176ARG	N	172LEU	O	1.96
176ARG	N	173ALA	O	77.35
176ARG	N	174ARG	O	0.48
175LYS	NZ	171GLU	OE1	0.74
175LYS	NZ	171GLU	OE2	0.68
175LYS	NZ	171GLU	O	0.84
175LYS	NZ	175LYS	O	0.01
175LYS	NZ	299GLU	OE1	1.14
175LYS	NZ	299GLU	OE2	1.63
175LYS	NZ	299GLU	O	0.43
175LYS	NZ	300HIS	ND1	0.06
175LYS	N	171GLU	O	6.00
175LYS	N	172LEU	O	53.46
175LYS	N	173ALA	O	0.07
174ARG	NH2	205GLY	O	0.13
174ARG	NH2	208ASP	OD1	99.86
174ARG	NH2	208ASP	OD2	1.48
174ARG	NH1	171GLU	OE1	15.18
174ARG	NH1	171GLU	OE2	15.84
174ARG	NE	208ASP	OD1	7.60

76LYS	N	9ASP	OD2	0.01	183VAL	N	234VAL	O	78.39	38VAL	N	37GLU	OE1	4.89	174ARG	NE	208ASP	OD2	96.26
76LYS	N	74GLY	O	0.11	182SER	N	212GLU	O	97.69	38VAL	N	37GLU	OE2	4.87	174ARG	N	170PHE	O	39.18
74GLY	N	9ASP	OD1	22.62	181VAL	N	232VAL	O	91.19	37GLU	N	3VAL	O	46.88	174ARG	N	171GLU	O	21.08
74GLY	N	9ASP	OD2	23.90	180VAL	N	179HIS	ND1	0.25	36TYR	OH	17GLU	OE1	0.02	173ALA	N	169ALA	O	73.11
74GLY	N	77TRP	O	0.40	180VAL	N	210ALA	O	85.51	36TYR	OH	17GLU	OE2	0.01	173ALA	N	170PHE	O	2.70
73GLY	N	9ASP	OD1	27.13	179HIS	NE2	211LEU	O	0.01	36TYR	N	34LEU	O	0.02	172LEU	N	168VAL	O	85.87
73GLY	N	9ASP	OD2	29.51	179HIS	NE2	212GLU	OE1	24.84	35ALA	N	1MET	O	58.92	172LEU	N	169ALA	O	2.82
73GLY	N	9ASP	O	6.49	179HIS	NE2	212GLU	OE2	69.27	35ALA	N	33GLY	O	0.12	171GLU	N	167ARG	O	44.02
71SER	OG	9ASP	O	11.13	179HIS	NE2	229ARG	NH1	0.03	34LEU	N	27ASP	OD1	75.98	171GLU	N	168VAL	O	13.09
71SER	OG	71SER	O	1.24	179HIS	NE2	229ARG	NH2	0.12	34LEU	N	27ASP	OD2	8.93	170PHE	N	166ALA	O	93.79
71SER	OG	72VAL	N	0.00	179HIS	N	177ARG	O	0.18	33GLY	N	27ASP	OD1	98.74	170PHE	N	167ARG	O	0.65
71SER	OG	72VAL	O	0.08	179HIS	N	231ASP	OD2	0.60	33GLY	N	27ASP	OD2	0.08	169ALA	N	165VAL	O	66.56
71SER	OG	273HIS	ND1	0.00	178LYS	NZ	174ARG	O	0.76	32LEU	N	26LEU	O	0.01	169ALA	N	166ALA	O	4.52
71SER	OG	273HIS	NE2	0.48	178LYS	NZ	176ARG	O	0.04	32LEU	N	27ASP	OD1	79.40	168VAL	N	164ARG	O	26.39
71SER	OG	273HIS	O	0.84	178LYS	NZ	177ARG	O	0.00	32LEU	N	27ASP	O	0.03	168VAL	N	165VAL	O	27.56
71SER	OG	274GLY	O	0.61	178LYS	NZ	208ASP	OD1	51.41	31GLY	N	26LEU	O	93.84	168VAL	N	166ALA	O	0.10
71SER	OG	275SER	OG	0.34	178LYS	NZ	208ASP	OD2	50.83	31GLY	N	27ASP	O	0.05	167ARG	NH2	163GLU	OE1	11.92
71SER	N	7PRO	O	0.97	178LYS	NZ	208ASP	O	0.06	30GLU	N	26LEU	O	69.41	167ARG	NH2	163GLU	OE2	21.92
71SER	N	273HIS	NE2	0.00	178LYS	N	173ALA	O	11.58	30GLU	N	27ASP	O	0.09	167ARG	NH2	202VAL	O	0.00
70GLY	N	6LEU	O	14.10	178LYS	N	176ARG	O	0.13	30GLU	N	28GLU	O	0.02	167ARG	NH1	163GLU	OE1	4.32
70GLY	N	7PRO	O	0.00	178LYS	N	231ASP	OD1	0.01	29ALA	N	25ALA	O	84.84	167ARG	NH1	163GLU	OE2	4.93
69LEU	N	269PHE	O	92.25	178LYS	N	231ASP	OD2	0.69	29ALA	N	26LEU	O	3.37	167ARG	NH1	163GLU	O	0.07
68LEU	N	4ALA	O	99.40	177ARG	NH2	125GLY	O	4.72	29ALA	N	27ASP	O	0.07	167ARG	NH1	202VAL	O	0.00
67VAL	N	267PRO	O	89.70	177ARG	NH2	127ASP	OD1	2.02	28GLU	N	24ARG	O	40.82	167ARG	NH1	205GLY	O	0.00
66ALA	N	2LYS	O	2.09	177ARG	NH2	127ASP	OD2	3.10	28GLU	N	25ALA	O	18.61	167ARG	NH1	206TYR	OH	4.56
65GLU	N	2LYS	O	98.70	177ARG	NH2	127ASP	O	2.66	28GLU	N	26LEU	O	0.02	167ARG	NE	163GLU	OE1	24.38
64ALA	N	60GLY	O	17.89	177ARG	NH2	228ALA	O	18.76	27ASP	N	23LEU	O	86.02	167ARG	NE	163GLU	OE2	55.97
64ALA	N	61VAL	O	17.87	177ARG	NH2	229ARG	O	0.08	27ASP	N	24ARG	O	0.74	167ARG	NE	163GLU	O	0.03
64ALA	N	62GLU	O	4.00	177ARG	NH2	230PHE	O	1.21	26LEU	N	22VAL	O	87.04	167ARG	NE	206TYR	OH	0.10
63GLU	N	59LYS	O	7.62	177ARG	NH2	231ASP	OD1	3.23	26LEU	N	23LEU	O	2.16	167ARG	N	163GLU	O	84.23
63GLU	N	60GLY	O	22.16	177ARG	NH2	231ASP	OD2	3.73	25ALA	N	21LYS	O	42.91	167ARG	N	164ARG	O	2.02
63GLU	N	61VAL	O	0.71	177ARG	NH1	125GLY	O	0.86	25ALA	N	22VAL	O	13.67	166ALA	N	162VAL	O	66.33
62GLU	N	58ARG	O	1.98	177ARG	NH1	127ASP	OD1	2.46	25ALA	N	23LEU	O	0.00	166ALA	N	163GLU	O	3.44
62GLU	N	59LYS	O	1.15	177ARG	NH1	127ASP	OD2	16.72	24ARG	NH2	17GLU	OE1	28.80	165VAL	N	161GLU	O	19.36
62GLU	N	60GLY	O	0.01	177ARG	NH1	127ASP	O	1.20	24ARG	NH2	17GLU	OE2	18.05	165VAL	N	162VAL	O	9.77
61VAL	N	57THR	O	74.39	177ARG	NH1	179HIS	ND1	0.01	24ARG	NH2	17GLU	O	0.09	165VAL	N	163GLU	O	0.02
61VAL	N	58ARG	O	0.03	177ARG	NH1	228ALA	O	33.87	24ARG	NH2	36TYR	OH	8.42	164ARG	NH2	98ASP	OD1	0.08
60GLY	N	56PRO	O	3.38	177ARG	NH1	229ARG	O	4.58	24ARG	NH1	17GLU	OE1	14.84	164ARG	NH2	98ASP	OD2	3.65
60GLY	N	57THR	O	69.95	177ARG	NH1	230PHE	O	0.49	24ARG	NH1	17GLU	OE2	27.26	164ARG	NH2	98ASP	O	0.04
60GLY	N	58ARG	O	0.30	177ARG	NH1	231ASP	OD1	22.35	24ARG	NH1	20LEU	O	0.00	164ARG	NH2	161GLU	OE1	0.01
59LYS	NZ	55GLU	OE1	39.41	177ARG	NH1	231ASP	OD2	0.02	24ARG	NH1	36TYR	OH	35.09	164ARG	NH2	161GLU	OE2	3.99
59LYS	NZ	55GLU	OE2	46.24	177ARG	NE	231ASP	OD1	4.93	24ARG	NE	20LEU	O	0.96	164ARG	NH2	163GLU	OE1	24.64
59LYS	NZ	55GLU	O	0.15	177ARG	NE	231ASP	OD2	2.65	24ARG	NE	36TYR	OH	9.50	164ARG	NH2	163GLU	OE2	42.09

59LYS	N	55GLU	O	44.35
59LYS	N	56PRO	O	21.44
59LYS	N	57THR	O	0.03
58ARG	NH2	51GLU	OE1	13.76
58ARG	NH2	51GLU	OE2	17.26
58ARG	NH2	53PHE	O	1.27
58ARG	NH2	55GLU	OE1	0.62
58ARG	NH2	55GLU	OE2	0.28
58ARG	NH2	92SER	OG	8.85
58ARG	NH2	96SER	OG	0.00
58ARG	NH1	51GLU	OE1	8.37
58ARG	NH1	51GLU	OE2	12.53
58ARG	NH1	53PHE	O	15.73
58ARG	NH1	55GLU	OE1	2.28
58ARG	NH1	55GLU	OE2	3.35
58ARG	NH1	92SER	OG	1.97
58ARG	NE	51GLU	OE1	0.16
58ARG	NE	51GLU	OE2	0.22
58ARG	NE	53PHE	O	48.20
58ARG	NE	92SER	OG	0.02
58ARG	N	54PRO	O	90.38
58ARG	N	55GLU	O	3.10
57THR	OG1	40PRO	O	98.21
57THR	N	54PRO	O	9.49
55GLU	N	51GLU	OE2	0.00
55GLU	N	55GLU	OE1	0.24
55GLU	N	55GLU	OE2	0.13
53PHE	N	51GLU	OE1	0.81
53PHE	N	51GLU	OE2	0.84
53PHE	N	51GLU	O	0.02
51GLU	N	45ALA	O	4.06
51GLU	N	49PHE	O	1.03
51GLU	N	51GLU	OE1	0.01
51GLU	N	51GLU	OE2	0.01
50GLY	N	45ALA	O	62.09
50GLY	N	46ILE	O	6.44
49PHE	N	45ALA	O	82.38
49PHE	N	46ILE	O	0.12
49PHE	N	47ASP	O	0.02
48ALA	N	44ALA	O	78.73
48ALA	N	45ALA	O	3.36
48ALA	N	46ILE	O	0.62
47ASP	N	43GLY	O	56.97

177ARG	N	177ARG	NE	0.00
177ARG	N	231ASP	OD1	24.78
177ARG	N	231ASP	OD2	51.65
176ARG	NH2	127ASP	OD1	48.80
176ARG	NH2	127ASP	OD2	44.69
176ARG	NH2	127ASP	O	0.03
176ARG	NH2	128VAL	O	0.00
176ARG	NH1	127ASP	OD1	9.33
176ARG	NH1	127ASP	OD2	14.03
176ARG	NH1	127ASP	O	68.49
176ARG	NH1	177ARG	NH1	0.01
176ARG	NH1	231ASP	OD1	60.66
176ARG	NH1	231ASP	OD2	34.44
176ARG	NH1	231ASP	O	0.08
176ARG	NE	172ALA	O	0.01
176ARG	NE	299GLU	OE1	0.00
176ARG	N	172ALA	O	0.44
176ARG	N	173ALA	O	78.54
176ARG	N	174ARG	O	0.46
175LYS	NZ	171GLU	OE1	0.85
175LYS	NZ	171GLU	OE2	0.91
175LYS	NZ	171GLU	O	0.68
175LYS	NZ	299GLU	OE1	30.65
175LYS	NZ	299GLU	OE2	29.61
175LYS	NZ	299GLU	O	0.75
175LYS	NZ	300HIS	ND1	1.44
175LYS	NZ	300HIS	O	0.33
175LYS	N	171GLU	O	1.35
175LYS	N	172ALA	O	49.59
175LYS	N	173ALA	O	0.15
174ARG	NH2	170PHE	O	0.00
174ARG	NH2	171GLU	OE1	39.89
174ARG	NH2	171GLU	OE2	32.78
174ARG	NH2	206TYR	OH	7.17
174ARG	NH2	207PRO	O	0.67
174ARG	NH2	208ASP	OD1	0.54
174ARG	NH2	208ASP	OD2	0.15
174ARG	NH1	170PHE	O	0.02
174ARG	NH1	171GLU	OE1	15.52
174ARG	NH1	171GLU	OE2	19.51
174ARG	NH1	171GLU	O	0.00
174ARG	NH1	206TYR	OH	9.22
174ARG	NH1	207PRO	O	1.57

24ARG	N	20LEU	O	97.80
24ARG	N	21LYS	O	0.10
23LEU	N	19ALA	O	70.96
23LEU	N	20LEU	O	1.00
22VAL	N	18ALA	O	24.52
22VAL	N	19ALA	O	18.75
21LYS	NZ	17GLU	OE1	0.70
21LYS	NZ	17GLU	OE2	1.45
21LYS	NZ	333THR	O	0.05
21LYS	NZ	334GLU	OE1	35.52
21LYS	NZ	334GLU	OE2	39.62
21LYS	NZ	334GLU	O	0.12
21LYS	NZ	337THR	OG1	15.58
21LYS	N	17GLU	O	77.24
21LYS	N	18ALA	O	3.36
20LEU	N	16THR	O	89.69
20LEU	N	17GLU	O	1.28
19ALA	N	15VAL	O	54.35
19ALA	N	16THR	O	13.60
18ALA	N	14GLU	O	67.26
18ALA	N	15VAL	O	2.39
17GLU	N	13PRO	O	82.09
17GLU	N	14GLU	O	1.95
16THR	OG1	7PRO	O	0.82
16THR	OG1	12GLY	O	95.24
16THR	N	12GLY	O	74.04
16THR	N	13PRO	O	2.57
15VAL	N	11ILE	O	94.01
15VAL	N	12GLY	O	1.34
14GLU	N	11ILE	O	20.10
14GLU	N	12GLY	O	0.00
14GLU	N	14GLU	OE1	0.01
12GLY	N	9ASP	O	2.50
12GLY	N	71SER	OG	0.46
12GLY	N	71SER	O	0.01
11ILE	N	9ASP	O	0.03
11ILE	N	275SER	O	0.64
11ILE	N	276ALA	O	0.03
11ILE	N	277PRO	O	0.00
10GLY	N	9ASP	OD1	0.35
10GLY	N	9ASP	OD2	0.62
9ASP	N	71SER	O	88.60
8GLY	N	41PHE	O	24.10

164ARG	NH2	167ARG	NE	0.00
164ARG	NH2	167ARG	O	0.02
164ARG	NH2	264ARG	NE	0.02
164ARG	NH2	264ARG	NH1	0.00
164ARG	NH2	264ARG	NH2	0.01
164ARG	NH1	98ASP	OD1	2.62
164ARG	NH1	98ASP	OD2	0.31
164ARG	NH1	160PRO	O	0.12
164ARG	NH1	161GLU	OE1	11.10
164ARG	NH1	161GLU	OE2	22.64
164ARG	NH1	163GLU	OE1	2.16
164ARG	NH1	163GLU	OE2	0.01
164ARG	NH1	264ARG	NH2	0.01
164ARG	NE	163GLU	OE1	43.80
164ARG	NE	163GLU	OE2	6.23
164ARG	NE	164ARG	O	0.04
164ARG	N	160PRO	O	72.53
164ARG	N	161GLU	O	3.35
163GLU	N	159LYS	O	80.38
163GLU	N	160PRO	O	1.94
162VAL	N	158SER	O	38.91
162VAL	N	159LYS	O	20.02
162VAL	N	160PRO	O	0.02
161GLU	N	158SER	O	36.15
161GLU	N	159LYS	O	0.03
161GLU	N	161GLU	OE1	1.07
161GLU	N	161GLU	OE2	0.38
159LYS	NZ	148GLU	OE1	24.15
159LYS	NZ	148GLU	OE2	15.77
159LYS	NZ	148GLU	O	2.38
159LYS	NZ	163GLU	OE1	3.65
159LYS	NZ	163GLU	OE2	3.44
159LYS	NZ	198THR	OG1	4.85
159LYS	NZ	201GLU	OE1	14.38
159LYS	NZ	201GLU	OE2	15.54
159LYS	N	149ALA	O	95.89
158SER	OG	149ALA	O	6.93
158SER	OG	150GLU	OE1	0.44
158SER	OG	150GLU	OE2	0.44
158SER	OG	158SER	O	0.06
158SER	OG	159LYS	N	0.00
158SER	OG	161GLU	OE1	2.98
158SER	OG	161GLU	OE2	0.39

47ASP	N	44ALA	O	8.12
46ILE	N	42GLY	O	6.36
46ILE	N	43GLY	O	34.26
45ALA	N	41PHE	O	0.01
45ALA	N	42GLY	O	7.99
44ALA	N	9ASP	OD1	1.46
44ALA	N	9ASP	OD2	0.82
43GLY	N	8GLY	O	0.09
43GLY	N	9ASP	OD1	2.04
43GLY	N	9ASP	OD2	6.16
43GLY	N	41PHE	O	0.02
42GLY	N	52PRO	O	43.24
39PHE	N	5VAL	O	95.58
39PHE	N	37GLU	O	0.15
38VAL	N	37GLU	OE1	3.39
38VAL	N	37GLU	OE2	4.03
37GLU	N	3VAL	O	45.32
36TYR	OH	17GLU	OE1	0.00
36TYR	OH	17GLU	OE2	0.04
36TYR	N	34LEU	O	0.02
35ALA	N	1MET	O	55.89
35ALA	N	33GLY	O	0.26
34LEU	N	27ASP	OD1	56.78
34LEU	N	27ASP	OD2	16.48
33GLY	N	27ASP	OD1	93.35
33GLY	N	27ASP	OD2	0.15
32LEU	N	27ASP	OD1	77.27
32LEU	N	27ASP	O	0.04
31GLY	N	26LEU	O	89.43
31GLY	N	27ASP	O	0.14
30GLU	N	26LEU	O	66.87
30GLU	N	27ASP	O	0.38
30GLU	N	28GLU	O	0.02
29ALA	N	25ALA	O	82.49
29ALA	N	26LEU	O	3.07
29ALA	N	27ASP	O	0.16
28GLU	N	24ARG	O	50.33
28GLU	N	25ALA	O	13.40
28GLU	N	26LEU	O	0.01
27ASP	N	23LEU	O	81.38
27ASP	N	24ARG	O	1.45
26LEU	N	22VAL	O	80.99
26LEU	N	23LEU	O	3.78

174ARG	NH1	208ASP	OD1	0.10
174ARG	NH1	208ASP	OD2	0.04
174ARG	NE	170PHE	O	0.08
174ARG	NE	171GLU	OE1	16.42
174ARG	NE	171GLU	OE2	21.41
174ARG	NE	171GLU	O	0.04
174ARG	NE	206TYR	OH	5.31
174ARG	NE	207PRO	O	0.26
174ARG	NE	208ASP	OD1	0.95
174ARG	NE	208ASP	OD2	0.51
174ARG	N	170PHE	O	29.89
174ARG	N	171GLU	O	26.73
174ARG	N	172ALA	O	0.00
173ALA	N	169ALA	O	72.27
173ALA	N	170PHE	O	3.84
172ALA	N	168VAL	O	70.61
172ALA	N	169ALA	O	5.43
171GLU	N	167ARG	O	63.03
171GLU	N	168VAL	O	5.85
171GLU	N	169ALA	O	0.01
170PHE	N	166ALA	O	72.56
170PHE	N	167ARG	O	3.68
169ALA	N	165VAL	O	23.45
169ALA	N	166ALA	O	24.04
168VAL	N	164ARG	O	25.74
168VAL	N	165VAL	O	14.40
168VAL	N	166ALA	O	0.01
167ARG	NH2	160PRO	O	0.00
167ARG	NH2	163GLU	OE1	33.95
167ARG	NH2	163GLU	OE2	39.06
167ARG	NH2	171GLU	OE1	0.01
167ARG	NH2	205GLY	O	0.01
167ARG	NH2	206TYR	OH	0.00
167ARG	NH1	163GLU	OE1	2.11
167ARG	NH1	163GLU	OE2	3.14
167ARG	NH1	163GLU	O	0.03
167ARG	NH1	171GLU	OE1	0.08
167ARG	NH1	171GLU	OE2	0.00
167ARG	NH1	202VAL	O	0.03
167ARG	NH1	206TYR	OH	1.53
167ARG	NE	163GLU	OE1	42.06
167ARG	NE	163GLU	OE2	30.79
167ARG	NE	163GLU	O	0.11

6LEU	N	68LEU	O	99.22
5VAL	N	37GLU	O	91.12
4ALA	N	66ALA	O	97.13
3VAL	N	35ALA	O	89.21
2LYS	NZ	36TYR	O	0.01
2LYS	NZ	37GLU	OE1	1.90
2LYS	NZ	37GLU	OE2	2.01
2LYS	NZ	63GLU	OE1	42.87
2LYS	NZ	63GLU	OE2	34.59
2LYS	NZ	63GLU	O	0.83
2LYS	N	65GLU	OE1	0.20
2LYS	N	65GLU	OE2	0.77

158SER	N	161GLU	OE1	1.03
158SER	N	161GLU	OE2	0.24
157TYR	OH	133GLU	OE1	64.53
157TYR	OH	133GLU	OE2	11.41
157TYR	OH	136GLY	O	1.59
157TYR	OH	155GLU	OE1	0.54
157TYR	OH	155GLU	OE2	1.62
157TYR	N	151ALA	O	83.15
156ARG	NH2	150GLU	OE1	49.45
156ARG	NH2	150GLU	OE2	43.98
156ARG	NH2	142GLU	O	0.15
156ARG	NH1	150GLU	OE2	0.16
156ARG	NH1	142GLU	OE1	0.46
156ARG	NH1	142GLU	OE2	0.16
156ARG	NH1	142GLU	O	92.73
156ARG	NE	150GLU	OE1	45.99
156ARG	NE	150GLU	OE2	51.85
156ARG	NE	142GLU	O	0.02
156ARG	N	137GLY	O	0.47
156ARG	N	155GLU	OE1	0.84
156ARG	N	155GLU	OE2	0.76
155GLU	N	153ASN	O	90.46
154THR	OG1	143PRO	O	81.59
154THR	OG1	153ASN	O	0.32
154THR	N	141GLY	O	0.40
154THR	N	143PRO	O	1.50
153ASN	ND2	188VAL	O	0.00
153ASN	ND2	190GLU	OE1	33.99
153ASN	ND2	190GLU	OE2	63.61
153ASN	ND2	138ILE	O	0.11
153ASN	ND2	152TRP	O	0.22
153ASN	N	155GLU	O	97.20
152TRP	NE1	143PRO	O	0.11
152TRP	NE1	144ARG	O	0.22
152TRP	NE1	154THR	OG1	0.01
152TRP	N	145GLY	O	98.84
151ALA	N	157TYR	O	91.66
150GLU	N	147SER	O	4.47
150GLU	N	148GLU	O	0.00
149ALA	N	147SER	O	1.24
148GLU	N	148GLU	OE1	0.11
148GLU	N	148GLU	OE2	0.18
147SER	OG	146MET	O	0.22

25ALA	N	21LYS	O	50.23
25ALA	N	22VAL	O	10.58
25ALA	N	23LEU	O	0.00
24ARG	NH2	17GLU	OE1	15.03
24ARG	NH2	17GLU	OE2	30.23
24ARG	NH2	17GLU	O	0.12
24ARG	NH2	27ASP	OD2	1.99
24ARG	NH2	28GLU	OE2	0.01
24ARG	NH2	34LEU	O	1.79
24ARG	NH2	36TYR	OH	6.15
24ARG	NH1	17GLU	OE1	14.49
24ARG	NH1	17GLU	OE2	11.31
24ARG	NH1	20LEU	O	0.00
24ARG	NH1	24ARG	O	0.23
24ARG	NH1	28GLU	OE2	0.47
24ARG	NH1	34LEU	O	0.05
24ARG	NH1	36TYR	OH	43.75
24ARG	NE	20LEU	O	0.72
24ARG	NE	36TYR	OH	4.31
24ARG	N	20LEU	O	96.63
24ARG	N	21LYS	O	0.17
23LEU	N	19ALA	O	71.59
23LEU	N	20LEU	O	1.42
22VAL	N	18ALA	O	47.80
22VAL	N	19ALA	O	7.44
21LYS	NZ	17GLU	OE1	0.04
21LYS	NZ	17GLU	OE2	1.58
21LYS	NZ	17GLU	O	0.02
21LYS	NZ	334GLU	OE11	22.72
21LYS	NZ	334GLU	OE21	22.42
21LYS	N	17GLU	O	79.82
21LYS	N	18ALA	O	2.88
20LEU	N	16THR	O	83.81
20LEU	N	17GLU	O	2.70
19ALA	N	15VAL	O	61.14
19ALA	N	16THR	O	8.49
18ALA	N	14GLU	O	63.15
18ALA	N	15VAL	O	2.34
17GLU	N	13PRO	O	72.93
17GLU	N	14GLU	O	2.90
17GLU	N	15VAL	O	0.00
16THR	OG1	7PRO	O	7.89
16THR	OG1	12GLY	O	87.24

167ARG	N	163GLU	O	78.20
167ARG	N	164ARG	O	3.79
167ARG	N	165VAL	O	0.00
166ALA	N	162VAL	O	63.13
166ALA	N	163GLU	O	6.72
165VAL	N	161GLU	O	8.14
165VAL	N	162VAL	O	23.40
164ARG	NH2	98ASP	OD1	8.16
164ARG	NH2	98ASP	OD2	63.24
164ARG	NH2	98ASP	O	0.50
164ARG	NH2	161GLU	OE1	2.63
164ARG	NH2	161GLU	OE2	5.36
164ARG	NH2	264ARG	NE	0.07
164ARG	NH2	264ARG	NH1	0.01
164ARG	NH2	264ARG	NH2	0.02
164ARG	NH1	98ASP	OD1	59.39
164ARG	NH1	98ASP	OD2	12.07
164ARG	NH1	98ASP	O	0.00
164ARG	NH1	99LEU	O	0.00
164ARG	NH1	161GLU	OE1	23.28
164ARG	NH1	161GLU	OE2	19.96
164ARG	NH1	264ARG	NH2	0.01
164ARG	NE	160PRO	O	0.06
164ARG	NE	264ARG	NH1	0.00
164ARG	N	160PRO	O	40.41
164ARG	N	161GLU	O	20.07
163GLU	N	159LYS	O	61.18
163GLU	N	160PRO	O	2.30
162VAL	N	158SER	O	45.77
162VAL	N	159LYS	O	15.59
162VAL	N	160PRO	O	0.08
161GLU	N	158SER	O	48.33
161GLU	N	159LYS	O	0.04
161GLU	N	161GLU	OE1	0.10
161GLU	N	161GLU	OE2	0.72
159LYS	NZ	148GLU	OE1	17.44
159LYS	NZ	148GLU	OE2	19.40
159LYS	NZ	148GLU	O	1.69
159LYS	NZ	163GLU	OE1	4.86
159LYS	NZ	163GLU	OE2	3.16
159LYS	NZ	198THR	OG1	10.89
159LYS	NZ	201GLU	OE1	31.19
159LYS	NZ	201GLU	OE2	32.35

147SER	OG	150GLU	OE1	0.00
147SER	OG	150GLU	OE2	0.02
147SER	OG	150GLU	O	63.68
147SER	N	147SER	O	0.00
147SER	N	150GLU	O	46.32
145GLY	N	190GLU	OE1	0.02
145GLY	N	152TRP	NE1	0.05
145GLY	N	152TRP	O	87.48
144ARG	NH2	188VAL	O	51.00
144ARG	NH2	190GLU	OE1	32.83
144ARG	NH2	190GLU	OE2	63.92
144ARG	NH2	138ILE	O	0.03
144ARG	NH2	139TYR	O	0.38
144ARG	NH2	153ASN	OD1	0.03
144ARG	NH2	153ASN	ND2	0.01
144ARG	NH1	188VAL	O	0.22
144ARG	NH1	138ILE	O	0.32
144ARG	NH1	139TYR	O	76.57
144ARG	NH1	140PHE	O	0.02
144ARG	NH1	141GLY	O	0.32
144ARG	NH1	142GLU	OE1	0.03
144ARG	NH1	142GLU	OE2	0.03
144ARG	NE	190GLU	OE1	64.79
144ARG	NE	190GLU	OE2	43.30
144ARG	NE	153ASN	ND2	0.03
144ARG	N	142GLU	OE1	0.33
144ARG	N	142GLU	OE2	0.31
142GLU	N	140PHE	O	0.01
142GLU	N	142GLU	OE1	0.26
142GLU	N	142GLU	OE2	0.07
141GLY	N	137GLY	O	0.93
141GLY	N	138ILE	O	22.58
141GLY	N	139TYR	O	0.01
141GLY	N	144ARG	NH1	0.12
140PHE	N	136GLY	O	0.15
140PHE	N	137GLY	O	93.48
139TYR	OH	237ASN	OD1	0.05
139TYR	OH	237ASN	O	0.06
139TYR	OH	238ILE	O	1.53
139TYR	OH	241ASP	OD1	42.51
139TYR	OH	241ASP	OD2	20.95
139TYR	N	137GLY	O	0.00
139TYR	N	237ASN	OD1	0.97

16THR	N	12GLY	O	84.34
16THR	N	13PRO	O	1.42
15VAL	N	11ILE	O	88.60
15VAL	N	12GLY	O	2.08
14GLU	N	11ILE	O	29.49
14GLU	N	12GLY	O	0.00
12GLY	N	9ASP	O	2.53
12GLY	N	71SER	OG	7.88
12GLY	N	71SER	O	0.29
12GLY	N	275SER	OG	0.89
11ILE	N	9ASP	O	0.00
11ILE	N	71SER	OG	0.02
11ILE	N	275SER	OG	87.66
11ILE	N	275SER	O	5.25
10GLY	N	9ASP	OD1	0.14
10GLY	N	9ASP	OD2	0.13
9ASP	N	71SER	OG	0.00
9ASP	N	71SER	O	95.62
8GLY	N	41PHE	O	22.92
6LEU	N	68LEU	O	98.51
5VAL	N	37GLU	O	93.67
4ALA	N	66ALA	O	96.02
3VAL	N	1MET	O	0.01
3VAL	N	35ALA	O	90.80
2LYS	NZ	37GLU	OE1	0.52
2LYS	NZ	37GLU	OE2	0.51
2LYS	NZ	63GLU	OE1	35.97
2LYS	NZ	63GLU	OE2	32.77
2LYS	NZ	63GLU	O	2.88
2LYS	N	65GLU	OE1	0.94
2LYS	N	65GLU	OE2	0.03

159LYS	N	149ALA	O	91.87
158SER	OG	149ALA	O	5.26
158SER	OG	150GLU	OE1	1.18
158SER	OG	150GLU	OE2	2.55
158SER	OG	157TYR	O	0.04
158SER	OG	158SER	O	0.05
158SER	OG	161GLU	OE1	2.83
158SER	OG	161GLU	OE2	4.56
158SER	N	161GLU	OE1	4.41
158SER	N	161GLU	OE2	4.43
157TYR	OH	133GLU	OE1	59.05
157TYR	OH	136GLY	O	0.43
157TYR	OH	155GLU	OE1	0.05
157TYR	OH	155GLU	OE2	0.98
157TYR	N	151ALA	O	97.39
156ARG	NH2	150GLU	OE1	37.89
156ARG	NH2	150GLU	OE2	37.27
156ARG	NH2	142GLU	O	1.83
156ARG	NH2	158SER	OG	0.01
156ARG	NH1	150GLU	OE1	1.86
156ARG	NH1	150GLU	OE2	3.26
156ARG	NH1	142GLU	OE1	0.23
156ARG	NH1	142GLU	OE2	0.04
156ARG	NH1	142GLU	O	13.91
156ARG	NH1	158SER	OG	0.02
156ARG	NH1	161GLU	OE1	0.01
156ARG	NE	150GLU	OE1	47.93
156ARG	NE	150GLU	OE2	42.73
156ARG	NE	142GLU	O	0.44
156ARG	NE	156ARG	O	0.02
156ARG	N	155GLU	OE1	39.19
156ARG	N	155GLU	OE2	33.50
155GLU	N	153ASN	O	98.54
154THR	OG1	153ASN	O	0.00
154THR	OG1	143PRO	N	0.01
154THR	OG1	143PRO	O	67.85
154THR	OG1	153ASN	O	2.43
154THR	N	138ILE	O	0.02
154THR	N	141GLY	O	0.26
154THR	N	143PRO	O	7.01
153ASN	ND2	190GLU	OE1	5.75
153ASN	ND2	190GLU	OE2	4.69
153ASN	ND2	138ILE	O	3.98

139TYR	N	237ASN	ND2	0.39
138ILE	N	155GLU	OE1	48.28
138ILE	N	155GLU	OE2	42.66
137GLY	N	140PHE	O	0.00
137GLY	N	156ARG	O	43.96
136GLY	N	133GLU	OE1	67.00
136GLY	N	133GLU	OE2	12.26
136GLY	N	157TYR	OH	0.23
136GLY	N	237ASN	OD1	0.01
136GLY	N	237ASN	ND2	0.02
135THR	OG1	98ASP	OD2	0.01
135THR	OG1	99LEU	O	4.29
135THR	OG1	100PHE	O	0.05
135THR	OG1	133GLU	OE1	17.46
135THR	OG1	133GLU	OE2	64.95
135THR	OG1	135THR	O	0.01
135THR	OG1	161GLU	OE1	0.12
135THR	OG1	161GLU	OE2	0.17
135THR	N	100PHE	O	0.00
135THR	N	133GLU	OE1	69.60
135THR	N	133GLU	OE2	54.95
135THR	N	157TYR	OH	0.01
134LEU	N	100PHE	O	25.36
134LEU	N	133GLU	OE1	22.86
134LEU	N	133GLU	OE2	15.51
133GLU	N	235THR	O	51.65
133GLU	N	236GLY	O	5.54
132ARG	NH2	102ASN	OD1	0.26
132ARG	NH2	139TYR	OH	0.57
132ARG	NH2	237ASN	OD1	29.16
132ARG	NH2	237ASN	O	8.70
132ARG	NH2	241ASP	N	0.00
132ARG	NH2	241ASP	OD1	2.23
132ARG	NH2	241ASP	OD2	3.90
132ARG	NH2	244SER	OG	0.00
132ARG	NH1	102ASN	OD1	1.21
132ARG	NH1	133GLU	O	1.46
132ARG	NH1	237ASN	OD1	0.48
132ARG	NH1	237ASN	O	0.88
132ARG	NH1	240GLY	O	0.44
132ARG	NH1	241ASP	OD1	1.99
132ARG	NH1	241ASP	OD2	2.26
132ARG	NH1	244SER	OG	23.93

153ASN	ND2	139TYR	O	0.04
153ASN	ND2	141GLY	O	0.01
153ASN	ND2	152TRP	O	0.22
153ASN	ND2	154THR	N	0.00
153ASN	ND2	154THR	O	0.01
153ASN	N	155GLU	O	98.36
152TRP	NE1	143PRO	O	0.16
152TRP	NE1	144ARG	O	0.12
152TRP	N	145GLY	O	98.35
151ALA	N	157TYR	O	86.62
150GLU	N	147SER	O	4.92
149ALA	N	147SER	O	2.53
148GLU	N	148GLU	OE1	0.68
148GLU	N	148GLU	OE2	0.60
147SER	OG	146MET	O	0.09
147SER	OG	148GLU	OE2	0.02
147SER	OG	150GLU	O	62.79
147SER	N	150GLU	O	47.29
145GLY	N	152TRP	NE1	0.62
145GLY	N	152TRP	O	82.39
144ARG	NH2	188VAL	O	3.91
144ARG	NH2	190GLU	OE1	27.49
144ARG	NH2	190GLU	OE2	33.22
144ARG	NH2	138ILE	O	0.00
144ARG	NH2	139TYR	O	0.48
144ARG	NH2	140PHE	O	0.00
144ARG	NH2	142GLU	OE1	4.34
144ARG	NH2	142GLU	OE2	4.39
144ARG	NH2	153ASN	OD1	0.04
144ARG	NH1	188VAL	O	1.68
144ARG	NH1	190GLU	OE1	1.59
144ARG	NH1	190GLU	OE2	0.03
144ARG	NH1	138ILE	O	0.16
144ARG	NH1	139TYR	O	19.20
144ARG	NH1	141GLY	O	1.82
144ARG	NH1	142GLU	OE1	3.36
144ARG	NH1	142GLU	OE2	2.32
144ARG	NE	190GLU	OE1	42.73
144ARG	NE	190GLU	OE2	28.19
144ARG	NE	139TYR	O	0.13
144ARG	NE	141GLY	O	0.21
144ARG	NE	142GLU	OE1	6.55
144ARG	NE	142GLU	OE2	4.92

132ARG	NE	102ASN	OD1	4.36
132ARG	NE	133GLU	O	8.99
132ARG	NE	237ASN	OD1	4.51
132ARG	NE	237ASN	O	3.38
132ARG	NE	240GLY	O	0.02
132ARG	NE	244SER	OG	0.01
132ARG	N	102ASN	O	96.78
132ARG	N	130ILE	O	0.02
131VAL	N	233VAL	O	97.71
130ILE	N	104ARG	O	95.02
129LEU	N	176ARG	NH1	0.00
129LEU	N	230PHE	O	0.01
129LEU	N	231ASP	O	87.44
128VAL	N	106ALA	O	84.84
127ASP	N	125GLY	O	0.00
126VAL	N	108VAL	O	0.00
126VAL	N	123ALA	O	3.66
126VAL	N	124ARG	O	0.10
125GLY	N	123ALA	O	0.04
124ARG	NH2	113GLU	OE1	9.64
124ARG	NH2	113GLU	OE2	11.34
124ARG	NH2	120GLU	OE1	19.51
124ARG	NH2	120GLU	OE2	17.35
124ARG	NH2	121GLU	OE1	23.40
124ARG	NH2	121GLU	OE2	29.96
124ARG	NH1	113GLU	OE1	11.72
124ARG	NH1	113GLU	OE2	4.67
124ARG	NH1	120GLU	OE1	10.62
124ARG	NH1	120GLU	OE2	6.24
124ARG	NH1	120GLU	O	0.03
124ARG	NH1	121GLU	OE1	20.15
124ARG	NH1	121GLU	OE2	20.57
124ARG	NE	113GLU	OE1	13.13
124ARG	NE	113GLU	OE2	10.85
124ARG	NE	120GLU	OE1	0.04
124ARG	NE	120GLU	O	0.02
124ARG	NE	121GLU	OE1	13.78
124ARG	NE	121GLU	OE2	9.71
124ARG	NE	121GLU	O	0.05
124ARG	N	120GLU	O	14.81
124ARG	N	121GLU	O	10.22
124ARG	N	122ILE	O	6.88
123ALA	N	119LYS	O	64.98

144ARG	NE	144ARG	O	0.07
144ARG	N	142GLU	OE1	1.45
144ARG	N	142GLU	OE2	1.44
144ARG	N	144ARG	NE	0.01
144ARG	N	144ARG	NH2	0.04
142GLU	N	140PHE	O	0.01
142GLU	N	142GLU	OE1	0.45
142GLU	N	142GLU	OE2	0.32
142GLU	N	154THR	O	0.00
142GLU	N	156ARG	NH1	0.00
141GLY	N	137GLY	O	0.18
141GLY	N	138ILE	O	27.90
141GLY	N	139TYR	O	0.85
141GLY	N	144ARG	NH1	0.01
141GLY	N	154THR	O	0.00
140PHE	N	136GLY	O	0.02
140PHE	N	137GLY	O	88.84
140PHE	N	138ILE	O	0.18
139TYR	OH	87GLU	OE1	25.12
139TYR	OH	87GLU	OE2	12.07
139TYR	OH	87GLU	O	0.19
139TYR	OH	136GLY	O	1.52
139TYR	OH	237ASN	OD1	11.39
139TYR	OH	237ASN	ND2	0.24
139TYR	OH	237ASN	O	0.88
139TYR	OH	238ILE	O	0.02
139TYR	OH	241ASP	OD1	0.02
139TYR	OH	241ASP	OD2	2.54
139TYR	N	137GLY	O	2.33
138ILE	N	154THR	O	0.02
138ILE	N	155GLU	OE1	1.87
138ILE	N	155GLU	OE2	7.19
138ILE	N	237ASN	OD1	0.20
137GLY	N	135THR	O	0.11
137GLY	N	139TYR	OH	0.41
137GLY	N	140PHE	O	0.02
137GLY	N	155GLU	OE1	0.29
137GLY	N	155GLU	OE2	0.23
137GLY	N	156ARG	O	0.10
137GLY	N	237ASN	ND2	0.00
136GLY	N	133GLU	OE1	1.62
136GLY	N	133GLU	OE2	31.09
136GLY	N	133GLU	O	0.00

123ALA	N	120GLU	O	8.52
122ILE	N	119LYS	O	78.02
122ILE	N	120GLU	O	0.05
121GLU	N	119LYS	O	0.02
121GLU	N	121GLU	OE1	0.47
121GLU	N	121GLU	OE2	0.39
120GLU	N	120GLU	OE1	19.22
120GLU	N	120GLU	OE2	17.61
119LYS	NZ	113GLU	O	10.12
119LYS	NZ	114ARG	O	25.31
119LYS	NZ	115LEU	O	0.01
119LYS	NZ	116SER	O	25.17
119LYS	NZ	117PRO	O	0.01
119LYS	NZ	118LEU	O	23.96
119LYS	NZ	120GLU	OE1	15.34
119LYS	NZ	120GLU	OE2	12.40
119LYS	N	117PRO	O	82.09
118LEU	N	116SER	O	0.02
116SER	OG	118LEU	N	0.00
116SER	OG	118LEU	O	0.08
116SER	OG	249VAL	O	33.77
116SER	OG	250LEU	O	55.04
116SER	OG	251PRO	O	0.00
116SER	N	112LEU	O	2.21
116SER	N	113GLU	O	67.07
116SER	N	114ARG	O	0.14
115LEU	N	111GLY	O	0.01
115LEU	N	112LEU	O	61.92
115LEU	N	113GLU	O	0.08
114ARG	NH2	111GLY	O	7.66
114ARG	NH2	120GLU	OE1	7.30
114ARG	NH2	120GLU	OE2	7.90
114ARG	NH2	319LEU	O	0.03
114ARG	NH2	320LEU	O	0.03
114ARG	NH2	327LEU	O	0.05
114ARG	NH1	111GLY	O	1.79
114ARG	NH1	113GLU	OE2	0.00
114ARG	NH1	120GLU	OE1	0.20
114ARG	NH1	120GLU	OE2	1.40
114ARG	NH1	320LEU	O	0.00
114ARG	NH1	327LEU	O	0.03
114ARG	NE	111GLY	O	2.40
114ARG	NE	114ARG	O	0.01

136GLY	N	134LEU	O	4.04
136GLY	N	237ASN	ND2	0.86
135THR	OG1	99LEU	O	0.01
135THR	OG1	100PHE	O	0.50
135THR	OG1	133GLU	OE1	6.75
135THR	OG1	133GLU	OE2	89.67
135THR	OG1	136GLY	N	0.02
135THR	OG1	161GLU	OE1	0.02
135THR	OG1	161GLU	OE2	0.01
135THR	OG1	161GLU	O	0.01
135THR	N	100PHE	O	0.09
135THR	N	133GLU	OE1	5.52
135THR	N	133GLU	OE2	87.98
135THR	N	237ASN	ND2	0.08
134LEU	N	100PHE	O	88.64
134LEU	N	133GLU	OE1	0.64
134LEU	N	133GLU	OE2	0.22
133GLU	N	235THR	O	55.92
133GLU	N	236GLY	O	6.30
132ARG	NH2	87GLU	OE1	0.01
132ARG	NH2	87GLU	OE2	0.01
132ARG	NH2	133GLU	O	0.23
132ARG	NH2	139TYR	OH	0.13
132ARG	NH2	237ASN	OD1	66.05
132ARG	NH2	237ASN	ND2	0.53
132ARG	NH2	237ASN	O	2.17
132ARG	NH2	241ASP	OD1	0.54
132ARG	NH2	241ASP	OD2	0.61
132ARG	NH1	102ASN	OD1	8.61
132ARG	NH1	241ASP	OD1	3.34
132ARG	NH1	241ASP	OD2	4.32
132ARG	NH1	244SER	OG	2.09
132ARG	NE	133GLU	O	21.56
132ARG	NE	237ASN	OD1	9.61
132ARG	NE	237ASN	ND2	0.14
132ARG	NE	237ASN	O	39.31
132ARG	NE	240GLY	O	0.00
132ARG	NE	241ASP	N	0.02
132ARG	N	102ASN	O	97.41
132ARG	N	130ILE	O	0.05
131VAL	N	233VAL	O	97.39
130ILE	N	104ARG	O	94.40
129LEU	N	176ARG	NH1	0.00

114ARG	NE	120GLU	OE1	12.83
114ARG	NE	120GLU	OE2	17.10
114ARG	N	111GLY	O	0.29
114ARG	N	112LEU	O	0.05
114ARG	N	113GLU	OE1	0.22
114ARG	N	113GLU	OE2	0.03
113GLU	N	109PHE	O	0.01
113GLU	N	111GLY	O	0.06
113GLU	N	113GLU	OE1	39.19
113GLU	N	113GLU	OE2	50.42
112LEU	N	109PHE	O	75.46
112LEU	N	110PRO	O	0.04
112LEU	N	113GLU	OE1	0.82
112LEU	N	113GLU	OE2	0.58
111GLY	N	113GLU	OE1	52.56
111GLY	N	113GLU	OE2	49.23
109PHE	N	107LYS	O	0.06
108VAL	N	126VAL	O	55.38
107LYS	NZ	127ASP	OD1	5.14
107LYS	NZ	127ASP	OD2	3.60
107LYS	NZ	176ARG	NH2	0.00
107LYS	NZ	299GLU	OE1	0.00
107LYS	NZ	309ARG	NE	0.02
107LYS	NZ	309ARG	NH2	0.06
107LYS	NZ	312GLU	OE1	38.45
107LYS	NZ	312GLU	OE2	45.65
107LYS	N	105PRO	O	0.03
106ALA	N	128VAL	O	80.45
104ARG	NH2	244SER	O	0.82
104ARG	NH2	245ASP	OD1	13.26
104ARG	NH2	245ASP	OD2	10.67
104ARG	NH2	248SER	OG	78.82
104ARG	NH2	254LEU	O	0.01
104ARG	NH1	102ASN	OD1	0.08
104ARG	NH1	132ARG	NE	0.02
104ARG	NH1	132ARG	NH1	0.00
104ARG	NH1	132ARG	NH2	0.02
104ARG	NH1	241ASP	OD1	1.39
104ARG	NH1	245ASP	OD1	1.49
104ARG	NH1	245ASP	OD2	0.50
104ARG	NH1	259SER	OG	0.03
104ARG	NE	244SER	O	0.12
104ARG	NE	248SER	OG	0.32

129LEU	N	231ASP	O	91.16
128VAL	N	106ALA	O	95.90
126VAL	N	123ALA	O	11.54
126VAL	N	124ARG	O	0.03
125GLY	N	123ALA	O	0.03
124ARG	NH2	113GLU	OE1	20.57
124ARG	NH2	113GLU	OE2	21.59
124ARG	NH2	120GLU	OE1	37.65
124ARG	NH2	120GLU	OE2	32.19
124ARG	NH2	121GLU	OE1	6.71
124ARG	NH2	121GLU	OE2	7.65
124ARG	NH1	113GLU	OE1	0.01
124ARG	NH1	113GLU	OE2	0.07
124ARG	NH1	120GLU	OE1	7.43
124ARG	NH1	120GLU	OE2	9.40
124ARG	NH1	120GLU	O	0.09
124ARG	NH1	121GLU	OE1	29.01
124ARG	NH1	121GLU	OE2	25.89
124ARG	NE	113GLU	OE1	23.03
124ARG	NE	113GLU	OE2	19.04
124ARG	NE	120GLU	OE1	6.76
124ARG	NE	120GLU	OE2	5.29
124ARG	NE	120GLU	O	0.03
124ARG	N	120GLU	O	32.55
124ARG	N	121GLU	O	6.91
124ARG	N	122ILE	O	0.67
123ALA	N	119LYS	O	73.24
123ALA	N	120GLU	O	8.76
122ILE	N	119LYS	O	74.68
122ILE	N	120GLU	O	0.03
121GLU	N	120GLU	OE1	0.06
121GLU	N	120GLU	OE2	0.07
121GLU	N	121GLU	OE1	2.45
121GLU	N	121GLU	OE2	2.36
120GLU	N	120GLU	OE1	1.56
120GLU	N	120GLU	OE2	0.70
119LYS	NZ	113GLU	O	0.04
119LYS	NZ	114ARG	O	21.99
119LYS	NZ	115LEU	O	0.01
119LYS	NZ	116SER	O	33.59
119LYS	NZ	118LEU	O	0.33
119LYS	NZ	120GLU	OE1	6.34
119LYS	NZ	120GLU	OE2	2.26

104ARG	N	130ILE	O	96.86
103LEU	N	102ASN	OD1	0.01
103LEU	N	260ALA	O	99.03
102ASN	ND2	99LEU	O	0.05
102ASN	ND2	100PHE	O	0.32
102ASN	ND2	101ALA	O	0.00
102ASN	ND2	102ASN	O	0.00
102ASN	ND2	103LEU	N	0.00
102ASN	ND2	132ARG	NE	0.04
102ASN	ND2	132ARG	O	0.00
102ASN	ND2	259SER	OG	5.36
102ASN	ND2	260ALA	O	0.47
102ASN	ND2	261SER	N	0.04
102ASN	ND2	261SER	OG	67.15
102ASN	N	100PHE	O	0.01
102ASN	N	132ARG	O	93.20
101ALA	N	262LEU	O	42.55
100PHE	N	98ASP	O	0.24
100PHE	N	262LEU	O	88.45
99LEU	N	94ARG	O	45.27
99LEU	N	97GLN	O	8.66
99LEU	N	98ASP	OD1	0.13
99LEU	N	98ASP	OD2	0.00
98ASP	N	62GLU	OE1	0.00
98ASP	N	62GLU	OE2	0.55
98ASP	N	94ARG	O	7.19
98ASP	N	95LYS	O	11.62
98ASP	N	96SER	O	0.65
98ASP	N	97GLN	OE1	0.86
98ASP	N	264ARG	O	0.16
97GLN	NE2	58ARG	O	0.02
97GLN	NE2	61VAL	O	2.89
97GLN	NE2	62GLU	OE1	17.04
97GLN	NE2	62GLU	OE2	17.33
97GLN	NE2	62GLU	O	0.14
97GLN	NE2	93LEU	O	2.33
97GLN	NE2	94ARG	O	0.89
97GLN	NE2	96SER	O	0.34
97GLN	NE2	264ARG	O	0.01
97GLN	NE2	266THR	N	0.00
97GLN	NE2	266THR	OG1	5.42
97GLN	NE2	266THR	O	0.44
97GLN	N	92SER	O	0.99

119LYS	N	117PRO	O	87.29
118LEU	N	116SER	O	0.02
116SER	OG	113GLU	O	2.54
116SER	OG	116SER	O	0.01
116SER	OG	118LEU	O	0.37
116SER	OG	249VAL	O	0.36
116SER	OG	250LEU	O	91.62
116SER	N	112LEU	O	3.27
116SER	N	113GLU	O	53.23
116SER	N	114ARG	O	0.14
115LEU	N	111GLY	O	0.09
115LEU	N	112LEU	O	66.67
114ARG	NH2	111GLY	O	2.88
114ARG	NH2	113GLU	OE1	0.00
114ARG	NH2	120GLU	OE1	16.19
114ARG	NH2	120GLU	OE2	16.08
114ARG	NH2	327LEU	O	0.01
114ARG	NH1	111GLY	O	0.52
114ARG	NH1	120GLU	OE1	0.10
114ARG	NH1	120GLU	OE2	0.19
114ARG	NH1	320LEU	O	0.00
114ARG	NE	111GLY	O	1.36
114ARG	NE	120GLU	OE1	27.11
114ARG	NE	120GLU	OE2	24.32
114ARG	N	111GLY	O	0.46
114ARG	N	112LEU	O	0.00
114ARG	N	113GLU	OE1	0.11
114ARG	N	113GLU	OE2	0.08
113GLU	N	109PHE	O	0.94
113GLU	N	111GLY	O	0.06
113GLU	N	113GLU	OE1	42.12
113GLU	N	113GLU	OE2	42.01
112LEU	N	109PHE	O	77.06
112LEU	N	110PRO	O	0.18
112LEU	N	113GLU	OE1	0.30
112LEU	N	113GLU	OE2	0.28
111GLY	N	113GLU	OE1	44.74
111GLY	N	113GLU	OE2	53.43
109PHE	N	107LYS	O	0.02
108VAL	N	126VAL	O	70.07
107LYS	NZ	127ASP	OD1	4.02
107LYS	NZ	127ASP	OD2	2.40
107LYS	NZ	312GLU	OE1	51.67

97GLN	N	93LEU	O	12.89
97GLN	N	94ARG	O	16.44
97GLN	N	95LYS	O	0.33
97GLN	N	97GLN	OE1	1.46
97GLN	N	97GLN	NE2	0.05
96SER	OG	91LEU	O	0.00
96SER	OG	92SER	O	37.98
96SER	OG	93LEU	O	26.29
96SER	OG	94ARG	O	0.00
96SER	OG	95LYS	O	2.79
96SER	OG	96SER	O	0.11
96SER	OG	97GLN	N	0.02
96SER	OG	97GLN	OE1	1.18
96SER	OG	97GLN	NE2	0.23
96SER	N	91LEU	O	0.16
96SER	N	92SER	O	46.13
96SER	N	93LEU	O	11.02
96SER	N	94ARG	O	0.34
96SER	N	97GLN	OE1	0.20
96SER	N	97GLN	O	0.37
95LYS	NZ	51GLU	OE1	0.44
95LYS	NZ	51GLU	OE2	0.72
95LYS	NZ	91LEU	O	0.01
95LYS	NZ	92SER	O	0.07
95LYS	NZ	95LYS	O	0.05
95LYS	NZ	98ASP	OD1	8.19
95LYS	NZ	98ASP	OD2	7.78
95LYS	NZ	135THR	OG1	0.00
95LYS	NZ	135THR	O	2.21
95LYS	NZ	140PHE	O	0.16
95LYS	NZ	142GLU	OE1	0.38
95LYS	NZ	142GLU	OE2	0.51
95LYS	N	91LEU	O	49.08
95LYS	N	92SER	O	23.62
95LYS	N	93LEU	O	1.45
95LYS	N	97GLN	OE1	0.00
94ARG	NH2	90LEU	O	0.22
94ARG	NH2	270GLU	OE1	65.98
94ARG	NH2	270GLU	OE2	36.73
94ARG	NH1	261SER	OG	1.29
94ARG	NH1	270GLU	OE1	31.79
94ARG	NH1	270GLU	OE2	63.33
94ARG	NE	90LEU	O	9.70

107LYS	NZ	312GLU	OE2	51.35
106ALA	N	128VAL	O	95.90
104ARG	NH2	102ASN	OD1	0.48
104ARG	NH2	241ASP	OD1	0.02
104ARG	NH2	241ASP	OD2	0.05
104ARG	NH2	241ASP	O	0.00
104ARG	NH2	245ASP	OD1	1.04
104ARG	NH2	245ASP	OD2	0.98
104ARG	NH2	259SER	OG	0.10
104ARG	NH2	270GLU	OE1	0.01
104ARG	NH2	270GLU	OE2	0.05
104ARG	NH2	275SER	OG	0.04
104ARG	NH1	240GLY	O	0.00
104ARG	NH1	241ASP	OD1	1.49
104ARG	NH1	241ASP	OD2	0.14
104ARG	NH1	244SER	OG	3.89
104ARG	NH1	244SER	O	0.06
104ARG	NH1	245ASP	OD1	0.45
104ARG	NH1	245ASP	OD2	0.24
104ARG	NH1	275SER	OG	0.01
104ARG	NE	102ASN	OD1	83.01
104ARG	NE	259SER	OG	0.06
104ARG	N	130ILE	O	99.24
103LEU	N	102ASN	OD1	0.00
103LEU	N	260ALA	O	99.10
102ASN	ND2	104ARG	NH2	0.01
102ASN	ND2	259SER	OG	0.28
102ASN	ND2	260ALA	O	0.86
102ASN	ND2	261SER	N	0.08
102ASN	ND2	261SER	OG	64.11
102ASN	ND2	270GLU	OE2	0.00
102ASN	N	132ARG	O	97.00
101ALA	N	262LEU	O	55.90
100PHE	N	98ASP	O	0.33
100PHE	N	262LEU	O	81.35
99LEU	N	94ARG	O	88.74
98ASP	N	93LEU	O	0.04
98ASP	N	94ARG	O	6.68
98ASP	N	95LYS	O	20.73
98ASP	N	96SER	O	0.05
98ASP	N	264ARG	O	0.00
97GLN	NE2	61VAL	O	0.31
97GLN	NE2	62GLU	OE1	35.95

94ARG	NE	97GLN	OE1	0.02
94ARG	N	90LEU	O	4.55
94ARG	N	91LEU	O	42.75
94ARG	N	92SER	O	0.93
93LEU	N	89GLY	O	13.36
93LEU	N	90LEU	O	16.06
93LEU	N	91LEU	O	0.01
92SER	OG	51GLU	OE1	0.03
92SER	OG	51GLU	OE2	0.01
92SER	OG	87GLU	O	3.63
92SER	OG	88THR	O	2.30
92SER	OG	89GLY	O	2.17
92SER	OG	90LEU	O	0.01
92SER	OG	91LEU	O	0.06
92SER	OG	92SER	O	0.01
92SER	OG	93LEU	N	0.01
92SER	N	86PRO	O	0.04
92SER	N	87GLU	O	0.01
92SER	N	88THR	O	1.96
92SER	N	89GLY	O	12.32
92SER	N	90LEU	O	0.03
91LEU	N	86PRO	O	14.22
91LEU	N	87GLU	O	2.78
91LEU	N	88THR	O	0.07
91LEU	N	89GLY	O	4.96
90LEU	N	85ARG	O	9.66
90LEU	N	86PRO	O	24.03
90LEU	N	87GLU	O	7.21
90LEU	N	88THR	O	0.13
89GLY	N	85ARG	O	38.50
89GLY	N	86PRO	O	22.20
89GLY	N	87GLU	O	0.01
88THR	OG1	187ASN	O	0.48
88THR	OG1	82ARG	O	74.12
88THR	OG1	83LYS	O	0.03
88THR	OG1	85ARG	O	0.57
88THR	OG1	87GLU	OE1	16.36
88THR	OG1	89GLY	N	0.03
88THR	N	82ARG	O	0.01
88THR	N	85ARG	O	50.17
88THR	N	86PRO	O	0.06
88THR	N	87GLU	OE1	12.62
88THR	N	87GLU	OE2	0.02

97GLN	NE2	62GLU	OE2	34.11
97GLN	NE2	93LEU	O	0.27
97GLN	NE2	96SER	O	0.06
97GLN	NE2	266THR	N	0.00
97GLN	NE2	266THR	OG1	1.14
97GLN	NE2	266THR	O	0.16
97GLN	N	93LEU	O	61.79
97GLN	N	94ARG	O	1.33
96SER	OG	92SER	O	80.94
96SER	OG	93LEU	O	9.74
96SER	OG	97GLN	OE1	0.00
96SER	N	92SER	O	79.74
96SER	N	93LEU	O	3.89
96SER	N	94ARG	O	0.24
95LYS	NZ	91LEU	O	0.02
95LYS	NZ	95LYS	O	0.02
95LYS	NZ	98ASP	OD1	0.42
95LYS	NZ	98ASP	OD2	0.24
95LYS	NZ	135THR	O	1.46
95LYS	NZ	136GLY	O	0.02
95LYS	NZ	161GLU	OE1	0.01
95LYS	N	91LEU	O	81.32
95LYS	N	92SER	O	3.26
95LYS	N	93LEU	O	0.01
94ARG	NH2	87GLU	OE1	9.61
94ARG	NH2	87GLU	OE2	19.53
94ARG	NH2	270GLU	OE1	2.36
94ARG	NH2	270GLU	OE2	0.72
94ARG	NH1	87GLU	OE1	49.51
94ARG	NH1	87GLU	OE2	32.07
94ARG	NH1	134LEU	O	0.01
94ARG	NH1	139TYR	OH	0.83
94ARG	NE	90LEU	O	0.02
94ARG	N	90LEU	O	85.51
94ARG	N	91LEU	O	2.01
93LEU	N	89GLY	O	36.49
93LEU	N	90LEU	O	14.54
92SER	OG	51GLU	OE2	0.00
92SER	OG	88THR	O	10.23
92SER	OG	89GLY	O	10.18
92SER	OG	92SER	O	0.04
92SER	OG	93LEU	N	0.02
92SER	N	88THR	O	7.10

87GLU	N	85ARG	O	1.40
87GLU	N	87GLU	OE1	29.29
87GLU	N	87GLU	OE2	32.88
85ARG	NH2	74GLY	O	0.66
85ARG	NH2	77TRP	O	0.02
85ARG	NH2	78ASP	OD1	4.87
85ARG	NH2	78ASP	OD2	5.57
85ARG	NH2	78ASP	O	0.28
85ARG	NH2	79GLY	O	0.02
85ARG	NH2	80LEU	O	3.96
85ARG	NH2	87GLU	OE1	11.30
85ARG	NH2	87GLU	OE2	31.70
85ARG	NH1	74GLY	O	0.03
85ARG	NH1	77TRP	O	5.29
85ARG	NH1	78ASP	OD1	18.59
85ARG	NH1	78ASP	OD2	21.95
85ARG	NH1	78ASP	O	1.48
85ARG	NH1	80LEU	O	0.99
85ARG	NH1	87GLU	OE1	0.62
85ARG	NH1	87GLU	OE2	0.10
85ARG	NE	77TRP	O	0.59
85ARG	NE	78ASP	O	0.00
85ARG	NE	80LEU	O	27.60
85ARG	NE	81PRO	O	0.00
85ARG	NE	87GLU	OE1	34.51
85ARG	NE	87GLU	OE2	15.04
85ARG	NE	88THR	OG1	0.01
85ARG	N	81PRO	O	21.74
85ARG	N	82ARG	O	46.21
85ARG	N	83LYS	O	0.01
85ARG	N	88THR	OG1	0.09
84ILE	N	81PRO	O	96.42
84ILE	N	82ARG	O	0.03
83LYS	NZ	188VAL	O	0.01
83LYS	NZ	190GLU	OE1	1.20
83LYS	NZ	190GLU	OE2	0.03
83LYS	NZ	50GLY	O	0.53
83LYS	NZ	51GLU	OE1	1.00
83LYS	NZ	51GLU	OE2	0.90
83LYS	NZ	144ARG	NE	0.00
83LYS	NZ	144ARG	NH2	0.00
83LYS	N	81PRO	O	0.10
82ARG	NH2	186ALA	O	0.27

92SER	N	89GLY	O	20.86
92SER	N	90LEU	O	0.00
91LEU	N	86PRO	O	0.00
91LEU	N	87GLU	O	75.70
91LEU	N	88THR	O	0.20
91LEU	N	89GLY	O	0.01
90LEU	N	86PRO	O	68.17
90LEU	N	87GLU	O	9.61
89GLY	N	43GLY	O	0.01
89GLY	N	85SER	O	0.66
89GLY	N	86PRO	O	80.09
89GLY	N	87GLU	O	0.21
88THR	OG1	43GLY	O	0.00
88THR	OG1	44ALA	O	0.01
88THR	OG1	82ARG	O	1.48
88THR	OG1	83LYS	O	1.69
88THR	OG1	85SER	O	72.19
88THR	OG1	86PRO	O	0.00
88THR	OG1	89GLY	N	0.01
88THR	N	85SER	O	64.27
88THR	N	86PRO	O	0.00
87GLU	N	85SER	O	0.14
87GLU	N	87GLU	OE1	0.97
87GLU	N	87GLU	OE2	0.43
85SER	OG	77TRP	O	0.01
85SER	OG	80LEU	O	0.00
85SER	OG	81PRO	O	0.72
85SER	OG	82ARG	O	38.68
85SER	OG	83LYS	O	0.02
85SER	OG	84ILE	O	0.04
85SER	OG	85SER	O	0.06
85SER	OG	87GLU	OE1	0.76
85SER	OG	87GLU	OE2	0.11
85SER	OG	88THR	OG1	10.21
85SER	N	81PRO	O	0.64
85SER	N	82ARG	O	47.92
85SER	N	83LYS	O	0.50
85SER	N	88THR	OG1	5.76
84ILE	N	81PRO	O	93.04
84ILE	N	82ARG	O	0.03
83LYS	N	81PRO	O	2.94
82ARG	NH2	186ALA	O	0.26
82ARG	NH2	187ASN	OD1	0.01

82ARG	NH2	187ASN	O	0.92
82ARG	NH2	188VAL	O	0.02
82ARG	NH2	190GLU	OE1	0.77
82ARG	NH2	190GLU	OE2	0.09
82ARG	NH2	193GLU	OE1	30.08
82ARG	NH2	193GLU	OE2	28.35
82ARG	NH2	87GLU	OE1	0.06
82ARG	NH2	87GLU	OE2	1.44
82ARG	NH2	144ARG	NE	0.02
82ARG	NH2	144ARG	NH1	0.00
82ARG	NH2	144ARG	NH2	0.03
82ARG	NH1	186ALA	O	0.31
82ARG	NH1	187ASN	O	0.96
82ARG	NH1	188VAL	O	0.01
82ARG	NH1	190GLU	OE1	0.54
82ARG	NH1	190GLU	OE2	0.24
82ARG	NH1	193GLU	OE1	20.88
82ARG	NH1	193GLU	OE2	20.54
82ARG	NH1	82ARG	O	0.00
82ARG	NH1	85ARG	NH1	0.01
82ARG	NH1	88THR	OG1	0.37
82ARG	NH1	88THR	O	0.22
82ARG	NH1	144ARG	NE	0.00
82ARG	NE	186ALA	O	0.01
82ARG	NE	187ASN	O	0.04
82ARG	NE	193GLU	OE1	4.10
82ARG	NE	193GLU	OE2	2.37
82ARG	NE	87GLU	OE2	0.06
82ARG	NE	88THR	OG1	0.02
80LEU	N	76LYS	O	0.06
80LEU	N	77TRP	O	43.61
80LEU	N	78ASP	O	0.33
80LEU	N	85ARG	NE	0.00
80LEU	N	85ARG	NH2	0.01
79GLY	N	75PRO	O	0.08
79GLY	N	76LYS	O	2.32
79GLY	N	77TRP	O	0.09
79GLY	N	78ASP	OD1	0.07
79GLY	N	78ASP	OD2	0.25
79GLY	N	80LEU	O	0.02
79GLY	N	85ARG	NE	0.01
79GLY	N	85ARG	NH1	1.66
79GLY	N	85ARG	NH2	10.00

82ARG	NH2	187ASN	O	0.41
82ARG	NH2	188VAL	O	0.01
82ARG	NH2	189LEU	O	0.05
82ARG	NH2	190GLU	OE1	21.07
82ARG	NH2	190GLU	OE2	43.82
82ARG	NH2	193GLU	OE1	27.88
82ARG	NH2	193GLU	OE2	33.66
82ARG	NH2	87GLU	OE1	1.89
82ARG	NH2	87GLU	OE2	4.16
82ARG	NH2	87GLU	O	0.00
82ARG	NH2	139TYR	O	0.01
82ARG	NH2	144ARG	NE	0.00
82ARG	NH1	186ALA	O	0.02
82ARG	NH1	187ASN	OD1	0.03
82ARG	NH1	187ASN	O	0.06
82ARG	NH1	188VAL	O	9.07
82ARG	NH1	189LEU	O	0.01
82ARG	NH1	190GLU	OE1	45.72
82ARG	NH1	190GLU	OE2	23.49
82ARG	NH1	193GLU	OE1	5.61
82ARG	NH1	193GLU	OE2	6.88
82ARG	NH1	85SER	OG	0.75
82ARG	NH1	87GLU	OE1	1.98
82ARG	NH1	87GLU	OE2	2.14
82ARG	NH1	144ARG	NE	0.02
82ARG	NH1	144ARG	NH2	0.04
82ARG	NE	186ALA	O	0.04
82ARG	NE	187ASN	O	0.28
82ARG	NE	193GLU	OE1	28.04
82ARG	NE	193GLU	OE2	22.21
82ARG	NE	82ARG	O	0.02
82ARG	NE	85SER	OG	0.00
82ARG	NE	87GLU	OE1	1.40
82ARG	NE	87GLU	OE2	1.26
80LEU	N	76LYS	O	1.19
80LEU	N	77TRP	O	39.56
80LEU	N	78ASP	O	0.38
79GLY	N	75PRO	O	0.62
79GLY	N	76LYS	O	6.09
79GLY	N	77TRP	O	0.11
79GLY	N	78ASP	OD1	0.05
79GLY	N	78ASP	OD2	0.04
79GLY	N	80LEU	O	0.01

78ASP	N	74GLY	O	17.50
78ASP	N	75PRO	O	31.04
78ASP	N	76LYS	O	1.97
77TRP	NE1	42GLY	O	0.29
77TRP	NE1	47ASP	OD1	0.19
77TRP	NE1	47ASP	OD2	0.00
77TRP	NE1	84ILE	O	56.16
77TRP	N	74GLY	O	45.40
77TRP	N	75PRO	O	0.79
77TRP	N	78ASP	O	0.00
76LYS	NZ	9ASP	OD1	32.74
76LYS	NZ	9ASP	OD2	21.77
76LYS	NZ	9ASP	O	0.00
76LYS	NZ	43GLY	O	0.26
76LYS	NZ	47ASP	OD1	0.66
76LYS	NZ	47ASP	OD2	0.68
76LYS	NZ	76LYS	O	0.04
76LYS	NZ	278ASP	OD1	0.96
76LYS	NZ	278ASP	OD2	0.91
76LYS	NZ	280ALA	O	0.06
76LYS	NZ	281GLY	O	0.13
76LYS	NZ	282LYS	O	0.04
76LYS	N	74GLY	O	3.45
74GLY	N	9ASP	OD1	0.02
74GLY	N	77TRP	O	0.00
74GLY	N	85ARG	NH1	0.00
74GLY	N	87GLU	OE1	6.89
74GLY	N	87GLU	OE2	6.12
74GLY	N	275SER	OG	0.16
74GLY	N	275SER	O	0.04
73GLY	N	9ASP	OD1	0.00
73GLY	N	9ASP	OD2	1.42
73GLY	N	9ASP	O	19.94
73GLY	N	275SER	OG	0.08
72VAL	N	275SER	OG	4.42
71SER	OG	9ASP	O	13.51
71SER	OG	11ILE	O	0.04
71SER	OG	70GLY	O	0.18
71SER	OG	71SER	O	0.66
71SER	OG	72VAL	N	0.00
71SER	OG	271PRO	O	0.00
71SER	OG	273HIS	NE2	2.71
71SER	OG	273HIS	O	0.10

78ASP	N	74GLY	O	21.88
78ASP	N	75PRO	O	32.33
78ASP	N	76LYS	O	0.70
78ASP	N	78ASP	OD1	0.01
78ASP	N	78ASP	OD2	0.01
77TRP	NE1	9ASP	OD1	0.08
77TRP	NE1	9ASP	OD2	0.21
77TRP	NE1	42GLY	O	0.32
77TRP	NE1	43GLY	O	0.02
77TRP	NE1	47ASP	OD1	0.06
77TRP	NE1	47ASP	OD2	0.00
77TRP	NE1	81PRO	O	0.00
77TRP	NE1	84ILE	O	18.83
77TRP	N	74GLY	O	66.48
77TRP	N	75PRO	O	0.62
76LYS	NZ	9ASP	OD1	41.80
76LYS	NZ	9ASP	OD2	45.41
76LYS	NZ	9ASP	O	0.68
76LYS	NZ	42GLY	O	0.04
76LYS	NZ	43GLY	O	0.04
76LYS	NZ	47ASP	OD1	0.27
76LYS	NZ	47ASP	OD2	0.17
76LYS	NZ	73GLY	O	0.06
76LYS	NZ	76LYS	O	0.00
76LYS	NZ	77TRP	NE1	0.06
76LYS	NZ	281GLY	O	0.01
76LYS	N	74GLY	O	1.93
76LYS	N	78ASP	O	0.30
74GLY	N	9ASP	OD2	0.00
74GLY	N	72VAL	O	0.00
74GLY	N	274GLY	O	0.01
74GLY	N	275SER	OG	0.12
74GLY	N	275SER	O	0.04
74GLY	N	280ALA	O	0.26
73GLY	N	9ASP	OD2	0.21
73GLY	N	9ASP	O	2.53
73GLY	N	42GLY	O	0.02
73GLY	N	71SER	O	2.61
73GLY	N	275SER	OG	0.04
72VAL	N	70GLY	O	0.02
72VAL	N	72VAL	O	0.01
71SER	OG	9ASP	O	1.76
71SER	OG	11ILE	O	1.36

71SER	OG	274GLY	O	9.79
71SER	OG	275SER	OG	0.05
71SER	N	7PRO	O	7.77
71SER	N	273HIS	NE2	3.48
70GLY	N	6LEU	O	23.60
69LEU	N	67VAL	O	0.01
69LEU	N	269PHE	O	94.25
68LEU	N	4ALA	O	98.71
67VAL	N	267PRO	O	86.06
66ALA	N	2LYS	O	6.95
66ALA	N	64ALA	O	0.00
65GLU	N	2LYS	O	97.54
65GLU	N	65GLU	OE2	0.02
64ALA	N	60GLY	O	14.65
64ALA	N	61VAL	O	14.57
64ALA	N	62GLU	O	4.65
64ALA	N	63GLU	OE1	0.01
64ALA	N	63GLU	OE2	0.00
64ALA	N	266THR	OG1	0.00
63GLU	N	59LYS	O	4.51
63GLU	N	60GLY	O	47.05
63GLU	N	61VAL	O	1.26
63GLU	N	63GLU	OE2	0.00
63GLU	N	64ALA	O	0.01
62GLU	N	58ARG	O	8.78
62GLU	N	59LYS	O	3.01
62GLU	N	60GLY	O	1.08
62GLU	N	62GLU	OE1	0.04
61VAL	N	57THR	O	73.77
61VAL	N	58ARG	O	0.87
61VAL	N	59LYS	O	0.01
60GLY	N	56PRO	O	4.75
60GLY	N	57THR	O	63.85
60GLY	N	58ARG	O	0.56
59LYS	NZ	55GLU	OE1	27.41
59LYS	NZ	55GLU	OE2	33.73
59LYS	NZ	55GLU	O	0.11
59LYS	NZ	62GLU	OE1	0.47
59LYS	NZ	62GLU	OE2	0.40
59LYS	NZ	62GLU	O	0.00
59LYS	NZ	63GLU	OE1	0.50
59LYS	NZ	63GLU	OE2	0.02
59LYS	N	55GLU	O	54.47

71SER	OG	70GLY	O	6.44
71SER	OG	71SER	O	1.76
71SER	OG	270GLU	OE1	11.66
71SER	OG	270GLU	OE2	6.27
71SER	OG	271PRO	O	0.09
71SER	OG	273HIS	O	8.39
71SER	OG	274GLY	N	0.02
71SER	OG	274GLY	O	0.51
71SER	OG	275SER	N	0.00
71SER	OG	275SER	OG	4.32
71SER	N	7PRO	O	2.83
70GLY	N	6LEU	O	26.42
70GLY	N	7PRO	O	0.40
69LEU	N	67VAL	O	0.00
69LEU	N	269PHE	O	95.38
68LEU	N	4ALA	O	98.08
67VAL	N	267PRO	O	90.05
66ALA	N	2LYS	O	6.10
65GLU	N	2LYS	O	97.41
65GLU	N	65GLU	OE1	0.05
65GLU	N	65GLU	OE2	0.01
64ALA	N	60GLY	O	19.00
64ALA	N	61VAL	O	12.75
64ALA	N	62GLU	O	5.12
63GLU	N	59LYS	O	10.31
63GLU	N	60GLY	O	35.32
63GLU	N	61VAL	O	0.66
63GLU	N	63GLU	OE1	0.00
62GLU	N	58ARG	O	11.36
62GLU	N	59LYS	O	2.22
62GLU	N	60GLY	O	0.05
61VAL	N	57THR	O	86.25
61VAL	N	58ARG	O	0.37
60GLY	N	56PRO	O	7.71
60GLY	N	57THR	O	56.06
60GLY	N	58ARG	O	0.44
59LYS	NZ	55GLU	OE1	40.74
59LYS	NZ	55GLU	OE2	27.16
59LYS	NZ	55GLU	O	0.08
59LYS	NZ	59LYS	O	0.00
59LYS	NZ	62GLU	OE1	0.15
59LYS	NZ	62GLU	OE2	0.14
59LYS	NZ	63GLU	OE1	0.29

59LYS	N	56PRO	O	14.27
59LYS	N	57THR	O	0.03
58ARG	NH2	51GLU	OE1	12.45
58ARG	NH2	51GLU	OE2	12.13
58ARG	NH2	53PHE	O	0.43
58ARG	NH2	55GLU	OE1	44.98
58ARG	NH2	55GLU	OE2	37.93
58ARG	NH2	92SER	OG	0.03
58ARG	NH1	51GLU	OE1	29.28
58ARG	NH1	51GLU	OE2	28.57
58ARG	NH1	53PHE	O	15.34
58ARG	NH1	54PRO	O	0.00
58ARG	NH1	55GLU	OE1	4.66
58ARG	NH1	55GLU	OE2	3.62
58ARG	NH1	92SER	OG	0.83
58ARG	NH1	96SER	OG	0.01
58ARG	NE	51GLU	OE1	1.01
58ARG	NE	51GLU	OE2	0.87
58ARG	NE	53PHE	O	3.80
58ARG	NE	55GLU	OE1	4.45
58ARG	NE	55GLU	OE2	3.43
58ARG	N	54PRO	O	86.62
58ARG	N	55GLU	O	4.78
57THR	OG1	40PRO	O	95.81
57THR	N	54PRO	O	16.22
55GLU	N	51GLU	OE1	0.01
55GLU	N	51GLU	OE2	0.00
55GLU	N	55GLU	OE1	0.01
53PHE	N	51GLU	OE1	0.12
53PHE	N	51GLU	OE2	0.34
53PHE	N	51GLU	O	0.04
51GLU	N	45ALA	O	6.02
51GLU	N	49PHE	O	2.08
51GLU	N	51GLU	OE1	0.01
51GLU	N	51GLU	OE2	0.03
50GLY	N	45ALA	O	63.14
50GLY	N	46ILE	O	4.02
50GLY	N	47ASP	O	0.04
49PHE	N	44ALA	O	2.36
49PHE	N	45ALA	O	73.78
49PHE	N	46ILE	O	0.10
49PHE	N	47ASP	O	0.02
48ALA	N	43GLY	O	0.08

59LYS	NZ	63GLU	OE2	0.12
59LYS	N	55GLU	O	67.99
59LYS	N	56PRO	O	8.03
58ARG	NH2	51GLU	OE1	9.16
58ARG	NH2	51GLU	OE2	13.03
58ARG	NH2	53PHE	O	0.28
58ARG	NH2	55GLU	OE1	30.22
58ARG	NH2	55GLU	OE2	46.75
58ARG	NH2	92SER	OG	1.75
58ARG	NH1	51GLU	OE1	31.63
58ARG	NH1	51GLU	OE2	28.07
58ARG	NH1	53PHE	O	10.11
58ARG	NH1	55GLU	OE1	3.04
58ARG	NH1	55GLU	OE2	1.06
58ARG	NH1	92SER	OG	1.93
58ARG	NE	51GLU	OE1	0.03
58ARG	NE	51GLU	OE2	0.01
58ARG	NE	53PHE	O	2.54
58ARG	NE	54PRO	O	0.00
58ARG	NE	55GLU	OE1	3.59
58ARG	NE	55GLU	OE2	4.11
58ARG	NE	92SER	OG	0.08
58ARG	N	54PRO	O	93.00
58ARG	N	55GLU	O	1.88
57THR	OG1	40PRO	O	92.53
57THR	OG1	54PRO	O	0.17
57THR	OG1	58ARG	N	0.00
57THR	N	54PRO	O	10.27
55GLU	N	51GLU	OE1	0.03
55GLU	N	51GLU	OE2	0.04
55GLU	N	55GLU	OE1	0.04
55GLU	N	55GLU	OE2	0.07
53PHE	N	51GLU	OE1	0.41
53PHE	N	51GLU	OE2	0.20
53PHE	N	51GLU	O	0.09
51GLU	N	45ALA	O	2.82
51GLU	N	49PHE	O	1.85
51GLU	N	51GLU	OE1	0.08
51GLU	N	51GLU	OE2	0.06
50GLY	N	45ALA	O	26.60
50GLY	N	46ILE	O	1.45
50GLY	N	47ASP	O	0.02
50GLY	N	51GLU	OE1	0.01

48ALA	N	44ALA	O	79.66
48ALA	N	45ALA	O	3.38
48ALA	N	46ILE	O	0.44
48ALA	N	49PHE	O	0.02
47ASP	N	43GLY	O	31.42
47ASP	N	44ALA	O	24.20
47ASP	N	45ALA	O	0.50
47ASP	N	49PHE	O	0.39
46ILE	N	42GLY	O	7.83
46ILE	N	43GLY	O	26.39
46ILE	N	44ALA	O	0.00
45ALA	N	41PHE	O	0.01
45ALA	N	42GLY	O	8.12
45ALA	N	43GLY	O	0.06
44ALA	N	9ASP	OD1	1.05
44ALA	N	9ASP	OD2	0.65
44ALA	N	41PHE	O	0.01
44ALA	N	42GLY	O	0.02
43GLY	N	8GLY	O	0.18
43GLY	N	9ASP	OD1	6.99
43GLY	N	9ASP	OD2	6.89
43GLY	N	41PHE	O	0.20
42GLY	N	52PRO	O	34.13
41PHE	N	39PHE	O	0.01
41PHE	N	42GLY	O	0.03
39PHE	N	5VAL	O	93.16
39PHE	N	37GLU	O	0.12
38VAL	N	37GLU	OE1	5.05
38VAL	N	37GLU	OE2	4.55
37GLU	N	3VAL	O	58.00
36TYR	OH	20LEU	O	0.00
36TYR	OH	23LEU	O	0.03
36TYR	OH	27ASP	OD1	0.03
36TYR	N	34LEU	O	0.05
35ALA	N	1MET	O	54.84
35ALA	N	33GLY	O	0.24
34LEU	N	27ASP	OD1	65.42
34LEU	N	27ASP	OD2	8.17
33GLY	N	27ASP	OD1	84.66
33GLY	N	27ASP	OD2	6.00
32LEU	N	26LEU	O	0.02
32LEU	N	27ASP	OD1	67.54
32LEU	N	27ASP	OD2	5.49

49PHE	N	44ALA	O	1.96
49PHE	N	45ALA	O	28.76
49PHE	N	46ILE	O	16.40
49PHE	N	47ASP	O	4.56
48ALA	N	44ALA	O	33.57
48ALA	N	45ALA	O	0.94
48ALA	N	46ILE	O	0.23
48ALA	N	47ASP	OD1	0.16
48ALA	N	47ASP	OD2	0.19
48ALA	N	49PHE	O	0.01
47ASP	N	43GLY	O	16.02
47ASP	N	44ALA	O	9.94
47ASP	N	45ALA	O	0.93
47ASP	N	47ASP	OD1	0.01
47ASP	N	47ASP	OD2	0.03
47ASP	N	49PHE	O	0.73
46ILE	N	42GLY	O	1.76
46ILE	N	43GLY	O	18.02
46ILE	N	44ALA	O	0.06
46ILE	N	49PHE	O	2.51
46ILE	N	51GLU	O	0.50
45ALA	N	41PHE	O	0.02
45ALA	N	42GLY	O	5.12
45ALA	N	43GLY	O	0.12
45ALA	N	47ASP	O	0.00
44ALA	N	8GLY	O	0.08
44ALA	N	9ASP	OD1	2.29
44ALA	N	9ASP	OD2	3.33
44ALA	N	41PHE	O	0.02
44ALA	N	42GLY	O	0.09
44ALA	N	77TRP	NE1	0.14
43GLY	N	8GLY	O	0.12
43GLY	N	9ASP	OD1	1.56
43GLY	N	9ASP	OD2	2.42
43GLY	N	41PHE	O	0.12
43GLY	N	45ALA	O	0.17
43GLY	N	77TRP	NE1	0.00
42GLY	N	52PRO	O	2.66
41PHE	N	39PHE	O	0.00
41PHE	N	45ALA	O	0.00
39PHE	N	5VAL	O	95.34
39PHE	N	37GLU	O	0.05
38VAL	N	37GLU	OE1	6.11

32LEU	N	27ASP	O	0.28
31GLY	N	26LEU	O	87.43
31GLY	N	27ASP	O	0.44
31GLY	N	30GLU	OE1	0.01
31GLY	N	30GLU	OE2	0.05
30GLU	N	26LEU	O	60.42
30GLU	N	27ASP	O	0.73
30GLU	N	28GLU	O	0.10
30GLU	N	30GLU	OE2	0.00
29ALA	N	25ALA	O	79.10
29ALA	N	26LEU	O	4.95
29ALA	N	27ASP	O	0.37
28GLU	N	24ARG	O	49.12
28GLU	N	25ALA	O	16.25
28GLU	N	26LEU	O	0.13
28GLU	N	30GLU	O	0.00
27ASP	N	23LEU	O	75.24
27ASP	N	24ARG	O	3.80
26LEU	N	22VAL	O	79.94
26LEU	N	23LEU	O	3.94
25ALA	N	21LYS	O	43.76
25ALA	N	22VAL	O	10.61
24ARG	NH2	17GLU	OE1	26.51
24ARG	NH2	17GLU	OE2	33.89
24ARG	NH2	17GLU	O	0.03
24ARG	NH2	28GLU	OE1	1.60
24ARG	NH2	28GLU	OE2	2.35
24ARG	NH2	36TYR	OH	23.36
24ARG	NH1	17GLU	OE1	36.90
24ARG	NH1	17GLU	OE2	27.70
24ARG	NH1	17GLU	O	0.00
24ARG	NH1	20LEU	O	0.00
24ARG	NH1	27ASP	OD2	0.14
24ARG	NH1	28GLU	OE1	2.90
24ARG	NH1	28GLU	OE2	3.28
24ARG	NH1	34LEU	O	0.12
24ARG	NH1	36TYR	OH	2.67
24ARG	NE	17GLU	OE2	0.00
24ARG	NE	20LEU	O	0.24
24ARG	NE	24ARG	O	0.01
24ARG	NE	28GLU	OE1	0.11
24ARG	NE	28GLU	OE2	0.03
24ARG	NE	36TYR	OH	46.43

38VAL	N	37GLU	OE2	6.79
37GLU	N	3VAL	O	58.88
36TYR	OH	17GLU	OE1	0.11
36TYR	OH	17GLU	OE2	0.12
36TYR	OH	20LEU	O	0.15
36TYR	OH	23LEU	O	0.03
36TYR	OH	27ASP	OD2	0.01
36TYR	N	34LEU	O	0.03
35ALA	N	1MET	O	61.24
35ALA	N	33GLY	O	0.09
34LEU	N	27ASP	OD1	49.77
34LEU	N	27ASP	OD2	30.62
34LEU	N	32LEU	O	0.00
33GLY	N	27ASP	OD1	68.66
33GLY	N	27ASP	OD2	30.73
32LEU	N	27ASP	OD1	60.57
32LEU	N	27ASP	OD2	28.39
32LEU	N	27ASP	O	0.14
31GLY	N	26LEU	O	85.34
31GLY	N	27ASP	O	0.61
31GLY	N	30GLU	OE2	0.00
30GLU	N	25ALA	O	0.00
30GLU	N	26LEU	O	66.33
30GLU	N	27ASP	O	0.56
30GLU	N	28GLU	O	0.02
29ALA	N	25ALA	O	74.31
29ALA	N	26LEU	O	5.63
29ALA	N	27ASP	O	0.10
28GLU	N	24ARG	O	69.53
28GLU	N	25ALA	O	6.66
27ASP	N	23LEU	O	76.09
27ASP	N	24ARG	O	4.20
26LEU	N	22VAL	O	69.53
26LEU	N	23LEU	O	6.18
26LEU	N	24ARG	O	0.00
25ALA	N	21LYS	O	69.61
25ALA	N	22VAL	O	3.65
24ARG	NH2	17GLU	OE1	0.32
24ARG	NH2	17GLU	OE2	0.22
24ARG	NH2	27ASP	OD1	1.62
24ARG	NH2	27ASP	OD2	4.32
24ARG	NH2	27ASP	O	0.11
24ARG	NH2	28GLU	OE1	42.11

24ARG	N	20LEU	O	96.56
24ARG	N	21LYS	O	0.24
23LEU	N	19ALA	O	74.49
23LEU	N	20LEU	O	1.81
22VAL	N	18ALA	O	14.15
22VAL	N	19ALA	O	25.27
21LYS	NZ	14GLU	OE1	0.00
21LYS	NZ	17GLU	OE1	2.67
21LYS	NZ	17GLU	OE2	0.58
21LYS	NZ	17GLU	O	0.02
21LYS	NZ	333THR	O	0.76
21LYS	NZ	334GLU	OE1	16.62
21LYS	NZ	334GLU	OE2	18.85
21LYS	NZ	334GLU	O	0.01
21LYS	NZ	337THR	OG1	21.91
21LYS	N	17GLU	O	83.16
21LYS	N	18ALA	O	1.61
20LEU	N	16THR	O	87.83
20LEU	N	17GLU	O	1.23
19ALA	N	15VAL	O	46.79
19ALA	N	16THR	O	18.99
18ALA	N	14GLU	O	63.45
18ALA	N	15VAL	O	3.05
17GLU	N	13PRO	O	74.15
17GLU	N	14GLU	O	2.62
16THR	OG1	6LEU	O	0.01
16THR	OG1	7PRO	O	3.02
16THR	OG1	12GLY	O	90.09
16THR	N	12GLY	O	77.37
16THR	N	13PRO	O	2.06
15VAL	N	11ILE	O	60.38
15VAL	N	12GLY	O	2.97
14GLU	N	10GLY	O	0.02
14GLU	N	11ILE	O	39.23
14GLU	N	12GLY	O	0.02
14GLU	N	14GLU	OE1	0.01
12GLY	N	9ASP	O	1.09
12GLY	N	71SER	OG	15.78
12GLY	N	71SER	O	0.01
11ILE	N	9ASP	O	0.47
11ILE	N	71SER	OG	11.29
11ILE	N	275SER	OG	0.02
11ILE	N	275SER	O	0.43

24ARG	NH2	28GLU	OE2	52.21
24ARG	NH2	34LEU	O	0.00
24ARG	NH2	36TYR	OH	0.84
24ARG	NH1	17GLU	OE1	0.22
24ARG	NH1	27ASP	OD1	29.44
24ARG	NH1	27ASP	OD2	61.47
24ARG	NH1	34LEU	O	0.15
24ARG	NH1	36TYR	OH	1.45
24ARG	NE	24ARG	O	0.01
24ARG	NE	28GLU	OE1	1.85
24ARG	NE	28GLU	OE2	1.98
24ARG	NE	36TYR	OH	0.82
24ARG	N	20LEU	O	93.22
24ARG	N	21LYS	O	0.45
23LEU	N	19ALA	O	46.40
23LEU	N	20LEU	O	6.10
22VAL	N	18ALA	O	31.39
22VAL	N	19ALA	O	10.49
21LYS	NZ	14GLU	OE1	0.02
21LYS	NZ	17GLU	OE1	4.67
21LYS	NZ	17GLU	OE2	6.70
21LYS	NZ	17GLU	O	0.05
21LYS	NZ	333THR	O	0.59
21LYS	NZ	334GLU	OE1	31.67
21LYS	NZ	334GLU	OE2	33.55
21LYS	NZ	334GLU	O	0.01
21LYS	NZ	337THR	OG1	2.75
21LYS	N	17GLU	O	56.28
21LYS	N	18ALA	O	7.71
20LEU	N	16THR	O	75.02
20LEU	N	17GLU	O	5.42
19ALA	N	15VAL	O	46.23
19ALA	N	16THR	O	15.98
19ALA	N	17GLU	O	0.00
18ALA	N	14GLU	O	29.32
18ALA	N	15VAL	O	13.92
18ALA	N	16THR	O	0.00
17GLU	N	13PRO	O	67.91
17GLU	N	14GLU	O	6.29
16THR	OG1	7PRO	O	1.23
16THR	OG1	12GLY	O	92.70
16THR	N	12GLY	O	64.96
16THR	N	13PRO	O	3.54

11ILE	N	276ALA	O	0.05
10GLY	N	9ASP	OD1	2.88
10GLY	N	9ASP	OD2	2.28
9ASP	N	71SER	O	81.51
8GLY	N	41PHE	O	35.55
6LEU	N	68LEU	O	98.43
5VAL	N	3VAL	O	0.00
5VAL	N	37GLU	O	93.68
4ALA	N	66ALA	O	95.32
3VAL	N	1MET	O	0.00
3VAL	N	35ALA	O	89.19
2LYS	NZ	36TYR	O	0.05
2LYS	NZ	37GLU	OE1	5.28
2LYS	NZ	37GLU	OE2	3.08
2LYS	NZ	59LYS	O	0.07
2LYS	NZ	60GLY	O	0.00
2LYS	NZ	63GLU	OE1	31.61
2LYS	NZ	63GLU	OE2	32.75
2LYS	NZ	63GLU	O	5.58
2LYS	NZ	65GLU	OE1	0.27
2LYS	NZ	65GLU	OE2	1.69
2LYS	N	65GLU	OE1	1.40
2LYS	N	65GLU	OE2	4.70

15VAL	N	11ILE	O	74.85
15VAL	N	12GLY	O	3.32
14GLU	N	10GLY	O	0.05
14GLU	N	11ILE	O	26.00
12GLY	N	9ASP	O	0.97
12GLY	N	10GLY	O	0.86
12GLY	N	71SER	OG	3.21
12GLY	N	71SER	O	1.94
12GLY	N	275SER	OG	3.14
11ILE	N	9ASP	O	0.56
11ILE	N	71SER	OG	0.71
11ILE	N	71SER	O	0.04
11ILE	N	73GLY	O	5.18
11ILE	N	275SER	OG	16.24
11ILE	N	275SER	O	0.12
11ILE	N	280ALA	O	0.00
10GLY	N	9ASP	OD1	1.92
10GLY	N	9ASP	OD2	2.31
9ASP	N	42GLY	O	0.12
9ASP	N	71SER	O	27.65
8GLY	N	41PHE	O	30.98
6LEU	N	68LEU	O	98.04
5VAL	N	37GLU	O	93.99
4ALA	N	66ALA	O	95.42
3VAL	N	1MET	O	0.00
3VAL	N	35ALA	O	86.45
2LYS	NZ	36TYR	O	0.00
2LYS	NZ	37GLU	OE1	2.14
2LYS	NZ	37GLU	OE2	3.58
2LYS	NZ	63GLU	OE1	31.49
2LYS	NZ	63GLU	OE2	36.94
2LYS	NZ	63GLU	O	7.97
2LYS	NZ	65GLU	OE1	1.78
2LYS	NZ	65GLU	OE2	5.11
2LYS	N	65GLU	OE1	8.00
2LYS	N	65GLU	OE2	8.13
