# Supplementary material for: Deciphering the Dynamics of Non-Covalent Interactions Affecting Thermal Stability of a Protein: Molecular Dynamics Study on Point Mutant of Thermus thermophilus Isopropylmalate Dehydrogenase
Source: PLoS One. 2015 Dec 11;10(12):e0144294. doi: 10.1371/journal.pone.0144294 (PMC4689552; doi:10.1371/journal.pone.0144294)
Supplement: S3 Table — The color formatting pattern is as followed in S1 Table. (PDF) [file pone.0144294.s005.pdf]

S3 Table. Percentage of time HBs existed between MM of the *wt* and *mut* at 300 K and 337 K.

1) *Wt* 300 K

Drnona	D	Arnona	A	percen
1MET	N	33GLY	O	88.96
345ALA	N	341LEU	O	1.80
345ALA	N	342ARG	O	7.17
345ALA	N	343HIS	O	6.62
344LEU	N	340VAL	O	29.19
344LEU	N	341LEU	O	17.19
344LEU	N	342ARG	O	0.19
343HIS	N	339THR	O	33.51
343HIS	N	340VAL	O	42.31
343HIS	N	341LEU	O	0.05
342ARG	N	338ALA	O	27.41
342ARG	N	339THR	O	30.68
342ARG	N	340VAL	O	0.38
341LEU	N	337THR	O	96.30
341LEU	N	338ALA	O	0.28
340VAL	N	336PHE	O	66.23
340VAL	N	337THR	O	6.13
339THR	N	335ALA	O	37.76
339THR	N	336PHE	O	22.74
339THR	N	337THR	O	0.22
338ALA	N	334GLU	O	66.57
338ALA	N	335ALA	O	4.57
337THR	N	332GLY	O	0.36
337THR	N	333THR	O	24.22
337THR	N	334GLU	O	10.75
337THR	N	335ALA	O	2.39
336PHE	N	332GLY	O	92.68
336PHE	N	333THR	O	0.32
336PHE	N	334GLU	O	0.31
335ALA	N	332GLY	O	7.89
335ALA	N	333THR	O	0.02
334GLU	N	332GLY	O	0.70
333THR	N	283GLY	O	10.66
333THR	N	285ALA	O	3.62
332GLY	N	283GLY	O	0.03
332GLY	N	330SER	O	5.21
332GLY	N	335ALA	O	0.03
332GLY	N	336PHE	N	0.00
331ALA	N	323PRO	O	0.15
331ALA	N	329GLY	O	0.18

2) *Wt* 337 K

Drnona	D	Arnona	A	percen
1MET	N	32LEU	O	0.05
1MET	N	33GLY	O	65.50
345ALA	N	341LEU	O	5.80
345ALA	N	342ARG	O	4.94
345ALA	N	343HIS	O	15.23
345ALA	N	345ALA	O	0.00
344LEU	N	340VAL	O	29.93
344LEU	N	341LEU	O	27.91
344LEU	N	342ARG	O	0.08
343HIS	N	339THR	O	10.62
343HIS	N	340VAL	O	60.19
343HIS	N	341LEU	O	0.27
342ARG	N	338ALA	O	33.79
342ARG	N	339THR	O	13.64
342ARG	N	340VAL	O	0.90
341LEU	N	337THR	O	95.95
341LEU	N	338ALA	O	0.42
340VAL	N	336PHE	O	31.81
340VAL	N	337THR	O	21.14
340VAL	N	338ALA	O	0.00
339THR	N	335ALA	O	61.34
339THR	N	336PHE	O	13.14
339THR	N	337THR	O	0.02
338ALA	N	334GLU	O	75.65
338ALA	N	335ALA	O	2.15
338ALA	N	336PHE	O	0.01
337THR	N	332GLY	O	0.01
337THR	N	333THR	O	39.56
337THR	N	334GLU	O	23.99
337THR	N	335ALA	O	6.55
336PHE	N	332GLY	O	84.83
336PHE	N	333THR	O	2.52
336PHE	N	334GLU	O	0.23
335ALA	N	332GLY	O	11.34
334GLU	N	332GLY	O	0.05
333THR	N	280ALA	O	0.00
333THR	N	281GLY	O	0.13
333THR	N	282LYS	O	0.02
333THR	N	283GLY	O	26.11
333THR	N	285ALA	O	1.29

3) *Mut* 300 K

Drnona	D	Arnona	A	percen
1MET	N	1MET	O	0.00
1MET	N	33GLY	O	57.77
345ALA	N	341LEU	O	4.20
345ALA	N	342ARG	O	13.72
345ALA	N	343HIS	O	11.17
344LEU	N	340VAL	O	36.51
344LEU	N	341LEU	O	17.30
344LEU	N	342ARG	O	0.17
343HIS	N	339THR	O	34.66
343HIS	N	340VAL	O	39.86
343HIS	N	341LEU	O	0.11
342ARG	N	338ALA	O	29.89
342ARG	N	339THR	O	27.81
342ARG	N	340VAL	O	0.68
341LEU	N	337THR	O	94.42
341LEU	N	338ALA	O	1.04
340VAL	N	336PHE	O	74.22
340VAL	N	337THR	O	6.15
340VAL	N	338ALA	O	0.01
339THR	N	335ALA	O	41.07
339THR	N	336PHE	O	20.31
339THR	N	337THR	O	0.00
338ALA	N	334GLU	O	63.42
338ALA	N	335ALA	O	5.29
338ALA	N	336PHE	O	0.04
337THR	N	333THR	O	80.44
337THR	N	334GLU	O	1.64
336PHE	N	332GLY	O	84.32
336PHE	N	333THR	O	2.65
336PHE	N	334GLU	O	0.00
335ALA	N	332GLY	O	9.26
333THR	N	283GLY	O	45.71
333THR	N	285ALA	O	0.04
331ALA	N	282LYS	O	0.01
331ALA	N	283GLY	O	0.02
331ALA	N	323PRO	O	59.85
331ALA	N	329GLY	O	0.01
330SER	N	323PRO	O	2.43
329GLY	N	324PRO	O	28.34
329GLY	N	325PRO	O	0.25

4) *Mut* 337 K

Drnona	D	Arnona	A	percen
1MET	N	1MET	O	0.00
1MET	N	32LEU	O	0.01
1MET	N	33GLY	O	66.65
345ALA	N	341LEU	O	3.92
345ALA	N	342ARG	O	5.63
345ALA	N	343HIS	O	10.81
345ALA	N	345ALA	O	0.01
344LEU	N	340VAL	O	33.15
344LEU	N	341LEU	O	19.68
344LEU	N	342ARG	O	0.14
343HIS	N	339THR	O	27.94
343HIS	N	340VAL	O	48.59
343HIS	N	341LEU	O	0.16
342ARG	N	338ALA	O	24.27
342ARG	N	339THR	O	31.00
342ARG	N	340VAL	O	0.78
342ARG	N	344LEU	N	0.00
341LEU	N	337THR	O	91.93
341LEU	N	338ALA	O	0.58
340VAL	N	336PHE	O	75.72
340VAL	N	337THR	O	6.21
340VAL	N	338ALA	O	0.00
339THR	N	335ALA	O	35.50
339THR	N	336PHE	O	25.87
339THR	N	337THR	O	0.06
338ALA	N	334GLU	O	58.77
338ALA	N	335ALA	O	3.98
338ALA	N	336PHE	O	0.02
337THR	N	332GLY	O	0.11
337THR	N	333THR	O	54.74
337THR	N	334GLU	O	2.12
337THR	N	335ALA	O	0.59
336PHE	N	332GLY	O	90.14
336PHE	N	333THR	O	1.84
336PHE	N	334GLU	O	0.05
335ALA	N	332GLY	O	8.54
335ALA	N	333THR	O	0.10
334GLU	N	283GLY	O	0.01
334GLU	N	332GLY	O	1.87
333THR	N	281GLY	O	5.79

330SER	N	323PRO	O	0.03
329GLY	N	283GLY	O	0.08
329GLY	N	324PRO	O	0.14
329GLY	N	325PRO	O	1.18
328GLY	N	282LYS	O	13.06
328GLY	N	324PRO	O	8.19
328GLY	N	325PRO	O	6.51
328GLY	N	326ASP	O	2.45
327LEU	N	324PRO	O	16.98
327LEU	N	328GLY	O	0.16
326ASP	N	282LYS	O	0.07
326ASP	N	324PRO	O	0.13
326ASP	N	328GLY	N	0.00
326ASP	N	328GLY	O	52.76
322THR	N	318ALA	O	86.94
322THR	N	319LEU	O	0.08
321GLU	N	317LYS	O	14.57
321GLU	N	318ALA	O	48.95
321GLU	N	319LEU	O	0.16
320LEU	N	316ALA	O	31.72
320LEU	N	317LYS	O	20.20
320LEU	N	318ALA	O	0.02
319LEU	N	315VAL	O	77.64
319LEU	N	316ALA	O	5.78
318ALA	N	314ALA	O	54.72
318ALA	N	315VAL	O	7.97
317LYS	N	313ASP	O	77.30
317LYS	N	314ALA	O	6.59
317LYS	N	315VAL	O	0.02
316ALA	N	312GLU	O	27.51
316ALA	N	313ASP	O	9.28
315VAL	N	311VAL	O	92.39
315VAL	N	312GLU	O	0.42
314ALA	N	310LYS	O	77.49
314ALA	N	311VAL	O	2.73
313ASP	N	309ARG	O	53.59
313ASP	N	310LYS	O	10.00
313ASP	N	311VAL	O	0.00
312GLU	N	308ALA	O	80.87
312GLU	N	309ARG	O	0.73
311VAL	N	307LEU	O	55.34
311VAL	N	308ALA	O	13.82
310LYS	N	306GLU	O	27.71

332GLY	N	283GLY	O	0.00
332GLY	N	335ALA	O	0.18
331ALA	N	283GLY	O	0.04
331ALA	N	323PRO	O	20.94
331ALA	N	329GLY	O	0.10
330SER	N	323PRO	O	1.91
330SER	N	328GLY	O	0.00
329GLY	N	323PRO	O	0.00
329GLY	N	324PRO	O	12.62
329GLY	N	325PRO	O	7.06
329GLY	N	327LEU	O	0.02
328GLY	N	324PRO	O	46.49
328GLY	N	325PRO	O	22.54
328GLY	N	326ASP	O	0.06
327LEU	N	324PRO	O	70.54
327LEU	N	325PRO	O	0.85
326ASP	N	254LEU	O	2.63
326ASP	N	284ILE	O	0.41
326ASP	N	324PRO	O	0.46
326ASP	N	328GLY	N	0.00
326ASP	N	329GLY	O	0.01
322THR	N	318ALA	O	78.96
322THR	N	319LEU	O	0.07
321GLU	N	317LYS	O	29.37
321GLU	N	318ALA	O	36.72
321GLU	N	319LEU	O	0.17
320LEU	N	316ALA	O	25.72
320LEU	N	317LYS	O	26.53
320LEU	N	318ALA	O	0.07
319LEU	N	315VAL	O	84.14
319LEU	N	316ALA	O	3.16
318ALA	N	314ALA	O	45.71
318ALA	N	315VAL	O	16.45
317LYS	N	313ASP	O	79.08
317LYS	N	314ALA	O	4.70
317LYS	N	315VAL	O	0.02
316ALA	N	312GLU	O	69.81
316ALA	N	313ASP	O	2.55
315VAL	N	311VAL	O	84.72
315VAL	N	312GLU	O	2.35
314ALA	N	310LYS	O	77.07
314ALA	N	311VAL	O	2.83
313ASP	N	309ARG	O	73.29

328GLY	N	324PRO	O	64.22
328GLY	N	325PRO	O	21.06
327LEU	N	324PRO	O	73.83
327LEU	N	325PRO	O	0.00
326ASP	N	284ILE	O	3.28
326ASP	N	324PRO	O	0.24
326ASP	N	328GLY	N	0.01
322THR	N	318ALA	O	97.64
322THR	N	319LEU	O	0.00
321GLU	N	317LYS	O	43.39
321GLU	N	318ALA	O	24.80
321GLU	N	319LEU	O	0.19
320LEU	N	316ALA	O	23.69
320LEU	N	317LYS	O	36.72
320LEU	N	318ALA	O	0.01
319LEU	N	315VAL	O	80.60
319LEU	N	316ALA	O	3.33
318ALA	N	314ALA	O	58.53
318ALA	N	315VAL	O	8.07
318ALA	N	316ALA	O	0.00
317LYS	N	313ASP	O	73.28
317LYS	N	314ALA	O	6.80
317LYS	N	315VAL	O	0.01
316ALA	N	312GLU	O	38.63
316ALA	N	313ASP	O	4.78
315VAL	N	311VAL	O	87.35
315VAL	N	312GLU	O	0.62
314ALA	N	310LYS	O	80.16
314ALA	N	311VAL	O	2.31
313ASP	N	309ARG	O	65.40
313ASP	N	310LYS	O	6.52
312GLU	N	308ALA	O	81.88
312GLU	N	309ARG	O	1.03
311VAL	N	307LEU	O	71.47
311VAL	N	308ALA	O	5.79
310LYS	N	306GLU	O	44.68
310LYS	N	307LEU	O	13.10
309ARG	N	305VAL	O	75.12
309ARG	N	306GLU	O	3.56
308ALA	N	304LEU	O	79.11
308ALA	N	305VAL	O	2.75
307LEU	N	304LEU	O	18.76
306GLU	N	304LEU	O	0.00

333THR	N	282LYS	O	0.00
333THR	N	283GLY	O	9.37
333THR	N	285ALA	O	3.97
332GLY	N	330SER	O	0.18
332GLY	N	335ALA	O	0.02
332GLY	N	336PHE	N	0.00
331ALA	N	282LYS	O	0.01
331ALA	N	283GLY	O	3.08
331ALA	N	284ILE	O	0.01
331ALA	N	323PRO	O	32.31
331ALA	N	329GLY	O	0.34
330SER	N	323PRO	O	4.42
329GLY	N	323PRO	O	0.39
329GLY	N	324PRO	O	36.99
329GLY	N	325PRO	O	0.72
329GLY	N	327LEU	O	0.01
328GLY	N	324PRO	O	27.71
328GLY	N	325PRO	O	13.65
328GLY	N	326ASP	O	0.27
327LEU	N	324PRO	O	51.45
327LEU	N	325PRO	O	14.63
326ASP	N	324PRO	O	0.04
326ASP	N	328GLY	O	0.37
326ASP	N	329GLY	O	0.06
322THR	N	318ALA	O	94.24
322THR	N	319LEU	O	0.06
321GLU	N	317LYS	O	19.61
321GLU	N	318ALA	O	41.78
321GLU	N	319LEU	O	0.66
320LEU	N	316ALA	O	32.23
320LEU	N	317LYS	O	21.46
319LEU	N	315VAL	O	85.63
319LEU	N	316ALA	O	2.63
318ALA	N	314ALA	O	56.48
318ALA	N	315VAL	O	11.38
318ALA	N	316ALA	O	0.00
317LYS	N	313ASP	O	77.91
317LYS	N	314ALA	O	5.01
317LYS	N	315VAL	O	0.05
316ALA	N	312GLU	O	81.71
316ALA	N	313ASP	O	1.32
315VAL	N	311VAL	O	86.32
315VAL	N	312GLU	O	2.26

310LYS	N	307LEU	O	20.19
310LYS	N	308ALA	O	0.00
309ARG	N	305VAL	O	81.17
309ARG	N	306GLU	O	3.54
308ALA	N	304LEU	O	64.89
308ALA	N	305VAL	O	9.17
307LEU	N	304LEU	O	28.10
307LEU	N	305VAL	O	0.00
305VAL	N	303GLY	O	37.83
304LEU	N	298LEU	O	77.20
304LEU	N	299GLU	O	0.00
304LEU	N	302PHE	O	0.01
303GLY	N	298LEU	O	53.65
303GLY	N	299GLU	O	0.86
303GLY	N	300HIS	O	0.06
302PHE	N	297MET	O	68.24
302PHE	N	298LEU	O	1.53
302PHE	N	299GLU	O	0.01
302PHE	N	300HIS	O	0.03
301ALA	N	296MET	O	0.01
301ALA	N	297MET	O	61.29
301ALA	N	298LEU	O	0.00
301ALA	N	299GLU	O	0.02
300HIS	N	296MET	O	94.25
300HIS	N	297MET	O	0.67
299GLU	N	295ALA	O	86.91
299GLU	N	296MET	O	1.39
298LEU	N	294ALA	O	47.46
298LEU	N	295ALA	O	13.04
297MET	N	293SER	O	72.95
297MET	N	294ALA	O	1.31
296MET	N	292LEU	O	84.14
296MET	N	293SER	O	1.69
295ALA	N	291ILE	O	39.37
295ALA	N	292LEU	O	12.82
294ALA	N	290ALA	O	89.63
294ALA	N	291ILE	O	1.53
293SER	N	289ALA	O	49.14
293SER	N	290ALA	O	7.48
292LEU	N	288THR	O	95.11
292LEU	N	289ALA	O	0.14
291ILE	N	287PRO	O	95.02
291ILE	N	288THR	O	0.20

313ASP	N	310LYS	O	3.90
312GLU	N	308ALA	O	78.70
312GLU	N	309ARG	O	1.52
312GLU	N	310LYS	O	0.00
311VAL	N	307LEU	O	66.66
311VAL	N	308ALA	O	7.52
310LYS	N	306GLU	O	37.26
310LYS	N	307LEU	O	16.00
309ARG	N	305VAL	O	76.89
309ARG	N	306GLU	O	3.66
308ALA	N	304LEU	O	69.13
308ALA	N	305VAL	O	5.50
307LEU	N	304LEU	O	24.04
307LEU	N	305VAL	O	0.01
305VAL	N	303GLY	O	48.43
304LEU	N	298LEU	O	72.44
304LEU	N	302PHE	O	0.02
303GLY	N	298LEU	O	50.27
303GLY	N	299GLU	O	3.78
303GLY	N	300HIS	O	0.03
302PHE	N	297MET	O	47.62
302PHE	N	298LEU	O	1.26
302PHE	N	299GLU	O	0.61
302PHE	N	300HIS	O	0.20
301ALA	N	296MET	O	0.01
301ALA	N	297MET	O	63.56
301ALA	N	299GLU	O	0.04
300HIS	N	296MET	O	94.35
300HIS	N	297MET	O	0.87
299GLU	N	295ALA	O	75.30
299GLU	N	296MET	O	4.50
298LEU	N	294ALA	O	62.16
298LEU	N	295ALA	O	10.81
297MET	N	293SER	O	67.13
297MET	N	294ALA	O	2.90
296MET	N	292LEU	O	85.86
296MET	N	293SER	O	1.04
295ALA	N	291ILE	O	62.96
295ALA	N	292LEU	O	6.42
294ALA	N	290ALA	O	65.57
294ALA	N	291ILE	O	9.55
293SER	N	289ALA	O	44.26
293SER	N	290ALA	O	7.36

305VAL	N	303GLY	O	58.77
304LEU	N	298LEU	O	75.65
304LEU	N	302PHE	O	0.04
303GLY	N	298LEU	O	34.35
303GLY	N	299GLU	O	7.91
303GLY	N	300HIS	O	0.06
302PHE	N	297MET	O	57.29
302PHE	N	298LEU	O	1.23
302PHE	N	299GLU	O	0.26
302PHE	N	300HIS	O	0.06
301ALA	N	297MET	O	68.17
301ALA	N	299GLU	O	0.03
300HIS	N	296MET	O	96.08
300HIS	N	297MET	O	0.44
299GLU	N	295ALA	O	88.29
299GLU	N	296MET	O	1.16
298LEU	N	294ALA	O	82.04
298LEU	N	295ALA	O	1.71
297MET	N	293SER	O	75.30
297MET	N	294ALA	O	1.85
296MET	N	292LEU	O	87.60
296MET	N	293SER	O	0.70
295ALA	N	291ILE	O	76.00
295ALA	N	292LEU	O	1.87
294ALA	N	290ALA	O	70.29
294ALA	N	291ILE	O	7.91
293SER	N	289ALA	O	36.69
293SER	N	290ALA	O	9.62
292LEU	N	288THR	O	98.10
292LEU	N	289ALA	O	0.01
291ILE	N	287PRO	O	85.57
291ILE	N	288THR	O	2.00
290ALA	N	287PRO	O	59.09
289ALA	N	255GLY	O	0.28
288THR	N	286ASN	O	0.06
286ASN	N	284ILE	O	2.18
285ALA	N	279ILE	O	9.39
285ALA	N	283GLY	O	1.02
284ILE	N	279ILE	O	2.28
284ILE	N	282LYS	O	0.38
283GLY	N	279ILE	O	1.62
283GLY	N	281GLY	O	0.00
282LYS	N	278ASP	O	0.02

314ALA	N	310LYS	O	75.96
314ALA	N	311VAL	O	2.72
313ASP	N	309ARG	O	80.06
313ASP	N	310LYS	O	2.28
313ASP	N	311VAL	O	0.00
312GLU	N	308ALA	O	76.62
312GLU	N	309ARG	O	1.37
312GLU	N	310LYS	O	0.00
311VAL	N	307LEU	O	58.25
311VAL	N	308ALA	O	10.98
310LYS	N	306GLU	O	37.87
310LYS	N	307LEU	O	15.77
310LYS	N	308ALA	O	0.00
309ARG	N	305VAL	O	77.75
309ARG	N	306GLU	O	3.54
308ALA	N	304LEU	O	70.05
308ALA	N	305VAL	O	5.28
307LEU	N	304LEU	O	26.31
307LEU	N	305VAL	O	0.01
305VAL	N	303GLY	O	46.18
304LEU	N	298LEU	O	76.40
304LEU	N	302PHE	O	0.03
303GLY	N	298LEU	O	55.24
303GLY	N	299GLU	O	3.92
303GLY	N	300HIS	O	0.04
302PHE	N	297MET	O	41.17
302PHE	N	298LEU	O	1.96
302PHE	N	299GLU	O	3.72
302PHE	N	300HIS	O	0.31
301ALA	N	296MET	O	0.01
301ALA	N	297MET	O	61.92
301ALA	N	299GLU	O	0.15
300HIS	N	296MET	O	94.56
300HIS	N	297MET	O	0.65
299GLU	N	295ALA	O	63.05
299GLU	N	296MET	O	10.53
298LEU	N	294ALA	O	55.64
298LEU	N	295ALA	O	13.19
297MET	N	293SER	O	69.89
297MET	N	294ALA	O	3.48
296MET	N	292LEU	O	81.53
296MET	N	293SER	O	1.86
295ALA	N	291ILE	O	65.47

290ALA	N	287PRO	O	23.43
289ALA	N	255GLY	O	17.94
288THR	N	255GLY	O	0.00
288THR	N	286ASN	O	0.01
286ASN	N	284ILE	O	0.14
285ALA	N	283GLY	O	9.28
284ILE	N	279ILE	O	0.01
284ILE	N	282LYS	O	0.84
284ILE	N	326ASP	O	15.75
284ILE	N	328GLY	O	0.26
284ILE	N	329GLY	O	0.02
283GLY	N	278ASP	O	0.64
283GLY	N	279ILE	O	12.50
283GLY	N	280ALA	O	0.14
283GLY	N	281GLY	O	3.86
283GLY	N	327LEU	O	0.02
283GLY	N	328GLY	N	0.00
282LYS	N	278ASP	O	27.60
282LYS	N	279ILE	O	29.72
282LYS	N	280ALA	O	0.15
282LYS	N	283GLY	O	0.05
281GLY	N	277PRO	O	2.17
281GLY	N	278ASP	O	51.85
281GLY	N	279ILE	O	0.86
281GLY	N	283GLY	O	0.03
280ALA	N	276ALA	O	28.85
280ALA	N	277PRO	O	25.30
279ILE	N	276ALA	O	55.95
278ASP	N	276ALA	O	0.57
278ASP	N	280ALA	O	0.03
276ALA	N	274GLY	O	0.53
275SER	N	72VAL	O	0.32
275SER	N	73GLY	O	2.13
273HIS	N	271PRO	O	10.49
272VAL	N	257LEU	O	98.41
270GLU	N	259SER	O	94.04
269PHE	N	67VAL	O	96.26
268VAL	N	261SER	O	95.80
266THR	N	97GLN	O	0.00
265GLY	N	97GLN	O	71.98
265GLY	N	263GLY	O	0.02
264ARG	N	97GLN	O	21.99
264ARG	N	98ASP	O	55.04

292LEU	N	288THR	O	96.74
292LEU	N	289ALA	O	0.09
291ILE	N	287PRO	O	72.18
291ILE	N	288THR	O	4.30
290ALA	N	287PRO	O	64.12
289ALA	N	255GLY	O	0.27
288THR	N	286ASN	O	0.45
286ASN	N	284ILE	O	0.45
285ALA	N	279ILE	O	13.09
285ALA	N	283GLY	O	7.48
285ALA	N	331ALA	O	0.22
284ILE	N	279ILE	O	52.61
284ILE	N	280ALA	O	0.03
284ILE	N	282LYS	O	0.43
283GLY	N	279ILE	O	35.65
283GLY	N	280ALA	O	2.14
283GLY	N	281GLY	O	0.00
282LYS	N	278ASP	O	28.76
282LYS	N	279ILE	O	34.23
282LYS	N	280ALA	O	0.15
281GLY	N	276ALA	O	0.00
281GLY	N	277PRO	O	6.83
281GLY	N	278ASP	O	53.04
281GLY	N	279ILE	O	0.21
280ALA	N	276ALA	O	34.81
280ALA	N	277PRO	O	21.36
280ALA	N	278ASP	O	0.09
279ILE	N	276ALA	O	10.85
279ILE	N	277PRO	O	14.86
278ASP	N	10GLY	O	0.01
278ASP	N	276ALA	O	0.41
276ALA	N	274GLY	O	0.14
276ALA	N	325PRO	O	9.66
275SER	N	72VAL	O	0.09
275SER	N	73GLY	O	1.93
275SER	N	273HIS	O	0.02
274GLY	N	255GLY	O	6.57
273HIS	N	254LEU	O	0.00
273HIS	N	271PRO	O	2.62
272VAL	N	257LEU	O	47.91
272VAL	N	270GLU	O	0.02
270GLU	N	259SER	O	91.35
270GLU	N	268VAL	O	0.02

282LYS	N	279ILE	O	19.73
282LYS	N	280ALA	O	2.02
281GLY	N	279ILE	O	0.08
280ALA	N	276ALA	O	5.94
280ALA	N	277PRO	O	60.23
280ALA	N	278ASP	O	0.06
279ILE	N	276ALA	O	10.36
279ILE	N	277PRO	O	0.14
278ASP	N	276ALA	O	0.25
276ALA	N	274GLY	O	4.34
275SER	N	72VAL	O	0.00
273HIS	N	255GLY	O	0.00
273HIS	N	271PRO	O	9.24
272VAL	N	257LEU	O	60.62
270GLU	N	259SER	O	98.81
269PHE	N	67VAL	O	96.42
268VAL	N	261SER	O	96.78
266THR	N	97GLN	O	0.19
265GLY	N	97GLN	O	23.35
265GLY	N	98ASP	O	0.11
265GLY	N	263GLY	O	1.49
264ARG	N	97GLN	O	6.04
264ARG	N	98ASP	O	74.49
262LEU	N	101ALA	O	99.10
261SER	N	268VAL	O	97.68
260ALA	N	103LEU	O	94.82
259SER	N	270GLU	O	10.32
257LEU	N	254LEU	O	0.58
257LEU	N	255GLY	O	0.06
257LEU	N	272VAL	O	0.02
256LEU	N	253SER	O	3.36
256LEU	N	254LEU	O	0.81
255GLY	N	253SER	O	0.11
253SER	N	249VAL	O	0.10
253SER	N	251PRO	O	1.01
252GLY	N	248SER	O	0.00
252GLY	N	249VAL	O	85.44
252GLY	N	250LEU	O	0.01
250LEU	N	247ALA	O	46.49
249VAL	N	245ASP	O	0.05
249VAL	N	246LEU	O	19.07
249VAL	N	247ALA	O	0.05
248SER	N	244SER	O	8.15

295ALA	N	292LEU	O	4.52
295ALA	N	293SER	O	0.00
294ALA	N	290ALA	O	71.80
294ALA	N	291ILE	O	7.16
293SER	N	289ALA	O	42.83
293SER	N	290ALA	O	10.00
292LEU	N	288THR	O	90.57
292LEU	N	289ALA	O	0.34
291ILE	N	287PRO	O	71.99
291ILE	N	288THR	O	3.61
290ALA	N	287PRO	O	41.04
289ALA	N	255GLY	O	15.41
289ALA	N	286ASN	O	0.05
288THR	N	286ASN	O	2.33
286ASN	N	284ILE	O	0.06
285ALA	N	279ILE	O	16.58
285ALA	N	283GLY	O	1.76
284ILE	N	279ILE	O	21.30
284ILE	N	282LYS	O	0.54
283GLY	N	279ILE	O	14.43
283GLY	N	280ALA	O	0.06
283GLY	N	281GLY	O	0.12
282LYS	N	278ASP	O	4.10
282LYS	N	279ILE	O	36.60
282LYS	N	280ALA	O	2.14
281GLY	N	278ASP	O	2.85
281GLY	N	279ILE	O	0.16
281GLY	N	283GLY	N	0.00
280ALA	N	276ALA	O	20.02
280ALA	N	277PRO	O	3.22
280ALA	N	278ASP	O	0.37
279ILE	N	276ALA	O	2.90
279ILE	N	277PRO	O	69.11
279ILE	N	280ALA	O	0.00
278ASP	N	276ALA	O	0.05
278ASP	N	280ALA	O	0.23
276ALA	N	274GLY	O	2.64
275SER	N	73GLY	O	0.00
273HIS	N	271PRO	O	0.33
272VAL	N	257LEU	O	36.61
270GLU	N	259SER	O	80.76
270GLU	N	268VAL	O	0.39
269PHE	N	67VAL	O	96.53

262LEU	N	101ALA	O	90.12
261SER	N	268VAL	O	92.16
260ALA	N	103LEU	O	94.45
259SER	N	270GLU	O	5.08
257LEU	N	254LEU	O	0.28
257LEU	N	255GLY	O	0.00
256LEU	N	253SER	O	0.75
256LEU	N	254LEU	O	0.74
255GLY	N	253SER	O	11.60
253SER	N	115LEU	O	0.01
253SER	N	248SER	O	0.00
253SER	N	251PRO	O	4.00
252GLY	N	248SER	O	1.11
252GLY	N	249VAL	O	13.76
252GLY	N	250LEU	O	0.00
250LEU	N	246LEU	O	0.44
250LEU	N	247ALA	O	34.36
249VAL	N	245ASP	O	2.81
249VAL	N	246LEU	O	36.96
249VAL	N	247ALA	O	0.01
248SER	N	244SER	O	21.58
248SER	N	245ASP	O	31.85
248SER	N	246LEU	O	0.07
247ALA	N	243LEU	O	73.88
247ALA	N	244SER	O	4.69
246LEU	N	242ILE	O	88.37
246LEU	N	243LEU	O	1.00
245ASP	N	241ASP	O	51.59
245ASP	N	242ILE	O	6.53
245ASP	N	243LEU	O	0.00
244SER	N	240GLY	O	54.93
244SER	N	241ASP	O	6.68
243LEU	N	239PHE	O	28.23
243LEU	N	240GLY	O	5.20
242ILE	N	238ILE	O	3.44
242ILE	N	239PHE	O	4.62
241ASP	N	237ASN	O	25.93
241ASP	N	238ILE	O	1.49
240GLY	N	236GLY	O	1.08
240GLY	N	237ASN	O	25.80
240GLY	N	238ILE	O	0.05
239PHE	N	236GLY	O	0.06
238ILE	N	236GLY	O	0.00

269PHE	N	67VAL	O	94.78
268VAL	N	261SER	O	93.06
266THR	N	61VAL	O	0.00
266THR	N	97GLN	O	0.15
266THR	N	263GLY	O	0.04
266THR	N	264ARG	O	0.02
265GLY	N	97GLN	O	15.48
265GLY	N	98ASP	O	1.68
265GLY	N	263GLY	O	3.72
264ARG	N	97GLN	O	8.20
264ARG	N	98ASP	O	76.84
263GLY	N	265GLY	O	0.02
263GLY	N	266THR	O	0.21
262LEU	N	101ALA	O	96.36
262LEU	N	260ALA	O	0.01
261SER	N	268VAL	O	92.78
260ALA	N	103LEU	O	93.38
259SER	N	270GLU	O	8.20
257LEU	N	253SER	O	2.15
257LEU	N	254LEU	O	1.32
257LEU	N	255GLY	O	0.30
257LEU	N	272VAL	O	10.38
256LEU	N	253SER	O	57.61
256LEU	N	254LEU	O	0.12
255GLY	N	253SER	O	1.01
254LEU	N	248SER	O	0.51
254LEU	N	252GLY	O	62.46
253SER	N	248SER	O	4.24
253SER	N	251PRO	O	0.39
252GLY	N	248SER	O	15.46
252GLY	N	249VAL	O	24.49
250LEU	N	246LEU	O	21.32
250LEU	N	247ALA	O	7.39
249VAL	N	245ASP	O	6.48
249VAL	N	246LEU	O	60.14
248SER	N	244SER	O	8.10
248SER	N	245ASP	O	58.53
248SER	N	246LEU	O	0.68
247ALA	N	243LEU	O	80.96
247ALA	N	244SER	O	2.50
246LEU	N	242ILE	O	35.35
246LEU	N	243LEU	O	1.81
246LEU	N	244SER	O	0.02

248SER	N	245ASP	O	55.79
248SER	N	246LEU	O	0.07
247ALA	N	243LEU	O	71.63
247ALA	N	244SER	O	3.68
246LEU	N	242ILE	O	81.30
246LEU	N	243LEU	O	0.91
245ASP	N	241ASP	O	49.53
245ASP	N	242ILE	O	3.11
244SER	N	240GLY	O	78.30
244SER	N	241ASP	O	8.67
244SER	N	242ILE	O	0.00
243LEU	N	239PHE	O	10.13
243LEU	N	240GLY	O	4.28
242ILE	N	238ILE	O	9.88
242ILE	N	239PHE	O	3.64
242ILE	N	240GLY	O	0.00
241ASP	N	237ASN	O	91.89
241ASP	N	238ILE	O	1.45
240GLY	N	236GLY	O	2.99
240GLY	N	237ASN	O	69.02
240GLY	N	238ILE	O	0.37
239PHE	N	236GLY	O	0.22
237ASN	N	136GLY	O	11.94
235THR	N	131VAL	O	63.96
234VAL	N	181VAL	O	90.94
233VAL	N	129LEU	O	91.47
232VAL	N	179HIS	O	9.60
231ASP	N	229ARG	O	0.00
230PHE	N	227PRO	O	38.63
230PHE	N	228ALA	O	1.96
229ARG	N	226SER	O	28.81
229ARG	N	227PRO	O	10.60
228ALA	N	226SER	O	0.02
226SER	N	222HIS	O	97.84
226SER	N	223LEU	O	0.12
225ARG	N	221MET	O	20.63
225ARG	N	222HIS	O	60.61
225ARG	N	223LEU	O	0.17
224VAL	N	220ALA	O	0.59
224VAL	N	221MET	O	66.27
224VAL	N	222HIS	O	0.02
223LEU	N	219MET	O	89.61
223LEU	N	220ALA	O	0.75

268VAL	N	261SER	O	68.43
266THR	N	263GLY	O	0.01
266THR	N	264ARG	O	0.07
265GLY	N	97GLN	O	28.55
265GLY	N	98ASP	O	0.08
265GLY	N	263GLY	O	0.68
264ARG	N	97GLN	O	18.75
264ARG	N	98ASP	O	41.24
263GLY	N	265GLY	O	0.04
263GLY	N	266THR	O	0.59
262LEU	N	101ALA	O	78.22
261SER	N	268VAL	O	60.91
260ALA	N	103LEU	O	90.39
259SER	N	270GLU	O	4.43
257LEU	N	253SER	O	0.00
257LEU	N	254LEU	O	21.02
257LEU	N	255GLY	O	1.47
257LEU	N	272VAL	O	0.11
256LEU	N	253SER	O	16.99
256LEU	N	254LEU	O	12.67
255GLY	N	253SER	O	0.26
254LEU	N	252GLY	O	0.00
253SER	N	248SER	O	2.49
253SER	N	251PRO	O	0.12
252GLY	N	248SER	O	53.45
252GLY	N	249VAL	O	19.41
250LEU	N	246LEU	O	0.02
250LEU	N	247ALA	O	36.97
249VAL	N	245ASP	O	0.14
249VAL	N	246LEU	O	76.66
249VAL	N	247ALA	O	0.04
248SER	N	244SER	O	2.44
248SER	N	245ASP	O	60.75
248SER	N	246LEU	O	0.36
247ALA	N	243LEU	O	57.93
247ALA	N	244SER	O	1.84
246LEU	N	242ILE	O	82.52
246LEU	N	243LEU	O	2.78
245ASP	N	241ASP	O	24.22
245ASP	N	242ILE	O	31.32
245ASP	N	243LEU	O	0.00
244SER	N	240GLY	O	64.39
244SER	N	241ASP	O	8.92

237ASN	N	136GLY	O	0.03	245ASP	N	241ASP	O	8.79	222HIS	N	218ALA	O	86.09	244SER	N	242ILE	O	0.01
235THR	N	131VAL	O	61.08	245ASP	N	242ILE	O	4.32	222HIS	N	219MET	O	1.50	243LEU	N	239PHE	O	42.72
234VAL	N	181VAL	O	92.05	244SER	N	240GLY	O	39.76	222HIS	N	220ALA	O	0.00	243LEU	N	240GLY	O	1.56
233VAL	N	129LEU	O	96.43	244SER	N	241ASP	O	25.20	221MET	N	217ASP	O	14.16	242ILE	N	238ILE	O	13.33
232VAL	N	179HIS	O	4.35	244SER	N	242ILE	O	0.02	221MET	N	218ALA	O	31.11	242ILE	N	239PHE	O	7.79
231ASP	N	229ARG	O	0.07	243LEU	N	239PHE	O	29.88	221MET	N	219MET	O	0.02	242ILE	N	240GLY	O	0.01
230PHE	N	227PRO	O	63.28	243LEU	N	240GLY	O	12.86	220ALA	N	216VAL	O	76.15	241ASP	N	237ASN	O	61.94
230PHE	N	228ALA	O	2.62	243LEU	N	241ASP	O	0.02	220ALA	N	217ASP	O	2.88	241ASP	N	238ILE	O	4.24
229ARG	N	226SER	O	18.80	242ILE	N	238ILE	O	52.37	219MET	N	215TYR	O	93.74	240GLY	N	236GLY	O	36.20
229ARG	N	227PRO	O	1.29	242ILE	N	239PHE	O	3.35	219MET	N	216VAL	O	0.69	240GLY	N	237ASN	O	28.24
228ALA	N	226SER	O	0.07	242ILE	N	240GLY	O	0.01	218ALA	N	215TYR	O	31.05	240GLY	N	238ILE	O	0.02
226SER	N	222HIS	O	94.72	241ASP	N	236GLY	O	0.14	218ALA	N	216VAL	O	0.35	239PHE	N	236GLY	O	26.40
226SER	N	223LEU	O	0.55	241ASP	N	237ASN	O	32.98	216VAL	N	184ASP	O	79.96	238ILE	N	236GLY	O	0.00
225ARG	N	221MET	O	34.97	241ASP	N	238ILE	O	22.56	214GLN	N	182SER	O	83.73	237ASN	N	136GLY	O	0.04
225ARG	N	222HIS	O	37.37	241ASP	N	239PHE	O	0.01	212GLU	N	180VAL	O	72.08	235THR	N	131VAL	O	56.09
225ARG	N	223LEU	O	0.92	240GLY	N	236GLY	O	55.27	210ALA	N	178LYS	O	94.72	234VAL	N	181VAL	O	93.70
224VAL	N	220ALA	O	7.60	240GLY	N	237ASN	O	12.19	209VAL	N	206TYR	O	64.97	233VAL	N	129LEU	O	92.90
224VAL	N	221MET	O	25.91	240GLY	N	238ILE	O	0.02	209VAL	N	207PRO	O	0.12	232VAL	N	179HIS	O	12.18
223LEU	N	219MET	O	83.26	239PHE	N	236GLY	O	1.57	208ASP	N	206TYR	O	0.34	231ASP	N	179HIS	O	0.00
223LEU	N	220ALA	O	1.96	238ILE	N	236GLY	O	0.16	206TYR	N	202VAL	O	28.67	231ASP	N	229ARG	O	0.08
222HIS	N	218ALA	O	70.09	237ASN	N	133GLU	O	0.04	206TYR	N	203GLY	O	33.97	230PHE	N	227PRO	O	52.78
222HIS	N	219MET	O	9.92	237ASN	N	136GLY	O	0.63	205GLY	N	201GLU	O	1.02	230PHE	N	228ALA	O	1.81
222HIS	N	220ALA	O	0.00	236GLY	N	240GLY	N	0.00	205GLY	N	202VAL	O	71.08	229ARG	N	226SER	O	27.92
221MET	N	217ASP	O	34.49	235THR	N	131VAL	O	84.04	205GLY	N	203GLY	O	0.11	229ARG	N	227PRO	O	5.02
221MET	N	218ALA	O	11.44	234VAL	N	181VAL	O	97.69	204ARG	N	200GLU	O	44.39	228ALA	N	122ILE	O	0.02
221MET	N	219MET	O	0.00	233VAL	N	129LEU	O	95.80	204ARG	N	201GLU	O	22.38	228ALA	N	226SER	O	0.06
220ALA	N	216VAL	O	89.18	233VAL	N	231ASP	O	0.00	204ARG	N	202VAL	O	0.00	226SER	N	222HIS	O	88.10
220ALA	N	217ASP	O	1.00	232VAL	N	179HIS	O	29.11	203GLY	N	199VAL	O	58.85	226SER	N	223LEU	O	1.41
219MET	N	215TYR	O	90.80	231ASP	N	229ARG	O	0.10	203GLY	N	200GLU	O	6.58	225ARG	N	221MET	O	33.03
219MET	N	216VAL	O	1.19	230PHE	N	227PRO	O	57.20	202VAL	N	198THR	O	41.68	225ARG	N	222HIS	O	40.42
218ALA	N	215TYR	O	24.30	230PHE	N	228ALA	O	2.09	202VAL	N	199VAL	O	13.36	225ARG	N	223LEU	O	1.93
218ALA	N	216VAL	O	0.01	229ARG	N	226SER	O	16.97	202VAL	N	200GLU	O	0.00	224VAL	N	220ALA	O	2.96
216VAL	N	184ASP	O	96.88	229ARG	N	227PRO	O	3.77	201GLU	N	197LYS	O	27.30	224VAL	N	221MET	O	33.84
214GLN	N	182SER	O	92.25	228ALA	N	226SER	O	0.08	201GLU	N	198THR	O	23.26	224VAL	N	222HIS	O	0.02
214GLN	N	212GLU	O	0.03	226SER	N	222HIS	O	93.01	200GLU	N	196ARG	O	28.37	223LEU	N	219MET	O	89.76
212GLU	N	180VAL	O	71.91	226SER	N	223LEU	O	1.36	200GLU	N	197LYS	O	7.19	223LEU	N	220ALA	O	0.56
210ALA	N	178LYS	O	96.86	225ARG	N	221MET	O	8.49	200GLU	N	198THR	O	0.00	222HIS	N	218ALA	O	73.87
209VAL	N	206TYR	O	52.50	225ARG	N	222HIS	O	54.35	199VAL	N	195TRP	O	95.71	222HIS	N	219MET	O	4.49
209VAL	N	207PRO	O	0.42	225ARG	N	223LEU	O	3.25	199VAL	N	196ARG	O	0.41	222HIS	N	220ALA	O	0.00
208ASP	N	206TYR	O	0.74	224VAL	N	220ALA	O	13.01	198THR	N	194PHE	O	55.05	221MET	N	217ASP	O	13.05
206TYR	N	202VAL	O	8.11	224VAL	N	221MET	O	11.60	198THR	N	195TRP	O	24.99	221MET	N	218ALA	O	32.24
206TYR	N	203GLY	O	62.57	223LEU	N	219MET	O	77.73	198THR	N	196ARG	O	0.02	221MET	N	219MET	O	0.09
206TYR	N	204ARG	O	0.14	223LEU	N	220ALA	O	3.70	197LYS	N	193GLU	O	46.74	220ALA	N	216VAL	O	64.32

205GLY	N	201GLU	O	0.27	222HIS	N	218ALA	O	46.84	197LYS	N	194PHE	O	12.61	220ALA	N	217ASP	O	4.13
205GLY	N	202VAL	O	51.16	222HIS	N	219MET	O	18.43	197LYS	N	195TRP	O	0.02	219MET	N	215TYR	O	75.36
205GLY	N	203GLY	O	0.12	222HIS	N	220ALA	O	0.02	196ARG	N	192GLY	O	95.74	219MET	N	216VAL	O	12.87
204ARG	N	200GLU	O	35.85	221MET	N	217ASP	O	64.43	196ARG	N	193GLU	O	0.26	219MET	N	217ASP	O	0.16
204ARG	N	201GLU	O	19.69	221MET	N	218ALA	O	2.41	195TRP	N	191VAL	O	61.58	218ALA	N	215TYR	O	29.46
203GLY	N	199VAL	O	40.31	220ALA	N	216VAL	O	89.49	195TRP	N	192GLY	O	2.04	218ALA	N	216VAL	O	0.07
203GLY	N	200GLU	O	18.74	220ALA	N	217ASP	O	0.77	194PHE	N	190GLU	O	92.66	217ASP	N	215TYR	O	0.12
203GLY	N	201GLU	O	0.01	219MET	N	215TYR	O	80.16	194PHE	N	191VAL	O	0.06	216VAL	N	184ASP	O	91.63
202VAL	N	198THR	O	64.70	219MET	N	216VAL	O	3.64	193GLU	N	189LEU	O	69.99	214GLN	N	182SER	O	81.04
202VAL	N	199VAL	O	6.57	218ALA	N	215TYR	O	19.71	193GLU	N	190GLU	O	5.27	214GLN	N	212GLU	O	0.19
201GLU	N	197LYS	O	51.49	216VAL	N	184ASP	O	97.36	192GLY	N	189LEU	O	57.11	213HIS	N	211LEU	O	0.08
201GLU	N	198THR	O	13.19	214GLN	N	182SER	O	69.14	192GLY	N	190GLU	O	0.03	212GLU	N	180VAL	O	77.28
200GLU	N	196ARG	O	28.85	212GLU	N	180VAL	O	54.86	189LEU	N	185LYS	O	74.73	212GLU	N	210ALA	O	0.00
200GLU	N	197LYS	O	8.09	211LEU	N	209VAL	O	0.01	189LEU	N	186ALA	O	5.43	211LEU	N	209VAL	O	0.02
200GLU	N	198THR	O	0.00	210ALA	N	178LYS	O	94.26	188VAL	N	185LYS	O	79.59	210ALA	N	178LYS	O	96.52
199VAL	N	195TRP	O	97.45	209VAL	N	206TYR	O	4.64	188VAL	N	186ALA	O	0.06	209VAL	N	206TYR	O	54.15
199VAL	N	196ARG	O	0.24	209VAL	N	207PRO	O	44.54	187ASN	N	185LYS	O	0.10	209VAL	N	207PRO	O	0.41
198THR	N	194PHE	O	64.35	208ASP	N	206TYR	O	0.03	184ASP	N	182SER	O	0.06	208ASP	N	206TYR	O	0.12
198THR	N	195TRP	O	15.51	206TYR	N	202VAL	O	27.35	184ASP	N	214GLN	O	82.16	206TYR	N	202VAL	O	19.43
197LYS	N	193GLU	O	44.68	206TYR	N	203GLY	O	26.27	183VAL	N	234VAL	O	68.55	206TYR	N	203GLY	O	41.21
197LYS	N	194PHE	O	14.90	206TYR	N	204ARG	O	0.07	182SER	N	212GLU	O	96.14	206TYR	N	204ARG	O	0.04
197LYS	N	195TRP	O	0.02	205GLY	N	201GLU	O	0.68	181VAL	N	232VAL	O	92.84	205GLY	N	201GLU	O	0.78
196ARG	N	192GLY	O	91.57	205GLY	N	202VAL	O	43.18	180VAL	N	178LYS	O	0.02	205GLY	N	202VAL	O	62.40
196ARG	N	193GLU	O	1.19	205GLY	N	203GLY	O	0.18	180VAL	N	210ALA	O	95.48	205GLY	N	203GLY	O	0.19
195TRP	N	191VAL	O	63.56	204ARG	N	199VAL	O	0.04	179HIS	N	177ARG	O	83.64	204ARG	N	200GLU	O	40.84
195TRP	N	192GLY	O	1.74	204ARG	N	200GLU	O	36.06	178LYS	N	173ALA	O	0.88	204ARG	N	201GLU	O	20.09
194PHE	N	190GLU	O	92.08	204ARG	N	201GLU	O	24.15	178LYS	N	176ARG	O	0.12	204ARG	N	202VAL	O	0.02
194PHE	N	191VAL	O	0.09	204ARG	N	202VAL	O	0.12	177ARG	N	173ALA	O	0.08	203GLY	N	199VAL	O	46.42
193GLU	N	189LEU	O	72.85	203GLY	N	199VAL	O	54.16	176ARG	N	172LEU	O	11.26	203GLY	N	200GLU	O	12.66
193GLU	N	190GLU	O	4.98	203GLY	N	200GLU	O	13.90	176ARG	N	173ALA	O	58.39	202VAL	N	198THR	O	70.89
192GLY	N	189LEU	O	47.18	202VAL	N	198THR	O	40.37	176ARG	N	174ARG	O	0.18	202VAL	N	199VAL	O	4.56
192GLY	N	190GLU	O	0.04	202VAL	N	199VAL	O	20.99	175LYS	N	171GLU	O	1.23	201GLU	N	197LYS	O	53.82
189LEU	N	185LYS	O	55.87	202VAL	N	200GLU	O	0.12	175LYS	N	172LEU	O	66.03	201GLU	N	198THR	O	14.04
189LEU	N	186ALA	O	10.71	201GLU	N	197LYS	O	30.88	175LYS	N	173ALA	O	0.36	200GLU	N	196ARG	O	26.05
188VAL	N	185LYS	O	76.05	201GLU	N	198THR	O	25.19	175LYS	N	176ARG	O	0.11	200GLU	N	197LYS	O	11.74
188VAL	N	186ALA	O	0.04	201GLU	N	199VAL	O	0.02	174ARG	N	170PHE	O	7.18	200GLU	N	198THR	O	0.02
187ASN	N	185LYS	O	0.26	200GLU	N	196ARG	O	66.67	174ARG	N	171GLU	O	57.85	199VAL	N	195TRP	O	93.78
184ASP	N	182SER	O	0.17	200GLU	N	197LYS	O	2.08	174ARG	N	172LEU	O	0.15	199VAL	N	196ARG	O	0.22
184ASP	N	214GLN	O	97.11	199VAL	N	195TRP	O	95.73	173ALA	N	169ALA	O	56.77	199VAL	N	197LYS	O	0.00
184ASP	N	216VAL	N	0.00	199VAL	N	196ARG	O	1.09	173ALA	N	170PHE	O	4.58	198THR	N	194PHE	O	53.79
183VAL	N	234VAL	O	44.77	198THR	N	194PHE	O	54.38	172LEU	N	168VAL	O	91.07	198THR	N	195TRP	O	19.22
182SER	N	212GLU	O	98.91	198THR	N	195TRP	O	24.69	172LEU	N	169ALA	O	1.74	198THR	N	196ARG	O	0.03
181VAL	N	232VAL	O	92.92	198THR	N	196ARG	O	0.02	171GLU	N	167ARG	O	30.54	197LYS	N	193GLU	O	50.49

180VAL	N	210ALA	O	95.21
178LYS	N	173ALA	O	12.20
178LYS	N	176ARG	O	0.08
176ARG	N	172ALA	O	0.65
176ARG	N	173ALA	O	83.72
176ARG	N	174ARG	O	0.32
175LYS	N	171GLU	O	1.14
175LYS	N	172ALA	O	63.35
175LYS	N	173ALA	O	0.05
174ARG	N	170PHE	O	37.38
174ARG	N	171GLU	O	21.28
173ALA	N	169ALA	O	76.91
173ALA	N	170PHE	O	2.96
172ALA	N	168VAL	O	65.11
172ALA	N	169ALA	O	4.84
171GLU	N	167ARG	O	79.64
171GLU	N	168VAL	O	1.03
170PHE	N	166ALA	O	49.14
170PHE	N	167ARG	O	14.07
169ALA	N	165VAL	O	5.73
169ALA	N	166ALA	O	36.89
168VAL	N	164ARG	O	26.87
168VAL	N	165VAL	O	0.31
167ARG	N	163GLU	O	82.00
167ARG	N	164ARG	O	4.66
166ALA	N	162VAL	O	45.41
166ALA	N	163GLU	O	4.03
165VAL	N	161GLU	O	4.63
165VAL	N	162VAL	O	7.82
164ARG	N	160PRO	O	23.63
164ARG	N	161GLU	O	37.88
163GLU	N	159LYS	O	47.07
163GLU	N	160PRO	O	1.10
162VAL	N	158SER	O	78.10
162VAL	N	159LYS	O	7.82
161GLU	N	158SER	O	58.66
161GLU	N	159LYS	O	0.08
159LYS	N	149ALA	O	94.14
157TYR	N	151ALA	O	95.64
155GLU	N	153ASN	O	99.41
154THR	N	141GLY	O	0.48
154THR	N	143PRO	O	10.09
153ASN	N	155GLU	O	99.00

197LYS	N	193GLU	O	73.29
197LYS	N	194PHE	O	4.22
196ARG	N	192GLY	O	97.83
196ARG	N	193GLU	O	0.04
195TRP	N	191VAL	O	50.92
195TRP	N	192GLY	O	3.10
194PHE	N	190GLU	O	92.17
194PHE	N	191VAL	O	0.10
194PHE	N	192GLY	O	0.01
193GLU	N	189LEU	O	84.40
193GLU	N	190GLU	O	3.03
192GLY	N	189LEU	O	52.15
192GLY	N	190GLU	O	0.14
189LEU	N	185LYS	O	81.41
189LEU	N	186ALA	O	2.04
188VAL	N	185LYS	O	75.55
188VAL	N	186ALA	O	0.02
187ASN	N	185LYS	O	0.96
184ASP	N	182SER	O	0.00
184ASP	N	214GLN	O	95.64
183VAL	N	234VAL	O	83.39
182SER	N	212GLU	O	91.03
181VAL	N	232VAL	O	90.34
180VAL	N	178LYS	O	0.08
180VAL	N	210ALA	O	90.75
179HIS	N	177ARG	O	0.05
178LYS	N	173ALA	O	15.28
178LYS	N	176ARG	O	0.16
177ARG	N	173ALA	O	0.04
176ARG	N	172ALA	O	1.38
176ARG	N	173ALA	O	74.15
176ARG	N	174ARG	O	0.49
175LYS	N	171GLU	O	1.84
175LYS	N	172ALA	O	53.82
175LYS	N	173ALA	O	0.44
174ARG	N	170PHE	O	35.41
174ARG	N	171GLU	O	19.47
173ALA	N	169ALA	O	76.86
173ALA	N	170PHE	O	3.37
172ALA	N	168VAL	O	56.29
172ALA	N	169ALA	O	8.78
172ALA	N	170PHE	O	0.00
171GLU	N	167ARG	O	68.43

171GLU	N	168VAL	O	20.18
170PHE	N	166ALA	O	93.44
170PHE	N	167ARG	O	0.61
169ALA	N	165VAL	O	79.87
169ALA	N	166ALA	O	2.53
168VAL	N	164ARG	O	19.61
168VAL	N	165VAL	O	25.08
168VAL	N	166ALA	O	0.18
167ARG	N	163GLU	O	93.24
167ARG	N	164ARG	O	0.84
166ALA	N	162VAL	O	59.81
166ALA	N	163GLU	O	3.67
165VAL	N	161GLU	O	2.56
165VAL	N	162VAL	O	8.74
165VAL	N	163GLU	O	0.03
164ARG	N	160PRO	O	72.97
164ARG	N	161GLU	O	4.58
163GLU	N	159LYS	O	52.40
163GLU	N	160PRO	O	5.76
162VAL	N	158SER	O	36.24
162VAL	N	159LYS	O	14.96
162VAL	N	160PRO	O	0.01
161GLU	N	158SER	O	41.71
161GLU	N	159LYS	O	0.02
159LYS	N	149ALA	O	86.70
158SER	N	156ARG	O	0.01
157TYR	N	151ALA	O	97.22
156ARG	N	154THR	O	0.03
155GLU	N	153ASN	O	97.14
154THR	N	141GLY	O	0.28
154THR	N	143PRO	O	3.47
153ASN	N	155GLU	O	97.69
152TRP	N	145GLY	O	99.44
151ALA	N	157TYR	O	95.25
150GLU	N	147SER	O	3.92
149ALA	N	147SER	O	0.64
147SER	N	150GLU	O	43.97
145GLY	N	152TRP	O	90.14
141GLY	N	137GLY	O	0.12
141GLY	N	138ILE	O	44.34
141GLY	N	139TYR	O	0.00
140PHE	N	137GLY	O	99.50
140PHE	N	138ILE	O	0.01

197LYS	N	194PHE	O	14.32
196ARG	N	192GLY	O	86.41
196ARG	N	193GLU	O	2.80
195TRP	N	191VAL	O	51.19
195TRP	N	192GLY	O	3.66
194PHE	N	190GLU	O	92.51
194PHE	N	191VAL	O	0.12
193GLU	N	189LEU	O	78.92
193GLU	N	190GLU	O	4.08
192GLY	N	189LEU	O	56.14
192GLY	N	190GLU	O	0.05
189LEU	N	185LYS	O	24.03
189LEU	N	186ALA	O	33.50
189LEU	N	187ASN	O	0.00
188VAL	N	185LYS	O	37.89
188VAL	N	186ALA	O	0.09
187ASN	N	185LYS	O	0.93
185LYS	N	192GLY	O	0.00
184ASP	N	182SER	O	0.01
184ASP	N	214GLN	O	83.17
184ASP	N	216VAL	N	0.00
183VAL	N	234VAL	O	54.46
182SER	N	212GLU	O	94.19
181VAL	N	232VAL	O	92.51
180VAL	N	210ALA	O	88.85
179HIS	N	177ARG	O	0.00
178LYS	N	173ALA	O	34.23
178LYS	N	176ARG	O	0.08
177ARG	N	173ALA	O	0.11
176ARG	N	172LEU	O	6.58
176ARG	N	173ALA	O	70.49
176ARG	N	174ARG	O	0.40
175LYS	N	171GLU	O	11.78
175LYS	N	172LEU	O	48.38
175LYS	N	173ALA	O	0.06
174ARG	N	170PHE	O	39.77
174ARG	N	171GLU	O	23.78
173ALA	N	169ALA	O	64.19
173ALA	N	170PHE	O	4.19
172LEU	N	168VAL	O	88.60
172LEU	N	169ALA	O	1.71
171GLU	N	167ARG	O	58.60
171GLU	N	168VAL	O	8.58

152TRP	N	145GLY	O	99.48
151ALA	N	157TYR	O	84.44
150GLU	N	147SER	O	3.41
149ALA	N	147SER	O	2.16
147SER	N	150GLU	O	44.17
145GLY	N	152TRP	O	91.05
142GLU	N	140PHE	O	0.02
142GLU	N	154THR	O	0.01
141GLY	N	137GLY	O	0.05
141GLY	N	138ILE	O	40.42
141GLY	N	139TYR	O	0.15
140PHE	N	137GLY	O	95.74
140PHE	N	138ILE	O	0.02
139TYR	N	137GLY	O	0.02
138ILE	N	237ASN	O	2.27
137GLY	N	156ARG	O	0.08
136GLY	N	134LEU	O	0.12
135THR	N	99LEU	O	0.01
135THR	N	100PHE	O	0.01
134LEU	N	100PHE	O	18.89
133GLU	N	235THR	O	0.23
133GLU	N	236GLY	O	82.30
132ARG	N	102ASN	O	92.81
131VAL	N	233VAL	O	96.17
130ILE	N	104ARG	O	88.37
130ILE	N	128VAL	O	0.00
129LEU	N	230PHE	O	0.01
129LEU	N	231ASP	O	90.87
128VAL	N	106ALA	O	93.20
127ASP	N	125GLY	O	0.02
127ASP	N	227PRO	O	0.00
126VAL	N	123ALA	O	21.66
126VAL	N	124ARG	O	0.03
125GLY	N	122ILE	O	20.54
125GLY	N	123ALA	O	0.60
124ARG	N	120GLU	O	46.67
124ARG	N	121GLU	O	5.99
124ARG	N	122ILE	O	0.25
123ALA	N	119LYS	O	79.82
123ALA	N	120GLU	O	4.16
122ILE	N	119LYS	O	75.04
122ILE	N	120GLU	O	0.00
119LYS	N	117PRO	O	82.98

171GLU	N	168VAL	O	3.71
171GLU	N	169ALA	O	0.00
170PHE	N	166ALA	O	90.17
170PHE	N	167ARG	O	1.64
169ALA	N	165VAL	O	29.64
169ALA	N	166ALA	O	18.74
168VAL	N	164ARG	O	40.43
168VAL	N	165VAL	O	3.66
167ARG	N	163GLU	O	82.14
167ARG	N	164ARG	O	3.88
166ALA	N	162VAL	O	45.32
166ALA	N	163GLU	O	11.25
165VAL	N	161GLU	O	15.01
165VAL	N	162VAL	O	8.36
165VAL	N	163GLU	O	0.04
164ARG	N	160PRO	O	62.70
164ARG	N	161GLU	O	6.56
163GLU	N	159LYS	O	66.69
163GLU	N	160PRO	O	5.20
163GLU	N	161GLU	O	0.01
162VAL	N	158SER	O	32.47
162VAL	N	159LYS	O	19.96
161GLU	N	158SER	O	45.28
161GLU	N	159LYS	O	0.06
159LYS	N	149ALA	O	91.22
157TYR	N	151ALA	O	88.04
156ARG	N	137GLY	O	0.23
156ARG	N	140PHE	O	1.62
155GLU	N	153ASN	O	96.60
154THR	N	138ILE	O	0.06
154THR	N	141GLY	O	2.15
154THR	N	143PRO	O	2.40
153ASN	N	155GLU	O	97.29
152TRP	N	145GLY	O	98.78
151ALA	N	157TYR	O	93.28
150GLU	N	147SER	O	6.60
149ALA	N	147SER	O	1.80
148GLU	N	146MET	O	0.01
148GLU	N	150GLU	O	0.09
147SER	N	150GLU	O	52.66
145GLY	N	152TRP	O	88.36
142GLU	N	140PHE	O	0.06
141GLY	N	137GLY	O	0.65

139TYR	N	137GLY	O	0.06
137GLY	N	135THR	O	0.00
137GLY	N	140PHE	O	0.00
137GLY	N	156ARG	O	0.25
136GLY	N	133GLU	O	0.02
135THR	N	100PHE	O	0.03
134LEU	N	100PHE	O	73.87
133GLU	N	235THR	O	12.96
133GLU	N	236GLY	O	52.36
132ARG	N	102ASN	O	97.55
131VAL	N	233VAL	O	97.16
130ILE	N	104ARG	O	96.57
129LEU	N	231ASP	O	85.23
128VAL	N	106ALA	O	97.92
126VAL	N	123ALA	O	4.99
126VAL	N	124ARG	O	0.02
125GLY	N	123ALA	O	0.02
124ARG	N	120GLU	O	19.82
124ARG	N	121GLU	O	7.65
124ARG	N	122ILE	O	0.68
123ALA	N	119LYS	O	76.20
123ALA	N	120GLU	O	6.85
122ILE	N	119LYS	O	73.47
122ILE	N	120GLU	O	0.01
119LYS	N	117PRO	O	81.47
118LEU	N	116SER	O	0.00
116SER	N	112LEU	O	6.90
116SER	N	113GLU	O	45.20
116SER	N	114ARG	O	0.02
115LEU	N	112LEU	O	63.29
115LEU	N	113GLU	O	0.00
114ARG	N	111GLY	O	0.20
114ARG	N	112LEU	O	0.00
113GLU	N	111GLY	O	0.08
112LEU	N	109PHE	O	88.90
112LEU	N	110PRO	O	0.01
109PHE	N	107LYS	O	0.02
108VAL	N	126VAL	O	76.11
106ALA	N	128VAL	O	97.39
104ARG	N	130ILE	O	99.55
103LEU	N	260ALA	O	99.54
102ASN	N	100PHE	O	0.00
102ASN	N	132ARG	O	98.95

170PHE	N	166ALA	O	57.96
170PHE	N	167ARG	O	10.89
169ALA	N	165VAL	O	35.42
169ALA	N	166ALA	O	17.44
168VAL	N	164ARG	O	32.43
168VAL	N	165VAL	O	9.19
168VAL	N	166ALA	O	0.05
167ARG	N	163GLU	O	89.06
167ARG	N	164ARG	O	2.34
166ALA	N	162VAL	O	45.35
166ALA	N	163GLU	O	7.70
165VAL	N	161GLU	O	4.64
165VAL	N	162VAL	O	9.43
165VAL	N	163GLU	O	0.02
164ARG	N	160PRO	O	39.59
164ARG	N	161GLU	O	19.18
163GLU	N	159LYS	O	36.73
163GLU	N	160PRO	O	10.61
163GLU	N	161GLU	O	0.00
162VAL	N	158SER	O	63.31
162VAL	N	159LYS	O	10.49
162VAL	N	160PRO	O	0.01
161GLU	N	158SER	O	36.87
161GLU	N	159LYS	O	0.01
159LYS	N	149ALA	O	94.29
157TYR	N	151ALA	O	94.31
156ARG	N	154THR	O	0.00
155GLU	N	153ASN	O	97.56
154THR	N	141GLY	O	0.22
154THR	N	143PRO	O	10.20
153ASN	N	155GLU	O	97.82
152TRP	N	145GLY	O	98.52
151ALA	N	157TYR	O	83.22
150GLU	N	147SER	O	4.40
149ALA	N	147SER	O	2.09
147SER	N	150GLU	O	46.47
145GLY	N	152TRP	O	88.11
141GLY	N	137GLY	O	2.56
141GLY	N	138ILE	O	45.33
141GLY	N	139TYR	O	0.16
140PHE	N	137GLY	O	96.22
140PHE	N	138ILE	O	0.04
139TYR	N	137GLY	O	0.04

116SER	N	112LEU	O	4.21	141GLY	N	138ILE	O	11.69	101ALA	N	262LEU	O	54.23	137GLY	N	156ARG	O	0.08
116SER	N	113GLU	O	49.89	141GLY	N	139TYR	O	0.77	100PHE	N	98ASP	O	0.04	136GLY	N	133GLU	O	0.04
116SER	N	114ARG	O	0.16	141GLY	N	154THR	O	0.47	100PHE	N	262LEU	O	94.93	136GLY	N	134LEU	O	0.30
115LEU	N	112LEU	O	83.16	140PHE	N	136GLY	O	0.03	99LEU	N	94ARG	O	94.12	135THR	N	99LEU	O	0.00
114ARG	N	111GLY	O	0.06	140PHE	N	137GLY	O	92.61	98ASP	N	93LEU	O	0.00	135THR	N	100PHE	O	0.00
113GLU	N	109PHE	O	0.01	140PHE	N	138ILE	O	0.02	98ASP	N	94ARG	O	10.84	134LEU	N	99LEU	O	0.06
113GLU	N	111GLY	O	0.00	140PHE	N	154THR	O	0.25	98ASP	N	95LYS	O	23.60	134LEU	N	100PHE	O	9.99
112LEU	N	109PHE	O	56.48	139TYR	N	137GLY	O	18.04	98ASP	N	96SER	O	0.01	133GLU	N	235THR	O	3.32
112LEU	N	110PRO	O	0.02	139TYR	N	154THR	O	0.01	97GLN	N	93LEU	O	72.13	133GLU	N	236GLY	O	29.80
109PHE	N	107LYS	O	0.20	137GLY	N	135THR	O	0.30	97GLN	N	94ARG	O	1.04	132ARG	N	102ASN	O	84.39
108VAL	N	126VAL	O	86.50	137GLY	N	156ARG	O	8.97	96SER	N	92SER	O	93.50	131VAL	N	233VAL	O	91.74
107LYS	N	105PRO	O	0.00	136GLY	N	133GLU	O	0.00	96SER	N	93LEU	O	1.73	130ILE	N	104ARG	O	85.56
106ALA	N	128VAL	O	97.48	136GLY	N	134LEU	O	0.22	96SER	N	94ARG	O	0.08	129LEU	N	231ASP	O	90.68
104ARG	N	130ILE	O	96.74	135THR	N	100PHE	O	0.03	95LYS	N	91LEU	O	70.74	128VAL	N	106ALA	O	83.56
103LEU	N	260ALA	O	99.62	134LEU	N	100PHE	O	68.63	95LYS	N	92SER	O	5.31	126VAL	N	108VAL	O	0.67
102ASN	N	100PHE	O	0.00	133GLU	N	235THR	O	58.37	95LYS	N	93LEU	O	0.00	126VAL	N	123ALA	O	6.16
102ASN	N	132ARG	O	98.90	133GLU	N	236GLY	O	6.39	94ARG	N	90LEU	O	92.64	126VAL	N	124ARG	O	0.04
101ALA	N	262LEU	O	34.25	132ARG	N	102ASN	O	97.35	94ARG	N	91LEU	O	0.50	125GLY	N	108VAL	O	2.11
100PHE	N	98ASP	O	0.47	132ARG	N	130ILE	O	0.04	93LEU	N	89GLY	O	81.05	125GLY	N	123ALA	O	0.09
100PHE	N	262LEU	O	80.34	131VAL	N	233VAL	O	98.32	93LEU	N	90LEU	O	1.64	124ARG	N	120GLU	O	24.33
99LEU	N	93LEU	O	0.01	130ILE	N	104ARG	O	96.55	92SER	N	88THR	O	31.95	124ARG	N	121GLU	O	8.52
99LEU	N	94ARG	O	93.50	130ILE	N	128VAL	O	0.01	92SER	N	89GLY	O	5.85	124ARG	N	122ILE	O	2.99
98ASP	N	93LEU	O	0.07	129LEU	N	231ASP	O	92.25	92SER	N	90LEU	O	0.34	123ALA	N	119LYS	O	64.66
98ASP	N	94ARG	O	3.38	128VAL	N	106ALA	O	83.79	91LEU	N	87GLU	O	89.82	123ALA	N	120GLU	O	11.11
98ASP	N	95LYS	O	24.56	127ASP	N	125GLY	O	0.10	91LEU	N	88THR	O	0.64	122ILE	N	119LYS	O	74.79
97GLN	N	93LEU	O	62.22	126VAL	N	122ILE	O	0.02	90LEU	N	86PRO	O	47.50	122ILE	N	120GLU	O	0.04
97GLN	N	94ARG	O	0.82	126VAL	N	123ALA	O	13.68	90LEU	N	87GLU	O	21.09	121GLU	N	119LYS	O	0.01
96SER	N	92SER	O	86.52	126VAL	N	124ARG	O	0.16	89GLY	N	85ARG	O	0.00	119LYS	N	117PRO	O	87.93
96SER	N	93LEU	O	2.31	125GLY	N	121GLU	O	0.04	89GLY	N	86PRO	O	92.94	118LEU	N	116SER	O	0.00
96SER	N	94ARG	O	0.12	125GLY	N	122ILE	O	35.86	89GLY	N	87GLU	O	0.12	116SER	N	112LEU	O	19.50
95LYS	N	91LEU	O	80.38	125GLY	N	123ALA	O	0.47	88THR	N	82ARG	O	0.02	116SER	N	113GLU	O	37.55
95LYS	N	92SER	O	3.76	124ARG	N	120GLU	O	59.41	88THR	N	85ARG	O	88.26	116SER	N	114ARG	O	0.28
95LYS	N	93LEU	O	0.00	124ARG	N	121GLU	O	3.41	87GLU	N	85ARG	O	0.52	115LEU	N	111GLY	O	2.32
94ARG	N	90LEU	O	78.36	124ARG	N	122ILE	O	0.20	85ARG	N	81PRO	O	3.93	115LEU	N	112LEU	O	59.85
94ARG	N	91LEU	O	2.78	123ALA	N	119LYS	O	75.30	85ARG	N	82ARG	O	53.83	115LEU	N	113GLU	O	0.02
93LEU	N	89GLY	O	53.93	123ALA	N	120GLU	O	5.08	85ARG	N	83LYS	O	0.01	114ARG	N	111GLY	O	9.18
93LEU	N	90LEU	O	10.49	122ILE	N	119LYS	O	79.88	84ILE	N	81PRO	O	96.73	114ARG	N	112LEU	O	0.02
92SER	N	88THR	O	14.13	122ILE	N	120GLU	O	0.04	84ILE	N	82ARG	O	0.00	113GLU	N	109PHE	O	1.40
92SER	N	89GLY	O	9.46	121GLU	N	119LYS	O	0.02	83LYS	N	81PRO	O	0.30	113GLU	N	111GLY	O	0.04
92SER	N	90LEU	O	0.00	119LYS	N	117PRO	O	84.36	80LEU	N	76LYS	O	1.66	112LEU	N	109PHE	O	82.72
91LEU	N	87GLU	O	73.05	116SER	N	112LEU	O	1.84	80LEU	N	77TRP	O	47.63	112LEU	N	110PRO	O	0.15
91LEU	N	88THR	O	0.04	116SER	N	113GLU	O	44.10	80LEU	N	78ASP	O	1.06	109PHE	N	107LYS	O	0.34
91LEU	N	89GLY	O	0.21	116SER	N	114ARG	O	0.66	79GLY	N	75PRO	O	0.00	108VAL	N	126VAL	O	69.95

90LEU	N	86PRO	O	59.91
90LEU	N	87GLU	O	21.22
90LEU	N	88THR	O	0.00
89GLY	N	85SER	O	7.99
89GLY	N	86PRO	O	64.05
89GLY	N	87GLU	O	0.35
88THR	N	85SER	O	30.15
88THR	N	86PRO	O	8.32
87GLU	N	85SER	O	1.89
85SER	N	81PRO	O	1.40
85SER	N	82ARG	O	70.35
85SER	N	83LYS	O	0.06
84ILE	N	81PRO	O	94.92
84ILE	N	82ARG	O	0.00
83LYS	N	81PRO	O	4.12
80LEU	N	76LYS	O	0.20
80LEU	N	77TRP	O	36.61
80LEU	N	78ASP	O	0.14
79GLY	N	75PRO	O	0.37
79GLY	N	76LYS	O	3.01
79GLY	N	77TRP	O	0.06
78ASP	N	74GLY	O	22.84
78ASP	N	75PRO	O	31.54
78ASP	N	76LYS	O	0.24
77TRP	N	74GLY	O	74.58
77TRP	N	75PRO	O	0.04
76LYS	N	74GLY	O	0.05
73GLY	N	9ASP	O	1.85
71SER	N	7PRO	O	1.11
70GLY	N	6LEU	O	34.20
70GLY	N	7PRO	O	0.00
69LEU	N	67VAL	O	0.01
69LEU	N	269PHE	O	97.26
68LEU	N	4ALA	O	99.21
67VAL	N	267PRO	O	88.78
66ALA	N	2LYS	O	3.33
65GLU	N	2LYS	O	99.22
64ALA	N	60GLY	O	11.93
64ALA	N	61VAL	O	8.58
64ALA	N	62GLU	O	8.37
63GLU	N	59LYS	O	4.66
63GLU	N	60GLY	O	42.98
63GLU	N	61VAL	O	0.23

115LEU	N	111GLY	O	0.66
115LEU	N	112LEU	O	65.63
115LEU	N	113GLU	O	0.09
114ARG	N	111GLY	O	2.80
114ARG	N	112LEU	O	0.03
113GLU	N	109PHE	O	1.46
113GLU	N	111GLY	O	0.01
112LEU	N	109PHE	O	68.58
112LEU	N	110PRO	O	0.95
111GLY	N	109PHE	O	0.00
108VAL	N	126VAL	O	49.34
107LYS	N	105PRO	O	0.09
106ALA	N	104ARG	O	0.02
106ALA	N	128VAL	O	96.17
104ARG	N	130ILE	O	97.14
103LEU	N	260ALA	O	99.08
102ASN	N	132ARG	O	97.69
101ALA	N	262LEU	O	56.45
100PHE	N	98ASP	O	0.01
100PHE	N	262LEU	O	94.10
99LEU	N	94ARG	O	30.57
99LEU	N	97GLN	O	13.21
98ASP	N	93LEU	O	0.00
98ASP	N	94ARG	O	13.44
98ASP	N	95LYS	O	5.09
98ASP	N	96SER	O	0.59
98ASP	N	264ARG	O	0.29
97GLN	N	93LEU	O	86.34
97GLN	N	94ARG	O	1.08
97GLN	N	95LYS	O	0.01
96SER	N	92SER	O	60.80
96SER	N	93LEU	O	18.94
96SER	N	94ARG	O	0.05
96SER	N	97GLN	O	0.20
95LYS	N	91LEU	O	36.67
95LYS	N	92SER	O	26.21
95LYS	N	93LEU	O	5.14
95LYS	N	97GLN	O	0.01
94ARG	N	90LEU	O	94.10
94ARG	N	91LEU	O	0.38
93LEU	N	89GLY	O	75.98
93LEU	N	90LEU	O	3.32
92SER	N	88THR	O	28.46

79GLY	N	76LYS	O	18.24
79GLY	N	77TRP	O	0.01
78ASP	N	74GLY	O	27.07
78ASP	N	75PRO	O	16.19
78ASP	N	76LYS	O	0.05
77TRP	N	74GLY	O	59.12
77TRP	N	75PRO	O	17.96
76LYS	N	74GLY	O	0.24
73GLY	N	9ASP	O	1.23
71SER	N	7PRO	O	2.90
70GLY	N	6LEU	O	10.15
69LEU	N	269PHE	O	96.87
68LEU	N	4ALA	O	99.39
67VAL	N	267PRO	O	88.22
66ALA	N	2LYS	O	9.32
65GLU	N	2LYS	O	98.36
64ALA	N	60GLY	O	25.13
64ALA	N	61VAL	O	11.90
64ALA	N	62GLU	O	4.22
63GLU	N	59LYS	O	12.61
63GLU	N	60GLY	O	30.73
63GLU	N	61VAL	O	0.46
62GLU	N	58ARG	O	7.64
62GLU	N	59LYS	O	2.08
62GLU	N	60GLY	O	0.00
61VAL	N	57THR	O	91.97
61VAL	N	58ARG	O	0.21
60GLY	N	56PRO	O	5.57
60GLY	N	57THR	O	57.85
60GLY	N	58ARG	O	0.36
59LYS	N	55GLU	O	66.78
59LYS	N	56PRO	O	6.05
58ARG	N	54PRO	O	93.84
58ARG	N	55GLU	O	1.58
57THR	N	54PRO	O	8.14
53PHE	N	51GLU	O	0.00
51GLU	N	45ALA	O	4.83
51GLU	N	49PHE	O	1.40
50GLY	N	45ALA	O	76.92
50GLY	N	46ILE	O	3.42
50GLY	N	47ASP	O	0.02
49PHE	N	45ALA	O	81.07
49PHE	N	46ILE	O	0.10

107LYS	N	105PRO	O	0.38
106ALA	N	104ARG	O	0.01
106ALA	N	128VAL	O	95.72
104ARG	N	102ASN	O	0.01
104ARG	N	130ILE	O	88.65
103LEU	N	101ALA	O	0.00
103LEU	N	260ALA	O	98.23
102ASN	N	100PHE	O	0.01
102ASN	N	132ARG	O	98.23
102ASN	N	133GLU	O	0.00
101ALA	N	99LEU	O	0.01
101ALA	N	262LEU	O	39.59
100PHE	N	98ASP	O	0.33
100PHE	N	262LEU	O	70.88
99LEU	N	94ARG	O	87.68
99LEU	N	97GLN	O	0.00
98ASP	N	94ARG	O	20.14
98ASP	N	95LYS	O	14.56
98ASP	N	96SER	O	0.10
97GLN	N	93LEU	O	76.35
97GLN	N	94ARG	O	2.14
96SER	N	92SER	O	71.99
96SER	N	93LEU	O	8.06
96SER	N	94ARG	O	0.03
95LYS	N	91LEU	O	70.57
95LYS	N	92SER	O	5.38
95LYS	N	93LEU	O	0.01
94ARG	N	90LEU	O	91.97
94ARG	N	91LEU	O	0.76
93LEU	N	89GLY	O	71.31
93LEU	N	90LEU	O	3.84
92SER	N	88THR	O	47.10
92SER	N	89GLY	O	3.03
92SER	N	90LEU	O	0.03
91LEU	N	87GLU	O	88.78
91LEU	N	88THR	O	0.18
90LEU	N	86PRO	O	38.17
90LEU	N	87GLU	O	27.84
90LEU	N	88THR	O	0.00
89GLY	N	85ARG	O	0.02
89GLY	N	86PRO	O	90.37
89GLY	N	87GLU	O	0.02
88THR	N	82ARG	O	0.10

62GLU	N	58ARG	O	1.37
62GLU	N	59LYS	O	2.03
62GLU	N	60GLY	O	0.01
61VAL	N	57THR	O	85.98
61VAL	N	58ARG	O	0.06
60GLY	N	56PRO	O	2.38
60GLY	N	57THR	O	70.86
60GLY	N	58ARG	O	0.13
59LYS	N	55GLU	O	54.47
59LYS	N	56PRO	O	11.26
58ARG	N	54PRO	O	94.22
58ARG	N	55GLU	O	1.05
57THR	N	54PRO	O	9.10
53PHE	N	51GLU	O	0.02
51GLU	N	45ALA	O	10.64
51GLU	N	49PHE	O	0.84
50GLY	N	45ALA	O	80.87
50GLY	N	46ILE	O	1.48
50GLY	N	47ASP	O	0.02
49PHE	N	44ALA	O	0.58
49PHE	N	45ALA	O	73.54
49PHE	N	46ILE	O	0.04
49PHE	N	47ASP	O	0.06
48ALA	N	43GLY	O	0.17
48ALA	N	44ALA	O	91.42
48ALA	N	45ALA	O	1.00
48ALA	N	46ILE	O	0.07
47ASP	N	43GLY	O	20.35
47ASP	N	44ALA	O	47.77
47ASP	N	45ALA	O	0.46
46ILE	N	42GLY	O	6.22
46ILE	N	43GLY	O	10.04
45ALA	N	41PHE	O	0.00
45ALA	N	42GLY	O	6.45
45ALA	N	43GLY	O	0.01
44ALA	N	41PHE	O	0.01
44ALA	N	42GLY	O	0.12
43GLY	N	8GLY	O	0.25
43GLY	N	41PHE	O	0.04
42GLY	N	52PRO	O	13.69
41PHE	N	39PHE	O	0.00
39PHE	N	5VAL	O	95.92
39PHE	N	37GLU	O	0.08

92SER	N	89GLY	O	8.45
92SER	N	90LEU	O	0.27
91LEU	N	87GLU	O	53.94
91LEU	N	88THR	O	3.85
90LEU	N	86PRO	O	20.68
90LEU	N	87GLU	O	41.49
89GLY	N	85SER	O	0.19
89GLY	N	86PRO	O	79.62
89GLY	N	87GLU	O	0.20
88THR	N	82ARG	O	0.06
88THR	N	85SER	O	78.74
88THR	N	86PRO	O	0.02
87GLU	N	72VAL	O	0.22
87GLU	N	85SER	O	0.31
85SER	N	81PRO	O	3.42
85SER	N	82ARG	O	70.59
85SER	N	83LYS	O	0.12
84ILE	N	81PRO	O	56.05
84ILE	N	82ARG	O	1.26
83LYS	N	81PRO	O	1.72
83LYS	N	85SER	O	0.79
82ARG	N	80LEU	O	0.01
80LEU	N	76LYS	O	7.51
80LEU	N	77TRP	O	29.75
80LEU	N	78ASP	O	1.38
79GLY	N	75PRO	O	0.06
79GLY	N	76LYS	O	6.18
79GLY	N	77TRP	O	0.06
79GLY	N	80LEU	O	0.06
78ASP	N	74GLY	O	3.21
78ASP	N	75PRO	O	22.18
78ASP	N	76LYS	O	4.50
78ASP	N	80LEU	O	0.02
77TRP	N	73GLY	O	0.00
77TRP	N	74GLY	O	41.77
77TRP	N	75PRO	O	0.38
76LYS	N	74GLY	O	4.28
73GLY	N	9ASP	O	9.67
73GLY	N	71SER	O	0.00
72VAL	N	273HIS	O	0.78
71SER	N	7PRO	O	6.49
70GLY	N	6LEU	O	13.72
70GLY	N	7PRO	O	0.06

49PHE	N	47ASP	O	0.04
48ALA	N	44ALA	O	77.52
48ALA	N	45ALA	O	3.39
48ALA	N	46ILE	O	0.65
47ASP	N	43GLY	O	58.73
47ASP	N	44ALA	O	10.01
47ASP	N	45ALA	O	0.00
46ILE	N	42GLY	O	34.51
46ILE	N	43GLY	O	18.76
45ALA	N	41PHE	O	0.08
45ALA	N	42GLY	O	25.45
45ALA	N	43GLY	O	0.01
44ALA	N	41PHE	O	0.07
44ALA	N	42GLY	O	0.00
43GLY	N	8GLY	O	0.01
42GLY	N	52PRO	O	80.30
39PHE	N	5VAL	O	97.15
39PHE	N	37GLU	O	0.01
37GLU	N	3VAL	O	42.21
35ALA	N	1MET	O	64.19
35ALA	N	33GLY	O	0.02
33GLY	N	27ASP	O	0.00
32LEU	N	26LEU	O	0.06
32LEU	N	27ASP	O	0.96
31GLY	N	26LEU	O	91.94
31GLY	N	27ASP	O	0.74
30GLU	N	25ALA	O	0.00
30GLU	N	26LEU	O	67.50
30GLU	N	27ASP	O	0.14
30GLU	N	28GLU	O	0.06
29ALA	N	25ALA	O	84.52
29ALA	N	26LEU	O	3.16
29ALA	N	27ASP	O	0.18
28GLU	N	24ARG	O	45.94
28GLU	N	25ALA	O	17.15
28GLU	N	26LEU	O	0.02
27ASP	N	23LEU	O	83.91
27ASP	N	24ARG	O	1.39
26LEU	N	22VAL	O	83.46
26LEU	N	23LEU	O	2.94
25ALA	N	21LYS	O	49.71
25ALA	N	22VAL	O	10.45
24ARG	N	20LEU	O	98.07

88THR	N	85ARG	O	84.85
87GLU	N	85ARG	O	0.06
85ARG	N	81PRO	O	1.77
85ARG	N	82ARG	O	74.62
85ARG	N	83LYS	O	0.01
84ILE	N	81PRO	O	96.58
84ILE	N	82ARG	O	0.02
83LYS	N	81PRO	O	0.02
80LEU	N	77TRP	O	50.57
80LEU	N	78ASP	O	0.36
79GLY	N	75PRO	O	0.00
79GLY	N	76LYS	O	0.02
79GLY	N	77TRP	O	0.04
78ASP	N	74GLY	O	5.28
78ASP	N	75PRO	O	45.95
78ASP	N	76LYS	O	0.55
77TRP	N	74GLY	O	50.55
77TRP	N	75PRO	O	6.46
76LYS	N	74GLY	O	1.42
74GLY	N	72VAL	O	0.00
73GLY	N	9ASP	O	10.78
73GLY	N	42GLY	O	0.00
73GLY	N	71SER	O	0.07
72VAL	N	70GLY	O	0.01
71SER	N	7PRO	O	3.09
70GLY	N	6LEU	O	25.76
69LEU	N	67VAL	O	0.01
69LEU	N	269PHE	O	90.09
68LEU	N	4ALA	O	98.57
67VAL	N	267PRO	O	86.15
66ALA	N	2LYS	O	6.77
65GLU	N	2LYS	O	96.74
65GLU	N	66ALA	O	0.00
64ALA	N	60GLY	O	19.23
64ALA	N	61VAL	O	7.90
64ALA	N	62GLU	O	7.34
63GLU	N	59LYS	O	7.75
63GLU	N	60GLY	O	40.87
63GLU	N	61VAL	O	0.70
62GLU	N	58ARG	O	3.06
62GLU	N	59LYS	O	4.30
62GLU	N	60GLY	O	0.15
61VAL	N	56PRO	O	0.00

37GLU	N	3VAL	O	55.32	69LEU	N	269PHE	O	86.24	24ARG	N	21LYS	O	0.11	61VAL	N	57THR	O	75.40
36TYR	N	34LEU	O	0.02	68LEU	N	4ALA	O	98.48	23LEU	N	19ALA	O	70.36	61VAL	N	58ARG	O	0.19
35ALA	N	1MET	O	51.95	67VAL	N	267PRO	O	87.65	23LEU	N	20LEU	O	1.91	60GLY	N	56PRO	O	8.26
35ALA	N	33GLY	O	0.24	66ALA	N	2LYS	O	5.66	22VAL	N	18ALA	O	21.27	60GLY	N	57THR	O	58.71
32LEU	N	26LEU	O	0.01	66ALA	N	64ALA	O	0.00	22VAL	N	19ALA	O	21.37	60GLY	N	58ARG	O	0.91
32LEU	N	27ASP	O	0.03	65GLU	N	2LYS	O	92.70	21LYS	N	17GLU	O	88.87	59LYS	N	55GLU	O	45.42
31GLY	N	26LEU	O	93.92	64ALA	N	60GLY	O	14.40	21LYS	N	18ALA	O	0.82	59LYS	N	56PRO	O	18.19
31GLY	N	27ASP	O	0.02	64ALA	N	61VAL	O	14.72	20LEU	N	16THR	O	92.57	59LYS	N	57THR	O	0.02
30GLU	N	26LEU	O	63.89	64ALA	N	62GLU	O	5.00	20LEU	N	17GLU	O	0.79	58ARG	N	54PRO	O	75.69
30GLU	N	27ASP	O	0.09	63GLU	N	59LYS	O	4.38	19ALA	N	15VAL	O	44.13	58ARG	N	55GLU	O	9.18
30GLU	N	28GLU	O	0.04	63GLU	N	60GLY	O	44.87	19ALA	N	16THR	O	20.76	57THR	N	54PRO	O	15.49
29ALA	N	25ALA	O	85.50	63GLU	N	61VAL	O	0.60	18ALA	N	14GLU	O	67.86	53PHE	N	51GLU	O	0.00
29ALA	N	26LEU	O	2.80	62GLU	N	58ARG	O	0.84	18ALA	N	15VAL	O	2.30	51GLU	N	45ALA	O	20.88
29ALA	N	27ASP	O	0.11	62GLU	N	59LYS	O	3.13	17GLU	N	13PRO	O	77.03	51GLU	N	49PHE	O	1.08
28GLU	N	24ARG	O	50.84	62GLU	N	60GLY	O	0.06	17GLU	N	14GLU	O	2.07	50GLY	N	45ALA	O	63.70
28GLU	N	25ALA	O	14.13	61VAL	N	57THR	O	76.32	16THR	N	12GLY	O	81.47	50GLY	N	46ILE	O	6.39
28GLU	N	26LEU	O	0.02	61VAL	N	58ARG	O	0.06	16THR	N	13PRO	O	1.82	50GLY	N	47ASP	O	0.00
27ASP	N	23LEU	O	86.24	61VAL	N	59LYS	O	0.00	15VAL	N	11ILE	O	76.75	49PHE	N	45ALA	O	84.07
27ASP	N	24ARG	O	1.13	60GLY	N	56PRO	O	4.60	15VAL	N	12GLY	O	2.02	49PHE	N	46ILE	O	0.18
26LEU	N	22VAL	O	85.80	60GLY	N	57THR	O	64.47	14GLU	N	11ILE	O	39.62	49PHE	N	47ASP	O	0.00
26LEU	N	23LEU	O	2.91	60GLY	N	58ARG	O	0.42	12GLY	N	9ASP	O	1.06	48ALA	N	44ALA	O	86.68
25ALA	N	21LYS	O	53.41	59LYS	N	55GLU	O	48.91	12GLY	N	71SER	O	0.00	48ALA	N	45ALA	O	1.42
25ALA	N	22VAL	O	9.03	59LYS	N	56PRO	O	15.74	11ILE	N	9ASP	O	0.07	48ALA	N	46ILE	O	0.04
24ARG	N	20LEU	O	96.07	59LYS	N	57THR	O	0.01	11ILE	N	275SER	O	2.90	47ASP	N	43GLY	O	57.01
24ARG	N	21LYS	O	0.30	58ARG	N	54PRO	O	86.64	11ILE	N	276ALA	O	0.04	47ASP	N	44ALA	O	11.78
23LEU	N	19ALA	O	78.52	58ARG	N	55GLU	O	4.14	9ASP	N	71SER	O	93.56	47ASP	N	45ALA	O	0.00
23LEU	N	20LEU	O	1.45	57THR	N	54PRO	O	13.39	8GLY	N	41PHE	O	1.93	46ILE	N	42GLY	O	1.51
22VAL	N	18ALA	O	30.49	53PHE	N	51GLU	O	0.04	6LEU	N	68LEU	O	99.65	46ILE	N	43GLY	O	18.55
22VAL	N	19ALA	O	15.85	51GLU	N	45ALA	O	4.98	5VAL	N	37GLU	O	95.45	46ILE	N	44ALA	O	0.00
21LYS	N	17GLU	O	65.38	51GLU	N	46ILE	O	0.06	4ALA	N	66ALA	O	96.98	45ALA	N	42GLY	O	0.86
21LYS	N	18ALA	O	8.02	51GLU	N	49PHE	O	1.40	3VAL	N	1MET	O	0.01	45ALA	N	43GLY	O	0.01
20LEU	N	16THR	O	88.28	50GLY	N	45ALA	O	63.60	3VAL	N	35ALA	O	92.96	44ALA	N	42GLY	O	0.01
20LEU	N	17GLU	O	1.33	50GLY	N	46ILE	O	6.40	1MET	N	32LEU	O	0.00	42GLY	N	52PRO	O	2.62
19ALA	N	15VAL	O	65.19	50GLY	N	47ASP	O	0.02	1MET	N	33GLY	O	94.35	41PHE	N	39PHE	O	0.12
19ALA	N	16THR	O	8.02	49PHE	N	44ALA	O	0.05	345ALA	N	341LEU	O	0.90	39PHE	N	5VAL	O	94.87
18ALA	N	14GLU	O	59.81	49PHE	N	45ALA	O	79.46	345ALA	N	342ARG	O	10.66	39PHE	N	37GLU	O	0.12
18ALA	N	15VAL	O	3.37	49PHE	N	46ILE	O	0.28	345ALA	N	343HIS	O	8.02	38VAL	N	36TYR	O	0.01
17GLU	N	13PRO	O	83.60	49PHE	N	47ASP	O	0.03	344LEU	N	340VAL	O	45.87	37GLU	N	3VAL	O	55.96
17GLU	N	14GLU	O	1.62	48ALA	N	44ALA	O	81.20	344LEU	N	341LEU	O	12.49	37GLU	N	35ALA	O	0.09
16THR	N	12GLY	O	78.68	48ALA	N	45ALA	O	3.08	344LEU	N	342ARG	O	0.08	36TYR	N	34LEU	O	0.09
16THR	N	13PRO	O	1.73	48ALA	N	46ILE	O	0.42	343HIS	N	339THR	O	44.97	35ALA	N	1MET	O	66.95
15VAL	N	11ILE	O	84.13	47ASP	N	43GLY	O	36.07	343HIS	N	340VAL	O	32.45	35ALA	N	33GLY	O	0.50
15VAL	N	12GLY	O	2.44	47ASP	N	44ALA	O	17.19	342ARG	N	338ALA	O	26.37	34LEU	N	32LEU	O	0.48

14GLU	N	11ILE	O	27.51	47ASP	N	45ALA	O	0.03	342ARG	N	339THR	O	31.42	32LEU	N	26LEU	O	0.05
12GLY	N	9ASP	O	1.43	46ILE	N	42GLY	O	6.75	342ARG	N	340VAL	O	0.84	32LEU	N	27ASP	O	0.85
11ILE	N	9ASP	O	0.02	46ILE	N	43GLY	O	30.67	342ARG	N	344LEU	O	0.00	32LEU	N	30GLU	O	0.90
11ILE	N	275SER	O	33.79	45ALA	N	41PHE	O	0.01	341LEU	N	337THR	O	97.78	31GLY	N	26LEU	O	78.36
9ASP	N	71SER	O	93.50	45ALA	N	42GLY	O	5.22	341LEU	N	338ALA	O	0.08	31GLY	N	27ASP	O	1.02
8GLY	N	41PHE	O	51.79	45ALA	N	43GLY	O	0.04	340VAL	N	336PHE	O	89.83	31GLY	N	28GLU	O	0.02
6LEU	N	68LEU	O	99.35	44ALA	N	41PHE	O	0.00	340VAL	N	337THR	O	1.81	30GLU	N	25ALA	O	0.24
5VAL	N	37GLU	O	95.27	43GLY	N	8GLY	O	0.90	339THR	N	335ALA	O	45.75	30GLU	N	26LEU	O	60.63
4ALA	N	66ALA	O	97.72	43GLY	N	41PHE	O	0.16	339THR	N	336PHE	O	19.77	30GLU	N	27ASP	O	0.66
3VAL	N	35ALA	O	87.03	42GLY	N	52PRO	O	47.28	339THR	N	337THR	O	0.01	30GLU	N	28GLU	O	0.08
1MET	N	33GLY	O	90.94	39PHE	N	5VAL	O	92.92	338ALA	N	334GLU	O	78.21	29ALA	N	25ALA	O	78.12
345ALA	N	341LEU	O	1.78	39PHE	N	37GLU	O	0.13	338ALA	N	335ALA	O	1.73	29ALA	N	26LEU	O	5.65
345ALA	N	342ARG	O	11.86	37GLU	N	3VAL	O	56.22	337THR	N	333THR	O	65.03	29ALA	N	27ASP	O	0.29
345ALA	N	343HIS	O	6.76	37GLU	N	35ALA	O	0.04	337THR	N	334GLU	O	0.88	29ALA	N	30GLU	O	0.02
344LEU	N	340VAL	O	32.65	36TYR	N	34LEU	O	0.05	336PHE	N	332GLY	O	92.93	28GLU	N	24ARG	O	45.87
344LEU	N	341LEU	O	17.08	35ALA	N	1MET	O	62.51	336PHE	N	333THR	O	1.01	28GLU	N	25ALA	O	17.01
344LEU	N	342ARG	O	0.23	35ALA	N	33GLY	O	0.10	336PHE	N	334GLU	O	0.00	28GLU	N	26LEU	O	0.09
343HIS	N	339THR	O	50.47	34LEU	N	32LEU	O	0.05	335ALA	N	332GLY	O	2.72	28GLU	N	30GLU	O	0.04
343HIS	N	340VAL	O	26.99	32LEU	N	26LEU	O	0.06	334GLU	N	283GLY	O	0.12	27ASP	N	23LEU	O	77.79
342ARG	N	338ALA	O	35.08	32LEU	N	27ASP	O	0.08	334GLU	N	332GLY	O	0.01	27ASP	N	24ARG	O	3.04
342ARG	N	339THR	O	22.75	32LEU	N	30GLU	O	0.33	333THR	N	283GLY	O	66.07	27ASP	N	25ALA	O	0.22
342ARG	N	340VAL	O	0.20	31GLY	N	26LEU	O	87.56	333THR	N	285ALA	O	1.08	26LEU	N	22VAL	O	81.28
341LEU	N	337THR	O	97.99	31GLY	N	27ASP	O	0.12	332GLY	N	285ALA	O	0.00	26LEU	N	23LEU	O	3.23
341LEU	N	338ALA	O	0.05	30GLU	N	26LEU	O	60.09	331ALA	N	323PRO	O	18.66	26LEU	N	24ARG	O	0.00
340VAL	N	336PHE	O	88.15	30GLU	N	27ASP	O	0.50	331ALA	N	329GLY	O	0.06	25ALA	N	21LYS	O	43.49
340VAL	N	337THR	O	2.42	30GLU	N	28GLU	O	0.09	330SER	N	323PRO	O	7.96	25ALA	N	22VAL	O	11.61
339THR	N	335ALA	O	42.55	29ALA	N	25ALA	O	79.19	329GLY	N	324PRO	O	26.56	24ARG	N	20LEU	O	96.56
339THR	N	336PHE	O	21.32	29ALA	N	26LEU	O	5.06	329GLY	N	325PRO	O	0.19	24ARG	N	21LYS	O	0.35
338ALA	N	334GLU	O	85.92	29ALA	N	27ASP	O	0.21	329GLY	N	327LEU	O	0.00	23LEU	N	19ALA	O	56.17
338ALA	N	335ALA	O	0.95	28GLU	N	24ARG	O	46.59	328GLY	N	324PRO	O	64.43	23LEU	N	20LEU	O	4.42
337THR	N	333THR	O	83.47	28GLU	N	25ALA	O	17.52	328GLY	N	325PRO	O	22.92	22VAL	N	18ALA	O	8.36
337THR	N	334GLU	O	1.15	28GLU	N	26LEU	O	0.04	328GLY	N	326ASP	O	0.01	22VAL	N	19ALA	O	29.57
336PHE	N	332GLY	O	90.48	27ASP	N	23LEU	O	81.01	327LEU	N	324PRO	O	74.93	22VAL	N	20LEU	O	0.00
336PHE	N	333THR	O	0.60	27ASP	N	24ARG	O	2.10	327LEU	N	325PRO	O	0.02	21LYS	N	17GLU	O	84.10
335ALA	N	332GLY	O	1.20	26LEU	N	22VAL	O	78.81	326ASP	N	284ILE	O	0.44	21LYS	N	18ALA	O	1.47
333THR	N	283GLY	O	57.18	26LEU	N	23LEU	O	4.40	326ASP	N	324PRO	O	0.03	20LEU	N	16THR	O	87.67
333THR	N	285ALA	O	0.06	25ALA	N	21LYS	O	45.93	322THR	N	318ALA	O	96.40	20LEU	N	17GLU	O	1.75
331ALA	N	323PRO	O	36.15	25ALA	N	22VAL	O	11.74	322THR	N	319LEU	O	0.05	19ALA	N	15VAL	O	45.37
331ALA	N	329GLY	O	0.01	24ARG	N	20LEU	O	95.76	321GLU	N	317LYS	O	27.67	19ALA	N	16THR	O	21.67
330SER	N	323PRO	O	1.71	24ARG	N	21LYS	O	0.36	321GLU	N	318ALA	O	30.88	18ALA	N	14GLU	O	53.89
329GLY	N	324PRO	O	38.61	23LEU	N	19ALA	O	65.04	321GLU	N	319LEU	O	0.50	18ALA	N	15VAL	O	5.59
329GLY	N	325PRO	O	0.10	23LEU	N	20LEU	O	2.80	320LEU	N	316ALA	O	51.11	18ALA	N	16THR	O	0.01
328GLY	N	324PRO	O	51.19	22VAL	N	18ALA	O	15.06	320LEU	N	317LYS	O	12.86	17GLU	N	13PRO	O	76.18

328GLY	N	325PRO	O	30.38	22VAL	N	19ALA	O	22.73	319LEU	N	315VAL	O	82.17	17GLU	N	14GLU	O	2.90
328GLY	N	326ASP	O	0.02	22VAL	N	20LEU	O	0.00	319LEU	N	316ALA	O	3.94	16THR	N	12GLY	O	74.87
327LEU	N	324PRO	O	69.94	21LYS	N	17GLU	O	82.04	318ALA	N	314ALA	O	54.30	16THR	N	13PRO	O	3.22
327LEU	N	325PRO	O	0.08	21LYS	N	18ALA	O	2.57	318ALA	N	315VAL	O	9.31	15VAL	N	11ILE	O	52.30
326ASP	N	324PRO	O	0.19	20LEU	N	16THR	O	82.42	317LYS	N	313ASP	O	87.66	15VAL	N	12GLY	O	5.92
322THR	N	318ALA	O	98.46	20LEU	N	17GLU	O	2.96	317LYS	N	314ALA	O	1.81	14GLU	N	11ILE	O	44.35
322THR	N	319LEU	O	0.02	19ALA	N	15VAL	O	48.65	316ALA	N	312GLU	O	84.05	14GLU	N	12GLY	O	0.01
321GLU	N	317LYS	O	34.27	19ALA	N	16THR	O	15.17	316ALA	N	313ASP	O	1.01	12GLY	N	9ASP	O	0.54
321GLU	N	318ALA	O	24.27	18ALA	N	14GLU	O	75.78	315VAL	N	311VAL	O	92.58	12GLY	N	10GLY	O	0.00
321GLU	N	319LEU	O	0.16	18ALA	N	15VAL	O	1.55	315VAL	N	312GLU	O	1.17	12GLY	N	71SER	O	2.82
320LEU	N	316ALA	O	48.20	17GLU	N	13PRO	O	47.70	314ALA	N	310LYS	O	78.46	11ILE	N	9ASP	O	0.39
320LEU	N	317LYS	O	11.41	17GLU	N	14GLU	O	3.15	314ALA	N	311VAL	O	2.50	11ILE	N	71SER	O	0.04
319LEU	N	315VAL	O	86.69	16THR	N	12GLY	O	83.70	313ASP	N	309ARG	O	77.40	11ILE	N	73GLY	O	0.28
319LEU	N	316ALA	O	2.35	16THR	N	13PRO	O	1.03	313ASP	N	310LYS	O	2.42	11ILE	N	274GLY	O	0.02
318ALA	N	314ALA	O	50.11	15VAL	N	11ILE	O	86.61	312GLU	N	308ALA	O	89.70	11ILE	N	275SER	O	1.86
318ALA	N	315VAL	O	10.38	15VAL	N	12GLY	O	3.58	312GLU	N	309ARG	O	0.20	11ILE	N	276ALA	O	0.01
317LYS	N	313ASP	O	79.97	14GLU	N	11ILE	O	49.94	311VAL	N	307LEU	O	70.31	9ASP	N	71SER	O	77.46
317LYS	N	314ALA	O	3.29	14GLU	N	12GLY	O	0.02	311VAL	N	308ALA	O	7.33	8GLY	N	41PHE	O	25.61
316ALA	N	312GLU	O	36.89	12GLY	N	9ASP	O	0.43	310LYS	N	306GLU	O	27.98	6LEU	N	68LEU	O	98.56
316ALA	N	313ASP	O	5.58	12GLY	N	10GLY	O	0.00	310LYS	N	307LEU	O	20.91	5VAL	N	37GLU	O	93.07
315VAL	N	311VAL	O	92.86	12GLY	N	71SER	O	0.01	309ARG	N	305VAL	O	72.28	4ALA	N	66ALA	O	93.09
315VAL	N	312GLU	O	0.29	11ILE	N	9ASP	O	0.01	309ARG	N	306GLU	O	4.18	3VAL	N	1MET	O	0.00
314ALA	N	310LYS	O	73.29	11ILE	N	275SER	O	8.02	308ALA	N	304LEU	O	61.52	3VAL	N	35ALA	O	87.38
314ALA	N	311VAL	O	3.23	9ASP	N	71SER	O	88.48	308ALA	N	305VAL	O	9.08	1MET	N	33GLY	O	78.25
313ASP	N	309ARG	O	63.90	8GLY	N	41PHE	O	38.93	307LEU	N	304LEU	O	25.86	345ALA	N	341LEU	O	2.88
313ASP	N	310LYS	O	5.62	6LEU	N	4ALA	O	0.00	307LEU	N	305VAL	O	0.01	345ALA	N	342ARG	O	4.66
313ASP	N	311VAL	O	0.00	6LEU	N	68LEU	O	98.08	306GLU	N	304LEU	O	0.00	345ALA	N	343HIS	O	16.62
312GLU	N	308ALA	O	79.19	5VAL	N	37GLU	O	95.21	305VAL	N	303GLY	O	38.72	345ALA	N	345ALA	O	0.00
312GLU	N	309ARG	O	1.02	4ALA	N	66ALA	O	92.68	304LEU	N	298LEU	O	77.58	345ALA	N	345ALA	O	0.00
311VAL	N	307LEU	O	46.59	3VAL	N	1MET	O	0.02	304LEU	N	302PHE	O	0.00	344LEU	N	340VAL	O	34.53
311VAL	N	308ALA	O	13.65	3VAL	N	35ALA	O	87.75	303GLY	N	298LEU	O	58.35	344LEU	N	341LEU	O	23.68
310LYS	N	306GLU	O	22.26	1MET	N	1MET	O	0.00	303GLY	N	299GLU	O	0.48	344LEU	N	342ARG	O	0.03
310LYS	N	307LEU	O	25.49	1MET	N	32LEU	O	0.00	303GLY	N	300HIS	O	0.03	343HIS	N	339THR	O	12.22
309ARG	N	305VAL	O	84.86	1MET	N	33GLY	O	85.09	302PHE	N	297MET	O	70.12	343HIS	N	340VAL	O	58.31
309ARG	N	306GLU	O	2.29	345ALA	N	341LEU	O	5.46	302PHE	N	298LEU	O	1.14	343HIS	N	341LEU	O	0.02
308ALA	N	304LEU	O	63.13	345ALA	N	342ARG	O	3.53	302PHE	N	299GLU	O	0.04	342ARG	N	338ALA	O	30.32
308ALA	N	305VAL	O	7.77	345ALA	N	343HIS	O	22.34	302PHE	N	300HIS	O	0.04	342ARG	N	339THR	O	14.07
307LEU	N	304LEU	O	32.45	344LEU	N	340VAL	O	32.53	301ALA	N	296MET	O	0.00	342ARG	N	340VAL	O	1.29
307LEU	N	305VAL	O	0.04	344LEU	N	341LEU	O	28.26	301ALA	N	297MET	O	61.43	341LEU	N	337THR	O	97.45
305VAL	N	303GLY	O	30.61	344LEU	N	342ARG	O	0.04	301ALA	N	298LEU	O	0.00	341LEU	N	338ALA	O	0.27
304LEU	N	298LEU	O	76.56	343HIS	N	339THR	O	5.32	301ALA	N	299GLU	O	0.02	340VAL	N	336PHE	O	21.66
304LEU	N	299GLU	O	0.00	343HIS	N	340VAL	O	67.74	300HIS	N	296MET	O	94.86	340VAL	N	337THR	O	22.87
304LEU	N	302PHE	O	0.01	343HIS	N	341LEU	O	0.06	300HIS	N	297MET	O	0.64	340VAL	N	338ALA	O	0.00

303GLY	N	298LEU	O	63.44	342ARG	N	338ALA	O	29.82	299GLU	N	295ALA	O	83.32	339THR	N	335ALA	O	64.82
303GLY	N	299GLU	O	0.89	342ARG	N	339THR	O	14.47	299GLU	N	296MET	O	1.66	339THR	N	336PHE	O	8.79
303GLY	N	300HIS	O	0.03	342ARG	N	340VAL	O	0.82	298LEU	N	294ALA	O	56.78	339THR	N	337THR	O	0.00
302PHE	N	297MET	O	52.77	342ARG	N	344LEU	O	0.00	298LEU	N	295ALA	O	11.53	338ALA	N	334GLU	O	72.45
302PHE	N	298LEU	O	2.64	341LEU	N	337THR	O	96.12	297MET	N	293SER	O	71.71	338ALA	N	335ALA	O	1.46
302PHE	N	299GLU	O	0.86	341LEU	N	338ALA	O	0.30	297MET	N	294ALA	O	1.87	337THR	N	333THR	O	26.38
302PHE	N	300HIS	O	0.18	340VAL	N	336PHE	O	46.77	296MET	N	292LEU	O	86.00	337THR	N	334GLU	O	28.81
301ALA	N	297MET	O	72.09	340VAL	N	337THR	O	17.59	296MET	N	293SER	O	0.92	337THR	N	335ALA	O	10.39
301ALA	N	298LEU	O	0.02	340VAL	N	338ALA	O	0.00	295ALA	N	291ILE	O	53.85	336PHE	N	332GLY	O	82.20
301ALA	N	299GLU	O	0.02	339THR	N	335ALA	O	39.57	295ALA	N	292LEU	O	7.04	336PHE	N	333THR	O	2.60
300HIS	N	296MET	O	91.62	339THR	N	336PHE	O	23.78	294ALA	N	290ALA	O	79.22	336PHE	N	334GLU	O	0.28
300HIS	N	297MET	O	1.21	339THR	N	337THR	O	0.01	294ALA	N	291ILE	O	4.34	335ALA	N	332GLY	O	20.03
299GLU	N	295ALA	O	77.62	338ALA	N	334GLU	O	74.36	293SER	N	289ALA	O	41.55	334GLU	N	283GLY	O	0.05
299GLU	N	296MET	O	4.14	338ALA	N	335ALA	O	3.06	293SER	N	290ALA	O	10.24	334GLU	N	332GLY	O	0.19
298LEU	N	294ALA	O	54.03	337THR	N	332GLY	O	0.08	292LEU	N	288THR	O	80.66	333THR	N	283GLY	O	30.77
298LEU	N	295ALA	O	12.90	337THR	N	333THR	O	31.12	292LEU	N	289ALA	O	0.46	333THR	N	285ALA	O	2.05
297MET	N	293SER	O	64.71	337THR	N	334GLU	O	13.56	291ILE	N	287PRO	O	94.92	332GLY	N	283GLY	O	0.08
297MET	N	294ALA	O	2.88	337THR	N	335ALA	O	0.40	291ILE	N	288THR	O	0.58	332GLY	N	285ALA	O	0.02
296MET	N	292LEU	O	88.26	336PHE	N	332GLY	O	74.87	290ALA	N	287PRO	O	27.19	332GLY	N	330SER	O	0.01
296MET	N	293SER	O	0.90	336PHE	N	333THR	O	5.82	289ALA	N	255GLY	O	0.17	332GLY	N	335ALA	O	0.18
295ALA	N	291ILE	O	50.63	336PHE	N	334GLU	O	0.03	288THR	N	286ASN	O	2.52	331ALA	N	284ILE	O	0.02
295ALA	N	292LEU	O	8.55	335ALA	N	332GLY	O	8.23	286ASN	N	284ILE	O	0.03	331ALA	N	323PRO	O	6.66
295ALA	N	293SER	O	0.00	335ALA	N	333THR	O	0.01	285ALA	N	279ILE	O	0.07	331ALA	N	329GLY	O	0.23
294ALA	N	290ALA	O	74.91	334GLU	N	332GLY	O	0.42	285ALA	N	283GLY	O	3.18	330SER	N	323PRO	O	1.69
294ALA	N	291ILE	O	8.98	333THR	N	283GLY	O	4.52	284ILE	N	279ILE	O	1.07	330SER	N	328GLY	O	0.00
293SER	N	289ALA	O	36.81	333THR	N	285ALA	O	0.07	284ILE	N	282LYS	O	0.34	329GLY	N	323PRO	O	0.19
293SER	N	290ALA	O	11.89	332GLY	N	285ALA	O	0.01	283GLY	N	279ILE	O	0.41	329GLY	N	324PRO	O	26.15
292LEU	N	288THR	O	98.68	332GLY	N	330SER	O	0.14	283GLY	N	281GLY	O	0.42	329GLY	N	325PRO	O	7.91
292LEU	N	289ALA	O	0.01	332GLY	N	335ALA	O	0.01	282LYS	N	278ASP	O	0.00	328GLY	N	324PRO	O	12.39
291ILE	N	287PRO	O	95.04	331ALA	N	323PRO	O	6.22	282LYS	N	279ILE	O	38.93	328GLY	N	325PRO	O	19.38
291ILE	N	288THR	O	0.52	331ALA	N	329GLY	O	0.06	282LYS	N	280ALA	O	0.86	328GLY	N	326ASP	O	0.51
290ALA	N	287PRO	O	53.29	330SER	N	323PRO	O	23.64	281GLY	N	10GLY	O	0.02	327LEU	N	324PRO	O	58.41
288THR	N	286ASN	O	0.02	330SER	N	328GLY	O	0.01	281GLY	N	279ILE	O	0.25	327LEU	N	325PRO	O	10.19
286ASN	N	284ILE	O	0.01	329GLY	N	323PRO	O	0.02	280ALA	N	276ALA	O	0.05	326ASP	N	324PRO	O	0.36
285ALA	N	283GLY	O	2.10	329GLY	N	324PRO	O	14.95	280ALA	N	277PRO	O	4.59	326ASP	N	328GLY	O	0.17
285ALA	N	331ALA	O	0.00	329GLY	N	325PRO	O	0.44	280ALA	N	278ASP	O	3.56	326ASP	N	329GLY	O	0.06
284ILE	N	279ILE	O	0.08	328GLY	N	324PRO	O	74.39	279ILE	N	276ALA	O	0.51	322THR	N	318ALA	O	67.11
284ILE	N	282LYS	O	0.22	328GLY	N	325PRO	O	12.50	279ILE	N	277PRO	O	18.78	322THR	N	319LEU	O	0.16
283GLY	N	279ILE	O	3.06	328GLY	N	326ASP	O	0.01	278ASP	N	276ALA	O	0.73	321GLU	N	317LYS	O	19.46
282LYS	N	278ASP	O	0.33	327LEU	N	324PRO	O	65.79	276ALA	N	73GLY	O	0.01	321GLU	N	318ALA	O	42.39
282LYS	N	279ILE	O	42.64	327LEU	N	325PRO	O	0.01	276ALA	N	273HIS	O	0.00	321GLU	N	319LEU	O	0.14
282LYS	N	280ALA	O	1.37	326ASP	N	282LYS	O	0.08	276ALA	N	274GLY	O	1.82	320LEU	N	316ALA	O	32.39
281GLY	N	10GLY	O	0.01	326ASP	N	283GLY	O	0.01	275SER	N	273HIS	O	5.48	320LEU	N	317LYS	O	17.16

281GLY	N	277PRO	O	0.02
281GLY	N	278ASP	O	0.79
281GLY	N	279ILE	O	0.45
280ALA	N	276ALA	O	31.96
280ALA	N	277PRO	O	18.87
280ALA	N	278ASP	O	0.06
279ILE	N	276ALA	O	29.28
279ILE	N	277PRO	O	0.03
278ASP	N	276ALA	O	0.10
276ALA	N	274GLY	O	0.70
275SER	N	72VAL	O	0.19
273HIS	N	255GLY	O	0.09
273HIS	N	271PRO	O	6.83
272VAL	N	257LEU	O	91.14
270GLU	N	259SER	O	91.20
270GLU	N	268VAL	O	0.01
269PHE	N	67VAL	O	97.48
268VAL	N	261SER	O	96.02
266THR	N	97GLN	O	0.59
265GLY	N	97GLN	O	26.45
265GLY	N	98ASP	O	1.05
265GLY	N	263GLY	O	2.66
264ARG	N	97GLN	O	9.52
264ARG	N	98ASP	O	79.58
262LEU	N	101ALA	O	97.57
261SER	N	268VAL	O	93.86
260ALA	N	103LEU	O	94.86
259SER	N	270GLU	O	5.01
257LEU	N	253SER	O	0.88
257LEU	N	254LEU	O	7.20
257LEU	N	255GLY	O	2.28
257LEU	N	272VAL	O	2.52
256LEU	N	253SER	O	17.50
256LEU	N	254LEU	O	1.55
255GLY	N	253SER	O	0.16
254LEU	N	248SER	O	0.05
254LEU	N	252GLY	O	9.10
253SER	N	248SER	O	1.33
253SER	N	251PRO	O	0.62
252GLY	N	248SER	O	45.63
252GLY	N	249VAL	O	15.20
250LEU	N	246LEU	O	10.30
250LEU	N	247ALA	O	61.21
326ASP	N	285ALA	O	0.01
326ASP	N	324PRO	O	0.42
322THR	N	318ALA	O	92.18
322THR	N	319LEU	O	0.06
321GLU	N	317LYS	O	27.11
321GLU	N	318ALA	O	33.40
321GLU	N	319LEU	O	0.18
320LEU	N	316ALA	O	40.00
320LEU	N	317LYS	O	16.55
320LEU	N	318ALA	O	0.01
319LEU	N	315VAL	O	84.58
319LEU	N	316ALA	O	3.29
318ALA	N	314ALA	O	43.13
318ALA	N	315VAL	O	16.70
318ALA	N	316ALA	O	0.00
317LYS	N	313ASP	O	86.03
317LYS	N	314ALA	O	2.62
317LYS	N	315VAL	O	0.02
316ALA	N	312GLU	O	76.56
316ALA	N	313ASP	O	1.95
315VAL	N	311VAL	O	84.26
315VAL	N	312GLU	O	2.49
314ALA	N	310LYS	O	82.27
314ALA	N	311VAL	O	1.58
313ASP	N	309ARG	O	73.93
313ASP	N	310LYS	O	4.23
312GLU	N	308ALA	O	72.73
312GLU	N	309ARG	O	2.06
311VAL	N	307LEU	O	64.63
311VAL	N	308ALA	O	8.80
310LYS	N	306GLU	O	37.63
310LYS	N	307LEU	O	15.88
310LYS	N	308ALA	O	0.00
309ARG	N	305VAL	O	75.63
309ARG	N	306GLU	O	3.62
308ALA	N	304LEU	O	70.98
308ALA	N	305VAL	O	5.00
307LEU	N	304LEU	O	24.19
307LEU	N	305VAL	O	0.01
305VAL	N	303GLY	O	39.48
304LEU	N	298LEU	O	73.23
304LEU	N	302PHE	O	0.01
303GLY	N	298LEU	O	58.39
273HIS	N	271PRO	O	0.26
272VAL	N	257LEU	O	37.17
270GLU	N	259SER	O	97.73
269PHE	N	67VAL	O	96.54
268VAL	N	261SER	O	97.16
265GLY	N	97GLN	O	68.25
265GLY	N	98ASP	O	0.03
265GLY	N	263GLY	O	0.01
264ARG	N	97GLN	O	8.27
264ARG	N	98ASP	O	79.76
262LEU	N	101ALA	O	97.62
261SER	N	268VAL	O	94.83
260ALA	N	103LEU	O	87.50
259SER	N	270GLU	O	9.54
257LEU	N	254LEU	O	10.35
257LEU	N	255GLY	O	0.18
257LEU	N	272VAL	O	0.06
256LEU	N	253SER	O	18.36
256LEU	N	254LEU	O	4.60
255GLY	N	253SER	O	0.09
253SER	N	248SER	O	1.29
253SER	N	251PRO	O	0.06
252GLY	N	248SER	O	65.30
252GLY	N	249VAL	O	2.44
250LEU	N	246LEU	O	0.00
250LEU	N	247ALA	O	52.20
249VAL	N	245ASP	O	0.06
249VAL	N	246LEU	O	70.19
248SER	N	244SER	O	2.94
248SER	N	245ASP	O	54.31
248SER	N	246LEU	O	0.16
247ALA	N	243LEU	O	85.00
247ALA	N	244SER	O	1.02
246LEU	N	242ILE	O	91.60
246LEU	N	243LEU	O	0.72
245ASP	N	241ASP	O	12.69
245ASP	N	242ILE	O	16.69
244SER	N	240GLY	O	62.86
244SER	N	241ASP	O	4.17
243LEU	N	239PHE	O	91.99
243LEU	N	240GLY	O	0.14
242ILE	N	238ILE	O	9.92
242ILE	N	239PHE	O	27.57
320LEU	N	318ALA	O	0.04
319LEU	N	315VAL	O	84.30
319LEU	N	316ALA	O	3.74
318ALA	N	314ALA	O	41.11
318ALA	N	315VAL	O	18.27
318ALA	N	316ALA	O	0.02
317LYS	N	313ASP	O	86.77
317LYS	N	314ALA	O	2.52
317LYS	N	315VAL	O	0.00
316ALA	N	312GLU	O	82.11
316ALA	N	313ASP	O	1.38
315VAL	N	311VAL	O	81.82
315VAL	N	312GLU	O	3.80
314ALA	N	310LYS	O	78.74
314ALA	N	311VAL	O	1.97
313ASP	N	309ARG	O	75.19
313ASP	N	310LYS	O	2.69
312GLU	N	308ALA	O	84.11
312GLU	N	309ARG	O	0.62
311VAL	N	307LEU	O	73.46
311VAL	N	308ALA	O	5.99
310LYS	N	306GLU	O	28.44
310LYS	N	307LEU	O	23.26
309ARG	N	305VAL	O	59.87
309ARG	N	306GLU	O	6.70
308ALA	N	304LEU	O	68.71
308ALA	N	305VAL	O	5.43
307LEU	N	304LEU	O	21.55
305VAL	N	303GLY	O	43.47
304LEU	N	298LEU	O	75.87
304LEU	N	302PHE	O	0.00
303GLY	N	298LEU	O	53.54
303GLY	N	299GLU	O	0.93
303GLY	N	300HIS	O	0.03
302PHE	N	297MET	O	68.33
302PHE	N	298LEU	O	1.12
302PHE	N	299GLU	O	0.10
302PHE	N	300HIS	O	0.14
301ALA	N	296MET	O	0.02
301ALA	N	297MET	O	60.91
301ALA	N	298LEU	O	0.00
301ALA	N	299GLU	O	0.02
300HIS	N	296MET	O	93.43

250LEU	N	248SER	O	0.01
249VAL	N	245ASP	O	1.78
249VAL	N	246LEU	O	31.90
249VAL	N	247ALA	O	0.06
248SER	N	244SER	O	8.63
248SER	N	245ASP	O	57.46
248SER	N	246LEU	O	0.32
247ALA	N	243LEU	O	76.39
247ALA	N	244SER	O	2.76
246LEU	N	242ILE	O	92.80
246LEU	N	243LEU	O	0.98
245ASP	N	241ASP	O	12.44
245ASP	N	242ILE	O	26.21
244SER	N	240GLY	O	77.97
244SER	N	241ASP	O	2.08
243LEU	N	239PHE	O	87.44
243LEU	N	240GLY	O	0.09
242ILE	N	238ILE	O	5.10
242ILE	N	239PHE	O	27.29
241ASP	N	237ASN	O	89.18
241ASP	N	238ILE	O	0.52
240GLY	N	236GLY	O	15.14
240GLY	N	237ASN	O	7.99
240GLY	N	238ILE	O	0.03
239PHE	N	236GLY	O	5.05
237ASN	N	133GLU	O	0.00
235THR	N	131VAL	O	54.53
234VAL	N	181VAL	O	89.80
233VAL	N	129LEU	O	96.39
233VAL	N	231ASP	O	0.00
232VAL	N	179HIS	O	2.82
230PHE	N	227PRO	O	64.27
230PHE	N	228ALA	O	0.04
229ARG	N	226SER	O	29.60
229ARG	N	227PRO	O	1.32
228ALA	N	122ILE	O	0.01
228ALA	N	226SER	O	0.00
226SER	N	222HIS	O	96.18
226SER	N	223LEU	O	0.51
225ARG	N	221MET	O	7.85
225ARG	N	222HIS	O	62.01
225ARG	N	223LEU	O	1.90
224VAL	N	220ALA	O	6.08
303GLY	N	299GLU	O	1.15
303GLY	N	300HIS	O	0.08
302PHE	N	297MET	O	48.60
302PHE	N	298LEU	O	2.13
302PHE	N	299GLU	O	0.50
302PHE	N	300HIS	O	0.62
301ALA	N	296MET	O	0.03
301ALA	N	297MET	O	65.83
301ALA	N	299GLU	O	0.02
300HIS	N	296MET	O	90.13
300HIS	N	297MET	O	1.82
299GLU	N	295ALA	O	76.55
299GLU	N	296MET	O	3.96
298LEU	N	294ALA	O	61.78
298LEU	N	295ALA	O	9.63
297MET	N	293SER	O	65.16
297MET	N	294ALA	O	3.79
296MET	N	292LEU	O	86.12
296MET	N	293SER	O	1.14
295ALA	N	291ILE	O	61.12
295ALA	N	292LEU	O	5.24
294ALA	N	290ALA	O	66.34
294ALA	N	291ILE	O	11.52
293SER	N	289ALA	O	30.27
293SER	N	290ALA	O	15.74
292LEU	N	288THR	O	93.25
292LEU	N	289ALA	O	0.18
291ILE	N	287PRO	O	90.78
291ILE	N	288THR	O	1.61
290ALA	N	287PRO	O	41.10
289ALA	N	255GLY	O	9.52
288THR	N	286ASN	O	1.22
286ASN	N	283GLY	O	0.26
286ASN	N	284ILE	O	1.88
285ALA	N	279ILE	O	1.50
285ALA	N	283GLY	O	2.49
285ALA	N	331ALA	O	0.00
284ILE	N	278ASP	O	0.01
284ILE	N	279ILE	O	23.96
284ILE	N	281GLY	O	0.00
284ILE	N	282LYS	O	0.68
283GLY	N	277PRO	O	0.56
283GLY	N	278ASP	O	0.88
242ILE	N	240GLY	O	0.00
241ASP	N	237ASN	O	91.88
241ASP	N	238ILE	O	0.90
240GLY	N	236GLY	O	18.58
240GLY	N	237ASN	O	8.40
240GLY	N	238ILE	O	0.02
239PHE	N	236GLY	O	4.39
237ASN	N	133GLU	O	0.08
235THR	N	131VAL	O	77.16
234VAL	N	181VAL	O	92.78
233VAL	N	129LEU	O	95.78
232VAL	N	179HIS	O	2.08
230PHE	N	227PRO	O	80.96
230PHE	N	228ALA	O	0.05
229ARG	N	226SER	O	28.57
229ARG	N	227PRO	O	0.71
228ALA	N	226SER	O	0.02
226SER	N	222HIS	O	96.65
226SER	N	223LEU	O	0.26
225ARG	N	221MET	O	1.09
225ARG	N	222HIS	O	70.30
225ARG	N	223LEU	O	3.06
224VAL	N	220ALA	O	3.18
224VAL	N	221MET	O	21.76
224VAL	N	222HIS	O	0.01
223LEU	N	219MET	O	66.06
223LEU	N	220ALA	O	9.05
222HIS	N	218ALA	O	43.56
222HIS	N	219MET	O	18.78
222HIS	N	220ALA	O	0.00
221MET	N	217ASP	O	47.31
221MET	N	218ALA	O	5.34
220ALA	N	216VAL	O	90.38
220ALA	N	217ASP	O	0.91
219MET	N	215TYR	O	87.35
219MET	N	216VAL	O	1.88
218ALA	N	215TYR	O	23.14
216VAL	N	184ASP	O	98.40
214GLN	N	182SER	O	93.26
212GLU	N	180VAL	O	64.41
211LEU	N	209VAL	O	0.00
210ALA	N	178LYS	O	95.32
209VAL	N	206TYR	O	59.98
300HIS	N	297MET	O	1.16
299GLU	N	295ALA	O	84.14
299GLU	N	296MET	O	1.76
298LEU	N	294ALA	O	73.25
298LEU	N	295ALA	O	4.82
297MET	N	293SER	O	69.40
297MET	N	294ALA	O	3.20
296MET	N	292LEU	O	81.69
296MET	N	293SER	O	1.53
295ALA	N	291ILE	O	71.67
295ALA	N	292LEU	O	2.88
294ALA	N	290ALA	O	69.25
294ALA	N	291ILE	O	7.06
293SER	N	289ALA	O	38.46
293SER	N	290ALA	O	9.63
292LEU	N	288THR	O	94.59
292LEU	N	289ALA	O	0.18
291ILE	N	287PRO	O	63.66
291ILE	N	288THR	O	3.56
290ALA	N	287PRO	O	66.78
289ALA	N	255GLY	O	51.33
288THR	N	255GLY	O	0.00
288THR	N	286ASN	O	0.36
286ASN	N	284ILE	O	0.00
285ALA	N	279ILE	O	0.02
285ALA	N	283GLY	O	3.70
285ALA	N	331ALA	O	1.39
284ILE	N	279ILE	O	16.33
284ILE	N	282LYS	O	0.76
283GLY	N	279ILE	O	15.89
283GLY	N	280ALA	O	0.17
283GLY	N	281GLY	O	0.03
282LYS	N	278ASP	O	9.37
282LYS	N	279ILE	O	26.09
282LYS	N	280ALA	O	0.39
281GLY	N	10GLY	O	0.01
281GLY	N	277PRO	O	0.24
281GLY	N	278ASP	O	7.57
281GLY	N	279ILE	O	0.32
281GLY	N	284ILE	O	0.10
280ALA	N	10GLY	O	30.04
280ALA	N	276ALA	O	23.10
280ALA	N	277PRO	O	2.11

224VAL	N	221MET	O	16.48
223LEU	N	219MET	O	77.46
223LEU	N	220ALA	O	3.62
222HIS	N	218ALA	O	43.55
222HIS	N	219MET	O	22.50
222HIS	N	220ALA	O	0.01
221MET	N	217ASP	O	53.76
221MET	N	218ALA	O	4.73
220ALA	N	216VAL	O	97.29
220ALA	N	217ASP	O	0.13
219MET	N	215TYR	O	85.72
219MET	N	216VAL	O	2.60
219MET	N	217ASP	O	0.00
218ALA	N	215TYR	O	29.75
218ALA	N	216VAL	O	0.01
216VAL	N	184ASP	O	97.91
214GLN	N	182SER	O	78.21
212GLU	N	180VAL	O	56.49
210ALA	N	178LYS	O	97.06
209VAL	N	206TYR	O	9.50
209VAL	N	207PRO	O	41.71
208ASP	N	206TYR	O	0.08
206TYR	N	202VAL	O	39.90
206TYR	N	203GLY	O	25.83
206TYR	N	204ARG	O	0.03
205GLY	N	201GLU	O	0.60
205GLY	N	202VAL	O	63.75
205GLY	N	203GLY	O	0.20
204ARG	N	200GLU	O	25.16
204ARG	N	201GLU	O	36.99
204ARG	N	202VAL	O	0.01
203GLY	N	199VAL	O	47.87
203GLY	N	200GLU	O	15.66
202VAL	N	198THR	O	41.01
202VAL	N	199VAL	O	20.38
201GLU	N	197LYS	O	24.96
201GLU	N	198THR	O	27.50
200GLU	N	196ARG	O	37.75
200GLU	N	197LYS	O	4.50
199VAL	N	195TRP	O	98.47
199VAL	N	196ARG	O	0.26
198THR	N	194PHE	O	64.38
198THR	N	195TRP	O	17.71

283GLY	N	279ILE	O	25.26
283GLY	N	280ALA	O	0.34
283GLY	N	281GLY	O	2.04
282LYS	N	277PRO	O	12.94
282LYS	N	278ASP	O	16.80
282LYS	N	279ILE	O	18.34
282LYS	N	280ALA	O	0.67
282LYS	N	283GLY	O	0.00
282LYS	N	284ILE	O	0.40
281GLY	N	10GLY	O	0.00
281GLY	N	276ALA	O	0.00
281GLY	N	277PRO	O	7.76
281GLY	N	278ASP	O	14.20
281GLY	N	279ILE	O	0.51
281GLY	N	283GLY	O	2.68
280ALA	N	276ALA	O	46.07
280ALA	N	277PRO	O	24.29
280ALA	N	278ASP	O	0.42
280ALA	N	283GLY	O	0.02
279ILE	N	276ALA	O	40.82
279ILE	N	277PRO	O	3.78
279ILE	N	280ALA	O	0.02
278ASP	N	276ALA	O	0.40
278ASP	N	280ALA	O	1.70
276ALA	N	274GLY	O	28.39
275SER	N	72VAL	O	4.04
275SER	N	73GLY	O	0.59
275SER	N	272VAL	O	0.07
275SER	N	273HIS	O	1.90
274GLY	N	272VAL	O	0.14
274GLY	N	279ILE	O	15.20
273HIS	N	255GLY	O	0.07
273HIS	N	271PRO	O	7.29
272VAL	N	255GLY	O	0.00
272VAL	N	257LEU	O	47.51
270GLU	N	259SER	O	98.27
269PHE	N	67VAL	O	95.50
268VAL	N	261SER	O	93.54
266THR	N	97GLN	O	0.00
265GLY	N	97GLN	O	55.27
265GLY	N	98ASP	O	0.06
265GLY	N	263GLY	O	0.10
264ARG	N	97GLN	O	15.51

209VAL	N	207PRO	O	0.56
208ASP	N	206TYR	O	0.07
206TYR	N	202VAL	O	12.22
206TYR	N	203GLY	O	57.33
206TYR	N	204ARG	O	0.02
205GLY	N	201GLU	O	0.39
205GLY	N	202VAL	O	69.18
205GLY	N	203GLY	O	0.07
204ARG	N	200GLU	O	35.88
204ARG	N	201GLU	O	24.72
204ARG	N	202VAL	O	0.00
203GLY	N	199VAL	O	50.91
203GLY	N	200GLU	O	10.52
202VAL	N	198THR	O	78.98
202VAL	N	199VAL	O	3.86
201GLU	N	197LYS	O	65.26
201GLU	N	198THR	O	8.34
200GLU	N	196ARG	O	35.91
200GLU	N	197LYS	O	7.76
199VAL	N	195TRP	O	96.95
199VAL	N	196ARG	O	0.12
198THR	N	194PHE	O	73.25
198THR	N	195TRP	O	10.30
197LYS	N	193GLU	O	64.22
197LYS	N	194PHE	O	8.36
196ARG	N	192GLY	O	96.53
196ARG	N	193GLU	O	0.68
195TRP	N	191VAL	O	60.37
195TRP	N	192GLY	O	1.62
194PHE	N	190GLU	O	96.16
194PHE	N	191VAL	O	0.05
193GLU	N	189LEU	O	84.75
193GLU	N	190GLU	O	2.01
192GLY	N	189LEU	O	48.37
192GLY	N	190GLU	O	0.01
189LEU	N	185LYS	O	24.04
189LEU	N	186ALA	O	30.13
188VAL	N	185LYS	O	53.84
188VAL	N	186ALA	O	0.05
187ASN	N	185LYS	O	0.10
184ASP	N	182SER	O	0.04
184ASP	N	214GLN	O	95.95
183VAL	N	234VAL	O	72.53

280ALA	N	278ASP	O	0.56
279ILE	N	10GLY	O	4.99
279ILE	N	276ALA	O	4.17
279ILE	N	277PRO	O	43.65
278ASP	N	276ALA	O	0.42
278ASP	N	280ALA	O	0.22
276ALA	N	274GLY	O	2.48
276ALA	N	279ILE	O	0.00
275SER	N	273HIS	O	0.02
274GLY	N	254LEU	O	0.14
274GLY	N	272VAL	O	0.05
273HIS	N	271PRO	O	0.78
272VAL	N	257LEU	O	77.77
270GLU	N	259SER	O	96.05
270GLU	N	268VAL	O	0.01
269PHE	N	67VAL	O	96.78
268VAL	N	261SER	O	93.68
266THR	N	97GLN	O	0.18
266THR	N	263GLY	O	0.10
266THR	N	264ARG	O	0.05
265GLY	N	97GLN	O	20.68
265GLY	N	98ASP	O	0.03
265GLY	N	263GLY	O	1.97
264ARG	N	97GLN	O	21.02
264ARG	N	98ASP	O	56.65
263GLY	N	266THR	O	0.52
262LEU	N	101ALA	O	96.43
261SER	N	268VAL	O	92.57
260ALA	N	103LEU	O	90.90
259SER	N	270GLU	O	6.48
257LEU	N	254LEU	O	1.37
257LEU	N	255GLY	O	0.26
257LEU	N	272VAL	O	1.77
256LEU	N	253SER	O	7.64
256LEU	N	254LEU	O	0.90
255GLY	N	253SER	O	0.81
255GLY	N	272VAL	O	39.97
253SER	N	248SER	O	0.33
253SER	N	251PRO	O	0.14
252GLY	N	248SER	O	55.05
252GLY	N	249VAL	O	10.15
252GLY	N	250LEU	O	0.00
250LEU	N	246LEU	O	1.20

197LYS	N	193GLU	O	64.61
197LYS	N	194PHE	O	6.48
196ARG	N	192GLY	O	99.54
196ARG	N	193GLU	O	0.00
195TRP	N	191VAL	O	77.90
195TRP	N	192GLY	O	0.48
194PHE	N	190GLU	O	94.80
194PHE	N	191VAL	O	0.09
193GLU	N	189LEU	O	80.31
193GLU	N	190GLU	O	3.20
192GLY	N	189LEU	O	45.96
192GLY	N	190GLU	O	0.01
189LEU	N	185LYS	O	78.70
189LEU	N	186ALA	O	0.80
188VAL	N	185LYS	O	91.18
187ASN	N	185LYS	O	2.34
184ASP	N	214GLN	O	98.99
183VAL	N	234VAL	O	89.67
182SER	N	212GLU	O	98.63
181VAL	N	232VAL	O	90.37
180VAL	N	210ALA	O	92.80
179HIS	N	177ARG	O	0.01
178LYS	N	173ALA	O	19.52
178LYS	N	176ARG	O	0.06
176ARG	N	172ALA	O	1.84
176ARG	N	173ALA	O	83.99
176ARG	N	174ARG	O	0.20
175LYS	N	171GLU	O	5.34
175LYS	N	172ALA	O	50.84
175LYS	N	173ALA	O	0.04
174ARG	N	170PHE	O	54.33
174ARG	N	171GLU	O	11.73
173ALA	N	169ALA	O	83.52
173ALA	N	170PHE	O	2.01
172ALA	N	168VAL	O	58.53
172ALA	N	169ALA	O	6.97
171GLU	N	167ARG	O	82.92
171GLU	N	168VAL	O	1.37
170PHE	N	166ALA	O	91.36
170PHE	N	167ARG	O	1.32
169ALA	N	165VAL	O	26.38
169ALA	N	166ALA	O	22.81
169ALA	N	167ARG	O	0.00

264ARG	N	98ASP	O	62.07
262LEU	N	101ALA	O	92.32
261SER	N	268VAL	O	92.42
260ALA	N	103LEU	O	93.41
259SER	N	270GLU	O	8.41
257LEU	N	254LEU	O	0.88
257LEU	N	255GLY	O	0.18
257LEU	N	272VAL	O	0.00
256LEU	N	253SER	O	1.81
256LEU	N	254LEU	O	0.20
255GLY	N	248SER	O	0.01
255GLY	N	253SER	O	0.10
253SER	N	248SER	O	0.10
253SER	N	249VAL	O	0.07
253SER	N	251PRO	O	0.54
252GLY	N	248SER	O	1.89
252GLY	N	249VAL	O	85.41
252GLY	N	250LEU	O	0.02
250LEU	N	246LEU	O	0.08
250LEU	N	247ALA	O	61.97
250LEU	N	248SER	O	0.01
249VAL	N	245ASP	O	0.03
249VAL	N	246LEU	O	10.53
249VAL	N	247ALA	O	0.02
248SER	N	244SER	O	23.78
248SER	N	245ASP	O	35.55
248SER	N	246LEU	O	0.17
247ALA	N	243LEU	O	63.99
247ALA	N	244SER	O	6.46
246LEU	N	242ILE	O	89.22
246LEU	N	243LEU	O	0.71
246LEU	N	244SER	O	0.00
245ASP	N	241ASP	O	68.14
245ASP	N	242ILE	O	6.48
244SER	N	240GLY	O	62.88
244SER	N	241ASP	O	6.64
243LEU	N	239PHE	O	57.45
243LEU	N	240GLY	O	0.44
242ILE	N	238ILE	O	45.63
242ILE	N	239PHE	O	5.83
241ASP	N	236GLY	O	0.00
241ASP	N	237ASN	O	25.99
241ASP	N	238ILE	O	26.78

182SER	N	212GLU	O	98.94
181VAL	N	232VAL	O	87.88
180VAL	N	210ALA	O	90.65
178LYS	N	173ALA	O	26.29
178LYS	N	176ARG	O	0.12
176ARG	N	172LEU	O	1.61
176ARG	N	173ALA	O	82.43
176ARG	N	174ARG	O	0.21
175LYS	N	171GLU	O	3.19
175LYS	N	172LEU	O	60.41
175LYS	N	173ALA	O	0.05
174ARG	N	170PHE	O	33.33
174ARG	N	171GLU	O	26.02
173ALA	N	169ALA	O	68.96
173ALA	N	170PHE	O	3.63
172LEU	N	168VAL	O	90.50
172LEU	N	169ALA	O	1.35
171GLU	N	167ARG	O	68.55
171GLU	N	168VAL	O	6.07
170PHE	N	166ALA	O	71.88
170PHE	N	167ARG	O	4.72
169ALA	N	165VAL	O	43.20
169ALA	N	166ALA	O	15.59
168VAL	N	164ARG	O	33.07
168VAL	N	165VAL	O	6.52
168VAL	N	166ALA	O	0.02
167ARG	N	163GLU	O	91.94
167ARG	N	164ARG	O	1.21
166ALA	N	162VAL	O	57.74
166ALA	N	163GLU	O	3.89
165VAL	N	161GLU	O	7.20
165VAL	N	162VAL	O	9.78
165VAL	N	163GLU	O	0.01
164ARG	N	160PRO	O	47.08
164ARG	N	161GLU	O	15.35
163GLU	N	159LYS	O	58.93
163GLU	N	160PRO	O	7.44
162VAL	N	158SER	O	62.72
162VAL	N	159LYS	O	9.87
162VAL	N	160PRO	O	0.00
161GLU	N	158SER	O	35.76
159LYS	N	149ALA	O	96.82
157TYR	N	151ALA	O	96.22

250LEU	N	247ALA	O	41.47
249VAL	N	245ASP	O	1.39
249VAL	N	246LEU	O	53.69
249VAL	N	247ALA	O	0.05
248SER	N	244SER	O	4.23
248SER	N	245ASP	O	63.00
248SER	N	246LEU	O	0.14
247ALA	N	243LEU	O	80.36
247ALA	N	244SER	O	1.86
246LEU	N	242ILE	O	87.10
246LEU	N	243LEU	O	0.77
245ASP	N	241ASP	O	19.74
245ASP	N	242ILE	O	19.93
244SER	N	240GLY	O	57.11
244SER	N	241ASP	O	6.40
244SER	N	242ILE	O	0.01
243LEU	N	239PHE	O	74.71
243LEU	N	240GLY	O	0.16
242ILE	N	238ILE	O	63.21
242ILE	N	239PHE	O	4.08
241ASP	N	237ASN	O	58.98
241ASP	N	238ILE	O	11.42
240GLY	N	236GLY	O	68.24
240GLY	N	237ASN	O	11.57
239PHE	N	236GLY	O	2.40
235THR	N	131VAL	O	63.25
234VAL	N	181VAL	O	89.97
233VAL	N	129LEU	O	90.16
232VAL	N	179HIS	O	4.82
231ASP	N	179HIS	O	0.00
231ASP	N	229ARG	O	0.07
230PHE	N	227PRO	O	62.64
230PHE	N	228ALA	O	0.33
229ARG	N	226SER	O	29.88
229ARG	N	227PRO	O	2.57
228ALA	N	226SER	O	0.04
226SER	N	222HIS	O	88.30
226SER	N	223LEU	O	1.80
225ARG	N	221MET	O	21.76
225ARG	N	222HIS	O	47.59
225ARG	N	223LEU	O	2.44
224VAL	N	220ALA	O	2.69
224VAL	N	221MET	O	25.26

168VAL	N	164ARG	O	33.66
168VAL	N	165VAL	O	3.16
167ARG	N	163GLU	O	87.18
167ARG	N	164ARG	O	2.42
166ALA	N	162VAL	O	77.72
166ALA	N	163GLU	O	3.11
165VAL	N	161GLU	O	19.57
165VAL	N	162VAL	O	12.53
164ARG	N	160PRO	O	64.89
164ARG	N	161GLU	O	5.36
163GLU	N	159LYS	O	71.33
163GLU	N	160PRO	O	1.49
162VAL	N	158SER	O	46.81
162VAL	N	159LYS	O	19.94
161GLU	N	158SER	O	40.85
159LYS	N	149ALA	O	95.89
157TYR	N	151ALA	O	82.91
156ARG	N	137GLY	O	0.00
155GLU	N	153ASN	O	96.39
154THR	N	138ILE	O	0.00
154THR	N	141GLY	O	12.49
154THR	N	143PRO	O	2.59
153ASN	N	155GLU	O	97.27
152TRP	N	145GLY	O	99.34
151ALA	N	157TYR	O	93.30
150GLU	N	147SER	O	8.89
150GLU	N	148GLU	O	0.01
149ALA	N	147SER	O	2.29
148GLU	N	146MET	O	0.01
148GLU	N	150GLU	O	0.08
147SER	N	150GLU	O	56.46
145GLY	N	152TRP	O	70.65
142GLU	N	140PHE	O	0.40
141GLY	N	137GLY	O	2.63
141GLY	N	138ILE	O	18.43
141GLY	N	139TYR	O	0.01
140PHE	N	136GLY	O	0.02
140PHE	N	137GLY	O	96.84
139TYR	N	137GLY	O	0.01
137GLY	N	156ARG	O	6.56
135THR	N	100PHE	O	0.01
134LEU	N	100PHE	O	73.60
133GLU	N	235THR	O	13.34

241ASP	N	239PHE	O	0.01
240GLY	N	236GLY	O	64.08
240GLY	N	237ASN	O	4.03
240GLY	N	238ILE	O	0.01
239PHE	N	236GLY	O	6.13
238ILE	N	236GLY	O	0.02
237ASN	N	136GLY	O	0.17
235THR	N	131VAL	O	81.95
234VAL	N	181VAL	O	88.61
233VAL	N	129LEU	O	94.56
232VAL	N	179HIS	O	2.32
230PHE	N	227PRO	O	63.87
230PHE	N	228ALA	O	0.07
229ARG	N	226SER	O	42.39
229ARG	N	227PRO	O	1.76
228ALA	N	226SER	O	0.00
226SER	N	222HIS	O	96.28
226SER	N	223LEU	O	0.47
225ARG	N	221MET	O	7.80
225ARG	N	222HIS	O	56.96
225ARG	N	223LEU	O	1.00
224VAL	N	220ALA	O	11.49
224VAL	N	221MET	O	13.26
224VAL	N	222HIS	O	0.02
223LEU	N	219MET	O	72.33
223LEU	N	220ALA	O	4.86
222HIS	N	218ALA	O	44.09
222HIS	N	219MET	O	17.61
221MET	N	217ASP	O	54.34
221MET	N	218ALA	O	3.38
220ALA	N	216VAL	O	95.34
220ALA	N	217ASP	O	0.18
219MET	N	215TYR	O	81.14
219MET	N	216VAL	O	4.02
218ALA	N	215TYR	O	28.49
218ALA	N	216VAL	O	0.01
216VAL	N	184ASP	O	97.35
214GLN	N	182SER	O	77.16
212GLU	N	180VAL	O	47.76
210ALA	N	178LYS	O	85.85
209VAL	N	206TYR	O	2.89
209VAL	N	207PRO	O	36.49
208ASP	N	206TYR	O	0.03

156ARG	N	137GLY	O	0.01
156ARG	N	154THR	O	0.03
155GLU	N	153ASN	O	98.72
154THR	N	141GLY	O	0.78
154THR	N	143PRO	O	10.67
153ASN	N	155GLU	O	98.32
152TRP	N	145GLY	O	99.34
151ALA	N	157TYR	O	94.06
150GLU	N	147SER	O	9.24
150GLU	N	148GLU	O	0.02
149ALA	N	147SER	O	0.80
147SER	N	150GLU	O	58.77
145GLY	N	152TRP	O	89.38
142GLU	N	140PHE	O	0.09
141GLY	N	137GLY	O	0.61
141GLY	N	138ILE	O	37.00
141GLY	N	139TYR	O	0.02
140PHE	N	137GLY	O	96.45
140PHE	N	138ILE	O	0.01
139TYR	N	137GLY	O	0.02
137GLY	N	140PHE	O	0.00
137GLY	N	156ARG	O	2.44
134LEU	N	100PHE	O	10.97
133GLU	N	235THR	O	11.98
133GLU	N	236GLY	O	63.26
132ARG	N	102ASN	O	96.32
131VAL	N	233VAL	O	97.85
130ILE	N	104ARG	O	97.48
129LEU	N	231ASP	O	90.51
128VAL	N	106ALA	O	83.21
126VAL	N	123ALA	O	2.98
126VAL	N	124ARG	O	0.10
125GLY	N	123ALA	O	0.02
124ARG	N	120GLU	O	38.85
124ARG	N	121GLU	O	7.51
124ARG	N	122ILE	O	1.74
123ALA	N	119LYS	O	55.71
123ALA	N	120GLU	O	9.14
122ILE	N	119LYS	O	85.53
122ILE	N	120GLU	O	0.01
121GLU	N	119LYS	O	0.01
119LYS	N	117PRO	O	84.54
116SER	N	112LEU	O	8.73

224VAL	N	222HIS	O	0.01
223LEU	N	219MET	O	83.79
223LEU	N	220ALA	O	1.90
222HIS	N	218ALA	O	63.31
222HIS	N	219MET	O	7.53
222HIS	N	220ALA	O	0.01
221MET	N	217ASP	O	32.37
221MET	N	218ALA	O	15.42
221MET	N	219MET	O	0.03
220ALA	N	216VAL	O	88.07
220ALA	N	217ASP	O	1.37
219MET	N	215TYR	O	83.69
219MET	N	216VAL	O	3.96
218ALA	N	215TYR	O	31.81
216VAL	N	184ASP	O	95.75
214GLN	N	182SER	O	72.93
212GLU	N	180VAL	O	66.39
211LEU	N	209VAL	O	0.00
210ALA	N	178LYS	O	96.34
209VAL	N	206TYR	O	53.06
209VAL	N	207PRO	O	0.55
208ASP	N	206TYR	O	0.16
206TYR	N	202VAL	O	18.68
206TYR	N	203GLY	O	42.74
206TYR	N	204ARG	O	0.02
205GLY	N	201GLU	O	0.39
205GLY	N	202VAL	O	75.07
205GLY	N	203GLY	O	0.16
204ARG	N	200GLU	O	36.65
204ARG	N	201GLU	O	22.52
204ARG	N	202VAL	O	0.02
203GLY	N	199VAL	O	56.47
203GLY	N	200GLU	O	10.59
202VAL	N	198THR	O	35.24
202VAL	N	199VAL	O	16.76
202VAL	N	200GLU	O	0.00
201GLU	N	197LYS	O	28.23
201GLU	N	198THR	O	27.29
201GLU	N	199VAL	O	0.00
200GLU	N	196ARG	O	38.85
200GLU	N	197LYS	O	5.03
199VAL	N	195TRP	O	96.52
199VAL	N	196ARG	O	0.36

133GLU	N	236GLY	O	63.77
132ARG	N	102ASN	O	93.11
132ARG	N	130ILE	O	0.00
131VAL	N	233VAL	O	96.62
130ILE	N	104ARG	O	96.89
129LEU	N	231ASP	O	89.36
128VAL	N	106ALA	O	89.81
126VAL	N	123ALA	O	18.23
126VAL	N	124ARG	O	0.07
124ARG	N	120GLU	O	52.87
124ARG	N	121GLU	O	3.55
124ARG	N	122ILE	O	3.31
123ALA	N	119LYS	O	70.90
123ALA	N	120GLU	O	9.29
122ILE	N	119LYS	O	78.23
122ILE	N	120GLU	O	0.04
121GLU	N	119LYS	O	0.00
119LYS	N	117PRO	O	88.22
116SER	N	112LEU	O	0.30
116SER	N	113GLU	O	71.35
116SER	N	114ARG	O	0.13
115LEU	N	112LEU	O	56.84
115LEU	N	113GLU	O	0.11
114ARG	N	112LEU	O	0.01
113GLU	N	111GLY	O	0.01
112LEU	N	109PHE	O	92.32
112LEU	N	110PRO	O	0.01
111GLY	N	109PHE	O	0.01
109PHE	N	112LEU	O	0.04
108VAL	N	126VAL	O	33.85
107LYS	N	105PRO	O	0.03
106ALA	N	128VAL	O	87.82
104ARG	N	130ILE	O	95.64
103LEU	N	260ALA	O	99.18
102ASN	N	100PHE	O	0.01
102ASN	N	132ARG	O	98.49
101ALA	N	262LEU	O	46.94
100PHE	N	98ASP	O	0.34
100PHE	N	262LEU	O	80.34
99LEU	N	94ARG	O	58.40
99LEU	N	97GLN	O	8.96
98ASP	N	94ARG	O	3.25
98ASP	N	95LYS	O	13.33
206TYR	N	202VAL	O	6.38
206TYR	N	203GLY	O	37.19
206TYR	N	204ARG	O	0.25
205GLY	N	200GLU	O	0.00
205GLY	N	201GLU	O	0.36
205GLY	N	202VAL	O	10.12
205GLY	N	203GLY	O	0.18
204ARG	N	199VAL	O	2.01
204ARG	N	200GLU	O	66.12
204ARG	N	201GLU	O	2.60
204ARG	N	202VAL	O	0.01
203GLY	N	199VAL	O	49.32
203GLY	N	200GLU	O	12.95
203GLY	N	201GLU	O	0.01
203GLY	N	204ARG	O	0.02
202VAL	N	198THR	O	66.59
202VAL	N	199VAL	O	6.18
202VAL	N	200GLU	O	0.03
201GLU	N	197LYS	O	57.23
201GLU	N	198THR	O	11.26
201GLU	N	199VAL	O	0.07
200GLU	N	196ARG	O	76.50
200GLU	N	197LYS	O	1.54
199VAL	N	195TRP	O	97.09
199VAL	N	196ARG	O	0.60
198THR	N	194PHE	O	67.43
198THR	N	195TRP	O	14.71
198THR	N	196ARG	O	0.00
197LYS	N	193GLU	O	83.73
197LYS	N	194PHE	O	1.91
196ARG	N	192GLY	O	98.90
196ARG	N	193GLU	O	0.02
195TRP	N	191VAL	O	49.53
195TRP	N	192GLY	O	2.44
194PHE	N	190GLU	O	95.46
194PHE	N	191VAL	O	0.03
193GLU	N	189LEU	O	86.06
193GLU	N	190GLU	O	2.52
192GLY	N	189LEU	O	55.92
192GLY	N	190GLU	O	0.21
189LEU	N	185LYS	O	76.49
189LEU	N	186ALA	O	2.60
188VAL	N	185LYS	O	81.99
116SER	N	113GLU	O	49.35
116SER	N	114ARG	O	0.16
115LEU	N	111GLY	O	0.03
115LEU	N	112LEU	O	66.81
115LEU	N	113GLU	O	0.02
114ARG	N	111GLY	O	7.21
114ARG	N	112LEU	O	0.05
113GLU	N	109PHE	O	0.00
113GLU	N	111GLY	O	0.01
112LEU	N	109PHE	O	81.74
112LEU	N	110PRO	O	0.03
109PHE	N	107LYS	O	0.04
108VAL	N	126VAL	O	72.44
107LYS	N	105PRO	O	0.25
106ALA	N	104ARG	O	0.00
106ALA	N	128VAL	O	94.79
104ARG	N	130ILE	O	99.14
103LEU	N	101ALA	O	0.00
103LEU	N	260ALA	O	99.60
102ASN	N	100PHE	O	0.01
102ASN	N	132ARG	O	98.70
101ALA	N	262LEU	O	73.85
100PHE	N	98ASP	O	0.28
100PHE	N	262LEU	O	90.30
99LEU	N	94ARG	O	97.18
98ASP	N	94ARG	O	4.26
98ASP	N	95LYS	O	16.16
98ASP	N	96SER	O	0.00
97GLN	N	93LEU	O	72.39
97GLN	N	94ARG	O	0.34
96SER	N	92SER	O	92.92
96SER	N	93LEU	O	1.53
95LYS	N	91LEU	O	78.01
95LYS	N	92SER	O	3.82
94ARG	N	90LEU	O	87.34
94ARG	N	91LEU	O	1.06
93LEU	N	89GLY	O	87.37
93LEU	N	90LEU	O	1.24
92SER	N	88THR	O	33.67
92SER	N	89GLY	O	7.11
92SER	N	90LEU	O	0.00
91LEU	N	87GLU	O	86.87
91LEU	N	88THR	O	0.60
198THR	N	194PHE	O	52.80
198THR	N	195TRP	O	26.89
198THR	N	196ARG	O	0.01
197LYS	N	193GLU	O	62.77
197LYS	N	194PHE	O	6.95
196ARG	N	192GLY	O	97.05
196ARG	N	193GLU	O	0.07
195TRP	N	191VAL	O	61.27
195TRP	N	192GLY	O	2.53
194PHE	N	190GLU	O	92.34
194PHE	N	191VAL	O	0.08
193GLU	N	189LEU	O	77.74
193GLU	N	190GLU	O	3.98
192GLY	N	189LEU	O	52.66
192GLY	N	190GLU	O	0.03
189LEU	N	185LYS	O	67.37
189LEU	N	186ALA	O	1.65
188VAL	N	185LYS	O	83.09
188VAL	N	186ALA	O	0.03
187ASN	N	185LYS	O	1.42
184ASP	N	214GLN	O	95.76
183VAL	N	234VAL	O	75.49
182SER	N	212GLU	O	97.40
181VAL	N	232VAL	O	88.07
180VAL	N	210ALA	O	89.86
179HIS	N	177ARG	O	0.00
178LYS	N	173ALA	O	24.44
178LYS	N	176ARG	O	0.06
177ARG	N	173ALA	O	0.01
176ARG	N	172LEU	O	1.96
176ARG	N	173ALA	O	77.35
176ARG	N	174ARG	O	0.48
175LYS	N	171GLU	O	6.00
175LYS	N	172LEU	O	53.46
175LYS	N	173ALA	O	0.07
174ARG	N	170PHE	O	39.18
174ARG	N	171GLU	O	21.08
173ALA	N	169ALA	O	73.11
173ALA	N	170PHE	O	2.70
172LEU	N	168VAL	O	85.87
172LEU	N	169ALA	O	2.82
171GLU	N	167ARG	O	44.02
171GLU	N	168VAL	O	13.09

98ASP	N	96SER	O	0.55
98ASP	N	264ARG	O	0.20
97GLN	N	93LEU	O	85.81
97GLN	N	94ARG	O	0.27
97GLN	N	95LYS	O	0.00
96SER	N	92SER	O	76.86
96SER	N	93LEU	O	12.78
96SER	N	94ARG	O	0.04
96SER	N	97GLN	O	0.20
95LYS	N	91LEU	O	49.70
95LYS	N	92SER	O	16.22
95LYS	N	93LEU	O	4.14
94ARG	N	90LEU	O	95.24
94ARG	N	91LEU	O	0.26
93LEU	N	89GLY	O	81.41
93LEU	N	90LEU	O	2.75
92SER	N	88THR	O	30.52
92SER	N	89GLY	O	8.16
92SER	N	90LEU	O	0.25
91LEU	N	87GLU	O	91.18
91LEU	N	88THR	O	0.18
90LEU	N	86PRO	O	58.53
90LEU	N	87GLU	O	13.82
89GLY	N	85SER	O	2.06
89GLY	N	86PRO	O	83.06
89GLY	N	87GLU	O	0.10
88THR	N	82ARG	O	0.02
88THR	N	85SER	O	84.47
87GLU	N	85SER	O	0.02
85SER	N	81PRO	O	1.10
85SER	N	82ARG	O	71.36
85SER	N	83LYS	O	0.00
84ILE	N	81PRO	O	71.61
84ILE	N	82ARG	O	0.02
83LYS	N	81PRO	O	1.36
80LEU	N	77TRP	O	25.67
80LEU	N	78ASP	O	0.95
79GLY	N	76LYS	O	0.00
79GLY	N	77TRP	O	0.03
78ASP	N	74GLY	O	0.68
78ASP	N	75PRO	O	39.34
78ASP	N	76LYS	O	1.46
77TRP	N	74GLY	O	7.21

188VAL	N	186ALA	O	0.04
187ASN	N	185LYS	O	2.50
184ASP	N	214GLN	O	96.94
183VAL	N	234VAL	O	78.39
182SER	N	212GLU	O	97.69
181VAL	N	232VAL	O	91.19
180VAL	N	210ALA	O	85.51
179HIS	N	177ARG	O	0.18
178LYS	N	173ALA	O	11.58
178LYS	N	176ARG	O	0.13
176ARG	N	172ALA	O	0.44
176ARG	N	173ALA	O	78.54
176ARG	N	174ARG	O	0.46
175LYS	N	171GLU	O	1.35
175LYS	N	172ALA	O	49.59
175LYS	N	173ALA	O	0.15
174ARG	N	170PHE	O	29.89
174ARG	N	171GLU	O	26.73
174ARG	N	172ALA	O	0.00
173ALA	N	169ALA	O	72.27
173ALA	N	170PHE	O	3.84
172ALA	N	168VAL	O	70.61
172ALA	N	169ALA	O	5.43
171GLU	N	167ARG	O	63.03
171GLU	N	168VAL	O	5.85
171GLU	N	169ALA	O	0.01
170PHE	N	166ALA	O	72.56
170PHE	N	167ARG	O	3.68
169ALA	N	165VAL	O	23.45
169ALA	N	166ALA	O	24.04
168VAL	N	164ARG	O	25.74
168VAL	N	165VAL	O	14.40
168VAL	N	166ALA	O	0.01
167ARG	N	163GLU	O	78.20
167ARG	N	164ARG	O	3.79
167ARG	N	165VAL	O	0.00
166ALA	N	162VAL	O	63.13
166ALA	N	163GLU	O	6.72
165VAL	N	161GLU	O	8.14
165VAL	N	162VAL	O	23.40
164ARG	N	160PRO	O	40.41
164ARG	N	161GLU	O	20.07
163GLU	N	159LYS	O	61.18

90LEU	N	86PRO	O	65.87
90LEU	N	87GLU	O	10.93
89GLY	N	85ARG	O	0.56
89GLY	N	86PRO	O	84.47
89GLY	N	87GLU	O	0.18
88THR	N	85ARG	O	76.94
87GLU	N	85ARG	O	1.63
85ARG	N	81PRO	O	0.20
85ARG	N	82ARG	O	75.50
85ARG	N	83LYS	O	0.04
84ILE	N	81PRO	O	94.27
84ILE	N	82ARG	O	0.04
83LYS	N	81PRO	O	0.06
80LEU	N	76LYS	O	0.27
80LEU	N	77TRP	O	64.59
80LEU	N	78ASP	O	0.44
79GLY	N	75PRO	O	1.93
79GLY	N	76LYS	O	3.08
79GLY	N	77TRP	O	0.04
78ASP	N	74GLY	O	9.74
78ASP	N	75PRO	O	45.87
78ASP	N	76LYS	O	0.38
77TRP	N	73GLY	O	0.00
77TRP	N	74GLY	O	40.51
77TRP	N	75PRO	O	0.84
76LYS	N	74GLY	O	8.82
74GLY	N	274GLY	O	0.00
74GLY	N	275SER	O	2.82
73GLY	N	9ASP	O	11.68
71SER	N	7PRO	O	21.11
70GLY	N	6LEU	O	33.47
70GLY	N	7PRO	O	0.01
69LEU	N	269PHE	O	95.30
68LEU	N	4ALA	O	99.18
67VAL	N	267PRO	O	91.25
66ALA	N	2LYS	O	3.06
65GLU	N	2LYS	O	99.00
64ALA	N	60GLY	O	20.89
64ALA	N	61VAL	O	8.94
64ALA	N	62GLU	O	4.71
63GLU	N	59LYS	O	12.66
63GLU	N	60GLY	O	30.33
63GLU	N	61VAL	O	0.22

170PHE	N	166ALA	O	93.79
170PHE	N	167ARG	O	0.65
169ALA	N	165VAL	O	66.56
169ALA	N	166ALA	O	4.52
168VAL	N	164ARG	O	26.39
168VAL	N	165VAL	O	27.56
168VAL	N	166ALA	O	0.10
167ARG	N	163GLU	O	84.23
167ARG	N	164ARG	O	2.02
166ALA	N	162VAL	O	66.33
166ALA	N	163GLU	O	3.44
165VAL	N	161GLU	O	19.36
165VAL	N	162VAL	O	9.77
165VAL	N	163GLU	O	0.02
164ARG	N	160PRO	O	72.53
164ARG	N	161GLU	O	3.35
163GLU	N	159LYS	O	80.38
163GLU	N	160PRO	O	1.94
162VAL	N	158SER	O	38.91
162VAL	N	159LYS	O	20.02
162VAL	N	160PRO	O	0.02
161GLU	N	158SER	O	36.15
161GLU	N	159LYS	O	0.03
159LYS	N	149ALA	O	95.89
157TYR	N	151ALA	O	83.15
156ARG	N	137GLY	O	0.47
155GLU	N	153ASN	O	90.46
154THR	N	141GLY	O	0.40
154THR	N	143PRO	O	1.50
153ASN	N	155GLU	O	97.20
152TRP	N	145GLY	O	98.84
151ALA	N	157TYR	O	91.66
150GLU	N	147SER	O	4.47
150GLU	N	148GLU	O	0.00
149ALA	N	147SER	O	1.24
147SER	N	147SER	O	0.00
147SER	N	150GLU	O	46.32
145GLY	N	152TRP	O	87.48
142GLU	N	140PHE	O	0.01
141GLY	N	137GLY	O	0.93
141GLY	N	138ILE	O	22.58
141GLY	N	139TYR	O	0.01
140PHE	N	136GLY	O	0.15

77TRP	N	75PRO	O	0.09	163GLU	N	160PRO	O	2.30	62GLU	N	58ARG	O	4.24	140PHE	N	137GLY	O	93.48
76LYS	N	74GLY	O	0.11	162VAL	N	158SER	O	45.77	62GLU	N	59LYS	O	2.27	139TYR	N	137GLY	O	0.00
74GLY	N	77TRP	O	0.40	162VAL	N	159LYS	O	15.59	62GLU	N	60GLY	O	0.01	137GLY	N	140PHE	O	0.00
73GLY	N	9ASP	O	6.49	162VAL	N	160PRO	O	0.08	61VAL	N	57THR	O	86.74	137GLY	N	156ARG	O	43.96
71SER	N	7PRO	O	0.97	161GLU	N	158SER	O	48.33	61VAL	N	58ARG	O	0.22	135THR	N	100PHE	O	0.00
70GLY	N	6LEU	O	14.10	161GLU	N	159LYS	O	0.04	60GLY	N	56PRO	O	5.54	134LEU	N	100PHE	O	25.36
70GLY	N	7PRO	O	0.00	159LYS	N	149ALA	O	91.87	60GLY	N	57THR	O	60.43	133GLU	N	235THR	O	51.65
69LEU	N	269PHE	O	92.25	157TYR	N	151ALA	O	97.39	60GLY	N	58ARG	O	0.26	133GLU	N	236GLY	O	5.54
68LEU	N	4ALA	O	99.40	155GLU	N	153ASN	O	98.54	59LYS	N	55GLU	O	58.61	132ARG	N	102ASN	O	96.78
67VAL	N	267PRO	O	89.70	154THR	N	138ILE	O	0.02	59LYS	N	56PRO	O	7.83	132ARG	N	130ILE	O	0.02
66ALA	N	2LYS	O	2.09	154THR	N	141GLY	O	0.26	59LYS	N	57THR	O	0.00	131VAL	N	233VAL	O	97.71
65GLU	N	2LYS	O	98.70	154THR	N	143PRO	O	7.01	58ARG	N	54PRO	O	89.62	130ILE	N	104ARG	O	95.02
64ALA	N	60GLY	O	17.89	153ASN	N	155GLU	O	98.36	58ARG	N	55GLU	O	3.34	129LEU	N	230PHE	O	0.01
64ALA	N	61VAL	O	17.87	152TRP	N	145GLY	O	98.35	57THR	N	54PRO	O	12.09	129LEU	N	231ASP	O	87.44
64ALA	N	62GLU	O	4.00	151ALA	N	157TYR	O	86.62	53PHE	N	51GLU	O	0.01	128VAL	N	106ALA	O	84.84
63GLU	N	59LYS	O	7.62	150GLU	N	147SER	O	4.92	51GLU	N	45ALA	O	4.25	127ASP	N	125GLY	O	0.00
63GLU	N	60GLY	O	22.16	149ALA	N	147SER	O	2.53	51GLU	N	49PHE	O	1.67	126VAL	N	108VAL	O	0.00
63GLU	N	61VAL	O	0.71	147SER	N	150GLU	O	47.29	50GLY	N	45ALA	O	71.76	126VAL	N	123ALA	O	3.66
62GLU	N	58ARG	O	1.98	145GLY	N	152TRP	O	82.39	50GLY	N	46ILE	O	3.42	126VAL	N	124ARG	O	0.10
62GLU	N	59LYS	O	1.15	142GLU	N	140PHE	O	0.01	50GLY	N	47ASP	O	0.02	125GLY	N	123ALA	O	0.04
62GLU	N	60GLY	O	0.01	142GLU	N	154THR	O	0.00	49PHE	N	44ALA	O	0.03	124ARG	N	120GLU	O	14.81
61VAL	N	57THR	O	74.39	141GLY	N	137GLY	O	0.18	49PHE	N	45ALA	O	82.60	124ARG	N	121GLU	O	10.22
61VAL	N	58ARG	O	0.03	141GLY	N	138ILE	O	27.90	49PHE	N	46ILE	O	0.08	124ARG	N	122ILE	O	6.88
60GLY	N	56PRO	O	3.38	141GLY	N	139TYR	O	0.85	49PHE	N	47ASP	O	0.02	123ALA	N	119LYS	O	64.98
60GLY	N	57THR	O	69.95	141GLY	N	154THR	O	0.00	48ALA	N	44ALA	O	86.32	123ALA	N	120GLU	O	8.52
60GLY	N	58ARG	O	0.30	140PHE	N	136GLY	O	0.02	48ALA	N	45ALA	O	2.35	122ILE	N	119LYS	O	78.02
59LYS	N	55GLU	O	44.35	140PHE	N	137GLY	O	88.84	48ALA	N	46ILE	O	0.23	122ILE	N	120GLU	O	0.05
59LYS	N	56PRO	O	21.44	140PHE	N	138ILE	O	0.18	47ASP	N	43GLY	O	41.61	121GLU	N	119LYS	O	0.02
59LYS	N	57THR	O	0.03	139TYR	N	137GLY	O	2.33	47ASP	N	44ALA	O	17.23	119LYS	N	117PRO	O	82.09
58ARG	N	54PRO	O	90.38	138ILE	N	154THR	O	0.02	47ASP	N	45ALA	O	0.01	118LEU	N	116SER	O	0.02
58ARG	N	55GLU	O	3.10	137GLY	N	135THR	O	0.11	46ILE	N	42GLY	O	7.30	116SER	N	112LEU	O	2.21
57THR	N	54PRO	O	9.49	137GLY	N	140PHE	O	0.02	46ILE	N	43GLY	O	29.89	116SER	N	113GLU	O	67.07
53PHE	N	51GLU	O	0.02	137GLY	N	156ARG	O	0.10	45ALA	N	41PHE	O	0.02	116SER	N	114ARG	O	0.14
51GLU	N	45ALA	O	4.06	136GLY	N	133GLU	O	0.00	45ALA	N	42GLY	O	4.29	115LEU	N	111GLY	O	0.01
51GLU	N	49PHE	O	1.03	136GLY	N	134LEU	O	4.04	45ALA	N	43GLY	O	0.00	115LEU	N	112LEU	O	61.92
50GLY	N	45ALA	O	62.09	135THR	N	100PHE	O	0.09	44ALA	N	42GLY	O	0.00	115LEU	N	113GLU	O	0.08
50GLY	N	46ILE	O	6.44	134LEU	N	100PHE	O	88.64	43GLY	N	8GLY	O	0.11	114ARG	N	111GLY	O	0.29
49PHE	N	45ALA	O	82.38	133GLU	N	235THR	O	55.92	43GLY	N	41PHE	O	0.18	114ARG	N	112LEU	O	0.05
49PHE	N	46ILE	O	0.12	133GLU	N	236GLY	O	6.30	42GLY	N	52PRO	O	34.89	113GLU	N	109PHE	O	0.01
49PHE	N	47ASP	O	0.02	132ARG	N	102ASN	O	97.41	39PHE	N	5VAL	O	92.46	113GLU	N	111GLY	O	0.06
48ALA	N	44ALA	O	78.73	132ARG	N	130ILE	O	0.05	39PHE	N	37GLU	O	0.11	112LEU	N	109PHE	O	75.46
48ALA	N	45ALA	O	3.36	131VAL	N	233VAL	O	97.39	37GLU	N	3VAL	O	46.88	112LEU	N	110PRO	O	0.04
48ALA	N	46ILE	O	0.62	130ILE	N	104ARG	O	94.40	36TYR	N	34LEU	O	0.02	109PHE	N	107LYS	O	0.06

47ASP	N	43GLY	O	56.97
47ASP	N	44ALA	O	8.12
46ILE	N	42GLY	O	6.36
46ILE	N	43GLY	O	34.26
45ALA	N	41PHE	O	0.01
45ALA	N	42GLY	O	7.99
43GLY	N	8GLY	O	0.09
43GLY	N	41PHE	O	0.02
42GLY	N	52PRO	O	43.24
39PHE	N	5VAL	O	95.58
39PHE	N	37GLU	O	0.15
37GLU	N	3VAL	O	45.32
36TYR	N	34LEU	O	0.02
35ALA	N	1MET	O	55.89
35ALA	N	33GLY	O	0.26
32LEU	N	27ASP	O	0.04
31GLY	N	26LEU	O	89.43
31GLY	N	27ASP	O	0.14
30GLU	N	26LEU	O	66.87
30GLU	N	27ASP	O	0.38
30GLU	N	28GLU	O	0.02
29ALA	N	25ALA	O	82.49
29ALA	N	26LEU	O	3.07
29ALA	N	27ASP	O	0.16
28GLU	N	24ARG	O	50.33
28GLU	N	25ALA	O	13.40
28GLU	N	26LEU	O	0.01
27ASP	N	23LEU	O	81.38
27ASP	N	24ARG	O	1.45
26LEU	N	22VAL	O	80.99
26LEU	N	23LEU	O	3.78
25ALA	N	21LYS	O	50.23
25ALA	N	22VAL	O	10.58
25ALA	N	23LEU	O	0.00
24ARG	N	20LEU	O	96.63
24ARG	N	21LYS	O	0.17
23LEU	N	19ALA	O	71.59
23LEU	N	20LEU	O	1.42
22VAL	N	18ALA	O	47.80
22VAL	N	19ALA	O	7.44
21LYS	N	17GLU	O	79.82
21LYS	N	18ALA	O	2.88
20LEU	N	16THR	O	83.81

129LEU	N	231ASP	O	91.16
128VAL	N	106ALA	O	95.90
126VAL	N	123ALA	O	11.54
126VAL	N	124ARG	O	0.03
125GLY	N	123ALA	O	0.03
124ARG	N	120GLU	O	32.55
124ARG	N	121GLU	O	6.91
124ARG	N	122ILE	O	0.67
123ALA	N	119LYS	O	73.24
123ALA	N	120GLU	O	8.76
122ILE	N	119LYS	O	74.68
122ILE	N	120GLU	O	0.03
119LYS	N	117PRO	O	87.29
118LEU	N	116SER	O	0.02
116SER	N	112LEU	O	3.27
116SER	N	113GLU	O	53.23
116SER	N	114ARG	O	0.14
115LEU	N	111GLY	O	0.09
115LEU	N	112LEU	O	66.67
114ARG	N	111GLY	O	0.46
114ARG	N	112LEU	O	0.00
113GLU	N	109PHE	O	0.94
113GLU	N	111GLY	O	0.06
112LEU	N	109PHE	O	77.06
112LEU	N	110PRO	O	0.18
109PHE	N	107LYS	O	0.02
108VAL	N	126VAL	O	70.07
106ALA	N	128VAL	O	95.90
104ARG	N	130ILE	O	99.24
103LEU	N	260ALA	O	99.10
102ASN	N	132ARG	O	97.00
101ALA	N	262LEU	O	55.90
100PHE	N	98ASP	O	0.33
100PHE	N	262LEU	O	81.35
99LEU	N	94ARG	O	88.74
98ASP	N	93LEU	O	0.04
98ASP	N	94ARG	O	6.68
98ASP	N	95LYS	O	20.73
98ASP	N	96SER	O	0.05
98ASP	N	264ARG	O	0.00
97GLN	N	93LEU	O	61.79
97GLN	N	94ARG	O	1.33
96SER	N	92SER	O	79.74

35ALA	N	1MET	O	58.92
35ALA	N	33GLY	O	0.12
32LEU	N	26LEU	O	0.01
32LEU	N	27ASP	O	0.03
31GLY	N	26LEU	O	93.84
31GLY	N	27ASP	O	0.05
30GLU	N	26LEU	O	69.41
30GLU	N	27ASP	O	0.09
30GLU	N	28GLU	O	0.02
29ALA	N	25ALA	O	84.84
29ALA	N	26LEU	O	3.37
29ALA	N	27ASP	O	0.07
28GLU	N	24ARG	O	40.82
28GLU	N	25ALA	O	18.61
28GLU	N	26LEU	O	0.02
27ASP	N	23LEU	O	86.02
27ASP	N	24ARG	O	0.74
26LEU	N	22VAL	O	87.04
26LEU	N	23LEU	O	2.16
25ALA	N	21LYS	O	42.91
25ALA	N	22VAL	O	13.67
25ALA	N	23LEU	O	0.00
24ARG	N	20LEU	O	97.80
24ARG	N	21LYS	O	0.10
23LEU	N	19ALA	O	70.96
23LEU	N	20LEU	O	1.00
22VAL	N	18ALA	O	24.52
22VAL	N	19ALA	O	18.75
21LYS	N	17GLU	O	77.24
21LYS	N	18ALA	O	3.36
20LEU	N	16THR	O	89.69
20LEU	N	17GLU	O	1.28
19ALA	N	15VAL	O	54.35
19ALA	N	16THR	O	13.60
18ALA	N	14GLU	O	67.26
18ALA	N	15VAL	O	2.39
17GLU	N	13PRO	O	82.09
17GLU	N	14GLU	O	1.95
16THR	N	12GLY	O	74.04
16THR	N	13PRO	O	2.57
15VAL	N	11ILE	O	94.01
15VAL	N	12GLY	O	1.34
14GLU	N	11ILE	O	20.10

108VAL	N	126VAL	O	55.38
107LYS	N	105PRO	O	0.03
106ALA	N	128VAL	O	80.45
104ARG	N	130ILE	O	96.86
103LEU	N	260ALA	O	99.03
102ASN	N	100PHE	O	0.01
102ASN	N	132ARG	O	93.20
101ALA	N	262LEU	O	42.55
100PHE	N	98ASP	O	0.24
100PHE	N	262LEU	O	88.45
99LEU	N	94ARG	O	45.27
99LEU	N	97GLN	O	8.66
98ASP	N	94ARG	O	7.19
98ASP	N	95LYS	O	11.62
98ASP	N	96SER	O	0.65
98ASP	N	264ARG	O	0.16
97GLN	N	92SER	O	0.99
97GLN	N	93LEU	O	12.89
97GLN	N	94ARG	O	16.44
97GLN	N	95LYS	O	0.33
96SER	N	91LEU	O	0.16
96SER	N	92SER	O	46.13
96SER	N	93LEU	O	11.02
96SER	N	94ARG	O	0.34
96SER	N	97GLN	O	0.37
95LYS	N	91LEU	O	49.08
95LYS	N	92SER	O	23.62
95LYS	N	93LEU	O	1.45
94ARG	N	90LEU	O	4.55
94ARG	N	91LEU	O	42.75
94ARG	N	92SER	O	0.93
93LEU	N	89GLY	O	13.36
93LEU	N	90LEU	O	16.06
93LEU	N	91LEU	O	0.01
92SER	N	86PRO	O	0.04
92SER	N	87GLU	O	0.01
92SER	N	88THR	O	1.96
92SER	N	89GLY	O	12.32
92SER	N	90LEU	O	0.03
91LEU	N	86PRO	O	14.22
91LEU	N	87GLU	O	2.78
91LEU	N	88THR	O	0.07
91LEU	N	89GLY	O	4.96

20LEU	N	17GLU	O	2.70
19ALA	N	15VAL	O	61.14
19ALA	N	16THR	O	8.49
18ALA	N	14GLU	O	63.15
18ALA	N	15VAL	O	2.34
17GLU	N	13PRO	O	72.93
17GLU	N	14GLU	O	2.90
17GLU	N	15VAL	O	0.00
16THR	N	12GLY	O	84.34
16THR	N	13PRO	O	1.42
15VAL	N	11ILE	O	88.60
15VAL	N	12GLY	O	2.08
14GLU	N	11ILE	O	29.49
14GLU	N	12GLY	O	0.00
12GLY	N	9ASP	O	2.53
12GLY	N	71SER	O	0.29
11ILE	N	9ASP	O	0.00
11ILE	N	275SER	O	5.25
9ASP	N	71SER	O	95.62
8GLY	N	41PHE	O	22.92
6LEU	N	68LEU	O	98.51
5VAL	N	37GLU	O	93.67
4ALA	N	66ALA	O	96.02
3VAL	N	1MET	O	0.01
3VAL	N	35ALA	O	90.80

96SER	N	93LEU	O	3.89
96SER	N	94ARG	O	0.24
95LYS	N	91LEU	O	81.32
95LYS	N	92SER	O	3.26
95LYS	N	93LEU	O	0.01
94ARG	N	90LEU	O	85.51
94ARG	N	91LEU	O	2.01
93LEU	N	89GLY	O	36.49
93LEU	N	90LEU	O	14.54
92SER	N	88THR	O	7.10
92SER	N	89GLY	O	20.86
92SER	N	90LEU	O	0.00
91LEU	N	86PRO	O	0.00
91LEU	N	87GLU	O	75.70
91LEU	N	88THR	O	0.20
91LEU	N	89GLY	O	0.01
90LEU	N	86PRO	O	68.17
90LEU	N	87GLU	O	9.61
89GLY	N	43GLY	O	0.01
89GLY	N	85SER	O	0.66
89GLY	N	86PRO	O	80.09
89GLY	N	87GLU	O	0.21
88THR	N	85SER	O	64.27
88THR	N	86PRO	O	0.00
87GLU	N	85SER	O	0.14
85SER	N	81PRO	O	0.64
85SER	N	82ARG	O	47.92
85SER	N	83LYS	O	0.50
84ILE	N	81PRO	O	93.04
84ILE	N	82ARG	O	0.03
83LYS	N	81PRO	O	2.94
80LEU	N	76LYS	O	1.19
80LEU	N	77TRP	O	39.56
80LEU	N	78ASP	O	0.38
79GLY	N	75PRO	O	0.62
79GLY	N	76LYS	O	6.09
79GLY	N	77TRP	O	0.11
79GLY	N	80LEU	O	0.01
78ASP	N	74GLY	O	21.88
78ASP	N	75PRO	O	32.33
78ASP	N	76LYS	O	0.70
77TRP	N	74GLY	O	66.48
77TRP	N	75PRO	O	0.62

14GLU	N	12GLY	O	0.00
12GLY	N	9ASP	O	2.50
12GLY	N	71SER	O	0.01
11ILE	N	9ASP	O	0.03
11ILE	N	275SER	O	0.64
11ILE	N	276ALA	O	0.03
11ILE	N	277PRO	O	0.00
9ASP	N	71SER	O	88.60
8GLY	N	41PHE	O	24.10
6LEU	N	68LEU	O	99.22
5VAL	N	37GLU	O	91.12
4ALA	N	66ALA	O	97.13
3VAL	N	35ALA	O	89.21

90LEU	N	85ARG	O	9.66
90LEU	N	86PRO	O	24.03
90LEU	N	87GLU	O	7.21
90LEU	N	88THR	O	0.13
89GLY	N	85ARG	O	38.50
89GLY	N	86PRO	O	22.20
89GLY	N	87GLU	O	0.01
88THR	N	82ARG	O	0.01
88THR	N	85ARG	O	50.17
88THR	N	86PRO	O	0.06
87GLU	N	85ARG	O	1.40
85ARG	N	81PRO	O	21.74
85ARG	N	82ARG	O	46.21
85ARG	N	83LYS	O	0.01
84ILE	N	81PRO	O	96.42
84ILE	N	82ARG	O	0.03
83LYS	N	81PRO	O	0.10
80LEU	N	76LYS	O	0.06
80LEU	N	77TRP	O	43.61
80LEU	N	78ASP	O	0.33
79GLY	N	75PRO	O	0.08
79GLY	N	76LYS	O	2.32
79GLY	N	77TRP	O	0.09
79GLY	N	80LEU	O	0.02
78ASP	N	74GLY	O	17.50
78ASP	N	75PRO	O	31.04
78ASP	N	76LYS	O	1.97
77TRP	N	74GLY	O	45.40
77TRP	N	75PRO	O	0.79
77TRP	N	78ASP	O	0.00
76LYS	N	74GLY	O	3.45
74GLY	N	77TRP	O	0.00
74GLY	N	275SER	O	0.04
73GLY	N	9ASP	O	19.94
71SER	N	7PRO	O	7.77
70GLY	N	6LEU	O	23.60
69LEU	N	67VAL	O	0.01
69LEU	N	269PHE	O	94.25
68LEU	N	4ALA	O	98.71
67VAL	N	267PRO	O	86.06
66ALA	N	2LYS	O	6.95
66ALA	N	64ALA	O	0.00
65GLU	N	2LYS	O	97.54

76LYS	N	74GLY	O	1.93
76LYS	N	78ASP	O	0.30
74GLY	N	72VAL	O	0.00
74GLY	N	274GLY	O	0.01
74GLY	N	275SER	O	0.04
74GLY	N	280ALA	O	0.26
73GLY	N	9ASP	O	2.53
73GLY	N	42GLY	O	0.02
73GLY	N	71SER	O	2.61
72VAL	N	70GLY	O	0.02
72VAL	N	72VAL	O	0.01
71SER	N	7PRO	O	2.83
70GLY	N	6LEU	O	26.42
70GLY	N	7PRO	O	0.40
69LEU	N	67VAL	O	0.00
69LEU	N	269PHE	O	95.38
68LEU	N	4ALA	O	98.08
67VAL	N	267PRO	O	90.05
66ALA	N	2LYS	O	6.10
65GLU	N	2LYS	O	97.41
64ALA	N	60GLY	O	19.00
64ALA	N	61VAL	O	12.75
64ALA	N	62GLU	O	5.12
63GLU	N	59LYS	O	10.31
63GLU	N	60GLY	O	35.32
63GLU	N	61VAL	O	0.66
62GLU	N	58ARG	O	11.36
62GLU	N	59LYS	O	2.22
62GLU	N	60GLY	O	0.05
61VAL	N	57THR	O	86.25
61VAL	N	58ARG	O	0.37
60GLY	N	56PRO	O	7.71
60GLY	N	57THR	O	56.06
60GLY	N	58ARG	O	0.44
59LYS	N	55GLU	O	67.99
59LYS	N	56PRO	O	8.03
58ARG	N	54PRO	O	93.00
58ARG	N	55GLU	O	1.88
57THR	N	54PRO	O	10.27
53PHE	N	51GLU	O	0.09
51GLU	N	45ALA	O	2.82
51GLU	N	49PHE	O	1.85
50GLY	N	45ALA	O	26.60

64ALA	N	60GLY	O	14.65
64ALA	N	61VAL	O	14.57
64ALA	N	62GLU	O	4.65
63GLU	N	59LYS	O	4.51
63GLU	N	60GLY	O	47.05
63GLU	N	61VAL	O	1.26
63GLU	N	64ALA	O	0.01
62GLU	N	58ARG	O	8.78
62GLU	N	59LYS	O	3.01
62GLU	N	60GLY	O	1.08
61VAL	N	57THR	O	73.77
61VAL	N	58ARG	O	0.87
61VAL	N	59LYS	O	0.01
60GLY	N	56PRO	O	4.75
60GLY	N	57THR	O	63.85
60GLY	N	58ARG	O	0.56
59LYS	N	55GLU	O	54.47
59LYS	N	56PRO	O	14.27
59LYS	N	57THR	O	0.03
58ARG	N	54PRO	O	86.62
58ARG	N	55GLU	O	4.78
57THR	N	54PRO	O	16.22
53PHE	N	51GLU	O	0.04
51GLU	N	45ALA	O	6.02
51GLU	N	49PHE	O	2.08
50GLY	N	45ALA	O	63.14
50GLY	N	46ILE	O	4.02
50GLY	N	47ASP	O	0.04
49PHE	N	44ALA	O	2.36
49PHE	N	45ALA	O	73.78
49PHE	N	46ILE	O	0.10
49PHE	N	47ASP	O	0.02
48ALA	N	43GLY	O	0.08
48ALA	N	44ALA	O	79.66
48ALA	N	45ALA	O	3.38
48ALA	N	46ILE	O	0.44
48ALA	N	49PHE	O	0.02
47ASP	N	43GLY	O	31.42
47ASP	N	44ALA	O	24.20
47ASP	N	45ALA	O	0.50
47ASP	N	49PHE	O	0.39
46ILE	N	42GLY	O	7.83
46ILE	N	43GLY	O	26.39

50GLY	N	46ILE	O	1.45
50GLY	N	47ASP	O	0.02
49PHE	N	44ALA	O	1.96
49PHE	N	45ALA	O	28.76
49PHE	N	46ILE	O	16.40
49PHE	N	47ASP	O	4.56
48ALA	N	44ALA	O	33.57
48ALA	N	45ALA	O	0.94
48ALA	N	46ILE	O	0.23
48ALA	N	49PHE	O	0.01
47ASP	N	43GLY	O	16.02
47ASP	N	44ALA	O	9.94
47ASP	N	45ALA	O	0.93
47ASP	N	49PHE	O	0.73
46ILE	N	42GLY	O	1.76
46ILE	N	43GLY	O	18.02
46ILE	N	44ALA	O	0.06
46ILE	N	49PHE	O	2.51
46ILE	N	51GLU	O	0.50
45ALA	N	41PHE	O	0.02
45ALA	N	42GLY	O	5.12
45ALA	N	43GLY	O	0.12
45ALA	N	47ASP	O	0.00
44ALA	N	8GLY	O	0.08
44ALA	N	41PHE	O	0.02
44ALA	N	42GLY	O	0.09
43GLY	N	8GLY	O	0.12
43GLY	N	41PHE	O	0.12
43GLY	N	45ALA	O	0.17
42GLY	N	52PRO	O	2.66
41PHE	N	39PHE	O	0.00
41PHE	N	45ALA	O	0.00
39PHE	N	5VAL	O	95.34
39PHE	N	37GLU	O	0.05
37GLU	N	3VAL	O	58.88
36TYR	N	34LEU	O	0.03
35ALA	N	1MET	O	61.24
35ALA	N	33GLY	O	0.09
34LEU	N	32LEU	O	0.00
32LEU	N	27ASP	O	0.14
31GLY	N	26LEU	O	85.34
31GLY	N	27ASP	O	0.61
30GLU	N	25ALA	O	0.00

46ILE	N	44ALA	O	0.00
45ALA	N	41PHE	O	0.01
45ALA	N	42GLY	O	8.12
45ALA	N	43GLY	O	0.06
44ALA	N	41PHE	O	0.01
44ALA	N	42GLY	O	0.02
43GLY	N	8GLY	O	0.18
43GLY	N	41PHE	O	0.20
42GLY	N	52PRO	O	34.13
41PHE	N	39PHE	O	0.01
41PHE	N	42GLY	O	0.03
39PHE	N	5VAL	O	93.16
39PHE	N	37GLU	O	0.12
37GLU	N	3VAL	O	58.00
36TYR	N	34LEU	O	0.05
35ALA	N	1MET	O	54.84
35ALA	N	33GLY	O	0.24
32LEU	N	26LEU	O	0.02
32LEU	N	27ASP	O	0.28
31GLY	N	26LEU	O	87.43
31GLY	N	27ASP	O	0.44
30GLU	N	26LEU	O	60.42
30GLU	N	27ASP	O	0.73
30GLU	N	28GLU	O	0.10
29ALA	N	25ALA	O	79.10
29ALA	N	26LEU	O	4.95
29ALA	N	27ASP	O	0.37
28GLU	N	24ARG	O	49.12
28GLU	N	25ALA	O	16.25
28GLU	N	26LEU	O	0.13
28GLU	N	30GLU	O	0.00
27ASP	N	23LEU	O	75.24
27ASP	N	24ARG	O	3.80
26LEU	N	22VAL	O	79.94
26LEU	N	23LEU	O	3.94
25ALA	N	21LYS	O	43.76
25ALA	N	22VAL	O	10.61
24ARG	N	20LEU	O	96.56
24ARG	N	21LYS	O	0.24
23LEU	N	19ALA	O	74.49
23LEU	N	20LEU	O	1.81
22VAL	N	18ALA	O	14.15
22VAL	N	19ALA	O	25.27

30GLU	N	26LEU	O	66.33
30GLU	N	27ASP	O	0.56
30GLU	N	28GLU	O	0.02
29ALA	N	25ALA	O	74.31
29ALA	N	26LEU	O	5.63
29ALA	N	27ASP	O	0.10
28GLU	N	24ARG	O	69.53
28GLU	N	25ALA	O	6.66
27ASP	N	23LEU	O	76.09
27ASP	N	24ARG	O	4.20
26LEU	N	22VAL	O	69.53
26LEU	N	23LEU	O	6.18
26LEU	N	24ARG	O	0.00
25ALA	N	21LYS	O	69.61
25ALA	N	22VAL	O	3.65
24ARG	N	20LEU	O	93.22
24ARG	N	21LYS	O	0.45
23LEU	N	19ALA	O	46.40
23LEU	N	20LEU	O	6.10
22VAL	N	18ALA	O	31.39
22VAL	N	19ALA	O	10.49
21LYS	N	17GLU	O	56.28
21LYS	N	18ALA	O	7.71
20LEU	N	16THR	O	75.02
20LEU	N	17GLU	O	5.42
19ALA	N	15VAL	O	46.23
19ALA	N	16THR	O	15.98
19ALA	N	17GLU	O	0.00
18ALA	N	14GLU	O	29.32
18ALA	N	15VAL	O	13.92
18ALA	N	16THR	O	0.00
17GLU	N	13PRO	O	67.91
17GLU	N	14GLU	O	6.29
16THR	N	12GLY	O	64.96
16THR	N	13PRO	O	3.54
15VAL	N	11ILE	O	74.85
15VAL	N	12GLY	O	3.32
14GLU	N	10GLY	O	0.05
14GLU	N	11ILE	O	26.00
12GLY	N	9ASP	O	0.97
12GLY	N	10GLY	O	0.86
12GLY	N	71SER	O	1.94
11ILE	N	9ASP	O	0.56

21LYS	N	17GLU	O	83.16
21LYS	N	18ALA	O	1.61
20LEU	N	16THR	O	87.83
20LEU	N	17GLU	O	1.23
19ALA	N	15VAL	O	46.79
19ALA	N	16THR	O	18.99
18ALA	N	14GLU	O	63.45
18ALA	N	15VAL	O	3.05
17GLU	N	13PRO	O	74.15
17GLU	N	14GLU	O	2.62
16THR	N	12GLY	O	77.37
16THR	N	13PRO	O	2.06
15VAL	N	11ILE	O	60.38
15VAL	N	12GLY	O	2.97
14GLU	N	10GLY	O	0.02
14GLU	N	11ILE	O	39.23
14GLU	N	12GLY	O	0.02
12GLY	N	9ASP	O	1.09
12GLY	N	71SER	O	0.01
11ILE	N	9ASP	O	0.47
11ILE	N	275SER	O	0.43
11ILE	N	276ALA	O	0.05
9ASP	N	71SER	O	81.51
8GLY	N	41PHE	O	35.55
6LEU	N	68LEU	O	98.43
5VAL	N	3VAL	O	0.00
5VAL	N	37GLU	O	93.68
4ALA	N	66ALA	O	95.32
3VAL	N	1MET	O	0.00
3VAL	N	35ALA	O	89.19

11ILE	N	71SER	O	0.04
11ILE	N	73GLY	O	5.18
11ILE	N	275SER	O	0.12
11ILE	N	280ALA	O	0.00
9ASP	N	42GLY	O	0.12
9ASP	N	71SER	O	27.65
8GLY	N	41PHE	O	30.98
6LEU	N	68LEU	O	98.04
5VAL	N	37GLU	O	93.99
4ALA	N	66ALA	O	95.42
3VAL	N	1MET	O	0.00
3VAL	N	35ALA	O	86.45
