# Supplementary material for: Deciphering the Dynamics of Non-Covalent Interactions Affecting Thermal Stability of a Protein: Molecular Dynamics Study on Point Mutant of Thermus thermophilus Isopropylmalate Dehydrogenase
Source: PLoS One. 2015 Dec 11;10(12):e0144294. doi: 10.1371/journal.pone.0144294 (PMC4689552; doi:10.1371/journal.pone.0144294)
Supplement: S4 Table — The color formatting indicates the percentage of time interaction existed is as in S1 Table. (PDF) [file pone.0144294.s006.pdf]

S4 Table. **Unique HBs between MS of *wt* and *mut* at 300 K and 337 K**. The color formatting that indicates the percentage of time interaction existed is as in Table S1.

1) *Wt* 300 K				
**Drnona**	**D**	**Arnona**	**A**	**percen**
1MET	N	65GLU	OE1	0.50
1MET	N	65GLU	OE2	0.02
340VAL	N	339THR	OG1	0.18
338ALA	N	337THR	OG1	0.06
335ALA	N	337THR	OG1	0.01
334GLU	N	333THR	OG1	0.32
331ALA	N	322THR	OG1	0.00
331ALA	N	330SER	OG	0.04
330SER	N	322THR	OG1	0.00
327LEU	N	326ASP	OD1	2.28
327LEU	N	326ASP	OD2	4.36
326ASP	N	326ASP	OD1	0.02
326ASP	N	326ASP	OD2	0.02
307LEU	N	306GLU	OE1	0.01
307LEU	N	306GLU	OE2	0.02
306GLU	N	306GLU	OE1	14.44
306GLU	N	306GLU	OE2	9.94
305VAL	N	306GLU	OE1	0.08
305VAL	N	306GLU	OE2	0.06
301ALA	N	300HIS	ND1	0.01
289ALA	N	286ASN	OD1	0.12
288THR	N	286ASN	OD1	84.70
288THR	N	286ASN	ND2	0.01
285ALA	N	333THR	OG1	0.12
281GLY	N	14GLU	OE2	0.00
279ILE	N	278ASP	OD1	0.01
279ILE	N	278ASP	OD2	0.01
278ASP	N	278ASP	OD1	0.04
278ASP	N	278ASP	OD2	0.02
276ALA	N	275SER	OG	0.02
274GLY	N	273HIS	ND1	0.07
270GLU	N	259SER	OG	0.01
266THR	N	97GLN	OE1	91.37
260ALA	N	259SER	OG	0.07
259SER	N	293SER	OG	7.24
255GLY	N	253SER	OG	31.48

2) *Wt* 337 K				
**Drnona**	**D**	**Arnona**	**A**	**percen**
1MET	N	65GLU	OE1	9.34
1MET	N	65GLU	OE2	8.34
340VAL	N	339THR	OG1	0.22
338ALA	N	337THR	OG1	0.27
335ALA	N	334GLU	OE1	0.07
334GLU	N	333THR	OG1	0.90
334GLU	N	334GLU	OE1	0.48
334GLU	N	334GLU	OE2	0.50
332GLY	N	334GLU	OE1	0.00
332GLY	N	334GLU	OE2	0.03
331ALA	N	322THR	OG1	0.01
330SER	N	322THR	OG1	0.24
329GLY	N	278ASP	OD1	0.88
329GLY	N	278ASP	OD2	0.02
326ASP	N	326ASP	OD1	0.60
326ASP	N	326ASP	OD2	0.66
306GLU	N	306GLU	OE1	2.08
306GLU	N	306GLU	OE2	0.51
305VAL	N	306GLU	OE1	0.05
305VAL	N	306GLU	OE2	0.01
290ALA	N	273HIS	NE2	0.03
289ALA	N	286ASN	OD1	0.01
289ALA	N	288THR	OG1	0.02
288THR	N	286ASN	OD1	75.46
286ASN	N	273HIS	NE2	0.20
286ASN	N	275SER	OG	0.06
286ASN	N	326ASP	OD1	0.04
286ASN	N	326ASP	OD2	0.00
285ALA	N	333THR	OG1	2.88
283GLY	N	14GLU	OE2	0.02
283GLY	N	333THR	OG1	0.00
281GLY	N	14GLU	OE2	0.01
279ILE	N	278ASP	OD2	0.01
278ASP	N	278ASP	OD1	0.05
278ASP	N	278ASP	OD2	0.01
276ALA	N	275SER	OG	8.58

3) *Mut* 300 K				
**Drnona**	**D**	**Arnona**	**A**	**percen**
1MET	N	65GLU	OE1	15.32
1MET	N	65GLU	OE2	24.08
345ALA	N	310LYS	NZ	0.00
338ALA	N	337THR	OG1	0.07
334GLU	N	333THR	OG1	0.07
326ASP	N	326ASP	OD1	0.01
326ASP	N	326ASP	OD2	0.02
306GLU	N	306GLU	OE2	0.00
290ALA	N	273HIS	NE2	0.02
289ALA	N	273HIS	NE2	0.04
288THR	N	286ASN	OD1	62.41
288THR	N	286ASN	ND2	0.04
288THR	N	288THR	OG1	0.00
286ASN	N	326ASP	OD1	0.03
286ASN	N	326ASP	OD2	0.38
285ALA	N	333THR	OG1	4.19
284ILE	N	333THR	OG1	0.00
281GLY	N	14GLU	OE2	0.02
279ILE	N	278ASP	OD1	0.02
278ASP	N	278ASP	OD1	0.11
278ASP	N	278ASP	OD2	0.02
276ALA	N	275SER	OG	0.05
275SER	N	71SER	OG	0.02
274GLY	N	71SER	OG	1.56
274GLY	N	273HIS	ND1	0.04
273HIS	N	71SER	OG	0.15
266THR	N	97GLN	OE1	91.74
264ARG	N	98ASP	OD1	0.00
264ARG	N	98ASP	OD2	0.00
260ALA	N	102ASN	OD1	0.01
259SER	N	293SER	OG	0.18
255GLY	N	253SER	OG	4.54
255GLY	N	326ASP	OD1	0.00
255GLY	N	326ASP	OD2	0.04
254LEU	N	253SER	OG	0.29
254LEU	N	326ASP	OD1	0.02

4) *Mut* 337 K			
**Drnona**	**D**	**Arnona**	**A**
1MET	N	65GLU	OE1
1MET	N	65GLU	OE2
345ALA	N	310LYS	NZ
340VAL	N	339THR	OG1
338ALA	N	337THR	OG1
335ALA	N	337THR	OG1
334GLU	N	333THR	OG1
334GLU	N	334GLU	OE1
334GLU	N	334GLU	OE2
332GLY	N	286ASN	OD1
332GLY	N	322THR	OG1
331ALA	N	330SER	OG
306GLU	N	306GLU	OE1
306GLU	N	306GLU	OE2
305VAL	N	306GLU	OE1
301ALA	N	300HIS	ND1
290ALA	N	273HIS	NE2
290ALA	N	286ASN	OD1
289ALA	N	273HIS	NE2
289ALA	N	286ASN	OD1
289ALA	N	286ASN	ND2
289ALA	N	288THR	OG1
288THR	N	286ASN	OD1
288THR	N	286ASN	ND2
288THR	N	288THR	OG1
285ALA	N	333THR	OG1
283GLY	N	14GLU	OE1
283GLY	N	14GLU	OE2
282LYS	N	14GLU	OE1
282LYS	N	14GLU	OE2
281GLY	N	14GLU	OE1
281GLY	N	14GLU	OE2
281GLY	N	333THR	OG1
278ASP	N	278ASP	OD1
278ASP	N	278ASP	OD2
276ALA	N	275SER	OG

255GLY	N	326ASP	OD1	0.16
255GLY	N	326ASP	OD2	0.02
254LEU	N	253SER	OG	0.00
254LEU	N	326ASP	OD1	5.05
254LEU	N	326ASP	OD2	4.04
249VAL	N	248SER	OG	0.00
245ASP	N	245ASP	OD2	0.00
242ILE	N	241ASP	OD1	3.30
241ASP	N	237ASN	OD1	0.09
241ASP	N	241ASP	OD1	0.24
240GLY	N	237ASN	OD1	0.00
237ASN	N	157TYR	OH	1.67
236GLY	N	235THR	OG1	24.58
232VAL	N	231ASP	OD2	0.77
228ALA	N	226SER	OG	0.14
218ALA	N	217ASP	OD1	3.01
218ALA	N	217ASP	OD2	1.58
217ASP	N	217ASP	OD1	17.66
217ASP	N	217ASP	OD2	22.19
215TYR	N	214GLN	OE1	0.20
215TYR	N	214GLN	NE2	0.00
213HIS	N	212GLU	OE1	1.48
213HIS	N	212GLU	OE2	1.73
209VAL	N	208ASP	OD1	0.01
208ASP	N	208ASP	OD1	2.94
208ASP	N	208ASP	OD2	1.57
191VAL	N	153ASN	OD1	95.60
190GLU	N	190GLU	OE1	6.73
190GLU	N	190GLU	OE2	2.10
190GLU	N	144ARG	NH2	0.01
190GLU	N	153ASN	OD1	35.92
190GLU	N	153ASN	ND2	0.51
188VAL	N	187ASN	OD1	0.13
187ASN	N	187ASN	OD1	2.68
187ASN	N	187ASN	ND2	0.50
186ALA	N	184ASP	OD1	19.57
186ALA	N	184ASP	OD2	31.30
185LYS	N	184ASP	OD1	0.00
185LYS	N	184ASP	OD2	1.31

276ALA	N	326ASP	OD1	0.14
276ALA	N	326ASP	OD2	1.17
275SER	N	326ASP	OD1	6.42
275SER	N	326ASP	OD2	17.63
274GLY	N	273HIS	ND1	0.19
273HIS	N	273HIS	ND1	0.05
270GLU	N	293SER	OG	0.01
268VAL	N	261SER	OG	0.03
266THR	N	62GLU	OE1	7.34
266THR	N	62GLU	OE2	7.21
266THR	N	97GLN	OE1	27.61
266THR	N	97GLN	NE2	0.90
264ARG	N	98ASP	OD1	0.75
264ARG	N	98ASP	OD2	0.82
260ALA	N	102ASN	OD1	0.04
260ALA	N	259SER	OG	0.04
259SER	N	293SER	OG	1.94
256LEU	N	253SER	OG	0.06
255GLY	N	253SER	OG	1.11
249VAL	N	248SER	OG	0.01
245ASP	N	244SER	OG	0.63
242ILE	N	241ASP	OD2	0.01
238ILE	N	237ASN	ND2	0.00
237ASN	N	133GLU	OE1	0.00
237ASN	N	157TYR	OH	16.31
237ASN	N	237ASN	ND2	0.01
236GLY	N	235THR	OG1	22.62
232VAL	N	231ASP	OD1	0.17
232VAL	N	231ASP	OD2	0.10
231ASP	N	179HIS	ND1	0.00
229ARG	N	226SER	OG	0.07
228ALA	N	226SER	OG	0.95
218ALA	N	217ASP	OD1	4.34
218ALA	N	217ASP	OD2	1.09
217ASP	N	217ASP	OD1	30.59
217ASP	N	217ASP	OD2	7.10
215TYR	N	214GLN	OE1	7.00
215TYR	N	214GLN	NE2	0.04
214GLN	N	184ASP	OD1	0.04

254LEU	N	326ASP	OD2	1.75
254LEU	N	225ARG	NH1	0.01
245ASP	N	244SER	OG	0.09
242ILE	N	241ASP	OD1	0.01
242ILE	N	241ASP	OD2	0.02
237ASN	N	157TYR	OH	8.90
236GLY	N	235THR	OG1	37.03
232VAL	N	231ASP	OD2	0.00
218ALA	N	217ASP	OD1	5.42
218ALA	N	217ASP	OD2	0.23
217ASP	N	217ASP	OD1	30.69
217ASP	N	217ASP	OD2	0.00
215TYR	N	214GLN	OE1	9.31
215TYR	N	214GLN	NE2	0.20
213HIS	N	212GLU	OE1	9.49
213HIS	N	212GLU	OE2	10.40
213HIS	N	213HIS	ND1	0.07
209VAL	N	208ASP	OD1	0.10
209VAL	N	208ASP	OD2	0.08
208ASP	N	208ASP	OD1	18.73
208ASP	N	208ASP	OD2	21.77
191VAL	N	153ASN	OD1	97.82
190GLU	N	190GLU	OE1	6.06
190GLU	N	190GLU	OE2	2.88
190GLU	N	153ASN	OD1	28.81
190GLU	N	153ASN	ND2	1.01
188VAL	N	187ASN	OD1	0.16
187ASN	N	187ASN	OD1	2.91
187ASN	N	187ASN	ND2	0.24
186ALA	N	184ASP	OD1	29.62
186ALA	N	184ASP	OD2	46.68
185LYS	N	184ASP	OD1	0.01
185LYS	N	184ASP	OD2	2.32
183VAL	N	182SER	OG	0.36
183VAL	N	195TRP	NE1	0.00
180VAL	N	179HIS	ND1	0.00
179HIS	N	231ASP	OD2	0.63
178LYS	N	231ASP	OD2	0.46
177ARG	N	177ARG	NE	1.22

274GLY	N	273HIS	ND1
274GLY	N	286ASN	ND2
273HIS	N	273HIS	ND1
270GLU	N	259SER	OG
268VAL	N	261SER	OG
266THR	N	62GLU	OE1
266THR	N	62GLU	OE2
266THR	N	97GLN	OE1
266THR	N	97GLN	NE2
265GLY	N	264ARG	NE
265GLY	N	264ARG	NH1
264ARG	N	98ASP	OD1
260ALA	N	102ASN	OD1
260ALA	N	259SER	OG
259SER	N	293SER	OG
256LEU	N	253SER	OG
255GLY	N	253SER	OG
255GLY	N	286ASN	ND2
255GLY	N	326ASP	OD1
255GLY	N	326ASP	OD2
254LEU	N	253SER	OG
254LEU	N	326ASP	OD1
254LEU	N	326ASP	OD2
249VAL	N	248SER	OG
245ASP	N	244SER	OG
241ASP	N	237ASN	OD1
240GLY	N	235THR	OG1
238ILE	N	155GLU	OE2
238ILE	N	157TYR	OH
237ASN	N	155GLU	OE2
237ASN	N	157TYR	OH
237ASN	N	237ASN	OD1
237ASN	N	237ASN	ND2
236GLY	N	235THR	OG1
232VAL	N	231ASP	OD1
232VAL	N	231ASP	OD2
231ASP	N	179HIS	ND1
229ARG	N	226SER	OG
219MET	N	214GLN	OE1

180VAL	N	179HIS	ND1	0.04
179HIS	N	231ASP	OD2	0.14
178LYS	N	231ASP	OD2	0.11
177ARG	N	231ASP	OD1	0.12
177ARG	N	231ASP	OD2	93.14
161GLU	N	161GLU	OE2	0.04
158SER	N	161GLU	OE1	2.28
158SER	N	161GLU	OE2	0.58
156ARG	N	155GLU	OE1	64.35
150GLU	N	147SER	OG	58.01
149ALA	N	147SER	OG	13.16
148GLU	N	147SER	OG	0.98
148GLU	N	148GLU	OE1	0.23
148GLU	N	148GLU	OE2	0.08
145GLY	N	152TRP	NE1	0.08
144ARG	N	142GLU	OE1	0.76
144ARG	N	142GLU	OE2	0.93
142GLU	N	142GLU	OE1	0.01
141GLY	N	144ARG	NH1	0.00
138ILE	N	155GLU	OE1	4.04
138ILE	N	155GLU	OE2	56.96
137GLY	N	155GLU	OE1	0.11
137GLY	N	155GLU	OE2	0.01
136GLY	N	133GLU	OE1	32.94
136GLY	N	133GLU	OE2	35.00
135THR	N	133GLU	OE1	38.45
135THR	N	133GLU	OE2	58.56
134LEU	N	133GLU	OE1	25.95
134LEU	N	133GLU	OE2	43.81
121GLU	N	121GLU	OE1	0.71
121GLU	N	121GLU	OE2	0.08
120GLU	N	120GLU	OE1	1.93
120GLU	N	120GLU	OE2	2.18
118LEU	N	116SER	OG	62.15
114ARG	N	113GLU	OE1	0.02
113GLU	N	113GLU	OE1	97.08
112LEU	N	113GLU	OE1	10.11
111GLY	N	113GLU	OE1	90.65
111GLY	N	113GLU	OE2	8.72

213HIS	N	212GLU	OE1	7.62
213HIS	N	212GLU	OE2	7.93
211LEU	N	204ARG	NH1	0.02
208ASP	N	208ASP	OD1	0.08
208ASP	N	208ASP	OD2	0.04
191VAL	N	153ASN	OD1	92.95
190GLU	N	190GLU	OE1	2.84
190GLU	N	190GLU	OE2	2.32
190GLU	N	153ASN	OD1	28.36
190GLU	N	153ASN	ND2	0.21
188VAL	N	187ASN	OD1	0.30
187ASN	N	187ASN	OD1	4.82
187ASN	N	187ASN	ND2	0.02
186ALA	N	184ASP	OD1	2.36
186ALA	N	184ASP	OD2	97.30
185LYS	N	184ASP	OD1	0.03
185LYS	N	184ASP	OD2	0.21
180VAL	N	179HIS	ND1	0.02
179HIS	N	231ASP	OD1	4.66
179HIS	N	231ASP	OD2	6.81
178LYS	N	231ASP	OD1	6.91
178LYS	N	231ASP	OD2	6.78
177ARG	N	177ARG	NE	0.06
177ARG	N	231ASP	OD1	36.55
177ARG	N	231ASP	OD2	36.09
161GLU	N	158SER	OG	0.02
161GLU	N	161GLU	OE1	0.08
161GLU	N	161GLU	OE2	0.08
159LYS	N	158SER	OG	0.03
158SER	N	161GLU	OE1	0.36
158SER	N	161GLU	OE2	0.65
156ARG	N	155GLU	OE1	0.82
156ARG	N	155GLU	OE2	2.67
150GLU	N	147SER	OG	43.02
149ALA	N	147SER	OG	13.95
149ALA	N	148GLU	OE1	0.04
149ALA	N	148GLU	OE2	0.07
148GLU	N	147SER	OG	4.11
148GLU	N	148GLU	OE1	0.51

177ARG	N	177ARG	NH2	0.08
177ARG	N	231ASP	OD1	3.14
177ARG	N	231ASP	OD2	6.82
161GLU	N	158SER	OG	1.60
161GLU	N	161GLU	OE1	0.02
158SER	N	161GLU	OE1	19.76
158SER	N	161GLU	OE2	18.63
156ARG	N	155GLU	OE1	6.39
150GLU	N	147SER	OG	53.83
149ALA	N	147SER	OG	9.39
148GLU	N	147SER	OG	1.58
145GLY	N	152TRP	NE1	0.04
144ARG	N	142GLU	OE1	0.18
144ARG	N	142GLU	OE2	0.28
142GLU	N	142GLU	OE1	0.03
142GLU	N	142GLU	OE2	0.01
141GLY	N	144ARG	NH1	0.10
138ILE	N	155GLU	OE1	88.73
138ILE	N	155GLU	OE2	9.73
136GLY	N	133GLU	OE1	42.02
136GLY	N	133GLU	OE2	29.13
135THR	N	133GLU	OE1	62.27
135THR	N	133GLU	OE2	36.61
134LEU	N	133GLU	OE1	10.01
134LEU	N	133GLU	OE2	1.26
121GLU	N	120GLU	OE2	0.00
121GLU	N	121GLU	OE1	2.53
121GLU	N	121GLU	OE2	8.81
118LEU	N	116SER	OG	32.18
114ARG	N	113GLU	OE2	0.01
113GLU	N	113GLU	OE1	13.11
113GLU	N	113GLU	OE2	76.81
112LEU	N	113GLU	OE1	0.14
112LEU	N	113GLU	OE2	0.38
111GLY	N	113GLU	OE1	52.92
111GLY	N	113GLU	OE2	54.40
97GLN	N	96SER	OG	0.08
93LEU	N	92SER	OG	0.14
87GLU	N	87GLU	OE1	27.84

218ALA	N	217ASP	OD1
218ALA	N	217ASP	OD2
218ALA	N	245ASP	OD1
218ALA	N	245ASP	OD2
217ASP	N	217ASP	OD1
217ASP	N	217ASP	OD2
215TYR	N	213HIS	ND1
215TYR	N	214GLN	OE1
215TYR	N	214GLN	NE2
214GLN	N	182SER	OG
214GLN	N	184ASP	OD1
213HIS	N	212GLU	OE1
213HIS	N	212GLU	OE2
209VAL	N	208ASP	OD1
209VAL	N	208ASP	OD2
208ASP	N	208ASP	OD1
208ASP	N	208ASP	OD2
191VAL	N	153ASN	OD1
191VAL	N	153ASN	ND2
190GLU	N	190GLU	OE1
190GLU	N	190GLU	OE2
190GLU	N	153ASN	OD1
190GLU	N	153ASN	ND2
188VAL	N	187ASN	OD1
187ASN	N	187ASN	OD1
187ASN	N	187ASN	ND2
186ALA	N	184ASP	OD1
186ALA	N	184ASP	OD2
186ALA	N	187ASN	OD1
186ALA	N	196ARG	NH2
185LYS	N	184ASP	OD1
185LYS	N	184ASP	OD2
183VAL	N	182SER	OG
180VAL	N	179HIS	ND1
179HIS	N	231ASP	OD2
178LYS	N	231ASP	OD2
177ARG	N	177ARG	NE
177ARG	N	231ASP	OD1
177ARG	N	231ASP	OD2

97GLN	N	96SER	OG	0.12
93LEU	N	92SER	OG	0.41
88THR	N	85SER	OG	10.96
87GLU	N	85SER	OG	4.84
87GLU	N	87GLU	OE1	0.85
87GLU	N	87GLU	OE2	0.46
85SER	N	88THR	OG1	2.60
79GLY	N	78ASP	OD1	0.03
79GLY	N	78ASP	OD2	0.01
73GLY	N	9ASP	OD1	0.00
73GLY	N	9ASP	OD2	1.46
73GLY	N	77TRP	NE1	6.95
73GLY	N	275SER	OG	0.06
65GLU	N	65GLU	OE1	0.00
55GLU	N	51GLU	OE1	0.00
55GLU	N	51GLU	OE2	0.00
55GLU	N	55GLU	OE1	0.01
55GLU	N	55GLU	OE2	0.04
53PHE	N	51GLU	OE1	0.06
53PHE	N	51GLU	OE2	0.03
51GLU	N	51GLU	OE1	0.00
51GLU	N	51GLU	OE2	0.00
48ALA	N	47ASP	OD1	0.00
44ALA	N	9ASP	OD1	1.14
44ALA	N	9ASP	OD2	1.41
43GLY	N	9ASP	OD1	0.34
43GLY	N	9ASP	OD2	3.42
38VAL	N	37GLU	OE1	3.68
38VAL	N	37GLU	OE2	3.48
34LEU	N	27ASP	OD1	27.29
34LEU	N	27ASP	OD2	56.27
33GLY	N	27ASP	OD1	33.00
33GLY	N	27ASP	OD2	62.98
32LEU	N	27ASP	OD1	26.17
32LEU	N	27ASP	OD2	40.20
30GLU	N	30GLU	OE2	0.02
12GLY	N	71SER	OG	5.48
12GLY	N	275SER	OG	51.51
11ILE	N	71SER	OG	0.00

148GLU	N	148GLU	OE2	0.40
145GLY	N	152TRP	NE1	0.09
145GLY	N	190GLU	OE2	0.00
144ARG	N	142GLU	OE1	1.08
144ARG	N	142GLU	OE2	1.34
142GLU	N	142GLU	OE1	0.70
142GLU	N	142GLU	OE2	0.42
141GLY	N	144ARG	NH1	0.08
139TYR	N	237ASN	ND2	0.00
138ILE	N	155GLU	OE1	9.72
138ILE	N	155GLU	OE2	24.31
137GLY	N	155GLU	OE1	0.86
137GLY	N	155GLU	OE2	5.54
137GLY	N	157TYR	OH	1.00
136GLY	N	133GLU	OE1	14.44
136GLY	N	133GLU	OE2	42.53
136GLY	N	135THR	OG1	0.02
136GLY	N	139TYR	OH	0.12
136GLY	N	157TYR	OH	0.20
136GLY	N	237ASN	ND2	0.26
135THR	N	133GLU	OE1	61.99
135THR	N	133GLU	OE2	43.80
134LEU	N	133GLU	OE1	11.26
134LEU	N	133GLU	OE2	3.61
121GLU	N	120GLU	OE2	0.00
121GLU	N	121GLU	OE1	0.88
121GLU	N	121GLU	OE2	1.26
120GLU	N	120GLU	OE1	9.57
120GLU	N	120GLU	OE2	18.90
118LEU	N	116SER	OG	77.19
114ARG	N	113GLU	OE1	9.51
114ARG	N	113GLU	OE2	10.61
113GLU	N	113GLU	OE1	20.20
113GLU	N	113GLU	OE2	45.67
112LEU	N	113GLU	OE1	0.27
112LEU	N	113GLU	OE2	0.56
111GLY	N	113GLU	OE1	46.00
111GLY	N	113GLU	OE2	52.72
111GLY	N	114ARG	NE	0.04

87GLU	N	87GLU	OE2	8.79
82ARG	N	82ARG	NE	0.02
79GLY	N	78ASP	OD1	0.00
79GLY	N	78ASP	OD2	0.04
78ASP	N	78ASP	OD2	0.39
76LYS	N	78ASP	OD2	4.68
74GLY	N	275SER	OG	2.86
73GLY	N	9ASP	OD1	1.94
73GLY	N	9ASP	OD2	20.12
72VAL	N	71SER	OG	0.01
65GLU	N	65GLU	OE2	0.01
55GLU	N	51GLU	OE1	0.00
55GLU	N	55GLU	OE1	0.01
55GLU	N	55GLU	OE2	0.03
53PHE	N	51GLU	OE1	0.30
53PHE	N	51GLU	OE2	0.48
51GLU	N	51GLU	OE1	0.01
44ALA	N	9ASP	OD1	9.20
44ALA	N	9ASP	OD2	2.04
43GLY	N	9ASP	OD1	7.48
43GLY	N	9ASP	OD2	28.01
38VAL	N	37GLU	OE1	6.37
38VAL	N	37GLU	OE2	6.37
34LEU	N	27ASP	OD1	71.55
34LEU	N	27ASP	OD2	6.56
33GLY	N	27ASP	OD1	98.87
33GLY	N	27ASP	OD2	0.09
32LEU	N	27ASP	OD1	67.95
14GLU	N	14GLU	OE1	0.01
12GLY	N	71SER	OG	0.10
10GLY	N	9ASP	OD1	4.26
10GLY	N	9ASP	OD2	3.10
2LYS	N	65GLU	OE1	17.55
2LYS	N	65GLU	OE2	28.13
338ALA	N	337THR	OG1	0.04
335ALA	N	337THR	OG1	0.00
334GLU	N	333THR	OG1	0.42
334GLU	N	334GLU	OE2	0.01
332GLY	N	286ASN	OD1	0.02

161GLU	N	158SER	OG
161GLU	N	161GLU	OE1
161GLU	N	161GLU	OE2
158SER	N	156ARG	NH2
158SER	N	161GLU	OE1
158SER	N	161GLU	OE2
156ARG	N	155GLU	OE1
150GLU	N	147SER	OG
149ALA	N	147SER	OG
149ALA	N	148GLU	OE1
149ALA	N	148GLU	OE2
148GLU	N	147SER	OG
148GLU	N	148GLU	OE1
148GLU	N	148GLU	OE2
145GLY	N	152TRP	NE1
144ARG	N	142GLU	OE1
144ARG	N	142GLU	OE2
142GLU	N	142GLU	OE1
142GLU	N	142GLU	OE2
141GLY	N	144ARG	NH1
139TYR	N	237ASN	OD1
138ILE	N	155GLU	OE1
138ILE	N	155GLU	OE2
137GLY	N	155GLU	OE1
137GLY	N	155GLU	OE2
136GLY	N	133GLU	OE1
136GLY	N	133GLU	OE2
136GLY	N	237ASN	ND2
135THR	N	133GLU	OE1
135THR	N	133GLU	OE2
134LEU	N	133GLU	OE1
134LEU	N	133GLU	OE2
133GLU	N	237ASN	OD1
121GLU	N	120GLU	OE1
121GLU	N	120GLU	OE2
121GLU	N	121GLU	OE1
121GLU	N	121GLU	OE2
120GLU	N	120GLU	OE1
120GLU	N	120GLU	OE2

11ILE	N	275SER	OG	43.22
10GLY	N	9ASP	OD1	0.29
10GLY	N	9ASP	OD2	0.03
2LYS	N	65GLU	OE1	1.33
2LYS	N	65GLU	OE2	0.02
1MET	N	65GLU	OE1	0.95
1MET	N	65GLU	OE2	0.02
331ALA	N1021	330SER	OG1	0.00
326ASP	N1015	326ASP	OD1	0.02
307LEU	N	306GLU	OE1	0.03
307LEU	N	306GLU	OE2	0.02
306GLU	N	306GLU	OE1	23.12
306GLU	N	306GLU	OE2	22.67
305VAL	N	306GLU	OE1	0.24
305VAL	N	306GLU	OE2	0.31
288THR	N	286ASN	OD1	68.87
285ALA	N	333THR	OG1	3.10
281GLY	N	14GLU	OE2	0.00
278ASP	N	278ASP	OD1	0.02
276ALA	N	275SER	OG	0.03
275SER	N	71SER	OG	3.02
274GLY	N	273HIS	ND1	0.00
273HIS	N	273HIS	ND1	0.01
268VAL	N	261SER	OG	0.06
266THR	N	62GLU	OE1	25.24
266THR	N	62GLU	OE2	28.60
266THR	N	97GLN	OE1	37.62
266THR	N	97GLN	NE2	0.17
264ARG	N	98ASP	OD2	0.15
260ALA	N	102ASN	OD1	0.01
260ALA	N	259SER	OG	0.01
259SER	N	293SER	OG	2.77
256LEU	N	253SER	OG	0.05
255GLY	N	253SER	OG	13.33
255GLY	N	326ASP	OD11(	0.01
254LEU	N	326ASP	OD11(	0.06
249VAL	N	248SER	OG	0.05
245ASP	N	245ASP	OD2	0.01
237ASN	N	157TYR	OH	24.78

111GLY	N	114ARG	NH2	0.32
107LYS	N	253SER	OG	0.00
103LEU	N	102ASN	OD1	0.01
99LEU	N	98ASP	OD1	0.02
98ASP	N	62GLU	OE2	0.04
97GLN	N	96SER	OG	0.00
93LEU	N	92SER	OG	0.11
88THR	N	85SER	OG	0.20
88THR	N	87GLU	OE1	0.02
88THR	N	87GLU	OE2	0.11
87GLU	N	85SER	OG	18.23
87GLU	N	87GLU	OE1	23.44
87GLU	N	87GLU	OE2	18.43
85SER	N	88THR	OG1	0.32
83LYS	N	85SER	OG	0.00
83LYS	N	88THR	OG1	1.57
82ARG	N	215TYR	OH	3.90
79GLY	N	78ASP	OD1	0.04
79GLY	N	78ASP	OD2	0.04
79GLY	N	82ARG	NH1	0.04
79GLY	N	82ARG	NH2	0.00
78ASP	N	78ASP	OD1	0.01
76LYS	N	77TRP	NE1	0.11
74GLY	N	9ASP	OD2	0.01
74GLY	N	77TRP	NE1	0.14
74GLY	N	78ASP	OD2	0.02
74GLY	N	82ARG	NE	0.00
74GLY	N	82ARG	NH1	0.03
74GLY	N	82ARG	NH2	0.08
74GLY	N	87GLU	OE1	0.62
74GLY	N	87GLU	OE2	0.27
73GLY	N	9ASP	OD1	0.09
73GLY	N	9ASP	OD2	0.99
73GLY	N	71SER	OG	0.01
65GLU	N	65GLU	OE1	0.01
65GLU	N	65GLU	OE2	0.17
62GLU	N	62GLU	OE1	0.01
62GLU	N	62GLU	OE2	0.07
61VAL	N	62GLU	OE2	0.01

331ALA	N	286ASN	OD1	0.22
331ALA	N	330SER	OG1	0.01
327LEU	N	326ASP	OD1	0.00
326ASP	N	326ASP	OD1	0.00
326ASP	N	326ASP	OD2	0.00
307LEU	N	306GLU	OE2	0.01
306GLU	N	306GLU	OE1	10.01
306GLU	N	306GLU	OE2	6.03
305VAL	N	306GLU	OE1	0.07
305VAL	N	306GLU	OE2	0.04
289ALA	N	286ASN	ND2	0.02
289ALA	N	288THR	OG1	0.24
288THR	N	286ASN	OD1	27.52
288THR	N	286ASN	ND2	0.07
288THR	N	288THR	OG1	0.00
286ASN	N	286ASN	ND2	0.02
286ASN	N	326ASP	OD1	0.42
285ALA	N	14GLU	OE2	0.00
285ALA	N	333THR	OG1	6.78
283GLY	N	14GLU	OE1	0.09
283GLY	N	14GLU	OE2	1.52
282LYS	N	14GLU	OE1	0.08
282LYS	N	14GLU	OE2	2.07
281GLY	N	14GLU	OE1	1.41
281GLY	N	14GLU	OE2	5.30
280ALA	N	278ASP	OD1	0.60
280ALA	N	278ASP	OD2	0.68
279ILE	N	278ASP	OD2	0.00
278ASP	N	278ASP	OD2	0.00
276ALA	N	275SER	OG	0.22
273HIS	N	273HIS	ND1	0.01
270GLU	N	293SER	OG	0.06
266THR	N	97GLN	OE1	91.84
260ALA	N	102ASN	OD1	0.00
259SER	N	293SER	OG	2.71
256LEU	N	253SER	OG	0.01
255GLY	N	253SER	OG	11.58
254LEU	N	253SER	OG	0.03
254LEU	N	326ASP	OD11(	0.00

118LEU	N	116SER	OG
114ARG	N	113GLU	OE1
114ARG	N	113GLU	OE2
113GLU	N	113GLU	OE1
113GLU	N	113GLU	OE2
112LEU	N	113GLU	OE1
112LEU	N	113GLU	OE2
111GLY	N	113GLU	OE1
111GLY	N	113GLU	OE2
111GLY	N	114ARG	NH2
103LEU	N	102ASN	OD1
97GLN	N	96SER	OG
93LEU	N	92SER	OG
87GLU	N	87GLU	OE1
87GLU	N	87GLU	OE2
82ARG	N	85ARG	NH1
79GLY	N	78ASP	OD1
79GLY	N	78ASP	OD2
78ASP	N	78ASP	OD2
76LYS	N	78ASP	OD1
76LYS	N	78ASP	OD2
74GLY	N	275SER	OG
73GLY	N	9ASP	OD1
73GLY	N	9ASP	OD2
73GLY	N	71SER	OG
73GLY	N	275SER	OG
72VAL	N	71SER	OG
71SER	N	273HIS	NE2
65GLU	N	65GLU	OE1
65GLU	N	65GLU	OE2
57THR	N	57THR	OG1
55GLU	N	51GLU	OE1
55GLU	N	51GLU	OE2
55GLU	N	55GLU	OE1
55GLU	N	55GLU	OE2
55GLU	N	58ARG	NH2
53PHE	N	51GLU	OE1
53PHE	N	51GLU	OE2
44ALA	N	9ASP	OD1

237ASN	N	237ASN	ND2	0.01	55GLU	N	51GLU	OE1	0.02	245ASP	N	245ASP	OD2	0.02	44ALA	N	9ASP	OD2
236GLY	N	235THR	OG1	18.99	55GLU	N	51GLU	OE2	0.02	240GLY	N	235THR	OG1	0.02	43GLY	N	9ASP	OD1
232VAL	N	231ASP	OD2	3.38	55GLU	N	55GLU	OE1	0.05	238ILE	N	155GLU	OE2	0.01	43GLY	N	9ASP	OD2
229ARG	N	226SER	OG	0.08	55GLU	N	55GLU	OE2	0.00	237ASN	N	157TYR	OH	8.40	38VAL	N	37GLU	OE1
228ALA	N	226SER	OG	0.15	53PHE	N	51GLU	OE1	0.44	236GLY	N	235THR	OG1	23.38	38VAL	N	37GLU	OE2
218ALA	N	217ASP	OD1	0.60	53PHE	N	51GLU	OE2	0.30	232VAL	N	231ASP	OD2	3.10	34LEU	N	27ASP	OD1
218ALA	N	217ASP	OD2	1.32	51GLU	N	51GLU	OE1	0.04	229ARG	N	226SER	OG	0.02	34LEU	N	27ASP	OD2
217ASP	N	217ASP	OD1	12.57	51GLU	N	51GLU	OE2	0.02	226SER	N	222HIS	ND1	0.01	33GLY	N	27ASP	OD1
217ASP	N	217ASP	OD2	13.87	44ALA	N	9ASP	OD1	0.20	218ALA	N	217ASP	OD1	0.02	33GLY	N	27ASP	OD2
215TYR	N	214GLN	OE1	0.53	44ALA	N	9ASP	OD2	1.10	218ALA	N	217ASP	OD2	5.23	32LEU	N	27ASP	OD1
214GLN	N	184ASP	OD2	0.06	43GLY	N	9ASP	OD1	1.13	217ASP	N	217ASP	OD1	40.61	32LEU	N	27ASP	OD2
211LEU	N	204ARG	NH1	0.01	43GLY	N	9ASP	OD2	2.40	215TYR	N	214GLN	OE1	0.30	31GLY	N	30GLU	OE1
208ASP	N	208ASP	OD1	0.02	38VAL	N	37GLU	OE1	5.22	209VAL	N	208ASP	OD1	0.10	31GLY	N	30GLU	OE2
208ASP	N	208ASP	OD2	0.02	38VAL	N	37GLU	OE2	5.10	208ASP	N	208ASP	OD1	48.61	30GLU	N	30GLU	OE2
191VAL	N	153ASN	OD1	96.80	34LEU	N	27ASP	OD1	37.08	191VAL	N	153ASN	OD1	97.87	14GLU	N	14GLU	OE1
190GLU	N	153ASN	OD1	22.76	34LEU	N	27ASP	OD2	29.03	190GLU	N	144ARG	NH2	0.00	14GLU	N	14GLU	OE2
190GLU	N	153ASN	ND2	0.64	33GLY	N	27ASP	OD1	45.66	190GLU	N	153ASN	OD1	10.36	12GLY	N	71SER	OG
190GLU	N	190GLU	OE1	1.80	33GLY	N	27ASP	OD2	37.90	190GLU	N	153ASN	ND2	1.13	11ILE	N	71SER	OG
190GLU	N	190GLU	OE2	0.07	32LEU	N	27ASP	OD1	32.68	190GLU	N	190GLU	OE1	20.94	11ILE	N	275SER	OG
188VAL	N	187ASN	OD1	0.59	32LEU	N	27ASP	OD2	26.97	190GLU	N	190GLU	OE2	21.50	10GLY	N	9ASP	OD1
187ASN	N	187ASN	OD1	5.27	32LEU	N	30GLU	OE1	0.00	188VAL	N	187ASN	OD1	0.03	10GLY	N	9ASP	OD2
187ASN	N	187ASN	ND2	0.01	31GLY	N	30GLU	OE1	0.83	187ASN	N	187ASN	OD1	0.90	2LYS	N	65GLU	OE1
186ALA	N	184ASP	OD1	99.92	31GLY	N	30GLU	OE2	0.66	187ASN	N	187ASN	ND2	1.13	2LYS	N	65GLU	OE2
185LYS	N	184ASP	OD1	0.16	30GLU	N	30GLU	OE2	0.00	186ALA	N	184ASP	OD1	11.17	1MET	N	65GLU	OE1
180VAL	N	179HIS	ND1	0.06	17GLU	N	16THR	OG1	0.05	186ALA	N	184ASP	OD2	42.56	1MET	N	65GLU	OE2
179HIS	N	231ASP	OD2	1.15	14GLU	N	14GLU	OE1	0.01	185LYS	N	184ASP	OD1	0.14	340VAL	N	339THR	OG11
178LYS	N	231ASP	OD2	1.13	12GLY	N	71SER	OG	28.16	185LYS	N	184ASP	OD2	0.01	338ALA	N	337THR	OG11
177ARG	N	177ARG	NE	0.00	12GLY	N	275SER	OG	0.00	180VAL	N	179HIS	ND1	0.07	335ALA	N	337THR	OG11
177ARG	N	231ASP	OD1	0.28	11ILE	N	71SER	OG	1.53	179HIS	N	231ASP	OD2	0.01	334GLU	N	333THR	OG11
177ARG	N	231ASP	OD2	71.54	11ILE	N	275SER	OG	7.97	178LYS	N	231ASP	OD2	0.07	334GLU	N	334GLU	OE11
161GLU	N	158SER	OG	0.14	10GLY	N	9ASP	OD1	0.44	177ARG	N	177ARG	NE	0.28	334GLU	N	334GLU	OE21
158SER	N	161GLU	OE1	1.01	10GLY	N	9ASP	OD2	0.18	177ARG	N	231ASP	OD1	0.02	333THR	N	333THR	OG11
158SER	N	161GLU	OE2	0.97	9ASP	N	71SER	OG	0.02	177ARG	N	231ASP	OD2	63.71	332GLY	N	286ASN	OD1
156ARG	N	155GLU	OE1	3.28	2LYS	N	65GLU	OE1	11.09	161GLU	N	158SER	OG	0.02	331ALA	N	286ASN	OD1
156ARG	N	155GLU	OE2	1.50	2LYS	N	65GLU	OE2	14.49	161GLU	N	161GLU	OE2	0.12	330SER	N	322THR	OG11
150GLU	N	147SER	OG	31.11	1MET	N	65GLU	OE1	4.57	158SER	N	161GLU	OE1	0.63	326ASP	N	326ASP	OD11
149ALA	N	147SER	OG	4.94	1MET	N	65GLU	OE2	1.12	158SER	N	161GLU	OE2	6.08	307LEU	N	306GLU	OE1
148GLU	N	147SER	OG	1.36	340VAL	N	339THR	OG11	0.11	156ARG	N	155GLU	OE1	51.34	307LEU	N	306GLU	OE2
148GLU	N	148GLU	OE1	0.17	338ALA	N	337THR	OG11	0.11	150GLU	N	147SER	OG	32.38	306GLU	N	306GLU	OE1

148GLU	N	148GLU	OE2	0.16	337THR	N	337THR	OG11	0.00	149ALA	N	147SER	OG	7.58	306GLU	N	306GLU	OE2
145GLY	N	152TRP	NE1	1.08	335ALA	N	337THR	OG11	0.02	148GLU	N	147SER	OG	3.66	305VAL	N	306GLU	OE1
144ARG	N	142GLU	OE1	1.63	334GLU	N	333THR	OG11	0.54	148GLU	N	148GLU	OE1	0.10	305VAL	N	306GLU	OE2
144ARG	N	142GLU	OE2	1.28	334GLU	N	334GLU	OE11	0.13	148GLU	N	148GLU	OE2	0.09	290ALA	N	273HIS	NE2
142GLU	N	142GLU	OE1	0.01	332GLY	N	286ASN	OD1	0.03	145GLY	N	152TRP	NE1	0.14	289ALA	N	286ASN	OD1
142GLU	N	142GLU	OE2	0.25	332GLY	N	330SER	OG10	9.02	144ARG	N	142GLU	OE1	0.52	289ALA	N	286ASN	ND2
142GLU	N	156ARG	NE	0.06	331ALA	N	322THR	OG11	14.85	144ARG	N	142GLU	OE2	0.42	289ALA	N	288THR	OG1
142GLU	N	156ARG	NH1	0.06	331ALA	N	330SER	OG10	0.03	141GLY	N	144ARG	NH1	0.00	288THR	N	286ASN	OD1
142GLU	N	156ARG	NH2	0.30	327LEU	N	253SER	OG	0.17	138ILE	N	155GLU	OE1	19.06	288THR	N	286ASN	ND2
141GLY	N	144ARG	NH1	0.12	327LEU	N	326ASP	OD11	0.01	138ILE	N	155GLU	OE2	64.03	285ALA	N	333THR	OG
138ILE	N	155GLU	OE1	66.37	326ASP	N	253SER	OG	0.02	137GLY	N	155GLU	OE1	0.69	283GLY	N	14GLU	OE1
138ILE	N	155GLU	OE2	15.27	326ASP	N	326ASP	OD11	0.07	137GLY	N	155GLU	OE2	0.00	283GLY	N	14GLU	OE2
137GLY	N	155GLU	OE1	0.06	326ASP	N	326ASP	OD21	0.04	136GLY	N	133GLU	OE1	52.73	282LYS	N	14GLU	OE1
136GLY	N	133GLU	OE1	57.55	306GLU	N	306GLU	OE1	1.63	136GLY	N	133GLU	OE2	11.24	282LYS	N	14GLU	OE2
136GLY	N	133GLU	OE2	34.36	306GLU	N	306GLU	OE2	1.44	136GLY	N	237ASN	ND2	0.07	281GLY	N	14GLU	OE1
136GLY	N	157TYR	OH	0.07	305VAL	N	306GLU	OE1	0.02	135THR	N	133GLU	OE1	13.83	281GLY	N	14GLU	OE2
136GLY	N	237ASN	ND2	0.40	305VAL	N	306GLU	OE2	0.00	135THR	N	133GLU	OE2	92.27	278ASP	N	278ASP	OD1
135THR	N	133GLU	OE1	65.17	290ALA	N	273HIS	NE2	0.04	134LEU	N	133GLU	OE2	62.96	278ASP	N	278ASP	OD2
135THR	N	133GLU	OE2	40.85	289ALA	N	273HIS	NE2	0.03	121GLU	N	120GLU	OE1	0.31	276ALA	N	275SER	OG
134LEU	N	133GLU	OE1	10.38	289ALA	N	286ASN	OD1	0.01	121GLU	N	120GLU	OE2	0.62	274GLY	N	273HIS	ND1
134LEU	N	133GLU	OE2	2.92	288THR	N	286ASN	OD1	64.29	121GLU	N	121GLU	OE1	1.28	273HIS	N	273HIS	ND1
122ILE	N	121GLU	OE2	0.00	288THR	N	286ASN	ND2	0.13	121GLU	N	121GLU	OE2	0.98	270GLU	N	293SER	OG
121GLU	N	121GLU	OE1	6.27	288THR	N	288THR	OG1	0.00	120GLU	N	120GLU	OE1	20.24	268VAL	N	261SER	OG
121GLU	N	121GLU	OE2	3.92	286ASN	N	273HIS	NE2	47.77	120GLU	N	120GLU	OE2	11.56	266THR	N	62GLU	OE1
120GLU	N	120GLU	OE1	7.17	285ALA	N	333THR	OG11	0.32	118LEU	N	116SER	OG	49.68	266THR	N	62GLU	OE2
120GLU	N	120GLU	OE2	9.38	284ILE	N	278ASP	OD1	0.02	114ARG	N	113GLU	OE2	0.04	266THR	N	97GLN	OE1
118LEU	N	116SER	OG	40.75	284ILE	N	278ASP	OD2	0.00	113GLU	N	113GLU	OE1	18.60	266THR	N	97GLN	NE2
114ARG	N	113GLU	OE1	0.01	283GLY	N	14GLU	OE2	0.18	113GLU	N	113GLU	OE2	73.11	265GLY	N	97GLN	OE1
113GLU	N	113GLU	OE1	88.82	283GLY	N	278ASP	OD2	0.00	112LEU	N	113GLU	OE1	0.19	265GLY	N	98ASP	OD1
113GLU	N	113GLU	OE2	3.68	282LYS	N	14GLU	OE2	0.22	112LEU	N	113GLU	OE2	1.21	265GLY	N	98ASP	OD2
112LEU	N	113GLU	OE1	0.11	281GLY	N	14GLU	OE1	0.11	111GLY	N	113GLU	OE1	37.64	264ARG	N	97GLN	OE1
112LEU	N	113GLU	OE2	0.02	281GLY	N	14GLU	OE2	0.69	111GLY	N	113GLU	OE2	64.34	264ARG	N	98ASP	OD1
111GLY	N	113GLU	OE1	29.52	279ILE	N	278ASP	OD1	0.17	97GLN	N	96SER	OG	0.10	264ARG	N	98ASP	OD2
111GLY	N	113GLU	OE2	62.95	278ASP	N	278ASP	OD1	0.62	87GLU	N	87GLU	OE1	2.73	260ALA	N	102ASN	OD1
103LEU	N	102ASN	OD1	0.00	278ASP	N	278ASP	OD2	0.55	87GLU	N	87GLU	OE2	2.18	260ALA	N	259SER	OG
97GLN	N	96SER	OG	0.04	276ALA	N	275SER	OG	0.17	85ARG	N	88THR	OG1	0.02	259SER	N	293SER	OG
93LEU	N	92SER	OG	0.17	275SER	N	71SER	OG	1.22	80LEU	N	85ARG	NE	0.00	256LEU	N	253SER	OG
88THR	N	85SER	OG	1.46	275SER	N	275SER	OG	0.01	80LEU	N	85ARG	NH2	0.01	255GLY	N	253SER	OG
87GLU	N	85SER	OG	10.04	274GLY	N	273HIS	ND1	0.98	79GLY	N	78ASP	OD2	0.00	255GLY	N	326ASP	OD1

87GLU	N	87GLU	OE1	1.77
87GLU	N	87GLU	OE2	0.09
85SER	N	88THR	OG1	3.48
79GLY	N	78ASP	OD1	0.08
79GLY	N	78ASP	OD2	0.10
78ASP	N	78ASP	OD1	0.01
76LYS	N	9ASP	OD1	0.32
76LYS	N	9ASP	OD2	0.01
74GLY	N	9ASP	OD1	22.62
74GLY	N	9ASP	OD2	23.90
73GLY	N	9ASP	OD1	27.13
73GLY	N	9ASP	OD2	29.51
72VAL	N	71SER	OG	0.04
71SER	N	273HIS	NE2	0.00
55GLU	N	51GLU	OE2	0.00
55GLU	N	55GLU	OE1	0.24
55GLU	N	55GLU	OE2	0.13
53PHE	N	51GLU	OE1	0.81
53PHE	N	51GLU	OE2	0.84
51GLU	N	51GLU	OE1	0.01
51GLU	N	51GLU	OE2	0.01
44ALA	N	9ASP	OD1	1.46
44ALA	N	9ASP	OD2	0.82
43GLY	N	9ASP	OD1	2.04
43GLY	N	9ASP	OD2	6.16
38VAL	N	37GLU	OE1	3.39
38VAL	N	37GLU	OE2	4.03
34LEU	N	27ASP	OD1	56.78
34LEU	N	27ASP	OD2	16.48
33GLY	N	27ASP	OD1	93.35
33GLY	N	27ASP	OD2	0.15
32LEU	N	27ASP	OD1	77.27
12GLY	N	71SER	OG	7.89
12GLY	N	275SER	OG	0.89
11ILE	N	71SER	OG	0.02
11ILE	N	275SER	OG	87.66
10GLY	N	9ASP	OD1	0.14
10GLY	N	9ASP	OD2	0.13
9ASP	N	71SER	OG	0.00

270GLU	N	293SER	OG	0.02
268VAL	N	261SER	OG	0.18
266THR	N	97GLN	OE1	83.72
266THR	N	97GLN	NE2	0.16
264ARG	N	98ASP	OD2	0.00
262LEU	N	261SER	OG	0.00
260ALA	N	102ASN	OD1	0.00
259SER	N	293SER	OG	1.30
256LEU	N	253SER	OG	0.01
255GLY	N	253SER	OG	1.01
255GLY	N	326ASP	OD11	0.01
254LEU	N	225ARG	NH1	0.04
254LEU	N	253SER	OG	2.15
254LEU	N	326ASP	OD11	3.44
254LEU	N	326ASP	OD21	24.80
249VAL	N	248SER	OG	0.09
241ASP	N	237ASN	OD1	0.00
240GLY	N	235THR	OG1	0.06
237ASN	N	133GLU	OE1	38.75
237ASN	N	157TYR	OH	1.07
236GLY	N	235THR	OG1	22.61
232VAL	N	231ASP	OD1	1.05
232VAL	N	231ASP	OD2	1.34
229ARG	N	226SER	OG	0.24
228ALA	N	226SER	OG	0.04
218ALA	N	217ASP	OD1	2.79
218ALA	N	217ASP	OD2	1.26
217ASP	N	217ASP	OD1	16.48
217ASP	N	217ASP	OD2	7.33
215TYR	N	214GLN	OE1	1.68
211LEU	N	204ARG	NH1	0.02
208ASP	N	208ASP	OD1	0.00
191VAL	N	153ASN	OD1	96.73
191VAL	N	153ASN	ND2	0.00
190GLU	N	153ASN	OD1	14.07
190GLU	N	153ASN	ND2	0.42
190GLU	N	190GLU	OE1	7.76
190GLU	N	190GLU	OE2	8.50
188VAL	N	187ASN	OD1	0.21

78ASP	N	78ASP	OD1	0.02
78ASP	N	78ASP	OD2	0.04
76LYS	N	78ASP	OD2	0.00
73GLY	N	9ASP	OD2	2.01
73GLY	N	71SER	OG	0.98
55GLU	N	55GLU	OE1	0.13
55GLU	N	55GLU	OE2	0.04
53PHE	N	51GLU	OE1	0.41
53PHE	N	51GLU	OE2	0.70
51GLU	N	51GLU	OE1	0.00
51GLU	N	51GLU	OE2	0.04
44ALA	N	9ASP	OD1	1.51
44ALA	N	9ASP	OD2	0.67
43GLY	N	9ASP	OD1	1.39
43GLY	N	9ASP	OD2	3.75
38VAL	N	37GLU	OE1	4.89
38VAL	N	37GLU	OE2	4.87
34LEU	N	27ASP	OD1	75.98
34LEU	N	27ASP	OD2	8.93
33GLY	N	27ASP	OD1	98.74
33GLY	N	27ASP	OD2	0.08
32LEU	N	27ASP	OD1	79.40
14GLU	N	14GLU	OE1	0.01
12GLY	N	71SER	OG	0.49
10GLY	N	9ASP	OD1	0.35
10GLY	N	9ASP	OD2	0.62
2LYS	N	65GLU	OE1	0.20
2LYS	N	65GLU	OE2	0.77
343HIS	NE2	317LYS	O	0.61
343HIS	NE2	318ALA	N	0.00
342ARG	NH2	338ALA	O	0.02
342ARG	NH2	345ALA	O2	0.00
342ARG	NH1	338ALA	O	0.18
342ARG	NH1	339THR	O	0.00
342ARG	NH1	342ARG	O	0.05
342ARG	NE	338ALA	O	0.36
342ARG	NE	342ARG	O	0.25
339THR	OG1	335ALA	O	55.99
339THR	OG1	336PHE	O	1.80

254LEU	N	253SER	OG
254LEU	N	326ASP	OD1
254LEU	N	326ASP	OD2
245ASP	N	244SER	OG
245ASP	N	245ASP	OD1
240GLY	N	235THR	OG1
238ILE	N	237ASN	OD1
237ASN	N	157TYR	OH
236GLY	N	235THR	OG1
232VAL	N	231ASP	OD1
232VAL	N	231ASP	OD2
231ASP	N	179HIS	ND1
229ARG	N	226SER	OG
228ALA	N	226SER	OG
218ALA	N	217ASP	OD1
218ALA	N	217ASP	OD2
217ASP	N	217ASP	OD1
217ASP	N	217ASP	OD2
215TYR	N	214GLN	OE1
215TYR	N	214GLN	NE2
214GLN	N	184ASP	OD2
213HIS	N	212GLU	OE1
213HIS	N	212GLU	OE2
209VAL	N	208ASP	OD1
208ASP	N	208ASP	OD1
191VAL	N	153ASN	OD1
190GLU	N	153ASN	OD1
190GLU	N	153ASN	ND2
190GLU	N	190GLU	OE1
190GLU	N	190GLU	OE2
188VAL	N	187ASN	OD1
187ASN	N	187ASN	OD1
187ASN	N	187ASN	ND2
186ALA	N	184ASP	OD1
185LYS	N	184ASP	OD1
180VAL	N	179HIS	ND1
179HIS	N	231ASP	OD2
178LYS	N	231ASP	OD1
178LYS	N	231ASP	OD2

2LYS	N	65GLU	OE1	0.94
2LYS	N	65GLU	OE2	0.03
343HIS	NE2	310LYS	O	0.01
343HIS	NE2	317LYS	O	0.26
342ARG	NH2	338ALA	O	0.08
342ARG	NH2	345ALA	O1	0.00
342ARG	NH2	345ALA	O2	0.53
342ARG	NH1	338ALA	O	0.17
342ARG	NE	338ALA	O	0.92
342ARG	NE	342ARG	O	0.04
342ARG	NE	345ALA	O1	0.41
342ARG	NE	345ALA	O2	0.01
339THR	OG1	335ALA	O	62.11
339THR	OG1	336PHE	O	3.65
339THR	OG1	339THR	O	0.10
337THR	OG1	18ALA	O	0.02
337THR	OG1	333THR	O	75.24
337THR	OG1	334GLU	N	0.02
337THR	OG1	334GLU	O	2.78
333THR	OG1	283GLY	O	2.91
333THR	OG1	285ALA	O	1.01
333THR	OG1	332GLY	O	0.00
330SER	OG	329GLY	O	0.08
330SER	OG	330SER	O	0.10
322THR	OG1	318ALA	O	5.84
322THR	OG1	321GLU	O	0.03
322THR	OG1	322THR	N	0.00
322THR	OG1	322THR	O	3.82
322THR	OG1	323PRO	N	0.00
322THR	OG1	323PRO	O	0.36
317LYS	NZ	313ASP	O	0.18
317LYS	NZ	343HIS	O	0.38
317LYS	NZ	345ALA	O2	0.03
310LYS	NZ	306GLU	O	0.01
310LYS	NZ	344LEU	O	2.12
310LYS	NZ	345ALA	O1	25.53
310LYS	NZ	345ALA	O2	21.79
309ARG	NH2	175LYS	O	17.21
293SER	OG	259SER	O	0.30

187ASN	N	187ASN	OD1	4.55
187ASN	N	187ASN	ND2	0.01
187ASN	N	215TYR	OH	0.00
186ALA	N	184ASP	OD1	99.76
185LYS	N	184ASP	OD1	0.10
180VAL	N	179HIS	ND1	0.25
179HIS	N	231ASP	OD2	0.60
178LYS	N	231ASP	OD1	0.01
178LYS	N	231ASP	OD2	0.69
177ARG	N	177ARG	NE	0.00
177ARG	N	231ASP	OD1	24.78
177ARG	N	231ASP	OD2	51.65
161GLU	N	158SER	OG	0.11
161GLU	N	161GLU	OE1	0.10
161GLU	N	161GLU	OE2	0.72
159LYS	N	158SER	OG	0.01
158SER	N	161GLU	OE1	4.41
158SER	N	161GLU	OE2	4.43
156ARG	N	155GLU	OE1	39.19
156ARG	N	155GLU	OE2	33.50
150GLU	N	147SER	OG	53.35
149ALA	N	147SER	OG	17.21
148GLU	N	147SER	OG	1.24
148GLU	N	148GLU	OE1	0.68
148GLU	N	148GLU	OE2	0.60
145GLY	N	152TRP	NE1	0.62
144ARG	N	142GLU	OE1	1.45
144ARG	N	142GLU	OE2	1.44
144ARG	N	144ARG	NE	0.01
144ARG	N	144ARG	NH2	0.04
142GLU	N	142GLU	OE1	0.45
142GLU	N	142GLU	OE2	0.32
142GLU	N	156ARG	NH1	0.00
141GLY	N	144ARG	NH1	0.01
138ILE	N	155GLU	OE1	1.87
138ILE	N	155GLU	OE2	7.19
138ILE	N	237ASN	OD1	0.20
137GLY	N	135THR	OG1	0.02
137GLY	N	139TYR	OH	0.41

339THR	OG1	338ALA	O	0.00
339THR	OG1	340VAL	N	0.00
337THR	OG1	18ALA	O	0.00
337THR	OG1	333THR	O	52.79
337THR	OG1	334GLU	O	1.93
337THR	OG1	337THR	O	0.01
333THR	OG1	283GLY	O	0.19
333THR	OG1	285ALA	O	69.71
330SER	OG	329GLY	O	0.36
330SER	OG	330SER	O	0.34
322THR	OG1	318ALA	O	19.39
322THR	OG1	322THR	O	11.79
322THR	OG1	330SER	O	0.19
322THR	OG1	335ALA	O	0.00
317LYS	NZ	313ASP	O	0.16
317LYS	NZ	343HIS	O	0.00
310LYS	NZ	306GLU	O	0.04
310LYS	NZ	343HIS	O	0.12
310LYS	NZ	344LEU	O	2.12
310LYS	NZ	345ALA	O1	13.17
310LYS	NZ	345ALA	O2	13.43
309ARG	NE	305VAL	O	0.01
293SER	OG	259SER	O	0.02
293SER	OG	270GLU	O	0.01
293SER	OG	289ALA	O	98.24
293SER	OG	293SER	O	0.01
288THR	OG1	256LEU	O	0.02
288THR	OG1	289ALA	N	0.02
286ASN	ND2	255GLY	O	0.03
286ASN	ND2	327LEU	N	0.14
286ASN	ND2	331ALA	O	0.03
282LYS	NZ	278ASP	O	19.03
282LYS	NZ	280ALA	O	0.14
275SER	OG	72VAL	O	6.35
275SER	OG	73GLY	N	0.10
275SER	OG	73GLY	O	2.72
275SER	OG	275SER	O	0.45
273HIS	ND1	271PRO	O	0.12
273HIS	ND1	273HIS	O	0.46

177ARG	N	177ARG	NE
177ARG	N	231ASP	OD1
177ARG	N	231ASP	OD2
161GLU	N	158SER	OG
161GLU	N	161GLU	OE1
161GLU	N	161GLU	OE2
159LYS	N	158SER	OG
158SER	N	161GLU	OE1
158SER	N	161GLU	OE2
156ARG	N	155GLU	OE1
156ARG	N	155GLU	OE2
150GLU	N	147SER	OG
149ALA	N	147SER	OG
148GLU	N	147SER	OG
148GLU	N	148GLU	OE1
148GLU	N	148GLU	OE2
145GLY	N	190GLU	OE1
145GLY	N	152TRP	NE1
144ARG	N	142GLU	OE1
144ARG	N	142GLU	OE2
142GLU	N	142GLU	OE1
142GLU	N	142GLU	OE2
141GLY	N	144ARG	NH1
139TYR	N	237ASN	OD1
139TYR	N	237ASN	ND2
138ILE	N	155GLU	OE1
138ILE	N	155GLU	OE2
136GLY	N	133GLU	OE1
136GLY	N	133GLU	OE2
136GLY	N	157TYR	OH
136GLY	N	237ASN	OD1
136GLY	N	237ASN	ND2
135THR	N	133GLU	OE1
135THR	N	133GLU	OE2
135THR	N	157TYR	OH
134LEU	N	133GLU	OE1
134LEU	N	133GLU	OE2
129LEU	N	176ARG	NH1
121GLU	N	121GLU	OE1

293SER	OG	270GLU	N	0.00	137GLY	N	155GLU	OE1	0.29	273HIS	ND1	274GLY	O	0.66	121GLU	N	121GLU	OE2
293SER	OG	270GLU	O	0.06	137GLY	N	155GLU	OE2	0.23	266THR	OG1	61VAL	O	75.03	120GLU	N	120GLU	OE1
293SER	OG	289ALA	O	96.25	137GLY	N	237ASN	ND2	0.06	266THR	OG1	62GLU	O	0.00	120GLU	N	120GLU	OE2
293SER	OG	293SER	O	0.01	136GLY	N	133GLU	OE1	1.62	266THR	OG1	64ALA	O	0.62	118LEU	N	116SER	OG
288THR	OG1	255GLY	O	7.92	136GLY	N	133GLU	OE2	31.09	266THR	OG1	265GLY	O	0.01	114ARG	N	113GLU	OE1
288THR	OG1	289ALA	N	0.02	136GLY	N	135THR	OG1	0.46	264ARG	NH2	96SER	O	0.17	114ARG	N	113GLU	OE2
286ASN	ND2	255GLY	O	0.27	136GLY	N	237ASN	ND2	0.86	264ARG	NH1	96SER	O	0.00	113GLU	N	113GLU	OE1
286ASN	ND2	325PRO	O	4.84	135THR	N	133GLU	OE1	5.52	264ARG	NH1	98ASP	O	0.02	113GLU	N	113GLU	OE2
286ASN	ND2	331ALA	O	10.29	135THR	N	133GLU	OE2	87.98	264ARG	NE	98ASP	O	0.01	112LEU	N	113GLU	OE1
282LYS	NZ	278ASP	O	2.98	135THR	N	237ASN	ND2	0.24	261SER	OG	101ALA	O	1.66	112LEU	N	113GLU	OE2
282LYS	NZ	326ASP	O	1.10	134LEU	N	133GLU	OE1	0.64	261SER	OG	102ASN	N	0.00	111GLY	N	113GLU	OE1
282LYS	NZ	327LEU	O	0.62	134LEU	N	133GLU	OE2	0.22	261SER	OG	260ALA	O	0.76	111GLY	N	113GLU	OE2
282LYS	NZ	328GLY	O	0.00	129LEU	N	176ARG	NH1	0.00	261SER	OG	268VAL	O	0.02	103LEU	N	102ASN	OD1
275SER	OG	9ASP	O	61.55	121GLU	N	120GLU	OE1	0.06	259SER	OG	259SER	O	0.01	99LEU	N	98ASP	OD1
275SER	OG	10GLY	N	0.00	121GLU	N	120GLU	OE2	0.07	259SER	OG	270GLU	O	88.98	99LEU	N	98ASP	OD2
275SER	OG	71SER	O	5.00	121GLU	N	121GLU	OE1	2.45	253SER	OG	252GLY	O	0.08	98ASP	N	62GLU	OE1
275SER	OG	72VAL	O	0.02	121GLU	N	121GLU	OE2	2.36	253SER	OG	253SER	O	0.12	98ASP	N	62GLU	OE2
275SER	OG	73GLY	O	7.35	120GLU	N	120GLU	OE1	1.56	253SER	OG	326ASP	O	6.02	98ASP	N	97GLN	OE1
275SER	OG	74GLY	N	0.06	120GLU	N	120GLU	OE2	0.70	253SER	OG	327LEU	N	0.02	98ASP	N	97GLN	NE2
275SER	OG	274GLY	O	4.12	118LEU	N	116SER	OG	32.77	248SER	OG	244SER	O	64.37	97GLN	N	96SER	OG
275SER	OG	275SER	O	0.07	116SER	N	119LYS	NZ	0.00	248SER	OG	245ASP	O	1.36	97GLN	N	97GLN	OE1
273HIS	ND1	254LEU	O	0.02	114ARG	N	113GLU	OE1	0.11	248SER	OG	248SER	O	0.55	97GLN	N	97GLN	NE2
273HIS	ND1	272VAL	O	0.00	114ARG	N	113GLU	OE2	0.08	248SER	OG	254LEU	O	16.26	96SER	N	97GLN	OE1
273HIS	ND1	273HIS	O	0.64	113GLU	N	113GLU	OE1	42.12	244SER	OG	240GLY	O	71.45	95LYS	N	97GLN	OE1
273HIS	ND1	274GLY	O	82.15	113GLU	N	113GLU	OE2	42.01	244SER	OG	241ASP	N	0.00	93LEU	N	92SER	OG
266THR	OG1	61VAL	O	86.48	112LEU	N	113GLU	OE1	0.30	244SER	OG	241ASP	O	12.59	88THR	N	87GLU	OE1
266THR	OG1	62GLU	O	0.00	112LEU	N	113GLU	OE2	0.28	237ASN	ND2	133GLU	O	0.20	88THR	N	87GLU	OE2
266THR	OG1	64ALA	O	0.53	111GLY	N	113GLU	OE1	44.74	237ASN	ND2	134LEU	N	0.02	87GLU	N	87GLU	OE1
264ARG	NH2	96SER	O	7.64	111GLY	N	113GLU	OE2	53.43	237ASN	ND2	134LEU	O	36.37	87GLU	N	87GLU	OE2
264ARG	NH1	96SER	O	0.88	103LEU	N	102ASN	OD1	0.00	237ASN	ND2	135THR	N	9.97	85ARG	N	88THR	OG1
264ARG	NH1	264ARG	O	0.10	97GLN	N	96SER	OG	0.34	237ASN	ND2	136GLY	N	0.02	80LEU	N	85ARG	NE
264ARG	NE	264ARG	O	0.00	93LEU	N	92SER	OG	0.24	237ASN	ND2	136GLY	O	89.43	80LEU	N	85ARG	NH2
261SER	OG	101ALA	O	4.18	88THR	N	85SER	OG	11.67	237ASN	ND2	137GLY	N	0.71	79GLY	N	78ASP	OD1
261SER	OG	102ASN	N	0.01	87GLU	N	85SER	OG	7.12	237ASN	ND2	137GLY	O	2.18	79GLY	N	78ASP	OD2
261SER	OG	260ALA	O	0.63	87GLU	N	87GLU	OE1	0.97	235THR	OG1	183VAL	O	4.79	79GLY	N	85ARG	NE
261SER	OG	261SER	O	0.00	87GLU	N	87GLU	OE2	0.43	235THR	OG1	236GLY	O	1.62	79GLY	N	85ARG	NH1
261SER	OG	262LEU	N	0.00	85SER	N	88THR	OG1	5.77	235THR	OG1	239PHE	O	5.43	79GLY	N	85ARG	NH2
261SER	OG	262LEU	O	0.02	79GLY	N	78ASP	OD1	0.05	235THR	OG1	240GLY	N	0.08	74GLY	N	9ASP	OD1
261SER	OG	268VAL	O	0.58	79GLY	N	78ASP	OD2	0.04	229ARG	NH1	228ALA	O	0.06	74GLY	N	85ARG	NH1

259SER	OG	103LEU	O	17.89
259SER	OG	258PRO	O	3.63
259SER	OG	259SER	O	0.01
259SER	OG	260ALA	O	0.09
259SER	OG	270GLU	O	67.17
253SER	OG	115LEU	O	0.00
253SER	OG	252GLY	O	0.01
253SER	OG	253SER	O	0.48
253SER	OG	325PRO	N	0.00
253SER	OG	325PRO	O	14.64
248SER	OG	244SER	O	71.15
248SER	OG	245ASP	O	14.64
248SER	OG	247ALA	O	0.00
248SER	OG	248SER	O	0.05
244SER	OG	240GLY	O	44.67
244SER	OG	241ASP	O	1.38
244SER	OG	245ASP	N	0.56
237ASN	ND2	133GLU	O	2.12
237ASN	ND2	134LEU	N	0.01
237ASN	ND2	134LEU	O	3.97
237ASN	ND2	135THR	N	0.40
237ASN	ND2	136GLY	N	0.00
237ASN	ND2	136GLY	O	89.70
237ASN	ND2	137GLY	O	0.00
237ASN	ND2	236GLY	O	0.02
235THR	OG1	183VAL	O	10.70
235THR	OG1	236GLY	O	0.40
235THR	OG1	239PHE	O	1.21
235THR	OG1	240GLY	N	0.00
229ARG	NH2	229ARG	O	0.01
229ARG	NH1	228ALA	O	0.06
229ARG	NH1	229ARG	O	9.73
229ARG	NE	229ARG	O	10.34
229ARG	NE	230PHE	N	0.00
226SER	OG	222HIS	O	5.39
226SER	OG	225ARG	O	1.50
226SER	OG	226SER	O	5.77
226SER	OG	228ALA	O	0.61
225ARG	NH2	218ALA	O	0.01

78ASP	N	78ASP	OD1	0.01
78ASP	N	78ASP	OD2	0.01
74GLY	N	9ASP	OD2	0.00
74GLY	N	275SER	OG	0.14
73GLY	N	9ASP	OD2	0.21
73GLY	N	71SER	OG	0.74
73GLY	N	275SER	OG	0.05
72VAL	N	71SER	OG	0.73
65GLU	N	65GLU	OE1	0.05
65GLU	N	65GLU	OE2	0.01
63GLU	N	63GLU	OE1	0.00
55GLU	N	51GLU	OE1	0.03
55GLU	N	51GLU	OE2	0.04
55GLU	N	55GLU	OE1	0.04
55GLU	N	55GLU	OE2	0.07
53PHE	N	51GLU	OE1	0.41
53PHE	N	51GLU	OE2	0.20
51GLU	N	51GLU	OE1	0.08
51GLU	N	51GLU	OE2	0.06
50GLY	N	51GLU	OE1	0.01
48ALA	N	47ASP	OD1	0.16
48ALA	N	47ASP	OD2	0.19
47ASP	N	47ASP	OD1	0.01
47ASP	N	47ASP	OD2	0.03
44ALA	N	9ASP	OD1	2.29
44ALA	N	9ASP	OD2	3.33
44ALA	N	77TRP	NE1	0.14
43GLY	N	9ASP	OD1	1.56
43GLY	N	9ASP	OD2	2.42
43GLY	N	77TRP	NE1	0.02
38VAL	N	37GLU	OE1	6.11
38VAL	N	37GLU	OE2	6.79
34LEU	N	27ASP	OD1	49.77
34LEU	N	27ASP	OD2	30.62
33GLY	N	27ASP	OD1	68.66
33GLY	N	27ASP	OD2	30.73
32LEU	N	27ASP	OD1	60.57
32LEU	N	27ASP	OD2	28.39
31GLY	N	30GLU	OE2	0.00

229ARG	NH1	229ARG	O	0.02
229ARG	NE	228ALA	O	0.10
229ARG	NE	229ARG	O	0.71
226SER	OG	222HIS	O	2.63
226SER	OG	226SER	O	7.68
225ARG	NH2	115LEU	O	0.04
225ARG	NH2	252GLY	O	30.79
225ARG	NH2	326ASP	O	0.33
225ARG	NH1	252GLY	O	29.37
225ARG	NE	252GLY	O	0.14
222HIS	NE2	226SER	O	0.01
222HIS	NE2	229ARG	O	0.44
214GLN	NE2	213HIS	O	9.85
214GLN	NE2	214GLN	N	0.03
214GLN	NE2	215TYR	O	2.67
214GLN	NE2	218ALA	O	0.05
214GLN	NE2	219MET	N	0.16
213HIS	NE2	196ARG	O	0.35
206TYR	OH	167ARG	O	0.10
204ARG	NH2	197LYS	O	0.00
204ARG	NE	200GLU	O	0.02
198THR	OG1	194PHE	O	93.95
198THR	OG1	195TRP	O	0.99
198THR	OG1	199VAL	N	0.00
197LYS	NZ	193GLU	O	0.08
197LYS	NZ	147SER	O	0.26
197LYS	NZ	148GLU	O	0.27
196ARG	NH2	214GLN	O	0.00
195TRP	NE1	183VAL	O	0.20
195TRP	NE1	234VAL	O	0.22
182SER	OG	181VAL	O	0.00
182SER	OG	183VAL	O	15.28
182SER	OG	212GLU	O	0.74
182SER	OG	234VAL	O	3.09
179HIS	NE2	211LEU	O	0.02
179HIS	NE2	229ARG	O	7.54
178LYS	NZ	174ARG	O	0.07
178LYS	NZ	176ARG	O	0.92
178LYS	NZ	208ASP	O	0.16

74GLY	N	87GLU	OE1
74GLY	N	87GLU	OE2
74GLY	N	275SER	OG
73GLY	N	9ASP	OD1
73GLY	N	9ASP	OD2
73GLY	N	275SER	OG
72VAL	N	71SER	OG
72VAL	N	275SER	OG
71SER	N	273HIS	NE2
65GLU	N	65GLU	OE2
64ALA	N	63GLU	OE1
64ALA	N	63GLU	OE2
64ALA	N	266THR	OG1
63GLU	N	63GLU	OE2
62GLU	N	62GLU	OE1
55GLU	N	51GLU	OE1
55GLU	N	51GLU	OE2
55GLU	N	55GLU	OE1
53PHE	N	51GLU	OE1
53PHE	N	51GLU	OE2
51GLU	N	51GLU	OE1
51GLU	N	51GLU	OE2
44ALA	N	9ASP	OD1
44ALA	N	9ASP	OD2
43GLY	N	9ASP	OD1
43GLY	N	9ASP	OD2
38VAL	N	37GLU	OE1
38VAL	N	37GLU	OE2
34LEU	N	27ASP	OD1
34LEU	N	27ASP	OD2
33GLY	N	27ASP	OD1
33GLY	N	27ASP	OD2
32LEU	N	27ASP	OD1
32LEU	N	27ASP	OD2
31GLY	N	30GLU	OE1
31GLY	N	30GLU	OE2
30GLU	N	30GLU	OE2
14GLU	N	14GLU	OE1
12GLY	N	71SER	OG

225ARG	NH2	221MET	O	0.08	12GLY	N	71SER	OG	3.60	177ARG	NH2	127ASP	O	1.15	11ILE	N	71SER	OG
225ARG	NH2	222HIS	N	0.08	12GLY	N	275SER	OG	3.14	177ARG	NH2	229ARG	O	0.02	11ILE	N	275SER	OG
225ARG	NH2	115LEU	O	0.00	11ILE	N	71SER	OG	0.71	177ARG	NH2	230PHE	O	0.46	10GLY	N	9ASP	OD1
225ARG	NH2	252GLY	O	42.35	11ILE	N	275SER	OG	16.24	177ARG	NH1	228ALA	O	0.04	10GLY	N	9ASP	OD2
225ARG	NH2	326ASP	O1016	1.77	10GLY	N	9ASP	OD1	1.92	177ARG	NH1	229ARG	O	24.94	2LYS	N	65GLU	OE1
225ARG	NH1	115LEU	O	0.14	10GLY	N	9ASP	OD2	2.31	176ARG	NH2	127ASP	O	0.01	2LYS	N	65GLU	OE2
225ARG	NH1	252GLY	O	0.61	2LYS	N	65GLU	OE1	8.00	176ARG	NH1	127ASP	O	45.69	343HIS	NE2	317LYS	O
225ARG	NH1	326ASP	O1016	0.01	2LYS	N	65GLU	OE2	8.13	176ARG	NH1	128VAL	N	0.02	343HIS	NE2	318ALA	N
225ARG	NE	221MET	O	11.52	343HIS	NE2	310LYS	O	0.01	176ARG	NH1	128VAL	O	0.00	342ARG	NH2	338ALA	O
225ARG	NE	225ARG	O	0.52	343HIS	NE2	317LYS	O	0.14	176ARG	NH1	231ASP	O	0.10	342ARG	NH2	345ALA	O1
225ARG	NE	252GLY	O	0.02	342ARG	NH2	317LYS	O	0.02	175LYS	NZ	171GLU	O	0.00	342ARG	NH2	345ALA	O2
215TYR	OH	186ALA	O	0.03	342ARG	NH2	338ALA	O	0.00	175LYS	NZ	299GLU	O	0.28	342ARG	NH1	338ALA	O
214GLN	NE2	213HIS	O	1.14	342ARG	NH2	339THR	N	0.00	174ARG	NH2	205GLY	O	0.03	342ARG	NH1	339THR	O
214GLN	NE2	214GLN	N	0.00	342ARG	NH2	341LEU	O	0.00	167ARG	NH2	201GLU	O	0.01	342ARG	NH1	342ARG	O
214GLN	NE2	215TYR	O	0.04	342ARG	NH2	345ALA	O1	0.26	167ARG	NE	163GLU	O	0.01	342ARG	NH1	345ALA	O1
214GLN	NE2	218ALA	O	0.01	342ARG	NH2	345ALA	O2	0.53	164ARG	NH2	135THR	O	0.00	342ARG	NH1	345ALA	O2
214GLN	NE2	219MET	N	0.01	342ARG	NH1	338ALA	O	0.11	164ARG	NH2	136GLY	N	0.01	342ARG	NE	338ALA	O
213HIS	NE2	196ARG	O	0.02	342ARG	NH1	341LEU	O	0.01	164ARG	NH1	160PRO	O	0.01	342ARG	NE	339THR	O
206TYR	OH	167ARG	O	0.76	342ARG	NH1	342ARG	O	0.11	164ARG	NE	161GLU	O	2.69	342ARG	NE	341LEU	O
204ARG	NH2	197LYS	O	0.00	342ARG	NH1	345ALA	O1	0.04	159LYS	NZ	197LYS	O	0.00	342ARG	NE	342ARG	N
204ARG	NH2	200GLU	O	0.00	342ARG	NH1	345ALA	O2	0.09	159LYS	NZ	148GLU	O	1.27	342ARG	NE	342ARG	O
204ARG	NH1	200GLU	O	0.12	342ARG	NE	338ALA	O	0.17	158SER	OG	158SER	O	0.01	339THR	OG1	335ALA	O
204ARG	NH1	201GLU	O	0.04	342ARG	NE	339THR	O	0.18	157TYR	OH	136GLY	O	22.53	339THR	OG1	336PHE	O
204ARG	NE	200GLU	O	0.09	342ARG	NE	341LEU	O	0.01	157TYR	OH	137GLY	N	0.04	339THR	OG1	339THR	O
204ARG	NE	204ARG	N	0.00	342ARG	NE	342ARG	O	0.18	157TYR	OH	161GLU	O	0.03	337THR	OG1	18ALA	O
198THR	OG1	162VAL	O	0.00	342ARG	NE	345ALA	O2	0.04	156ARG	NH2	142GLU	O	29.95	337THR	OG1	332GLY	O
198THR	OG1	194PHE	O	91.17	339THR	OG1	314ALA	O	0.00	156ARG	NH1	142GLU	O	4.89	337THR	OG1	333THR	O
198THR	OG1	195TRP	N	0.00	339THR	OG1	335ALA	O	73.70	156ARG	NE	142GLU	O	52.97	337THR	OG1	334GLU	O
198THR	OG1	195TRP	O	0.99	339THR	OG1	336PHE	O	2.40	154THR	OG1	143PRO	O	81.72	333THR	OG1	14GLU	O
198THR	OG1	199VAL	N	0.03	339THR	OG1	339THR	O	0.06	154THR	OG1	153ASN	O	0.16	333THR	OG1	281GLY	O
197LYS	NZ	193GLU	O	0.18	337THR	OG1	18ALA	O	0.01	153ASN	ND2	138ILE	O	3.03	333THR	OG1	282LYS	O
197LYS	NZ	147SER	O	1.22	337THR	OG1	333THR	O	79.68	153ASN	ND2	141GLY	O	0.00	333THR	OG1	283GLY	N
197LYS	NZ	148GLU	O	0.70	337THR	OG1	334GLU	O	1.58	153ASN	ND2	152TRP	O	0.02	333THR	OG1	283GLY	O
196ARG	NH2	213HIS	O	0.00	333THR	OG1	280ALA	O	2.12	153ASN	ND2	154THR	N	0.01	333THR	OG1	285ALA	O
196ARG	NH2	214GLN	O	0.00	333THR	OG1	281GLY	O	0.01	153ASN	ND2	154THR	O	0.00	333THR	OG1	332GLY	O
196ARG	NH2	215TYR	N	0.00	333THR	OG1	283GLY	O	0.99	153ASN	ND2	188VAL	O	0.50	330SER	OG	283GLY	O
195TRP	NE1	183VAL	O	0.23	333THR	OG1	285ALA	O	18.38	152TRP	NE1	143PRO	O	0.03	330SER	OG	329GLY	O
195TRP	NE1	234VAL	O	0.02	333THR	OG1	332GLY	O	0.00	152TRP	NE1	144ARG	N	0.02	330SER	OG	330SER	O
182SER	OG	183VAL	N	0.02	330SER	OG	328GLY	O	0.00	152TRP	NE1	144ARG	O	0.24	322THR	OG1	318ALA	O

182SER	OG	183VAL	O	1.30	330SER	OG	329GLY	O	0.98	147SER	OG	146MET	O	0.14	322THR	OG1	321GLU	O
182SER	OG	212GLU	O	0.01	330SER	OG	330SER	O	0.46	147SER	OG	150GLU	O	70.71	322THR	OG1	322THR	O
182SER	OG	234VAL	O	0.02	330SER	OG	331ALA	N	0.01	144ARG	NH2	139TYR	O	0.61	322THR	OG1	323PRO	O
179HIS	NE2	211LEU	O	0.00	322THR	OG1	318ALA	O	10.88	144ARG	NH2	187ASN	O	0.14	322THR	OG1	330SER	O
179HIS	NE2	229ARG	O	2.10	322THR	OG1	321GLU	O	0.14	144ARG	NH2	188VAL	O	55.85	317LYS	NZ	313ASP	O
178LYS	NZ	174ARG	O	0.05	322THR	OG1	322THR	O	5.55	144ARG	NH1	138ILE	O	0.02	317LYS	NZ	343HIS	O
178LYS	NZ	176ARG	O	0.14	322THR	OG1	323PRO	N	0.00	144ARG	NH1	139TYR	O	80.86	310LYS	NZ	29ALA	O
178LYS	NZ	208ASP	O	0.09	322THR	OG1	323PRO	O	0.20	144ARG	NH1	140PHE	N	0.32	310LYS	NZ	306GLU	O
177ARG	NH2	125GLY	O	0.18	322THR	OG1	331ALA	O	0.00	144ARG	NH1	141GLY	O	0.18	310LYS	NZ	343HIS	O
177ARG	NH2	127ASP	O	13.47	322THR	OG1	335ALA	O	0.00	144ARG	NH1	188VAL	O	0.20	310LYS	NZ	344LEU	O
177ARG	NH2	228ALA	O	7.11	317LYS	NZ	313ASP	O	0.54	139TYR	OH	237ASN	O	0.06	310LYS	NZ	345ALA	O1
177ARG	NH2	229ARG	O	0.05	317LYS	NZ	342ARG	O	0.03	139TYR	OH	238ILE	O	9.92	310LYS	NZ	345ALA	O2
177ARG	NH2	230PHE	O	7.78	317LYS	NZ	343HIS	O	0.00	135THR	OG1	99LEU	O	0.12	309ARG	NH1	305VAL	O
177ARG	NH1	125GLY	O	0.08	310LYS	NZ	306GLU	O	0.09	135THR	OG1	100PHE	O	0.01	309ARG	NH1	309ARG	O
177ARG	NH1	228ALA	O	37.10	310LYS	NZ	343HIS	O	0.37	135THR	OG1	136GLY	N	0.02	309ARG	NE	305VAL	O
177ARG	NH1	229ARG	O	1.51	310LYS	NZ	344LEU	O	0.95	132ARG	NH2	133GLU	O	0.00	300HIS	NE2	65GLU	O
177ARG	NH1	230PHE	N	0.00	310LYS	NZ	345ALA	O1	8.26	132ARG	NH2	237ASN	O	0.00	300HIS	NE2	261SER	O
177ARG	NH1	230PHE	O	3.61	310LYS	NZ	345ALA	O2	8.85	132ARG	NE	133GLU	O	78.81	293SER	OG	259SER	O
176ARG	NH2	127ASP	O	0.00	309ARG	NH2	175LYS	O	6.16	132ARG	NE	237ASN	O	0.20	293SER	OG	270GLU	N
176ARG	NH1	127ASP	O	23.13	309ARG	NH1	305VAL	O	0.01	124ARG	NH2	120GLU	O	0.00	293SER	OG	270GLU	O
176ARG	NH1	231ASP	O	0.10	309ARG	NE	305VAL	O	0.02	124ARG	NE	120GLU	O	0.02	293SER	OG	271PRO	N
175LYS	NZ	171GLU	O	3.74	300HIS	NE2	168VAL	O	0.08	119LYS	NZ	113GLU	O	7.57	293SER	OG	289ALA	O
175LYS	NZ	299GLU	O	4.62	300HIS	NE2	172ALA	N	0.00	119LYS	NZ	114ARG	O	22.27	293SER	OG	293SER	O
175LYS	NZ	300HIS	O	0.01	300HIS	NE2	172ALA	O	0.00	119LYS	NZ	116SER	O	27.90	288THR	OG1	255GLY	O
174ARG	NH1	205GLY	O	0.01	300HIS	NE2	261SER	O	0.00	119LYS	NZ	118LEU	O	10.50	288THR	OG1	286ASN	O
174ARG	NE	171GLU	O	0.20	293SER	OG	259SER	O	0.68	116SER	OG	250LEU	O	96.51	286ASN	ND2	255GLY	O
167ARG	NH1	163GLU	O	0.03	293SER	OG	269PHE	O	0.00	114ARG	NH2	111GLY	O	0.84	286ASN	ND2	274GLY	O
164ARG	NH2	98ASP	O	0.52	293SER	OG	270GLU	O	0.21	114ARG	NH1	111GLY	O	0.10	286ASN	ND2	286ASN	N
164ARG	NH2	264ARG	N	0.02	293SER	OG	271PRO	N	0.01	114ARG	NE	111GLY	O	0.01	286ASN	ND2	322THR	O
164ARG	NE	160PRO	O	0.00	293SER	OG	289ALA	O	94.11	104ARG	NH1	244SER	O	0.71	286ASN	ND2	323PRO	O
159LYS	NZ	197LYS	O	0.00	293SER	OG	293SER	O	0.04	102ASN	ND2	103LEU	N	0.01	286ASN	ND2	325PRO	O
159LYS	NZ	148GLU	O	1.47	288THR	OG1	255GLY	O	0.05	102ASN	ND2	260ALA	O	2.46	286ASN	ND2	331ALA	O
158SER	OG	156ARG	O	0.01	288THR	OG1	256LEU	O	0.02	102ASN	ND2	261SER	N	0.01	286ASN	ND2	332GLY	O
158SER	OG	157TYR	O	0.03	286ASN	ND2	255GLY	O	0.04	97GLN	NE2	61VAL	O	0.04	282LYS	NZ	278ASP	O
158SER	OG	158SER	O	0.08	286ASN	ND2	283GLY	O	0.01	97GLN	NE2	62GLU	N	0.01	282LYS	NZ	280ALA	O
158SER	OG	149ALA	O	2.19	286ASN	ND2	322THR	O	0.08	97GLN	NE2	96SER	O	0.02	282LYS	NZ	330SER	O
157TYR	OH	136GLY	O	1.56	286ASN	ND2	323PRO	O	0.20	97GLN	NE2	97GLN	N	0.00	275SER	OG	9ASP	O
157TYR	OH	191VAL	O	0.01	286ASN	ND2	327LEU	N	0.02	96SER	OG	92SER	O	85.92	275SER	OG	71SER	O
156ARG	NH2	142GLU	O	31.86	286ASN	ND2	331ALA	O	0.12	96SER	OG	93LEU	N	0.01	275SER	OG	72VAL	O

156ARG	NE	142GLU	O	9.52	282LYS	NZ	277PRO	O	1.22	96SER	OG	93LEU	O	7.95	275SER	OG	73GLY	O
154THR	OG1	141GLY	O	0.00	282LYS	NZ	278ASP	O	2.02	95LYS	NZ	91LEU	O	0.01	275SER	OG	274GLY	O
154THR	OG1	143PRO	O	68.78	282LYS	NZ	280ALA	O	0.21	95LYS	NZ	135THR	O	0.24	275SER	OG	275SER	O
154THR	OG1	153ASN	O	0.44	282LYS	NZ	281GLY	O	0.01	94ARG	NH1	134LEU	O	0.08	275SER	OG	276ALA	O
153ASN	ND2	138ILE	O	3.06	282LYS	NZ	282LYS	O	0.01	94ARG	NE	90LEU	O	0.00	273HIS	ND1	255GLY	O
153ASN	ND2	141GLY	O	0.01	282LYS	NZ	329GLY	O	1.08	92SER	OG	88THR	O	35.93	273HIS	ND1	271PRO	O
153ASN	ND2	143PRO	O	0.02	275SER	OG	9ASP	O	0.20	92SER	OG	89GLY	O	1.08	273HIS	ND1	272VAL	N
153ASN	ND2	152TRP	O	0.01	275SER	OG	72VAL	N	0.00	92SER	OG	91LEU	O	0.00	273HIS	ND1	272VAL	O
153ASN	ND2	154THR	N	0.02	275SER	OG	72VAL	O	0.01	92SER	OG	92SER	O	0.04	273HIS	ND1	273HIS	O
153ASN	ND2	154THR	O	0.02	275SER	OG	73GLY	N	0.00	88THR	OG1	82ARG	O	1.01	273HIS	ND1	274GLY	O
152TRP	NE1	143PRO	O	0.01	275SER	OG	73GLY	O	0.12	88THR	OG1	83LYS	O	0.04	273HIS	ND1	275SER	N
152TRP	NE1	144ARG	O	0.02	275SER	OG	273HIS	O	0.00	88THR	OG1	85ARG	O	85.67	273HIS	ND1	286ASN	O
147SER	OG	146MET	O	0.08	275SER	OG	274GLY	O	5.97	88THR	OG1	86PRO	N	0.00	266THR	OG1	61VAL	O
147SER	OG	150GLU	O	73.77	275SER	OG	275SER	O	0.60	88THR	OG1	89GLY	N	0.01	266THR	OG1	62GLU	O
144ARG	NH2	139TYR	O	0.24	275SER	OG	276ALA	O	2.37	85ARG	NH2	72VAL	O	0.02	266THR	OG1	64ALA	O
144ARG	NH2	187ASN	O	0.01	275SER	OG	280ALA	N	0.00	85ARG	NH2	74GLY	O	0.00	266THR	OG1	65GLU	O
144ARG	NH2	188VAL	O	1.32	275SER	OG	284ILE	O	0.04	85ARG	NH1	77TRP	O	15.62	266THR	OG1	266THR	O
144ARG	NH1	138ILE	O	0.03	275SER	OG	325PRO	O	0.01	85ARG	NH1	78ASP	O	7.88	266THR	OG1	267PRO	O
144ARG	NH1	139TYR	O	2.62	273HIS	ND1	70GLY	O	0.08	85ARG	NH1	80LEU	O	7.11	264ARG	NH2	96SER	O
144ARG	NH1	141GLY	O	0.04	273HIS	ND1	254LEU	O	0.02	82ARG	NH2	80LEU	O	0.04	264ARG	NH2	97GLN	O
144ARG	NH1	187ASN	O	0.00	273HIS	ND1	255GLY	O	1.32	82ARG	NH2	186ALA	O	0.00	264ARG	NH1	96SER	O
144ARG	NH1	188VAL	O	0.58	273HIS	ND1	271PRO	O	0.17	82ARG	NH2	187ASN	O	0.12	264ARG	NH1	97GLN	O
139TYR	OH	237ASN	O	1.96	273HIS	ND1	272VAL	O	0.49	82ARG	NH1	139TYR	O	0.24	264ARG	NH1	98ASP	O
139TYR	OH	238ILE	N	0.00	273HIS	ND1	273HIS	O	1.62	82ARG	NH1	186ALA	O	0.00	264ARG	NH1	264ARG	O
139TYR	OH	238ILE	O	52.66	273HIS	ND1	274GLY	O	3.47	82ARG	NH1	187ASN	O	0.08	264ARG	NE	96SER	O
135THR	OG1	99LEU	O	15.91	273HIS	ND1	286ASN	O	1.54	82ARG	NE	80LEU	O	0.00	264ARG	NE	97GLN	O
135THR	OG1	100PHE	O	0.06	266THR	OG1	61VAL	O	41.21	82ARG	NE	187ASN	O	0.01	264ARG	NE	264ARG	O
135THR	OG1	136GLY	N	0.02	266THR	OG1	62GLU	O	0.02	77TRP	NE1	43GLY	O	0.08	261SER	OG	101ALA	O
132ARG	NH2	133GLU	O	1.56	266THR	OG1	64ALA	O	10.57	77TRP	NE1	84ILE	O	1.38	261SER	OG	102ASN	N
132ARG	NH1	240GLY	O	0.00	266THR	OG1	65GLU	O	0.76	71SER	OG	9ASP	O	0.20	261SER	OG	259SER	O
132ARG	NE	133GLU	O	35.23	266THR	OG1	66ALA	N	0.00	71SER	OG	70GLY	O	0.00	261SER	OG	260ALA	O
132ARG	NE	237ASN	O	1.40	266THR	OG1	265GLY	O	0.01	71SER	OG	71SER	O	0.13	261SER	OG	261SER	O
119LYS	NZ	113GLU	O	2.00	266THR	OG1	266THR	O	0.38	71SER	OG	72VAL	O	0.02	261SER	OG	262LEU	O
119LYS	NZ	114ARG	O	22.22	266THR	OG1	267PRO	N	0.00	71SER	OG	271PRO	O	0.04	261SER	OG	268VAL	O
119LYS	NZ	116SER	O	26.90	266THR	OG1	267PRO	O	1.31	71SER	OG	273HIS	O	4.88	259SER	OG	103LEU	O
119LYS	NZ	118LEU	O	9.32	264ARG	NH2	98ASP	O	0.01	71SER	OG	274GLY	O	11.45	259SER	OG	258PRO	O
116SER	OG	118LEU	O	0.02	264ARG	NH2	99LEU	O	0.28	59LYS	NZ	55GLU	O	0.06	259SER	OG	259SER	N
116SER	OG	249VAL	O	3.82	264ARG	NH1	98ASP	O	1.06	58ARG	NH2	53PHE	O	1.06	259SER	OG	259SER	O
116SER	OG	250LEU	O	91.97	264ARG	NH1	99LEU	O	0.01	58ARG	NH1	53PHE	O	1.66	259SER	OG	260ALA	O

116SER	OG	251PRO	O	0.00	264ARG	NE	98ASP	O	0.25	58ARG	NE	53PHE	O	21.34	259SER	OG	270GLU	O
114ARG	NH2	111GLY	O	2.58	264ARG	NE	99LEU	O	0.57	57THR	OG1	40PRO	O	98.32	259SER	OG	271PRO	O
114ARG	NH2	319LEU	O	0.02	261SER	OG	99LEU	O	0.00	24ARG	NH2	17GLU	O	0.02	253SER	OG	252GLY	O
114ARG	NH2	327LEU	O	0.03	261SER	OG	101ALA	O	3.07	24ARG	NH2	21LYS	N	0.06	253SER	OG	325PRO	O
114ARG	NH1	111GLY	O	0.21	261SER	OG	102ASN	N	0.02	24ARG	NE	20LEU	O	0.16	253SER	OG	326ASP	O
114ARG	NE	111GLY	O	1.36	261SER	OG	260ALA	O	2.03	24ARG	NE	21LYS	N	0.00	248SER	OG	244SER	O
104ARG	NH2	241ASP	O	0.00	261SER	OG	261SER	O	0.09	21LYS	NZ	334GLU	O	0.02	248SER	OG	245ASP	O
104ARG	NH1	244SER	O	1.19	261SER	OG	262LEU	N	0.01	21LYS	NZ	337THR	O	0.02	248SER	OG	246LEU	O
102ASN	ND2	260ALA	O	0.67	261SER	OG	268VAL	O	3.39	16THR	OG1	7PRO	O	2.65	248SER	OG	248SER	N
102ASN	ND2	261SER	N	0.01	259SER	OG	103LEU	O	11.70	16THR	OG1	12GLY	O	89.34	248SER	OG	253SER	O
97GLN	NE2	61VAL	O	0.05	259SER	OG	258PRO	O	0.56	2LYS	NZ	36TYR	O	0.01	248SER	OG	254LEU	O
97GLN	NE2	62GLU	N	0.00	259SER	OG	259SER	O	0.07	2LYS	NZ	63GLU	O	2.09	244SER	OG	240GLY	O
97GLN	NE2	96SER	O	0.03	259SER	OG	260ALA	O	0.02	343HIS	NE2	317LYS	O	0.92	244SER	OG	241ASP	O
96SER	OG	92SER	O	83.31	259SER	OG	270GLU	O	73.87	343HIS	NE2	318ALA	N	0.01	244SER	OG	244SER	O
96SER	OG	93LEU	N	0.01	253SER	OG	106ALA	O	0.00	342ARG	NH2	338ALA	O	0.00	237ASN	ND2	133GLU	N
96SER	OG	93LEU	O	9.59	253SER	OG	107LYS	O	57.99	342ARG	NH1	338ALA	O	0.01	237ASN	ND2	133GLU	O
95LYS	NZ	91LEU	O	0.04	253SER	OG	248SER	O	1.12	342ARG	NH1	342ARG	O	0.17	237ASN	ND2	134LEU	N
95LYS	NZ	95LYS	O	0.01	253SER	OG	251PRO	O	0.02	342ARG	NE1	338ALA	O	0.04	237ASN	ND2	134LEU	O
95LYS	NZ	135THR	O	0.02	253SER	OG	252GLY	O	0.06	342ARG	NE1	342ARG	O	0.02	237ASN	ND2	135THR	N
94ARG	NH2	87GLU	O	0.01	253SER	OG	253SER	N	0.00	339THR	OG1	335ALA	O	73.35	237ASN	ND2	136GLY	O
94ARG	NH2	134LEU	O	0.00	253SER	OG	253SER	O	2.36	339THR	OG1	336PHE	O	1.50	237ASN	ND2	137GLY	N
94ARG	NH1	134LEU	O	0.78	253SER	OG	254LEU	N	0.00	339THR	OG1	338ALA	O	0.00	237ASN	ND2	137GLY	O
94ARG	NE	90LEU	O	0.06	253SER	OG	256LEU	O	0.91	339THR	OG1	340VAL	N	0.02	237ASN	ND2	236GLY	O
92SER	OG	88THR	O	20.40	248SER	OG	105PRO	O	0.00	337THR	OG1	18ALA	O	0.03	237ASN	ND2	237ASN	O
92SER	OG	89GLY	O	0.88	248SER	OG	244SER	O	30.46	337THR	OG1	333THR	O	75.07	235THR	OG1	183VAL	O
92SER	OG	92SER	O	0.02	248SER	OG	245ASP	O	43.87	337THR	OG1	334GLU	O	1.03	235THR	OG1	236GLY	O
88THR	OG1	82ARG	O	32.65	248SER	OG	247ALA	O	0.00	333THR	OG1	283GLY	O	6.97	235THR	OG1	239PHE	O
88THR	OG1	83LYS	O	0.68	248SER	OG	248SER	O	0.04	333THR	OG1	285ALA	O	75.85	235THR	OG1	240GLY	O
88THR	OG1	85SER	O	32.15	248SER	OG	253SER	O	0.20	330SER	OG1	329GLY	O	1.22	229ARG	NH2	228ALA	O
88THR	OG1	86PRO	O	0.03	248SER	OG	254LEU	N	0.00	330SER	OG1	330SER	O	0.47	229ARG	NH2	229ARG	O
88THR	OG1	89GLY	N	0.02	248SER	OG	256LEU	O	0.16	330SER	OG1	331ALA	O	0.00	229ARG	NH1	228ALA	O
85SER	OG	77TRP	O	0.09	244SER	OG	240GLY	O	74.69	322THR	OG1	318ALA	O	18.03	229ARG	NH1	229ARG	O
85SER	OG	80LEU	O	0.04	244SER	OG	241ASP	O	11.38	322THR	OG1	322THR	O	15.95	229ARG	NE	226SER	O
85SER	OG	81PRO	O	20.55	244SER	OG	243LEU	O	0.01	322THR	OG1	330SER	O	0.10	229ARG	NE	228ALA	O
85SER	OG	82ARG	N	0.01	237ASN	ND2	133GLU	O	3.90	317LYS	NZ1	313ASP	O	0.14	229ARG	NE	229ARG	N
85SER	OG	82ARG	O	24.02	237ASN	ND2	134LEU	N	0.06	317LYS	NZ1	343HIS	O	0.02	229ARG	NE	229ARG	O
85SER	OG	84ILE	O	0.03	237ASN	ND2	134LEU	O	16.01	310LYS	NZ	343HIS	O	0.14	226SER	OG	222HIS	O
85SER	OG	85SER	O	0.04	237ASN	ND2	135THR	N	2.79	310LYS	NZ	344LEU	O	0.48	226SER	OG	225ARG	O
85SER	OG	86PRO	N	0.00	237ASN	ND2	135THR	O	0.00	310LYS	NZ	345ALA	O	13.88	226SER	OG	226SER	O

85SER	OG	86PRO	O	0.02	237ASN	ND2	136GLY	O	79.87	310LYS	NZ	345ALA	O	22.27	225ARG	NH2	115LEU	O
83LYS	NZ	50GLY	O	0.06	237ASN	ND2	137GLY	N	0.04	309ARG	NH1	305VAL	O	0.03	225ARG	NH2	252GLY	O
82ARG	NH2	77TRP	O	0.01	237ASN	ND2	137GLY	O	0.04	293SER	OG	259SER	O	1.77	225ARG	NH2	326ASP	O102
82ARG	NH2	80LEU	O	0.69	237ASN	ND2	237ASN	O	0.02	293SER	OG	270GLU	O	0.28	225ARG	NH1	115LEU	O
82ARG	NH2	186ALA	O	0.08	235THR	OG1	183VAL	O	14.13	293SER	OG	289ALA	O	92.56	225ARG	NH1	252GLY	O
82ARG	NH2	187ASN	O	0.19	235THR	OG1	236GLY	O	2.28	293SER	OG	290ALA	O	0.08	225ARG	NH1	326ASP	O102
82ARG	NH1	82ARG	O	0.00	235THR	OG1	239PHE	O	1.98	293SER	OG	293SER	O	0.03	225ARG	NE	252GLY	O
82ARG	NH1	186ALA	O	0.01	235THR	OG1	240GLY	N	0.09	288THR	OG1	255GLY	O	46.10	222HIS	NE2	229ARG	O
82ARG	NH1	187ASN	O	0.14	229ARG	NH2	228ALA	O	0.00	288THR	OG1	256LEU	O	0.02	215TYR	OH	186ALA	O
82ARG	NE	82ARG	N	0.00	229ARG	NH2	229ARG	O	0.01	286ASN	ND2	255GLY	O	2.45	215TYR	OH	87GLU	O
77TRP	NE1	9ASP	O	0.00	229ARG	NH1	228ALA	O	0.21	286ASN	ND2	284ILE	O	0.12	214GLN	NE2	182SER	O
77TRP	NE1	43GLY	O	0.01	229ARG	NH1	229ARG	O	0.36	286ASN	ND2	322THR	O	0.25	214GLN	NE2	212GLU	O
77TRP	NE1	73GLY	O	0.00	229ARG	NE	228ALA	O	0.14	286ASN	ND2	326ASP	O	0.00	214GLN	NE2	213HIS	O
76LYS	NZ	43GLY	O	0.11	229ARG	NE	229ARG	O	1.44	286ASN	ND2	331ALA	N	0.00	214GLN	NE2	214GLN	N
71SER	OG	9ASP	O	9.86	226SER	OG	222HIS	O	5.07	286ASN	ND2	331ALA	O	0.94	214GLN	NE2	215TYR	O
71SER	OG	71SER	O	2.63	226SER	OG	225ARG	O	2.58	282LYS	NZ	278ASP	O	1.35	214GLN	NE2	218ALA	O
71SER	OG	273HIS	O	0.00	226SER	OG	226SER	O	4.44	282LYS	NZ	280ALA	O	0.47	214GLN	NE2	219MET	N
71SER	OG	274GLY	O	9.89	226SER	OG	228ALA	O	0.02	275SER	OG	72VAL	O	0.18	213HIS	NE2	196ARG	O
59LYS	NZ	55GLU	O	0.02	225ARG	NH2	218ALA	O	0.01	275SER	OG	73GLY	N	0.02	206TYR	OH	167ARG	O
58ARG	NH1	53PHE	O	2.24	225ARG	NH2	221MET	O	0.14	275SER	OG	73GLY	O	0.83	204ARG	NH2	200GLU	O
57THR	OG1	40PRO	N	0.00	225ARG	NH2	222HIS	N	0.06	275SER	OG	74GLY	N	0.03	204ARG	NE	200GLU	O
57THR	OG1	40PRO	O	97.35	225ARG	NH2	115LEU	O	0.00	275SER	OG	274GLY	O	0.60	198THR	OG1	194PHE	O
24ARG	NH2	17GLU	O	0.02	225ARG	NH2	252GLY	O	6.16	275SER	OG	275SER	O	0.73	198THR	OG1	195TRP	O
24ARG	NH2	21LYS	N	0.18	225ARG	NH2	278ASP	O	0.00	275SER	OG	276ALA	O	0.02	198THR	OG1	198THR	N
24ARG	NH2	34LEU	O	0.74	225ARG	NH2	326ASP	O101	0.24	273HIS	ND1	271PRO	O	0.03	198THR	OG1	198THR	O
24ARG	NH1	34LEU	O	0.21	225ARG	NH1	252GLY	O	6.30	273HIS	ND1	272VAL	O	0.04	197LYS	NZ	197LYS	O
24ARG	NE	20LEU	O	0.60	225ARG	NH1	326ASP	O101	0.03	273HIS	ND1	273HIS	O	0.06	197LYS	NZ	147SER	O
24ARG	NE	21LYS	N	0.01	225ARG	NE	221MET	O	5.28	273HIS	ND1	274GLY	O	0.03	197LYS	NZ	148GLU	O
24ARG	NE	24ARG	O	0.00	225ARG	NE	252GLY	O	0.13	273HIS	ND1	275SER	O	0.01	196ARG	NH2	213HIS	O
21LYS	NZ	17GLU	O	0.02	222HIS	NE2	226SER	O	0.00	273HIS	ND1	286ASN	O	0.00	196ARG	NH2	214GLN	O
21LYS	NZ	333THR	O	0.75	222HIS	NE2	229ARG	O	0.01	266THR	OG1	61VAL	O	83.76	195TRP	NE1	183VAL	O
21LYS	NZ	334GLU	O	0.32	215TYR	OH	186ALA	O	0.94	266THR	OG1	64ALA	O	0.52	195TRP	NE1	234VAL	O
16THR	OG1	7PRO	O	1.77	214GLN	NE2	212GLU	O	0.02	264ARG	NH2	96SER	O	26.19	187ASN	ND2	87GLU	O
16THR	OG1	12GLY	O	94.75	214GLN	NE2	213HIS	O	33.96	264ARG	NH1	96SER	O	2.32	182SER	OG	182SER	O
2LYS	NZ	63GLU	O	0.78	214GLN	NE2	214GLN	N	0.13	264ARG	NH1	264ARG	O	0.28	182SER	OG	183VAL	O
343HIS	NE2	317LYS	O	1.02	214GLN	NE2	215TYR	O	0.54	264ARG	NE	97GLN	O	0.01	182SER	OG	212GLU	O
343HIS	NE2	318ALA	N	0.00	214GLN	NE2	218ALA	O	0.36	264ARG	NE	264ARG	O	0.06	182SER	OG	234VAL	O
342ARG	NH1	338ALA	O	0.00	214GLN	NE2	219MET	N	0.16	261SER	OG	101ALA	O	1.58	179HIS	NE2	211LEU	O
342ARG	NH1	342ARG	O	0.45	206TYR	OH	167ARG	O	0.65	261SER	OG	260ALA	O	0.49	179HIS	NE2	229ARG	O

342ARG	NH1	345ALA	O	0.12
342ARG	NE	338ALA	O	0.02
342ARG	NE	342ARG	O	0.32
339THR	OG1	335ALA	O	78.57
339THR	OG1	336PHE	O	0.03
339THR	OG1	339THR	O	0.00
337THR	OG1	333THR	O	93.98
337THR	OG1	334GLU	O	0.00
333THR	OG1	283GLY	O	0.67
333THR	OG1	285ALA	O	90.50
330SER	OG	329GLY	O	1.00
330SER	OG	330SER	O	0.24
322THR	OG1	318ALA	O	19.50
322THR	OG1	322THR	O	17.26
317LYS	NZ	313ASP	O	0.07
317LYS	NZ1	343HIS	O	0.06
310LYS	NZ	29ALA	O	0.03
310LYS	NZ	306GLU	O	0.10
310LYS	NZ	343HIS	O1040	0.08
310LYS	NZ	344LEU	O1041	2.39
310LYS	NZ	345ALA	O1104	23.96
310LYS	NZ	345ALA	O2104	24.35
309ARG	NH2	175LYS	O	9.52
300HIS	NE2	65GLU	O	0.00
300HIS	NE2	261SER	O	0.01
293SER	OG	259SER	O	0.06
293SER	OG	270GLU	O	0.00
293SER	OG	289ALA	O	98.46
293SER	OG	293SER	O	0.00
286ASN	ND2	322THR	O1011	0.08
286ASN	ND2	331ALA	O1022	0.00
282LYS	NZ	278ASP	O	10.31
282LYS	NZ	280ALA	O	0.03
275SER	OG	9ASP	O	5.37
275SER	OG	71SER	O	0.02
275SER	OG	72VAL	O	0.21
275SER	OG	73GLY	O	87.29
275SER	OG	74GLY	N	0.00
275SER	OG	274GLY	O	0.01

206TYR	OH	170PHE	O	0.96
206TYR	OH	171GLU	N	0.01
206TYR	OH	202VAL	O	0.02
204ARG	NH2	197LYS	O	0.12
204ARG	NH2	211LEU	O	0.26
204ARG	NH1	201GLU	N	0.00
204ARG	NH1	211LEU	O	6.77
204ARG	NE	200GLU	O	0.02
198THR	OG1	194PHE	O	95.71
198THR	OG1	195TRP	N	0.00
198THR	OG1	195TRP	O	0.58
198THR	OG1	199VAL	N	0.00
197LYS	NZ	193GLU	O	0.15
197LYS	NZ	147SER	O	0.31
197LYS	NZ	148GLU	O	0.06
196ARG	NH2	186ALA	O	0.35
196ARG	NH2	213HIS	O	0.02
196ARG	NH2	214GLN	O	0.04
196ARG	NH2	215TYR	N	0.02
195TRP	NE1	183VAL	O	0.29
195TRP	NE1	234VAL	O	0.06
187ASN	ND2	185LYS	O	0.00
187ASN	ND2	186ALA	O	0.02
182SER	OG	183VAL	O	0.02
182SER	OG	212GLU	O	0.00
182SER	OG	234VAL	O	0.00
179HIS	NE2	211LEU	O	0.02
179HIS	NE2	212GLU	N	0.01
179HIS	NE2	229ARG	O	13.14
179HIS	NE2	230PHE	N	0.00
178LYS	NZ	174ARG	O	0.07
178LYS	NZ	176ARG	O	0.06
178LYS	NZ	177ARG	O	0.01
178LYS	NZ	208ASP	O	0.25
177ARG	NH2	125GLY	O	4.02
177ARG	NH2	127ASP	O	10.93
177ARG	NH2	228ALA	O	2.86
177ARG	NH2	229ARG	O	1.15
177ARG	NH2	230PHE	N	0.00

261SER	OG	268VAL	O	0.05
259SER	OG	259SER	O	0.04
259SER	OG	270GLU	O	90.02
259SER	OG	271PRO	O	0.00
253SER	OG	251PRO	O	0.00
253SER	OG	252GLY	O	0.70
253SER	OG	253SER	O	0.02
253SER	OG	326ASP	O	0.03
248SER	OG	244SER	O	69.89
248SER	OG	245ASP	O	18.07
248SER	OG	247ALA	O	0.00
248SER	OG	248SER	O	0.04
248SER	OG	253SER	O	0.27
244SER	OG	240GLY	O	74.22
244SER	OG	241ASP	O	0.01
244SER	OG	245ASP	N	0.08
237ASN	ND2	133GLU	O	24.15
237ASN	ND2	134LEU	N	0.09
237ASN	ND2	134LEU	O	3.01
237ASN	ND2	135THR	N	0.89
237ASN	ND2	136GLY	O	57.53
235THR	OG1	183VAL	O	7.17
235THR	OG1	236GLY	O	2.31
235THR	OG1	239PHE	O	0.53
229ARG	NH2	229ARG	O	0.00
229ARG	NE	229ARG	O	38.74
229ARG	NE	230PHE	N	0.00
226SER	OG	222HIS	O	1.01
226SER	OG	225ARG	O	3.45
226SER	OG	226SER	O	3.15
225ARG	NH2	252GLY	O	30.21
225ARG	NH2	326ASP	O	1.15
225ARG	NH2	221MET	O	0.00
225ARG	NH1	252GLY	O	0.88
225ARG	NH1	326ASP	O	0.02
225ARG	NH1	221MET	O	0.56
225ARG	NE	252GLY	O	0.21
222HIS	NE2	226SER	O	0.05
215TYR	OH	78ASP	O	0.49

178LYS	NZ	174ARG	O
178LYS	NZ	176ARG	O
178LYS	NZ	177ARG	O
178LYS	NZ	208ASP	O
177ARG	NH2	125GLY	O
177ARG	NH2	127ASP	N
177ARG	NH2	127ASP	O
177ARG	NH2	228ALA	O
177ARG	NH2	229ARG	O
177ARG	NH2	230PHE	O
177ARG	NH1	125GLY	O
177ARG	NH1	127ASP	O
177ARG	NH1	228ALA	O
177ARG	NH1	229ARG	O
177ARG	NH1	230PHE	O
176ARG	NH2	127ASP	O
176ARG	NH2	128VAL	O
176ARG	NH1	127ASP	O
176ARG	NH1	128VAL	N
176ARG	NH1	231ASP	O
175LYS	NZ	171GLU	O
175LYS	NZ	174ARG	O
175LYS	NZ	175LYS	O
175LYS	NZ	299GLU	O
175LYS	NZ	300HIS	O
174ARG	NH2	205GLY	O
167ARG	NH2	201GLU	O
167ARG	NH2	202VAL	O
167ARG	NH1	163GLU	O
167ARG	NH1	201GLU	O
167ARG	NH1	202VAL	O
167ARG	NE	163GLU	O
164ARG	NH2	98ASP	O
164ARG	NH2	99LEU	O
164ARG	NH1	98ASP	O
164ARG	NE	99LEU	O
164ARG	NE	160PRO	O
164ARG	NE	161GLU	O
159LYS	NZ	197LYS	O

275SER	OG	275SER	O	0.00
275SER	OG	276ALA	O	0.03
273HIS	ND1	255GLY	O	5.43
273HIS	ND1	271PRO	O	0.02
273HIS	ND1	273HIS	O	0.81
273HIS	ND1	274GLY	O	3.64
273HIS	ND1	286ASN	O	2.66
266THR	OG1	61VAL	O	31.23
266THR	OG1	64ALA	O	0.13
266THR	OG1	265GLY	O	0.05
264ARG	NH2	96SER	O	1.28
264ARG	NH2	98ASP	O	0.00
264ARG	NH2	99LEU	O	0.25
264ARG	NH1	96SER	O	1.18
264ARG	NE	98ASP	O	0.42
264ARG	NE	99LEU	O	0.01
264ARG	NE	264ARG	O	0.03
261SER	OG	101ALA	O	0.09
261SER	OG	260ALA	O	0.55
261SER	OG	261SER	O	0.14
261SER	OG	268VAL	O	5.52
259SER	OG	103LEU	O	2.91
259SER	OG	258PRO	O	0.02
259SER	OG	259SER	O	0.05
259SER	OG	260ALA	O	0.02
259SER	OG	270GLU	O	85.25
253SER	OG	107LYS	O	8.62
253SER	OG	251PRO	O	0.11
253SER	OG	252GLY	O	0.20
253SER	OG	253SER	O	0.08
253SER	OG	326ASP	O1016	0.01
248SER	OG	244SER	O	39.94
248SER	OG	245ASP	O	12.99
248SER	OG	248SER	O	0.06
248SER	OG	253SER	O	0.65
248SER	OG	254LEU	O	0.15
244SER	OG	240GLY	O	89.65
244SER	OG	244SER	O	0.04
237ASN	ND2	133GLU	O	0.23

177ARG	NH2	230PHE	O	9.92
177ARG	NH1	125GLY	O	0.81
177ARG	NH1	127ASP	O	0.36
177ARG	NH1	177ARG	O	0.26
177ARG	NH1	228ALA	O	22.88
177ARG	NH1	229ARG	O	1.16
177ARG	NH1	230PHE	N	0.01
177ARG	NH1	230PHE	O	4.70
177ARG	NE	230PHE	O	0.02
176ARG	NH2	127ASP	O	0.06
176ARG	NH2	128VAL	O	0.01
176ARG	NH1	127ASP	O	41.11
176ARG	NH1	231ASP	O	0.10
175LYS	NZ	171GLU	O	2.34
175LYS	NZ	299GLU	O	6.14
175LYS	NZ	300HIS	O	0.01
175LYS	NZ	303GLY	O	0.22
174ARG	NH2	207PRO	O	0.18
174ARG	NH1	170PHE	O	0.02
174ARG	NH1	205GLY	O	0.00
174ARG	NH1	207PRO	O	0.16
174ARG	NE	170PHE	O	0.02
174ARG	NE	171GLU	O	0.08
174ARG	NE	207PRO	O	0.06
167ARG	NH2	202VAL	O	0.00
167ARG	NH2	205GLY	O	0.00
167ARG	NH1	163GLU	O	0.00
167ARG	NH1	202VAL	O	0.10
167ARG	NH1	205GLY	O	0.07
167ARG	NE	163GLU	O	0.03
167ARG	NE	167ARG	O	0.02
164ARG	NH2	160PRO	O	0.00
164ARG	NH1	160PRO	O	0.01
164ARG	NH1	161GLU	O	0.01
164ARG	NE	160PRO	O	0.02
159LYS	NZ	197LYS	O	0.02
159LYS	NZ	148GLU	O	3.07
158SER	OG	157TYR	O	0.00
158SER	OG	158SER	O	0.06

215TYR	OH	186ALA	O	0.01
214GLN	NE2	213HIS	O	0.08
214GLN	NE2	218ALA	O	0.00
213HIS	NE2	196ARG	O	1.08
213HIS	NE2	200GLU	N	0.01
206TYR	OH	167ARG	O	0.39
204ARG	NE	200GLU	O	0.05
198THR	OG1	194PHE	O	92.57
198THR	OG1	195TRP	O	0.01
197LYS	NZ	148GLU	O	0.08
196ARG	NH2	213HIS	O	0.02
196ARG	NH2	214GLN	O	0.01
195TRP	NE1	183VAL	O	0.03
182SER	OG	183VAL	O	0.03
182SER	OG	212GLU	O	0.01
182SER	OG	234VAL	O	0.00
179HIS	NE2	212GLU	N	0.00
179HIS	NE2	229ARG	O	0.16
178LYS	NZ	174ARG	O	1.84
178LYS	NZ	176ARG	O	1.42
178LYS	NZ	177ARG	O	0.03
178LYS	NZ	208ASP	O	0.19
177ARG	NH2	125GLY	O	0.03
177ARG	NH2	127ASP	O	0.97
177ARG	NH2	228ALA	O	26.93
177ARG	NH2	229ARG	O	0.29
177ARG	NH2	230PHE	O	0.74
177ARG	NH1	228ALA	O	33.25
177ARG	NH1	229ARG	O	10.67
177ARG	NH1	230PHE	N	0.00
177ARG	NH1	230PHE	O	0.48
176ARG	NH2	127ASP	O	0.06
176ARG	NH1	127ASP	O	91.12
176ARG	NH1	231ASP	O	0.14
175LYS	NZ	171GLU	O	0.49
175LYS	NZ	299GLU	O	0.48
174ARG	NH2	205GLY	O	0.17
167ARG	NH1	163GLU	O	0.01
167ARG	NE	163GLU	O	0.02

159LYS	NZ	148GLU	O
158SER	OG	157TYR	O
158SER	OG	158SER	O
158SER	OG	149ALA	O
157TYR	OH	136GLY	O
157TYR	OH	137GLY	N
156ARG	NH2	142GLU	O
156ARG	NH1	142GLU	O
156ARG	NH1	156ARG	O
156ARG	NE	142GLU	O
154THR	OG1	143PRO	N
154THR	OG1	143PRO	O
154THR	OG1	153ASN	O
153ASN	ND2	138ILE	O
153ASN	ND2	152TRP	O
152TRP	NE1	143PRO	O
152TRP	NE1	144ARG	N
152TRP	NE1	144ARG	O
147SER	OG	146MET	O
147SER	OG	147SER	O
147SER	OG	150GLU	O
144ARG	NH2	139TYR	O
144ARG	NH2	140PHE	O
144ARG	NH2	187ASN	O
144ARG	NH2	188VAL	O
144ARG	NH2	189LEU	N
144ARG	NH1	138ILE	O
144ARG	NH1	139TYR	O
144ARG	NH1	140PHE	N
144ARG	NH1	140PHE	O
144ARG	NH1	141GLY	O
144ARG	NH1	188VAL	O
144ARG	NE	141GLY	O
144ARG	NE	144ARG	O
139TYR	OH	136GLY	O
139TYR	OH	237ASN	O
139TYR	OH	238ILE	O
135THR	OG1	99LEU	O
135THR	OG1	100PHE	O

237ASN	ND2	134LEU	N	0.38
237ASN	ND2	134LEU	O	16.70
237ASN	ND2	135THR	N	1.69
237ASN	ND2	136GLY	O	90.14
237ASN	ND2	137GLY	N	0.00
237ASN	ND2	137GLY	O	0.01
237ASN	ND2	237ASN	O	0.06
235THR	OG1	183VAL	O	70.94
235THR	OG1	236GLY	O	0.43
235THR	OG1	239PHE	O	0.49
235THR	OG1	240GLY	N	0.02
229ARG	NH2	229ARG	O	0.03
229ARG	NH1	229ARG	O	0.24
229ARG	NE	229ARG	O	38.96
226SER	OG	122ILE	O	0.22
226SER	OG	222HIS	O	1.15
226SER	OG	225ARG	O	7.99
226SER	OG	226SER	O	2.81
225ARG	NH2	115LEU	O	0.25
225ARG	NH2	248SER	O	0.02
225ARG	NH2	252GLY	O	0.19
225ARG	NH2	218ALA	O	0.00
225ARG	NH2	221MET	O	0.09
225ARG	NH2	222HIS	N	0.09
225ARG	NH1	115LEU	O	3.87
225ARG	NH1	252GLY	O	0.05
225ARG	NH1	221MET	O	0.30
225ARG	NE	221MET	O	21.20
225ARG	NE	225ARG	O	2.17
215TYR	OH	186ALA	O	0.27
214GLN	NE2	213HIS	O	6.74
214GLN	NE2	214GLN	N	0.04
214GLN	NE2	215TYR	N	0.00
214GLN	NE2	215TYR	O	0.02
206TYR	OH	167ARG	O	0.69
206TYR	OH	170PHE	O	0.89
206TYR	OH	171GLU	N	0.02
204ARG	NH2	197LYS	O	0.00
204ARG	NH2	211LEU	O	0.14

158SER	OG	149ALA	O	11.70
157TYR	OH	136GLY	O	3.19
156ARG	NH2	142GLU	O	3.00
156ARG	NH1	142GLU	O	30.49
156ARG	NE	142GLU	O	2.71
154THR	OG1	143PRO	N	0.01
154THR	OG1	143PRO	O	76.10
154THR	OG1	153ASN	O	1.38
154THR	OG1	153ASN	O	0.00
153ASN	ND2	138ILE	O	11.65
153ASN	ND2	141GLY	O	0.21
153ASN	ND2	152TRP	O	0.10
153ASN	ND2	154THR	N	0.04
153ASN	ND2	154THR	O	0.03
153ASN	ND2	188VAL	O	0.01
152TRP	NE1	143PRO	O	0.04
152TRP	NE1	144ARG	N	0.02
152TRP	NE1	144ARG	O	0.27
147SER	OG	146MET	O	0.35
147SER	OG	147SER	O	0.00
147SER	OG	150GLU	O	51.40
144ARG	NH2	138ILE	O	0.00
144ARG	NH2	139TYR	O	1.04
144ARG	NH2	188VAL	O	16.91
144ARG	NH1	138ILE	O	0.16
144ARG	NH1	139TYR	O	27.82
144ARG	NH1	140PHE	N	0.07
144ARG	NH1	141GLY	O	0.20
144ARG	NH1	188VAL	O	0.29
144ARG	NE	139TYR	O	0.82
144ARG	NE	144ARG	O	0.01
139TYR	OH	134LEU	O	0.03
139TYR	OH	136GLY	O	0.41
139TYR	OH	237ASN	O	3.77
139TYR	OH	238ILE	O	0.54
135THR	OG1	99LEU	O	1.22
135THR	OG1	100PHE	O	0.56
135THR	OG1	134LEU	O	0.04
135THR	OG1	135THR	O	0.24

164ARG	NH2	98ASP	O	0.08
164ARG	NH1	98ASP	O	0.00
159LYS	NZ	148GLU	O	2.09
158SER	OG	149ALA	O	2.65
158SER	OG	158SER	O	0.01
156ARG	NH2	142GLU	O	9.24
156ARG	NH1	142GLU	O	6.66
156ARG	NE	142GLU	O	16.82
154THR	OG1	143PRO	O	78.70
154THR	OG1	153ASN	O	0.23
154THR	OG1	154THR	N	0.00
153ASN	ND2	188VAL	O	0.00
153ASN	ND2	138ILE	O	2.68
153ASN	ND2	141GLY	O	0.04
153ASN	ND2	154THR	N	0.00
152TRP	NE1	143PRO	O	0.03
152TRP	NE1	144ARG	N	0.00
152TRP	NE1	144ARG	O	0.07
147SER	OG	146MET	O	0.27
147SER	OG	150GLU	O	58.02
144ARG	NH2	188VAL	O	2.55
144ARG	NH2	139TYR	O	0.06
144ARG	NH2	140PHE	O	0.00
144ARG	NH1	188VAL	O	0.04
144ARG	NH1	139TYR	O	4.52
144ARG	NH1	140PHE	N	0.00
144ARG	NH1	140PHE	O	0.02
144ARG	NH1	144ARG	O	0.02
144ARG	NE	139TYR	O	0.00
144ARG	NE	141GLY	O	0.17
144ARG	NE	144ARG	N	0.00
144ARG	NE	144ARG	O	0.01
139TYR	OH	237ASN	O	4.13
139TYR	OH	238ILE	O	68.43
135THR	OG1	99LEU	O	0.28
135THR	OG1	100PHE	O	0.01
135THR	OG1	136GLY	N	0.01
132ARG	NH2	133GLU	O	0.65
132ARG	NH2	237ASN	O	0.01

135THR	OG1	135THR	N
135THR	OG1	135THR	O
135THR	OG1	136GLY	N
132ARG	NH2	133GLU	O
132ARG	NH2	237ASN	O
132ARG	NH2	241ASP	O
132ARG	NH1	133GLU	O
132ARG	NH1	237ASN	O
132ARG	NH1	240GLY	O
132ARG	NH1	241ASP	O
132ARG	NE	133GLU	O
132ARG	NE	236GLY	O
132ARG	NE	237ASN	O
132ARG	NE	240GLY	O
124ARG	NH2	120GLU	O
124ARG	NH1	120GLU	O
124ARG	NH1	124ARG	O
124ARG	NE	120GLU	O
124ARG	NE	121GLU	O
119LYS	NZ	113GLU	O
119LYS	NZ	114ARG	O
119LYS	NZ	115LEU	O
119LYS	NZ	116SER	O
119LYS	NZ	117PRO	O
119LYS	NZ	118LEU	O
116SER	OG	113GLU	O
116SER	OG	118LEU	O
116SER	OG	249VAL	O
116SER	OG	250LEU	O
116SER	OG	251PRO	O
114ARG	NH2	111GLY	O
114ARG	NH2	319LEU	O
114ARG	NH1	111GLY	O
114ARG	NE	111GLY	N
114ARG	NE	111GLY	O
104ARG	NH1	244SER	O
104ARG	NH1	245ASP	N
104ARG	NE	258PRO	O
102ASN	ND2	102ASN	O

204ARG	NH1	197LYS	O	0.00
204ARG	NH1	211LEU	O	1.01
204ARG	NE	200GLU	O	0.02
204ARG	NE	201GLU	O	0.01
198THR	OG1	194PHE	O	98.01
198THR	OG1	195TRP	O	0.00
197LYS	NZ	147SER	O	0.18
197LYS	NZ	148GLU	O	0.04
197LYS	NZ	193GLU	O	0.07
196ARG	NH2	213HIS	O	0.02
196ARG	NH2	214GLN	O	0.00
196ARG	NH2	215TYR	N	0.02
195TRP	NE1	183VAL	O	0.00
187ASN	ND2	185LYS	O	0.00
179HIS	NE2	211LEU	O	0.00
179HIS	NE2	229ARG	O	0.12
178LYS	NZ	174ARG	O	0.01
178LYS	NZ	176ARG	O	0.02
178LYS	NZ	208ASP	O	0.02
177ARG	NH2	125GLY	O	2.84
177ARG	NH2	127ASP	O	3.08
177ARG	NH2	228ALA	O	19.64
177ARG	NH2	229ARG	O	0.63
177ARG	NH2	230PHE	O	2.38
177ARG	NH1	125GLY	O	0.04
177ARG	NH1	127ASP	O	0.10
177ARG	NH1	228ALA	O	25.09
177ARG	NH1	229ARG	O	8.06
177ARG	NH1	230PHE	N	0.00
177ARG	NH1	230PHE	O	0.24
177ARG	NE	230PHE	O	0.00
176ARG	NH2	127ASP	O	0.02
176ARG	NH1	127ASP	O	88.95
176ARG	NH1	231ASP	O	0.06
175LYS	NZ	171GLU	O	1.48
175LYS	NZ	299GLU	O	7.84
175LYS	NZ	300HIS	O	0.01
174ARG	NH1	207PRO	O	0.00
174ARG	NE	171GLU	O	0.01

132ARG	NH2	133GLU	O	0.02
132ARG	NH2	237ASN	O	1.16
132ARG	NH1	240GLY	O	0.08
132ARG	NE	133GLU	O	26.49
132ARG	NE	236GLY	O	0.11
132ARG	NE	237ASN	O	14.42
132ARG	NE	241ASP	N	0.00
124ARG	NH1	120GLU	O	0.02
124ARG	NE	120GLU	O	0.02
124ARG	NE	124ARG	O	0.01
119LYS	NZ	113GLU	O	0.21
119LYS	NZ	114ARG	O	45.52
119LYS	NZ	115LEU	O	0.07
119LYS	NZ	116SER	O	32.95
119LYS	NZ	118LEU	O	0.70
116SER	OG	112LEU	O	0.02
116SER	OG	113GLU	O	0.03
116SER	OG	118LEU	O	0.12
116SER	OG	249VAL	O	10.22
116SER	OG	250LEU	O	80.57
116SER	OG	251PRO	O	0.04
116SER	OG	252GLY	N	0.09
114ARG	NH2	111GLY	O	5.65
114ARG	NH2	327LEU	O	1.08
114ARG	NH1	111GLY	O	1.79
114ARG	NH1	327LEU	O	0.20
114ARG	NE	111GLY	O	3.60
107LYS	NZ	108VAL	O	0.10
104ARG	NH2	272VAL	O	0.11
104ARG	NH1	105PRO	O	0.00
104ARG	NH1	244SER	O	24.87
104ARG	NE	244SER	O	0.00
102ASN	ND2	103LEU	N	0.02
102ASN	ND2	103LEU	O	0.00
102ASN	ND2	260ALA	O	1.68
102ASN	ND2	261SER	N	0.02
97GLN	NE2	61VAL	O	3.57
97GLN	NE2	62GLU	N	0.05
97GLN	NE2	93LEU	O	0.17

132ARG	NH1	240GLY	O	1.04
132ARG	NE	133GLU	O	15.28
132ARG	NE	237ASN	O	1.19
119LYS	NZ	113GLU	O	0.13
119LYS	NZ	114ARG	O	54.89
119LYS	NZ	115LEU	O	0.01
119LYS	NZ	116SER	O	28.00
119LYS	NZ	118LEU	O	0.25
116SER	OG	118LEU	O	0.07
116SER	OG	249VAL	O	54.90
116SER	OG	250LEU	O	35.54
114ARG	NH2	111GLY	O	3.78
114ARG	NH2	319LEU	O	0.00
114ARG	NH2	320LEU	O	0.38
114ARG	NH2	327LEU	O	0.09
114ARG	NH1	111GLY	O	1.45
114ARG	NH1	320LEU	O	0.39
114ARG	NE	111GLY	O	3.94
104ARG	NH2	244SER	O	0.23
102ASN	ND2	260ALA	O	0.38
102ASN	ND2	261SER	N	0.00
97GLN	NE2	61VAL	O	0.02
97GLN	NE2	93LEU	O	0.00
97GLN	NE2	96SER	O	0.06
96SER	OG	92SER	O	88.20
96SER	OG	93LEU	N	0.01
96SER	OG	93LEU	O	5.42
94ARG	NH2	134LEU	O	2.12
94ARG	NH2	135THR	O	0.10
94ARG	NH1	134LEU	O	0.46
94ARG	NH1	135THR	O	0.02
94ARG	NE	90LEU	O	0.43
94ARG	NE	94ARG	O	0.05
94ARG	NE	99LEU	O	0.01
94ARG	NE	134LEU	O	0.00
92SER	OG	88THR	O	37.97
92SER	OG	89GLY	O	0.46
92SER	OG	92SER	O	0.05
92SER	OG	93LEU	N	0.02

102ASN	ND2	103LEU	N
102ASN	ND2	260ALA	O
102ASN	ND2	261SER	N
97GLN	NE2	58ARG	O
97GLN	NE2	61VAL	O
97GLN	NE2	62GLU	N
97GLN	NE2	93LEU	O
97GLN	NE2	96SER	O
97GLN	NE2	97GLN	N
97GLN	NE2	97GLN	O
97GLN	NE2	264ARG	O
97GLN	NE2	266THR	O
97GLN	NE2	267PRO	O
96SER	OG	92SER	O
96SER	OG	93LEU	O
96SER	OG	96SER	O
95LYS	NZ	91LEU	O
95LYS	NZ	95LYS	O
95LYS	NZ	135THR	O
95LYS	NZ	140PHE	O
94ARG	NH1	90LEU	O
94ARG	NH1	134LEU	O
94ARG	NE	90LEU	O
92SER	OG	88THR	O
92SER	OG	89GLY	O
92SER	OG	92SER	O
88THR	OG1	82ARG	O
88THR	OG1	83LYS	O
88THR	OG1	85ARG	O
88THR	OG1	88THR	O
85ARG	NH2	78ASP	O
85ARG	NH2	187ASN	O
85ARG	NH1	77TRP	O
85ARG	NH1	78ASP	O
85ARG	NH1	80LEU	O
85ARG	NH1	187ASN	O
82ARG	NH2	139TYR	O
82ARG	NH2	187ASN	O
82ARG	NH2	188VAL	O

167ARG	NH2	201GLU	O	0.00	97GLN	NE2	96SER	O	0.30	88THR	OG1	42GLY	O	0.02	82ARG	NH1	139TYR	O
167ARG	NH1	202VAL	O	0.05	97GLN	NE2	97GLN	N	0.02	88THR	OG1	82ARG	O	15.42	82ARG	NH1	187ASN	O
167ARG	NH1	205GLY	O	0.01	97GLN	NE2	97GLN	O	0.03	88THR	OG1	83LYS	O	0.21	82ARG	NH1	188VAL	O
167ARG	NE	163GLU	O	0.12	97GLN	NE2	264ARG	O	0.01	88THR	OG1	85ARG	O	64.69	82ARG	NE	187ASN	O
164ARG	NH2	160PRO	O	0.01	97GLN	NE2	266THR	O	17.55	85ARG	NH2	77TRP	O	0.26	77TRP	NE1	43GLY	O
164ARG	NH2	161GLU	N	0.00	97GLN	NE2	267PRO	O	0.05	85ARG	NH2	78ASP	O	1.77	77TRP	NE1	46ILE	O
164ARG	NE	160PRO	O	0.42	96SER	OG	92SER	O	42.21	85ARG	NH2	80LEU	O	55.98	77TRP	NE1	84ILE	O
159LYS	NZ	148GLU	O	0.96	96SER	OG	93LEU	N	0.00	85ARG	NH1	78ASP	O	0.04	76LYS	NZ	10GLY	N
158SER	OG	149ALA	O	1.38	96SER	OG	93LEU	O	0.96	85ARG	NE	77TRP	O	22.00	76LYS	NZ	75PRO	O
158SER	OG	158SER	O	0.01	96SER	OG	95LYS	O	0.02	85ARG	NE	80LEU	O	60.27	76LYS	NZ	76LYS	O
157TYR	OH	136GLY	O	8.51	96SER	OG	96SER	O	0.14	85ARG	NE	81PRO	O	0.08	71SER	OG	9ASP	O
157TYR	OH	137GLY	N	0.02	95LYS	NZ	91LEU	O	0.13	82ARG	NH2	187ASN	O	3.58	71SER	OG	70GLY	O
156ARG	NH2	140PHE	O	0.76	95LYS	NZ	95LYS	O	0.05	82ARG	NH1	187ASN	O	6.00	71SER	OG	71SER	O
156ARG	NH2	142GLU	O	1.27	94ARG	NH1	90LEU	O	0.01	77TRP	NE1	42GLY	O	1.74	71SER	OG	72VAL	O
156ARG	NH1	142GLU	O	69.38	92SER	OG	88THR	O	23.17	77TRP	NE1	43GLY	N	0.02	71SER	OG	271PRO	O
156ARG	NE	140PHE	O	0.05	92SER	OG	89GLY	O	1.37	77TRP	NE1	43GLY	O	0.03	71SER	OG	273HIS	O
156ARG	NE	142GLU	O	1.18	92SER	OG	91LEU	O	0.00	77TRP	NE1	84ILE	O	4.98	71SER	OG	274GLY	O
154THR	OG1	143PRO	O	88.60	92SER	OG	92SER	O	0.08	76LYS	NZ	73GLY	O	0.19	59LYS	NZ	55GLU	O
154THR	OG1	153ASN	O	0.19	88THR	OG1	82ARG	O	2.25	71SER	OG	9ASP	O	0.37	58ARG	NH2	53PHE	O
153ASN	ND2	188VAL	O	0.01	88THR	OG1	83LYS	O	0.23	71SER	OG	70GLY	O	0.12	58ARG	NH1	53PHE	O
153ASN	ND2	138ILE	O	68.59	88THR	OG1	85SER	O	80.88	71SER	OG	71SER	O	0.06	58ARG	NH1	55GLU	N
153ASN	ND2	141GLY	O	0.84	85SER	OG	72VAL	O	0.05	71SER	OG	73GLY	O	0.01	58ARG	NE	53PHE	O
153ASN	ND2	152TRP	O	0.01	85SER	OG	77TRP	O	0.06	71SER	OG	271PRO	O	0.20	57THR	OG1	40PRO	O
153ASN	ND2	154THR	N	0.08	85SER	OG	80LEU	O	0.06	71SER	OG	273HIS	O	0.23	57THR	OG1	54PRO	N
153ASN	ND2	154THR	O	0.07	85SER	OG	81PRO	O	1.98	59LYS	NZ	55GLU	O	0.02	57THR	OG1	54PRO	O
152TRP	NE1	144ARG	O	0.05	85SER	OG	82ARG	N	0.01	58ARG	NH2	53PHE	O	2.60	57THR	OG1	58ARG	N
147SER	OG	146MET	O	0.03	85SER	OG	82ARG	O	11.58	58ARG	NH1	53PHE	O	0.12	36TYR	OH	23LEU	O
147SER	OG	147SER	O	0.02	85SER	OG	84ILE	O	0.00	58ARG	NH1	55GLU	N	0.00	36TYR	OH	24ARG	N
147SER	OG	150GLU	O	65.72	85SER	OG	85SER	O	0.02	58ARG	NE	53PHE	O	44.29	36TYR	OH	34LEU	O
144ARG	NH2	188VAL	O	13.79	83LYS	NZ	186ALA	O	0.92	58ARG	NE	54PRO	O	0.01	24ARG	NH2	17GLU	O
144ARG	NH2	189LEU	N	0.00	83LYS	NZ	187ASN	O	0.02	57THR	OG1	40PRO	O	97.65	24ARG	NH2	21LYS	N
144ARG	NH2	139TYR	O	0.27	83LYS	NZ	189LEU	O	0.13	24ARG	NH2	17GLU	O	0.09	24ARG	NH1	17GLU	O
144ARG	NH1	188VAL	O	0.40	82ARG	NH2	74GLY	O	0.00	24ARG	NH2	21LYS	N	0.49	24ARG	NE	20LEU	O
144ARG	NH1	138ILE	O	0.06	82ARG	NH2	77TRP	O	0.70	24ARG	NH1	20LEU	O	0.00	21LYS	NZ	17GLU	O
144ARG	NH1	139TYR	O	18.44	82ARG	NH2	78ASP	O	0.36	24ARG	NE	20LEU	O	0.96	21LYS	NZ	333THR	O
144ARG	NH1	140PHE	N	0.04	82ARG	NH2	80LEU	O	0.00	24ARG	NE	21LYS	N	0.00	21LYS	NZ	334GLU	O
144ARG	NH1	140PHE	O	0.02	82ARG	NH1	77TRP	O	0.17	21LYS	NZ	333THR	O	0.05	16THR	OG1	6LEU	O
144ARG	NH1	141GLY	O	7.56	82ARG	NH1	78ASP	O	0.12	21LYS	NZ	334GLU	O	0.12	16THR	OG1	7PRO	O
144ARG	NE	139TYR	O	0.32	82ARG	NH1	80LEU	O	0.13	16THR	OG1	7PRO	O	0.82	16THR	OG1	12GLY	O

139TYR	OH	237ASN	O	3.96
139TYR	OH	238ILE	N	0.00
139TYR	OH	238ILE	O	68.07
135THR	OG1	99LEU	O	5.91
135THR	OG1	100PHE	O	0.01
135THR	OG1	136GLY	N	0.00
132ARG	NH2	133GLU	O	0.04
132ARG	NH2	134LEU	O	0.00
132ARG	NH2	237ASN	O	0.01
132ARG	NE	133GLU	O	57.51
132ARG	NE	236GLY	O	0.01
132ARG	NE	237ASN	O	1.07
124ARG	NH1	120GLU	O	0.01
124ARG	NE	120GLU	O	0.01
124ARG	NE	121GLU	O	0.00
119LYS	NZ	113GLU	O	0.06
119LYS	NZ	114ARG	O	35.55
119LYS	NZ	116SER	O	27.35
119LYS	NZ	118LEU	O	0.24
116SER	OG	113GLU	O	1.48
116SER	OG	118LEU	O	0.01
116SER	OG	249VAL	O	44.79
116SER	OG	250LEU	N	0.00
116SER	OG	250LEU	O	47.37
116SER	OG	251PRO	O	0.00
114ARG	NH2	111GLY	O	3.10
114ARG	NH2	320LEU	O	0.01
114ARG	NH2	327LEU	O	0.44
114ARG	NH2	328GLY	O	0.00
114ARG	NH1	111GLY	O	4.40
114ARG	NH1	320LEU	O	0.00
114ARG	NE	111GLY	O	1.54
104ARG	NH1	244SER	O	9.83
102ASN	ND2	260ALA	O	1.36
102ASN	ND2	261SER	N	0.01
97GLN	NE2	61VAL	O	1.36
97GLN	NE2	62GLU	N	0.00
97GLN	NE2	93LEU	O	0.21
97GLN	NE2	96SER	O	0.04

82ARG	NH1	187ASN	O	0.01
82ARG	NE	77TRP	O	0.16
82ARG	NE	78ASP	O	0.16
77TRP	NE1	9ASP	O	0.01
77TRP	NE1	72VAL	O	0.28
77TRP	NE1	73GLY	O	0.03
77TRP	NE1	74GLY	O	0.22
77TRP	NE1	80LEU	O	0.28
77TRP	NE1	81PRO	O	40.37
77TRP	NE1	82ARG	N	0.04
77TRP	NE1	84ILE	O	0.73
76LYS	NZ	9ASP	O	0.04
76LYS	NZ	43GLY	O	0.02
76LYS	NZ	73GLY	O	0.56
76LYS	NZ	74GLY	O	0.06
76LYS	NZ	275SER	O	1.02
71SER	OG	9ASP	O	29.23
71SER	OG	70GLY	O	0.02
71SER	OG	71SER	O	1.44
71SER	OG	73GLY	O	0.05
71SER	OG	271PRO	O	0.03
71SER	OG	273HIS	O	5.04
71SER	OG	274GLY	N	0.06
71SER	OG	274GLY	O	0.14
59LYS	NZ	55GLU	O	0.05
59LYS	NZ	59LYS	O	0.03
58ARG	NH2	53PHE	O	0.58
58ARG	NH1	53PHE	O	12.76
58ARG	NE	53PHE	O	7.10
58ARG	NE	58ARG	O	0.00
57THR	OG1	40PRO	O	97.00
57THR	OG1	54PRO	O	0.00
36TYR	OH	23LEU	O	0.23
24ARG	NH2	17GLU	O	0.03
24ARG	NH2	21LYS	N	0.12
24ARG	NH2	34LEU	O	0.00
24ARG	NH1	17GLU	O	0.00
24ARG	NH1	21LYS	N	0.00
24ARG	NH1	24ARG	O	0.30

16THR	OG1	12GLY	O	95.24
2LYS	NZ	36TYR	O	0.01
2LYS	NZ	63GLU	O	0.83

16THR	OG1	16THR	O
2LYS	NZ	36TYR	O
2LYS	NZ	63GLU	O
343HIS	NE2	310LYS	O
343HIS	NE2	317LYS	O
343HIS	NE2	318ALA	N
342ARG	NH2	317LYS	O
342ARG	NH2	338ALA	O
342ARG	NH2	345ALA	O
342ARG	NH2	345ALA	O
342ARG	NH1	338ALA	O
342ARG	NH1	339THR	O
342ARG	NH1	342ARG	O
342ARG	NH1	345ALA	O
342ARG	NH1	345ALA	O
342ARG	NE1	338ALA	O
342ARG	NE1	339THR	O
342ARG	NE1	342ARG	O
342ARG	NE1	345ALA	O
339THR	OG1	314ALA	O
339THR	OG1	318ALA	O
339THR	OG1	335ALA	O
339THR	OG1	336PHE	N
339THR	OG1	336PHE	O
337THR	OG1	18ALA	O
337THR	OG1	333THR	O
337THR	OG1	334GLU	O
333THR	OG1	283GLY	O
333THR	OG1	285ALA	O
330SER	OG1	284ILE	O
330SER	OG1	328GLY	O
330SER	OG1	329GLY	O
330SER	OG1	330SER	O
330SER	OG1	331ALA	N
330SER	OG1	331ALA	O
322THR	OG1	318ALA	O
322THR	OG1	321GLU	O
322THR	OG1	322THR	O
322THR	OG1	323PRO	N

97GLN	NE2	97GLN	O	0.00
97GLN	NE2	266THR	O	24.78
96SER	OG	92SER	O	86.21
96SER	OG	93LEU	N	0.00
96SER	OG	93LEU	O	6.72
95LYS	NZ	91LEU	O	0.03
95LYS	NZ	95LYS	O	0.02
94ARG	NH1	134LEU	O	0.00
94ARG	NE	90LEU	O	0.00
92SER	OG	88THR	O	38.90
92SER	OG	89GLY	O	1.42
92SER	OG	92SER	O	0.04
88THR	OG1	82ARG	O	9.24
88THR	OG1	83LYS	O	0.07
88THR	OG1	85SER	O	82.38
88THR	OG1	89GLY	N	0.00
85SER	OG	77TRP	O	0.04
85SER	OG	81PRO	O	7.16
85SER	OG	82ARG	N	0.00
85SER	OG	82ARG	O	47.50
85SER	OG	84ILE	O	0.11
85SER	OG	85SER	O	0.06
82ARG	NH2	187ASN	O	0.16
82ARG	NH1	187ASN	O	3.18
77TRP	NE1	43GLY	O	0.01
77TRP	NE1	80LEU	O	0.00
77TRP	NE1	81PRO	O	23.31
77TRP	NE1	84ILE	O	26.24
76LYS	NZ	43GLY	O	0.12
71SER	OG	9ASP	O	11.13
71SER	OG	71SER	O	1.24
71SER	OG	72VAL	O	0.08
71SER	OG	273HIS	O	0.84
71SER	OG	274GLY	O	0.61
59LYS	NZ	55GLU	O	0.15
58ARG	NH2	53PHE	O	1.27
58ARG	NH1	53PHE	O	15.73
58ARG	NE	53PHE	O	48.20
57THR	OG1	40PRO	O	98.21

24ARG	NH1	34LEU	O	0.01
24ARG	NE	20LEU	O	0.40
21LYS	NZ	17GLU	O	0.02
21LYS	NZ	333THR	O	0.01
21LYS	NZ	334GLU	O	0.01
16THR	OG1	7PRO	O	0.73
16THR	OG1	12GLY	O	89.49
16THR	OG1	13PRO	O	0.28
16THR	OG1	69LEU	O	0.00
2LYS	NZ	35ALA	O	0.01
2LYS	NZ	36TYR	O	0.02
2LYS	NZ	63GLU	O	8.74
343HIS	NE2	310LYS	O	0.00
343HIS	NE2	317LYS	O	0.01
342ARG	NH2	317LYS	O	0.04
342ARG	NH2	338ALA	O	0.02
342ARG	NH2	339THR	N	0.00
342ARG	NH2	345ALA	O	0.02
342ARG	NH2	345ALA	O	0.05
342ARG	NH1	338ALA	O	0.12
342ARG	NH1	342ARG	O	0.03
342ARG	NE	338ALA	O	0.10
342ARG	NE	339THR	O	0.48
342ARG	NE	342ARG	O	0.08
339THR	OG1	314ALA	O	0.01
339THR	OG1	335ALA	O	60.64
339THR	OG1	336PHE	O	10.17
339THR	OG1	339THR	O	0.00
337THR	OG1	18ALA	O	0.06
337THR	OG1	332GLY	O	0.03
337THR	OG1	333THR	O	59.55
337THR	OG1	334GLU	N	0.00
337THR	OG1	334GLU	O	4.83
337THR	OG1	336PHE	O	0.04
333THR	OG1	14GLU	O	0.00
333THR	OG1	281GLY	O	0.02
333THR	OG1	283GLY	O	0.36
333THR	OG1	285ALA	O	7.70
330SER	OG	323PRO	O	13.29

322THR	OG1	323PRO	O
322THR	OG1	330SER	O
317LYS	NZ	110PRO	O
317LYS	NZ	313ASP	O
310LYS	NZ	306GLU	O
310LYS	NZ	343HIS	O
310LYS	NZ	344LEU	O
310LYS	NZ	345ALA	O
310LYS	NZ	345ALA	O
309ARG	NH1	305VAL	O
300HIS	NE2	261SER	O
293SER	OG	259SER	O
293SER	OG	270GLU	O
293SER	OG	271PRO	N
293SER	OG	289ALA	O
293SER	OG	293SER	O
288THR	OG1	255GLY	O
288THR	OG1	256LEU	O
286ASN	ND2	255GLY	O
286ASN	ND2	284ILE	O
286ASN	ND2	322THR	O
286ASN	ND2	323PRO	O
286ASN	ND2	325PRO	O
286ASN	ND2	331ALA	O
286ASN	ND2	332GLY	O
282LYS	NZ	277PRO	O
282LYS	NZ	278ASP	O
282LYS	NZ	279ILE	O
282LYS	NZ	280ALA	O
282LYS	NZ	281GLY	O
275SER	OG	9ASP	O
275SER	OG	72VAL	O
275SER	OG	73GLY	O
275SER	OG	274GLY	O
275SER	OG	275SER	O
275SER	OG	276ALA	O
275SER	OG	284ILE	O
273HIS	ND1	254LEU	O
273HIS	ND1	255GLY	O

24ARG	NH2	17GLU	O	0.12
24ARG	NH2	21LYS	N	0.40
24ARG	NH2	34LEU	O	1.79
24ARG	NH1	20LEU	O	0.00
24ARG	NH1	24ARG	O	0.23
24ARG	NH1	34LEU	O	0.05
24ARG	NE	20LEU	O	0.72
24ARG	NE	21LYS	N	0.01
21LYS	NZ	17GLU	O	0.02
16THR	OG1	7PRO	O	7.89
16THR	OG1	12GLY	O	87.24
2LYS	NZ	63GLU	O	2.88

330SER	OG	329GLY	O	0.77
330SER	OG	330SER	O	0.41
322THR	OG	318ALA	O	17.93
322THR	OG1	321GLU	O	0.08
322THR	OG1	322THR	O	13.04
322THR	OG1	323PRO	N	0.00
322THR	OG1	323PRO	O	0.20
322THR	OG1	329GLY	O	0.01
322THR	OG1	330SER	O	1.07
322THR	OG1	335ALA	O	0.00
317LYS	NZ	313ASP	O	0.11
310LYS	NZ	306GLU	O	0.07
310LYS	NZ	343HIS	O	0.31
310LYS	NZ	344LEU	O	0.89
310LYS	NZ	345ALA	N	0.00
310LYS	NZ	345ALA	O	7.56
310LYS	NZ	345ALA	O	6.53
309ARG	NH2	175LYS	O	1.29
309ARG	NH2	305VAL	O	0.00
309ARG	NH1	175LYS	O	0.02
309ARG	NH1	305VAL	O	0.04
309ARG	NE	305VAL	O	0.02
309ARG	NE	309ARG	O	0.00
300HIS	NE2	65GLU	O	0.06
300HIS	NE2	261SER	O	0.15
293SER	OG	259SER	O	0.04
293SER	OG	270GLU	O	0.04
293SER	OG	289ALA	O	94.39
293SER	OG	290ALA	O	2.14
293SER	OG	293SER	O	0.02
293SER	OG	294ALA	N	0.00
288THR	OG1	255GLY	O	15.58
288THR	OG1	256LEU	O	0.14
288THR	OG1	286ASN	O	0.02
288THR	OG1	287PRO	O	0.12
288THR	OG1	289ALA	N	0.02
286ASN	ND2	254LEU	O	0.03
286ASN	ND2	255GLY	O	0.32
286ASN	ND2	322THR	O	0.02

273HIS	ND1	271PRO	O
273HIS	ND1	272VAL	O
273HIS	ND1	273HIS	O
273HIS	ND1	274GLY	O
273HIS	ND1	286ASN	O
266THR	OG1	61VAL	O
266THR	OG1	62GLU	O
266THR	OG1	64ALA	O
266THR	OG1	65GLU	O
266THR	OG1	66ALA	N
266THR	OG1	265GLY	O
266THR	OG1	266THR	O
266THR	OG1	267PRO	N
266THR	OG1	267PRO	O
264ARG	NH2	96SER	O
264ARG	NH2	99LEU	O
264ARG	NH1	96SER	O
264ARG	NH1	98ASP	O
264ARG	NH1	99LEU	O
264ARG	NE	97GLN	O
264ARG	NE	98ASP	O
261SER	OG	101ALA	O
261SER	OG	102ASN	N
261SER	OG	260ALA	O
261SER	OG	261SER	O
261SER	OG	262LEU	N
261SER	OG	262LEU	O
261SER	OG	268VAL	O
259SER	OG	103LEU	O
259SER	OG	258PRO	O
259SER	OG	259SER	N
259SER	OG	259SER	O
259SER	OG	270GLU	O
259SER	OG	271PRO	O
253SER	OG	252GLY	O
253SER	OG	253SER	O
253SER	OG	254LEU	O
253SER	OG	326ASP	O
248SER	OG	244SER	O

	286ASN	ND2	331ALA	O	0.03
	286ASN	ND2	332GLY	O	0.04
	282LYS	NZ	277PRO	O	0.20
	282LYS	NZ	278ASP	O	2.90
	282LYS	NZ	279ILE	O	0.00
	282LYS	NZ	280ALA	O	0.04
	282LYS	NZ	281GLY	O	0.02
	282LYS	NZ	282LYS	O	0.15
	282LYS	NZ	325PRO	O	0.01
	282LYS	NZ	326ASP	O	0.32
	275SER	OG	9ASP	O	9.10
	275SER	OG	71SER	O	0.30
	275SER	OG	72VAL	N	0.00
	275SER	OG	72VAL	O	0.20
	275SER	OG	73GLY	O	1.44
	275SER	OG	272VAL	O	0.53
	275SER	OG	273HIS	O	0.02
	275SER	OG	274GLY	O	0.60
	275SER	OG	275SER	O	0.36
	273HIS	ND1	254LEU	O	2.90
	273HIS	ND1	255GLY	N	0.00
	273HIS	ND1	255GLY	O	2.04
	273HIS	ND1	271PRO	O	0.00
	273HIS	ND1	272VAL	O	2.82
	273HIS	ND1	273HIS	O	3.23
	273HIS	ND1	274GLY	O	3.20
	273HIS	ND1	279ILE	O	8.22
	273HIS	ND1	280ALA	O	0.00
	266THR	OG1	61VAL	O	73.29
	266THR	OG1	62GLU	O	0.01
	266THR	OG1	64ALA	O	1.01
	266THR	OG1	265GLY	O	0.02
	266THR	OG1	266THR	O	0.50
	266THR	OG1	267PRO	O	0.00
	264ARG	NH2	96SER	O	3.16
	264ARG	NH2	97GLN	O	0.00
	264ARG	NH1	96SER	O	0.90
	264ARG	NH1	97GLN	O	0.14
	264ARG	NH1	264ARG	O	0.24

	248SER	OG	245ASP	O
	248SER	OG	247ALA	O
	248SER	OG	248SER	O
	248SER	OG	253SER	O
	248SER	OG	254LEU	O
	244SER	OG	240GLY	O
	244SER	OG	241ASP	O
	244SER	OG	244SER	O
	237ASN	ND2	133GLU	O
	237ASN	ND2	134LEU	N
	237ASN	ND2	134LEU	O
	237ASN	ND2	135THR	N
	237ASN	ND2	136GLY	O
	237ASN	ND2	137GLY	N
	237ASN	ND2	137GLY	O
	235THR	OG1	183VAL	O
	235THR	OG1	236GLY	O
	235THR	OG1	239PHE	O
	229ARG	NH2	229ARG	O
	229ARG	NH1	228ALA	O
	229ARG	NH1	229ARG	O
	229ARG	NE	228ALA	O
	229ARG	NE	229ARG	O
	226SER	OG	122ILE	O
	226SER	OG	222HIS	O
	226SER	OG	225ARG	O
	226SER	OG	226SER	O
	225ARG	NH2	252GLY	O
	225ARG	NH2	326ASP	O
	225ARG	NH1	252GLY	O
	225ARG	NH1	326ASP	O
	225ARG	NH1	221MET	O
	225ARG	NE	252GLY	O
	225ARG	NE	221MET	O
	215TYR	OH	186ALA	O
	214GLN	NE2	213HIS	O
	214GLN	NE2	214GLN	N
	214GLN	NE2	215TYR	O
	214GLN	NE2	218ALA	O

264ARG	NE	97GLN	O	0.01
264ARG	NE	264ARG	O	0.28
261SER	OG	101ALA	O	7.41
261SER	OG	260ALA	O	0.32
261SER	OG	261SER	O	0.05
261SER	OG	268VAL	O	1.51
259SER	OG	103LEU	O	0.04
259SER	OG	259SER	O	0.04
259SER	OG	270GLU	O	86.42
259SER	OG	271PRO	O	0.02
253SER	OG	252GLY	O	0.76
253SER	OG	253SER	O	0.30
253SER	OG	254LEU	O	0.21
253SER	OG	326ASP	O	0.03
248SER	OG	244SER	O	1.16
248SER	OG	245ASP	O	93.65
248SER	OG	254LEU	O	0.00
244SER	OG	104ARG	O	0.00
244SER	OG	240GLY	O	77.18
244SER	OG	244SER	O	0.02
237ASN	ND2	133GLU	O	13.20
237ASN	ND2	134LEU	O	0.72
237ASN	ND2	136GLY	O	13.72
237ASN	ND2	137GLY	O	0.09
237ASN	ND2	238ILE	N	0.00
235THR	OG1	183VAL	O	31.41
235THR	OG1	236GLY	O	0.60
235THR	OG1	239PHE	O	0.09
229ARG	NH2	229ARG	O	0.01
229ARG	NH1	229ARG	O	0.02
229ARG	NE	229ARG	O	48.20
226SER	OG	122ILE	O	0.00
226SER	OG	222HIS	O	1.11
226SER	OG	225ARG	O	6.55
226SER	OG	226SER	O	5.69
226SER	OG	228ALA	O	0.00
225ARG	NH2	115LEU	O	0.04
225ARG	NH2	252GLY	O	0.44
225ARG	NH2	275SER	O	0.00

214GLN	NE2	219MET	N
213HIS	NE2	196ARG	O
213HIS	NE2	200GLU	N
206TYR	OH	167ARG	O
204ARG	NH2	197LYS	O
204ARG	NH2	203GLY	O
204ARG	NH1	200GLU	O
204ARG	NE	200GLU	O
204ARG	NE	203GLY	O
198THR	OG1	194PHE	O
198THR	OG1	195TRP	O
197LYS	NZ	147SER	O
197LYS	NZ	148GLU	O
197LYS	NZ	193GLU	O
197LYS	NZ	197LYS	O
196ARG	NH2	213HIS	O
196ARG	NH2	214GLN	N
196ARG	NH2	214GLN	O
196ARG	NH2	215TYR	N
195TRP	NE1	183VAL	O
195TRP	NE1	234VAL	O
187ASN	ND2	187ASN	O
185LYS	NZ	241ASP	O
182SER	OG	183VAL	O
182SER	OG	234VAL	O
179HIS	NE2	211LEU	O
179HIS	NE2	212GLU	N
179HIS	NE2	229ARG	O
178LYS	NZ	174ARG	O
178LYS	NZ	176ARG	O
178LYS	NZ	208ASP	O
177ARG	NH2	125GLY	O
177ARG	NH2	127ASP	N
177ARG	NH2	127ASP	O
177ARG	NH2	228ALA	O
177ARG	NH2	229ARG	O
177ARG	NH2	230PHE	O
177ARG	NH1	125GLY	O
177ARG	NH1	228ALA	O

225ARG	NH2	326ASP	O	4.35
225ARG	NH2	327LEU	O	0.02
225ARG	NH2	221MET	O	0.01
225ARG	NH1	252GLY	O	0.00
225ARG	NH1	326ASP	O	0.23
225ARG	NH1	221MET	O	0.34
225ARG	NE	221MET	O	0.22
222HIS	NE2	226SER	O	0.01
215TYR	OH	77TRP	O	0.12
215TYR	OH	186ALA	O	0.86
214GLN	NE2	213HIS	O	0.92
214GLN	NE2	214GLN	N	0.04
214GLN	NE2	215TYR	N	0.01
214GLN	NE2	215TYR	O	0.02
214GLN	NE2	218ALA	O	0.01
206TYR	OH	167ARG	O	0.17
206TYR	OH	170PHE	O	1.97
206TYR	OH	171GLU	N	0.02
204ARG	NH2	211LEU	O	1.18
204ARG	NH1	203GLY	O	0.01
204ARG	NH1	211LEU	O	22.18
204ARG	NE	200GLU	O	0.01
198THR	OG1	194PHE	O	95.83
198THR	OG1	195TRP	O	0.01
197LYS	NZ	147SER	O	0.02
197LYS	NZ	148GLU	O	0.35
197LYS	NZ	193GLU	O	0.01
196ARG	NH2	213HIS	O	0.02
196ARG	NH2	214GLN	N	0.01
196ARG	NH2	214GLN	O	0.04
196ARG	NH2	215TYR	N	0.02
196ARG	NH1	213HIS	O	0.00
195TRP	NE1	183VAL	O	1.14
195TRP	NE1	234VAL	O	0.04
187ASN	ND2	187ASN	O	0.00
179HIS	NE2	211LEU	O	0.01
178LYS	NZ	174ARG	O	0.76
178LYS	NZ	176ARG	O	0.04
178LYS	NZ	177ARG	O	0.00

177ARG	NH1	229ARG	O
177ARG	NH1	230PHE	N
177ARG	NH1	230PHE	O
176ARG	NH2	127ASP	O
176ARG	NH1	127ASP	O
176ARG	NH1	128VAL	N
176ARG	NH1	231ASP	O
175LYS	NZ	171GLU	O
175LYS	NZ	175LYS	O
175LYS	NZ	299GLU	O
174ARG	NH2	205GLY	O
167ARG	NH2	202VAL	O
167ARG	NH1	163GLU	O
167ARG	NH1	202VAL	O
167ARG	NH1	205GLY	O
167ARG	NE	163GLU	O
164ARG	NH2	98ASP	O
164ARG	NH2	167ARG	O
164ARG	NH1	160PRO	O
164ARG	NE	164ARG	N
164ARG	NE	164ARG	O
159LYS	NZ	148GLU	O
158SER	OG	149ALA	O
158SER	OG	158SER	O
157TYR	OH	136GLY	O
157TYR	OH	137GLY	N
156ARG	NH2	142GLU	O
156ARG	NH1	142GLU	O
156ARG	NE	142GLU	O
154THR	OG1	143PRO	O
154THR	OG1	153ASN	O
153ASN	ND2	188VAL	O
153ASN	ND2	138ILE	O
153ASN	ND2	152TRP	O
152TRP	NE1	143PRO	O
152TRP	NE1	144ARG	N
152TRP	NE1	144ARG	O
147SER	OG	146MET	O
147SER	OG	150GLU	O

178LYS	NZ	208ASP	O	0.06
177ARG	NH2	125GLY	O	4.72
177ARG	NH2	127ASP	N	0.02
177ARG	NH2	127ASP	O	2.66
177ARG	NH2	228ALA	O	18.76
177ARG	NH2	229ARG	O	0.08
177ARG	NH2	230PHE	O	1.21
177ARG	NH1	125GLY	O	0.86
177ARG	NH1	127ASP	O	1.20
177ARG	NH1	228ALA	O	33.87
177ARG	NH1	229ARG	O	4.58
177ARG	NH1	230PHE	O	0.49
176ARG	NH2	127ASP	O	0.03
176ARG	NH2	128VAL	O	0.00
176ARG	NH1	127ASP	O	68.49
176ARG	NH1	128VAL	N	0.04
176ARG	NH1	231ASP	O	0.08
176ARG	NE	172ALA	O	0.01
175LYS	NZ	171GLU	O	0.68
175LYS	NZ	299GLU	O	0.75
175LYS	NZ	300HIS	O	0.33
174ARG	NH2	170PHE	O	0.00
174ARG	NH2	207PRO	O	0.67
174ARG	NH1	170PHE	O	0.02
174ARG	NH1	171GLU	O	0.00
174ARG	NH1	207PRO	O	1.57
174ARG	NE	170PHE	O	0.08
174ARG	NE	171GLU	O	0.04
174ARG	NE	207PRO	O	0.26
167ARG	NH2	160PRO	O	0.00
167ARG	NH2	205GLY	O	0.01
167ARG	NH1	163GLU	O	0.03
167ARG	NH1	202VAL	O	0.03
167ARG	NE	163GLU	O	0.11
164ARG	NH2	98ASP	O	0.50
164ARG	NH1	98ASP	O	0.00
164ARG	NH1	99LEU	O	0.00
164ARG	NE	160PRO	O	0.06
159LYS	NZ	148GLU	O	1.69

144ARG	NH2	188VAL	O
144ARG	NH2	138ILE	O
144ARG	NH2	139TYR	O
144ARG	NH1	188VAL	O
144ARG	NH1	138ILE	O
144ARG	NH1	139TYR	O
144ARG	NH1	140PHE	N
144ARG	NH1	140PHE	O
144ARG	NH1	141GLY	O
139TYR	OH	237ASN	O
139TYR	OH	238ILE	O
135THR	OG1	99LEU	O
135THR	OG1	100PHE	O
135THR	OG1	135THR	N
135THR	OG1	135THR	O
132ARG	NH2	237ASN	O
132ARG	NH2	241ASP	N
132ARG	NH1	133GLU	O
132ARG	NH1	237ASN	O
132ARG	NH1	240GLY	O
132ARG	NE	133GLU	O
132ARG	NE	237ASN	O
132ARG	NE	240GLY	O
124ARG	NH1	120GLU	O
124ARG	NE	120GLU	O
124ARG	NE	121GLU	O
119LYS	NZ	113GLU	O
119LYS	NZ	114ARG	O
119LYS	NZ	115LEU	O
119LYS	NZ	116SER	O
119LYS	NZ	117PRO	O
119LYS	NZ	118LEU	O
116SER	OG	118LEU	O
116SER	OG	249VAL	O
116SER	OG	250LEU	O
116SER	OG	251PRO	O
114ARG	NH2	111GLY	O
114ARG	NH2	319LEU	O
114ARG	NH2	320LEU	O

158SER	OG	149ALA	O	5.26
158SER	OG	157TYR	O	0.04
158SER	OG	158SER	O	0.05
157TYR	OH	136GLY	O	0.43
156ARG	NH2	142GLU	O	1.83
156ARG	NH1	142GLU	O	13.91
156ARG	NE	142GLU	O	0.44
156ARG	NE	156ARG	O	0.02
154THR	OG1	153ASN	O	0.00
154THR	OG1	143PRO	N	0.01
154THR	OG1	143PRO	O	67.85
154THR	OG1	153ASN	O	2.43
154THR	OG1	154THR	N	0.00
153ASN	ND2	138ILE	O	3.98
153ASN	ND2	139TYR	O	0.04
153ASN	ND2	141GLY	O	0.01
153ASN	ND2	152TRP	O	0.22
153ASN	ND2	154THR	N	0.00
153ASN	ND2	154THR	O	0.01
152TRP	NE1	143PRO	O	0.16
152TRP	NE1	144ARG	N	0.02
152TRP	NE1	144ARG	O	0.12
147SER	OG	146MET	O	0.09
147SER	OG	150GLU	O	62.79
144ARG	NH2	188VAL	O	3.91
144ARG	NH2	189LEU	N	0.00
144ARG	NH2	138ILE	O	0.00
144ARG	NH2	139TYR	O	0.48
144ARG	NH2	140PHE	O	0.00
144ARG	NH1	188VAL	O	1.68
144ARG	NH1	138ILE	O	0.16
144ARG	NH1	139TYR	O	19.20
144ARG	NH1	140PHE	N	0.01
144ARG	NH1	141GLY	O	1.82
144ARG	NE	139TYR	O	0.13
144ARG	NE	141GLY	O	0.21
144ARG	NE	144ARG	O	0.07
139TYR	OH	87GLU	O	0.19
139TYR	OH	136GLY	O	1.52

114ARG	NH2	327LEU	O
114ARG	NH1	111GLY	O
114ARG	NH1	320LEU	O
114ARG	NH1	327LEU	O
114ARG	NE	111GLY	O
114ARG	NE	114ARG	O
104ARG	NH2	244SER	O
104ARG	NH2	254LEU	O
104ARG	NE	244SER	O
102ASN	ND2	99LEU	O
102ASN	ND2	100PHE	O
102ASN	ND2	101ALA	O
102ASN	ND2	102ASN	O
102ASN	ND2	103LEU	N
102ASN	ND2	132ARG	O
102ASN	ND2	260ALA	O
102ASN	ND2	261SER	N
97GLN	NE2	58ARG	O
97GLN	NE2	61VAL	O
97GLN	NE2	62GLU	N
97GLN	NE2	62GLU	O
97GLN	NE2	93LEU	O
97GLN	NE2	94ARG	O
97GLN	NE2	96SER	O
97GLN	NE2	264ARG	O
97GLN	NE2	266THR	O
96SER	OG	91LEU	O
96SER	OG	92SER	O
96SER	OG	93LEU	O
96SER	OG	94ARG	O
96SER	OG	95LYS	O
96SER	OG	96SER	O
95LYS	NZ	91LEU	O
95LYS	NZ	92SER	O
95LYS	NZ	95LYS	O
95LYS	NZ	135THR	O
95LYS	NZ	140PHE	O
94ARG	NH2	90LEU	O
94ARG	NE	90LEU	O

139TYR	OH	237ASN	O	0.88
139TYR	OH	238ILE	O	0.02
135THR	OG1	99LEU	O	0.01
135THR	OG1	100PHE	O	0.50
135THR	OG1	161GLU	O	0.01
132ARG	NH2	133GLU	O	0.23
132ARG	NH2	237ASN	O	2.17
132ARG	NE	133GLU	O	21.56
132ARG	NE	237ASN	O	39.31
132ARG	NE	240GLY	O	0.00
132ARG	NE	241ASP	N	0.02
124ARG	NH1	120GLU	O	0.09
124ARG	NE	120GLU	O	0.03
119LYS	NZ	113GLU	O	0.04
119LYS	NZ	114ARG	O	21.99
119LYS	NZ	115LEU	O	0.01
119LYS	NZ	116SER	O	33.59
119LYS	NZ	118LEU	O	0.33
116SER	OG	113GLU	O	2.54
116SER	OG	116SER	O	0.01
116SER	OG	118LEU	O	0.37
116SER	OG	249VAL	O	0.36
116SER	OG	250LEU	O	91.62
114ARG	NH2	111GLY	O	2.88
114ARG	NH2	327LEU	O	0.01
114ARG	NH1	111GLY	O	0.52
114ARG	NH1	320LEU	O	0.00
114ARG	NE	111GLY	O	1.36
104ARG	NH2	241ASP	O	0.00
104ARG	NH1	240GLY	O	0.00
104ARG	NH1	244SER	O	0.06
102ASN	ND2	260ALA	O	0.86
102ASN	ND2	261SER	N	0.08
97GLN	NE2	61VAL	O	0.31
97GLN	NE2	62GLU	N	0.01
97GLN	NE2	93LEU	O	0.27
97GLN	NE2	96SER	O	0.06
97GLN	NE2	97GLN	N	0.01
97GLN	NE2	266THR	O	0.16

92SER	OG	87GLU	O
92SER	OG	88THR	O
92SER	OG	89GLY	N
92SER	OG	89GLY	O
92SER	OG	90LEU	O
92SER	OG	91LEU	O
92SER	OG	92SER	O
88THR	OG1	187ASN	O
88THR	OG1	82ARG	O
88THR	OG1	83LYS	O
88THR	OG1	85ARG	O
88THR	OG1	89GLY	N
85ARG	NH2	74GLY	O
85ARG	NH2	77TRP	O
85ARG	NH2	78ASP	O
85ARG	NH2	79GLY	O
85ARG	NH2	80LEU	O
85ARG	NH1	74GLY	O
85ARG	NH1	77TRP	O
85ARG	NH1	78ASP	N
85ARG	NH1	78ASP	O
85ARG	NH1	80LEU	O
85ARG	NE	77TRP	O
85ARG	NE	78ASP	O
85ARG	NE	80LEU	O
85ARG	NE	81PRO	O
83LYS	NZ	188VAL	O
83LYS	NZ	50GLY	O
82ARG	NH2	186ALA	O
82ARG	NH2	187ASN	O
82ARG	NH2	188VAL	O
82ARG	NH1	186ALA	O
82ARG	NH1	187ASN	O
82ARG	NH1	188VAL	O
82ARG	NH1	82ARG	O
82ARG	NH1	88THR	O
82ARG	NE	186ALA	O
82ARG	NE	187ASN	O
77TRP	NE1	42GLY	O

96SER	OG	92SER	O	80.94
96SER	OG	93LEU	N	0.01
96SER	OG	93LEU	O	9.74
95LYS	NZ	91LEU	O	0.02
95LYS	NZ	95LYS	O	0.02
95LYS	NZ	135THR	O	1.46
95LYS	NZ	136GLY	O	0.02
94ARG	NH1	134LEU	O	0.01
94ARG	NE	90LEU	O	0.02
92SER	OG	88THR	O	10.23
92SER	OG	89GLY	O	10.18
92SER	OG	92SER	O	0.04
88THR	OG1	43GLY	O	0.00
88THR	OG1	44ALA	O	0.01
88THR	OG1	82ARG	O	1.48
88THR	OG1	83LYS	O	1.69
88THR	OG1	84ILE	N	0.00
88THR	OG1	85SER	O	72.19
88THR	OG1	86PRO	O	0.00
88THR	OG1	89GLY	N	0.01
85SER	OG	77TRP	O	0.01
85SER	OG	80LEU	O	0.00
85SER	OG	81PRO	O	0.72
85SER	OG	82ARG	O	38.68
85SER	OG	83LYS	O	0.02
85SER	OG	84ILE	O	0.04
85SER	OG	85SER	O	0.06
82ARG	NH2	186ALA	O	0.26
82ARG	NH2	187ASN	O	0.41
82ARG	NH2	188VAL	O	0.01
82ARG	NH2	189LEU	O	0.05
82ARG	NH2	190GLU	N	0.02
82ARG	NH2	87GLU	O	0.00
82ARG	NH2	139TYR	O	0.01
82ARG	NH1	186ALA	O	0.02
82ARG	NH1	187ASN	O	0.06
82ARG	NH1	188VAL	O	9.07
82ARG	NH1	189LEU	O	0.01
82ARG	NH1	190GLU	N	0.01

77TRP	NE1	84ILE	O
76LYS	NZ	9ASP	O
76LYS	NZ	10GLY	N
76LYS	NZ	43GLY	O
76LYS	NZ	76LYS	O
76LYS	NZ	280ALA	O
76LYS	NZ	281GLY	O
76LYS	NZ	282LYS	O
71SER	OG	9ASP	O
71SER	OG	10GLY	N
71SER	OG	11ILE	O
71SER	OG	70GLY	O
71SER	OG	71SER	O
71SER	OG	271PRO	O
71SER	OG	273HIS	O
71SER	OG	274GLY	O
59LYS	NZ	55GLU	O
59LYS	NZ	62GLU	O
58ARG	NH2	53PHE	O
58ARG	NH2	55GLU	N
58ARG	NH1	53PHE	O
58ARG	NH1	54PRO	O
58ARG	NE	53PHE	O
57THR	OG1	40PRO	O
36TYR	OH	20LEU	O
36TYR	OH	23LEU	O
24ARG	NH2	17GLU	O
24ARG	NH2	21LYS	N
24ARG	NH1	17GLU	O
24ARG	NH1	20LEU	O
24ARG	NH1	34LEU	O
24ARG	NE	20LEU	O
24ARG	NE	21LYS	N
24ARG	NE	24ARG	O
21LYS	NZ	17GLU	O
21LYS	NZ	333THR	O
21LYS	NZ	334GLU	O
16THR	OG1	6LEU	O
16THR	OG1	7PRO	O

82ARG	NE	186ALA	O	0.04
82ARG	NE	187ASN	O	0.28
82ARG	NE	82ARG	O	0.02
77TRP	NE1	42GLY	O	0.32
77TRP	NE1	43GLY	O	0.02
77TRP	NE1	81PRO	O	0.00
77TRP	NE1	84ILE	O	18.83
76LYS	NZ	9ASP	O	0.68
76LYS	NZ	10GLY	N	0.01
76LYS	NZ	42GLY	O	0.04
76LYS	NZ	43GLY	O	0.04
76LYS	NZ	73GLY	O	0.06
76LYS	NZ	76LYS	O	0.00
76LYS	NZ	281GLY	O	0.01
71SER	OG	9ASP	O	1.76
71SER	OG	11ILE	O	1.36
71SER	OG	70GLY	O	6.44
71SER	OG	71SER	O	1.76
71SER	OG	271PRO	O	0.09
71SER	OG	273HIS	O	8.39
71SER	OG	274GLY	N	0.02
71SER	OG	274GLY	O	0.51
59LYS	NZ	55GLU	O	0.08
59LYS	NZ	59LYS	O	0.00
58ARG	NH2	53PHE	O	0.28
58ARG	NH1	53PHE	O	10.11
58ARG	NE	53PHE	O	2.54
58ARG	NE	54PRO	O	0.00
57THR	OG1	40PRO	O	92.53
57THR	OG1	54PRO	O	0.17
57THR	OG1	58ARG	N	0.00
36TYR	OH	20LEU	O	0.15
36TYR	OH	23LEU	O	0.03
24ARG	NH2	27ASP	O	0.11
24ARG	NH2	34LEU	O	0.00
24ARG	NH1	34LEU	O	0.15
24ARG	NE	24ARG	O	0.01
21LYS	NZ	17GLU	O	0.05
21LYS	NZ	333THR	O	0.59

16THR	OG1	12GLY	O
2LYS	NZ	36TYR	O
2LYS	NZ	59LYS	O
2LYS	NZ	60GLY	O
2LYS	NZ	63GLU	O

21LYS	NZ	334GLU	O	0.01
16THR	OG1	7PRO	O	1.23
16THR	OG1	12GLY	O	92.70
2LYS	NZ	36TYR	O	0.00
2LYS	NZ	63GLU	O	7.97

percen
7.49
6.86
0.00
0.22
0.10
0.02
1.08
0.05
0.02
0.00
0.00
0.01
0.24
0.24
0.00
0.00
0.02
0.00
0.01
7.01
0.02
0.05
56.25
0.02
0.01
0.83
0.15
0.44
0.04
0.12
1.72
2.54
0.07
0.00
0.00
0.15

10.22
0.02
0.02
0.02
22.84
7.13
4.04
29.15
0.85
0.01
0.01
0.00
0.00
0.04
20.02
0.10
18.40
0.02
0.12
0.08
0.06
3.64
5.30
0.01
0.14
0.01
0.02
0.13
0.09
0.00
14.58
0.02
0.02
24.59
0.16
0.63
0.24
0.00
0.00

3.43
0.63
0.16
13.67
15.31
3.23
0.00
6.08
0.06
0.00
0.02
5.78
5.84
0.16
0.34
18.57
24.39
98.77
0.00
19.39
35.31
5.54
1.23
0.80
2.02
0.20
28.58
32.93
0.00
0.01
0.12
0.65
0.30
0.02
1.48
2.54
0.25
10.64
44.19

0.32
0.08
0.82
0.00
13.89
9.57
40.48
50.34
16.38
0.20
0.17
2.48
6.24
8.68
0.21
1.66
1.67
0.02
0.04
0.03
0.00
28.84
40.19
0.44
0.01
59.02
0.67
0.02
98.43
1.08
80.79
0.04
1.82
0.02
0.10
0.42
0.58
15.06
20.53

52.70
3.79
7.43
37.33
41.94
0.56
0.48
46.12
59.04
0.04
0.01
0.03
0.02
0.16
0.28
0.00
0.05
0.05
0.01
0.01
0.07
1.02
0.01
0.81
0.33
0.08
0.64
43.73
0.10
0.20
0.00
0.03
0.04
0.25
0.28
0.00
0.13
0.05
0.37

0.25
1.09
2.36
4.59
5.10
10.15
41.50
11.17
58.82
7.96
42.26
2.32
2.80
0.00
0.00
0.00
2.17
0.04
0.02
0.80
0.84
11.25
10.63
0.36
2.66
0.19
0.38
0.01
1.17
0.94
0.49
0.00
0.44
0.02
0.15
0.02
0.04
0.02
4.86

4.21
0.01
0.02
0.01
0.61
0.02
0.15
66.33
0.02
0.12
4.56
26.60
4.24
25.31
13.23
33.30
0.00
0.01
0.26
0.97
0.01
0.01
0.16
0.14
0.13
52.49
1.52
9.36
0.03
0.11
0.02
0.22
1.91
0.01
0.02
1.81
0.01
8.87
0.01

5.30
0.28
1.32
0.02
0.02
0.16
0.01
88.38
15.86
2.69
0.01
0.12
0.03
0.06
3.64
3.24
14.25
13.95
6.35
0.02
0.02
1.71
1.90
0.49
44.03
92.63
20.84
1.24
11.19
17.26
0.57
6.27
0.03
99.72
0.38
0.08
0.01
0.02
0.02

0.25
58.95
4.53
0.11
1.07
0.38
0.01
1.03
0.24
0.84
0.76
50.19
11.62
1.22
0.11
0.18
0.02
0.05
0.33
0.31
0.26
0.07
0.12
0.97
0.39
48.28
42.66
67.00
12.26
0.23
0.01
0.09
69.60
54.95
0.01
22.86
15.51
0.00
0.47

0.39
19.22
17.61
49.98
0.22
0.03
39.19
50.42
0.82
0.58
52.56
49.23
0.01
0.13
0.00
0.00
0.55
0.86
0.91
1.00
1.46
0.07
0.20
0.00
1.38
12.62
0.02
29.29
32.88
0.09
0.00
0.01
0.07
0.25
0.01
1.66
10.00
0.02
0.00

6.89
6.12
0.22
0.00
1.42
0.18
0.01
4.42
3.48
0.02
0.01
0.00
0.00
0.00
0.04
0.01
0.00
0.01
0.12
0.34
0.01
0.03
1.05
0.65
6.99
6.89
5.05
4.55
65.42
8.17
84.66
6.00
67.54
5.49
0.01
0.05
0.00
0.01
15.93

11.29
0.02
2.88
2.28
1.40
4.70
0.72
0.00
0.04
0.27
0.86
0.39
0.00
0.16
0.52
0.62
0.82
0.01
0.01
0.00
0.08
63.67
4.80
0.01
0.04
0.00
67.29
1.14
0.00
3.50
0.00
0.00
1.96
13.20
0.07
0.72
0.88
0.61
14.61

0.33
15.72
0.23
0.01
0.34
0.17
0.00
0.06
0.27
1.66
20.48
19.95
0.01
0.00
0.01
0.06
15.12
1.12
0.01
0.24
0.00
92.45
0.04
21.98
0.00
2.11
0.01
0.00
0.50
0.01
2.52
20.17
0.61
5.23
0.04
0.02
1.24
0.03
0.78

1.13
0.40
0.68
0.04
0.06
37.50
0.00
0.31
0.40
4.52
0.00
0.04
36.32
0.00
7.61
0.49
3.16
0.04
8.48
0.38
1.67
0.03
0.26
0.08
3.93
0.98
0.29
2.54
0.02
0.00
0.87
0.27
0.00
33.55
11.34
7.06
0.00
0.08
0.02

69.19
0.00
0.17
0.00
0.01
29.76
50.02
0.02
0.00
0.01
0.06
57.06
11.52
0.04
0.02
23.83
0.15
4.18
3.26
55.16
0.00
0.01
0.04
0.01
13.58
1.70
0.40
0.01
0.01
0.01
0.25
2.84
0.09
0.06
0.01
1.12
10.41
4.33
5.09

0.00
7.47
3.23
0.00
58.61
0.04
0.19
0.04
0.08
0.00
0.01
0.06
9.58
0.04
0.10
0.08
0.03
0.43
0.38
0.00
0.13
84.37
0.03
0.00
0.00
0.00
0.85
1.54
0.00
0.01
6.89
0.10
34.35
0.27
0.18
18.61
3.67
0.01
7.91

1.02
1.37
0.04
0.33
0.72
0.02
2.94
4.56
0.04
1.34
0.44
1.79
15.24
1.11
0.51
0.07
0.00
71.17
0.01
0.12
0.99
0.00
0.00
0.55
0.16
0.34
0.02
0.02
0.03
0.00
0.02
0.02
0.57
0.96
0.50
0.00
0.11
0.43
0.01

2.16
0.02
0.06
1.34
9.49
0.06
4.32
21.64
0.43
2.16
0.01
81.40
0.11
1.97
0.00
0.12
0.04
0.18
0.05
0.01
62.43
1.08
0.01
0.00
24.88
0.00
0.06
47.42
0.04
0.01
1.22
2.29
0.06
0.00
0.01
2.30
5.66
38.13
0.41

0.00
0.02
0.02
0.27
0.06
0.02
32.05
1.14
0.01
0.00
18.12
0.01
0.53
0.43
0.01
0.03
0.00
0.07
0.03
3.03
19.86
0.00
33.59
0.01
19.11
0.86
0.01
9.94
82.14
0.02
1.51
0.00
0.12
0.00
0.32
50.87
0.00
0.98
0.01

0.01
0.66
0.01
0.05
13.13
0.00
0.20
0.10
0.03
0.06
0.08
17.59
0.04
52.50
6.70
0.07
0.00
0.08
0.54
0.00
0.01
5.84
0.01
36.07
0.12
0.06
13.90
2.94
1.40
0.80
0.00
45.89
0.26
0.10
90.43
0.38
0.01
10.00
0.20

0.64
0.96
0.00
0.14
0.22
0.01
0.94
0.00
0.00
0.01
0.46
0.09
0.21
1.72
0.06
0.27
1.93
0.21
5.10
0.12
0.01
19.04
86.22
0.01
2.73
0.00
1.02
0.00
0.01
0.00
0.02
0.00
0.06
0.04
7.20
0.03
0.02
3.89
85.64

0.03
0.02
5.10
0.01
0.08
0.00
0.04
0.01
0.06
0.20
0.09
0.01
0.06
0.02
0.00
0.15
0.25
0.09
0.00
0.00
0.00
76.11
0.00
0.28
0.02
85.12
0.90
11.78
1.04
0.00
0.00
0.45
0.49
0.00
0.05
3.32
0.05
4.70
0.01

0.44
0.22
0.14
0.08
0.12
0.12
0.56
7.89
9.80
0.19
0.00
0.55
0.08
0.00
95.14
0.04
12.85
0.01
1.76
0.00
0.15
0.01
0.81
1.74
0.02
0.09
15.33
0.06
0.60
0.00
0.02
0.72
11.33
2.54
1.11
0.12
0.01
0.64
0.01

8.02
0.03
0.09
4.89
1.36
38.67
0.01
9.40
3.46
0.08
0.03
2.75
0.00
3.56
0.04
0.00
0.00
0.58
0.72
0.02
0.01
3.87
0.02
0.40
0.03
0.02
0.01
1.81
2.03
2.48
0.00
0.04
83.16
0.00
0.05
0.00
0.44
0.05
77.98

0.89
0.01
0.01
0.05
0.01
87.35
0.53
0.03
0.00
0.11
14.52
0.60
87.02
0.02
0.05
34.03
4.57
1.08
0.00
0.04
4.29
0.02
17.68
0.00
10.72
3.99
5.22
18.48
2.44
56.21
0.12
0.00
0.08
0.02
0.14
13.52
0.15
0.23
0.12

0.07
0.83
0.00
0.11
0.01
0.00
0.12
0.07
0.08
96.44
0.01
0.20
0.00
0.22
0.05
0.00
0.00
0.02
0.00
0.01
0.01
0.00
0.00
0.00
0.01
0.01
0.00
2.44
3.19
1.54
0.33
0.83
0.01
1.86
15.74
0.40
0.89
0.28
30.61

5.69
0.03
0.54
0.04
79.36
0.00
0.11
0.84
0.01
0.43
0.13
0.00
0.07
0.00
0.00
0.03
0.04
0.02
0.12
0.01
0.04
2.38
6.93
0.06
1.59
0.00
0.15
92.73
0.02
81.59
0.32
0.00
0.11
0.22
0.11
0.09
0.22
0.22
63.68

51.00
0.03
0.38
0.22
0.32
76.57
0.17
0.02
0.32
0.06
1.53
4.29
0.05
0.00
0.01
8.70
0.00
1.46
0.88
0.44
8.99
3.38
0.02
0.03
0.02
0.05
10.12
25.31
0.01
25.17
0.01
23.96
0.08
33.77
55.04
0.00
7.66
0.03
0.03

0.05
1.79
0.00
0.03
2.40
0.01
0.82
0.01
0.12
0.05
0.32
0.00
0.00
0.00
0.00
0.47
0.04
0.02
2.89
0.01
0.14
2.33
0.89
0.34
0.01
0.44
0.00
37.98
26.29
0.00
2.79
0.11
0.01
0.07
0.05
2.21
0.16
0.22
9.70

3.63
2.30
0.01
2.17
0.01
0.06
0.01
0.48
74.12
0.03
0.57
0.03
0.66
0.02
0.28
0.02
3.96
0.03
5.29
0.00
1.48
0.99
0.59
0.00
27.60
0.00
0.01
0.53
0.27
0.92
0.02
0.31
0.96
0.01
0.00
0.22
0.01
0.04
0.29

56.16
0.00
0.00
0.26
0.04
0.06
0.13
0.04
13.51
0.00
0.04
0.18
0.66
0.00
0.10
9.79
0.11
0.00
0.43
0.00
15.34
0.00
3.80
95.81
0.00
0.03
0.03
0.08
0.00
0.00
0.12
0.24
0.00
0.01
0.02
0.76
0.01
0.01
3.02

90.09
0.05
0.07
0.00
5.58
