# Supplementary material for: Deciphering the Dynamics of Non-Covalent Interactions Affecting Thermal Stability of a Protein: Molecular Dynamics Study on Point Mutant of Thermus thermophilus Isopropylmalate Dehydrogenase
Source: PLoS One. 2015 Dec 11;10(12):e0144294. doi: 10.1371/journal.pone.0144294 (PMC4689552; doi:10.1371/journal.pone.0144294)
Supplement: S5 Table — The color formatting indicates the percentage of time interaction existed is as in S1 Table. (PDF) [file pone.0144294.s007.pdf]

S5 Table. Unique HBs between SS of *wt* and *mut* at 300 K and 337 K.

1) *Wt* 300 K

Drnona	D	Arnona	A	percen
343HIS	NE2	313ASP	OD1	15.50
343HIS	NE2	313ASP	OD2	0.01
343HIS	NE2	321GLU	OE1	9.17
343HIS	NE2	321GLU	OE2	32.62
342ARG	NH2	321GLU	OE1	41.75
342ARG	NH2	321GLU	OE2	20.54
342ARG	NH2	343HIS	NE2	0.00
342ARG	NH1	321GLU	OE1	16.25
342ARG	NH1	321GLU	OE2	30.87
342ARG	NH1	343HIS	NE2	0.00
342ARG	NE	321GLU	OE1	3.64
342ARG	NE	321GLU	OE2	2.02
342ARG	NE	343HIS	NE2	0.00
339THR	OG1	321GLU	OE1	6.39
339THR	OG1	321GLU	OE2	2.37
339THR	OG1	342ARG	NE	0.02
339THR	OG1	342ARG	NH1	0.18
339THR	OG1	342ARG	NH2	1.00
339THR	OG1	343HIS	NE2	0.00
337THR	OG1	334GLU	OE1	0.02
333THR	OG1	14GLU	OE1	0.90
333THR	OG1	14GLU	OE2	0.50
333THR	OG1	334GLU	OE1	0.04
333THR	OG1	334GLU	OE2	0.08
322THR	OG1	330SER	OG	0.06
317LYS	NZ	313ASP	OD1	77.89
317LYS	NZ	313ASP	OD2	25.69
317LYS	NZ	343HIS	ND1	0.01
317LYS	NZ	343HIS	NE2	0.01
310LYS	NZ	30GLU	OE1	8.20
310LYS	NZ	30GLU	OE2	8.76
310LYS	NZ	306GLU	OE2	0.03
310LYS	NZ	313ASP	OD1	0.10
309ARG	NH2	299GLU	OE1	0.84
309ARG	NH2	299GLU	OE2	29.92
309ARG	NH2	306GLU	OE1	16.40
309ARG	NH2	306GLU	OE2	23.28
309ARG	NH2	312GLU	OE1	0.52
309ARG	NH2	312GLU	OE2	9.40
309ARG	NH1	299GLU	OE1	31.53

2) *Wt* 337 K

Drnona	D	Arnona	A	percen
343HIS	NE2	313ASP	OD1	39.48
343HIS	NE2	313ASP	OD2	26.72
343HIS	NE2	321GLU	OE1	0.85
343HIS	NE2	321GLU	OE2	1.54
342ARG	NH2	321GLU	OE1	25.86
342ARG	NH2	321GLU	OE2	27.66
342ARG	NH2	343HIS	ND1	0.02
342ARG	NH2	343HIS	NE2	0.01
342ARG	NH1	321GLU	OE1	21.38
342ARG	NH1	321GLU	OE2	18.69
339THR	OG1	321GLU	OE1	0.02
339THR	OG1	342ARG	NH2	0.02
339THR	OG1	343HIS	NE2	0.00
337THR	OG1	334GLU	OE2	0.02
333THR	OG1	14GLU	OE1	2.50
333THR	OG1	14GLU	OE2	1.94
333THR	OG1	334GLU	OE1	0.72
333THR	OG1	334GLU	OE2	0.84
322THR	OG1	330SER	OG	0.01
322THR	OG1	339THR	OG1	0.02
317LYS	NZ	313ASP	OD1	54.19
317LYS	NZ	313ASP	OD2	50.61
317LYS	NZ	321GLU	OE1	0.00
317LYS	NZ	321GLU	OE2	0.03
317LYS	NZ	343HIS	ND1	0.19
317LYS	NZ	343HIS	NE2	0.01
310LYS	NZ	30GLU	OE1	5.85
310LYS	NZ	30GLU	OE2	6.33
310LYS	NZ	306GLU	OE1	0.50
310LYS	NZ	306GLU	OE2	0.95
310LYS	NZ	313ASP	OD1	0.02
310LYS	NZ	313ASP	OD2	0.02
310LYS	NZ	343HIS	ND1	0.07
310LYS	NZ	343HIS	NE2	0.00
309ARG	NH2	299GLU	OE1	2.55
309ARG	NH2	299GLU	OE2	13.46
309ARG	NH2	306GLU	OE1	31.42
309ARG	NH2	306GLU	OE2	30.98
309ARG	NH1	299GLU	OE1	14.82
309ARG	NH1	299GLU	OE2	4.24

3) *Mut* 300 K

Drnona	D	Arnona	A	percen
343HIS	NE2	313ASP	OD2	29.42
343HIS	NE2	321GLU	OE1	5.81
343HIS	NE2	321GLU	OE2	9.63
342ARG	NH2	321GLU	OE1	30.01
342ARG	NH2	321GLU	OE2	24.83
342ARG	NH2	343HIS	NE2	0.06
342ARG	NH1	321GLU	OE1	14.63
342ARG	NH1	321GLU	OE2	22.60
342ARG	NE	321GLU	OE1	3.84
342ARG	NE	321GLU	OE2	0.07
339THR	OG1	321GLU	OE1	0.01
339THR	OG1	321GLU	OE2	0.02
339THR	OG1	342ARG	NH1	0.00
339THR	OG1	342ARG	NH2	0.03
337THR	OG1	334GLU	OE1	0.00
337THR	OG1	334GLU	OE2	0.13
333THR	OG1	14GLU	OE1	0.71
333THR	OG1	14GLU	OE2	0.19
322THR	OG1	339THR	OG1	0.06
317LYS	NZ	313ASP	OD1	36.91
317LYS	NZ	313ASP	OD2	69.11
317LYS	NZ	343HIS	ND1	0.05
317LYS	NZ	343HIS	NE2	0.01
310LYS	NZ	30GLU	OE1	1.39
310LYS	NZ	30GLU	OE2	4.26
310LYS	NZ	306GLU	OE1	0.65
310LYS	NZ	306GLU	OE2	0.10
310LYS	NZ	313ASP	OD2	0.00
309ARG	NH2	299GLU	OE1	0.37
309ARG	NH2	299GLU	OE2	4.03
309ARG	NH2	306GLU	OE1	51.46
309ARG	NH2	306GLU	OE2	40.73
309ARG	NH1	299GLU	OE1	4.83
309ARG	NH1	299GLU	OE2	2.28
309ARG	NH1	306GLU	OE1	0.52
309ARG	NH1	306GLU	OE2	10.43
309ARG	NH1	312GLU	OE1	0.03
309ARG	NH1	312GLU	OE2	0.42
309ARG	NH1	313ASP	OD2	0.00
309ARG	NE	306GLU	OE1	19.43

4) *Mut* 337 K

Drnona	D	Arnona	A	percen
343HIS	NE2	313ASP	OD1	11.68
343HIS	NE2	313ASP	OD2	1.62
343HIS	NE2	321GLU	OE1	10.14
343HIS	NE2	321GLU	OE2	10.36
342ARG	NH2	321GLU	OE1	22.94
342ARG	NH2	321GLU	OE2	23.57
342ARG	NH2	343HIS	ND1	0.01
342ARG	NH2	343HIS	NE2	0.04
342ARG	NH1	321GLU	OE1	20.61
342ARG	NH1	321GLU	OE2	21.35
342ARG	NH1	343HIS	ND1	0.02
342ARG	NH1	343HIS	NE2	0.00
342ARG	NE	321GLU	OE1	0.01
342ARG	NE	321GLU	OE2	0.38
339THR	OG1	321GLU	OE1	0.16
339THR	OG1	321GLU	OE2	0.11
339THR	OG1	342ARG	NH1	0.04
339THR	OG1	342ARG	NH2	0.07
339THR	OG1	343HIS	NE2	0.00
337THR	OG1	334GLU	OE2	0.00
333THR	OG1	14GLU	OE1	7.76
333THR	OG1	14GLU	OE2	4.65
333THR	OG1	334GLU	OE1	0.15
333THR	OG1	334GLU	OE2	0.28
330SER	OG	278ASP	OD1	0.00
330SER	OG	278ASP	OD2	0.01
322THR	OG1	321GLU	OE1	0.05
322THR	OG1	339THR	OG1	0.03
317LYS	NZ	313ASP	OD1	53.18
317LYS	NZ	313ASP	OD2	47.01
317LYS	NZ	321GLU	OE1	0.26
317LYS	NZ	321GLU	OE2	1.11
317LYS	NZ	343HIS	ND1	0.10
317LYS	NZ	343HIS	NE2	0.01
310LYS	NZ	30GLU	OE1	11.72
310LYS	NZ	30GLU	OE2	8.60
310LYS	NZ	306GLU	OE1	0.04
310LYS	NZ	306GLU	OE2	0.52
310LYS	NZ	313ASP	OD1	0.00
310LYS	NZ	313ASP	OD2	0.05

309ARG	NH1	299GLU	OE2	2.71
309ARG	NH1	306GLU	OE2	0.02
309ARG	NH1	312GLU	OE1	23.02
309ARG	NH1	312GLU	OE2	17.71
309ARG	NE	306GLU	OE1	14.28
309ARG	NE	306GLU	OE2	8.38
300HIS	NE2	299GLU	OE2	0.07
288THR	OG1	286ASN	OD1	86.26
286ASN	ND2	288THR	OG1	1.90
286ASN	ND2	326ASP	OD1	0.05
282LYS	NZ	278ASP	OD1	34.76
282LYS	NZ	278ASP	OD2	41.61
282LYS	NZ	326ASP	OD1	3.27
282LYS	NZ	326ASP	OD2	0.16
275SER	OG	9ASP	OD2	0.02
266THR	OG1	97GLN	OE1	4.87
264ARG	NH2	98ASP	OD1	5.71
264ARG	NH2	98ASP	OD2	10.41
264ARG	NH2	161GLU	OE1	0.02
264ARG	NH2	161GLU	OE2	0.00
264ARG	NH1	98ASP	OD1	6.08
264ARG	NH1	98ASP	OD2	2.14
264ARG	NE	98ASP	OD1	32.15
264ARG	NE	98ASP	OD2	28.26
261SER	OG	270GLU	OE1	0.10
261SER	OG	270GLU	OE2	0.01
259SER	OG	102ASN	OD1	0.19
259SER	OG	270GLU	OE1	0.02
259SER	OG	270GLU	OE2	0.00
253SER	OG	326ASP	OD1	12.20
253SER	OG	326ASP	OD2	7.24
244SER	OG	245ASP	OD1	1.05
244SER	OG	245ASP	OD2	2.46
237ASN	ND2	133GLU	OE1	0.03
237ASN	ND2	133GLU	OE2	0.00
229ARG	NH2	212GLU	OE1	29.19
229ARG	NH2	212GLU	OE2	31.85
229ARG	NH2	214GLN	OE1	0.21
229ARG	NH1	179HIS	ND1	0.60
229ARG	NH1	212GLU	OE1	31.51
229ARG	NH1	212GLU	OE2	28.32
229ARG	NH1	214GLN	OE1	0.60
229ARG	NE	214GLN	OE1	0.02

309ARG	NH1	306GLU	OE1	10.12
309ARG	NH1	306GLU	OE2	7.27
309ARG	NH1	312GLU	OE1	0.89
309ARG	NH1	312GLU	OE2	1.17
309ARG	NH1	313ASP	OD1	0.02
309ARG	NH1	313ASP	OD2	0.01
309ARG	NE	299GLU	OE1	0.06
309ARG	NE	306GLU	OE1	13.24
309ARG	NE	306GLU	OE2	15.41
300HIS	NE2	299GLU	OE2	0.03
288THR	OG1	286ASN	OD1	94.83
286ASN	ND2	288THR	OG1	0.90
286ASN	ND2	326ASP	OD1	8.44
286ASN	ND2	326ASP	OD2	5.29
282LYS	NZ	278ASP	OD1	43.23
282LYS	NZ	278ASP	OD2	43.89
282LYS	NZ	330SER	OG	0.00
275SER	OG	273HIS	NE2	0.04
275SER	OG	326ASP	OD1	0.04
273HIS	ND1	275SER	OG	0.05
266THR	OG1	62GLU	OE1	7.42
266THR	OG1	62GLU	OE2	7.85
266THR	OG1	97GLN	OE1	5.56
264ARG	NH2	98ASP	OD1	38.90
264ARG	NH2	98ASP	OD2	28.39
264ARG	NH2	161GLU	OE1	0.47
264ARG	NH2	161GLU	OE2	2.72
264ARG	NH1	98ASP	OD1	4.58
264ARG	NH1	98ASP	OD2	8.27
264ARG	NH1	133GLU	OE1	0.05
264ARG	NH1	161GLU	OE1	3.03
264ARG	NH1	161GLU	OE2	2.02
264ARG	NE	98ASP	OD1	34.29
264ARG	NE	98ASP	OD2	46.43
261SER	OG	102ASN	OD1	0.49
261SER	OG	270GLU	OE1	10.81
261SER	OG	270GLU	OE2	0.82
259SER	OG	102ASN	OD1	0.02
259SER	OG	270GLU	OE1	0.06
259SER	OG	270GLU	OE2	0.02
253SER	OG	326ASP	OD1	0.06
253SER	OG	326ASP	OD2	0.03
248SER	OG	245ASP	OD1	0.23

309ARG	NE	306GLU	OE2	23.94
288THR	OG1	286ASN	OD1	86.10
286ASN	ND2	288THR	OG1	7.37
286ASN	ND2	326ASP	OD1	22.97
286ASN	ND2	326ASP	OD2	11.58
282LYS	NZ	278ASP	OD1	21.77
282LYS	NZ	278ASP	OD2	22.26
266THR	OG1	97GLN	OE1	13.30
264ARG	NH2	98ASP	OD1	18.90
264ARG	NH2	98ASP	OD2	26.88
264ARG	NH1	98ASP	OD1	5.94
264ARG	NH1	98ASP	OD2	15.68
264ARG	NE	98ASP	OD1	25.06
264ARG	NE	98ASP	OD2	47.58
261SER	OG	102ASN	OD1	0.16
261SER	OG	270GLU	OE1	1.64
261SER	OG	270GLU	OE2	1.26
253SER	OG	326ASP	OD1	20.24
253SER	OG	326ASP	OD2	39.19
244SER	OG	245ASP	OD1	1.10
244SER	OG	245ASP	OD2	1.57
237ASN	ND2	133GLU	OE2	0.00
229ARG	NH2	212GLU	OE1	0.98
229ARG	NH2	212GLU	OE2	1.46
229ARG	NH1	212GLU	OE2	0.04
229ARG	NH1	222HIS	ND1	0.02
229ARG	NE	212GLU	OE1	0.00
229ARG	NE	212GLU	OE2	0.00
226SER	OG	222HIS	ND1	14.71
226SER	OG	229ARG	NE	0.01
226SER	OG	229ARG	NH1	0.01
225ARG	NH2	222HIS	ND1	3.32
225ARG	NH2	248SER	OG	0.02
225ARG	NH2	253SER	OG	0.02
225ARG	NH2	326ASP	OD1	44.00
225ARG	NH2	326ASP	OD2	21.72
225ARG	NH1	222HIS	ND1	0.02
225ARG	NH1	326ASP	OD1	11.02
225ARG	NH1	326ASP	OD2	36.50
225ARG	NE	222HIS	ND1	0.16
225ARG	NE	226SER	OG	0.01
222HIS	NE2	214GLN	OE1	0.39
222HIS	NE2	226SER	OG	0.03

310LYS	NZ	343HIS	ND1	0.00
309ARG	NH2	299GLU	OE1	2.76
309ARG	NH2	299GLU	OE2	5.10
309ARG	NH2	306GLU	OE1	29.89
309ARG	NH2	306GLU	OE2	31.59
309ARG	NH2	312GLU	OE1	0.04
309ARG	NH2	312GLU	OE2	1.45
309ARG	NH2	313ASP	OD2	0.16
309ARG	NH1	299GLU	OE1	6.21
309ARG	NH1	299GLU	OE2	5.00
309ARG	NH1	306GLU	OE1	17.04
309ARG	NH1	306GLU	OE2	10.31
309ARG	NH1	312GLU	OE1	3.30
309ARG	NH1	312GLU	OE2	7.12
309ARG	NH1	313ASP	OD1	0.01
309ARG	NH1	313ASP	OD2	0.00
309ARG	NE	299GLU	OE2	0.01
309ARG	NE	306GLU	OE1	10.68
309ARG	NE	306GLU	OE2	13.05
309ARG	NE	312GLU	OE1	0.52
309ARG	NE	312GLU	OE2	0.07
309ARG	NE	313ASP	OD2	0.00
288THR	OG1	286ASN	OD1	66.52
286ASN	ND2	288THR	OG1	3.17
286ASN	ND2	326ASP	OD1	0.56
286ASN	ND2	326ASP	OD2	0.06
282LYS	NZ	278ASP	OD1	44.93
282LYS	NZ	278ASP	OD2	44.58
275SER	OG	326ASP	OD1	0.18
273HIS	ND1	275SER	OG	0.00
273HIS	ND1	286ASN	OD1	0.13
266THR	OG1	62GLU	OE1	6.67
266THR	OG1	62GLU	OE2	5.52
266THR	OG1	97GLN	OE1	7.88
264ARG	NH2	62GLU	OE1	13.56
264ARG	NH2	62GLU	OE2	9.01
264ARG	NH2	97GLN	OE1	0.04
264ARG	NH2	98ASP	OD1	7.83
264ARG	NH2	98ASP	OD2	0.73
264ARG	NH2	163GLU	OE1	0.00
264ARG	NH2	163GLU	OE2	0.13
264ARG	NH2	266THR	OG1	0.00
264ARG	NH1	62GLU	OE1	9.29

226SER	OG	222HIS	ND1	0.77
226SER	OG	229ARG	NE	0.01
225ARG	NH2	222HIS	ND1	2.76
225ARG	NH2	326ASP	OD1	46.44
225ARG	NH2	326ASP	OD2	5.14
225ARG	NH1	222HIS	ND1	0.78
225ARG	NH1	326ASP	OD1	6.28
225ARG	NH1	326ASP	OD2	40.90
225ARG	NE	222HIS	ND1	2.08
222HIS	NE2	214GLN	OE1	0.15
222HIS	NE2	229ARG	NE	0.08
222HIS	NE2	229ARG	NH1	0.40
222HIS	NE2	229ARG	NH2	0.00
215TYR	OH	187ASN	OD1	0.10
214GLN	NE2	212GLU	OE1	31.47
214GLN	NE2	212GLU	OE2	29.35
214GLN	NE2	222HIS	ND1	12.98
214GLN	NE2	222HIS	NE2	0.00
214GLN	NE2	229ARG	NE	0.10
214GLN	NE2	229ARG	NH1	0.01
214GLN	NE2	229ARG	NH2	0.03
213HIS	NE2	200GLU	OE1	4.14
213HIS	NE2	200GLU	OE2	4.19
206TYR	OH	171GLU	OE1	50.37
206TYR	OH	171GLU	OE2	37.37
204ARG	NH2	200GLU	OE1	14.65
204ARG	NH2	200GLU	OE2	61.81
204ARG	NH2	201GLU	OE1	21.79
204ARG	NH2	201GLU	OE2	30.01
204ARG	NH1	200GLU	OE1	0.51
204ARG	NH1	200GLU	OE2	0.28
204ARG	NH1	201GLU	OE1	34.23
204ARG	NH1	201GLU	OE2	32.21
204ARG	NE	200GLU	OE1	56.73
204ARG	NE	200GLU	OE2	18.18
197LYS	NZ	193GLU	OE1	2.94
197LYS	NZ	193GLU	OE2	2.23
197LYS	NZ	201GLU	OE1	0.02
197LYS	NZ	201GLU	OE2	0.28
197LYS	NZ	148GLU	OE1	18.08
197LYS	NZ	148GLU	OE2	23.75
196ARG	NH2	184ASP	OD1	93.68
196ARG	NH2	184ASP	OD2	7.04

248SER	OG	245ASP	OD2	0.08
244SER	OG	241ASP	OD2	0.00
244SER	OG	245ASP	OD1	0.28
244SER	OG	245ASP	OD2	0.36
237ASN	ND2	133GLU	OE1	0.03
237ASN	ND2	133GLU	OE2	3.96
229ARG	NH2	179HIS	ND1	0.02
229ARG	NH2	212GLU	OE1	22.34
229ARG	NH2	212GLU	OE2	15.87
229ARG	NH2	214GLN	OE1	1.64
229ARG	NH2	278ASP	OD1	0.02
229ARG	NH2	278ASP	OD2	0.56
229ARG	NH1	179HIS	ND1	0.01
229ARG	NH1	212GLU	OE1	3.80
229ARG	NH1	212GLU	OE2	5.80
229ARG	NH1	214GLN	OE1	0.66
229ARG	NH1	222HIS	ND1	0.42
229ARG	NH1	278ASP	OD1	0.13
229ARG	NH1	278ASP	OD2	0.78
229ARG	NE	179HIS	ND1	0.00
229ARG	NE	212GLU	OE1	1.23
229ARG	NE	212GLU	OE2	1.06
229ARG	NE	214GLN	OE1	0.07
229ARG	NE	222HIS	ND1	0.01
226SER	OG	222HIS	ND1	1.46
226SER	OG	229ARG	NE	0.00
225ARG	NH2	222HIS	ND1	0.53
225ARG	NH2	253SER	OG	0.00
225ARG	NH2	278ASP	OD1	24.01
225ARG	NH2	278ASP	OD2	16.16
225ARG	NH2	326ASP	OD1	74.46
225ARG	NH2	326ASP	OD2	14.03
225ARG	NH1	222HIS	ND1	0.00
225ARG	NH1	253SER	OG	0.01
225ARG	NH1	278ASP	OD2	0.03
225ARG	NH1	326ASP	OD1	9.33
225ARG	NH1	326ASP	OD2	70.50
225ARG	NE	222HIS	ND1	0.10
225ARG	NE	278ASP	OD1	0.72
225ARG	NE	278ASP	OD2	0.49
222HIS	NE2	214GLN	OE1	0.20
222HIS	NE2	226SER	OG	0.07
222HIS	NE2	229ARG	NE	0.47

222HIS	NE2	229ARG	NE	0.26
222HIS	NE2	229ARG	NH1	0.18
222HIS	NE2	229ARG	NH2	0.01
215TYR	OH	184ASP	OD1	0.00
215TYR	OH	187ASN	OD1	0.21
215TYR	OH	275SER	OG	0.01
214GLN	NE2	212GLU	OE1	2.63
214GLN	NE2	212GLU	OE2	4.12
214GLN	NE2	222HIS	ND1	5.65
214GLN	NE2	222HIS	NE2	0.32
214GLN	NE2	229ARG	NE	0.01
214GLN	NE2	229ARG	NH2	0.01
213HIS	NE2	200GLU	OE1	0.56
213HIS	NE2	200GLU	OE2	0.34
213HIS	NE2	215TYR	OH	1.68
206TYR	OH	171GLU	OE1	67.16
206TYR	OH	171GLU	OE2	26.79
204ARG	NH2	200GLU	OE1	21.76
204ARG	NH2	200GLU	OE2	74.71
204ARG	NH2	201GLU	OE1	47.23
204ARG	NH2	201GLU	OE2	23.90
204ARG	NH1	201GLU	OE1	26.81
204ARG	NH1	201GLU	OE2	44.41
204ARG	NE	200GLU	OE1	72.86
204ARG	NE	200GLU	OE2	27.00
197LYS	NZ	193GLU	OE1	2.15
197LYS	NZ	193GLU	OE2	2.21
197LYS	NZ	148GLU	OE1	26.38
197LYS	NZ	148GLU	OE2	23.78
196ARG	NH2	184ASP	OD1	94.25
196ARG	NH2	184ASP	OD2	5.32
196ARG	NH2	213HIS	NE2	0.00
196ARG	NH2	215TYR	OH	3.66
196ARG	NH1	193GLU	OE1	0.00
196ARG	NH1	215TYR	OH	0.01
196ARG	NE	184ASP	OD1	7.49
196ARG	NE	184ASP	OD2	93.26
196ARG	NE	213HIS	ND1	0.00
187ASN	ND2	215TYR	OH	0.04
185LYS	NZ	187ASN	OD1	0.32
185LYS	NZ	217ASP	OD1	73.22
185LYS	NZ	217ASP	OD2	2.73
185LYS	NZ	139TYR	OH	0.43

264ARG	NH1	62GLU	OE2	13.99
264ARG	NH1	98ASP	OD1	21.90
264ARG	NH1	98ASP	OD2	3.19
264ARG	NE	62GLU	OE2	0.00
264ARG	NE	98ASP	OD1	26.71
264ARG	NE	98ASP	OD2	0.89
261SER	OG	102ASN	OD1	1.03
261SER	OG	270GLU	OE2	0.02
259SER	OG	102ASN	OD1	0.20
253SER	OG	326ASP	OD1	16.86
253SER	OG	326ASP	OD2	77.29
253SER	OG	225ARG	NH2	0.00
248SER	OG	245ASP	OD1	0.07
244SER	OG	245ASP	OD1	0.60
244SER	OG	245ASP	OD2	1.24
237ASN	ND2	133GLU	OE1	0.13
237ASN	ND2	155GLU	OE2	0.00
237ASN	ND2	241ASP	OD1	0.10
237ASN	ND2	241ASP	OD2	0.30
229ARG	NH2	179HIS	ND1	0.00
229ARG	NH2	212GLU	OE1	7.28
229ARG	NH2	212GLU	OE2	5.45
229ARG	NH2	214GLN	OE1	0.08
229ARG	NH2	222HIS	ND1	0.06
229ARG	NH1	179HIS	ND1	0.18
229ARG	NH1	212GLU	OE1	2.72
229ARG	NH1	212GLU	OE2	5.19
229ARG	NH1	214GLN	OE1	0.00
229ARG	NH1	222HIS	ND1	0.02
229ARG	NE	212GLU	OE1	0.22
229ARG	NE	212GLU	OE2	0.16
229ARG	NE	214GLN	OE1	0.01
229ARG	NE	222HIS	ND1	0.00
226SER	OG	222HIS	ND1	8.33
226SER	OG	229ARG	NE	0.01
226SER	OG	229ARG	NH1	0.00
226SER	OG	229ARG	NH2	0.00
225ARG	NH2	222HIS	ND1	0.27
225ARG	NH2	253SER	OG	0.04
225ARG	NH2	326ASP	OD1	73.65
225ARG	NH2	326ASP	OD2	16.52
225ARG	NH1	222HIS	ND1	0.00
225ARG	NH1	253SER	OG	0.24

196ARG	NH2	215TYR	OH	0.36
196ARG	NH1	193GLU	OE2	0.06
196ARG	NH1	215TYR	OH	0.02
196ARG	NE	184ASP	OD1	13.45
196ARG	NE	184ASP	OD2	91.45
195TRP	NE1	184ASP	OD2	0.00
187ASN	ND2	215TYR	OH	0.02
185LYS	NZ	187ASN	OD1	0.18
185LYS	NZ	187ASN	ND2	0.01
185LYS	NZ	217ASP	OD1	49.25
185LYS	NZ	217ASP	OD2	49.61
185LYS	NZ	139TYR	OH	0.03
185LYS	NZ	241ASP	OD1	37.27
185LYS	NZ	241ASP	OD2	62.17
182SER	OG	184ASP	OD1	0.42
182SER	OG	184ASP	OD2	1.39
182SER	OG	213HIS	ND1	4.39
179HIS	NE2	212GLU	OE1	31.05
179HIS	NE2	212GLU	OE2	30.67
179HIS	NE2	214GLN	OE1	0.00
179HIS	NE2	229ARG	NH1	0.02
179HIS	NE2	229ARG	NH2	0.08
178LYS	NZ	208ASP	OD1	45.17
178LYS	NZ	208ASP	OD2	51.13
177ARG	NH2	127ASP	OD1	16.13
177ARG	NH2	127ASP	OD2	9.50
177ARG	NH2	231ASP	OD1	27.75
177ARG	NH2	231ASP	OD2	0.00
177ARG	NH1	127ASP	OD2	0.14
177ARG	NE	231ASP	OD1	18.99
177ARG	NE	231ASP	OD2	8.04
176ARG	NH2	127ASP	OD1	7.05
176ARG	NH2	127ASP	OD2	17.65
176ARG	NH2	299GLU	OE1	0.01
176ARG	NH2	299GLU	OE2	0.01
176ARG	NH1	127ASP	OD1	22.99
176ARG	NH1	127ASP	OD2	47.43
176ARG	NH1	231ASP	OD1	94.57
176ARG	NH1	231ASP	OD2	0.58
176ARG	NE	299GLU	OE2	0.07
175LYS	NZ	171GLU	OE1	1.81
175LYS	NZ	171GLU	OE2	0.91
175LYS	NZ	299GLU	OE1	35.89

222HIS	NE2	229ARG	NH1	0.26
222HIS	NE2	229ARG	NH2	0.04
222HIS	NE2	278ASP	OD1	0.12
215TYR	OH	78ASP	OD1	9.15
215TYR	OH	78ASP	OD2	6.11
214GLN	NE2	212GLU	OE1	5.74
214GLN	NE2	212GLU	OE2	7.26
214GLN	NE2	222HIS	ND1	6.06
214GLN	NE2	222HIS	NE2	0.03
214GLN	NE2	229ARG	NE	0.00
214GLN	NE2	229ARG	NH1	0.05
214GLN	NE2	229ARG	NH2	0.18
213HIS	NE2	200GLU	OE1	50.43
213HIS	NE2	200GLU	OE2	32.24
206TYR	OH	171GLU	OE1	22.50
206TYR	OH	171GLU	OE2	29.92
204ARG	NH2	200GLU	OE1	23.92
204ARG	NH2	200GLU	OE2	11.57
204ARG	NH2	201GLU	OE1	25.62
204ARG	NH2	201GLU	OE2	33.83
204ARG	NH2	213HIS	NE2	0.19
204ARG	NH1	200GLU	OE1	5.92
204ARG	NH1	200GLU	OE2	10.18
204ARG	NH1	201GLU	OE1	2.69
204ARG	NH1	201GLU	OE2	2.72
204ARG	NH1	212GLU	OE1	0.02
204ARG	NH1	212GLU	OE2	0.03
204ARG	NE	200GLU	OE1	10.56
204ARG	NE	200GLU	OE2	23.30
204ARG	NE	201GLU	OE1	25.25
204ARG	NE	201GLU	OE2	19.24
197LYS	NZ	193GLU	OE1	4.82
197LYS	NZ	193GLU	OE2	3.80
197LYS	NZ	201GLU	OE1	2.09
197LYS	NZ	201GLU	OE2	2.46
197LYS	NZ	148GLU	OE1	21.65
197LYS	NZ	148GLU	OE2	19.58
196ARG	NH2	184ASP	OD1	18.30
196ARG	NH2	184ASP	OD2	89.63
196ARG	NH2	215TYR	OH	0.10
196ARG	NH2	78ASP	OD1	0.00
196ARG	NH1	193GLU	OE2	0.00
196ARG	NH1	215TYR	OH	0.91

185LYS	NZ	241ASP	OD1	53.71
185LYS	NZ	241ASP	OD2	14.40
182SER	OG	184ASP	OD1	0.77
182SER	OG	184ASP	OD2	1.78
182SER	OG	213HIS	ND1	3.23
179HIS	NE2	212GLU	OE1	10.05
179HIS	NE2	212GLU	OE2	9.34
178LYS	NZ	208ASP	OD1	42.97
178LYS	NZ	208ASP	OD2	35.93
177ARG	NH2	127ASP	OD2	3.60
177ARG	NH2	179HIS	ND1	0.06
177ARG	NH2	231ASP	OD1	36.49
177ARG	NH2	231ASP	OD2	2.64
177ARG	NH1	127ASP	OD2	1.17
177ARG	NH1	179HIS	ND1	0.06
177ARG	NH1	231ASP	OD1	0.00
177ARG	NH1	231ASP	OD2	18.64
177ARG	NE	179HIS	ND1	0.02
177ARG	NE	231ASP	OD1	0.34
177ARG	NE	231ASP	OD2	37.21
176ARG	NH2	127ASP	OD1	61.18
176ARG	NH2	127ASP	OD2	0.91
176ARG	NH1	127ASP	OD1	35.15
176ARG	NH1	127ASP	OD2	14.10
176ARG	NH1	177ARG	NH2	0.00
176ARG	NH1	231ASP	OD1	69.40
176ARG	NH1	231ASP	OD2	0.00
175LYS	NZ	171GLU	OE1	0.19
175LYS	NZ	171GLU	OE2	0.02
175LYS	NZ	299GLU	OE1	52.68
175LYS	NZ	299GLU	OE2	39.88
175LYS	NZ	300HIS	ND1	5.91
174ARG	NH2	208ASP	OD1	39.07
174ARG	NH2	208ASP	OD2	45.15
174ARG	NH1	171GLU	OE1	6.84
174ARG	NH1	171GLU	OE2	29.83
174ARG	NE	208ASP	OD1	46.51
174ARG	NE	208ASP	OD2	40.74
167ARG	NH2	163GLU	OE1	21.19
167ARG	NH2	163GLU	OE2	27.51
167ARG	NH2	171GLU	OE2	0.02
167ARG	NH2	201GLU	OE2	0.04
167ARG	NH1	163GLU	OE1	1.90

225ARG	NH1	326ASP	OD1	9.18
225ARG	NH1	326ASP	OD2	75.98
225ARG	NE	222HIS	ND1	0.38
225ARG	NE	226SER	OG	0.00
222HIS	NE2	214GLN	OE1	0.74
222HIS	NE2	226SER	OG	0.01
222HIS	NE2	229ARG	NE	0.75
222HIS	NE2	229ARG	NH1	0.65
222HIS	NE2	229ARG	NH2	0.20
215TYR	OH	187ASN	OD1	21.11
215TYR	OH	217ASP	OD1	0.23
215TYR	OH	78ASP	OD1	2.26
215TYR	OH	78ASP	OD2	0.75
215TYR	OH	275SER	OG	0.18
214GLN	NE2	212GLU	OE1	6.93
214GLN	NE2	212GLU	OE2	9.68
214GLN	NE2	222HIS	ND1	7.00
214GLN	NE2	222HIS	NE2	0.17
214GLN	NE2	229ARG	NH1	0.07
214GLN	NE2	229ARG	NH2	0.02
213HIS	NE2	184ASP	OD1	5.08
213HIS	NE2	184ASP	OD2	27.29
213HIS	NE2	200GLU	OE1	0.61
213HIS	NE2	200GLU	OE2	0.37
206TYR	OH	171GLU	OE1	41.65
206TYR	OH	171GLU	OE2	50.63
204ARG	NH2	200GLU	OE1	48.77
204ARG	NH2	200GLU	OE2	44.88
204ARG	NH2	201GLU	OE1	34.84
204ARG	NH2	201GLU	OE2	34.16
204ARG	NH1	200GLU	OE1	0.00
204ARG	NH1	201GLU	OE1	29.50
204ARG	NH1	201GLU	OE2	31.97
204ARG	NE	200GLU	OE1	45.45
204ARG	NE	200GLU	OE2	50.84
197LYS	NZ	193GLU	OE1	0.23
197LYS	NZ	193GLU	OE2	1.30
197LYS	NZ	200GLU	OE1	0.54
197LYS	NZ	200GLU	OE2	0.04
197LYS	NZ	201GLU	OE1	3.95
197LYS	NZ	201GLU	OE2	3.44
197LYS	NZ	148GLU	OE1	30.19
197LYS	NZ	148GLU	OE2	30.16

175LYS	NZ	299GLU	OE2	37.74
175LYS	NZ	300HIS	ND1	4.91
174ARG	NH2	171GLU	OE1	48.43
174ARG	NH2	171GLU	OE2	50.15
174ARG	NH2	208ASP	OD1	0.02
174ARG	NH2	208ASP	OD2	4.19
174ARG	NH1	208ASP	OD1	4.77
174ARG	NH1	208ASP	OD2	6.21
174ARG	NE	171GLU	OE1	25.62
174ARG	NE	171GLU	OE2	30.53
167ARG	NH2	163GLU	OE1	29.91
167ARG	NH2	163GLU	OE2	37.64
167ARG	NH1	163GLU	OE1	38.54
167ARG	NH1	163GLU	OE2	26.34
167ARG	NH1	206TYR	OH	0.35
167ARG	NE	163GLU	OE1	1.71
167ARG	NE	163GLU	OE2	0.82
167ARG	NE	206TYR	OH	1.26
164ARG	NH2	98ASP	OD1	43.88
164ARG	NH2	98ASP	OD2	37.47
164ARG	NH2	161GLU	OE1	0.04
164ARG	NH2	161GLU	OE2	0.11
164ARG	NH2	264ARG	NE	0.12
164ARG	NH2	264ARG	NH1	0.02
164ARG	NH2	264ARG	NH2	0.01
164ARG	NH1	98ASP	OD1	22.07
164ARG	NH1	98ASP	OD2	44.06
164ARG	NH1	133GLU	OE2	1.31
164ARG	NH1	161GLU	OE1	16.36
164ARG	NH1	161GLU	OE2	18.98
164ARG	NH1	264ARG	NH2	0.02
159LYS	NZ	163GLU	OE1	15.57
159LYS	NZ	163GLU	OE2	12.60
159LYS	NZ	198THR	OG1	0.49
159LYS	NZ	201GLU	OE1	11.26
159LYS	NZ	201GLU	OE2	6.06
159LYS	NZ	148GLU	OE1	12.97
159LYS	NZ	148GLU	OE2	12.73
158SER	OG	161GLU	OE1	0.47
158SER	OG	161GLU	OE2	0.20
158SER	OG	150GLU	OE1	11.52
158SER	OG	150GLU	OE2	9.79
157TYR	OH	155GLU	OE1	0.01

196ARG	NH1	78ASP	OD1	0.79
196ARG	NH1	78ASP	OD2	0.68
196ARG	NE	184ASP	OD1	96.82
196ARG	NE	184ASP	OD2	3.79
187ASN	ND2	215TYR	OH	1.22
187ASN	ND2	217ASP	OD1	0.05
187ASN	ND2	217ASP	OD2	0.11
187ASN	ND2	82ARG	NH1	0.02
187ASN	ND2	87GLU	OE1	0.00
185LYS	NZ	187ASN	OD1	1.75
185LYS	NZ	187ASN	ND2	0.08
185LYS	NZ	217ASP	OD1	69.20
185LYS	NZ	217ASP	OD2	15.72
185LYS	NZ	139TYR	OH	0.03
185LYS	NZ	241ASP	OD1	64.09
185LYS	NZ	241ASP	OD2	17.36
182SER	OG	184ASP	OD1	4.09
182SER	OG	184ASP	OD2	0.34
182SER	OG	195TRP	NE1	0.00
182SER	OG	213HIS	ND1	0.26
179HIS	NE2	212GLU	OE1	8.59
179HIS	NE2	212GLU	OE2	6.78
179HIS	NE2	229ARG	NE	0.18
179HIS	NE2	229ARG	NH1	0.12
179HIS	NE2	229ARG	NH2	0.58
178LYS	NZ	208ASP	OD1	47.95
178LYS	NZ	208ASP	OD2	53.82
177ARG	NH2	127ASP	OD1	29.73
177ARG	NH2	127ASP	OD2	5.62
177ARG	NH2	179HIS	ND1	0.06
177ARG	NH2	231ASP	OD1	24.49
177ARG	NH2	231ASP	OD2	13.60
177ARG	NH2	309ARG	NH2	0.00
177ARG	NH1	127ASP	OD1	5.05
177ARG	NH1	127ASP	OD2	0.70
177ARG	NH1	179HIS	ND1	0.00
177ARG	NH1	231ASP	OD1	1.95
177ARG	NH1	231ASP	OD2	0.95
177ARG	NE	127ASP	OD1	0.18
177ARG	NE	127ASP	OD2	0.04
177ARG	NE	231ASP	OD1	10.68
177ARG	NE	231ASP	OD2	25.34
176ARG	NH2	127ASP	OD1	2.55

167ARG	NH1	163GLU	OE2	0.21
167ARG	NH1	206TYR	OH	1.12
167ARG	NE	163GLU	OE1	40.67
167ARG	NE	163GLU	OE2	43.71
167ARG	NE	206TYR	OH	0.18
164ARG	NH2	133GLU	OE1	42.45
164ARG	NH2	133GLU	OE2	63.97
164ARG	NH2	161GLU	OE1	24.31
164ARG	NH2	161GLU	OE2	23.66
164ARG	NH1	98ASP	OD1	0.02
164ARG	NH1	161GLU	OE1	5.25
164ARG	NH1	161GLU	OE2	4.34
164ARG	NH1	264ARG	NH2	0.04
164ARG	NE	133GLU	OE1	33.27
164ARG	NE	133GLU	OE2	46.03
159LYS	NZ	163GLU	OE1	5.83
159LYS	NZ	163GLU	OE2	11.45
159LYS	NZ	198THR	OG1	3.91
159LYS	NZ	201GLU	OE1	8.44
159LYS	NZ	201GLU	OE2	7.10
159LYS	NZ	148GLU	OE1	6.65
159LYS	NZ	148GLU	OE2	6.87
158SER	OG	161GLU	OE1	24.41
158SER	OG	161GLU	OE2	24.10
158SER	OG	150GLU	OE1	0.01
158SER	OG	150GLU	OE2	0.42
157TYR	OH	133GLU	OE1	0.01
157TYR	OH	133GLU	OE2	0.04
157TYR	OH	155GLU	OE1	3.36
157TYR	OH	155GLU	OE2	39.22
156ARG	NH2	150GLU	OE1	2.78
156ARG	NH2	150GLU	OE2	4.22
156ARG	NH2	152TRP	NE1	0.04
156ARG	NH1	150GLU	OE1	28.23
156ARG	NH1	150GLU	OE2	61.30
156ARG	NH1	152TRP	NE1	0.00
156ARG	NE	150GLU	OE1	5.92
156ARG	NE	150GLU	OE2	3.75
154THR	OG1	154THR	OG1	1.88
153ASN	ND2	190GLU	OE1	40.21
153ASN	ND2	190GLU	OE2	45.05
144ARG	NH2	190GLU	OE1	47.41
144ARG	NH2	190GLU	OE2	47.67

196ARG	NH2	184ASP	OD1	71.17
196ARG	NH2	184ASP	OD2	31.86
196ARG	NH2	213HIS	ND1	0.00
196ARG	NH2	215TYR	OH	0.72
196ARG	NH2	78ASP	OD1	0.56
196ARG	NH2	78ASP	OD2	0.24
196ARG	NH1	193GLU	OE1	0.22
196ARG	NH1	193GLU	OE2	0.07
196ARG	NH1	213HIS	ND1	0.00
196ARG	NH1	215TYR	OH	0.45
196ARG	NH1	78ASP	OD1	0.26
196ARG	NH1	78ASP	OD2	0.43
196ARG	NH1	82ARG	NE	0.00
196ARG	NH1	82ARG	NH1	0.00
196ARG	NE	184ASP	OD1	33.89
196ARG	NE	184ASP	OD2	68.32
187ASN	ND2	215TYR	OH	4.21
187ASN	ND2	85ARG	NH2	0.03
187ASN	ND2	87GLU	OE1	0.06
187ASN	ND2	87GLU	OE2	5.48
187ASN	ND2	88THR	OG1	0.01
187ASN	ND2	92SER	OG	0.08
185LYS	NZ	187ASN	OD1	3.03
185LYS	NZ	187ASN	ND2	0.06
185LYS	NZ	215TYR	OH	9.45
185LYS	NZ	217ASP	OD1	48.54
185LYS	NZ	217ASP	OD2	25.65
185LYS	NZ	139TYR	OH	5.38
185LYS	NZ	241ASP	OD1	48.91
185LYS	NZ	241ASP	OD2	27.27
182SER	OG	184ASP	OD1	0.00
182SER	OG	184ASP	OD2	0.12
182SER	OG	213HIS	ND1	14.60
182SER	OG	213HIS	NE2	0.00
179HIS	NE2	212GLU	OE1	14.52
179HIS	NE2	212GLU	OE2	10.68
179HIS	NE2	229ARG	NE	0.02
179HIS	NE2	229ARG	NH1	0.01
179HIS	NE2	229ARG	NH2	0.09
178LYS	NZ	208ASP	OD1	34.33
178LYS	NZ	208ASP	OD2	21.35
177ARG	NH2	127ASP	OD1	1.49
177ARG	NH2	127ASP	OD2	33.56

157TYR	OH	155GLU	OE2	0.34
156ARG	NH2	150GLU	OE1	0.66
156ARG	NH2	150GLU	OE2	0.38
156ARG	NH2	152TRP	NE1	0.02
156ARG	NH1	150GLU	OE1	56.90
156ARG	NH1	150GLU	OE2	39.56
156ARG	NE	150GLU	OE1	0.06
156ARG	NE	150GLU	OE2	0.56
154THR	OG1	154THR	OG1	4.35
153ASN	ND2	190GLU	OE1	6.18
153ASN	ND2	190GLU	OE2	0.68
144ARG	NH2	190GLU	OE1	43.81
144ARG	NH2	190GLU	OE2	42.78
144ARG	NH1	142GLU	OE1	0.24
144ARG	NH1	142GLU	OE2	0.50
144ARG	NE	153ASN	ND2	0.01
144ARG	NE	190GLU	OE1	44.76
144ARG	NE	190GLU	OE2	37.12
139TYR	OH	237ASN	OD1	0.22
139TYR	OH	237ASN	ND2	0.10
139TYR	OH	241ASP	OD1	0.66
139TYR	OH	241ASP	OD2	19.96
135THR	OG1	98ASP	OD2	0.00
135THR	OG1	133GLU	OE1	13.88
135THR	OG1	133GLU	OE2	34.07
135THR	OG1	161GLU	OE1	0.02
135THR	OG1	161GLU	OE2	0.02
132ARG	NH2	102ASN	OD1	0.01
132ARG	NH2	237ASN	OD1	44.72
132ARG	NH2	237ASN	ND2	0.15
132ARG	NH2	241ASP	OD1	8.61
132ARG	NH2	241ASP	OD2	9.44
132ARG	NH1	102ASN	OD1	0.08
132ARG	NH1	241ASP	OD1	4.24
132ARG	NH1	241ASP	OD2	2.63
132ARG	NH1	244SER	OG	48.21
132ARG	NE	102ASN	OD1	0.14
132ARG	NE	237ASN	OD1	4.26
132ARG	NE	237ASN	ND2	0.01
124ARG	NH2	113GLU	OE1	0.03
124ARG	NH2	113GLU	OE2	2.38
124ARG	NH2	120GLU	OE1	17.17
124ARG	NH2	120GLU	OE2	20.28

176ARG	NH2	127ASP	OD2	27.12
176ARG	NH2	299GLU	OE1	0.00
176ARG	NH1	127ASP	OD1	12.37
176ARG	NH1	127ASP	OD2	36.36
176ARG	NH1	177ARG	NH2	0.00
176ARG	NH1	231ASP	OD1	50.88
176ARG	NH1	231ASP	OD2	42.51
176ARG	NE	299GLU	OE2	0.10
175LYS	NZ	171GLU	OE1	1.12
175LYS	NZ	171GLU	OE2	0.55
175LYS	NZ	299GLU	OE1	35.38
175LYS	NZ	299GLU	OE2	33.34
175LYS	NZ	300HIS	ND1	5.57
174ARG	NH2	171GLU	OE1	46.30
174ARG	NH2	171GLU	OE2	45.36
174ARG	NH2	206TYR	OH	0.90
174ARG	NH2	208ASP	OD2	0.25
174ARG	NH1	171GLU	OE1	9.96
174ARG	NH1	171GLU	OE2	13.90
174ARG	NH1	206TYR	OH	7.28
174ARG	NH1	208ASP	OD1	0.87
174ARG	NH1	208ASP	OD2	0.08
174ARG	NE	171GLU	OE1	23.94
174ARG	NE	171GLU	OE2	19.29
174ARG	NE	206TYR	OH	3.62
174ARG	NE	208ASP	OD2	0.29
167ARG	NH2	163GLU	OE1	35.55
167ARG	NH2	163GLU	OE2	29.20
167ARG	NH2	171GLU	OE2	1.60
167ARG	NH2	206TYR	OH	0.03
167ARG	NH1	163GLU	OE1	5.59
167ARG	NH1	163GLU	OE2	7.71
167ARG	NH1	206TYR	OH	2.87
167ARG	NE	163GLU	OE1	32.47
167ARG	NE	163GLU	OE2	34.91
167ARG	NE	171GLU	OE2	0.00
167ARG	NE	206TYR	OH	1.76
164ARG	NH2	133GLU	OE1	0.02
164ARG	NH2	133GLU	OE2	0.08
164ARG	NH2	161GLU	OE1	32.77
164ARG	NH2	161GLU	OE2	27.55
164ARG	NH1	133GLU	OE1	0.31
164ARG	NH1	133GLU	OE2	0.82

144ARG	NH1	142GLU	OE1	0.01
144ARG	NH1	142GLU	OE2	0.55
144ARG	NE	190GLU	OE1	50.77
144ARG	NE	190GLU	OE2	59.61
139TYR	OH	241ASP	OD1	54.49
139TYR	OH	241ASP	OD2	32.74
135THR	OG1	133GLU	OE1	61.20
135THR	OG1	133GLU	OE2	36.43
135THR	OG1	164ARG	NH2	0.03
132ARG	NH2	102ASN	OD1	0.01
132ARG	NH2	237ASN	OD1	98.16
132ARG	NH2	241ASP	OD1	0.28
132ARG	NH2	241ASP	OD2	9.80
132ARG	NH2	245ASP	OD1	0.02
132ARG	NH2	245ASP	OD2	0.02
132ARG	NH1	102ASN	OD1	0.03
132ARG	NH1	241ASP	OD1	6.10
132ARG	NH1	241ASP	OD2	13.97
132ARG	NH1	244SER	OG	5.48
132ARG	NH1	245ASP	OD1	0.07
132ARG	NH1	245ASP	OD2	0.64
132ARG	NE	102ASN	OD1	0.08
132ARG	NE	237ASN	OD1	4.01
132ARG	NE	237ASN	ND2	0.04
124ARG	NH2	113GLU	OE1	1.88
124ARG	NH2	113GLU	OE2	5.08
124ARG	NH2	120GLU	OE1	24.58
124ARG	NH2	120GLU	OE2	33.44
124ARG	NH2	121GLU	OE1	22.92
124ARG	NH2	121GLU	OE2	21.36
124ARG	NH1	113GLU	OE1	0.03
124ARG	NH1	120GLU	OE1	7.21
124ARG	NH1	120GLU	OE2	8.57
124ARG	NH1	121GLU	OE1	37.19
124ARG	NH1	121GLU	OE2	34.15
124ARG	NE	113GLU	OE1	4.81
124ARG	NE	113GLU	OE2	1.10
124ARG	NE	120GLU	OE1	14.88
124ARG	NE	120GLU	OE2	7.71
119LYS	NZ	120GLU	OE1	4.42
119LYS	NZ	120GLU	OE2	11.73
114ARG	NH2	120GLU	OE1	22.30
114ARG	NH2	120GLU	OE2	10.97

177ARG	NH2	231ASP	OD1	31.22
177ARG	NH2	231ASP	OD2	25.37
177ARG	NH1	127ASP	OD1	0.03
177ARG	NH1	127ASP	OD2	9.13
177ARG	NH1	179HIS	ND1	0.06
177ARG	NH1	231ASP	OD1	13.15
177ARG	NH1	231ASP	OD2	0.03
177ARG	NE	231ASP	OD1	32.14
177ARG	NE	231ASP	OD2	25.53
176ARG	NH2	127ASP	OD1	69.77
176ARG	NH2	127ASP	OD2	6.41
176ARG	NH1	127ASP	OD1	10.70
176ARG	NH1	127ASP	OD2	7.68
176ARG	NH1	177ARG	NH1	0.00
176ARG	NH1	177ARG	NH2	0.00
176ARG	NH1	231ASP	OD1	62.55
176ARG	NH1	231ASP	OD2	32.83
175LYS	NZ	171GLU	OE1	1.02
175LYS	NZ	171GLU	OE2	0.59
175LYS	NZ	299GLU	OE1	29.66
175LYS	NZ	299GLU	OE2	25.06
175LYS	NZ	300HIS	ND1	1.42
174ARG	NH2	208ASP	OD1	41.52
174ARG	NH2	208ASP	OD2	57.11
174ARG	NH1	171GLU	OE1	14.87
174ARG	NH1	171GLU	OE2	11.34
174ARG	NE	208ASP	OD1	58.33
174ARG	NE	208ASP	OD2	44.35
167ARG	NH2	163GLU	OE1	36.11
167ARG	NH2	163GLU	OE2	27.67
167ARG	NH2	171GLU	OE1	0.01
167ARG	NH2	201GLU	OE2	0.17
167ARG	NH2	206TYR	OH	0.01
167ARG	NH1	163GLU	OE1	7.46
167ARG	NH1	163GLU	OE2	5.49
167ARG	NH1	206TYR	OH	2.43
167ARG	NE	163GLU	OE1	33.69
167ARG	NE	163GLU	OE2	34.03
167ARG	NE	171GLU	OE1	0.01
167ARG	NE	206TYR	OH	0.41
164ARG	NH2	98ASP	OD1	18.97
164ARG	NH2	98ASP	OD2	50.27
164ARG	NH2	133GLU	OE1	5.44

124ARG	NH2	121GLU	OE1	49.21
124ARG	NH2	121GLU	OE2	40.22
124ARG	NH1	113GLU	OE1	0.06
124ARG	NH1	113GLU	OE2	40.25
124ARG	NH1	120GLU	OE1	28.25
124ARG	NH1	120GLU	OE2	27.95
124ARG	NH1	121GLU	OE1	7.30
124ARG	NH1	121GLU	OE2	7.67
124ARG	NE	113GLU	OE2	0.42
124ARG	NE	120GLU	OE1	3.12
124ARG	NE	120GLU	OE2	0.05
124ARG	NE	121GLU	OE1	18.58
124ARG	NE	121GLU	OE2	18.97
119LYS	NZ	120GLU	OE1	9.28
119LYS	NZ	120GLU	OE2	4.16
114ARG	NH2	120GLU	OE1	1.91
114ARG	NH2	120GLU	OE2	1.93
114ARG	NH1	113GLU	OE2	0.02
114ARG	NE	120GLU	OE1	12.64
114ARG	NE	120GLU	OE2	12.53
107LYS	NZ	127ASP	OD1	5.11
107LYS	NZ	127ASP	OD2	5.66
107LYS	NZ	309ARG	NH1	0.00
107LYS	NZ	312GLU	OE1	51.99
107LYS	NZ	312GLU	OE2	45.61
104ARG	NH2	102ASN	OD1	0.32
104ARG	NH2	245ASP	OD1	0.64
104ARG	NH2	245ASP	OD2	1.54
104ARG	NH2	259SER	OG	0.14
104ARG	NH1	245ASP	OD1	13.61
104ARG	NH1	245ASP	OD2	6.92
104ARG	NH1	248SER	OG	19.60
104ARG	NE	102ASN	OD1	66.21
104ARG	NE	259SER	OG	2.17
102ASN	ND2	104ARG	NE	0.18
102ASN	ND2	104ARG	NH2	0.05
102ASN	ND2	259SER	OG	2.90
102ASN	ND2	261SER	OG	56.01
97GLN	NE2	62GLU	OE1	34.44
97GLN	NE2	62GLU	OE2	46.69
95LYS	NZ	98ASP	OD1	0.06
95LYS	NZ	98ASP	OD2	0.67
94ARG	NH2	87GLU	OE1	20.08

164ARG	NH1	161GLU	OE1	42.43
164ARG	NH1	161GLU	OE2	49.74
164ARG	NH1	264ARG	NH1	0.02
164ARG	NE	163GLU	OE2	0.00
164ARG	NE	167ARG	NH2	0.00
159LYS	NZ	163GLU	OE1	2.09
159LYS	NZ	163GLU	OE2	3.56
159LYS	NZ	198THR	OG1	5.67
159LYS	NZ	201GLU	OE1	11.80
159LYS	NZ	201GLU	OE2	12.26
159LYS	NZ	148GLU	OE1	21.60
159LYS	NZ	148GLU	OE2	22.98
158SER	OG	161GLU	OE1	0.17
158SER	OG	161GLU	OE2	0.18
158SER	OG	150GLU	OE1	1.23
158SER	OG	150GLU	OE2	0.90
157TYR	OH	133GLU	OE1	14.07
157TYR	OH	133GLU	OE2	42.99
157TYR	OH	155GLU	OE1	5.73
157TYR	OH	155GLU	OE2	13.29
156ARG	NH2	142GLU	OE1	0.01
156ARG	NH2	150GLU	OE1	50.16
156ARG	NH2	150GLU	OE2	38.03
156ARG	NH2	152TRP	NE1	0.02
156ARG	NH1	142GLU	OE1	0.05
156ARG	NH1	158SER	OG	0.00
156ARG	NH1	150GLU	OE1	2.50
156ARG	NH1	150GLU	OE2	4.75
156ARG	NH1	152TRP	NE1	0.00
156ARG	NE	150GLU	OE1	38.39
156ARG	NE	150GLU	OE2	50.10
154THR	OG1	154THR	OG1	0.19
153ASN	ND2	190GLU	OE1	15.57
153ASN	ND2	190GLU	OE2	18.26
147SER	OG	148GLU	OE1	0.17
147SER	OG	148GLU	OE2	0.29
144ARG	NH2	153ASN	OD1	0.01
144ARG	NH2	190GLU	OE1	33.50
144ARG	NH2	190GLU	OE2	35.71
144ARG	NH1	142GLU	OE1	1.88
144ARG	NH1	142GLU	OE2	2.57
144ARG	NE	153ASN	ND2	0.02
144ARG	NE	190GLU	OE1	43.51

114ARG	NH1	120GLU	OE1	0.15
114ARG	NE	120GLU	OE1	14.94
114ARG	NE	120GLU	OE2	25.80
107LYS	NZ	127ASP	OD1	1.58
107LYS	NZ	127ASP	OD2	2.26
107LYS	NZ	312GLU	OE1	44.73
107LYS	NZ	312GLU	OE2	55.74
104ARG	NH2	102ASN	OD1	0.06
104ARG	NH2	132ARG	NH1	0.00
104ARG	NH2	244SER	OG	0.58
104ARG	NH2	245ASP	OD1	16.25
104ARG	NH2	245ASP	OD2	25.55
104ARG	NH2	248SER	OG	3.26
104ARG	NH2	270GLU	OE2	0.00
104ARG	NH1	244SER	OG	10.57
104ARG	NH1	245ASP	OD1	8.26
104ARG	NH1	245ASP	OD2	8.83
104ARG	NH1	248SER	OG	50.73
104ARG	NE	102ASN	OD1	16.37
104ARG	NE	244SER	OG	0.23
102ASN	ND2	104ARG	NE	0.01
102ASN	ND2	132ARG	NE	0.01
102ASN	ND2	259SER	OG	0.05
102ASN	ND2	261SER	OG	71.42
102ASN	ND2	270GLU	OE1	0.03
102ASN	ND2	270GLU	OE2	0.04
97GLN	NE2	62GLU	OE1	45.28
97GLN	NE2	62GLU	OE2	39.59
97GLN	NE2	266THR	OG1	0.00
94ARG	NH2	87GLU	OE2	0.04
94ARG	NH2	261SER	OG	0.02
94ARG	NH2	270GLU	OE1	3.68
94ARG	NH2	270GLU	OE2	10.00
94ARG	NH1	87GLU	OE2	0.07
94ARG	NE	261SER	OG	0.93
94ARG	NE	270GLU	OE1	0.00
88THR	OG1	51GLU	OE1	0.00
85ARG	NH2	78ASP	OD1	1.00
85ARG	NH2	78ASP	OD2	0.33
85ARG	NH2	87GLU	OE1	32.57
85ARG	NH2	87GLU	OE2	65.04
85ARG	NH1	78ASP	OD1	2.29
85ARG	NH1	78ASP	OD2	0.13

164ARG	NH2	133GLU	OE2	24.14
164ARG	NH2	161GLU	OE1	9.32
164ARG	NH2	161GLU	OE2	8.20
164ARG	NH1	98ASP	OD1	51.67
164ARG	NH1	98ASP	OD2	20.18
164ARG	NH1	161GLU	OE1	0.16
164ARG	NH1	161GLU	OE2	0.48
164ARG	NH1	264ARG	NE	0.01
164ARG	NE	133GLU	OE2	18.20
164ARG	NE	161GLU	OE1	0.88
164ARG	NE	161GLU	OE2	0.75
159LYS	NZ	163GLU	OE1	2.80
159LYS	NZ	163GLU	OE2	8.88
159LYS	NZ	198THR	OG1	4.75
159LYS	NZ	201GLU	OE1	6.76
159LYS	NZ	201GLU	OE2	6.70
159LYS	NZ	148GLU	OE1	23.47
159LYS	NZ	148GLU	OE2	22.05
158SER	OG	161GLU	OE1	16.24
158SER	OG	161GLU	OE2	13.16
158SER	OG	150GLU	OE1	2.24
158SER	OG	150GLU	OE2	1.48
157TYR	OH	133GLU	OE1	1.33
157TYR	OH	133GLU	OE2	0.10
157TYR	OH	155GLU	OE1	0.46
157TYR	OH	155GLU	OE2	4.72
156ARG	NH2	158SER	OG	0.04
156ARG	NH2	161GLU	OE1	0.49
156ARG	NH2	161GLU	OE2	0.60
156ARG	NH2	150GLU	OE1	36.15
156ARG	NH2	150GLU	OE2	34.05
156ARG	NH2	152TRP	NE1	0.05
156ARG	NH1	142GLU	OE1	0.20
156ARG	NH1	142GLU	OE2	0.03
156ARG	NH1	158SER	OG	0.01
156ARG	NH1	161GLU	OE1	1.04
156ARG	NH1	161GLU	OE2	0.72
156ARG	NH1	150GLU	OE1	5.64
156ARG	NH1	150GLU	OE2	0.86
156ARG	NH1	152TRP	NE1	0.00
156ARG	NE	150GLU	OE1	41.68
156ARG	NE	150GLU	OE2	45.91
154THR	OG1	154THR	OG1	3.02

94ARG	NH2	87GLU	OE2	11.36
94ARG	NH2	261SER	OG	0.63
94ARG	NH2	270GLU	OE1	0.20
94ARG	NH2	270GLU	OE2	0.52
94ARG	NH1	87GLU	OE1	27.35
94ARG	NH1	87GLU	OE2	34.65
94ARG	NE	261SER	OG	0.04
92SER	OG	51GLU	OE1	0.10
85SER	OG	87GLU	OE1	0.03
85SER	OG	88THR	OG1	8.47
82ARG	NH2	78ASP	OD1	0.03
82ARG	NH2	78ASP	OD2	0.01
82ARG	NH2	85SER	OG	0.14
82ARG	NH2	87GLU	OE1	5.70
82ARG	NH2	87GLU	OE2	6.01
82ARG	NH2	190GLU	OE1	0.01
82ARG	NH2	193GLU	OE1	3.46
82ARG	NH2	193GLU	OE2	6.15
82ARG	NH2	215TYR	OH	0.02
82ARG	NH1	85SER	OG	0.18
82ARG	NH1	87GLU	OE1	3.95
82ARG	NH1	87GLU	OE2	5.65
82ARG	NH1	144ARG	NH2	0.00
82ARG	NH1	190GLU	OE2	0.02
82ARG	NH1	193GLU	OE1	6.89
82ARG	NH1	193GLU	OE2	2.82
82ARG	NE	87GLU	OE1	3.60
82ARG	NE	87GLU	OE2	0.85
82ARG	NE	88THR	OG1	0.00
77TRP	NE1	9ASP	OD1	51.62
77TRP	NE1	9ASP	OD2	34.82
77TRP	NE1	47ASP	OD1	0.32
77TRP	NE1	47ASP	OD2	0.21
77TRP	NE1	275SER	OG	0.00
76LYS	NZ	9ASP	OD1	18.84
76LYS	NZ	9ASP	OD2	35.39
76LYS	NZ	47ASP	OD1	0.20
76LYS	NZ	47ASP	OD2	0.46
76LYS	NZ	77TRP	NE1	0.02
71SER	OG	273HIS	ND1	0.03
71SER	OG	275SER	OG	49.10
59LYS	NZ	55GLU	OE1	37.89
59LYS	NZ	55GLU	OE2	17.07

144ARG	NE	190GLU	OE2	48.23
139TYR	OH	237ASN	OD1	10.68
139TYR	OH	237ASN	ND2	0.35
139TYR	OH	241ASP	OD1	6.54
139TYR	OH	241ASP	OD2	16.48
135THR	OG1	133GLU	OE1	57.03
135THR	OG1	133GLU	OE2	27.28
135THR	OG1	157TYR	OH	0.14
135THR	OG1	161GLU	OE1	0.01
132ARG	NH2	139TYR	OH	0.22
132ARG	NH2	237ASN	OD1	82.40
132ARG	NH2	237ASN	ND2	0.07
132ARG	NH2	241ASP	OD1	5.21
132ARG	NH2	241ASP	OD2	11.49
132ARG	NH1	102ASN	OD1	2.01
132ARG	NH1	241ASP	OD1	8.49
132ARG	NH1	241ASP	OD2	14.16
132ARG	NH1	244SER	OG	9.06
132ARG	NE	102ASN	OD1	0.07
132ARG	NE	237ASN	OD1	22.22
132ARG	NE	237ASN	ND2	0.05
124ARG	NH2	113GLU	OE1	9.62
124ARG	NH2	113GLU	OE2	13.30
124ARG	NH2	120GLU	OE1	11.74
124ARG	NH2	120GLU	OE2	10.83
124ARG	NH2	121GLU	OE1	24.65
124ARG	NH2	121GLU	OE2	22.83
124ARG	NH1	113GLU	OE1	0.81
124ARG	NH1	113GLU	OE2	1.34
124ARG	NH1	120GLU	OE1	10.02
124ARG	NH1	120GLU	OE2	14.20
124ARG	NH1	121GLU	OE1	16.34
124ARG	NH1	121GLU	OE2	18.54
124ARG	NE	113GLU	OE1	23.59
124ARG	NE	113GLU	OE2	8.38
124ARG	NE	120GLU	OE1	0.66
124ARG	NE	120GLU	OE2	0.44
124ARG	NE	121GLU	OE1	4.42
124ARG	NE	121GLU	OE2	3.88
119LYS	NZ	121GLU	OE1	0.05
119LYS	NZ	121GLU	OE2	0.06
119LYS	NZ	120GLU	OE1	0.14
119LYS	NZ	120GLU	OE2	0.21

85ARG	NE	87GLU	OE1	67.20
85ARG	NE	87GLU	OE2	23.78
83LYS	NZ	193GLU	OE2	0.00
82ARG	NH2	87GLU	OE1	2.07
82ARG	NH2	87GLU	OE2	2.04
82ARG	NH2	187ASN	OD1	0.09
82ARG	NH2	193GLU	OE1	10.26
82ARG	NH2	193GLU	OE2	7.43
82ARG	NH2	215TYR	OH	2.58
82ARG	NH1	87GLU	OE1	5.52
82ARG	NH1	87GLU	OE2	7.98
82ARG	NH1	88THR	OG1	0.00
82ARG	NH1	187ASN	OD1	0.04
82ARG	NH1	193GLU	OE1	0.32
82ARG	NH1	193GLU	OE2	0.27
82ARG	NH1	215TYR	OH	0.18
82ARG	NE	87GLU	OE1	5.42
82ARG	NE	87GLU	OE2	3.10
82ARG	NE	187ASN	OD1	0.14
82ARG	NE	193GLU	OE1	0.05
82ARG	NE	193GLU	OE2	6.46
77TRP	NE1	47ASP	OD1	0.10
77TRP	NE1	47ASP	OD2	0.00
76LYS	NZ	9ASP	OD1	0.59
76LYS	NZ	9ASP	OD2	2.30
76LYS	NZ	47ASP	OD1	1.54
76LYS	NZ	47ASP	OD2	1.06
76LYS	NZ	77TRP	NE1	0.00
71SER	OG	270GLU	OE2	0.03
71SER	OG	273HIS	NE2	0.02
71SER	OG	275SER	OG	0.30
59LYS	NZ	55GLU	OE1	33.53
59LYS	NZ	55GLU	OE2	25.65
59LYS	NZ	63GLU	OE2	0.03
58ARG	NH2	51GLU	OE1	11.97
58ARG	NH2	51GLU	OE2	14.48
58ARG	NH2	55GLU	OE1	35.02
58ARG	NH2	55GLU	OE2	30.04
58ARG	NH2	92SER	OG	1.71
58ARG	NH1	51GLU	OE1	21.07
58ARG	NH1	51GLU	OE2	19.04
58ARG	NH1	55GLU	OE1	3.90
58ARG	NH1	55GLU	OE2	8.85

153ASN	ND2	190GLU	OE1	24.61
153ASN	ND2	190GLU	OE2	31.95
147SER	OG	148GLU	OE1	0.18
147SER	OG	148GLU	OE2	0.10
147SER	OG	150GLU	OE2	0.04
144ARG	NH2	142GLU	OE1	7.54
144ARG	NH2	142GLU	OE2	11.50
144ARG	NH2	153ASN	ND2	0.00
144ARG	NH2	190GLU	OE1	33.57
144ARG	NH2	190GLU	OE2	32.76
144ARG	NH1	87GLU	OE1	0.02
144ARG	NH1	87GLU	OE2	0.00
144ARG	NH1	142GLU	OE1	2.38
144ARG	NH1	142GLU	OE2	0.91
144ARG	NH1	190GLU	OE1	1.74
144ARG	NH1	190GLU	OE2	0.51
144ARG	NE	142GLU	OE1	13.48
144ARG	NE	142GLU	OE2	12.66
144ARG	NE	153ASN	ND2	0.00
144ARG	NE	190GLU	OE1	35.27
144ARG	NE	190GLU	OE2	38.28
139TYR	OH	237ASN	OD1	0.04
139TYR	OH	241ASP	OD1	36.15
139TYR	OH	241ASP	OD2	33.41
135THR	OG1	133GLU	OE1	26.64
135THR	OG1	133GLU	OE2	1.48
135THR	OG1	161GLU	OE1	0.02
135THR	OG1	161GLU	OE2	0.04
135THR	OG1	164ARG	NH2	0.02
132ARG	NH2	102ASN	OD1	0.04
132ARG	NH2	139TYR	OH	0.02
132ARG	NH2	237ASN	OD1	26.15
132ARG	NH2	241ASP	OD1	6.32
132ARG	NH2	241ASP	OD2	3.18
132ARG	NH2	244SER	OG	5.93
132ARG	NH2	245ASP	OD1	0.02
132ARG	NH2	245ASP	OD2	0.48
132ARG	NH1	102ASN	OD1	0.04
132ARG	NH1	139TYR	OH	0.14
132ARG	NH1	237ASN	OD1	34.91
132ARG	NH1	237ASN	ND2	0.03
132ARG	NH1	241ASP	OD1	39.62
132ARG	NH1	241ASP	OD2	5.24

58ARG	NH2	51GLU	OE1	14.16
58ARG	NH2	51GLU	OE2	8.20
58ARG	NH2	55GLU	OE1	35.45
58ARG	NH2	55GLU	OE2	57.39
58ARG	NH1	51GLU	OE1	21.43
58ARG	NH1	51GLU	OE2	28.99
58ARG	NH1	55GLU	OE1	2.79
58ARG	NH1	55GLU	OE2	6.67
58ARG	NH1	92SER	OG	10.03
58ARG	NE	55GLU	OE1	8.08
58ARG	NE	55GLU	OE2	7.71
24ARG	NH2	17GLU	OE1	13.06
24ARG	NH2	17GLU	OE2	21.63
24ARG	NH2	27ASP	OD2	1.35
24ARG	NH2	36TYR	OH	16.89
24ARG	NH1	17GLU	OE1	15.97
24ARG	NH1	17GLU	OE2	11.94
24ARG	NH1	27ASP	OD1	0.02
24ARG	NH1	27ASP	OD2	0.04
24ARG	NH1	36TYR	OH	15.27
24ARG	NE	27ASP	OD2	0.00
24ARG	NE	36TYR	OH	35.38
21LYS	NZ	17GLU	OE1	0.25
21LYS	NZ	17GLU	OE2	0.29
21LYS	NZ	334GLU	OE1	22.78
21LYS	NZ	334GLU	OE2	21.52
21LYS	NZ	337THR	OG1	13.51
2LYS	NZ	37GLU	OE1	0.42
2LYS	NZ	37GLU	OE2	0.64
2LYS	NZ	63GLU	OE1	29.05
2LYS	NZ	63GLU	OE2	40.71
343HIS	NE2	321GLU	OE1	10.05
343HIS	NE2	321GLU	OE2	15.16
342ARG	NH2	321GLU	OE2	21.62
342ARG	NH2	321GLU	OE2	29.48
342ARG	NH2	343HIS	ND1	0.01
342ARG	NH2	343HIS	NE2	0.04
342ARG	NH1	321GLU	OE1	33.68
342ARG	NH1	321GLU	OE2	20.91
342ARG	NH1	343HIS	ND1	0.04
339THR	OG1	321GLU	OE1	0.04
339THR	OG1	321GLU	OE2	0.02
339THR	OG1	342ARG	NH1	0.02

114ARG	NH2	113GLU	OE1	19.00
114ARG	NH2	113GLU	OE2	15.86
114ARG	NH2	120GLU	OE1	4.13
114ARG	NH2	120GLU	OE2	2.27
114ARG	NH1	120GLU	OE1	0.92
114ARG	NH1	120GLU	OE2	0.06
114ARG	NE	113GLU	OE1	18.19
114ARG	NE	113GLU	OE2	22.35
114ARG	NE	120GLU	OE1	5.24
114ARG	NE	120GLU	OE2	8.63
107LYS	NZ	127ASP	OD1	2.69
107LYS	NZ	127ASP	OD2	1.29
107LYS	NZ	312GLU	OE1	50.20
107LYS	NZ	312GLU	OE2	53.43
104ARG	NH2	102ASN	OD1	2.17
104ARG	NH2	132ARG	NH1	0.01
104ARG	NH2	241ASP	OD1	0.09
104ARG	NH2	241ASP	OD2	0.25
104ARG	NH2	244SER	OG	0.13
104ARG	NH2	245ASP	OD1	8.63
104ARG	NH2	245ASP	OD2	12.19
104ARG	NH2	248SER	OG	0.25
104ARG	NH2	259SER	OG	0.36
104ARG	NH2	270GLU	OE1	0.00
104ARG	NH1	241ASP	OD1	0.59
104ARG	NH1	244SER	OG	3.07
104ARG	NH1	245ASP	OD1	34.52
104ARG	NH1	245ASP	OD2	29.63
104ARG	NH1	248SER	OG	9.62
104ARG	NE	102ASN	OD1	12.18
104ARG	NE	244SER	OG	0.06
104ARG	NE	259SER	OG	1.88
102ASN	ND2	104ARG	NE	0.46
102ASN	ND2	104ARG	NH1	0.03
102ASN	ND2	104ARG	NH2	0.14
102ASN	ND2	132ARG	NH1	0.00
102ASN	ND2	259SER	OG	10.06
102ASN	ND2	261SER	OG	58.01
102ASN	ND2	270GLU	OE1	0.03
102ASN	ND2	270GLU	OE2	0.20
97GLN	NE2	62GLU	OE1	22.80
97GLN	NE2	62GLU	OE2	19.06
97GLN	NE2	266THR	OG1	28.11

58ARG	NH1	92SER	OG	1.67
58ARG	NE	55GLU	OE1	3.33
58ARG	NE	55GLU	OE2	2.25
58ARG	NE	92SER	OG	0.00
36TYR	OH	17GLU	OE1	0.00
36TYR	OH	17GLU	OE2	0.00
24ARG	NH2	17GLU	OE1	42.21
24ARG	NH2	17GLU	OE2	34.23
24ARG	NH2	36TYR	OH	12.60
24ARG	NH1	17GLU	OE1	33.33
24ARG	NH1	17GLU	OE2	35.08
24ARG	NH1	36TYR	OH	58.63
24ARG	NE	36TYR	OH	12.07
21LYS	NZ	17GLU	OE1	0.98
21LYS	NZ	17GLU	OE2	0.00
21LYS	NZ	334GLU	OE1	28.09
21LYS	NZ	334GLU	OE2	36.07
21LYS	NZ	337THR	OG1	10.57
2LYS	NZ	37GLU	OE1	0.00
2LYS	NZ	37GLU	OE2	0.06
2LYS	NZ	63GLU	OE1	39.37
2LYS	NZ	63GLU	OE2	28.47
2LYS	NZ	65GLU	OE1	0.05
2LYS	NZ	65GLU	OE2	0.05
343HIS	NE2	321GLU	OE1	5.45
343HIS	NE2	321GLU	OE2	12.02
342ARG	NH2	321GLU	OE1	26.52
342ARG	NH2	321GLU	OE2	31.24
342ARG	NH2	343HIS	ND1	0.00
342ARG	NH2	343HIS	NE2	0.05
342ARG	NH1	321GLU	OE1	28.63
342ARG	NH1	321GLU	OE2	19.74
342ARG	NH1	343HIS	NE2	0.00
342ARG	NE	321GLU	OE1	0.74
342ARG	NE	321GLU	OE2	0.63
339THR	OG1	321GLU	OE1	0.07
339THR	OG1	321GLU	OE2	0.10
339THR	OG1	342ARG	NH1	0.02
339THR	OG1	342ARG	NH2	0.03
337THR	OG1	334GLU	OE1	0.01
333THR	OG1	14GLU	OE1	0.40
333THR	OG1	14GLU	OE2	1.15
333THR	OG1	334GLU	OE1	0.24

132ARG	NH1	244SER	OG	0.53
132ARG	NH1	245ASP	OD1	0.00
132ARG	NH1	245ASP	OD2	0.00
132ARG	NE	102ASN	OD1	0.26
132ARG	NE	237ASN	OD1	3.85
132ARG	NE	237ASN	ND2	0.04
132ARG	NE	244SER	OG	29.69
124ARG	NH2	113GLU	OE1	5.37
124ARG	NH2	113GLU	OE2	1.93
124ARG	NH2	120GLU	OE1	10.27
124ARG	NH2	120GLU	OE2	11.04
124ARG	NH2	121GLU	OE1	33.54
124ARG	NH2	121GLU	OE2	39.25
124ARG	NH1	113GLU	OE1	9.34
124ARG	NH1	113GLU	OE2	16.34
124ARG	NH1	120GLU	OE1	13.53
124ARG	NH1	120GLU	OE2	12.87
124ARG	NH1	121GLU	OE1	11.25
124ARG	NH1	121GLU	OE2	13.44
124ARG	NE	113GLU	OE1	4.35
124ARG	NE	113GLU	OE2	2.54
124ARG	NE	121GLU	OE1	21.10
124ARG	NE	121GLU	OE2	14.38
119LYS	NZ	121GLU	OE1	0.01
119LYS	NZ	121GLU	OE2	0.05
119LYS	NZ	114ARG	NH2	0.00
119LYS	NZ	120GLU	OE1	15.64
119LYS	NZ	120GLU	OE2	18.37
114ARG	NH2	113GLU	OE1	12.34
114ARG	NH2	113GLU	OE2	6.16
114ARG	NH2	120GLU	OE1	10.01
114ARG	NH2	120GLU	OE2	9.32
114ARG	NH2	124ARG	NH2	0.00
114ARG	NH1	113GLU	OE2	0.02
114ARG	NH1	120GLU	OE1	0.16
114ARG	NH1	120GLU	OE2	0.08
114ARG	NE	113GLU	OE1	10.54
114ARG	NE	113GLU	OE2	12.02
114ARG	NE	120GLU	OE1	16.92
114ARG	NE	120GLU	OE2	18.86
107LYS	NZ	127ASP	OD1	0.78
107LYS	NZ	127ASP	OD2	0.50
107LYS	NZ	299GLU	OE1	0.16

339THR	OG1	342ARG	NH2	0.03
333THR	OG1	14GLU	OE1	0.00
322THR	OG1	339THR	OG1	0.01
317LYS	NZ	313ASP	OD1	88.24
317LYS	NZ	313ASP	OD2	15.34
310LYS	NZ	30GLU	OE1	4.20
310LYS	NZ	30GLU	OE2	5.77
310LYS	NZ	306GLU	OE1	0.00
310LYS	NZ	313ASP	OD1	3.18
309ARG	NH2	299GLU	OE1	8.87
309ARG	NH2	299GLU	OE2	17.00
309ARG	NH2	306GLU	OE1	17.53
309ARG	NH2	306GLU	OE2	29.70
309ARG	NH1	299GLU	OE1	19.02
309ARG	NH1	299GLU	OE2	12.35
309ARG	NH1	312GLU	OE1	17.73
309ARG	NH1	312GLU	OE2	13.06
309ARG	NE	306GLU	OE1	13.44
309ARG	NE	306GLU	OE2	12.35
300HIS	NE2	299GLU	OE2	0.02
288THR	OG1	286ASN	OD1	95.95
286ASN	ND2	288THR	OG1	0.38
282LYS	NZ	278ASP	OD1	30.39
282LYS	NZ	278ASP	OD2	33.40
266THR	OG1	62GLU	OE1	28.47
266THR	OG1	62GLU	OE2	32.47
266THR	OG1	97GLN	OE1	7.14
264ARG	NH2	62GLU	OE1	0.00
264ARG	NH2	62GLU	OE2	0.61
264ARG	NH2	97GLN	OE1	0.03
264ARG	NH2	98ASP	OD1	32.17
264ARG	NH2	98ASP	OD2	34.57
264ARG	NH2	161GLU	OE1	0.14
264ARG	NH2	161GLU	OE2	0.94
264ARG	NH1	62GLU	OE2	0.02
264ARG	NH1	98ASP	OD1	7.60
264ARG	NH1	98ASP	OD2	0.36
264ARG	NH1	161GLU	OE1	17.90
264ARG	NH1	161GLU	OE2	3.69
264ARG	NE	98ASP	OD1	51.64
264ARG	NE	98ASP	OD2	31.06
261SER	OG	102ASN	OD1	0.06
261SER	OG	270GLU	OE1	0.01

96SER	OG	62GLU	OE1	0.15
96SER	OG	62GLU	OE2	0.02
96SER	OG	97GLN	OE1	0.27
95LYS	NZ	98ASP	OD1	2.74
95LYS	NZ	98ASP	OD2	1.96
94ARG	NH2	102ASN	OD1	0.08
94ARG	NH2	261SER	OG	0.96
94ARG	NH2	270GLU	OE1	47.51
94ARG	NH2	270GLU	OE2	40.80
94ARG	NH1	87GLU	OE2	0.00
94ARG	NH1	270GLU	OE1	0.04
94ARG	NH1	270GLU	OE2	1.29
94ARG	NE	261SER	OG	0.11
94ARG	NE	270GLU	OE1	0.05
94ARG	NE	270GLU	OE2	0.07
92SER	OG	51GLU	OE1	0.00
92SER	OG	51GLU	OE2	0.02
88THR	OG1	87GLU	OE1	0.05
88THR	OG1	87GLU	OE2	0.13
85SER	OG	87GLU	OE1	24.97
85SER	OG	87GLU	OE2	17.72
85SER	OG	88THR	OG1	0.04
83LYS	NZ	51GLU	OE1	0.18
83LYS	NZ	51GLU	OE2	0.36
83LYS	NZ	190GLU	OE1	0.08
83LYS	NZ	190GLU	OE2	1.45
83LYS	NZ	193GLU	OE1	10.90
83LYS	NZ	193GLU	OE2	4.51
82ARG	NH2	78ASP	OD1	2.45
82ARG	NH2	78ASP	OD2	1.41
82ARG	NH2	85SER	OG	1.10
82ARG	NH2	87GLU	OE1	31.23
82ARG	NH2	87GLU	OE2	23.17
82ARG	NH2	217ASP	OD1	0.13
82ARG	NH2	217ASP	OD2	1.01
82ARG	NH1	78ASP	OD1	0.18
82ARG	NH1	78ASP	OD2	0.58
82ARG	NH1	85SER	OG	0.72
82ARG	NH1	87GLU	OE1	4.44
82ARG	NH1	87GLU	OE2	11.53
82ARG	NH1	187ASN	OD1	0.00
82ARG	NH1	215TYR	OH	1.26
82ARG	NH1	217ASP	OD1	3.02

333THR	OG1	334GLU	OE2	0.06
322THR	OG1	339THR	OG1	0.06
317LYS	NZ	313ASP	OD1	16.07
317LYS	NZ	313ASP	OD2	85.53
317LYS	NZ	343HIS	ND1	0.02
310LYS	NZ	30GLU	OE1	2.95
310LYS	NZ	30GLU	OE2	6.75
310LYS	NZ	306GLU	OE1	0.16
310LYS	NZ	306GLU	OE2	0.10
309ARG	NH2	299GLU	OE1	52.61
309ARG	NH2	299GLU	OE2	31.95
309ARG	NH2	312GLU	OE1	86.09
309ARG	NH2	312GLU	OE2	12.64
309ARG	NH1	299GLU	OE1	35.92
309ARG	NH1	299GLU	OE2	56.90
309ARG	NE	312GLU	OE1	14.40
309ARG	NE	312GLU	OE2	87.01
300HIS	NE2	299GLU	OE1	0.01
288THR	OG1	286ASN	OD1	33.68
286ASN	ND2	288THR	OG1	16.68
286ASN	ND2	326ASP	OD1	10.26
286ASN	ND2	326ASP	OD2	2.00
282LYS	NZ	278ASP	OD1	2.48
282LYS	NZ	278ASP	OD2	1.84
266THR	OG1	97GLN	OE1	6.41
264ARG	NH2	62GLU	OE1	5.06
264ARG	NH2	62GLU	OE2	0.65
264ARG	NH2	98ASP	OD1	0.55
264ARG	NH2	98ASP	OD2	5.58
264ARG	NH1	62GLU	OE1	0.38
264ARG	NH1	62GLU	OE2	5.02
264ARG	NH1	98ASP	OD1	0.73
264ARG	NH1	98ASP	OD2	8.90
264ARG	NE	98ASP	OD1	2.28
264ARG	NE	98ASP	OD2	17.87
261SER	OG	102ASN	OD1	0.01
253SER	OG	326ASP	OD1	15.89
253SER	OG	326ASP	OD2	49.52
244SER	OG	245ASP	OD2	13.43
237ASN	ND2	133GLU	OE1	2.13
229ARG	NH2	212GLU	OE1	41.49
229ARG	NH2	212GLU	OE2	59.88
229ARG	NH1	212GLU	OE1	59.45

107LYS	NZ	299GLU	OE2	0.19
107LYS	NZ	309ARG	NH1	0.01
107LYS	NZ	312GLU	OE1	48.17
107LYS	NZ	312GLU	OE2	55.79
104ARG	NH2	102ASN	OD1	0.21
104ARG	NH2	244SER	OG	0.02
104ARG	NH2	245ASP	OD1	1.16
104ARG	NH2	245ASP	OD2	0.36
104ARG	NH2	259SER	OG	2.52
104ARG	NH1	244SER	OG	0.18
104ARG	NH1	245ASP	OD1	33.30
104ARG	NH1	245ASP	OD2	49.72
104ARG	NH1	248SER	OG	25.95
104ARG	NE	102ASN	OD1	7.46
104ARG	NE	244SER	OG	0.01
104ARG	NE	259SER	OG	7.72
102ASN	ND2	104ARG	NE	0.15
102ASN	ND2	104ARG	NH2	0.13
102ASN	ND2	132ARG	NE	0.06
102ASN	ND2	132ARG	NH1	0.11
102ASN	ND2	132ARG	NH2	0.05
102ASN	ND2	259SER	OG	12.92
102ASN	ND2	261SER	OG	35.92
97GLN	NE2	62GLU	OE1	15.66
97GLN	NE2	62GLU	OE2	12.08
97GLN	NE2	266THR	OG1	10.97
96SER	OG	51GLU	OE1	0.01
96SER	OG	97GLN	OE1	0.09
95LYS	NZ	98ASP	OD1	0.67
95LYS	NZ	98ASP	OD2	4.06
95LYS	NZ	135THR	OG1	1.37
95LYS	NZ	161GLU	OE1	0.05
95LYS	NZ	161GLU	OE2	0.02
94ARG	NH2	270GLU	OE1	33.81
94ARG	NH2	270GLU	OE2	49.63
94ARG	NE	261SER	OG	0.00
94ARG	NE	270GLU	OE1	0.04
94ARG	NE	270GLU	OE2	0.16
92SER	OG	51GLU	OE1	0.01
92SER	OG	51GLU	OE2	0.02
92SER	OG	96SER	OG	0.00
85ARG	NH2	78ASP	OD2	0.04
85ARG	NH2	87GLU	OE1	53.78

261SER	OG	270GLU	OE2	0.01
259SER	OG	102ASN	OD1	0.01
259SER	OG	270GLU	OE2	0.01
253SER	OG	326ASP	OD1	50.63
253SER	OG	326ASP	OD2	12.14
244SER	OG	245ASP	OD2	0.00
237ASN	ND2	133GLU	OE1	0.97
237ASN	ND2	133GLU	OE2	2.09
229ARG	NH2	212GLU	OE1	97.44
229ARG	NH2	212GLU	OE2	1.10
229ARG	NH2	214GLN	OE1	0.26
229ARG	NH1	212GLU	OE1	3.44
229ARG	NH1	212GLU	OE2	97.65
229ARG	NE	222HIS	ND1	0.30
226SER	OG	222HIS	ND1	0.58
225ARG	NH2	326ASP	OD1	1.38
225ARG	NH2	326ASP	OD2	1.64
225ARG	NH2	222HIS	ND1	13.48
225ARG	NH1	326ASP	OD1	1.58
225ARG	NH1	326ASP	OD2	0.34
225ARG	NH1	222HIS	ND1	0.14
225ARG	NE	222HIS	ND1	9.24
225ARG	NE	226SER	OG	0.00
222HIS	NE2	214GLN	OE1	0.51
222HIS	NE2	226SER	OG	0.04
222HIS	NE2	229ARG	NE	0.07
222HIS	NE2	229ARG	NH1	0.10
222HIS	NE2	229ARG	NH2	0.01
214GLN	NE2	212GLU	OE1	18.51
214GLN	NE2	212GLU	OE2	61.67
214GLN	NE2	222HIS	ND1	0.12
214GLN	NE2	222HIS	NE2	0.01
214GLN	NE2	229ARG	NE	0.01
214GLN	NE2	229ARG	NH1	0.02
214GLN	NE2	229ARG	NH2	0.26
213HIS	NE2	200GLU	OE1	41.63
213HIS	NE2	200GLU	OE2	44.39
206TYR	OH	171GLU	OE1	40.81
206TYR	OH	171GLU	OE2	23.64
204ARG	NH2	200GLU	OE1	11.29
204ARG	NH2	200GLU	OE2	5.13
204ARG	NH2	201GLU	OE1	1.87
204ARG	NH2	201GLU	OE2	4.95

82ARG	NH1	217ASP	OD2	6.85
82ARG	NE	78ASP	OD1	0.27
82ARG	NE	78ASP	OD2	0.02
82ARG	NE	85SER	OG	0.01
82ARG	NE	87GLU	OE1	20.74
82ARG	NE	87GLU	OE2	28.18
77TRP	NE1	9ASP	OD1	7.72
77TRP	NE1	9ASP	OD2	12.30
77TRP	NE1	47ASP	OD1	0.25
77TRP	NE1	47ASP	OD2	0.04
77TRP	NE1	85SER	OG	0.00
76LYS	NZ	9ASP	OD1	48.52
76LYS	NZ	9ASP	OD2	50.93
76LYS	NZ	47ASP	OD1	0.16
76LYS	NZ	47ASP	OD2	0.27
76LYS	NZ	77TRP	NE1	0.17
71SER	OG	270GLU	OE1	0.05
71SER	OG	273HIS	NE2	0.23
71SER	OG	275SER	OG	0.16
59LYS	NZ	55GLU	OE1	36.36
59LYS	NZ	55GLU	OE2	24.16
59LYS	NZ	62GLU	OE2	0.00
59LYS	NZ	63GLU	OE1	0.65
59LYS	NZ	63GLU	OE2	0.04
58ARG	NH2	51GLU	OE1	13.03
58ARG	NH2	51GLU	OE2	11.59
58ARG	NH2	55GLU	OE1	33.09
58ARG	NH2	55GLU	OE2	45.21
58ARG	NH2	92SER	OG	0.93
58ARG	NH2	96SER	OG	0.00
58ARG	NH1	51GLU	OE1	29.73
58ARG	NH1	51GLU	OE2	29.76
58ARG	NH1	55GLU	OE1	4.34
58ARG	NH1	55GLU	OE2	6.47
58ARG	NH1	92SER	OG	0.31
58ARG	NH1	96SER	OG	0.27
58ARG	NE	51GLU	OE1	0.02
58ARG	NE	51GLU	OE2	0.04
58ARG	NE	55GLU	OE1	5.50
58ARG	NE	55GLU	OE2	5.98
36TYR	OH	17GLU	OE1	0.08
36TYR	OH	17GLU	OE2	0.12
36TYR	OH	27ASP	OD1	0.21

229ARG	NH1	212GLU	OE2	41.53
229ARG	NH1	214GLN	OE1	0.02
226SER	OG	222HIS	ND1	7.65
225ARG	NH2	326ASP	OD1	10.53
225ARG	NH2	326ASP	OD2	9.14
225ARG	NH2	222HIS	ND1	0.41
225ARG	NH1	326ASP	OD1	7.95
225ARG	NH1	326ASP	OD2	7.97
225ARG	NH1	222HIS	ND1	8.11
225ARG	NE	222HIS	ND1	0.03
225ARG	NE	226SER	OG	0.00
222HIS	NE2	214GLN	OE1	0.32
222HIS	NE2	226SER	OG	0.05
215TYR	OH	78ASP	OD1	1.78
215TYR	OH	78ASP	OD2	3.16
215TYR	OH	187ASN	OD1	0.01
214GLN	NE2	212GLU	OE1	57.08
214GLN	NE2	212GLU	OE2	39.54
214GLN	NE2	222HIS	NE2	0.00
214GLN	NE2	229ARG	NH1	0.02
213HIS	NE2	200GLU	OE1	0.99
213HIS	NE2	200GLU	OE2	0.14
206TYR	OH	171GLU	OE1	56.00
206TYR	OH	171GLU	OE2	32.23
204ARG	NH2	200GLU	OE1	17.97
204ARG	NH2	200GLU	OE2	81.99
204ARG	NH2	201GLU	OE1	37.42
204ARG	NH2	201GLU	OE2	18.91
204ARG	NH1	201GLU	OE1	21.55
204ARG	NH1	201GLU	OE2	32.67
204ARG	NE	200GLU	OE1	80.56
204ARG	NE	200GLU	OE2	19.41
197LYS	NZ	148GLU	OE1	35.87
197LYS	NZ	148GLU	OE2	33.84
197LYS	NZ	193GLU	OE1	0.65
197LYS	NZ	193GLU	OE2	0.04
197LYS	NZ	201GLU	OE1	16.00
197LYS	NZ	201GLU	OE2	10.32
196ARG	NH2	184ASP	OD1	10.26
196ARG	NH2	184ASP	OD2	87.53
196ARG	NH2	215TYR	OH	2.58
196ARG	NH1	215TYR	OH	0.20
196ARG	NE	184ASP	OD1	95.07

85ARG	NH2	87GLU	OE2	45.75
85ARG	NE	87GLU	OE1	42.51
85ARG	NE	87GLU	OE2	52.91
82ARG	NH2	87GLU	OE1	7.50
82ARG	NH2	87GLU	OE2	6.34
82ARG	NH2	187ASN	OD1	0.00
82ARG	NH2	190GLU	OE1	0.60
82ARG	NH2	190GLU	OE2	13.50
82ARG	NH1	87GLU	OE1	0.08
82ARG	NH1	190GLU	OE1	16.99
82ARG	NH1	190GLU	OE2	5.42
82ARG	NE	85ARG	NH2	0.04
82ARG	NE	87GLU	OE1	50.37
82ARG	NE	87GLU	OE2	42.07
77TRP	NE1	47ASP	OD1	9.54
77TRP	NE1	47ASP	OD2	35.61
76LYS	NZ	9ASP	OD1	24.38
76LYS	NZ	9ASP	OD2	24.14
76LYS	NZ	47ASP	OD1	12.57
76LYS	NZ	47ASP	OD2	7.91
76LYS	NZ	77TRP	NE1	0.23
71SER	OG	270GLU	OE1	0.74
71SER	OG	270GLU	OE2	1.53
71SER	OG	273HIS	ND1	0.04
71SER	OG	273HIS	NE2	5.92
71SER	OG	275SER	OG	1.72
59LYS	NZ	55GLU	OE1	30.14
59LYS	NZ	55GLU	OE2	31.59
59LYS	NZ	62GLU	OE1	0.25
59LYS	NZ	62GLU	OE2	0.40
59LYS	NZ	63GLU	OE1	0.11
59LYS	NZ	63GLU	OE2	0.16
58ARG	NH2	51GLU	OE1	43.38
58ARG	NH2	51GLU	OE2	36.90
58ARG	NH2	55GLU	OE1	11.48
58ARG	NH2	55GLU	OE2	5.58
58ARG	NH2	92SER	OG	0.30
58ARG	NH2	96SER	OG	0.18
58ARG	NH1	51GLU	OE1	4.09
58ARG	NH1	51GLU	OE2	6.38
58ARG	NH1	55GLU	OE1	24.15
58ARG	NH1	55GLU	OE2	28.59
58ARG	NH1	92SER	OG	0.38

204ARG	NH2	213HIS	NE2	0.01
204ARG	NH1	201GLU	OE1	36.62
204ARG	NH1	201GLU	OE2	27.00
204ARG	NE	200GLU	OE1	9.09
204ARG	NE	200GLU	OE2	11.21
204ARG	NE	201GLU	OE1	0.01
204ARG	NE	201GLU	OE2	0.18
197LYS	NZ	148GLU	OE1	21.80
197LYS	NZ	148GLU	OE2	25.13
197LYS	NZ	193GLU	OE1	3.21
197LYS	NZ	193GLU	OE2	2.86
197LYS	NZ	201GLU	OE1	1.52
197LYS	NZ	201GLU	OE2	0.84
196ARG	NH2	184ASP	OD1	93.52
196ARG	NH2	184ASP	OD2	13.52
196ARG	NH2	215TYR	OH	0.02
196ARG	NH1	215TYR	OH	0.75
196ARG	NE	184ASP	OD1	1.41
196ARG	NE	184ASP	OD2	99.18
187ASN	ND2	215TYR	OH	0.09
187ASN	ND2	217ASP	OD1	0.03
187ASN	ND2	217ASP	OD2	0.01
185LYS	NZ	241ASP	OD1	17.78
185LYS	NZ	241ASP	OD2	78.77
185LYS	NZ	187ASN	OD1	0.03
185LYS	NZ	217ASP	OD1	46.47
185LYS	NZ	217ASP	OD2	52.33
179HIS	NE2	212GLU	OE1	62.23
179HIS	NE2	212GLU	OE2	34.13
179HIS	NE2	229ARG	NH1	0.02
179HIS	NE2	229ARG	NH2	0.09
178LYS	NZ	208ASP	OD1	83.10
178LYS	NZ	208ASP	OD2	18.15
177ARG	NH2	127ASP	OD2	6.03
177ARG	NH2	231ASP	OD1	10.70
177ARG	NH2	231ASP	OD2	0.01
177ARG	NH1	127ASP	OD2	13.16
177ARG	NH1	179HIS	ND1	0.01
177ARG	NH1	231ASP	OD1	12.92
177ARG	NH1	231ASP	OD2	0.03
177ARG	NE	231ASP	OD1	6.76
177ARG	NE	231ASP	OD2	4.74
176ARG	NH2	127ASP	OD1	92.64

36TYR	OH	27ASP	OD2	2.60
24ARG	NH2	17GLU	OE1	34.92
24ARG	NH2	17GLU	OE2	28.44
24ARG	NH2	27ASP	OD2	2.92
24ARG	NH2	28GLU	OE1	0.90
24ARG	NH2	28GLU	OE2	1.83
24ARG	NH2	36TYR	OH	17.86
24ARG	NH1	17GLU	OE1	25.61
24ARG	NH1	17GLU	OE2	36.63
24ARG	NH1	28GLU	OE1	1.05
24ARG	NH1	28GLU	OE2	2.38
24ARG	NH1	36TYR	OH	13.46
24ARG	NE	28GLU	OE1	0.61
24ARG	NE	28GLU	OE2	0.06
24ARG	NE	36TYR	OH	30.18
21LYS	NZ	17GLU	OE1	3.92
21LYS	NZ	17GLU	OE2	5.83
21LYS	NZ	334GLU	OE1	18.86
21LYS	NZ	334GLU	OE2	21.58
21LYS	NZ	337THR	OG1	15.07
2LYS	NZ	37GLU	OE1	2.70
2LYS	NZ	37GLU	OE2	5.87
2LYS	NZ	63GLU	OE1	26.53
2LYS	NZ	63GLU	OE2	33.58
2LYS	NZ	65GLU	OE1	2.07
2LYS	NZ	65GLU	OE2	2.86
343HIS	NE2	313ASP	OD1	43.24
343HIS	NE2	313ASP	OD2	29.83
343HIS	NE2	321GLU	OE1	0.10
343HIS	NE2	321GLU	OE2	0.18
342ARG	NH2	321GLU	OE1	29.97
342ARG	NH2	321GLU	OE2	31.63
342ARG	NH1	321GLU	OE1	25.24
342ARG	NH1	321GLU	OE2	19.99
342ARG	NH1	343HIS	ND1	0.00
339THR	OG1	321GLU	OE1	0.01
339THR	OG1	342ARG	NH1	0.01
339THR	OG1	342ARG	NH2	0.02
337THR	OG1	334GLU	OE1	0.07
337THR	OG1	334GLU	OE2	0.16
333THR	OG1	14GLU	OE1	2.06
333THR	OG1	14GLU	OE2	1.99
333THR	OG1	334GLU	OE1	0.09

196ARG	NE	184ASP	OD2	10.54
187ASN	ND2	215TYR	OH	0.04
187ASN	ND2	217ASP	OD1	0.03
185LYS	NZ	241ASP	OD1	67.62
185LYS	NZ	241ASP	OD2	34.81
185LYS	NZ	187ASN	OD1	0.01
185LYS	NZ	217ASP	OD1	99.68
185LYS	NZ	217ASP	OD2	0.07
182SER	OG	184ASP	OD1	0.09
182SER	OG	184ASP	OD2	0.44
182SER	OG	213HIS	ND1	0.23
179HIS	NE2	212GLU	OE1	37.13
179HIS	NE2	212GLU	OE2	52.17
179HIS	NE2	229ARG	NH1	0.07
179HIS	NE2	229ARG	NH2	0.08
178LYS	NZ	208ASP	OD2	18.85
177ARG	NH2	127ASP	OD2	1.49
177ARG	NH2	179HIS	ND1	0.06
177ARG	NH2	231ASP	OD1	27.97
177ARG	NH2	231ASP	OD2	0.34
177ARG	NH1	179HIS	ND1	0.07
177ARG	NE	231ASP	OD1	9.24
177ARG	NE	231ASP	OD2	21.14
176ARG	NH2	127ASP	OD1	96.26
176ARG	NH2	127ASP	OD2	1.49
176ARG	NH1	127ASP	OD1	2.95
176ARG	NH1	127ASP	OD2	2.36
176ARG	NH1	231ASP	OD1	96.46
176ARG	NH1	231ASP	OD2	0.38
175LYS	NZ	171GLU	OE1	0.41
175LYS	NZ	171GLU	OE2	0.56
175LYS	NZ	299GLU	OE1	1.46
175LYS	NZ	299GLU	OE2	1.05
175LYS	NZ	300HIS	ND1	1.47
174ARG	NH2	208ASP	OD1	99.92
174ARG	NH2	208ASP	OD2	1.34
174ARG	NH1	171GLU	OE1	14.37
174ARG	NH1	171GLU	OE2	22.19
174ARG	NE	208ASP	OD1	4.22
174ARG	NE	208ASP	OD2	98.15
167ARG	NH2	163GLU	OE1	35.31
167ARG	NH2	163GLU	OE2	46.50
167ARG	NH2	171GLU	OE1	0.12

58ARG	NH1	96SER	OG	0.88
58ARG	NH1	97GLN	OE1	0.01
58ARG	NE	51GLU	OE1	2.58
58ARG	NE	51GLU	OE2	2.09
58ARG	NE	96SER	OG	0.20
36TYR	OH	27ASP	OD1	4.08
36TYR	OH	27ASP	OD2	3.55
24ARG	NH2	17GLU	OE1	35.53
24ARG	NH2	17GLU	OE2	44.37
24ARG	NH2	36TYR	OH	16.71
24ARG	NH1	17GLU	OE1	48.46
24ARG	NH1	17GLU	OE2	36.19
24ARG	NH1	36TYR	OH	12.10
24ARG	NE	36TYR	OH	37.07
21LYS	NZ	17GLU	OE1	5.21
21LYS	NZ	17GLU	OE2	3.55
21LYS	NZ	334GLU	OE1	24.81
21LYS	NZ	334GLU	OE2	34.65
21LYS	NZ	337THR	OG1	11.80
2LYS	NZ	37GLU	OE1	5.91
2LYS	NZ	37GLU	OE2	3.70
2LYS	NZ	63GLU	OE1	29.35
2LYS	NZ	63GLU	OE2	27.47
2LYS	NZ	65GLU	OE1	1.81
2LYS	NZ	65GLU	OE2	1.33
343HIS	NE2	313ASP	OD1	46.71
343HIS	NE2	313ASP	OD2	24.36
343HIS	NE2	321GLU	OE1	1.85
343HIS	NE2	321GLU	OE2	0.47
342ARG	NH2	321GLU	OE1	36.77
342ARG	NH2	321GLU	OE2	35.64
342ARG	NH2	343HIS	ND1	0.00
342ARG	NH2	343HIS	NE2	0.00
342ARG	NH1	321GLU	OE1	25.63
342ARG	NH1	321GLU	OE2	24.20
342ARG	NH1	343HIS	ND1	0.00
342ARG	NE	321GLU	OE2	0.00
342ARG	NE	343HIS	NE2	0.00
339THR	OG1	321GLU	OE1	0.04
339THR	OG1	321GLU	OE2	0.11
339THR	OG1	342ARG	NH1	0.02
337THR	OG1	334GLU	OE1	0.01
337THR	OG1	334GLU	OE2	0.07

176ARG	NH2	127ASP	OD2	1.10
176ARG	NH1	127ASP	OD1	3.96
176ARG	NH1	127ASP	OD2	3.00
176ARG	NH1	177ARG	NH1	0.00
176ARG	NH1	177ARG	NH2	0.02
176ARG	NH1	231ASP	OD1	92.68
176ARG	NH1	231ASP	OD2	1.88
175LYS	NZ	171GLU	OE1	1.14
175LYS	NZ	171GLU	OE2	0.94
175LYS	NZ	299GLU	OE1	23.69
175LYS	NZ	299GLU	OE2	36.19
175LYS	NZ	300HIS	ND1	3.59
174ARG	NH2	171GLU	OE1	49.71
174ARG	NH2	171GLU	OE2	54.91
174ARG	NH2	206TYR	OH	0.04
174ARG	NH1	206TYR	OH	0.05
174ARG	NH1	208ASP	OD1	0.30
174ARG	NH1	208ASP	OD2	0.00
174ARG	NE	171GLU	OE1	31.89
174ARG	NE	171GLU	OE2	36.43
174ARG	NE	206TYR	OH	0.86
167ARG	NH2	163GLU	OE1	25.76
167ARG	NH2	163GLU	OE2	20.47
167ARG	NH2	201GLU	OE1	0.00
167ARG	NH2	201GLU	OE2	0.01
167ARG	NH2	206TYR	OH	0.11
167ARG	NH1	163GLU	OE1	12.25
167ARG	NH1	163GLU	OE2	12.72
167ARG	NH1	206TYR	OH	1.82
167ARG	NE	163GLU	OE1	19.32
167ARG	NE	163GLU	OE2	19.58
167ARG	NE	171GLU	OE1	0.01
167ARG	NE	206TYR	OH	1.71
164ARG	NH2	98ASP	OD1	0.97
164ARG	NH2	98ASP	OD2	19.83
164ARG	NH2	133GLU	OE2	0.11
164ARG	NH2	161GLU	OE1	14.86
164ARG	NH2	161GLU	OE2	41.49
164ARG	NH1	98ASP	OD1	20.27
164ARG	NH1	98ASP	OD2	0.09
164ARG	NH1	161GLU	OE1	6.91
164ARG	NH1	161GLU	OE2	9.95
164ARG	NH1	264ARG	NE	0.01

333THR	OG1	334GLU	OE2	0.09
322THR	OG1	330SER	OG	0.05
322THR	OG1	339THR	OG1	0.00
317LYS	NZ	313ASP	OD1	58.03
317LYS	NZ	313ASP	OD2	48.87
317LYS	NZ	343HIS	ND1	0.14
317LYS	NZ	343HIS	NE2	0.02
310LYS	NZ	30GLU	OE1	2.44
310LYS	NZ	30GLU	OE2	1.39
310LYS	NZ	306GLU	OE1	0.11
310LYS	NZ	306GLU	OE2	0.42
310LYS	NZ	343HIS	ND1	0.12
310LYS	NZ	343HIS	NE2	0.00
309ARG	NH2	299GLU	OE1	4.71
309ARG	NH2	299GLU	OE2	3.05
309ARG	NH2	306GLU	OE1	31.08
309ARG	NH2	306GLU	OE2	35.58
309ARG	NH2	313ASP	OD1	0.07
309ARG	NH1	299GLU	OE1	3.80
309ARG	NH1	299GLU	OE2	4.33
309ARG	NH1	306GLU	OE1	10.92
309ARG	NH1	306GLU	OE2	8.80
309ARG	NH1	312GLU	OE1	1.03
309ARG	NH1	312GLU	OE2	3.54
309ARG	NH1	313ASP	OD1	0.17
309ARG	NH1	313ASP	OD2	0.05
309ARG	NE	299GLU	OE1	0.07
309ARG	NE	299GLU	OE2	0.04
309ARG	NE	306GLU	OE1	19.17
309ARG	NE	306GLU	OE2	16.12
288THR	OG1	286ASN	OD1	71.20
286ASN	ND2	273HIS	NE2	0.14
286ASN	ND2	288THR	OG1	13.27
286ASN	ND2	326ASP	OD1	0.82
286ASN	ND2	326ASP	OD2	0.07
282LYS	NZ	78ASP	OD1	1.37
282LYS	NZ	78ASP	OD2	0.79
282LYS	NZ	278ASP	OD1	32.29
282LYS	NZ	278ASP	OD2	31.55
282LYS	NZ	326ASP	OD1	20.97
282LYS	NZ	330SER	OG	0.01
275SER	OG	217ASP	OD2	0.10
275SER	OG	245ASP	OD1	1.46

167ARG	NH2	206TYR	OH	0.04
167ARG	NH1	163GLU	OE1	5.99
167ARG	NH1	163GLU	OE2	6.55
167ARG	NH1	171GLU	OE2	0.00
167ARG	NH1	206TYR	OH	0.15
167ARG	NE	163GLU	OE1	36.89
167ARG	NE	163GLU	OE2	27.57
167ARG	NE	171GLU	OE1	0.02
167ARG	NE	206TYR	OH	0.98
164ARG	NH2	98ASP	OD1	6.19
164ARG	NH2	98ASP	OD2	93.95
164ARG	NH2	264ARG	NE	0.04
164ARG	NH2	264ARG	NH2	0.01
164ARG	NH1	98ASP	OD1	89.44
164ARG	NH1	98ASP	OD2	9.29
164ARG	NH1	161GLU	OE1	0.14
164ARG	NH1	161GLU	OE2	0.38
159LYS	NZ	148GLU	OE1	24.85
159LYS	NZ	148GLU	OE2	27.61
159LYS	NZ	163GLU	OE1	1.08
159LYS	NZ	163GLU	OE2	6.55
159LYS	NZ	198THR	OG1	3.43
159LYS	NZ	201GLU	OE1	12.86
159LYS	NZ	201GLU	OE2	18.27
158SER	OG	150GLU	OE1	0.55
158SER	OG	150GLU	OE2	0.84
158SER	OG	161GLU	OE1	0.73
158SER	OG	161GLU	OE2	7.63
157TYR	OH	133GLU	OE1	21.24
157TYR	OH	155GLU	OE2	0.03
156ARG	NH2	150GLU	OE1	18.92
156ARG	NH2	150GLU	OE2	23.46
156ARG	NH2	158SER	OG	0.01
156ARG	NH1	150GLU	OE1	25.21
156ARG	NH1	150GLU	OE2	40.97
156ARG	NE	150GLU	OE1	19.00
156ARG	NE	150GLU	OE2	12.33
153ASN	ND2	190GLU	OE1	12.33
153ASN	ND2	190GLU	OE2	5.66
147SER	OG	148GLU	OE2	0.00
147SER	OG	150GLU	OE1	0.02
144ARG	NH2	190GLU	OE1	21.94
144ARG	NH2	190GLU	OE2	16.52

333THR	OG1	14GLU	OE1	0.83
333THR	OG1	14GLU	OE2	1.07
333THR	OG1	334GLU	OE1	1.28
333THR	OG1	334GLU	OE2	0.76
322THR	OG1	339THR	OG1	0.00
317LYS	NZ	313ASP	OD1	46.53
317LYS	NZ	313ASP	OD2	53.54
317LYS	NZ	321GLU	OE1	0.51
317LYS	NZ	321GLU	OE2	0.38
317LYS	NZ	343HIS	ND1	0.63
317LYS	NZ	343HIS	NE2	0.01
310LYS	NZ	30GLU	OE1	3.90
310LYS	NZ	30GLU	OE2	4.12
310LYS	NZ	306GLU	OE1	1.49
310LYS	NZ	306GLU	OE2	1.16
310LYS	NZ	313ASP	OD1	0.01
310LYS	NZ	343HIS	ND1	0.01
310LYS	NZ	343HIS	NE2	0.00
309ARG	NH2	299GLU	OE1	35.86
309ARG	NH2	299GLU	OE2	27.49
309ARG	NH2	312GLU	OE1	42.39
309ARG	NH2	312GLU	OE2	53.91
309ARG	NH1	299GLU	OE1	45.33
309ARG	NH1	299GLU	OE2	51.50
309ARG	NE	312GLU	OE1	56.03
309ARG	NE	312GLU	OE2	40.87
288THR	OG1	286ASN	OD1	78.41
286ASN	ND2	288THR	OG1	3.10
286ASN	ND2	326ASP	OD1	0.58
286ASN	ND2	326ASP	OD2	0.02
282LYS	NZ	278ASP	OD1	31.65
282LYS	NZ	278ASP	OD2	36.39
282LYS	NZ	330SER	OG	0.00
275SER	OG	87GLU	OE1	0.66
275SER	OG	87GLU	OE2	1.10
273HIS	ND1	286ASN	OD1	0.01
273HIS	ND1	286ASN	ND2	0.00
266THR	OG1	62GLU	OE1	0.19
266THR	OG1	62GLU	OE2	0.26
266THR	OG1	97GLN	OE1	6.80
264ARG	NH2	98ASP	OD1	3.94
264ARG	NH2	98ASP	OD2	29.77
264ARG	NH2	161GLU	OE1	1.02

164ARG	NH1	264ARG	NH2	0.01
164ARG	NE	161GLU	OE1	18.29
164ARG	NE	161GLU	OE2	4.81
159LYS	NZ	148GLU	OE1	29.50
159LYS	NZ	148GLU	OE2	22.31
159LYS	NZ	163GLU	OE1	0.79
159LYS	NZ	163GLU	OE2	0.72
159LYS	NZ	198THR	OG1	5.18
159LYS	NZ	201GLU	OE1	12.20
159LYS	NZ	201GLU	OE2	11.32
158SER	OG	150GLU	OE1	0.04
158SER	OG	150GLU	OE2	0.03
158SER	OG	161GLU	OE1	0.49
158SER	OG	161GLU	OE2	0.08
157TYR	OH	133GLU	OE1	40.23
157TYR	OH	133GLU	OE2	42.16
157TYR	OH	155GLU	OE1	0.01
156ARG	NH2	150GLU	OE1	44.27
156ARG	NH2	150GLU	OE2	49.12
156ARG	NH2	142GLU	OE1	0.50
156ARG	NH2	142GLU	OE2	0.66
156ARG	NH1	150GLU	OE1	0.24
156ARG	NH1	150GLU	OE2	2.75
156ARG	NH1	142GLU	OE1	0.78
156ARG	NE	150GLU	OE1	47.02
156ARG	NE	150GLU	OE2	41.34
153ASN	ND2	190GLU	OE1	14.72
153ASN	ND2	190GLU	OE2	4.98
147SER	OG	150GLU	OE1	0.09
144ARG	NH2	190GLU	OE1	26.98
144ARG	NH2	190GLU	OE2	22.28
144ARG	NH2	142GLU	OE1	0.00
144ARG	NH1	142GLU	OE1	5.80
144ARG	NH1	142GLU	OE2	4.47
144ARG	NE	190GLU	OE1	13.11
144ARG	NE	190GLU	OE2	16.92
139TYR	OH	241ASP	OD1	2.64
139TYR	OH	241ASP	OD2	16.08
135THR	OG1	133GLU	OE1	46.89
135THR	OG1	133GLU	OE2	38.97
132ARG	NH2	237ASN	OD1	87.73
132ARG	NH2	237ASN	ND2	0.12
132ARG	NH2	241ASP	OD1	20.23

275SER	OG	245ASP	OD2	0.70
266THR	OG1	97GLN	OE1	9.78
264ARG	NH2	62GLU	OE1	0.47
264ARG	NH2	62GLU	OE2	0.01
264ARG	NH2	98ASP	OD1	5.56
264ARG	NH2	98ASP	OD2	10.98
264ARG	NH2	161GLU	OE1	0.05
264ARG	NH2	161GLU	OE2	0.14
264ARG	NH1	62GLU	OE2	0.40
264ARG	NH1	98ASP	OD1	1.56
264ARG	NH1	98ASP	OD2	10.44
264ARG	NH1	161GLU	OE1	0.00
264ARG	NH1	161GLU	OE2	0.02
264ARG	NE	98ASP	OD1	15.94
264ARG	NE	98ASP	OD2	32.29
253SER	OG	326ASP	OD1	18.79
253SER	OG	326ASP	OD2	65.23
248SER	OG	245ASP	OD1	0.01
244SER	OG	245ASP	OD2	0.01
237ASN	ND2	133GLU	OE1	1.82
237ASN	ND2	133GLU	OE2	50.11
229ARG	NH2	212GLU	OE1	43.56
229ARG	NH2	212GLU	OE2	57.64
229ARG	NH2	214GLN	OE1	0.05
229ARG	NH1	212GLU	OE1	57.69
229ARG	NH1	212GLU	OE2	44.00
229ARG	NH1	214GLN	OE1	0.01
229ARG	NE	222HIS	ND1	0.62
226SER	OG	222HIS	ND1	1.26
225ARG	NH2	326ASP	OD1	9.22
225ARG	NH2	326ASP	OD2	15.75
225ARG	NH2	222HIS	ND1	0.91
225ARG	NH2	226SER	OG	0.03
225ARG	NH1	326ASP	OD1	10.22
225ARG	NH1	326ASP	OD2	6.64
225ARG	NH1	222HIS	ND1	0.44
225ARG	NE	222HIS	ND1	0.36
225ARG	NE	226SER	OG	0.09
222HIS	NE2	214GLN	OE1	0.31
222HIS	NE2	225ARG	NH2	0.00
222HIS	NE2	226SER	OG	0.08
214GLN	NE2	78ASP	OD1	0.04
214GLN	NE2	78ASP	OD2	0.02

144ARG	NH2	142GLU	OE1	19.32
144ARG	NH2	142GLU	OE2	13.14
144ARG	NH1	190GLU	OE1	1.38
144ARG	NH1	190GLU	OE2	0.74
144ARG	NH1	142GLU	OE1	5.30
144ARG	NH1	142GLU	OE2	6.40
144ARG	NE	190GLU	OE1	8.64
144ARG	NE	190GLU	OE2	21.94
144ARG	NE	142GLU	OE1	20.32
144ARG	NE	142GLU	OE2	24.25
144ARG	NE	153ASN	ND2	0.00
139TYR	OH	237ASN	OD1	1.45
139TYR	OH	237ASN	ND2	0.02
139TYR	OH	241ASP	OD1	4.15
139TYR	OH	241ASP	OD2	11.59
135THR	OG1	133GLU	OE1	3.32
135THR	OG1	133GLU	OE2	83.85
135THR	OG1	161GLU	OE1	0.06
135THR	OG1	161GLU	OE2	0.07
132ARG	NH2	102ASN	OD1	0.18
132ARG	NH2	237ASN	OD1	43.19
132ARG	NH2	237ASN	ND2	0.10
132ARG	NH2	241ASP	OD1	1.49
132ARG	NH2	241ASP	OD2	15.30
132ARG	NH1	102ASN	OD1	0.26
132ARG	NH1	241ASP	OD1	0.40
132ARG	NH1	241ASP	OD2	0.46
132ARG	NH1	244SER	OG	60.10
132ARG	NH1	245ASP	OD2	0.01
132ARG	NE	102ASN	OD1	5.27
132ARG	NE	237ASN	OD1	17.32
132ARG	NE	237ASN	ND2	0.04
124ARG	NH2	113GLU	OE1	7.22
124ARG	NH2	113GLU	OE2	16.30
124ARG	NH2	120GLU	OE1	21.30
124ARG	NH2	120GLU	OE2	11.80
124ARG	NH2	121GLU	OE1	12.25
124ARG	NH2	121GLU	OE2	8.08
124ARG	NH1	113GLU	OE1	1.91
124ARG	NH1	113GLU	OE2	0.54
124ARG	NH1	120GLU	OE1	2.36
124ARG	NH1	120GLU	OE2	0.73
124ARG	NH1	121GLU	OE1	21.81

264ARG	NH2	161GLU	OE2	1.23
264ARG	NH1	98ASP	OD1	26.04
264ARG	NH1	98ASP	OD2	23.90
264ARG	NH1	161GLU	OE1	0.20
264ARG	NH1	161GLU	OE2	0.92
264ARG	NE	98ASP	OD1	32.49
264ARG	NE	98ASP	OD2	12.53
261SER	OG	102ASN	OD1	0.68
261SER	OG	270GLU	OE2	0.01
259SER	OG	102ASN	OD1	0.05
253SER	OG	326ASP	OD1	3.44
253SER	OG	326ASP	OD2	78.68
248SER	OG	245ASP	OD1	0.01
248SER	OG	245ASP	OD2	0.01
244SER	OG	245ASP	OD1	0.11
244SER	OG	245ASP	OD2	0.01
237ASN	ND2	133GLU	OE1	0.83
237ASN	ND2	133GLU	OE2	0.02
229ARG	NH2	179HIS	ND1	0.01
229ARG	NH2	212GLU	OE1	30.26
229ARG	NH2	212GLU	OE2	34.72
229ARG	NH2	214GLN	OE1	0.94
229ARG	NH2	222HIS	ND1	0.12
229ARG	NH1	212GLU	OE1	32.24
229ARG	NH1	212GLU	OE2	27.97
229ARG	NH1	214GLN	OE1	0.08
229ARG	NH1	222HIS	ND1	1.02
229ARG	NE	212GLU	OE1	0.32
229ARG	NE	212GLU	OE2	0.15
229ARG	NE	222HIS	ND1	0.15
226SER	OG	222HIS	ND1	1.73
225ARG	NH2	326ASP	OD1	70.28
225ARG	NH2	326ASP	OD2	24.91
225ARG	NH2	222HIS	ND1	3.78
225ARG	NH1	326ASP	OD1	11.16
225ARG	NH1	326ASP	OD2	61.61
225ARG	NH1	222HIS	ND1	0.43
225ARG	NE	222HIS	ND1	2.51
225ARG	NE	226SER	OG	0.01
222HIS	NE2	214GLN	OE1	0.10
222HIS	NE2	226SER	OG	0.02
222HIS	NE2	229ARG	NE	0.57
222HIS	NE2	229ARG	NH1	0.34

132ARG	NH2	241ASP	OD2	16.31
132ARG	NH1	102ASN	OD1	0.58
132ARG	NH1	241ASP	OD1	4.10
132ARG	NH1	241ASP	OD2	2.86
132ARG	NH1	244SER	OG	1.29
132ARG	NE	237ASN	OD1	20.34
132ARG	NE	237ASN	ND2	0.03
124ARG	NH2	113GLU	OE1	17.66
124ARG	NH2	113GLU	OE2	0.70
124ARG	NH2	120GLU	OE1	14.00
124ARG	NH2	120GLU	OE2	3.86
124ARG	NH2	121GLU	OE1	28.66
124ARG	NH2	121GLU	OE2	36.72
124ARG	NH1	113GLU	OE1	1.17
124ARG	NH1	113GLU	OE2	2.08
124ARG	NH1	120GLU	OE1	4.20
124ARG	NH1	120GLU	OE2	5.34
124ARG	NH1	121GLU	OE1	20.97
124ARG	NH1	121GLU	OE2	12.90
124ARG	NE	113GLU	OE1	0.74
124ARG	NE	113GLU	OE2	14.74
124ARG	NE	120GLU	OE1	0.08
124ARG	NE	120GLU	OE2	0.00
124ARG	NE	121GLU	OE1	4.19
124ARG	NE	121GLU	OE2	4.09
119LYS	NZ	120GLU	OE1	0.31
119LYS	NZ	120GLU	OE2	0.27
119LYS	NZ	121GLU	OE2	0.01
114ARG	NH2	120GLU	OE1	8.08
114ARG	NH2	120GLU	OE2	6.04
114ARG	NH1	120GLU	OE1	0.01
114ARG	NH1	120GLU	OE2	0.01
114ARG	NE	120GLU	OE1	4.53
114ARG	NE	120GLU	OE2	14.01
107LYS	NZ	127ASP	OD1	11.08
107LYS	NZ	127ASP	OD2	1.02
107LYS	NZ	309ARG	NE	0.00
107LYS	NZ	309ARG	NH1	0.03
107LYS	NZ	312GLU	OE1	52.80
107LYS	NZ	312GLU	OE2	54.43
104ARG	NH2	102ASN	OD1	0.50
104ARG	NH2	132ARG	NH1	0.02
104ARG	NH2	245ASP	OD1	0.24

214GLN	NE2	212GLU	OE1	66.53
214GLN	NE2	212GLU	OE2	24.19
214GLN	NE2	222HIS	ND1	0.04
214GLN	NE2	222HIS	NE2	0.00
214GLN	NE2	229ARG	NE	0.00
214GLN	NE2	229ARG	NH1	0.04
214GLN	NE2	229ARG	NH2	0.07
213HIS	NE2	200GLU	OE1	47.27
213HIS	NE2	200GLU	OE2	49.33
206TYR	OH	171GLU	OE1	10.85
206TYR	OH	171GLU	OE2	13.58
204ARG	NH2	200GLU	OE1	41.19
204ARG	NH2	200GLU	OE2	45.63
204ARG	NH2	201GLU	OE1	0.02
204ARG	NH2	213HIS	NE2	0.21
204ARG	NH1	200GLU	OE1	0.17
204ARG	NH1	201GLU	OE1	0.69
204ARG	NH1	201GLU	OE2	0.24
204ARG	NE	200GLU	OE1	49.70
204ARG	NE	200GLU	OE2	48.04
197LYS	NZ	148GLU	OE1	30.12
197LYS	NZ	148GLU	OE2	30.16
197LYS	NZ	193GLU	OE1	4.92
197LYS	NZ	193GLU	OE2	3.98
197LYS	NZ	201GLU	OE1	7.82
197LYS	NZ	201GLU	OE2	7.48
196ARG	NH2	184ASP	OD1	90.88
196ARG	NH2	184ASP	OD2	15.65
196ARG	NH2	215TYR	OH	1.27
196ARG	NH1	193GLU	OE2	0.02
196ARG	NH1	215TYR	OH	1.08
196ARG	NE	184ASP	OD1	1.95
196ARG	NE	184ASP	OD2	99.02
187ASN	ND2	215TYR	OH	0.09
187ASN	ND2	217ASP	OD1	0.00
187ASN	ND2	217ASP	OD2	0.01
185LYS	NZ	241ASP	OD1	55.53
185LYS	NZ	241ASP	OD2	31.65
185LYS	NZ	187ASN	OD1	0.32
185LYS	NZ	187ASN	ND2	0.00
185LYS	NZ	217ASP	OD1	72.85
185LYS	NZ	217ASP	OD2	23.46
179HIS	NE2	212GLU	OE1	24.84

124ARG	NH1	121GLU	OE2	32.51
124ARG	NE	113GLU	OE1	27.51
124ARG	NE	113GLU	OE2	2.93
124ARG	NE	120GLU	OE1	3.10
124ARG	NE	120GLU	OE2	0.35
124ARG	NE	121GLU	OE1	2.73
124ARG	NE	121GLU	OE2	0.27
119LYS	NZ	121GLU	OE1	0.00
119LYS	NZ	121GLU	OE2	0.06
114ARG	NH2	120GLU	OE1	7.51
114ARG	NH2	120GLU	OE2	3.81
114ARG	NH1	120GLU	OE1	0.06
114ARG	NH1	120GLU	OE2	0.00
114ARG	NE	120GLU	OE1	9.94
114ARG	NE	120GLU	OE2	18.32
107LYS	NZ	127ASP	OD1	0.37
107LYS	NZ	127ASP	OD2	0.65
107LYS	NZ	299GLU	OE1	0.02
107LYS	NZ	299GLU	OE2	0.01
107LYS	NZ	309ARG	NE	0.08
107LYS	NZ	309ARG	NH2	0.08
107LYS	NZ	312GLU	OE1	42.93
107LYS	NZ	312GLU	OE2	67.66
104ARG	NH2	245ASP	OD1	13.14
104ARG	NH2	245ASP	OD2	5.37
104ARG	NH2	248SER	OG	0.56
104ARG	NH1	102ASN	OD1	42.66
104ARG	NH1	132ARG	NH2	0.02
104ARG	NH1	245ASP	OD1	0.34
104ARG	NH1	245ASP	OD2	0.39
104ARG	NE	244SER	OG	0.01
102ASN	ND2	132ARG	NE	0.00
102ASN	ND2	259SER	OG	0.11
102ASN	ND2	261SER	OG	74.38
97GLN	NE2	62GLU	OE1	44.91
97GLN	NE2	62GLU	OE2	42.78
94ARG	NH2	87GLU	OE1	0.00
94ARG	NH2	270GLU	OE1	5.18
94ARG	NH2	270GLU	OE2	0.27
94ARG	NH1	261SER	OG	3.95
94ARG	NH1	270GLU	OE1	0.01
94ARG	NH1	270GLU	OE2	6.52
85ARG	NH2	78ASP	OD1	0.00

222HIS	NE2	229ARG	NH2	0.08
215TYR	OH	78ASP	OD1	0.98
215TYR	OH	78ASP	OD2	1.60
215TYR	OH	187ASN	OD1	0.00
214GLN	NE2	212GLU	OE1	22.15
214GLN	NE2	212GLU	OE2	27.51
214GLN	NE2	222HIS	ND1	4.25
214GLN	NE2	222HIS	NE2	0.01
214GLN	NE2	229ARG	NE	0.01
214GLN	NE2	229ARG	NH1	0.01
214GLN	NE2	229ARG	NH2	0.03
213HIS	NE2	200GLU	OE1	10.82
213HIS	NE2	200GLU	OE2	15.12
206TYR	OH	171GLU	OE1	39.83
206TYR	OH	171GLU	OE2	45.55
204ARG	NH2	200GLU	OE1	40.20
204ARG	NH2	200GLU	OE2	32.40
204ARG	NH2	201GLU	OE1	23.01
204ARG	NH2	201GLU	OE2	18.10
204ARG	NH1	200GLU	OE1	4.02
204ARG	NH1	200GLU	OE2	4.19
204ARG	NH1	201GLU	OE1	26.95
204ARG	NH1	201GLU	OE2	30.15
204ARG	NE	200GLU	OE1	32.45
204ARG	NE	200GLU	OE2	38.59
197LYS	NZ	148GLU	OE1	13.34
197LYS	NZ	148GLU	OE2	15.22
197LYS	NZ	193GLU	OE1	11.23
197LYS	NZ	193GLU	OE2	11.90
197LYS	NZ	200GLU	OE1	0.01
197LYS	NZ	200GLU	OE2	0.24
197LYS	NZ	201GLU	OE1	0.68
197LYS	NZ	201GLU	OE2	0.84
196ARG	NH2	184ASP	OD1	88.52
196ARG	NH2	184ASP	OD2	18.33
196ARG	NH2	215TYR	OH	1.61
196ARG	NH1	78ASP	OD1	0.04
196ARG	NH1	78ASP	OD2	0.02
196ARG	NH1	215TYR	OH	1.63
196ARG	NE	184ASP	OD1	2.73
196ARG	NE	184ASP	OD2	98.54
187ASN	ND2	215TYR	OH	0.11
187ASN	ND2	217ASP	OD1	0.09

104ARG	NH2	245ASP	OD2	0.30
104ARG	NH2	259SER	OG	0.12
104ARG	NH2	270GLU	OE1	0.00
104ARG	NH2	270GLU	OE2	0.06
104ARG	NH1	245ASP	OD1	25.49
104ARG	NH1	245ASP	OD2	31.27
104ARG	NH1	248SER	OG	12.12
104ARG	NE	102ASN	OD1	27.66
104ARG	NE	244SER	OG	0.01
104ARG	NE	259SER	OG	0.91
102ASN	ND2	104ARG	NE	0.01
102ASN	ND2	104ARG	NH2	0.06
102ASN	ND2	259SER	OG	9.31
102ASN	ND2	261SER	OG	44.49
97GLN	NE2	62GLU	OE1	40.35
97GLN	NE2	62GLU	OE2	40.00
96SER	OG	97GLN	OE1	0.07
95LYS	NZ	98ASP	OD1	0.03
95LYS	NZ	98ASP	OD2	0.08
94ARG	NH2	87GLU	OE1	0.11
94ARG	NH2	87GLU	OE2	0.04
94ARG	NH2	270GLU	OE1	40.58
94ARG	NH2	270GLU	OE2	42.92
94ARG	NH1	87GLU	OE1	0.21
94ARG	NH1	87GLU	OE2	0.80
94ARG	NE	270GLU	OE1	0.36
88THR	OG1	87GLU	OE1	0.21
85SER	OG	87GLU	OE1	0.02
85SER	OG	88THR	OG1	0.60
82ARG	NH2	87GLU	OE1	50.35
82ARG	NH2	87GLU	OE2	40.33
82ARG	NH1	187ASN	OD1	0.00
82ARG	NH1	87GLU	OE1	0.07
82ARG	NH1	87GLU	OE2	0.03
82ARG	NE	87GLU	OE1	34.17
82ARG	NE	87GLU	OE2	44.02
77TRP	NE1	47ASP	OD1	0.57
77TRP	NE1	47ASP	OD2	0.28
76LYS	NZ	47ASP	OD1	10.69
76LYS	NZ	47ASP	OD2	8.17
71SER	OG	273HIS	ND1	0.00
71SER	OG	273HIS	NE2	0.48
71SER	OG	275SER	OG	0.34

179HIS	NE2	212GLU	OE2	69.27
179HIS	NE2	229ARG	NH1	0.03
179HIS	NE2	229ARG	NH2	0.12
178LYS	NZ	208ASP	OD1	51.41
178LYS	NZ	208ASP	OD2	50.83
177ARG	NH2	127ASP	OD1	2.02
177ARG	NH2	127ASP	OD2	3.10
177ARG	NH2	231ASP	OD1	3.23
177ARG	NH2	231ASP	OD2	3.73
177ARG	NH1	127ASP	OD1	2.46
177ARG	NH1	127ASP	OD2	16.72
177ARG	NH1	179HIS	ND1	0.01
177ARG	NH1	231ASP	OD1	22.35
177ARG	NH1	231ASP	OD2	0.02
177ARG	NE	231ASP	OD1	4.93
177ARG	NE	231ASP	OD2	2.65
176ARG	NH2	127ASP	OD1	48.80
176ARG	NH2	127ASP	OD2	44.69
176ARG	NH1	127ASP	OD1	9.33
176ARG	NH1	127ASP	OD2	14.03
176ARG	NH1	177ARG	NH1	0.01
176ARG	NH1	231ASP	OD1	60.66
176ARG	NH1	231ASP	OD2	34.44
176ARG	NE	299GLU	OE1	0.00
175LYS	NZ	171GLU	OE1	0.85
175LYS	NZ	171GLU	OE2	0.91
175LYS	NZ	299GLU	OE1	30.65
175LYS	NZ	299GLU	OE2	29.61
175LYS	NZ	300HIS	ND1	1.44
174ARG	NH2	171GLU	OE1	39.89
174ARG	NH2	171GLU	OE2	32.78
174ARG	NH2	206TYR	OH	7.17
174ARG	NH2	208ASP	OD1	0.54
174ARG	NH2	208ASP	OD2	0.15
174ARG	NH1	171GLU	OE1	15.52
174ARG	NH1	171GLU	OE2	19.51
174ARG	NH1	206TYR	OH	9.22
174ARG	NH1	208ASP	OD1	0.10
174ARG	NH1	208ASP	OD2	0.04
174ARG	NE	171GLU	OE1	16.42
174ARG	NE	171GLU	OE2	21.41
174ARG	NE	206TYR	OH	5.31
174ARG	NE	208ASP	OD1	0.95

85ARG	NH2	78ASP	OD2	0.01
85ARG	NH1	78ASP	OD1	0.26
85ARG	NH1	78ASP	OD2	0.14
85ARG	NH1	87GLU	OE1	0.80
85ARG	NH1	87GLU	OE2	2.07
82ARG	NH2	187ASN	OD1	0.01
82ARG	NH2	87GLU	OE1	38.89
82ARG	NH2	87GLU	OE2	35.21
82ARG	NH1	187ASN	OD1	0.03
82ARG	NE	85ARG	NH1	0.00
82ARG	NE	87GLU	OE1	36.10
82ARG	NE	87GLU	OE2	42.91
82ARG	NE	88THR	OG1	0.00
77TRP	NE1	9ASP	OD1	0.00
77TRP	NE1	47ASP	OD1	0.54
77TRP	NE1	47ASP	OD2	0.01
76LYS	NZ	9ASP	OD1	54.77
76LYS	NZ	9ASP	OD2	45.58
76LYS	NZ	47ASP	OD1	0.48
76LYS	NZ	47ASP	OD2	0.50
76LYS	NZ	77TRP	NE1	0.01
76LYS	NZ	278ASP	OD2	0.06
71SER	OG	270GLU	OE1	0.01
71SER	OG	273HIS	NE2	3.34
59LYS	NZ	55GLU	OE1	34.60
59LYS	NZ	55GLU	OE2	29.31
59LYS	NZ	62GLU	OE1	0.36
59LYS	NZ	62GLU	OE2	0.02
59LYS	NZ	63GLU	OE2	0.04
58ARG	NH2	51GLU	OE1	12.69
58ARG	NH2	51GLU	OE2	24.24
58ARG	NH2	55GLU	OE1	11.26
58ARG	NH2	55GLU	OE2	7.06
58ARG	NH2	92SER	OG	7.94
58ARG	NH1	51GLU	OE1	5.00
58ARG	NH1	51GLU	OE2	4.92
58ARG	NH1	55GLU	OE1	10.62
58ARG	NH1	55GLU	OE2	10.02
58ARG	NH1	92SER	OG	3.82
58ARG	NE	51GLU	OE1	2.94
58ARG	NE	51GLU	OE2	1.26
58ARG	NE	55GLU	OE1	1.52
58ARG	NE	55GLU	OE2	7.66

187ASN	ND2	217ASP	OD2	0.29
185LYS	NZ	241ASP	OD1	22.32
185LYS	NZ	241ASP	OD2	50.08
185LYS	NZ	245ASP	OD1	4.56
185LYS	NZ	245ASP	OD2	0.04
185LYS	NZ	187ASN	OD1	0.91
185LYS	NZ	217ASP	OD1	42.73
185LYS	NZ	217ASP	OD2	40.65
182SER	OG	184ASP	OD2	0.08
182SER	OG	213HIS	ND1	0.12
179HIS	NE2	212GLU	OE1	31.96
179HIS	NE2	212GLU	OE2	25.09
179HIS	NE2	229ARG	NE	0.00
179HIS	NE2	229ARG	NH1	0.01
179HIS	NE2	229ARG	NH2	0.19
178LYS	NZ	208ASP	OD2	36.03
177ARG	NH2	127ASP	OD1	14.16
177ARG	NH2	127ASP	OD2	4.03
177ARG	NH2	179HIS	ND1	0.03
177ARG	NH2	231ASP	OD1	6.54
177ARG	NH2	231ASP	OD2	40.26
177ARG	NH1	127ASP	OD1	0.38
177ARG	NH1	127ASP	OD2	0.13
177ARG	NH1	179HIS	ND1	0.06
177ARG	NH1	231ASP	OD2	0.07
177ARG	NE	231ASP	OD1	32.08
177ARG	NE	231ASP	OD2	15.87
176ARG	NH2	127ASP	OD1	17.31
176ARG	NH2	127ASP	OD2	73.56
176ARG	NH1	127ASP	OD1	6.00
176ARG	NH1	127ASP	OD2	5.39
176ARG	NH1	231ASP	OD1	8.25
176ARG	NH1	231ASP	OD2	87.40
175LYS	NZ	171GLU	OE1	0.74
175LYS	NZ	171GLU	OE2	0.68
175LYS	NZ	299GLU	OE1	1.14
175LYS	NZ	299GLU	OE2	1.63
175LYS	NZ	300HIS	ND1	0.06
174ARG	NH2	208ASP	OD1	99.86
174ARG	NH2	208ASP	OD2	1.48
174ARG	NH1	171GLU	OE1	15.18
174ARG	NH1	171GLU	OE2	15.84
174ARG	NE	208ASP	OD1	7.60

59LYS	NZ	55GLU	OE1	39.41
59LYS	NZ	55GLU	OE2	46.24
58ARG	NH2	51GLU	OE1	13.76
58ARG	NH2	51GLU	OE2	17.26
58ARG	NH2	55GLU	OE1	0.62
58ARG	NH2	55GLU	OE2	0.28
58ARG	NH2	92SER	OG	8.85
58ARG	NH2	96SER	OG	0.00
58ARG	NH1	51GLU	OE1	8.37
58ARG	NH1	51GLU	OE2	12.53
58ARG	NH1	55GLU	OE1	2.28
58ARG	NH1	55GLU	OE2	3.35
58ARG	NH1	92SER	OG	1.97
58ARG	NE	51GLU	OE1	0.16
58ARG	NE	51GLU	OE2	0.22
58ARG	NE	92SER	OG	0.02
36TYR	OH	17GLU	OE1	0.00
36TYR	OH	17GLU	OE2	0.04
24ARG	NH2	17GLU	OE1	15.03
24ARG	NH2	17GLU	OE2	30.23
24ARG	NH2	27ASP	OD2	1.99
24ARG	NH2	28GLU	OE2	0.01
24ARG	NH2	36TYR	OH	6.15
24ARG	NH1	17GLU	OE1	14.49
24ARG	NH1	17GLU	OE2	11.31
24ARG	NH1	28GLU	OE2	0.47
24ARG	NH1	36TYR	OH	43.75
24ARG	NE	36TYR	OH	4.31
21LYS	NZ	17GLU	OE1	0.04
21LYS	NZ	17GLU	OE2	1.58
21LYS	NZ	334GLU	OE1	22.72
21LYS	NZ	334GLU	OE2	22.42
2LYS	NZ	37GLU	OE1	0.52
2LYS	NZ	37GLU	OE2	0.51
2LYS	NZ	63GLU	OE1	35.97
2LYS	NZ	63GLU	OE2	32.77

174ARG	NE	208ASP	OD2	0.51
167ARG	NH2	163GLU	OE1	33.95
167ARG	NH2	163GLU	OE2	39.06
167ARG	NH2	171GLU	OE1	0.01
167ARG	NH2	206TYR	OH	0.00
167ARG	NH1	163GLU	OE1	2.11
167ARG	NH1	163GLU	OE2	3.14
167ARG	NH1	171GLU	OE1	0.08
167ARG	NH1	171GLU	OE2	0.00
167ARG	NH1	206TYR	OH	1.53
167ARG	NE	163GLU	OE1	42.06
167ARG	NE	163GLU	OE2	30.79
164ARG	NH2	98ASP	OD1	8.16
164ARG	NH2	98ASP	OD2	63.24
164ARG	NH2	161GLU	OE1	2.63
164ARG	NH2	161GLU	OE2	5.36
164ARG	NH2	264ARG	NE	0.07
164ARG	NH2	264ARG	NH1	0.01
164ARG	NH2	264ARG	NH2	0.02
164ARG	NH1	98ASP	OD1	59.39
164ARG	NH1	98ASP	OD2	12.07
164ARG	NH1	161GLU	OE1	23.28
164ARG	NH1	161GLU	OE2	19.96
164ARG	NH1	264ARG	NH2	0.01
164ARG	NE	264ARG	NH1	0.00
159LYS	NZ	148GLU	OE1	17.44
159LYS	NZ	148GLU	OE2	19.40
159LYS	NZ	163GLU	OE1	4.86
159LYS	NZ	163GLU	OE2	3.16
159LYS	NZ	198THR	OG1	10.89
159LYS	NZ	201GLU	OE1	31.19
159LYS	NZ	201GLU	OE2	32.35
158SER	OG	150GLU	OE1	1.18
158SER	OG	150GLU	OE2	2.55
158SER	OG	161GLU	OE1	2.83
158SER	OG	161GLU	OE2	4.56
157TYR	OH	133GLU	OE1	59.05
157TYR	OH	155GLU	OE1	0.05
157TYR	OH	155GLU	OE2	0.98
156ARG	NH2	150GLU	OE1	37.89
156ARG	NH2	150GLU	OE2	37.27
156ARG	NH2	158SER	OG	0.01
156ARG	NH1	150GLU	OE1	1.86

58ARG	NE	92SER	OG	0.16
36TYR	OH	17GLU	OE1	0.02
36TYR	OH	17GLU	OE2	0.01
24ARG	NH2	17GLU	OE1	28.80
24ARG	NH2	17GLU	OE2	18.05
24ARG	NH2	36TYR	OH	8.42
24ARG	NH1	17GLU	OE1	14.84
24ARG	NH1	17GLU	OE2	27.26
24ARG	NH1	36TYR	OH	35.09
24ARG	NE	36TYR	OH	9.50
21LYS	NZ	17GLU	OE1	0.70
21LYS	NZ	17GLU	OE2	1.45
21LYS	NZ	334GLU	OE1	35.52
21LYS	NZ	334GLU	OE2	39.62
21LYS	NZ	337THR	OG1	15.58
2LYS	NZ	37GLU	OE1	1.90
2LYS	NZ	37GLU	OE2	2.01
2LYS	NZ	63GLU	OE1	42.87
2LYS	NZ	63GLU	OE2	34.59

174ARG	NE	208ASP	OD2	96.26
167ARG	NH2	163GLU	OE1	11.92
167ARG	NH2	163GLU	OE2	21.92
167ARG	NH1	163GLU	OE1	4.32
167ARG	NH1	163GLU	OE2	4.93
167ARG	NH1	206TYR	OH	4.56
167ARG	NE	163GLU	OE1	24.38
167ARG	NE	163GLU	OE2	55.97
167ARG	NE	206TYR	OH	0.10
164ARG	NH2	98ASP	OD1	0.08
164ARG	NH2	98ASP	OD2	3.65
164ARG	NH2	161GLU	OE1	0.01
164ARG	NH2	161GLU	OE2	3.99
164ARG	NH2	163GLU	OE1	24.64
164ARG	NH2	163GLU	OE2	42.09
164ARG	NH2	167ARG	NE	0.00
164ARG	NH2	264ARG	NE	0.02
164ARG	NH2	264ARG	NH1	0.00
164ARG	NH2	264ARG	NH2	0.01
164ARG	NH1	98ASP	OD1	2.62
164ARG	NH1	98ASP	OD2	0.31
164ARG	NH1	161GLU	OE1	11.10
164ARG	NH1	161GLU	OE2	22.64
164ARG	NH1	163GLU	OE1	2.16
164ARG	NH1	163GLU	OE2	0.01
164ARG	NH1	264ARG	NH2	0.01
164ARG	NE	163GLU	OE1	43.80
164ARG	NE	163GLU	OE2	6.23
159LYS	NZ	148GLU	OE1	24.15
159LYS	NZ	148GLU	OE2	15.77
159LYS	NZ	163GLU	OE1	3.65
159LYS	NZ	163GLU	OE2	3.44
159LYS	NZ	198THR	OG1	4.85
159LYS	NZ	201GLU	OE1	14.38
159LYS	NZ	201GLU	OE2	15.54
158SER	OG	150GLU	OE1	0.44
158SER	OG	150GLU	OE2	0.44
158SER	OG	161GLU	OE1	2.98
158SER	OG	161GLU	OE2	0.39
157TYR	OH	133GLU	OE1	64.53
157TYR	OH	133GLU	OE2	11.41
157TYR	OH	155GLU	OE1	0.54
157TYR	OH	155GLU	OE2	1.62

156ARG	NH1	150GLU	OE2	3.26
156ARG	NH1	142GLU	OE1	0.23
156ARG	NH1	142GLU	OE2	0.04
156ARG	NH1	158SER	OG	0.02
156ARG	NH1	161GLU	OE1	0.01
156ARG	NE	150GLU	OE1	47.93
156ARG	NE	150GLU	OE2	42.73
153ASN	ND2	190GLU	OE1	5.75
153ASN	ND2	190GLU	OE2	4.69
147SER	OG	148GLU	OE2	0.02
144ARG	NH2	190GLU	OE1	27.49
144ARG	NH2	190GLU	OE2	33.22
144ARG	NH2	142GLU	OE1	4.34
144ARG	NH2	142GLU	OE2	4.39
144ARG	NH2	153ASN	OD1	0.04
144ARG	NH1	190GLU	OE1	1.59
144ARG	NH1	190GLU	OE2	0.03
144ARG	NH1	142GLU	OE1	3.36
144ARG	NH1	142GLU	OE2	2.32
144ARG	NE	190GLU	OE1	42.73
144ARG	NE	190GLU	OE2	28.19
144ARG	NE	142GLU	OE1	6.55
144ARG	NE	142GLU	OE2	4.92
139TYR	OH	87GLU	OE1	25.12
139TYR	OH	87GLU	OE2	12.07
139TYR	OH	237ASN	OD1	11.39
139TYR	OH	237ASN	ND2	0.24
139TYR	OH	241ASP	OD1	0.02
139TYR	OH	241ASP	OD2	2.54
135THR	OG1	133GLU	OE1	6.75
135THR	OG1	133GLU	OE2	89.67
135THR	OG1	161GLU	OE1	0.02
135THR	OG1	161GLU	OE2	0.01
132ARG	NH2	87GLU	OE1	0.01
132ARG	NH2	87GLU	OE2	0.01
132ARG	NH2	139TYR	OH	0.13
132ARG	NH2	237ASN	OD1	66.05
132ARG	NH2	237ASN	ND2	0.53
132ARG	NH2	241ASP	OD1	0.54
132ARG	NH2	241ASP	OD2	0.61
132ARG	NH1	102ASN	OD1	8.61
132ARG	NH1	241ASP	OD1	3.34
132ARG	NH1	241ASP	OD2	4.32

156ARG	NH2	150GLU	OE1	49.45
156ARG	NH2	150GLU	OE2	43.98
156ARG	NH1	150GLU	OE2	0.16
156ARG	NH1	142GLU	OE1	0.46
156ARG	NH1	142GLU	OE2	0.16
156ARG	NE	150GLU	OE1	45.99
156ARG	NE	150GLU	OE2	51.85
153ASN	ND2	190GLU	OE1	33.99
153ASN	ND2	190GLU	OE2	63.61
152TRP	NE1	154THR	OG1	0.01
147SER	OG	150GLU	OE1	0.00
147SER	OG	150GLU	OE2	0.02
144ARG	NH2	190GLU	OE1	32.83
144ARG	NH2	190GLU	OE2	63.92
144ARG	NH2	153ASN	OD1	0.03
144ARG	NH2	153ASN	ND2	0.01
144ARG	NH1	142GLU	OE1	0.03
144ARG	NH1	142GLU	OE2	0.03
144ARG	NE	190GLU	OE1	64.79
144ARG	NE	190GLU	OE2	43.30
144ARG	NE	153ASN	ND2	0.03
139TYR	OH	237ASN	OD1	0.05
139TYR	OH	241ASP	OD1	42.51
139TYR	OH	241ASP	OD2	20.95
135THR	OG1	98ASP	OD2	0.01
135THR	OG1	133GLU	OE1	17.46
135THR	OG1	133GLU	OE2	64.95
135THR	OG1	161GLU	OE1	0.12
135THR	OG1	161GLU	OE2	0.17
132ARG	NH2	102ASN	OD1	0.26
132ARG	NH2	139TYR	OH	0.57
132ARG	NH2	237ASN	OD1	29.16
132ARG	NH2	241ASP	OD1	2.23
132ARG	NH2	241ASP	OD2	3.90
132ARG	NH2	244SER	OG	0.00
132ARG	NH1	102ASN	OD1	1.21
132ARG	NH1	237ASN	OD1	0.48
132ARG	NH1	241ASP	OD1	1.99
132ARG	NH1	241ASP	OD2	2.26
132ARG	NH1	244SER	OG	23.93
132ARG	NE	102ASN	OD1	4.36
132ARG	NE	237ASN	OD1	4.51
132ARG	NE	244SER	OG	0.01

132ARG	NH1	244SER	OG	2.09
132ARG	NE	237ASN	OD1	9.61
132ARG	NE	237ASN	ND2	0.14
124ARG	NH2	113GLU	OE1	20.57
124ARG	NH2	113GLU	OE2	21.59
124ARG	NH2	120GLU	OE1	37.65
124ARG	NH2	120GLU	OE2	32.19
124ARG	NH2	121GLU	OE1	6.71
124ARG	NH2	121GLU	OE2	7.65
124ARG	NH1	113GLU	OE1	0.01
124ARG	NH1	113GLU	OE2	0.07
124ARG	NH1	120GLU	OE1	7.43
124ARG	NH1	120GLU	OE2	9.40
124ARG	NH1	121GLU	OE1	29.01
124ARG	NH1	121GLU	OE2	25.89
124ARG	NE	113GLU	OE1	23.03
124ARG	NE	113GLU	OE2	19.04
124ARG	NE	120GLU	OE1	6.76
124ARG	NE	120GLU	OE2	5.29
119LYS	NZ	120GLU	OE1	6.34
119LYS	NZ	120GLU	OE2	2.26
114ARG	NH2	113GLU	OE1	0.00
114ARG	NH2	120GLU	OE1	16.19
114ARG	NH2	120GLU	OE2	16.08
114ARG	NH1	120GLU	OE1	0.10
114ARG	NH1	120GLU	OE2	0.19
114ARG	NE	120GLU	OE1	27.11
114ARG	NE	120GLU	OE2	24.32
107LYS	NZ	127ASP	OD1	4.02
107LYS	NZ	127ASP	OD2	2.40
107LYS	NZ	312GLU	OE1	51.67
107LYS	NZ	312GLU	OE2	51.35
104ARG	NH2	102ASN	OD1	0.48
104ARG	NH2	241ASP	OD1	0.02
104ARG	NH2	241ASP	OD2	0.05
104ARG	NH2	245ASP	OD1	1.04
104ARG	NH2	245ASP	OD2	0.98
104ARG	NH2	259SER	OG	0.10
104ARG	NH2	270GLU	OE1	0.01
104ARG	NH2	270GLU	OE2	0.05
104ARG	NH2	275SER	OG	0.04
104ARG	NH1	241ASP	OD1	1.49
104ARG	NH1	241ASP	OD2	0.14

124ARG	NH2	113GLU	OE1	9.64
124ARG	NH2	113GLU	OE2	11.34
124ARG	NH2	120GLU	OE1	19.51
124ARG	NH2	120GLU	OE2	17.35
124ARG	NH2	121GLU	OE1	23.40
124ARG	NH2	121GLU	OE2	29.96
124ARG	NH1	113GLU	OE1	11.72
124ARG	NH1	113GLU	OE2	4.67
124ARG	NH1	120GLU	OE1	10.62
124ARG	NH1	120GLU	OE2	6.24
124ARG	NH1	121GLU	OE1	20.15
124ARG	NH1	121GLU	OE2	20.57
124ARG	NE	113GLU	OE1	13.13
124ARG	NE	113GLU	OE2	10.85
124ARG	NE	120GLU	OE1	0.04
124ARG	NE	121GLU	OE1	13.78
124ARG	NE	121GLU	OE2	9.71
119LYS	NZ	120GLU	OE1	15.34
119LYS	NZ	120GLU	OE2	12.40
114ARG	NH2	120GLU	OE1	7.30
114ARG	NH2	120GLU	OE2	7.90
114ARG	NH1	113GLU	OE2	0.00
114ARG	NH1	120GLU	OE1	0.20
114ARG	NH1	120GLU	OE2	1.40
114ARG	NE	120GLU	OE1	12.83
114ARG	NE	120GLU	OE2	17.10
107LYS	NZ	127ASP	OD1	5.14
107LYS	NZ	127ASP	OD2	3.60
107LYS	NZ	176ARG	NH2	0.00
107LYS	NZ	299GLU	OE1	0.00
107LYS	NZ	309ARG	NE	0.02
107LYS	NZ	309ARG	NH2	0.06
107LYS	NZ	312GLU	OE1	38.45
107LYS	NZ	312GLU	OE2	45.65
104ARG	NH2	245ASP	OD1	13.26
104ARG	NH2	245ASP	OD2	10.67
104ARG	NH2	248SER	OG	78.82
104ARG	NH1	102ASN	OD1	0.08
104ARG	NH1	132ARG	NE	0.02
104ARG	NH1	132ARG	NH1	0.00
104ARG	NH1	132ARG	NH2	0.02
104ARG	NH1	241ASP	OD1	1.39
104ARG	NH1	245ASP	OD1	1.49

104ARG	NH1	244SER	OG	3.89
104ARG	NH1	245ASP	OD1	0.45
104ARG	NH1	245ASP	OD2	0.24
104ARG	NH1	275SER	OG	0.01
104ARG	NE	102ASN	OD1	83.01
104ARG	NE	259SER	OG	0.06
102ASN	ND2	104ARG	NH2	0.01
102ASN	ND2	259SER	OG	0.28
102ASN	ND2	261SER	OG	64.11
102ASN	ND2	270GLU	OE2	0.00
97GLN	NE2	62GLU	OE1	35.95
97GLN	NE2	62GLU	OE2	34.11
97GLN	NE2	266THR	OG1	1.14
96SER	OG	97GLN	OE1	0.00
95LYS	NZ	98ASP	OD1	0.42
95LYS	NZ	98ASP	OD2	0.24
95LYS	NZ	161GLU	OE1	0.01
94ARG	NH2	87GLU	OE1	9.61
94ARG	NH2	87GLU	OE2	19.53
94ARG	NH2	270GLU	OE1	2.36
94ARG	NH2	270GLU	OE2	0.72
94ARG	NH1	87GLU	OE1	49.51
94ARG	NH1	87GLU	OE2	32.07
94ARG	NH1	139TYR	OH	0.83
92SER	OG	51GLU	OE2	0.00
85SER	OG	87GLU	OE1	0.76
85SER	OG	87GLU	OE2	0.11
85SER	OG	88THR	OG1	10.21
82ARG	NH2	187ASN	OD1	0.01
82ARG	NH2	190GLU	OE1	21.07
82ARG	NH2	190GLU	OE2	43.82
82ARG	NH2	193GLU	OE1	27.88
82ARG	NH2	193GLU	OE2	33.66
82ARG	NH2	87GLU	OE1	1.89
82ARG	NH2	87GLU	OE2	4.16
82ARG	NH2	144ARG	NE	0.00
82ARG	NH1	187ASN	OD1	0.03
82ARG	NH1	190GLU	OE1	45.72
82ARG	NH1	190GLU	OE2	23.49
82ARG	NH1	193GLU	OE1	5.61
82ARG	NH1	193GLU	OE2	6.88
82ARG	NH1	85SER	OG	0.75
82ARG	NH1	87GLU	OE1	1.98

104ARG	NH1	245ASP	OD2	0.50
104ARG	NH1	259SER	OG	0.03
104ARG	NE	248SER	OG	0.32
102ASN	ND2	132ARG	NE	0.04
102ASN	ND2	259SER	OG	5.36
102ASN	ND2	261SER	OG	67.15
97GLN	NE2	62GLU	OE1	17.04
97GLN	NE2	62GLU	OE2	17.33
97GLN	NE2	266THR	OG1	5.42
96SER	OG	97GLN	OE1	1.18
96SER	OG	97GLN	NE2	0.23
95LYS	NZ	51GLU	OE1	0.44
95LYS	NZ	51GLU	OE2	0.72
95LYS	NZ	98ASP	OD1	8.19
95LYS	NZ	98ASP	OD2	7.78
95LYS	NZ	135THR	OG1	0.00
95LYS	NZ	142GLU	OE1	0.38
95LYS	NZ	142GLU	OE2	0.51
94ARG	NH2	270GLU	OE1	65.98
94ARG	NH2	270GLU	OE2	36.73
94ARG	NH1	261SER	OG	1.29
94ARG	NH1	270GLU	OE1	31.79
94ARG	NH1	270GLU	OE2	63.33
94ARG	NE	97GLN	OE1	0.02
92SER	OG	51GLU	OE1	0.03
92SER	OG	51GLU	OE2	0.01
88THR	OG1	87GLU	OE1	16.36
85ARG	NH2	78ASP	OD1	4.87
85ARG	NH2	78ASP	OD2	5.57
85ARG	NH2	87GLU	OE1	11.30
85ARG	NH2	87GLU	OE2	31.70
85ARG	NH1	78ASP	OD1	18.59
85ARG	NH1	78ASP	OD2	21.95
85ARG	NH1	87GLU	OE1	0.62
85ARG	NH1	87GLU	OE2	0.10
85ARG	NE	87GLU	OE1	34.51
85ARG	NE	87GLU	OE2	15.04
85ARG	NE	88THR	OG1	0.01
83LYS	NZ	190GLU	OE1	1.20
83LYS	NZ	190GLU	OE2	0.03
83LYS	NZ	51GLU	OE1	1.00
83LYS	NZ	51GLU	OE2	0.90
83LYS	NZ	144ARG	NE	0.00

82ARG	NH1	87GLU	OE2	2.14
82ARG	NH1	144ARG	NE	0.02
82ARG	NH1	144ARG	NH2	0.04
82ARG	NE	193GLU	OE1	28.04
82ARG	NE	193GLU	OE2	22.21
82ARG	NE	85SER	OG	0.00
82ARG	NE	87GLU	OE1	1.40
82ARG	NE	87GLU	OE2	1.26
77TRP	NE1	9ASP	OD1	0.08
77TRP	NE1	9ASP	OD2	0.21
77TRP	NE1	47ASP	OD1	0.06
77TRP	NE1	47ASP	OD2	0.00
76LYS	NZ	9ASP	OD1	41.80
76LYS	NZ	9ASP	OD2	45.41
76LYS	NZ	47ASP	OD1	0.27
76LYS	NZ	47ASP	OD2	0.17
76LYS	NZ	77TRP	NE1	0.06
71SER	OG	270GLU	OE1	11.66
71SER	OG	270GLU	OE2	6.27
71SER	OG	275SER	OG	4.32
59LYS	NZ	55GLU	OE1	40.74
59LYS	NZ	55GLU	OE2	27.16
59LYS	NZ	62GLU	OE1	0.15
59LYS	NZ	62GLU	OE2	0.14
59LYS	NZ	63GLU	OE1	0.29
59LYS	NZ	63GLU	OE2	0.12
58ARG	NH2	51GLU	OE1	9.16
58ARG	NH2	51GLU	OE2	13.03
58ARG	NH2	55GLU	OE1	30.22
58ARG	NH2	55GLU	OE2	46.75
58ARG	NH2	92SER	OG	1.75
58ARG	NH1	51GLU	OE1	31.63
58ARG	NH1	51GLU	OE2	28.07
58ARG	NH1	55GLU	OE1	3.04
58ARG	NH1	55GLU	OE2	1.06
58ARG	NH1	92SER	OG	1.93
58ARG	NE	51GLU	OE1	0.03
58ARG	NE	51GLU	OE2	0.01
58ARG	NE	55GLU	OE1	3.59
58ARG	NE	55GLU	OE2	4.11
58ARG	NE	92SER	OG	0.08
36TYR	OH	17GLU	OE1	0.11
36TYR	OH	17GLU	OE2	0.12

83LYS	NZ	144ARG	NH2	0.00
82ARG	NH2	190GLU	OE1	0.77
82ARG	NH2	190GLU	OE2	0.09
82ARG	NH2	193GLU	OE1	30.08
82ARG	NH2	193GLU	OE2	28.35
82ARG	NH2	87GLU	OE1	0.06
82ARG	NH2	87GLU	OE2	1.44
82ARG	NH2	144ARG	NE	0.02
82ARG	NH2	144ARG	NH1	0.00
82ARG	NH2	144ARG	NH2	0.03
82ARG	NH1	190GLU	OE1	0.54
82ARG	NH1	190GLU	OE2	0.24
82ARG	NH1	193GLU	OE1	20.88
82ARG	NH1	193GLU	OE2	20.54
82ARG	NH1	85ARG	NH1	0.01
82ARG	NH1	88THR	OG1	0.37
82ARG	NH1	144ARG	NE	0.00
82ARG	NE	193GLU	OE1	4.10
82ARG	NE	193GLU	OE2	2.37
82ARG	NE	87GLU	OE2	0.06
82ARG	NE	88THR	OG1	0.02
77TRP	NE1	47ASP	OD1	0.19
77TRP	NE1	47ASP	OD2	0.00
76LYS	NZ	9ASP	OD1	32.74
76LYS	NZ	9ASP	OD2	21.77
76LYS	NZ	47ASP	OD1	0.66
76LYS	NZ	47ASP	OD2	0.68
76LYS	NZ	278ASP	OD1	0.96
76LYS	NZ	278ASP	OD2	0.91
71SER	OG	273HIS	NE2	2.71
71SER	OG	275SER	OG	0.05
59LYS	NZ	55GLU	OE1	27.41
59LYS	NZ	55GLU	OE2	33.73
59LYS	NZ	62GLU	OE1	0.47
59LYS	NZ	62GLU	OE2	0.40
59LYS	NZ	63GLU	OE1	0.50
59LYS	NZ	63GLU	OE2	0.02
58ARG	NH2	51GLU	OE1	12.45
58ARG	NH2	51GLU	OE2	12.13
58ARG	NH2	55GLU	OE1	44.98
58ARG	NH2	55GLU	OE2	37.93
58ARG	NH2	92SER	OG	0.03
58ARG	NH1	51GLU	OE1	29.28

36TYR	OH	27ASP	OD2	0.01
24ARG	NH2	17GLU	OE1	0.32
24ARG	NH2	17GLU	OE2	0.22
24ARG	NH2	27ASP	OD1	1.62
24ARG	NH2	27ASP	OD2	4.32
24ARG	NH2	28GLU	OE1	42.11
24ARG	NH2	28GLU	OE2	52.21
24ARG	NH2	36TYR	OH	0.84
24ARG	NH1	17GLU	OE1	0.22
24ARG	NH1	27ASP	OD1	29.44
24ARG	NH1	27ASP	OD2	61.47
24ARG	NH1	36TYR	OH	1.45
24ARG	NE	28GLU	OE1	1.85
24ARG	NE	28GLU	OE2	1.98
24ARG	NE	36TYR	OH	0.82
21LYS	NZ	14GLU	OE1	0.02
21LYS	NZ	17GLU	OE1	4.67
21LYS	NZ	17GLU	OE2	6.70
21LYS	NZ	334GLU	OE1	31.67
21LYS	NZ	334GLU	OE2	33.55
21LYS	NZ	337THR	OG1	2.75
2LYS	NZ	37GLU	OE1	2.14
2LYS	NZ	37GLU	OE2	3.58
2LYS	NZ	63GLU	OE1	31.49
2LYS	NZ	63GLU	OE2	36.94
2LYS	NZ	65GLU	OE1	1.78
2LYS	NZ	65GLU	OE2	5.11

58ARG	NH1	51GLU	OE2	28.57
58ARG	NH1	55GLU	OE1	4.66
58ARG	NH1	55GLU	OE2	3.62
58ARG	NH1	92SER	OG	0.83
58ARG	NH1	96SER	OG	0.01
58ARG	NE	51GLU	OE1	1.01
58ARG	NE	51GLU	OE2	0.87
58ARG	NE	55GLU	OE1	4.45
58ARG	NE	55GLU	OE2	3.43
36TYR	OH	27ASP	OD1	0.03
24ARG	NH2	17GLU	OE1	26.51
24ARG	NH2	17GLU	OE2	33.89
24ARG	NH2	28GLU	OE1	1.60
24ARG	NH2	28GLU	OE2	2.35
24ARG	NH2	36TYR	OH	23.36
24ARG	NH1	17GLU	OE1	36.90
24ARG	NH1	17GLU	OE2	27.70
24ARG	NH1	27ASP	OD2	0.14
24ARG	NH1	28GLU	OE1	2.90
24ARG	NH1	28GLU	OE2	3.28
24ARG	NH1	36TYR	OH	2.67
24ARG	NE	17GLU	OE2	0.00
24ARG	NE	28GLU	OE1	0.11
24ARG	NE	28GLU	OE2	0.03
24ARG	NE	36TYR	OH	46.43
21LYS	NZ	14GLU	OE1	0.00
21LYS	NZ	17GLU	OE1	2.67
21LYS	NZ	17GLU	OE2	0.58
21LYS	NZ	334GLU	OE1	16.62
21LYS	NZ	334GLU	OE2	18.85
21LYS	NZ	337THR	OG1	21.91
2LYS	NZ	37GLU	OE1	5.28
2LYS	NZ	37GLU	OE2	3.08
2LYS	NZ	63GLU	OE1	31.61
2LYS	NZ	63GLU	OE2	32.75
2LYS	NZ	65GLU	OE1	0.27
2LYS	NZ	65GLU	OE2	1.69
