# Supplementary material for: Deciphering the Dynamics of Non-Covalent Interactions Affecting Thermal Stability of a Protein: Molecular Dynamics Study on Point Mutant of Thermus thermophilus Isopropylmalate Dehydrogenase
Source: PLoS One. 2015 Dec 11;10(12):e0144294. doi: 10.1371/journal.pone.0144294 (PMC4689552; doi:10.1371/journal.pone.0144294)
Supplement: S6 Table — The color formatting indicates the percentage of time interaction existed is as in S1 Table. (PDF) [file pone.0144294.s008.pdf]

S6 Table. Percentage existence of HBs between PP of *wt* and *mut* at 300 K and 337 K.

1) *Wt* 300K

Drnona	D	Arnona	A	percen
339THR	OG1	339THR	O	0.10
339THR	N	337THR	O	0.22
337THR	OG1	333THR	O	75.24
337THR	N	333THR	O	24.22
330SER	OG	330SER	O	0.10
322THR	OG1	322THR	O	3.82
322THR	OG1	330SER	OG	0.06
293SER	OG	259SER	O	0.30
293SER	OG	293SER	O	0.01
288THR	OG1	286ASN	OD1	86.26
288THR	N	286ASN	OD1	84.70
288THR	N	286ASN	O	0.01
286ASN	ND2	288THR	OG1	1.90
275SER	OG	71SER	O	5.00
275SER	OG	275SER	O	0.07
266THR	OG1	97GLN	OE1	4.87
266THR	N	97GLN	OE1	91.37
266THR	N	97GLN	O	0.00
261SER	OG	261SER	O	0.00
259SER	OG	102ASN	OD1	0.19
259SER	OG	259SER	O	0.01
259SER	N	293SER	OG	7.24
253SER	OG	253SER	O	0.48
253SER	N	248SER	O	0.00
248SER	OG	244SER	O	71.15
248SER	OG	248SER	O	0.05
248SER	N	244SER	O	21.58
226SER	OG	226SER	O	5.77
215TYR	OH	187ASN	OD1	0.10
215TYR	N	214GLN	OE1	0.20
214GLN	NE2	215TYR	O	0.04
214GLN	N	182SER	O	92.25
187ASN	ND2	215TYR	OH	0.02
187ASN	N	187ASN	OD1	2.68
187ASN	N	187ASN	ND2	0.50
158SER	OG	157TYR	O	0.03
158SER	OG	158SER	O	0.08
154THR	OG1	153ASN	O	0.44
154THR	OG1	154THR	OG1	4.35
153ASN	ND2	154THR	N	0.02

2) *Wt* 337 K

Drnona	D	Arnona	A	percen
339THR	OG1	339THR	O	0.06
339THR	N	337THR	O	0.02
337THR	OG1	333THR	O	79.68
337THR	N	333THR	O	39.56
330SER	OG	330SER	O	0.46
322THR	OG1	322THR	O	5.55
322THR	OG1	330SER	OG	0.01
322THR	OG1	339THR	OG1	0.02
293SER	OG	259SER	O	0.68
293SER	OG	293SER	O	0.04
288THR	OG1	286ASN	OD1	94.83
288THR	N	286ASN	OD1	75.46
288THR	N	286ASN	O	0.45
286ASN	ND2	288THR	OG1	0.90
286ASN	ND2	322THR	O	0.08
275SER	OG	275SER	O	0.60
266THR	OG1	97GLN	OE1	5.56
266THR	OG1	266THR	O	0.38
266THR	N	97GLN	OE1	27.61
266THR	N	97GLN	O	0.15
261SER	OG	102ASN	OD1	0.49
261SER	OG	261SER	O	0.09
259SER	OG	102ASN	OD1	0.02
259SER	OG	259SER	O	0.07
259SER	N	293SER	OG	1.94
253SER	OG	248SER	O	1.12
253SER	OG	253SER	O	2.36
253SER	N	248SER	O	4.24
248SER	OG	244SER	O	30.46
248SER	OG	248SER	O	0.04
248SER	OG	253SER	O	0.20
248SER	N	244SER	O	8.10
237ASN	ND2	135THR	O	0.00
237ASN	ND2	237ASN	O	0.02
237ASN	N	237ASN	ND2	0.01
226SER	OG	226SER	O	4.44
215TYR	N	214GLN	OE1	7.00
214GLN	NE2	215TYR	N	0.02
214GLN	NE2	215TYR	O	0.54
214GLN	N	182SER	O	69.14

3) *Mut* 300 K

Drnona	D	Arnona	A	percen
339THR	N	337THR	O	0.00
337THR	OG1	333THR	O	52.79
337THR	OG1	337THR	O	0.01
337THR	N	333THR	O	80.44
330SER	OG	330SER	O	0.34
322THR	OG1	322THR	O	11.79
322THR	OG1	330SER	O	0.19
322THR	OG1	339THR	OG1	0.06
293SER	OG	259SER	O	0.02
293SER	OG	293SER	O	0.01
288THR	OG1	286ASN	OD1	86.10
288THR	N	286ASN	OD1	62.41
288THR	N	286ASN	O	0.06
288THR	N	288THR	OG1	0.00
286ASN	ND2	288THR	OG1	7.37
275SER	OG	275SER	O	0.45
266THR	OG1	97GLN	OE1	13.30
266THR	N	97GLN	OE1	91.74
266THR	N	97GLN	O	0.19
261SER	OG	102ASN	OD1	0.16
259SER	OG	259SER	O	0.01
259SER	N	293SER	OG	0.18
253SER	OG	253SER	O	0.12
248SER	OG	244SER	O	64.37
248SER	OG	248SER	O	0.55
248SER	N	244SER	O	8.15
226SER	OG	226SER	O	7.68
215TYR	OH	187ASN	OD1	0.21
215TYR	OH	275SER	OG	0.01
215TYR	N	214GLN	OE1	9.31
214GLN	NE2	215TYR	N	0.00
214GLN	NE2	215TYR	O	2.67
214GLN	N	182SER	O	83.73
187ASN	ND2	215TYR	OH	0.04
187ASN	N	187ASN	OD1	2.91
187ASN	N	187ASN	ND2	0.24
158SER	OG	158SER	O	0.01
154THR	OG1	153ASN	O	0.16
154THR	OG1	154THR	OG1	1.88
153ASN	ND2	154THR	N	0.01

4) *Mut* 337 K

Drnona	D	Arnona	A	percen
339THR	OG1	339THR	O	0.01
339THR	N	337THR	O	0.06
337THR	OG1	333THR	O	67.29
337THR	N	333THR	O	54.74
330SER	OG	330SER	O	0.61
322THR	OG1	322THR	O	15.72
322THR	OG1	330SER	O	0.01
322THR	OG1	339THR	OG1	0.03
293SER	OG	259SER	O	1.12
293SER	OG	293SER	O	0.04
288THR	OG1	286ASN	OD1	66.52
288THR	OG1	286ASN	O	0.00
288THR	N	286ASN	OD1	56.25
288THR	N	286ASN	O	2.33
288THR	N	288THR	OG1	0.01
286ASN	ND2	288THR	OG1	3.17
286ASN	ND2	322THR	O	0.50
275SER	OG	71SER	O	0.03
275SER	OG	275SER	O	0.68
266THR	OG1	97GLN	OE1	7.88
266THR	OG1	266THR	O	3.16
266THR	N	97GLN	OE1	29.15
261SER	OG	102ASN	OD1	1.03
261SER	OG	259SER	O	0.00
261SER	OG	261SER	O	0.27
259SER	OG	102ASN	OD1	0.20
259SER	OG	259SER	O	0.08
259SER	N	293SER	OG	20.02
253SER	N	248SER	O	2.49
248SER	OG	244SER	O	29.76
248SER	OG	253SER	O	0.01
248SER	N	244SER	O	2.44
244SER	OG	244SER	O	0.04
237ASN	ND2	237ASN	O	0.01
237ASN	N	237ASN	OD1	0.02
237ASN	N	237ASN	ND2	0.02
226SER	OG	226SER	O	5.09
215TYR	OH	187ASN	OD1	21.11
215TYR	OH	275SER	OG	0.18
215TYR	N	214GLN	OE1	6.08

153ASN	ND2	154THR	O	0.02
139TYR	OH	237ASN	OD1	0.22
139TYR	OH	237ASN	ND2	0.10
139TYR	OH	237ASN	O	1.96
102ASN	ND2	259SER	OG	2.90
102ASN	ND2	261SER	N	0.01
102ASN	ND2	261SER	OG	56.01
97GLN	NE2	96SER	O	0.03
96SER	OG	92SER	O	83.31
96SER	N	92SER	O	86.52
92SER	OG	88THR	O	20.40
92SER	OG	92SER	O	0.02
92SER	N	88THR	O	14.13
88THR	OG1	85SER	O	32.15
88THR	N	85SER	O	30.15
85SER	OG	85SER	O	0.04
85SER	OG	88THR	OG1	8.47
85SER	N	88THR	OG1	2.60
71SER	OG	71SER	O	2.63
71SER	OG	275SER	OG	49.10
339THR	OG1	339THR	O	0.00
337THR	OG1	333THR	O	93.98
337THR	N	333THR	O	83.47
330SER	OG1	330SER	O	0.24
322THR	OG1	322THR	O	17.26
322THR	OG1	339THR	OG1	0.01
293SER	OG	259SER	O	0.06
293SER	OG	293SER	O	0.00
288THR	OG1	286ASN	OD1	95.95
288THR	N	286ASN	OD1	68.87
288THR	N	286ASN	O	0.02
286ASN	ND2	288THR	OG1	0.38
286ASN	ND2	322THR	O10	0.08
275SER	OG	71SER	O	0.02
275SER	OG	275SER	O	0.00
266THR	OG1	97GLN	OE1	7.14
266THR	N	97GLN	OE1	37.62
266THR	N	97GLN	O	0.59
261SER	OG	102ASN	OD1	0.06
261SER	OG	261SER	O	0.14
259SER	OG	102ASN	OD1	0.01
259SER	OG	259SER	O	0.05
259SER	N	293SER	OG	2.77

187ASN	ND2	215TYR	OH	1.22
187ASN	N	187ASN	OD1	4.82
187ASN	N	187ASN	ND2	0.02
158SEF	OG	157TYR	O	0.00
158SEF	OG	158SER	O	0.06
154THF	OG1	153ASN	O	1.38
154THF	OG1	153ASN	O	0.00
154THF	OG1	154THR	OG1	0.19
153ASN	ND2	154THR	N	0.04
153ASN	ND2	154THR	O	0.03
147SEF	OG	147SER	O	0.00
139TYR	OH	237ASN	OD1	10.68
139TYR	OH	237ASN	ND2	0.35
139TYR	OH	237ASN	O	3.77
139TYR	N	154THR	O	0.01
139TYR	N	237ASN	ND2	0.00
135THF	OG1	135THR	O	0.24
135THF	OG1	157TYR	OH	0.14
102ASN	ND2	259SER	OG	10.06
102ASN	ND2	261SER	N	0.02
102ASN	ND2	261SER	OG	58.01
97GLN	NE2	96SER	O	0.30
97GLN	NE2	97GLN	O	0.03
97GLN	NE2	266THR	N	0.03
97GLN	NE2	266THR	OG1	28.11
97GLN	NE2	266THR	O	17.55
96SER	OG	92SER	O	42.21
96SER	OG	96SER	O	0.14
96SER	OG	97GLN	OE1	0.27
96SER	N	92SER	O	60.80
96SER	N	97GLN	O	0.20
92SER	OG	88THR	O	23.17
92SER	OG	92SER	O	0.08
92SER	N	88THR	O	28.46
88THR	OG1	85SER	O	80.88
88THR	N	85SER	O	78.74
85SER	OG	85SER	O	0.02
85SER	OG	88THR	OG1	0.04
85SER	N	88THR	OG1	0.27
71SER	OG	71SER	O	1.44
71SER	OG	275SER	OG	0.16
339THF	OG1	339THR	O	0.00
339THF	N	337THR	O	0.01

153ASN	ND2	154THF	O	0.00
139TYR	OH	237ASN	O	0.06
102ASN	ND2	259SEF	OG	0.05
102ASN	ND2	261SEF	N	0.01
102ASN	ND2	261SEF	OG	71.42
97GLN	NE2	96SER	O	0.02
97GLN	NE2	266THF	OG:	0.00
96SER	OG	92SER	O	85.92
96SER	N	92SER	O	93.50
92SER	OG	88THR	O	35.93
92SER	OG	92SER	O	0.04
92SER	N	88THR	O	31.95
71SER	OG	71SER	O	0.13
71SER	OG	275SEF	N	0.00
71SER	OG	275SEF	OG	0.30
339THR	N	337THF	O	0.01
337THR	OG1	333THF	O	75.07
337THR	N	333THF	O	65.03
330SER	OG	330SEF	O	0.47
322THR	OG1	322THF	O	15.95
322THR	OG1	330SEF	O	0.10
322THR	OG1	339THF	OG:	0.06
293SER	OG	259SEF	O	1.77
293SER	OG	293SEF	O	0.03
288THR	OG1	286ASN	OD1	33.68
288THR	N	286ASN	OD1	27.52
288THR	N	286ASN	O	2.52
288THR	N	288THF	OG:	0.00
286ASN	ND2	288THF	OG:	16.68
286ASN	ND2	322THF	O	0.25
286ASN	N	286ASN	ND2	0.01
275SER	OG	275SEF	O	0.73
266THR	OG1	97GLN	OE1	6.41
266THR	N	97GLN	OE1	91.84
261SER	OG	102ASN	OD1	0.01
259SER	OG	259SEF	O	0.04
259SER	N	293SEF	OG	2.71
253SER	OG	253SEF	O	0.02
253SER	N	248SEF	O	1.29
248SER	OG	244SEF	O	69.89
248SER	OG	248SEF	O	0.04
248SER	OG	253SEF	O	0.27
248SER	N	244SEF	O	2.94

214GLN	NE2	182SER	O	0.01
214GLN	NE2	215TYR	N	0.01
214GLN	NE2	215TYR	O	0.10
214GLN	N	182SER	O	81.04
198THR	OG1	198THR	O	0.00
187ASN	ND2	215TYR	OH	4.21
187ASN	ND2	88THR	OG1	0.01
187ASN	ND2	92SER	OG	0.08
187ASN	N	187ASN	OD1	2.02
187ASN	N	187ASN	ND2	0.20
182SER	OG	182SER	O	0.27
158SER	OG	157TYR	O	0.02
158SER	OG	158SER	O	0.06
154THR	OG1	153ASN	O	0.11
154THR	OG1	154THR	OG1	3.02
147SER	OG	147SER	O	0.01
139TYR	OH	237ASN	OD1	0.04
139TYR	OH	237ASN	O	2.30
139TYR	N	237ASN	OD1	0.00
135THR	OG1	135THR	O	0.02
102ASN	ND2	102ASN	O	0.01
102ASN	ND2	259SER	OG	12.92
102ASN	ND2	261SER	N	0.01
102ASN	ND2	261SER	OG	35.92
97GLN	NE2	96SER	O	0.10
97GLN	NE2	97GLN	O	0.06
97GLN	NE2	266THR	N	0.03
97GLN	NE2	266THR	OG1	10.97
97GLN	NE2	266THR	O	17.59
96SER	OG	92SER	O	52.50
96SER	OG	96SER	O	0.07
96SER	OG	97GLN	N	0.00
96SER	OG	97GLN	OE1	0.09
96SER	N	92SER	O	71.99
92SER	OG	88THR	O	36.07
92SER	OG	92SER	O	0.06
92SER	OG	96SER	OG	0.00
92SER	N	88THR	O	47.10
88THR	OG1	88THR	O	0.80
71SER	OG	71SER	O	0.21
71SER	OG	275SER	OG	1.72
57THR	N	57THR	OG1	0.00
16THR	OG1	16THR	O	0.03

253SER	OG	253SER	O	0.08
253SER	N	248SER	O	1.33
248SER	OG	244SER	O	39.94
248SER	OG	248SER	O	0.06
248SER	OG	253SER	O	0.65
248SER	N	244SER	O	8.63
244SER	OG	244SER	O	0.04
237ASN	ND2	237ASN	O	0.06
237ASN	N	237ASN	ND2	0.01
226SER	OG	226SER	O	2.81
215TYR	N	214GLN	OE1	0.53
214GLN	NE2	215TYR	N	0.00
214GLN	NE2	215TYR	O	0.02
214GLN	N	182SER	O	78.21
187ASN	ND2	215TYR	OH	0.09
187ASN	N	187ASN	OD1	5.27
187ASN	N	187ASN	ND2	0.01
158SER	OG	158SER	O	0.01
154THR	OG1	153ASN	O	0.19
153ASN	ND2	154THR	N	0.08
153ASN	ND2	154THR	O	0.07
147SER	OG	147SER	O	0.02
139TYR	OH	237ASN	O	3.96
102ASN	ND2	259SER	OG	9.31
102ASN	ND2	261SER	N	0.01
102ASN	ND2	261SER	OG	44.49
97GLN	NE2	96SER	O	0.04
97GLN	NE2	97GLN	O	0.00
97GLN	NE2	266THR	N	0.00
97GLN	NE2	266THR	O	24.78
96SER	OG	92SER	O	86.21
96SER	OG	97GLN	N	0.01
96SER	OG	97GLN	OE1	0.07
96SER	N	92SER	O	76.86
96SER	N	97GLN	O	0.20
92SER	OG	88THR	O	38.90
92SER	OG	92SER	O	0.04
92SER	N	88THR	O	30.52
88THR	OG1	85SER	O	82.38
88THR	N	85SER	O	84.47
85SER	OG	85SER	O	0.06
85SER	OG	88THR	OG1	0.60
85SER	N	88THR	OG1	3.44

337THR	OG1	333THR	O	59.55
337THR	N	333THR	O	31.12
337THR	N	337THR	OG1	0.00
330SER	OG	330SER	O	0.41
322THR	OG1	322THR	O	13.04
322THR	OG1	330SER	OG	0.05
322THR	OG1	330SER	O	1.07
322THR	OG1	339THR	OG1	0.00
293SER	OG	259SER	O	0.04
293SER	OG	293SER	O	0.02
288THR	OG1	286ASN	OD1	71.20
288THR	OG1	286ASN	O	0.02
288THR	N	286ASN	OD1	64.29
288THR	N	286ASN	O	1.22
288THR	N	288THR	OG1	0.00
286ASN	ND2	288THR	OG1	13.27
286ASN	ND2	322THR	O	0.02
275SER	OG	71SER	O	0.30
275SER	OG	275SER	O	0.36
275SER	N	275SER	OG	0.01
266THR	OG1	97GLN	OE1	9.78
266THR	OG1	266THR	O	0.50
266THR	N	97GLN	OE1	83.72
266THR	N	97GLN	O	0.00
261SER	OG	261SER	O	0.05
259SER	OG	259SER	O	0.04
259SER	N	293SER	OG	1.30
253SER	OG	253SER	O	0.30
253SER	N	248SER	O	0.10
248SER	OG	244SER	O	1.16
248SER	N	244SER	O	23.78
244SER	OG	244SER	O	0.02
226SER	OG	226SER	O	5.69
215TYR	N	214GLN	OE1	1.68
214GLN	NE2	215TYR	N	0.01
214GLN	NE2	215TYR	O	0.02
214GLN	N	182SER	O	77.16
187ASN	ND2	187ASN	O	0.00
187ASN	ND2	215TYR	OH	0.09
187ASN	N	187ASN	OD1	4.55
187ASN	N	187ASN	ND2	0.01
187ASN	N	215TYR	OH	0.00
158SER	OG	157TYR	O	0.04

226SER	OG	226SER	O	3.15
215TYR	OH	187ASN	OD1	0.01
215TYR	N	214GLN	OE1	0.30
214GLN	N	182SER	O	93.26
187ASN	ND2	215TYR	OH	0.04
187ASN	N	187ASN	OD1	0.90
187ASN	N	187ASN	ND2	1.13
158SER	OG	158SER	O	0.01
154THR	OG1	153ASN	O	0.23
153ASN	ND2	154THR	N	0.00
139TYR	OH	237ASN	OD1	1.45
139TYR	OH	237ASN	ND2	0.02
139TYR	OH	237ASN	O	4.13
102ASN	ND2	259SER	OG	0.11
102ASN	ND2	261SER	N	0.00
102ASN	ND2	261SER	OG	74.38
97GLN	NE2	96SER	O	0.06
96SER	OG	92SER	O	88.20
96SER	N	92SER	O	92.92
92SER	OG	88THR	O	37.97
92SER	OG	92SER	O	0.05
92SER	N	88THR	O	33.67
71SER	OG	71SER	O	0.06

339THR	N	337THR	O	0.00
337THR	OG1	333THR	O	85.12
337THR	N	333THR	O	26.38
333THR	N	333THR	OG1	0.00
330SER	OG	330SER	O	0.49
322THR	OG1	322THR	O	4.70
322THR	OG1	330SER	O	0.22
322THR	OG1	339THR	OG1	0.00
293SER	OG	259SER	O	0.55
293SER	OG	293SER	O	0.04
288THR	OG1	286ASN	OD1	78.41
288THR	N	286ASN	OD1	66.33
288THR	N	286ASN	O	0.36
286ASN	ND2	288THR	OG1	3.10
286ASN	ND2	322THR	O	0.15
275SER	OG	275SER	O	1.11
266THR	OG1	97GLN	OE1	6.80
266THR	OG1	266THR	O	2.75
266THR	N	97GLN	OE1	52.49
266THR	N	97GLN	O	0.18
261SER	OG	102ASN	OD1	0.68
261SER	OG	261SER	O	0.03
259SER	OG	102ASN	OD1	0.05
259SER	OG	259SER	O	0.04
259SER	N	293SER	OG	1.81
253SER	OG	253SER	O	0.00
253SER	N	248SER	O	0.33
248SER	OG	244SER	O	77.98
248SER	OG	248SER	O	0.01
248SER	OG	253SER	O	0.05
248SER	N	244SER	O	4.23
244SER	OG	244SER	O	0.03
226SER	OG	226SER	O	5.22
215TYR	OH	187ASN	OD1	0.00
215TYR	N	214GLN	OE1	6.35
214GLN	NE2	215TYR	N	0.00
214GLN	NE2	215TYR	O	0.23
214GLN	N	182SER	O	72.93
187ASN	ND2	187ASN	O	0.00
187ASN	ND2	215TYR	OH	0.11
187ASN	N	187ASN	OD1	6.27
187ASN	N	187ASN	ND2	0.03
158SER	OG	158SER	O	0.06

71SER	OG	71SER	O	1.24
71SER	OG	275SER	OG	0.34

158SER	OG	158SER	O	0.05
154THR	OG1	153ASN	O	0.00
154THR	OG1	153ASN	O	2.43
153ASN	ND2	139TYR	O	0.04
153ASN	ND2	154THR	N	0.00
153ASN	ND2	154THR	O	0.01
139TYR	OH	237ASN	OD1	11.39
139TYR	OH	237ASN	ND2	0.24
139TYR	OH	237ASN	O	0.88
135THR	N	237ASN	ND2	0.08
116SER	OG	116SER	O	0.01
102ASN	ND2	259SER	OG	0.28
102ASN	ND2	261SER	N	0.08
102ASN	ND2	261SER	OG	64.11
97GLN	NE2	96SER	O	0.06
97GLN	NE2	266THR	N	0.00
97GLN	NE2	266THR	OG1	1.14
97GLN	NE2	266THR	O	0.16
96SER	OG	92SER	O	80.94
96SER	OG	97GLN	OE1	0.00
96SER	N	92SER	O	79.74
92SER	OG	88THR	O	10.23
92SER	OG	92SER	O	0.04
92SER	N	88THR	O	7.10
88THR	OG1	85SER	O	72.19
88THR	N	85SER	O	64.27
85SER	OG	85SER	O	0.06
85SER	OG	88THR	OG1	10.21
85SER	N	88THR	OG1	5.76
71SER	OG	71SER	O	1.76
71SER	OG	275SER	N	0.00
71SER	OG	275SER	OG	4.32

154THR	OG1	153ASN	O	0.32
147SER	N	147SER	O	0.00
139TYR	OH	237ASN	OD1	0.05
139TYR	OH	237ASN	O	0.06
139TYR	N	237ASN	OD1	0.97
139TYR	N	237ASN	ND2	0.39
135THR	OG1	135THR	O	0.01
135THR	N	157TYR	OH	0.01
102ASN	ND2	102ASN	O	0.00
102ASN	ND2	259SER	OG	5.36
102ASN	ND2	261SER	N	0.04
102ASN	ND2	261SER	OG	67.15
97GLN	NE2	96SER	O	0.34
97GLN	NE2	266THR	N	0.00
97GLN	NE2	266THR	OG1	5.42
97GLN	NE2	266THR	O	0.44
97GLN	N	92SER	O	0.99
97GLN	N	97GLN	OE1	1.46
97GLN	N	97GLN	NE2	0.05
96SER	OG	92SER	O	37.98
96SER	OG	96SER	O	0.11
96SER	OG	97GLN	N	0.02
96SER	OG	97GLN	OE1	1.18
96SER	OG	97GLN	NE2	0.23
96SER	N	92SER	O	46.13
96SER	N	97GLN	OE1	0.20
96SER	N	97GLN	O	0.37
92SER	OG	88THR	O	2.30
92SER	OG	92SER	O	0.01
92SER	N	88THR	O	1.96
88THR	OG1	187ASN	O	0.48
71SER	OG	71SER	O	0.66
71SER	OG	275SER	OG	0.05
