# Supplementary material for: Deciphering the Dynamics of Non-Covalent Interactions Affecting Thermal Stability of a Protein: Molecular Dynamics Study on Point Mutant of Thermus thermophilus Isopropylmalate Dehydrogenase
Source: PLoS One. 2015 Dec 11;10(12):e0144294. doi: 10.1371/journal.pone.0144294 (PMC4689552; doi:10.1371/journal.pone.0144294)
Supplement: S7 Table — The color formatting indicates the percentage of time interaction existed is as in S1 Table. (PDF) [file pone.0144294.s009.pdf]

S7 Table. Percentage of existence of unique HBs between CC of *wt* and *mut* at 300 K and 337 K.

1) *Wt* 300 K

Drnona	D	Arnona	A	percen
342ARC	NH2	321GLL	OE1	41.75
342ARC	NH1	321GLL	OE2	20.54
342ARC	NH1	321GLL	OE1	16.25
342ARC	NE	321GLL	OE2	30.87
342ARC	NE	321GLL	OE1	3.64
342ARC	NE	321GLL	OE2	2.02
326ASP	N	342ARC	O	0.04
326ASP	N	282LYS	O	0.07
326ASP	N	326ASF	OD1	0.02
321GLU	N	326ASF	OD2	0.02
317LYS	NZ	317LYS	O	14.57
317LYS	NZ	313ASF	OD1	77.89
317LYS	NZ	313ASF	OD2	25.69
317LYS	N	313ASF	O	0.18
313ASP	N	313ASF	O	77.30
313ASP	N	309ARC	O	53.59
312GLU	N	310LYS	O	10.00
310LYS	NZ	309ARC	O	0.73
310LYS	NZ	30GLU	OE1	8.20
310LYS	NZ	30GLU	OE2	8.76
310LYS	NZ	306GLL	OE2	0.03
310LYS	NZ	306GLL	O	0.01
310LYS	N	313ASF	OD1	0.10
309ARC	NH2	306GLL	O	27.71
309ARC	NH2	175LYS	O	17.21
309ARC	NH2	299GLL	OE1	0.84
309ARC	NH2	299GLL	OE2	29.92
309ARC	NH2	306GLL	OE1	16.40
309ARC	NH2	306GLL	OE2	23.28
309ARC	NH2	312GLL	OE1	0.52
309ARC	NH1	312GLL	OE2	9.40
309ARC	NH1	299GLL	OE1	31.53
309ARC	NH1	299GLL	OE2	2.71
309ARC	NH1	306GLL	OE2	0.02
309ARC	NH1	312GLL	OE1	23.02
309ARC	NE	312GLL	OE2	17.71
309ARC	NE	306GLL	OE1	14.28
309ARC	N	306GLL	OE2	8.38
306GLU	N	306GLL	O	3.54
306GLU	N	306GLL	OE1	14.44

2) *Wt* 337 K

Drnona	D	Arnona	A	percen
342ARG	NH2	317LYS	O	0.02
342ARG	NH2	321GLU	OE1	25.86
342ARG	NH2	321GLU	OE2	27.66
342ARG	NH1	321GLU	OE1	21.38
342ARG	NH1	321GLU	OE2	18.69
342ARG	NH1	342ARG	O	0.11
342ARG	NE	342ARG	O	0.18
334GLU	N	334GLU	OE1	0.48
334GLU	N	334GLU	OE2	0.50
326ASP	N	326ASP	OD1	0.60
326ASP	N	326ASP	OD2	0.66
321GLU	N	317LYS	O	29.37
317LYS	NZ	313ASP	OD1	54.19
317LYS	NZ	313ASP	OD2	50.61
317LYS	NZ	313ASP	O	0.54
317LYS	NZ	321GLU	OE1	0.00
317LYS	NZ	321GLU	OE2	0.03
317LYS	NZ	342ARG	O	0.03
317LYS	N	313ASP	O	79.08
313ASP	N	309ARG	O	73.29
313ASP	N	310LYS	O	3.90
312GLU	N	309ARG	O	1.52
312GLU	N	310LYS	O	0.00
310LYS	NZ	30GLU	OE1	5.85
310LYS	NZ	30GLU	OE2	6.33
310LYS	NZ	306GLU	OE1	0.50
310LYS	NZ	306GLU	OE2	0.95
310LYS	NZ	306GLU	O	0.09
310LYS	NZ	313ASP	OD1	0.02
310LYS	NZ	313ASP	OD2	0.02
310LYS	N	306GLU	O	37.26
309ARG	NH2	175LYS	O	6.16
309ARG	NH2	299GLU	OE1	2.55
309ARG	NH2	299GLU	OE2	13.46
309ARG	NH2	306GLU	OE1	31.42
309ARG	NH2	306GLU	OE2	30.98
309ARG	NH1	299GLU	OE1	14.82
309ARG	NH1	299GLU	OE2	4.24
309ARG	NH1	306GLU	OE1	10.12
309ARG	NH1	306GLU	OE2	7.27

3) *Mut* 300 K

Drnona	D	Arnona	A	percen
342ARG	NH2	321GLU	OE1	30.01
342ARG	NH2	321GLU	OE2	24.83
342ARG	NH1	321GLU	OE1	14.63
342ARG	NH1	321GLU	OE2	22.60
342ARG	NH1	342ARG	O	0.05
342ARG	NE	321GLU	OE1	3.84
342ARG	NE	321GLU	OE2	0.07
342ARG	NE	342ARG	O	0.25
326ASP	N	326ASP	OD1	0.01
326ASP	N	326ASP	OD2	0.02
321GLU	N	317LYS	O	43.39
317LYS	NZ	313ASP	OD1	36.91
317LYS	NZ	313ASP	OD2	69.11
317LYS	NZ	313ASP	O	0.16
317LYS	N	313ASP	O	73.28
313ASP	N	309ARG	O	65.40
313ASP	N	310LYS	O	6.52
312GLU	N	309ARG	O	1.03
310LYS	NZ	30GLU	OE1	1.39
310LYS	NZ	30GLU	OE2	4.26
310LYS	NZ	306GLU	OE1	0.65
310LYS	NZ	306GLU	OE2	0.10
310LYS	NZ	306GLU	O	0.04
310LYS	NZ	313ASP	OD2	0.00
310LYS	N	306GLU	O	44.68
309ARG	NH2	299GLU	OE1	0.37
309ARG	NH2	299GLU	OE2	4.03
309ARG	NH2	306GLU	OE1	51.46
309ARG	NH2	306GLU	OE2	40.73
309ARG	NH1	299GLU	OE1	4.83
309ARG	NH1	299GLU	OE2	2.28
309ARG	NH1	306GLU	OE1	0.52
309ARG	NH1	306GLU	OE2	10.43
309ARG	NH1	312GLU	OE1	0.03
309ARG	NH1	312GLU	OE2	0.42
309ARG	NH1	313ASP	OD2	0.00
309ARG	NE	306GLU	OE1	19.43
309ARG	NE	306GLU	OE2	23.94
309ARG	N	306GLU	O	3.56
306GLU	N	306GLU	OE2	0.00

4) *Mut* 337 K

Drnona	D	Arnona	A	percen
342ARG	NH2	321GLU	OE1	22.94
342ARG	NH2	321GLU	OE2	23.57
342ARG	NH1	321GLU	OE1	20.61
342ARG	NH1	321GLU	OE2	21.35
342ARG	NH1	342ARG	O	0.16
342ARG	NE	321GLU	OE1	0.01
342ARG	NE	321GLU	OE2	0.38
342ARG	NE	342ARG	O	0.08
334GLU	N	334GLU	OE1	0.05
334GLU	N	334GLU	OE2	0.02
321GLU	N	317LYS	O	19.61
317LYS	NZ	313ASP	OD1	53.18
317LYS	NZ	313ASP	OD2	47.01
317LYS	NZ	313ASP	O	0.34
317LYS	NZ	321GLU	OE1	0.26
317LYS	NZ	321GLU	OE2	1.11
317LYS	N	313ASP	O	77.91
313ASP	N	309ARG	O	80.06
313ASP	N	310LYS	O	2.28
312GLU	N	309ARG	O	1.37
312GLU	N	310LYS	O	0.00
310LYS	NZ	30GLU	OE1	11.72
310LYS	NZ	30GLU	OE2	8.60
310LYS	NZ	306GLU	OE1	0.04
310LYS	NZ	306GLU	OE2	0.52
310LYS	NZ	306GLU	O	0.06
310LYS	NZ	313ASP	OD1	0.00
310LYS	NZ	313ASP	OD2	0.05
310LYS	N	306GLU	O	37.87
309ARG	NH2	299GLU	OE1	2.76
309ARG	NH2	299GLU	OE2	5.10
309ARG	NH2	306GLU	OE1	29.89
309ARG	NH2	306GLU	OE2	31.59
309ARG	NH2	312GLU	OE1	0.04
309ARG	NH2	312GLU	OE2	1.45
309ARG	NH2	313ASP	OD2	0.16
309ARG	NH1	299GLU	OE1	6.21
309ARG	NH1	299GLU	OE2	5.00
309ARG	NH1	306GLU	OE1	17.04
309ARG	NH1	306GLU	OE2	10.31

282LYS	NZ	306GLL	OE2	9.94
282LYS	NZ	278ASF	OD1	34.76
282LYS	NZ	278ASF	OD2	41.61
282LYS	NZ	278ASF	O	2.98
282LYS	NZ	326ASF	OD1	3.27
282LYS	NZ	326ASF	OD2	0.16
282LYS	N	326ASF	O	1.10
278ASP	N	278ASF	O	27.60
278ASP	N	278ASF	OD1	0.04
264ARC	NH2	278ASF	OD2	0.02
264ARC	NH2	98ASP	OD1	5.71
264ARC	NH2	98ASP	OD2	10.41
264ARC	NH2	161GLL	OE1	0.02
264ARC	NH1	161GLL	OE2	0.00
264ARC	NH1	98ASP	OD1	6.08
264ARC	NH1	98ASP	OD2	2.14
264ARC	NE	264ARC	O	0.10
264ARC	NE	98ASP	OD1	32.15
264ARC	NE	98ASP	OD2	28.26
264ARC	N	264ARC	O	0.00
245ASP	N	98ASP	O	55.04
245ASP	N	241ASF	O	51.59
241ASP	N	245ASF	OD2	0.00
231ASP	N	241ASF	OD1	0.24
229ARC	NH2	229ARC	O	0.07
229ARC	NH2	212GLL	OE1	29.19
229ARC	NH2	212GLL	OE2	31.85
229ARC	NH1	229ARC	O	0.01
229ARC	NH1	212GLL	OE1	31.51
229ARC	NH1	212GLL	OE2	28.32
229ARC	NE	229ARC	O	9.73
225ARC	NH2	229ARC	O	10.34
225ARC	NH2	326ASF	OD1	46.44
225ARC	NH2	326ASF	OD2	5.14
225ARC	NH1	326ASF	O10	1.77
225ARC	NH1	326ASF	OD1	6.28
225ARC	NH1	326ASF	OD2	40.90
225ARC	NE	326ASF	O10	0.01
217ASP	N	225ARC	O	0.52
217ASP	N	217ASF	OD1	17.66
208ASP	N	217ASF	OD2	22.19
208ASP	N	208ASF	OD1	2.94
204ARC	NH2	208ASF	OD2	1.57

309ARG	NH1	312GLU	OE1	0.89
309ARG	NH1	312GLU	OE2	1.17
309ARG	NH1	313ASP	OD1	0.02
309ARG	NH1	313ASP	OD2	0.01
309ARG	NE	299GLU	OE1	0.06
309ARG	NE	306GLU	OE1	13.24
309ARG	NE	306GLU	OE2	15.41
309ARG	N	306GLU	O	3.66
306GLU	N	306GLU	OE1	2.08
306GLU	N	306GLU	OE2	0.51
282LYS	NZ	278ASP	OD1	43.23
282LYS	NZ	278ASP	OD2	43.89
282LYS	NZ	278ASP	O	2.02
282LYS	NZ	282LYS	O	0.01
282LYS	N	278ASP	O	28.76
278ASP	N	278ASP	OD1	0.05
278ASP	N	278ASP	OD2	0.01
264ARG	NH2	98ASP	OD1	38.90
264ARG	NH2	98ASP	OD2	28.39
264ARG	NH2	98ASP	O	0.01
264ARG	NH2	161GLU	OE1	0.47
264ARG	NH2	161GLU	OE2	2.72
264ARG	NH1	98ASP	OD1	4.58
264ARG	NH1	98ASP	OD2	8.27
264ARG	NH1	98ASP	O	1.06
264ARG	NH1	133GLU	OE1	0.05
264ARG	NH1	161GLU	OE1	3.03
264ARG	NH1	161GLU	OE2	2.02
264ARG	NE	98ASP	OD1	34.29
264ARG	NE	98ASP	OD2	46.43
264ARG	NE	98ASP	O	0.25
264ARG	N	98ASP	OD1	0.75
264ARG	N	98ASP	OD2	0.82
264ARG	N	98ASP	O	76.84
245ASP	N	241ASP	O	8.79
231ASP	N	229ARG	O	0.10
229ARG	NH2	212GLU	OE1	22.34
229ARG	NH2	212GLU	OE2	15.87
229ARG	NH2	229ARG	O	0.01
229ARG	NH2	278ASP	OD1	0.02
229ARG	NH2	278ASP	OD2	0.56
229ARG	NH1	212GLU	OE1	3.80
229ARG	NH1	212GLU	OE2	5.80

282LYS	NZ	278ASP	OD1	21.77
282LYS	NZ	278ASP	OD2	22.26
282LYS	NZ	278ASP	O	19.03
282LYS	N	278ASP	O	0.02
278ASP	N	278ASP	OD1	0.11
278ASP	N	278ASP	OD2	0.02
264ARG	NH2	98ASP	OD1	18.90
264ARG	NH2	98ASP	OD2	26.88
264ARG	NH1	98ASP	OD1	5.94
264ARG	NH1	98ASP	OD2	15.68
264ARG	NH1	98ASP	O	0.02
264ARG	NE	98ASP	OD1	25.06
264ARG	NE	98ASP	OD2	47.58
264ARG	NE	98ASP	O	0.01
264ARG	N	98ASP	OD1	0.00
264ARG	N	98ASP	OD2	0.00
264ARG	N	98ASP	O	74.49
245ASP	N	241ASP	O	49.53
231ASP	N	229ARG	O	0.00
229ARG	NH2	212GLU	OE1	0.98
229ARG	NH2	212GLU	OE2	1.46
229ARG	NH1	212GLU	OE2	0.04
229ARG	NH1	229ARG	O	0.02
229ARG	NE	212GLU	OE1	0.00
229ARG	NE	212GLU	OE2	0.00
229ARG	NE	229ARG	O	0.71
225ARG	NH2	326ASP	OD1	44.00
225ARG	NH2	326ASP	OD2	21.72
225ARG	NH2	326ASP	O10	0.33
225ARG	NH1	326ASP	OD1	11.02
225ARG	NH1	326ASP	OD2	36.50
217ASP	N	217ASP	OD1	30.69
217ASP	N	217ASP	OD2	0.00
208ASP	N	208ASP	OD1	18.73
208ASP	N	208ASP	OD2	21.77
204ARG	NH2	197LYS	O	0.00
204ARG	NH2	200GLU	OE1	21.76
204ARG	NH2	200GLU	OE2	74.71
204ARG	NH2	201GLU	OE1	47.23
204ARG	NH2	201GLU	OE2	23.90
204ARG	NH1	201GLU	OE1	26.81
204ARG	NH1	201GLU	OE2	44.41
204ARG	NE	200GLU	OE1	72.86

309ARG	NH1	309ARG	O	0.00
309ARG	NH1	312GLU	OE1	3.30
309ARG	NH1	312GLU	OE2	7.12
309ARG	NH1	313ASP	OD1	0.01
309ARG	NH1	313ASP	OD2	0.00
309ARG	NE	299GLU	OE2	0.01
309ARG	NE	306GLU	OE1	10.68
309ARG	NE	306GLU	OE2	13.05
309ARG	NE	312GLU	OE1	0.52
309ARG	NE	312GLU	OE2	0.07
309ARG	NE	313ASP	OD2	0.00
309ARG	N	306GLU	O	3.54
306GLU	N	306GLU	OE1	0.24
306GLU	N	306GLU	OE2	0.24
282LYS	NZ	278ASP	OD1	44.93
282LYS	NZ	278ASP	OD2	44.58
282LYS	NZ	278ASP	O	5.23
282LYS	N	14GLU	OE1	0.04
282LYS	N	14GLU	OE2	0.12
282LYS	N	278ASP	O	4.10
278ASP	N	278ASP	OD1	0.00
278ASP	N	278ASP	OD2	0.00
264ARG	NH2	62GLU	OE1	13.56
264ARG	NH2	62GLU	OE2	9.01
264ARG	NH2	98ASP	OD1	7.83
264ARG	NH2	98ASP	OD2	0.73
264ARG	NH2	163GLU	OE1	0.00
264ARG	NH2	163GLU	OE2	0.13
264ARG	NH1	62GLU	OE1	9.29
264ARG	NH1	62GLU	OE2	13.99
264ARG	NH1	98ASP	OD1	21.90
264ARG	NH1	98ASP	OD2	3.19
264ARG	NH1	98ASP	O	0.26
264ARG	NH1	264ARG	O	0.08
264ARG	NE	62GLU	OE2	0.00
264ARG	NE	98ASP	OD1	26.71
264ARG	NE	98ASP	OD2	0.89
264ARG	NE	264ARG	O	0.29
264ARG	N	98ASP	OD1	0.00
264ARG	N	98ASP	O	41.24
245ASP	N	241ASP	O	24.22
231ASP	N	229ARG	O	0.08
229ARG	NH2	212GLU	OE1	7.28

204ARG	NH2	197LYS	O	0.00
204ARG	NH2	200GLU	OE1	14.65
204ARG	NH2	200GLU	OE2	61.81
204ARG	NH2	200GLU	O	0.00
204ARG	NH2	201GLU	OE1	21.79
204ARG	NH1	201GLU	OE2	30.01
204ARG	NH1	200GLU	OE1	0.51
204ARG	NH1	200GLU	OE2	0.28
204ARG	NH1	200GLU	O	0.12
204ARG	NH1	201GLU	OE1	34.23
204ARG	NH1	201GLU	OE2	32.21
204ARG	NE	201GLU	O	0.04
204ARG	NE	200GLU	OE1	56.73
204ARG	NE	200GLU	OE2	18.18
204ARG	N	200GLU	O	0.09
204ARG	N	200GLU	O	35.85
201GLU	N	201GLU	O	19.69
200GLU	N	197LYS	O	51.49
200GLU	N	196ARG	O	28.85
197LYS	NZ	197LYS	O	8.09
197LYS	NZ	193GLU	OE1	2.94
197LYS	NZ	193GLU	OE2	2.23
197LYS	NZ	193GLU	O	0.18
197LYS	NZ	201GLU	OE1	0.02
197LYS	NZ	201GLU	OE2	0.28
197LYS	NZ	148GLU	OE1	18.08
197LYS	NZ	148GLU	OE2	23.75
197LYS	N	148GLU	O	0.70
196ARG	NH2	193GLU	O	44.68
196ARG	NH2	184ASP	OD1	93.68
196ARG	NH1	184ASP	OD2	7.04
196ARG	NE	193GLU	OE2	0.06
196ARG	NE	184ASP	OD1	13.45
196ARG	N	184ASP	OD2	91.45
193GLU	N	193GLU	O	1.19
190GLU	N	190GLU	O	4.98
190GLU	N	190GLU	OE1	6.73
190GLU	N	190GLU	OE2	2.10
185LYS	NZ	144ARG	NH2	0.01
185LYS	NZ	217ASP	OD1	49.25
185LYS	NZ	217ASP	OD2	49.61
185LYS	NZ	241ASP	OD1	37.27
185LYS	N	241ASP	OD2	62.17

229ARG	NH1	229ARG	O	0.36
229ARG	NH1	278ASP	OD1	0.13
229ARG	NH1	278ASP	OD2	0.78
229ARG	NE	212GLU	OE1	1.23
229ARG	NE	212GLU	OE2	1.06
229ARG	NE	229ARG	O	1.44
225ARG	NH2	278ASP	OD1	24.01
225ARG	NH2	278ASP	OD2	16.16
225ARG	NH2	278ASP	O	0.00
225ARG	NH2	326ASP	OD1	74.46
225ARG	NH2	326ASP	OD2	14.03
225ARG	NH2	326ASP	O	0.24
225ARG	NH1	278ASP	OD2	0.03
225ARG	NH1	326ASP	OD1	9.33
225ARG	NH1	326ASP	OD2	70.50
225ARG	NH1	326ASP	O	0.03
225ARG	NE	278ASP	OD1	0.72
225ARG	NE	278ASP	OD2	0.49
217ASP	N	217ASP	OD1	30.59
217ASP	N	217ASP	OD2	7.10
208ASP	N	208ASP	OD1	0.08
208ASP	N	208ASP	OD2	0.04
204ARG	NH2	197LYS	O	0.12
204ARG	NH2	200GLU	OE1	23.92
204ARG	NH2	200GLU	OE2	11.57
204ARG	NH2	201GLU	OE1	25.62
204ARG	NH2	201GLU	OE2	33.83
204ARG	NH1	200GLU	OE1	5.92
204ARG	NH1	200GLU	OE2	10.18
204ARG	NH1	201GLU	OE1	2.69
204ARG	NH1	201GLU	OE2	2.72
204ARG	NH1	212GLU	OE1	0.02
204ARG	NH1	212GLU	OE2	0.03
204ARG	NE	200GLU	OE1	10.56
204ARG	NE	200GLU	OE2	23.30
204ARG	NE	200GLU	O	0.02
204ARG	NE	201GLU	OE1	25.25
204ARG	NE	201GLU	OE2	19.24
204ARG	N	200GLU	O	36.06
204ARG	N	201GLU	O	24.15
201GLU	N	197LYS	O	30.88
200GLU	N	196ARG	O	66.67
200GLU	N	197LYS	O	2.08

204ARG	NE	200GLU	OE2	27.00
204ARG	NE	200GLU	O	0.02
204ARG	N	200GLU	O	44.39
204ARG	N	201GLU	O	22.38
201GLU	N	197LYS	O	27.30
200GLU	N	196ARG	O	28.37
200GLU	N	197LYS	O	7.19
197LYS	NZ	193GLU	OE1	2.15
197LYS	NZ	193GLU	OE2	2.21
197LYS	NZ	193GLU	O	0.08
197LYS	NZ	148GLU	OE1	26.38
197LYS	NZ	148GLU	OE2	23.78
197LYS	NZ	148GLU	O	0.27
197LYS	N	193GLU	O	46.74
196ARG	NH2	184ASP	OD1	94.25
196ARG	NH2	184ASP	OD2	5.32
196ARG	NH1	193GLU	OE1	0.00
196ARG	NE	184ASP	OD1	7.49
196ARG	NE	184ASP	OD2	93.26
196ARG	N	193GLU	O	0.26
193GLU	N	190GLU	O	5.27
190GLU	N	190GLU	OE1	6.06
190GLU	N	190GLU	OE2	2.88
185LYS	NZ	217ASP	OD1	73.22
185LYS	NZ	217ASP	OD2	2.73
185LYS	NZ	241ASP	OD1	53.71
185LYS	NZ	241ASP	OD2	14.40
185LYS	N	184ASP	OD1	0.01
185LYS	N	184ASP	OD2	2.32
178LYS	NZ	174ARG	O	0.07
178LYS	NZ	176ARG	O	0.92
178LYS	NZ	208ASP	OD1	42.97
178LYS	NZ	208ASP	OD2	35.93
178LYS	NZ	208ASP	O	0.16
178LYS	N	176ARG	O	0.12
178LYS	N	231ASP	OD2	0.46
177ARG	NH2	127ASP	OD2	3.60
177ARG	NH2	127ASP	O	1.15
177ARG	NH2	229ARG	O	0.02
177ARG	NH2	231ASP	OD1	36.49
177ARG	NH2	231ASP	OD2	2.64
177ARG	NH1	127ASP	OD2	1.17
177ARG	NH1	229ARG	O	24.94

229ARG	NH2	212GLU	OE2	5.45
229ARG	NH2	229ARG	O	0.01
229ARG	NH1	212GLU	OE1	2.72
229ARG	NH1	212GLU	OE2	5.19
229ARG	NH1	229ARG	O	2.84
229ARG	NE	212GLU	OE1	0.22
229ARG	NE	212GLU	OE2	0.16
229ARG	NE	229ARG	O	1.12
225ARG	NH2	326ASP	OD1	73.65
225ARG	NH2	326ASP	OD2	16.52
225ARG	NH2	326ASP	O	3.23
225ARG	NH1	326ASP	OD1	9.18
225ARG	NH1	326ASP	OD2	75.98
225ARG	NH1	326ASP	O	0.04
217ASP	N	217ASP	OD1	15.31
217ASP	N	217ASP	OD2	3.23
208ASP	N	208ASP	OD1	18.57
208ASP	N	208ASP	OD2	24.39
204ARG	NH2	200GLU	OE1	48.77
204ARG	NH2	200GLU	OE2	44.88
204ARG	NH2	200GLU	O	0.00
204ARG	NH2	201GLU	OE1	34.84
204ARG	NH2	201GLU	OE2	34.16
204ARG	NH1	200GLU	OE1	0.00
204ARG	NH1	201GLU	OE1	29.50
204ARG	NH1	201GLU	OE2	31.97
204ARG	NE	200GLU	OE1	45.45
204ARG	NE	200GLU	OE2	50.84
204ARG	NE	200GLU	O	0.13
204ARG	N	200GLU	O	40.84
204ARG	N	201GLU	O	20.09
201GLU	N	197LYS	O	53.82
200GLU	N	196ARG	O	26.05
200GLU	N	197LYS	O	11.74
197LYS	NZ	193GLU	OE1	0.23
197LYS	NZ	193GLU	OE2	1.30
197LYS	NZ	197LYS	O	0.00
197LYS	NZ	200GLU	OE1	0.54
197LYS	NZ	200GLU	OE2	0.04
197LYS	NZ	201GLU	OE1	3.95
197LYS	NZ	201GLU	OE2	3.44
197LYS	NZ	148GLU	OE1	30.19
197LYS	NZ	148GLU	OE2	30.16

185LYS	N	184ASF	OD1	0.00
178LYS	NZ	184ASF	OD2	1.31
178LYS	NZ	174ARC	O	0.05
178LYS	NZ	176ARC	O	0.14
178LYS	NZ	208ASF	OD1	45.17
178LYS	NZ	208ASF	OD2	51.13
178LYS	N	208ASF	O	0.09
178LYS	N	176ARC	O	0.08
177ARC	NH2	231ASF	OD2	0.11
177ARC	NH2	127ASF	OD1	16.13
177ARC	NH2	127ASF	OD2	9.50
177ARC	NH2	127ASF	O	13.47
177ARC	NH2	229ARC	O	0.05
177ARC	NH2	231ASF	OD1	27.75
177ARC	NH1	231ASF	OD2	0.00
177ARC	NH1	127ASF	OD2	0.14
177ARC	NE	229ARC	O	1.51
177ARC	NE	231ASF	OD1	18.99
177ARC	N	231ASF	OD2	8.04
177ARC	N	231ASF	OD1	0.12
176ARC	NH2	231ASF	OD2	93.14
176ARC	NH2	127ASF	OD1	7.05
176ARC	NH2	127ASF	OD2	17.65
176ARC	NH2	127ASF	O	0.00
176ARC	NH2	299GLL	OE1	0.01
176ARC	NH1	299GLL	OE2	0.01
176ARC	NH1	127ASF	OD1	22.99
176ARC	NH1	127ASF	OD2	47.43
176ARC	NH1	127ASF	O	23.13
176ARC	NH1	231ASF	OD1	94.57
176ARC	NH1	231ASF	OD2	0.58
176ARC	NE	231ASF	O	0.10
176ARC	N	299GLL	OE2	0.07
175LYS	NZ	174ARC	O	0.32
175LYS	NZ	171GLL	OE1	1.81
175LYS	NZ	171GLL	OE2	0.91
175LYS	NZ	171GLL	O	3.74
175LYS	NZ	299GLL	OE1	35.89
175LYS	NZ	299GLL	OE2	37.74
175LYS	N	299GLL	O	4.62
174ARC	NH2	171GLL	O	1.14
174ARC	NH2	171GLL	OE1	48.43
174ARC	NH2	171GLL	OE2	50.15

197LYS	NZ	193GLU	OE1	4.82
197LYS	NZ	193GLU	OE2	3.80
197LYS	NZ	193GLU	O	0.15
197LYS	NZ	201GLU	OE1	2.09
197LYS	NZ	201GLU	OE2	2.46
197LYS	NZ	148GLU	OE1	21.65
197LYS	NZ	148GLU	OE2	19.58
197LYS	NZ	148GLU	O	0.06
197LYS	N	193GLU	O	73.29
196ARG	NH2	184ASP	OD1	18.30
196ARG	NH2	184ASP	OD2	89.63
196ARG	NH2	78ASP	OD1	0.00
196ARG	NH1	193GLU	OE2	0.00
196ARG	NH1	78ASP	OD1	0.79
196ARG	NH1	78ASP	OD2	0.68
196ARG	NE	184ASP	OD1	96.82
196ARG	NE	184ASP	OD2	3.79
196ARG	N	193GLU	O	0.04
193GLU	N	190GLU	O	3.03
190GLU	N	190GLU	OE1	2.84
190GLU	N	190GLU	OE2	2.32
185LYS	NZ	217ASP	OD1	69.20
185LYS	NZ	217ASP	OD2	15.72
185LYS	NZ	241ASP	OD1	64.09
185LYS	NZ	241ASP	OD2	17.36
185LYS	N	184ASP	OD1	0.03
185LYS	N	184ASP	OD2	0.21
178LYS	NZ	174ARG	O	0.07
178LYS	NZ	176ARG	O	0.06
178LYS	NZ	177ARG	O	0.01
178LYS	NZ	208ASP	OD1	47.95
178LYS	NZ	208ASP	OD2	53.82
178LYS	NZ	208ASP	O	0.25
178LYS	N	176ARG	O	0.16
178LYS	N	231ASP	OD1	6.91
178LYS	N	231ASP	OD2	6.78
177ARG	NH2	127ASP	OD1	29.73
177ARG	NH2	127ASP	OD2	5.62
177ARG	NH2	127ASP	O	10.93
177ARG	NH2	229ARG	O	1.15
177ARG	NH2	231ASP	OD1	24.49
177ARG	NH2	231ASP	OD2	13.60
177ARG	NH2	309ARG	NH2	0.00

177ARG	NH1	231ASP	OD1	0.00
177ARG	NH1	231ASP	OD2	18.64
177ARG	NE	231ASP	OD1	0.34
177ARG	NE	231ASP	OD2	37.21
177ARG	N	177ARG	NE	1.22
177ARG	N	177ARG	NH2	0.08
177ARG	N	231ASP	OD1	3.14
177ARG	N	231ASP	OD2	6.82
176ARG	NH2	127ASP	OD1	61.18
176ARG	NH2	127ASP	OD2	0.91
176ARG	NH2	127ASP	O	0.01
176ARG	NH1	127ASP	OD1	35.15
176ARG	NH1	127ASP	OD2	14.10
176ARG	NH1	127ASP	O	45.69
176ARG	NH1	177ARG	NH2	0.00
176ARG	NH1	231ASP	OD1	69.40
176ARG	NH1	231ASP	OD2	0.00
176ARG	NH1	231ASP	O	0.10
176ARG	N	174ARG	O	0.18
175LYS	NZ	171GLU	OE1	0.19
175LYS	NZ	171GLU	OE2	0.02
175LYS	NZ	171GLU	O	0.00
175LYS	NZ	299GLU	OE1	52.68
175LYS	NZ	299GLU	OE2	39.88
175LYS	NZ	299GLU	O	0.28
175LYS	N	171GLU	O	1.23
175LYS	N	176ARG	O	0.11
174ARG	NH2	208ASP	OD1	39.07
174ARG	NH2	208ASP	OD2	45.15
174ARG	NH1	171GLU	OE1	6.84
174ARG	NH1	171GLU	OE2	29.83
174ARG	NE	208ASP	OD1	46.51
174ARG	NE	208ASP	OD2	40.74
174ARG	N	171GLU	O	57.85
171GLU	N	167ARG	O	30.54
167ARG	NH2	163GLU	OE1	21.19
167ARG	NH2	163GLU	OE2	27.51
167ARG	NH2	171GLU	OE2	0.02
167ARG	NH2	201GLU	OE2	0.04
167ARG	NH2	201GLU	O	0.01
167ARG	NH1	163GLU	OE1	1.90
167ARG	NH1	163GLU	OE2	0.21
167ARG	NE	163GLU	OE1	40.67

197LYS	NZ	148GLU	O	1.54
197LYS	N	193GLU	O	50.49
196ARG	NH2	184ASP	OD1	71.17
196ARG	NH2	184ASP	OD2	31.86
196ARG	NH2	78ASP	OD1	0.56
196ARG	NH2	78ASP	OD2	0.24
196ARG	NH1	193GLU	OE1	0.22
196ARG	NH1	193GLU	OE2	0.07
196ARG	NH1	78ASP	OD1	0.26
196ARG	NH1	78ASP	OD2	0.43
196ARG	NH1	82ARG	NE	0.00
196ARG	NH1	82ARG	NH1	0.00
196ARG	NE	184ASP	OD1	33.89
196ARG	NE	184ASP	OD2	68.32
196ARG	N	193GLU	O	2.80
193GLU	N	190GLU	O	4.08
190GLU	N	190GLU	OE1	19.39
190GLU	N	190GLU	OE2	35.31
185LYS	NZ	217ASP	OD1	48.54
185LYS	NZ	217ASP	OD2	25.65
185LYS	NZ	241ASP	OD1	48.91
185LYS	NZ	241ASP	OD2	27.27
185LYS	N	184ASP	OD1	0.12
185LYS	N	184ASP	OD2	0.65
178LYS	NZ	174ARG	O	1.02
178LYS	NZ	176ARG	O	1.37
178LYS	NZ	177ARG	O	0.04
178LYS	NZ	208ASP	OD1	34.33
178LYS	NZ	208ASP	OD2	21.35
178LYS	NZ	208ASP	O	0.33
178LYS	N	176ARG	O	0.08
178LYS	N	231ASP	OD2	2.54
177ARG	NH2	127ASP	OD1	1.49
177ARG	NH2	127ASP	OD2	33.56
177ARG	NH2	127ASP	O	2.94
177ARG	NH2	229ARG	O	0.04
177ARG	NH2	231ASP	OD1	31.22
177ARG	NH2	231ASP	OD2	25.37
177ARG	NH1	127ASP	OD1	0.03
177ARG	NH1	127ASP	OD2	9.13
177ARG	NH1	127ASP	O	1.79
177ARG	NH1	229ARG	O	1.11
177ARG	NH1	231ASP	OD1	13.15

174ARG	NH2	208ASP	OD1	0.02	177ARG	NH1	127ASP	OD1	5.05	167ARG	NE	163GLU	OE2	43.71	177ARG	NH1	231ASP	OD2	0.03
174ARG	NH1	208ASP	OD2	4.19	177ARG	NH1	127ASP	OD2	0.70	167ARG	NE	163GLU	O	0.01	177ARG	NE	231ASP	OD1	32.14
174ARG	NH1	208ASP	OD1	4.77	177ARG	NH1	127ASP	O	0.36	167ARG	N	163GLU	O	93.24	177ARG	NE	231ASP	OD2	25.53
174ARG	NE	208ASP	OD2	6.21	177ARG	NH1	177ARG	O	0.26	167ARG	N	164ARG	O	0.84	177ARG	N	177ARG	NE	0.25
174ARG	NE	171GLU	OE1	25.62	177ARG	NH1	229ARG	O	1.16	164ARG	NH2	133GLU	OE1	42.45	177ARG	N	231ASP	OD1	10.64
174ARG	NE	171GLU	OE2	30.53	177ARG	NH1	231ASP	OD1	1.95	164ARG	NH2	133GLU	OE2	63.97	177ARG	N	231ASP	OD2	44.19
174ARG	N	171GLU	O	0.20	177ARG	NH1	231ASP	OD2	0.95	164ARG	NH2	161GLU	OE1	24.31	176ARG	NH2	127ASP	OD1	69.77
171GLU	N	171GLU	O	21.28	177ARG	NE	127ASP	OD1	0.18	164ARG	NH2	161GLU	OE2	23.66	176ARG	NH2	127ASP	OD2	6.41
167ARG	NH2	167ARG	O	79.64	177ARG	NE	127ASP	OD2	0.04	164ARG	NH1	98ASP	OD1	0.02	176ARG	NH2	127ASP	O	0.07
167ARG	NH2	163GLU	OE1	29.91	177ARG	NE	231ASP	OD1	10.68	164ARG	NH1	161GLU	OE1	5.25	176ARG	NH1	127ASP	OD1	10.70
167ARG	NH1	163GLU	OE2	37.64	177ARG	NE	231ASP	OD2	25.34	164ARG	NH1	161GLU	OE2	4.34	176ARG	NH1	127ASP	OD2	7.68
167ARG	NH1	163GLU	OE1	38.54	177ARG	N	177ARG	NE	0.06	164ARG	NH1	264ARG	NH2	0.04	176ARG	NH1	127ASP	O	71.17
167ARG	NH1	163GLU	OE2	26.34	177ARG	N	231ASP	OD1	36.55	164ARG	NE	133GLU	OE1	33.27	176ARG	NH1	177ARG	NH1	0.00
167ARG	NE	163GLU	O	0.03	177ARG	N	231ASP	OD2	36.09	164ARG	NE	133GLU	OE2	46.03	176ARG	NH1	177ARG	NH2	0.00
167ARG	NE	163GLU	OE1	1.71	176ARG	NH2	127ASP	OD1	2.55	164ARG	NE	161GLU	O	2.69	176ARG	NH1	231ASP	OD1	62.55
167ARG	N	163GLU	OE2	0.82	176ARG	NH2	127ASP	OD2	27.12	164ARG	N	161GLU	O	4.58	176ARG	NH1	231ASP	OD2	32.83
167ARG	N	163GLU	O	82.00	176ARG	NH2	127ASP	O	0.06	163GLU	N	159LYS	O	52.40	176ARG	NH1	231ASP	O	0.12
164ARG	NH2	164ARG	O	4.66	176ARG	NH2	299GLU	OE1	0.00	161GLU	N	159LYS	O	0.02	176ARG	N	174ARG	O	0.40
164ARG	NH2	98ASP	OD1	43.88	176ARG	NH1	127ASP	OD1	12.37	161GLU	N	161GLU	OE1	0.02	175LYS	NZ	171GLU	OE1	1.02
164ARG	NH2	98ASP	OD2	37.47	176ARG	NH1	127ASP	OD2	36.36	159LYS	NZ	163GLU	OE1	5.83	175LYS	NZ	171GLU	OE2	0.59
164ARG	NH2	98ASP	O	0.52	176ARG	NH1	127ASP	O	41.11	159LYS	NZ	163GLU	OE2	11.45	175LYS	NZ	171GLU	O	0.99
164ARG	NH2	161GLU	OE1	0.04	176ARG	NH1	177ARG	NH2	0.00	159LYS	NZ	197LYS	O	0.00	175LYS	NZ	174ARG	O	0.00
164ARG	NH2	161GLU	OE2	0.11	176ARG	NH1	231ASP	OD1	50.88	159LYS	NZ	201GLU	OE1	8.44	175LYS	NZ	175LYS	O	0.00
164ARG	NH2	264ARG	N	0.02	176ARG	NH1	231ASP	OD2	42.51	159LYS	NZ	201GLU	OE2	7.10	175LYS	NZ	299GLU	OE1	29.66
164ARG	NH2	264ARG	NE	0.12	176ARG	NH1	231ASP	O	0.10	159LYS	NZ	148GLU	OE1	6.65	175LYS	NZ	299GLU	OE2	25.06
164ARG	NH2	264ARG	NH1	0.02	176ARG	NE	299GLU	OE2	0.10	159LYS	NZ	148GLU	OE2	6.87	175LYS	NZ	299GLU	O	0.55
164ARG	NH1	264ARG	NH2	0.01	176ARG	N	174ARG	O	0.49	159LYS	NZ	148GLU	O	1.27	175LYS	N	171GLU	O	11.78
164ARG	NH1	98ASP	OD1	22.07	175LYS	NZ	171GLU	OE1	1.12	156ARG	NH2	142GLU	O	29.95	174ARG	NH2	208ASP	OD1	41.52
164ARG	NH1	98ASP	OD2	44.06	175LYS	NZ	171GLU	OE2	0.55	156ARG	NH2	150GLU	OE1	2.78	174ARG	NH2	208ASP	OD2	57.11
164ARG	NH1	133GLU	OE2	1.31	175LYS	NZ	171GLU	O	2.34	156ARG	NH2	150GLU	OE2	4.22	174ARG	NH1	171GLU	OE1	14.87
164ARG	NH1	161GLU	OE1	16.36	175LYS	NZ	299GLU	OE1	35.38	156ARG	NH1	142GLU	O	4.89	174ARG	NH1	171GLU	OE2	11.34
164ARG	NH1	161GLU	OE2	18.98	175LYS	NZ	299GLU	OE2	33.34	156ARG	NH1	150GLU	OE1	28.23	174ARG	NE	208ASP	OD1	58.33
164ARG	N	264ARG	NH2	0.02	175LYS	NZ	299GLU	O	6.14	156ARG	NH1	150GLU	OE2	61.30	174ARG	NE	208ASP	OD2	44.35
163GLU	N	161GLU	O	37.88	175LYS	N	171GLU	O	1.84	156ARG	NE	142GLU	O	52.97	174ARG	N	171GLU	O	23.78
161GLU	N	159LYS	O	47.07	174ARG	NH2	171GLU	OE1	46.30	156ARG	NE	150GLU	OE1	5.92	171GLU	N	167ARG	O	58.60
161GLU	N	159LYS	O	0.08	174ARG	NH2	171GLU	OE2	45.36	156ARG	NE	150GLU	OE2	3.75	167ARG	NH2	163GLU	OE1	36.11
159LYS	NZ	161GLU	OE2	0.04	174ARG	NH2	208ASP	OD2	0.25	156ARG	N	155GLU	OE1	6.39	167ARG	NH2	163GLU	OE2	27.67
159LYS	NZ	163GLU	OE1	15.57	174ARG	NH1	171GLU	OE1	9.96	144ARG	NH2	190GLU	OE1	47.41	167ARG	NH2	171GLU	OE1	0.01
159LYS	NZ	163GLU	OE2	12.60	174ARG	NH1	171GLU	OE2	13.90	144ARG	NH2	190GLU	OE2	47.67	167ARG	NH2	201GLU	OE2	0.17
159LYS	NZ	197LYS	O	0.00	174ARG	NH1	208ASP	OD1	0.87	144ARG	NH1	142GLU	OE1	0.01	167ARG	NH2	201GLU	O	0.02
159LYS	NZ	201GLU	OE1	11.26	174ARG	NH1	208ASP	OD2	0.08	144ARG	NH1	142GLU	OE2	0.55	167ARG	NH1	163GLU	OE1	7.46
159LYS	NZ	201GLU	OE2	6.06	174ARG	NE	171GLU	OE1	23.94	144ARG	NE	190GLU	OE1	50.77	167ARG	NH1	163GLU	OE2	5.49
159LYS	NZ	148GLU	OE1	12.97	174ARG	NE	171GLU	OE2	19.29	144ARG	NE	190GLU	OE2	59.61	167ARG	NH1	163GLU	O	0.03

159LYS	NZ	148GLL	OE2	12.73
156ARC	NH2	148GLL	O	1.47
156ARC	NH2	142GLL	O	31.86
156ARC	NH2	150GLL	OE1	0.66
156ARC	NH1	150GLL	OE2	0.38
156ARC	NH1	150GLL	OE1	56.90
156ARC	NE	150GLL	OE2	39.56
156ARC	NE	142GLL	O	9.52
156ARC	NE	150GLL	OE1	0.06
156ARC	N	150GLL	OE2	0.56
148GLU	N	155GLL	OE1	64.35
148GLU	N	148GLL	OE1	0.23
144ARC	NH2	148GLL	OE2	0.08
144ARC	NH2	190GLL	OE1	43.81
144ARC	NH1	190GLL	OE2	42.78
144ARC	NH1	142GLL	OE1	0.24
144ARC	NE	142GLL	OE2	0.50
144ARC	NE	190GLL	OE1	44.76
144ARC	N	190GLL	OE2	37.12
144ARC	N	142GLL	OE1	0.76
142GLU	N	142GLL	OE2	0.93
132ARC	NH2	142GLL	OE1	0.01
132ARC	NH2	133GLL	O	1.56
132ARC	NH2	241ASF	OD1	8.61
132ARC	NH1	241ASF	OD2	9.44
132ARC	NH1	241ASF	OD1	4.24
132ARC	NE	241ASF	OD2	2.63
124ARC	NH2	133GLL	O	35.23
124ARC	NH2	113GLL	OE1	0.03
124ARC	NH2	113GLL	OE2	2.38
124ARC	NH2	120GLL	OE1	17.17
124ARC	NH2	120GLL	OE2	20.28
124ARC	NH2	121GLL	OE1	49.21
124ARC	NH1	121GLL	OE2	40.22
124ARC	NH1	113GLL	OE1	0.06
124ARC	NH1	113GLL	OE2	40.25
124ARC	NH1	120GLL	OE1	28.25
124ARC	NH1	120GLL	OE2	27.95
124ARC	NH1	121GLL	OE1	7.30
124ARC	NE	121GLL	OE2	7.67
124ARC	NE	113GLL	OE2	0.42
124ARC	NE	120GLL	OE1	3.12
124ARC	NE	120GLL	OE2	0.05

174ARG	NE	171GLU	O	0.08
174ARG	NE	208ASP	OD2	0.29
174ARG	N	171GLU	O	19.47
171GLU	N	167ARG	O	68.43
167ARG	NH2	163GLU	OE1	35.55
167ARG	NH2	163GLU	OE2	29.20
167ARG	NH2	171GLU	OE2	1.60
167ARG	NH1	163GLU	OE1	5.59
167ARG	NH1	163GLU	OE2	7.71
167ARG	NH1	163GLU	O	0.00
167ARG	NE	163GLU	OE1	32.47
167ARG	NE	163GLU	OE2	34.91
167ARG	NE	163GLU	O	0.03
167ARG	NE	167ARG	O	0.02
167ARG	NE	171GLU	OE2	0.00
167ARG	N	163GLU	O	82.14
167ARG	N	164ARG	O	3.88
164ARG	NH2	133GLU	OE1	0.02
164ARG	NH2	133GLU	OE2	0.08
164ARG	NH2	161GLU	OE1	32.77
164ARG	NH2	161GLU	OE2	27.55
164ARG	NH1	133GLU	OE1	0.31
164ARG	NH1	133GLU	OE2	0.82
164ARG	NH1	161GLU	OE1	42.43
164ARG	NH1	161GLU	OE2	49.74
164ARG	NH1	161GLU	O	0.01
164ARG	NH1	264ARG	NH1	0.02
164ARG	NE	163GLU	OE2	0.00
164ARG	NE	167ARG	NH2	0.00
164ARG	N	161GLU	O	6.56
163GLU	N	159LYS	O	66.69
163GLU	N	161GLU	O	0.01
161GLU	N	159LYS	O	0.06
161GLU	N	161GLU	OE1	0.08
161GLU	N	161GLU	OE2	0.08
159LYS	NZ	163GLU	OE1	2.09
159LYS	NZ	163GLU	OE2	3.56
159LYS	NZ	197LYS	O	0.02
159LYS	NZ	201GLU	OE1	11.80
159LYS	NZ	201GLU	OE2	12.26
159LYS	NZ	148GLU	OE1	21.60
159LYS	NZ	148GLU	OE2	22.98
159LYS	NZ	148GLU	O	3.07

144ARG	N	142GLU	OE1	0.18
144ARG	N	142GLU	OE2	0.28
142GLU	N	142GLU	OE1	0.03
142GLU	N	142GLU	OE2	0.01
132ARG	NH2	133GLU	O	0.00
132ARG	NH2	241ASP	OD1	0.28
132ARG	NH2	241ASP	OD2	9.80
132ARG	NH2	245ASP	OD1	0.02
132ARG	NH2	245ASP	OD2	0.02
132ARG	NH1	241ASP	OD1	6.10
132ARG	NH1	241ASP	OD2	13.97
132ARG	NH1	245ASP	OD1	0.07
132ARG	NH1	245ASP	OD2	0.64
132ARG	NE	133GLU	O	78.81
124ARG	NH2	113GLU	OE1	1.88
124ARG	NH2	113GLU	OE2	5.08
124ARG	NH2	120GLU	OE1	24.58
124ARG	NH2	120GLU	OE2	33.44
124ARG	NH2	120GLU	O	0.00
124ARG	NH2	121GLU	OE1	22.92
124ARG	NH2	121GLU	OE2	21.36
124ARG	NH1	113GLU	OE1	0.03
124ARG	NH1	120GLU	OE1	7.21
124ARG	NH1	120GLU	OE2	8.57
124ARG	NH1	121GLU	OE1	37.19
124ARG	NH1	121GLU	OE2	34.15
124ARG	NE	113GLU	OE1	4.81
124ARG	NE	113GLU	OE2	1.10
124ARG	NE	120GLU	OE1	14.88
124ARG	NE	120GLU	OE2	7.71
124ARG	NE	120GLU	O	0.02
124ARG	N	120GLU	O	19.82
124ARG	N	121GLU	O	7.65
121GLU	N	120GLU	OE2	0.00
121GLU	N	121GLU	OE1	2.53
121GLU	N	121GLU	OE2	8.81
119LYS	NZ	113GLU	O	7.57
119LYS	NZ	114ARG	O	22.27
119LYS	NZ	120GLU	OE1	4.42
119LYS	NZ	120GLU	OE2	11.73
114ARG	NH2	120GLU	OE1	22.30
114ARG	NH2	120GLU	OE2	10.97
114ARG	NH1	120GLU	OE1	0.15

167ARG	NH1	201GLU	O	0.00
167ARG	NE	163GLU	OE1	33.69
167ARG	NE	163GLU	OE2	34.03
167ARG	NE	163GLU	O	0.02
167ARG	NE	171GLU	OE1	0.01
167ARG	N	163GLU	O	89.06
167ARG	N	164ARG	O	2.34
164ARG	NH2	98ASP	OD1	18.97
164ARG	NH2	98ASP	OD2	50.27
164ARG	NH2	98ASP	O	0.57
164ARG	NH2	133GLU	OE1	5.44
164ARG	NH2	133GLU	OE2	24.14
164ARG	NH2	161GLU	OE1	9.32
164ARG	NH2	161GLU	OE2	8.20
164ARG	NH1	98ASP	OD1	51.67
164ARG	NH1	98ASP	OD2	20.18
164ARG	NH1	98ASP	O	0.50
164ARG	NH1	161GLU	OE1	0.16
164ARG	NH1	161GLU	OE2	0.48
164ARG	NH1	264ARG	NE	0.01
164ARG	NE	133GLU	OE2	18.20
164ARG	NE	161GLU	OE1	0.88
164ARG	NE	161GLU	OE2	0.75
164ARG	NE	161GLU	O	0.43
164ARG	N	161GLU	O	19.18
163GLU	N	159LYS	O	36.73
163GLU	N	161GLU	O	0.00
161GLU	N	159LYS	O	0.01
161GLU	N	161GLU	OE1	0.08
161GLU	N	161GLU	OE2	0.82
159LYS	NZ	163GLU	OE1	2.80
159LYS	NZ	163GLU	OE2	8.88
159LYS	NZ	197LYS	O	0.01
159LYS	NZ	201GLU	OE1	6.76
159LYS	NZ	201GLU	OE2	6.70
159LYS	NZ	148GLU	OE1	23.47
159LYS	NZ	148GLU	OE2	22.05
159LYS	NZ	148GLU	O	2.16
156ARG	NH2	142GLU	O	4.32
156ARG	NH2	161GLU	OE1	0.49
156ARG	NH2	161GLU	OE2	0.60
156ARG	NH2	150GLU	OE1	36.15
156ARG	NH2	150GLU	OE2	34.05

124ARG	NE	121GLU	OE1	18.58
124ARG	N	121GLU	OE2	18.97
124ARG	N	120GLU	O	46.67
121GLU	N	121GLU	O	5.99
121GLU	N	121GLU	OE1	0.71
120GLU	N	121GLU	OE2	0.08
120GLU	N	120GLU	OE1	1.93
119LYS	NZ	120GLU	OE2	2.18
119LYS	NZ	113GLU	O	2.00
119LYS	NZ	114ARG	O	22.22
119LYS	NZ	120GLU	OE1	9.28
114ARG	NH2	120GLU	OE2	4.16
114ARG	NH2	120GLU	OE1	1.91
114ARG	NH1	120GLU	OE2	1.93
114ARG	NE	113GLU	OE2	0.02
114ARG	NE	120GLU	OE1	12.64
114ARG	N	120GLU	OE2	12.53
113GLU	N	113GLU	OE1	0.02
107LYS	NZ	113GLU	OE1	97.08
107LYS	NZ	127ASP	OD1	5.11
107LYS	NZ	127ASP	OD2	5.66
107LYS	NZ	309ARG	NH1	0.00
107LYS	NZ	312GLU	OE1	51.99
104ARG	NH2	312GLU	OE2	45.61
104ARG	NH2	241ASP	O	0.00
104ARG	NH2	245ASP	OD1	0.64
104ARG	NH1	245ASP	OD2	1.54
104ARG	NH1	245ASP	OD1	13.61
98ASP	N	245ASP	OD2	6.92
98ASP	N	94ARG	O	3.38
95LYS	NZ	95LYS	O	24.56
95LYS	NZ	95LYS	O	0.01
95LYS	NZ	98ASP	OD1	0.06
94ARG	NH2	98ASP	OD2	0.67
94ARG	NH2	87GLU	OE1	20.08
94ARG	NH2	87GLU	OE2	11.36
94ARG	NH2	87GLU	O	0.01
94ARG	NH2	270GLU	OE1	0.20
94ARG	NH1	270GLU	OE2	0.52
94ARG	NH1	87GLU	OE1	27.35
87GLU	N	87GLU	OE2	34.65
87GLU	N	87GLU	OE1	0.85
82ARG	NH2	87GLU	OE2	0.46

156ARG	NH2	142GLU	OE1	0.01
156ARG	NH2	142GLU	O	3.00
156ARG	NH2	150GLU	OE1	50.16
156ARG	NH2	150GLU	OE2	38.03
156ARG	NH1	142GLU	OE1	0.05
156ARG	NH1	142GLU	O	30.49
156ARG	NH1	150GLU	OE1	2.50
156ARG	NH1	150GLU	OE2	4.75
156ARG	NE	142GLU	O	2.71
156ARG	NE	150GLU	OE1	38.39
156ARG	NE	150GLU	OE2	50.10
156ARG	N	155GLU	OE1	0.82
156ARG	N	155GLU	OE2	2.67
148GLU	N	148GLU	OE1	0.51
148GLU	N	148GLU	OE2	0.40
148GLU	N	150GLU	O	0.09
144ARG	NH2	190GLU	OE1	33.50
144ARG	NH2	190GLU	OE2	35.71
144ARG	NH1	142GLU	OE1	1.88
144ARG	NH1	142GLU	OE2	2.57
144ARG	NE	144ARG	O	0.01
144ARG	NE	190GLU	OE1	43.51
144ARG	NE	190GLU	OE2	48.23
144ARG	N	142GLU	OE1	1.08
144ARG	N	142GLU	OE2	1.34
142GLU	N	142GLU	OE1	0.70
142GLU	N	142GLU	OE2	0.42
132ARG	NH2	133GLU	O	0.02
132ARG	NH2	241ASP	OD1	5.21
132ARG	NH2	241ASP	OD2	11.49
132ARG	NH1	241ASP	OD1	8.49
132ARG	NH1	241ASP	OD2	14.16
132ARG	NE	133GLU	O	26.49
132ARG	NE	241ASP	N	0.00
124ARG	NH2	113GLU	OE1	9.62
124ARG	NH2	113GLU	OE2	13.30
124ARG	NH2	120GLU	OE1	11.74
124ARG	NH2	120GLU	OE2	10.83
124ARG	NH2	121GLU	OE1	24.65
124ARG	NH2	121GLU	OE2	22.83
124ARG	NH1	113GLU	OE1	0.81
124ARG	NH1	113GLU	OE2	1.34
124ARG	NH1	120GLU	OE1	10.02

114ARG	NE	120GLU	OE1	14.94
114ARG	NE	120GLU	OE2	25.80
114ARG	N	113GLU	OE2	0.01
113GLU	N	113GLU	OE1	13.11
113GLU	N	113GLU	OE2	76.81
107LYS	NZ	127ASP	OD1	1.58
107LYS	NZ	127ASP	OD2	2.26
107LYS	NZ	312GLU	OE1	44.73
107LYS	NZ	312GLU	OE2	55.74
104ARG	NH2	132ARG	NH1	0.00
104ARG	NH2	245ASP	OD1	16.25
104ARG	NH2	245ASP	OD2	25.55
104ARG	NH2	270GLU	OE2	0.00
104ARG	NH1	245ASP	OD1	8.26
104ARG	NH1	245ASP	OD2	8.83
98ASP	N	94ARG	O	10.84
98ASP	N	95LYS	O	23.60
94ARG	NH2	87GLU	OE2	0.04
94ARG	NH2	270GLU	OE1	3.68
94ARG	NH2	270GLU	OE2	10.00
94ARG	NH1	87GLU	OE2	0.07
94ARG	NE	270GLU	OE1	0.00
87GLU	N	85ARG	O	0.52
87GLU	N	87GLU	OE1	27.84
87GLU	N	87GLU	OE2	8.79
85ARG	NH2	78ASP	OD1	1.00
85ARG	NH2	78ASP	OD2	0.33
85ARG	NH2	87GLU	OE1	32.57
85ARG	NH2	87GLU	OE2	65.04
85ARG	NH1	78ASP	OD1	2.29
85ARG	NH1	78ASP	OD2	0.13
85ARG	NH1	78ASP	O	7.88
85ARG	NE	87GLU	OE1	67.20
85ARG	NE	87GLU	OE2	23.78
85ARG	N	82ARG	O	53.83
85ARG	N	83LYS	O	0.01
83LYS	NZ	193GLU	OE2	0.00
82ARG	NH2	87GLU	OE1	2.07
82ARG	NH2	87GLU	OE2	2.04
82ARG	NH2	193GLU	OE1	10.26
82ARG	NH2	193GLU	OE2	7.43
82ARG	NH1	87GLU	OE1	5.52
82ARG	NH1	87GLU	OE2	7.98

156ARG	NH1	142GLU	OE1	0.20
156ARG	NH1	142GLU	OE2	0.03
156ARG	NH1	142GLU	O	21.64
156ARG	NH1	156ARG	O	0.43
156ARG	NH1	161GLU	OE1	1.04
156ARG	NH1	161GLU	OE2	0.72
156ARG	NH1	150GLU	OE1	5.64
156ARG	NH1	150GLU	OE2	0.86
156ARG	NE	142GLU	O	2.16
156ARG	NE	150GLU	OE1	41.68
156ARG	NE	150GLU	OE2	45.91
156ARG	N	155GLU	OE1	40.48
148GLU	N	148GLU	OE1	6.24
148GLU	N	148GLU	OE2	8.68
144ARG	NH2	142GLU	OE1	7.54
144ARG	NH2	142GLU	OE2	11.50
144ARG	NH2	190GLU	OE1	33.57
144ARG	NH2	190GLU	OE2	32.76
144ARG	NH1	87GLU	OE1	0.02
144ARG	NH1	87GLU	OE2	0.00
144ARG	NH1	142GLU	OE1	2.38
144ARG	NH1	142GLU	OE2	0.91
144ARG	NH1	190GLU	OE1	1.74
144ARG	NH1	190GLU	OE2	0.51
144ARG	NE	142GLU	OE1	13.48
144ARG	NE	142GLU	OE2	12.66
144ARG	NE	144ARG	O	0.00
144ARG	NE	190GLU	OE1	35.27
144ARG	NE	190GLU	OE2	38.28
144ARG	N	142GLU	OE1	1.66
144ARG	N	142GLU	OE2	1.67
142GLU	N	142GLU	OE1	0.02
142GLU	N	142GLU	OE2	0.04
132ARG	NH2	133GLU	O	0.27
132ARG	NH2	241ASP	OD1	6.32
132ARG	NH2	241ASP	OD2	3.18
132ARG	NH2	241ASP	O	0.02
132ARG	NH2	245ASP	OD1	0.02
132ARG	NH2	245ASP	OD2	0.48
132ARG	NH1	133GLU	O	32.05
132ARG	NH1	241ASP	OD1	39.62
132ARG	NH1	241ASP	OD2	5.24
132ARG	NH1	241ASP	O	0.00

82ARG	NH2	78ASP	OD1	0.03
82ARG	NH2	78ASP	OD2	0.01
82ARG	NH2	87GLU	OE1	5.70
82ARG	NH2	87GLU	OE2	6.01
82ARG	NH2	190GLU	OE1	0.01
82ARG	NH2	193GLU	OE1	3.46
82ARG	NH1	193GLU	OE2	6.15
82ARG	NH1	82ARG	O	0.00
82ARG	NH1	87GLU	OE1	3.95
82ARG	NH1	87GLU	OE2	5.65
82ARG	NH1	144ARG	NH2	0.00
82ARG	NH1	190GLU	OE2	0.02
82ARG	NH1	193GLU	OE1	6.89
82ARG	NE	193GLU	OE2	2.82
82ARG	NE	87GLU	OE1	3.60
78ASP	N	87GLU	OE2	0.85
76LYS	NZ	76LYS	O	0.24
76LYS	NZ	9ASP	OD1	18.84
76LYS	NZ	9ASP	OD2	35.39
76LYS	NZ	47ASP	OD1	0.20
65GLU	N	47ASP	OD2	0.46
65GLU	N	2LYS	O	99.22
63GLU	N	65GLU	OE1	0.00
62GLU	N	59LYS	O	4.66
62GLU	N	58ARG	O	1.37
59LYS	NZ	59LYS	O	2.03
59LYS	NZ	55GLU	OE1	37.89
59LYS	NZ	55GLU	OE2	17.07
59LYS	N	55GLU	O	0.02
58ARG	NH2	55GLU	O	54.47
58ARG	NH2	51GLU	OE1	14.16
58ARG	NH2	51GLU	OE2	8.20
58ARG	NH2	55GLU	OE1	35.45
58ARG	NH1	55GLU	OE2	57.39
58ARG	NH1	51GLU	OE1	21.43
58ARG	NH1	51GLU	OE2	28.99
58ARG	NH1	55GLU	OE1	2.79
58ARG	NE	55GLU	OE2	6.67
58ARG	NE	55GLU	OE1	8.08
58ARG	N	55GLU	OE2	7.71
55GLU	N	55GLU	O	1.05
55GLU	N	51GLU	OE1	0.00
55GLU	N	51GLU	OE2	0.00

124ARG	NH1	120GLU	OE2	14.20
124ARG	NH1	120GLU	O	0.02
124ARG	NH1	121GLU	OE1	16.34
124ARG	NH1	121GLU	OE2	18.54
124ARG	NE	113GLU	OE1	23.59
124ARG	NE	113GLU	OE2	8.38
124ARG	NE	120GLU	OE1	0.66
124ARG	NE	120GLU	OE2	0.44
124ARG	NE	120GLU	O	0.02
124ARG	NE	121GLU	OE1	4.42
124ARG	NE	121GLU	OE2	3.88
124ARG	NE	124ARG	O	0.01
124ARG	N	120GLU	O	59.41
124ARG	N	121GLU	O	3.41
121GLU	N	119LYS	O	0.02
121GLU	N	120GLU	OE2	0.00
121GLU	N	121GLU	OE1	0.88
121GLU	N	121GLU	OE2	1.26
120GLU	N	120GLU	OE1	9.57
120GLU	N	120GLU	OE2	18.90
119LYS	NZ	121GLU	OE1	0.05
119LYS	NZ	121GLU	OE2	0.06
119LYS	NZ	113GLU	O	0.21
119LYS	NZ	114ARG	O	45.52
119LYS	NZ	120GLU	OE1	0.14
119LYS	NZ	120GLU	OE2	0.21
114ARG	NH2	113GLU	OE1	19.00
114ARG	NH2	113GLU	OE2	15.86
114ARG	NH2	120GLU	OE1	4.13
114ARG	NH2	120GLU	OE2	2.27
114ARG	NH1	120GLU	OE1	0.92
114ARG	NH1	120GLU	OE2	0.06
114ARG	NE	113GLU	OE1	18.19
114ARG	NE	113GLU	OE2	22.35
114ARG	NE	120GLU	OE1	5.24
114ARG	NE	120GLU	OE2	8.63
114ARG	N	113GLU	OE1	9.51
114ARG	N	113GLU	OE2	10.61
113GLU	N	113GLU	OE1	20.20
113GLU	N	113GLU	OE2	45.67
107LYS	NZ	127ASP	OD1	2.69
107LYS	NZ	127ASP	OD2	1.29
107LYS	NZ	312GLU	OE1	50.20

82ARG	NH1	193GLU	OE1	0.32
82ARG	NH1	193GLU	OE2	0.27
82ARG	NE	87GLU	OE1	5.42
82ARG	NE	87GLU	OE2	3.10
82ARG	NE	193GLU	OE1	0.05
82ARG	NE	193GLU	OE2	6.46
82ARG	N	82ARG	NE	0.00
78ASP	N	76LYS	O	0.05
78ASP	N	78ASP	OD2	0.39
76LYS	NZ	9ASP	OD1	0.59
76LYS	NZ	9ASP	OD2	2.30
76LYS	NZ	47ASP	OD1	1.54
76LYS	NZ	47ASP	OD2	1.06
76LYS	N	78ASP	OD2	4.68
65GLU	N	2LYS	O	98.36
65GLU	N	65GLU	OE2	0.01
63GLU	N	59LYS	O	12.61
62GLU	N	58ARG	O	7.64
62GLU	N	59LYS	O	2.08
59LYS	NZ	55GLU	OE1	33.53
59LYS	NZ	55GLU	OE2	25.65
59LYS	NZ	55GLU	O	0.06
59LYS	NZ	63GLU	OE2	0.03
59LYS	N	55GLU	O	66.78
58ARG	NH2	51GLU	OE1	11.97
58ARG	NH2	51GLU	OE2	14.48
58ARG	NH2	55GLU	OE1	35.02
58ARG	NH2	55GLU	OE2	30.04
58ARG	NH1	51GLU	OE1	21.07
58ARG	NH1	51GLU	OE2	19.04
58ARG	NH1	55GLU	OE1	3.90
58ARG	NH1	55GLU	OE2	8.85
58ARG	NE	55GLU	OE1	3.33
58ARG	NE	55GLU	OE2	2.25
58ARG	N	55GLU	O	1.58
55GLU	N	51GLU	OE1	0.00
55GLU	N	55GLU	OE1	0.01
55GLU	N	55GLU	OE2	0.03
51GLU	N	51GLU	OE1	0.01
30GLU	N	27ASP	O	0.14
30GLU	N	28GLU	O	0.06
28GLU	N	24ARG	O	45.94
27ASP	N	24ARG	O	1.39

132ARG	NH1	245ASP	OD1	0.00
132ARG	NH1	245ASP	OD2	0.00
132ARG	NE	133GLU	O	18.12
124ARG	NH2	113GLU	OE1	5.37
124ARG	NH2	113GLU	OE2	1.93
124ARG	NH2	120GLU	OE1	10.27
124ARG	NH2	120GLU	OE2	11.04
124ARG	NH2	120GLU	O	0.01
124ARG	NH2	121GLU	OE1	33.54
124ARG	NH2	121GLU	OE2	39.25
124ARG	NH1	113GLU	OE1	9.34
124ARG	NH1	113GLU	OE2	16.34
124ARG	NH1	120GLU	OE1	13.53
124ARG	NH1	120GLU	OE2	12.87
124ARG	NH1	120GLU	O	0.03
124ARG	NH1	121GLU	OE1	11.25
124ARG	NH1	121GLU	OE2	13.44
124ARG	NH1	124ARG	O	0.00
124ARG	NE	113GLU	OE1	4.35
124ARG	NE	113GLU	OE2	2.54
124ARG	NE	120GLU	O	0.07
124ARG	NE	121GLU	OE1	21.10
124ARG	NE	121GLU	OE2	14.38
124ARG	NE	121GLU	O	0.03
124ARG	N	120GLU	O	24.33
124ARG	N	121GLU	O	8.52
121GLU	N	119LYS	O	0.01
121GLU	N	120GLU	OE1	0.02
121GLU	N	120GLU	OE2	0.10
121GLU	N	121GLU	OE1	0.42
121GLU	N	121GLU	OE2	0.58
120GLU	N	120GLU	OE1	15.06
120GLU	N	120GLU	OE2	20.53
119LYS	NZ	121GLU	OE1	0.01
119LYS	NZ	121GLU	OE2	0.05
119LYS	NZ	113GLU	O	3.03
119LYS	NZ	114ARG	NH2	0.00
119LYS	NZ	114ARG	O	19.86
119LYS	NZ	120GLU	OE1	15.64
119LYS	NZ	120GLU	OE2	18.37
114ARG	NH2	113GLU	OE1	12.34
114ARG	NH2	113GLU	OE2	6.16
114ARG	NH2	120GLU	OE1	10.01

55GLU	N	55GLU	OE1	0.01
51GLU	N	55GLU	OE2	0.04
51GLU	N	51GLU	OE1	0.00
30GLU	N	51GLU	OE2	0.00
30GLU	N	27ASP	O	0.09
30GLU	N	28GLU	O	0.04
28GLU	N	30GLU	OE2	0.02
27ASP	N	24ARG	O	50.84
24ARG	NH2	24ARG	O	1.13
24ARG	NH2	17GLU	OE1	13.06
24ARG	NH2	17GLU	OE2	21.63
24ARG	NH2	17GLU	O	0.02
24ARG	NH1	27ASP	OD2	1.35
24ARG	NH1	17GLU	OE1	15.97
24ARG	NH1	17GLU	OE2	11.94
24ARG	NH1	27ASP	OD1	0.02
24ARG	NE	27ASP	OD2	0.04
24ARG	NE	24ARG	O	0.00
24ARG	N	27ASP	OD2	0.00
21LYS	NZ	21LYS	O	0.30
21LYS	NZ	17GLU	OE1	0.25
21LYS	NZ	17GLU	OE2	0.29
21LYS	NZ	17GLU	O	0.02
21LYS	NZ	334GLL	OE1	22.78
21LYS	NZ	334GLL	OE2	21.52
21LYS	N	334GLL	O	0.32
17GLU	N	17GLU	O	65.38
2LYS	NZ	14GLU	O	1.62
2LYS	NZ	37GLU	OE1	0.42
2LYS	NZ	37GLU	OE2	0.64
2LYS	NZ	63GLU	OE1	29.05
2LYS	NZ	63GLU	OE2	40.71
2LYS	N	63GLU	O	0.78
2LYS	N	65GLU	OE1	1.33
342ARC	NH2	65GLU	OE2	0.02
342ARC	NH2	321GLL	OE1	21.62
342ARC	NH1	321GLL	OE2	29.48
342ARC	NH1	321GLL	OE1	33.68
342ARC	NH1	321GLL	OE2	20.91
342ARC	NE1(	342ARC	O	0.45
326ASP	N10:	342ARC	O	0.32
321GLU	N10(	326ASF	OD1	0.02
317LYS	NZ1(	317LYS	O	34.27

107LYS	NZ	312GLU	OE2	53.43
104ARG	NH2	132ARG	NH1	0.01
104ARG	NH2	241ASP	OD1	0.09
104ARG	NH2	241ASP	OD2	0.25
104ARG	NH2	245ASP	OD1	8.63
104ARG	NH2	245ASP	OD2	12.19
104ARG	NH2	270GLU	OE1	0.00
104ARG	NH1	241ASP	OD1	0.59
104ARG	NH1	245ASP	OD1	34.52
104ARG	NH1	245ASP	OD2	29.63
98ASP	N	62GLU	OE2	0.04
98ASP	N	94ARG	O	13.44
98ASP	N	95LYS	O	5.09
98ASP	N	264ARG	O	0.29
95LYS	NZ	95LYS	O	0.05
95LYS	NZ	98ASP	OD1	2.74
95LYS	NZ	98ASP	OD2	1.96
94ARG	NH2	270GLU	OE1	47.51
94ARG	NH2	270GLU	OE2	40.80
94ARG	NH1	87GLU	OE2	0.00
94ARG	NH1	270GLU	OE1	0.04
94ARG	NH1	270GLU	OE2	1.29
94ARG	NE	270GLU	OE1	0.05
94ARG	NE	270GLU	OE2	0.07
87GLU	N	87GLU	OE1	23.44
87GLU	N	87GLU	OE2	18.43
83LYS	NZ	51GLU	OE1	0.18
83LYS	NZ	51GLU	OE2	0.36
83LYS	NZ	190GLU	OE1	0.08
83LYS	NZ	190GLU	OE2	1.45
83LYS	NZ	193GLU	OE1	10.90
83LYS	NZ	193GLU	OE2	4.51
82ARG	NH2	78ASP	OD1	2.45
82ARG	NH2	78ASP	OD2	1.41
82ARG	NH2	78ASP	O	0.36
82ARG	NH2	87GLU	OE1	31.23
82ARG	NH2	87GLU	OE2	23.17
82ARG	NH2	217ASP	OD1	0.13
82ARG	NH2	217ASP	OD2	1.01
82ARG	NH1	78ASP	OD1	0.18
82ARG	NH1	78ASP	OD2	0.58
82ARG	NH1	78ASP	O	0.12
82ARG	NH1	87GLU	OE1	4.44

24ARG	NH2	17GLU	OE1	42.21
24ARG	NH2	17GLU	OE2	34.23
24ARG	NH2	17GLU	O	0.02
24ARG	NH1	17GLU	OE1	33.33
24ARG	NH1	17GLU	OE2	35.08
24ARG	N	21LYS	O	0.11
21LYS	NZ	17GLU	OE1	0.98
21LYS	NZ	17GLU	OE2	0.00
21LYS	NZ	334GLU	OE1	28.09
21LYS	NZ	334GLU	OE2	36.07
21LYS	NZ	334GLU	O	0.02
21LYS	N	17GLU	O	88.87
17GLU	N	14GLU	O	2.07
14GLU	N	14GLU	OE1	0.01
2LYS	NZ	37GLU	OE1	0.00
2LYS	NZ	37GLU	OE2	0.06
2LYS	NZ	63GLU	OE1	39.37
2LYS	NZ	63GLU	OE2	28.47
2LYS	NZ	63GLU	O	2.09
2LYS	NZ	65GLU	OE1	0.05
2LYS	NZ	65GLU	OE2	0.05
2LYS	N	65GLU	OE1	17.55
2LYS	N	65GLU	OE2	28.13
342ARG	NH2	321GLU	OE1	26.52
342ARG	NH2	321GLU	OE2	31.24
342ARG	NH1	321GLU	OE1	28.63
342ARG	NH1	321GLU	OE2	19.74
342ARG	NH1	342ARG	O	0.17
342ARG	NE	321GLU	OE1	0.74
342ARG	NE	321GLU	OE2	0.63
342ARG	NE	342ARG	O	0.02
334GLU	N	334GLU	OE2	0.01
326ASP	N	326ASP	OD1	0.00
326ASP	N	326ASP	OD2	0.00
321GLU	N	317LYS	O	27.67
317LYS	NZ	313ASP	OD1	16.07
317LYS	NZ	313ASP	OD2	85.53
317LYS	NZ	313ASP	O	0.14
317LYS	N	313ASP	O	87.66
313ASP	N	309ARG	O	77.40
313ASP	N	310LYS	O	2.42
312GLU	N	309ARG	O	0.20
310LYS	NZ	30GLU	OE1	2.95

114ARG	NH2	120GLU	OE2	9.32
114ARG	NH2	124ARG	NH2	0.00
114ARG	NH1	113GLU	OE2	0.02
114ARG	NH1	120GLU	OE1	0.16
114ARG	NH1	120GLU	OE2	0.08
114ARG	NE	113GLU	OE1	10.54
114ARG	NE	113GLU	OE2	12.02
114ARG	NE	120GLU	OE1	16.92
114ARG	NE	120GLU	OE2	18.86
114ARG	N	113GLU	OE1	3.79
114ARG	N	113GLU	OE2	7.43
113GLU	N	113GLU	OE1	37.33
113GLU	N	113GLU	OE2	41.94
107LYS	NZ	127ASP	OD1	0.78
107LYS	NZ	127ASP	OD2	0.50
107LYS	NZ	299GLU	OE1	0.16
107LYS	NZ	299GLU	OE2	0.19
107LYS	NZ	309ARG	NH1	0.01
107LYS	NZ	312GLU	OE1	48.17
107LYS	NZ	312GLU	OE2	55.79
104ARG	NH2	245ASP	OD1	1.16
104ARG	NH2	245ASP	OD2	0.36
104ARG	NH1	245ASP	N	0.00
104ARG	NH1	245ASP	OD1	33.30
104ARG	NH1	245ASP	OD2	49.72
98ASP	N	94ARG	O	20.14
98ASP	N	95LYS	O	14.56
95LYS	NZ	95LYS	O	0.08
95LYS	NZ	98ASP	OD1	0.67
95LYS	NZ	98ASP	OD2	4.06
95LYS	NZ	161GLU	OE1	0.05
95LYS	NZ	161GLU	OE2	0.02
94ARG	NH2	270GLU	OE1	33.81
94ARG	NH2	270GLU	OE2	49.63
94ARG	NE	270GLU	OE1	0.04
94ARG	NE	270GLU	OE2	0.16
87GLU	N	85ARG	O	0.06
87GLU	N	87GLU	OE1	0.16
87GLU	N	87GLU	OE2	0.28
85ARG	NH2	78ASP	OD2	0.04
85ARG	NH2	78ASP	O	0.00
85ARG	NH2	87GLU	OE1	53.78
85ARG	NH2	87GLU	OE2	45.75

317LYS	NZ1(	313ASF	OD1	88.24
317LYS	NZ1(	313ASF	OD2	15.34
317LYS	N10(	313ASF	O	0.07
313ASP	N	313ASF	O	79.97
313ASP	N	309ARC	O	63.90
312GLU	N	310LYS	O	5.62
310LYS	NZ	309ARC	O	1.02
310LYS	NZ	30GLU	OE1	4.20
310LYS	NZ	30GLU	OE2	5.77
310LYS	NZ	306GLL	OE1	0.00
310LYS	NZ	306GLL	O	0.10
310LYS	N	313ASF	OD1	3.18
309ARC	NH2	306GLL	O	22.26
309ARC	NH2	175LYS	O	9.52
309ARC	NH2	299GLL	OE1	8.87
309ARC	NH2	299GLL	OE2	17.00
309ARC	NH2	306GLL	OE1	17.53
309ARC	NH1	306GLL	OE2	29.70
309ARC	NH1	299GLL	OE1	19.02
309ARC	NH1	299GLL	OE2	12.35
309ARC	NH1	312GLL	OE1	17.73
309ARC	NE	312GLL	OE2	13.06
309ARC	NE	306GLL	OE1	13.44
309ARC	N	306GLL	OE2	12.35
306GLU	N	306GLL	O	2.29
306GLU	N	306GLL	OE1	23.12
282LYS	NZ	306GLL	OE2	22.67
282LYS	NZ	278ASF	OD1	30.39
282LYS	NZ	278ASF	OD2	33.40
282LYS	N	278ASF	O	10.31
278ASP	N	278ASF	O	0.33
264ARC	NH2	278ASF	OD1	0.02
264ARC	NH2	62GLU	OE1	0.00
264ARC	NH2	62GLU	OE2	0.61
264ARC	NH2	98ASP	OD1	32.17
264ARC	NH2	98ASP	OD2	34.57
264ARC	NH2	98ASP	O	0.00
264ARC	NH2	161GLL	OE1	0.14
264ARC	NH1	161GLL	OE2	0.94
264ARC	NH1	62GLU	OE2	0.02
264ARC	NH1	98ASP	OD1	7.60
264ARC	NH1	98ASP	OD2	0.36
264ARC	NH1	161GLL	OE1	17.90

82ARG	NH1	87GLU	OE2	11.53
82ARG	NH1	217ASP	OD1	3.02
82ARG	NH1	217ASP	OD2	6.85
82ARG	NE	78ASP	OD1	0.27
82ARG	NE	78ASP	OD2	0.02
82ARG	NE	78ASP	O	0.16
82ARG	NE	87GLU	OE1	20.74
82ARG	NE	87GLU	OE2	28.18
78ASP	N	76LYS	O	4.50
78ASP	N	78ASP	OD1	0.01
76LYS	NZ	9ASP	OD1	48.52
76LYS	NZ	9ASP	OD2	50.93
76LYS	NZ	9ASP	O	0.04
76LYS	NZ	47ASP	OD1	0.16
76LYS	NZ	47ASP	OD2	0.27
65GLU	N	2LYS	O	92.70
65GLU	N	65GLU	OE1	0.01
65GLU	N	65GLU	OE2	0.17
63GLU	N	59LYS	O	4.38
62GLU	N	58ARG	O	0.84
62GLU	N	59LYS	O	3.13
62GLU	N	62GLU	OE1	0.01
62GLU	N	62GLU	OE2	0.07
59LYS	NZ	55GLU	OE1	36.36
59LYS	NZ	55GLU	OE2	24.16
59LYS	NZ	55GLU	O	0.05
59LYS	NZ	59LYS	O	0.03
59LYS	NZ	62GLU	OE2	0.00
59LYS	NZ	63GLU	OE1	0.65
59LYS	NZ	63GLU	OE2	0.04
59LYS	N	55GLU	O	48.91
58ARG	NH2	51GLU	OE1	13.03
58ARG	NH2	51GLU	OE2	11.59
58ARG	NH2	55GLU	OE1	33.09
58ARG	NH2	55GLU	OE2	45.21
58ARG	NH1	51GLU	OE1	29.73
58ARG	NH1	51GLU	OE2	29.76
58ARG	NH1	55GLU	OE1	4.34
58ARG	NH1	55GLU	OE2	6.47
58ARG	NE	51GLU	OE1	0.02
58ARG	NE	51GLU	OE2	0.04
58ARG	NE	55GLU	OE1	5.50
58ARG	NE	55GLU	OE2	5.98

310LYS	NZ	30GLU	OE2	6.75
310LYS	NZ	306GLU	OE1	0.16
310LYS	NZ	306GLU	OE2	0.10
310LYS	N	306GLU	O	27.98
309ARG	NH2	299GLU	OE1	52.61
309ARG	NH2	299GLU	OE2	31.95
309ARG	NH2	312GLU	OE1	86.09
309ARG	NH2	312GLU	OE2	12.64
309ARG	NH1	299GLU	OE1	35.92
309ARG	NH1	299GLU	OE2	56.90
309ARG	NE	312GLU	OE1	14.40
309ARG	NE	312GLU	OE2	87.01
309ARG	N	306GLU	O	4.18
306GLU	N	306GLU	OE1	10.01
306GLU	N	306GLU	OE2	6.03
282LYS	NZ	278ASP	OD1	2.48
282LYS	NZ	278ASP	OD2	1.84
282LYS	NZ	278ASP	O	1.35
282LYS	N	14GLU	OE1	0.08
282LYS	N	14GLU	OE2	2.07
282LYS	N	278ASP	O	0.00
278ASP	N	278ASP	OD2	0.00
264ARG	NH2	62GLU	OE1	5.06
264ARG	NH2	62GLU	OE2	0.65
264ARG	NH2	98ASP	OD1	0.55
264ARG	NH2	98ASP	OD2	5.58
264ARG	NH1	62GLU	OE1	0.38
264ARG	NH1	62GLU	OE2	5.02
264ARG	NH1	98ASP	OD1	0.73
264ARG	NH1	98ASP	OD2	8.90
264ARG	NH1	264ARG	O	0.28
264ARG	NE	98ASP	OD1	2.28
264ARG	NE	98ASP	OD2	17.87
264ARG	NE	264ARG	O	0.06
264ARG	N	98ASP	O	79.76
245ASP	N	241ASP	O	12.69
245ASP	N	245ASP	OD2	0.02
229ARG	NH2	212GLU	OE1	41.49
229ARG	NH2	212GLU	OE2	59.88
229ARG	NH2	229ARG	O	0.00
229ARG	NH1	212GLU	OE1	59.45
229ARG	NH1	212GLU	OE2	41.53
229ARG	NE	229ARG	O	38.74

85ARG	NH1	78ASP	O	0.10
85ARG	NE	87GLU	OE1	42.51
85ARG	NE	87GLU	OE2	52.91
85ARG	N	82ARG	O	74.62
85ARG	N	83LYS	O	0.01
82ARG	NH2	87GLU	OE1	7.50
82ARG	NH2	87GLU	OE2	6.34
82ARG	NH2	190GLU	OE1	0.60
82ARG	NH2	190GLU	OE2	13.50
82ARG	NH1	87GLU	OE1	0.08
82ARG	NH1	190GLU	OE1	16.99
82ARG	NH1	190GLU	OE2	5.42
82ARG	NE	85ARG	NH2	0.04
82ARG	NE	87GLU	OE1	50.37
82ARG	NE	87GLU	OE2	42.07
82ARG	N	85ARG	NH1	0.00
78ASP	N	76LYS	O	0.55
78ASP	N	78ASP	OD2	0.01
76LYS	NZ	9ASP	OD1	24.38
76LYS	NZ	9ASP	OD2	24.14
76LYS	NZ	47ASP	OD1	12.57
76LYS	NZ	47ASP	OD2	7.91
76LYS	NZ	76LYS	O	0.01
76LYS	N	78ASP	OD1	0.01
76LYS	N	78ASP	OD2	0.07
65GLU	N	2LYS	O	96.74
65GLU	N	65GLU	OE1	0.10
65GLU	N	65GLU	OE2	0.20
63GLU	N	59LYS	O	7.75
62GLU	N	58ARG	O	3.06
62GLU	N	59LYS	O	4.30
59LYS	NZ	55GLU	OE1	30.14
59LYS	NZ	55GLU	OE2	31.59
59LYS	NZ	55GLU	O	0.21
59LYS	NZ	62GLU	OE1	0.25
59LYS	NZ	62GLU	OE2	0.40
59LYS	NZ	63GLU	OE1	0.11
59LYS	NZ	63GLU	OE2	0.16
59LYS	N	55GLU	O	45.42
58ARG	NH2	51GLU	OE1	43.38
58ARG	NH2	51GLU	OE2	36.90
58ARG	NH2	55GLU	OE1	11.48
58ARG	NH2	55GLU	OE2	5.58

264ARC	NE	161GLL	OE2	3.69
264ARC	NE	98ASP	OD1	51.64
264ARC	NE	98ASP	OD2	31.06
264ARC	NE	98ASP	O	0.42
264ARC	N	264ARC	O	0.03
264ARC	N	98ASP	OD2	0.15
245ASP	N	98ASP	O	79.58
245ASP	N	241ASF	O	12.44
229ARC	NH2	245ASF	OD2	0.01
229ARC	NH2	212GLL	OE1	97.44
229ARC	NH2	212GLL	OE2	1.10
229ARC	NH1	229ARC	O	0.03
229ARC	NH1	212GLL	OE1	3.44
229ARC	NH1	212GLL	OE2	97.65
229ARC	NE	229ARC	O	0.24
225ARC	NH2	229ARC	O	38.96
225ARC	NH2	326ASF	OD1	1.38
225ARC	NH1	326ASF	OD2	1.64
225ARC	NH1	326ASF	OD1	1.58
225ARC	NE	326ASF	OD2	0.34
217ASP	N	225ARC	O	2.17
217ASP	N	217ASF	OD1	12.57
208ASP	N	217ASF	OD2	13.87
208ASP	N	208ASF	OD1	0.02
204ARC	NH2	208ASF	OD2	0.02
204ARC	NH2	197LYS	O	0.00
204ARC	NH2	200GLL	OE1	11.29
204ARC	NH2	200GLL	OE2	5.13
204ARC	NH2	201GLL	OE1	1.87
204ARC	NH1	201GLL	OE2	4.95
204ARC	NH1	197LYS	O	0.00
204ARC	NH1	201GLL	OE1	36.62
204ARC	NE	201GLL	OE2	27.00
204ARC	NE	200GLL	OE1	9.09
204ARC	NE	200GLL	OE2	11.21
204ARC	NE	200GLL	O	0.02
204ARC	NE	201GLL	OE1	0.01
204ARC	NE	201GLL	OE2	0.18
204ARC	N	201GLL	O	0.01
204ARC	N	200GLL	O	25.16
201GLL	N	201GLL	O	36.99
200GLL	N	197LYS	O	24.96
200GLL	N	196ARC	O	37.75

58ARG	NE	58ARG	O	0.00
58ARG	N	55GLU	O	4.14
55GLU	N	51GLU	OE1	0.02
55GLU	N	51GLU	OE2	0.02
55GLU	N	55GLU	OE1	0.05
55GLU	N	55GLU	OE2	0.00
51GLU	N	51GLU	OE1	0.04
51GLU	N	51GLU	OE2	0.02
30GLU	N	27ASP	O	0.50
30GLU	N	28GLU	O	0.09
30GLU	N	30GLU	OE2	0.00
28GLU	N	24ARG	O	46.59
27ASP	N	24ARG	O	2.10
24ARG	NH2	17GLU	OE1	34.92
24ARG	NH2	17GLU	OE2	28.44
24ARG	NH2	17GLU	O	0.03
24ARG	NH2	27ASP	OD2	2.92
24ARG	NH2	28GLU	OE1	0.90
24ARG	NH2	28GLU	OE2	1.83
24ARG	NH1	17GLU	OE1	25.61
24ARG	NH1	17GLU	OE2	36.63
24ARG	NH1	17GLU	O	0.00
24ARG	NH1	24ARG	O	0.30
24ARG	NH1	28GLU	OE1	1.05
24ARG	NH1	28GLU	OE2	2.38
24ARG	NE	28GLU	OE1	0.61
24ARG	NE	28GLU	OE2	0.06
24ARG	N	21LYS	O	0.36
21LYS	NZ	17GLU	OE1	3.92
21LYS	NZ	17GLU	OE2	5.83
21LYS	NZ	17GLU	O	0.02
21LYS	NZ	334GLU	OE1	18.86
21LYS	NZ	334GLU	OE2	21.58
21LYS	NZ	334GLU	O	0.01
21LYS	N	17GLU	O	82.04
17GLU	N	14GLU	O	3.15
14GLU	N	14GLU	OE1	0.01
2LYS	NZ	37GLU	OE1	2.70
2LYS	NZ	37GLU	OE2	5.87
2LYS	NZ	63GLU	OE1	26.53
2LYS	NZ	63GLU	OE2	33.58
2LYS	NZ	63GLU	O	8.74
2LYS	NZ	65GLU	OE1	2.07

225ARG	NH2	326ASP	OD1	10.53
225ARG	NH2	326ASP	OD2	9.14
225ARG	NH2	326ASP	O	1.15
225ARG	NH1	326ASP	OD1	7.95
225ARG	NH1	326ASP	OD2	7.97
225ARG	NH1	326ASP	O	0.02
217ASP	N	217ASP	OD1	40.61
208ASP	N	208ASP	OD1	48.61
204ARG	NH2	200GLU	OE1	17.97
204ARG	NH2	200GLU	OE2	81.99
204ARG	NH2	201GLU	OE1	37.42
204ARG	NH2	201GLU	OE2	18.91
204ARG	NH1	201GLU	OE1	21.55
204ARG	NH1	201GLU	OE2	32.67
204ARG	NE	200GLU	OE1	80.56
204ARG	NE	200GLU	OE2	19.41
204ARG	NE	200GLU	O	0.05
204ARG	N	200GLU	O	35.88
204ARG	N	201GLU	O	24.72
201GLU	N	197LYS	O	65.26
200GLU	N	196ARG	O	35.91
200GLU	N	197LYS	O	7.76
197LYS	NZ	148GLU	OE1	35.87
197LYS	NZ	148GLU	OE2	33.84
197LYS	NZ	148GLU	O	0.08
197LYS	NZ	193GLU	OE1	0.65
197LYS	NZ	193GLU	OE2	0.04
197LYS	NZ	201GLU	OE1	16.00
197LYS	NZ	201GLU	OE2	10.32
197LYS	N	193GLU	O	64.22
196ARG	NH2	184ASP	OD1	10.26
196ARG	NH2	184ASP	OD2	87.53
196ARG	NE	184ASP	OD1	95.07
196ARG	NE	184ASP	OD2	10.54
196ARG	N	193GLU	O	0.68
193GLU	N	190GLU	O	2.01
190GLU	N	190GLU	OE1	20.94
190GLU	N	190GLU	OE2	21.50
185LYS	NZ	241ASP	OD1	67.62
185LYS	NZ	241ASP	OD2	34.81
185LYS	NZ	217ASP	OD1	99.68
185LYS	NZ	217ASP	OD2	0.07
185LYS	N	184ASP	OD1	0.14

58ARG	NH1	51GLU	OE1	4.09
58ARG	NH1	51GLU	OE2	6.38
58ARG	NH1	55GLU	OE1	24.15
58ARG	NH1	55GLU	OE2	28.59
58ARG	NE	51GLU	OE1	2.58
58ARG	NE	51GLU	OE2	2.09
58ARG	N	55GLU	O	9.18
55GLU	N	51GLU	OE1	0.03
55GLU	N	51GLU	OE2	0.04
55GLU	N	55GLU	OE1	0.25
55GLU	N	55GLU	OE2	0.28
55GLU	N	58ARG	NH2	0.00
30GLU	N	27ASP	O	0.66
30GLU	N	28GLU	O	0.08
30GLU	N	30GLU	OE2	0.00
28GLU	N	24ARG	O	45.87
28GLU	N	30GLU	O	0.04
27ASP	N	24ARG	O	3.04
24ARG	NH2	17GLU	OE1	35.53
24ARG	NH2	17GLU	OE2	44.37
24ARG	NH2	17GLU	O	0.00
24ARG	NH1	17GLU	OE1	48.46
24ARG	NH1	17GLU	OE2	36.19
24ARG	NH1	17GLU	O	0.00
24ARG	N	21LYS	O	0.35
21LYS	NZ	17GLU	OE1	5.21
21LYS	NZ	17GLU	OE2	3.55
21LYS	NZ	17GLU	O	0.04
21LYS	NZ	334GLU	OE1	24.81
21LYS	NZ	334GLU	OE2	34.65
21LYS	NZ	334GLU	O	0.03
21LYS	N	17GLU	O	84.10
17GLU	N	14GLU	O	2.90
14GLU	N	14GLU	OE1	0.00
14GLU	N	14GLU	OE2	0.00
2LYS	NZ	37GLU	OE1	5.91
2LYS	NZ	37GLU	OE2	3.70
2LYS	NZ	63GLU	OE1	29.35
2LYS	NZ	63GLU	OE2	27.47
2LYS	NZ	63GLU	O	5.10
2LYS	NZ	65GLU	OE1	1.81
2LYS	NZ	65GLU	OE2	1.33
2LYS	N	65GLU	OE1	11.25

197LYS	NZ	197LYS	O	4.50
197LYS	NZ	148GLU	OE1	21.80
197LYS	NZ	148GLU	OE2	25.13
197LYS	NZ	148GLU	O	0.04
197LYS	NZ	193GLU	OE1	3.21
197LYS	NZ	193GLU	OE2	2.86
197LYS	NZ	193GLU	O	0.07
197LYS	NZ	201GLU	OE1	1.52
197LYS	N	201GLU	OE2	0.84
196ARG	NH2	193GLU	O	64.61
196ARG	NH2	184ASP	OD1	93.52
196ARG	NE	184ASP	OD2	13.52
196ARG	NE	184ASP	OD1	1.41
196ARG	N	184ASP	OD2	99.18
193GLU	N	193GLU	O	0.00
190GLU	N	190GLU	O	3.20
190GLU	N	190GLU	OE1	1.80
185LYS	NZ	190GLU	OE2	0.07
185LYS	NZ	241ASP	OD1	17.78
185LYS	NZ	241ASP	OD2	78.77
185LYS	NZ	217ASP	OD1	46.47
185LYS	N	217ASP	OD2	52.33
178LYS	NZ	184ASP	OD1	0.16
178LYS	NZ	174ARG	O	0.01
178LYS	NZ	176ARG	O	0.02
178LYS	NZ	208ASP	OD1	83.10
178LYS	NZ	208ASP	OD2	18.15
178LYS	N	208ASP	O	0.02
178LYS	N	176ARG	O	0.06
177ARG	NH2	231ASP	OD2	1.13
177ARG	NH2	127ASP	OD2	6.03
177ARG	NH2	127ASP	O	3.08
177ARG	NH2	229ARG	O	0.63
177ARG	NH2	231ASP	OD1	10.70
177ARG	NH1	231ASP	OD2	0.01
177ARG	NH1	127ASP	OD2	13.16
177ARG	NH1	127ASP	O	0.10
177ARG	NH1	229ARG	O	8.06
177ARG	NH1	231ASP	OD1	12.92
177ARG	NE	231ASP	OD2	0.03
177ARG	NE	231ASP	OD1	6.76
177ARG	N	231ASP	OD2	4.74
177ARG	N	177ARG	NE	0.00

2LYS	NZ	65GLU	OE2	2.86
2LYS	N	65GLU	OE1	11.09
2LYS	N	65GLU	OE2	14.49
342ARG	NH21	317LYS	O	0.04
342ARG	NH21	321GLU	OE1	29.97
342ARG	NH21	321GLU	OE2	31.63
342ARG	NH11	321GLU	OE1	25.24
342ARG	NH11	321GLU	OE2	19.99
342ARG	NH11	342ARG	O	0.03
342ARG	NE10	342ARG	O	0.08
334GLU	N102	334GLU	OE1	0.13
326ASP	N101	282LYS	O	0.08
326ASP	N101	326ASP	OD1	0.07
326ASP	N101	326ASP	OD2	0.04
321GLU	N100	317LYS	O	27.11
317LYS	NZ10	313ASP	OD1	58.03
317LYS	NZ10	313ASP	OD2	48.87
317LYS	NZ10	313ASP	O	0.11
317LYS	N100	313ASP	O	86.03
313ASP	N	309ARG	O	73.93
313ASP	N	310LYS	O	4.23
312GLU	N	309ARG	O	2.06
310LYS	NZ	30GLU	OE1	2.44
310LYS	NZ	30GLU	OE2	1.39
310LYS	NZ	306GLU	OE1	0.11
310LYS	NZ	306GLU	OE2	0.42
310LYS	NZ	306GLU	O	0.07
310LYS	N	306GLU	O	37.63
309ARG	NH2	175LYS	O	1.29
309ARG	NH2	299GLU	OE1	4.71
309ARG	NH2	299GLU	OE2	3.05
309ARG	NH2	306GLU	OE1	31.08
309ARG	NH2	306GLU	OE2	35.58
309ARG	NH2	313ASP	OD1	0.07
309ARG	NH1	175LYS	O	0.02
309ARG	NH1	299GLU	OE1	3.80
309ARG	NH1	299GLU	OE2	4.33
309ARG	NH1	306GLU	OE1	10.92
309ARG	NH1	306GLU	OE2	8.80
309ARG	NH1	312GLU	OE1	1.03
309ARG	NH1	312GLU	OE2	3.54
309ARG	NH1	313ASP	OD1	0.17
309ARG	NH1	313ASP	OD2	0.05

185LYS	N	184ASP	OD2	0.01
178LYS	NZ	174ARG	O	1.84
178LYS	NZ	176ARG	O	1.42
178LYS	NZ	177ARG	O	0.03
178LYS	NZ	208ASP	OD2	18.85
178LYS	NZ	208ASP	O	0.19
178LYS	N	176ARG	O	0.12
178LYS	N	231ASP	OD2	0.07
177ARG	NH2	127ASP	OD2	1.49
177ARG	NH2	127ASP	O	0.97
177ARG	NH2	229ARG	O	0.29
177ARG	NH2	231ASP	OD1	27.97
177ARG	NH2	231ASP	OD2	0.34
177ARG	NH1	229ARG	O	10.67
177ARG	NE	231ASP	OD1	9.24
177ARG	NE	231ASP	OD2	21.14
177ARG	N	177ARG	NE	0.28
177ARG	N	231ASP	OD1	0.02
177ARG	N	231ASP	OD2	63.71
176ARG	NH2	127ASP	OD1	96.26
176ARG	NH2	127ASP	OD2	1.49
176ARG	NH2	127ASP	O	0.06
176ARG	NH1	127ASP	OD1	2.95
176ARG	NH1	127ASP	OD2	2.36
176ARG	NH1	127ASP	O	91.12
176ARG	NH1	231ASP	OD1	96.46
176ARG	NH1	231ASP	OD2	0.38
176ARG	NH1	231ASP	O	0.14
176ARG	N	174ARG	O	0.21
175LYS	NZ	171GLU	OE1	0.41
175LYS	NZ	171GLU	OE2	0.56
175LYS	NZ	171GLU	O	0.49
175LYS	NZ	299GLU	OE1	1.46
175LYS	NZ	299GLU	OE2	1.05
175LYS	NZ	299GLU	O	0.48
175LYS	N	171GLU	O	3.19
174ARG	NH2	208ASP	OD1	99.92
174ARG	NH2	208ASP	OD2	1.34
174ARG	NH1	171GLU	OE1	14.37
174ARG	NH1	171GLU	OE2	22.19
174ARG	NE	208ASP	OD1	4.22
174ARG	NE	208ASP	OD2	98.15
174ARG	N	171GLU	O	26.02

2LYS	N	65GLU	OE2	10.63
342ARG	NH2	317LYS	O	0.04
342ARG	NH2	321GLU	OE1	36.77
342ARG	NH2	321GLU	OE2	35.64
342ARG	NH1	321GLU	OE1	25.63
342ARG	NH1	321GLU	OE2	24.20
342ARG	NH1	342ARG	O	0.06
342ARG	NE	321GLU	OE2	0.00
342ARG	NE	342ARG	O	0.09
334GLU	N	334GLU	OE1	0.94
334GLU	N	334GLU	OE2	0.49
326ASP	N	326ASP	OD1	0.02
321GLU	N	317LYS	O	19.46
317LYS	NZ	313ASP	OD1	46.53
317LYS	NZ	313ASP	OD2	53.54
317LYS	NZ	313ASP	O	0.08
317LYS	NZ	321GLU	OE1	0.51
317LYS	NZ	321GLU	OE2	0.38
317LYS	N	313ASP	O	86.77
313ASP	N	309ARG	O	75.19
313ASP	N	310LYS	O	2.69
312GLU	N	309ARG	O	0.62
310LYS	NZ	30GLU	OE1	3.90
310LYS	NZ	30GLU	OE2	4.12
310LYS	NZ	306GLU	OE1	1.49
310LYS	NZ	306GLU	OE2	1.16
310LYS	NZ	306GLU	O	0.12
310LYS	NZ	313ASP	OD1	0.01
310LYS	N	306GLU	O	28.44
309ARG	NH2	299GLU	OE1	35.86
309ARG	NH2	299GLU	OE2	27.49
309ARG	NH2	312GLU	OE1	42.39
309ARG	NH2	312GLU	OE2	53.91
309ARG	NH1	299GLU	OE1	45.33
309ARG	NH1	299GLU	OE2	51.50
309ARG	NE	312GLU	OE1	56.03
309ARG	NE	312GLU	OE2	40.87
309ARG	N	306GLU	O	6.70
306GLU	N	306GLU	OE1	4.86
306GLU	N	306GLU	OE2	4.21
282LYS	NZ	278ASP	OD1	31.65
282LYS	NZ	278ASP	OD2	36.39
282LYS	NZ	278ASP	O	15.33

177ARG	N	231ASP	OD1	0.28
176ARG	NH2	231ASP	OD2	71.54
176ARG	NH2	127ASP	OD1	92.64
176ARG	NH2	127ASP	OD2	1.10
176ARG	NH1	127ASP	O	0.02
176ARG	NH1	127ASP	OD1	3.96
176ARG	NH1	127ASP	OD2	3.00
176ARG	NH1	127ASP	O	88.95
176ARG	NH1	177ARG	NH1	0.00
176ARG	NH1	177ARG	NH2	0.02
176ARG	NH1	231ASP	OD1	92.68
176ARG	NH1	231ASP	OD2	1.88
176ARG	N	231ASP	O	0.06
175LYS	NZ	174ARG	O	0.20
175LYS	NZ	171GLU	OE1	1.14
175LYS	NZ	171GLU	OE2	0.94
175LYS	NZ	171GLU	O	1.48
175LYS	NZ	299GLU	OE1	23.69
175LYS	NZ	299GLU	OE2	36.19
175LYS	N	299GLU	O	7.84
174ARG	NH2	171GLU	O	5.34
174ARG	NH2	171GLU	OE1	49.71
174ARG	NH1	171GLU	OE2	54.91
174ARG	NH1	208ASP	OD1	0.30
174ARG	NE	208ASP	OD2	0.00
174ARG	NE	171GLU	OE1	31.89
174ARG	NE	171GLU	OE2	36.43
174ARG	N	171GLU	O	0.01
171GLU	N	171GLU	O	11.73
167ARG	NH2	167ARG	O	82.92
167ARG	NH2	163GLU	OE1	25.76
167ARG	NH2	163GLU	OE2	20.47
167ARG	NH2	201GLU	OE1	0.00
167ARG	NH2	201GLU	OE2	0.01
167ARG	NH1	201GLU	O	0.00
167ARG	NH1	163GLU	OE1	12.25
167ARG	NE	163GLU	OE2	12.72
167ARG	NE	163GLU	OE1	19.32
167ARG	NE	163GLU	OE2	19.58
167ARG	NE	163GLU	O	0.12
167ARG	N	171GLU	OE1	0.01
167ARG	N	163GLU	O	87.18
164ARG	NH2	164ARG	O	2.42

309ARG	NE	299GLU	OE1	0.07
309ARG	NE	299GLU	OE2	0.04
309ARG	NE	306GLU	OE1	19.17
309ARG	NE	306GLU	OE2	16.12
309ARG	NE	309ARG	O	0.00
309ARG	N	306GLU	O	3.62
306GLU	N	306GLU	OE1	1.63
306GLU	N	306GLU	OE2	1.44
282LYS	NZ	78ASP	OD1	1.37
282LYS	NZ	78ASP	OD2	0.79
282LYS	NZ	278ASP	OD1	32.29
282LYS	NZ	278ASP	OD2	31.55
282LYS	NZ	278ASP	O	2.90
282LYS	NZ	282LYS	O	0.15
282LYS	NZ	326ASP	OD1	20.97
282LYS	NZ	326ASP	O	0.32
282LYS	N	14GLU	OE2	0.22
282LYS	N	278ASP	O	16.80
278ASP	N	278ASP	OD1	0.62
278ASP	N	278ASP	OD2	0.55
264ARG	NH2	62GLU	OE1	0.47
264ARG	NH2	62GLU	OE2	0.01
264ARG	NH2	98ASP	OD1	5.56
264ARG	NH2	98ASP	OD2	10.98
264ARG	NH2	161GLU	OE1	0.05
264ARG	NH2	161GLU	OE2	0.14
264ARG	NH1	62GLU	OE2	0.40
264ARG	NH1	98ASP	OD1	1.56
264ARG	NH1	98ASP	OD2	10.44
264ARG	NH1	161GLU	OE1	0.00
264ARG	NH1	161GLU	OE2	0.02
264ARG	NH1	264ARG	O	0.24
264ARG	NE	98ASP	OD1	15.94
264ARG	NE	98ASP	OD2	32.29
264ARG	NE	264ARG	O	0.28
264ARG	N	98ASP	OD2	0.00
264ARG	N	98ASP	O	62.07
245ASP	N	241ASP	O	68.14
229ARG	NH2	212GLU	OE1	43.56
229ARG	NH2	212GLU	OE2	57.64
229ARG	NH2	229ARG	O	0.01
229ARG	NH1	212GLU	OE1	57.69
229ARG	NH1	212GLU	OE2	44.00

171GLU	N	167ARG	O	68.55
167ARG	NH2	163GLU	OE1	35.31
167ARG	NH2	163GLU	OE2	46.50
167ARG	NH2	171GLU	OE1	0.12
167ARG	NH1	163GLU	OE1	5.99
167ARG	NH1	163GLU	OE2	6.55
167ARG	NH1	163GLU	O	0.01
167ARG	NH1	171GLU	OE2	0.00
167ARG	NE	163GLU	OE1	36.89
167ARG	NE	163GLU	OE2	27.57
167ARG	NE	163GLU	O	0.02
167ARG	NE	171GLU	OE1	0.02
167ARG	N	163GLU	O	91.94
167ARG	N	164ARG	O	1.21
164ARG	NH2	98ASP	OD1	6.19
164ARG	NH2	98ASP	OD2	93.95
164ARG	NH2	98ASP	O	0.08
164ARG	NH2	264ARG	NE	0.04
164ARG	NH2	264ARG	NH2	0.01
164ARG	NH1	98ASP	OD1	89.44
164ARG	NH1	98ASP	OD2	9.29
164ARG	NH1	98ASP	O	0.00
164ARG	NH1	161GLU	OE1	0.14
164ARG	NH1	161GLU	OE2	0.38
164ARG	N	161GLU	O	15.35
163GLU	N	159LYS	O	58.93
161GLU	N	161GLU	OE2	0.12
159LYS	NZ	148GLU	OE1	24.85
159LYS	NZ	148GLU	OE2	27.61
159LYS	NZ	148GLU	O	2.09
159LYS	NZ	163GLU	OE1	1.08
159LYS	NZ	163GLU	OE2	6.55
159LYS	NZ	201GLU	OE1	12.86
159LYS	NZ	201GLU	OE2	18.27
156ARG	NH2	150GLU	OE1	18.92
156ARG	NH2	150GLU	OE2	23.46
156ARG	NH2	142GLU	O	9.24
156ARG	NH1	150GLU	OE1	25.21
156ARG	NH1	150GLU	OE2	40.97
156ARG	NH1	142GLU	O	6.66
156ARG	NE	150GLU	OE1	19.00
156ARG	NE	150GLU	OE2	12.33
156ARG	NE	142GLU	O	16.82

282LYS	N	14GLU	OE1	4.24
282LYS	N	14GLU	OE2	25.31
282LYS	N	278ASP	O	9.37
278ASP	N	278ASP	OD1	0.00
278ASP	N	278ASP	OD2	0.01
264ARG	NH2	98ASP	OD1	3.94
264ARG	NH2	98ASP	OD2	29.77
264ARG	NH2	161GLU	OE1	1.02
264ARG	NH2	161GLU	OE2	1.23
264ARG	NH1	98ASP	OD1	26.04
264ARG	NH1	98ASP	OD2	23.90
264ARG	NH1	98ASP	O	0.58
264ARG	NH1	161GLU	OE1	0.20
264ARG	NH1	161GLU	OE2	0.92
264ARG	NE	98ASP	OD1	32.49
264ARG	NE	98ASP	OD2	12.53
264ARG	NE	98ASP	O	0.01
264ARG	N	98ASP	OD1	0.22
264ARG	N	98ASP	OD2	1.91
264ARG	N	98ASP	O	56.65
245ASP	N	241ASP	O	19.74
245ASP	N	245ASP	OD1	0.02
231ASP	N	229ARG	O	0.07
229ARG	NH2	212GLU	OE1	30.26
229ARG	NH2	212GLU	OE2	34.72
229ARG	NH2	229ARG	O	0.00
229ARG	NH1	212GLU	OE1	32.24
229ARG	NH1	212GLU	OE2	27.97
229ARG	NH1	229ARG	O	4.29
229ARG	NE	212GLU	OE1	0.32
229ARG	NE	212GLU	OE2	0.15
229ARG	NE	229ARG	O	17.68
225ARG	NH2	326ASP	OD1	70.28
225ARG	NH2	326ASP	OD2	24.91
225ARG	NH2	326ASP	O	2.44
225ARG	NH1	326ASP	OD1	11.16
225ARG	NH1	326ASP	OD2	61.61
225ARG	NH1	326ASP	O	0.12
217ASP	N	217ASP	OD1	14.25
217ASP	N	217ASP	OD2	13.95
208ASP	N	208ASP	OD1	44.03
204ARG	NH2	197LYS	O	0.01
204ARG	NH2	200GLU	OE1	40.20

164ARC	NH2	98ASP	OD1	0.97	229ARG	NH1	229ARG	O	0.02	156ARG	N	155GLU	OE1	51.34	204ARG	NH2	200GLU	OE2	32.40
164ARC	NH2	98ASP	OD2	19.83	229ARG	NE	229ARG	O	48.20	150GLU	N	148GLU	O	0.02	204ARG	NH2	201GLU	OE1	23.01
164ARC	NH2	133GLL	OE2	0.11	225ARG	NH2	326ASP	OD1	9.22	148GLU	N	148GLU	OE1	0.10	204ARG	NH2	201GLU	OE2	18.10
164ARC	NH2	161GLL	OE1	14.86	225ARG	NH2	326ASP	OD2	15.75	148GLU	N	148GLU	OE2	0.09	204ARG	NH1	200GLU	OE1	4.02
164ARC	NH1	161GLL	OE2	41.49	225ARG	NH2	326ASP	O	4.35	144ARG	NH2	190GLU	OE1	21.94	204ARG	NH1	200GLU	OE2	4.19
164ARC	NH1	98ASP	OD1	20.27	225ARG	NH1	326ASP	OD1	10.22	144ARG	NH2	190GLU	OE2	16.52	204ARG	NH1	200GLU	O	0.12
164ARC	NH1	98ASP	OD2	0.09	225ARG	NH1	326ASP	OD2	6.64	144ARG	NH2	142GLU	OE1	19.32	204ARG	NH1	201GLU	OE1	26.95
164ARC	NH1	161GLL	OE1	6.91	225ARG	NH1	326ASP	O	0.23	144ARG	NH2	142GLU	OE2	13.14	204ARG	NH1	201GLU	OE2	30.15
164ARC	NH1	161GLL	OE2	9.95	217ASP	N	217ASP	OD1	16.48	144ARG	NH1	190GLU	OE1	1.38	204ARG	NE	200GLU	OE1	32.45
164ARC	NH1	264ARC	NE	0.01	217ASP	N	217ASP	OD2	7.33	144ARG	NH1	190GLU	OE2	0.74	204ARG	NE	200GLU	OE2	38.59
164ARC	NE	264ARC	NH2	0.01	208ASP	N	208ASP	OD1	0.00	144ARG	NH1	142GLU	OE1	5.30	204ARG	NE	200GLU	O	0.07
164ARC	NE	161GLL	OE1	18.29	204ARG	NH2	200GLU	OE1	41.19	144ARG	NH1	142GLU	OE2	6.40	204ARG	N	200GLU	O	36.65
164ARC	N	161GLL	OE2	4.81	204ARG	NH2	200GLU	OE2	45.63	144ARG	NH1	144ARG	O	0.02	204ARG	N	201GLU	O	22.52
163GLU	N	161GLL	O	5.36	204ARG	NH2	201GLU	OE1	0.02	144ARG	NE	190GLU	OE1	8.64	201GLU	N	197LYS	O	28.23
159LYS	NZ	159LYS	O	71.33	204ARG	NH1	200GLU	OE1	0.17	144ARG	NE	190GLU	OE2	21.94	200GLU	N	196ARG	O	38.85
159LYS	NZ	148GLL	OE1	29.50	204ARG	NH1	201GLU	OE1	0.69	144ARG	NE	142GLU	OE1	20.32	200GLU	N	197LYS	O	5.03
159LYS	NZ	148GLL	OE2	22.31	204ARG	NH1	201GLU	OE2	0.24	144ARG	NE	142GLU	OE2	24.25	197LYS	NZ	148GLU	OE1	13.34
159LYS	NZ	148GLL	O	0.96	204ARG	NE	200GLU	OE1	49.70	144ARG	NE	144ARG	O	0.01	197LYS	NZ	148GLU	OE2	15.22
159LYS	NZ	163GLL	OE1	0.79	204ARG	NE	200GLU	OE2	48.04	144ARG	N	142GLU	OE1	0.52	197LYS	NZ	148GLU	O	0.00
159LYS	NZ	163GLL	OE2	0.72	204ARG	NE	200GLU	O	0.01	144ARG	N	142GLU	OE2	0.42	197LYS	NZ	193GLU	OE1	11.23
159LYS	NZ	201GLL	OE1	12.20	204ARG	N	200GLU	O	66.12	132ARG	NH2	133GLU	O	0.65	197LYS	NZ	193GLU	OE2	11.90
156ARC	NH2	201GLL	OE2	11.32	204ARG	N	201GLU	O	2.60	132ARG	NH2	241ASP	OD1	1.49	197LYS	NZ	193GLU	O	0.22
156ARC	NH2	150GLL	OE1	44.27	201GLU	N	197LYS	O	57.23	132ARG	NH2	241ASP	OD2	15.30	197LYS	NZ	197LYS	O	0.05
156ARC	NH2	150GLL	OE2	49.12	200GLU	N	196ARG	O	76.50	132ARG	NH1	241ASP	OD1	0.40	197LYS	NZ	200GLU	OE1	0.01
156ARC	NH2	142GLL	OE1	0.50	200GLU	N	197LYS	O	1.54	132ARG	NH1	241ASP	OD2	0.46	197LYS	NZ	200GLU	OE2	0.24
156ARC	NH2	142GLL	OE2	0.66	197LYS	NZ	148GLU	OE1	30.12	132ARG	NH1	245ASP	OD2	0.01	197LYS	NZ	201GLU	OE1	0.68
156ARC	NH1	142GLL	O	1.27	197LYS	NZ	148GLU	OE2	30.16	132ARG	NE	133GLU	O	15.28	197LYS	NZ	201GLU	OE2	0.84
156ARC	NH1	150GLL	OE1	0.24	197LYS	NZ	148GLU	O	0.35	124ARG	NH2	113GLU	OE1	7.22	197LYS	N	193GLU	O	62.77
156ARC	NH1	150GLL	OE2	2.75	197LYS	NZ	193GLU	OE1	4.92	124ARG	NH2	113GLU	OE2	16.30	196ARG	NH2	184ASP	OD1	88.52
156ARC	NH1	142GLL	OE1	0.78	197LYS	NZ	193GLU	OE2	3.98	124ARG	NH2	120GLU	OE1	21.30	196ARG	NH2	184ASP	OD2	18.33
156ARC	NE	142GLL	O	69.38	197LYS	NZ	193GLU	O	0.01	124ARG	NH2	120GLU	OE2	11.80	196ARG	NH1	78ASP	OD1	0.04
156ARC	NE	150GLL	OE1	47.02	197LYS	NZ	201GLU	OE1	7.82	124ARG	NH2	121GLU	OE1	12.25	196ARG	NH1	78ASP	OD2	0.02
156ARC	NE	150GLL	OE2	41.34	197LYS	NZ	201GLU	OE2	7.48	124ARG	NH2	121GLU	OE2	8.08	196ARG	NE	184ASP	OD1	2.73
156ARC	N	142GLL	O	1.18	197LYS	N	193GLU	O	83.73	124ARG	NH1	113GLU	OE1	1.91	196ARG	NE	184ASP	OD2	98.54
156ARC	N	155GLL	OE1	3.28	196ARG	NH2	184ASP	OD1	90.88	124ARG	NH1	113GLU	OE2	0.54	196ARG	N	193GLU	O	0.07
150GLU	N	155GLL	OE2	1.50	196ARG	NH2	184ASP	OD2	15.65	124ARG	NH1	120GLU	OE1	2.36	193GLU	N	190GLU	O	3.98
148GLU	N	148GLL	O	0.01	196ARG	NH1	193GLU	OE2	0.02	124ARG	NH1	120GLU	OE2	0.73	190GLU	N	190GLU	OE1	11.19
148GLU	N	148GLL	OE1	0.17	196ARG	NE	184ASP	OD1	1.95	124ARG	NH1	121GLU	OE1	21.81	190GLU	N	190GLU	OE2	17.26
148GLU	N	148GLL	OE2	0.16	196ARG	NE	184ASP	OD2	99.02	124ARG	NH1	121GLU	OE2	32.51	185LYS	NZ	241ASP	OD1	22.32
144ARC	NH2	150GLL	O	0.08	196ARG	N	193GLU	O	0.02	124ARG	NE	113GLU	OE1	27.51	185LYS	NZ	241ASP	OD2	50.08
144ARC	NH2	190GLL	OE1	26.98	193GLU	N	190GLU	O	2.52	124ARG	NE	113GLU	OE2	2.93	185LYS	NZ	241ASP	O	0.00
144ARC	NH2	190GLL	OE2	22.28	190GLU	N	190GLU	OE1	7.76	124ARG	NE	120GLU	OE1	3.10	185LYS	NZ	245ASP	OD1	4.56
144ARC	NH1	142GLL	OE1	0.00	190GLU	N	190GLU	OE2	8.50	124ARG	NE	120GLU	OE2	0.35	185LYS	NZ	245ASP	OD2	0.04

144ARC	NH1	142GLL	OE1	5.80	185LYS	NZ	241ASP	OD1	55.53	124ARG	NE	121GLU	OE1	2.73	185LYS	NZ	217ASP	OD1	42.73
144ARC	NE	142GLL	OE2	4.47	185LYS	NZ	241ASP	OD2	31.65	124ARG	NE	121GLU	OE2	0.27	185LYS	NZ	217ASP	OD2	40.65
144ARC	NE	190GLL	OE1	13.11	185LYS	NZ	217ASP	OD1	72.85	124ARG	N	120GLU	O	38.85	185LYS	N	184ASP	OD1	0.38
144ARC	N	190GLL	OE2	16.92	185LYS	NZ	217ASP	OD2	23.46	124ARG	N	121GLU	O	7.51	178LYS	NZ	174ARG	O	3.19
144ARC	N	142GLL	OE1	1.63	185LYS	N	184ASP	OD1	0.10	121GLU	N	119LYS	O	0.01	178LYS	NZ	176ARG	O	1.54
142GLU	N	142GLL	OE2	1.28	178LYS	NZ	174ARG	O	0.76	121GLU	N	120GLU	OE1	0.31	178LYS	NZ	208ASP	OD2	36.03
142GLU	N	142GLL	OE1	0.01	178LYS	NZ	176ARG	O	0.04	121GLU	N	120GLU	OE2	0.62	178LYS	NZ	208ASP	O	0.33
142GLU	N	142GLL	OE2	0.25	178LYS	NZ	177ARG	O	0.00	121GLU	N	121GLU	OE1	1.28	178LYS	N	176ARG	O	0.06
142GLU	N	156ARC	NE	0.06	178LYS	NZ	208ASP	OD1	51.41	121GLU	N	121GLU	OE2	0.98	178LYS	N	231ASP	OD1	0.02
142GLU	N	156ARC	NH1	0.06	178LYS	NZ	208ASP	OD2	50.83	120GLU	N	120GLU	OE1	20.24	178LYS	N	231ASP	OD2	0.02
132ARC	NH2	156ARC	NH2	0.30	178LYS	NZ	208ASP	O	0.06	120GLU	N	120GLU	OE2	11.56	177ARG	NH2	127ASP	OD1	14.16
132ARC	NH2	133GLL	O	0.04	178LYS	N	176ARG	O	0.13	119LYS	NZ	113GLU	O	0.13	177ARG	NH2	127ASP	OD2	4.03
132ARC	NH2	241ASF	OD1	20.23	178LYS	N	231ASP	OD1	0.01	119LYS	NZ	114ARG	O	54.89	177ARG	NH2	127ASP	O	1.86
132ARC	NH1	241ASF	OD2	16.31	178LYS	N	231ASP	OD2	0.69	119LYS	NZ	121GLU	OE1	0.00	177ARG	NH2	229ARG	O	0.40
132ARC	NH1	241ASF	OD1	4.10	177ARG	NH2	127ASP	OD1	2.02	119LYS	NZ	121GLU	OE2	0.06	177ARG	NH2	231ASP	OD1	6.54
132ARC	NE	241ASF	OD2	2.86	177ARG	NH2	127ASP	OD2	3.10	114ARG	NH2	120GLU	OE1	7.51	177ARG	NH2	231ASP	OD2	40.26
124ARC	NH2	133GLL	O	57.51	177ARG	NH2	127ASP	O	2.66	114ARG	NH2	120GLU	OE2	3.81	177ARG	NH1	127ASP	OD1	0.38
124ARC	NH2	113GLL	OE1	17.66	177ARG	NH2	229ARG	O	0.08	114ARG	NH1	120GLU	OE1	0.06	177ARG	NH1	127ASP	OD2	0.13
124ARC	NH2	113GLL	OE2	0.70	177ARG	NH2	231ASP	OD1	3.23	114ARG	NH1	120GLU	OE2	0.00	177ARG	NH1	229ARG	O	5.69
124ARC	NH2	120GLL	OE1	14.00	177ARG	NH2	231ASP	OD2	3.73	114ARG	NE	120GLU	OE1	9.94	177ARG	NH1	231ASP	OD2	0.07
124ARC	NH2	120GLL	OE2	3.86	177ARG	NH1	127ASP	OD1	2.46	114ARG	NE	120GLU	OE2	18.32	177ARG	NE	231ASP	OD1	32.08
124ARC	NH2	121GLL	OE1	28.66	177ARG	NH1	127ASP	OD2	16.72	114ARG	N	113GLU	OE2	0.04	177ARG	NE	231ASP	OD2	15.87
124ARC	NH1	121GLL	OE2	36.72	177ARG	NH1	127ASP	O	1.20	113GLU	N	113GLU	OE1	18.60	177ARG	N	177ARG	NE	0.25
124ARC	NH1	113GLL	OE1	1.17	177ARG	NH1	229ARG	O	4.58	113GLU	N	113GLU	OE2	73.11	177ARG	N	231ASP	OD1	58.95
124ARC	NH1	113GLL	OE2	2.08	177ARG	NH1	231ASP	OD1	22.35	107LYS	NZ	127ASP	OD1	0.37	177ARG	N	231ASP	OD2	4.53
124ARC	NH1	120GLL	OE1	4.20	177ARG	NH1	231ASP	OD2	0.02	107LYS	NZ	127ASP	OD2	0.65	176ARG	NH2	127ASP	OD1	17.31
124ARC	NH1	120GLL	OE2	5.34	177ARG	NE	231ASP	OD1	4.93	107LYS	NZ	299GLU	OE1	0.02	176ARG	NH2	127ASP	OD2	73.56
124ARC	NH1	120GLL	O	0.01	177ARG	NE	231ASP	OD2	2.65	107LYS	NZ	299GLU	OE2	0.01	176ARG	NH2	127ASP	O	0.04
124ARC	NH1	121GLL	OE1	20.97	177ARG	N	177ARG	NE	0.00	107LYS	NZ	309ARG	NE	0.08	176ARG	NH1	127ASP	OD1	6.00
124ARC	NE	121GLL	OE2	12.90	177ARG	N	231ASP	OD1	24.78	107LYS	NZ	309ARG	NH2	0.08	176ARG	NH1	127ASP	OD2	5.39
124ARC	NE	113GLL	OE1	0.74	177ARG	N	231ASP	OD2	51.65	107LYS	NZ	312GLU	OE1	42.93	176ARG	NH1	127ASP	O	79.36
124ARC	NE	113GLL	OE2	14.74	176ARG	NH2	127ASP	OD1	48.80	107LYS	NZ	312GLU	OE2	67.66	176ARG	NH1	231ASP	OD1	8.25
124ARC	NE	120GLL	OE1	0.08	176ARG	NH2	127ASP	OD2	44.69	104ARG	NH2	245ASP	OD1	13.14	176ARG	NH1	231ASP	OD2	87.40
124ARC	NE	120GLL	OE2	0.00	176ARG	NH2	127ASP	O	0.03	104ARG	NH2	245ASP	OD2	5.37	176ARG	NH1	231ASP	O	0.11
124ARC	NE	120GLL	O	0.01	176ARG	NH1	127ASP	OD1	9.33	104ARG	NH1	132ARG	NH2	0.02	176ARG	N	174ARG	O	0.48
124ARC	NE	121GLL	OE1	4.19	176ARG	NH1	127ASP	OD2	14.03	104ARG	NH1	245ASP	OD1	0.34	175LYS	NZ	171GLU	OE1	0.74
124ARC	NE	121GLL	OE2	4.09	176ARG	NH1	127ASP	O	68.49	104ARG	NH1	245ASP	OD2	0.39	175LYS	NZ	171GLU	OE2	0.68
124ARC	N	121GLL	O	0.00	176ARG	NH1	177ARG	NH1	0.01	98ASP	N	94ARG	O	4.26	175LYS	NZ	171GLU	O	0.84
124ARC	N	120GLL	O	52.87	176ARG	NH1	231ASP	OD1	60.66	98ASP	N	95LYS	O	16.16	175LYS	NZ	175LYS	O	0.01
121GLU	N	121GLL	O	3.55	176ARG	NH1	231ASP	OD2	34.44	94ARG	NH2	87GLU	OE1	0.00	175LYS	NZ	299GLU	OE1	1.14
121GLU	N	119LYS	O	0.00	176ARG	NH1	231ASP	O	0.08	94ARG	NH2	270GLU	OE1	5.18	175LYS	NZ	299GLU	OE2	1.63
121GLU	N	121GLL	OE1	6.27	176ARG	NE	299GLU	OE1	0.00	94ARG	NH2	270GLU	OE2	0.27	175LYS	NZ	299GLU	O	0.43
120GLU	N	121GLL	OE2	3.92	176ARG	N	174ARG	O	0.46	94ARG	NH1	270GLU	OE1	0.01	175LYS	N	171GLU	O	6.00

120GLU	N	120GLL	OE1	7.17	175LYS	NZ	171GLU	OE1	0.85	94ARG	NH1	270GLU	OE2	6.52	174ARG	NH2	208ASP	OD1	99.86
119LYS	NZ	120GLL	OE2	9.38	175LYS	NZ	171GLU	OE2	0.91	94ARG	NE	94ARG	O	0.05	174ARG	NH2	208ASP	OD2	1.48
119LYS	NZ	113GLL	O	0.06	175LYS	NZ	171GLU	O	0.68	87GLU	N	85ARG	O	1.63	174ARG	NH1	171GLU	OE1	15.18
119LYS	NZ	114ARC	O	35.55	175LYS	NZ	299GLU	OE1	30.65	87GLU	N	87GLU	OE1	2.73	174ARG	NH1	171GLU	OE2	15.84
119LYS	NZ	120GLL	OE1	0.31	175LYS	NZ	299GLU	OE2	29.61	87GLU	N	87GLU	OE2	2.18	174ARG	NE	208ASP	OD1	7.60
119LYS	NZ	120GLL	OE2	0.27	175LYS	NZ	299GLU	O	0.75	85ARG	NH2	78ASP	OD1	0.00	174ARG	NE	208ASP	OD2	96.26
114ARC	NH2	121GLL	OE2	0.01	175LYS	N	171GLU	O	1.35	85ARG	NH2	78ASP	OD2	0.01	174ARG	N	171GLU	O	21.08
114ARC	NH2	120GLL	OE1	8.08	174ARG	NH2	171GLU	OE1	39.89	85ARG	NH2	78ASP	O	1.77	171GLU	N	167ARG	O	44.02
114ARC	NH1	120GLL	OE2	6.04	174ARG	NH2	171GLU	OE2	32.78	85ARG	NH1	78ASP	OD1	0.26	167ARG	NH2	163GLU	OE1	11.92
114ARC	NH1	120GLL	OE1	0.01	174ARG	NH2	208ASP	OD1	0.54	85ARG	NH1	78ASP	OD2	0.14	167ARG	NH2	163GLU	OE2	21.92
114ARC	NE	120GLL	OE2	0.01	174ARG	NH2	208ASP	OD2	0.15	85ARG	NH1	78ASP	O	0.04	167ARG	NH1	163GLU	OE1	4.32
114ARC	NE	120GLL	OE1	4.53	174ARG	NH1	171GLU	OE1	15.52	85ARG	NH1	87GLU	OE1	0.80	167ARG	NH1	163GLU	OE2	4.93
114ARC	N	120GLL	OE2	14.01	174ARG	NH1	171GLU	OE2	19.51	85ARG	NH1	87GLU	OE2	2.07	167ARG	NH1	163GLU	O	0.07
113GLU	N	113GLL	OE1	0.01	174ARG	NH1	171GLU	O	0.00	85ARG	N	82ARG	O	75.50	167ARG	NE	163GLU	OE1	24.38
113GLU	N	113GLL	OE1	88.82	174ARG	NH1	208ASP	OD1	0.10	85ARG	N	83LYS	O	0.04	167ARG	NE	163GLU	OE2	55.97
107LYS	NZ	113GLL	OE2	3.68	174ARG	NH1	208ASP	OD2	0.04	82ARG	NH2	87GLU	OE1	38.89	167ARG	NE	163GLU	O	0.03
107LYS	NZ	127ASF	OD1	11.08	174ARG	NE	171GLU	OE1	16.42	82ARG	NH2	87GLU	OE2	35.21	167ARG	N	163GLU	O	84.23
107LYS	NZ	127ASF	OD2	1.02	174ARG	NE	171GLU	OE2	21.41	82ARG	NE	85ARG	NH1	0.00	167ARG	N	164ARG	O	2.02
107LYS	NZ	309ARC	NE	0.00	174ARG	NE	171GLU	O	0.04	82ARG	NE	87GLU	OE1	36.10	164ARG	NH2	98ASP	OD1	0.08
107LYS	NZ	309ARC	NH1	0.03	174ARG	NE	208ASP	OD1	0.95	82ARG	NE	87GLU	OE2	42.91	164ARG	NH2	98ASP	OD2	3.65
107LYS	NZ	312GLL	OE1	52.80	174ARG	NE	208ASP	OD2	0.51	78ASP	N	76LYS	O	0.38	164ARG	NH2	98ASP	O	0.04
104ARC	NH2	312GLL	OE2	54.43	174ARG	N	171GLU	O	26.73	78ASP	N	78ASP	OD1	0.02	164ARG	NH2	161GLU	OE1	0.01
104ARC	NH2	132ARC	NH1	0.02	171GLU	N	167ARG	O	63.03	78ASP	N	78ASP	OD2	0.04	164ARG	NH2	161GLU	OE2	3.99
104ARC	NH2	245ASF	OD1	0.24	167ARG	NH2	163GLU	OE1	33.95	76LYS	NZ	9ASP	OD1	54.77	164ARG	NH2	163GLU	OE1	24.64
104ARC	NH2	245ASF	OD2	0.30	167ARG	NH2	163GLU	OE2	39.06	76LYS	NZ	9ASP	OD2	45.58	164ARG	NH2	163GLU	OE2	42.09
104ARC	NH2	270GLL	OE1	0.00	167ARG	NH2	171GLU	OE1	0.01	76LYS	NZ	47ASP	OD1	0.48	164ARG	NH2	167ARG	NE	0.00
104ARC	NH1	270GLL	OE2	0.06	167ARG	NH1	163GLU	OE1	2.11	76LYS	NZ	47ASP	OD2	0.50	164ARG	NH2	167ARG	O	0.02
104ARC	NH1	245ASF	OD1	25.49	167ARG	NH1	163GLU	OE2	3.14	76LYS	NZ	278ASP	OD2	0.06	164ARG	NH2	264ARG	NE	0.02
98ASP	N	245ASF	OD2	31.27	167ARG	NH1	163GLU	O	0.03	76LYS	N	78ASP	OD2	0.00	164ARG	NH2	264ARG	NH1	0.00
98ASP	N	94ARG	O	3.25	167ARG	NH1	171GLU	OE1	0.08	65GLU	N	2LYS	O	99.00	164ARG	NH2	264ARG	NH2	0.01
98ASP	N	95LYS	O	13.33	167ARG	NH1	171GLU	OE2	0.00	63GLU	N	59LYS	O	12.66	164ARG	NH1	98ASP	OD1	2.62
95LYS	NZ	264ARC	O	0.20	167ARG	NE	163GLU	OE1	42.06	62GLU	N	58ARG	O	4.24	164ARG	NH1	98ASP	OD2	0.31
95LYS	NZ	95LYS	O	0.02	167ARG	NE	163GLU	OE2	30.79	62GLU	N	59LYS	O	2.27	164ARG	NH1	161GLU	OE1	11.10
95LYS	NZ	98ASP	OD1	0.03	167ARG	NE	163GLU	O	0.11	59LYS	NZ	55GLU	OE1	34.60	164ARG	NH1	161GLU	OE2	22.64
94ARG	NH2	98ASP	OD2	0.08	167ARG	N	163GLU	O	78.20	59LYS	NZ	55GLU	OE2	29.31	164ARG	NH1	163GLU	OE1	2.16
94ARG	NH2	87GLU	OE1	0.11	167ARG	N	164ARG	O	3.79	59LYS	NZ	55GLU	O	0.02	164ARG	NH1	163GLU	OE2	0.01
94ARG	NH2	87GLU	OE2	0.04	164ARG	NH2	98ASP	OD1	8.16	59LYS	NZ	62GLU	OE1	0.36	164ARG	NH1	264ARG	NH2	0.01
94ARG	NH2	270GLL	OE1	40.58	164ARG	NH2	98ASP	OD2	63.24	59LYS	NZ	62GLU	OE2	0.02	164ARG	NE	163GLU	OE1	43.80
94ARG	NH1	270GLL	OE2	42.92	164ARG	NH2	98ASP	O	0.50	59LYS	NZ	63GLU	OE2	0.04	164ARG	NE	163GLU	OE2	6.23
94ARG	NH1	87GLU	OE1	0.21	164ARG	NH2	161GLU	OE1	2.63	59LYS	N	55GLU	O	58.61	164ARG	NE	164ARG	O	0.04
94ARG	NE	87GLU	OE2	0.80	164ARG	NH2	161GLU	OE2	5.36	58ARG	NH2	51GLU	OE1	12.69	164ARG	N	161GLU	O	3.35
87GLU	N	270GLL	OE1	0.36	164ARG	NH2	264ARG	NE	0.07	58ARG	NH2	51GLU	OE2	24.24	163GLU	N	159LYS	O	80.38
87GLU	N	87GLU	OE1	1.77	164ARG	NH2	264ARG	NH1	0.01	58ARG	NH2	55GLU	OE1	11.26	161GLU	N	159LYS	O	0.03

82ARG	NH2	87GLU	OE2	0.09
82ARG	NH2	87GLU	OE1	50.35
82ARG	NH1	87GLU	OE2	40.33
82ARG	NH1	87GLU	OE1	0.07
82ARG	NE	87GLU	OE2	0.03
82ARG	NE	87GLU	OE1	34.17
78ASP	N	87GLU	OE2	44.02
78ASP	N	76LYS	O	1.46
76LYS	NZ	78ASP	OD1	0.01
76LYS	NZ	47ASP	OD1	10.69
76LYS	N	47ASP	OD2	8.17
76LYS	N	9ASP	OD1	0.32
65GLU	N	9ASP	OD2	0.01
63GLU	N	2LYS	O	98.70
62GLU	N	59LYS	O	7.62
62GLU	N	58ARG	O	1.98
59LYS	NZ	59LYS	O	1.15
59LYS	NZ	55GLU	OE1	39.41
59LYS	NZ	55GLU	OE2	46.24
59LYS	N	55GLU	O	0.15
58ARG	NH2	55GLU	O	44.35
58ARG	NH2	51GLU	OE1	13.76
58ARG	NH2	51GLU	OE2	17.26
58ARG	NH2	55GLU	OE1	0.62
58ARG	NH1	55GLU	OE2	0.28
58ARG	NH1	51GLU	OE1	8.37
58ARG	NH1	51GLU	OE2	12.53
58ARG	NH1	55GLU	OE1	2.28
58ARG	NE	55GLU	OE2	3.35
58ARG	NE	51GLU	OE1	0.16
58ARG	N	51GLU	OE2	0.22
55GLU	N	55GLU	O	3.10
55GLU	N	51GLU	OE2	0.00
55GLU	N	55GLU	OE1	0.24
51GLU	N	55GLU	OE2	0.13
51GLU	N	51GLU	OE1	0.01
30GLU	N	51GLU	OE2	0.01
30GLU	N	27ASP	O	0.38
28GLU	N	28GLU	O	0.02
27ASP	N	24ARG	O	50.33
24ARG	NH2	24ARG	O	1.45
24ARG	NH2	17GLU	OE1	15.03
24ARG	NH2	17GLU	OE2	30.23

164ARG	NH2	264ARG	NH2	0.02
164ARG	NH1	98ASP	OD1	59.39
164ARG	NH1	98ASP	OD2	12.07
164ARG	NH1	98ASP	O	0.00
164ARG	NH1	161GLU	OE1	23.28
164ARG	NH1	161GLU	OE2	19.96
164ARG	NH1	264ARG	NH2	0.01
164ARG	NE	264ARG	NH1	0.00
164ARG	N	161GLU	O	20.07
163GLU	N	159LYS	O	61.18
161GLU	N	159LYS	O	0.04
161GLU	N	161GLU	OE1	0.10
161GLU	N	161GLU	OE2	0.72
159LYS	NZ	148GLU	OE1	17.44
159LYS	NZ	148GLU	OE2	19.40
159LYS	NZ	148GLU	O	1.69
159LYS	NZ	163GLU	OE1	4.86
159LYS	NZ	163GLU	OE2	3.16
159LYS	NZ	201GLU	OE1	31.19
159LYS	NZ	201GLU	OE2	32.35
156ARG	NH2	150GLU	OE1	37.89
156ARG	NH2	150GLU	OE2	37.27
156ARG	NH2	142GLU	O	1.83
156ARG	NH1	150GLU	OE1	1.86
156ARG	NH1	150GLU	OE2	3.26
156ARG	NH1	142GLU	OE1	0.23
156ARG	NH1	142GLU	OE2	0.04
156ARG	NH1	142GLU	O	13.91
156ARG	NH1	161GLU	OE1	0.01
156ARG	NE	150GLU	OE1	47.93
156ARG	NE	150GLU	OE2	42.73
156ARG	NE	142GLU	O	0.44
156ARG	NE	156ARG	O	0.02
156ARG	N	155GLU	OE1	39.19
156ARG	N	155GLU	OE2	33.50
148GLU	N	148GLU	OE1	0.68
148GLU	N	148GLU	OE2	0.60
144ARG	NH2	190GLU	OE1	27.49
144ARG	NH2	190GLU	OE2	33.22
144ARG	NH2	142GLU	OE1	4.34
144ARG	NH2	142GLU	OE2	4.39
144ARG	NH1	190GLU	OE1	1.59
144ARG	NH1	190GLU	OE2	0.03

58ARG	NH2	55GLU	OE2	7.06
58ARG	NH1	51GLU	OE1	5.00
58ARG	NH1	51GLU	OE2	4.92
58ARG	NH1	55GLU	OE1	10.62
58ARG	NH1	55GLU	OE2	10.02
58ARG	NE	51GLU	OE1	2.94
58ARG	NE	51GLU	OE2	1.26
58ARG	NE	55GLU	OE1	1.52
58ARG	NE	55GLU	OE2	7.66
58ARG	N	55GLU	O	3.34
55GLU	N	55GLU	OE1	0.13
55GLU	N	55GLU	OE2	0.04
51GLU	N	51GLU	OE1	0.00
51GLU	N	51GLU	OE2	0.04
30GLU	N	27ASP	O	0.09
30GLU	N	28GLU	O	0.02
28GLU	N	24ARG	O	40.82
27ASP	N	24ARG	O	0.74
24ARG	NH2	17GLU	OE1	28.80
24ARG	NH2	17GLU	OE2	18.05
24ARG	NH2	17GLU	O	0.09
24ARG	NH1	17GLU	OE1	14.84
24ARG	NH1	17GLU	OE2	27.26
24ARG	N	21LYS	O	0.10
21LYS	NZ	17GLU	OE1	0.70
21LYS	NZ	17GLU	OE2	1.45
21LYS	NZ	334GLU	OE1	35.52
21LYS	NZ	334GLU	OE2	39.62
21LYS	NZ	334GLU	O10[illegible]	0.12
21LYS	N	17GLU	O	77.24
17GLU	N	14GLU	O	1.95
14GLU	N	14GLU	OE1	0.01
2LYS	NZ	37GLU	OE1	1.90
2LYS	NZ	37GLU	OE2	2.01
2LYS	NZ	63GLU	OE1	42.87
2LYS	NZ	63GLU	OE2	34.59
2LYS	NZ	63GLU	O	0.83
2LYS	N	65GLU	OE1	0.20
2LYS	N	65GLU	OE2	0.77

161GLU	N	161GLU	OE1	1.07
161GLU	N	161GLU	OE2	0.38
159LYS	NZ	148GLU	OE1	24.15
159LYS	NZ	148GLU	OE2	15.77
159LYS	NZ	148GLU	O	2.38
159LYS	NZ	163GLU	OE1	3.65
159LYS	NZ	163GLU	OE2	3.44
159LYS	NZ	201GLU	OE1	14.38
159LYS	NZ	201GLU	OE2	15.54
156ARG	NH2	150GLU	OE1	49.45
156ARG	NH2	150GLU	OE2	43.98
156ARG	NH2	142GLU	O	0.15
156ARG	NH1	150GLU	OE2	0.16
156ARG	NH1	142GLU	OE1	0.46
156ARG	NH1	142GLU	OE2	0.16
156ARG	NH1	142GLU	O	92.73
156ARG	NE	150GLU	OE1	45.99
156ARG	NE	150GLU	OE2	51.85
156ARG	NE	142GLU	O	0.02
156ARG	N	155GLU	OE1	0.84
156ARG	N	155GLU	OE2	0.76
150GLU	N	148GLU	O	0.00
148GLU	N	148GLU	OE1	0.11
148GLU	N	148GLU	OE2	0.18
144ARG	NH2	190GLU	OE1	32.83
144ARG	NH2	190GLU	OE2	63.92
144ARG	NH1	142GLU	OE1	0.03
144ARG	NH1	142GLU	OE2	0.03
144ARG	NE	190GLU	OE1	64.79
144ARG	NE	190GLU	OE2	43.30
144ARG	N	142GLU	OE1	0.33
144ARG	N	142GLU	OE2	0.31
142GLU	N	142GLU	OE1	0.26
142GLU	N	142GLU	OE2	0.07
132ARG	NH2	241ASP	N	0.00
132ARG	NH2	241ASP	OD1	2.23
132ARG	NH2	241ASP	OD2	3.90
132ARG	NH1	133GLU	O	1.46
132ARG	NH1	241ASP	OD1	1.99
132ARG	NH1	241ASP	OD2	2.26
132ARG	NE	133GLU	O	8.99
124ARG	NH2	113GLU	OE1	9.64
124ARG	NH2	113GLU	OE2	11.34

24ARG	NH2	17GLU	O	0.12
24ARG	NH2	27ASP	OD2	1.99
24ARG	NH1	28GLU	OE2	0.01
24ARG	NH1	17GLU	OE1	14.49
24ARG	NH1	17GLU	OE2	11.31
24ARG	NH1	24ARG	O	0.23
24ARG	N	28GLU	OE2	0.47
21LYS	NZ	21LYS	O	0.17
21LYS	NZ	17GLU	OE1	0.04
21LYS	NZ	17GLU	OE2	1.58
21LYS	NZ	17GLU	O	0.02
21LYS	NZ	334GLU	OE1	22.72
21LYS	N	334GLU	OE2	22.42
17GLU	N	17GLU	O	79.82
2LYS	NZ	14GLU	O	2.90
2LYS	NZ	37GLU	OE1	0.52
2LYS	NZ	37GLU	OE2	0.51
2LYS	NZ	63GLU	OE1	35.97
2LYS	NZ	63GLU	OE2	32.77
2LYS	N	63GLU	O	2.88
2LYS	N	65GLU	OE1	0.94
2LYS	N	65GLU	OE2	0.03

144ARG	NH1	142GLU	OE1	3.36
144ARG	NH1	142GLU	OE2	2.32
144ARG	NE	190GLU	OE1	42.73
144ARG	NE	190GLU	OE2	28.19
144ARG	NE	142GLU	OE1	6.55
144ARG	NE	142GLU	OE2	4.92
144ARG	NE	144ARG	O	0.07
144ARG	N	142GLU	OE1	1.45
144ARG	N	142GLU	OE2	1.44
144ARG	N	144ARG	NE	0.01
144ARG	N	144ARG	NH2	0.04
142GLU	N	142GLU	OE1	0.45
142GLU	N	142GLU	OE2	0.32
142GLU	N	156ARG	NH1	0.00
132ARG	NH2	87GLU	OE1	0.01
132ARG	NH2	87GLU	OE2	0.01
132ARG	NH2	133GLU	O	0.23
132ARG	NH2	241ASP	OD1	0.54
132ARG	NH2	241ASP	OD2	0.61
132ARG	NH1	241ASP	OD1	3.34
132ARG	NH1	241ASP	OD2	4.32
132ARG	NE	133GLU	O	21.56
132ARG	NE	241ASP	N	0.02
124ARG	NH2	113GLU	OE1	20.57
124ARG	NH2	113GLU	OE2	21.59
124ARG	NH2	120GLU	OE1	37.65
124ARG	NH2	120GLU	OE2	32.19
124ARG	NH2	121GLU	OE1	6.71
124ARG	NH2	121GLU	OE2	7.65
124ARG	NH1	113GLU	OE1	0.01
124ARG	NH1	113GLU	OE2	0.07
124ARG	NH1	120GLU	OE1	7.43
124ARG	NH1	120GLU	OE2	9.40
124ARG	NH1	120GLU	O	0.09
124ARG	NH1	121GLU	OE1	29.01
124ARG	NH1	121GLU	OE2	25.89
124ARG	NE	113GLU	OE1	23.03
124ARG	NE	113GLU	OE2	19.04
124ARG	NE	120GLU	OE1	6.76
124ARG	NE	120GLU	OE2	5.29
124ARG	NE	120GLU	O	0.03
124ARG	N	120GLU	O	32.55
124ARG	N	121GLU	O	6.91

124ARG	NH2	120GLU	OE1	19.51
124ARG	NH2	120GLU	OE2	17.35
124ARG	NH2	121GLU	OE1	23.40
124ARG	NH2	121GLU	OE2	29.96
124ARG	NH1	113GLU	OE1	11.72
124ARG	NH1	113GLU	OE2	4.67
124ARG	NH1	120GLU	OE1	10.62
124ARG	NH1	120GLU	OE2	6.24
124ARG	NH1	120GLU	O	0.03
124ARG	NH1	121GLU	OE1	20.15
124ARG	NH1	121GLU	OE2	20.57
124ARG	NE	113GLU	OE1	13.13
124ARG	NE	113GLU	OE2	10.85
124ARG	NE	120GLU	OE1	0.04
124ARG	NE	120GLU	O	0.02
124ARG	NE	121GLU	OE1	13.78
124ARG	NE	121GLU	OE2	9.71
124ARG	NE	121GLU	O	0.05
124ARG	N	120GLU	O	14.81
124ARG	N	121GLU	O	10.22
121GLU	N	119LYS	O	0.02
121GLU	N	121GLU	OE1	0.47
121GLU	N	121GLU	OE2	0.39
120GLU	N	120GLU	OE1	19.22
120GLU	N	120GLU	OE2	17.61
119LYS	NZ	113GLU	O	10.12
119LYS	NZ	114ARG	O	25.31
119LYS	NZ	120GLU	OE1	15.34
119LYS	NZ	120GLU	OE2	12.40
114ARG	NH2	120GLU	OE1	7.30
114ARG	NH2	120GLU	OE2	7.90
114ARG	NH1	113GLU	OE2	0.00
114ARG	NH1	120GLU	OE1	0.20
114ARG	NH1	120GLU	OE2	1.40
114ARG	NE	114ARG	O	0.01
114ARG	NE	120GLU	OE1	12.83
114ARG	NE	120GLU	OE2	17.10
114ARG	N	113GLU	OE1	0.22
114ARG	N	113GLU	OE2	0.03
113GLU	N	113GLU	OE1	39.19
113GLU	N	113GLU	OE2	50.42
107LYS	NZ	127ASP	OD1	5.14
107LYS	NZ	127ASP	OD2	3.60

121GLU	N	120GLU	OE1	0.06
121GLU	N	120GLU	OE2	0.07
121GLU	N	121GLU	OE1	2.45
121GLU	N	121GLU	OE2	2.36
120GLU	N	120GLU	OE1	1.56
120GLU	N	120GLU	OE2	0.70
119LYS	NZ	113GLU	O	0.04
119LYS	NZ	114ARG	O	21.99
119LYS	NZ	120GLU	OE1	6.34
119LYS	NZ	120GLU	OE2	2.26
114ARG	NH2	113GLU	OE1	0.00
114ARG	NH2	120GLU	OE1	16.19
114ARG	NH2	120GLU	OE2	16.08
114ARG	NH1	120GLU	OE1	0.10
114ARG	NH1	120GLU	OE2	0.19
114ARG	NE	120GLU	OE1	27.11
114ARG	NE	120GLU	OE2	24.32
114ARG	N	113GLU	OE1	0.11
114ARG	N	113GLU	OE2	0.08
113GLU	N	113GLU	OE1	42.12
113GLU	N	113GLU	OE2	42.01
107LYS	NZ	127ASP	OD1	4.02
107LYS	NZ	127ASP	OD2	2.40
107LYS	NZ	312GLU	OE1	51.67
107LYS	NZ	312GLU	OE2	51.35
104ARG	NH2	241ASP	OD1	0.02
104ARG	NH2	241ASP	OD2	0.05
104ARG	NH2	241ASP	O	0.00
104ARG	NH2	245ASP	OD1	1.04
104ARG	NH2	245ASP	OD2	0.98
104ARG	NH2	270GLU	OE1	0.01
104ARG	NH2	270GLU	OE2	0.05
104ARG	NH1	241ASP	OD1	1.49
104ARG	NH1	241ASP	OD2	0.14
104ARG	NH1	245ASP	OD1	0.45
104ARG	NH1	245ASP	OD2	0.24
98ASP	N	94ARG	O	6.68
98ASP	N	95LYS	O	20.73
98ASP	N	264ARG	O	0.00
95LYS	NZ	95LYS	O	0.02
95LYS	NZ	98ASP	OD1	0.42
95LYS	NZ	98ASP	OD2	0.24
95LYS	NZ	161GLU	OE1	0.01

107LYS	NZ	176ARG	NH2	0.00
107LYS	NZ	299GLU	OE1	0.00
107LYS	NZ	309ARG	NE	0.02
107LYS	NZ	309ARG	NH2	0.06
107LYS	NZ	312GLU	OE1	38.45
107LYS	NZ	312GLU	OE2	45.65
104ARG	NH2	245ASP	OD1	13.26
104ARG	NH2	245ASP	OD2	10.67
104ARG	NH1	132ARG	NE	0.02
104ARG	NH1	132ARG	NH1	0.00
104ARG	NH1	132ARG	NH2	0.02
104ARG	NH1	241ASP	OD1	1.39
104ARG	NH1	245ASP	OD1	1.49
104ARG	NH1	245ASP	OD2	0.50
98ASP	N	62GLU	OE1	0.00
98ASP	N	62GLU	OE2	0.55
98ASP	N	94ARG	O	7.19
98ASP	N	95LYS	O	11.62
98ASP	N	264ARG	O	0.16
95LYS	NZ	51GLU	OE1	0.44
95LYS	NZ	51GLU	OE2	0.72
95LYS	NZ	95LYS	O	0.05
95LYS	NZ	98ASP	OD1	8.19
95LYS	NZ	98ASP	OD2	7.78
95LYS	NZ	142GLU	OE1	0.38
95LYS	NZ	142GLU	OE2	0.51
94ARG	NH2	270GLU	OE1	65.98
94ARG	NH2	270GLU	OE2	36.73
94ARG	NH1	270GLU	OE1	31.79
94ARG	NH1	270GLU	OE2	63.33
87GLU	N	85ARG	O	1.40
87GLU	N	87GLU	OE1	29.29
87GLU	N	87GLU	OE2	32.88
85ARG	NH2	78ASP	OD1	4.87
85ARG	NH2	78ASP	OD2	5.57
85ARG	NH2	78ASP	O	0.28
85ARG	NH2	87GLU	OE1	11.30
85ARG	NH2	87GLU	OE2	31.70
85ARG	NH1	78ASP	OD1	18.59
85ARG	NH1	78ASP	OD2	21.95
85ARG	NH1	78ASP	O	1.48
85ARG	NH1	87GLU	OE1	0.62
85ARG	NH1	87GLU	OE2	0.10

94ARG	NH2	87GLU	OE1	9.61
94ARG	NH2	87GLU	OE2	19.53
94ARG	NH2	270GLU	OE1	2.36
94ARG	NH2	270GLU	OE2	0.72
94ARG	NH1	87GLU	OE1	49.51
94ARG	NH1	87GLU	OE2	32.07
87GLU	N	87GLU	OE1	0.97
87GLU	N	87GLU	OE2	0.43
82ARG	NH2	190GLU	OE1	21.07
82ARG	NH2	190GLU	OE2	43.82
82ARG	NH2	193GLU	OE1	27.88
82ARG	NH2	193GLU	OE2	33.66
82ARG	NH2	87GLU	OE1	1.89
82ARG	NH2	87GLU	OE2	4.16
82ARG	NH2	87GLU	O	0.00
82ARG	NH2	144ARG	NE	0.00
82ARG	NH1	190GLU	OE1	45.72
82ARG	NH1	190GLU	OE2	23.49
82ARG	NH1	193GLU	OE1	5.61
82ARG	NH1	193GLU	OE2	6.88
82ARG	NH1	87GLU	OE1	1.98
82ARG	NH1	87GLU	OE2	2.14
82ARG	NH1	144ARG	NE	0.02
82ARG	NH1	144ARG	NH2	0.04
82ARG	NE	193GLU	OE1	28.04
82ARG	NE	193GLU	OE2	22.21
82ARG	NE	82ARG	O	0.02
82ARG	NE	87GLU	OE1	1.40
82ARG	NE	87GLU	OE2	1.26
78ASP	N	76LYS	O	0.70
78ASP	N	78ASP	OD1	0.01
78ASP	N	78ASP	OD2	0.01
76LYS	NZ	9ASP	OD1	41.80
76LYS	NZ	9ASP	OD2	45.41
76LYS	NZ	9ASP	O	0.68
76LYS	NZ	47ASP	OD1	0.27
76LYS	NZ	47ASP	OD2	0.17
76LYS	NZ	76LYS	O	0.00
76LYS	N	78ASP	O	0.30
65GLU	N	2LYS	O	97.41
65GLU	N	65GLU	OE1	0.05
65GLU	N	65GLU	OE2	0.01
63GLU	N	59LYS	O	10.31

85ARG	NE	78ASP	O	0.00
85ARG	NE	87GLU	OE1	34.51
85ARG	NE	87GLU	OE2	15.04
85ARG	N	82ARG	O	46.21
85ARG	N	83LYS	O	0.01
83LYS	NZ	190GLU	OE1	1.20
83LYS	NZ	190GLU	OE2	0.03
83LYS	NZ	51GLU	OE1	1.00
83LYS	NZ	51GLU	OE2	0.90
83LYS	NZ	144ARG	NE	0.00
83LYS	NZ	144ARG	NH2	0.00
82ARG	NH2	190GLU	OE1	0.77
82ARG	NH2	190GLU	OE2	0.09
82ARG	NH2	193GLU	OE1	30.08
82ARG	NH2	193GLU	OE2	28.35
82ARG	NH2	87GLU	OE1	0.06
82ARG	NH2	87GLU	OE2	1.44
82ARG	NH2	144ARG	NE	0.02
82ARG	NH2	144ARG	NH1	0.00
82ARG	NH2	144ARG	NH2	0.03
82ARG	NH1	190GLU	OE1	0.54
82ARG	NH1	190GLU	OE2	0.24
82ARG	NH1	193GLU	OE1	20.88
82ARG	NH1	193GLU	OE2	20.54
82ARG	NH1	82ARG	O	0.00
82ARG	NH1	85ARG	NH1	0.01
82ARG	NH1	144ARG	NE	0.00
82ARG	NE	193GLU	OE1	4.10
82ARG	NE	193GLU	OE2	2.37
82ARG	NE	87GLU	OE2	0.06
78ASP	N	76LYS	O	1.97
76LYS	NZ	9ASP	OD1	32.74
76LYS	NZ	9ASP	OD2	21.77
76LYS	NZ	9ASP	O	0.00
76LYS	NZ	47ASP	OD1	0.66
76LYS	NZ	47ASP	OD2	0.68
76LYS	NZ	76LYS	O	0.04
76LYS	NZ	278ASP	OD1	0.96
76LYS	NZ	278ASP	OD2	0.91
76LYS	NZ	282LYS	O	0.04
65GLU	N	2LYS	O	97.54
65GLU	N	65GLU	OE2	0.02
63GLU	N	59LYS	O	4.51

63GLU	N	63GLU	OE1	0.00
62GLU	N	58ARG	O	11.36
62GLU	N	59LYS	O	2.22
59LYS	NZ	55GLU	OE1	40.74
59LYS	NZ	55GLU	OE2	27.16
59LYS	NZ	55GLU	O	0.08
59LYS	NZ	59LYS	O	0.00
59LYS	NZ	62GLU	OE1	0.15
59LYS	NZ	62GLU	OE2	0.14
59LYS	NZ	63GLU	OE1	0.29
59LYS	NZ	63GLU	OE2	0.12
59LYS	N	55GLU	O	67.99
58ARG	NH2	51GLU	OE1	9.16
58ARG	NH2	51GLU	OE2	13.03
58ARG	NH2	55GLU	OE1	30.22
58ARG	NH2	55GLU	OE2	46.75
58ARG	NH1	51GLU	OE1	31.63
58ARG	NH1	51GLU	OE2	28.07
58ARG	NH1	55GLU	OE1	3.04
58ARG	NH1	55GLU	OE2	1.06
58ARG	NE	51GLU	OE1	0.03
58ARG	NE	51GLU	OE2	0.01
58ARG	NE	55GLU	OE1	3.59
58ARG	NE	55GLU	OE2	4.11
58ARG	N	55GLU	O	1.88
55GLU	N	51GLU	OE1	0.03
55GLU	N	51GLU	OE2	0.04
55GLU	N	55GLU	OE1	0.04
55GLU	N	55GLU	OE2	0.07
51GLU	N	51GLU	OE1	0.08
51GLU	N	51GLU	OE2	0.06
47ASP	N	47ASP	OD1	0.01
47ASP	N	47ASP	OD2	0.03
30GLU	N	27ASP	O	0.56
30GLU	N	28GLU	O	0.02
28GLU	N	24ARG	O	69.53
27ASP	N	24ARG	O	4.20
24ARG	NH2	17GLU	OE1	0.32
24ARG	NH2	17GLU	OE2	0.22
24ARG	NH2	27ASP	OD1	1.62
24ARG	NH2	27ASP	OD2	4.32
24ARG	NH2	27ASP	O	0.11
24ARG	NH2	28GLU	OE1	42.11

63GLU	N	63GLU	OE2	0.00
62GLU	N	58ARG	O	8.78
62GLU	N	59LYS	O	3.01
62GLU	N	62GLU	OE1	0.04
59LYS	NZ	55GLU	OE1	27.41
59LYS	NZ	55GLU	OE2	33.73
59LYS	NZ	55GLU	O	0.11
59LYS	NZ	62GLU	OE1	0.47
59LYS	NZ	62GLU	OE2	0.40
59LYS	NZ	62GLU	O	0.00
59LYS	NZ	63GLU	OE1	0.50
59LYS	NZ	63GLU	OE2	0.02
59LYS	N	55GLU	O	54.47
58ARG	NH2	51GLU	OE1	12.45
58ARG	NH2	51GLU	OE2	12.13
58ARG	NH2	55GLU	OE1	44.98
58ARG	NH2	55GLU	OE2	37.93
58ARG	NH1	51GLU	OE1	29.28
58ARG	NH1	51GLU	OE2	28.57
58ARG	NH1	55GLU	OE1	4.66
58ARG	NH1	55GLU	OE2	3.62
58ARG	NE	51GLU	OE1	1.01
58ARG	NE	51GLU	OE2	0.87
58ARG	NE	55GLU	OE1	4.45
58ARG	NE	55GLU	OE2	3.43
58ARG	N	55GLU	O	4.78
55GLU	N	51GLU	OE1	0.01
55GLU	N	51GLU	OE2	0.00
55GLU	N	55GLU	OE1	0.01
51GLU	N	51GLU	OE1	0.01
51GLU	N	51GLU	OE2	0.03
30GLU	N	27ASP	O	0.73
30GLU	N	28GLU	O	0.10
30GLU	N	30GLU	OE2	0.00
28GLU	N	24ARG	O	49.12
28GLU	N	30GLU	O	0.00
27ASP	N	24ARG	O	3.80
24ARG	NH2	17GLU	OE1	26.51
24ARG	NH2	17GLU	OE2	33.89
24ARG	NH2	17GLU	O	0.03
24ARG	NH2	28GLU	OE1	1.60
24ARG	NH2	28GLU	OE2	2.35
24ARG	NH1	17GLU	OE1	36.90

24ARG	NH2	28GLU	OE2	52.21
24ARG	NH1	17GLU	OE1	0.22
24ARG	NH1	27ASP	OD1	29.44
24ARG	NH1	27ASP	OD2	61.47
24ARG	NE	24ARG	O	0.01
24ARG	NE	28GLU	OE1	1.85
24ARG	NE	28GLU	OE2	1.98
24ARG	N	21LYS	O	0.45
21LYS	NZ	14GLU	OE1	0.02
21LYS	NZ	17GLU	OE1	4.67
21LYS	NZ	17GLU	OE2	6.70
21LYS	NZ	17GLU	O	0.05
21LYS	NZ	334GLU	OE1	31.67
21LYS	NZ	334GLU	OE2	33.55
21LYS	NZ	334GLU	O	0.01
21LYS	N	17GLU	O	56.28
17GLU	N	14GLU	O	6.29
2LYS	NZ	37GLU	OE1	2.14
2LYS	NZ	37GLU	OE2	3.58
2LYS	NZ	63GLU	OE1	31.49
2LYS	NZ	63GLU	OE2	36.94
2LYS	NZ	63GLU	O	7.97
2LYS	NZ	65GLU	OE1	1.78
2LYS	NZ	65GLU	OE2	5.11
2LYS	N	65GLU	OE1	8.00
2LYS	N	65GLU	OE2	8.13

24ARG	NH1	17GLU	OE2	27.70
24ARG	NH1	17GLU	O	0.00
24ARG	NH1	27ASP	OD2	0.14
24ARG	NH1	28GLU	OE1	2.90
24ARG	NH1	28GLU	OE2	3.28
24ARG	NE	17GLU	OE2	0.00
24ARG	NE	24ARG	O	0.01
24ARG	NE	28GLU	OE1	0.11
24ARG	NE	28GLU	OE2	0.03
24ARG	N	21LYS	O	0.24
21LYS	NZ	14GLU	OE1	0.00
21LYS	NZ	17GLU	OE1	2.67
21LYS	NZ	17GLU	OE2	0.58
21LYS	NZ	17GLU	O	0.02
21LYS	NZ	334GLU	OE1	16.62
21LYS	NZ	334GLU	OE2	18.85
21LYS	NZ	334GLU	O	0.01
21LYS	N	17GLU	O	83.16
17GLU	N	14GLU	O	2.62
14GLU	N	14GLU	OE1	0.01
2LYS	NZ	37GLU	OE1	5.28
2LYS	NZ	37GLU	OE2	3.08
2LYS	NZ	59LYS	O	0.07
2LYS	NZ	63GLU	OE1	31.61
2LYS	NZ	63GLU	OE2	32.75
2LYS	NZ	63GLU	O	5.58
2LYS	NZ	65GLU	OE1	0.27
2LYS	NZ	65GLU	OE2	1.69
2LYS	N	65GLU	OE1	1.40
2LYS	N	65GLU	OE2	4.70
