# Supplementary material for: Deciphering the Dynamics of Non-Covalent Interactions Affecting Thermal Stability of a Protein: Molecular Dynamics Study on Point Mutant of Thermus thermophilus Isopropylmalate Dehydrogenase
Source: PLoS One. 2015 Dec 11;10(12):e0144294. doi: 10.1371/journal.pone.0144294 (PMC4689552; doi:10.1371/journal.pone.0144294)
Supplement: S8 Table — The color formatting indicates the percentage of time interaction existed is as in S1 Table. (PDF) [file pone.0144294.s010.pdf]

S8 Table. **Unique intra hydrophobic contacts and the percentage of their existence in *wt* and *mut* at 300 K and 337 K.**

1) *Wt* 300 K

Drnona	D	Arnona	A	percen
1MET	CA	1MET	CB	100.00
1MET	CA	1MET	CG	100.00
1MET	CA	1MET	CE	0.00
1MET	CA	1MET	C	100.00
1MET	CA	65GLU	CD	0.01
1MET	CA	302PHE	CE1	0.02
1MET	CB	1MET	CG	100.00
1MET	CB	1MET	CE	31.99
1MET	CB	1MET	C	100.00
1MET	CB	33GLY	C	0.01
1MET	CB	65GLU	CB	0.00
1MET	CB	65GLU	CG	0.00
1MET	CB	65GLU	CD	0.00
1MET	CB	302PHE	CD1	0.04
1MET	CB	302PHE	CD2	0.16
1MET	CB	302PHE	CE1	1.33
1MET	CB	302PHE	CE2	10.21
1MET	CB	302PHE	CZ	3.95
1MET	CG	1MET	CE	100.00
1MET	CG	1MET	C	47.04
1MET	CG	65GLU	CB	3.16
1MET	CG	65GLU	CG	0.20
1MET	CG	65GLU	CD	0.10
1MET	CG	302PHE	CG	0.02
1MET	CG	302PHE	CD1	0.10
1MET	CG	302PHE	CD2	0.35
1MET	CG	302PHE	CE1	0.23
1MET	CG	302PHE	CE2	1.88
1MET	CG	302PHE	CZ	1.16
1MET	CE	1MET	C	0.01
1MET	CE	3VAL	CG2	1.19
1MET	CE	32LEU	CD1	0.05
1MET	CE	32LEU	CD2	0.10
1MET	CE	32LEU	C	0.01
1MET	CE	33GLY	C	0.01
1MET	CE	34LEU	CA	0.00
1MET	CE	34LEU	CD1	0.00
1MET	CE	65GLU	CB	0.17
1MET	CE	65GLU	CG	0.50
1MET	CE	65GLU	CD	0.03
1MET	CE	65GLU	C	0.04
1MET	CE	66ALA	CB	0.48
1MET	CE	297MET	CE	0.00
1MET	CE	298LEU	CD1	0.00
1MET	CE	301ALA	CB	1.74
1MET	CE	301ALA	C	0.41
1MET	CE	302PHE	CA	0.03
1MET	CE	302PHE	CB	0.03
1MET	CE	302PHE	CG	0.64
1MET	CE	302PHE	CD1	2.62
1MET	CE	302PHE	CD2	4.70
1MET	CE	302PHE	CE1	5.29
1MET	CE	302PHE	CE2	1.86
1MET	CE	302PHE	CZ	1.18
1MET	CE	304LEU	CD1	0.04
1MET	CE	304LEU	CD2	0.01
1MET	C	2LYS	CA	100.00
1MET	C	2LYS	CB	4.76
1MET	C	2LYS	CG	0.12

2) Wt 337 K

Drnona	D	Arnona	A	percen
1MET	CA	1MET	CB	100.00
1MET	CA	1MET	CG	100.00
1MET	CA	1MET	C	100.00
1MET	CA	65GLU	CD	0.18
1MET	CA	302PHE	CE1	0.01
1MET	CA	302PHE	CE2	0.18
1MET	CA	302PHE	CZ	0.09
1MET	CB	1MET	CG	100.00
1MET	CB	1MET	CE	25.70
1MET	CB	1MET	C	100.00
1MET	CB	32LEU	CG	0.00
1MET	CB	32LEU	CD1	0.01
1MET	CB	32LEU	CD2	0.02
1MET	CB	33GLY	C	0.01
1MET	CB	34LEU	CD2	0.02
1MET	CB	65GLU	CB	0.01
1MET	CB	65GLU	CG	0.01
1MET	CB	302PHE	CG	0.00
1MET	CB	302PHE	CD1	0.12
1MET	CB	302PHE	CD2	0.05
1MET	CB	302PHE	CE1	4.87
1MET	CB	302PHE	CE2	1.22
1MET	CB	302PHE	CZ	2.03
1MET	CG	1MET	CE	100.00
1MET	CG	1MET	C	83.05
1MET	CG	3VAL	CG1	0.02
1MET	CG	3VAL	CG2	0.06
1MET	CG	32LEU	CD2	0.00
1MET	CG	34LEU	CG	0.00
1MET	CG	34LEU	CD1	0.02
1MET	CG	34LEU	CD2	0.06
1MET	CG	65GLU	CB	3.47
1MET	CG	65GLU	CG	0.28
1MET	CG	65GLU	CD	0.08
1MET	CG	302PHE	CB	0.00
1MET	CG	302PHE	CG	0.28
1MET	CG	302PHE	CD1	0.87
1MET	CG	302PHE	CD2	0.74
1MET	CG	302PHE	CE1	3.22
1MET	CG	302PHE	CE2	1.64
1MET	CG	302PHE	CZ	2.74
1MET	CE	1MET	C	0.02
1MET	CE	2LYS	C	0.00
1MET	CE	3VAL	CG1	0.10
1MET	CE	3VAL	CG2	1.33
1MET	CE	23LEU	CD1	0.02
1MET	CE	23LEU	CD2	0.10
1MET	CE	32LEU	CB	0.01
1MET	CE	32LEU	CG	0.01
1MET	CE	32LEU	CD1	0.04
1MET	CE	32LEU	CD2	0.08
1MET	CE	32LEU	C	0.08
1MET	CE	33GLY	C	0.05
1MET	CE	34LEU	CA	0.00
1MET	CE	34LEU	CD1	0.14
1MET	CE	34LEU	CD2	0.14
1MET	CE	65GLU	CB	0.18
1MET	CE	65GLU	CG	0.40
1MET	CE	65GLU	CD	0.02

3) *Mut* 300 K

Drnon	D	Arnona	A	percen
1MET	CA	1MET	CB	100.00
1MET	CA	1MET	CG	100.00
1MET	CA	1MET	C	100.00
1MET	CA	65GLU	CD	0.33
1MET	CA	302PHE	CD1	0.00
1MET	CA	302PHE	CE1	0.84
1MET	CA	302PHE	CE2	0.00
1MET	CA	302PHE	CZ	0.14
1MET	CB	1MET	CG	100.00
1MET	CB	1MET	CE	39.85
1MET	CB	1MET	C	100.00
1MET	CB	32LEU	CD2	0.01
1MET	CB	33GLY	C	0.09
1MET	CB	302PHE	CD1	0.01
1MET	CB	302PHE	CD2	0.04
1MET	CB	302PHE	CE1	0.44
1MET	CB	302PHE	CE2	4.27
1MET	CB	302PHE	CZ	0.71
1MET	CG	1MET	CE	100.00
1MET	CG	1MET	C	92.01
1MET	CG	3VAL	CG2	0.08
1MET	CG	65GLU	CB	4.84
1MET	CG	65GLU	CG	0.41
1MET	CG	65GLU	CD	0.03
1MET	CG	297MET	CE	0.01
1MET	CG	302PHE	CG	0.35
1MET	CG	302PHE	CD1	0.39
1MET	CG	302PHE	CD2	1.09
1MET	CG	302PHE	CE1	1.40
1MET	CG	302PHE	CE2	5.58
1MET	CG	302PHE	CZ	4.22
1MET	CE	1MET	C	0.00
1MET	CE	2LYS	C	0.00
1MET	CE	3VAL	CG2	1.88
1MET	CE	23LEU	CD1	0.04
1MET	CE	23LEU	CD2	0.06
1MET	CE	32LEU	CB	0.10
1MET	CE	32LEU	CD1	0.02
1MET	CE	32LEU	CD2	0.03
1MET	CE	32LEU	C	0.06
1MET	CE	34LEU	CA	0.00
1MET	CE	34LEU	CD1	0.22
1MET	CE	65GLU	CB	0.15
1MET	CE	65GLU	CG	0.25
1MET	CE	65GLU	CD	0.01
1MET	CE	65GLU	C	0.03
1MET	CE	66ALA	CB	1.19
1MET	CE	297MET	CG	0.01
1MET	CE	297MET	CE	0.04
1MET	CE	298LEU	CG	0.01
1MET	CE	298LEU	CD1	2.66
1MET	CE	301ALA	CB	1.25
1MET	CE	301ALA	C	0.25
1MET	CE	302PHE	CA	0.02
1MET	CE	302PHE	CB	0.14
1MET	CE	302PHE	CG	1.19
1MET	CE	302PHE	CD1	2.58
1MET	CE	302PHE	CD2	3.33
1MET	CE	302PHE	CE1	8.27

1MET	C	2LYS	C	96.35
1MET	C	302PHE	CZ	0.04
2LYS	CA	2LYS	CB	100.00
2LYS	CA	2LYS	CG	100.00
2LYS	CA	2LYS	CD	0.67
2LYS	CA	2LYS	C	100.00
2LYS	CA	35ALA	CB	0.01
2LYS	CB	2LYS	CG	100.00
2LYS	CB	2LYS	CD	100.00
2LYS	CB	2LYS	CE	12.32
2LYS	CB	2LYS	C	100.00
2LYS	CB	35ALA	CB	0.00
2LYS	CG	2LYS	CD	100.00
2LYS	CG	2LYS	CE	100.00
2LYS	CG	2LYS	C	31.79
2LYS	CG	35ALA	CB	0.03
2LYS	CG	36TYR	C	0.00
2LYS	CD	2LYS	CE	100.00
2LYS	CD	2LYS	C	0.01
2LYS	CD	35ALA	CB	0.00
2LYS	CD	37GLU	CB	0.00
2LYS	CD	63GLU	CG	0.03
2LYS	CD	63GLU	CD	0.03
2LYS	CE	36TYR	C	0.01
2LYS	CE	37GLU	CB	0.23
2LYS	CE	37GLU	CG	0.00
2LYS	CE	37GLU	CD	0.01
2LYS	CE	63GLU	CB	0.00
2LYS	CE	63GLU	CG	0.02
2LYS	CE	63GLU	CD	0.04
2LYS	C	3VAL	CA	100.00
2LYS	C	3VAL	CB	26.22
2LYS	C	3VAL	CG2	18.02
2LYS	C	3VAL	C	82.38
2LYS	C	64ALA	CB	0.02
3VAL	CA	3VAL	CB	100.00
3VAL	CA	3VAL	CG1	100.00
3VAL	CA	3VAL	CG2	100.00
3VAL	CA	3VAL	C	100.00
3VAL	CA	64ALA	CB	0.01
3VAL	CB	3VAL	CG1	100.00
3VAL	CB	3VAL	CG2	100.00
3VAL	CB	3VAL	C	100.00
3VAL	CB	34LEU	CD1	0.04
3VAL	CB	36TYR	CB	0.00
3VAL	CG1	3VAL	CG2	100.00
3VAL	CG1	3VAL	C	100.00
3VAL	CG1	4ALA	C	0.02
3VAL	CG1	5VAL	CG2	0.27
3VAL	CG1	68LEU	CB	1.46
3VAL	CG1	68LEU	CG	0.31
3VAL	CG1	68LEU	CD1	1.77
3VAL	CG1	297MET	CE	0.48
3VAL	CG2	34LEU	CG	0.01
3VAL	CG2	34LEU	CD1	3.32
3VAL	CG2	34LEU	CD2	0.01
3VAL	CG2	297MET	CE	1.21
3VAL	CG2	302PHE	CE1	0.95
3VAL	CG2	302PHE	CE2	0.10
3VAL	CG2	302PHE	CZ	4.67
3VAL	C	4ALA	CA	100.00
3VAL	C	4ALA	CB	1.94

1MET	CE	65GLU	C	0.03
1MET	CE	66ALA	CB	1.14
1MET	CE	297MET	CG	0.19
1MET	CE	297MET	CE	0.05
1MET	CE	297MET	C	0.00
1MET	CE	298LEU	CG	0.00
1MET	CE	298LEU	CD1	0.10
1MET	CE	298LEU	CD2	0.75
1MET	CE	301ALA	CB	2.26
1MET	CE	301ALA	C	0.30
1MET	CE	302PHE	CA	0.02
1MET	CE	302PHE	CB	0.07
1MET	CE	302PHE	CG	0.65
1MET	CE	302PHE	CD1	2.01
1MET	CE	302PHE	CD2	2.24
1MET	CE	302PHE	CE1	1.56
1MET	CE	302PHE	CE2	5.86
1MET	CE	302PHE	CZ	1.54
1MET	CE	304LEU	CG	0.00
1MET	CE	304LEU	CD1	0.72
1MET	CE	304LEU	CD2	0.01
1MET	C	2LYS	CA	100.00
1MET	C	2LYS	CB	14.15
1MET	C	2LYS	CG	0.07
1MET	C	2LYS	C	89.20
1MET	C	34LEU	CD1	0.00
1MET	C	35ALA	CB	0.00
1MET	C	302PHE	CE2	0.02
1MET	C	302PHE	CZ	0.01
2LYS	CA	2LYS	CB	100.00
2LYS	CA	2LYS	CG	100.00
2LYS	CA	2LYS	CD	1.52
2LYS	CA	2LYS	C	100.00
2LYS	CA	34LEU	CD1	0.00
2LYS	CA	35ALA	CB	0.03
2LYS	CB	2LYS	CG	100.00
2LYS	CB	2LYS	CD	100.00
2LYS	CB	2LYS	CE	21.40
2LYS	CB	2LYS	C	100.00
2LYS	CB	35ALA	CB	0.01
2LYS	CB	64ALA	CA	0.01
2LYS	CB	64ALA	CB	0.01
2LYS	CB	65GLU	CD	0.05
2LYS	CG	2LYS	CD	100.00
2LYS	CG	2LYS	CE	100.00
2LYS	CG	2LYS	C	69.74
2LYS	CG	35ALA	CB	0.08
2LYS	CG	36TYR	C	0.00
2LYS	CG	37GLU	CB	0.00
2LYS	CG	64ALA	CA	0.00
2LYS	CG	64ALA	CB	0.00
2LYS	CG	65GLU	CD	0.02
2LYS	CD	2LYS	CE	100.00
2LYS	CD	2LYS	C	0.14
2LYS	CD	35ALA	CB	0.02
2LYS	CD	35ALA	C	0.00
2LYS	CD	37GLU	CB	0.01
2LYS	CD	37GLU	CG	0.01
2LYS	CD	37GLU	CD	0.00
2LYS	CD	63GLU	CG	0.01
2LYS	CD	63GLU	CD	0.08
2LYS	CD	63GLU	C	0.00

1MET	CE	302PHE	CE2	2.64
1MET	CE	302PHE	CZ	2.82
1MET	CE	304LEU	CD1	3.14
1MET	CE	304LEU	CD2	0.05
1MET	C	2LYS	CA	100.00
1MET	C	2LYS	CB	1.60
1MET	C	2LYS	CG	0.03
1MET	C	2LYS	C	99.24
1MET	C	302PHE	CE1	0.02
1MET	C	302PHE	CE2	0.00
1MET	C	302PHE	CZ	0.21
2LYS	CA	2LYS	CB	100.00
2LYS	CA	2LYS	CG	100.00
2LYS	CA	2LYS	CD	0.86
2LYS	CA	2LYS	C	100.00
2LYS	CB	2LYS	CG	100.00
2LYS	CB	2LYS	CD	100.00
2LYS	CB	2LYS	CE	21.46
2LYS	CB	2LYS	C	100.00
2LYS	CB	64ALA	CA	0.00
2LYS	CB	65GLU	CD	0.10
2LYS	CG	2LYS	CD	100.00
2LYS	CG	2LYS	CE	100.00
2LYS	CG	2LYS	C	34.87
2LYS	CG	35ALA	CB	0.02
2LYS	CG	64ALA	CB	0.00
2LYS	CG	65GLU	CD	0.06
2LYS	CD	2LYS	CE	100.00
2LYS	CD	2LYS	C	0.00
2LYS	CD	35ALA	CB	0.00
2LYS	CD	63GLU	CG	0.04
2LYS	CD	63GLU	CD	0.02
2LYS	CE	36TYR	C	0.00
2LYS	CE	37GLU	CB	0.04
2LYS	CE	63GLU	CG	0.02
2LYS	CE	63GLU	CD	0.03
2LYS	CE	65GLU	CD	0.04
2LYS	C	3VAL	CA	100.00
2LYS	C	3VAL	CB	25.37
2LYS	C	3VAL	CG2	16.44
2LYS	C	3VAL	C	83.23
2LYS	C	64ALA	CB	0.01
3VAL	CA	3VAL	CB	100.00
3VAL	CA	3VAL	CG1	100.00
3VAL	CA	3VAL	CG2	100.00
3VAL	CA	3VAL	C	100.00
3VAL	CB	3VAL	CG1	100.00
3VAL	CB	3VAL	CG2	100.00
3VAL	CB	3VAL	C	100.00
3VAL	CB	36TYR	CB	0.01
3VAL	CG1	3VAL	CG2	100.00
3VAL	CG1	3VAL	C	99.94
3VAL	CG1	4ALA	C	0.02
3VAL	CG1	5VAL	CG2	0.49
3VAL	CG1	34LEU	CD1	0.04
3VAL	CG1	36TYR	CB	0.06
3VAL	CG1	66ALA	C	0.01
3VAL	CG1	68LEU	CB	0.68
3VAL	CG1	68LEU	CG	0.03
3VAL	CG1	68LEU	CD1	0.22
3VAL	CG1	68LEU	CD2	0.02
3VAL	CG1	297MET	CE	0.84

3VAL	C	4ALA	C	99.29
3VAL	C	64ALA	CB	0.32
4ALA	CA	4ALA	CB	100.00
4ALA	CA	4ALA	C	100.00
4ALA	CA	39PHE	CD1	0.03
4ALA	CA	39PHE	CE1	0.11
4ALA	CB	4ALA	C	100.00
4ALA	CB	6LEU	CD1	0.17
4ALA	CB	39PHE	CD1	1.42
4ALA	CB	39PHE	CE1	6.64
4ALA	CB	61VAL	CG1	0.07
4ALA	CB	64ALA	CB	11.84
4ALA	CB	67VAL	CG2	0.04
4ALA	C	5VAL	CA	100.00
4ALA	C	5VAL	CB	14.13
4ALA	C	5VAL	CG2	7.52
4ALA	C	5VAL	C	96.43
4ALA	C	6LEU	CD1	0.06
5VAL	CA	5VAL	CB	100.00
5VAL	CA	5VAL	CG1	100.00
5VAL	CA	5VAL	CG2	100.00
5VAL	CA	5VAL	C	100.00
5VAL	CB	5VAL	CG1	100.00
5VAL	CB	5VAL	CG2	100.00
5VAL	CB	5VAL	C	100.00
5VAL	CB	38VAL	CG2	0.01
5VAL	CG1	5VAL	CG2	100.00
5VAL	CG1	5VAL	C	100.00
5VAL	CG1	7PRO	CB	0.00
5VAL	CG1	16THR	CG2	16.23
5VAL	CG1	20LEU	CD1	0.63
5VAL	CG1	20LEU	CD2	0.00
5VAL	CG1	38VAL	CG2	0.02
5VAL	CG2	20LEU	CD1	0.88
5VAL	CG2	20LEU	CD2	0.36
5VAL	CG2	36TYR	CB	0.10
5VAL	CG2	36TYR	CG	0.02
5VAL	CG2	36TYR	CD1	0.90
5VAL	CG2	36TYR	CD2	4.13
5VAL	CG2	36TYR	CE2	0.00
5VAL	CG2	68LEU	CD1	0.02
5VAL	CG2	68LEU	CD2	0.02
5VAL	C	6LEU	CA	100.00
5VAL	C	6LEU	CB	18.14
5VAL	C	6LEU	CD1	26.63
5VAL	C	6LEU	C	95.08
5VAL	C	7PRO	CD	0.26
6LEU	CA	6LEU	CB	100.00
6LEU	CA	6LEU	CG	100.00
6LEU	CA	6LEU	CD1	99.98
6LEU	CA	6LEU	C	100.00
6LEU	CA	7PRO	CD	100.00
6LEU	CA	41PHE	CB	0.01
6LEU	CB	6LEU	CG	100.00
6LEU	CB	6LEU	CD1	100.00
6LEU	CB	6LEU	CD2	100.00
6LEU	CB	6LEU	C	100.00
6LEU	CB	41PHE	CB	0.80
6LEU	CB	41PHE	CG	0.14
6LEU	CB	41PHE	CD2	0.87
6LEU	CB	69LEU	CD1	5.57
6LEU	CB	69LEU	CD2	0.13

2LYS	CE	35ALA	CB	0.03
2LYS	CE	36TYR	C	0.01
2LYS	CE	37GLU	CB	1.04
2LYS	CE	37GLU	CG	0.09
2LYS	CE	37GLU	CD	0.06
2LYS	CE	63GLU	CB	0.01
2LYS	CE	63GLU	CG	0.04
2LYS	CE	63GLU	CD	0.18
2LYS	CE	65GLU	CD	0.01
2LYS	C	3VAL	CA	100.00
2LYS	C	3VAL	CB	30.84
2LYS	C	3VAL	CG1	3.31
2LYS	C	3VAL	CG2	13.30
2LYS	C	3VAL	C	77.47
2LYS	C	34LEU	CD1	0.01
2LYS	C	64ALA	CB	0.02
3VAL	CA	3VAL	CB	100.00
3VAL	CA	3VAL	CG1	100.00
3VAL	CA	3VAL	CG2	100.00
3VAL	CA	3VAL	C	100.00
3VAL	CA	64ALA	CB	0.02
3VAL	CB	3VAL	CG1	100.00
3VAL	CB	3VAL	CG2	100.00
3VAL	CB	3VAL	C	100.00
3VAL	CB	34LEU	CD1	0.02
3VAL	CB	36TYR	CB	0.00
3VAL	CG1	3VAL	CG2	100.00
3VAL	CG1	3VAL	C	90.29
3VAL	CG1	4ALA	C	0.05
3VAL	CG1	5VAL	CG2	0.49
3VAL	CG1	20LEU	CD2	0.02
3VAL	CG1	34LEU	CG	0.01
3VAL	CG1	34LEU	CD1	0.31
3VAL	CG1	34LEU	CD2	0.04
3VAL	CG1	36TYR	CA	0.02
3VAL	CG1	36TYR	CB	0.42
3VAL	CG1	36TYR	CD2	0.31
3VAL	CG1	36TYR	CE2	0.04
3VAL	CG1	66ALA	CB	0.01
3VAL	CG1	66ALA	C	0.00
3VAL	CG1	68LEU	CB	1.07
3VAL	CG1	68LEU	CG	0.04
3VAL	CG1	68LEU	CD1	0.31
3VAL	CG1	68LEU	CD2	0.00
3VAL	CG1	297MET	CE	0.88
3VAL	CG1	302PHE	CE1	0.15
3VAL	CG1	302PHE	CE2	0.04
3VAL	CG1	302PHE	CZ	0.35
3VAL	CG2	3VAL	C	20.14
3VAL	CG2	4ALA	C	0.01
3VAL	CG2	5VAL	CG2	0.02
3VAL	CG2	34LEU	CG	0.00
3VAL	CG2	34LEU	CD1	2.92
3VAL	CG2	34LEU	CD2	0.51
3VAL	CG2	36TYR	CA	0.01
3VAL	CG2	36TYR	CB	0.43
3VAL	CG2	36TYR	CD2	0.16
3VAL	CG2	36TYR	CE2	0.00
3VAL	CG2	66ALA	C	0.01
3VAL	CG2	67VAL	C	0.01
3VAL	CG2	68LEU	CB	0.23
3VAL	CG2	297MET	CE	1.04

3VAL	CG2	3VAL	C	0.94
3VAL	CG2	34LEU	CD1	2.79
3VAL	CG2	34LEU	CD2	0.02
3VAL	CG2	297MET	CE	1.08
3VAL	CG2	302PHE	CE1	0.05
3VAL	CG2	302PHE	CE2	0.07
3VAL	CG2	302PHE	CZ	0.44
3VAL	C	4ALA	CA	100.00
3VAL	C	4ALA	CB	1.74
3VAL	C	4ALA	C	99.34
3VAL	C	36TYR	CB	0.00
3VAL	C	64ALA	CB	0.46
4ALA	CA	4ALA	CB	100.00
4ALA	CA	4ALA	C	100.00
4ALA	CA	39PHE	CE1	0.03
4ALA	CA	64ALA	CB	0.00
4ALA	CB	4ALA	C	100.00
4ALA	CB	6LEU	CD1	0.23
4ALA	CB	39PHE	CD1	0.40
4ALA	CB	39PHE	CE1	3.48
4ALA	CB	61VAL	CG1	0.12
4ALA	CB	61VAL	CG2	0.00
4ALA	CB	64ALA	CB	14.96
4ALA	CB	67VAL	CG1	0.00
4ALA	CB	67VAL	CG2	0.03
4ALA	C	5VAL	CA	100.00
4ALA	C	5VAL	CB	21.86
4ALA	C	5VAL	CG2	11.61
4ALA	C	5VAL	C	94.08
4ALA	C	6LEU	CD1	0.11
5VAL	CA	5VAL	CB	100.00
5VAL	CA	5VAL	CG1	100.00
5VAL	CA	5VAL	CG2	100.00
5VAL	CA	5VAL	C	100.00
5VAL	CB	5VAL	CG1	100.00
5VAL	CB	5VAL	CG2	100.00
5VAL	CB	5VAL	C	100.00
5VAL	CB	38VAL	CG1	0.01
5VAL	CB	38VAL	CG2	0.00
5VAL	CG1	5VAL	CG2	100.00
5VAL	CG1	5VAL	C	100.00
5VAL	CG1	7PRO	CD	0.00
5VAL	CG1	16THR	CG2	44.48
5VAL	CG1	20LEU	CD1	0.19
5VAL	CG1	38VAL	CG1	0.01
5VAL	CG1	38VAL	CG2	0.02
5VAL	CG2	20LEU	CD1	0.38
5VAL	CG2	20LEU	CD2	0.14
5VAL	CG2	36TYR	CB	0.23
5VAL	CG2	36TYR	CG	0.01
5VAL	CG2	36TYR	CD1	3.96
5VAL	CG2	38VAL	CG2	0.00
5VAL	C	6LEU	CA	100.00
5VAL	C	6LEU	CB	22.65
5VAL	C	6LEU	CG	0.01
5VAL	C	6LEU	CD1	41.39
5VAL	C	6LEU	C	93.64
5VAL	C	7PRO	CD	1.14
6LEU	CA	6LEU	CB	100.00
6LEU	CA	6LEU	CG	100.00
6LEU	CA	6LEU	CD1	99.95
6LEU	CA	6LEU	C	100.00

6LEU	CG	6LEU	CD1	100.00
6LEU	CG	6LEU	CD2	100.00
6LEU	CG	41PHE	CB	1.03
6LEU	CG	41PHE	CG	0.06
6LEU	CG	41PHE	CD1	0.01
6LEU	CG	41PHE	CD2	0.01
6LEU	CG	57THR	CG2	3.02
6LEU	CD1	6LEU	CD2	100.00
6LEU	CD1	39PHE	CB	0.02
6LEU	CD1	39PHE	CD1	0.04
6LEU	CD1	57THR	CG2	2.62
6LEU	CD1	61VAL	CG1	7.00
6LEU	CD1	67VAL	CG1	1.33
6LEU	CD2	41PHE	CB	0.30
6LEU	CD2	41PHE	CG	1.31
6LEU	CD2	41PHE	CD1	1.58
6LEU	CD2	41PHE	CD2	0.04
6LEU	CD2	41PHE	CE1	0.06
6LEU	CD2	53PHE	CD1	0.03
6LEU	CD2	53PHE	CE1	0.67
6LEU	CD2	53PHE	CZ	0.07
6LEU	CD2	57THR	CG2	4.94
6LEU	CD2	61VAL	CG1	0.43
6LEU	CD2	67VAL	CG1	0.17
6LEU	CD2	69LEU	CD1	2.74
6LEU	CD2	69LEU	CD2	0.27
6LEU	CD2	93LEU	CD1	1.09
6LEU	CD2	93LEU	CD2	0.45
6LEU	C	7PRO	CA	100.00
6LEU	C	7PRO	CB	0.20
6LEU	C	7PRO	CD	100.00
6LEU	C	7PRO	C	100.00
6LEU	C	41PHE	CD2	0.41
7PRO	CA	7PRO	CB	100.00
7PRO	CA	7PRO	CG	100.00
7PRO	CA	7PRO	CD	100.00
7PRO	CA	7PRO	C	100.00
7PRO	CB	7PRO	CG	100.00
7PRO	CB	7PRO	CD	100.00
7PRO	CB	7PRO	C	100.00
7PRO	CB	13PRO	CA	0.06
7PRO	CB	13PRO	CB	0.12
7PRO	CG	7PRO	CD	100.00
7PRO	CG	7PRO	C	92.50
7PRO	CG	38VAL	CG1	0.88
7PRO	CD	7PRO	C	72.27
7PRO	CD	38VAL	CG1	0.02
7PRO	C	8GLY	CA	100.00
7PRO	C	8GLY	C	88.70
7PRO	C	13PRO	CG	0.00
8GLY	CA	8GLY	C	100.00
8GLY	CA	41PHE	CD2	1.51
8GLY	CA	41PHE	CE2	0.49
8GLY	CA	42GLY	CA	0.02
8GLY	CA	70GLY	C	0.04
8GLY	CA	72VAL	CB	0.01
8GLY	CA	72VAL	CG1	6.24
8GLY	CA	72VAL	CG2	0.29
8GLY	C	9ASP	CA	100.00
8GLY	C	9ASP	CB	98.72
8GLY	C	9ASP	CG	0.42
8GLY	C	9ASP	C	1.19

3VAL	CG2	302PHE	CE1	0.08
3VAL	CG2	302PHE	CE2	0.13
3VAL	CG2	302PHE	CZ	1.02
3VAL	C	4ALA	CA	100.00
3VAL	C	4ALA	CB	2.59
3VAL	C	4ALA	C	98.77
3VAL	C	64ALA	CB	0.56
4ALA	CA	4ALA	CB	100.00
4ALA	CA	4ALA	C	100.00
4ALA	CA	39PHE	CD1	0.06
4ALA	CA	39PHE	CE1	0.10
4ALA	CB	4ALA	C	100.00
4ALA	CB	6LEU	CD1	0.24
4ALA	CB	39PHE	CD1	1.97
4ALA	CB	39PHE	CE1	6.86
4ALA	CB	39PHE	CZ	0.00
4ALA	CB	61VAL	CA	0.01
4ALA	CB	61VAL	CB	0.00
4ALA	CB	61VAL	CG1	0.07
4ALA	CB	64ALA	CB	14.26
4ALA	CB	67VAL	CG1	0.00
4ALA	CB	67VAL	CG2	0.05
4ALA	C	5VAL	CA	100.00
4ALA	C	5VAL	CB	15.86
4ALA	C	5VAL	CG2	9.88
4ALA	C	5VAL	C	94.83
4ALA	C	6LEU	CD1	0.13
5VAL	CA	5VAL	CB	100.00
5VAL	CA	5VAL	CG1	100.00
5VAL	CA	5VAL	CG2	100.00
5VAL	CA	5VAL	C	100.00
5VAL	CB	5VAL	CG1	100.00
5VAL	CB	5VAL	CG2	100.00
5VAL	CB	5VAL	C	100.00
5VAL	CB	38VAL	CG1	0.00
5VAL	CB	38VAL	CG2	0.02
5VAL	CG1	5VAL	CG2	100.00
5VAL	CG1	5VAL	C	100.00
5VAL	CG1	6LEU	C	0.00
5VAL	CG1	16THR	CB	0.37
5VAL	CG1	16THR	CG2	0.22
5VAL	CG1	20LEU	CD1	0.50
5VAL	CG1	20LEU	CD2	0.04
5VAL	CG1	38VAL	CG1	0.03
5VAL	CG1	38VAL	CG2	0.02
5VAL	CG1	68LEU	CD2	0.03
5VAL	CG2	20LEU	CD1	1.43
5VAL	CG2	20LEU	CD2	0.79
5VAL	CG2	36TYR	CB	0.15
5VAL	CG2	36TYR	CG	0.01
5VAL	CG2	36TYR	CD1	0.99
5VAL	CG2	36TYR	CD2	0.60
5VAL	CG2	38VAL	CG2	0.01
5VAL	CG2	68LEU	CD2	0.02
5VAL	C	6LEU	CA	100.00
5VAL	C	6LEU	CB	19.03
5VAL	C	6LEU	CG	0.28
5VAL	C	6LEU	CD1	21.39
5VAL	C	6LEU	CD2	0.09
5VAL	C	6LEU	C	93.65
5VAL	C	7PRO	CD	0.04
6LEU	CA	6LEU	CB	100.00

6LEU	CA	7PRO	CD	100.00
6LEU	CB	6LEU	CG	100.00
6LEU	CB	6LEU	CD1	100.00
6LEU	CB	6LEU	CD2	100.00
6LEU	CB	6LEU	C	100.00
6LEU	CB	41PHE	CB	0.18
6LEU	CB	41PHE	CG	0.02
6LEU	CB	41PHE	CD2	0.24
6LEU	CB	41PHE	CE2	0.01
6LEU	CB	69LEU	CD1	3.12
6LEU	CB	69LEU	CD2	0.02
6LEU	CG	6LEU	CD1	100.00
6LEU	CG	6LEU	CD2	100.00
6LEU	CG	41PHE	CB	0.33
6LEU	CG	41PHE	CG	0.02
6LEU	CG	41PHE	CD1	0.00
6LEU	CG	41PHE	CD2	0.00
6LEU	CG	57THR	CG2	1.95
6LEU	CD1	6LEU	CD2	100.00
6LEU	CD1	39PHE	CB	0.04
6LEU	CD1	39PHE	CD1	0.07
6LEU	CD1	57THR	CG2	1.04
6LEU	CD1	61VAL	CG1	4.06
6LEU	CD1	61VAL	CG2	0.26
6LEU	CD1	67VAL	CG1	0.91
6LEU	CD2	41PHE	CB	1.00
6LEU	CD2	41PHE	CG	6.58
6LEU	CD2	41PHE	CD1	5.00
6LEU	CD2	41PHE	CD2	0.30
6LEU	CD2	41PHE	CE1	0.25
6LEU	CD2	41PHE	CE2	0.01
6LEU	CD2	53PHE	CD1	0.01
6LEU	CD2	53PHE	CE1	0.08
6LEU	CD2	57THR	CG2	5.56
6LEU	CD2	61VAL	CG1	0.48
6LEU	CD2	61VAL	CG2	0.04
6LEU	CD2	67VAL	CG1	0.04
6LEU	CD2	69LEU	CD1	7.72
6LEU	CD2	93LEU	CD1	2.15
6LEU	CD2	93LEU	CD2	0.34
6LEU	C	7PRO	CA	100.00
6LEU	C	7PRO	CB	0.12
6LEU	C	7PRO	CD	100.00
6LEU	C	7PRO	C	100.00
6LEU	C	41PHE	CD2	0.06
6LEU	C	41PHE	CE2	0.00
7PRO	CA	7PRO	CB	100.00
7PRO	CA	7PRO	CG	100.00
7PRO	CA	7PRO	CD	100.00
7PRO	CA	7PRO	C	100.00
7PRO	CB	7PRO	CG	100.00
7PRO	CB	7PRO	CD	100.00
7PRO	CB	7PRO	C	100.00
7PRO	CB	12GLY	C	0.01
7PRO	CB	13PRO	CA	0.58
7PRO	CB	13PRO	CB	0.09
7PRO	CB	13PRO	CG	0.00
7PRO	CB	16THR	CB	0.11
7PRO	CB	16THR	CG2	0.04
7PRO	CB	38VAL	CG1	0.01
7PRO	CG	7PRO	CD	100.00
7PRO	CG	7PRO	C	90.22

8GLY	C	13PRO	CG	0.58
8GLY	C	13PRO	CD	0.61
8GLY	C	42GLY	CA	2.96
8GLY	C	72VAL	CG1	0.15
8GLY	C	72VAL	CG2	0.00
9ASP	CA	9ASP	CB	100.00
9ASP	CA	9ASP	CG	100.00
9ASP	CA	9ASP	C	100.00
9ASP	CA	13PRO	CG	0.02
9ASP	CA	13PRO	CD	0.12
9ASP	CB	9ASP	CG	100.00
9ASP	CB	9ASP	C	100.00
9ASP	CB	42GLY	CA	0.46
9ASP	CB	43GLY	CA	0.02
9ASP	CB	72VAL	CG2	0.09
9ASP	CB	77TRP	CZ2	0.47
9ASP	CB	77TRP	CZ3	0.09
9ASP	CG	9ASP	C	97.00
9ASP	CG	43GLY	CA	0.01
9ASP	CG	76LYS	CD	0.00
9ASP	CG	76LYS	CE	0.81
9ASP	CG	77TRP	CZ2	0.10
9ASP	CG	77TRP	CZ3	0.06
9ASP	C	10GLY	CA	100.00
9ASP	C	10GLY	C	80.46
9ASP	C	13PRO	CD	0.94
9ASP	C	275SER	CB	0.00
10GLY	CA	10GLY	C	100.00
10GLY	CA	275SER	CB	0.02
10GLY	CA	280ALA	CB	0.34
10GLY	C	11ILE	CA	100.00
10GLY	C	11ILE	CB	0.17
10GLY	C	11ILE	CG1	0.03
10GLY	C	11ILE	CG2	0.02
10GLY	C	11ILE	CD	0.00
10GLY	C	11ILE	C	99.92
10GLY	C	13PRO	CD	0.01
10GLY	C	280ALA	CB	3.82
11ILE	CA	11ILE	CB	100.00
11ILE	CA	11ILE	CG1	100.00
11ILE	CA	11ILE	CG2	100.00
11ILE	CA	11ILE	CD	33.75
11ILE	CA	11ILE	C	100.00
11ILE	CB	11ILE	CG1	100.00
11ILE	CB	11ILE	CG2	100.00
11ILE	CB	11ILE	CD	100.00
11ILE	CB	11ILE	C	100.00
11ILE	CB	273HIS	CE1	0.02
11ILE	CB	285ALA	CB	0.02
11ILE	CG1	11ILE	CG2	100.00
11ILE	CG1	11ILE	CD	100.00
11ILE	CG1	11ILE	C	33.26
11ILE	CG1	273HIS	CE1	0.18
11ILE	CG1	275SER	CA	0.05
11ILE	CG1	275SER	CB	0.01
11ILE	CG1	275SER	C	0.08
11ILE	CG1	276ALA	CB	0.00
11ILE	CG1	279ILE	CG2	0.01
11ILE	CG1	279ILE	CD	0.01
11ILE	CG1	280ALA	CA	0.00
11ILE	CG1	280ALA	CB	0.05
11ILE	CG1	285ALA	CB	0.00

6LEU	CA	6LEU	CG	100.00
6LEU	CA	6LEU	CD1	84.25
6LEU	CA	6LEU	CD2	15.60
6LEU	CA	6LEU	C	100.00
6LEU	CA	7PRO	CD	100.00
6LEU	CB	6LEU	CG	100.00
6LEU	CB	6LEU	CD1	100.00
6LEU	CB	6LEU	CD2	100.00
6LEU	CB	6LEU	C	100.00
6LEU	CB	41PHE	CB	0.51
6LEU	CB	41PHE	CG	0.04
6LEU	CB	41PHE	CD2	1.35
6LEU	CB	69LEU	CD1	2.61
6LEU	CB	69LEU	CD2	0.77
6LEU	CG	6LEU	CD1	100.00
6LEU	CG	6LEU	CD2	100.00
6LEU	CG	6LEU	C	0.00
6LEU	CG	41PHE	CB	0.67
6LEU	CG	41PHE	CG	0.11
6LEU	CG	41PHE	CD1	0.02
6LEU	CG	41PHE	CD2	0.00
6LEU	CG	57THR	CG2	1.23
6LEU	CG	67VAL	CG1	0.06
6LEU	CD1	6LEU	CD2	100.00
6LEU	CD1	39PHE	CB	0.04
6LEU	CD1	39PHE	CD1	0.06
6LEU	CD1	39PHE	CE1	0.00
6LEU	CD1	41PHE	CB	0.00
6LEU	CD1	41PHE	CG	0.00
6LEU	CD1	41PHE	CD1	0.03
6LEU	CD1	41PHE	CD2	0.00
6LEU	CD1	41PHE	CE1	0.00
6LEU	CD1	57THR	CG2	1.07
6LEU	CD1	61VAL	CG1	5.07
6LEU	CD1	67VAL	CG1	5.62
6LEU	CD1	69LEU	CD1	0.10
6LEU	CD1	69LEU	CD2	0.23
6LEU	CD1	93LEU	CD1	1.35
6LEU	CD1	93LEU	CD2	1.93
6LEU	CD2	39PHE	CB	0.08
6LEU	CD2	39PHE	CD1	0.00
6LEU	CD2	41PHE	CB	0.48
6LEU	CD2	41PHE	CG	2.18
6LEU	CD2	41PHE	CD1	3.02
6LEU	CD2	41PHE	CD2	0.12
6LEU	CD2	41PHE	CE1	0.24
6LEU	CD2	41PHE	CE2	0.02
6LEU	CD2	41PHE	CZ	0.01
6LEU	CD2	53PHE	CE1	0.05
6LEU	CD2	57THR	CG2	10.29
6LEU	CD2	61VAL	CG1	0.93
6LEU	CD2	67VAL	CG1	0.18
6LEU	CD2	69LEU	CD1	1.04
6LEU	CD2	69LEU	CD2	1.30
6LEU	CD2	93LEU	CD1	8.69
6LEU	CD2	93LEU	CD2	3.16
6LEU	C	7PRO	CA	100.00
6LEU	C	7PRO	CB	0.10
6LEU	C	7PRO	CG	0.00
6LEU	C	7PRO	CD	100.00
6LEU	C	7PRO	C	100.00
6LEU	C	41PHE	CD2	0.12

7PRO	CG	38VAL	CG1	1.19
7PRO	CD	7PRO	C	62.52
7PRO	CD	38VAL	CG1	0.08
7PRO	C	8GLY	CA	100.00
7PRO	C	8GLY	C	82.26
7PRO	C	12GLY	C	0.00
7PRO	C	70GLY	CA	0.01
8GLY	CA	8GLY	C	100.00
8GLY	CA	12GLY	CA	0.00
8GLY	CA	41PHE	CD2	0.03
8GLY	CA	41PHE	CE2	0.01
8GLY	CA	70GLY	CA	0.03
8GLY	CA	70GLY	C	0.52
8GLY	CA	71SER	C	0.02
8GLY	CA	72VAL	CG1	3.16
8GLY	CA	72VAL	CG2	0.00
8GLY	C	9ASP	CA	100.00
8GLY	C	9ASP	CB	71.79
8GLY	C	9ASP	CG	0.28
8GLY	C	9ASP	C	27.47
8GLY	C	12GLY	CA	0.00
8GLY	C	13PRO	CG	0.01
8GLY	C	13PRO	CD	0.04
8GLY	C	42GLY	CA	0.04
8GLY	C	72VAL	CG1	0.06
9ASP	CA	9ASP	CB	100.00
9ASP	CA	9ASP	CG	100.00
9ASP	CA	9ASP	C	100.00
9ASP	CA	13PRO	CD	0.02
9ASP	CB	9ASP	CG	100.00
9ASP	CB	9ASP	C	100.00
9ASP	CB	42GLY	CA	0.01
9ASP	CB	73GLY	C	0.02
9ASP	CB	77TRP	CZ2	0.00
9ASP	CG	9ASP	C	69.36
9ASP	CG	42GLY	CA	0.00
9ASP	CG	43GLY	CA	0.02
9ASP	CG	44ALA	CB	0.00
9ASP	CG	77TRP	CZ2	0.00
9ASP	CG	77TRP	CZ3	0.01
9ASP	C	10GLY	CA	100.00
9ASP	C	10GLY	C	82.32
9ASP	C	13PRO	CD	0.02
9ASP	C	275SER	CB	0.02
10GLY	CA	10GLY	C	100.00
10GLY	CA	275SER	C	0.31
10GLY	CA	276ALA	C	0.10
10GLY	CA	277PRO	CA	0.02
10GLY	CA	277PRO	CB	0.02
10GLY	CA	277PRO	CG	0.00
10GLY	CA	280ALA	CB	0.29
10GLY	C	11ILE	CA	100.00
10GLY	C	11ILE	CB	1.83
10GLY	C	11ILE	CG1	0.07
10GLY	C	11ILE	CG2	1.16
10GLY	C	11ILE	C	98.32
10GLY	C	277PRO	CA	0.00
10GLY	C	280ALA	CB	7.79
11ILE	CA	11ILE	CB	100.00
11ILE	CA	11ILE	CG1	100.00
11ILE	CA	11ILE	CG2	100.00
11ILE	CA	11ILE	CD	1.03

11ILE	CG2	11ILE	CD	98.16
11ILE	CG2	11ILE	C	66.73
11ILE	CG2	15VAL	CG1	6.56
11ILE	CG2	273HIS	CD2	0.01
11ILE	CG2	273HIS	CE1	3.90
11ILE	CG2	276ALA	CB	0.05
11ILE	CG2	279ILE	CB	0.01
11ILE	CG2	279ILE	CG2	0.60
11ILE	CG2	279ILE	CD	0.02
11ILE	CG2	279ILE	C	0.10
11ILE	CG2	280ALA	CA	0.36
11ILE	CG2	280ALA	CB	0.26
11ILE	CG2	285ALA	CB	6.57
11ILE	CD	11ILE	C	0.50
11ILE	CD	15VAL	CG1	0.04
11ILE	CD	15VAL	CG2	0.00
11ILE	CD	273HIS	CG	0.04
11ILE	CD	273HIS	CD2	0.02
11ILE	CD	273HIS	CE1	6.40
11ILE	CD	275SER	CA	0.01
11ILE	CD	275SER	C	0.05
11ILE	CD	276ALA	CB	0.23
11ILE	CD	279ILE	CB	0.20
11ILE	CD	279ILE	CG1	0.03
11ILE	CD	279ILE	CG2	1.37
11ILE	CD	279ILE	CD	0.84
11ILE	CD	279ILE	C	0.01
11ILE	CD	280ALA	CA	0.02
11ILE	CD	280ALA	CB	1.31
11ILE	CD	285ALA	CB	0.28
11ILE	C	12GLY	CA	100.00
11ILE	C	12GLY	C	100.00
11ILE	C	13PRO	CD	8.73
12GLY	CA	12GLY	C	100.00
12GLY	CA	13PRO	CD	100.00
12GLY	CA	15VAL	CG2	0.10
12GLY	CA	70GLY	CA	0.00
12GLY	C	13PRO	CA	100.00
12GLY	C	13PRO	CB	0.01
12GLY	C	13PRO	CD	100.00
12GLY	C	13PRO	C	100.00
13PRO	CA	13PRO	CB	100.00
13PRO	CA	13PRO	CG	100.00
13PRO	CA	13PRO	CD	100.00
13PRO	CA	13PRO	C	100.00
13PRO	CB	13PRO	CG	100.00
13PRO	CB	13PRO	CD	100.00
13PRO	CB	13PRO	C	100.00
13PRO	CG	13PRO	CD	100.00
13PRO	CG	13PRO	C	45.69
13PRO	CD	13PRO	C	36.14
13PRO	C	14GLU	CA	100.00
13PRO	C	14GLU	CB	0.02
13PRO	C	14GLU	C	100.00
14GLU	CA	14GLU	CB	100.00
14GLU	CA	14GLU	CG	100.00
14GLU	CA	14GLU	CD	97.38
14GLU	CA	14GLU	C	100.00
14GLU	CB	14GLU	CG	100.00
14GLU	CB	14GLU	CD	100.00
14GLU	CB	14GLU	C	100.00
14GLU	CB	285ALA	CB	0.00

7PRO	CA	7PRO	CB	100.00
7PRO	CA	7PRO	CG	100.00
7PRO	CA	7PRO	CD	100.00
7PRO	CA	7PRO	C	100.00
7PRO	CB	7PRO	CG	100.00
7PRO	CB	7PRO	CD	100.00
7PRO	CB	7PRO	C	100.00
7PRO	CB	12GLY	C	0.03
7PRO	CB	13PRO	CA	1.38
7PRO	CB	13PRO	CB	0.38
7PRO	CB	13PRO	CG	0.01
7PRO	CG	7PRO	CD	100.00
7PRO	CG	7PRO	C	87.37
7PRO	CG	38VAL	CG1	0.54
7PRO	CD	7PRO	C	69.90
7PRO	CD	38VAL	CG1	0.06
7PRO	CD	41PHE	CB	0.00
7PRO	C	8GLY	CA	100.00
7PRO	C	8GLY	C	81.43
7PRO	C	12GLY	CA	0.01
7PRO	C	12GLY	C	0.06
7PRO	C	41PHE	CD2	0.00
8GLY	CA	8GLY	C	100.00
8GLY	CA	41PHE	CD2	2.27
8GLY	CA	41PHE	CE2	0.36
8GLY	CA	41PHE	C	0.00
8GLY	CA	42GLY	CA	0.06
8GLY	CA	69LEU	CD2	0.00
8GLY	CA	70GLY	C	0.03
8GLY	CA	72VAL	CB	0.04
8GLY	CA	72VAL	CG1	5.10
8GLY	CA	72VAL	CG2	0.44
8GLY	C	9ASP	CA	100.00
8GLY	C	9ASP	CB	93.92
8GLY	C	9ASP	CG	0.06
8GLY	C	9ASP	C	8.51
8GLY	C	13PRO	CG	0.01
8GLY	C	13PRO	CD	0.19
8GLY	C	42GLY	CA	2.10
8GLY	C	72VAL	CG1	0.06
8GLY	C	72VAL	CG2	0.00
9ASP	CA	9ASP	CB	100.00
9ASP	CA	9ASP	CG	100.00
9ASP	CA	9ASP	C	100.00
9ASP	CA	13PRO	CD	0.03
9ASP	CB	9ASP	CG	100.00
9ASP	CB	9ASP	C	100.00
9ASP	CB	42GLY	CA	0.29
9ASP	CB	42GLY	C	0.03
9ASP	CB	43GLY	CA	0.13
9ASP	CB	43GLY	C	0.00
9ASP	CB	72VAL	CG1	0.01
9ASP	CB	72VAL	CG2	0.01
9ASP	CB	77TRP	CZ2	0.01
9ASP	CB	86PRO	CB	0.07
9ASP	CB	86PRO	CG	0.92
9ASP	CG	9ASP	C	98.42
9ASP	CG	43GLY	CA	0.05
9ASP	CG	76LYS	CD	0.01
9ASP	CG	76LYS	CE	0.53
9ASP	CG	77TRP	CZ2	0.02
9ASP	CG	77TRP	CZ3	0.02

11ILE	CA	11ILE	C	100.00
11ILE	CA	280ALA	CB	0.00
11ILE	CB	11ILE	CG1	100.00
11ILE	CB	11ILE	CG2	100.00
11ILE	CB	11ILE	CD	100.00
11ILE	CB	11ILE	C	100.00
11ILE	CB	285ALA	CB	0.08
11ILE	CG1	11ILE	CG2	100.00
11ILE	CG1	11ILE	CD	100.00
11ILE	CG1	11ILE	C	89.26
11ILE	CG1	15VAL	CG1	0.05
11ILE	CG1	15VAL	CG2	0.03
11ILE	CG1	71SER	CB	0.00
11ILE	CG1	273HIS	CE1	0.00
11ILE	CG1	279ILE	CG1	0.00
11ILE	CG1	279ILE	CD	0.00
11ILE	CG1	280ALA	CB	0.00
11ILE	CG1	285ALA	CB	0.04
11ILE	CG2	11ILE	CD	98.33
11ILE	CG2	11ILE	C	10.70
11ILE	CG2	15VAL	CG1	1.41
11ILE	CG2	15VAL	CG2	0.04
11ILE	CG2	273HIS	CE1	0.14
11ILE	CG2	276ALA	CB	0.02
11ILE	CG2	279ILE	CB	0.29
11ILE	CG2	279ILE	CG1	0.90
11ILE	CG2	279ILE	CG2	3.14
11ILE	CG2	279ILE	CD	2.12
11ILE	CG2	279ILE	C	0.52
11ILE	CG2	280ALA	CA	0.31
11ILE	CG2	280ALA	CB	0.87
11ILE	CG2	285ALA	CB	10.04
11ILE	CD	11ILE	C	0.60
11ILE	CD	15VAL	CG1	0.34
11ILE	CD	15VAL	CG2	0.09
11ILE	CD	273HIS	CB	0.01
11ILE	CD	273HIS	CG	0.80
11ILE	CD	273HIS	CD2	0.15
11ILE	CD	273HIS	CE1	1.72
11ILE	CD	274GLY	C	0.78
11ILE	CD	275SER	CA	0.04
11ILE	CD	275SER	C	0.13
11ILE	CD	276ALA	CB	0.02
11ILE	CD	279ILE	CG1	0.07
11ILE	CD	279ILE	CD	0.40
11ILE	CD	285ALA	CA	0.00
11ILE	CD	285ALA	CB	0.28
11ILE	C	12GLY	CA	100.00
11ILE	C	12GLY	C	100.00
11ILE	C	13PRO	CD	4.81
11ILE	C	15VAL	CG2	0.18
12GLY	CA	12GLY	C	100.00
12GLY	CA	13PRO	CD	100.00
12GLY	CA	15VAL	CG2	0.30
12GLY	C	13PRO	CA	100.00
12GLY	C	13PRO	CB	0.02
12GLY	C	13PRO	CD	100.00
12GLY	C	13PRO	C	100.00
12GLY	C	15VAL	CG2	0.02
13PRC	CA	13PRO	CB	100.00
13PRC	CA	13PRO	CG	100.00
13PRC	CA	13PRO	CD	100.00

14GLU	CB	333THR	CG2	0.01
14GLU	CG	14GLU	CD	100.00
14GLU	CG	14GLU	C	76.48
14GLU	CG	285ALA	CB	0.08
14GLU	CG	333THR	CB	0.24
14GLU	CG	333THR	CG2	0.57
14GLU	CD	14GLU	C	0.02
14GLU	C	15VAL	CA	100.00
14GLU	C	15VAL	CB	0.06
14GLU	C	15VAL	CG1	0.00
14GLU	C	15VAL	C	99.98
14GLU	C	333THR	CG2	0.06
15VAL	CA	15VAL	CB	100.00
15VAL	CA	15VAL	CG1	100.00
15VAL	CA	15VAL	CG2	100.00
15VAL	CA	15VAL	C	100.00
15VAL	CA	333THR	CG2	0.00
15VAL	CB	15VAL	CG1	100.00
15VAL	CB	15VAL	CG2	100.00
15VAL	CB	15VAL	C	100.00
15VAL	CB	290ALA	CB	0.20
15VAL	CG1	15VAL	CG2	100.00
15VAL	CG1	285ALA	CB	0.28
15VAL	CG1	285ALA	C	0.00
15VAL	CG1	286ASN	C	0.08
15VAL	CG1	287PRO	CA	0.29
15VAL	CG1	333THR	CG2	2.25
15VAL	CG2	15VAL	C	100.00
15VAL	CG2	273HIS	CE1	0.13
15VAL	CG2	290ALA	CB	0.54
15VAL	C	16THR	CA	100.00
15VAL	C	16THR	CB	0.01
15VAL	C	16THR	C	100.00
15VAL	C	290ALA	CB	1.58
16THR	CA	16THR	CB	100.00
16THR	CA	16THR	CG2	100.00
16THR	CA	16THR	C	100.00
16THR	CB	16THR	CG2	100.00
16THR	CB	16THR	C	100.00
16THR	CG2	16THR	C	100.00
16THR	CG2	20LEU	CD1	0.05
16THR	CG2	68LEU	CG	0.04
16THR	CG2	68LEU	CD1	0.01
16THR	CG2	68LEU	CD2	2.00
16THR	C	17GLU	CA	100.00
16THR	C	17GLU	C	100.00
17GLU	CA	17GLU	CB	100.00
17GLU	CA	17GLU	CG	100.00
17GLU	CA	17GLU	CD	95.24
17GLU	CA	17GLU	C	100.00
17GLU	CB	17GLU	CG	100.00
17GLU	CB	17GLU	CD	100.00
17GLU	CB	17GLU	C	100.00
17GLU	CG	17GLU	CD	100.00
17GLU	CG	17GLU	C	99.92
17GLU	CG	21LYS	CE	0.01
17GLU	CD	17GLU	C	3.55
17GLU	CD	21LYS	CE	0.02
17GLU	C	18ALA	CA	100.00
17GLU	C	18ALA	C	100.00
18ALA	CA	18ALA	CB	100.00
18ALA	CA	18ALA	C	100.00

9ASP	CG	86PRO	CB	0.14
9ASP	CG	86PRO	CG	0.29
9ASP	C	10GLY	CA	100.00
9ASP	C	10GLY	C	75.78
9ASP	C	13PRO	CD	0.06
10GLY	CA	10GLY	C	100.00
10GLY	CA	74GLY	CA	0.00
10GLY	CA	76LYS	CD	0.16
10GLY	CA	275SER	CB	0.01
10GLY	CA	276ALA	C	0.00
10GLY	CA	277PRO	CB	0.21
10GLY	CA	277PRO	CD	0.02
10GLY	CA	280ALA	CB	0.06
10GLY	C	11ILE	CA	100.00
10GLY	C	11ILE	CB	1.64
10GLY	C	11ILE	CG1	0.03
10GLY	C	11ILE	CG2	0.88
10GLY	C	11ILE	C	98.58
10GLY	C	277PRO	CA	0.09
10GLY	C	277PRO	CB	0.01
10GLY	C	280ALA	CA	0.01
10GLY	C	280ALA	CB	0.80
11ILE	CA	11ILE	CB	100.00
11ILE	CA	11ILE	CG1	100.00
11ILE	CA	11ILE	CG2	100.00
11ILE	CA	11ILE	CD	0.93
11ILE	CA	11ILE	C	100.00
11ILE	CA	280ALA	CB	0.05
11ILE	CB	11ILE	CG1	100.00
11ILE	CB	11ILE	CG2	100.00
11ILE	CB	11ILE	CD	100.00
11ILE	CB	11ILE	C	100.00
11ILE	CB	15VAL	CG1	0.01
11ILE	CB	280ALA	CB	0.02
11ILE	CB	285ALA	CB	0.24
11ILE	CG1	11ILE	CG2	100.00
11ILE	CG1	11ILE	CD	100.00
11ILE	CG1	11ILE	C	93.65
11ILE	CG1	15VAL	CG1	0.35
11ILE	CG1	15VAL	CG2	0.00
11ILE	CG1	71SER	CB	0.98
11ILE	CG1	273HIS	CE1	0.02
11ILE	CG1	274GLY	CA	0.00
11ILE	CG1	274GLY	C	0.01
11ILE	CG1	275SER	CB	0.07
11ILE	CG1	277PRO	CA	0.00
11ILE	CG1	277PRO	CB	0.00
11ILE	CG1	279ILE	CG1	0.00
11ILE	CG1	280ALA	CB	0.02
11ILE	CG2	11ILE	CD	98.08
11ILE	CG2	11ILE	C	6.27
11ILE	CG2	15VAL	CG1	1.85
11ILE	CG2	15VAL	CG2	0.02
11ILE	CG2	71SER	CB	0.00
11ILE	CG2	273HIS	CD2	0.16
11ILE	CG2	273HIS	CE1	0.20
11ILE	CG2	275SER	CB	0.34
11ILE	CG2	275SER	C	0.03
11ILE	CG2	276ALA	CA	0.00
11ILE	CG2	276ALA	C	0.08
11ILE	CG2	277PRO	CA	0.02
11ILE	CG2	279ILE	CB	0.00

13PRC	CA	13PRO	C	100.00
13PRC	CB	13PRO	CG	100.00
13PRC	CB	13PRO	CD	100.00
13PRC	CB	13PRO	C	100.00
13PRC	CG	13PRO	CD	100.00
13PRC	CG	13PRO	C	53.31
13PRC	CD	13PRO	C	36.19
13PRC	C	14GLU	CA	100.00
13PRC	C	14GLU	CB	0.04
13PRC	C	14GLU	C	99.99
14GLL	CA	14GLU	CB	100.00
14GLL	CA	14GLU	CG	100.00
14GLL	CA	14GLU	CD	97.56
14GLL	CA	14GLU	C	100.00
14GLL	CB	14GLU	CG	100.00
14GLL	CB	14GLU	CD	100.00
14GLL	CB	14GLU	C	100.00
14GLL	CG	14GLU	CD	100.00
14GLL	CG	14GLU	C	98.98
14GLL	CG	285ALA	CB	0.01
14GLL	CG	333THR	CB	0.02
14GLL	CG	333THR	CG2	0.24
14GLL	CD	14GLU	C	0.04
14GLL	C	15VAL	CA	100.00
14GLL	C	15VAL	CB	0.12
14GLL	C	15VAL	CG1	0.03
14GLL	C	15VAL	C	99.91
14GLL	C	333THR	CG2	0.00
15VAL	CA	15VAL	CB	100.00
15VAL	CA	15VAL	CG1	100.00
15VAL	CA	15VAL	CG2	100.00
15VAL	CA	15VAL	C	100.00
15VAL	CA	18ALA	CB	0.02
15VAL	CB	15VAL	CG1	100.00
15VAL	CB	15VAL	CG2	100.00
15VAL	CB	15VAL	C	100.00
15VAL	CB	273HIS	CE1	0.03
15VAL	CB	287PRO	CB	0.02
15VAL	CB	290ALA	CB	0.07
15VAL	CG1	15VAL	CG2	100.00
15VAL	CG1	273HIS	CE1	0.12
15VAL	CG1	285ALA	CB	4.06
15VAL	CG1	285ALA	C	0.01
15VAL	CG1	286ASN	C	0.02
15VAL	CG1	287PRO	CA	0.82
15VAL	CG1	287PRO	CB	0.03
15VAL	CG1	333THR	CG2	0.02
15VAL	CG2	15VAL	C	100.00
15VAL	CG2	273HIS	CD2	0.00
15VAL	CG2	273HIS	CE1	9.16
15VAL	CG2	290ALA	CB	0.18
15VAL	C	16THR	CA	100.00
15VAL	C	16THR	CB	0.01
15VAL	C	16THR	C	100.00
15VAL	C	290ALA	CB	0.23
16THF	CA	16THR	CB	100.00
16THF	CA	16THR	CG2	100.00
16THF	CA	16THR	C	100.00
16THF	CB	16THR	CG2	100.00
16THF	CB	16THR	C	100.00
16THF	CG2	16THR	C	100.00
16THF	CG2	20LEU	CD1	0.28

18ALA	CA	337THR	CG2	0.07	11ILE	CG2	279ILE	CG1	0.68	16THF	CG2	68LEU	CG	0.03
18ALA	CB	18ALA	C	100.00	11ILE	CG2	279ILE	CG2	0.11	16THF	CG2	68LEU	CD1	0.02
18ALA	CB	287PRO	CB	17.87	11ILE	CG2	279ILE	CD	1.09	16THF	CG2	68LEU	CD2	2.08
18ALA	CB	287PRO	CG	0.08	11ILE	CG2	279ILE	C	0.00	16THF	C	17GLU	CA	100.00
18ALA	CB	333THR	CB	0.00	11ILE	CG2	280ALA	CA	0.04	16THF	C	17GLU	C	100.00
18ALA	CB	333THR	CG2	0.10	11ILE	CG2	280ALA	CB	1.75	17GLL	CA	17GLU	CB	100.00
18ALA	CB	337THR	CG2	4.83	11ILE	CG2	285ALA	CA	0.01	17GLL	CA	17GLU	CG	100.00
18ALA	C	19ALA	CA	100.00	11ILE	CG2	285ALA	CB	8.11	17GLL	CA	17GLU	CD	99.66
18ALA	C	19ALA	CB	0.02	11ILE	CD	11ILE	C	0.78	17GLL	CA	17GLU	C	100.00
18ALA	C	19ALA	C	99.99	11ILE	CD	15VAL	CG1	0.85	17GLL	CB	17GLU	CG	100.00
18ALA	C	21LYS	CB	0.00	11ILE	CD	15VAL	CG2	0.04	17GLL	CB	17GLU	CD	100.00
18ALA	C	291ILE	CG1	0.01	11ILE	CD	71SER	CB	0.03	17GLL	CB	17GLU	C	100.00
19ALA	CA	19ALA	CB	100.00	11ILE	CD	75PRO	CD	0.00	17GLL	CG	17GLU	CD	100.00
19ALA	CA	19ALA	C	100.00	11ILE	CD	273HIS	CB	0.38	17GLL	CG	17GLU	C	94.19
19ALA	CA	291ILE	CG1	0.04	11ILE	CD	273HIS	CG	2.37	17GLL	CG	21LYS	CE	0.03
19ALA	CB	19ALA	C	100.00	11ILE	CD	273HIS	CD2	4.15	17GLL	CD	17GLU	C	7.61
19ALA	CB	68LEU	CD2	0.00	11ILE	CD	273HIS	CE1	2.26	17GLL	CD	20LEU	CD1	0.00
19ALA	CB	290ALA	CB	0.17	11ILE	CD	273HIS	C	0.02	17GLL	C	18ALA	CA	100.00
19ALA	CB	290ALA	C	3.70	11ILE	CD	274GLY	CA	0.15	17GLL	C	18ALA	C	100.00
19ALA	CB	291ILE	CA	0.01	11ILE	CD	274GLY	C	0.16	17GLL	C	21LYS	CE	0.00
19ALA	CB	291ILE	CG1	0.02	11ILE	CD	275SER	CB	0.24	18ALA	CA	18ALA	CB	100.00
19ALA	CB	294ALA	CB	0.21	11ILE	CD	275SER	C	0.01	18ALA	CA	18ALA	C	100.00
19ALA	C	20LEU	CA	100.00	11ILE	CD	276ALA	C	0.01	18ALA	CA	21LYS	CB	0.00
19ALA	C	20LEU	CB	0.00	11ILE	CD	279ILE	CD	0.03	18ALA	CA	337THR	CG2	0.17
19ALA	C	20LEU	C	100.00	11ILE	CD	280ALA	CB	0.01	18ALA	CB	18ALA	C	100.00
20LEU	CA	20LEU	CB	100.00	11ILE	CD	285ALA	CB	0.34	18ALA	CB	287PRO	CB	21.52
20LEU	CA	20LEU	CG	100.00	11ILE	C	12GLY	CA	100.00	18ALA	CB	287PRO	CG	0.11
20LEU	CA	20LEU	CD1	9.76	11ILE	C	12GLY	C	100.00	18ALA	CB	291ILE	CG1	0.00
20LEU	CA	20LEU	CD2	90.05	11ILE	C	13PRO	CD	7.89	18ALA	CB	291ILE	CD	0.02
20LEU	CA	20LEU	C	100.00	11ILE	C	15VAL	CG2	0.18	18ALA	CB	333THR	CG2	1.94
20LEU	CB	20LEU	CG	100.00	12GLY	CA	12GLY	C	100.00	18ALA	CB	337THR	CG2	10.19
20LEU	CB	20LEU	CD1	100.00	12GLY	CA	13PRO	CD	100.00	18ALA	C	19ALA	CA	100.00
20LEU	CB	20LEU	CD2	100.00	12GLY	CA	15VAL	CG2	0.72	18ALA	C	19ALA	CB	0.12
20LEU	CB	20LEU	C	100.00	12GLY	CA	70GLY	CA	0.03	18ALA	C	19ALA	C	99.87
20LEU	CB	24ARG	CZ	2.98	12GLY	CA	70GLY	C	0.01	18ALA	C	21LYS	CG	0.00
20LEU	CB	36TYR	CZ	0.00	12GLY	CA	71SER	CB	0.00	18ALA	C	291ILE	CG1	0.01
20LEU	CG	20LEU	CD1	100.00	12GLY	C	13PRO	CA	100.00	18ALA	C	291ILE	CD	0.10
20LEU	CG	20LEU	CD2	100.00	12GLY	C	13PRO	CB	0.02	19ALA	CA	19ALA	CB	100.00
20LEU	CG	20LEU	C	9.26	12GLY	C	13PRO	CD	100.00	19ALA	CA	19ALA	C	100.00
20LEU	CG	24ARG	CZ	0.17	12GLY	C	13PRO	C	100.00	19ALA	CA	291ILE	CG1	0.08
20LEU	CG	36TYR	CE1	0.00	12GLY	C	15VAL	CG2	0.03	19ALA	CA	291ILE	CD	0.05
20LEU	CG	36TYR	CZ	0.00	13PRO	CA	13PRO	CB	100.00	19ALA	CB	19ALA	C	100.00
20LEU	CG	68LEU	CD1	0.02	13PRO	CA	13PRO	CG	100.00	19ALA	CB	68LEU	CD2	0.04
20LEU	CG	68LEU	CD2	0.15	13PRO	CA	13PRO	CD	100.00	19ALA	CB	290ALA	CB	0.14
20LEU	CD1	20LEU	CD2	100.00	13PRO	CA	13PRO	C	100.00	19ALA	CB	290ALA	C	6.65
20LEU	CD1	20LEU	C	0.14	13PRO	CB	13PRO	CG	100.00	19ALA	CB	291ILE	CA	0.14
20LEU	CD1	23LEU	CD1	0.01	13PRO	CB	13PRO	CD	100.00	19ALA	CB	291ILE	CG1	0.07
20LEU	CD1	24ARG	CG	0.00	13PRO	CB	13PRO	C	100.00	19ALA	CB	294ALA	CB	0.01
20LEU	CD1	24ARG	CD	0.01	13PRO	CG	13PRO	CD	100.00	19ALA	C	20LEU	CA	100.00
20LEU	CD1	36TYR	CB	0.01	13PRO	CG	13PRO	C	46.10	19ALA	C	20LEU	C	100.00
20LEU	CD1	36TYR	CG	0.02	13PRO	CD	13PRO	C	31.85	20LEU	CA	20LEU	CB	100.00
20LEU	CD1	36TYR	CD1	0.11	13PRO	C	14GLU	CA	100.00	20LEU	CA	20LEU	CG	100.00
20LEU	CD1	36TYR	CD2	0.05	13PRO	C	14GLU	CB	0.51	20LEU	CA	20LEU	CD1	3.03
20LEU	CD1	36TYR	CE1	0.61	13PRO	C	14GLU	CG	0.01	20LEU	CA	20LEU	CD2	96.79
20LEU	CD1	36TYR	CE2	0.87	13PRO	C	14GLU	C	99.81	20LEU	CA	20LEU	C	100.00
20LEU	CD1	36TYR	CZ	0.07	14GLU	CA	14GLU	CB	100.00	20LEU	CB	20LEU	CG	100.00
20LEU	CD1	38VAL	CG2	0.00	14GLU	CA	14GLU	CG	100.00	20LEU	CB	20LEU	CD1	100.00
20LEU	CD1	68LEU	CD1	0.00	14GLU	CA	14GLU	CD	98.00	20LEU	CB	20LEU	CD2	100.00
20LEU	CD1	68LEU	CD2	0.13	14GLU	CA	14GLU	C	100.00	20LEU	CB	20LEU	C	100.00
20LEU	CD2	20LEU	C	0.06	14GLU	CB	14GLU	CG	100.00	20LEU	CB	24ARG	CZ	2.35
20LEU	CD2	23LEU	CD1	0.06	14GLU	CB	14GLU	CD	100.00	20LEU	CG	20LEU	CD1	100.00
20LEU	CD2	24ARG	CZ	0.25	14GLU	CB	14GLU	C	100.00	20LEU	CG	20LEU	CD2	100.00

20LEU	CD2	34LEU	CD2	0.00
20LEU	CD2	36TYR	CB	0.01
20LEU	CD2	36TYR	CG	0.32
20LEU	CD2	36TYR	CD1	0.74
20LEU	CD2	36TYR	CD2	0.70
20LEU	CD2	36TYR	CE1	1.03
20LEU	CD2	36TYR	CE2	0.76
20LEU	CD2	36TYR	CZ	0.48
20LEU	CD2	38VAL	CG2	0.03
20LEU	CD2	68LEU	CD1	1.27
20LEU	CD2	68LEU	CD2	1.02
20LEU	C	21LYS	CA	100.00
20LEU	C	21LYS	C	100.00
20LEU	C	24ARG	CD	0.00
20LEU	C	24ARG	CZ	0.01
21LYS	CA	21LYS	CB	100.00
21LYS	CA	21LYS	CG	100.00
21LYS	CA	21LYS	CD	0.48
21LYS	CA	21LYS	C	100.00
21LYS	CA	24ARG	CZ	0.04
21LYS	CB	21LYS	CG	100.00
21LYS	CB	21LYS	CD	100.00
21LYS	CB	21LYS	CE	13.04
21LYS	CB	21LYS	C	100.00
21LYS	CB	337THR	CG2	0.13
21LYS	CB	341LEU	CD1	0.00
21LYS	CG	21LYS	CD	100.00
21LYS	CG	21LYS	CE	100.00
21LYS	CG	21LYS	C	60.63
21LYS	CG	337THR	CG2	0.01
21LYS	CG	341LEU	CD1	0.02
21LYS	CG	341LEU	CD2	0.00
21LYS	CD	21LYS	CE	100.00
21LYS	CD	21LYS	C	0.00
21LYS	CD	337THR	CB	0.03
21LYS	CD	337THR	CG2	0.10
21LYS	CD	341LEU	CD1	0.15
21LYS	CD	341LEU	CD2	0.00
21LYS	CE	334GLU	CD	0.01
21LYS	CE	337THR	CB	0.02
21LYS	CE	341LEU	CD1	0.05
21LYS	CE	341LEU	CD2	0.01
21LYS	C	22VAL	CA	100.00
21LYS	C	22VAL	CB	0.10
21LYS	C	22VAL	CG2	0.01
21LYS	C	22VAL	C	99.98
21LYS	C	341LEU	CD2	0.03
22VAL	CA	22VAL	CB	100.00
22VAL	CA	22VAL	CG1	100.00
22VAL	CA	22VAL	CG2	100.00
22VAL	CA	22VAL	C	100.00
22VAL	CA	341LEU	CD1	0.01
22VAL	CA	341LEU	CD2	0.04
22VAL	CA	344LEU	CD1	0.00
22VAL	CB	22VAL	CG1	100.00
22VAL	CB	22VAL	CG2	100.00
22VAL	CB	22VAL	C	100.00
22VAL	CG1	22VAL	CG2	100.00
22VAL	CG1	22VAL	C	100.00
22VAL	CG1	26LEU	CD1	0.30
22VAL	CG1	291ILE	CG2	0.00
22VAL	CG1	311VAL	CG1	0.08
14GLU	CB	280ALA	CB	0.04
14GLU	CB	285ALA	CB	0.02
14GLU	CB	333THR	CG2	0.00
14GLU	CG	14GLU	CD	100.00
14GLU	CG	14GLU	C	92.90
14GLU	CG	280ALA	CB	0.03
14GLU	CG	280ALA	C	0.02
14GLU	CG	281GLY	CA	0.01
14GLU	CG	285ALA	CB	0.04
14GLU	CG	333THR	CB	0.09
14GLU	CG	333THR	CG2	1.30
14GLU	CD	14GLU	C	0.08
14GLU	CD	283GLY	CA	0.01
14GLU	CD	333THR	CB	0.01
14GLU	CD	333THR	CG2	0.00
14GLU	C	15VAL	CA	100.00
14GLU	C	15VAL	CB	0.31
14GLU	C	15VAL	CG1	0.08
14GLU	C	15VAL	C	99.80
14GLU	C	333THR	CG2	0.00
15VAL	CA	15VAL	CB	100.00
15VAL	CA	15VAL	CG1	100.00
15VAL	CA	15VAL	CG2	100.00
15VAL	CA	15VAL	C	100.00
15VAL	CA	18ALA	CB	0.02
15VAL	CB	15VAL	CG1	100.00
15VAL	CB	15VAL	CG2	100.00
15VAL	CB	15VAL	C	100.00
15VAL	CB	273HIS	CE1	0.11
15VAL	CB	287PRO	CB	0.02
15VAL	CB	290ALA	CB	0.26
15VAL	CG1	15VAL	CG2	100.00
15VAL	CG1	15VAL	C	0.00
15VAL	CG1	273HIS	CE1	0.51
15VAL	CG1	280ALA	CB	0.01
15VAL	CG1	285ALA	CB	1.24
15VAL	CG1	286ASN	C	0.01
15VAL	CG1	287PRO	CA	0.72
15VAL	CG1	287PRO	CB	0.05
15VAL	CG1	333THR	CG2	0.15
15VAL	CG2	15VAL	C	99.99
15VAL	CG2	70GLY	CA	0.15
15VAL	CG2	271PRO	CG	0.00
15VAL	CG2	273HIS	CE1	3.01
15VAL	CG2	290ALA	CB	0.67
15VAL	C	16THR	CA	100.00
15VAL	C	16THR	CB	0.04
15VAL	C	16THR	CG2	0.00
15VAL	C	16THR	C	99.99
15VAL	C	290ALA	CB	0.57
16THR	CA	16THR	CB	100.00
16THR	CA	16THR	CG2	100.00
16THR	CA	16THR	C	100.00
16THR	CA	19ALA	CB	0.00
16THR	CA	68LEU	CD2	0.00
16THR	CB	16THR	CG2	100.00
16THR	CB	16THR	C	100.00
16THR	CB	68LEU	CD2	0.07
16THR	CG2	16THR	C	0.13
16THR	CG2	68LEU	CG	0.00
16THR	CG2	68LEU	CD1	0.06
16THR	CG2	68LEU	CD2	0.43
20LEU	CG	20LEU	C	2.34
20LEU	CG	24ARG	CZ	0.12
20LEU	CG	68LEU	CD1	0.01
20LEU	CG	68LEU	CD2	0.06
20LEU	CD1	20LEU	CD2	100.00
20LEU	CD1	20LEU	C	0.04
20LEU	CD1	36TYR	CG	0.00
20LEU	CD1	36TYR	CD1	0.15
20LEU	CD1	36TYR	CD2	0.00
20LEU	CD1	36TYR	CE1	4.33
20LEU	CD1	36TYR	CE2	0.01
20LEU	CD1	36TYR	CZ	0.31
20LEU	CD1	68LEU	CD1	0.00
20LEU	CD1	68LEU	CD2	0.10
20LEU	CD2	20LEU	C	0.02
20LEU	CD2	23LEU	CD1	0.02
20LEU	CD2	23LEU	CD2	0.02
20LEU	CD2	24ARG	CZ	0.38
20LEU	CD2	34LEU	CD2	0.00
20LEU	CD2	36TYR	CB	0.02
20LEU	CD2	36TYR	CG	0.31
20LEU	CD2	36TYR	CD1	0.77
20LEU	CD2	36TYR	CD2	0.62
20LEU	CD2	36TYR	CE1	0.58
20LEU	CD2	36TYR	CE2	0.82
20LEU	CD2	36TYR	CZ	0.32
20LEU	CD2	68LEU	CD1	0.26
20LEU	CD2	68LEU	CD2	0.53
20LEU	C	21LYS	CA	100.00
20LEU	C	21LYS	C	100.00
20LEU	C	24ARG	CZ	0.05
21LYS	CA	21LYS	CB	100.00
21LYS	CA	21LYS	CG	100.00
21LYS	CA	21LYS	CD	0.03
21LYS	CA	21LYS	C	100.00
21LYS	CA	24ARG	CZ	0.27
21LYS	CB	21LYS	CG	100.00
21LYS	CB	21LYS	CD	100.00
21LYS	CB	21LYS	CE	28.56
21LYS	CB	21LYS	C	100.00
21LYS	CB	24ARG	CZ	0.01
21LYS	CB	337THR	CG2	0.06
21LYS	CB	341LEU	CD1	0.00
21LYS	CB	341LEU	CD2	0.01
21LYS	CG	21LYS	CD	100.00
21LYS	CG	21LYS	CE	100.00
21LYS	CG	21LYS	C	53.00
21LYS	CG	24ARG	CZ	0.00
21LYS	CG	341LEU	CD1	0.01
21LYS	CG	341LEU	CD2	0.00
21LYS	CD	21LYS	CE	100.00
21LYS	CD	21LYS	C	0.02
21LYS	CD	337THR	CB	0.12
21LYS	CD	337THR	CG2	0.14
21LYS	CD	341LEU	CD1	0.11
21LYS	CE	337THR	CB	0.02
21LYS	CE	341LEU	CD1	0.14
21LYS	CE	341LEU	CD2	0.01
21LYS	C	22VAL	CA	100.00
21LYS	C	22VAL	CB	0.06
21LYS	C	22VAL	CG2	0.01
21LYS	C	22VAL	C	99.98

22VAL	CG1	311VAL	CG2	0.60
22VAL	CG1	340VAL	CG1	0.18
22VAL	CG1	341LEU	CD2	0.02
22VAL	CG1	344LEU	CD1	3.96
22VAL	CG1	344LEU	CD2	0.71
22VAL	CG2	291ILE	CG2	0.00
22VAL	CG2	291ILE	CD	0.04
22VAL	CG2	337THR	CB	0.03
22VAL	CG2	337THR	CG2	1.77
22VAL	CG2	340VAL	CG1	0.39
22VAL	CG2	341LEU	CD1	0.16
22VAL	CG2	341LEU	CD2	0.28
22VAL	C	23LEU	CA	100.00
22VAL	C	23LEU	C	100.00
23LEU	CA	23LEU	CB	100.00
23LEU	CA	23LEU	CG	100.00
23LEU	CA	23LEU	CD2	99.67
23LEU	CA	23LEU	C	100.00
23LEU	CB	23LEU	CG	100.00
23LEU	CB	23LEU	CD1	100.00
23LEU	CB	23LEU	CD2	100.00
23LEU	CB	23LEU	C	100.00
23LEU	CB	32LEU	CD1	0.03
23LEU	CB	34LEU	CD1	0.02
23LEU	CB	34LEU	CD2	1.08
23LEU	CG	23LEU	CD1	100.00
23LEU	CG	23LEU	CD2	100.00
23LEU	CG	23LEU	C	0.02
23LEU	CG	294ALA	CB	0.00
23LEU	CD1	23LEU	CD2	100.00
23LEU	CD1	34LEU	CD1	0.06
23LEU	CD1	34LEU	CD2	0.01
23LEU	CD1	68LEU	CD1	0.31
23LEU	CD1	68LEU	CD2	0.06
23LEU	CD1	294ALA	CB	1.42
23LEU	CD1	297MET	CE	4.36
23LEU	CD2	23LEU	C	0.02
23LEU	CD2	32LEU	CD1	2.87
23LEU	CD2	32LEU	CD2	0.18
23LEU	CD2	34LEU	CD1	0.04
23LEU	CD2	297MET	CE	1.86
23LEU	CD2	298LEU	CG	0.01
23LEU	CD2	298LEU	CD1	2.35
23LEU	CD2	298LEU	CD2	0.75
23LEU	C	24ARG	CA	100.00
23LEU	C	24ARG	C	100.00
23LEU	C	34LEU	CD1	0.01
23LEU	C	34LEU	CD2	1.68
24ARG	CA	24ARG	CB	100.00
24ARG	CA	24ARG	CG	100.00
24ARG	CA	24ARG	CD	6.66
24ARG	CA	24ARG	C	100.00
24ARG	CA	34LEU	CD2	0.36
24ARG	CB	24ARG	CG	100.00
24ARG	CB	24ARG	CD	100.00
24ARG	CB	24ARG	CZ	1.56
24ARG	CB	24ARG	C	100.00
24ARG	CB	34LEU	CD2	0.00
24ARG	CG	24ARG	CD	100.00
24ARG	CG	24ARG	CZ	18.34
24ARG	CG	24ARG	C	22.61
24ARG	CG	34LEU	CD2	1.62

16THR	CG2	69LEU	C	2.41
16THR	CG2	70GLY	CA	12.19
16THR	C	17GLU	CA	100.00
16THR	C	17GLU	C	100.00
16THR	C	20LEU	CD1	0.03
17GLU	CA	17GLU	CB	100.00
17GLU	CA	17GLU	CG	100.00
17GLU	CA	17GLU	CD	92.27
17GLU	CA	17GLU	C	100.00
17GLU	CB	17GLU	CG	100.00
17GLU	CB	17GLU	CD	100.00
17GLU	CB	17GLU	C	100.00
17GLU	CG	17GLU	CD	100.00
17GLU	CG	17GLU	C	95.70
17GLU	CG	21LYS	CE	0.04
17GLU	CD	17GLU	C	8.68
17GLU	CD	20LEU	CD1	0.00
17GLU	CD	21LYS	CD	0.00
17GLU	CD	21LYS	CE	0.04
17GLU	C	18ALA	CA	100.00
17GLU	C	18ALA	CB	0.00
17GLU	C	18ALA	C	100.00
17GLU	C	21LYS	CE	0.00
18ALA	CA	18ALA	CB	100.00
18ALA	CA	18ALA	C	100.00
18ALA	CA	337THR	CG2	0.45
18ALA	CB	18ALA	C	100.00
18ALA	CB	287PRO	CB	18.83
18ALA	CB	287PRO	CG	0.47
18ALA	CB	291ILE	CG1	0.01
18ALA	CB	291ILE	CD	0.00
18ALA	CB	333THR	CB	0.01
18ALA	CB	333THR	CG2	0.49
18ALA	CB	337THR	CG2	9.77
18ALA	C	19ALA	CA	100.00
18ALA	C	19ALA	CB	0.12
18ALA	C	19ALA	C	99.90
18ALA	C	291ILE	CG1	0.01
18ALA	C	291ILE	CD	0.06
19ALA	CA	19ALA	CB	100.00
19ALA	CA	19ALA	C	100.00
19ALA	CA	291ILE	CG1	0.04
19ALA	CA	291ILE	CD	0.02
19ALA	CB	19ALA	C	100.00
19ALA	CB	68LEU	CD2	0.30
19ALA	CB	290ALA	CB	0.21
19ALA	CB	290ALA	C	3.35
19ALA	CB	291ILE	CA	0.05
19ALA	CB	291ILE	CG1	0.03
19ALA	CB	294ALA	CB	0.09
19ALA	C	20LEU	CA	100.00
19ALA	C	20LEU	CB	0.02
19ALA	C	20LEU	C	99.99
19ALA	C	23LEU	CD1	0.01
20LEU	CA	20LEU	CB	100.00
20LEU	CA	20LEU	CG	100.00
20LEU	CA	20LEU	CD1	16.02
20LEU	CA	20LEU	CD2	88.42
20LEU	CA	20LEU	C	100.00
20LEU	CB	20LEU	CG	100.00
20LEU	CB	20LEU	CD1	100.00
20LEU	CB	20LEU	CD2	100.00

21LYS	C	341LEU	CD2	0.42
22VAL	CA	22VAL	CB	100.00
22VAL	CA	22VAL	CG1	100.00
22VAL	CA	22VAL	CG2	100.00
22VAL	CA	22VAL	C	100.00
22VAL	CA	341LEU	CD2	0.19
22VAL	CA	344LEU	CD1	0.00
22VAL	CB	22VAL	CG1	100.00
22VAL	CB	22VAL	CG2	100.00
22VAL	CB	22VAL	C	100.00
22VAL	CG1	22VAL	CG2	100.00
22VAL	CG1	22VAL	C	100.00
22VAL	CG1	26LEU	CD1	0.29
22VAL	CG1	291ILE	CG2	0.03
22VAL	CG1	294ALA	CB	0.01
22VAL	CG1	311VAL	CG1	0.27
22VAL	CG1	311VAL	CG2	2.38
22VAL	CG1	340VAL	CG1	0.20
22VAL	CG1	341LEU	CD2	0.00
22VAL	CG1	344LEU	CD1	4.08
22VAL	CG1	344LEU	CD2	0.70
22VAL	CG2	22VAL	C	0.15
22VAL	CG2	291ILE	CG1	0.00
22VAL	CG2	291ILE	CG2	0.06
22VAL	CG2	291ILE	CD	0.01
22VAL	CG2	337THR	CB	0.00
22VAL	CG2	337THR	CG2	0.07
22VAL	CG2	340VAL	CG1	1.93
22VAL	CG2	341LEU	CD1	0.08
22VAL	CG2	341LEU	CD2	0.25
22VAL	CG2	344LEU	CD1	0.06
22VAL	CG2	344LEU	CD2	0.00
22VAL	C	23LEU	CA	100.00
22VAL	C	23LEU	CB	0.01
22VAL	C	23LEU	C	100.00
23LEU	CA	23LEU	CB	100.00
23LEU	CA	23LEU	CG	100.00
23LEU	CA	23LEU	CD1	25.62
23LEU	CA	23LEU	CD2	74.18
23LEU	CA	23LEU	C	100.00
23LEU	CA	32LEU	CD1	0.00
23LEU	CA	32LEU	CD2	0.00
23LEU	CB	23LEU	CG	100.00
23LEU	CB	23LEU	CD1	100.00
23LEU	CB	23LEU	CD2	100.00
23LEU	CB	23LEU	C	100.00
23LEU	CB	32LEU	CD1	0.03
23LEU	CB	32LEU	CD2	0.02
23LEU	CB	34LEU	CD1	0.01
23LEU	CB	34LEU	CD2	1.53
23LEU	CG	23LEU	CD1	100.00
23LEU	CG	23LEU	CD2	100.00
23LEU	CG	23LEU	C	13.90
23LEU	CG	32LEU	CD1	0.05
23LEU	CG	34LEU	CD1	0.03
23LEU	CG	34LEU	CD2	0.03
23LEU	CG	294ALA	CB	0.02
23LEU	CG	298LEU	CD1	0.00
23LEU	CD1	23LEU	CD2	100.00
23LEU	CD1	23LEU	C	0.01
23LEU	CD1	26LEU	CD1	0.00
23LEU	CD1	32LEU	CD1	0.78

24ARG	CG	36TYR	CE1	0.00
24ARG	CG	36TYR	CE2	0.02
24ARG	CG	36TYR	CZ	0.01
24ARG	CD	24ARG	CZ	100.00
24ARG	CD	24ARG	C	0.12
24ARG	CD	34LEU	CD2	0.02
24ARG	CD	36TYR	CE1	0.02
24ARG	CD	36TYR	CE2	0.15
24ARG	CD	36TYR	CZ	0.07
24ARG	CZ	34LEU	CD2	0.09
24ARG	CZ	36TYR	CD1	0.00
24ARG	CZ	36TYR	CD2	0.01
24ARG	CZ	36TYR	CE1	0.53
24ARG	CZ	36TYR	CE2	1.08
24ARG	CZ	36TYR	CZ	0.08
24ARG	C	25ALA	CA	100.00
24ARG	C	25ALA	CB	0.01
24ARG	C	25ALA	C	99.99
25ALA	CA	25ALA	CB	100.00
25ALA	CA	25ALA	C	100.00
25ALA	CB	25ALA	C	100.00
25ALA	CB	341LEU	CG	0.08
25ALA	CB	341LEU	CD1	0.67
25ALA	CB	341LEU	CD2	32.90
25ALA	CB	344LEU	CB	0.00
25ALA	CB	344LEU	CD1	0.56
25ALA	CB	344LEU	CD2	0.65
25ALA	CB	345ALA	CB	0.00
25ALA	C	26LEU	CA	100.00
25ALA	C	26LEU	CB	0.02
25ALA	C	26LEU	C	99.96
25ALA	C	344LEU	CD1	0.01
25ALA	C	344LEU	CD2	0.00
26LEU	CA	26LEU	CB	100.00
26LEU	CA	26LEU	CG	100.00
26LEU	CA	26LEU	CD1	17.06
26LEU	CA	26LEU	CD2	82.61
26LEU	CA	26LEU	C	100.00
26LEU	CB	26LEU	CG	100.00
26LEU	CB	26LEU	CD1	100.00
26LEU	CB	26LEU	CD2	100.00
26LEU	CB	26LEU	C	100.00
26LEU	CB	32LEU	CG	0.00
26LEU	CB	32LEU	CD1	0.81
26LEU	CB	32LEU	CD2	2.95
26LEU	CG	26LEU	CD1	100.00
26LEU	CG	26LEU	CD2	100.00
26LEU	CG	26LEU	C	16.88
26LEU	CG	32LEU	CD1	0.00
26LEU	CG	32LEU	CD2	0.04
26LEU	CG	341LEU	CD2	0.01
26LEU	CG	344LEU	CD1	0.00
26LEU	CG	344LEU	CD2	0.01
26LEU	CD1	26LEU	CD2	100.00
26LEU	CD1	26LEU	C	0.28
26LEU	CD1	30GLU	CB	0.26
26LEU	CD1	32LEU	CD1	0.00
26LEU	CD1	298LEU	CD1	1.52
26LEU	CD1	298LEU	CD2	0.22
26LEU	CD1	307LEU	CG	0.00
26LEU	CD1	307LEU	CD1	0.03
26LEU	CD1	307LEU	CD2	0.03

20LEU	CB	20LEU	C	100.00
20LEU	CB	24ARG	CZ	5.36
20LEU	CB	36TYR	CE1	0.00
20LEU	CB	36TYR	CE2	0.00
20LEU	CG	20LEU	CD1	100.00
20LEU	CG	20LEU	CD2	100.00
20LEU	CG	20LEU	C	9.53
20LEU	CG	24ARG	CZ	0.09
20LEU	CG	36TYR	CD1	0.01
20LEU	CG	36TYR	CE1	0.01
20LEU	CG	36TYR	CE2	0.01
20LEU	CG	36TYR	CZ	0.00
20LEU	CG	68LEU	CD1	0.00
20LEU	CG	68LEU	CD2	0.09
20LEU	CD1	20LEU	CD2	100.00
20LEU	CD1	20LEU	C	0.52
20LEU	CD1	23LEU	CD1	0.01
20LEU	CD1	34LEU	CD1	0.01
20LEU	CD1	34LEU	CD2	0.02
20LEU	CD1	36TYR	CB	0.14
20LEU	CD1	36TYR	CG	0.56
20LEU	CD1	36TYR	CD1	0.62
20LEU	CD1	36TYR	CD2	0.87
20LEU	CD1	36TYR	CE1	2.15
20LEU	CD1	36TYR	CE2	1.60
20LEU	CD1	36TYR	CZ	0.27
20LEU	CD1	38VAL	CG1	0.00
20LEU	CD1	38VAL	CG2	0.46
20LEU	CD1	68LEU	CD1	0.01
20LEU	CD1	68LEU	CD2	0.30
20LEU	CD2	20LEU	C	0.17
20LEU	CD2	23LEU	CD1	0.06
20LEU	CD2	23LEU	CD2	0.01
20LEU	CD2	24ARG	CD	0.00
20LEU	CD2	24ARG	CZ	0.23
20LEU	CD2	34LEU	CD1	0.01
20LEU	CD2	34LEU	CD2	0.01
20LEU	CD2	36TYR	CB	0.22
20LEU	CD2	36TYR	CG	2.26
20LEU	CD2	36TYR	CD1	2.63
20LEU	CD2	36TYR	CD2	4.45
20LEU	CD2	36TYR	CE1	1.04
20LEU	CD2	36TYR	CE2	1.81
20LEU	CD2	36TYR	CZ	0.45
20LEU	CD2	38VAL	CG2	0.18
20LEU	CD2	68LEU	CD1	0.16
20LEU	CD2	68LEU	CD2	0.91
20LEU	C	21LYS	CA	100.00
20LEU	C	21LYS	C	100.00
20LEU	C	24ARG	CG	0.00
20LEU	C	24ARG	CD	0.00
20LEU	C	24ARG	CZ	0.06
21LYS	CA	21LYS	CB	100.00
21LYS	CA	21LYS	CG	100.00
21LYS	CA	21LYS	CD	0.81
21LYS	CA	21LYS	CE	0.00
21LYS	CA	21LYS	C	100.00
21LYS	CA	24ARG	CZ	0.11
21LYS	CB	21LYS	CG	100.00
21LYS	CB	21LYS	CD	100.00
21LYS	CB	21LYS	CE	13.16
21LYS	CB	21LYS	C	100.00

23LEU	CD1	34LEU	CD1	0.06
23LEU	CD1	34LEU	CD2	0.00
23LEU	CD1	68LEU	CD1	0.07
23LEU	CD1	68LEU	CD2	0.02
23LEU	CD1	294ALA	CB	1.86
23LEU	CD1	297MET	CE	2.87
23LEU	CD1	298LEU	CD1	0.88
23LEU	CD1	298LEU	CD2	0.06
23LEU	CD2	32LEU	CD1	0.94
23LEU	CD2	32LEU	CD2	0.44
23LEU	CD2	34LEU	CD1	1.26
23LEU	CD2	34LEU	CD2	0.13
23LEU	CD2	294ALA	CB	0.07
23LEU	CD2	297MET	CE	0.82
23LEU	CD2	298LEU	CG	0.13
23LEU	CD2	298LEU	CD1	3.65
23LEU	CD2	298LEU	CD2	0.76
23LEU	CD2	302PHE	CE1	0.01
23LEU	C	24ARG	CA	100.00
23LEU	C	24ARG	C	100.00
23LEU	C	32LEU	CD1	0.01
23LEU	C	34LEU	CD1	0.03
23LEU	C	34LEU	CD2	1.30
24ARG	CA	24ARG	CB	100.00
24ARG	CA	24ARG	CG	100.00
24ARG	CA	24ARG	C	100.00
24ARG	CA	34LEU	CD2	0.24
24ARG	CB	24ARG	CG	100.00
24ARG	CB	24ARG	CD	100.00
24ARG	CB	24ARG	CZ	0.58
24ARG	CB	24ARG	C	100.00
24ARG	CB	34LEU	CD2	0.00
24ARG	CG	24ARG	CD	100.00
24ARG	CG	24ARG	CZ	16.50
24ARG	CG	24ARG	C	6.98
24ARG	CG	34LEU	CD1	0.01
24ARG	CG	34LEU	CD2	3.42
24ARG	CD	24ARG	CZ	100.00
24ARG	CD	36TYR	CE1	0.01
24ARG	CD	36TYR	CE2	0.47
24ARG	CD	36TYR	CZ	0.09
24ARG	CZ	36TYR	CE2	0.01
24ARG	C	25ALA	CA	100.00
24ARG	C	25ALA	CB	0.00
24ARG	C	25ALA	C	99.99
25ALA	CA	25ALA	CB	100.00
25ALA	CA	25ALA	C	100.00
25ALA	CB	25ALA	C	100.00
25ALA	CB	341LEU	CG	0.01
25ALA	CB	341LEU	CD1	0.44
25ALA	CB	341LEU	CD2	29.76
25ALA	CB	344LEU	CD1	0.42
25ALA	CB	344LEU	CD2	0.25
25ALA	CB	345ALA	CB	0.05
25ALA	C	26LEU	CA	100.00
25ALA	C	26LEU	CB	0.03
25ALA	C	26LEU	C	99.97
26LEU	CA	26LEU	CB	100.00
26LEU	CA	26LEU	CG	100.00
26LEU	CA	26LEU	CD1	14.04
26LEU	CA	26LEU	CD2	85.71
26LEU	CA	26LEU	C	100.00

26LEU	CD1	310LYS	CD	0.00
26LEU	CD1	310LYS	CE	0.02
26LEU	CD1	311VAL	CG2	4.93
26LEU	CD1	341LEU	CD2	0.00
26LEU	CD1	344LEU	CD1	0.01
26LEU	CD1	344LEU	CD2	0.05
26LEU	CD2	26LEU	C	0.29
26LEU	CD2	30GLU	CB	0.09
26LEU	CD2	32LEU	CD1	0.03
26LEU	CD2	32LEU	CD2	0.08
26LEU	CD2	298LEU	CD1	0.64
26LEU	CD2	307LEU	CG	0.00
26LEU	CD2	307LEU	CD1	0.23
26LEU	CD2	307LEU	CD2	0.14
26LEU	CD2	310LYS	CG	0.00
26LEU	CD2	310LYS	CD	0.10
26LEU	CD2	310LYS	CE	0.07
26LEU	CD2	311VAL	CG1	0.12
26LEU	CD2	311VAL	CG2	0.96
26LEU	CD2	341LEU	CD2	0.03
26LEU	CD2	344LEU	CG	0.02
26LEU	CD2	344LEU	CD1	0.14
26LEU	CD2	344LEU	CD2	0.46
26LEU	C	27ASP	CA	100.00
26LEU	C	27ASP	CB	0.02
26LEU	C	27ASP	C	99.99
26LEU	C	32LEU	CD1	0.04
26LEU	C	32LEU	CD2	0.48
27ASP	CA	27ASP	CB	100.00
27ASP	CA	27ASP	CG	100.00
27ASP	CA	27ASP	C	100.00
27ASP	CA	31GLY	CA	0.04
27ASP	CA	32LEU	CB	1.04
27ASP	CA	32LEU	CD2	0.15
27ASP	CB	27ASP	CG	100.00
27ASP	CB	27ASP	C	100.00
27ASP	CB	32LEU	CB	0.15
27ASP	CB	32LEU	CG	0.01
27ASP	CB	32LEU	CD1	0.02
27ASP	CB	32LEU	CD2	0.03
27ASP	CB	34LEU	CB	0.01
27ASP	CG	27ASP	C	100.00
27ASP	CG	32LEU	CD1	0.00
27ASP	CG	34LEU	CB	6.93
27ASP	C	28GLU	CA	100.00
27ASP	C	28GLU	CB	0.74
27ASP	C	28GLU	C	99.45
28GLU	CA	28GLU	CB	100.00
28GLU	CA	28GLU	CG	100.00
28GLU	CA	28GLU	CD	94.21
28GLU	CA	28GLU	C	100.00
28GLU	CB	28GLU	CG	100.00
28GLU	CB	28GLU	CD	100.00
28GLU	CB	28GLU	C	100.00
28GLU	CG	28GLU	CD	100.00
28GLU	CG	28GLU	C	99.33
28GLU	CD	28GLU	C	1.64
28GLU	C	29ALA	CA	100.00
28GLU	C	29ALA	CB	6.60
28GLU	C	29ALA	C	92.88
29ALA	CA	29ALA	CB	100.00
29ALA	CA	29ALA	C	100.00

21LYS	CB	337THR	CG2	0.10
21LYS	CB	341LEU	CD2	0.00
21LYS	CG	21LYS	CD	100.00
21LYS	CG	21LYS	CE	100.00
21LYS	CG	21LYS	C	47.30
21LYS	CG	24ARG	CZ	0.02
21LYS	CG	341LEU	CD1	0.01
21LYS	CG	341LEU	CD2	0.02
21LYS	CD	21LYS	CE	100.00
21LYS	CD	337THR	CB	0.11
21LYS	CD	337THR	CG2	0.14
21LYS	CD	341LEU	CD1	0.02
21LYS	CD	341LEU	CD2	0.01
21LYS	CE	334GLU	CD	0.02
21LYS	CE	337THR	CB	0.04
21LYS	CE	341LEU	CD1	0.07
21LYS	CE	341LEU	CD2	0.01
21LYS	C	22VAL	CA	100.00
21LYS	C	22VAL	CB	0.11
21LYS	C	22VAL	CG2	0.05
21LYS	C	22VAL	C	99.94
21LYS	C	341LEU	CD2	0.20
22VAL	CA	22VAL	CB	100.00
22VAL	CA	22VAL	CG1	100.00
22VAL	CA	22VAL	CG2	100.00
22VAL	CA	22VAL	C	100.00
22VAL	CA	341LEU	CD1	0.01
22VAL	CA	341LEU	CD2	0.16
22VAL	CA	344LEU	CD1	0.01
22VAL	CB	22VAL	CG1	100.00
22VAL	CB	22VAL	CG2	100.00
22VAL	CB	22VAL	C	100.00
22VAL	CG1	22VAL	CG2	100.00
22VAL	CG1	22VAL	C	100.00
22VAL	CG1	26LEU	CD1	0.34
22VAL	CG1	291ILE	CG2	0.12
22VAL	CG1	294ALA	CB	0.01
22VAL	CG1	311VAL	CG1	0.90
22VAL	CG1	311VAL	CG2	2.20
22VAL	CG1	340VAL	CG1	0.79
22VAL	CG1	341LEU	CD2	0.00
22VAL	CG1	344LEU	CD1	4.40
22VAL	CG1	344LEU	CD2	0.25
22VAL	CG2	22VAL	C	0.16
22VAL	CG2	291ILE	CG2	0.19
22VAL	CG2	291ILE	CD	0.05
22VAL	CG2	311VAL	CG2	0.00
22VAL	CG2	337THR	CG2	0.22
22VAL	CG2	340VAL	CG1	1.84
22VAL	CG2	341LEU	CD1	0.26
22VAL	CG2	341LEU	CD2	0.12
22VAL	CG2	344LEU	CD1	0.06
22VAL	CG2	344LEU	CD2	0.00
22VAL	C	23LEU	CA	100.00
22VAL	C	23LEU	C	100.00
22VAL	C	26LEU	CD1	0.00
23LEU	CA	23LEU	CB	100.00
23LEU	CA	23LEU	CG	100.00
23LEU	CA	23LEU	CD1	7.29
23LEU	CA	23LEU	CD2	92.50
23LEU	CA	23LEU	C	100.00
23LEU	CA	32LEU	CD1	0.01

26LEU	CB	26LEU	CG	100.00
26LEU	CB	26LEU	CD1	100.00
26LEU	CB	26LEU	CD2	100.00
26LEU	CB	26LEU	C	100.00
26LEU	CB	32LEU	CG	0.00
26LEU	CB	32LEU	CD1	1.16
26LEU	CB	32LEU	CD2	0.72
26LEU	CB	344LEU	CD2	0.00
26LEU	CG	26LEU	CD1	100.00
26LEU	CG	26LEU	CD2	100.00
26LEU	CG	26LEU	C	13.10
26LEU	CG	32LEU	CD2	0.18
26LEU	CG	307LEU	CD2	0.01
26LEU	CG	344LEU	CD1	0.05
26LEU	CG	344LEU	CD2	0.04
26LEU	CD1	26LEU	CD2	100.00
26LEU	CD1	26LEU	C	0.12
26LEU	CD1	30GLU	CB	0.12
26LEU	CD1	30GLU	CG	0.02
26LEU	CD1	298LEU	CD1	2.99
26LEU	CD1	298LEU	CD2	0.57
26LEU	CD1	307LEU	CD1	0.05
26LEU	CD1	307LEU	CD2	0.06
26LEU	CD1	310LYS	CD	0.01
26LEU	CD1	310LYS	CE	0.01
26LEU	CD1	311VAL	CG2	4.21
26LEU	CD1	341LEU	CD2	0.01
26LEU	CD1	344LEU	CB	0.01
26LEU	CD1	344LEU	CG	0.00
26LEU	CD1	344LEU	CD1	0.02
26LEU	CD1	344LEU	CD2	0.05
26LEU	CD2	26LEU	C	0.20
26LEU	CD2	30GLU	CB	0.04
26LEU	CD2	30GLU	CG	0.10
26LEU	CD2	32LEU	CD2	0.20
26LEU	CD2	298LEU	CD1	0.40
26LEU	CD2	298LEU	CD2	0.09
26LEU	CD2	307LEU	CB	0.00
26LEU	CD2	307LEU	CG	0.03
26LEU	CD2	307LEU	CD1	0.15
26LEU	CD2	307LEU	CD2	0.29
26LEU	CD2	310LYS	CD	0.00
26LEU	CD2	311VAL	CG2	1.09
26LEU	CD2	344LEU	CB	0.02
26LEU	CD2	344LEU	CG	0.01
26LEU	CD2	344LEU	CD1	0.16
26LEU	CD2	344LEU	CD2	0.34
26LEU	C	27ASP	CA	100.00
26LEU	C	27ASP	CB	0.02
26LEU	C	27ASP	C	99.97
26LEU	C	32LEU	CD1	0.16
26LEU	C	32LEU	CD2	0.33
27ASF	CA	27ASP	CB	100.00
27ASF	CA	27ASP	CG	100.00
27ASF	CA	27ASP	C	100.00
27ASF	CA	31GLY	CA	0.05
27ASF	CA	32LEU	CB	0.72
27ASF	CA	32LEU	CD1	0.01
27ASF	CA	32LEU	CD2	0.14
27ASF	CB	27ASP	CG	100.00
27ASF	CB	27ASP	C	100.00
27ASF	CB	32LEU	CB	0.04

29ALA	CB	29ALA	C	100.00	23LEU	CA	32LEU	CD2	0.00	27ASF	CB	32LEU	CG	0.01
29ALA	CB	344LEU	CD2	0.00	23LEU	CB	23LEU	CG	100.00	27ASF	CB	32LEU	CD1	0.01
29ALA	CB	344LEU	C	0.00	23LEU	CB	23LEU	CD1	100.00	27ASF	CB	32LEU	CD2	0.02
29ALA	CB	345ALA	CA	0.01	23LEU	CB	23LEU	CD2	100.00	27ASF	CB	34LEU	CB	0.01
29ALA	CB	345ALA	CB	0.15	23LEU	CB	23LEU	C	100.00	27ASF	CB	34LEU	CD1	0.02
29ALA	CB	345ALA	C	0.02	23LEU	CB	32LEU	CD1	0.07	27ASF	CG	27ASP	C	100.00
29ALA	C	30GLU	CA	100.00	23LEU	CB	32LEU	CD2	0.02	27ASF	CG	32LEU	CB	0.00
29ALA	C	30GLU	CB	62.88	23LEU	CB	34LEU	CD1	0.08	27ASF	CG	34LEU	CB	4.82
29ALA	C	30GLU	C	43.18	23LEU	CB	34LEU	CD2	1.66	27ASF	C	28GLU	CA	100.00
30GLU	CA	30GLU	CB	100.00	23LEU	CB	36TYR	CD1	0.00	27ASF	C	28GLU	CB	1.12
30GLU	CA	30GLU	CG	100.00	23LEU	CB	36TYR	CE1	0.03	27ASF	C	28GLU	C	99.22
30GLU	CA	30GLU	CD	98.00	23LEU	CB	36TYR	CE2	0.04	28GLL	CA	28GLU	CB	100.00
30GLU	CA	30GLU	C	100.00	23LEU	CB	36TYR	CZ	0.03	28GLL	CA	28GLU	CG	100.00
30GLU	CB	30GLU	CG	100.00	23LEU	CG	23LEU	CD1	100.00	28GLL	CA	28GLU	CD	93.65
30GLU	CB	30GLU	CD	100.00	23LEU	CG	23LEU	CD2	100.00	28GLL	CA	28GLU	C	100.00
30GLU	CB	30GLU	C	100.00	23LEU	CG	23LEU	C	2.88	28GLL	CB	28GLU	CG	100.00
30GLU	CB	307LEU	CD1	0.10	23LEU	CG	32LEU	CD1	0.05	28GLL	CB	28GLU	CD	100.00
30GLU	CB	307LEU	CD2	0.03	23LEU	CG	32LEU	CD2	0.01	28GLL	CB	28GLU	C	100.00
30GLU	CG	30GLU	CD	100.00	23LEU	CG	34LEU	CD1	0.01	28GLL	CG	28GLU	CD	100.00
30GLU	CG	30GLU	C	99.05	23LEU	CG	34LEU	CD2	0.00	28GLL	CG	28GLU	C	95.55
30GLU	CG	307LEU	CD1	1.62	23LEU	CG	294ALA	CB	0.01	28GLL	CD	28GLU	C	1.82
30GLU	CG	307LEU	CD2	0.92	23LEU	CD1	23LEU	CD2	100.00	28GLL	C	29ALA	CA	100.00
30GLU	CD	30GLU	C	1.10	23LEU	CD1	23LEU	C	0.01	28GLL	C	29ALA	CB	5.28
30GLU	CD	307LEU	CD1	0.03	23LEU	CD1	32LEU	CD1	0.08	28GLL	C	29ALA	C	94.31
30GLU	CD	307LEU	CD2	0.05	23LEU	CD1	32LEU	CD2	0.18	29ALA	CA	29ALA	CB	100.00
30GLU	CD	310LYS	CD	0.01	23LEU	CD1	34LEU	CD1	0.11	29ALA	CA	29ALA	C	100.00
30GLU	CD	310LYS	CE	0.08	23LEU	CD1	34LEU	CD2	0.07	29ALA	CB	29ALA	C	100.00
30GLU	C	31GLY	CA	100.00	23LEU	CD1	36TYR	CD1	0.02	29ALA	CB	344LEU	CD1	0.01
30GLU	C	31GLY	C	64.64	23LEU	CD1	36TYR	CD2	0.01	29ALA	CB	344LEU	C	0.00
31GLY	CA	31GLY	C	100.00	23LEU	CD1	36TYR	CE2	0.08	29ALA	CB	345ALA	CA	0.03
31GLY	C	32LEU	CA	100.00	23LEU	CD1	36TYR	CZ	0.01	29ALA	CB	345ALA	CB	0.61
31GLY	C	32LEU	CB	53.16	23LEU	CD1	68LEU	CD1	0.13	29ALA	CB	345ALA	C	0.06
31GLY	C	32LEU	CG	0.97	23LEU	CD1	68LEU	CD2	0.03	29ALA	C	30GLU	CA	100.00
31GLY	C	32LEU	CD1	0.12	23LEU	CD1	294ALA	CB	1.29	29ALA	C	30GLU	CB	56.95
31GLY	C	32LEU	CD2	0.06	23LEU	CD1	297MET	CE	2.06	29ALA	C	30GLU	CG	0.26
31GLY	C	32LEU	C	46.89	23LEU	CD1	298LEU	CD1	0.04	29ALA	C	30GLU	CD	0.00
32LEU	CA	32LEU	CB	100.00	23LEU	CD2	23LEU	C	0.02	29ALA	C	30GLU	C	48.40
32LEU	CA	32LEU	CG	100.00	23LEU	CD2	26LEU	CD1	0.01	30GLL	CA	30GLU	CB	100.00
32LEU	CA	32LEU	CD1	15.42	23LEU	CD2	26LEU	CD2	0.00	30GLL	CA	30GLU	CG	100.00
32LEU	CA	32LEU	CD2	71.25	23LEU	CD2	32LEU	CD1	1.45	30GLL	CA	30GLU	CD	97.92
32LEU	CA	32LEU	C	100.00	23LEU	CD2	32LEU	CD2	0.20	30GLL	CA	30GLU	C	100.00
32LEU	CA	304LEU	CD1	0.18	23LEU	CD2	34LEU	CD1	0.32	30GLL	CB	30GLU	CG	100.00
32LEU	CA	304LEU	CD2	0.00	23LEU	CD2	34LEU	CD2	0.06	30GLL	CB	30GLU	CD	100.00
32LEU	CB	32LEU	CG	100.00	23LEU	CD2	36TYR	CB	0.01	30GLL	CB	30GLU	C	100.00
32LEU	CB	32LEU	CD1	100.00	23LEU	CD2	36TYR	CG	0.02	30GLL	CB	32LEU	CD2	0.04
32LEU	CB	32LEU	CD2	100.00	23LEU	CD2	36TYR	CD1	0.05	30GLL	CB	307LEU	CD1	0.09
32LEU	CB	32LEU	C	100.00	23LEU	CD2	36TYR	CD2	0.01	30GLL	CB	307LEU	CD2	0.11
32LEU	CB	298LEU	CD2	0.03	23LEU	CD2	294ALA	CB	0.05	30GLL	CG	30GLU	CD	100.00
32LEU	CB	302PHE	CD1	0.00	23LEU	CD2	297MET	CE	0.56	30GLL	CG	30GLU	C	86.10
32LEU	CB	302PHE	CD2	0.01	23LEU	CD2	298LEU	CG	0.12	30GLL	CG	32LEU	CD2	0.06
32LEU	CB	302PHE	CE1	0.13	23LEU	CD2	298LEU	CD1	2.15	30GLL	CG	304LEU	CD2	0.01
32LEU	CB	302PHE	CE2	0.48	23LEU	CD2	298LEU	CD2	0.59	30GLL	CG	307LEU	CD1	0.31
32LEU	CB	302PHE	CZ	0.08	23LEU	CD2	302PHE	CE1	0.00	30GLL	CG	307LEU	CD2	0.30
32LEU	CB	304LEU	CD1	4.33	23LEU	CD2	311VAL	CG2	0.00	30GLL	CD	30GLU	C	1.49
32LEU	CB	304LEU	CD2	0.06	23LEU	C	24ARG	CA	100.00	30GLL	CD	307LEU	CD1	0.08
32LEU	CB	307LEU	CD1	0.23	23LEU	C	24ARG	CB	0.00	30GLL	CD	307LEU	CD2	0.05
32LEU	CB	307LEU	CD2	0.08	23LEU	C	24ARG	C	100.00	30GLL	C	31GLY	CA	100.00
32LEU	CG	32LEU	CD1	100.00	23LEU	C	32LEU	CD2	0.00	30GLL	C	31GLY	C	67.61
32LEU	CG	32LEU	CD2	100.00	23LEU	C	34LEU	CD1	0.01	31GLY	CA	31GLY	C	100.00
32LEU	CG	32LEU	C	43.52	23LEU	C	34LEU	CD2	1.72	31GLY	C	32LEU	CA	100.00
32LEU	CG	298LEU	CD2	0.06	24ARG	CA	24ARG	CB	100.00	31GLY	C	32LEU	CB	47.43
32LEU	CG	302PHE	CE2	0.02	24ARG	CA	24ARG	CG	100.00	31GLY	C	32LEU	CG	1.57

32LEU	CG	302PHE	CZ	0.02
32LEU	CG	304LEU	CD1	0.02
32LEU	CG	304LEU	CD2	0.00
32LEU	CG	307LEU	CD1	0.02
32LEU	CG	307LEU	CD2	0.22
32LEU	CD1	32LEU	CD2	100.00
32LEU	CD1	32LEU	C	7.30
32LEU	CD1	33GLY	C	0.00
32LEU	CD1	34LEU	CA	0.00
32LEU	CD1	34LEU	CB	0.06
32LEU	CD1	34LEU	CD1	0.08
32LEU	CD1	298LEU	CD1	0.21
32LEU	CD1	298LEU	CD2	2.09
32LEU	CD1	302PHE	CD2	0.86
32LEU	CD1	302PHE	CE1	0.08
32LEU	CD1	302PHE	CE2	5.86
32LEU	CD1	302PHE	CZ	2.07
32LEU	CD1	304LEU	CD1	1.54
32LEU	CD1	307LEU	CD1	0.15
32LEU	CD1	307LEU	CD2	0.16
32LEU	CD2	32LEU	C	15.80
32LEU	CD2	33GLY	C	0.00
32LEU	CD2	34LEU	CD1	0.05
32LEU	CD2	298LEU	CD1	0.86
32LEU	CD2	298LEU	CD2	2.96
32LEU	CD2	302PHE	CG	0.00
32LEU	CD2	302PHE	CD1	0.01
32LEU	CD2	302PHE	CD2	1.39
32LEU	CD2	302PHE	CE1	0.04
32LEU	CD2	302PHE	CE2	7.08
32LEU	CD2	302PHE	CZ	0.41
32LEU	CD2	304LEU	CD1	3.53
32LEU	CD2	304LEU	CD2	0.02
32LEU	CD2	307LEU	CG	0.02
32LEU	CD2	307LEU	CD1	6.18
32LEU	CD2	307LEU	CD2	4.15
32LEU	C	33GLY	CA	100.00
32LEU	C	33GLY	C	83.84
32LEU	C	302PHE	CE1	0.08
32LEU	C	302PHE	CE2	0.35
32LEU	C	302PHE	CZ	0.02
33GLY	CA	33GLY	C	100.00
33GLY	C	34LEU	CA	100.00
33GLY	C	34LEU	CB	0.80
33GLY	C	34LEU	C	99.68
33GLY	C	302PHE	CE2	0.02
34LEU	CA	34LEU	CB	100.00
34LEU	CA	34LEU	CG	100.00
34LEU	CA	34LEU	CD1	96.22
34LEU	CA	34LEU	CD2	1.36
34LEU	CA	34LEU	C	100.00
34LEU	CB	34LEU	CG	100.00
34LEU	CB	34LEU	CD1	100.00
34LEU	CB	34LEU	CD2	100.00
34LEU	CB	34LEU	C	100.00
34LEU	CB	302PHE	CZ	0.00
34LEU	CG	34LEU	CD1	100.00
34LEU	CG	34LEU	CD2	100.00
34LEU	CG	34LEU	C	99.36
34LEU	CG	36TYR	CD1	0.04
34LEU	CG	36TYR	CD2	0.02
34LEU	CG	36TYR	CE1	0.00
24ARG	CA	24ARG	CD	2.68
24ARG	CA	24ARG	CZ	0.02
24ARG	CA	24ARG	C	100.00
24ARG	CA	34LEU	CD2	0.54
24ARG	CB	24ARG	CG	100.00
24ARG	CB	24ARG	CD	100.00
24ARG	CB	24ARG	CZ	1.44
24ARG	CB	24ARG	C	100.00
24ARG	CB	34LEU	CD2	0.03
24ARG	CG	24ARG	CD	100.00
24ARG	CG	24ARG	CZ	11.78
24ARG	CG	24ARG	C	12.53
24ARG	CG	34LEU	CD1	0.02
24ARG	CG	34LEU	CD2	1.66
24ARG	CG	36TYR	CE1	0.12
24ARG	CG	36TYR	CE2	0.22
24ARG	CG	36TYR	CZ	0.04
24ARG	CD	24ARG	CZ	100.00
24ARG	CD	24ARG	C	0.77
24ARG	CD	36TYR	CD1	0.00
24ARG	CD	36TYR	CE1	0.38
24ARG	CD	36TYR	CE2	0.23
24ARG	CD	36TYR	CZ	0.26
24ARG	CZ	24ARG	C	0.02
24ARG	CZ	25ALA	CA	0.03
24ARG	CZ	28GLU	CD	0.00
24ARG	CZ	34LEU	CD2	0.00
24ARG	CZ	36TYR	CE1	0.03
24ARG	CZ	36TYR	CE2	0.08
24ARG	CZ	36TYR	CZ	0.01
24ARG	C	25ALA	CA	100.00
24ARG	C	25ALA	CB	0.03
24ARG	C	25ALA	C	99.97
25ALA	CA	25ALA	CB	100.00
25ALA	CA	25ALA	C	100.00
25ALA	CB	25ALA	C	100.00
25ALA	CB	341LEU	CG	0.21
25ALA	CB	341LEU	CD1	0.64
25ALA	CB	341LEU	CD2	25.28
25ALA	CB	344LEU	CB	0.03
25ALA	CB	344LEU	CD1	0.79
25ALA	CB	344LEU	CD2	0.10
25ALA	CB	345ALA	CB	0.08
25ALA	C	26LEU	CA	100.00
25ALA	C	26LEU	CB	0.08
25ALA	C	26LEU	C	99.95
25ALA	C	344LEU	CD2	0.01
25ALA	C	345ALA	CB	0.02
26LEU	CA	26LEU	CB	100.00
26LEU	CA	26LEU	CG	100.00
26LEU	CA	26LEU	CD1	23.19
26LEU	CA	26LEU	CD2	76.49
26LEU	CA	26LEU	C	100.00
26LEU	CA	344LEU	CD2	0.03
26LEU	CB	26LEU	CG	100.00
26LEU	CB	26LEU	CD1	100.00
26LEU	CB	26LEU	CD2	100.00
26LEU	CB	26LEU	C	100.00
26LEU	CB	32LEU	CD1	0.98
26LEU	CB	32LEU	CD2	1.26
26LEU	CB	34LEU	CD1	0.02
26LEU	CB	344LEU	CD1	0.00
31GLY	C	32LEU	CD1	0.82
31GLY	C	32LEU	CD2	0.06
31GLY	C	32LEU	C	51.82
32LEU	CA	32LEU	CB	100.00
32LEU	CA	32LEU	CG	100.00
32LEU	CA	32LEU	CD1	26.00
32LEU	CA	32LEU	CD2	64.98
32LEU	CA	32LEU	C	100.00
32LEU	CA	302PHE	CE1	0.00
32LEU	CA	302PHE	CZ	0.00
32LEU	CA	304LEU	CD1	0.11
32LEU	CB	32LEU	CG	100.00
32LEU	CB	32LEU	CD1	100.00
32LEU	CB	32LEU	CD2	100.00
32LEU	CB	32LEU	C	100.00
32LEU	CB	298LEU	CD2	0.03
32LEU	CB	302PHE	CD1	0.00
32LEU	CB	302PHE	CE1	0.15
32LEU	CB	302PHE	CE2	0.31
32LEU	CB	302PHE	CZ	0.06
32LEU	CB	304LEU	CD1	2.92
32LEU	CB	304LEU	CD2	0.01
32LEU	CB	307LEU	CD1	0.00
32LEU	CG	32LEU	CD1	100.00
32LEU	CG	32LEU	CD2	100.00
32LEU	CG	32LEU	C	32.61
32LEU	CG	298LEU	CD1	0.01
32LEU	CG	298LEU	CD2	0.04
32LEU	CG	302PHE	CD2	0.02
32LEU	CG	302PHE	CE1	0.00
32LEU	CG	302PHE	CE2	0.52
32LEU	CG	302PHE	CZ	0.18
32LEU	CG	304LEU	CD1	0.06
32LEU	CG	307LEU	CD2	0.01
32LEU	CD1	32LEU	CD2	100.00
32LEU	CD1	32LEU	C	3.49
32LEU	CD1	34LEU	CB	0.04
32LEU	CD1	34LEU	CD1	0.02
32LEU	CD1	298LEU	CG	0.00
32LEU	CD1	298LEU	CD1	0.06
32LEU	CD1	298LEU	CD2	4.97
32LEU	CD1	302PHE	CB	0.00
32LEU	CD1	302PHE	CG	0.32
32LEU	CD1	302PHE	CD1	0.05
32LEU	CD1	302PHE	CD2	3.26
32LEU	CD1	302PHE	CE1	0.25
32LEU	CD1	302PHE	CE2	5.29
32LEU	CD1	302PHE	CZ	3.11
32LEU	CD1	304LEU	CD1	4.79
32LEU	CD1	304LEU	CD2	0.01
32LEU	CD1	307LEU	CD1	0.24
32LEU	CD1	307LEU	CD2	0.45
32LEU	CD2	32LEU	C	4.86
32LEU	CD2	33GLY	C	0.00
32LEU	CD2	34LEU	CB	0.01
32LEU	CD2	34LEU	CD1	0.02
32LEU	CD2	298LEU	CD1	0.27
32LEU	CD2	298LEU	CD2	3.65
32LEU	CD2	302PHE	CG	0.01
32LEU	CD2	302PHE	CD1	0.01
32LEU	CD2	302PHE	CD2	0.26
32LEU	CD2	302PHE	CE1	0.09

34LEU	CG	302PHE	CE2	0.00	26LEU	CB	344LEU	CD2	0.01	32LEU	CD2	302PHE	CE2	1.04
34LEU	CG	302PHE	CZ	0.00	26LEU	CG	26LEU	CD1	100.00	32LEU	CD2	302PHE	CZ	0.47
34LEU	CD1	34LEU	CD2	100.00	26LEU	CG	26LEU	CD2	100.00	32LEU	CD2	304LEU	CD1	4.84
34LEU	CD1	34LEU	C	0.49	26LEU	CG	26LEU	C	21.62	32LEU	CD2	304LEU	CD2	0.54
34LEU	CD1	36TYR	CD1	0.08	26LEU	CG	32LEU	CD2	0.15	32LEU	CD2	307LEU	CD1	5.38
34LEU	CD1	36TYR	CD2	0.05	26LEU	CG	344LEU	CD1	0.01	32LEU	CD2	307LEU	CD2	3.38
34LEU	CD1	297MET	CE	0.01	26LEU	CG	344LEU	CD2	0.03	32LEU	C	33GLY	CA	100.00
34LEU	CD1	302PHE	CE1	0.02	26LEU	CD1	26LEU	CD2	100.00	32LEU	C	33GLY	C	82.88
34LEU	CD1	302PHE	CE2	0.02	26LEU	CD1	26LEU	C	0.37	32LEU	C	302PHE	CE1	0.08
34LEU	CD1	302PHE	CZ	0.20	26LEU	CD1	30GLU	CB	0.30	32LEU	C	302PHE	CE2	0.14
34LEU	CD2	34LEU	C	0.80	26LEU	CD1	30GLU	CG	0.02	32LEU	C	302PHE	CZ	0.02
34LEU	CD2	36TYR	CB	0.00	26LEU	CD1	32LEU	CD1	0.03	33GLY	CA	33GLY	C	100.00
34LEU	CD2	36TYR	CD1	1.36	26LEU	CD1	32LEU	CD2	0.01	33GLY	C	34LEU	CA	100.00
34LEU	CD2	36TYR	CD2	0.46	26LEU	CD1	298LEU	CD1	2.84	33GLY	C	34LEU	CB	0.69
34LEU	CD2	36TYR	CE1	0.28	26LEU	CD1	298LEU	CD2	0.28	33GLY	C	34LEU	C	99.71
34LEU	CD2	36TYR	CE2	0.10	26LEU	CD1	307LEU	CD1	0.02	33GLY	C	302PHE	CE1	0.01
34LEU	C	35ALA	CA	100.00	26LEU	CD1	307LEU	CD2	0.04	33GLY	C	302PHE	CE2	0.00
34LEU	C	35ALA	CB	64.24	26LEU	CD1	310LYS	CB	0.00	34LEU	CA	34LEU	CB	100.00
34LEU	C	35ALA	C	45.09	26LEU	CD1	310LYS	CD	0.02	34LEU	CA	34LEU	CG	100.00
35ALA	CA	35ALA	CB	100.00	26LEU	CD1	310LYS	CE	0.01	34LEU	CA	34LEU	CD1	97.73
35ALA	CA	35ALA	C	100.00	26LEU	CD1	311VAL	CG1	0.06	34LEU	CA	34LEU	CD2	1.20
35ALA	CB	35ALA	C	100.00	26LEU	CD1	311VAL	CG2	5.40	34LEU	CA	34LEU	C	100.00
35ALA	C	36TYR	CA	100.00	26LEU	CD1	344LEU	CD1	0.10	34LEU	CB	34LEU	CG	100.00
35ALA	C	36TYR	CB	99.33	26LEU	CD1	344LEU	CD2	0.26	34LEU	CB	34LEU	CD1	100.00
35ALA	C	36TYR	CG	0.03	26LEU	CD2	26LEU	C	0.30	34LEU	CB	34LEU	CD2	100.00
35ALA	C	36TYR	CD1	0.29	26LEU	CD2	30GLU	CB	0.05	34LEU	CB	34LEU	C	100.00
35ALA	C	36TYR	CD2	0.12	26LEU	CD2	30GLU	CG	0.02	34LEU	CG	34LEU	CD1	100.00
35ALA	C	36TYR	C	1.19	26LEU	CD2	32LEU	CD1	0.04	34LEU	CG	34LEU	CD2	100.00
36TYR	CA	36TYR	CB	100.00	26LEU	CD2	32LEU	CD2	0.24	34LEU	CG	34LEU	C	99.19
36TYR	CA	36TYR	CG	100.00	26LEU	CD2	298LEU	CD1	1.04	34LEU	CG	36TYR	CD2	0.03
36TYR	CA	36TYR	CD1	74.98	26LEU	CD2	298LEU	CD2	0.24	34LEU	CG	36TYR	CE2	0.00
36TYR	CA	36TYR	CD2	71.44	26LEU	CD2	307LEU	CG	0.02	34LEU	CD1	34LEU	CD2	100.00
36TYR	CA	36TYR	C	100.00	26LEU	CD2	307LEU	CD1	0.18	34LEU	CD1	34LEU	C	1.06
36TYR	CB	36TYR	CG	100.00	26LEU	CD2	307LEU	CD2	0.38	34LEU	CD1	36TYR	CD2	0.10
36TYR	CB	36TYR	CD1	100.00	26LEU	CD2	310LYS	CD	0.00	34LEU	CD1	298LEU	CD1	0.00
36TYR	CB	36TYR	CD2	100.00	26LEU	CD2	310LYS	CE	0.01	34LEU	CD1	302PHE	CE2	0.02
36TYR	CB	36TYR	C	100.00	26LEU	CD2	311VAL	CG1	0.02	34LEU	CD1	302PHE	CZ	0.06
36TYR	CG	36TYR	CD1	100.00	26LEU	CD2	311VAL	CG2	1.53	34LEU	CD2	34LEU	C	0.32
36TYR	CG	36TYR	CD2	100.00	26LEU	CD2	344LEU	CG	0.00	34LEU	CD2	36TYR	CD2	1.05
36TYR	CG	36TYR	CE1	100.00	26LEU	CD2	344LEU	CD1	0.16	34LEU	CD2	36TYR	CE1	0.00
36TYR	CG	36TYR	CE2	100.00	26LEU	CD2	344LEU	CD2	0.47	34LEU	CD2	36TYR	CE2	0.16
36TYR	CG	36TYR	CZ	100.00	26LEU	C	27ASP	CA	100.00	34LEU	C	35ALA	CA	100.00
36TYR	CG	36TYR	C	99.88	26LEU	C	27ASP	CB	0.03	34LEU	C	35ALA	CB	73.09
36TYR	CD1	36TYR	CD2	100.00	26LEU	C	27ASP	C	99.98	34LEU	C	35ALA	C	34.85
36TYR	CD1	36TYR	CE1	100.00	26LEU	C	32LEU	CD1	0.07	35ALA	CA	35ALA	CB	100.00
36TYR	CD1	36TYR	CE2	100.00	26LEU	C	32LEU	CD2	0.45	35ALA	CA	35ALA	C	100.00
36TYR	CD1	36TYR	CZ	100.00	26LEU	C	34LEU	CD1	0.00	35ALA	CB	35ALA	C	100.00
36TYR	CD1	36TYR	C	15.36	27ASP	CA	27ASP	CB	100.00	35ALA	C	36TYR	CA	100.00
36TYR	CD1	38VAL	CG2	0.14	27ASP	CA	27ASP	CG	100.00	35ALA	C	36TYR	CB	99.67
36TYR	CD2	36TYR	CE1	100.00	27ASP	CA	27ASP	C	100.00	35ALA	C	36TYR	CG	0.02
36TYR	CD2	36TYR	CE2	100.00	27ASP	CA	31GLY	CA	0.07	35ALA	C	36TYR	CD2	0.40
36TYR	CD2	36TYR	CZ	100.00	27ASP	CA	32LEU	CB	0.35	35ALA	C	36TYR	C	0.59
36TYR	CD2	36TYR	C	41.66	27ASP	CA	32LEU	CD1	0.04	36TYR	CA	36TYR	CB	100.00
36TYR	CD2	38VAL	CG2	0.32	27ASP	CA	32LEU	CD2	0.32	36TYR	CA	36TYR	CG	100.00
36TYR	CE1	36TYR	CE2	100.00	27ASP	CA	34LEU	CD1	0.00	36TYR	CA	36TYR	CD1	69.21
36TYR	CE1	36TYR	CZ	100.00	27ASP	CB	27ASP	CG	100.00	36TYR	CA	36TYR	CD2	81.60
36TYR	CE1	38VAL	CG1	0.00	27ASP	CB	27ASP	C	100.00	36TYR	CA	36TYR	C	100.00
36TYR	CE1	38VAL	CG2	0.45	27ASP	CB	32LEU	CB	0.06	36TYR	CB	36TYR	CG	100.00
36TYR	CE2	36TYR	CZ	100.00	27ASP	CB	32LEU	CG	0.00	36TYR	CB	36TYR	CD1	100.00
36TYR	CE2	38VAL	CG2	1.42	27ASP	CB	32LEU	CD1	0.01	36TYR	CB	36TYR	CD2	100.00
36TYR	C	37GLU	CA	100.00	27ASP	CB	32LEU	CD2	0.08	36TYR	CB	36TYR	C	100.00
36TYR	C	37GLU	CB	79.87	27ASP	CB	34LEU	CB	0.06	36TYR	CG	36TYR	CD1	100.00

36TYR	C	37GLU	C	34.28
37GLU	CA	37GLU	CB	100.00
37GLU	CA	37GLU	CG	100.00
37GLU	CA	37GLU	CD	97.77
37GLU	CA	37GLU	C	100.00
37GLU	CB	37GLU	CG	100.00
37GLU	CB	37GLU	CD	100.00
37GLU	CB	37GLU	C	100.00
37GLU	CB	39PHE	CE1	3.05
37GLU	CB	39PHE	CE2	0.01
37GLU	CB	39PHE	CZ	1.67
37GLU	CG	37GLU	CD	100.00
37GLU	CG	37GLU	C	99.49
37GLU	CG	39PHE	CD1	0.06
37GLU	CG	39PHE	CD2	0.03
37GLU	CG	39PHE	CE1	5.42
37GLU	CG	39PHE	CE2	4.98
37GLU	CG	39PHE	CZ	19.83
37GLU	CD	37GLU	C	0.01
37GLU	CD	39PHE	CE2	0.03
37GLU	CD	39PHE	CZ	0.07
37GLU	C	38VAL	CA	100.00
37GLU	C	38VAL	CB	2.55
37GLU	C	38VAL	CG2	1.36
37GLU	C	38VAL	C	99.36
37GLU	C	39PHE	CD1	0.01
37GLU	C	39PHE	CE1	0.02
38VAL	CA	38VAL	CB	100.00
38VAL	CA	38VAL	CG1	100.00
38VAL	CA	38VAL	CG2	100.00
38VAL	CA	38VAL	C	100.00
38VAL	CB	38VAL	CG1	100.00
38VAL	CB	38VAL	CG2	100.00
38VAL	CB	38VAL	C	100.00
38VAL	CG1	38VAL	CG2	100.00
38VAL	CG1	38VAL	C	99.75
38VAL	CG2	38VAL	C	0.25
38VAL	C	39PHE	CA	100.00
38VAL	C	39PHE	CB	15.94
38VAL	C	39PHE	CG	2.62
38VAL	C	39PHE	CD1	0.72
38VAL	C	39PHE	C	84.47
39PHE	CA	39PHE	CB	100.00
39PHE	CA	39PHE	CG	100.00
39PHE	CA	39PHE	CD1	50.72
39PHE	CA	39PHE	CD2	98.25
39PHE	CA	39PHE	C	100.00
39PHE	CA	40PRO	CD	100.00
39PHE	CB	39PHE	CG	100.00
39PHE	CB	39PHE	CD1	100.00
39PHE	CB	39PHE	CD2	100.00
39PHE	CB	39PHE	C	100.00
39PHE	CB	40PRO	CD	22.28
39PHE	CB	57THR	CA	0.03
39PHE	CB	57THR	CG2	0.91
39PHE	CG	39PHE	CD1	100.00
39PHE	CG	39PHE	CD2	100.00
39PHE	CG	39PHE	CE1	100.00
39PHE	CG	39PHE	CE2	100.00
39PHE	CG	39PHE	CZ	100.00
39PHE	CG	57THR	CG2	0.01
39PHE	CD1	39PHE	CD2	100.00
27ASP	CB	34LEU	CD1	0.00
27ASP	CB	34LEU	CD2	0.05
27ASP	CB	36TYR	CE1	0.00
27ASP	CG	27ASP	C	99.99
27ASP	CG	32LEU	CD1	0.01
27ASP	CG	34LEU	CB	5.53
27ASP	CG	34LEU	CD2	0.01
27ASP	CG	36TYR	CE1	0.02
27ASP	C	28GLU	CA	100.00
27ASP	C	28GLU	CB	1.90
27ASP	C	28GLU	C	98.53
27ASP	C	31GLY	CA	0.00
28GLU	CA	28GLU	CB	100.00
28GLU	CA	28GLU	CG	100.00
28GLU	CA	28GLU	CD	90.80
28GLU	CA	28GLU	C	100.00
28GLU	CB	28GLU	CG	100.00
28GLU	CB	28GLU	CD	100.00
28GLU	CB	28GLU	C	100.00
28GLU	CG	28GLU	CD	100.00
28GLU	CG	28GLU	C	95.78
28GLU	CD	28GLU	C	2.14
28GLU	C	29ALA	CA	100.00
28GLU	C	29ALA	CB	9.61
28GLU	C	29ALA	C	89.49
29ALA	CA	29ALA	CB	100.00
29ALA	CA	29ALA	C	100.00
29ALA	CB	29ALA	C	100.00
29ALA	CB	344LEU	CD2	0.02
29ALA	CB	344LEU	C	0.11
29ALA	CB	345ALA	CA	0.06
29ALA	CB	345ALA	CB	0.50
29ALA	CB	345ALA	C	0.14
29ALA	C	30GLU	CA	100.00
29ALA	C	30GLU	CB	66.73
29ALA	C	30GLU	CG	0.38
29ALA	C	30GLU	CD	0.01
29ALA	C	30GLU	C	38.27
30GLU	CA	30GLU	CB	100.00
30GLU	CA	30GLU	CG	100.00
30GLU	CA	30GLU	CD	97.62
30GLU	CA	30GLU	C	100.00
30GLU	CB	30GLU	CG	100.00
30GLU	CB	30GLU	CD	100.00
30GLU	CB	30GLU	C	100.00
30GLU	CB	32LEU	CD1	0.01
30GLU	CB	307LEU	CD1	0.02
30GLU	CB	307LEU	CD2	0.03
30GLU	CG	30GLU	CD	100.00
30GLU	CG	30GLU	C	89.57
30GLU	CG	32LEU	CG	0.01
30GLU	CG	32LEU	CD1	0.00
30GLU	CG	32LEU	CD2	0.06
30GLU	CG	307LEU	CG	0.02
30GLU	CG	307LEU	CD1	0.52
30GLU	CG	307LEU	CD2	1.20
30GLU	CD	30GLU	C	1.42
30GLU	CD	32LEU	CB	0.00
30GLU	CD	307LEU	CD1	0.05
30GLU	CD	307LEU	CD2	0.05
30GLU	CD	310LYS	CD	0.01
30GLU	CD	310LYS	CE	0.04
36TYR	CG	36TYR	CD2	100.00
36TYR	CG	36TYR	CE1	100.00
36TYR	CG	36TYR	CE2	100.00
36TYR	CG	36TYR	CZ	100.00
36TYR	CG	36TYR	C	99.97
36TYR	CD1	36TYR	CD2	100.00
36TYR	CD1	36TYR	CE1	100.00
36TYR	CD1	36TYR	CE2	100.00
36TYR	CD1	36TYR	CZ	100.00
36TYR	CD1	36TYR	C	60.90
36TYR	CD1	38VAL	CG2	0.35
36TYR	CD2	36TYR	CE1	100.00
36TYR	CD2	36TYR	CE2	100.00
36TYR	CD2	36TYR	CZ	100.00
36TYR	CD2	36TYR	C	0.09
36TYR	CE1	36TYR	CE2	100.00
36TYR	CE1	36TYR	CZ	100.00
36TYR	CE1	38VAL	CG2	1.37
36TYR	CE2	36TYR	CZ	100.00
36TYR	C	37GLU	CA	100.00
36TYR	C	37GLU	CB	89.14
36TYR	C	37GLU	CG	0.02
36TYR	C	37GLU	C	20.99
37GLL	CA	37GLU	CB	100.00
37GLL	CA	37GLU	CG	100.00
37GLL	CA	37GLU	CD	97.21
37GLL	CA	37GLU	C	100.00
37GLL	CB	37GLU	CG	100.00
37GLL	CB	37GLU	CD	100.00
37GLL	CB	37GLU	C	100.00
37GLL	CB	39PHE	CE1	2.98
37GLL	CB	39PHE	CE2	0.01
37GLL	CB	39PHE	CZ	3.17
37GLL	CG	37GLU	CD	100.00
37GLL	CG	37GLU	C	98.90
37GLL	CG	39PHE	CD1	0.00
37GLL	CG	39PHE	CD2	0.04
37GLL	CG	39PHE	CE1	3.97
37GLL	CG	39PHE	CE2	5.34
37GLL	CG	39PHE	CZ	27.89
37GLL	CD	37GLU	C	0.02
37GLL	CD	39PHE	CE2	0.10
37GLL	CD	39PHE	CZ	0.23
37GLL	C	38VAL	CA	100.00
37GLL	C	38VAL	CB	4.78
37GLL	C	38VAL	CG1	0.03
37GLL	C	38VAL	CG2	2.86
37GLL	C	38VAL	C	98.09
37GLL	C	39PHE	CD1	0.01
37GLL	C	39PHE	CE1	0.24
38VAL	CA	38VAL	CB	100.00
38VAL	CA	38VAL	CG1	100.00
38VAL	CA	38VAL	CG2	100.00
38VAL	CA	38VAL	C	100.00
38VAL	CB	38VAL	CG1	100.00
38VAL	CB	38VAL	CG2	100.00
38VAL	CB	38VAL	C	100.00
38VAL	CG1	38VAL	CG2	100.00
38VAL	CG1	38VAL	C	98.88
38VAL	CG2	38VAL	C	1.12
38VAL	C	39PHE	CA	100.00
38VAL	C	39PHE	CB	44.14

39PHE	CD1	39PHE	CE1	100.00	30GLU	C	31GLY	CA	100.00	38VAL	C	39PHE	CG	13.84
39PHE	CD1	39PHE	CE2	100.00	30GLU	C	31GLY	C	62.97	38VAL	C	39PHE	CD1	2.52
39PHE	CD1	39PHE	CZ	100.00	30GLU	C	32LEU	CD1	0.01	38VAL	C	39PHE	CD2	0.05
39PHE	CD1	60GLY	CA	0.01	31GLY	CA	31GLY	C	100.00	38VAL	C	39PHE	C	52.17
39PHE	CD2	39PHE	CE1	100.00	31GLY	C	32LEU	CA	100.00	39PHE	CA	39PHE	CB	100.00
39PHE	CD2	39PHE	CE2	100.00	31GLY	C	32LEU	CB	52.03	39PHE	CA	39PHE	CG	100.00
39PHE	CD2	39PHE	CZ	100.00	31GLY	C	32LEU	CG	2.08	39PHE	CA	39PHE	CD1	43.74
39PHE	CD2	56PRO	C	0.28	31GLY	C	32LEU	CD1	0.24	39PHE	CA	39PHE	CD2	97.23
39PHE	CD2	57THR	CA	0.62	31GLY	C	32LEU	CD2	0.34	39PHE	CA	39PHE	C	100.00
39PHE	CD2	60GLY	CA	0.02	31GLY	C	32LEU	C	47.65	39PHE	CA	40PRO	CD	100.00
39PHE	CE1	39PHE	CE2	100.00	32LEU	CA	32LEU	CB	100.00	39PHE	CB	39PHE	CG	100.00
39PHE	CE1	39PHE	CZ	100.00	32LEU	CA	32LEU	CG	100.00	39PHE	CB	39PHE	CD1	100.00
39PHE	CE1	60GLY	CA	3.46	32LEU	CA	32LEU	CD1	23.80	39PHE	CB	39PHE	CD2	100.00
39PHE	CE1	60GLY	C	1.64	32LEU	CA	32LEU	CD2	66.61	39PHE	CB	39PHE	C	100.00
39PHE	CE2	39PHE	CZ	100.00	32LEU	CA	32LEU	C	100.00	39PHE	CB	40PRO	CD	15.03
39PHE	CE2	56PRO	C	0.09	32LEU	CA	302PHE	CE2	0.00	39PHE	CB	57THR	CA	0.02
39PHE	CE2	60GLY	CA	11.13	32LEU	CA	304LEU	CD1	0.16	39PHE	CB	57THR	CG2	1.05
39PHE	CE2	60GLY	C	0.01	32LEU	CB	32LEU	CG	100.00	39PHE	CG	39PHE	CD1	100.00
39PHE	CZ	60GLY	CA	35.20	32LEU	CB	32LEU	CD1	100.00	39PHE	CG	39PHE	CD2	100.00
39PHE	CZ	60GLY	C	1.14	32LEU	CB	32LEU	CD2	100.00	39PHE	CG	39PHE	CE1	100.00
39PHE	C	40PRO	CA	100.00	32LEU	CB	32LEU	C	100.00	39PHE	CG	39PHE	CE2	100.00
39PHE	C	40PRO	CB	0.03	32LEU	CB	34LEU	CB	0.01	39PHE	CG	39PHE	CZ	100.00
39PHE	C	40PRO	CD	100.00	32LEU	CB	34LEU	CD1	0.04	39PHE	CD1	39PHE	CD2	100.00
39PHE	C	40PRO	C	100.00	32LEU	CB	34LEU	CD2	0.00	39PHE	CD1	39PHE	CE1	100.00
40PRO	CA	40PRO	CB	100.00	32LEU	CB	298LEU	CD1	0.04	39PHE	CD1	39PHE	CE2	100.00
40PRO	CA	40PRO	CG	100.00	32LEU	CB	298LEU	CD2	0.01	39PHE	CD1	39PHE	CZ	100.00
40PRO	CA	40PRO	CD	100.00	32LEU	CB	302PHE	CD1	0.01	39PHE	CD1	60GLY	CA	0.01
40PRO	CA	40PRO	C	100.00	32LEU	CB	302PHE	CE1	0.60	39PHE	CD2	39PHE	CE1	100.00
40PRO	CB	40PRO	CG	100.00	32LEU	CB	302PHE	CE2	0.06	39PHE	CD2	39PHE	CE2	100.00
40PRO	CB	40PRO	CD	100.00	32LEU	CB	302PHE	CZ	0.12	39PHE	CD2	39PHE	CZ	100.00
40PRO	CB	40PRO	C	100.00	32LEU	CB	304LEU	CD1	2.75	39PHE	CD2	56PRO	C	0.50
40PRO	CB	44ALA	CB	0.04	32LEU	CB	304LEU	CD2	0.03	39PHE	CD2	57THR	CA	1.15
40PRO	CB	44ALA	C	0.01	32LEU	CB	307LEU	CD1	0.13	39PHE	CE1	39PHE	CE2	100.00
40PRO	CB	45ALA	CB	0.09	32LEU	CB	307LEU	CD2	0.04	39PHE	CE1	39PHE	CZ	100.00
40PRO	CB	49PHE	CD1	0.08	32LEU	CG	32LEU	CD1	100.00	39PHE	CE1	60GLY	CA	5.07
40PRO	CB	49PHE	CE1	2.96	32LEU	CG	32LEU	CD2	100.00	39PHE	CE1	60GLY	C	0.76
40PRO	CB	49PHE	CZ	0.05	32LEU	CG	32LEU	C	38.35	39PHE	CE2	39PHE	CZ	100.00
40PRO	CB	54PRO	CG	0.01	32LEU	CG	34LEU	CB	0.01	39PHE	CE2	56PRO	C	0.28
40PRO	CG	40PRO	CD	100.00	32LEU	CG	34LEU	CD1	0.01	39PHE	CE2	60GLY	CA	4.97
40PRO	CG	40PRO	C	89.60	32LEU	CG	298LEU	CD2	0.04	39PHE	CZ	60GLY	CA	33.33
40PRO	CG	49PHE	CD1	0.11	32LEU	CG	302PHE	CE1	0.10	39PHE	CZ	60GLY	C	0.14
40PRO	CG	49PHE	CE1	14.56	32LEU	CG	302PHE	CE2	0.09	39PHE	C	40PRO	CA	100.00
40PRO	CG	49PHE	CE2	0.01	32LEU	CG	302PHE	CZ	0.11	39PHE	C	40PRO	CB	0.06
40PRO	CG	49PHE	CZ	2.42	32LEU	CG	304LEU	CD1	0.02	39PHE	C	40PRO	CD	100.00
40PRO	CG	54PRO	CG	0.05	32LEU	CG	307LEU	CD1	0.03	39PHE	C	40PRO	C	100.00
40PRO	CG	56PRO	CG	0.47	32LEU	CG	307LEU	CD2	0.03	40PRC	CA	40PRO	CB	100.00
40PRO	CD	40PRO	C	78.77	32LEU	CD1	32LEU	CD2	100.00	40PRC	CA	40PRO	CG	100.00
40PRO	CD	56PRO	CG	0.00	32LEU	CD1	32LEU	C	4.85	40PRC	CA	40PRO	CD	100.00
40PRO	C	41PHE	CA	100.00	32LEU	CD1	34LEU	CA	0.00	40PRC	CA	40PRO	C	100.00
40PRO	C	41PHE	CB	99.79	32LEU	CD1	34LEU	CB	0.13	40PRC	CB	40PRO	CG	100.00
40PRO	C	41PHE	C	1.14	32LEU	CD1	34LEU	CG	0.01	40PRC	CB	40PRO	CD	100.00
40PRO	C	45ALA	CB	0.08	32LEU	CD1	34LEU	CD1	0.16	40PRC	CB	40PRO	C	100.00
41PHE	CA	41PHE	CB	100.00	32LEU	CD1	34LEU	CD2	0.04	40PRC	CB	44ALA	CB	0.01
41PHE	CA	41PHE	CG	100.00	32LEU	CD1	36TYR	CE2	0.01	40PRC	CB	45ALA	CB	0.00
41PHE	CA	41PHE	CD1	93.41	32LEU	CD1	298LEU	CG	0.01	40PRC	CB	49PHE	CG	0.00
41PHE	CA	41PHE	CD2	61.65	32LEU	CD1	298LEU	CD1	0.18	40PRC	CB	49PHE	CD2	0.39
41PHE	CA	41PHE	C	100.00	32LEU	CD1	298LEU	CD2	2.79	40PRC	CB	49PHE	CE2	2.35
41PHE	CA	45ALA	CB	0.03	32LEU	CD1	302PHE	CG	0.08	40PRC	CB	49PHE	CZ	0.06
41PHE	CB	41PHE	CG	100.00	32LEU	CD1	302PHE	CD1	0.93	40PRC	CG	40PRO	CD	100.00
41PHE	CB	41PHE	CD1	100.00	32LEU	CD1	302PHE	CD2	0.31	40PRC	CG	40PRO	C	97.74
41PHE	CB	41PHE	CD2	100.00	32LEU	CD1	302PHE	CE1	3.55	40PRC	CG	49PHE	CG	0.00
41PHE	CB	41PHE	C	100.00	32LEU	CD1	302PHE	CE2	0.72	40PRC	CG	49PHE	CD1	0.01

41PHE	CG	41PHE	CD1	100.00
41PHE	CG	41PHE	CD2	100.00
41PHE	CG	41PHE	CE1	100.00
41PHE	CG	41PHE	CE2	100.00
41PHE	CG	41PHE	CZ	100.00
41PHE	CG	41PHE	C	100.00
41PHE	CD1	41PHE	CD2	100.00
41PHE	CD1	41PHE	CE1	100.00
41PHE	CD1	41PHE	CE2	100.00
41PHE	CD1	41PHE	CZ	100.00
41PHE	CD1	41PHE	C	0.38
41PHE	CD1	52PRO	C	0.01
41PHE	CD1	53PHE	CD1	0.09
41PHE	CD1	53PHE	CE1	0.01
41PHE	CD1	93LEU	CD1	0.00
41PHE	CD2	41PHE	CE1	100.00
41PHE	CD2	41PHE	CE2	100.00
41PHE	CD2	41PHE	CZ	100.00
41PHE	CD2	41PHE	C	61.40
41PHE	CD2	69LEU	CD1	1.14
41PHE	CD2	69LEU	CD2	0.04
41PHE	CD2	72VAL	CG1	0.51
41PHE	CD2	72VAL	CG2	0.06
41PHE	CE1	41PHE	CE2	100.00
41PHE	CE1	41PHE	CZ	100.00
41PHE	CE1	53PHE	CB	0.01
41PHE	CE1	53PHE	CG	0.01
41PHE	CE1	53PHE	CD1	0.28
41PHE	CE1	53PHE	CE1	0.01
41PHE	CE1	86PRO	CB	0.05
41PHE	CE1	89GLY	C	0.12
41PHE	CE1	90LEU	CD1	0.07
41PHE	CE1	90LEU	CD2	0.57
41PHE	CE1	93LEU	CD1	0.35
41PHE	CE1	93LEU	CD2	0.06
41PHE	CE2	41PHE	CZ	100.00
41PHE	CE2	69LEU	CG	0.06
41PHE	CE2	69LEU	CD1	28.45
41PHE	CE2	69LEU	CD2	1.04
41PHE	CE2	72VAL	CB	0.00
41PHE	CE2	72VAL	CG1	11.88
41PHE	CE2	72VAL	CG2	2.76
41PHE	CE2	90LEU	CD2	0.15
41PHE	CZ	69LEU	CG	0.01
41PHE	CZ	69LEU	CD1	3.50
41PHE	CZ	69LEU	CD2	0.14
41PHE	CZ	72VAL	CB	0.00
41PHE	CZ	72VAL	CG1	3.21
41PHE	CZ	72VAL	CG2	1.04
41PHE	CZ	86PRO	CB	0.08
41PHE	CZ	89GLY	C	0.18
41PHE	CZ	90LEU	CG	0.07
41PHE	CZ	90LEU	CD1	0.06
41PHE	CZ	90LEU	CD2	4.24
41PHE	CZ	93LEU	CB	0.00
41PHE	CZ	93LEU	CD1	0.07
41PHE	CZ	93LEU	CD2	0.12
41PHE	C	42GLY	CA	100.00
41PHE	C	42GLY	C	24.81
41PHE	C	45ALA	CB	0.04
42GLY	CA	42GLY	C	100.00
42GLY	CA	72VAL	CB	0.04
32LEU	CD1	302PHE	CZ	1.40
32LEU	CD1	304LEU	CD1	2.41
32LEU	CD1	304LEU	CD2	0.05
32LEU	CD1	307LEU	CD1	0.50
32LEU	CD1	307LEU	CD2	0.20
32LEU	CD2	32LEU	C	5.06
32LEU	CD2	33GLY	C	0.01
32LEU	CD2	34LEU	CB	0.10
32LEU	CD2	34LEU	CD1	0.04
32LEU	CD2	34LEU	CD2	0.00
32LEU	CD2	36TYR	CD1	0.01
32LEU	CD2	36TYR	CE1	0.04
32LEU	CD2	298LEU	CD1	0.27
32LEU	CD2	298LEU	CD2	4.26
32LEU	CD2	302PHE	CG	0.01
32LEU	CD2	302PHE	CD1	0.16
32LEU	CD2	302PHE	CD2	0.08
32LEU	CD2	302PHE	CE1	0.79
32LEU	CD2	302PHE	CE2	0.58
32LEU	CD2	302PHE	CZ	0.41
32LEU	CD2	304LEU	CD1	5.09
32LEU	CD2	304LEU	CD2	0.15
32LEU	CD2	307LEU	CB	0.00
32LEU	CD2	307LEU	CG	0.06
32LEU	CD2	307LEU	CD1	5.01
32LEU	CD2	307LEU	CD2	3.04
32LEU	C	33GLY	CA	100.00
32LEU	C	33GLY	C	80.46
32LEU	C	34LEU	CD1	0.00
32LEU	C	302PHE	CE1	0.19
32LEU	C	302PHE	CE2	0.02
32LEU	C	302PHE	CZ	0.04
33GLY	CA	33GLY	C	100.00
33GLY	CA	302PHE	CE2	0.00
33GLY	C	34LEU	CA	100.00
33GLY	C	34LEU	CB	1.85
33GLY	C	34LEU	CG	0.04
33GLY	C	34LEU	CD1	0.06
33GLY	C	34LEU	CD2	0.00
33GLY	C	34LEU	C	98.76
33GLY	C	302PHE	CE1	0.01
33GLY	C	302PHE	CE2	0.01
33GLY	C	302PHE	CZ	0.04
34LEU	CA	34LEU	CB	100.00
34LEU	CA	34LEU	CG	100.00
34LEU	CA	34LEU	CD1	86.36
34LEU	CA	34LEU	CD2	13.66
34LEU	CA	34LEU	C	100.00
34LEU	CB	34LEU	CG	100.00
34LEU	CB	34LEU	CD1	100.00
34LEU	CB	34LEU	CD2	100.00
34LEU	CB	34LEU	C	100.00
34LEU	CB	36TYR	CD1	0.00
34LEU	CB	36TYR	CE1	0.01
34LEU	CB	36TYR	CE2	0.26
34LEU	CB	36TYR	CZ	0.10
34LEU	CG	34LEU	CD1	100.00
34LEU	CG	34LEU	CD2	100.00
34LEU	CG	34LEU	C	89.75
34LEU	CG	36TYR	CD1	0.01
34LEU	CG	36TYR	CD2	0.03
34LEU	CG	298LEU	CD1	0.01
40PRC	CG	49PHE	CD2	0.34
40PRC	CG	49PHE	CE1	0.02
40PRC	CG	49PHE	CE2	14.30
40PRC	CG	49PHE	CZ	2.06
40PRC	CG	54PRO	CG	0.05
40PRC	CG	56PRO	CG	0.01
40PRC	CD	40PRO	C	93.22
40PRC	C	41PHE	CA	100.00
40PRC	C	41PHE	CB	99.93
40PRC	C	41PHE	C	0.10
40PRC	C	45ALA	CB	0.03
40PRC	C	54PRO	CG	0.00
41PHE	CA	41PHE	CB	100.00
41PHE	CA	41PHE	CG	100.00
41PHE	CA	41PHE	CD1	97.41
41PHE	CA	41PHE	CD2	27.07
41PHE	CA	41PHE	C	100.00
41PHE	CA	45ALA	CB	0.06
41PHE	CB	41PHE	CG	100.00
41PHE	CB	41PHE	CD1	100.00
41PHE	CB	41PHE	CD2	100.00
41PHE	CB	41PHE	C	100.00
41PHE	CG	41PHE	CD1	100.00
41PHE	CG	41PHE	CD2	100.00
41PHE	CG	41PHE	CE1	100.00
41PHE	CG	41PHE	CE2	100.00
41PHE	CG	41PHE	CZ	100.00
41PHE	CG	41PHE	C	100.00
41PHE	CD1	41PHE	CD2	100.00
41PHE	CD1	41PHE	CE1	100.00
41PHE	CD1	41PHE	CE2	100.00
41PHE	CD1	41PHE	CZ	100.00
41PHE	CD1	41PHE	C	1.32
41PHE	CD1	53PHE	CB	0.00
41PHE	CD1	53PHE	CD1	0.26
41PHE	CD1	72VAL	CG1	0.00
41PHE	CD2	41PHE	CE1	100.00
41PHE	CD2	41PHE	CE2	100.00
41PHE	CD2	41PHE	CZ	100.00
41PHE	CD2	41PHE	C	27.40
41PHE	CD2	42GLY	CA	0.02
41PHE	CD2	69LEU	CD1	2.07
41PHE	CD2	69LEU	CD2	0.01
41PHE	CD2	72VAL	CG1	17.07
41PHE	CD2	72VAL	CG2	0.02
41PHE	CE1	41PHE	CE2	100.00
41PHE	CE1	41PHE	CZ	100.00
41PHE	CE1	53PHE	CA	0.00
41PHE	CE1	53PHE	CB	0.10
41PHE	CE1	53PHE	CG	0.04
41PHE	CE1	53PHE	CD1	2.55
41PHE	CE1	69LEU	CD1	0.01
41PHE	CE1	72VAL	CG1	0.06
41PHE	CE1	72VAL	CG2	0.01
41PHE	CE1	89GLY	CA	0.02
41PHE	CE1	89GLY	C	1.01
41PHE	CE1	90LEU	CD2	0.06
41PHE	CE1	93LEU	CD1	0.58
41PHE	CE1	93LEU	CD2	0.04
41PHE	CE2	41PHE	CZ	100.00
41PHE	CE2	69LEU	CG	0.19
41PHE	CE2	69LEU	CD1	16.23

42GLY	CA	72VAL	CG1	0.90
42GLY	CA	72VAL	CG2	0.08
42GLY	CA	77TRP	CZ2	0.09
42GLY	C	43GLY	CA	100.00
42GLY	C	43GLY	C	87.12
42GLY	C	46ILE	CD	0.01
42GLY	C	72VAL	CG2	0.02
42GLY	C	77TRP	CZ2	9.85
42GLY	C	86PRO	CB	0.00
43GLY	CA	43GLY	C	100.00
43GLY	CA	46ILE	CG1	0.02
43GLY	CA	46ILE	CG2	0.02
43GLY	CA	46ILE	CD	0.06
43GLY	CA	76LYS	CD	0.00
43GLY	CA	77TRP	CG	0.00
43GLY	CA	77TRP	CD1	0.06
43GLY	CA	77TRP	CD2	0.18
43GLY	CA	77TRP	CE2	0.53
43GLY	CA	77TRP	CE3	0.05
43GLY	CA	77TRP	CZ2	6.14
43GLY	CA	77TRP	CZ3	0.02
43GLY	CA	86PRO	CG	0.01
43GLY	C	44ALA	CA	100.00
43GLY	C	44ALA	CB	1.82
43GLY	C	44ALA	C	98.34
43GLY	C	46ILE	CG1	0.81
43GLY	C	46ILE	CD	0.00
43GLY	C	77TRP	CD1	0.02
43GLY	C	77TRP	CD2	0.06
43GLY	C	77TRP	CE2	0.55
43GLY	C	77TRP	CE3	0.01
43GLY	C	77TRP	CZ2	0.74
43GLY	C	77TRP	CZ3	0.04
44ALA	CA	44ALA	CB	100.00
44ALA	CA	44ALA	C	100.00
44ALA	CA	47ASP	CB	0.01
44ALA	CA	77TRP	CZ2	0.03
44ALA	CA	77TRP	CZ3	0.00
44ALA	CB	44ALA	C	100.00
44ALA	CB	77TRP	CZ2	0.00
44ALA	CB	77TRP	CZ3	0.00
44ALA	C	45ALA	CA	100.00
44ALA	C	45ALA	CB	0.04
44ALA	C	45ALA	C	99.95
44ALA	C	48ALA	CB	0.05
45ALA	CA	45ALA	CB	100.00
45ALA	CA	45ALA	C	100.00
45ALA	CA	49PHE	CD1	0.02
45ALA	CA	49PHE	CE1	0.00
45ALA	CB	45ALA	C	100.00
45ALA	CB	49PHE	CD1	0.00
45ALA	CB	52PRO	CA	0.02
45ALA	CB	52PRO	C	0.01
45ALA	CB	54PRO	CG	0.04
45ALA	CB	54PRO	CD	0.02
45ALA	C	46ILE	CA	100.00
45ALA	C	46ILE	CB	0.02
45ALA	C	46ILE	CG2	0.01
45ALA	C	46ILE	C	99.99
46ILE	CA	46ILE	CB	100.00
46ILE	CA	46ILE	CG1	100.00
46ILE	CA	46ILE	CG2	100.00

34LEU	CG	302PHE	CD1	0.01
34LEU	CG	302PHE	CD2	0.03
34LEU	CG	302PHE	CE1	0.01
34LEU	CG	302PHE	CE2	0.01
34LEU	CG	302PHE	CZ	0.00
34LEU	CD1	34LEU	CD2	100.00
34LEU	CD1	34LEU	C	4.18
34LEU	CD1	35ALA	C	0.01
34LEU	CD1	36TYR	CD1	0.02
34LEU	CD1	36TYR	CD2	0.29
34LEU	CD1	36TYR	CE2	0.04
34LEU	CD1	297MET	CE	0.03
34LEU	CD1	298LEU	CD1	0.20
34LEU	CD1	298LEU	CD2	0.15
34LEU	CD1	302PHE	CG	0.05
34LEU	CD1	302PHE	CD1	0.28
34LEU	CD1	302PHE	CD2	0.26
34LEU	CD1	302PHE	CE1	0.77
34LEU	CD1	302PHE	CE2	0.20
34LEU	CD1	302PHE	CZ	0.54
34LEU	CD1	304LEU	CD1	0.08
34LEU	CD2	34LEU	C	2.13
34LEU	CD2	35ALA	C	0.02
34LEU	CD2	36TYR	CD1	0.65
34LEU	CD2	36TYR	CD2	0.99
34LEU	CD2	36TYR	CE1	0.08
34LEU	CD2	36TYR	CE2	0.19
34LEU	CD2	297MET	CE	0.03
34LEU	CD2	298LEU	CD1	0.09
34LEU	CD2	298LEU	CD2	0.11
34LEU	CD2	302PHE	CG	0.07
34LEU	CD2	302PHE	CD1	0.19
34LEU	CD2	302PHE	CD2	0.22
34LEU	CD2	302PHE	CE1	0.79
34LEU	CD2	302PHE	CE2	0.47
34LEU	CD2	302PHE	CZ	0.76
34LEU	C	35ALA	CA	100.00
34LEU	C	35ALA	CB	58.73
34LEU	C	35ALA	C	48.63
34LEU	C	36TYR	CD1	0.00
35ALA	CA	35ALA	CB	100.00
35ALA	CA	35ALA	C	100.00
35ALA	CB	35ALA	C	100.00
35ALA	C	36TYR	CA	100.00
35ALA	C	36TYR	CB	93.95
35ALA	C	36TYR	CG	0.96
35ALA	C	36TYR	CD1	0.84
35ALA	C	36TYR	CD2	1.00
35ALA	C	36TYR	C	6.94
36TYR	CA	36TYR	CB	100.00
36TYR	CA	36TYR	CG	100.00
36TYR	CA	36TYR	CD1	72.09
36TYR	CA	36TYR	CD2	75.64
36TYR	CA	36TYR	C	100.00
36TYR	CB	36TYR	CG	100.00
36TYR	CB	36TYR	CD1	100.00
36TYR	CB	36TYR	CD2	100.00
36TYR	CB	36TYR	C	100.00
36TYR	CG	36TYR	CD1	100.00
36TYR	CG	36TYR	CD2	100.00
36TYR	CG	36TYR	CE1	100.00
36TYR	CG	36TYR	CE2	100.00

41PHE	CE2	69LEU	CD2	0.10
41PHE	CE2	72VAL	CG1	22.36
41PHE	CE2	72VAL	CG2	1.98
41PHE	CE2	90LEU	CD1	0.04
41PHE	CE2	90LEU	CD2	0.88
41PHE	CE2	93LEU	CD1	0.00
41PHE	CZ	53PHE	CB	0.00
41PHE	CZ	69LEU	CG	0.02
41PHE	CZ	69LEU	CD1	1.31
41PHE	CZ	69LEU	CD2	0.01
41PHE	CZ	72VAL	CG1	1.07
41PHE	CZ	72VAL	CG2	0.58
41PHE	CZ	89GLY	C	1.80
41PHE	CZ	90LEU	CA	0.01
41PHE	CZ	90LEU	CG	0.01
41PHE	CZ	90LEU	CD1	0.16
41PHE	CZ	90LEU	CD2	3.77
41PHE	CZ	93LEU	CB	0.00
41PHE	CZ	93LEU	CD1	0.51
41PHE	CZ	93LEU	CD2	0.68
41PHE	C	42GLY	CA	100.00
41PHE	C	42GLY	C	57.72
41PHE	C	45ALA	CB	0.39
41PHE	C	72VAL	CG1	0.01
42GLY	CA	42GLY	C	100.00
42GLY	CA	72VAL	CB	0.73
42GLY	CA	72VAL	CG1	18.02
42GLY	CA	72VAL	CG2	0.01
42GLY	CA	86PRO	CB	0.08
42GLY	C	43GLY	CA	100.00
42GLY	C	43GLY	C	96.17
42GLY	C	46ILE	CG1	0.00
42GLY	C	46ILE	CD	0.10
42GLY	C	86PRO	CB	1.00
42GLY	C	86PRO	CG	0.21
43GLY	CA	43GLY	C	100.00
43GLY	CA	46ILE	CG1	0.00
43GLY	CA	46ILE	CG2	0.03
43GLY	CA	46ILE	CD	0.09
43GLY	CA	77TRP	CG	0.01
43GLY	CA	77TRP	CD1	0.17
43GLY	CA	77TRP	CD2	0.21
43GLY	CA	77TRP	CE2	1.37
43GLY	CA	77TRP	CE3	0.14
43GLY	CA	77TRP	CZ2	5.17
43GLY	CA	77TRP	CZ3	2.63
43GLY	CA	86PRO	CB	0.03
43GLY	CA	86PRO	CG	0.26
43GLY	C	44ALA	CA	100.00
43GLY	C	44ALA	CB	0.01
43GLY	C	44ALA	C	99.99
43GLY	C	46ILE	CG2	0.02
43GLY	C	77TRP	CD2	0.02
43GLY	C	77TRP	CE2	1.08
43GLY	C	77TRP	CE3	0.01
43GLY	C	77TRP	CZ2	6.02
43GLY	C	77TRP	CZ3	0.30
44ALA	CA	44ALA	CB	100.00
44ALA	CA	44ALA	C	100.00
44ALA	CA	47ASP	CB	0.00
44ALA	CA	77TRP	CZ2	0.01
44ALA	CA	77TRP	CZ3	0.00

46ILE	CA	46ILE	CD	6.06	36TYR	CG	36TYR	CZ	100.00	44ALA	CB	44ALA	C	100.00
46ILE	CA	46ILE	C	100.00	36TYR	CG	36TYR	C	94.22	44ALA	C	45ALA	CA	100.00
46ILE	CA	50GLY	CA	0.00	36TYR	CD1	36TYR	CD2	100.00	44ALA	C	45ALA	CB	0.18
46ILE	CA	50GLY	C	0.01	36TYR	CD1	36TYR	CE1	100.00	44ALA	C	45ALA	C	99.86
46ILE	CB	46ILE	CG1	100.00	36TYR	CD1	36TYR	CE2	100.00	45ALA	CA	45ALA	CB	100.00
46ILE	CB	46ILE	CG2	100.00	36TYR	CD1	36TYR	CZ	100.00	45ALA	CA	45ALA	C	100.00
46ILE	CB	46ILE	CD	100.00	36TYR	CD1	36TYR	C	31.90	45ALA	CA	49PHE	CD2	0.04
46ILE	CB	46ILE	C	100.00	36TYR	CD1	38VAL	CG1	0.01	45ALA	CA	49PHE	CE1	0.01
46ILE	CB	84ILE	CG2	0.15	36TYR	CD1	38VAL	CG2	0.26	45ALA	CA	49PHE	CZ	0.01
46ILE	CG1	46ILE	CG2	100.00	36TYR	CD2	36TYR	CE1	100.00	45ALA	CB	45ALA	C	100.00
46ILE	CG1	46ILE	CD	100.00	36TYR	CD2	36TYR	CE2	100.00	45ALA	CB	49PHE	CD2	0.02
46ILE	CG1	46ILE	C	61.21	36TYR	CD2	36TYR	CZ	100.00	45ALA	CB	49PHE	CE1	0.00
46ILE	CG1	51GLU	C	0.17	36TYR	CD2	36TYR	C	21.42	45ALA	CB	49PHE	CE2	0.02
46ILE	CG1	52PRO	CA	0.09	36TYR	CD2	38VAL	CG1	0.00	45ALA	CB	49PHE	CZ	0.06
46ILE	CG1	52PRO	CB	0.17	36TYR	CD2	38VAL	CG2	0.20	45ALA	CB	52PRO	CA	0.02
46ILE	CG1	52PRO	CD	0.02	36TYR	CE1	36TYR	CE2	100.00	45ALA	CB	52PRO	C	0.08
46ILE	CG1	77TRP	CZ3	0.10	36TYR	CE1	36TYR	CZ	100.00	45ALA	CB	54PRO	CG	0.24
46ILE	CG2	46ILE	CD	98.13	36TYR	CE1	38VAL	CG1	0.01	45ALA	CB	54PRO	CD	0.30
46ILE	CG2	46ILE	C	39.47	36TYR	CE1	38VAL	CG2	0.95	45ALA	C	46ILE	CA	100.00
46ILE	CG2	50GLY	C	0.01	36TYR	CE2	36TYR	CZ	100.00	45ALA	C	46ILE	C	100.00
46ILE	CG2	51GLU	CA	0.00	36TYR	CE2	38VAL	CG1	0.00	46ILE	CA	46ILE	CB	100.00
46ILE	CG2	51GLU	C	0.25	36TYR	CE2	38VAL	CG2	0.51	46ILE	CA	46ILE	CG1	100.00
46ILE	CG2	52PRO	CA	2.25	36TYR	C	37GLU	CA	100.00	46ILE	CA	46ILE	CG2	100.00
46ILE	CG2	52PRO	CB	2.41	36TYR	C	37GLU	CB	83.72	46ILE	CA	46ILE	CD	0.04
46ILE	CG2	52PRO	CG	0.06	36TYR	C	37GLU	CG	0.06	46ILE	CA	46ILE	C	100.00
46ILE	CG2	52PRO	CD	0.24	36TYR	C	37GLU	C	28.11	46ILE	CA	50GLY	CA	0.00
46ILE	CG2	77TRP	CD1	0.04	37GLU	CA	37GLU	CB	100.00	46ILE	CA	50GLY	C	0.00
46ILE	CG2	77TRP	CE2	0.01	37GLU	CA	37GLU	CG	100.00	46ILE	CB	46ILE	CG1	100.00
46ILE	CG2	77TRP	CZ3	0.49	37GLU	CA	37GLU	CD	92.18	46ILE	CB	46ILE	CG2	100.00
46ILE	CG2	80LEU	CD1	0.02	37GLU	CA	37GLU	C	100.00	46ILE	CB	46ILE	CD	100.00
46ILE	CG2	84ILE	CA	0.00	37GLU	CB	37GLU	CG	100.00	46ILE	CB	46ILE	C	100.00
46ILE	CG2	84ILE	CB	0.27	37GLU	CB	37GLU	CD	100.00	46ILE	CB	77TRP	CD1	0.00
46ILE	CG2	84ILE	CG2	0.70	37GLU	CB	37GLU	C	100.00	46ILE	CB	77TRP	CZ2	0.03
46ILE	CG2	84ILE	CD	0.22	37GLU	CB	39PHE	CD1	0.00	46ILE	CG1	46ILE	CG2	100.00
46ILE	CD	46ILE	C	0.94	37GLU	CB	39PHE	CE1	3.96	46ILE	CG1	46ILE	CD	100.00
46ILE	CD	51GLU	C	0.06	37GLU	CB	39PHE	CE2	0.04	46ILE	CG1	46ILE	C	1.14
46ILE	CD	52PRO	CA	0.06	37GLU	CB	39PHE	CZ	1.54	46ILE	CG1	51GLU	C	0.17
46ILE	CD	52PRO	CB	0.03	37GLU	CG	37GLU	CD	100.00	46ILE	CG1	52PRO	CA	0.15
46ILE	CD	52PRO	CG	0.03	37GLU	CG	37GLU	C	96.59	46ILE	CG1	52PRO	CB	1.64
46ILE	CD	52PRO	CD	0.17	37GLU	CG	39PHE	CG	0.00	46ILE	CG1	52PRO	CG	0.02
46ILE	CD	72VAL	CG2	0.02	37GLU	CG	39PHE	CD1	0.12	46ILE	CG1	52PRO	CD	0.21
46ILE	CD	77TRP	CG	0.01	37GLU	CG	39PHE	CD2	0.13	46ILE	CG2	46ILE	CD	99.99
46ILE	CD	77TRP	CD1	0.04	37GLU	CG	39PHE	CE1	9.11	46ILE	CG2	46ILE	C	100.00
46ILE	CD	77TRP	CD2	0.00	37GLU	CG	39PHE	CE2	4.24	46ILE	CG2	50GLY	C	0.01
46ILE	CD	77TRP	CE2	0.02	37GLU	CG	39PHE	CZ	18.20	46ILE	CG2	77TRP	CD1	0.09
46ILE	CD	77TRP	CE3	0.08	37GLU	CD	37GLU	C	0.02	46ILE	CG2	77TRP	CE2	0.03
46ILE	CD	77TRP	CZ2	0.01	37GLU	CD	39PHE	CE1	0.02	46ILE	CG2	77TRP	CZ2	0.92
46ILE	CD	77TRP	CZ3	5.46	37GLU	CD	39PHE	CE2	0.22	46ILE	CG2	80LEU	CD1	0.00
46ILE	CD	80LEU	CD1	3.02	37GLU	CD	39PHE	CZ	0.68	46ILE	CG2	84ILE	CG2	0.01
46ILE	CD	80LEU	CD2	0.66	37GLU	C	38VAL	CA	100.00	46ILE	CD	52PRO	CB	0.06
46ILE	CD	84ILE	CG2	0.25	37GLU	C	38VAL	CB	5.49	46ILE	CD	52PRO	CD	0.02
46ILE	CD	84ILE	CD	0.02	37GLU	C	38VAL	CG1	0.07	46ILE	CD	77TRP	CD1	0.17
46ILE	CD	84ILE	C	0.06	37GLU	C	38VAL	CG2	3.32	46ILE	CD	77TRP	CD2	0.00
46ILE	CD	85SER	CA	0.03	37GLU	C	38VAL	C	98.16	46ILE	CD	77TRP	CE2	0.16
46ILE	CD	85SER	C	0.06	37GLU	C	39PHE	CD1	0.01	46ILE	CD	77TRP	CZ2	1.67
46ILE	CD	86PRO	CG	0.00	37GLU	C	39PHE	CE1	0.07	46ILE	CD	77TRP	CZ3	0.00
46ILE	CD	86PRO	CD	0.06	38VAL	CA	38VAL	CB	100.00	46ILE	CD	80LEU	CD1	0.00
46ILE	C	47ASP	CA	100.00	38VAL	CA	38VAL	CG1	100.00	46ILE	CD	84ILE	C	0.04
46ILE	C	47ASP	CB	20.22	38VAL	CA	38VAL	CG2	100.00	46ILE	CD	85ARG	CA	0.01
46ILE	C	47ASP	C	81.63	38VAL	CA	38VAL	C	100.00	46ILE	CD	85ARG	C	0.04
46ILE	C	50GLY	CA	0.12	38VAL	CB	38VAL	CG1	100.00	46ILE	CD	86PRO	CA	0.03
46ILE	C	50GLY	C	0.00	38VAL	CB	38VAL	CG2	100.00	46ILE	CD	86PRO	CB	0.07

47ASP	CA	47ASP	CB	100.00	38VAL	CB	38VAL	C	100.00	46ILE	CD	86PRO	CG	0.15
47ASP	CA	47ASP	CG	100.00	38VAL	CG1	38VAL	CG2	100.00	46ILE	CD	86PRO	CD	0.47
47ASP	CA	47ASP	C	100.00	38VAL	CG1	38VAL	C	98.16	46ILE	C	47ASP	CA	100.00
47ASP	CB	47ASP	CG	100.00	38VAL	CG2	38VAL	C	1.80	46ILE	C	47ASP	CB	0.60
47ASP	CB	47ASP	C	100.00	38VAL	C	39PHE	CA	100.00	46ILE	C	47ASP	C	99.61
47ASP	CB	77TRP	CZ2	0.02	38VAL	C	39PHE	CB	18.46	47ASF	CA	47ASP	CB	100.00
47ASP	CG	47ASP	C	98.78	38VAL	C	39PHE	CG	3.62	47ASF	CA	47ASP	CG	100.00
47ASP	C	48ALA	CA	100.00	38VAL	C	39PHE	CD1	1.29	47ASF	CA	47ASP	C	100.00
47ASP	C	48ALA	CB	1.29	38VAL	C	39PHE	CD2	0.02	47ASF	CB	47ASP	CG	100.00
47ASP	C	48ALA	C	98.66	38VAL	C	39PHE	C	81.74	47ASF	CB	47ASP	C	100.00
48ALA	CA	48ALA	CB	100.00	39PHE	CA	39PHE	CB	100.00	47ASF	CB	77TRP	CZ2	0.01
48ALA	CA	48ALA	C	100.00	39PHE	CA	39PHE	CG	100.00	47ASF	CG	47ASP	C	98.73
48ALA	CB	48ALA	C	100.00	39PHE	CA	39PHE	CD1	53.65	47ASF	CG	76LYS	CE	0.00
48ALA	CB	49PHE	CG	0.01	39PHE	CA	39PHE	CD2	95.61	47ASF	C	48ALA	CA	100.00
48ALA	CB	49PHE	CD1	5.36	39PHE	CA	39PHE	C	100.00	47ASF	C	48ALA	CB	4.44
48ALA	CB	49PHE	CE1	0.68	39PHE	CA	40PRO	CD	100.00	47ASF	C	48ALA	C	95.15
48ALA	CB	49PHE	CE2	0.00	39PHE	CB	39PHE	CG	100.00	48ALA	CA	48ALA	CB	100.00
48ALA	CB	49PHE	CZ	0.01	39PHE	CB	39PHE	CD1	100.00	48ALA	CA	48ALA	C	100.00
48ALA	C	49PHE	CA	100.00	39PHE	CB	39PHE	CD2	100.00	48ALA	CB	48ALA	C	100.00
48ALA	C	49PHE	CB	31.86	39PHE	CB	39PHE	C	100.00	48ALA	CB	49PHE	CG	0.01
48ALA	C	49PHE	CG	8.36	39PHE	CB	40PRO	CD	15.74	48ALA	CB	49PHE	CD1	0.22
48ALA	C	49PHE	CD1	6.57	39PHE	CB	57THR	CA	0.01	48ALA	CB	49PHE	CD2	9.72
48ALA	C	49PHE	CD2	0.14	39PHE	CB	57THR	CG2	1.42	48ALA	CB	49PHE	CE1	0.07
48ALA	C	49PHE	C	65.10	39PHE	CG	39PHE	CD1	100.00	48ALA	CB	49PHE	CE2	1.50
49PHE	CA	49PHE	CB	100.00	39PHE	CG	39PHE	CD2	100.00	48ALA	CB	49PHE	CZ	0.04
49PHE	CA	49PHE	CG	100.00	39PHE	CG	39PHE	CE1	100.00	48ALA	C	49PHE	CA	100.00
49PHE	CA	49PHE	CD1	78.18	39PHE	CG	39PHE	CE2	100.00	48ALA	C	49PHE	CB	38.36
49PHE	CA	49PHE	CD2	82.66	39PHE	CG	39PHE	CZ	100.00	48ALA	C	49PHE	CG	11.43
49PHE	CA	49PHE	C	100.00	39PHE	CG	39PHE	C	0.01	48ALA	C	49PHE	CD1	0.77
49PHE	CB	49PHE	CG	100.00	39PHE	CG	57THR	CG2	0.01	48ALA	C	49PHE	CD2	6.74
49PHE	CB	49PHE	CD1	100.00	39PHE	CG	61VAL	CG1	0.01	48ALA	C	49PHE	C	56.12
49PHE	CB	49PHE	CD2	100.00	39PHE	CD1	39PHE	CD2	100.00	49PHE	CA	49PHE	CB	100.00
49PHE	CB	49PHE	C	100.00	39PHE	CD1	39PHE	CE1	100.00	49PHE	CA	49PHE	CG	100.00
49PHE	CB	54PRO	CB	16.21	39PHE	CD1	39PHE	CE2	100.00	49PHE	CA	49PHE	CD1	80.49
49PHE	CB	54PRO	CG	0.06	39PHE	CD1	39PHE	CZ	100.00	49PHE	CA	49PHE	CD2	72.95
49PHE	CG	49PHE	CD1	100.00	39PHE	CD1	39PHE	C	0.00	49PHE	CA	49PHE	C	100.00
49PHE	CG	49PHE	CD2	100.00	39PHE	CD1	61VAL	CG1	0.04	49PHE	CB	49PHE	CG	100.00
49PHE	CG	49PHE	CE1	100.00	39PHE	CD2	39PHE	CE1	100.00	49PHE	CB	49PHE	CD1	100.00
49PHE	CG	49PHE	CE2	100.00	39PHE	CD2	39PHE	CE2	100.00	49PHE	CB	49PHE	CD2	100.00
49PHE	CG	49PHE	CZ	100.00	39PHE	CD2	39PHE	CZ	100.00	49PHE	CB	49PHE	C	100.00
49PHE	CG	49PHE	C	0.83	39PHE	CD2	56PRO	C	0.24	49PHE	CB	54PRO	CB	6.79
49PHE	CG	54PRO	CB	23.88	39PHE	CD2	57THR	CA	0.77	49PHE	CB	54PRO	CG	0.05
49PHE	CG	54PRO	CG	0.52	39PHE	CD2	57THR	CG2	0.00	49PHE	CG	49PHE	CD1	100.00
49PHE	CD1	49PHE	CD2	100.00	39PHE	CD2	60GLY	CA	0.04	49PHE	CG	49PHE	CD2	100.00
49PHE	CD1	49PHE	CE1	100.00	39PHE	CD2	61VAL	CG1	0.01	49PHE	CG	49PHE	CE1	100.00
49PHE	CD1	49PHE	CE2	100.00	39PHE	CE1	39PHE	CE2	100.00	49PHE	CG	49PHE	CE2	100.00
49PHE	CD1	49PHE	CZ	100.00	39PHE	CE1	39PHE	CZ	100.00	49PHE	CG	49PHE	CZ	100.00
49PHE	CD1	54PRO	CB	9.12	39PHE	CE1	60GLY	CA	3.51	49PHE	CG	49PHE	C	2.86
49PHE	CD1	54PRO	CG	4.28	39PHE	CE1	60GLY	C	1.69	49PHE	CG	54PRO	CB	16.20
49PHE	CD2	49PHE	CE1	100.00	39PHE	CE1	61VAL	CG1	0.01	49PHE	CG	54PRO	CG	0.21
49PHE	CD2	49PHE	CE2	100.00	39PHE	CE2	39PHE	CZ	100.00	49PHE	CD1	49PHE	CD2	100.00
49PHE	CD2	49PHE	CZ	100.00	39PHE	CE2	56PRO	C	0.11	49PHE	CD1	49PHE	CE1	100.00
49PHE	CD2	49PHE	C	0.42	39PHE	CE2	60GLY	CA	11.55	49PHE	CD1	49PHE	CE2	100.00
49PHE	CD2	54PRO	CB	6.39	39PHE	CE2	60GLY	C	0.02	49PHE	CD1	49PHE	CZ	100.00
49PHE	CD2	54PRO	CG	0.10	39PHE	CE2	61VAL	CG1	0.02	49PHE	CD1	49PHE	C	1.13
49PHE	CD2	56PRO	CG	0.01	39PHE	CZ	60GLY	CA	30.01	49PHE	CD1	54PRO	CB	6.21
49PHE	CD2	56PRO	CD	0.00	39PHE	CZ	60GLY	C	1.48	49PHE	CD1	54PRO	CG	0.09
49PHE	CE1	49PHE	CE2	100.00	39PHE	CZ	61VAL	CG1	0.02	49PHE	CD1	56PRO	CG	0.01
49PHE	CE1	49PHE	CZ	100.00	39PHE	C	40PRO	CA	100.00	49PHE	CD1	56PRO	CD	0.00
49PHE	CE1	54PRO	CB	0.25	39PHE	C	40PRO	CB	0.06	49PHE	CD2	49PHE	CE1	100.00
49PHE	CE1	54PRO	CG	0.55	39PHE	C	40PRO	CD	100.00	49PHE	CD2	49PHE	CE2	100.00
49PHE	CE1	56PRO	CG	0.38	39PHE	C	40PRO	C	100.00	49PHE	CD2	49PHE	CZ	100.00

49PHE	CE1	56PRO	CD	0.13	40PRO	CA	40PRO	CB	100.00	49PHE	CD2	49PHE	C	0.30
49PHE	CE2	49PHE	CZ	100.00	40PRO	CA	40PRO	CG	100.00	49PHE	CD2	54PRO	CB	11.34
49PHE	CE2	54PRO	CB	0.09	40PRO	CA	40PRO	CD	100.00	49PHE	CD2	54PRO	CG	4.47
49PHE	CE2	54PRO	CG	0.01	40PRO	CA	40PRO	C	100.00	49PHE	CE1	49PHE	CE2	100.00
49PHE	CE2	56PRO	CG	4.17	40PRO	CA	44ALA	CB	0.00	49PHE	CE1	49PHE	CZ	100.00
49PHE	CE2	56PRO	CD	4.90	40PRO	CB	40PRO	CG	100.00	49PHE	CE1	54PRO	CB	0.28
49PHE	CZ	54PRO	CB	0.00	40PRO	CB	40PRO	CD	100.00	49PHE	CE1	54PRO	CG	0.01
49PHE	CZ	56PRO	CG	5.66	40PRO	CB	40PRO	C	100.00	49PHE	CE1	55GLU	CB	0.00
49PHE	CZ	56PRO	CD	1.98	40PRO	CB	44ALA	CB	0.01	49PHE	CE1	56PRO	CG	1.77
49PHE	C	50GLY	CA	100.00	40PRO	CB	44ALA	C	0.01	49PHE	CE1	56PRO	CD	2.01
49PHE	C	50GLY	C	53.45	40PRO	CB	45ALA	CB	0.11	49PHE	CE2	49PHE	CZ	100.00
50GLY	CA	50GLY	C	100.00	40PRO	CB	48ALA	CB	0.00	49PHE	CE2	54PRO	CB	0.65
50GLY	C	51GLU	CA	100.00	40PRO	CB	49PHE	CD1	0.15	49PHE	CE2	54PRO	CG	1.30
50GLY	C	51GLU	CB	63.64	40PRO	CB	49PHE	CD2	0.29	49PHE	CE2	56PRO	CG	0.29
50GLY	C	51GLU	CG	1.25	40PRO	CB	49PHE	CE1	0.78	49PHE	CE2	56PRO	CD	0.09
50GLY	C	51GLU	C	39.63	40PRO	CB	49PHE	CE2	2.22	49PHE	CZ	54PRO	CB	0.02
50GLY	C	84ILE	CG2	0.16	40PRO	CB	49PHE	CZ	0.04	49PHE	CZ	56PRO	CG	4.12
51GLU	CA	51GLU	CB	100.00	40PRO	CB	54PRO	CG	0.01	49PHE	CZ	56PRO	CD	2.20
51GLU	CA	51GLU	CG	100.00	40PRO	CG	40PRO	CD	100.00	49PHE	C	50GLY	CA	100.00
51GLU	CA	51GLU	CD	44.65	40PRO	CG	40PRO	C	90.24	49PHE	C	50GLY	C	47.60
51GLU	CA	51GLU	C	100.00	40PRO	CG	49PHE	CG	0.02	50GLY	CA	50GLY	C	100.00
51GLU	CA	52PRO	CD	100.00	40PRO	CG	49PHE	CD1	0.16	50GLY	C	51GLU	CA	100.00
51GLU	CB	51GLU	CG	100.00	40PRO	CG	49PHE	CD2	0.38	50GLY	C	51GLU	CB	45.53
51GLU	CB	51GLU	CD	100.00	40PRO	CG	49PHE	CE1	3.09	50GLY	C	51GLU	CG	0.88
51GLU	CB	51GLU	C	100.00	40PRO	CG	49PHE	CE2	11.21	50GLY	C	51GLU	CD	0.01
51GLU	CB	52PRO	CD	10.45	40PRO	CG	49PHE	CZ	1.78	50GLY	C	51GLU	C	58.55
51GLU	CB	53PHE	C	0.01	40PRO	CG	54PRO	CG	0.10	51GLL	CA	51GLU	CB	100.00
51GLU	CB	54PRO	CA	0.04	40PRO	CG	56PRO	CG	0.19	51GLL	CA	51GLU	CG	100.00
51GLU	CB	84ILE	CG2	0.00	40PRO	CD	40PRO	C	80.96	51GLL	CA	51GLU	CD	60.47
51GLU	CG	51GLU	CD	100.00	40PRO	CD	56PRO	CG	0.01	51GLL	CA	51GLU	C	100.00
51GLU	CG	51GLU	C	80.26	40PRO	C	41PHE	CA	100.00	51GLL	CA	52PRO	CD	100.00
51GLU	CG	52PRO	CD	1.64	40PRO	C	41PHE	CB	99.89	51GLL	CB	51GLU	CG	100.00
51GLU	CG	53PHE	C	0.32	40PRO	C	41PHE	C	0.52	51GLL	CB	51GLU	CD	100.00
51GLU	CG	54PRO	CA	0.16	40PRO	C	45ALA	CB	0.14	51GLL	CB	51GLU	C	100.00
51GLU	CG	54PRO	CB	0.00	40PRO	C	54PRO	CG	0.01	51GLL	CB	52PRO	CD	6.70
51GLU	CG	84ILE	CG2	0.00	41PHE	CA	41PHE	CB	100.00	51GLL	CB	54PRO	CA	0.06
51GLU	CD	51GLU	C	0.02	41PHE	CA	41PHE	CG	100.00	51GLL	CG	51GLU	CD	100.00
51GLU	CD	52PRO	CD	0.08	41PHE	CA	41PHE	CD1	92.41	51GLL	CG	51GLU	C	85.27
51GLU	CD	54PRO	CA	0.00	41PHE	CA	41PHE	CD2	45.59	51GLL	CG	52PRO	CD	1.61
51GLU	CD	84ILE	CG2	0.00	41PHE	CA	41PHE	C	100.00	51GLL	CG	53PHE	C	0.88
51GLU	C	52PRO	CA	100.00	41PHE	CA	45ALA	CB	0.11	51GLL	CG	54PRO	CA	0.14
51GLU	C	52PRO	CB	0.02	41PHE	CB	41PHE	CG	100.00	51GLL	CG	54PRO	CB	0.00
51GLU	C	52PRO	CD	100.00	41PHE	CB	41PHE	CD1	100.00	51GLL	CD	51GLU	C	0.14
51GLU	C	52PRO	C	100.00	41PHE	CB	41PHE	CD2	100.00	51GLL	CD	52PRO	CD	0.16
52PRO	CA	52PRO	CB	100.00	41PHE	CB	41PHE	C	100.00	51GLL	CD	58ARG	CZ	0.00
52PRO	CA	52PRO	CG	100.00	41PHE	CG	41PHE	CD1	100.00	51GLL	C	52PRO	CA	100.00
52PRO	CA	52PRO	CD	100.00	41PHE	CG	41PHE	CD2	100.00	51GLL	C	52PRO	CB	0.02
52PRO	CA	52PRO	C	100.00	41PHE	CG	41PHE	CE1	100.00	51GLL	C	52PRO	CD	100.00
52PRO	CB	52PRO	CG	100.00	41PHE	CG	41PHE	CE2	100.00	51GLL	C	52PRO	C	100.00
52PRO	CB	52PRO	CD	100.00	41PHE	CG	41PHE	CZ	100.00	52PRC	CA	52PRO	CB	100.00
52PRO	CB	52PRO	C	100.00	41PHE	CG	41PHE	C	99.96	52PRC	CA	52PRO	CG	100.00
52PRO	CB	77TRP	CZ3	0.02	41PHE	CD1	41PHE	CD2	100.00	52PRC	CA	52PRO	CD	100.00
52PRO	CB	86PRO	CA	0.03	41PHE	CD1	41PHE	CE1	100.00	52PRC	CA	52PRO	C	100.00
52PRO	CB	89GLY	CA	0.05	41PHE	CD1	41PHE	CE2	100.00	52PRC	CB	52PRO	CG	100.00
52PRO	CB	90LEU	CD1	0.01	41PHE	CD1	41PHE	CZ	100.00	52PRC	CB	52PRO	CD	100.00
52PRO	CB	90LEU	CD2	0.05	41PHE	CD1	41PHE	C	0.78	52PRC	CB	52PRO	C	100.00
52PRO	CG	52PRO	CD	100.00	41PHE	CD1	52PRO	C	0.00	52PRC	CB	86PRO	CA	0.16
52PRO	CG	52PRO	C	88.16	41PHE	CD1	53PHE	CD1	0.26	52PRC	CB	86PRO	CB	0.32
52PRO	CG	84ILE	CA	0.01	41PHE	CD1	93LEU	CD1	0.02	52PRC	CB	89GLY	CA	0.06
52PRO	CG	84ILE	CB	0.01	41PHE	CD2	41PHE	CE1	100.00	52PRC	CG	52PRO	CD	100.00
52PRO	CG	84ILE	CG2	0.04	41PHE	CD2	41PHE	CE2	100.00	52PRC	CG	52PRO	C	90.39
52PRO	CG	84ILE	C	0.07	41PHE	CD2	41PHE	CZ	100.00	52PRC	CG	86PRO	CA	0.00

52PRO	CG	85SER	C	0.06
52PRO	CG	86PRO	CA	0.02
52PRO	CG	88THR	CB	0.01
52PRO	CG	88THR	CG2	0.02
52PRO	CG	88THR	C	0.17
52PRO	CG	89GLY	CA	0.96
52PRO	CG	89GLY	C	0.03
52PRO	CG	90LEU	CD1	0.03
52PRO	CG	90LEU	CD2	0.18
52PRO	CD	52PRO	C	69.89
52PRO	CD	84ILE	CA	0.00
52PRO	CD	84ILE	CB	0.02
52PRO	CD	84ILE	CG2	2.68
52PRO	CD	84ILE	C	0.00
52PRO	C	53PHE	CA	100.00
52PRO	C	53PHE	CB	80.56
52PRO	C	53PHE	CD1	0.00
52PRO	C	53PHE	C	49.12
52PRO	C	54PRO	CD	12.82
52PRO	C	90LEU	CD2	0.01
53PHE	CA	53PHE	CB	100.00
53PHE	CA	53PHE	CG	100.00
53PHE	CA	53PHE	CD1	95.24
53PHE	CA	53PHE	CD2	16.15
53PHE	CA	53PHE	C	100.00
53PHE	CA	54PRO	CD	100.00
53PHE	CB	53PHE	CG	100.00
53PHE	CB	53PHE	CD1	100.00
53PHE	CB	53PHE	CD2	100.00
53PHE	CB	53PHE	C	100.00
53PHE	CB	89GLY	CA	5.82
53PHE	CB	89GLY	C	0.13
53PHE	CB	90LEU	CD1	0.04
53PHE	CB	90LEU	CD2	0.17
53PHE	CB	92SER	CB	0.14
53PHE	CB	93LEU	CD1	0.00
53PHE	CB	93LEU	CD2	0.00
53PHE	CG	53PHE	CD1	100.00
53PHE	CG	53PHE	CD2	100.00
53PHE	CG	53PHE	CE1	100.00
53PHE	CG	53PHE	CE2	100.00
53PHE	CG	53PHE	CZ	100.00
53PHE	CG	53PHE	C	99.76
53PHE	CG	89GLY	CA	0.86
53PHE	CG	90LEU	CD1	0.00
53PHE	CG	92SER	CB	0.28
53PHE	CG	93LEU	CG	0.00
53PHE	CG	93LEU	CD1	0.02
53PHE	CG	93LEU	CD2	0.01
53PHE	CD1	53PHE	CD2	100.00
53PHE	CD1	53PHE	CE1	100.00
53PHE	CD1	53PHE	CE2	100.00
53PHE	CD1	53PHE	CZ	100.00
53PHE	CD1	53PHE	C	0.34
53PHE	CD1	57THR	CG2	4.04
53PHE	CD1	89GLY	CA	0.28
53PHE	CD1	89GLY	C	0.03
53PHE	CD1	90LEU	CD2	0.01
53PHE	CD1	93LEU	CB	0.03
53PHE	CD1	93LEU	CG	0.05
53PHE	CD1	93LEU	CD1	0.38
53PHE	CD1	93LEU	CD2	0.04

41PHE	CD2	41PHE	C	48.09
41PHE	CD2	42GLY	CA	0.01
41PHE	CD2	69LEU	CD1	0.57
41PHE	CD2	69LEU	CD2	0.18
41PHE	CD2	72VAL	CG1	0.33
41PHE	CD2	72VAL	CG2	0.03
41PHE	CE1	41PHE	CE2	100.00
41PHE	CE1	41PHE	CZ	100.00
41PHE	CE1	53PHE	CB	0.04
41PHE	CE1	53PHE	CG	0.02
41PHE	CE1	53PHE	CD1	0.90
41PHE	CE1	89GLY	CA	0.02
41PHE	CE1	89GLY	C	1.74
41PHE	CE1	90LEU	CA	0.00
41PHE	CE1	90LEU	CD1	0.00
41PHE	CE1	90LEU	CD2	0.00
41PHE	CE1	93LEU	CB	0.00
41PHE	CE1	93LEU	CD1	0.74
41PHE	CE1	93LEU	CD2	0.40
41PHE	CE2	41PHE	CZ	100.00
41PHE	CE2	69LEU	CG	0.04
41PHE	CE2	69LEU	CD1	12.57
41PHE	CE2	69LEU	CD2	8.36
41PHE	CE2	72VAL	CB	0.06
41PHE	CE2	72VAL	CG1	9.92
41PHE	CE2	72VAL	CG2	1.89
41PHE	CE2	90LEU	CD1	0.05
41PHE	CE2	90LEU	CD2	0.15
41PHE	CE2	93LEU	CD1	0.01
41PHE	CE2	93LEU	CD2	0.04
41PHE	CZ	69LEU	CD1	0.66
41PHE	CZ	69LEU	CD2	1.17
41PHE	CZ	72VAL	CG1	1.83
41PHE	CZ	72VAL	CG2	0.64
41PHE	CZ	86PRO	CB	0.01
41PHE	CZ	89GLY	C	1.19
41PHE	CZ	90LEU	CA	0.01
41PHE	CZ	90LEU	CB	0.00
41PHE	CZ	90LEU	CG	0.00
41PHE	CZ	90LEU	CD1	0.28
41PHE	CZ	90LEU	CD2	1.04
41PHE	CZ	93LEU	CD1	0.39
41PHE	CZ	93LEU	CD2	1.31
41PHE	C	42GLY	CA	100.00
41PHE	C	42GLY	C	38.85
41PHE	C	45ALA	CB	0.17
42GLY	CA	42GLY	C	100.00
42GLY	CA	72VAL	CG1	0.77
42GLY	CA	72VAL	CG2	0.28
42GLY	CA	86PRO	CB	0.13
42GLY	CA	86PRO	CG	0.00
42GLY	C	43GLY	CA	100.00
42GLY	C	43GLY	C	82.41
42GLY	C	46ILE	CD	0.02
42GLY	C	86PRO	CA	0.00
42GLY	C	86PRO	CB	3.95
42GLY	C	86PRO	CG	0.30
43GLY	CA	43GLY	C	100.00
43GLY	CA	46ILE	CG1	0.00
43GLY	CA	46ILE	CG2	0.03
43GLY	CA	46ILE	CD	0.17
43GLY	CA	77TRP	CG	0.00

52PRC	CG	88THR	C	0.58
52PRC	CG	89GLY	CA	2.52
52PRC	CD	52PRO	C	68.76
52PRC	C	53PHE	CA	100.00
52PRC	C	53PHE	CB	81.84
52PRC	C	53PHE	C	48.21
52PRC	C	54PRO	CD	9.70
53PHE	CA	53PHE	CB	100.00
53PHE	CA	53PHE	CG	100.00
53PHE	CA	53PHE	CD1	98.82
53PHE	CA	53PHE	CD2	4.48
53PHE	CA	53PHE	C	100.00
53PHE	CA	54PRO	CD	100.00
53PHE	CB	53PHE	CG	100.00
53PHE	CB	53PHE	CD1	100.00
53PHE	CB	53PHE	CD2	100.00
53PHE	CB	53PHE	C	100.00
53PHE	CB	89GLY	CA	3.30
53PHE	CB	89GLY	C	0.01
53PHE	CB	92SER	CB	0.08
53PHE	CG	53PHE	CD1	100.00
53PHE	CG	53PHE	CD2	100.00
53PHE	CG	53PHE	CE1	100.00
53PHE	CG	53PHE	CE2	100.00
53PHE	CG	53PHE	CZ	100.00
53PHE	CG	53PHE	C	99.98
53PHE	CG	58ARG	CG	0.00
53PHE	CG	92SER	CB	0.06
53PHE	CD1	53PHE	CD2	100.00
53PHE	CD1	53PHE	CE1	100.00
53PHE	CD1	53PHE	CE2	100.00
53PHE	CD1	53PHE	CZ	100.00
53PHE	CD1	53PHE	C	4.00
53PHE	CD1	57THR	CG2	2.38
53PHE	CD1	58ARG	CG	0.02
53PHE	CD1	93LEU	CB	0.01
53PHE	CD1	93LEU	CG	0.01
53PHE	CD1	93LEU	CD1	0.03
53PHE	CD1	93LEU	CD2	0.02
53PHE	CD2	53PHE	CE1	100.00
53PHE	CD2	53PHE	CE2	100.00
53PHE	CD2	53PHE	CZ	100.00
53PHE	CD2	53PHE	C	1.56
53PHE	CD2	58ARG	CB	0.01
53PHE	CD2	58ARG	CG	2.23
53PHE	CD2	58ARG	CD	0.07
53PHE	CD2	58ARG	CZ	2.86
53PHE	CD2	92SER	CB	8.31
53PHE	CD2	92SER	C	0.09
53PHE	CD2	93LEU	CA	0.03
53PHE	CD2	93LEU	CB	0.00
53PHE	CE1	53PHE	CE2	100.00
53PHE	CE1	53PHE	CZ	100.00
53PHE	CE1	57THR	CG2	1.06
53PHE	CE1	57THR	C	0.21
53PHE	CE1	58ARG	CA	1.14
53PHE	CE1	58ARG	CG	0.32
53PHE	CE1	61VAL	CG1	0.00
53PHE	CE1	61VAL	CG2	0.16
53PHE	CE1	93LEU	CA	0.02
53PHE	CE1	93LEU	CB	0.28
53PHE	CE1	93LEU	CG	0.58

53PHE	CD2	53PHE	CE1	100.00	43GLY	CA	77TRP	CD1	0.02	53PHE	CE1	93LEU	CD1	6.28
53PHE	CD2	53PHE	CE2	100.00	43GLY	CA	77TRP	CD2	0.05	53PHE	CE1	93LEU	CD2	0.58
53PHE	CD2	53PHE	CZ	100.00	43GLY	CA	77TRP	CE2	0.14	53PHE	CE2	53PHE	CZ	100.00
53PHE	CD2	53PHE	C	6.96	43GLY	CA	77TRP	CZ2	0.71	53PHE	CE2	57THR	CG2	0.03
53PHE	CD2	58ARG	CB	0.12	43GLY	CA	77TRP	CZ3	0.02	53PHE	CE2	57THR	C	0.01
53PHE	CD2	58ARG	CG	0.05	43GLY	CA	86PRO	CA	0.00	53PHE	CE2	58ARG	CA	0.72
53PHE	CD2	58ARG	CD	0.02	43GLY	CA	86PRO	CB	0.12	53PHE	CE2	58ARG	CB	2.00
53PHE	CD2	89GLY	CA	0.52	43GLY	CA	86PRO	CG	0.08	53PHE	CE2	58ARG	CG	11.18
53PHE	CD2	89GLY	C	0.02	43GLY	C	44ALA	CA	100.00	53PHE	CE2	58ARG	CD	0.44
53PHE	CD2	92SER	CB	13.49	43GLY	C	44ALA	CB	0.21	53PHE	CE2	58ARG	CZ	0.10
53PHE	CD2	92SER	C	0.11	43GLY	C	44ALA	C	99.78	53PHE	CE2	92SER	CB	0.09
53PHE	CD2	93LEU	CA	0.07	43GLY	C	46ILE	CG2	0.07	53PHE	CE2	92SER	C	0.47
53PHE	CD2	93LEU	CB	0.01	43GLY	C	46ILE	CD	0.01	53PHE	CE2	93LEU	CA	3.46
53PHE	CD2	93LEU	CD2	0.00	43GLY	C	76LYS	CD	0.01	53PHE	CE2	93LEU	CB	0.07
53PHE	CE1	53PHE	CE2	100.00	43GLY	C	77TRP	CD2	0.01	53PHE	CE2	93LEU	CG	0.01
53PHE	CE1	53PHE	CZ	100.00	43GLY	C	77TRP	CE2	0.19	53PHE	CE2	93LEU	CD1	0.06
53PHE	CE1	57THR	CG2	2.48	43GLY	C	77TRP	CE3	0.00	53PHE	CE2	96SER	CB	0.04
53PHE	CE1	57THR	C	0.11	43GLY	C	77TRP	CZ2	0.38	53PHE	CZ	57THR	CG2	0.02
53PHE	CE1	58ARG	CA	0.39	43GLY	C	77TRP	CZ3	0.00	53PHE	CZ	58ARG	CA	4.72
53PHE	CE1	58ARG	CB	0.01	44ALA	CA	44ALA	CB	100.00	53PHE	CZ	58ARG	CB	0.32
53PHE	CE1	58ARG	CG	0.00	44ALA	CA	44ALA	C	100.00	53PHE	CZ	58ARG	CG	2.59
53PHE	CE1	61VAL	CG1	0.01	44ALA	CA	47ASP	CB	0.00	53PHE	CZ	58ARG	CD	0.00
53PHE	CE1	61VAL	CG2	0.08	44ALA	CA	77TRP	CZ2	0.04	53PHE	CZ	61VAL	CG1	0.01
53PHE	CE1	89GLY	CA	0.00	44ALA	CB	44ALA	C	100.00	53PHE	CZ	61VAL	CG2	1.33
53PHE	CE1	89GLY	C	0.02	44ALA	C	45ALA	CA	100.00	53PHE	CZ	93LEU	CA	0.63
53PHE	CE1	90LEU	CD2	0.01	44ALA	C	45ALA	CB	0.11	53PHE	CZ	93LEU	CB	0.26
53PHE	CE1	93LEU	CA	0.02	44ALA	C	45ALA	C	99.91	53PHE	CZ	93LEU	CG	0.14
53PHE	CE1	93LEU	CB	0.79	44ALA	C	48ALA	CB	0.01	53PHE	CZ	93LEU	CD1	6.06
53PHE	CE1	93LEU	CG	0.70	45ALA	CA	45ALA	CB	100.00	53PHE	CZ	93LEU	CD2	0.26
53PHE	CE1	93LEU	CD1	4.60	45ALA	CA	45ALA	C	100.00	53PHE	CZ	96SER	CB	0.04
53PHE	CE1	93LEU	CD2	0.57	45ALA	CA	49PHE	CD1	0.01	53PHE	C	54PRO	CA	100.00
53PHE	CE2	53PHE	CZ	100.00	45ALA	CA	49PHE	CD2	0.02	53PHE	C	54PRO	CB	1.37
53PHE	CE2	57THR	CG2	0.02	45ALA	CA	49PHE	CE1	0.00	53PHE	C	54PRO	CD	100.00
53PHE	CE2	57THR	C	0.01	45ALA	CA	49PHE	CE2	0.01	53PHE	C	54PRO	C	100.00
53PHE	CE2	58ARG	CA	3.82	45ALA	CA	49PHE	CZ	0.01	54PRC	CA	54PRO	CB	100.00
53PHE	CE2	58ARG	CB	5.49	45ALA	CB	45ALA	C	100.00	54PRC	CA	54PRO	CG	100.00
53PHE	CE2	58ARG	CG	2.73	45ALA	CB	49PHE	CD1	0.01	54PRC	CA	54PRO	CD	100.00
53PHE	CE2	58ARG	CD	0.24	45ALA	CB	49PHE	CE2	0.02	54PRC	CA	54PRO	C	100.00
53PHE	CE2	89GLY	CA	0.00	45ALA	CB	49PHE	CZ	0.04	54PRC	CB	54PRO	CG	100.00
53PHE	CE2	92SER	CB	1.28	45ALA	CB	52PRO	CA	0.02	54PRC	CB	54PRO	CD	100.00
53PHE	CE2	92SER	C	0.65	45ALA	CB	52PRO	C	0.03	54PRC	CB	54PRO	C	100.00
53PHE	CE2	93LEU	CA	2.77	45ALA	CB	54PRO	CG	0.08	54PRC	CG	54PRO	CD	100.00
53PHE	CE2	93LEU	CB	0.19	45ALA	CB	54PRO	CD	0.05	54PRC	CG	54PRO	C	99.93
53PHE	CE2	93LEU	CD1	0.01	45ALA	C	46ILE	CA	100.00	54PRC	CG	57THR	CB	0.00
53PHE	CE2	96SER	CB	0.00	45ALA	C	46ILE	CB	0.06	54PRC	CD	54PRO	C	98.47
53PHE	CZ	57THR	CG2	0.01	45ALA	C	46ILE	CG1	0.04	54PRC	CD	57THR	CB	0.08
53PHE	CZ	57THR	C	0.00	45ALA	C	46ILE	C	99.97	54PRC	C	55GLU	CA	100.00
53PHE	CZ	58ARG	CA	5.65	45ALA	C	51GLU	C	0.00	54PRC	C	55GLU	C	100.00
53PHE	CZ	58ARG	CB	0.74	46ILE	CA	46ILE	CB	100.00	54PRC	C	56PRO	CD	23.16
53PHE	CZ	58ARG	CG	0.14	46ILE	CA	46ILE	CG1	100.00	54PRC	C	58ARG	CB	0.01
53PHE	CZ	61VAL	CG1	0.01	46ILE	CA	46ILE	CG2	100.00	54PRC	C	58ARG	CG	0.00
53PHE	CZ	61VAL	CG2	0.50	46ILE	CA	46ILE	CD	8.02	54PRC	C	58ARG	CD	0.02
53PHE	CZ	92SER	CB	0.01	46ILE	CA	46ILE	C	100.00	54PRC	C	58ARG	CZ	0.01
53PHE	CZ	92SER	C	0.00	46ILE	CA	50GLY	CA	0.04	55GLL	CA	55GLU	CB	100.00
53PHE	CZ	93LEU	CA	1.00	46ILE	CA	50GLY	C	0.01	55GLL	CA	55GLU	CG	100.00
53PHE	CZ	93LEU	CB	1.12	46ILE	CA	51GLU	C	0.00	55GLL	CA	55GLU	CD	93.64
53PHE	CZ	93LEU	CG	0.24	46ILE	CB	46ILE	CG1	100.00	55GLL	CA	55GLU	C	100.00
53PHE	CZ	93LEU	CD1	3.40	46ILE	CB	46ILE	CG2	100.00	55GLL	CA	56PRO	CD	100.00
53PHE	CZ	93LEU	CD2	0.37	46ILE	CB	46ILE	CD	100.00	55GLL	CA	58ARG	CZ	0.06
53PHE	CZ	96SER	CB	0.00	46ILE	CB	46ILE	C	100.00	55GLL	CB	55GLU	CG	100.00
53PHE	C	54PRO	CA	100.00	46ILE	CB	77TRP	CZ2	0.00	55GLL	CB	55GLU	CD	100.00
53PHE	C	54PRO	CB	1.54	46ILE	CG1	46ILE	CG2	100.00	55GLL	CB	55GLU	C	100.00

53PHE	C	54PRO	CD	100.00	46ILE	CG1	46ILE	CD	100.00	55GLU	CB	56PRO	CD	72.30
53PHE	C	54PRO	C	99.99	46ILE	CG1	46ILE	C	3.70	55GLU	CG	55GLU	CD	100.00
54PRO	CA	54PRO	CB	100.00	46ILE	CG1	51GLU	C	0.48	55GLU	CG	55GLU	C	96.60
54PRO	CA	54PRO	CG	100.00	46ILE	CG1	52PRO	CA	0.56	55GLU	CG	56PRO	CD	0.17
54PRO	CA	54PRO	CD	100.00	46ILE	CG1	52PRO	CB	0.70	55GLU	CG	59LYS	CE	0.08
54PRO	CA	54PRO	C	100.00	46ILE	CG1	52PRO	CG	0.00	55GLU	CD	55GLU	C	3.53
54PRO	CB	54PRO	CG	100.00	46ILE	CG1	52PRO	CD	0.08	55GLU	CD	58ARG	CD	0.00
54PRO	CB	54PRO	CD	100.00	46ILE	CG2	46ILE	CD	98.94	55GLU	CD	58ARG	CZ	0.02
54PRO	CB	54PRO	C	100.00	46ILE	CG2	46ILE	C	99.99	55GLU	CD	59LYS	CG	0.00
54PRO	CG	54PRO	CD	100.00	46ILE	CG2	50GLY	C	0.05	55GLU	CD	59LYS	CD	0.06
54PRO	CG	54PRO	C	99.94	46ILE	CG2	77TRP	CD2	0.01	55GLU	CD	59LYS	CE	0.72
54PRO	CD	54PRO	C	98.55	46ILE	CG2	77TRP	CE2	0.01	55GLU	C	56PRO	CA	100.00
54PRO	CD	57THR	CB	0.04	46ILE	CG2	77TRP	CE3	0.02	55GLU	C	56PRO	CB	0.02
54PRO	C	55GLU	CA	100.00	46ILE	CG2	77TRP	CZ2	0.34	55GLU	C	56PRO	CD	100.00
54PRO	C	55GLU	C	100.00	46ILE	CG2	77TRP	CZ3	0.16	55GLU	C	56PRO	C	100.00
54PRO	C	56PRO	CD	23.29	46ILE	CG2	80LEU	CD1	0.03	55GLU	C	59LYS	CE	0.00
54PRO	C	58ARG	CB	0.02	46ILE	CG2	80LEU	CD2	0.02	56PRO	CA	56PRO	CB	100.00
54PRO	C	58ARG	CZ	0.08	46ILE	CG2	84ILE	CB	0.01	56PRO	CA	56PRO	CG	100.00
55GLU	CA	55GLU	CB	100.00	46ILE	CG2	84ILE	CG1	0.01	56PRO	CA	56PRO	CD	100.00
55GLU	CA	55GLU	CG	100.00	46ILE	CG2	84ILE	CG2	0.32	56PRO	CA	56PRO	C	100.00
55GLU	CA	55GLU	CD	98.19	46ILE	CG2	84ILE	CD	0.55	56PRO	CB	56PRO	CG	100.00
55GLU	CA	55GLU	C	100.00	46ILE	CD	46ILE	C	0.01	56PRO	CB	56PRO	CD	100.00
55GLU	CA	56PRO	CD	100.00	46ILE	CD	50GLY	C	0.02	56PRO	CB	56PRO	C	100.00
55GLU	CA	58ARG	CZ	0.39	46ILE	CD	51GLU	C	0.16	56PRO	CG	56PRO	CD	100.00
55GLU	CB	55GLU	CG	100.00	46ILE	CD	52PRO	CA	0.16	56PRO	CG	56PRO	C	92.07
55GLU	CB	55GLU	CD	100.00	46ILE	CD	52PRO	CB	0.10	56PRO	CD	56PRO	C	77.96
55GLU	CB	55GLU	C	100.00	46ILE	CD	52PRO	CG	0.03	56PRO	C	57THR	CA	100.00
55GLU	CB	56PRO	CD	68.30	46ILE	CD	52PRO	CD	0.31	56PRO	C	57THR	CB	0.01
55GLU	CG	55GLU	CD	100.00	46ILE	CD	77TRP	CD1	0.01	56PRO	C	57THR	C	100.00
55GLU	CG	55GLU	C	95.72	46ILE	CD	77TRP	CD2	0.00	57THR	CA	57THR	CB	100.00
55GLU	CG	56PRO	CD	0.09	46ILE	CD	77TRP	CE3	0.02	57THR	CA	57THR	CG2	100.00
55GLU	CG	59LYS	CE	0.01	46ILE	CD	77TRP	CZ2	0.32	57THR	CA	57THR	C	100.00
55GLU	CD	55GLU	C	2.28	46ILE	CD	77TRP	CZ3	0.50	57THR	CB	57THR	CG2	100.00
55GLU	CD	58ARG	CZ	0.03	46ILE	CD	80LEU	CD1	0.05	57THR	CB	57THR	C	100.00
55GLU	CD	59LYS	CD	0.03	46ILE	CD	84ILE	CA	0.01	57THR	CG2	57THR	C	100.00
55GLU	CD	59LYS	CE	0.74	46ILE	CD	84ILE	CB	0.01	57THR	CG2	61VAL	CG1	0.00
55GLU	C	56PRO	CA	100.00	46ILE	CD	84ILE	CG2	0.01	57THR	C	58ARG	CA	100.00
55GLU	C	56PRO	CB	0.02	46ILE	CD	84ILE	C	0.15	57THR	C	58ARG	C	100.00
55GLU	C	56PRO	CD	100.00	46ILE	CD	85SER	CA	0.01	58ARG	CA	58ARG	CB	100.00
55GLU	C	56PRO	C	100.00	46ILE	CD	85SER	C	0.27	58ARG	CA	58ARG	CG	100.00
56PRO	CA	56PRO	CB	100.00	46ILE	CD	86PRO	CA	0.06	58ARG	CA	58ARG	CD	0.05
56PRO	CA	56PRO	CG	100.00	46ILE	CD	86PRO	CB	0.04	58ARG	CA	58ARG	C	100.00
56PRO	CA	56PRO	CD	100.00	46ILE	CD	86PRO	CG	0.32	58ARG	CB	58ARG	CG	100.00
56PRO	CA	56PRO	C	100.00	46ILE	CD	86PRO	CD	1.06	58ARG	CB	58ARG	CD	100.00
56PRO	CB	56PRO	CG	100.00	46ILE	C	47ASP	CA	100.00	58ARG	CB	58ARG	CZ	1.93
56PRO	CB	56PRO	CD	100.00	46ILE	C	47ASP	CB	1.27	58ARG	CB	58ARG	C	100.00
56PRO	CB	56PRO	C	100.00	46ILE	C	47ASP	C	98.99	58ARG	CG	58ARG	CD	100.00
56PRO	CG	56PRO	CD	100.00	46ILE	C	50GLY	CA	0.01	58ARG	CG	58ARG	CZ	20.08
56PRO	CG	56PRO	C	90.28	47ASP	CA	47ASP	CB	100.00	58ARG	CG	58ARG	C	71.80
56PRO	CD	56PRO	C	75.83	47ASP	CA	47ASP	CG	100.00	58ARG	CD	58ARG	CZ	100.00
56PRO	C	57THR	CA	100.00	47ASP	CA	47ASP	C	100.00	58ARG	CD	58ARG	C	0.03
56PRO	C	57THR	CB	0.02	47ASP	CB	47ASP	CG	100.00	58ARG	CZ	96SER	CB	0.02
56PRO	C	57THR	C	100.00	47ASP	CB	47ASP	C	100.00	58ARG	C	59LYS	CA	100.00
57THR	CA	57THR	CB	100.00	47ASP	CG	47ASP	C	97.08	58ARG	C	59LYS	CB	3.28
57THR	CA	57THR	CG2	100.00	47ASP	CG	77TRP	CZ2	0.00	58ARG	C	59LYS	CG	0.14
57THR	CA	57THR	C	100.00	47ASP	C	48ALA	CA	100.00	58ARG	C	59LYS	C	96.70
57THR	CB	57THR	CG2	100.00	47ASP	C	48ALA	CB	3.33	59LYS	CA	59LYS	CB	100.00
57THR	CB	57THR	C	100.00	47ASP	C	48ALA	C	96.31	59LYS	CA	59LYS	CG	100.00
57THR	CG2	57THR	C	100.00	48ALA	CA	48ALA	CB	100.00	59LYS	CA	59LYS	CD	12.23
57THR	CG2	61VAL	CG1	0.01	48ALA	CA	48ALA	C	100.00	59LYS	CA	59LYS	C	100.00
57THR	C	58ARG	CA	100.00	48ALA	CB	48ALA	C	100.00	59LYS	CB	59LYS	CG	100.00
57THR	C	58ARG	CB	0.00	48ALA	CB	49PHE	CG	0.03	59LYS	CB	59LYS	CD	100.00

57THR	C	58ARG	C	100.00	48ALA	CB	49PHE	CD1	3.00	59LYS	CB	59LYS	CE	12.95
58ARG	CA	58ARG	CB	100.00	48ALA	CB	49PHE	CD2	9.74	59LYS	CB	59LYS	C	100.00
58ARG	CA	58ARG	CG	100.00	48ALA	CB	49PHE	CE1	0.45	59LYS	CG	59LYS	CD	100.00
58ARG	CA	58ARG	CD	0.02	48ALA	CB	49PHE	CE2	1.50	59LYS	CG	59LYS	CE	100.00
58ARG	CA	58ARG	C	100.00	48ALA	CB	49PHE	CZ	0.03	59LYS	CG	59LYS	C	14.26
58ARG	CB	58ARG	CG	100.00	48ALA	C	49PHE	CA	100.00	59LYS	CD	59LYS	CE	100.00
58ARG	CB	58ARG	CD	100.00	48ALA	C	49PHE	CB	44.94	59LYS	CD	59LYS	C	0.07
58ARG	CB	58ARG	CZ	2.41	48ALA	C	49PHE	CG	14.89	59LYS	C	60GLY	CA	100.00
58ARG	CB	58ARG	C	100.00	48ALA	C	49PHE	CD1	2.78	59LYS	C	60GLY	C	83.41
58ARG	CG	58ARG	CD	100.00	48ALA	C	49PHE	CD2	6.28	59LYS	C	62GLU	CB	0.04
58ARG	CG	58ARG	CZ	18.46	48ALA	C	49PHE	C	49.98	59LYS	C	62GLU	CG	0.00
58ARG	CG	58ARG	C	98.05	49PHE	CA	49PHE	CB	100.00	60GLY	CA	60GLY	C	100.00
58ARG	CD	58ARG	CZ	100.00	49PHE	CA	49PHE	CG	100.00	60GLY	CA	63GLU	CB	0.00
58ARG	CD	58ARG	C	0.01	49PHE	CA	49PHE	CD1	81.32	60GLY	C	61VAL	CA	100.00
58ARG	CZ	59LYS	CG	0.00	49PHE	CA	49PHE	CD2	73.51	60GLY	C	61VAL	CB	0.06
58ARG	CZ	59LYS	CE	0.00	49PHE	CA	49PHE	C	100.00	60GLY	C	61VAL	CG1	0.02
58ARG	C	59LYS	CA	100.00	49PHE	CB	49PHE	CG	100.00	60GLY	C	61VAL	C	99.96
58ARG	C	59LYS	CB	17.07	49PHE	CB	49PHE	CD1	100.00	61VAL	CA	61VAL	CB	100.00
58ARG	C	59LYS	CG	0.18	49PHE	CB	49PHE	CD2	100.00	61VAL	CA	61VAL	CG1	100.00
58ARG	C	59LYS	C	83.32	49PHE	CB	49PHE	C	100.00	61VAL	CA	61VAL	CG2	100.00
58ARG	C	61VAL	CG2	0.00	49PHE	CB	54PRO	CB	15.65	61VAL	CA	61VAL	C	100.00
59LYS	CA	59LYS	CB	100.00	49PHE	CB	54PRO	CG	0.14	61VAL	CB	61VAL	CG1	100.00
59LYS	CA	59LYS	CG	100.00	49PHE	CG	49PHE	CD1	100.00	61VAL	CB	61VAL	CG2	100.00
59LYS	CA	59LYS	CD	7.20	49PHE	CG	49PHE	CD2	100.00	61VAL	CB	61VAL	C	100.00
59LYS	CA	59LYS	C	100.00	49PHE	CG	49PHE	CE1	100.00	61VAL	CB	67VAL	CG2	1.06
59LYS	CB	59LYS	CG	100.00	49PHE	CG	49PHE	CE2	100.00	61VAL	CG1	61VAL	CG2	100.00
59LYS	CB	59LYS	CD	100.00	49PHE	CG	49PHE	CZ	100.00	61VAL	CG1	61VAL	C	14.60
59LYS	CB	59LYS	CE	13.54	49PHE	CG	49PHE	C	2.06	61VAL	CG1	67VAL	CG1	0.61
59LYS	CB	59LYS	C	100.00	49PHE	CG	54PRO	CB	16.75	61VAL	CG1	67VAL	CG2	7.58
59LYS	CG	59LYS	CD	100.00	49PHE	CG	54PRO	CG	0.27	61VAL	CG1	93LEU	CD1	0.09
59LYS	CG	59LYS	CE	100.00	49PHE	CG	56PRO	CG	0.00	61VAL	CG1	93LEU	CD2	0.00
59LYS	CG	59LYS	C	42.49	49PHE	CD1	49PHE	CD2	100.00	61VAL	CG1	97GLN	CG	0.29
59LYS	CD	59LYS	CE	100.00	49PHE	CD1	49PHE	CE1	100.00	61VAL	CG1	97GLN	CD	0.36
59LYS	CD	59LYS	C	0.01	49PHE	CD1	49PHE	CE2	100.00	61VAL	CG1	266THR	CB	0.01
59LYS	C	60GLY	CA	100.00	49PHE	CD1	49PHE	CZ	100.00	61VAL	CG1	266THR	CG2	0.00
59LYS	C	60GLY	C	79.84	49PHE	CD1	49PHE	C	0.56	61VAL	CG2	61VAL	C	85.40
59LYS	C	62GLU	CB	0.05	49PHE	CD1	54PRO	CA	0.01	61VAL	CG2	67VAL	CG1	0.01
59LYS	C	62GLU	CG	0.00	49PHE	CD1	54PRO	CB	5.80	61VAL	CG2	67VAL	CG2	0.12
60GLY	CA	60GLY	C	100.00	49PHE	CD1	54PRO	CG	1.04	61VAL	CG2	93LEU	CD1	0.12
60GLY	C	61VAL	CA	100.00	49PHE	CD1	56PRO	CG	0.06	61VAL	CG2	97GLN	CG	0.30
60GLY	C	61VAL	CB	0.15	49PHE	CD1	56PRO	CD	0.01	61VAL	CG2	97GLN	CD	0.29
60GLY	C	61VAL	CG1	0.06	49PHE	CD2	49PHE	CE1	100.00	61VAL	C	62GLU	CA	100.00
60GLY	C	61VAL	C	99.86	49PHE	CD2	49PHE	CE2	100.00	61VAL	C	62GLU	CB	0.30
61VAL	CA	61VAL	CB	100.00	49PHE	CD2	49PHE	CZ	100.00	61VAL	C	62GLU	C	99.90
61VAL	CA	61VAL	CG1	100.00	49PHE	CD2	49PHE	C	0.53	62GLU	CA	62GLU	CB	100.00
61VAL	CA	61VAL	CG2	100.00	49PHE	CD2	51GLU	CB	0.00	62GLU	CA	62GLU	CG	100.00
61VAL	CA	61VAL	C	100.00	49PHE	CD2	54PRO	CB	8.71	62GLU	CA	62GLU	CD	96.06
61VAL	CA	64ALA	CB	0.00	49PHE	CD2	54PRO	CG	3.26	62GLU	CA	62GLU	C	100.00
61VAL	CA	67VAL	CG2	0.00	49PHE	CD2	56PRO	CG	0.04	62GLU	CB	62GLU	CG	100.00
61VAL	CB	61VAL	CG1	100.00	49PHE	CD2	56PRO	CD	0.01	62GLU	CB	62GLU	CD	100.00
61VAL	CB	61VAL	CG2	100.00	49PHE	CE1	49PHE	CE2	100.00	62GLU	CB	62GLU	C	100.00
61VAL	CB	61VAL	C	100.00	49PHE	CE1	49PHE	CZ	100.00	62GLU	CG	62GLU	CD	100.00
61VAL	CB	67VAL	CG2	1.43	49PHE	CE1	54PRO	CA	0.00	62GLU	CG	62GLU	C	97.99
61VAL	CB	93LEU	CD1	0.04	49PHE	CE1	54PRO	CB	0.17	62GLU	CD	62GLU	C	0.62
61VAL	CG1	61VAL	CG2	100.00	49PHE	CE1	54PRO	CG	0.14	62GLU	C	63GLU	CA	100.00
61VAL	CG1	67VAL	CG1	0.56	49PHE	CE1	55GLU	CB	0.04	62GLU	C	63GLU	CB	16.44
61VAL	CG1	67VAL	CG2	9.70	49PHE	CE1	56PRO	CG	3.01	62GLU	C	63GLU	CG	0.10
61VAL	CG1	93LEU	CD1	0.11	49PHE	CE1	56PRO	CD	2.52	62GLU	C	63GLU	C	86.40
61VAL	CG1	93LEU	CD2	0.02	49PHE	CE2	49PHE	CZ	100.00	63GLU	CA	63GLU	CB	100.00
61VAL	CG2	61VAL	C	100.00	49PHE	CE2	51GLU	CB	0.01	63GLU	CA	63GLU	CG	100.00
61VAL	CG2	93LEU	CD1	0.33	49PHE	CE2	54PRO	CB	0.30	63GLU	CA	63GLU	CD	84.65
61VAL	CG2	93LEU	CD2	0.04	49PHE	CE2	54PRO	CG	0.55	63GLU	CA	63GLU	C	100.00

61VAL	CG2	97GLN	CG	0.14	49PHE	CE2	55GLU	CB	0.03	63GLL	CB	63GLU	CG	100.00
61VAL	CG2	97GLN	CD	0.12	49PHE	CE2	56PRO	CG	1.06	63GLL	CB	63GLU	CD	100.00
61VAL	C	62GLU	CA	100.00	49PHE	CE2	56PRO	CD	0.88	63GLL	CB	63GLU	C	100.00
61VAL	C	62GLU	CB	0.49	49PHE	CZ	54PRO	CB	0.03	63GLL	CG	63GLU	CD	100.00
61VAL	C	62GLU	C	99.57	49PHE	CZ	54PRO	CG	0.01	63GLL	CG	63GLU	C	80.52
61VAL	C	64ALA	CB	0.00	49PHE	CZ	55GLU	CB	0.07	63GLL	CD	63GLU	C	2.74
62GLU	CA	62GLU	CB	100.00	49PHE	CZ	56PRO	CG	5.65	63GLL	C	64ALA	CA	100.00
62GLU	CA	62GLU	CG	100.00	49PHE	CZ	56PRO	CD	2.28	63GLL	C	64ALA	CB	22.86
62GLU	CA	62GLU	CD	95.50	49PHE	C	50GLY	CA	100.00	63GLL	C	64ALA	C	77.11
62GLU	CA	62GLU	C	100.00	49PHE	C	50GLY	C	50.95	64ALA	CA	64ALA	CB	100.00
62GLU	CB	62GLU	CG	100.00	50GLY	CA	50GLY	C	100.00	64ALA	CA	64ALA	C	100.00
62GLU	CB	62GLU	CD	100.00	50GLY	C	51GLU	CA	100.00	64ALA	CB	64ALA	C	100.00
62GLU	CB	62GLU	C	100.00	50GLY	C	51GLU	CB	64.59	64ALA	C	65GLU	CA	100.00
62GLU	CG	62GLU	CD	100.00	50GLY	C	51GLU	CG	2.77	64ALA	C	65GLU	CB	10.61
62GLU	CG	62GLU	C	83.74	50GLY	C	51GLU	CD	0.04	64ALA	C	65GLU	CG	0.17
62GLU	CD	62GLU	C	0.66	50GLY	C	51GLU	C	38.88	64ALA	C	65GLU	CD	0.01
62GLU	C	63GLU	CA	100.00	50GLY	C	84ILE	CG2	0.01	64ALA	C	65GLU	C	90.17
62GLU	C	63GLU	CB	32.98	51GLU	CA	51GLU	CB	100.00	64ALA	C	266THR	CG2	0.33
62GLU	C	63GLU	CG	0.22	51GLU	CA	51GLU	CG	100.00	65GLL	CA	65GLU	CB	100.00
62GLU	C	63GLU	C	72.97	51GLU	CA	51GLU	CD	39.85	65GLL	CA	65GLU	CG	100.00
63GLU	CA	63GLU	CB	100.00	51GLU	CA	51GLU	C	100.00	65GLL	CA	65GLU	CD	56.68
63GLU	CA	63GLU	CG	100.00	51GLU	CA	52PRO	CD	100.00	65GLL	CA	65GLU	C	100.00
63GLU	CA	63GLU	CD	84.97	51GLU	CB	51GLU	CG	100.00	65GLL	CA	266THR	CG2	0.01
63GLU	CA	63GLU	C	100.00	51GLU	CB	51GLU	CD	100.00	65GLL	CB	65GLU	CG	100.00
63GLU	CB	63GLU	CG	100.00	51GLU	CB	51GLU	C	100.00	65GLL	CB	65GLU	CD	100.00
63GLU	CB	63GLU	CD	100.00	51GLU	CB	52PRO	CD	12.69	65GLL	CB	65GLU	C	100.00
63GLU	CB	63GLU	C	100.00	51GLU	CB	53PHE	C	0.02	65GLL	CB	302PHE	CE1	1.94
63GLU	CG	63GLU	CD	100.00	51GLU	CB	54PRO	CA	0.07	65GLL	CB	302PHE	CZ	1.14
63GLU	CG	63GLU	C	86.59	51GLU	CB	54PRO	CB	0.01	65GLL	CG	65GLU	CD	100.00
63GLU	CD	63GLU	C	2.05	51GLU	CB	84ILE	CG2	0.02	65GLL	CG	65GLU	C	42.69
63GLU	C	64ALA	CA	100.00	51GLU	CB	84ILE	CD	0.01	65GLL	CG	302PHE	CD1	0.02
63GLU	C	64ALA	CB	50.61	51GLU	CG	51GLU	CD	100.00	65GLL	CG	302PHE	CE1	0.12
63GLU	C	64ALA	C	49.55	51GLU	CG	51GLU	C	71.05	65GLL	CD	302PHE	CE1	0.04
64ALA	CA	64ALA	CB	100.00	51GLU	CG	52PRO	CD	1.73	65GLL	C	66ALA	CA	100.00
64ALA	CA	64ALA	C	100.00	51GLU	CG	53PHE	C	0.49	65GLL	C	66ALA	CB	99.90
64ALA	CB	64ALA	C	100.00	51GLU	CG	54PRO	CA	0.22	65GLL	C	66ALA	C	0.14
64ALA	C	65GLU	CA	100.00	51GLU	CG	54PRO	CB	0.01	65GLL	C	266THR	CG2	8.30
64ALA	C	65GLU	CB	7.72	51GLU	CG	84ILE	CG2	0.02	66ALA	CA	66ALA	CB	100.00
64ALA	C	65GLU	CG	0.01	51GLU	CD	51GLU	C	0.11	66ALA	CA	66ALA	C	100.00
64ALA	C	65GLU	CD	0.00	51GLU	CD	52PRO	CD	0.13	66ALA	CA	266THR	CG2	6.17
64ALA	C	65GLU	C	93.54	51GLU	CD	53PHE	C	0.01	66ALA	CB	66ALA	C	100.00
64ALA	C	266THR	CG2	0.29	51GLU	CD	54PRO	CA	0.00	66ALA	CB	269PHE	CE1	0.80
65GLU	CA	65GLU	CB	100.00	51GLU	CD	83LYS	CE	0.03	66ALA	CB	297MET	CG	4.19
65GLU	CA	65GLU	CG	100.00	51GLU	CD	92SER	CB	0.00	66ALA	CB	297MET	CE	0.38
65GLU	CA	65GLU	CD	98.30	51GLU	C	52PRO	CA	100.00	66ALA	CB	301ALA	CB	0.18
65GLU	CA	65GLU	C	100.00	51GLU	C	52PRO	CB	0.09	66ALA	CB	302PHE	CE1	0.16
65GLU	CB	65GLU	CG	100.00	51GLU	C	52PRO	CD	100.00	66ALA	CB	302PHE	CE2	0.74
65GLU	CB	65GLU	CD	100.00	51GLU	C	52PRO	C	100.00	66ALA	CB	302PHE	CZ	0.94
65GLU	CB	65GLU	C	100.00	52PRO	CA	52PRO	CB	100.00	66ALA	C	67VAL	CA	100.00
65GLU	CB	302PHE	CE1	0.22	52PRO	CA	52PRO	CG	100.00	66ALA	C	67VAL	CB	29.70
65GLU	CB	302PHE	CE2	0.01	52PRO	CA	52PRO	CD	100.00	66ALA	C	67VAL	CG2	20.10
65GLU	CB	302PHE	CZ	0.00	52PRO	CA	52PRO	C	100.00	66ALA	C	67VAL	C	81.64
65GLU	CG	65GLU	CD	100.00	52PRO	CB	52PRO	CG	100.00	66ALA	C	266THR	CG2	1.60
65GLU	CG	65GLU	C	90.85	52PRO	CB	52PRO	CD	100.00	66ALA	C	297MET	CE	0.00
65GLU	CG	301ALA	CB	0.00	52PRO	CB	52PRO	C	100.00	67VAL	CA	67VAL	CB	100.00
65GLU	CG	302PHE	CD1	0.18	52PRO	CB	86PRO	CA	0.39	67VAL	CA	67VAL	CG1	100.00
65GLU	CG	302PHE	CD2	0.00	52PRO	CB	86PRO	CB	0.60	67VAL	CA	67VAL	CG2	100.00
65GLU	CG	302PHE	CE1	1.24	52PRO	CB	89GLY	CA	0.19	67VAL	CA	67VAL	C	100.00
65GLU	CG	302PHE	CE2	0.04	52PRO	CG	52PRO	CD	100.00	67VAL	CB	67VAL	CG1	100.00
65GLU	CG	302PHE	CZ	0.01	52PRO	CG	52PRO	C	90.21	67VAL	CB	67VAL	CG2	100.00
65GLU	CD	65GLU	C	0.00	52PRO	CG	84ILE	C	0.01	67VAL	CB	67VAL	C	100.00
65GLU	C	66ALA	CA	100.00	52PRO	CG	88THR	CG2	0.00	67VAL	CB	268VAL	CG1	0.00

65GLU	C	66ALA	CB	99.92	52PRO	CG	88THR	C	0.68	67VAL	CB	268VAL	CG2	0.13
65GLU	C	66ALA	C	0.04	52PRO	CG	89GLY	CA	3.06	67VAL	CG1	67VAL	CG2	100.00
65GLU	C	266THR	CG2	5.91	52PRO	CD	52PRO	C	68.93	67VAL	CG1	67VAL	C	100.00
66ALA	CA	66ALA	CB	100.00	52PRO	CD	83LYS	CG	0.00	67VAL	CG1	68LEU	C	0.09
66ALA	CA	66ALA	C	100.00	52PRO	CD	84ILE	CB	0.00	67VAL	CG1	69LEU	CD1	0.92
66ALA	CA	266THR	CG2	8.74	52PRO	CD	84ILE	CG2	0.04	67VAL	CG1	69LEU	CD2	0.00
66ALA	CB	66ALA	C	100.00	52PRO	C	53PHE	CA	100.00	67VAL	CG1	268VAL	CG1	0.11
66ALA	CB	267PRO	CG	0.00	52PRO	C	53PHE	CB	81.33	67VAL	CG1	268VAL	CG2	0.02
66ALA	CB	269PHE	CD1	0.02	52PRO	C	53PHE	C	45.45	67VAL	CG2	266THR	CG2	5.91
66ALA	CB	269PHE	CE1	2.27	52PRO	C	54PRO	CD	10.93	67VAL	CG2	268VAL	CG2	0.42
66ALA	CB	297MET	CG	13.14	53PHE	CA	53PHE	CB	100.00	67VAL	C	68LEU	CA	100.00
66ALA	CB	297MET	CE	0.16	53PHE	CA	53PHE	CG	100.00	67VAL	C	68LEU	CB	6.67
66ALA	CB	301ALA	CB	0.41	53PHE	CA	53PHE	CD1	98.62	67VAL	C	68LEU	C	98.88
66ALA	CB	302PHE	CE1	0.30	53PHE	CA	53PHE	CD2	8.02	67VAL	C	297MET	CE	1.94
66ALA	CB	302PHE	CZ	0.03	53PHE	CA	53PHE	C	100.00	68LEU	CA	68LEU	CB	100.00
66ALA	C	67VAL	CA	100.00	53PHE	CA	54PRO	CD	100.00	68LEU	CA	68LEU	CG	100.00
66ALA	C	67VAL	CB	21.06	53PHE	CB	53PHE	CG	100.00	68LEU	CA	68LEU	CD1	80.91
66ALA	C	67VAL	CG2	13.22	53PHE	CB	53PHE	CD1	100.00	68LEU	CA	68LEU	CD2	18.81
66ALA	C	67VAL	C	87.74	53PHE	CB	53PHE	CD2	100.00	68LEU	CA	68LEU	C	100.00
66ALA	C	266THR	CG2	3.18	53PHE	CB	53PHE	C	100.00	68LEU	CA	297MET	CE	0.02
67VAL	CA	67VAL	CB	100.00	53PHE	CB	89GLY	CA	1.72	68LEU	CB	68LEU	CG	100.00
67VAL	CA	67VAL	CG1	100.00	53PHE	CB	89GLY	C	0.00	68LEU	CB	68LEU	CD1	100.00
67VAL	CA	67VAL	CG2	100.00	53PHE	CB	92SER	CB	0.43	68LEU	CB	68LEU	CD2	100.00
67VAL	CA	67VAL	C	100.00	53PHE	CG	53PHE	CD1	100.00	68LEU	CB	68LEU	C	100.00
67VAL	CB	67VAL	CG1	100.00	53PHE	CG	53PHE	CD2	100.00	68LEU	CB	297MET	CE	0.07
67VAL	CB	67VAL	CG2	100.00	53PHE	CG	53PHE	CE1	100.00	68LEU	CG	68LEU	CD1	100.00
67VAL	CB	67VAL	C	100.00	53PHE	CG	53PHE	CE2	100.00	68LEU	CG	68LEU	CD2	100.00
67VAL	CB	268VAL	CG1	0.00	53PHE	CG	53PHE	CZ	100.00	68LEU	CG	68LEU	C	85.06
67VAL	CB	268VAL	CG2	0.12	53PHE	CG	53PHE	C	99.94	68LEU	CG	297MET	CE	0.20
67VAL	CG1	67VAL	CG2	100.00	53PHE	CG	57THR	CG2	0.00	68LEU	CD1	68LEU	CD2	100.00
67VAL	CG1	67VAL	C	100.00	53PHE	CG	58ARG	CG	0.01	68LEU	CD1	68LEU	C	1.62
67VAL	CG1	68LEU	C	0.08	53PHE	CG	92SER	CB	0.15	68LEU	CD1	269PHE	CB	0.00
67VAL	CG1	69LEU	CD1	0.14	53PHE	CD1	53PHE	CD2	100.00	68LEU	CD1	293SER	CB	0.37
67VAL	CG1	69LEU	CD2	0.06	53PHE	CD1	53PHE	CE1	100.00	68LEU	CD1	293SER	C	0.04
67VAL	CG1	268VAL	CG1	0.10	53PHE	CD1	53PHE	CE2	100.00	68LEU	CD1	294ALA	CA	0.03
67VAL	CG1	268VAL	CG2	0.01	53PHE	CD1	53PHE	CZ	100.00	68LEU	CD1	294ALA	CB	0.01
67VAL	CG2	266THR	CB	0.01	53PHE	CD1	53PHE	C	4.04	68LEU	CD1	297MET	CE	0.44
67VAL	CG2	266THR	CG2	7.34	53PHE	CD1	57THR	CB	0.00	68LEU	CD2	68LEU	C	4.16
67VAL	CG2	268VAL	CG2	0.22	53PHE	CD1	57THR	CG2	3.00	68LEU	CD2	293SER	CB	0.01
67VAL	C	68LEU	CA	100.00	53PHE	CD1	58ARG	CG	0.05	68LEU	CD2	293SER	C	0.01
67VAL	C	68LEU	CB	5.89	53PHE	CD1	89GLY	CA	0.00	68LEU	CD2	294ALA	CA	0.00
67VAL	C	68LEU	CD1	0.00	53PHE	CD1	93LEU	CB	0.02	68LEU	CD2	294ALA	CB	0.00
67VAL	C	68LEU	C	99.12	53PHE	CD1	93LEU	CG	0.01	68LEU	CD2	297MET	CE	0.14
67VAL	C	297MET	CE	0.20	53PHE	CD1	93LEU	CD1	0.14	68LEU	C	69LEU	CA	100.00
68LEU	CA	68LEU	CB	100.00	53PHE	CD1	93LEU	CD2	0.02	68LEU	C	69LEU	CB	23.66
68LEU	CA	68LEU	CG	100.00	53PHE	CD2	53PHE	CE1	100.00	68LEU	C	69LEU	C	84.65
68LEU	CA	68LEU	CD1	61.63	53PHE	CD2	53PHE	CE2	100.00	69LEU	CA	69LEU	CB	100.00
68LEU	CA	68LEU	CD2	38.02	53PHE	CD2	53PHE	CZ	100.00	69LEU	CA	69LEU	CG	100.00
68LEU	CA	68LEU	C	100.00	53PHE	CD2	53PHE	C	3.38	69LEU	CA	69LEU	CD1	95.42
68LEU	CB	68LEU	CG	100.00	53PHE	CD2	58ARG	CB	0.02	69LEU	CA	69LEU	CD2	1.42
68LEU	CB	68LEU	CD1	100.00	53PHE	CD2	58ARG	CG	2.83	69LEU	CA	69LEU	C	100.00
68LEU	CB	68LEU	CD2	100.00	53PHE	CD2	58ARG	CD	0.16	69LEU	CB	69LEU	CG	100.00
68LEU	CB	68LEU	C	100.00	53PHE	CD2	58ARG	CZ	1.21	69LEU	CB	69LEU	CD1	100.00
68LEU	CB	297MET	CE	0.01	53PHE	CD2	92SER	CB	7.88	69LEU	CB	69LEU	CD2	100.00
68LEU	CG	68LEU	CD1	100.00	53PHE	CD2	92SER	C	0.22	69LEU	CB	69LEU	C	100.00
68LEU	CG	68LEU	CD2	100.00	53PHE	CD2	93LEU	CA	0.03	69LEU	CB	268VAL	CG1	3.00
68LEU	CG	68LEU	C	63.81	53PHE	CD2	93LEU	CB	0.00	69LEU	CB	270GLU	CB	0.05
68LEU	CG	297MET	CE	0.06	53PHE	CE1	53PHE	CE2	100.00	69LEU	CG	69LEU	CD1	100.00
68LEU	CD1	68LEU	CD2	100.00	53PHE	CE1	53PHE	CZ	100.00	69LEU	CG	69LEU	CD2	100.00
68LEU	CD1	68LEU	C	0.60	53PHE	CE1	57THR	CG2	3.57	69LEU	CG	69LEU	C	96.80
68LEU	CD1	269PHE	CB	0.01	53PHE	CE1	57THR	C	0.25	69LEU	CG	268VAL	CG1	0.03
68LEU	CD1	294ALA	CA	0.03	53PHE	CE1	58ARG	CA	1.00	69LEU	CD1	69LEU	CD2	100.00

68LEU	CD1	294ALA	CB	0.05	53PHE	CE1	58ARG	CB	0.02	69LEU	CD1	69LEU	C	0.17
68LEU	CD1	297MET	CE	0.73	53PHE	CE1	58ARG	CG	0.26	69LEU	CD1	90LEU	CD1	0.10
68LEU	CD2	68LEU	C	2.73	53PHE	CE1	61VAL	CG1	0.01	69LEU	CD1	90LEU	CD2	0.05
68LEU	CD2	294ALA	CA	0.00	53PHE	CE1	61VAL	CG2	0.04	69LEU	CD1	93LEU	CD1	0.69
68LEU	CD2	294ALA	CB	0.06	53PHE	CE1	93LEU	CA	0.12	69LEU	CD1	93LEU	CD2	7.46
68LEU	CD2	297MET	CE	0.18	53PHE	CE1	93LEU	CB	0.48	69LEU	CD1	268VAL	CG1	0.37
68LEU	C	69LEU	CA	100.00	53PHE	CE1	93LEU	CG	1.02	69LEU	CD1	270GLU	CD	0.00
68LEU	C	69LEU	CB	28.55	53PHE	CE1	93LEU	CD1	10.18	69LEU	CD2	69LEU	C	0.81
68LEU	C	69LEU	C	83.01	53PHE	CE1	93LEU	CD2	0.66	69LEU	CD2	70GLY	C	0.10
69LEU	CA	69LEU	CB	100.00	53PHE	CE2	53PHE	CZ	100.00	69LEU	CD2	90LEU	CD1	0.87
69LEU	CA	69LEU	CG	100.00	53PHE	CE2	57THR	C	0.00	69LEU	CD2	90LEU	CD2	1.09
69LEU	CA	69LEU	CD1	90.08	53PHE	CE2	58ARG	CA	1.01	69LEU	CD2	93LEU	CD1	0.01
69LEU	CA	69LEU	CD2	6.34	53PHE	CE2	58ARG	CB	1.78	69LEU	CD2	93LEU	CD2	0.79
69LEU	CA	69LEU	C	100.00	53PHE	CE2	58ARG	CG	8.39	69LEU	CD2	268VAL	CG1	11.59
69LEU	CB	69LEU	CG	100.00	53PHE	CE2	58ARG	CD	0.50	69LEU	CD2	270GLU	CB	0.13
69LEU	CB	69LEU	CD1	100.00	53PHE	CE2	58ARG	CZ	0.09	69LEU	CD2	270GLU	CG	0.65
69LEU	CB	69LEU	CD2	100.00	53PHE	CE2	92SER	CB	0.13	69LEU	CD2	270GLU	CD	3.57
69LEU	CB	69LEU	C	100.00	53PHE	CE2	92SER	C	0.59	69LEU	C	70GLY	CA	100.00
69LEU	CB	268VAL	CG1	3.06	53PHE	CE2	93LEU	CA	3.08	69LEU	C	70GLY	C	17.19
69LEU	CB	270GLU	CB	0.02	53PHE	CE2	93LEU	CB	0.05	69LEU	C	271PRO	CD	0.02
69LEU	CB	270GLU	CG	0.04	53PHE	CE2	93LEU	CD1	0.01	70GLY	CA	70GLY	C	100.00
69LEU	CG	69LEU	CD1	100.00	53PHE	CE2	96SER	CB	1.33	70GLY	CA	271PRO	CD	0.01
69LEU	CG	69LEU	CD2	100.00	53PHE	CZ	57THR	CG2	0.01	70GLY	C	71SER	CA	100.00
69LEU	CG	69LEU	C	94.30	53PHE	CZ	57THR	C	0.03	70GLY	C	71SER	CB	6.22
69LEU	CG	268VAL	CG1	0.10	53PHE	CZ	58ARG	CA	4.73	70GLY	C	71SER	C	94.19
69LEU	CG	270GLU	CG	0.00	53PHE	CZ	58ARG	CB	0.78	71SEF	CA	71SER	CB	100.00
69LEU	CD1	69LEU	CD2	100.00	53PHE	CZ	58ARG	CG	1.11	71SEF	CA	71SER	C	100.00
69LEU	CD1	69LEU	C	0.40	53PHE	CZ	61VAL	CG1	0.00	71SEF	CB	71SER	C	100.00
69LEU	CD1	90LEU	CD2	0.20	53PHE	CZ	61VAL	CG2	0.24	71SEF	CB	273HIS	CB	0.08
69LEU	CD1	93LEU	CD1	0.28	53PHE	CZ	93LEU	CA	0.98	71SEF	CB	273HIS	CE1	0.02
69LEU	CD1	93LEU	CD2	1.57	53PHE	CZ	93LEU	CB	0.18	71SEF	CB	273HIS	C	0.17
69LEU	CD1	268VAL	CG1	1.87	53PHE	CZ	93LEU	CG	0.12	71SEF	CB	274GLY	CA	0.01
69LEU	CD1	270GLU	CB	0.01	53PHE	CZ	93LEU	CD1	5.10	71SEF	CB	274GLY	C	1.22
69LEU	CD1	270GLU	CG	0.03	53PHE	CZ	93LEU	CD2	0.18	71SEF	CB	275SER	CB	0.01
69LEU	CD1	270GLU	CD	0.05	53PHE	CZ	96SER	CB	0.30	71SEF	C	72VAL	CA	100.00
69LEU	CD2	69LEU	C	1.23	53PHE	C	54PRO	CA	100.00	71SEF	C	72VAL	CB	76.00
69LEU	CD2	70GLY	C	0.01	53PHE	C	54PRO	CB	2.14	71SEF	C	72VAL	CG1	43.70
69LEU	CD2	90LEU	CD2	0.91	53PHE	C	54PRO	CD	100.00	71SEF	C	72VAL	CG2	0.14
69LEU	CD2	93LEU	CD1	0.07	53PHE	C	54PRO	C	99.98	71SEF	C	72VAL	C	23.52
69LEU	CD2	93LEU	CD2	0.42	54PRO	CA	54PRO	CB	100.00	71SEF	C	275SER	CB	0.01
69LEU	CD2	268VAL	CG1	9.54	54PRO	CA	54PRO	CG	100.00	72VAL	CA	72VAL	CB	100.00
69LEU	CD2	270GLU	CB	0.08	54PRO	CA	54PRO	CD	100.00	72VAL	CA	72VAL	CG1	100.00
69LEU	CD2	270GLU	CG	0.52	54PRO	CA	54PRO	C	100.00	72VAL	CA	72VAL	CG2	100.00
69LEU	CD2	270GLU	CD	3.82	54PRO	CB	54PRO	CG	100.00	72VAL	CA	72VAL	C	100.00
69LEU	C	70GLY	CA	100.00	54PRO	CB	54PRO	CD	100.00	72VAL	CB	72VAL	CG1	100.00
69LEU	C	70GLY	C	44.09	54PRO	CB	54PRO	C	100.00	72VAL	CB	72VAL	CG2	100.00
69LEU	C	270GLU	CG	0.00	54PRO	CB	56PRO	CD	0.00	72VAL	CB	72VAL	C	100.00
70GLY	CA	70GLY	C	100.00	54PRO	CG	54PRO	CD	100.00	72VAL	CB	86PRO	CB	0.10
70GLY	CA	271PRO	CD	0.00	54PRO	CG	54PRO	C	99.86	72VAL	CG1	72VAL	CG2	100.00
70GLY	C	71SER	CA	100.00	54PRO	CD	54PRO	C	98.12	72VAL	CG1	72VAL	C	0.35
70GLY	C	71SER	CB	4.83	54PRO	CD	57THR	CB	0.06	72VAL	CG1	86PRO	CB	0.06
70GLY	C	71SER	C	95.81	54PRO	C	55GLU	CA	100.00	72VAL	CG1	86PRO	CG	0.04
70GLY	C	271PRO	CG	0.01	54PRO	C	55GLU	C	100.00	72VAL	CG1	86PRO	C	0.03
71SER	CA	71SER	CB	100.00	54PRO	C	56PRO	CD	24.76	72VAL	CG2	72VAL	C	99.49
71SER	CA	71SER	C	100.00	54PRO	C	58ARG	CB	0.00	72VAL	CG2	86PRO	CB	3.16
71SER	CB	71SER	C	100.00	54PRO	C	58ARG	CG	0.02	72VAL	CG2	86PRO	CG	0.05
71SER	CB	273HIS	CE1	0.64	54PRO	C	58ARG	CD	0.02	72VAL	CG2	86PRO	C	0.06
71SER	CB	274GLY	CA	0.04	54PRO	C	58ARG	CZ	0.74	72VAL	CG2	90LEU	CG	0.04
71SER	CB	274GLY	C	2.63	55GLU	CA	55GLU	CB	100.00	72VAL	CG2	90LEU	CD1	2.11
71SER	CB	275SER	CA	0.05	55GLU	CA	55GLU	CG	100.00	72VAL	CG2	90LEU	CD2	0.32
71SER	CB	275SER	CB	0.15	55GLU	CA	55GLU	CD	95.23	72VAL	C	73GLY	CA	100.00
71SER	C	72VAL	CA	100.00	55GLU	CA	55GLU	C	100.00	72VAL	C	73GLY	C	37.11

71SER	C	72VAL	CB	83.34	55GLU	CA	56PRO	CD	100.00	72VAL	C	86PRO	CG	0.01
71SER	C	72VAL	CG1	21.00	55GLU	CA	58ARG	CZ	0.85	72VAL	C	87GLU	CG	0.00
71SER	C	72VAL	CG2	4.11	55GLU	CB	55GLU	CG	100.00	72VAL	C	275SER	CB	3.38
71SER	C	72VAL	C	15.45	55GLU	CB	55GLU	CD	100.00	73GLY	CA	73GLY	C	100.00
71SER	C	275SER	CB	0.59	55GLU	CB	55GLU	C	100.00	73GLY	CA	77TRP	CG	0.00
72VAL	CA	72VAL	CB	100.00	55GLU	CB	56PRO	CD	67.41	73GLY	CA	77TRP	CE3	0.13
72VAL	CA	72VAL	CG1	100.00	55GLU	CG	55GLU	CD	100.00	73GLY	CA	77TRP	CZ3	0.01
72VAL	CA	72VAL	CG2	100.00	55GLU	CG	55GLU	C	92.18	73GLY	CA	85ARG	CZ	0.01
72VAL	CA	72VAL	C	100.00	55GLU	CG	56PRO	CD	0.06	73GLY	CA	86PRO	CB	0.04
72VAL	CA	275SER	CB	0.07	55GLU	CG	59LYS	CD	0.00	73GLY	CA	86PRO	CG	1.14
72VAL	CB	72VAL	CG1	100.00	55GLU	CG	59LYS	CE	0.03	73GLY	CA	87GLU	CG	0.00
72VAL	CB	72VAL	CG2	100.00	55GLU	CD	55GLU	C	3.94	73GLY	CA	87GLU	CD	0.06
72VAL	CB	72VAL	C	100.00	55GLU	CD	58ARG	CD	0.00	73GLY	CA	275SER	CB	0.04
72VAL	CB	77TRP	CE2	0.00	55GLU	CD	58ARG	CZ	0.05	73GLY	C	74GLY	CA	100.00
72VAL	CB	77TRP	CZ2	1.54	55GLU	CD	59LYS	CD	0.05	73GLY	C	74GLY	C	28.22
72VAL	CB	77TRP	CZ3	0.00	55GLU	CD	59LYS	CE	0.82	73GLY	C	77TRP	CB	0.08
72VAL	CB	86PRO	CB	0.10	55GLU	C	56PRO	CA	100.00	73GLY	C	77TRP	CE3	3.46
72VAL	CB	86PRO	CG	0.04	55GLU	C	56PRO	CB	0.09	73GLY	C	77TRP	CZ3	0.60
72VAL	CG1	72VAL	CG2	100.00	55GLU	C	56PRO	CD	100.00	73GLY	C	275SER	CB	1.19
72VAL	CG1	72VAL	C	30.45	55GLU	C	56PRO	C	100.00	74GLY	CA	74GLY	C	100.00
72VAL	CG1	77TRP	CD2	0.12	56PRO	CA	56PRO	CB	100.00	74GLY	CA	75PRO	CD	100.00
72VAL	CG1	77TRP	CE2	1.34	56PRO	CA	56PRO	CG	100.00	74GLY	CA	77TRP	CE3	0.02
72VAL	CG1	77TRP	CE3	0.08	56PRO	CA	56PRO	CD	100.00	74GLY	CA	77TRP	CZ3	0.01
72VAL	CG1	77TRP	CZ2	8.09	56PRO	CA	56PRO	C	100.00	74GLY	CA	275SER	CB	0.04
72VAL	CG1	77TRP	CZ3	0.17	56PRO	CA	59LYS	CG	0.00	74GLY	CA	277PRO	CG	0.02
72VAL	CG1	86PRO	CB	0.74	56PRO	CB	56PRO	CG	100.00	74GLY	CA	277PRO	CD	0.40
72VAL	CG1	86PRO	CG	0.30	56PRO	CB	56PRO	CD	100.00	74GLY	C	75PRO	CA	100.00
72VAL	CG1	90LEU	CG	0.01	56PRO	CB	56PRO	C	100.00	74GLY	C	75PRO	CB	0.02
72VAL	CG1	90LEU	CD1	0.18	56PRO	CG	56PRO	CD	100.00	74GLY	C	75PRO	CD	100.00
72VAL	CG1	90LEU	CD2	0.04	56PRO	CG	56PRO	C	88.62	74GLY	C	75PRO	C	100.00
72VAL	CG2	72VAL	C	91.47	56PRO	CD	56PRO	C	73.56	74GLY	C	77TRP	CE3	0.05
72VAL	CG2	77TRP	CD1	0.00	56PRO	C	57THR	CA	100.00	74GLY	C	78ASP	CB	0.03
72VAL	CG2	77TRP	CD2	0.01	56PRO	C	57THR	CB	0.07	75PRC	CA	75PRO	CB	100.00
72VAL	CG2	77TRP	CE2	1.29	56PRO	C	57THR	C	99.99	75PRC	CA	75PRO	CG	100.00
72VAL	CG2	77TRP	CE3	0.00	57THR	CA	57THR	CB	100.00	75PRC	CA	75PRO	CD	100.00
72VAL	CG2	77TRP	CZ2	4.56	57THR	CA	57THR	CG2	100.00	75PRC	CA	75PRO	C	100.00
72VAL	CG2	77TRP	CZ3	0.04	57THR	CA	57THR	C	100.00	75PRC	CA	78ASP	CB	0.08
72VAL	CG2	86PRO	CB	7.66	57THR	CB	57THR	CG2	100.00	75PRC	CB	75PRO	CG	100.00
72VAL	CG2	86PRO	CG	1.64	57THR	CB	57THR	C	100.00	75PRC	CB	75PRO	CD	100.00
72VAL	CG2	90LEU	CG	0.02	57THR	CG2	57THR	C	99.99	75PRC	CB	75PRO	C	100.00
72VAL	CG2	90LEU	CD1	1.16	57THR	CG2	61VAL	CG1	0.05	75PRC	CG	75PRO	CD	100.00
72VAL	CG2	90LEU	CD2	0.05	57THR	C	58ARG	CA	100.00	75PRC	CG	75PRO	C	50.66
72VAL	C	73GLY	CA	100.00	57THR	C	58ARG	CB	0.02	75PRC	CG	276ALA	CB	0.03
72VAL	C	73GLY	C	31.31	57THR	C	58ARG	C	100.00	75PRC	CG	277PRO	CB	0.27
72VAL	C	86PRO	CB	0.02	58ARG	CA	58ARG	CB	100.00	75PRC	CG	277PRO	CG	0.96
72VAL	C	86PRO	CG	0.02	58ARG	CA	58ARG	CG	100.00	75PRC	CG	277PRO	CD	2.47
72VAL	C	274GLY	CA	0.00	58ARG	CA	58ARG	CD	0.02	75PRC	CD	75PRO	C	42.17
72VAL	C	274GLY	C	0.00	58ARG	CA	58ARG	C	100.00	75PRC	CD	77TRP	CE3	0.00
72VAL	C	275SER	CB	5.63	58ARG	CA	61VAL	CG2	0.00	75PRC	CD	276ALA	CA	0.06
73GLY	CA	73GLY	C	100.00	58ARG	CB	58ARG	CG	100.00	75PRC	CD	276ALA	CB	0.14
73GLY	CA	77TRP	CB	0.10	58ARG	CB	58ARG	CD	100.00	75PRC	CD	277PRO	CB	0.12
73GLY	CA	77TRP	CG	2.00	58ARG	CB	58ARG	CZ	2.26	75PRC	CD	277PRO	CG	0.93
73GLY	CA	77TRP	CD1	15.54	58ARG	CB	58ARG	C	100.00	75PRC	CD	277PRO	CD	7.80
73GLY	CA	77TRP	CD2	0.01	58ARG	CG	58ARG	CD	100.00	75PRC	C	76LYS	CA	100.00
73GLY	CA	86PRO	CB	0.10	58ARG	CG	58ARG	CZ	27.48	75PRC	C	76LYS	CB	42.37
73GLY	CA	86PRO	CG	0.64	58ARG	CG	58ARG	C	62.36	75PRC	C	76LYS	CG	3.06
73GLY	CA	275SER	CB	0.02	58ARG	CG	96SER	CB	0.00	75PRC	C	76LYS	CD	0.08
73GLY	C	74GLY	CA	100.00	58ARG	CD	58ARG	CZ	100.00	75PRC	C	76LYS	C	97.29
73GLY	C	74GLY	C	19.98	58ARG	CD	58ARG	C	0.01	75PRC	C	78ASP	CB	0.00
73GLY	C	77TRP	CB	0.05	58ARG	CZ	58ARG	C	0.01	76LYS	CA	76LYS	CB	100.00
73GLY	C	77TRP	CG	0.10	58ARG	CZ	59LYS	CG	0.02	76LYS	CA	76LYS	CG	100.00
73GLY	C	77TRP	CD1	40.78	58ARG	C	59LYS	CA	100.00	76LYS	CA	76LYS	CD	2.50

73GLY	C	77TRP	CE3	0.07	58ARG	C	59LYS	CB	17.90	76LYS	CA	76LYS	CE	0.01
73GLY	C	77TRP	CZ3	0.00	58ARG	C	59LYS	CG	0.77	76LYS	CA	76LYS	C	100.00
73GLY	C	275SER	CB	0.30	58ARG	C	59LYS	CD	0.02	76LYS	CA	77TRP	CE3	0.00
73GLY	C	275SER	C	0.02	58ARG	C	59LYS	C	82.01	76LYS	CB	76LYS	CG	100.00
74GLY	CA	74GLY	C	100.00	58ARG	C	61VAL	CG2	0.02	76LYS	CB	76LYS	CD	100.00
74GLY	CA	75PRO	CD	100.00	59LYS	CA	59LYS	CB	100.00	76LYS	CB	76LYS	CE	17.79
74GLY	CA	275SER	CB	0.00	59LYS	CA	59LYS	CG	100.00	76LYS	CB	76LYS	C	100.00
74GLY	CA	275SER	C	0.00	59LYS	CA	59LYS	CD	7.62	76LYS	CB	77TRP	CD2	0.21
74GLY	CA	276ALA	CA	0.01	59LYS	CA	59LYS	CE	0.01	76LYS	CB	77TRP	CE2	0.26
74GLY	CA	276ALA	CB	0.27	59LYS	CA	59LYS	C	100.00	76LYS	CB	77TRP	CE3	2.91
74GLY	CA	277PRO	CD	0.02	59LYS	CA	62GLU	CB	0.01	76LYS	CB	77TRP	CZ2	0.02
74GLY	C	75PRO	CA	100.00	59LYS	CA	62GLU	CG	0.01	76LYS	CB	77TRP	CZ3	0.69
74GLY	C	75PRO	CB	0.01	59LYS	CB	59LYS	CG	100.00	76LYS	CG	76LYS	CD	100.00
74GLY	C	75PRO	CD	100.00	59LYS	CB	59LYS	CD	100.00	76LYS	CG	76LYS	CE	100.00
74GLY	C	75PRO	C	100.00	59LYS	CB	59LYS	CE	14.38	76LYS	CG	76LYS	C	71.54
75PRO	CA	75PRO	CB	100.00	59LYS	CB	59LYS	C	100.00	76LYS	CG	77TRP	CD1	0.00
75PRO	CA	75PRO	CG	100.00	59LYS	CG	59LYS	CD	100.00	76LYS	CG	77TRP	CD2	0.04
75PRO	CA	75PRO	CD	100.00	59LYS	CG	59LYS	CE	100.00	76LYS	CG	77TRP	CE2	0.06
75PRO	CA	75PRO	C	100.00	59LYS	CG	59LYS	C	31.94	76LYS	CG	77TRP	CE3	0.88
75PRO	CA	78ASP	CB	0.01	59LYS	CD	59LYS	CE	100.00	76LYS	CG	77TRP	CZ2	0.01
75PRO	CB	75PRO	CG	100.00	59LYS	CD	59LYS	C	0.30	76LYS	CG	77TRP	CZ3	0.51
75PRO	CB	75PRO	CD	100.00	59LYS	CE	59LYS	C	0.00	76LYS	CG	80LEU	CD1	0.02
75PRO	CB	75PRO	C	100.00	59LYS	C	60GLY	CA	100.00	76LYS	CG	277PRO	CG	0.00
75PRO	CB	277PRO	CG	0.00	59LYS	C	60GLY	C	69.70	76LYS	CD	76LYS	CE	100.00
75PRO	CG	75PRO	CD	100.00	59LYS	C	62GLU	CB	0.08	76LYS	CD	76LYS	C	0.13
75PRO	CG	75PRO	C	52.39	59LYS	C	62GLU	CG	0.05	76LYS	CD	77TRP	CD2	0.01
75PRO	CG	277PRO	CB	0.06	60GLY	CA	60GLY	C	100.00	76LYS	CD	77TRP	CE2	0.06
75PRO	CG	277PRO	CG	0.33	60GLY	CA	63GLU	CB	0.02	76LYS	CD	77TRP	CE3	0.00
75PRO	CG	277PRO	CD	0.44	60GLY	C	61VAL	CA	100.00	76LYS	CD	77TRP	CZ2	0.50
75PRO	CD	75PRO	C	32.23	60GLY	C	61VAL	CB	0.36	76LYS	CD	77TRP	CZ3	0.56
75PRO	CD	276ALA	CB	0.53	60GLY	C	61VAL	CG1	0.10	76LYS	CD	277PRO	CG	0.00
75PRO	CD	277PRO	CB	0.30	60GLY	C	61VAL	C	99.71	76LYS	CE	77TRP	CD1	0.00
75PRO	CD	277PRO	CG	1.49	60GLY	C	63GLU	CB	0.02	76LYS	CE	77TRP	CE2	0.14
75PRO	CD	277PRO	CD	7.76	61VAL	CA	61VAL	CB	100.00	76LYS	CE	77TRP	CE3	0.04
75PRO	C	76LYS	CA	100.00	61VAL	CA	61VAL	CG1	100.00	76LYS	CE	77TRP	CZ2	1.17
75PRO	C	76LYS	CB	0.52	61VAL	CA	61VAL	CG2	100.00	76LYS	CE	77TRP	CZ3	1.52
75PRO	C	76LYS	CG	0.04	61VAL	CA	61VAL	C	100.00	76LYS	CE	80LEU	CD1	0.01
75PRO	C	76LYS	C	99.57	61VAL	CA	64ALA	CB	0.00	76LYS	C	77TRP	CA	100.00
76LYS	CA	76LYS	CB	100.00	61VAL	CA	67VAL	CG2	0.02	76LYS	C	77TRP	CB	46.71
76LYS	CA	76LYS	CG	100.00	61VAL	CB	61VAL	CG1	100.00	76LYS	C	77TRP	CG	17.71
76LYS	CA	76LYS	CD	4.11	61VAL	CB	61VAL	CG2	100.00	76LYS	C	77TRP	CD1	0.20
76LYS	CA	76LYS	C	100.00	61VAL	CB	61VAL	C	100.00	76LYS	C	77TRP	CD2	2.05
76LYS	CB	76LYS	CG	100.00	61VAL	CB	67VAL	CG2	0.58	76LYS	C	77TRP	CE3	0.75
76LYS	CB	76LYS	CD	100.00	61VAL	CB	97GLN	CG	0.00	76LYS	C	77TRP	C	47.53
76LYS	CB	76LYS	CE	33.96	61VAL	CB	97GLN	CD	0.01	76LYS	C	80LEU	CD1	0.04
76LYS	CB	76LYS	C	100.00	61VAL	CG1	61VAL	CG2	100.00	76LYS	C	80LEU	CD2	0.22
76LYS	CB	77TRP	CD1	0.02	61VAL	CG1	67VAL	CG1	0.50	77TRP	CA	77TRP	CB	100.00
76LYS	CB	77TRP	CD2	0.06	61VAL	CG1	67VAL	CG2	3.06	77TRP	CA	77TRP	CG	100.00
76LYS	CB	77TRP	CE2	0.06	61VAL	CG1	93LEU	CD1	0.27	77TRP	CA	77TRP	CD1	90.59
76LYS	CB	77TRP	CE3	0.05	61VAL	CG1	93LEU	CD2	0.01	77TRP	CA	77TRP	CD2	8.95
76LYS	CB	77TRP	CZ2	0.01	61VAL	CG2	61VAL	C	99.86	77TRP	CA	77TRP	CE3	0.00
76LYS	CB	77TRP	CZ3	0.01	61VAL	CG2	93LEU	CD1	0.10	77TRP	CA	77TRP	C	100.00
76LYS	CG	76LYS	CD	100.00	61VAL	CG2	93LEU	CD2	0.00	77TRP	CA	80LEU	CD1	0.10
76LYS	CG	76LYS	CE	100.00	61VAL	CG2	97GLN	CG	1.11	77TRP	CB	77TRP	CG	100.00
76LYS	CG	76LYS	C	58.66	61VAL	CG2	97GLN	CD	0.35	77TRP	CB	77TRP	CD1	100.00
76LYS	CG	77TRP	CD1	0.90	61VAL	C	62GLU	CA	100.00	77TRP	CB	77TRP	CD2	100.00
76LYS	CG	77TRP	CE2	0.04	61VAL	C	62GLU	CB	2.46	77TRP	CB	77TRP	CE3	97.84
76LYS	CG	77TRP	CE3	0.00	61VAL	C	62GLU	CG	0.01	77TRP	CB	77TRP	C	100.00
76LYS	CG	77TRP	CZ2	0.01	61VAL	C	62GLU	C	97.77	77TRP	CB	80LEU	CD1	0.00
76LYS	CG	77TRP	CZ3	0.01	61VAL	C	64ALA	CB	0.00	77TRP	CB	85ARG	CB	0.13
76LYS	CG	277PRO	CB	0.01	61VAL	C	67VAL	CG2	0.00	77TRP	CB	85ARG	CG	0.07
76LYS	CD	76LYS	CE	100.00	61VAL	C	97GLN	CD	0.00	77TRP	CB	85ARG	CD	0.02

76LYS	CD	76LYS	C	0.38
76LYS	CD	77TRP	CD1	0.06
76LYS	CD	77TRP	CE2	0.09
76LYS	CD	77TRP	CZ2	0.17
76LYS	CD	277PRO	CB	0.01
76LYS	CE	77TRP	CD1	0.09
76LYS	CE	77TRP	CE2	0.07
76LYS	CE	77TRP	CZ2	0.44
76LYS	C	77TRP	CA	100.00
76LYS	C	77TRP	CB	7.17
76LYS	C	77TRP	CG	3.08
76LYS	C	77TRP	CD1	0.68
76LYS	C	77TRP	CD2	0.11
76LYS	C	77TRP	C	90.44
76LYS	C	80LEU	CD1	0.00
77TRP	CA	77TRP	CB	100.00
77TRP	CA	77TRP	CG	100.00
77TRP	CA	77TRP	CD1	86.24
77TRP	CA	77TRP	CD2	23.63
77TRP	CA	77TRP	CE3	0.26
77TRP	CA	77TRP	C	100.00
77TRP	CA	80LEU	CD1	0.84
77TRP	CB	77TRP	CG	100.00
77TRP	CB	77TRP	CD1	100.00
77TRP	CB	77TRP	CD2	100.00
77TRP	CB	77TRP	CE3	98.56
77TRP	CB	77TRP	C	100.00
77TRP	CB	80LEU	CD1	0.07
77TRP	CB	86PRO	CG	0.09
77TRP	CB	86PRO	CD	0.09
77TRP	CG	77TRP	CD1	100.00
77TRP	CG	77TRP	CD2	100.00
77TRP	CG	77TRP	CE2	100.00
77TRP	CG	77TRP	CE3	100.00
77TRP	CG	80LEU	CD1	0.00
77TRP	CG	86PRO	CG	0.12
77TRP	CG	86PRO	CD	0.20
77TRP	CD1	77TRP	CD2	100.00
77TRP	CD1	77TRP	CE2	100.00
77TRP	CD1	80LEU	CD1	0.14
77TRP	CD1	86PRO	CG	0.20
77TRP	CD1	86PRO	CD	0.02
77TRP	CD2	77TRP	CE2	100.00
77TRP	CD2	77TRP	CE3	100.00
77TRP	CD2	77TRP	CZ2	100.00
77TRP	CD2	77TRP	CZ3	100.00
77TRP	CD2	80LEU	CD1	0.05
77TRP	CD2	86PRO	CB	0.00
77TRP	CD2	86PRO	CG	0.15
77TRP	CD2	86PRO	CD	0.44
77TRP	CE2	77TRP	CE3	100.00
77TRP	CE2	77TRP	CZ2	100.00
77TRP	CE2	77TRP	CZ3	100.00
77TRP	CE2	86PRO	CG	0.12
77TRP	CE2	86PRO	CD	0.06
77TRP	CE3	77TRP	CZ2	100.00
77TRP	CE3	77TRP	CZ3	100.00
77TRP	CE3	80LEU	CD1	7.60
77TRP	CE3	80LEU	CD2	0.92
77TRP	CE3	85SER	C	0.08
77TRP	CE3	86PRO	CA	0.04
77TRP	CE3	86PRO	CB	0.59

62GLU	CA	62GLU	CB	100.00
62GLU	CA	62GLU	CG	100.00
62GLU	CA	62GLU	CD	88.24
62GLU	CA	62GLU	C	100.00
62GLU	CB	62GLU	CG	100.00
62GLU	CB	62GLU	CD	100.00
62GLU	CB	62GLU	C	100.00
62GLU	CB	265GLY	CA	0.00
62GLU	CG	62GLU	CD	100.00
62GLU	CG	62GLU	C	65.42
62GLU	CD	62GLU	C	2.18
62GLU	CD	265GLY	CA	0.01
62GLU	CD	266THR	CB	0.06
62GLU	CD	266THR	CG2	0.03
62GLU	C	63GLU	CA	100.00
62GLU	C	63GLU	CB	29.99
62GLU	C	63GLU	CG	0.19
62GLU	C	63GLU	C	73.42
63GLU	CA	63GLU	CB	100.00
63GLU	CA	63GLU	CG	100.00
63GLU	CA	63GLU	CD	70.85
63GLU	CA	63GLU	C	100.00
63GLU	CB	63GLU	CG	100.00
63GLU	CB	63GLU	CD	100.00
63GLU	CB	63GLU	C	100.00
63GLU	CG	63GLU	CD	100.00
63GLU	CG	63GLU	C	82.36
63GLU	CD	63GLU	C	7.20
63GLU	C	64ALA	CA	100.00
63GLU	C	64ALA	CB	32.87
63GLU	C	64ALA	C	67.43
64ALA	CA	64ALA	CB	100.00
64ALA	CA	64ALA	C	100.00
64ALA	CB	64ALA	C	100.00
64ALA	CB	67VAL	CG2	0.22
64ALA	CB	266THR	CG2	0.01
64ALA	C	65GLU	CA	100.00
64ALA	C	65GLU	CB	6.15
64ALA	C	65GLU	CG	0.12
64ALA	C	65GLU	CD	0.06
64ALA	C	65GLU	C	94.50
64ALA	C	266THR	CG2	0.21
65GLU	CA	65GLU	CB	100.00
65GLU	CA	65GLU	CG	100.00
65GLU	CA	65GLU	CD	83.66
65GLU	CA	65GLU	C	100.00
65GLU	CA	266THR	CG2	0.03
65GLU	CB	65GLU	CG	100.00
65GLU	CB	65GLU	CD	100.00
65GLU	CB	65GLU	C	100.00
65GLU	CB	302PHE	CE1	0.03
65GLU	CB	302PHE	CE2	0.86
65GLU	CB	302PHE	CZ	0.39
65GLU	CG	65GLU	CD	100.00
65GLU	CG	65GLU	C	66.61
65GLU	CG	301ALA	CB	0.00
65GLU	CG	302PHE	CD1	0.03
65GLU	CG	302PHE	CD2	0.07
65GLU	CG	302PHE	CE1	0.14
65GLU	CG	302PHE	CE2	0.69
65GLU	CG	302PHE	CZ	0.22
65GLU	CD	65GLU	C	0.03

77TRP	CB	85ARG	CZ	0.01
77TRP	CB	86PRO	CG	0.14
77TRP	CB	86PRO	CD	0.76
77TRP	CG	77TRP	CD1	100.00
77TRP	CG	77TRP	CD2	100.00
77TRP	CG	77TRP	CE2	100.00
77TRP	CG	77TRP	CE3	100.00
77TRP	CG	77TRP	C	0.16
77TRP	CG	85ARG	CB	0.01
77TRP	CG	85ARG	CG	0.01
77TRP	CG	86PRO	CG	0.08
77TRP	CG	86PRO	CD	3.58
77TRP	CD1	77TRP	CD2	100.00
77TRP	CD1	77TRP	CE2	100.00
77TRP	CD1	77TRP	C	0.01
77TRP	CD1	80LEU	CB	0.01
77TRP	CD1	80LEU	CG	0.02
77TRP	CD1	80LEU	CD1	3.64
77TRP	CD1	80LEU	CD2	1.03
77TRP	CD1	84ILE	CG2	0.00
77TRP	CD1	84ILE	CD	0.00
77TRP	CD1	85ARG	CA	0.21
77TRP	CD1	85ARG	CB	0.38
77TRP	CD1	85ARG	CG	0.44
77TRP	CD1	86PRO	CG	0.15
77TRP	CD1	86PRO	CD	6.21
77TRP	CD2	77TRP	CE2	100.00
77TRP	CD2	77TRP	CE3	100.00
77TRP	CD2	77TRP	CZ2	100.00
77TRP	CD2	77TRP	CZ3	100.00
77TRP	CD2	80LEU	CD1	0.02
77TRP	CD2	86PRO	CG	0.33
77TRP	CD2	86PRO	CD	1.52
77TRP	CE2	77TRP	CE3	100.00
77TRP	CE2	77TRP	CZ2	100.00
77TRP	CE2	77TRP	CZ3	100.00
77TRP	CE2	80LEU	CD1	0.03
77TRP	CE2	86PRO	CG	0.20
77TRP	CE2	86PRO	CD	0.64
77TRP	CE3	77TRP	CZ2	100.00
77TRP	CE3	77TRP	CZ3	100.00
77TRP	CE3	80LEU	CD1	0.01
77TRP	CE3	86PRO	CG	0.61
77TRP	CE3	86PRO	CD	0.38
77TRP	CZ2	77TRP	CZ3	100.00
77TRP	CZ2	80LEU	CD1	0.00
77TRP	CZ2	86PRO	CG	0.13
77TRP	CZ2	86PRO	CD	0.04
77TRP	CZ3	86PRO	CG	0.30
77TRP	CZ3	86PRO	CD	0.03
77TRP	C	78ASP	CA	100.00
77TRP	C	78ASP	CB	0.88
77TRP	C	78ASP	C	99.17
77TRP	C	85ARG	CZ	0.02
78ASP	CA	78ASP	CB	100.00
78ASP	CA	78ASP	CG	100.00
78ASP	CA	78ASP	C	100.00
78ASP	CA	85ARG	CZ	0.11
78ASP	CB	78ASP	CG	100.00
78ASP	CB	78ASP	C	100.00
78ASP	CG	78ASP	C	99.92
78ASP	C	79GLY	CA	100.00

77TRP	CE3	86PRO	CG	1.61	65GLU	CD	302PHE	CE2	0.02	78ASF	C	79GLY	C	54.04
77TRP	CE3	86PRO	CD	6.50	65GLU	CD	302PHE	CZ	0.00	79GLY	CA	79GLY	C	100.00
77TRP	CZ2	77TRP	CZ3	100.00	65GLU	C	66ALA	CA	100.00	79GLY	C	80LEU	CA	100.00
77TRP	CZ2	86PRO	CG	0.04	65GLU	C	66ALA	CB	99.64	79GLY	C	80LEU	CB	6.95
77TRP	CZ2	86PRO	CD	0.01	65GLU	C	66ALA	C	0.36	79GLY	C	80LEU	CG	0.69
77TRP	CZ3	80LEU	CD1	0.18	65GLU	C	266THR	CB	0.03	79GLY	C	80LEU	CD1	0.22
77TRP	CZ3	80LEU	CD2	0.24	65GLU	C	266THR	CG2	3.89	79GLY	C	80LEU	CD2	0.22
77TRP	CZ3	84ILE	CG2	0.02	66ALA	CA	66ALA	CB	100.00	79GLY	C	80LEU	C	92.68
77TRP	CZ3	84ILE	C	0.01	66ALA	CA	66ALA	C	100.00	80LEU	CA	80LEU	CB	100.00
77TRP	CZ3	85SER	CA	0.01	66ALA	CA	266THR	CG2	2.21	80LEU	CA	80LEU	CG	100.00
77TRP	CZ3	85SER	C	0.56	66ALA	CB	66ALA	C	100.00	80LEU	CA	80LEU	CD1	16.18
77TRP	CZ3	86PRO	CA	0.60	66ALA	CB	267PRO	CG	0.00	80LEU	CA	80LEU	CD2	86.71
77TRP	CZ3	86PRO	CB	0.56	66ALA	CB	269PHE	CD1	0.10	80LEU	CA	80LEU	C	100.00
77TRP	CZ3	86PRO	CG	0.93	66ALA	CB	269PHE	CE1	1.79	80LEU	CA	81PRO	CD	100.00
77TRP	CZ3	86PRO	CD	1.24	66ALA	CB	297MET	CG	4.27	80LEU	CB	80LEU	CG	100.00
77TRP	C	78ASP	CA	100.00	66ALA	CB	297MET	CE	0.66	80LEU	CB	80LEU	CD1	100.00
77TRP	C	78ASP	CB	1.46	66ALA	CB	301ALA	CB	0.18	80LEU	CB	80LEU	CD2	100.00
77TRP	C	78ASP	C	98.96	66ALA	CB	302PHE	CE1	0.18	80LEU	CB	80LEU	C	100.00
77TRP	C	80LEU	CD1	0.05	66ALA	CB	302PHE	CE2	0.09	80LEU	CB	81PRO	CD	36.03
77TRP	C	80LEU	CD2	0.00	66ALA	CB	302PHE	CZ	0.24	80LEU	CB	84ILE	CG2	0.00
78ASP	CA	78ASP	CB	100.00	66ALA	C	67VAL	CA	100.00	80LEU	CB	84ILE	CD	0.00
78ASP	CA	78ASP	CG	100.00	66ALA	C	67VAL	CB	34.46	80LEU	CB	85ARG	CB	0.00
78ASP	CA	78ASP	C	100.00	66ALA	C	67VAL	CG2	24.39	80LEU	CB	85ARG	CG	0.07
78ASP	CB	78ASP	CG	100.00	66ALA	C	67VAL	C	79.80	80LEU	CG	80LEU	CD1	100.00
78ASP	CB	78ASP	C	100.00	66ALA	C	266THR	CG2	0.38	80LEU	CG	80LEU	CD2	100.00
78ASP	CG	78ASP	C	99.17	66ALA	C	297MET	CE	0.03	80LEU	CG	80LEU	C	1.33
78ASP	C	79GLY	CA	100.00	67VAL	CA	67VAL	CB	100.00	80LEU	CG	81PRO	CD	0.07
78ASP	C	79GLY	C	43.14	67VAL	CA	67VAL	CG1	100.00	80LEU	CG	84ILE	CD	0.03
78ASP	C	80LEU	CD1	0.00	67VAL	CA	67VAL	CG2	100.00	80LEU	CD1	80LEU	CD2	100.00
79GLY	CA	79GLY	C	100.00	67VAL	CA	67VAL	C	100.00	80LEU	CD1	80LEU	C	0.16
79GLY	C	80LEU	CA	100.00	67VAL	CB	67VAL	CG1	100.00	80LEU	CD1	81PRO	CD	0.04
79GLY	C	80LEU	CB	2.08	67VAL	CB	67VAL	CG2	100.00	80LEU	CD1	84ILE	CG2	0.09
79GLY	C	80LEU	CG	0.13	67VAL	CB	67VAL	C	100.00	80LEU	CD1	84ILE	CD	0.10
79GLY	C	80LEU	CD1	0.10	67VAL	CB	69LEU	CD1	0.00	80LEU	CD1	85ARG	CG	0.06
79GLY	C	80LEU	CD2	0.02	67VAL	CB	268VAL	CG1	0.06	80LEU	CD1	86PRO	CD	0.00
79GLY	C	80LEU	C	97.71	67VAL	CB	268VAL	CG2	0.05	80LEU	CD2	81PRO	CD	0.04
80LEU	CA	80LEU	CB	100.00	67VAL	CG1	67VAL	CG2	100.00	80LEU	CD2	84ILE	CG1	0.01
80LEU	CA	80LEU	CG	100.00	67VAL	CG1	67VAL	C	100.00	80LEU	CD2	84ILE	CG2	0.01
80LEU	CA	80LEU	CD1	11.80	67VAL	CG1	68LEU	C	0.03	80LEU	CD2	84ILE	CD	0.40
80LEU	CA	80LEU	CD2	87.64	67VAL	CG1	69LEU	CB	0.01	80LEU	CD2	84ILE	C	0.01
80LEU	CA	80LEU	C	100.00	67VAL	CG1	69LEU	CG	0.00	80LEU	C	81PRO	CA	100.00
80LEU	CA	81PRO	CD	100.00	67VAL	CG1	69LEU	CD1	0.41	80LEU	C	81PRO	CB	0.04
80LEU	CB	80LEU	CG	100.00	67VAL	CG1	69LEU	CD2	0.68	80LEU	C	81PRO	CD	100.00
80LEU	CB	80LEU	CD1	100.00	67VAL	CG1	268VAL	CG1	0.12	80LEU	C	81PRO	C	100.00
80LEU	CB	80LEU	CD2	100.00	67VAL	CG1	268VAL	CG2	0.09	81PRC	CA	81PRO	CB	100.00
80LEU	CB	80LEU	C	100.00	67VAL	CG2	97GLN	CD	0.05	81PRC	CA	81PRO	CG	100.00
80LEU	CB	81PRO	CD	27.31	67VAL	CG2	266THR	CB	0.01	81PRC	CA	81PRO	CD	100.00
80LEU	CB	84ILE	CG2	0.01	67VAL	CG2	266THR	CG2	2.80	81PRC	CA	81PRO	C	100.00
80LEU	CB	84ILE	CD	0.01	67VAL	CG2	268VAL	CG1	0.08	81PRC	CB	81PRO	CG	100.00
80LEU	CG	80LEU	CD1	100.00	67VAL	CG2	268VAL	CG2	0.13	81PRC	CB	81PRO	CD	100.00
80LEU	CG	80LEU	CD2	100.00	67VAL	C	68LEU	CA	100.00	81PRC	CB	81PRO	C	100.00
80LEU	CG	80LEU	C	3.15	67VAL	C	68LEU	CB	15.98	81PRC	CB	83LYS	CG	0.09
80LEU	CG	81PRO	CD	0.05	67VAL	C	68LEU	CG	0.00	81PRC	CB	83LYS	CE	0.04
80LEU	CG	84ILE	CD	0.03	67VAL	C	68LEU	C	91.36	81PRC	CG	81PRO	CD	100.00
80LEU	CD1	80LEU	CD2	100.00	67VAL	C	297MET	CE	1.40	81PRC	CG	81PRO	C	90.54
80LEU	CD1	80LEU	C	0.71	68LEU	CA	68LEU	CB	100.00	81PRC	CG	83LYS	CG	0.04
80LEU	CD1	81PRO	CD	0.00	68LEU	CA	68LEU	CG	100.00	81PRC	CG	83LYS	CD	0.00
80LEU	CD1	84ILE	CG2	0.07	68LEU	CA	68LEU	CD1	88.44	81PRC	CG	83LYS	CE	1.83
80LEU	CD1	84ILE	CD	0.06	68LEU	CA	68LEU	CD2	9.84	81PRC	CG	84ILE	CG1	0.05
80LEU	CD1	84ILE	C	0.01	68LEU	CA	68LEU	C	100.00	81PRC	CG	84ILE	CD	0.12
80LEU	CD1	85SER	CB	0.01	68LEU	CA	297MET	CE	0.05	81PRC	CD	81PRO	C	84.77
80LEU	CD1	86PRO	CD	0.01	68LEU	CB	68LEU	CG	100.00	81PRC	CD	84ILE	CG1	0.02

80LEU	CD2	80LEU	C	0.10
80LEU	CD2	81PRO	CD	0.04
80LEU	CD2	84ILE	CG1	0.00
80LEU	CD2	84ILE	CD	0.38
80LEU	CD2	84ILE	C	0.00
80LEU	CD2	85SER	CA	0.01
80LEU	CD2	85SER	CB	0.00
80LEU	C	81PRO	CA	100.00
80LEU	C	81PRO	CB	0.07
80LEU	C	81PRO	CD	100.00
80LEU	C	81PRO	C	100.00
81PRO	CA	81PRO	CB	100.00
81PRO	CA	81PRO	CG	100.00
81PRO	CA	81PRO	CD	100.00
81PRO	CA	81PRO	C	100.00
81PRO	CB	81PRO	CG	100.00
81PRO	CB	81PRO	CD	100.00
81PRO	CB	81PRO	C	100.00
81PRO	CB	83LYS	CG	0.02
81PRO	CB	83LYS	CE	0.01
81PRO	CG	81PRO	CD	100.00
81PRO	CG	81PRO	C	94.22
81PRO	CG	83LYS	CG	0.06
81PRO	CG	83LYS	CD	0.00
81PRO	CG	83LYS	CE	1.92
81PRO	CG	84ILE	CG1	0.04
81PRO	CG	84ILE	CD	0.05
81PRO	CD	81PRO	C	89.64
81PRO	CD	84ILE	CG1	0.04
81PRO	CD	84ILE	CD	0.02
81PRO	C	82ARG	CA	100.00
81PRO	C	82ARG	C	100.00
82ARG	CA	82ARG	CB	100.00
82ARG	CA	82ARG	CG	100.00
82ARG	CA	82ARG	CD	27.69
82ARG	CA	82ARG	CZ	0.01
82ARG	CA	82ARG	C	100.00
82ARG	CA	85SER	CB	0.04
82ARG	CB	82ARG	CG	100.00
82ARG	CB	82ARG	CD	100.00
82ARG	CB	82ARG	CZ	0.57
82ARG	CB	82ARG	C	100.00
82ARG	CG	82ARG	CD	100.00
82ARG	CG	82ARG	CZ	20.05
82ARG	CG	82ARG	C	60.41
82ARG	CG	88THR	CB	0.10
82ARG	CG	88THR	CG2	0.37
82ARG	CD	82ARG	CZ	100.00
82ARG	CD	82ARG	C	1.33
82ARG	CD	88THR	CG2	0.39
82ARG	CZ	85SER	CB	0.04
82ARG	CZ	87GLU	CG	0.10
82ARG	CZ	87GLU	CD	0.06
82ARG	CZ	88THR	CB	0.01
82ARG	CZ	88THR	CG2	3.15
82ARG	CZ	187ASN	CA	0.22
82ARG	C	83LYS	CA	100.00
82ARG	C	83LYS	CB	4.17
82ARG	C	83LYS	CG	0.10
82ARG	C	83LYS	C	95.68
82ARG	C	88THR	CG2	0.03
83LYS	CA	83LYS	CB	100.00

68LEU	CB	68LEU	CD1	100.00
68LEU	CB	68LEU	CD2	100.00
68LEU	CB	68LEU	C	100.00
68LEU	CB	297MET	CE	0.11
68LEU	CG	68LEU	CD1	100.00
68LEU	CG	68LEU	CD2	100.00
68LEU	CG	68LEU	C	94.78
68LEU	CG	297MET	CE	0.03
68LEU	CD1	68LEU	CD2	100.00
68LEU	CD1	68LEU	C	2.00
68LEU	CD1	269PHE	CB	0.16
68LEU	CD1	293SER	CB	0.72
68LEU	CD1	293SER	C	0.16
68LEU	CD1	294ALA	CA	0.09
68LEU	CD1	294ALA	CB	0.09
68LEU	CD1	297MET	CE	0.84
68LEU	CD2	68LEU	C	3.58
68LEU	CD2	269PHE	CB	0.03
68LEU	CD2	293SER	CB	0.02
68LEU	CD2	294ALA	CA	0.00
68LEU	CD2	294ALA	CB	0.02
68LEU	CD2	297MET	CE	0.06
68LEU	C	69LEU	CA	100.00
68LEU	C	69LEU	CB	28.20
68LEU	C	69LEU	CG	0.11
68LEU	C	69LEU	CD1	0.13
68LEU	C	69LEU	CD2	0.01
68LEU	C	69LEU	C	77.67
69LEU	CA	69LEU	CB	100.00
69LEU	CA	69LEU	CG	100.00
69LEU	CA	69LEU	CD1	55.82
69LEU	CA	69LEU	CD2	39.88
69LEU	CA	69LEU	C	100.00
69LEU	CB	69LEU	CG	100.00
69LEU	CB	69LEU	CD1	100.00
69LEU	CB	69LEU	CD2	100.00
69LEU	CB	69LEU	C	100.00
69LEU	CB	268VAL	CG1	0.58
69LEU	CB	268VAL	CG2	0.14
69LEU	CB	270GLU	CA	0.00
69LEU	CB	270GLU	CB	0.06
69LEU	CB	270GLU	CG	0.02
69LEU	CB	271PRO	CD	0.01
69LEU	CG	69LEU	CD1	100.00
69LEU	CG	69LEU	CD2	100.00
69LEU	CG	69LEU	C	54.87
69LEU	CG	90LEU	CD2	0.00
69LEU	CG	93LEU	CD2	0.01
69LEU	CG	268VAL	CG1	0.32
69LEU	CG	268VAL	CG2	0.00
69LEU	CG	270GLU	CD	0.00
69LEU	CD1	69LEU	CD2	100.00
69LEU	CD1	69LEU	C	0.40
69LEU	CD1	70GLY	C	0.00
69LEU	CD1	72VAL	CG1	0.00
69LEU	CD1	72VAL	CG2	0.01
69LEU	CD1	90LEU	CG	0.00
69LEU	CD1	90LEU	CD1	1.64
69LEU	CD1	90LEU	CD2	1.30
69LEU	CD1	93LEU	CD1	0.82
69LEU	CD1	93LEU	CD2	4.64
69LEU	CD1	268VAL	CB	0.09

81PRC	CD	84ILE	CD	0.02
81PRC	C	82ARG	CA	100.00
81PRC	C	82ARG	C	100.00
81PRC	C	85ARG	CB	0.00
81PRC	C	85ARG	CD	0.02
82ARC	CA	82ARG	CB	100.00
82ARC	CA	82ARG	CG	100.00
82ARC	CA	82ARG	CD	29.88
82ARC	CA	82ARG	C	100.00
82ARC	CA	85ARG	CB	0.01
82ARC	CA	85ARG	CG	0.09
82ARC	CA	85ARG	CD	0.02
82ARC	CA	85ARG	CZ	0.88
82ARC	CB	82ARG	CG	100.00
82ARC	CB	82ARG	CD	100.00
82ARC	CB	82ARG	CZ	0.10
82ARC	CB	82ARG	C	100.00
82ARC	CB	85ARG	CZ	0.06
82ARC	CG	82ARG	CD	100.00
82ARC	CG	82ARG	CZ	26.01
82ARC	CG	82ARG	C	22.32
82ARC	CG	85ARG	CD	0.00
82ARC	CG	85ARG	CZ	0.21
82ARC	CG	87GLU	CB	0.23
82ARC	CG	87GLU	CG	0.02
82ARC	CG	87GLU	CD	0.06
82ARC	CG	88THR	CG2	0.08
82ARC	CG	91LEU	CD1	0.04
82ARC	CG	187ASN	CB	0.14
82ARC	CD	82ARG	CZ	100.00
82ARC	CD	82ARG	C	0.12
82ARC	CD	85ARG	CZ	0.35
82ARC	CD	87GLU	CG	0.27
82ARC	CD	87GLU	CD	5.06
82ARC	CD	88THR	CG2	0.00
82ARC	CD	91LEU	CD1	0.03
82ARC	CD	187ASN	CA	0.02
82ARC	CD	187ASN	CB	0.20
82ARC	CD	187ASN	CG	0.00
82ARC	CD	188VAL	CG1	0.04
82ARC	CZ	85ARG	CD	0.18
82ARC	CZ	85ARG	CZ	7.20
82ARC	CZ	87GLU	CB	0.02
82ARC	CZ	87GLU	CG	0.34
82ARC	CZ	87GLU	CD	0.02
82ARC	CZ	88THR	CG2	0.07
82ARC	CZ	91LEU	CD1	0.09
82ARC	CZ	91LEU	CD2	0.00
82ARC	CZ	140PHE	CE1	0.16
82ARC	CZ	140PHE	CZ	0.17
82ARC	CZ	186ALA	CB	0.00
82ARC	CZ	186ALA	C	0.04
82ARC	CZ	187ASN	CA	2.14
82ARC	CZ	187ASN	CB	12.30
82ARC	CZ	187ASN	CG	1.75
82ARC	CZ	187ASN	C	0.01
82ARC	CZ	188VAL	CG1	0.66
82ARC	C	83LYS	CA	100.00
82ARC	C	83LYS	CB	0.34
82ARC	C	83LYS	CG	0.00
82ARC	C	83LYS	C	99.63
82ARC	C	88THR	CG2	0.01

83LYS	CA	83LYS	CG	100.00
83LYS	CA	83LYS	CD	0.06
83LYS	CA	83LYS	C	100.00
83LYS	CA	88THR	CG2	0.00
83LYS	CB	83LYS	CG	100.00
83LYS	CB	83LYS	CD	100.00
83LYS	CB	83LYS	CE	12.10
83LYS	CB	83LYS	C	100.00
83LYS	CG	83LYS	CD	100.00
83LYS	CG	83LYS	CE	100.00
83LYS	CG	83LYS	C	98.94
83LYS	CG	84ILE	CG1	0.02
83LYS	CG	84ILE	CD	0.00
83LYS	CD	83LYS	CE	100.00
83LYS	CD	83LYS	C	0.11
83LYS	C	84ILE	CA	100.00
83LYS	C	84ILE	CB	91.90
83LYS	C	84ILE	CG1	1.62
83LYS	C	84ILE	CG2	59.20
83LYS	C	84ILE	CD	0.00
83LYS	C	84ILE	C	8.31
83LYS	C	88THR	CG2	0.02
83LYS	C	89GLY	CA	3.78
84ILE	CA	84ILE	CB	100.00
84ILE	CA	84ILE	CG1	100.00
84ILE	CA	84ILE	CG2	100.00
84ILE	CA	84ILE	CD	0.36
84ILE	CA	84ILE	C	100.00
84ILE	CB	84ILE	CG1	100.00
84ILE	CB	84ILE	CG2	100.00
84ILE	CB	84ILE	CD	100.00
84ILE	CB	84ILE	C	100.00
84ILE	CG1	84ILE	CG2	100.00
84ILE	CG1	84ILE	CD	100.00
84ILE	CG1	84ILE	C	94.90
84ILE	CG2	84ILE	CD	99.28
84ILE	CG2	84ILE	C	4.21
84ILE	CD	84ILE	C	0.26
84ILE	C	85SER	CA	100.00
84ILE	C	85SER	CB	4.81
84ILE	C	85SER	C	96.33
85SER	CA	85SER	CB	100.00
85SER	CA	85SER	C	100.00
85SER	CA	86PRO	CD	100.00
85SER	CB	85SER	C	100.00
85SER	CB	86PRO	CD	33.43
85SER	CB	88THR	CG2	0.00
85SER	C	86PRO	CA	100.00
85SER	C	86PRO	CB	0.04
85SER	C	86PRO	CD	100.00
85SER	C	86PRO	C	100.00
86PRO	CA	86PRO	CB	100.00
86PRO	CA	86PRO	CG	100.00
86PRO	CA	86PRO	CD	100.00
86PRO	CA	86PRO	C	100.00
86PRO	CA	90LEU	CD1	0.02
86PRO	CB	86PRO	CG	100.00
86PRO	CB	86PRO	CD	100.00
86PRO	CB	86PRO	C	100.00
86PRO	CB	90LEU	CD1	0.05
86PRO	CG	86PRO	CD	100.00
86PRO	CG	86PRO	C	69.99

69LEU	CD1	268VAL	CG1	9.25
69LEU	CD1	268VAL	CG2	0.18
69LEU	CD1	270GLU	CB	0.05
69LEU	CD1	270GLU	CG	0.42
69LEU	CD1	270GLU	CD	0.25
69LEU	CD2	69LEU	C	0.65
69LEU	CD2	70GLY	C	0.08
69LEU	CD2	72VAL	CG1	0.11
69LEU	CD2	72VAL	CG2	0.02
69LEU	CD2	90LEU	CG	0.00
69LEU	CD2	90LEU	CD1	1.57
69LEU	CD2	90LEU	CD2	1.30
69LEU	CD2	93LEU	CD1	0.20
69LEU	CD2	93LEU	CD2	1.90
69LEU	CD2	268VAL	CG1	3.83
69LEU	CD2	268VAL	CG2	0.07
69LEU	CD2	270GLU	CB	0.10
69LEU	CD2	270GLU	CG	0.50
69LEU	CD2	270GLU	CD	1.04
69LEU	CD2	271PRO	CD	0.00
69LEU	C	70GLY	CA	100.00
69LEU	C	70GLY	C	55.26
69LEU	C	271PRO	CG	0.00
69LEU	C	271PRO	CD	0.26
70GLY	CA	70GLY	C	100.00
70GLY	CA	271PRO	CG	0.09
70GLY	CA	271PRO	CD	0.02
70GLY	C	71SER	CA	100.00
70GLY	C	71SER	CB	25.91
70GLY	C	71SER	C	75.40
70GLY	C	271PRO	CG	0.18
70GLY	C	271PRO	CD	0.01
70GLY	C	273HIS	CE1	0.01
71SER	CA	71SER	CB	100.00
71SER	CA	71SER	C	100.00
71SER	CA	273HIS	CD2	0.12
71SER	CA	273HIS	CE1	0.04
71SER	CB	71SER	C	100.00
71SER	CB	271PRO	CG	0.00
71SER	CB	273HIS	CB	0.01
71SER	CB	273HIS	CD2	0.38
71SER	CB	273HIS	CE1	0.42
71SER	CB	273HIS	C	0.12
71SER	CB	274GLY	CA	1.64
71SER	CB	274GLY	C	0.00
71SER	CB	275SER	CB	0.03
71SER	C	72VAL	CA	100.00
71SER	C	72VAL	CB	83.76
71SER	C	72VAL	CG1	23.12
71SER	C	72VAL	CG2	7.20
71SER	C	72VAL	C	18.04
72VAL	CA	72VAL	CB	100.00
72VAL	CA	72VAL	CG1	100.00
72VAL	CA	72VAL	CG2	100.00
72VAL	CA	72VAL	C	100.00
72VAL	CB	72VAL	CG1	100.00
72VAL	CB	72VAL	CG2	100.00
72VAL	CB	72VAL	C	100.00
72VAL	CB	86PRO	CB	0.10
72VAL	CB	86PRO	CG	0.00
72VAL	CB	86PRO	C	0.00
72VAL	CB	87GLU	CB	0.01

83LYS	CA	83LYS	CB	100.00
83LYS	CA	83LYS	CG	100.00
83LYS	CA	83LYS	CD	1.21
83LYS	CA	83LYS	C	100.00
83LYS	CA	88THR	CG2	0.25
83LYS	CB	83LYS	CG	100.00
83LYS	CB	83LYS	CD	100.00
83LYS	CB	83LYS	CE	11.57
83LYS	CB	83LYS	C	100.00
83LYS	CG	83LYS	CD	100.00
83LYS	CG	83LYS	CE	100.00
83LYS	CG	83LYS	C	94.65
83LYS	CG	84ILE	CG1	0.05
83LYS	CD	83LYS	CE	100.00
83LYS	CD	83LYS	C	0.04
83LYS	CE	84ILE	CG1	0.00
83LYS	C	84ILE	CA	100.00
83LYS	C	84ILE	CB	92.12
83LYS	C	84ILE	CG1	3.91
83LYS	C	84ILE	CG2	60.71
83LYS	C	84ILE	CD	0.00
83LYS	C	84ILE	C	8.27
83LYS	C	88THR	CG2	4.63
84ILE	CA	84ILE	CB	100.00
84ILE	CA	84ILE	CG1	100.00
84ILE	CA	84ILE	CG2	100.00
84ILE	CA	84ILE	CD	0.70
84ILE	CA	84ILE	C	100.00
84ILE	CB	84ILE	CG1	100.00
84ILE	CB	84ILE	CG2	100.00
84ILE	CB	84ILE	CD	100.00
84ILE	CB	84ILE	C	100.00
84ILE	CG1	84ILE	CG2	100.00
84ILE	CG1	84ILE	CD	100.00
84ILE	CG1	84ILE	C	89.93
84ILE	CG2	84ILE	CD	99.15
84ILE	CG2	84ILE	C	9.09
84ILE	CD	84ILE	C	0.42
84ILE	C	85ARG	CA	100.00
84ILE	C	85ARG	CB	18.37
84ILE	C	85ARG	CG	0.94
84ILE	C	85ARG	C	80.41
85ARC	CA	85ARG	CB	100.00
85ARC	CA	85ARG	CG	100.00
85ARC	CA	85ARG	C	100.00
85ARC	CA	86PRO	CD	100.00
85ARC	CB	85ARG	CG	100.00
85ARC	CB	85ARG	CD	100.00
85ARC	CB	85ARG	C	100.00
85ARC	CB	86PRO	CD	58.39
85ARC	CG	85ARG	CD	100.00
85ARC	CG	85ARG	CZ	4.29
85ARC	CG	85ARG	C	10.30
85ARC	CG	86PRO	CD	1.21
85ARC	CG	87GLU	CB	0.01
85ARC	CD	85ARG	CZ	100.00
85ARC	CZ	87GLU	CD	0.04
85ARC	C	86PRO	CA	100.00
85ARC	C	86PRO	CD	100.00
85ARC	C	86PRO	C	100.00
86PRC	CA	86PRO	CB	100.00
86PRC	CA	86PRO	CG	100.00

86PRO	CD	86PRO	C	58.38	72VAL	CB	90LEU	CD1	0.01	86PRC	CA	86PRO	CD	100.00
86PRO	C	87GLU	CA	100.00	72VAL	CG1	72VAL	CG2	100.00	86PRC	CA	86PRO	C	100.00
86PRO	C	87GLU	CB	20.23	72VAL	CG1	72VAL	C	36.63	86PRC	CB	86PRO	CG	100.00
86PRO	C	87GLU	C	95.80	72VAL	CG1	77TRP	CZ3	0.00	86PRC	CB	86PRO	CD	100.00
86PRO	C	90LEU	CB	0.00	72VAL	CG1	86PRO	CB	1.14	86PRC	CB	86PRO	C	100.00
86PRO	C	90LEU	CD1	0.28	72VAL	CG1	86PRO	CG	0.14	86PRC	CG	86PRO	CD	100.00
86PRO	C	90LEU	CD2	0.04	72VAL	CG1	86PRO	C	0.45	86PRC	CG	86PRO	C	30.87
87GLU	CA	87GLU	CB	100.00	72VAL	CG1	87GLU	CA	0.11	86PRC	CD	86PRO	C	16.34
87GLU	CA	87GLU	CG	100.00	72VAL	CG1	87GLU	CB	0.02	86PRC	C	87GLU	CA	100.00
87GLU	CA	87GLU	CD	76.42	72VAL	CG1	87GLU	CG	0.01	86PRC	C	87GLU	C	100.00
87GLU	CA	87GLU	C	100.00	72VAL	CG1	90LEU	CB	0.04	86PRC	C	90LEU	CD1	0.01
87GLU	CA	90LEU	CD2	0.00	72VAL	CG1	90LEU	CG	0.30	87GLL	CA	87GLU	CB	100.00
87GLU	CB	87GLU	CG	100.00	72VAL	CG1	90LEU	CD1	0.73	87GLL	CA	87GLU	CG	100.00
87GLU	CB	87GLU	CD	100.00	72VAL	CG1	90LEU	CD2	0.52	87GLL	CA	87GLU	CD	81.26
87GLU	CB	87GLU	C	100.00	72VAL	CG2	72VAL	C	87.63	87GLL	CA	87GLU	C	100.00
87GLU	CG	87GLU	CD	100.00	72VAL	CG2	77TRP	CZ2	0.01	87GLL	CA	90LEU	CD1	0.02
87GLU	CG	87GLU	C	95.24	72VAL	CG2	86PRO	CB	2.00	87GLL	CA	90LEU	CD2	0.01
87GLU	CG	91LEU	CD1	0.56	72VAL	CG2	86PRO	CG	0.68	87GLL	CB	87GLU	CG	100.00
87GLU	CG	140PHE	CZ	0.01	72VAL	CG2	86PRO	C	1.00	87GLL	CB	87GLU	CD	100.00
87GLU	CG	188VAL	CG1	0.01	72VAL	CG2	87GLU	CA	0.29	87GLL	CB	87GLU	C	100.00
87GLU	CD	87GLU	C	2.21	72VAL	CG2	87GLU	CB	0.14	87GLL	CG	87GLU	CD	100.00
87GLU	CD	90LEU	CD1	0.00	72VAL	CG2	87GLU	CG	0.02	87GLL	CG	87GLU	C	13.34
87GLU	CD	91LEU	CD1	0.04	72VAL	CG2	90LEU	CB	0.14	87GLL	CG	90LEU	CD1	0.02
87GLU	CD	94ARG	CZ	0.00	72VAL	CG2	90LEU	CG	0.41	87GLL	CG	91LEU	CD1	0.02
87GLU	CD	140PHE	CZ	0.00	72VAL	CG2	90LEU	CD1	1.97	87GLL	CD	90LEU	CD1	0.02
87GLU	C	88THR	CA	100.00	72VAL	CG2	90LEU	CD2	0.80	87GLL	C	88THR	CA	100.00
87GLU	C	88THR	CB	0.64	72VAL	C	73GLY	CA	100.00	87GLL	C	88THR	C	100.00
87GLU	C	88THR	CG2	0.24	72VAL	C	73GLY	C	36.15	87GLL	C	91LEU	CD1	0.07
87GLU	C	88THR	C	99.48	72VAL	C	87GLU	CB	0.01	88THF	CA	88THR	CB	100.00
87GLU	C	90LEU	CD2	0.00	72VAL	C	274GLY	CA	0.04	88THF	CA	88THR	CG2	100.00
87GLU	C	91LEU	CD1	0.01	72VAL	C	275SER	CB	0.01	88THF	CA	88THR	C	100.00
88THR	CA	88THR	CB	100.00	73GLY	CA	73GLY	C	100.00	88THF	CA	91LEU	CB	0.00
88THR	CA	88THR	CG2	100.00	73GLY	CA	77TRP	CD1	1.03	88THF	CA	91LEU	CD1	0.01
88THR	CA	88THR	C	100.00	73GLY	CA	77TRP	CE3	0.00	88THF	CB	88THR	CG2	100.00
88THR	CA	91LEU	CB	0.00	73GLY	CA	77TRP	CZ3	0.02	88THF	CB	88THR	C	100.00
88THR	CA	91LEU	CD1	0.01	73GLY	CA	86PRO	CB	0.02	88THF	CG2	88THR	C	0.93
88THR	CB	88THR	CG2	100.00	73GLY	CA	86PRO	CG	0.03	88THF	C	89GLY	CA	100.00
88THR	CB	88THR	C	100.00	73GLY	CA	87GLU	CA	0.00	88THF	C	89GLY	C	91.41
88THR	CB	188VAL	CG1	0.00	73GLY	CA	87GLU	CB	0.10	88THF	C	91LEU	CB	0.01
88THR	CG2	88THR	C	14.70	73GLY	CA	87GLU	CG	0.01	89GLY	CA	89GLY	C	100.00
88THR	CG2	91LEU	CD1	0.01	73GLY	CA	87GLU	CD	0.16	89GLY	C	90LEU	CA	100.00
88THR	CG2	140PHE	CZ	0.00	73GLY	CA	274GLY	C	0.02	89GLY	C	90LEU	CB	0.08
88THR	CG2	188VAL	CG1	0.92	73GLY	CA	275SER	C	0.00	89GLY	C	90LEU	C	99.96
88THR	C	89GLY	CA	100.00	73GLY	C	74GLY	CA	100.00	90LEU	CA	90LEU	CB	100.00
88THR	C	89GLY	C	78.56	73GLY	C	74GLY	C	28.62	90LEU	CA	90LEU	CG	100.00
88THR	C	91LEU	CB	0.02	73GLY	C	75PRO	CD	0.01	90LEU	CA	90LEU	CD1	18.40
88THR	C	91LEU	CG	0.00	73GLY	C	76LYS	CE	0.00	90LEU	CA	90LEU	CD2	79.98
88THR	C	91LEU	CD1	0.10	73GLY	C	77TRP	CB	0.00	90LEU	CA	90LEU	C	100.00
88THR	C	91LEU	CD2	0.02	73GLY	C	77TRP	CD1	0.72	90LEU	CB	90LEU	CG	100.00
89GLY	CA	89GLY	C	100.00	73GLY	C	77TRP	CE3	0.06	90LEU	CB	90LEU	CD1	100.00
89GLY	CA	91LEU	CD1	0.00	73GLY	C	77TRP	CZ3	0.04	90LEU	CB	90LEU	CD2	100.00
89GLY	C	90LEU	CA	100.00	73GLY	C	82ARG	CZ	0.00	90LEU	CB	90LEU	C	100.00
89GLY	C	90LEU	CB	5.34	73GLY	C	274GLY	CA	0.04	90LEU	CB	94ARG	CZ	0.28
89GLY	C	90LEU	CG	0.79	73GLY	C	274GLY	C	0.02	90LEU	CG	90LEU	CD1	100.00
89GLY	C	90LEU	CD1	0.12	73GLY	C	275SER	CB	0.00	90LEU	CG	90LEU	CD2	100.00
89GLY	C	90LEU	CD2	0.19	73GLY	C	275SER	C	0.02	90LEU	CG	90LEU	C	17.96
89GLY	C	90LEU	C	93.19	73GLY	C	277PRO	CG	0.00	90LEU	CG	94ARG	CZ	0.38
89GLY	C	91LEU	CD1	0.00	74GLY	CA	74GLY	C	100.00	90LEU	CD1	90LEU	CD2	100.00
90LEU	CA	90LEU	CB	100.00	74GLY	CA	75PRO	CD	100.00	90LEU	CD1	90LEU	C	0.89
90LEU	CA	90LEU	CG	100.00	74GLY	CA	76LYS	CE	0.00	90LEU	CD1	94ARG	CZ	0.14
90LEU	CA	90LEU	CD1	3.14	74GLY	CA	77TRP	CE3	0.00	90LEU	CD2	90LEU	C	1.47
90LEU	CA	90LEU	CD2	97.44	74GLY	CA	82ARG	CZ	0.01	90LEU	CD2	93LEU	CD2	0.00

90LEU	CA	90LEU	C	100.00	74GLY	CA	274GLY	CA	0.00	90LEU	CD2	94ARG	CZ	0.87
90LEU	CA	93LEU	CB	0.00	74GLY	CA	274GLY	C	0.01	90LEU	CD2	270GLU	CD	0.00
90LEU	CB	90LEU	CG	100.00	74GLY	CA	275SER	CB	0.00	90LEU	C	91LEU	CA	100.00
90LEU	CB	90LEU	CD1	100.00	74GLY	CA	275SER	C	0.03	90LEU	C	91LEU	CB	0.00
90LEU	CB	90LEU	CD2	100.00	74GLY	CA	276ALA	CA	0.01	90LEU	C	91LEU	C	100.00
90LEU	CB	90LEU	C	100.00	74GLY	CA	276ALA	CB	0.02	91LEU	CA	91LEU	CB	100.00
90LEU	CB	94ARG	CZ	4.09	74GLY	CA	277PRO	CG	0.09	91LEU	CA	91LEU	CG	100.00
90LEU	CG	90LEU	CD1	100.00	74GLY	CA	277PRO	CD	0.04	91LEU	CA	91LEU	CD1	15.58
90LEU	CG	90LEU	CD2	100.00	74GLY	C	75PRO	CA	100.00	91LEU	CA	91LEU	CD2	83.84
90LEU	CG	90LEU	C	1.19	74GLY	C	75PRO	CB	0.24	91LEU	CA	91LEU	C	100.00
90LEU	CD1	90LEU	CD2	100.00	74GLY	C	75PRO	CG	0.00	91LEU	CB	91LEU	CG	100.00
90LEU	CD1	90LEU	C	0.01	74GLY	C	75PRO	CD	100.00	91LEU	CB	91LEU	CD1	100.00
90LEU	CD1	93LEU	CB	0.01	74GLY	C	75PRO	C	100.00	91LEU	CB	91LEU	CD2	100.00
90LEU	CD1	93LEU	CD1	0.01	74GLY	C	76LYS	CG	0.01	91LEU	CB	91LEU	C	100.00
90LEU	CD1	93LEU	CD2	0.00	74GLY	C	76LYS	CE	0.01	91LEU	CG	91LEU	CD1	100.00
90LEU	CD1	94ARG	CZ	0.00	74GLY	C	77TRP	CE3	0.01	91LEU	CG	91LEU	CD2	100.00
90LEU	CD2	90LEU	C	0.04	74GLY	C	78ASP	CB	0.01	91LEU	CG	91LEU	C	10.02
90LEU	CD2	93LEU	CB	0.01	74GLY	C	78ASP	CG	0.00	91LEU	CG	95LYS	CD	0.00
90LEU	CD2	93LEU	CD1	0.01	75PRO	CA	75PRO	CB	100.00	91LEU	CD1	91LEU	CD2	100.00
90LEU	CD2	93LEU	CD2	0.08	75PRO	CA	75PRO	CG	100.00	91LEU	CD1	91LEU	C	0.38
90LEU	CD2	94ARG	CZ	0.01	75PRO	CA	75PRO	CD	100.00	91LEU	CD1	94ARG	CZ	0.02
90LEU	C	91LEU	CA	100.00	75PRO	CA	75PRO	C	100.00	91LEU	CD1	95LYS	CD	0.00
90LEU	C	91LEU	CB	0.02	75PRO	CA	78ASP	CB	0.02	91LEU	CD1	95LYS	CE	0.03
90LEU	C	91LEU	C	99.99	75PRO	CB	75PRO	CG	100.00	91LEU	CD1	140PHE	CE1	0.32
90LEU	C	93LEU	CD2	0.00	75PRO	CB	75PRO	CD	100.00	91LEU	CD1	140PHE	CE2	0.02
90LEU	C	94ARG	CD	0.02	75PRO	CB	75PRO	C	100.00	91LEU	CD1	140PHE	CZ	0.20
90LEU	C	94ARG	CZ	0.03	75PRO	CB	78ASP	CB	0.01	91LEU	CD2	91LEU	C	0.89
91LEU	CA	91LEU	CB	100.00	75PRO	CB	276ALA	C	0.10	91LEU	CD2	94ARG	CZ	0.55
91LEU	CA	91LEU	CG	100.00	75PRO	CB	277PRO	CA	0.08	91LEU	CD2	95LYS	CD	0.00
91LEU	CA	91LEU	CD1	34.36	75PRO	CB	277PRO	CB	0.05	91LEU	CD2	95LYS	CE	0.02
91LEU	CA	91LEU	CD2	64.83	75PRO	CB	277PRO	CG	0.03	91LEU	CD2	140PHE	CD1	0.00
91LEU	CA	91LEU	C	100.00	75PRO	CB	277PRO	CD	0.20	91LEU	CD2	140PHE	CE1	0.94
91LEU	CA	94ARG	CZ	0.01	75PRO	CB	280ALA	CB	0.04	91LEU	CD2	140PHE	CE2	0.01
91LEU	CB	91LEU	CG	100.00	75PRO	CG	75PRO	CD	100.00	91LEU	CD2	140PHE	CZ	0.93
91LEU	CB	91LEU	CD1	100.00	75PRO	CG	75PRO	C	74.19	91LEU	C	92SER	CA	100.00
91LEU	CB	91LEU	CD2	100.00	75PRO	CG	274GLY	CA	0.00	91LEU	C	92SER	CB	0.66
91LEU	CB	91LEU	C	100.00	75PRO	CG	274GLY	C	0.01	91LEU	C	92SER	C	99.43
91LEU	CG	91LEU	CD1	100.00	75PRO	CG	275SER	CB	0.04	91LEU	C	95LYS	CE	0.01
91LEU	CG	91LEU	CD2	100.00	75PRO	CG	275SER	C	0.28	92SEF	CA	92SER	CB	100.00
91LEU	CG	91LEU	C	34.97	75PRO	CG	276ALA	CA	0.06	92SEF	CA	92SER	C	100.00
91LEU	CG	95LYS	CD	0.01	75PRO	CG	276ALA	CB	0.02	92SEF	CB	92SER	C	100.00
91LEU	CG	95LYS	CE	0.01	75PRO	CG	276ALA	C	0.31	92SEF	C	93LEU	CA	100.00
91LEU	CD1	91LEU	CD2	100.00	75PRO	CG	277PRO	CA	0.02	92SEF	C	93LEU	C	100.00
91LEU	CD1	91LEU	C	2.07	75PRO	CG	277PRO	CB	0.04	93LEU	CA	93LEU	CB	100.00
91LEU	CD1	94ARG	CZ	0.16	75PRO	CG	277PRO	CG	0.06	93LEU	CA	93LEU	CG	100.00
91LEU	CD1	95LYS	CG	0.02	75PRO	CG	277PRO	CD	0.10	93LEU	CA	93LEU	CD1	82.88
91LEU	CD1	95LYS	CD	0.07	75PRO	CG	280ALA	CB	0.55	93LEU	CA	93LEU	CD2	14.21
91LEU	CD1	95LYS	CE	0.09	75PRO	CG	281GLY	CA	0.01	93LEU	CA	93LEU	C	100.00
91LEU	CD1	135THR	CG2	0.05	75PRO	CD	75PRO	C	62.31	93LEU	CB	93LEU	CG	100.00
91LEU	CD1	140PHE	CD1	0.01	75PRO	CD	274GLY	CA	0.00	93LEU	CB	93LEU	CD1	100.00
91LEU	CD1	140PHE	CE1	0.30	75PRO	CD	274GLY	C	0.02	93LEU	CB	93LEU	CD2	100.00
91LEU	CD1	140PHE	CZ	0.05	75PRO	CD	275SER	CB	0.02	93LEU	CB	93LEU	C	100.00
91LEU	CD2	91LEU	C	3.75	75PRO	CD	276ALA	CB	0.08	93LEU	CG	93LEU	CD1	100.00
91LEU	CD2	94ARG	CD	0.02	75PRO	CD	277PRO	CB	0.01	93LEU	CG	93LEU	CD2	100.00
91LEU	CD2	94ARG	CZ	0.80	75PRO	CD	277PRO	CG	0.04	93LEU	CG	93LEU	C	97.26
91LEU	CD2	95LYS	CG	0.00	75PRO	CD	277PRO	CD	0.01	93LEU	CD1	93LEU	CD2	100.00
91LEU	CD2	95LYS	CD	0.04	75PRO	C	76LYS	CA	100.00	93LEU	CD1	93LEU	C	12.50
91LEU	CD2	95LYS	CE	0.01	75PRO	C	76LYS	CB	44.00	93LEU	CD1	97GLN	CG	0.26
91LEU	CD2	140PHE	CD1	0.01	75PRO	C	76LYS	CG	2.28	93LEU	CD1	268VAL	CG2	0.01
91LEU	CD2	140PHE	CE1	0.36	75PRO	C	76LYS	C	55.98	93LEU	CD2	93LEU	C	11.89
91LEU	CD2	140PHE	CE2	0.01	75PRO	C	277PRO	CG	0.01	93LEU	CD2	97GLN	CG	0.03
91LEU	CD2	140PHE	CZ	0.22	76LYS	CA	76LYS	CB	100.00	93LEU	CD2	99LEU	CD1	0.11

91LEU	C	92SER	CA	100.00	76LYS	CA	76LYS	CG	100.00	93LEU	CD2	99LEU	CD2	0.00
91LEU	C	92SER	CB	0.05	76LYS	CA	76LYS	CD	1.77	93LEU	CD2	268VAL	CG1	0.02
91LEU	C	92SER	C	99.98	76LYS	CA	76LYS	C	100.00	93LEU	CD2	268VAL	CG2	0.66
92SER	CA	92SER	CB	100.00	76LYS	CA	277PRO	CG	0.01	93LEU	C	94ARG	CA	100.00
92SER	CA	92SER	C	100.00	76LYS	CB	76LYS	CG	100.00	93LEU	C	94ARG	C	100.00
92SER	CA	95LYS	CG	0.00	76LYS	CB	76LYS	CD	100.00	93LEU	C	99LEU	CD1	0.03
92SER	CB	92SER	C	100.00	76LYS	CB	76LYS	CE	31.61	94ARG	CA	94ARG	CB	100.00
92SER	C	93LEU	CA	100.00	76LYS	CB	76LYS	C	100.00	94ARG	CA	94ARG	CG	100.00
92SER	C	93LEU	CB	0.01	76LYS	CB	77TRP	CD1	0.20	94ARG	CA	94ARG	CD	0.00
92SER	C	93LEU	C	100.00	76LYS	CB	77TRP	CD2	0.03	94ARG	CA	94ARG	C	100.00
93LEU	CA	93LEU	CB	100.00	76LYS	CB	77TRP	CE2	0.07	94ARG	CA	99LEU	CD1	0.09
93LEU	CA	93LEU	CG	100.00	76LYS	CB	77TRP	CE3	0.39	94ARG	CB	94ARG	CG	100.00
93LEU	CA	93LEU	CD1	79.81	76LYS	CB	77TRP	CZ2	0.00	94ARG	CB	94ARG	CD	100.00
93LEU	CA	93LEU	CD2	17.85	76LYS	CB	77TRP	CZ3	0.14	94ARG	CB	94ARG	C	100.00
93LEU	CA	93LEU	C	100.00	76LYS	CB	80LEU	CD1	0.02	94ARG	CB	99LEU	CB	0.00
93LEU	CB	93LEU	CG	100.00	76LYS	CB	86PRO	CG	0.00	94ARG	CB	135THR	CG2	0.16
93LEU	CB	93LEU	CD1	100.00	76LYS	CB	277PRO	CG	0.01	94ARG	CG	94ARG	CD	100.00
93LEU	CB	93LEU	CD2	100.00	76LYS	CG	76LYS	CD	100.00	94ARG	CG	94ARG	CZ	0.44
93LEU	CB	93LEU	C	100.00	76LYS	CG	76LYS	CE	100.00	94ARG	CG	94ARG	C	6.23
93LEU	CB	99LEU	CD1	0.00	76LYS	CG	76LYS	C	94.27	94ARG	CG	99LEU	CD1	0.08
93LEU	CG	93LEU	CD1	100.00	76LYS	CG	77TRP	CG	0.00	94ARG	CG	99LEU	CD2	0.42
93LEU	CG	93LEU	CD2	100.00	76LYS	CG	77TRP	CD1	1.05	94ARG	CG	134LEU	CD2	0.01
93LEU	CG	93LEU	C	92.18	76LYS	CG	77TRP	CD2	0.02	94ARG	CD	94ARG	CZ	100.00
93LEU	CD1	93LEU	CD2	100.00	76LYS	CG	77TRP	CE2	0.26	94ARG	CD	134LEU	CD1	0.04
93LEU	CD1	93LEU	C	10.75	76LYS	CG	77TRP	CE3	0.00	94ARG	CD	134LEU	CD2	0.12
93LEU	CD1	94ARG	CG	0.01	76LYS	CG	77TRP	CZ2	0.10	94ARG	CD	135THR	CG2	0.01
93LEU	CD1	97GLN	CG	0.18	76LYS	CG	77TRP	CZ3	0.02	94ARG	CZ	134LEU	CG	0.00
93LEU	CD1	268VAL	CG2	0.06	76LYS	CG	80LEU	CD1	0.06	94ARG	CZ	134LEU	CD1	0.71
93LEU	CD2	93LEU	C	10.30	76LYS	CG	86PRO	CG	0.00	94ARG	CZ	134LEU	CD2	8.19
93LEU	CD2	97GLN	CG	0.05	76LYS	CD	76LYS	CE	100.00	94ARG	C	95LYS	CA	100.00
93LEU	CD2	99LEU	CD1	0.09	76LYS	CD	76LYS	C	1.08	94ARG	C	95LYS	CB	0.34
93LEU	CD2	268VAL	CG1	0.06	76LYS	CD	77TRP	CD1	0.05	94ARG	C	95LYS	C	99.75
93LEU	CD2	268VAL	CG2	0.66	76LYS	CD	77TRP	CE2	0.19	95LYS	CA	95LYS	CB	100.00
93LEU	C	94ARG	CA	100.00	76LYS	CD	77TRP	CE3	0.01	95LYS	CA	95LYS	CG	100.00
93LEU	C	94ARG	CB	0.01	76LYS	CD	77TRP	CZ2	0.66	95LYS	CA	95LYS	CD	22.82
93LEU	C	94ARG	C	99.99	76LYS	CD	77TRP	CZ3	0.01	95LYS	CA	95LYS	CE	0.05
93LEU	C	99LEU	CD1	0.04	76LYS	CD	80LEU	CD1	0.01	95LYS	CA	95LYS	C	100.00
94ARG	CA	94ARG	CB	100.00	76LYS	CD	86PRO	CG	0.02	95LYS	CB	95LYS	CG	100.00
94ARG	CA	94ARG	CG	100.00	76LYS	CD	277PRO	CB	0.00	95LYS	CB	95LYS	CD	100.00
94ARG	CA	94ARG	CD	0.17	76LYS	CD	277PRO	CG	0.02	95LYS	CB	95LYS	CE	10.57
94ARG	CA	94ARG	C	100.00	76LYS	CD	277PRO	CD	0.00	95LYS	CB	95LYS	C	100.00
94ARG	CA	99LEU	CG	0.00	76LYS	CE	77TRP	CD1	0.05	95LYS	CG	95LYS	CD	100.00
94ARG	CA	99LEU	CD1	0.48	76LYS	CE	77TRP	CE2	0.75	95LYS	CG	95LYS	CE	100.00
94ARG	CB	94ARG	CG	100.00	76LYS	CE	77TRP	CE3	0.02	95LYS	CG	95LYS	C	69.45
94ARG	CB	94ARG	CD	100.00	76LYS	CE	77TRP	CZ2	1.70	95LYS	CG	135THR	CG2	0.02
94ARG	CB	94ARG	CZ	0.03	76LYS	CE	77TRP	CZ3	0.03	95LYS	CD	95LYS	CE	100.00
94ARG	CB	94ARG	C	100.00	76LYS	CE	80LEU	CD1	0.04	95LYS	CD	95LYS	C	1.22
94ARG	CB	99LEU	CD1	0.01	76LYS	CE	80LEU	CD2	0.00	95LYS	CE	135THR	CG2	0.00
94ARG	CB	135THR	CG2	0.75	76LYS	CE	86PRO	CG	0.02	95LYS	C	96SER	CA	100.00
94ARG	CG	94ARG	CD	100.00	76LYS	C	77TRP	CA	100.00	95LYS	C	96SER	CB	0.68
94ARG	CG	94ARG	CZ	0.64	76LYS	C	77TRP	CB	43.73	95LYS	C	96SER	C	99.52
94ARG	CG	94ARG	C	7.29	76LYS	C	77TRP	CG	9.75	96SER	CA	96SER	CB	100.00
94ARG	CG	99LEU	CD1	0.09	76LYS	C	77TRP	CD1	0.85	96SER	CA	96SER	C	100.00
94ARG	CG	99LEU	CD2	0.00	76LYS	C	77TRP	CD2	0.38	96SER	CB	96SER	C	100.00
94ARG	CG	134LEU	CD1	0.01	76LYS	C	77TRP	CE3	0.06	96SER	C	97GLN	CA	100.00
94ARG	CG	134LEU	CD2	0.02	76LYS	C	77TRP	C	56.18	96SER	C	97GLN	CB	9.11
94ARG	CG	135THR	CG2	0.07	76LYS	C	80LEU	CD2	0.00	96SER	C	97GLN	CG	1.09
94ARG	CD	94ARG	CZ	100.00	77TRP	CA	77TRP	CB	100.00	96SER	C	97GLN	CD	0.30
94ARG	CD	94ARG	C	0.34	77TRP	CA	77TRP	CG	100.00	96SER	C	97GLN	C	90.58
94ARG	CD	134LEU	CD1	0.38	77TRP	CA	77TRP	CD1	40.15	97GLN	CA	97GLN	CB	100.00
94ARG	CD	134LEU	CD2	0.31	77TRP	CA	77TRP	CD2	70.12	97GLN	CA	97GLN	CG	100.00
94ARG	CD	135THR	CG2	0.15	77TRP	CA	77TRP	CE3	3.64	97GLN	CA	97GLN	CD	99.99

94ARG	CZ	134LEU	CG	0.06
94ARG	CZ	134LEU	CD1	3.12
94ARG	CZ	134LEU	CD2	2.08
94ARG	CZ	135THR	CG2	0.02
94ARG	CZ	140PHE	CE1	0.00
94ARG	C	95LYS	CA	100.00
94ARG	C	95LYS	CB	0.48
94ARG	C	95LYS	CG	0.02
94ARG	C	95LYS	C	99.54
94ARG	C	135THR	CG2	1.07
95LYS	CA	95LYS	CB	100.00
95LYS	CA	95LYS	CG	100.00
95LYS	CA	95LYS	CD	18.10
95LYS	CA	95LYS	CE	0.12
95LYS	CA	95LYS	C	100.00
95LYS	CA	135THR	CG2	0.00
95LYS	CB	95LYS	CG	100.00
95LYS	CB	95LYS	CD	100.00
95LYS	CB	95LYS	CE	11.11
95LYS	CB	95LYS	C	100.00
95LYS	CG	95LYS	CD	100.00
95LYS	CG	95LYS	CE	100.00
95LYS	CG	95LYS	C	53.91
95LYS	CG	135THR	CG2	0.02
95LYS	CD	95LYS	CE	100.00
95LYS	CD	95LYS	C	0.79
95LYS	CE	95LYS	C	0.01
95LYS	C	96SER	CA	100.00
95LYS	C	96SER	CB	0.72
95LYS	C	96SER	C	99.42
96SER	CA	96SER	CB	100.00
96SER	CA	96SER	C	100.00
96SER	CB	96SER	C	100.00
96SER	C	97GLN	CA	100.00
96SER	C	97GLN	CB	6.98
96SER	C	97GLN	CG	0.90
96SER	C	97GLN	CD	0.26
96SER	C	97GLN	C	92.53
97GLN	CA	97GLN	CB	100.00
97GLN	CA	97GLN	CG	100.00
97GLN	CA	97GLN	CD	99.99
97GLN	CA	97GLN	C	100.00
97GLN	CB	97GLN	CG	100.00
97GLN	CB	97GLN	CD	100.00
97GLN	CB	97GLN	C	100.00
97GLN	CB	99LEU	CG	0.00
97GLN	CB	99LEU	CD1	0.36
97GLN	CB	99LEU	CD2	2.55
97GLN	CG	97GLN	CD	100.00
97GLN	CD	266THR	CB	0.05
97GLN	C	98ASP	CA	100.00
97GLN	C	98ASP	CB	99.71
97GLN	C	98ASP	C	100.00
97GLN	C	99LEU	CD1	0.05
97GLN	C	99LEU	CD2	0.00
97GLN	C	264ARG	CZ	0.02
98ASP	CA	98ASP	CB	100.00
98ASP	CA	98ASP	CG	100.00
98ASP	CA	98ASP	C	100.00
98ASP	CB	98ASP	CG	100.00
98ASP	CB	98ASP	C	100.00
98ASP	CB	264ARG	CB	0.20

77TRP	CA	77TRP	C	100.00
77TRP	CA	80LEU	CD1	0.03
77TRP	CA	80LEU	CD2	0.00
77TRP	CB	77TRP	CG	100.00
77TRP	CB	77TRP	CD1	100.00
77TRP	CB	77TRP	CD2	100.00
77TRP	CB	77TRP	CE3	97.81
77TRP	CB	77TRP	C	100.00
77TRP	CB	80LEU	CD1	0.02
77TRP	CB	82ARG	CB	0.01
77TRP	CB	82ARG	CG	0.03
77TRP	CB	82ARG	CD	0.02
77TRP	CB	82ARG	CZ	0.06
77TRP	CB	85SER	CB	0.03
77TRP	CB	86PRO	CG	0.03
77TRP	CB	86PRO	CD	0.25
77TRP	CG	77TRP	CD1	100.00
77TRP	CG	77TRP	CD2	100.00
77TRP	CG	77TRP	CE2	100.00
77TRP	CG	77TRP	CE3	100.00
77TRP	CG	77TRP	C	38.70
77TRP	CG	80LEU	CB	0.23
77TRP	CG	80LEU	CD1	0.00
77TRP	CG	80LEU	CD2	0.00
77TRP	CG	85SER	CA	0.01
77TRP	CG	85SER	CB	0.93
77TRP	CG	86PRO	CG	0.05
77TRP	CG	86PRO	CD	0.60
77TRP	CD1	77TRP	CD2	100.00
77TRP	CD1	77TRP	CE2	100.00
77TRP	CD1	77TRP	C	1.69
77TRP	CD1	80LEU	CB	5.55
77TRP	CD1	80LEU	CD1	0.15
77TRP	CD1	80LEU	CD2	0.00
77TRP	CD1	80LEU	C	0.84
77TRP	CD1	81PRO	C	0.36
77TRP	CD1	82ARG	CA	0.10
77TRP	CD1	82ARG	CG	0.03
77TRP	CD1	82ARG	CD	0.02
77TRP	CD1	82ARG	CZ	0.04
77TRP	CD1	84ILE	CG1	0.02
77TRP	CD1	84ILE	CD	0.00
77TRP	CD1	84ILE	C	0.00
77TRP	CD1	85SER	CA	1.45
77TRP	CD1	85SER	CB	7.65
77TRP	CD1	86PRO	CG	0.13
77TRP	CD1	86PRO	CD	0.27
77TRP	CD2	77TRP	CE2	100.00
77TRP	CD2	77TRP	CE3	100.00
77TRP	CD2	77TRP	CZ2	100.00
77TRP	CD2	77TRP	CZ3	100.00
77TRP	CD2	80LEU	CB	0.55
77TRP	CD2	80LEU	CG	0.01
77TRP	CD2	80LEU	CD1	0.91
77TRP	CD2	80LEU	CD2	0.16
77TRP	CD2	85SER	CA	0.05
77TRP	CD2	85SER	CB	4.34
77TRP	CD2	86PRO	CG	0.12
77TRP	CD2	86PRO	CD	4.52
77TRP	CE2	77TRP	CE3	100.00
77TRP	CE2	77TRP	CZ2	100.00
77TRP	CE2	77TRP	CZ3	100.00

97GLN	CA	97GLN	C	100.00
97GLN	CB	97GLN	CG	100.00
97GLN	CB	97GLN	CD	100.00
97GLN	CB	97GLN	C	100.00
97GLN	CB	99LEU	CG	0.18
97GLN	CB	99LEU	CD1	4.16
97GLN	CB	99LEU	CD2	1.07
97GLN	CG	97GLN	CD	100.00
97GLN	CD	266THR	CB	0.01
97GLN	C	98ASP	CA	100.00
97GLN	C	98ASP	CB	99.90
97GLN	C	98ASP	C	99.88
97GLN	C	99LEU	CD1	0.86
97GLN	C	265GLY	CA	0.02
98ASP	CA	98ASP	CB	100.00
98ASP	CA	98ASP	CG	100.00
98ASP	CA	98ASP	C	100.00
98ASP	CB	98ASP	CG	100.00
98ASP	CB	98ASP	C	100.00
98ASP	CB	264ARG	CB	0.02
98ASP	CB	264ARG	CZ	0.06
98ASP	CG	98ASP	C	99.51
98ASP	CG	100PHE	CE2	0.01
98ASP	CG	264ARG	CB	0.01
98ASP	CG	264ARG	CG	0.00
98ASP	CG	264ARG	CD	0.19
98ASP	CG	264ARG	CZ	0.04
98ASP	C	99LEU	CA	100.00
98ASP	C	99LEU	CB	0.59
98ASP	C	99LEU	CG	0.08
98ASP	C	99LEU	CD1	0.01
98ASP	C	99LEU	CD2	0.00
98ASP	C	99LEU	C	99.24
98ASP	C	100PHE	CD2	0.03
98ASP	C	263GLY	CA	0.38
99LEU	CA	99LEU	CB	100.00
99LEU	CA	99LEU	CG	100.00
99LEU	CA	99LEU	CD1	24.46
99LEU	CA	99LEU	CD2	75.51
99LEU	CA	99LEU	C	100.00
99LEU	CA	263GLY	CA	3.28
99LEU	CB	99LEU	CG	100.00
99LEU	CB	99LEU	CD1	100.00
99LEU	CB	99LEU	CD2	100.00
99LEU	CB	99LEU	C	100.00
99LEU	CB	134LEU	CD1	0.09
99LEU	CB	134LEU	CD2	0.03
99LEU	CG	99LEU	CD1	100.00
99LEU	CG	99LEU	CD2	100.00
99LEU	CG	99LEU	C	0.06
99LEU	CG	261SER	CB	0.00
99LEU	CD1	99LEU	CD2	100.00
99LEU	CD1	261SER	CB	0.75
99LEU	CD1	263GLY	CA	0.01
99LEU	CD1	268VAL	CB	0.00
99LEU	CD1	268VAL	CG2	14.18
99LEU	CD2	261SER	CB	7.98
99LEU	CD2	261SER	C	0.02
99LEU	CD2	262LEU	CA	0.03
99LEU	CD2	262LEU	C	1.69
99LEU	CD2	263GLY	CA	0.38
99LEU	CD2	263GLY	C	0.01

98ASP	CB	264ARG	CD	0.02
98ASP	CB	264ARG	CZ	0.28
98ASP	CG	98ASP	C	99.92
98ASP	CG	264ARG	CG	0.00
98ASP	CG	264ARG	CD	0.03
98ASP	CG	264ARG	CZ	0.00
98ASP	C	99LEU	CA	100.00
98ASP	C	99LEU	CB	0.05
98ASP	C	99LEU	CG	0.00
98ASP	C	99LEU	C	99.99
98ASP	C	263GLY	CA	0.37
99LEU	CA	99LEU	CB	100.00
99LEU	CA	99LEU	CG	100.00
99LEU	CA	99LEU	CD1	3.98
99LEU	CA	99LEU	CD2	95.96
99LEU	CA	99LEU	C	100.00
99LEU	CA	263GLY	CA	2.56
99LEU	CB	99LEU	CG	100.00
99LEU	CB	99LEU	CD1	100.00
99LEU	CB	99LEU	CD2	100.00
99LEU	CB	99LEU	C	100.00
99LEU	CB	134LEU	CD1	0.01
99LEU	CB	134LEU	CD2	4.30
99LEU	CG	99LEU	CD1	100.00
99LEU	CG	99LEU	CD2	100.00
99LEU	CG	99LEU	C	0.01
99LEU	CD1	99LEU	CD2	100.00
99LEU	CD1	134LEU	CD2	0.25
99LEU	CD1	261SER	CB	1.01
99LEU	CD1	268VAL	CB	0.00
99LEU	CD1	268VAL	CG2	8.94
99LEU	CD2	261SER	CB	0.60
99LEU	CD2	261SER	C	0.06
99LEU	CD2	262LEU	C	0.64
99LEU	CD2	263GLY	CA	0.87
99LEU	CD2	268VAL	CG2	0.25
99LEU	C	100PHE	CA	100.00
99LEU	C	100PHE	CB	71.30
99LEU	C	100PHE	CG	0.03
99LEU	C	100PHE	CD2	0.17
99LEU	C	100PHE	C	28.65
99LEU	C	134LEU	CD1	0.02
99LEU	C	134LEU	CD2	0.07
99LEU	C	135THR	CG2	0.01
100PHE	CA	100PHE	CB	100.00
100PHE	CA	100PHE	CG	100.00
100PHE	CA	100PHE	CD1	77.61
100PHE	CA	100PHE	CD2	48.75
100PHE	CA	100PHE	C	100.00
100PHE	CA	133GLU	CD	0.01
100PHE	CA	134LEU	CD1	0.00
100PHE	CA	134LEU	CD2	0.02
100PHE	CA	135THR	CG2	0.01
100PHE	CB	100PHE	CG	100.00
100PHE	CB	100PHE	CD1	100.00
100PHE	CB	100PHE	CD2	100.00
100PHE	CB	100PHE	C	100.00
100PHE	CB	133GLU	CD	0.02
100PHE	CB	135THR	CG2	0.00
100PHE	CB	164ARG	CB	0.01
100PHE	CB	164ARG	CD	0.04
100PHE	CB	164ARG	CZ	4.35

77TRP	CE2	80LEU	CB	6.79
77TRP	CE2	80LEU	CG	0.02
77TRP	CE2	80LEU	CD1	0.29
77TRP	CE2	80LEU	CD2	0.11
77TRP	CE2	83LYS	CG	0.00
77TRP	CE2	84ILE	CG1	0.02
77TRP	CE2	84ILE	C	0.01
77TRP	CE2	85SER	CA	0.90
77TRP	CE2	85SER	CB	2.34
77TRP	CE2	86PRO	CG	0.81
77TRP	CE2	86PRO	CD	6.47
77TRP	CE3	77TRP	CZ2	100.00
77TRP	CE3	77TRP	CZ3	100.00
77TRP	CE3	80LEU	CB	2.88
77TRP	CE3	80LEU	CD1	3.18
77TRP	CE3	80LEU	CD2	0.60
77TRP	CE3	80LEU	C	0.00
77TRP	CE3	81PRO	C	0.08
77TRP	CE3	82ARG	CA	0.00
77TRP	CE3	84ILE	CG1	0.00
77TRP	CE3	84ILE	CD	0.04
77TRP	CE3	85SER	CA	0.26
77TRP	CE3	85SER	CB	1.42
77TRP	CE3	86PRO	CG	0.08
77TRP	CE3	86PRO	CD	3.22
77TRP	CZ2	77TRP	CZ3	100.00
77TRP	CZ2	80LEU	CB	1.19
77TRP	CZ2	80LEU	CG	0.04
77TRP	CZ2	80LEU	CD1	1.25
77TRP	CZ2	80LEU	CD2	0.36
77TRP	CZ2	83LYS	CG	0.00
77TRP	CZ2	84ILE	CB	0.02
77TRP	CZ2	84ILE	CG1	0.19
77TRP	CZ2	84ILE	CG2	0.08
77TRP	CZ2	84ILE	CD	0.37
77TRP	CZ2	84ILE	C	0.44
77TRP	CZ2	85SER	CA	0.85
77TRP	CZ2	85SER	CB	0.06
77TRP	CZ2	86PRO	CG	0.43
77TRP	CZ2	86PRO	CD	9.23
77TRP	CZ3	80LEU	CB	1.04
77TRP	CZ3	80LEU	CD1	1.60
77TRP	CZ3	80LEU	CD2	0.61
77TRP	CZ3	84ILE	CG1	0.36
77TRP	CZ3	84ILE	CG2	0.06
77TRP	CZ3	84ILE	CD	0.34
77TRP	CZ3	84ILE	C	0.11
77TRP	CZ3	85SER	CA	0.21
77TRP	CZ3	85SER	CB	0.08
77TRP	CZ3	86PRO	CG	0.13
77TRP	CZ3	86PRO	CD	1.73
77TRP	C	78ASP	CA	100.00
77TRP	C	78ASP	CB	13.92
77TRP	C	78ASP	CG	0.01
77TRP	C	78ASP	C	86.85
77TRP	C	80LEU	CD1	0.02
77TRP	C	80LEU	CD2	0.01
77TRP	C	82ARG	CZ	0.01
78ASP	CA	78ASP	CB	100.00
78ASP	CA	78ASP	CG	100.00
78ASP	CA	78ASP	C	100.00
78ASP	CA	82ARG	CZ	0.02

99LEU	CD2	265GLY	CA	0.31
99LEU	CD2	265GLY	C	0.28
99LEU	CD2	268VAL	CG2	0.82
99LEU	C	100PHE	CA	100.00
99LEU	C	100PHE	CB	95.76
99LEU	C	100PHE	CG	1.60
99LEU	C	100PHE	CD2	3.16
99LEU	C	100PHE	C	4.50
99LEU	C	134LEU	CD1	0.06
99LEU	C	134LEU	CD2	0.02
100PH	CA	100PHE	CB	100.00
100PH	CA	100PHE	CG	100.00
100PH	CA	100PHE	CD1	91.24
100PH	CA	100PHE	CD2	56.48
100PH	CA	100PHE	C	100.00
100PH	CA	135THR	CG2	0.00
100PH	CB	100PHE	CG	100.00
100PH	CB	100PHE	CD1	100.00
100PH	CB	100PHE	CD2	100.00
100PH	CB	100PHE	C	100.00
100PH	CB	133GLU	CD	0.02
100PH	CB	135THR	CG2	0.02
100PH	CB	164ARG	CG	0.00
100PH	CB	164ARG	CD	0.93
100PH	CB	164ARG	CZ	4.58
100PH	CG	100PHE	CD1	100.00
100PH	CG	100PHE	CD2	100.00
100PH	CG	100PHE	CE1	100.00
100PH	CG	100PHE	CE2	100.00
100PH	CG	100PHE	CZ	100.00
100PH	CG	100PHE	C	98.22
100PH	CG	164ARG	CG	1.47
100PH	CG	164ARG	CD	5.88
100PH	CG	164ARG	CZ	0.50
100PH	CD1	100PHE	CD2	100.00
100PH	CD1	100PHE	CE1	100.00
100PH	CD1	100PHE	CE2	100.00
100PH	CD1	100PHE	CZ	100.00
100PH	CD1	100PHE	C	58.55
100PH	CD1	101ALA	CB	6.78
100PH	CD1	164ARG	CB	1.17
100PH	CD1	164ARG	CG	28.62
100PH	CD1	164ARG	CD	12.43
100PH	CD1	164ARG	C	0.01
100PH	CD1	165VAL	CG1	0.01
100PH	CD2	100PHE	CE1	100.00
100PH	CD2	100PHE	CE2	100.00
100PH	CD2	100PHE	CZ	100.00
100PH	CD2	164ARG	CG	0.02
100PH	CD2	164ARG	CD	0.83
100PH	CD2	164ARG	CZ	0.16
100PH	CD2	262LEU	C	0.01
100PH	CD2	263GLY	CA	0.42
100PH	CD2	263GLY	C	0.00
100PH	CD2	264ARG	CG	0.01
100PH	CD2	264ARG	CZ	0.20
100PH	CE1	100PHE	CE2	100.00
100PH	CE1	100PHE	CZ	100.00
100PH	CE1	101ALA	CB	3.14
100PH	CE1	164ARG	CB	2.32
100PH	CE1	164ARG	CG	25.05
100PH	CE1	164ARG	CD	1.55

100PHE CG	100PHE	CD1	100.00	78ASP CB	78ASP	CG	100.00	100PH CE1	164ARG	C	1.52
100PHE CG	100PHE	CD2	100.00	78ASP CB	78ASP	C	100.00	100PH CE1	168VAL	CG2	1.42
100PHE CG	100PHE	CE1	100.00	78ASP CG	78ASP	C	98.02	100PH CE1	262LEU	CD1	0.00
100PHE CG	100PHE	CE2	100.00	78ASP CG	82ARG	CZ	0.00	100PH CE1	262LEU	CD2	0.02
100PHE CG	100PHE	CZ	100.00	78ASP CG	277PRO	CG	0.02	100PH CE1	263GLY	CA	0.76
100PHE CG	100PHE	C	99.66	78ASP C	79GLY	CA	100.00	100PH CE2	100PHE	CZ	100.00
100PHE CG	164ARG	CB	0.24	78ASP C	79GLY	C	53.54	100PH CE2	164ARG	CG	0.02
100PHE CG	164ARG	CG	0.05	78ASP C	80LEU	CD2	0.00	100PH CE2	164ARG	CD	0.04
100PHE CG	164ARG	CD	0.47	79GLY CA	79GLY	C	100.00	100PH CE2	262LEU	CB	0.09
100PHE CG	164ARG	CZ	5.42	79GLY C	80LEU	CA	100.00	100PH CE2	262LEU	CD2	0.01
100PHE CG	263GLY	CA	0.00	79GLY C	80LEU	CB	16.49	100PH CE2	262LEU	C	0.15
100PHE CD1	100PHE	CD2	100.00	79GLY C	80LEU	CG	2.54	100PH CE2	263GLY	CA	8.38
100PHE CD1	100PHE	CE1	100.00	79GLY C	80LEU	CD1	0.43	100PH CE2	263GLY	C	7.24
100PHE CD1	100PHE	CE2	100.00	79GLY C	80LEU	CD2	0.32	100PH CE2	264ARG	CG	0.20
100PHE CD1	100PHE	CZ	100.00	79GLY C	80LEU	C	83.82	100PH CE2	264ARG	CD	0.00
100PHE CD1	100PHE	C	67.81	80LEU CA	80LEU	CB	100.00	100PH CE2	264ARG	CZ	7.42
100PHE CD1	101ALA	CB	8.23	80LEU CA	80LEU	CG	100.00	100PH CZ	164ARG	CB	0.15
100PHE CD1	133GLU	CG	0.07	80LEU CA	80LEU	CD1	22.95	100PH CZ	164ARG	CG	0.82
100PHE CD1	164ARG	CB	12.04	80LEU CA	80LEU	CD2	78.91	100PH CZ	164ARG	CD	0.07
100PHE CD1	164ARG	CG	0.49	80LEU CA	80LEU	C	100.00	100PH CZ	168VAL	CG2	0.39
100PHE CD1	164ARG	CD	0.59	80LEU CA	81PRO	CD	100.00	100PH CZ	262LEU	CB	0.28
100PHE CD1	164ARG	CZ	0.70	80LEU CB	80LEU	CG	100.00	100PH CZ	262LEU	CD1	0.06
100PHE CD1	164ARG	C	0.74	80LEU CB	80LEU	CD1	100.00	100PH CZ	262LEU	CD2	0.22
100PHE CD1	165VAL	CG2	0.00	80LEU CB	80LEU	CD2	100.00	100PH CZ	263GLY	CA	8.03
100PHE CD1	262LEU	C	0.01	80LEU CB	80LEU	C	100.00	100PH CZ	263GLY	C	0.50
100PHE CD1	263GLY	CA	0.01	80LEU CB	81PRO	CD	39.33	100PH CZ	264ARG	CZ	0.23
100PHE CD2	100PHE	CE1	100.00	80LEU CB	84ILE	CD	0.01	100PH C	101ALA	CA	100.00
100PHE CD2	100PHE	CE2	100.00	80LEU CB	85SER	CB	0.00	100PH C	101ALA	CB	8.29
100PHE CD2	100PHE	CZ	100.00	80LEU CG	80LEU	CD1	100.00	100PH C	101ALA	C	94.94
100PHE CD2	100PHE	C	0.04	80LEU CG	80LEU	CD2	100.00	100PH C	133GLU	CD	0.01
100PHE CD2	164ARG	CB	0.01	80LEU CG	80LEU	C	1.37	100PH C	134LEU	CB	0.05
100PHE CD2	164ARG	CG	0.05	80LEU CG	81PRO	CD	0.04	100PH C	134LEU	CD1	0.07
100PHE CD2	164ARG	CD	0.66	80LEU CG	84ILE	CG2	0.00	101AL CA	101ALA	CB	100.00
100PHE CD2	164ARG	CZ	36.24	80LEU CG	84ILE	CD	0.01	101AL CA	101ALA	C	100.00
100PHE CD2	262LEU	C	0.07	80LEU CD1	80LEU	CD2	100.00	101AL CA	165VAL	CG1	0.01
100PHE CD2	263GLY	CA	1.92	80LEU CD1	80LEU	C	0.08	101AL CB	101ALA	C	100.00
100PHE CD2	263GLY	C	0.33	80LEU CD1	81PRO	CD	0.02	101AL CB	103LEU	CD1	0.08
100PHE CE1	100PHE	CE2	100.00	80LEU CD1	84ILE	CG2	0.04	101AL CB	131VAL	CG1	0.02
100PHE CE1	100PHE	CZ	100.00	80LEU CD1	84ILE	CD	0.05	101AL CB	165VAL	CG1	0.09
100PHE CE1	101ALA	CB	3.83	80LEU CD1	86PRO	CD	0.00	101AL CB	165VAL	CG2	0.00
100PHE CE1	164ARG	CB	3.68	80LEU CD2	80LEU	C	0.07	101AL CB	168VAL	CB	0.03
100PHE CE1	164ARG	CG	1.97	80LEU CD2	81PRO	CD	0.21	101AL CB	168VAL	CG1	0.73
100PHE CE1	164ARG	CD	0.78	80LEU CD2	84ILE	CG1	0.00	101AL CB	168VAL	CG2	0.23
100PHE CE1	164ARG	CZ	0.20	80LEU CD2	84ILE	CG2	0.01	101AL CB	262LEU	CD1	0.04
100PHE CE1	164ARG	C	4.88	80LEU CD2	84ILE	CD	0.12	101AL CB	262LEU	CD2	0.01
100PHE CE1	165VAL	CA	0.00	80LEU CD2	85SER	CA	0.00	101AL C	102ASN	CA	100.00
100PHE CE1	165VAL	CG1	0.02	80LEU C	81PRO	CA	100.00	101AL C	102ASN	CB	12.32
100PHE CE1	168VAL	CG1	4.40	80LEU C	81PRO	CB	0.13	101AL C	102ASN	C	91.74
100PHE CE1	168VAL	CG2	0.04	80LEU C	81PRO	CD	100.00	101AL C	103LEU	CD1	0.01
100PHE CE1	262LEU	CB	0.02	80LEU C	81PRO	C	100.00	101AL C	134LEU	CD1	0.20
100PHE CE1	262LEU	CD2	0.02	81PRO CA	81PRO	CB	100.00	102AS CA	102ASN	CB	100.00
100PHE CE1	262LEU	C	0.00	81PRO CA	81PRO	CG	100.00	102AS CA	102ASN	CG	100.00
100PHE CE1	263GLY	CA	0.03	81PRO CA	81PRO	CD	100.00	102AS CA	102ASN	C	100.00
100PHE CE1	263GLY	C	0.01	81PRO CA	81PRO	C	100.00	102AS CB	102ASN	CG	100.00
100PHE CE2	100PHE	CZ	100.00	81PRO CB	81PRO	CG	100.00	102AS CB	102ASN	C	100.00
100PHE CE2	101ALA	CB	0.00	81PRO CB	81PRO	CD	100.00	102AS CB	132ARG	CD	0.31
100PHE CE2	164ARG	CG	0.01	81PRO CB	81PRO	C	100.00	102AS CB	134LEU	CG	0.46
100PHE CE2	164ARG	CD	0.12	81PRO CB	83LYS	CG	0.11	102AS CB	134LEU	CD1	0.23
100PHE CE2	164ARG	CZ	3.83	81PRO CB	83LYS	CD	0.00	102AS CB	134LEU	CD2	2.03
100PHE CE2	262LEU	CB	0.19	81PRO CB	83LYS	CE	0.08	102AS CG	102ASN	C	99.98
100PHE CE2	262LEU	CD2	0.00	81PRO CB	84ILE	CG1	0.02	102AS CG	104ARG	CG	0.02
100PHE CE2	262LEU	C	1.31	81PRO CB	84ILE	CD	0.00	102AS CG	134LEU	CD1	0.34

100PHE CE2	263GLY	CA	5.44	81PRO CG	81PRO	CD	100.00	102AS CG	134LEU	CD2	0.43
100PHE CE2	263GLY	C	19.75	81PRO CG	81PRO	C	85.37	102AS C	103LEU	CA	100.00
100PHE CE2	264ARG	CA	0.00	81PRO CG	83LYS	CG	0.03	102AS C	103LEU	CB	2.70
100PHE CE2	264ARG	CG	0.03	81PRO CG	83LYS	CD	0.01	102AS C	103LEU	CG	0.14
100PHE CE2	264ARG	CZ	0.00	81PRO CG	83LYS	CE	0.43	102AS C	103LEU	CD1	2.32
100PHE CZ	101ALA	CB	0.23	81PRO CG	84ILE	CG1	0.13	102AS C	103LEU	CD2	0.02
100PHE CZ	164ARG	CB	0.09	81PRO CG	84ILE	CG2	0.01	102AS C	103LEU	C	98.08
100PHE CZ	164ARG	CG	0.68	81PRO CG	84ILE	CD	0.21	103LE CA	103LEU	CB	100.00
100PHE CZ	164ARG	CD	0.17	81PRO CD	81PRO	C	78.28	103LE CA	103LEU	CG	100.00
100PHE CZ	164ARG	CZ	0.16	81PRO CD	84ILE	CG1	0.00	103LE CA	103LEU	CD1	60.05
100PHE CZ	164ARG	C	0.00	81PRO CD	84ILE	CG2	0.03	103LE CA	103LEU	CD2	39.73
100PHE CZ	168VAL	CG1	2.20	81PRO CD	84ILE	CD	0.02	103LE CA	103LEU	C	100.00
100PHE CZ	168VAL	CG2	0.01	81PRO C	82ARG	CA	100.00	103LE CA	129LEU	CD1	0.00
100PHE CZ	262LEU	CB	0.56	81PRO C	82ARG	CB	0.13	103LE CA	129LEU	CD2	0.00
100PHE CZ	262LEU	CD1	0.00	81PRO C	82ARG	CG	0.02	103LE CB	103LEU	CG	100.00
100PHE CZ	262LEU	CD2	0.13	81PRO C	82ARG	C	99.96	103LE CB	103LEU	CD1	100.00
100PHE CZ	262LEU	C	0.01	81PRO C	85SER	CB	0.02	103LE CB	103LEU	CD2	100.00
100PHE CZ	263GLY	CA	0.23	82ARG CA	82ARG	CB	100.00	103LE CB	103LEU	C	100.00
100PHE CZ	263GLY	C	1.04	82ARG CA	82ARG	CG	100.00	103LE CB	129LEU	CD1	0.05
100PHE C	101ALA	CA	100.00	82ARG CA	82ARG	CD	20.17	103LE CB	129LEU	CD2	0.02
100PHE C	101ALA	CB	59.97	82ARG CA	82ARG	CZ	0.04	103LE CB	260ALA	CB	0.00
100PHE C	101ALA	C	41.37	82ARG CA	82ARG	C	100.00	103LE CB	296MET	CE	0.15
100PHE C	133GLU	CG	0.00	82ARG CB	82ARG	CG	100.00	103LE CG	103LEU	CD1	100.00
100PHE C	133GLU	CD	0.03	82ARG CB	82ARG	CD	100.00	103LE CG	103LEU	CD2	100.00
100PHE C	134LEU	CB	0.18	82ARG CB	82ARG	CZ	0.62	103LE CG	103LEU	C	0.02
100PHE C	134LEU	CD1	0.01	82ARG CB	82ARG	C	100.00	103LE CG	129LEU	CD2	0.01
100PHE C	134LEU	CD2	0.78	82ARG CB	88THR	CG2	0.22	103LE CG	172LEU	CD1	0.06
101ALA CA	101ALA	CB	100.00	82ARG CB	187ASN	CB	0.01	103LE CG	262LEU	CD1	0.09
101ALA CA	101ALA	C	100.00	82ARG CB	215TYR	CE1	0.03	103LE CD1	103LEU	CD2	100.00
101ALA CA	134LEU	CB	0.02	82ARG CB	215TYR	CE2	0.33	103LE CD1	129LEU	CG	0.00
101ALA CA	134LEU	CD2	0.02	82ARG CB	215TYR	CZ	2.48	103LE CD1	129LEU	CD1	0.01
101ALA CA	165VAL	CG1	0.00	82ARG CG	82ARG	CD	100.00	103LE CD1	168VAL	CG1	0.01
101ALA CB	101ALA	C	100.00	82ARG CG	82ARG	CZ	15.41	103LE CD1	172LEU	CD1	0.60
101ALA CB	103LEU	CD1	0.79	82ARG CG	82ARG	C	63.33	103LE CD1	172LEU	CD2	0.09
101ALA CB	131VAL	CG1	0.01	82ARG CG	85SER	CB	0.02	103LE CD1	260ALA	CB	0.01
101ALA CB	165VAL	CB	0.00	82ARG CG	87GLU	CB	0.01	103LE CD1	262LEU	CB	0.03
101ALA CB	165VAL	CG1	0.02	82ARG CG	87GLU	CD	0.02	103LE CD1	262LEU	CG	0.26
101ALA CB	165VAL	CG2	0.26	82ARG CG	88THR	CG2	0.40	103LE CD1	262LEU	CD1	4.76
101ALA CB	168VAL	CG2	5.63	82ARG CG	91LEU	CD1	0.01	103LE CD1	262LEU	CD2	0.53
101ALA C	102ASN	CA	100.00	82ARG CG	187ASN	CB	0.06	103LE CD1	296MET	CE	0.31
101ALA C	102ASN	CB	39.33	82ARG CG	187ASN	CG	0.02	103LE CD1	300HIS	CD2	0.01
101ALA C	102ASN	C	72.85	82ARG CD	82ARG	CZ	100.00	103LE CD1	300HIS	CE1	0.35
101ALA C	103LEU	CD1	0.09	82ARG CD	82ARG	C	0.93	103LE CD2	129LEU	CD1	0.01
101ALA C	134LEU	CB	0.04	82ARG CD	85SER	CB	0.10	103LE CD2	129LEU	CD2	0.02
101ALA C	134LEU	CD2	0.34	82ARG CD	87GLU	CD	0.01	103LE CD2	131VAL	CG1	0.04
102ASN CA	102ASN	CB	100.00	82ARG CD	88THR	CG2	0.04	103LE CD2	131VAL	CG2	0.49
102ASN CA	102ASN	CG	100.00	82ARG CD	91LEU	CD1	0.04	103LE CD2	168VAL	CG1	0.20
102ASN CA	102ASN	C	100.00	82ARG CD	187ASN	CB	0.02	103LE CD2	172LEU	CD1	6.41
102ASN CB	102ASN	CG	100.00	82ARG CD	187ASN	CG	0.07	103LE CD2	172LEU	CD2	0.30
102ASN CB	102ASN	C	100.00	82ARG CD	215TYR	CD2	0.15	103LE CD2	262LEU	CD1	1.92
102ASN CB	132ARG	CG	0.00	82ARG CD	215TYR	CE1	0.01	103LE CD2	262LEU	CD2	0.16
102ASN CB	132ARG	CD	0.00	82ARG CD	215TYR	CE2	0.76	103LE CD2	296MET	CE	0.47
102ASN CB	133GLU	C	0.00	82ARG CD	215TYR	CZ	0.33	103LE CD2	300HIS	CD2	0.00
102ASN CB	134LEU	CB	0.02	82ARG CZ	85SER	CB	0.21	103LE CD2	300HIS	CE1	0.34
102ASN CB	134LEU	CG	0.04	82ARG CZ	87GLU	CG	0.02	103LE C	104ARG	CA	100.00
102ASN CB	134LEU	CD1	0.47	82ARG CZ	87GLU	CD	0.08	103LE C	104ARG	CB	39.78
102ASN CB	134LEU	CD2	0.33	82ARG CZ	88THR	CG2	0.42	103LE C	104ARG	CG	5.68
102ASN CG	102ASN	C	99.81	82ARG CZ	91LEU	CD1	0.61	103LE C	104ARG	C	74.94
102ASN CG	104ARG	CG	0.00	82ARG CZ	91LEU	CD2	0.03	103LE C	105PRO	CD	6.04
102ASN CG	134LEU	CD1	0.26	82ARG CZ	187ASN	CB	0.01	103LE C	129LEU	CD1	0.00
102ASN CG	134LEU	CD2	0.08	82ARG CZ	187ASN	CG	0.05	104AR CA	104ARG	CB	100.00
102ASN C	103LEU	CA	100.00	82ARG C	83LYS	CA	100.00	104AR CA	104ARG	CG	100.00

102ASN C	103LEU CB	5.30	82ARG C	83LYS CB	5.99	104AR CA	104ARG CD	76.04
102ASN C	103LEU CG	0.23	82ARG C	83LYS CG	0.50	104AR CA	104ARG C	100.00
102ASN C	103LEU CD1	4.61	82ARG C	83LYS C	97.47	104AR CA	105PRO CD	100.00
102ASN C	103LEU CD2	0.03	82ARG C	88THR CG2	0.25	104AR CB	104ARG CG	100.00
102ASN C	103LEU C	96.37	82ARG C	187ASN CB	0.12	104AR CB	104ARG CD	100.00
103LEU CA	103LEU CB	100.00	83LYS CA	83LYS CB	100.00	104AR CB	104ARG C	100.00
103LEU CA	103LEU CG	100.00	83LYS CA	83LYS CG	100.00	104AR CB	244SER CB	2.58
103LEU CA	103LEU CD1	83.98	83LYS CA	83LYS CD	1.58	104AR CG	104ARG CD	100.00
103LEU CA	103LEU CD2	14.10	83LYS CA	83LYS C	100.00	104AR CG	104ARG CZ	13.61
103LEU CA	103LEU C	100.00	83LYS CA	88THR CG2	0.07	104AR CG	104ARG C	0.02
103LEU CA	129LEU CD1	0.02	83LYS CB	83LYS CG	100.00	104AR CG	244SER CB	1.87
103LEU CB	103LEU CG	100.00	83LYS CB	83LYS CD	100.00	104AR CG	259SER CB	0.07
103LEU CB	103LEU CD1	100.00	83LYS CB	83LYS CE	13.62	104AR CD	104ARG CZ	100.00
103LEU CB	103LEU CD2	100.00	83LYS CB	83LYS C	100.00	104AR CD	244SER CB	1.10
103LEU CB	103LEU C	100.00	83LYS CG	83LYS CD	100.00	104AR CD	259SER CB	2.32
103LEU CB	129LEU CD1	0.18	83LYS CG	83LYS CE	100.00	104AR CD	272VAL CG2	0.00
103LEU CB	129LEU CD2	0.02	83LYS CG	83LYS C	87.59	104AR CZ	132ARG CZ	0.04
103LEU CB	296MET CE	0.72	83LYS CG	84ILE CG1	0.03	104AR CZ	134LEU CD1	0.01
103LEU CG	103LEU CD1	100.00	83LYS CG	84ILE CG2	0.01	104AR CZ	244SER CB	0.74
103LEU CG	103LEU CD2	100.00	83LYS CG	84ILE CD	0.01	104AR CZ	244SER C	0.20
103LEU CG	103LEU C	0.06	83LYS CG	88THR CG2	0.01	104AR CZ	245ASP CG	0.00
103LEU CG	129LEU CD1	0.00	83LYS CD	83LYS CE	100.00	104AR CZ	248SER CB	0.00
103LEU CG	129LEU CD2	0.00	83LYS CD	83LYS C	0.25	104AR CZ	259SER CB	0.22
103LEU CG	131VAL CG1	0.00	83LYS CD	84ILE CG2	0.01	104AR CZ	272VAL CG1	0.19
103LEU CG	131VAL CG2	0.01	83LYS CD	193GLU CD	0.02	104AR CZ	272VAL CG2	0.35
103LEU CG	262LEU CD1	0.06	83LYS CE	84ILE CG2	0.01	104AR C	105PRO CA	100.00
103LEU CG	262LEU CD2	0.01	83LYS CE	187ASN C	0.01	104AR C	105PRO CB	0.07
103LEU CD1	103LEU CD2	100.00	83LYS CE	193GLU CD	0.03	104AR C	105PRO CD	100.00
103LEU CD1	103LEU C	0.00	83LYS C	84ILE CA	100.00	104AR C	105PRO C	100.00
103LEU CD1	131VAL CG1	0.08	83LYS C	84ILE CB	72.03	104AR C	129LEU CD1	0.75
103LEU CD1	131VAL CG2	0.06	83LYS C	84ILE CG1	1.56	104AR C	129LEU CD2	0.04
103LEU CD1	168VAL CG2	0.01	83LYS C	84ILE CG2	38.03	105PR CA	105PRO CB	100.00
103LEU CD1	262LEU CB	0.06	83LYS C	84ILE CD	0.04	105PR CA	105PRO CG	100.00
103LEU CD1	262LEU CG	0.32	83LYS C	84ILE C	27.28	105PR CA	105PRO CD	100.00
103LEU CD1	262LEU CD1	4.46	83LYS C	88THR CG2	0.24	105PR CA	105PRO C	100.00
103LEU CD1	262LEU CD2	0.18	84ILE CA	84ILE CB	100.00	105PR CA	129LEU CD1	0.17
103LEU CD1	296MET CE	0.19	84ILE CA	84ILE CG1	100.00	105PR CA	129LEU CD2	0.01
103LEU CD1	300HIS CD2	0.04	84ILE CA	84ILE CG2	100.00	105PR CB	105PRO CG	100.00
103LEU CD1	300HIS CE1	0.27	84ILE CA	84ILE CD	5.80	105PR CB	105PRO CD	100.00
103LEU CD2	103LEU C	0.00	84ILE CA	84ILE C	100.00	105PR CB	105PRO C	100.00
103LEU CD2	129LEU CD1	0.01	84ILE CB	84ILE CG1	100.00	105PR CB	129LEU CD1	0.09
103LEU CD2	129LEU CD2	0.04	84ILE CB	84ILE CG2	100.00	105PR CB	129LEU CD2	0.03
103LEU CD2	131VAL CG1	0.10	84ILE CB	84ILE CD	100.00	105PR CG	105PRO CD	100.00
103LEU CD2	131VAL CG2	0.84	84ILE CB	84ILE C	100.00	105PR CG	105PRO C	96.88
103LEU CD2	262LEU CG	0.00	84ILE CG1	84ILE CG2	100.00	105PR CG	129LEU CD1	0.02
103LEU CD2	262LEU CD1	0.69	84ILE CG1	84ILE CD	100.00	105PR CG	258PRO CG	0.22
103LEU CD2	262LEU CD2	0.15	84ILE CG1	84ILE C	91.62	105PR CG	258PRO C	0.00
103LEU CD2	296MET CE	0.21	84ILE CG2	84ILE CD	94.96	105PR CG	296MET CE	0.33
103LEU CD2	300HIS CD2	0.04	84ILE CG2	84ILE C	9.63	105PR CD	105PRO C	93.84
103LEU CD2	300HIS CE1	2.07	84ILE CD	84ILE C	2.23	105PR CD	129LEU CD1	0.12
103LEU C	104ARG CA	100.00	84ILE C	85SER CA	100.00	105PR CD	129LEU CD2	0.01
103LEU C	104ARG CB	70.82	84ILE C	85SER CB	8.24	105PR CD	259SER CA	0.00
103LEU C	104ARG CG	27.26	84ILE C	85SER C	93.17	105PR CD	296MET CE	0.12
103LEU C	104ARG CD	0.00	85SER CA	85SER CB	100.00	105PR C	106ALA CA	100.00
103LEU C	104ARG C	39.86	85SER CA	85SER C	100.00	105PR C	106ALA CB	90.03
103LEU C	105PRO CD	3.68	85SER CA	86PRO CD	100.00	105PR C	106ALA C	17.21
103LEU C	129LEU CD1	0.01	85SER CB	85SER C	100.00	106AL CA	106ALA CB	100.00
104ARG CA	104ARG CB	100.00	85SER CB	86PRO CD	9.33	106AL CA	106ALA C	100.00
104ARG CA	104ARG CG	100.00	85SER CB	87GLU CB	0.02	106AL CA	250LEU CD1	0.00
104ARG CA	104ARG CD	11.45	85SER CB	87GLU CG	0.00	106AL CA	257LEU CD1	0.42
104ARG CA	104ARG C	100.00	85SER CB	87GLU CD	0.95	106AL CA	257LEU CD2	0.04
104ARG CA	105PRO CD	100.00	85SER CB	88THR CG2	0.02	106AL CB	106ALA C	100.00

104ARG CB	104ARG	CG	100.00	85SER C	86PRO	CA	100.00	106AL CB	128VAL	CG1	0.12
104ARG CB	104ARG	CD	100.00	85SER C	86PRO	CB	0.04	106AL CB	128VAL	CG2	0.10
104ARG CB	104ARG	C	100.00	85SER C	86PRO	CD	100.00	106AL CB	130ILE	CD	0.27
104ARG CB	244SER	CB	0.09	85SER C	86PRO	C	100.00	106AL CB	247ALA	CB	3.94
104ARG CG	104ARG	CD	100.00	86PRO CA	86PRO	CB	100.00	106AL CB	247ALA	C	0.03
104ARG CG	104ARG	CZ	4.59	86PRO CA	86PRO	CG	100.00	106AL CB	250LEU	CD1	0.36
104ARG CG	104ARG	C	22.70	86PRO CA	86PRO	CD	100.00	106AL CB	257LEU	CD1	1.01
104ARG CG	244SER	CB	0.03	86PRO CA	86PRO	C	100.00	106AL CB	257LEU	CD2	0.03
104ARG CG	259SER	CA	0.00	86PRO CB	86PRO	CG	100.00	106AL C	107LYS	CA	100.00
104ARG CG	259SER	CB	0.26	86PRO CB	86PRO	CD	100.00	106AL C	107LYS	CB	94.26
104ARG CD	104ARG	CZ	100.00	86PRO CB	86PRO	C	100.00	106AL C	107LYS	CG	0.19
104ARG CD	132ARG	CD	0.00	86PRO CB	90LEU	CD1	0.00	106AL C	107LYS	C	7.82
104ARG CD	132ARG	CZ	1.31	86PRO CG	86PRO	CD	100.00	106AL C	250LEU	CD1	0.42
104ARG CD	244SER	CB	0.03	86PRO CG	86PRO	C	45.92	106AL C	250LEU	CD2	0.01
104ARG CD	259SER	CB	0.02	86PRO CD	86PRO	C	32.13	107LY CA	107LYS	CB	100.00
104ARG CD	272VAL	CG2	0.04	86PRO C	87GLU	CA	100.00	107LY CA	107LYS	CG	100.00
104ARG CZ	132ARG	CZ	7.66	86PRO C	87GLU	CB	0.26	107LY CA	107LYS	CD	0.02
104ARG CZ	244SER	CB	2.78	86PRO C	87GLU	C	99.74	107LY CA	107LYS	C	100.00
104ARG CZ	244SER	C	0.02	86PRO C	90LEU	CD1	0.00	107LY CA	126VAL	CG1	0.00
104ARG CZ	272VAL	CG1	4.24	87GLU CA	87GLU	CB	100.00	107LY CB	107LYS	CG	100.00
104ARG CZ	272VAL	CG2	4.44	87GLU CA	87GLU	CG	100.00	107LY CB	107LYS	CD	100.00
104ARG C	105PRO	CA	100.00	87GLU CA	87GLU	CD	39.64	107LY CB	107LYS	CE	47.11
104ARG C	105PRO	CB	0.30	87GLU CA	87GLU	C	100.00	107LY CB	107LYS	C	100.00
104ARG C	105PRO	CD	100.00	87GLU CA	90LEU	CB	0.01	107LY CB	109PHE	CE1	0.01
104ARG C	105PRO	C	100.00	87GLU CA	90LEU	CD1	0.01	107LY CB	109PHE	CE2	0.00
104ARG C	129LEU	CD1	0.89	87GLU CB	87GLU	CG	100.00	107LY CB	109PHE	CZ	0.00
104ARG C	129LEU	CD2	0.02	87GLU CB	87GLU	CD	100.00	107LY CB	127ASP	CA	0.26
105PRO CA	105PRO	CB	100.00	87GLU CB	87GLU	C	100.00	107LY CB	127ASP	CB	0.17
105PRO CA	105PRO	CG	100.00	87GLU CG	87GLU	CD	100.00	107LY CB	127ASP	CG	0.02
105PRO CA	105PRO	CD	100.00	87GLU CG	87GLU	C	98.19	107LY CB	257LEU	CD2	0.02
105PRO CA	105PRO	C	100.00	87GLU CG	90LEU	CD1	0.03	107LY CG	107LYS	CD	100.00
105PRO CA	129LEU	CD1	0.02	87GLU CG	90LEU	CD2	0.08	107LY CG	107LYS	CE	100.00
105PRO CB	105PRO	CG	100.00	87GLU CG	91LEU	CD1	0.09	107LY CG	107LYS	C	99.53
105PRO CB	105PRO	CD	100.00	87GLU CD	87GLU	C	1.48	107LY CG	108VAL	C	0.10
105PRO CB	105PRO	C	100.00	87GLU CD	88THR	CG2	0.00	107LY CG	109PHE	CD1	0.02
105PRO CB	129LEU	CD1	0.38	87GLU CD	90LEU	CD1	0.00	107LY CG	109PHE	CD2	0.01
105PRO CB	129LEU	CD2	0.01	87GLU CD	91LEU	CD1	0.00	107LY CG	109PHE	CE1	0.53
105PRO CG	105PRO	CD	100.00	87GLU C	88THR	CA	100.00	107LY CG	109PHE	CE2	0.50
105PRO CG	105PRO	C	94.53	87GLU C	88THR	CB	0.12	107LY CG	109PHE	CZ	0.79
105PRO CG	129LEU	CD1	0.20	87GLU C	88THR	CG2	0.02	107LY CG	257LEU	CD2	0.68
105PRO CG	129LEU	CD2	0.02	87GLU C	88THR	C	99.92	107LY CD	107LYS	CE	100.00
105PRO CG	258PRO	CG	0.98	87GLU C	91LEU	CD1	0.01	107LY CD	109PHE	CD1	0.04
105PRO CG	258PRO	C	0.01	88THR CA	88THR	CB	100.00	107LY CD	109PHE	CD2	0.08
105PRO CG	296MET	CG	0.00	88THR CA	88THR	CG2	100.00	107LY CD	109PHE	CE1	1.71
105PRO CG	296MET	CE	1.80	88THR CA	88THR	C	100.00	107LY CD	109PHE	CE2	1.14
105PRO CD	105PRO	C	94.24	88THR CA	91LEU	CB	0.00	107LY CD	109PHE	CZ	0.75
105PRO CD	129LEU	CD1	1.24	88THR CA	91LEU	CD1	0.05	107LY CD	127ASP	CG	0.32
105PRO CD	129LEU	CD2	0.01	88THR CA	91LEU	CD2	0.01	107LY CD	257LEU	CD2	0.00
105PRO CD	296MET	CE	2.16	88THR CB	88THR	CG2	100.00	107LY CD	312GLU	CD	0.01
105PRO C	106ALA	CA	100.00	88THR CB	88THR	C	100.00	107LY CE	109PHE	CD1	0.10
105PRO C	106ALA	CB	45.68	88THR CG2	88THR	C	4.00	107LY CE	109PHE	CD2	0.08
105PRO C	106ALA	C	61.14	88THR CG2	91LEU	CD1	0.02	107LY CE	109PHE	CE1	1.11
106ALA CA	106ALA	CB	100.00	88THR C	89GLY	CA	100.00	107LY CE	109PHE	CE2	0.72
106ALA CA	106ALA	C	100.00	88THR C	89GLY	C	92.43	107LY CE	109PHE	CZ	0.26
106ALA CA	257LEU	CD2	0.04	88THR C	91LEU	CD1	0.02	107LY CE	127ASP	CG	0.36
106ALA CB	106ALA	C	100.00	88THR C	91LEU	CD2	0.05	107LY CE	257LEU	CD2	0.00
106ALA CB	126VAL	CG1	0.15	89GLY CA	89GLY	C	100.00	107LY CE	312GLU	CD	0.07
106ALA CB	128VAL	CG1	0.04	89GLY CA	92SER	CB	0.01	107LY C	108VAL	CA	100.00
106ALA CB	128VAL	CG2	0.04	89GLY C	90LEU	CA	100.00	107LY C	108VAL	CB	16.98
106ALA CB	130ILE	CD	0.04	89GLY C	90LEU	CB	0.01	107LY C	108VAL	CG2	11.06
106ALA CB	247ALA	CB	0.38	89GLY C	90LEU	C	100.00	107LY C	108VAL	C	94.01
106ALA CB	247ALA	C	0.30	90LEU CA	90LEU	CB	100.00	107LY C	126VAL	CG1	0.01

106ALA	CB	248SER	CA	0.01	90LEU	CA	90LEU	CG	100.00	107LY	C	250LEU	CB	0.02
106ALA	CB	250LEU	CD1	0.02	90LEU	CA	90LEU	CD1	59.43	108VA	CA	108VAL	CB	100.00
106ALA	CB	250LEU	CD2	0.00	90LEU	CA	90LEU	CD2	41.91	108VA	CA	108VAL	CG1	100.00
106ALA	CB	251PRO	CG	0.04	90LEU	CA	90LEU	C	100.00	108VA	CA	108VAL	CG2	100.00
106ALA	CB	257LEU	CD2	0.66	90LEU	CA	93LEU	CB	0.00	108VA	CA	108VAL	C	100.00
106ALA	C	107LYS	CA	100.00	90LEU	CB	90LEU	CG	100.00	108VA	CB	108VAL	CG1	100.00
106ALA	C	107LYS	CB	94.94	90LEU	CB	90LEU	CD1	100.00	108VA	CB	108VAL	CG2	100.00
106ALA	C	107LYS	CG	0.43	90LEU	CB	90LEU	CD2	100.00	108VA	CB	108VAL	C	100.00
106ALA	C	107LYS	C	7.01	90LEU	CB	90LEU	C	100.00	108VA	CG1	108VAL	CG2	100.00
106ALA	C	126VAL	CG1	0.09	90LEU	CB	94ARG	CZ	0.66	108VA	CG1	108VAL	C	100.00
107LYS	CA	107LYS	CB	100.00	90LEU	CG	90LEU	CD1	100.00	108VA	CG1	112LEU	CB	0.04
107LYS	CA	107LYS	CG	100.00	90LEU	CG	90LEU	CD2	100.00	108VA	CG1	112LEU	C	1.65
107LYS	CA	107LYS	CD	1.58	90LEU	CG	90LEU	C	62.61	108VA	CG1	113GLU	CA	1.10
107LYS	CA	107LYS	C	100.00	90LEU	CG	94ARG	CZ	2.13	108VA	CG1	113GLU	CB	0.02
107LYS	CB	107LYS	CG	100.00	90LEU	CD1	90LEU	CD2	100.00	108VA	CG1	113GLU	CG	0.29
107LYS	CB	107LYS	CD	100.00	90LEU	CD1	90LEU	C	4.62	108VA	CG1	113GLU	CD	0.03
107LYS	CB	107LYS	CE	20.38	90LEU	CD1	91LEU	CD2	0.00	108VA	CG1	116SER	CB	0.01
107LYS	CB	107LYS	C	100.00	90LEU	CD1	93LEU	CD2	0.02	108VA	CG1	123ALA	CB	0.00
107LYS	CB	109PHE	CE1	0.04	90LEU	CD1	94ARG	CG	0.00	108VA	CG1	123ALA	C	0.00
107LYS	CB	109PHE	CE2	0.26	90LEU	CD1	94ARG	CD	0.05	108VA	CG2	126VAL	CB	1.06
107LYS	CB	109PHE	CZ	0.16	90LEU	CD1	94ARG	CZ	2.11	108VA	CG2	126VAL	CG1	0.72
107LYS	CB	127ASP	CA	0.11	90LEU	CD1	270GLU	CD	0.01	108VA	CG2	126VAL	CG2	0.16
107LYS	CB	127ASP	CB	0.06	90LEU	CD2	90LEU	C	2.51	108VA	CG2	250LEU	CB	0.36
107LYS	CB	127ASP	CG	0.01	90LEU	CD2	91LEU	CD2	0.00	108VA	CG2	250LEU	CD2	0.80
107LYS	CG	107LYS	CD	100.00	90LEU	CD2	93LEU	CD1	0.01	108VA	CG2	250LEU	C	1.30
107LYS	CG	107LYS	CE	100.00	90LEU	CD2	93LEU	CD2	0.02	108VA	CG2	251PRO	CA	0.04
107LYS	CG	107LYS	C	97.85	90LEU	CD2	94ARG	CD	0.02	108VA	CG2	251PRO	CD	0.00
107LYS	CG	108VAL	C	0.04	90LEU	CD2	94ARG	CZ	6.06	108VA	C	109PHE	CA	100.00
107LYS	CG	109PHE	CD1	0.04	90LEU	CD2	270GLU	CD	0.03	108VA	C	109PHE	CB	0.60
107LYS	CG	109PHE	CD2	0.02	90LEU	C	91LEU	CA	100.00	108VA	C	109PHE	CG	0.05
107LYS	CG	109PHE	CE1	0.59	90LEU	C	91LEU	C	100.00	108VA	C	109PHE	CD1	0.01
107LYS	CG	109PHE	CE2	0.67	90LEU	C	94ARG	CD	0.00	108VA	C	109PHE	CD2	0.06
107LYS	CG	109PHE	CZ	0.96	91LEU	CA	91LEU	CB	100.00	108VA	C	109PHE	C	99.62
107LYS	CG	127ASP	CB	0.10	91LEU	CA	91LEU	CG	100.00	109PH	CA	109PHE	CB	100.00
107LYS	CG	127ASP	CG	0.01	91LEU	CA	91LEU	CD1	22.26	109PH	CA	109PHE	CG	100.00
107LYS	CD	107LYS	CE	100.00	91LEU	CA	91LEU	CD2	77.14	109PH	CA	109PHE	CD1	74.92
107LYS	CD	107LYS	C	0.02	91LEU	CA	91LEU	C	100.00	109PH	CA	109PHE	CD2	71.77
107LYS	CD	109PHE	CD1	0.03	91LEU	CB	91LEU	CG	100.00	109PH	CA	109PHE	C	100.00
107LYS	CD	109PHE	CE1	2.63	91LEU	CB	91LEU	CD1	100.00	109PH	CA	110PRO	CD	100.00
107LYS	CD	109PHE	CE2	0.10	91LEU	CB	91LEU	CD2	100.00	109PH	CB	109PHE	CG	100.00
107LYS	CD	109PHE	CZ	1.12	91LEU	CB	91LEU	C	100.00	109PH	CB	109PHE	CD1	100.00
107LYS	CD	127ASP	CB	0.03	91LEU	CG	91LEU	CD1	100.00	109PH	CB	109PHE	CD2	100.00
107LYS	CD	127ASP	CG	0.55	91LEU	CG	91LEU	CD2	100.00	109PH	CB	109PHE	C	100.00
107LYS	CD	312GLU	CD	0.15	91LEU	CG	91LEU	C	13.66	109PH	CB	110PRO	CD	21.02
107LYS	CE	109PHE	CD1	0.03	91LEU	CG	95LYS	CD	0.00	109PH	CB	112LEU	CB	0.02
107LYS	CE	109PHE	CE1	0.61	91LEU	CD1	91LEU	CD2	100.00	109PH	CB	112LEU	CG	0.00
107LYS	CE	109PHE	CZ	0.11	91LEU	CD1	91LEU	C	0.73	109PH	CB	112LEU	CD1	2.09
107LYS	CE	110PRO	CD	0.00	91LEU	CD1	95LYS	CG	0.00	109PH	CB	112LEU	CD2	0.02
107LYS	CE	127ASP	CB	0.04	91LEU	CD1	95LYS	CE	0.02	109PH	CB	316ALA	CB	0.12
107LYS	CE	127ASP	CG	1.10	91LEU	CD1	139TYR	CE1	0.01	109PH	CB	319LEU	CD1	0.01
107LYS	CE	312GLU	CD	0.12	91LEU	CD1	140PHE	CE1	0.04	109PH	CG	109PHE	CD1	100.00
107LYS	C	108VAL	CA	100.00	91LEU	CD1	140PHE	CZ	0.04	109PH	CG	109PHE	CD2	100.00
107LYS	C	108VAL	CB	7.69	91LEU	CD1	188VAL	CG1	0.03	109PH	CG	109PHE	CE1	100.00
107LYS	C	108VAL	CG2	4.50	91LEU	CD2	91LEU	C	2.23	109PH	CG	109PHE	CE2	100.00
107LYS	C	108VAL	C	97.00	91LEU	CD2	94ARG	CZ	0.00	109PH	CG	109PHE	CZ	100.00
107LYS	C	250LEU	CB	0.00	91LEU	CD2	95LYS	CD	0.01	109PH	CG	112LEU	CD1	0.01
107LYS	C	251PRO	CB	2.06	91LEU	CD2	95LYS	CE	0.03	109PH	CG	251PRO	CB	0.06
107LYS	C	251PRO	CG	0.02	91LEU	CD2	139TYR	CE1	0.01	109PH	CG	316ALA	CB	0.31
108VAL	CA	108VAL	CB	100.00	91LEU	CD2	140PHE	CE1	0.08	109PH	CG	319LEU	CD1	0.26
108VAL	CA	108VAL	CG1	100.00	91LEU	CD2	140PHE	CE2	0.01	109PH	CD1	109PHE	CD2	100.00
108VAL	CA	108VAL	CG2	100.00	91LEU	CD2	140PHE	CZ	0.25	109PH	CD1	109PHE	CE1	100.00
108VAL	CA	108VAL	C	100.00	91LEU	CD2	188VAL	CG1	0.00	109PH	CD1	109PHE	CE2	100.00

108VAL CA	251PRO CB	1.69	91LEU C	92SER CA	100.00	109PH CD1	109PHE CZ	100.00
108VAL CB	108VAL CG1	100.00	91LEU C	92SER CB	0.72	109PH CD1	110PRO CD	0.00
108VAL CB	108VAL CG2	100.00	91LEU C	92SER C	99.45	109PH CD1	112LEU CD1	0.05
108VAL CB	108VAL C	100.00	91LEU C	95LYS CE	0.02	109PH CD1	112LEU CD2	0.02
108VAL CB	251PRO CB	0.45	92SER CA	92SER CB	100.00	109PH CD1	251PRO CB	1.50
108VAL CG1	108VAL CG2	100.00	92SER CA	92SER C	100.00	109PH CD1	256LEU CD2	0.16
108VAL CG1	108VAL C	100.00	92SER CB	92SER C	100.00	109PH CD1	312GLU CG	0.14
108VAL CG1	112LEU CB	0.02	92SER C	93LEU CA	100.00	109PH CD1	312GLU CD	0.02
108VAL CG1	112LEU CD1	0.19	92SER C	93LEU C	100.00	109PH CD1	316ALA CA	0.02
108VAL CG1	112LEU C	0.05	93LEU CA	93LEU CB	100.00	109PH CD1	316ALA CB	1.38
108VAL CG1	113GLU CA	1.21	93LEU CA	93LEU CG	100.00	109PH CD1	319LEU CD1	0.92
108VAL CG1	113GLU CB	0.17	93LEU CA	93LEU CD1	71.98	109PH CD1	319LEU CD2	0.05
108VAL CG1	113GLU CG	2.42	93LEU CA	93LEU CD2	27.38	109PH CD2	109PHE CE1	100.00
108VAL CG1	123ALA C	0.01	93LEU CA	93LEU C	100.00	109PH CD2	109PHE CE2	100.00
108VAL CG1	124ARG CA	0.04	93LEU CB	93LEU CG	100.00	109PH CD2	109PHE CZ	100.00
108VAL CG1	124ARG C	0.75	93LEU CB	93LEU CD1	100.00	109PH CD2	112LEU CD1	0.24
108VAL CG1	125GLY C	0.00	93LEU CB	93LEU CD2	100.00	109PH CD2	112LEU CD2	0.02
108VAL CG1	126VAL CB	0.11	93LEU CB	93LEU C	100.00	109PH CD2	251PRO CB	1.28
108VAL CG1	126VAL CG2	0.00	93LEU CG	93LEU CD1	100.00	109PH CD2	256LEU CD2	0.30
108VAL CG2	108VAL C	52.99	93LEU CG	93LEU CD2	100.00	109PH CD2	312GLU CG	0.00
108VAL CG2	112LEU CB	0.04	93LEU CG	93LEU C	98.88	109PH CD2	316ALA CA	0.00
108VAL CG2	112LEU CD1	0.22	93LEU CD1	93LEU CD2	100.00	109PH CD2	316ALA CB	0.81
108VAL CG2	113GLU CB	0.00	93LEU CD1	93LEU C	15.27	109PH CD2	319LEU CD1	0.87
108VAL CG2	113GLU CG	3.43	93LEU CD1	94ARG CG	0.01	109PH CD2	319LEU CD2	0.02
108VAL CG2	113GLU CD	0.00	93LEU CD1	97GLN CG	1.10	109PH CE1	109PHE CE2	100.00
108VAL CG2	126VAL CB	2.69	93LEU CD1	97GLN CD	0.00	109PH CE1	109PHE CZ	100.00
108VAL CG2	126VAL CG1	1.42	93LEU CD1	99LEU CD1	0.00	109PH CE1	251PRO CB	1.53
108VAL CG2	126VAL CG2	0.36	93LEU CD1	268VAL CG1	0.02	109PH CE1	251PRO CG	0.04
108VAL CG2	250LEU CB	0.14	93LEU CD1	268VAL CG2	0.02	109PH CE1	256LEU CB	0.02
108VAL CG2	250LEU CD2	0.02	93LEU CD2	93LEU C	23.73	109PH CE1	256LEU CG	0.00
108VAL CG2	250LEU C	0.77	93LEU CD2	94ARG CG	0.00	109PH CE1	256LEU CD1	0.08
108VAL CG2	251PRO CA	0.94	93LEU CD2	97GLN CG	0.23	109PH CE1	256LEU CD2	0.91
108VAL CG2	251PRO CB	0.26	93LEU CD2	97GLN CD	0.00	109PH CE1	257LEU CD2	0.28
108VAL CG2	251PRO CG	0.01	93LEU CD2	99LEU CD1	0.23	109PH CE1	288THR CG2	0.00
108VAL CG2	251PRO CD	0.08	93LEU CD2	268VAL CG1	0.17	109PH CE1	292LEU CD1	0.55
108VAL C	109PHE CA	100.00	93LEU CD2	268VAL CG2	0.31	109PH CE1	292LEU CD2	0.10
108VAL C	109PHE CB	41.00	93LEU C	94ARG CA	100.00	109PH CE1	312GLU CG	0.79
108VAL C	109PHE CG	17.59	93LEU C	94ARG CB	0.02	109PH CE1	312GLU CD	0.10
108VAL C	109PHE CD1	0.22	93LEU C	94ARG C	99.99	109PH CE1	315VAL CG1	0.01
108VAL C	109PHE CD2	14.05	93LEU C	97GLN CG	0.00	109PH CE1	315VAL CG2	0.04
108VAL C	109PHE C	64.65	94ARG CA	94ARG CB	100.00	109PH CE1	316ALA CA	0.00
109PHE CA	109PHE CB	100.00	94ARG CA	94ARG CG	100.00	109PH CE1	316ALA CB	0.04
109PHE CA	109PHE CG	100.00	94ARG CA	94ARG CD	0.21	109PH CE1	319LEU CD1	1.13
109PHE CA	109PHE CD1	87.40	94ARG CA	94ARG C	100.00	109PH CE1	319LEU CD2	0.03
109PHE CA	109PHE CD2	69.80	94ARG CA	99LEU CD1	0.31	109PH CE2	109PHE CZ	100.00
109PHE CA	109PHE C	100.00	94ARG CB	94ARG CG	100.00	109PH CE2	251PRO CB	1.20
109PHE CA	110PRO CD	100.00	94ARG CB	94ARG CD	100.00	109PH CE2	251PRO CG	0.01
109PHE CB	109PHE CG	100.00	94ARG CB	94ARG CZ	0.02	109PH CE2	256LEU CB	0.04
109PHE CB	109PHE CD1	100.00	94ARG CB	94ARG C	100.00	109PH CE2	256LEU CG	0.00
109PHE CB	109PHE CD2	100.00	94ARG CB	99LEU CD1	0.44	109PH CE2	256LEU CD1	0.02
109PHE CB	109PHE C	100.00	94ARG CB	135THR CG2	0.01	109PH CE2	256LEU CD2	1.94
109PHE CB	110PRO CD	30.64	94ARG CG	94ARG CD	100.00	109PH CE2	257LEU CD2	0.36
109PHE CB	112LEU CG	0.05	94ARG CG	94ARG CZ	1.86	109PH CE2	288THR CG2	0.01
109PHE CB	112LEU CD1	0.22	94ARG CG	94ARG C	0.50	109PH CE2	292LEU CD1	0.86
109PHE CB	112LEU CD2	0.88	94ARG CG	99LEU CB	0.01	109PH CE2	292LEU CD2	0.02
109PHE CB	316ALA CB	0.22	94ARG CG	99LEU CD1	1.86	109PH CE2	312GLU CG	0.21
109PHE CB	319LEU CD1	9.24	94ARG CG	99LEU CD2	0.06	109PH CE2	312GLU CD	0.03
109PHE CB	319LEU CD2	0.24	94ARG CD	94ARG CZ	100.00	109PH CE2	315VAL CG1	0.00
109PHE CG	109PHE CD1	100.00	94ARG CD	99LEU CD1	0.09	109PH CE2	315VAL CG2	0.08
109PHE CG	109PHE CD2	100.00	94ARG CD	99LEU CD2	0.01	109PH CE2	316ALA CB	0.05
109PHE CG	109PHE CE1	100.00	94ARG CD	134LEU CD1	0.01	109PH CE2	319LEU CD1	0.92
109PHE CG	109PHE CE2	100.00	94ARG CD	134LEU CD2	0.01	109PH CE2	319LEU CD2	0.02

109PHE CG	109PHE CZ	100.00	94ARG CZ	99LEU CD1	0.02	109PH CZ	251PRO CB	0.06
109PHE CG	256LEU CD1	0.05	94ARG CZ	134LEU CD1	0.21	109PH CZ	256LEU CB	0.01
109PHE CG	256LEU CD2	0.08	94ARG CZ	134LEU CD2	2.72	109PH CZ	256LEU CG	0.02
109PHE CG	315VAL CG2	0.00	94ARG C	95LYS CA	100.00	109PH CZ	256LEU CD1	0.00
109PHE CG	316ALA CB	0.09	94ARG C	95LYS CB	19.52	109PH CZ	256LEU CD2	0.16
109PHE CG	319LEU CD1	0.45	94ARG C	95LYS CG	0.75	109PH CZ	257LEU CD2	0.57
109PHE CD1	109PHE CD2	100.00	94ARG C	95LYS C	79.92	109PH CZ	288THR CG2	0.24
109PHE CD1	109PHE CE1	100.00	95LYS CA	95LYS CB	100.00	109PH CZ	292LEU CD1	10.89
109PHE CD1	109PHE CE2	100.00	95LYS CA	95LYS CG	100.00	109PH CZ	292LEU CD2	0.22
109PHE CD1	109PHE CZ	100.00	95LYS CA	95LYS CD	25.02	109PH CZ	312GLU CG	0.05
109PHE CD1	256LEU CD1	0.03	95LYS CA	95LYS CE	0.22	109PH CZ	315VAL CG1	0.01
109PHE CD1	256LEU CD2	0.00	95LYS CA	95LYS C	100.00	109PH CZ	315VAL CG2	0.03
109PHE CD1	312GLU CB	0.00	95LYS CB	95LYS CG	100.00	109PH CZ	319LEU CD1	0.22
109PHE CD1	315VAL CG2	0.39	95LYS CB	95LYS CD	100.00	109PH C	110PRO CA	100.00
109PHE CD1	316ALA CA	0.02	95LYS CB	95LYS CE	10.67	109PH C	110PRO CB	0.02
109PHE CD1	316ALA CB	2.46	95LYS CB	95LYS C	100.00	109PH C	110PRO CD	100.00
109PHE CD1	319LEU CD1	0.26	95LYS CG	95LYS CD	100.00	109PH C	110PRO C	100.00
109PHE CD2	109PHE CE1	100.00	95LYS CG	95LYS CE	100.00	110PR CA	110PRO CB	100.00
109PHE CD2	109PHE CE2	100.00	95LYS CG	95LYS C	66.57	110PR CA	110PRO CG	100.00
109PHE CD2	109PHE CZ	100.00	95LYS CD	95LYS CE	100.00	110PR CA	110PRO CD	100.00
109PHE CD2	112LEU CD1	0.05	95LYS CD	95LYS C	1.50	110PR CA	110PRO C	100.00
109PHE CD2	112LEU CD2	0.07	95LYS CE	98ASP CG	0.02	110PR CA	113GLU CD	0.01
109PHE CD2	251PRO CB	0.01	95LYS CE	140PHE CE1	0.05	110PR CB	110PRO CG	100.00
109PHE CD2	256LEU CB	0.15	95LYS CE	140PHE CZ	0.02	110PR CB	110PRO CD	100.00
109PHE CD2	256LEU CD1	2.32	95LYS C	96SER CA	100.00	110PR CB	110PRO C	100.00
109PHE CD2	256LEU CD2	0.73	95LYS C	96SER CB	5.27	110PR CG	110PRO CD	100.00
109PHE CD2	315VAL CG2	0.01	95LYS C	96SER C	95.14	110PR CG	110PRO C	90.38
109PHE CD2	316ALA CA	0.00	95LYS C	97GLN C	0.02	110PR CG	316ALA CB	0.01
109PHE CD2	316ALA CB	0.03	96SER CA	96SER CB	100.00	110PR CD	110PRO C	83.73
109PHE CD2	319LEU CD1	2.49	96SER CA	96SER C	100.00	110PR CD	316ALA CB	0.01
109PHE CE1	109PHE CE2	100.00	96SER CB	96SER C	100.00	110PR C	111GLY CA	100.00
109PHE CE1	109PHE CZ	100.00	96SER C	97GLN CA	100.00	110PR C	111GLY C	55.45
109PHE CE1	256LEU CD1	0.01	96SER C	97GLN CB	6.44	111GL CA	111GLY C	100.00
109PHE CE1	288THR CG2	0.01	96SER C	97GLN CG	0.98	111GL CA	320LEU CD1	0.12
109PHE CE1	292LEU CD1	0.02	96SER C	97GLN CD	0.09	111GL CA	320LEU CD2	0.19
109PHE CE1	292LEU CD2	0.03	96SER C	97GLN C	95.94	111GL C	112LEU CA	100.00
109PHE CE1	312GLU CB	0.07	97GLN CA	97GLN CB	100.00	111GL C	112LEU CB	47.99
109PHE CE1	312GLU CG	1.69	97GLN CA	97GLN CG	100.00	111GL C	112LEU CG	13.71
109PHE CE1	312GLU CD	0.12	97GLN CA	97GLN CD	98.66	111GL C	112LEU CD1	7.88
109PHE CE1	315VAL CG2	5.27	97GLN CA	97GLN C	100.00	111GL C	112LEU CD2	0.21
109PHE CE1	316ALA CA	0.00	97GLN CB	97GLN CG	100.00	111GL C	112LEU C	44.64
109PHE CE1	316ALA CB	0.06	97GLN CB	97GLN CD	100.00	111GL C	320LEU CD1	0.05
109PHE CE1	319LEU CD1	0.04	97GLN CB	97GLN C	100.00	111GL C	320LEU CD2	0.18
109PHE CE2	109PHE CZ	100.00	97GLN CB	99LEU CG	0.10	112LE CA	112LEU CB	100.00
109PHE CE2	251PRO CB	0.04	97GLN CB	99LEU CD1	0.39	112LE CA	112LEU CG	100.00
109PHE CE2	256LEU CB	0.55	97GLN CB	99LEU CD2	3.11	112LE CA	112LEU CD1	41.15
109PHE CE2	256LEU CD1	0.58	97GLN CB	268VAL CG2	0.03	112LE CA	112LEU CD2	44.44
109PHE CE2	256LEU CD2	0.35	97GLN CG	97GLN CD	100.00	112LE CA	112LEU C	100.00
109PHE CE2	292LEU CD1	2.13	97GLN CG	97GLN C	1.17	112LE CA	115LEU CD1	0.06
109PHE CE2	292LEU CD2	0.01	97GLN CG	99LEU CD2	0.01	112LE CA	115LEU CD2	0.00
109PHE CE2	312GLU CG	0.07	97GLN CG	266THR CG2	0.01	112LE CB	112LEU CG	100.00
109PHE CE2	315VAL CG2	0.19	97GLN CG	268VAL CG1	0.10	112LE CB	112LEU CD1	100.00
109PHE CE2	319LEU CD1	0.13	97GLN CG	268VAL CG2	0.80	112LE CB	112LEU CD2	100.00
109PHE CZ	256LEU CB	0.01	97GLN CD	266THR CB	0.02	112LE CB	112LEU C	100.00
109PHE CZ	256LEU CD2	0.02	97GLN CD	266THR CG2	0.10	112LE CB	115LEU CD1	0.07
109PHE CZ	292LEU CD1	8.00	97GLN CD	268VAL CG1	0.24	112LE CG	112LEU CD1	100.00
109PHE CZ	292LEU CD2	1.72	97GLN CD	268VAL CG2	0.90	112LE CG	112LEU CD2	100.00
109PHE CZ	312GLU CG	0.39	97GLN C	98ASP CA	100.00	112LE CG	112LEU C	4.20
109PHE CZ	312GLU CD	0.01	97GLN C	98ASP CB	53.15	112LE CG	115LEU CD1	0.06
109PHE CZ	315VAL CG2	0.36	97GLN C	98ASP C	98.93	112LE CG	115LEU CD2	0.00
109PHE CZ	319LEU CD1	0.00	97GLN C	99LEU CD1	0.04	112LE CG	320LEU CD1	0.01
109PHE C	110PRO CA	100.00	97GLN C	99LEU CD2	0.04	112LE CG	320LEU CD2	0.16

109PHE	C	110PRO	CB	0.05
109PHE	C	110PRO	CD	100.00
109PHE	C	110PRO	C	100.00
109PHE	C	112LEU	CD2	0.02
110PRO	CA	110PRO	CB	100.00
110PRO	CA	110PRO	CG	100.00
110PRO	CA	110PRO	CD	100.00
110PRO	CA	110PRO	C	100.00
110PRO	CA	113GLU	CD	0.04
110PRO	CB	110PRO	CG	100.00
110PRO	CB	110PRO	CD	100.00
110PRO	CB	110PRO	C	100.00
110PRO	CB	316ALA	CB	0.00
110PRO	CB	320LEU	CG	0.02
110PRO	CB	320LEU	CD1	0.16
110PRO	CB	320LEU	CD2	0.09
110PRO	CG	110PRO	CD	100.00
110PRO	CG	110PRO	C	77.75
110PRO	CG	316ALA	CB	0.32
110PRO	CG	316ALA	C	0.00
110PRO	CG	320LEU	CG	0.01
110PRO	CG	320LEU	CD1	0.38
110PRO	CG	320LEU	CD2	0.06
110PRO	CD	110PRO	C	73.19
110PRO	CD	316ALA	CA	0.00
110PRO	CD	316ALA	CB	1.06
110PRO	C	111GLY	CA	100.00
110PRO	C	111GLY	C	42.55
110PRO	C	112LEU	CD2	0.00
110PRO	C	319LEU	CD2	0.01
110PRO	C	320LEU	CD1	0.02
110PRO	C	320LEU	CD2	0.50
111GLY	CA	111GLY	C	100.00
111GLY	CA	114ARG	CZ	0.02
111GLY	CA	319LEU	CD2	0.01
111GLY	CA	319LEU	C	0.00
111GLY	CA	320LEU	CG	0.03
111GLY	CA	320LEU	CD1	0.49
111GLY	CA	320LEU	CD2	2.18
111GLY	C	112LEU	CA	100.00
111GLY	C	112LEU	CB	53.49
111GLY	C	112LEU	CG	3.48
111GLY	C	112LEU	CD1	0.53
111GLY	C	112LEU	CD2	1.04
111GLY	C	112LEU	C	41.02
111GLY	C	114ARG	CZ	0.10
111GLY	C	319LEU	CD1	0.01
111GLY	C	319LEU	CD2	0.04
111GLY	C	320LEU	CD1	0.03
111GLY	C	320LEU	CD2	0.10
111GLY	C	324PRO	CG	0.01
112LEU	CA	112LEU	CB	100.00
112LEU	CA	112LEU	CG	100.00
112LEU	CA	112LEU	CD1	32.92
112LEU	CA	112LEU	CD2	46.81
112LEU	CA	112LEU	C	100.00
112LEU	CA	115LEU	CD1	0.01
112LEU	CB	112LEU	CG	100.00
112LEU	CB	112LEU	CD1	100.00
112LEU	CB	112LEU	CD2	100.00
112LEU	CB	112LEU	C	100.00
112LEU	CB	115LEU	CD1	0.15
97GLN	C	263GLY	CA	0.00
98ASP	CA	98ASP	CB	100.00
98ASP	CA	98ASP	CG	100.00
98ASP	CA	98ASP	C	100.00
98ASP	CB	98ASP	CG	100.00
98ASP	CB	98ASP	C	100.00
98ASP	CB	264ARG	CB	0.00
98ASP	CB	264ARG	CD	0.00
98ASP	CG	98ASP	C	99.82
98ASP	CG	264ARG	CB	0.02
98ASP	CG	264ARG	CG	0.00
98ASP	CG	264ARG	CD	0.26
98ASP	CG	264ARG	CZ	0.06
98ASP	C	99LEU	CA	100.00
98ASP	C	99LEU	CB	3.25
98ASP	C	99LEU	CG	1.24
98ASP	C	99LEU	CD1	0.02
98ASP	C	99LEU	CD2	0.01
98ASP	C	99LEU	C	96.89
98ASP	C	263GLY	CA	0.22
99LEU	CA	99LEU	CB	100.00
99LEU	CA	99LEU	CG	100.00
99LEU	CA	99LEU	CD1	6.38
99LEU	CA	99LEU	CD2	94.01
99LEU	CA	99LEU	C	100.00
99LEU	CA	263GLY	CA	2.25
99LEU	CB	99LEU	CG	100.00
99LEU	CB	99LEU	CD1	100.00
99LEU	CB	99LEU	CD2	100.00
99LEU	CB	99LEU	C	100.00
99LEU	CB	134LEU	CD1	2.44
99LEU	CB	134LEU	CD2	0.58
99LEU	CB	135THR	CG2	0.02
99LEU	CG	99LEU	CD1	100.00
99LEU	CG	99LEU	CD2	100.00
99LEU	CG	99LEU	C	2.62
99LEU	CG	135THR	CG2	0.00
99LEU	CG	261SER	CB	0.01
99LEU	CD1	99LEU	CD2	100.00
99LEU	CD1	99LEU	C	0.04
99LEU	CD1	101ALA	C	0.02
99LEU	CD1	134LEU	CD1	0.09
99LEU	CD1	134LEU	CD2	0.18
99LEU	CD1	261SER	CA	0.01
99LEU	CD1	261SER	CB	2.98
99LEU	CD1	261SER	C	0.08
99LEU	CD1	262LEU	C	0.04
99LEU	CD1	263GLY	CA	0.01
99LEU	CD1	268VAL	CG1	0.11
99LEU	CD1	268VAL	CG2	2.02
99LEU	CD2	99LEU	C	0.34
99LEU	CD2	100PHE	C	0.01
99LEU	CD2	101ALA	C	0.00
99LEU	CD2	134LEU	CB	0.07
99LEU	CD2	134LEU	CD1	0.09
99LEU	CD2	134LEU	CD2	0.01
99LEU	CD2	135THR	CG2	0.01
99LEU	CD2	261SER	CB	2.56
99LEU	CD2	261SER	C	0.44
99LEU	CD2	262LEU	CA	0.04
99LEU	CD2	262LEU	C	1.48
99LEU	CD2	263GLY	CA	0.42
112LE	CD1	112LEU	CD2	100.00
112LE	CD1	114ARG	CZ	0.00
112LE	CD1	115LEU	CB	0.00
112LE	CD1	115LEU	CD1	0.17
112LE	CD1	115LEU	CD2	0.01
112LE	CD1	251PRO	CB	0.46
112LE	CD1	256LEU	CD1	0.06
112LE	CD1	256LEU	CD2	0.70
112LE	CD1	316ALA	CA	0.02
112LE	CD1	316ALA	CB	0.22
112LE	CD1	319LEU	CB	1.72
112LE	CD1	319LEU	CD1	0.59
112LE	CD1	319LEU	CD2	0.10
112LE	CD1	319LEU	C	0.01
112LE	CD1	320LEU	CG	0.05
112LE	CD1	320LEU	CD1	0.41
112LE	CD1	320LEU	CD2	0.59
112LE	CD1	327LEU	CD1	0.00
112LE	CD1	327LEU	CD2	0.01
112LE	CD2	114ARG	CZ	0.02
112LE	CD2	115LEU	CG	0.00
112LE	CD2	115LEU	CD1	2.02
112LE	CD2	115LEU	CD2	0.25
112LE	CD2	251PRO	CA	0.01
112LE	CD2	251PRO	CB	0.20
112LE	CD2	256LEU	CD1	0.26
112LE	CD2	256LEU	CD2	0.42
112LE	CD2	316ALA	CB	0.00
112LE	CD2	319LEU	CB	2.14
112LE	CD2	319LEU	CG	0.03
112LE	CD2	319LEU	CD1	0.60
112LE	CD2	319LEU	CD2	0.18
112LE	CD2	319LEU	C	0.04
112LE	CD2	320LEU	CA	0.02
112LE	CD2	320LEU	CG	0.04
112LE	CD2	320LEU	CD1	0.22
112LE	CD2	320LEU	CD2	1.92
112LE	CD2	327LEU	CD1	0.32
112LE	CD2	327LEU	CD2	0.51
112LE	C	113GLU	CA	100.00
112LE	C	113GLU	C	100.00
112LE	C	115LEU	CD1	0.00
113GL	CA	113GLU	CB	100.00
113GL	CA	113GLU	CG	100.00
113GL	CA	113GLU	CD	76.41
113GL	CA	113GLU	C	100.00
113GL	CA	123ALA	CB	0.02
113GL	CB	113GLU	CG	100.00
113GL	CB	113GLU	CD	100.00
113GL	CB	113GLU	C	100.00
113GL	CB	120GLU	CG	2.06
113GL	CB	120GLU	CD	0.00
113GL	CB	123ALA	CB	0.06
113GL	CG	113GLU	CD	100.00
113GL	CG	123ALA	CB	0.02
113GL	CG	123ALA	C	0.14
113GL	CG	124ARG	CA	0.80
113GL	CG	124ARG	CB	0.01
113GL	CG	124ARG	CG	0.20
113GL	CG	124ARG	CZ	0.01
113GL	CD	124ARG	CG	0.00
113GL	CD	124ARG	CZ	0.11

112LEU CB	319LEU CD1	0.00	99LEU CD2	265GLY CA	0.04	113GL C	114ARG CA	100.00
112LEU CB	324PRO CG	1.95	99LEU CD2	265GLY C	0.10	113GL C	114ARG CB	0.14
112LEU CB	324PRO CD	0.93	99LEU CD2	268VAL CB	0.00	113GL C	114ARG CG	0.02
112LEU CG	112LEU CD1	100.00	99LEU CD2	268VAL CG1	0.06	113GL C	114ARG C	99.86
112LEU CG	112LEU CD2	100.00	99LEU CD2	268VAL CG2	0.50	113GL C	120GLU CB	0.00
112LEU CG	112LEU C	53.05	99LEU C	100PHE CA	100.00	113GL C	120GLU CG	0.08
112LEU CG	115LEU CD1	0.01	99LEU C	100PHE CB	82.52	114AR CA	114ARG CB	100.00
112LEU CG	319LEU CD1	0.01	99LEU C	100PHE CG	0.04	114AR CA	114ARG CG	100.00
112LEU CG	319LEU CD2	0.02	99LEU C	100PHE CD2	0.16	114AR CA	114ARG CD	3.83
112LEU CG	320LEU CD2	0.04	99LEU C	100PHE C	16.10	114AR CA	114ARG C	100.00
112LEU CD1	112LEU CD2	100.00	99LEU C	134LEU CD1	0.09	114AR CA	120GLU CG	0.06
112LEU CD1	112LEU C	8.82	99LEU C	134LEU CD2	0.01	114AR CA	120GLU CD	0.01
112LEU CD1	115LEU CD1	0.59	99LEU C	135THR CG2	0.03	114AR CB	114ARG CG	100.00
112LEU CD1	115LEU CD2	0.10	100PHI CA	100PHE CB	100.00	114AR CB	114ARG CD	100.00
112LEU CD1	251PRO CA	0.02	100PHI CA	100PHE CG	100.00	114AR CB	114ARG CZ	1.64
112LEU CD1	251PRO CB	0.93	100PHI CA	100PHE CD1	84.07	114AR CB	114ARG C	100.00
112LEU CD1	251PRO C	0.08	100PHI CA	100PHE CD2	52.58	114AR CB	115LEU CD2	0.00
112LEU CD1	252GLY C	0.14	100PHI CA	100PHE C	100.00	114AR CB	120GLU CD	0.04
112LEU CD1	253SER CB	0.26	100PHI CA	135THR CG2	0.48	114AR CG	114ARG CD	100.00
112LEU CD1	256LEU CD1	1.68	100PHI CB	100PHE CG	100.00	114AR CG	114ARG CZ	25.66
112LEU CD1	256LEU CD2	2.88	100PHI CB	100PHE CD1	100.00	114AR CG	114ARG C	72.20
112LEU CD1	316ALA CA	0.00	100PHI CB	100PHE CD2	100.00	114AR CG	115LEU CD1	0.00
112LEU CD1	319LEU CB	0.89	100PHI CB	100PHE C	100.00	114AR CG	120GLU CG	0.23
112LEU CD1	319LEU CD1	0.68	100PHI CB	133GLU CD	0.01	114AR CG	120GLU CD	1.52
112LEU CD1	319LEU CD2	0.16	100PHI CB	135THR CG2	2.36	114AR CD	114ARG CZ	100.00
112LEU CD1	319LEU C	0.01	100PHI CB	164ARG CD	0.28	114AR CD	114ARG C	1.24
112LEU CD1	320LEU CD1	0.01	100PHI CB	264ARG CZ	0.50	114AR CD	120GLU CD	0.01
112LEU CD1	320LEU CD2	0.19	100PHI CG	100PHE CD1	100.00	114AR CD	320LEU CD2	0.00
112LEU CD1	324PRO CB	0.02	100PHI CG	100PHE CD2	100.00	114AR CZ	115LEU CG	0.02
112LEU CD1	324PRO CG	0.23	100PHI CG	100PHE CE1	100.00	114AR CZ	115LEU CD1	0.31
112LEU CD1	324PRO CD	0.02	100PHI CG	100PHE CE2	100.00	114AR CZ	115LEU CD2	0.96
112LEU CD2	112LEU C	0.03	100PHI CG	100PHE CZ	100.00	114AR CZ	120GLU CB	0.02
112LEU CD2	114ARG CZ	0.04	100PHI CG	100PHE C	99.72	114AR CZ	120GLU CG	0.23
112LEU CD2	115LEU CD1	0.30	100PHI CG	164ARG CB	0.00	114AR CZ	120GLU CD	0.02
112LEU CD2	115LEU CD2	0.02	100PHI CG	164ARG CG	0.03	114AR CZ	320LEU CD1	0.01
112LEU CD2	251PRO CB	0.87	100PHI CG	164ARG CD	9.26	114AR CZ	320LEU CD2	0.02
112LEU CD2	251PRO C	0.15	100PHI CG	164ARG CZ	0.00	114AR C	115LEU CA	100.00
112LEU CD2	252GLY CA	0.01	100PHI CG	264ARG CZ	0.00	114AR C	115LEU CB	1.86
112LEU CD2	252GLY C	0.02	100PHI CD1	100PHE CD2	100.00	114AR C	115LEU CG	0.12
112LEU CD2	253SER CB	0.86	100PHI CD1	100PHE CE1	100.00	114AR C	115LEU CD1	0.17
112LEU CD2	256LEU CD1	0.44	100PHI CD1	100PHE CE2	100.00	114AR C	115LEU CD2	0.07
112LEU CD2	256LEU CD2	2.63	100PHI CD1	100PHE CZ	100.00	114AR C	115LEU C	97.31
112LEU CD2	316ALA CB	0.02	100PHI CD1	100PHE C	78.54	115LEI CA	115LEU CB	100.00
112LEU CD2	319LEU CB	1.00	100PHI CD1	101ALA CB	10.57	115LEI CA	115LEU CG	100.00
112LEU CD2	319LEU CD1	0.84	100PHI CD1	133GLU CD	0.00	115LEI CA	115LEU CD1	28.08
112LEU CD2	319LEU CD2	0.47	100PHI CD1	164ARG CB	0.25	115LEI CA	115LEU CD2	76.12
112LEU CD2	319LEU C	0.01	100PHI CD1	164ARG CG	2.16	115LEI CA	115LEU C	100.00
112LEU CD2	320LEU CD1	0.03	100PHI CD1	164ARG CD	17.42	115LEI CB	115LEU CG	100.00
112LEU CD2	320LEU CD2	0.22	100PHI CD1	164ARG C	0.00	115LEI CB	115LEU CD1	100.00
112LEU CD2	324PRO CB	0.00	100PHI CD1	165VAL CG1	0.04	115LEI CB	115LEU CD2	100.00
112LEU CD2	324PRO CG	0.40	100PHI CD1	165VAL CG2	0.07	115LEI CB	115LEU C	100.00
112LEU CD2	324PRO CD	0.05	100PHI CD1	262LEU C	0.01	115LEI CB	252GLY CA	0.00
112LEU C	113GLU CA	100.00	100PHI CD1	263GLY CA	0.01	115LEI CB	327LEU CD1	0.01
112LEU C	113GLU C	100.00	100PHI CD2	100PHE CE1	100.00	115LEI CB	327LEU CD2	0.04
112LEU C	115LEU CD1	0.01	100PHI CD2	100PHE CE2	100.00	115LEI CG	115LEU CD1	100.00
113GLU CA	113GLU CB	100.00	100PHI CD2	100PHE CZ	100.00	115LEI CG	115LEU CD2	100.00
113GLU CA	113GLU CG	100.00	100PHI CD2	100PHE C	0.00	115LEI CG	115LEU C	4.55
113GLU CA	113GLU CD	54.90	100PHI CD2	164ARG CG	0.01	115LEI CG	327LEU CD1	0.05
113GLU CA	113GLU C	100.00	100PHI CD2	164ARG CD	3.24	115LEI CG	327LEU CD2	0.06
113GLU CA	123ALA CB	0.02	100PHI CD2	164ARG CZ	0.03	115LEI CD1	115LEU CD2	100.00
113GLU CB	113GLU CG	100.00	100PHI CD2	262LEU C	0.56	115LEI CD1	115LEU C	0.00
113GLU CB	113GLU CD	100.00	100PHI CD2	263GLY CA	3.90	115LEI CD1	319LEU CD2	0.03

113GLU	CB	113GLU	C	100.00	100PH	CD2	263GLY	C	2.04	115LE	CD1	320LEU	CD1	0.01
113GLU	CB	120GLU	CB	0.00	100PH	CD2	264ARG	CG	0.47	115LE	CD1	320LEU	CD2	0.33
113GLU	CB	120GLU	CG	2.61	100PH	CD2	264ARG	CD	0.00	115LE	CD1	327LEU	CB	0.00
113GLU	CB	123ALA	CB	0.02	100PH	CD2	264ARG	CZ	1.35	115LE	CD1	327LEU	CG	0.08
113GLU	CG	113GLU	CD	100.00	100PH	CE1	100PHE	CE2	100.00	115LE	CD1	327LEU	CD1	0.35
113GLU	CG	123ALA	CB	0.00	100PH	CE1	100PHE	CZ	100.00	115LE	CD1	327LEU	CD2	0.92
113GLU	CG	123ALA	C	0.00	100PH	CE1	101ALA	CB	3.75	115LE	CD2	115LEU	C	0.08
113GLU	CG	124ARG	CA	0.13	100PH	CE1	164ARG	CB	0.34	115LE	CD2	319LEU	CD2	0.08
113GLU	CG	124ARG	CB	0.00	100PH	CE1	164ARG	CG	6.18	115LE	CD2	320LEU	CD1	0.00
113GLU	CG	124ARG	CG	0.11	100PH	CE1	164ARG	CD	8.45	115LE	CD2	320LEU	CD2	0.01
113GLU	CD	124ARG	CG	0.01	100PH	CE1	164ARG	C	0.18	115LE	CD2	327LEU	CA	0.00
113GLU	C	114ARG	CA	100.00	100PH	CE1	165VAL	CA	0.00	115LE	CD2	327LEU	CB	0.02
113GLU	C	114ARG	CB	0.36	100PH	CE1	165VAL	CG1	0.01	115LE	CD2	327LEU	CG	0.14
113GLU	C	114ARG	CG	0.06	100PH	CE1	165VAL	CG2	0.02	115LE	CD2	327LEU	CD1	0.54
113GLU	C	114ARG	C	99.44	100PH	CE1	168VAL	CG1	0.38	115LE	CD2	327LEU	CD2	0.53
113GLU	C	120GLU	CB	0.02	100PH	CE1	168VAL	CG2	0.28	115LE	C	116SER	CA	100.00
113GLU	C	120GLU	CG	0.16	100PH	CE1	262LEU	CB	0.43	115LE	C	116SER	CB	0.06
114ARG	CA	114ARG	CB	100.00	100PH	CE1	262LEU	CD1	0.12	115LE	C	116SER	C	99.97
114ARG	CA	114ARG	CG	100.00	100PH	CE1	262LEU	CD2	0.04	115LE	C	252GLY	CA	0.18
114ARG	CA	114ARG	CD	1.36	100PH	CE1	263GLY	CA	0.06	116SE	CA	116SER	CB	100.00
114ARG	CA	114ARG	C	100.00	100PH	CE1	263GLY	C	0.05	116SE	CA	116SER	C	100.00
114ARG	CA	120GLU	CG	0.42	100PH	CE2	100PHE	CZ	100.00	116SE	CA	117PRO	CD	100.00
114ARG	CA	120GLU	CD	0.03	100PH	CE2	164ARG	CG	0.05	116SE	CA	252GLY	CA	0.00
114ARG	CB	114ARG	CG	100.00	100PH	CE2	164ARG	CD	3.48	116SE	CB	116SER	C	100.00
114ARG	CB	114ARG	CD	100.00	100PH	CE2	164ARG	CZ	0.04	116SE	CB	117PRO	CD	1.18
114ARG	CB	114ARG	CZ	0.33	100PH	CE2	262LEU	CB	2.31	116SE	CB	123ALA	CB	6.78
114ARG	CB	114ARG	C	100.00	100PH	CE2	262LEU	CD1	0.00	116SE	C	117PRO	CA	100.00
114ARG	CB	120GLU	CG	0.00	100PH	CE2	262LEU	CD2	0.02	116SE	C	117PRO	CB	0.07
114ARG	CB	120GLU	CD	0.03	100PH	CE2	262LEU	C	1.76	116SE	C	117PRO	CD	100.00
114ARG	CG	114ARG	CD	100.00	100PH	CE2	263GLY	CA	6.16	116SE	C	117PRO	C	100.00
114ARG	CG	114ARG	CZ	13.92	100PH	CE2	263GLY	C	15.27	117PR	CA	117PRO	CB	100.00
114ARG	CG	114ARG	C	70.01	100PH	CE2	264ARG	CA	0.00	117PR	CA	117PRO	CG	100.00
114ARG	CG	115LEU	CG	0.01	100PH	CE2	264ARG	CG	0.43	117PR	CA	117PRO	CD	100.00
114ARG	CG	115LEU	CD1	0.07	100PH	CE2	264ARG	CD	0.00	117PR	CA	117PRO	C	100.00
114ARG	CG	115LEU	CD2	0.09	100PH	CE2	264ARG	CZ	0.62	117PR	CA	122ILE	CD	0.44
114ARG	CG	120GLU	CG	0.04	100PH	CZ	101ALA	CB	0.00	117PR	CB	117PRO	CG	100.00
114ARG	CG	120GLU	CD	1.30	100PH	CZ	164ARG	CB	0.03	117PR	CB	117PRO	CD	100.00
114ARG	CD	114ARG	CZ	100.00	100PH	CZ	164ARG	CG	1.38	117PR	CB	117PRO	C	100.00
114ARG	CD	114ARG	C	0.19	100PH	CZ	164ARG	CD	5.75	117PR	CB	118LEU	CD2	0.03
114ARG	CD	115LEU	CD2	0.01	100PH	CZ	168VAL	CG1	0.40	117PR	CB	122ILE	CD	10.46
114ARG	CD	120GLU	CD	0.00	100PH	CZ	168VAL	CG2	0.12	117PR	CB	224VAL	CG1	0.11
114ARG	CZ	115LEU	CD1	0.02	100PH	CZ	262LEU	CB	3.26	117PR	CG	117PRO	CD	100.00
114ARG	CZ	115LEU	CD2	0.36	100PH	CZ	262LEU	CG	0.01	117PR	CG	117PRO	C	97.24
114ARG	CZ	120GLU	CD	0.05	100PH	CZ	262LEU	CD1	0.16	117PR	CG	118LEU	CD1	0.01
114ARG	CZ	320LEU	CD2	0.15	100PH	CZ	262LEU	CD2	0.30	117PR	CG	118LEU	CD2	0.00
114ARG	C	115LEU	CA	100.00	100PH	CZ	262LEU	C	0.01	117PR	CG	249VAL	CG1	0.03
114ARG	C	115LEU	CB	1.04	100PH	CZ	263GLY	CA	0.68	117PR	CG	249VAL	CG2	0.96
114ARG	C	115LEU	CG	0.06	100PH	CZ	263GLY	C	0.32	117PR	CG	224VAL	CG1	0.28
114ARG	C	115LEU	CD1	0.01	100PH	CZ	264ARG	CG	0.00	117PR	CD	117PRO	C	88.90
114ARG	C	115LEU	CD2	0.01	100PH	C	101ALA	CA	100.00	117PR	C	118LEU	CA	100.00
114ARG	C	115LEU	C	98.01	100PH	C	101ALA	CB	75.30	117PR	C	118LEU	CB	0.02
115LEU	CA	115LEU	CB	100.00	100PH	C	101ALA	C	27.38	117PR	C	118LEU	CD1	0.00
115LEU	CA	115LEU	CG	100.00	100PH	C	134LEU	CB	1.12	117PR	C	118LEU	C	99.96
115LEU	CA	115LEU	CD1	6.85	100PH	C	134LEU	CG	0.01	117PR	C	118LEU	CD1	0.00
115LEU	CA	115LEU	CD2	92.30	100PH	C	134LEU	CD1	0.12	117PR	C	118LEU	CD2	0.14
115LEU	CA	115LEU	C	100.00	101AL	CA	101ALA	CB	100.00	117PR	C	119LYS	CG	0.00
115LEU	CB	115LEU	CG	100.00	101AL	CA	101ALA	C	100.00	117PR	C	119LYS	CD	0.06
115LEU	CB	115LEU	CD1	100.00	101AL	CA	134LEU	CB	0.00	118LE	CA	118LEU	CB	100.00
115LEU	CB	115LEU	CD2	100.00	101AL	CA	134LEU	CD1	0.02	118LE	CA	118LEU	CG	100.00
115LEU	CB	115LEU	C	100.00	101AL	CB	101ALA	C	100.00	118LE	CA	118LEU	CD1	13.90
115LEU	CB	252GLY	CA	0.03	101AL	CB	103LEU	CD1	0.36	118LE	CA	118LEU	CD2	86.91
115LEU	CG	115LEU	CD1	100.00	101AL	CB	131VAL	CG1	0.03	118LE	CA	118LEU	C	100.00

115LEU	CG	115LEU	CD2	100.00
115LEU	CG	115LEU	C	4.13
115LEU	CD1	115LEU	CD2	100.00
115LEU	CD1	115LEU	C	0.00
115LEU	CD1	253SER	CB	0.02
115LEU	CD1	256LEU	CD1	0.00
115LEU	CD1	323PRO	CB	0.02
115LEU	CD1	324PRO	CG	2.97
115LEU	CD1	324PRO	CD	0.58
115LEU	CD1	327LEU	CD1	0.12
115LEU	CD1	327LEU	CD2	0.04
115LEU	CD2	115LEU	C	0.09
115LEU	CD2	252GLY	CA	0.00
115LEU	CD2	252GLY	C	0.15
115LEU	CD2	253SER	CB	0.01
115LEU	CD2	324PRO	CG	0.32
115LEU	CD2	324PRO	CD	0.01
115LEU	CD2	326ASP	CB	0.03
115LEU	CD2	326ASP	CG	0.02
115LEU	CD2	327LEU	CD1	0.73
115LEU	CD2	327LEU	CD2	0.57
115LEU	C	116SER	CA	100.00
115LEU	C	116SER	CB	0.34
115LEU	C	116SER	C	99.82
115LEU	C	252GLY	CA	0.96
116SER	CA	116SER	CB	100.00
116SER	CA	116SER	C	100.00
116SER	CA	117PRO	CD	100.00
116SER	CA	252GLY	CA	0.45
116SER	CB	116SER	C	100.00
116SER	CB	117PRO	CD	1.25
116SER	CB	123ALA	CB	15.34
116SER	CB	252GLY	CA	0.19
116SER	C	117PRO	CA	100.00
116SER	C	117PRO	CB	0.08
116SER	C	117PRO	CD	100.00
116SER	C	117PRO	C	100.00
117PRO	CA	117PRO	CB	100.00
117PRO	CA	117PRO	CG	100.00
117PRO	CA	117PRO	CD	100.00
117PRO	CA	117PRO	C	100.00
117PRO	CA	122ILE	CD	1.32
117PRO	CB	117PRO	CG	100.00
117PRO	CB	117PRO	CD	100.00
117PRO	CB	117PRO	C	100.00
117PRO	CB	118LEU	CD2	0.00
117PRO	CB	122ILE	CD	5.92
117PRO	CB	224VAL	CG1	0.00
117PRO	CB	225ARG	CA	0.01
117PRO	CB	225ARG	CB	0.01
117PRO	CB	225ARG	CZ	0.08
117PRO	CG	117PRO	CD	100.00
117PRO	CG	117PRO	C	98.02
117PRO	CG	249VAL	CG1	5.36
117PRO	CG	249VAL	CG2	0.02
117PRO	CG	250LEU	CD2	0.04
117PRO	CG	224VAL	CG1	0.44
117PRO	CG	224VAL	C	0.00
117PRO	CG	225ARG	CA	0.03
117PRO	CG	225ARG	CB	0.02
117PRO	CG	225ARG	CG	0.18
117PRO	CG	225ARG	CD	0.00

101AL/	CB	165VAL	CG1	0.23
101AL/	CB	165VAL	CG2	0.34
101AL/	CB	168VAL	CG1	0.00
101AL/	CB	168VAL	CG2	0.57
101AL/	CB	262LEU	CB	0.00
101AL/	CB	262LEU	CD1	0.14
101AL/	C	102ASN	CA	100.00
101AL/	C	102ASN	CB	33.17
101AL/	C	102ASN	C	72.86
101AL/	C	103LEU	CD1	0.04
101AL/	C	134LEU	CG	0.02
101AL/	C	134LEU	CD1	4.88
101AL/	C	134LEU	CD2	0.01
101AL/	C	262LEU	CD2	0.01
102ASI	CA	102ASN	CB	100.00
102ASI	CA	102ASN	CG	100.00
102ASI	CA	102ASN	C	100.00
102ASI	CB	102ASN	CG	100.00
102ASI	CB	102ASN	C	100.00
102ASI	CB	132ARG	CG	0.02
102ASI	CB	132ARG	CD	0.19
102ASI	CB	134LEU	CG	0.13
102ASI	CB	134LEU	CD1	0.81
102ASI	CB	134LEU	CD2	1.10
102ASI	CG	102ASN	C	99.88
102ASI	CG	104ARG	CG	0.02
102ASI	CG	132ARG	CZ	0.00
102ASI	CG	134LEU	CD1	0.01
102ASI	CG	134LEU	CD2	0.00
102ASI	CG	259SER	CB	0.01
102ASI	C	103LEU	CA	100.00
102ASI	C	103LEU	CB	10.52
102ASI	C	103LEU	CG	0.74
102ASI	C	103LEU	CD1	4.37
102ASI	C	103LEU	CD2	0.16
102ASI	C	103LEU	C	90.61
103LEL	CA	103LEU	CB	100.00
103LEL	CA	103LEU	CG	100.00
103LEL	CA	103LEU	CD1	49.33
103LEL	CA	103LEU	CD2	47.91
103LEL	CA	103LEU	C	100.00
103LEL	CA	129LEU	CD1	0.00
103LEL	CA	129LEU	CD2	0.00
103LEL	CB	103LEU	CG	100.00
103LEL	CB	103LEU	CD1	100.00
103LEL	CB	103LEU	CD2	100.00
103LEL	CB	103LEU	C	100.00
103LEL	CB	129LEU	CD1	0.05
103LEL	CB	129LEU	CD2	0.00
103LEL	CB	260ALA	CB	0.01
103LEL	CB	262LEU	CD1	0.02
103LEL	CB	296MET	CE	0.70
103LEL	CG	103LEU	CD1	100.00
103LEL	CG	103LEU	CD2	100.00
103LEL	CG	103LEU	C	1.70
103LEL	CG	129LEU	CD1	0.02
103LEL	CG	131VAL	CG2	0.00
103LEL	CG	262LEU	CD1	0.16
103LEL	CG	262LEU	CD2	0.03
103LEL	CD1	103LEU	CD2	100.00
103LEL	CD1	103LEU	C	0.21
103LEL	CD1	129LEU	CG	0.02

118LE	CB	118LEU	CG	100.00
118LE	CB	118LEU	CD1	100.00
118LE	CB	118LEU	CD2	100.00
118LE	CB	118LEU	C	100.00
118LE	CB	122ILE	CG2	0.01
118LE	CG	118LEU	CD1	100.00
118LE	CG	118LEU	CD2	100.00
118LE	CG	118LEU	C	0.81
118LE	CG	224VAL	CG1	0.00
118LE	CG	250LEU	CD2	0.04
118LE	CG	118LEU	CD2	0.00
118LE	CD1	118LEU	CD2	100.00
118LE	CD1	122ILE	CG2	0.44
118LE	CD1	123ALA	CA	0.00
118LE	CD1	126VAL	CG2	0.00
118LE	CD1	224VAL	CG1	0.01
118LE	CD1	224VAL	C	0.03
118LE	CD1	227PRO	CG	4.18
118LE	CD1	227PRO	CD	0.72
118LE	CD1	250LEU	CG	0.00
118LE	CD1	250LEU	CD1	0.01
118LE	CD1	250LEU	CD2	15.86
118LE	CD1	117PRO	C	0.00
118LE	CD1	118LEU	CD2	0.23
118LE	CD2	122ILE	CG2	0.45
118LE	CD2	122ILE	CD	0.00
118LE	CD2	123ALA	CA	0.00
118LE	CD2	224VAL	CA	0.01
118LE	CD2	224VAL	CG1	0.23
118LE	CD2	224VAL	C	0.78
118LE	CD2	227PRO	CG	0.96
118LE	CD2	227PRO	CD	0.75
118LE	CD2	250LEU	CD2	0.48
118LE	CD2	117PRO	C	0.13
118LE	CD2	118LEU	CG	0.01
118LE	CD2	118LEU	CD1	0.06
118LE	CD2	118LEU	CD2	1.47
118LE	CD2	224VAL	CG1	0.00
118LE	C	119LYS	CA	100.00
118LE	C	119LYS	CB	0.00
118LE	C	119LYS	C	100.00
118LE	C	119LYS	CG	0.01
119LY	CA	119LYS	CB	100.00
119LY	CA	119LYS	CG	100.00
119LY	CA	119LYS	CD	0.66
119LY	CA	119LYS	C	100.00
119LY	CB	119LYS	CG	100.00
119LY	CB	119LYS	CD	100.00
119LY	CB	119LYS	CE	24.83
119LY	CB	119LYS	C	100.00
119LY	CB	121GLU	CD	0.01
119LY	CB	122ILE	CG1	0.01
119LY	CB	122ILE	CD	0.16
119LY	CG	119LYS	CD	100.00
119LY	CG	119LYS	CE	100.00
119LY	CG	119LYS	C	3.70
119LY	CG	122ILE	CG1	0.01
119LY	CG	122ILE	CD	0.20
119LY	CG	117PRO	C	0.01
119LY	CG	118LEU	C	0.06
119LY	CG	119LYS	CA	0.09
119LY	CG	119LYS	CB	0.00

117PRO	CD	117PRO	C	92.34	103LEU	CD1	129LEU	CD1	0.07	119LYS	CG	119LYS	CG	0.06
117PRO	CD	249VAL	CG1	0.14	103LEU	CD1	131VAL	CG1	0.08	119LYS	CD	119LYS	CE	100.00
117PRO	CD	249VAL	CG2	0.00	103LEU	CD1	131VAL	CG2	0.13	119LYS	CD	122ILE	CD	0.02
117PRO	CD	225ARG	CZ	0.03	103LEU	CD1	168VAL	CG1	0.10	119LYS	CD	117PRO	C	0.05
117PRO	C	118LEU	CA	100.00	103LEU	CD1	168VAL	CG2	0.01	119LYS	CE	122ILE	CD	0.05
117PRO	C	118LEU	CB	0.01	103LEU	CD1	260ALA	CB	0.02	119LYS	CE	117PRO	C	0.08
117PRO	C	118LEU	C	100.00	103LEU	CD1	262LEU	CB	0.05	119LYS	CE	118LEU	C	0.01
117PRO	C	118LEU	CD1	0.00	103LEU	CD1	262LEU	CG	0.55	119LYS	CE	120GLU	CD	0.66
117PRO	C	118LEU	CD2	0.09	103LEU	CD1	262LEU	CD1	3.53	119LYS	C	120GLU	CA	100.00
117PRO	C	119LYS	CD	0.02	103LEU	CD1	262LEU	CD2	0.48	119LYS	C	120GLU	C	100.00
117PRO	C	119LYS	CE	0.04	103LEU	CD1	296MET	CE	0.46	120GLU	CA	120GLU	CB	100.00
118LEU	CA	118LEU	CB	100.00	103LEU	CD1	300HIS	CG	0.01	120GLU	CA	120GLU	CG	100.00
118LEU	CA	118LEU	CG	100.00	103LEU	CD1	300HIS	CD2	0.11	120GLU	CA	120GLU	CD	21.12
118LEU	CA	118LEU	CD1	19.32	103LEU	CD1	300HIS	CE1	1.04	120GLU	CA	120GLU	C	100.00
118LEU	CA	118LEU	CD2	80.08	103LEU	CD2	103LEU	C	0.12	120GLU	CB	120GLU	CG	100.00
118LEU	CA	118LEU	C	100.00	103LEU	CD2	129LEU	CG	0.00	120GLU	CB	120GLU	CD	100.00
118LEU	CB	118LEU	CG	100.00	103LEU	CD2	129LEU	CD1	0.06	120GLU	CB	120GLU	C	100.00
118LEU	CB	118LEU	CD1	100.00	103LEU	CD2	129LEU	CD2	0.06	120GLU	CG	120GLU	CD	100.00
118LEU	CB	118LEU	CD2	100.00	103LEU	CD2	131VAL	CG1	0.32	120GLU	CG	120GLU	C	91.77
118LEU	CB	118LEU	C	100.00	103LEU	CD2	131VAL	CG2	1.62	120GLU	CG	124ARG	CZ	0.04
118LEU	CB	122ILE	CG2	0.01	103LEU	CD2	168VAL	CG1	0.08	120GLU	CD	120GLU	C	18.74
118LEU	CB	123ALA	CB	0.01	103LEU	CD2	172ALA	CB	0.00	120GLU	CD	124ARG	CZ	0.05
118LEU	CG	118LEU	CD1	100.00	103LEU	CD2	262LEU	CG	0.01	120GLU	C	121GLU	CA	100.00
118LEU	CG	118LEU	CD2	100.00	103LEU	CD2	262LEU	CD1	0.36	120GLU	C	121GLU	CB	0.09
118LEU	CG	118LEU	C	0.60	103LEU	CD2	262LEU	CD2	0.02	120GLU	C	121GLU	C	99.97
118LEU	CG	250LEU	CD2	0.02	103LEU	CD2	296MET	CE	0.17	121GLU	CA	121GLU	CB	100.00
118LEU	CG	118LEU	CD2	0.00	103LEU	CD2	300HIS	CG	0.02	121GLU	CA	121GLU	CG	100.00
118LEU	CD1	118LEU	CD2	100.00	103LEU	CD2	300HIS	CD2	0.04	121GLU	CA	121GLU	CD	93.36
118LEU	CD1	122ILE	CG2	0.40	103LEU	CD2	300HIS	CE1	1.22	121GLU	CA	121GLU	C	100.00
118LEU	CD1	123ALA	CA	0.01	103LEU	C	104ARG	CA	100.00	121GLU	CA	124ARG	CZ	0.88
118LEU	CD1	126VAL	CG2	0.05	103LEU	C	104ARG	CB	54.98	121GLU	CB	121GLU	CG	100.00
118LEU	CD1	224VAL	CG1	0.03	103LEU	C	104ARG	CG	4.40	121GLU	CB	121GLU	CD	100.00
118LEU	CD1	227PRO	CG	1.35	103LEU	C	104ARG	C	55.25	121GLU	CB	121GLU	C	100.00
118LEU	CD1	227PRO	CD	0.13	103LEU	C	105PRO	CD	2.33	121GLU	CB	124ARG	CZ	0.07
118LEU	CD1	249VAL	CG1	0.10	103LEU	C	129LEU	CD1	0.01	121GLU	CG	121GLU	CD	100.00
118LEU	CD1	250LEU	CA	0.01	104ARG	CA	104ARG	CB	100.00	121GLU	CG	121GLU	C	94.16
118LEU	CD1	250LEU	CB	0.07	104ARG	CA	104ARG	CG	100.00	121GLU	CD	121GLU	C	0.36
118LEU	CD1	250LEU	CG	0.01	104ARG	CA	104ARG	CD	33.43	121GLU	CD	124ARG	CD	0.08
118LEU	CD1	250LEU	CD1	0.03	104ARG	CA	104ARG	C	100.00	121GLU	CD	124ARG	CZ	0.32
118LEU	CD1	250LEU	CD2	5.77	104ARG	CA	105PRO	CD	100.00	121GLU	C	122ILE	CA	100.00
118LEU	CD1	117PRO	CB	0.00	104ARG	CA	259SER	CB	0.03	121GLU	C	122ILE	CB	2.56
118LEU	CD1	117PRO	C	0.00	104ARG	CB	104ARG	CG	100.00	121GLU	C	122ILE	CG1	0.63
118LEU	CD1	118LEU	CD1	0.02	104ARG	CB	104ARG	CD	100.00	121GLU	C	122ILE	CG2	0.78
118LEU	CD1	118LEU	CD2	0.38	104ARG	CB	104ARG	C	100.00	121GLU	C	122ILE	CD	0.00
118LEU	CD1	224VAL	CG1	0.35	104ARG	CB	244SER	CB	0.56	121GLU	C	122ILE	C	97.52
118LEU	CD2	122ILE	CG2	0.48	104ARG	CB	257LEU	CD2	0.00	122ILE	CA	122ILE	CB	100.00
118LEU	CD2	122ILE	CD	0.03	104ARG	CB	259SER	CB	0.06	122ILE	CA	122ILE	CG1	100.00
118LEU	CD2	122ILE	C	0.00	104ARG	CG	104ARG	CD	100.00	122ILE	CA	122ILE	CG2	100.00
118LEU	CD2	224VAL	CB	0.00	104ARG	CG	104ARG	CZ	12.42	122ILE	CA	122ILE	CD	0.40
118LEU	CD2	224VAL	CG1	0.68	104ARG	CG	104ARG	C	36.42	122ILE	CA	122ILE	C	100.00
118LEU	CD2	224VAL	C	0.03	104ARG	CG	244SER	CB	0.27	122ILE	CB	122ILE	CG1	100.00
118LEU	CD2	227PRO	CG	0.26	104ARG	CG	257LEU	CD1	0.05	122ILE	CB	122ILE	CG2	100.00
118LEU	CD2	227PRO	CD	0.12	104ARG	CG	257LEU	CD2	0.19	122ILE	CB	122ILE	CD	100.00
118LEU	CD2	250LEU	CD2	1.18	104ARG	CG	259SER	CB	0.51	122ILE	CB	122ILE	C	100.00
118LEU	CD2	117PRO	CB	0.01	104ARG	CD	104ARG	CZ	100.00	122ILE	CG1	122ILE	CG2	100.00
118LEU	CD2	117PRO	C	0.16	104ARG	CD	104ARG	C	0.01	122ILE	CG1	122ILE	CD	100.00
118LEU	CD2	118LEU	CG	0.00	104ARG	CD	130ILE	CD	0.00	122ILE	CG1	122ILE	C	3.37
118LEU	CD2	118LEU	CD1	0.28	104ARG	CD	132ARG	CZ	0.01	122ILE	CG2	122ILE	CD	99.17
118LEU	CD2	118LEU	CD2	0.76	104ARG	CD	244SER	CA	0.00	122ILE	CG2	122ILE	C	96.62
118LEU	CD2	224VAL	CG1	0.03	104ARG	CD	244SER	CB	1.14	122ILE	CG2	225ARG	C	0.11
118LEU	C	119LYS	CA	100.00	104ARG	CD	244SER	C	0.01	122ILE	CG2	226SER	CA	0.00
118LEU	C	119LYS	C	100.00	104ARG	CD	257LEU	CD1	0.03	122ILE	CG2	227PRO	CG	0.00

118LEU C	119LYS	CG	0.15	104AR(CD	257LEU	CD2	0.22	122ILE CG2	227PRO	CD	1.51
119LYS CA	119LYS	CB	100.00	104AR(CD	259SER	CB	0.60	122ILE CD	122ILE	C	0.02
119LYS CA	119LYS	CG	100.00	104AR(CD	272VAL	CG1	0.08	122ILE CD	117PRO	CA	1.57
119LYS CA	119LYS	CD	0.49	104AR(CD	272VAL	CG2	0.02	122ILE CD	117PRO	CB	12.66
119LYS CA	119LYS	C	100.00	104AR(CZ	132ARG	CZ	0.20	122ILE C	123ALA	CA	100.00
119LYS CA	119LYS	CG	0.07	104AR(CZ	244SER	CB	4.04	122ILE C	123ALA	CB	0.13
119LYS CB	119LYS	CG	100.00	104AR(CZ	244SER	C	0.05	122ILE C	123ALA	C	99.83
119LYS CB	119LYS	CD	100.00	104AR(CZ	245ASP	CG	0.04	123AL. CA	123ALA	CB	100.00
119LYS CB	119LYS	CE	22.44	104AR(CZ	257LEU	CD1	0.00	123AL. CA	123ALA	C	100.00
119LYS CB	119LYS	C	100.00	104AR(CZ	257LEU	CD2	0.06	123AL. CA	126VAL	CG2	0.03
119LYS CB	122ILE	CG1	0.02	104AR(CZ	259SER	CB	0.00	123AL. CB	123ALA	C	100.00
119LYS CB	122ILE	CD	0.20	104AR(CZ	272VAL	CB	0.01	123AL. C	124ARG	CA	100.00
119LYS CG	119LYS	CD	100.00	104AR(CZ	272VAL	CG1	3.74	123AL. C	124ARG	CB	1.18
119LYS CG	119LYS	CE	100.00	104AR(CZ	272VAL	CG2	4.44	123AL. C	124ARG	CG	0.03
119LYS CG	119LYS	C	9.74	104AR(C	105PRO	CA	100.00	123AL. C	124ARG	C	99.38
119LYS CG	122ILE	CG1	0.28	104AR(C	105PRO	CB	0.36	123AL. C	126VAL	CG2	1.52
119LYS CG	122ILE	CD	0.32	104AR(C	105PRO	CD	100.00	124AR CA	124ARG	CB	100.00
119LYS CG	118LEU	C	0.02	104AR(C	105PRO	C	99.99	124AR CA	124ARG	CG	100.00
119LYS CG	119LYS	CA	0.01	104AR(C	129LEU	CD1	0.55	124AR CA	124ARG	CD	0.02
119LYS CG	119LYS	CB	0.00	104AR(C	129LEU	CD2	0.02	124AR CA	124ARG	C	100.00
119LYS CG	119LYS	CG	0.11	105PR(CA	105PRO	CB	100.00	124AR CB	124ARG	CG	100.00
119LYS CD	119LYS	CE	100.00	105PR(CA	105PRO	CG	100.00	124AR CB	124ARG	CD	100.00
119LYS CD	122ILE	CD	0.03	105PR(CA	105PRO	CD	100.00	124AR CB	124ARG	CZ	10.27
119LYS CD	117PRO	CA	0.00	105PR(CA	105PRO	C	100.00	124AR CB	124ARG	C	100.00
119LYS CD	117PRO	C	0.02	105PR(CA	129LEU	CB	0.01	124AR CG	124ARG	CD	100.00
119LYS CE	122ILE	CG1	0.02	105PR(CA	129LEU	CD1	0.10	124AR CG	124ARG	CZ	40.57
119LYS CE	122ILE	CD	0.05	105PR(CA	129LEU	CD2	0.00	124AR CG	124ARG	C	72.33
119LYS CE	117PRO	C	0.08	105PR(CB	105PRO	CG	100.00	124AR CD	124ARG	CZ	100.00
119LYS CE	118LEU	C	0.00	105PR(CB	105PRO	CD	100.00	124AR CD	124ARG	C	0.01
119LYS CE	120GLU	CD	0.32	105PR(CB	105PRO	C	100.00	124AR C	125GLY	CA	100.00
119LYS C	120GLU	CA	100.00	105PR(CB	107LYS	CD	0.01	124AR C	125GLY	C	53.12
119LYS C	120GLU	C	100.00	105PR(CB	107LYS	CE	0.02	125GL CA	125GLY	C	100.00
120GLU CA	120GLU	CB	100.00	105PR(CB	129LEU	CD1	0.21	125GL CA	228ALA	CB	0.04
120GLU CA	120GLU	CG	100.00	105PR(CG	105PRO	CD	100.00	125GL C	126VAL	CA	100.00
120GLU CA	120GLU	CD	15.32	105PR(CG	105PRO	C	98.04	125GL C	126VAL	CB	2.50
120GLU CA	120GLU	C	100.00	105PR(CG	107LYS	CD	0.00	125GL C	126VAL	CG2	1.10
120GLU CB	120GLU	CG	100.00	105PR(CG	107LYS	CE	0.00	125GL C	126VAL	C	98.82
120GLU CB	120GLU	CD	100.00	105PR(CG	129LEU	CD1	0.01	125GL C	228ALA	CB	5.74
120GLU CB	120GLU	C	100.00	105PR(CG	129LEU	CD2	0.00	126VA CA	126VAL	CB	100.00
120GLU CB	124ARG	CZ	0.00	105PR(CG	258PRO	CG	0.93	126VA CA	126VAL	CG1	100.00
120GLU CG	120GLU	CD	100.00	105PR(CG	258PRO	CD	0.02	126VA CA	126VAL	CG2	100.00
120GLU CG	120GLU	C	89.98	105PR(CG	258PRO	C	0.01	126VA CA	126VAL	C	100.00
120GLU CG	124ARG	CZ	0.14	105PR(CG	296MET	CE	0.81	126VA CB	126VAL	CG1	100.00
120GLU CD	120GLU	C	7.18	105PR(CD	105PRO	C	96.67	126VA CB	126VAL	CG2	100.00
120GLU CD	124ARG	CZ	0.20	105PR(CD	129LEU	CD1	0.14	126VA CB	126VAL	C	100.00
120GLU CD	119LYS	CE	0.07	105PR(CD	258PRO	CG	0.00	126VA CG1	126VAL	CG2	100.00
120GLU C	121GLU	CA	100.00	105PR(CD	296MET	CE	2.54	126VA CG1	126VAL	C	100.00
120GLU C	121GLU	CB	0.09	105PR(C	106ALA	CA	100.00	126VA CG1	128VAL	CG1	0.01
120GLU C	121GLU	C	99.94	105PR(C	106ALA	CB	46.76	126VA CG1	128VAL	CG2	0.10
120GLU C	124ARG	CZ	0.06	105PR(C	106ALA	C	60.28	126VA CG1	227PRO	CB	0.26
121GLU CA	121GLU	CB	100.00	105PR(C	257LEU	CD1	0.08	126VA CG1	227PRO	CG	0.54
121GLU CA	121GLU	CG	100.00	106AL/ CA	106ALA	CB	100.00	126VA CG1	250LEU	CD1	4.14
121GLU CA	121GLU	CD	99.23	106AL/ CA	106ALA	C	100.00	126VA CG1	250LEU	CD2	1.19
121GLU CA	121GLU	C	100.00	106AL/ CA	257LEU	CD1	0.40	126VA CG2	227PRO	CB	0.17
121GLU CA	124ARG	CB	0.00	106AL/ CA	257LEU	CD2	0.11	126VA CG2	227PRO	CG	1.44
121GLU CA	124ARG	CZ	0.03	106AL/ CB	106ALA	C	100.00	126VA CG2	228ALA	CA	0.00
121GLU CB	121GLU	CG	100.00	106AL/ CB	126VAL	CG1	0.98	126VA CG2	228ALA	CB	0.11
121GLU CB	121GLU	CD	100.00	106AL/ CB	128VAL	CB	0.00	126VA C	127ASP	CA	100.00
121GLU CB	121GLU	C	100.00	106AL/ CB	128VAL	CG1	0.20	126VA C	127ASP	CB	100.00
121GLU CG	121GLU	CD	100.00	106AL/ CB	128VAL	CG2	0.27	126VA C	127ASP	C	0.20
121GLU CG	121GLU	C	94.88	106AL/ CB	130ILE	CD	0.18	127AS CA	127ASP	CB	100.00
121GLU CG	124ARG	CZ	0.01	106AL/ CB	247ALA	CB	1.43	127AS CA	127ASP	CG	100.00

121GLU	CD	121GLU	C	0.26
121GLU	CD	124ARG	CD	0.08
121GLU	CD	124ARG	CZ	0.05
121GLU	C	122ILE	CA	100.00
121GLU	C	122ILE	CB	2.08
121GLU	C	122ILE	CG1	0.46
121GLU	C	122ILE	CG2	0.64
121GLU	C	122ILE	CD	0.02
121GLU	C	122ILE	C	98.07
122ILE	CA	122ILE	CB	100.00
122ILE	CA	122ILE	CG1	100.00
122ILE	CA	122ILE	CG2	100.00
122ILE	CA	122ILE	CD	2.68
122ILE	CA	122ILE	C	100.00
122ILE	CB	122ILE	CG1	100.00
122ILE	CB	122ILE	CG2	100.00
122ILE	CB	122ILE	CD	100.00
122ILE	CB	122ILE	C	100.00
122ILE	CG1	122ILE	CG2	100.00
122ILE	CG1	122ILE	CD	100.00
122ILE	CG1	122ILE	C	7.57
122ILE	CG2	122ILE	CD	99.28
122ILE	CG2	122ILE	C	92.42
122ILE	CG2	225ARG	C	0.08
122ILE	CG2	227PRO	CG	0.02
122ILE	CG2	227PRO	CD	11.12
122ILE	CG2	250LEU	CD2	0.02
122ILE	CD	122ILE	C	0.05
122ILE	CD	225ARG	CZ	0.02
122ILE	CD	227PRO	CD	0.00
122ILE	CD	117PRO	CA	1.16
122ILE	CD	117PRO	CB	9.83
122ILE	CD	117PRO	C	0.00
122ILE	C	123ALA	CA	100.00
122ILE	C	123ALA	CB	0.10
122ILE	C	123ALA	C	99.94
123ALA	CA	123ALA	CB	100.00
123ALA	CA	123ALA	C	100.00
123ALA	CA	126VAL	CG2	0.03
123ALA	CB	123ALA	C	100.00
123ALA	C	124ARG	CA	100.00
123ALA	C	124ARG	CB	0.58
123ALA	C	124ARG	CG	0.00
123ALA	C	124ARG	C	99.66
123ALA	C	126VAL	CG2	0.48
124ARG	CA	124ARG	CB	100.00
124ARG	CA	124ARG	CG	100.00
124ARG	CA	124ARG	CD	1.20
124ARG	CA	124ARG	C	100.00
124ARG	CB	124ARG	CG	100.00
124ARG	CB	124ARG	CD	100.00
124ARG	CB	124ARG	CZ	21.88
124ARG	CB	124ARG	C	100.00
124ARG	CG	124ARG	CD	100.00
124ARG	CG	124ARG	CZ	47.33
124ARG	CG	124ARG	C	76.15
124ARG	CD	124ARG	CZ	100.00
124ARG	CD	124ARG	C	0.04
124ARG	C	125GLY	CA	100.00
124ARG	C	125GLY	C	44.83
125GLY	CA	125GLY	C	100.00
125GLY	CA	228ALA	CA	0.00

106AL	CB	247ALA	C	0.67
106AL	CB	248SER	CA	0.00
106AL	CB	251PRO	CG	0.01
106AL	CB	257LEU	CD1	0.50
106AL	CB	257LEU	CD2	0.23
106AL	C	107LYS	CA	100.00
106AL	C	107LYS	CB	92.95
106AL	C	107LYS	CG	1.90
106AL	C	107LYS	C	8.10
106AL	C	126VAL	CG1	0.09
106AL	C	251PRO	CG	0.00
107LY	CA	107LYS	CB	100.00
107LY	CA	107LYS	CG	100.00
107LY	CA	107LYS	CD	3.69
107LY	CA	107LYS	C	100.00
107LY	CB	107LYS	CG	100.00
107LY	CB	107LYS	CD	100.00
107LY	CB	107LYS	CE	24.77
107LY	CB	107LYS	C	100.00
107LY	CB	109PHE	CE2	0.16
107LY	CB	109PHE	CZ	0.06
107LY	CB	127ASP	CA	0.00
107LY	CB	127ASP	CB	0.03
107LY	CB	127ASP	CG	0.02
107LY	CB	257LEU	CD2	0.08
107LY	CG	107LYS	CD	100.00
107LY	CG	107LYS	CE	100.00
107LY	CG	107LYS	C	92.32
107LY	CG	108VAL	C	0.01
107LY	CG	109PHE	CE1	0.11
107LY	CG	109PHE	CE2	0.62
107LY	CG	109PHE	CZ	0.54
107LY	CG	127ASP	CB	0.02
107LY	CG	127ASP	CG	0.01
107LY	CG	257LEU	CD2	0.15
107LY	CD	107LYS	CE	100.00
107LY	CD	107LYS	C	0.42
107LY	CD	109PHE	CE1	0.02
107LY	CD	109PHE	CE2	0.14
107LY	CD	109PHE	CZ	0.20
107LY	CD	127ASP	CB	0.00
107LY	CD	127ASP	CG	0.47
107LY	CD	312GLU	CD	0.38
107LY	CE	109PHE	CD1	0.00
107LY	CE	109PHE	CD2	0.01
107LY	CE	109PHE	CE1	0.17
107LY	CE	109PHE	CE2	0.12
107LY	CE	109PHE	CZ	0.46
107LY	CE	127ASP	CG	0.50
107LY	CE	258PRO	CG	0.03
107LY	CE	258PRO	CD	0.00
107LY	CE	312GLU	CG	0.00
107LY	CE	312GLU	CD	1.44
107LY	C	108VAL	CA	100.00
107LY	C	108VAL	CB	5.98
107LY	C	108VAL	CG1	0.01
107LY	C	108VAL	CG2	0.04
107LY	C	108VAL	C	96.35
107LY	C	251PRO	CB	5.84
107LY	C	251PRO	CG	0.06
108VA	CA	108VAL	CB	100.00
108VA	CA	108VAL	CG1	100.00

127AS	CA	127ASP	C	100.00
127AS	CB	127ASP	CG	100.00
127AS	CB	127ASP	C	100.00
127AS	CG	127ASP	C	100.00
127AS	C	128VAL	CA	100.00
127AS	C	128VAL	CB	44.67
127AS	C	128VAL	CG1	3.92
127AS	C	128VAL	CG2	7.45
127AS	C	128VAL	C	58.46
128VA	CA	128VAL	CB	100.00
128VA	CA	128VAL	CG1	100.00
128VA	CA	128VAL	CG2	100.00
128VA	CA	128VAL	C	100.00
128VA	CB	128VAL	CG1	100.00
128VA	CB	128VAL	CG2	100.00
128VA	CB	128VAL	C	100.00
128VA	CB	230PHE	CB	0.00
128VA	CB	230PHE	CD2	0.00
128VA	CB	230PHE	CE1	0.04
128VA	CB	230PHE	CE2	0.02
128VA	CB	230PHE	CZ	0.00
128VA	CG1	128VAL	CG2	100.00
128VA	CG1	128VAL	C	91.41
128VA	CG1	129LEU	C	0.00
128VA	CG1	130ILE	CG1	0.25
128VA	CG1	130ILE	CD	0.54
128VA	CG1	223LEU	CD1	0.00
128VA	CG1	230PHE	CB	0.99
128VA	CG1	230PHE	CD1	0.60
128VA	CG1	230PHE	CD2	0.13
128VA	CG1	230PHE	CE1	1.92
128VA	CG1	230PHE	CE2	0.26
128VA	CG1	230PHE	CZ	0.02
128VA	CG1	230PHE	C	0.01
128VA	CG1	233VAL	CG2	1.46
128VA	CG1	250LEU	CD1	0.01
128VA	CG2	128VAL	C	41.44
128VA	CG2	129LEU	C	0.02
128VA	CG2	130ILE	CG1	0.00
128VA	CG2	130ILE	CD	0.09
128VA	CG2	227PRO	CB	0.01
128VA	CG2	230PHE	CB	2.02
128VA	CG2	230PHE	CD1	0.93
128VA	CG2	230PHE	CD2	0.37
128VA	CG2	230PHE	CE1	1.60
128VA	CG2	230PHE	CE2	0.06
128VA	CG2	230PHE	C	0.06
128VA	CG2	231ASP	C	0.01
128VA	CG2	233VAL	CG2	1.62
128VA	CG2	250LEU	CD1	0.00
128VA	C	129LEU	CA	100.00
128VA	C	129LEU	CB	70.18
128VA	C	129LEU	C	41.83
128VA	C	176ARG	CZ	0.00
129LE	CA	129LEU	CB	100.00
129LE	CA	129LEU	CG	100.00
129LE	CA	129LEU	CD1	85.46
129LE	CA	129LEU	CD2	13.77
129LE	CA	129LEU	C	100.00
129LE	CB	129LEU	CG	100.00
129LE	CB	129LEU	CD1	100.00
129LE	CB	129LEU	CD2	100.00

125GLY	CA	228ALA	CB	0.08	108VAI	CA	108VAL	CG2	100.00	129LE	CB	129LEU	C	100.00
125GLY	C	126VAL	CA	100.00	108VAI	CA	108VAL	C	100.00	129LE	CB	176ARG	CD	0.02
125GLY	C	126VAL	CB	1.18	108VAI	CA	251PRO	CB	3.06	129LE	CB	176ARG	CZ	3.92
125GLY	C	126VAL	CG2	0.59	108VAI	CB	108VAL	CG1	100.00	129LE	CG	129LEU	CD1	100.00
125GLY	C	126VAL	C	99.42	108VAI	CB	108VAL	CG2	100.00	129LE	CG	129LEU	CD2	100.00
125GLY	C	227PRO	CB	0.00	108VAI	CB	108VAL	C	100.00	129LE	CG	129LEU	C	97.75
125GLY	C	227PRO	CG	0.02	108VAI	CB	126VAL	CG2	0.00	129LE	CG	131VAL	CG2	0.03
125GLY	C	228ALA	CB	1.81	108VAI	CB	251PRO	CB	1.51	129LE	CG	176ARG	CZ	0.01
126VAL	CA	126VAL	CB	100.00	108VAI	CG1	108VAL	CG2	100.00	129LE	CD1	129LEU	CD2	100.00
126VAL	CA	126VAL	CG1	100.00	108VAI	CG1	108VAL	C	91.69	129LE	CD1	129LEU	C	6.42
126VAL	CA	126VAL	CG2	100.00	108VAI	CG1	109PHE	C	0.02	129LE	CD1	130ILE	C	0.00
126VAL	CA	126VAL	C	100.00	108VAI	CG1	113GLU	CA	0.02	129LE	CD1	131VAL	CG2	0.15
126VAL	CA	227PRO	CB	0.00	108VAI	CG1	113GLU	CB	0.54	129LE	CD1	172LEU	CB	0.00
126VAL	CB	126VAL	CG1	100.00	108VAI	CG1	113GLU	CG	0.04	129LE	CD1	172LEU	CD1	0.03
126VAL	CB	126VAL	CG2	100.00	108VAI	CG1	113GLU	CD	0.00	129LE	CD1	172LEU	CD2	0.14
126VAL	CB	126VAL	C	100.00	108VAI	CG1	123ALA	C	0.00	129LE	CD1	176ARG	CD	0.01
126VAL	CG1	126VAL	CG2	100.00	108VAI	CG1	124ARG	CA	0.09	129LE	CD1	176ARG	CZ	0.14
126VAL	CG1	126VAL	C	100.00	108VAI	CG1	124ARG	C	0.88	129LE	CD2	129LEU	C	10.10
126VAL	CG1	128VAL	CG1	0.09	108VAI	CG1	125GLY	CA	0.00	129LE	CD2	130ILE	C	0.05
126VAL	CG1	128VAL	CG2	0.04	108VAI	CG1	125GLY	C	0.00	129LE	CD2	131VAL	CG2	1.90
126VAL	CG1	227PRO	CB	0.02	108VAI	CG1	126VAL	CB	0.02	129LE	CD2	172LEU	CB	0.26
126VAL	CG1	227PRO	CG	0.08	108VAI	CG1	126VAL	CG2	0.02	129LE	CD2	172LEU	CG	0.02
126VAL	CG1	250LEU	CB	0.17	108VAI	CG1	126VAL	C	0.01	129LE	CD2	172LEU	CD1	1.31
126VAL	CG1	250LEU	CD1	1.08	108VAI	CG1	251PRO	CA	0.00	129LE	CD2	172LEU	CD2	0.63
126VAL	CG1	250LEU	CD2	0.03	108VAI	CG1	251PRO	CB	0.20	129LE	CD2	176ARG	CD	0.16
126VAL	CG1	251PRO	CB	0.04	108VAI	CG2	108VAL	C	86.23	129LE	CD2	176ARG	CZ	0.06
126VAL	CG1	251PRO	CG	0.03	108VAI	CG2	109PHE	C	0.01	129LE	C	130ILE	CA	100.00
126VAL	CG1	251PRO	CD	0.02	108VAI	CG2	112LEU	C	0.05	129LE	C	130ILE	CB	10.41
126VAL	CG2	227PRO	CB	1.54	108VAI	CG2	113GLU	CA	0.60	129LE	C	130ILE	CG1	5.24
126VAL	CG2	227PRO	CG	13.29	108VAI	CG2	113GLU	CB	5.65	129LE	C	130ILE	CD	0.05
126VAL	CG2	228ALA	CA	0.02	108VAI	CG2	113GLU	CG	7.55	129LE	C	130ILE	C	97.14
126VAL	CG2	228ALA	CB	0.01	108VAI	CG2	113GLU	CD	0.00	130ILE	CA	130ILE	CB	100.00
126VAL	CG2	250LEU	CB	2.34	108VAI	CG2	123ALA	C	0.01	130ILE	CA	130ILE	CG1	100.00
126VAL	CG2	250LEU	CD2	0.02	108VAI	CG2	124ARG	CA	0.06	130ILE	CA	130ILE	CG2	100.00
126VAL	C	127ASP	CA	100.00	108VAI	CG2	124ARG	C	0.21	130ILE	CA	130ILE	CD	20.56
126VAL	C	127ASP	CB	99.90	108VAI	CG2	126VAL	CB	0.02	130ILE	CA	130ILE	C	100.00
126VAL	C	127ASP	C	0.86	108VAI	CG2	126VAL	CG1	0.00	130ILE	CB	130ILE	CG1	100.00
127ASP	CA	127ASP	CB	100.00	108VAI	CG2	126VAL	CG2	0.01	130ILE	CB	130ILE	CG2	100.00
127ASP	CA	127ASP	CG	100.00	108VAI	CG2	126VAL	C	0.00	130ILE	CB	130ILE	CD	100.00
127ASP	CA	127ASP	C	100.00	108VAI	CG2	250LEU	C	0.00	130ILE	CB	130ILE	C	100.00
127ASP	CB	127ASP	CG	100.00	108VAI	CG2	251PRO	CA	0.04	130ILE	CG1	130ILE	CG2	100.00
127ASP	CB	127ASP	C	100.00	108VAI	CG2	251PRO	CB	1.19	130ILE	CG1	130ILE	CD	100.00
127ASP	CG	127ASP	C	100.00	108VAI	C	109PHE	CA	100.00	130ILE	CG1	233VAL	CB	0.03
127ASP	CG	176ARG	CZ	0.00	108VAI	C	109PHE	CB	26.20	130ILE	CG1	233VAL	CG1	0.00
127ASP	C	128VAL	CA	100.00	108VAI	C	109PHE	CG	5.48	130ILE	CG1	233VAL	CG2	0.06
127ASP	C	128VAL	CB	76.33	108VAI	C	109PHE	CD1	1.15	130ILE	CG1	247ALA	CB	0.04
127ASP	C	128VAL	CG1	3.86	108VAI	C	109PHE	CD2	5.60	130ILE	CG2	130ILE	CD	78.31
127ASP	C	128VAL	CG2	2.75	108VAI	C	109PHE	C	78.40	130ILE	CG2	130ILE	C	100.00
127ASP	C	128VAL	C	30.13	108VAI	C	110PRO	CD	0.02	130ILE	CG2	131VAL	C	0.00
128VAL	CA	128VAL	CB	100.00	109PHI	CA	109PHE	CB	100.00	130ILE	CG2	233VAL	CG1	0.01
128VAL	CA	128VAL	CG1	100.00	109PHI	CA	109PHE	CG	100.00	130ILE	CG2	235THR	CG2	3.52
128VAL	CA	128VAL	CG2	100.00	109PHI	CA	109PHE	CD1	70.33	130ILE	CG2	243LEU	CD2	0.03
128VAL	CA	128VAL	C	100.00	109PHI	CA	109PHE	CD2	74.17	130ILE	CG2	244SER	CB	0.08
128VAL	CB	128VAL	CG1	100.00	109PHI	CA	109PHE	C	100.00	130ILE	CD	223LEU	CD1	0.00
128VAL	CB	128VAL	CG2	100.00	109PHI	CA	110PRO	CD	100.00	130ILE	CD	223LEU	CD2	0.00
128VAL	CB	128VAL	C	100.00	109PHI	CB	109PHE	CG	100.00	130ILE	CD	233VAL	CB	0.12
128VAL	CG1	128VAL	CG2	100.00	109PHI	CB	109PHE	CD1	100.00	130ILE	CD	233VAL	CG1	0.02
128VAL	CG1	128VAL	C	90.89	109PHI	CB	109PHE	CD2	100.00	130ILE	CD	233VAL	CG2	0.22
128VAL	CG1	129LEU	C	0.00	109PHI	CB	109PHE	C	100.00	130ILE	CD	243LEU	CD2	0.14
128VAL	CG1	130ILE	CG1	0.02	109PHI	CB	110PRO	CD	7.29	130ILE	CD	243LEU	C	0.02
128VAL	CG1	130ILE	CD	1.20	109PHI	CB	112LEU	CB	0.08	130ILE	CD	244SER	CA	0.89
128VAL	CG1	223LEU	CD1	0.00	109PHI	CB	112LEU	CG	0.02	130ILE	CD	244SER	CB	0.37

128VAL	CG1	230PHE	CB	0.40	109PHE	CB	112LEU	CD1	1.83	130ILE	CD	247ALA	CB	6.05
128VAL	CG1	230PHE	C	0.01	109PHE	CB	112LEU	CD2	0.18	130ILE	C	131VAL	CA	100.00
128VAL	CG1	233VAL	CG2	0.29	109PHE	CB	316ALA	CB	0.05	130ILE	C	131VAL	CB	6.08
128VAL	CG1	247ALA	CB	0.05	109PHE	CB	319LEU	CD1	0.56	130ILE	C	131VAL	CG2	3.92
128VAL	CG1	250LEU	CD1	0.16	109PHE	CB	319LEU	CD2	0.04	130ILE	C	131VAL	C	97.85
128VAL	CG2	128VAL	C	86.50	109PHE	CG	109PHE	CD1	100.00	131VA	CA	131VAL	CB	100.00
128VAL	CG2	130ILE	CG1	0.00	109PHE	CG	109PHE	CD2	100.00	131VA	CA	131VAL	CG1	100.00
128VAL	CG2	130ILE	CD	0.73	109PHE	CG	109PHE	CE1	100.00	131VA	CA	131VAL	CG2	100.00
128VAL	CG2	230PHE	CB	2.82	109PHE	CG	109PHE	CE2	100.00	131VA	CA	131VAL	C	100.00
128VAL	CG2	230PHE	CD2	0.00	109PHE	CG	109PHE	CZ	100.00	131VA	CB	131VAL	CG1	100.00
128VAL	CG2	230PHE	C	0.12	109PHE	CG	112LEU	CD1	0.01	131VA	CB	131VAL	CG2	100.00
128VAL	CG2	231ASP	C	0.00	109PHE	CG	112LEU	CD2	0.00	131VA	CB	131VAL	C	100.00
128VAL	CG2	233VAL	CG2	5.26	109PHE	CG	253SER	CB	0.00	131VA	CB	234VAL	CG2	0.01
128VAL	CG2	247ALA	CB	0.02	109PHE	CG	256LEU	CD1	0.19	131VA	CG1	131VAL	CG2	100.00
128VAL	C	129LEU	CA	100.00	109PHE	CG	316ALA	CB	0.40	131VA	CG1	131VAL	C	100.00
128VAL	C	129LEU	CB	28.89	109PHE	CG	319LEU	CD1	1.70	131VA	CG1	165VAL	CB	0.02
128VAL	C	129LEU	C	76.90	109PHE	CG	319LEU	CD2	0.06	131VA	CG1	165VAL	CG1	0.24
129LEU	CA	129LEU	CB	100.00	109PHE	CD1	109PHE	CD2	100.00	131VA	CG1	165VAL	CG2	3.84
129LEU	CA	129LEU	CG	100.00	109PHE	CD1	109PHE	CE1	100.00	131VA	CG1	168VAL	CG1	0.81
129LEU	CA	129LEU	CD1	92.70	109PHE	CD1	109PHE	CE2	100.00	131VA	CG1	169ALA	CB	0.01
129LEU	CA	129LEU	CD2	6.32	109PHE	CD1	109PHE	CZ	100.00	131VA	CG1	234VAL	CG2	0.06
129LEU	CA	129LEU	C	100.00	109PHE	CD1	110PRO	CD	0.00	131VA	CG2	169ALA	CB	0.18
129LEU	CB	129LEU	CG	100.00	109PHE	CD1	112LEU	CD1	0.06	131VA	CG2	172LEU	CD1	3.31
129LEU	CB	129LEU	CD1	100.00	109PHE	CD1	112LEU	CD2	0.03	131VA	CG2	172LEU	CD2	0.18
129LEU	CB	129LEU	CD2	100.00	109PHE	CD1	253SER	CB	0.50	131VA	CG2	232VAL	CG1	0.15
129LEU	CB	129LEU	C	100.00	109PHE	CD1	256LEU	CD1	0.50	131VA	C	132ARG	CA	100.00
129LEU	CB	176ARG	CZ	0.99	109PHE	CD1	256LEU	CD2	0.01	131VA	C	132ARG	CB	70.06
129LEU	CG	129LEU	CD1	100.00	109PHE	CD1	316ALA	CA	0.14	131VA	C	132ARG	CG	0.00
129LEU	CG	129LEU	CD2	100.00	109PHE	CD1	316ALA	CB	2.20	131VA	C	132ARG	C	41.29
129LEU	CG	129LEU	C	99.06	109PHE	CD1	319LEU	CD1	4.12	132AR	CA	132ARG	CB	100.00
129LEU	CG	131VAL	CG2	0.15	109PHE	CD1	319LEU	CD2	0.25	132AR	CA	132ARG	CG	100.00
129LEU	CD1	129LEU	CD2	100.00	109PHE	CD2	109PHE	CE1	100.00	132AR	CA	132ARG	CD	27.74
129LEU	CD1	129LEU	C	6.82	109PHE	CD2	109PHE	CE2	100.00	132AR	CA	132ARG	C	100.00
129LEU	CD1	131VAL	CG2	0.12	109PHE	CD2	109PHE	CZ	100.00	132AR	CA	235THR	CG2	0.02
129LEU	CD1	172ALA	CB	0.09	109PHE	CD2	112LEU	CD1	0.04	132AR	CB	132ARG	CG	100.00
129LEU	CD1	176ARG	CZ	0.10	109PHE	CD2	112LEU	CD2	0.01	132AR	CB	132ARG	CD	100.00
129LEU	CD1	296MET	CE	0.26	109PHE	CD2	253SER	CB	0.71	132AR	CB	132ARG	C	100.00
129LEU	CD2	129LEU	C	4.80	109PHE	CD2	256LEU	CD1	6.62	132AR	CB	235THR	CG2	0.14
129LEU	CD2	130ILE	C	0.06	109PHE	CD2	256LEU	CD2	0.01	132AR	CG	132ARG	CD	100.00
129LEU	CD2	131VAL	CG2	3.34	109PHE	CD2	312GLU	CG	0.00	132AR	CG	132ARG	CZ	38.19
129LEU	CD2	172ALA	CB	16.96	109PHE	CD2	316ALA	CA	0.13	132AR	CG	132ARG	C	99.99
129LEU	CD2	176ARG	CD	0.01	109PHE	CD2	316ALA	CB	2.43	132AR	CG	133GLU	C	0.03
129LEU	CD2	176ARG	CZ	0.07	109PHE	CD2	319LEU	CD1	3.11	132AR	CG	240GLY	CA	0.00
129LEU	CD2	232VAL	CG1	0.01	109PHE	CD2	319LEU	CD2	0.08	132AR	CG	240GLY	C	0.24
129LEU	CD2	232VAL	CG2	0.01	109PHE	CE1	109PHE	CE2	100.00	132AR	CD	132ARG	CZ	100.00
129LEU	CD2	296MET	CE	0.01	109PHE	CE1	109PHE	CZ	100.00	132AR	CD	132ARG	C	46.66
129LEU	C	130ILE	CA	100.00	109PHE	CE1	253SER	CB	0.82	132AR	CD	133GLU	C	0.88
129LEU	C	130ILE	CB	9.63	109PHE	CE1	256LEU	CB	0.66	132AR	CD	134LEU	CD1	0.01
129LEU	C	130ILE	CG1	5.13	109PHE	CE1	256LEU	CD1	1.01	132AR	CD	134LEU	CD2	0.20
129LEU	C	130ILE	CD	0.58	109PHE	CE1	256LEU	CD2	0.05	132AR	CD	240GLY	C	0.00
129LEU	C	130ILE	C	97.44	109PHE	CE1	257LEU	CD2	0.03	132AR	CD	244SER	CB	0.00
130ILE	CA	130ILE	CB	100.00	109PHE	CE1	288THR	CG2	0.06	132AR	CZ	134LEU	CG	0.60
130ILE	CA	130ILE	CG1	100.00	109PHE	CE1	292LEU	CD1	0.11	132AR	CZ	134LEU	CD1	2.22
130ILE	CA	130ILE	CG2	100.00	109PHE	CE1	292LEU	CD2	0.16	132AR	CZ	134LEU	CD2	3.19
130ILE	CA	130ILE	CD	76.09	109PHE	CE1	312GLU	CB	0.01	132AR	CZ	240GLY	C	0.01
130ILE	CA	130ILE	C	100.00	109PHE	CE1	312GLU	CG	0.20	132AR	CZ	241ASP	CA	1.51
130ILE	CB	130ILE	CG1	100.00	109PHE	CE1	312GLU	CD	0.01	132AR	CZ	241ASP	CB	1.57
130ILE	CB	130ILE	CG2	100.00	109PHE	CE1	315VAL	CG1	0.16	132AR	C	133GLU	CA	100.00
130ILE	CB	130ILE	CD	100.00	109PHE	CE1	315VAL	CG2	0.12	132AR	C	133GLU	CB	1.50
130ILE	CB	130ILE	C	100.00	109PHE	CE1	316ALA	CA	0.09	132AR	C	133GLU	C	99.37
130ILE	CG1	130ILE	CG2	100.00	109PHE	CE1	316ALA	CB	0.18	132AR	C	165VAL	CG1	0.19
130ILE	CG1	130ILE	CD	100.00	109PHE	CE1	319LEU	CB	0.00	132AR	C	165VAL	CG2	0.03

130ILE	CG1	233VAL	CB	0.02	109PHI	CE1	319LEU	CD1	3.20	133GL	CA	133GLU	CB	100.00
130ILE	CG1	233VAL	CG1	0.00	109PHI	CE1	319LEU	CD2	0.05	133GL	CA	133GLU	CG	100.00
130ILE	CG1	233VAL	CG2	0.01	109PHI	CE2	109PHE	CZ	100.00	133GL	CA	133GLU	CD	98.72
130ILE	CG1	243LEU	CD2	0.00	109PHI	CE2	253SER	CB	0.69	133GL	CA	133GLU	C	100.00
130ILE	CG1	244SER	CA	0.00	109PHI	CE2	256LEU	CB	0.50	133GL	CA	165VAL	CG1	0.07
130ILE	CG1	244SER	CB	0.00	109PHI	CE2	256LEU	CD1	3.88	133GL	CB	133GLU	CG	100.00
130ILE	CG1	247ALA	CB	0.34	109PHI	CE2	256LEU	CD2	0.06	133GL	CB	133GLU	CD	100.00
130ILE	CG2	130ILE	CD	65.29	109PHI	CE2	257LEU	CD1	0.00	133GL	CB	133GLU	C	100.00
130ILE	CG2	130ILE	C	100.00	109PHI	CE2	257LEU	CD2	0.04	133GL	CB	165VAL	CG1	0.03
130ILE	CG2	132ARG	CB	0.04	109PHI	CE2	288THR	CG2	0.06	133GL	CB	165VAL	CG2	0.00
130ILE	CG2	235THR	CG2	0.32	109PHI	CE2	292LEU	CD1	0.38	133GL	CB	236GLY	CA	0.04
130ILE	CG2	243LEU	CB	0.01	109PHI	CE2	292LEU	CD2	0.05	133GL	CB	236GLY	C	0.38
130ILE	CG2	243LEU	CG	0.00	109PHI	CE2	312GLU	CB	0.01	133GL	CG	133GLU	CD	100.00
130ILE	CG2	243LEU	CD2	0.06	109PHI	CE2	312GLU	CG	0.24	133GL	CG	133GLU	C	12.94
130ILE	CG2	244SER	CA	0.00	109PHI	CE2	312GLU	CD	0.02	133GL	CG	136GLY	CA	0.05
130ILE	CG2	244SER	CB	0.15	109PHI	CE2	315VAL	CG1	0.24	133GL	CG	136GLY	C	0.02
130ILE	CD	233VAL	CB	0.80	109PHI	CE2	315VAL	CG2	0.04	133GL	CG	165VAL	CB	1.00
130ILE	CD	233VAL	CG1	0.18	109PHI	CE2	316ALA	CA	0.06	133GL	CG	165VAL	CG1	1.39
130ILE	CD	233VAL	CG2	1.52	109PHI	CE2	316ALA	CB	0.16	133GL	CG	165VAL	CG2	2.27
130ILE	CD	243LEU	CB	0.05	109PHI	CE2	319LEU	CD1	2.30	133GL	CD	133GLU	C	0.68
130ILE	CD	243LEU	CG	0.04	109PHI	CE2	319LEU	CD2	0.04	133GL	CD	136GLY	CA	0.02
130ILE	CD	243LEU	CD2	0.08	109PHI	CZ	253SER	CB	0.01	133GL	C	134LEU	CA	100.00
130ILE	CD	243LEU	C	0.32	109PHI	CZ	256LEU	CB	0.67	133GL	C	134LEU	CB	14.48
130ILE	CD	244SER	CA	0.12	109PHI	CZ	256LEU	CD1	0.28	133GL	C	134LEU	CG	0.20
130ILE	CD	244SER	CB	0.00	109PHI	CZ	256LEU	CD2	0.00	133GL	C	134LEU	C	78.85
130ILE	CD	247ALA	CB	10.01	109PHI	CZ	257LEU	CD1	0.00	134LE	CA	134LEU	CB	100.00
130ILE	C	131VAL	CA	100.00	109PHI	CZ	257LEU	CD2	0.04	134LE	CA	134LEU	CG	100.00
130ILE	C	131VAL	CB	6.67	109PHI	CZ	288THR	CG2	0.60	134LE	CA	134LEU	CD1	8.05
130ILE	C	131VAL	CG2	4.38	109PHI	CZ	292LEU	CD1	7.50	134LE	CA	134LEU	CD2	91.44
130ILE	C	131VAL	C	97.15	109PHI	CZ	292LEU	CD2	1.06	134LE	CA	134LEU	C	100.00
131VAL	CA	131VAL	CB	100.00	109PHI	CZ	312GLU	CG	0.14	134LE	CB	134LEU	CG	100.00
131VAL	CA	131VAL	CG1	100.00	109PHI	CZ	312GLU	CD	0.01	134LE	CB	134LEU	CD1	100.00
131VAL	CA	131VAL	CG2	100.00	109PHI	CZ	315VAL	CG1	0.36	134LE	CB	134LEU	CD2	100.00
131VAL	CA	131VAL	C	100.00	109PHI	CZ	315VAL	CG2	0.04	134LE	CB	134LEU	C	100.00
131VAL	CB	131VAL	CG1	100.00	109PHI	CZ	316ALA	CB	0.00	134LE	CB	135THR	CG2	0.08
131VAL	CB	131VAL	CG2	100.00	109PHI	CZ	319LEU	CD1	0.88	134LE	CG	134LEU	CD1	100.00
131VAL	CB	131VAL	C	100.00	109PHI	C	110PRO	CA	100.00	134LE	CG	134LEU	CD2	100.00
131VAL	CB	234VAL	CG2	0.02	109PHI	C	110PRO	CB	0.11	134LE	CG	134LEU	C	8.56
131VAL	CG1	131VAL	CG2	100.00	109PHI	C	110PRO	CD	100.00	134LE	CG	135THR	CG2	0.00
131VAL	CG1	131VAL	C	100.00	109PHI	C	110PRO	C	100.00	134LE	CD1	134LEU	CD2	100.00
131VAL	CG1	165VAL	CG1	0.08	110PR(	CA	110PRO	CB	100.00	134LE	CD1	134LEU	C	0.04
131VAL	CG1	165VAL	CG2	0.29	110PR(	CA	110PRO	CG	100.00	134LE	CD1	261SER	CB	0.06
131VAL	CG1	168VAL	CG2	1.47	110PR(	CA	110PRO	CD	100.00	134LE	CD2	134LEU	C	0.18
131VAL	CG1	169ALA	CB	0.03	110PR(	CA	110PRO	C	100.00	134LE	CD2	261SER	CB	0.12
131VAL	CG1	234VAL	CG2	0.28	110PR(	CA	113GLU	CD	0.03	134LE	C	135THR	CA	100.00
131VAL	CG2	169ALA	CA	0.00	110PR(	CB	110PRO	CG	100.00	134LE	C	135THR	CB	75.26
131VAL	CG2	169ALA	CB	0.32	110PR(	CB	110PRO	CD	100.00	134LE	C	135THR	CG2	50.36
131VAL	CG2	232VAL	CG1	0.19	110PR(	CB	110PRO	C	100.00	134LE	C	135THR	C	25.15
131VAL	C	132ARG	CA	100.00	110PR(	CB	113GLU	CD	0.00	134LE	C	140PHE	CD2	0.02
131VAL	C	132ARG	CB	89.90	110PR(	CB	320LEU	CG	0.00	134LE	C	140PHE	CE2	0.10
131VAL	C	132ARG	CG	0.12	110PR(	CB	320LEU	CD1	0.02	134LE	C	140PHE	CZ	0.00
131VAL	C	132ARG	C	13.55	110PR(	CB	320LEU	CD2	0.00	135TH	CA	135THR	CB	100.00
132ARG	CA	132ARG	CB	100.00	110PR(	CG	110PRO	CD	100.00	135TH	CA	135THR	CG2	100.00
132ARG	CA	132ARG	CG	100.00	110PR(	CG	110PRO	C	90.06	135TH	CA	135THR	C	100.00
132ARG	CA	132ARG	CD	7.17	110PR(	CG	316ALA	CB	0.07	135TH	CA	140PHE	CG	0.02
132ARG	CA	132ARG	C	100.00	110PR(	CG	316ALA	C	0.00	135TH	CA	140PHE	CD1	0.26
132ARG	CA	235THR	CG2	0.02	110PR(	CG	320LEU	CD1	0.08	135TH	CA	140PHE	CD2	0.02
132ARG	CB	132ARG	CG	100.00	110PR(	CD	110PRO	C	85.02	135TH	CA	140PHE	CE2	0.02
132ARG	CB	132ARG	CD	100.00	110PR(	CD	316ALA	CB	0.48	135TH	CA	140PHE	CZ	0.00
132ARG	CB	132ARG	C	100.00	110PR(	C	111GLY	CA	100.00	135TH	CB	135THR	CG2	100.00
132ARG	CB	235THR	CG2	0.70	110PR(	C	111GLY	C	60.37	135TH	CB	135THR	C	100.00
132ARG	CG	132ARG	CD	100.00	110PR(	C	112LEU	CD1	0.00	135TH	CB	140PHE	CD1	0.04

132ARG	CG	132ARG	CZ	9.85	110PR(	C	320LEU	CD1	0.05	135TH CB	140PHE	CE1	0.01
132ARG	CG	132ARG	C	97.32	110PR(	C	320LEU	CD2	0.09	135TH CG2	140PHE	CD1	0.01
132ARG	CG	133GLU	C	0.07	111GL\	CA	111GLY	C	100.00	135TH CG2	140PHE	CD2	0.00
132ARG	CG	134LEU	CD2	0.00	111GL\	CA	113GLU	CD	0.04	135TH CG2	140PHE	CE1	0.03
132ARG	CG	235THR	CG2	0.02	111GL\	CA	114ARG	CZ	1.72	135TH CG2	140PHE	CE2	0.01
132ARG	CG	240GLY	CA	0.02	111GL\	CA	320LEU	CG	0.00	135TH CG2	140PHE	CZ	0.03
132ARG	CG	240GLY	C	0.34	111GL\	CA	320LEU	CD1	0.46	135TH C	136GLY	CA	100.00
132ARG	CD	132ARG	CZ	100.00	111GL\	CA	320LEU	CD2	0.34	135TH C	136GLY	C	72.30
132ARG	CD	132ARG	C	9.91	111GL\	C	112LEU	CA	100.00	135TH C	140PHE	CB	0.01
132ARG	CD	133GLU	C	0.09	111GL\	C	112LEU	CB	41.77	135TH C	140PHE	CG	0.01
132ARG	CD	134LEU	CD1	0.08	111GL\	C	112LEU	CG	11.60	135TH C	140PHE	CD1	0.00
132ARG	CD	134LEU	CD2	0.27	111GL\	C	112LEU	CD1	3.20	135TH C	140PHE	CD2	0.94
132ARG	CD	240GLY	CA	0.00	111GL\	C	112LEU	CD2	0.34	135TH C	140PHE	CE2	0.12
132ARG	CD	240GLY	C	0.16	111GL\	C	112LEU	C	53.84	136GL CA	136GLY	C	100.00
132ARG	CD	241ASP	CA	0.04	111GL\	C	113GLU	CG	0.00	136GL CA	140PHE	CD2	0.02
132ARG	CD	244SER	CB	0.05	111GL\	C	113GLU	CD	0.05	136GL CA	157TYR	CE1	0.02
132ARG	CZ	134LEU	CB	0.03	111GL\	C	114ARG	CD	0.01	136GL CA	157TYR	CZ	0.01
132ARG	CZ	134LEU	CG	0.69	111GL\	C	114ARG	CZ	0.01	136GL CA	161GLU	CG	1.26
132ARG	CZ	134LEU	CD1	8.08	111GL\	C	320LEU	CD1	0.04	136GL CA	161GLU	CD	2.25
132ARG	CZ	134LEU	CD2	2.03	111GL\	C	320LEU	CD2	0.12	136GL C	137GLY	CA	100.00
132ARG	CZ	240GLY	CA	0.02	112LEl	CA	112LEU	CB	100.00	136GL C	137GLY	C	34.15
132ARG	CZ	240GLY	C	0.86	112LEl	CA	112LEU	CG	100.00	136GL C	140PHE	CD2	0.24
132ARG	CZ	241ASP	CA	2.55	112LEl	CA	112LEU	CD1	35.05	136GL C	157TYR	CE1	6.46
132ARG	CZ	241ASP	CB	15.64	112LEl	CA	112LEU	CD2	56.16	136GL C	157TYR	CE2	0.60
132ARG	CZ	241ASP	CG	0.04	112LEl	CA	112LEU	C	100.00	136GL C	157TYR	CZ	1.43
132ARG	C	133GLU	CA	100.00	112LEl	CA	115LEU	CD1	0.03	137GL CA	137GLY	C	100.00
132ARG	C	133GLU	CB	1.58	112LEl	CA	327LEU	CD2	0.00	137GL CA	155GLU	CD	0.23
132ARG	C	133GLU	C	99.10	112LEl	CB	112LEU	CG	100.00	137GL CA	157TYR	CE1	0.44
132ARG	C	165VAL	CG1	0.02	112LEl	CB	112LEU	CD1	100.00	137GL CA	157TYR	CE2	2.57
132ARG	C	165VAL	CG2	0.74	112LEl	CB	112LEU	CD2	100.00	137GL CA	157TYR	CZ	2.38
133GLU	CA	133GLU	CB	100.00	112LEl	CB	112LEU	C	100.00	137GL CA	237ASN	CB	0.00
133GLU	CA	133GLU	CG	100.00	112LEl	CB	115LEU	CD1	0.12	137GL C	138ILE	CA	100.00
133GLU	CA	133GLU	CD	96.78	112LEl	CB	320LEU	CD2	0.00	137GL C	138ILE	C	100.00
133GLU	CA	133GLU	C	100.00	112LEl	CB	327LEU	CD1	0.01	137GL C	155GLU	CD	0.08
133GLU	CA	165VAL	CG2	0.00	112LEl	CB	327LEU	CD2	0.04	138ILE CA	138ILE	CB	100.00
133GLU	CB	133GLU	CG	100.00	112LEl	CG	112LEU	CD1	100.00	138ILE CA	138ILE	CG1	100.00
133GLU	CB	133GLU	CD	100.00	112LEl	CG	112LEU	CD2	100.00	138ILE CA	138ILE	CG2	100.00
133GLU	CB	133GLU	C	100.00	112LEl	CG	112LEU	C	7.02	138ILE CA	138ILE	CD	0.27
133GLU	CB	165VAL	CG1	0.00	112LEl	CG	114ARG	CZ	0.00	138ILE CA	138ILE	C	100.00
133GLU	CB	236GLY	CA	0.01	112LEl	CG	115LEU	CD1	0.14	138ILE CB	138ILE	CG1	100.00
133GLU	CB	236GLY	C	0.50	112LEl	CG	256LEU	CD2	0.00	138ILE CB	138ILE	CG2	100.00
133GLU	CB	237ASN	CB	6.93	112LEl	CG	320LEU	CG	0.00	138ILE CB	138ILE	CD	100.00
133GLU	CB	237ASN	CG	0.01	112LEl	CG	320LEU	CD1	0.01	138ILE CB	138ILE	C	100.00
133GLU	CG	133GLU	CD	100.00	112LEl	CG	320LEU	CD2	0.09	138ILE CB	155GLU	CA	0.16
133GLU	CG	133GLU	C	17.40	112LEl	CG	324PRO	CG	0.01	138ILE CB	155GLU	CD	0.13
133GLU	CG	136GLY	CA	0.09	112LEl	CG	324PRO	CD	0.00	138ILE CG1	138ILE	CG2	100.00
133GLU	CG	165VAL	CB	0.03	112LEl	CG	327LEU	CD1	0.11	138ILE CG1	138ILE	CD	100.00
133GLU	CG	165VAL	CG1	0.30	112LEl	CG	327LEU	CD2	0.06	138ILE CG1	138ILE	C	100.00
133GLU	CG	165VAL	CG2	0.98	112LEl	CD1	112LEU	CD2	100.00	138ILE CG1	153ASN	CB	0.80
133GLU	CD	133GLU	C	1.24	112LEl	CD1	112LEU	C	0.00	138ILE CG1	154THR	C	0.94
133GLU	CD	136GLY	CA	0.93	112LEl	CD1	114ARG	CZ	0.17	138ILE CG1	155GLU	CA	0.00
133GLU	CD	165VAL	CG2	0.02	112LEl	CD1	115LEU	CG	0.00	138ILE CG1	138ILE	CD	0.00
133GLU	C	134LEU	CA	100.00	112LEl	CD1	115LEU	CD1	0.19	138ILE CG1	189LEU	CD1	0.02
133GLU	C	134LEU	CB	84.28	112LEl	CD1	115LEU	CD2	0.02	138ILE CG1	189LEU	CD2	0.54
133GLU	C	134LEU	CG	0.10	112LEl	CD1	252GLY	C	0.00	138ILE CG2	138ILE	CD	99.65
133GLU	C	134LEU	CD1	0.01	112LEl	CD1	253SER	CA	0.02	138ILE CG2	138ILE	C	99.80
133GLU	C	134LEU	CD2	0.04	112LEl	CD1	253SER	CB	0.03	138ILE CG2	139TYR	CG	0.01
133GLU	C	134LEU	C	15.75	112LEl	CD1	256LEU	CD1	1.34	138ILE CG2	139TYR	CD1	0.72
133GLU	C	237ASN	CB	0.02	112LEl	CD1	256LEU	CD2	1.31	138ILE CG2	139TYR	CD2	0.06
133GLU	C	237ASN	CG	0.00	112LEl	CD1	316ALA	CB	0.00	138ILE CG2	139TYR	CE1	0.21
134LEU	CA	134LEU	CB	100.00	112LEl	CD1	319LEU	CB	2.05	138ILE CG2	139TYR	CE2	0.12
134LEU	CA	134LEU	CG	100.00	112LEl	CD1	319LEU	CG	0.03	138ILE CG2	139TYR	CZ	0.09

134LEU CA	134LEU CD1	76.88	112LEL CD1	319LEU	CD1	0.64	138ILE CG2	155GLU	CD	0.07
134LEU CA	134LEU CD2	19.29	112LEL CD1	319LEU	CD2	0.23	138ILE CG2	238ILE	CB	3.38
134LEU CA	134LEU C	100.00	112LEL CD1	319LEU	C	0.01	138ILE CG2	238ILE	CG1	0.40
134LEU CA	237ASN CG	0.00	112LEL CD1	320LEU	CG	0.01	138ILE CG2	238ILE	CG2	6.23
134LEU CB	134LEU CG	100.00	112LEL CD1	320LEU	CD1	0.15	138ILE CG2	238ILE	CD	5.06
134LEU CB	134LEU CD1	100.00	112LEL CD1	320LEU	CD2	0.88	138ILE CD	138ILE	C	0.01
134LEU CB	134LEU CD2	100.00	112LEL CD1	324PRO	CG	0.10	138ILE CD	153ASN	CB	0.17
134LEU CB	134LEU C	100.00	112LEL CD1	324PRO	CD	0.11	138ILE CD	153ASN	C	0.15
134LEU CG	134LEU CD1	100.00	112LEL CD1	327LEU	CD1	0.10	138ILE CD	154THR	C	0.06
134LEU CG	134LEU CD2	100.00	112LEL CD1	327LEU	CD2	0.13	138ILE CD	238ILE	CD	0.06
134LEU CG	134LEU C	91.99	112LEL CD2	112LEU	C	0.04	138ILE CD	138ILE	CD	4.45
134LEU CD1	134LEU CD2	100.00	112LEL CD2	114ARG	CZ	0.44	138ILE CD	155GLU	CB	0.65
134LEU CD1	134LEU C	1.20	112LEL CD2	115LEU	CG	0.00	138ILE CD	189LEU	CD1	0.02
134LEU CD1	135THR CG2	0.03	112LEL CD2	115LEU	CD1	0.94	138ILE CD	189LEU	CD2	0.22
134LEU CD1	140PHE CZ	0.00	112LEL CD2	115LEU	CD2	0.05	138ILE CD	238ILE	CD	0.22
134LEU CD1	261SER CB	0.02	112LEL CD2	252GLY	C	0.10	138ILE C	139TYR	CA	100.00
134LEU CD2	134LEU C	3.27	112LEL CD2	253SER	CA	0.02	138ILE C	139TYR	C	100.00
134LEU CD2	135THR CG2	0.02	112LEL CD2	256LEU	CD1	1.83	138ILE C	189LEU	CD2	0.01
134LEU CD2	140PHE CZ	0.00	112LEL CD2	256LEU	CD2	1.41	139TY CA	139TYR	CB	100.00
134LEU CD2	261SER CB	2.90	112LEL CD2	319LEU	CB	0.30	139TY CA	139TYR	CG	100.00
134LEU C	135THR CA	100.00	112LEL CD2	319LEU	CG	0.00	139TY CA	139TYR	CD1	100.00
134LEU C	135THR CB	74.28	112LEL CD2	319LEU	CD1	0.09	139TY CA	139TYR	CD2	0.28
134LEU C	135THR CG2	45.31	112LEL CD2	319LEU	CD2	0.14	139TY CA	139TYR	C	100.00
134LEU C	135THR C	27.25	112LEL CD2	319LEU	C	0.02	139TY CA	188VAL	CG1	0.02
134LEU C	140PHE CE2	0.09	112LEL CD2	320LEU	CG	0.01	139TY CB	139TYR	CG	100.00
135THR CA	135THR CB	100.00	112LEL CD2	320LEU	CD1	0.05	139TY CB	139TYR	CD1	100.00
135THR CA	135THR CG2	100.00	112LEL CD2	320LEU	CD2	0.62	139TY CB	139TYR	CD2	100.00
135THR CA	135THR C	100.00	112LEL CD2	323PRO	CB	0.02	139TY CB	139TYR	C	100.00
135THR CA	140PHE CD2	0.00	112LEL CD2	324PRO	CB	0.01	139TY CB	140PHE	CD2	7.84
135THR CA	140PHE CE1	0.01	112LEL CD2	324PRO	CG	0.82	139TY CB	140PHE	CE1	0.00
135THR CA	140PHE CE2	0.04	112LEL CD2	324PRO	CD	2.83	139TY CB	140PHE	CE2	3.50
135THR CA	140PHE CZ	0.01	112LEL CD2	327LEU	CG	0.00	139TY CB	140PHE	CZ	0.03
135THR CB	135THR CG2	100.00	112LEL CD2	327LEU	CD1	0.56	139TY CB	237ASN	CG	0.02
135THR CB	135THR C	100.00	112LEL CD2	327LEU	CD2	0.75	139TY CB	188VAL	CB	0.01
135THR C	136GLY CA	100.00	112LEL C	113GLU	CA	100.00	139TY CB	188VAL	CG1	0.25
135THR C	136GLY C	47.55	112LEL C	113GLU	CB	0.03	139TY CB	188VAL	CG2	0.03
135THR C	140PHE CG	0.02	112LEL C	113GLU	C	100.00	139TY CG	139TYR	CD1	100.00
135THR C	140PHE CD1	0.07	112LEL C	114ARG	CG	0.01	139TY CG	139TYR	CD2	100.00
135THR C	140PHE CD2	2.41	113GLI CA	113GLU	CB	100.00	139TY CG	139TYR	CE1	100.00
135THR C	140PHE CE1	0.03	113GLI CA	113GLU	CG	100.00	139TY CG	139TYR	CE2	100.00
135THR C	140PHE CE2	0.70	113GLI CA	113GLU	CD	73.11	139TY CG	139TYR	CZ	100.00
135THR C	140PHE CZ	0.43	113GLI CA	113GLU	C	100.00	139TY CG	188VAL	CB	0.15
136GLY CA	136GLY C	100.00	113GLI CA	123ALA	CB	0.00	139TY CG	188VAL	CG1	0.73
136GLY CA	140PHE CG	0.00	113GLI CB	113GLU	CG	100.00	139TY CG	188VAL	CG2	5.52
136GLY CA	140PHE CD2	0.05	113GLI CB	113GLU	CD	100.00	139TY CD1	139TYR	CD2	100.00
136GLY CA	140PHE CE2	0.01	113GLI CB	113GLU	C	100.00	139TY CD1	139TYR	CE1	100.00
136GLY CA	157TYR CE1	0.00	113GLI CB	120GLU	CB	0.03	139TY CD1	139TYR	CE2	100.00
136GLY CA	161GLU CD	0.00	113GLI CB	120GLU	CG	1.52	139TY CD1	139TYR	CZ	100.00
136GLY C	137GLY CA	100.00	113GLI CB	120GLU	CD	0.01	139TY CD1	238ILE	CG1	0.00
136GLY C	137GLY C	39.54	113GLI CB	123ALA	CB	0.11	139TY CD1	188VAL	CB	0.60
136GLY C	140PHE CB	0.02	113GLI CB	123ALA	C	0.03	139TY CD1	188VAL	CG1	0.54
136GLY C	140PHE CG	0.00	113GLI CB	124ARG	CA	0.00	139TY CD1	188VAL	CG2	26.51
136GLY C	140PHE CD1	0.08	113GLI CB	124ARG	CB	0.00	139TY CD1	189LEU	CG	0.02
136GLY C	140PHE CD2	1.37	113GLI CB	124ARG	CG	0.02	139TY CD1	189LEU	CD1	0.13
136GLY C	140PHE CE1	0.02	113GLI CG	113GLU	CD	100.00	139TY CD1	189LEU	CD2	1.20
136GLY C	140PHE CE2	0.28	113GLI CG	113GLU	C	47.44	139TY CD2	139TYR	CE1	100.00
136GLY C	140PHE CZ	0.26	113GLI CG	120GLU	CG	0.62	139TY CD2	139TYR	CE2	100.00
136GLY C	157TYR CE1	0.25	113GLI CG	120GLU	CD	0.01	139TY CD2	139TYR	CZ	100.00
136GLY C	157TYR CE2	0.00	113GLI CG	123ALA	C	0.00	139TY CD2	237ASN	CB	0.56
136GLY C	157TYR CZ	0.06	113GLI CG	124ARG	CA	0.06	139TY CD2	237ASN	CG	1.81
137GLY CA	137GLY C	100.00	113GLI CG	124ARG	CB	0.02	139TY CD2	188VAL	CG1	0.05
137GLY CA	155GLU CD	0.04	113GLI CG	124ARG	CG	0.52	139TY CD2	188VAL	CG2	0.76

137GLY	CA	157TYR	CE1	0.00	113GLU	CG	124ARG	CD	0.01	139TY CE1	139TYR	CE2	100.00
137GLY	CA	157TYR	CE2	0.18	113GLU	CG	124ARG	CZ	0.01	139TY CE1	139TYR	CZ	100.00
137GLY	CA	157TYR	CZ	0.22	113GLU	CD	113GLU	C	0.56	139TY CE1	238ILE	CA	0.03
137GLY	CA	237ASN	CB	0.24	113GLU	CD	114ARG	CG	0.08	139TY CE1	238ILE	CB	0.34
137GLY	CA	237ASN	CG	0.00	113GLU	CD	114ARG	CD	0.00	139TY CE1	238ILE	CG1	1.84
137GLY	CA	237ASN	C	0.19	113GLU	CD	114ARG	CZ	0.01	139TY CE1	238ILE	CD	0.64
137GLY	C	138ILE	CA	100.00	113GLU	CD	124ARG	CG	0.04	139TY CE1	185LYS	CG	0.04
137GLY	C	138ILE	C	100.00	113GLU	CD	124ARG	CZ	0.41	139TY CE1	185LYS	CD	0.01
137GLY	C	139TYR	CD2	0.00	113GLU	C	114ARG	CA	100.00	139TY CE1	185LYS	CE	0.11
137GLY	C	155GLU	CD	0.81	113GLU	C	114ARG	CB	0.09	139TY CE1	188VAL	CG1	0.05
138ILE	CA	138ILE	CB	100.00	113GLU	C	114ARG	CG	0.02	139TY CE1	188VAL	CG2	11.37
138ILE	CA	138ILE	CG1	100.00	113GLU	C	114ARG	C	99.86	139TY CE1	189LEU	CG	0.03
138ILE	CA	138ILE	CG2	100.00	113GLU	C	120GLU	CB	0.00	139TY CE1	189LEU	CD1	0.11
138ILE	CA	138ILE	CD	1.26	113GLU	C	120GLU	CG	0.04	139TY CE1	189LEU	CD2	0.16
138ILE	CA	138ILE	C	100.00	114ARG	CA	114ARG	CB	100.00	139TY CE2	139TYR	CZ	100.00
138ILE	CA	155GLU	CD	0.12	114ARG	CA	114ARG	CG	100.00	139TY CE2	237ASN	CG	0.01
138ILE	CB	138ILE	CG1	100.00	114ARG	CA	114ARG	CD	3.00	139TY CE2	238ILE	CA	0.06
138ILE	CB	138ILE	CG2	100.00	114ARG	CA	114ARG	C	100.00	139TY CE2	241ASP	CB	0.39
138ILE	CB	138ILE	CD	100.00	114ARG	CA	120GLU	CG	0.02	139TY CE2	241ASP	CG	2.41
138ILE	CB	138ILE	C	100.00	114ARG	CA	120GLU	CD	0.03	139TY CE2	185LYS	CE	0.01
138ILE	CB	155GLU	CA	0.00	114ARG	CB	114ARG	CG	100.00	139TY CE2	188VAL	CG1	0.00
138ILE	CB	155GLU	CD	0.02	114ARG	CB	114ARG	CD	100.00	139TY CE2	188VAL	CG2	0.12
138ILE	CG1	138ILE	CG2	100.00	114ARG	CB	114ARG	CZ	0.60	139TY CZ	238ILE	CA	0.42
138ILE	CG1	138ILE	CD	100.00	114ARG	CB	114ARG	C	100.00	139TY CZ	238ILE	CB	0.00
138ILE	CG1	138ILE	C	100.00	114ARG	CB	115LEU	CD1	0.01	139TY CZ	238ILE	CG1	0.10
138ILE	CG1	153ASN	CB	0.35	114ARG	CB	115LEU	CD2	0.00	139TY CZ	238ILE	CD	0.00
138ILE	CG1	154THR	C	0.14	114ARG	CB	120GLU	CD	0.04	139TY CZ	185LYS	CG	0.01
138ILE	CG1	138ILE	CD	0.08	114ARG	CG	114ARG	CD	100.00	139TY CZ	185LYS	CD	0.12
138ILE	CG1	189LEU	CD1	0.03	114ARG	CG	114ARG	CZ	16.55	139TY CZ	185LYS	CE	1.22
138ILE	CG1	189LEU	CD2	0.04	114ARG	CG	114ARG	C	34.72	139TY CZ	188VAL	CG2	0.50
138ILE	CG2	138ILE	CD	98.97	114ARG	CG	115LEU	CD1	0.02	139TY C	140PHE	CA	100.00
138ILE	CG2	138ILE	C	99.67	114ARG	CG	115LEU	CD2	0.02	139TY C	140PHE	CB	94.77
138ILE	CG2	139TYR	CG	0.10	114ARG	CG	120GLU	CG	0.20	139TY C	140PHE	CG	72.61
138ILE	CG2	139TYR	CD1	2.71	114ARG	CG	120GLU	CD	1.73	139TY C	140PHE	CD1	2.47
138ILE	CG2	139TYR	CD2	1.65	114ARG	CD	114ARG	CZ	100.00	139TY C	140PHE	CD2	26.69
138ILE	CG2	139TYR	CE1	9.29	114ARG	CD	114ARG	C	0.17	139TY C	140PHE	C	3.99
138ILE	CG2	139TYR	CE2	5.11	114ARG	CD	120GLU	CG	0.03	140PH CA	140PHE	CB	100.00
138ILE	CG2	139TYR	CZ	7.13	114ARG	CD	120GLU	CD	0.01	140PH CA	140PHE	CG	100.00
138ILE	CG2	155GLU	CD	0.00	114ARG	CD	327LEU	CD1	0.01	140PH CA	140PHE	CD1	93.88
138ILE	CG2	238ILE	CA	0.05	114ARG	CZ	115LEU	CD1	0.01	140PH CA	140PHE	CD2	51.71
138ILE	CG2	238ILE	CB	0.34	114ARG	CZ	115LEU	CD2	0.12	140PH CA	140PHE	C	100.00
138ILE	CG2	238ILE	CG1	0.48	114ARG	CZ	120GLU	CD	0.00	140PH CB	140PHE	CG	100.00
138ILE	CG2	238ILE	CG2	1.14	114ARG	CZ	320LEU	CD1	0.01	140PH CB	140PHE	CD1	100.00
138ILE	CG2	238ILE	CD	1.80	114ARG	CZ	320LEU	CD2	0.01	140PH CB	140PHE	CD2	100.00
138ILE	CG2	189LEU	CD1	0.01	114ARG	CZ	327LEU	CD1	0.02	140PH CB	140PHE	C	100.00
138ILE	CD	138ILE	C	0.35	114ARG	CZ	327LEU	CD2	0.01	140PH CG	140PHE	CD1	100.00
138ILE	CD	153ASN	CB	0.12	114ARG	C	115LEU	CA	100.00	140PH CG	140PHE	CD2	100.00
138ILE	CD	153ASN	C	0.04	114ARG	C	115LEU	CB	4.22	140PH CG	140PHE	CE1	100.00
138ILE	CD	154THR	C	0.16	114ARG	C	115LEU	CG	0.40	140PH CG	140PHE	CE2	100.00
138ILE	CD	155GLU	CB	0.00	114ARG	C	115LEU	CD1	0.16	140PH CG	140PHE	CZ	100.00
138ILE	CD	238ILE	CD	0.46	114ARG	C	115LEU	CD2	0.07	140PH CG	140PHE	C	3.54
138ILE	CD	138ILE	CG1	0.01	114ARG	C	115LEU	C	93.60	140PH CD1	140PHE	CD2	100.00
138ILE	CD	138ILE	CD	6.33	115LEU	CA	115LEU	CB	100.00	140PH CD1	140PHE	CE1	100.00
138ILE	CD	155GLU	CB	1.04	115LEU	CA	115LEU	CG	100.00	140PH CD1	140PHE	CE2	100.00
138ILE	CD	189LEU	CD1	0.48	115LEU	CA	115LEU	CD1	11.74	140PH CD1	140PHE	CZ	100.00
138ILE	CD	189LEU	CD2	0.03	115LEU	CA	115LEU	CD2	88.01	140PH CD1	140PHE	C	0.12
138ILE	CD	238ILE	CD	1.43	115LEU	CA	115LEU	C	100.00	140PH CD2	140PHE	CE1	100.00
138ILE	C	139TYR	CA	100.00	115LEU	CB	115LEU	CG	100.00	140PH CD2	140PHE	CE2	100.00
138ILE	C	139TYR	CB	0.02	115LEU	CB	115LEU	CD1	100.00	140PH CD2	140PHE	CZ	100.00
138ILE	C	139TYR	C	99.97	115LEU	CB	115LEU	CD2	100.00	140PH CE1	140PHE	CE2	100.00
138ILE	C	189LEU	CD2	0.00	115LEU	CB	115LEU	C	100.00	140PH CE1	140PHE	CZ	100.00
139TYR	CA	139TYR	CB	100.00	115LEU	CB	252GLY	CA	0.04	140PH CE1	188VAL	CG1	0.01

139TYR	CA	139TYR	CG	100.00	115LE	CB	252GLY	C	0.02	140PH	CE2	140PHE	CZ	100.00
139TYR	CA	139TYR	CD1	99.20	115LE	CB	254LEU	CD1	0.03	140PH	CE2	188VAL	CG1	0.01
139TYR	CA	139TYR	CD2	10.90	115LE	CB	327LEU	CD1	0.15	140PH	CZ	188VAL	CG1	0.22
139TYR	CA	139TYR	C	100.00	115LE	CB	327LEU	CD2	0.51	140PH	C	141GLY	CA	100.00
139TYR	CA	188VAL	CG1	0.01	115LE	CG	115LEU	CD1	100.00	140PH	C	141GLY	C	56.00
139TYR	CA	188VAL	CG2	0.00	115LE	CG	115LEU	CD2	100.00	141GL	CA	141GLY	C	100.00
139TYR	CB	139TYR	CG	100.00	115LE	CG	115LEU	C	6.02	141GL	C	142GLU	CA	100.00
139TYR	CB	139TYR	CD1	100.00	115LE	CG	252GLY	CA	0.00	141GL	C	142GLU	CB	32.59
139TYR	CB	139TYR	CD2	100.00	115LE	CG	252GLY	C	0.00	141GL	C	142GLU	CG	1.68
139TYR	CB	139TYR	C	100.00	115LE	CG	254LEU	CD1	0.00	141GL	C	142GLU	CD	0.07
139TYR	CB	140PHE	CG	0.00	115LE	CG	327LEU	CD1	0.00	141GL	C	142GLU	C	70.39
139TYR	CB	140PHE	CD1	0.08	115LE	CD1	115LEU	CD2	100.00	142GL	CA	142GLU	CB	100.00
139TYR	CB	140PHE	CD2	1.28	115LE	CD1	115LEU	C	0.03	142GL	CA	142GLU	CG	100.00
139TYR	CB	140PHE	CE1	0.10	115LE	CD1	253SER	CB	0.00	142GL	CA	142GLU	CD	86.90
139TYR	CB	140PHE	CE2	1.02	115LE	CD1	254LEU	CD1	0.00	142GL	CA	142GLU	C	100.00
139TYR	CB	140PHE	CZ	0.04	115LE	CD1	256LEU	CD1	0.02	142GL	CA	143PRO	CA	100.00
139TYR	CB	188VAL	CB	0.00	115LE	CD1	323PRO	CB	0.79	142GL	CA	143PRO	C	44.94
139TYR	CB	188VAL	CG1	0.02	115LE	CD1	323PRO	CG	0.01	142GL	CB	142GLU	CG	100.00
139TYR	CB	188VAL	CG2	0.68	115LE	CD1	324PRO	CG	1.25	142GL	CB	142GLU	CD	100.00
139TYR	CB	188VAL	C	0.02	115LE	CD1	324PRO	CD	0.40	142GL	CB	142GLU	C	100.00
139TYR	CG	139TYR	CD1	100.00	115LE	CD1	326ASP	CB	0.00	142GL	CB	143PRO	CA	9.46
139TYR	CG	139TYR	CD2	100.00	115LE	CD1	327LEU	CB	0.06	142GL	CB	143PRO	C	0.03
139TYR	CG	139TYR	CE1	100.00	115LE	CD1	327LEU	CG	0.05	142GL	CG	142GLU	CD	100.00
139TYR	CG	139TYR	CE2	100.00	115LE	CD1	327LEU	CD1	0.82	142GL	CG	142GLU	C	54.00
139TYR	CG	139TYR	CZ	100.00	115LE	CD1	327LEU	CD2	0.84	142GL	CG	143PRO	CA	2.44
139TYR	CG	139TYR	C	0.76	115LE	CD2	115LEU	C	0.06	142GL	CG	144ARG	CG	0.01
139TYR	CG	188VAL	CB	0.11	115LE	CD2	252GLY	C	0.12	142GL	CG	144ARG	CD	0.02
139TYR	CG	188VAL	CG1	0.32	115LE	CD2	256LEU	CD1	0.01	142GL	CD	142GLU	C	0.86
139TYR	CG	188VAL	CG2	4.97	115LE	CD2	323PRO	CB	0.16	142GL	CD	143PRO	CA	0.48
139TYR	CD1	139TYR	CD2	100.00	115LE	CD2	324PRO	CG	0.25	142GL	CD	144ARG	CG	0.12
139TYR	CD1	139TYR	CE1	100.00	115LE	CD2	324PRO	CD	0.04	142GL	CD	144ARG	CD	0.10
139TYR	CD1	139TYR	CE2	100.00	115LE	CD2	326ASP	CB	0.04	142GL	C	143PRO	CA	100.00
139TYR	CD1	139TYR	CZ	100.00	115LE	CD2	326ASP	CG	0.03	142GL	C	143PRO	CB	0.04
139TYR	CD1	185LYS	CD	0.06	115LE	CD2	327LEU	CB	0.04	142GL	C	143PRO	CD	100.00
139TYR	CD1	188VAL	CB	0.74	115LE	CD2	327LEU	CG	0.04	142GL	C	143PRO	C	99.98
139TYR	CD1	188VAL	CG1	0.66	115LE	CD2	327LEU	CD1	0.35	142GL	C	154THR	CB	0.20
139TYR	CD1	188VAL	CG2	25.89	115LE	CD2	327LEU	CD2	0.42	143PR	CA	143PRO	CB	100.00
139TYR	CD1	188VAL	C	0.50	115LE	C	116SER	CA	100.00	143PR	CA	143PRO	CG	100.00
139TYR	CD1	189LEU	CB	0.01	115LE	C	116SER	CB	0.69	143PR	CA	143PRO	CD	100.00
139TYR	CD1	189LEU	CG	0.01	115LE	C	116SER	C	99.51	143PR	CA	143PRO	C	100.00
139TYR	CD1	189LEU	CD1	1.93	115LE	C	252GLY	CA	1.48	143PR	CB	143PRO	CG	100.00
139TYR	CD1	189LEU	CD2	0.62	115LE	C	254LEU	CD1	0.01	143PR	CB	143PRO	CD	100.00
139TYR	CD2	139TYR	CE1	100.00	116SE	CA	116SER	CB	100.00	143PR	CB	143PRO	C	100.00
139TYR	CD2	139TYR	CE2	100.00	116SE	CA	116SER	C	100.00	143PR	CB	152TRP	CD1	0.02
139TYR	CD2	139TYR	CZ	100.00	116SE	CA	117PRO	CD	100.00	143PR	CG	143PRO	CD	100.00
139TYR	CD2	139TYR	C	0.18	116SE	CA	252GLY	CA	0.37	143PR	CG	143PRO	C	97.76
139TYR	CD2	140PHE	CD1	0.01	116SE	CB	116SER	C	100.00	143PR	CG	154THR	CB	0.00
139TYR	CD2	140PHE	CD2	0.13	116SE	CB	117PRO	CD	1.83	143PR	CD	143PRO	C	93.64
139TYR	CD2	140PHE	CE1	0.03	116SE	CB	123ALA	CB	9.06	143PR	CD	154THR	CB	0.13
139TYR	CD2	140PHE	CE2	0.20	116SE	CB	251PRO	C	0.01	143PR	CD	156ARG	CZ	0.00
139TYR	CD2	140PHE	CZ	0.02	116SE	CB	252GLY	CA	0.34	143PR	C	144ARG	CA	100.00
139TYR	CD2	237ASN	CG	1.04	116SE	C	117PRO	CA	100.00	143PR	C	144ARG	CB	94.23
139TYR	CD2	188VAL	CB	0.01	116SE	C	117PRO	CB	0.13	143PR	C	144ARG	CG	1.75
139TYR	CD2	188VAL	CG1	0.07	116SE	C	117PRO	CD	100.00	143PR	C	144ARG	C	7.42
139TYR	CD2	188VAL	CG2	0.40	116SE	C	117PRO	C	100.00	143PR	C	152TRP	CD1	0.04
139TYR	CE1	139TYR	CE2	100.00	117PR	CA	117PRO	CB	100.00	144AR	CA	144ARG	CB	100.00
139TYR	CE1	139TYR	CZ	100.00	117PR	CA	117PRO	CG	100.00	144AR	CA	144ARG	CG	100.00
139TYR	CE1	238ILE	CB	0.09	117PR	CA	117PRO	CD	100.00	144AR	CA	144ARG	CD	0.02
139TYR	CE1	238ILE	CG1	0.90	117PR	CA	117PRO	C	100.00	144AR	CA	144ARG	C	100.00
139TYR	CE1	238ILE	CD	0.73	117PR	CA	122ILE	CD	1.60	144AR	CA	152TRP	CD1	1.14
139TYR	CE1	185LYS	CG	3.92	117PR	CB	117PRO	CG	100.00	144AR	CB	144ARG	CG	100.00
139TYR	CE1	185LYS	CD	6.49	117PR	CB	117PRO	CD	100.00	144AR	CB	144ARG	CD	100.00

139TYR	CE1	185LYS	CE	0.14	117PR(	CB	117PRO	C	100.00	144AR	CB	144ARG	CZ	0.05
139TYR	CE1	188VAL	CG1	0.25	117PR(	CB	118LEU	CD2	0.01	144AR	CB	144ARG	C	100.00
139TYR	CE1	188VAL	CG2	4.89	117PR(	CB	122ILE	CG2	0.00	144AR	CB	190GLU	CD	0.03
139TYR	CE1	189LEU	CG	0.01	117PR(	CB	122ILE	CD	9.90	144AR	CG	144ARG	CD	100.00
139TYR	CE1	189LEU	CD1	1.05	117PR(	CB	225ARG	CA	0.02	144AR	CG	144ARG	CZ	6.70
139TYR	CE1	189LEU	CD2	0.08	117PR(	CG	117PRO	CD	100.00	144AR	CG	144ARG	C	93.22
139TYR	CE2	139TYR	CZ	100.00	117PR(	CG	117PRO	C	96.72	144AR	CG	190GLU	CB	0.01
139TYR	CE2	140PHE	CD2	0.01	117PR(	CG	118LEU	CD2	0.03	144AR	CG	190GLU	CD	0.05
139TYR	CE2	140PHE	CE2	0.13	117PR(	CG	249VAL	CG1	12.08	144AR	CD	144ARG	CZ	100.00
139TYR	CE2	237ASN	CG	0.04	117PR(	CG	249VAL	CG2	0.01	144AR	CD	144ARG	C	0.02
139TYR	CE2	237ASN	C	0.01	117PR(	CG	224VAL	CG1	0.09	144AR	CD	190GLU	CD	0.01
139TYR	CE2	238ILE	CA	0.02	117PR(	CG	224VAL	CG2	0.00	144AR	CZ	188VAL	CA	0.01
139TYR	CE2	241ASP	CB	0.03	117PR(	CG	225ARG	CA	0.04	144AR	CZ	188VAL	C	0.14
139TYR	CE2	241ASP	CG	0.37	117PR(	CG	225ARG	CG	0.00	144AR	CZ	190GLU	CD	0.03
139TYR	CE2	185LYS	CD	0.00	117PR(	CD	117PRO	C	90.06	144AR	C	145GLY	CA	100.00
139TYR	CE2	185LYS	CE	0.06	117PR(	CD	249VAL	CG1	2.37	144AR	C	145GLY	C	4.11
139TYR	CE2	188VAL	CG1	0.01	117PR(	CD	249VAL	CG2	0.00	144AR	C	152TRP	CD1	3.24
139TYR	CE2	188VAL	CG2	0.04	117PR(	C	118LEU	CA	100.00	144AR	C	152TRP	CE2	0.33
139TYR	CZ	238ILE	CA	0.05	117PR(	C	118LEU	CB	0.21	144AR	C	152TRP	CZ2	0.04
139TYR	CZ	238ILE	CG1	0.02	117PR(	C	118LEU	CG	0.01	145GL	CA	145GLY	C	100.00
139TYR	CZ	238ILE	CD	0.01	117PR(	C	118LEU	CD1	0.03	145GL	CA	152TRP	CD1	0.06
139TYR	CZ	185LYS	CG	0.02	117PR(	C	118LEU	CD2	0.02	145GL	CA	152TRP	CD2	0.24
139TYR	CZ	185LYS	CD	2.63	117PR(	C	118LEU	C	99.85	145GL	CA	152TRP	CE2	7.74
139TYR	CZ	185LYS	CE	0.85	117PR(	C	118LEU	CD1	0.01	145GL	CA	152TRP	CE3	0.04
139TYR	CZ	188VAL	CG2	0.16	117PR(	C	118LEU	CD2	0.17	145GL	CA	152TRP	CZ2	1.85
139TYR	C	140PHE	CA	100.00	117PR(	C	119LYS	CB	0.01	145GL	CA	152TRP	CZ3	0.04
139TYR	C	140PHE	CB	74.37	117PR(	C	119LYS	CD	0.04	145GL	CA	190GLU	CG	0.02
139TYR	C	140PHE	CG	43.64	117PR(	C	119LYS	CE	0.04	145GL	C	146MET	CA	100.00
139TYR	C	140PHE	CD1	11.99	118LEl	CA	118LEU	CB	100.00	145GL	C	146MET	CB	94.80
139TYR	C	140PHE	CD2	5.62	118LEl	CA	118LEU	CG	100.00	145GL	C	146MET	CG	0.34
139TYR	C	140PHE	C	21.01	118LEl	CA	118LEU	CD1	46.31	145GL	C	146MET	C	7.26
140PHE	CA	140PHE	CB	100.00	118LEl	CA	118LEU	CD2	71.40	145GL	C	152TRP	CD2	0.03
140PHE	CA	140PHE	CG	100.00	118LEl	CA	118LEU	C	100.00	145GL	C	152TRP	CE2	0.55
140PHE	CA	140PHE	CD1	90.74	118LEl	CB	118LEU	CG	100.00	145GL	C	152TRP	CE3	0.36
140PHE	CA	140PHE	CD2	33.13	118LEl	CB	118LEU	CD1	100.00	145GL	C	152TRP	CZ2	0.44
140PHE	CA	140PHE	C	100.00	118LEl	CB	118LEU	CD2	100.00	145GL	C	152TRP	CZ3	0.64
140PHE	CB	140PHE	CG	100.00	118LEl	CB	118LEU	C	100.00	145GL	C	190GLU	CB	0.14
140PHE	CB	140PHE	CD1	100.00	118LEl	CB	122ILE	CG2	0.01	145GL	C	190GLU	CG	0.20
140PHE	CB	140PHE	CD2	100.00	118LEl	CB	122ILE	CD	0.00	146ME	CA	146MET	CB	100.00
140PHE	CB	140PHE	C	100.00	118LEl	CG	118LEU	CD1	100.00	146ME	CA	146MET	CG	100.00
140PHE	CG	140PHE	CD1	100.00	118LEl	CG	118LEU	CD2	100.00	146ME	CA	146MET	CE	0.00
140PHE	CG	140PHE	CD2	100.00	118LEl	CG	118LEU	C	4.93	146ME	CA	146MET	C	100.00
140PHE	CG	140PHE	CE1	100.00	118LEl	CG	122ILE	CG2	0.01	146ME	CA	151ALA	CB	0.12
140PHE	CG	140PHE	CE2	100.00	118LEl	CG	122ILE	CD	0.00	146ME	CA	152TRP	CZ3	0.14
140PHE	CG	140PHE	CZ	100.00	118LEl	CG	250LEU	CD1	0.02	146ME	CB	146MET	CG	100.00
140PHE	CG	140PHE	C	6.55	118LEl	CG	250LEU	CD2	0.06	146ME	CB	146MET	CE	24.28
140PHE	CD1	140PHE	CD2	100.00	118LEl	CG	118LEU	CD2	0.04	146ME	CB	146MET	C	100.00
140PHE	CD1	140PHE	CE1	100.00	118LEl	CD1	118LEU	CD2	100.00	146ME	CB	151ALA	CA	0.03
140PHE	CD1	140PHE	CE2	100.00	118LEl	CD1	118LEU	C	0.00	146ME	CB	151ALA	CB	2.58
140PHE	CD1	140PHE	CZ	100.00	118LEl	CD1	122ILE	CB	0.00	146ME	CB	190GLU	CG	0.98
140PHE	CD1	140PHE	C	0.03	118LEl	CD1	122ILE	CG2	0.54	146ME	CG	146MET	CE	100.00
140PHE	CD2	140PHE	CE1	100.00	118LEl	CD1	122ILE	CD	0.02	146ME	CG	146MET	C	96.37
140PHE	CD2	140PHE	CE2	100.00	118LEl	CD1	224VAL	CG1	0.23	146ME	CG	151ALA	CB	0.12
140PHE	CD2	140PHE	CZ	100.00	118LEl	CD1	227PRO	CG	0.39	146ME	CG	190GLU	CB	0.03
140PHE	CD2	140PHE	C	0.75	118LEl	CD1	227PRO	CD	0.10	146ME	CG	190GLU	CG	0.02
140PHE	CE1	140PHE	CE2	100.00	118LEl	CD1	249VAL	CG1	0.02	146ME	CG	194PHE	CD1	0.02
140PHE	CE1	140PHE	CZ	100.00	118LEl	CD1	250LEU	CA	0.02	146ME	CE	146MET	C	0.01
140PHE	CE1	188VAL	CG1	0.36	118LEl	CD1	250LEU	CB	0.05	146ME	CE	151ALA	CB	0.01
140PHE	CE1	188VAL	CG2	0.01	118LEl	CD1	250LEU	CG	0.09	146ME	CE	190GLU	CB	0.00
140PHE	CE2	140PHE	CZ	100.00	118LEl	CD1	250LEU	CD1	0.56	146ME	CE	190GLU	CG	0.04
140PHE	CE2	188VAL	CG1	0.03	118LEl	CD1	250LEU	CD2	2.56	146ME	CE	190GLU	C	0.01
140PHE	CZ	237ASN	CG	0.00	118LEl	CD1	117PRO	CB	0.04	146ME	CE	193GLU	CB	0.20

140PHE CZ	188VAL CG1	0.27	118LEU CD1	117PRO C	0.12	146ME CE	193GLU CG	1.81
140PHE CZ	188VAL CG2	0.00	118LEU CD1	118LEU CG	0.00	146ME CE	193GLU C	0.13
140PHE C	141GLY CA	100.00	118LEU CD1	118LEU CD1	0.18	146ME CE	194PHE CA	0.60
140PHE C	141GLY C	65.09	118LEU CD1	118LEU CD2	0.61	146ME CE	194PHE CB	0.00
141GLY CA	141GLY C	100.00	118LEU CD1	224VAL CG1	0.20	146ME CE	194PHE CD1	0.00
141GLY C	142GLU CA	100.00	118LEU CD1	224VAL CG2	0.01	146ME CE	197LYS CD	0.56
141GLY C	142GLU CB	40.59	118LEU CD2	122ILE CB	0.02	146ME CE	197LYS CE	0.09
141GLY C	142GLU CG	0.21	118LEU CD2	122ILE CG1	0.00	146ME C	147SER CA	100.00
141GLY C	142GLU CD	0.02	118LEU CD2	122ILE CG2	1.18	146ME C	147SER CB	94.47
141GLY C	142GLU C	66.32	118LEU CD2	122ILE CD	0.03	146ME C	147SER C	10.41
142GLU CA	142GLU CB	100.00	118LEU CD2	122ILE C	0.00	146ME C	152TRP CZ3	0.11
142GLU CA	142GLU CG	100.00	118LEU CD2	123ALA CA	0.01	147SE CA	147SER CB	100.00
142GLU CA	142GLU CD	92.96	118LEU CD2	224VAL CA	0.00	147SE CA	147SER C	100.00
142GLU CA	142GLU C	100.00	118LEU CD2	224VAL CB	0.01	147SE CB	147SER C	100.00
142GLU CA	143PRO CA	100.00	118LEU CD2	224VAL CG1	0.17	147SE CB	152TRP CZ3	0.00
142GLU CA	143PRO C	46.64	118LEU CD2	227PRO CG	0.01	147SE C	148GLU CA	100.00
142GLU CB	142GLU CG	100.00	118LEU CD2	227PRO CD	0.00	147SE C	148GLU CB	0.77
142GLU CB	142GLU CD	100.00	118LEU CD2	249VAL CG1	0.00	147SE C	148GLU C	99.54
142GLU CB	142GLU C	100.00	118LEU CD2	250LEU CB	0.03	148GL CA	148GLU CB	100.00
142GLU CB	143PRO CA	0.84	118LEU CD2	250LEU CG	0.03	148GL CA	148GLU CG	100.00
142GLU CG	142GLU CD	100.00	118LEU CD2	250LEU CD1	0.13	148GL CA	148GLU CD	99.24
142GLU CG	142GLU C	93.92	118LEU CD2	250LEU CD2	1.08	148GL CA	148GLU C	100.00
142GLU CG	143PRO CA	1.41	118LEU CD2	117PRO CB	0.02	148GL CB	148GLU CG	100.00
142GLU CD	142GLU C	0.95	118LEU CD2	117PRO C	0.17	148GL CB	148GLU CD	100.00
142GLU CD	143PRO CA	0.70	118LEU CD2	118LEU CG	0.02	148GL CB	148GLU C	100.00
142GLU CD	144ARG CD	0.00	118LEU CD2	118LEU CD1	0.37	148GL CG	148GLU CD	100.00
142GLU C	143PRO CA	100.00	118LEU CD2	118LEU CD2	1.00	148GL CG	148GLU C	95.96
142GLU C	143PRO CB	0.06	118LEU CD2	224VAL CG1	0.22	148GL CD	148GLU C	1.62
142GLU C	143PRO CD	100.00	118LEU C	119LYS CA	100.00	148GL CD	159LYS CD	0.00
142GLU C	143PRO C	99.97	118LEU C	119LYS CB	0.00	148GL CD	159LYS CE	0.00
142GLU C	154THR CB	0.08	118LEU C	119LYS C	100.00	148GL CD	197LYS CE	0.12
143PRO CA	143PRO CB	100.00	118LEU C	119LYS CG	0.05	148GL C	149ALA CA	100.00
143PRO CA	143PRO CG	100.00	118LEU C	119LYS CD	0.01	148GL C	149ALA CB	23.82
143PRO CA	143PRO CD	100.00	118LEU C	119LYS CE	0.00	148GL C	149ALA C	74.73
143PRO CA	143PRO C	100.00	119LYS CA	119LYS CB	100.00	148GL C	194PHE CE1	0.10
143PRO CB	143PRO CG	100.00	119LYS CA	119LYS CG	100.00	148GL C	194PHE CZ	0.01
143PRO CB	143PRO CD	100.00	119LYS CA	119LYS CD	0.55	149AL CA	149ALA CB	100.00
143PRO CB	143PRO C	100.00	119LYS CA	119LYS C	100.00	149AL CA	149ALA C	100.00
143PRO CB	152TRP CD1	0.05	119LYS CA	119LYS CG	0.01	149AL CA	159LYS CB	0.02
143PRO CG	143PRO CD	100.00	119LYS CB	119LYS CG	100.00	149AL CA	159LYS CG	0.00
143PRO CG	143PRO C	97.25	119LYS CB	119LYS CD	100.00	149AL CA	194PHE CE1	0.04
143PRO CG	152TRP CD1	0.09	119LYS CB	119LYS CE	15.61	149AL CA	194PHE CZ	0.01
143PRO CD	143PRO C	90.08	119LYS CB	119LYS C	100.00	149AL CB	149ALA C	100.00
143PRO CD	154THR CB	0.26	119LYS CB	122ILE CD	0.20	149AL CB	150GLU CD	0.02
143PRO C	144ARG CA	100.00	119LYS CB	117PRO C	0.00	149AL CB	159LYS CB	0.07
143PRO C	144ARG CB	58.29	119LYS CG	119LYS CD	100.00	149AL CB	159LYS CD	0.05
143PRO C	144ARG CG	1.40	119LYS CG	119LYS CE	100.00	149AL C	150GLU CA	100.00
143PRO C	144ARG C	48.45	119LYS CG	119LYS C	10.99	149AL C	150GLU CB	99.92
144ARG CA	144ARG CB	100.00	119LYS CG	121GLU CB	0.00	149AL C	150GLU CG	3.06
144ARG CA	144ARG CG	100.00	119LYS CG	122ILE CG1	0.05	149AL C	150GLU CD	0.00
144ARG CA	144ARG CD	0.14	119LYS CG	122ILE CD	0.22	149AL C	150GLU C	0.07
144ARG CA	144ARG C	100.00	119LYS CG	117PRO C	0.01	149AL C	194PHE CE1	2.46
144ARG CA	152TRP CD1	0.06	119LYS CG	118LEU C	0.01	149AL C	194PHE CE2	0.01
144ARG CB	144ARG CG	100.00	119LYS CG	119LYS CG	0.13	149AL C	194PHE CZ	9.23
144ARG CB	144ARG CD	100.00	119LYS CD	119LYS CE	100.00	150GL CA	150GLU CB	100.00
144ARG CB	144ARG CZ	0.98	119LYS CD	119LYS C	0.03	150GL CA	150GLU CG	100.00
144ARG CB	144ARG C	100.00	119LYS CD	121GLU CB	0.00	150GL CA	150GLU CD	0.38
144ARG CB	190GLU CD	0.04	119LYS CD	121GLU CG	0.00	150GL CA	150GLU C	100.00
144ARG CG	144ARG CD	100.00	119LYS CD	122ILE CD	0.02	150GL CA	194PHE CE1	2.30
144ARG CG	144ARG CZ	27.49	119LYS CD	117PRO C	0.02	150GL CA	194PHE CE2	0.02
144ARG CG	144ARG C	64.64	119LYS CD	118LEU C	0.01	150GL CA	194PHE CZ	2.09
144ARG CG	153ASN CG	0.00	119LYS CE	122ILE CG1	0.01	150GL CB	150GLU CG	100.00

144ARG	CG	190GLU	CB	0.03	119LYS	CE	122ILE	CD	0.02	150GL	CB	150GLU	CD	100.00
144ARG	CG	190GLU	CG	0.01	119LYS	CE	117PRO	C	0.00	150GL	CB	150GLU	C	100.00
144ARG	CG	190GLU	CD	0.56	119LYS	CE	120GLU	CD	0.04	150GL	CB	152TRP	CZ3	0.02
144ARG	CD	144ARG	CZ	100.00	119LYS	C	120GLU	CA	100.00	150GL	CB	158SER	CB	0.10
144ARG	CD	144ARG	C	0.08	119LYS	C	120GLU	CB	0.00	150GL	CG	150GLU	CD	100.00
144ARG	CD	190GLU	CD	0.07	119LYS	C	120GLU	C	100.00	150GL	CG	150GLU	C	91.96
144ARG	CZ	190GLU	CD	0.06	120GLU	CA	120GLU	CB	100.00	150GL	CG	152TRP	CE3	0.02
144ARG	C	145GLY	CA	100.00	120GLU	CA	120GLU	CG	100.00	150GL	CG	152TRP	CZ3	0.09
144ARG	C	145GLY	C	21.58	120GLU	CA	120GLU	CD	27.90	150GL	CG	156ARG	CG	0.01
144ARG	C	152TRP	CD1	0.19	120GLU	CA	120GLU	C	100.00	150GL	CG	156ARG	CD	0.00
144ARG	C	152TRP	CE2	0.02	120GLU	CB	120GLU	CG	100.00	150GL	CG	157TYR	C	0.02
144ARG	C	190GLU	CG	0.02	120GLU	CB	120GLU	CD	100.00	150GL	CG	158SER	CA	0.08
145GLY	CA	145GLY	C	100.00	120GLU	CB	120GLU	C	100.00	150GL	CG	158SER	CB	0.40
145GLY	CA	152TRP	CD1	0.42	120GLU	CB	124ARG	CZ	1.13	150GL	CD	152TRP	CE3	0.02
145GLY	CA	152TRP	CD2	0.27	120GLU	CG	120GLU	CD	100.00	150GL	CD	152TRP	CZ3	0.08
145GLY	CA	152TRP	CE2	6.26	120GLU	CG	120GLU	C	60.37	150GL	CD	156ARG	CG	0.47
145GLY	CA	152TRP	CE3	0.03	120GLU	CG	124ARG	CZ	0.49	150GL	CD	156ARG	CD	0.67
145GLY	CA	152TRP	CZ2	0.60	120GLU	CD	120GLU	C	0.51	150GL	CD	156ARG	CZ	0.42
145GLY	CA	152TRP	CZ3	0.02	120GLU	CD	124ARG	CZ	0.11	150GL	CD	158SER	CA	0.01
145GLY	CA	190GLU	CG	0.00	120GLU	CD	119LYS	CE	0.70	150GL	CD	158SER	CB	0.07
145GLY	C	146MET	CA	100.00	120GLU	C	121GLU	CA	100.00	150GL	C	151ALA	CA	100.00
145GLY	C	146MET	CB	96.42	120GLU	C	121GLU	CB	0.07	150GL	C	151ALA	CB	93.35
145GLY	C	146MET	CG	0.20	120GLU	C	121GLU	C	99.98	150GL	C	151ALA	C	5.64
145GLY	C	146MET	C	5.90	120GLU	C	124ARG	CD	0.00	150GL	C	152TRP	CE3	0.00
145GLY	C	152TRP	CD2	0.00	120GLU	C	124ARG	CZ	0.04	150GL	C	152TRP	CZ3	0.09
145GLY	C	152TRP	CE2	0.20	121GLU	CA	121GLU	CB	100.00	150GL	C	194PHE	CD1	0.14
145GLY	C	152TRP	CE3	0.16	121GLU	CA	121GLU	CG	100.00	150GL	C	194PHE	CE1	9.17
145GLY	C	152TRP	CZ2	0.06	121GLU	CA	121GLU	CD	96.58	150GL	C	194PHE	CE2	0.02
145GLY	C	152TRP	CZ3	0.08	121GLU	CA	121GLU	C	100.00	150GL	C	194PHE	CZ	0.16
145GLY	C	190GLU	CB	0.01	121GLU	CA	124ARG	CZ	0.04	151AL	CA	151ALA	CB	100.00
145GLY	C	190GLU	CG	0.28	121GLU	CB	121GLU	CG	100.00	151AL	CA	151ALA	C	100.00
146MET	CA	146MET	CB	100.00	121GLU	CB	121GLU	CD	100.00	151AL	CA	152TRP	CE3	3.90
146MET	CA	146MET	CG	100.00	121GLU	CB	121GLU	C	100.00	151AL	CB	151ALA	C	100.00
146MET	CA	146MET	CE	0.00	121GLU	CG	121GLU	CD	100.00	151AL	CB	190GLU	C	0.17
146MET	CA	146MET	C	100.00	121GLU	CG	121GLU	C	79.08	151AL	CB	191VAL	CA	0.05
146MET	CA	151ALA	CB	0.07	121GLU	CD	121GLU	C	0.30	151AL	CB	191VAL	CG1	0.01
146MET	CB	146MET	CG	100.00	121GLU	CD	124ARG	CD	0.02	151AL	CB	191VAL	CG2	0.11
146MET	CB	146MET	CE	25.35	121GLU	CD	124ARG	CZ	0.06	151AL	CB	194PHE	CB	31.12
146MET	CB	146MET	C	100.00	121GLU	C	122ILE	CA	100.00	151AL	CB	194PHE	CG	25.01
146MET	CB	151ALA	CB	0.20	121GLU	C	122ILE	CB	3.84	151AL	CB	194PHE	CD1	12.46
146MET	CB	190GLU	CG	1.59	121GLU	C	122ILE	CG1	0.72	151AL	CB	194PHE	CD2	1.90
146MET	CG	146MET	CE	100.00	121GLU	C	122ILE	CG2	1.53	151AL	C	152TRP	CA	100.00
146MET	CG	146MET	C	94.82	121GLU	C	122ILE	CD	0.03	151AL	C	152TRP	CB	99.03
146MET	CG	151ALA	CB	0.22	121GLU	C	122ILE	C	96.24	151AL	C	152TRP	CG	0.00
146MET	CG	190GLU	CB	0.00	122ILE	CA	122ILE	CB	100.00	151AL	C	152TRP	CD2	0.00
146MET	CG	190GLU	CG	0.09	122ILE	CA	122ILE	CG1	100.00	151AL	C	152TRP	CE3	17.18
146MET	CG	194PHE	CD1	0.14	122ILE	CA	122ILE	CG2	100.00	151AL	C	152TRP	C	0.54
146MET	CG	194PHE	CE1	0.00	122ILE	CA	122ILE	CD	3.87	151AL	C	191VAL	CG1	0.47
146MET	CE	146MET	C	0.02	122ILE	CA	122ILE	C	100.00	151AL	C	191VAL	CG2	1.61
146MET	CE	151ALA	CB	0.01	122ILE	CB	122ILE	CG1	100.00	152TR	CA	152TRP	CB	100.00
146MET	CE	190GLU	CG	0.04	122ILE	CB	122ILE	CG2	100.00	152TR	CA	152TRP	CG	100.00
146MET	CE	190GLU	CD	0.06	122ILE	CB	122ILE	CD	100.00	152TR	CA	152TRP	CD1	41.43
146MET	CE	193GLU	CB	0.07	122ILE	CB	122ILE	C	100.00	152TR	CA	152TRP	CD2	32.80
146MET	CE	193GLU	CG	1.70	122ILE	CG1	122ILE	CG2	100.00	152TR	CA	152TRP	C	100.00
146MET	CE	193GLU	CD	0.08	122ILE	CG1	122ILE	CD	100.00	152TR	CA	191VAL	CG1	0.32
146MET	CE	193GLU	C	0.06	122ILE	CG1	122ILE	C	7.67	152TR	CA	191VAL	CG2	3.74
146MET	CE	194PHE	CA	1.05	122ILE	CG2	122ILE	CD	96.32	152TR	CB	152TRP	CG	100.00
146MET	CE	194PHE	CB	0.03	122ILE	CG2	122ILE	C	92.30	152TR	CB	152TRP	CD1	100.00
146MET	CE	194PHE	CD1	0.01	122ILE	CG2	224VAL	CA	0.01	152TR	CB	152TRP	CD2	100.00
146MET	CE	197LYS	CD	1.08	122ILE	CG2	224VAL	CB	0.00	152TR	CB	152TRP	CE3	97.98
146MET	CE	197LYS	CE	0.05	122ILE	CG2	224VAL	C	0.13	152TR	CB	152TRP	C	100.00
146MET	C	147SER	CA	100.00	122ILE	CG2	225ARG	CA	0.00	152TR	CB	154THR	CG2	0.01

146MET C	147SER CB	91.11	122ILE CG2	225ARG C	0.05	152TR CB	156ARG CA	0.00
146MET C	147SER C	15.24	122ILE CG2	227PRO CG	0.28	152TR CB	156ARG CB	0.31
146MET C	152TRP CZ3	0.08	122ILE CG2	227PRO CD	5.04	152TR CB	156ARG CG	0.15
147SER CA	147SER CB	100.00	122ILE CG2	250LEU CD1	0.07	152TR CB	156ARG CD	0.01
147SER CA	147SER C	100.00	122ILE CG2	250LEU CD2	0.30	152TR CB	156ARG CZ	2.03
147SER CB	147SER C	100.00	122ILE CD	122ILE C	0.11	152TR CG	152TRP CD1	100.00
147SER CB	152TRP CZ3	0.00	122ILE CD	224VAL C	0.03	152TR CG	152TRP CD2	100.00
147SER C	148GLU CA	100.00	122ILE CD	225ARG CA	0.00	152TR CG	152TRP CE2	100.00
147SER C	148GLU CB	1.14	122ILE CD	225ARG C	0.03	152TR CG	152TRP CE3	100.00
147SER C	148GLU C	99.42	122ILE CD	117PRO CA	0.52	152TR CG	152TRP C	99.92
148GLU CA	148GLU CB	100.00	122ILE CD	117PRO CB	13.27	152TR CG	156ARG CZ	1.14
148GLU CA	148GLU CG	100.00	122ILE C	123ALA CA	100.00	152TR CD1	152TRP CD2	100.00
148GLU CA	148GLU CD	98.64	122ILE C	123ALA CB	0.18	152TR CD1	152TRP CE2	100.00
148GLU CA	148GLU C	100.00	122ILE C	123ALA C	99.84	152TR CD1	152TRP C	16.28
148GLU CB	148GLU CG	100.00	123AL/ CA	123ALA CB	100.00	152TR CD1	154THR CG2	0.02
148GLU CB	148GLU CD	100.00	123AL/ CA	123ALA C	100.00	152TR CD1	156ARG CZ	0.02
148GLU CB	148GLU C	100.00	123AL/ CA	126VAL CG2	0.02	152TR CD2	152TRP CE2	100.00
148GLU CG	148GLU CD	100.00	123AL/ CB	123ALA C	100.00	152TR CD2	152TRP CE3	100.00
148GLU CG	148GLU C	93.69	123AL/ C	124ARG CA	100.00	152TR CD2	152TRP CZ2	100.00
148GLU CD	148GLU C	1.88	123AL/ C	124ARG CB	1.40	152TR CD2	152TRP CZ3	100.00
148GLU CD	159LYS CD	0.00	123AL/ C	124ARG CG	0.06	152TR CD2	156ARG CZ	0.86
148GLU CD	159LYS CE	0.02	123AL/ C	124ARG C	98.93	152TR CE2	152TRP CE3	100.00
148GLU CD	197LYS CE	0.06	123AL/ C	126VAL CG2	0.53	152TR CE2	152TRP CZ2	100.00
148GLU C	149ALA CA	100.00	124AR(CA	124ARG CB	100.00	152TR CE2	152TRP CZ3	100.00
148GLU C	149ALA CB	25.20	124AR(CA	124ARG CG	100.00	152TR CE2	156ARG CZ	0.02
148GLU C	149ALA C	73.16	124AR(CA	124ARG CD	0.03	152TR CE3	152TRP CZ2	100.00
148GLU C	159LYS CD	0.03	124AR(CA	124ARG C	100.00	152TR CE3	152TRP CZ3	100.00
148GLU C	194PHE CE1	0.04	124AR(CB	124ARG CG	100.00	152TR CE3	156ARG CD	0.04
148GLU C	194PHE CZ	0.00	124AR(CB	124ARG CD	100.00	152TR CE3	156ARG CZ	4.33
149ALA CA	149ALA CB	100.00	124AR(CB	124ARG CZ	5.36	152TR CZ2	152TRP CZ3	100.00
149ALA CA	149ALA C	100.00	124AR(CB	124ARG C	100.00	152TR CZ3	156ARG CZ	1.36
149ALA CA	194PHE CE1	0.01	124AR(CG	124ARG CD	100.00	152TR C	153ASN CA	100.00
149ALA CA	194PHE CZ	0.00	124AR(CG	124ARG CZ	21.93	152TR C	153ASN CB	93.42
149ALA CB	149ALA C	100.00	124AR(CG	124ARG C	67.60	152TR C	153ASN CG	17.63
149ALA CB	159LYS CB	0.04	124AR(CD	124ARG CZ	100.00	152TR C	153ASN C	3.82
149ALA CB	159LYS CD	0.01	124AR(CD	124ARG C	0.01	152TR C	191VAL CG1	0.28
149ALA C	150GLU CA	100.00	124AR(C	125GLY CA	100.00	152TR C	191VAL CG2	6.58
149ALA C	150GLU CB	99.86	124AR(C	125GLY C	55.01	153AS CA	153ASN CB	100.00
149ALA C	150GLU CG	0.23	125GL\ CA	125GLY C	100.00	153AS CA	153ASN CG	100.00
149ALA C	150GLU C	0.11	125GL\ CA	228ALA CB	0.16	153AS CA	153ASN C	100.00
149ALA C	194PHE CE1	1.03	125GL\ C	126VAL CA	100.00	153AS CB	153ASN CG	100.00
149ALA C	194PHE CE2	0.01	125GL\ C	126VAL CB	1.71	153AS CB	153ASN C	100.00
149ALA C	194PHE CZ	8.06	125GL\ C	126VAL CG2	0.85	153AS CB	189LEU CD1	0.04
150GLU CA	150GLU CB	100.00	125GL\ C	126VAL C	99.13	153AS CB	189LEU CD2	0.14
150GLU CA	150GLU CG	100.00	125GL\ C	227PRO CB	0.00	153AS CB	191VAL CG1	0.00
150GLU CA	150GLU CD	0.11	125GL\ C	227PRO CG	0.34	153AS CB	191VAL CG2	0.08
150GLU CA	150GLU C	100.00	125GL\ C	228ALA CB	3.58	153AS CG	153ASN C	0.08
150GLU CA	158SER CB	0.00	126VAI CA	126VAL CB	100.00	153AS CG	189LEU CD1	0.30
150GLU CA	194PHE CE1	0.78	126VAI CA	126VAL CG1	100.00	153AS CG	189LEU CD2	5.60
150GLU CA	194PHE CE2	0.08	126VAI CA	126VAL CG2	100.00	153AS C	154THR CA	100.00
150GLU CA	194PHE CZ	4.13	126VAI CA	126VAL C	100.00	153AS C	154THR CB	64.63
150GLU CB	150GLU CG	100.00	126VAI CA	228ALA CB	0.01	153AS C	154THR C	47.53
150GLU CB	150GLU CD	100.00	126VAI CB	126VAL CG1	100.00	154TH CA	154THR CB	100.00
150GLU CB	150GLU C	100.00	126VAI CB	126VAL CG2	100.00	154TH CA	154THR CG2	100.00
150GLU CB	152TRP CZ3	0.00	126VAI CB	126VAL C	100.00	154TH CA	154THR C	100.00
150GLU CB	158SER CA	0.00	126VAI CG1	126VAL CG2	100.00	154TH CB	154THR CG2	100.00
150GLU CB	158SER CB	0.02	126VAI CG1	126VAL C	99.90	154TH CB	154THR C	100.00
150GLU CG	150GLU CD	100.00	126VAI CG1	128VAL CG1	0.07	154TH CG2	154THR C	100.00
150GLU CG	150GLU C	96.65	126VAI CG1	128VAL CG2	0.60	154TH CG2	155GLU C	0.00
150GLU CG	152TRP CZ3	0.04	126VAI CG1	227PRO CB	0.01	154TH CG2	156ARG CB	0.02
150GLU CG	156ARG CD	0.00	126VAI CG1	227PRO CG	0.00	154TH CG2	156ARG CG	0.31
150GLU CG	157TYR C	1.06	126VAI CG1	250LEU CB	0.02	154TH CG2	152TRP CB	0.10

150GLU	CG	158SER	CA	0.08
150GLU	CG	158SER	CB	0.20
150GLU	CD	152TRP	CE3	0.02
150GLU	CD	152TRP	CZ3	0.12
150GLU	CD	156ARG	CG	0.02
150GLU	CD	156ARG	CD	0.04
150GLU	CD	156ARG	CZ	0.60
150GLU	CD	158SER	CA	0.00
150GLU	CD	158SER	CB	0.02
150GLU	C	151ALA	CA	100.00
150GLU	C	151ALA	CB	88.55
150GLU	C	151ALA	C	12.24
150GLU	C	152TRP	CE3	0.01
150GLU	C	152TRP	CZ3	0.15
150GLU	C	194PHE	CD1	0.02
150GLU	C	194PHE	CD2	0.00
150GLU	C	194PHE	CE1	7.08
150GLU	C	194PHE	CE2	0.09
150GLU	C	194PHE	CZ	1.04
151ALA	CA	151ALA	CB	100.00
151ALA	CA	151ALA	C	100.00
151ALA	CA	152TRP	CE3	2.28
151ALA	CB	151ALA	C	100.00
151ALA	CB	190GLU	C	0.01
151ALA	CB	191VAL	CA	0.04
151ALA	CB	191VAL	CG2	0.32
151ALA	CB	194PHE	CB	27.46
151ALA	CB	194PHE	CG	35.76
151ALA	CB	194PHE	CD1	5.82
151ALA	CB	194PHE	CD2	4.44
151ALA	CB	194PHE	CE1	0.00
151ALA	CB	194PHE	CE2	0.01
151ALA	C	152TRP	CA	100.00
151ALA	C	152TRP	CB	99.48
151ALA	C	152TRP	CG	0.03
151ALA	C	152TRP	CD2	0.02
151ALA	C	152TRP	CE3	18.70
151ALA	C	152TRP	C	0.15
151ALA	C	191VAL	CG2	3.75
152TRP	CA	152TRP	CB	100.00
152TRP	CA	152TRP	CG	100.00
152TRP	CA	152TRP	CD1	53.55
152TRP	CA	152TRP	CD2	22.06
152TRP	CA	152TRP	C	100.00
152TRP	CA	191VAL	CG2	9.35
152TRP	CB	152TRP	CG	100.00
152TRP	CB	152TRP	CD1	100.00
152TRP	CB	152TRP	CD2	100.00
152TRP	CB	152TRP	CE3	98.21
152TRP	CB	152TRP	C	100.00
152TRP	CB	154THR	CG2	0.22
152TRP	CB	156ARG	CB	1.70
152TRP	CB	156ARG	CG	0.02
152TRP	CB	156ARG	CD	0.01
152TRP	CB	156ARG	CZ	0.68
152TRP	CG	152TRP	CD1	100.00
152TRP	CG	152TRP	CD2	100.00
152TRP	CG	152TRP	CE2	100.00
152TRP	CG	152TRP	CE3	100.00
152TRP	CG	152TRP	C	99.68
152TRP	CG	156ARG	CZ	0.06
152TRP	CD1	152TRP	CD2	100.00

126VAl	CG1	250LEU	CD2	0.02
126VAl	CG1	251PRO	CB	0.27
126VAl	CG1	251PRO	CG	0.19
126VAl	CG1	251PRO	CD	0.03
126VAl	CG2	126VAL	C	0.09
126VAl	CG2	227PRO	CB	1.10
126VAl	CG2	227PRO	CG	7.62
126VAl	CG2	227PRO	C	0.01
126VAl	CG2	228ALA	CA	0.02
126VAl	CG2	228ALA	CB	0.02
126VAl	CG2	250LEU	CB	0.18
126VAl	CG2	250LEU	CG	0.03
126VAl	CG2	250LEU	CD1	0.03
126VAl	CG2	250LEU	CD2	0.14
126VAl	CG2	251PRO	CA	0.01
126VAl	CG2	251PRO	CB	0.02
126VAl	CG2	251PRO	CG	0.00
126VAl	C	127ASP	CA	100.00
126VAl	C	127ASP	CB	99.68
126VAl	C	127ASP	C	1.69
127ASI	CA	127ASP	CB	100.00
127ASI	CA	127ASP	CG	100.00
127ASI	CA	127ASP	C	100.00
127ASI	CB	127ASP	CG	100.00
127ASI	CB	127ASP	C	100.00
127ASI	CB	177ARG	CZ	0.02
127ASI	CG	127ASP	C	99.96
127ASI	CG	176ARG	CZ	0.01
127ASI	CG	177ARG	CZ	0.02
127ASI	C	128VAL	CA	100.00
127ASI	C	128VAL	CB	48.50
127ASI	C	128VAL	CG1	3.24
127ASI	C	128VAL	CG2	8.01
127ASI	C	128VAL	C	55.88
128VAl	CA	128VAL	CB	100.00
128VAl	CA	128VAL	CG1	100.00
128VAl	CA	128VAL	CG2	100.00
128VAl	CA	128VAL	C	100.00
128VAl	CB	128VAL	CG1	100.00
128VAl	CB	128VAL	CG2	100.00
128VAl	CB	128VAL	C	100.00
128VAl	CB	130ILE	CD	0.00
128VAl	CB	230PHE	CB	0.00
128VAl	CG1	128VAL	CG2	100.00
128VAl	CG1	128VAL	C	90.02
128VAl	CG1	129LEU	C	0.00
128VAl	CG1	130ILE	CG1	0.48
128VAl	CG1	130ILE	CD	2.28
128VAl	CG1	230PHE	CB	0.97
128VAl	CG1	230PHE	C	0.04
128VAl	CG1	233VAL	CG1	0.05
128VAl	CG1	233VAL	CG2	5.76
128VAl	CG1	247ALA	CB	0.01
128VAl	CG1	250LEU	CD1	0.00
128VAl	CG2	128VAL	C	46.47
128VAl	CG2	129LEU	C	0.01
128VAl	CG2	130ILE	CG1	0.02
128VAl	CG2	130ILE	CD	0.25
128VAl	CG2	230PHE	CB	1.81
128VAl	CG2	230PHE	CD1	0.00
128VAl	CG2	230PHE	C	0.05
128VAl	CG2	231ASP	C	0.03

154TH	CG2	152TRP	CD1	0.02
154TH	CG2	153ASN	C	0.02
154TH	C	155GLU	CA	100.00
154TH	C	155GLU	CB	0.50
154TH	C	155GLU	C	99.88
155GL	CA	155GLU	CB	100.00
155GL	CA	155GLU	CG	100.00
155GL	CA	155GLU	CD	88.36
155GL	CA	155GLU	C	100.00
155GL	CB	155GLU	CG	100.00
155GL	CB	155GLU	CD	100.00
155GL	CB	155GLU	C	100.00
155GL	CB	138ILE	CD	1.12
155GL	CG	155GLU	CD	100.00
155GL	CG	155GLU	C	99.99
155GL	CG	157TYR	CD1	0.02
155GL	CG	157TYR	CD2	0.00
155GL	CG	157TYR	CE1	0.66
155GL	CG	157TYR	CE2	1.10
155GL	CG	157TYR	CZ	0.00
155GL	CG	191VAL	CG1	0.02
155GL	CG	191VAL	CG2	1.65
155GL	CD	155GLU	C	0.02
155GL	CD	157TYR	CD1	0.03
155GL	CD	157TYR	CE1	3.44
155GL	CD	157TYR	CE2	5.96
155GL	CD	157TYR	CZ	0.57
155GL	C	156ARG	CA	100.00
155GL	C	156ARG	CB	93.87
155GL	C	156ARG	CG	2.78
155GL	C	156ARG	C	11.08
156AR	CA	156ARG	CB	100.00
156AR	CA	156ARG	CG	100.00
156AR	CA	156ARG	CD	0.12
156AR	CA	156ARG	C	100.00
156AR	CB	156ARG	CG	100.00
156AR	CB	156ARG	CD	100.00
156AR	CB	156ARG	CZ	0.28
156AR	CB	156ARG	C	100.00
156AR	CB	152TRP	CB	0.01
156AR	CG	156ARG	CD	100.00
156AR	CG	156ARG	CZ	82.98
156AR	CG	156ARG	C	93.19
156AR	CG	150GLU	CG	0.00
156AR	CG	150GLU	CD	0.53
156AR	CG	152TRP	CB	0.03
156AR	CD	156ARG	CZ	100.00
156AR	CD	150GLU	CD	0.69
156AR	CZ	150GLU	CD	0.00
156AR	CZ	152TRP	CB	3.42
156AR	CZ	152TRP	CG	0.66
156AR	CZ	152TRP	CD2	0.40
156AR	CZ	152TRP	CE3	1.97
156AR	CZ	152TRP	CZ3	0.09
156AR	C	157TYR	CA	100.00
156AR	C	157TYR	CB	81.44
156AR	C	157TYR	CG	29.56
156AR	C	157TYR	CD1	1.24
156AR	C	157TYR	CD2	14.28
156AR	C	157TYR	C	21.12
157TY	CA	157TYR	CB	100.00
157TY	CA	157TYR	CG	100.00

152TRP CD1	152TRP CE2	100.00	128VAL CG2	233VAL	CG1	0.17	157TY CA	157TYR	CD1	93.54
152TRP CD1	152TRP C	16.78	128VAL CG2	233VAL	CG2	5.51	157TY CA	157TYR	CD2	43.84
152TRP CD1	154THR CG2	0.01	128VAL C	129LEU	CA	100.00	157TY CA	157TYR	C	100.00
152TRP CD2	152TRP CE2	100.00	128VAL C	129LEU	CB	35.60	157TY CA	162VAL	CG1	0.01
152TRP CD2	152TRP CE3	100.00	128VAL C	129LEU	C	75.09	157TY CB	157TYR	CG	100.00
152TRP CD2	152TRP CZ2	100.00	129LEU CA	129LEU	CB	100.00	157TY CB	157TYR	CD1	100.00
152TRP CD2	152TRP CZ3	100.00	129LEU CA	129LEU	CG	100.00	157TY CB	157TYR	CD2	100.00
152TRP CD2	156ARG CZ	0.18	129LEU CA	129LEU	CD1	95.75	157TY CB	157TYR	C	100.00
152TRP CE2	152TRP CE3	100.00	129LEU CA	129LEU	CD2	3.59	157TY CB	161GLU	CB	0.00
152TRP CE2	152TRP CZ2	100.00	129LEU CA	129LEU	C	100.00	157TY CB	162VAL	CG1	0.70
152TRP CE2	152TRP CZ3	100.00	129LEU CB	129LEU	CG	100.00	157TY CB	162VAL	CG2	1.04
152TRP CE3	152TRP CZ2	100.00	129LEU CB	129LEU	CD1	100.00	157TY CB	194PHE	CD2	0.28
152TRP CE3	152TRP CZ3	100.00	129LEU CB	129LEU	CD2	100.00	157TY CB	194PHE	CE2	0.15
152TRP CE3	156ARG CD	0.00	129LEU CB	129LEU	C	100.00	157TY CG	157TYR	CD1	100.00
152TRP CE3	156ARG CZ	6.42	129LEU CB	176ARG	CZ	3.57	157TY CG	157TYR	CD2	100.00
152TRP CZ2	152TRP CZ3	100.00	129LEU CG	129LEU	CD1	100.00	157TY CG	157TYR	CE1	100.00
152TRP CZ3	156ARG CZ	1.19	129LEU CG	129LEU	CD2	100.00	157TY CG	157TYR	CE2	100.00
152TRP C	153ASN CA	100.00	129LEU CG	129LEU	C	98.92	157TY CG	157TYR	CZ	100.00
152TRP C	153ASN CB	69.76	129LEU CG	131VAL	CG2	0.11	157TY CG	157TYR	C	0.04
152TRP C	153ASN CG	1.52	129LEU CG	176ARG	CZ	0.01	157TY CG	162VAL	CG1	3.55
152TRP C	153ASN C	18.52	129LEU CD1	129LEU	CD2	100.00	157TY CG	162VAL	CG2	0.23
152TRP C	191VAL CG2	14.74	129LEU CD1	129LEU	C	5.26	157TY CG	191VAL	CB	0.08
153ASN CA	153ASN CB	100.00	129LEU CD1	131VAL	CG2	0.17	157TY CG	191VAL	CG1	0.02
153ASN CA	153ASN CG	100.00	129LEU CD1	172ALA	CB	0.04	157TY CG	195TRP	CE3	0.01
153ASN CA	153ASN C	100.00	129LEU CD1	176ARG	CZ	0.10	157TY CG	195TRP	CZ3	0.01
153ASN CB	153ASN CG	100.00	129LEU CD1	232VAL	CG1	0.00	157TY CD1	157TYR	CD2	100.00
153ASN CB	153ASN C	100.00	129LEU CD1	296MET	CE	0.08	157TY CD1	157TYR	CE1	100.00
153ASN CB	189LEU CD1	0.29	129LEU CD1	300HIS	CE1	0.01	157TY CD1	157TYR	CE2	100.00
153ASN CB	189LEU CD2	0.03	129LEU CD2	129LEU	C	2.19	157TY CD1	157TYR	CZ	100.00
153ASN CB	191VAL CG2	0.05	129LEU CD2	130ILE	C	0.01	157TY CD1	161GLU	CB	0.25
153ASN CG	153ASN C	0.11	129LEU CD2	131VAL	CG2	2.18	157TY CD1	161GLU	CG	1.98
153ASN CG	189LEU CD1	13.58	129LEU CD2	172ALA	CB	7.92	157TY CD1	161GLU	CD	0.01
153ASN CG	189LEU CD2	0.69	129LEU CD2	176ARG	CD	0.03	157TY CD1	162VAL	CG1	4.65
153ASN CG	190GLU CB	0.02	129LEU CD2	176ARG	CZ	0.15	157TY CD1	162VAL	CG2	4.08
153ASN CG	191VAL CG2	0.00	129LEU CD2	232VAL	CG1	0.01	157TY CD1	191VAL	CB	0.43
153ASN C	154THR CA	100.00	129LEU CD2	232VAL	CG2	0.04	157TY CD1	191VAL	CG1	0.13
153ASN C	154THR CB	45.05	129LEU CD2	300HIS	CE1	0.11	157TY CD1	195TRP	CE3	0.02
153ASN C	154THR C	67.76	129LEU C	130ILE	CA	100.00	157TY CD1	195TRP	CZ3	0.27
153ASN C	154THR CG2	0.01	129LEU C	130ILE	CB	3.29	157TY CD2	157TYR	CE1	100.00
154THR CA	154THR CB	100.00	129LEU C	130ILE	CG1	1.23	157TY CD2	157TYR	CE2	100.00
154THR CA	154THR CG2	100.00	129LEU C	130ILE	CD	0.06	157TY CD2	157TYR	CZ	100.00
154THR CA	154THR C	100.00	129LEU C	130ILE	C	99.44	157TY CD2	157TYR	C	0.01
154THR CB	154THR CG2	100.00	130ILE CA	130ILE	CB	100.00	157TY CD2	162VAL	CG1	2.45
154THR CB	154THR C	100.00	130ILE CA	130ILE	CG1	100.00	157TY CD2	162VAL	CG2	0.10
154THR CG2	154THR C	100.00	130ILE CA	130ILE	CG2	100.00	157TY CD2	191VAL	CB	0.20
154THR CG2	155GLU C	0.02	130ILE CA	130ILE	CD	37.63	157TY CD2	191VAL	CG1	0.16
154THR CG2	156ARG CG	0.03	130ILE CA	130ILE	C	100.00	157TY CD2	195TRP	CE3	0.18
154THR CG2	156ARG CZ	0.01	130ILE CA	233VAL	CG1	0.00	157TY CD2	195TRP	CZ3	0.02
154THR C	155GLU CA	100.00	130ILE CA	233VAL	CG2	0.08	157TY CE1	157TYR	CE2	100.00
154THR C	155GLU CB	6.88	130ILE CB	130ILE	CG1	100.00	157TY CE1	157TYR	CZ	100.00
154THR C	155GLU C	97.01	130ILE CB	130ILE	CG2	100.00	157TY CE1	161GLU	CB	0.01
155GLU CA	155GLU CB	100.00	130ILE CB	130ILE	CD	100.00	157TY CE1	161GLU	CG	0.50
155GLU CA	155GLU CG	100.00	130ILE CB	130ILE	C	100.00	157TY CE1	161GLU	C	0.02
155GLU CA	155GLU CD	99.74	130ILE CG1	130ILE	CG2	100.00	157TY CE1	162VAL	CG1	0.06
155GLU CA	155GLU C	100.00	130ILE CG1	130ILE	CD	100.00	157TY CE1	162VAL	CG2	0.00
155GLU CB	155GLU CG	100.00	130ILE CG1	233VAL	CB	0.00	157TY CE1	191VAL	CB	0.00
155GLU CB	155GLU CD	100.00	130ILE CG1	233VAL	CG1	0.01	157TY CE1	191VAL	CG2	0.01
155GLU CB	155GLU C	100.00	130ILE CG1	233VAL	CG2	0.19	157TY CE1	195TRP	CE3	0.12
155GLU CB	238ILE CD	0.00	130ILE CG1	243LEU	CD1	0.01	157TY CE1	195TRP	CZ3	3.56
155GLU CB	138ILE CD	0.26	130ILE CG1	243LEU	CD2	0.02	157TY CE2	157TYR	CZ	100.00
155GLU CG	155GLU CD	100.00	130ILE CG1	247ALA	CB	0.18	157TY CE2	162VAL	CG1	0.15
155GLU CG	155GLU C	100.00	130ILE CG2	130ILE	CD	78.69	157TY CE2	191VAL	CB	0.04

155GLU CG	157TYR	CD1	0.41	130ILE CG2	130ILE	C	100.00	157TY CE2	191VAL	CG1	0.00
155GLU CG	157TYR	CD2	0.06	130ILE CG2	131VAL	C	0.00	157TY CE2	191VAL	CG2	0.00
155GLU CG	157TYR	CE1	5.93	130ILE CG2	132ARG	CB	0.01	157TY CE2	195TRP	CB	0.00
155GLU CG	157TYR	CE2	1.10	130ILE CG2	132ARG	CG	0.01	157TY CE2	195TRP	CE3	1.22
155GLU CG	191VAL	CG1	0.04	130ILE CG2	132ARG	CD	0.03	157TY CE2	195TRP	CZ3	0.14
155GLU CG	191VAL	CG2	0.01	130ILE CG2	233VAL	CG1	0.01	157TY CZ	161GLU	CG	0.01
155GLU CG	238ILE	CD	0.02	130ILE CG2	235THR	CG2	7.48	157TY CZ	162VAL	CG1	0.01
155GLU CD	155GLU	C	0.05	130ILE CG2	240GLY	CA	0.02	157TY CZ	191VAL	CG2	0.00
155GLU CD	157TYR	CD1	0.34	130ILE CG2	243LEU	CB	0.00	157TY CZ	195TRP	CE3	0.26
155GLU CD	157TYR	CD2	0.04	130ILE CG2	243LEU	CD2	0.02	157TY CZ	195TRP	CZ3	0.80
155GLU CD	157TYR	CE1	24.55	130ILE CG2	244SER	CB	0.01	157TY C	158SER	CA	100.00
155GLU CD	157TYR	CE2	3.91	130ILE CD	233VAL	CB	0.18	157TY C	158SER	CB	38.29
155GLU CD	157TYR	CZ	0.03	130ILE CD	233VAL	CG1	0.22	157TY C	158SER	C	67.42
155GLU C	156ARG	CA	100.00	130ILE CD	233VAL	CG2	0.22	157TY C	194PHE	CE2	0.37
155GLU C	156ARG	CB	97.99	130ILE CD	243LEU	CB	0.10	157TY C	150GLU	CG	0.01
155GLU C	156ARG	CG	5.79	130ILE CD	243LEU	CG	0.02	158SE CA	158SER	CB	100.00
155GLU C	156ARG	C	4.41	130ILE CD	243LEU	CD1	0.02	158SE CA	158SER	C	100.00
156ARG CA	156ARG	CB	100.00	130ILE CD	243LEU	CD2	0.11	158SE CA	194PHE	CE2	0.38
156ARG CA	156ARG	CG	100.00	130ILE CD	243LEU	C	0.15	158SE CA	194PHE	CZ	0.08
156ARG CA	156ARG	CD	0.31	130ILE CD	244SER	CA	0.24	158SE CA	150GLU	CG	0.04
156ARG CA	156ARG	C	100.00	130ILE CD	244SER	CB	0.02	158SE CB	158SER	C	100.00
156ARG CB	156ARG	CG	100.00	130ILE CD	247ALA	CB	7.84	158SE CB	150GLU	CB	0.03
156ARG CB	156ARG	CD	100.00	130ILE C	131VAL	CA	100.00	158SE CB	150GLU	CG	0.02
156ARG CB	156ARG	CZ	2.00	130ILE C	131VAL	CB	10.59	158SE CB	150GLU	CD	0.02
156ARG CB	156ARG	C	100.00	130ILE C	131VAL	CG2	8.15	158SE C	159LYS	CA	100.00
156ARG CB	150GLU	CD	0.00	130ILE C	131VAL	C	95.56	158SE C	159LYS	C	100.00
156ARG CB	152TRP	CB	0.01	131VAl CA	131VAL	CB	100.00	158SE C	160PRO	CD	17.33
156ARG CG	156ARG	CD	100.00	131VAl CA	131VAL	CG1	100.00	158SE C	194PHE	CE2	3.96
156ARG CG	156ARG	CZ	83.44	131VAl CA	131VAL	CG2	100.00	158SE C	194PHE	CZ	0.62
156ARG CG	156ARG	C	90.19	131VAl CA	131VAL	C	100.00	159LY CA	159LYS	CB	100.00
156ARG CG	150GLU	CG	0.12	131VAl CB	131VAL	CG1	100.00	159LY CA	159LYS	CG	100.00
156ARG CG	150GLU	CD	3.48	131VAl CB	131VAL	CG2	100.00	159LY CA	159LYS	CD	19.06
156ARG CG	152TRP	CB	0.03	131VAl CB	131VAL	C	100.00	159LY CA	159LYS	C	100.00
156ARG CD	156ARG	CZ	100.00	131VAl CB	234VAL	CG2	0.00	159LY CA	160PRO	CD	100.00
156ARG CD	156ARG	C	0.08	131VAl CG1	131VAL	CG2	100.00	159LY CA	194PHE	CZ	0.38
156ARG CD	150GLU	CD	2.88	131VAl CG1	131VAL	C	99.99	159LY CB	159LYS	CG	100.00
156ARG CZ	158SER	CB	0.05	131VAl CG1	165VAL	CG1	0.31	159LY CB	159LYS	CD	100.00
156ARG CZ	150GLU	CD	0.00	131VAl CG1	165VAL	CG2	3.10	159LY CB	159LYS	CE	4.82
156ARG CZ	152TRP	CB	1.81	131VAl CG1	168VAL	CG1	0.21	159LY CB	159LYS	C	100.00
156ARG CZ	152TRP	CG	0.69	131VAl CG1	168VAL	CG2	0.26	159LY CB	160PRO	CD	77.29
156ARG CZ	152TRP	CD2	0.42	131VAl CG1	169ALA	CB	0.04	159LY CB	194PHE	CZ	0.00
156ARG CZ	152TRP	CE2	0.00	131VAl CG1	234VAL	CG1	0.00	159LY CB	149ALA	CB	0.12
156ARG CZ	152TRP	CE3	2.12	131VAl CG1	234VAL	CG2	0.04	159LY CG	159LYS	CD	100.00
156ARG CZ	152TRP	CZ3	0.05	131VAl CG2	169ALA	CB	0.30	159LY CG	159LYS	CE	100.00
156ARG C	157TYR	CA	100.00	131VAl CG2	232VAL	CG1	1.33	159LY CG	159LYS	C	80.67
156ARG C	157TYR	CB	8.53	131VAl C	132ARG	CA	100.00	159LY CG	160PRO	CD	0.10
156ARG C	157TYR	CG	0.72	131VAl C	132ARG	CB	45.49	159LY CG	194PHE	CE1	0.01
156ARG C	157TYR	CD1	2.68	131VAl C	132ARG	CG	0.06	159LY CG	194PHE	CE2	0.01
156ARG C	157TYR	CD2	1.63	131VAl C	132ARG	C	65.17	159LY CG	194PHE	CZ	0.31
156ARG C	157TYR	C	93.48	131VAl C	165VAL	CG2	0.01	159LY CG	198THR	CG2	0.68
157TYR CA	157TYR	CB	100.00	132AR(CA	132ARG	CB	100.00	159LY CG	149ALA	CB	0.01
157TYR CA	157TYR	CG	100.00	132AR(CA	132ARG	CG	100.00	159LY CD	159LYS	CE	100.00
157TYR CA	157TYR	CD1	85.32	132AR(CA	132ARG	CD	15.26	159LY CD	159LYS	C	9.88
157TYR CA	157TYR	CD2	16.94	132AR(CA	132ARG	C	100.00	159LY CD	160PRO	CD	0.03
157TYR CA	157TYR	C	100.00	132AR(CA	235THR	CG2	0.05	159LY CD	163GLU	CD	0.01
157TYR CA	162VAL	CG1	0.00	132AR(CB	132ARG	CG	100.00	159LY CD	194PHE	CE1	0.00
157TYR CA	162VAL	CG2	0.43	132AR(CB	132ARG	CD	100.00	159LY CD	194PHE	CZ	0.10
157TYR CB	157TYR	CG	100.00	132AR(CB	132ARG	C	100.00	159LY CD	198THR	CG2	0.16
157TYR CB	157TYR	CD1	100.00	132AR(CB	235THR	CG2	0.06	159LY CD	149ALA	CA	0.00
157TYR CB	157TYR	CD2	100.00	132AR(CB	236GLY	C	0.00	159LY CD	149ALA	CB	0.07
157TYR CB	157TYR	C	100.00	132AR(CG	132ARG	CD	100.00	159LY CE	159LYS	C	0.01
157TYR CB	162VAL	CG1	0.16	132AR(CG	132ARG	CZ	35.46	159LY CE	163GLU	CD	0.02

157TYR	CB	162VAL	CG2	1.11	132AR(	CG	132ARG	C	99.00	159LY:	CE	194PHE	CE1	0.05
157TYR	CB	194PHE	CD2	2.59	132AR(	CG	133GLU	C	0.01	159LY:	CE	194PHE	CZ	0.02
157TYR	CB	194PHE	CE2	0.30	132AR(	CG	134LEU	CD2	0.02	159LY:	CE	198THR	CG2	0.00
157TYR	CG	157TYR	CD1	100.00	132AR(	CG	235THR	CG2	0.01	159LY:	CE	201GLU	CG	0.02
157TYR	CG	157TYR	CD2	100.00	132AR(	CG	236GLY	C	0.02	159LY:	CE	201GLU	CD	0.38
157TYR	CG	157TYR	CE1	100.00	132AR(	CG	240GLY	CA	0.06	159LY:	CE	148GLU	CD	0.05
157TYR	CG	157TYR	CE2	100.00	132AR(	CG	240GLY	C	0.41	159LY:	CE	149ALA	CB	0.01
157TYR	CG	157TYR	CZ	100.00	132AR(	CD	132ARG	CZ	100.00	159LY:	C	160PRO	CA	100.00
157TYR	CG	157TYR	C	0.00	132AR(	CD	132ARG	C	26.48	159LY:	C	160PRO	CB	0.08
157TYR	CG	162VAL	CG1	0.74	132AR(	CD	133GLU	C	0.17	159LY:	C	160PRO	CD	100.00
157TYR	CG	162VAL	CG2	0.94	132AR(	CD	134LEU	CG	0.00	159LY:	C	160PRO	C	100.00
157TYR	CG	191VAL	CB	0.26	132AR(	CD	134LEU	CD1	0.30	160PR	CA	160PRO	CB	100.00
157TYR	CG	191VAL	CG1	0.07	132AR(	CD	134LEU	CD2	1.03	160PR	CA	160PRO	CG	100.00
157TYR	CD1	157TYR	CD2	100.00	132AR(	CD	240GLY	CA	0.00	160PR	CA	160PRO	CD	100.00
157TYR	CD1	157TYR	CE1	100.00	132AR(	CD	240GLY	C	0.32	160PR	CA	160PRO	C	100.00
157TYR	CD1	157TYR	CE2	100.00	132AR(	CZ	134LEU	CG	0.16	160PR	CA	163GLU	CB	0.03
157TYR	CD1	157TYR	CZ	100.00	132AR(	CZ	134LEU	CD1	2.55	160PR	CB	160PRO	CG	100.00
157TYR	CD1	161GLU	CB	0.00	132AR(	CZ	134LEU	CD2	4.01	160PR	CB	160PRO	CD	100.00
157TYR	CD1	161GLU	CG	0.02	132AR(	CZ	237ASN	CA	0.01	160PR	CB	160PRO	C	100.00
157TYR	CD1	161GLU	CD	0.00	132AR(	CZ	240GLY	C	0.05	160PR	CG	160PRO	CD	100.00
157TYR	CD1	162VAL	CG1	1.29	132AR(	CZ	241ASP	CA	0.55	160PR	CG	160PRO	C	85.79
157TYR	CD1	162VAL	CG2	0.41	132AR(	CZ	241ASP	CB	2.77	160PR	CD	160PRO	C	71.75
157TYR	CD1	191VAL	CB	3.02	132AR(	CZ	241ASP	CG	0.25	160PR	C	161GLU	CA	100.00
157TYR	CD1	191VAL	CG1	1.32	132AR(	C	133GLU	CA	100.00	160PR	C	161GLU	CB	0.08
157TYR	CD1	191VAL	CG2	0.01	132AR(	C	133GLU	CB	0.44	160PR	C	161GLU	C	99.96
157TYR	CD1	191VAL	C	0.00	132AR(	C	133GLU	C	99.88	160PR	C	163GLU	CB	0.02
157TYR	CD1	195TRP	CE3	0.06	132AR(	C	165VAL	CG2	0.08	160PR	C	164ARG	CZ	0.01
157TYR	CD1	195TRP	CZ3	0.00	133GL(	CA	133GLU	CB	100.00	161GL	CA	161GLU	CB	100.00
157TYR	CD2	157TYR	CE1	100.00	133GL(	CA	133GLU	CG	100.00	161GL	CA	161GLU	CG	100.00
157TYR	CD2	157TYR	CE2	100.00	133GL(	CA	133GLU	CD	96.14	161GL	CA	161GLU	CD	43.56
157TYR	CD2	157TYR	CZ	100.00	133GL(	CA	133GLU	C	100.00	161GL	CA	161GLU	C	100.00
157TYR	CD2	162VAL	CB	0.00	133GL(	CA	165VAL	CG1	0.00	161GL	CA	164ARG	CZ	0.37
157TYR	CD2	162VAL	CG1	2.34	133GL(	CA	165VAL	CG2	0.04	161GL	CB	161GLU	CG	100.00
157TYR	CD2	162VAL	CG2	6.14	133GL(	CB	133GLU	CG	100.00	161GL	CB	161GLU	CD	100.00
157TYR	CD2	191VAL	CB	1.83	133GL(	CB	133GLU	CD	100.00	161GL	CB	161GLU	C	100.00
157TYR	CD2	191VAL	CG1	0.44	133GL(	CB	133GLU	C	100.00	161GL	CB	164ARG	CZ	0.00
157TYR	CD2	191VAL	CG2	0.01	133GL(	CB	165VAL	CG1	0.01	161GL	CG	161GLU	CD	100.00
157TYR	CD2	191VAL	C	0.01	133GL(	CB	165VAL	CG2	0.08	161GL	CG	161GLU	C	75.05
157TYR	CD2	194PHE	CD2	0.02	133GL(	CB	236GLY	CA	0.05	161GL	CG	164ARG	CZ	1.38
157TYR	CD2	195TRP	CE3	0.08	133GL(	CB	236GLY	C	0.20	161GL	CD	164ARG	CZ	1.74
157TYR	CD2	195TRP	CZ3	0.01	133GL(	CG	133GLU	CD	100.00	161GL	C	162VAL	CA	100.00
157TYR	CE1	157TYR	CE2	100.00	133GL(	CG	133GLU	C	71.57	161GL	C	162VAL	CB	4.16
157TYR	CE1	157TYR	CZ	100.00	133GL(	CG	136GLY	CA	0.07	161GL	C	162VAL	CG1	2.18
157TYR	CE1	162VAL	CG1	0.01	133GL(	CG	157TYR	CE1	0.00	161GL	C	162VAL	CG2	0.24
157TYR	CE1	162VAL	CG2	0.01	133GL(	CG	165VAL	CB	0.06	161GL	C	162VAL	C	96.06
157TYR	CE1	191VAL	CB	0.15	133GL(	CG	165VAL	CG1	2.97	161GL	C	164ARG	CG	0.01
157TYR	CE1	191VAL	CG2	0.08	133GL(	CG	165VAL	CG2	2.96	161GL	C	164ARG	CD	0.00
157TYR	CE1	195TRP	CB	0.02	133GL(	CD	133GLU	C	43.85	162VA	CA	162VAL	CB	100.00
157TYR	CE1	195TRP	CG	0.00	133GL(	CD	135THR	CB	0.04	162VA	CA	162VAL	CG1	100.00
157TYR	CE1	195TRP	CD2	0.02	133GL(	CD	135THR	C	0.03	162VA	CA	162VAL	CG2	100.00
157TYR	CE1	195TRP	CE3	3.08	133GL(	CD	136GLY	CA	0.05	162VA	CA	162VAL	C	100.00
157TYR	CE1	195TRP	CZ3	0.03	133GL(	CD	157TYR	CE1	1.00	162VA	CA	165VAL	CG1	0.01
157TYR	CE2	157TYR	CZ	100.00	133GL(	CD	157TYR	CE2	0.06	162VA	CA	195TRP	CZ3	0.01
157TYR	CE2	162VAL	CG1	0.11	133GL(	CD	162VAL	CG1	0.01	162VA	CB	162VAL	CG1	100.00
157TYR	CE2	162VAL	CG2	0.22	133GL(	CD	165VAL	CG2	0.02	162VA	CB	162VAL	CG2	100.00
157TYR	CE2	191VAL	CB	0.10	133GL(	C	134LEU	CA	100.00	162VA	CB	162VAL	C	100.00
157TYR	CE2	191VAL	CG1	0.00	133GL(	C	134LEU	CB	42.02	162VA	CB	195TRP	CE3	0.11
157TYR	CE2	191VAL	CG2	0.02	133GL(	C	134LEU	CG	2.45	162VA	CB	195TRP	CZ3	0.02
157TYR	CE2	191VAL	C	0.04	133GL(	C	134LEU	CD1	0.03	162VA	CB	198THR	CB	0.00
157TYR	CE2	195TRP	CB	0.03	133GL(	C	134LEU	CD2	0.02	162VA	CB	198THR	CG2	1.19
157TYR	CE2	195TRP	CE3	8.92	133GL(	C	134LEU	C	44.25	162VA	CG1	162VAL	CG2	100.00
157TYR	CE2	195TRP	CZ3	0.05	133GL(	C	237ASN	CB	0.00	162VA	CG1	162VAL	C	69.61

157TYR	CZ	162VAL	CG1	0.00	134LEL	CA	134LEU	CB	100.00	162VA	CG1	194PHE	CD2	0.02
157TYR	CZ	191VAL	CB	0.01	134LEL	CA	134LEU	CG	100.00	162VA	CG1	194PHE	CE2	0.02
157TYR	CZ	191VAL	CG2	0.08	134LEL	CA	134LEU	CD1	21.27	162VA	CG1	195TRP	CA	0.00
157TYR	CZ	195TRP	CE3	0.13	134LEL	CA	134LEU	CD2	77.88	162VA	CG1	195TRP	CG	0.00
157TYR	CZ	195TRP	CZ3	0.03	134LEL	CA	134LEU	C	100.00	162VA	CG1	195TRP	CD2	4.07
157TYR	C	158SER	CA	100.00	134LEL	CA	237ASN	CG	0.01	162VA	CG1	195TRP	CE2	0.24
157TYR	C	158SER	CB	53.72	134LEL	CB	134LEU	CG	100.00	162VA	CG1	195TRP	CE3	13.06
157TYR	C	158SER	C	55.40	134LEL	CB	134LEU	CD1	100.00	162VA	CG1	195TRP	CZ2	0.01
157TYR	C	162VAL	CG2	0.11	134LEL	CB	134LEU	CD2	100.00	162VA	CG1	195TRP	CZ3	1.50
157TYR	C	194PHE	CE2	0.67	134LEL	CB	134LEU	C	100.00	162VA	CG1	198THR	CB	0.49
158SER	CA	158SER	CB	100.00	134LEL	CB	135THR	CG2	0.06	162VA	CG1	198THR	CG2	2.79
158SER	CA	158SER	C	100.00	134LEL	CG	134LEU	CD1	100.00	162VA	CG1	198THR	C	0.08
158SER	CA	194PHE	CE2	0.03	134LEL	CG	134LEU	CD2	100.00	162VA	CG1	199VAL	CA	0.00
158SER	CA	194PHE	CZ	0.00	134LEL	CG	134LEU	C	20.64	162VA	CG1	199VAL	CB	0.00
158SER	CA	150GLU	CB	0.01	134LEL	CD1	134LEU	CD2	100.00	162VA	CG1	199VAL	CG1	6.54
158SER	CA	150GLU	CG	0.02	134LEL	CD1	134LEU	C	0.10	162VA	CG1	199VAL	CG2	1.89
158SER	CB	158SER	C	100.00	134LEL	CD1	261SER	CB	0.21	162VA	CG2	162VAL	C	30.30
158SER	CB	161GLU	CG	0.02	134LEL	CD2	134LEU	C	0.64	162VA	CG2	194PHE	CD2	0.06
158SER	CB	161GLU	CD	0.10	134LEL	CD2	135THR	CG2	0.01	162VA	CG2	194PHE	CE2	1.24
158SER	CB	150GLU	CB	0.06	134LEL	CD2	139TYR	CE1	0.00	162VA	CG2	195TRP	CE3	5.39
158SER	CB	150GLU	CG	0.24	134LEL	CD2	139TYR	CE2	0.01	162VA	CG2	195TRP	CZ3	0.58
158SER	CB	150GLU	CD	0.03	134LEL	CD2	237ASN	CG	0.03	162VA	CG2	198THR	CB	0.34
158SER	C	159LYS	CA	100.00	134LEL	CD2	261SER	CB	0.01	162VA	CG2	198THR	CG2	0.68
158SER	C	159LYS	C	100.00	134LEL	C	135THR	CA	100.00	162VA	CG2	198THR	C	0.00
158SER	C	160PRO	CD	17.26	134LEL	C	135THR	CB	70.37	162VA	C	163GLU	CA	100.00
158SER	C	162VAL	CG1	0.14	134LEL	C	135THR	CG2	46.05	162VA	C	163GLU	CB	0.10
158SER	C	162VAL	CG2	0.06	134LEL	C	135THR	C	32.23	162VA	C	163GLU	C	99.95
158SER	C	194PHE	CE2	0.38	134LEL	C	140PHE	CD2	0.01	162VA	C	165VAL	CG1	0.04
158SER	C	194PHE	CZ	0.03	134LEL	C	140PHE	CE2	0.08	162VA	C	198THR	CG2	0.17
159LYS	CA	159LYS	CB	100.00	134LEL	C	140PHE	CZ	0.01	163GL	CA	163GLU	CB	100.00
159LYS	CA	159LYS	CG	100.00	134LEL	C	237ASN	CG	0.56	163GL	CA	163GLU	CG	100.00
159LYS	CA	159LYS	CD	8.87	135THI	CA	135THR	CB	100.00	163GL	CA	163GLU	CD	22.16
159LYS	CA	159LYS	CE	0.01	135THI	CA	135THR	CG2	100.00	163GL	CA	163GLU	C	100.00
159LYS	CA	159LYS	C	100.00	135THI	CA	135THR	C	100.00	163GL	CA	166ALA	CB	0.01
159LYS	CA	160PRO	CD	100.00	135THI	CA	140PHE	CG	0.00	163GL	CA	198THR	CG2	1.78
159LYS	CA	162VAL	CG1	0.01	135THI	CA	140PHE	CD1	0.02	163GL	CB	163GLU	CG	100.00
159LYS	CA	194PHE	CE2	0.00	135THI	CA	140PHE	CD2	0.03	163GL	CB	163GLU	CD	100.00
159LYS	CA	194PHE	CZ	0.27	135THI	CA	140PHE	CE1	0.23	163GL	CB	163GLU	C	100.00
159LYS	CB	159LYS	CG	100.00	135THI	CA	140PHE	CE2	0.23	163GL	CB	198THR	CG2	0.59
159LYS	CB	159LYS	CD	100.00	135THI	CA	140PHE	CZ	0.47	163GL	CB	202VAL	CG2	0.02
159LYS	CB	159LYS	CE	5.21	135THI	CB	135THR	CG2	100.00	163GL	CG	163GLU	CD	100.00
159LYS	CB	159LYS	C	100.00	135THI	CB	135THR	C	100.00	163GL	CG	163GLU	C	92.28
159LYS	CB	160PRO	CD	78.28	135THI	CB	140PHE	CE1	0.08	163GL	CG	167ARG	CG	0.04
159LYS	CB	194PHE	CZ	0.06	135THI	CB	140PHE	CE2	0.27	163GL	CG	167ARG	CD	0.34
159LYS	CB	149ALA	CA	0.02	135THI	CB	140PHE	CZ	0.14	163GL	CG	198THR	CG2	0.43
159LYS	CB	149ALA	CB	0.15	135THI	CB	161GLU	CG	0.02	163GL	CG	202VAL	CG1	0.01
159LYS	CB	149ALA	C	0.02	135THI	CG2	135THR	C	5.55	163GL	CG	202VAL	CG2	7.24
159LYS	CG	159LYS	CD	100.00	135THI	CG2	140PHE	CE1	0.03	163GL	CD	163GLU	C	13.51
159LYS	CG	159LYS	CE	100.00	135THI	CG2	140PHE	CE2	0.20	163GL	CD	167ARG	CG	0.44
159LYS	CG	159LYS	C	95.27	135THI	CG2	140PHE	CZ	0.17	163GL	CD	167ARG	CD	0.09
159LYS	CG	160PRO	CD	0.12	135THI	CG2	161GLU	CG	0.20	163GL	CD	167ARG	CZ	0.06
159LYS	CG	163GLU	CD	0.04	135THI	CG2	161GLU	CD	0.21	163GL	CD	201GLU	CG	0.03
159LYS	CG	194PHE	CZ	0.14	135THI	CG2	264ARG	CZ	0.01	163GL	CD	202VAL	CG2	0.32
159LYS	CG	198THR	CG2	0.26	135THI	C	136GLY	CA	100.00	163GL	C	164ARG	CA	100.00
159LYS	CD	159LYS	CE	100.00	135THI	C	136GLY	C	42.75	163GL	C	164ARG	CB	0.00
159LYS	CD	159LYS	C	6.91	135THI	C	140PHE	CB	0.08	163GL	C	164ARG	C	100.00
159LYS	CD	163GLU	CD	0.00	135THI	C	140PHE	CG	0.69	163GL	C	166ALA	CB	0.00
159LYS	CD	194PHE	CZ	0.02	135THI	C	140PHE	CD1	0.68	164AR	CA	164ARG	CB	100.00
159LYS	CD	198THR	CG2	0.01	135THI	C	140PHE	CD2	2.52	164AR	CA	164ARG	CG	100.00
159LYS	CD	149ALA	CB	0.28	135THI	C	140PHE	CE1	0.20	164AR	CA	164ARG	C	100.00
159LYS	CE	163GLU	CD	0.00	135THI	C	140PHE	CE2	0.39	164AR	CA	167ARG	CZ	0.02
159LYS	CE	198THR	CG2	0.01	135THI	C	140PHE	CZ	0.06	164AR	CB	164ARG	CG	100.00

159LYS	CE	201GLU	CD	0.06	135THI	C	157TYR	CE1	0.00	164AR	CB	164ARG	CD	100.00
159LYS	CE	149ALA	CB	0.01	135THI	C	157TYR	CZ	0.01	164AR	CB	164ARG	CZ	3.17
159LYS	C	160PRO	CA	100.00	135THI	C	237ASN	CB	0.06	164AR	CB	164ARG	C	100.00
159LYS	C	160PRO	CB	0.16	136GL	CA	136GLY	C	100.00	164AR	CG	164ARG	CD	100.00
159LYS	C	160PRO	CD	100.00	136GL	CA	140PHE	CB	0.21	164AR	CG	164ARG	CZ	12.83
159LYS	C	160PRO	C	100.00	136GL	CA	140PHE	CG	0.16	164AR	CG	164ARG	C	83.95
160PRO	CA	160PRO	CB	100.00	136GL	CA	140PHE	CD1	0.16	164AR	CG	165VAL	CG2	0.04
160PRO	CA	160PRO	CG	100.00	136GL	CA	140PHE	CD2	0.14	164AR	CD	164ARG	CZ	100.00
160PRO	CA	160PRO	CD	100.00	136GL	CA	157TYR	CE1	1.56	164AR	CD	264ARG	CZ	0.01
160PRO	CA	160PRO	C	100.00	136GL	CA	157TYR	CE2	0.16	164AR	C	165VAL	CA	100.00
160PRO	CA	163GLU	CG	0.00	136GL	CA	157TYR	CZ	1.91	164AR	C	165VAL	CB	2.24
160PRO	CB	160PRO	CG	100.00	136GL	CA	237ASN	CB	0.01	164AR	C	165VAL	CG1	0.26
160PRO	CB	160PRO	CD	100.00	136GL	C	137GLY	CA	100.00	164AR	C	165VAL	C	98.35
160PRO	CB	160PRO	C	100.00	136GL	C	137GLY	C	55.79	165VA	CA	165VAL	CB	100.00
160PRO	CG	160PRO	CD	100.00	136GL	C	140PHE	CB	0.79	165VA	CA	165VAL	CG1	100.00
160PRO	CG	160PRO	C	94.47	136GL	C	140PHE	CG	0.01	165VA	CA	165VAL	CG2	100.00
160PRO	CD	160PRO	C	80.89	136GL	C	140PHE	CD1	1.40	165VA	CA	165VAL	C	100.00
160PRO	C	161GLU	CA	100.00	136GL	C	140PHE	CD2	2.70	165VA	CA	168VAL	CG1	0.01
160PRO	C	161GLU	CB	3.53	136GL	C	140PHE	CE1	0.01	165VA	CB	165VAL	CG1	100.00
160PRO	C	161GLU	CG	0.08	136GL	C	140PHE	CE2	0.04	165VA	CB	165VAL	CG2	100.00
160PRO	C	161GLU	C	97.07	136GL	C	157TYR	CD1	0.01	165VA	CB	165VAL	C	100.00
160PRO	C	163GLU	CB	0.01	136GL	C	157TYR	CE1	1.73	165VA	CB	234VAL	CG2	0.06
160PRO	C	163GLU	CG	0.32	136GL	C	157TYR	CE2	0.54	165VA	CG1	165VAL	CG2	100.00
161GLU	CA	161GLU	CB	100.00	136GL	C	157TYR	CZ	3.12	165VA	CG1	165VAL	C	69.67
161GLU	CA	161GLU	CG	100.00	136GL	C	237ASN	CB	0.28	165VA	CG1	195TRP	CZ2	0.02
161GLU	CA	161GLU	CD	91.08	136GL	C	237ASN	CG	0.01	165VA	CG1	195TRP	CZ3	0.31
161GLU	CA	161GLU	C	100.00	137GL	CA	137GLY	C	100.00	165VA	CG1	234VAL	CG2	0.10
161GLU	CA	164ARG	CZ	0.06	137GL	CA	139TYR	CE2	0.01	165VA	CG2	165VAL	C	96.45
161GLU	CB	161GLU	CG	100.00	137GL	CA	139TYR	CZ	0.02	165VA	CG2	195TRP	CZ2	0.01
161GLU	CB	161GLU	CD	100.00	137GL	CA	155GLU	CD	0.52	165VA	CG2	234VAL	CG2	0.84
161GLU	CB	161GLU	C	100.00	137GL	CA	157TYR	CD2	0.05	165VA	C	166ALA	CA	100.00
161GLU	CG	161GLU	CD	100.00	137GL	CA	157TYR	CE1	0.47	165VA	C	166ALA	CB	0.01
161GLU	CG	161GLU	C	23.85	137GL	CA	157TYR	CE2	4.54	165VA	C	166ALA	C	100.00
161GLU	CD	161GLU	C	0.06	137GL	CA	157TYR	CZ	1.15	165VA	C	168VAL	CG1	0.01
161GLU	CD	164ARG	CD	0.40	137GL	CA	237ASN	CB	0.32	165VA	C	234VAL	CG2	0.48
161GLU	CD	164ARG	CZ	0.00	137GL	CA	237ASN	CG	0.26	166AL	CA	166ALA	CB	100.00
161GLU	C	162VAL	CA	100.00	137GL	CA	238ILE	CG1	0.97	166AL	CA	166ALA	C	100.00
161GLU	C	162VAL	CB	0.25	137GL	CA	238ILE	CG2	0.00	166AL	CA	234VAL	CG2	0.01
161GLU	C	162VAL	CG1	0.03	137GL	CA	238ILE	CD	0.80	166AL	CB	166ALA	C	100.00
161GLU	C	162VAL	CG2	0.04	137GL	C	138ILE	CA	100.00	166AL	CB	170PHE	CD1	0.01
161GLU	C	162VAL	C	99.86	137GL	C	138ILE	CB	0.02	166AL	CB	170PHE	CE1	0.02
161GLU	C	164ARG	CG	0.00	137GL	C	138ILE	CG2	0.02	166AL	CB	170PHE	CE2	0.02
162VAL	CA	162VAL	CB	100.00	137GL	C	138ILE	C	100.00	166AL	CB	170PHE	CZ	0.01
162VAL	CA	162VAL	CG1	100.00	137GL	C	139TYR	CD2	0.07	166AL	CB	195TRP	CZ3	0.00
162VAL	CA	162VAL	CG2	100.00	137GL	C	139TYR	CE2	0.00	166AL	CB	199VAL	CG1	0.43
162VAL	CA	162VAL	C	100.00	137GL	C	155GLU	CB	0.02	166AL	CB	202VAL	CG1	0.18
162VAL	CB	162VAL	CG1	100.00	137GL	C	155GLU	CG	0.48	166AL	CB	202VAL	CG2	0.50
162VAL	CB	162VAL	CG2	100.00	137GL	C	155GLU	CD	1.32	166AL	C	167ARG	CA	100.00
162VAL	CB	162VAL	C	100.00	137GL	C	238ILE	CD	0.02	166AL	C	167ARG	CB	0.01
162VAL	CB	195TRP	CZ3	0.06	138ILE	CA	138ILE	CB	100.00	166AL	C	167ARG	C	99.99
162VAL	CB	198THR	CG2	0.08	138ILE	CA	138ILE	CG1	100.00	166AL	C	170PHE	CD1	0.11
162VAL	CG1	162VAL	CG2	100.00	138ILE	CA	138ILE	CG2	100.00	166AL	C	170PHE	CD2	0.14
162VAL	CG1	162VAL	C	68.61	138ILE	CA	138ILE	CD	10.16	166AL	C	170PHE	CE2	0.00
162VAL	CG1	194PHE	CE2	0.32	138ILE	CA	138ILE	C	100.00	167AR	CA	167ARG	CB	100.00
162VAL	CG1	194PHE	CZ	0.01	138ILE	CA	154THR	C	0.00	167AR	CA	167ARG	CG	100.00
162VAL	CG1	195TRP	CD2	0.06	138ILE	CA	155GLU	CA	0.06	167AR	CA	167ARG	CD	12.66
162VAL	CG1	195TRP	CE3	10.62	138ILE	CA	155GLU	CB	0.05	167AR	CA	167ARG	C	100.00
162VAL	CG1	195TRP	CZ3	1.25	138ILE	CA	155GLU	CD	0.05	167AR	CA	202VAL	CG1	0.06
162VAL	CG1	198THR	CB	0.99	138ILE	CB	138ILE	CG1	100.00	167AR	CB	167ARG	CG	100.00
162VAL	CG1	198THR	CG2	7.02	138ILE	CB	138ILE	CG2	100.00	167AR	CB	167ARG	CD	100.00
162VAL	CG1	199VAL	CG1	0.11	138ILE	CB	138ILE	CD	100.00	167AR	CB	167ARG	CZ	2.55
162VAL	CG1	199VAL	CG2	0.42	138ILE	CB	138ILE	C	100.00	167AR	CB	167ARG	C	100.00

162VAL CG2	162VAL C	48.38	138ILE CB	155GLU CA	0.04	167AR CB	202VAL CG1	0.02
162VAL CG2	194PHE CE2	0.08	138ILE CB	155GLU CD	0.11	167AR CG	167ARG CD	100.00
162VAL CG2	195TRP CE3	1.94	138ILE CB	238ILE CD	0.00	167AR CG	167ARG CZ	63.02
162VAL CG2	195TRP CZ3	0.67	138ILE CG1	138ILE CG2	100.00	167AR CG	167ARG C	27.20
162VAL CG2	198THR CB	0.76	138ILE CG1	138ILE CD	100.00	167AR CG	202VAL CG1	1.85
162VAL CG2	198THR CG2	0.69	138ILE CG1	138ILE C	99.68	167AR CG	202VAL CG2	0.36
162VAL C	163GLU CA	100.00	138ILE CG1	153ASN CB	3.06	167AR CG	206TYR CE1	0.02
162VAL C	163GLU CB	0.01	138ILE CG1	153ASN CG	0.01	167AR CD	167ARG CZ	100.00
162VAL C	163GLU C	100.00	138ILE CG1	153ASN C	0.02	167AR CD	167ARG C	0.09
162VAL C	165VAL CG1	0.01	138ILE CG1	154THR C	0.18	167AR CD	202VAL CG1	0.03
162VAL C	198THR CG2	0.54	138ILE CG1	155GLU CB	0.01	167AR CZ	171GLU CG	0.01
163GLU CA	163GLU CB	100.00	138ILE CG1	155GLU CD	0.02	167AR CZ	171GLU CD	0.00
163GLU CA	163GLU CG	100.00	138ILE CG1	138ILE CD	0.00	167AR CZ	202VAL CG1	0.15
163GLU CA	163GLU CD	8.22	138ILE CG1	155GLU CB	0.01	167AR CZ	202VAL CG2	0.20
163GLU CA	163GLU C	100.00	138ILE CG1	189LEU CD1	0.11	167AR CZ	206TYR CE1	0.04
163GLU CA	198THR CG2	1.61	138ILE CG1	189LEU CD2	0.60	167AR C	168VAL CA	100.00
163GLU CA	202VAL CG1	0.00	138ILE CG2	138ILE CD	88.36	167AR C	168VAL CB	0.68
163GLU CA	202VAL CG2	0.02	138ILE CG2	138ILE C	99.55	167AR C	168VAL CG2	0.28
163GLU CB	163GLU CG	100.00	138ILE CG2	139TYR CD1	0.28	167AR C	168VAL C	99.58
163GLU CB	163GLU CD	100.00	138ILE CG2	139TYR CD2	0.16	168VA CA	168VAL CB	100.00
163GLU CB	163GLU C	100.00	138ILE CG2	139TYR CE1	0.03	168VA CA	168VAL CG1	100.00
163GLU CB	167ARG CZ	0.54	138ILE CG2	139TYR CE2	0.00	168VA CA	168VAL CG2	100.00
163GLU CB	198THR CG2	0.18	138ILE CG2	153ASN CB	0.01	168VA CA	168VAL C	100.00
163GLU CB	202VAL CG1	0.21	138ILE CG2	155GLU CD	0.08	168VA CB	168VAL CG1	100.00
163GLU CB	202VAL CG2	0.61	138ILE CG2	238ILE CB	0.32	168VA CB	168VAL CG2	100.00
163GLU CG	163GLU CD	100.00	138ILE CG2	238ILE CG1	3.04	168VA CB	168VAL C	100.00
163GLU CG	163GLU C	75.38	138ILE CG2	238ILE CG2	1.35	168VA CG1	168VAL CG2	100.00
163GLU CG	167ARG CD	0.00	138ILE CG2	238ILE CD	3.77	168VA CG1	168VAL C	100.00
163GLU CG	167ARG CZ	0.02	138ILE CG2	189LEU CG	0.14	168VA CG1	172LEU CD1	6.26
163GLU CG	198THR CG2	4.39	138ILE CG2	189LEU CD1	2.08	168VA CG1	172LEU CD2	0.00
163GLU CG	202VAL CG1	1.20	138ILE CG2	189LEU CD2	6.41	168VA CG1	262LEU CD1	1.29
163GLU CG	202VAL CG2	4.18	138ILE CG2	239PHE CZ	0.01	168VA CG1	262LEU CD2	0.23
163GLU CD	163GLU C	12.79	138ILE CD	138ILE C	0.08	168VA CG2	168VAL C	0.96
163GLU CD	167ARG CD	0.00	138ILE CD	153ASN CB	0.44	168VA CG2	172LEU CD1	0.00
163GLU CD	167ARG CZ	0.09	138ILE CD	153ASN C	0.14	168VA CG2	262LEU CD1	0.11
163GLU CD	201GLU CG	0.02	138ILE CD	154THR C	0.14	168VA CG2	262LEU CD2	0.12
163GLU CD	202VAL CG1	0.04	138ILE CD	155GLU CB	0.03	168VA C	169ALA CA	100.00
163GLU CD	202VAL CG2	0.05	138ILE CD	155GLU CG	0.02	168VA C	169ALA CB	0.00
163GLU C	164ARG CA	100.00	138ILE CD	238ILE CD	0.00	168VA C	169ALA C	100.00
163GLU C	164ARG CB	0.01	138ILE CD	138ILE CG1	0.02	168VA C	172LEU CD1	0.01
163GLU C	164ARG C	100.00	138ILE CD	138ILE CG2	1.40	169AL. CA	169ALA CB	100.00
164ARG CA	164ARG CB	100.00	138ILE CD	138ILE CD	1.50	169AL. CA	169ALA C	100.00
164ARG CA	164ARG CG	100.00	138ILE CD	154THR C	0.00	169AL. CA	232VAL CG1	0.01
164ARG CA	164ARG C	100.00	138ILE CD	155GLU CB	0.97	169AL. CB	169ALA C	100.00
164ARG CA	167ARG CZ	0.09	138ILE CD	189LEU CG	0.00	169AL. CB	180VAL CG1	0.02
164ARG CB	164ARG CG	100.00	138ILE CD	189LEU CD1	0.05	169AL. CB	180VAL CG2	0.01
164ARG CB	164ARG CD	100.00	138ILE CD	189LEU CD2	0.32	169AL. CB	232VAL CG1	0.57
164ARG CB	164ARG C	100.00	138ILE CD	191VAL CG2	0.00	169AL. CB	234VAL CB	5.55
164ARG CG	164ARG CD	100.00	138ILE CD	238ILE CG2	0.01	169AL. CB	234VAL CG1	0.69
164ARG CG	164ARG CZ	2.23	138ILE CD	238ILE CD	0.14	169AL. CB	234VAL CG2	11.80
164ARG CG	164ARG C	21.98	138ILE CD	239PHE CZ	0.01	169AL. C	170PHE CA	100.00
164ARG CG	167ARG CZ	0.00	138ILE C	139TYR CA	100.00	169AL. C	170PHE CD2	0.00
164ARG CD	164ARG CZ	100.00	138ILE C	139TYR CB	41.55	169AL. C	170PHE C	100.00
164ARG CZ	264ARG CZ	3.15	138ILE C	139TYR CG	0.00	169AL. C	180VAL CG1	0.00
164ARG C	165VAL CA	100.00	138ILE C	139TYR CD2	0.16	169AL. C	180VAL CG2	0.03
164ARG C	165VAL CB	0.11	138ILE C	139TYR C	56.92	170PH CA	170PHE CB	100.00
164ARG C	165VAL CG2	0.00	138ILE C	189LEU CD1	0.01	170PH CA	170PHE CG	100.00
164ARG C	165VAL C	99.97	138ILE C	189LEU CD2	0.17	170PH CA	170PHE CD1	68.66
165VAL CA	165VAL CB	100.00	139TYF CA	139TYR CB	100.00	170PH CA	170PHE CD2	64.05
165VAL CA	165VAL CG1	100.00	139TYF CA	139TYR CG	100.00	170PH CA	170PHE C	100.00
165VAL CA	165VAL CG2	100.00	139TYF CA	139TYR CD1	64.51	170PH CA	180VAL CG2	0.00
165VAL CA	165VAL C	100.00	139TYF CA	139TYR CD2	57.88	170PH CB	170PHE CG	100.00

165VAL	CA	168VAL	CG2	0.01
165VAL	CB	165VAL	CG1	100.00
165VAL	CB	165VAL	CG2	100.00
165VAL	CB	165VAL	C	100.00
165VAL	CG1	165VAL	CG2	100.00
165VAL	CG1	165VAL	C	96.50
165VAL	CG1	195TRP	CZ3	0.66
165VAL	CG1	234VAL	CG2	0.06
165VAL	CG2	165VAL	C	67.03
165VAL	CG2	195TRP	CZ3	0.08
165VAL	CG2	234VAL	CG2	0.01
165VAL	C	166ALA	CA	100.00
165VAL	C	166ALA	CB	0.02
165VAL	C	166ALA	C	100.00
165VAL	C	168VAL	CG2	0.18
166ALA	CA	166ALA	CB	100.00
166ALA	CA	166ALA	C	100.00
166ALA	CA	234VAL	CG2	0.01
166ALA	CB	166ALA	C	100.00
166ALA	CB	170PHE	CZ	0.00
166ALA	CB	195TRP	CZ3	0.04
166ALA	CB	198THR	CG2	0.19
166ALA	CB	199VAL	CA	0.00
166ALA	CB	199VAL	CG1	0.33
166ALA	CB	199VAL	CG2	0.12
166ALA	CB	202VAL	CG1	0.06
166ALA	CB	202VAL	CG2	2.27
166ALA	C	167ARG	CA	100.00
166ALA	C	167ARG	CB	0.01
166ALA	C	167ARG	C	99.99
166ALA	C	170PHE	CD1	0.08
166ALA	C	170PHE	CD2	0.10
166ALA	C	202VAL	CG2	0.19
167ARG	CA	167ARG	CB	100.00
167ARG	CA	167ARG	CG	100.00
167ARG	CA	167ARG	CD	0.25
167ARG	CA	167ARG	C	100.00
167ARG	CA	170PHE	CD2	0.00
167ARG	CA	202VAL	CG1	0.54
167ARG	CA	202VAL	CG2	0.15
167ARG	CB	167ARG	CG	100.00
167ARG	CB	167ARG	CD	100.00
167ARG	CB	167ARG	CZ	17.65
167ARG	CB	167ARG	C	100.00
167ARG	CB	202VAL	CG1	0.08
167ARG	CB	202VAL	CG2	0.00
167ARG	CG	167ARG	CD	100.00
167ARG	CG	167ARG	CZ	49.93
167ARG	CG	167ARG	C	82.70
167ARG	CG	202VAL	CB	0.03
167ARG	CG	202VAL	CG1	0.72
167ARG	CG	202VAL	CG2	0.02
167ARG	CG	206TYR	CE1	0.25
167ARG	CD	167ARG	CZ	100.00
167ARG	CD	206TYR	CE1	0.00
167ARG	CZ	202VAL	CB	0.00
167ARG	CZ	202VAL	CG1	0.88
167ARG	CZ	202VAL	CG2	0.04
167ARG	CZ	206TYR	CE1	0.00
167ARG	C	168VAL	CA	100.00
167ARG	C	168VAL	CB	0.02
167ARG	C	168VAL	CG1	0.00

139TYF	CA	139TYR	C	100.00
139TYF	CA	144ARG	CZ	0.00
139TYF	CA	188VAL	CG1	0.01
139TYF	CA	189LEU	CD2	0.01
139TYF	CB	139TYR	CG	100.00
139TYF	CB	139TYR	CD1	100.00
139TYF	CB	139TYR	CD2	100.00
139TYF	CB	139TYR	C	100.00
139TYF	CB	140PHE	CD1	0.00
139TYF	CB	140PHE	CD2	3.00
139TYF	CB	140PHE	CE2	1.38
139TYF	CB	140PHE	CZ	0.02
139TYF	CB	144ARG	CZ	0.15
139TYF	CB	237ASN	CG	0.08
139TYF	CB	188VAL	CB	0.01
139TYF	CB	188VAL	CG1	0.12
139TYF	CB	188VAL	CG2	0.14
139TYF	CB	188VAL	C	0.02
139TYF	CB	189LEU	CA	0.00
139TYF	CB	189LEU	CD1	0.01
139TYF	CB	189LEU	CD2	0.08
139TYF	CG	139TYR	CD1	100.00
139TYF	CG	139TYR	CD2	100.00
139TYF	CG	139TYR	CE1	100.00
139TYF	CG	139TYR	CE2	100.00
139TYF	CG	139TYR	CZ	100.00
139TYF	CG	139TYR	C	74.96
139TYF	CG	237ASN	CB	0.01
139TYF	CG	237ASN	CG	0.03
139TYF	CG	238ILE	CD	0.01
139TYF	CG	188VAL	CB	0.22
139TYF	CG	188VAL	CG1	0.72
139TYF	CG	188VAL	CG2	1.93
139TYF	CG	189LEU	CD1	0.01
139TYF	CD1	139TYR	CD2	100.00
139TYF	CD1	139TYR	CE1	100.00
139TYF	CD1	139TYR	CE2	100.00
139TYF	CD1	139TYR	CZ	100.00
139TYF	CD1	139TYR	C	29.51
139TYF	CD1	140PHE	CA	0.00
139TYF	CD1	140PHE	CB	0.04
139TYF	CD1	140PHE	CG	0.06
139TYF	CD1	140PHE	CD1	0.96
139TYF	CD1	140PHE	CD2	1.44
139TYF	CD1	140PHE	CE1	0.93
139TYF	CD1	140PHE	CE2	2.00
139TYF	CD1	140PHE	CZ	0.22
139TYF	CD1	144ARG	CZ	0.01
139TYF	CD1	237ASN	CG	0.16
139TYF	CD1	238ILE	CD	0.08
139TYF	CD1	188VAL	CB	1.31
139TYF	CD1	188VAL	CG1	1.41
139TYF	CD1	188VAL	CG2	3.59
139TYF	CD1	189LEU	CG	0.01
139TYF	CD1	189LEU	CD1	0.06
139TYF	CD1	189LEU	CD2	0.26
139TYF	CD2	139TYR	CE1	100.00
139TYF	CD2	139TYR	CE2	100.00
139TYF	CD2	139TYR	CZ	100.00
139TYF	CD2	139TYR	C	0.80
139TYF	CD2	140PHE	CB	0.00
139TYF	CD2	140PHE	CD1	0.05

170PH	CB	170PHE	CD1	100.00
170PH	CB	170PHE	CD2	100.00
170PH	CB	170PHE	C	100.00
170PH	CB	202VAL	CG1	0.17
170PH	CB	206TYR	CB	0.10
170PH	CB	206TYR	CG	6.78
170PH	CB	206TYR	CD1	11.49
170PH	CB	206TYR	CD2	3.10
170PH	CB	206TYR	CE1	3.40
170PH	CB	206TYR	CE2	0.48
170PH	CB	206TYR	CZ	0.58
170PH	CG	170PHE	CD1	100.00
170PH	CG	170PHE	CD2	100.00
170PH	CG	170PHE	CE1	100.00
170PH	CG	170PHE	CE2	100.00
170PH	CG	170PHE	CZ	100.00
170PH	CG	180VAL	CG2	0.00
170PH	CG	202VAL	CG1	3.01
170PH	CG	206TYR	CB	0.04
170PH	CG	206TYR	CG	0.04
170PH	CG	206TYR	CD1	0.63
170PH	CG	206TYR	CE1	0.01
170PH	CG	209VAL	CG1	0.05
170PH	CD1	170PHE	CD2	100.00
170PH	CD1	170PHE	CE1	100.00
170PH	CD1	170PHE	CE2	100.00
170PH	CD1	170PHE	CZ	100.00
170PH	CD1	180VAL	CG1	0.09
170PH	CD1	180VAL	CG2	0.30
170PH	CD1	202VAL	CB	0.01
170PH	CD1	202VAL	CG1	8.14
170PH	CD1	202VAL	CG2	0.01
170PH	CD1	203GLY	CA	0.01
170PH	CD1	206TYR	CB	9.40
170PH	CD1	206TYR	CG	0.57
170PH	CD1	206TYR	CD1	0.73
170PH	CD1	206TYR	CD2	0.01
170PH	CD1	206TYR	CE1	0.06
170PH	CD1	209VAL	CB	1.38
170PH	CD1	209VAL	CG1	18.38
170PH	CD1	209VAL	CG2	0.00
170PH	CD2	170PHE	CE1	100.00
170PH	CD2	170PHE	CE2	100.00
170PH	CD2	170PHE	CZ	100.00
170PH	CD2	180VAL	CB	0.00
170PH	CD2	180VAL	CG1	0.09
170PH	CD2	180VAL	CG2	0.30
170PH	CD2	202VAL	CB	0.01
170PH	CD2	202VAL	CG1	10.76
170PH	CD2	202VAL	CG2	0.02
170PH	CD2	206TYR	CB	6.36
170PH	CD2	206TYR	CG	0.32
170PH	CD2	206TYR	CD1	0.56
170PH	CD2	206TYR	CD2	0.00
170PH	CD2	206TYR	CE1	0.02
170PH	CD2	209VAL	CB	1.13
170PH	CD2	209VAL	CG1	15.18
170PH	CD2	209VAL	CG2	0.01
170PH	CE1	170PHE	CE2	100.00
170PH	CE1	170PHE	CZ	100.00
170PH	CE1	180VAL	CB	0.09
170PH	CE1	180VAL	CG1	1.18

167ARG	C	168VAL	C	99.99
168VAL	CA	168VAL	CB	100.00
168VAL	CA	168VAL	CG1	100.00
168VAL	CA	168VAL	CG2	100.00
168VAL	CA	168VAL	C	100.00
168VAL	CA	171GLU	CB	0.01
168VAL	CB	168VAL	CG1	100.00
168VAL	CB	168VAL	CG2	100.00
168VAL	CB	168VAL	C	100.00
168VAL	CB	262LEU	CD1	0.58
168VAL	CB	262LEU	CD2	0.06
168VAL	CG1	168VAL	CG2	100.00
168VAL	CG1	262LEU	CD1	1.96
168VAL	CG1	262LEU	CD2	0.82
168VAL	CG2	168VAL	C	99.94
168VAL	CG2	262LEU	CD1	0.14
168VAL	C	169ALA	CA	100.00
168VAL	C	169ALA	CB	0.01
168VAL	C	169ALA	C	100.00
169ALA	CA	169ALA	CB	100.00
169ALA	CA	169ALA	C	100.00
169ALA	CB	169ALA	C	100.00
169ALA	CB	180VAL	CG1	0.05
169ALA	CB	232VAL	CG1	0.25
169ALA	CB	234VAL	CB	2.93
169ALA	CB	234VAL	CG1	0.54
169ALA	CB	234VAL	CG2	11.18
169ALA	C	170PHE	CA	100.00
169ALA	C	170PHE	C	100.00
169ALA	C	180VAL	CG1	0.01
169ALA	C	180VAL	CG2	0.16
170PHE	CA	170PHE	CB	100.00
170PHE	CA	170PHE	CG	100.00
170PHE	CA	170PHE	CD1	70.05
170PHE	CA	170PHE	CD2	75.80
170PHE	CA	170PHE	C	100.00
170PHE	CA	180VAL	CG2	0.01
170PHE	CB	170PHE	CG	100.00
170PHE	CB	170PHE	CD1	100.00
170PHE	CB	170PHE	CD2	100.00
170PHE	CB	170PHE	C	100.00
170PHE	CB	202VAL	CG1	0.07
170PHE	CB	206TYR	CB	0.23
170PHE	CB	206TYR	CG	11.05
170PHE	CB	206TYR	CD1	25.12
170PHE	CB	206TYR	CD2	2.45
170PHE	CB	206TYR	CE1	7.91
170PHE	CB	206TYR	CE2	0.64
170PHE	CB	206TYR	CZ	1.56
170PHE	CG	170PHE	CD1	100.00
170PHE	CG	170PHE	CD2	100.00
170PHE	CG	170PHE	CE1	100.00
170PHE	CG	170PHE	CE2	100.00
170PHE	CG	170PHE	CZ	100.00
170PHE	CG	170PHE	C	0.01
170PHE	CG	180VAL	CG2	0.00
170PHE	CG	202VAL	CG1	0.83
170PHE	CG	202VAL	CG2	0.02
170PHE	CG	203GLY	CA	0.01
170PHE	CG	206TYR	CB	0.08
170PHE	CG	206TYR	CG	0.07
170PHE	CG	206TYR	CD1	2.45

139TYF	CD2	140PHE	CD2	0.11
139TYF	CD2	140PHE	CE1	0.02
139TYF	CD2	140PHE	CE2	0.34
139TYF	CD2	140PHE	CZ	0.13
139TYF	CD2	237ASN	CB	0.14
139TYF	CD2	237ASN	CG	1.53
139TYF	CD2	238ILE	CG1	0.06
139TYF	CD2	238ILE	CD	1.73
139TYF	CD2	188VAL	CB	0.43
139TYF	CD2	188VAL	CG1	0.38
139TYF	CD2	188VAL	CG2	2.91
139TYF	CD2	189LEU	CG	0.00
139TYF	CD2	189LEU	CD1	0.96
139TYF	CD2	189LEU	CD2	0.97
139TYF	CE1	139TYR	CE2	100.00
139TYF	CE1	139TYR	CZ	100.00
139TYF	CE1	140PHE	CB	0.03
139TYF	CE1	140PHE	CG	0.02
139TYF	CE1	140PHE	CD1	1.91
139TYF	CE1	140PHE	CD2	2.56
139TYF	CE1	140PHE	CE1	9.23
139TYF	CE1	140PHE	CE2	19.06
139TYF	CE1	140PHE	CZ	6.57
139TYF	CE1	237ASN	CG	0.05
139TYF	CE1	238ILE	CA	0.06
139TYF	CE1	238ILE	CB	0.27
139TYF	CE1	238ILE	CG1	0.76
139TYF	CE1	238ILE	CD	0.64
139TYF	CE1	241ASP	CG	0.01
139TYF	CE1	185LYS	CG	0.03
139TYF	CE1	185LYS	CD	0.06
139TYF	CE1	188VAL	CB	1.13
139TYF	CE1	188VAL	CG1	5.03
139TYF	CE1	188VAL	CG2	1.97
139TYF	CE1	189LEU	CG	0.01
139TYF	CE1	189LEU	CD1	0.15
139TYF	CE2	139TYR	CZ	100.00
139TYF	CE2	140PHE	CB	0.00
139TYF	CE2	140PHE	CD1	0.05
139TYF	CE2	140PHE	CD2	0.02
139TYF	CE2	140PHE	CE1	0.12
139TYF	CE2	140PHE	CE2	0.44
139TYF	CE2	140PHE	CZ	0.14
139TYF	CE2	237ASN	CB	0.02
139TYF	CE2	237ASN	CG	1.13
139TYF	CE2	237ASN	C	0.02
139TYF	CE2	238ILE	CA	0.04
139TYF	CE2	238ILE	CG1	0.16
139TYF	CE2	238ILE	CG2	0.01
139TYF	CE2	238ILE	CD	5.89
139TYF	CE2	241ASP	CB	0.05
139TYF	CE2	241ASP	CG	0.48
139TYF	CE2	188VAL	CB	0.30
139TYF	CE2	188VAL	CG1	0.35
139TYF	CE2	188VAL	CG2	3.92
139TYF	CE2	189LEU	CD1	0.01
139TYF	CE2	189LEU	CD2	0.00
139TYF	CZ	140PHE	CD1	0.17
139TYF	CZ	140PHE	CD2	0.15
139TYF	CZ	140PHE	CE1	2.22
139TYF	CZ	140PHE	CE2	5.17
139TYF	CZ	140PHE	CZ	0.60

170PH	CE1	180VAL	CG2	0.16
170PH	CE1	199VAL	CG1	0.04
170PH	CE1	202VAL	CB	0.02
170PH	CE1	202VAL	CG1	1.00
170PH	CE1	202VAL	C	0.05
170PH	CE1	203GLY	CA	2.78
170PH	CE1	206TYR	CB	0.07
170PH	CE1	206TYR	CD1	0.00
170PH	CE1	209VAL	CB	0.08
170PH	CE1	209VAL	CG1	4.58
170PH	CE1	211LEU	CD1	0.36
170PH	CE1	211LEU	CD2	0.04
170PH	CE2	170PHE	CZ	100.00
170PH	CE2	180VAL	CB	0.08
170PH	CE2	180VAL	CG1	1.18
170PH	CE2	180VAL	CG2	0.12
170PH	CE2	199VAL	CG1	0.06
170PH	CE2	202VAL	CB	0.01
170PH	CE2	202VAL	CG1	1.32
170PH	CE2	202VAL	CG2	0.01
170PH	CE2	202VAL	C	0.07
170PH	CE2	203GLY	CA	2.50
170PH	CE2	206TYR	CB	0.05
170PH	CE2	206TYR	CG	0.00
170PH	CE2	206TYR	CD1	0.00
170PH	CE2	209VAL	CB	0.08
170PH	CE2	209VAL	CG1	3.29
170PH	CE2	211LEU	CB	0.00
170PH	CE2	211LEU	CD1	0.35
170PH	CE2	211LEU	CD2	0.04
170PH	CZ	180VAL	CB	0.02
170PH	CZ	180VAL	CG1	0.32
170PH	CZ	180VAL	CG2	0.21
170PH	CZ	199VAL	CG1	0.02
170PH	CZ	202VAL	CG1	0.04
170PH	CZ	203GLY	CA	10.26
170PH	CZ	211LEU	CB	0.06
170PH	CZ	211LEU	CG	0.03
170PH	CZ	211LEU	CD1	2.08
170PH	CZ	211LEU	CD2	0.21
170PH	C	171GLU	CA	100.00
170PH	C	171GLU	CB	0.09
170PH	C	171GLU	C	99.96
170PH	C	206TYR	CD2	0.15
170PH	C	206TYR	CE2	3.43
171GL	CA	171GLU	CB	100.00
171GL	CA	171GLU	CG	100.00
171GL	CA	171GLU	CD	98.50
171GL	CA	171GLU	C	100.00
171GL	CA	206TYR	CE2	0.24
171GL	CB	171GLU	CG	100.00
171GL	CB	171GLU	CD	100.00
171GL	CB	171GLU	C	100.00
171GL	CG	171GLU	CD	100.00
171GL	CG	171GLU	C	99.01
171GL	CD	171GLU	C	0.14
171GL	CD	174ARG	CD	0.09
171GL	CD	175LYS	CE	0.01
171GL	CD	206TYR	CE2	0.20
171GL	C	172LEU	CA	100.00
171GL	C	172LEU	CB	0.01
171GL	C	172LEU	C	99.99

170PHE	CG	206TYR	CE1	0.08	139TYF	CZ	237ASN	CB	0.00	172LEI	CA	172LEU	CB	100.00
170PHE	CD1	170PHE	CD2	100.00	139TYF	CZ	237ASN	CG	0.07	172LEI	CA	172LEU	CG	100.00
170PHE	CD1	170PHE	CE1	100.00	139TYF	CZ	238ILE	CA	0.14	172LEI	CA	172LEU	CD1	5.29
170PHE	CD1	170PHE	CE2	100.00	139TYF	CZ	238ILE	CB	0.03	172LEI	CA	172LEU	CD2	94.74
170PHE	CD1	170PHE	CZ	100.00	139TYF	CZ	238ILE	CG1	0.04	172LEI	CA	172LEU	C	100.00
170PHE	CD1	180VAL	CG2	0.60	139TYF	CZ	238ILE	CD	0.31	172LEI	CB	172LEU	CG	100.00
170PHE	CD1	202VAL	CG1	2.32	139TYF	CZ	185LYS	CG	0.01	172LEI	CB	172LEU	CD1	100.00
170PHE	CD1	202VAL	CG2	0.40	139TYF	CZ	185LYS	CD	0.14	172LEI	CB	172LEU	CD2	100.00
170PHE	CD1	203GLY	CA	0.10	139TYF	CZ	185LYS	CE	0.13	172LEI	CB	172LEU	C	100.00
170PHE	CD1	206TYR	CB	4.24	139TYF	CZ	188VAL	CB	0.44	172LEI	CB	232VAL	CG2	0.01
170PHE	CD1	206TYR	CG	0.27	139TYF	CZ	188VAL	CG1	1.28	172LEI	CG	172LEU	CD1	100.00
170PHE	CD1	206TYR	CD1	0.78	139TYF	CZ	188VAL	CG2	0.84	172LEI	CG	172LEU	CD2	100.00
170PHE	CD1	206TYR	CD2	0.01	139TYF	C	140PHE	CA	100.00	172LEI	CG	172LEU	C	3.25
170PHE	CD1	206TYR	CE1	0.14	139TYF	C	140PHE	CB	54.74	172LEI	CD1	172LEU	CD2	100.00
170PHE	CD1	206TYR	CE2	0.00	139TYF	C	140PHE	CG	22.21	172LEI	CD1	172LEU	C	0.08
170PHE	CD1	209VAL	CB	0.31	139TYF	C	140PHE	CD1	2.97	172LEI	CD1	175LYS	CD	0.00
170PHE	CD1	209VAL	CG1	8.86	139TYF	C	140PHE	CD2	5.06	172LEI	CD1	175LYS	CE	0.08
170PHE	CD2	170PHE	CE1	100.00	139TYF	C	140PHE	C	43.47	172LEI	CD1	300HIS	CE1	0.05
170PHE	CD2	170PHE	CE2	100.00	140PHI	CA	140PHE	CB	100.00	172LEI	CD2	172LEU	C	0.22
170PHE	CD2	170PHE	CZ	100.00	140PHI	CA	140PHE	CG	100.00	172LEI	CD2	175LYS	CD	0.08
170PHE	CD2	180VAL	CG2	1.12	140PHI	CA	140PHE	CD1	79.50	172LEI	CD2	175LYS	CE	0.42
170PHE	CD2	202VAL	CB	0.00	140PHI	CA	140PHE	CD2	53.22	172LEI	CD2	232VAL	CG1	0.02
170PHE	CD2	202VAL	CG1	6.02	140PHI	CA	140PHE	C	100.00	172LEI	CD2	232VAL	CG2	0.01
170PHE	CD2	202VAL	CG2	0.38	140PHI	CB	140PHE	CG	100.00	172LEI	CD2	300HIS	CD2	0.00
170PHE	CD2	203GLY	CA	0.34	140PHI	CB	140PHE	CD1	100.00	172LEI	CD2	300HIS	CE1	0.55
170PHE	CD2	206TYR	CB	3.94	140PHI	CB	140PHE	CD2	100.00	172LEI	C	173ALA	CA	100.00
170PHE	CD2	206TYR	CG	0.41	140PHI	CB	140PHE	C	100.00	172LEI	C	173ALA	CB	0.01
170PHE	CD2	206TYR	CD1	0.64	140PHI	CG	140PHE	CD1	100.00	172LEI	C	173ALA	C	100.00
170PHE	CD2	206TYR	CD2	0.01	140PHI	CG	140PHE	CD2	100.00	172LEI	C	232VAL	CG2	0.01
170PHE	CD2	206TYR	CE1	0.02	140PHI	CG	140PHE	CE1	100.00	173AL	CA	173ALA	CB	100.00
170PHE	CD2	209VAL	CB	0.11	140PHI	CG	140PHE	CE2	100.00	173AL	CA	173ALA	C	100.00
170PHE	CD2	209VAL	CG1	9.30	140PHI	CG	140PHE	CZ	100.00	173AL	CA	232VAL	CG2	0.33
170PHE	CE1	170PHE	CE2	100.00	140PHI	CG	140PHE	C	10.35	173AL	CB	173ALA	C	100.00
170PHE	CE1	170PHE	CZ	100.00	140PHI	CD1	140PHE	CD2	100.00	173AL	CB	180VAL	CG1	0.43
170PHE	CE1	180VAL	CB	0.01	140PHI	CD1	140PHE	CE1	100.00	173AL	CB	180VAL	CG2	15.97
170PHE	CE1	180VAL	CG1	0.07	140PHI	CD1	140PHE	CE2	100.00	173AL	CB	209VAL	CG1	9.16
170PHE	CE1	180VAL	CG2	0.08	140PHI	CD1	140PHE	CZ	100.00	173AL	CB	209VAL	CG2	0.16
170PHE	CE1	199VAL	CG1	0.01	140PHI	CD1	140PHE	C	0.24	173AL	CB	232VAL	CB	0.00
170PHE	CE1	199VAL	CG2	0.01	140PHI	CD2	140PHE	CE1	100.00	173AL	CB	232VAL	CG2	0.15
170PHE	CE1	202VAL	CB	0.00	140PHI	CD2	140PHE	CE2	100.00	173AL	C	174ARG	CA	100.00
170PHE	CE1	202VAL	CG1	1.05	140PHI	CD2	140PHE	CZ	100.00	173AL	C	174ARG	CB	0.99
170PHE	CE1	202VAL	CG2	1.98	140PHI	CD2	140PHE	C	0.45	173AL	C	174ARG	CG	0.17
170PHE	CE1	202VAL	C	0.03	140PHI	CE1	140PHE	CE2	100.00	173AL	C	174ARG	C	98.99
170PHE	CE1	203GLY	CA	4.48	140PHI	CE1	140PHE	CZ	100.00	174AR	CA	174ARG	CB	100.00
170PHE	CE1	203GLY	C	0.00	140PHI	CE1	237ASN	CG	0.01	174AR	CA	174ARG	CG	100.00
170PHE	CE1	206TYR	CB	0.07	140PHI	CE1	188VAL	CG1	0.03	174AR	CA	174ARG	CD	1.46
170PHE	CE1	209VAL	CB	0.03	140PHI	CE1	188VAL	CG2	0.06	174AR	CA	174ARG	C	100.00
170PHE	CE1	209VAL	CG1	1.77	140PHI	CE2	140PHE	CZ	100.00	174AR	CA	209VAL	CG2	0.00
170PHE	CE1	211LEU	CD1	0.29	140PHI	CE2	237ASN	CG	0.05	174AR	CB	174ARG	CG	100.00
170PHE	CE1	211LEU	CD2	0.01	140PHI	CE2	188VAL	CG1	0.00	174AR	CB	174ARG	CD	100.00
170PHE	CE2	170PHE	CZ	100.00	140PHI	CZ	237ASN	CG	0.00	174AR	CB	174ARG	CZ	0.12
170PHE	CE2	180VAL	CB	0.03	140PHI	CZ	188VAL	CG1	0.05	174AR	CB	174ARG	C	100.00
170PHE	CE2	180VAL	CG1	0.26	140PHI	CZ	188VAL	CG2	0.05	174AR	CG	174ARG	CD	100.00
170PHE	CE2	180VAL	CG2	0.24	140PHI	C	141GLY	CA	100.00	174AR	CG	174ARG	CZ	3.59
170PHE	CE2	199VAL	CG1	0.03	140PHI	C	141GLY	C	53.67	174AR	CG	174ARG	C	3.18
170PHE	CE2	199VAL	CG2	0.00	140PHI	C	144ARG	CZ	0.03	174AR	CG	209VAL	CG2	1.27
170PHE	CE2	202VAL	CB	0.02	141GL	CA	141GLY	C	100.00	174AR	CD	174ARG	CZ	100.00
170PHE	CE2	202VAL	CG1	1.16	141GL	CA	156ARG	CB	0.11	174AR	CD	206TYR	CD2	0.12
170PHE	CE2	202VAL	CG2	0.73	141GL	CA	156ARG	CG	0.02	174AR	CD	206TYR	CE2	0.10
170PHE	CE2	202VAL	C	0.01	141GL	CA	156ARG	CD	0.13	174AR	CZ	206TYR	CG	0.01
170PHE	CE2	203GLY	CA	8.68	141GL	C	142GLU	CA	100.00	174AR	CZ	206TYR	CD1	0.00
170PHE	CE2	206TYR	CB	0.09	141GL	C	142GLU	CB	12.34	174AR	CZ	206TYR	CD2	62.05

170PHE CE2	209VAL	CB	0.01	141GL C	142GLU	CG	0.24	174AR CZ	206TYR	CE2	15.18
170PHE CE2	209VAL	CG1	0.94	141GL C	142GLU	CD	0.07	174AR C	175LYS	CA	100.00
170PHE CE2	211LEU	CB	0.01	141GL C	142GLU	C	91.28	174AR C	175LYS	CB	38.27
170PHE CE2	211LEU	CD1	0.31	141GL C	144ARG	CD	0.00	174AR C	175LYS	CG	0.36
170PHE CE2	211LEU	CD2	0.02	141GL C	144ARG	CZ	0.00	174AR C	175LYS	C	60.99
170PHE CZ	180VAL	CB	0.00	141GL C	154THR	CB	0.01	174AR C	178LYS	CE	0.00
170PHE CZ	180VAL	CG1	0.02	141GL C	154THR	CG2	0.00	175LY CA	175LYS	CB	100.00
170PHE CZ	180VAL	CG2	0.04	142GL CA	142GLU	CB	100.00	175LY CA	175LYS	CG	100.00
170PHE CZ	199VAL	CG1	0.06	142GL CA	142GLU	CG	100.00	175LY CA	175LYS	CD	1.55
170PHE CZ	202VAL	CG1	0.01	142GL CA	142GLU	CD	87.91	175LY CA	175LYS	C	100.00
170PHE CZ	202VAL	CG2	0.03	142GL CA	142GLU	C	100.00	175LY CB	175LYS	CG	100.00
170PHE CZ	202VAL	C	0.01	142GL CA	143PRO	CA	100.00	175LY CB	175LYS	CD	100.00
170PHE CZ	203GLY	CA	21.50	142GL CA	143PRO	C	44.00	175LY CB	175LYS	CE	24.04
170PHE CZ	211LEU	CB	0.10	142GL CB	142GLU	CG	100.00	175LY CB	175LYS	C	100.00
170PHE CZ	211LEU	CG	0.01	142GL CB	142GLU	CD	100.00	175LY CG	175LYS	CD	100.00
170PHE CZ	211LEU	CD1	2.08	142GL CB	142GLU	C	100.00	175LY CG	175LYS	CE	100.00
170PHE CZ	211LEU	CD2	0.31	142GL CB	143PRO	CA	5.00	175LY CG	175LYS	C	71.89
170PHE C	171GLU	CA	100.00	142GL CB	143PRO	C	0.01	175LY CD	175LYS	CE	100.00
170PHE C	171GLU	CB	0.01	142GL CG	142GLU	CD	100.00	175LY CD	175LYS	C	0.02
170PHE C	171GLU	C	100.00	142GL CG	142GLU	C	66.11	175LY CE	299GLU	CD	0.14
170PHE C	206TYR	CD2	0.10	142GL CG	143PRO	CA	2.81	175LY CE	300HIS	CE1	0.01
170PHE C	206TYR	CE2	2.40	142GL CG	144ARG	CG	0.00	175LY CE	305VAL	CG1	0.00
171GLU CA	171GLU	CB	100.00	142GL CG	144ARG	CD	0.02	175LY C	176ARG	CA	100.00
171GLU CA	171GLU	CG	100.00	142GL CD	142GLU	C	0.66	175LY C	176ARG	CB	74.29
171GLU CA	171GLU	CD	99.55	142GL CD	143PRO	CA	0.55	175LY C	176ARG	CG	37.27
171GLU CA	171GLU	C	100.00	142GL CD	144ARG	CB	0.01	175LY C	176ARG	C	33.48
171GLU CA	206TYR	CE2	0.22	142GL CD	144ARG	CG	0.09	176AR CA	176ARG	CB	100.00
171GLU CB	171GLU	CG	100.00	142GL CD	144ARG	CD	0.07	176AR CA	176ARG	CG	100.00
171GLU CB	171GLU	CD	100.00	142GL C	143PRO	CA	100.00	176AR CA	176ARG	CD	0.80
171GLU CB	171GLU	C	100.00	142GL C	143PRO	CB	0.14	176AR CA	176ARG	C	100.00
171GLU CG	171GLU	CD	100.00	142GL C	143PRO	CD	100.00	176AR CA	177ARG	CZ	0.48
171GLU CG	171GLU	C	79.37	142GL C	143PRO	C	99.85	176AR CB	176ARG	CG	100.00
171GLU CG	206TYR	CE2	0.01	142GL C	154THR	CB	0.07	176AR CB	176ARG	CD	100.00
171GLU CG	206TYR	CZ	0.01	142GL C	154THR	CG2	0.00	176AR CB	176ARG	C	100.00
171GLU CD	171GLU	C	2.00	143PR CA	143PRO	CB	100.00	176AR CB	177ARG	CZ	0.14
171GLU CD	175LYS	CE	0.01	143PR CA	143PRO	CG	100.00	176AR CB	231ASP	CG	0.03
171GLU CD	206TYR	CE2	0.52	143PR CA	143PRO	CD	100.00	176AR CB	232VAL	CG2	0.50
171GLU CD	206TYR	CZ	0.00	143PR CA	143PRO	C	100.00	176AR CG	176ARG	CD	100.00
171GLU C	172ALA	CA	100.00	143PR CB	143PRO	CG	100.00	176AR CG	176ARG	CZ	66.13
171GLU C	172ALA	CB	0.00	143PR CB	143PRO	CD	100.00	176AR CG	176ARG	C	0.11
171GLU C	172ALA	C	99.99	143PR CB	143PRO	C	100.00	176AR CG	232VAL	CG2	0.01
172ALA CA	172ALA	CB	100.00	143PR CB	152TRP	CD1	0.38	176AR CD	176ARG	CZ	100.00
172ALA CA	172ALA	C	100.00	143PR CB	152TRP	CZ2	0.00	176AR CD	231ASP	CG	0.10
172ALA CA	175LYS	CD	0.00	143PR CG	143PRO	CD	100.00	176AR CD	232VAL	CG1	0.04
172ALA CA	300HIS	CE1	0.01	143PR CG	143PRO	C	97.20	176AR CD	232VAL	CG2	9.16
172ALA CB	172ALA	C	100.00	143PR CG	152TRP	CD1	0.42	176AR C	177ARG	CA	100.00
172ALA CB	300HIS	CE1	0.20	143PR CG	152TRP	CZ2	0.00	176AR C	177ARG	CB	1.57
172ALA C	173ALA	CA	100.00	143PR CG	143PRO	CG	0.01	176AR C	177ARG	CG	0.08
172ALA C	173ALA	CB	0.00	143PR CG	143PRO	CD	0.02	176AR C	177ARG	CD	0.37
172ALA C	173ALA	C	100.00	143PR CG	154THR	CG2	0.00	176AR C	177ARG	CZ	0.87
173ALA CA	173ALA	CB	100.00	143PR CD	143PRO	C	90.95	176AR C	177ARG	C	98.74
173ALA CA	173ALA	C	100.00	143PR CD	154THR	CB	0.02	177AR CA	177ARG	CB	100.00
173ALA CA	232VAL	CG2	0.39	143PR CD	143PRO	CG	0.03	177AR CA	177ARG	CG	100.00
173ALA CB	173ALA	C	100.00	143PR C	144ARG	CA	100.00	177AR CA	177ARG	CD	32.41
173ALA CB	180VAL	CG1	0.16	143PR C	144ARG	CB	82.05	177AR CA	177ARG	CZ	0.01
173ALA CB	180VAL	CG2	23.96	143PR C	144ARG	CG	1.25	177AR CA	177ARG	C	100.00
173ALA CB	209VAL	CG1	9.76	143PR C	144ARG	CD	0.01	177AR CB	177ARG	CG	100.00
173ALA CB	209VAL	CG2	0.07	143PR C	144ARG	C	22.78	177AR CB	177ARG	CD	100.00
173ALA CB	232VAL	CG2	0.13	143PR C	152TRP	CD1	0.30	177AR CB	177ARG	CZ	0.08
173ALA C	174ARG	CA	100.00	144AR CA	144ARG	CB	100.00	177AR CB	177ARG	C	100.00
173ALA C	174ARG	C	100.00	144AR CA	144ARG	CG	100.00	177AR CB	231ASP	CB	0.35
173ALA C	209VAL	CG2	0.02	144AR CA	144ARG	CD	3.21	177AR CB	231ASP	CG	6.11

174ARG CA	174ARG CB	100.00	144AR(CA	144ARG C	100.00	177AR CG	177ARG CD	100.00
174ARG CA	174ARG CG	100.00	144AR(CA	152TRP CD1	1.32	177AR CG	177ARG CZ	12.98
174ARG CA	174ARG CD	0.46	144AR(CB	144ARG CG	100.00	177AR CG	177ARG C	57.62
174ARG CA	174ARG C	100.00	144AR(CB	144ARG CD	100.00	177AR CG	179HIS CB	0.06
174ARG CA	178LYS CG	0.00	144AR(CB	144ARG CZ	0.38	177AR CD	177ARG CZ	100.00
174ARG CA	209VAL CG2	0.13	144AR(CB	144ARG C	100.00	177AR CD	177ARG C	0.38
174ARG CB	174ARG CG	100.00	144AR(CB	153ASN CG	0.00	177AR CD	179HIS CB	0.31
174ARG CB	174ARG CD	100.00	144AR(CB	190GLU CD	0.01	177AR CD	231ASP CB	0.00
174ARG CB	174ARG CZ	5.22	144AR(CG	144ARG CD	100.00	177AR CD	231ASP CG	1.15
174ARG CB	174ARG C	100.00	144AR(CG	144ARG CZ	11.08	177AR CZ	179HIS CB	2.63
174ARG CB	209VAL CG2	0.06	144AR(CG	144ARG C	85.50	177AR CZ	179HIS CG	3.08
174ARG CG	174ARG CD	100.00	144AR(CG	190GLU CG	0.00	177AR CZ	179HIS CD2	4.58
174ARG CG	174ARG CZ	32.97	144AR(CG	190GLU CD	1.17	177AR CZ	179HIS CE1	0.02
174ARG CG	174ARG C	44.45	144AR(CD	144ARG CZ	100.00	177AR CZ	231ASP CG	0.00
174ARG CG	209VAL CG2	1.19	144AR(CD	144ARG C	0.20	177AR C	178LYS CA	100.00
174ARG CD	174ARG CZ	100.00	144AR(CD	190GLU CD	0.01	177AR C	178LYS CB	3.15
174ARG CD	174ARG C	0.70	144AR(CZ	144ARG C	0.00	177AR C	178LYS CG	1.03
174ARG CD	206TYR CD2	0.08	144AR(CZ	190GLU CD	0.03	177AR C	178LYS CD	0.01
174ARG CD	206TYR CE2	0.44	144AR(C	145GLY CA	100.00	177AR C	178LYS C	99.91
174ARG CD	208ASP CG	0.01	144AR(C	145GLY C	7.95	178LY: CA	178LYS CB	100.00
174ARG CD	209VAL CG2	0.01	144AR(C	152TRP CD1	1.92	178LY: CA	178LYS CG	100.00
174ARG CZ	175LYS CE	0.00	144AR(C	152TRP CE2	0.24	178LY: CA	178LYS CD	2.12
174ARG CZ	206TYR CD2	3.31	144AR(C	152TRP CZ2	0.02	178LY: CA	178LYS C	100.00
174ARG CZ	206TYR CE2	16.25	145GL' CA	145GLY C	100.00	178LY: CB	178LYS CG	100.00
174ARG CZ	206TYR CZ	0.00	145GL' CA	152TRP CD1	0.27	178LY: CB	178LYS CD	100.00
174ARG CZ	209VAL CG2	0.03	145GL' CA	152TRP CD2	0.26	178LY: CB	178LYS CE	5.15
174ARG C	175LYS CA	100.00	145GL' CA	152TRP CE2	7.38	178LY: CB	178LYS C	100.00
174ARG C	175LYS CB	31.36	145GL' CA	152TRP CE3	0.06	178LY: CB	209VAL CG2	0.01
174ARG C	175LYS CG	0.41	145GL' CA	152TRP CZ2	1.51	178LY: CG	178LYS CD	100.00
174ARG C	175LYS CD	0.01	145GL' CA	152TRP CZ3	0.10	178LY: CG	178LYS CE	100.00
174ARG C	175LYS CE	0.00	145GL' CA	190GLU CG	0.04	178LY: CD	178LYS CE	100.00
174ARG C	175LYS C	67.00	145GL' C	146MET CA	100.00	178LY: CD	208ASP CB	0.01
175LYS CA	175LYS CB	100.00	145GL' C	146MET CB	94.83	178LY: CD	208ASP CG	0.08
175LYS CA	175LYS CG	100.00	145GL' C	146MET CG	0.31	178LY: CD	209VAL CG2	0.00
175LYS CA	175LYS CD	7.17	145GL' C	146MET C	7.20	178LY: CE	208ASP CG	0.02
175LYS CA	175LYS C	100.00	145GL' C	152TRP CD1	0.00	178LY: C	179HIS CA	100.00
175LYS CB	175LYS CG	100.00	145GL' C	152TRP CD2	0.05	178LY: C	179HIS CB	93.54
175LYS CB	175LYS CD	100.00	145GL' C	152TRP CE2	0.50	178LY: C	179HIS CG	0.00
175LYS CB	175LYS CE	6.88	145GL' C	152TRP CE3	0.26	178LY: C	179HIS C	9.13
175LYS CB	175LYS C	100.00	145GL' C	152TRP CZ2	0.30	178LY: C	209VAL CG1	0.58
175LYS CB	305VAL CG1	0.01	145GL' C	152TRP CZ3	0.32	179HIS CA	179HIS CB	100.00
175LYS CB	305VAL CG2	0.08	145GL' C	190GLU CB	0.04	179HIS CA	179HIS CG	100.00
175LYS CG	175LYS CD	100.00	145GL' C	190GLU CG	0.54	179HIS CA	179HIS CD2	28.02
175LYS CG	175LYS CE	100.00	146ME' CA	146MET CB	100.00	179HIS CA	179HIS C	100.00
175LYS CG	175LYS C	50.51	146ME' CA	146MET CG	100.00	179HIS CB	179HIS CG	100.00
175LYS CG	299GLU CG	0.00	146ME' CA	146MET C	100.00	179HIS CB	179HIS CD2	100.00
175LYS CG	300HIS CE1	0.02	146ME' CA	151ALA CB	0.12	179HIS CB	179HIS CE1	0.18
175LYS CG	305VAL CG2	1.43	146ME' CA	152TRP CZ3	0.12	179HIS CB	179HIS C	100.00
175LYS CD	175LYS CE	100.00	146ME' CB	146MET CG	100.00	179HIS CB	210ALA CB	0.00
175LYS CD	175LYS C	0.38	146ME' CB	146MET CE	25.65	179HIS CG	179HIS CD2	100.00
175LYS CD	299GLU CG	0.10	146ME' CB	146MET C	100.00	179HIS CG	179HIS CE1	100.00
175LYS CD	299GLU CD	0.02	146ME' CB	151ALA CA	0.08	179HIS CG	179HIS C	99.71
175LYS CD	300HIS CE1	0.29	146ME' CB	151ALA CB	1.22	179HIS CG	210ALA CB	0.02
175LYS CD	305VAL CG2	0.11	146ME' CB	190GLU CG	2.52	179HIS CD2	179HIS CE1	100.00
175LYS CE	299GLU CG	0.01	146ME' CG	146MET CE	100.00	179HIS CD2	179HIS C	6.80
175LYS CE	299GLU CD	0.02	146ME' CG	146MET C	94.90	179HIS CD2	180VAL C	0.01
175LYS CE	300HIS CE1	0.13	146ME' CG	151ALA CA	0.00	179HIS CD2	181VAL CG2	0.75
175LYS CE	305VAL CG2	0.03	146ME' CG	151ALA CB	0.32	179HIS CD2	210ALA CB	1.26
175LYS C	176ARG CA	100.00	146ME' CG	190GLU CG	0.11	179HIS CD2	210ALA C	0.02
175LYS C	176ARG CB	85.80	146ME' CG	194PHE CD1	0.13	179HIS CD2	211LEU C	0.00
175LYS C	176ARG CG	42.95	146ME' CE	146MET C	0.00	179HIS CD2	212GLU CB	0.03
175LYS C	176ARG C	21.10	146ME' CE	151ALA CB	0.00	179HIS CD2	212GLU CG	0.00

176ARG	CA	176ARG	CB	100.00	146ME	CE	190GLU	CA	0.02	179HI	CD2	229ARG	CZ	0.01
176ARG	CA	176ARG	CG	100.00	146ME	CE	190GLU	CB	0.01	179HI	CD2	230PHE	C	0.02
176ARG	CA	176ARG	CD	0.00	146ME	CE	190GLU	CG	0.04	179HI	CD2	231ASP	CB	0.02
176ARG	CA	176ARG	C	100.00	146ME	CE	190GLU	CD	0.01	179HI	CE1	180VAL	C	0.01
176ARG	CB	176ARG	CG	100.00	146ME	CE	190GLU	C	0.01	179HI	CE1	181VAL	CG1	0.00
176ARG	CB	176ARG	CD	100.00	146ME	CE	193GLU	CB	0.08	179HI	CE1	181VAL	CG2	6.28
176ARG	CB	176ARG	C	100.00	146ME	CE	193GLU	CG	1.89	179HI	CE1	210ALA	CB	0.07
176ARG	CB	231ASP	CG	0.22	146ME	CE	193GLU	CD	0.20	179HI	CE1	211LEU	C	0.06
176ARG	CG	176ARG	CD	100.00	146ME	CE	193GLU	C	0.04	179HI	CE1	212GLU	CB	12.21
176ARG	CG	176ARG	CZ	61.34	146ME	CE	194PHE	CA	0.87	179HI	CE1	212GLU	CG	0.81
176ARG	CG	176ARG	C	0.03	146ME	CE	194PHE	CB	0.04	179HI	CE1	212GLU	CD	0.10
176ARG	CG	231ASP	CG	0.02	146ME	CE	194PHE	CD1	0.00	179HI	CE1	229ARG	CZ	0.05
176ARG	CD	176ARG	CZ	100.00	146ME	CE	197LYS	CB	0.00	179HI	CE1	230PHE	CD2	0.00
176ARG	CD	231ASP	CG	0.09	146ME	CE	197LYS	CG	0.01	179HI	CE1	230PHE	CE2	0.00
176ARG	CD	232VAL	CG2	26.89	146ME	CE	197LYS	CD	1.36	179HI	C	180VAL	CA	100.00
176ARG	C	177ARG	CA	100.00	146ME	CE	197LYS	CE	0.01	179HI	C	180VAL	CB	35.35
176ARG	C	177ARG	CB	1.46	146ME	C	147SER	CA	100.00	179HI	C	180VAL	CG1	0.58
176ARG	C	177ARG	CG	0.39	146ME	C	147SER	CB	90.02	179HI	C	180VAL	CG2	14.77
176ARG	C	177ARG	C	98.02	146ME	C	147SER	C	16.42	179HI	C	180VAL	C	73.51
177ARG	CA	177ARG	CB	100.00	146ME	C	152TRP	CZ3	0.16	180VA	CA	180VAL	CB	100.00
177ARG	CA	177ARG	CG	100.00	147SE	CA	147SER	CB	100.00	180VA	CA	180VAL	CG1	100.00
177ARG	CA	177ARG	C	100.00	147SE	CA	147SER	C	100.00	180VA	CA	180VAL	CG2	100.00
177ARG	CB	177ARG	CG	100.00	147SE	CB	147SER	C	100.00	180VA	CA	180VAL	C	100.00
177ARG	CB	177ARG	CD	100.00	147SE	CB	152TRP	CZ3	0.05	180VA	CB	180VAL	CG1	100.00
177ARG	CB	177ARG	CZ	0.03	147SE	C	148GLU	CA	100.00	180VA	CB	180VAL	CG2	100.00
177ARG	CB	177ARG	C	100.00	147SE	C	148GLU	CB	3.40	180VA	CB	180VAL	C	100.00
177ARG	CB	179HIS	CD2	0.10	147SE	C	148GLU	C	98.81	180VA	CB	211LEU	CD1	0.06
177ARG	CB	231ASP	CG	0.46	148GL	CA	148GLU	CB	100.00	180VA	CB	232VAL	CG1	0.00
177ARG	CG	177ARG	CD	100.00	148GL	CA	148GLU	CG	100.00	180VA	CG1	180VAL	CG2	100.00
177ARG	CG	177ARG	CZ	2.32	148GL	CA	148GLU	CD	97.64	180VA	CG1	180VAL	C	97.43
177ARG	CG	177ARG	C	0.97	148GL	CA	148GLU	C	100.00	180VA	CG1	181VAL	C	0.02
177ARG	CG	231ASP	CG	2.72	148GL	CB	148GLU	CG	100.00	180VA	CG1	209VAL	CG1	0.81
177ARG	CD	177ARG	CZ	100.00	148GL	CB	148GLU	CD	100.00	180VA	CG1	211LEU	CD1	1.50
177ARG	CD	231ASP	CB	0.41	148GL	CB	148GLU	C	100.00	180VA	CG1	211LEU	CD2	0.00
177ARG	CD	231ASP	CG	0.56	148GL	CG	148GLU	CD	100.00	180VA	CG1	232VAL	CG1	0.06
177ARG	CZ	231ASP	CA	0.00	148GL	CG	148GLU	C	92.12	180VA	CG1	234VAL	CB	0.00
177ARG	CZ	231ASP	CB	17.29	148GL	CD	148GLU	C	2.25	180VA	CG1	234VAL	CG1	16.22
177ARG	CZ	231ASP	CG	3.16	148GL	CD	149ALA	CB	0.01	180VA	CG2	180VAL	C	13.71
177ARG	C	178LYS	CA	100.00	148GL	CD	159LYS	CE	0.00	180VA	CG2	209VAL	CG1	15.14
177ARG	C	178LYS	CB	99.99	148GL	CD	197LYS	CE	0.20	180VA	CG2	211LEU	CD1	0.03
177ARG	C	178LYS	CG	25.67	148GL	C	149ALA	CA	100.00	180VA	CG2	232VAL	CB	0.02
177ARG	C	178LYS	CD	0.06	148GL	C	149ALA	CB	38.41	180VA	CG2	232VAL	CG1	0.56
177ARG	C	178LYS	CE	0.00	148GL	C	149ALA	C	59.56	180VA	CG2	232VAL	CG2	0.00
177ARG	C	178LYS	C	99.05	148GL	C	159LYS	CD	0.01	180VA	CG2	234VAL	CG1	0.96
177ARG	C	179HIS	CD2	0.01	148GL	C	194PHE	CE1	0.08	180VA	C	181VAL	CA	100.00
178LYS	CA	178LYS	CB	100.00	148GL	C	194PHE	CZ	0.00	180VA	C	181VAL	CB	14.26
178LYS	CA	178LYS	CG	100.00	149AL	CA	149ALA	CB	100.00	180VA	C	181VAL	CG1	0.00
178LYS	CA	178LYS	CD	0.41	149AL	CA	149ALA	C	100.00	180VA	C	181VAL	CG2	5.89
178LYS	CA	178LYS	C	100.00	149AL	CA	159LYS	CB	0.02	180VA	C	181VAL	C	92.90
178LYS	CB	178LYS	CG	100.00	149AL	CA	194PHE	CE1	0.03	180VA	C	211LEU	CD1	0.00
178LYS	CB	178LYS	CD	100.00	149AL	CA	194PHE	CZ	0.04	181VA	CA	181VAL	CB	100.00
178LYS	CB	178LYS	CE	27.52	149AL	CB	149ALA	C	100.00	181VA	CA	181VAL	CG1	100.00
178LYS	CB	178LYS	C	100.00	149AL	CB	150GLU	CG	0.00	181VA	CA	181VAL	CG2	100.00
178LYS	CB	209VAL	CG2	0.00	149AL	CB	159LYS	CB	0.11	181VA	CA	181VAL	C	100.00
178LYS	CG	178LYS	CD	100.00	149AL	CB	159LYS	CD	0.04	181VA	CB	181VAL	CG1	100.00
178LYS	CG	178LYS	CE	100.00	149AL	C	150GLU	CA	100.00	181VA	CB	181VAL	CG2	100.00
178LYS	CG	178LYS	C	1.96	149AL	C	150GLU	CB	99.71	181VA	CB	181VAL	C	100.00
178LYS	CG	209VAL	CG2	0.01	149AL	C	150GLU	CG	3.40	181VA	CB	219MET	CE	0.01
178LYS	CD	178LYS	CE	100.00	149AL	C	150GLU	CD	0.00	181VA	CB	230PHE	CE2	0.01
178LYS	CD	208ASP	CG	0.34	149AL	C	150GLU	C	0.32	181VA	CG1	181VAL	CG2	100.00
178LYS	CE	208ASP	CG	0.20	149AL	C	159LYS	CB	0.01	181VA	CG1	181VAL	C	99.96
178LYS	CE	209VAL	CG2	0.00	149AL	C	194PHE	CE1	2.71	181VA	CG1	183VAL	CG2	0.00

178LYS	C	179HIS	CA	100.00	149AL	C	194PHE	CE2	0.02	181VA	CG1	212GLU	CB	0.00
178LYS	C	179HIS	CB	93.73	149AL	C	194PHE	CZ	11.56	181VA	CG1	212GLU	CG	0.00
178LYS	C	179HIS	CD2	0.01	150GL	CA	150GLU	CB	100.00	181VA	CG1	213HIS	C	0.06
178LYS	C	179HIS	C	8.02	150GL	CA	150GLU	CG	100.00	181VA	CG1	214GLN	CB	5.90
178LYS	C	209VAL	CG1	0.64	150GL	CA	150GLU	CD	1.60	181VA	CG1	214GLN	CG	1.82
179HIS	CA	179HIS	CB	100.00	150GL	CA	150GLU	C	100.00	181VA	CG1	214GLN	CD	0.27
179HIS	CA	179HIS	CG	100.00	150GL	CA	194PHE	CE1	1.48	181VA	CG1	219MET	CG	0.00
179HIS	CA	179HIS	CD2	57.04	150GL	CA	194PHE	CE2	0.05	181VA	CG1	219MET	CE	5.89
179HIS	CA	179HIS	C	100.00	150GL	CA	194PHE	CZ	3.86	181VA	CG1	230PHE	CB	0.01
179HIS	CB	179HIS	CG	100.00	150GL	CB	150GLU	CG	100.00	181VA	CG1	230PHE	CD1	0.01
179HIS	CB	179HIS	CD2	100.00	150GL	CB	150GLU	CD	100.00	181VA	CG1	230PHE	CD2	0.24
179HIS	CB	179HIS	CE1	0.21	150GL	CB	150GLU	C	100.00	181VA	CG1	230PHE	CE1	0.00
179HIS	CB	179HIS	C	100.00	150GL	CB	152TRP	CZ3	0.05	181VA	CG1	230PHE	CE2	0.12
179HIS	CB	210ALA	CB	0.01	150GL	CB	158SER	CB	0.05	181VA	CG1	230PHE	CZ	0.01
179HIS	CG	179HIS	CD2	100.00	150GL	CG	150GLU	CD	100.00	181VA	CG1	233VAL	CG1	0.00
179HIS	CG	179HIS	CE1	100.00	150GL	CG	150GLU	C	88.79	181VA	CG1	233VAL	CG2	0.12
179HIS	CG	179HIS	C	99.93	150GL	CG	152TRP	CE3	0.05	181VA	CG2	181VAL	C	5.59
179HIS	CD2	179HIS	CE1	100.00	150GL	CG	152TRP	CZ3	0.22	181VA	CG2	212GLU	CB	0.01
179HIS	CD2	179HIS	C	8.34	150GL	CG	156ARG	CG	0.02	181VA	CG2	212GLU	CG	0.06
179HIS	CD2	180VAL	C	0.02	150GL	CG	156ARG	CD	0.00	181VA	CG2	213HIS	C	0.00
179HIS	CD2	181VAL	CG1	0.00	150GL	CG	156ARG	CZ	0.01	181VA	CG2	214GLN	CB	0.45
179HIS	CD2	181VAL	CG2	0.70	150GL	CG	157TYR	C	0.06	181VA	CG2	214GLN	CG	0.00
179HIS	CD2	210ALA	CB	0.92	150GL	CG	158SER	CA	0.10	181VA	CG2	214GLN	CD	0.01
179HIS	CD2	210ALA	C	0.00	150GL	CG	158SER	CB	0.40	181VA	CG2	219MET	CE	0.04
179HIS	CD2	212GLU	CB	0.01	150GL	CD	152TRP	CE3	0.07	181VA	CG2	230PHE	CB	1.90
179HIS	CD2	229ARG	CZ	0.00	150GL	CD	152TRP	CZ3	0.25	181VA	CG2	230PHE	CG	0.05
179HIS	CD2	231ASP	CB	0.00	150GL	CD	156ARG	CG	0.13	181VA	CG2	230PHE	CD1	0.40
179HIS	CE1	180VAL	C	0.01	150GL	CD	156ARG	CD	0.69	181VA	CG2	230PHE	CD2	0.41
179HIS	CE1	181VAL	CG2	21.68	150GL	CD	156ARG	CZ	1.00	181VA	CG2	230PHE	CE1	0.02
179HIS	CE1	210ALA	CB	0.02	150GL	CD	158SER	CA	0.02	181VA	CG2	230PHE	CE2	0.03
179HIS	CE1	211LEU	C	0.14	150GL	CD	158SER	CB	0.11	181VA	CG2	230PHE	CZ	0.00
179HIS	CE1	212GLU	CB	5.44	150GL	C	151ALA	CA	100.00	181VA	C	182SER	CA	100.00
179HIS	CE1	212GLU	CG	0.07	150GL	C	151ALA	CB	92.12	181VA	C	182SER	CB	11.42
179HIS	CE1	212GLU	CD	0.17	150GL	C	151ALA	C	6.67	181VA	C	182SER	C	92.75
179HIS	CE1	229ARG	CZ	4.65	150GL	C	152TRP	CE3	0.01	182SE	CA	182SER	CB	100.00
179HIS	CE1	231ASP	CB	2.34	150GL	C	152TRP	CZ3	0.20	182SE	CA	182SER	C	100.00
179HIS	C	180VAL	CA	100.00	150GL	C	194PHE	CD1	0.07	182SE	CA	234VAL	CG1	0.54
179HIS	C	180VAL	CB	28.19	150GL	C	194PHE	CE1	10.87	182SE	CB	182SER	C	100.00
179HIS	C	180VAL	CG1	0.32	150GL	C	194PHE	CE2	0.14	182SE	CB	195TRP	CD1	0.01
179HIS	C	180VAL	CG2	16.52	150GL	C	194PHE	CZ	1.38	182SE	CB	211LEU	CD1	0.89
179HIS	C	180VAL	C	79.14	151AL	CA	151ALA	CB	100.00	182SE	CB	211LEU	CD2	0.12
180VAL	CA	180VAL	CB	100.00	151AL	CA	151ALA	C	100.00	182SE	CB	213HIS	CE1	1.08
180VAL	CA	180VAL	CG1	100.00	151AL	CA	152TRP	CE3	3.30	182SE	CB	234VAL	CG1	0.41
180VAL	CA	180VAL	CG2	100.00	151AL	CB	151ALA	C	100.00	182SE	C	183VAL	CA	100.00
180VAL	CA	180VAL	C	100.00	151AL	CB	190GLU	C	0.03	182SE	C	183VAL	CB	3.39
180VAL	CB	180VAL	CG1	100.00	151AL	CB	191VAL	CA	0.01	182SE	C	183VAL	CG2	2.68
180VAL	CB	180VAL	CG2	100.00	151AL	CB	191VAL	CG1	0.15	182SE	C	183VAL	C	98.12
180VAL	CB	180VAL	C	100.00	151AL	CB	194PHE	CB	21.68	182SE	C	219MET	CE	0.21
180VAL	CB	211LEU	CD1	0.03	151AL	CB	194PHE	CG	34.49	183VA	CA	183VAL	CB	100.00
180VAL	CB	211LEU	CD2	0.01	151AL	CB	194PHE	CD1	12.32	183VA	CA	183VAL	CG1	100.00
180VAL	CG1	180VAL	CG2	100.00	151AL	CB	194PHE	CD2	3.68	183VA	CA	183VAL	CG2	100.00
180VAL	CG1	180VAL	C	99.27	151AL	CB	194PHE	CE1	0.06	183VA	CA	183VAL	C	100.00
180VAL	CG1	181VAL	C	0.01	151AL	CB	194PHE	CE2	0.02	183VA	CA	219MET	CE	0.40
180VAL	CG1	209VAL	CG1	0.01	151AL	C	152TRP	CA	100.00	183VA	CB	183VAL	CG1	100.00
180VAL	CG1	211LEU	CD1	2.27	151AL	C	152TRP	CB	99.22	183VA	CB	183VAL	CG2	100.00
180VAL	CG1	211LEU	CD2	0.28	151AL	C	152TRP	CG	0.06	183VA	CB	183VAL	C	100.00
180VAL	CG1	232VAL	CG1	0.05	151AL	C	152TRP	CD2	0.02	183VA	CB	219MET	CE	0.01
180VAL	CG1	234VAL	CG1	19.70	151AL	C	152TRP	CE3	19.00	183VA	CB	235THR	CB	0.08
180VAL	CG2	180VAL	C	0.72	151AL	C	152TRP	C	0.47	183VA	CB	239PHE	CD2	0.00
180VAL	CG2	209VAL	CG1	10.50	151AL	C	191VAL	CG1	2.43	183VA	CG1	183VAL	CG2	100.00
180VAL	CG2	211LEU	CD1	0.02	151AL	C	191VAL	CG2	0.01	183VA	CG1	183VAL	C	91.39
180VAL	CG2	211LEU	CD2	0.00	152TR	CA	152TRP	CB	100.00	183VA	CG1	195TRP	CD1	0.03

180VAL	CG2	232VAL	CG1	0.04	152TRI	CA	152TRP	CG	100.00	183VA	CG1	195TRP	CD2	0.00
180VAL	C	181VAL	CA	100.00	152TRI	CA	152TRP	CD1	49.89	183VA	CG1	195TRP	CE2	0.95
180VAL	C	181VAL	CB	12.53	152TRI	CA	152TRP	CD2	26.93	183VA	CG1	195TRP	CZ2	0.42
180VAL	C	181VAL	CG1	0.02	152TRI	CA	152TRP	C	100.00	183VA	CG1	216VAL	CA	0.62
180VAL	C	181VAL	CG2	4.28	152TRI	CA	191VAL	CG1	3.97	183VA	CG1	216VAL	CB	5.86
180VAL	C	181VAL	C	95.04	152TRI	CA	191VAL	CG2	0.02	183VA	CG1	216VAL	CG1	0.07
181VAL	CA	181VAL	CB	100.00	152TRI	CB	152TRP	CG	100.00	183VA	CG1	216VAL	CG2	13.84
181VAL	CA	181VAL	CG1	100.00	152TRI	CB	152TRP	CD1	100.00	183VA	CG1	219MET	CB	0.02
181VAL	CA	181VAL	CG2	100.00	152TRI	CB	152TRP	CD2	100.00	183VA	CG1	219MET	CG	0.03
181VAL	CA	181VAL	C	100.00	152TRI	CB	152TRP	CE3	97.18	183VA	CG1	219MET	CE	0.90
181VAL	CB	181VAL	CG1	100.00	152TRI	CB	152TRP	C	100.00	183VA	CG1	235THR	CA	0.12
181VAL	CB	181VAL	CG2	100.00	152TRI	CB	154THR	CG2	0.02	183VA	CG1	235THR	CB	0.08
181VAL	CB	181VAL	C	100.00	152TRI	CB	156ARG	CB	0.24	183VA	CG1	239PHE	CB	0.07
181VAL	CG1	181VAL	CG2	100.00	152TRI	CB	156ARG	CG	0.05	183VA	CG1	239PHE	CD1	0.00
181VAL	CG1	181VAL	C	99.86	152TRI	CB	156ARG	CD	0.02	183VA	CG1	239PHE	CD2	0.29
181VAL	CG1	182SER	C	0.07	152TRI	CB	156ARG	CZ	1.29	183VA	CG1	243LEU	CD1	0.02
181VAL	CG1	183VAL	CG1	0.01	152TRI	CG	152TRP	CD1	100.00	183VA	CG1	243LEU	CD2	0.04
181VAL	CG1	183VAL	CG2	0.04	152TRI	CG	152TRP	CD2	100.00	183VA	CG2	183VAL	C	25.00
181VAL	CG1	212GLU	CG	0.00	152TRI	CG	152TRP	CE2	100.00	183VA	CG2	195TRP	CD1	0.03
181VAL	CG1	212GLU	CD	0.00	152TRI	CG	152TRP	CE3	100.00	183VA	CG2	195TRP	CE2	0.17
181VAL	CG1	214GLN	CB	0.60	152TRI	CG	152TRP	C	99.65	183VA	CG2	195TRP	CZ2	0.04
181VAL	CG1	214GLN	CG	4.74	152TRI	CG	156ARG	CZ	0.34	183VA	CG2	214GLN	CB	0.00
181VAL	CG1	214GLN	CD	0.60	152TRI	CD1	152TRP	CD2	100.00	183VA	CG2	216VAL	CA	0.01
181VAL	CG1	219MET	CG	0.01	152TRI	CD1	152TRP	CE2	100.00	183VA	CG2	216VAL	CB	0.50
181VAL	CG1	219MET	CE	0.73	152TRI	CD1	152TRP	C	19.29	183VA	CG2	216VAL	CG2	0.72
181VAL	CG1	230PHE	CD1	0.02	152TRI	CD1	154THR	CG2	0.07	183VA	CG2	219MET	CG	0.12
181VAL	CG1	230PHE	CE1	0.04	152TRI	CD1	156ARG	CZ	0.02	183VA	CG2	219MET	CE	1.21
181VAL	CG1	233VAL	CG1	0.00	152TRI	CD2	152TRP	CE2	100.00	183VA	CG2	233VAL	CG1	28.34
181VAL	CG1	233VAL	CG2	0.03	152TRI	CD2	152TRP	CE3	100.00	183VA	CG2	235THR	CA	0.01
181VAL	CG2	181VAL	C	1.29	152TRI	CD2	152TRP	CZ2	100.00	183VA	CG2	235THR	CB	1.04
181VAL	CG2	212GLU	CB	0.00	152TRI	CD2	152TRP	CZ3	100.00	183VA	CG2	235THR	CG2	0.17
181VAL	CG2	212GLU	CD	0.01	152TRI	CD2	156ARG	CZ	0.42	183VA	CG2	239PHE	CD2	1.04
181VAL	CG2	214GLN	CB	0.02	152TRI	CE2	152TRP	CE3	100.00	183VA	CG2	243LEU	CD2	0.24
181VAL	CG2	214GLN	CG	0.22	152TRI	CE2	152TRP	CZ2	100.00	183VA	C	184ASP	CA	100.00
181VAL	CG2	214GLN	CD	0.04	152TRI	CE2	152TRP	CZ3	100.00	183VA	C	184ASP	CB	63.94
181VAL	CG2	229ARG	CZ	0.50	152TRI	CE3	152TRP	CZ2	100.00	183VA	C	184ASP	C	39.05
181VAL	CG2	230PHE	CD1	0.19	152TRI	CE3	152TRP	CZ3	100.00	184AS	CA	184ASP	CB	100.00
181VAL	CG2	230PHE	CE1	0.01	152TRI	CE3	156ARG	CD	0.04	184AS	CA	184ASP	CG	100.00
181VAL	CG2	233VAL	CG2	0.03	152TRI	CE3	156ARG	CZ	8.24	184AS	CA	184ASP	C	100.00
181VAL	C	182SER	CA	100.00	152TRI	CZ2	152TRP	CZ3	100.00	184AS	CA	239PHE	CE2	0.01
181VAL	C	182SER	CB	5.54	152TRI	CZ3	156ARG	CZ	2.34	184AS	CB	184ASP	CG	100.00
181VAL	C	182SER	C	98.58	152TRI	C	153ASN	CA	100.00	184AS	CB	184ASP	C	100.00
182SER	CA	182SER	CB	100.00	152TRI	C	153ASN	CB	88.88	184AS	CB	195TRP	CD1	0.02
182SER	CA	182SER	C	100.00	152TRI	C	153ASN	CG	7.98	184AS	CB	213HIS	CB	0.02
182SER	CA	234VAL	CG1	0.47	152TRI	C	153ASN	C	7.04	184AS	CB	213HIS	CG	0.00
182SER	CB	182SER	C	100.00	152TRI	C	191VAL	CG1	5.37	184AS	CB	213HIS	CD2	0.06
182SER	CB	195TRP	CD1	0.00	152TRI	C	191VAL	CG2	0.03	184AS	CB	213HIS	C	0.00
182SER	CB	199VAL	CG1	0.00	153ASI	CA	153ASN	CB	100.00	184AS	CB	214GLN	C	0.41
182SER	CB	211LEU	CD1	0.36	153ASI	CA	153ASN	CG	100.00	184AS	CB	215TYR	CD1	0.02
182SER	CB	211LEU	CD2	0.07	153ASI	CA	153ASN	C	100.00	184AS	CG	184ASP	C	100.00
182SER	CB	213HIS	CE1	0.30	153ASI	CB	153ASN	CG	100.00	184AS	CG	186ALA	CB	0.44
182SER	CB	234VAL	CG1	0.24	153ASI	CB	153ASN	C	100.00	184AS	CG	196ARG	CD	0.10
182SER	C	183VAL	CA	100.00	153ASI	CB	189LEU	CD1	0.00	184AS	CG	196ARG	CZ	0.22
182SER	C	183VAL	CB	6.43	153ASI	CB	191VAL	CG1	0.07	184AS	CG	213HIS	CD2	0.11
182SER	C	183VAL	CG1	0.30	153ASI	CB	191VAL	CG2	0.03	184AS	CG	213HIS	CE1	0.00
182SER	C	183VAL	CG2	3.19	153ASI	CG	153ASN	C	0.14	184AS	CG	215TYR	CD1	0.07
182SER	C	183VAL	C	97.12	153ASI	CG	189LEU	CD1	0.34	184AS	CG	215TYR	CE1	0.86
183VAL	CA	183VAL	CB	100.00	153ASI	CG	189LEU	CD2	1.27	184AS	CG	215TYR	CE2	0.02
183VAL	CA	183VAL	CG1	100.00	153ASI	CG	190GLU	CB	0.06	184AS	C	185LYS	CA	100.00
183VAL	CA	183VAL	CG2	100.00	153ASI	CG	191VAL	CG2	0.03	184AS	C	185LYS	CB	99.95
183VAL	CA	183VAL	C	100.00	153ASI	C	154THR	CA	100.00	184AS	C	185LYS	CG	0.02
183VAL	CB	183VAL	CG1	100.00	153ASI	C	154THR	CB	59.72	184AS	C	185LYS	C	0.19

183VAL	CB	183VAL	CG2	100.00	153ASI	C	154THR	C	51.86	184AS	C	215TYR	CD1	0.00
183VAL	CB	183VAL	C	100.00	154THF	CA	154THR	CB	100.00	184AS	C	239PHE	CE2	0.60
183VAL	CB	216VAL	CB	0.00	154THF	CA	154THR	CG2	100.00	185LY:	CA	185LYS	CB	100.00
183VAL	CB	216VAL	CG2	0.39	154THF	CA	154THR	C	100.00	185LY:	CA	185LYS	CG	100.00
183VAL	CB	235THR	CB	0.01	154THF	CB	154THR	CG2	100.00	185LY:	CA	185LYS	CD	75.26
183VAL	CG1	183VAL	CG2	100.00	154THF	CB	154THR	C	100.00	185LY:	CA	185LYS	CE	0.04
183VAL	CG1	183VAL	C	19.52	154THF	CG2	154THR	C	100.00	185LY:	CA	185LYS	C	100.00
183VAL	CG1	215TYR	C	0.00	154THF	CG2	155GLU	C	0.09	185LY:	CB	185LYS	CG	100.00
183VAL	CG1	216VAL	CA	0.79	154THF	CG2	156ARG	CB	0.04	185LY:	CB	185LYS	CD	100.00
183VAL	CG1	216VAL	CB	1.03	154THF	CG2	156ARG	CG	0.14	185LY:	CB	185LYS	CE	1.21
183VAL	CG1	216VAL	CG2	2.46	154THF	CG2	156ARG	CD	0.01	185LY:	CB	185LYS	C	100.00
183VAL	CG1	219MET	CB	0.18	154THF	CG2	152TRP	CB	0.21	185LY:	CB	189LEU	CD1	0.48
183VAL	CG1	219MET	CG	2.05	154THF	CG2	152TRP	CG	0.00	185LY:	CB	216VAL	CG1	0.10
183VAL	CG1	219MET	CE	1.76	154THF	CG2	152TRP	CD1	0.06	185LY:	CB	239PHE	CE1	0.07
183VAL	CG1	233VAL	CG1	7.23	154THF	CG2	153ASN	C	0.01	185LY:	CB	239PHE	CE2	0.98
183VAL	CG1	235THR	CB	0.01	154THF	C	155GLU	CA	100.00	185LY:	CB	239PHE	CZ	1.00
183VAL	CG1	239PHE	CB	0.01	154THF	C	155GLU	CB	6.08	185LY:	CG	185LYS	CD	100.00
183VAL	CG1	239PHE	CD2	0.02	154THF	C	155GLU	C	97.26	185LY:	CG	185LYS	CE	100.00
183VAL	CG1	243LEU	CD1	0.14	155GLI	CA	155GLU	CB	100.00	185LY:	CG	185LYS	C	93.65
183VAL	CG1	243LEU	CD2	0.10	155GLI	CA	155GLU	CG	100.00	185LY:	CG	188VAL	CG1	0.61
183VAL	CG2	183VAL	C	79.89	155GLI	CA	155GLU	CD	51.62	185LY:	CG	188VAL	CG2	4.44
183VAL	CG2	216VAL	CB	0.00	155GLI	CA	155GLU	C	100.00	185LY:	CG	189LEU	CG	0.01
183VAL	CG2	216VAL	CG2	1.38	155GLI	CB	155GLU	CG	100.00	185LY:	CG	189LEU	CD1	0.96
183VAL	CG2	219MET	CG	0.26	155GLI	CB	155GLU	CD	100.00	185LY:	CG	216VAL	CG1	0.20
183VAL	CG2	219MET	CE	0.10	155GLI	CB	155GLU	C	100.00	185LY:	CG	239PHE	CZ	0.01
183VAL	CG2	233VAL	CG1	6.10	155GLI	CB	138ILE	CG2	0.00	185LY:	CG	139TYR	CE1	0.31
183VAL	CG2	233VAL	CG2	0.00	155GLI	CB	138ILE	CD	1.56	185LY:	CD	185LYS	CE	100.00
183VAL	CG2	235THR	CA	0.01	155GLI	CB	153ASN	CB	0.02	185LY:	CD	185LYS	C	0.04
183VAL	CG2	235THR	CB	16.97	155GLI	CG	155GLU	CD	100.00	185LY:	CD	188VAL	CG1	0.18
183VAL	CG2	235THR	CG2	0.03	155GLI	CG	155GLU	C	94.84	185LY:	CD	188VAL	CG2	1.31
183VAL	CG2	239PHE	CD2	0.00	155GLI	CG	157TYR	CD2	0.03	185LY:	CD	215TYR	CD1	0.02
183VAL	CG2	239PHE	CE2	0.12	155GLI	CG	157TYR	CE1	0.62	185LY:	CD	215TYR	CD2	0.02
183VAL	CG2	243LEU	CD1	0.20	155GLI	CG	157TYR	CE2	2.98	185LY:	CD	216VAL	CG1	5.33
183VAL	CG2	243LEU	CD2	0.03	155GLI	CG	157TYR	CZ	0.02	185LY:	CD	216VAL	CG2	0.04
183VAL	C	184ASP	CA	100.00	155GLI	CG	191VAL	CG1	3.95	185LY:	CD	139TYR	CD1	0.38
183VAL	C	184ASP	CB	35.17	155GLI	CG	191VAL	CG2	0.56	185LY:	CD	139TYR	CE1	2.88
183VAL	C	184ASP	C	67.32	155GLI	CG	138ILE	CG2	0.02	185LY:	CD	139TYR	CZ	0.04
184ASP	CA	184ASP	CB	100.00	155GLI	CG	138ILE	CD	0.01	185LY:	CE	188VAL	CG1	0.22
184ASP	CA	184ASP	CG	100.00	155GLI	CD	155GLU	C	0.13	185LY:	CE	188VAL	CG2	4.07
184ASP	CA	184ASP	C	100.00	155GLI	CD	157TYR	CD1	0.01	185LY:	CE	215TYR	CG	0.00
184ASP	CA	239PHE	CE2	0.00	155GLI	CD	157TYR	CD2	0.02	185LY:	CE	215TYR	CD1	0.00
184ASP	CB	184ASP	CG	100.00	155GLI	CD	157TYR	CE1	1.24	185LY:	CE	215TYR	CD2	0.01
184ASP	CB	184ASP	C	100.00	155GLI	CD	157TYR	CE2	0.82	185LY:	CE	216VAL	CG1	0.71
184ASP	CB	195TRP	CD1	0.22	155GLI	CD	157TYR	CZ	0.20	185LY:	CE	217ASP	CG	0.43
184ASP	CB	213HIS	CB	0.00	155GLI	CD	191VAL	CG1	0.18	185LY:	CE	139TYR	CD1	0.25
184ASP	CB	213HIS	C	0.02	155GLI	CD	191VAL	CG2	0.27	185LY:	CE	139TYR	CE1	8.78
184ASP	CB	214GLN	C	0.25	155GLI	CD	238ILE	CD	0.00	185LY:	CE	139TYR	CE2	0.03
184ASP	CG	184ASP	C	100.00	155GLI	CD	239PHE	CE1	0.00	185LY:	CE	139TYR	CZ	1.80
184ASP	CG	186ALA	CB	0.62	155GLI	C	156ARG	CA	100.00	185LY:	CE	241ASP	CG	0.00
184ASP	CG	196ARG	CZ	0.24	155GLI	C	156ARG	CB	97.72	185LY:	C	186ALA	CA	100.00
184ASP	CG	213HIS	CB	0.00	155GLI	C	156ARG	CG	3.53	185LY:	C	186ALA	CB	0.01
184ASP	CG	213HIS	CE1	0.00	155GLI	C	156ARG	C	4.50	185LY:	C	186ALA	C	100.00
184ASP	CG	215TYR	CE1	0.02	156AR(	CA	156ARG	CB	100.00	186AL.	CA	186ALA	CB	100.00
184ASP	CG	215TYR	CE2	0.00	156AR(	CA	156ARG	CG	100.00	186AL.	CA	186ALA	C	100.00
184ASP	C	185LYS	CA	100.00	156AR(	CA	156ARG	CD	0.72	186AL.	CA	192GLY	C	1.98
184ASP	C	185LYS	CB	99.99	156AR(	CA	156ARG	C	100.00	186AL.	CB	186ALA	C	100.00
184ASP	C	185LYS	CG	0.02	156AR(	CA	191VAL	CG1	0.00	186AL.	CB	192GLY	CA	0.03
184ASP	C	185LYS	C	0.18	156AR(	CA	191VAL	CG2	0.02	186AL.	CB	192GLY	C	27.15
184ASP	C	239PHE	CE2	0.13	156AR(	CA	152TRP	CB	0.01	186AL.	CB	193GLU	CA	1.52
185LYS	CA	185LYS	CB	100.00	156AR(	CB	156ARG	CG	100.00	186AL.	CB	193GLU	CB	0.00
185LYS	CA	185LYS	CG	100.00	156AR(	CB	156ARG	CD	100.00	186AL.	CB	196ARG	CG	0.40
185LYS	CA	185LYS	CD	83.87	156AR(	CB	156ARG	CZ	0.17	186AL.	CB	196ARG	CD	15.27

185LYS	CA	185LYS	CE	0.08	156AR(	CB	156ARG	C	100.00	186AL	CB	196ARG	CZ	4.91
185LYS	CA	185LYS	C	100.00	156AR(	CB	152TRP	CB	1.11	186AL	C	187ASN	CA	100.00
185LYS	CB	185LYS	CG	100.00	156AR(	CG	156ARG	CD	100.00	186AL	C	187ASN	CB	0.04
185LYS	CB	185LYS	CD	100.00	156AR(	CG	156ARG	CZ	16.98	186AL	C	187ASN	C	99.93
185LYS	CB	185LYS	CE	6.62	156AR(	CG	156ARG	C	94.53	187AS	CA	187ASN	CB	100.00
185LYS	CB	185LYS	C	100.00	156AR(	CG	150GLU	CD	0.19	187AS	CA	187ASN	CG	100.00
185LYS	CB	189LEU	CD1	0.77	156AR(	CG	152TRP	CB	0.06	187AS	CA	187ASN	C	100.00
185LYS	CB	216VAL	CG1	0.08	156AR(	CD	156ARG	CZ	100.00	187AS	CB	187ASN	CG	100.00
185LYS	CB	239PHE	CE1	0.05	156AR(	CD	156ARG	C	0.80	187AS	CB	187ASN	C	100.00
185LYS	CB	239PHE	CE2	0.22	156AR(	CD	150GLU	CG	0.00	187AS	CB	215TYR	CE1	0.11
185LYS	CB	239PHE	CZ	0.36	156AR(	CD	150GLU	CD	0.18	187AS	CB	215TYR	CE2	0.02
185LYS	CG	185LYS	CD	100.00	156AR(	CD	152TRP	CB	0.05	187AS	CB	215TYR	CZ	0.06
185LYS	CG	185LYS	CE	100.00	156AR(	CD	152TRP	CD2	0.00	187AS	CG	187ASN	C	99.98
185LYS	CG	185LYS	C	93.13	156AR(	CD	152TRP	CE3	0.31	187AS	CG	188VAL	CG1	0.01
185LYS	CG	188VAL	CG2	1.74	156AR(	CZ	158SER	CB	0.12	187AS	CG	188VAL	CG2	0.01
185LYS	CG	189LEU	CD1	0.78	156AR(	CZ	150GLU	CD	0.89	187AS	CG	215TYR	CD1	0.02
185LYS	CG	215TYR	CD1	0.01	156AR(	CZ	152TRP	CB	0.54	187AS	CG	215TYR	CE1	2.08
185LYS	CG	216VAL	CG1	0.28	156AR(	CZ	152TRP	CG	0.16	187AS	CG	215TYR	CE2	0.98
185LYS	CG	239PHE	CE2	0.00	156AR(	CZ	152TRP	CD2	0.80	187AS	CG	215TYR	CZ	0.16
185LYS	CG	239PHE	CZ	0.01	156AR(	CZ	152TRP	CE2	0.03	187AS	C	188VAL	CA	100.00
185LYS	CG	139TYR	CE1	0.83	156AR(	CZ	152TRP	CE3	12.71	187AS	C	188VAL	CB	98.18
185LYS	CD	185LYS	CE	100.00	156AR(	CZ	152TRP	CZ2	0.00	187AS	C	188VAL	CG1	47.02
185LYS	CD	185LYS	C	0.01	156AR(	CZ	152TRP	CZ3	6.53	187AS	C	188VAL	CG2	1.01
185LYS	CD	188VAL	CG2	3.70	156AR(	C	157TYR	CA	100.00	187AS	C	188VAL	C	1.20
185LYS	CD	216VAL	CG1	1.68	156AR(	C	157TYR	CB	81.63	188VA	CA	188VAL	CB	100.00
185LYS	CD	239PHE	CZ	0.01	156AR(	C	157TYR	CG	9.51	188VA	CA	188VAL	CG1	100.00
185LYS	CD	139TYR	CE1	1.68	156AR(	C	157TYR	CD1	1.19	188VA	CA	188VAL	CG2	100.00
185LYS	CD	139TYR	CZ	0.73	156AR(	C	157TYR	CD2	3.56	188VA	CA	188VAL	C	100.00
185LYS	CD	242ILE	CD	0.00	156AR(	C	157TYR	C	17.18	188VA	CB	188VAL	CG1	100.00
185LYS	CE	187ASN	CG	0.00	156AR(	C	191VAL	CG1	0.03	188VA	CB	188VAL	CG2	100.00
185LYS	CE	188VAL	CG2	0.94	156AR(	C	191VAL	CG2	0.00	188VA	CB	188VAL	C	100.00
185LYS	CE	216VAL	CG1	1.25	157TYF	CA	157TYR	CB	100.00	188VA	CB	139TYR	CB	0.03
185LYS	CE	217ASP	CG	0.90	157TYF	CA	157TYR	CG	100.00	188VA	CB	139TYR	CG	0.00
185LYS	CE	139TYR	CD1	0.00	157TYF	CA	157TYR	CD1	96.86	188VA	CB	139TYR	CD1	0.05
185LYS	CE	139TYR	CE1	3.70	157TYF	CA	157TYR	CD2	28.51	188VA	CB	140PHE	CE2	0.00
185LYS	CE	139TYR	CE2	0.04	157TYF	CA	157TYR	C	100.00	188VA	CG1	188VAL	CG2	100.00
185LYS	CE	139TYR	CZ	5.33	157TYF	CB	157TYR	CG	100.00	188VA	CG1	188VAL	C	17.20
185LYS	CE	241ASP	CG	1.00	157TYF	CB	157TYR	CD1	100.00	188VA	CG1	82ARG	CD	0.00
185LYS	CE	242ILE	CD	0.00	157TYF	CB	157TYR	CD2	100.00	188VA	CG1	82ARG	CZ	0.11
185LYS	C	186ALA	CA	100.00	157TYF	CB	157TYR	C	100.00	188VA	CG1	139TYR	CB	0.10
185LYS	C	186ALA	CB	0.00	157TYF	CB	162VAL	CG1	0.27	188VA	CG1	139TYR	CG	0.09
185LYS	C	186ALA	C	100.00	157TYF	CB	162VAL	CG2	1.07	188VA	CG1	139TYR	CD1	1.41
186ALA	CA	186ALA	CB	100.00	157TYF	CB	191VAL	CG1	0.06	188VA	CG1	139TYR	CE1	1.00
186ALA	CA	186ALA	C	100.00	157TYF	CB	191VAL	CG2	0.04	188VA	CG1	140PHE	CE1	0.24
186ALA	CA	192GLY	CA	0.00	157TYF	CB	194PHE	CD2	0.08	188VA	CG1	140PHE	CE2	0.89
186ALA	CA	192GLY	C	2.80	157TYF	CB	194PHE	CE2	0.21	188VA	CG1	140PHE	CZ	2.12
186ALA	CB	186ALA	C	100.00	157TYF	CG	157TYR	CD1	100.00	188VA	CG2	188VAL	C	97.66
186ALA	CB	192GLY	C	17.94	157TYF	CG	157TYR	CD2	100.00	188VA	CG2	139TYR	CB	0.51
186ALA	CB	193GLU	CA	1.24	157TYF	CG	157TYR	CE1	100.00	188VA	CG2	139TYR	CG	0.34
186ALA	CB	196ARG	CG	0.05	157TYF	CG	157TYR	CE2	100.00	188VA	CG2	139TYR	CD1	13.20
186ALA	CB	196ARG	CD	8.94	157TYF	CG	157TYR	CZ	100.00	188VA	CG2	139TYR	CD2	0.04
186ALA	CB	196ARG	CZ	1.57	157TYF	CG	157TYR	C	1.36	188VA	CG2	139TYR	CE1	1.40
186ALA	C	187ASN	CA	100.00	157TYF	CG	162VAL	CG1	1.25	188VA	CG2	139TYR	CE2	0.00
186ALA	C	187ASN	CB	0.27	157TYF	CG	162VAL	CG2	0.80	188VA	CG2	139TYR	CZ	0.01
186ALA	C	187ASN	C	99.70	157TYF	CG	191VAL	CB	0.01	188VA	CG2	140PHE	CE1	0.06
186ALA	C	215TYR	CE1	0.00	157TYF	CG	191VAL	CG1	0.01	188VA	CG2	140PHE	CE2	0.13
187ASN	CA	187ASN	CB	100.00	157TYF	CG	191VAL	CG2	0.06	188VA	CG2	140PHE	CZ	0.12
187ASN	CA	187ASN	CG	100.00	157TYF	CG	195TRP	CZ3	0.02	188VA	C	189LEU	CA	100.00
187ASN	CA	187ASN	C	100.00	157TYF	CD1	157TYR	CD2	100.00	188VA	C	189LEU	CB	24.14
187ASN	CB	187ASN	CG	100.00	157TYF	CD1	157TYR	CE1	100.00	188VA	C	189LEU	CG	4.00
187ASN	CB	187ASN	C	100.00	157TYF	CD1	157TYR	CE2	100.00	188VA	C	189LEU	CD2	0.02
187ASN	CB	215TYR	CE1	0.11	157TYF	CD1	157TYR	CZ	100.00	188VA	C	189LEU	C	74.85

187ASN CB	215TYR CZ	0.05	157TYF CD1	157TYR C	0.01	189LE CA	189LEU CB	100.00
187ASN CG	187ASN C	99.98	157TYF CD1	161GLU CB	0.13	189LE CA	189LEU CG	100.00
187ASN CG	188VAL CG2	0.02	157TYF CD1	161GLU CG	0.03	189LE CA	189LEU CD2	99.96
187ASN CG	215TYR CD1	0.08	157TYF CD1	162VAL CG1	4.43	189LE CA	189LEU C	100.00
187ASN CG	215TYR CE1	1.02	157TYF CD1	162VAL CG2	10.73	189LE CB	189LEU CG	100.00
187ASN CG	215TYR CE2	0.23	157TYF CD1	191VAL CB	0.01	189LE CB	189LEU CD1	100.00
187ASN CG	215TYR CZ	0.12	157TYF CD1	191VAL CG1	0.02	189LE CB	189LEU CD2	100.00
187ASN C	188VAL CA	100.00	157TYF CD1	195TRP CE3	0.01	189LE CB	189LEU C	100.00
187ASN C	188VAL CB	97.76	157TYF CD1	195TRP CZ3	0.19	189LE CB	191VAL CG2	0.42
187ASN C	188VAL CG1	51.57	157TYF CD2	157TYR CE1	100.00	189LE CB	192GLY CA	0.01
187ASN C	188VAL CG2	1.66	157TYF CD2	157TYR CE2	100.00	189LE CG	189LEU CD1	100.00
187ASN C	188VAL C	1.25	157TYF CD2	157TYR CZ	100.00	189LE CG	189LEU CD2	100.00
188VAL CA	188VAL CB	100.00	157TYF CD2	161GLU CB	0.03	189LE CG	139TYR CD1	0.02
188VAL CA	188VAL CG1	100.00	157TYF CD2	162VAL CG1	0.21	189LE CG	139TYR CE1	0.00
188VAL CA	188VAL CG2	100.00	157TYF CD2	162VAL CG2	0.40	189LE CD1	189LEU CD2	100.00
188VAL CA	188VAL C	100.00	157TYF CD2	191VAL CB	0.14	189LE CD1	191VAL CG2	0.60
188VAL CB	188VAL CG1	100.00	157TYF CD2	191VAL CG1	0.46	189LE CD1	239PHE CE2	0.34
188VAL CB	188VAL CG2	100.00	157TYF CD2	191VAL CG2	1.22	189LE CD1	239PHE CZ	0.78
188VAL CB	188VAL C	100.00	157TYF CD2	191VAL C	0.01	189LE CD1	138ILE CG1	0.00
188VAL CB	139TYR CG	0.04	157TYF CD2	195TRP CB	0.00	189LE CD1	138ILE CD	0.02
188VAL CB	139TYR CD1	1.96	157TYF CD2	195TRP CE3	0.29	189LE CD1	139TYR CD1	0.08
188VAL CG1	188VAL CG2	100.00	157TYF CD2	195TRP CZ3	0.12	189LE CD1	139TYR CE1	0.02
188VAL CG1	188VAL C	1.86	157TYF CE1	157TYR CE2	100.00	189LE CD2	191VAL CG2	0.02
188VAL CG1	139TYR CA	0.00	157TYF CE1	157TYR CZ	100.00	189LE CD2	138ILE CG1	0.20
188VAL CG1	139TYR CB	0.02	157TYF CE1	161GLU CB	0.43	189LE CD2	138ILE CD	0.07
188VAL CG1	139TYR CG	0.15	157TYF CE1	161GLU CG	0.03	189LE CD2	138ILE C	0.09
188VAL CG1	139TYR CD1	0.51	157TYF CE1	162VAL CG1	0.27	189LE CD2	139TYR CA	0.08
188VAL CG1	139TYR CD2	0.00	157TYF CE1	162VAL CG2	0.49	189LE CD2	139TYR CB	0.01
188VAL CG1	139TYR CE1	0.02	157TYF CE1	191VAL CG2	0.00	189LE CD2	139TYR CD1	2.81
188VAL CG1	140PHE CE1	0.12	157TYF CE1	195TRP CE3	0.15	189LE CD2	139TYR CE1	0.02
188VAL CG1	140PHE CE2	0.06	157TYF CE1	195TRP CZ3	1.57	189LE CD2	153ASN CB	0.08
188VAL CG1	140PHE CZ	0.62	157TYF CE2	157TYR CZ	100.00	189LE CD2	153ASN CG	5.40
188VAL CG2	188VAL C	96.58	157TYF CE2	161GLU CB	0.76	189LE C	190GLU CA	100.00
188VAL CG2	139TYR CB	0.09	157TYF CE2	161GLU C	0.06	189LE C	190GLU CB	0.01
188VAL CG2	139TYR CG	1.12	157TYF CE2	162VAL CG1	0.01	189LE C	190GLU C	100.00
188VAL CG2	139TYR CD1	52.59	157TYF CE2	162VAL CG2	0.02	190GL CA	190GLU CB	100.00
188VAL CG2	139TYR CD2	0.06	157TYF CE2	191VAL CB	0.04	190GL CA	190GLU CG	100.00
188VAL CG2	139TYR CE1	10.27	157TYF CE2	191VAL CG1	0.06	190GL CA	190GLU CD	98.63
188VAL CG2	139TYR CE2	0.02	157TYF CE2	191VAL CG2	0.04	190GL CA	190GLU C	100.00
188VAL CG2	139TYR CZ	0.15	157TYF CE2	191VAL C	0.01	190GL CA	146MET CG	0.00
188VAL C	189LEU CA	100.00	157TYF CE2	195TRP CB	0.01	190GL CB	190GLU CG	100.00
188VAL C	189LEU CB	29.12	157TYF CE2	195TRP CE3	2.56	190GL CB	190GLU CD	100.00
188VAL C	189LEU CG	4.08	157TYF CE2	195TRP CZ3	1.27	190GL CB	190GLU C	100.00
188VAL C	189LEU CD2	0.01	157TYF CZ	161GLU CB	0.88	190GL CB	145GLY C	0.08
188VAL C	189LEU C	67.24	157TYF CZ	162VAL CG1	0.02	190GL CB	146MET CG	0.01
189LEU CA	189LEU CB	100.00	157TYF CZ	162VAL CG2	0.01	190GL CB	153ASN CG	0.00
189LEU CA	189LEU CG	100.00	157TYF CZ	191VAL CG1	0.00	190GL CG	190GLU CD	100.00
189LEU CA	189LEU CD1	0.14	157TYF CZ	191VAL CG2	0.00	190GL CG	190GLU C	7.44
189LEU CA	189LEU CD2	99.79	157TYF CZ	195TRP CE3	0.49	190GL CG	144ARG CG	0.06
189LEU CA	189LEU C	100.00	157TYF CZ	195TRP CZ3	1.66	190GL CG	144ARG CD	0.01
189LEU CB	189LEU CG	100.00	157TYF CZ	236GLY CA	0.01	190GL CG	144ARG C	0.01
189LEU CB	189LEU CD1	100.00	157TYF C	158SER CA	100.00	190GL CG	145GLY CA	0.02
189LEU CB	189LEU CD2	100.00	157TYF C	158SER CB	46.63	190GL CG	145GLY C	0.88
189LEU CB	189LEU C	100.00	157TYF C	158SER C	59.64	190GL CG	146MET CB	3.54
189LEU CB	191VAL CG2	0.06	157TYF C	194PHE CE2	0.52	190GL CG	146MET CG	0.03
189LEU CB	192GLY CA	0.04	157TYF C	150GLU CG	0.17	190GL CG	146MET CE	0.01
189LEU CG	189LEU CD1	100.00	158SEF CA	158SER CB	100.00	190GL CD	144ARG CB	0.01
189LEU CG	189LEU CD2	100.00	158SEF CA	158SER C	100.00	190GL CD	144ARG CG	0.16
189LEU CG	189LEU C	0.04	158SEF CA	194PHE CE2	0.08	190GL CD	144ARG CD	0.14
189LEU CG	139TYR CD1	0.24	158SEF CA	194PHE CZ	0.01	190GL CD	144ARG CZ	0.02
189LEU CG	139TYR CE1	0.22	158SEF CA	150GLU CB	0.01	190GL C	191VAL CA	100.00
189LEU CD1	189LEU CD2	100.00	158SEF CA	150GLU CG	0.10	190GL C	191VAL C	100.00

189LEU	CD1	191VAL	CG1	0.00
189LEU	CD1	191VAL	CG2	0.06
189LEU	CD1	238ILE	CD	0.04
189LEU	CD1	239PHE	CE1	0.03
189LEU	CD1	239PHE	CE2	0.61
189LEU	CD1	239PHE	CZ	0.30
189LEU	CD1	139TYR	CD1	0.02
189LEU	CD1	139TYR	CE1	0.05
189LEU	CD1	153ASN	CG	0.01
189LEU	CD1	238ILE	CD	0.00
189LEU	CD2	191VAL	CG2	0.06
189LEU	CD2	238ILE	CD	0.02
189LEU	CD2	138ILE	CG1	3.84
189LEU	CD2	138ILE	CG2	0.13
189LEU	CD2	138ILE	CD	0.28
189LEU	CD2	138ILE	C	0.05
189LEU	CD2	139TYR	CD1	16.74
189LEU	CD2	139TYR	CE1	1.41
189LEU	CD2	153ASN	CG	1.56
189LEU	C	190GLU	CA	100.00
189LEU	C	190GLU	CB	0.01
189LEU	C	190GLU	CD	0.00
189LEU	C	190GLU	C	100.00
190GLU	CA	190GLU	CB	100.00
190GLU	CA	190GLU	CG	100.00
190GLU	CA	190GLU	CD	99.07
190GLU	CA	190GLU	C	100.00
190GLU	CB	190GLU	CG	100.00
190GLU	CB	190GLU	CD	100.00
190GLU	CB	190GLU	C	100.00
190GLU	CB	144ARG	CZ	0.00
190GLU	CB	145GLY	CA	0.01
190GLU	CB	145GLY	C	0.26
190GLU	CB	146MET	CG	0.00
190GLU	CB	146MET	CE	0.00
190GLU	CG	190GLU	CD	100.00
190GLU	CG	190GLU	C	3.04
190GLU	CG	144ARG	CB	0.00
190GLU	CG	144ARG	CG	0.02
190GLU	CG	144ARG	C	0.12
190GLU	CG	145GLY	CA	0.11
190GLU	CG	145GLY	C	1.36
190GLU	CG	146MET	CB	1.24
190GLU	CG	146MET	CG	0.04
190GLU	CG	146MET	CE	0.02
190GLU	CD	144ARG	CB	0.00
190GLU	CD	144ARG	CG	0.01
190GLU	CD	145GLY	C	0.00
190GLU	CD	146MET	CB	0.00
190GLU	C	191VAL	CA	100.00
190GLU	C	191VAL	C	100.00
190GLU	C	146MET	CE	0.00
190GLU	C	151ALA	CB	0.12
191VAL	CA	191VAL	CB	100.00
191VAL	CA	191VAL	CG1	100.00
191VAL	CA	191VAL	CG2	100.00
191VAL	CA	191VAL	C	100.00
191VAL	CA	151ALA	CB	0.05
191VAL	CB	191VAL	CG1	100.00
191VAL	CB	191VAL	CG2	100.00
191VAL	CB	191VAL	C	100.00
191VAL	CG1	191VAL	CG2	100.00

158SER	CA	150GLU	CD	0.03
158SER	CB	158SER	C	100.00
158SER	CB	160PRO	CD	0.00
158SER	CB	161GLU	CB	0.00
158SER	CB	161GLU	CG	0.10
158SER	CB	161GLU	CD	0.04
158SER	CB	150GLU	CB	0.08
158SER	CB	150GLU	CG	0.33
158SER	CB	150GLU	CD	0.07
158SER	C	159LYS	CA	100.00
158SER	C	159LYS	C	100.00
158SER	C	160PRO	CD	22.78
158SER	C	194PHE	CE2	0.81
158SER	C	194PHE	CZ	0.14
159LYS	CA	159LYS	CB	100.00
159LYS	CA	159LYS	CG	100.00
159LYS	CA	159LYS	CD	9.86
159LYS	CA	159LYS	CE	0.00
159LYS	CA	159LYS	C	100.00
159LYS	CA	160PRO	CD	100.00
159LYS	CA	162VAL	CG2	0.00
159LYS	CA	194PHE	CE2	0.02
159LYS	CA	194PHE	CZ	1.12
159LYS	CB	159LYS	CG	100.00
159LYS	CB	159LYS	CD	100.00
159LYS	CB	159LYS	CE	7.95
159LYS	CB	159LYS	C	100.00
159LYS	CB	160PRO	CD	64.34
159LYS	CB	194PHE	CE2	0.02
159LYS	CB	194PHE	CZ	1.01
159LYS	CB	198THR	CG2	0.01
159LYS	CB	149ALA	CA	0.01
159LYS	CB	149ALA	CB	0.06
159LYS	CB	149ALA	C	0.01
159LYS	CG	159LYS	CD	100.00
159LYS	CG	159LYS	CE	100.00
159LYS	CG	159LYS	C	87.74
159LYS	CG	160PRO	CD	0.43
159LYS	CG	194PHE	CE1	0.17
159LYS	CG	194PHE	CE2	0.10
159LYS	CG	194PHE	CZ	2.60
159LYS	CG	198THR	CG2	2.49
159LYS	CD	159LYS	CE	100.00
159LYS	CD	159LYS	C	2.89
159LYS	CD	160PRO	CD	0.01
159LYS	CD	194PHE	CE1	0.06
159LYS	CD	194PHE	CZ	0.24
159LYS	CD	198THR	CG2	0.10
159LYS	CD	148GLU	CD	0.00
159LYS	CD	148GLU	C	0.00
159LYS	CD	149ALA	CB	0.04
159LYS	CE	163GLU	CD	0.01
159LYS	CE	194PHE	CD1	0.01
159LYS	CE	194PHE	CE1	0.20
159LYS	CE	194PHE	CZ	0.07
159LYS	CE	198THR	CG2	0.07
159LYS	CE	201GLU	CG	0.01
159LYS	CE	201GLU	CD	0.24
159LYS	CE	148GLU	CG	0.00
159LYS	CE	148GLU	CD	0.04
159LYS	CE	149ALA	CB	0.01
159LYS	C	160PRO	CA	100.00

190GL	C	151ALA	CB	0.05
191VA	CA	191VAL	CB	100.00
191VA	CA	191VAL	CG1	100.00
191VA	CA	191VAL	CG2	100.00
191VA	CA	191VAL	C	100.00
191VA	CA	194PHE	CB	0.00
191VA	CA	151ALA	CB	0.04
191VA	CB	191VAL	CG1	100.00
191VA	CB	191VAL	CG2	100.00
191VA	CB	191VAL	C	100.00
191VA	CG1	191VAL	CG2	100.00
191VA	CG1	191VAL	C	0.52
191VA	CG1	151ALA	CB	0.26
191VA	CG1	151ALA	C	6.08
191VA	CG1	152TRP	CA	7.00
191VA	CG1	152TRP	C	6.57
191VA	CG1	153ASN	CB	0.02
191VA	CG2	191VAL	C	99.46
191VA	CG2	151ALA	CB	0.00
191VA	CG2	151ALA	C	0.03
191VA	CG2	152TRP	CA	0.08
191VA	CG2	152TRP	C	0.10
191VA	CG2	153ASN	CB	0.02
191VA	C	192GLY	CA	100.00
191VA	C	192GLY	C	98.49
191VA	C	194PHE	CB	0.01
192GL	CA	192GLY	C	100.00
192GL	CA	195TRP	CB	0.02
192GL	C	193GLU	CA	100.00
192GL	C	193GLU	C	100.00
193GL	CA	193GLU	CB	100.00
193GL	CA	193GLU	CG	100.00
193GL	CA	193GLU	CD	95.73
193GL	CA	193GLU	C	100.00
193GL	CB	193GLU	CG	100.00
193GL	CB	193GLU	CD	100.00
193GL	CB	193GLU	C	100.00
193GL	CB	146MET	CE	0.12
193GL	CG	193GLU	CD	100.00
193GL	CG	193GLU	C	90.96
193GL	CG	146MET	CE	1.73
193GL	CD	193GLU	C	0.32
193GL	CD	197LYS	CE	0.05
193GL	CD	146MET	CE	0.02
193GL	C	194PHE	CA	100.00
193GL	C	194PHE	C	100.00
193GL	C	146MET	CE	0.12
194PH	CA	194PHE	CB	100.00
194PH	CA	194PHE	CG	100.00
194PH	CA	194PHE	CD1	95.00
194PH	CA	194PHE	CD2	52.41
194PH	CA	194PHE	C	100.00
194PH	CA	146MET	CE	0.64
194PH	CB	194PHE	CG	100.00
194PH	CB	194PHE	CD1	100.00
194PH	CB	194PHE	CD2	100.00
194PH	CB	194PHE	C	100.00
194PH	CB	146MET	CE	0.01
194PH	CB	151ALA	CB	20.28
194PH	CG	194PHE	CD1	100.00
194PH	CG	194PHE	CD2	100.00
194PH	CG	194PHE	CE1	100.00

191VAL	CG1	191VAL	C	3.12	159LYS	C	160PRO	CB	0.08	194PH	CG	194PHE	CE2	100.00
191VAL	CG1	151ALA	CB	0.12	159LYS	C	160PRO	CD	100.00	194PH	CG	194PHE	CZ	100.00
191VAL	CG1	151ALA	C	4.52	159LYS	C	160PRO	C	100.00	194PH	CG	194PHE	C	100.00
191VAL	CG1	152TRP	CA	5.07	160PR(	CA	160PRO	CB	100.00	194PH	CG	151ALA	CB	22.83
191VAL	CG1	152TRP	C	7.21	160PR(	CA	160PRO	CG	100.00	194PH	CD1	194PHE	CD2	100.00
191VAL	CG1	153ASN	CB	0.01	160PR(	CA	160PRO	CD	100.00	194PH	CD1	194PHE	CE1	100.00
191VAL	CG2	191VAL	C	97.08	160PR(	CA	160PRO	C	100.00	194PH	CD1	194PHE	CE2	100.00
191VAL	CG2	238ILE	CD	0.00	160PR(	CA	163GLU	CB	0.06	194PH	CD1	194PHE	CZ	100.00
191VAL	CG2	151ALA	C	0.10	160PR(	CB	160PRO	CG	100.00	194PH	CD1	194PHE	C	0.44
191VAL	CG2	152TRP	CA	0.41	160PR(	CB	160PRO	CD	100.00	194PH	CD1	197LYS	CD	0.01
191VAL	CG2	152TRP	C	0.42	160PR(	CB	160PRO	C	100.00	194PH	CD1	197LYS	CE	0.03
191VAL	CG2	153ASN	CB	0.02	160PR(	CB	164ARG	CZ	1.56	194PH	CD1	146MET	CG	0.04
191VAL	C	192GLY	CA	100.00	160PR(	CG	160PRO	CD	100.00	194PH	CD1	150GLU	C	0.03
191VAL	C	192GLY	C	98.62	160PR(	CG	160PRO	C	80.94	194PH	CD1	151ALA	CB	4.83
192GLY	CA	192GLY	C	100.00	160PR(	CG	164ARG	CZ	0.04	194PH	CD2	194PHE	CE1	100.00
192GLY	CA	195TRP	CB	0.01	160PR(	CD	160PRO	C	66.16	194PH	CD2	194PHE	CE2	100.00
192GLY	C	193GLU	CA	100.00	160PR(	C	161GLU	CA	100.00	194PH	CD2	194PHE	CZ	100.00
192GLY	C	193GLU	C	100.00	160PR(	C	161GLU	CB	0.14	194PH	CD2	194PHE	C	59.15
193GLU	CA	193GLU	CB	100.00	160PR(	C	161GLU	C	99.92	194PH	CD2	198THR	CG2	0.01
193GLU	CA	193GLU	CG	100.00	160PR(	C	163GLU	CB	0.02	194PH	CD2	151ALA	CB	2.27
193GLU	CA	193GLU	CD	94.47	160PR(	C	163GLU	CG	0.00	194PH	CE1	194PHE	CE2	100.00
193GLU	CA	193GLU	C	100.00	160PR(	C	164ARG	CD	0.00	194PH	CE1	194PHE	CZ	100.00
193GLU	CB	193GLU	CG	100.00	160PR(	C	164ARG	CZ	0.00	194PH	CE1	197LYS	CE	0.01
193GLU	CB	193GLU	CD	100.00	161GLI	CA	161GLU	CB	100.00	194PH	CE1	198THR	CG2	0.32
193GLU	CB	193GLU	C	100.00	161GLI	CA	161GLU	CG	100.00	194PH	CE1	148GLU	C	0.02
193GLU	CB	146MET	CE	0.50	161GLI	CA	161GLU	CD	97.42	194PH	CE1	149ALA	CA	0.04
193GLU	CG	193GLU	CD	100.00	161GLI	CA	161GLU	C	100.00	194PH	CE1	149ALA	C	3.37
193GLU	CG	193GLU	C	88.58	161GLI	CA	164ARG	CZ	0.03	194PH	CE1	150GLU	CA	0.69
193GLU	CG	146MET	CE	2.44	161GLI	CB	161GLU	CG	100.00	194PH	CE1	150GLU	C	4.24
193GLU	CD	193GLU	C	0.18	161GLI	CB	161GLU	CD	100.00	194PH	CE2	194PHE	CZ	100.00
193GLU	CD	197LYS	CE	0.05	161GLI	CB	161GLU	C	100.00	194PH	CE2	198THR	CG2	0.95
193GLU	CD	146MET	CE	0.02	161GLI	CG	161GLU	CD	100.00	194PH	CE2	150GLU	CA	0.02
193GLU	C	194PHE	CA	100.00	161GLI	CG	161GLU	C	6.89	194PH	CE2	150GLU	C	0.02
193GLU	C	194PHE	CB	0.00	161GLI	CG	164ARG	CZ	0.01	194PH	CE2	151ALA	CB	0.00
193GLU	C	194PHE	C	100.00	161GLI	CD	161GLU	C	0.06	194PH	CZ	198THR	CG2	1.62
193GLU	C	146MET	CE	0.10	161GLI	CD	164ARG	CD	0.00	194PH	CZ	149ALA	CA	0.03
194PHE	CA	194PHE	CB	100.00	161GLI	CD	164ARG	CZ	0.28	194PH	CZ	149ALA	C	14.95
194PHE	CA	194PHE	CG	100.00	161GLI	C	162VAL	CA	100.00	194PH	CZ	150GLU	CA	1.78
194PHE	CA	194PHE	CD1	91.77	161GLI	C	162VAL	CB	1.08	194PH	CZ	150GLU	C	0.30
194PHE	CA	194PHE	CD2	62.66	161GLI	C	162VAL	CG1	0.49	194PH	C	195TRP	CA	100.00
194PHE	CA	194PHE	C	100.00	161GLI	C	162VAL	CG2	0.11	194PH	C	195TRP	C	100.00
194PHE	CA	146MET	CE	0.57	161GLI	C	162VAL	C	99.05	195TR	CA	195TRP	CB	100.00
194PHE	CB	194PHE	CG	100.00	162VAI	CA	162VAL	CB	100.00	195TR	CA	195TRP	CG	100.00
194PHE	CB	194PHE	CD1	100.00	162VAI	CA	162VAL	CG1	100.00	195TR	CA	195TRP	CD1	83.25
194PHE	CB	194PHE	CD2	100.00	162VAI	CA	162VAL	CG2	100.00	195TR	CA	195TRP	CD2	14.82
194PHE	CB	194PHE	C	100.00	162VAI	CA	162VAL	C	100.00	195TR	CA	195TRP	CE3	0.20
194PHE	CB	146MET	CE	0.06	162VAI	CA	165VAL	CG1	0.04	195TR	CA	195TRP	C	100.00
194PHE	CB	151ALA	CB	22.24	162VAI	CA	165VAL	CG2	0.00	195TR	CB	195TRP	CG	100.00
194PHE	CG	194PHE	CD1	100.00	162VAI	CA	195TRP	CE3	0.00	195TR	CB	195TRP	CD1	100.00
194PHE	CG	194PHE	CD2	100.00	162VAI	CB	162VAL	CG1	100.00	195TR	CB	195TRP	CD2	100.00
194PHE	CG	194PHE	CE1	100.00	162VAI	CB	162VAL	CG2	100.00	195TR	CB	195TRP	CE3	97.44
194PHE	CG	194PHE	CE2	100.00	162VAI	CB	162VAL	C	100.00	195TR	CB	195TRP	C	100.00
194PHE	CG	194PHE	CZ	100.00	162VAI	CB	195TRP	CE3	0.19	195TR	CG	195TRP	CD1	100.00
194PHE	CG	194PHE	C	100.00	162VAI	CB	195TRP	CZ3	0.12	195TR	CG	195TRP	CD2	100.00
194PHE	CG	151ALA	CB	16.78	162VAI	CB	198THR	CG2	0.53	195TR	CG	195TRP	CE2	100.00
194PHE	CD1	194PHE	CD2	100.00	162VAI	CG1	162VAL	CG2	100.00	195TR	CG	195TRP	CE3	100.00
194PHE	CD1	194PHE	CE1	100.00	162VAI	CG1	162VAL	C	84.76	195TR	CG	195TRP	C	100.00
194PHE	CD1	194PHE	CE2	100.00	162VAI	CG1	195TRP	CA	0.00	195TR	CD1	195TRP	CD2	100.00
194PHE	CD1	194PHE	CZ	100.00	162VAI	CG1	195TRP	CG	0.06	195TR	CD1	195TRP	CE2	100.00
194PHE	CD1	194PHE	C	1.35	162VAI	CG1	195TRP	CD2	5.69	195TR	CD1	195TRP	C	86.62
194PHE	CD1	197LYS	CD	0.00	162VAI	CG1	195TRP	CE2	0.20	195TR	CD1	199VAL	CG1	1.54
194PHE	CD1	197LYS	CE	0.20	162VAI	CG1	195TRP	CE3	17.60	195TR	CD1	199VAL	CG2	0.22

194PHE	CD1	146MET CG	0.02	162VAl	CG1	195TRP CZ2	0.02	195TR	CD1	213HIS CE1	0.00
194PHE	CD1	146MET CE	0.07	162VAl	CG1	195TRP CZ3	1.99	195TR	CD2	195TRP CE2	100.00
194PHE	CD1	150GLU C	0.05	162VAl	CG1	198THR CB	0.77	195TR	CD2	195TRP CE3	100.00
194PHE	CD1	151ALA CB	8.57	162VAl	CG1	198THR CG2	4.97	195TR	CD2	195TRP CZ2	100.00
194PHE	CD2	194PHE CE1	100.00	162VAl	CG1	199VAL CG1	1.33	195TR	CD2	195TRP CZ3	100.00
194PHE	CD2	194PHE CE2	100.00	162VAl	CG1	199VAL CG2	3.28	195TR	CD2	195TRP C	0.08
194PHE	CD2	194PHE CZ	100.00	162VAl	CG2	162VAL C	15.19	195TR	CD2	199VAL CG1	0.02
194PHE	CD2	194PHE C	62.30	162VAl	CG2	194PHE CD2	0.03	195TR	CE2	195TRP CE3	100.00
194PHE	CD2	198THR CG2	0.05	162VAl	CG2	194PHE CE2	0.29	195TR	CE2	195TRP CZ2	100.00
194PHE	CD2	150GLU C	0.00	162VAl	CG2	195TRP CE3	5.71	195TR	CE2	195TRP CZ3	100.00
194PHE	CD2	151ALA CB	1.17	162VAl	CG2	195TRP CZ3	1.39	195TR	CE2	199VAL CG1	1.61
194PHE	CE1	194PHE CE2	100.00	162VAl	CG2	198THR CB	0.22	195TR	CE2	199VAL CG2	0.17
194PHE	CE1	194PHE CZ	100.00	162VAl	CG2	198THR CG2	0.84	195TR	CE3	195TRP CZ2	100.00
194PHE	CE1	197LYS CD	0.01	162VAl	C	163GLU CA	100.00	195TR	CE3	195TRP CZ3	100.00
194PHE	CE1	197LYS CE	0.01	162VAl	C	163GLU CB	0.56	195TR	CE3	198THR CG2	0.01
194PHE	CE1	198THR CG2	0.03	162VAl	C	163GLU C	99.58	195TR	CE3	199VAL CG1	0.01
194PHE	CE1	148GLU C	0.06	162VAl	C	165VAL CG1	0.08	195TR	CZ2	195TRP CZ3	100.00
194PHE	CE1	149ALA CA	0.05	162VAl	C	165VAL CG2	0.00	195TR	CZ2	199VAL CG1	1.10
194PHE	CE1	149ALA C	3.84	162VAl	C	198THR CG2	0.07	195TR	CZ2	199VAL CG2	0.01
194PHE	CE1	150GLU CA	1.42	163GLl	CA	163GLU CB	100.00	195TR	CZ2	234VAL CG2	0.04
194PHE	CE1	150GLU C	7.75	163GLl	CA	163GLU CG	100.00	195TR	CZ2	234VAL C	0.01
194PHE	CE1	151ALA CB	0.00	163GLl	CA	163GLU CD	17.66	195TR	CZ2	235THR CA	1.23
194PHE	CE2	194PHE CZ	100.00	163GLl	CA	163GLU C	100.00	195TR	CZ2	235THR C	1.85
194PHE	CE2	198THR CG2	1.40	163GLl	CA	198THR CG2	0.20	195TR	CZ3	198THR CG2	0.03
194PHE	CE2	149ALA C	0.01	163GLl	CB	163GLU CG	100.00	195TR	CZ3	199VAL CG1	0.03
194PHE	CE2	150GLU CA	0.04	163GLl	CB	163GLU CD	100.00	195TR	CZ3	235THR C	0.01
194PHE	CE2	150GLU C	0.01	163GLl	CB	163GLU C	100.00	195TR	C	196ARG CA	100.00
194PHE	CZ	198THR CG2	0.32	163GLl	CB	167ARG CZ	0.03	195TR	C	196ARG C	100.00
194PHE	CZ	148GLU C	0.00	163GLl	CB	198THR CG2	0.70	196AR	CA	196ARG CB	100.00
194PHE	CZ	149ALA CA	0.03	163GLl	CB	202VAL CG1	0.00	196AR	CA	196ARG CG	100.00
194PHE	CZ	149ALA C	16.06	163GLl	CB	202VAL CG2	0.01	196AR	CA	196ARG CD	2.02
194PHE	CZ	150GLU CA	1.32	163GLl	CG	163GLU CD	100.00	196AR	CA	196ARG C	100.00
194PHE	CZ	150GLU C	0.16	163GLl	CG	163GLU C	92.49	196AR	CA	199VAL CG1	0.01
194PHE	C	195TRP CA	100.00	163GLl	CG	167ARG CG	0.04	196AR	CA	199VAL CG2	0.11
194PHE	C	195TRP C	100.00	163GLl	CG	167ARG CD	0.08	196AR	CA	213HIS CE1	0.28
195TRP	CA	195TRP CB	100.00	163GLl	CG	167ARG CZ	0.04	196AR	CB	196ARG CG	100.00
195TRP	CA	195TRP CG	100.00	163GLl	CG	198THR CG2	0.81	196AR	CB	196ARG CD	100.00
195TRP	CA	195TRP CD1	51.69	163GLl	CG	202VAL CB	0.08	196AR	CB	196ARG CZ	0.03
195TRP	CA	195TRP CD2	47.73	163GLl	CG	202VAL CG1	0.46	196AR	CB	196ARG C	100.00
195TRP	CA	195TRP CE3	0.71	163GLl	CG	202VAL CG2	8.83	196AR	CB	213HIS CD2	0.00
195TRP	CA	195TRP C	100.00	163GLl	CD	163GLU C	15.56	196AR	CB	213HIS CE1	0.10
195TRP	CB	195TRP CG	100.00	163GLl	CD	167ARG CG	0.22	196AR	CG	196ARG CD	100.00
195TRP	CB	195TRP CD1	100.00	163GLl	CD	167ARG CD	0.61	196AR	CG	196ARG CZ	26.40
195TRP	CB	195TRP CD2	100.00	163GLl	CD	167ARG CZ	0.25	196AR	CG	196ARG C	28.19
195TRP	CB	195TRP CE3	96.63	163GLl	CD	202VAL CG1	0.05	196AR	CG	213HIS CG	0.16
195TRP	CB	195TRP C	100.00	163GLl	CD	202VAL CG2	0.51	196AR	CG	213HIS CD2	0.30
195TRP	CG	195TRP CD1	100.00	163GLl	C	164ARG CA	100.00	196AR	CG	213HIS CE1	4.10
195TRP	CG	195TRP CD2	100.00	163GLl	C	164ARG CB	0.00	196AR	CD	196ARG CZ	100.00
195TRP	CG	195TRP CE2	100.00	163GLl	C	164ARG C	100.00	196AR	CD	213HIS CB	0.01
195TRP	CG	195TRP CE3	100.00	164AR(	CA	164ARG CB	100.00	196AR	CD	213HIS CG	0.12
195TRP	CG	195TRP C	100.00	164AR(	CA	164ARG CG	100.00	196AR	CD	213HIS CD2	0.31
195TRP	CG	199VAL CG2	0.01	164AR(	CA	164ARG CD	0.01	196AR	CD	213HIS CE1	0.53
195TRP	CD1	195TRP CD2	100.00	164AR(	CA	164ARG C	100.00	196AR	CZ	213HIS CB	6.12
195TRP	CD1	195TRP CE2	100.00	164AR(	CA	167ARG CZ	0.06	196AR	CZ	213HIS CG	4.00
195TRP	CD1	195TRP C	66.55	164AR(	CB	164ARG CG	100.00	196AR	CZ	213HIS CD2	9.14
195TRP	CD1	199VAL CG1	0.06	164AR(	CB	164ARG CD	100.00	196AR	CZ	213HIS CE1	6.43
195TRP	CD1	199VAL CG2	0.05	164AR(	CB	164ARG CZ	18.62	196AR	CZ	215TYR CE1	0.08
195TRP	CD1	213HIS CE1	0.01	164AR(	CB	164ARG C	100.00	196AR	CZ	215TYR CZ	0.00
195TRP	CD2	195TRP CE2	100.00	164AR(	CG	164ARG CD	100.00	196AR	C	197LYS CA	100.00
195TRP	CD2	195TRP CE3	100.00	164AR(	CG	164ARG CZ	35.60	196AR	C	197LYS CB	1.53
195TRP	CD2	195TRP CZ2	100.00	164AR(	CG	164ARG C	80.75	196AR	C	197LYS CG	0.02
195TRP	CD2	195TRP CZ3	100.00	164AR(	CG	167ARG CZ	0.12	196AR	C	197LYS C	98.35

195TRP	CD2	195TRP	C	0.29
195TRP	CD2	199VAL	CG1	0.10
195TRP	CD2	199VAL	CG2	2.76
195TRP	CE2	195TRP	CE3	100.00
195TRP	CE2	195TRP	CZ2	100.00
195TRP	CE2	195TRP	CZ3	100.00
195TRP	CE2	199VAL	CB	0.01
195TRP	CE2	199VAL	CG1	6.05
195TRP	CE2	199VAL	CG2	6.08
195TRP	CE3	195TRP	CZ2	100.00
195TRP	CE3	195TRP	CZ3	100.00
195TRP	CE3	198THR	CB	0.02
195TRP	CE3	198THR	CG2	0.04
195TRP	CE3	199VAL	CG1	0.03
195TRP	CE3	199VAL	CG2	0.80
195TRP	CZ2	195TRP	CZ3	100.00
195TRP	CZ2	199VAL	CB	0.00
195TRP	CZ2	199VAL	CG1	4.08
195TRP	CZ2	199VAL	CG2	1.60
195TRP	CZ2	234VAL	CG1	0.00
195TRP	CZ2	234VAL	CG2	0.37
195TRP	CZ2	234VAL	C	0.04
195TRP	CZ2	235THR	CA	1.73
195TRP	CZ2	235THR	C	2.16
195TRP	CZ3	198THR	CG2	0.10
195TRP	CZ3	199VAL	CG1	0.06
195TRP	CZ3	199VAL	CG2	0.23
195TRP	CZ3	235THR	C	0.00
195TRP	CZ3	236GLY	CA	0.00
195TRP	C	196ARG	CA	100.00
195TRP	C	196ARG	C	100.00
195TRP	C	199VAL	CG1	0.00
196ARG	CA	196ARG	CB	100.00
196ARG	CA	196ARG	CG	100.00
196ARG	CA	196ARG	CD	1.03
196ARG	CA	196ARG	C	100.00
196ARG	CA	199VAL	CG1	0.00
196ARG	CA	199VAL	CG2	0.04
196ARG	CA	213HIS	CE1	0.21
196ARG	CB	196ARG	CG	100.00
196ARG	CB	196ARG	CD	100.00
196ARG	CB	196ARG	C	100.00
196ARG	CB	213HIS	CE1	0.20
196ARG	CG	196ARG	CD	100.00
196ARG	CG	196ARG	CZ	33.15
196ARG	CG	196ARG	C	55.14
196ARG	CG	213HIS	CB	0.00
196ARG	CG	213HIS	CG	0.52
196ARG	CG	213HIS	CD2	0.49
196ARG	CG	213HIS	CE1	4.36
196ARG	CD	196ARG	CZ	100.00
196ARG	CD	213HIS	CB	0.02
196ARG	CD	213HIS	CG	0.22
196ARG	CD	213HIS	CD2	0.26
196ARG	CD	213HIS	CE1	0.18
196ARG	CZ	213HIS	CB	8.90
196ARG	CZ	213HIS	CG	1.55
196ARG	CZ	213HIS	CD2	3.26
196ARG	CZ	213HIS	CE1	0.03
196ARG	CZ	215TYR	CE1	0.29
196ARG	C	197LYS	CA	100.00
196ARG	C	197LYS	CB	0.76
164AR(	CD	164ARG	CZ	100.00
164AR(	CD	164ARG	C	0.01
164AR(	CD	264ARG	CZ	0.02
164AR(	CZ	264ARG	CD	0.06
164AR(	CZ	264ARG	CZ	0.54
164AR(	C	165VAL	CA	100.00
164AR(	C	165VAL	CB	0.52
164AR(	C	165VAL	CG1	0.06
164AR(	C	165VAL	CG2	0.01
164AR(	C	165VAL	C	99.64
165VAI	CA	165VAL	CB	100.00
165VAI	CA	165VAL	CG1	100.00
165VAI	CA	165VAL	CG2	100.00
165VAI	CA	165VAL	C	100.00
165VAI	CA	168VAL	CG2	0.03
165VAI	CB	165VAL	CG1	100.00
165VAI	CB	165VAL	CG2	100.00
165VAI	CB	165VAL	C	100.00
165VAI	CG1	165VAL	CG2	100.00
165VAI	CG1	165VAL	C	92.02
165VAI	CG1	195TRP	CZ2	0.09
165VAI	CG1	195TRP	CZ3	0.11
165VAI	CG1	234VAL	CG1	0.26
165VAI	CG1	234VAL	CG2	0.16
165VAI	CG2	165VAL	C	81.48
165VAI	CG2	195TRP	CZ2	0.01
165VAI	CG2	234VAL	CG1	0.11
165VAI	CG2	234VAL	CG2	0.06
165VAI	C	166ALA	CA	100.00
165VAI	C	166ALA	CB	0.04
165VAI	C	166ALA	C	99.99
165VAI	C	168VAL	CG1	0.00
165VAI	C	168VAL	CG2	0.10
165VAI	C	234VAL	CG1	0.01
165VAI	C	234VAL	CG2	0.00
166AL/	CA	166ALA	CB	100.00
166AL/	CA	166ALA	C	100.00
166AL/	CA	234VAL	CG1	0.02
166AL/	CB	166ALA	C	100.00
166AL/	CB	170PHE	CE1	0.09
166AL/	CB	170PHE	CE2	0.03
166AL/	CB	170PHE	CZ	0.10
166AL/	CB	199VAL	CB	0.00
166AL/	CB	199VAL	CG1	0.16
166AL/	CB	199VAL	CG2	0.08
166AL/	CB	202VAL	CB	0.00
166AL/	CB	202VAL	CG1	0.82
166AL/	CB	202VAL	CG2	0.66
166AL/	C	167ARG	CA	100.00
166AL/	C	167ARG	C	100.00
166AL/	C	170PHE	CD1	0.24
166AL/	C	170PHE	CD2	0.12
166AL/	C	170PHE	CE1	0.04
166AL/	C	170PHE	CE2	0.02
166AL/	C	202VAL	CG1	0.02
167AR(	CA	167ARG	CB	100.00
167AR(	CA	167ARG	CG	100.00
167AR(	CA	167ARG	CD	11.75
167AR(	CA	167ARG	C	100.00
167AR(	CA	170PHE	CD1	0.00
167AR(	CA	170PHE	CD2	0.01
167AR(	CA	202VAL	CG1	0.10
196AR	C	199VAL	CG1	0.00
196AR	C	199VAL	CG2	0.02
196AR	C	213HIS	CE1	0.00
197LY:	CA	197LYS	CB	100.00
197LY:	CA	197LYS	CG	100.00
197LY:	CA	197LYS	CD	1.92
197LY:	CA	197LYS	C	100.00
197LY:	CA	200GLU	CB	0.00
197LY:	CB	197LYS	CG	100.00
197LY:	CB	197LYS	CD	100.00
197LY:	CB	197LYS	CE	12.49
197LY:	CB	197LYS	C	100.00
197LY:	CG	197LYS	CD	100.00
197LY:	CG	197LYS	CE	100.00
197LY:	CG	197LYS	C	87.41
197LY:	CD	197LYS	CE	100.00
197LY:	CD	197LYS	C	1.00
197LY:	CD	146MET	CE	0.86
197LY:	CE	146MET	CE	0.09
197LY:	CE	148GLU	CD	0.22
197LY:	C	198THR	CA	100.00
197LY:	C	198THR	CB	6.30
197LY:	C	198THR	CG2	2.06
197LY:	C	198THR	C	94.79
197LY:	C	200GLU	CB	0.04
198TH	CA	198THR	CB	100.00
198TH	CA	198THR	CG2	100.00
198TH	CA	198THR	C	100.00
198TH	CB	198THR	CG2	100.00
198TH	CB	198THR	C	100.00
198TH	CG2	198THR	C	77.37
198TH	C	199VAL	CA	100.00
198TH	C	199VAL	CB	0.08
198TH	C	199VAL	CG1	0.03
198TH	C	199VAL	CG2	0.00
198TH	C	199VAL	C	99.92
199VA	CA	199VAL	CB	100.00
199VA	CA	199VAL	CG1	100.00
199VA	CA	199VAL	CG2	100.00
199VA	CA	199VAL	C	100.00
199VA	CA	202VAL	CG1	0.02
199VA	CA	202VAL	CG2	0.00
199VA	CB	199VAL	CG1	100.00
199VA	CB	199VAL	CG2	100.00
199VA	CB	199VAL	C	100.00
199VA	CB	211LEU	CD1	0.06
199VA	CB	211LEU	CD2	0.15
199VA	CG1	199VAL	CG2	100.00
199VA	CG1	199VAL	C	12.20
199VA	CG1	211LEU	CD1	1.09
199VA	CG1	211LEU	CD2	0.12
199VA	CG1	213HIS	CE1	0.10
199VA	CG2	199VAL	C	90.06
199VA	CG2	211LEU	CD1	0.39
199VA	CG2	211LEU	CD2	4.66
199VA	CG2	213HIS	CE1	0.84
199VA	C	200GLU	CA	100.00
199VA	C	200GLU	CB	0.02
199VA	C	200GLU	C	100.00
199VA	C	202VAL	CG1	0.00
199VA	C	211LEU	CD1	0.02
199VA	C	211LEU	CD2	0.34

196ARG C	197LYS	CG	0.01	167AR(CB	167ARG	CG	100.00	200GL CA	200GLU	CB	100.00
196ARG C	197LYS	C	99.28	167AR(CB	167ARG	CD	100.00	200GL CA	200GLU	CG	100.00
196ARG C	199VAL	CG2	0.02	167AR(CB	167ARG	CZ	1.87	200GL CA	200GLU	CD	65.48
197LYS CA	197LYS	CB	100.00	167AR(CB	167ARG	C	100.00	200GL CA	200GLU	C	100.00
197LYS CA	197LYS	CG	100.00	167AR(CB	202VAL	CG1	0.02	200GL CA	211LEU	CD1	0.01
197LYS CA	197LYS	CD	5.17	167AR(CG	167ARG	CD	100.00	200GL CA	211LEU	CD2	0.75
197LYS CA	197LYS	CE	0.01	167AR(CG	167ARG	CZ	43.59	200GL CB	200GLU	CG	100.00
197LYS CA	197LYS	C	100.00	167AR(CG	167ARG	C	32.85	200GL CB	200GLU	CD	100.00
197LYS CB	197LYS	CG	100.00	167AR(CG	202VAL	CG1	1.36	200GL CB	200GLU	C	100.00
197LYS CB	197LYS	CD	100.00	167AR(CG	202VAL	CG2	0.23	200GL CB	211LEU	CD2	0.08
197LYS CB	197LYS	CE	11.94	167AR(CG	206TYR	CE2	0.11	200GL CG	200GLU	CD	100.00
197LYS CB	197LYS	C	100.00	167AR(CG	206TYR	CZ	0.02	200GL CG	200GLU	C	98.73
197LYS CG	197LYS	CD	100.00	167AR(CD	167ARG	CZ	100.00	200GL CG	211LEU	CD2	0.05
197LYS CG	197LYS	CE	100.00	167AR(CD	167ARG	C	1.10	200GL CD	200GLU	C	62.20
197LYS CG	197LYS	C	75.08	167AR(CD	202VAL	CG1	0.06	200GL CD	204ARG	CD	0.00
197LYS CD	197LYS	CE	100.00	167AR(CD	202VAL	CG2	0.01	200GL CD	204ARG	CZ	0.02
197LYS CD	197LYS	C	1.34	167AR(CD	206TYR	CE2	0.01	200GL C	201GLU	CA	100.00
197LYS CD	146MET	CE	0.62	167AR(CD	206TYR	CZ	0.00	200GL C	201GLU	C	100.00
197LYS CE	146MET	CE	0.12	167AR(CZ	168VAL	CG1	0.01	200GL C	204ARG	CZ	0.01
197LYS CE	148GLU	CG	0.00	167AR(CZ	202VAL	CA	0.00	201GL CA	201GLU	CB	100.00
197LYS CE	148GLU	CD	0.12	167AR(CZ	202VAL	CB	0.01	201GL CA	201GLU	CG	100.00
197LYS C	198THR	CA	100.00	167AR(CZ	202VAL	CG1	0.92	201GL CA	201GLU	CD	83.95
197LYS C	198THR	CB	1.08	167AR(CZ	202VAL	CG2	3.24	201GL CA	201GLU	C	100.00
197LYS C	198THR	CG2	0.13	167AR(CZ	206TYR	CE2	0.05	201GL CA	204ARG	CZ	1.26
197LYS C	198THR	C	99.30	167AR(C	168VAL	CA	100.00	201GL CB	201GLU	CG	100.00
198THR CA	198THR	CB	100.00	167AR(C	168VAL	CB	0.08	201GL CB	201GLU	CD	100.00
198THR CA	198THR	CG2	100.00	167AR(C	168VAL	CG1	0.02	201GL CB	201GLU	C	100.00
198THR CA	198THR	C	100.00	167AR(C	168VAL	C	99.95	201GL CB	204ARG	CZ	0.01
198THR CB	198THR	CG2	100.00	168VAI CA	168VAL	CB	100.00	201GL CG	201GLU	CD	100.00
198THR CB	198THR	C	100.00	168VAI CA	168VAL	CG1	100.00	201GL CG	201GLU	C	92.67
198THR CG2	198THR	C	74.98	168VAI CA	168VAL	CG2	100.00	201GL CG	204ARG	CZ	0.14
198THR CG2	202VAL	CG2	0.00	168VAI CA	168VAL	C	100.00	201GL CD	201GLU	C	0.52
198THR C	199VAL	CA	100.00	168VAI CB	168VAL	CG1	100.00	201GL CD	204ARG	CZ	0.81
198THR C	199VAL	CB	0.04	168VAI CB	168VAL	CG2	100.00	201GL C	202VAL	CA	100.00
198THR C	199VAL	CG2	0.02	168VAI CB	168VAL	C	100.00	201GL C	202VAL	CB	0.14
198THR C	199VAL	C	100.00	168VAI CB	262LEU	CD1	0.33	201GL C	202VAL	CG2	0.05
199VAL CA	199VAL	CB	100.00	168VAI CB	262LEU	CD2	0.03	201GL C	202VAL	C	99.92
199VAL CA	199VAL	CG1	100.00	168VAI CG1	168VAL	CG2	100.00	202VA CA	202VAL	CB	100.00
199VAL CA	199VAL	CG2	100.00	168VAI CG1	168VAL	C	27.74	202VA CA	202VAL	CG1	100.00
199VAL CA	199VAL	C	100.00	168VAI CG1	262LEU	CD1	2.54	202VA CA	202VAL	CG2	100.00
199VAL CA	202VAL	CG2	0.04	168VAI CG1	262LEU	CD2	0.49	202VA CA	202VAL	C	100.00
199VAL CB	199VAL	CG1	100.00	168VAI CG2	168VAL	C	72.95	202VA CB	202VAL	CG1	100.00
199VAL CB	199VAL	CG2	100.00	168VAI CG2	262LEU	CD1	1.15	202VA CB	202VAL	CG2	100.00
199VAL CB	199VAL	C	100.00	168VAI CG2	262LEU	CD2	0.16	202VA CB	202VAL	C	100.00
199VAL CB	211LEU	CD1	0.02	168VAI C	169ALA	CA	100.00	202VA CG1	202VAL	CG2	100.00
199VAL CB	211LEU	CD2	0.02	168VAI C	169ALA	CB	0.07	202VA CG1	202VAL	C	99.91
199VAL CG1	199VAL	CG2	100.00	168VAI C	169ALA	C	99.96	202VA CG1	206TYR	CD1	2.41
199VAL CG1	199VAL	C	48.18	169AL/ CA	169ALA	CB	100.00	202VA CG1	206TYR	CE1	0.60
199VAL CG1	211LEU	CD1	1.97	169AL/ CA	169ALA	C	100.00	202VA CG2	202VAL	C	1.62
199VAL CG1	211LEU	CD2	0.42	169AL/ CA	232VAL	CG1	0.05	202VA CG2	206TYR	CD1	0.01
199VAL CG1	213HIS	CE1	0.09	169AL/ CB	169ALA	C	100.00	202VA CG2	206TYR	CE1	0.00
199VAL CG2	199VAL	C	61.38	169AL/ CB	180VAL	CG1	0.02	202VA C	203GLY	CA	100.00
199VAL CG2	211LEU	CD1	0.18	169AL/ CB	180VAL	CG2	0.02	202VA C	203GLY	C	99.56
199VAL CG2	211LEU	CD2	1.48	169AL/ CB	232VAL	CG1	1.95	203GL CA	203GLY	C	100.00
199VAL CG2	213HIS	CE1	1.12	169AL/ CB	234VAL	CB	0.19	203GL CA	211LEU	CD2	0.00
199VAL CG2	234VAL	CG1	0.00	169AL/ CB	234VAL	CG1	0.84	203GL C	204ARG	CA	100.00
199VAL C	200GLU	CA	100.00	169AL/ CB	234VAL	CG2	49.22	203GL C	204ARG	CB	1.75
199VAL C	200GLU	CB	0.08	169AL/ C	170PHE	CA	100.00	203GL C	204ARG	CG	0.17
199VAL C	200GLU	C	99.98	169AL/ C	170PHE	C	100.00	203GL C	204ARG	C	97.54
199VAL C	202VAL	CG2	0.02	169AL/ C	180VAL	CG2	0.01	204AR CA	204ARG	CB	100.00
199VAL C	211LEU	CD1	0.01	170PHI CA	170PHE	CB	100.00	204AR CA	204ARG	CG	100.00
199VAL C	211LEU	CD2	0.26	170PHI CA	170PHE	CG	100.00	204AR CA	204ARG	C	100.00

200GLU CA	200GLU CB	100.00	170PHE CA	170PHE CD1	70.46	204AR CB	204ARG CG	100.00
200GLU CA	200GLU CG	100.00	170PHE CA	170PHE CD2	74.06	204AR CB	204ARG CD	100.00
200GLU CA	200GLU CD	70.68	170PHE CA	170PHE C	100.00	204AR CB	204ARG CZ	7.35
200GLU CA	200GLU C	100.00	170PHE CA	209VAL CG2	0.00	204AR CB	204ARG C	100.00
200GLU CA	211LEU CD1	0.00	170PHE CB	170PHE CG	100.00	204AR CG	204ARG CD	100.00
200GLU CA	211LEU CD2	0.85	170PHE CB	170PHE CD1	100.00	204AR CG	204ARG CZ	61.76
200GLU CB	200GLU CG	100.00	170PHE CB	170PHE CD2	100.00	204AR CG	204ARG C	2.11
200GLU CB	200GLU CD	100.00	170PHE CB	170PHE C	100.00	204AR CD	204ARG CZ	100.00
200GLU CB	200GLU C	100.00	170PHE CB	202VAL CG1	0.02	204AR C	205GLY CA	100.00
200GLU CB	211LEU CD2	0.08	170PHE CB	206TYR CB	0.07	204AR C	205GLY C	71.99
200GLU CG	200GLU CD	100.00	170PHE CB	206TYR CG	2.59	205GL CA	205GLY C	100.00
200GLU CG	200GLU C	93.50	170PHE CB	206TYR CD1	2.82	205GL C	206TYR CA	100.00
200GLU CG	204ARG CZ	0.57	170PHE CB	206TYR CD2	3.91	205GL C	206TYR CB	68.70
200GLU CG	211LEU CD2	0.08	170PHE CB	206TYR CE1	2.38	205GL C	206TYR CG	11.61
200GLU CG	213HIS CE1	0.01	170PHE CB	206TYR CE2	2.22	205GL C	206TYR CD1	27.05
200GLU CD	200GLU C	46.11	170PHE CB	206TYR CZ	1.96	205GL C	206TYR CD2	0.02
200GLU CD	204ARG CZ	0.02	170PHE CB	209VAL CG2	0.03	205GL C	206TYR C	36.18
200GLU CD	211LEU CD2	0.17	170PHE CG	170PHE CD1	100.00	205GL C	207PRO CD	24.27
200GLU C	201GLU CA	100.00	170PHE CG	170PHE CD2	100.00	206TY CA	206TYR CB	100.00
200GLU C	201GLU CB	0.03	170PHE CG	170PHE CE1	100.00	206TY CA	206TYR CG	100.00
200GLU C	201GLU C	99.96	170PHE CG	170PHE CE2	100.00	206TY CA	206TYR CD1	83.82
200GLU C	204ARG CZ	0.00	170PHE CG	170PHE CZ	100.00	206TY CA	206TYR CD2	47.79
201GLU CA	201GLU CB	100.00	170PHE CG	170PHE C	0.02	206TY CA	206TYR C	100.00
201GLU CA	201GLU CG	100.00	170PHE CG	180VAL CG2	0.01	206TY CA	207PRO CD	100.00
201GLU CA	201GLU CD	79.96	170PHE CG	202VAL CG1	1.26	206TY CB	206TYR CG	100.00
201GLU CA	201GLU C	100.00	170PHE CG	206TYR CB	0.04	206TY CB	206TYR CD1	100.00
201GLU CA	204ARG CZ	1.51	170PHE CG	206TYR CG	0.06	206TY CB	206TYR CD2	100.00
201GLU CB	201GLU CG	100.00	170PHE CG	206TYR CD1	0.86	206TY CB	206TYR C	100.00
201GLU CB	201GLU CD	100.00	170PHE CG	206TYR CD2	0.40	206TY CB	209VAL CG2	0.07
201GLU CB	201GLU C	100.00	170PHE CG	206TYR CE1	0.22	206TY CG	206TYR CD1	100.00
201GLU CB	204ARG CZ	0.02	170PHE CG	206TYR CE2	0.03	206TY CG	206TYR CD2	100.00
201GLU CG	201GLU CD	100.00	170PHE CG	206TYR CZ	0.02	206TY CG	206TYR CE1	100.00
201GLU CG	201GLU C	97.22	170PHE CG	209VAL CG1	0.02	206TY CG	206TYR CE2	100.00
201GLU CG	204ARG CZ	0.14	170PHE CG	209VAL CG2	0.06	206TY CG	206TYR CZ	100.00
201GLU CD	201GLU C	0.63	170PHE CD1	170PHE CD2	100.00	206TY CD1	206TYR CD2	100.00
201GLU CD	204ARG CZ	0.55	170PHE CD1	170PHE CE1	100.00	206TY CD1	206TYR CE1	100.00
201GLU C	202VAL CA	100.00	170PHE CD1	170PHE CE2	100.00	206TY CD1	206TYR CE2	100.00
201GLU C	202VAL CB	0.58	170PHE CD1	170PHE CZ	100.00	206TY CD1	206TYR CZ	100.00
201GLU C	202VAL CG1	0.25	170PHE CD1	180VAL CG1	0.09	206TY CD2	206TYR CE1	100.00
201GLU C	202VAL CG2	0.05	170PHE CD1	180VAL CG2	0.40	206TY CD2	206TYR CE2	100.00
201GLU C	202VAL C	99.40	170PHE CD1	202VAL CG1	6.97	206TY CD2	206TYR CZ	100.00
202VAL CA	202VAL CB	100.00	170PHE CD1	202VAL CG2	0.00	206TY CD2	209VAL CG2	0.04
202VAL CA	202VAL CG1	100.00	170PHE CD1	203GLY CA	0.00	206TY CE1	206TYR CE2	100.00
202VAL CA	202VAL CG2	100.00	170PHE CD1	206TYR CB	6.06	206TY CE1	206TYR CZ	100.00
202VAL CA	202VAL C	100.00	170PHE CD1	206TYR CG	2.18	206TY CE2	206TYR CZ	100.00
202VAL CB	202VAL CG1	100.00	170PHE CD1	206TYR CD1	1.27	206TY C	207PRO CA	100.00
202VAL CB	202VAL CG2	100.00	170PHE CD1	206TYR CD2	1.93	206TY C	207PRO CB	0.26
202VAL CB	202VAL C	100.00	170PHE CD1	206TYR CE1	0.36	206TY C	207PRO CD	100.00
202VAL CG1	202VAL CG2	100.00	170PHE CD1	206TYR CE2	0.35	206TY C	207PRO C	100.00
202VAL CG1	202VAL C	56.07	170PHE CD1	206TYR CZ	0.09	207PR CA	207PRO CB	100.00
202VAL CG1	206TYR CD1	0.40	170PHE CD1	209VAL CB	0.42	207PR CA	207PRO CG	100.00
202VAL CG1	206TYR CE1	0.13	170PHE CD1	209VAL CG1	9.02	207PR CA	207PRO CD	100.00
202VAL CG2	202VAL C	44.30	170PHE CD1	209VAL CG2	0.22	207PR CA	207PRO C	100.00
202VAL C	203GLY CA	100.00	170PHE CD2	170PHE CE1	100.00	207PR CB	207PRO CG	100.00
202VAL C	203GLY C	94.29	170PHE CD2	170PHE CE2	100.00	207PR CB	207PRO CD	100.00
203GLY CA	203GLY C	100.00	170PHE CD2	170PHE CZ	100.00	207PR CB	207PRO C	100.00
203GLY CA	211LEU CD2	0.01	170PHE CD2	180VAL CG1	0.05	207PR CG	207PRO CD	100.00
203GLY C	204ARG CA	100.00	170PHE CD2	180VAL CG2	0.61	207PR CG	207PRO C	98.30
203GLY C	204ARG CB	0.30	170PHE CD2	202VAL CB	0.01	207PR CD	207PRO C	87.76
203GLY C	204ARG CG	0.04	170PHE CD2	202VAL CG1	5.43	207PR C	208ASP CA	100.00
203GLY C	204ARG CZ	0.00	170PHE CD2	202VAL CG2	0.04	207PR C	208ASP CB	5.32
203GLY C	204ARG C	99.53	170PHE CD2	203GLY CA	0.01	207PR C	208ASP C	94.50

204ARG CA	204ARG CB	100.00	170PHI CD2	206TYR	CB	7.27	208AS CA	208ASP	CB	100.00
204ARG CA	204ARG CG	100.00	170PHI CD2	206TYR	CG	3.38	208AS CA	208ASP	CG	100.00
204ARG CA	204ARG CD	1.76	170PHI CD2	206TYR	CD1	2.05	208AS CA	208ASP	C	100.00
204ARG CA	204ARG CZ	0.06	170PHI CD2	206TYR	CD2	3.00	208AS CB	208ASP	CG	100.00
204ARG CA	204ARG C	100.00	170PHI CD2	206TYR	CE1	0.44	208AS CB	208ASP	C	100.00
204ARG CB	204ARG CG	100.00	170PHI CD2	206TYR	CE2	0.56	208AS CG	208ASP	C	99.94
204ARG CB	204ARG CD	100.00	170PHI CD2	206TYR	CZ	0.08	208AS CG	209VAL	CG2	0.16
204ARG CB	204ARG CZ	5.50	170PHI CD2	209VAL	CB	0.30	208AS C	209VAL	CA	100.00
204ARG CB	204ARG C	100.00	170PHI CD2	209VAL	CG1	13.38	208AS C	209VAL	CB	19.81
204ARG CG	204ARG CD	100.00	170PHI CD2	209VAL	CG2	0.54	208AS C	209VAL	CG2	12.49
204ARG CG	204ARG CZ	41.49	170PHI CE1	170PHE	CE2	100.00	208AS C	209VAL	C	88.08
204ARG CG	204ARG C	18.77	170PHI CE1	170PHE	CZ	100.00	209VA CA	209VAL	CB	100.00
204ARG CD	204ARG CZ	100.00	170PHI CE1	180VAL	CB	0.31	209VA CA	209VAL	CG1	100.00
204ARG C	205GLY CA	100.00	170PHI CE1	180VAL	CG1	1.92	209VA CA	209VAL	CG2	100.00
204ARG C	205GLY C	73.12	170PHI CE1	180VAL	CG2	0.50	209VA CA	209VAL	C	100.00
205GLY CA	205GLY C	100.00	170PHI CE1	199VAL	CG1	0.02	209VA CB	209VAL	CG1	100.00
205GLY C	206TYR CA	100.00	170PHI CE1	199VAL	CG2	0.00	209VA CB	209VAL	CG2	100.00
205GLY C	206TYR CB	76.71	170PHI CE1	202VAL	CB	0.04	209VA CB	209VAL	C	100.00
205GLY C	206TYR CG	21.89	170PHI CE1	202VAL	CG1	0.95	209VA CG1	209VAL	CG2	100.00
205GLY C	206TYR CD1	24.73	170PHI CE1	202VAL	CG2	0.01	209VA CG1	209VAL	C	100.00
205GLY C	206TYR CD2	0.02	170PHI CE1	202VAL	C	0.06	209VA C	210ALA	CA	100.00
205GLY C	206TYR C	29.56	170PHI CE1	203GLY	CA	5.80	209VA C	210ALA	CB	3.92
205GLY C	207PRO CD	21.56	170PHI CE1	203GLY	C	0.00	209VA C	210ALA	C	96.38
206TYR CA	206TYR CB	100.00	170PHI CE1	206TYR	CB	0.50	210AL CA	210ALA	CB	100.00
206TYR CA	206TYR CG	100.00	170PHI CE1	206TYR	CG	0.05	210AL CA	210ALA	C	100.00
206TYR CA	206TYR CD1	74.05	170PHI CE1	206TYR	CD1	0.02	210AL CB	210ALA	C	100.00
206TYR CA	206TYR CD2	68.81	170PHI CE1	206TYR	CD2	0.04	210AL C	211LEU	CA	100.00
206TYR CA	206TYR C	100.00	170PHI CE1	206TYR	CE1	0.02	210AL C	211LEU	CB	35.46
206TYR CA	207PRO CD	100.00	170PHI CE1	209VAL	CB	0.16	210AL C	211LEU	CG	0.00
206TYR CB	206TYR CG	100.00	170PHI CE1	209VAL	CG1	4.45	210AL C	211LEU	CD1	0.06
206TYR CB	206TYR CD1	100.00	170PHI CE1	209VAL	CG2	0.01	210AL C	211LEU	C	72.31
206TYR CB	206TYR CD2	100.00	170PHI CE1	211LEU	CD1	0.15	211LE CA	211LEU	CB	100.00
206TYR CB	206TYR C	100.00	170PHI CE1	211LEU	CD2	0.01	211LE CA	211LEU	CG	100.00
206TYR CB	209VAL CG2	0.04	170PHI CE2	170PHE	CZ	100.00	211LE CA	211LEU	CD1	91.49
206TYR CG	206TYR CD1	100.00	170PHI CE2	180VAL	CB	0.07	211LE CA	211LEU	CD2	5.70
206TYR CG	206TYR CD2	100.00	170PHI CE2	180VAL	CG1	0.97	211LE CA	211LEU	C	100.00
206TYR CG	206TYR CE1	100.00	170PHI CE2	180VAL	CG2	0.54	211LE CB	211LEU	CG	100.00
206TYR CG	206TYR CE2	100.00	170PHI CE2	199VAL	CG1	0.01	211LE CB	211LEU	CD1	100.00
206TYR CG	206TYR CZ	100.00	170PHI CE2	202VAL	CB	0.03	211LE CB	211LEU	CD2	100.00
206TYR CD1	206TYR CD2	100.00	170PHI CE2	202VAL	CG1	1.21	211LE CB	211LEU	C	100.00
206TYR CD1	206TYR CE1	100.00	170PHI CE2	202VAL	CG2	0.04	211LE CG	211LEU	CD1	100.00
206TYR CD1	206TYR CE2	100.00	170PHI CE2	202VAL	C	0.06	211LE CG	211LEU	CD2	100.00
206TYR CD1	206TYR CZ	100.00	170PHI CE2	203GLY	CA	4.97	211LE CG	211LEU	C	97.47
206TYR CD2	206TYR CE1	100.00	170PHI CE2	206TYR	CB	0.39	211LE CG	213HIS	CE1	0.28
206TYR CD2	206TYR CE2	100.00	170PHI CE2	206TYR	CG	0.32	211LE CD1	211LEU	CD2	100.00
206TYR CD2	206TYR CZ	100.00	170PHI CE2	206TYR	CD1	0.12	211LE CD1	211LEU	C	3.26
206TYR CD2	209VAL CG2	0.05	170PHI CE2	206TYR	CD2	0.23	211LE CD1	213HIS	CD2	0.00
206TYR CE1	206TYR CE2	100.00	170PHI CE2	206TYR	CE1	0.01	211LE CD1	213HIS	CE1	0.12
206TYR CE1	206TYR CZ	100.00	170PHI CE2	206TYR	CE2	0.01	211LE CD2	211LEU	C	4.17
206TYR CE2	206TYR CZ	100.00	170PHI CE2	206TYR	CZ	0.00	211LE CD2	212GLU	C	0.04
206TYR C	207PRO CA	100.00	170PHI CE2	209VAL	CB	0.24	211LE CD2	213HIS	CG	0.00
206TYR C	207PRO CB	0.20	170PHI CE2	209VAL	CG1	8.38	211LE CD2	213HIS	CD2	0.13
206TYR C	207PRO CD	100.00	170PHI CE2	209VAL	CG2	0.06	211LE CD2	213HIS	CE1	4.24
206TYR C	207PRO C	100.00	170PHI CE2	211LEU	CB	0.00	211LE C	212GLU	CA	100.00
207PRO CA	207PRO CB	100.00	170PHI CE2	211LEU	CG	0.00	211LE C	212GLU	CB	69.75
207PRO CA	207PRO CG	100.00	170PHI CE2	211LEU	CD1	0.15	211LE C	212GLU	CG	0.01
207PRO CA	207PRO CD	100.00	170PHI CE2	211LEU	CD2	0.06	211LE C	212GLU	C	38.78
207PRO CA	207PRO C	100.00	170PHI CZ	180VAL	CB	0.08	212GL CA	212GLU	CB	100.00
207PRO CB	207PRO CG	100.00	170PHI CZ	180VAL	CG1	0.38	212GL CA	212GLU	CG	100.00
207PRO CB	207PRO CD	100.00	170PHI CZ	180VAL	CG2	0.25	212GL CA	212GLU	CD	90.91
207PRO CB	207PRO C	100.00	170PHI CZ	199VAL	CG1	0.03	212GL CA	212GLU	C	100.00
207PRO CG	207PRO CD	100.00	170PHI CZ	202VAL	CB	0.01	212GL CB	212GLU	CG	100.00

207PRO CG	207PRO C	96.75	170PHI CZ	202VAL CG1	0.04	212GL CB	212GLU CD	100.00
207PRO CD	207PRO C	80.80	170PHI CZ	202VAL C	0.03	212GL CB	212GLU C	100.00
207PRO C	208ASP CA	100.00	170PHI CZ	203GLY CA	28.22	212GL CG	212GLU CD	100.00
207PRO C	208ASP CB	23.04	170PHI CZ	203GLY C	0.11	212GL CG	212GLU C	99.47
207PRO C	208ASP C	79.16	170PHI CZ	206TYR CD1	0.00	212GL CG	214GLN CG	0.00
208ASP CA	208ASP CB	100.00	170PHI CZ	209VAL CG1	0.04	212GL CD	212GLU C	0.02
208ASP CA	208ASP CG	100.00	170PHI CZ	211LEU CB	0.07	212GL CD	214GLN CG	0.00
208ASP CA	208ASP C	100.00	170PHI CZ	211LEU CG	0.05	212GL C	213HIS CA	100.00
208ASP CB	208ASP CG	100.00	170PHI CZ	211LEU CD1	0.96	212GL C	213HIS CB	38.37
208ASP CB	208ASP C	100.00	170PHI CZ	211LEU CD2	0.17	212GL C	213HIS CG	5.00
208ASP CG	208ASP C	98.19	170PHI C	171GLU CA	100.00	212GL C	213HIS CD2	0.62
208ASP C	209VAL CA	100.00	170PHI C	171GLU CB	0.12	212GL C	213HIS C	63.36
208ASP C	209VAL CB	16.23	170PHI C	171GLU C	99.94	213HIS CA	213HIS CB	100.00
208ASP C	209VAL CG2	10.99	170PHI C	173ALA CB	0.00	213HIS CA	213HIS CG	100.00
208ASP C	209VAL C	89.22	170PHI C	206TYR CD1	0.07	213HIS CA	213HIS CD2	13.28
209VAL CA	209VAL CB	100.00	170PHI C	206TYR CE1	0.74	213HIS CA	213HIS C	100.00
209VAL CA	209VAL CG1	100.00	170PHI C	206TYR CE2	0.08	213HIS CB	213HIS CG	100.00
209VAL CA	209VAL CG2	100.00	170PHI C	206TYR CZ	0.01	213HIS CB	213HIS CD2	100.00
209VAL CA	209VAL C	100.00	171GLI CA	171GLU CB	100.00	213HIS CB	213HIS CE1	0.14
209VAL CB	209VAL CG1	100.00	171GLI CA	171GLU CG	100.00	213HIS CB	213HIS C	100.00
209VAL CB	209VAL CG2	100.00	171GLI CA	171GLU CD	99.16	213HIS CG	213HIS CD2	100.00
209VAL CB	209VAL C	100.00	171GLI CA	171GLU C	100.00	213HIS CG	213HIS CE1	100.00
209VAL CG1	209VAL CG2	100.00	171GLI CA	174ARG CD	0.00	213HIS CG	213HIS C	19.68
209VAL CG1	209VAL C	100.00	171GLI CA	206TYR CE1	0.14	213HIS CD2	213HIS CE1	100.00
209VAL C	210ALA CA	100.00	171GLI CB	171GLU CG	100.00	213HIS CD2	213HIS C	3.35
209VAL C	210ALA CB	4.97	171GLI CB	171GLU CD	100.00	213HIS CD2	214GLN C	0.00
209VAL C	210ALA C	95.24	171GLI CB	171GLU C	100.00	213HIS CD2	215TYR CD1	0.00
210ALA CA	210ALA CB	100.00	171GLI CB	206TYR CE1	0.01	213HIS CD2	215TYR CE1	0.56
210ALA CA	210ALA C	100.00	171GLI CG	171GLU CD	100.00	213HIS CD2	215TYR CE2	0.04
210ALA CB	210ALA C	100.00	171GLI CG	171GLU C	82.13	213HIS CD2	215TYR CZ	0.08
210ALA C	211LEU CA	100.00	171GLI CG	206TYR CE2	0.01	213HIS CE1	215TYR CD2	0.00
210ALA C	211LEU CB	34.95	171GLI CG	206TYR CZ	0.00	213HIS CE1	215TYR CE1	0.01
210ALA C	211LEU C	71.10	171GLI CD	171GLU C	1.44	213HIS CE1	215TYR CE2	0.02
211LEU CA	211LEU CB	100.00	171GLI CD	174ARG CD	0.08	213HIS CE1	215TYR CZ	0.00
211LEU CA	211LEU CG	100.00	171GLI CD	174ARG CZ	0.22	213HIS C	214GLN CA	100.00
211LEU CA	211LEU CD1	92.94	171GLI CD	175LYS CE	0.00	213HIS C	214GLN CB	90.76
211LEU CA	211LEU CD2	7.34	171GLI CD	206TYR CE1	0.10	213HIS C	214GLN CG	3.00
211LEU CA	211LEU C	100.00	171GLI CD	206TYR CE2	0.08	213HIS C	214GLN C	12.51
211LEU CB	211LEU CG	100.00	171GLI CD	206TYR CZ	0.00	214GL CA	214GLN CB	100.00
211LEU CB	211LEU CD1	100.00	171GLI C	172ALA CA	100.00	214GL CA	214GLN CG	100.00
211LEU CB	211LEU CD2	100.00	171GLI C	172ALA CB	0.03	214GL CA	214GLN CD	66.87
211LEU CB	211LEU C	100.00	171GLI C	172ALA C	99.96	214GL CA	214GLN C	100.00
211LEU CG	211LEU CD1	100.00	172AL/ CA	172ALA CB	100.00	214GL CB	214GLN CG	100.00
211LEU CG	211LEU CD2	100.00	172AL/ CA	172ALA C	100.00	214GL CB	214GLN CD	100.00
211LEU CG	211LEU C	99.84	172AL/ CA	175LYS CG	0.01	214GL CB	214GLN C	100.00
211LEU CG	213HIS CE1	0.11	172AL/ CA	300HIS CE1	0.00	214GL CB	219MET CG	0.07
211LEU CD1	211LEU CD2	100.00	172AL/ CB	172ALA C	100.00	214GL CB	219MET CE	0.17
211LEU CD1	211LEU C	7.05	172AL/ CB	300HIS CE1	0.08	214GL CG	214GLN CD	100.00
211LEU CD1	213HIS CD2	0.00	172AL/ C	173ALA CA	100.00	214GL CG	214GLN C	80.36
211LEU CD1	213HIS CE1	1.70	172AL/ C	173ALA CB	0.02	214GL CG	218ALA CB	0.02
211LEU CD2	211LEU C	3.59	172AL/ C	173ALA C	99.98	214GL CG	219MET CA	0.05
211LEU CD2	212GLU C	0.02	173AL/ CA	173ALA CB	100.00	214GL CG	219MET CB	0.28
211LEU CD2	213HIS CD2	0.16	173AL/ CA	173ALA C	100.00	214GL CG	219MET CG	0.25
211LEU CD2	213HIS CE1	3.74	173AL/ CA	232VAL CG2	0.93	214GL CG	219MET CE	0.26
211LEU C	212GLU CA	100.00	173AL/ CB	173ALA C	100.00	214GL CG	230PHE CE1	0.00
211LEU C	212GLU CB	71.92	173AL/ CB	179HIS C	0.01	214GL CD	214GLN C	0.16
211LEU C	212GLU C	37.26	173AL/ CB	180VAL CB	0.00	214GL CD	215TYR CD2	0.00
212GLU CA	212GLU CB	100.00	173AL/ CB	180VAL CG1	0.25	214GL CD	218ALA CB	0.42
212GLU CA	212GLU CG	100.00	173AL/ CB	180VAL CG2	29.81	214GL CD	219MET CA	0.04
212GLU CA	212GLU CD	37.18	173AL/ CB	209VAL CG1	6.25	214GL CD	219MET CB	0.01
212GLU CA	212GLU C	100.00	173AL/ CB	209VAL CG2	0.06	214GL CD	219MET CG	0.02
212GLU CB	212GLU CG	100.00	173AL/ CB	232VAL CG2	1.66	214GL CD	219MET CE	0.03

212GLU CB	212GLU	CD	100.00	173AL C	174ARG	CA	100.00	214GL CD	222HIS	CG	0.00
212GLU CB	212GLU	C	100.00	173AL C	174ARG	CB	0.04	214GL CD	222HIS	CD2	0.24
212GLU CG	212GLU	CD	100.00	173AL C	174ARG	C	99.99	214GL CD	222HIS	CE1	0.01
212GLU CG	212GLU	C	99.52	173AL C	178LYS	CB	0.00	214GL CD	230PHE	CE2	0.01
212GLU CG	214GLN	CG	0.26	173AL C	178LYS	CG	0.00	214GL C	215TYR	CA	100.00
212GLU CG	214GLN	CD	0.15	173AL C	209VAL	CG1	0.04	214GL C	215TYR	CB	0.02
212GLU CD	212GLU	C	0.02	173AL C	209VAL	CG2	0.01	214GL C	215TYR	CG	0.00
212GLU CD	214GLN	CG	0.01	174AR CA	174ARG	CB	100.00	214GL C	215TYR	C	99.98
212GLU C	213HIS	CA	100.00	174AR CA	174ARG	CG	100.00	214GL C	219MET	CE	0.00
212GLU C	213HIS	CB	13.72	174AR CA	174ARG	CD	0.54	215TY CA	215TYR	CB	100.00
212GLU C	213HIS	CG	2.58	174AR CA	174ARG	C	100.00	215TY CA	215TYR	CG	100.00
212GLU C	213HIS	CD2	0.28	174AR CA	178LYS	CG	0.00	215TY CA	215TYR	CD1	76.78
212GLU C	213HIS	C	89.04	174AR CA	178LYS	CE	0.00	215TY CA	215TYR	CD2	37.04
213HIS CA	213HIS	CB	100.00	174AR CA	209VAL	CG1	0.00	215TY CA	215TYR	C	100.00
213HIS CA	213HIS	CG	100.00	174AR CA	209VAL	CG2	0.28	215TY CB	215TYR	CG	100.00
213HIS CA	213HIS	CD2	11.00	174AR CB	174ARG	CG	100.00	215TY CB	215TYR	CD1	100.00
213HIS CA	213HIS	C	100.00	174AR CB	174ARG	CD	100.00	215TY CB	215TYR	CD2	100.00
213HIS CB	213HIS	CG	100.00	174AR CB	174ARG	CZ	8.24	215TY CB	215TYR	C	100.00
213HIS CB	213HIS	CD2	100.00	174AR CB	174ARG	C	100.00	215TY CB	218ALA	CB	0.18
213HIS CB	213HIS	CE1	0.10	174AR CB	209VAL	CG2	0.14	215TY CG	215TYR	CD1	100.00
213HIS CB	213HIS	C	100.00	174AR CG	174ARG	CD	100.00	215TY CG	215TYR	CD2	100.00
213HIS CG	213HIS	CD2	100.00	174AR CG	174ARG	CZ	30.21	215TY CG	215TYR	CE1	100.00
213HIS CG	213HIS	CE1	100.00	174AR CG	174ARG	C	55.97	215TY CG	215TYR	CE2	100.00
213HIS CD2	213HIS	CE1	100.00	174AR CG	206TYR	CE2	0.00	215TY CG	215TYR	CZ	100.00
213HIS C	214GLN	CA	100.00	174AR CG	208ASP	CB	0.01	215TY CD1	215TYR	CD2	100.00
213HIS C	214GLN	CB	55.54	174AR CG	209VAL	CG1	0.06	215TY CD1	215TYR	CE1	100.00
213HIS C	214GLN	CG	11.13	174AR CG	209VAL	CG2	0.92	215TY CD1	215TYR	CE2	100.00
213HIS C	214GLN	C	47.69	174AR CD	174ARG	CZ	100.00	215TY CD1	215TYR	CZ	100.00
214GLN CA	214GLN	CB	100.00	174AR CD	174ARG	C	0.70	215TY CD2	215TYR	CE1	100.00
214GLN CA	214GLN	CG	100.00	174AR CD	206TYR	CD1	0.01	215TY CD2	215TYR	CE2	100.00
214GLN CA	214GLN	CD	46.82	174AR CD	206TYR	CD2	0.01	215TY CD2	215TYR	CZ	100.00
214GLN CA	214GLN	C	100.00	174AR CD	206TYR	CE1	0.04	215TY CE1	215TYR	CE2	100.00
214GLN CB	214GLN	CG	100.00	174AR CD	206TYR	CE2	0.02	215TY CE1	215TYR	CZ	100.00
214GLN CB	214GLN	CD	100.00	174AR CD	206TYR	CZ	0.00	215TY CE1	275SER	CB	0.01
214GLN CB	214GLN	C	100.00	174AR CD	208ASP	CG	0.01	215TY CE2	215TYR	CZ	100.00
214GLN CB	219MET	CG	0.13	174AR CD	209VAL	CG2	0.04	215TY CE2	275SER	CB	0.08
214GLN CB	219MET	CE	0.02	174AR CZ	175LYS	CE	0.06	215TY CZ	275SER	CB	0.00
214GLN CG	214GLN	CD	100.00	174AR CZ	206TYR	CD1	3.89	215TY C	216VAL	CA	100.00
214GLN CG	214GLN	C	15.28	174AR CZ	206TYR	CD2	0.18	215TY C	216VAL	C	100.00
214GLN CG	219MET	CA	0.00	174AR CZ	206TYR	CE1	9.97	216VA CA	216VAL	CB	100.00
214GLN CG	219MET	CB	0.02	174AR CZ	206TYR	CE2	0.44	216VA CA	216VAL	CG1	100.00
214GLN CG	219MET	CG	0.01	174AR CZ	206TYR	CZ	0.16	216VA CA	216VAL	CG2	100.00
214GLN CG	230PHE	CE1	0.00	174AR CZ	207PRO	CB	0.01	216VA CA	216VAL	C	100.00
214GLN CD	214GLN	C	0.08	174AR CZ	207PRO	CG	0.01	216VA CA	219MET	CB	0.01
214GLN CD	219MET	CA	0.02	174AR C	175LYS	CA	100.00	216VA CB	216VAL	CG1	100.00
214GLN CD	219MET	CB	0.07	174AR C	175LYS	CB	40.61	216VA CB	216VAL	CG2	100.00
214GLN CD	219MET	CG	0.06	174AR C	175LYS	CG	0.53	216VA CB	216VAL	C	100.00
214GLN CD	222HIS	CD2	0.00	174AR C	175LYS	CD	0.01	216VA CB	239PHE	CD2	0.00
214GLN CD	230PHE	CE1	0.09	174AR C	175LYS	C	57.85	216VA CG1	216VAL	CG2	100.00
214GLN C	215TYR	CA	100.00	174AR C	178LYS	CE	0.00	216VA CG1	216VAL	C	99.52
214GLN C	215TYR	CB	0.01	175LY CA	175LYS	CB	100.00	216VA CG1	239PHE	CB	0.00
214GLN C	215TYR	C	100.00	175LY CA	175LYS	CG	100.00	216VA CG1	239PHE	CD1	0.03
215TYR CA	215TYR	CB	100.00	175LY CA	175LYS	CD	3.00	216VA CG1	239PHE	CD2	0.08
215TYR CA	215TYR	CG	100.00	175LY CA	175LYS	C	100.00	216VA CG1	239PHE	CE1	0.22
215TYR CA	215TYR	CD1	73.25	175LY CB	175LYS	CG	100.00	216VA CG1	239PHE	CE2	0.30
215TYR CA	215TYR	CD2	39.93	175LY CB	175LYS	CD	100.00	216VA CG1	239PHE	CZ	0.45
215TYR CA	215TYR	C	100.00	175LY CB	175LYS	CE	13.78	216VA CG1	242ILE	CD	0.52
215TYR CB	215TYR	CG	100.00	175LY CB	175LYS	C	100.00	216VA CG2	216VAL	C	100.00
215TYR CB	215TYR	CD1	100.00	175LY CB	305VAL	CG1	0.00	216VA CG2	239PHE	CB	1.85
215TYR CB	215TYR	CD2	100.00	175LY CB	305VAL	CG2	0.03	216VA CG2	239PHE	CG	3.61
215TYR CB	215TYR	C	100.00	175LY CG	175LYS	CD	100.00	216VA CG2	239PHE	CD1	2.18
215TYR CB	218ALA	CB	0.08	175LY CG	175LYS	CE	100.00	216VA CG2	239PHE	CD2	0.74

215TYR	CG	215TYR	CD1	100.00	175LYS	CG	175LYS	C	71.28	216VA	CG2	239PHE	CE1	0.02
215TYR	CG	215TYR	CD2	100.00	175LYS	CG	305VAL	CG2	0.30	216VA	CG2	239PHE	CE2	0.01
215TYR	CG	215TYR	CE1	100.00	175LYS	CD	175LYS	CE	100.00	216VA	CG2	239PHE	CZ	0.00
215TYR	CG	215TYR	CE2	100.00	175LYS	CD	175LYS	C	0.10	216VA	CG2	243LEU	CD1	0.26
215TYR	CG	215TYR	CZ	100.00	175LYS	CD	299GLU	CG	0.03	216VA	CG2	243LEU	CD2	0.15
215TYR	CD1	215TYR	CD2	100.00	175LYS	CD	299GLU	CD	0.01	216VA	CG2	242ILE	CG2	0.00
215TYR	CD1	215TYR	CE1	100.00	175LYS	CD	300HIS	CE1	0.01	216VA	CG2	242ILE	CD	1.45
215TYR	CD1	215TYR	CE2	100.00	175LYS	CD	305VAL	CG1	0.02	216VA	C	217ASP	CA	100.00
215TYR	CD1	215TYR	CZ	100.00	175LYS	CD	305VAL	CG2	0.02	216VA	C	217ASP	CB	0.02
215TYR	CD2	215TYR	CE1	100.00	175LYS	CE	299GLU	CG	0.02	216VA	C	217ASP	C	99.97
215TYR	CD2	215TYR	CE2	100.00	175LYS	CE	299GLU	CD	0.09	217AS	CA	217ASP	CB	100.00
215TYR	CD2	215TYR	CZ	100.00	175LYS	CE	300HIS	CE1	0.04	217AS	CA	217ASP	CG	100.00
215TYR	CE1	215TYR	CE2	100.00	175LYS	CE	305VAL	CG1	0.01	217AS	CA	217ASP	C	100.00
215TYR	CE1	215TYR	CZ	100.00	175LYS	CE	305VAL	CG2	0.02	217AS	CA	242ILE	CD	0.00
215TYR	CE2	215TYR	CZ	100.00	175LYS	C	176ARG	CA	100.00	217AS	CB	217ASP	CG	100.00
215TYR	C	216VAL	CA	100.00	175LYS	C	176ARG	CB	74.03	217AS	CB	217ASP	C	100.00
215TYR	C	216VAL	C	100.00	175LYS	C	176ARG	CG	33.81	217AS	CB	241ASP	CG	0.01
216VAL	CA	216VAL	CB	100.00	175LYS	C	176ARG	C	33.21	217AS	CB	242ILE	CA	0.15
216VAL	CA	216VAL	CG1	100.00	176AR(	CA	176ARG	CB	100.00	217AS	CB	242ILE	CG1	0.02
216VAL	CA	216VAL	CG2	100.00	176AR(	CA	176ARG	CG	100.00	217AS	CB	242ILE	CD	0.01
216VAL	CA	216VAL	C	100.00	176AR(	CA	176ARG	CD	0.08	217AS	CB	245ASP	CB	0.03
216VAL	CB	216VAL	CG1	100.00	176AR(	CA	176ARG	C	100.00	217AS	CB	245ASP	CG	0.04
216VAL	CB	216VAL	CG2	100.00	176AR(	CA	177ARG	CZ	0.02	217AS	CG	217ASP	C	96.12
216VAL	CB	216VAL	C	100.00	176AR(	CB	176ARG	CG	100.00	217AS	C	218ALA	CA	100.00
216VAL	CB	239PHE	CD2	0.00	176AR(	CB	176ARG	CD	100.00	217AS	C	218ALA	CB	0.79
216VAL	CB	239PHE	CE2	0.02	176AR(	CB	176ARG	C	100.00	217AS	C	218ALA	C	99.21
216VAL	CG1	216VAL	CG2	100.00	176AR(	CB	177ARG	CZ	0.01	217AS	C	245ASP	CB	0.02
216VAL	CG1	216VAL	C	99.94	176AR(	CB	231ASP	CG	0.48	217AS	C	245ASP	CG	0.00
216VAL	CG1	239PHE	CD1	0.04	176AR(	CG	176ARG	CD	100.00	218AL.	CA	218ALA	CB	100.00
216VAL	CG1	239PHE	CD2	0.04	176AR(	CG	176ARG	CZ	59.41	218AL.	CA	218ALA	C	100.00
216VAL	CG1	239PHE	CE1	0.10	176AR(	CG	176ARG	C	0.36	218AL.	CB	218ALA	C	100.00
216VAL	CG1	239PHE	CE2	0.31	176AR(	CG	231ASP	CG	0.02	218AL.	C	219MET	CA	100.00
216VAL	CG1	239PHE	CZ	0.15	176AR(	CG	232VAL	CG2	0.00	218AL.	C	219MET	C	100.00
216VAL	CG1	242ILE	CG1	0.01	176AR(	CD	176ARG	CZ	100.00	218AL.	C	222HIS	CD2	1.15
216VAL	CG1	242ILE	CD	0.86	176AR(	CD	231ASP	CG	0.20	219ME	CA	219MET	CB	100.00
216VAL	CG2	216VAL	C	100.00	176AR(	CD	232VAL	CG2	17.76	219ME	CA	219MET	CG	100.00
216VAL	CG2	239PHE	CB	0.16	176AR(	C	177ARG	CA	100.00	219ME	CA	219MET	CE	0.06
216VAL	CG2	239PHE	CG	0.09	176AR(	C	177ARG	CB	7.52	219ME	CA	219MET	C	100.00
216VAL	CG2	239PHE	CD1	0.14	176AR(	C	177ARG	CG	2.58	219ME	CA	222HIS	CB	0.12
216VAL	CG2	239PHE	CD2	1.50	176AR(	C	177ARG	CD	0.05	219ME	CA	222HIS	CD2	0.02
216VAL	CG2	239PHE	CE1	0.00	176AR(	C	177ARG	C	91.46	219ME	CB	219MET	CG	100.00
216VAL	CG2	239PHE	CE2	1.54	177AR(	CA	177ARG	CB	100.00	219ME	CB	219MET	CE	28.91
216VAL	CG2	239PHE	CZ	0.02	177AR(	CA	177ARG	CG	100.00	219ME	CB	219MET	C	100.00
216VAL	CG2	243LEU	CD1	0.30	177AR(	CA	177ARG	CD	1.81	219ME	CB	243LEU	CD2	0.01
216VAL	CG2	243LEU	CD2	0.02	177AR(	CA	177ARG	C	100.00	219ME	CG	219MET	CE	100.00
216VAL	CG2	242ILE	CG1	0.01	177AR(	CB	177ARG	CG	100.00	219ME	CG	219MET	C	99.52
216VAL	CG2	242ILE	CD	0.86	177AR(	CB	177ARG	CD	100.00	219ME	CG	223LEU	CG	0.00
216VAL	C	217ASP	CA	100.00	177AR(	CB	177ARG	CZ	0.54	219ME	CG	223LEU	CD1	0.02
216VAL	C	217ASP	CB	0.01	177AR(	CB	177ARG	C	100.00	219ME	CG	230PHE	CE1	0.68
216VAL	C	217ASP	C	99.99	177AR(	CB	231ASP	CB	0.03	219ME	CG	230PHE	CE2	1.33
217ASP	CA	217ASP	CB	100.00	177AR(	CB	231ASP	CG	1.75	219ME	CG	230PHE	CZ	2.04
217ASP	CA	217ASP	CG	100.00	177AR(	CG	177ARG	CD	100.00	219ME	CG	243LEU	CD1	0.02
217ASP	CA	217ASP	C	100.00	177AR(	CG	177ARG	CZ	17.63	219ME	CG	243LEU	CD2	0.42
217ASP	CA	242ILE	CG1	0.01	177AR(	CG	177ARG	C	5.53	219ME	CE	219MET	C	0.06
217ASP	CA	242ILE	CG2	0.00	177AR(	CG	231ASP	CB	0.01	219ME	CE	222HIS	CD2	0.06
217ASP	CA	242ILE	CD	0.00	177AR(	CG	231ASP	CG	0.96	219ME	CE	223LEU	CG	0.01
217ASP	CB	217ASP	CG	100.00	177AR(	CD	177ARG	CZ	100.00	219ME	CE	223LEU	CD1	0.14
217ASP	CB	217ASP	C	100.00	177AR(	CD	177ARG	C	0.44	219ME	CE	223LEU	CD2	0.04
217ASP	CB	241ASP	CG	0.00	177AR(	CD	179HIS	CB	0.06	219ME	CE	230PHE	CG	0.29
217ASP	CB	241ASP	C	0.00	177AR(	CD	231ASP	CB	0.04	219ME	CE	230PHE	CD1	1.00
217ASP	CB	242ILE	CA	0.22	177AR(	CD	231ASP	CG	0.36	219ME	CE	230PHE	CD2	1.12
217ASP	CB	242ILE	CG1	0.01	177AR(	CZ	179HIS	CB	0.06	219ME	CE	230PHE	CE1	5.91

217ASP	CB	245ASP	CB	0.16	177AR(	CZ	179HIS	CG	0.01	219ME	CE	230PHE	CE2	4.49
217ASP	CB	245ASP	CG	0.14	177AR(	CZ	179HIS	CD2	0.02	219ME	CE	230PHE	CZ	6.40
217ASP	CG	217ASP	C	71.59	177AR(	CZ	231ASP	CA	0.01	219ME	CE	233VAL	CG1	3.18
217ASP	CG	241ASP	CG	0.01	177AR(	CZ	231ASP	CB	5.24	219ME	CE	233VAL	CG2	5.09
217ASP	CG	242ILE	CG1	0.15	177AR(	CZ	231ASP	CG	2.07	219ME	CE	243LEU	CD1	0.06
217ASP	CG	242ILE	CD	0.00	177AR(	C	178LYS	CA	100.00	219ME	CE	243LEU	CD2	0.40
217ASP	C	218ALA	CA	100.00	177AR(	C	178LYS	CB	100.00	219ME	C	220ALA	CA	100.00
217ASP	C	218ALA	CB	0.18	177AR(	C	178LYS	CG	34.93	219ME	C	220ALA	CB	0.01
217ASP	C	218ALA	C	99.84	177AR(	C	178LYS	CD	0.04	219ME	C	220ALA	C	100.00
217ASP	C	245ASP	CB	0.17	177AR(	C	178LYS	C	97.20	219ME	C	222HIS	CB	0.00
218ALA	CA	218ALA	CB	100.00	178LYS	CA	178LYS	CB	100.00	219ME	C	243LEU	CD2	0.01
218ALA	CA	218ALA	C	100.00	178LYS	CA	178LYS	CG	100.00	220AL	CA	220ALA	CB	100.00
218ALA	CA	254LEU	CD1	0.01	178LYS	CA	178LYS	CD	0.70	220AL	CA	220ALA	C	100.00
218ALA	CB	218ALA	C	100.00	178LYS	CA	178LYS	C	100.00	220AL	CA	243LEU	CD1	0.13
218ALA	CB	254LEU	CD1	0.34	178LYS	CA	209VAL	CG1	0.00	220AL	CA	243LEU	CD2	0.06
218ALA	C	219MET	CA	100.00	178LYS	CB	178LYS	CG	100.00	220AL	CA	246LEU	CD2	0.04
218ALA	C	219MET	CB	0.00	178LYS	CB	178LYS	CD	100.00	220AL	CB	220ALA	C	100.00
218ALA	C	219MET	C	100.00	178LYS	CB	178LYS	CE	25.47	220AL	CB	243LEU	CG	0.01
218ALA	C	222HIS	CD2	0.22	178LYS	CB	178LYS	C	100.00	220AL	CB	243LEU	CD1	0.29
219MET	CA	219MET	CB	100.00	178LYS	CB	209VAL	CG1	0.01	220AL	CB	243LEU	CD2	0.13
219MET	CA	219MET	CG	100.00	178LYS	CB	209VAL	CG2	0.01	220AL	CB	246LEU	CD1	0.01
219MET	CA	219MET	CE	0.01	178LYS	CG	178LYS	CD	100.00	220AL	CB	246LEU	CD2	0.12
219MET	CA	219MET	C	100.00	178LYS	CG	178LYS	CE	100.00	220AL	CB	242ILE	CG2	7.59
219MET	CA	222HIS	CB	0.03	178LYS	CG	178LYS	C	8.17	220AL	CB	246LEU	CA	0.04
219MET	CB	219MET	CG	100.00	178LYS	CG	209VAL	CG1	0.00	220AL	CB	246LEU	CB	0.10
219MET	CB	219MET	CE	9.77	178LYS	CG	209VAL	CG2	0.02	220AL	CB	246LEU	CG	0.01
219MET	CB	219MET	C	100.00	178LYS	CD	178LYS	CE	100.00	220AL	CB	246LEU	CD1	0.03
219MET	CG	219MET	CE	100.00	178LYS	CD	208ASP	CB	0.04	220AL	CB	246LEU	CD2	0.03
219MET	CG	219MET	C	99.88	178LYS	CD	208ASP	CG	0.70	220AL	C	221MET	CA	100.00
219MET	CG	230PHE	CE1	8.87	178LYS	CD	208ASP	C	0.00	220AL	C	221MET	CB	0.71
219MET	CG	230PHE	CE2	0.06	178LYS	CD	209VAL	CG2	0.00	220AL	C	221MET	C	99.53
219MET	CG	230PHE	CZ	4.82	178LYS	CE	208ASP	CB	0.00	220AL	C	246LEU	CD1	0.33
219MET	CG	243LEU	CD1	0.01	178LYS	CE	208ASP	CG	0.26	220AL	C	246LEU	CD2	0.19
219MET	CG	243LEU	CD2	0.00	178LYS	CE	209VAL	CG2	0.01	221ME	CA	221MET	CB	100.00
219MET	CE	219MET	C	1.00	178LYS	C	179HIS	CA	100.00	221ME	CA	221MET	CG	100.00
219MET	CE	220ALA	CA	0.01	178LYS	C	179HIS	CB	76.24	221ME	CA	221MET	C	100.00
219MET	CE	223LEU	CB	0.00	178LYS	C	179HIS	C	30.29	221ME	CA	224VAL	CG2	0.02
219MET	CE	223LEU	CG	0.02	178LYS	C	209VAL	CG1	0.45	221ME	CA	246LEU	CD2	0.00
219MET	CE	223LEU	CD1	0.58	179HIS	CA	179HIS	CB	100.00	221ME	CA	249VAL	CG1	0.03
219MET	CE	223LEU	CD2	0.08	179HIS	CA	179HIS	CG	100.00	221ME	CA	249VAL	CG2	0.14
219MET	CE	230PHE	CG	0.45	179HIS	CA	179HIS	CD2	35.02	221ME	CB	221MET	CG	100.00
219MET	CE	230PHE	CD1	6.14	179HIS	CA	179HIS	C	100.00	221ME	CB	221MET	CE	32.67
219MET	CE	230PHE	CD2	0.61	179HIS	CB	179HIS	CG	100.00	221ME	CB	221MET	C	100.00
219MET	CE	230PHE	CE1	12.50	179HIS	CB	179HIS	CD2	100.00	221ME	CB	225ARG	CZ	0.01
219MET	CE	230PHE	CE2	1.15	179HIS	CB	179HIS	CE1	0.30	221ME	CB	249VAL	CG1	0.00
219MET	CE	230PHE	CZ	1.79	179HIS	CB	179HIS	C	100.00	221ME	CB	249VAL	CG2	0.04
219MET	CE	233VAL	CG1	2.31	179HIS	CB	229ARG	CZ	0.00	221ME	CG	221MET	CE	100.00
219MET	CE	233VAL	CG2	5.57	179HIS	CB	231ASP	CB	0.02	221ME	CG	221MET	C	96.46
219MET	CE	243LEU	CD1	0.25	179HIS	CG	179HIS	CD2	100.00	221ME	CG	225ARG	CD	0.02
219MET	CE	243LEU	CD2	0.23	179HIS	CG	179HIS	CE1	100.00	221ME	CG	225ARG	CZ	0.03
219MET	C	220ALA	CA	100.00	179HIS	CG	179HIS	C	99.95	221ME	CG	245ASP	C	0.02
219MET	C	220ALA	C	100.00	179HIS	CG	210ALA	CB	0.02	221ME	CG	249VAL	CA	0.01
220ALA	CA	220ALA	CB	100.00	179HIS	CG	229ARG	CZ	0.03	221ME	CG	249VAL	CB	0.05
220ALA	CA	220ALA	C	100.00	179HIS	CD2	179HIS	CE1	100.00	221ME	CG	249VAL	CG1	4.62
220ALA	CA	223LEU	CB	0.00	179HIS	CD2	179HIS	C	6.49	221ME	CG	249VAL	CG2	3.89
220ALA	CA	243LEU	CD2	0.03	179HIS	CD2	180VAL	C	0.00	221ME	CG	254LEU	CD1	0.00
220ALA	CB	220ALA	C	100.00	179HIS	CD2	181VAL	CG2	1.02	221ME	CG	254LEU	CD2	0.02
220ALA	CB	243LEU	CD1	0.08	179HIS	CD2	210ALA	CB	1.22	221ME	CE	221MET	C	0.02
220ALA	CB	243LEU	CD2	0.57	179HIS	CD2	210ALA	C	0.03	221ME	CE	222HIS	CG	0.00
220ALA	CB	246LEU	CD1	0.01	179HIS	CD2	211LEU	C	0.00	221ME	CE	222HIS	CD2	0.02
220ALA	CB	246LEU	CD2	0.07	179HIS	CD2	229ARG	CZ	0.08	221ME	CE	222HIS	CE1	0.51
220ALA	CB	242ILE	CG2	3.44	179HIS	CD2	229ARG	C	0.01	221ME	CE	225ARG	CD	0.26

220ALA	CB	246LEU	CA	0.01	179HIS	CD2	230PHE	CA	0.04	221ME	CE	225ARG	CZ	14.30
220ALA	CB	246LEU	CB	0.07	179HIS	CD2	230PHE	C	0.02	221ME	CE	245ASP	CB	0.04
220ALA	CB	246LEU	CG	0.01	179HIS	CD2	231ASP	CB	0.15	221ME	CE	245ASP	CG	0.02
220ALA	CB	246LEU	CD1	0.21	179HIS	CE1	180VAL	C	0.02	221ME	CE	245ASP	C	0.00
220ALA	CB	246LEU	CD2	0.02	179HIS	CE1	181VAL	CB	0.00	221ME	CE	248SER	CB	0.26
220ALA	C	221MET	CA	100.00	179HIS	CE1	181VAL	CG1	0.03	221ME	CE	248SER	C	0.70
220ALA	C	221MET	CB	0.04	179HIS	CE1	181VAL	CG2	16.12	221ME	CE	249VAL	CA	0.00
220ALA	C	221MET	C	99.98	179HIS	CE1	210ALA	CB	0.03	221ME	CE	249VAL	CB	0.00
220ALA	C	246LEU	CB	0.01	179HIS	CE1	211LEU	C	0.09	221ME	CE	249VAL	CG1	0.15
220ALA	C	246LEU	CD1	1.27	179HIS	CE1	212GLU	CB	4.13	221ME	CE	249VAL	CG2	0.08
220ALA	C	246LEU	CD2	0.36	179HIS	CE1	212GLU	CG	0.06	221ME	CE	252GLY	C	0.28
221MET	CA	221MET	CB	100.00	179HIS	CE1	212GLU	CD	0.04	221ME	CE	253SER	CA	0.09
221MET	CA	221MET	CG	100.00	179HIS	CE1	229ARG	CG	0.01	221ME	CE	253SER	C	0.01
221MET	CA	221MET	C	100.00	179HIS	CE1	229ARG	CD	0.02	221ME	CE	254LEU	CA	0.00
221MET	CA	246LEU	CD1	0.00	179HIS	CE1	229ARG	CZ	2.58	221ME	CE	254LEU	CB	0.00
221MET	CA	246LEU	CD2	0.01	179HIS	C	180VAL	CA	100.00	221ME	CE	254LEU	CG	0.20
221MET	CA	249VAL	CG1	0.02	179HIS	C	180VAL	CB	28.55	221ME	CE	254LEU	CD1	0.61
221MET	CA	249VAL	CG2	0.02	179HIS	C	180VAL	CG1	0.32	221ME	CE	254LEU	CD2	0.44
221MET	CB	221MET	CG	100.00	179HIS	C	180VAL	CG2	13.24	221ME	C	222HIS	CA	100.00
221MET	CB	221MET	CE	35.18	179HIS	C	180VAL	C	77.90	221ME	C	222HIS	CB	0.04
221MET	CB	221MET	C	100.00	180VAL	CA	180VAL	CB	100.00	221ME	C	222HIS	C	99.97
221MET	CB	225ARG	CZ	0.01	180VAL	CA	180VAL	CG1	100.00	221ME	C	224VAL	CG2	0.01
221MET	CB	249VAL	CG2	0.00	180VAL	CA	180VAL	CG2	100.00	222HIS	CA	222HIS	CB	100.00
221MET	CB	254LEU	CD2	0.05	180VAL	CA	180VAL	C	100.00	222HIS	CA	222HIS	CG	100.00
221MET	CG	221MET	CE	100.00	180VAL	CB	180VAL	CG1	100.00	222HIS	CA	222HIS	CD2	13.71
221MET	CG	221MET	C	99.52	180VAL	CB	180VAL	CG2	100.00	222HIS	CA	222HIS	C	100.00
221MET	CG	225ARG	CZ	0.01	180VAL	CB	180VAL	C	100.00	222HIS	CA	225ARG	CB	0.00
221MET	CG	245ASP	C	0.05	180VAL	CB	211LEU	CD1	0.04	222HIS	CB	222HIS	CG	100.00
221MET	CG	248SER	C	0.01	180VAL	CG1	180VAL	CG2	100.00	222HIS	CB	222HIS	CD2	100.00
221MET	CG	249VAL	CB	0.33	180VAL	CG1	180VAL	C	98.86	222HIS	CB	222HIS	CE1	0.16
221MET	CG	249VAL	CG1	4.62	180VAL	CG1	181VAL	C	0.04	222HIS	CB	222HIS	C	100.00
221MET	CG	249VAL	CG2	1.51	180VAL	CG1	209VAL	CG1	0.76	222HIS	CB	230PHE	CE1	0.03
221MET	CG	254LEU	CD1	0.07	180VAL	CG1	211LEU	CD1	2.86	222HIS	CB	230PHE	CE2	0.30
221MET	CG	254LEU	CD2	0.39	180VAL	CG1	211LEU	CD2	0.08	222HIS	CB	230PHE	CZ	1.46
221MET	CE	221MET	C	0.11	180VAL	CG1	232VAL	CG1	0.06	222HIS	CG	222HIS	CD2	100.00
221MET	CE	222HIS	CE1	0.07	180VAL	CG1	234VAL	CG1	1.20	222HIS	CG	222HIS	CE1	100.00
221MET	CE	225ARG	CD	0.17	180VAL	CG1	234VAL	CG2	16.08	222HIS	CG	222HIS	C	88.38
221MET	CE	225ARG	CZ	3.38	180VAL	CG2	180VAL	C	8.90	222HIS	CG	230PHE	CE1	0.61
221MET	CE	245ASP	CB	0.08	180VAL	CG2	209VAL	CG1	14.21	222HIS	CG	230PHE	CE2	0.37
221MET	CE	245ASP	CG	0.07	180VAL	CG2	209VAL	CG2	0.02	222HIS	CG	230PHE	CZ	1.48
221MET	CE	248SER	CB	0.10	180VAL	CG2	211LEU	CD1	0.04	222HIS	CD2	222HIS	CE1	100.00
221MET	CE	248SER	C	0.60	180VAL	CG2	232VAL	CB	0.01	222HIS	CD2	222HIS	C	5.33
221MET	CE	249VAL	CA	0.00	180VAL	CG2	232VAL	CG1	0.23	222HIS	CD2	229ARG	CB	0.01
221MET	CE	249VAL	CG1	0.56	180VAL	CG2	234VAL	CG2	2.52	222HIS	CD2	229ARG	CD	0.00
221MET	CE	249VAL	CG2	0.01	180VAL	C	181VAL	CA	100.00	222HIS	CD2	229ARG	CZ	0.00
221MET	CE	252GLY	C	0.59	180VAL	C	181VAL	CB	25.14	222HIS	CD2	230PHE	CG	0.02
221MET	CE	253SER	CA	0.35	180VAL	C	181VAL	CG1	0.05	222HIS	CD2	230PHE	CD1	0.38
221MET	CE	253SER	C	0.11	180VAL	C	181VAL	CG2	9.25	222HIS	CD2	230PHE	CD2	1.43
221MET	CE	254LEU	CB	0.01	180VAL	C	181VAL	C	87.54	222HIS	CD2	230PHE	CE1	8.30
221MET	CE	254LEU	CG	0.07	180VAL	C	211LEU	CD1	0.00	222HIS	CD2	230PHE	CE2	13.76
221MET	CE	254LEU	CD1	1.22	181VAL	CA	181VAL	CB	100.00	222HIS	CD2	230PHE	CZ	10.31
221MET	CE	254LEU	CD2	1.11	181VAL	CA	181VAL	CG1	100.00	222HIS	CE1	225ARG	CD	0.00
221MET	C	222HIS	CA	100.00	181VAL	CA	181VAL	CG2	100.00	222HIS	CE1	225ARG	CZ	0.01
221MET	C	222HIS	CB	0.33	181VAL	CA	181VAL	C	100.00	222HIS	CE1	226SER	CB	0.02
221MET	C	222HIS	C	99.56	181VAL	CB	181VAL	CG1	100.00	222HIS	CE1	226SER	C	0.01
221MET	C	224VAL	CG2	0.01	181VAL	CB	181VAL	CG2	100.00	222HIS	CE1	227PRO	CA	0.00
222HIS	CA	222HIS	CB	100.00	181VAL	CB	181VAL	C	100.00	222HIS	CE1	229ARG	CB	0.12
222HIS	CA	222HIS	CG	100.00	181VAL	CB	233VAL	CG1	0.00	222HIS	CE1	229ARG	CG	0.06
222HIS	CA	222HIS	CD2	37.21	181VAL	CG1	181VAL	CG2	100.00	222HIS	CE1	229ARG	CD	0.22
222HIS	CA	222HIS	C	100.00	181VAL	CG1	181VAL	C	99.78	222HIS	CE1	229ARG	CZ	0.56
222HIS	CA	225ARG	CB	0.04	181VAL	CG1	182SER	C	0.07	222HIS	CE1	230PHE	CG	0.06
222HIS	CB	222HIS	CG	100.00	181VAL	CG1	183VAL	CG1	0.01	222HIS	CE1	230PHE	CD1	0.13

222HIS	CB	222HIS	CD2	100.00	181VAI	CG1	183VAL	CG2	0.53	222HI	CE1	230PHE	CD2	0.23
222HIS	CB	222HIS	CE1	0.22	181VAI	CG1	212GLU	CG	0.01	222HI	CE1	230PHE	CE1	0.42
222HIS	CB	222HIS	C	100.00	181VAI	CG1	213HIS	C	0.00	222HI	CE1	230PHE	CE2	1.75
222HIS	CB	230PHE	CE2	0.02	181VAI	CG1	214GLN	CB	2.96	222HI	CE1	230PHE	CZ	1.46
222HIS	CB	230PHE	CZ	0.07	181VAI	CG1	214GLN	CG	2.41	222HI	C	223LEU	CA	100.00
222HIS	CG	222HIS	CD2	100.00	181VAI	CG1	214GLN	CD	0.19	222HI	C	223LEU	C	100.00
222HIS	CG	222HIS	CE1	100.00	181VAI	CG1	219MET	CG	0.04	222HI	C	230PHE	CE1	0.05
222HIS	CG	222HIS	C	97.92	181VAI	CG1	219MET	CE	0.21	222HI	C	230PHE	CE2	0.00
222HIS	CG	230PHE	CE2	0.14	181VAI	CG1	230PHE	CD1	0.37	223LE	CA	223LEU	CB	100.00
222HIS	CG	230PHE	CZ	0.29	181VAI	CG1	230PHE	CE1	0.02	223LE	CA	223LEU	CG	100.00
222HIS	CD2	222HIS	CE1	100.00	181VAI	CG1	233VAL	CA	0.01	223LE	CA	223LEU	CD1	47.19
222HIS	CD2	222HIS	C	18.94	181VAI	CG1	233VAL	CG1	0.02	223LE	CA	223LEU	CD2	48.99
222HIS	CD2	225ARG	CZ	0.12	181VAI	CG1	233VAL	CG2	0.30	223LE	CA	223LEU	C	100.00
222HIS	CD2	226SER	CB	0.01	181VAI	CG2	181VAL	C	5.75	223LE	CA	230PHE	CE1	0.76
222HIS	CD2	229ARG	CB	0.15	181VAI	CG2	212GLU	CB	0.08	223LE	CA	230PHE	CE2	0.04
222HIS	CD2	229ARG	CG	0.34	181VAI	CG2	212GLU	CG	0.11	223LE	CA	230PHE	CZ	0.20
222HIS	CD2	229ARG	CD	0.64	181VAI	CG2	212GLU	CD	0.01	223LE	CB	223LEU	CG	100.00
222HIS	CD2	230PHE	CE2	3.94	181VAI	CG2	212GLU	C	0.00	223LE	CB	223LEU	CD1	100.00
222HIS	CD2	230PHE	CZ	4.56	181VAI	CG2	214GLN	CB	0.34	223LE	CB	223LEU	CD2	100.00
222HIS	CE1	225ARG	CB	0.00	181VAI	CG2	214GLN	CG	0.18	223LE	CB	223LEU	C	100.00
222HIS	CE1	225ARG	CG	0.00	181VAI	CG2	214GLN	CD	0.00	223LE	CB	230PHE	CZ	0.02
222HIS	CE1	225ARG	CD	0.02	181VAI	CG2	219MET	CE	0.01	223LE	CB	246LEU	CD2	0.04
222HIS	CE1	225ARG	CZ	0.01	181VAI	CG2	230PHE	CG	0.00	223LE	CG	223LEU	CD1	100.00
222HIS	CE1	226SER	CB	1.16	181VAI	CG2	230PHE	CD1	0.80	223LE	CG	223LEU	CD2	100.00
222HIS	CE1	229ARG	CB	0.03	181VAI	CG2	230PHE	CE1	0.08	223LE	CG	223LEU	C	52.32
222HIS	CE1	229ARG	CG	0.23	181VAI	CG2	233VAL	CG1	0.00	223LE	CG	230PHE	CD1	0.00
222HIS	CE1	229ARG	CD	1.88	181VAI	CG2	233VAL	CG2	0.00	223LE	CG	230PHE	CE1	0.18
222HIS	CE1	229ARG	CZ	0.73	181VAI	C	182SER	CA	100.00	223LE	CG	230PHE	CE2	0.04
222HIS	CE1	230PHE	CE2	0.01	181VAI	C	182SER	CB	23.42	223LE	CG	230PHE	CZ	0.12
222HIS	CE1	230PHE	CZ	0.02	181VAI	C	182SER	C	90.35	223LE	CD1	223LEU	CD2	100.00
222HIS	C	223LEU	CA	100.00	182SE	CA	182SER	CB	100.00	223LE	CD1	223LEU	C	0.66
222HIS	C	223LEU	C	100.00	182SE	CA	182SER	C	100.00	223LE	CD1	227PRO	CB	0.01
222HIS	C	230PHE	CE2	0.21	182SE	CA	234VAL	CG1	0.01	223LE	CD1	230PHE	CB	0.00
222HIS	C	230PHE	CZ	0.00	182SE	CA	234VAL	CG2	0.04	223LE	CD1	230PHE	CG	0.28
223LEU	CA	223LEU	CB	100.00	182SE	CB	182SER	C	100.00	223LE	CD1	230PHE	CD1	1.34
223LEU	CA	223LEU	CG	100.00	182SE	CB	199VAL	CG1	0.41	223LE	CD1	230PHE	CD2	0.43
223LEU	CA	223LEU	CD1	90.01	182SE	CB	199VAL	CG2	0.01	223LE	CD1	230PHE	CE1	0.49
223LEU	CA	223LEU	CD2	6.99	182SE	CB	211LEU	CD1	0.08	223LE	CD1	230PHE	CE2	0.14
223LEU	CA	223LEU	C	100.00	182SE	CB	211LEU	CD2	0.02	223LE	CD1	230PHE	CZ	0.79
223LEU	CA	230PHE	CD2	0.00	182SE	CB	234VAL	CG2	0.02	223LE	CD1	243LEU	CB	0.03
223LEU	CA	230PHE	CE2	2.84	182SE	C	183VAL	CA	100.00	223LE	CD1	243LEU	CG	0.74
223LEU	CA	230PHE	CZ	0.02	182SE	C	183VAL	CB	26.51	223LE	CD1	243LEU	CD1	1.98
223LEU	CB	223LEU	CG	100.00	182SE	C	183VAL	CG1	0.29	223LE	CD1	243LEU	CD2	0.48
223LEU	CB	223LEU	CD1	100.00	182SE	C	183VAL	CG2	15.49	223LE	CD1	246LEU	CD2	0.05
223LEU	CB	223LEU	CD2	100.00	182SE	C	183VAL	C	85.38	223LE	CD1	247ALA	CB	0.05
223LEU	CB	223LEU	C	100.00	183VAI	CA	183VAL	CB	100.00	223LE	CD1	250LEU	CD1	0.01
223LEU	CB	230PHE	CE2	0.01	183VAI	CA	183VAL	CG1	100.00	223LE	CD2	223LEU	C	2.38
223LEU	CB	230PHE	CZ	0.00	183VAI	CA	183VAL	CG2	100.00	223LE	CD2	227PRO	CB	0.01
223LEU	CB	246LEU	CD2	0.00	183VAI	CA	183VAL	C	100.00	223LE	CD2	230PHE	CB	0.01
223LEU	CG	223LEU	CD1	100.00	183VAI	CB	183VAL	CG1	100.00	223LE	CD2	230PHE	CG	0.13
223LEU	CG	223LEU	CD2	100.00	183VAI	CB	183VAL	CG2	100.00	223LE	CD2	230PHE	CD1	1.54
223LEU	CG	223LEU	C	95.44	183VAI	CB	183VAL	C	100.00	223LE	CD2	230PHE	CD2	0.66
223LEU	CG	230PHE	CD2	0.00	183VAI	CB	233VAL	CG1	0.00	223LE	CD2	230PHE	CE1	2.07
223LEU	CG	230PHE	CE2	0.00	183VAI	CB	235THR	CB	0.03	223LE	CD2	230PHE	CE2	0.62
223LEU	CG	230PHE	CZ	0.00	183VAI	CB	235THR	CG2	0.00	223LE	CD2	230PHE	CZ	0.94
223LEU	CG	250LEU	CD1	0.02	183VAI	CG1	183VAL	CG2	100.00	223LE	CD2	243LEU	CB	0.00
223LEU	CD1	223LEU	CD2	100.00	183VAI	CG1	183VAL	C	97.67	223LE	CD2	243LEU	CG	0.52
223LEU	CD1	223LEU	C	1.92	183VAI	CG1	214GLN	CG	0.00	223LE	CD2	243LEU	CD1	2.28
223LEU	CD1	227PRO	CB	0.15	183VAI	CG1	215TYR	C	0.02	223LE	CD2	243LEU	CD2	0.07
223LEU	CD1	230PHE	CB	0.25	183VAI	CG1	216VAL	CA	8.42	223LE	CD2	246LEU	CB	0.02
223LEU	CD1	230PHE	CG	1.00	183VAI	CG1	216VAL	CB	4.32	223LE	CD2	246LEU	CG	0.06
223LEU	CD1	230PHE	CD1	0.04	183VAI	CG1	216VAL	CG2	2.60	223LE	CD2	246LEU	CD1	0.03

223LEU	CD1	230PHE	CD2	6.47	183VAI	CG1	219MET	CB	0.16	223LEI	CD2	246LEU	CD2	0.31
223LEU	CD1	230PHE	CE1	0.02	183VAI	CG1	219MET	CG	0.13	223LEI	CD2	247ALA	CA	0.04
223LEU	CD1	230PHE	CE2	0.54	183VAI	CG1	219MET	CE	0.05	223LEI	CD2	247ALA	CB	0.43
223LEU	CD1	230PHE	CZ	0.00	183VAI	CG1	233VAL	CG1	0.55	223LEI	CD2	250LEU	CD1	0.00
223LEU	CD1	243LEU	CG	0.00	183VAI	CG1	235THR	CB	0.40	223LEI	C	224VAL	CA	100.00
223LEU	CD1	243LEU	CD1	0.13	183VAI	CG1	235THR	CG2	0.05	223LEI	C	224VAL	CB	64.62
223LEU	CD1	243LEU	CD2	0.10	183VAI	CG1	239PHE	CB	0.02	223LEI	C	224VAL	CG1	25.40
223LEU	CD1	246LEU	CB	0.00	183VAI	CG1	239PHE	CD1	0.00	223LEI	C	224VAL	CG2	4.54
223LEU	CD1	246LEU	CD2	0.05	183VAI	CG1	239PHE	CD2	0.05	223LEI	C	224VAL	C	40.90
223LEU	CD1	250LEU	CD1	0.18	183VAI	CG1	243LEU	CD1	0.22	224VA	CA	224VAL	CB	100.00
223LEU	CD1	250LEU	CD2	0.00	183VAI	CG2	183VAL	C	5.81	224VA	CA	224VAL	CG1	100.00
223LEU	CD2	223LEU	C	2.83	183VAI	CG2	215TYR	C	0.01	224VA	CA	224VAL	CG2	100.00
223LEU	CD2	227PRO	CB	0.01	183VAI	CG2	216VAL	CA	0.91	224VA	CA	224VAL	C	100.00
223LEU	CD2	230PHE	CG	0.04	183VAI	CG2	216VAL	CB	0.04	224VA	CB	224VAL	CG1	100.00
223LEU	CD2	230PHE	CD1	0.01	183VAI	CG2	216VAL	CG2	0.03	224VA	CB	224VAL	CG2	100.00
223LEU	CD2	230PHE	CD2	0.56	183VAI	CG2	219MET	CB	0.10	224VA	CB	224VAL	C	100.00
223LEU	CD2	230PHE	CE1	0.00	183VAI	CG2	219MET	CG	0.38	224VA	CB	224VAL	CG1	0.00
223LEU	CD2	230PHE	CE2	0.21	183VAI	CG2	219MET	CE	0.60	224VA	CB	249VAL	CG1	0.04
223LEU	CD2	230PHE	CZ	0.01	183VAI	CG2	233VAL	CB	0.01	224VA	CG1	224VAL	CG2	100.00
223LEU	CD2	243LEU	CG	0.01	183VAI	CG2	233VAL	CG1	18.27	224VA	CG1	224VAL	C	29.31
223LEU	CD2	243LEU	CD1	0.69	183VAI	CG2	233VAL	CG2	0.08	224VA	CG1	246LEU	CG	0.06
223LEU	CD2	243LEU	CD2	0.47	183VAI	CG2	234VAL	C	0.00	224VA	CG1	246LEU	CD1	0.20
223LEU	CD2	246LEU	CB	0.48	183VAI	CG2	235THR	CB	0.51	224VA	CG1	246LEU	CD2	0.13
223LEU	CD2	246LEU	CG	0.04	183VAI	CG2	235THR	CG2	0.00	224VA	CG1	249VAL	CG2	0.14
223LEU	CD2	246LEU	CD1	0.00	183VAI	CG2	243LEU	CD1	0.02	224VA	CG1	250LEU	CD1	0.00
223LEU	CD2	246LEU	CD2	1.04	183VAI	C	184ASP	CA	100.00	224VA	CG1	250LEU	CD2	0.02
223LEU	CD2	246LEU	C	0.05	183VAI	C	184ASP	CB	98.02	224VA	CG1	117PRO	CG	0.09
223LEU	CD2	247ALA	CB	0.01	183VAI	C	184ASP	CG	0.01	224VA	CG1	118LEU	CG	0.04
223LEU	CD2	250LEU	CD1	0.08	183VAI	C	184ASP	C	2.68	224VA	CG1	118LEU	CD1	0.10
223LEU	CD2	250LEU	CD2	0.00	184ASI	CA	184ASP	CB	100.00	224VA	CG1	118LEU	CD2	0.51
223LEU	C	224VAL	CA	100.00	184ASI	CA	184ASP	CG	100.00	224VA	CG1	224VAL	CG1	0.46
223LEU	C	224VAL	CB	9.72	184ASI	CA	184ASP	C	100.00	224VA	CG1	224VAL	CG2	0.00
223LEU	C	224VAL	CG1	3.84	184ASI	CA	239PHE	CE2	0.02	224VA	CG1	249VAL	CB	0.02
223LEU	C	224VAL	CG2	0.38	184ASI	CB	184ASP	CG	100.00	224VA	CG1	249VAL	CG1	1.14
223LEU	C	224VAL	C	92.57	184ASI	CB	184ASP	C	100.00	224VA	CG1	249VAL	CG2	0.23
224VAL	CA	224VAL	CB	100.00	184ASI	CB	195TRP	CD1	0.56	224VA	CG2	224VAL	C	65.27
224VAL	CA	224VAL	CG1	100.00	184ASI	CB	213HIS	C	0.00	224VA	CG2	246LEU	CG	0.00
224VAL	CA	224VAL	CG2	100.00	184ASI	CB	214GLN	C	0.00	224VA	CG2	246LEU	CD1	0.02
224VAL	CA	224VAL	C	100.00	184ASI	CG	184ASP	C	100.00	224VA	CG2	246LEU	CD2	0.22
224VAL	CB	224VAL	CG1	100.00	184ASI	CG	186ALA	CB	11.75	224VA	CG2	117PRO	CG	0.04
224VAL	CB	224VAL	CG2	100.00	184ASI	CG	196ARG	CG	0.00	224VA	CG2	224VAL	CG1	0.08
224VAL	CB	224VAL	C	100.00	184ASI	CG	196ARG	CD	0.00	224VA	CG2	246LEU	CD1	2.02
224VAL	CB	224VAL	CG1	0.00	184ASI	CG	196ARG	CZ	0.02	224VA	CG2	246LEU	CD2	0.22
224VAL	CB	249VAL	CG1	0.05	184ASI	C	185LYS	CA	100.00	224VA	CG2	249VAL	CG1	0.53
224VAL	CB	249VAL	CG2	0.01	184ASI	C	185LYS	CB	99.89	224VA	CG2	249VAL	CG2	2.15
224VAL	CG1	224VAL	CG2	100.00	184ASI	C	185LYS	CG	0.06	224VA	C	225ARG	CA	100.00
224VAL	CG1	224VAL	C	56.25	184ASI	C	185LYS	C	1.32	224VA	C	225ARG	CB	57.43
224VAL	CG1	246LEU	CG	0.01	184ASI	C	239PHE	CD2	0.00	224VA	C	225ARG	CG	0.34
224VAL	CG1	246LEU	CD1	0.04	184ASI	C	239PHE	CE1	0.34	224VA	C	225ARG	C	42.70
224VAL	CG1	246LEU	CD2	0.05	184ASI	C	239PHE	CE2	0.50	224VA	C	249VAL	CG2	0.15
224VAL	CG1	250LEU	CD2	0.08	185LYS	CA	185LYS	CB	100.00	225AR	CA	225ARG	CB	100.00
224VAL	CG1	117PRO	CB	0.00	185LYS	CA	185LYS	CG	100.00	225AR	CA	225ARG	CG	100.00
224VAL	CG1	117PRO	CG	0.35	185LYS	CA	185LYS	CD	66.02	225AR	CA	225ARG	CD	0.06
224VAL	CG1	118LEU	CG	0.00	185LYS	CA	185LYS	CE	0.04	225AR	CA	225ARG	C	100.00
224VAL	CG1	118LEU	CD1	0.18	185LYS	CA	185LYS	C	100.00	225AR	CA	117PRO	CB	0.02
224VAL	CG1	118LEU	CD2	0.06	185LYS	CA	215TYR	CD1	0.01	225AR	CA	117PRO	CG	0.08
224VAL	CG1	224VAL	CG1	0.58	185LYS	CA	215TYR	CD2	0.03	225AR	CB	225ARG	CG	100.00
224VAL	CG1	224VAL	CG2	0.06	185LYS	CB	185LYS	CG	100.00	225AR	CB	225ARG	CD	100.00
224VAL	CG1	246LEU	CD1	0.29	185LYS	CB	185LYS	CD	100.00	225AR	CB	225ARG	CZ	0.02
224VAL	CG1	246LEU	CD2	0.20	185LYS	CB	185LYS	CE	0.59	225AR	CB	225ARG	C	100.00
224VAL	CG1	249VAL	CG1	0.59	185LYS	CB	185LYS	C	100.00	225AR	CB	117PRO	CG	0.03
224VAL	CG1	249VAL	CG2	0.05	185LYS	CB	188VAL	CG2	0.02	225AR	CB	249VAL	CG2	0.04

224VAL CG1	249VAL C		0.00	185LYS CB	189LEU	CB	0.01	225AR CG	225ARG	CD	100.00
224VAL CG1	250LEU CD2		0.01	185LYS CB	189LEU	CD1	1.37	225AR CG	225ARG	CZ	2.64
224VAL CG2	224VAL C		43.42	185LYS CB	189LEU	CD2	0.01	225AR CG	225ARG	C	81.01
224VAL CG2	246LEU CG		0.02	185LYS CB	216VAL	CG1	0.00	225AR CG	117PRO	CG	0.07
224VAL CG2	246LEU CD1		0.15	185LYS CB	239PHE	CE1	0.90	225AR CG	249VAL	CG1	0.13
224VAL CG2	246LEU CD2		0.34	185LYS CB	239PHE	CE2	0.97	225AR CG	249VAL	CG2	0.13
224VAL CG2	250LEU CD2		0.00	185LYS CB	239PHE	CZ	1.59	225AR CD	225ARG	CZ	100.00
224VAL CG2	224VAL CG1		0.04	185LYS CG	185LYS	CD	100.00	225AR CD	225ARG	C	0.02
224VAL CG2	224VAL CG2		0.05	185LYS CG	185LYS	CE	100.00	225AR CD	249VAL	CG2	0.00
224VAL CG2	246LEU CD1		1.88	185LYS CG	185LYS	C	88.36	225AR CD	327LEU	CD1	0.00
224VAL CG2	246LEU CD2		0.37	185LYS CG	188VAL	CG1	0.01	225AR CZ	249VAL	CG1	0.01
224VAL CG2	249VAL CB		0.01	185LYS CG	188VAL	CG2	3.17	225AR CZ	252GLY	C	0.00
224VAL CG2	249VAL CG1		0.89	185LYS CG	189LEU	CB	0.00	225AR CZ	254LEU	CD1	0.03
224VAL CG2	249VAL CG2		3.20	185LYS CG	189LEU	CD1	0.18	225AR CZ	254LEU	CD2	0.00
224VAL CG2	250LEU CD1		0.01	185LYS CG	216VAL	CG1	0.09	225AR CZ	327LEU	CD1	0.00
224VAL CG2	250LEU CD2		0.03	185LYS CG	239PHE	CZ	0.00	225AR CZ	327LEU	CD2	0.09
224VAL C	225ARG CA		100.00	185LYS CG	139TYR	CE1	0.04	225AR C	226SER	CA	100.00
224VAL C	225ARG CB		17.03	185LYS CG	139TYR	CE2	0.04	225AR C	226SER	CB	100.00
224VAL C	225ARG CG		2.05	185LYS CG	139TYR	CZ	0.01	225AR C	226SER	C	0.02
224VAL C	225ARG C		81.62	185LYS CD	185LYS	CE	100.00	225AR C	227PRO	CD	0.87
224VAL C	249VAL CG2		0.00	185LYS CD	185LYS	C	0.06	226SE CA	226SER	CB	100.00
225ARG CA	225ARG CB		100.00	185LYS CD	188VAL	CG2	0.31	226SE CA	226SER	C	100.00
225ARG CA	225ARG CG		100.00	185LYS CD	215TYR	CB	0.00	226SE CA	227PRO	CD	100.00
225ARG CA	225ARG CD		3.80	185LYS CD	215TYR	CG	0.01	226SE CB	226SER	C	100.00
225ARG CA	225ARG C		100.00	185LYS CD	215TYR	CD2	0.00	226SE CB	229ARG	CG	0.03
225ARG CA	117PRO CB		0.02	185LYS CD	216VAL	CG1	1.55	226SE CB	229ARG	CD	0.17
225ARG CA	117PRO CG		0.02	185LYS CD	239PHE	CZ	0.01	226SE CB	229ARG	CZ	4.38
225ARG CA	249VAL CG2		0.00	185LYS CD	139TYR	CE1	0.01	226SE C	227PRO	CA	100.00
225ARG CB	225ARG CG		100.00	185LYS CD	139TYR	CE2	0.04	226SE C	227PRO	CB	0.04
225ARG CB	225ARG CD		100.00	185LYS CD	238ILE	CG1	0.12	226SE C	227PRO	CD	100.00
225ARG CB	225ARG CZ		0.67	185LYS CD	238ILE	CG2	0.25	226SE C	227PRO	C	100.00
225ARG CB	225ARG C		100.00	185LYS CD	238ILE	CD	0.18	227PR CA	227PRO	CB	100.00
225ARG CB	249VAL CG2		0.04	185LYS CE	188VAL	CG1	0.00	227PR CA	227PRO	CG	100.00
225ARG CG	225ARG CD		100.00	185LYS CE	188VAL	CG2	0.69	227PR CA	227PRO	CD	100.00
225ARG CG	225ARG CZ		4.18	185LYS CE	215TYR	CD1	0.00	227PR CA	227PRO	C	100.00
225ARG CG	225ARG C		71.59	185LYS CE	215TYR	CD2	0.00	227PR CA	230PHE	CD1	0.01
225ARG CG	117PRO CG		0.00	185LYS CE	216VAL	CG1	0.24	227PR CA	230PHE	CD2	0.01
225ARG CG	249VAL CB		0.01	185LYS CE	217ASP	CG	0.29	227PR CA	230PHE	CE1	0.12
225ARG CG	249VAL CG1		0.71	185LYS CE	139TYR	CE1	0.00	227PR CA	230PHE	CE2	0.32
225ARG CG	249VAL CG2		0.83	185LYS CE	139TYR	CE2	0.05	227PR CA	230PHE	CZ	0.67
225ARG CD	225ARG CZ		100.00	185LYS CE	139TYR	CZ	0.04	227PR CB	227PRO	CG	100.00
225ARG CD	225ARG C		0.03	185LYS CE	238ILE	CG1	0.00	227PR CB	227PRO	CD	100.00
225ARG CD	249VAL CB		0.00	185LYS CE	238ILE	CG2	0.04	227PR CB	227PRO	C	100.00
225ARG CD	249VAL CG1		0.48	185LYS CE	238ILE	CD	0.12	227PR CB	230PHE	CE1	0.01
225ARG CD	249VAL CG2		0.49	185LYS CE	241ASP	CG	0.02	227PR CB	230PHE	CZ	0.04
225ARG CD	254LEU CD1		0.03	185LYS CE	242ILE	CG1	0.03	227PR CB	250LEU	CD1	0.20
225ARG CD	254LEU CD2		0.15	185LYS CE	242ILE	CD	0.00	227PR CB	250LEU	CD2	0.05
225ARG CZ	117PRO CB		0.03	185LYS C	186ALA	CA	100.00	227PR CG	227PRO	CD	100.00
225ARG CZ	249VAL CG1		0.69	185LYS C	186ALA	CB	0.04	227PR CG	227PRO	C	86.83
225ARG CZ	249VAL CG2		0.03	185LYS C	186ALA	C	99.97	227PR CG	250LEU	CD1	0.26
225ARG CZ	254LEU CG		0.02	185LYS C	189LEU	CD1	0.02	227PR CG	250LEU	CD2	0.36
225ARG CZ	254LEU CD1		2.03	185LYS C	189LEU	CD2	0.00	227PR CD	227PRO	C	52.51
225ARG CZ	254LEU CD2		0.59	185LYS C	215TYR	CD1	0.00	227PR C	228ALA	CA	100.00
225ARG CZ	327LEU CD2		0.02	185LYS C	215TYR	CD2	0.00	227PR C	228ALA	CB	0.14
225ARG C	226SER CA		100.00	185LYS C	215TYR	CE1	0.01	227PR C	228ALA	C	99.88
225ARG C	226SER CB		100.00	185LYS C	215TYR	CE2	0.00	227PR C	230PHE	CE1	0.01
225ARG C	226SER C		0.18	186ALA CA	186ALA	CB	100.00	227PR C	230PHE	CE2	0.00
225ARG C	227PRO CD		0.25	186ALA CA	186ALA	C	100.00	228AL CA	228ALA	CB	100.00
226SER CA	226SER CB		100.00	186ALA CA	192GLY	C	1.85	228AL CA	228ALA	C	100.00
226SER CA	226SER C		100.00	186ALA CB	186ALA	C	100.00	228AL CB	228ALA	C	100.00
226SER CA	227PRO CD		100.00	186ALA CB	192GLY	CA	0.13	228AL CB	229ARG	CZ	0.08
226SER CB	226SER C		100.00	186ALA CB	192GLY	C	15.78	228AL C	229ARG	CA	100.00

226SER CB	229ARG CG	0.02	186AL CB	193GLU	CA	1.07	228AL C	229ARG	CB	19.21
226SER CB	229ARG CD	0.01	186AL CB	193GLU	CB	0.00	228AL C	229ARG	CG	11.71
226SER CB	229ARG CZ	0.00	186AL CB	196ARG	CG	0.05	228AL C	229ARG	CD	0.01
226SER C	227PRO CA	100.00	186AL CB	196ARG	CD	4.90	228AL C	229ARG	CZ	0.06
226SER C	227PRO CB	0.07	186AL CB	196ARG	CZ	3.58	228AL C	229ARG	C	78.54
226SER C	227PRO CD	100.00	186AL C	187ASN	CA	100.00	229AR CA	229ARG	CB	100.00
226SER C	227PRO C	100.00	186AL C	187ASN	CB	2.70	229AR CA	229ARG	CG	100.00
227PRO CA	227PRO CB	100.00	186AL C	187ASN	CG	0.04	229AR CA	229ARG	CD	24.59
227PRO CA	227PRO CG	100.00	186AL C	187ASN	C	97.13	229AR CA	229ARG	CZ	0.60
227PRO CA	227PRO CD	100.00	186AL C	215TYR	CE1	0.03	229AR CA	229ARG	C	100.00
227PRO CA	227PRO C	100.00	186AL C	215TYR	CE2	0.03	229AR CB	229ARG	CG	100.00
227PRO CA	230PHE CD2	0.07	187AS CA	187ASN	CB	100.00	229AR CB	229ARG	CD	100.00
227PRO CB	227PRO CG	100.00	187AS CA	187ASN	CG	100.00	229AR CB	229ARG	CZ	0.08
227PRO CB	227PRO CD	100.00	187AS CA	187ASN	C	100.00	229AR CB	229ARG	C	100.00
227PRO CB	227PRO C	100.00	187AS CA	82ARG	CZ	0.01	229AR CB	230PHE	CD1	0.02
227PRO CB	230PHE CD2	0.01	187AS CB	187ASN	CG	100.00	229AR CB	230PHE	CD2	0.04
227PRO CB	230PHE CE2	0.00	187AS CB	187ASN	C	100.00	229AR CB	230PHE	CE1	0.08
227PRO CB	250LEU CD1	1.52	187AS CB	215TYR	CD2	0.01	229AR CB	230PHE	CE2	0.02
227PRO CB	250LEU CD2	0.16	187AS CB	215TYR	CE1	0.53	229AR CG	229ARG	CD	100.00
227PRO CG	227PRO CD	100.00	187AS CB	215TYR	CE2	1.83	229AR CG	229ARG	CZ	17.62
227PRO CG	227PRO C	84.62	187AS CB	215TYR	CZ	1.68	229AR CG	229ARG	C	9.74
227PRO CG	250LEU CD1	0.24	187AS CB	82ARG	CZ	0.00	229AR CG	230PHE	CD1	0.00
227PRO CG	250LEU CD2	0.20	187AS CG	187ASN	C	99.66	229AR CG	230PHE	CE1	0.03
227PRO CD	227PRO C	63.55	187AS CG	188VAL	CG2	0.01	229AR CD	229ARG	CZ	100.00
227PRO C	228ALA CA	100.00	187AS CG	215TYR	CD1	0.12	229AR CD	229ARG	C	0.11
227PRO C	228ALA CB	0.02	187AS CG	215TYR	CD2	0.41	229AR CD	230PHE	CE1	0.03
227PRO C	228ALA C	99.98	187AS CG	215TYR	CE1	0.70	229AR CD	230PHE	CZ	0.00
227PRO C	230PHE CD2	0.01	187AS CG	215TYR	CE2	2.28	229AR CZ	230PHE	CZ	0.00
228ALA CA	228ALA CB	100.00	187AS CG	215TYR	CZ	0.72	229AR C	230PHE	CA	100.00
228ALA CA	228ALA C	100.00	187AS C	188VAL	CA	100.00	229AR C	230PHE	CB	51.39
228ALA CB	228ALA C	100.00	187AS C	188VAL	CB	97.14	229AR C	230PHE	CG	6.52
228ALA CB	229ARG CZ	0.08	187AS C	188VAL	CG1	56.04	229AR C	230PHE	CD1	4.51
228ALA C	229ARG CA	100.00	187AS C	188VAL	CG2	1.18	229AR C	230PHE	CD2	2.72
228ALA C	229ARG CB	16.41	187AS C	188VAL	C	2.36	229AR C	230PHE	C	48.57
228ALA C	229ARG CG	1.30	187AS C	82ARG	CG	0.04	230PH CA	230PHE	CB	100.00
228ALA C	229ARG CD	0.00	187AS C	82ARG	CD	5.43	230PH CA	230PHE	CG	100.00
228ALA C	229ARG CZ	0.12	188VA CA	188VAL	CB	100.00	230PH CA	230PHE	CD1	71.49
228ALA C	229ARG C	93.94	188VA CA	188VAL	CG1	100.00	230PH CA	230PHE	CD2	65.46
229ARG CA	229ARG CB	100.00	188VA CA	188VAL	CG2	100.00	230PH CA	230PHE	C	100.00
229ARG CA	229ARG CG	100.00	188VA CA	188VAL	C	100.00	230PH CB	230PHE	CG	100.00
229ARG CA	229ARG CD	33.34	188VA CA	82ARG	CD	0.10	230PH CB	230PHE	CD1	100.00
229ARG CA	229ARG CZ	0.69	188VA CB	188VAL	CG1	100.00	230PH CB	230PHE	CD2	100.00
229ARG CA	229ARG C	100.00	188VA CB	188VAL	CG2	100.00	230PH CB	230PHE	C	100.00
229ARG CB	229ARG CG	100.00	188VA CB	188VAL	C	100.00	230PH CB	233VAL	CG2	0.04
229ARG CB	229ARG CD	100.00	188VA CB	138ILE	CG1	0.00	230PH CG	230PHE	CD1	100.00
229ARG CB	229ARG CZ	0.46	188VA CB	138ILE	CD	0.10	230PH CG	230PHE	CD2	100.00
229ARG CB	229ARG C	100.00	188VA CB	139TYR	CB	0.20	230PH CG	230PHE	CE1	100.00
229ARG CB	230PHE CD2	0.10	188VA CB	139TYR	CG	1.53	230PH CG	230PHE	CE2	100.00
229ARG CB	230PHE CE2	0.08	188VA CB	139TYR	CD1	1.96	230PH CG	230PHE	CZ	100.00
229ARG CG	229ARG CD	100.00	188VA CB	139TYR	CD2	1.64	230PH CG	230PHE	C	34.87
229ARG CG	229ARG CZ	45.51	188VA CB	139TYR	CE1	0.08	230PH CG	233VAL	CG2	0.02
229ARG CG	229ARG C	71.95	188VA CB	139TYR	CE2	0.03	230PH CD1	230PHE	CD2	100.00
229ARG CG	230PHE CD1	0.07	188VA CB	139TYR	CZ	0.00	230PH CD1	230PHE	CE1	100.00
229ARG CG	230PHE CD2	0.20	188VA CB	140PHE	CZ	0.00	230PH CD1	230PHE	CE2	100.00
229ARG CG	230PHE CE1	0.51	188VA CG1	188VAL	CG2	100.00	230PH CD1	230PHE	CZ	100.00
229ARG CG	230PHE CE2	0.35	188VA CG1	188VAL	C	1.58	230PH CD1	230PHE	C	14.84
229ARG CG	230PHE CZ	0.18	188VA CG1	189LEU	CD2	0.00	230PH CD1	233VAL	CG2	9.20
229ARG CD	229ARG CZ	100.00	188VA CG1	82ARG	CD	0.13	230PH CD2	230PHE	CE1	100.00
229ARG CD	229ARG C	16.30	188VA CG1	82ARG	CZ	0.70	230PH CD2	230PHE	CE2	100.00
229ARG CD	230PHE CG	0.00	188VA CG1	87GLU	CG	0.02	230PH CD2	230PHE	CZ	100.00
229ARG CD	230PHE CD1	0.12	188VA CG1	87GLU	CD	0.02	230PH CD2	230PHE	C	2.27
229ARG CD	230PHE CD2	0.07	188VA CG1	139TYR	CA	0.00	230PH CD2	233VAL	CG2	3.00

229ARG CD	230PHE	CE1	2.12	188VAl CG1	139TYR	CB	0.16	230PH CE1	230PHE	CE2	100.00
229ARG CD	230PHE	CE2	0.74	188VAl CG1	139TYR	CG	0.50	230PH CE1	230PHE	CZ	100.00
229ARG CD	230PHE	CZ	2.48	188VAl CG1	139TYR	CD1	1.84	230PH CE1	233VAL	CG2	2.99
229ARG CZ	230PHE	CD1	0.26	188VAl CG1	139TYR	CD2	2.42	230PH CE2	230PHE	CZ	100.00
229ARG CZ	230PHE	CE1	1.48	188VAl CG1	139TYR	CE1	0.59	230PH CE2	233VAL	CG1	0.00
229ARG CZ	230PHE	CZ	0.01	188VAl CG1	139TYR	CE2	2.72	230PH CE2	233VAL	CG2	0.59
229ARG C	230PHE	CA	100.00	188VAl CG1	139TYR	CZ	0.74	230PH CZ	233VAL	CG1	0.00
229ARG C	230PHE	CB	16.84	188VAl CG1	140PHE	CE1	0.00	230PH CZ	233VAL	CG2	0.04
229ARG C	230PHE	CG	8.03	188VAl CG1	140PHE	CE2	0.01	230PH C	231ASP	CA	100.00
229ARG C	230PHE	CD1	0.20	188VAl CG1	140PHE	CZ	0.06	230PH C	231ASP	CB	70.57
229ARG C	230PHE	CD2	3.52	188VAl CG1	238ILE	CD	0.02	230PH C	231ASP	CG	4.81
229ARG C	230PHE	C	84.20	188VAl CG2	188VAL	C	96.72	230PH C	231ASP	C	28.66
230PHE CA	230PHE	CB	100.00	188VAl CG2	189LEU	CD1	0.09	231AS CA	231ASP	CB	100.00
230PHE CA	230PHE	CG	100.00	188VAl CG2	189LEU	CD2	0.02	231AS CA	231ASP	CG	100.00
230PHE CA	230PHE	CD1	98.03	188VAl CG2	82ARG	CD	0.00	231AS CA	231ASP	C	100.00
230PHE CA	230PHE	CD2	45.18	188VAl CG2	82ARG	CZ	0.08	231AS CB	231ASP	CG	100.00
230PHE CA	230PHE	C	100.00	188VAl CG2	138ILE	CG1	0.01	231AS CB	231ASP	C	100.00
230PHE CB	230PHE	CG	100.00	188VAl CG2	138ILE	CD	0.64	231AS CB	232VAL	CG2	0.00
230PHE CB	230PHE	CD1	100.00	188VAl CG2	139TYR	CB	0.45	231AS CG	231ASP	C	38.54
230PHE CB	230PHE	CD2	100.00	188VAl CG2	139TYR	CG	2.37	231AS C	232VAL	CA	100.00
230PHE CB	230PHE	C	100.00	188VAl CG2	139TYR	CD1	6.24	231AS C	232VAL	CB	44.51
230PHE CB	233VAL	CG2	0.04	188VAl CG2	139TYR	CD2	4.15	231AS C	232VAL	CG1	0.02
230PHE CG	230PHE	CD1	100.00	188VAl CG2	139TYR	CE1	0.78	231AS C	232VAL	CG2	39.60
230PHE CG	230PHE	CD2	100.00	188VAl CG2	139TYR	CE2	1.89	231AS C	232VAL	C	62.75
230PHE CG	230PHE	CE1	100.00	188VAl CG2	139TYR	CZ	0.18	232VA CA	232VAL	CB	100.00
230PHE CG	230PHE	CE2	100.00	188VAl CG2	238ILE	CD	0.09	232VA CA	232VAL	CG1	100.00
230PHE CG	230PHE	CZ	100.00	188VAl C	189LEU	CA	100.00	232VA CA	232VAL	CG2	100.00
230PHE CD1	230PHE	CD2	100.00	188VAl C	189LEU	CB	10.22	232VA CA	232VAL	C	100.00
230PHE CD1	230PHE	CE1	100.00	188VAl C	189LEU	CG	1.49	232VA CB	232VAL	CG1	100.00
230PHE CD1	230PHE	CE2	100.00	188VAl C	189LEU	CD1	0.08	232VA CB	232VAL	CG2	100.00
230PHE CD1	230PHE	CZ	100.00	188VAl C	189LEU	CD2	0.01	232VA CB	232VAL	C	100.00
230PHE CD1	233VAL	CG2	0.26	188VAl C	189LEU	C	90.77	232VA CG1	232VAL	CG2	100.00
230PHE CD2	230PHE	CE1	100.00	188VAl C	82ARG	CD	0.45	232VA CG1	232VAL	C	99.62
230PHE CD2	230PHE	CE2	100.00	188VAl C	82ARG	CZ	0.04	232VA CG2	232VAL	C	0.19
230PHE CD2	230PHE	CZ	100.00	188VAl C	138ILE	CD	0.24	232VA C	233VAL	CA	100.00
230PHE CE1	230PHE	CE2	100.00	188VAl C	139TYR	CB	0.16	232VA C	233VAL	CB	53.62
230PHE CE1	230PHE	CZ	100.00	188VAl C	139TYR	CD2	0.18	232VA C	233VAL	CG2	40.37
230PHE CE2	230PHE	CZ	100.00	189LEl CA	189LEU	CB	100.00	232VA C	233VAL	C	61.35
230PHE C	231ASP	CA	100.00	189LEl CA	189LEU	CG	100.00	233VA CA	233VAL	CB	100.00
230PHE C	231ASP	CB	82.63	189LEl CA	189LEU	CD1	22.48	233VA CA	233VAL	CG1	100.00
230PHE C	231ASP	C	21.50	189LEl CA	189LEU	CD2	76.30	233VA CA	233VAL	CG2	100.00
231ASP CA	231ASP	CB	100.00	189LEl CA	189LEU	C	100.00	233VA CA	233VAL	C	100.00
231ASP CA	231ASP	CG	100.00	189LEl CB	189LEU	CG	100.00	233VA CB	233VAL	CG1	100.00
231ASP CA	231ASP	C	100.00	189LEl CB	189LEU	CD1	100.00	233VA CB	233VAL	CG2	100.00
231ASP CB	231ASP	CG	100.00	189LEl CB	189LEU	CD2	100.00	233VA CB	233VAL	C	100.00
231ASP CB	231ASP	C	100.00	189LEl CB	189LEU	C	100.00	233VA CG1	233VAL	CG2	100.00
231ASP CG	231ASP	C	99.98	189LEl CB	191VAL	CG2	0.02	233VA CG1	233VAL	C	99.97
231ASP C	232VAL	CA	100.00	189LEl CB	192GLY	CA	0.08	233VA CG1	243LEU	CD2	0.06
231ASP C	232VAL	CB	61.33	189LEl CG	189LEU	CD1	100.00	233VA CG2	233VAL	C	0.00
231ASP C	232VAL	CG2	51.86	189LEl CG	189LEU	CD2	100.00	233VA C	234VAL	CA	100.00
231ASP C	232VAL	C	49.95	189LEl CG	189LEU	C	18.06	233VA C	234VAL	CB	77.37
232VAL CA	232VAL	CB	100.00	189LEl CG	191VAL	CG1	0.01	233VA C	234VAL	C	34.07
232VAL CA	232VAL	CG1	100.00	189LEl CG	191VAL	CG2	0.38	234VA CA	234VAL	CB	100.00
232VAL CA	232VAL	CG2	100.00	189LEl CG	138ILE	CG2	0.01	234VA CA	234VAL	CG1	100.00
232VAL CA	232VAL	C	100.00	189LEl CG	138ILE	CD	0.09	234VA CA	234VAL	CG2	100.00
232VAL CB	232VAL	CG1	100.00	189LEl CG	153ASN	CG	0.00	234VA CA	234VAL	C	100.00
232VAL CB	232VAL	CG2	100.00	189LEl CD1	189LEU	CD2	100.00	234VA CB	234VAL	CG1	100.00
232VAL CB	232VAL	C	100.00	189LEl CD1	189LEU	C	0.00	234VA CB	234VAL	CG2	100.00
232VAL CG1	232VAL	CG2	100.00	189LEl CD1	191VAL	CG1	0.02	234VA CB	234VAL	C	100.00
232VAL CG1	232VAL	C	100.00	189LEl CD1	191VAL	CG2	0.14	234VA CG1	234VAL	CG2	100.00
232VAL C	233VAL	CA	100.00	189LEl CD1	239PHE	CE1	0.04	234VA CG1	234VAL	C	99.85
232VAL C	233VAL	CB	39.36	189LEl CD1	239PHE	CE2	0.08	234VA CG2	234VAL	C	99.98

232VAL	C	233VAL	CG2	27.29	189LEU	CD1	239PHE	CZ	0.21	234VA	C	235THR	CA	100.00
232VAL	C	233VAL	C	76.47	189LEU	CD1	138ILE	CB	0.00	234VA	C	235THR	CB	99.99
233VAL	CA	233VAL	CB	100.00	189LEU	CD1	138ILE	CG1	0.08	234VA	C	235THR	CG2	1.66
233VAL	CA	233VAL	CG1	100.00	189LEU	CD1	138ILE	CG2	0.61	234VA	C	235THR	C	0.11
233VAL	CA	233VAL	CG2	100.00	189LEU	CD1	138ILE	CD	0.28	235TH	CA	235THR	CB	100.00
233VAL	CA	233VAL	C	100.00	189LEU	CD1	138ILE	C	0.01	235TH	CA	235THR	CG2	100.00
233VAL	CB	233VAL	CG1	100.00	189LEU	CD1	139TYR	CA	0.01	235TH	CA	235THR	C	100.00
233VAL	CB	233VAL	CG2	100.00	189LEU	CD1	139TYR	CB	0.01	235TH	CB	235THR	CG2	100.00
233VAL	CB	233VAL	C	100.00	189LEU	CD1	139TYR	CD1	0.05	235TH	CB	235THR	C	100.00
233VAL	CG1	233VAL	CG2	100.00	189LEU	CD1	139TYR	CD2	0.17	235TH	CG2	235THR	C	99.37
233VAL	CG1	233VAL	C	100.00	189LEU	CD1	139TYR	CE1	0.02	235TH	CG2	239PHE	CB	0.02
233VAL	CG1	243LEU	CD1	0.00	189LEU	CD1	139TYR	CE2	0.05	235TH	CG2	239PHE	C	1.42
233VAL	CG1	243LEU	CD2	0.37	189LEU	CD1	153ASN	CB	0.10	235TH	CG2	240GLY	CA	43.27
233VAL	C	234VAL	CA	100.00	189LEU	CD1	153ASN	CG	1.71	235TH	CG2	243LEU	CD2	0.02
233VAL	C	234VAL	CB	74.65	189LEU	CD1	238ILE	CD	0.12	235TH	C	236GLY	CA	100.00
233VAL	C	234VAL	C	42.38	189LEU	CD2	189LEU	C	0.02	235TH	C	236GLY	C	78.53
234VAL	CA	234VAL	CB	100.00	189LEU	CD2	191VAL	CB	0.01	236GL	CA	236GLY	C	100.00
234VAL	CA	234VAL	CG1	100.00	189LEU	CD2	191VAL	CG1	0.26	236GL	C	237ASN	CA	100.00
234VAL	CA	234VAL	CG2	100.00	189LEU	CD2	191VAL	CG2	0.84	236GL	C	237ASN	CB	10.52
234VAL	CA	234VAL	C	100.00	189LEU	CD2	191VAL	C	0.01	236GL	C	237ASN	C	90.52
234VAL	CB	234VAL	CG1	100.00	189LEU	CD2	192GLY	CA	0.01	237AS	CA	237ASN	CB	100.00
234VAL	CB	234VAL	CG2	100.00	189LEU	CD2	239PHE	CE1	0.00	237AS	CA	237ASN	CG	100.00
234VAL	CB	234VAL	C	100.00	189LEU	CD2	239PHE	CZ	0.03	237AS	CA	237ASN	C	100.00
234VAL	CG1	234VAL	CG2	100.00	189LEU	CD2	138ILE	CB	0.02	237AS	CB	237ASN	CG	100.00
234VAL	CG1	234VAL	C	99.91	189LEU	CD2	138ILE	CG1	0.64	237AS	CB	237ASN	C	100.00
234VAL	CG2	234VAL	C	99.98	189LEU	CD2	138ILE	CG2	4.35	237AS	CG	237ASN	C	24.86
234VAL	C	235THR	CA	100.00	189LEU	CD2	138ILE	CD	1.78	237AS	C	238ILE	CA	100.00
234VAL	C	235THR	CB	99.95	189LEU	CD2	138ILE	C	0.17	237AS	C	238ILE	CB	0.00
234VAL	C	235THR	CG2	0.31	189LEU	CD2	139TYR	CA	0.26	237AS	C	238ILE	C	100.00
234VAL	C	235THR	C	0.45	189LEU	CD2	139TYR	CB	0.22	238ILE	CA	238ILE	CB	100.00
235THR	CA	235THR	CB	100.00	189LEU	CD2	139TYR	CD1	0.06	238ILE	CA	238ILE	CG1	100.00
235THR	CA	235THR	CG2	100.00	189LEU	CD2	139TYR	CD2	0.06	238ILE	CA	238ILE	CG2	100.00
235THR	CA	235THR	C	100.00	189LEU	CD2	153ASN	CB	0.22	238ILE	CA	238ILE	CD	0.38
235THR	CB	235THR	CG2	100.00	189LEU	CD2	153ASN	CG	2.67	238ILE	CA	238ILE	C	100.00
235THR	CB	235THR	C	100.00	189LEU	CD2	238ILE	CD	0.13	238ILE	CB	238ILE	CG1	100.00
235THR	CB	243LEU	CD1	0.02	189LEU	C	190GLU	CA	100.00	238ILE	CB	238ILE	CG2	100.00
235THR	CB	243LEU	CD2	0.00	189LEU	C	190GLU	C	100.00	238ILE	CB	238ILE	CD	100.00
235THR	CG2	235THR	C	99.80	190GLU	CA	190GLU	CB	100.00	238ILE	CB	238ILE	C	100.00
235THR	CG2	239PHE	CB	0.02	190GLU	CA	190GLU	CG	100.00	238ILE	CG1	238ILE	CG2	100.00
235THR	CG2	239PHE	C	0.62	190GLU	CA	190GLU	CD	98.24	238ILE	CG1	238ILE	CD	100.00
235THR	CG2	240GLY	CA	23.61	190GLU	CA	190GLU	C	100.00	238ILE	CG1	238ILE	C	99.16
235THR	CG2	243LEU	CD1	0.03	190GLU	CA	146MET	CE	0.01	238ILE	CG1	239PHE	CD1	0.01
235THR	CG2	243LEU	CD2	0.06	190GLU	CB	190GLU	CG	100.00	238ILE	CG1	239PHE	CE1	0.11
235THR	C	236GLY	CA	100.00	190GLU	CB	190GLU	CD	100.00	238ILE	CG1	239PHE	CE2	0.02
235THR	C	236GLY	C	88.02	190GLU	CB	190GLU	C	100.00	238ILE	CG1	239PHE	CZ	0.01
236GLY	CA	236GLY	C	100.00	190GLU	CB	144ARG	CG	0.01	238ILE	CG1	242ILE	CD	0.31
236GLY	C	237ASN	CA	100.00	190GLU	CB	144ARG	CZ	0.04	238ILE	CG1	239PHE	CE1	13.53
236GLY	C	237ASN	CB	69.43	190GLU	CB	145GLY	C	0.00	238ILE	CG1	239PHE	CE2	0.01
236GLY	C	237ASN	C	32.48	190GLU	CB	153ASN	CG	0.32	238ILE	CG1	239PHE	CZ	5.88
237ASN	CA	237ASN	CB	100.00	190GLU	CG	190GLU	CD	100.00	238ILE	CG2	238ILE	CD	99.82
237ASN	CA	237ASN	CG	100.00	190GLU	CG	190GLU	C	25.20	238ILE	CG2	238ILE	C	99.53
237ASN	CA	237ASN	C	100.00	190GLU	CG	144ARG	CB	0.00	238ILE	CG2	239PHE	CG	0.06
237ASN	CB	237ASN	CG	100.00	190GLU	CG	144ARG	CG	0.00	238ILE	CG2	239PHE	CD1	3.34
237ASN	CB	237ASN	C	100.00	190GLU	CG	144ARG	C	0.03	238ILE	CG2	239PHE	CD2	1.12
237ASN	CG	237ASN	C	95.71	190GLU	CG	145GLY	CA	0.06	238ILE	CG2	239PHE	CE1	0.19
237ASN	C	238ILE	CA	100.00	190GLU	CG	145GLY	C	1.22	238ILE	CG2	239PHE	CE2	0.68
237ASN	C	238ILE	CB	29.98	190GLU	CG	146MET	CB	2.02	238ILE	CG2	239PHE	CZ	0.09
237ASN	C	238ILE	CG2	0.02	190GLU	CG	146MET	CG	0.10	238ILE	CG2	239PHE	CD1	0.00
237ASN	C	238ILE	C	75.24	190GLU	CG	146MET	CE	0.10	238ILE	CG2	239PHE	CE1	0.04
238ILE	CA	238ILE	CB	100.00	190GLU	CD	82ARG	CZ	0.61	238ILE	CD	238ILE	C	0.14
238ILE	CA	238ILE	CG1	100.00	190GLU	CD	144ARG	CB	0.02	238ILE	CD	239PHE	CD1	0.02
238ILE	CA	238ILE	CG2	100.00	190GLU	CD	144ARG	CG	0.26	238ILE	CD	239PHE	CE1	0.12

238ILE	CA	238ILE	CD	3.04	190GLU	CD	144ARG	CD	0.05	238ILE	CD	239PHE	CE2	0.02
238ILE	CA	238ILE	C	100.00	190GLU	CD	144ARG	CZ	0.13	238ILE	CD	239PHE	CZ	0.10
238ILE	CB	238ILE	CG1	100.00	190GLU	CD	146MET	CG	0.01	238ILE	CD	138ILE	CD	0.44
238ILE	CB	238ILE	CG2	100.00	190GLU	CD	146MET	CE	0.02	238ILE	CD	238ILE	CG1	0.47
238ILE	CB	238ILE	CD	100.00	190GLU	C	191VAL	CA	100.00	238ILE	CD	238ILE	CD	4.76
238ILE	CB	238ILE	C	100.00	190GLU	C	191VAL	CB	0.00	238ILE	CD	239PHE	CE1	4.10
238ILE	CG1	238ILE	CG2	100.00	190GLU	C	191VAL	C	100.00	238ILE	CD	239PHE	CE2	0.49
238ILE	CG1	238ILE	CD	100.00	190GLU	C	151ALA	CB	0.02	238ILE	CD	239PHE	CZ	7.94
238ILE	CG1	238ILE	C	99.98	191VAL	CA	191VAL	CB	100.00	238ILE	C	239PHE	CA	100.00
238ILE	CG1	239PHE	CD1	0.14	191VAL	CA	191VAL	CG1	100.00	238ILE	C	239PHE	CB	1.19
238ILE	CG1	239PHE	CE1	0.09	191VAL	CA	191VAL	CG2	100.00	238ILE	C	239PHE	CG	0.10
238ILE	CG1	239PHE	CE2	0.02	191VAL	CA	191VAL	C	100.00	238ILE	C	239PHE	CD1	0.59
238ILE	CG1	239PHE	CZ	0.02	191VAL	CA	194PHE	CB	0.01	238ILE	C	239PHE	C	99.08
238ILE	CG1	242ILE	CD	0.00	191VAL	CA	151ALA	CB	0.12	239PH	CA	239PHE	CB	100.00
238ILE	CG1	138ILE	CD	0.01	191VAL	CB	191VAL	CG1	100.00	239PH	CA	239PHE	CG	100.00
238ILE	CG1	238ILE	CD	0.05	191VAL	CB	191VAL	CG2	100.00	239PH	CA	239PHE	CD1	98.99
238ILE	CG1	239PHE	CE1	0.55	191VAL	CB	191VAL	C	100.00	239PH	CA	239PHE	CD2	1.51
238ILE	CG1	239PHE	CZ	0.24	191VAL	CG1	191VAL	CG2	100.00	239PH	CA	239PHE	C	100.00
238ILE	CG2	238ILE	CD	98.82	191VAL	CG1	191VAL	C	50.71	239PH	CA	242ILE	CG1	0.01
238ILE	CG2	238ILE	C	98.00	191VAL	CG1	151ALA	CB	0.10	239PH	CA	242ILE	CD	0.06
238ILE	CG2	239PHE	CD1	0.60	191VAL	CG1	151ALA	C	1.79	239PH	CB	239PHE	CG	100.00
238ILE	CG2	239PHE	CD2	0.47	191VAL	CG1	152TRP	CA	3.50	239PH	CB	239PHE	CD1	100.00
238ILE	CG2	239PHE	CE1	0.84	191VAL	CG1	152TRP	C	4.91	239PH	CB	239PHE	CD2	100.00
238ILE	CG2	239PHE	CE2	0.22	191VAL	CG1	153ASN	CB	0.03	239PH	CB	239PHE	C	100.00
238ILE	CG2	239PHE	CZ	0.19	191VAL	CG1	153ASN	CG	0.00	239PH	CB	242ILE	CD	0.01
238ILE	CD	238ILE	C	2.43	191VAL	CG2	191VAL	C	80.84	239PH	CG	239PHE	CD1	100.00
238ILE	CD	239PHE	CG	0.00	191VAL	CG2	151ALA	CB	0.08	239PH	CG	239PHE	CD2	100.00
238ILE	CD	239PHE	CD1	0.40	191VAL	CG2	151ALA	C	0.89	239PH	CG	239PHE	CE1	100.00
238ILE	CD	239PHE	CE1	0.40	191VAL	CG2	152TRP	CA	1.00	239PH	CG	239PHE	CE2	100.00
238ILE	CD	239PHE	CE2	0.06	191VAL	CG2	152TRP	C	1.81	239PH	CG	239PHE	CZ	100.00
238ILE	CD	239PHE	CZ	0.16	191VAL	CG2	153ASN	CB	0.02	239PH	CG	242ILE	CD	0.91
238ILE	CD	138ILE	CG1	0.18	191VAL	CG2	153ASN	CG	0.01	239PH	CD1	239PHE	CD2	100.00
238ILE	CD	138ILE	CD	6.07	191VAL	C	192GLY	CA	100.00	239PH	CD1	239PHE	CE1	100.00
238ILE	CD	189LEU	CD1	0.00	191VAL	C	192GLY	C	95.39	239PH	CD1	239PHE	CE2	100.00
238ILE	CD	238ILE	CG1	0.11	191VAL	C	194PHE	CB	0.01	239PH	CD1	239PHE	CZ	100.00
238ILE	CD	238ILE	CD	2.69	192GLY	CA	192GLY	C	100.00	239PH	CD1	242ILE	CD	0.00
238ILE	CD	239PHE	CE1	0.93	192GLY	CA	195TRP	CB	0.01	239PH	CD1	239PHE	CD1	0.05
238ILE	CD	239PHE	CZ	0.30	192GLY	C	193GLU	CA	100.00	239PH	CD1	239PHE	CE1	0.15
238ILE	C	239PHE	CA	100.00	192GLY	C	193GLU	CB	0.00	239PH	CD1	242ILE	CD	10.32
238ILE	C	239PHE	CB	57.57	192GLY	C	193GLU	C	100.00	239PH	CD2	239PHE	CE1	100.00
238ILE	C	239PHE	CG	3.97	192GLY	C	195TRP	CB	0.00	239PH	CD2	239PHE	CE2	100.00
238ILE	C	239PHE	CD1	2.30	193GLU	CA	193GLU	CB	100.00	239PH	CD2	239PHE	CZ	100.00
238ILE	C	239PHE	C	40.62	193GLU	CA	193GLU	CG	100.00	239PH	CD2	242ILE	CD	0.04
239PHE	CA	239PHE	CB	100.00	193GLU	CA	193GLU	CD	30.72	239PH	CE1	239PHE	CE2	100.00
239PHE	CA	239PHE	CG	100.00	193GLU	CA	193GLU	C	100.00	239PH	CE1	239PHE	CZ	100.00
239PHE	CA	239PHE	CD1	51.24	193GLU	CB	193GLU	CG	100.00	239PH	CE1	238ILE	CG1	0.02
239PHE	CA	239PHE	CD2	51.76	193GLU	CB	193GLU	CD	100.00	239PH	CE1	238ILE	CD	0.29
239PHE	CA	239PHE	C	100.00	193GLU	CB	193GLU	C	100.00	239PH	CE1	239PHE	CD1	0.10
239PHE	CA	242ILE	CD	0.36	193GLU	CB	146MET	CE	0.11	239PH	CE1	239PHE	CE1	0.07
239PHE	CB	239PHE	CG	100.00	193GLU	CG	193GLU	CD	100.00	239PH	CE1	242ILE	CD	6.35
239PHE	CB	239PHE	CD1	100.00	193GLU	CG	193GLU	C	93.49	239PH	CE2	239PHE	CZ	100.00
239PHE	CB	239PHE	CD2	100.00	193GLU	CG	197LYS	CE	0.09	239PH	CE2	242ILE	CD	0.02
239PHE	CB	239PHE	C	100.00	193GLU	CG	82ARG	CZ	0.02	239PH	CZ	139TYR	CE1	0.06
239PHE	CB	242ILE	CD	0.62	193GLU	CG	146MET	CE	0.74	239PH	CZ	139TYR	CZ	0.02
239PHE	CB	243LEU	CD1	0.19	193GLU	CD	193GLU	C	0.30	239PH	CZ	238ILE	CD	0.09
239PHE	CB	239PHE	CD1	0.00	193GLU	CD	197LYS	CE	0.03	239PH	CZ	242ILE	CD	0.10
239PHE	CB	242ILE	CD	0.92	193GLU	CD	82ARG	CD	0.00	239PH	C	240GLY	CA	100.00
239PHE	CG	239PHE	CD1	100.00	193GLU	CD	82ARG	CZ	0.36	239PH	C	240GLY	C	72.38
239PHE	CG	239PHE	CD2	100.00	193GLU	CD	146MET	CE	0.12	239PH	C	242ILE	CG1	0.02
239PHE	CG	239PHE	CE1	100.00	193GLU	C	194PHE	CA	100.00	239PH	C	242ILE	CD	0.05
239PHE	CG	239PHE	CE2	100.00	193GLU	C	194PHE	CB	0.03	239PH	C	243LEU	CD1	0.02
239PHE	CG	239PHE	CZ	100.00	193GLU	C	194PHE	C	99.98	240GL	CA	240GLY	C	100.00

239PHE	CG	239PHE	C	37.25	193GLI	C	197LYS	CE	0.01	240GL	CA	243LEU	CB	0.02
239PHE	CG	242ILE	CD	4.90	193GLI	C	146MET	CE	0.06	240GL	C	241ASP	CA	100.00
239PHE	CD1	239PHE	CD2	100.00	194PHI	CA	194PHE	CB	100.00	240GL	C	241ASP	CB	0.00
239PHE	CD1	239PHE	CE1	100.00	194PHI	CA	194PHE	CG	100.00	240GL	C	241ASP	C	100.00
239PHE	CD1	239PHE	CE2	100.00	194PHI	CA	194PHE	CD1	93.52	240GL	C	243LEU	CB	0.01
239PHE	CD1	239PHE	CZ	100.00	194PHI	CA	194PHE	CD2	48.11	241AS	CA	241ASP	CB	100.00
239PHE	CD1	242ILE	CD	0.00	194PHI	CA	194PHE	C	100.00	241AS	CA	241ASP	CG	100.00
239PHE	CD1	238ILE	CG1	0.00	194PHI	CA	146MET	CE	0.74	241AS	CA	241ASP	C	100.00
239PHE	CD1	238ILE	CD	0.12	194PHI	CB	194PHE	CG	100.00	241AS	CB	241ASP	CG	100.00
239PHE	CD1	239PHE	CD1	0.13	194PHI	CB	194PHE	CD1	100.00	241AS	CB	241ASP	C	100.00
239PHE	CD1	239PHE	CE1	0.10	194PHI	CB	194PHE	CD2	100.00	241AS	CG	241ASP	C	99.96
239PHE	CD1	242ILE	CG1	0.02	194PHI	CB	194PHE	C	100.00	241AS	CG	185LYS	CE	0.03
239PHE	CD1	242ILE	CD	17.83	194PHI	CB	146MET	CE	0.05	241AS	C	242ILE	CA	100.00
239PHE	CD2	239PHE	CE1	100.00	194PHI	CB	151ALA	CB	20.50	241AS	C	242ILE	CB	0.18
239PHE	CD2	239PHE	CE2	100.00	194PHI	CG	194PHE	CD1	100.00	241AS	C	242ILE	CG1	0.04
239PHE	CD2	239PHE	CZ	100.00	194PHI	CG	194PHE	CD2	100.00	241AS	C	242ILE	CG2	0.02
239PHE	CD2	239PHE	C	26.08	194PHI	CG	194PHE	CE1	100.00	241AS	C	242ILE	CD	0.02
239PHE	CD2	243LEU	CD1	0.00	194PHI	CG	194PHE	CE2	100.00	241AS	C	242ILE	C	99.93
239PHE	CD2	242ILE	CD	1.23	194PHI	CG	194PHE	CZ	100.00	242ILE	CA	242ILE	CB	100.00
239PHE	CE1	239PHE	CE2	100.00	194PHI	CG	194PHE	C	99.99	242ILE	CA	242ILE	CG1	100.00
239PHE	CE1	239PHE	CZ	100.00	194PHI	CG	151ALA	CB	36.03	242ILE	CA	242ILE	CG2	100.00
239PHE	CE1	238ILE	CG1	0.32	194PHI	CD1	194PHE	CD2	100.00	242ILE	CA	242ILE	CD	13.90
239PHE	CE1	238ILE	CD	0.37	194PHI	CD1	194PHE	CE1	100.00	242ILE	CA	242ILE	C	100.00
239PHE	CE1	238ILE	C	0.01	194PHI	CD1	194PHE	CE2	100.00	242ILE	CB	242ILE	CG1	100.00
239PHE	CE1	239PHE	CD1	0.25	194PHI	CD1	194PHE	CZ	100.00	242ILE	CB	242ILE	CG2	100.00
239PHE	CE1	239PHE	CE1	0.04	194PHI	CD1	194PHE	C	0.76	242ILE	CB	242ILE	CD	100.00
239PHE	CE1	242ILE	CG1	0.02	194PHI	CD1	197LYS	CD	0.00	242ILE	CB	242ILE	C	100.00
239PHE	CE1	242ILE	CD	5.28	194PHI	CD1	197LYS	CE	0.06	242ILE	CG1	242ILE	CG2	100.00
239PHE	CE2	239PHE	CZ	100.00	194PHI	CD1	198THR	CG2	0.00	242ILE	CG1	242ILE	CD	100.00
239PHE	CE2	242ILE	CD	0.61	194PHI	CD1	146MET	CG	0.33	242ILE	CG1	242ILE	C	64.30
239PHE	CZ	138ILE	CD	0.00	194PHI	CD1	146MET	CE	0.03	242ILE	CG1	243LEU	CD1	0.02
239PHE	CZ	238ILE	CG1	0.03	194PHI	CD1	150GLU	C	0.04	242ILE	CG1	216VAL	CG1	0.00
239PHE	CZ	238ILE	CD	0.13	194PHI	CD1	151ALA	CB	9.05	242ILE	CG1	217ASP	CA	0.01
239PHE	CZ	242ILE	CD	0.89	194PHI	CD2	194PHE	CE1	100.00	242ILE	CG1	217ASP	CB	0.00
239PHE	C	240GLY	CA	100.00	194PHI	CD2	194PHE	CE2	100.00	242ILE	CG1	239PHE	CE1	0.00
239PHE	C	240GLY	C	83.54	194PHI	CD2	194PHE	CZ	100.00	242ILE	CG2	242ILE	CD	81.87
239PHE	C	242ILE	CG1	0.01	194PHI	CD2	194PHE	C	60.92	242ILE	CG2	242ILE	C	35.68
239PHE	C	242ILE	CD	0.01	194PHI	CD2	198THR	CG2	0.00	242ILE	CG2	243LEU	CD2	0.00
239PHE	C	243LEU	CD1	0.00	194PHI	CD2	150GLU	C	0.00	242ILE	CG2	216VAL	CG1	0.66
240GLY	CA	240GLY	C	100.00	194PHI	CD2	151ALA	CB	5.67	242ILE	CG2	216VAL	CG2	0.04
240GLY	CA	243LEU	CB	0.00	194PHI	CE1	194PHE	CE2	100.00	242ILE	CG2	216VAL	C	0.00
240GLY	C	241ASP	CA	100.00	194PHI	CE1	194PHE	CZ	100.00	242ILE	CG2	217ASP	CA	0.03
240GLY	C	241ASP	CB	0.14	194PHI	CE1	197LYS	CE	0.01	242ILE	CG2	220ALA	CB	1.02
240GLY	C	241ASP	C	99.91	194PHI	CE1	198THR	CG2	0.10	242ILE	CG2	239PHE	CD1	0.00
241ASP	CA	241ASP	CB	100.00	194PHI	CE1	146MET	CG	0.01	242ILE	CG2	239PHE	CE1	1.41
241ASP	CA	241ASP	CG	100.00	194PHI	CE1	148GLU	C	0.07	242ILE	CG2	239PHE	CZ	0.00
241ASP	CA	241ASP	C	100.00	194PHI	CE1	149ALA	CA	0.01	242ILE	CD	242ILE	C	7.56
241ASP	CB	241ASP	CG	100.00	194PHI	CE1	149ALA	C	1.15	242ILE	CD	243LEU	CD1	0.10
241ASP	CB	241ASP	C	100.00	194PHI	CE1	150GLU	CA	0.88	242ILE	CD	216VAL	CG1	1.43
241ASP	CB	217ASP	CB	0.64	194PHI	CE1	150GLU	C	6.81	242ILE	CD	216VAL	CG2	0.52
241ASP	CB	217ASP	CG	0.19	194PHI	CE1	151ALA	CB	0.08	242ILE	CD	239PHE	CG	0.11
241ASP	CG	241ASP	C	93.58	194PHI	CE2	194PHE	CZ	100.00	242ILE	CD	239PHE	CD1	4.13
241ASP	CG	185LYS	CE	0.10	194PHI	CE2	198THR	CG2	0.12	242ILE	CD	239PHE	CD2	0.01
241ASP	C	242ILE	CA	100.00	194PHI	CE2	149ALA	C	0.04	242ILE	CD	239PHE	CE1	3.32
241ASP	C	242ILE	CB	0.00	194PHI	CE2	150GLU	CA	0.16	242ILE	CD	239PHE	CE2	0.14
241ASP	C	242ILE	C	99.99	194PHI	CE2	150GLU	C	0.18	242ILE	CD	239PHE	CZ	0.03
241ASP	C	217ASP	CB	0.02	194PHI	CE2	151ALA	CB	0.04	242ILE	CD	242ILE	CG2	0.06
242ILE	CA	242ILE	CB	100.00	194PHI	CZ	198THR	CG2	0.22	242ILE	CD	242ILE	CD	0.68
242ILE	CA	242ILE	CG1	100.00	194PHI	CZ	146MET	CG	0.00	242ILE	CD	246LEU	CD1	0.01
242ILE	CA	242ILE	CG2	100.00	194PHI	CZ	148GLU	C	0.02	242ILE	C	243LEU	CA	100.00
242ILE	CA	242ILE	CD	2.65	194PHI	CZ	149ALA	CA	0.01	242ILE	C	243LEU	CB	0.11
242ILE	CA	242ILE	C	100.00	194PHI	CZ	149ALA	C	9.02	242ILE	C	243LEU	CD1	0.01

242ILE	CA	217ASP	CB	0.02
242ILE	CB	242ILE	CG1	100.00
242ILE	CB	242ILE	CG2	100.00
242ILE	CB	242ILE	CD	100.00
242ILE	CB	242ILE	C	100.00
242ILE	CG1	242ILE	CG2	100.00
242ILE	CG1	242ILE	CD	100.00
242ILE	CG1	242ILE	C	8.06
242ILE	CG1	216VAL	CG1	0.14
242ILE	CG1	216VAL	CG2	0.03
242ILE	CG1	217ASP	CA	0.00
242ILE	CG1	217ASP	CB	0.02
242ILE	CG2	242ILE	CD	95.47
242ILE	CG2	242ILE	C	92.22
242ILE	CG2	246LEU	CD1	0.28
242ILE	CG2	216VAL	CG1	0.31
242ILE	CG2	216VAL	CG2	0.07
242ILE	CG2	217ASP	CA	0.00
242ILE	CG2	220ALA	CB	5.49
242ILE	CG2	239PHE	CD1	0.04
242ILE	CG2	239PHE	CE1	0.11
242ILE	CD	242ILE	C	0.26
242ILE	CD	243LEU	CD2	0.00
242ILE	CD	216VAL	CG1	1.86
242ILE	CD	216VAL	CG2	2.94
242ILE	CD	220ALA	CB	0.02
242ILE	CD	239PHE	CB	0.19
242ILE	CD	239PHE	CG	0.02
242ILE	CD	239PHE	CD1	6.80
242ILE	CD	239PHE	CE1	2.26
242ILE	CD	239PHE	CZ	0.01
242ILE	CD	242ILE	CG2	0.01
242ILE	CD	242ILE	CD	0.38
242ILE	CD	243LEU	CD1	0.00
242ILE	C	243LEU	CA	100.00
242ILE	C	243LEU	CB	0.02
242ILE	C	243LEU	C	99.99
243LEU	CA	243LEU	CB	100.00
243LEU	CA	243LEU	CG	100.00
243LEU	CA	243LEU	CD1	24.64
243LEU	CA	243LEU	CD2	74.81
243LEU	CA	243LEU	C	100.00
243LEU	CB	243LEU	CG	100.00
243LEU	CB	243LEU	CD1	100.00
243LEU	CB	243LEU	CD2	100.00
243LEU	CB	243LEU	C	100.00
243LEU	CG	243LEU	CD1	100.00
243LEU	CG	243LEU	CD2	100.00
243LEU	CG	243LEU	C	23.60
243LEU	CD1	243LEU	CD2	100.00
243LEU	CD1	243LEU	C	0.33
243LEU	CD1	246LEU	CD1	0.00
243LEU	CD2	243LEU	C	0.52
243LEU	CD2	246LEU	CD1	0.00
243LEU	CD2	246LEU	CD2	0.03
243LEU	C	244SER	CA	100.00
243LEU	C	244SER	CB	0.02
243LEU	C	244SER	C	100.00
244SER	CA	244SER	CB	100.00
244SER	CA	244SER	C	100.00
244SER	CB	244SER	C	100.00
244SER	C	245ASP	CA	100.00

194PHI	CZ	150GLU	CA	4.87
194PHI	CZ	150GLU	C	1.56
194PHI	CZ	151ALA	CB	0.00
194PHI	C	195TRP	CA	100.00
194PHI	C	195TRP	C	100.00
195TRI	CA	195TRP	CB	100.00
195TRI	CA	195TRP	CG	100.00
195TRI	CA	195TRP	CD1	91.36
195TRI	CA	195TRP	CD2	4.16
195TRI	CA	195TRP	CE3	0.00
195TRI	CA	195TRP	C	100.00
195TRI	CB	195TRP	CG	100.00
195TRI	CB	195TRP	CD1	100.00
195TRI	CB	195TRP	CD2	100.00
195TRI	CB	195TRP	CE3	96.66
195TRI	CB	195TRP	C	100.00
195TRI	CG	195TRP	CD1	100.00
195TRI	CG	195TRP	CD2	100.00
195TRI	CG	195TRP	CE2	100.00
195TRI	CG	195TRP	CE3	100.00
195TRI	CG	195TRP	C	99.99
195TRI	CD1	195TRP	CD2	100.00
195TRI	CD1	195TRP	CE2	100.00
195TRI	CD1	195TRP	CE3	0.01
195TRI	CD1	195TRP	C	92.02
195TRI	CD1	196ARG	CA	0.01
195TRI	CD1	199VAL	CG1	0.16
195TRI	CD1	199VAL	CG2	1.60
195TRI	CD2	195TRP	CE2	100.00
195TRI	CD2	195TRP	CE3	100.00
195TRI	CD2	195TRP	CZ2	100.00
195TRI	CD2	195TRP	CZ3	100.00
195TRI	CD2	195TRP	C	0.00
195TRI	CE2	195TRP	CE3	100.00
195TRI	CE2	195TRP	CZ2	100.00
195TRI	CE2	195TRP	CZ3	100.00
195TRI	CE2	199VAL	CG1	0.04
195TRI	CE2	199VAL	CG2	0.35
195TRI	CE3	195TRP	CZ2	100.00
195TRI	CE3	195TRP	CZ3	100.00
195TRI	CZ2	195TRP	CZ3	100.00
195TRI	CZ2	199VAL	CG1	0.00
195TRI	CZ2	199VAL	CG2	0.10
195TRI	CZ2	234VAL	CG1	0.55
195TRI	CZ2	234VAL	CG2	0.00
195TRI	CZ2	234VAL	C	0.01
195TRI	CZ2	235THR	CA	1.03
195TRI	CZ2	235THR	C	5.77
195TRI	CZ2	236GLY	CA	0.02
195TRI	CZ3	199VAL	CG2	0.01
195TRI	CZ3	236GLY	CA	0.06
195TRI	C	196ARG	CA	100.00
195TRI	C	196ARG	C	100.00
196AR(	CA	196ARG	CB	100.00
196AR(	CA	196ARG	CG	100.00
196AR(	CA	196ARG	CD	0.09
196AR(	CA	196ARG	C	100.00
196AR(	CA	199VAL	CG2	0.02
196AR(	CA	213HIS	CE1	0.12
196AR(	CB	196ARG	CG	100.00
196AR(	CB	196ARG	CD	100.00
196AR(	CB	196ARG	C	100.00

242ILE	C	243LEU	C	99.92
243LEI	CA	243LEU	CB	100.00
243LEI	CA	243LEU	CG	100.00
243LEI	CA	243LEU	CD1	90.04
243LEI	CA	243LEU	CD2	4.55
243LEI	CA	243LEU	C	100.00
243LEI	CB	243LEU	CG	100.00
243LEI	CB	243LEU	CD1	100.00
243LEI	CB	243LEU	CD2	100.00
243LEI	CB	243LEU	C	100.00
243LEI	CG	243LEU	CD1	100.00
243LEI	CG	243LEU	CD2	100.00
243LEI	CG	243LEU	C	68.07
243LEI	CD1	243LEU	CD2	100.00
243LEI	CD1	243LEU	C	0.14
243LEI	CD1	246LEU	CD2	0.01
243LEI	CD1	242ILE	CG2	0.00
243LEI	CD2	243LEU	C	1.38
243LEI	CD2	246LEU	CD1	0.00
243LEI	CD2	246LEU	CD2	0.02
243LEI	C	244SER	CA	100.00
243LEI	C	244SER	CB	0.27
243LEI	C	244SER	C	99.92
244SE	CA	244SER	CB	100.00
244SE	CA	244SER	C	100.00
244SE	CB	244SER	C	100.00
244SE	C	245ASP	CA	100.00
244SE	C	245ASP	CB	0.02
244SE	C	245ASP	C	99.98
245AS	CA	245ASP	CB	100.00
245AS	CA	245ASP	CG	100.00
245AS	CA	245ASP	C	100.00
245AS	CB	245ASP	CG	100.00
245AS	CB	245ASP	C	100.00
245AS	CB	217ASP	CB	0.16
245AS	CB	221MET	CE	0.72
245AS	CG	245ASP	C	0.42
245AS	CG	217ASP	CB	0.18
245AS	CG	221MET	CE	0.96
245AS	C	246LEU	CA	100.00
245AS	C	246LEU	C	100.00
245AS	C	221MET	CB	0.01
245AS	C	221MET	CG	0.41
245AS	C	221MET	CE	0.02
246LEI	CA	246LEU	CB	100.00
246LEI	CA	246LEU	CG	100.00
246LEI	CA	246LEU	CD1	94.01
246LEI	CA	246LEU	CD2	5.78
246LEI	CA	246LEU	C	100.00
246LEI	CB	246LEU	CG	100.00
246LEI	CB	246LEU	CD1	100.00
246LEI	CB	246LEU	CD2	100.00
246LEI	CB	246LEU	C	100.00
246LEI	CG	246LEU	CD1	100.00
246LEI	CG	246LEU	CD2	100.00
246LEI	CG	246LEU	C	98.24
246LEI	CG	246LEU	CD2	0.00
246LEI	CD1	246LEU	CD2	100.00
246LEI	CD1	246LEU	C	9.58
246LEI	CD1	220ALA	CB	0.02
246LEI	CD1	220ALA	C	0.94
246LEI	CD1	224VAL	CG1	0.61

244SER	C	245ASP	CB	0.04	196AR(	CB	213HIS	CE1	0.14	246LEI	CD1	224VAL	CG2	3.07
244SER	C	245ASP	C	99.96	196AR(	CG	196ARG	CD	100.00	246LEI	CD1	246LEU	CG	0.02
245ASP	CA	245ASP	CB	100.00	196AR(	CG	196ARG	CZ	0.36	246LEI	CD1	246LEU	CD1	1.27
245ASP	CA	245ASP	CG	100.00	196AR(	CG	196ARG	C	88.07	246LEI	CD1	246LEU	CD2	0.46
245ASP	CA	245ASP	C	100.00	196AR(	CG	213HIS	CB	0.01	246LEI	CD2	246LEU	C	3.31
245ASP	CB	245ASP	CG	100.00	196AR(	CG	213HIS	CG	3.02	246LEI	CD2	220ALA	CB	0.01
245ASP	CB	245ASP	C	100.00	196AR(	CG	213HIS	CD2	3.18	246LEI	CD2	220ALA	C	0.11
245ASP	CB	217ASP	CB	0.01	196AR(	CG	213HIS	CE1	5.98	246LEI	CD2	224VAL	CG1	0.02
245ASP	CB	221MET	CG	0.00	196AR(	CD	196ARG	CZ	100.00	246LEI	CD2	224VAL	CG2	0.11
245ASP	CB	221MET	CE	0.35	196AR(	CD	213HIS	CB	0.04	246LEI	CD2	246LEU	CG	0.02
245ASP	CG	245ASP	C	0.13	196AR(	CD	213HIS	CG	0.22	246LEI	CD2	246LEU	CD1	4.41
245ASP	CG	217ASP	CB	0.01	196AR(	CD	213HIS	CD2	0.49	246LEI	CD2	246LEU	CD2	0.44
245ASP	CG	221MET	CE	0.97	196AR(	CD	213HIS	CE1	0.02	246LEI	C	247ALA	CA	100.00
245ASP	C	246LEU	CA	100.00	196AR(	CZ	213HIS	CB	40.14	246LEI	C	247ALA	CB	0.13
245ASP	C	246LEU	CB	0.01	196AR(	CZ	213HIS	CG	0.08	246LEI	C	247ALA	C	99.87
245ASP	C	246LEU	C	100.00	196AR(	CZ	213HIS	CD2	0.02	247AL.	CA	247ALA	CB	100.00
245ASP	C	221MET	CG	0.09	196AR(	CZ	215TYR	CE1	0.35	247AL.	CA	247ALA	C	100.00
245ASP	C	221MET	CE	0.00	196AR(	CZ	215TYR	CE2	1.73	247AL.	CA	250LEU	CD1	0.06
246LEU	CA	246LEU	CB	100.00	196AR(	CZ	215TYR	CZ	0.02	247AL.	CB	247ALA	C	100.00
246LEU	CA	246LEU	CG	100.00	196AR(	C	197LYS	CA	100.00	247AL.	CB	250LEU	CD1	0.01
246LEU	CA	246LEU	CD1	65.99	196AR(	C	197LYS	CB	0.16	247AL.	C	248SER	CA	100.00
246LEU	CA	246LEU	CD2	31.42	196AR(	C	197LYS	C	99.87	247AL.	C	248SER	CB	0.28
246LEU	CA	246LEU	C	100.00	196AR(	C	199VAL	CG2	0.01	247AL.	C	248SER	C	99.78
246LEU	CB	246LEU	CG	100.00	197LYS	CA	197LYS	CB	100.00	248SE	CA	248SER	CB	100.00
246LEU	CB	246LEU	CD1	100.00	197LYS	CA	197LYS	CG	100.00	248SE	CA	248SER	C	100.00
246LEU	CB	246LEU	CD2	100.00	197LYS	CA	197LYS	CD	7.05	248SE	CA	257LEU	CD1	0.76
246LEU	CB	246LEU	C	100.00	197LYS	CA	197LYS	CE	0.05	248SE	CB	248SER	C	100.00
246LEU	CG	246LEU	CD1	100.00	197LYS	CA	197LYS	C	100.00	248SE	CB	254LEU	CD1	0.08
246LEU	CG	246LEU	CD2	100.00	197LYS	CA	200GLU	CB	0.01	248SE	CB	254LEU	CD2	0.19
246LEU	CG	246LEU	C	76.42	197LYS	CB	197LYS	CG	100.00	248SE	CB	257LEU	CD1	0.09
246LEU	CG	220ALA	CB	0.01	197LYS	CB	197LYS	CD	100.00	248SE	CB	221MET	CE	0.26
246LEU	CG	224VAL	CG1	0.01	197LYS	CB	197LYS	CE	10.39	248SE	C	249VAL	CA	100.00
246LEU	CG	224VAL	CG2	0.00	197LYS	CB	197LYS	C	100.00	248SE	C	249VAL	CB	0.33
246LEU	CD1	246LEU	CD2	100.00	197LYS	CG	197LYS	CD	100.00	248SE	C	249VAL	CG1	0.16
246LEU	CD1	246LEU	C	2.42	197LYS	CG	197LYS	CE	100.00	248SE	C	249VAL	C	99.60
246LEU	CD1	220ALA	CB	0.02	197LYS	CG	197LYS	C	79.33	248SE	C	254LEU	CD2	0.07
246LEU	CD1	220ALA	C	0.19	197LYS	CD	197LYS	CE	100.00	248SE	C	257LEU	CD1	0.01
246LEU	CD1	224VAL	CG1	1.20	197LYS	CD	197LYS	C	4.38	248SE	C	221MET	CE	0.02
246LEU	CD1	224VAL	CG2	3.92	197LYS	CD	146MET	CE	0.75	249VA	CA	249VAL	CB	100.00
246LEU	CD1	246LEU	CD1	0.57	197LYS	CE	197LYS	C	0.05	249VA	CA	249VAL	CG1	100.00
246LEU	CD1	246LEU	CD2	0.52	197LYS	CE	201GLU	CD	0.00	249VA	CA	249VAL	CG2	100.00
246LEU	CD2	246LEU	C	8.90	197LYS	CE	146MET	CG	0.00	249VA	CA	249VAL	C	100.00
246LEU	CD2	250LEU	CD1	0.01	197LYS	CE	146MET	CE	0.03	249VA	CA	254LEU	CD1	0.00
246LEU	CD2	220ALA	CB	0.03	197LYS	CE	148GLU	CD	0.19	249VA	CA	254LEU	CD2	0.17
246LEU	CD2	220ALA	C	0.28	197LYS	C	198THR	CA	100.00	249VA	CB	249VAL	CG1	100.00
246LEU	CD2	221MET	CA	0.00	197LYS	C	198THR	CB	0.75	249VA	CB	249VAL	CG2	100.00
246LEU	CD2	224VAL	CG1	0.15	197LYS	C	198THR	CG2	0.01	249VA	CB	249VAL	C	100.00
246LEU	CD2	224VAL	CG2	1.07	197LYS	C	198THR	C	99.64	249VA	CB	254LEU	CD2	0.00
246LEU	CD2	246LEU	CD1	1.95	198THF	CA	198THR	CB	100.00	249VA	CB	224VAL	CG2	0.03
246LEU	CD2	246LEU	CD2	0.16	198THF	CA	198THR	CG2	100.00	249VA	CB	225ARG	CZ	0.00
246LEU	C	247ALA	CA	100.00	198THF	CA	198THR	C	100.00	249VA	CG1	249VAL	CG2	100.00
246LEU	C	247ALA	CB	0.08	198THF	CB	198THR	CG2	100.00	249VA	CG1	249VAL	C	6.14
246LEU	C	247ALA	C	99.96	198THF	CB	198THR	C	100.00	249VA	CG1	254LEU	CA	0.02
246LEU	C	249VAL	CG1	0.02	198THF	CG2	198THR	C	94.72	249VA	CG1	254LEU	CB	0.06
247ALA	CA	247ALA	CB	100.00	198THF	CG2	202VAL	CG2	0.01	249VA	CG1	254LEU	CG	0.01
247ALA	CA	247ALA	C	100.00	198THF	C	199VAL	CA	100.00	249VA	CG1	254LEU	CD1	0.12
247ALA	CA	250LEU	CD1	0.44	198THF	C	199VAL	CB	0.05	249VA	CG1	254LEU	CD2	1.22
247ALA	CB	247ALA	C	100.00	198THF	C	199VAL	CG2	0.02	249VA	CG1	221MET	CA	0.44
247ALA	C	248SER	CA	100.00	198THF	C	199VAL	C	99.98	249VA	CG1	221MET	CB	0.57
247ALA	C	248SER	CB	0.48	199VAI	CA	199VAL	CB	100.00	249VA	CG1	221MET	CG	17.63
247ALA	C	248SER	C	99.44	199VAI	CA	199VAL	CG1	100.00	249VA	CG1	221MET	CE	0.02
247ALA	C	250LEU	CG	0.00	199VAI	CA	199VAL	CG2	100.00	249VA	CG1	224VAL	CG1	0.03

247ALA	C	250LEU	CD1	0.01	199VA	CA	199VAL	C	100.00	249VA	CG1	224VAL	CG2	0.66
247ALA	C	251PRO	CD	0.02	199VA	CA	202VAL	CG1	0.02	249VA	CG1	225ARG	CG	0.15
248SER	CA	248SER	CB	100.00	199VA	CA	202VAL	CG2	0.00	249VA	CG1	225ARG	CD	0.24
248SER	CA	248SER	C	100.00	199VA	CB	199VAL	CG1	100.00	249VA	CG1	225ARG	CZ	0.10
248SER	CA	257LEU	CD1	0.03	199VA	CB	199VAL	CG2	100.00	249VA	CG2	249VAL	C	93.81
248SER	CA	257LEU	CD2	0.02	199VA	CB	199VAL	C	100.00	249VA	CG2	250LEU	CD1	0.06
248SER	CB	248SER	C	100.00	199VA	CB	211LEU	CD2	0.00	249VA	CG2	250LEU	CD2	0.03
248SER	CB	254LEU	CD1	0.00	199VA	CG1	199VAL	CG2	100.00	249VA	CG2	254LEU	CD2	0.08
248SER	CB	254LEU	CD2	0.28	199VA	CG1	199VAL	C	84.95	249VA	CG2	221MET	CB	0.01
248SER	CB	257LEU	CD1	2.71	199VA	CG1	211LEU	CD1	3.72	249VA	CG2	221MET	CG	2.61
248SER	CB	257LEU	CD2	0.61	199VA	CG1	211LEU	CD2	0.32	249VA	CG2	221MET	CE	0.00
248SER	CB	221MET	CE	0.10	199VA	CG1	213HIS	CE1	0.30	249VA	CG2	224VAL	CG1	0.19
248SER	C	249VAL	CA	100.00	199VA	CG1	234VAL	CG1	0.01	249VA	CG2	224VAL	CG2	1.25
248SER	C	249VAL	CB	3.48	199VA	CG2	199VAL	C	15.50	249VA	C	250LEU	CA	100.00
248SER	C	249VAL	CG1	0.50	199VA	CG2	211LEU	CD1	0.00	249VA	C	250LEU	C	100.00
248SER	C	249VAL	CG2	0.05	199VA	CG2	211LEU	CD2	0.06	249VA	C	251PRO	CD	25.77
248SER	C	249VAL	C	97.29	199VA	CG2	213HIS	CE1	0.34	250LE	CA	250LEU	CB	100.00
248SER	C	251PRO	CD	0.24	199VA	C	200GLU	CA	100.00	250LE	CA	250LEU	CG	100.00
248SER	C	254LEU	CD2	0.40	199VA	C	200GLU	CB	0.01	250LE	CA	250LEU	CD1	2.43
248SER	C	221MET	CE	0.04	199VA	C	200GLU	C	100.00	250LE	CA	250LEU	CD2	97.51
249VAL	CA	249VAL	CB	100.00	199VA	C	202VAL	CG1	0.01	250LE	CA	250LEU	C	100.00
249VAL	CA	249VAL	CG1	100.00	199VA	C	211LEU	CD1	0.04	250LE	CA	251PRO	CD	100.00
249VAL	CA	249VAL	CG2	100.00	199VA	C	211LEU	CD2	0.41	250LE	CB	250LEU	CG	100.00
249VAL	CA	249VAL	C	100.00	200GL	CA	200GLU	CB	100.00	250LE	CB	250LEU	CD1	100.00
249VAL	CA	254LEU	CD2	0.05	200GL	CA	200GLU	CG	100.00	250LE	CB	250LEU	CD2	100.00
249VAL	CA	221MET	CE	0.00	200GL	CA	200GLU	CD	76.30	250LE	CB	250LEU	C	100.00
249VAL	CB	249VAL	CG1	100.00	200GL	CA	200GLU	C	100.00	250LE	CB	251PRO	CD	50.43
249VAL	CB	249VAL	CG2	100.00	200GL	CA	211LEU	CD1	0.05	250LE	CG	250LEU	CD1	100.00
249VAL	CB	249VAL	C	100.00	200GL	CA	211LEU	CD2	1.35	250LE	CG	250LEU	CD2	100.00
249VAL	CB	221MET	CG	0.30	200GL	CB	200GLU	CG	100.00	250LE	CG	250LEU	C	0.00
249VAL	CB	221MET	CE	0.02	200GL	CB	200GLU	CD	100.00	250LE	CD1	250LEU	CD2	100.00
249VAL	CB	224VAL	CG1	0.00	200GL	CB	200GLU	C	100.00	250LE	CD2	250LEU	C	0.00
249VAL	CB	224VAL	CG2	0.05	200GL	CB	211LEU	CD2	0.03	250LE	C	251PRO	CA	100.00
249VAL	CB	225ARG	CZ	0.00	200GL	CG	200GLU	CD	100.00	250LE	C	251PRO	CB	0.26
249VAL	CG1	249VAL	CG2	100.00	200GL	CG	200GLU	C	90.80	250LE	C	251PRO	CD	100.00
249VAL	CG1	249VAL	C	74.50	200GL	CG	204ARG	CB	0.01	250LE	C	251PRO	C	100.00
249VAL	CG1	250LEU	CD2	0.02	200GL	CG	204ARG	CG	0.00	251PR	CA	251PRO	CB	100.00
249VAL	CG1	254LEU	CD1	0.01	200GL	CG	204ARG	CZ	0.24	251PR	CA	251PRO	CG	100.00
249VAL	CG1	254LEU	CD2	0.03	200GL	CG	211LEU	CD1	0.01	251PR	CA	251PRO	CD	100.00
249VAL	CG1	221MET	CA	0.02	200GL	CG	211LEU	CD2	0.10	251PR	CA	251PRO	C	100.00
249VAL	CG1	221MET	CB	0.05	200GL	CG	213HIS	CE1	0.02	251PR	CB	251PRO	CG	100.00
249VAL	CG1	221MET	CG	5.83	200GL	CD	200GLU	C	2.10	251PR	CB	251PRO	CD	100.00
249VAL	CG1	221MET	CE	0.53	200GL	CD	204ARG	CB	0.06	251PR	CB	251PRO	C	100.00
249VAL	CG1	224VAL	CB	0.02	200GL	CD	204ARG	CG	0.48	251PR	CB	256LEU	CD1	0.13
249VAL	CG1	224VAL	CG1	0.94	200GL	CD	204ARG	CD	0.00	251PR	CB	256LEU	CD2	1.41
249VAL	CG1	224VAL	CG2	4.27	200GL	CD	204ARG	CZ	0.02	251PR	CG	251PRO	CD	100.00
249VAL	CG1	225ARG	CB	0.02	200GL	CD	211LEU	CD1	0.00	251PR	CG	251PRO	C	97.58
249VAL	CG1	225ARG	CG	2.88	200GL	CD	211LEU	CD2	0.16	251PR	CG	256LEU	CB	0.06
249VAL	CG1	225ARG	CD	0.50	200GL	CD	213HIS	CE1	0.00	251PR	CG	256LEU	CD1	0.10
249VAL	CG1	225ARG	CZ	0.34	200GL	C	201GLU	CA	100.00	251PR	CG	256LEU	CD2	1.28
249VAL	CG2	249VAL	C	52.54	200GL	C	201GLU	CB	0.20	251PR	CG	257LEU	CG	0.02
249VAL	CG2	250LEU	CD1	0.00	200GL	C	201GLU	C	99.87	251PR	CG	257LEU	CD1	0.51
249VAL	CG2	250LEU	CD2	0.02	200GL	C	204ARG	CB	0.00	251PR	CG	257LEU	CD2	0.01
249VAL	CG2	221MET	CA	0.04	200GL	C	204ARG	CZ	0.08	251PR	CD	251PRO	C	83.61
249VAL	CG2	221MET	CB	0.01	201GL	CA	201GLU	CB	100.00	251PR	CD	257LEU	CD1	0.08
249VAL	CG2	221MET	CG	9.81	201GL	CA	201GLU	CG	100.00	251PR	C	252GLY	CA	100.00
249VAL	CG2	221MET	CE	0.21	201GL	CA	201GLU	CD	79.28	251PR	C	252GLY	C	35.54
249VAL	CG2	224VAL	CB	0.00	201GL	CA	201GLU	C	100.00	251PR	C	256LEU	CD1	0.10
249VAL	CG2	224VAL	CG1	0.30	201GL	CA	204ARG	CZ	0.06	252GL	CA	252GLY	C	100.00
249VAL	CG2	224VAL	CG2	4.14	201GL	CB	201GLU	CG	100.00	252GL	CA	327LEU	CD1	0.12
249VAL	CG2	225ARG	CB	0.02	201GL	CB	201GLU	CD	100.00	252GL	CA	327LEU	CD2	0.07
249VAL	CG2	225ARG	CG	0.50	201GL	CB	201GLU	C	100.00	252GL	C	253SER	CA	100.00

249VAL	CG2	225ARG	CD	0.36	201GL	CG	201GLU	CD	100.00	252GL	C	253SER	CB	55.73
249VAL	CG2	225ARG	CZ	0.07	201GL	CG	201GLU	C	94.95	252GL	C	253SER	C	46.61
249VAL	C	250LEU	CA	100.00	201GL	CG	204ARG	CZ	0.01	252GL	C	327LEU	CB	0.00
249VAL	C	250LEU	CB	0.01	201GL	CD	201GLU	C	0.26	252GL	C	327LEU	CG	0.01
249VAL	C	250LEU	C	99.96	201GL	CD	204ARG	CD	0.03	252GL	C	327LEU	CD1	1.04
249VAL	C	251PRO	CD	17.77	201GL	CD	204ARG	CZ	0.07	252GL	C	327LEU	CD2	1.19
250LEU	CA	250LEU	CB	100.00	201GL	C	202VAL	CA	100.00	253SE	CA	253SER	CB	100.00
250LEU	CA	250LEU	CG	100.00	201GL	C	202VAL	CB	3.31	253SE	CA	253SER	C	100.00
250LEU	CA	250LEU	CD1	1.41	201GL	C	202VAL	CG1	0.06	253SE	CA	326ASP	CG	0.00
250LEU	CA	250LEU	CD2	98.43	201GL	C	202VAL	CG2	0.18	253SE	CB	253SER	C	100.00
250LEU	CA	250LEU	C	100.00	201GL	C	202VAL	C	97.33	253SE	CB	256LEU	CB	0.02
250LEU	CA	251PRO	CD	100.00	202VA	CA	202VAL	CB	100.00	253SE	CB	256LEU	CG	0.04
250LEU	CB	250LEU	CG	100.00	202VA	CA	202VAL	CG1	100.00	253SE	CB	256LEU	CD1	0.73
250LEU	CB	250LEU	CD1	100.00	202VA	CA	202VAL	CG2	100.00	253SE	CB	256LEU	CD2	0.02
250LEU	CB	250LEU	CD2	100.00	202VA	CA	202VAL	C	100.00	253SE	CB	324PRO	CG	0.00
250LEU	CB	250LEU	C	100.00	202VA	CB	202VAL	CG1	100.00	253SE	CB	326ASP	CB	0.03
250LEU	CB	251PRO	CD	44.67	202VA	CB	202VAL	CG2	100.00	253SE	CB	326ASP	CG	0.12
250LEU	CG	250LEU	CD1	100.00	202VA	CB	202VAL	C	100.00	253SE	CB	326ASP	C	0.02
250LEU	CG	250LEU	CD2	100.00	202VA	CG1	202VAL	CG2	100.00	253SE	CB	327LEU	CB	0.01
250LEU	CG	250LEU	C	0.29	202VA	CG1	202VAL	C	98.16	253SE	CB	327LEU	CG	0.02
250LEU	CD1	250LEU	CD2	100.00	202VA	CG1	206TYR	CD1	0.09	253SE	CB	327LEU	CD1	1.32
250LEU	CD1	250LEU	C	0.04	202VA	CG1	206TYR	CD2	1.47	253SE	CB	327LEU	CD2	0.67
250LEU	CD2	250LEU	C	0.07	202VA	CG1	206TYR	CE1	0.07	253SE	C	254LEU	CA	100.00
250LEU	CD2	224VAL	CG1	0.20	202VA	CG1	206TYR	CE2	0.37	253SE	C	254LEU	CB	0.26
250LEU	CD2	224VAL	CG2	0.03	202VA	CG2	202VAL	C	14.21	253SE	C	254LEU	CD1	0.04
250LEU	C	251PRO	CA	100.00	202VA	CG2	206TYR	CD2	0.01	253SE	C	254LEU	CD2	0.00
250LEU	C	251PRO	CB	0.05	202VA	CG2	206TYR	CE2	0.01	253SE	C	254LEU	C	99.55
250LEU	C	251PRO	CD	100.00	202VA	C	203GLY	CA	100.00	253SE	C	256LEU	CD1	0.03
250LEU	C	251PRO	C	100.00	202VA	C	203GLY	C	86.68	254LE	CA	254LEU	CB	100.00
251PRO	CA	251PRO	CB	100.00	203GL	CA	203GLY	C	100.00	254LE	CA	254LEU	CG	100.00
251PRO	CA	251PRO	CG	100.00	203GL	CA	206TYR	CB	0.00	254LE	CA	254LEU	CD1	10.67
251PRO	CA	251PRO	CD	100.00	203GL	CA	211LEU	CD1	0.01	254LE	CA	254LEU	CD2	80.80
251PRO	CA	251PRO	C	100.00	203GL	CA	211LEU	CD2	0.04	254LE	CA	254LEU	C	100.00
251PRO	CB	251PRO	CG	100.00	203GL	C	204ARG	CA	100.00	254LE	CA	257LEU	CD1	0.01
251PRO	CB	251PRO	CD	100.00	203GL	C	204ARG	CB	5.20	254LE	CB	254LEU	CG	100.00
251PRO	CB	251PRO	C	100.00	203GL	C	204ARG	CG	0.43	254LE	CB	254LEU	CD1	100.00
251PRO	CB	256LEU	CB	0.01	203GL	C	204ARG	CD	0.01	254LE	CB	254LEU	CD2	100.00
251PRO	CB	256LEU	CD1	3.39	203GL	C	204ARG	C	95.31	254LE	CB	254LEU	C	100.00
251PRO	CG	251PRO	CD	100.00	203GL	C	206TYR	CB	0.00	254LE	CB	272VAL	CG1	0.00
251PRO	CG	251PRO	C	66.67	203GL	C	211LEU	CD1	0.02	254LE	CB	221MET	CE	0.22
251PRO	CG	256LEU	CD1	1.44	203GL	C	211LEU	CD2	0.29	254LE	CG	254LEU	CD1	100.00
251PRO	CG	256LEU	CD2	0.00	204AR	CA	204ARG	CB	100.00	254LE	CG	254LEU	CD2	100.00
251PRO	CG	257LEU	CD1	0.02	204AR	CA	204ARG	CG	100.00	254LE	CG	254LEU	C	21.08
251PRO	CG	257LEU	CD2	0.39	204AR	CA	204ARG	CD	29.67	254LE	CG	221MET	CE	0.01
251PRO	CD	251PRO	C	54.62	204AR	CA	204ARG	C	100.00	254LE	CG	225ARG	CZ	1.50
251PRO	C	252GLY	CA	100.00	204AR	CB	204ARG	CG	100.00	254LE	CD1	254LEU	CD2	100.00
251PRO	C	252GLY	C	36.97	204AR	CB	204ARG	CD	100.00	254LE	CD1	254LEU	C	6.47
251PRO	C	256LEU	CD1	0.00	204AR	CB	204ARG	CZ	6.87	254LE	CD1	255GLY	CA	0.01
252GLY	CA	252GLY	C	100.00	204AR	CB	204ARG	C	100.00	254LE	CD1	272VAL	CG1	0.01
252GLY	C	253SER	CA	100.00	204AR	CB	211LEU	CD2	0.00	254LE	CD1	273HIS	CA	0.01
252GLY	C	253SER	CB	68.78	204AR	CG	204ARG	CD	100.00	254LE	CD1	221MET	CB	0.02
252GLY	C	253SER	C	32.97	204AR	CG	204ARG	CZ	46.94	254LE	CD1	221MET	CG	0.00
253SER	CA	253SER	CB	100.00	204AR	CG	204ARG	C	44.52	254LE	CD1	221MET	CE	0.36
253SER	CA	253SER	C	100.00	204AR	CG	211LEU	CD2	0.01	254LE	CD1	225ARG	CD	0.04
253SER	CB	253SER	C	100.00	204AR	CD	204ARG	CZ	100.00	254LE	CD1	225ARG	CZ	4.54
253SER	CB	256LEU	CG	0.08	204AR	CD	204ARG	C	0.01	254LE	CD2	254LEU	C	0.06
253SER	CB	256LEU	CD1	0.06	204AR	CZ	211LEU	CB	0.08	254LE	CD2	272VAL	CG1	0.04
253SER	CB	256LEU	CD2	0.47	204AR	CZ	211LEU	CG	0.01	254LE	CD2	221MET	CG	1.14
253SER	CB	324PRO	CB	0.06	204AR	CZ	211LEU	CD1	0.02	254LE	CD2	221MET	CE	0.41
253SER	CB	324PRO	CG	0.40	204AR	CZ	211LEU	CD2	0.92	254LE	CD2	225ARG	CD	0.77
253SER	CB	326ASP	CB	0.02	204AR	C	205GLY	CA	100.00	254LE	CD2	225ARG	CZ	6.26
253SER	CB	326ASP	CG	0.12	204AR	C	205GLY	C	54.60	254LE	C	255GLY	CA	100.00

253SER	CB	327LEU	CD1	0.04	205GL	CA	205GLY	C	100.00	254LE	C	255GLY	C	25.45
253SER	C	254LEU	CA	100.00	205GL	C	206TYR	CA	100.00	255GL	CA	255GLY	C	100.00
253SER	C	254LEU	CB	7.81	205GL	C	206TYR	CB	28.19	255GL	CA	272VAL	CG1	0.13
253SER	C	254LEU	CG	1.19	205GL	C	206TYR	CG	2.94	255GL	CA	272VAL	C	0.04
253SER	C	254LEU	CD1	0.29	205GL	C	206TYR	CD1	1.59	255GL	CA	273HIS	CA	0.06
253SER	C	254LEU	CD2	0.06	205GL	C	206TYR	CD2	3.68	255GL	CA	273HIS	CB	0.33
253SER	C	254LEU	C	90.58	205GL	C	206TYR	C	73.74	255GL	CA	273HIS	CD2	0.02
253SER	C	256LEU	CD1	0.04	205GL	C	207PRO	CD	5.19	255GL	C	256LEU	CA	100.00
253SER	C	256LEU	CD2	0.10	206TY	CA	206TYR	CB	100.00	255GL	C	256LEU	CB	40.52
254LEU	CA	254LEU	CB	100.00	206TY	CA	206TYR	CG	100.00	255GL	C	256LEU	CG	1.79
254LEU	CA	254LEU	CG	100.00	206TY	CA	206TYR	CD1	62.96	255GL	C	256LEU	CD1	0.04
254LEU	CA	254LEU	CD1	16.86	206TY	CA	206TYR	CD2	83.82	255GL	C	256LEU	C	55.36
254LEU	CA	254LEU	CD2	79.38	206TY	CA	206TYR	C	100.00	255GL	C	273HIS	CB	0.06
254LEU	CA	254LEU	C	100.00	206TY	CA	207PRO	CD	100.00	255GL	C	289ALA	CB	0.07
254LEU	CA	257LEU	CD1	0.36	206TY	CB	206TYR	CG	100.00	256LE	CA	256LEU	CB	100.00
254LEU	CB	254LEU	CG	100.00	206TY	CB	206TYR	CD1	100.00	256LE	CA	256LEU	CG	100.00
254LEU	CB	254LEU	CD1	100.00	206TY	CB	206TYR	CD2	100.00	256LE	CA	256LEU	CD1	31.17
254LEU	CB	254LEU	CD2	100.00	206TY	CB	206TYR	C	100.00	256LE	CA	256LEU	CD2	62.19
254LEU	CB	254LEU	C	100.00	206TY	CB	207PRO	CD	0.03	256LE	CA	256LEU	C	100.00
254LEU	CB	257LEU	CD1	0.16	206TY	CB	209VAL	CG1	0.00	256LE	CA	288THR	CG2	0.01
254LEU	CB	326ASP	CG	0.01	206TY	CB	209VAL	CG2	0.26	256LE	CA	289ALA	CB	0.00
254LEU	CG	254LEU	CD1	100.00	206TY	CG	206TYR	CD1	100.00	256LE	CB	256LEU	CG	100.00
254LEU	CG	254LEU	CD2	100.00	206TY	CG	206TYR	CD2	100.00	256LE	CB	256LEU	CD1	100.00
254LEU	CG	254LEU	C	2.18	206TY	CG	206TYR	CE1	100.00	256LE	CB	256LEU	CD2	100.00
254LEU	CG	257LEU	CD1	0.02	206TY	CG	206TYR	CE2	100.00	256LE	CB	256LEU	C	100.00
254LEU	CG	326ASP	CB	0.01	206TY	CG	206TYR	CZ	100.00	256LE	CB	288THR	CG2	0.00
254LEU	CD1	254LEU	CD2	100.00	206TY	CG	206TYR	C	39.30	256LE	CG	256LEU	CD1	100.00
254LEU	CD1	254LEU	C	0.00	206TY	CG	207PRO	CD	1.85	256LE	CG	256LEU	CD2	100.00
254LEU	CD1	257LEU	CD1	0.08	206TY	CD1	206TYR	CD2	100.00	256LE	CG	256LEU	C	41.31
254LEU	CD1	326ASP	CB	0.06	206TY	CD1	206TYR	CE1	100.00	256LE	CG	288THR	CG2	0.20
254LEU	CD1	221MET	CE	1.10	206TY	CD1	206TYR	CE2	100.00	256LE	CD1	256LEU	CD2	100.00
254LEU	CD1	225ARG	CZ	0.74	206TY	CD1	206TYR	CZ	100.00	256LE	CD1	256LEU	C	0.66
254LEU	CD2	254LEU	C	0.02	206TY	CD1	206TYR	C	2.43	256LE	CD1	286ASN	CG	0.19
254LEU	CD2	257LEU	CD1	0.88	206TY	CD1	207PRO	CD	0.10	256LE	CD1	288THR	CB	0.29
254LEU	CD2	273HIS	CB	0.05	206TY	CD1	209VAL	CG1	0.00	256LE	CD1	288THR	CG2	2.07
254LEU	CD2	279ILE	CG2	0.01	206TY	CD1	209VAL	CG2	0.10	256LE	CD1	319LEU	CD1	0.05
254LEU	CD2	221MET	CE	7.65	206TY	CD2	206TYR	CE1	100.00	256LE	CD1	319LEU	CD2	0.07
254LEU	CD2	225ARG	CZ	0.01	206TY	CD2	206TYR	CE2	100.00	256LE	CD1	327LEU	CD1	0.55
254LEU	C	255GLY	CA	100.00	206TY	CD2	206TYR	CZ	100.00	256LE	CD1	327LEU	CD2	0.05
254LEU	C	255GLY	C	52.73	206TY	CD2	206TYR	C	25.03	256LE	CD2	256LEU	C	5.76
254LEU	C	257LEU	CG	0.00	206TY	CD2	207PRO	CD	2.60	256LE	CD2	286ASN	CB	0.00
254LEU	C	257LEU	CD1	0.04	206TY	CD2	209VAL	CB	0.14	256LE	CD2	286ASN	CG	1.01
255GLY	CA	255GLY	C	100.00	206TY	CD2	209VAL	CG1	0.05	256LE	CD2	288THR	CB	0.13
255GLY	CA	286ASN	CB	0.05	206TY	CD2	209VAL	CG2	1.20	256LE	CD2	288THR	CG2	0.56
255GLY	CA	286ASN	CG	0.02	206TY	CE1	206TYR	CE2	100.00	256LE	CD2	292LEU	CD1	0.02
255GLY	C	256LEU	CA	100.00	206TY	CE1	206TYR	CZ	100.00	256LE	CD2	319LEU	CD1	0.25
255GLY	C	256LEU	CB	96.53	206TY	CE1	209VAL	CG2	0.14	256LE	CD2	319LEU	CD2	0.46
255GLY	C	256LEU	CG	6.16	206TY	CE2	206TYR	CZ	100.00	256LE	CD2	327LEU	CD1	0.14
255GLY	C	256LEU	CD1	1.16	206TY	CE2	209VAL	CB	0.01	256LE	C	257LEU	CA	100.00
255GLY	C	256LEU	CD2	0.08	206TY	CE2	209VAL	CG2	1.36	256LE	C	257LEU	CB	11.76
255GLY	C	256LEU	C	2.47	206TY	C	207PRO	CA	100.00	256LE	C	257LEU	CG	1.72
255GLY	C	286ASN	CG	0.01	206TY	C	207PRO	CB	0.35	256LE	C	257LEU	CD1	0.02
255GLY	C	289ALA	CB	0.33	206TY	C	207PRO	CD	100.00	256LE	C	257LEU	C	87.26
256LEU	CA	256LEU	CB	100.00	206TY	C	207PRO	C	100.00	256LE	C	289ALA	CA	0.01
256LEU	CA	256LEU	CG	100.00	207PR	CA	207PRO	CB	100.00	256LE	C	289ALA	CB	3.30
256LEU	CA	256LEU	CD1	74.95	207PR	CA	207PRO	CG	100.00	256LE	C	292LEU	CD1	0.01
256LEU	CA	256LEU	CD2	13.26	207PR	CA	207PRO	CD	100.00	257LE	CA	257LEU	CB	100.00
256LEU	CA	256LEU	C	100.00	207PR	CA	207PRO	C	100.00	257LE	CA	257LEU	CG	100.00
256LEU	CA	289ALA	CA	0.00	207PR	CB	207PRO	CG	100.00	257LE	CA	257LEU	CD1	0.52
256LEU	CA	289ALA	CB	0.04	207PR	CB	207PRO	CD	100.00	257LE	CA	257LEU	CD2	98.18
256LEU	CA	292LEU	CD1	0.02	207PR	CB	207PRO	C	100.00	257LE	CA	257LEU	C	100.00
256LEU	CB	256LEU	CG	100.00	207PR	CG	207PRO	CD	100.00	257LE	CA	258PRO	CD	100.00

256LEU	CB	256LEU	CD1	100.00	207PR(	CG	207PRO	C	96.96	257LEI	CB	257LEU	CG	100.00
256LEU	CB	256LEU	CD2	100.00	207PR(	CD	207PRO	C	92.61	257LEI	CB	257LEU	CD1	100.00
256LEU	CB	256LEU	C	100.00	207PR(	C	208ASP	CA	100.00	257LEI	CB	257LEU	CD2	100.00
256LEU	CB	288THR	CG2	8.01	207PR(	C	208ASP	CB	92.79	257LEI	CB	257LEU	C	100.00
256LEU	CB	292LEU	CD1	0.76	207PR(	C	208ASP	CG	0.48	257LEI	CB	258PRO	CD	14.79
256LEU	CG	256LEU	CD1	100.00	207PR(	C	208ASP	C	96.57	257LEI	CB	272VAL	CG2	0.04
256LEU	CG	256LEU	CD2	100.00	208ASI	CA	208ASP	CB	100.00	257LEI	CG	257LEU	CD1	100.00
256LEU	CG	256LEU	C	55.92	208ASI	CA	208ASP	CG	100.00	257LEI	CG	257LEU	CD2	100.00
256LEU	CD1	256LEU	CD2	100.00	208ASI	CA	208ASP	C	100.00	257LEI	CD1	257LEU	CD2	100.00
256LEU	CD1	256LEU	C	39.27	208ASI	CB	208ASP	CG	100.00	257LEI	CD1	272VAL	CB	0.00
256LEU	CD1	257LEU	CD2	0.03	208ASI	CB	208ASP	C	100.00	257LEI	C	258PRO	CA	100.00
256LEU	CD1	319LEU	CD1	0.11	208ASI	CG	208ASP	C	96.84	257LEI	C	258PRO	CB	0.85
256LEU	CD1	319LEU	CD2	0.03	208ASI	C	209VAL	CA	100.00	257LEI	C	258PRO	CD	100.00
256LEU	CD2	256LEU	C	1.20	208ASI	C	209VAL	CB	15.14	257LEI	C	258PRO	C	100.00
256LEU	CD2	288THR	CB	0.11	208ASI	C	209VAL	CG1	0.44	257LEI	C	272VAL	CG2	0.02
256LEU	CD2	288THR	CG2	0.29	208ASI	C	209VAL	CG2	10.76	257LEI	C	289ALA	CB	0.47
256LEU	CD2	292LEU	CD1	0.06	208ASI	C	209VAL	C	88.28	258PR	CA	258PRO	CB	100.00
256LEU	CD2	315VAL	CG2	0.04	209VAI	CA	209VAL	CB	100.00	258PR	CA	258PRO	CG	100.00
256LEU	CD2	319LEU	CD1	3.69	209VAI	CA	209VAL	CG1	100.00	258PR	CA	258PRO	CD	100.00
256LEU	CD2	319LEU	CD2	0.22	209VAI	CA	209VAL	CG2	100.00	258PR	CA	258PRO	C	100.00
256LEU	CD2	324PRO	CB	0.12	209VAI	CA	209VAL	C	100.00	258PR	CB	258PRO	CG	100.00
256LEU	CD2	324PRO	CG	0.16	209VAI	CB	209VAL	CG1	100.00	258PR	CB	258PRO	CD	100.00
256LEU	C	257LEU	CA	100.00	209VAI	CB	209VAL	CG2	100.00	258PR	CB	258PRO	C	100.00
256LEU	C	257LEU	CB	22.98	209VAI	CB	209VAL	C	100.00	258PR	CB	292LEU	C	1.35
256LEU	C	257LEU	CG	1.38	209VAI	CG1	209VAL	CG2	100.00	258PR	CB	293SER	CA	0.62
256LEU	C	257LEU	CD1	0.06	209VAI	CG1	209VAL	C	93.94	258PR	CB	296MET	CE	0.00
256LEU	C	257LEU	CD2	0.04	209VAI	CG1	210ALA	C	0.01	258PR	CG	258PRO	CD	100.00
256LEU	C	257LEU	C	84.17	209VAI	CG1	211LEU	CD1	0.01	258PR	CG	258PRO	C	100.00
256LEU	C	258PRO	CD	1.47	209VAI	CG1	211LEU	CD2	0.02	258PR	CG	292LEU	CB	0.14
256LEU	C	289ALA	CA	0.03	209VAI	CG2	209VAL	C	6.18	258PR	CG	296MET	CE	0.11
256LEU	C	289ALA	CB	0.11	209VAI	C	210ALA	CA	100.00	258PR	CD	258PRO	C	99.90
256LEU	C	292LEU	CD1	0.07	209VAI	C	210ALA	CB	7.29	258PR	CD	292LEU	CB	0.10
257LEU	CA	257LEU	CB	100.00	209VAI	C	210ALA	C	92.84	258PR	CD	292LEU	CD1	0.02
257LEU	CA	257LEU	CG	100.00	210AL/	CA	210ALA	CB	100.00	258PR	C	259SER	CA	100.00
257LEU	CA	257LEU	CD1	0.76	210AL/	CA	210ALA	C	100.00	258PR	C	259SER	CB	99.98
257LEU	CA	257LEU	CD2	97.91	210AL/	CB	210ALA	C	100.00	258PR	C	259SER	C	0.20
257LEU	CA	257LEU	C	100.00	210AL/	C	211LEU	CA	100.00	258PR	C	272VAL	CG2	0.22
257LEU	CA	258PRO	CD	100.00	210AL/	C	211LEU	CB	46.50	259SE	CA	259SER	CB	100.00
257LEU	CB	257LEU	CG	100.00	210AL/	C	211LEU	CG	0.00	259SE	CA	259SER	C	100.00
257LEU	CB	257LEU	CD1	100.00	210AL/	C	211LEU	CD1	0.02	259SE	CB	259SER	C	100.00
257LEU	CB	257LEU	CD2	100.00	210AL/	C	211LEU	C	59.44	259SE	CB	272VAL	CG2	0.49
257LEU	CB	257LEU	C	100.00	211LEI	CA	211LEU	CB	100.00	259SE	C	260ALA	CA	100.00
257LEU	CB	258PRO	CD	0.00	211LEI	CA	211LEU	CG	100.00	259SE	C	260ALA	CB	93.30
257LEU	CB	272VAL	CB	0.06	211LEI	CA	211LEU	CD1	95.06	259SE	C	260ALA	C	7.88
257LEU	CB	272VAL	CG2	0.01	211LEI	CA	211LEU	CD2	5.20	259SE	C	296MET	CE	0.04
257LEU	CG	257LEU	CD1	100.00	211LEI	CA	211LEU	C	100.00	260AL.	CA	260ALA	CB	100.00
257LEU	CG	257LEU	CD2	100.00	211LEI	CB	211LEU	CG	100.00	260AL.	CA	260ALA	C	100.00
257LEU	CD1	257LEU	CD2	100.00	211LEI	CB	211LEU	CD1	100.00	260AL.	CA	269PHE	CD2	0.00
257LEU	CD1	272VAL	CG1	0.01	211LEI	CB	211LEU	CD2	100.00	260AL.	CB	260ALA	C	100.00
257LEU	C	258PRO	CA	100.00	211LEI	CB	211LEU	C	100.00	260AL.	CB	262LEU	CD1	0.77
257LEU	C	258PRO	CB	0.98	211LEI	CG	211LEU	CD1	100.00	260AL.	CB	262LEU	CD2	0.02
257LEU	C	258PRO	CD	100.00	211LEI	CG	211LEU	CD2	100.00	260AL.	CB	269PHE	CD2	23.02
257LEU	C	258PRO	C	99.99	211LEI	CG	211LEU	C	97.07	260AL.	CB	269PHE	CE2	14.88
257LEU	C	289ALA	CB	0.11	211LEI	CG	213HIS	CE1	0.29	260AL.	CB	296MET	CE	9.31
258PRO	CA	258PRO	CB	100.00	211LEI	CD1	211LEU	CD2	100.00	260AL.	CB	300HIS	CD2	0.00
258PRO	CA	258PRO	CG	100.00	211LEI	CD1	211LEU	C	4.42	260AL.	C	261SER	CA	100.00
258PRO	CA	258PRO	CD	100.00	211LEI	CD1	212GLU	C	0.01	260AL.	C	261SER	CB	47.28
258PRO	CA	258PRO	C	100.00	211LEI	CD1	213HIS	CD2	0.01	260AL.	C	261SER	C	61.76
258PRO	CB	258PRO	CG	100.00	211LEI	CD1	213HIS	CE1	0.87	260AL.	C	262LEU	CD1	0.01
258PRO	CB	258PRO	CD	100.00	211LEI	CD2	211LEU	C	1.58	261SE	CA	261SER	CB	100.00
258PRO	CB	258PRO	C	100.00	211LEI	CD2	212GLU	C	0.08	261SE	CA	261SER	C	100.00
258PRO	CB	292LEU	CB	0.13	211LEI	CD2	213HIS	CD2	0.02	261SE	CB	261SER	C	100.00

258PRO	CB	292LEU	C	1.51
258PRO	CB	293SER	CA	0.81
258PRO	CB	293SER	CB	0.04
258PRO	CB	296MET	CE	0.05
258PRO	CG	258PRO	CD	100.00
258PRO	CG	258PRO	C	99.98
258PRO	CG	292LEU	CB	0.22
258PRO	CG	296MET	CE	0.12
258PRO	CD	258PRO	C	99.68
258PRO	C	259SER	CA	100.00
258PRO	C	259SER	CB	99.98
258PRO	C	259SER	C	0.11
258PRO	C	272VAL	CG2	0.45
259SER	CA	259SER	CB	100.00
259SER	CA	259SER	C	100.00
259SER	CB	259SER	C	100.00
259SER	CB	272VAL	CG2	19.41
259SER	C	260ALA	CA	100.00
259SER	C	260ALA	CB	71.55
259SER	C	260ALA	C	30.23
259SER	C	296MET	CE	0.09
260ALA	CA	260ALA	CB	100.00
260ALA	CA	260ALA	C	100.00
260ALA	CA	269PHE	CD2	0.07
260ALA	CA	269PHE	CE2	0.00
260ALA	CB	260ALA	C	100.00
260ALA	CB	262LEU	CD1	1.10
260ALA	CB	262LEU	CD2	0.03
260ALA	CB	269PHE	CA	0.00
260ALA	CB	269PHE	CG	0.00
260ALA	CB	269PHE	CD1	0.00
260ALA	CB	269PHE	CD2	23.48
260ALA	CB	269PHE	CE2	36.48
260ALA	CB	269PHE	CZ	0.01
260ALA	CB	296MET	CE	12.58
260ALA	CB	300HIS	CE1	0.01
260ALA	C	261SER	CA	100.00
260ALA	C	261SER	CB	36.10
260ALA	C	261SER	C	73.09
260ALA	C	262LEU	CD1	0.02
261SER	CA	261SER	CB	100.00
261SER	CA	261SER	C	100.00
261SER	CB	261SER	C	100.00
261SER	C	262LEU	CA	100.00
261SER	C	262LEU	CB	7.42
261SER	C	262LEU	CG	0.52
261SER	C	262LEU	CD1	2.33
261SER	C	262LEU	CD2	0.22
261SER	C	262LEU	C	92.80
262LEU	CA	262LEU	CB	100.00
262LEU	CA	262LEU	CG	100.00
262LEU	CA	262LEU	CD1	29.52
262LEU	CA	262LEU	CD2	70.91
262LEU	CA	262LEU	C	100.00
262LEU	CB	262LEU	CG	100.00
262LEU	CB	262LEU	CD1	100.00
262LEU	CB	262LEU	CD2	100.00
262LEU	CB	262LEU	C	100.00
262LEU	CG	262LEU	CD1	100.00
262LEU	CG	262LEU	CD2	100.00
262LEU	CG	262LEU	C	1.93
262LEU	CG	300HIS	CD2	0.00

211LE	CD2	213HIS	CE1	7.25
211LE	C	212GLU	CA	100.00
211LE	C	212GLU	CB	79.21
211LE	C	212GLU	CG	0.01
211LE	C	212GLU	C	27.97
212GL	CA	212GLU	CB	100.00
212GL	CA	212GLU	CG	100.00
212GL	CA	212GLU	CD	77.41
212GL	CA	212GLU	C	100.00
212GL	CB	212GLU	CG	100.00
212GL	CB	212GLU	CD	100.00
212GL	CB	212GLU	C	100.00
212GL	CG	212GLU	CD	100.00
212GL	CG	212GLU	C	98.91
212GL	CG	214GLN	CG	0.08
212GL	CG	214GLN	CD	0.02
212GL	CD	212GLU	C	0.07
212GL	CD	213HIS	C	0.00
212GL	CD	214GLN	CG	0.00
212GL	CD	229ARG	CZ	0.01
212GL	C	213HIS	CA	100.00
212GL	C	213HIS	CB	25.70
212GL	C	213HIS	CG	7.06
212GL	C	213HIS	CD2	0.35
212GL	C	213HIS	C	76.87
213HIS	CA	213HIS	CB	100.00
213HIS	CA	213HIS	CG	100.00
213HIS	CA	213HIS	CD2	6.24
213HIS	CA	213HIS	C	100.00
213HIS	CB	213HIS	CG	100.00
213HIS	CB	213HIS	CD2	100.00
213HIS	CB	213HIS	CE1	0.19
213HIS	CB	213HIS	C	100.00
213HIS	CG	213HIS	CD2	100.00
213HIS	CG	213HIS	CE1	100.00
213HIS	CD2	213HIS	CE1	100.00
213HIS	C	214GLN	CA	100.00
213HIS	C	214GLN	CB	89.89
213HIS	C	214GLN	CG	9.37
213HIS	C	214GLN	CD	0.01
213HIS	C	214GLN	C	9.64
214GL	CA	214GLN	CB	100.00
214GL	CA	214GLN	CG	100.00
214GL	CA	214GLN	CD	77.40
214GL	CA	214GLN	C	100.00
214GL	CB	214GLN	CG	100.00
214GL	CB	214GLN	CD	100.00
214GL	CB	214GLN	C	100.00
214GL	CB	219MET	CB	0.00
214GL	CB	219MET	CG	0.09
214GL	CG	214GLN	CD	100.00
214GL	CG	214GLN	C	57.06
214GL	CG	218ALA	CB	0.00
214GL	CG	219MET	CA	0.10
214GL	CG	219MET	CB	0.95
214GL	CG	219MET	CG	0.06
214GL	CD	214GLN	C	0.01
214GL	CD	218ALA	CB	0.22
214GL	CD	219MET	CA	0.23
214GL	CD	219MET	CB	0.04
214GL	CD	219MET	CG	0.04
214GL	CD	222HIS	CD2	0.01

261SE	C	262LEU	CA	100.00
261SE	C	262LEU	CB	19.47
261SE	C	262LEU	CG	1.34
261SE	C	262LEU	CD1	1.73
261SE	C	262LEU	CD2	0.29
261SE	C	262LEU	C	77.23
262LE	CA	262LEU	CB	100.00
262LE	CA	262LEU	CG	100.00
262LE	CA	262LEU	CD1	21.63
262LE	CA	262LEU	CD2	76.29
262LE	CA	262LEU	C	100.00
262LE	CB	262LEU	CG	100.00
262LE	CB	262LEU	CD1	100.00
262LE	CB	262LEU	CD2	100.00
262LE	CB	262LEU	C	100.00
262LE	CG	262LEU	CD1	100.00
262LE	CG	262LEU	CD2	100.00
262LE	CG	262LEU	C	9.95
262LE	CG	267PRO	CB	0.00
262LE	CG	300HIS	CE1	0.01
262LE	CD1	262LEU	CD2	100.00
262LE	CD1	262LEU	C	4.64
262LE	CD1	267PRO	CB	0.13
262LE	CD1	300HIS	CD2	0.05
262LE	CD1	300HIS	CE1	2.48
262LE	CD2	262LEU	C	0.66
262LE	CD2	267PRO	CA	0.00
262LE	CD2	267PRO	CB	5.07
262LE	CD2	300HIS	CD2	1.96
262LE	CD2	300HIS	CE1	4.08
262LE	C	263GLY	CA	100.00
262LE	C	263GLY	C	2.54
263GL	CA	263GLY	C	100.00
263GL	C	264ARG	CA	100.00
263GL	C	264ARG	CB	7.79
263GL	C	264ARG	CG	2.57
263GL	C	264ARG	CD	0.00
263GL	C	264ARG	C	91.62
264AR	CA	264ARG	CB	100.00
264AR	CA	264ARG	CG	100.00
264AR	CA	264ARG	CD	0.80
264AR	CA	264ARG	C	100.00
264AR	CB	264ARG	CG	100.00
264AR	CB	264ARG	CD	100.00
264AR	CB	264ARG	CZ	1.90
264AR	CB	264ARG	C	100.00
264AR	CG	264ARG	CD	100.00
264AR	CG	264ARG	CZ	42.24
264AR	CG	264ARG	C	7.87
264AR	CD	264ARG	CZ	100.00
264AR	C	265GLY	CA	100.00
264AR	C	265GLY	C	60.37
265GL	CA	265GLY	C	100.00
265GL	C	266THR	CA	100.00
265GL	C	266THR	CB	0.50
265GL	C	266THR	C	99.60
266TH	CA	266THR	CB	100.00
266TH	CA	266THR	CG2	100.00
266TH	CA	266THR	C	100.00
266TH	CA	267PRO	CD	100.00
266TH	CB	266THR	CG2	100.00
266TH	CB	266THR	C	100.00

262LEU	CG	300HIS	CE1	0.32	214GLI	CD	230PHE	CE1	0.02	266TH	CG2	266THR	C	100.00
262LEU	CD1	262LEU	CD2	100.00	214GLI	CD	230PHE	CZ	0.00	266TH	CG2	267PRO	CD	4.89
262LEU	CD1	262LEU	C	0.05	214GLI	C	215TYR	CA	100.00	266TH	C	267PRO	CA	100.00
262LEU	CD1	267PRO	CB	0.02	214GLI	C	215TYR	CB	0.31	266TH	C	267PRO	CB	0.06
262LEU	CD1	300HIS	CD2	0.04	214GLI	C	215TYR	CG	0.02	266TH	C	267PRO	CD	100.00
262LEU	CD1	300HIS	CE1	1.33	214GLI	C	215TYR	CD1	0.01	266TH	C	267PRO	C	100.00
262LEU	CD2	262LEU	C	0.68	214GLI	C	215TYR	CD2	0.02	267PR	CA	267PRO	CB	100.00
262LEU	CD2	267PRO	CB	1.69	214GLI	C	215TYR	C	99.79	267PR	CA	267PRO	CG	100.00
262LEU	CD2	300HIS	CD2	0.82	215TYF	CA	215TYR	CB	100.00	267PR	CA	267PRO	CD	100.00
262LEU	CD2	300HIS	CE1	7.56	215TYF	CA	215TYR	CG	100.00	267PR	CA	267PRO	C	100.00
262LEU	C	263GLY	CA	100.00	215TYF	CA	215TYR	CD1	18.59	267PR	CB	267PRO	CG	100.00
262LEU	C	263GLY	C	1.81	215TYF	CA	215TYR	CD2	84.16	267PR	CB	267PRO	CD	100.00
263GLY	CA	263GLY	C	100.00	215TYF	CA	215TYR	C	100.00	267PR	CB	267PRO	C	100.00
263GLY	C	264ARG	CA	100.00	215TYF	CB	215TYR	CG	100.00	267PR	CB	269PHE	CE1	1.36
263GLY	C	264ARG	CB	0.88	215TYF	CB	215TYR	CD1	100.00	267PR	CB	269PHE	CZ	0.27
263GLY	C	264ARG	CG	0.08	215TYF	CB	215TYR	CD2	100.00	267PR	CB	300HIS	CD2	0.02
263GLY	C	264ARG	C	99.05	215TYF	CB	215TYR	C	100.00	267PR	CB	300HIS	CE1	0.03
264ARG	CA	264ARG	CB	100.00	215TYF	CB	218ALA	CB	0.01	267PR	CG	267PRO	CD	100.00
264ARG	CA	264ARG	CG	100.00	215TYF	CG	215TYR	CD1	100.00	267PR	CG	267PRO	C	99.23
264ARG	CA	264ARG	CD	8.70	215TYF	CG	215TYR	CD2	100.00	267PR	CG	269PHE	CE1	0.17
264ARG	CA	264ARG	C	100.00	215TYF	CG	215TYR	CE1	100.00	267PR	CG	269PHE	CZ	0.04
264ARG	CB	264ARG	CG	100.00	215TYF	CG	215TYR	CE2	100.00	267PR	CG	300HIS	CD2	0.07
264ARG	CB	264ARG	CD	100.00	215TYF	CG	215TYR	CZ	100.00	267PR	CG	300HIS	CE1	0.01
264ARG	CB	264ARG	CZ	2.02	215TYF	CD1	215TYR	CD2	100.00	267PR	CG	301ALA	CB	0.04
264ARG	CB	264ARG	C	100.00	215TYF	CD1	215TYR	CE1	100.00	267PR	CD	267PRO	C	95.98
264ARG	CG	264ARG	CD	100.00	215TYF	CD1	215TYR	CE2	100.00	267PR	C	268VAL	CA	100.00
264ARG	CG	264ARG	CZ	35.44	215TYF	CD1	215TYR	CZ	100.00	267PR	C	268VAL	CB	43.30
264ARG	CG	264ARG	C	71.71	215TYF	CD2	215TYR	CE1	100.00	267PR	C	268VAL	CG2	29.29
264ARG	CD	264ARG	CZ	100.00	215TYF	CD2	215TYR	CE2	100.00	267PR	C	268VAL	C	74.02
264ARG	CD	264ARG	C	16.18	215TYF	CD2	215TYR	CZ	100.00	267PR	C	269PHE	CE1	0.05
264ARG	CZ	264ARG	C	0.10	215TYF	CE1	215TYR	CE2	100.00	268VA	CA	268VAL	CB	100.00
264ARG	CZ	265GLY	CA	0.07	215TYF	CE1	215TYR	CZ	100.00	268VA	CA	268VAL	CG1	100.00
264ARG	C	265GLY	CA	100.00	215TYF	CE1	78ASP	CG	0.01	268VA	CA	268VAL	CG2	100.00
264ARG	C	265GLY	C	78.92	215TYF	CE2	215TYR	CZ	100.00	268VA	CA	268VAL	C	100.00
265GLY	CA	265GLY	C	100.00	215TYF	C	216VAL	CA	100.00	268VA	CB	268VAL	CG1	100.00
265GLY	C	266THR	CA	100.00	215TYF	C	216VAL	C	100.00	268VA	CB	268VAL	CG2	100.00
265GLY	C	266THR	CB	0.13	216VAI	CA	216VAL	CB	100.00	268VA	CB	268VAL	C	100.00
265GLY	C	266THR	C	99.96	216VAI	CA	216VAL	CG1	100.00	268VA	CG1	268VAL	CG2	100.00
266THR	CA	266THR	CB	100.00	216VAI	CA	216VAL	CG2	100.00	268VA	CG1	268VAL	C	100.00
266THR	CA	266THR	CG2	100.00	216VAI	CA	216VAL	C	100.00	268VA	CG1	270GLU	CG	0.02
266THR	CA	266THR	C	100.00	216VAI	CB	216VAL	CG1	100.00	268VA	C	269PHE	CA	100.00
266THR	CA	267PRO	CD	100.00	216VAI	CB	216VAL	CG2	100.00	268VA	C	269PHE	CB	28.68
266THR	CB	266THR	CG2	100.00	216VAI	CB	216VAL	C	100.00	268VA	C	269PHE	CG	10.30
266THR	CB	266THR	C	100.00	216VAI	CB	239PHE	CG	0.00	268VA	C	269PHE	CD1	14.13
266THR	CG2	266THR	C	100.00	216VAI	CB	239PHE	CD1	0.20	268VA	C	269PHE	C	80.46
266THR	CG2	267PRO	CD	3.88	216VAI	CB	239PHE	CD2	0.13	269PH	CA	269PHE	CB	100.00
266THR	C	267PRO	CA	100.00	216VAI	CB	239PHE	CE1	0.08	269PH	CA	269PHE	CG	100.00
266THR	C	267PRO	CB	0.04	216VAI	CB	239PHE	CE2	0.09	269PH	CA	269PHE	CD1	93.51
266THR	C	267PRO	CD	100.00	216VAI	CG1	216VAL	CG2	100.00	269PH	CA	269PHE	CD2	93.86
266THR	C	267PRO	C	100.00	216VAI	CG1	216VAL	C	99.79	269PH	CA	269PHE	C	100.00
267PRO	CA	267PRO	CB	100.00	216VAI	CG1	239PHE	CG	0.02	269PH	CB	269PHE	CG	100.00
267PRO	CA	267PRO	CG	100.00	216VAI	CG1	239PHE	CD1	0.50	269PH	CB	269PHE	CD1	100.00
267PRO	CA	267PRO	CD	100.00	216VAI	CG1	239PHE	CD2	0.28	269PH	CB	269PHE	CD2	100.00
267PRO	CA	267PRO	C	100.00	216VAI	CG1	239PHE	CE1	2.67	269PH	CB	269PHE	C	100.00
267PRO	CB	267PRO	CG	100.00	216VAI	CG1	239PHE	CE2	1.47	269PH	CB	297MET	CE	0.08
267PRO	CB	267PRO	CD	100.00	216VAI	CG1	239PHE	CZ	1.15	269PH	CG	269PHE	CD1	100.00
267PRO	CB	267PRO	C	100.00	216VAI	CG1	242ILE	CG1	0.12	269PH	CG	269PHE	CD2	100.00
267PRO	CB	269PHE	CE1	1.08	216VAI	CG1	242ILE	CD	2.77	269PH	CG	269PHE	CE1	100.00
267PRO	CB	269PHE	CZ	0.18	216VAI	CG2	216VAL	C	100.00	269PH	CG	269PHE	CE2	100.00
267PRO	CB	300HIS	CD2	0.03	216VAI	CG2	239PHE	CB	0.80	269PH	CG	269PHE	CZ	100.00
267PRO	CB	300HIS	CE1	0.07	216VAI	CG2	239PHE	CG	1.74	269PH	CG	297MET	CA	0.01
267PRO	CG	267PRO	CD	100.00	216VAI	CG2	239PHE	CD1	1.10	269PH	CG	297MET	CB	0.45

267PRO CG	267PRO C	99.35	216VAL CG2	239PHE	CD2	0.78	269PH CG	297MET	CG	0.04
267PRO CG	269PHE CE1	0.32	216VAL CG2	239PHE	CE1	0.01	269PH CG	297MET	CE	0.05
267PRO CG	269PHE CZ	0.07	216VAL CG2	239PHE	CE2	0.01	269PH CD1	269PHE	CD2	100.00
267PRO CG	300HIS CD2	0.27	216VAL CG2	239PHE	C	0.00	269PH CD1	269PHE	CE1	100.00
267PRO CG	300HIS CE1	0.03	216VAL CG2	242ILE	CD	0.00	269PH CD1	269PHE	CE2	100.00
267PRO CG	301ALA CB	0.17	216VAL CG2	243LEU	CD1	0.13	269PH CD1	269PHE	CZ	100.00
267PRO CD	267PRO C	96.94	216VAL CG2	242ILE	CG1	0.02	269PH CD1	297MET	CA	0.04
267PRO C	268VAL CA	100.00	216VAL CG2	242ILE	CD	4.24	269PH CD1	297MET	CB	3.12
267PRO C	268VAL CB	46.46	216VAL C	217ASP	CA	100.00	269PH CD1	297MET	CG	4.20
267PRO C	268VAL CG2	33.03	216VAL C	217ASP	CB	0.02	269PH CD1	297MET	CE	1.59
267PRO C	268VAL C	70.35	216VAL C	217ASP	C	99.99	269PH CD2	269PHE	CE1	100.00
267PRO C	269PHE CE1	0.08	217ASP CA	217ASP	CB	100.00	269PH CD2	269PHE	CE2	100.00
268VAL CA	268VAL CB	100.00	217ASP CA	217ASP	CG	100.00	269PH CD2	269PHE	CZ	100.00
268VAL CA	268VAL CG1	100.00	217ASP CA	217ASP	C	100.00	269PH CD2	296MET	CB	0.49
268VAL CA	268VAL CG2	100.00	217ASP CA	220ALA	CB	0.01	269PH CD2	296MET	CG	0.04
268VAL CA	268VAL C	100.00	217ASP CB	217ASP	CG	100.00	269PH CD2	296MET	C	0.14
268VAL CB	268VAL CG1	100.00	217ASP CB	217ASP	C	100.00	269PH CD2	297MET	CA	0.44
268VAL CB	268VAL CG2	100.00	217ASP CB	241ASP	CG	0.03	269PH CD2	297MET	CB	0.02
268VAL CB	268VAL C	100.00	217ASP CB	241ASP	C	0.01	269PH CE1	269PHE	CE2	100.00
268VAL CG1	268VAL CG2	100.00	217ASP CB	242ILE	CA	0.18	269PH CE1	269PHE	CZ	100.00
268VAL CG1	268VAL C	100.00	217ASP CB	242ILE	CG1	0.02	269PH CE1	297MET	CA	0.30
268VAL C	269PHE CA	100.00	217ASP CB	245ASP	CB	0.01	269PH CE1	297MET	CB	0.14
268VAL C	269PHE CB	33.92	217ASP CB	245ASP	CG	0.02	269PH CE1	297MET	CG	1.11
268VAL C	269PHE CG	12.41	217ASP CG	217ASP	C	98.37	269PH CE1	301ALA	CB	0.39
268VAL C	269PHE CD1	13.74	217ASP CG	241ASP	CG	0.01	269PH CE2	269PHE	CZ	100.00
268VAL C	269PHE C	75.16	217ASP C	218ALA	CA	100.00	269PH CE2	296MET	CB	0.10
269PHE CA	269PHE CB	100.00	217ASP C	218ALA	CB	0.07	269PH CE2	296MET	CG	0.04
269PHE CA	269PHE CG	100.00	217ASP C	218ALA	C	99.95	269PH CE2	296MET	C	6.96
269PHE CA	269PHE CD1	93.03	217ASP C	245ASP	CB	0.00	269PH CE2	297MET	CA	5.56
269PHE CA	269PHE CD2	94.22	217ASP C	245ASP	CG	0.00	269PH CE2	300HIS	CB	0.06
269PHE CA	269PHE C	100.00	218ALA CA	218ALA	CB	100.00	269PH CE2	300HIS	CG	0.01
269PHE CB	269PHE CG	100.00	218ALA CA	218ALA	C	100.00	269PH CE2	300HIS	CD2	0.01
269PHE CB	269PHE CD1	100.00	218ALA CA	221MET	CE	0.02	269PH CZ	297MET	CA	1.74
269PHE CB	269PHE CD2	100.00	218ALA CB	218ALA	C	100.00	269PH CZ	300HIS	CB	0.43
269PHE CB	269PHE C	100.00	218ALA CB	276ALA	CA	0.01	269PH CZ	300HIS	CG	0.11
269PHE CB	297MET CE	0.06	218ALA CB	276ALA	CB	0.42	269PH CZ	300HIS	CD2	0.36
269PHE CG	269PHE CD1	100.00	218ALA CB	277PRO	CG	0.07	269PH CZ	301ALA	CB	0.40
269PHE CG	269PHE CD2	100.00	218ALA CB	277PRO	CD	0.03	269PH C	270GLU	CA	100.00
269PHE CG	269PHE CE1	100.00	218ALA C	219MET	CA	100.00	269PH C	270GLU	CB	97.35
269PHE CG	269PHE CE2	100.00	218ALA C	219MET	CB	0.02	269PH C	270GLU	CG	0.20
269PHE CG	269PHE CZ	100.00	218ALA C	219MET	C	99.97	269PH C	270GLU	C	4.29
269PHE CG	297MET CB	0.17	218ALA C	221MET	CG	0.04	270GL CA	270GLU	CB	100.00
269PHE CG	297MET CG	0.01	218ALA C	222HIS	CG	0.00	270GL CA	270GLU	CG	100.00
269PHE CG	297MET CE	0.02	218ALA C	222HIS	CD2	0.22	270GL CA	270GLU	C	100.00
269PHE CD1	269PHE CD2	100.00	219MET CA	219MET	CB	100.00	270GL CA	271PRO	CD	100.00
269PHE CD1	269PHE CE1	100.00	219MET CA	219MET	CG	100.00	270GL CB	270GLU	CG	100.00
269PHE CD1	269PHE CE2	100.00	219MET CA	219MET	CE	0.01	270GL CB	270GLU	CD	100.00
269PHE CD1	269PHE CZ	100.00	219MET CA	219MET	C	100.00	270GL CB	270GLU	C	100.00
269PHE CD1	297MET CA	0.01	219MET CA	230PHE	CZ	0.00	270GL CB	271PRO	CD	0.08
269PHE CD1	297MET CB	2.02	219MET CB	219MET	CG	100.00	270GL CG	270GLU	CD	100.00
269PHE CD1	297MET CG	3.70	219MET CB	219MET	CE	10.12	270GL CG	270GLU	C	99.09
269PHE CD1	297MET CE	0.70	219MET CB	219MET	C	100.00	270GL CG	271PRO	CD	1.48
269PHE CD2	269PHE CE1	100.00	219MET CB	230PHE	CZ	0.00	270GL C	271PRO	CA	100.00
269PHE CD2	269PHE CE2	100.00	219MET CG	219MET	CE	100.00	270GL C	271PRO	CB	0.16
269PHE CD2	269PHE CZ	100.00	219MET CG	219MET	C	99.65	270GL C	271PRO	CD	100.00
269PHE CD2	296MET CB	0.30	219MET CG	223LEU	CD1	0.02	270GL C	271PRO	C	100.00
269PHE CD2	296MET C	0.08	219MET CG	230PHE	CD1	0.07	271PR CA	271PRO	CB	100.00
269PHE CD2	297MET CA	0.26	219MET CG	230PHE	CD2	0.00	271PR CA	271PRO	CG	100.00
269PHE CD2	297MET CB	0.02	219MET CG	230PHE	CE1	8.59	271PR CA	271PRO	CD	100.00
269PHE CE1	269PHE CE2	100.00	219MET CG	230PHE	CE2	0.55	271PR CA	271PRO	C	100.00
269PHE CE1	269PHE CZ	100.00	219MET CG	230PHE	CZ	7.09	271PR CA	289ALA	CB	0.01
269PHE CE1	297MET CA	0.42	219MET CG	243LEU	CD1	0.04	271PR CB	271PRO	CG	100.00

269PHE	CE1	297MET	CB	0.28	219ME	CG	243LEU	CD2	0.02	271PR	CB	271PRO	CD	100.00
269PHE	CE1	297MET	CG	1.16	219ME	CE	219MET	C	1.04	271PR	CB	271PRO	C	100.00
269PHE	CE1	301ALA	CB	0.37	219ME	CE	220ALA	CA	0.02	271PR	CB	273HIS	CG	0.46
269PHE	CE2	269PHE	CZ	100.00	219ME	CE	223LEU	CB	0.02	271PR	CB	273HIS	CD2	0.05
269PHE	CE2	296MET	CB	0.25	219ME	CE	223LEU	CG	0.03	271PR	CB	273HIS	CE1	6.50
269PHE	CE2	296MET	CG	0.01	219ME	CE	223LEU	CD1	0.95	271PR	CB	289ALA	CB	19.06
269PHE	CE2	296MET	C	11.67	219ME	CE	223LEU	CD2	0.16	271PR	CB	289ALA	C	2.60
269PHE	CE2	297MET	CA	7.56	219ME	CE	230PHE	CB	0.00	271PR	CB	290ALA	CA	0.00
269PHE	CE2	297MET	CB	0.01	219ME	CE	230PHE	CG	1.03	271PR	CG	271PRO	CD	100.00
269PHE	CE2	300HIS	CB	0.05	219ME	CE	230PHE	CD1	4.36	271PR	CG	271PRO	C	99.52
269PHE	CE2	300HIS	CG	0.01	219ME	CE	230PHE	CD2	1.07	271PR	CG	273HIS	CG	0.03
269PHE	CZ	296MET	C	0.00	219ME	CE	230PHE	CE1	8.03	271PR	CG	273HIS	CE1	4.34
269PHE	CZ	297MET	CA	3.78	219ME	CE	230PHE	CE2	0.63	271PR	CD	271PRO	C	98.97
269PHE	CZ	297MET	CB	0.01	219ME	CE	230PHE	CZ	0.91	271PR	C	272VAL	CA	100.00
269PHE	CZ	297MET	CG	0.00	219ME	CE	233VAL	CB	0.03	271PR	C	272VAL	CB	3.52
269PHE	CZ	300HIS	CB	0.37	219ME	CE	233VAL	CG1	3.30	271PR	C	272VAL	CG2	2.48
269PHE	CZ	300HIS	CG	0.06	219ME	CE	233VAL	CG2	3.84	271PR	C	272VAL	C	98.54
269PHE	CZ	300HIS	CD2	0.26	219ME	CE	243LEU	CG	0.00	272VA	CA	272VAL	CB	100.00
269PHE	CZ	301ALA	CB	0.30	219ME	CE	243LEU	CD1	1.29	272VA	CA	272VAL	CG1	100.00
269PHE	C	270GLU	CA	100.00	219ME	CE	243LEU	CD2	0.79	272VA	CA	272VAL	CG2	100.00
269PHE	C	270GLU	CB	98.83	219ME	C	220ALA	CA	100.00	272VA	CA	272VAL	C	100.00
269PHE	C	270GLU	CG	0.08	219ME	C	220ALA	C	100.00	272VA	CB	272VAL	CG1	100.00
269PHE	C	270GLU	C	1.58	219ME	C	223LEU	CD1	0.00	272VA	CB	272VAL	CG2	100.00
270GLU	CA	270GLU	CB	100.00	220AL	CA	220ALA	CB	100.00	272VA	CB	272VAL	C	100.00
270GLU	CA	270GLU	CG	100.00	220AL	CA	220ALA	C	100.00	272VA	CG1	272VAL	CG2	100.00
270GLU	CA	270GLU	CD	0.10	220AL	CA	223LEU	CD2	0.01	272VA	CG1	272VAL	C	100.00
270GLU	CA	270GLU	C	100.00	220AL	CA	243LEU	CD2	0.05	272VA	C	273HIS	CA	100.00
270GLU	CA	271PRO	CD	100.00	220AL	CA	246LEU	CD2	0.00	272VA	C	273HIS	CB	85.27
270GLU	CB	270GLU	CG	100.00	220AL	CB	220ALA	C	100.00	272VA	C	273HIS	CG	7.86
270GLU	CB	270GLU	CD	100.00	220AL	CB	243LEU	CD1	0.05	272VA	C	273HIS	CD2	5.34
270GLU	CB	270GLU	C	100.00	220AL	CB	243LEU	CD2	0.26	272VA	C	273HIS	C	12.35
270GLU	CB	271PRO	CD	0.41	220AL	CB	246LEU	CD1	0.00	273HI	CA	273HIS	CB	100.00
270GLU	CG	270GLU	CD	100.00	220AL	CB	246LEU	CD2	0.02	273HI	CA	273HIS	CG	100.00
270GLU	CG	270GLU	C	99.93	220AL	CB	242ILE	CG2	1.46	273HI	CA	273HIS	CD2	93.64
270GLU	CG	271PRO	CD	3.25	220AL	CB	246LEU	CG	0.03	273HI	CA	273HIS	C	100.00
270GLU	CD	270GLU	C	0.03	220AL	CB	246LEU	CD1	0.10	273HI	CB	273HIS	CG	100.00
270GLU	C	271PRO	CA	100.00	220AL	CB	246LEU	CD2	0.12	273HI	CB	273HIS	CD2	100.00
270GLU	C	271PRO	CB	0.26	220AL	C	221MET	CA	100.00	273HI	CB	273HIS	CE1	0.03
270GLU	C	271PRO	CD	100.00	220AL	C	221MET	CB	0.04	273HI	CB	273HIS	C	100.00
270GLU	C	271PRO	C	100.00	220AL	C	221MET	C	99.98	273HI	CB	289ALA	CB	0.00
271PRO	CA	271PRO	CB	100.00	220AL	C	223LEU	CD2	0.00	273HI	CG	273HIS	CD2	100.00
271PRO	CA	271PRO	CG	100.00	220AL	C	246LEU	CD1	0.15	273HI	CG	273HIS	CE1	100.00
271PRO	CA	271PRO	CD	100.00	220AL	C	246LEU	CD2	0.27	273HI	CG	273HIS	C	6.10
271PRO	CA	271PRO	C	100.00	221ME	CA	221MET	CB	100.00	273HI	CG	289ALA	CB	0.00
271PRO	CB	271PRO	CG	100.00	221ME	CA	221MET	CG	100.00	273HI	CD2	273HIS	CE1	100.00
271PRO	CB	271PRO	CD	100.00	221ME	CA	221MET	C	100.00	273HI	CD2	273HIS	C	0.00
271PRO	CB	271PRO	C	100.00	221ME	CA	249VAL	CG1	0.02	273HI	CD2	289ALA	CB	1.02
271PRO	CB	273HIS	CD2	1.10	221ME	CA	249VAL	CG2	0.08	273HI	CE1	287PRO	C	0.00
271PRO	CB	273HIS	CE1	0.61	221ME	CB	221MET	CG	100.00	273HI	CE1	289ALA	CB	0.04
271PRO	CB	289ALA	CB	15.86	221ME	CB	221MET	CE	73.56	273HI	CE1	290ALA	CB	0.11
271PRO	CB	289ALA	C	0.67	221ME	CB	221MET	C	100.00	273HI	C	274GLY	CA	100.00
271PRO	CG	271PRO	CD	100.00	221ME	CB	245ASP	C	0.01	273HI	C	274GLY	C	44.29
271PRO	CG	271PRO	C	99.64	221ME	CB	249VAL	CG1	0.12	274GL	CA	274GLY	C	100.00
271PRO	CG	273HIS	CD2	0.09	221ME	CB	249VAL	CG2	0.07	274GL	C	275SER	CA	100.00
271PRO	CG	273HIS	CE1	3.59	221ME	CG	221MET	CE	100.00	274GL	C	275SER	CB	78.10
271PRO	CG	289ALA	C	0.00	221ME	CG	221MET	C	99.25	274GL	C	275SER	C	22.25
271PRO	CD	271PRO	C	98.38	221ME	CG	225ARG	CZ	0.05	274GL	C	279ILE	CD	0.04
271PRO	C	272VAL	CA	100.00	221ME	CG	245ASP	C	0.01	275SE	CA	275SER	CB	100.00
271PRO	C	272VAL	CB	2.90	221ME	CG	249VAL	CG1	1.78	275SE	CA	275SER	C	100.00
271PRO	C	272VAL	CG2	1.36	221ME	CG	249VAL	CG2	1.99	275SE	CB	275SER	C	100.00
271PRO	C	272VAL	C	99.47	221ME	CG	254LEU	CD1	0.21	275SE	C	276ALA	CA	100.00
272VAL	CA	272VAL	CB	100.00	221ME	CG	254LEU	CD2	3.00	275SE	C	276ALA	CB	6.70

272VAL	CA	272VAL	CG1	100.00	221ME	CE	221MET	C	0.23	275SE	C	276ALA	C	97.14
272VAL	CA	272VAL	CG2	100.00	221ME	CE	222HIS	CA	0.01	275SE	C	277PRO	CD	0.04
272VAL	CA	272VAL	C	100.00	221ME	CE	222HIS	CB	0.00	276AL	CA	276ALA	CB	100.00
272VAL	CB	272VAL	CG1	100.00	221ME	CE	222HIS	CG	0.00	276AL	CA	276ALA	C	100.00
272VAL	CB	272VAL	CG2	100.00	221ME	CE	222HIS	CD2	0.12	276AL	CA	277PRO	CD	100.00
272VAL	CB	272VAL	C	100.00	221ME	CE	222HIS	CE1	0.25	276AL	CB	276ALA	C	100.00
272VAL	CG1	272VAL	CG2	100.00	221ME	CE	225ARG	CG	0.00	276AL	CB	277PRO	CD	2.55
272VAL	CG1	272VAL	C	100.00	221ME	CE	225ARG	CD	0.02	276AL	CB	279ILE	CG1	0.76
272VAL	C	273HIS	CA	100.00	221ME	CE	225ARG	CZ	0.03	276AL	CB	279ILE	CG2	0.01
272VAL	C	273HIS	CB	99.62	221ME	CE	245ASP	CB	0.76	276AL	CB	279ILE	CD	1.25
272VAL	C	273HIS	CG	2.64	221ME	CE	245ASP	CG	3.27	276AL	C	277PRO	CA	100.00
272VAL	C	273HIS	CD2	0.38	221ME	CE	245ASP	C	0.00	276AL	C	277PRO	CB	0.27
272VAL	C	273HIS	C	0.72	221ME	CE	248SER	CB	0.02	276AL	C	277PRO	CD	100.00
273HIS	CA	273HIS	CB	100.00	221ME	CE	249VAL	CG1	0.04	276AL	C	277PRO	C	100.00
273HIS	CA	273HIS	CG	100.00	221ME	CE	249VAL	CG2	0.00	277PR	CA	277PRO	CB	100.00
273HIS	CA	273HIS	CD2	3.82	221ME	CE	254LEU	CB	0.01	277PR	CA	277PRO	CG	100.00
273HIS	CA	273HIS	C	100.00	221ME	CE	254LEU	CG	0.01	277PR	CA	277PRO	CD	100.00
273HIS	CB	273HIS	CG	100.00	221ME	CE	254LEU	CD1	1.59	277PR	CA	277PRO	C	100.00
273HIS	CB	273HIS	CD2	100.00	221ME	CE	254LEU	CD2	0.55	277PR	CA	280ALA	CB	0.02
273HIS	CB	273HIS	CE1	0.01	221ME	CE	275SER	CB	0.12	277PR	CB	277PRO	CG	100.00
273HIS	CB	273HIS	C	100.00	221ME	CE	275SER	C	0.01	277PR	CB	277PRO	CD	100.00
273HIS	CB	279ILE	CD	0.00	221ME	CE	276ALA	CA	0.01	277PR	CB	277PRO	C	100.00
273HIS	CG	273HIS	CD2	100.00	221ME	CE	276ALA	CB	1.41	277PR	CB	280ALA	CB	0.02
273HIS	CG	273HIS	CE1	100.00	221ME	CE	277PRO	CG	0.01	277PR	CG	277PRO	CD	100.00
273HIS	CG	273HIS	C	97.12	221ME	CE	277PRO	CD	0.00	277PR	CG	277PRO	C	94.75
273HIS	CD2	273HIS	CE1	100.00	221ME	CE	278ASP	CB	0.00	277PR	CD	277PRO	C	80.86
273HIS	CD2	273HIS	C	0.66	221ME	CE	279ILE	CD	0.10	277PR	C	278ASP	CA	100.00
273HIS	CD2	289ALA	CB	0.91	221ME	C	222HIS	CA	100.00	277PR	C	278ASP	CB	0.38
273HIS	CE1	289ALA	CB	0.00	221ME	C	222HIS	CB	1.40	277PR	C	278ASP	C	99.66
273HIS	C	274GLY	CA	100.00	221ME	C	222HIS	C	98.83	277PR	C	280ALA	CB	0.01
273HIS	C	274GLY	C	65.83	221ME	C	224VAL	CG1	0.00	278AS	CA	278ASP	CB	100.00
274GLY	CA	274GLY	C	100.00	221ME	C	224VAL	CG2	0.03	278AS	CA	278ASP	CG	100.00
274GLY	CA	279ILE	CD	0.88	221ME	C	249VAL	CG2	0.00	278AS	CA	278ASP	C	100.00
274GLY	C	275SER	CA	100.00	222HIS	CA	222HIS	CB	100.00	278AS	CB	278ASP	CG	100.00
274GLY	C	275SER	CB	67.97	222HIS	CA	222HIS	CG	100.00	278AS	CB	278ASP	C	100.00
274GLY	C	275SER	C	32.46	222HIS	CA	222HIS	CD2	33.45	278AS	CG	278ASP	C	98.77
274GLY	C	279ILE	CD	10.30	222HIS	CA	222HIS	C	100.00	278AS	CG	282LYS	CD	0.00
275SER	CA	275SER	CB	100.00	222HIS	CA	225ARG	CG	0.01	278AS	CG	282LYS	CE	0.27
275SER	CA	275SER	C	100.00	222HIS	CB	222HIS	CG	100.00	278AS	C	279ILE	CA	100.00
275SER	CA	280ALA	CB	0.02	222HIS	CB	222HIS	CD2	100.00	278AS	C	279ILE	CB	5.05
275SER	CB	275SER	C	100.00	222HIS	CB	222HIS	CE1	0.35	278AS	C	279ILE	CG1	0.34
275SER	CB	280ALA	CB	0.03	222HIS	CB	222HIS	C	100.00	278AS	C	279ILE	CG2	2.26
275SER	C	276ALA	CA	100.00	222HIS	CB	230PHE	CE2	0.17	278AS	C	279ILE	CD	0.02
275SER	C	276ALA	CB	26.18	222HIS	CB	230PHE	CZ	0.38	278AS	C	279ILE	C	94.89
275SER	C	276ALA	C	99.76	222HIS	CG	222HIS	CD2	100.00	278AS	C	282LYS	CE	0.01
275SER	C	277PRO	CD	0.07	222HIS	CG	222HIS	CE1	100.00	279ILE	CA	279ILE	CB	100.00
275SER	C	280ALA	CB	0.18	222HIS	CG	222HIS	C	93.12	279ILE	CA	279ILE	CG1	100.00
276ALA	CA	276ALA	CB	100.00	222HIS	CG	229ARG	CD	0.04	279ILE	CA	279ILE	CG2	100.00
276ALA	CA	276ALA	C	100.00	222HIS	CG	229ARG	CZ	0.09	279ILE	CA	279ILE	CD	23.14
276ALA	CA	277PRO	CD	100.00	222HIS	CG	230PHE	CE2	0.24	279ILE	CA	279ILE	C	100.00
276ALA	CB	276ALA	C	100.00	222HIS	CG	230PHE	CZ	0.46	279ILE	CA	284ILE	CD	0.00
276ALA	CB	277PRO	CD	16.00	222HIS	CD2	222HIS	CE1	100.00	279ILE	CB	279ILE	CG1	100.00
276ALA	CB	279ILE	CB	0.00	222HIS	CD2	222HIS	C	16.85	279ILE	CB	279ILE	CG2	100.00
276ALA	CB	279ILE	CG1	0.10	222HIS	CD2	225ARG	CB	0.01	279ILE	CB	279ILE	CD	100.00
276ALA	CB	279ILE	CD	0.69	222HIS	CD2	225ARG	CZ	0.00	279ILE	CB	279ILE	C	100.00
276ALA	C	277PRO	CA	100.00	222HIS	CD2	226SER	CB	0.05	279ILE	CB	284ILE	CG2	0.08
276ALA	C	277PRO	CB	0.02	222HIS	CD2	229ARG	CB	0.66	279ILE	CB	284ILE	CD	0.04
276ALA	C	277PRO	CD	100.00	222HIS	CD2	229ARG	CG	0.29	279ILE	CG1	279ILE	CG2	100.00
276ALA	C	277PRO	C	100.00	222HIS	CD2	229ARG	CD	0.43	279ILE	CG1	279ILE	CD	100.00
276ALA	C	280ALA	CB	0.01	222HIS	CD2	229ARG	CZ	0.74	279ILE	CG1	279ILE	C	55.62
277PRO	CA	277PRO	CB	100.00	222HIS	CD2	230PHE	CE1	0.03	279ILE	CG1	284ILE	CG1	0.01
277PRO	CA	277PRO	CG	100.00	222HIS	CD2	230PHE	CE2	4.89	279ILE	CG1	285ALA	CB	0.02

277PRO	CA	277PRO	CD	100.00	222HIS	CD2	230PHE	CZ	6.58	279ILE	CG2	279ILE	CD	94.63
277PRO	CA	277PRO	C	100.00	222HIS	CE1	225ARG	CB	0.02	279ILE	CG2	279ILE	C	44.31
277PRO	CB	277PRO	CG	100.00	222HIS	CE1	226SER	CB	0.76	279ILE	CG2	282LYS	CD	0.00
277PRO	CB	277PRO	CD	100.00	222HIS	CE1	229ARG	CB	0.17	279ILE	CG2	284ILE	CB	0.04
277PRO	CB	277PRO	C	100.00	222HIS	CE1	229ARG	CG	0.28	279ILE	CG2	284ILE	CG1	0.12
277PRO	CG	277PRO	CD	100.00	222HIS	CE1	229ARG	CD	1.14	279ILE	CG2	284ILE	CG2	2.52
277PRO	CG	277PRO	C	72.21	222HIS	CE1	229ARG	CZ	3.23	279ILE	CG2	284ILE	CD	0.23
277PRO	CD	277PRO	C	50.79	222HIS	CE1	230PHE	CE1	0.00	279ILE	CG2	284ILE	C	0.08
277PRO	C	278ASP	CA	100.00	222HIS	CE1	230PHE	CE2	0.13	279ILE	CG2	285ALA	CA	0.26
277PRO	C	278ASP	CB	0.67	222HIS	CE1	230PHE	CZ	0.16	279ILE	CG2	285ALA	CB	0.06
277PRO	C	278ASP	C	99.36	222HIS	CE1	277PRO	CG	0.02	279ILE	CD	279ILE	C	0.78
278ASP	CA	278ASP	CB	100.00	222HIS	CE1	277PRO	CD	0.01	279ILE	CD	282LYS	CB	0.00
278ASP	CA	278ASP	CG	100.00	222HIS	CE1	278ASP	CB	0.02	279ILE	CD	282LYS	CD	0.00
278ASP	CA	278ASP	C	100.00	222HIS	CE1	278ASP	CG	0.02	279ILE	CD	284ILE	CG2	0.00
278ASP	CB	278ASP	CG	100.00	222HIS	C	223LEU	CA	100.00	279ILE	CD	284ILE	CD	0.06
278ASP	CB	278ASP	C	100.00	222HIS	C	223LEU	CB	0.01	279ILE	CD	284ILE	C	0.01
278ASP	CB	327LEU	CD2	0.00	222HIS	C	223LEU	C	100.00	279ILE	CD	285ALA	CA	0.06
278ASP	CG	278ASP	C	98.13	222HIS	C	230PHE	CE2	0.09	279ILE	CD	285ALA	CB	0.08
278ASP	CG	282LYS	CG	0.01	222HIS	C	230PHE	CZ	0.01	279ILE	C	280ALA	CA	100.00
278ASP	CG	282LYS	CD	0.02	223LEU	CA	223LEU	CB	100.00	279ILE	C	280ALA	CB	1.10
278ASP	CG	282LYS	CE	1.16	223LEU	CA	223LEU	CG	100.00	279ILE	C	280ALA	C	99.08
278ASP	C	279ILE	CA	100.00	223LEU	CA	223LEU	CD1	77.02	279ILE	C	282LYS	CB	0.00
278ASP	C	279ILE	CB	2.65	223LEU	CA	223LEU	CD2	21.10	279ILE	C	282LYS	CE	0.01
278ASP	C	279ILE	CG1	0.32	223LEU	CA	223LEU	C	100.00	279ILE	C	285ALA	CB	0.15
278ASP	C	279ILE	CG2	1.22	223LEU	CA	230PHE	CD2	0.00	280AL	CA	280ALA	CB	100.00
278ASP	C	279ILE	CD	0.01	223LEU	CA	230PHE	CE2	1.54	280AL	CA	280ALA	C	100.00
278ASP	C	279ILE	C	97.44	223LEU	CA	230PHE	CZ	0.06	280AL	CA	285ALA	CB	0.23
278ASP	C	282LYS	CD	0.00	223LEU	CB	223LEU	CG	100.00	280AL	CB	280ALA	C	100.00
278ASP	C	282LYS	CE	0.01	223LEU	CB	223LEU	CD1	100.00	280AL	CB	285ALA	CB	0.04
279ILE	CA	279ILE	CB	100.00	223LEU	CB	223LEU	CD2	100.00	280AL	C	281GLY	CA	100.00
279ILE	CA	279ILE	CG1	100.00	223LEU	CB	223LEU	C	100.00	280AL	C	281GLY	C	60.69
279ILE	CA	279ILE	CG2	100.00	223LEU	CB	230PHE	CE2	0.02	281GL	CA	281GLY	C	100.00
279ILE	CA	279ILE	CD	7.68	223LEU	CB	230PHE	CZ	0.00	281GL	CA	333THR	CG2	0.02
279ILE	CA	279ILE	C	100.00	223LEU	CB	250LEU	CD1	0.01	281GL	C	282LYS	CA	100.00
279ILE	CA	282LYS	CB	0.00	223LEU	CB	250LEU	CD2	0.01	281GL	C	282LYS	CB	12.20
279ILE	CB	279ILE	CG1	100.00	223LEU	CG	223LEU	CD1	100.00	281GL	C	282LYS	CG	1.65
279ILE	CB	279ILE	CG2	100.00	223LEU	CG	223LEU	CD2	100.00	281GL	C	282LYS	CD	0.03
279ILE	CB	279ILE	CD	100.00	223LEU	CG	223LEU	C	78.91	281GL	C	282LYS	C	87.13
279ILE	CB	279ILE	C	100.00	223LEU	CG	230PHE	CE2	0.07	282LY	CA	282LYS	CB	100.00
279ILE	CG1	279ILE	CG2	100.00	223LEU	CG	230PHE	CZ	0.01	282LY	CA	282LYS	CG	100.00
279ILE	CG1	279ILE	CD	100.00	223LEU	CG	250LEU	CD1	1.01	282LY	CA	282LYS	CD	2.88
279ILE	CG1	279ILE	C	9.20	223LEU	CG	250LEU	CD2	0.22	282LY	CA	282LYS	CE	0.00
279ILE	CG2	279ILE	CD	97.96	223LEU	CD1	223LEU	CD2	100.00	282LY	CA	282LYS	C	100.00
279ILE	CG2	279ILE	C	90.78	223LEU	CD1	223LEU	C	1.48	282LY	CB	282LYS	CG	100.00
279ILE	CG2	283GLY	C	0.02	223LEU	CD1	227PRO	CB	0.08	282LY	CB	282LYS	CD	100.00
279ILE	CG2	284ILE	CG1	0.14	223LEU	CD1	230PHE	CB	0.02	282LY	CB	282LYS	CE	3.47
279ILE	CG2	284ILE	CD	0.03	223LEU	CD1	230PHE	CG	0.33	282LY	CB	282LYS	C	100.00
279ILE	CG2	284ILE	C	1.85	223LEU	CD1	230PHE	CD1	0.03	282LY	CB	284ILE	CG1	0.75
279ILE	CG2	285ALA	CA	0.50	223LEU	CD1	230PHE	CD2	5.84	282LY	CB	284ILE	CG2	0.02
279ILE	CG2	285ALA	CB	0.29	223LEU	CD1	230PHE	CE1	0.02	282LY	CB	284ILE	CD	0.80
279ILE	CD	279ILE	C	0.18	223LEU	CD1	230PHE	CE2	1.01	282LY	CG	282LYS	CD	100.00
279ILE	CD	282LYS	CE	0.00	223LEU	CD1	230PHE	CZ	0.02	282LY	CG	282LYS	CE	100.00
279ILE	CD	284ILE	CG1	0.00	223LEU	CD1	243LEU	CD1	0.29	282LY	CG	282LYS	C	11.57
279ILE	CD	284ILE	CD	0.04	223LEU	CD1	243LEU	CD2	0.80	282LY	CG	284ILE	CG1	0.02
279ILE	C	280ALA	CA	100.00	223LEU	CD1	246LEU	CG	0.00	282LY	CG	284ILE	CD	0.04
279ILE	C	280ALA	CB	7.38	223LEU	CD1	246LEU	CD1	0.01	282LY	CD	282LYS	CE	100.00
279ILE	C	280ALA	C	92.09	223LEU	CD1	246LEU	CD2	0.16	282LY	CD	284ILE	CG1	0.01
279ILE	C	282LYS	CB	0.00	223LEU	CD1	250LEU	CD1	0.75	282LY	CD	284ILE	CD	0.52
279ILE	C	283GLY	C	0.01	223LEU	CD1	250LEU	CD2	0.24	282LY	CE	284ILE	CD	0.13
280ALA	CA	280ALA	CB	100.00	223LEU	CD2	223LEU	C	0.38	282LY	C	283GLY	CA	100.00
280ALA	CA	280ALA	C	100.00	223LEU	CD2	227PRO	CB	0.04	282LY	C	283GLY	C	65.68
280ALA	CA	283GLY	C	0.00	223LEU	CD2	230PHE	CG	0.01	283GL	CA	283GLY	C	100.00

280ALA	CB	280ALA	C	100.00	223LEU	CD2	230PHE	CD1	0.00	283GL	CA	325PRO	CG	0.06
280ALA	CB	284ILE	CD	0.00	223LEU	CD2	230PHE	CD2	1.87	283GL	CA	325PRO	CD	0.01
280ALA	CB	284ILE	C	0.00	223LEU	CD2	230PHE	CE2	1.16	283GL	CA	331ALA	C	0.00
280ALA	CB	285ALA	CB	0.01	223LEU	CD2	230PHE	CZ	0.01	283GL	CA	332GLY	CA	0.02
280ALA	C	281GLY	CA	100.00	223LEU	CD2	243LEU	CG	0.00	283GL	C	284ILE	CA	100.00
280ALA	C	281GLY	C	50.98	223LEU	CD2	243LEU	CD1	0.63	283GL	C	284ILE	CB	46.36
280ALA	C	283GLY	C	0.01	223LEU	CD2	243LEU	CD2	1.52	283GL	C	284ILE	CG1	0.58
281GLY	CA	281GLY	C	100.00	223LEU	CD2	246LEU	CB	0.00	283GL	C	284ILE	CG2	31.09
281GLY	C	282LYS	CA	100.00	223LEU	CD2	246LEU	CG	0.04	283GL	C	284ILE	CD	0.00
281GLY	C	282LYS	CB	29.17	223LEU	CD2	246LEU	CD1	0.17	283GL	C	284ILE	C	51.80
281GLY	C	282LYS	CG	1.68	223LEU	CD2	246LEU	CD2	2.02	283GL	C	325PRO	CG	0.38
281GLY	C	282LYS	CD	0.00	223LEU	CD2	247ALA	CB	0.01	283GL	C	325PRO	CD	0.87
281GLY	C	282LYS	C	71.76	223LEU	CD2	250LEU	CD1	1.84	283GL	C	332GLY	CA	0.04
281GLY	C	328GLY	CA	0.00	223LEU	CD2	250LEU	CD2	0.44	284ILE	CA	284ILE	CB	100.00
282LYS	CA	282LYS	CB	100.00	223LEU	CD2	246LEU	CD1	0.01	284ILE	CA	284ILE	CG1	100.00
282LYS	CA	282LYS	CG	100.00	223LEU	C	224VAL	CA	100.00	284ILE	CA	284ILE	CG2	100.00
282LYS	CA	282LYS	CD	2.64	223LEU	C	224VAL	CB	7.26	284ILE	CA	284ILE	CD	13.83
282LYS	CA	282LYS	C	100.00	223LEU	C	224VAL	CG1	1.81	284ILE	CA	284ILE	C	100.00
282LYS	CA	327LEU	CD1	0.00	223LEU	C	224VAL	CG2	0.70	284ILE	CA	325PRO	CG	0.05
282LYS	CA	327LEU	CD2	0.00	223LEU	C	224VAL	C	94.38	284ILE	CB	284ILE	CG1	100.00
282LYS	CB	282LYS	CG	100.00	223LEU	C	250LEU	CD1	0.04	284ILE	CB	284ILE	CG2	100.00
282LYS	CB	282LYS	CD	100.00	223LEU	C	250LEU	CD2	0.05	284ILE	CB	284ILE	CD	100.00
282LYS	CB	282LYS	CE	6.52	224VAL	CA	224VAL	CB	100.00	284ILE	CB	284ILE	C	100.00
282LYS	CB	282LYS	C	100.00	224VAL	CA	224VAL	CG1	100.00	284ILE	CB	325PRO	CG	0.09
282LYS	CB	284ILE	CG1	0.20	224VAL	CA	224VAL	CG2	100.00	284ILE	CG1	284ILE	CG2	100.00
282LYS	CB	284ILE	CD	0.07	224VAL	CA	224VAL	C	100.00	284ILE	CG1	284ILE	CD	100.00
282LYS	CB	327LEU	CD1	0.02	224VAL	CB	224VAL	CG1	100.00	284ILE	CG1	284ILE	C	76.94
282LYS	CB	327LEU	CD2	0.01	224VAL	CB	224VAL	CG2	100.00	284ILE	CG1	325PRO	CB	0.01
282LYS	CG	282LYS	CD	100.00	224VAL	CB	224VAL	C	100.00	284ILE	CG1	325PRO	CG	0.10
282LYS	CG	282LYS	CE	100.00	224VAL	CB	246LEU	CD1	0.02	284ILE	CG1	325PRO	CD	0.01
282LYS	CG	282LYS	C	56.70	224VAL	CB	246LEU	CD2	0.01	284ILE	CG2	284ILE	CD	88.88
282LYS	CG	284ILE	CG1	0.00	224VAL	CB	249VAL	CG1	0.04	284ILE	CG2	284ILE	C	22.22
282LYS	CG	284ILE	CD	0.00	224VAL	CB	249VAL	CG2	0.02	284ILE	CG2	325PRO	CB	0.01
282LYS	CG	327LEU	CD1	0.08	224VAL	CG1	224VAL	CG2	100.00	284ILE	CG2	325PRO	CG	0.68
282LYS	CG	327LEU	CD2	0.08	224VAL	CG1	224VAL	C	36.21	284ILE	CG2	325PRO	CD	0.06
282LYS	CD	282LYS	CE	100.00	224VAL	CG1	246LEU	CG	0.00	284ILE	CG2	326ASP	CB	0.01
282LYS	CD	282LYS	C	0.27	224VAL	CG1	246LEU	CD1	0.01	284ILE	CG2	326ASP	CG	0.00
282LYS	CD	284ILE	CG1	0.01	224VAL	CG1	246LEU	CD2	0.08	284ILE	CG2	332GLY	CA	0.04
282LYS	CD	284ILE	CD	0.19	224VAL	CG1	250LEU	CG	0.00	284ILE	CD	284ILE	C	7.91
282LYS	CD	326ASP	C	0.01	224VAL	CG1	250LEU	CD1	0.06	284ILE	CD	325PRO	CB	0.01
282LYS	CD	327LEU	CD1	0.04	224VAL	CG1	250LEU	CD2	0.41	284ILE	CD	325PRO	CG	0.21
282LYS	CD	327LEU	CD2	0.28	224VAL	CG1	117PRO	CB	0.32	284ILE	CD	325PRO	C	0.06
282LYS	CE	284ILE	CD	0.01	224VAL	CG1	117PRO	CG	0.85	284ILE	CD	326ASP	CA	0.03
282LYS	CE	325PRO	C	0.01	224VAL	CG1	118LEU	CD1	0.03	284ILE	CD	326ASP	CB	0.02
282LYS	CE	326ASP	CG	0.01	224VAL	CG1	118LEU	CD2	0.00	284ILE	C	285ALA	CA	100.00
282LYS	CE	327LEU	CD1	0.00	224VAL	CG1	224VAL	CG1	0.63	284ILE	C	285ALA	CB	1.13
282LYS	CE	327LEU	CD2	0.10	224VAL	CG1	224VAL	CG2	0.07	284ILE	C	285ALA	C	99.13
282LYS	C	283GLY	CA	100.00	224VAL	CG1	246LEU	CG	0.01	284ILE	C	325PRO	CG	0.00
282LYS	C	283GLY	C	43.02	224VAL	CG1	246LEU	CD1	3.34	285AL	CA	285ALA	CB	100.00
282LYS	C	327LEU	CD1	0.00	224VAL	CG1	246LEU	CD2	0.77	285AL	CA	285ALA	C	100.00
282LYS	C	327LEU	CD2	0.00	224VAL	CG1	249VAL	CB	0.01	285AL	CA	326ASP	CB	0.00
282LYS	C	327LEU	C	0.33	224VAL	CG1	249VAL	CG1	1.18	285AL	CB	285ALA	C	100.00
282LYS	C	328GLY	CA	0.10	224VAL	CG1	249VAL	CG2	0.70	285AL	CB	333THR	CG2	0.12
283GLY	CA	283GLY	C	100.00	224VAL	CG2	224VAL	C	68.03	285AL	C	286ASN	CA	100.00
283GLY	CA	325PRO	CB	0.02	224VAL	CG2	246LEU	CG	0.00	285AL	C	286ASN	CB	11.56
283GLY	CA	325PRO	CG	0.01	224VAL	CG2	246LEU	CD1	0.02	285AL	C	286ASN	C	89.92
283GLY	CA	326ASP	C	0.00	224VAL	CG2	246LEU	CD2	0.16	285AL	C	324PRO	CG	0.00
283GLY	CA	327LEU	CA	0.02	224VAL	CG2	250LEU	CD1	0.01	285AL	C	333THR	CG2	0.14
283GLY	CA	327LEU	CD2	0.01	224VAL	CG2	250LEU	CD2	0.02	286AS	CA	286ASN	CB	100.00
283GLY	CA	327LEU	C	0.11	224VAL	CG2	117PRO	CG	0.01	286AS	CA	286ASN	CG	100.00
283GLY	CA	328GLY	CA	0.30	224VAL	CG2	224VAL	CG1	0.24	286AS	CA	286ASN	C	100.00
283GLY	CA	328GLY	C	3.12	224VAL	CG2	224VAL	CG2	0.02	286AS	CA	287PRO	CD	100.00

283GLY	C	284ILE	CA	100.00	224VAl	CG2	246LEU	CD1	2.30	286AS	CB	286ASN	CG	100.00
283GLY	C	284ILE	CB	75.84	224VAl	CG2	246LEU	CD2	0.64	286AS	CB	286ASN	C	100.00
283GLY	C	284ILE	CG1	4.56	224VAl	CG2	249VAL	CB	0.06	286AS	CB	287PRO	CD	0.01
283GLY	C	284ILE	CG2	21.50	224VAl	CG2	249VAL	CG1	0.72	286AS	CB	324PRO	CB	0.01
283GLY	C	284ILE	CD	0.16	224VAl	CG2	249VAL	CG2	4.31	286AS	CB	324PRO	CG	0.72
283GLY	C	284ILE	C	26.97	224VAl	C	225ARG	CA	100.00	286AS	CB	326ASP	CB	0.91
283GLY	C	329GLY	CA	0.01	224VAl	C	225ARG	CB	32.50	286AS	CB	326ASP	CG	0.12
283GLY	C	332GLY	CA	0.03	224VAl	C	225ARG	CG	2.79	286AS	CG	286ASN	C	96.26
283GLY	C	333THR	CG2	0.05	224VAl	C	225ARG	CD	0.00	286AS	CG	287PRO	CD	6.16
284ILE	CA	284ILE	CB	100.00	224VAl	C	225ARG	C	66.81	286AS	CG	288THR	CB	0.04
284ILE	CA	284ILE	CG1	100.00	225AR(	CA	225ARG	CB	100.00	286AS	CG	324PRO	CB	0.20
284ILE	CA	284ILE	CG2	100.00	225AR(	CA	225ARG	CG	100.00	286AS	CG	324PRO	CG	7.65
284ILE	CA	284ILE	CD	17.31	225AR(	CA	225ARG	CD	21.54	286AS	CG	336PHE	CD1	0.01
284ILE	CA	284ILE	C	100.00	225AR(	CA	225ARG	C	100.00	286AS	CG	336PHE	CE2	0.01
284ILE	CA	332GLY	CA	0.03	225AR(	CA	249VAL	CG1	0.00	286AS	CG	336PHE	CZ	0.00
284ILE	CB	284ILE	CG1	100.00	225AR(	CB	225ARG	CG	100.00	286AS	C	287PRO	CA	100.00
284ILE	CB	284ILE	CG2	100.00	225AR(	CB	225ARG	CD	100.00	286AS	C	287PRO	CB	2.74
284ILE	CB	284ILE	CD	100.00	225AR(	CB	225ARG	CZ	0.26	286AS	C	287PRO	CD	100.00
284ILE	CB	284ILE	C	100.00	225AR(	CB	225ARG	C	100.00	286AS	C	287PRO	C	99.90
284ILE	CB	325PRO	CB	0.10	225AR(	CB	117PRO	CB	0.00	287PR	CA	287PRO	CB	100.00
284ILE	CB	325PRO	CG	0.00	225AR(	CB	249VAL	CG1	0.06	287PR	CA	287PRO	CG	100.00
284ILE	CB	332GLY	CA	0.07	225AR(	CG	225ARG	CD	100.00	287PR	CA	287PRO	CD	100.00
284ILE	CG1	284ILE	CG2	100.00	225AR(	CG	225ARG	CZ	27.49	287PR	CA	287PRO	C	100.00
284ILE	CG1	284ILE	CD	100.00	225AR(	CG	225ARG	C	42.56	287PR	CB	287PRO	CG	100.00
284ILE	CG1	284ILE	C	90.81	225AR(	CG	249VAL	CB	0.00	287PR	CB	287PRO	CD	100.00
284ILE	CG1	325PRO	CB	0.82	225AR(	CG	249VAL	CG1	0.57	287PR	CB	287PRO	C	100.00
284ILE	CG1	325PRO	CG	0.12	225AR(	CG	249VAL	CG2	0.03	287PR	CB	291ILE	CD	1.42
284ILE	CG1	328GLY	CA	0.00	225AR(	CG	254LEU	CD2	0.00	287PR	CB	333THR	CG2	0.10
284ILE	CG1	328GLY	C	0.00	225AR(	CD	225ARG	CZ	100.00	287PR	CG	287PRO	CD	100.00
284ILE	CG1	329GLY	CA	0.01	225AR(	CD	225ARG	C	0.32	287PR	CG	287PRO	C	99.51
284ILE	CG1	329GLY	C	0.47	225AR(	CD	249VAL	CG1	2.07	287PR	CG	291ILE	CD	2.38
284ILE	CG1	330SER	CA	0.05	225AR(	CD	249VAL	CG2	0.01	287PR	CG	333THR	CG2	0.12
284ILE	CG1	330SER	C	0.21	225AR(	CD	254LEU	CG	0.02	287PR	CG	336PHE	CB	0.29
284ILE	CG1	331ALA	C	0.29	225AR(	CD	254LEU	CD1	1.34	287PR	CG	336PHE	CG	0.56
284ILE	CG1	332GLY	CA	1.36	225AR(	CD	254LEU	CD2	0.46	287PR	CG	336PHE	CD1	0.10
284ILE	CG2	284ILE	CD	85.74	225AR(	CZ	249VAL	CB	0.00	287PR	CG	336PHE	CD2	27.02
284ILE	CG2	284ILE	C	41.72	225AR(	CZ	249VAL	CG1	0.07	287PR	CG	336PHE	CE2	0.77
284ILE	CG2	325PRO	CA	0.07	225AR(	CZ	252GLY	CA	0.03	287PR	CG	337THR	CG2	0.11
284ILE	CG2	325PRO	CB	3.90	225AR(	CZ	252GLY	C	0.12	287PR	CD	287PRO	C	96.07
284ILE	CG2	325PRO	CG	0.32	225AR(	CZ	254LEU	CB	0.00	287PR	CD	333THR	CG2	0.01
284ILE	CG2	326ASP	C	0.01	225AR(	CZ	254LEU	CG	1.96	287PR	CD	336PHE	CG	0.06
284ILE	CG2	328GLY	CA	0.01	225AR(	CZ	254LEU	CD1	11.67	287PR	CD	336PHE	CD1	0.33
284ILE	CG2	328GLY	C	0.40	225AR(	CZ	254LEU	CD2	1.46	287PR	CD	336PHE	CD2	1.55
284ILE	CG2	329GLY	CA	0.01	225AR(	CZ	278ASP	CG	0.00	287PR	CD	336PHE	CE1	0.03
284ILE	CG2	329GLY	C	0.28	225AR(	CZ	279ILE	CG2	0.02	287PR	CD	336PHE	CE2	0.04
284ILE	CG2	330SER	CA	0.07	225AR(	CZ	279ILE	CD	0.02	287PR	C	288THR	CA	100.00
284ILE	CG2	330SER	CB	0.06	225AR(	CZ	327LEU	CD1	0.00	287PR	C	288THR	C	100.00
284ILE	CG2	330SER	C	0.34	225AR(	CZ	327LEU	CD2	0.02	287PR	C	291ILE	CD	0.02
284ILE	CG2	331ALA	C	2.43	225AR(	C	226SER	CA	100.00	288TH	CA	288THR	CB	100.00
284ILE	CG2	332GLY	CA	4.55	225AR(	C	226SER	CB	99.89	288TH	CA	288THR	CG2	100.00
284ILE	CG2	333THR	CG2	0.00	225AR(	C	226SER	C	0.44	288TH	CA	288THR	C	100.00
284ILE	CD	284ILE	C	9.88	225AR(	C	227PRO	CD	0.61	288TH	CA	315VAL	CG2	0.00
284ILE	CD	285ALA	C	0.07	226SEI	CA	226SER	CB	100.00	288TH	CB	288THR	CG2	100.00
284ILE	CD	286ASN	CB	0.01	226SEI	CA	226SER	C	100.00	288TH	CB	288THR	C	100.00
284ILE	CD	325PRO	CB	0.72	226SEI	CA	227PRO	CD	100.00	288TH	CG2	288THR	C	92.65
284ILE	CD	325PRO	CG	0.23	226SEI	CB	226SER	C	100.00	288TH	CG2	292LEU	CD1	4.59
284ILE	CD	327LEU	C	0.13	226SEI	CB	229ARG	CG	0.09	288TH	CG2	315VAL	CB	0.07
284ILE	CD	328GLY	CA	0.08	226SEI	CB	229ARG	CD	0.07	288TH	CG2	315VAL	CG1	1.11
284ILE	CD	328GLY	C	0.25	226SEI	CB	229ARG	CZ	0.46	288TH	CG2	315VAL	CG2	0.17
284ILE	CD	329GLY	CA	0.03	226SEI	C	227PRO	CA	100.00	288TH	CG2	319LEU	CD1	0.01
284ILE	CD	329GLY	C	2.37	226SEI	C	227PRO	CB	0.14	288TH	CG2	319LEU	CD2	0.01
284ILE	CD	330SER	CA	0.38	226SEI	C	227PRO	CD	100.00	288TH	CG2	336PHE	CE2	1.01

284ILE	CD	330SER	C	1.66
284ILE	CD	331ALA	C	1.35
284ILE	CD	332GLY	CA	1.18
284ILE	C	285ALA	CA	100.00
284ILE	C	285ALA	CB	7.26
284ILE	C	285ALA	C	92.81
284ILE	C	325PRO	CG	0.04
284ILE	C	332GLY	CA	0.01
285ALA	CA	285ALA	CB	100.00
285ALA	CA	285ALA	C	100.00
285ALA	CA	325PRO	CG	0.03
285ALA	CB	285ALA	C	100.00
285ALA	CB	333THR	CG2	1.29
285ALA	C	286ASN	CA	100.00
285ALA	C	286ASN	CB	7.58
285ALA	C	286ASN	C	95.13
285ALA	C	325PRO	CG	0.56
285ALA	C	333THR	CG2	0.03
286ASN	CA	286ASN	CB	100.00
286ASN	CA	286ASN	CG	100.00
286ASN	CA	286ASN	C	100.00
286ASN	CA	287PRO	CD	100.00
286ASN	CA	325PRO	CG	0.32
286ASN	CA	336PHE	CE2	0.00
286ASN	CB	286ASN	CG	100.00
286ASN	CB	286ASN	C	100.00
286ASN	CB	325PRO	CG	0.35
286ASN	CB	325PRO	CD	1.41
286ASN	CG	286ASN	C	99.35
286ASN	CG	287PRO	CD	2.96
286ASN	CG	288THR	CB	0.01
286ASN	CG	324PRO	CB	0.01
286ASN	CG	324PRO	CG	0.02
286ASN	CG	325PRO	CG	0.21
286ASN	CG	325PRO	CD	0.16
286ASN	CG	336PHE	CD1	0.01
286ASN	CG	336PHE	CD2	0.04
286ASN	CG	336PHE	CE1	0.02
286ASN	CG	336PHE	CE2	0.03
286ASN	CG	336PHE	CZ	0.02
286ASN	C	287PRO	CA	100.00
286ASN	C	287PRO	CB	1.29
286ASN	C	287PRO	CD	100.00
286ASN	C	287PRO	C	99.98
287PRO	CA	287PRO	CB	100.00
287PRO	CA	287PRO	CG	100.00
287PRO	CA	287PRO	CD	100.00
287PRO	CA	287PRO	C	100.00
287PRO	CB	287PRO	CG	100.00
287PRO	CB	287PRO	CD	100.00
287PRO	CB	287PRO	C	100.00
287PRO	CB	291ILE	CD	0.86
287PRO	CB	333THR	CG2	0.01
287PRO	CG	287PRO	CD	100.00
287PRO	CG	287PRO	C	99.96
287PRO	CG	291ILE	CD	0.38
287PRO	CG	333THR	CA	0.01
287PRO	CG	333THR	CG2	0.01
287PRO	CG	336PHE	CB	9.52
287PRO	CG	336PHE	CG	0.06
287PRO	CG	336PHE	CD1	1.59
287PRO	CG	336PHE	CD2	0.05

226SEI	C	227PRO	C	100.00
227PR(	CA	227PRO	CB	100.00
227PR(	CA	227PRO	CG	100.00
227PR(	CA	227PRO	CD	100.00
227PR(	CA	227PRO	C	100.00
227PR(	CA	230PHE	CD2	0.01
227PR(	CB	227PRO	CG	100.00
227PR(	CB	227PRO	CD	100.00
227PR(	CB	227PRO	C	100.00
227PR(	CB	250LEU	CD1	0.28
227PR(	CB	250LEU	CD2	0.14
227PR(	CG	227PRO	CD	100.00
227PR(	CG	227PRO	C	91.32
227PR(	CG	250LEU	CD1	0.02
227PR(	CG	250LEU	CD2	0.01
227PR(	CD	227PRO	C	71.20
227PR(	C	228ALA	CA	100.00
227PR(	C	228ALA	CB	0.10
227PR(	C	228ALA	C	99.92
228AL/	CA	228ALA	CB	100.00
228AL/	CA	228ALA	C	100.00
228AL/	CB	228ALA	C	100.00
228AL/	CB	229ARG	CZ	0.04
228AL/	C	229ARG	CA	100.00
228AL/	C	229ARG	CB	5.83
228AL/	C	229ARG	CG	1.25
228AL/	C	229ARG	CD	0.02
228AL/	C	229ARG	CZ	0.03
228AL/	C	229ARG	C	93.79
229AR(	CA	229ARG	CB	100.00
229AR(	CA	229ARG	CG	100.00
229AR(	CA	229ARG	CD	50.93
229AR(	CA	229ARG	CZ	0.30
229AR(	CA	229ARG	C	100.00
229AR(	CB	229ARG	CG	100.00
229AR(	CB	229ARG	CD	100.00
229AR(	CB	229ARG	CZ	0.51
229AR(	CB	229ARG	C	100.00
229AR(	CB	230PHE	CD2	0.05
229AR(	CB	230PHE	CE1	0.01
229AR(	CB	230PHE	CE2	0.06
229AR(	CB	230PHE	CZ	0.01
229AR(	CG	229ARG	CD	100.00
229AR(	CG	229ARG	CZ	19.63
229AR(	CG	229ARG	C	66.81
229AR(	CG	230PHE	CD1	0.11
229AR(	CG	230PHE	CD2	0.26
229AR(	CG	230PHE	CE1	0.92
229AR(	CG	230PHE	CE2	0.53
229AR(	CG	230PHE	CZ	0.40
229AR(	CD	229ARG	CZ	100.00
229AR(	CD	229ARG	C	3.24
229AR(	CD	230PHE	CD1	0.01
229AR(	CD	230PHE	CE1	0.30
229AR(	CD	230PHE	CE2	0.08
229AR(	CD	230PHE	CZ	0.35
229AR(	CZ	230PHE	CD1	0.03
229AR(	CZ	230PHE	CE1	0.21
229AR(	CZ	230PHE	CZ	0.01
229AR(	C	230PHE	CA	100.00
229AR(	C	230PHE	CB	25.15
229AR(	C	230PHE	CG	10.83

288TH	CG2	336PHE	CZ	2.33
288TH	C	289ALA	CA	100.00
288TH	C	289ALA	CB	0.00
288TH	C	289ALA	C	100.00
288TH	C	292LEU	CD1	0.02
289AL.	CA	289ALA	CB	100.00
289AL.	CA	289ALA	C	100.00
289AL.	CB	289ALA	C	100.00
289AL.	C	290ALA	CA	100.00
289AL.	C	290ALA	C	100.00
290AL.	CA	290ALA	CB	100.00
290AL.	CA	290ALA	C	100.00
290AL.	CB	290ALA	C	100.00
290AL.	C	291ILE	CA	100.00
290AL.	C	291ILE	C	100.00
291ILE	CA	291ILE	CB	100.00
291ILE	CA	291ILE	CG1	100.00
291ILE	CA	291ILE	CG2	100.00
291ILE	CA	291ILE	CD	0.73
291ILE	CA	291ILE	C	100.00
291ILE	CB	291ILE	CG1	100.00
291ILE	CB	291ILE	CG2	100.00
291ILE	CB	291ILE	CD	100.00
291ILE	CB	291ILE	C	100.00
291ILE	CB	315VAL	CG2	0.01
291ILE	CG1	291ILE	CG2	100.00
291ILE	CG1	291ILE	CD	100.00
291ILE	CG2	291ILE	CD	99.99
291ILE	CG2	291ILE	C	100.00
291ILE	CG2	311VAL	CG1	22.97
291ILE	CG2	311VAL	CG2	0.11
291ILE	CG2	315VAL	CB	0.02
291ILE	CG2	315VAL	CG1	19.25
291ILE	CG2	315VAL	CG2	5.60
291ILE	CD	315VAL	CG1	0.15
291ILE	CD	315VAL	CG2	0.07
291ILE	CD	336PHE	CB	0.00
291ILE	CD	336PHE	CD2	0.17
291ILE	CD	336PHE	CE2	0.40
291ILE	CD	337THR	CG2	0.21
291ILE	C	292LEU	CA	100.00
291ILE	C	292LEU	CB	0.00
291ILE	C	292LEU	C	100.00
292LE	CA	292LEU	CB	100.00
292LE	CA	292LEU	CG	100.00
292LE	CA	292LEU	CD1	0.52
292LE	CA	292LEU	CD2	99.88
292LE	CA	292LEU	C	100.00
292LE	CA	295ALA	CB	0.06
292LE	CB	292LEU	CG	100.00
292LE	CB	292LEU	CD1	100.00
292LE	CB	292LEU	CD2	100.00
292LE	CB	292LEU	C	100.00
292LE	CG	292LEU	CD1	100.00
292LE	CG	292LEU	CD2	100.00
292LE	CG	292LEU	C	0.00
292LE	CG	315VAL	CG1	0.00
292LE	CD1	292LEU	CD2	100.00
292LE	CD1	292LEU	C	0.00
292LE	CD1	315VAL	CG1	0.00
292LE	CD2	295ALA	CB	0.00
292LE	CD2	312GLU	CA	0.11

287PRO	CG	336PHE	CE1	0.00	229AR(	C	230PHE	CD1	1.93	292LEI	CD2	312GLU	CB	0.09
287PRO	CG	337THR	CG2	0.03	229AR(	C	230PHE	CD2	3.75	292LEI	CD2	312GLU	CG	4.24
287PRO	CD	287PRO	C	97.39	229AR(	C	230PHE	C	78.10	292LEI	CD2	312GLU	CD	0.34
287PRO	CD	332GLY	CA	0.00	230PHI	CA	230PHE	CB	100.00	292LEI	CD2	315VAL	CG1	1.12
287PRO	CD	332GLY	C	0.41	230PHI	CA	230PHE	CG	100.00	292LEI	CD2	315VAL	CG2	2.91
287PRO	CD	333THR	CG2	0.01	230PHI	CA	230PHE	CD1	95.43	292LEI	C	293SER	CA	100.00
287PRO	CD	336PHE	CB	1.70	230PHI	CA	230PHE	CD2	44.86	292LEI	C	293SER	C	100.00
287PRO	CD	336PHE	CG	0.46	230PHI	CA	230PHE	C	100.00	293SE	CA	293SER	CB	100.00
287PRO	CD	336PHE	CD1	0.21	230PHI	CB	230PHE	CG	100.00	293SE	CA	293SER	C	100.00
287PRO	CD	336PHE	CD2	1.64	230PHI	CB	230PHE	CD1	100.00	293SE	CB	293SER	C	100.00
287PRO	CD	336PHE	CE2	0.01	230PHI	CB	230PHE	CD2	100.00	293SE	C	294ALA	CA	100.00
287PRO	C	288THR	CA	100.00	230PHI	CB	230PHE	C	100.00	293SE	C	294ALA	CB	0.01
287PRO	C	288THR	CB	0.00	230PHI	CB	233VAL	CG1	0.06	293SE	C	294ALA	C	100.00
287PRO	C	288THR	C	100.00	230PHI	CB	233VAL	CG2	0.10	294AL.	CA	294ALA	CB	100.00
287PRO	C	291ILE	CD	0.07	230PHI	CG	230PHE	CD1	100.00	294AL.	CA	294ALA	C	100.00
288THR	CA	288THR	CB	100.00	230PHI	CG	230PHE	CD2	100.00	294AL.	CB	294ALA	C	100.00
288THR	CA	288THR	CG2	100.00	230PHI	CG	230PHE	CE1	100.00	294AL.	C	295ALA	CA	100.00
288THR	CA	288THR	C	100.00	230PHI	CG	230PHE	CE2	100.00	294AL.	C	295ALA	C	99.99
288THR	CB	288THR	CG2	100.00	230PHI	CG	230PHE	CZ	100.00	295AL.	CA	295ALA	CB	100.00
288THR	CB	288THR	C	100.00	230PHI	CD1	230PHE	CD2	100.00	295AL.	CA	295ALA	C	100.00
288THR	CG2	288THR	C	82.40	230PHI	CD1	230PHE	CE1	100.00	295AL.	CA	298LEU	CD1	0.00
288THR	CG2	292LEU	CD1	1.71	230PHI	CD1	230PHE	CE2	100.00	295AL.	CB	295ALA	C	100.00
288THR	CG2	315VAL	CB	0.14	230PHI	CD1	230PHE	CZ	100.00	295AL.	CB	308ALA	CA	0.00
288THR	CG2	315VAL	CG1	0.42	230PHI	CD1	233VAL	CG1	0.53	295AL.	CB	311VAL	CB	0.05
288THR	CG2	315VAL	CG2	0.10	230PHI	CD1	233VAL	CG2	0.46	295AL.	CB	311VAL	CG1	2.13
288THR	CG2	319LEU	CD1	0.00	230PHI	CD2	230PHE	CE1	100.00	295AL.	CB	311VAL	CG2	0.01
288THR	CG2	336PHE	CB	0.06	230PHI	CD2	230PHE	CE2	100.00	295AL.	CB	312GLU	CG	0.02
288THR	CG2	336PHE	CG	0.26	230PHI	CD2	230PHE	CZ	100.00	295AL.	C	296MET	CA	100.00
288THR	CG2	336PHE	CD1	3.04	230PHI	CE1	230PHE	CE2	100.00	295AL.	C	296MET	C	100.00
288THR	CG2	336PHE	CD2	0.01	230PHI	CE1	230PHE	CZ	100.00	296ME	CA	296MET	CB	100.00
288THR	CG2	336PHE	CE1	0.66	230PHI	CE1	233VAL	CG2	0.01	296ME	CA	296MET	CG	100.00
288THR	CG2	336PHE	CZ	0.03	230PHI	CE2	230PHE	CZ	100.00	296ME	CA	296MET	C	100.00
288THR	C	289ALA	CA	100.00	230PHI	C	231ASP	CA	100.00	296ME	CB	296MET	CG	100.00
288THR	C	289ALA	CB	0.00	230PHI	C	231ASP	CB	80.68	296ME	CB	296MET	CE	1.81
288THR	C	289ALA	C	100.00	230PHI	C	231ASP	C	24.18	296ME	CB	296MET	C	100.00
289ALA	CA	289ALA	CB	100.00	231ASI	CA	231ASP	CB	100.00	296ME	CG	296MET	CE	100.00
289ALA	CA	289ALA	C	100.00	231ASI	CA	231ASP	CG	100.00	296ME	CG	296MET	C	95.04
289ALA	CB	289ALA	C	100.00	231ASI	CA	231ASP	C	100.00	296ME	CG	300HIS	CD2	0.20
289ALA	C	290ALA	CA	100.00	231ASI	CB	231ASP	CG	100.00	296ME	CE	300HIS	CE1	0.02
289ALA	C	290ALA	C	100.00	231ASI	CB	231ASP	C	100.00	296ME	C	297MET	CA	100.00
290ALA	CA	290ALA	CB	100.00	231ASI	CG	231ASP	C	99.44	296ME	C	297MET	CB	0.00
290ALA	CA	290ALA	C	100.00	231ASI	CG	232VAL	CG2	0.04	296ME	C	297MET	C	100.00
290ALA	CB	290ALA	C	100.00	231ASI	C	232VAL	CA	100.00	296ME	C	299GLU	CB	0.00
290ALA	C	291ILE	CA	100.00	231ASI	C	232VAL	CB	47.57	297ME	CA	297MET	CB	100.00
290ALA	C	291ILE	C	100.00	231ASI	C	232VAL	CG2	38.86	297ME	CA	297MET	CG	100.00
291ILE	CA	291ILE	CB	100.00	231ASI	C	232VAL	C	64.80	297ME	CA	297MET	C	100.00
291ILE	CA	291ILE	CG1	100.00	232VAI	CA	232VAL	CB	100.00	297ME	CB	297MET	CG	100.00
291ILE	CA	291ILE	CG2	100.00	232VAI	CA	232VAL	CG1	100.00	297ME	CB	297MET	CE	86.68
291ILE	CA	291ILE	CD	0.00	232VAI	CA	232VAL	CG2	100.00	297ME	CB	297MET	C	100.00
291ILE	CA	291ILE	C	100.00	232VAI	CA	232VAL	C	100.00	297ME	CB	302PHE	CD2	0.00
291ILE	CB	291ILE	CG1	100.00	232VAI	CB	232VAL	CG1	100.00	297ME	CB	302PHE	CE2	0.01
291ILE	CB	291ILE	CG2	100.00	232VAI	CB	232VAL	CG2	100.00	297ME	CG	297MET	CE	100.00
291ILE	CB	291ILE	CD	100.00	232VAI	CB	232VAL	C	100.00	297ME	CG	297MET	C	99.78
291ILE	CB	291ILE	C	100.00	232VAI	CG1	232VAL	CG2	100.00	297ME	CG	301ALA	CB	0.13
291ILE	CG1	291ILE	CG2	100.00	232VAI	CG1	232VAL	C	99.99	297ME	CG	302PHE	CD2	0.00
291ILE	CG1	291ILE	CD	100.00	232VAI	CG1	234VAL	CG2	0.05	297ME	CG	302PHE	CE1	0.07
291ILE	CG2	291ILE	CD	100.00	232VAI	C	233VAL	CA	100.00	297ME	CG	302PHE	CE2	1.26
291ILE	CG2	291ILE	C	100.00	232VAI	C	233VAL	CB	56.10	297ME	CG	302PHE	CZ	0.36
291ILE	CG2	311VAL	CG1	25.54	232VAI	C	233VAL	CG1	5.61	297ME	CE	298LEU	CD1	0.24
291ILE	CG2	315VAL	CG1	9.86	232VAI	C	233VAL	CG2	30.55	297ME	CE	302PHE	CE1	0.13
291ILE	CD	315VAL	CG1	0.01	232VAI	C	233VAL	C	63.41	297ME	CE	302PHE	CE2	0.06
291ILE	CD	336PHE	CD1	0.01	233VAI	CA	233VAL	CB	100.00	297ME	CE	302PHE	CZ	0.03

291ILE	CD	336PHE	CE1	0.01	233VAI	CA	233VAL	CG1	100.00	297ME	C	298LEU	CA	100.00
291ILE	CD	337THR	CG2	0.09	233VAI	CA	233VAL	CG2	100.00	297ME	C	298LEU	C	100.00
291ILE	C	292LEU	CA	100.00	233VAI	CA	233VAL	C	100.00	297ME	C	302PHE	CD1	0.00
291ILE	C	292LEU	C	99.99	233VAI	CB	233VAL	CG1	100.00	297ME	C	302PHE	CD2	0.01
292LEU	CA	292LEU	CB	100.00	233VAI	CB	233VAL	CG2	100.00	298LEI	CA	298LEU	CB	100.00
292LEU	CA	292LEU	CG	100.00	233VAI	CB	233VAL	C	100.00	298LEI	CA	298LEU	CG	100.00
292LEU	CA	292LEU	CD1	0.09	233VAI	CG1	233VAL	CG2	100.00	298LEI	CA	298LEU	CD1	47.18
292LEU	CA	292LEU	CD2	99.94	233VAI	CG1	233VAL	C	86.34	298LEI	CA	298LEU	CD2	52.48
292LEU	CA	292LEU	C	100.00	233VAI	CG1	235THR	CG2	0.11	298LEI	CA	298LEU	C	100.00
292LEU	CA	295ALA	CB	0.06	233VAI	CG1	243LEU	CD1	0.01	298LEI	CA	302PHE	CD1	0.01
292LEU	CB	292LEU	CG	100.00	233VAI	CG1	243LEU	CD2	0.11	298LEI	CA	302PHE	CD2	0.00
292LEU	CB	292LEU	CD1	100.00	233VAI	CG2	233VAL	C	13.69	298LEI	CB	298LEU	CG	100.00
292LEU	CB	292LEU	CD2	100.00	233VAI	C	234VAL	CA	100.00	298LEI	CB	298LEU	CD1	100.00
292LEU	CB	292LEU	C	100.00	233VAI	C	234VAL	CB	14.60	298LEI	CB	298LEU	CD2	100.00
292LEU	CG	292LEU	CD1	100.00	233VAI	C	234VAL	CG1	0.15	298LEI	CB	298LEU	C	100.00
292LEU	CG	292LEU	CD2	100.00	233VAI	C	234VAL	CG2	3.97	298LEI	CB	308ALA	CA	0.01
292LEU	CG	292LEU	C	0.00	233VAI	C	234VAL	C	89.40	298LEI	CB	308ALA	CB	0.22
292LEU	CG	315VAL	CG2	0.00	234VAI	CA	234VAL	CB	100.00	298LEI	CG	298LEU	CD1	100.00
292LEU	CD1	292LEU	CD2	100.00	234VAI	CA	234VAL	CG1	100.00	298LEI	CG	298LEU	CD2	100.00
292LEU	CD2	312GLU	CA	0.01	234VAI	CA	234VAL	CG2	100.00	298LEI	CG	298LEU	C	17.53
292LEU	CD2	312GLU	CG	1.58	234VAI	CA	234VAL	C	100.00	298LEI	CG	304LEU	CD1	0.01
292LEU	CD2	315VAL	CG2	0.70	234VAI	CB	234VAL	CG1	100.00	298LEI	CD1	298LEU	CD2	100.00
292LEU	C	293SER	CA	100.00	234VAI	CB	234VAL	CG2	100.00	298LEI	CD1	298LEU	C	0.08
292LEU	C	293SER	C	100.00	234VAI	CB	234VAL	C	100.00	298LEI	CD1	302PHE	CD2	0.21
293SER	CA	293SER	CB	100.00	234VAI	CG1	234VAL	CG2	100.00	298LEI	CD1	302PHE	CE2	0.12
293SER	CA	293SER	C	100.00	234VAI	CG1	234VAL	C	98.95	298LEI	CD1	304LEU	CB	0.16
293SER	CB	293SER	C	100.00	234VAI	CG2	234VAL	C	13.82	298LEI	CD1	304LEU	CD1	0.54
293SER	C	294ALA	CA	100.00	234VAI	C	235THR	CA	100.00	298LEI	CD1	304LEU	CD2	0.00
293SER	C	294ALA	CB	0.02	234VAI	C	235THR	CB	99.99	298LEI	CD1	307LEU	CB	0.01
293SER	C	294ALA	C	99.98	234VAI	C	235THR	CG2	0.76	298LEI	CD1	307LEU	CG	0.00
293SER	C	296MET	CB	0.00	234VAI	C	235THR	C	0.34	298LEI	CD1	307LEU	CD2	0.01
294ALA	CA	294ALA	CB	100.00	235THI	CA	235THR	CB	100.00	298LEI	CD1	307LEU	C	0.03
294ALA	CA	294ALA	C	100.00	235THI	CA	235THR	CG2	100.00	298LEI	CD1	311VAL	CG2	0.37
294ALA	CA	297MET	CB	0.01	235THI	CA	235THR	C	100.00	298LEI	CD2	298LEU	C	0.07
294ALA	CA	297MET	CE	0.01	235THI	CB	235THR	CG2	100.00	298LEI	CD2	302PHE	CG	0.03
294ALA	CB	294ALA	C	100.00	235THI	CB	235THR	C	100.00	298LEI	CD2	302PHE	CD1	0.34
294ALA	C	295ALA	CA	100.00	235THI	CG2	235THR	C	99.23	298LEI	CD2	302PHE	CD2	0.19
294ALA	C	295ALA	CB	0.07	235THI	CG2	239PHE	CB	0.02	298LEI	CD2	302PHE	CE1	0.04
294ALA	C	295ALA	C	99.89	235THI	CG2	239PHE	C	0.38	298LEI	CD2	302PHE	CE2	0.01
294ALA	C	297MET	CB	0.01	235THI	CG2	240GLY	CA	29.83	298LEI	CD2	304LEU	CB	0.06
295ALA	CA	295ALA	CB	100.00	235THI	CG2	243LEU	CD1	0.02	298LEI	CD2	304LEU	CD1	0.29
295ALA	CA	295ALA	C	100.00	235THI	CG2	243LEU	CD2	0.01	298LEI	CD2	307LEU	CB	0.78
295ALA	CB	295ALA	C	100.00	235THI	C	236GLY	CA	100.00	298LEI	CD2	307LEU	CG	0.02
295ALA	CB	311VAL	CB	0.08	235THI	C	236GLY	C	62.69	298LEI	CD2	307LEU	CD1	0.02
295ALA	CB	311VAL	CG1	1.74	236GL	CA	236GLY	C	100.00	298LEI	CD2	307LEU	CD2	0.21
295ALA	CB	311VAL	CG2	0.59	236GL	C	237ASN	CA	100.00	298LEI	CD2	307LEU	C	0.08
295ALA	C	296MET	CA	100.00	236GL	C	237ASN	CB	0.91	298LEI	CD2	308ALA	CA	0.02
295ALA	C	296MET	C	100.00	236GL	C	237ASN	CG	0.00	298LEI	CD2	308ALA	CB	0.00
295ALA	C	308ALA	CB	0.01	236GL	C	237ASN	C	99.28	298LEI	CD2	311VAL	CG2	0.00
296MET	CA	296MET	CB	100.00	237ASI	CA	237ASN	CB	100.00	298LEI	C	299GLU	CA	100.00
296MET	CA	296MET	CG	100.00	237ASI	CA	237ASN	CG	100.00	298LEI	C	299GLU	CB	0.02
296MET	CA	296MET	C	100.00	237ASI	CA	237ASN	C	100.00	298LEI	C	299GLU	C	100.00
296MET	CB	296MET	CG	100.00	237ASI	CB	237ASN	CG	100.00	298LEI	C	308ALA	CB	0.01
296MET	CB	296MET	CE	7.36	237ASI	CB	237ASN	C	100.00	299GL	CA	299GLU	CB	100.00
296MET	CB	296MET	C	100.00	237ASI	CB	238ILE	CD	0.01	299GL	CA	299GLU	CG	100.00
296MET	CG	296MET	CE	100.00	237ASI	CG	237ASN	C	76.88	299GL	CA	299GLU	CD	69.00
296MET	CG	296MET	C	81.95	237ASI	CG	238ILE	CD	0.00	299GL	CA	299GLU	C	100.00
296MET	CG	300HIS	CD2	1.10	237ASI	C	238ILE	CA	100.00	299GL	CA	308ALA	CB	0.16
296MET	CG	300HIS	CE1	0.00	237ASI	C	238ILE	CB	17.46	299GL	CB	299GLU	CG	100.00
296MET	CE	300HIS	CD2	0.30	237ASI	C	238ILE	CG1	4.08	299GL	CB	299GLU	CD	100.00
296MET	CE	300HIS	CE1	0.13	237ASI	C	238ILE	CG2	0.00	299GL	CB	299GLU	C	100.00
296MET	C	297MET	CA	100.00	237ASI	C	238ILE	CD	0.40	299GL	CB	308ALA	CB	3.46

296MET	C	297MET	C	100.00	237ASI	C	238ILE	C	86.82	299GL	CG	299GLU	CD	100.00
297MET	CA	297MET	CB	100.00	237ASI	C	241ASP	CB	0.05	299GL	CG	299GLU	C	97.38
297MET	CA	297MET	CG	100.00	238ILE	CA	238ILE	CB	100.00	299GL	CG	300HIS	CD2	0.04
297MET	CA	297MET	C	100.00	238ILE	CA	238ILE	CG1	100.00	299GL	CG	300HIS	CE1	0.00
297MET	CB	297MET	CG	100.00	238ILE	CA	238ILE	CG2	100.00	299GL	CG	305VAL	CA	0.01
297MET	CB	297MET	CE	85.04	238ILE	CA	238ILE	CD	60.53	299GL	CG	305VAL	CB	0.01
297MET	CB	297MET	C	100.00	238ILE	CA	238ILE	C	100.00	299GL	CG	305VAL	CG1	0.11
297MET	CG	297MET	CE	100.00	238ILE	CA	241ASP	CB	0.02	299GL	CG	305VAL	CG2	0.21
297MET	CG	297MET	C	99.79	238ILE	CB	238ILE	CG1	100.00	299GL	CG	308ALA	CB	0.59
297MET	CG	301ALA	CB	0.11	238ILE	CB	238ILE	CG2	100.00	299GL	CD	299GLU	C	54.93
297MET	CG	302PHE	CD1	0.03	238ILE	CB	238ILE	CD	100.00	299GL	CD	300HIS	CD2	0.02
297MET	CG	302PHE	CE1	0.26	238ILE	CB	238ILE	C	100.00	299GL	CD	300HIS	CE1	0.01
297MET	CG	302PHE	CE2	0.04	238ILE	CB	239PHE	CE1	0.01	299GL	CD	305VAL	CG1	0.11
297MET	CG	302PHE	CZ	0.35	238ILE	CB	239PHE	CE2	0.01	299GL	CD	309ARG	CZ	0.03
297MET	CE	297MET	C	0.04	238ILE	CB	239PHE	CZ	0.00	299GL	C	300HIS	CA	100.00
297MET	CE	298LEU	CD1	0.01	238ILE	CG1	238ILE	CG2	100.00	299GL	C	300HIS	CB	1.32
297MET	CE	298LEU	CD2	0.12	238ILE	CG1	238ILE	CD	100.00	299GL	C	300HIS	C	98.88
297MET	CE	302PHE	CG	0.00	238ILE	CG1	238ILE	C	34.26	300HI	CA	300HIS	CB	100.00
297MET	CE	302PHE	CD1	0.04	238ILE	CG1	239PHE	CE1	0.00	300HI	CA	300HIS	CG	100.00
297MET	CE	302PHE	CE1	1.40	238ILE	CG1	242ILE	CD	0.08	300HI	CA	300HIS	CD2	39.67
297MET	CE	302PHE	CE2	0.14	238ILE	CG1	238ILE	CG2	0.01	300HI	CA	300HIS	C	100.00
297MET	CE	302PHE	CZ	0.41	238ILE	CG1	238ILE	CD	0.01	300HI	CB	300HIS	CG	100.00
297MET	C	298LEU	CA	100.00	238ILE	CG1	239PHE	CE1	0.31	300HI	CB	300HIS	CD2	100.00
297MET	C	298LEU	C	100.00	238ILE	CG1	239PHE	CE2	0.15	300HI	CB	300HIS	CE1	0.26
297MET	C	302PHE	CD1	0.00	238ILE	CG1	239PHE	CZ	0.12	300HI	CB	300HIS	C	100.00
298LEU	CA	298LEU	CB	100.00	238ILE	CG2	238ILE	CD	94.12	300HI	CG	300HIS	CD2	100.00
298LEU	CA	298LEU	CG	100.00	238ILE	CG2	238ILE	C	99.82	300HI	CG	300HIS	CE1	100.00
298LEU	CA	298LEU	CD1	21.44	238ILE	CG2	239PHE	CG	0.01	300HI	CG	300HIS	C	98.80
298LEU	CA	298LEU	CD2	78.38	238ILE	CG2	239PHE	CD1	0.88	300HI	CD2	300HIS	CE1	100.00
298LEU	CA	298LEU	C	100.00	238ILE	CG2	239PHE	CD2	0.23	300HI	CD2	300HIS	C	50.86
298LEU	CA	302PHE	CD1	0.02	238ILE	CG2	239PHE	CE1	0.14	300HI	CD2	301ALA	CA	0.02
298LEU	CB	298LEU	CG	100.00	238ILE	CG2	239PHE	CE2	0.16	300HI	C	301ALA	CA	100.00
298LEU	CB	298LEU	CD1	100.00	238ILE	CG2	239PHE	CZ	0.03	300HI	C	301ALA	CB	5.61
298LEU	CB	298LEU	CD2	100.00	238ILE	CG2	242ILE	CG2	0.01	300HI	C	301ALA	C	94.63
298LEU	CB	298LEU	C	100.00	238ILE	CG2	242ILE	CD	0.32	301AL	CA	301ALA	CB	100.00
298LEU	CB	308ALA	CB	0.09	238ILE	CG2	216VAL	CG1	0.00	301AL	CA	301ALA	C	100.00
298LEU	CG	298LEU	CD1	100.00	238ILE	CG2	239PHE	CD1	0.05	301AL	CB	301ALA	C	100.00
298LEU	CG	298LEU	CD2	100.00	238ILE	CG2	239PHE	CD2	0.08	301AL	CB	302PHE	CD1	0.29
298LEU	CG	298LEU	C	14.34	238ILE	CG2	239PHE	CE1	2.70	301AL	CB	302PHE	CD2	0.64
298LEU	CD1	298LEU	CD2	100.00	238ILE	CG2	239PHE	CE2	2.52	301AL	CB	302PHE	CE1	0.05
298LEU	CD1	298LEU	C	0.16	238ILE	CG2	239PHE	CZ	2.06	301AL	CB	302PHE	CE2	0.51
298LEU	CD1	302PHE	CB	0.02	238ILE	CD	238ILE	C	0.90	301AL	C	302PHE	CA	100.00
298LEU	CD1	302PHE	CG	0.18	238ILE	CD	239PHE	CD1	0.01	301AL	C	302PHE	CB	29.39
298LEU	CD1	302PHE	CD1	0.18	238ILE	CD	239PHE	CE1	0.01	301AL	C	302PHE	CG	16.78
298LEU	CD1	302PHE	CD2	0.34	238ILE	CD	242ILE	CD	0.11	301AL	C	302PHE	CD1	3.01
298LEU	CD1	302PHE	CE1	0.03	238ILE	CD	138ILE	CD	0.00	301AL	C	302PHE	CD2	1.50
298LEU	CD1	302PHE	CE2	0.06	238ILE	CD	185LYS	CB	0.00	301AL	C	302PHE	C	72.06
298LEU	CD1	304LEU	CB	0.02	238ILE	CD	185LYS	CG	0.02	302PH	CA	302PHE	CB	100.00
298LEU	CD1	304LEU	CD1	0.04	238ILE	CD	185LYS	CD	0.02	302PH	CA	302PHE	CG	100.00
298LEU	CD1	307LEU	CB	0.30	238ILE	CD	185LYS	CE	0.07	302PH	CA	302PHE	CD1	84.52
298LEU	CD1	307LEU	CD2	0.06	238ILE	CD	188VAL	CG2	0.20	302PH	CA	302PHE	CD2	43.27
298LEU	CD1	307LEU	C	0.08	238ILE	CD	189LEU	CD1	0.48	302PH	CA	302PHE	C	100.00
298LEU	CD1	311VAL	CG2	0.12	238ILE	CD	189LEU	CD2	0.10	302PH	CB	302PHE	CG	100.00
298LEU	CD2	298LEU	C	0.14	238ILE	CD	238ILE	CG2	0.10	302PH	CB	302PHE	CD1	100.00
298LEU	CD2	302PHE	CG	0.36	238ILE	CD	238ILE	CD	0.24	302PH	CB	302PHE	CD2	100.00
298LEU	CD2	302PHE	CD1	1.32	238ILE	CD	239PHE	CD1	0.00	302PH	CB	302PHE	C	100.00
298LEU	CD2	302PHE	CD2	1.97	238ILE	CD	239PHE	CD2	0.03	302PH	CB	304LEU	CG	0.03
298LEU	CD2	302PHE	CE1	0.33	238ILE	CD	239PHE	CE1	1.05	302PH	CB	304LEU	CD1	2.36
298LEU	CD2	302PHE	CE2	0.53	238ILE	CD	239PHE	CE2	0.83	302PH	CB	304LEU	CD2	0.08
298LEU	CD2	302PHE	CZ	0.00	238ILE	CD	239PHE	CZ	0.86	302PH	CG	302PHE	CD1	100.00
298LEU	CD2	304LEU	CB	0.03	238ILE	C	239PHE	CA	100.00	302PH	CG	302PHE	CD2	100.00
298LEU	CD2	304LEU	CD1	0.21	238ILE	C	239PHE	CB	0.11	302PH	CG	302PHE	CE1	100.00

298LEU	CD2	307LEU	CB	0.45	238ILE	C	239PHE	CG	0.00	302PH	CG	302PHE	CE2	100.00
298LEU	CD2	307LEU	CG	0.02	238ILE	C	239PHE	CD1	0.07	302PH	CG	302PHE	CZ	100.00
298LEU	CD2	307LEU	CD1	0.00	238ILE	C	239PHE	C	99.90	302PH	CG	302PHE	C	1.76
298LEU	CD2	307LEU	CD2	0.14	239PHI	CA	239PHE	CB	100.00	302PH	CG	304LEU	CD1	0.14
298LEU	CD2	307LEU	C	0.20	239PHI	CA	239PHE	CG	100.00	302PH	CD1	302PHE	CD2	100.00
298LEU	CD2	308ALA	CA	0.02	239PHI	CA	239PHE	CD1	48.04	302PH	CD1	302PHE	CE1	100.00
298LEU	CD2	311VAL	CG2	0.01	239PHI	CA	239PHE	CD2	53.85	302PH	CD1	302PHE	CE2	100.00
298LEU	C	299GLU	CA	100.00	239PHI	CA	239PHE	C	100.00	302PH	CD1	302PHE	CZ	100.00
298LEU	C	299GLU	C	100.00	239PHI	CB	239PHE	CG	100.00	302PH	CD1	302PHE	C	0.01
298LEU	C	308ALA	CB	0.00	239PHI	CB	239PHE	CD1	100.00	302PH	CD1	304LEU	CD1	0.08
299GLU	CA	299GLU	CB	100.00	239PHI	CB	239PHE	CD2	100.00	302PH	CD2	302PHE	CE1	100.00
299GLU	CA	299GLU	CG	100.00	239PHI	CB	239PHE	C	100.00	302PH	CD2	302PHE	CE2	100.00
299GLU	CA	299GLU	CD	48.82	239PHI	CB	243LEU	CD1	0.00	302PH	CD2	302PHE	CZ	100.00
299GLU	CA	299GLU	C	100.00	239PHI	CB	242ILE	CD	0.00	302PH	CD2	302PHE	C	1.02
299GLU	CA	308ALA	CB	0.02	239PHI	CG	239PHE	CD1	100.00	302PH	CD2	304LEU	CG	0.14
299GLU	CB	299GLU	CG	100.00	239PHI	CG	239PHE	CD2	100.00	302PH	CD2	304LEU	CD1	0.84
299GLU	CB	299GLU	CD	100.00	239PHI	CG	239PHE	CE1	100.00	302PH	CD2	304LEU	CD2	0.02
299GLU	CB	299GLU	C	100.00	239PHI	CG	239PHE	CE2	100.00	302PH	CE1	302PHE	CE2	100.00
299GLU	CB	300HIS	CD2	0.12	239PHI	CG	239PHE	CZ	100.00	302PH	CE1	302PHE	CZ	100.00
299GLU	CB	308ALA	CB	1.86	239PHI	CG	239PHE	C	0.00	302PH	CE1	304LEU	CD1	0.01
299GLU	CG	299GLU	CD	100.00	239PHI	CG	239PHE	CE1	0.00	302PH	CE2	302PHE	CZ	100.00
299GLU	CG	299GLU	C	98.05	239PHI	CG	239PHE	CE2	0.00	302PH	CE2	304LEU	CD1	0.13
299GLU	CG	300HIS	CD2	1.69	239PHI	CG	242ILE	CD	0.01	302PH	CE2	304LEU	CD2	0.01
299GLU	CG	300HIS	CE1	0.08	239PHI	CD1	239PHE	CD2	100.00	302PH	C	303GLY	CA	100.00
299GLU	CG	305VAL	CG1	0.06	239PHI	CD1	239PHE	CE1	100.00	302PH	C	303GLY	C	79.80
299GLU	CG	305VAL	CG2	0.02	239PHI	CD1	239PHE	CE2	100.00	302PH	C	304LEU	CD1	0.13
299GLU	CG	308ALA	CB	0.28	239PHI	CD1	239PHE	CZ	100.00	302PH	C	304LEU	CD2	0.00
299GLU	CD	299GLU	C	38.61	239PHI	CD1	238ILE	CG2	0.00	303GL	CA	303GLY	C	100.00
299GLU	CD	300HIS	CD2	0.89	239PHI	CD1	239PHE	CE2	0.04	303GL	C	304LEU	CA	100.00
299GLU	CD	300HIS	CE1	0.13	239PHI	CD1	242ILE	CD	0.52	303GL	C	304LEU	CB	6.06
299GLU	CD	305VAL	CG1	0.03	239PHI	CD2	239PHE	CE1	100.00	303GL	C	304LEU	CG	0.86
299GLU	CD	309ARG	CZ	1.25	239PHI	CD2	239PHE	CE2	100.00	303GL	C	304LEU	CD1	0.05
299GLU	C	300HIS	CA	100.00	239PHI	CD2	239PHE	CZ	100.00	303GL	C	304LEU	CD2	0.06
299GLU	C	300HIS	CB	5.48	239PHI	CD2	239PHE	CD1	0.00	303GL	C	304LEU	C	96.48
299GLU	C	300HIS	CG	0.84	239PHI	CD2	239PHE	CE1	0.08	303GL	C	305VAL	CG1	0.00
299GLU	C	300HIS	CD2	0.01	239PHI	CD2	239PHE	CE2	0.04	304LE	CA	304LEU	CB	100.00
299GLU	C	300HIS	C	93.16	239PHI	CD2	239PHE	CZ	0.01	304LE	CA	304LEU	CG	100.00
300HIS	CA	300HIS	CB	100.00	239PHI	CD2	242ILE	CD	0.30	304LE	CA	304LEU	CD1	6.61
300HIS	CA	300HIS	CG	100.00	239PHI	CE1	239PHE	CE2	100.00	304LE	CA	304LEU	CD2	91.15
300HIS	CA	300HIS	CD2	36.28	239PHI	CE1	239PHE	CZ	100.00	304LE	CA	304LEU	C	100.00
300HIS	CA	300HIS	C	100.00	239PHI	CE1	242ILE	CD	0.00	304LE	CB	304LEU	CG	100.00
300HIS	CB	300HIS	CG	100.00	239PHI	CE1	238ILE	CB	0.00	304LE	CB	304LEU	CD1	100.00
300HIS	CB	300HIS	CD2	100.00	239PHI	CE1	238ILE	CG1	0.12	304LE	CB	304LEU	CD2	100.00
300HIS	CB	300HIS	CE1	0.19	239PHI	CE1	238ILE	CG2	0.62	304LE	CB	304LEU	C	100.00
300HIS	CB	300HIS	C	100.00	239PHI	CE1	238ILE	CD	0.19	304LE	CB	307LEU	CB	0.02
300HIS	CG	300HIS	CD2	100.00	239PHI	CE1	239PHE	CD1	0.02	304LE	CB	307LEU	CG	0.00
300HIS	CG	300HIS	CE1	100.00	239PHI	CE1	239PHE	CD2	0.06	304LE	CB	307LEU	CD1	0.10
300HIS	CG	300HIS	C	77.12	239PHI	CE1	239PHE	CE1	0.12	304LE	CB	307LEU	CD2	0.00
300HIS	CD2	300HIS	CE1	100.00	239PHI	CE1	239PHE	CE2	0.22	304LE	CG	304LEU	CD1	100.00
300HIS	CD2	300HIS	C	37.36	239PHI	CE1	239PHE	CZ	0.06	304LE	CG	304LEU	CD2	100.00
300HIS	CD2	301ALA	CA	0.03	239PHI	CE1	242ILE	CD	0.53	304LE	CG	307LEU	CD1	0.00
300HIS	C	301ALA	CA	100.00	239PHI	CE2	239PHE	CZ	100.00	304LE	CD1	304LEU	CD2	100.00
300HIS	C	301ALA	CB	11.00	239PHI	CE2	238ILE	CG1	0.06	304LE	CD1	307LEU	CD1	0.13
300HIS	C	301ALA	C	89.50	239PHI	CE2	238ILE	CG2	0.77	304LE	CD1	307LEU	CD2	0.15
301ALA	CA	301ALA	CB	100.00	239PHI	CE2	238ILE	CD	0.08	304LE	CD2	307LEU	CD1	0.23
301ALA	CA	301ALA	C	100.00	239PHI	CE2	239PHE	CD1	0.08	304LE	CD2	307LEU	CD2	0.05
301ALA	CB	301ALA	C	100.00	239PHI	CE2	239PHE	CD2	0.03	304LE	C	305VAL	CA	100.00
301ALA	CB	302PHE	CD1	0.18	239PHI	CE2	239PHE	CE1	0.36	304LE	C	305VAL	CB	0.01
301ALA	CB	302PHE	CD2	0.01	239PHI	CE2	239PHE	CE2	0.29	304LE	C	305VAL	CG2	0.00
301ALA	CB	302PHE	CE1	0.01	239PHI	CE2	239PHE	CZ	0.06	304LE	C	305VAL	C	100.00
301ALA	C	302PHE	CA	100.00	239PHI	CE2	242ILE	CD	0.65	305VA	CA	305VAL	CB	100.00
301ALA	C	302PHE	CB	1.75	239PHI	CZ	138ILE	CD	0.01	305VA	CA	305VAL	CG1	100.00

301ALA C	302PHE CG	0.09	239PHI CZ	238ILE CB	0.01	305VA CA	305VAL CG2	100.00
301ALA C	302PHE CD1	0.80	239PHI CZ	238ILE CG1	0.06	305VA CA	305VAL C	100.00
301ALA C	302PHE CD2	0.08	239PHI CZ	238ILE CG2	0.95	305VA CA	308ALA CB	0.00
301ALA C	302PHE C	98.12	239PHI CZ	238ILE CD	0.26	305VA CB	305VAL CG1	100.00
302PHE CA	302PHE CB	100.00	239PHI CZ	239PHE CD1	0.00	305VA CB	305VAL CG2	100.00
302PHE CA	302PHE CG	100.00	239PHI CZ	239PHE CD2	0.01	305VA CB	305VAL C	100.00
302PHE CA	302PHE CD1	88.20	239PHI CZ	239PHE CE1	0.16	305VA CB	309ARG CZ	0.00
302PHE CA	302PHE CD2	29.27	239PHI CZ	239PHE CE2	0.12	305VA CG1	305VAL CG2	100.00
302PHE CA	302PHE C	100.00	239PHI CZ	239PHE CZ	0.06	305VA CG1	305VAL C	29.63
302PHE CB	302PHE CG	100.00	239PHI CZ	242ILE CD	0.02	305VA CG1	309ARG CZ	0.89
302PHE CB	302PHE CD1	100.00	239PHI C	240GLY CA	100.00	305VA CG2	305VAL C	88.88
302PHE CB	302PHE CD2	100.00	239PHI C	240GLY C	88.89	305VA CG2	309ARG CD	0.00
302PHE CB	302PHE C	100.00	239PHI C	242ILE CG1	0.05	305VA CG2	309ARG CZ	0.62
302PHE CB	304LEU CG	0.06	239PHI C	242ILE CD	0.00	305VA C	306GLU CA	100.00
302PHE CB	304LEU CD1	1.80	239PHI C	243LEU CD1	0.02	305VA C	306GLU CB	0.09
302PHE CG	302PHE CD1	100.00	240GLY CA	240GLY C	100.00	305VA C	306GLU C	99.98
302PHE CG	302PHE CD2	100.00	240GLY CA	243LEU CB	0.01	305VA C	309ARG CZ	0.15
302PHE CG	302PHE CE1	100.00	240GLY C	241ASP CA	100.00	306GL CA	306GLU CB	100.00
302PHE CG	302PHE CE2	100.00	240GLY C	241ASP CB	0.13	306GL CA	306GLU CG	100.00
302PHE CG	302PHE CZ	100.00	240GLY C	241ASP C	99.92	306GL CA	306GLU CD	98.61
302PHE CG	302PHE C	1.31	241ASI CA	241ASP CB	100.00	306GL CA	306GLU C	100.00
302PHE CG	304LEU CD1	0.05	241ASI CA	241ASP CG	100.00	306GL CA	309ARG CZ	0.01
302PHE CD1	302PHE CD2	100.00	241ASI CA	241ASP C	100.00	306GL CB	306GLU CG	100.00
302PHE CD1	302PHE CE1	100.00	241ASI CB	241ASP CG	100.00	306GL CB	306GLU CD	100.00
302PHE CD1	302PHE CE2	100.00	241ASI CB	241ASP C	100.00	306GL CB	306GLU C	100.00
302PHE CD1	302PHE CZ	100.00	241ASI CG	241ASP C	98.53	306GL CG	306GLU CD	100.00
302PHE CD1	302PHE C	0.33	241ASI CG	185LYS CE	0.06	306GL CG	306GLU C	94.69
302PHE CD1	304LEU CG	0.01	241ASI CG	217ASP CB	0.02	306GL CD	306GLU C	0.50
302PHE CD1	304LEU CD1	0.10	241ASI C	242ILE CA	100.00	306GL CD	309ARG CZ	0.06
302PHE CD2	302PHE CE1	100.00	241ASI C	242ILE CB	0.02	306GL CD	310LYS CE	0.00
302PHE CD2	302PHE CE2	100.00	241ASI C	242ILE CG1	0.00	306GL C	307LEU CA	100.00
302PHE CD2	302PHE CZ	100.00	241ASI C	242ILE C	99.98	306GL C	307LEU CB	0.03
302PHE CD2	302PHE C	0.22	241ASI C	217ASP CB	0.02	306GL C	307LEU CD1	0.01
302PHE CD2	304LEU CG	0.01	242ILE CA	242ILE CB	100.00	306GL C	307LEU C	99.99
302PHE CD2	304LEU CD1	0.95	242ILE CA	242ILE CG1	100.00	307LEI CA	307LEU CB	100.00
302PHE CD2	307LEU CD1	0.01	242ILE CA	242ILE CG2	100.00	307LEI CA	307LEU CG	100.00
302PHE CE1	302PHE CE2	100.00	242ILE CA	242ILE CD	0.84	307LEI CA	307LEU CD1	46.39
302PHE CE1	302PHE CZ	100.00	242ILE CA	242ILE C	100.00	307LEI CA	307LEU CD2	52.94
302PHE CE1	304LEU CD1	0.05	242ILE CA	217ASP CB	0.16	307LEI CA	307LEU C	100.00
302PHE CE2	302PHE CZ	100.00	242ILE CB	242ILE CG1	100.00	307LEI CA	310LYS CB	0.00
302PHE CE2	304LEU CD1	0.07	242ILE CB	242ILE CG2	100.00	307LEI CB	307LEU CG	100.00
302PHE CE2	307LEU CD1	0.00	242ILE CB	242ILE CD	100.00	307LEI CB	307LEU CD1	100.00
302PHE CZ	304LEU CD1	0.01	242ILE CB	242ILE C	100.00	307LEI CB	307LEU CD2	100.00
302PHE C	303GLY CA	100.00	242ILE CG1	242ILE CG2	100.00	307LEI CB	307LEU C	100.00
302PHE C	303GLY C	77.75	242ILE CG1	242ILE CD	100.00	307LEI CG	307LEU CD1	100.00
302PHE C	304LEU CD1	0.00	242ILE CG1	242ILE C	8.33	307LEI CG	307LEU CD2	100.00
303GLY CA	303GLY C	100.00	242ILE CG1	243LEU CD1	0.01	307LEI CG	307LEU C	23.21
303GLY C	304LEU CA	100.00	242ILE CG1	216VAL CG1	0.23	307LEI CD1	307LEU CD2	100.00
303GLY C	304LEU CB	12.47	242ILE CG1	216VAL CG2	0.03	307LEI CD1	307LEU C	0.26
303GLY C	304LEU CG	2.22	242ILE CG1	217ASP CA	0.00	307LEI CD1	310LYS CD	0.01
303GLY C	304LEU CD1	0.17	242ILE CG1	217ASP CB	0.00	307LEI CD1	345ALA C	0.03
303GLY C	304LEU CD2	0.05	242ILE CG2	242ILE CD	98.36	307LEI CD2	307LEU C	0.74
303GLY C	304LEU C	91.46	242ILE CG2	242ILE C	91.65	307LEI CD2	310LYS CD	0.02
304LEU CA	304LEU CB	100.00	242ILE CG2	243LEU CD1	0.00	307LEI CD2	311VAL CG2	0.01
304LEU CA	304LEU CG	100.00	242ILE CG2	243LEU CD2	0.01	307LEI C	308ALA CA	100.00
304LEU CA	304LEU CD1	2.64	242ILE CG2	246LEU CD1	0.04	307LEI C	308ALA CB	0.00
304LEU CA	304LEU CD2	96.50	242ILE CG2	246LEU CD2	0.02	307LEI C	308ALA C	100.00
304LEU CA	304LEU C	100.00	242ILE CG2	216VAL CG1	0.14	308AL CA	308ALA CB	100.00
304LEU CA	306GLU CD	0.00	242ILE CG2	216VAL CG2	0.04	308AL CA	308ALA C	100.00
304LEU CB	304LEU CG	100.00	242ILE CG2	217ASP CA	0.01	308AL CA	311VAL CG1	0.00
304LEU CB	304LEU CD1	100.00	242ILE CG2	220ALA CB	3.25	308AL CB	308ALA C	100.00
304LEU CB	304LEU CD2	100.00	242ILE CG2	239PHE CE1	0.01	308AL C	309ARG CA	100.00

304LEU	CB	304LEU	C	100.00	242ILE	CG2	239PHE	CE2	0.00	308AL	C	309ARG	CB	0.01
304LEU	CB	307LEU	CB	0.03	242ILE	CG2	242ILE	CG2	0.04	308AL	C	309ARG	C	99.99
304LEU	CB	307LEU	CD1	0.18	242ILE	CG2	242ILE	CD	0.00	308AL	C	311VAL	CG1	0.00
304LEU	CG	304LEU	CD1	100.00	242ILE	CG2	243LEU	CD1	0.06	309AR	CA	309ARG	CB	100.00
304LEU	CG	304LEU	CD2	100.00	242ILE	CG2	243LEU	CD2	0.02	309AR	CA	309ARG	CG	100.00
304LEU	CG	304LEU	C	0.00	242ILE	CD	242ILE	C	0.47	309AR	CA	309ARG	CD	6.74
304LEU	CD1	304LEU	CD2	100.00	242ILE	CD	243LEU	CG	0.01	309AR	CA	309ARG	C	100.00
304LEU	CD1	307LEU	CD1	0.44	242ILE	CD	243LEU	CD1	0.01	309AR	CB	309ARG	CG	100.00
304LEU	CD1	307LEU	CD2	0.23	242ILE	CD	243LEU	CD2	0.01	309AR	CB	309ARG	CD	100.00
304LEU	CD2	306GLU	CD	0.02	242ILE	CD	216VAL	CG1	0.75	309AR	CB	309ARG	CZ	4.17
304LEU	CD2	307LEU	CD1	0.20	242ILE	CD	216VAL	CG2	2.34	309AR	CB	309ARG	C	100.00
304LEU	CD2	307LEU	CD2	0.02	242ILE	CD	239PHE	CD1	0.41	309AR	CG	309ARG	CD	100.00
304LEU	C	305VAL	CA	100.00	242ILE	CD	239PHE	CD2	0.17	309AR	CG	309ARG	CZ	52.71
304LEU	C	305VAL	CB	0.01	242ILE	CD	239PHE	CE1	0.40	309AR	CG	309ARG	C	46.89
304LEU	C	305VAL	C	100.00	242ILE	CD	239PHE	CE2	0.09	309AR	CD	309ARG	CZ	100.00
304LEU	C	306GLU	CD	0.01	242ILE	CD	242ILE	CG2	0.21	309AR	CD	309ARG	C	0.21
305VAL	CA	305VAL	CB	100.00	242ILE	CD	242ILE	CD	1.02	309AR	CD	312GLU	CD	0.00
305VAL	CA	305VAL	CG1	100.00	242ILE	C	243LEU	CA	100.00	309AR	CZ	310LYS	CE	0.00
305VAL	CA	305VAL	CG2	100.00	242ILE	C	243LEU	CB	3.43	309AR	C	310LYS	CA	100.00
305VAL	CA	305VAL	C	100.00	242ILE	C	243LEU	CG	0.34	309AR	C	310LYS	CB	0.01
305VAL	CA	308ALA	CB	0.00	242ILE	C	243LEU	CD1	0.04	309AR	C	310LYS	C	100.00
305VAL	CB	305VAL	CG1	100.00	242ILE	C	243LEU	CD2	0.03	310LY	CA	310LYS	CB	100.00
305VAL	CB	305VAL	CG2	100.00	242ILE	C	243LEU	C	95.07	310LY	CA	310LYS	CG	100.00
305VAL	CB	305VAL	C	100.00	243LE	CA	243LEU	CB	100.00	310LY	CA	310LYS	CD	3.08
305VAL	CG1	305VAL	CG2	100.00	243LE	CA	243LEU	CG	100.00	310LY	CA	310LYS	C	100.00
305VAL	CG1	305VAL	C	68.65	243LE	CA	243LEU	CD1	16.24	310LY	CA	343HIS	CE1	0.01
305VAL	CG1	309ARG	CG	0.04	243LE	CA	243LEU	CD2	83.22	310LY	CB	310LYS	CG	100.00
305VAL	CG1	309ARG	CD	0.01	243LE	CA	243LEU	C	100.00	310LY	CB	310LYS	CD	100.00
305VAL	CG1	309ARG	CZ	3.00	243LE	CA	246LEU	CB	0.02	310LY	CB	310LYS	CE	6.72
305VAL	CG2	305VAL	C	31.30	243LE	CB	243LEU	CG	100.00	310LY	CB	310LYS	C	100.00
305VAL	CG2	309ARG	CZ	0.01	243LE	CB	243LEU	CD1	100.00	310LY	CB	343HIS	CE1	0.01
305VAL	C	306GLU	CA	100.00	243LE	CB	243LEU	CD2	100.00	310LY	CB	344LEU	CD1	0.21
305VAL	C	306GLU	CB	0.04	243LE	CB	243LEU	C	100.00	310LY	CB	344LEU	CD2	0.25
305VAL	C	306GLU	C	99.98	243LE	CG	243LEU	CD1	100.00	310LY	CG	310LYS	CD	100.00
305VAL	C	309ARG	CZ	0.07	243LE	CG	243LEU	CD2	100.00	310LY	CG	310LYS	CE	100.00
306GLU	CA	306GLU	CB	100.00	243LE	CG	243LEU	C	12.69	310LY	CG	310LYS	C	72.21
306GLU	CA	306GLU	CG	100.00	243LE	CD1	243LEU	CD2	100.00	310LY	CG	343HIS	CD2	0.03
306GLU	CA	306GLU	CD	89.91	243LE	CD1	243LEU	C	0.33	310LY	CG	343HIS	CE1	0.83
306GLU	CA	306GLU	C	100.00	243LE	CD1	246LEU	CD1	0.00	310LY	CG	344LEU	CB	0.02
306GLU	CA	309ARG	CZ	0.01	243LE	CD1	246LEU	CD2	0.02	310LY	CG	344LEU	CD1	0.07
306GLU	CB	306GLU	CG	100.00	243LE	CD1	250LEU	CD1	0.00	310LY	CG	344LEU	CD2	0.70
306GLU	CB	306GLU	CD	100.00	243LE	CD1	242ILE	CG2	0.00	310LY	CD	310LYS	CE	100.00
306GLU	CB	306GLU	C	100.00	243LE	CD1	242ILE	CD	0.00	310LY	CD	310LYS	C	0.03
306GLU	CG	306GLU	CD	100.00	243LE	CD2	243LEU	C	0.32	310LY	CD	344LEU	CB	0.03
306GLU	CG	306GLU	C	95.51	243LE	CD2	246LEU	CD1	0.06	310LY	CD	344LEU	CD1	0.02
306GLU	CG	307LEU	CD1	0.02	243LE	CD2	246LEU	CD2	0.26	310LY	CD	344LEU	CD2	0.17
306GLU	CG	307LEU	CD2	0.01	243LE	CD2	242ILE	CG2	0.01	310LY	CD	344LEU	C	0.02
306GLU	CD	306GLU	C	0.14	243LE	CD2	246LEU	CD1	0.02	310LY	CD	345ALA	C	0.01
306GLU	CD	307LEU	CD2	0.01	243LE	C	244SER	CA	100.00	310LY	CE	343HIS	CD2	0.00
306GLU	CD	309ARG	CZ	0.00	243LE	C	244SER	CB	0.21	310LY	CE	343HIS	CE1	0.06
306GLU	C	307LEU	CA	100.00	243LE	C	244SER	C	99.83	310LY	CE	344LEU	CB	0.00
306GLU	C	307LEU	CB	0.42	243LE	C	246LEU	CB	0.06	310LY	CE	344LEU	CD1	0.00
306GLU	C	307LEU	CG	0.02	243LE	C	246LEU	CD1	0.00	310LY	CE	344LEU	CD2	0.02
306GLU	C	307LEU	CD1	0.02	244SE	CA	244SER	CB	100.00	310LY	CE	344LEU	C	0.25
306GLU	C	307LEU	CD2	0.02	244SE	CA	244SER	C	100.00	310LY	CE	345ALA	CA	0.00
306GLU	C	307LEU	C	99.52	244SE	CB	244SER	C	100.00	310LY	CE	345ALA	C	0.66
307LEU	CA	307LEU	CB	100.00	244SE	C	245ASP	CA	100.00	310LY	C	311VAL	CA	100.00
307LEU	CA	307LEU	CG	100.00	244SE	C	245ASP	CB	1.75	310LY	C	311VAL	CB	0.02
307LEU	CA	307LEU	CD1	46.83	244SE	C	245ASP	CG	0.06	310LY	C	311VAL	C	100.00
307LEU	CA	307LEU	CD2	53.55	244SE	C	245ASP	C	97.26	310LY	C	344LEU	CD1	0.41
307LEU	CA	307LEU	C	100.00	245AS	CA	245ASP	CB	100.00	310LY	C	344LEU	CD2	0.83
307LEU	CB	307LEU	CG	100.00	245AS	CA	245ASP	CG	100.00	311VA	CA	311VAL	CB	100.00

307LEU CB	307LEU	CD1	100.00	245ASP CA	245ASP	C	100.00	311VA CA	311VAL	CG1	100.00
307LEU CB	307LEU	CD2	100.00	245ASP CB	245ASP	CG	100.00	311VA CA	311VAL	CG2	100.00
307LEU CB	307LEU	C	100.00	245ASP CB	245ASP	C	100.00	311VA CA	311VAL	C	100.00
307LEU CG	307LEU	CD1	100.00	245ASP CB	254LEU	CD1	0.02	311VA CA	344LEU	CD1	0.04
307LEU CG	307LEU	CD2	100.00	245ASP CB	221MET	CB	0.01	311VA CA	344LEU	CD2	0.02
307LEU CG	307LEU	C	31.73	245ASP CB	221MET	CG	0.28	311VA CB	311VAL	CG1	100.00
307LEU CD1	307LEU	CD2	100.00	245ASP CB	221MET	CE	0.50	311VA CB	311VAL	CG2	100.00
307LEU CD1	307LEU	C	0.18	245ASP CG	245ASP	C	1.66	311VA CB	311VAL	C	100.00
307LEU CD1	310LYS	CD	0.05	245ASP CG	254LEU	CD1	0.05	311VA CG1	311VAL	CG2	100.00
307LEU CD1	344LEU	CD2	0.02	245ASP CG	257LEU	CD2	0.00	311VA CG1	311VAL	C	100.00
307LEU CD2	307LEU	C	0.57	245ASP CG	272VAL	CG1	0.02	311VA CG1	315VAL	CG1	0.00
307LEU CD2	310LYS	CD	0.03	245ASP CG	221MET	CG	0.01	311VA CG1	315VAL	CG2	0.01
307LEU CD2	310LYS	CE	0.02	245ASP CG	221MET	CE	0.47	311VA CG2	311VAL	C	0.90
307LEU CD2	311VAL	CG2	0.01	245ASP C	246LEU	CA	100.00	311VA CG2	344LEU	CB	0.00
307LEU C	308ALA	CA	100.00	245ASP C	246LEU	CB	0.06	311VA CG2	344LEU	CD1	0.65
307LEU C	308ALA	C	100.00	245ASP C	246LEU	C	99.93	311VA CG2	344LEU	CD2	1.03
307LEU C	310LYS	CB	0.00	245ASP C	221MET	CB	0.03	311VA C	312GLU	CA	100.00
308ALA CA	308ALA	CB	100.00	245ASP C	221MET	CG	0.32	311VA C	312GLU	C	100.00
308ALA CA	308ALA	C	100.00	245ASP C	221MET	CE	0.02	312GL CA	312GLU	CB	100.00
308ALA CB	308ALA	C	100.00	246LEU CA	246LEU	CB	100.00	312GL CA	312GLU	CG	100.00
308ALA C	309ARG	CA	100.00	246LEU CA	246LEU	CG	100.00	312GL CA	312GLU	CD	2.96
308ALA C	309ARG	CB	0.01	246LEU CA	246LEU	CD1	51.42	312GL CA	312GLU	C	100.00
308ALA C	309ARG	C	100.00	246LEU CA	246LEU	CD2	37.84	312GL CA	315VAL	CG1	0.01
309ARG CA	309ARG	CB	100.00	246LEU CA	246LEU	C	100.00	312GL CA	315VAL	CG2	0.01
309ARG CA	309ARG	CG	100.00	246LEU CA	249VAL	CG2	0.00	312GL CB	312GLU	CG	100.00
309ARG CA	309ARG	CD	54.91	246LEU CA	221MET	CB	0.01	312GL CB	312GLU	CD	100.00
309ARG CA	309ARG	CZ	0.71	246LEU CA	221MET	CG	0.00	312GL CB	312GLU	C	100.00
309ARG CA	309ARG	C	100.00	246LEU CB	246LEU	CG	100.00	312GL CG	312GLU	CD	100.00
309ARG CA	312GLU	CB	0.01	246LEU CB	246LEU	CD1	100.00	312GL CG	312GLU	C	94.71
309ARG CB	309ARG	CG	100.00	246LEU CB	246LEU	CD2	100.00	312GL CD	312GLU	C	0.50
309ARG CB	309ARG	CD	100.00	246LEU CB	246LEU	C	100.00	312GL C	313ASP	CA	100.00
309ARG CB	309ARG	CZ	1.39	246LEU CB	224VAL	CG2	0.04	312GL C	313ASP	CB	0.02
309ARG CB	309ARG	C	100.00	246LEU CG	246LEU	CD1	100.00	312GL C	313ASP	C	100.00
309ARG CG	309ARG	CD	100.00	246LEU CG	246LEU	CD2	100.00	312GL C	315VAL	CG2	0.04
309ARG CG	309ARG	CZ	56.71	246LEU CG	246LEU	C	96.00	313AS CA	313ASP	CB	100.00
309ARG CG	309ARG	C	10.51	246LEU CG	250LEU	CD1	0.00	313AS CA	313ASP	CG	100.00
309ARG CD	309ARG	CZ	100.00	246LEU CG	224VAL	CG1	0.03	313AS CA	313ASP	C	100.00
309ARG CD	312GLU	CD	0.14	246LEU CG	224VAL	CG2	0.06	313AS CB	313ASP	CG	100.00
309ARG CZ	312GLU	CD	0.22	246LEU CD1	246LEU	CD2	100.00	313AS CB	313ASP	C	100.00
309ARG C	310LYS	CA	100.00	246LEU CD1	246LEU	C	9.15	313AS CB	343HIS	CE1	0.01
309ARG C	310LYS	CB	0.02	246LEU CD1	250LEU	CD1	0.22	313AS CG	313ASP	C	100.00
309ARG C	310LYS	C	100.00	246LEU CD1	250LEU	CD2	0.05	313AS CG	317LYS	CD	0.04
309ARG C	312GLU	CB	0.00	246LEU CD1	220ALA	CB	0.74	313AS CG	317LYS	CE	1.11
310LYS CA	310LYS	CB	100.00	246LEU CD1	220ALA	C	1.38	313AS CG	343HIS	CE1	0.01
310LYS CA	310LYS	CG	100.00	246LEU CD1	221MET	CA	0.04	313AS C	314ALA	CA	100.00
310LYS CA	310LYS	CD	8.01	246LEU CD1	224VAL	CB	0.16	313AS C	314ALA	CB	0.01
310LYS CA	310LYS	C	100.00	246LEU CD1	224VAL	CG1	1.95	313AS C	314ALA	C	100.00
310LYS CB	310LYS	CG	100.00	246LEU CD1	224VAL	CG2	6.56	314AL CA	314ALA	CB	100.00
310LYS CB	310LYS	CD	100.00	246LEU CD1	246LEU	CD1	0.92	314AL CA	314ALA	C	100.00
310LYS CB	310LYS	CE	5.10	246LEU CD1	246LEU	CD2	1.75	314AL CA	343HIS	CB	0.12
310LYS CB	310LYS	C	100.00	246LEU CD2	246LEU	C	37.07	314AL CA	343HIS	CG	0.31
310LYS CB	344LEU	CD1	0.07	246LEU CD2	250LEU	CG	0.01	314AL CA	343HIS	CD2	4.45
310LYS CB	344LEU	CD2	0.09	246LEU CD2	250LEU	CD1	0.72	314AL CA	343HIS	CE1	0.21
310LYS CG	310LYS	CD	100.00	246LEU CD2	250LEU	CD2	0.08	314AL CB	314ALA	C	100.00
310LYS CG	310LYS	CE	100.00	246LEU CD2	220ALA	CB	0.03	314AL CB	340VAL	CG1	0.77
310LYS CG	310LYS	C	78.99	246LEU CD2	220ALA	C	0.12	314AL CB	340VAL	CG2	0.01
310LYS CG	343HIS	CD2	0.01	246LEU CD2	221MET	CA	0.00	314AL CB	343HIS	CB	4.50
310LYS CG	343HIS	CE1	0.06	246LEU CD2	224VAL	CG1	0.16	314AL CB	343HIS	CG	2.20
310LYS CG	344LEU	CD1	0.13	246LEU CD2	224VAL	CG2	0.54	314AL CB	343HIS	CD2	2.26
310LYS CG	344LEU	CD2	0.59	246LEU CD2	246LEU	CD1	0.50	314AL CB	343HIS	CE1	0.16
310LYS CD	310LYS	CE	100.00	246LEU CD2	246LEU	CD2	0.43	314AL CB	343HIS	C	0.10
310LYS CD	310LYS	C	0.06	246LEU C	247ALA	CA	100.00	314AL CB	344LEU	CA	0.01

310LYS	CD	344LEU	CG	0.00	246LE	C	247ALA	CB	0.02	314AL	CB	344LEU	CB	0.01
310LYS	CD	344LEU	CD1	0.07	246LE	C	247ALA	C	100.00	314AL	CB	344LEU	CG	0.19
310LYS	CD	344LEU	CD2	0.18	246LE	C	249VAL	CG1	0.00	314AL	CB	344LEU	CD1	0.34
310LYS	CD	345ALA	C	0.00	247AL	CA	247ALA	CB	100.00	314AL	CB	344LEU	CD2	0.66
310LYS	CE	343HIS	CD2	0.00	247AL	CA	247ALA	C	100.00	314AL	C	315VAL	CA	100.00
310LYS	CE	343HIS	CE1	0.01	247AL	CA	250LEU	CD1	0.17	314AL	C	315VAL	CB	0.01
310LYS	CE	344LEU	CD1	0.02	247AL	CB	247ALA	C	100.00	314AL	C	315VAL	C	100.00
310LYS	CE	344LEU	CD2	0.02	247AL	CB	257LEU	CD1	0.01	314AL	C	340VAL	CG2	0.02
310LYS	CE	344LEU	C	0.16	247AL	CB	257LEU	CD2	0.04	315VA	CA	315VAL	CB	100.00
310LYS	CE	345ALA	C	1.08	247AL	C	248SER	CA	100.00	315VA	CA	315VAL	CG1	100.00
310LYS	C	311VAL	CA	100.00	247AL	C	248SER	CB	3.42	315VA	CA	315VAL	CG2	100.00
310LYS	C	311VAL	CB	0.01	247AL	C	248SER	C	97.04	315VA	CA	315VAL	C	100.00
310LYS	C	311VAL	C	100.00	247AL	C	251PRO	CD	0.26	315VA	CA	318ALA	CB	0.00
310LYS	C	344LEU	CD1	0.48	247AL	C	257LEU	CD1	0.01	315VA	CB	315VAL	CG1	100.00
310LYS	C	344LEU	CD2	0.41	248SE	CA	248SER	CB	100.00	315VA	CB	315VAL	CG2	100.00
311VAL	CA	311VAL	CB	100.00	248SE	CA	248SER	C	100.00	315VA	CB	315VAL	C	100.00
311VAL	CA	311VAL	CG1	100.00	248SE	CA	257LEU	CD1	0.06	315VA	CG1	315VAL	CG2	100.00
311VAL	CA	311VAL	CG2	100.00	248SE	CB	248SER	C	100.00	315VA	CG1	315VAL	C	26.97
311VAL	CA	311VAL	C	100.00	248SE	CB	253SER	C	0.01	315VA	CG1	319LEU	CD1	0.07
311VAL	CA	344LEU	CD1	0.03	248SE	CB	254LEU	CA	0.01	315VA	CG1	336PHE	CE2	0.01
311VAL	CB	311VAL	CG1	100.00	248SE	CB	254LEU	CB	0.08	315VA	CG1	340VAL	CG1	0.12
311VAL	CB	311VAL	CG2	100.00	248SE	CB	254LEU	CG	0.01	315VA	CG1	340VAL	CG2	0.32
311VAL	CB	311VAL	C	100.00	248SE	CB	254LEU	CD1	0.37	315VA	CG2	315VAL	C	82.22
311VAL	CG1	311VAL	CG2	100.00	248SE	CB	254LEU	CD2	0.61	315VA	CG2	336PHE	CE2	0.09
311VAL	CG1	311VAL	C	98.98	248SE	CB	257LEU	CD1	4.71	315VA	CG2	336PHE	CZ	0.03
311VAL	CG1	344LEU	CD1	0.02	248SE	CB	257LEU	CD2	0.15	315VA	CG2	340VAL	CG1	0.04
311VAL	CG2	311VAL	C	1.01	248SE	CB	221MET	CE	0.00	315VA	CG2	340VAL	CG2	0.06
311VAL	CG2	344LEU	CD1	0.42	248SE	C	249VAL	CA	100.00	315VA	C	316ALA	CA	100.00
311VAL	CG2	344LEU	CD2	0.18	248SE	C	249VAL	CB	0.95	315VA	C	316ALA	CB	0.00
311VAL	C	312GLU	CA	100.00	248SE	C	249VAL	CG1	0.31	315VA	C	316ALA	C	100.00
311VAL	C	312GLU	CB	0.00	248SE	C	249VAL	CG2	0.01	315VA	C	319LEU	CD1	0.00
311VAL	C	312GLU	C	100.00	248SE	C	249VAL	C	99.14	316AL	CA	316ALA	CB	100.00
312GLU	CA	312GLU	CB	100.00	248SE	C	251PRO	CD	0.94	316AL	CA	316ALA	C	100.00
312GLU	CA	312GLU	CG	100.00	248SE	C	254LEU	CB	0.04	316AL	CB	316ALA	C	100.00
312GLU	CA	312GLU	CD	0.22	248SE	C	254LEU	CD1	0.02	316AL	C	317LYS	CA	100.00
312GLU	CA	312GLU	C	100.00	248SE	C	254LEU	CD2	0.43	316AL	C	317LYS	CB	0.28
312GLU	CA	315VAL	CG2	0.06	249VA	CA	249VAL	CB	100.00	316AL	C	317LYS	CG	0.02
312GLU	CB	312GLU	CG	100.00	249VA	CA	249VAL	CG1	100.00	316AL	C	317LYS	CE	0.00
312GLU	CB	312GLU	CD	100.00	249VA	CA	249VAL	CG2	100.00	316AL	C	317LYS	C	99.70
312GLU	CB	312GLU	C	100.00	249VA	CA	249VAL	C	100.00	317LY	CA	317LYS	CB	100.00
312GLU	CG	312GLU	CD	100.00	249VA	CA	254LEU	CD1	0.02	317LY	CA	317LYS	CG	100.00
312GLU	CG	312GLU	C	69.25	249VA	CA	254LEU	CD2	0.17	317LY	CA	317LYS	CD	9.97
312GLU	CD	312GLU	C	0.08	249VA	CB	249VAL	CG1	100.00	317LY	CA	317LYS	CE	0.74
312GLU	C	313ASP	CA	100.00	249VA	CB	249VAL	CG2	100.00	317LY	CA	317LYS	C	100.00
312GLU	C	313ASP	CB	0.13	249VA	CB	249VAL	C	100.00	317LY	CB	317LYS	CG	100.00
312GLU	C	313ASP	C	99.92	249VA	CB	254LEU	CD2	0.00	317LY	CB	317LYS	CD	100.00
312GLU	C	315VAL	CG2	0.11	249VA	CB	224VAL	CG1	0.01	317LY	CB	317LYS	CE	2.93
313ASP	CA	313ASP	CB	100.00	249VA	CB	224VAL	CG2	0.14	317LY	CB	317LYS	C	100.00
313ASP	CA	313ASP	CG	100.00	249VA	CG1	249VAL	CG2	100.00	317LY	CB	343HIS	CG	0.42
313ASP	CA	313ASP	C	100.00	249VA	CG1	249VAL	C	90.36	317LY	CB	343HIS	CD2	5.30
313ASP	CB	313ASP	CG	100.00	249VA	CG1	254LEU	CD1	0.13	317LY	CB	343HIS	CE1	1.95
313ASP	CB	313ASP	C	100.00	249VA	CG1	254LEU	CD2	0.08	317LY	CG	317LYS	CD	100.00
313ASP	CG	313ASP	C	99.98	249VA	CG1	221MET	CB	0.00	317LY	CG	317LYS	CE	100.00
313ASP	CG	317LYS	CD	0.06	249VA	CG1	221MET	CG	0.90	317LY	CG	317LYS	C	0.28
313ASP	CG	317LYS	CE	0.98	249VA	CG1	221MET	CE	0.42	317LY	CG	343HIS	CE1	0.18
313ASP	CG	343HIS	CE1	0.01	249VA	CG1	224VAL	CB	0.01	317LY	CD	317LYS	CE	100.00
313ASP	C	314ALA	CA	100.00	249VA	CG1	224VAL	CG1	0.61	317LY	CD	343HIS	CG	0.02
313ASP	C	314ALA	CB	0.00	249VA	CG1	224VAL	CG2	1.18	317LY	CD	343HIS	CD2	0.30
313ASP	C	314ALA	C	100.00	249VA	CG1	224VAL	C	0.02	317LY	CD	343HIS	CE1	3.30
313ASP	C	316ALA	CB	0.00	249VA	CG1	225ARG	CA	0.09	317LY	CE	343HIS	CE1	0.21
313ASP	C	343HIS	CD2	0.01	249VA	CG1	225ARG	CB	0.08	317LY	C	318ALA	CA	100.00
314ALA	CA	314ALA	CB	100.00	249VA	CG1	225ARG	CG	2.87	317LY	C	318ALA	CB	0.04

314ALA	CA	314ALA	C	100.00
314ALA	CA	343HIS	CB	0.05
314ALA	CA	343HIS	CG	0.10
314ALA	CA	343HIS	CD2	5.42
314ALA	CA	343HIS	CE1	0.00
314ALA	CB	314ALA	C	100.00
314ALA	CB	340VAL	CG1	1.34
314ALA	CB	343HIS	CB	0.89
314ALA	CB	343HIS	CG	0.27
314ALA	CB	343HIS	CD2	1.28
314ALA	CB	343HIS	CE1	0.00
314ALA	CB	343HIS	C	0.83
314ALA	CB	344LEU	CA	0.09
314ALA	CB	344LEU	CB	0.07
314ALA	CB	344LEU	CG	0.64
314ALA	CB	344LEU	CD1	1.31
314ALA	CB	344LEU	CD2	1.95
314ALA	C	315VAL	CA	100.00
314ALA	C	315VAL	CB	0.00
314ALA	C	315VAL	C	100.00
314ALA	C	340VAL	CG2	0.01
315VAL	CA	315VAL	CB	100.00
315VAL	CA	315VAL	CG1	100.00
315VAL	CA	315VAL	CG2	100.00
315VAL	CA	315VAL	C	100.00
315VAL	CA	318ALA	CB	0.00
315VAL	CA	340VAL	CG2	0.00
315VAL	CB	315VAL	CG1	100.00
315VAL	CB	315VAL	CG2	100.00
315VAL	CB	315VAL	C	100.00
315VAL	CG1	315VAL	CG2	100.00
315VAL	CG1	340VAL	CG1	0.67
315VAL	CG1	340VAL	CG2	0.50
315VAL	CG2	315VAL	C	100.00
315VAL	C	316ALA	CA	100.00
315VAL	C	316ALA	CB	0.00
315VAL	C	316ALA	C	99.99
315VAL	C	318ALA	CB	0.01
315VAL	C	319LEU	CD1	0.04
316ALA	CA	316ALA	CB	100.00
316ALA	CA	316ALA	C	100.00
316ALA	CB	316ALA	C	100.00
316ALA	C	317LYS	CA	100.00
316ALA	C	317LYS	CB	0.34
316ALA	C	317LYS	CG	0.02
316ALA	C	317LYS	C	99.72
317LYS	CA	317LYS	CB	100.00
317LYS	CA	317LYS	CG	100.00
317LYS	CA	317LYS	CD	17.79
317LYS	CA	317LYS	CE	0.03
317LYS	CA	317LYS	C	100.00
317LYS	CA	320LEU	CD1	0.01
317LYS	CB	317LYS	CG	100.00
317LYS	CB	317LYS	CD	100.00
317LYS	CB	317LYS	CE	1.76
317LYS	CB	317LYS	C	100.00
317LYS	CB	343HIS	CB	0.21
317LYS	CB	343HIS	CG	9.08
317LYS	CB	343HIS	CD2	6.63
317LYS	CB	343HIS	CE1	1.52
317LYS	CG	317LYS	CD	100.00
317LYS	CG	317LYS	CE	100.00

249VAl	CG1	225ARG	CD	0.15
249VAl	CG1	225ARG	CZ	0.48
249VAl	CG2	249VAL	C	10.76
249VAl	CG2	250LEU	CD2	0.00
249VAl	CG2	254LEU	CB	0.01
249VAl	CG2	254LEU	CG	0.00
249VAl	CG2	254LEU	CD1	0.29
249VAl	CG2	254LEU	CD2	0.43
249VAl	CG2	221MET	CB	0.02
249VAl	CG2	221MET	CG	11.29
249VAl	CG2	221MET	CE	1.44
249VAl	CG2	224VAL	CB	0.02
249VAl	CG2	224VAL	CG1	0.16
249VAl	CG2	224VAL	CG2	1.03
249VAl	CG2	225ARG	CG	0.20
249VAl	CG2	225ARG	CD	0.03
249VAl	CG2	225ARG	CZ	0.00
249VAl	C	250LEU	CA	100.00
249VAl	C	250LEU	C	100.00
249VAl	C	251PRO	CD	7.32
250LEl	CA	250LEU	CB	100.00
250LEl	CA	250LEU	CG	100.00
250LEl	CA	250LEU	CD1	26.74
250LEl	CA	250LEU	CD2	72.39
250LEl	CA	250LEU	C	100.00
250LEl	CA	251PRO	CD	100.00
250LEl	CB	250LEU	CG	100.00
250LEl	CB	250LEU	CD1	100.00
250LEl	CB	250LEU	CD2	100.00
250LEl	CB	250LEU	C	100.00
250LEl	CB	251PRO	CD	79.29
250LEl	CG	250LEU	CD1	100.00
250LEl	CG	250LEU	CD2	100.00
250LEl	CG	250LEU	C	3.76
250LEl	CG	251PRO	CD	0.08
250LEl	CD1	250LEU	CD2	100.00
250LEl	CD1	250LEU	C	0.39
250LEl	CD1	224VAL	CB	0.00
250LEl	CD1	224VAL	CG1	0.04
250LEl	CD1	224VAL	CG2	0.08
250LEl	CD2	250LEU	C	0.28
250LEl	CD2	224VAL	CB	0.00
250LEl	CD2	224VAL	CG1	0.23
250LEl	CD2	224VAL	CG2	0.05
250LEl	C	251PRO	CA	100.00
250LEl	C	251PRO	CB	0.31
250LEl	C	251PRO	CD	100.00
250LEl	C	251PRO	C	99.99
251PR(	CA	251PRO	CB	100.00
251PR(	CA	251PRO	CG	100.00
251PR(	CA	251PRO	CD	100.00
251PR(	CA	251PRO	C	100.00
251PR(	CB	251PRO	CG	100.00
251PR(	CB	251PRO	CD	100.00
251PR(	CB	251PRO	C	100.00
251PR(	CG	251PRO	CD	100.00
251PR(	CG	251PRO	C	98.46
251PR(	CG	253SER	CB	0.04
251PR(	CG	257LEU	CD2	0.01
251PR(	CD	251PRO	C	90.80
251PR(	C	252GLY	CA	100.00
251PR(	C	252GLY	C	68.01

317LY:	C	318ALA	C	99.95
317LY:	C	339THR	CG2	0.01
317LY:	C	343HIS	CD2	0.24
317LY:	C	343HIS	CE1	0.11
318AL	CA	318ALA	CB	100.00
318AL	CA	318ALA	C	100.00
318AL	CA	339THR	CG2	0.83
318AL	CA	343HIS	CD2	0.03
318AL	CA	343HIS	CE1	0.02
318AL	CB	318ALA	C	100.00
318AL	CB	336PHE	CD1	0.10
318AL	CB	336PHE	CD2	0.09
318AL	CB	336PHE	CE1	11.45
318AL	CB	336PHE	CE2	1.81
318AL	CB	336PHE	CZ	13.26
318AL	CB	339THR	CB	0.03
318AL	CB	339THR	CG2	2.80
318AL	CB	339THR	C	0.00
318AL	CB	340VAL	CG2	14.60
318AL	C	319LEU	CA	100.00
318AL	C	319LEU	C	100.00
318AL	C	336PHE	CE1	5.54
318AL	C	336PHE	CE2	0.00
318AL	C	336PHE	CZ	0.14
319LE	CA	319LEU	CB	100.00
319LE	CA	319LEU	CG	100.00
319LE	CA	319LEU	CD1	7.95
319LE	CA	319LEU	CD2	91.26
319LE	CA	319LEU	C	100.00
319LE	CA	336PHE	CE1	0.00
319LE	CA	336PHE	CZ	0.04
319LE	CB	319LEU	CG	100.00
319LE	CB	319LEU	CD1	100.00
319LE	CB	319LEU	CD2	100.00
319LE	CB	319LEU	C	100.00
319LE	CB	327LEU	CD2	0.02
319LE	CG	319LEU	CD1	100.00
319LE	CG	319LEU	CD2	100.00
319LE	CG	319LEU	C	8.44
319LE	CG	327LEU	CD1	0.02
319LE	CG	327LEU	CD2	0.08
319LE	CG	336PHE	CE1	0.10
319LE	CG	336PHE	CZ	0.01
319LE	CD1	319LEU	CD2	100.00
319LE	CD1	319LEU	C	0.07
319LE	CD1	324PRO	CG	0.00
319LE	CD1	324PRO	CD	0.58
319LE	CD1	327LEU	CD1	0.05
319LE	CD1	327LEU	CD2	0.30
319LE	CD1	336PHE	CE1	0.02
319LE	CD1	336PHE	CZ	0.07
319LE	CD2	319LEU	C	0.24
319LE	CD2	324PRO	CG	0.41
319LE	CD2	324PRO	CD	4.82
319LE	CD2	327LEU	CD1	1.12
319LE	CD2	327LEU	CD2	1.23
319LE	CD2	336PHE	CE1	4.14
319LE	CD2	336PHE	CE2	0.01
319LE	CD2	336PHE	CZ	1.23
319LE	C	320LEU	CA	100.00
319LE	C	320LEU	CB	2.26
319LE	C	320LEU	CG	0.23

317LYS	CG	317LYS	C	0.40	252GLY	CA	252GLY	C	100.00	319LEU	C	320LEU	CD1	0.13
317LYS	CG	343HIS	CE1	0.76	252GLY	CA	254LEU	CD1	0.08	319LEU	C	320LEU	CD2	0.04
317LYS	CD	317LYS	CE	100.00	252GLY	CA	327LEU	CD1	0.00	319LEU	C	320LEU	C	96.69
317LYS	CD	320LEU	CD1	0.00	252GLY	CA	327LEU	CD2	0.00	320LEU	CA	320LEU	CB	100.00
317LYS	CD	343HIS	CB	0.00	252GLY	C	253SER	CA	100.00	320LEU	CA	320LEU	CG	100.00
317LYS	CD	343HIS	CG	0.02	252GLY	C	253SER	CB	8.69	320LEU	CA	320LEU	CD1	16.09
317LYS	CD	343HIS	CD2	0.02	252GLY	C	253SER	C	93.40	320LEU	CA	320LEU	CD2	83.16
317LYS	CD	343HIS	CE1	0.62	252GLY	C	254LEU	CD1	0.96	320LEU	CA	320LEU	C	100.00
317LYS	CE	343HIS	CE1	0.18	252GLY	C	254LEU	CD2	0.17	320LEU	CB	320LEU	CG	100.00
317LYS	C	318ALA	CA	100.00	252GLY	C	327LEU	CD2	0.01	320LEU	CB	320LEU	CD1	100.00
317LYS	C	318ALA	CB	0.05	253SER	CA	253SER	CB	100.00	320LEU	CB	320LEU	CD2	100.00
317LYS	C	318ALA	C	99.95	253SER	CA	253SER	C	100.00	320LEU	CB	320LEU	C	100.00
317LYS	C	343HIS	CD2	2.83	253SER	CA	256LEU	CG	0.14	320LEU	CG	320LEU	CD1	100.00
317LYS	C	343HIS	CE1	0.08	253SER	CA	256LEU	CD1	0.19	320LEU	CG	320LEU	CD2	100.00
318ALA	CA	318ALA	CB	100.00	253SER	CA	256LEU	CD2	0.08	320LEU	CG	320LEU	C	13.31
318ALA	CA	318ALA	C	100.00	253SER	CA	327LEU	CD1	0.00	320LEU	CD1	320LEU	CD2	100.00
318ALA	CA	321GLU	CB	0.01	253SER	CB	253SER	C	100.00	320LEU	CD1	320LEU	C	0.22
318ALA	CA	339THR	CG2	0.74	253SER	CB	256LEU	CB	0.02	320LEU	CD2	320LEU	C	0.66
318ALA	CA	343HIS	CD2	0.51	253SER	CB	256LEU	CG	0.03	320LEU	C	321GLU	CA	100.00
318ALA	CA	343HIS	CE1	0.00	253SER	CB	256LEU	CD1	2.10	320LEU	C	321GLU	CB	10.98
318ALA	CB	318ALA	C	100.00	253SER	CB	256LEU	CD2	0.02	320LEU	C	321GLU	CG	0.00
318ALA	CB	336PHE	CD1	0.80	253SER	CB	257LEU	CG	0.07	320LEU	C	321GLU	C	90.10
318ALA	CB	336PHE	CE1	2.86	253SER	CB	257LEU	CD1	0.56	321GL	CA	321GLU	CB	100.00
318ALA	CB	336PHE	CE2	3.02	253SER	CB	257LEU	CD2	1.30	321GL	CA	321GLU	CG	100.00
318ALA	CB	336PHE	CZ	14.20	253SER	CB	327LEU	CD1	0.02	321GL	CA	321GLU	CD	52.94
318ALA	CB	339THR	CB	0.18	253SER	CB	327LEU	CD2	0.04	321GL	CA	321GLU	C	100.00
318ALA	CB	339THR	CG2	5.28	253SER	C	254LEU	CA	100.00	321GL	CB	321GLU	CG	100.00
318ALA	CB	339THR	C	0.01	253SER	C	254LEU	CB	0.58	321GL	CB	321GLU	CD	100.00
318ALA	CB	340VAL	CG2	8.87	253SER	C	254LEU	CG	0.01	321GL	CB	321GLU	C	100.00
318ALA	CB	343HIS	CD2	0.02	253SER	C	254LEU	CD1	0.00	321GL	CB	339THR	CG2	0.25
318ALA	C	319LEU	CA	100.00	253SER	C	254LEU	C	99.64	321GL	CB	343HIS	CE1	0.00
318ALA	C	319LEU	CB	0.00	253SER	C	256LEU	CG	0.08	321GL	CG	321GLU	CD	100.00
318ALA	C	319LEU	C	99.99	253SER	C	256LEU	CD1	0.04	321GL	CG	321GLU	C	96.58
318ALA	C	336PHE	CE1	0.06	253SER	C	256LEU	CD2	1.18	321GL	CG	322THR	CG2	0.01
318ALA	C	336PHE	CE2	0.37	253SER	C	257LEU	CD1	0.09	321GL	CG	339THR	CG2	2.34
318ALA	C	336PHE	CZ	0.79	253SER	C	257LEU	CD2	0.01	321GL	CG	343HIS	CE1	0.00
319LEU	CA	319LEU	CB	100.00	254LEU	CA	254LEU	CB	100.00	321GL	CD	321GLU	C	0.14
319LEU	CA	319LEU	CG	100.00	254LEU	CA	254LEU	CG	100.00	321GL	CD	339THR	CG2	2.72
319LEU	CA	319LEU	CD1	2.68	254LEU	CA	254LEU	CD1	27.91	321GL	CD	342ARG	CZ	0.01
319LEU	CA	319LEU	CD2	96.86	254LEU	CA	254LEU	CD2	58.37	321GL	CD	343HIS	CE1	0.01
319LEU	CA	319LEU	C	100.00	254LEU	CA	254LEU	C	100.00	321GL	C	322THR	CA	100.00
319LEU	CB	319LEU	CG	100.00	254LEU	CA	257LEU	CD1	0.01	321GL	C	322THR	CB	95.86
319LEU	CB	319LEU	CD1	100.00	254LEU	CA	257LEU	CD2	0.02	321GL	C	322THR	CG2	54.94
319LEU	CB	319LEU	CD2	100.00	254LEU	CB	254LEU	CG	100.00	321GL	C	322THR	C	9.85
319LEU	CB	319LEU	C	100.00	254LEU	CB	254LEU	CD1	100.00	321GL	C	323PRO	CD	13.68
319LEU	CG	319LEU	CD1	100.00	254LEU	CB	254LEU	CD2	100.00	322TH	CA	322THR	CB	100.00
319LEU	CG	319LEU	CD2	100.00	254LEU	CB	254LEU	C	100.00	322TH	CA	322THR	CG2	100.00
319LEU	CG	319LEU	C	2.20	254LEU	CB	324PRO	CB	0.00	322TH	CA	322THR	C	100.00
319LEU	CG	336PHE	CE2	0.01	254LEU	CB	326ASP	CB	0.90	322TH	CA	323PRO	CD	100.00
319LEU	CG	336PHE	CZ	0.02	254LEU	CB	326ASP	CG	0.08	322TH	CA	331ALA	CB	0.52
319LEU	CD1	319LEU	CD2	100.00	254LEU	CB	327LEU	CD1	0.06	322TH	CB	322THR	CG2	100.00
319LEU	CD1	319LEU	C	0.02	254LEU	CB	327LEU	CD2	0.14	322TH	CB	322THR	C	100.00
319LEU	CD1	324PRO	CB	0.02	254LEU	CB	221MET	CE	0.10	322TH	CB	323PRO	CD	0.01
319LEU	CD1	324PRO	CG	0.00	254LEU	CG	254LEU	CD1	100.00	322TH	CB	331ALA	CB	1.02
319LEU	CD1	324PRO	CD	0.03	254LEU	CG	254LEU	CD2	100.00	322TH	CG2	331ALA	CB	0.11
319LEU	CD1	336PHE	CE1	0.02	254LEU	CG	254LEU	C	39.03	322TH	CG2	335ALA	CB	0.01
319LEU	CD1	336PHE	CE2	0.00	254LEU	CG	273HIS	CE1	0.00	322TH	CG2	339THR	CG2	0.02
319LEU	CD2	319LEU	C	0.36	254LEU	CG	327LEU	CD1	0.00	322TH	C	323PRO	CA	100.00
319LEU	CD2	324PRO	CA	0.04	254LEU	CG	327LEU	CD2	0.02	322TH	C	323PRO	CB	0.12
319LEU	CD2	324PRO	CB	1.77	254LEU	CG	221MET	CE	0.06	322TH	C	323PRO	CD	100.00
319LEU	CD2	324PRO	CG	1.71	254LEU	CD1	254LEU	CD2	100.00	322TH	C	323PRO	C	100.00
319LEU	CD2	324PRO	CD	1.14	254LEU	CD1	254LEU	C	6.23	322TH	C	331ALA	CB	12.20

319LEU CD2	327LEU CD2	0.02	254LEU CD1	272VAL CG1	0.01	323PR CA	323PRO CB	100.00
319LEU CD2	336PHE CD1	0.02	254LEU CD1	272VAL CG2	0.00	323PR CA	323PRO CG	100.00
319LEU CD2	336PHE CE1	0.77	254LEU CD1	273HIS CD2	0.01	323PR CA	323PRO CD	100.00
319LEU CD2	336PHE CE2	0.42	254LEU CD1	273HIS CE1	0.06	323PR CA	323PRO C	100.00
319LEU CD2	336PHE CZ	2.86	254LEU CD1	326ASP CB	0.01	323PR CA	324PRO CD	100.00
319LEU C	320LEU CA	100.00	254LEU CD1	326ASP CG	0.01	323PR CB	323PRO CG	100.00
319LEU C	320LEU CB	0.81	254LEU CD1	326ASP C	0.00	323PR CB	323PRO CD	100.00
319LEU C	320LEU CG	0.01	254LEU CD1	327LEU CA	0.00	323PR CB	323PRO C	100.00
319LEU C	320LEU CD1	0.04	254LEU CD1	327LEU CD1	0.30	323PR CB	324PRO CD	11.26
319LEU C	320LEU C	98.85	254LEU CD1	327LEU CD2	0.51	323PR CB	327LEU CB	0.34
320LEU CA	320LEU CB	100.00	254LEU CD1	221MET CG	0.01	323PR CB	327LEU CD1	0.28
320LEU CA	320LEU CG	100.00	254LEU CD1	221MET CE	1.32	323PR CB	327LEU CD2	0.88
320LEU CA	320LEU CD1	22.02	254LEU CD1	225ARG CD	0.02	323PR CB	327LEU C	0.00
320LEU CA	320LEU CD2	77.10	254LEU CD1	225ARG CZ	5.00	323PR CB	329GLY CA	0.00
320LEU CA	320LEU C	100.00	254LEU CD2	254LEU C	4.39	323PR CG	323PRO CD	100.00
320LEU CB	320LEU CG	100.00	254LEU CD2	272VAL CG1	0.01	323PR CG	323PRO C	96.41
320LEU CB	320LEU CD1	100.00	254LEU CD2	272VAL CG2	0.02	323PR CG	328GLY C	0.00
320LEU CB	320LEU CD2	100.00	254LEU CD2	273HIS CD2	0.01	323PR CG	329GLY CA	0.06
320LEU CB	320LEU C	100.00	254LEU CD2	273HIS CE1	0.02	323PR CD	323PRO C	91.22
320LEU CG	320LEU CD1	100.00	254LEU CD2	274GLY CA	0.01	323PR C	324PRO CA	100.00
320LEU CG	320LEU CD2	100.00	254LEU CD2	326ASP CB	0.02	323PR C	324PRO CB	0.21
320LEU CG	320LEU C	21.70	254LEU CD2	326ASP C	0.01	323PR C	324PRO CD	100.00
320LEU CD1	320LEU CD2	100.00	254LEU CD2	327LEU CD1	0.02	323PR C	324PRO C	100.00
320LEU CD1	320LEU C	0.38	254LEU CD2	327LEU CD2	0.09	323PR C	331ALA CB	3.16
320LEU CD2	320LEU C	0.53	254LEU CD2	221MET CG	0.07	324PR CA	324PRO CB	100.00
320LEU C	321GLU CA	100.00	254LEU CD2	221MET CE	4.83	324PR CA	324PRO CG	100.00
320LEU C	321GLU CB	6.04	254LEU CD2	225ARG CD	0.08	324PR CA	324PRO CD	100.00
320LEU C	321GLU CG	0.00	254LEU CD2	225ARG CZ	6.61	324PR CA	324PRO C	100.00
320LEU C	321GLU C	94.58	254LEU C	255GLY CA	100.00	324PR CA	325PRO CD	100.00
321GLU CA	321GLU CB	100.00	254LEU C	255GLY C	62.27	324PR CA	331ALA CB	0.09
321GLU CA	321GLU CG	100.00	254LEU C	256LEU CD2	0.06	324PR CB	324PRO CG	100.00
321GLU CA	321GLU CD	53.33	254LEU C	257LEU CD1	0.01	324PR CB	324PRO CD	100.00
321GLU CA	321GLU C	100.00	254LEU C	257LEU CD2	0.03	324PR CB	324PRO C	100.00
321GLU CB	321GLU CG	100.00	254LEU C	273HIS CE1	0.02	324PR CB	325PRO CD	59.28
321GLU CB	321GLU CD	100.00	254LEU C	326ASP CB	0.40	324PR CB	327LEU CD1	0.01
321GLU CB	321GLU C	100.00	255GLY CA	255GLY C	100.00	324PR CB	331ALA CB	0.00
321GLU CB	339THR CG2	0.39	255GLY CA	272VAL CG2	0.08	324PR CG	324PRO CD	100.00
321GLU CG	321GLU CD	100.00	255GLY CA	273HIS CA	0.03	324PR CG	324PRO C	93.26
321GLU CG	321GLU C	97.54	255GLY CA	273HIS CB	0.16	324PR CG	327LEU CB	0.01
321GLU CG	339THR CG2	2.10	255GLY CA	273HIS CG	0.31	324PR CG	327LEU CD1	0.36
321GLU CG	342ARG CZ	0.00	255GLY CA	273HIS CD2	0.20	324PR CG	327LEU CD2	0.02
321GLU CD	321GLU C	0.18	255GLY CA	273HIS CE1	1.56	324PR CG	336PHE CE1	0.01
321GLU CD	339THR CG2	1.32	255GLY CA	273HIS C	1.09	324PR CD	324PRO C	90.22
321GLU CD	342ARG CZ	0.04	255GLY CA	274GLY CA	0.56	324PR CD	327LEU CB	0.02
321GLU CD	343HIS CE1	0.05	255GLY CA	274GLY C	0.02	324PR CD	327LEU CD1	0.07
321GLU C	322THR CA	100.00	255GLY CA	325PRO CG	0.02	324PR CD	327LEU CD2	0.01
321GLU C	322THR CB	92.08	255GLY CA	326ASP CB	0.01	324PR CD	331ALA CB	0.00
321GLU C	322THR CG2	9.81	255GLY C	256LEU CA	100.00	324PR CD	336PHE CD1	0.00
321GLU C	322THR C	10.23	255GLY C	256LEU CB	59.29	324PR CD	336PHE CE1	0.03
321GLU C	323PRO CD	0.14	255GLY C	256LEU CG	3.03	324PR C	325PRO CA	100.00
322THR CA	322THR CB	100.00	255GLY C	256LEU CD1	0.37	324PR C	325PRO CB	0.00
322THR CA	322THR CG2	100.00	255GLY C	256LEU CD2	0.52	324PR C	325PRO CD	100.00
322THR CA	322THR C	100.00	255GLY C	256LEU C	37.15	324PR C	325PRO C	100.00
322THR CA	323PRO CD	100.00	255GLY C	272VAL CG2	0.39	324PR C	329GLY C	0.03
322THR CB	322THR CG2	100.00	255GLY C	273HIS CB	0.01	325PR CA	325PRO CB	100.00
322THR CB	322THR C	100.00	255GLY C	273HIS CE1	0.01	325PR CA	325PRO CG	100.00
322THR CB	323PRO CD	1.18	255GLY C	273HIS C	0.00	325PR CA	325PRO CD	100.00
322THR CB	331ALA CB	0.14	255GLY C	289ALA CB	0.04	325PR CA	325PRO C	100.00
322THR CB	336PHE CD1	0.04	256LEU CA	256LEU CB	100.00	325PR CA	328GLY CA	0.00
322THR CB	336PHE CD2	0.02	256LEU CA	256LEU CG	100.00	325PR CA	329GLY C	0.01
322THR CB	336PHE CE1	0.04	256LEU CA	256LEU CD1	12.27	325PR CB	325PRO CG	100.00
322THR CB	336PHE CE2	0.10	256LEU CA	256LEU CD2	54.13	325PR CB	325PRO CD	100.00

322THR CB	336PHE	CZ	0.02	256LEL CA	256LEU	C	100.00	325PR CB	325PRO	C	100.00
322THR CG2	322THR	C	75.36	256LEL CA	288THR	CG2	0.00	325PR CB	329GLY	C	0.02
322THR CG2	323PRO	CD	0.00	256LEL CA	289ALA	CB	0.03	325PR CB	330SER	CA	0.00
322THR CG2	331ALA	CB	3.30	256LEL CB	256LEU	CG	100.00	325PR CG	325PRO	CD	100.00
322THR CG2	335ALA	CB	0.02	256LEL CB	256LEU	CD1	100.00	325PR CG	325PRO	C	41.04
322THR CG2	336PHE	CG	0.00	256LEL CB	256LEU	CD2	100.00	325PR CG	331ALA	CB	0.00
322THR CG2	336PHE	CD2	2.26	256LEL CB	256LEU	C	100.00	325PR CD	325PRO	C	22.99
322THR CG2	336PHE	CE1	0.55	256LEL CB	288THR	CG2	0.03	325PR CD	330SER	C	0.02
322THR CG2	336PHE	CE2	2.36	256LEL CB	292LEU	CD1	0.03	325PR CD	331ALA	CB	0.07
322THR CG2	336PHE	CZ	0.75	256LEL CG	256LEU	CD1	100.00	325PR C	326ASP	CA	100.00
322THR CG2	339THR	CG2	0.07	256LEL CG	256LEU	CD2	100.00	325PR C	326ASP	CB	0.13
322THR C	323PRO	CA	100.00	256LEL CG	256LEU	C	48.07	325PR C	326ASP	C	99.89
322THR C	323PRO	CB	0.09	256LEL CG	288THR	CG2	0.02	326AS CA	326ASP	CB	100.00
322THR C	323PRO	CD	100.00	256LEL CD1	256LEU	CD2	100.00	326AS CA	326ASP	CG	100.00
322THR C	323PRO	C	100.00	256LEL CD1	256LEU	C	3.63	326AS CA	326ASP	C	100.00
322THR C	331ALA	CB	0.01	256LEL CD1	257LEU	CD2	0.01	326AS CB	326ASP	CG	100.00
322THR C	336PHE	CZ	0.02	256LEL CD1	286ASN	CG	0.02	326AS CB	326ASP	C	100.00
323PRO CA	323PRO	CB	100.00	256LEL CD1	288THR	CB	0.04	326AS CG	326ASP	C	94.80
323PRO CA	323PRO	CG	100.00	256LEL CD1	288THR	CG2	0.09	326AS C	327LEU	CA	100.00
323PRO CA	323PRO	CD	100.00	256LEL CD1	292LEU	CD1	1.65	326AS C	327LEU	CB	7.99
323PRO CA	323PRO	C	100.00	256LEL CD1	315VAL	CG1	0.01	326AS C	327LEU	CG	0.63
323PRO CA	324PRO	CD	100.00	256LEL CD1	319LEU	CD1	3.37	326AS C	327LEU	CD1	0.12
323PRO CB	323PRO	CG	100.00	256LEL CD1	319LEU	CD2	0.19	326AS C	327LEU	CD2	0.00
323PRO CB	323PRO	CD	100.00	256LEL CD1	324PRO	CB	0.01	326AS C	327LEU	C	90.75
323PRO CB	323PRO	C	100.00	256LEL CD1	324PRO	CG	0.00	327LE CA	327LEU	CB	100.00
323PRO CB	324PRO	CD	47.10	256LEL CD1	326ASP	CG	0.01	327LE CA	327LEU	CG	100.00
323PRO CB	327LEU	CD1	0.01	256LEL CD1	327LEU	CG	0.00	327LE CA	327LEU	CD1	63.49
323PRO CB	327LEU	CD2	0.12	256LEL CD1	327LEU	CD1	0.21	327LE CA	327LEU	CD2	32.39
323PRO CG	323PRO	CD	100.00	256LEL CD1	327LEU	CD2	0.07	327LE CA	327LEU	C	100.00
323PRO CG	323PRO	C	95.57	256LEL CD2	256LEU	C	0.08	327LE CB	327LEU	CG	100.00
323PRO CG	327LEU	CD2	0.00	256LEL CD2	286ASN	CB	0.04	327LE CB	327LEU	CD1	100.00
323PRO CG	329GLY	CA	0.01	256LEL CD2	286ASN	CG	0.18	327LE CB	327LEU	CD2	100.00
323PRO CD	323PRO	C	90.14	256LEL CD2	288THR	CB	0.14	327LE CB	327LEU	C	100.00
323PRO C	324PRO	CA	100.00	256LEL CD2	288THR	CG2	0.25	327LE CG	327LEU	CD1	100.00
323PRO C	324PRO	CB	0.09	256LEL CD2	292LEU	CD1	0.00	327LE CG	327LEU	CD2	100.00
323PRO C	324PRO	CD	100.00	256LEL CD2	319LEU	CD1	0.60	327LE CG	327LEU	C	44.74
323PRO C	324PRO	C	100.00	256LEL CD2	319LEU	CD2	0.74	327LE CD1	327LEU	CD2	100.00
323PRO C	336PHE	CZ	0.02	256LEL CD2	324PRO	CB	0.63	327LE CD1	327LEU	C	3.59
324PRO CA	324PRO	CB	100.00	256LEL CD2	324PRO	CG	0.64	327LE CD2	327LEU	C	2.00
324PRO CA	324PRO	CG	100.00	256LEL CD2	325PRO	CG	0.04	327LE C	328GLY	CA	100.00
324PRO CA	324PRO	CD	100.00	256LEL CD2	325PRO	CD	0.60	327LE C	328GLY	C	45.48
324PRO CA	324PRO	C	100.00	256LEL CD2	326ASP	CG	0.01	328GL CA	328GLY	C	100.00
324PRO CA	325PRO	CD	100.00	256LEL CD2	327LEU	CD1	0.06	328GL C	329GLY	CA	100.00
324PRO CA	336PHE	CE2	0.00	256LEL CD2	327LEU	CD2	0.01	328GL C	329GLY	C	31.21
324PRO CA	336PHE	CZ	0.06	256LEL C	257LEU	CA	100.00	329GL CA	329GLY	C	100.00
324PRO CB	324PRO	CG	100.00	256LEL C	257LEU	CB	22.34	329GL C	330SER	CA	100.00
324PRO CB	324PRO	CD	100.00	256LEL C	257LEU	CG	1.96	329GL C	330SER	CB	99.35
324PRO CB	324PRO	C	100.00	256LEL C	257LEU	CD1	0.03	329GL C	330SER	C	0.97
324PRO CB	325PRO	CD	24.11	256LEL C	257LEU	CD2	0.05	330SE CA	330SER	CB	100.00
324PRO CB	327LEU	CD1	0.01	256LEL C	257LEU	C	78.46	330SE CA	330SER	C	100.00
324PRO CB	336PHE	CZ	0.00	256LEL C	289ALA	CA	0.04	330SE CB	330SER	C	100.00
324PRO CG	324PRO	CD	100.00	256LEL C	289ALA	CB	1.19	330SE C	331ALA	CA	100.00
324PRO CG	324PRO	C	93.14	256LEL C	292LEU	CD1	0.08	330SE C	331ALA	CB	25.96
324PRO CG	327LEU	CB	0.00	257LEL CA	257LEU	CB	100.00	330SE C	331ALA	C	74.15
324PRO CG	327LEU	CD1	0.07	257LEL CA	257LEU	CG	100.00	331AL CA	331ALA	CB	100.00
324PRO CD	324PRO	C	88.40	257LEL CA	257LEU	CD1	19.86	331AL CA	331ALA	C	100.00
324PRO CD	327LEU	CD1	0.02	257LEL CA	257LEU	CD2	79.38	331AL CA	335ALA	CB	0.01
324PRO CD	336PHE	CE2	0.00	257LEL CA	257LEU	C	100.00	331AL CB	331ALA	C	100.00
324PRO C	325PRO	CA	100.00	257LEL CA	258PRO	CD	100.00	331AL CB	335ALA	CB	0.01
324PRO C	325PRO	CB	0.12	257LEL CB	257LEU	CG	100.00	331AL CB	336PHE	CD1	0.03
324PRO C	325PRO	CD	100.00	257LEL CB	257LEU	CD1	100.00	331AL CB	336PHE	CE1	0.09
324PRO C	325PRO	C	100.00	257LEL CB	257LEU	CD2	100.00	331AL CB	336PHE	CZ	0.03

325PRO	CA	325PRO CB	100.00	257LE	CB	257LEU C	100.00	331AL	C	332GLY CA	100.00
325PRO	CA	325PRO CG	100.00	257LE	CB	258PRO CD	7.01	331AL	C	332GLY C	55.13
325PRO	CA	325PRO CD	100.00	257LE	CB	272VAL CB	0.01	332GL	CA	332GLY C	100.00
325PRO	CA	325PRO C	100.00	257LE	CB	272VAL CG1	0.00	332GL	CA	335ALA CB	0.01
325PRO	CA	329GLY CA	0.00	257LE	CB	272VAL CG2	0.02	332GL	C	333THR CA	100.00
325PRO	CA	329GLY C	0.00	257LE	CG	257LEU CD1	100.00	332GL	C	333THR CB	0.41
325PRO	CB	325PRO CG	100.00	257LE	CG	257LEU CD2	100.00	332GL	C	333THR CG2	0.23
325PRO	CB	325PRO CD	100.00	257LE	CG	257LEU C	16.30	332GL	C	333THR C	99.67
325PRO	CB	325PRO C	100.00	257LE	CG	258PRO CD	0.00	333TH	CA	333THR CB	100.00
325PRO	CB	328GLY C	0.03	257LE	CD1	257LEU CD2	100.00	333TH	CA	333THR CG2	100.00
325PRO	CB	329GLY CA	0.10	257LE	CD1	257LEU C	0.18	333TH	CA	333THR C	100.00
325PRO	CB	329GLY C	0.06	257LE	CD1	258PRO CD	0.01	333TH	CB	333THR CG2	100.00
325PRO	CB	330SER CA	0.00	257LE	CD1	272VAL CG1	0.01	333TH	CB	333THR C	100.00
325PRO	CB	331ALA CB	0.02	257LE	CD2	257LEU C	0.18	333TH	CG2	333THR C	79.00
325PRO	CB	331ALA C	0.00	257LE	CD2	258PRO CD	0.01	333TH	CG2	337THR CG2	0.00
325PRO	CG	325PRO CD	100.00	257LE	CD2	272VAL CB	0.00	333TH	C	334GLU CA	100.00
325PRO	CG	325PRO C	89.42	257LE	CD2	272VAL CG1	0.06	333TH	C	334GLU CB	0.09
325PRO	CG	331ALA CB	0.00	257LE	CD2	272VAL CG2	0.02	333TH	C	334GLU C	99.93
325PRO	CG	331ALA C	0.00	257LE	C	258PRO CA	100.00	334GL	CA	334GLU CB	100.00
325PRO	CG	332GLY C	0.00	257LE	C	258PRO CB	1.43	334GL	CA	334GLU CG	100.00
325PRO	CG	336PHE CD2	0.01	257LE	C	258PRO CD	100.00	334GL	CA	334GLU CD	99.56
325PRO	CG	336PHE CE2	0.05	257LE	C	258PRO C	100.00	334GL	CA	334GLU C	100.00
325PRO	CG	336PHE CZ	0.03	257LE	C	272VAL CG1	0.00	334GL	CB	334GLU CG	100.00
325PRO	CD	325PRO C	81.88	257LE	C	272VAL CG2	0.00	334GL	CB	334GLU CD	100.00
325PRO	CD	331ALA CB	0.05	257LE	C	289ALA CB	0.16	334GL	CB	334GLU C	100.00
325PRO	CD	336PHE CG	0.00	258PR	CA	258PRO CB	100.00	334GL	CG	334GLU CD	100.00
325PRO	CD	336PHE CD1	0.00	258PR	CA	258PRO CG	100.00	334GL	CG	334GLU C	95.58
325PRO	CD	336PHE CD2	0.32	258PR	CA	258PRO CD	100.00	334GL	CD	334GLU C	0.41
325PRO	CD	336PHE CE1	0.03	258PR	CA	258PRO C	100.00	334GL	C	335ALA CA	100.00
325PRO	CD	336PHE CE2	0.50	258PR	CB	258PRO CG	100.00	334GL	C	335ALA CB	0.10
325PRO	CD	336PHE CZ	0.27	258PR	CB	258PRO CD	100.00	334GL	C	335ALA C	99.89
325PRO	C	326ASP CA	100.00	258PR	CB	258PRO C	100.00	335AL	CA	335ALA CB	100.00
325PRO	C	326ASP CB	76.81	258PR	CB	292LEU CB	0.02	335AL	CA	335ALA C	100.00
325PRO	C	326ASP CG	0.00	258PR	CB	292LEU C	2.11	335AL	CB	335ALA C	100.00
325PRO	C	326ASP C	24.36	258PR	CB	293SER CA	1.38	335AL	C	336PHE CA	100.00
326ASP	CA	326ASP CB	100.00	258PR	CB	293SER CB	0.07	335AL	C	336PHE CB	0.05
326ASP	CA	326ASP CG	100.00	258PR	CB	296MET CE	0.06	335AL	C	336PHE CG	0.00
326ASP	CA	326ASP C	100.00	258PR	CG	258PRO CD	100.00	335AL	C	336PHE CD1	0.02
326ASP	CB	326ASP CG	100.00	258PR	CG	258PRO C	99.97	335AL	C	336PHE C	99.92
326ASP	CB	326ASP C	100.00	258PR	CG	292LEU CB	0.17	336PH	CA	336PHE CB	100.00
326ASP	CB	327LEU CD2	0.00	258PR	CG	292LEU C	0.00	336PH	CA	336PHE CG	100.00
326ASP	CG	326ASP C	99.70	258PR	CG	296MET CE	0.22	336PH	CA	336PHE CD1	87.18
326ASP	CG	327LEU CD1	0.04	258PR	CD	258PRO C	99.65	336PH	CA	336PHE CD2	85.22
326ASP	CG	327LEU CD2	0.05	258PR	CD	292LEU CB	0.05	336PH	CA	336PHE C	100.00
326ASP	C	327LEU CA	100.00	258PR	CD	292LEU CD1	0.02	336PH	CB	336PHE CG	100.00
326ASP	C	327LEU CB	31.49	258PR	CD	292LEU CD2	0.02	336PH	CB	336PHE CD1	100.00
326ASP	C	327LEU CG	1.80	258PR	C	259SER CA	100.00	336PH	CB	336PHE CD2	100.00
326ASP	C	327LEU CD1	0.05	258PR	C	259SER CB	99.91	336PH	CB	336PHE C	100.00
326ASP	C	327LEU CD2	0.02	258PR	C	259SER C	0.73	336PH	CG	336PHE CD1	100.00
326ASP	C	327LEU C	69.29	258PR	C	272VAL CG1	0.12	336PH	CG	336PHE CD2	100.00
327LEU	CA	327LEU CB	100.00	258PR	C	272VAL CG2	0.10	336PH	CG	336PHE CE1	100.00
327LEU	CA	327LEU CG	100.00	259SE	CA	259SER CB	100.00	336PH	CG	336PHE CE2	100.00
327LEU	CA	327LEU CD1	41.02	259SE	CA	259SER C	100.00	336PH	CG	336PHE CZ	100.00
327LEU	CA	327LEU CD2	57.19	259SE	CB	259SER C	100.00	336PH	CG	336PHE C	98.67
327LEU	CA	327LEU C	100.00	259SE	CB	272VAL CG1	1.03	336PH	CD1	336PHE CD2	100.00
327LEU	CB	327LEU CG	100.00	259SE	CB	272VAL CG2	1.52	336PH	CD1	336PHE CE1	100.00
327LEU	CB	327LEU CD1	100.00	259SE	C	260ALA CA	100.00	336PH	CD1	336PHE CE2	100.00
327LEU	CB	327LEU CD2	100.00	259SE	C	260ALA CB	75.74	336PH	CD1	336PHE CZ	100.00
327LEU	CB	327LEU C	100.00	259SE	C	260ALA C	28.19	336PH	CD1	336PHE C	0.22
327LEU	CG	327LEU CD1	100.00	259SE	C	270GLU CG	0.01	336PH	CD1	340VAL CG2	0.00
327LEU	CG	327LEU CD2	100.00	259SE	C	296MET CE	0.07	336PH	CD2	336PHE CE1	100.00
327LEU	CG	327LEU C	40.16	260AL	CA	260ALA CB	100.00	336PH	CD2	336PHE CE2	100.00

327LEU	CD1	327LEU	CD2	100.00	260AL	CA	260ALA	C	100.00	336PH	CD2	336PHE	CZ	100.00
327LEU	CD1	327LEU	C	0.68	260AL	CA	269PHE	CD2	0.04	336PH	CD2	336PHE	C	82.96
327LEU	CD1	225ARG	CZ	0.01	260AL	CB	260ALA	C	100.00	336PH	CD2	340VAL	CG2	1.34
327LEU	CD2	327LEU	C	2.28	260AL	CB	262LEU	CG	0.01	336PH	CE1	336PHE	CE2	100.00
327LEU	C	328GLY	CA	100.00	260AL	CB	262LEU	CD1	0.30	336PH	CE1	336PHE	CZ	100.00
327LEU	C	328GLY	C	37.05	260AL	CB	262LEU	CD2	0.14	336PH	CE1	340VAL	CG2	0.01
328GLY	CA	328GLY	C	100.00	260AL	CB	269PHE	CA	0.00	336PH	CE2	336PHE	CZ	100.00
328GLY	C	329GLY	CA	100.00	260AL	CB	269PHE	CG	0.04	336PH	CE2	340VAL	CG2	9.78
328GLY	C	329GLY	C	37.83	260AL	CB	269PHE	CD2	20.44	336PH	CZ	340VAL	CG2	0.23
329GLY	CA	329GLY	C	100.00	260AL	CB	269PHE	CE1	0.00	336PH	C	337THR	CA	100.00
329GLY	C	330SER	CA	100.00	260AL	CB	269PHE	CE2	23.85	336PH	C	337THR	CB	1.24
329GLY	C	330SER	CB	94.54	260AL	CB	269PHE	CZ	0.17	336PH	C	337THR	CG2	0.90
329GLY	C	330SER	C	5.71	260AL	CB	296MET	CE	10.72	336PH	C	337THR	C	98.88
330SER	CA	330SER	CB	100.00	260AL	CB	300HIS	CD2	0.01	337TH	CA	337THR	CB	100.00
330SER	CA	330SER	C	100.00	260AL	CB	300HIS	CE1	0.02	337TH	CA	337THR	CG2	100.00
330SER	CB	330SER	C	100.00	260AL	C	261SER	CA	100.00	337TH	CA	337THR	C	100.00
330SER	C	331ALA	CA	100.00	260AL	C	261SER	CB	48.58	337TH	CA	340VAL	CG2	0.01
330SER	C	331ALA	CB	24.60	260AL	C	261SER	C	60.88	337TH	CB	337THR	CG2	100.00
330SER	C	331ALA	C	77.33	260AL	C	262LEU	CD1	0.02	337TH	CB	337THR	C	100.00
330SER	C	335ALA	CB	0.02	260AL	C	262LEU	CD2	0.01	337TH	CB	341LEU	CD1	0.00
331ALA	CA	331ALA	CB	100.00	261SE	CA	261SER	CB	100.00	337TH	CG2	337THR	C	25.53
331ALA	CA	331ALA	C	100.00	261SE	CA	261SER	C	100.00	337TH	CG2	341LEU	CD1	0.06
331ALA	CA	335ALA	CB	0.93	261SE	CB	261SER	C	100.00	337TH	C	338ALA	CA	100.00
331ALA	CA	336PHE	CD2	0.00	261SE	CB	268VAL	CG1	0.03	337TH	C	338ALA	CB	0.83
331ALA	CB	331ALA	C	100.00	261SE	CB	268VAL	CG2	0.20	337TH	C	338ALA	C	99.33
331ALA	CB	335ALA	CB	3.70	261SE	CB	270GLU	CD	0.00	337TH	C	341LEU	CG	0.00
331ALA	CB	335ALA	C	0.03	261SE	C	262LEU	CA	100.00	337TH	C	341LEU	CD1	0.10
331ALA	CB	336PHE	CG	0.00	261SE	C	262LEU	CB	9.34	338AL	CA	338ALA	CB	100.00
331ALA	CB	336PHE	CD2	1.09	261SE	C	262LEU	CG	1.22	338AL	CA	338ALA	C	100.00
331ALA	CB	336PHE	CE1	0.12	261SE	C	262LEU	CD1	0.78	338AL	CB	338ALA	C	100.00
331ALA	CB	336PHE	CE2	3.08	261SE	C	262LEU	CD2	0.20	338AL	CB	342ARG	CZ	0.38
331ALA	CB	336PHE	CZ	0.69	261SE	C	262LEU	C	89.60	338AL	C	339THR	CA	100.00
331ALA	C	332GLY	CA	100.00	262LE	CA	262LEU	CB	100.00	338AL	C	339THR	CB	0.56
331ALA	C	332GLY	C	37.75	262LE	CA	262LEU	CG	100.00	338AL	C	339THR	CG2	0.13
331ALA	C	335ALA	CB	0.03	262LE	CA	262LEU	CD1	18.09	338AL	C	339THR	C	99.58
331ALA	C	336PHE	CB	0.04	262LE	CA	262LEU	CD2	80.84	338AL	C	342ARG	CD	0.00
331ALA	C	336PHE	CD2	0.02	262LE	CA	262LEU	C	100.00	338AL	C	342ARG	CZ	0.04
331ALA	C	336PHE	CE2	0.08	262LE	CB	262LEU	CG	100.00	339TH	CA	339THR	CB	100.00
331ALA	C	336PHE	CZ	0.01	262LE	CB	262LEU	CD1	100.00	339TH	CA	339THR	CG2	100.00
332GLY	CA	332GLY	C	100.00	262LE	CB	262LEU	CD2	100.00	339TH	CA	339THR	C	100.00
332GLY	CA	336PHE	CE2	0.01	262LE	CB	262LEU	C	100.00	339TH	CA	342ARG	CB	0.00
332GLY	C	333THR	CA	100.00	262LE	CG	262LEU	CD1	100.00	339TH	CA	342ARG	CZ	0.06
332GLY	C	333THR	CB	0.01	262LE	CG	262LEU	CD2	100.00	339TH	CB	339THR	CG2	100.00
332GLY	C	333THR	C	100.00	262LE	CG	262LEU	C	5.22	339TH	CB	339THR	C	100.00
332GLY	C	336PHE	CB	0.01	262LE	CG	267PRO	CB	0.00	339TH	CG2	339THR	C	90.54
332GLY	C	336PHE	CD2	0.60	262LE	CG	300HIS	CE1	0.03	339TH	CG2	342ARG	CZ	0.22
332GLY	C	336PHE	CE2	0.04	262LE	CD1	262LEU	CD2	100.00	339TH	CG2	343HIS	CD2	0.42
333THR	CA	333THR	CB	100.00	262LE	CD1	262LEU	C	1.40	339TH	CG2	343HIS	CE1	0.40
333THR	CA	333THR	CG2	100.00	262LE	CD1	267PRO	CB	0.04	339TH	C	340VAL	CA	100.00
333THR	CA	333THR	C	100.00	262LE	CD1	300HIS	CD2	0.03	339TH	C	340VAL	CB	0.06
333THR	CB	333THR	CG2	100.00	262LE	CD1	300HIS	CE1	0.58	339TH	C	340VAL	CG2	0.02
333THR	CB	333THR	C	100.00	262LE	CD2	262LEU	C	0.40	339TH	C	340VAL	C	99.96
333THR	CG2	333THR	C	1.10	262LE	CD2	267PRO	CB	1.88	339TH	C	343HIS	CD2	0.02
333THR	C	334GLU	CA	100.00	262LE	CD2	300HIS	CD2	0.35	340VA	CA	340VAL	CB	100.00
333THR	C	334GLU	CB	11.76	262LE	CD2	300HIS	CE1	4.60	340VA	CA	340VAL	CG1	100.00
333THR	C	334GLU	CD	0.01	262LE	C	263GLY	CA	100.00	340VA	CA	340VAL	CG2	100.00
333THR	C	334GLU	C	90.96	262LE	C	263GLY	C	8.98	340VA	CA	340VAL	C	100.00
334GLU	CA	334GLU	CB	100.00	263GL	CA	263GLY	C	100.00	340VA	CB	340VAL	CG1	100.00
334GLU	CA	334GLU	CG	100.00	263GL	C	264ARG	CA	100.00	340VA	CB	340VAL	CG2	100.00
334GLU	CA	334GLU	CD	99.04	263GL	C	264ARG	CB	2.67	340VA	CB	340VAL	C	100.00
334GLU	CA	334GLU	C	100.00	263GL	C	264ARG	CG	0.81	340VA	CG1	340VAL	CG2	100.00
334GLU	CB	334GLU	CG	100.00	263GL	C	264ARG	CD	0.02	340VA	CG1	340VAL	C	100.00

334GLU CB	334GLU CD	100.00	263GL\ C	264ARG C	97.38	340VA CG1	344LEU CD1	0.84
334GLU CB	334GLU C	100.00	264AR(CA	264ARG CB	100.00	340VA CG1	344LEU CD2	0.06
334GLU CG	334GLU CD	100.00	264AR(CA	264ARG CG	100.00	340VA CG2	340VAL C	0.00
334GLU CG	334GLU C	97.45	264AR(CA	264ARG CD	2.29	340VA C	341LEU CA	100.00
334GLU CD	334GLU C	0.84	264AR(CA	264ARG C	100.00	340VA C	341LEU CB	0.01
334GLU C	335ALA CA	100.00	264AR(CB	264ARG CG	100.00	340VA C	341LEU C	100.00
334GLU C	335ALA CB	2.51	264AR(CB	264ARG CD	100.00	341LEI CA	341LEU CB	100.00
334GLU C	335ALA C	97.36	264AR(CB	264ARG CZ	1.03	341LEI CA	341LEU CG	100.00
335ALA CA	335ALA CB	100.00	264AR(CB	264ARG C	100.00	341LEI CA	341LEU CD1	3.75
335ALA CA	335ALA C	100.00	264AR(CG	264ARG CD	100.00	341LEI CA	341LEU CD2	96.28
335ALA CB	335ALA C	100.00	264AR(CG	264ARG CZ	38.21	341LEI CA	341LEU C	100.00
335ALA C	336PHE CA	100.00	264AR(CG	264ARG C	2.74	341LEI CB	341LEU CG	100.00
335ALA C	336PHE CB	3.42	264AR(CD	264ARG CZ	100.00	341LEI CB	341LEU CD1	100.00
335ALA C	336PHE CG	0.01	264AR(C	265GLY CA	100.00	341LEI CB	341LEU CD2	100.00
335ALA C	336PHE CD1	0.00	264AR(C	265GLY C	59.15	341LEI CB	341LEU C	100.00
335ALA C	336PHE CD2	0.00	265GL\ CA	265GLY C	100.00	341LEI CG	341LEU CD1	100.00
335ALA C	336PHE C	96.72	265GL\ C	266THR CA	100.00	341LEI CG	341LEU CD2	100.00
336PHE CA	336PHE CB	100.00	265GL\ C	266THR CB	15.83	341LEI CG	341LEU C	0.15
336PHE CA	336PHE CG	100.00	265GL\ C	266THR CG2	4.46	341LEI CD1	341LEU CD2	100.00
336PHE CA	336PHE CD1	82.50	265GL\ C	266THR C	85.04	341LEI CD1	341LEU C	0.00
336PHE CA	336PHE CD2	74.29	266THF CA	266THR CB	100.00	341LEI CD1	344LEU CD1	0.01
336PHE CA	336PHE C	100.00	266THF CA	266THR CG2	100.00	341LEI CD2	344LEU CD1	0.05
336PHE CA	339THR CG2	0.01	266THF CA	266THR C	100.00	341LEI CD2	344LEU CD2	0.09
336PHE CB	336PHE CG	100.00	266THF CA	267PRO CD	100.00	341LEI C	342ARG CA	100.00
336PHE CB	336PHE CD1	100.00	266THF CB	266THR CG2	100.00	341LEI C	342ARG CB	1.95
336PHE CB	336PHE CD2	100.00	266THF CB	266THR C	100.00	341LEI C	342ARG CG	0.29
336PHE CB	336PHE C	100.00	266THF CB	267PRO CD	13.73	341LEI C	342ARG C	97.95
336PHE CG	336PHE CD1	100.00	266THF CG2	266THR C	55.91	342AR CA	342ARG CB	100.00
336PHE CG	336PHE CD2	100.00	266THF CG2	267PRO CD	4.03	342AR CA	342ARG CG	100.00
336PHE CG	336PHE CE1	100.00	266THF C	267PRO CA	100.00	342AR CA	342ARG CD	3.62
336PHE CG	336PHE CE2	100.00	266THF C	267PRO CB	0.15	342AR CA	342ARG CZ	0.00
336PHE CG	336PHE CZ	100.00	266THF C	267PRO CD	100.00	342AR CA	342ARG C	100.00
336PHE CG	336PHE C	66.21	266THF C	267PRO C	100.00	342AR CB	342ARG CG	100.00
336PHE CD1	336PHE CD2	100.00	267PR(CA	267PRO CB	100.00	342AR CB	342ARG CD	100.00
336PHE CD1	336PHE CE1	100.00	267PR(CA	267PRO CG	100.00	342AR CB	342ARG CZ	0.99
336PHE CD1	336PHE CE2	100.00	267PR(CA	267PRO CD	100.00	342AR CB	342ARG C	100.00
336PHE CD1	336PHE CZ	100.00	267PR(CA	267PRO C	100.00	342AR CG	342ARG CD	100.00
336PHE CD1	336PHE C	39.93	267PR(CB	267PRO CG	100.00	342AR CG	342ARG CZ	17.83
336PHE CD1	340VAL CG2	2.87	267PR(CB	267PRO CD	100.00	342AR CG	342ARG C	74.99
336PHE CD2	336PHE CE1	100.00	267PR(CB	267PRO C	100.00	342AR CG	343HIS CD2	0.00
336PHE CD2	336PHE CE2	100.00	267PR(CB	269PHE CE1	1.48	342AR CD	342ARG CZ	100.00
336PHE CD2	336PHE CZ	100.00	267PR(CB	269PHE CZ	0.21	342AR CD	342ARG C	3.31
336PHE CD2	336PHE C	0.06	267PR(CB	300HIS CD2	0.02	342AR CZ	343HIS CD2	0.01
336PHE CD2	339THR CG2	0.35	267PR(CB	300HIS CE1	0.07	342AR CZ	343HIS CE1	0.29
336PHE CE1	336PHE CE2	100.00	267PR(CG	267PRO CD	100.00	342AR CZ	345ALA CB	0.03
336PHE CE1	336PHE CZ	100.00	267PR(CG	267PRO C	98.30	342AR C	343HIS CA	100.00
336PHE CE1	340VAL CG2	11.28	267PR(CG	269PHE CE1	1.02	342AR C	343HIS CB	2.61
336PHE CE2	336PHE CZ	100.00	267PR(CG	269PHE CZ	0.15	342AR C	343HIS CG	0.16
336PHE CE2	339THR CG2	0.10	267PR(CG	300HIS CD2	0.07	342AR C	343HIS CD2	0.04
336PHE CZ	340VAL CG2	0.07	267PR(CG	300HIS CE1	0.08	342AR C	343HIS C	96.92
336PHE C	337THR CA	100.00	267PR(CG	301ALA CB	0.12	342AR C	345ALA CB	0.00
336PHE C	337THR CB	11.92	267PR(CD	267PRO C	94.28	343HI$ CA	343HIS CB	100.00
336PHE C	337THR CG2	3.98	267PR(C	268VAL CA	100.00	343HI$ CA	343HIS CG	100.00
336PHE C	337THR C	90.04	267PR(C	268VAL CB	37.80	343HI$ CA	343HIS CD2	7.12
336PHE C	339THR CG2	0.02	267PR(C	268VAL CG1	2.87	343HI$ CA	343HIS C	100.00
336PHE C	340VAL CG2	0.34	267PR(C	268VAL CG2	20.63	343HI$ CB	343HIS CG	100.00
337THR CA	337THR CB	100.00	267PR(C	268VAL C	76.66	343HI$ CB	343HIS CD2	100.00
337THR CA	337THR CG2	100.00	267PR(C	269PHE CE1	0.03	343HI$ CB	343HIS CE1	0.15
337THR CA	337THR C	100.00	268VAI CA	268VAL CB	100.00	343HI$ CB	343HIS C	100.00
337THR CA	340VAL CG2	0.01	268VAI CA	268VAL CG1	100.00	343HI$ CG	343HIS CD2	100.00
337THR CB	337THR CG2	100.00	268VAI CA	268VAL CG2	100.00	343HI$ CG	343HIS CE1	100.00
337THR CB	337THR C	100.00	268VAI CA	268VAL C	100.00	343HI$ CG	343HIS C	34.90

337THR	CG2	337THR	C	55.87	268VAI	CB	268VAL	CG1	100.00	343HI	CD2	343HIS	CE1	100.00
337THR	CG2	341LEU	CD1	0.07	268VAI	CB	268VAL	CG2	100.00	343HI	CD2	343HIS	C	1.92
337THR	C	338ALA	CA	100.00	268VAI	CB	268VAL	C	100.00	343HI	C	344LEU	CA	100.00
337THR	C	338ALA	CB	0.00	268VAI	CG1	268VAL	CG2	100.00	343HI	C	344LEU	CB	15.43
337THR	C	338ALA	C	100.00	268VAI	CG1	268VAL	C	88.29	343HI	C	344LEU	CG	0.75
337THR	C	341LEU	CD1	0.11	268VAI	CG1	270GLU	CB	0.01	343HI	C	344LEU	CD2	0.02
338ALA	CA	338ALA	CB	100.00	268VAI	CG1	270GLU	CG	0.18	343HI	C	344LEU	C	83.44
338ALA	CA	338ALA	C	100.00	268VAI	CG2	268VAL	C	13.88	344LE	CA	344LEU	CB	100.00
338ALA	CB	338ALA	C	100.00	268VAI	C	269PHE	CA	100.00	344LE	CA	344LEU	CG	100.00
338ALA	CB	342ARG	CZ	0.05	268VAI	C	269PHE	CB	23.28	344LE	CA	344LEU	CD1	30.83
338ALA	C	339THR	CA	100.00	268VAI	C	269PHE	CG	5.24	344LE	CA	344LEU	CD2	66.40
338ALA	C	339THR	CB	1.44	268VAI	C	269PHE	CD1	11.14	344LE	CA	344LEU	C	100.00
338ALA	C	339THR	CG2	0.04	268VAI	C	269PHE	CD2	0.00	344LE	CB	344LEU	CG	100.00
338ALA	C	339THR	C	98.92	268VAI	C	269PHE	C	84.70	344LE	CB	344LEU	CD1	100.00
338ALA	C	342ARG	CZ	0.01	269PHI	CA	269PHE	CB	100.00	344LE	CB	344LEU	CD2	100.00
339THR	CA	339THR	CB	100.00	269PHI	CA	269PHE	CG	100.00	344LE	CB	344LEU	C	100.00
339THR	CA	339THR	CG2	100.00	269PHI	CA	269PHE	CD1	93.91	344LE	CG	344LEU	CD1	100.00
339THR	CA	339THR	C	100.00	269PHI	CA	269PHE	CD2	87.68	344LE	CG	344LEU	CD2	100.00
339THR	CA	342ARG	CZ	0.18	269PHI	CA	269PHE	C	100.00	344LE	CG	344LEU	C	30.58
339THR	CB	339THR	CG2	100.00	269PHI	CB	269PHE	CG	100.00	344LE	CD1	344LEU	CD2	100.00
339THR	CB	339THR	C	100.00	269PHI	CB	269PHE	CD1	100.00	344LE	CD1	344LEU	C	10.32
339THR	CB	342ARG	CZ	0.00	269PHI	CB	269PHE	CD2	100.00	344LE	CD1	345ALA	CB	0.01
339THR	CG2	339THR	C	79.48	269PHI	CB	269PHE	C	100.00	344LE	CD2	344LEU	C	0.42
339THR	CG2	342ARG	CZ	0.08	269PHI	CB	297MET	CE	0.33	344LE	C	345ALA	CA	100.00
339THR	CG2	343HIS	CD2	0.27	269PHI	CG	269PHE	CD1	100.00	344LE	C	345ALA	CB	48.30
339THR	CG2	343HIS	CE1	0.12	269PHI	CG	269PHE	CD2	100.00	344LE	C	345ALA	C	53.11
339THR	C	340VAL	CA	100.00	269PHI	CG	269PHE	CE1	100.00	345AL	CA	345ALA	CB	100.00
339THR	C	340VAL	CB	0.11	269PHI	CG	269PHE	CE2	100.00	345AL	CA	345ALA	C	100.00
339THR	C	340VAL	CG2	0.05	269PHI	CG	269PHE	CZ	100.00	345AL	CB	345ALA	C	100.00
339THR	C	340VAL	C	99.94	269PHI	CG	297MET	CB	0.09	1MET	CA	1MET	CB	100.00
339THR	C	343HIS	CD2	0.36	269PHI	CG	297MET	CG	0.06	1MET	CA	1MET	CG	100.00
340VAL	CA	340VAL	CB	100.00	269PHI	CG	297MET	CE	0.53	1MET	CA	1MET	C	100.00
340VAL	CA	340VAL	CG1	100.00	269PHI	CD1	269PHE	CD2	100.00	1MET	CA	302PHE	CE1	0.00
340VAL	CA	340VAL	CG2	100.00	269PHI	CD1	269PHE	CE1	100.00	1MET	CB	1MET	CG	100.00
340VAL	CA	340VAL	C	100.00	269PHI	CD1	269PHE	CE2	100.00	1MET	CB	1MET	CE	34.57
340VAL	CB	340VAL	CG1	100.00	269PHI	CD1	269PHE	CZ	100.00	1MET	CB	1MET	C	100.00
340VAL	CB	340VAL	CG2	100.00	269PHI	CD1	297MET	CA	0.00	1MET	CB	32LEU	CD2	0.05
340VAL	CB	340VAL	C	100.00	269PHI	CD1	297MET	CB	1.28	1MET	CB	65GLU	CG	0.00
340VAL	CG1	340VAL	CG2	100.00	269PHI	CD1	297MET	CG	2.08	1MET	CB	65GLU	CD	0.00
340VAL	CG1	340VAL	C	100.00	269PHI	CD1	297MET	CE	4.81	1MET	CB	302PHE	CD1	0.01
340VAL	CG1	344LEU	CD1	0.69	269PHI	CD2	269PHE	CE1	100.00	1MET	CB	302PHE	CD2	0.08
340VAL	C	341LEU	CA	100.00	269PHI	CD2	269PHE	CE2	100.00	1MET	CB	302PHE	CE1	1.47
340VAL	C	341LEU	CB	0.01	269PHI	CD2	269PHE	CZ	100.00	1MET	CB	302PHE	CE2	7.98
340VAL	C	341LEU	C	99.99	269PHI	CD2	296MET	CB	0.13	1MET	CB	302PHE	CZ	2.07
341LEU	CA	341LEU	CB	100.00	269PHI	CD2	296MET	CG	0.03	1MET	CG	1MET	CE	100.00
341LEU	CA	341LEU	CG	100.00	269PHI	CD2	296MET	C	0.04	1MET	CG	1MET	C	88.32
341LEU	CA	341LEU	CD1	4.86	269PHI	CD2	297MET	CA	0.11	1MET	CG	33GLY	C	0.01
341LEU	CA	341LEU	CD2	94.96	269PHI	CD2	297MET	CB	0.07	1MET	CG	65GLU	CB	7.47
341LEU	CA	341LEU	C	100.00	269PHI	CD2	297MET	CG	0.02	1MET	CG	65GLU	CG	0.42
341LEU	CB	341LEU	CG	100.00	269PHI	CE1	269PHE	CE2	100.00	1MET	CG	65GLU	CD	0.03
341LEU	CB	341LEU	CD1	100.00	269PHI	CE1	269PHE	CZ	100.00	1MET	CG	302PHE	CD1	0.06
341LEU	CB	341LEU	CD2	100.00	269PHI	CE1	297MET	CA	0.31	1MET	CG	302PHE	CD2	0.10
341LEU	CB	341LEU	C	100.00	269PHI	CE1	297MET	CB	0.37	1MET	CG	302PHE	CE1	0.40
341LEU	CG	341LEU	CD1	100.00	269PHI	CE1	297MET	CG	1.74	1MET	CG	302PHE	CE2	1.80
341LEU	CG	341LEU	CD2	100.00	269PHI	CE1	297MET	CE	0.02	1MET	CG	302PHE	CZ	0.90
341LEU	CG	341LEU	C	0.45	269PHI	CE1	300HIS	CD2	0.00	1MET	CE	1MET	C	0.02
341LEU	CD1	341LEU	CD2	100.00	269PHI	CE1	301ALA	CB	0.49	1MET	CE	2LYS	C	0.01
341LEU	CD1	341LEU	C	0.01	269PHI	CE2	269PHE	CZ	100.00	1MET	CE	3VAL	CG2	3.77
341LEU	CD1	344LEU	CD1	0.00	269PHI	CE2	296MET	CB	0.15	1MET	CE	32LEU	CD1	0.00
341LEU	CD2	341LEU	C	0.10	269PHI	CE2	296MET	CG	0.08	1MET	CE	32LEU	C	0.01
341LEU	CD2	344LEU	CD1	0.09	269PHI	CE2	296MET	C	7.58	1MET	CE	34LEU	CD1	0.01
341LEU	CD2	344LEU	CD2	0.17	269PHI	CE2	297MET	CA	9.54	1MET	CE	34LEU	CD2	0.01

341LEU	CD2	345ALA	CB	0.00
341LEU	C	342ARG	CA	100.00
341LEU	C	342ARG	CB	1.06
341LEU	C	342ARG	CG	0.07
341LEU	C	342ARG	CZ	0.00
341LEU	C	342ARG	C	98.92
342ARG	CA	342ARG	CB	100.00
342ARG	CA	342ARG	CG	100.00
342ARG	CA	342ARG	CD	2.45
342ARG	CA	342ARG	CZ	0.10
342ARG	CA	342ARG	C	100.00
342ARG	CB	342ARG	CG	100.00
342ARG	CB	342ARG	CD	100.00
342ARG	CB	342ARG	CZ	3.09
342ARG	CB	342ARG	C	100.00
342ARG	CG	342ARG	CD	100.00
342ARG	CG	342ARG	CZ	42.05
342ARG	CG	342ARG	C	71.61
342ARG	CG	343HIS	CD2	0.00
342ARG	CG	343HIS	CE1	0.01
342ARG	CD	342ARG	CZ	100.00
342ARG	CD	342ARG	C	1.35
342ARG	CD	343HIS	CD2	0.01
342ARG	CD	343HIS	CE1	0.01
342ARG	CZ	343HIS	CE1	0.70
342ARG	C	343HIS	CA	100.00
342ARG	C	343HIS	CB	8.07
342ARG	C	343HIS	CG	1.16
342ARG	C	343HIS	CD2	0.04
342ARG	C	343HIS	C	90.24
343HIS	CA	343HIS	CB	100.00
343HIS	CA	343HIS	CG	100.00
343HIS	CA	343HIS	CD2	5.52
343HIS	CA	343HIS	C	100.00
343HIS	CB	343HIS	CG	100.00
343HIS	CB	343HIS	CD2	100.00
343HIS	CB	343HIS	CE1	0.22
343HIS	CB	343HIS	C	100.00
343HIS	CG	343HIS	CD2	100.00
343HIS	CG	343HIS	CE1	100.00
343HIS	CG	343HIS	C	19.68
343HIS	CD2	343HIS	CE1	100.00
343HIS	CD2	343HIS	C	2.77
343HIS	C	344LEU	CA	100.00
343HIS	C	344LEU	CB	7.80
343HIS	C	344LEU	CG	1.21
343HIS	C	344LEU	CD1	0.00
343HIS	C	344LEU	CD2	0.02
343HIS	C	344LEU	C	91.65
344LEU	CA	344LEU	CB	100.00
344LEU	CA	344LEU	CG	100.00
344LEU	CA	344LEU	CD1	34.53
344LEU	CA	344LEU	CD2	63.71
344LEU	CA	344LEU	C	100.00
344LEU	CB	344LEU	CG	100.00
344LEU	CB	344LEU	CD1	100.00
344LEU	CB	344LEU	CD2	100.00
344LEU	CB	344LEU	C	100.00
344LEU	CG	344LEU	CD1	100.00
344LEU	CG	344LEU	CD2	100.00
344LEU	CG	344LEU	C	38.53
344LEU	CD1	344LEU	CD2	100.00

269PHI	CE2	297MET	CB	0.10
269PHI	CE2	297MET	CG	0.00
269PHI	CE2	300HIS	CB	0.04
269PHI	CE2	300HIS	CG	0.02
269PHI	CE2	300HIS	CD2	0.02
269PHI	CZ	297MET	CA	4.24
269PHI	CZ	297MET	CB	0.07
269PHI	CZ	297MET	CG	0.03
269PHI	CZ	300HIS	CB	0.24
269PHI	CZ	300HIS	CG	0.16
269PHI	CZ	300HIS	CD2	0.61
269PHI	CZ	301ALA	CB	0.62
269PHI	C	270GLU	CA	100.00
269PHI	C	270GLU	CB	82.71
269PHI	C	270GLU	CG	0.78
269PHI	C	270GLU	C	20.75
269PHI	C	271PRO	CD	0.00
270GLI	CA	270GLU	CB	100.00
270GLI	CA	270GLU	CG	100.00
270GLI	CA	270GLU	CD	0.02
270GLI	CA	270GLU	C	100.00
270GLI	CA	271PRO	CD	100.00
270GLI	CB	270GLU	CG	100.00
270GLI	CB	270GLU	CD	100.00
270GLI	CB	270GLU	C	100.00
270GLI	CB	271PRO	CD	1.59
270GLI	CG	270GLU	CD	100.00
270GLI	CG	270GLU	C	88.84
270GLI	CG	271PRO	CD	1.32
270GLI	CG	272VAL	CG1	0.04
270GLI	CD	270GLU	C	0.00
270GLI	CD	272VAL	CG1	0.02
270GLI	C	271PRO	CA	100.00
270GLI	C	271PRO	CB	0.35
270GLI	C	271PRO	CG	0.00
270GLI	C	271PRO	CD	100.00
270GLI	C	271PRO	C	100.00
271PR(	CA	271PRO	CB	100.00
271PR(	CA	271PRO	CG	100.00
271PR(	CA	271PRO	CD	100.00
271PR(	CA	271PRO	C	100.00
271PR(	CB	271PRO	CG	100.00
271PR(	CB	271PRO	CD	100.00
271PR(	CB	271PRO	C	100.00
271PR(	CB	273HIS	CG	0.02
271PR(	CB	273HIS	CD2	3.34
271PR(	CB	273HIS	CE1	5.09
271PR(	CB	289ALA	CB	11.65
271PR(	CB	289ALA	C	5.93
271PR(	CB	290ALA	CA	0.26
271PR(	CB	290ALA	CB	0.00
271PR(	CG	271PRO	CD	100.00
271PR(	CG	271PRO	C	98.60
271PR(	CG	273HIS	CD2	3.11
271PR(	CG	273HIS	CE1	4.34
271PR(	CG	290ALA	CB	0.00
271PR(	CD	271PRO	C	97.08
271PR(	C	272VAL	CA	100.00
271PR(	C	272VAL	CB	24.82
271PR(	C	272VAL	CG1	2.82
271PR(	C	272VAL	CG2	15.37
271PR(	C	272VAL	C	85.18

1MET	CE	65GLU	CB	0.46
1MET	CE	65GLU	CG	0.50
1MET	CE	65GLU	C	0.06
1MET	CE	66ALA	CB	1.59
1MET	CE	297MET	CG	0.06
1MET	CE	297MET	CE	0.07
1MET	CE	298LEU	CD2	0.05
1MET	CE	301ALA	CB	3.46
1MET	CE	301ALA	C	0.66
1MET	CE	302PHE	CA	0.01
1MET	CE	302PHE	CG	0.83
1MET	CE	302PHE	CD1	5.61
1MET	CE	302PHE	CD2	1.97
1MET	CE	302PHE	CE1	13.93
1MET	CE	302PHE	CE2	2.42
1MET	CE	302PHE	CZ	3.78
1MET	CE	304LEU	CD1	0.04
1MET	C	2LYS	CA	100.00
1MET	C	2LYS	CB	2.16
1MET	C	2LYS	CG	0.02
1MET	C	2LYS	C	98.62
2LYS	CA	2LYS	CB	100.00
2LYS	CA	2LYS	CG	100.00
2LYS	CA	2LYS	CD	7.71
2LYS	CA	2LYS	C	100.00
2LYS	CA	35ALA	CB	0.01
2LYS	CB	2LYS	CG	100.00
2LYS	CB	2LYS	CD	100.00
2LYS	CB	2LYS	CE	11.56
2LYS	CB	2LYS	C	100.00
2LYS	CG	2LYS	CD	100.00
2LYS	CG	2LYS	CE	100.00
2LYS	CG	2LYS	C	27.26
2LYS	CG	35ALA	CB	0.02
2LYS	CG	64ALA	CA	0.01
2LYS	CD	2LYS	CE	100.00
2LYS	CD	2LYS	C	0.02
2LYS	CD	35ALA	CB	0.02
2LYS	CD	37GLU	CB	0.00
2LYS	CD	63GLU	CG	0.03
2LYS	CD	63GLU	CD	0.04
2LYS	CE	35ALA	CB	0.01
2LYS	CE	36TYR	C	0.03
2LYS	CE	37GLU	CB	0.47
2LYS	CE	37GLU	CG	0.00
2LYS	CE	37GLU	CD	0.01
2LYS	CE	63GLU	CG	0.01
2LYS	CE	63GLU	CD	0.05
2LYS	C	3VAL	CA	100.00
2LYS	C	3VAL	CB	26.44
2LYS	C	3VAL	CG2	17.68
2LYS	C	3VAL	C	82.22
2LYS	C	64ALA	CB	0.02
3VAL	CA	3VAL	CB	100.00
3VAL	CA	3VAL	CG1	100.00
3VAL	CA	3VAL	CG2	100.00
3VAL	CA	3VAL	C	100.00
3VAL	CA	64ALA	CB	0.00
3VAL	CB	3VAL	CG1	100.00
3VAL	CB	3VAL	CG2	100.00
3VAL	CB	3VAL	C	100.00
3VAL	CB	34LEU	CD1	0.04

344LEU	CD1	344LEU	C	0.51	271PR(	C	273HIS	CD2	0.01	3VAL	CB	34LEU	CD2	0.00
344LEU	CD2	344LEU	C	2.08	271PR(	C	273HIS	CE1	0.00	3VAL	CB	36TYR	CB	0.00
344LEU	C	345ALA	CA	100.00	272VAI	CA	272VAL	CB	100.00	3VAL	CG1	3VAL	CG2	100.00
344LEU	C	345ALA	CB	57.61	272VAI	CA	272VAL	CG1	100.00	3VAL	CG1	3VAL	C	100.00
344LEU	C	345ALA	C	44.11	272VAI	CA	272VAL	CG2	100.00	3VAL	CG1	4ALA	C	0.02
345ALA	CA	345ALA	CB	100.00	272VAI	CA	272VAL	C	100.00	3VAL	CG1	5VAL	CG2	0.14
345ALA	CA	345ALA	C	100.00	272VAI	CB	272VAL	CG1	100.00	3VAL	CG1	36TYR	CB	0.01
345ALA	CB	345ALA	C	100.00	272VAI	CB	272VAL	CG2	100.00	3VAL	CG1	68LEU	CB	0.71
1MET	CA	1MET	CB	100.00	272VAI	CB	272VAL	C	100.00	3VAL	CG1	68LEU	CG	0.11
1MET	CA	1MET	CG	100.00	272VAI	CB	273HIS	CD2	0.00	3VAL	CG1	68LEU	CD1	0.69
1MET	CA	1MET	C	100.00	272VAI	CG1	272VAL	CG2	100.00	3VAL	CG1	297MET	CE	0.69
1MET	CA	65GLU	CD	0.02	272VAI	CG1	272VAL	C	60.89	3VAL	CG2	34LEU	CG	0.00
1MET	CA	302PHE	CE1	0.01	272VAI	CG1	273HIS	CD2	0.09	3VAL	CG2	34LEU	CD1	3.58
1MET	CA	302PHE	CE2	0.01	272VAI	CG1	273HIS	CE1	0.00	3VAL	CG2	34LEU	CD2	0.21
1MET	CA	302PHE	CZ	0.00	272VAI	CG2	272VAL	C	38.75	3VAL	CG2	297MET	CE	0.76
1MET	CB	1MET	CG	100.00	272VAI	CG2	273HIS	CD2	0.16	3VAL	CG2	302PHE	CE1	0.15
1MET	CB	1MET	CE	38.30	272VAI	CG2	273HIS	CE1	0.12	3VAL	CG2	302PHE	CE2	0.16
1MET	CB	1MET	C	100.00	272VAI	C	273HIS	CA	100.00	3VAL	CG2	302PHE	CZ	0.95
1MET	CB	32LEU	CD1	0.00	272VAI	C	273HIS	CB	26.56	3VAL	C	4ALA	CA	100.00
1MET	CB	32LEU	CD2	0.01	272VAI	C	273HIS	CG	2.52	3VAL	C	4ALA	CB	2.19
1MET	CB	65GLU	CG	0.00	272VAI	C	273HIS	CD2	1.62	3VAL	C	4ALA	C	99.03
1MET	CB	302PHE	CD1	0.01	272VAI	C	273HIS	C	73.20	3VAL	C	36TYR	CB	0.00
1MET	CB	302PHE	CD2	0.10	272VAI	C	289ALA	CB	0.19	3VAL	C	64ALA	CB	0.24
1MET	CB	302PHE	CE1	0.22	273HIS	CA	273HIS	CB	100.00	4ALA	CA	4ALA	CB	100.00
1MET	CB	302PHE	CE2	9.78	273HIS	CA	273HIS	CG	100.00	4ALA	CA	4ALA	C	100.00
1MET	CB	302PHE	CZ	2.51	273HIS	CA	273HIS	CD2	42.32	4ALA	CA	39PHE	CD1	0.00
1MET	CG	1MET	CE	100.00	273HIS	CA	273HIS	C	100.00	4ALA	CA	39PHE	CE1	0.09
1MET	CG	1MET	C	89.88	273HIS	CB	273HIS	CG	100.00	4ALA	CB	4ALA	C	100.00
1MET	CG	3VAL	CG2	0.01	273HIS	CB	273HIS	CD2	100.00	4ALA	CB	6LEU	CD1	0.04
1MET	CG	32LEU	CD1	0.00	273HIS	CB	273HIS	CE1	0.06	4ALA	CB	39PHE	CD1	0.84
1MET	CG	65GLU	CB	6.29	273HIS	CB	273HIS	C	100.00	4ALA	CB	39PHE	CE1	4.48
1MET	CG	65GLU	CG	0.56	273HIS	CB	289ALA	CB	0.03	4ALA	CB	60GLY	C	0.01
1MET	CG	65GLU	CD	0.02	273HIS	CG	273HIS	CD2	100.00	4ALA	CB	61VAL	CA	0.04
1MET	CG	302PHE	CG	0.02	273HIS	CG	273HIS	CE1	100.00	4ALA	CB	61VAL	CG1	0.12
1MET	CG	302PHE	CD1	0.10	273HIS	CG	273HIS	C	38.79	4ALA	CB	64ALA	CB	14.77
1MET	CG	302PHE	CD2	0.16	273HIS	CG	289ALA	CB	0.10	4ALA	CB	67VAL	CG1	0.00
1MET	CG	302PHE	CE1	0.22	273HIS	CD2	273HIS	CE1	100.00	4ALA	CB	67VAL	CG2	0.03
1MET	CG	302PHE	CE2	0.80	273HIS	CD2	273HIS	C	16.17	4ALA	C	5VAL	CA	100.00
1MET	CG	302PHE	CZ	0.82	273HIS	CD2	274GLY	CA	0.00	4ALA	C	5VAL	CB	26.44
1MET	CE	1MET	C	0.01	273HIS	CD2	274GLY	C	0.00	4ALA	C	5VAL	CG2	16.64
1MET	CE	2LYS	C	0.00	273HIS	CD2	289ALA	CB	0.56	4ALA	C	5VAL	C	90.90
1MET	CE	3VAL	CG2	2.55	273HIS	CE1	275SER	CB	0.02	4ALA	C	6LEU	CD1	0.02
1MET	CE	23LEU	CD1	0.00	273HIS	CE1	279ILE	CD	0.18	5VAL	CA	5VAL	CB	100.00
1MET	CE	23LEU	CD2	0.01	273HIS	CE1	285ALA	CA	0.03	5VAL	CA	5VAL	CG1	100.00
1MET	CE	32LEU	CB	0.03	273HIS	CE1	285ALA	CB	0.18	5VAL	CA	5VAL	CG2	100.00
1MET	CE	32LEU	CD1	0.17	273HIS	CE1	287PRO	C	0.00	5VAL	CA	5VAL	C	100.00
1MET	CE	33GLY	C	0.00	273HIS	CE1	289ALA	CB	1.18	5VAL	CB	5VAL	CG1	100.00
1MET	CE	34LEU	CA	0.00	273HIS	CE1	290ALA	CB	0.05	5VAL	CB	5VAL	CG2	100.00
1MET	CE	34LEU	CD1	0.03	273HIS	CE1	221MET	CE	0.01	5VAL	CB	5VAL	C	100.00
1MET	CE	34LEU	CD2	0.00	273HIS	C	274GLY	CA	100.00	5VAL	CB	38VAL	CG1	0.01
1MET	CE	65GLU	CB	0.14	273HIS	C	274GLY	C	46.59	5VAL	CB	38VAL	CG2	0.02
1MET	CE	65GLU	CG	0.47	274GL\	CA	274GLY	C	100.00	5VAL	CG1	5VAL	CG2	100.00
1MET	CE	65GLU	CD	0.02	274GL\	CA	325PRO	CG	0.00	5VAL	CG1	5VAL	C	100.00
1MET	CE	65GLU	C	0.03	274GL\	C	275SER	CA	100.00	5VAL	CG1	16THR	CG2	29.12
1MET	CE	66ALA	CB	1.87	274GL\	C	275SER	CB	88.12	5VAL	CG1	20LEU	CD1	0.88
1MET	CE	297MET	CB	0.08	274GL\	C	275SER	C	42.22	5VAL	CG1	38VAL	CG1	0.08
1MET	CE	297MET	CG	0.23	274GL\	C	325PRO	CB	0.00	5VAL	CG1	38VAL	CG2	0.07
1MET	CE	297MET	CE	0.04	274GL\	C	325PRO	CG	0.01	5VAL	CG2	20LEU	CD1	1.84
1MET	CE	297MET	C	0.02	275SEI	CA	275SER	CB	100.00	5VAL	CG2	20LEU	CD2	0.28
1MET	CE	298LEU	CA	0.00	275SEI	CA	275SER	C	100.00	5VAL	CG2	36TYR	CB	0.03
1MET	CE	298LEU	CD1	0.01	275SEI	CA	280ALA	CB	0.01	5VAL	CG2	36TYR	CG	0.00
1MET	CE	298LEU	CD2	0.15	275SEI	CA	326ASP	CB	0.01	5VAL	CG2	36TYR	CD1	0.74

1MET	CE	301ALA	CB	2.01
1MET	CE	301ALA	C	0.34
1MET	CE	302PHE	CA	0.02
1MET	CE	302PHE	CB	0.05
1MET	CE	302PHE	CG	0.59
1MET	CE	302PHE	CD1	3.69
1MET	CE	302PHE	CD2	1.32
1MET	CE	302PHE	CE1	13.61
1MET	CE	302PHE	CE2	0.57
1MET	CE	302PHE	CZ	1.98
1MET	CE	304LEU	CD1	0.08
1MET	C	2LYS	CA	100.00
1MET	C	2LYS	CB	4.16
1MET	C	2LYS	CG	0.04
1MET	C	2LYS	C	97.38
2LYS	CA	2LYS	CB	100.00
2LYS	CA	2LYS	CG	100.00
2LYS	CA	2LYS	CD	0.46
2LYS	CA	2LYS	C	100.00
2LYS	CA	35ALA	CB	0.02
2LYS	CB	2LYS	CG	100.00
2LYS	CB	2LYS	CD	100.00
2LYS	CB	2LYS	CE	20.66
2LYS	CB	2LYS	C	100.00
2LYS	CB	64ALA	CA	0.00
2LYS	CG	2LYS	CD	100.00
2LYS	CG	2LYS	CE	100.00
2LYS	CG	2LYS	C	56.05
2LYS	CG	35ALA	CB	0.01
2LYS	CG	35ALA	C	0.01
2LYS	CG	65GLU	CB	0.00
2LYS	CD	2LYS	CE	100.00
2LYS	CD	2LYS	C	0.00
2LYS	CD	63GLU	CG	0.00
2LYS	CD	63GLU	CD	0.01
2LYS	CE	37GLU	CB	0.27
2LYS	CE	37GLU	CG	0.00
2LYS	CE	37GLU	CD	0.01
2LYS	CE	63GLU	CG	0.01
2LYS	CE	63GLU	CD	0.04
2LYS	CE	63GLU	C	0.02
2LYS	CE	64ALA	CA	0.00
2LYS	C	3VAL	CA	100.00
2LYS	C	3VAL	CB	25.75
2LYS	C	3VAL	CG2	16.62
2LYS	C	3VAL	C	82.81
3VAL	CA	3VAL	CB	100.00
3VAL	CA	3VAL	CG1	100.00
3VAL	CA	3VAL	CG2	100.00
3VAL	CA	3VAL	C	100.00
3VAL	CA	64ALA	CB	0.01
3VAL	CB	3VAL	CG1	100.00
3VAL	CB	3VAL	CG2	100.00
3VAL	CB	3VAL	C	100.00
3VAL	CB	34LEU	CD1	0.02
3VAL	CG1	3VAL	CG2	100.00
3VAL	CG1	3VAL	C	100.00
3VAL	CG1	4ALA	C	0.03
3VAL	CG1	5VAL	CG2	0.30
3VAL	CG1	66ALA	C	0.01
3VAL	CG1	68LEU	CB	0.48
3VAL	CG1	68LEU	CG	0.04

275SEI	CA	326ASP	CG	0.00
275SEI	CB	275SER	C	100.00
275SEI	CB	279ILE	CG1	0.00
275SEI	CB	279ILE	CD	1.02
275SEI	CB	280ALA	CB	0.08
275SEI	CB	285ALA	CB	0.03
275SEI	CB	325PRO	CB	0.00
275SEI	C	276ALA	CA	100.00
275SEI	C	276ALA	CB	26.27
275SEI	C	276ALA	C	74.21
275SEI	C	280ALA	CB	0.00
275SEI	C	325PRO	CB	0.00
275SEI	C	326ASP	CB	0.01
276AL/	CA	276ALA	CB	100.00
276AL/	CA	276ALA	C	100.00
276AL/	CA	277PRO	CD	100.00
276AL/	CA	279ILE	CG1	0.00
276AL/	CB	276ALA	C	100.00
276AL/	CB	277PRO	CD	36.09
276AL/	CB	279ILE	CG1	0.38
276AL/	CB	279ILE	CD	0.46
276AL/	CB	325PRO	CB	0.04
276AL/	CB	325PRO	C	0.04
276AL/	CB	326ASP	CA	0.04
276AL/	CB	326ASP	CB	0.07
276AL/	CB	326ASP	CG	0.44
276AL/	CB	326ASP	C	0.01
276AL/	CB	327LEU	C	0.08
276AL/	CB	328GLY	CA	0.35
276AL/	C	277PRO	CA	100.00
276AL/	C	277PRO	CB	0.10
276AL/	C	277PRO	CD	100.00
276AL/	C	277PRO	C	100.00
276AL/	C	279ILE	CD	0.01
276AL/	C	280ALA	CB	0.01
277PR(	CA	277PRO	CB	100.00
277PR(	CA	277PRO	CG	100.00
277PR(	CA	277PRO	CD	100.00
277PR(	CA	277PRO	C	100.00
277PR(	CA	280ALA	CB	0.03
277PR(	CB	277PRO	CG	100.00
277PR(	CB	277PRO	CD	100.00
277PR(	CB	277PRO	C	100.00
277PR(	CG	277PRO	CD	100.00
277PR(	CG	277PRO	C	72.30
277PR(	CD	277PRO	C	56.31
277PR(	CD	328GLY	CA	0.00
277PR(	C	278ASP	CA	100.00
277PR(	C	278ASP	CB	23.53
277PR(	C	278ASP	C	98.85
277PR(	C	280ALA	CB	0.00
278ASI	CA	278ASP	CB	100.00
278ASI	CA	278ASP	CG	100.00
278ASI	CA	278ASP	C	100.00
278ASI	CB	278ASP	CG	100.00
278ASI	CB	278ASP	C	100.00
278ASI	CG	278ASP	C	97.65
278ASI	CG	279ILE	CG2	0.01
278ASI	CG	282LYS	CG	0.01
278ASI	CG	282LYS	CD	0.04
278ASI	CG	282LYS	CE	1.16
278ASI	C	279ILE	CA	100.00

5VAL	CG2	36TYR	CD2	2.84
5VAL	CG2	38VAL	CG2	0.01
5VAL	CG2	68LEU	CD1	0.00
5VAL	C	6LEU	CA	100.00
5VAL	C	6LEU	CB	19.47
5VAL	C	6LEU	CG	0.95
5VAL	C	6LEU	CD1	9.02
5VAL	C	6LEU	CD2	0.22
5VAL	C	6LEU	C	89.70
5VAL	C	7PRO	CD	0.44
6LEU	CA	6LEU	CB	100.00
6LEU	CA	6LEU	CG	100.00
6LEU	CA	6LEU	CD1	26.76
6LEU	CA	6LEU	CD2	73.19
6LEU	CA	6LEU	C	100.00
6LEU	CA	7PRO	CD	100.00
6LEU	CA	41PHE	CD1	0.00
6LEU	CA	41PHE	CD2	0.01
6LEU	CA	41PHE	CE1	0.01
6LEU	CB	6LEU	CG	100.00
6LEU	CB	6LEU	CD1	100.00
6LEU	CB	6LEU	CD2	100.00
6LEU	CB	6LEU	C	100.00
6LEU	CB	41PHE	CB	0.10
6LEU	CB	41PHE	CG	0.03
6LEU	CB	41PHE	CD1	0.58
6LEU	CB	41PHE	CD2	0.36
6LEU	CB	41PHE	CE1	0.04
6LEU	CB	41PHE	CE2	0.02
6LEU	CB	41PHE	CZ	0.00
6LEU	CB	69LEU	CD1	3.50
6LEU	CB	69LEU	CD2	0.03
6LEU	CG	6LEU	CD1	100.00
6LEU	CG	6LEU	CD2	100.00
6LEU	CG	6LEU	C	0.01
6LEU	CG	41PHE	CB	0.14
6LEU	CG	41PHE	CG	0.08
6LEU	CG	41PHE	CD1	0.01
6LEU	CG	41PHE	CD2	0.00
6LEU	CG	57THR	CG2	0.26
6LEU	CG	67VAL	CG1	0.96
6LEU	CD1	6LEU	CD2	100.00
6LEU	CD1	39PHE	CB	0.01
6LEU	CD1	39PHE	CD1	0.00
6LEU	CD1	41PHE	CG	0.02
6LEU	CD1	41PHE	CD1	0.02
6LEU	CD1	41PHE	CD2	0.04
6LEU	CD1	41PHE	CE1	0.00
6LEU	CD1	41PHE	CE2	0.03
6LEU	CD1	41PHE	CZ	0.00
6LEU	CD1	53PHE	CE1	0.02
6LEU	CD1	53PHE	CZ	0.00
6LEU	CD1	57THR	CG2	0.22
6LEU	CD1	61VAL	CG1	1.26
6LEU	CD1	67VAL	CG1	21.74
6LEU	CD1	69LEU	CD1	0.26
6LEU	CD1	69LEU	CD2	0.04
6LEU	CD1	93LEU	CD1	0.64
6LEU	CD1	93LEU	CD2	5.20
6LEU	CD2	39PHE	CB	0.05
6LEU	CD2	41PHE	CB	0.59
6LEU	CD2	41PHE	CG	0.87

3VAL	CG1	68LEU	CD1	0.18	278ASP	C	279ILE	CB	4.77	6LEU	CD2	41PHE	CD1	1.36
3VAL	CG1	297MET	CE	1.28	278ASP	C	279ILE	CG1	0.32	6LEU	CD2	41PHE	CD2	0.07
3VAL	CG2	34LEU	CD1	3.22	278ASP	C	279ILE	CG2	2.48	6LEU	CD2	41PHE	CE1	0.14
3VAL	CG2	34LEU	CD2	0.16	278ASP	C	279ILE	CD	0.01	6LEU	CD2	41PHE	CE2	0.00
3VAL	CG2	297MET	CE	1.03	278ASP	C	279ILE	C	95.49	6LEU	CD2	53PHE	CE1	0.09
3VAL	CG2	302PHE	CE1	0.26	278ASP	C	282LYS	CE	0.01	6LEU	CD2	57THR	CG2	40.71
3VAL	CG2	302PHE	CE2	0.00	279ILE	CA	279ILE	CB	100.00	6LEU	CD2	61VAL	CG1	0.19
3VAL	CG2	302PHE	CZ	1.11	279ILE	CA	279ILE	CG1	100.00	6LEU	CD2	67VAL	CG1	0.05
3VAL	C	4ALA	CA	100.00	279ILE	CA	279ILE	CG2	100.00	6LEU	CD2	69LEU	CD1	0.32
3VAL	C	4ALA	CB	1.82	279ILE	CA	279ILE	CD	4.82	6LEU	CD2	69LEU	CD2	0.01
3VAL	C	4ALA	C	99.12	279ILE	CA	279ILE	C	100.00	6LEU	CD2	93LEU	CD1	0.53
3VAL	C	64ALA	CB	0.52	279ILE	CA	284ILE	CG1	0.00	6LEU	CD2	93LEU	CD2	0.44
4ALA	CA	4ALA	CB	100.00	279ILE	CB	279ILE	CG1	100.00	6LEU	C	7PRO	CA	100.00
4ALA	CA	4ALA	C	100.00	279ILE	CB	279ILE	CG2	100.00	6LEU	C	7PRO	CB	0.12
4ALA	CA	39PHE	CD1	0.00	279ILE	CB	279ILE	CD	100.00	6LEU	C	7PRO	CD	100.00
4ALA	CA	39PHE	CE1	0.04	279ILE	CB	279ILE	C	100.00	6LEU	C	7PRO	C	100.00
4ALA	CB	4ALA	C	100.00	279ILE	CB	284ILE	CB	0.01	6LEU	C	41PHE	CD1	0.06
4ALA	CB	6LEU	CD1	0.06	279ILE	CB	284ILE	CG1	0.03	6LEU	C	41PHE	CD2	0.11
4ALA	CB	39PHE	CD1	0.76	279ILE	CB	284ILE	CG2	0.02	6LEU	C	41PHE	CE1	0.06
4ALA	CB	39PHE	CE1	4.51	279ILE	CB	284ILE	CD	0.06	6LEU	C	41PHE	CE2	0.03
4ALA	CB	60GLY	C	0.01	279ILE	CG1	279ILE	CG2	100.00	6LEU	C	41PHE	CZ	0.00
4ALA	CB	61VAL	CA	0.01	279ILE	CG1	279ILE	CD	100.00	7PRO	CA	7PRO	CB	100.00
4ALA	CB	61VAL	CG1	0.06	279ILE	CG1	279ILE	C	93.12	7PRO	CA	7PRO	CG	100.00
4ALA	CB	64ALA	CB	17.22	279ILE	CG1	284ILE	CG2	0.00	7PRO	CA	7PRO	CD	100.00
4ALA	CB	67VAL	CG2	0.02	279ILE	CG1	285ALA	CA	0.00	7PRO	CA	7PRO	C	100.00
4ALA	C	5VAL	CA	100.00	279ILE	CG1	285ALA	CB	0.08	7PRO	CB	7PRO	CG	100.00
4ALA	C	5VAL	CB	21.87	279ILE	CG1	325PRO	CB	0.00	7PRO	CB	7PRO	CD	100.00
4ALA	C	5VAL	CG2	12.64	279ILE	CG2	279ILE	CD	94.79	7PRO	CB	7PRO	C	100.00
4ALA	C	5VAL	C	93.07	279ILE	CG2	279ILE	C	6.54	7PRO	CB	13PRO	CA	0.77
4ALA	C	6LEU	CD1	0.02	279ILE	CG2	282LYS	CD	0.01	7PRO	CB	13PRO	CB	0.52
5VAL	CA	5VAL	CB	100.00	279ILE	CG2	284ILE	CB	0.05	7PRO	CB	13PRO	CG	0.00
5VAL	CA	5VAL	CG1	100.00	279ILE	CG2	284ILE	CG1	0.07	7PRO	CB	16THR	CB	0.00
5VAL	CA	5VAL	CG2	100.00	279ILE	CG2	284ILE	CG2	0.80	7PRO	CB	16THR	CG2	0.00
5VAL	CA	5VAL	C	100.00	279ILE	CG2	284ILE	CD	0.36	7PRO	CB	38VAL	CG1	0.00
5VAL	CB	5VAL	CG1	100.00	279ILE	CG2	284ILE	C	0.04	7PRO	CG	7PRO	CD	100.00
5VAL	CB	5VAL	CG2	100.00	279ILE	CG2	285ALA	CA	0.25	7PRO	CG	7PRO	C	93.53
5VAL	CB	5VAL	C	100.00	279ILE	CG2	285ALA	CB	0.04	7PRO	CG	38VAL	CG1	0.94
5VAL	CB	38VAL	CG2	0.00	279ILE	CG2	325PRO	CB	0.06	7PRO	CG	38VAL	CG2	0.02
5VAL	CG1	5VAL	CG2	100.00	279ILE	CG2	325PRO	CG	0.00	7PRO	CD	7PRO	C	76.64
5VAL	CG1	5VAL	C	99.99	279ILE	CG2	325PRO	C	0.00	7PRO	CD	38VAL	CG1	0.12
5VAL	CG1	6LEU	C	0.00	279ILE	CG2	326ASP	CB	0.00	7PRO	CD	38VAL	CG2	0.04
5VAL	CG1	16THR	CG2	25.93	279ILE	CG2	328GLY	CA	0.22	7PRO	C	8GLY	CA	100.00
5VAL	CG1	20LEU	CD1	0.85	279ILE	CG2	328GLY	C	0.41	7PRO	C	8GLY	C	88.59
5VAL	CG1	20LEU	CD2	0.04	279ILE	CG2	329GLY	C	0.14	7PRO	C	13PRO	CG	0.00
5VAL	CG1	38VAL	CG1	0.02	279ILE	CG2	330SER	CB	0.00	8GLY	CA	8GLY	C	100.00
5VAL	CG1	38VAL	CG2	0.03	279ILE	CD	279ILE	C	2.59	8GLY	CA	41PHE	CD1	0.44
5VAL	CG1	68LEU	CD2	0.01	279ILE	CD	284ILE	CG2	0.15	8GLY	CA	41PHE	CD2	0.58
5VAL	CG2	20LEU	CG	0.00	279ILE	CD	284ILE	CD	0.05	8GLY	CA	41PHE	CE1	0.23
5VAL	CG2	20LEU	CD1	1.22	279ILE	CD	285ALA	CA	0.06	8GLY	CA	41PHE	CE2	0.41
5VAL	CG2	20LEU	CD2	0.45	279ILE	CD	285ALA	CB	0.46	8GLY	CA	41PHE	CZ	0.02
5VAL	CG2	36TYR	CB	0.14	279ILE	CD	325PRO	CA	0.02	8GLY	CA	42GLY	CA	0.06
5VAL	CG2	36TYR	CG	0.04	279ILE	CD	325PRO	CB	1.39	8GLY	CA	70GLY	C	0.02
5VAL	CG2	36TYR	CD1	4.56	279ILE	CD	325PRO	CG	0.05	8GLY	CA	72VAL	CB	0.07
5VAL	CG2	68LEU	CD2	0.00	279ILE	CD	325PRO	C	0.14	8GLY	CA	72VAL	CG1	0.94
5VAL	C	6LEU	CA	100.00	279ILE	CD	326ASP	CA	0.03	8GLY	CA	72VAL	CG2	0.41
5VAL	C	6LEU	CB	15.34	279ILE	CD	326ASP	CB	0.01	8GLY	C	9ASP	CA	100.00
5VAL	C	6LEU	CG	0.29	279ILE	CD	326ASP	CG	0.11	8GLY	C	9ASP	CB	97.96
5VAL	C	6LEU	CD1	21.00	279ILE	CD	328GLY	CA	0.09	8GLY	C	9ASP	CG	0.26
5VAL	C	6LEU	CD2	0.05	279ILE	CD	328GLY	C	0.06	8GLY	C	9ASP	C	2.16
5VAL	C	6LEU	C	95.41	279ILE	CD	329GLY	C	0.00	8GLY	C	12GLY	CA	0.00
5VAL	C	7PRO	CD	0.12	279ILE	C	280ALA	CA	100.00	8GLY	C	13PRO	CG	0.20
6LEU	CA	6LEU	CB	100.00	279ILE	C	280ALA	CB	0.26	8GLY	C	13PRO	CD	0.14

6LEU	CA	6LEU	CG	100.00
6LEU	CA	6LEU	CD1	82.56
6LEU	CA	6LEU	CD2	17.34
6LEU	CA	6LEU	C	100.00
6LEU	CA	7PRO	CD	100.00
6LEU	CB	6LEU	CG	100.00
6LEU	CB	6LEU	CD1	100.00
6LEU	CB	6LEU	CD2	100.00
6LEU	CB	6LEU	C	100.00
6LEU	CB	41PHE	CB	0.19
6LEU	CB	41PHE	CG	0.04
6LEU	CB	41PHE	CD2	0.60
6LEU	CB	41PHE	CE2	0.00
6LEU	CB	69LEU	CD1	4.47
6LEU	CB	69LEU	CD2	0.09
6LEU	CG	6LEU	CD1	100.00
6LEU	CG	6LEU	CD2	100.00
6LEU	CG	41PHE	CB	0.44
6LEU	CG	41PHE	CG	0.06
6LEU	CG	41PHE	CD1	0.01
6LEU	CG	57THR	CG2	1.54
6LEU	CG	67VAL	CG1	0.06
6LEU	CD1	6LEU	CD2	100.00
6LEU	CD1	39PHE	CB	0.03
6LEU	CD1	39PHE	CG	0.00
6LEU	CD1	39PHE	CD1	0.03
6LEU	CD1	41PHE	CD1	0.01
6LEU	CD1	41PHE	CE1	0.01
6LEU	CD1	57THR	CG2	1.45
6LEU	CD1	61VAL	CG1	9.39
6LEU	CD1	67VAL	CG1	4.96
6LEU	CD1	69LEU	CD1	0.04
6LEU	CD1	69LEU	CD2	0.30
6LEU	CD1	93LEU	CG	0.01
6LEU	CD1	93LEU	CD1	0.66
6LEU	CD1	93LEU	CD2	3.56
6LEU	CD2	39PHE	CB	0.03
6LEU	CD2	41PHE	CB	0.44
6LEU	CD2	41PHE	CG	1.76
6LEU	CD2	41PHE	CD1	3.40
6LEU	CD2	41PHE	CD2	0.06
6LEU	CD2	41PHE	CE1	0.28
6LEU	CD2	53PHE	CE1	0.01
6LEU	CD2	57THR	CG2	12.41
6LEU	CD2	61VAL	CG1	1.41
6LEU	CD2	67VAL	CG1	0.02
6LEU	CD2	69LEU	CD1	1.28
6LEU	CD2	69LEU	CD2	0.32
6LEU	CD2	93LEU	CG	0.02
6LEU	CD2	93LEU	CD1	5.23
6LEU	CD2	93LEU	CD2	3.36
6LEU	C	7PRO	CA	100.00
6LEU	C	7PRO	CB	0.13
6LEU	C	7PRO	CD	100.00
6LEU	C	7PRO	C	100.00
6LEU	C	41PHE	CD2	0.04
7PRO	CA	7PRO	CB	100.00
7PRO	CA	7PRO	CG	100.00
7PRO	CA	7PRO	CD	100.00
7PRO	CA	7PRO	C	100.00
7PRO	CB	7PRO	CG	100.00
7PRO	CB	7PRO	CD	100.00
279ILE	C	280ALA	C	99.77
279ILE	C	282LYS	CD	0.00
279ILE	C	285ALA	CB	0.13
280ALA	CA	280ALA	CB	100.00
280ALA	CA	280ALA	C	100.00
280ALA	CA	285ALA	CB	0.26
280ALA	CB	280ALA	C	100.00
280ALA	CB	285ALA	CB	0.15
280ALA	C	281GLY	CA	100.00
280ALA	C	281GLY	C	72.24
280ALA	C	282LYS	CD	0.01
280ALA	C	333THR	CG2	0.00
281GLY	CA	281GLY	C	100.00
281GLY	CA	333THR	CG2	0.02
281GLY	C	282LYS	CA	100.00
281GLY	C	282LYS	CB	23.39
281GLY	C	282LYS	CG	2.42
281GLY	C	282LYS	CD	0.10
281GLY	C	282LYS	C	75.72
281GLY	C	333THR	CG2	0.14
282LYS	CA	282LYS	CB	100.00
282LYS	CA	282LYS	CG	100.00
282LYS	CA	282LYS	CD	6.10
282LYS	CA	282LYS	C	100.00
282LYS	CA	333THR	CG2	0.00
282LYS	CB	282LYS	CG	100.00
282LYS	CB	282LYS	CD	100.00
282LYS	CB	282LYS	CE	7.18
282LYS	CB	282LYS	C	100.00
282LYS	CB	284ILE	CG1	0.25
282LYS	CB	284ILE	CD	1.39
282LYS	CG	282LYS	CD	100.00
282LYS	CG	282LYS	CE	100.00
282LYS	CG	282LYS	C	19.84
282LYS	CG	284ILE	CG1	0.04
282LYS	CG	284ILE	CD	0.12
282LYS	CD	282LYS	CE	100.00
282LYS	CD	282LYS	C	0.03
282LYS	CD	284ILE	CG1	0.01
282LYS	CD	284ILE	CD	0.26
282LYS	CE	284ILE	CD	0.11
282LYS	C	283GLY	CA	100.00
282LYS	C	283GLY	C	61.17
282LYS	C	284ILE	CD	0.02
282LYS	C	333THR	CG2	0.04
283GLY	CA	283GLY	C	100.00
283GLY	CA	325PRO	CG	0.00
283GLY	CA	332GLY	CA	0.07
283GLY	CA	332GLY	C	0.02
283GLY	CA	333THR	CG2	0.15
283GLY	C	284ILE	CA	100.00
283GLY	C	284ILE	CB	25.37
283GLY	C	284ILE	CG1	3.40
283GLY	C	284ILE	CG2	13.64
283GLY	C	284ILE	CD	0.05
283GLY	C	284ILE	C	77.32
283GLY	C	285ALA	C	0.01
283GLY	C	325PRO	CG	0.17
283GLY	C	325PRO	CD	0.15
283GLY	C	332GLY	CA	0.07
283GLY	C	333THR	CG2	0.03
284ILE	CA	284ILE	CB	100.00
8GLY	C	42GLY	CA	1.67
8GLY	C	72VAL	CG1	0.01
8GLY	C	72VAL	CG2	0.04
9ASP	CA	9ASP	CB	100.00
9ASP	CA	9ASP	CG	100.00
9ASP	CA	9ASP	C	100.00
9ASP	CA	13PRO	CG	0.02
9ASP	CA	13PRO	CD	0.06
9ASP	CB	9ASP	CG	100.00
9ASP	CB	9ASP	C	100.00
9ASP	CB	42GLY	CA	0.01
9ASP	CB	43GLY	CA	0.00
9ASP	CB	72VAL	CG1	0.00
9ASP	CB	72VAL	CG2	0.12
9ASP	CB	77TRP	CZ2	0.03
9ASP	CB	77TRP	CZ3	0.03
9ASP	CG	9ASP	C	97.17
9ASP	CG	43GLY	CA	0.02
9ASP	CG	76LYS	CD	0.01
9ASP	CG	76LYS	CE	0.31
9ASP	CG	77TRP	CZ2	0.11
9ASP	CG	77TRP	CZ3	0.18
9ASP	C	10GLY	CA	100.00
9ASP	C	10GLY	C	79.67
9ASP	C	13PRO	CD	0.31
9ASP	C	275SER	CB	0.01
10GLY	CA	10GLY	C	100.00
10GLY	CA	277PRO	CA	0.00
10GLY	CA	277PRO	CB	0.14
10GLY	CA	277PRO	CG	0.13
10GLY	CA	280ALA	CB	1.05
10GLY	C	11ILE	CA	100.00
10GLY	C	11ILE	CB	0.57
10GLY	C	11ILE	CG2	0.22
10GLY	C	11ILE	C	99.42
10GLY	C	277PRO	CB	0.00
10GLY	C	280ALA	CB	11.74
11ILE	CA	11ILE	CB	100.00
11ILE	CA	11ILE	CG1	100.00
11ILE	CA	11ILE	CG2	100.00
11ILE	CA	11ILE	CD	3.64
11ILE	CA	11ILE	C	100.00
11ILE	CA	280ALA	CB	0.01
11ILE	CB	11ILE	CG1	100.00
11ILE	CB	11ILE	CG2	100.00
11ILE	CB	11ILE	CD	100.00
11ILE	CB	11ILE	C	100.00
11ILE	CB	273HIS	CE1	0.00
11ILE	CB	285ALA	CB	0.15
11ILE	CG1	11ILE	CG2	100.00
11ILE	CG1	11ILE	CD	100.00
11ILE	CG1	11ILE	C	99.48
11ILE	CG1	15VAL	CG1	0.01
11ILE	CG1	71SER	CB	0.08
11ILE	CG1	273HIS	CE1	0.08
11ILE	CG1	275SER	C	0.01
11ILE	CG1	276ALA	CB	0.01
11ILE	CG1	277PRO	CB	0.02
11ILE	CG1	277PRO	CG	0.06
11ILE	CG2	11ILE	CD	88.27
11ILE	CG2	11ILE	C	0.45
11ILE	CG2	15VAL	CG1	0.09

7PRO	CB	7PRO	C	100.00
7PRO	CB	13PRO	CA	0.39
7PRO	CB	13PRO	CB	0.33
7PRO	CB	16THR	CB	0.00
7PRO	CB	16THR	CG2	0.01
7PRO	CB	38VAL	CG1	0.00
7PRO	CG	7PRO	CD	100.00
7PRO	CG	7PRO	C	89.76
7PRO	CG	38VAL	CG1	1.04
7PRO	CD	7PRO	C	68.73
7PRO	CD	38VAL	CG1	0.07
7PRO	C	8GLY	CA	100.00
7PRO	C	8GLY	C	80.76
7PRO	C	13PRO	CD	0.01
8GLY	CA	8GLY	C	100.00
8GLY	CA	41PHE	CD2	0.87
8GLY	CA	41PHE	CE2	0.26
8GLY	CA	42GLY	CA	0.00
8GLY	CA	70GLY	C	0.24
8GLY	CA	72VAL	CG1	1.15
8GLY	CA	72VAL	CG2	0.02
8GLY	C	9ASP	CA	100.00
8GLY	C	9ASP	CB	95.90
8GLY	C	9ASP	CG	0.26
8GLY	C	9ASP	C	4.42
8GLY	C	13PRO	CG	0.04
8GLY	C	13PRO	CD	0.25
8GLY	C	42GLY	CA	0.32
8GLY	C	72VAL	CG1	0.01
9ASP	CA	9ASP	CB	100.00
9ASP	CA	9ASP	CG	100.00
9ASP	CA	9ASP	C	100.00
9ASP	CA	13PRO	CD	0.00
9ASP	CB	9ASP	CG	100.00
9ASP	CB	9ASP	C	100.00
9ASP	CB	42GLY	CA	0.52
9ASP	CB	72VAL	CA	0.00
9ASP	CB	72VAL	CG1	0.01
9ASP	CB	77TRP	CZ3	0.02
9ASP	CB	86PRO	CG	0.02
9ASP	CG	9ASP	C	95.79
9ASP	CG	42GLY	CA	0.00
9ASP	CG	43GLY	CA	0.03
9ASP	CG	44ALA	CB	0.01
9ASP	CG	74GLY	CA	0.28
9ASP	CG	77TRP	CZ3	0.07
9ASP	CG	86PRO	CB	0.01
9ASP	CG	86PRO	CG	0.07
9ASP	C	10GLY	CA	100.00
9ASP	C	10GLY	C	87.20
9ASP	C	13PRO	CD	0.05
9ASP	C	74GLY	CA	0.01
9ASP	C	275SER	CB	0.00
10GLY	CA	10GLY	C	100.00
10GLY	CA	73GLY	C	0.16
10GLY	CA	74GLY	CA	27.03
10GLY	CA	75PRO	CD	0.01
10GLY	CA	280ALA	CB	8.00
10GLY	C	11ILE	CA	100.00
10GLY	C	11ILE	CB	0.04
10GLY	C	11ILE	CG2	0.02
10GLY	C	11ILE	C	99.98

284ILE	CA	284ILE	CG1	100.00
284ILE	CA	284ILE	CG2	100.00
284ILE	CA	284ILE	CD	10.80
284ILE	CA	284ILE	C	100.00
284ILE	CA	325PRO	CG	0.02
284ILE	CA	332GLY	CA	0.02
284ILE	CB	284ILE	CG1	100.00
284ILE	CB	284ILE	CG2	100.00
284ILE	CB	284ILE	CD	100.00
284ILE	CB	284ILE	C	100.00
284ILE	CB	325PRO	CG	0.10
284ILE	CB	332GLY	CA	0.00
284ILE	CG1	284ILE	CG2	100.00
284ILE	CG1	284ILE	CD	100.00
284ILE	CG1	284ILE	C	28.43
284ILE	CG1	325PRO	CB	0.02
284ILE	CG1	325PRO	CG	0.06
284ILE	CG1	330SER	C	0.00
284ILE	CG1	331ALA	C	0.00
284ILE	CG2	284ILE	CD	94.54
284ILE	CG2	284ILE	C	71.03
284ILE	CG2	325PRO	CB	2.89
284ILE	CG2	325PRO	CG	2.07
284ILE	CG2	325PRO	CD	0.02
284ILE	CG2	325PRO	C	0.00
284ILE	CG2	326ASP	CB	0.04
284ILE	CG2	326ASP	CG	0.00
284ILE	CG2	329GLY	CA	0.01
284ILE	CG2	329GLY	C	0.15
284ILE	CG2	330SER	CA	0.03
284ILE	CG2	330SER	CB	0.01
284ILE	CG2	330SER	C	0.21
284ILE	CG2	331ALA	C	1.21
284ILE	CG2	332GLY	CA	1.27
284ILE	CD	284ILE	C	0.91
284ILE	CD	325PRO	CB	0.08
284ILE	CD	325PRO	CG	0.19
284ILE	CD	329GLY	C	0.00
284ILE	CD	330SER	CA	0.04
284ILE	CD	330SER	CB	0.08
284ILE	CD	330SER	C	0.08
284ILE	CD	331ALA	C	0.00
284ILE	C	285ALA	CA	100.00
284ILE	C	285ALA	CB	33.55
284ILE	C	285ALA	C	67.79
284ILE	C	325PRO	CG	0.01
284ILE	C	332GLY	CA	0.02
285AL/	CA	285ALA	CB	100.00
285AL/	CA	285ALA	C	100.00
285AL/	CA	325PRO	CG	0.02
285AL/	CB	285ALA	C	100.00
285AL/	CB	333THR	CG2	0.20
285AL/	C	286ASN	CA	100.00
285AL/	C	286ASN	CB	10.88
285AL/	C	286ASN	C	92.86
285AL/	C	325PRO	CG	0.04
285AL/	C	332GLY	CA	0.01
285AL/	C	333THR	CG2	0.03
286ASI	CA	286ASN	CB	100.00
286ASI	CA	286ASN	CG	100.00
286ASI	CA	286ASN	C	100.00
286ASI	CA	287PRO	CD	100.00

11ILE	CG2	71SER	CB	0.00
11ILE	CG2	277PRO	CB	0.10
11ILE	CG2	277PRO	CG	0.16
11ILE	CG2	279ILE	CG1	5.47
11ILE	CG2	279ILE	CD	0.57
11ILE	CG2	279ILE	C	0.44
11ILE	CG2	280ALA	CA	0.20
11ILE	CG2	280ALA	CB	0.27
11ILE	CG2	285ALA	CB	7.15
11ILE	CD	11ILE	C	4.68
11ILE	CD	15VAL	CG1	0.07
11ILE	CD	15VAL	CG2	0.03
11ILE	CD	71SER	CB	0.30
11ILE	CD	273HIS	CB	0.03
11ILE	CD	273HIS	CG	0.54
11ILE	CD	273HIS	CD2	1.89
11ILE	CD	273HIS	CE1	15.05
11ILE	CD	274GLY	C	0.02
11ILE	CD	275SER	CA	0.00
11ILE	CD	275SER	C	0.00
11ILE	CD	276ALA	CA	0.00
11ILE	CD	276ALA	CB	0.66
11ILE	CD	277PRO	CB	0.05
11ILE	CD	277PRO	CG	1.92
11ILE	CD	277PRO	CD	0.75
11ILE	CD	285ALA	CB	0.18
11ILE	C	12GLY	CA	100.00
11ILE	C	12GLY	C	100.00
11ILE	C	13PRO	CD	3.60
11ILE	C	15VAL	CG2	0.01
12GLY	CA	12GLY	C	100.00
12GLY	CA	13PRO	CD	100.00
12GLY	CA	15VAL	CG2	0.05
12GLY	CA	71SER	CB	0.11
12GLY	C	13PRO	CA	100.00
12GLY	C	13PRO	CB	0.01
12GLY	C	13PRO	CG	0.00
12GLY	C	13PRO	CD	100.00
12GLY	C	13PRO	C	100.00
13PRC	CA	13PRO	CB	100.00
13PRC	CA	13PRO	CG	100.00
13PRC	CA	13PRO	CD	100.00
13PRC	CA	13PRO	C	100.00
13PRC	CB	13PRO	CG	100.00
13PRC	CB	13PRO	CD	100.00
13PRC	CB	13PRO	C	100.00
13PRC	CG	13PRO	CD	100.00
13PRC	CG	13PRO	C	36.85
13PRC	CD	13PRO	C	26.80
13PRC	C	14GLU	CA	100.00
13PRC	C	14GLU	CB	0.06
13PRC	C	14GLU	C	99.98
14GLL	CA	14GLU	CB	100.00
14GLL	CA	14GLU	CG	100.00
14GLL	CA	14GLU	CD	94.10
14GLL	CA	14GLU	C	100.00
14GLL	CB	14GLU	CG	100.00
14GLL	CB	14GLU	CD	100.00
14GLL	CB	14GLU	C	100.00
14GLL	CB	280ALA	CB	0.01
14GLL	CB	285ALA	CB	0.01
14GLL	CG	14GLU	CD	100.00

10GLY	C	280ALA	CA	0.00	286ASI	CA	324PRO	CG	0.00	14GLL	CG	14GLU	C	77.53
10GLY	C	280ALA	CB	10.53	286ASI	CB	286ASN	CG	100.00	14GLL	CG	280ALA	CB	0.01
11ILE	CA	11ILE	CB	100.00	286ASI	CB	286ASN	C	100.00	14GLL	CG	283GLY	CA	0.02
11ILE	CA	11ILE	CG1	100.00	286ASI	CB	287PRO	CD	0.00	14GLL	CG	285ALA	CB	0.07
11ILE	CA	11ILE	CG2	100.00	286ASI	CB	324PRO	CB	0.07	14GLL	CG	333THR	CB	0.01
11ILE	CA	11ILE	CD	2.92	286ASI	CB	324PRO	CG	0.31	14GLL	CG	333THR	CG2	0.92
11ILE	CA	11ILE	C	100.00	286ASI	CB	325PRO	CB	0.34	14GLL	CD	281GLY	CA	0.00
11ILE	CB	11ILE	CG1	100.00	286ASI	CB	325PRO	CG	0.16	14GLL	CD	283GLY	CA	0.02
11ILE	CB	11ILE	CG2	100.00	286ASI	CB	325PRO	CD	0.10	14GLL	C	15VAL	CA	100.00
11ILE	CB	11ILE	CD	100.00	286ASI	CB	326ASP	CB	0.32	14GLL	C	15VAL	CB	0.05
11ILE	CB	11ILE	C	100.00	286ASI	CB	326ASP	CG	0.04	14GLL	C	15VAL	CG1	0.02
11ILE	CB	15VAL	CG1	0.00	286ASI	CG	286ASN	C	99.58	14GLL	C	15VAL	C	99.98
11ILE	CB	285ALA	CB	0.29	286ASI	CG	287PRO	CD	5.76	14GLL	C	333THR	CG2	0.08
11ILE	CG1	11ILE	CG2	100.00	286ASI	CG	288THR	CB	0.02	15VAL	CA	15VAL	CB	100.00
11ILE	CG1	11ILE	CD	100.00	286ASI	CG	319LEU	CD2	0.01	15VAL	CA	15VAL	CG1	100.00
11ILE	CG1	11ILE	C	100.00	286ASI	CG	324PRO	CB	0.90	15VAL	CA	15VAL	CG2	100.00
11ILE	CG1	15VAL	CG2	0.00	286ASI	CG	324PRO	CG	2.67	15VAL	CA	15VAL	C	100.00
11ILE	CG1	71SER	CB	0.14	286ASI	CG	325PRO	CB	0.07	15VAL	CB	15VAL	CG1	100.00
11ILE	CG1	273HIS	CE1	0.03	286ASI	CG	325PRO	CG	0.86	15VAL	CB	15VAL	CG2	100.00
11ILE	CG1	275SER	CA	0.03	286ASI	CG	325PRO	CD	0.02	15VAL	CB	15VAL	C	100.00
11ILE	CG1	275SER	CB	4.78	286ASI	CG	336PHE	CD2	0.01	15VAL	CB	287PRO	CB	0.01
11ILE	CG1	275SER	C	0.00	286ASI	CG	336PHE	CE1	0.08	15VAL	CB	290ALA	CB	0.18
11ILE	CG1	285ALA	CB	0.01	286ASI	CG	336PHE	CE2	0.12	15VAL	CG1	15VAL	CG2	100.00
11ILE	CG2	11ILE	CD	88.29	286ASI	CG	336PHE	CZ	0.09	15VAL	CG1	273HIS	CE1	0.02
11ILE	CG2	276ALA	CB	0.00	286ASI	C	287PRO	CA	100.00	15VAL	CG1	285ALA	CB	3.15
11ILE	CG2	279ILE	CB	0.01	286ASI	C	287PRO	CB	2.61	15VAL	CG1	285ALA	C	0.02
11ILE	CG2	279ILE	CG1	0.47	286ASI	C	287PRO	CD	100.00	15VAL	CG1	286ASN	C	0.05
11ILE	CG2	279ILE	CG2	0.84	286ASI	C	287PRO	C	99.82	15VAL	CG1	287PRO	CA	0.38
11ILE	CG2	279ILE	CD	1.62	287PR(	CA	287PRO	CB	100.00	15VAL	CG1	287PRO	CB	0.01
11ILE	CG2	279ILE	C	0.43	287PR(	CA	287PRO	CG	100.00	15VAL	CG1	333THR	CG2	0.09
11ILE	CG2	280ALA	CA	0.37	287PR(	CA	287PRO	CD	100.00	15VAL	CG2	15VAL	C	100.00
11ILE	CG2	280ALA	CB	0.09	287PR(	CA	287PRO	C	100.00	15VAL	CG2	273HIS	CE1	0.22
11ILE	CG2	285ALA	CB	9.14	287PR(	CB	287PRO	CG	100.00	15VAL	CG2	290ALA	CB	0.46
11ILE	CD	11ILE	C	5.26	287PR(	CB	287PRO	CD	100.00	15VAL	C	16THR	CA	100.00
11ILE	CD	15VAL	CG1	0.17	287PR(	CB	287PRO	C	100.00	15VAL	C	16THR	CB	0.02
11ILE	CD	15VAL	CG2	0.29	287PR(	CB	291ILE	CD	1.06	15VAL	C	16THR	C	100.00
11ILE	CD	71SER	CB	0.00	287PR(	CB	333THR	CG2	0.03	15VAL	C	18ALA	CB	0.00
11ILE	CD	273HIS	CG	0.10	287PR(	CG	287PRO	CD	100.00	15VAL	C	290ALA	CB	0.76
11ILE	CD	273HIS	CD2	2.13	287PR(	CG	287PRO	C	99.60	16THF	CA	16THR	CB	100.00
11ILE	CD	273HIS	CE1	5.39	287PR(	CG	291ILE	CG1	0.00	16THF	CA	16THR	CG2	100.00
11ILE	CD	274GLY	C	0.03	287PR(	CG	291ILE	CD	1.58	16THF	CA	16THR	C	100.00
11ILE	CD	275SER	CA	0.07	287PR(	CG	333THR	CG2	0.10	16THF	CB	16THR	CG2	100.00
11ILE	CD	275SER	CB	0.13	287PR(	CG	336PHE	CB	16.13	16THF	CB	16THR	C	100.00
11ILE	CD	276ALA	CB	0.01	287PR(	CG	336PHE	CG	0.29	16THF	CG2	16THR	C	100.00
11ILE	CD	279ILE	CG2	0.00	287PR(	CG	336PHE	CD1	11.07	16THF	CG2	20LEU	CD1	0.05
11ILE	CD	279ILE	CD	0.02	287PR(	CG	336PHE	CD2	0.02	16THF	CG2	68LEU	CG	0.04
11ILE	CD	285ALA	CB	1.36	287PR(	CG	336PHE	CE1	0.34	16THF	CG2	68LEU	CD1	0.00
11ILE	C	12GLY	CA	100.00	287PR(	CG	336PHE	CE2	0.00	16THF	CG2	68LEU	CD2	2.92
11ILE	C	12GLY	C	100.00	287PR(	CG	337THR	CG2	0.35	16THF	C	17GLU	CA	100.00
11ILE	C	13PRO	CD	3.27	287PR(	CD	287PRO	C	94.63	16THF	C	17GLU	C	100.00
11ILE	C	15VAL	CG2	0.06	287PR(	CD	332GLY	C	0.02	17GLL	CA	17GLU	CB	100.00
12GLY	CA	12GLY	C	100.00	287PR(	CD	333THR	CG2	0.08	17GLL	CA	17GLU	CG	100.00
12GLY	CA	13PRO	CD	100.00	287PR(	CD	336PHE	CB	11.36	17GLL	CA	17GLU	CD	94.97
12GLY	CA	15VAL	CG2	0.04	287PR(	CD	336PHE	CG	0.16	17GLL	CA	17GLU	C	100.00
12GLY	C	13PRO	CA	100.00	287PR(	CD	336PHE	CD1	1.08	17GLL	CB	17GLU	CG	100.00
12GLY	C	13PRO	CB	0.01	287PR(	CD	336PHE	CD2	1.18	17GLL	CB	17GLU	CD	100.00
12GLY	C	13PRO	CD	100.00	287PR(	CD	336PHE	CE1	0.02	17GLL	CB	17GLU	C	100.00
12GLY	C	13PRO	C	100.00	287PR(	CD	336PHE	CE2	0.02	17GLL	CG	17GLU	CD	100.00
13PRO	CA	13PRO	CB	100.00	287PR(	C	288THR	CA	100.00	17GLL	CG	17GLU	C	96.67
13PRO	CA	13PRO	CG	100.00	287PR(	C	288THR	C	100.00	17GLL	CG	21LYS	CE	0.04
13PRO	CA	13PRO	CD	100.00	287PR(	C	291ILE	CD	0.06	17GLL	CD	17GLU	C	5.53
13PRO	CA	13PRO	C	100.00	288THI	CA	288THR	CB	100.00	17GLL	CD	21LYS	CE	0.00

13PRO	CB	13PRO	CG	100.00
13PRO	CB	13PRO	CD	100.00
13PRO	CB	13PRO	C	100.00
13PRO	CG	13PRO	CD	100.00
13PRO	CG	13PRO	C	41.29
13PRO	CD	13PRO	C	28.91
13PRO	C	14GLU	CA	100.00
13PRO	C	14GLU	CB	0.10
13PRO	C	14GLU	C	99.97
14GLU	CA	14GLU	CB	100.00
14GLU	CA	14GLU	CG	100.00
14GLU	CA	14GLU	CD	96.39
14GLU	CA	14GLU	C	100.00
14GLU	CB	14GLU	CG	100.00
14GLU	CB	14GLU	CD	100.00
14GLU	CB	14GLU	C	100.00
14GLU	CG	14GLU	CD	100.00
14GLU	CG	14GLU	C	95.90
14GLU	CG	333THR	CB	0.02
14GLU	CG	333THR	CG2	1.45
14GLU	CD	14GLU	C	0.01
14GLU	CD	283GLY	CA	0.01
14GLU	CD	333THR	CG2	0.00
14GLU	C	15VAL	CA	100.00
14GLU	C	15VAL	CB	0.10
14GLU	C	15VAL	CG1	0.01
14GLU	C	15VAL	C	99.96
14GLU	C	333THR	CG2	0.01
15VAL	CA	15VAL	CB	100.00
15VAL	CA	15VAL	CG1	100.00
15VAL	CA	15VAL	CG2	100.00
15VAL	CA	15VAL	C	100.00
15VAL	CA	18ALA	CB	0.00
15VAL	CB	15VAL	CG1	100.00
15VAL	CB	15VAL	CG2	100.00
15VAL	CB	15VAL	C	100.00
15VAL	CB	287PRO	CA	0.00
15VAL	CB	287PRO	CB	0.09
15VAL	CB	290ALA	CB	0.06
15VAL	CG1	15VAL	CG2	100.00
15VAL	CG1	273HIS	CE1	0.01
15VAL	CG1	285ALA	CB	1.44
15VAL	CG1	286ASN	C	0.01
15VAL	CG1	287PRO	CA	0.40
15VAL	CG1	287PRO	CB	0.02
15VAL	CG1	333THR	CG2	0.14
15VAL	CG2	15VAL	C	100.00
15VAL	CG2	273HIS	CE1	1.99
15VAL	CG2	290ALA	CB	0.34
15VAL	C	16THR	CA	100.00
15VAL	C	16THR	CB	0.02
15VAL	C	16THR	C	100.00
15VAL	C	18ALA	CB	0.01
15VAL	C	290ALA	CB	0.23
16THR	CA	16THR	CB	100.00
16THR	CA	16THR	CG2	100.00
16THR	CA	16THR	C	100.00
16THR	CB	16THR	CG2	100.00
16THR	CB	16THR	C	100.00
16THR	CG2	16THR	C	100.00
16THR	CG2	20LEU	CD1	0.03
16THR	CG2	68LEU	CG	0.01

288THR	CA	288THR	CG2	100.00
288THR	CA	288THR	C	100.00
288THR	CA	315VAL	CG1	0.00
288THR	CB	288THR	CG2	100.00
288THR	CB	288THR	C	100.00
288THR	CB	315VAL	CG1	0.17
288THR	CB	315VAL	CG2	0.02
288THR	CG2	288THR	C	46.02
288THR	CG2	292LEU	CD1	3.18
288THR	CG2	315VAL	CB	0.05
288THR	CG2	315VAL	CG1	2.58
288THR	CG2	315VAL	CG2	0.23
288THR	CG2	319LEU	CD1	0.01
288THR	CG2	319LEU	CD2	0.04
288THR	CG2	336PHE	CB	0.04
288THR	CG2	336PHE	CG	0.32
288THR	CG2	336PHE	CD1	9.59
288THR	CG2	336PHE	CD2	0.03
288THR	CG2	336PHE	CE1	2.47
288THR	CG2	336PHE	CE2	0.02
288THR	CG2	336PHE	CZ	0.63
288THR	C	289ALA	CA	100.00
288THR	C	289ALA	CB	0.00
288THR	C	289ALA	C	100.00
288THR	C	292LEU	CD1	0.00
289ALA	CA	289ALA	CB	100.00
289ALA	CA	289ALA	C	100.00
289ALA	CB	289ALA	C	100.00
289ALA	C	290ALA	CA	100.00
289ALA	C	290ALA	CB	0.05
289ALA	C	290ALA	C	99.98
290ALA	CA	290ALA	CB	100.00
290ALA	CA	290ALA	C	100.00
290ALA	CA	293SER	CB	0.00
290ALA	CB	290ALA	C	100.00
290ALA	C	291ILE	CA	100.00
290ALA	C	291ILE	CG1	0.00
290ALA	C	291ILE	C	100.00
291ILE	CA	291ILE	CB	100.00
291ILE	CA	291ILE	CG1	100.00
291ILE	CA	291ILE	CG2	100.00
291ILE	CA	291ILE	CD	0.80
291ILE	CA	291ILE	C	100.00
291ILE	CB	291ILE	CG1	100.00
291ILE	CB	291ILE	CG2	100.00
291ILE	CB	291ILE	CD	100.00
291ILE	CB	291ILE	C	100.00
291ILE	CB	315VAL	CG2	0.01
291ILE	CG1	291ILE	CG2	100.00
291ILE	CG1	291ILE	CD	100.00
291ILE	CG2	291ILE	CD	99.95
291ILE	CG2	291ILE	C	100.00
291ILE	CG2	311VAL	CG1	13.09
291ILE	CG2	315VAL	CB	0.01
291ILE	CG2	315VAL	CG1	3.72
291ILE	CG2	315VAL	CG2	18.16
291ILE	CD	315VAL	CG1	0.03
291ILE	CD	315VAL	CG2	0.02
291ILE	CD	336PHE	CD1	0.08
291ILE	CD	336PHE	CE1	0.13
291ILE	CD	337THR	CG2	0.31
291ILE	C	292LEU	CA	100.00

17GLU	CD	24ARG	CZ	0.00
17GLU	C	18ALA	CA	100.00
17GLU	C	18ALA	C	100.00
18ALA	CA	18ALA	CB	100.00
18ALA	CA	18ALA	C	100.00
18ALA	CA	337THR	CG2	0.08
18ALA	CB	18ALA	C	100.00
18ALA	CB	287PRO	CB	15.90
18ALA	CB	287PRO	CG	0.14
18ALA	CB	291ILE	CG1	0.00
18ALA	CB	333THR	CB	0.01
18ALA	CB	333THR	CG2	0.46
18ALA	CB	337THR	CG2	4.56
18ALA	C	19ALA	CA	100.00
18ALA	C	19ALA	CB	0.05
18ALA	C	19ALA	C	99.95
18ALA	C	291ILE	CG1	0.02
18ALA	C	291ILE	CD	0.02
18ALA	C	337THR	CG2	0.00
19ALA	CA	19ALA	CB	100.00
19ALA	CA	19ALA	C	100.00
19ALA	CA	291ILE	CG1	0.02
19ALA	CA	291ILE	CD	0.01
19ALA	CB	19ALA	C	100.00
19ALA	CB	68LEU	CD2	0.02
19ALA	CB	290ALA	CB	0.02
19ALA	CB	290ALA	C	5.77
19ALA	CB	291ILE	CA	0.03
19ALA	CB	291ILE	CG1	0.01
19ALA	CB	294ALA	CB	0.09
19ALA	C	20LEU	CA	100.00
19ALA	C	20LEU	C	99.99
20LEU	CA	20LEU	CB	100.00
20LEU	CA	20LEU	CG	100.00
20LEU	CA	20LEU	CD1	1.93
20LEU	CA	20LEU	CD2	97.80
20LEU	CA	20LEU	C	100.00
20LEU	CB	20LEU	CG	100.00
20LEU	CB	20LEU	CD1	100.00
20LEU	CB	20LEU	CD2	100.00
20LEU	CB	20LEU	C	100.00
20LEU	CB	24ARG	CZ	1.01
20LEU	CB	36TYR	CE1	0.00
20LEU	CB	36TYR	CE2	0.00
20LEU	CB	36TYR	CZ	0.00
20LEU	CG	20LEU	CD1	100.00
20LEU	CG	20LEU	CD2	100.00
20LEU	CG	20LEU	C	1.73
20LEU	CG	68LEU	CD1	0.03
20LEU	CG	68LEU	CD2	0.38
20LEU	CD1	20LEU	CD2	100.00
20LEU	CD1	20LEU	C	0.02
20LEU	CD1	36TYR	CD1	0.05
20LEU	CD1	36TYR	CD2	0.09
20LEU	CD1	36TYR	CE1	0.84
20LEU	CD1	36TYR	CE2	1.65
20LEU	CD1	36TYR	CZ	0.05
20LEU	CD1	38VAL	CG2	0.01
20LEU	CD1	68LEU	CD2	0.08
20LEU	CD2	20LEU	C	0.05
20LEU	CD2	23LEU	CD1	0.04
20LEU	CD2	34LEU	CD2	0.00

16THR	CG2	68LEU	CD2	1.78	291ILE	C	292LEU	C	100.00	20LEU	CD2	36TYR	CB	0.02
16THR	C	17GLU	CA	100.00	292LE	CA	292LEU	CB	100.00	20LEU	CD2	36TYR	CG	0.58
16THR	C	17GLU	CB	0.00	292LE	CA	292LEU	CG	100.00	20LEU	CD2	36TYR	CD1	0.78
16THR	C	17GLU	C	100.00	292LE	CA	292LEU	CD1	1.25	20LEU	CD2	36TYR	CD2	1.17
17GLU	CA	17GLU	CB	100.00	292LE	CA	292LEU	CD2	98.80	20LEU	CD2	36TYR	CE1	0.47
17GLU	CA	17GLU	CG	100.00	292LE	CA	292LEU	C	100.00	20LEU	CD2	36TYR	CE2	0.54
17GLU	CA	17GLU	CD	97.35	292LE	CA	295ALA	CB	0.07	20LEU	CD2	36TYR	CZ	0.19
17GLU	CA	17GLU	C	100.00	292LE	CB	292LEU	CG	100.00	20LEU	CD2	68LEU	CD1	1.15
17GLU	CB	17GLU	CG	100.00	292LE	CB	292LEU	CD1	100.00	20LEU	CD2	68LEU	CD2	1.48
17GLU	CB	17GLU	CD	100.00	292LE	CB	292LEU	CD2	100.00	20LEU	C	21LYS	CA	100.00
17GLU	CB	17GLU	C	100.00	292LE	CB	292LEU	C	100.00	20LEU	C	21LYS	C	100.00
17GLU	CG	17GLU	CD	100.00	292LE	CG	292LEU	CD1	100.00	20LEU	C	24ARG	CD	0.01
17GLU	CG	17GLU	C	93.08	292LE	CG	292LEU	CD2	100.00	20LEU	C	24ARG	CZ	0.01
17GLU	CG	21LYS	CE	0.04	292LE	CG	292LEU	C	0.23	21LYS	CA	21LYS	CB	100.00
17GLU	CD	17GLU	C	2.44	292LE	CG	315VAL	CG1	0.01	21LYS	CA	21LYS	CG	100.00
17GLU	CD	20LEU	CD1	0.00	292LE	CD1	292LEU	CD2	100.00	21LYS	CA	21LYS	CD	0.04
17GLU	CD	20LEU	CD2	0.00	292LE	CD1	292LEU	C	0.00	21LYS	CA	21LYS	C	100.00
17GLU	C	18ALA	CA	100.00	292LE	CD1	295ALA	CB	0.00	21LYS	CA	24ARG	CG	0.01
17GLU	C	18ALA	C	100.00	292LE	CD1	312GLU	CG	0.00	21LYS	CA	24ARG	CZ	2.14
18ALA	CA	18ALA	CB	100.00	292LE	CD1	315VAL	CG1	0.34	21LYS	CB	21LYS	CG	100.00
18ALA	CA	18ALA	C	100.00	292LE	CD1	315VAL	CG2	0.02	21LYS	CB	21LYS	CD	100.00
18ALA	CA	21LYS	CD	0.00	292LE	CD2	292LEU	C	0.01	21LYS	CB	21LYS	CE	19.40
18ALA	CA	337THR	CG2	0.05	292LE	CD2	295ALA	CB	0.02	21LYS	CB	21LYS	C	100.00
18ALA	CB	18ALA	C	100.00	292LE	CD2	311VAL	CG1	0.00	21LYS	CB	24ARG	CZ	0.04
18ALA	CB	287PRO	CB	13.74	292LE	CD2	312GLU	CA	0.09	21LYS	CB	337THR	CG2	0.20
18ALA	CB	287PRO	CG	0.31	292LE	CD2	312GLU	CB	0.06	21LYS	CG	21LYS	CD	100.00
18ALA	CB	291ILE	CD	0.00	292LE	CD2	312GLU	CG	2.50	21LYS	CG	21LYS	CE	100.00
18ALA	CB	333THR	CG2	0.19	292LE	CD2	312GLU	CD	0.07	21LYS	CG	21LYS	C	58.31
18ALA	CB	337THR	CG2	1.96	292LE	CD2	315VAL	CB	0.00	21LYS	CG	24ARG	CZ	0.18
18ALA	C	19ALA	CA	100.00	292LE	CD2	315VAL	CG1	0.58	21LYS	CG	337THR	CG2	0.00
18ALA	C	19ALA	CB	0.00	292LE	CD2	315VAL	CG2	0.57	21LYS	CG	341LEU	CD1	0.02
18ALA	C	19ALA	C	100.00	292LE	C	293SER	CA	100.00	21LYS	CG	341LEU	CD2	0.00
18ALA	C	291ILE	CG1	0.03	292LE	C	293SER	C	100.00	21LYS	CD	21LYS	CE	100.00
18ALA	C	291ILE	CD	0.01	293SE	CA	293SER	CB	100.00	21LYS	CD	337THR	CB	0.13
19ALA	CA	19ALA	CB	100.00	293SE	CA	293SER	C	100.00	21LYS	CD	337THR	CG2	0.23
19ALA	CA	19ALA	C	100.00	293SE	CA	296MET	CB	0.00	21LYS	CD	341LEU	CD1	0.09
19ALA	CA	291ILE	CG1	0.14	293SE	CB	293SER	C	100.00	21LYS	CE	337THR	CB	0.02
19ALA	CA	291ILE	CD	0.01	293SE	C	294ALA	CA	100.00	21LYS	CE	337THR	CG2	0.01
19ALA	CB	19ALA	C	100.00	293SE	C	294ALA	CB	0.05	21LYS	CE	341LEU	CD1	0.06
19ALA	CB	68LEU	CD2	0.01	293SE	C	294ALA	C	99.97	21LYS	CE	341LEU	CD2	0.00
19ALA	CB	290ALA	CB	0.12	293SE	C	297MET	CE	0.00	21LYS	C	22VAL	CA	100.00
19ALA	CB	290ALA	C	12.65	294AL	CA	294ALA	CB	100.00	21LYS	C	22VAL	CB	0.06
19ALA	CB	291ILE	CA	0.12	294AL	CA	294ALA	C	100.00	21LYS	C	22VAL	CG2	0.01
19ALA	CB	291ILE	CG1	0.00	294AL	CB	294ALA	C	100.00	21LYS	C	22VAL	C	99.98
19ALA	CB	294ALA	CB	0.06	294AL	C	295ALA	CA	100.00	21LYS	C	341LEU	CD2	0.02
19ALA	C	20LEU	CA	100.00	294AL	C	295ALA	CB	0.07	22VAL	CA	22VAL	CB	100.00
19ALA	C	20LEU	CB	0.00	294AL	C	295ALA	C	99.91	22VAL	CA	22VAL	CG1	100.00
19ALA	C	20LEU	C	99.99	294AL	C	297MET	CG	0.00	22VAL	CA	22VAL	CG2	100.00
20LEU	CA	20LEU	CB	100.00	295AL	CA	295ALA	CB	100.00	22VAL	CA	22VAL	C	100.00
20LEU	CA	20LEU	CG	100.00	295AL	CA	295ALA	C	100.00	22VAL	CA	341LEU	CD2	0.03
20LEU	CA	20LEU	CD1	11.52	295AL	CA	311VAL	CG2	0.01	22VAL	CB	22VAL	CG1	100.00
20LEU	CA	20LEU	CD2	91.87	295AL	CB	295ALA	C	100.00	22VAL	CB	22VAL	CG2	100.00
20LEU	CA	20LEU	C	100.00	295AL	CB	308ALA	CA	0.00	22VAL	CB	22VAL	C	100.00
20LEU	CB	20LEU	CG	100.00	295AL	CB	311VAL	CB	0.02	22VAL	CG1	22VAL	CG2	100.00
20LEU	CB	20LEU	CD1	100.00	295AL	CB	311VAL	CG1	2.68	22VAL	CG1	22VAL	C	100.00
20LEU	CB	20LEU	CD2	100.00	295AL	CB	311VAL	CG2	0.52	22VAL	CG1	26LEU	CD1	0.43
20LEU	CB	20LEU	C	100.00	295AL	CB	312GLU	CG	0.09	22VAL	CG1	291ILE	CG2	0.01
20LEU	CB	24ARG	CZ	2.14	295AL	C	296MET	CA	100.00	22VAL	CG1	311VAL	CG1	0.12
20LEU	CB	36TYR	CE1	0.00	295AL	C	296MET	C	100.00	22VAL	CG1	311VAL	CG2	0.58
20LEU	CG	20LEU	CD1	100.00	295AL	C	308ALA	CB	0.01	22VAL	CG1	340VAL	CG1	0.15
20LEU	CG	20LEU	CD2	100.00	296ME	CA	296MET	CB	100.00	22VAL	CG1	341LEU	CD2	0.00
20LEU	CG	20LEU	C	7.35	296ME	CA	296MET	CG	100.00	22VAL	CG1	344LEU	CD1	4.28

20LEU	CG	24ARG	CD	0.00	296ME	CA	296MET	C	100.00	22VAL	CG1	344LEU	CD2	0.46
20LEU	CG	24ARG	CZ	0.16	296ME	CB	296MET	CG	100.00	22VAL	CG2	291ILE	CG2	0.07
20LEU	CG	36TYR	CE1	0.04	296ME	CB	296MET	CE	2.95	22VAL	CG2	291ILE	CD	0.01
20LEU	CG	68LEU	CD2	0.06	296ME	CB	296MET	C	100.00	22VAL	CG2	337THR	CB	0.02
20LEU	CD1	20LEU	CD2	100.00	296ME	CG	296MET	CE	100.00	22VAL	CG2	337THR	CG2	0.34
20LEU	CD1	20LEU	C	0.40	296ME	CG	296MET	C	94.86	22VAL	CG2	340VAL	CG1	0.80
20LEU	CD1	23LEU	CD2	0.02	296ME	CG	300HIS	CD2	1.02	22VAL	CG2	341LEU	CD1	0.12
20LEU	CD1	24ARG	CZ	0.02	296ME	CG	300HIS	CE1	0.01	22VAL	CG2	341LEU	CD2	0.06
20LEU	CD1	36TYR	CB	0.01	296ME	CE	300HIS	CD2	0.20	22VAL	C	23LEU	CA	100.00
20LEU	CD1	36TYR	CG	0.27	296ME	CE	300HIS	CE1	0.06	22VAL	C	23LEU	C	100.00
20LEU	CD1	36TYR	CD1	0.51	296ME	C	297MET	CA	100.00	22VAL	C	26LEU	CD1	0.00
20LEU	CD1	36TYR	CD2	0.19	296ME	C	297MET	CB	0.00	23LEU	CA	23LEU	CB	100.00
20LEU	CD1	36TYR	CE1	3.43	296ME	C	297MET	C	100.00	23LEU	CA	23LEU	CG	100.00
20LEU	CD1	36TYR	CE2	0.12	297ME	CA	297MET	CB	100.00	23LEU	CA	23LEU	CD1	0.01
20LEU	CD1	36TYR	CZ	0.30	297ME	CA	297MET	CG	100.00	23LEU	CA	23LEU	CD2	99.79
20LEU	CD1	38VAL	CG2	0.04	297ME	CA	297MET	C	100.00	23LEU	CA	23LEU	C	100.00
20LEU	CD1	68LEU	CD2	0.15	297ME	CB	297MET	CG	100.00	23LEU	CA	32LEU	CD1	0.00
20LEU	CD2	20LEU	C	0.12	297ME	CB	297MET	CE	81.36	23LEU	CB	23LEU	CG	100.00
20LEU	CD2	23LEU	CD1	0.02	297ME	CB	297MET	C	100.00	23LEU	CB	23LEU	CD1	100.00
20LEU	CD2	23LEU	CD2	0.02	297ME	CG	297MET	CE	100.00	23LEU	CB	23LEU	CD2	100.00
20LEU	CD2	24ARG	CZ	0.07	297ME	CG	297MET	C	98.87	23LEU	CB	23LEU	C	100.00
20LEU	CD2	34LEU	CD2	0.00	297ME	CG	301ALA	CB	0.15	23LEU	CB	32LEU	CD1	0.02
20LEU	CD2	36TYR	CB	0.00	297ME	CG	302PHE	CD2	0.01	23LEU	CB	34LEU	CD1	0.04
20LEU	CD2	36TYR	CG	0.28	297ME	CG	302PHE	CE1	0.71	23LEU	CB	34LEU	CD2	1.24
20LEU	CD2	36TYR	CD1	1.50	297ME	CG	302PHE	CE2	0.10	23LEU	CG	23LEU	CD1	100.00
20LEU	CD2	36TYR	CD2	0.28	297ME	CG	302PHE	CZ	0.39	23LEU	CG	23LEU	CD2	100.00
20LEU	CD2	36TYR	CE1	1.47	297ME	CE	297MET	C	0.01	23LEU	CG	23LEU	C	0.03
20LEU	CD2	36TYR	CE2	0.49	297ME	CE	298LEU	CD1	0.10	23LEU	CG	294ALA	CB	0.06
20LEU	CD2	36TYR	CZ	0.46	297ME	CE	298LEU	CD2	0.05	23LEU	CD1	23LEU	CD2	100.00
20LEU	CD2	38VAL	CG2	0.01	297ME	CE	302PHE	CD2	0.01	23LEU	CD1	32LEU	CD1	0.00
20LEU	CD2	68LEU	CD1	0.14	297ME	CE	302PHE	CE1	0.12	23LEU	CD1	34LEU	CD1	0.06
20LEU	CD2	68LEU	CD2	1.87	297ME	CE	302PHE	CE2	0.24	23LEU	CD1	68LEU	CD1	0.06
20LEU	C	21LYS	CA	100.00	297ME	CE	302PHE	CZ	0.08	23LEU	CD1	68LEU	CD2	0.03
20LEU	C	21LYS	C	100.00	297ME	C	298LEU	CA	100.00	23LEU	CD1	294ALA	CB	3.70
20LEU	C	24ARG	CD	0.01	297ME	C	298LEU	CB	0.00	23LEU	CD1	297MET	CE	7.39
20LEU	C	24ARG	CZ	0.04	297ME	C	298LEU	C	100.00	23LEU	CD1	298LEU	CD1	0.01
21LYS	CA	21LYS	CB	100.00	297ME	C	302PHE	CD1	0.01	23LEU	CD2	23LEU	C	0.03
21LYS	CA	21LYS	CG	100.00	298LE	CA	298LEU	CB	100.00	23LEU	CD2	32LEU	CD1	2.06
21LYS	CA	21LYS	CD	0.06	298LE	CA	298LEU	CG	100.00	23LEU	CD2	32LEU	CD2	0.11
21LYS	CA	21LYS	C	100.00	298LE	CA	298LEU	CD1	21.38	23LEU	CD2	34LEU	CD1	0.00
21LYS	CA	24ARG	CZ	0.11	298LE	CA	298LEU	CD2	78.36	23LEU	CD2	294ALA	CB	0.02
21LYS	CB	21LYS	CG	100.00	298LE	CA	298LEU	C	100.00	23LEU	CD2	297MET	CE	0.63
21LYS	CB	21LYS	CD	100.00	298LE	CA	302PHE	CB	0.01	23LEU	CD2	298LEU	CG	0.16
21LYS	CB	21LYS	CE	12.88	298LE	CA	302PHE	CD2	0.02	23LEU	CD2	298LEU	CD1	3.45
21LYS	CB	21LYS	C	100.00	298LE	CA	302PHE	CE1	0.00	23LEU	CD2	298LEU	CD2	1.60
21LYS	CB	337THR	CG2	0.12	298LE	CB	298LEU	CG	100.00	23LEU	CD2	302PHE	CE1	0.01
21LYS	CG	21LYS	CD	100.00	298LE	CB	298LEU	CD1	100.00	23LEU	CD2	302PHE	CE2	0.00
21LYS	CG	21LYS	CE	100.00	298LE	CB	298LEU	CD2	100.00	23LEU	C	24ARG	CA	100.00
21LYS	CG	21LYS	C	36.81	298LE	CB	298LEU	C	100.00	23LEU	C	24ARG	CB	0.01
21LYS	CG	24ARG	CZ	0.02	298LE	CB	308ALA	CA	0.02	23LEU	C	24ARG	C	100.00
21LYS	CG	337THR	CG2	0.02	298LE	CB	308ALA	CB	0.44	23LEU	C	32LEU	CD1	0.00
21LYS	CG	341LEU	CD1	0.05	298LE	CG	298LEU	CD1	100.00	23LEU	C	34LEU	CD1	0.03
21LYS	CD	21LYS	CE	100.00	298LE	CG	298LEU	CD2	100.00	23LEU	C	34LEU	CD2	1.41
21LYS	CD	337THR	CG2	0.57	298LE	CG	298LEU	C	11.02	24AR	CA	24ARG	CB	100.00
21LYS	CD	341LEU	CD1	0.16	298LE	CG	302PHE	CD2	0.00	24AR	CA	24ARG	CG	100.00
21LYS	CE	337THR	CG2	0.02	298LE	CD1	298LEU	CD2	100.00	24AR	CA	24ARG	CD	0.02
21LYS	CE	341LEU	CD1	0.12	298LE	CD1	298LEU	C	0.16	24AR	CA	24ARG	C	100.00
21LYS	CE	341LEU	CD2	0.00	298LE	CD1	302PHE	CB	0.01	24AR	CA	34LEU	CD2	1.39
21LYS	C	22VAL	CA	100.00	298LE	CD1	302PHE	CG	0.04	24AR	CB	24ARG	CG	100.00
21LYS	C	22VAL	CB	0.02	298LE	CD1	302PHE	CD1	0.10	24AR	CB	24ARG	CD	100.00
21LYS	C	22VAL	C	100.00	298LE	CD1	302PHE	CD2	0.07	24AR	CB	24ARG	CZ	0.84
21LYS	C	341LEU	CD2	0.04	298LE	CD1	302PHE	CE1	0.05	24AR	CB	24ARG	C	100.00

22VAL	CA	22VAL	CB	100.00	298LEU	CD1	304LEU	CB	0.02	24ARG	CB	34LEU	CD1	0.00
22VAL	CA	22VAL	CG1	100.00	298LEU	CD1	304LEU	CD1	0.11	24ARG	CB	34LEU	CD2	0.47
22VAL	CA	22VAL	CG2	100.00	298LEU	CD1	307LEU	CB	0.26	24ARG	CG	24ARG	CD	100.00
22VAL	CA	22VAL	C	100.00	298LEU	CD1	307LEU	CD1	0.00	24ARG	CG	24ARG	CZ	9.95
22VAL	CA	341LEU	CD2	0.07	298LEU	CD1	307LEU	CD2	0.06	24ARG	CG	24ARG	C	44.68
22VAL	CB	22VAL	CG1	100.00	298LEU	CD1	307LEU	C	0.10	24ARG	CG	34LEU	CD1	0.00
22VAL	CB	22VAL	CG2	100.00	298LEU	CD1	311VAL	CG2	0.47	24ARG	CG	34LEU	CD2	2.28
22VAL	CB	22VAL	C	100.00	298LEU	CD2	298LEU	C	0.18	24ARG	CG	36TYR	CE1	0.02
22VAL	CG1	22VAL	CG2	100.00	298LEU	CD2	302PHE	CG	0.12	24ARG	CG	36TYR	CE2	0.01
22VAL	CG1	22VAL	C	100.00	298LEU	CD2	302PHE	CD1	0.48	24ARG	CD	24ARG	CZ	100.00
22VAL	CG1	26LEU	CD1	0.41	298LEU	CD2	302PHE	CD2	0.78	24ARG	CD	36TYR	CE1	0.13
22VAL	CG1	291ILE	CG2	0.14	298LEU	CD2	302PHE	CE1	0.15	24ARG	CD	36TYR	CE2	0.12
22VAL	CG1	311VAL	CG1	0.28	298LEU	CD2	302PHE	CE2	0.18	24ARG	CD	36TYR	CZ	0.07
22VAL	CG1	311VAL	CG2	2.56	298LEU	CD2	302PHE	CZ	0.00	24ARG	CZ	36TYR	CE1	0.06
22VAL	CG1	340VAL	CG1	0.33	298LEU	CD2	304LEU	CB	0.07	24ARG	CZ	36TYR	CE2	0.00
22VAL	CG1	344LEU	CD1	1.81	298LEU	CD2	304LEU	CD1	0.86	24ARG	CZ	36TYR	CZ	0.04
22VAL	CG1	344LEU	CD2	0.72	298LEU	CD2	304LEU	CD2	0.01	24ARG	C	25ALA	CA	100.00
22VAL	CG2	291ILE	CG1	0.00	298LEU	CD2	307LEU	CB	0.55	24ARG	C	25ALA	CB	0.02
22VAL	CG2	291ILE	CG2	0.04	298LEU	CD2	307LEU	CG	0.02	24ARG	C	25ALA	C	99.97
22VAL	CG2	291ILE	CD	0.03	298LEU	CD2	307LEU	CD1	0.02	25ALA	CA	25ALA	CB	100.00
22VAL	CG2	337THR	CG2	0.35	298LEU	CD2	307LEU	CD2	0.08	25ALA	CA	25ALA	C	100.00
22VAL	CG2	340VAL	CG1	2.74	298LEU	CD2	307LEU	C	0.06	25ALA	CB	25ALA	C	100.00
22VAL	CG2	341LEU	CD1	0.12	298LEU	CD2	308ALA	CA	0.03	25ALA	CB	341LEU	CG	0.01
22VAL	CG2	341LEU	CD2	0.37	298LEU	CD2	308ALA	CB	0.00	25ALA	CB	341LEU	CD1	0.50
22VAL	CG2	344LEU	CD1	0.01	298LEU	CD2	311VAL	CG2	0.01	25ALA	CB	341LEU	CD2	33.31
22VAL	C	23LEU	CA	100.00	298LEU	C	299GLU	CA	100.00	25ALA	CB	344LEU	CD1	0.05
22VAL	C	23LEU	C	99.99	298LEU	C	299GLU	CB	0.10	25ALA	CB	344LEU	CD2	0.21
23LEU	CA	23LEU	CB	100.00	298LEU	C	299GLU	C	99.97	25ALA	C	26LEU	CA	100.00
23LEU	CA	23LEU	CG	100.00	298LEU	C	308ALA	CB	0.01	25ALA	C	26LEU	CB	0.01
23LEU	CA	23LEU	CD1	53.39	299GLU	CA	299GLU	CB	100.00	25ALA	C	26LEU	C	99.99
23LEU	CA	23LEU	CD2	46.48	299GLU	CA	299GLU	CG	100.00	26LEU	CA	26LEU	CB	100.00
23LEU	CA	23LEU	C	100.00	299GLU	CA	299GLU	CD	49.49	26LEU	CA	26LEU	CG	100.00
23LEU	CB	23LEU	CG	100.00	299GLU	CA	299GLU	C	100.00	26LEU	CA	26LEU	CD1	14.83
23LEU	CB	23LEU	CD1	100.00	299GLU	CA	305VAL	CG1	0.00	26LEU	CA	26LEU	CD2	84.77
23LEU	CB	23LEU	CD2	100.00	299GLU	CA	308ALA	CB	0.11	26LEU	CA	26LEU	C	100.00
23LEU	CB	23LEU	C	100.00	299GLU	CB	299GLU	CG	100.00	26LEU	CA	29ALA	CB	0.00
23LEU	CB	32LEU	CD1	0.00	299GLU	CB	299GLU	CD	100.00	26LEU	CB	26LEU	CG	100.00
23LEU	CB	34LEU	CD1	0.02	299GLU	CB	299GLU	C	100.00	26LEU	CB	26LEU	CD1	100.00
23LEU	CB	34LEU	CD2	0.55	299GLU	CB	300HIS	CD2	0.06	26LEU	CB	26LEU	CD2	100.00
23LEU	CG	23LEU	CD1	100.00	299GLU	CB	308ALA	CB	2.55	26LEU	CB	26LEU	C	100.00
23LEU	CG	23LEU	CD2	100.00	299GLU	CG	299GLU	CD	100.00	26LEU	CB	32LEU	CD1	0.36
23LEU	CG	23LEU	C	50.86	299GLU	CG	299GLU	C	96.08	26LEU	CB	32LEU	CD2	3.89
23LEU	CG	32LEU	CD1	0.19	299GLU	CG	300HIS	CD2	0.95	26LEU	CG	26LEU	CD1	100.00
23LEU	CG	34LEU	CD1	0.03	299GLU	CG	300HIS	CE1	0.02	26LEU	CG	26LEU	CD2	100.00
23LEU	CG	34LEU	CD2	0.02	299GLU	CG	305VAL	CA	0.00	26LEU	CG	26LEU	C	13.20
23LEU	CG	294ALA	CB	0.00	299GLU	CG	305VAL	CB	0.01	26LEU	CG	30GLU	CG	0.00
23LEU	CD1	23LEU	CD2	100.00	299GLU	CG	305VAL	CG1	0.24	26LEU	CG	32LEU	CD1	0.00
23LEU	CD1	23LEU	C	0.07	299GLU	CG	305VAL	CG2	0.35	26LEU	CG	32LEU	CD2	0.07
23LEU	CD1	26LEU	CD1	0.00	299GLU	CG	308ALA	CB	0.75	26LEU	CG	344LEU	CD2	0.02
23LEU	CD1	26LEU	CD2	0.01	299GLU	CD	299GLU	C	35.77	26LEU	CD1	26LEU	CD2	100.00
23LEU	CD1	32LEU	CD1	1.76	299GLU	CD	300HIS	CD2	0.49	26LEU	CD1	26LEU	C	0.09
23LEU	CD1	32LEU	CD2	0.49	299GLU	CD	300HIS	CE1	0.05	26LEU	CD1	30GLU	CB	0.15
23LEU	CD1	34LEU	CD1	0.10	299GLU	CD	305VAL	CG1	0.11	26LEU	CD1	30GLU	CG	0.10
23LEU	CD1	68LEU	CD1	0.02	299GLU	CD	305VAL	CG2	0.15	26LEU	CD1	32LEU	CD1	0.01
23LEU	CD1	68LEU	CD2	0.01	299GLU	CD	309ARG	CZ	0.18	26LEU	CD1	32LEU	CD2	0.00
23LEU	CD1	294ALA	CB	1.18	299GLU	C	300HIS	CA	100.00	26LEU	CD1	298LEU	CD1	2.44
23LEU	CD1	294ALA	C	0.00	299GLU	C	300HIS	CB	4.10	26LEU	CD1	298LEU	CD2	0.11
23LEU	CD1	297MET	CE	1.93	299GLU	C	300HIS	CG	0.24	26LEU	CD1	307LEU	CD1	0.00
23LEU	CD1	298LEU	CG	0.11	299GLU	C	300HIS	CD2	0.02	26LEU	CD1	307LEU	CD2	0.06
23LEU	CD1	298LEU	CD1	1.92	299GLU	C	300HIS	C	95.16	26LEU	CD1	310LYS	CD	0.01
23LEU	CD1	298LEU	CD2	0.34	300HIS	CA	300HIS	CB	100.00	26LEU	CD1	310LYS	CE	0.00
23LEU	CD2	23LEU	C	0.02	300HIS	CA	300HIS	CG	100.00	26LEU	CD1	311VAL	CG2	6.66

23LEU	CD2	32LEU	CD1	0.27	300HIS	CA	300HIS	CD2	38.34	26LEU	CD1	344LEU	CD1	0.05
23LEU	CD2	32LEU	CD2	0.07	300HIS	CA	300HIS	C	100.00	26LEU	CD1	344LEU	CD2	0.06
23LEU	CD2	34LEU	CD1	6.56	300HIS	CB	300HIS	CG	100.00	26LEU	CD2	26LEU	C	0.36
23LEU	CD2	34LEU	CD2	0.16	300HIS	CB	300HIS	CD2	100.00	26LEU	CD2	30GLU	CB	0.04
23LEU	CD2	294ALA	CB	0.02	300HIS	CB	300HIS	CE1	0.31	26LEU	CD2	30GLU	CG	0.12
23LEU	CD2	297MET	CE	2.13	300HIS	CB	300HIS	C	100.00	26LEU	CD2	32LEU	CD1	0.02
23LEU	CD2	298LEU	CG	0.01	300HIS	CG	300HIS	CD2	100.00	26LEU	CD2	32LEU	CD2	0.12
23LEU	CD2	298LEU	CD1	1.68	300HIS	CG	300HIS	CE1	100.00	26LEU	CD2	298LEU	CD1	1.04
23LEU	CD2	298LEU	CD2	0.28	300HIS	CG	300HIS	C	89.94	26LEU	CD2	298LEU	CD2	0.03
23LEU	C	24ARG	CA	100.00	300HIS	CD2	300HIS	CE1	100.00	26LEU	CD2	307LEU	CD1	0.08
23LEU	C	24ARG	C	100.00	300HIS	CD2	300HIS	C	38.47	26LEU	CD2	307LEU	CD2	0.20
23LEU	C	32LEU	CD1	0.00	300HIS	CD2	301ALA	CA	0.04	26LEU	CD2	310LYS	CD	0.07
23LEU	C	34LEU	CD1	0.00	300HIS	CD2	301ALA	CB	0.03	26LEU	CD2	310LYS	CE	0.03
23LEU	C	34LEU	CD2	0.80	300HIS	C	301ALA	CA	100.00	26LEU	CD2	311VAL	CG1	0.01
24ARG	CA	24ARG	CB	100.00	300HIS	C	301ALA	CB	20.02	26LEU	CD2	311VAL	CG2	0.86
24ARG	CA	24ARG	CG	100.00	300HIS	C	301ALA	C	80.40	26LEU	CD2	341LEU	CD2	0.00
24ARG	CA	24ARG	CD	1.46	301AL/	CA	301ALA	CB	100.00	26LEU	CD2	344LEU	CD1	0.04
24ARG	CA	24ARG	C	100.00	301AL/	CA	301ALA	C	100.00	26LEU	CD2	344LEU	CD2	0.35
24ARG	CA	34LEU	CD2	1.22	301AL/	CB	301ALA	C	100.00	26LEU	C	27ASP	CA	100.00
24ARG	CB	24ARG	CG	100.00	301AL/	CB	302PHE	CD1	0.38	26LEU	C	27ASP	CB	0.02
24ARG	CB	24ARG	CD	100.00	301AL/	CB	302PHE	CD2	0.30	26LEU	C	27ASP	C	100.00
24ARG	CB	24ARG	CZ	0.67	301AL/	CB	302PHE	CE1	0.26	26LEU	C	32LEU	CD1	0.02
24ARG	CB	24ARG	C	100.00	301AL/	CB	302PHE	CE2	0.08	26LEU	C	32LEU	CD2	0.98
24ARG	CB	34LEU	CD2	0.19	301AL/	CB	302PHE	CZ	0.01	27ASF	CA	27ASP	CB	100.00
24ARG	CG	24ARG	CD	100.00	301AL/	C	302PHE	CA	100.00	27ASF	CA	27ASP	CG	100.00
24ARG	CG	24ARG	CZ	14.98	301AL/	C	302PHE	CB	32.27	27ASF	CA	27ASP	C	100.00
24ARG	CG	24ARG	C	24.42	301AL/	C	302PHE	CG	7.38	27ASF	CA	31GLY	CA	0.05
24ARG	CG	34LEU	CD2	3.67	301AL/	C	302PHE	CD1	1.06	27ASF	CA	32LEU	CB	0.10
24ARG	CG	36TYR	CE2	0.02	301AL/	C	302PHE	CD2	2.44	27ASF	CA	32LEU	CD1	0.01
24ARG	CD	24ARG	CZ	100.00	301AL/	C	302PHE	C	68.53	27ASF	CA	32LEU	CD2	0.30
24ARG	CD	24ARG	C	0.19	302PHI	CA	302PHE	CB	100.00	27ASF	CB	27ASP	CG	100.00
24ARG	CD	36TYR	CE2	0.56	302PHI	CA	302PHE	CG	100.00	27ASF	CB	27ASP	C	100.00
24ARG	CD	36TYR	CZ	0.16	302PHI	CA	302PHE	CD1	50.40	27ASF	CB	32LEU	CB	0.02
24ARG	CZ	34LEU	CD2	0.05	302PHI	CA	302PHE	CD2	74.13	27ASF	CB	32LEU	CD1	0.01
24ARG	CZ	36TYR	CE2	0.56	302PHI	CA	302PHE	C	100.00	27ASF	CB	32LEU	CD2	0.02
24ARG	CZ	36TYR	CZ	0.04	302PHI	CB	302PHE	CG	100.00	27ASF	CB	34LEU	CB	0.02
24ARG	C	25ALA	CA	100.00	302PHI	CB	302PHE	CD1	100.00	27ASF	CB	34LEU	CD1	0.02
24ARG	C	25ALA	CB	0.01	302PHI	CB	302PHE	CD2	100.00	27ASF	CB	34LEU	CD2	0.00
24ARG	C	25ALA	C	99.98	302PHI	CB	302PHE	C	100.00	27ASF	CG	27ASP	C	100.00
25ALA	CA	25ALA	CB	100.00	302PHI	CB	304LEU	CG	0.07	27ASF	CG	34LEU	CB	5.83
25ALA	CA	25ALA	C	100.00	302PHI	CB	304LEU	CD1	1.92	27ASF	C	28GLU	CA	100.00
25ALA	CB	25ALA	C	100.00	302PHI	CB	304LEU	CD2	0.02	27ASF	C	28GLU	CB	0.83
25ALA	CB	341LEU	CD1	0.17	302PHI	CG	302PHE	CD1	100.00	27ASF	C	28GLU	C	99.52
25ALA	CB	341LEU	CD2	23.43	302PHI	CG	302PHE	CD2	100.00	28GLL	CA	28GLU	CB	100.00
25ALA	CB	344LEU	CD1	0.14	302PHI	CG	302PHE	CE1	100.00	28GLL	CA	28GLU	CG	100.00
25ALA	CB	344LEU	CD2	2.22	302PHI	CG	302PHE	CE2	100.00	28GLL	CA	28GLU	CD	93.43
25ALA	C	26LEU	CA	100.00	302PHI	CG	302PHE	CZ	100.00	28GLL	CA	28GLU	C	100.00
25ALA	C	26LEU	CB	0.03	302PHI	CG	302PHE	C	16.62	28GLL	CB	28GLU	CG	100.00
25ALA	C	26LEU	CG	0.00	302PHI	CG	304LEU	CD1	0.71	28GLL	CB	28GLU	CD	100.00
25ALA	C	26LEU	C	99.99	302PHI	CD1	302PHE	CD2	100.00	28GLL	CB	28GLU	C	100.00
25ALA	C	344LEU	CD2	0.04	302PHI	CD1	302PHE	CE1	100.00	28GLL	CG	28GLU	CD	100.00
26LEU	CA	26LEU	CB	100.00	302PHI	CD1	302PHE	CE2	100.00	28GLL	CG	28GLU	C	98.55
26LEU	CA	26LEU	CG	100.00	302PHI	CD1	302PHE	CZ	100.00	28GLL	CD	28GLU	C	1.26
26LEU	CA	26LEU	CD1	23.47	302PHI	CD1	302PHE	C	5.62	28GLL	C	29ALA	CA	100.00
26LEU	CA	26LEU	CD2	76.15	302PHI	CD1	304LEU	CG	0.48	28GLL	C	29ALA	CB	4.16
26LEU	CA	26LEU	C	100.00	302PHI	CD1	304LEU	CD1	2.15	28GLL	C	29ALA	C	95.27
26LEU	CA	29ALA	CB	0.00	302PHI	CD1	304LEU	CD2	0.08	29ALA	CA	29ALA	CB	100.00
26LEU	CB	26LEU	CG	100.00	302PHI	CD2	302PHE	CE1	100.00	29ALA	CA	29ALA	C	100.00
26LEU	CB	26LEU	CD1	100.00	302PHI	CD2	302PHE	CE2	100.00	29ALA	CB	29ALA	C	100.00
26LEU	CB	26LEU	CD2	100.00	302PHI	CD2	302PHE	CZ	100.00	29ALA	CB	30GLU	CD	0.00
26LEU	CB	26LEU	C	100.00	302PHI	CD2	302PHE	C	3.06	29ALA	CB	345ALA	C	0.04
26LEU	CB	32LEU	CD1	0.12	302PHI	CD2	304LEU	CB	0.02	29ALA	C	30GLU	CA	100.00

26LEU	CB	32LEU	CD2	7.83	302PHI	CD2	304LEU	CG	0.26	29ALA	C	30GLU	CB	58.91
26LEU	CB	307LEU	CD2	0.00	302PHI	CD2	304LEU	CD1	1.61	29ALA	C	30GLU	CG	0.43
26LEU	CG	26LEU	CD1	100.00	302PHI	CD2	304LEU	CD2	0.05	29ALA	C	30GLU	CD	0.00
26LEU	CG	26LEU	CD2	100.00	302PHI	CE1	302PHE	CE2	100.00	29ALA	C	30GLU	C	46.74
26LEU	CG	26LEU	C	22.92	302PHI	CE1	302PHE	CZ	100.00	30GLL	CA	30GLU	CB	100.00
26LEU	CG	32LEU	CD2	0.10	302PHI	CE1	304LEU	CG	0.02	30GLL	CA	30GLU	CG	100.00
26LEU	CG	307LEU	CD1	0.01	302PHI	CE1	304LEU	CD1	0.76	30GLL	CA	30GLU	CD	98.11
26LEU	CG	307LEU	CD2	0.01	302PHI	CE1	304LEU	CD2	0.04	30GLL	CA	30GLU	C	100.00
26LEU	CG	344LEU	CD2	0.05	302PHI	CE1	307LEU	CD1	0.00	30GLL	CB	30GLU	CG	100.00
26LEU	CD1	26LEU	CD2	100.00	302PHI	CE2	302PHE	CZ	100.00	30GLL	CB	30GLU	CD	100.00
26LEU	CD1	26LEU	C	0.12	302PHI	CE2	304LEU	CB	0.01	30GLL	CB	30GLU	C	100.00
26LEU	CD1	30GLU	CB	0.11	302PHI	CE2	304LEU	CG	0.01	30GLL	CB	32LEU	CD1	0.01
26LEU	CD1	32LEU	CD1	0.00	302PHI	CE2	304LEU	CD1	0.82	30GLL	CB	32LEU	CD2	0.00
26LEU	CD1	298LEU	CD1	2.16	302PHI	CE2	304LEU	CD2	0.05	30GLL	CB	307LEU	CD1	0.05
26LEU	CD1	298LEU	CD2	0.72	302PHI	CE2	307LEU	CD2	0.01	30GLL	CB	307LEU	CD2	0.19
26LEU	CD1	307LEU	CG	0.00	302PHI	CZ	304LEU	CD1	0.21	30GLL	CG	30GLU	CD	100.00
26LEU	CD1	307LEU	CD1	0.08	302PHI	C	303GLY	CA	100.00	30GLL	CG	30GLU	C	84.63
26LEU	CD1	307LEU	CD2	0.03	302PHI	C	303GLY	C	69.87	30GLL	CG	307LEU	CD1	0.20
26LEU	CD1	310LYS	CB	0.00	302PHI	C	304LEU	CD1	0.05	30GLL	CG	307LEU	CD2	0.38
26LEU	CD1	310LYS	CG	0.01	302PHI	C	304LEU	CD2	0.01	30GLL	CD	30GLU	C	1.16
26LEU	CD1	310LYS	CD	0.08	303GL\	CA	303GLY	C	100.00	30GLL	CD	307LEU	CD1	0.02
26LEU	CD1	310LYS	CE	0.01	303GL\	C	304LEU	CA	100.00	30GLL	CD	307LEU	CD2	0.13
26LEU	CD1	311VAL	CG2	7.58	303GL\	C	304LEU	CB	8.82	30GLL	CD	310LYS	CE	0.01
26LEU	CD1	344LEU	CD1	0.06	303GL\	C	304LEU	CG	1.11	30GLL	C	31GLY	CA	100.00
26LEU	CD1	344LEU	CD2	0.05	303GL\	C	304LEU	CD1	0.13	30GLL	C	31GLY	C	67.14
26LEU	CD2	26LEU	C	0.18	303GL\	C	304LEU	CD2	0.10	31GLY	CA	31GLY	C	100.00
26LEU	CD2	30GLU	CB	0.02	303GL\	C	304LEU	C	94.42	31GLY	C	32LEU	CA	100.00
26LEU	CD2	32LEU	CD2	0.28	303GL\	C	305VAL	CG1	0.01	31GLY	C	32LEU	CB	46.05
26LEU	CD2	298LEU	CD1	2.54	303GL\	C	305VAL	CG2	0.02	31GLY	C	32LEU	CG	1.14
26LEU	CD2	298LEU	CD2	0.18	304LEI	CA	304LEU	CB	100.00	31GLY	C	32LEU	CD1	0.06
26LEU	CD2	307LEU	CB	0.01	304LEI	CA	304LEU	CG	100.00	31GLY	C	32LEU	CD2	0.12
26LEU	CD2	307LEU	CG	0.03	304LEI	CA	304LEU	CD1	6.98	31GLY	C	32LEU	C	52.35
26LEU	CD2	307LEU	CD1	0.25	304LEI	CA	304LEU	CD2	91.76	32LEU	CA	32LEU	CB	100.00
26LEU	CD2	307LEU	CD2	0.15	304LEI	CA	304LEU	C	100.00	32LEU	CA	32LEU	CG	100.00
26LEU	CD2	310LYS	CD	0.08	304LEI	CB	304LEU	CG	100.00	32LEU	CA	32LEU	CD1	15.42
26LEU	CD2	310LYS	CE	0.04	304LEI	CB	304LEU	CD1	100.00	32LEU	CA	32LEU	CD2	66.17
26LEU	CD2	311VAL	CG1	0.01	304LEI	CB	304LEU	CD2	100.00	32LEU	CA	32LEU	C	100.00
26LEU	CD2	311VAL	CG2	1.82	304LEI	CB	304LEU	C	100.00	32LEU	CA	302PHE	CE1	0.00
26LEU	CD2	344LEU	CG	0.04	304LEI	CB	307LEU	CB	0.02	32LEU	CA	304LEU	CD1	0.27
26LEU	CD2	344LEU	CD1	0.10	304LEI	CB	307LEU	CG	0.00	32LEU	CB	32LEU	CG	100.00
26LEU	CD2	344LEU	CD2	0.78	304LEI	CB	307LEU	CD1	0.18	32LEU	CB	32LEU	CD1	100.00
26LEU	C	27ASP	CA	100.00	304LEI	CG	304LEU	CD1	100.00	32LEU	CB	32LEU	CD2	100.00
26LEU	C	27ASP	CB	0.01	304LEI	CG	304LEU	CD2	100.00	32LEU	CB	32LEU	C	100.00
26LEU	C	27ASP	C	100.00	304LEI	CG	304LEU	C	1.08	32LEU	CB	34LEU	CD2	0.01
26LEU	C	32LEU	CD1	0.00	304LEI	CG	307LEU	CB	0.00	32LEU	CB	298LEU	CD1	0.01
26LEU	C	32LEU	CD2	1.40	304LEI	CD1	304LEU	CD2	100.00	32LEU	CB	298LEU	CD2	0.04
27ASP	CA	27ASP	CB	100.00	304LEI	CD1	304LEU	C	0.05	32LEU	CB	302PHE	CD2	0.02
27ASP	CA	27ASP	CG	100.00	304LEI	CD1	307LEU	CD1	0.42	32LEU	CB	302PHE	CE1	0.24
27ASP	CA	27ASP	C	100.00	304LEI	CD1	307LEU	CD2	0.21	32LEU	CB	302PHE	CE2	1.45
27ASP	CA	31GLY	CA	0.07	304LEI	CD2	304LEU	C	0.06	32LEU	CB	302PHE	CZ	0.31
27ASP	CA	32LEU	CB	0.12	304LEI	CD2	306GLU	CB	0.00	32LEU	CB	304LEU	CD1	7.27
27ASP	CA	32LEU	CD2	0.86	304LEI	CD2	306GLU	CD	0.00	32LEU	CB	304LEU	CD2	0.03
27ASP	CB	27ASP	CG	100.00	304LEI	CD2	307LEU	CB	0.02	32LEU	CB	307LEU	CD1	0.11
27ASP	CB	27ASP	C	100.00	304LEI	CD2	307LEU	CG	0.00	32LEU	CB	307LEU	CD2	0.02
27ASP	CB	32LEU	CB	0.02	304LEI	CD2	307LEU	CD1	0.43	32LEU	CG	32LEU	CD1	100.00
27ASP	CB	32LEU	CG	0.02	304LEI	CD2	307LEU	CD2	0.08	32LEU	CG	32LEU	CD2	100.00
27ASP	CB	32LEU	CD1	0.01	304LEI	C	305VAL	CA	100.00	32LEU	CG	32LEU	C	34.76
27ASP	CB	32LEU	CD2	0.20	304LEI	C	305VAL	CB	0.07	32LEU	CG	298LEU	CD1	0.04
27ASP	CB	34LEU	CB	0.01	304LEI	C	305VAL	CG1	0.01	32LEU	CG	298LEU	CD2	0.00
27ASP	CB	34LEU	CD2	0.06	304LEI	C	305VAL	CG2	0.01	32LEU	CG	302PHE	CD2	0.01
27ASP	CG	27ASP	C	100.00	304LEI	C	305VAL	C	99.96	32LEU	CG	302PHE	CE1	0.00
27ASP	CG	32LEU	CD1	0.00	305VAI	CA	305VAL	CB	100.00	32LEU	CG	302PHE	CE2	0.02

27ASP	CG	34LEU	CB	4.98	305VAl	CA	305VAL	CG1	100.00	32LEU	CG	304LEU	CD1	0.00
27ASP	C	28GLU	CA	100.00	305VAl	CA	305VAL	CG2	100.00	32LEU	CG	307LEU	CD1	0.00
27ASP	C	28GLU	CB	0.67	305VAl	CA	305VAL	C	100.00	32LEU	CD1	32LEU	CD2	100.00
27ASP	C	28GLU	CG	0.00	305VAl	CA	308ALA	CB	0.02	32LEU	CD1	32LEU	C	5.91
27ASP	C	28GLU	C	99.51	305VAl	CB	305VAL	CG1	100.00	32LEU	CD1	34LEU	CB	0.12
27ASP	C	31GLY	CA	0.00	305VAl	CB	305VAL	CG2	100.00	32LEU	CD1	34LEU	CD1	0.06
28GLU	CA	28GLU	CB	100.00	305VAl	CB	305VAL	C	100.00	32LEU	CD1	34LEU	CD2	0.00
28GLU	CA	28GLU	CG	100.00	305VAl	CB	309ARG	CZ	0.23	32LEU	CD1	298LEU	CG	0.00
28GLU	CA	28GLU	CD	95.16	305VAl	CG1	305VAL	CG2	100.00	32LEU	CD1	298LEU	CD1	0.46
28GLU	CA	28GLU	C	100.00	305VAl	CG1	305VAL	C	53.32	32LEU	CD1	298LEU	CD2	1.04
28GLU	CB	28GLU	CG	100.00	305VAl	CG1	306GLU	CD	0.00	32LEU	CD1	302PHE	CB	0.00
28GLU	CB	28GLU	CD	100.00	305VAl	CG1	309ARG	CG	0.01	32LEU	CD1	302PHE	CG	0.08
28GLU	CB	28GLU	C	100.00	305VAl	CG1	309ARG	CD	0.01	32LEU	CD1	302PHE	CD1	0.07
28GLU	CG	28GLU	CD	100.00	305VAl	CG1	309ARG	CZ	1.76	32LEU	CD1	302PHE	CD2	0.44
28GLU	CG	28GLU	C	98.13	305VAl	CG2	305VAL	C	68.45	32LEU	CD1	302PHE	CE1	0.76
28GLU	CD	28GLU	C	1.40	305VAl	CG2	309ARG	CG	0.03	32LEU	CD1	302PHE	CE2	5.33
28GLU	C	29ALA	CA	100.00	305VAl	CG2	309ARG	CZ	2.88	32LEU	CD1	302PHE	CZ	4.04
28GLU	C	29ALA	CB	4.18	305VAl	C	306GLU	CA	100.00	32LEU	CD1	304LEU	CD1	0.68
28GLU	C	29ALA	C	95.39	305VAl	C	306GLU	CB	0.08	32LEU	CD1	304LEU	CD2	0.05
29ALA	CA	29ALA	CB	100.00	305VAl	C	306GLU	C	99.95	32LEU	CD1	307LEU	CD1	0.01
29ALA	CA	29ALA	C	100.00	305VAl	C	308ALA	CB	0.00	32LEU	CD1	307LEU	CD2	0.05
29ALA	CB	29ALA	C	100.00	305VAl	C	309ARG	CZ	0.10	32LEU	CD2	32LEU	C	2.22
29ALA	CB	344LEU	CD2	0.00	306GLl	CA	306GLU	CB	100.00	32LEU	CD2	34LEU	CB	0.01
29ALA	CB	345ALA	CB	0.16	306GLl	CA	306GLU	CG	100.00	32LEU	CD2	34LEU	CD1	0.03
29ALA	C	30GLU	CA	100.00	306GLl	CA	306GLU	CD	96.88	32LEU	CD2	298LEU	CG	0.01
29ALA	C	30GLU	CB	70.15	306GLl	CA	306GLU	C	100.00	32LEU	CD2	298LEU	CD1	0.75
29ALA	C	30GLU	CG	0.01	306GLl	CA	309ARG	CZ	0.02	32LEU	CD2	298LEU	CD2	7.02
29ALA	C	30GLU	C	35.85	306GLl	CB	306GLU	CG	100.00	32LEU	CD2	302PHE	CD1	0.01
30GLU	CA	30GLU	CB	100.00	306GLl	CB	306GLU	CD	100.00	32LEU	CD2	302PHE	CD2	0.05
30GLU	CA	30GLU	CG	100.00	306GLl	CB	306GLU	C	100.00	32LEU	CD2	302PHE	CE1	0.08
30GLU	CA	30GLU	CD	98.46	306GLl	CG	306GLU	CD	100.00	32LEU	CD2	302PHE	CE2	0.14
30GLU	CA	30GLU	C	100.00	306GLl	CG	306GLU	C	96.38	32LEU	CD2	302PHE	CZ	0.07
30GLU	CB	30GLU	CG	100.00	306GLl	CG	307LEU	CD1	0.04	32LEU	CD2	304LEU	CD1	6.30
30GLU	CB	30GLU	CD	100.00	306GLl	CD	306GLU	C	0.36	32LEU	CD2	304LEU	CD2	0.05
30GLU	CB	30GLU	C	100.00	306GLl	CD	309ARG	CD	0.01	32LEU	CD2	307LEU	CG	0.00
30GLU	CB	307LEU	CD1	0.14	306GLl	CD	309ARG	CZ	0.09	32LEU	CD2	307LEU	CD1	7.53
30GLU	CB	307LEU	CD2	0.03	306GLl	CD	310LYS	CE	0.00	32LEU	CD2	307LEU	CD2	6.77
30GLU	CG	30GLU	CD	100.00	306GLl	C	307LEU	CA	100.00	32LEU	C	33GLY	CA	100.00
30GLU	CG	30GLU	C	98.58	306GLl	C	307LEU	CB	0.17	32LEU	C	33GLY	C	85.60
30GLU	CG	32LEU	CG	0.00	306GLl	C	307LEU	CD1	0.03	32LEU	C	302PHE	CE1	0.12
30GLU	CG	32LEU	CD2	0.03	306GLl	C	307LEU	CD2	0.02	32LEU	C	302PHE	CE2	0.57
30GLU	CG	307LEU	CG	0.04	306GLl	C	307LEU	C	99.85	32LEU	C	302PHE	CZ	0.04
30GLU	CG	307LEU	CD1	0.78	307LEl	CA	307LEU	CB	100.00	33GLY	CA	33GLY	C	100.00
30GLU	CG	307LEU	CD2	0.70	307LEl	CA	307LEU	CG	100.00	33GLY	C	34LEU	CA	100.00
30GLU	CD	30GLU	C	1.49	307LEl	CA	307LEU	CD1	41.87	33GLY	C	34LEU	CB	0.84
30GLU	CD	307LEU	CD1	0.01	307LEl	CA	307LEU	CD2	60.85	33GLY	C	34LEU	C	99.77
30GLU	CD	310LYS	CD	0.01	307LEl	CA	307LEU	C	100.00	33GLY	C	302PHE	CE1	0.01
30GLU	CD	310LYS	CE	0.03	307LEl	CA	310LYS	CB	0.00	33GLY	C	302PHE	CE2	0.00
30GLU	C	31GLY	CA	100.00	307LEl	CB	307LEU	CG	100.00	33GLY	C	302PHE	CZ	0.00
30GLU	C	31GLY	C	59.72	307LEl	CB	307LEU	CD1	100.00	34LEU	CA	34LEU	CB	100.00
31GLY	CA	31GLY	C	100.00	307LEl	CB	307LEU	CD2	100.00	34LEU	CA	34LEU	CG	100.00
31GLY	C	32LEU	CA	100.00	307LEl	CB	307LEU	C	100.00	34LEU	CA	34LEU	CD1	96.50
31GLY	C	32LEU	CB	78.84	307LEl	CG	307LEU	CD1	100.00	34LEU	CA	34LEU	CD2	3.28
31GLY	C	32LEU	CG	0.43	307LEl	CG	307LEU	CD2	100.00	34LEU	CA	34LEU	C	100.00
31GLY	C	32LEU	CD1	0.00	307LEl	CG	307LEU	C	19.46	34LEU	CB	34LEU	CG	100.00
31GLY	C	32LEU	CD2	0.16	307LEl	CD1	307LEU	CD2	100.00	34LEU	CB	34LEU	CD1	100.00
31GLY	C	32LEU	C	20.58	307LEl	CD1	307LEU	C	0.26	34LEU	CB	34LEU	CD2	100.00
32LEU	CA	32LEU	CB	100.00	307LEl	CD1	310LYS	CD	0.02	34LEU	CB	34LEU	C	100.00
32LEU	CA	32LEU	CG	100.00	307LEl	CD1	310LYS	CE	0.00	34LEU	CG	34LEU	CD1	100.00
32LEU	CA	32LEU	CD1	24.60	307LEl	CD2	307LEU	C	1.20	34LEU	CG	34LEU	CD2	100.00
32LEU	CA	32LEU	CD2	28.27	307LEl	CD2	310LYS	CD	0.02	34LEU	CG	34LEU	C	99.39
32LEU	CA	32LEU	C	100.00	307LEl	CD2	310LYS	CE	0.02	34LEU	CG	36TYR	CD1	0.05

32LEU	CA	302PHE	CE1	0.01
32LEU	CA	302PHE	CZ	0.02
32LEU	CA	304LEU	CD1	0.40
32LEU	CA	304LEU	CD2	0.02
32LEU	CB	32LEU	CG	100.00
32LEU	CB	32LEU	CD1	100.00
32LEU	CB	32LEU	CD2	100.00
32LEU	CB	32LEU	C	100.00
32LEU	CB	34LEU	CD2	0.00
32LEU	CB	298LEU	CD1	0.15
32LEU	CB	298LEU	CD2	0.08
32LEU	CB	302PHE	CD2	0.02
32LEU	CB	302PHE	CE1	0.13
32LEU	CB	302PHE	CE2	0.36
32LEU	CB	302PHE	CZ	0.09
32LEU	CB	304LEU	CD1	12.32
32LEU	CB	304LEU	CD2	0.10
32LEU	CB	307LEU	CD1	0.20
32LEU	CB	307LEU	CD2	0.18
32LEU	CG	32LEU	CD1	100.00
32LEU	CG	32LEU	CD2	100.00
32LEU	CG	32LEU	C	75.18
32LEU	CG	298LEU	CD1	0.01
32LEU	CG	298LEU	CD2	0.01
32LEU	CG	307LEU	CD2	0.00
32LEU	CD1	32LEU	CD2	100.00
32LEU	CD1	32LEU	C	13.56
32LEU	CD1	33GLY	C	0.01
32LEU	CD1	34LEU	CA	0.01
32LEU	CD1	34LEU	CB	0.24
32LEU	CD1	34LEU	CD1	0.03
32LEU	CD1	298LEU	CG	0.00
32LEU	CD1	298LEU	CD1	0.52
32LEU	CD1	298LEU	CD2	0.97
32LEU	CD1	302PHE	CD1	0.05
32LEU	CD1	302PHE	CD2	0.27
32LEU	CD1	302PHE	CE1	0.30
32LEU	CD1	302PHE	CE2	12.66
32LEU	CD1	302PHE	CZ	9.08
32LEU	CD1	304LEU	CD1	0.31
32LEU	CD1	304LEU	CD2	0.00
32LEU	CD1	307LEU	CD1	0.07
32LEU	CD1	307LEU	CD2	0.01
32LEU	CD2	32LEU	C	2.50
32LEU	CD2	33GLY	C	0.00
32LEU	CD2	34LEU	CB	0.00
32LEU	CD2	34LEU	CD1	0.00
32LEU	CD2	298LEU	CD1	0.83
32LEU	CD2	298LEU	CD2	1.14
32LEU	CD2	302PHE	CG	0.01
32LEU	CD2	302PHE	CD1	0.00
32LEU	CD2	302PHE	CD2	0.03
32LEU	CD2	302PHE	CE1	0.02
32LEU	CD2	302PHE	CE2	0.07
32LEU	CD2	304LEU	CD1	1.83
32LEU	CD2	304LEU	CD2	0.02
32LEU	CD2	307LEU	CB	0.00
32LEU	CD2	307LEU	CD1	3.58
32LEU	CD2	307LEU	CD2	1.91
32LEU	C	33GLY	CA	100.00
32LEU	C	33GLY	C	84.72
32LEU	C	302PHE	CE2	0.34

307LEU	CD2	311VAL	CG2	0.01
307LEU	C	308ALA	CA	100.00
307LEU	C	308ALA	CB	0.00
307LEU	C	308ALA	C	99.98
307LEU	C	310LYS	CB	0.01
308ALA	CA	308ALA	CB	100.00
308ALA	CA	308ALA	C	100.00
308ALA	CB	308ALA	C	100.00
308ALA	C	309ARG	CA	100.00
308ALA	C	309ARG	CB	0.01
308ALA	C	309ARG	C	99.99
309ARG	CA	309ARG	CB	100.00
309ARG	CA	309ARG	CG	100.00
309ARG	CA	309ARG	CD	18.36
309ARG	CA	309ARG	C	100.00
309ARG	CA	312GLU	CB	0.01
309ARG	CB	309ARG	CG	100.00
309ARG	CB	309ARG	CD	100.00
309ARG	CB	309ARG	CZ	4.16
309ARG	CB	309ARG	C	100.00
309ARG	CG	309ARG	CD	100.00
309ARG	CG	309ARG	CZ	48.49
309ARG	CG	309ARG	C	34.61
309ARG	CD	309ARG	CZ	100.00
309ARG	CD	309ARG	C	0.20
309ARG	CD	312GLU	CD	0.01
309ARG	CZ	310LYS	CG	0.02
309ARG	CZ	310LYS	CE	0.01
309ARG	C	310LYS	CA	100.00
309ARG	C	310LYS	CB	0.03
309ARG	C	310LYS	C	99.98
310LYS	CA	310LYS	CB	100.00
310LYS	CA	310LYS	CG	100.00
310LYS	CA	310LYS	CD	5.82
310LYS	CA	310LYS	CE	0.01
310LYS	CA	310LYS	C	100.00
310LYS	CB	310LYS	CG	100.00
310LYS	CB	310LYS	CD	100.00
310LYS	CB	310LYS	CE	7.90
310LYS	CB	310LYS	C	100.00
310LYS	CB	344LEU	CD1	0.01
310LYS	CB	344LEU	CD2	0.21
310LYS	CG	310LYS	CD	100.00
310LYS	CG	310LYS	CE	100.00
310LYS	CG	310LYS	C	76.15
310LYS	CG	343HIS	CD2	0.08
310LYS	CG	343HIS	CE1	0.76
310LYS	CG	344LEU	CB	0.02
310LYS	CG	344LEU	CD1	0.05
310LYS	CG	344LEU	CD2	1.29
310LYS	CD	310LYS	CE	100.00
310LYS	CD	310LYS	C	0.05
310LYS	CD	343HIS	CD2	0.01
310LYS	CD	343HIS	CE1	0.00
310LYS	CD	344LEU	CB	0.01
310LYS	CD	344LEU	CD1	0.02
310LYS	CD	344LEU	CD2	0.41
310LYS	CD	344LEU	C	0.01
310LYS	CE	343HIS	CG	0.00
310LYS	CE	343HIS	CD2	0.04
310LYS	CE	343HIS	CE1	0.08
310LYS	CE	344LEU	CB	0.01

34LEU	CG	36TYR	CD2	0.02
34LEU	CG	36TYR	CE1	0.00
34LEU	CD1	34LEU	CD2	100.00
34LEU	CD1	34LEU	C	2.22
34LEU	CD1	35ALA	C	0.00
34LEU	CD1	36TYR	CD1	0.26
34LEU	CD1	36TYR	CD2	0.01
34LEU	CD1	36TYR	CE1	0.01
34LEU	CD1	297MET	CE	0.01
34LEU	CD1	298LEU	CD2	0.00
34LEU	CD1	302PHE	CE1	0.01
34LEU	CD1	302PHE	CE2	0.01
34LEU	CD1	302PHE	CZ	0.24
34LEU	CD2	34LEU	C	0.84
34LEU	CD2	35ALA	C	0.00
34LEU	CD2	36TYR	CD1	1.91
34LEU	CD2	36TYR	CD2	0.57
34LEU	CD2	36TYR	CE1	0.30
34LEU	CD2	36TYR	CE2	0.06
34LEU	CD2	36TYR	CZ	0.00
34LEU	CD2	302PHE	CE2	0.02
34LEU	CD2	302PHE	CZ	0.04
34LEU	C	35ALA	CA	100.00
34LEU	C	35ALA	CB	60.28
34LEU	C	35ALA	C	49.47
35ALA	CA	35ALA	CB	100.00
35ALA	CA	35ALA	C	100.00
35ALA	CB	35ALA	C	100.00
35ALA	C	36TYR	CA	100.00
35ALA	C	36TYR	CB	98.86
35ALA	C	36TYR	CG	0.01
35ALA	C	36TYR	CD1	0.14
35ALA	C	36TYR	CD2	0.07
35ALA	C	36TYR	C	1.96
36TYR	CA	36TYR	CB	100.00
36TYR	CA	36TYR	CG	100.00
36TYR	CA	36TYR	CD1	74.01
36TYR	CA	36TYR	CD2	71.46
36TYR	CA	36TYR	C	100.00
36TYR	CB	36TYR	CG	100.00
36TYR	CB	36TYR	CD1	100.00
36TYR	CB	36TYR	CD2	100.00
36TYR	CB	36TYR	C	100.00
36TYR	CG	36TYR	CD1	100.00
36TYR	CG	36TYR	CD2	100.00
36TYR	CG	36TYR	CE1	100.00
36TYR	CG	36TYR	CE2	100.00
36TYR	CG	36TYR	CZ	100.00
36TYR	CG	36TYR	C	99.97
36TYR	CD1	36TYR	CD2	100.00
36TYR	CD1	36TYR	CE1	100.00
36TYR	CD1	36TYR	CE2	100.00
36TYR	CD1	36TYR	CZ	100.00
36TYR	CD1	36TYR	C	15.85
36TYR	CD1	38VAL	CG1	0.00
36TYR	CD1	38VAL	CG2	0.09
36TYR	CD2	36TYR	CE1	100.00
36TYR	CD2	36TYR	CE2	100.00
36TYR	CD2	36TYR	CZ	100.00
36TYR	CD2	36TYR	C	40.79
36TYR	CD2	37GLU	C	0.00
36TYR	CD2	38VAL	CG2	0.10

32LEU	C	302PHE	CZ	0.01	310LYS	CE	344LEU	CD1	0.00	36TYR	CE1	36TYR	CE2	100.00
33GLY	CA	33GLY	C	100.00	310LYS	CE	344LEU	CD2	0.07	36TYR	CE1	36TYR	CZ	100.00
33GLY	C	34LEU	CA	100.00	310LYS	CE	344LEU	C	0.04	36TYR	CE1	38VAL	CG1	0.01
33GLY	C	34LEU	CB	1.18	310LYS	CE	345ALA	C	0.17	36TYR	CE1	38VAL	CG2	0.36
33GLY	C	34LEU	C	99.41	310LYS	C	311VAL	CA	100.00	36TYR	CE2	36TYR	CZ	100.00
33GLY	C	302PHE	CE2	0.06	310LYS	C	311VAL	CB	0.00	36TYR	CE2	38VAL	CG2	0.74
34LEU	CA	34LEU	CB	100.00	310LYS	C	311VAL	C	99.99	36TYR	C	37GLU	CA	100.00
34LEU	CA	34LEU	CG	100.00	310LYS	C	344LEU	CD1	0.06	36TYR	C	37GLU	CB	86.67
34LEU	CA	34LEU	CD1	99.12	310LYS	C	344LEU	CD2	0.34	36TYR	C	37GLU	C	25.01
34LEU	CA	34LEU	CD2	1.72	311VAL	CA	311VAL	CB	100.00	37GLU	CA	37GLU	CB	100.00
34LEU	CA	34LEU	C	100.00	311VAL	CA	311VAL	CG1	100.00	37GLU	CA	37GLU	CG	100.00
34LEU	CB	34LEU	CG	100.00	311VAL	CA	311VAL	CG2	100.00	37GLU	CA	37GLU	CD	94.76
34LEU	CB	34LEU	CD1	100.00	311VAL	CA	311VAL	C	100.00	37GLU	CA	37GLU	C	100.00
34LEU	CB	34LEU	CD2	100.00	311VAL	CA	344LEU	CD1	0.01	37GLU	CB	37GLU	CG	100.00
34LEU	CB	34LEU	C	100.00	311VAL	CA	344LEU	CD2	0.02	37GLU	CB	37GLU	CD	100.00
34LEU	CG	34LEU	CD1	100.00	311VAL	CB	311VAL	CG1	100.00	37GLU	CB	37GLU	C	100.00
34LEU	CG	34LEU	CD2	100.00	311VAL	CB	311VAL	CG2	100.00	37GLU	CB	39PHE	CD1	0.01
34LEU	CG	34LEU	C	99.78	311VAL	CB	311VAL	C	100.00	37GLU	CB	39PHE	CE1	3.50
34LEU	CG	36TYR	CD2	0.13	311VAL	CG1	311VAL	CG2	100.00	37GLU	CB	39PHE	CE2	0.01
34LEU	CG	36TYR	CE2	0.02	311VAL	CG1	311VAL	C	98.35	37GLU	CB	39PHE	CZ	2.10
34LEU	CD1	34LEU	CD2	100.00	311VAL	CG1	315VAL	CG2	0.01	37GLU	CG	37GLU	CD	100.00
34LEU	CD1	34LEU	C	2.56	311VAL	CG1	344LEU	CD1	0.00	37GLU	CG	37GLU	C	99.32
34LEU	CD1	36TYR	CD2	0.76	311VAL	CG2	311VAL	C	1.64	37GLU	CG	39PHE	CD1	0.02
34LEU	CD1	36TYR	CE2	0.00	311VAL	CG2	344LEU	CD1	0.25	37GLU	CG	39PHE	CD2	0.04
34LEU	CD1	297MET	CE	0.00	311VAL	CG2	344LEU	CD2	0.59	37GLU	CG	39PHE	CE1	4.89
34LEU	CD1	302PHE	CE1	0.00	311VAL	C	312GLU	CA	100.00	37GLU	CG	39PHE	CE2	5.10
34LEU	CD1	302PHE	CZ	0.10	311VAL	C	312GLU	CB	0.02	37GLU	CG	39PHE	CZ	20.62
34LEU	CD2	34LEU	C	0.24	311VAL	C	312GLU	C	100.00	37GLU	CD	37GLU	C	0.02
34LEU	CD2	36TYR	CD2	1.79	312GLU	CA	312GLU	CB	100.00	37GLU	CD	39PHE	CE2	0.26
34LEU	CD2	36TYR	CE2	0.27	312GLU	CA	312GLU	CG	100.00	37GLU	CD	39PHE	CZ	0.51
34LEU	CD2	302PHE	CE2	0.00	312GLU	CA	312GLU	CD	1.28	37GLU	C	38VAL	CA	100.00
34LEU	CD2	302PHE	CZ	0.08	312GLU	CA	312GLU	C	100.00	37GLU	C	38VAL	CB	4.08
34LEU	C	35ALA	CA	100.00	312GLU	CA	315VAL	CG2	0.01	37GLU	C	38VAL	CG1	0.02
34LEU	C	35ALA	CB	63.69	312GLU	CB	312GLU	CG	100.00	37GLU	C	38VAL	CG2	1.31
34LEU	C	35ALA	C	45.50	312GLU	CB	312GLU	CD	100.00	37GLU	C	38VAL	C	98.59
35ALA	CA	35ALA	CB	100.00	312GLU	CB	312GLU	C	100.00	37GLU	C	39PHE	CD1	0.01
35ALA	CA	35ALA	C	100.00	312GLU	CG	312GLU	CD	100.00	37GLU	C	39PHE	CE1	0.06
35ALA	CB	35ALA	C	100.00	312GLU	CG	312GLU	C	85.80	38VAL	CA	38VAL	CB	100.00
35ALA	C	36TYR	CA	100.00	312GLU	CD	312GLU	C	0.25	38VAL	CA	38VAL	CG1	100.00
35ALA	C	36TYR	CB	98.90	312GLU	C	313ASP	CA	100.00	38VAL	CA	38VAL	CG2	100.00
35ALA	C	36TYR	CG	0.02	312GLU	C	313ASP	CB	0.02	38VAL	CA	38VAL	C	100.00
35ALA	C	36TYR	CD2	0.34	312GLU	C	313ASP	C	99.99	38VAL	CB	38VAL	CG1	100.00
35ALA	C	36TYR	C	1.74	312GLU	C	315VAL	CG2	0.01	38VAL	CB	38VAL	CG2	100.00
36TYR	CA	36TYR	CB	100.00	313ASP	CA	313ASP	CB	100.00	38VAL	CB	38VAL	C	100.00
36TYR	CA	36TYR	CG	100.00	313ASP	CA	313ASP	CG	100.00	38VAL	CG1	38VAL	CG2	100.00
36TYR	CA	36TYR	CD1	60.99	313ASP	CA	313ASP	C	100.00	38VAL	CG1	38VAL	C	96.45
36TYR	CA	36TYR	CD2	85.83	313ASP	CA	316ALA	CB	0.01	38VAL	CG2	38VAL	C	8.76
36TYR	CA	36TYR	C	100.00	313ASP	CB	313ASP	CG	100.00	38VAL	C	39PHE	CA	100.00
36TYR	CB	36TYR	CG	100.00	313ASP	CB	313ASP	C	100.00	38VAL	C	39PHE	CB	18.76
36TYR	CB	36TYR	CD1	100.00	313ASP	CB	343HIS	CE1	0.00	38VAL	C	39PHE	CG	4.04
36TYR	CB	36TYR	CD2	100.00	313ASP	CG	313ASP	C	99.99	38VAL	C	39PHE	CD1	1.41
36TYR	CB	36TYR	C	100.00	313ASP	CG	317LYS	CD	0.04	38VAL	C	39PHE	CD2	0.00
36TYR	CG	36TYR	CD1	100.00	313ASP	CG	317LYS	CE	0.52	38VAL	C	39PHE	C	82.56
36TYR	CG	36TYR	CD2	100.00	313ASP	CG	343HIS	CE1	0.05	39PHE	CA	39PHE	CB	100.00
36TYR	CG	36TYR	CE1	100.00	313ASP	C	314ALA	CA	100.00	39PHE	CA	39PHE	CG	100.00
36TYR	CG	36TYR	CE2	100.00	313ASP	C	314ALA	C	100.00	39PHE	CA	39PHE	CD1	56.79
36TYR	CG	36TYR	CZ	100.00	313ASP	C	316ALA	CB	0.00	39PHE	CA	39PHE	CD2	97.63
36TYR	CG	36TYR	C	99.97	313ASP	C	317LYS	CE	0.00	39PHE	CA	39PHE	C	100.00
36TYR	CD1	36TYR	CD2	100.00	313ASP	C	343HIS	CD2	0.04	39PHE	CA	40PRO	CD	100.00
36TYR	CD1	36TYR	CE1	100.00	313ASP	C	343HIS	CE1	0.01	39PHE	CB	39PHE	CG	100.00
36TYR	CD1	36TYR	CE2	100.00	314ALA	CA	314ALA	CB	100.00	39PHE	CB	39PHE	CD1	100.00
36TYR	CD1	36TYR	CZ	100.00	314ALA	CA	314ALA	C	100.00	39PHE	CB	39PHE	CD2	100.00

36TYR	CD1	36TYR	C	54.54	314ALA	CA	343HIS	CB	0.04	39PHE	CB	39PHE	C	100.00
36TYR	CD1	38VAL	CG1	0.02	314ALA	CA	343HIS	CG	0.55	39PHE	CB	40PRO	CD	23.51
36TYR	CD1	38VAL	CG2	0.69	314ALA	CA	343HIS	CD2	14.90	39PHE	CB	57THR	CA	0.01
36TYR	CD2	36TYR	CE1	100.00	314ALA	CA	343HIS	CE1	0.13	39PHE	CB	57THR	CG2	0.84
36TYR	CD2	36TYR	CE2	100.00	314ALA	CB	314ALA	C	100.00	39PHE	CG	39PHE	CD1	100.00
36TYR	CD2	36TYR	CZ	100.00	314ALA	CB	340VAL	CG1	2.72	39PHE	CG	39PHE	CD2	100.00
36TYR	CD2	36TYR	C	0.09	314ALA	CB	340VAL	CG2	0.00	39PHE	CG	39PHE	CE1	100.00
36TYR	CE1	36TYR	CE2	100.00	314ALA	CB	343HIS	CB	3.83	39PHE	CG	39PHE	CE2	100.00
36TYR	CE1	36TYR	CZ	100.00	314ALA	CB	343HIS	CG	3.81	39PHE	CG	39PHE	CZ	100.00
36TYR	CE1	38VAL	CG1	0.03	314ALA	CB	343HIS	CD2	5.09	39PHE	CD1	39PHE	CD2	100.00
36TYR	CE1	38VAL	CG2	3.40	314ALA	CB	343HIS	CE1	0.12	39PHE	CD1	39PHE	CE1	100.00
36TYR	CE2	36TYR	CZ	100.00	314ALA	CB	343HIS	C	0.11	39PHE	CD1	39PHE	CE2	100.00
36TYR	C	37GLU	CA	100.00	314ALA	CB	344LEU	CA	0.00	39PHE	CD1	39PHE	CZ	100.00
36TYR	C	37GLU	CB	81.66	314ALA	CB	344LEU	CB	0.01	39PHE	CD1	60GLY	CA	0.02
36TYR	C	37GLU	C	32.86	314ALA	CB	344LEU	CG	0.16	39PHE	CD2	39PHE	CE1	100.00
37GLU	CA	37GLU	CB	100.00	314ALA	CB	344LEU	CD1	0.61	39PHE	CD2	39PHE	CE2	100.00
37GLU	CA	37GLU	CG	100.00	314ALA	CB	344LEU	CD2	0.71	39PHE	CD2	39PHE	CZ	100.00
37GLU	CA	37GLU	CD	97.54	314ALA	C	315VAL	CA	100.00	39PHE	CD2	56PRO	C	0.26
37GLU	CA	37GLU	C	100.00	314ALA	C	315VAL	CB	0.01	39PHE	CD2	57THR	CA	0.40
37GLU	CB	37GLU	CG	100.00	314ALA	C	315VAL	C	100.00	39PHE	CD2	60GLY	CA	0.03
37GLU	CB	37GLU	CD	100.00	314ALA	C	340VAL	CG2	0.02	39PHE	CE1	39PHE	CE2	100.00
37GLU	CB	37GLU	C	100.00	315VAL	CA	315VAL	CB	100.00	39PHE	CE1	39PHE	CZ	100.00
37GLU	CB	39PHE	CE1	3.34	315VAL	CA	315VAL	CG1	100.00	39PHE	CE1	60GLY	CA	4.82
37GLU	CB	39PHE	CE2	0.04	315VAL	CA	315VAL	CG2	100.00	39PHE	CE1	60GLY	C	1.78
37GLU	CB	39PHE	CZ	2.13	315VAL	CA	315VAL	C	100.00	39PHE	CE2	39PHE	CZ	100.00
37GLU	CG	37GLU	CD	100.00	315VAL	CA	318ALA	CB	0.00	39PHE	CE2	56PRO	C	0.04
37GLU	CG	37GLU	C	99.46	315VAL	CB	315VAL	CG1	100.00	39PHE	CE2	60GLY	CA	10.88
37GLU	CG	39PHE	CD1	0.04	315VAL	CB	315VAL	CG2	100.00	39PHE	CZ	60GLY	CA	40.49
37GLU	CG	39PHE	CD2	0.10	315VAL	CB	315VAL	C	100.00	39PHE	CZ	60GLY	C	0.66
37GLU	CG	39PHE	CE1	5.25	315VAL	CG1	315VAL	CG2	100.00	39PHE	C	40PRO	CA	100.00
37GLU	CG	39PHE	CE2	6.10	315VAL	CG1	315VAL	C	79.74	39PHE	C	40PRO	CB	0.03
37GLU	CG	39PHE	CZ	19.86	315VAL	CG1	319LEU	CD1	2.57	39PHE	C	40PRO	CD	100.00
37GLU	CD	37GLU	C	0.02	315VAL	CG1	336PHE	CZ	0.01	39PHE	C	40PRO	C	100.00
37GLU	CD	39PHE	CE1	0.00	315VAL	CG1	340VAL	CG1	0.08	40PRO	CA	40PRO	CB	100.00
37GLU	CD	39PHE	CE2	0.02	315VAL	CG1	340VAL	CG2	0.10	40PRO	CA	40PRO	CG	100.00
37GLU	CD	39PHE	CZ	0.13	315VAL	CG2	315VAL	C	23.42	40PRO	CA	40PRO	CD	100.00
37GLU	C	38VAL	CA	100.00	315VAL	CG2	336PHE	CE1	0.02	40PRO	CA	40PRO	C	100.00
37GLU	C	38VAL	CB	2.29	315VAL	CG2	336PHE	CZ	0.02	40PRO	CB	40PRO	CG	100.00
37GLU	C	38VAL	CG1	0.02	315VAL	CG2	340VAL	CG1	0.20	40PRO	CB	40PRO	CD	100.00
37GLU	C	38VAL	CG2	1.12	315VAL	CG2	340VAL	CG2	0.24	40PRO	CB	40PRO	C	100.00
37GLU	C	38VAL	C	99.44	315VAL	C	316ALA	CA	100.00	40PRO	CB	44ALA	CB	0.00
37GLU	C	39PHE	CD1	0.00	315VAL	C	316ALA	CB	0.01	40PRO	CB	44ALA	C	0.00
37GLU	C	39PHE	CE1	0.03	315VAL	C	316ALA	C	100.00	40PRO	CB	45ALA	CA	0.00
38VAL	CA	38VAL	CB	100.00	315VAL	C	319LEU	CD1	0.00	40PRO	CB	45ALA	CB	0.10
38VAL	CA	38VAL	CG1	100.00	316ALA	CA	316ALA	CB	100.00	40PRO	CB	49PHE	CD1	0.22
38VAL	CA	38VAL	CG2	100.00	316ALA	CA	316ALA	C	100.00	40PRO	CB	49PHE	CD2	0.08
38VAL	CA	38VAL	C	100.00	316ALA	CB	316ALA	C	100.00	40PRO	CB	49PHE	CE1	2.34
38VAL	CB	38VAL	CG1	100.00	316ALA	C	317LYS	CA	100.00	40PRO	CB	49PHE	CE2	1.64
38VAL	CB	38VAL	CG2	100.00	316ALA	C	317LYS	CB	0.39	40PRO	CB	49PHE	CZ	0.10
38VAL	CB	38VAL	C	100.00	316ALA	C	317LYS	CG	0.04	40PRO	CB	54PRO	CG	0.03
38VAL	CG1	38VAL	CG2	100.00	316ALA	C	317LYS	CD	0.00	40PRO	CG	40PRO	CD	100.00
38VAL	CG1	38VAL	C	98.65	316ALA	C	317LYS	CE	0.01	40PRO	CG	40PRO	C	89.77
38VAL	CG2	38VAL	C	1.34	316ALA	C	317LYS	C	99.56	40PRO	CG	49PHE	CD1	0.11
38VAL	C	39PHE	CA	100.00	316ALA	C	320LEU	CD1	0.00	40PRO	CG	49PHE	CD2	0.08
38VAL	C	39PHE	CB	13.61	316ALA	C	320LEU	CD2	0.00	40PRO	CG	49PHE	CE1	8.75
38VAL	C	39PHE	CG	2.53	317LYS	CA	317LYS	CB	100.00	40PRO	CG	49PHE	CE2	7.75
38VAL	C	39PHE	CD1	0.68	317LYS	CA	317LYS	CG	100.00	40PRO	CG	49PHE	CZ	2.41
38VAL	C	39PHE	C	86.82	317LYS	CA	317LYS	CD	28.63	40PRO	CG	54PRO	CG	0.08
39PHE	CA	39PHE	CB	100.00	317LYS	CA	317LYS	CE	0.58	40PRO	CG	56PRO	CG	0.16
39PHE	CA	39PHE	CG	100.00	317LYS	CA	317LYS	C	100.00	40PRO	CD	40PRO	C	80.88
39PHE	CA	39PHE	CD1	49.63	317LYS	CA	320LEU	CD1	0.01	40PRO	C	41PHE	CA	100.00
39PHE	CA	39PHE	CD2	97.80	317LYS	CB	317LYS	CG	100.00	40PRO	C	41PHE	CB	99.94

39PHE	CA	39PHE	C	100.00
39PHE	CA	40PRO	CD	100.00
39PHE	CB	39PHE	CG	100.00
39PHE	CB	39PHE	CD1	100.00
39PHE	CB	39PHE	CD2	100.00
39PHE	CB	39PHE	C	100.00
39PHE	CB	40PRO	CD	14.96
39PHE	CB	57THR	CA	0.01
39PHE	CB	57THR	CG2	1.26
39PHE	CG	39PHE	CD1	100.00
39PHE	CG	39PHE	CD2	100.00
39PHE	CG	39PHE	CE1	100.00
39PHE	CG	39PHE	CE2	100.00
39PHE	CG	39PHE	CZ	100.00
39PHE	CG	57THR	CG2	0.00
39PHE	CG	61VAL	CG1	0.00
39PHE	CD1	39PHE	CD2	100.00
39PHE	CD1	39PHE	CE1	100.00
39PHE	CD1	39PHE	CE2	100.00
39PHE	CD1	39PHE	CZ	100.00
39PHE	CD1	60GLY	CA	0.02
39PHE	CD1	61VAL	CG1	0.01
39PHE	CD2	39PHE	CE1	100.00
39PHE	CD2	39PHE	CE2	100.00
39PHE	CD2	39PHE	CZ	100.00
39PHE	CD2	56PRO	C	0.30
39PHE	CD2	57THR	CA	0.66
39PHE	CD2	60GLY	CA	0.00
39PHE	CE1	39PHE	CE2	100.00
39PHE	CE1	39PHE	CZ	100.00
39PHE	CE1	60GLY	CA	4.63
39PHE	CE1	60GLY	C	1.04
39PHE	CE2	39PHE	CZ	100.00
39PHE	CE2	56PRO	C	0.05
39PHE	CE2	60GLY	CA	7.60
39PHE	CZ	60GLY	CA	34.95
39PHE	CZ	60GLY	C	0.35
39PHE	CZ	63GLU	CG	0.00
39PHE	C	40PRO	CA	100.00
39PHE	C	40PRO	CB	0.04
39PHE	C	40PRO	CD	100.00
39PHE	C	40PRO	C	100.00
40PRO	CA	40PRO	CB	100.00
40PRO	CA	40PRO	CG	100.00
40PRO	CA	40PRO	CD	100.00
40PRO	CA	40PRO	C	100.00
40PRO	CB	40PRO	CG	100.00
40PRO	CB	40PRO	CD	100.00
40PRO	CB	40PRO	C	100.00
40PRO	CB	44ALA	CB	0.01
40PRO	CB	45ALA	CB	0.09
40PRO	CB	49PHE	CD1	0.55
40PRO	CB	49PHE	CE1	3.93
40PRO	CB	49PHE	CZ	0.02
40PRO	CB	54PRO	CG	0.02
40PRO	CG	40PRO	CD	100.00
40PRO	CG	40PRO	C	90.30
40PRO	CG	49PHE	CG	0.01
40PRO	CG	49PHE	CD1	0.50
40PRO	CG	49PHE	CE1	19.61
40PRO	CG	49PHE	CE2	0.02
40PRO	CG	49PHE	CZ	2.40

317LYS	CB	317LYS	CD	100.00
317LYS	CB	317LYS	CE	2.89
317LYS	CB	317LYS	C	100.00
317LYS	CB	343HIS	CG	0.17
317LYS	CB	343HIS	CD2	1.28
317LYS	CB	343HIS	CE1	0.36
317LYS	CG	317LYS	CD	100.00
317LYS	CG	317LYS	CE	100.00
317LYS	CG	317LYS	C	1.55
317LYS	CG	343HIS	CE1	0.76
317LYS	CD	317LYS	CE	100.00
317LYS	CD	317LYS	C	0.02
317LYS	CD	320LEU	CD1	0.00
317LYS	CD	343HIS	CG	0.05
317LYS	CD	343HIS	CD2	0.60
317LYS	CD	343HIS	CE1	6.88
317LYS	CE	343HIS	CD2	0.00
317LYS	CE	343HIS	CE1	1.38
317LYS	C	318ALA	CA	100.00
317LYS	C	318ALA	CB	0.10
317LYS	C	318ALA	C	99.89
317LYS	C	343HIS	CD2	0.12
317LYS	C	343HIS	CE1	0.00
318ALA	CA	318ALA	CB	100.00
318ALA	CA	318ALA	C	100.00
318ALA	CA	339THR	CB	0.01
318ALA	CA	339THR	CG2	0.38
318ALA	CA	343HIS	CD2	0.01
318ALA	CB	318ALA	C	100.00
318ALA	CB	336PHE	CD1	0.32
318ALA	CB	336PHE	CD2	0.09
318ALA	CB	336PHE	CE1	1.17
318ALA	CB	336PHE	CE2	6.50
318ALA	CB	336PHE	CZ	5.48
318ALA	CB	339THR	CB	0.57
318ALA	CB	339THR	CG2	1.37
318ALA	CB	339THR	C	0.01
318ALA	CB	340VAL	CG2	7.66
318ALA	C	319LEU	CA	100.00
318ALA	C	319LEU	CB	0.03
318ALA	C	319LEU	C	99.98
318ALA	C	336PHE	CE1	0.19
318ALA	C	336PHE	CE2	2.82
318ALA	C	336PHE	CZ	0.06
319LEU	CA	319LEU	CB	100.00
319LEU	CA	319LEU	CG	100.00
319LEU	CA	319LEU	CD1	3.20
319LEU	CA	319LEU	CD2	95.99
319LEU	CA	319LEU	C	100.00
319LEU	CA	336PHE	CE2	0.01
319LEU	CA	336PHE	CZ	0.01
319LEU	CB	319LEU	CG	100.00
319LEU	CB	319LEU	CD1	100.00
319LEU	CB	319LEU	CD2	100.00
319LEU	CB	319LEU	C	100.00
319LEU	CB	327LEU	CD2	0.00
319LEU	CG	319LEU	CD1	100.00
319LEU	CG	319LEU	CD2	100.00
319LEU	CG	319LEU	C	3.26
319LEU	CG	327LEU	CD1	0.01
319LEU	CG	327LEU	CD2	0.00
319LEU	CG	336PHE	CE2	0.04

40PRO	C	41PHE	C	0.29
40PRO	C	45ALA	CB	0.14
41PHE	CA	41PHE	CB	100.00
41PHE	CA	41PHE	CG	100.00
41PHE	CA	41PHE	CD1	75.78
41PHE	CA	41PHE	CD2	74.31
41PHE	CA	41PHE	C	100.00
41PHE	CA	45ALA	CB	0.13
41PHE	CB	41PHE	CG	100.00
41PHE	CB	41PHE	CD1	100.00
41PHE	CB	41PHE	CD2	100.00
41PHE	CB	41PHE	C	100.00
41PHE	CB	53PHE	CD1	0.00
41PHE	CG	41PHE	CD1	100.00
41PHE	CG	41PHE	CD2	100.00
41PHE	CG	41PHE	CE1	100.00
41PHE	CG	41PHE	CE2	100.00
41PHE	CG	41PHE	CZ	100.00
41PHE	CG	41PHE	C	99.98
41PHE	CG	53PHE	CD1	0.00
41PHE	CD1	41PHE	CD2	100.00
41PHE	CD1	41PHE	CE1	100.00
41PHE	CD1	41PHE	CE2	100.00
41PHE	CD1	41PHE	CZ	100.00
41PHE	CD1	41PHE	C	34.49
41PHE	CD1	42GLY	CA	0.02
41PHE	CD1	53PHE	CB	0.00
41PHE	CD1	53PHE	CD1	0.11
41PHE	CD1	69LEU	CD1	0.21
41PHE	CD2	41PHE	CE1	100.00
41PHE	CD2	41PHE	CE2	100.00
41PHE	CD2	41PHE	CZ	100.00
41PHE	CD2	41PHE	C	24.55
41PHE	CD2	42GLY	CA	0.02
41PHE	CD2	53PHE	CD1	0.41
41PHE	CD2	69LEU	CD1	0.32
41PHE	CD2	69LEU	CD2	0.00
41PHE	CD2	72VAL	CG1	0.04
41PHE	CD2	72VAL	CG2	0.10
41PHE	CE1	41PHE	CE2	100.00
41PHE	CE1	41PHE	CZ	100.00
41PHE	CE1	53PHE	CB	0.05
41PHE	CE1	53PHE	CG	0.02
41PHE	CE1	53PHE	CD1	0.78
41PHE	CE1	69LEU	CD1	10.40
41PHE	CE1	69LEU	CD2	0.19
41PHE	CE1	72VAL	CB	0.01
41PHE	CE1	72VAL	CG1	1.35
41PHE	CE1	72VAL	CG2	0.06
41PHE	CE1	86PRO	CB	0.04
41PHE	CE1	89GLY	C	0.16
41PHE	CE1	90LEU	CD1	0.06
41PHE	CE1	90LEU	CD2	0.42
41PHE	CE1	93LEU	CD1	0.02
41PHE	CE1	93LEU	CD2	0.01
41PHE	CE2	41PHE	CZ	100.00
41PHE	CE2	53PHE	CB	0.04
41PHE	CE2	53PHE	CG	0.04
41PHE	CE2	53PHE	CD1	0.79
41PHE	CE2	69LEU	CG	0.01
41PHE	CE2	69LEU	CD1	7.84
41PHE	CE2	69LEU	CD2	0.16

40PRO	CG	54PRO	CG	0.14	319LEL	CG	336PHE	CZ	0.01	41PHE	CE2	72VAL	CG1	2.76
40PRO	CG	56PRO	CG	0.12	319LEL	CD1	319LEU	CD2	100.00	41PHE	CE2	72VAL	CG2	1.04
40PRO	CD	40PRO	C	80.50	319LEL	CD1	319LEU	C	0.02	41PHE	CE2	86PRO	CB	0.12
40PRO	C	41PHE	CA	100.00	319LEL	CD1	324PRO	CG	0.01	41PHE	CE2	89GLY	CA	0.00
40PRO	C	41PHE	CB	99.92	319LEL	CD1	324PRO	CD	0.17	41PHE	CE2	89GLY	C	0.16
40PRO	C	41PHE	C	0.20	319LEL	CD1	327LEU	CD1	0.09	41PHE	CE2	90LEU	CD1	0.07
40PRO	C	45ALA	CB	0.08	319LEL	CD1	327LEU	CD2	0.05	41PHE	CE2	90LEU	CD2	0.38
40PRO	C	54PRO	CG	0.00	319LEL	CD1	336PHE	CE2	0.05	41PHE	CE2	93LEU	CD1	0.04
41PHE	CA	41PHE	CB	100.00	319LEL	CD1	336PHE	CZ	0.04	41PHE	CE2	93LEU	CD2	0.02
41PHE	CA	41PHE	CG	100.00	319LEL	CD2	319LEU	C	0.04	41PHE	CZ	69LEU	CD1	1.66
41PHE	CA	41PHE	CD1	92.64	319LEL	CD2	324PRO	CA	0.46	41PHE	CZ	69LEU	CD2	0.08
41PHE	CA	41PHE	CD2	54.45	319LEL	CD2	324PRO	CB	0.14	41PHE	CZ	72VAL	CG1	2.86
41PHE	CA	41PHE	C	100.00	319LEL	CD2	324PRO	CG	0.46	41PHE	CZ	72VAL	CG2	0.17
41PHE	CA	45ALA	CB	0.11	319LEL	CD2	324PRO	CD	1.98	41PHE	CZ	86PRO	CB	0.06
41PHE	CB	41PHE	CG	100.00	319LEL	CD2	325PRO	CD	0.00	41PHE	CZ	89GLY	C	0.83
41PHE	CB	41PHE	CD1	100.00	319LEL	CD2	327LEU	CD1	0.89	41PHE	CZ	90LEU	CG	0.30
41PHE	CB	41PHE	CD2	100.00	319LEL	CD2	327LEU	CD2	0.55	41PHE	CZ	90LEU	CD1	0.35
41PHE	CB	41PHE	C	100.00	319LEL	CD2	336PHE	CD1	0.01	41PHE	CZ	90LEU	CD2	9.04
41PHE	CG	41PHE	CD1	100.00	319LEL	CD2	336PHE	CE1	2.20	41PHE	CZ	93LEU	CD1	0.06
41PHE	CG	41PHE	CD2	100.00	319LEL	CD2	336PHE	CE2	1.57	41PHE	CZ	93LEU	CD2	0.32
41PHE	CG	41PHE	CE1	100.00	319LEL	CD2	336PHE	CZ	0.93	41PHE	C	42GLY	CA	100.00
41PHE	CG	41PHE	CE2	100.00	319LEL	C	320LEU	CA	100.00	41PHE	C	42GLY	C	35.73
41PHE	CG	41PHE	CZ	100.00	319LEL	C	320LEU	CB	2.22	41PHE	C	45ALA	CB	0.36
41PHE	CG	41PHE	C	100.00	319LEL	C	320LEU	CG	0.15	42GLY	CA	42GLY	C	100.00
41PHE	CD1	41PHE	CD2	100.00	319LEL	C	320LEU	CD1	0.03	42GLY	CA	72VAL	CG1	0.53
41PHE	CD1	41PHE	CE1	100.00	319LEL	C	320LEU	CD2	0.03	42GLY	CA	72VAL	CG2	1.65
41PHE	CD1	41PHE	CE2	100.00	319LEL	C	320LEU	C	96.96	42GLY	CA	86PRO	CB	0.05
41PHE	CD1	41PHE	CZ	100.00	320LEL	CA	320LEU	CB	100.00	42GLY	C	43GLY	CA	100.00
41PHE	CD1	41PHE	C	1.32	320LEL	CA	320LEU	CG	100.00	42GLY	C	43GLY	C	79.95
41PHE	CD1	53PHE	CD1	0.20	320LEL	CA	320LEU	CD1	32.82	42GLY	C	46ILE	CD	0.05
41PHE	CD2	41PHE	CE1	100.00	320LEL	CA	320LEU	CD2	65.74	42GLY	C	72VAL	CG1	0.01
41PHE	CD2	41PHE	CE2	100.00	320LEL	CA	320LEU	C	100.00	42GLY	C	72VAL	CG2	0.03
41PHE	CD2	41PHE	CZ	100.00	320LEL	CB	320LEU	CG	100.00	42GLY	C	77TRP	CZ2	0.08
41PHE	CD2	41PHE	C	57.68	320LEL	CB	320LEU	CD1	100.00	42GLY	C	86PRO	CB	0.44
41PHE	CD2	42GLY	CA	0.02	320LEL	CB	320LEU	CD2	100.00	42GLY	C	86PRO	CG	0.05
41PHE	CD2	69LEU	CD1	0.90	320LEL	CB	320LEU	C	100.00	43GLY	CA	43GLY	C	100.00
41PHE	CD2	69LEU	CD2	0.03	320LEL	CG	320LEU	CD1	100.00	43GLY	CA	46ILE	CD	0.08
41PHE	CD2	72VAL	CG1	1.37	320LEL	CG	320LEU	CD2	100.00	43GLY	CA	76LYS	CD	0.01
41PHE	CD2	72VAL	CG2	0.01	320LEL	CG	320LEU	C	31.51	43GLY	CA	77TRP	CD1	0.02
41PHE	CE1	41PHE	CE2	100.00	320LEL	CD1	320LEU	CD2	100.00	43GLY	CA	77TRP	CD2	0.29
41PHE	CE1	41PHE	CZ	100.00	320LEL	CD1	320LEU	C	0.57	43GLY	CA	77TRP	CE2	0.98
41PHE	CE1	53PHE	CB	0.06	320LEL	CD2	320LEU	C	0.89	43GLY	CA	77TRP	CE3	0.04
41PHE	CE1	53PHE	CG	0.04	320LEL	C	321GLU	CA	100.00	43GLY	CA	77TRP	CZ2	1.76
41PHE	CE1	53PHE	CD1	0.97	320LEL	C	321GLU	CB	5.41	43GLY	CA	77TRP	CZ3	0.01
41PHE	CE1	72VAL	CG1	0.01	320LEL	C	321GLU	CG	0.00	43GLY	CA	86PRO	CG	0.00
41PHE	CE1	86PRO	CB	0.02	320LEL	C	321GLU	C	95.20	43GLY	C	44ALA	CA	100.00
41PHE	CE1	89GLY	CA	0.01	320LEL	C	323PRO	CD	0.01	43GLY	C	44ALA	CB	0.06
41PHE	CE1	89GLY	C	1.35	321GLL	CA	321GLU	CB	100.00	43GLY	C	44ALA	C	99.96
41PHE	CE1	90LEU	CG	0.00	321GLL	CA	321GLU	CG	100.00	43GLY	C	46ILE	CG2	0.00
41PHE	CE1	90LEU	CD1	0.00	321GLL	CA	321GLU	CD	74.13	43GLY	C	77TRP	CD2	0.00
41PHE	CE1	90LEU	CD2	0.00	321GLL	CA	321GLU	C	100.00	43GLY	C	77TRP	CE2	0.20
41PHE	CE1	93LEU	CD1	0.26	321GLL	CB	321GLU	CG	100.00	43GLY	C	77TRP	CZ2	0.74
41PHE	CE1	93LEU	CD2	0.15	321GLL	CB	321GLU	CD	100.00	44ALA	CA	44ALA	CB	100.00
41PHE	CE2	41PHE	CZ	100.00	321GLL	CB	321GLU	C	100.00	44ALA	CA	44ALA	C	100.00
41PHE	CE2	69LEU	CG	0.02	321GLL	CB	339THR	CG2	0.22	44ALA	CB	44ALA	C	100.00
41PHE	CE2	69LEU	CD1	23.24	321GLL	CG	321GLU	CD	100.00	44ALA	C	45ALA	CA	100.00
41PHE	CE2	69LEU	CD2	2.82	321GLL	CG	321GLU	C	97.38	44ALA	C	45ALA	CB	0.02
41PHE	CE2	72VAL	CG1	8.01	321GLL	CG	322THR	CG2	0.00	44ALA	C	45ALA	C	99.96
41PHE	CE2	72VAL	CG2	0.58	321GLL	CG	339THR	CG2	1.60	45ALA	CA	45ALA	CB	100.00
41PHE	CE2	90LEU	CG	0.00	321GLL	CD	321GLU	C	0.19	45ALA	CA	45ALA	C	100.00
41PHE	CE2	90LEU	CD1	0.33	321GLL	CD	339THR	CG2	0.94	45ALA	CA	49PHE	CD1	0.01
41PHE	CE2	90LEU	CD2	0.39	321GLL	CD	342ARG	CZ	0.08	45ALA	CA	49PHE	CD2	0.00

41PHE	CE2	93LEU	CD2	0.01
41PHE	CZ	69LEU	CD1	1.10
41PHE	CZ	69LEU	CD2	0.58
41PHE	CZ	72VAL	CG1	2.82
41PHE	CZ	72VAL	CG2	0.15
41PHE	CZ	86PRO	CB	0.04
41PHE	CZ	89GLY	CA	0.00
41PHE	CZ	89GLY	C	1.11
41PHE	CZ	90LEU	CA	0.02
41PHE	CZ	90LEU	CG	0.14
41PHE	CZ	90LEU	CD1	0.58
41PHE	CZ	90LEU	CD2	3.07
41PHE	CZ	93LEU	CB	0.00
41PHE	CZ	93LEU	CD1	0.16
41PHE	CZ	93LEU	CD2	0.45
41PHE	C	42GLY	CA	100.00
41PHE	C	42GLY	C	37.37
41PHE	C	45ALA	CB	0.32
42GLY	CA	42GLY	C	100.00
42GLY	CA	72VAL	CB	0.01
42GLY	CA	72VAL	CG1	1.26
42GLY	CA	86PRO	CB	0.02
42GLY	C	43GLY	CA	100.00
42GLY	C	43GLY	C	86.59
42GLY	C	46ILE	CG1	0.00
42GLY	C	46ILE	CD	0.13
42GLY	C	72VAL	CG1	0.01
42GLY	C	86PRO	CB	0.90
42GLY	C	86PRO	CG	0.94
43GLY	CA	43GLY	C	100.00
43GLY	CA	46ILE	CD	0.13
43GLY	CA	76LYS	CB	0.01
43GLY	CA	76LYS	CG	0.01
43GLY	CA	77TRP	CB	0.16
43GLY	CA	77TRP	CG	0.49
43GLY	CA	77TRP	CD1	0.11
43GLY	CA	77TRP	CD2	1.73
43GLY	CA	77TRP	CE2	1.20
43GLY	CA	77TRP	CE3	2.31
43GLY	CA	77TRP	CZ2	0.22
43GLY	CA	77TRP	CZ3	2.17
43GLY	CA	80LEU	CD1	0.00
43GLY	CA	86PRO	CB	0.00
43GLY	CA	86PRO	CG	0.72
43GLY	C	44ALA	CA	100.00
43GLY	C	44ALA	CB	0.03
43GLY	C	44ALA	C	99.98
43GLY	C	76LYS	CB	0.02
43GLY	C	76LYS	CG	0.72
43GLY	C	76LYS	CD	0.07
43GLY	C	76LYS	CE	0.02
43GLY	C	77TRP	CB	0.04
43GLY	C	77TRP	CG	0.03
43GLY	C	77TRP	CD1	0.04
43GLY	C	77TRP	CD2	1.82
43GLY	C	77TRP	CE2	1.27
43GLY	C	77TRP	CE3	2.68
43GLY	C	77TRP	CZ2	0.70
43GLY	C	77TRP	CZ3	1.93
44ALA	CA	44ALA	CB	100.00
44ALA	CA	44ALA	C	100.00
44ALA	CA	47ASP	CB	0.01

321GLU	C	322THR	CA	100.00
321GLU	C	322THR	CB	97.72
321GLU	C	322THR	CG2	26.62
321GLU	C	322THR	C	5.14
321GLU	C	323PRO	CD	5.76
322THF	CA	322THR	CB	100.00
322THF	CA	322THR	CG2	100.00
322THF	CA	322THR	C	100.00
322THF	CA	323PRO	CD	100.00
322THF	CA	331ALA	CB	0.18
322THF	CB	322THR	CG2	100.00
322THF	CB	322THR	C	100.00
322THF	CB	323PRO	CD	1.27
322THF	CB	331ALA	CB	0.60
322THF	CG2	322THR	C	53.35
322THF	CG2	323PRO	CD	0.03
322THF	CG2	331ALA	CB	6.81
322THF	CG2	335ALA	CB	0.02
322THF	CG2	336PHE	CD1	0.07
322THF	CG2	336PHE	CD2	0.19
322THF	CG2	336PHE	CE1	2.00
322THF	CG2	336PHE	CE2	2.82
322THF	CG2	336PHE	CZ	7.18
322THF	CG2	339THR	CG2	0.09
322THF	C	323PRO	CA	100.00
322THF	C	323PRO	CB	0.20
322THF	C	323PRO	CD	100.00
322THF	C	323PRO	C	100.00
322THF	C	331ALA	CB	3.78
323PR(	CA	323PRO	CB	100.00
323PR(	CA	323PRO	CG	100.00
323PR(	CA	323PRO	CD	100.00
323PR(	CA	323PRO	C	100.00
323PR(	CA	324PRO	CD	100.00
323PR(	CB	323PRO	CG	100.00
323PR(	CB	323PRO	CD	100.00
323PR(	CB	323PRO	C	100.00
323PR(	CB	324PRO	CD	32.09
323PR(	CB	327LEU	CB	0.11
323PR(	CB	327LEU	CD1	0.09
323PR(	CB	327LEU	CD2	0.53
323PR(	CB	329GLY	CA	0.01
323PR(	CG	323PRO	CD	100.00
323PR(	CG	323PRO	C	95.22
323PR(	CG	327LEU	CB	0.00
323PR(	CG	327LEU	CD2	0.01
323PR(	CG	328GLY	C	0.02
323PR(	CG	329GLY	CA	0.12
323PR(	CG	330SER	CB	0.01
323PR(	CD	323PRO	C	87.46
323PR(	C	324PRO	CA	100.00
323PR(	C	324PRO	CB	0.26
323PR(	C	324PRO	CD	100.00
323PR(	C	324PRO	C	100.00
323PR(	C	331ALA	CB	1.06
323PR(	C	336PHE	CZ	0.01
324PR(	CA	324PRO	CB	100.00
324PR(	CA	324PRO	CG	100.00
324PR(	CA	324PRO	CD	100.00
324PR(	CA	324PRO	C	100.00
324PR(	CA	325PRO	CD	100.00
324PR(	CA	331ALA	CB	0.10

45ALA	CB	45ALA	C	100.00
45ALA	CB	49PHE	CD1	0.02
45ALA	CB	52PRO	CA	0.01
45ALA	CB	52PRO	C	0.07
45ALA	CB	54PRO	CG	0.05
45ALA	CB	54PRO	CD	0.06
45ALA	C	46ILE	CA	100.00
45ALA	C	46ILE	C	100.00
46ILE	CA	46ILE	CB	100.00
46ILE	CA	46ILE	CG1	100.00
46ILE	CA	46ILE	CG2	100.00
46ILE	CA	46ILE	CD	0.68
46ILE	CA	46ILE	C	100.00
46ILE	CA	50GLY	CA	0.03
46ILE	CB	46ILE	CG1	100.00
46ILE	CB	46ILE	CG2	100.00
46ILE	CB	46ILE	CD	100.00
46ILE	CB	46ILE	C	100.00
46ILE	CG1	46ILE	CG2	100.00
46ILE	CG1	46ILE	CD	100.00
46ILE	CG1	46ILE	C	0.48
46ILE	CG1	51GLU	C	0.21
46ILE	CG1	52PRO	CA	0.37
46ILE	CG1	52PRO	CB	1.11
46ILE	CG1	52PRO	CG	0.01
46ILE	CG1	52PRO	CD	0.02
46ILE	CG2	46ILE	CD	99.80
46ILE	CG2	46ILE	C	100.00
46ILE	CG2	50GLY	C	0.00
46ILE	CG2	77TRP	CD1	0.11
46ILE	CG2	80LEU	CD1	0.29
46ILE	CG2	80LEU	CD2	0.22
46ILE	CG2	84ILE	CA	0.01
46ILE	CG2	84ILE	CB	0.07
46ILE	CG2	84ILE	CG2	0.26
46ILE	CG2	84ILE	CD	0.09
46ILE	CD	51GLU	C	0.00
46ILE	CD	52PRO	CA	0.00
46ILE	CD	52PRO	CB	0.04
46ILE	CD	52PRO	CG	0.02
46ILE	CD	52PRO	CD	0.07
46ILE	CD	77TRP	CD1	2.79
46ILE	CD	77TRP	CE2	0.04
46ILE	CD	77TRP	CZ2	0.33
46ILE	CD	80LEU	CD1	0.15
46ILE	CD	80LEU	CD2	0.05
46ILE	CD	84ILE	CA	0.04
46ILE	CD	84ILE	CB	0.00
46ILE	CD	84ILE	C	0.22
46ILE	CD	85ARG	C	0.04
46ILE	CD	86PRO	CA	0.02
46ILE	CD	86PRO	CB	0.02
46ILE	CD	86PRO	CG	0.04
46ILE	CD	86PRO	CD	0.43
46ILE	C	47ASP	CA	100.00
46ILE	C	47ASP	CB	0.69
46ILE	C	47ASP	C	99.45
46ILE	C	50GLY	CA	0.00
47ASF	CA	47ASP	CB	100.00
47ASF	CA	47ASP	CG	100.00
47ASF	CA	47ASP	C	100.00
47ASF	CB	47ASP	CG	100.00

44ALA	CA	76LYS	CG	0.02	324PR(	CA	336PHE	CE2	0.00	47ASF	CB	47ASP	C	100.00
44ALA	CA	76LYS	CD	0.00	324PR(	CA	336PHE	CZ	0.17	47ASF	CG	47ASP	C	98.34
44ALA	CA	76LYS	CE	0.02	324PR(	CB	324PRO	CG	100.00	47ASF	C	48ALA	CA	100.00
44ALA	CA	77TRP	CE2	0.00	324PR(	CB	324PRO	CD	100.00	47ASF	C	48ALA	CB	1.86
44ALA	CA	77TRP	CE3	0.01	324PR(	CB	324PRO	C	100.00	47ASF	C	48ALA	C	97.82
44ALA	CA	77TRP	CZ2	0.12	324PR(	CB	325PRO	CD	53.29	48ALA	CA	48ALA	CB	100.00
44ALA	CA	77TRP	CZ3	0.45	324PR(	CB	327LEU	CD1	0.08	48ALA	CA	48ALA	C	100.00
44ALA	CB	44ALA	C	100.00	324PR(	CB	327LEU	CD2	0.02	48ALA	CB	48ALA	C	100.00
44ALA	CB	76LYS	CG	0.00	324PR(	CB	336PHE	CE2	0.00	48ALA	CB	49PHE	CG	0.01
44ALA	CB	76LYS	CD	0.03	324PR(	CB	336PHE	CZ	0.03	48ALA	CB	49PHE	CD1	5.16
44ALA	CB	76LYS	CE	0.01	324PR(	CG	324PRO	CD	100.00	48ALA	CB	49PHE	CD2	4.90
44ALA	CB	77TRP	CE2	0.01	324PR(	CG	324PRO	C	81.74	48ALA	CB	49PHE	CE1	0.72
44ALA	CB	77TRP	CZ2	0.05	324PR(	CG	327LEU	CB	0.01	48ALA	CB	49PHE	CE2	0.51
44ALA	CB	77TRP	CZ3	0.32	324PR(	CG	327LEU	CG	0.01	48ALA	CB	49PHE	CZ	0.01
44ALA	C	45ALA	CA	100.00	324PR(	CG	327LEU	CD1	1.10	48ALA	C	49PHE	CA	100.00
44ALA	C	45ALA	CB	0.02	324PR(	CG	327LEU	CD2	0.16	48ALA	C	49PHE	CB	36.30
44ALA	C	45ALA	C	99.98	324PR(	CG	336PHE	CE2	0.01	48ALA	C	49PHE	CG	10.54
45ALA	CA	45ALA	CB	100.00	324PR(	CG	336PHE	CZ	0.00	48ALA	C	49PHE	CD1	4.08
45ALA	CA	45ALA	C	100.00	324PR(	CD	324PRO	C	77.03	48ALA	C	49PHE	CD2	3.75
45ALA	CA	49PHE	CD1	0.02	324PR(	CD	327LEU	CB	0.00	48ALA	C	49PHE	C	58.66
45ALA	CB	45ALA	C	100.00	324PR(	CD	327LEU	CD1	0.05	49PHE	CA	49PHE	CB	100.00
45ALA	CB	49PHE	CD1	0.01	324PR(	CD	327LEU	CD2	0.03	49PHE	CA	49PHE	CG	100.00
45ALA	CB	52PRO	CA	0.02	324PR(	CD	336PHE	CD2	0.01	49PHE	CA	49PHE	CD1	79.30
45ALA	CB	52PRO	C	0.06	324PR(	CD	336PHE	CE1	0.00	49PHE	CA	49PHE	CD2	79.52
45ALA	CB	54PRO	CG	0.12	324PR(	CD	336PHE	CE2	0.03	49PHE	CA	49PHE	C	100.00
45ALA	CB	54PRO	CD	0.08	324PR(	CD	336PHE	CZ	0.01	49PHE	CB	49PHE	CG	100.00
45ALA	C	46ILE	CA	100.00	324PR(	C	325PRO	CA	100.00	49PHE	CB	49PHE	CD1	100.00
45ALA	C	46ILE	C	100.00	324PR(	C	325PRO	CB	0.04	49PHE	CB	49PHE	CD2	100.00
46ILE	CA	46ILE	CB	100.00	324PR(	C	325PRO	CD	100.00	49PHE	CB	49PHE	C	100.00
46ILE	CA	46ILE	CG1	100.00	324PR(	C	325PRO	C	100.00	49PHE	CB	54PRO	CB	13.43
46ILE	CA	46ILE	CG2	100.00	324PR(	C	329GLY	CA	0.03	49PHE	CB	54PRO	CG	0.15
46ILE	CA	46ILE	CD	0.27	324PR(	C	329GLY	C	0.01	49PHE	CG	49PHE	CD1	100.00
46ILE	CA	46ILE	C	100.00	325PR(	CA	325PRO	CB	100.00	49PHE	CG	49PHE	CD2	100.00
46ILE	CA	50GLY	CA	0.01	325PR(	CA	325PRO	CG	100.00	49PHE	CG	49PHE	CE1	100.00
46ILE	CA	50GLY	C	0.00	325PR(	CA	325PRO	CD	100.00	49PHE	CG	49PHE	CE2	100.00
46ILE	CB	46ILE	CG1	100.00	325PR(	CA	325PRO	C	100.00	49PHE	CG	49PHE	CZ	100.00
46ILE	CB	46ILE	CG2	100.00	325PR(	CA	328GLY	CA	0.01	49PHE	CG	49PHE	C	0.67
46ILE	CB	46ILE	CD	100.00	325PR(	CA	328GLY	C	0.00	49PHE	CG	54PRO	CB	18.18
46ILE	CB	46ILE	C	100.00	325PR(	CA	329GLY	CA	0.01	49PHE	CG	54PRO	CG	0.85
46ILE	CB	77TRP	CD1	0.01	325PR(	CA	329GLY	C	0.08	49PHE	CD1	49PHE	CD2	100.00
46ILE	CB	77TRP	CE2	0.02	325PR(	CB	325PRO	CG	100.00	49PHE	CD1	49PHE	CE1	100.00
46ILE	CB	77TRP	CZ2	0.60	325PR(	CB	325PRO	CD	100.00	49PHE	CD1	49PHE	CE2	100.00
46ILE	CB	77TRP	CZ3	0.06	325PR(	CB	325PRO	C	100.00	49PHE	CD1	49PHE	CZ	100.00
46ILE	CG1	46ILE	CG2	100.00	325PR(	CB	329GLY	C	0.02	49PHE	CD1	49PHE	C	0.14
46ILE	CG1	46ILE	CD	100.00	325PR(	CG	325PRO	CD	100.00	49PHE	CD1	54PRO	CB	7.36
46ILE	CG1	46ILE	C	0.02	325PR(	CG	325PRO	C	59.84	49PHE	CD1	54PRO	CG	2.84
46ILE	CG1	51GLU	C	0.13	325PR(	CG	336PHE	CZ	0.00	49PHE	CD1	56PRO	CG	0.01
46ILE	CG1	52PRO	CA	1.00	325PR(	CD	325PRO	C	41.33	49PHE	CD1	56PRO	CD	0.01
46ILE	CG1	52PRO	CB	2.70	325PR(	CD	330SER	C	0.00	49PHE	CD2	49PHE	CE1	100.00
46ILE	CG1	52PRO	CG	0.02	325PR(	CD	331ALA	CB	0.01	49PHE	CD2	49PHE	CE2	100.00
46ILE	CG1	52PRO	CD	0.08	325PR(	CD	331ALA	C	0.00	49PHE	CD2	49PHE	CZ	100.00
46ILE	CG2	46ILE	CD	99.72	325PR(	CD	336PHE	CE2	0.11	49PHE	CD2	49PHE	C	0.14
46ILE	CG2	46ILE	C	100.00	325PR(	CD	336PHE	CZ	0.03	49PHE	CD2	54PRO	CB	6.30
46ILE	CG2	50GLY	C	0.06	325PR(	C	326ASP	CA	100.00	49PHE	CD2	54PRO	CG	2.96
46ILE	CG2	77TRP	CD1	0.25	325PR(	C	326ASP	CB	3.38	49PHE	CD2	56PRO	CG	0.01
46ILE	CG2	77TRP	CD2	0.00	325PR(	C	326ASP	CG	0.00	49PHE	CD2	56PRO	CD	0.01
46ILE	CG2	77TRP	CE2	1.64	325PR(	C	326ASP	C	99.27	49PHE	CE1	49PHE	CE2	100.00
46ILE	CG2	77TRP	CE3	0.12	325PR(	C	328GLY	CA	0.00	49PHE	CE1	49PHE	CZ	100.00
46ILE	CG2	77TRP	CZ2	16.10	326ASF	CA	326ASP	CB	100.00	49PHE	CE1	54PRO	CB	0.16
46ILE	CG2	77TRP	CZ3	3.34	326ASF	CA	326ASP	CG	100.00	49PHE	CE1	54PRO	CG	0.46
46ILE	CG2	80LEU	CD1	0.05	326ASF	CA	326ASP	C	100.00	49PHE	CE1	55GLU	CB	0.00
46ILE	CG2	80LEU	CD2	0.00	326ASF	CB	326ASP	CG	100.00	49PHE	CE1	56PRO	CG	1.97

46ILE	CG2	84ILE	CG2	0.06
46ILE	CG2	84ILE	CD	0.55
46ILE	CD	46ILE	C	0.00
46ILE	CD	52PRO	CA	0.01
46ILE	CD	52PRO	CB	0.09
46ILE	CD	52PRO	CG	0.05
46ILE	CD	52PRO	CD	0.02
46ILE	CD	77TRP	CG	0.16
46ILE	CD	77TRP	CD1	1.46
46ILE	CD	77TRP	CD2	0.59
46ILE	CD	77TRP	CE2	1.08
46ILE	CD	77TRP	CE3	0.86
46ILE	CD	77TRP	CZ2	1.44
46ILE	CD	77TRP	CZ3	1.66
46ILE	CD	80LEU	CD1	1.65
46ILE	CD	80LEU	CD2	0.14
46ILE	CD	84ILE	CA	0.00
46ILE	CD	84ILE	CG2	0.01
46ILE	CD	84ILE	C	0.21
46ILE	CD	85SER	CA	0.14
46ILE	CD	85SER	C	0.27
46ILE	CD	86PRO	CB	0.00
46ILE	CD	86PRO	CG	0.12
46ILE	CD	86PRO	CD	2.40
46ILE	C	47ASP	CA	100.00
46ILE	C	47ASP	CB	0.29
46ILE	C	47ASP	C	99.83
46ILE	C	77TRP	CZ3	0.02
47ASP	CA	47ASP	CB	100.00
47ASP	CA	47ASP	CG	100.00
47ASP	CA	47ASP	C	100.00
47ASP	CA	77TRP	CZ3	0.23
47ASP	CB	47ASP	CG	100.00
47ASP	CB	47ASP	C	100.00
47ASP	CB	76LYS	CE	0.34
47ASP	CB	77TRP	CD1	0.04
47ASP	CB	77TRP	CE2	0.03
47ASP	CB	77TRP	CE3	0.01
47ASP	CB	77TRP	CZ2	1.26
47ASP	CB	77TRP	CZ3	0.30
47ASP	CG	47ASP	C	98.83
47ASP	CG	76LYS	CD	0.00
47ASP	CG	76LYS	CE	1.08
47ASP	CG	77TRP	CD1	0.00
47ASP	CG	77TRP	CZ2	0.28
47ASP	CG	77TRP	CZ3	0.01
47ASP	C	48ALA	CA	100.00
47ASP	C	48ALA	CB	3.76
47ASP	C	48ALA	C	95.74
48ALA	CA	48ALA	CB	100.00
48ALA	CA	48ALA	C	100.00
48ALA	CB	48ALA	C	100.00
48ALA	CB	49PHE	CG	0.01
48ALA	CB	49PHE	CD1	10.94
48ALA	CB	49PHE	CD2	0.03
48ALA	CB	49PHE	CE1	1.28
48ALA	CB	49PHE	CE2	0.01
48ALA	CB	49PHE	CZ	0.03
48ALA	C	49PHE	CA	100.00
48ALA	C	49PHE	CB	31.53
48ALA	C	49PHE	CG	8.45
48ALA	C	49PHE	CD1	5.53

326ASF	CB	326ASP	C	100.00
326ASF	CB	327LEU	CD1	0.00
326ASF	CB	327LEU	CD2	0.00
326ASF	CG	326ASP	C	69.45
326ASF	CG	327LEU	CD2	0.00
326ASF	C	327LEU	CA	100.00
326ASF	C	327LEU	CB	11.61
326ASF	C	327LEU	CG	1.22
326ASF	C	327LEU	CD1	0.46
326ASF	C	327LEU	CD2	0.24
326ASF	C	327LEU	C	86.44
327LEL	CA	327LEU	CB	100.00
327LEL	CA	327LEU	CG	100.00
327LEL	CA	327LEU	CD1	47.79
327LEL	CA	327LEU	CD2	51.19
327LEL	CA	327LEU	C	100.00
327LEL	CB	327LEU	CG	100.00
327LEL	CB	327LEU	CD1	100.00
327LEL	CB	327LEU	CD2	100.00
327LEL	CB	327LEU	C	100.00
327LEL	CG	327LEU	CD1	100.00
327LEL	CG	327LEU	CD2	100.00
327LEL	CG	327LEU	C	23.75
327LEL	CD1	327LEU	CD2	100.00
327LEL	CD1	327LEU	C	1.52
327LEL	CD2	327LEU	C	1.80
327LEL	C	328GLY	CA	100.00
327LEL	C	328GLY	C	43.90
328GL\	CA	328GLY	C	100.00
328GL\	C	329GLY	CA	100.00
328GL\	C	329GLY	C	30.50
329GL\	CA	329GLY	C	100.00
329GL\	C	330SER	CA	100.00
329GL\	C	330SER	CB	97.55
329GL\	C	330SER	C	2.70
330SEF	CA	330SER	CB	100.00
330SEF	CA	330SER	C	100.00
330SEF	CB	330SER	C	100.00
330SEF	C	331ALA	CA	100.00
330SEF	C	331ALA	CB	23.36
330SEF	C	331ALA	C	77.04
331AL/	CA	331ALA	CB	100.00
331AL/	CA	331ALA	C	100.00
331AL/	CA	335ALA	CB	0.11
331AL/	CB	331ALA	C	100.00
331AL/	CB	335ALA	CB	3.65
331AL/	CB	336PHE	CD2	0.30
331AL/	CB	336PHE	CE1	0.01
331AL/	CB	336PHE	CE2	8.97
331AL/	CB	336PHE	CZ	1.42
331AL/	C	332GLY	CA	100.00
331AL/	C	332GLY	C	39.60
331AL/	C	335ALA	CB	0.00
331AL/	C	336PHE	CD2	0.01
331AL/	C	336PHE	CE2	0.11
332GL\	CA	332GLY	C	100.00
332GL\	CA	335ALA	CB	0.00
332GL\	C	333THR	CA	100.00
332GL\	C	333THR	CB	1.68
332GL\	C	333THR	CG2	0.78
332GL\	C	333THR	C	98.74
332GL\	C	336PHE	CB	0.01

49PHE	CE1	56PRO	CD	1.26
49PHE	CE2	49PHE	CZ	100.00
49PHE	CE2	54PRO	CB	0.15
49PHE	CE2	54PRO	CG	0.36
49PHE	CE2	55GLU	CB	0.01
49PHE	CE2	56PRO	CG	1.46
49PHE	CE2	56PRO	CD	1.86
49PHE	CZ	54PRO	CB	0.01
49PHE	CZ	54PRO	CG	0.01
49PHE	CZ	55GLU	CB	0.02
49PHE	CZ	56PRO	CG	4.30
49PHE	CZ	56PRO	CD	1.87
49PHE	C	50GLY	CA	100.00
49PHE	C	50GLY	C	50.84
50GLY	CA	50GLY	C	100.00
50GLY	C	51GLU	CA	100.00
50GLY	C	51GLU	CB	48.10
50GLY	C	51GLU	CG	1.62
50GLY	C	51GLU	CD	0.05
50GLY	C	51GLU	C	54.75
50GLY	C	84ILE	CG2	0.01
51GLL	CA	51GLU	CB	100.00
51GLL	CA	51GLU	CG	100.00
51GLL	CA	51GLU	CD	66.19
51GLL	CA	51GLU	C	100.00
51GLL	CA	52PRO	CD	100.00
51GLL	CB	51GLU	CG	100.00
51GLL	CB	51GLU	CD	100.00
51GLL	CB	51GLU	C	100.00
51GLL	CB	52PRO	CD	7.20
51GLL	CB	53PHE	C	0.01
51GLL	CB	54PRO	CA	0.05
51GLL	CB	84ILE	CG2	0.04
51GLL	CG	51GLU	CD	100.00
51GLL	CG	51GLU	C	56.40
51GLL	CG	52PRO	CD	1.79
51GLL	CG	53PHE	C	0.51
51GLL	CG	54PRO	CA	0.20
51GLL	CG	54PRO	CB	0.00
51GLL	CG	84ILE	CG2	0.00
51GLL	CD	51GLU	C	0.05
51GLL	CD	52PRO	CD	0.09
51GLL	CD	53PHE	C	0.01
51GLL	CD	54PRO	CA	0.01
51GLL	CD	58ARG	CZ	0.00
51GLL	C	52PRO	CA	100.00
51GLL	C	52PRO	CB	0.05
51GLL	C	52PRO	CD	100.00
51GLL	C	52PRO	C	100.00
52PRC	CA	52PRO	CB	100.00
52PRC	CA	52PRO	CG	100.00
52PRC	CA	52PRO	CD	100.00
52PRC	CA	52PRO	C	100.00
52PRC	CB	52PRO	CG	100.00
52PRC	CB	52PRO	CD	100.00
52PRC	CB	52PRO	C	100.00
52PRC	CB	77TRP	CD1	0.04
52PRC	CB	86PRO	CA	0.27
52PRC	CB	86PRO	CB	0.45
52PRC	CB	86PRO	CG	0.02
52PRC	CB	89GLY	CA	0.05
52PRC	CG	52PRO	CD	100.00

48ALA	C	49PHE	CD2	0.38
48ALA	C	49PHE	C	62.98
49PHE	CA	49PHE	CB	100.00
49PHE	CA	49PHE	CG	100.00
49PHE	CA	49PHE	CD1	77.06
49PHE	CA	49PHE	CD2	80.71
49PHE	CA	49PHE	C	100.00
49PHE	CB	49PHE	CG	100.00
49PHE	CB	49PHE	CD1	100.00
49PHE	CB	49PHE	CD2	100.00
49PHE	CB	49PHE	C	100.00
49PHE	CB	54PRO	CB	9.62
49PHE	CB	54PRO	CG	0.09
49PHE	CG	49PHE	CD1	100.00
49PHE	CG	49PHE	CD2	100.00
49PHE	CG	49PHE	CE1	100.00
49PHE	CG	49PHE	CE2	100.00
49PHE	CG	49PHE	CZ	100.00
49PHE	CG	49PHE	C	2.42
49PHE	CG	54PRO	CB	15.38
49PHE	CG	54PRO	CG	0.37
49PHE	CD1	49PHE	CD2	100.00
49PHE	CD1	49PHE	CE1	100.00
49PHE	CD1	49PHE	CE2	100.00
49PHE	CD1	49PHE	CZ	100.00
49PHE	CD1	54PRO	CB	7.62
49PHE	CD1	54PRO	CG	4.10
49PHE	CD2	49PHE	CE1	100.00
49PHE	CD2	49PHE	CE2	100.00
49PHE	CD2	49PHE	CZ	100.00
49PHE	CD2	49PHE	C	1.30
49PHE	CD2	51GLU	CB	0.00
49PHE	CD2	54PRO	CB	4.64
49PHE	CD2	54PRO	CG	0.10
49PHE	CD2	56PRO	CG	0.00
49PHE	CD2	56PRO	CD	0.01
49PHE	CE1	49PHE	CE2	100.00
49PHE	CE1	49PHE	CZ	100.00
49PHE	CE1	54PRO	CB	0.25
49PHE	CE1	54PRO	CG	0.74
49PHE	CE1	55GLU	CB	0.03
49PHE	CE1	56PRO	CG	0.29
49PHE	CE1	56PRO	CD	0.11
49PHE	CE2	49PHE	CZ	100.00
49PHE	CE2	51GLU	CG	0.01
49PHE	CE2	54PRO	CB	0.19
49PHE	CE2	54PRO	CG	0.01
49PHE	CE2	55GLU	CB	0.01
49PHE	CE2	56PRO	CG	1.96
49PHE	CE2	56PRO	CD	2.96
49PHE	CZ	54PRO	CB	0.02
49PHE	CZ	55GLU	CB	0.07
49PHE	CZ	56PRO	CG	3.58
49PHE	CZ	56PRO	CD	2.02
49PHE	C	50GLY	CA	100.00
49PHE	C	50GLY	C	53.46
50GLY	CA	50GLY	C	100.00
50GLY	C	51GLU	CA	100.00
50GLY	C	51GLU	CB	62.53
50GLY	C	51GLU	CG	1.18
50GLY	C	51GLU	CD	0.01
50GLY	C	51GLU	C	42.37

332GLY	C	336PHE	CD2	0.18
332GLY	C	336PHE	CE2	0.00
333THR	CA	333THR	CB	100.00
333THR	CA	333THR	CG2	100.00
333THR	CA	333THR	C	100.00
333THR	CB	333THR	CG2	100.00
333THR	CB	333THR	C	100.00
333THR	CG2	333THR	C	22.14
333THR	CG2	337THR	CG2	0.01
333THR	C	334GLU	CA	100.00
333THR	C	334GLU	CB	0.63
333THR	C	334GLU	C	99.50
334GLU	CA	334GLU	CB	100.00
334GLU	CA	334GLU	CG	100.00
334GLU	CA	334GLU	CD	98.11
334GLU	CA	334GLU	C	100.00
334GLU	CB	334GLU	CG	100.00
334GLU	CB	334GLU	CD	100.00
334GLU	CB	334GLU	C	100.00
334GLU	CG	334GLU	CD	100.00
334GLU	CG	334GLU	C	88.27
334GLU	CD	334GLU	C	0.84
334GLU	C	335ALA	CA	100.00
334GLU	C	335ALA	CB	0.13
334GLU	C	335ALA	C	99.90
335ALA	CA	335ALA	CB	100.00
335ALA	CA	335ALA	C	100.00
335ALA	CB	335ALA	C	100.00
335ALA	C	336PHE	CA	100.00
335ALA	C	336PHE	CB	0.83
335ALA	C	336PHE	CG	0.08
335ALA	C	336PHE	CD1	0.00
335ALA	C	336PHE	CD2	0.00
335ALA	C	336PHE	C	99.06
336PHE	CA	336PHE	CB	100.00
336PHE	CA	336PHE	CG	100.00
336PHE	CA	336PHE	CD1	91.93
336PHE	CA	336PHE	CD2	48.56
336PHE	CA	336PHE	C	100.00
336PHE	CB	336PHE	CG	100.00
336PHE	CB	336PHE	CD1	100.00
336PHE	CB	336PHE	CD2	100.00
336PHE	CB	336PHE	C	100.00
336PHE	CG	336PHE	CD1	100.00
336PHE	CG	336PHE	CD2	100.00
336PHE	CG	336PHE	CE1	100.00
336PHE	CG	336PHE	CE2	100.00
336PHE	CG	336PHE	CZ	100.00
336PHE	CG	336PHE	C	47.63
336PHE	CD1	336PHE	CD2	100.00
336PHE	CD1	336PHE	CE1	100.00
336PHE	CD1	336PHE	CE2	100.00
336PHE	CD1	336PHE	CZ	100.00
336PHE	CD1	336PHE	C	40.56
336PHE	CD1	337THR	CG2	0.00
336PHE	CD1	340VAL	CG2	0.51
336PHE	CD2	336PHE	CE1	100.00
336PHE	CD2	336PHE	CE2	100.00
336PHE	CD2	336PHE	CZ	100.00
336PHE	CD2	336PHE	C	0.08
336PHE	CE1	336PHE	CE2	100.00
336PHE	CE1	336PHE	CZ	100.00

52PRO	CG	52PRO	C	89.55
52PRO	CG	80LEU	CD1	0.00
52PRO	CG	84ILE	CA	0.01
52PRO	CG	84ILE	C	0.12
52PRO	CG	85ARG	C	0.09
52PRO	CG	86PRO	CA	0.09
52PRO	CG	86PRO	CB	0.01
52PRO	CG	86PRO	CD	0.00
52PRO	CG	88THR	CG2	0.01
52PRO	CG	88THR	C	0.19
52PRO	CG	89GLY	CA	0.78
52PRO	CD	52PRO	C	70.84
52PRO	CD	84ILE	CA	0.05
52PRO	C	53PHE	CA	100.00
52PRO	C	53PHE	CB	88.35
52PRO	C	53PHE	C	37.95
52PRO	C	54PRO	CD	11.02
53PHE	CA	53PHE	CB	100.00
53PHE	CA	53PHE	CG	100.00
53PHE	CA	53PHE	CD1	96.27
53PHE	CA	53PHE	CD2	12.88
53PHE	CA	53PHE	C	100.00
53PHE	CA	54PRO	CD	100.00
53PHE	CB	53PHE	CG	100.00
53PHE	CB	53PHE	CD1	100.00
53PHE	CB	53PHE	CD2	100.00
53PHE	CB	53PHE	C	100.00
53PHE	CB	89GLY	CA	6.48
53PHE	CB	89GLY	C	0.01
53PHE	CB	92SER	CB	0.15
53PHE	CG	53PHE	CD1	100.00
53PHE	CG	53PHE	CD2	100.00
53PHE	CG	53PHE	CE1	100.00
53PHE	CG	53PHE	CE2	100.00
53PHE	CG	53PHE	CZ	100.00
53PHE	CG	53PHE	C	99.79
53PHE	CG	57THR	CG2	0.00
53PHE	CG	58ARG	CG	0.04
53PHE	CG	89GLY	CA	0.47
53PHE	CG	92SER	CB	0.06
53PHE	CD1	53PHE	CD2	100.00
53PHE	CD1	53PHE	CE1	100.00
53PHE	CD1	53PHE	CE2	100.00
53PHE	CD1	53PHE	CZ	100.00
53PHE	CD1	53PHE	C	2.53
53PHE	CD1	57THR	CG2	8.24
53PHE	CD1	58ARG	CG	0.08
53PHE	CD1	89GLY	CA	0.13
53PHE	CD1	89GLY	C	0.01
53PHE	CD1	93LEU	CD1	0.01
53PHE	CD2	53PHE	CE1	100.00
53PHE	CD2	53PHE	CE2	100.00
53PHE	CD2	53PHE	CZ	100.00
53PHE	CD2	53PHE	C	4.12
53PHE	CD2	57THR	CG2	0.00
53PHE	CD2	58ARG	CB	0.03
53PHE	CD2	58ARG	CG	7.39
53PHE	CD2	58ARG	CD	0.14
53PHE	CD2	58ARG	CZ	4.24
53PHE	CD2	89GLY	CA	0.24
53PHE	CD2	89GLY	C	0.01
53PHE	CD2	92SER	CB	10.58

50GLY	C	84ILE	CG2	0.01
51GLU	CA	51GLU	CB	100.00
51GLU	CA	51GLU	CG	100.00
51GLU	CA	51GLU	CD	75.74
51GLU	CA	51GLU	C	100.00
51GLU	CA	52PRO	CD	100.00
51GLU	CB	51GLU	CG	100.00
51GLU	CB	51GLU	CD	100.00
51GLU	CB	51GLU	C	100.00
51GLU	CB	52PRO	CD	5.22
51GLU	CB	53PHE	C	0.00
51GLU	CB	54PRO	CA	0.05
51GLU	CB	84ILE	CG2	0.00
51GLU	CG	51GLU	CD	100.00
51GLU	CG	51GLU	C	79.61
51GLU	CG	52PRO	CD	2.07
51GLU	CG	53PHE	C	1.36
51GLU	CG	54PRO	CA	0.20
51GLU	CG	54PRO	CB	0.01
51GLU	CD	51GLU	C	0.14
51GLU	CD	52PRO	CD	0.25
51GLU	CD	53PHE	C	0.00
51GLU	CD	58ARG	CZ	0.01
51GLU	C	52PRO	CA	100.00
51GLU	C	52PRO	CB	0.03
51GLU	C	52PRO	CD	100.00
51GLU	C	52PRO	C	100.00
52PRO	CA	52PRO	CB	100.00
52PRO	CA	52PRO	CG	100.00
52PRO	CA	52PRO	CD	100.00
52PRO	CA	52PRO	C	100.00
52PRO	CB	52PRO	CG	100.00
52PRO	CB	52PRO	CD	100.00
52PRO	CB	52PRO	C	100.00
52PRO	CB	85SER	C	0.01
52PRO	CB	86PRO	CA	0.36
52PRO	CB	86PRO	CB	0.08
52PRO	CB	88THR	C	0.00
52PRO	CB	89GLY	CA	0.03
52PRO	CG	52PRO	CD	100.00
52PRO	CG	52PRO	C	92.57
52PRO	CG	84ILE	CG1	0.00
52PRO	CG	84ILE	C	0.02
52PRO	CG	85SER	C	0.02
52PRO	CG	86PRO	CA	0.01
52PRO	CG	88THR	C	0.24
52PRO	CG	89GLY	CA	2.71
52PRO	CD	52PRO	C	73.03
52PRO	CD	84ILE	CA	0.00
52PRO	CD	84ILE	CG1	0.03
52PRO	CD	84ILE	CG2	0.04
52PRO	CD	84ILE	CD	0.00
52PRO	C	53PHE	CA	100.00
52PRO	C	53PHE	CB	88.67
52PRO	C	53PHE	C	40.82
52PRO	C	54PRO	CD	11.28
53PHE	CA	53PHE	CB	100.00
53PHE	CA	53PHE	CG	100.00
53PHE	CA	53PHE	CD1	99.44
53PHE	CA	53PHE	CD2	2.89
53PHE	CA	53PHE	C	100.00
53PHE	CA	54PRO	CD	100.00

336PHI	CE1	340VAL	CG2	3.56
336PHI	CE2	336PHE	CZ	100.00
336PHI	CZ	340VAL	CG2	0.05
336PHI	C	337THR	CA	100.00
336PHI	C	337THR	CB	29.28
336PHI	C	337THR	CG2	7.48
336PHI	C	337THR	C	75.46
336PHI	C	340VAL	CG2	0.35
337THI	CA	337THR	CB	100.00
337THI	CA	337THR	CG2	100.00
337THI	CA	337THR	C	100.00
337THI	CA	340VAL	CG2	0.02
337THI	CB	337THR	CG2	100.00
337THI	CB	337THR	C	100.00
337THI	CG2	337THR	C	19.08
337THI	CG2	341LEU	CD1	0.02
337THI	C	338ALA	CA	100.00
337THI	C	338ALA	CB	0.01
337THI	C	338ALA	C	100.00
337THI	C	341LEU	CD1	0.20
338AL/	CA	338ALA	CB	100.00
338AL/	CA	338ALA	C	100.00
338AL/	CB	338ALA	C	100.00
338AL/	CB	342ARG	CZ	0.19
338AL/	C	339THR	CA	100.00
338AL/	C	339THR	CB	0.62
338AL/	C	339THR	CG2	0.08
338AL/	C	339THR	C	99.54
338AL/	C	342ARG	CZ	0.02
339THI	CA	339THR	CB	100.00
339THI	CA	339THR	CG2	100.00
339THI	CA	339THR	C	100.00
339THI	CA	342ARG	CZ	0.05
339THI	CB	339THR	CG2	100.00
339THI	CB	339THR	C	100.00
339THI	CB	342ARG	CZ	0.00
339THI	CG2	339THR	C	43.64
339THI	CG2	342ARG	CZ	0.49
339THI	CG2	343HIS	CD2	0.10
339THI	CG2	343HIS	CE1	0.00
339THI	C	340VAL	CA	100.00
339THI	C	340VAL	CB	0.46
339THI	C	340VAL	CG2	0.22
339THI	C	340VAL	C	99.74
339THI	C	342ARG	CB	0.01
339THI	C	343HIS	CD2	0.04
340VAI	CA	340VAL	CB	100.00
340VAI	CA	340VAL	CG1	100.00
340VAI	CA	340VAL	CG2	100.00
340VAI	CA	340VAL	C	100.00
340VAI	CB	340VAL	CG1	100.00
340VAI	CB	340VAL	CG2	100.00
340VAI	CB	340VAL	C	100.00
340VAI	CG1	340VAL	CG2	100.00
340VAI	CG1	340VAL	C	100.00
340VAI	CG1	344LEU	CD1	1.06
340VAI	CG1	344LEU	CD2	0.02
340VAI	C	341LEU	CA	100.00
340VAI	C	341LEU	CB	0.02
340VAI	C	341LEU	C	99.96
341LEL	CA	341LEU	CB	100.00
341LEL	CA	341LEU	CG	100.00

53PHE	CD2	92SER	C	0.01
53PHE	CE1	53PHE	CE2	100.00
53PHE	CE1	53PHE	CZ	100.00
53PHE	CE1	57THR	CG2	6.41
53PHE	CE1	57THR	C	0.20
53PHE	CE1	58ARG	CA	0.22
53PHE	CE1	58ARG	CG	0.36
53PHE	CE1	61VAL	CG2	0.05
53PHE	CE1	93LEU	CA	0.01
53PHE	CE1	93LEU	CB	0.29
53PHE	CE1	93LEU	CG	0.12
53PHE	CE1	93LEU	CD1	2.87
53PHE	CE1	93LEU	CD2	0.12
53PHE	CE2	53PHE	CZ	100.00
53PHE	CE2	57THR	CG2	0.06
53PHE	CE2	58ARG	CA	0.72
53PHE	CE2	58ARG	CB	1.03
53PHE	CE2	58ARG	CG	15.21
53PHE	CE2	58ARG	CD	0.32
53PHE	CE2	58ARG	CZ	0.12
53PHE	CE2	61VAL	CG2	0.00
53PHE	CE2	89GLY	CA	0.00
53PHE	CE2	89GLY	C	0.00
53PHE	CE2	92SER	CB	0.58
53PHE	CE2	92SER	C	0.48
53PHE	CE2	93LEU	CA	2.04
53PHE	CE2	93LEU	CB	0.04
53PHE	CE2	93LEU	CG	0.00
53PHE	CE2	93LEU	CD1	0.00
53PHE	CE2	96SER	CB	0.01
53PHE	CZ	57THR	CG2	0.12
53PHE	CZ	58ARG	CA	1.10
53PHE	CZ	58ARG	CB	0.12
53PHE	CZ	58ARG	CG	1.23
53PHE	CZ	61VAL	CG1	0.01
53PHE	CZ	61VAL	CG2	0.58
53PHE	CZ	93LEU	CA	0.79
53PHE	CZ	93LEU	CB	0.56
53PHE	CZ	93LEU	CG	0.22
53PHE	CZ	93LEU	CD1	4.84
53PHE	CZ	93LEU	CD2	0.10
53PHE	CZ	96SER	CB	0.00
53PHE	C	54PRO	CA	100.00
53PHE	C	54PRO	CB	1.28
53PHE	C	54PRO	CD	100.00
53PHE	C	54PRO	C	99.99
54PRC	CA	54PRO	CB	100.00
54PRC	CA	54PRO	CG	100.00
54PRC	CA	54PRO	CD	100.00
54PRC	CA	54PRO	C	100.00
54PRC	CB	54PRO	CG	100.00
54PRC	CB	54PRO	CD	100.00
54PRC	CB	54PRO	C	100.00
54PRC	CG	54PRO	CD	100.00
54PRC	CG	54PRO	C	99.88
54PRC	CD	54PRO	C	98.02
54PRC	CD	57THR	CB	0.12
54PRC	C	55GLU	CA	100.00
54PRC	C	55GLU	C	100.00
54PRC	C	56PRO	CD	20.42
54PRC	C	58ARG	CG	0.02
54PRC	C	58ARG	CD	0.15

53PHE	CB	53PHE	CG	100.00	341LEL	CA	341LEU	CD1	14.36	54PRC	C	58ARG	CZ	0.16
53PHE	CB	53PHE	CD1	100.00	341LEL	CA	341LEU	CD2	85.59	55GLL	CA	55GLU	CB	100.00
53PHE	CB	53PHE	CD2	100.00	341LEL	CA	341LEU	C	100.00	55GLL	CA	55GLU	CG	100.00
53PHE	CB	53PHE	C	100.00	341LEL	CA	344LEU	CD1	0.00	55GLL	CA	55GLU	CD	93.24
53PHE	CB	89GLY	CA	2.48	341LEL	CA	344LEU	CD2	0.00	55GLL	CA	55GLU	C	100.00
53PHE	CB	89GLY	C	0.01	341LEL	CB	341LEU	CG	100.00	55GLL	CA	56PRO	CD	100.00
53PHE	CB	92SER	CB	0.01	341LEL	CB	341LEU	CD1	100.00	55GLL	CA	58ARG	CZ	0.85
53PHE	CG	53PHE	CD1	100.00	341LEL	CB	341LEU	CD2	100.00	55GLL	CB	55GLU	CG	100.00
53PHE	CG	53PHE	CD2	100.00	341LEL	CB	341LEU	C	100.00	55GLL	CB	55GLU	CD	100.00
53PHE	CG	53PHE	CE1	100.00	341LEL	CB	342ARG	CZ	0.03	55GLL	CB	55GLU	C	100.00
53PHE	CG	53PHE	CE2	100.00	341LEL	CG	341LEU	CD1	100.00	55GLL	CB	56PRO	CD	75.12
53PHE	CG	53PHE	CZ	100.00	341LEL	CG	341LEU	CD2	100.00	55GLL	CG	55GLU	CD	100.00
53PHE	CG	53PHE	C	99.91	341LEL	CG	341LEU	C	0.91	55GLL	CG	55GLU	C	91.63
53PHE	CG	58ARG	CG	0.04	341LEL	CD1	341LEU	CD2	100.00	55GLL	CG	56PRO	CD	0.29
53PHE	CG	89GLY	CA	0.01	341LEL	CD1	341LEU	C	0.01	55GLL	CG	59LYS	CE	0.03
53PHE	CG	92SER	CB	0.01	341LEL	CD1	344LEU	CD1	0.02	55GLL	CD	55GLU	C	6.66
53PHE	CD1	53PHE	CD2	100.00	341LEL	CD1	345ALA	CB	0.00	55GLL	CD	58ARG	CZ	0.05
53PHE	CD1	53PHE	CE1	100.00	341LEL	CD2	341LEU	C	0.20	55GLL	CD	59LYS	CG	0.00
53PHE	CD1	53PHE	CE2	100.00	341LEL	CD2	344LEU	CD1	0.09	55GLL	CD	59LYS	CD	0.07
53PHE	CD1	53PHE	CZ	100.00	341LEL	CD2	344LEU	CD2	0.02	55GLL	CD	59LYS	CE	0.72
53PHE	CD1	53PHE	C	5.18	341LEL	CD2	345ALA	CB	0.02	55GLL	C	56PRO	CA	100.00
53PHE	CD1	57THR	CG2	5.59	341LEL	C	342ARG	CA	100.00	55GLL	C	56PRO	CB	0.02
53PHE	CD1	58ARG	CG	0.11	341LEL	C	342ARG	CB	1.28	55GLL	C	56PRO	CD	100.00
53PHE	CD1	89GLY	CA	0.00	341LEL	C	342ARG	CG	0.04	55GLL	C	56PRO	C	100.00
53PHE	CD1	93LEU	CD1	0.02	341LEL	C	342ARG	CZ	0.01	56PRC	CA	56PRO	CB	100.00
53PHE	CD1	93LEU	CD2	0.00	341LEL	C	342ARG	C	98.68	56PRC	CA	56PRO	CG	100.00
53PHE	CD2	53PHE	CE1	100.00	342AR(	CA	342ARG	CB	100.00	56PRC	CA	56PRO	CD	100.00
53PHE	CD2	53PHE	CE2	100.00	342AR(	CA	342ARG	CG	100.00	56PRC	CA	56PRO	C	100.00
53PHE	CD2	53PHE	CZ	100.00	342AR(	CA	342ARG	CD	5.11	56PRC	CB	56PRO	CG	100.00
53PHE	CD2	53PHE	C	0.50	342AR(	CA	342ARG	CZ	0.33	56PRC	CB	56PRO	CD	100.00
53PHE	CD2	58ARG	CG	9.24	342AR(	CA	342ARG	C	100.00	56PRC	CB	56PRO	C	100.00
53PHE	CD2	58ARG	CD	0.20	342AR(	CB	342ARG	CG	100.00	56PRC	CG	56PRO	CD	100.00
53PHE	CD2	58ARG	CZ	9.54	342AR(	CB	342ARG	CD	100.00	56PRC	CG	56PRO	C	91.84
53PHE	CD2	89GLY	CA	0.01	342AR(	CB	342ARG	CZ	0.37	56PRC	CD	56PRO	C	77.90
53PHE	CD2	92SER	CB	6.62	342AR(	CB	342ARG	C	100.00	56PRC	C	57THR	CA	100.00
53PHE	CD2	92SER	C	0.03	342AR(	CG	342ARG	CD	100.00	56PRC	C	57THR	CB	0.03
53PHE	CE1	53PHE	CE2	100.00	342AR(	CG	342ARG	CZ	7.23	56PRC	C	57THR	C	100.00
53PHE	CE1	53PHE	CZ	100.00	342AR(	CG	342ARG	C	79.87	57THF	CA	57THR	CB	100.00
53PHE	CE1	57THR	CB	0.00	342AR(	CD	342ARG	CZ	100.00	57THF	CA	57THR	CG2	100.00
53PHE	CE1	57THR	CG2	3.19	342AR(	CD	342ARG	C	0.40	57THF	CA	57THR	C	100.00
53PHE	CE1	57THR	C	0.39	342AR(	CD	343HIS	CE1	0.01	57THF	CB	57THR	CG2	100.00
53PHE	CE1	58ARG	CA	0.40	342AR(	CZ	343HIS	CE1	0.07	57THF	CB	57THR	C	100.00
53PHE	CE1	58ARG	CB	0.00	342AR(	CZ	345ALA	CB	0.02	57THF	CG2	57THR	C	99.99
53PHE	CE1	58ARG	CG	0.67	342AR(	C	343HIS	CA	100.00	57THF	C	58ARG	CA	100.00
53PHE	CE1	61VAL	CG1	0.02	342AR(	C	343HIS	CB	5.07	57THF	C	58ARG	C	100.00
53PHE	CE1	61VAL	CG2	0.02	342AR(	C	343HIS	CG	0.14	58ARC	CA	58ARG	CB	100.00
53PHE	CE1	93LEU	CA	0.03	342AR(	C	343HIS	CD2	0.00	58ARC	CA	58ARG	CG	100.00
53PHE	CE1	93LEU	CB	0.28	342AR(	C	343HIS	C	94.76	58ARC	CA	58ARG	CD	0.02
53PHE	CE1	93LEU	CG	0.26	342AR(	C	345ALA	CB	0.01	58ARC	CA	58ARG	C	100.00
53PHE	CE1	93LEU	CD1	6.14	343HIS	CA	343HIS	CB	100.00	58ARC	CB	58ARG	CG	100.00
53PHE	CE1	93LEU	CD2	0.33	343HIS	CA	343HIS	CG	100.00	58ARC	CB	58ARG	CD	100.00
53PHE	CE2	53PHE	CZ	100.00	343HIS	CA	343HIS	CD2	8.11	58ARC	CB	58ARG	CZ	1.87
53PHE	CE2	58ARG	CA	0.10	343HIS	CA	343HIS	C	100.00	58ARC	CB	58ARG	C	100.00
53PHE	CE2	58ARG	CB	0.14	343HIS	CB	343HIS	CG	100.00	58ARC	CG	58ARG	CD	100.00
53PHE	CE2	58ARG	CG	24.72	343HIS	CB	343HIS	CD2	100.00	58ARC	CG	58ARG	CZ	34.93
53PHE	CE2	58ARG	CD	0.44	343HIS	CB	343HIS	CE1	0.33	58ARC	CG	58ARG	C	32.25
53PHE	CE2	58ARG	CZ	0.52	343HIS	CB	343HIS	C	100.00	58ARC	CD	58ARG	CZ	100.00
53PHE	CE2	92SER	CB	0.16	343HIS	CG	343HIS	CD2	100.00	58ARC	CZ	58ARG	C	0.00
53PHE	CE2	92SER	C	0.48	343HIS	CG	343HIS	CE1	100.00	58ARC	CZ	59LYS	CG	0.01
53PHE	CE2	93LEU	CA	3.34	343HIS	CG	343HIS	C	86.58	58ARC	CZ	92SER	CB	0.01
53PHE	CE2	93LEU	CB	0.04	343HIS	CD2	343HIS	CE1	100.00	58ARC	CZ	96SER	CB	0.04
53PHE	CE2	93LEU	CD1	0.02	343HIS	CD2	343HIS	C	7.69	58ARC	C	59LYS	CA	100.00

53PHE	CE2	96SER	CB	0.01	343HIS	CD2	344LEU	CD2	0.03	58ARG	C	59LYS	CB	2.90
53PHE	CZ	57THR	CG2	0.02	343HIS	CE1	344LEU	CD2	0.00	58ARG	C	59LYS	CG	0.27
53PHE	CZ	58ARG	CA	0.61	343HIS	C	344LEU	CA	100.00	58ARG	C	59LYS	C	97.44
53PHE	CZ	58ARG	CB	0.05	343HIS	C	344LEU	CB	13.88	58ARG	C	61VAL	CG2	0.00
53PHE	CZ	58ARG	CG	4.09	343HIS	C	344LEU	CG	1.59	59LYS	CA	59LYS	CB	100.00
53PHE	CZ	61VAL	CG1	0.00	343HIS	C	344LEU	CD1	0.06	59LYS	CA	59LYS	CG	100.00
53PHE	CZ	61VAL	CG2	0.21	343HIS	C	344LEU	CD2	0.05	59LYS	CA	59LYS	CD	9.10
53PHE	CZ	93LEU	CA	0.81	343HIS	C	344LEU	C	85.14	59LYS	CA	59LYS	CE	0.04
53PHE	CZ	93LEU	CB	0.21	344LEU	CA	344LEU	CB	100.00	59LYS	CA	59LYS	C	100.00
53PHE	CZ	93LEU	CG	0.06	344LEU	CA	344LEU	CG	100.00	59LYS	CA	62GLU	CB	0.00
53PHE	CZ	93LEU	CD1	6.40	344LEU	CA	344LEU	CD1	13.75	59LYS	CB	59LYS	CG	100.00
53PHE	CZ	93LEU	CD2	0.30	344LEU	CA	344LEU	CD2	84.36	59LYS	CB	59LYS	CD	100.00
53PHE	C	54PRO	CA	100.00	344LEU	CA	344LEU	C	100.00	59LYS	CB	59LYS	CE	9.76
53PHE	C	54PRO	CB	1.35	344LEU	CB	344LEU	CG	100.00	59LYS	CB	59LYS	C	100.00
53PHE	C	54PRO	CD	100.00	344LEU	CB	344LEU	CD1	100.00	59LYS	CG	59LYS	CD	100.00
53PHE	C	54PRO	C	99.99	344LEU	CB	344LEU	CD2	100.00	59LYS	CG	59LYS	CE	100.00
54PRO	CA	54PRO	CB	100.00	344LEU	CB	344LEU	C	100.00	59LYS	CG	59LYS	C	14.07
54PRO	CA	54PRO	CG	100.00	344LEU	CG	344LEU	CD1	100.00	59LYS	CD	59LYS	CE	100.00
54PRO	CA	54PRO	CD	100.00	344LEU	CG	344LEU	CD2	100.00	59LYS	CD	59LYS	C	0.09
54PRO	CA	54PRO	C	100.00	344LEU	CG	344LEU	C	11.25	59LYS	CD	62GLU	CD	0.00
54PRO	CA	58ARG	CZ	0.00	344LEU	CD1	344LEU	CD2	100.00	59LYS	C	60GLY	CA	100.00
54PRO	CB	54PRO	CG	100.00	344LEU	CD1	344LEU	C	4.39	59LYS	C	60GLY	C	83.81
54PRO	CB	54PRO	CD	100.00	344LEU	CD1	345ALA	CB	0.02	59LYS	C	62GLU	CB	0.01
54PRO	CB	54PRO	C	100.00	344LEU	CD2	344LEU	C	0.24	60GLY	CA	60GLY	C	100.00
54PRO	CG	54PRO	CD	100.00	344LEU	C	345ALA	CA	100.00	60GLY	C	61VAL	CA	100.00
54PRO	CG	54PRO	C	99.94	344LEU	C	345ALA	CB	62.52	60GLY	C	61VAL	CB	0.02
54PRO	CD	54PRO	C	98.33	344LEU	C	345ALA	C	40.76	60GLY	C	61VAL	CG1	0.01
54PRO	CD	57THR	CB	0.12	345ALA	CA	345ALA	CB	100.00	60GLY	C	61VAL	C	99.99
54PRO	C	55GLU	CA	100.00	345ALA	CA	345ALA	C	100.00	61VAL	CA	61VAL	CB	100.00
54PRO	C	55GLU	C	100.00	345ALA	CB	345ALA	C	100.00	61VAL	CA	61VAL	CG1	100.00
54PRO	C	56PRO	CD	20.88	1MET	CA	1MET	CB	100.00	61VAL	CA	61VAL	CG2	100.00
54PRO	C	58ARG	CG	0.02	1MET	CA	1MET	CG	100.00	61VAL	CA	61VAL	C	100.00
54PRO	C	58ARG	CD	0.29	1MET	CA	1MET	CE	0.02	61VAL	CA	67VAL	CG2	0.00
54PRO	C	58ARG	CZ	0.10	1MET	CA	1MET	C	100.00	61VAL	CB	61VAL	CG1	100.00
55GLU	CA	55GLU	CB	100.00	1MET	CA	65GLU	CD	0.09	61VAL	CB	61VAL	CG2	100.00
55GLU	CA	55GLU	CG	100.00	1MET	CA	302PHE	CE1	0.03	61VAL	CB	61VAL	C	100.00
55GLU	CA	55GLU	CD	93.44	1MET	CA	302PHE	CE2	0.01	61VAL	CB	67VAL	CG2	1.04
55GLU	CA	55GLU	C	100.00	1MET	CA	302PHE	CZ	0.04	61VAL	CB	93LEU	CD1	0.01
55GLU	CA	56PRO	CD	100.00	1MET	CB	1MET	CG	100.00	61VAL	CG1	61VAL	CG2	100.00
55GLU	CA	58ARG	CZ	0.13	1MET	CB	1MET	CE	21.38	61VAL	CG1	61VAL	C	1.27
55GLU	CB	55GLU	CG	100.00	1MET	CB	1MET	C	100.00	61VAL	CG1	67VAL	CG1	0.56
55GLU	CB	55GLU	CD	100.00	1MET	CB	32LEU	CD1	0.00	61VAL	CG1	67VAL	CG2	5.30
55GLU	CB	55GLU	C	100.00	1MET	CB	32LEU	CD2	0.00	61VAL	CG1	93LEU	CD1	0.12
55GLU	CB	56PRO	CD	81.36	1MET	CB	33GLY	C	0.00	61VAL	CG1	93LEU	CD2	0.01
55GLU	CG	55GLU	CD	100.00	1MET	CB	65GLU	CB	0.01	61VAL	CG1	97GLN	CG	0.02
55GLU	CG	55GLU	C	85.01	1MET	CB	302PHE	CD1	0.04	61VAL	CG1	97GLN	CD	0.02
55GLU	CG	56PRO	CD	0.32	1MET	CB	302PHE	CD2	0.03	61VAL	CG2	61VAL	C	98.72
55GLU	CG	59LYS	CE	0.04	1MET	CB	302PHE	CE1	2.64	61VAL	CG2	93LEU	CD1	0.33
55GLU	CD	55GLU	C	8.74	1MET	CB	302PHE	CE2	4.72	61VAL	CG2	97GLN	CG	0.04
55GLU	CD	58ARG	CD	0.01	1MET	CB	302PHE	CZ	2.62	61VAL	CG2	97GLN	CD	0.15
55GLU	CD	58ARG	CZ	0.00	1MET	CG	1MET	CE	100.00	61VAL	C	62GLU	CA	100.00
55GLU	CD	59LYS	CG	0.00	1MET	CG	1MET	C	87.81	61VAL	C	62GLU	CB	0.58
55GLU	CD	59LYS	CD	0.06	1MET	CG	3VAL	CG2	0.02	61VAL	C	62GLU	CG	0.02
55GLU	CD	59LYS	CE	0.86	1MET	CG	32LEU	CD2	0.00	61VAL	C	62GLU	C	99.39
55GLU	C	56PRO	CA	100.00	1MET	CG	34LEU	CD2	0.02	62GLU	CA	62GLU	CB	100.00
55GLU	C	56PRO	CB	0.03	1MET	CG	65GLU	CB	5.35	62GLU	CA	62GLU	CG	100.00
55GLU	C	56PRO	CD	100.00	1MET	CG	65GLU	CG	0.42	62GLU	CA	62GLU	CD	97.07
55GLU	C	56PRO	C	100.00	1MET	CG	65GLU	CD	0.08	62GLU	CA	62GLU	C	100.00
56PRO	CA	56PRO	CB	100.00	1MET	CG	301ALA	CB	0.00	62GLU	CB	62GLU	CG	100.00
56PRO	CA	56PRO	CG	100.00	1MET	CG	302PHE	CG	0.04	62GLU	CB	62GLU	CD	100.00
56PRO	CA	56PRO	CD	100.00	1MET	CG	302PHE	CD1	0.27	62GLU	CB	62GLU	C	100.00
56PRO	CA	56PRO	C	100.00	1MET	CG	302PHE	CD2	0.22	62GLU	CG	62GLU	CD	100.00

56PRO	CB	56PRO	CG	100.00	1MET	CG	302PHE	CE1	1.86	62GLL	CG	62GLU	C	80.97
56PRO	CB	56PRO	CD	100.00	1MET	CG	302PHE	CE2	2.97	62GLL	CD	62GLU	C	0.24
56PRO	CB	56PRO	C	100.00	1MET	CG	302PHE	CZ	3.24	62GLL	C	63GLU	CA	100.00
56PRO	CG	56PRO	CD	100.00	1MET	CE	1MET	C	0.01	62GLL	C	63GLU	CB	20.35
56PRO	CG	56PRO	C	92.62	1MET	CE	3VAL	CG2	1.82	62GLL	C	63GLU	CG	0.16
56PRO	CD	56PRO	C	78.46	1MET	CE	23LEU	CD1	0.02	62GLL	C	63GLU	CD	0.00
56PRO	C	57THR	CA	100.00	1MET	CE	23LEU	CD2	0.01	62GLL	C	63GLU	C	83.06
56PRO	C	57THR	CB	0.01	1MET	CE	32LEU	CB	0.00	63GLL	CA	63GLU	CB	100.00
56PRO	C	57THR	C	100.00	1MET	CE	32LEU	CD1	0.04	63GLL	CA	63GLU	CG	100.00
57THR	CA	57THR	CB	100.00	1MET	CE	32LEU	CD2	0.02	63GLL	CA	63GLU	CD	77.10
57THR	CA	57THR	CG2	100.00	1MET	CE	32LEU	C	0.02	63GLL	CA	63GLU	C	100.00
57THR	CA	57THR	C	100.00	1MET	CE	33GLY	CA	0.00	63GLL	CB	63GLU	CG	100.00
57THR	CB	57THR	CG2	100.00	1MET	CE	33GLY	C	0.02	63GLL	CB	63GLU	CD	100.00
57THR	CB	57THR	C	100.00	1MET	CE	34LEU	CD1	0.06	63GLL	CB	63GLU	C	100.00
57THR	CG2	57THR	C	99.99	1MET	CE	34LEU	CD2	0.01	63GLL	CG	63GLU	CD	100.00
57THR	CG2	61VAL	CG1	0.06	1MET	CE	65GLU	CB	0.41	63GLL	CG	63GLU	C	86.34
57THR	C	58ARG	CA	100.00	1MET	CE	65GLU	CG	0.57	63GLL	CD	63GLU	C	4.13
57THR	C	58ARG	C	100.00	1MET	CE	65GLU	CD	0.00	63GLL	C	64ALA	CA	100.00
58ARG	CA	58ARG	CB	100.00	1MET	CE	65GLU	C	0.03	63GLL	C	64ALA	CB	28.94
58ARG	CA	58ARG	CG	100.00	1MET	CE	66ALA	CB	1.96	63GLL	C	64ALA	C	71.27
58ARG	CA	58ARG	CD	0.16	1MET	CE	297MET	CB	0.00	64ALA	CA	64ALA	CB	100.00
58ARG	CA	58ARG	C	100.00	1MET	CE	297MET	CG	0.16	64ALA	CA	64ALA	C	100.00
58ARG	CB	58ARG	CG	100.00	1MET	CE	297MET	CE	0.09	64ALA	CB	64ALA	C	100.00
58ARG	CB	58ARG	CD	100.00	1MET	CE	298LEU	CD1	0.00	64ALA	CB	67VAL	CG2	0.01
58ARG	CB	58ARG	CZ	0.57	1MET	CE	298LEU	CD2	0.31	64ALA	CB	266THR	CG2	0.00
58ARG	CB	58ARG	C	100.00	1MET	CE	301ALA	CB	4.31	64ALA	C	65GLU	CA	100.00
58ARG	CG	58ARG	CD	100.00	1MET	CE	301ALA	C	0.48	64ALA	C	65GLU	CB	11.56
58ARG	CG	58ARG	CZ	36.34	1MET	CE	302PHE	CA	0.02	64ALA	C	65GLU	CG	0.09
58ARG	CG	58ARG	C	9.54	1MET	CE	302PHE	CB	0.04	64ALA	C	65GLU	CD	0.01
58ARG	CD	58ARG	CZ	100.00	1MET	CE	302PHE	CG	0.70	64ALA	C	65GLU	C	90.81
58ARG	CZ	96SER	CB	0.04	1MET	CE	302PHE	CD1	2.79	64ALA	C	266THR	CG2	0.47
58ARG	C	59LYS	CA	100.00	1MET	CE	302PHE	CD2	2.20	65GLL	CA	65GLU	CB	100.00
58ARG	C	59LYS	CB	18.25	1MET	CE	302PHE	CE1	7.70	65GLL	CA	65GLU	CG	100.00
58ARG	C	59LYS	CG	1.64	1MET	CE	302PHE	CE2	4.10	65GLL	CA	65GLU	CD	98.18
58ARG	C	59LYS	CD	0.00	1MET	CE	302PHE	CZ	3.29	65GLL	CA	65GLU	C	100.00
58ARG	C	59LYS	C	81.42	1MET	CE	304LEU	CD1	0.23	65GLL	CA	266THR	CG2	0.01
58ARG	C	61VAL	CG2	0.01	1MET	C	2LYS	CA	100.00	65GLL	CB	65GLU	CG	100.00
59LYS	CA	59LYS	CB	100.00	1MET	C	2LYS	CB	11.12	65GLL	CB	65GLU	CD	100.00
59LYS	CA	59LYS	CG	100.00	1MET	C	2LYS	CG	0.24	65GLL	CB	65GLU	C	100.00
59LYS	CA	59LYS	CD	11.86	1MET	C	2LYS	C	92.22	65GLL	CB	302PHE	CE1	0.01
59LYS	CA	59LYS	CE	0.03	1MET	C	302PHE	CZ	0.02	65GLL	CG	65GLU	CD	100.00
59LYS	CA	59LYS	C	100.00	2LYS	CA	2LYS	CB	100.00	65GLL	CG	65GLU	C	83.30
59LYS	CA	62GLU	CB	0.00	2LYS	CA	2LYS	CG	100.00	65GLL	CG	302PHE	CD1	0.02
59LYS	CB	59LYS	CG	100.00	2LYS	CA	2LYS	CD	1.42	65GLL	CG	302PHE	CE1	0.24
59LYS	CB	59LYS	CD	100.00	2LYS	CA	2LYS	C	100.00	65GLL	CG	302PHE	CZ	0.01
59LYS	CB	59LYS	CE	6.41	2LYS	CA	35ALA	CB	0.00	65GLL	CD	65GLU	C	0.00
59LYS	CB	59LYS	C	100.00	2LYS	CB	2LYS	CG	100.00	65GLL	C	66ALA	CA	100.00
59LYS	CG	59LYS	CD	100.00	2LYS	CB	2LYS	CD	100.00	65GLL	C	66ALA	CB	99.88
59LYS	CG	59LYS	CE	100.00	2LYS	CB	2LYS	CE	20.46	65GLL	C	66ALA	C	0.14
59LYS	CG	59LYS	C	14.32	2LYS	CB	2LYS	C	100.00	65GLL	C	266THR	CG2	9.78
59LYS	CD	59LYS	CE	100.00	2LYS	CB	65GLU	CD	0.00	66ALA	CA	66ALA	CB	100.00
59LYS	CD	59LYS	C	0.06	2LYS	CG	2LYS	CD	100.00	66ALA	CA	66ALA	C	100.00
59LYS	C	60GLY	CA	100.00	2LYS	CG	2LYS	CE	100.00	66ALA	CA	266THR	CG2	7.53
59LYS	C	60GLY	C	55.76	2LYS	CG	2LYS	C	49.68	66ALA	CB	66ALA	C	100.00
60GLY	CA	60GLY	C	100.00	2LYS	CG	35ALA	CB	0.02	66ALA	CB	269PHE	CD1	0.04
60GLY	CA	63GLU	CB	0.02	2LYS	CD	2LYS	CE	100.00	66ALA	CB	269PHE	CE1	2.41
60GLY	C	61VAL	CA	100.00	2LYS	CD	2LYS	C	0.11	66ALA	CB	297MET	CG	3.94
60GLY	C	61VAL	CB	0.42	2LYS	CD	35ALA	CB	0.01	66ALA	CB	297MET	CE	0.28
60GLY	C	61VAL	CG1	0.09	2LYS	CD	63GLU	CG	0.06	66ALA	CB	301ALA	CB	0.24
60GLY	C	61VAL	C	99.62	2LYS	CD	63GLU	CD	0.09	66ALA	CB	302PHE	CE1	0.08
60GLY	C	63GLU	CB	0.04	2LYS	CD	63GLU	C	0.00	66ALA	CB	302PHE	CE2	0.02
61VAL	CA	61VAL	CB	100.00	2LYS	CE	35ALA	CB	0.01	66ALA	CB	302PHE	CZ	0.03

61VAL	CA	61VAL	CG1	100.00	2LYS	CE	36TYR	C	0.01	66ALA	C	67VAL	CA	100.00
61VAL	CA	61VAL	CG2	100.00	2LYS	CE	37GLU	CB	0.78	66ALA	C	67VAL	CB	19.74
61VAL	CA	61VAL	C	100.00	2LYS	CE	37GLU	CG	0.01	66ALA	C	67VAL	CG2	11.53
61VAL	CA	67VAL	CG2	0.04	2LYS	CE	37GLU	CD	0.05	66ALA	C	67VAL	C	89.73
61VAL	CB	61VAL	CG1	100.00	2LYS	CE	63GLU	CA	0.00	66ALA	C	266THR	CG2	3.67
61VAL	CB	61VAL	CG2	100.00	2LYS	CE	63GLU	CG	0.03	67VAL	CA	67VAL	CB	100.00
61VAL	CB	61VAL	C	100.00	2LYS	CE	63GLU	CD	0.12	67VAL	CA	67VAL	CG1	100.00
61VAL	CB	67VAL	CG2	0.82	2LYS	CE	63GLU	C	0.01	67VAL	CA	67VAL	CG2	100.00
61VAL	CB	93LEU	CD1	0.00	2LYS	CE	64ALA	CA	0.01	67VAL	CA	67VAL	C	100.00
61VAL	CG1	61VAL	CG2	100.00	2LYS	C	3VAL	CA	100.00	67VAL	CB	67VAL	CG1	100.00
61VAL	CG1	67VAL	CG1	0.36	2LYS	C	3VAL	CB	24.40	67VAL	CB	67VAL	CG2	100.00
61VAL	CG1	67VAL	CG2	2.83	2LYS	C	3VAL	CG2	16.51	67VAL	CB	67VAL	C	100.00
61VAL	CG1	93LEU	CD1	0.28	2LYS	C	3VAL	C	83.51	67VAL	CB	268VAL	CG2	0.08
61VAL	CG1	93LEU	CD2	0.06	2LYS	C	64ALA	CB	0.02	67VAL	CG1	67VAL	CG2	100.00
61VAL	CG2	61VAL	C	99.80	3VAL	CA	3VAL	CB	100.00	67VAL	CG1	67VAL	C	100.00
61VAL	CG2	93LEU	CD1	0.11	3VAL	CA	3VAL	CG1	100.00	67VAL	CG1	68LEU	C	0.04
61VAL	CG2	93LEU	CD2	0.01	3VAL	CA	3VAL	CG2	100.00	67VAL	CG1	69LEU	CD1	0.05
61VAL	CG2	97GLN	CG	0.25	3VAL	CA	3VAL	C	100.00	67VAL	CG1	69LEU	CD2	0.01
61VAL	CG2	97GLN	CD	0.96	3VAL	CB	3VAL	CG1	100.00	67VAL	CG1	268VAL	CG1	0.04
61VAL	C	62GLU	CA	100.00	3VAL	CB	3VAL	CG2	100.00	67VAL	CG1	268VAL	CG2	0.00
61VAL	C	62GLU	CB	2.75	3VAL	CB	3VAL	C	100.00	67VAL	CG2	266THR	CB	0.01
61VAL	C	62GLU	CG	0.01	3VAL	CB	34LEU	CD1	0.00	67VAL	CG2	266THR	CG2	9.62
61VAL	C	62GLU	C	98.20	3VAL	CG1	3VAL	CG2	100.00	67VAL	CG2	268VAL	CG2	0.20
61VAL	C	67VAL	CG2	0.01	3VAL	CG1	3VAL	C	100.00	67VAL	C	68LEU	CA	100.00
62GLU	CA	62GLU	CB	100.00	3VAL	CG1	4ALA	C	0.06	67VAL	C	68LEU	CB	7.56
62GLU	CA	62GLU	CG	100.00	3VAL	CG1	5VAL	CG2	0.32	67VAL	C	68LEU	C	98.69
62GLU	CA	62GLU	CD	97.58	3VAL	CG1	66ALA	CB	0.00	67VAL	C	297MET	CE	0.20
62GLU	CA	62GLU	C	100.00	3VAL	CG1	66ALA	C	0.00	68LEU	CA	68LEU	CB	100.00
62GLU	CB	62GLU	CG	100.00	3VAL	CG1	68LEU	CB	1.79	68LEU	CA	68LEU	CG	100.00
62GLU	CB	62GLU	CD	100.00	3VAL	CG1	68LEU	CG	0.15	68LEU	CA	68LEU	CD1	82.55
62GLU	CB	62GLU	C	100.00	3VAL	CG1	68LEU	CD1	0.78	68LEU	CA	68LEU	CD2	17.17
62GLU	CG	62GLU	CD	100.00	3VAL	CG1	297MET	CE	0.90	68LEU	CA	68LEU	C	100.00
62GLU	CG	62GLU	C	91.70	3VAL	CG2	34LEU	CD1	3.99	68LEU	CB	68LEU	CG	100.00
62GLU	CD	62GLU	C	0.51	3VAL	CG2	34LEU	CD2	1.07	68LEU	CB	68LEU	CD1	100.00
62GLU	CD	265GLY	CA	0.04	3VAL	CG2	297MET	CE	0.91	68LEU	CB	68LEU	CD2	100.00
62GLU	CD	266THR	CB	0.39	3VAL	CG2	302PHE	CE1	0.06	68LEU	CB	68LEU	C	100.00
62GLU	C	63GLU	CA	100.00	3VAL	CG2	302PHE	CE2	0.04	68LEU	CB	297MET	CE	0.01
62GLU	C	63GLU	CB	12.51	3VAL	CG2	302PHE	CZ	0.44	68LEU	CG	68LEU	CD1	100.00
62GLU	C	63GLU	CG	0.07	3VAL	C	4ALA	CA	100.00	68LEU	CG	68LEU	CD2	100.00
62GLU	C	63GLU	C	88.85	3VAL	C	4ALA	CB	2.12	68LEU	CG	68LEU	C	83.38
63GLU	CA	63GLU	CB	100.00	3VAL	C	4ALA	C	99.04	68LEU	CG	297MET	CE	0.05
63GLU	CA	63GLU	CG	100.00	3VAL	C	64ALA	CB	0.28	68LEU	CD1	68LEU	CD2	100.00
63GLU	CA	63GLU	CD	80.67	4ALA	CA	4ALA	CB	100.00	68LEU	CD1	68LEU	C	0.90
63GLU	CA	63GLU	C	100.00	4ALA	CA	4ALA	C	100.00	68LEU	CD1	269PHE	CB	0.03
63GLU	CB	63GLU	CG	100.00	4ALA	CA	39PHE	CD1	0.05	68LEU	CD1	293SER	CB	0.04
63GLU	CB	63GLU	CD	100.00	4ALA	CA	39PHE	CE1	0.18	68LEU	CD1	294ALA	CA	0.04
63GLU	CB	63GLU	C	100.00	4ALA	CB	4ALA	C	100.00	68LEU	CD1	294ALA	CB	0.02
63GLU	CG	63GLU	CD	100.00	4ALA	CB	6LEU	CD1	0.11	68LEU	CD1	297MET	CE	0.37
63GLU	CG	63GLU	C	80.16	4ALA	CB	39PHE	CD1	1.85	68LEU	CD2	68LEU	C	0.92
63GLU	CD	63GLU	C	9.18	4ALA	CB	39PHE	CE1	4.89	68LEU	CD2	269PHE	CB	0.01
63GLU	C	64ALA	CA	100.00	4ALA	CB	60GLY	C	0.02	68LEU	CD2	293SER	CB	0.00
63GLU	C	64ALA	CB	18.90	4ALA	CB	61VAL	CA	0.01	68LEU	CD2	294ALA	CB	0.00
63GLU	C	64ALA	C	80.96	4ALA	CB	61VAL	CG1	0.12	68LEU	CD2	297MET	CE	0.12
64ALA	CA	64ALA	CB	100.00	4ALA	CB	61VAL	CG2	0.14	68LEU	C	69LEU	CA	100.00
64ALA	CA	64ALA	C	100.00	4ALA	CB	64ALA	CB	14.23	68LEU	C	69LEU	CB	30.81
64ALA	CB	64ALA	C	100.00	4ALA	CB	67VAL	CG1	0.00	68LEU	C	69LEU	C	80.42
64ALA	CB	66ALA	C	0.01	4ALA	CB	67VAL	CG2	0.11	69LEU	CA	69LEU	CB	100.00
64ALA	CB	266THR	CG2	0.00	4ALA	C	5VAL	CA	100.00	69LEU	CA	69LEU	CG	100.00
64ALA	C	65GLU	CA	100.00	4ALA	C	5VAL	CB	18.53	69LEU	CA	69LEU	CD1	94.27
64ALA	C	65GLU	CB	5.94	4ALA	C	5VAL	CG2	11.03	69LEU	CA	69LEU	CD2	4.13
64ALA	C	65GLU	CG	0.02	4ALA	C	5VAL	C	94.07	69LEU	CA	69LEU	C	100.00
64ALA	C	65GLU	CD	0.01	4ALA	C	6LEU	CD1	0.06	69LEU	CB	69LEU	CG	100.00

64ALA	C	65GLU	C	95.73
64ALA	C	266THR	CG2	0.06
65GLU	CA	65GLU	CB	100.00
65GLU	CA	65GLU	CG	100.00
65GLU	CA	65GLU	CD	97.85
65GLU	CA	65GLU	C	100.00
65GLU	CA	266THR	CG2	0.01
65GLU	CB	65GLU	CG	100.00
65GLU	CB	65GLU	CD	100.00
65GLU	CB	65GLU	C	100.00
65GLU	CB	302PHE	CE1	0.03
65GLU	CB	302PHE	CE2	0.00
65GLU	CB	302PHE	CZ	0.01
65GLU	CG	65GLU	CD	100.00
65GLU	CG	65GLU	C	91.76
65GLU	CG	301ALA	CB	0.02
65GLU	CG	302PHE	CD1	0.00
65GLU	CG	302PHE	CE1	0.15
65GLU	CG	302PHE	CE2	0.01
65GLU	CG	302PHE	CZ	0.03
65GLU	CD	65GLU	C	0.02
65GLU	CD	302PHE	CE1	0.00
65GLU	C	66ALA	CA	100.00
65GLU	C	66ALA	CB	99.92
65GLU	C	66ALA	C	0.11
65GLU	C	266THR	CG2	8.25
66ALA	CA	66ALA	CB	100.00
66ALA	CA	66ALA	C	100.00
66ALA	CA	266THR	CG2	4.94
66ALA	CB	66ALA	C	100.00
66ALA	CB	267PRO	CG	0.00
66ALA	CB	269PHE	CD1	0.12
66ALA	CB	269PHE	CE1	3.24
66ALA	CB	297MET	CG	5.56
66ALA	CB	297MET	CE	0.55
66ALA	CB	301ALA	CB	0.15
66ALA	CB	302PHE	CE2	0.01
66ALA	CB	302PHE	CZ	0.02
66ALA	C	67VAL	CA	100.00
66ALA	C	67VAL	CB	17.10
66ALA	C	67VAL	CG2	10.06
66ALA	C	67VAL	C	91.36
66ALA	C	266THR	CG2	0.84
66ALA	C	297MET	CE	0.00
67VAL	CA	67VAL	CB	100.00
67VAL	CA	67VAL	CG1	100.00
67VAL	CA	67VAL	CG2	100.00
67VAL	CA	67VAL	C	100.00
67VAL	CB	67VAL	CG1	100.00
67VAL	CB	67VAL	CG2	100.00
67VAL	CB	67VAL	C	100.00
67VAL	CB	268VAL	CG1	0.00
67VAL	CB	268VAL	CG2	0.10
67VAL	CG1	67VAL	CG2	100.00
67VAL	CG1	67VAL	C	100.00
67VAL	CG1	68LEU	C	0.01
67VAL	CG1	69LEU	CD1	0.08
67VAL	CG1	69LEU	CD2	0.06
67VAL	CG1	268VAL	CG1	0.02
67VAL	CG2	266THR	CG2	2.58
67VAL	CG2	268VAL	CG2	0.14
67VAL	C	68LEU	CA	100.00

5VAL	CA	5VAL	CB	100.00
5VAL	CA	5VAL	CG1	100.00
5VAL	CA	5VAL	CG2	100.00
5VAL	CA	5VAL	C	100.00
5VAL	CB	5VAL	CG1	100.00
5VAL	CB	5VAL	CG2	100.00
5VAL	CB	5VAL	C	100.00
5VAL	CB	38VAL	CG2	0.00
5VAL	CG1	5VAL	CG2	100.00
5VAL	CG1	5VAL	C	99.99
5VAL	CG1	7PRO	CB	0.00
5VAL	CG1	16THR	CG2	26.90
5VAL	CG1	20LEU	CD1	0.29
5VAL	CG1	38VAL	CG1	0.01
5VAL	CG1	38VAL	CG2	0.08
5VAL	CG2	16THR	CG2	0.00
5VAL	CG2	20LEU	CD1	1.35
5VAL	CG2	20LEU	CD2	1.09
5VAL	CG2	36TYR	CB	0.17
5VAL	CG2	36TYR	CG	0.02
5VAL	CG2	36TYR	CD1	1.28
5VAL	CG2	36TYR	CD2	0.60
5VAL	CG2	36TYR	C	0.01
5VAL	CG2	38VAL	CG2	0.01
5VAL	CG2	68LEU	CD2	0.00
5VAL	C	6LEU	CA	100.00
5VAL	C	6LEU	CB	19.92
5VAL	C	6LEU	CG	0.60
5VAL	C	6LEU	CD1	15.66
5VAL	C	6LEU	CD2	0.42
5VAL	C	6LEU	C	91.88
5VAL	C	7PRO	CD	0.54
6LEU	CA	6LEU	CB	100.00
6LEU	CA	6LEU	CG	100.00
6LEU	CA	6LEU	CD1	56.97
6LEU	CA	6LEU	CD2	42.90
6LEU	CA	6LEU	C	100.00
6LEU	CA	7PRO	CD	100.00
6LEU	CA	41PHE	CB	0.00
6LEU	CB	6LEU	CG	100.00
6LEU	CB	6LEU	CD1	100.00
6LEU	CB	6LEU	CD2	100.00
6LEU	CB	6LEU	C	100.00
6LEU	CB	7PRO	CD	0.00
6LEU	CB	41PHE	CB	0.40
6LEU	CB	41PHE	CG	0.06
6LEU	CB	41PHE	CD2	0.44
6LEU	CB	41PHE	CE2	0.01
6LEU	CB	69LEU	CD1	3.63
6LEU	CB	69LEU	CD2	0.32
6LEU	CG	6LEU	CD1	100.00
6LEU	CG	6LEU	CD2	100.00
6LEU	CG	6LEU	C	0.01
6LEU	CG	41PHE	CB	0.71
6LEU	CG	41PHE	CG	0.03
6LEU	CG	41PHE	CD2	0.00
6LEU	CG	57THR	CG2	0.39
6LEU	CG	67VAL	CG1	1.00
6LEU	CG	69LEU	CD2	0.00
6LEU	CD1	6LEU	CD2	100.00
6LEU	CD1	39PHE	CB	0.01
6LEU	CD1	39PHE	CD1	0.01

69LEU	CB	69LEU	CD1	100.00
69LEU	CB	69LEU	CD2	100.00
69LEU	CB	69LEU	C	100.00
69LEU	CB	268VAL	CG1	3.06
69LEU	CG	69LEU	CD1	100.00
69LEU	CG	69LEU	CD2	100.00
69LEU	CG	69LEU	C	96.33
69LEU	CG	268VAL	CG1	0.08
69LEU	CD1	69LEU	CD2	100.00
69LEU	CD1	69LEU	C	0.88
69LEU	CD1	90LEU	CD1	0.11
69LEU	CD1	90LEU	CD2	0.21
69LEU	CD1	93LEU	CD1	0.08
69LEU	CD1	93LEU	CD2	1.05
69LEU	CD1	268VAL	CG1	1.20
69LEU	CD1	270GLU	CG	0.00
69LEU	CD1	270GLU	CD	0.05
69LEU	CD2	69LEU	C	0.51
69LEU	CD2	70GLY	C	0.02
69LEU	CD2	90LEU	CD1	0.57
69LEU	CD2	90LEU	CD2	2.73
69LEU	CD2	93LEU	CD2	0.14
69LEU	CD2	268VAL	CG1	7.49
69LEU	CD2	270GLU	CG	0.90
69LEU	CD2	270GLU	CD	4.50
69LEU	C	70GLY	CA	100.00
69LEU	C	70GLY	C	55.40
70GLY	CA	70GLY	C	100.00
70GLY	CA	271PRO	CG	0.00
70GLY	CA	271PRO	CD	0.01
70GLY	C	71SER	CA	100.00
70GLY	C	71SER	CB	33.99
70GLY	C	71SER	C	66.50
71SEF	CA	71SER	CB	100.00
71SEF	CA	71SER	C	100.00
71SEF	CB	71SER	C	100.00
71SEF	CB	273HIS	CB	0.00
71SEF	CB	273HIS	CD2	1.00
71SEF	CB	273HIS	CE1	0.34
71SEF	CB	274GLY	C	0.18
71SEF	CB	276ALA	CB	0.30
71SEF	C	72VAL	CA	100.00
71SEF	C	72VAL	CB	94.26
71SEF	C	72VAL	CG1	3.12
71SEF	C	72VAL	CG2	2.33
71SEF	C	72VAL	C	10.67
72VAL	CA	72VAL	CB	100.00
72VAL	CA	72VAL	CG1	100.00
72VAL	CA	72VAL	CG2	100.00
72VAL	CA	72VAL	C	100.00
72VAL	CB	72VAL	CG1	100.00
72VAL	CB	72VAL	CG2	100.00
72VAL	CB	72VAL	C	100.00
72VAL	CB	77TRP	CZ2	0.00
72VAL	CB	86PRO	CB	0.08
72VAL	CB	86PRO	CG	0.00
72VAL	CG1	72VAL	CG2	100.00
72VAL	CG1	72VAL	C	92.88
72VAL	CG1	77TRP	CE2	0.00
72VAL	CG1	77TRP	CE3	0.00
72VAL	CG1	77TRP	CZ2	0.14
72VAL	CG1	77TRP	CZ3	0.38

67VAL	C	68LEU	CB	6.77	6LEU	CD1	41PHE	CG	0.04	72VAL	CG1	86PRO	CB	1.40
67VAL	C	68LEU	C	98.25	6LEU	CD1	41PHE	CD1	0.04	72VAL	CG1	86PRO	CG	0.92
67VAL	C	297MET	CE	0.90	6LEU	CD1	41PHE	CD2	0.14	72VAL	CG1	86PRO	C	0.02
68LEU	CA	68LEU	CB	100.00	6LEU	CD1	41PHE	CE1	0.02	72VAL	CG1	90LEU	CG	0.00
68LEU	CA	68LEU	CG	100.00	6LEU	CD1	41PHE	CE2	0.01	72VAL	CG1	90LEU	CD1	0.87
68LEU	CA	68LEU	CD1	92.18	6LEU	CD1	57THR	CG2	0.67	72VAL	CG1	90LEU	CD2	0.06
68LEU	CA	68LEU	CD2	7.46	6LEU	CD1	61VAL	CG1	1.37	72VAL	CG2	72VAL	C	93.05
68LEU	CA	68LEU	C	100.00	6LEU	CD1	61VAL	CG2	1.31	72VAL	CG2	77TRP	CE2	0.01
68LEU	CA	297MET	CE	0.04	6LEU	CD1	67VAL	CG1	14.41	72VAL	CG2	77TRP	CZ2	1.20
68LEU	CB	68LEU	CG	100.00	6LEU	CD1	69LEU	CB	0.00	72VAL	CG2	77TRP	CZ3	2.71
68LEU	CB	68LEU	CD1	100.00	6LEU	CD1	69LEU	CD1	0.17	72VAL	CG2	86PRO	CB	4.74
68LEU	CB	68LEU	CD2	100.00	6LEU	CD1	69LEU	CD2	0.91	72VAL	CG2	86PRO	CG	7.99
68LEU	CB	68LEU	C	100.00	6LEU	CD1	93LEU	CD1	0.52	72VAL	CG2	86PRO	C	0.00
68LEU	CB	297MET	CE	0.10	6LEU	CD1	93LEU	CD2	1.26	72VAL	CG2	90LEU	CG	0.00
68LEU	CG	68LEU	CD1	100.00	6LEU	CD2	39PHE	CB	0.16	72VAL	CG2	90LEU	CD1	0.31
68LEU	CG	68LEU	CD2	100.00	6LEU	CD2	41PHE	CB	1.12	72VAL	CG2	90LEU	CD2	0.01
68LEU	CG	68LEU	C	94.42	6LEU	CD2	41PHE	CG	1.37	72VAL	C	73GLY	CA	100.00
68LEU	CG	297MET	CE	0.04	6LEU	CD2	41PHE	CD1	1.36	72VAL	C	73GLY	C	30.10
68LEU	CD1	68LEU	CD2	100.00	6LEU	CD2	41PHE	CD2	0.08	72VAL	C	77TRP	CE3	0.10
68LEU	CD1	68LEU	C	1.26	6LEU	CD2	41PHE	CE1	0.05	72VAL	C	77TRP	CZ3	1.92
68LEU	CD1	269PHE	CB	0.06	6LEU	CD2	53PHE	CE1	0.30	72VAL	C	86PRO	CG	0.00
68LEU	CD1	293SER	CB	0.28	6LEU	CD2	53PHE	CZ	0.14	72VAL	C	275SER	CB	0.10
68LEU	CD1	293SER	C	0.06	6LEU	CD2	57THR	CG2	16.57	72VAL	C	276ALA	CB	0.07
68LEU	CD1	294ALA	CA	0.06	6LEU	CD2	61VAL	CG1	0.14	73GLY	CA	73GLY	C	100.00
68LEU	CD1	294ALA	CB	0.03	6LEU	CD2	61VAL	CG2	0.07	73GLY	CA	77TRP	CD2	0.01
68LEU	CD1	297MET	CE	0.44	6LEU	CD2	67VAL	CG1	0.08	73GLY	CA	77TRP	CE3	1.50
68LEU	CD2	68LEU	C	1.96	6LEU	CD2	69LEU	CD1	0.52	73GLY	CA	77TRP	CZ3	0.76
68LEU	CD2	269PHE	CB	0.00	6LEU	CD2	69LEU	CD2	0.17	73GLY	CA	86PRO	CB	0.05
68LEU	CD2	293SER	CB	0.00	6LEU	CD2	93LEU	CD1	1.67	73GLY	CA	86PRO	CG	0.09
68LEU	CD2	293SER	C	0.00	6LEU	CD2	93LEU	CD2	0.73	73GLY	CA	87GLU	CG	0.01
68LEU	CD2	297MET	CE	0.02	6LEU	C	7PRO	CA	100.00	73GLY	CA	275SER	CA	0.00
68LEU	C	69LEU	CA	100.00	6LEU	C	7PRO	CB	0.28	73GLY	CA	275SER	CB	0.00
68LEU	C	69LEU	CB	40.34	6LEU	C	7PRO	CD	100.00	73GLY	CA	275SER	C	0.03
68LEU	C	69LEU	C	71.81	6LEU	C	7PRO	C	100.00	73GLY	CA	276ALA	CB	0.18
69LEU	CA	69LEU	CB	100.00	6LEU	C	41PHE	CD2	0.05	73GLY	CA	277PRO	CD	0.04
69LEU	CA	69LEU	CG	100.00	6LEU	C	41PHE	CE2	0.00	73GLY	C	74GLY	CA	100.00
69LEU	CA	69LEU	CD1	84.23	7PRO	CA	7PRO	CB	100.00	73GLY	C	74GLY	C	24.48
69LEU	CA	69LEU	CD2	14.98	7PRO	CA	7PRO	CG	100.00	73GLY	C	77TRP	CD2	0.00
69LEU	CA	69LEU	C	100.00	7PRO	CA	7PRO	CD	100.00	73GLY	C	77TRP	CE3	2.62
69LEU	CB	69LEU	CG	100.00	7PRO	CA	7PRO	C	100.00	73GLY	C	77TRP	CZ3	1.84
69LEU	CB	69LEU	CD1	100.00	7PRO	CB	7PRO	CG	100.00	73GLY	C	275SER	CB	0.04
69LEU	CB	69LEU	CD2	100.00	7PRO	CB	7PRO	CD	100.00	73GLY	C	275SER	C	0.00
69LEU	CB	69LEU	C	100.00	7PRO	CB	7PRO	C	100.00	73GLY	C	276ALA	CB	1.16
69LEU	CB	268VAL	CG1	1.70	7PRO	CB	12GLY	C	0.00	73GLY	C	277PRO	CG	0.11
69LEU	CB	270GLU	CB	0.01	7PRO	CB	13PRO	CA	0.39	73GLY	C	277PRO	CD	0.29
69LEU	CB	270GLU	CG	0.01	7PRO	CB	13PRO	CB	0.43	74GLY	CA	74GLY	C	100.00
69LEU	CG	69LEU	CD1	100.00	7PRO	CB	13PRO	CG	0.02	74GLY	CA	75PRO	CD	100.00
69LEU	CG	69LEU	CD2	100.00	7PRO	CB	16THR	CG2	0.00	74GLY	CA	77TRP	CE3	0.01
69LEU	CG	69LEU	C	85.25	7PRO	CB	38VAL	CG1	0.01	74GLY	CA	77TRP	CZ3	0.01
69LEU	CG	93LEU	CD2	0.04	7PRO	CG	7PRO	CD	100.00	74GLY	CA	275SER	CB	0.01
69LEU	CG	268VAL	CG1	0.11	7PRO	CG	7PRO	C	93.68	74GLY	CA	276ALA	CB	0.46
69LEU	CD1	69LEU	CD2	100.00	7PRO	CG	38VAL	CG1	1.03	74GLY	CA	277PRO	CG	0.01
69LEU	CD1	69LEU	C	0.36	7PRO	CG	45ALA	CB	0.01	74GLY	CA	277PRO	CD	0.06
69LEU	CD1	90LEU	CG	0.00	7PRO	CD	7PRO	C	77.65	74GLY	C	75PRO	CA	100.00
69LEU	CD1	90LEU	CD1	0.70	7PRO	CD	38VAL	CG1	0.14	74GLY	C	75PRO	CB	0.25
69LEU	CD1	90LEU	CD2	0.41	7PRO	CD	41PHE	CB	0.00	74GLY	C	75PRO	CD	100.00
69LEU	CD1	93LEU	CG	0.00	7PRO	C	8GLY	CA	100.00	74GLY	C	75PRO	C	100.00
69LEU	CD1	93LEU	CD1	0.82	7PRO	C	8GLY	C	82.76	74GLY	C	78ASP	CB	0.01
69LEU	CD1	93LEU	CD2	4.59	7PRO	C	12GLY	CA	0.01	75PRC	CA	75PRO	CB	100.00
69LEU	CD1	268VAL	CG1	4.20	7PRO	C	12GLY	C	0.01	75PRC	CA	75PRO	CG	100.00
69LEU	CD1	270GLU	CG	0.18	7PRO	C	13PRO	CG	0.00	75PRC	CA	75PRO	CD	100.00
69LEU	CD1	270GLU	CD	0.14	7PRO	C	13PRO	CD	0.01	75PRC	CA	75PRO	C	100.00

69LEU	CD2	69LEU	C	0.39
69LEU	CD2	70GLY	C	0.00
69LEU	CD2	72VAL	CG2	0.00
69LEU	CD2	90LEU	CG	0.00
69LEU	CD2	90LEU	CD1	1.64
69LEU	CD2	90LEU	CD2	0.79
69LEU	CD2	93LEU	CD1	0.15
69LEU	CD2	93LEU	CD2	2.32
69LEU	CD2	268VAL	CG1	9.36
69LEU	CD2	270GLU	CB	0.00
69LEU	CD2	270GLU	CG	0.83
69LEU	CD2	270GLU	CD	4.02
69LEU	C	70GLY	CA	100.00
69LEU	C	70GLY	C	27.35
69LEU	C	271PRO	CD	0.01
70GLY	CA	70GLY	C	100.00
70GLY	CA	271PRO	CD	0.01
70GLY	C	71SER	CA	100.00
70GLY	C	71SER	CB	4.64
70GLY	C	71SER	C	95.59
70GLY	C	271PRO	CG	0.02
71SER	CA	71SER	CB	100.00
71SER	CA	71SER	C	100.00
71SER	CB	71SER	C	100.00
71SER	CB	273HIS	CD2	0.11
71SER	CB	273HIS	CE1	0.05
71SER	CB	274GLY	CA	0.01
71SER	CB	274GLY	C	0.74
71SER	CB	275SER	CA	0.03
71SER	CB	275SER	CB	0.59
71SER	C	72VAL	CA	100.00
71SER	C	72VAL	CB	45.17
71SER	C	72VAL	CG1	19.96
71SER	C	72VAL	CG2	2.92
71SER	C	72VAL	C	58.34
71SER	C	275SER	CB	0.00
72VAL	CA	72VAL	CB	100.00
72VAL	CA	72VAL	CG1	100.00
72VAL	CA	72VAL	CG2	100.00
72VAL	CA	72VAL	C	100.00
72VAL	CB	72VAL	CG1	100.00
72VAL	CB	72VAL	CG2	100.00
72VAL	CB	72VAL	C	100.00
72VAL	CB	86PRO	CB	0.26
72VAL	CB	86PRO	CG	0.04
72VAL	CG1	72VAL	CG2	100.00
72VAL	CG1	72VAL	C	6.68
72VAL	CG1	86PRO	CB	1.18
72VAL	CG1	86PRO	CG	0.32
72VAL	CG1	86PRO	C	0.02
72VAL	CG1	87GLU	CB	0.00
72VAL	CG1	90LEU	CD1	0.25
72VAL	CG1	90LEU	CD2	0.00
72VAL	CG2	72VAL	C	93.12
72VAL	CG2	86PRO	CB	0.65
72VAL	CG2	86PRO	CG	0.07
72VAL	CG2	87GLU	CB	0.00
72VAL	CG2	87GLU	CG	0.01
72VAL	CG2	90LEU	CG	0.03
72VAL	CG2	90LEU	CD1	1.24
72VAL	CG2	90LEU	CD2	0.52
72VAL	C	73GLY	CA	100.00

8GLY	CA	8GLY	C	100.00
8GLY	CA	12GLY	CA	0.00
8GLY	CA	41PHE	CD2	0.49
8GLY	CA	41PHE	CE2	0.09
8GLY	CA	42GLY	CA	0.05
8GLY	CA	69LEU	CD1	0.01
8GLY	CA	70GLY	C	0.01
8GLY	CA	72VAL	CG1	7.31
8GLY	CA	72VAL	CG2	0.15
8GLY	C	9ASP	CA	100.00
8GLY	C	9ASP	CB	98.32
8GLY	C	9ASP	CG	0.02
8GLY	C	9ASP	C	3.42
8GLY	C	12GLY	CA	0.00
8GLY	C	13PRO	CG	0.31
8GLY	C	13PRO	CD	0.55
8GLY	C	42GLY	CA	1.13
8GLY	C	72VAL	CG1	0.04
9ASP	CA	9ASP	CB	100.00
9ASP	CA	9ASP	CG	100.00
9ASP	CA	9ASP	C	100.00
9ASP	CA	13PRO	CG	0.01
9ASP	CA	13PRO	CD	0.17
9ASP	CB	9ASP	CG	100.00
9ASP	CB	9ASP	C	100.00
9ASP	CB	42GLY	CA	0.13
9ASP	CB	42GLY	C	0.05
9ASP	CB	43GLY	CA	0.10
9ASP	CB	44ALA	CB	0.01
9ASP	CB	72VAL	CG1	0.00
9ASP	CB	72VAL	CG2	0.01
9ASP	CB	77TRP	CE3	0.04
9ASP	CB	77TRP	CZ2	0.15
9ASP	CB	77TRP	CZ3	0.14
9ASP	CG	9ASP	C	98.86
9ASP	CG	43GLY	CA	0.05
9ASP	CG	43GLY	C	0.00
9ASP	CG	44ALA	CB	0.07
9ASP	CG	45ALA	CB	0.06
9ASP	CG	76LYS	CG	0.04
9ASP	CG	76LYS	CD	0.16
9ASP	CG	76LYS	CE	0.38
9ASP	CG	77TRP	CZ2	0.07
9ASP	CG	77TRP	CZ3	0.03
9ASP	C	10GLY	CA	100.00
9ASP	C	10GLY	C	74.85
9ASP	C	12GLY	CA	0.00
9ASP	C	13PRO	CD	0.30
9ASP	C	76LYS	CG	0.01
9ASP	C	76LYS	CD	0.00
9ASP	C	76LYS	CE	0.02
9ASP	C	77TRP	CZ2	0.05
9ASP	C	77TRP	CZ3	0.00
10GLY	CA	10GLY	C	100.00
10GLY	CA	74GLY	CA	0.01
10GLY	CA	76LYS	CD	0.01
10GLY	CA	76LYS	CE	0.02
10GLY	CA	77TRP	CZ2	0.02
10GLY	CA	77TRP	CZ3	0.09
10GLY	CA	275SER	CB	0.00
10GLY	CA	275SER	C	0.02
10GLY	CA	276ALA	C	0.01

75PRC	CA	78ASP	CB	0.07
75PRC	CB	75PRO	CG	100.00
75PRC	CB	75PRO	CD	100.00
75PRC	CB	75PRO	C	100.00
75PRC	CB	78ASP	CB	0.00
75PRC	CB	278ASP	CB	0.00
75PRC	CG	75PRO	CD	100.00
75PRC	CG	75PRO	C	67.60
75PRC	CG	277PRO	CG	0.00
75PRC	CG	277PRO	C	0.00
75PRC	CG	278ASP	CA	0.00
75PRC	CG	278ASP	CB	0.35
75PRC	CG	278ASP	CG	0.43
75PRC	CD	75PRO	C	55.17
75PRC	CD	276ALA	CB	0.00
75PRC	CD	277PRO	CB	0.01
75PRC	CD	277PRO	CG	0.02
75PRC	CD	277PRO	CD	0.04
75PRC	CD	277PRO	C	0.02
75PRC	CD	278ASP	CB	0.33
75PRC	CD	278ASP	CG	0.64
75PRC	C	76LYS	CA	100.00
75PRC	C	76LYS	CB	14.68
75PRC	C	76LYS	CG	0.90
75PRC	C	76LYS	C	85.16
75PRC	C	78ASP	CB	0.00
76LYS	CA	76LYS	CB	100.00
76LYS	CA	76LYS	CG	100.00
76LYS	CA	76LYS	CD	0.02
76LYS	CA	76LYS	C	100.00
76LYS	CA	77TRP	CE3	0.00
76LYS	CB	76LYS	CG	100.00
76LYS	CB	76LYS	CD	100.00
76LYS	CB	76LYS	CE	32.36
76LYS	CB	76LYS	C	100.00
76LYS	CB	77TRP	CD1	0.01
76LYS	CB	77TRP	CD2	0.59
76LYS	CB	77TRP	CE2	1.56
76LYS	CB	77TRP	CE3	0.74
76LYS	CB	77TRP	CZ2	0.13
76LYS	CB	77TRP	CZ3	0.20
76LYS	CB	80LEU	CD1	0.00
76LYS	CG	76LYS	CD	100.00
76LYS	CG	76LYS	CE	100.00
76LYS	CG	76LYS	C	84.66
76LYS	CG	77TRP	CD2	1.06
76LYS	CG	77TRP	CE2	1.24
76LYS	CG	77TRP	CE3	5.92
76LYS	CG	77TRP	CZ2	0.31
76LYS	CG	77TRP	CZ3	1.57
76LYS	CD	76LYS	CE	100.00
76LYS	CD	76LYS	C	0.02
76LYS	CD	77TRP	CD2	0.03
76LYS	CD	77TRP	CE2	0.13
76LYS	CD	77TRP	CE3	0.56
76LYS	CD	77TRP	CZ2	1.74
76LYS	CD	77TRP	CZ3	1.64
76LYS	CE	77TRP	CD2	0.01
76LYS	CE	77TRP	CE2	0.31
76LYS	CE	77TRP	CE3	0.96
76LYS	CE	77TRP	CZ2	3.50
76LYS	CE	77TRP	CZ3	9.27

72VAL	C	73GLY	C	23.04	10GLY	CA	280ALA	CB	0.04	76LYS	C	77TRP	CA	100.00
72VAL	C	275SER	CB	0.39	10GLY	CA	281GLY	CA	0.00	76LYS	C	77TRP	CB	48.11
73GLY	CA	73GLY	C	100.00	10GLY	C	11ILE	CA	100.00	76LYS	C	77TRP	CG	22.07
73GLY	CA	78ASP	CB	0.01	10GLY	C	11ILE	CB	2.61	76LYS	C	77TRP	CD1	1.33
73GLY	CA	86PRO	CB	0.02	10GLY	C	11ILE	CG1	0.20	76LYS	C	77TRP	CD2	1.11
73GLY	CA	86PRO	CG	0.10	10GLY	C	11ILE	CG2	0.99	76LYS	C	77TRP	CE3	0.11
73GLY	C	74GLY	CA	100.00	10GLY	C	11ILE	CD	0.02	76LYS	C	77TRP	C	45.55
73GLY	C	74GLY	C	32.95	10GLY	C	11ILE	C	97.66	77TRP	CA	77TRP	CB	100.00
73GLY	C	77TRP	CE3	0.14	10GLY	C	13PRO	CD	0.02	77TRP	CA	77TRP	CG	100.00
73GLY	C	78ASP	CB	0.02	10GLY	C	280ALA	CB	0.22	77TRP	CA	77TRP	CD1	93.93
73GLY	C	275SER	CB	0.04	10GLY	C	281GLY	CA	0.01	77TRP	CA	77TRP	CD2	3.91
73GLY	C	277PRO	CG	0.00	11ILE	CA	11ILE	CB	100.00	77TRP	CA	77TRP	C	100.00
73GLY	C	277PRO	CD	0.01	11ILE	CA	11ILE	CG1	100.00	77TRP	CA	80LEU	CD1	0.06
74GLY	CA	74GLY	C	100.00	11ILE	CA	11ILE	CG2	100.00	77TRP	CB	77TRP	CG	100.00
74GLY	CA	75PRO	CD	100.00	11ILE	CA	11ILE	CD	10.69	77TRP	CB	77TRP	CD1	100.00
74GLY	CA	77TRP	CE3	0.00	11ILE	CA	11ILE	C	100.00	77TRP	CB	77TRP	CD2	100.00
74GLY	CA	277PRO	CB	0.00	11ILE	CA	280ALA	CB	0.01	77TRP	CB	77TRP	CE3	98.12
74GLY	CA	280ALA	CB	0.02	11ILE	CB	11ILE	CG1	100.00	77TRP	CB	77TRP	C	100.00
74GLY	C	75PRO	CA	100.00	11ILE	CB	11ILE	CG2	100.00	77TRP	CB	80LEU	CD1	0.01
74GLY	C	75PRO	CB	0.52	11ILE	CB	11ILE	CD	100.00	77TRP	CB	80LEU	CD2	0.00
74GLY	C	75PRO	CD	100.00	11ILE	CB	11ILE	C	100.00	77TRP	CB	85ARG	CG	0.04
74GLY	C	75PRO	C	99.98	11ILE	CB	273HIS	CE1	0.01	77TRP	CB	85ARG	CD	0.02
74GLY	C	277PRO	CB	0.29	11ILE	CB	285ALA	CB	0.12	77TRP	CB	86PRO	CG	0.20
74GLY	C	277PRO	CG	0.02	11ILE	CG1	11ILE	CG2	100.00	77TRP	CB	86PRO	CD	0.37
75PRO	CA	75PRO	CB	100.00	11ILE	CG1	11ILE	CD	100.00	77TRP	CG	77TRP	CD1	100.00
75PRO	CA	75PRO	CG	100.00	11ILE	CG1	11ILE	C	44.70	77TRP	CG	77TRP	CD2	100.00
75PRO	CA	75PRO	CD	100.00	11ILE	CG1	15VAL	CG1	0.00	77TRP	CG	77TRP	CE2	100.00
75PRO	CA	75PRO	C	100.00	11ILE	CG1	71SER	CB	0.31	77TRP	CG	77TRP	CE3	100.00
75PRO	CA	78ASP	CB	0.02	11ILE	CG1	72VAL	C	0.02	77TRP	CG	77TRP	C	0.02
75PRO	CA	277PRO	CB	0.48	11ILE	CG1	73GLY	C	0.02	77TRP	CG	86PRO	CG	0.11
75PRO	CB	75PRO	CG	100.00	11ILE	CG1	273HIS	CD2	0.01	77TRP	CG	86PRO	CD	3.33
75PRO	CB	75PRO	CD	100.00	11ILE	CG1	273HIS	CE1	0.01	77TRP	CD1	77TRP	CD2	100.00
75PRO	CB	75PRO	C	100.00	11ILE	CG1	274GLY	C	0.03	77TRP	CD1	77TRP	CE2	100.00
75PRO	CB	277PRO	CB	0.06	11ILE	CG1	275SER	CA	0.04	77TRP	CD1	80LEU	CB	0.00
75PRO	CG	75PRO	CD	100.00	11ILE	CG1	275SER	CB	0.02	77TRP	CD1	80LEU	CD1	2.72
75PRO	CG	75PRO	C	94.88	11ILE	CG1	279ILE	CD	0.04	77TRP	CD1	80LEU	CD2	0.98
75PRO	CG	277PRO	CB	0.02	11ILE	CG1	279ILE	C	0.00	77TRP	CD1	84ILE	CD	0.02
75PRO	CG	277PRO	CG	0.01	11ILE	CG1	280ALA	CA	0.01	77TRP	CD1	85ARG	CA	0.18
75PRO	CG	280ALA	CB	0.04	11ILE	CG1	280ALA	CB	0.01	77TRP	CD1	85ARG	CG	0.21
75PRO	CD	75PRO	C	79.19	11ILE	CG1	280ALA	C	0.02	77TRP	CD1	86PRO	CG	0.22
75PRO	CD	277PRO	CB	0.17	11ILE	CG1	281GLY	CA	0.06	77TRP	CD1	86PRO	CD	4.87
75PRO	CD	277PRO	CG	0.24	11ILE	CG1	285ALA	CB	0.16	77TRP	CD2	77TRP	CE2	100.00
75PRO	CD	280ALA	CB	3.08	11ILE	CG2	11ILE	CD	93.19	77TRP	CD2	77TRP	CE3	100.00
75PRO	C	76LYS	CA	100.00	11ILE	CG2	11ILE	C	55.19	77TRP	CD2	77TRP	CZ2	100.00
75PRO	C	76LYS	CB	4.10	11ILE	CG2	15VAL	CG1	9.31	77TRP	CD2	77TRP	CZ3	100.00
75PRO	C	76LYS	CG	0.04	11ILE	CG2	15VAL	CG2	0.22	77TRP	CD2	86PRO	CG	2.69
75PRO	C	76LYS	C	96.20	11ILE	CG2	74GLY	CA	0.00	77TRP	CD2	86PRO	CD	6.09
75PRO	C	78ASP	CB	0.01	11ILE	CG2	273HIS	CB	0.00	77TRP	CE2	77TRP	CE3	100.00
76LYS	CA	76LYS	CB	100.00	11ILE	CG2	273HIS	CG	0.02	77TRP	CE2	77TRP	CZ2	100.00
76LYS	CA	76LYS	CG	100.00	11ILE	CG2	273HIS	CD2	1.94	77TRP	CE2	77TRP	CZ3	100.00
76LYS	CA	76LYS	CD	40.40	11ILE	CG2	273HIS	CE1	0.70	77TRP	CE2	86PRO	CG	3.04
76LYS	CA	76LYS	CE	0.06	11ILE	CG2	279ILE	CG1	0.07	77TRP	CE2	86PRO	CD	12.36
76LYS	CA	76LYS	C	100.00	11ILE	CG2	279ILE	CG2	0.03	77TRP	CE3	77TRP	CZ2	100.00
76LYS	CB	76LYS	CG	100.00	11ILE	CG2	279ILE	CD	0.21	77TRP	CE3	77TRP	CZ3	100.00
76LYS	CB	76LYS	CD	100.00	11ILE	CG2	280ALA	CA	0.06	77TRP	CE3	86PRO	CG	4.21
76LYS	CB	76LYS	CE	5.42	11ILE	CG2	280ALA	CB	0.16	77TRP	CE3	86PRO	CD	1.65
76LYS	CB	76LYS	C	100.00	11ILE	CG2	280ALA	C	0.13	77TRP	CZ2	77TRP	CZ3	100.00
76LYS	CB	77TRP	CD2	0.04	11ILE	CG2	281GLY	CA	1.18	77TRP	CZ2	86PRO	CG	4.75
76LYS	CB	77TRP	CE2	0.03	11ILE	CG2	284ILE	C	0.00	77TRP	CZ2	86PRO	CD	2.59
76LYS	CB	77TRP	CE3	1.39	11ILE	CG2	285ALA	CA	0.08	77TRP	CZ3	86PRO	CG	3.34
76LYS	CB	77TRP	CZ2	0.00	11ILE	CG2	285ALA	CB	4.30	77TRP	CZ3	86PRO	CD	0.30
76LYS	CB	77TRP	CZ3	0.26	11ILE	CG2	285ALA	C	0.00	77TRP	C	78ASP	CA	100.00

76LYS	CG	76LYS	CD	100.00	11ILE	CG2	333THR	CG2	0.00	77TRP	C	78ASP	CB	1.11
76LYS	CG	76LYS	CE	100.00	11ILE	CD	11ILE	C	3.21	77TRP	C	78ASP	C	98.93
76LYS	CG	76LYS	C	82.10	11ILE	CD	15VAL	CG1	0.11	77TRP	C	80LEU	CD1	0.00
76LYS	CG	77TRP	CD2	0.06	11ILE	CD	15VAL	CG2	0.05	77TRP	C	85ARG	CD	0.00
76LYS	CG	77TRP	CE2	0.12	11ILE	CD	71SER	CB	0.15	78ASF	CA	78ASP	CB	100.00
76LYS	CG	77TRP	CE3	0.39	11ILE	CD	73GLY	CA	0.00	78ASF	CA	78ASP	CG	100.00
76LYS	CG	77TRP	CZ2	0.02	11ILE	CD	73GLY	C	0.33	78ASF	CA	78ASP	C	100.00
76LYS	CG	77TRP	CZ3	0.75	11ILE	CD	74GLY	CA	0.03	78ASF	CA	85ARG	CZ	0.12
76LYS	CD	76LYS	CE	100.00	11ILE	CD	75PRO	CD	0.01	78ASF	CB	78ASP	CG	100.00
76LYS	CD	76LYS	C	1.88	11ILE	CD	273HIS	CB	0.28	78ASF	CB	78ASP	C	100.00
76LYS	CD	77TRP	CE2	0.03	11ILE	CD	273HIS	CG	1.11	78ASF	CG	78ASP	C	98.55
76LYS	CD	77TRP	CE3	0.01	11ILE	CD	273HIS	CD2	3.17	78ASF	CG	85ARG	CZ	0.00
76LYS	CD	77TRP	CZ2	0.27	11ILE	CD	273HIS	CE1	1.63	78ASF	C	79GLY	CA	100.00
76LYS	CD	77TRP	CZ3	0.97	11ILE	CD	273HIS	C	0.02	78ASF	C	79GLY	C	67.82
76LYS	CE	77TRP	CE2	0.02	11ILE	CD	274GLY	CA	0.46	78ASF	C	85ARG	CZ	0.06
76LYS	CE	77TRP	CZ2	0.54	11ILE	CD	274GLY	C	0.77	79GLY	CA	79GLY	C	100.00
76LYS	CE	77TRP	CZ3	0.11	11ILE	CD	275SER	CA	0.16	79GLY	C	80LEU	CA	100.00
76LYS	C	77TRP	CA	100.00	11ILE	CD	275SER	CB	0.13	79GLY	C	80LEU	CB	9.01
76LYS	C	77TRP	CB	57.59	11ILE	CD	276ALA	CB	0.00	79GLY	C	80LEU	CG	0.91
76LYS	C	77TRP	CG	8.02	11ILE	CD	279ILE	CG1	0.02	79GLY	C	80LEU	CD1	0.24
76LYS	C	77TRP	CD1	0.21	11ILE	CD	279ILE	CG2	0.89	79GLY	C	80LEU	CD2	0.08
76LYS	C	77TRP	CD2	0.52	11ILE	CD	279ILE	CD	0.27	79GLY	C	80LEU	C	90.45
76LYS	C	77TRP	CE3	0.91	11ILE	CD	279ILE	C	0.22	80LEU	CA	80LEU	CB	100.00
76LYS	C	77TRP	C	42.54	11ILE	CD	280ALA	CA	0.12	80LEU	CA	80LEU	CG	100.00
77TRP	CA	77TRP	CB	100.00	11ILE	CD	280ALA	CB	0.32	80LEU	CA	80LEU	CD1	18.50
77TRP	CA	77TRP	CG	100.00	11ILE	CD	280ALA	C	0.12	80LEU	CA	80LEU	CD2	81.93
77TRP	CA	77TRP	CD1	63.63	11ILE	CD	281GLY	CA	0.22	80LEU	CA	80LEU	C	100.00
77TRP	CA	77TRP	CD2	56.70	11ILE	CD	283GLY	CA	0.00	80LEU	CA	81PRO	CD	100.00
77TRP	CA	77TRP	CE3	0.69	11ILE	CD	283GLY	C	0.00	80LEU	CB	80LEU	CG	100.00
77TRP	CA	77TRP	C	100.00	11ILE	CD	284ILE	CG2	0.02	80LEU	CB	80LEU	CD1	100.00
77TRP	CA	80LEU	CD1	0.15	11ILE	CD	284ILE	C	0.01	80LEU	CB	80LEU	CD2	100.00
77TRP	CB	77TRP	CG	100.00	11ILE	CD	285ALA	CA	0.01	80LEU	CB	80LEU	C	100.00
77TRP	CB	77TRP	CD1	100.00	11ILE	CD	285ALA	CB	1.84	80LEU	CB	81PRO	CD	25.88
77TRP	CB	77TRP	CD2	100.00	11ILE	C	12GLY	CA	100.00	80LEU	CG	80LEU	CD1	100.00
77TRP	CB	77TRP	CE3	98.55	11ILE	C	12GLY	C	98.41	80LEU	CG	80LEU	CD2	100.00
77TRP	CB	77TRP	C	100.00	11ILE	C	13PRO	CD	5.40	80LEU	CG	80LEU	C	4.76
77TRP	CB	80LEU	CD1	0.09	11ILE	C	15VAL	CG2	0.05	80LEU	CG	81PRO	CD	0.09
77TRP	CB	80LEU	CD2	0.06	12GLY	CA	12GLY	C	100.00	80LEU	CG	84ILE	CD	0.04
77TRP	CB	85SER	CB	0.01	12GLY	CA	13PRO	CD	100.00	80LEU	CD1	80LEU	CD2	100.00
77TRP	CB	86PRO	CG	0.06	12GLY	CA	15VAL	CG2	0.08	80LEU	CD1	81PRO	CD	0.01
77TRP	CB	86PRO	CD	0.35	12GLY	CA	70GLY	CA	0.02	80LEU	CD1	84ILE	CG2	0.02
77TRP	CG	77TRP	CD1	100.00	12GLY	CA	70GLY	C	0.16	80LEU	CD1	84ILE	CD	0.18
77TRP	CG	77TRP	CD2	100.00	12GLY	CA	71SER	CB	0.02	80LEU	CD2	80LEU	C	0.16
77TRP	CG	77TRP	CE2	100.00	12GLY	C	13PRO	CA	100.00	80LEU	CD2	81PRO	CD	0.02
77TRP	CG	77TRP	CE3	100.00	12GLY	C	13PRO	CB	0.03	80LEU	CD2	84ILE	CG1	0.01
77TRP	CG	77TRP	C	55.69	12GLY	C	13PRO	CD	100.00	80LEU	CD2	84ILE	CG2	0.04
77TRP	CG	80LEU	CB	0.05	12GLY	C	13PRO	C	100.00	80LEU	CD2	84ILE	CD	0.36
77TRP	CG	80LEU	CD1	0.18	13PRO	CA	13PRO	CB	100.00	80LEU	CD2	84ILE	C	0.05
77TRP	CG	80LEU	CD2	0.07	13PRO	CA	13PRO	CG	100.00	80LEU	CD2	85ARG	CG	0.02
77TRP	CG	85SER	CB	0.05	13PRO	CA	13PRO	CD	100.00	80LEU	C	81PRO	CA	100.00
77TRP	CG	86PRO	CG	0.01	13PRO	CA	13PRO	C	100.00	80LEU	C	81PRO	CB	0.04
77TRP	CG	86PRO	CD	1.04	13PRO	CB	13PRO	CG	100.00	80LEU	C	81PRO	CD	100.00
77TRP	CD1	77TRP	CD2	100.00	13PRO	CB	13PRO	CD	100.00	80LEU	C	81PRO	C	100.00
77TRP	CD1	77TRP	CE2	100.00	13PRO	CB	13PRO	C	100.00	81PRC	CA	81PRO	CB	100.00
77TRP	CD1	77TRP	C	0.70	13PRO	CG	13PRO	CD	100.00	81PRC	CA	81PRO	CG	100.00
77TRP	CD1	80LEU	CB	3.83	13PRO	CG	13PRO	C	54.21	81PRC	CA	81PRO	CD	100.00
77TRP	CD1	80LEU	CG	0.02	13PRO	CD	13PRO	C	40.56	81PRC	CA	81PRO	C	100.00
77TRP	CD1	80LEU	CD1	2.06	13PRO	C	14GLU	CA	100.00	81PRC	CB	81PRO	CG	100.00
77TRP	CD1	80LEU	CD2	1.20	13PRO	C	14GLU	CB	0.04	81PRC	CB	81PRO	CD	100.00
77TRP	CD1	80LEU	C	0.14	13PRO	C	14GLU	C	99.97	81PRC	CB	81PRO	C	100.00
77TRP	CD1	84ILE	CD	0.02	14GLU	CA	14GLU	CB	100.00	81PRC	CB	83LYS	CG	0.06
77TRP	CD1	85SER	CA	3.23	14GLU	CA	14GLU	CG	100.00	81PRC	CB	83LYS	CE	0.06

77TRP	CD1	85SER	CB	16.17	14GLU	CA	14GLU	CD	95.66	81PRC	CG	81PRO	CD	100.00
77TRP	CD1	86PRO	CG	0.01	14GLU	CA	14GLU	C	100.00	81PRC	CG	81PRO	C	90.54
77TRP	CD1	86PRO	CD	1.59	14GLU	CB	14GLU	CG	100.00	81PRC	CG	83LYS	CG	0.05
77TRP	CD2	77TRP	CE2	100.00	14GLU	CB	14GLU	CD	100.00	81PRC	CG	83LYS	CE	2.10
77TRP	CD2	77TRP	CE3	100.00	14GLU	CB	14GLU	C	100.00	81PRC	CG	84ILE	CG1	0.02
77TRP	CD2	77TRP	CZ2	100.00	14GLU	CB	280ALA	CB	0.01	81PRC	CG	84ILE	CD	0.05
77TRP	CD2	77TRP	CZ3	100.00	14GLU	CB	285ALA	CB	0.02	81PRC	CD	81PRO	C	84.64
77TRP	CD2	80LEU	CB	0.50	14GLU	CB	333THR	CG2	0.00	81PRC	CD	84ILE	CG1	0.01
77TRP	CD2	80LEU	CD1	2.51	14GLU	CG	14GLU	CD	100.00	81PRC	CD	84ILE	CD	0.01
77TRP	CD2	80LEU	CD2	0.49	14GLU	CG	14GLU	C	88.82	81PRC	C	82ARG	CA	100.00
77TRP	CD2	86PRO	CD	0.28	14GLU	CG	280ALA	CB	0.00	81PRC	C	82ARG	C	100.00
77TRP	CE2	77TRP	CE3	100.00	14GLU	CG	281GLY	CA	0.00	81PRC	C	83LYS	CG	0.00
77TRP	CE2	77TRP	CZ2	100.00	14GLU	CG	283GLY	CA	0.00	81PRC	C	85ARG	CZ	0.01
77TRP	CE2	77TRP	CZ3	100.00	14GLU	CG	285ALA	CB	0.48	82ARC	CA	82ARG	CB	100.00
77TRP	CE2	80LEU	CB	4.31	14GLU	CG	333THR	CB	0.08	82ARC	CA	82ARG	CG	100.00
77TRP	CE2	80LEU	CG	0.16	14GLU	CG	333THR	CG2	0.72	82ARC	CA	82ARG	CD	1.12
77TRP	CE2	80LEU	CD1	4.16	14GLU	CD	14GLU	C	0.29	82ARC	CA	82ARG	C	100.00
77TRP	CE2	80LEU	CD2	1.76	14GLU	CD	280ALA	CA	0.01	82ARC	CA	85ARG	CB	0.00
77TRP	CE2	84ILE	CG2	0.00	14GLU	CD	281GLY	CA	0.02	82ARC	CA	85ARG	CG	0.05
77TRP	CE2	85SER	CA	0.17	14GLU	CD	333THR	CG2	0.01	82ARC	CA	85ARG	CD	0.03
77TRP	CE2	85SER	CB	0.01	14GLU	C	15VAL	CA	100.00	82ARC	CA	85ARG	CZ	1.38
77TRP	CE2	86PRO	CD	0.02	14GLU	C	15VAL	CB	0.43	82ARC	CB	82ARG	CG	100.00
77TRP	CE3	77TRP	CZ2	100.00	14GLU	C	15VAL	CG1	0.19	82ARC	CB	82ARG	CD	100.00
77TRP	CE3	77TRP	CZ3	100.00	14GLU	C	15VAL	C	99.73	82ARC	CB	82ARG	CZ	0.20
77TRP	CE3	80LEU	CD1	1.39	14GLU	C	333THR	CG2	0.06	82ARC	CB	82ARG	C	100.00
77TRP	CE3	80LEU	CD2	0.58	15VAL	CA	15VAL	CB	100.00	82ARC	CB	85ARG	CZ	0.17
77TRP	CE3	86PRO	CG	0.05	15VAL	CA	15VAL	CG1	100.00	82ARC	CG	82ARG	CD	100.00
77TRP	CE3	86PRO	CD	0.12	15VAL	CA	15VAL	CG2	100.00	82ARC	CG	82ARG	CZ	21.56
77TRP	CZ2	77TRP	CZ3	100.00	15VAL	CA	15VAL	C	100.00	82ARC	CG	82ARG	C	67.56
77TRP	CZ2	80LEU	CB	0.11	15VAL	CB	15VAL	CG1	100.00	82ARC	CG	85ARG	CZ	2.82
77TRP	CZ2	80LEU	CG	0.00	15VAL	CB	15VAL	CG2	100.00	82ARC	CG	87GLU	CG	0.01
77TRP	CZ2	80LEU	CD1	0.54	15VAL	CB	15VAL	C	100.00	82ARC	CG	87GLU	CD	0.04
77TRP	CZ2	80LEU	CD2	1.17	15VAL	CB	273HIS	CE1	0.03	82ARC	CG	88THR	CG2	0.76
77TRP	CZ2	81PRO	CD	0.01	15VAL	CB	287PRO	CA	0.00	82ARC	CD	82ARG	CZ	100.00
77TRP	CZ2	84ILE	CB	0.03	15VAL	CB	287PRO	CB	0.02	82ARC	CD	82ARG	C	0.94
77TRP	CZ2	84ILE	CG1	0.85	15VAL	CB	290ALA	CB	0.21	82ARC	CD	85ARG	CZ	0.00
77TRP	CZ2	84ILE	CG2	0.09	15VAL	CG1	15VAL	CG2	100.00	82ARC	CD	87GLU	CD	0.01
77TRP	CZ2	84ILE	CD	0.71	15VAL	CG1	15VAL	C	6.40	82ARC	CD	88THR	CG2	0.16
77TRP	CZ2	84ILE	C	0.12	15VAL	CG1	271PRO	CG	0.00	82ARC	CD	140PHE	CE2	0.11
77TRP	CZ2	85SER	CA	0.01	15VAL	CG1	273HIS	CD2	0.00	82ARC	CD	140PHE	CZ	0.02
77TRP	CZ3	80LEU	CG	0.00	15VAL	CG1	273HIS	CE1	0.06	82ARC	CZ	85ARG	CZ	0.00
77TRP	CZ3	80LEU	CD1	0.50	15VAL	CG1	285ALA	CA	0.02	82ARC	CZ	87GLU	CG	0.01
77TRP	CZ3	80LEU	CD2	0.43	15VAL	CG1	285ALA	CB	0.88	82ARC	CZ	87GLU	CD	0.02
77TRP	CZ3	86PRO	CG	0.02	15VAL	CG1	285ALA	C	0.55	82ARC	CZ	88THR	CG2	0.49
77TRP	CZ3	86PRO	CD	0.01	15VAL	CG1	286ASN	C	0.27	82ARC	CZ	91LEU	CD1	0.08
77TRP	C	78ASP	CA	100.00	15VAL	CG1	287PRO	CA	0.50	82ARC	CZ	140PHE	CE1	0.02
77TRP	C	78ASP	CB	8.81	15VAL	CG1	287PRO	CB	0.02	82ARC	CZ	140PHE	CE2	0.89
77TRP	C	78ASP	C	91.79	15VAL	CG1	290ALA	CB	1.15	82ARC	CZ	140PHE	CZ	0.40
77TRP	C	80LEU	CD1	0.55	15VAL	CG1	333THR	CG2	0.67	82ARC	C	83LYS	CA	100.00
77TRP	C	80LEU	CD2	0.05	15VAL	CG2	15VAL	C	93.74	82ARC	C	83LYS	CB	0.04
78ASP	CA	78ASP	CB	100.00	15VAL	CG2	70GLY	CA	0.07	82ARC	C	83LYS	CG	0.00
78ASP	CA	78ASP	CG	100.00	15VAL	CG2	271PRO	CG	0.02	82ARC	C	83LYS	C	99.94
78ASP	CA	78ASP	C	100.00	15VAL	CG2	273HIS	CD2	0.02	82ARC	C	88THR	CG2	0.06
78ASP	CB	78ASP	CG	100.00	15VAL	CG2	273HIS	CE1	0.67	83LYS	CA	83LYS	CB	100.00
78ASP	CB	78ASP	C	100.00	15VAL	CG2	285ALA	CB	0.31	83LYS	CA	83LYS	CG	100.00
78ASP	CB	277PRO	CB	0.04	15VAL	CG2	285ALA	C	0.00	83LYS	CA	83LYS	CD	0.00
78ASP	CB	277PRO	CG	0.50	15VAL	CG2	290ALA	CB	0.47	83LYS	CA	83LYS	C	100.00
78ASP	CB	277PRO	CD	0.01	15VAL	C	16THR	CA	100.00	83LYS	CA	88THR	CG2	0.06
78ASP	CG	78ASP	C	99.98	15VAL	C	16THR	CB	0.02	83LYS	CB	83LYS	CG	100.00
78ASP	CG	277PRO	CB	0.14	15VAL	C	16THR	C	99.99	83LYS	CB	83LYS	CD	100.00
78ASP	CG	277PRO	CG	3.16	15VAL	C	290ALA	CB	0.99	83LYS	CB	83LYS	CE	12.20
78ASP	CG	277PRO	CD	0.03	16THR	CA	16THR	CB	100.00	83LYS	CB	83LYS	C	100.00

78ASP	C	79GLY	CA	100.00	16THR	CA	16THR	CG2	100.00	83LYS	CG	83LYS	CD	100.00
78ASP	C	79GLY	C	50.19	16THR	CA	16THR	C	100.00	83LYS	CG	83LYS	CE	100.00
79GLY	CA	79GLY	C	100.00	16THR	CA	19ALA	CB	0.00	83LYS	CG	83LYS	C	99.02
79GLY	C	80LEU	CA	100.00	16THR	CB	16THR	CG2	100.00	83LYS	CG	84ILE	CG1	0.03
79GLY	C	80LEU	CB	13.19	16THR	CB	16THR	C	100.00	83LYS	CG	84ILE	CG2	0.00
79GLY	C	80LEU	CG	2.42	16THR	CG2	16THR	C	100.00	83LYS	CG	84ILE	CD	0.00
79GLY	C	80LEU	CD1	0.52	16THR	CG2	20LEU	CD1	0.12	83LYS	CD	83LYS	CE	100.00
79GLY	C	80LEU	CD2	0.19	16THR	CG2	68LEU	CB	0.01	83LYS	CD	83LYS	C	0.02
79GLY	C	80LEU	C	86.81	16THR	CG2	68LEU	CG	0.08	83LYS	CE	84ILE	CG2	0.00
80LEU	CA	80LEU	CB	100.00	16THR	CG2	68LEU	CD1	0.01	83LYS	C	84ILE	CA	100.00
80LEU	CA	80LEU	CG	100.00	16THR	CG2	68LEU	CD2	3.45	83LYS	C	84ILE	CB	87.30
80LEU	CA	80LEU	CD1	38.06	16THR	C	17GLU	CA	100.00	83LYS	C	84ILE	CG1	0.86
80LEU	CA	80LEU	CD2	64.77	16THR	C	17GLU	CB	0.01	83LYS	C	84ILE	CG2	55.38
80LEU	CA	80LEU	C	100.00	16THR	C	17GLU	C	100.00	83LYS	C	84ILE	C	11.84
80LEU	CA	81PRO	CD	100.00	17GLU	CA	17GLU	CB	100.00	83LYS	C	88THR	CG2	0.43
80LEU	CB	80LEU	CG	100.00	17GLU	CA	17GLU	CG	100.00	84ILE	CA	84ILE	CB	100.00
80LEU	CB	80LEU	CD1	100.00	17GLU	CA	17GLU	CD	78.69	84ILE	CA	84ILE	CG1	100.00
80LEU	CB	80LEU	CD2	100.00	17GLU	CA	17GLU	C	100.00	84ILE	CA	84ILE	CG2	100.00
80LEU	CB	80LEU	C	100.00	17GLU	CB	17GLU	CG	100.00	84ILE	CA	84ILE	CD	1.44
80LEU	CB	81PRO	CD	39.57	17GLU	CB	17GLU	CD	100.00	84ILE	CA	84ILE	C	100.00
80LEU	CB	84ILE	CD	0.01	17GLU	CB	17GLU	C	100.00	84ILE	CB	84ILE	CG1	100.00
80LEU	CB	85SER	CA	0.00	17GLU	CG	17GLU	CD	100.00	84ILE	CB	84ILE	CG2	100.00
80LEU	CB	85SER	CB	0.01	17GLU	CG	17GLU	C	93.70	84ILE	CB	84ILE	CD	100.00
80LEU	CG	80LEU	CD1	100.00	17GLU	CG	21LYS	CE	0.10	84ILE	CB	84ILE	C	100.00
80LEU	CG	80LEU	CD2	100.00	17GLU	CD	17GLU	C	3.00	84ILE	CG1	84ILE	CG2	100.00
80LEU	CG	80LEU	C	1.55	17GLU	CD	21LYS	CD	0.01	84ILE	CG1	84ILE	CD	100.00
80LEU	CG	81PRO	CD	0.01	17GLU	CD	21LYS	CE	0.36	84ILE	CG1	84ILE	C	95.71
80LEU	CD1	80LEU	CD2	100.00	17GLU	C	18ALA	CA	100.00	84ILE	CG2	84ILE	CD	98.09
80LEU	CD1	80LEU	C	0.59	17GLU	C	18ALA	CB	0.03	84ILE	CG2	84ILE	C	3.44
80LEU	CD1	81PRO	CD	0.02	17GLU	C	18ALA	C	99.97	84ILE	CD	84ILE	C	1.11
80LEU	CD1	84ILE	CD	0.05	18ALA	CA	18ALA	CB	100.00	84ILE	C	85ARG	CA	100.00
80LEU	CD1	84ILE	C	0.02	18ALA	CA	18ALA	C	100.00	84ILE	C	85ARG	CB	4.47
80LEU	CD1	85SER	CA	0.01	18ALA	CA	21LYS	CB	0.00	84ILE	C	85ARG	CG	1.29
80LEU	CD1	85SER	CB	0.01	18ALA	CA	337THR	CG2	0.07	84ILE	C	85ARG	C	95.65
80LEU	CD1	86PRO	CD	0.09	18ALA	CB	18ALA	C	100.00	85ARC	CA	85ARG	CB	100.00
80LEU	CD2	80LEU	C	0.01	18ALA	CB	287PRO	CB	15.03	85ARC	CA	85ARG	CG	100.00
80LEU	CD2	81PRO	CD	0.12	18ALA	CB	287PRO	CG	0.95	85ARC	CA	85ARG	CD	1.98
80LEU	CD2	84ILE	CG1	0.00	18ALA	CB	291ILE	CG1	0.15	85ARC	CA	85ARG	C	100.00
80LEU	CD2	84ILE	CG2	0.00	18ALA	CB	291ILE	CD	0.03	85ARC	CA	86PRO	CD	100.00
80LEU	CD2	84ILE	CD	0.66	18ALA	CB	333THR	CG2	0.05	85ARC	CB	85ARG	CG	100.00
80LEU	CD2	86PRO	CD	0.00	18ALA	CB	337THR	CG2	3.81	85ARC	CB	85ARG	CD	100.00
80LEU	C	81PRO	CA	100.00	18ALA	C	19ALA	CA	100.00	85ARC	CB	85ARG	C	100.00
80LEU	C	81PRO	CB	0.07	18ALA	C	19ALA	CB	0.09	85ARC	CB	86PRO	CD	24.25
80LEU	C	81PRO	CD	100.00	18ALA	C	19ALA	C	99.95	85ARC	CB	87GLU	CB	0.06
80LEU	C	81PRO	C	100.00	18ALA	C	21LYS	CB	0.00	85ARC	CB	87GLU	CG	0.13
81PRO	CA	81PRO	CB	100.00	18ALA	C	291ILE	CG1	0.01	85ARC	CB	87GLU	CD	0.12
81PRO	CA	81PRO	CG	100.00	18ALA	C	291ILE	CD	0.10	85ARC	CG	85ARG	CD	100.00
81PRO	CA	81PRO	CD	100.00	19ALA	CA	19ALA	CB	100.00	85ARC	CG	85ARG	CZ	1.63
81PRO	CA	81PRO	C	100.00	19ALA	CA	19ALA	C	100.00	85ARC	CG	85ARG	C	0.52
81PRO	CB	81PRO	CG	100.00	19ALA	CA	22VAL	CG2	0.01	85ARC	CG	86PRO	CD	0.00
81PRO	CB	81PRO	CD	100.00	19ALA	CA	291ILE	CG1	0.01	85ARC	CG	87GLU	CD	0.11
81PRO	CB	81PRO	C	100.00	19ALA	CA	291ILE	CD	0.02	85ARC	CD	85ARG	CZ	100.00
81PRO	CB	83LYS	CG	0.07	19ALA	CA	294ALA	CB	0.00	85ARC	CD	87GLU	CD	0.02
81PRO	CB	83LYS	CD	0.00	19ALA	CB	19ALA	C	100.00	85ARC	C	86PRO	CA	100.00
81PRO	CB	83LYS	CE	0.02	19ALA	CB	68LEU	CD1	0.08	85ARC	C	86PRO	CB	0.01
81PRO	CG	81PRO	CD	100.00	19ALA	CB	68LEU	CD2	0.40	85ARC	C	86PRO	CD	100.00
81PRO	CG	81PRO	C	90.18	19ALA	CB	290ALA	CB	0.27	85ARC	C	86PRO	C	100.00
81PRO	CG	83LYS	CG	0.04	19ALA	CB	290ALA	C	5.80	86PRC	CA	86PRO	CB	100.00
81PRO	CG	83LYS	CD	0.03	19ALA	CB	291ILE	CA	0.01	86PRC	CA	86PRO	CG	100.00
81PRO	CG	83LYS	CE	1.51	19ALA	CB	291ILE	CG1	0.01	86PRC	CA	86PRO	CD	100.00
81PRO	CG	84ILE	CG1	0.01	19ALA	CB	294ALA	CB	0.01	86PRC	CA	86PRO	C	100.00
81PRO	CG	84ILE	CD	0.01	19ALA	C	20LEU	CA	100.00	86PRC	CB	86PRO	CG	100.00

81PRO	CD	81PRO	C	81.60
81PRO	CD	84ILE	CG1	0.02
81PRO	CD	84ILE	CD	0.02
81PRO	C	82ARG	CA	100.00
81PRO	C	82ARG	CB	0.06
81PRO	C	82ARG	CG	0.00
81PRO	C	82ARG	C	99.97
82ARG	CA	82ARG	CB	100.00
82ARG	CA	82ARG	CG	100.00
82ARG	CA	82ARG	CD	1.44
82ARG	CA	82ARG	C	100.00
82ARG	CA	85SER	CB	0.12
82ARG	CB	82ARG	CG	100.00
82ARG	CB	82ARG	CD	100.00
82ARG	CB	82ARG	CZ	0.27
82ARG	CB	82ARG	C	100.00
82ARG	CG	82ARG	CD	100.00
82ARG	CG	82ARG	CZ	6.98
82ARG	CG	82ARG	C	81.62
82ARG	CG	87GLU	CG	0.01
82ARG	CG	88THR	CG2	0.10
82ARG	CG	91LEU	CD1	0.01
82ARG	CD	82ARG	CZ	100.00
82ARG	CD	82ARG	C	0.76
82ARG	CD	87GLU	CG	0.01
82ARG	CD	87GLU	CD	0.01
82ARG	CD	88THR	CG2	0.02
82ARG	CD	91LEU	CD1	0.01
82ARG	CZ	87GLU	CG	0.11
82ARG	CZ	87GLU	CD	0.02
82ARG	CZ	88THR	CG2	0.21
82ARG	CZ	91LEU	CD1	0.01
82ARG	C	83LYS	CA	100.00
82ARG	C	83LYS	CB	1.06
82ARG	C	83LYS	CG	0.07
82ARG	C	83LYS	C	98.89
82ARG	C	85SER	CB	0.01
82ARG	C	88THR	CG2	0.08
83LYS	CA	83LYS	CB	100.00
83LYS	CA	83LYS	CG	100.00
83LYS	CA	83LYS	CD	0.03
83LYS	CA	83LYS	C	100.00
83LYS	CA	88THR	CG2	0.01
83LYS	CB	83LYS	CG	100.00
83LYS	CB	83LYS	CD	100.00
83LYS	CB	83LYS	CE	10.45
83LYS	CB	83LYS	C	100.00
83LYS	CG	83LYS	CD	100.00
83LYS	CG	83LYS	CE	100.00
83LYS	CG	83LYS	C	96.66
83LYS	CG	84ILE	CG1	0.01
83LYS	CG	84ILE	CD	0.01
83LYS	CD	83LYS	CE	100.00
83LYS	CD	83LYS	C	0.05
83LYS	C	84ILE	CA	100.00
83LYS	C	84ILE	CB	94.82
83LYS	C	84ILE	CG1	1.57
83LYS	C	84ILE	CG2	61.41
83LYS	C	84ILE	CD	0.01
83LYS	C	84ILE	C	5.40
83LYS	C	88THR	CG2	0.84
84ILE	CA	84ILE	CB	100.00

19ALA	C	20LEU	CB	0.04
19ALA	C	20LEU	C	99.96
19ALA	C	22VAL	CG2	0.06
20LEU	CA	20LEU	CB	100.00
20LEU	CA	20LEU	CG	100.00
20LEU	CA	20LEU	CD1	4.42
20LEU	CA	20LEU	CD2	95.28
20LEU	CA	20LEU	C	100.00
20LEU	CB	20LEU	CG	100.00
20LEU	CB	20LEU	CD1	100.00
20LEU	CB	20LEU	CD2	100.00
20LEU	CB	20LEU	C	100.00
20LEU	CB	24ARG	CZ	0.01
20LEU	CB	36TYR	CE1	0.02
20LEU	CB	36TYR	CE2	0.01
20LEU	CB	36TYR	CZ	0.02
20LEU	CG	20LEU	CD1	100.00
20LEU	CG	20LEU	CD2	100.00
20LEU	CG	20LEU	C	3.02
20LEU	CG	36TYR	CD1	0.01
20LEU	CG	36TYR	CD2	0.00
20LEU	CG	36TYR	CE1	0.02
20LEU	CG	36TYR	CE2	0.00
20LEU	CG	68LEU	CD1	0.01
20LEU	CG	68LEU	CD2	0.52
20LEU	CD1	20LEU	CD2	100.00
20LEU	CD1	20LEU	C	0.05
20LEU	CD1	36TYR	CG	0.05
20LEU	CD1	36TYR	CD1	0.28
20LEU	CD1	36TYR	CD2	0.17
20LEU	CD1	36TYR	CE1	1.60
20LEU	CD1	36TYR	CE2	0.92
20LEU	CD1	36TYR	CZ	0.17
20LEU	CD1	38VAL	CG2	0.01
20LEU	CD1	68LEU	CD1	0.00
20LEU	CD1	68LEU	CD2	0.16
20LEU	CD2	20LEU	C	0.09
20LEU	CD2	23LEU	CD1	0.01
20LEU	CD2	23LEU	CD2	0.00
20LEU	CD2	36TYR	CB	0.12
20LEU	CD2	36TYR	CG	1.39
20LEU	CD2	36TYR	CD1	1.51
20LEU	CD2	36TYR	CD2	1.55
20LEU	CD2	36TYR	CE1	0.96
20LEU	CD2	36TYR	CE2	0.60
20LEU	CD2	36TYR	CZ	0.32
20LEU	CD2	38VAL	CG2	0.01
20LEU	CD2	68LEU	CD1	0.79
20LEU	CD2	68LEU	CD2	3.18
20LEU	C	21LYS	CA	100.00
20LEU	C	21LYS	C	100.00
21LYS	CA	21LYS	CB	100.00
21LYS	CA	21LYS	CG	100.00
21LYS	CA	21LYS	CD	1.02
21LYS	CA	21LYS	C	100.00
21LYS	CB	21LYS	CG	100.00
21LYS	CB	21LYS	CD	100.00
21LYS	CB	21LYS	CE	25.75
21LYS	CB	21LYS	C	100.00
21LYS	CB	337THR	CG2	0.34
21LYS	CB	341LEU	CD2	0.02
21LYS	CG	21LYS	CD	100.00

86PRC	CB	86PRO	CD	100.00
86PRC	CB	86PRO	C	100.00
86PRC	CB	90LEU	CD1	0.00
86PRC	CG	86PRO	CD	100.00
86PRC	CG	86PRO	C	45.43
86PRC	CD	86PRO	C	27.40
86PRC	C	87GLU	CA	100.00
86PRC	C	87GLU	C	100.00
86PRC	C	90LEU	CD1	0.01
87GLL	CA	87GLU	CB	100.00
87GLL	CA	87GLU	CG	100.00
87GLL	CA	87GLU	CD	19.75
87GLL	CA	87GLU	C	100.00
87GLL	CA	90LEU	CD1	0.01
87GLL	CB	87GLU	CG	100.00
87GLL	CB	87GLU	CD	100.00
87GLL	CB	87GLU	C	100.00
87GLL	CG	87GLU	CD	100.00
87GLL	CG	87GLU	C	92.36
87GLL	CG	88THR	CG2	0.00
87GLL	CG	91LEU	CD1	0.44
87GLL	CG	91LEU	CD2	0.00
87GLL	CD	87GLU	C	2.52
87GLL	CD	88THR	CG2	0.03
87GLL	CD	90LEU	CD1	0.01
87GLL	CD	90LEU	CD2	0.00
87GLL	CD	91LEU	CD1	0.02
87GLL	C	88THR	CA	100.00
87GLL	C	88THR	CB	0.03
87GLL	C	88THR	CG2	0.00
87GLL	C	88THR	C	99.98
87GLL	C	91LEU	CD1	0.02
88THF	CA	88THR	CB	100.00
88THF	CA	88THR	CG2	100.00
88THF	CA	88THR	C	100.00
88THF	CB	88THR	CG2	100.00
88THF	CB	88THR	C	100.00
88THF	CG2	88THR	C	13.68
88THF	C	89GLY	CA	100.00
88THF	C	89GLY	C	92.93
89GLY	CA	89GLY	C	100.00
89GLY	CA	92SER	CB	0.00
89GLY	C	90LEU	CA	100.00
89GLY	C	90LEU	CB	0.01
89GLY	C	90LEU	C	99.98
90LEU	CA	90LEU	CB	100.00
90LEU	CA	90LEU	CG	100.00
90LEU	CA	90LEU	CD1	7.86
90LEU	CA	90LEU	CD2	91.30
90LEU	CA	90LEU	C	100.00
90LEU	CB	90LEU	CG	100.00
90LEU	CB	90LEU	CD1	100.00
90LEU	CB	90LEU	CD2	100.00
90LEU	CB	90LEU	C	100.00
90LEU	CG	90LEU	CD1	100.00
90LEU	CG	90LEU	CD2	100.00
90LEU	CG	90LEU	C	5.70
90LEU	CG	94ARG	CZ	0.04
90LEU	CD1	90LEU	CD2	100.00
90LEU	CD1	90LEU	C	0.33
90LEU	CD1	94ARG	CZ	0.04
90LEU	CD2	90LEU	C	0.32

84ILE	CA	84ILE	CG1	100.00	21LYS	CG	21LYS	CE	100.00	90LEU	CD2	93LEU	CD2	0.03
84ILE	CA	84ILE	CG2	100.00	21LYS	CG	21LYS	C	53.51	90LEU	CD2	94ARG	CZ	0.03
84ILE	CA	84ILE	CD	1.38	21LYS	CG	337THR	CG2	0.03	90LEU	CD2	270GLU	CD	0.00
84ILE	CA	84ILE	C	100.00	21LYS	CG	341LEU	CD1	0.02	90LEU	C	91LEU	CA	100.00
84ILE	CA	88THR	CG2	0.00	21LYS	CG	341LEU	CD2	0.01	90LEU	C	91LEU	CB	0.00
84ILE	CB	84ILE	CG1	100.00	21LYS	CD	21LYS	CE	100.00	90LEU	C	91LEU	CD1	0.00
84ILE	CB	84ILE	CG2	100.00	21LYS	CD	21LYS	C	0.01	90LEU	C	91LEU	C	100.00
84ILE	CB	84ILE	CD	100.00	21LYS	CD	337THR	CB	0.03	91LEU	CA	91LEU	CB	100.00
84ILE	CB	84ILE	C	100.00	21LYS	CD	337THR	CG2	0.10	91LEU	CA	91LEU	CG	100.00
84ILE	CG1	84ILE	CG2	100.00	21LYS	CD	341LEU	CD1	0.20	91LEU	CA	91LEU	CD1	26.52
84ILE	CG1	84ILE	CD	100.00	21LYS	CD	341LEU	CD2	0.00	91LEU	CA	91LEU	CD2	76.70
84ILE	CG1	84ILE	C	96.35	21LYS	CE	334GLU	CD	0.01	91LEU	CA	91LEU	C	100.00
84ILE	CG2	84ILE	CD	98.10	21LYS	CE	337THR	CB	0.00	91LEU	CA	94ARG	CZ	0.00
84ILE	CG2	84ILE	C	4.14	21LYS	CE	337THR	CG2	0.01	91LEU	CB	91LEU	CG	100.00
84ILE	CD	84ILE	C	0.89	21LYS	CE	341LEU	CD1	0.08	91LEU	CB	91LEU	CD1	100.00
84ILE	C	85SER	CA	100.00	21LYS	CE	341LEU	CD2	0.00	91LEU	CB	91LEU	CD2	100.00
84ILE	C	85SER	CB	11.78	21LYS	C	22VAL	CA	100.00	91LEU	CB	91LEU	C	100.00
84ILE	C	85SER	C	89.68	21LYS	C	22VAL	CB	0.08	91LEU	CG	91LEU	CD1	100.00
85SER	CA	85SER	CB	100.00	21LYS	C	22VAL	CG1	0.03	91LEU	CG	91LEU	CD2	100.00
85SER	CA	85SER	C	100.00	21LYS	C	22VAL	C	99.94	91LEU	CG	91LEU	C	23.46
85SER	CA	86PRO	CD	100.00	21LYS	C	341LEU	CD1	0.00	91LEU	CG	94ARG	CZ	0.34
85SER	CB	85SER	C	100.00	21LYS	C	341LEU	CD2	0.27	91LEU	CG	95LYS	CD	0.00
85SER	CB	86PRO	CD	46.52	22VAL	CA	22VAL	CB	100.00	91LEU	CG	95LYS	CE	0.01
85SER	C	86PRO	CA	100.00	22VAL	CA	22VAL	CG1	100.00	91LEU	CD1	91LEU	CD2	100.00
85SER	C	86PRO	CD	100.00	22VAL	CA	22VAL	CG2	100.00	91LEU	CD1	91LEU	C	3.80
85SER	C	86PRO	C	100.00	22VAL	CA	22VAL	C	100.00	91LEU	CD1	94ARG	CD	0.00
86PRO	CA	86PRO	CB	100.00	22VAL	CA	341LEU	CD1	0.00	91LEU	CD1	94ARG	CZ	3.72
86PRO	CA	86PRO	CG	100.00	22VAL	CA	341LEU	CD2	0.06	91LEU	CD1	95LYS	CG	0.00
86PRO	CA	86PRO	CD	100.00	22VAL	CA	344LEU	CD1	0.02	91LEU	CD1	95LYS	CD	0.05
86PRO	CA	86PRO	C	100.00	22VAL	CB	22VAL	CG1	100.00	91LEU	CD1	95LYS	CE	0.04
86PRO	CB	86PRO	CG	100.00	22VAL	CB	22VAL	CG2	100.00	91LEU	CD1	140PHE	CE1	0.03
86PRO	CB	86PRO	CD	100.00	22VAL	CB	22VAL	C	100.00	91LEU	CD1	140PHE	CZ	0.03
86PRO	CB	86PRO	C	100.00	22VAL	CB	340VAL	CG1	0.00	91LEU	CD2	91LEU	C	3.33
86PRO	CB	90LEU	CD1	0.00	22VAL	CB	344LEU	CD1	0.48	91LEU	CD2	94ARG	CD	0.00
86PRO	CG	86PRO	CD	100.00	22VAL	CB	344LEU	CD2	0.01	91LEU	CD2	94ARG	CZ	19.86
86PRO	CG	86PRO	C	20.55	22VAL	CG1	22VAL	CG2	100.00	91LEU	CD2	95LYS	CD	0.04
86PRO	CD	86PRO	C	11.35	22VAL	CG1	22VAL	C	55.10	91LEU	CD2	95LYS	CE	0.03
86PRO	C	87GLU	CA	100.00	22VAL	CG1	26LEU	CD1	0.23	91LEU	CD2	140PHE	CE1	0.01
86PRO	C	87GLU	C	100.00	22VAL	CG1	291ILE	CG1	0.02	91LEU	CD2	140PHE	CZ	0.01
87GLU	CA	87GLU	CB	100.00	22VAL	CG1	291ILE	CG2	0.08	91LEU	C	92SER	CA	100.00
87GLU	CA	87GLU	CG	100.00	22VAL	CG1	291ILE	CD	0.04	91LEU	C	92SER	CB	0.05
87GLU	CA	87GLU	CD	8.63	22VAL	CG1	294ALA	CB	0.02	91LEU	C	92SER	C	99.98
87GLU	CA	87GLU	C	100.00	22VAL	CG1	311VAL	CG1	0.33	91LEU	C	95LYS	CD	0.00
87GLU	CA	90LEU	CD1	0.01	22VAL	CG1	311VAL	CG2	0.68	92SER	CA	92SER	CB	100.00
87GLU	CB	87GLU	CG	100.00	22VAL	CG1	337THR	CA	0.01	92SER	CA	92SER	C	100.00
87GLU	CB	87GLU	CD	100.00	22VAL	CG1	337THR	CB	0.10	92SER	CB	92SER	C	100.00
87GLU	CB	87GLU	C	100.00	22VAL	CG1	337THR	CG2	0.38	92SER	C	93LEU	CA	100.00
87GLU	CG	87GLU	CD	100.00	22VAL	CG1	340VAL	CG1	2.49	92SER	C	93LEU	C	100.00
87GLU	CG	87GLU	C	96.95	22VAL	CG1	341LEU	CG	0.01	93LEU	CA	93LEU	CB	100.00
87GLU	CG	90LEU	CD1	0.01	22VAL	CG1	341LEU	CD1	0.06	93LEU	CA	93LEU	CG	100.00
87GLU	CG	90LEU	CD2	0.01	22VAL	CG1	341LEU	CD2	0.28	93LEU	CA	93LEU	CD1	93.12
87GLU	CG	91LEU	CD1	0.31	22VAL	CG1	344LEU	CD1	2.12	93LEU	CA	93LEU	CD2	6.29
87GLU	CG	91LEU	CD2	0.00	22VAL	CG1	344LEU	CD2	0.13	93LEU	CA	93LEU	C	100.00
87GLU	CD	87GLU	C	0.69	22VAL	CG2	22VAL	C	45.01	93LEU	CB	93LEU	CG	100.00
87GLU	CD	91LEU	CD1	0.01	22VAL	CG2	23LEU	CD1	0.01	93LEU	CB	93LEU	CD1	100.00
87GLU	C	88THR	CA	100.00	22VAL	CG2	291ILE	CG2	0.16	93LEU	CB	93LEU	CD2	100.00
87GLU	C	88THR	CB	0.06	22VAL	CG2	291ILE	CD	0.01	93LEU	CB	93LEU	C	100.00
87GLU	C	88THR	CG2	0.02	22VAL	CG2	294ALA	CB	0.47	93LEU	CG	93LEU	CD1	100.00
87GLU	C	88THR	C	99.97	22VAL	CG2	337THR	CB	0.02	93LEU	CG	93LEU	CD2	100.00
87GLU	C	91LEU	CD1	0.00	22VAL	CG2	337THR	CG2	0.89	93LEU	CG	93LEU	C	98.58
88THR	CA	88THR	CB	100.00	22VAL	CG2	340VAL	CG1	0.56	93LEU	CD1	93LEU	CD2	100.00
88THR	CA	88THR	CG2	100.00	22VAL	CG2	341LEU	CG	0.00	93LEU	CD1	93LEU	C	8.40

88THR	CA	88THR	C	100.00	22VAL	CG2	341LEU	CD1	0.12	93LEU	CD1	97GLN	CG	0.22
88THR	CA	91LEU	CB	0.00	22VAL	CG2	341LEU	CD2	0.12	93LEU	CD1	268VAL	CG2	0.05
88THR	CA	91LEU	CD1	0.00	22VAL	CG2	344LEU	CD1	0.04	93LEU	CD2	93LEU	C	4.92
88THR	CB	88THR	CG2	100.00	22VAL	C	23LEU	CA	100.00	93LEU	CD2	97GLN	CG	0.04
88THR	CB	88THR	C	100.00	22VAL	C	23LEU	CB	0.12	93LEU	CD2	99LEU	CD1	0.01
88THR	CG2	88THR	C	10.01	22VAL	C	23LEU	C	99.96	93LEU	CD2	268VAL	CG1	0.07
88THR	C	89GLY	CA	100.00	22VAL	C	26LEU	CD1	0.01	93LEU	CD2	268VAL	CG2	0.81
88THR	C	89GLY	C	93.54	23LEU	CA	23LEU	CB	100.00	93LEU	C	94ARG	CA	100.00
89GLY	CA	89GLY	C	100.00	23LEU	CA	23LEU	CG	100.00	93LEU	C	94ARG	CB	0.00
89GLY	CA	92SER	CB	0.01	23LEU	CA	23LEU	CD1	24.38	93LEU	C	94ARG	C	100.00
89GLY	C	90LEU	CA	100.00	23LEU	CA	23LEU	CD2	75.31	93LEU	C	99LEU	CD1	0.00
89GLY	C	90LEU	CB	0.00	23LEU	CA	23LEU	C	100.00	94ARG	CA	94ARG	CB	100.00
89GLY	C	90LEU	C	100.00	23LEU	CA	32LEU	CD1	0.01	94ARG	CA	94ARG	CG	100.00
89GLY	C	92SER	CB	0.00	23LEU	CB	23LEU	CG	100.00	94ARG	CA	94ARG	CD	0.00
90LEU	CA	90LEU	CB	100.00	23LEU	CB	23LEU	CD1	100.00	94ARG	CA	94ARG	C	100.00
90LEU	CA	90LEU	CG	100.00	23LEU	CB	23LEU	CD2	100.00	94ARG	CA	99LEU	CD1	0.40
90LEU	CA	90LEU	CD1	63.94	23LEU	CB	23LEU	C	100.00	94ARG	CB	94ARG	CG	100.00
90LEU	CA	90LEU	CD2	35.02	23LEU	CB	32LEU	CD1	0.03	94ARG	CB	94ARG	CD	100.00
90LEU	CA	90LEU	C	100.00	23LEU	CB	34LEU	CG	0.00	94ARG	CB	94ARG	CZ	0.16
90LEU	CB	90LEU	CG	100.00	23LEU	CB	34LEU	CD1	0.14	94ARG	CB	94ARG	C	100.00
90LEU	CB	90LEU	CD1	100.00	23LEU	CB	34LEU	CD2	2.47	94ARG	CB	99LEU	CD1	0.00
90LEU	CB	90LEU	CD2	100.00	23LEU	CB	36TYR	CD2	0.00	94ARG	CG	94ARG	CD	100.00
90LEU	CB	90LEU	C	100.00	23LEU	CB	36TYR	CE2	0.01	94ARG	CG	94ARG	CZ	6.35
90LEU	CB	94ARG	CZ	0.66	23LEU	CB	36TYR	CZ	0.00	94ARG	CG	94ARG	C	82.44
90LEU	CG	90LEU	CD1	100.00	23LEU	CG	23LEU	CD1	100.00	94ARG	CG	99LEU	CB	0.00
90LEU	CG	90LEU	CD2	100.00	23LEU	CG	23LEU	CD2	100.00	94ARG	CG	99LEU	CD1	0.02
90LEU	CG	90LEU	C	64.37	23LEU	CG	23LEU	C	20.99	94ARG	CG	134LEU	CD1	0.26
90LEU	CG	94ARG	CZ	3.68	23LEU	CG	32LEU	CD1	0.11	94ARG	CG	134LEU	CD2	0.02
90LEU	CD1	90LEU	CD2	100.00	23LEU	CG	32LEU	CD2	0.02	94ARG	CG	135THR	CG2	0.40
90LEU	CD1	90LEU	C	1.87	23LEU	CG	34LEU	CD1	0.06	94ARG	CD	94ARG	CZ	100.00
90LEU	CD1	93LEU	CD1	0.01	23LEU	CG	34LEU	CD2	0.08	94ARG	CD	94ARG	C	0.02
90LEU	CD1	93LEU	CD2	0.01	23LEU	CG	294ALA	CB	0.15	94ARG	CD	134LEU	CD1	0.29
90LEU	CD1	94ARG	CD	0.01	23LEU	CG	298LEU	CD1	0.00	94ARG	CD	134LEU	CD2	0.13
90LEU	CD1	94ARG	CZ	2.62	23LEU	CD1	23LEU	CD2	100.00	94ARG	CD	135THR	CG2	0.38
90LEU	CD2	90LEU	C	1.68	23LEU	CD1	23LEU	C	0.03	94ARG	CD	261SER	CB	0.04
90LEU	CD2	94ARG	CD	0.00	23LEU	CD1	32LEU	CD1	1.38	94ARG	CZ	95LYS	CG	0.00
90LEU	CD2	94ARG	CZ	5.41	23LEU	CD1	32LEU	CD2	0.24	94ARG	CZ	95LYS	CE	0.00
90LEU	CD2	270GLU	CD	0.00	23LEU	CD1	34LEU	CD1	0.08	94ARG	CZ	134LEU	CG	0.12
90LEU	C	91LEU	CA	100.00	23LEU	CD1	34LEU	CD2	0.03	94ARG	CZ	134LEU	CD1	0.47
90LEU	C	91LEU	CB	0.01	23LEU	CD1	36TYR	CD2	0.00	94ARG	CZ	134LEU	CD2	0.22
90LEU	C	91LEU	C	100.00	23LEU	CD1	36TYR	CE2	0.00	94ARG	CZ	135THR	CA	1.77
90LEU	C	94ARG	CD	0.02	23LEU	CD1	68LEU	CD1	0.06	94ARG	CZ	135THR	CB	0.03
91LEU	CA	91LEU	CB	100.00	23LEU	CD1	68LEU	CD2	0.04	94ARG	CZ	135THR	CG2	0.47
91LEU	CA	91LEU	CG	100.00	23LEU	CD1	294ALA	CB	2.00	94ARG	CZ	140PHE	CE2	0.00
91LEU	CA	91LEU	CD1	23.18	23LEU	CD1	297MET	CE	4.10	94ARG	CZ	140PHE	CZ	0.00
91LEU	CA	91LEU	CD2	76.79	23LEU	CD1	298LEU	CG	0.18	94ARG	C	95LYS	CA	100.00
91LEU	CA	91LEU	C	100.00	23LEU	CD1	298LEU	CD1	0.76	94ARG	C	95LYS	CB	0.22
91LEU	CB	91LEU	CG	100.00	23LEU	CD1	298LEU	CD2	0.25	94ARG	C	95LYS	C	99.81
91LEU	CB	91LEU	CD1	100.00	23LEU	CD1	302PHE	CE1	0.00	95LYS	CA	95LYS	CB	100.00
91LEU	CB	91LEU	CD2	100.00	23LEU	CD2	23LEU	C	0.02	95LYS	CA	95LYS	CG	100.00
91LEU	CB	91LEU	C	100.00	23LEU	CD2	32LEU	CD1	1.00	95LYS	CA	95LYS	CD	10.50
91LEU	CG	91LEU	CD1	100.00	23LEU	CD2	32LEU	CD2	0.04	95LYS	CA	95LYS	CE	0.02
91LEU	CG	91LEU	CD2	100.00	23LEU	CD2	34LEU	CD1	2.45	95LYS	CA	95LYS	C	100.00
91LEU	CG	91LEU	C	20.18	23LEU	CD2	34LEU	CD2	0.09	95LYS	CB	95LYS	CG	100.00
91LEU	CG	95LYS	CE	0.02	23LEU	CD2	68LEU	CD1	0.00	95LYS	CB	95LYS	CD	100.00
91LEU	CD1	91LEU	CD2	100.00	23LEU	CD2	294ALA	CB	0.26	95LYS	CB	95LYS	CE	10.48
91LEU	CD1	91LEU	C	1.46	23LEU	CD2	297MET	CE	1.71	95LYS	CB	95LYS	C	100.00
91LEU	CD1	94ARG	CZ	0.01	23LEU	CD2	298LEU	CG	0.37	95LYS	CG	95LYS	CD	100.00
91LEU	CD1	95LYS	CD	0.05	23LEU	CD2	298LEU	CD1	2.08	95LYS	CG	95LYS	CE	100.00
91LEU	CD1	95LYS	CE	0.05	23LEU	CD2	298LEU	CD2	0.90	95LYS	CG	95LYS	C	65.18
91LEU	CD1	140PHE	CE2	0.07	23LEU	CD2	302PHE	CE1	0.02	95LYS	CD	95LYS	CE	100.00
91LEU	CD1	140PHE	CZ	0.04	23LEU	CD2	302PHE	CE2	0.01	95LYS	CD	95LYS	C	0.79

91LEU	CD2	91LEU	C	2.15	23LEU	CD2	302PHE	CZ	0.01	95LYS	CE	95LYS	C	0.01
91LEU	CD2	94ARG	CZ	0.04	23LEU	C	24ARG	CA	100.00	95LYS	C	96SER	CA	100.00
91LEU	CD2	95LYS	CG	0.00	23LEU	C	24ARG	CB	0.01	95LYS	C	96SER	CB	0.38
91LEU	CD2	95LYS	CD	0.02	23LEU	C	24ARG	C	99.99	95LYS	C	96SER	C	99.77
91LEU	CD2	95LYS	CE	0.01	23LEU	C	34LEU	CD1	0.02	96SEF	CA	96SER	CB	100.00
91LEU	CD2	140PHE	CE2	0.12	23LEU	C	34LEU	CD2	3.71	96SEF	CA	96SER	C	100.00
91LEU	CD2	140PHE	CZ	0.07	23LEU	C	36TYR	CE2	0.00	96SEF	CB	96SER	C	100.00
91LEU	C	92SER	CA	100.00	24ARG	CA	24ARG	CB	100.00	96SEF	C	97GLN	CA	100.00
91LEU	C	92SER	CB	0.85	24ARG	CA	24ARG	CG	100.00	96SEF	C	97GLN	CB	3.48
91LEU	C	92SER	C	99.32	24ARG	CA	24ARG	CD	95.36	96SEF	C	97GLN	CG	0.32
91LEU	C	95LYS	CE	0.01	24ARG	CA	24ARG	CZ	0.04	96SEF	C	97GLN	CD	0.06
92SER	CA	92SER	CB	100.00	24ARG	CA	24ARG	C	100.00	96SEF	C	97GLN	C	96.65
92SER	CA	92SER	C	100.00	24ARG	CA	34LEU	CD1	0.00	97GLN	CA	97GLN	CB	100.00
92SER	CB	92SER	C	100.00	24ARG	CA	34LEU	CD2	1.19	97GLN	CA	97GLN	CG	100.00
92SER	C	93LEU	CA	100.00	24ARG	CB	24ARG	CG	100.00	97GLN	CA	97GLN	CD	100.00
92SER	C	93LEU	C	100.00	24ARG	CB	24ARG	CD	100.00	97GLN	CA	97GLN	C	100.00
93LEU	CA	93LEU	CB	100.00	24ARG	CB	24ARG	CZ	0.00	97GLN	CB	97GLN	CG	100.00
93LEU	CA	93LEU	CG	100.00	24ARG	CB	24ARG	C	100.00	97GLN	CB	97GLN	CD	100.00
93LEU	CA	93LEU	CD1	84.23	24ARG	CB	34LEU	CD2	0.02	97GLN	CB	97GLN	C	100.00
93LEU	CA	93LEU	CD2	18.09	24ARG	CG	24ARG	CD	100.00	97GLN	CB	99LEU	CG	0.05
93LEU	CA	93LEU	C	100.00	24ARG	CG	24ARG	CZ	6.44	97GLN	CB	99LEU	CD1	0.10
93LEU	CB	93LEU	CG	100.00	24ARG	CG	24ARG	C	97.02	97GLN	CB	99LEU	CD2	1.56
93LEU	CB	93LEU	CD1	100.00	24ARG	CG	34LEU	CD2	0.06	97GLN	CG	97GLN	CD	100.00
93LEU	CB	93LEU	CD2	100.00	24ARG	CD	24ARG	CZ	100.00	97GLN	CD	266THR	CB	0.02
93LEU	CB	93LEU	C	100.00	24ARG	CD	24ARG	C	0.17	97GLN	C	98ASP	CA	100.00
93LEU	CG	93LEU	CD1	100.00	24ARG	CD	36TYR	CE1	0.01	97GLN	C	98ASP	CB	99.77
93LEU	CG	93LEU	CD2	100.00	24ARG	CD	36TYR	CE2	0.01	97GLN	C	98ASP	C	100.00
93LEU	CG	93LEU	C	99.16	24ARG	CZ	27ASP	CB	0.15	97GLN	C	264ARG	CZ	0.03
93LEU	CD1	93LEU	CD2	100.00	24ARG	CZ	36TYR	CE2	0.12	98ASF	CA	98ASP	CB	100.00
93LEU	CD1	93LEU	C	14.90	24ARG	CZ	36TYR	CZ	0.01	98ASF	CA	98ASP	CG	100.00
93LEU	CD1	97GLN	CG	0.63	24ARG	C	25ALA	CA	100.00	98ASF	CA	98ASP	C	100.00
93LEU	CD1	268VAL	CG1	0.03	24ARG	C	25ALA	CB	0.01	98ASF	CB	98ASP	CG	100.00
93LEU	CD1	268VAL	CG2	0.09	24ARG	C	25ALA	C	100.00	98ASF	CB	98ASP	C	100.00
93LEU	CD2	93LEU	C	13.70	25ALA	CA	25ALA	CB	100.00	98ASF	CB	264ARG	CB	0.12
93LEU	CD2	94ARG	CG	0.00	25ALA	CA	25ALA	C	100.00	98ASF	CB	264ARG	CZ	0.63
93LEU	CD2	97GLN	CG	0.08	25ALA	CA	28GLU	CB	0.00	98ASF	CG	98ASP	C	99.99
93LEU	CD2	99LEU	CD1	0.01	25ALA	CB	25ALA	C	100.00	98ASF	CG	164ARG	CZ	0.01
93LEU	CD2	268VAL	CG1	0.04	25ALA	CB	341LEU	CB	0.10	98ASF	CG	264ARG	CZ	0.01
93LEU	CD2	268VAL	CG2	0.15	25ALA	CB	341LEU	CG	0.06	98ASF	C	99LEU	CA	100.00
93LEU	C	94ARG	CA	100.00	25ALA	CB	341LEU	CD1	1.04	98ASF	C	99LEU	CB	0.24
93LEU	C	94ARG	C	100.00	25ALA	CB	341LEU	CD2	26.59	98ASF	C	99LEU	CG	0.02
94ARG	CA	94ARG	CB	100.00	25ALA	CB	344LEU	CB	0.02	98ASF	C	99LEU	C	99.77
94ARG	CA	94ARG	CG	100.00	25ALA	CB	344LEU	CD1	0.30	98ASF	C	100PHE	CD2	0.00
94ARG	CA	94ARG	CD	0.11	25ALA	CB	344LEU	CD2	0.06	98ASF	C	263GLY	CA	0.11
94ARG	CA	94ARG	C	100.00	25ALA	CB	345ALA	CB	0.04	99LEU	CA	99LEU	CB	100.00
94ARG	CA	99LEU	CD1	0.47	25ALA	C	26LEU	CA	100.00	99LEU	CA	99LEU	CG	100.00
94ARG	CB	94ARG	CG	100.00	25ALA	C	26LEU	CB	0.04	99LEU	CA	99LEU	CD2	99.99
94ARG	CB	94ARG	CD	100.00	25ALA	C	26LEU	C	99.95	99LEU	CA	99LEU	C	100.00
94ARG	CB	94ARG	CZ	0.00	25ALA	C	344LEU	CD1	0.00	99LEU	CA	263GLY	CA	4.00
94ARG	CB	94ARG	C	100.00	25ALA	C	345ALA	CB	0.00	99LEU	CB	99LEU	CG	100.00
94ARG	CB	99LEU	CD1	0.20	26LEU	CA	26LEU	CB	100.00	99LEU	CB	99LEU	CD1	100.00
94ARG	CB	135THR	CG2	0.03	26LEU	CA	26LEU	CG	100.00	99LEU	CB	99LEU	CD2	100.00
94ARG	CG	94ARG	CD	100.00	26LEU	CA	26LEU	CD1	7.46	99LEU	CB	99LEU	C	100.00
94ARG	CG	94ARG	CZ	1.37	26LEU	CA	26LEU	CD2	92.70	99LEU	CB	134LEU	CD1	0.03
94ARG	CG	94ARG	C	0.32	26LEU	CA	26LEU	C	100.00	99LEU	CB	134LEU	CD2	0.90
94ARG	CG	99LEU	CD1	1.02	26LEU	CA	344LEU	CD2	0.00	99LEU	CG	99LEU	CD1	100.00
94ARG	CG	134LEU	CD2	0.01	26LEU	CB	26LEU	CG	100.00	99LEU	CG	99LEU	CD2	100.00
94ARG	CD	94ARG	CZ	100.00	26LEU	CB	26LEU	CD1	100.00	99LEU	CD1	99LEU	CD2	100.00
94ARG	CD	99LEU	CD1	0.01	26LEU	CB	26LEU	CD2	100.00	99LEU	CD1	261SER	CB	0.84
94ARG	CD	134LEU	CD1	0.00	26LEU	CB	26LEU	C	100.00	99LEU	CD1	268VAL	CB	0.02
94ARG	CD	134LEU	CD2	0.14	26LEU	CB	32LEU	CG	0.00	99LEU	CD1	268VAL	CG2	9.00
94ARG	CZ	134LEU	CG	0.01	26LEU	CB	32LEU	CD1	0.95	99LEU	CD2	261SER	CB	0.04

94ARG	CZ	134LEU	CD1	0.27	26LEU	CB	32LEU	CD2	1.80	99LEU	CD2	261SER	C	0.01
94ARG	CZ	134LEU	CD2	0.33	26LEU	CB	344LEU	CD2	0.00	99LEU	CD2	262LEU	CA	0.00
94ARG	C	95LYS	CA	100.00	26LEU	CG	26LEU	CD1	100.00	99LEU	CD2	262LEU	C	0.82
94ARG	C	95LYS	CB	14.91	26LEU	CG	26LEU	CD2	100.00	99LEU	CD2	263GLY	CA	0.08
94ARG	C	95LYS	CG	1.56	26LEU	CG	26LEU	C	7.53	99LEU	CD2	265GLY	C	0.05
94ARG	C	95LYS	C	84.47	26LEU	CG	32LEU	CD1	0.00	99LEU	CD2	268VAL	CG2	0.10
95LYS	CA	95LYS	CB	100.00	26LEU	CG	32LEU	CD2	0.04	99LEU	C	100PHE	CA	100.00
95LYS	CA	95LYS	CG	100.00	26LEU	CG	344LEU	CD1	0.01	99LEU	C	100PHE	CB	91.91
95LYS	CA	95LYS	CD	23.95	26LEU	CG	344LEU	CD2	0.02	99LEU	C	100PHE	CG	0.05
95LYS	CA	95LYS	CE	0.12	26LEU	CD1	26LEU	CD2	100.00	99LEU	C	100PHE	CD2	0.16
95LYS	CA	95LYS	C	100.00	26LEU	CD1	26LEU	C	0.19	99LEU	C	100PHE	C	7.15
95LYS	CB	95LYS	CG	100.00	26LEU	CD1	30GLU	CB	0.05	99LEU	C	134LEU	CD1	0.18
95LYS	CB	95LYS	CD	100.00	26LEU	CD1	30GLU	CG	0.04	99LEU	C	134LEU	CD2	0.09
95LYS	CB	95LYS	CE	11.31	26LEU	CD1	32LEU	CD2	0.00	100PH	CA	100PHE	CB	100.00
95LYS	CB	95LYS	C	100.00	26LEU	CD1	298LEU	CD1	6.31	100PH	CA	100PHE	CG	100.00
95LYS	CG	95LYS	CD	100.00	26LEU	CD1	298LEU	CD2	0.41	100PH	CA	100PHE	CD1	97.66
95LYS	CG	95LYS	CE	100.00	26LEU	CD1	307LEU	CB	0.00	100PH	CA	100PHE	CD2	17.72
95LYS	CG	95LYS	C	61.30	26LEU	CD1	307LEU	CD1	0.02	100PH	CA	100PHE	C	100.00
95LYS	CD	95LYS	CE	100.00	26LEU	CD1	307LEU	CD2	0.09	100PH	CA	134LEU	CD1	0.18
95LYS	CD	95LYS	C	0.96	26LEU	CD1	310LYS	CD	0.01	100PH	CA	134LEU	CD2	0.53
95LYS	CE	95LYS	C	0.00	26LEU	CD1	311VAL	CG1	0.01	100PH	CA	135THR	CG2	0.16
95LYS	CE	98ASP	CG	0.00	26LEU	CD1	311VAL	CG2	5.13	100PH	CB	100PHE	CG	100.00
95LYS	C	96SER	CA	100.00	26LEU	CD1	344LEU	CB	0.01	100PH	CB	100PHE	CD1	100.00
95LYS	C	96SER	CB	13.11	26LEU	CD1	344LEU	CD1	0.02	100PH	CB	100PHE	CD2	100.00
95LYS	C	96SER	C	87.27	26LEU	CD1	344LEU	CD2	0.07	100PH	CB	100PHE	C	100.00
96SER	CA	96SER	CB	100.00	26LEU	CD2	26LEU	C	0.90	100PH	CB	135THR	CG2	0.69
96SER	CA	96SER	C	100.00	26LEU	CD2	30GLU	CB	0.11	100PH	CB	164ARG	CB	0.06
96SER	CB	96SER	C	100.00	26LEU	CD2	30GLU	CG	0.05	100PH	CB	164ARG	CG	0.01
96SER	C	97GLN	CA	100.00	26LEU	CD2	32LEU	CG	0.00	100PH	CB	164ARG	CD	0.24
96SER	C	97GLN	CB	2.78	26LEU	CD2	32LEU	CD1	0.01	100PH	CB	164ARG	CZ	1.04
96SER	C	97GLN	CG	0.32	26LEU	CD2	32LEU	CD2	0.08	100PH	CG	100PHE	CD1	100.00
96SER	C	97GLN	CD	0.05	26LEU	CD2	298LEU	CD1	0.88	100PH	CG	100PHE	CD2	100.00
96SER	C	97GLN	C	97.97	26LEU	CD2	298LEU	CD2	0.11	100PH	CG	100PHE	CE1	100.00
97GLN	CA	97GLN	CB	100.00	26LEU	CD2	307LEU	CG	0.04	100PH	CG	100PHE	CE2	100.00
97GLN	CA	97GLN	CG	100.00	26LEU	CD2	307LEU	CD1	0.28	100PH	CG	100PHE	CZ	100.00
97GLN	CA	97GLN	CD	99.95	26LEU	CD2	307LEU	CD2	0.33	100PH	CG	100PHE	C	98.71
97GLN	CA	97GLN	C	100.00	26LEU	CD2	310LYS	CD	0.01	100PH	CG	164ARG	CB	0.22
97GLN	CB	97GLN	CG	100.00	26LEU	CD2	311VAL	CG2	0.29	100PH	CG	164ARG	CG	0.40
97GLN	CB	97GLN	CD	100.00	26LEU	CD2	341LEU	CD2	0.09	100PH	CG	164ARG	CD	0.62
97GLN	CB	97GLN	C	100.00	26LEU	CD2	344LEU	CB	0.04	100PH	CG	164ARG	CZ	0.35
97GLN	CB	99LEU	CG	0.09	26LEU	CD2	344LEU	CD1	0.13	100PH	CG	263GLY	CA	0.02
97GLN	CB	99LEU	CD1	0.06	26LEU	CD2	344LEU	CD2	0.54	100PH	CD1	100PHE	CD2	100.00
97GLN	CB	99LEU	CD2	3.14	26LEU	C	27ASP	CA	100.00	100PH	CD1	100PHE	CE1	100.00
97GLN	CG	97GLN	CD	100.00	26LEU	C	27ASP	CB	0.02	100PH	CD1	100PHE	CE2	100.00
97GLN	CG	268VAL	CG2	0.02	26LEU	C	27ASP	C	99.99	100PH	CD1	100PHE	CZ	100.00
97GLN	CD	266THR	CB	0.02	26LEU	C	32LEU	CG	0.00	100PH	CD1	100PHE	C	84.12
97GLN	CD	268VAL	CG2	0.02	26LEU	C	32LEU	CD1	0.12	100PH	CD1	101ALA	CB	1.46
97GLN	C	98ASP	CA	100.00	26LEU	C	32LEU	CD2	0.45	100PH	CD1	164ARG	CB	8.96
97GLN	C	98ASP	CB	69.50	27ASP	CA	27ASP	CB	100.00	100PH	CD1	164ARG	CG	1.30
97GLN	C	98ASP	C	99.53	27ASP	CA	27ASP	CG	100.00	100PH	CD1	164ARG	CD	0.14
97GLN	C	99LEU	CD2	0.01	27ASP	CA	27ASP	C	100.00	100PH	CD1	164ARG	C	0.23
98ASP	CA	98ASP	CB	100.00	27ASP	CA	31GLY	CA	0.06	100PH	CD1	165VAL	CG1	0.02
98ASP	CA	98ASP	CG	100.00	27ASP	CA	32LEU	CB	0.06	100PH	CD1	165VAL	CG2	0.03
98ASP	CA	98ASP	C	100.00	27ASP	CA	32LEU	CD1	0.01	100PH	CD1	168VAL	CG2	0.16
98ASP	CB	98ASP	CG	100.00	27ASP	CA	32LEU	CD2	0.17	100PH	CD1	262LEU	C	0.00
98ASP	CB	98ASP	C	100.00	27ASP	CB	27ASP	CG	100.00	100PH	CD1	263GLY	CA	0.06
98ASP	CB	264ARG	CB	0.06	27ASP	CB	27ASP	C	100.00	100PH	CD2	100PHE	CE1	100.00
98ASP	CB	264ARG	CD	0.00	27ASP	CB	32LEU	CB	0.04	100PH	CD2	100PHE	CE2	100.00
98ASP	CB	264ARG	CZ	0.34	27ASP	CB	32LEU	CG	0.00	100PH	CD2	100PHE	CZ	100.00
98ASP	CG	98ASP	C	99.62	27ASP	CB	32LEU	CD1	0.04	100PH	CD2	164ARG	CG	0.05
98ASP	CG	264ARG	CD	0.01	27ASP	CB	32LEU	CD2	0.03	100PH	CD2	164ARG	CD	1.13
98ASP	CG	264ARG	CZ	0.06	27ASP	CB	34LEU	CB	0.16	100PH	CD2	164ARG	CZ	66.97

98ASP	C	99LEU	CA	100.00	27ASP	CB	34LEU	CD1	0.02	100PH	CD2	263GLY	CA	1.02
98ASP	C	99LEU	CB	0.73	27ASP	CG	27ASP	C	100.00	100PH	CD2	263GLY	C	0.46
98ASP	C	99LEU	CG	0.19	27ASP	CG	32LEU	CB	0.00	100PH	CE1	100PHE	CE2	100.00
98ASP	C	99LEU	CD2	0.00	27ASP	CG	34LEU	CB	6.40	100PH	CE1	100PHE	CZ	100.00
98ASP	C	99LEU	C	99.41	27ASP	CG	34LEU	CG	0.00	100PH	CE1	101ALA	CB	0.57
98ASP	C	263GLY	CA	0.14	27ASP	CG	34LEU	CD1	0.01	100PH	CE1	164ARG	CB	1.76
99LEU	CA	99LEU	CB	100.00	27ASP	C	28GLU	CA	100.00	100PH	CE1	164ARG	CG	0.70
99LEU	CA	99LEU	CG	100.00	27ASP	C	28GLU	CB	0.28	100PH	CE1	164ARG	CD	0.02
99LEU	CA	99LEU	CD2	99.97	27ASP	C	28GLU	C	99.79	100PH	CE1	164ARG	C	1.57
99LEU	CA	99LEU	C	100.00	27ASP	C	31GLY	CA	0.00	100PH	CE1	165VAL	CG1	0.00
99LEU	CA	263GLY	CA	1.76	28GLU	CA	28GLU	CB	100.00	100PH	CE1	165VAL	CG2	0.00
99LEU	CB	99LEU	CG	100.00	28GLU	CA	28GLU	CG	100.00	100PH	CE1	168VAL	CG1	0.89
99LEU	CB	99LEU	CD1	100.00	28GLU	CA	28GLU	CD	97.06	100PH	CE1	168VAL	CG2	6.02
99LEU	CB	99LEU	CD2	100.00	28GLU	CA	28GLU	C	100.00	100PH	CE1	262LEU	CB	0.04
99LEU	CB	99LEU	C	100.00	28GLU	CB	28GLU	CG	100.00	100PH	CE1	262LEU	CD1	0.01
99LEU	CB	134LEU	CD1	0.89	28GLU	CB	28GLU	CD	100.00	100PH	CE1	262LEU	CD2	0.28
99LEU	CB	134LEU	CD2	0.70	28GLU	CB	28GLU	C	100.00	100PH	CE1	262LEU	C	0.01
99LEU	CB	135THR	CG2	0.00	28GLU	CG	28GLU	CD	100.00	100PH	CE1	263GLY	CA	0.25
99LEU	CB	261SER	CB	0.00	28GLU	CG	28GLU	C	96.74	100PH	CE1	263GLY	C	0.11
99LEU	CG	99LEU	CD1	100.00	28GLU	CD	28GLU	C	0.09	100PH	CE2	100PHE	CZ	100.00
99LEU	CG	99LEU	CD2	100.00	28GLU	C	29ALA	CA	100.00	100PH	CE2	164ARG	CG	0.01
99LEU	CD1	99LEU	CD2	100.00	28GLU	C	29ALA	CB	6.68	100PH	CE2	164ARG	CD	0.12
99LEU	CD1	134LEU	CD1	0.02	28GLU	C	29ALA	C	92.19	100PH	CE2	164ARG	CZ	10.02
99LEU	CD1	134LEU	CD2	0.08	29ALA	CA	29ALA	CB	100.00	100PH	CE2	262LEU	CB	0.00
99LEU	CD1	261SER	CB	3.51	29ALA	CA	29ALA	C	100.00	100PH	CE2	262LEU	CD1	0.01
99LEU	CD1	268VAL	CB	0.04	29ALA	CB	29ALA	C	100.00	100PH	CE2	262LEU	C	0.05
99LEU	CD1	268VAL	CG1	0.00	29ALA	CB	344LEU	CD	0.01	100PH	CE2	263GLY	CA	7.07
99LEU	CD1	268VAL	CG2	2.94	29ALA	CB	344LEU	C	0.04	100PH	CE2	263GLY	C	37.35
99LEU	CD2	261SER	CB	1.18	29ALA	CB	345ALA	CA	0.06	100PH	CE2	264ARG	CA	0.06
99LEU	CD2	261SER	C	0.24	29ALA	CB	345ALA	CB	0.28	100PH	CE2	264ARG	CB	0.00
99LEU	CD2	262LEU	C	0.53	29ALA	CB	345ALA	C	0.18	100PH	CZ	164ARG	CB	0.04
99LEU	CD2	263GLY	CA	0.78	29ALA	C	30GLU	CA	100.00	100PH	CZ	164ARG	CG	0.20
99LEU	CD2	265GLY	C	0.00	29ALA	C	30GLU	CB	54.01	100PH	CZ	164ARG	CD	0.03
99LEU	CD2	268VAL	CG2	0.46	29ALA	C	30GLU	CG	0.55	100PH	CZ	168VAL	CG1	0.28
99LEU	C	100PHE	CA	100.00	29ALA	C	30GLU	CD	0.01	100PH	CZ	168VAL	CG2	0.11
99LEU	C	100PHE	CB	64.50	29ALA	C	30GLU	C	51.12	100PH	CZ	262LEU	CB	0.02
99LEU	C	100PHE	CG	0.03	30GLU	CA	30GLU	CB	100.00	100PH	CZ	262LEU	CG	0.00
99LEU	C	100PHE	CD2	0.20	30GLU	CA	30GLU	CG	100.00	100PH	CZ	262LEU	CD1	0.09
99LEU	C	100PHE	C	35.23	30GLU	CA	30GLU	CD	97.45	100PH	CZ	262LEU	CD2	0.22
99LEU	C	134LEU	CD1	0.10	30GLU	CA	30GLU	C	100.00	100PH	CZ	263GLY	CA	3.01
99LEU	C	135THR	CG2	0.00	30GLU	CB	30GLU	CG	100.00	100PH	CZ	263GLY	C	7.47
100PHE	CA	100PHE	CB	100.00	30GLU	CB	30GLU	CD	100.00	100PH	C	101ALA	CA	100.00
100PHE	CA	100PHE	CG	100.00	30GLU	CB	30GLU	C	100.00	100PH	C	101ALA	CB	80.27
100PHE	CA	100PHE	CD1	74.85	30GLU	CB	32LEU	CD1	0.02	100PH	C	101ALA	C	24.17
100PHE	CA	100PHE	CD2	60.92	30GLU	CB	32LEU	CD2	0.04	100PH	C	134LEU	CB	0.48
100PHE	CA	100PHE	C	100.00	30GLU	CB	307LEU	CD1	0.03	100PH	C	134LEU	CG	0.01
100PHE	CA	135THR	CG2	0.03	30GLU	CB	307LEU	CD2	0.03	100PH	C	134LEU	CD1	0.39
100PHE	CA	264ARG	CZ	0.00	30GLU	CG	30GLU	CD	100.00	100PH	C	134LEU	CD2	13.31
100PHE	CB	100PHE	CG	100.00	30GLU	CG	30GLU	C	79.33	100PH	C	165VAL	CG1	0.03
100PHE	CB	100PHE	CD1	100.00	30GLU	CG	32LEU	CD1	0.01	101AL	CA	101ALA	CB	100.00
100PHE	CB	100PHE	CD2	100.00	30GLU	CG	32LEU	CD2	0.04	101AL	CA	101ALA	C	100.00
100PHE	CB	100PHE	C	100.00	30GLU	CG	304LEU	CD2	0.00	101AL	CA	134LEU	CD2	0.39
100PHE	CB	133GLU	CD	0.01	30GLU	CG	307LEU	CG	0.00	101AL	CA	165VAL	CG1	0.02
100PHE	CB	135THR	CG2	0.07	30GLU	CG	307LEU	CD1	0.31	101AL	CB	101ALA	C	100.00
100PHE	CB	164ARG	CB	0.02	30GLU	CG	307LEU	CD2	0.38	101AL	CB	103LEU	CD1	0.04
100PHE	CB	164ARG	CD	0.08	30GLU	CD	30GLU	C	1.65	101AL	CB	131VAL	CG1	0.10
100PHE	CB	164ARG	CZ	0.88	30GLU	CD	307LEU	CD1	0.04	101AL	CB	165VAL	CG1	0.05
100PHE	CB	264ARG	CZ	10.68	30GLU	CD	307LEU	CD2	0.06	101AL	CB	165VAL	CG2	0.06
100PHE	CG	100PHE	CD1	100.00	30GLU	CD	310LYS	CE	0.00	101AL	CB	168VAL	CB	0.11
100PHE	CG	100PHE	CD2	100.00	30GLU	C	31GLY	CA	100.00	101AL	CB	168VAL	CG1	3.10
100PHE	CG	100PHE	CE1	100.00	30GLU	C	31GLY	C	64.52	101AL	CB	168VAL	CG2	1.62
100PHE	CG	100PHE	CE2	100.00	31GLY	CA	31GLY	C	100.00	101AL	CB	262LEU	CD1	0.02

100PHE	CG	100PHE	CZ	100.00	31GLY	C	32LEU	CA	100.00	101AL	CB	262LEU	CD2	0.01
100PHE	CG	100PHE	C	99.46	31GLY	C	32LEU	CB	37.92	101AL	C	102ASN	CA	100.00
100PHE	CG	164ARG	CB	0.11	31GLY	C	32LEU	CG	2.68	101AL	C	102ASN	CB	18.14
100PHE	CG	164ARG	CG	0.04	31GLY	C	32LEU	CD1	0.43	101AL	C	102ASN	C	89.28
100PHE	CG	164ARG	CD	4.79	31GLY	C	32LEU	CD2	0.16	101AL	C	103LEU	CD1	0.02
100PHE	CG	164ARG	CZ	0.36	31GLY	C	32LEU	C	59.64	101AL	C	103LEU	CD2	0.00
100PHE	CG	264ARG	CZ	0.00	32LEU	CA	32LEU	CB	100.00	101AL	C	134LEU	CD1	0.03
100PHE	CD1	100PHE	CD2	100.00	32LEU	CA	32LEU	CG	100.00	101AL	C	134LEU	CD2	3.10
100PHE	CD1	100PHE	CE1	100.00	32LEU	CA	32LEU	CD1	10.61	102AS	CA	102ASN	CB	100.00
100PHE	CD1	100PHE	CE2	100.00	32LEU	CA	32LEU	CD2	79.83	102AS	CA	102ASN	CG	100.00
100PHE	CD1	100PHE	CZ	100.00	32LEU	CA	32LEU	C	100.00	102AS	CA	102ASN	C	100.00
100PHE	CD1	100PHE	C	69.26	32LEU	CA	302PHE	CE1	0.02	102AS	CB	102ASN	CG	100.00
100PHE	CD1	101ALA	CB	16.41	32LEU	CA	302PHE	CE2	0.03	102AS	CB	102ASN	C	100.00
100PHE	CD1	164ARG	CB	1.26	32LEU	CA	302PHE	CZ	0.02	102AS	CB	132ARG	CG	0.01
100PHE	CD1	164ARG	CG	2.66	32LEU	CA	304LEU	CD1	0.03	102AS	CB	132ARG	CD	0.06
100PHE	CD1	164ARG	CD	7.46	32LEU	CB	32LEU	CG	100.00	102AS	CB	132ARG	CZ	0.00
100PHE	CD1	164ARG	CZ	0.01	32LEU	CB	32LEU	CD1	100.00	102AS	CB	134LEU	CG	0.06
100PHE	CD1	164ARG	C	0.33	32LEU	CB	32LEU	CD2	100.00	102AS	CB	134LEU	CD1	0.14
100PHE	CD1	165VAL	CA	0.00	32LEU	CB	32LEU	C	100.00	102AS	CB	134LEU	CD2	1.32
100PHE	CD1	165VAL	CG2	0.10	32LEU	CB	34LEU	CB	0.00	102AS	CG	102ASN	C	100.00
100PHE	CD1	168VAL	CG2	0.00	32LEU	CB	34LEU	CD1	0.00	102AS	CG	132ARG	CZ	0.01
100PHE	CD2	100PHE	CE1	100.00	32LEU	CB	34LEU	CD2	0.03	102AS	CG	134LEU	CD1	0.14
100PHE	CD2	100PHE	CE2	100.00	32LEU	CB	302PHE	CD1	0.03	102AS	CG	134LEU	CD2	1.63
100PHE	CD2	100PHE	CZ	100.00	32LEU	CB	302PHE	CD2	0.04	102AS	C	103LEU	CA	100.00
100PHE	CD2	100PHE	C	0.01	32LEU	CB	302PHE	CE1	0.75	102AS	C	103LEU	CB	2.18
100PHE	CD2	164ARG	CB	0.00	32LEU	CB	302PHE	CE2	1.62	102AS	C	103LEU	CG	0.07
100PHE	CD2	164ARG	CG	0.03	32LEU	CB	302PHE	CZ	0.53	102AS	C	103LEU	CD1	1.63
100PHE	CD2	164ARG	CD	1.81	32LEU	CB	304LEU	CD1	2.34	102AS	C	103LEU	CD2	0.08
100PHE	CD2	164ARG	CZ	15.32	32LEU	CB	304LEU	CD2	0.03	102AS	C	103LEU	C	98.36
100PHE	CD2	262LEU	C	1.51	32LEU	CB	307LEU	CD1	0.01	103LE	CA	103LEU	CB	100.00
100PHE	CD2	263GLY	CA	1.64	32LEU	CB	307LEU	CD2	0.02	103LE	CA	103LEU	CG	100.00
100PHE	CD2	263GLY	C	2.48	32LEU	CG	32LEU	CD1	100.00	103LE	CA	103LEU	CD1	36.93
100PHE	CD2	264ARG	CG	0.56	32LEU	CG	32LEU	CD2	100.00	103LE	CA	103LEU	CD2	63.16
100PHE	CD2	264ARG	CZ	0.31	32LEU	CG	32LEU	C	18.48	103LE	CA	103LEU	C	100.00
100PHE	CE1	100PHE	CE2	100.00	32LEU	CG	298LEU	CD1	0.04	103LE	CB	103LEU	CG	100.00
100PHE	CE1	100PHE	CZ	100.00	32LEU	CG	298LEU	CD2	0.00	103LE	CB	103LEU	CD1	100.00
100PHE	CE1	101ALA	CB	6.53	32LEU	CG	302PHE	CE1	0.02	103LE	CB	103LEU	CD2	100.00
100PHE	CE1	164ARG	CB	2.72	32LEU	CG	302PHE	CE2	0.01	103LE	CB	103LEU	C	100.00
100PHE	CE1	164ARG	CG	6.88	32LEU	CG	302PHE	CZ	0.01	103LE	CB	129LEU	CD1	0.03
100PHE	CE1	164ARG	CD	5.24	32LEU	CG	304LEU	CD1	0.02	103LE	CB	129LEU	CD2	0.01
100PHE	CE1	164ARG	CZ	0.00	32LEU	CG	307LEU	CD1	0.02	103LE	CB	296MET	CE	0.52
100PHE	CE1	164ARG	C	2.78	32LEU	CG	307LEU	CD2	0.00	103LE	CG	103LEU	CD1	100.00
100PHE	CE1	165VAL	CG2	0.06	32LEU	CD1	32LEU	CD2	100.00	103LE	CG	103LEU	CD2	100.00
100PHE	CE1	168VAL	CG1	1.32	32LEU	CD1	32LEU	C	3.55	103LE	CG	103LEU	C	0.90
100PHE	CE1	168VAL	CG2	1.23	32LEU	CD1	33GLY	C	0.01	103LE	CG	129LEU	CD1	0.01
100PHE	CE1	262LEU	CB	0.02	32LEU	CD1	34LEU	CA	0.00	103LE	CG	129LEU	CD2	0.02
100PHE	CE1	262LEU	C	0.00	32LEU	CD1	34LEU	CB	0.07	103LE	CG	172LEU	CD1	0.02
100PHE	CE1	263GLY	CA	0.05	32LEU	CD1	34LEU	CG	0.00	103LE	CG	262LEU	CD1	0.11
100PHE	CE2	100PHE	CZ	100.00	32LEU	CD1	34LEU	CD1	0.18	103LE	CG	262LEU	CD2	0.08
100PHE	CE2	164ARG	CG	0.12	32LEU	CD1	34LEU	CD2	0.02	103LE	CG	296MET	CE	0.00
100PHE	CE2	164ARG	CD	1.20	32LEU	CD1	298LEU	CG	0.00	103LE	CD1	103LEU	CD2	100.00
100PHE	CE2	164ARG	CZ	3.03	32LEU	CD1	298LEU	CD1	0.18	103LE	CD1	103LEU	C	0.02
100PHE	CE2	262LEU	CB	4.39	32LEU	CD1	298LEU	CD2	0.91	103LE	CD1	129LEU	CB	0.00
100PHE	CE2	262LEU	C	3.78	32LEU	CD1	302PHE	CG	0.02	103LE	CD1	129LEU	CD1	0.01
100PHE	CE2	263GLY	CA	3.86	32LEU	CD1	302PHE	CD1	0.07	103LE	CD1	131VAL	CG2	0.03
100PHE	CE2	263GLY	C	16.58	32LEU	CD1	302PHE	CD2	0.15	103LE	CD1	168VAL	CG1	0.02
100PHE	CE2	264ARG	CG	0.05	32LEU	CD1	302PHE	CE1	0.63	103LE	CD1	172LEU	CD1	0.36
100PHE	CE2	264ARG	CZ	0.03	32LEU	CD1	302PHE	CE2	2.31	103LE	CD1	172LEU	CD2	0.02
100PHE	CZ	101ALA	CB	0.00	32LEU	CD1	302PHE	CZ	2.04	103LE	CD1	260ALA	CB	0.01
100PHE	CZ	164ARG	CB	0.11	32LEU	CD1	304LEU	CD1	0.41	103LE	CD1	262LEU	CB	0.04
100PHE	CZ	164ARG	CG	1.08	32LEU	CD1	304LEU	CD2	0.00	103LE	CD1	262LEU	CG	0.12
100PHE	CZ	164ARG	CD	2.36	32LEU	CD1	307LEU	CD1	0.20	103LE	CD1	262LEU	CD1	3.14

100PHE	CZ	168VAL	CG1	1.14	32LEU	CD1	307LEU	CD2	0.06	103LE	CD1	262LEU	CD2	0.25
100PHE	CZ	168VAL	CG2	0.11	32LEU	CD2	32LEU	C	1.42	103LE	CD1	296MET	CE	0.76
100PHE	CZ	262LEU	CB	1.98	32LEU	CD2	34LEU	CB	0.00	103LE	CD1	300HIS	CD2	0.01
100PHE	CZ	262LEU	CD1	0.04	32LEU	CD2	34LEU	CD1	0.03	103LE	CD1	300HIS	CE1	0.54
100PHE	CZ	262LEU	CD2	0.02	32LEU	CD2	298LEU	CG	0.01	103LE	CD2	103LEU	C	0.31
100PHE	CZ	263GLY	CA	0.49	32LEU	CD2	298LEU	CD1	0.52	103LE	CD2	104ARG	C	0.00
100PHE	CZ	263GLY	C	1.27	32LEU	CD2	298LEU	CD2	7.02	103LE	CD2	129LEU	CG	0.01
100PHE	C	101ALA	CA	100.00	32LEU	CD2	302PHE	CG	0.00	103LE	CD2	129LEU	CD1	0.02
100PHE	C	101ALA	CB	66.88	32LEU	CD2	302PHE	CD1	0.06	103LE	CD2	129LEU	CD2	0.01
100PHE	C	101ALA	C	35.44	32LEU	CD2	302PHE	CD2	0.05	103LE	CD2	131VAL	CA	0.00
100PHE	C	134LEU	CB	0.12	32LEU	CD2	302PHE	CE1	0.19	103LE	CD2	131VAL	CG1	0.05
100PHE	C	134LEU	CG	0.00	32LEU	CD2	302PHE	CE2	0.27	103LE	CD2	131VAL	CG2	0.97
100PHE	C	134LEU	CD1	0.06	32LEU	CD2	302PHE	CZ	0.07	103LE	CD2	168VAL	CG1	0.11
101ALA	CA	101ALA	CB	100.00	32LEU	CD2	304LEU	CD1	11.51	103LE	CD2	172LEU	CD1	6.21
101ALA	CA	101ALA	C	100.00	32LEU	CD2	304LEU	CD2	0.15	103LE	CD2	172LEU	CD2	0.15
101ALA	CB	101ALA	C	100.00	32LEU	CD2	307LEU	CG	0.02	103LE	CD2	262LEU	CG	0.01
101ALA	CB	103LEU	CD1	0.10	32LEU	CD2	307LEU	CD1	5.32	103LE	CD2	262LEU	CD1	0.65
101ALA	CB	131VAL	CG1	0.02	32LEU	CD2	307LEU	CD2	4.15	103LE	CD2	262LEU	CD2	0.06
101ALA	CB	165VAL	CG1	0.02	32LEU	C	33GLY	CA	100.00	103LE	CD2	296MET	CE	0.49
101ALA	CB	165VAL	CG2	0.08	32LEU	C	33GLY	C	83.47	103LE	CD2	300HIS	CD2	0.03
101ALA	CB	168VAL	CB	0.07	32LEU	C	302PHE	CD1	0.00	103LE	CD2	300HIS	CE1	0.66
101ALA	CB	168VAL	CG1	0.80	32LEU	C	302PHE	CE1	0.15	103LE	C	104ARG	CA	100.00
101ALA	CB	168VAL	CG2	1.44	32LEU	C	302PHE	CE2	0.29	103LE	C	104ARG	CB	44.51
101ALA	CB	262LEU	CD1	0.12	32LEU	C	302PHE	CZ	0.06	103LE	C	104ARG	CG	9.98
101ALA	C	102ASN	CA	100.00	33GLY	CA	33GLY	C	100.00	103LE	C	104ARG	CD	0.18
101ALA	C	102ASN	CB	36.27	33GLY	C	34LEU	CA	100.00	103LE	C	104ARG	C	66.89
101ALA	C	102ASN	C	69.57	33GLY	C	34LEU	CB	2.58	103LE	C	105PRO	CD	4.33
101ALA	C	103LEU	CD1	0.03	33GLY	C	34LEU	CD2	0.00	103LE	C	129LEU	CD2	0.00
101ALA	C	134LEU	CG	0.01	33GLY	C	34LEU	C	98.76	104AR	CA	104ARG	CB	100.00
101ALA	C	134LEU	CD1	5.82	33GLY	C	302PHE	CE1	0.00	104AR	CA	104ARG	CG	100.00
101ALA	C	134LEU	CD2	0.00	33GLY	C	302PHE	CE2	0.01	104AR	CA	104ARG	CD	99.30
101ALA	C	262LEU	CD1	0.00	33GLY	C	302PHE	CZ	0.01	104AR	CA	104ARG	C	100.00
101ALA	C	262LEU	CD2	0.01	34LEU	CA	34LEU	CB	100.00	104AR	CA	105PRO	CD	100.00
102ASN	CA	102ASN	CB	100.00	34LEU	CA	34LEU	CG	100.00	104AR	CB	104ARG	CG	100.00
102ASN	CA	102ASN	CG	100.00	34LEU	CA	34LEU	CD1	90.69	104AR	CB	104ARG	CD	100.00
102ASN	CA	102ASN	C	100.00	34LEU	CA	34LEU	CD2	16.02	104AR	CB	104ARG	CZ	0.00
102ASN	CB	102ASN	CG	100.00	34LEU	CA	34LEU	C	100.00	104AR	CB	104ARG	C	100.00
102ASN	CB	102ASN	C	100.00	34LEU	CB	34LEU	CG	100.00	104AR	CB	244SER	CB	0.30
102ASN	CB	132ARG	CG	0.00	34LEU	CB	34LEU	CD1	100.00	104AR	CG	104ARG	CD	100.00
102ASN	CB	132ARG	CD	0.04	34LEU	CB	34LEU	CD2	100.00	104AR	CG	104ARG	CZ	61.31
102ASN	CB	134LEU	CG	0.07	34LEU	CB	34LEU	C	100.00	104AR	CG	132ARG	CD	0.78
102ASN	CB	134LEU	CD1	0.25	34LEU	CB	36TYR	CD2	0.00	104AR	CG	132ARG	CZ	1.10
102ASN	CB	134LEU	CD2	0.88	34LEU	CG	34LEU	CD1	100.00	104AR	CG	244SER	CB	0.28
102ASN	CG	102ASN	C	99.51	34LEU	CG	34LEU	CD2	100.00	104AR	CD	104ARG	CZ	100.00
102ASN	CG	104ARG	CG	0.01	34LEU	CG	34LEU	C	96.87	104AR	CD	132ARG	CZ	0.31
102ASN	CG	132ARG	CZ	0.00	34LEU	CG	36TYR	CD1	0.01	104AR	CD	244SER	CB	0.02
102ASN	CG	134LEU	CD1	0.04	34LEU	CG	36TYR	CD2	0.02	104AR	CD	257LEU	CD2	0.02
102ASN	CG	134LEU	CD2	0.00	34LEU	CG	302PHE	CE2	0.00	104AR	CD	259SER	CB	6.98
102ASN	C	103LEU	CA	100.00	34LEU	CG	302PHE	CZ	0.01	104AR	CD	272VAL	CG2	0.09
102ASN	C	103LEU	CB	10.12	34LEU	CD1	34LEU	CD2	100.00	104AR	CZ	132ARG	CZ	1.34
102ASN	C	103LEU	CG	0.38	34LEU	CD1	34LEU	C	9.79	104AR	CZ	244SER	CB	10.04
102ASN	C	103LEU	CD1	3.48	34LEU	CD1	35ALA	C	0.02	104AR	CZ	259SER	CB	0.05
102ASN	C	103LEU	CD2	0.09	34LEU	CD1	36TYR	CD1	0.04	104AR	CZ	272VAL	CG1	0.17
102ASN	C	103LEU	C	90.76	34LEU	CD1	36TYR	CD2	0.28	104AR	CZ	272VAL	CG2	2.72
103LEU	CA	103LEU	CB	100.00	34LEU	CD1	36TYR	CE2	0.04	104AR	C	105PRO	CA	100.00
103LEU	CA	103LEU	CG	100.00	34LEU	CD1	297MET	CE	0.03	104AR	C	105PRO	CB	0.11
103LEU	CA	103LEU	CD1	46.13	34LEU	CD1	298LEU	CD2	0.02	104AR	C	105PRO	CD	100.00
103LEU	CA	103LEU	CD2	49.88	34LEU	CD1	302PHE	CE1	0.06	104AR	C	105PRO	C	100.00
103LEU	CA	103LEU	C	100.00	34LEU	CD1	302PHE	CE2	0.08	104AR	C	129LEU	CD1	0.21
103LEU	CA	129LEU	CD1	0.00	34LEU	CD1	302PHE	CZ	0.42	104AR	C	129LEU	CD2	0.04
103LEU	CB	103LEU	CG	100.00	34LEU	CD1	304LEU	CD1	0.00	105PR	CA	105PRO	CB	100.00
103LEU	CB	103LEU	CD1	100.00	34LEU	CD2	34LEU	C	2.68	105PR	CA	105PRO	CG	100.00

103LEU CB	103LEU	CD2	100.00	34LEU CD2	35ALA	C	0.02	105PR CA	105PRO	CD	100.00
103LEU CB	103LEU	C	100.00	34LEU CD2	36TYR	CD1	0.38	105PR CA	105PRO	C	100.00
103LEU CB	129LEU	CD1	0.01	34LEU CD2	36TYR	CD2	0.69	105PR CA	129LEU	CD1	0.10
103LEU CB	129LEU	CD2	0.00	34LEU CD2	36TYR	CE1	0.18	105PR CA	129LEU	CD2	0.05
103LEU CB	131VAL	CG2	0.00	34LEU CD2	36TYR	CE2	0.28	105PR CB	105PRO	CG	100.00
103LEU CB	260ALA	CB	0.01	34LEU CD2	297MET	CE	0.01	105PR CB	105PRO	CD	100.00
103LEU CB	262LEU	CD1	0.00	34LEU CD2	298LEU	CD1	0.00	105PR CB	105PRO	C	100.00
103LEU CB	296MET	CE	1.42	34LEU CD2	298LEU	CD2	0.04	105PR CB	129LEU	CD1	0.10
103LEU CG	103LEU	CD1	100.00	34LEU CD2	302PHE	CD1	0.01	105PR CB	129LEU	CD2	0.08
103LEU CG	103LEU	CD2	100.00	34LEU CD2	302PHE	CE1	0.15	105PR CG	105PRO	CD	100.00
103LEU CG	103LEU	C	4.93	34LEU CD2	302PHE	CE2	0.02	105PR CG	105PRO	C	98.22
103LEU CG	129LEU	CD2	0.01	34LEU CD2	302PHE	CZ	0.22	105PR CG	129LEU	CD1	0.00
103LEU CG	131VAL	CG2	0.01	34LEU C	35ALA	CA	100.00	105PR CG	129LEU	CD2	0.00
103LEU CG	262LEU	CD1	0.15	34LEU C	35ALA	CB	66.31	105PR CG	258PRO	CG	1.81
103LEU CD1	103LEU	CD2	100.00	34LEU C	35ALA	C	40.17	105PR CG	258PRO	CD	0.04
103LEU CD1	103LEU	C	0.74	35ALA CA	35ALA	CB	100.00	105PR CG	296MET	CE	0.86
103LEU CD1	129LEU	CG	0.00	35ALA CA	35ALA	C	100.00	105PR CD	105PRO	C	96.27
103LEU CD1	129LEU	CD1	0.01	35ALA CB	35ALA	C	100.00	105PR CD	129LEU	CD1	0.04
103LEU CD1	129LEU	CD2	0.02	35ALA C	36TYR	CA	100.00	105PR CD	257LEU	CD1	0.00
103LEU CD1	130ILE	C	0.00	35ALA C	36TYR	CB	98.55	105PR CD	296MET	CE	0.56
103LEU CD1	131VAL	CG1	0.04	35ALA C	36TYR	CG	0.12	105PR C	106ALA	CA	100.00
103LEU CD1	131VAL	CG2	0.19	35ALA C	36TYR	CD1	0.20	105PR C	106ALA	CB	61.38
103LEU CD1	168VAL	CG1	0.09	35ALA C	36TYR	CD2	0.49	105PR C	106ALA	C	49.09
103LEU CD1	260ALA	CB	0.04	35ALA C	36TYR	C	2.48	105PR C	257LEU	CD1	0.02
103LEU CD1	260ALA	C	0.00	36TYR CA	36TYR	CB	100.00	105PR C	257LEU	CD2	0.07
103LEU CD1	262LEU	CB	0.00	36TYR CA	36TYR	CG	100.00	106AL CA	106ALA	CB	100.00
103LEU CD1	262LEU	CG	0.40	36TYR CA	36TYR	CD1	70.44	106AL CA	106ALA	C	100.00
103LEU CD1	262LEU	CD1	4.18	36TYR CA	36TYR	CD2	70.13	106AL CA	257LEU	CD1	0.05
103LEU CD1	262LEU	CD2	0.31	36TYR CA	36TYR	C	100.00	106AL CA	257LEU	CD2	0.38
103LEU CD1	296MET	CE	0.67	36TYR CB	36TYR	CG	100.00	106AL CB	106ALA	C	100.00
103LEU CD1	300HIS	CD2	0.06	36TYR CB	36TYR	CD1	100.00	106AL CB	126VAL	CG1	0.00
103LEU CD1	300HIS	CE1	0.91	36TYR CB	36TYR	CD2	100.00	106AL CB	128VAL	CB	0.00
103LEU CD2	103LEU	C	0.20	36TYR CB	36TYR	C	100.00	106AL CB	128VAL	CG1	0.06
103LEU CD2	104ARG	C	0.00	36TYR CG	36TYR	CD1	100.00	106AL CB	128VAL	CG2	0.00
103LEU CD2	129LEU	CD1	0.01	36TYR CG	36TYR	CD2	100.00	106AL CB	130ILE	CD	0.48
103LEU CD2	129LEU	CD2	0.11	36TYR CG	36TYR	CE1	100.00	106AL CB	247ALA	CB	1.96
103LEU CD2	131VAL	CG1	0.11	36TYR CG	36TYR	CE2	100.00	106AL CB	247ALA	C	0.01
103LEU CD2	131VAL	CG2	2.65	36TYR CG	36TYR	CZ	100.00	106AL CB	250LEU	CD1	0.05
103LEU CD2	168VAL	CG1	0.06	36TYR CG	36TYR	C	99.12	106AL CB	250LEU	CD2	0.00
103LEU CD2	260ALA	CB	0.01	36TYR CD1	36TYR	CD2	100.00	106AL CB	257LEU	CD1	0.04
103LEU CD2	260ALA	C	0.01	36TYR CD1	36TYR	CE1	100.00	106AL CB	257LEU	CD2	0.64
103LEU CD2	262LEU	CG	0.02	36TYR CD1	36TYR	CE2	100.00	106AL C	107LYS	CA	100.00
103LEU CD2	262LEU	CD1	0.53	36TYR CD1	36TYR	CZ	100.00	106AL C	107LYS	CB	69.69
103LEU CD2	262LEU	CD2	0.04	36TYR CD1	36TYR	C	39.20	106AL C	107LYS	CG	0.07
103LEU CD2	296MET	CE	0.12	36TYR CD1	38VAL	CG2	0.12	106AL C	107LYS	C	35.99
103LEU CD2	300HIS	CD2	0.04	36TYR CD2	36TYR	CE1	100.00	106AL C	126VAL	CG1	0.02
103LEU CD2	300HIS	CE1	1.27	36TYR CD2	36TYR	CE2	100.00	106AL C	251PRO	CG	0.00
103LEU C	104ARG	CA	100.00	36TYR CD2	36TYR	CZ	100.00	107LY CA	107LYS	CB	100.00
103LEU C	104ARG	CB	63.57	36TYR CD2	36TYR	C	17.85	107LY CA	107LYS	CG	100.00
103LEU C	104ARG	CG	2.36	36TYR CD2	38VAL	CG2	0.02	107LY CA	107LYS	CD	0.05
103LEU C	104ARG	C	45.97	36TYR CE1	36TYR	CE2	100.00	107LY CA	107LYS	C	100.00
103LEU C	105PRO	CD	1.31	36TYR CE1	36TYR	CZ	100.00	107LY CA	126VAL	CG1	0.00
103LEU C	129LEU	CD1	0.00	36TYR CE1	38VAL	CG2	0.53	107LY CB	107LYS	CG	100.00
104ARG CA	104ARG	CB	100.00	36TYR CE2	36TYR	CZ	100.00	107LY CB	107LYS	CD	100.00
104ARG CA	104ARG	CG	100.00	36TYR CE2	38VAL	CG2	0.08	107LY CB	107LYS	CE	8.16
104ARG CA	104ARG	CD	25.55	36TYR CZ	38VAL	CG2	0.00	107LY CB	107LYS	C	100.00
104ARG CA	104ARG	C	100.00	36TYR C	37GLU	CA	100.00	107LY CB	109PHE	CD1	0.00
104ARG CA	105PRO	CD	100.00	36TYR C	37GLU	CB	88.27	107LY CB	109PHE	CE1	0.18
104ARG CB	104ARG	CG	100.00	36TYR C	37GLU	CG	0.04	107LY CB	109PHE	CE2	0.00
104ARG CB	104ARG	CD	100.00	36TYR C	37GLU	CD	0.01	107LY CB	109PHE	CZ	0.02
104ARG CB	104ARG	C	100.00	36TYR C	37GLU	C	21.14	107LY CG	107LYS	CD	100.00
104ARG CB	105PRO	CD	0.32	37GLU CA	37GLU	CB	100.00	107LY CG	107LYS	CE	100.00

104ARG CB	244SER CB	0.00	37GLU CA	37GLU CG	100.00	107LY CG	107LYS C	99.39
104ARG CB	247ALA CB	0.00	37GLU CA	37GLU CD	92.35	107LY CG	108VAL C	0.02
104ARG CG	104ARG CD	100.00	37GLU CA	37GLU C	100.00	107LY CG	109PHE CD1	0.13
104ARG CG	104ARG CZ	5.05	37GLU CB	37GLU CG	100.00	107LY CG	109PHE CD2	0.00
104ARG CG	104ARG C	59.49	37GLU CB	37GLU CD	100.00	107LY CG	109PHE CE1	0.66
104ARG CG	105PRO CD	1.46	37GLU CB	37GLU C	100.00	107LY CG	109PHE CE2	0.02
104ARG CG	244SER CB	0.00	37GLU CB	39PHE CE1	2.66	107LY CG	109PHE CZ	0.02
104ARG CG	257LEU CD1	0.02	37GLU CB	39PHE CE2	0.00	107LY CG	127ASP CB	0.01
104ARG CG	257LEU CD2	0.06	37GLU CB	39PHE CZ	2.24	107LY CG	127ASP CG	0.01
104ARG CG	259SER CB	0.07	37GLU CG	37GLU CD	100.00	107LY CG	257LEU CD2	0.00
104ARG CD	104ARG CZ	100.00	37GLU CG	37GLU C	98.38	107LY CD	107LYS CE	100.00
104ARG CD	244SER CB	0.10	37GLU CG	39PHE CD1	0.02	107LY CD	107LYS C	0.05
104ARG CD	257LEU CD1	0.04	37GLU CG	39PHE CD2	0.02	107LY CD	109PHE CD1	0.06
104ARG CD	257LEU CD2	1.65	37GLU CG	39PHE CE1	5.44	107LY CD	109PHE CD2	0.01
104ARG CD	259SER CB	0.17	37GLU CG	39PHE CE2	5.13	107LY CD	109PHE CE1	1.27
104ARG CD	272VAL CG2	0.02	37GLU CG	39PHE CZ	23.64	107LY CD	109PHE CE2	0.19
104ARG CZ	244SER CB	18.28	37GLU CD	37GLU C	0.04	107LY CD	109PHE CZ	0.07
104ARG CZ	244SER C	0.00	37GLU CD	39PHE CE2	0.20	107LY CD	127ASP CB	0.01
104ARG CZ	257LEU CD1	0.00	37GLU CD	39PHE CZ	0.64	107LY CD	127ASP CG	0.80
104ARG CZ	259SER CB	0.80	37GLU C	38VAL CA	100.00	107LY CD	312GLU CD	0.05
104ARG CZ	272VAL CG1	6.79	37GLU C	38VAL CB	4.77	107LY CE	109PHE CD1	0.07
104ARG CZ	272VAL CG2	0.80	37GLU C	38VAL CG2	2.86	107LY CE	109PHE CE1	0.24
104ARG C	105PRO CA	100.00	37GLU C	38VAL C	98.20	107LY CE	109PHE CE2	0.06
104ARG C	105PRO CB	0.26	37GLU C	39PHE CD1	0.02	107LY CE	109PHE CZ	0.00
104ARG C	105PRO CD	100.00	37GLU C	39PHE CE1	0.21	107LY CE	127ASP CG	0.66
104ARG C	105PRO C	100.00	38VAL CA	38VAL CB	100.00	107LY CE	309ARG CZ	0.02
104ARG C	129LEU CD1	0.27	38VAL CA	38VAL CG1	100.00	107LY CE	312GLU CD	0.25
105PRO CA	105PRO CB	100.00	38VAL CA	38VAL CG2	100.00	107LY C	108VAL CA	100.00
105PRO CA	105PRO CG	100.00	38VAL CA	38VAL C	100.00	107LY C	108VAL CB	30.08
105PRO CA	105PRO CD	100.00	38VAL CB	38VAL CG1	100.00	107LY C	108VAL CG1	0.00
105PRO CA	105PRO C	100.00	38VAL CB	38VAL CG2	100.00	107LY C	108VAL CG2	23.71
105PRO CA	129LEU CD1	0.02	38VAL CB	38VAL C	100.00	107LY C	108VAL C	84.46
105PRO CA	129LEU CD2	0.01	38VAL CG1	38VAL CG2	100.00	107LY C	126VAL CG2	0.04
105PRO CB	105PRO CG	100.00	38VAL CG1	38VAL C	99.94	108VA CA	108VAL CB	100.00
105PRO CB	105PRO CD	100.00	38VAL CG2	38VAL C	0.05	108VA CA	108VAL CG1	100.00
105PRO CB	105PRO C	100.00	38VAL C	39PHE CA	100.00	108VA CA	108VAL CG2	100.00
105PRO CB	107LYS CD	0.01	38VAL C	39PHE CB	36.51	108VA CA	108VAL C	100.00
105PRO CB	129LEU CD1	0.02	38VAL C	39PHE CG	10.93	108VA CA	112LEU CD2	0.00
105PRO CB	129LEU CD2	0.01	38VAL C	39PHE CD1	2.81	108VA CB	108VAL CG1	100.00
105PRO CG	105PRO CD	100.00	38VAL C	39PHE CD2	0.10	108VA CB	108VAL CG2	100.00
105PRO CG	105PRO C	99.12	38VAL C	39PHE C	64.92	108VA CB	108VAL C	100.00
105PRO CG	129LEU CD1	0.02	39PHE CA	39PHE CB	100.00	108VA CB	126VAL CG2	0.41
105PRO CG	258PRO CG	4.25	39PHE CA	39PHE CG	100.00	108VA CG1	108VAL CG2	100.00
105PRO CG	258PRO CD	0.41	39PHE CA	39PHE CD1	49.36	108VA CG1	108VAL C	100.00
105PRO CG	258PRO C	0.00	39PHE CA	39PHE CD2	97.44	108VA CG1	112LEU CB	0.88
105PRO CG	296MET CE	0.16	39PHE CA	39PHE C	100.00	108VA CG1	112LEU C	0.44
105PRO CD	105PRO C	97.89	39PHE CA	40PRO CD	100.00	108VA CG1	113GLU CA	0.15
105PRO CD	129LEU CD1	0.07	39PHE CB	39PHE CG	100.00	108VA CG1	113GLU CB	0.03
105PRO CD	258PRO CG	0.05	39PHE CB	39PHE CD1	100.00	108VA CG1	113GLU CG	1.06
105PRO CD	296MET CE	0.55	39PHE CB	39PHE CD2	100.00	108VA CG1	113GLU CD	0.06
105PRO C	106ALA CA	100.00	39PHE CB	39PHE C	100.00	108VA CG1	123ALA C	0.00
105PRO C	106ALA CB	77.30	39PHE CB	40PRO CD	18.89	108VA CG1	126VAL CG2	1.01
105PRO C	106ALA C	31.98	39PHE CB	57THR CA	0.07	108VA CG2	108VAL C	10.09
105PRO C	130ILE CD	0.03	39PHE CB	57THR CG2	0.51	108VA CG2	113GLU CA	0.15
106ALA CA	106ALA CB	100.00	39PHE CG	39PHE CD1	100.00	108VA CG2	113GLU CB	0.01
106ALA CA	106ALA C	100.00	39PHE CG	39PHE CD2	100.00	108VA CG2	113GLU CG	0.59
106ALA CA	251PRO CG	0.02	39PHE CG	39PHE CE1	100.00	108VA CG2	123ALA CB	0.01
106ALA CA	257LEU CD1	0.00	39PHE CG	39PHE CE2	100.00	108VA CG2	126VAL CB	1.05
106ALA CA	257LEU CD2	0.00	39PHE CG	39PHE CZ	100.00	108VA CG2	126VAL CG1	0.78
106ALA CB	106ALA C	100.00	39PHE CG	60GLY CA	0.01	108VA CG2	126VAL CG2	0.47
106ALA CB	126VAL CG1	0.00	39PHE CD1	39PHE CD2	100.00	108VA CG2	250LEU CB	0.61
106ALA CB	128VAL CB	0.00	39PHE CD1	39PHE CE1	100.00	108VA CG2	250LEU CD2	0.01

106ALA	CB	128VAL	CG1	0.00
106ALA	CB	128VAL	CG2	0.02
106ALA	CB	130ILE	CG1	0.01
106ALA	CB	130ILE	CD	1.30
106ALA	CB	247ALA	CA	0.06
106ALA	CB	247ALA	CB	2.84
106ALA	CB	247ALA	C	0.12
106ALA	CB	250LEU	CD1	0.35
106ALA	CB	251PRO	CG	0.04
106ALA	CB	251PRO	CD	0.00
106ALA	CB	257LEU	CD1	0.00
106ALA	CB	257LEU	CD2	0.03
106ALA	C	107LYS	CA	100.00
106ALA	C	107LYS	CB	82.93
106ALA	C	107LYS	CG	0.56
106ALA	C	107LYS	C	21.48
106ALA	C	126VAL	CG1	0.02
106ALA	C	128VAL	CG2	0.00
106ALA	C	250LEU	CD1	0.36
106ALA	C	250LEU	CD2	0.00
106ALA	C	251PRO	CG	0.02
107LYS	CA	107LYS	CB	100.00
107LYS	CA	107LYS	CG	100.00
107LYS	CA	107LYS	CD	0.03
107LYS	CA	107LYS	C	100.00
107LYS	CB	107LYS	CG	100.00
107LYS	CB	107LYS	CD	100.00
107LYS	CB	107LYS	CE	14.20
107LYS	CB	107LYS	C	100.00
107LYS	CB	109PHE	CE2	0.01
107LYS	CB	109PHE	CZ	0.01
107LYS	CB	127ASP	CA	0.03
107LYS	CB	127ASP	CB	0.02
107LYS	CB	127ASP	CG	0.01
107LYS	CB	257LEU	CD2	0.06
107LYS	CG	107LYS	CD	100.00
107LYS	CG	107LYS	CE	100.00
107LYS	CG	107LYS	C	95.92
107LYS	CG	109PHE	CZ	0.00
107LYS	CG	127ASP	CB	0.02
107LYS	CG	127ASP	CG	0.03
107LYS	CG	257LEU	CD2	0.12
107LYS	CD	107LYS	CE	100.00
107LYS	CD	107LYS	C	0.02
107LYS	CD	109PHE	CE1	0.05
107LYS	CD	109PHE	CZ	0.02
107LYS	CD	127ASP	CG	0.93
107LYS	CD	312GLU	CD	0.06
107LYS	CE	109PHE	CE1	0.01
107LYS	CE	127ASP	CG	0.41
107LYS	CE	312GLU	CD	1.23
107LYS	C	108VAL	CA	100.00
107LYS	C	108VAL	CB	0.30
107LYS	C	108VAL	CG1	0.10
107LYS	C	108VAL	CG2	0.00
107LYS	C	108VAL	C	99.83
107LYS	C	250LEU	CB	0.08
107LYS	C	251PRO	CB	3.92
107LYS	C	251PRO	CG	0.00
108VAL	CA	108VAL	CB	100.00
108VAL	CA	108VAL	CG1	100.00
108VAL	CA	108VAL	CG2	100.00

39PHE	CD1	39PHE	CE2	100.00
39PHE	CD1	39PHE	CZ	100.00
39PHE	CD1	60GLY	CA	0.06
39PHE	CD1	60GLY	C	0.01
39PHE	CD1	61VAL	CG2	0.00
39PHE	CD2	39PHE	CE1	100.00
39PHE	CD2	39PHE	CE2	100.00
39PHE	CD2	39PHE	CZ	100.00
39PHE	CD2	56PRO	C	0.93
39PHE	CD2	57THR	CA	0.29
39PHE	CD2	57THR	CB	0.00
39PHE	CD2	57THR	CG2	0.00
39PHE	CD2	60GLY	CA	0.08
39PHE	CE1	39PHE	CE2	100.00
39PHE	CE1	39PHE	CZ	100.00
39PHE	CE1	60GLY	CA	7.05
39PHE	CE1	60GLY	C	2.22
39PHE	CE2	39PHE	CZ	100.00
39PHE	CE2	56PRO	C	0.16
39PHE	CE2	60GLY	CA	11.45
39PHE	CZ	60GLY	CA	36.29
39PHE	CZ	60GLY	C	0.66
39PHE	C	40PRO	CA	100.00
39PHE	C	40PRO	CB	0.05
39PHE	C	40PRO	CD	100.00
39PHE	C	40PRO	C	100.00
40PRO	CA	40PRO	CB	100.00
40PRO	CA	40PRO	CG	100.00
40PRO	CA	40PRO	CD	100.00
40PRO	CA	40PRO	C	100.00
40PRO	CA	45ALA	CB	0.02
40PRO	CB	40PRO	CG	100.00
40PRO	CB	40PRO	CD	100.00
40PRO	CB	40PRO	C	100.00
40PRO	CB	44ALA	CB	0.13
40PRO	CB	44ALA	C	0.01
40PRO	CB	45ALA	CB	0.17
40PRO	CB	45ALA	C	0.01
40PRO	CB	46ILE	CG1	0.01
40PRO	CB	46ILE	CG2	0.06
40PRO	CB	46ILE	CD	0.10
40PRO	CB	49PHE	CD1	0.14
40PRO	CB	49PHE	CD2	0.03
40PRO	CB	49PHE	CE1	1.32
40PRO	CB	49PHE	CE2	0.48
40PRO	CB	49PHE	CZ	0.16
40PRO	CB	54PRO	CG	0.02
40PRO	CG	40PRO	CD	100.00
40PRO	CG	40PRO	C	87.43
40PRO	CG	49PHE	CD1	0.12
40PRO	CG	49PHE	CD2	0.04
40PRO	CG	49PHE	CE1	6.60
40PRO	CG	49PHE	CE2	2.12
40PRO	CG	49PHE	CZ	2.70
40PRO	CG	54PRO	CG	0.04
40PRO	CG	56PRO	CG	0.37
40PRO	CD	40PRO	C	75.28
40PRO	CD	56PRO	CG	0.01
40PRO	C	41PHE	CA	100.00
40PRO	C	41PHE	CB	99.85
40PRO	C	41PHE	C	0.73
40PRO	C	45ALA	CB	0.05

108VA	CG2	250LEU	C	24.21
108VA	CG2	251PRO	CA	0.23
108VA	CG2	251PRO	CB	0.00
108VA	CG2	251PRO	CD	0.11
108VA	C	109PHE	CA	100.00
108VA	C	109PHE	CB	3.11
108VA	C	109PHE	CG	0.27
108VA	C	109PHE	CD1	0.50
108VA	C	109PHE	CD2	0.07
108VA	C	109PHE	C	97.32
109PH	CA	109PHE	CB	100.00
109PH	CA	109PHE	CG	100.00
109PH	CA	109PHE	CD1	79.38
109PH	CA	109PHE	CD2	35.91
109PH	CA	109PHE	C	100.00
109PH	CA	110PRO	CD	100.00
109PH	CB	109PHE	CG	100.00
109PH	CB	109PHE	CD1	100.00
109PH	CB	109PHE	CD2	100.00
109PH	CB	109PHE	C	100.00
109PH	CB	110PRO	CD	26.40
109PH	CB	112LEU	CB	0.01
109PH	CB	112LEU	CG	0.02
109PH	CB	112LEU	CD1	1.30
109PH	CB	112LEU	CD2	0.18
109PH	CB	312GLU	CD1	0.00
109PH	CB	316ALA	CB	4.36
109PH	CG	109PHE	CD1	100.00
109PH	CG	109PHE	CD2	100.00
109PH	CG	109PHE	CE1	100.00
109PH	CG	109PHE	CE2	100.00
109PH	CG	109PHE	CZ	100.00
109PH	CG	109PHE	C	3.26
109PH	CG	112LEU	CD1	0.01
109PH	CG	312GLU	CG	0.03
109PH	CG	316ALA	CB	1.46
109PH	CG	319LEU	CD1	0.18
109PH	CG	319LEU	CD2	0.48
109PH	CD1	109PHE	CD2	100.00
109PH	CD1	109PHE	CE1	100.00
109PH	CD1	109PHE	CE2	100.00
109PH	CD1	109PHE	CZ	100.00
109PH	CD1	112LEU	CD1	0.08
109PH	CD1	112LEU	CD2	0.01
109PH	CD1	312GLU	CB	0.27
109PH	CD1	312GLU	CG	2.23
109PH	CD1	312GLU	CD	0.30
109PH	CD1	316ALA	CA	0.05
109PH	CD1	316ALA	CB	2.33
109PH	CD1	319LEU	CD1	0.44
109PH	CD1	319LEU	CD2	0.10
109PH	CD2	109PHE	CE1	100.00
109PH	CD2	109PHE	CE2	100.00
109PH	CD2	109PHE	CZ	100.00
109PH	CD2	109PHE	C	0.64
109PH	CD2	112LEU	CG	0.01
109PH	CD2	112LEU	CD1	1.45
109PH	CD2	112LEU	CD2	3.40
109PH	CD2	251PRO	CB	0.02
109PH	CD2	312GLU	CG	0.07
109PH	CD2	312GLU	C	0.01
109PH	CD2	316ALA	CA	0.04

108VAL	CA	108VAL	C	100.00
108VAL	CA	251PRO	CB	0.01
108VAL	CB	108VAL	CG1	100.00
108VAL	CB	108VAL	CG2	100.00
108VAL	CB	108VAL	C	100.00
108VAL	CB	113GLU	CG	0.04
108VAL	CG1	108VAL	CG2	100.00
108VAL	CG1	108VAL	C	0.23
108VAL	CG1	113GLU	CA	0.01
108VAL	CG1	113GLU	CB	0.00
108VAL	CG1	113GLU	CG	0.02
108VAL	CG1	113GLU	CD	0.00
108VAL	CG1	123ALA	CA	0.00
108VAL	CG1	123ALA	CB	0.25
108VAL	CG1	123ALA	C	0.07
108VAL	CG1	126VAL	CB	0.03
108VAL	CG1	126VAL	CG1	0.01
108VAL	CG1	126VAL	CG2	0.03
108VAL	CG1	250LEU	CB	0.00
108VAL	CG1	250LEU	CD2	0.33
108VAL	CG1	250LEU	C	0.02
108VAL	CG1	251PRO	CA	0.98
108VAL	CG1	251PRO	CB	4.28
108VAL	CG2	108VAL	C	99.71
108VAL	CG2	113GLU	CG	2.44
108VAL	CG2	251PRO	CA	0.01
108VAL	CG2	251PRO	CB	0.04
108VAL	C	109PHE	CA	100.00
108VAL	C	109PHE	CB	1.42
108VAL	C	109PHE	CG	0.43
108VAL	C	109PHE	CD2	0.31
108VAL	C	109PHE	C	99.04
109PHE	CA	109PHE	CB	100.00
109PHE	CA	109PHE	CG	100.00
109PHE	CA	109PHE	CD1	91.51
109PHE	CA	109PHE	CD2	64.91
109PHE	CA	109PHE	C	100.00
109PHE	CA	110PRO	CD	100.00
109PHE	CB	109PHE	CG	100.00
109PHE	CB	109PHE	CD1	100.00
109PHE	CB	109PHE	CD2	100.00
109PHE	CB	109PHE	C	100.00
109PHE	CB	110PRO	CD	2.29
109PHE	CB	112LEU	CB	0.25
109PHE	CB	112LEU	CG	0.00
109PHE	CB	112LEU	CD1	1.27
109PHE	CB	112LEU	CD2	0.03
109PHE	CB	316ALA	CB	0.01
109PHE	CB	319LEU	CD1	0.03
109PHE	CG	109PHE	CD1	100.00
109PHE	CG	109PHE	CD2	100.00
109PHE	CG	109PHE	CE1	100.00
109PHE	CG	109PHE	CE2	100.00
109PHE	CG	109PHE	CZ	100.00
109PHE	CG	112LEU	CD1	0.00
109PHE	CG	316ALA	CB	0.01
109PHE	CG	319LEU	CD1	1.42
109PHE	CG	319LEU	CD2	0.08
109PHE	CD1	109PHE	CD2	100.00
109PHE	CD1	109PHE	CE1	100.00
109PHE	CD1	109PHE	CE2	100.00
109PHE	CD1	109PHE	CZ	100.00
40PRO	C	46ILE	CG1	0.02
40PRO	C	46ILE	CD	0.40
41PHE	CA	41PHE	CB	100.00
41PHE	CA	41PHE	CG	100.00
41PHE	CA	41PHE	CD1	94.00
41PHE	CA	41PHE	CD2	52.86
41PHE	CA	41PHE	C	100.00
41PHE	CA	45ALA	CB	0.05
41PHE	CA	46ILE	CG1	0.14
41PHE	CA	46ILE	CG2	0.01
41PHE	CA	46ILE	CD	0.17
41PHE	CB	41PHE	CG	100.00
41PHE	CB	41PHE	CD1	100.00
41PHE	CB	41PHE	CD2	100.00
41PHE	CB	41PHE	C	100.00
41PHE	CG	41PHE	CD1	100.00
41PHE	CG	41PHE	CD2	100.00
41PHE	CG	41PHE	CE1	100.00
41PHE	CG	41PHE	CE2	100.00
41PHE	CG	41PHE	CZ	100.00
41PHE	CG	41PHE	C	99.70
41PHE	CD1	41PHE	CD2	100.00
41PHE	CD1	41PHE	CE1	100.00
41PHE	CD1	41PHE	CE2	100.00
41PHE	CD1	41PHE	CZ	100.00
41PHE	CD1	41PHE	C	0.41
41PHE	CD1	53PHE	CD1	0.30
41PHE	CD1	53PHE	CE1	0.14
41PHE	CD1	57THR	CG2	0.01
41PHE	CD1	93LEU	CD1	0.01
41PHE	CD2	41PHE	CE1	100.00
41PHE	CD2	41PHE	CE2	100.00
41PHE	CD2	41PHE	CZ	100.00
41PHE	CD2	41PHE	C	39.32
41PHE	CD2	42GLY	CA	0.01
41PHE	CD2	69LEU	CD1	1.07
41PHE	CD2	69LEU	CD2	0.16
41PHE	CD2	72VAL	CG1	1.58
41PHE	CD2	72VAL	CG2	0.04
41PHE	CE1	41PHE	CE2	100.00
41PHE	CE1	41PHE	CZ	100.00
41PHE	CE1	43GLY	CA	0.04
41PHE	CE1	52PRO	C	0.00
41PHE	CE1	53PHE	CB	0.02
41PHE	CE1	53PHE	CG	0.02
41PHE	CE1	53PHE	CD1	0.94
41PHE	CE1	53PHE	CE1	0.20
41PHE	CE1	72VAL	CG1	0.00
41PHE	CE1	86PRO	CB	0.06
41PHE	CE1	89GLY	CA	0.03
41PHE	CE1	89GLY	C	0.24
41PHE	CE1	90LEU	CG	0.00
41PHE	CE1	90LEU	CD1	0.02
41PHE	CE1	90LEU	CD2	0.12
41PHE	CE1	93LEU	CG	0.00
41PHE	CE1	93LEU	CD1	0.55
41PHE	CE1	93LEU	CD2	0.08
41PHE	CE2	41PHE	CZ	100.00
41PHE	CE2	42GLY	C	0.00
41PHE	CE2	43GLY	CA	0.24
41PHE	CE2	69LEU	CG	0.00
41PHE	CE2	69LEU	CD1	7.13
109PH	CD2	316ALA	CB	3.06
109PH	CD2	319LEU	CD1	1.22
109PH	CD2	319LEU	CD2	1.55
109PH	CE1	109PHE	CE2	100.00
109PH	CE1	109PHE	CZ	100.00
109PH	CE1	112LEU	CD1	0.17
109PH	CE1	112LEU	CD2	0.18
109PH	CE1	256LEU	CD2	0.00
109PH	CE1	288THR	CG2	0.01
109PH	CE1	292LEU	CD1	0.23
109PH	CE1	292LEU	CD2	0.18
109PH	CE1	312GLU	CB	0.88
109PH	CE1	312GLU	CG	6.30
109PH	CE1	312GLU	CD	1.36
109PH	CE1	315VAL	CG1	0.57
109PH	CE1	316ALA	CA	0.01
109PH	CE1	316ALA	CB	0.11
109PH	CE1	319LEU	CD1	0.49
109PH	CE1	319LEU	CD2	0.02
109PH	CE2	109PHE	CZ	100.00
109PH	CE2	112LEU	CG	0.02
109PH	CE2	112LEU	CD1	0.73
109PH	CE2	112LEU	CD2	0.58
109PH	CE2	251PRO	CB	0.14
109PH	CE2	256LEU	CB	0.08
109PH	CE2	256LEU	CD1	0.06
109PH	CE2	256LEU	CD2	0.01
109PH	CE2	257LEU	CD2	0.02
109PH	CE2	288THR	CG2	0.01
109PH	CE2	292LEU	CD1	1.69
109PH	CE2	292LEU	CD2	0.82
109PH	CE2	312GLU	CB	0.24
109PH	CE2	312GLU	CG	1.85
109PH	CE2	312GLU	CD	0.16
109PH	CE2	312GLU	C	0.01
109PH	CE2	315VAL	CG1	0.08
109PH	CE2	316ALA	CA	0.00
109PH	CE2	316ALA	CB	0.09
109PH	CE2	319LEU	CD1	2.51
109PH	CE2	319LEU	CD2	0.58
109PH	CZ	112LEU	CD1	0.20
109PH	CZ	112LEU	CD2	0.21
109PH	CZ	256LEU	CB	0.01
109PH	CZ	257LEU	CD2	0.04
109PH	CZ	288THR	CG2	0.07
109PH	CZ	292LEU	CD1	2.33
109PH	CZ	292LEU	CD2	6.52
109PH	CZ	312GLU	CB	0.08
109PH	CZ	312GLU	CG	0.73
109PH	CZ	312GLU	CD	0.01
109PH	CZ	312GLU	C	0.01
109PH	CZ	315VAL	CG1	0.75
109PH	CZ	319LEU	CD1	0.37
109PH	CZ	319LEU	CD2	0.04
109PH	C	110PRO	CA	100.00
109PH	C	110PRO	CB	0.02
109PH	C	110PRO	CD	100.00
109PH	C	110PRO	C	100.00
110PR	CA	110PRO	CB	100.00
110PR	CA	110PRO	CG	100.00
110PR	CA	110PRO	CD	100.00
110PR	CA	110PRO	C	100.00

109PHE	CD1	315VAL	CG2	0.01
109PHE	CD1	316ALA	CA	0.01
109PHE	CD1	316ALA	CB	0.33
109PHE	CD1	319LEU	CD1	7.26
109PHE	CD1	319LEU	CD2	0.09
109PHE	CD2	109PHE	CE1	100.00
109PHE	CD2	109PHE	CE2	100.00
109PHE	CD2	109PHE	CZ	100.00
109PHE	CD2	112LEU	CD1	0.03
109PHE	CD2	112LEU	CD2	0.01
109PHE	CD2	115LEU	CD1	0.18
109PHE	CD2	251PRO	CB	0.06
109PHE	CD2	253SER	CB	0.08
109PHE	CD2	256LEU	CD1	0.14
109PHE	CD2	256LEU	CD2	0.00
109PHE	CD2	319LEU	CD1	2.51
109PHE	CD2	319LEU	CD2	0.36
109PHE	CE1	109PHE	CE2	100.00
109PHE	CE1	109PHE	CZ	100.00
109PHE	CE1	251PRO	CB	0.00
109PHE	CE1	288THR	CG2	0.62
109PHE	CE1	292LEU	CD1	0.52
109PHE	CE1	312GLU	CG	0.00
109PHE	CE1	315VAL	CG2	1.34
109PHE	CE1	319LEU	CD1	9.17
109PHE	CE1	319LEU	CD2	0.07
109PHE	CE2	109PHE	CZ	100.00
109PHE	CE2	115LEU	CD1	0.01
109PHE	CE2	251PRO	CA	0.05
109PHE	CE2	251PRO	CB	4.37
109PHE	CE2	253SER	CB	0.13
109PHE	CE2	256LEU	CB	1.54
109PHE	CE2	256LEU	CG	0.02
109PHE	CE2	256LEU	CD1	2.55
109PHE	CE2	256LEU	CD2	0.72
109PHE	CE2	257LEU	CD2	0.02
109PHE	CE2	319LEU	CD1	5.25
109PHE	CE2	319LEU	CD2	0.42
109PHE	CZ	251PRO	CB	0.10
109PHE	CZ	256LEU	CB	1.46
109PHE	CZ	256LEU	CG	0.01
109PHE	CZ	256LEU	CD1	0.10
109PHE	CZ	256LEU	CD2	0.26
109PHE	CZ	257LEU	CD2	0.01
109PHE	CZ	288THR	CG2	1.91
109PHE	CZ	292LEU	CD1	2.26
109PHE	CZ	315VAL	CG2	0.02
109PHE	CZ	319LEU	CD1	6.64
109PHE	CZ	319LEU	CD2	0.10
109PHE	C	110PRO	CA	100.00
109PHE	C	110PRO	CB	0.01
109PHE	C	110PRO	CD	100.00
109PHE	C	110PRO	C	100.00
110PRO	CA	110PRO	CB	100.00
110PRO	CA	110PRO	CG	100.00
110PRO	CA	110PRO	CD	100.00
110PRO	CA	110PRO	C	100.00
110PRO	CA	113GLU	CD	0.01
110PRO	CB	110PRO	CG	100.00
110PRO	CB	110PRO	CD	100.00
110PRO	CB	110PRO	C	100.00
110PRO	CG	110PRO	CD	100.00

41PHE	CE2	69LEU	CD2	4.70
41PHE	CE2	72VAL	CB	0.00
41PHE	CE2	72VAL	CG1	8.29
41PHE	CE2	72VAL	CG2	1.66
41PHE	CE2	77TRP	CZ3	0.00
41PHE	CE2	90LEU	CD1	0.03
41PHE	CE2	90LEU	CD2	0.98
41PHE	CE2	93LEU	CD1	0.02
41PHE	CZ	43GLY	CA	0.37
41PHE	CZ	69LEU	CD1	0.32
41PHE	CZ	69LEU	CD2	0.40
41PHE	CZ	72VAL	CB	0.00
41PHE	CZ	72VAL	CG1	0.48
41PHE	CZ	72VAL	CG2	0.34
41PHE	CZ	86PRO	CB	0.08
41PHE	CZ	89GLY	C	0.68
41PHE	CZ	90LEU	CG	0.06
41PHE	CZ	90LEU	CD1	0.05
41PHE	CZ	90LEU	CD2	4.72
41PHE	CZ	93LEU	CG	0.00
41PHE	CZ	93LEU	CD1	0.60
41PHE	CZ	93LEU	CD2	0.17
41PHE	C	42GLY	CA	100.00
41PHE	C	42GLY	C	23.00
41PHE	C	45ALA	CB	0.26
41PHE	C	46ILE	CG1	0.02
41PHE	C	46ILE	CG2	0.09
41PHE	C	46ILE	CD	0.03
42GLY	CA	42GLY	C	100.00
42GLY	CA	46ILE	CG1	0.01
42GLY	CA	46ILE	CG2	0.73
42GLY	CA	46ILE	CD	0.00
42GLY	CA	72VAL	CG1	0.47
42GLY	CA	72VAL	CG2	0.01
42GLY	CA	77TRP	CE3	0.00
42GLY	CA	77TRP	CZ2	0.01
42GLY	CA	77TRP	CZ3	0.28
42GLY	C	43GLY	CA	100.00
42GLY	C	43GLY	C	64.15
42GLY	C	46ILE	CG1	0.02
42GLY	C	46ILE	CG2	0.01
42GLY	C	46ILE	CD	0.02
42GLY	C	72VAL	CG1	0.36
42GLY	C	72VAL	CG2	0.06
42GLY	C	77TRP	CE3	0.02
42GLY	C	77TRP	CZ2	0.29
42GLY	C	77TRP	CZ3	0.54
42GLY	C	86PRO	CB	0.05
42GLY	C	86PRO	CG	0.01
43GLY	CA	43GLY	C	100.00
43GLY	CA	46ILE	CG1	0.02
43GLY	CA	46ILE	CD	0.10
43GLY	CA	72VAL	CB	0.02
43GLY	CA	72VAL	CG1	0.89
43GLY	CA	72VAL	CG2	0.80
43GLY	CA	77TRP	CG	0.01
43GLY	CA	77TRP	CD1	0.02
43GLY	CA	77TRP	CD2	0.07
43GLY	CA	77TRP	CE2	0.24
43GLY	CA	77TRP	CE3	0.03
43GLY	CA	77TRP	CZ2	0.73
43GLY	CA	77TRP	CZ3	0.10

110PR	CA	113GLU	CD	0.02
110PR	CB	110PRO	CG	100.00
110PR	CB	110PRO	CD	100.00
110PR	CB	110PRO	C	100.00
110PR	CB	124ARG	CZ	0.04
110PR	CG	110PRO	CD	100.00
110PR	CG	110PRO	C	84.75
110PR	CG	316ALA	CB	0.00
110PR	CD	110PRO	C	78.76
110PR	CD	316ALA	CB	0.07
110PR	C	111GLY	CA	100.00
110PR	C	111GLY	C	45.78
110PR	C	112LEU	CD1	0.01
110PR	C	316ALA	CB	0.04
111GL	CA	111GLY	C	100.00
111GL	CA	113GLU	CD	0.00
111GL	CA	114ARG	CZ	0.02
111GL	CA	316ALA	CB	0.01
111GL	CA	320LEU	CG	0.05
111GL	CA	320LEU	CD1	0.29
111GL	CA	320LEU	CD2	0.18
111GL	C	112LEU	CA	100.00
111GL	C	112LEU	CB	44.04
111GL	C	112LEU	CG	10.23
111GL	C	112LEU	CD1	3.32
111GL	C	112LEU	CD2	0.34
111GL	C	112LEU	C	51.82
111GL	C	113GLU	CD	0.01
111GL	C	316ALA	CB	0.01
111GL	C	320LEU	CD1	0.15
111GL	C	320LEU	CD2	0.12
112LE	CA	112LEU	CB	100.00
112LE	CA	112LEU	CG	100.00
112LE	CA	112LEU	CD1	46.73
112LE	CA	112LEU	CD2	43.35
112LE	CA	112LEU	C	100.00
112LE	CA	115LEU	CD1	0.05
112LE	CA	115LEU	CD2	0.02
112LE	CB	112LEU	CG	100.00
112LE	CB	112LEU	CD1	100.00
112LE	CB	112LEU	CD2	100.00
112LE	CB	112LEU	C	100.00
112LE	CB	115LEU	CD1	0.08
112LE	CB	320LEU	CD1	0.00
112LE	CB	320LEU	CD2	0.02
112LE	CG	112LEU	CD1	100.00
112LE	CG	112LEU	CD2	100.00
112LE	CG	112LEU	C	25.89
112LE	CG	115LEU	CD1	0.09
112LE	CG	319LEU	CD1	0.00
112LE	CG	319LEU	CD2	0.01
112LE	CG	320LEU	CG	0.02
112LE	CG	320LEU	CD1	0.04
112LE	CG	320LEU	CD2	0.06
112LE	CD1	112LEU	CD2	100.00
112LE	CD1	112LEU	C	0.12
112LE	CD1	114ARG	CZ	0.00
112LE	CD1	115LEU	CB	0.00
112LE	CD1	115LEU	CD1	1.35
112LE	CD1	115LEU	CD2	0.09
112LE	CD1	251PRO	CB	0.07
112LE	CD1	256LEU	CD1	0.01

110PRO	CG	110PRO	C	94.42	43GLY	CA	86PRO	CA	0.01	112LE	CD1	316ALA	CA	0.29
110PRO	CG	312GLU	CB	0.00	43GLY	CA	86PRO	CB	0.44	112LE	CD1	316ALA	CB	1.60
110PRO	CG	312GLU	CG	0.02	43GLY	CA	90LEU	CD1	0.00	112LE	CD1	316ALA	C	0.02
110PRO	CG	312GLU	CD	0.01	43GLY	C	44ALA	CA	100.00	112LE	CD1	319LEU	CB	3.96
110PRO	CG	312GLU	C	0.08	43GLY	C	44ALA	CB	5.10	112LE	CD1	319LEU	CG	0.02
110PRO	CG	316ALA	CB	0.27	43GLY	C	44ALA	C	94.69	112LE	CD1	319LEU	CD1	5.47
110PRO	CG	320LEU	CD1	0.04	43GLY	C	46ILE	CG1	0.02	112LE	CD1	319LEU	CD2	0.12
110PRO	CD	110PRO	C	86.92	43GLY	C	46ILE	CD	0.01	112LE	CD1	319LEU	C	0.04
110PRO	CD	312GLU	CG	0.05	43GLY	C	72VAL	CG2	0.00	112LE	CD1	320LEU	CA	0.00
110PRO	CD	316ALA	CB	0.85	43GLY	C	76LYS	CD	0.00	112LE	CD1	320LEU	CG	0.07
110PRO	C	111GLY	CA	100.00	43GLY	C	77TRP	CD1	0.01	112LE	CD1	320LEU	CD1	0.20
110PRO	C	111GLY	C	72.38	43GLY	C	77TRP	CE2	0.05	112LE	CD1	320LEU	CD2	0.24
110PRO	C	316ALA	CB	0.01	43GLY	C	77TRP	CZ2	0.28	112LE	CD2	112LEU	C	0.09
111GLY	CA	111GLY	C	100.00	43GLY	C	86PRO	CA	0.02	112LE	CD2	114ARG	CZ	0.17
111GLY	CA	113GLU	CD	0.02	43GLY	C	86PRO	CB	0.17	112LE	CD2	115LEU	CD1	1.68
111GLY	CA	320LEU	CD1	0.03	43GLY	C	89GLY	CA	0.01	112LE	CD2	115LEU	CD2	0.05
111GLY	CA	320LEU	CD2	0.02	43GLY	C	90LEU	CD1	0.00	112LE	CD2	251PRO	CA	0.09
111GLY	C	112LEU	CA	100.00	44ALA	CA	44ALA	CB	100.00	112LE	CD2	251PRO	CB	2.80
111GLY	C	112LEU	CB	90.82	44ALA	CA	44ALA	C	100.00	112LE	CD2	256LEU	CD1	0.06
111GLY	C	112LEU	CG	22.37	44ALA	CA	47ASP	CB	0.00	112LE	CD2	256LEU	CD2	0.03
111GLY	C	112LEU	CD1	3.10	44ALA	CA	52PRO	CB	0.04	112LE	CD2	316ALA	CA	0.01
111GLY	C	112LEU	CD2	0.05	44ALA	CA	77TRP	CZ2	0.01	112LE	CD2	316ALA	CB	0.10
111GLY	C	112LEU	C	5.64	44ALA	CA	89GLY	CA	0.02	112LE	CD2	319LEU	CB	2.34
111GLY	C	113GLU	CD	0.01	44ALA	CB	44ALA	C	100.00	112LE	CD2	319LEU	CG	0.04
111GLY	C	320LEU	CD1	0.01	44ALA	CB	52PRO	CB	0.02	112LE	CD2	319LEU	CD1	1.00
111GLY	C	320LEU	CD2	0.05	44ALA	CB	77TRP	CD1	0.29	112LE	CD2	319LEU	CD2	0.12
112LEU	CA	112LEU	CB	100.00	44ALA	CB	77TRP	CD2	0.00	112LE	CD2	319LEU	C	0.12
112LEU	CA	112LEU	CG	100.00	44ALA	CB	77TRP	CE2	0.03	112LE	CD2	320LEU	CA	0.04
112LEU	CA	112LEU	CD1	41.40	44ALA	CB	77TRP	CE3	0.00	112LE	CD2	320LEU	CG	0.08
112LEU	CA	112LEU	CD2	43.95	44ALA	CB	77TRP	CZ2	0.04	112LE	CD2	320LEU	CD1	0.18
112LEU	CA	112LEU	C	100.00	44ALA	CB	77TRP	CZ3	0.02	112LE	CD2	320LEU	CD2	2.27
112LEU	CA	115LEU	CD1	0.02	44ALA	CB	80LEU	CD1	0.06	112LE	CD2	327LEU	CD1	0.04
112LEU	CB	112LEU	CG	100.00	44ALA	CB	80LEU	CD2	0.02	112LE	CD2	327LEU	CD2	0.05
112LEU	CB	112LEU	CD1	100.00	44ALA	CB	84ILE	CA	0.04	112LE	C	113GLU	CA	100.00
112LEU	CB	112LEU	CD2	100.00	44ALA	CB	84ILE	CG2	0.07	112LE	C	113GLU	C	100.00
112LEU	CB	112LEU	C	100.00	44ALA	CB	84ILE	C	0.84	112LE	C	115LEU	CD1	0.01
112LEU	CB	115LEU	CD1	0.47	44ALA	CB	85SER	C	0.78	112LE	C	115LEU	CD2	0.00
112LEU	CB	115LEU	CD2	0.00	44ALA	CB	86PRO	CA	0.06	113GL	CA	113GLU	CB	100.00
112LEU	CG	112LEU	CD1	100.00	44ALA	CB	89GLY	CA	0.05	113GL	CA	113GLU	CG	100.00
112LEU	CG	112LEU	CD2	100.00	44ALA	C	45ALA	CA	100.00	113GL	CA	113GLU	CD	63.70
112LEU	CG	112LEU	C	26.14	44ALA	C	45ALA	CB	44.34	113GL	CA	113GLU	C	100.00
112LEU	CG	114ARG	CZ	0.01	44ALA	C	45ALA	C	55.78	113GL	CA	123ALA	CB	0.04
112LEU	CG	115LEU	CD1	0.53	44ALA	C	46ILE	CD	0.02	113GL	CB	113GLU	CG	100.00
112LEU	CG	115LEU	CD2	0.01	44ALA	C	48ALA	CB	0.01	113GL	CB	113GLU	CD	100.00
112LEU	CG	320LEU	CG	0.00	44ALA	C	52PRO	CB	0.21	113GL	CB	113GLU	C	100.00
112LEU	CG	320LEU	CD1	0.02	44ALA	C	52PRO	CG	0.01	113GL	CB	120GLU	CB	0.31
112LEU	CG	320LEU	CD2	0.32	45ALA	CA	45ALA	CB	100.00	113GL	CB	120GLU	CG	4.33
112LEU	CD1	112LEU	CD2	100.00	45ALA	CA	45ALA	C	100.00	113GL	CB	120GLU	CD	0.04
112LEU	CD1	112LEU	C	0.30	45ALA	CA	48ALA	CB	0.00	113GL	CB	123ALA	CB	0.17
112LEU	CD1	114ARG	CB	0.02	45ALA	CA	49PHE	CD1	0.03	113GL	CG	113GLU	CD	100.00
112LEU	CD1	114ARG	CG	0.06	45ALA	CA	49PHE	CE1	0.00	113GL	CG	123ALA	CB	0.39
112LEU	CD1	114ARG	CD	0.06	45ALA	CA	49PHE	CE2	0.06	113GL	CG	123ALA	C	0.12
112LEU	CD1	114ARG	CZ	2.63	45ALA	CA	49PHE	CZ	0.02	113GL	CG	124ARG	CA	0.70
112LEU	CD1	115LEU	CG	0.09	45ALA	CB	45ALA	C	100.00	113GL	CG	124ARG	CB	0.03
112LEU	CD1	115LEU	CD1	0.08	45ALA	CB	49PHE	CD1	0.01	113GL	CG	124ARG	CG	0.18
112LEU	CD1	115LEU	CD2	0.06	45ALA	CB	49PHE	CE1	0.11	113GL	CG	124ARG	CZ	0.03
112LEU	CD1	256LEU	CD1	0.04	45ALA	CB	49PHE	CE2	0.16	113GL	CD	120GLU	CG	0.00
112LEU	CD1	316ALA	CA	0.01	45ALA	CB	49PHE	CZ	0.45	113GL	CD	124ARG	CG	0.04
112LEU	CD1	316ALA	CB	0.11	45ALA	CB	50GLY	CA	0.00	113GL	CD	124ARG	CD	0.01
112LEU	CD1	316ALA	C	0.04	45ALA	CB	50GLY	C	0.03	113GL	CD	124ARG	CZ	0.30
112LEU	CD1	319LEU	CB	8.98	45ALA	CB	52PRO	CA	0.02	113GL	C	114ARG	CA	100.00
112LEU	CD1	319LEU	CG	0.05	45ALA	CB	52PRO	C	0.02	113GL	C	114ARG	CB	0.34

112LEU	CD1	319LEU	CD1	0.16	45ALA	CB	54PRO	CG	0.08	113GL	C	114ARG	CG	0.04
112LEU	CD1	319LEU	CD2	0.08	45ALA	CB	54PRO	CD	0.07	113GL	C	114ARG	C	99.58
112LEU	CD1	319LEU	C	0.26	45ALA	CB	77TRP	CE2	0.00	113GL	C	120GLU	CB	0.00
112LEU	CD1	320LEU	CA	0.03	45ALA	CB	77TRP	CZ2	0.01	113GL	C	120GLU	CG	0.15
112LEU	CD1	320LEU	CB	0.01	45ALA	C	46ILE	CA	100.00	113GL	C	120GLU	CD	0.00
112LEU	CD1	320LEU	CG	0.10	45ALA	C	46ILE	CB	30.67	114AR	CA	114ARG	CB	100.00
112LEU	CD1	320LEU	CD1	0.90	45ALA	C	46ILE	CG1	19.13	114AR	CA	114ARG	CG	100.00
112LEU	CD1	320LEU	CD2	1.17	45ALA	C	46ILE	CG2	3.14	114AR	CA	114ARG	CD	2.05
112LEU	CD1	327LEU	CD1	0.07	45ALA	C	46ILE	CD	0.24	114AR	CA	114ARG	C	100.00
112LEU	CD1	327LEU	CD2	0.14	45ALA	C	46ILE	C	74.44	114AR	CA	120GLU	CG	0.03
112LEU	CD2	112LEU	C	0.01	45ALA	C	49PHE	CB	0.00	114AR	CA	120GLU	CD	0.01
112LEU	CD2	114ARG	CG	0.01	46ILE	CA	46ILE	CB	100.00	114AR	CB	114ARG	CG	100.00
112LEU	CD2	114ARG	CD	0.01	46ILE	CA	46ILE	CG1	100.00	114AR	CB	114ARG	CD	100.00
112LEU	CD2	114ARG	CZ	2.72	46ILE	CA	46ILE	CG2	100.00	114AR	CB	114ARG	CZ	0.68
112LEU	CD2	115LEU	CG	0.03	46ILE	CA	46ILE	CD	15.74	114AR	CB	114ARG	C	100.00
112LEU	CD2	115LEU	CD1	1.84	46ILE	CA	46ILE	C	100.00	114AR	CB	120GLU	CD	0.02
112LEU	CD2	115LEU	CD2	0.08	46ILE	CA	50GLY	CA	0.02	114AR	CB	320LEU	CD2	0.01
112LEU	CD2	256LEU	CD1	0.10	46ILE	CB	46ILE	CG1	100.00	114AR	CG	114ARG	CD	100.00
112LEU	CD2	256LEU	CD2	0.00	46ILE	CB	46ILE	CG2	100.00	114AR	CG	114ARG	CZ	18.87
112LEU	CD2	319LEU	CB	8.10	46ILE	CB	46ILE	CD	100.00	114AR	CG	114ARG	C	60.53
112LEU	CD2	319LEU	CG	0.00	46ILE	CB	46ILE	C	100.00	114AR	CG	115LEU	CD1	0.01
112LEU	CD2	319LEU	CD1	0.06	46ILE	CB	49PHE	CB	0.01	114AR	CG	120GLU	CG	0.08
112LEU	CD2	319LEU	CD2	0.03	46ILE	CB	49PHE	CD1	0.13	114AR	CG	120GLU	CD	2.20
112LEU	CD2	319LEU	C	1.79	46ILE	CB	49PHE	CD2	0.09	114AR	CD	114ARG	CZ	100.00
112LEU	CD2	320LEU	CA	0.14	46ILE	CG1	46ILE	CG2	100.00	114AR	CD	114ARG	C	0.49
112LEU	CD2	320LEU	CG	0.03	46ILE	CG1	46ILE	CD	100.00	114AR	CD	120GLU	CG	0.00
112LEU	CD2	320LEU	CD1	0.34	46ILE	CG1	46ILE	C	12.79	114AR	CD	120GLU	CD	0.00
112LEU	CD2	320LEU	CD2	1.86	46ILE	CG1	49PHE	CB	0.03	114AR	CD	320LEU	CD1	0.00
112LEU	CD2	327LEU	CD1	0.31	46ILE	CG1	49PHE	CD1	0.01	114AR	CD	320LEU	CD2	0.09
112LEU	CD2	327LEU	CD2	0.45	46ILE	CG1	49PHE	CD2	0.01	114AR	CZ	115LEU	CG	0.01
112LEU	C	113GLU	CA	100.00	46ILE	CG1	51GLU	C	0.04	114AR	CZ	115LEU	CD1	0.08
112LEU	C	113GLU	CB	0.01	46ILE	CG1	52PRO	CA	0.19	114AR	CZ	115LEU	CD2	0.34
112LEU	C	113GLU	C	99.98	46ILE	CG1	52PRO	CB	0.15	114AR	CZ	120GLU	CG	0.02
112LEU	C	114ARG	CG	0.00	46ILE	CG1	52PRO	CD	0.02	114AR	CZ	120GLU	CD	0.01
112LEU	C	115LEU	CD1	0.00	46ILE	CG1	54PRO	CD	0.00	114AR	CZ	320LEU	CG	0.01
113GLU	CA	113GLU	CB	100.00	46ILE	CG1	77TRP	CZ2	0.03	114AR	CZ	320LEU	CD1	0.54
113GLU	CA	113GLU	CG	100.00	46ILE	CG1	80LEU	CD1	0.00	114AR	CZ	320LEU	CD2	2.11
113GLU	CA	113GLU	CD	76.63	46ILE	CG1	84ILE	CG2	0.00	114AR	C	115LEU	CA	100.00
113GLU	CA	113GLU	C	100.00	46ILE	CG2	46ILE	CD	92.23	114AR	C	115LEU	CB	1.02
113GLU	CB	113GLU	CG	100.00	46ILE	CG2	46ILE	C	86.19	114AR	C	115LEU	CG	0.08
113GLU	CB	113GLU	CD	100.00	46ILE	CG2	49PHE	CB	0.06	114AR	C	115LEU	CD1	0.06
113GLU	CB	113GLU	C	100.00	46ILE	CG2	49PHE	CG	0.01	114AR	C	115LEU	CD2	0.01
113GLU	CB	120GLU	CB	0.00	46ILE	CG2	49PHE	CD1	1.20	114AR	C	115LEU	C	98.79
113GLU	CB	120GLU	CG	0.62	46ILE	CG2	49PHE	CD2	0.93	115LE	CA	115LEU	CB	100.00
113GLU	CB	123ALA	CB	0.00	46ILE	CG2	49PHE	CE1	0.07	115LE	CA	115LEU	CG	100.00
113GLU	CB	124ARG	CG	0.02	46ILE	CG2	49PHE	CE2	0.06	115LE	CA	115LEU	CD1	17.56
113GLU	CG	113GLU	CD	100.00	46ILE	CG2	49PHE	C	0.04	115LE	CA	115LEU	CD2	82.44
113GLU	CG	123ALA	CB	0.02	46ILE	CG2	50GLY	CA	0.03	115LE	CA	115LEU	C	100.00
113GLU	CG	123ALA	C	0.00	46ILE	CG2	50GLY	C	0.09	115LE	CB	115LEU	CG	100.00
113GLU	CG	124ARG	CA	0.10	46ILE	CG2	51GLU	C	0.01	115LE	CB	115LEU	CD1	100.00
113GLU	CG	124ARG	CG	0.16	46ILE	CG2	52PRO	CA	0.14	115LE	CB	115LEU	CD2	100.00
113GLU	CD	124ARG	CA	0.00	46ILE	CG2	52PRO	CB	0.17	115LE	CB	115LEU	C	100.00
113GLU	CD	124ARG	CB	0.00	46ILE	CG2	52PRO	CG	0.02	115LE	CB	252GLY	CA	0.01
113GLU	CD	124ARG	CG	0.13	46ILE	CG2	52PRO	CD	0.02	115LE	CB	327LEU	CD1	0.19
113GLU	CD	124ARG	CD	0.02	46ILE	CG2	77TRP	CD1	0.01	115LE	CB	327LEU	CD2	0.04
113GLU	CD	124ARG	CZ	0.29	46ILE	CG2	77TRP	CE2	0.00	115LE	CG	115LEU	CD1	100.00
113GLU	C	114ARG	CA	100.00	46ILE	CG2	77TRP	CZ2	0.04	115LE	CG	115LEU	CD2	100.00
113GLU	C	114ARG	CB	0.01	46ILE	CG2	80LEU	CD1	0.03	115LE	CG	115LEU	C	1.36
113GLU	C	114ARG	C	99.99	46ILE	CG2	84ILE	CA	0.00	115LE	CG	320LEU	CD2	0.01
113GLU	C	120GLU	CG	0.02	46ILE	CG2	84ILE	CB	0.03	115LE	CG	327LEU	CD1	0.12
114ARG	CA	114ARG	CB	100.00	46ILE	CG2	84ILE	CG2	0.87	115LE	CG	327LEU	CD2	0.02
114ARG	CA	114ARG	CG	100.00	46ILE	CG2	84ILE	CD	0.16	115LE	CD1	115LEU	CD2	100.00

114ARG CA	114ARG CD	0.80	46ILE CG2	84ILE C	0.00	115LE CD1	256LEU CD1	0.07
114ARG CA	114ARG C	100.00	46ILE CD	46ILE C	0.38	115LE CD1	256LEU CD2	0.04
114ARG CA	120GLU CB	0.02	46ILE CD	49PHE CB	0.46	115LE CD1	319LEU CB	0.05
114ARG CA	120GLU CG	0.20	46ILE CD	49PHE CG	0.05	115LE CD1	319LEU CD1	0.03
114ARG CA	120GLU CD	0.10	46ILE CD	49PHE CD1	0.15	115LE CD1	319LEU CD2	0.24
114ARG CB	114ARG CG	100.00	46ILE CD	49PHE CD2	0.19	115LE CD1	320LEU CD1	0.00
114ARG CB	114ARG CD	100.00	46ILE CD	49PHE CE1	0.04	115LE CD1	320LEU CD2	0.31
114ARG CB	114ARG CZ	0.92	46ILE CD	49PHE CE2	0.00	115LE CD1	323PRO CB	0.02
114ARG CB	114ARG C	100.00	46ILE CD	49PHE CZ	0.02	115LE CD1	324PRO CG	0.01
114ARG CB	120GLU CB	0.02	46ILE CD	49PHE C	0.00	115LE CD1	324PRO CD	0.01
114ARG CB	120GLU CG	0.08	46ILE CD	50GLY C	0.00	115LE CD1	327LEU CB	0.00
114ARG CB	120GLU CD	0.25	46ILE CD	51GLU C	0.03	115LE CD1	327LEU CG	0.00
114ARG CG	114ARG CD	100.00	46ILE CD	52PRO CA	0.04	115LE CD1	327LEU CD1	0.40
114ARG CG	114ARG CZ	26.85	46ILE CD	52PRO CB	0.07	115LE CD1	327LEU CD2	0.32
114ARG CG	114ARG C	90.74	46ILE CD	52PRO CG	0.00	115LE CD2	115LEU C	0.04
114ARG CG	120GLU CG	0.24	46ILE CD	52PRO CD	0.01	115LE CD2	253SER CB	0.01
114ARG CG	120GLU CD	0.06	46ILE CD	52PRO C	0.09	115LE CD2	256LEU CD1	0.08
114ARG CD	114ARG CZ	100.00	46ILE CD	54PRO CG	1.41	115LE CD2	256LEU CD2	0.02
114ARG CD	114ARG C	0.21	46ILE CD	54PRO CD	5.56	115LE CD2	319LEU CD2	0.07
114ARG CZ	115LEU CG	0.01	46ILE CD	77TRP CD1	0.06	115LE CD2	320LEU CD1	0.00
114ARG CZ	115LEU CD1	0.06	46ILE CD	77TRP CD2	0.02	115LE CD2	320LEU CD2	0.02
114ARG CZ	115LEU CD2	0.17	46ILE CD	77TRP CE2	0.40	115LE CD2	323PRO CB	0.08
114ARG CZ	120GLU CG	0.06	46ILE CD	77TRP CE3	0.01	115LE CD2	324PRO CD	0.07
114ARG CZ	120GLU CD	0.04	46ILE CD	77TRP CZ2	1.34	115LE CD2	327LEU CB	0.00
114ARG CZ	320LEU CD1	0.14	46ILE CD	77TRP CZ3	0.01	115LE CD2	327LEU CG	0.02
114ARG CZ	320LEU CD2	0.08	46ILE CD	80LEU CD1	0.42	115LE CD2	327LEU CD1	0.66
114ARG CZ	327LEU CD1	0.00	46ILE CD	80LEU CD2	0.21	115LE CD2	327LEU CD2	0.14
114ARG CZ	327LEU CD2	0.00	46ILE CD	84ILE CB	0.01	115LE C	116SER CA	100.00
114ARG C	115LEU CA	100.00	46ILE CD	84ILE CG2	0.17	115LE C	116SER CB	0.17
114ARG C	115LEU CB	0.75	46ILE CD	84ILE CD	0.02	115LE C	116SER C	99.94
114ARG C	115LEU CG	0.10	46ILE CD	84ILE C	0.06	115LE C	252GLY CA	0.03
114ARG C	115LEU CD1	0.02	46ILE CD	85SER CA	0.01	116SE CA	116SER CB	100.00
114ARG C	115LEU CD2	0.00	46ILE CD	85SER C	0.33	116SE CA	116SER C	100.00
114ARG C	115LEU C	98.92	46ILE CD	86PRO CA	0.01	116SE CA	117PRO CD	100.00
115LEU CA	115LEU CB	100.00	46ILE CD	86PRO CG	0.00	116SE CB	116SER C	100.00
115LEU CA	115LEU CG	100.00	46ILE CD	86PRO CD	0.09	116SE CB	117PRO CD	0.63
115LEU CA	115LEU CD1	11.43	46ILE C	47ASP CA	100.00	116SE CB	123ALA CB	1.75
115LEU CA	115LEU CD2	88.20	46ILE C	47ASP CB	13.63	116SE C	117PRO CA	100.00
115LEU CA	115LEU C	100.00	46ILE C	47ASP C	86.87	116SE C	117PRO CB	0.10
115LEU CB	115LEU CG	100.00	46ILE C	50GLY CA	0.02	116SE C	117PRO CD	100.00
115LEU CB	115LEU CD1	100.00	47ASP CA	47ASP CB	100.00	116SE C	117PRO C	100.00
115LEU CB	115LEU CD2	100.00	47ASP CA	47ASP CG	100.00	117PR CA	117PRO CB	100.00
115LEU CB	115LEU C	100.00	47ASP CA	47ASP C	100.00	117PR CA	117PRO CG	100.00
115LEU CB	252GLY CA	0.04	47ASP CA	84ILE CG2	0.00	117PR CA	117PRO CD	100.00
115LEU CB	252GLY C	0.01	47ASP CB	47ASP CG	100.00	117PR CA	117PRO C	100.00
115LEU CB	327LEU CG	0.00	47ASP CB	47ASP C	100.00	117PR CB	117PRO CG	100.00
115LEU CB	327LEU CD1	0.01	47ASP CB	77TRP CZ2	0.00	117PR CB	117PRO CD	100.00
115LEU CB	327LEU CD2	0.03	47ASP CB	84ILE CG2	0.00	117PR CB	117PRO C	100.00
115LEU CG	115LEU CD1	100.00	47ASP CG	47ASP C	97.63	117PR CG	117PRO CD	100.00
115LEU CG	115LEU CD2	100.00	47ASP CG	48ALA CB	0.00	117PR CG	117PRO C	99.22
115LEU CG	115LEU C	8.98	47ASP C	48ALA CA	100.00	117PR CG	249VAL CB	0.00
115LEU CG	252GLY CA	0.00	47ASP C	48ALA CB	38.95	117PR CG	249VAL CG1	4.37
115LEU CD1	115LEU CD2	100.00	47ASP C	48ALA C	92.69	117PR CG	249VAL CG2	4.06
115LEU CD1	115LEU C	0.00	48ALA CA	48ALA CB	100.00	117PR CD	117PRO C	96.22
115LEU CD1	253SER CB	0.00	48ALA CA	48ALA C	100.00	117PR CD	249VAL CB	0.00
115LEU CD1	319LEU CD1	0.00	48ALA CB	48ALA C	100.00	117PR CD	249VAL CG1	0.38
115LEU CD1	319LEU CD2	0.18	48ALA CB	49PHE CD1	2.68	117PR CD	249VAL CG2	0.03
115LEU CD1	323PRO CB	0.02	48ALA CB	49PHE CD2	0.62	117PR CD	252GLY CA	0.22
115LEU CD1	327LEU CG	0.10	48ALA CB	49PHE CE1	0.44	117PR C	118LEU CA	100.00
115LEU CD1	327LEU CD1	1.38	48ALA CB	49PHE CE2	0.07	117PR C	118LEU CB	0.01
115LEU CD1	327LEU CD2	1.02	48ALA CB	49PHE CZ	0.01	117PR C	118LEU C	99.98
115LEU CD2	115LEU C	0.01	48ALA C	49PHE CA	100.00	118LE CA	118LEU CB	100.00

115LEU	CD2	252GLY	C	0.11	48ALA	C	49PHE	CB	47.33	118LE	CA	118LEU	CG	100.00
115LEU	CD2	253SER	CB	0.02	48ALA	C	49PHE	CG	14.80	118LE	CA	118LEU	CD1	4.34
115LEU	CD2	327LEU	CB	0.00	48ALA	C	49PHE	CD1	6.58	118LE	CA	118LEU	CD2	93.03
115LEU	CD2	327LEU	CG	0.02	48ALA	C	49PHE	CD2	3.96	118LE	CA	118LEU	C	100.00
115LEU	CD2	327LEU	CD1	0.45	48ALA	C	49PHE	C	51.76	118LE	CB	118LEU	CG	100.00
115LEU	CD2	327LEU	CD2	0.56	49PHE	CA	49PHE	CB	100.00	118LE	CB	118LEU	CD1	100.00
115LEU	C	116SER	CA	100.00	49PHE	CA	49PHE	CG	100.00	118LE	CB	118LEU	CD2	100.00
115LEU	C	116SER	CB	0.28	49PHE	CA	49PHE	CD1	69.06	118LE	CB	118LEU	C	100.00
115LEU	C	116SER	C	99.92	49PHE	CA	49PHE	CD2	78.30	118LE	CB	122ILE	CG2	0.01
115LEU	C	252GLY	CA	0.91	49PHE	CA	49PHE	C	100.00	118LE	CG	118LEU	CD1	100.00
116SER	CA	116SER	CB	100.00	49PHE	CB	49PHE	CG	100.00	118LE	CG	118LEU	CD2	100.00
116SER	CA	116SER	C	100.00	49PHE	CB	49PHE	CD1	100.00	118LE	CG	118LEU	C	0.12
116SER	CA	117PRO	CD	100.00	49PHE	CB	49PHE	CD2	100.00	118LE	CG	122ILE	CG2	0.00
116SER	CA	252GLY	CA	0.01	49PHE	CB	49PHE	C	100.00	118LE	CG	249VAL	CG1	0.02
116SER	CB	116SER	C	100.00	49PHE	CB	54PRO	CB	4.17	118LE	CD1	118LEU	CD2	100.00
116SER	CB	117PRO	CD	1.69	49PHE	CB	54PRO	CG	0.04	118LE	CD1	118LEU	C	0.00
116SER	CB	123ALA	CB	4.09	49PHE	CG	49PHE	CD1	100.00	118LE	CD1	122ILE	CG2	0.27
116SER	C	117PRO	CA	100.00	49PHE	CG	49PHE	CD2	100.00	118LE	CD1	224VAL	CB	0.01
116SER	C	117PRO	CB	0.10	49PHE	CG	49PHE	CE1	100.00	118LE	CD1	224VAL	CG1	0.26
116SER	C	117PRO	CD	100.00	49PHE	CG	49PHE	CE2	100.00	118LE	CD1	227PRO	CG	0.01
116SER	C	117PRO	C	100.00	49PHE	CG	49PHE	CZ	100.00	118LE	CD1	249VAL	CB	0.00
117PRO	CA	117PRO	CB	100.00	49PHE	CG	49PHE	C	5.26	118LE	CD1	249VAL	CG1	0.54
117PRO	CA	117PRO	CG	100.00	49PHE	CG	54PRO	CB	10.58	118LE	CD1	249VAL	CG2	0.86
117PRO	CA	117PRO	CD	100.00	49PHE	CG	54PRO	CG	0.58	118LE	CD1	250LEU	CD1	0.18
117PRO	CA	117PRO	C	100.00	49PHE	CD1	49PHE	CD2	100.00	118LE	CD1	250LEU	CD2	5.94
117PRO	CB	117PRO	CG	100.00	49PHE	CD1	49PHE	CE1	100.00	118LE	CD2	122ILE	CG2	0.14
117PRO	CB	117PRO	CD	100.00	49PHE	CD1	49PHE	CE2	100.00	118LE	CD2	122ILE	CD	0.02
117PRO	CB	117PRO	C	100.00	49PHE	CD1	49PHE	CZ	100.00	118LE	CD2	224VAL	CA	0.01
117PRO	CG	117PRO	CD	100.00	49PHE	CD1	49PHE	C	1.46	118LE	CD2	224VAL	CB	0.03
117PRO	CG	117PRO	C	98.28	49PHE	CD1	51GLU	CB	0.06	118LE	CD2	224VAL	CG1	0.60
117PRO	CG	118LEU	CD1	0.00	49PHE	CD1	54PRO	CB	12.61	118LE	CD2	224VAL	C	0.01
117PRO	CG	249VAL	CB	0.01	49PHE	CD1	54PRO	CG	3.79	118LE	CD2	227PRO	CG	0.00
117PRO	CG	249VAL	CG1	2.30	49PHE	CD2	49PHE	CE1	100.00	118LE	CD2	249VAL	CG2	0.01
117PRO	CG	249VAL	CG2	0.31	49PHE	CD2	49PHE	CE2	100.00	118LE	CD2	250LEU	CD2	0.12
117PRO	CG	250LEU	CD2	0.00	49PHE	CD2	49PHE	CZ	100.00	118LE	C	119LYS	CA	100.00
117PRO	CD	117PRO	C	93.00	49PHE	CD2	49PHE	C	0.19	118LE	C	119LYS	CB	0.01
117PRO	CD	249VAL	CG1	0.04	49PHE	CD2	51GLU	CB	0.04	118LE	C	119LYS	C	99.99
117PRO	CD	249VAL	CG2	0.00	49PHE	CD2	54PRO	CB	11.49	119LY	CA	119LYS	CB	100.00
117PRO	C	118LEU	CA	100.00	49PHE	CD2	54PRO	CG	1.64	119LY	CA	119LYS	CG	100.00
117PRO	C	118LEU	CB	0.02	49PHE	CE1	49PHE	CE2	100.00	119LY	CA	119LYS	CD	0.50
117PRO	C	118LEU	CD1	0.00	49PHE	CE1	49PHE	CZ	100.00	119LY	CA	119LYS	C	100.00
117PRO	C	118LEU	CD2	0.00	49PHE	CE1	51GLU	CB	0.92	119LY	CB	119LYS	CG	100.00
117PRO	C	118LEU	C	99.98	49PHE	CE1	54PRO	CB	1.80	119LY	CB	119LYS	CD	100.00
118LEU	CA	118LEU	CB	100.00	49PHE	CE1	54PRO	CG	0.98	119LY	CB	119LYS	CE	20.58
118LEU	CA	118LEU	CG	100.00	49PHE	CE1	55GLU	CB	0.01	119LY	CB	119LYS	C	100.00
118LEU	CA	118LEU	CD1	21.58	49PHE	CE1	56PRO	CG	0.71	119LY	CB	122ILE	CG1	0.01
118LEU	CA	118LEU	CD2	77.96	49PHE	CE1	56PRO	CD	0.33	119LY	CB	122ILE	CD	0.11
118LEU	CA	118LEU	C	100.00	49PHE	CE2	49PHE	CZ	100.00	119LY	CG	119LYS	CD	100.00
118LEU	CB	118LEU	CG	100.00	49PHE	CE2	51GLU	CB	0.02	119LY	CG	119LYS	CE	100.00
118LEU	CB	118LEU	CD1	100.00	49PHE	CE2	54PRO	CB	1.77	119LY	CG	119LYS	C	17.20
118LEU	CB	118LEU	CD2	100.00	49PHE	CE2	54PRO	CG	0.63	119LY	CG	122ILE	CG1	0.10
118LEU	CB	118LEU	C	100.00	49PHE	CE2	55GLU	CB	0.02	119LY	CG	122ILE	CD	0.41
118LEU	CG	118LEU	CD1	100.00	49PHE	CE2	56PRO	CG	1.18	119LY	CD	119LYS	CE	100.00
118LEU	CG	118LEU	CD2	100.00	49PHE	CE2	56PRO	CD	1.45	119LY	CE	121GLU	CB	0.00
118LEU	CG	118LEU	C	0.42	49PHE	CZ	51GLU	CB	0.12	119LY	CE	122ILE	CD	0.01
118LEU	CD1	118LEU	CD2	100.00	49PHE	CZ	54PRO	CB	0.11	119LY	C	120GLU	CA	100.00
118LEU	CD1	122ILE	CG2	0.06	49PHE	CZ	54PRO	CG	0.02	119LY	C	120GLU	C	100.00
118LEU	CD1	122ILE	CD	0.01	49PHE	CZ	55GLU	CB	0.03	120GL	CA	120GLU	CB	100.00
118LEU	CD1	123ALA	CA	0.00	49PHE	CZ	56PRO	CG	3.95	120GL	CA	120GLU	CG	100.00
118LEU	CD1	224VAL	CA	0.00	49PHE	CZ	56PRO	CD	3.41	120GL	CA	120GLU	CD	38.33
118LEU	CD1	224VAL	C	0.02	49PHE	C	50GLY	CA	100.00	120GL	CA	120GLU	C	100.00
118LEU	CD1	227PRO	CG	2.80	49PHE	C	50GLY	C	54.08	120GL	CB	120GLU	CG	100.00

118LEU	CD1	227PRO	CD	0.50
118LEU	CD1	249VAL	CG1	0.00
118LEU	CD1	249VAL	CG2	0.17
118LEU	CD1	250LEU	CG	0.01
118LEU	CD1	250LEU	CD1	0.20
118LEU	CD1	250LEU	CD2	5.02
118LEU	CD2	122ILE	CG2	0.15
118LEU	CD2	122ILE	CD	0.10
118LEU	CD2	224VAL	CA	0.01
118LEU	CD2	224VAL	CB	0.00
118LEU	CD2	224VAL	CG1	0.14
118LEU	CD2	224VAL	C	0.11
118LEU	CD2	225ARG	C	0.00
118LEU	CD2	227PRO	CG	1.07
118LEU	CD2	227PRO	CD	0.66
118LEU	CD2	250LEU	CD1	0.06
118LEU	CD2	250LEU	CD2	0.32
118LEU	C	119LYS	CA	100.00
118LEU	C	119LYS	C	100.00
119LYS	CA	119LYS	CB	100.00
119LYS	CA	119LYS	CG	100.00
119LYS	CA	119LYS	CD	0.08
119LYS	CA	119LYS	C	100.00
119LYS	CB	119LYS	CG	100.00
119LYS	CB	119LYS	CD	100.00
119LYS	CB	119LYS	CE	27.33
119LYS	CB	119LYS	C	100.00
119LYS	CB	121GLU	CD	0.03
119LYS	CB	122ILE	CG1	0.03
119LYS	CB	122ILE	CD	0.13
119LYS	CG	119LYS	CD	100.00
119LYS	CG	119LYS	CE	100.00
119LYS	CG	119LYS	C	5.52
119LYS	CG	121GLU	CG	0.00
119LYS	CG	122ILE	CG1	0.01
119LYS	CG	122ILE	CD	0.06
119LYS	CD	119LYS	CE	100.00
119LYS	CD	122ILE	CD	0.05
119LYS	CE	122ILE	CD	0.13
119LYS	C	120GLU	CA	100.00
119LYS	C	120GLU	C	100.00
120GLU	CA	120GLU	CB	100.00
120GLU	CA	120GLU	CG	100.00
120GLU	CA	120GLU	CD	21.50
120GLU	CA	120GLU	C	100.00
120GLU	CA	123ALA	CB	0.00
120GLU	CB	120GLU	CG	100.00
120GLU	CB	120GLU	CD	100.00
120GLU	CB	120GLU	C	100.00
120GLU	CB	124ARG	CZ	0.09
120GLU	CG	120GLU	CD	100.00
120GLU	CG	120GLU	C	67.27
120GLU	CG	124ARG	CZ	0.46
120GLU	CD	120GLU	C	0.83
120GLU	CD	124ARG	CZ	0.17
120GLU	C	121GLU	CA	100.00
120GLU	C	121GLU	CB	0.03
120GLU	C	121GLU	C	99.98
120GLU	C	124ARG	CZ	0.09
121GLU	CA	121GLU	CB	100.00
121GLU	CA	121GLU	CG	100.00
121GLU	CA	121GLU	CD	91.20
50GLY	CA	50GLY	C	100.00
50GLY	C	51GLU	CA	100.00
50GLY	C	51GLU	CB	37.46
50GLY	C	51GLU	CG	0.69
50GLY	C	51GLU	CD	0.00
50GLY	C	51GLU	C	66.18
50GLY	C	84ILE	CD	0.00
51GLU	CA	51GLU	CB	100.00
51GLU	CA	51GLU	CG	100.00
51GLU	CA	51GLU	CD	50.41
51GLU	CA	51GLU	C	100.00
51GLU	CA	52PRO	CD	100.00
51GLU	CB	51GLU	CG	100.00
51GLU	CB	51GLU	CD	100.00
51GLU	CB	51GLU	C	100.00
51GLU	CB	52PRO	CD	4.55
51GLU	CB	54PRO	CA	0.06
51GLU	CB	54PRO	CB	0.01
51GLU	CB	84ILE	CG2	0.00
51GLU	CG	51GLU	CD	100.00
51GLU	CG	51GLU	C	81.76
51GLU	CG	52PRO	CD	1.93
51GLU	CG	53PHE	C	0.48
51GLU	CG	54PRO	CA	0.18
51GLU	CG	54PRO	CB	0.01
51GLU	CG	58ARG	CZ	0.00
51GLU	CD	51GLU	C	0.06
51GLU	CD	52PRO	CD	0.08
51GLU	CD	53PHE	C	0.00
51GLU	CD	54PRO	CA	0.01
51GLU	CD	54PRO	CB	0.00
51GLU	CD	58ARG	CZ	0.01
51GLU	C	52PRO	CA	100.00
51GLU	C	52PRO	CB	0.22
51GLU	C	52PRO	CD	100.00
51GLU	C	52PRO	C	100.00
52PRO	CA	52PRO	CB	100.00
52PRO	CA	52PRO	CG	100.00
52PRO	CA	52PRO	CD	100.00
52PRO	CA	52PRO	C	100.00
52PRO	CB	52PRO	CG	100.00
52PRO	CB	52PRO	CD	100.00
52PRO	CB	52PRO	C	100.00
52PRO	CB	77TRP	CZ2	0.08
52PRO	CB	85SER	C	0.01
52PRO	CB	86PRO	CA	0.03
52PRO	CB	86PRO	CB	0.01
52PRO	CB	88THR	CB	0.03
52PRO	CB	88THR	C	0.07
52PRO	CB	89GLY	CA	1.54
52PRO	CG	52PRO	CD	100.00
52PRO	CG	52PRO	C	88.84
52PRO	CG	84ILE	CB	0.01
52PRO	CG	84ILE	CG2	0.03
52PRO	CG	84ILE	C	0.02
52PRO	CG	85SER	C	0.01
52PRO	CG	86PRO	CA	0.01
52PRO	CG	88THR	CB	0.00
52PRO	CG	88THR	C	0.26
52PRO	CG	89GLY	CA	1.10
52PRO	CG	89GLY	C	0.00
52PRO	CG	92SER	CB	0.01
120GL	CB	120GLU	CD	100.00
120GL	CB	120GLU	C	100.00
120GL	CB	124ARG	CZ	0.43
120GL	CG	120GLU	CD	100.00
120GL	CG	120GLU	C	57.61
120GL	CG	124ARG	CZ	0.66
120GL	CD	120GLU	C	2.08
120GL	CD	124ARG	CZ	0.10
120GL	C	121GLU	CA	100.00
120GL	C	121GLU	CB	0.04
120GL	C	121GLU	C	100.00
120GL	C	124ARG	CD	0.00
121GL	CA	121GLU	CB	100.00
121GL	CA	121GLU	CG	100.00
121GL	CA	121GLU	CD	96.91
121GL	CA	121GLU	C	100.00
121GL	CA	124ARG	CB	0.00
121GL	CA	124ARG	CZ	0.01
121GL	CB	121GLU	CG	100.00
121GL	CB	121GLU	CD	100.00
121GL	CB	121GLU	C	100.00
121GL	CG	121GLU	CD	100.00
121GL	CG	121GLU	C	90.28
121GL	CD	121GLU	C	0.20
121GL	CD	124ARG	CD	0.06
121GL	CD	124ARG	CZ	0.04
121GL	C	122ILE	CA	100.00
121GL	C	122ILE	CB	0.50
121GL	C	122ILE	CG1	0.29
121GL	C	122ILE	CD	0.00
121GL	C	122ILE	C	99.64
122ILE	CA	122ILE	CB	100.00
122ILE	CA	122ILE	CG1	100.00
122ILE	CA	122ILE	CG2	100.00
122ILE	CA	122ILE	CD	8.26
122ILE	CA	122ILE	C	100.00
122ILE	CB	122ILE	CG1	100.00
122ILE	CB	122ILE	CG2	100.00
122ILE	CB	122ILE	CD	100.00
122ILE	CB	122ILE	C	100.00
122ILE	CG1	122ILE	CG2	100.00
122ILE	CG1	122ILE	CD	100.00
122ILE	CG2	122ILE	CD	99.52
122ILE	CG2	122ILE	C	100.00
122ILE	CG2	224VAL	C	0.79
122ILE	CG2	225ARG	CA	0.01
122ILE	CG2	225ARG	C	0.38
122ILE	CG2	227PRO	CG	0.10
122ILE	CG2	227PRO	CD	12.12
122ILE	CG2	250LEU	CD2	0.05
122ILE	CD	225ARG	C	0.00
122ILE	C	123ALA	CA	100.00
122ILE	C	123ALA	CB	0.47
122ILE	C	123ALA	C	99.56
123AL	CA	123ALA	CB	100.00
123AL	CA	123ALA	C	100.00
123AL	CA	126VAL	CG2	0.01
123AL	CA	250LEU	CD2	0.01
123AL	CB	123ALA	C	100.00
123AL	C	124ARG	CA	100.00
123AL	C	124ARG	CB	4.30
123AL	C	124ARG	CG	0.41

121GLU CA	121GLU C	100.00	52PRO CD	52PRO C	75.40	123AL C	124ARG C	96.36
121GLU CA	124ARG CZ	0.02	52PRO CD	84ILE CG2	0.04	123AL C	126VAL CG2	1.27
121GLU CB	121GLU CG	100.00	52PRO C	53PHE CA	100.00	124AR CA	124ARG CB	100.00
121GLU CB	121GLU CD	100.00	52PRO C	53PHE CB	90.49	124AR CA	124ARG CG	100.00
121GLU CB	121GLU C	100.00	52PRO C	53PHE C	33.03	124AR CA	124ARG CD	0.32
121GLU CG	121GLU CD	100.00	52PRO C	54PRO CD	7.23	124AR CA	124ARG C	100.00
121GLU CG	121GLU C	87.08	52PRO C	89GLY CA	0.00	124AR CB	124ARG CG	100.00
121GLU CG	122ILE CG1	0.00	53PHE CA	53PHE CB	100.00	124AR CB	124ARG CD	100.00
121GLU CD	121GLU C	0.58	53PHE CA	53PHE CG	100.00	124AR CB	124ARG CZ	6.60
121GLU CD	124ARG CD	0.04	53PHE CA	53PHE CD1	97.58	124AR CB	124ARG C	100.00
121GLU CD	124ARG CZ	0.10	53PHE CA	53PHE CD2	14.30	124AR CG	124ARG CD	100.00
121GLU C	122ILE CA	100.00	53PHE CA	53PHE C	100.00	124AR CG	124ARG CZ	18.33
121GLU C	122ILE CB	11.06	53PHE CA	54PRO CD	100.00	124AR CG	124ARG C	74.44
121GLU C	122ILE CG1	0.69	53PHE CB	53PHE CG	100.00	124AR CD	124ARG CZ	100.00
121GLU C	122ILE CG2	5.26	53PHE CB	53PHE CD1	100.00	124AR C	125GLY CA	100.00
121GLU C	122ILE CD	0.01	53PHE CB	53PHE CD2	100.00	124AR C	125GLY C	56.94
121GLU C	122ILE C	88.87	53PHE CB	53PHE C	100.00	124AR C	126VAL CG2	0.00
121GLU C	124ARG CG	0.00	53PHE CB	89GLY CA	0.51	125GL CA	125GLY C	100.00
122ILE CA	122ILE CB	100.00	53PHE CB	89GLY C	0.01	125GL CA	228ALA CB	0.14
122ILE CA	122ILE CG1	100.00	53PHE CB	92SER CB	8.50	125GL C	126VAL CA	100.00
122ILE CA	122ILE CG2	100.00	53PHE CG	53PHE CD1	100.00	125GL C	126VAL CB	2.40
122ILE CA	122ILE CD	0.62	53PHE CG	53PHE CD2	100.00	125GL C	126VAL CG1	0.08
122ILE CA	122ILE C	100.00	53PHE CG	53PHE CE1	100.00	125GL C	126VAL CG2	1.01
122ILE CB	122ILE CG1	100.00	53PHE CG	53PHE CE2	100.00	125GL C	126VAL C	99.03
122ILE CB	122ILE CG2	100.00	53PHE CG	53PHE CZ	100.00	125GL C	228ALA CB	5.29
122ILE CB	122ILE CD	100.00	53PHE CG	53PHE C	98.97	126VA CA	126VAL CB	100.00
122ILE CB	122ILE C	100.00	53PHE CG	54PRO CD	0.01	126VA CA	126VAL CG1	100.00
122ILE CG1	122ILE CG2	100.00	53PHE CG	89GLY CA	0.20	126VA CA	126VAL CG2	100.00
122ILE CG1	122ILE CD	100.00	53PHE CG	92SER CB	3.15	126VA CA	126VAL C	100.00
122ILE CG1	122ILE C	40.64	53PHE CG	93LEU CD1	0.00	126VA CA	228ALA CB	0.00
122ILE CG2	122ILE CD	99.33	53PHE CG	93LEU CD2	0.00	126VA CB	126VAL CG1	100.00
122ILE CG2	122ILE C	59.29	53PHE CD1	53PHE CD2	100.00	126VA CB	126VAL CG2	100.00
122ILE CG2	225ARG C	0.01	53PHE CD1	53PHE CE1	100.00	126VA CB	126VAL C	100.00
122ILE CG2	226SER CB	0.00	53PHE CD1	53PHE CE2	100.00	126VA CG1	126VAL CG2	100.00
122ILE CG2	227PRO CG	0.14	53PHE CD1	53PHE CZ	100.00	126VA CG1	126VAL C	89.56
122ILE CG2	227PRO CD	6.42	53PHE CD1	53PHE C	0.78	126VA CG1	128VAL CB	0.00
122ILE CD	122ILE C	0.09	53PHE CD1	57THR CB	0.00	126VA CG1	128VAL CG1	0.29
122ILE CD	224VAL C	0.01	53PHE CD1	57THR CG2	2.82	126VA CG1	128VAL CG2	1.19
122ILE CD	225ARG C	0.05	53PHE CD1	58ARG CG	0.02	126VA CG1	227PRO CB	0.60
122ILE CD	227PRO CD	0.02	53PHE CD1	89GLY CA	0.26	126VA CG1	227PRO CG	1.99
122ILE C	123ALA CA	100.00	53PHE CD1	89GLY C	0.05	126VA CG1	227PRO C	0.06
122ILE C	123ALA CB	0.29	53PHE CD1	92SER CB	0.09	126VA CG1	228ALA CA	0.01
122ILE C	123ALA C	99.62	53PHE CD1	93LEU CG	0.02	126VA CG1	228ALA CB	0.00
123ALA CA	123ALA CB	100.00	53PHE CD1	93LEU CD1	0.38	126VA CG1	250LEU CD1	2.48
123ALA CA	123ALA C	100.00	53PHE CD1	93LEU CD2	0.81	126VA CG1	250LEU CD2	0.11
123ALA CA	126VAL CG2	0.04	53PHE CD2	53PHE CE1	100.00	126VA CG2	126VAL C	10.43
123ALA CA	250LEU CD2	0.00	53PHE CD2	53PHE CE2	100.00	126VA CG2	227PRO CB	0.75
123ALA CB	123ALA C	100.00	53PHE CD2	53PHE CZ	100.00	126VA CG2	227PRO CG	7.20
123ALA CB	250LEU CD2	0.00	53PHE CD2	53PHE C	3.60	126VA CG2	227PRO C	0.00
123ALA C	124ARG CA	100.00	53PHE CD2	57THR CG2	0.01	126VA CG2	228ALA CB	0.02
123ALA C	124ARG CB	4.22	53PHE CD2	58ARG CA	0.00	126VA CG2	250LEU CD1	0.02
123ALA C	124ARG C	96.12	53PHE CD2	58ARG CB	0.18	126VA C	127ASP CA	100.00
123ALA C	126VAL CG2	1.54	53PHE CD2	58ARG CG	4.40	126VA C	127ASP CB	99.90
124ARG CA	124ARG CB	100.00	53PHE CD2	58ARG CD	0.17	126VA C	127ASP C	0.69
124ARG CA	124ARG CG	100.00	53PHE CD2	58ARG CZ	0.65	127AS CA	127ASP CB	100.00
124ARG CA	124ARG CD	0.28	53PHE CD2	89GLY CA	0.03	127AS CA	127ASP CG	100.00
124ARG CA	124ARG C	100.00	53PHE CD2	92SER CB	13.63	127AS CA	127ASP C	100.00
124ARG CB	124ARG CG	100.00	53PHE CD2	92SER C	0.30	127AS CB	127ASP CG	100.00
124ARG CB	124ARG CD	100.00	53PHE CD2	93LEU CA	0.00	127AS CB	127ASP C	100.00
124ARG CB	124ARG CZ	10.52	53PHE CD2	96SER CB	0.00	127AS CG	127ASP C	100.00
124ARG CB	124ARG C	100.00	53PHE CE1	53PHE CE2	100.00	127AS C	128VAL CA	100.00
124ARG CG	124ARG CD	100.00	53PHE CE1	53PHE CZ	100.00	127AS C	128VAL CB	7.03

124ARG CG	124ARG	CZ	41.28	53PHE CE1	57THR	CG2	7.43	127AS C	128VAL	CG1	0.01
124ARG CG	124ARG	C	70.13	53PHE CE1	57THR	C	0.10	127AS C	128VAL	CG2	2.58
124ARG CD	124ARG	CZ	100.00	53PHE CE1	58ARG	CA	0.27	127AS C	128VAL	C	95.47
124ARG C	125GLY	CA	100.00	53PHE CE1	58ARG	CB	0.00	128VA CA	128VAL	CB	100.00
124ARG C	125GLY	C	54.12	53PHE CE1	58ARG	CG	0.06	128VA CA	128VAL	CG1	100.00
125GLY CA	125GLY	C	100.00	53PHE CE1	61VAL	CG1	0.00	128VA CA	128VAL	CG2	100.00
125GLY CA	228ALA	CB	0.16	53PHE CE1	61VAL	CG2	0.07	128VA CA	128VAL	C	100.00
125GLY C	126VAL	CA	100.00	53PHE CE1	89GLY	CA	0.01	128VA CB	128VAL	CG1	100.00
125GLY C	126VAL	CB	1.68	53PHE CE1	93LEU	CA	0.16	128VA CB	128VAL	CG2	100.00
125GLY C	126VAL	CG2	0.81	53PHE CE1	93LEU	CB	0.58	128VA CB	128VAL	C	100.00
125GLY C	126VAL	C	99.07	53PHE CE1	93LEU	CG	0.87	128VA CG1	128VAL	CG2	100.00
125GLY C	228ALA	CA	0.00	53PHE CE1	93LEU	CD1	4.88	128VA CG1	128VAL	C	99.22
125GLY C	228ALA	CB	6.54	53PHE CE1	93LEU	CD2	3.05	128VA CG1	129LEU	C	0.00
126VAL CA	126VAL	CB	100.00	53PHE CE2	53PHE	CZ	100.00	128VA CG1	130ILE	CG1	0.24
126VAL CA	126VAL	CG1	100.00	53PHE CE2	57THR	CG2	0.18	128VA CG1	130ILE	CD	0.38
126VAL CA	126VAL	CG2	100.00	53PHE CE2	57THR	C	0.04	128VA CG1	230PHE	CB	0.32
126VAL CA	126VAL	C	100.00	53PHE CE2	58ARG	CA	3.18	128VA CG1	230PHE	C	0.00
126VAL CA	228ALA	CB	0.00	53PHE CE2	58ARG	CB	3.04	128VA CG1	233VAL	CG1	0.00
126VAL CB	126VAL	CG1	100.00	53PHE CE2	58ARG	CG	6.24	128VA CG1	233VAL	CG2	2.59
126VAL CB	126VAL	CG2	100.00	53PHE CE2	58ARG	CD	0.34	128VA CG1	250LEU	CD2	0.01
126VAL CB	126VAL	C	100.00	53PHE CE2	58ARG	CZ	0.07	128VA CG2	128VAL	C	10.63
126VAL CB	250LEU	CD2	0.03	53PHE CE2	61VAL	CG2	0.00	128VA CG2	129LEU	C	0.00
126VAL CG1	126VAL	CG2	100.00	53PHE CE2	92SER	CB	0.34	128VA CG2	130ILE	CD	0.00
126VAL CG1	126VAL	C	100.00	53PHE CE2	92SER	C	0.48	128VA CG2	230PHE	CB	0.51
126VAL CG1	128VAL	CG1	0.00	53PHE CE2	93LEU	CA	0.81	128VA CG2	230PHE	C	0.04
126VAL CG1	128VAL	CG2	2.46	53PHE CE2	93LEU	CB	0.02	128VA CG2	233VAL	CG2	0.75
126VAL CG1	227PRO	CB	0.13	53PHE CE2	93LEU	CD1	0.02	128VA CG2	250LEU	CD1	0.07
126VAL CG1	227PRO	CG	0.04	53PHE CE2	93LEU	CD2	0.00	128VA C	129LEU	CA	100.00
126VAL CG1	227PRO	C	0.02	53PHE CE2	96SER	CB	0.60	128VA C	129LEU	CB	55.59
126VAL CG1	250LEU	CB	0.07	53PHE CZ	57THR	CB	0.00	128VA C	129LEU	CG	0.03
126VAL CG1	250LEU	CD1	5.51	53PHE CZ	57THR	CG2	0.56	128VA C	129LEU	C	58.07
126VAL CG1	250LEU	CD2	1.35	53PHE CZ	57THR	C	0.00	129LE CA	129LEU	CB	100.00
126VAL CG1	251PRO	CG	0.00	53PHE CZ	58ARG	CA	2.42	129LE CA	129LEU	CG	100.00
126VAL CG2	227PRO	CB	0.92	53PHE CZ	58ARG	CB	0.15	129LE CA	129LEU	CD1	78.30
126VAL CG2	227PRO	CG	6.01	53PHE CZ	58ARG	CG	0.32	129LE CA	129LEU	CD2	22.16
126VAL CG2	227PRO	C	0.02	53PHE CZ	61VAL	CG1	0.02	129LE CA	129LEU	C	100.00
126VAL CG2	228ALA	CA	0.04	53PHE CZ	61VAL	CG2	0.62	129LE CB	129LEU	CG	100.00
126VAL CG2	228ALA	CB	0.22	53PHE CZ	92SER	CB	0.00	129LE CB	129LEU	CD1	100.00
126VAL CG2	250LEU	CD2	0.02	53PHE CZ	93LEU	CA	0.82	129LE CB	129LEU	CD2	100.00
126VAL C	127ASP	CA	100.00	53PHE CZ	93LEU	CB	0.29	129LE CB	129LEU	C	100.00
126VAL C	127ASP	CB	99.96	53PHE CZ	93LEU	CG	0.17	129LE CB	176ARG	CZ	0.36
126VAL C	127ASP	C	0.43	53PHE CZ	93LEU	CD1	3.54	129LE CG	129LEU	CD1	100.00
127ASP CA	127ASP	CB	100.00	53PHE CZ	93LEU	CD2	0.76	129LE CG	129LEU	CD2	100.00
127ASP CA	127ASP	CG	100.00	53PHE CZ	96SER	CB	0.08	129LE CG	129LEU	C	86.71
127ASP CA	127ASP	C	100.00	53PHE C	54PRO	CA	100.00	129LE CG	172LEU	CD1	0.00
127ASP CB	127ASP	CG	100.00	53PHE C	54PRO	CB	1.50	129LE CG	176ARG	CZ	0.20
127ASP CB	127ASP	C	100.00	53PHE C	54PRO	CD	100.00	129LE CD1	129LEU	CD2	100.00
127ASP CG	127ASP	C	100.00	53PHE C	54PRO	C	99.99	129LE CD1	129LEU	C	5.72
127ASP CG	176ARG	CZ	0.02	54PRO CA	54PRO	CB	100.00	129LE CD1	130ILE	C	0.01
127ASP C	128VAL	CA	100.00	54PRO CA	54PRO	CG	100.00	129LE CD1	131VAL	CG2	0.20
127ASP C	128VAL	CB	7.58	54PRO CA	54PRO	CD	100.00	129LE CD1	172LEU	CD1	0.05
127ASP C	128VAL	CG1	0.12	54PRO CA	54PRO	C	100.00	129LE CD1	172LEU	CD2	0.10
127ASP C	128VAL	CG2	4.16	54PRO CB	54PRO	CG	100.00	129LE CD1	176ARG	CD	0.12
127ASP C	128VAL	C	95.01	54PRO CB	54PRO	CD	100.00	129LE CD1	176ARG	CZ	2.07
128VAL CA	128VAL	CB	100.00	54PRO CB	54PRO	C	100.00	129LE CD1	296MET	CE	0.01
128VAL CA	128VAL	CG1	100.00	54PRO CB	56PRO	CD	0.01	129LE CD1	300HIS	CE1	0.01
128VAL CA	128VAL	CG2	100.00	54PRO CG	54PRO	CD	100.00	129LE CD2	129LEU	C	7.40
128VAL CA	128VAL	C	100.00	54PRO CG	54PRO	C	98.64	129LE CD2	131VAL	CG2	0.68
128VAL CB	128VAL	CG1	100.00	54PRO CD	54PRO	C	95.76	129LE CD2	172LEU	CB	0.03
128VAL CB	128VAL	CG2	100.00	54PRO CD	57THR	CB	0.08	129LE CD2	172LEU	CG	0.00
128VAL CB	128VAL	C	100.00	54PRO CD	57THR	CG2	0.00	129LE CD2	172LEU	CD1	0.51
128VAL CB	130ILE	CD	0.00	54PRO C	55GLU	CA	100.00	129LE CD2	172LEU	CD2	0.55

128VAL	CG1	128VAL	CG2	100.00	54PRO	C	55GLU	C	100.00	129LE	CD2	176ARG	CD	0.02
128VAL	CG1	128VAL	C	98.95	54PRO	C	56PRO	CD	24.22	129LE	CD2	176ARG	CZ	0.15
128VAL	CG1	129LEU	C	0.01	54PRO	C	58ARG	CB	0.02	129LE	CD2	232VAL	CG2	0.05
128VAL	CG1	130ILE	CG1	0.40	54PRO	C	58ARG	CG	0.01	129LE	CD2	296MET	CE	0.02
128VAL	CG1	130ILE	CD	3.38	54PRO	C	58ARG	CD	0.02	129LE	CD2	300HIS	CE1	0.01
128VAL	CG1	230PHE	CB	1.16	54PRO	C	58ARG	CZ	0.44	129LE	C	130ILE	CA	100.00
128VAL	CG1	230PHE	C	0.00	55GLU	CA	55GLU	CB	100.00	129LE	C	130ILE	CB	7.12
128VAL	CG1	231ASP	C	0.01	55GLU	CA	55GLU	CG	100.00	129LE	C	130ILE	CG1	3.18
128VAL	CG1	233VAL	CG1	0.01	55GLU	CA	55GLU	CD	95.19	129LE	C	130ILE	CD	0.00
128VAL	CG1	233VAL	CG2	5.19	55GLU	CA	55GLU	C	100.00	129LE	C	130ILE	C	98.19
128VAL	CG1	250LEU	CD2	0.00	55GLU	CA	56PRO	CD	100.00	130ILE	CA	130ILE	CB	100.00
128VAL	CG2	128VAL	C	2.38	55GLU	CA	58ARG	CZ	0.43	130ILE	CA	130ILE	CG1	100.00
128VAL	CG2	130ILE	CD	0.02	55GLU	CB	55GLU	CG	100.00	130ILE	CA	130ILE	CG2	100.00
128VAL	CG2	230PHE	CB	0.23	55GLU	CB	55GLU	CD	100.00	130ILE	CA	130ILE	CD	8.84
128VAL	CG2	230PHE	C	0.03	55GLU	CB	55GLU	C	100.00	130ILE	CA	130ILE	C	100.00
128VAL	CG2	233VAL	CG2	0.33	55GLU	CB	56PRO	CD	72.21	130ILE	CA	233VAL	CG2	0.02
128VAL	CG2	250LEU	CD1	0.20	55GLU	CG	55GLU	CD	100.00	130ILE	CB	130ILE	CG1	100.00
128VAL	C	129LEU	CA	100.00	55GLU	CG	55GLU	C	87.02	130ILE	CB	130ILE	CG2	100.00
128VAL	C	129LEU	CB	43.56	55GLU	CG	56PRO	CD	0.20	130ILE	CB	130ILE	CD	100.00
128VAL	C	129LEU	C	68.77	55GLU	CG	59LYS	CD	0.00	130ILE	CB	130ILE	C	100.00
129LEU	CA	129LEU	CB	100.00	55GLU	CG	59LYS	CE	0.03	130ILE	CG1	130ILE	CG2	100.00
129LEU	CA	129LEU	CG	100.00	55GLU	CD	55GLU	C	2.58	130ILE	CG1	130ILE	CD	100.00
129LEU	CA	129LEU	CD1	88.99	55GLU	CD	58ARG	CZ	0.02	130ILE	CG1	233VAL	CG1	0.03
129LEU	CA	129LEU	CD2	11.25	55GLU	CD	59LYS	CG	0.01	130ILE	CG1	233VAL	CG2	0.28
129LEU	CA	129LEU	C	100.00	55GLU	CD	59LYS	CD	0.11	130ILE	CG1	243LEU	CD2	0.00
129LEU	CB	129LEU	CG	100.00	55GLU	CD	59LYS	CE	0.87	130ILE	CG2	130ILE	CD	86.54
129LEU	CB	129LEU	CD1	100.00	55GLU	C	56PRO	CA	100.00	130ILE	CG2	130ILE	C	100.00
129LEU	CB	129LEU	CD2	100.00	55GLU	C	56PRO	CB	0.05	130ILE	CG2	131VAL	C	0.00
129LEU	CB	129LEU	C	100.00	55GLU	C	56PRO	CD	100.00	130ILE	CG2	132ARG	CG	0.01
129LEU	CB	176ARG	CZ	0.76	55GLU	C	56PRO	C	100.00	130ILE	CG2	132ARG	CD	0.00
129LEU	CG	129LEU	CD1	100.00	55GLU	C	59LYS	CE	0.01	130ILE	CG2	233VAL	CG1	0.00
129LEU	CG	129LEU	CD2	100.00	56PRO	CA	56PRO	CB	100.00	130ILE	CG2	235THR	CG2	2.92
129LEU	CG	129LEU	C	92.75	56PRO	CA	56PRO	CG	100.00	130ILE	CG2	243LEU	CD2	0.12
129LEU	CG	131VAL	CG2	0.02	56PRO	CA	56PRO	CD	100.00	130ILE	CD	233VAL	CB	0.02
129LEU	CG	176ARG	CZ	0.18	56PRO	CA	56PRO	C	100.00	130ILE	CD	233VAL	CG1	0.01
129LEU	CD1	129LEU	CD2	100.00	56PRO	CA	59LYS	CD	0.00	130ILE	CD	233VAL	CG2	0.02
129LEU	CD1	129LEU	C	4.04	56PRO	CB	56PRO	CG	100.00	130ILE	CD	243LEU	CG	0.00
129LEU	CD1	130ILE	C	0.01	56PRO	CB	56PRO	CD	100.00	130ILE	CD	243LEU	CD1	0.00
129LEU	CD1	131VAL	CG2	0.24	56PRO	CB	56PRO	C	100.00	130ILE	CD	243LEU	CD2	0.10
129LEU	CD1	172ALA	CB	0.01	56PRO	CG	56PRO	CD	100.00	130ILE	CD	243LEU	C	0.08
129LEU	CD1	176ARG	CD	0.05	56PRO	CG	56PRO	C	88.38	130ILE	CD	244SER	CA	0.22
129LEU	CD1	176ARG	CZ	0.33	56PRO	CD	56PRO	C	73.19	130ILE	CD	247ALA	CB	8.74
129LEU	CD1	296MET	CE	0.04	56PRO	C	57THR	CA	100.00	130ILE	C	131VAL	CA	100.00
129LEU	CD2	129LEU	C	2.12	56PRO	C	57THR	CB	0.02	130ILE	C	131VAL	CB	4.07
129LEU	CD2	130ILE	C	0.00	56PRO	C	57THR	CG2	0.00	130ILE	C	131VAL	CG2	2.64
129LEU	CD2	131VAL	CG2	0.71	56PRO	C	57THR	C	99.99	130ILE	C	131VAL	C	98.79
129LEU	CD2	172ALA	CB	5.37	57THR	CA	57THR	CB	100.00	131VA	CA	131VAL	CB	100.00
129LEU	CD2	176ARG	CD	0.02	57THR	CA	57THR	CG2	100.00	131VA	CA	131VAL	CG1	100.00
129LEU	CD2	176ARG	CZ	0.04	57THR	CA	57THR	C	100.00	131VA	CA	131VAL	CG2	100.00
129LEU	CD2	232VAL	CG2	0.01	57THR	CB	57THR	CG2	100.00	131VA	CA	131VAL	C	100.00
129LEU	CD2	300HIS	CE1	0.00	57THR	CB	57THR	C	100.00	131VA	CB	131VAL	CG1	100.00
129LEU	C	130ILE	CA	100.00	57THR	CG2	57THR	C	99.64	131VA	CB	131VAL	CG2	100.00
129LEU	C	130ILE	CB	11.16	57THR	CG2	61VAL	CG1	0.00	131VA	CB	131VAL	C	100.00
129LEU	C	130ILE	CG1	6.30	57THR	CG2	61VAL	CG2	0.03	131VA	CB	234VAL	CG2	0.01
129LEU	C	130ILE	CD	0.15	57THR	C	58ARG	CA	100.00	131VA	CG1	131VAL	CG2	100.00
129LEU	C	130ILE	C	95.82	57THR	C	58ARG	CB	0.01	131VA	CG1	131VAL	C	100.00
130ILE	CA	130ILE	CB	100.00	57THR	C	58ARG	C	100.00	131VA	CG1	165VAL	CG1	0.16
130ILE	CA	130ILE	CG1	100.00	58ARG	CA	58ARG	CB	100.00	131VA	CG1	165VAL	CG2	0.70
130ILE	CA	130ILE	CG2	100.00	58ARG	CA	58ARG	CG	100.00	131VA	CG1	168VAL	CG1	0.54
130ILE	CA	130ILE	CD	47.18	58ARG	CA	58ARG	CD	0.01	131VA	CG1	168VAL	CG2	0.07
130ILE	CA	130ILE	C	100.00	58ARG	CA	58ARG	C	100.00	131VA	CG1	234VAL	CG2	0.15
130ILE	CA	233VAL	CG2	0.02	58ARG	CB	58ARG	CG	100.00	131VA	CG2	169ALA	CB	0.25

130ILE	CB	130ILE	CG1	100.00
130ILE	CB	130ILE	CG2	100.00
130ILE	CB	130ILE	CD	100.00
130ILE	CB	130ILE	C	100.00
130ILE	CG1	130ILE	CG2	100.00
130ILE	CG1	130ILE	CD	100.00
130ILE	CG1	233VAL	CB	0.01
130ILE	CG1	233VAL	CG1	0.02
130ILE	CG1	233VAL	CG2	0.16
130ILE	CG1	247ALA	CB	1.71
130ILE	CG2	130ILE	CD	81.74
130ILE	CG2	130ILE	C	100.00
130ILE	CG2	131VAL	C	0.00
130ILE	CG2	132ARG	CB	0.00
130ILE	CG2	233VAL	CG1	0.00
130ILE	CG2	233VAL	CG2	0.01
130ILE	CG2	235THR	CG2	0.47
130ILE	CG2	243LEU	CB	0.02
130ILE	CG2	243LEU	CD1	0.00
130ILE	CG2	243LEU	CD2	0.12
130ILE	CD	233VAL	CB	0.24
130ILE	CD	233VAL	CG1	0.30
130ILE	CD	233VAL	CG2	0.39
130ILE	CD	243LEU	CG	0.00
130ILE	CD	243LEU	CD1	0.00
130ILE	CD	243LEU	CD2	0.03
130ILE	CD	243LEU	C	0.02
130ILE	CD	244SER	CA	0.00
130ILE	CD	247ALA	CB	16.96
130ILE	C	131VAL	CA	100.00
130ILE	C	131VAL	CB	5.63
130ILE	C	131VAL	CG2	4.18
130ILE	C	131VAL	C	97.62
131VAL	CA	131VAL	CB	100.00
131VAL	CA	131VAL	CG1	100.00
131VAL	CA	131VAL	CG2	100.00
131VAL	CA	131VAL	C	100.00
131VAL	CB	131VAL	CG1	100.00
131VAL	CB	131VAL	CG2	100.00
131VAL	CB	131VAL	C	100.00
131VAL	CB	234VAL	CG2	0.05
131VAL	CG1	131VAL	CG2	100.00
131VAL	CG1	131VAL	C	100.00
131VAL	CG1	165VAL	CG1	0.25
131VAL	CG1	165VAL	CG2	1.48
131VAL	CG1	168VAL	CG1	0.03
131VAL	CG1	168VAL	CG2	0.59
131VAL	CG1	169ALA	CB	0.01
131VAL	CG1	234VAL	CG2	0.82
131VAL	CG2	169ALA	CB	0.19
131VAL	CG2	232VAL	CG1	0.16
131VAL	C	132ARG	CA	100.00
131VAL	C	132ARG	CB	81.65
131VAL	C	132ARG	CG	0.00
131VAL	C	132ARG	C	25.78
132ARG	CA	132ARG	CB	100.00
132ARG	CA	132ARG	CG	100.00
132ARG	CA	132ARG	CD	27.60
132ARG	CA	132ARG	C	100.00
132ARG	CB	132ARG	CG	100.00
132ARG	CB	132ARG	CD	100.00
132ARG	CB	132ARG	CZ	0.00

58ARG	CB	58ARG	CD	100.00
58ARG	CB	58ARG	CZ	1.74
58ARG	CB	58ARG	C	100.00
58ARG	CG	58ARG	CD	100.00
58ARG	CG	58ARG	CZ	14.16
58ARG	CG	58ARG	C	73.00
58ARG	CG	96SER	CB	0.01
58ARG	CD	58ARG	CZ	100.00
58ARG	CD	58ARG	C	0.00
58ARG	CD	96SER	CB	0.00
58ARG	CZ	92SER	CB	0.00
58ARG	CZ	96SER	CB	0.01
58ARG	C	59LYS	CA	100.00
58ARG	C	59LYS	CB	7.14
58ARG	C	59LYS	CG	0.27
58ARG	C	59LYS	CD	0.01
58ARG	C	59LYS	C	92.81
59LYS	CA	59LYS	CB	100.00
59LYS	CA	59LYS	CG	100.00
59LYS	CA	59LYS	CD	10.07
59LYS	CA	59LYS	CE	0.09
59LYS	CA	59LYS	C	100.00
59LYS	CA	62GLU	CB	0.00
59LYS	CB	59LYS	CG	100.00
59LYS	CB	59LYS	CD	100.00
59LYS	CB	59LYS	CE	11.42
59LYS	CB	59LYS	C	100.00
59LYS	CG	59LYS	CD	100.00
59LYS	CG	59LYS	CE	100.00
59LYS	CG	59LYS	C	17.78
59LYS	CD	59LYS	CE	100.00
59LYS	CD	59LYS	C	0.08
59LYS	CD	63GLU	CG	0.00
59LYS	CE	62GLU	CD	0.01
59LYS	CE	63GLU	CB	0.00
59LYS	C	60GLY	CA	100.00
59LYS	C	60GLY	C	81.19
59LYS	C	62GLU	CB	0.05
59LYS	C	62GLU	CG	0.01
59LYS	C	63GLU	CG	0.00
60GLY	CA	60GLY	C	100.00
60GLY	CA	63GLU	CB	0.00
60GLY	CA	63GLU	CG	0.00
60GLY	C	61VAL	CA	100.00
60GLY	C	61VAL	CB	0.42
60GLY	C	61VAL	CG1	0.15
60GLY	C	61VAL	CG2	0.01
60GLY	C	61VAL	C	99.68
61VAL	CA	61VAL	CB	100.00
61VAL	CA	61VAL	CG1	100.00
61VAL	CA	61VAL	CG2	100.00
61VAL	CA	61VAL	C	100.00
61VAL	CA	67VAL	CG2	0.00
61VAL	CB	61VAL	CG1	100.00
61VAL	CB	61VAL	CG2	100.00
61VAL	CB	61VAL	C	100.00
61VAL	CB	67VAL	CG2	0.58
61VAL	CB	93LEU	CD1	0.01
61VAL	CG1	61VAL	CG2	100.00
61VAL	CG1	61VAL	C	42.48
61VAL	CG1	67VAL	CG1	0.42
61VAL	CG1	67VAL	CG2	8.17

131VA	CG2	172LEU	CD1	3.53
131VA	CG2	172LEU	CD2	0.36
131VA	CG2	232VAL	CG1	0.07
131VA	CG2	232VAL	CG2	0.02
131VA	C	132ARG	CA	100.00
131VA	C	132ARG	CB	76.11
131VA	C	132ARG	CG	0.41
131VA	C	132ARG	C	28.58
132AR	CA	132ARG	CB	100.00
132AR	CA	132ARG	CG	100.00
132AR	CA	132ARG	CD	2.79
132AR	CA	132ARG	C	100.00
132AR	CA	235THR	CG2	0.01
132AR	CB	132ARG	CG	100.00
132AR	CB	132ARG	CD	100.00
132AR	CB	132ARG	C	100.00
132AR	CB	235THR	CG2	0.12
132AR	CG	132ARG	CD	100.00
132AR	CG	132ARG	CZ	4.92
132AR	CG	132ARG	C	89.10
132AR	CG	133GLU	C	0.03
132AR	CG	134LEU	CD1	0.01
132AR	CG	235THR	CG2	0.06
132AR	CG	240GLY	CA	0.04
132AR	CG	240GLY	C	0.09
132AR	CD	132ARG	CZ	100.00
132AR	CD	132ARG	C	4.47
132AR	CD	134LEU	CD1	0.12
132AR	CD	134LEU	CD2	0.01
132AR	CD	240GLY	CA	0.01
132AR	CD	240GLY	C	0.20
132AR	CZ	134LEU	CB	0.22
132AR	CZ	134LEU	CG	0.02
132AR	CZ	134LEU	CD1	2.97
132AR	CZ	134LEU	CD2	0.48
132AR	CZ	240GLY	C	0.62
132AR	CZ	241ASP	CA	4.36
132AR	CZ	241ASP	CB	0.74
132AR	CZ	241ASP	CG	0.04
132AR	C	133GLU	CA	100.00
132AR	C	133GLU	CB	1.32
132AR	C	133GLU	C	99.50
132AR	C	165VAL	CG1	0.07
132AR	C	165VAL	CG2	2.45
133GL	CA	133GLU	CB	100.00
133GL	CA	133GLU	CG	100.00
133GL	CA	133GLU	CD	99.23
133GL	CA	133GLU	C	100.00
133GL	CA	165VAL	CG1	0.10
133GL	CA	165VAL	CG2	0.05
133GL	CB	133GLU	CG	100.00
133GL	CB	133GLU	CD	100.00
133GL	CB	133GLU	C	100.00
133GL	CB	165VAL	CG1	0.02
133GL	CB	165VAL	CG2	0.01
133GL	CB	236GLY	CA	0.20
133GL	CB	236GLY	C	4.37
133GL	CB	237ASN	CG	0.00
133GL	CG	133GLU	CD	100.00
133GL	CG	133GLU	C	76.66
133GL	CG	136GLY	CA	0.49
133GL	CG	136GLY	C	0.05

132ARG CB	132ARG	C	100.00	61VAL CG1	93LEU	CD1	0.50	133GL CG	157TYR	CE1	0.00
132ARG CB	235THR	CG2	0.02	61VAL CG1	93LEU	CD2	0.10	133GL CG	165VAL	CG1	2.18
132ARG CB	240GLY	CA	0.00	61VAL CG1	97GLN	CG	3.06	133GL CG	165VAL	CG2	1.30
132ARG CB	240GLY	C	0.00	61VAL CG1	97GLN	CD	1.92	133GL CD	133GLU	C	40.35
132ARG CG	132ARG	CD	100.00	61VAL CG1	266THR	CB	0.02	133GL CD	135THR	C	0.02
132ARG CG	132ARG	CZ	34.11	61VAL CG1	266THR	CG2	0.00	133GL CD	136GLY	CA	0.00
132ARG CG	132ARG	C	99.85	61VAL CG2	61VAL	C	57.52	133GL CD	157TYR	CE1	0.46
132ARG CG	133GLU	C	0.06	61VAL CG2	67VAL	CG1	0.00	133GL CD	165VAL	CG2	0.04
132ARG CG	240GLY	CA	0.24	61VAL CG2	67VAL	CG2	0.36	133GL C	134LEU	CA	100.00
132ARG CG	240GLY	C	0.94	61VAL CG2	93LEU	CD1	0.20	133GL C	134LEU	CB	92.26
132ARG CD	132ARG	CZ	100.00	61VAL CG2	93LEU	CD2	0.02	133GL C	134LEU	CG	0.20
132ARG CD	132ARG	C	42.30	61VAL CG2	97GLN	CG	0.06	133GL C	134LEU	CD2	0.16
132ARG CD	133GLU	C	0.15	61VAL CG2	97GLN	CD	0.31	133GL C	134LEU	C	6.68
132ARG CD	134LEU	CD1	0.02	61VAL C	62GLU	CA	100.00	134LE CA	134LEU	CB	100.00
132ARG CD	134LEU	CD2	1.23	61VAL C	62GLU	CB	0.58	134LE CA	134LEU	CG	100.00
132ARG CD	240GLY	C	0.45	61VAL C	62GLU	CG	0.00	134LE CA	134LEU	CD1	42.30
132ARG CD	241ASP	CA	0.02	61VAL C	62GLU	C	99.52	134LE CA	134LEU	CD2	54.95
132ARG CZ	134LEU	CG	0.69	62GLU CA	62GLU	CB	100.00	134LE CA	134LEU	C	100.00
132ARG CZ	134LEU	CD1	1.79	62GLU CA	62GLU	CG	100.00	134LE CA	237ASN	CG	0.02
132ARG CZ	134LEU	CD2	3.64	62GLU CA	62GLU	CD	92.80	134LE CB	134LEU	CG	100.00
132ARG CZ	241ASP	CA	0.73	62GLU CA	62GLU	C	100.00	134LE CB	134LEU	CD1	100.00
132ARG CZ	241ASP	CB	0.88	62GLU CB	62GLU	CG	100.00	134LE CB	134LEU	CD2	100.00
132ARG CZ	241ASP	CG	0.14	62GLU CB	62GLU	CD	100.00	134LE CB	134LEU	C	100.00
132ARG C	133GLU	CA	100.00	62GLU CB	62GLU	C	100.00	134LE CB	135THR	CG2	0.05
132ARG C	133GLU	CB	0.61	62GLU CG	62GLU	CD	100.00	134LE CB	237ASN	CG	0.11
132ARG C	133GLU	C	99.88	62GLU CG	62GLU	C	83.10	134LE CG	134LEU	CD1	100.00
132ARG C	165VAL	CG1	0.04	62GLU CD	62GLU	C	0.96	134LE CG	134LEU	CD2	100.00
132ARG C	165VAL	CG2	0.04	62GLU C	63GLU	CA	100.00	134LE CG	134LEU	C	90.22
133GLU CA	133GLU	CB	100.00	62GLU C	63GLU	CB	31.08	134LE CD1	134LEU	CD2	100.00
133GLU CA	133GLU	CG	100.00	62GLU C	63GLU	CG	0.04	134LE CD1	134LEU	C	18.80
133GLU CA	133GLU	CD	96.83	62GLU C	63GLU	C	73.10	134LE CD1	135THR	CG2	0.52
133GLU CA	133GLU	C	100.00	63GLU CA	63GLU	CB	100.00	134LE CD1	140PHE	CE2	0.00
133GLU CB	133GLU	CG	100.00	63GLU CA	63GLU	CG	100.00	134LE CD1	237ASN	CG	0.03
133GLU CB	133GLU	CD	100.00	63GLU CA	63GLU	CD	68.79	134LE CD1	261SER	CB	0.20
133GLU CB	133GLU	C	100.00	63GLU CA	63GLU	C	100.00	134LE CD2	134LEU	C	1.04
133GLU CB	165VAL	CG1	0.01	63GLU CB	63GLU	CG	100.00	134LE CD2	135THR	CG2	0.01
133GLU CB	165VAL	CG2	0.05	63GLU CB	63GLU	CD	100.00	134LE CD2	237ASN	CG	0.02
133GLU CB	236GLY	CA	0.35	63GLU CB	63GLU	C	100.00	134LE CD2	261SER	CB	0.24
133GLU CB	236GLY	C	4.09	63GLU CG	63GLU	CD	100.00	134LE C	135THR	CA	100.00
133GLU CG	133GLU	CD	100.00	63GLU CG	63GLU	C	89.53	134LE C	135THR	CB	26.52
133GLU CG	133GLU	C	84.42	63GLU CD	63GLU	C	3.96	134LE C	135THR	CG2	15.15
133GLU CG	157TYR	CE1	0.00	63GLU C	64ALA	CA	100.00	134LE C	135THR	C	74.94
133GLU CG	165VAL	CB	0.02	63GLU C	64ALA	CB	33.95	134LE C	140PHE	CE1	0.02
133GLU CG	165VAL	CG1	1.04	63GLU C	64ALA	C	66.26	134LE C	140PHE	CE2	0.05
133GLU CG	165VAL	CG2	8.18	64ALA CA	64ALA	CB	100.00	134LE C	140PHE	CZ	0.02
133GLU CD	133GLU	C	58.42	64ALA CA	64ALA	C	100.00	135TH CA	135THR	CB	100.00
133GLU CD	136GLY	CA	0.00	64ALA CB	64ALA	C	100.00	135TH CA	135THR	CG2	100.00
133GLU CD	157TYR	CE1	0.58	64ALA CB	266THR	CG2	0.01	135TH CA	135THR	C	100.00
133GLU C	134LEU	CA	100.00	64ALA C	65GLU	CA	100.00	135TH CA	140PHE	CE2	0.00
133GLU C	134LEU	CB	35.71	64ALA C	65GLU	CB	9.27	135TH CB	135THR	CG2	100.00
133GLU C	134LEU	CG	1.15	64ALA C	65GLU	CG	0.06	135TH CB	135THR	C	100.00
133GLU C	134LEU	CD2	0.01	64ALA C	65GLU	CD	0.05	135TH C	136GLY	CA	100.00
133GLU C	134LEU	C	46.01	64ALA C	65GLU	C	91.91	135TH C	136GLY	C	30.95
133GLU C	237ASN	CG	0.00	64ALA C	266THR	CG2	0.39	135TH C	140PHE	CG	0.00
134LEU CA	134LEU	CB	100.00	65GLU CA	65GLU	CB	100.00	135TH C	140PHE	CD2	1.03
134LEU CA	134LEU	CG	100.00	65GLU CA	65GLU	CG	100.00	135TH C	140PHE	CE1	0.43
134LEU CA	134LEU	CD1	25.73	65GLU CA	65GLU	CD	92.23	135TH C	140PHE	CE2	0.73
134LEU CA	134LEU	CD2	73.67	65GLU CA	65GLU	C	100.00	135TH C	140PHE	CZ	0.79
134LEU CA	134LEU	C	100.00	65GLU CA	266THR	CG2	0.00	136GL CA	136GLY	C	100.00
134LEU CB	134LEU	CG	100.00	65GLU CB	65GLU	CG	100.00	136GL CA	140PHE	CE1	0.21
134LEU CB	134LEU	CD1	100.00	65GLU CB	65GLU	CD	100.00	136GL CA	140PHE	CE2	0.06
134LEU CB	134LEU	CD2	100.00	65GLU CB	65GLU	C	100.00	136GL CA	140PHE	CZ	0.09

134LEU	CB	134LEU	C	100.00	65GLU	CB	302PHE	CE1	0.22	136GL	CA	157TYR	CE1	0.51
134LEU	CB	135THR	CG2	0.30	65GLU	CB	302PHE	CE2	0.17	136GL	CA	157TYR	CZ	0.05
134LEU	CG	134LEU	CD1	100.00	65GLU	CB	302PHE	CZ	0.27	136GL	C	137GLY	CA	100.00
134LEU	CG	134LEU	CD2	100.00	65GLU	CG	65GLU	CD	100.00	136GL	C	137GLY	C	41.41
134LEU	CG	134LEU	C	27.16	65GLU	CG	65GLU	C	73.09	136GL	C	140PHE	CD1	0.01
134LEU	CG	135THR	CG2	0.01	65GLU	CG	301ALA	CB	0.02	136GL	C	140PHE	CD2	0.02
134LEU	CD1	134LEU	CD2	100.00	65GLU	CG	302PHE	CD1	0.03	136GL	C	140PHE	CE1	2.83
134LEU	CD1	134LEU	C	0.24	65GLU	CG	302PHE	CD2	0.00	136GL	C	140PHE	CE2	1.32
134LEU	CD1	261SER	CB	0.03	65GLU	CG	302PHE	CE1	0.51	136GL	C	140PHE	CZ	2.12
134LEU	CD2	134LEU	C	1.06	65GLU	CG	302PHE	CE2	0.02	136GL	C	157TYR	CE1	0.38
134LEU	CD2	135THR	CG2	0.02	65GLU	CG	302PHE	CZ	0.03	136GL	C	157TYR	CE2	0.08
134LEU	CD2	261SER	CB	0.18	65GLU	CD	65GLU	C	0.01	136GL	C	157TYR	CZ	3.14
134LEU	C	135THR	CA	100.00	65GLU	C	66ALA	CA	100.00	136GL	C	237ASN	CG	0.00
134LEU	C	135THR	CB	73.41	65GLU	C	66ALA	CB	99.59	137GL	CA	137GLY	C	100.00
134LEU	C	135THR	CG2	44.02	65GLU	C	66ALA	C	0.38	137GL	CA	139TYR	CE2	0.00
134LEU	C	135THR	C	28.53	65GLU	C	266THR	CG2	7.30	137GL	CA	140PHE	CE2	0.00
134LEU	C	140PHE	CE1	0.02	66ALA	CA	66ALA	CB	100.00	137GL	CA	155GLU	CD	0.16
134LEU	C	140PHE	CE2	0.03	66ALA	CA	66ALA	C	100.00	137GL	CA	157TYR	CD2	0.08
134LEU	C	237ASN	CG	0.02	66ALA	CA	266THR	CG2	7.52	137GL	CA	157TYR	CE2	4.48
135THR	CA	135THR	CB	100.00	66ALA	CB	66ALA	C	100.00	137GL	CA	157TYR	CZ	0.16
135THR	CA	135THR	CG2	100.00	66ALA	CB	68LEU	CD1	0.00	137GL	CA	237ASN	CB	1.70
135THR	CA	135THR	C	100.00	66ALA	CB	269PHE	CD1	0.06	137GL	CA	237ASN	CG	0.06
135THR	CA	140PHE	CE1	0.01	66ALA	CB	269PHE	CE1	2.41	137GL	C	138ILE	CA	100.00
135THR	CA	140PHE	CE2	0.01	66ALA	CB	297MET	CB	0.00	137GL	C	138ILE	CB	0.00
135THR	CB	135THR	CG2	100.00	66ALA	CB	297MET	CG	4.54	137GL	C	138ILE	C	100.00
135THR	CB	135THR	C	100.00	66ALA	CB	297MET	CE	0.47	137GL	C	139TYR	CD2	0.02
135THR	C	136GLY	CA	100.00	66ALA	CB	301ALA	CB	0.24	137GL	C	155GLU	CD	0.60
135THR	C	136GLY	C	49.12	66ALA	CB	302PHE	CE1	0.04	138ILE	CA	138ILE	CB	100.00
135THR	C	140PHE	CG	0.01	66ALA	CB	302PHE	CE2	0.03	138ILE	CA	138ILE	CG1	100.00
135THR	C	140PHE	CD1	0.68	66ALA	CB	302PHE	CZ	0.05	138ILE	CA	138ILE	CG2	100.00
135THR	C	140PHE	CD2	2.25	66ALA	C	67VAL	CA	100.00	138ILE	CA	138ILE	CD	1.74
135THR	C	140PHE	CE1	0.07	66ALA	C	67VAL	CB	21.53	138ILE	CA	138ILE	C	100.00
135THR	C	140PHE	CE2	0.38	66ALA	C	67VAL	CG2	13.14	138ILE	CA	155GLU	CD	0.01
135THR	C	140PHE	CZ	0.08	66ALA	C	67VAL	C	87.72	138ILE	CB	138ILE	CG1	100.00
136GLY	CA	136GLY	C	100.00	66ALA	C	266THR	CG2	3.52	138ILE	CB	138ILE	CG2	100.00
136GLY	CA	140PHE	CD1	0.00	67VAL	CA	67VAL	CB	100.00	138ILE	CB	138ILE	CD	100.00
136GLY	CA	140PHE	CE2	0.00	67VAL	CA	67VAL	CG1	100.00	138ILE	CB	138ILE	C	100.00
136GLY	CA	157TYR	CE1	1.04	67VAL	CA	67VAL	CG2	100.00	138ILE	CB	155GLU	CA	0.02
136GLY	CA	157TYR	CZ	0.12	67VAL	CA	67VAL	C	100.00	138ILE	CB	155GLU	CD	0.00
136GLY	CA	161GLU	CG	0.00	67VAL	CB	67VAL	CG1	100.00	138ILE	CG1	138ILE	CG2	100.00
136GLY	CA	161GLU	CD	0.01	67VAL	CB	67VAL	CG2	100.00	138ILE	CG1	138ILE	CD	100.00
136GLY	C	137GLY	CA	100.00	67VAL	CB	67VAL	C	100.00	138ILE	CG1	138ILE	C	99.99
136GLY	C	137GLY	C	80.20	67VAL	CB	268VAL	CG2	0.14	138ILE	CG1	153ASN	CB	0.66
136GLY	C	140PHE	CB	0.00	67VAL	CG1	67VAL	CG2	100.00	138ILE	CG1	154THR	C	0.28
136GLY	C	140PHE	CD1	0.01	67VAL	CG1	67VAL	C	100.00	138ILE	CG2	138ILE	CD	98.20
136GLY	C	140PHE	CD2	0.02	67VAL	CG1	68LEU	C	0.23	138ILE	CG2	138ILE	C	99.86
136GLY	C	140PHE	CE1	0.02	67VAL	CG1	69LEU	CD1	0.06	138ILE	CG2	139TYR	CG	0.02
136GLY	C	140PHE	CE2	0.04	67VAL	CG1	69LEU	CD2	0.07	138ILE	CG2	139TYR	CD1	1.52
136GLY	C	140PHE	CZ	0.02	67VAL	CG1	268VAL	CG1	0.20	138ILE	CG2	139TYR	CD2	0.63
136GLY	C	157TYR	CE1	2.03	67VAL	CG1	268VAL	CG2	0.02	138ILE	CG2	139TYR	CE1	2.00
136GLY	C	157TYR	CE2	0.80	67VAL	CG2	266THR	CB	0.03	138ILE	CG2	139TYR	CE2	1.18
136GLY	C	157TYR	CZ	2.98	67VAL	CG2	266THR	CG2	8.60	138ILE	CG2	139TYR	CZ	0.71
136GLY	C	237ASN	CB	0.04	67VAL	CG2	268VAL	CG2	0.27	138ILE	CG2	238ILE	CA	0.00
136GLY	C	237ASN	CG	0.01	67VAL	C	68LEU	CA	100.00	138ILE	CG2	238ILE	CB	0.04
137GLY	CA	137GLY	C	100.00	67VAL	C	68LEU	CB	21.11	138ILE	CG2	238ILE	CG1	1.25
137GLY	CA	155GLU	CD	0.49	67VAL	C	68LEU	CD1	0.01	138ILE	CG2	238ILE	CG2	0.05
137GLY	CA	157TYR	CD2	0.02	67VAL	C	68LEU	C	90.21	138ILE	CG2	238ILE	CD	4.07
137GLY	CA	157TYR	CE2	3.53	67VAL	C	297MET	CE	0.36	138ILE	CD	138ILE	C	0.24
137GLY	CA	157TYR	CZ	0.98	68LEU	CA	68LEU	CB	100.00	138ILE	CD	153ASN	CB	0.07
137GLY	CA	237ASN	CB	0.01	68LEU	CA	68LEU	CG	100.00	138ILE	CD	153ASN	C	0.12
137GLY	C	138ILE	CA	100.00	68LEU	CA	68LEU	CD1	83.54	138ILE	CD	154THR	C	0.35
137GLY	C	138ILE	C	100.00	68LEU	CA	68LEU	CD2	16.10	138ILE	CD	155GLU	CB	0.01

137GLY C	155GLU CD	0.07	68LEU CA	68LEU C	100.00	138ILE CD	238ILE CD	0.34
138ILE CA	138ILE CB	100.00	68LEU CA	297MET CE	0.01	138ILE C	139TYR CA	100.00
138ILE CA	138ILE CG1	100.00	68LEU CB	68LEU CG	100.00	138ILE C	139TYR CB	0.04
138ILE CA	138ILE CG2	100.00	68LEU CB	68LEU CD1	100.00	138ILE C	139TYR CG	0.00
138ILE CA	138ILE CD	43.13	68LEU CB	68LEU CD2	100.00	138ILE C	139TYR C	99.92
138ILE CA	138ILE C	100.00	68LEU CB	68LEU C	100.00	139TY CA	139TYR CB	100.00
138ILE CA	154THR C	0.00	68LEU CB	297MET CE	0.04	139TY CA	139TYR CG	100.00
138ILE CA	155GLU CA	0.02	68LEU CG	68LEU CD1	100.00	139TY CA	139TYR CD1	98.70
138ILE CB	138ILE CG1	100.00	68LEU CG	68LEU CD2	100.00	139TY CA	139TYR CD2	9.62
138ILE CB	138ILE CG2	100.00	68LEU CG	68LEU C	84.26	139TY CA	139TYR C	100.00
138ILE CB	138ILE CD	100.00	68LEU CG	297MET CE	0.14	139TY CB	139TYR CG	100.00
138ILE CB	138ILE C	100.00	68LEU CD1	68LEU CD2	100.00	139TY CB	139TYR CD1	100.00
138ILE CB	155GLU CA	0.05	68LEU CD1	68LEU C	0.87	139TY CB	139TYR CD2	100.00
138ILE CB	155GLU CD	0.02	68LEU CD1	269PHE CB	0.14	139TY CB	139TYR C	100.00
138ILE CB	238ILE CD	0.04	68LEU CD1	293SER CB	0.11	139TY CB	140PHE CD1	1.17
138ILE CG1	138ILE CG2	100.00	68LEU CD1	293SER C	0.02	139TY CB	140PHE CD2	1.36
138ILE CG1	138ILE CD	100.00	68LEU CD1	294ALA CA	0.03	139TY CB	140PHE CE1	0.72
138ILE CG1	138ILE C	100.00	68LEU CD1	294ALA CB	0.01	139TY CB	140PHE CE2	0.74
138ILE CG1	153ASN CB	2.69	68LEU CD1	297MET CE	0.89	139TY CB	140PHE CZ	0.08
138ILE CG1	153ASN CG	0.00	68LEU CD2	68LEU C	1.23	139TY CG	139TYR CD1	100.00
138ILE CG1	154THR C	0.14	68LEU CD2	269PHE CB	0.01	139TY CG	139TYR CD2	100.00
138ILE CG2	138ILE CD	55.86	68LEU CD2	293SER CB	0.00	139TY CG	139TYR CE1	100.00
138ILE CG2	138ILE C	99.96	68LEU CD2	294ALA CA	0.00	139TY CG	139TYR CE2	100.00
138ILE CG2	139TYR CG	0.16	68LEU CD2	294ALA CB	0.01	139TY CG	139TYR CZ	100.00
138ILE CG2	139TYR CD1	2.76	68LEU CD2	297MET CE	0.22	139TY CG	139TYR C	0.36
138ILE CG2	139TYR CD2	1.55	68LEU C	69LEU CA	100.00	139TY CD1	139TYR CD2	100.00
138ILE CG2	139TYR CE1	3.10	68LEU C	69LEU CB	22.77	139TY CD1	139TYR CE1	100.00
138ILE CG2	139TYR CE2	1.47	68LEU C	69LEU CG	0.00	139TY CD1	139TYR CE2	100.00
138ILE CG2	139TYR CZ	1.10	68LEU C	69LEU CD1	0.02	139TY CD1	139TYR CZ	100.00
138ILE CG2	155GLU CD	0.03	68LEU C	69LEU CD2	0.00	139TY CD2	139TYR CE1	100.00
138ILE CG2	238ILE CA	0.01	68LEU C	69LEU C	85.80	139TY CD2	139TYR CE2	100.00
138ILE CG2	238ILE CB	0.48	69LEU CA	69LEU CB	100.00	139TY CD2	139TYR CZ	100.00
138ILE CG2	238ILE CG1	1.75	69LEU CA	69LEU CG	100.00	139TY CD2	139TYR C	0.06
138ILE CG2	238ILE CG2	1.16	69LEU CA	69LEU CD1	73.54	139TY CD2	140PHE CG	0.00
138ILE CG2	238ILE CD	3.71	69LEU CA	69LEU CD2	24.22	139TY CD2	140PHE CD1	0.06
138ILE CD	138ILE C	0.02	69LEU CA	69LEU C	100.00	139TY CD2	140PHE CD2	0.01
138ILE CD	153ASN CB	4.31	69LEU CB	69LEU CG	100.00	139TY CD2	140PHE CE1	0.15
138ILE CD	153ASN C	2.78	69LEU CB	69LEU CD1	100.00	139TY CD2	140PHE CE2	0.08
138ILE CD	154THR C	1.13	69LEU CB	69LEU CD2	100.00	139TY CD2	140PHE CZ	0.04
138ILE CD	155GLU CB	0.01	69LEU CB	69LEU C	100.00	139TY CD2	237ASN CB	0.00
138ILE CD	238ILE CD	0.12	69LEU CB	268VAL CG1	3.24	139TY CD2	237ASN CG	0.21
138ILE C	139TYR CA	100.00	69LEU CB	270GLU CG	0.04	139TY CE1	139TYR CE2	100.00
138ILE C	139TYR CB	0.00	69LEU CG	69LEU CD1	100.00	139TY CE1	139TYR CZ	100.00
138ILE C	139TYR C	100.00	69LEU CG	69LEU CD2	100.00	139TY CE1	238ILE CG1	0.05
138ILE C	141GLY CA	0.01	69LEU CG	69LEU C	74.84	139TY CE1	238ILE CD	0.44
139TYR CA	139TYR CB	100.00	69LEU CG	268VAL CG1	0.68	139TY CE2	139TYR CZ	100.00
139TYR CA	139TYR CG	100.00	69LEU CD1	69LEU CD2	100.00	139TY CE2	140PHE CD1	0.01
139TYR CA	139TYR CD1	99.97	69LEU CD1	69LEU C	0.70	139TY CE2	140PHE CE1	0.06
139TYR CA	139TYR CD2	1.84	69LEU CD1	71SER CA	0.00	139TY CE2	140PHE CE2	0.00
139TYR CA	139TYR C	100.00	69LEU CD1	90LEU CD1	0.06	139TY CE2	140PHE CZ	0.00
139TYR CB	139TYR CG	100.00	69LEU CD1	90LEU CD2	0.73	139TY CE2	237ASN CG	0.14
139TYR CB	139TYR CD1	100.00	69LEU CD1	93LEU CD1	0.34	139TY CE2	238ILE CA	0.14
139TYR CB	139TYR CD2	100.00	69LEU CD1	93LEU CD2	0.96	139TY CE2	241ASP CB	1.30
139TYR CB	139TYR C	100.00	69LEU CD1	268VAL CG1	9.08	139TY CE2	241ASP CG	0.03
139TYR CB	140PHE CG	0.04	69LEU CD1	270GLU CG	0.26	139TY CZ	238ILE CA	0.02
139TYR CB	140PHE CD1	1.48	69LEU CD1	270GLU CD	0.67	139TY CZ	238ILE CB	0.00
139TYR CB	140PHE CD2	5.22	69LEU CD2	69LEU C	0.52	139TY CZ	238ILE CG1	0.04
139TYR CB	140PHE CE1	0.95	69LEU CD2	70GLY C	0.02	139TY CZ	238ILE CD	0.06
139TYR CB	140PHE CE2	2.68	69LEU CD2	71SER CA	0.02	139TY CZ	241ASP CB	0.01
139TYR CB	140PHE CZ	0.16	69LEU CD2	72VAL CG1	0.01	139TY C	140PHE CA	100.00
139TYR CG	139TYR CD1	100.00	69LEU CD2	90LEU CD1	0.13	139TY C	140PHE CB	96.47
139TYR CG	139TYR CD2	100.00	69LEU CD2	90LEU CD2	1.09	139TY C	140PHE CG	43.65

139TYR CG	139TYR CE1	100.00
139TYR CG	139TYR CE2	100.00
139TYR CG	139TYR CZ	100.00
139TYR CG	139TYR C	0.00
139TYR CD1	139TYR CD2	100.00
139TYR CD1	139TYR CE1	100.00
139TYR CD1	139TYR CE2	100.00
139TYR CD1	139TYR CZ	100.00
139TYR CD2	139TYR CE1	100.00
139TYR CD2	139TYR CE2	100.00
139TYR CD2	139TYR CZ	100.00
139TYR CD2	140PHE CD1	0.00
139TYR CD2	140PHE CE1	0.00
139TYR CD2	237ASN CB	0.05
139TYR CD2	237ASN CG	2.06
139TYR CE1	139TYR CE2	100.00
139TYR CE1	139TYR CZ	100.00
139TYR CE1	238ILE CA	0.00
139TYR CE1	238ILE CB	0.06
139TYR CE1	238ILE CG1	0.28
139TYR CE1	238ILE CD	0.64
139TYR CE2	139TYR CZ	100.00
139TYR CE2	237ASN CB	0.23
139TYR CE2	237ASN CG	0.22
139TYR CE2	237ASN C	0.12
139TYR CE2	238ILE CA	0.40
139TYR CE2	241ASP CB	0.10
139TYR CE2	241ASP CG	0.04
139TYR CZ	238ILE CA	0.06
139TYR CZ	238ILE CB	0.02
139TYR CZ	238ILE CG1	0.01
139TYR CZ	238ILE CD	0.08
139TYR C	140PHE CA	100.00
139TYR C	140PHE CB	91.80
139TYR C	140PHE CG	70.88
139TYR C	140PHE CD1	16.67
139TYR C	140PHE CD2	6.70
139TYR C	140PHE C	6.17
139TYR C	144ARG CD	0.01
140PHE CA	140PHE CB	100.00
140PHE CA	140PHE CG	100.00
140PHE CA	140PHE CD1	77.24
140PHE CA	140PHE CD2	38.07
140PHE CA	140PHE C	100.00
140PHE CA	144ARG CD	0.03
140PHE CA	144ARG CZ	1.61
140PHE CB	140PHE CG	100.00
140PHE CB	140PHE CD1	100.00
140PHE CB	140PHE CD2	100.00
140PHE CB	140PHE C	100.00
140PHE CB	144ARG CZ	0.00
140PHE CG	140PHE CD1	100.00
140PHE CG	140PHE CD2	100.00
140PHE CG	140PHE CE1	100.00
140PHE CG	140PHE CE2	100.00
140PHE CG	140PHE CZ	100.00
140PHE CG	140PHE C	3.42
140PHE CD1	140PHE CD2	100.00
140PHE CD1	140PHE CE1	100.00
140PHE CD1	140PHE CE2	100.00
140PHE CD1	140PHE CZ	100.00
140PHE CD1	140PHE C	0.12

69LEU CD2	93LEU	CD1	0.08
69LEU CD2	93LEU	CD2	0.31
69LEU CD2	268VAL	CG1	4.51
69LEU CD2	270GLU	CB	0.01
69LEU CD2	270GLU	CG	0.66
69LEU CD2	270GLU	CD	3.04
69LEU C	70GLY	CA	100.00
69LEU C	70GLY	C	58.20
69LEU C	271PRO	CD	0.00
70GLY CA	70GLY	C	100.00
70GLY CA	271PRO	CG	0.10
70GLY CA	271PRO	CD	0.18
70GLY C	71SER	CA	100.00
70GLY C	71SER	CB	49.65
70GLY C	71SER	C	89.31
70GLY C	271PRO	CG	1.68
70GLY C	271PRO	CD	0.10
71SER CA	71SER	CB	100.00
71SER CA	71SER	C	100.00
71SER CA	271PRO	CG	0.00
71SER CB	71SER	C	100.00
71SER CB	270GLU	CG	0.14
71SER CB	273HIS	CB	1.16
71SER CB	273HIS	CG	0.01
71SER CB	273HIS	CD2	0.24
71SER CB	273HIS	CE1	0.02
71SER CB	273HIS	C	0.21
71SER CB	274GLY	CA	0.19
71SER CB	274GLY	C	0.09
71SER CB	275SER	CA	0.00
71SER CB	275SER	CB	0.04
71SER C	72VAL	CA	100.00
71SER C	72VAL	CB	81.60
71SER C	72VAL	CG1	27.54
71SER C	72VAL	CG2	2.72
71SER C	72VAL	C	20.53
71SER C	275SER	CB	0.00
72VAL CA	72VAL	CB	100.00
72VAL CA	72VAL	CG1	100.00
72VAL CA	72VAL	CG2	100.00
72VAL CA	72VAL	C	100.00
72VAL CA	77TRP	CE3	0.01
72VAL CA	77TRP	CZ2	0.00
72VAL CA	77TRP	CZ3	0.07
72VAL CB	72VAL	CG1	100.00
72VAL CB	72VAL	CG2	100.00
72VAL CB	72VAL	C	100.00
72VAL CB	77TRP	CE2	0.00
72VAL CB	77TRP	CZ2	0.18
72VAL CB	77TRP	CZ3	0.37
72VAL CB	86PRO	CB	0.18
72VAL CB	86PRO	CG	0.03
72VAL CG1	72VAL	CG2	100.00
72VAL CG1	72VAL	C	28.80
72VAL CG1	77TRP	CD1	0.02
72VAL CG1	77TRP	CE2	0.11
72VAL CG1	77TRP	CZ2	1.43
72VAL CG1	77TRP	CZ3	0.40
72VAL CG1	86PRO	CB	0.82
72VAL CG1	86PRO	CG	0.25
72VAL CG1	90LEU	CG	0.03
72VAL CG1	90LEU	CD1	0.27

139TY C	140PHE	CD1	9.44
139TY C	140PHE	CD2	7.85
139TY C	140PHE	C	3.05
140PH CA	140PHE	CB	100.00
140PH CA	140PHE	CG	100.00
140PH CA	140PHE	CD1	67.71
140PH CA	140PHE	CD2	64.52
140PH CA	140PHE	C	100.00
140PH CA	144ARG	CZ	0.36
140PH CB	140PHE	CG	100.00
140PH CB	140PHE	CD1	100.00
140PH CB	140PHE	CD2	100.00
140PH CB	140PHE	C	100.00
140PH CB	144ARG	CZ	0.06
140PH CG	140PHE	CD1	100.00
140PH CG	140PHE	CD2	100.00
140PH CG	140PHE	CE1	100.00
140PH CG	140PHE	CE2	100.00
140PH CG	140PHE	CZ	100.00
140PH CG	140PHE	C	36.04
140PH CD1	140PHE	CD2	100.00
140PH CD1	140PHE	CE1	100.00
140PH CD1	140PHE	CE2	100.00
140PH CD1	140PHE	CZ	100.00
140PH CD1	140PHE	C	6.57
140PH CD2	140PHE	CE1	100.00
140PH CD2	140PHE	CE2	100.00
140PH CD2	140PHE	CZ	100.00
140PH CD2	140PHE	C	1.30
140PH CE1	140PHE	CE2	100.00
140PH CE1	140PHE	CZ	100.00
140PH CE2	140PHE	CZ	100.00
140PH C	141GLY	CA	100.00
140PH C	141GLY	C	61.49
140PH C	144ARG	CZ	0.43
141GL CA	141GLY	C	100.00
141GL C	142GLU	CA	100.00
141GL C	142GLU	CB	26.54
141GL C	142GLU	CG	0.56
141GL C	142GLU	CD	0.13
141GL C	142GLU	C	77.72
141GL C	144ARG	CD	0.00
141GL C	144ARG	CZ	0.08
142GL CA	142GLU	CB	100.00
142GL CA	142GLU	CG	100.00
142GL CA	142GLU	CD	97.98
142GL CA	142GLU	C	100.00
142GL CA	143PRO	CA	100.00
142GL CA	143PRO	C	47.03
142GL CB	142GLU	CG	100.00
142GL CB	142GLU	CD	100.00
142GL CB	142GLU	C	100.00
142GL CB	143PRO	CA	4.94
142GL CG	142GLU	CD	100.00
142GL CG	142GLU	C	56.48
142GL CG	143PRO	CA	1.46
142GL CD	142GLU	C	0.04
142GL CD	143PRO	CA	0.24
142GL CD	144ARG	CG	0.34
142GL CD	144ARG	CD	0.05
142GL C	143PRO	CA	100.00
142GL C	143PRO	CB	0.04

140PHE	CD1	144ARG	CZ	0.00
140PHE	CD2	140PHE	CE1	100.00
140PHE	CD2	140PHE	CE2	100.00
140PHE	CD2	140PHE	CZ	100.00
140PHE	CD2	140PHE	C	0.34
140PHE	CD2	144ARG	CZ	0.04
140PHE	CE1	140PHE	CE2	100.00
140PHE	CE1	140PHE	CZ	100.00
140PHE	CE2	140PHE	CZ	100.00
140PHE	CE2	144ARG	CZ	0.00
140PHE	CE2	237ASN	CG	0.00
140PHE	C	141GLY	CA	100.00
140PHE	C	141GLY	C	68.28
140PHE	C	144ARG	CD	0.80
140PHE	C	144ARG	CZ	0.42
141GLY	CA	141GLY	C	100.00
141GLY	CA	156ARG	CD	0.01
141GLY	CA	156ARG	CZ	0.01
141GLY	C	142GLU	CA	100.00
141GLY	C	142GLU	CB	6.34
141GLY	C	142GLU	CG	0.23
141GLY	C	142GLU	CD	0.02
141GLY	C	142GLU	C	94.98
141GLY	C	144ARG	CG	0.00
141GLY	C	144ARG	CD	0.58
142GLU	CA	142GLU	CB	100.00
142GLU	CA	142GLU	CG	100.00
142GLU	CA	142GLU	CD	92.98
142GLU	CA	142GLU	C	100.00
142GLU	CA	143PRO	CA	100.00
142GLU	CA	143PRO	C	52.79
142GLU	CB	142GLU	CG	100.00
142GLU	CB	142GLU	CD	100.00
142GLU	CB	142GLU	C	100.00
142GLU	CB	143PRO	CA	3.23
142GLU	CB	143PRO	C	0.03
142GLU	CB	144ARG	CD	0.01
142GLU	CB	156ARG	CZ	0.01
142GLU	CG	142GLU	CD	100.00
142GLU	CG	142GLU	C	82.24
142GLU	CG	143PRO	CA	2.36
142GLU	CG	144ARG	CG	0.00
142GLU	CG	144ARG	CD	0.00
142GLU	CD	142GLU	C	0.48
142GLU	CD	143PRO	CA	0.42
142GLU	CD	144ARG	CG	0.02
142GLU	CD	144ARG	CD	0.03
142GLU	CD	156ARG	CZ	0.01
142GLU	C	143PRO	CA	100.00
142GLU	C	143PRO	CB	0.03
142GLU	C	143PRO	CD	100.00
142GLU	C	143PRO	C	100.00
142GLU	C	154THR	CB	0.09
143PRO	CA	143PRO	CB	100.00
143PRO	CA	143PRO	CG	100.00
143PRO	CA	143PRO	CD	100.00
143PRO	CA	143PRO	C	100.00
143PRO	CB	143PRO	CG	100.00
143PRO	CB	143PRO	CD	100.00
143PRO	CB	143PRO	C	100.00
143PRO	CB	152TRP	CD1	0.28
143PRO	CG	143PRO	CD	100.00

72VAL	CG1	90LEU	CD2	0.04
72VAL	CG2	72VAL	C	92.54
72VAL	CG2	77TRP	CD1	0.02
72VAL	CG2	77TRP	CD2	0.00
72VAL	CG2	77TRP	CE2	0.67
72VAL	CG2	77TRP	CE3	0.00
72VAL	CG2	77TRP	CZ2	4.24
72VAL	CG2	77TRP	CZ3	0.22
72VAL	CG2	86PRO	CB	3.17
72VAL	CG2	86PRO	CG	0.43
72VAL	CG2	87GLU	CA	0.00
72VAL	CG2	90LEU	CG	0.12
72VAL	CG2	90LEU	CD1	1.68
72VAL	CG2	90LEU	CD2	0.37
72VAL	C	73GLY	CA	100.00
72VAL	C	73GLY	C	35.63
72VAL	C	77TRP	CG	0.00
72VAL	C	77TRP	CD2	0.04
72VAL	C	77TRP	CE3	0.44
72VAL	C	77TRP	CZ3	1.22
72VAL	C	86PRO	CB	0.12
72VAL	C	86PRO	CG	0.09
72VAL	C	275SER	CB	0.48
73GLY	CA	73GLY	C	100.00
73GLY	CA	77TRP	CB	0.01
73GLY	CA	77TRP	CG	0.04
73GLY	CA	77TRP	CD2	0.72
73GLY	CA	77TRP	CE3	12.94
73GLY	CA	77TRP	CZ3	1.29
73GLY	CA	86PRO	CB	0.23
73GLY	CA	86PRO	CG	1.37
73GLY	CA	87GLU	CG	0.01
73GLY	CA	87GLU	CD	0.01
73GLY	CA	274GLY	CA	0.02
73GLY	CA	274GLY	C	0.00
73GLY	CA	275SER	CB	0.03
73GLY	C	74GLY	CA	100.00
73GLY	C	74GLY	C	29.16
73GLY	C	77TRP	CB	0.02
73GLY	C	77TRP	CD2	0.02
73GLY	C	77TRP	CE3	24.46
73GLY	C	77TRP	CZ3	5.03
73GLY	C	274GLY	CA	0.06
73GLY	C	274GLY	C	0.00
73GLY	C	275SER	CA	0.04
73GLY	C	275SER	CB	0.46
74GLY	CA	74GLY	C	100.00
74GLY	CA	75PRO	CD	100.00
74GLY	CA	77TRP	CE3	0.04
74GLY	CA	77TRP	CZ3	0.01
74GLY	CA	275SER	CA	0.01
74GLY	CA	275SER	CB	0.70
74GLY	CA	275SER	C	0.01
74GLY	CA	277PRO	CD	0.02
74GLY	CA	280ALA	CB	0.78
74GLY	CA	280ALA	C	0.01
74GLY	CA	281GLY	C	0.02
74GLY	C	75PRO	CA	100.00
74GLY	C	75PRO	CB	0.08
74GLY	C	75PRO	CD	100.00
74GLY	C	75PRO	C	100.00
74GLY	C	77TRP	CE3	0.06

142GL	C	143PRO	CG	0.00
142GL	C	143PRO	CD	100.00
142GL	C	143PRO	C	99.98
142GL	C	154THR	CB	0.04
143PR	CA	143PRO	CB	100.00
143PR	CA	143PRO	CG	100.00
143PR	CA	143PRO	CD	100.00
143PR	CA	143PRO	C	100.00
143PR	CB	143PRO	CG	100.00
143PR	CB	143PRO	CD	100.00
143PR	CB	143PRO	C	100.00
143PR	CB	152TRP	CD1	0.03
143PR	CG	143PRO	CD	100.00
143PR	CG	143PRO	C	97.96
143PR	CG	152TRP	CD1	0.01
143PR	CD	143PRO	C	92.84
143PR	CD	154THR	CB	0.40
143PR	CD	154THR	CG2	0.00
143PR	CD	156ARG	CD	0.00
143PR	C	144ARG	CA	100.00
143PR	C	144ARG	CB	83.34
143PR	C	144ARG	CG	3.57
143PR	C	144ARG	CD	0.13
143PR	C	144ARG	C	19.47
143PR	C	152TRP	CD1	0.02
144AR	CA	144ARG	CB	100.00
144AR	CA	144ARG	CG	100.00
144AR	CA	144ARG	CD	11.69
144AR	CA	144ARG	C	100.00
144AR	CA	152TRP	CD1	1.06
144AR	CB	144ARG	CG	100.00
144AR	CB	144ARG	CD	100.00
144AR	CB	144ARG	CZ	0.23
144AR	CB	144ARG	C	100.00
144AR	CB	153ASN	CG	0.01
144AR	CG	144ARG	CD	100.00
144AR	CG	144ARG	CZ	13.79
144AR	CG	144ARG	C	83.80
144AR	CD	144ARG	CZ	100.00
144AR	CD	144ARG	C	0.75
144AR	CZ	144ARG	C	0.02
144AR	CZ	145GLY	CA	0.00
144AR	C	145GLY	CA	100.00
144AR	C	145GLY	C	8.87
144AR	C	152TRP	CD1	1.11
144AR	C	152TRP	CE2	0.16
144AR	C	152TRP	CE3	0.00
144AR	C	152TRP	CZ2	0.04
144AR	C	152TRP	CZ3	0.00
145GL	CA	145GLY	C	100.00
145GL	CA	152TRP	CD1	0.07
145GL	CA	152TRP	CD2	0.09
145GL	CA	152TRP	CE2	8.32
145GL	CA	152TRP	CE3	0.04
145GL	CA	152TRP	CZ2	1.53
145GL	CA	152TRP	CZ3	0.10
145GL	C	146MET	CA	100.00
145GL	C	146MET	CB	94.52
145GL	C	146MET	CG	0.12
145GL	C	146MET	C	8.46
145GL	C	152TRP	CD2	0.04
145GL	C	152TRP	CE2	0.33

143PRO CG	143PRO C	96.32	74GLY C	78ASP	CB	0.04	145GL C	152TRP	CE3	0.17
143PRO CD	143PRO C	91.55	74GLY C	277PRO	CB	0.01	145GL C	152TRP	CZ2	0.23
143PRO CD	154THR CB	0.07	74GLY C	280ALA	C	0.00	145GL C	152TRP	CZ3	0.31
143PRO C	144ARG CA	100.00	75PRO CA	75PRO	CB	100.00	146ME CA	146MET	CB	100.00
143PRO C	144ARG CB	98.37	75PRO CA	75PRO	CG	100.00	146ME CA	146MET	CG	100.00
143PRO C	144ARG CG	0.94	75PRO CA	75PRO	CD	100.00	146ME CA	146MET	C	100.00
143PRO C	144ARG C	2.73	75PRO CA	75PRO	C	100.00	146ME CA	151ALA	CB	0.06
143PRO C	152TRP CD1	0.56	75PRO CA	78ASP	CB	0.08	146ME CA	152TRP	CZ3	0.02
144ARG CA	144ARG CB	100.00	75PRO CA	277PRO	CB	0.00	146ME CB	146MET	CG	100.00
144ARG CA	144ARG CG	100.00	75PRO CB	75PRO	CG	100.00	146ME CB	146MET	CE	30.65
144ARG CA	144ARG CD	2.62	75PRO CB	75PRO	CD	100.00	146ME CB	146MET	C	100.00
144ARG CA	144ARG C	100.00	75PRO CB	75PRO	C	100.00	146ME CB	151ALA	CB	0.54
144ARG CA	152TRP CD1	2.09	75PRO CB	277PRO	CB	0.00	146ME CG	146MET	CE	100.00
144ARG CB	144ARG CG	100.00	75PRO CB	280ALA	CB	0.08	146ME CG	146MET	C	97.15
144ARG CB	144ARG CD	100.00	75PRO CB	281GLY	CA	0.00	146ME CG	151ALA	CB	0.15
144ARG CB	144ARG CZ	0.67	75PRO CB	282LYS	CG	0.03	146ME C	147SER	CA	100.00
144ARG CB	144ARG C	100.00	75PRO CB	282LYS	CE	0.02	146ME C	147SER	CB	88.23
144ARG CB	153ASN CG	0.07	75PRO CG	75PRO	CD	100.00	146ME C	147SER	C	18.54
144ARG CG	144ARG CD	100.00	75PRO CG	75PRO	C	45.00	146ME C	152TRP	CZ3	0.03
144ARG CG	144ARG CZ	58.41	75PRO CG	276ALA	CB	0.00	147SE CA	147SER	CB	100.00
144ARG CG	144ARG C	96.94	75PRO CG	276ALA	C	0.04	147SE CA	147SER	C	100.00
144ARG CD	144ARG CZ	100.00	75PRO CG	277PRO	CA	0.24	147SE CB	147SER	C	100.00
144ARG CD	144ARG C	0.04	75PRO CG	277PRO	CB	0.24	147SE CB	152TRP	CZ3	0.03
144ARG C	145GLY CA	100.00	75PRO CG	277PRO	CG	0.08	147SE C	148GLU	CA	100.00
144ARG C	145GLY C	3.44	75PRO CG	277PRO	CD	0.14	147SE C	148GLU	CB	0.28
144ARG C	152TRP CD1	1.58	75PRO CG	280ALA	CB	0.54	147SE C	148GLU	C	99.89
144ARG C	152TRP CE2	0.19	75PRO CG	280ALA	C	0.08	148GL CA	148GLU	CB	100.00
144ARG C	152TRP CZ2	0.00	75PRO CG	281GLY	CA	0.20	148GL CA	148GLU	CG	100.00
145GLY CA	145GLY C	100.00	75PRO CG	281GLY	C	0.06	148GL CA	148GLU	CD	98.16
145GLY CA	152TRP CD2	0.06	75PRO CG	282LYS	CA	0.00	148GL CA	148GLU	C	100.00
145GLY CA	152TRP CE2	10.30	75PRO CG	282LYS	CB	0.04	148GL CB	148GLU	CG	100.00
145GLY CA	152TRP CE3	0.01	75PRO CG	282LYS	CG	0.05	148GL CB	148GLU	CD	100.00
145GLY CA	152TRP CZ2	2.46	75PRO CG	282LYS	CD	0.01	148GL CB	148GLU	C	100.00
145GLY CA	152TRP CZ3	0.03	75PRO CG	282LYS	CE	0.01	148GL CG	148GLU	CD	100.00
145GLY C	146MET CA	100.00	75PRO CD	75PRO	C	33.69	148GL CG	148GLU	C	81.64
145GLY C	146MET CB	93.04	75PRO CD	275SER	CA	0.01	148GL CD	148GLU	C	2.94
145GLY C	146MET CG	0.11	75PRO CD	275SER	CB	0.16	148GL C	149ALA	CA	100.00
145GLY C	146MET C	9.50	75PRO CD	275SER	C	0.11	148GL C	149ALA	CB	52.65
145GLY C	152TRP CD2	0.08	75PRO CD	276ALA	CA	0.00	148GL C	149ALA	C	48.30
145GLY C	152TRP CE2	1.52	75PRO CD	276ALA	CB	0.00	149AL CA	149ALA	CB	100.00
145GLY C	152TRP CE3	0.21	75PRO CD	276ALA	C	0.00	149AL CA	149ALA	C	100.00
145GLY C	152TRP CZ2	0.82	75PRO CD	277PRO	CA	0.00	149AL CB	149ALA	C	100.00
145GLY C	152TRP CZ3	0.33	75PRO CD	277PRO	CB	0.05	149AL CB	150GLU	CB	0.00
146MET CA	146MET CB	100.00	75PRO CD	277PRO	CG	0.21	149AL C	150GLU	CA	100.00
146MET CA	146MET CG	100.00	75PRO CD	277PRO	CD	0.70	149AL C	150GLU	CB	99.96
146MET CA	146MET C	100.00	75PRO CD	280ALA	CB	1.18	149AL C	150GLU	CG	0.13
146MET CA	151ALA CB	0.07	75PRO CD	280ALA	C	0.03	149AL C	150GLU	C	0.07
146MET CA	152TRP CZ3	0.08	75PRO CD	281GLY	CA	0.03	150GL CA	150GLU	CB	100.00
146MET CB	146MET CG	100.00	75PRO CD	281GLY	C	0.04	150GL CA	150GLU	CG	100.00
146MET CB	146MET CE	29.91	75PRO CD	282LYS	CA	0.00	150GL CA	150GLU	CD	0.22
146MET CB	146MET C	100.00	75PRO C	76LYS	CA	100.00	150GL CA	150GLU	C	100.00
146MET CB	151ALA CA	0.02	75PRO C	76LYS	CB	10.48	150GL CB	150GLU	CG	100.00
146MET CB	151ALA CB	1.14	75PRO C	76LYS	CG	0.46	150GL CB	150GLU	CD	100.00
146MET CG	146MET CE	100.00	75PRO C	76LYS	C	93.85	150GL CB	150GLU	C	100.00
146MET CG	146MET C	96.92	75PRO C	77TRP	CE3	0.01	150GL CB	152TRP	CZ3	0.00
146MET CG	151ALA CB	0.24	75PRO C	78ASP	CB	0.01	150GL CG	150GLU	CD	100.00
146MET CE	146MET C	0.00	76LYS CA	76LYS	CB	100.00	150GL CG	150GLU	C	98.45
146MET CE	148GLU CD	0.01	76LYS CA	76LYS	CG	100.00	150GL CG	152TRP	CE3	0.01
146MET CE	151ALA CB	0.00	76LYS CA	76LYS	CD	1.79	150GL CG	152TRP	CZ3	0.08
146MET C	147SER CA	100.00	76LYS CA	76LYS	C	100.00	150GL CD	152TRP	CE3	0.04
146MET C	147SER CB	92.87	76LYS CB	76LYS	CG	100.00	150GL CD	152TRP	CZ3	0.22
146MET C	147SER C	12.44	76LYS CB	76LYS	CD	100.00	150GL C	151ALA	CA	100.00

146MET C	152TRP CZ3	0.12	76LYS CB	76LYS CE	23.07	150GL C	151ALA CB	95.74
147SER CA	147SER CB	100.00	76LYS CB	76LYS C	100.00	150GL C	151ALA C	3.36
147SER CA	147SER C	100.00	76LYS CB	77TRP CD1	0.01	150GL C	152TRP CZ3	0.10
147SER CB	147SER C	100.00	76LYS CB	77TRP CD2	0.22	151AL CA	151ALA CB	100.00
147SER CB	152TRP CZ3	0.01	76LYS CB	77TRP CE2	0.23	151AL CA	151ALA C	100.00
147SER C	148GLU CA	100.00	76LYS CB	77TRP CE3	3.67	151AL CA	152TRP CE3	3.70
147SER C	148GLU CB	3.16	76LYS CB	77TRP CZ2	0.03	151AL CB	151ALA C	100.00
147SER C	148GLU C	99.64	76LYS CB	77TRP CZ3	0.74	151AL C	152TRP CA	100.00
148GLU CA	148GLU CB	100.00	76LYS CG	76LYS CD	100.00	151AL C	152TRP CB	99.62
148GLU CA	148GLU CG	100.00	76LYS CG	76LYS CE	100.00	151AL C	152TRP CG	0.02
148GLU CA	148GLU CD	96.51	76LYS CG	76LYS C	74.95	151AL C	152TRP CD2	0.01
148GLU CA	148GLU C	100.00	76LYS CG	77TRP CD1	0.03	151AL C	152TRP CE3	25.69
148GLU CB	148GLU CG	100.00	76LYS CG	77TRP CD2	0.18	151AL C	152TRP C	0.18
148GLU CB	148GLU CD	100.00	76LYS CG	77TRP CE2	0.08	152TR CA	152TRP CB	100.00
148GLU CB	148GLU C	100.00	76LYS CG	77TRP CE3	1.52	152TR CA	152TRP CG	100.00
148GLU CG	148GLU CD	100.00	76LYS CG	77TRP CZ2	0.01	152TR CA	152TRP CD1	46.89
148GLU CG	148GLU C	75.10	76LYS CG	77TRP CZ3	0.80	152TR CA	152TRP CD2	31.57
148GLU CD	148GLU C	8.80	76LYS CG	80LEU CD1	0.02	152TR CA	152TRP C	100.00
148GLU C	149ALA CA	100.00	76LYS CD	76LYS CE	100.00	152TR CB	152TRP CG	100.00
148GLU C	149ALA CB	44.97	76LYS CD	76LYS C	0.69	152TR CB	152TRP CD1	100.00
148GLU C	149ALA C	52.83	76LYS CD	77TRP CD1	0.00	152TR CB	152TRP CD2	100.00
149ALA CA	149ALA CB	100.00	76LYS CD	77TRP CD2	0.06	152TR CB	152TRP CE3	97.92
149ALA CA	149ALA C	100.00	76LYS CD	77TRP CE2	0.24	152TR CB	152TRP C	100.00
149ALA CB	149ALA C	100.00	76LYS CD	77TRP CE3	0.04	152TR CG	152TRP CD1	100.00
149ALA CB	150GLU CG	0.00	76LYS CD	77TRP CZ2	0.52	152TR CG	152TRP CD2	100.00
149ALA CB	150GLU CD	0.01	76LYS CD	77TRP CZ3	0.28	152TR CG	152TRP CE2	100.00
149ALA C	150GLU CA	100.00	76LYS CE	76LYS C	0.02	152TR CG	152TRP CE3	100.00
149ALA C	150GLU CB	99.94	76LYS CE	77TRP CD1	0.00	152TR CG	152TRP C	99.89
149ALA C	150GLU CG	0.86	76LYS CE	77TRP CE2	0.38	152TR CD1	152TRP CD2	100.00
149ALA C	150GLU CD	0.02	76LYS CE	77TRP CE3	0.03	152TR CD1	152TRP CE2	100.00
149ALA C	150GLU C	0.15	76LYS CE	77TRP CZ2	2.10	152TR CD1	152TRP CE3	0.00
150GLU CA	150GLU CB	100.00	76LYS CE	77TRP CZ3	0.94	152TR CD1	152TRP C	17.37
150GLU CA	150GLU CG	100.00	76LYS CE	80LEU CD1	0.01	152TR CD2	152TRP CE2	100.00
150GLU CA	150GLU CD	0.26	76LYS CE	277PRO CB	0.01	152TR CD2	152TRP CE3	100.00
150GLU CA	150GLU C	100.00	76LYS C	77TRP CA	100.00	152TR CD2	152TRP CZ2	100.00
150GLU CB	150GLU CG	100.00	76LYS C	77TRP CB	32.86	152TR CD2	152TRP CZ3	100.00
150GLU CB	150GLU CD	100.00	76LYS C	77TRP CG	10.69	152TR CE2	152TRP CE3	100.00
150GLU CB	150GLU C	100.00	76LYS C	77TRP CD1	0.67	152TR CE2	152TRP CZ2	100.00
150GLU CB	152TRP CZ3	0.00	76LYS C	77TRP CD2	1.25	152TR CE2	152TRP CZ3	100.00
150GLU CG	150GLU CD	100.00	76LYS C	77TRP CE3	0.57	152TR CE3	152TRP CZ2	100.00
150GLU CG	150GLU C	94.75	76LYS C	77TRP C	61.33	152TR CE3	152TRP CZ3	100.00
150GLU CG	152TRP CE3	0.01	76LYS C	80LEU CD1	0.00	152TR CZ2	152TRP CZ3	100.00
150GLU CG	152TRP CZ3	0.08	76LYS C	80LEU CD2	0.02	152TR C	153ASN CA	100.00
150GLU CD	152TRP CE3	0.06	77TRP CA	77TRP CB	100.00	152TR C	153ASN CB	89.52
150GLU CD	152TRP CZ3	0.08	77TRP CA	77TRP CG	100.00	152TR C	153ASN CG	6.09
150GLU C	151ALA CA	100.00	77TRP CA	77TRP CD1	79.87	152TR C	153ASN C	5.99
150GLU C	151ALA CB	93.94	77TRP CA	77TRP CD2	17.60	153AS CA	153ASN CB	100.00
150GLU C	151ALA C	5.35	77TRP CA	77TRP CE3	0.00	153AS CA	153ASN CG	100.00
150GLU C	152TRP CZ3	0.19	77TRP CA	77TRP C	100.00	153AS CA	153ASN C	100.00
151ALA CA	151ALA CB	100.00	77TRP CA	80LEU CD1	0.44	153AS CB	153ASN CG	100.00
151ALA CA	151ALA C	100.00	77TRP CB	77TRP CG	100.00	153AS CB	153ASN C	100.00
151ALA CA	152TRP CE3	6.18	77TRP CB	77TRP CD1	100.00	153AS CG	153ASN C	0.02
151ALA CB	151ALA C	100.00	77TRP CB	77TRP CD2	100.00	153AS C	154THR CA	100.00
151ALA C	152TRP CA	100.00	77TRP CB	77TRP CE3	97.68	153AS C	154THR CB	54.58
151ALA C	152TRP CB	99.27	77TRP CB	77TRP C	100.00	153AS C	154THR C	54.38
151ALA C	152TRP CG	0.04	77TRP CB	80LEU CD1	0.05	154TH CA	154THR CB	100.00
151ALA C	152TRP CD2	0.02	77TRP CB	85SER CB	0.08	154TH CA	154THR CG2	100.00
151ALA C	152TRP CE3	28.70	77TRP CB	86PRO CG	0.31	154TH CA	154THR C	100.00
151ALA C	152TRP C	0.47	77TRP CB	86PRO CD	0.12	154TH CB	154THR CG2	100.00
152TRP CA	152TRP CB	100.00	77TRP CG	77TRP CD1	100.00	154TH CB	154THR C	100.00
152TRP CA	152TRP CG	100.00	77TRP CG	77TRP CD2	100.00	154TH CG2	154THR C	100.00
152TRP CA	152TRP CD1	52.17	77TRP CG	77TRP CE2	100.00	154TH CG2	155GLU C	0.04

152TRP CA	152TRP CD2	35.93	77TRP CG	77TRP	CE3	100.00	154TH CG2	156ARG	CB	0.02
152TRP CA	152TRP C	100.00	77TRP CG	77TRP	C	13.55	154TH CG2	156ARG	CG	0.62
152TRP CB	152TRP CG	100.00	77TRP CG	80LEU	CD1	0.03	154TH C	155GLU	CA	100.00
152TRP CB	152TRP CD1	100.00	77TRP CG	85SER	CB	0.30	154TH C	155GLU	CB	5.67
152TRP CB	152TRP CD2	100.00	77TRP CG	86PRO	CB	0.00	154TH C	155GLU	C	97.36
152TRP CB	152TRP CE3	97.94	77TRP CG	86PRO	CG	1.72	155GL CA	155GLU	CB	100.00
152TRP CB	152TRP C	100.00	77TRP CG	86PRO	CD	0.50	155GL CA	155GLU	CG	100.00
152TRP CG	152TRP CD1	100.00	77TRP CD1	77TRP	CD2	100.00	155GL CA	155GLU	CD	98.11
152TRP CG	152TRP CD2	100.00	77TRP CD1	77TRP	CE2	100.00	155GL CA	155GLU	C	100.00
152TRP CG	152TRP CE2	100.00	77TRP CD1	77TRP	C	5.10	155GL CB	155GLU	CG	100.00
152TRP CG	152TRP CE3	100.00	77TRP CD1	80LEU	CB	0.06	155GL CB	155GLU	CD	100.00
152TRP CG	152TRP C	99.91	77TRP CD1	80LEU	CD1	2.50	155GL CB	155GLU	C	100.00
152TRP CD1	152TRP CD2	100.00	77TRP CD1	80LEU	CD2	0.84	155GL CG	155GLU	CD	100.00
152TRP CD1	152TRP CE2	100.00	77TRP CD1	82ARG	CA	0.00	155GL CG	155GLU	C	100.00
152TRP CD1	152TRP C	16.88	77TRP CD1	84ILE	CG2	0.29	155GL CG	157TYR	CD2	0.08
152TRP CD2	152TRP CE2	100.00	77TRP CD1	84ILE	CD	0.02	155GL CG	157TYR	CE1	0.04
152TRP CD2	152TRP CE3	100.00	77TRP CD1	84ILE	C	0.04	155GL CG	157TYR	CE2	2.56
152TRP CD2	152TRP CZ2	100.00	77TRP CD1	85SER	CA	0.60	155GL CG	189LEU	CD2	0.00
152TRP CD2	152TRP CZ3	100.00	77TRP CD1	85SER	CB	0.36	155GL CG	191VAL	CG1	0.32
152TRP CE2	152TRP CE3	100.00	77TRP CD1	85SER	C	0.57	155GL CG	191VAL	CG2	0.10
152TRP CE2	152TRP CZ2	100.00	77TRP CD1	86PRO	CA	0.03	155GL CG	238ILE	CD	0.03
152TRP CE2	152TRP CZ3	100.00	77TRP CD1	86PRO	CB	0.24	155GL CD	155GLU	C	0.04
152TRP CE3	152TRP CZ2	100.00	77TRP CD1	86PRO	CG	4.77	155GL CD	157TYR	CD1	0.01
152TRP CE3	152TRP CZ3	100.00	77TRP CD1	86PRO	CD	4.57	155GL CD	157TYR	CD2	0.04
152TRP CZ2	152TRP CZ3	100.00	77TRP CD2	77TRP	CE2	100.00	155GL CD	157TYR	CE1	1.18
152TRP C	153ASN CA	100.00	77TRP CD2	77TRP	CE3	100.00	155GL CD	157TYR	CE2	15.59
152TRP C	153ASN CB	96.50	77TRP CD2	77TRP	CZ2	100.00	155GL CD	238ILE	CG2	0.00
152TRP C	153ASN CG	6.08	77TRP CD2	77TRP	CZ3	100.00	155GL CD	238ILE	CD	0.06
152TRP C	153ASN C	1.58	77TRP CD2	80LEU	CG	0.01	155GL C	156ARG	CA	100.00
153ASN CA	153ASN CB	100.00	77TRP CD2	80LEU	CD1	0.27	155GL C	156ARG	CB	93.99
153ASN CA	153ASN CG	100.00	77TRP CD2	80LEU	CD2	0.01	155GL C	156ARG	CG	18.28
153ASN CA	153ASN C	100.00	77TRP CD2	84ILE	CG2	0.00	155GL C	156ARG	C	7.20
153ASN CB	153ASN CG	100.00	77TRP CD2	85SER	CA	0.00	156AR CA	156ARG	CB	100.00
153ASN CB	153ASN C	100.00	77TRP CD2	85SER	CB	0.04	156AR CA	156ARG	CG	100.00
153ASN CG	153ASN C	0.20	77TRP CD2	86PRO	CB	0.00	156AR CA	156ARG	CD	3.31
153ASN C	154THR CA	100.00	77TRP CD2	86PRO	CG	0.37	156AR CA	156ARG	C	100.00
153ASN C	154THR CB	64.48	77TRP CD2	86PRO	CD	1.03	156AR CB	156ARG	CG	100.00
153ASN C	154THR C	54.27	77TRP CE2	77TRP	CE3	100.00	156AR CB	156ARG	CD	100.00
154THR CA	154THR CB	100.00	77TRP CE2	77TRP	CZ2	100.00	156AR CB	156ARG	CZ	1.06
154THR CA	154THR CG2	100.00	77TRP CE2	77TRP	CZ3	100.00	156AR CB	156ARG	C	100.00
154THR CA	154THR C	100.00	77TRP CE2	80LEU	CD1	0.29	156AR CG	156ARG	CD	100.00
154THR CB	154THR CG2	100.00	77TRP CE2	80LEU	CD2	0.03	156AR CG	156ARG	CZ	59.44
154THR CB	154THR C	100.00	77TRP CE2	84ILE	CG2	0.01	156AR CG	156ARG	C	67.94
154THR CG2	154THR C	100.00	77TRP CE2	85SER	CB	0.02	156AR CD	156ARG	CZ	100.00
154THR CG2	155GLU C	0.01	77TRP CE2	85SER	C	0.08	156AR CD	156ARG	C	3.54
154THR CG2	156ARG CB	0.01	77TRP CE2	86PRO	CA	0.05	156AR CZ	158SER	CB	0.89
154THR CG2	156ARG CG	0.08	77TRP CE2	86PRO	CB	0.02	156AR C	157TYR	CA	100.00
154THR CG2	156ARG CD	0.01	77TRP CE2	86PRO	CG	0.27	156AR C	157TYR	CB	27.87
154THR C	155GLU CA	100.00	77TRP CE2	86PRO	CD	2.42	156AR C	157TYR	CG	1.52
154THR C	155GLU CB	2.50	77TRP CE3	77TRP	CZ2	100.00	156AR C	157TYR	CD1	0.18
154THR C	155GLU C	99.06	77TRP CE3	77TRP	CZ3	100.00	156AR C	157TYR	CD2	9.42
155GLU CA	155GLU CB	100.00	77TRP CE3	80LEU	CD1	0.04	156AR C	157TYR	C	71.81
155GLU CA	155GLU CG	100.00	77TRP CE3	85SER	CB	0.02	157TY CA	157TYR	CB	100.00
155GLU CA	155GLU CD	98.74	77TRP CE3	86PRO	CA	0.00	157TY CA	157TYR	CG	100.00
155GLU CA	155GLU C	100.00	77TRP CE3	86PRO	CB	0.03	157TY CA	157TYR	CD1	24.36
155GLU CB	155GLU CG	100.00	77TRP CE3	86PRO	CG	0.27	157TY CA	157TYR	CD2	83.82
155GLU CB	155GLU CD	100.00	77TRP CE3	86PRO	CD	0.75	157TY CA	157TYR	C	100.00
155GLU CB	155GLU C	100.00	77TRP CZ2	77TRP	CZ3	100.00	157TY CA	162VAL	CG1	0.00
155GLU CG	155GLU CD	100.00	77TRP CZ2	80LEU	CD1	0.02	157TY CB	157TYR	CG	100.00
155GLU CG	155GLU C	99.98	77TRP CZ2	84ILE	CG2	0.04	157TY CB	157TYR	CD1	100.00
155GLU CG	191VAL CB	0.00	77TRP CZ2	85SER	CA	0.01	157TY CB	157TYR	CD2	100.00
155GLU CG	191VAL CG1	0.06	77TRP CZ2	85SER	C	0.50	157TY CB	157TYR	C	100.00

155GLU CG	191VAL	CG2	0.05	77TRP CZ2	86PRO	CA	0.87	157TY CB	162VAL	CG1	0.28
155GLU CG	238ILE	CD	0.15	77TRP CZ2	86PRO	CB	0.08	157TY CB	162VAL	CG2	0.30
155GLU CD	155GLU	C	0.64	77TRP CZ2	86PRO	CG	0.37	157TY CB	191VAL	CG2	0.20
155GLU CD	157TYR	CD2	0.08	77TRP CZ2	86PRO	CD	1.29	157TY CB	194PHE	CD2	1.29
155GLU CD	157TYR	CE2	0.99	77TRP CZ3	80LEU	CD1	0.00	157TY CB	194PHE	CE2	0.28
155GLU CD	238ILE	CG2	0.25	77TRP CZ3	86PRO	CA	0.01	157TY CG	157TYR	CD1	100.00
155GLU CD	238ILE	CD	0.67	77TRP CZ3	86PRO	CB	0.03	157TY CG	157TYR	CD2	100.00
155GLU C	156ARG	CA	100.00	77TRP CZ3	86PRO	CG	0.56	157TY CG	157TYR	CE1	100.00
155GLU C	156ARG	CB	99.83	77TRP CZ3	86PRO	CD	0.84	157TY CG	157TYR	CE2	100.00
155GLU C	156ARG	CG	1.08	77TRP C	78ASP	CA	100.00	157TY CG	157TYR	CZ	100.00
155GLU C	156ARG	C	0.36	77TRP C	78ASP	CB	5.19	157TY CG	162VAL	CG1	2.38
156ARG CA	156ARG	CB	100.00	77TRP C	78ASP	CG	0.00	157TY CG	162VAL	CG2	0.09
156ARG CA	156ARG	CG	100.00	77TRP C	78ASP	C	95.40	157TY CG	191VAL	CG1	1.61
156ARG CA	156ARG	CD	0.04	77TRP C	80LEU	CD1	0.04	157TY CG	191VAL	CG2	2.74
156ARG CA	156ARG	C	100.00	77TRP C	80LEU	CD2	0.01	157TY CG	195TRP	CZ3	0.10
156ARG CB	156ARG	CG	100.00	78ASP CA	78ASP	CB	100.00	157TY CD1	157TYR	CD2	100.00
156ARG CB	156ARG	CD	100.00	78ASP CA	78ASP	CG	100.00	157TY CD1	157TYR	CE1	100.00
156ARG CB	156ARG	CZ	0.24	78ASP CA	78ASP	C	100.00	157TY CD1	157TYR	CE2	100.00
156ARG CB	156ARG	C	100.00	78ASP CB	78ASP	CG	100.00	157TY CD1	157TYR	CZ	100.00
156ARG CG	156ARG	CD	100.00	78ASP CB	78ASP	C	100.00	157TY CD1	161GLU	CB	0.14
156ARG CG	156ARG	CZ	4.28	78ASP CB	277PRO	CG	0.04	157TY CD1	161GLU	CG	0.05
156ARG CG	156ARG	C	98.66	78ASP CB	282LYS	CE	0.00	157TY CD1	161GLU	CD	0.06
156ARG CD	156ARG	CZ	100.00	78ASP CG	78ASP	C	98.81	157TY CD1	162VAL	CG1	2.42
156ARG CD	156ARG	C	0.04	78ASP CG	277PRO	CG	0.15	157TY CD1	162VAL	CG2	2.36
156ARG C	157TYR	CA	100.00	78ASP CG	277PRO	CD	0.01	157TY CD1	191VAL	CG1	5.16
156ARG C	157TYR	CB	95.29	78ASP CG	282LYS	CE	0.06	157TY CD1	191VAL	CG2	1.94
156ARG C	157TYR	CG	11.88	78ASP C	79GLY	CA	100.00	157TY CD1	195TRP	CE3	0.26
156ARG C	157TYR	CD2	11.20	78ASP C	79GLY	C	48.19	157TY CD1	195TRP	CZ3	0.18
156ARG C	157TYR	C	4.61	78ASP C	80LEU	CD1	0.02	157TY CD2	157TYR	CE1	100.00
156ARG C	191VAL	CG1	0.02	79GLY CA	79GLY	C	100.00	157TY CD2	157TYR	CE2	100.00
157TYR CA	157TYR	CB	100.00	79GLY C	80LEU	CA	100.00	157TY CD2	157TYR	CZ	100.00
157TYR CA	157TYR	CG	100.00	79GLY C	80LEU	CB	6.46	157TY CD2	162VAL	CG1	0.94
157TYR CA	157TYR	CD1	92.50	79GLY C	80LEU	CG	0.78	157TY CD2	162VAL	CG2	0.10
157TYR CA	157TYR	CD2	33.43	79GLY C	80LEU	CD1	0.30	157TY CD2	191VAL	CG1	4.17
157TYR CA	157TYR	C	100.00	79GLY C	80LEU	CD2	0.12	157TY CD2	191VAL	CG2	0.69
157TYR CB	157TYR	CG	100.00	79GLY C	80LEU	C	93.39	157TY CD2	195TRP	CE3	0.10
157TYR CB	157TYR	CD1	100.00	80LEU CA	80LEU	CB	100.00	157TY CD2	195TRP	CZ3	0.41
157TYR CB	157TYR	CD2	100.00	80LEU CA	80LEU	CG	100.00	157TY CE1	157TYR	CE2	100.00
157TYR CB	157TYR	C	100.00	80LEU CA	80LEU	CD1	24.62	157TY CE1	157TYR	CZ	100.00
157TYR CB	162VAL	CG2	4.09	80LEU CA	80LEU	CD2	77.56	157TY CE1	162VAL	CG1	0.02
157TYR CB	191VAL	CG1	0.03	80LEU CA	80LEU	C	100.00	157TY CE1	191VAL	CG1	9.59
157TYR CB	194PHE	CE2	0.00	80LEU CA	81PRO	CD	100.00	157TY CE1	191VAL	CG2	0.03
157TYR CG	157TYR	CD1	100.00	80LEU CB	80LEU	CG	100.00	157TY CE1	195TRP	CB	0.01
157TYR CG	157TYR	CD2	100.00	80LEU CB	80LEU	CD1	100.00	157TY CE1	195TRP	CG	0.00
157TYR CG	157TYR	CE1	100.00	80LEU CB	80LEU	CD2	100.00	157TY CE1	195TRP	CD2	0.01
157TYR CG	157TYR	CE2	100.00	80LEU CB	80LEU	C	100.00	157TY CE1	195TRP	CE3	12.74
157TYR CG	157TYR	CZ	100.00	80LEU CB	81PRO	CD	29.24	157TY CE1	195TRP	CZ3	0.50
157TYR CG	162VAL	CG2	1.03	80LEU CB	84ILE	CG2	0.01	157TY CE2	157TYR	CZ	100.00
157TYR CG	195TRP	CZ3	0.00	80LEU CB	84ILE	CD	0.02	157TY CE2	162VAL	CG1	0.04
157TYR CD1	157TYR	CD2	100.00	80LEU CG	80LEU	CD1	100.00	157TY CE2	191VAL	CG1	6.13
157TYR CD1	157TYR	CE1	100.00	80LEU CG	80LEU	CD2	100.00	157TY CE2	191VAL	CG2	0.01
157TYR CD1	157TYR	CE2	100.00	80LEU CG	80LEU	C	3.00	157TY CE2	195TRP	CD2	0.01
157TYR CD1	157TYR	CZ	100.00	80LEU CG	81PRO	CD	0.05	157TY CE2	195TRP	CE3	1.59
157TYR CD1	161GLU	CB	0.05	80LEU CG	84ILE	CD	0.05	157TY CE2	195TRP	CZ3	0.86
157TYR CD1	161GLU	CG	1.36	80LEU CD1	80LEU	CD2	100.00	157TY CZ	191VAL	CG1	5.06
157TYR CD1	161GLU	CD	0.04	80LEU CD1	80LEU	C	0.05	157TY CZ	195TRP	CE3	0.08
157TYR CD1	161GLU	C	0.00	80LEU CD1	81PRO	CD	0.00	157TY CZ	195TRP	CZ3	0.48
157TYR CD1	162VAL	CG2	3.31	80LEU CD1	84ILE	CG1	0.01	157TY C	158SER	CA	100.00
157TYR CD1	195TRP	CZ3	0.06	80LEU CD1	84ILE	CG2	0.48	157TY C	158SER	CB	68.91
157TYR CD2	157TYR	CE1	100.00	80LEU CD1	84ILE	CD	0.20	157TY C	158SER	C	37.37
157TYR CD2	157TYR	CE2	100.00	80LEU CD1	86PRO	CD	0.02	157TY C	194PHE	CE2	0.71
157TYR CD2	157TYR	CZ	100.00	80LEU CD2	80LEU	C	0.13	158SE CA	158SER	CB	100.00

157TYR	CD2	162VAL	CG2	0.01	80LEU	CD2	81PRO	CD	0.08	158SE	CA	158SER	C	100.00
157TYR	CD2	191VAL	CG1	1.32	80LEU	CD2	84ILE	CG1	0.01	158SE	CA	194PHE	CE2	0.17
157TYR	CD2	195TRP	CE3	0.21	80LEU	CD2	84ILE	CG2	0.07	158SE	CA	194PHE	CZ	0.03
157TYR	CD2	195TRP	CZ3	0.59	80LEU	CD2	84ILE	CD	0.50	158SE	CB	158SER	C	100.00
157TYR	CE1	157TYR	CE2	100.00	80LEU	CD2	84ILE	C	0.02	158SE	CB	161GLU	CG	0.12
157TYR	CE1	157TYR	CZ	100.00	80LEU	CD2	85SER	CA	0.01	158SE	C	159LYS	CA	100.00
157TYR	CE1	161GLU	CG	0.18	80LEU	C	81PRO	CA	100.00	158SE	C	159LYS	C	100.00
157TYR	CE1	195TRP	CZ3	0.24	80LEU	C	81PRO	CB	0.08	158SE	C	160PRO	CD	17.19
157TYR	CE2	157TYR	CZ	100.00	80LEU	C	81PRO	CD	100.00	158SE	C	194PHE	CE2	1.75
157TYR	CE2	195TRP	CE3	0.08	80LEU	C	81PRO	C	100.00	158SE	C	194PHE	CZ	0.14
157TYR	CE2	195TRP	CZ3	2.98	81PRO	CA	81PRO	CB	100.00	159LY	CA	159LYS	CB	100.00
157TYR	CZ	195TRP	CZ3	0.16	81PRO	CA	81PRO	CG	100.00	159LY	CA	159LYS	CG	100.00
157TYR	C	158SER	CA	100.00	81PRO	CA	81PRO	CD	100.00	159LY	CA	159LYS	CD	7.31
157TYR	C	158SER	CB	23.85	81PRO	CA	81PRO	C	100.00	159LY	CA	159LYS	C	100.00
157TYR	C	158SER	C	79.56	81PRO	CB	81PRO	CG	100.00	159LY	CA	160PRO	CD	100.00
157TYR	C	194PHE	CE2	0.25	81PRO	CB	81PRO	CD	100.00	159LY	CA	162VAL	CG2	0.00
158SER	CA	158SER	CB	100.00	81PRO	CB	81PRO	C	100.00	159LY	CA	194PHE	CE2	0.03
158SER	CA	158SER	C	100.00	81PRO	CB	83LYS	CG	0.08	159LY	CA	194PHE	CZ	0.52
158SER	CA	194PHE	CE2	0.02	81PRO	CB	83LYS	CE	0.01	159LY	CB	159LYS	CG	100.00
158SER	CA	194PHE	CZ	0.01	81PRO	CG	81PRO	CD	100.00	159LY	CB	159LYS	CD	100.00
158SER	CB	158SER	C	100.00	81PRO	CG	81PRO	C	88.66	159LY	CB	159LYS	CE	6.24
158SER	CB	161GLU	CD	0.04	81PRO	CG	83LYS	CG	0.13	159LY	CB	159LYS	C	100.00
158SER	C	159LYS	CA	100.00	81PRO	CG	83LYS	CD	0.02	159LY	CB	160PRO	CD	72.67
158SER	C	159LYS	C	100.00	81PRO	CG	83LYS	CE	1.60	159LY	CB	194PHE	CE2	0.00
158SER	C	160PRO	CD	18.96	81PRO	CG	84ILE	CG1	0.07	159LY	CB	194PHE	CZ	0.27
158SER	C	194PHE	CE2	0.69	81PRO	CG	84ILE	CD	0.06	159LY	CG	159LYS	CD	100.00
158SER	C	194PHE	CZ	0.09	81PRO	CD	81PRO	C	81.66	159LY	CG	159LYS	CE	100.00
159LYS	CA	159LYS	CB	100.00	81PRO	CD	84ILE	CG1	0.10	159LY	CG	159LYS	C	95.29
159LYS	CA	159LYS	CG	100.00	81PRO	CD	84ILE	CG2	0.01	159LY	CG	160PRO	CD	0.09
159LYS	CA	159LYS	CD	6.36	81PRO	CD	84ILE	CD	0.03	159LY	CG	163GLU	CD	0.07
159LYS	CA	159LYS	CE	0.03	81PRO	C	82ARG	CA	100.00	159LY	CG	194PHE	CE1	0.00
159LYS	CA	159LYS	C	100.00	81PRO	C	82ARG	CB	0.01	159LY	CG	194PHE	CE2	0.00
159LYS	CA	160PRO	CD	100.00	81PRO	C	82ARG	C	100.00	159LY	CG	194PHE	CZ	0.26
159LYS	CA	194PHE	CE2	0.01	82ARG	CA	82ARG	CB	100.00	159LY	CG	198THR	CG2	0.86
159LYS	CA	194PHE	CZ	0.77	82ARG	CA	82ARG	CG	100.00	159LY	CD	159LYS	CE	100.00
159LYS	CB	159LYS	CG	100.00	82ARG	CA	82ARG	CD	6.50	159LY	CD	159LYS	C	0.71
159LYS	CB	159LYS	CD	100.00	82ARG	CA	82ARG	C	100.00	159LY	CD	160PRO	CD	0.01
159LYS	CB	159LYS	CE	7.36	82ARG	CA	85SER	CB	0.02	159LY	CD	163GLU	CD	0.00
159LYS	CB	159LYS	C	100.00	82ARG	CA	88THR	CG2	0.00	159LY	CD	194PHE	CZ	0.22
159LYS	CB	160PRO	CD	71.27	82ARG	CB	82ARG	CG	100.00	159LY	CD	198THR	CG2	0.04
159LYS	CB	194PHE	CZ	0.66	82ARG	CB	82ARG	CD	100.00	159LY	CE	163GLU	CD	0.00
159LYS	CG	159LYS	CD	100.00	82ARG	CB	82ARG	CZ	0.72	159LY	CE	198THR	CG2	0.02
159LYS	CG	159LYS	CE	100.00	82ARG	CB	82ARG	C	100.00	159LY	CE	201GLU	CD	0.58
159LYS	CG	159LYS	C	93.30	82ARG	CB	88THR	CG2	0.01	159LY	C	160PRO	CA	100.00
159LYS	CG	160PRO	CD	0.04	82ARG	CG	82ARG	CD	100.00	159LY	C	160PRO	CB	0.12
159LYS	CG	194PHE	CE1	0.11	82ARG	CG	82ARG	CZ	14.31	159LY	C	160PRO	CD	100.00
159LYS	CG	194PHE	CE2	0.02	82ARG	CG	82ARG	C	28.95	159LY	C	160PRO	C	100.00
159LYS	CG	194PHE	CZ	0.93	82ARG	CG	85SER	CB	0.00	160PR	CA	160PRO	CB	100.00
159LYS	CG	198THR	CG2	2.72	82ARG	CG	88THR	CG2	0.19	160PR	CA	160PRO	CG	100.00
159LYS	CD	159LYS	CE	100.00	82ARG	CD	82ARG	CZ	100.00	160PR	CA	160PRO	CD	100.00
159LYS	CD	159LYS	C	0.54	82ARG	CD	82ARG	C	0.45	160PR	CA	160PRO	C	100.00
159LYS	CD	160PRO	CD	0.00	82ARG	CD	87GLU	CG	0.00	160PR	CA	163GLU	CB	0.00
159LYS	CD	163GLU	CD	0.00	82ARG	CD	87GLU	CD	0.01	160PR	CB	160PRO	CG	100.00
159LYS	CD	194PHE	CE1	0.04	82ARG	CD	88THR	CG2	0.06	160PR	CB	160PRO	CD	100.00
159LYS	CD	194PHE	CZ	0.19	82ARG	CD	140PHE	CZ	0.01	160PR	CB	160PRO	C	100.00
159LYS	CD	198THR	CG2	0.08	82ARG	CZ	85SER	CB	0.13	160PR	CG	160PRO	CD	100.00
159LYS	CE	194PHE	CE1	0.04	82ARG	CZ	87GLU	CB	0.01	160PR	CG	160PRO	C	92.73
159LYS	CE	194PHE	CZ	0.01	82ARG	CZ	87GLU	CG	0.22	160PR	CD	160PRO	C	81.22
159LYS	CE	198THR	CG2	0.02	82ARG	CZ	87GLU	CD	0.04	160PR	C	161GLU	CA	100.00
159LYS	CE	201GLU	CD	0.23	82ARG	CZ	88THR	CG2	0.22	160PR	C	161GLU	CB	0.07
159LYS	C	160PRO	CA	100.00	82ARG	CZ	91LEU	CD1	0.00	160PR	C	161GLU	CG	0.00
159LYS	C	160PRO	CB	0.06	82ARG	CZ	139TYR	CB	0.04	160PR	C	161GLU	C	99.95

159LYS C	160PRO CD	100.00
159LYS C	160PRO C	100.00
160PRO CA	160PRO CB	100.00
160PRO CA	160PRO CG	100.00
160PRO CA	160PRO CD	100.00
160PRO CA	160PRO C	100.00
160PRO CA	163GLU CB	0.04
160PRO CB	160PRO CG	100.00
160PRO CB	160PRO CD	100.00
160PRO CB	160PRO C	100.00
160PRO CB	164ARG CZ	0.09
160PRO CG	160PRO CD	100.00
160PRO CG	160PRO C	87.50
160PRO CD	160PRO C	72.47
160PRO C	161GLU CA	100.00
160PRO C	161GLU CB	0.11
160PRO C	161GLU C	99.96
160PRO C	163GLU CB	0.02
160PRO C	164ARG CZ	0.38
161GLU CA	161GLU CB	100.00
161GLU CA	161GLU CG	100.00
161GLU CA	161GLU CD	94.75
161GLU CA	161GLU C	100.00
161GLU CA	164ARG CZ	0.10
161GLU CB	161GLU CG	100.00
161GLU CB	161GLU CD	100.00
161GLU CB	161GLU C	100.00
161GLU CG	161GLU CD	100.00
161GLU CG	161GLU C	78.65
161GLU CG	164ARG CZ	0.00
161GLU C	162VAL CA	100.00
161GLU C	162VAL CB	0.14
161GLU C	162VAL CG2	0.06
161GLU C	162VAL C	99.90
162VAL CA	162VAL CB	100.00
162VAL CA	162VAL CG1	100.00
162VAL CA	162VAL CG2	100.00
162VAL CA	162VAL C	100.00
162VAL CA	195TRP CZ3	0.00
162VAL CB	162VAL CG1	100.00
162VAL CB	162VAL CG2	100.00
162VAL CB	162VAL C	100.00
162VAL CB	198THR CG2	0.56
162VAL CG1	162VAL CG2	100.00
162VAL CG1	162VAL C	100.00
162VAL CG1	195TRP CD2	4.21
162VAL CG1	195TRP CE2	0.02
162VAL CG1	195TRP CE3	18.39
162VAL CG1	195TRP CZ3	1.59
162VAL CG1	198THR CB	0.86
162VAL CG1	198THR CG2	7.48
162VAL CG1	199VAL CG1	4.26
162VAL CG1	199VAL CG2	2.10
162VAL CG2	194PHE CE2	0.13
162VAL CG2	195TRP CE3	6.13
162VAL CG2	195TRP CZ3	1.52
162VAL C	163GLU CA	100.00
162VAL C	163GLU CB	0.01
162VAL C	163GLU C	100.00
162VAL C	165VAL CG1	0.01
162VAL C	165VAL CG2	0.00
163GLU CA	163GLU CB	100.00

82ARG CZ	140PHE	CE1	0.01
82ARG CZ	144ARG	CZ	0.12
82ARG C	83LYS	CA	100.00
82ARG C	83LYS	CB	2.42
82ARG C	83LYS	CG	0.08
82ARG C	83LYS	C	97.38
82ARG C	88THR	CG2	0.02
83LYS CA	83LYS	CB	100.00
83LYS CA	83LYS	CG	100.00
83LYS CA	83LYS	CD	1.38
83LYS CA	83LYS	CE	0.00
83LYS CA	83LYS	C	100.00
83LYS CA	88THR	CG2	0.01
83LYS CB	83LYS	CG	100.00
83LYS CB	83LYS	CD	100.00
83LYS CB	83LYS	CE	16.02
83LYS CB	83LYS	C	100.00
83LYS CG	83LYS	CD	100.00
83LYS CG	83LYS	CE	100.00
83LYS CG	83LYS	C	94.42
83LYS CG	84ILE	CG1	0.04
83LYS CG	84ILE	CG2	0.00
83LYS CG	84ILE	CD	0.05
83LYS CD	83LYS	CE	100.00
83LYS CD	83LYS	C	0.14
83LYS CD	84ILE	CG2	0.00
83LYS CD	84ILE	CD	0.00
83LYS CE	84ILE	CG2	0.01
83LYS C	84ILE	CA	100.00
83LYS C	84ILE	CB	76.18
83LYS C	84ILE	CG1	5.11
83LYS C	84ILE	CG2	45.73
83LYS C	84ILE	CD	0.08
83LYS C	84ILE	C	26.03
83LYS C	88THR	CG2	0.18
84ILE CA	84ILE	CB	100.00
84ILE CA	84ILE	CG1	100.00
84ILE CA	84ILE	CG2	100.00
84ILE CA	84ILE	CD	12.85
84ILE CA	84ILE	C	100.00
84ILE CB	84ILE	CG1	100.00
84ILE CB	84ILE	CG2	100.00
84ILE CB	84ILE	CD	100.00
84ILE CB	84ILE	C	100.00
84ILE CG1	84ILE	CG2	100.00
84ILE CG1	84ILE	CD	100.00
84ILE CG1	84ILE	C	71.84
84ILE CG2	84ILE	CD	97.97
84ILE CG2	84ILE	C	27.14
84ILE CD	84ILE	C	0.50
84ILE C	85SER	CA	100.00
84ILE C	85SER	CB	16.23
84ILE C	85SER	C	85.15
85SER CA	85SER	CB	100.00
85SER CA	85SER	C	100.00
85SER CA	86PRO	CD	100.00
85SER CB	85SER	C	100.00
85SER CB	86PRO	CD	38.41
85SER CB	87GLU	CD	0.00
85SER C	86PRO	CA	100.00
85SER C	86PRO	CB	0.05
85SER C	86PRO	CD	100.00

160PR C	163GLU	CB	0.01
161GL CA	161GLU	CB	100.00
161GL CA	161GLU	CG	100.00
161GL CA	161GLU	CD	89.26
161GL CA	161GLU	C	100.00
161GL CB	161GLU	CG	100.00
161GL CB	161GLU	CD	100.00
161GL CB	161GLU	C	100.00
161GL CG	161GLU	CD	100.00
161GL CG	161GLU	C	4.91
161GL CD	161GLU	C	0.01
161GL C	162VAL	CA	100.00
161GL C	162VAL	CB	2.76
161GL C	162VAL	CG1	1.24
161GL C	162VAL	CG2	0.04
161GL C	162VAL	C	97.22
162VA CA	162VAL	CB	100.00
162VA CA	162VAL	CG1	100.00
162VA CA	162VAL	CG2	100.00
162VA CA	162VAL	C	100.00
162VA CA	165VAL	CG1	0.02
162VA CB	162VAL	CG1	100.00
162VA CB	162VAL	CG2	100.00
162VA CB	162VAL	C	100.00
162VA CB	195TRP	CE3	0.00
162VA CB	195TRP	CZ3	0.03
162VA CB	198THR	CG2	0.20
162VA CG1	162VAL	CG2	100.00
162VA CG1	162VAL	C	68.51
162VA CG1	195TRP	CD2	1.64
162VA CG1	195TRP	CE2	0.19
162VA CG1	195TRP	CE3	10.47
162VA CG1	195TRP	CZ2	0.03
162VA CG1	195TRP	CZ3	1.21
162VA CG1	198THR	CB	1.18
162VA CG1	198THR	CG2	9.81
162VA CG1	199VAL	CG1	0.36
162VA CG1	199VAL	CG2	0.77
162VA CG2	162VAL	C	31.44
162VA CG2	194PHE	CE2	0.15
162VA CG2	195TRP	CD2	0.00
162VA CG2	195TRP	CE3	2.19
162VA CG2	195TRP	CZ3	1.52
162VA CG2	198THR	CG2	0.83
162VA C	163GLU	CA	100.00
162VA C	163GLU	CB	0.06
162VA C	163GLU	C	99.99
162VA C	165VAL	CG1	0.09
162VA C	198THR	CG2	0.42
163GL CA	163GLU	CB	100.00
163GL CA	163GLU	CG	100.00
163GL CA	163GLU	CD	26.79
163GL CA	163GLU	C	100.00
163GL CA	198THR	CG2	0.75
163GL CA	202VAL	CG2	0.00
163GL CB	163GLU	CG	100.00
163GL CB	163GLU	CD	100.00
163GL CB	163GLU	C	100.00
163GL CB	167ARG	CZ	0.32
163GL CB	198THR	CG2	0.09
163GL CB	202VAL	CG2	0.04
163GL CG	163GLU	CD	100.00

163GLU	CA	163GLU	CG	100.00
163GLU	CA	163GLU	CD	15.56
163GLU	CA	163GLU	C	100.00
163GLU	CA	198THR	CG2	0.03
163GLU	CA	202VAL	CG2	0.01
163GLU	CB	163GLU	CG	100.00
163GLU	CB	163GLU	CD	100.00
163GLU	CB	163GLU	C	100.00
163GLU	CB	167ARG	CZ	0.14
163GLU	CB	198THR	CG2	0.06
163GLU	CG	163GLU	CD	100.00
163GLU	CG	163GLU	C	90.70
163GLU	CG	167ARG	CG	0.01
163GLU	CG	167ARG	CD	0.07
163GLU	CG	167ARG	CZ	0.21
163GLU	CG	198THR	CG2	0.43
163GLU	CG	201GLU	CG	0.00
163GLU	CG	202VAL	CG1	0.16
163GLU	CG	202VAL	CG2	8.60
163GLU	CD	163GLU	C	12.44
163GLU	CD	167ARG	CG	0.02
163GLU	CD	167ARG	CD	2.07
163GLU	CD	167ARG	CZ	0.21
163GLU	CD	201GLU	CG	0.00
163GLU	CD	202VAL	CG2	0.21
163GLU	C	164ARG	CA	100.00
163GLU	C	164ARG	CB	0.01
163GLU	C	164ARG	C	100.00
163GLU	C	167ARG	CG	0.00
163GLU	C	167ARG	CZ	0.00
164ARG	CA	164ARG	CB	100.00
164ARG	CA	164ARG	CG	100.00
164ARG	CA	164ARG	CD	0.05
164ARG	CA	164ARG	C	100.00
164ARG	CA	167ARG	CZ	0.08
164ARG	CB	164ARG	CG	100.00
164ARG	CB	164ARG	CD	100.00
164ARG	CB	164ARG	CZ	5.28
164ARG	CB	164ARG	C	100.00
164ARG	CG	164ARG	CD	100.00
164ARG	CG	164ARG	CZ	44.25
164ARG	CG	164ARG	C	46.06
164ARG	CG	167ARG	CZ	0.26
164ARG	CD	164ARG	CZ	100.00
164ARG	CD	264ARG	CG	0.01
164ARG	CD	264ARG	CD	0.00
164ARG	CZ	264ARG	CG	0.42
164ARG	CZ	264ARG	CD	2.97
164ARG	CZ	264ARG	CZ	0.10
164ARG	C	165VAL	CA	100.00
164ARG	C	165VAL	CB	0.07
164ARG	C	165VAL	CG2	0.01
164ARG	C	165VAL	C	99.97
164ARG	C	167ARG	CB	0.00
165VAL	CA	165VAL	CB	100.00
165VAL	CA	165VAL	CG1	100.00
165VAL	CA	165VAL	CG2	100.00
165VAL	CA	165VAL	C	100.00
165VAL	CA	168VAL	CG2	0.02
165VAL	CB	165VAL	CG1	100.00
165VAL	CB	165VAL	CG2	100.00
165VAL	CB	165VAL	C	100.00

85SER	C	86PRO	C	100.00
86PRO	CA	86PRO	CB	100.00
86PRO	CA	86PRO	CG	100.00
86PRO	CA	86PRO	CD	100.00
86PRO	CA	86PRO	C	100.00
86PRO	CB	86PRO	CG	100.00
86PRO	CB	86PRO	CD	100.00
86PRO	CB	86PRO	C	100.00
86PRO	CB	90LEU	CD1	0.08
86PRO	CG	86PRO	CD	100.00
86PRO	CG	86PRO	C	53.25
86PRO	CD	86PRO	C	42.36
86PRO	C	87GLU	CA	100.00
86PRO	C	87GLU	CB	0.10
86PRO	C	87GLU	C	99.92
86PRO	C	90LEU	CD1	0.18
87GLU	CA	87GLU	CB	100.00
87GLU	CA	87GLU	CG	100.00
87GLU	CA	87GLU	CD	90.96
87GLU	CA	87GLU	C	100.00
87GLU	CA	90LEU	CD1	0.01
87GLU	CB	87GLU	CG	100.00
87GLU	CB	87GLU	CD	100.00
87GLU	CB	87GLU	C	100.00
87GLU	CG	87GLU	CD	100.00
87GLU	CG	87GLU	C	88.32
87GLU	CG	91LEU	CD1	0.16
87GLU	CG	91LEU	CD2	0.02
87GLU	CG	139TYR	CG	0.05
87GLU	CG	139TYR	CD1	0.03
87GLU	CG	139TYR	CD2	0.12
87GLU	CG	139TYR	CE1	0.70
87GLU	CG	139TYR	CE2	1.55
87GLU	CG	139TYR	CZ	1.08
87GLU	CG	140PHE	CE1	0.10
87GLU	CG	140PHE	CE2	0.17
87GLU	CG	140PHE	CZ	0.53
87GLU	CD	87GLU	C	0.92
87GLU	CD	90LEU	CD1	0.02
87GLU	CD	94ARG	CZ	0.02
87GLU	CD	139TYR	CE1	0.09
87GLU	CD	139TYR	CE2	0.12
87GLU	CD	139TYR	CZ	0.00
87GLU	C	88THR	CA	100.00
87GLU	C	88THR	CB	0.26
87GLU	C	88THR	CG2	0.06
87GLU	C	88THR	C	99.79
87GLU	C	91LEU	CD1	0.02
87GLU	C	140PHE	CZ	0.02
88THR	CA	88THR	CB	100.00
88THR	CA	88THR	CG2	100.00
88THR	CA	88THR	C	100.00
88THR	CA	91LEU	CB	0.01
88THR	CA	91LEU	CD1	0.01
88THR	CA	140PHE	CZ	0.00
88THR	CB	88THR	CG2	100.00
88THR	CB	88THR	C	100.00
88THR	CG2	88THR	C	0.94
88THR	CG2	139TYR	CD1	0.03
88THR	CG2	139TYR	CD2	0.00
88THR	CG2	139TYR	CE1	0.01
88THR	CG2	140PHE	CD2	0.01

163GL	CG	163GLU	C	90.25
163GL	CG	167ARG	CG	0.00
163GL	CG	167ARG	CD	0.00
163GL	CG	167ARG	CZ	0.01
163GL	CG	198THR	CG2	1.70
163GL	CG	202VAL	CG1	0.08
163GL	CG	202VAL	CG2	10.00
163GL	CD	163GLU	C	29.59
163GL	CD	167ARG	CG	0.04
163GL	CD	167ARG	CD	0.06
163GL	CD	167ARG	CZ	0.15
163GL	CD	202VAL	CG1	0.00
163GL	CD	202VAL	CG2	0.27
163GL	C	164ARG	CA	100.00
163GL	C	164ARG	C	100.00
164AR	CA	164ARG	CB	100.00
164AR	CA	164ARG	CG	100.00
164AR	CA	164ARG	CD	0.00
164AR	CA	164ARG	C	100.00
164AR	CA	167ARG	CZ	0.02
164AR	CB	164ARG	CG	100.00
164AR	CB	164ARG	CD	100.00
164AR	CB	164ARG	C	100.00
164AR	CG	164ARG	CD	100.00
164AR	CG	164ARG	CZ	0.39
164AR	CG	164ARG	C	14.29
164AR	CG	167ARG	CZ	0.02
164AR	CD	164ARG	CZ	100.00
164AR	C	165VAL	CA	100.00
164AR	C	165VAL	CB	0.48
164AR	C	165VAL	CG1	0.06
164AR	C	165VAL	CG2	0.03
164AR	C	165VAL	C	99.71
164AR	C	168VAL	CG2	0.00
165VA	CA	165VAL	CB	100.00
165VA	CA	165VAL	CG1	100.00
165VA	CA	165VAL	CG2	100.00
165VA	CA	165VAL	C	100.00
165VA	CA	168VAL	CG1	0.02
165VA	CB	165VAL	CG1	100.00
165VA	CB	165VAL	CG2	100.00
165VA	CB	165VAL	C	100.00
165VA	CG1	165VAL	CG2	100.00
165VA	CG1	165VAL	C	73.35
165VA	CG1	195TRP	CZ2	0.03
165VA	CG1	195TRP	CZ3	0.43
165VA	CG1	234VAL	CG2	0.49
165VA	CG2	165VAL	C	77.63
165VA	CG2	195TRP	CZ2	0.06
165VA	CG2	195TRP	CZ3	0.38
165VA	CG2	234VAL	CG2	0.10
165VA	C	166ALA	CA	100.00
165VA	C	166ALA	CB	0.01
165VA	C	166ALA	C	100.00
165VA	C	168VAL	CG1	0.05
165VA	C	168VAL	CG2	0.01
165VA	C	234VAL	CG2	0.02
166AL	CA	166ALA	CB	100.00
166AL	CA	166ALA	C	100.00
166AL	CA	234VAL	CG2	0.01
166AL	CB	166ALA	C	100.00
166AL	CB	198THR	CG2	0.05

165VAL CG1	165VAL	CG2	100.00	88THR CG2	140PHE	CE1	0.31	166AL CB	199VAL	CG1	0.15
165VAL CG1	165VAL	C	98.12	88THR CG2	140PHE	CE2	0.10	166AL CB	199VAL	CG2	0.12
165VAL CG1	195TRP	CZ2	0.04	88THR CG2	140PHE	CZ	0.52	166AL CB	202VAL	CG1	0.37
165VAL CG1	234VAL	CG2	2.21	88THR C	89GLY	CA	100.00	166AL CB	202VAL	CG2	0.22
165VAL CG2	165VAL	C	35.31	88THR C	89GLY	C	82.24	166AL C	167ARG	CA	100.00
165VAL CG2	195TRP	CZ3	0.00	88THR C	91LEU	CB	0.09	166AL C	167ARG	C	100.00
165VAL CG2	234VAL	CG2	0.05	88THR C	91LEU	CG	0.00	166AL C	170PHE	CD2	0.59
165VAL C	166ALA	CA	100.00	88THR C	91LEU	CD1	0.01	166AL C	170PHE	CE2	0.01
165VAL C	166ALA	CB	0.01	89GLY CA	89GLY	C	100.00	166AL C	202VAL	CG1	0.01
165VAL C	166ALA	C	100.00	89GLY C	90LEU	CA	100.00	167AR CA	167ARG	CB	100.00
165VAL C	168VAL	CG2	0.07	89GLY C	90LEU	CB	0.41	167AR CA	167ARG	CG	100.00
165VAL C	234VAL	CG2	0.00	89GLY C	90LEU	CG	0.02	167AR CA	167ARG	CD	1.16
166ALA CA	166ALA	CB	100.00	89GLY C	90LEU	C	99.37	167AR CA	167ARG	C	100.00
166ALA CA	166ALA	C	100.00	90LEU CA	90LEU	CB	100.00	167AR CA	202VAL	CG1	0.58
166ALA CB	166ALA	C	100.00	90LEU CA	90LEU	CG	100.00	167AR CA	202VAL	CG2	0.00
166ALA CB	170PHE	CE2	0.00	90LEU CA	90LEU	CD1	5.92	167AR CA	206TYR	CE1	0.00
166ALA CB	199VAL	CB	0.00	90LEU CA	90LEU	CD2	93.40	167AR CB	167ARG	CG	100.00
166ALA CB	199VAL	CG1	0.60	90LEU CA	90LEU	C	100.00	167AR CB	167ARG	CD	100.00
166ALA CB	199VAL	CG2	0.07	90LEU CB	90LEU	CG	100.00	167AR CB	167ARG	CZ	4.43
166ALA CB	202VAL	CG1	0.24	90LEU CB	90LEU	CD1	100.00	167AR CB	167ARG	C	100.00
166ALA CB	202VAL	CG2	0.88	90LEU CB	90LEU	CD2	100.00	167AR CB	202VAL	CG1	0.09
166ALA C	167ARG	CA	100.00	90LEU CB	90LEU	C	100.00	167AR CB	202VAL	CG2	0.01
166ALA C	167ARG	C	100.00	90LEU CB	94ARG	CZ	4.10	167AR CB	206TYR	CE1	0.00
166ALA C	170PHE	CD1	0.12	90LEU CG	90LEU	CD1	100.00	167AR CG	167ARG	CD	100.00
166ALA C	170PHE	CD2	0.07	90LEU CG	90LEU	CD2	100.00	167AR CG	167ARG	CZ	42.14
166ALA C	202VAL	CG1	0.01	90LEU CG	90LEU	C	5.48	167AR CG	167ARG	C	73.07
167ARG CA	167ARG	CB	100.00	90LEU CG	94ARG	CZ	0.22	167AR CG	202VAL	CG1	0.84
167ARG CA	167ARG	CG	100.00	90LEU CD1	90LEU	CD2	100.00	167AR CG	202VAL	CG2	0.12
167ARG CA	167ARG	CD	3.14	90LEU CD1	90LEU	C	0.10	167AR CG	206TYR	CE1	0.12
167ARG CA	167ARG	C	100.00	90LEU CD1	93LEU	CB	0.01	167AR CG	206TYR	CZ	0.01
167ARG CA	202VAL	CG1	0.04	90LEU CD1	93LEU	CD2	0.00	167AR CD	167ARG	CZ	100.00
167ARG CB	167ARG	CG	100.00	90LEU CD1	94ARG	CZ	0.16	167AR CD	167ARG	C	0.04
167ARG CB	167ARG	CD	100.00	90LEU CD2	90LEU	C	0.34	167AR CD	202VAL	CG1	0.00
167ARG CB	167ARG	CZ	3.17	90LEU CD2	93LEU	CD1	0.07	167AR CD	206TYR	CE1	0.00
167ARG CB	167ARG	C	100.00	90LEU CD2	93LEU	CD2	0.16	167AR CZ	202VAL	CA	0.00
167ARG CB	202VAL	CG1	0.01	90LEU CD2	94ARG	CG	0.00	167AR CZ	202VAL	CG1	0.03
167ARG CG	167ARG	CD	100.00	90LEU CD2	94ARG	CZ	0.45	167AR CZ	202VAL	CG2	0.02
167ARG CG	167ARG	CZ	33.01	90LEU C	91LEU	CA	100.00	167AR CZ	206TYR	CE1	0.01
167ARG CG	167ARG	C	32.01	90LEU C	91LEU	CB	0.00	167AR C	168VAL	CA	100.00
167ARG CG	202VAL	CG1	2.14	90LEU C	91LEU	C	100.00	167AR C	168VAL	CB	0.13
167ARG CG	202VAL	CG2	0.01	90LEU C	94ARG	CG	0.00	167AR C	168VAL	CG1	0.01
167ARG CG	206TYR	CE1	0.04	90LEU C	94ARG	CD	0.02	167AR C	168VAL	CG2	0.02
167ARG CG	206TYR	CE2	0.08	91LEU CA	91LEU	CB	100.00	167AR C	168VAL	C	99.88
167ARG CG	206TYR	CZ	0.01	91LEU CA	91LEU	CG	100.00	168VA CA	168VAL	CB	100.00
167ARG CD	167ARG	CZ	100.00	91LEU CA	91LEU	CD1	52.93	168VA CA	168VAL	CG1	100.00
167ARG CD	167ARG	C	0.02	91LEU CA	91LEU	CD2	46.27	168VA CA	168VAL	CG2	100.00
167ARG CD	202VAL	CG1	0.02	91LEU CA	91LEU	C	100.00	168VA CA	168VAL	C	100.00
167ARG CD	206TYR	CE1	0.01	91LEU CB	91LEU	CG	100.00	168VA CB	168VAL	CG1	100.00
167ARG CD	206TYR	CE2	0.01	91LEU CB	91LEU	CD1	100.00	168VA CB	168VAL	CG2	100.00
167ARG CD	206TYR	CZ	0.01	91LEU CB	91LEU	CD2	100.00	168VA CB	168VAL	C	100.00
167ARG CZ	168VAL	CG1	0.00	91LEU CB	91LEU	C	100.00	168VA CB	262LEU	CD1	0.02
167ARG CZ	202VAL	CG1	0.19	91LEU CB	139TYR	CE2	0.00	168VA CB	262LEU	CD2	0.03
167ARG CZ	202VAL	CG2	0.01	91LEU CB	140PHE	CE1	0.00	168VA CG1	168VAL	CG2	100.00
167ARG CZ	206TYR	CD2	0.01	91LEU CB	140PHE	CE2	0.01	168VA CG1	168VAL	C	86.71
167ARG CZ	206TYR	CE1	0.18	91LEU CB	140PHE	CZ	0.01	168VA CG1	172LEU	CD1	4.56
167ARG CZ	206TYR	CE2	0.13	91LEU CG	91LEU	CD1	100.00	168VA CG1	172LEU	CD2	0.00
167ARG CZ	206TYR	CZ	0.00	91LEU CG	91LEU	CD2	100.00	168VA CG1	262LEU	CD1	1.37
167ARG C	168VAL	CA	100.00	91LEU CG	91LEU	C	57.93	168VA CG1	262LEU	CD2	0.59
167ARG C	168VAL	CB	0.04	91LEU CG	95LYS	CD	0.00	168VA CG2	168VAL	C	24.06
167ARG C	168VAL	CG1	0.00	91LEU CG	95LYS	CE	0.00	168VA CG2	172LEU	CD1	0.01
167ARG C	168VAL	C	99.97	91LEU CG	139TYR	CE2	0.00	168VA CG2	262LEU	CD1	0.66
168VAL CA	168VAL	CB	100.00	91LEU CG	139TYR	CZ	0.00	168VA CG2	262LEU	CD2	0.20

168VAL	CA	168VAL	CG1	100.00	91LEU	CG	140PHE	CD1	0.01	168VA	C	169ALA	CA	100.00
168VAL	CA	168VAL	CG2	100.00	91LEU	CG	140PHE	CE1	0.02	168VA	C	169ALA	C	100.00
168VAL	CA	168VAL	C	100.00	91LEU	CG	140PHE	CE2	0.00	169AL	CA	169ALA	CB	100.00
168VAL	CB	168VAL	CG1	100.00	91LEU	CG	140PHE	CZ	0.02	169AL	CA	169ALA	C	100.00
168VAL	CB	168VAL	CG2	100.00	91LEU	CD1	91LEU	CD2	100.00	169AL	CA	232VAL	CG1	0.00
168VAL	CB	168VAL	C	100.00	91LEU	CD1	91LEU	C	5.38	169AL	CB	169ALA	C	100.00
168VAL	CB	262LEU	CD1	0.85	91LEU	CD1	94ARG	CZ	0.01	169AL	CB	180VAL	CG1	0.10
168VAL	CB	262LEU	CD2	0.03	91LEU	CD1	95LYS	CG	0.10	169AL	CB	180VAL	CG2	0.05
168VAL	CG1	168VAL	CG2	100.00	91LEU	CD1	95LYS	CD	0.10	169AL	CB	232VAL	CG1	0.19
168VAL	CG1	168VAL	C	18.29	91LEU	CD1	95LYS	CE	0.08	169AL	CB	234VAL	CB	6.15
168VAL	CG1	262LEU	CD1	2.38	91LEU	CD1	136GLY	CA	0.02	169AL	CB	234VAL	CG1	2.44
168VAL	CG1	262LEU	CD2	0.73	91LEU	CD1	139TYR	CE1	0.06	169AL	CB	234VAL	CG2	12.43
168VAL	CG2	168VAL	C	81.79	91LEU	CD1	139TYR	CE2	0.07	169AL	C	170PHE	CA	100.00
168VAL	CG2	262LEU	CD1	1.32	91LEU	CD1	139TYR	CZ	0.01	169AL	C	170PHE	C	100.00
168VAL	CG2	262LEU	CD2	0.03	91LEU	CD1	140PHE	CB	0.03	169AL	C	180VAL	CG1	0.02
168VAL	C	169ALA	CA	100.00	91LEU	CD1	140PHE	CG	0.12	169AL	C	180VAL	CG2	0.32
168VAL	C	169ALA	CB	0.02	91LEU	CD1	140PHE	CD1	0.90	170PH	CA	170PHE	CB	100.00
168VAL	C	169ALA	C	99.98	91LEU	CD1	140PHE	CD2	0.50	170PH	CA	170PHE	CG	100.00
169ALA	CA	169ALA	CB	100.00	91LEU	CD1	140PHE	CE1	1.15	170PH	CA	170PHE	CD1	84.95
169ALA	CA	169ALA	C	100.00	91LEU	CD1	140PHE	CE2	1.01	170PH	CA	170PHE	CD2	45.13
169ALA	CA	232VAL	CG1	0.00	91LEU	CD1	140PHE	CZ	1.80	170PH	CA	170PHE	C	100.00
169ALA	CB	169ALA	C	100.00	91LEU	CD2	91LEU	C	4.55	170PH	CA	180VAL	CG2	0.02
169ALA	CB	180VAL	CG1	0.04	91LEU	CD2	94ARG	CD	0.00	170PH	CB	170PHE	CG	100.00
169ALA	CB	232VAL	CG1	0.09	91LEU	CD2	94ARG	CZ	0.42	170PH	CB	170PHE	CD1	100.00
169ALA	CB	234VAL	CB	2.98	91LEU	CD2	95LYS	CG	0.01	170PH	CB	170PHE	CD2	100.00
169ALA	CB	234VAL	CG1	1.57	91LEU	CD2	95LYS	CD	0.03	170PH	CB	170PHE	C	100.00
169ALA	CB	234VAL	CG2	3.19	91LEU	CD2	95LYS	CE	0.06	170PH	CB	202VAL	CG1	0.04
169ALA	C	170PHE	CA	100.00	91LEU	CD2	139TYR	CG	0.00	170PH	CB	206TYR	CB	0.63
169ALA	C	170PHE	C	100.00	91LEU	CD2	139TYR	CD2	0.11	170PH	CB	206TYR	CG	23.01
169ALA	C	180VAL	CG2	0.12	91LEU	CD2	139TYR	CE1	0.03	170PH	CB	206TYR	CD1	15.34
170PHE	CA	170PHE	CB	100.00	91LEU	CD2	139TYR	CE2	0.42	170PH	CB	206TYR	CD2	11.25
170PHE	CA	170PHE	CG	100.00	91LEU	CD2	139TYR	CZ	0.04	170PH	CB	206TYR	CE1	2.96
170PHE	CA	170PHE	CD1	75.12	91LEU	CD2	140PHE	CB	0.06	170PH	CB	206TYR	CE2	0.89
170PHE	CA	170PHE	CD2	77.19	91LEU	CD2	140PHE	CG	0.39	170PH	CB	206TYR	CZ	0.57
170PHE	CA	170PHE	C	100.00	91LEU	CD2	140PHE	CD1	0.78	170PH	CB	209VAL	CG2	0.00
170PHE	CA	180VAL	CG2	0.06	91LEU	CD2	140PHE	CD2	1.01	170PH	CG	170PHE	CD1	100.00
170PHE	CB	170PHE	CG	100.00	91LEU	CD2	140PHE	CE1	1.40	170PH	CG	170PHE	CD2	100.00
170PHE	CB	170PHE	CD1	100.00	91LEU	CD2	140PHE	CE2	1.78	170PH	CG	170PHE	CE1	100.00
170PHE	CB	170PHE	CD2	100.00	91LEU	CD2	140PHE	CZ	2.00	170PH	CG	170PHE	CE2	100.00
170PHE	CB	170PHE	C	100.00	91LEU	C	92SER	CA	100.00	170PH	CG	170PHE	CZ	100.00
170PHE	CB	202VAL	CG1	0.01	91LEU	C	92SER	CB	0.08	170PH	CG	180VAL	CG1	0.04
170PHE	CB	206TYR	CB	0.02	91LEU	C	92SER	C	99.96	170PH	CG	180VAL	CG2	0.00
170PHE	CB	206TYR	CG	2.94	92SER	CA	92SER	CB	100.00	170PH	CG	202VAL	CG1	1.46
170PHE	CB	206TYR	CD1	5.13	92SER	CA	92SER	C	100.00	170PH	CG	206TYR	CB	0.18
170PHE	CB	206TYR	CD2	3.38	92SER	CB	92SER	C	100.00	170PH	CG	206TYR	CG	0.22
170PHE	CB	206TYR	CE1	3.25	92SER	C	93LEU	CA	100.00	170PH	CG	206TYR	CD1	2.43
170PHE	CB	206TYR	CE2	2.14	92SER	C	93LEU	CB	0.02	170PH	CG	206TYR	CE1	0.05
170PHE	CB	206TYR	CZ	1.78	92SER	C	93LEU	CG	0.00	170PH	CG	209VAL	CG1	0.01
170PHE	CG	170PHE	CD1	100.00	92SER	C	93LEU	C	99.98	170PH	CD1	170PHE	CD2	100.00
170PHE	CG	170PHE	CD2	100.00	93LEU	CA	93LEU	CB	100.00	170PH	CD1	170PHE	CE1	100.00
170PHE	CG	170PHE	CE1	100.00	93LEU	CA	93LEU	CG	100.00	170PH	CD1	170PHE	CE2	100.00
170PHE	CG	170PHE	CE2	100.00	93LEU	CA	93LEU	CD1	73.40	170PH	CD1	170PHE	CZ	100.00
170PHE	CG	170PHE	CZ	100.00	93LEU	CA	93LEU	CD2	24.09	170PH	CD1	180VAL	CG1	0.91
170PHE	CG	170PHE	C	0.02	93LEU	CA	93LEU	C	100.00	170PH	CD1	180VAL	CG2	2.57
170PHE	CG	180VAL	CG2	0.03	93LEU	CB	93LEU	CG	100.00	170PH	CD1	202VAL	CG1	0.12
170PHE	CG	202VAL	CG1	1.44	93LEU	CB	93LEU	CD1	100.00	170PH	CD1	202VAL	CG2	0.00
170PHE	CG	206TYR	CB	0.02	93LEU	CB	93LEU	CD2	100.00	170PH	CD1	206TYR	CB	12.35
170PHE	CG	206TYR	CG	0.09	93LEU	CB	93LEU	C	100.00	170PH	CD1	206TYR	CG	0.86
170PHE	CG	206TYR	CD1	1.05	93LEU	CB	99LEU	CD1	0.01	170PH	CD1	206TYR	CD1	0.34
170PHE	CG	206TYR	CD2	1.42	93LEU	CG	93LEU	CD1	100.00	170PH	CD1	206TYR	CD2	0.02
170PHE	CG	206TYR	CE1	0.10	93LEU	CG	93LEU	CD2	100.00	170PH	CD1	209VAL	CB	0.68
170PHE	CG	206TYR	CE2	0.26	93LEU	CG	93LEU	C	81.81	170PH	CD1	209VAL	CG1	17.85

170PHE CG	206TYR	CZ	0.00	93LEU CG	268VAL	CG2	0.01	170PH CD1	209VAL	CG2	1.25
170PHE CG	209VAL	CG1	0.01	93LEU CD1	93LEU	CD2	100.00	170PH CD2	170PHE	CE1	100.00
170PHE CD1	170PHE	CD2	100.00	93LEU CD1	93LEU	C	7.94	170PH CD2	170PHE	CE2	100.00
170PHE CD1	170PHE	CE1	100.00	93LEU CD1	97GLN	CG	0.20	170PH CD2	170PHE	CZ	100.00
170PHE CD1	170PHE	CE2	100.00	93LEU CD1	99LEU	CD1	0.00	170PH CD2	180VAL	CG1	0.34
170PHE CD1	170PHE	CZ	100.00	93LEU CD1	268VAL	CG1	0.00	170PH CD2	180VAL	CG2	0.02
170PHE CD1	180VAL	CG2	1.24	93LEU CD1	268VAL	CG2	0.07	170PH CD2	202VAL	CB	0.01
170PHE CD1	202VAL	CG1	6.12	93LEU CD2	93LEU	C	8.89	170PH CD2	202VAL	CG1	14.80
170PHE CD1	203GLY	CA	0.01	93LEU CD2	97GLN	CG	0.03	170PH CD2	202VAL	CG2	0.07
170PHE CD1	206TYR	CB	13.29	93LEU CD2	99LEU	CD1	0.04	170PH CD2	203GLY	CA	0.02
170PHE CD1	206TYR	CG	4.12	93LEU CD2	268VAL	CG1	0.14	170PH CD2	206TYR	CB	0.01
170PHE CD1	206TYR	CD1	2.04	93LEU CD2	268VAL	CG2	0.89	170PH CD2	206TYR	CG	0.01
170PHE CD1	206TYR	CD2	1.71	93LEU C	94ARG	CA	100.00	170PH CD2	206TYR	CD1	2.25
170PHE CD1	206TYR	CE1	0.04	93LEU C	94ARG	CB	0.01	170PH CD2	206TYR	CE1	0.22
170PHE CD1	206TYR	CE2	0.16	93LEU C	94ARG	C	100.00	170PH CD2	206TYR	CZ	0.00
170PHE CD1	209VAL	CB	0.59	93LEU C	99LEU	CD1	0.18	170PH CE1	170PHE	CE2	100.00
170PHE CD1	209VAL	CG1	21.57	94ARG CA	94ARG	CB	100.00	170PH CE1	170PHE	CZ	100.00
170PHE CD1	209VAL	CG2	0.00	94ARG CA	94ARG	CG	100.00	170PH CE1	180VAL	CB	0.00
170PHE CD2	170PHE	CE1	100.00	94ARG CA	94ARG	CD	0.08	170PH CE1	180VAL	CG1	2.83
170PHE CD2	170PHE	CE2	100.00	94ARG CA	94ARG	C	100.00	170PH CE1	180VAL	CG2	0.50
170PHE CD2	170PHE	CZ	100.00	94ARG CA	99LEU	CG	0.00	170PH CE1	202VAL	CG1	0.02
170PHE CD2	180VAL	CG2	1.38	94ARG CA	99LEU	CD1	0.52	170PH CE1	202VAL	CG2	0.00
170PHE CD2	202VAL	CB	0.01	94ARG CB	94ARG	CG	100.00	170PH CE1	203GLY	CA	5.40
170PHE CD2	202VAL	CG1	12.86	94ARG CB	94ARG	CD	100.00	170PH CE1	206TYR	CB	0.15
170PHE CD2	202VAL	CG2	0.02	94ARG CB	94ARG	CZ	0.02	170PH CE1	209VAL	CB	0.06
170PHE CD2	206TYR	CB	6.21	94ARG CB	94ARG	C	100.00	170PH CE1	209VAL	CG1	2.42
170PHE CD2	206TYR	CG	1.21	94ARG CB	99LEU	CD1	0.06	170PH CE1	209VAL	CG2	0.02
170PHE CD2	206TYR	CD1	1.06	94ARG CG	94ARG	CD	100.00	170PH CE1	211LEU	CD1	0.02
170PHE CD2	206TYR	CD2	0.40	94ARG CG	94ARG	CZ	0.84	170PH CE2	170PHE	CZ	100.00
170PHE CD2	206TYR	CE1	0.32	94ARG CG	94ARG	C	7.02	170PH CE2	180VAL	CB	0.01
170PHE CD2	206TYR	CE2	0.18	94ARG CG	99LEU	CB	0.01	170PH CE2	180VAL	CG1	1.19
170PHE CD2	206TYR	CZ	0.01	94ARG CG	99LEU	CD1	0.44	170PH CE2	180VAL	CG2	0.01
170PHE CD2	209VAL	CB	0.19	94ARG CG	99LEU	CD2	0.01	170PH CE2	199VAL	CG1	0.02
170PHE CD2	209VAL	CG1	9.28	94ARG CG	134LEU	CD2	0.03	170PH CE2	199VAL	CG2	0.02
170PHE CD2	209VAL	CG2	0.50	94ARG CD	94ARG	CZ	100.00	170PH CE2	202VAL	CB	0.03
170PHE CE1	170PHE	CE2	100.00	94ARG CD	134LEU	CD1	0.03	170PH CE2	202VAL	CG1	4.79
170PHE CE1	170PHE	CZ	100.00	94ARG CD	134LEU	CD2	0.90	170PH CE2	202VAL	CG2	0.03
170PHE CE1	180VAL	CB	0.01	94ARG CD	135THR	CG2	0.01	170PH CE2	202VAL	C	0.18
170PHE CE1	180VAL	CG1	0.03	94ARG CZ	134LEU	CG	0.00	170PH CE2	203GLY	CA	7.83
170PHE CE1	180VAL	CG2	0.44	94ARG CZ	134LEU	CD1	0.41	170PH CE2	211LEU	CD1	0.01
170PHE CE1	202VAL	CB	0.06	94ARG CZ	134LEU	CD2	0.75	170PH CE2	211LEU	CD2	0.03
170PHE CE1	202VAL	CG1	1.29	94ARG CZ	135THR	CG2	0.02	170PH CZ	180VAL	CG1	1.17
170PHE CE1	202VAL	C	0.07	94ARG C	95LYS	CA	100.00	170PH CZ	180VAL	CG2	0.09
170PHE CE1	203GLY	CA	5.08	94ARG C	95LYS	CB	0.76	170PH CZ	199VAL	CG1	0.06
170PHE CE1	206TYR	CB	0.81	94ARG C	95LYS	CG	0.03	170PH CZ	199VAL	CG2	0.01
170PHE CE1	206TYR	CG	0.01	94ARG C	95LYS	C	99.34	170PH CZ	202VAL	CB	0.00
170PHE CE1	206TYR	CD1	0.01	95LYS CA	95LYS	CB	100.00	170PH CZ	202VAL	CG1	0.04
170PHE CE1	206TYR	CD2	0.02	95LYS CA	95LYS	CG	100.00	170PH CZ	203GLY	CA	26.83
170PHE CE1	209VAL	CB	0.14	95LYS CA	95LYS	CD	28.74	170PH CZ	211LEU	CB	0.01
170PHE CE1	209VAL	CG1	6.53	95LYS CA	95LYS	CE	0.20	170PH CZ	211LEU	CG	0.02
170PHE CE1	211LEU	CB	0.01	95LYS CA	95LYS	C	100.00	170PH CZ	211LEU	CD1	0.56
170PHE CE1	211LEU	CD1	0.81	95LYS CB	95LYS	CG	100.00	170PH CZ	211LEU	CD2	0.73
170PHE CE2	170PHE	CZ	100.00	95LYS CB	95LYS	CD	100.00	170PH C	171GLU	CA	100.00
170PHE CE2	180VAL	CB	0.02	95LYS CB	95LYS	CE	14.61	170PH C	171GLU	CB	0.03
170PHE CE2	180VAL	CG1	0.12	95LYS CB	95LYS	C	100.00	170PH C	171GLU	C	99.98
170PHE CE2	180VAL	CG2	0.28	95LYS CG	95LYS	CD	100.00	170PH C	206TYR	CD2	0.18
170PHE CE2	199VAL	CG1	0.02	95LYS CG	95LYS	CE	100.00	170PH C	206TYR	CE2	0.99
170PHE CE2	199VAL	CG2	0.01	95LYS CG	95LYS	C	60.14	171GL CA	171GLU	CB	100.00
170PHE CE2	202VAL	CB	0.09	95LYS CG	140PHE	CZ	0.00	171GL CA	171GLU	CG	100.00
170PHE CE2	202VAL	CG1	2.51	95LYS CD	95LYS	CE	100.00	171GL CA	171GLU	CD	98.47
170PHE CE2	202VAL	CG2	0.02	95LYS CD	95LYS	C	1.42	171GL CA	171GLU	C	100.00
170PHE CE2	202VAL	C	0.06	95LYS CD	140PHE	CE1	0.01	171GL CA	206TYR	CE2	0.14

170PHE CE2	203GLY CA	2.92	95LYS CD	140PHE	CE2	0.00	171GL CB	171GLU	CG	100.00
170PHE CE2	206TYR CB	0.22	95LYS CD	140PHE	CZ	0.02	171GL CB	171GLU	CD	100.00
170PHE CE2	206TYR CG	0.01	95LYS CE	140PHE	CE1	0.00	171GL CB	171GLU	C	100.00
170PHE CE2	206TYR CD1	0.02	95LYS CE	140PHE	CE2	0.02	171GL CG	171GLU	CD	100.00
170PHE CE2	206TYR CD2	0.00	95LYS CE	140PHE	CZ	0.07	171GL CG	171GLU	C	74.79
170PHE CE2	209VAL CB	0.08	95LYS C	96SER	CA	100.00	171GL CG	206TYR	CE2	0.02
170PHE CE2	209VAL CG1	1.74	95LYS C	96SER	CB	1.56	171GL CG	206TYR	CZ	0.00
170PHE CE2	209VAL CG2	0.02	95LYS C	96SER	C	98.58	171GL CD	171GLU	C	0.62
170PHE CE2	211LEU CB	0.01	96SER CA	96SER	CB	100.00	171GL CD	174ARG	CD	0.03
170PHE CE2	211LEU CD1	0.08	96SER CA	96SER	C	100.00	171GL CD	175LYS	CE	0.01
170PHE CZ	180VAL CB	0.01	96SER CB	96SER	C	100.00	171GL CD	206TYR	CE2	0.41
170PHE CZ	180VAL CG2	0.06	96SER C	97GLN	CA	100.00	171GL C	172LEU	CA	100.00
170PHE CZ	199VAL CG1	0.00	96SER C	97GLN	CB	8.60	171GL C	172LEU	CB	0.01
170PHE CZ	202VAL CB	0.00	96SER C	97GLN	CG	1.38	171GL C	172LEU	CG	0.00
170PHE CZ	202VAL CG1	0.04	96SER C	97GLN	CD	0.35	171GL C	172LEU	C	99.99
170PHE CZ	202VAL C	0.07	96SER C	97GLN	C	91.42	172LE CA	172LEU	CB	100.00
170PHE CZ	203GLY CA	30.67	97GLN CA	97GLN	CB	100.00	172LE CA	172LEU	CG	100.00
170PHE CZ	203GLY C	0.02	97GLN CA	97GLN	CG	100.00	172LE CA	172LEU	CD1	7.11
170PHE CZ	206TYR CB	0.00	97GLN CA	97GLN	CD	99.99	172LE CA	172LEU	CD2	92.86
170PHE CZ	209VAL CB	0.00	97GLN CA	97GLN	C	100.00	172LE CA	172LEU	C	100.00
170PHE CZ	209VAL CG1	0.01	97GLN CB	97GLN	CG	100.00	172LE CB	172LEU	CG	100.00
170PHE CZ	211LEU CB	0.20	97GLN CB	97GLN	CD	100.00	172LE CB	172LEU	CD1	100.00
170PHE CZ	211LEU CD1	1.55	97GLN CB	97GLN	C	100.00	172LE CB	172LEU	CD2	100.00
170PHE CZ	211LEU CD2	0.01	97GLN CB	99LEU	CG	0.04	172LE CB	172LEU	C	100.00
170PHE C	171GLU CA	100.00	97GLN CB	99LEU	CD1	0.10	172LE CB	232VAL	CG2	0.00
170PHE C	171GLU CB	0.03	97GLN CB	99LEU	CD2	2.34	172LE CG	172LEU	CD1	100.00
170PHE C	171GLU C	99.99	97GLN CG	97GLN	CD	100.00	172LE CG	172LEU	CD2	100.00
170PHE C	206TYR CD1	0.02	97GLN CD	266THR	CB	0.06	172LE CG	172LEU	C	6.33
170PHE C	206TYR CD2	0.00	97GLN C	98ASP	CA	100.00	172LE CG	232VAL	CG2	0.00
170PHE C	206TYR CE1	0.32	97GLN C	98ASP	CB	99.40	172LE CD1	172LEU	CD2	100.00
170PHE C	206TYR CE2	0.27	97GLN C	98ASP	C	99.98	172LE CD1	172LEU	C	0.30
170PHE C	206TYR CZ	0.01	97GLN C	99LEU	CD1	0.04	172LE CD1	175LYS	CE	0.02
171GLU CA	171GLU CB	100.00	97GLN C	99LEU	CD2	0.00	172LE CD1	176ARG	CG	0.00
171GLU CA	171GLU CG	100.00	97GLN C	264ARG	CZ	0.02	172LE CD1	300HIS	CE1	0.23
171GLU CA	171GLU CD	99.66	98ASP CA	98ASP	CB	100.00	172LE CD2	172LEU	C	0.29
171GLU CA	171GLU C	100.00	98ASP CA	98ASP	CG	100.00	172LE CD2	175LYS	CD	0.02
171GLU CA	206TYR CE1	0.02	98ASP CA	98ASP	C	100.00	172LE CD2	175LYS	CE	0.25
171GLU CA	206TYR CE2	0.00	98ASP CB	98ASP	CG	100.00	172LE CD2	232VAL	CG1	0.01
171GLU CB	171GLU CG	100.00	98ASP CB	98ASP	C	100.00	172LE CD2	232VAL	CG2	0.04
171GLU CB	171GLU CD	100.00	98ASP CB	264ARG	CB	0.17	172LE CD2	300HIS	CD2	0.02
171GLU CB	171GLU C	100.00	98ASP CB	264ARG	CG	0.02	172LE CD2	300HIS	CE1	1.15
171GLU CG	171GLU CD	100.00	98ASP CB	264ARG	CD	0.03	172LE C	173ALA	CA	100.00
171GLU CG	171GLU C	81.11	98ASP CB	264ARG	CZ	0.76	172LE C	173ALA	CB	0.00
171GLU CG	206TYR CE2	0.01	98ASP CG	98ASP	C	99.76	172LE C	173ALA	C	100.00
171GLU CG	206TYR CZ	0.01	98ASP CG	264ARG	CB	0.01	172LE C	175LYS	CG	0.01
171GLU CD	171GLU C	0.96	98ASP CG	264ARG	CG	0.00	173AL CA	173ALA	CB	100.00
171GLU CD	174ARG CZ	0.00	98ASP CG	264ARG	CD	0.03	173AL CA	173ALA	C	100.00
171GLU CD	175LYS CE	0.01	98ASP CG	264ARG	CZ	0.02	173AL CA	232VAL	CG1	0.01
171GLU CD	206TYR CE1	0.02	98ASP C	99LEU	CA	100.00	173AL CA	232VAL	CG2	0.17
171GLU CD	206TYR CE2	0.14	98ASP C	99LEU	CB	0.13	173AL CB	173ALA	C	100.00
171GLU C	172ALA CA	100.00	98ASP C	99LEU	CG	0.01	173AL CB	179HIS	C	0.02
171GLU C	172ALA CB	0.02	98ASP C	99LEU	CD2	0.00	173AL CB	180VAL	CG1	0.42
171GLU C	172ALA C	99.99	98ASP C	99LEU	C	99.91	173AL CB	180VAL	CG2	19.26
171GLU C	174ARG CG	0.00	98ASP C	263GLY	CA	0.34	173AL CB	209VAL	CG1	10.08
172ALA CA	172ALA CB	100.00	99LEU CA	99LEU	CB	100.00	173AL CB	209VAL	CG2	0.06
172ALA CA	172ALA C	100.00	99LEU CA	99LEU	CG	100.00	173AL CB	232VAL	CG1	0.13
172ALA CB	172ALA C	100.00	99LEU CA	99LEU	CD1	0.61	173AL CB	232VAL	CG2	0.08
172ALA CB	232VAL CG1	0.01	99LEU CA	99LEU	CD2	99.36	173AL C	174ARG	CA	100.00
172ALA CB	232VAL CG2	0.02	99LEU CA	99LEU	C	100.00	173AL C	174ARG	CB	0.00
172ALA CB	300HIS CE1	0.04	99LEU CA	263GLY	CA	3.27	173AL C	174ARG	C	100.00
172ALA C	173ALA CA	100.00	99LEU CB	99LEU	CG	100.00	173AL C	209VAL	CG1	0.03
172ALA C	173ALA CB	0.02	99LEU CB	99LEU	CD1	100.00	174AR CA	174ARG	CB	100.00

172ALA	C	173ALA	C	99.99	99LEU	CB	99LEU	CD2	100.00	174AR	CA	174ARG	CG	100.00
172ALA	C	232VAL	CG2	0.01	99LEU	CB	99LEU	C	100.00	174AR	CA	174ARG	CD	0.43
173ALA	CA	173ALA	CB	100.00	99LEU	CB	134LEU	CD1	0.02	174AR	CA	174ARG	C	100.00
173ALA	CA	173ALA	C	100.00	99LEU	CB	134LEU	CD2	0.28	174AR	CA	209VAL	CG1	0.00
173ALA	CA	232VAL	CG2	0.54	99LEU	CG	99LEU	CD1	100.00	174AR	CA	209VAL	CG2	0.06
173ALA	CB	173ALA	C	100.00	99LEU	CG	99LEU	CD2	100.00	174AR	CB	174ARG	CG	100.00
173ALA	CB	179HIS	C	0.03	99LEU	CD1	99LEU	CD2	100.00	174AR	CB	174ARG	CD	100.00
173ALA	CB	180VAL	CG1	0.03	99LEU	CD1	134LEU	CD2	0.12	174AR	CB	174ARG	C	100.00
173ALA	CB	180VAL	CG2	30.46	99LEU	CD1	261SER	CB	2.70	174AR	CB	209VAL	CG2	0.08
173ALA	CB	209VAL	CG1	7.65	99LEU	CD1	268VAL	CG2	8.21	174AR	CG	174ARG	CD	100.00
173ALA	CB	209VAL	CG2	0.00	99LEU	CD2	261SER	CB	0.39	174AR	CG	174ARG	CZ	0.74
173ALA	CB	232VAL	CG2	0.31	99LEU	CD2	261SER	C	0.14	174AR	CG	174ARG	C	23.00
173ALA	C	174ARG	CA	100.00	99LEU	CD2	262LEU	CA	0.01	174AR	CG	209VAL	CG1	0.40
173ALA	C	174ARG	CB	0.01	99LEU	CD2	262LEU	C	2.95	174AR	CG	209VAL	CG2	5.11
173ALA	C	174ARG	C	100.00	99LEU	CD2	263GLY	CA	0.66	174AR	CD	174ARG	CZ	100.00
173ALA	C	209VAL	CG1	0.00	99LEU	CD2	265GLY	CA	0.02	174AR	CD	206TYR	CD2	0.02
174ARG	CA	174ARG	CB	100.00	99LEU	CD2	265GLY	C	0.04	174AR	CZ	206TYR	CD2	66.45
174ARG	CA	174ARG	CG	100.00	99LEU	CD2	268VAL	CG2	0.03	174AR	CZ	206TYR	CE2	21.28
174ARG	CA	174ARG	CD	0.47	99LEU	C	100PHE	CA	100.00	174AR	C	175LYS	CA	100.00
174ARG	CA	174ARG	C	100.00	99LEU	C	100PHE	CB	78.49	174AR	C	175LYS	CB	28.94
174ARG	CA	209VAL	CG1	0.04	99LEU	C	100PHE	CG	0.07	174AR	C	175LYS	CG	0.52
174ARG	CA	209VAL	CG2	0.36	99LEU	C	100PHE	CD2	0.10	174AR	C	175LYS	CD	0.00
174ARG	CB	174ARG	CG	100.00	99LEU	C	100PHE	C	19.59	174AR	C	175LYS	C	70.65
174ARG	CB	174ARG	CD	100.00	99LEU	C	134LEU	CD1	0.02	174AR	C	178LYS	CE	0.00
174ARG	CB	174ARG	CZ	7.17	99LEU	C	134LEU	CD2	0.00	175LY	CA	175LYS	CB	100.00
174ARG	CB	174ARG	C	100.00	100PHI	CA	100PHE	CB	100.00	175LY	CA	175LYS	CG	100.00
174ARG	CB	209VAL	CG1	0.02	100PHI	CA	100PHE	CG	100.00	175LY	CA	175LYS	CD	2.59
174ARG	CB	209VAL	CG2	0.24	100PHI	CA	100PHE	CD1	95.17	175LY	CA	175LYS	CE	0.01
174ARG	CG	174ARG	CD	100.00	100PHI	CA	100PHE	CD2	29.48	175LY	CA	175LYS	C	100.00
174ARG	CG	174ARG	CZ	29.08	100PHI	CA	100PHE	C	100.00	175LY	CB	175LYS	CG	100.00
174ARG	CG	174ARG	C	75.69	100PHI	CA	134LEU	C	0.01	175LY	CB	175LYS	CD	100.00
174ARG	CG	209VAL	CG1	0.01	100PHI	CA	135THR	CG2	0.36	175LY	CB	175LYS	CE	14.70
174ARG	CG	209VAL	CG2	0.60	100PHI	CB	100PHE	CG	100.00	175LY	CB	175LYS	C	100.00
174ARG	CD	174ARG	CZ	100.00	100PHI	CB	100PHE	CD1	100.00	175LY	CG	175LYS	CD	100.00
174ARG	CD	174ARG	C	0.80	100PHI	CB	100PHE	CD2	100.00	175LY	CG	175LYS	CE	100.00
174ARG	CD	206TYR	CD2	0.01	100PHI	CB	100PHE	C	100.00	175LY	CG	175LYS	C	50.83
174ARG	CD	206TYR	CE1	0.03	100PHI	CB	135THR	CG2	3.30	175LY	CD	175LYS	CE	100.00
174ARG	CD	206TYR	CE2	0.02	100PHI	CB	164ARG	CB	0.00	175LY	CD	175LYS	C	0.00
174ARG	CD	208ASP	CG	0.00	100PHI	CB	164ARG	CG	0.01	175LY	CD	300HIS	CE1	0.00
174ARG	CD	209VAL	CG2	0.03	100PHI	CB	164ARG	CD	0.10	175LY	CE	300HIS	CE1	0.02
174ARG	CZ	175LYS	CD	0.00	100PHI	CB	164ARG	CZ	1.42	175LY	CE	305VAL	CG1	0.00
174ARG	CZ	175LYS	CE	0.03	100PHI	CG	100PHE	CD1	100.00	175LY	C	176ARG	CA	100.00
174ARG	CZ	206TYR	CD1	2.23	100PHI	CG	100PHE	CD2	100.00	175LY	C	176ARG	CB	82.42
174ARG	CZ	206TYR	CD2	1.96	100PHI	CG	100PHE	CE1	100.00	175LY	C	176ARG	CG	40.98
174ARG	CZ	206TYR	CE1	8.29	100PHI	CG	100PHE	CE2	100.00	175LY	C	176ARG	C	26.65
174ARG	CZ	206TYR	CE2	8.02	100PHI	CG	100PHE	CZ	100.00	176AR	CA	176ARG	CB	100.00
174ARG	CZ	206TYR	CZ	0.20	100PHI	CG	100PHE	C	98.43	176AR	CA	176ARG	CG	100.00
174ARG	C	175LYS	CA	100.00	100PHI	CG	164ARG	CB	0.56	176AR	CA	176ARG	C	100.00
174ARG	C	175LYS	CB	26.93	100PHI	CG	164ARG	CG	0.16	176AR	CB	176ARG	CG	100.00
174ARG	C	175LYS	CG	0.55	100PHI	CG	164ARG	CD	2.32	176AR	CB	176ARG	CD	100.00
174ARG	C	175LYS	C	71.14	100PHI	CG	164ARG	CZ	0.32	176AR	CB	176ARG	C	100.00
175LYS	CA	175LYS	CB	100.00	100PHI	CG	263GLY	CA	0.02	176AR	CB	177ARG	CZ	0.02
175LYS	CA	175LYS	CG	100.00	100PHI	CD1	100PHE	CD2	100.00	176AR	CB	231ASP	CG	0.05
175LYS	CA	175LYS	CD	0.88	100PHI	CD1	100PHE	CE1	100.00	176AR	CG	176ARG	CD	100.00
175LYS	CA	175LYS	C	100.00	100PHI	CD1	100PHE	CE2	100.00	176AR	CG	176ARG	CZ	33.32
175LYS	CB	175LYS	CG	100.00	100PHI	CD1	100PHE	CZ	100.00	176AR	CG	176ARG	C	0.16
175LYS	CB	175LYS	CD	100.00	100PHI	CD1	100PHE	C	80.72	176AR	CD	176ARG	CZ	100.00
175LYS	CB	175LYS	CE	9.92	100PHI	CD1	101ALA	CB	3.90	176AR	CD	231ASP	CG	0.03
175LYS	CB	175LYS	C	100.00	100PHI	CD1	164ARG	CB	6.95	176AR	CD	232VAL	CB	0.01
175LYS	CB	305VAL	CG2	0.02	100PHI	CD1	164ARG	CG	1.12	176AR	CD	232VAL	CG1	0.03
175LYS	CG	175LYS	CD	100.00	100PHI	CD1	164ARG	CD	1.80	176AR	CD	232VAL	CG2	26.52
175LYS	CG	175LYS	CE	100.00	100PHI	CD1	164ARG	CZ	0.00	176AR	C	177ARG	CA	100.00

175LYS	CG	175LYS	C	65.60	100PHI	CD1	164ARG	C	0.34	176AR	C	177ARG	CB	9.02
175LYS	CG	305VAL	CG2	0.18	100PHI	CD1	165VAL	CG1	0.01	176AR	C	177ARG	CG	2.63
175LYS	CD	175LYS	CE	100.00	100PHI	CD1	165VAL	CG2	0.11	176AR	C	177ARG	CZ	0.01
175LYS	CD	175LYS	C	0.05	100PHI	CD1	168VAL	CG2	0.01	176AR	C	177ARG	C	90.19
175LYS	CD	299GLU	CG	0.03	100PHI	CD1	262LEU	CD1	0.00	177AR	CA	177ARG	CB	100.00
175LYS	CD	300HIS	CE1	0.12	100PHI	CD1	263GLY	CA	0.02	177AR	CA	177ARG	CG	100.00
175LYS	CD	305VAL	CG2	0.11	100PHI	CD2	100PHE	CE1	100.00	177AR	CA	177ARG	CD	0.13
175LYS	CE	299GLU	CG	0.00	100PHI	CD2	100PHE	CE2	100.00	177AR	CA	177ARG	C	100.00
175LYS	CE	299GLU	CD	0.02	100PHI	CD2	100PHE	CZ	100.00	177AR	CB	177ARG	CG	100.00
175LYS	CE	300HIS	CE1	0.01	100PHI	CD2	164ARG	CB	0.08	177AR	CB	177ARG	CD	100.00
175LYS	CE	303GLY	CA	0.00	100PHI	CD2	164ARG	CG	0.07	177AR	CB	177ARG	CZ	0.02
175LYS	CE	305VAL	CG2	0.02	100PHI	CD2	164ARG	CD	4.11	177AR	CB	177ARG	C	100.00
175LYS	C	176ARG	CA	100.00	100PHI	CD2	164ARG	CZ	34.75	177AR	CB	231ASP	CB	0.02
175LYS	C	176ARG	CB	81.81	100PHI	CD2	262LEU	C	0.05	177AR	CB	231ASP	CG	1.25
175LYS	C	176ARG	CG	38.32	100PHI	CD2	263GLY	CA	2.57	177AR	CG	177ARG	CD	100.00
175LYS	C	176ARG	C	24.85	100PHI	CD2	263GLY	C	0.23	177AR	CG	177ARG	CZ	18.98
176ARG	CA	176ARG	CB	100.00	100PHI	CD2	264ARG	CG	0.00	177AR	CG	177ARG	C	6.89
176ARG	CA	176ARG	CG	100.00	100PHI	CE1	100PHE	CE2	100.00	177AR	CG	231ASP	CG	2.71
176ARG	CA	176ARG	C	100.00	100PHI	CE1	100PHE	CZ	100.00	177AR	CD	177ARG	CZ	100.00
176ARG	CA	177ARG	CZ	0.02	100PHI	CE1	101ALA	CB	0.94	177AR	CD	231ASP	CB	0.73
176ARG	CB	176ARG	CG	100.00	100PHI	CE1	164ARG	CB	2.72	177AR	CD	231ASP	CG	0.98
176ARG	CB	176ARG	CD	100.00	100PHI	CE1	164ARG	CG	1.53	177AR	CZ	231ASP	CB	9.08
176ARG	CB	176ARG	C	100.00	100PHI	CE1	164ARG	CD	0.56	177AR	CZ	231ASP	CG	0.42
176ARG	CB	177ARG	CZ	0.04	100PHI	CE1	164ARG	C	3.62	177AR	C	178LYS	CA	100.00
176ARG	CB	231ASP	CG	0.12	100PHI	CE1	165VAL	CG1	0.01	177AR	C	178LYS	CB	100.00
176ARG	CG	176ARG	CD	100.00	100PHI	CE1	165VAL	CG2	0.03	177AR	C	178LYS	CG	31.63
176ARG	CG	176ARG	CZ	51.41	100PHI	CE1	168VAL	CG1	1.47	177AR	C	178LYS	CD	1.38
176ARG	CG	176ARG	C	0.07	100PHI	CE1	168VAL	CG2	2.77	177AR	C	178LYS	CE	0.00
176ARG	CG	177ARG	CZ	0.01	100PHI	CE1	262LEU	CB	0.04	177AR	C	178LYS	C	99.36
176ARG	CD	176ARG	CZ	100.00	100PHI	CE1	262LEU	CG	0.00	178LY	CA	178LYS	CB	100.00
176ARG	CD	232VAL	CG2	32.17	100PHI	CE1	262LEU	CD1	0.41	178LY	CA	178LYS	CG	100.00
176ARG	C	177ARG	CA	100.00	100PHI	CE1	262LEU	CD2	0.31	178LY	CA	178LYS	CD	3.50
176ARG	C	177ARG	CB	3.02	100PHI	CE1	262LEU	C	0.01	178LY	CA	178LYS	C	100.00
176ARG	C	177ARG	CG	0.89	100PHI	CE1	263GLY	CA	0.42	178LY	CA	209VAL	CG1	0.00
176ARG	C	177ARG	CD	0.02	100PHI	CE2	100PHE	CZ	100.00	178LY	CB	178LYS	CG	100.00
176ARG	C	177ARG	CZ	0.02	100PHI	CE2	164ARG	CB	0.04	178LY	CB	178LYS	CD	100.00
176ARG	C	177ARG	C	96.67	100PHI	CE2	164ARG	CG	0.14	178LY	CB	178LYS	CE	7.50
177ARG	CA	177ARG	CB	100.00	100PHI	CE2	164ARG	CD	1.34	178LY	CB	178LYS	C	100.00
177ARG	CA	177ARG	CG	100.00	100PHI	CE2	164ARG	CZ	9.36	178LY	CB	209VAL	CG1	0.02
177ARG	CA	177ARG	CD	7.87	100PHI	CE2	262LEU	CB	0.17	178LY	CB	209VAL	CG2	0.00
177ARG	CA	177ARG	CZ	0.01	100PHI	CE2	262LEU	CD1	0.01	178LY	CG	178LYS	CD	100.00
177ARG	CA	177ARG	C	100.00	100PHI	CE2	262LEU	CD2	0.04	178LY	CG	178LYS	CE	100.00
177ARG	CB	177ARG	CG	100.00	100PHI	CE2	262LEU	C	0.42	178LY	CG	178LYS	C	0.66
177ARG	CB	177ARG	CD	100.00	100PHI	CE2	263GLY	CA	14.41	178LY	CG	209VAL	CG1	0.01
177ARG	CB	177ARG	CZ	0.04	100PHI	CE2	263GLY	C	24.92	178LY	CD	178LYS	CE	100.00
177ARG	CB	177ARG	C	100.00	100PHI	CE2	264ARG	CB	0.00	178LY	CE	208ASP	C	0.00
177ARG	CB	231ASP	CB	0.03	100PHI	CE2	264ARG	CG	0.09	178LY	C	179HIS	CA	100.00
177ARG	CB	231ASP	CG	2.11	100PHI	CZ	164ARG	CB	0.15	178LY	C	179HIS	CB	93.70
177ARG	CG	177ARG	CD	100.00	100PHI	CZ	164ARG	CG	0.54	178LY	C	179HIS	C	7.92
177ARG	CG	177ARG	CZ	18.15	100PHI	CZ	164ARG	CD	0.60	178LY	C	209VAL	CG1	1.02
177ARG	CG	177ARG	C	9.94	100PHI	CZ	164ARG	CZ	0.01	179HI	CA	179HIS	CB	100.00
177ARG	CG	231ASP	CG	3.34	100PHI	CZ	167ARG	CZ	0.00	179HI	CA	179HIS	CG	100.00
177ARG	CD	177ARG	CZ	100.00	100PHI	CZ	168VAL	CG1	0.48	179HI	CA	179HIS	CD2	56.80
177ARG	CD	177ARG	C	0.01	100PHI	CZ	168VAL	CG2	0.30	179HI	CA	179HIS	C	100.00
177ARG	CD	231ASP	CB	0.59	100PHI	CZ	262LEU	CB	0.28	179HI	CA	209VAL	CG1	0.00
177ARG	CD	231ASP	CG	5.56	100PHI	CZ	262LEU	CD1	0.34	179HI	CB	179HIS	CG	100.00
177ARG	CZ	179HIS	CD2	0.00	100PHI	CZ	262LEU	CD2	0.71	179HI	CB	179HIS	CD2	100.00
177ARG	CZ	231ASP	CB	6.72	100PHI	CZ	262LEU	C	0.00	179HI	CB	179HIS	CE1	0.28
177ARG	CZ	231ASP	CG	0.19	100PHI	CZ	263GLY	CA	4.54	179HI	CB	179HIS	C	100.00
177ARG	C	178LYS	CA	100.00	100PHI	CZ	263GLY	C	2.21	179HI	CG	179HIS	CD2	100.00
177ARG	C	178LYS	CB	100.00	100PHI	C	101ALA	CA	100.00	179HI	CG	179HIS	CE1	100.00
177ARG	C	178LYS	CG	26.06	100PHI	C	101ALA	CB	87.52	179HI	CG	179HIS	C	99.99

177ARG	C	178LYS	CD	0.04	100PHI	C	101ALA	C	15.53	179HIS	CG	210ALA	CB	0.01
177ARG	C	178LYS	C	99.02	100PHI	C	134LEU	CB	1.22	179HIS	CD2	179HIS	CE1	100.00
178LYS	CA	178LYS	CB	100.00	100PHI	C	134LEU	CD1	0.01	179HIS	CD2	179HIS	C	11.40
178LYS	CA	178LYS	CG	100.00	100PHI	C	134LEU	C	0.33	179HIS	CD2	180VAL	C	0.02
178LYS	CA	178LYS	CD	1.07	100PHI	C	165VAL	CG1	0.01	179HIS	CD2	181VAL	CG2	0.64
178LYS	CA	178LYS	C	100.00	100PHI	C	165VAL	CG2	0.02	179HIS	CD2	210ALA	CB	1.15
178LYS	CB	178LYS	CG	100.00	101AL/	CA	101ALA	CB	100.00	179HIS	CD2	229ARG	CZ	0.00
178LYS	CB	178LYS	CD	100.00	101AL/	CA	101ALA	C	100.00	179HIS	CE1	180VAL	C	0.05
178LYS	CB	178LYS	CE	21.63	101AL/	CA	134LEU	CB	0.20	179HIS	CE1	181VAL	CG2	24.31
178LYS	CB	178LYS	C	100.00	101AL/	CB	101ALA	C	100.00	179HIS	CE1	210ALA	CB	0.00
178LYS	CB	209VAL	CG1	0.00	101AL/	CB	103LEU	CD1	0.22	179HIS	CE1	211LEU	C	0.18
178LYS	CB	209VAL	CG2	0.00	101AL/	CB	103LEU	CD2	0.00	179HIS	CE1	212GLU	CB	5.96
178LYS	CG	178LYS	CD	100.00	101AL/	CB	131VAL	CG1	0.18	179HIS	CE1	212GLU	CD	0.16
178LYS	CG	178LYS	CE	100.00	101AL/	CB	165VAL	CG1	0.02	179HIS	CE1	229ARG	CZ	2.42
178LYS	CG	178LYS	C	0.38	101AL/	CB	165VAL	CG2	0.07	179HIS	C	180VAL	CA	100.00
178LYS	CD	178LYS	CE	100.00	101AL/	CB	168VAL	CB	0.00	179HIS	C	180VAL	CB	53.65
178LYS	CD	208ASP	CG	0.27	101AL/	CB	168VAL	CG1	0.33	179HIS	C	180VAL	CG1	0.74
178LYS	CE	208ASP	CG	0.13	101AL/	CB	168VAL	CG2	1.01	179HIS	C	180VAL	CG2	16.80
178LYS	C	179HIS	CA	100.00	101AL/	CB	262LEU	CD1	0.11	179HIS	C	180VAL	C	53.21
178LYS	C	179HIS	CB	96.20	101AL/	CB	262LEU	CD2	0.10	179HIS	C	209VAL	CG1	0.00
178LYS	C	179HIS	C	5.79	101AL/	C	102ASN	CA	100.00	180VA	CA	180VAL	CB	100.00
178LYS	C	209VAL	CG1	0.56	101AL/	C	102ASN	CB	46.84	180VA	CA	180VAL	CG1	100.00
179HIS	CA	179HIS	CB	100.00	101AL/	C	102ASN	C	66.05	180VA	CA	180VAL	CG2	100.00
179HIS	CA	179HIS	CG	100.00	101AL/	C	103LEU	CD1	0.03	180VA	CA	180VAL	C	100.00
179HIS	CA	179HIS	CD2	23.79	101AL/	C	134LEU	CB	0.21	180VA	CB	180VAL	CG1	100.00
179HIS	CA	179HIS	C	100.00	101AL/	C	134LEU	CD1	0.28	180VA	CB	180VAL	CG2	100.00
179HIS	CB	179HIS	CG	100.00	101AL/	C	134LEU	CD2	0.01	180VA	CB	180VAL	C	100.00
179HIS	CB	179HIS	CD2	100.00	101AL/	C	262LEU	CD1	0.00	180VA	CB	209VAL	CG1	0.00
179HIS	CB	179HIS	CE1	0.20	101AL/	C	262LEU	CD2	0.04	180VA	CB	211LEU	CD1	0.01
179HIS	CB	179HIS	C	100.00	102ASI	CA	102ASN	CB	100.00	180VA	CG1	180VAL	CG2	100.00
179HIS	CG	179HIS	CD2	100.00	102ASI	CA	102ASN	CG	100.00	180VA	CG1	180VAL	C	97.26
179HIS	CG	179HIS	CE1	100.00	102ASI	CA	102ASN	C	100.00	180VA	CG1	181VAL	C	0.02
179HIS	CG	179HIS	C	99.99	102ASI	CA	261SER	CB	0.00	180VA	CG1	209VAL	CG1	1.23
179HIS	CG	210ALA	CB	0.00	102ASI	CB	102ASN	CG	100.00	180VA	CG1	211LEU	CD1	1.27
179HIS	CD2	179HIS	CE1	100.00	102ASI	CB	102ASN	C	100.00	180VA	CG1	211LEU	CD2	0.10
179HIS	CD2	179HIS	C	15.52	102ASI	CB	132ARG	CD	0.16	180VA	CG1	232VAL	CG1	0.04
179HIS	CD2	180VAL	C	0.03	102ASI	CB	132ARG	CZ	0.01	180VA	CG1	234VAL	CG1	15.60
179HIS	CD2	181VAL	CG2	3.90	102ASI	CB	134LEU	CB	0.02	180VA	CG2	180VAL	C	28.48
179HIS	CD2	210ALA	CB	0.05	102ASI	CB	134LEU	CG	0.02	180VA	CG2	209VAL	CG1	7.26
179HIS	CD2	231ASP	CB	0.01	102ASI	CB	134LEU	CD1	0.18	180VA	CG2	209VAL	CG2	0.02
179HIS	CE1	181VAL	CG2	1.04	102ASI	CB	134LEU	CD2	0.22	180VA	CG2	211LEU	CA	0.00
179HIS	CE1	210ALA	CB	0.01	102ASI	CG	102ASN	C	99.90	180VA	CG2	211LEU	CD1	0.04
179HIS	CE1	211LEU	C	1.41	102ASI	CG	132ARG	CZ	0.01	180VA	CG2	232VAL	CG1	0.39
179HIS	CE1	212GLU	CB	15.87	102ASI	CG	134LEU	CD1	0.01	180VA	CG2	234VAL	CG1	1.96
179HIS	CE1	212GLU	CD	0.01	102ASI	CG	260ALA	C	0.00	180VA	C	181VAL	CA	100.00
179HIS	C	180VAL	CA	100.00	102ASI	C	103LEU	CA	100.00	180VA	C	181VAL	CB	16.72
179HIS	C	180VAL	CB	12.61	102ASI	C	103LEU	CB	2.94	180VA	C	181VAL	CG2	5.56
179HIS	C	180VAL	CG1	0.02	102ASI	C	103LEU	CG	0.08	180VA	C	181VAL	C	92.96
179HIS	C	180VAL	CG2	6.72	102ASI	C	103LEU	CD1	1.86	181VA	CA	181VAL	CB	100.00
179HIS	C	180VAL	C	91.74	102ASI	C	103LEU	CD2	0.14	181VA	CA	181VAL	CG1	100.00
180VAL	CA	180VAL	CB	100.00	102ASI	C	103LEU	C	98.29	181VA	CA	181VAL	CG2	100.00
180VAL	CA	180VAL	CG1	100.00	103LEL	CA	103LEU	CB	100.00	181VA	CA	181VAL	C	100.00
180VAL	CA	180VAL	CG2	100.00	103LEL	CA	103LEU	CG	100.00	181VA	CB	181VAL	CG1	100.00
180VAL	CA	180VAL	C	100.00	103LEL	CA	103LEU	CD1	50.43	181VA	CB	181VAL	CG2	100.00
180VAL	CB	180VAL	CG1	100.00	103LEL	CA	103LEU	CD2	48.70	181VA	CB	181VAL	C	100.00
180VAL	CB	180VAL	CG2	100.00	103LEL	CA	103LEU	C	100.00	181VA	CB	219MET	CE	0.00
180VAL	CB	180VAL	C	100.00	103LEL	CA	129LEU	CD1	0.00	181VA	CB	233VAL	CG1	0.00
180VAL	CB	211LEU	CD1	0.17	103LEL	CB	103LEU	CG	100.00	181VA	CG1	181VAL	CG2	100.00
180VAL	CG1	180VAL	CG2	100.00	103LEL	CB	103LEU	CD1	100.00	181VA	CG1	181VAL	C	99.98
180VAL	CG1	180VAL	C	99.86	103LEL	CB	103LEU	CD2	100.00	181VA	CG1	182SER	C	0.03
180VAL	CG1	181VAL	C	0.06	103LEL	CB	103LEU	C	100.00	181VA	CG1	183VAL	CG1	0.02
180VAL	CG1	211LEU	CD1	4.43	103LEL	CB	129LEU	CD1	0.03	181VA	CG1	183VAL	CG2	0.11

180VAL	CG1	232VAL	CG1	0.01
180VAL	CG1	234VAL	CG1	19.12
180VAL	CG2	180VAL	C	0.11
180VAL	CG2	209VAL	CG1	4.73
180VAL	CG2	211LEU	CD1	0.01
180VAL	CG2	232VAL	CG1	0.01
180VAL	C	181VAL	CA	100.00
180VAL	C	181VAL	CB	16.06
180VAL	C	181VAL	CG2	6.27
180VAL	C	181VAL	C	96.06
181VAL	CA	181VAL	CB	100.00
181VAL	CA	181VAL	CG1	100.00
181VAL	CA	181VAL	CG2	100.00
181VAL	CA	181VAL	C	100.00
181VAL	CB	181VAL	CG1	100.00
181VAL	CB	181VAL	CG2	100.00
181VAL	CB	181VAL	C	100.00
181VAL	CG1	181VAL	CG2	100.00
181VAL	CG1	181VAL	C	100.00
181VAL	CG1	183VAL	CG2	0.32
181VAL	CG1	214GLN	CB	1.42
181VAL	CG1	214GLN	CG	11.42
181VAL	CG1	214GLN	CD	0.74
181VAL	CG1	219MET	CE	0.24
181VAL	CG1	230PHE	CD1	0.01
181VAL	CG1	230PHE	CE1	0.02
181VAL	CG1	233VAL	CG1	0.01
181VAL	CG2	212GLU	CG	0.01
181VAL	CG2	212GLU	CD	0.08
181VAL	CG2	229ARG	CZ	0.02
181VAL	CG2	230PHE	CD1	0.14
181VAL	CG2	230PHE	CE1	0.00
181VAL	C	182SER	CA	100.00
181VAL	C	182SER	CB	6.51
181VAL	C	182SER	C	98.18
182SER	CA	182SER	CB	100.00
182SER	CA	182SER	C	100.00
182SER	CA	234VAL	CG1	1.71
182SER	CB	182SER	C	100.00
182SER	CB	199VAL	CG1	0.06
182SER	CB	211LEU	CD1	0.13
182SER	CB	211LEU	CD2	0.02
182SER	CB	234VAL	CG1	0.74
182SER	C	183VAL	CA	100.00
182SER	C	183VAL	CB	33.89
182SER	C	183VAL	CG2	25.55
182SER	C	183VAL	C	82.59
183VAL	CA	183VAL	CB	100.00
183VAL	CA	183VAL	CG1	100.00
183VAL	CA	183VAL	CG2	100.00
183VAL	CA	183VAL	C	100.00
183VAL	CB	183VAL	CG1	100.00
183VAL	CB	183VAL	CG2	100.00
183VAL	CB	183VAL	C	100.00
183VAL	CB	235THR	CB	0.00
183VAL	CG1	183VAL	CG2	100.00
183VAL	CG1	183VAL	C	99.99
183VAL	CG1	216VAL	CA	11.17
183VAL	CG1	216VAL	CB	2.70
183VAL	CG1	216VAL	CG2	2.62
183VAL	CG1	219MET	CB	0.11
183VAL	CG1	219MET	CG	0.13

103LEU	CB	129LEU	CD2	0.01
103LEU	CB	262LEU	CD1	0.06
103LEU	CB	296MET	CE	0.44
103LEU	CG	103LEU	CD1	100.00
103LEU	CG	103LEU	CD2	100.00
103LEU	CG	103LEU	C	3.32
103LEU	CG	129LEU	CD1	0.01
103LEU	CG	129LEU	CD2	0.00
103LEU	CG	131VAL	CG2	0.00
103LEU	CG	262LEU	CD1	0.15
103LEU	CG	262LEU	CD2	0.40
103LEU	CG	296MET	CE	0.01
103LEU	CD1	103LEU	CD2	100.00
103LEU	CD1	103LEU	C	0.17
103LEU	CD1	129LEU	CG	0.01
103LEU	CD1	129LEU	CD1	0.06
103LEU	CD1	129LEU	CD2	0.03
103LEU	CD1	131VAL	CG1	0.04
103LEU	CD1	131VAL	CG2	0.18
103LEU	CD1	168VAL	CG1	0.40
103LEU	CD1	168VAL	CG2	0.01
103LEU	CD1	260ALA	CB	0.00
103LEU	CD1	260ALA	C	0.00
103LEU	CD1	261SER	CA	0.00
103LEU	CD1	262LEU	CB	0.19
103LEU	CD1	262LEU	CG	0.32
103LEU	CD1	262LEU	CD1	3.76
103LEU	CD1	262LEU	CD2	1.44
103LEU	CD1	296MET	CE	0.42
103LEU	CD1	300HIS	CD2	0.06
103LEU	CD1	300HIS	CE1	0.54
103LEU	CD2	103LEU	C	0.37
103LEU	CD2	129LEU	CD1	0.05
103LEU	CD2	129LEU	CD2	0.07
103LEU	CD2	131VAL	CG1	0.06
103LEU	CD2	131VAL	CG2	1.00
103LEU	CD2	168VAL	CG1	0.58
103LEU	CD2	168VAL	CG2	0.00
103LEU	CD2	262LEU	CB	0.02
103LEU	CD2	262LEU	CG	0.03
103LEU	CD2	262LEU	CD1	0.77
103LEU	CD2	262LEU	CD2	0.19
103LEU	CD2	296MET	CE	0.30
103LEU	CD2	300HIS	CD2	0.04
103LEU	CD2	300HIS	CE1	0.50
103LEU	C	104ARG	CA	100.00
103LEU	C	104ARG	CB	60.17
103LEU	C	104ARG	CG	19.76
103LEU	C	104ARG	C	54.49
103LEU	C	105PRO	CD	5.20
104ARG	CA	104ARG	CB	100.00
104ARG	CA	104ARG	CG	100.00
104ARG	CA	104ARG	CD	50.63
104ARG	CA	104ARG	C	100.00
104ARG	CA	105PRO	CD	100.00
104ARG	CB	104ARG	CG	100.00
104ARG	CB	104ARG	CD	100.00
104ARG	CB	104ARG	CZ	0.00
104ARG	CB	104ARG	C	100.00
104ARG	CB	244SER	CB	0.06
104ARG	CG	104ARG	CD	100.00
104ARG	CG	104ARG	CZ	5.39

181VA	CG1	212GLU	CG	0.00
181VA	CG1	214GLN	CB	2.62
181VA	CG1	214GLN	CG	12.27
181VA	CG1	214GLN	CD	0.62
181VA	CG1	219MET	CG	0.00
181VA	CG1	219MET	CE	3.17
181VA	CG1	230PHE	CD1	0.04
181VA	CG1	230PHE	CE1	0.01
181VA	CG1	233VAL	CG1	0.01
181VA	CG1	233VAL	CG2	0.04
181VA	CG2	181VAL	C	2.54
181VA	CG2	212GLU	CB	0.01
181VA	CG2	212GLU	CG	0.00
181VA	CG2	212GLU	CD	0.05
181VA	CG2	214GLN	CB	0.11
181VA	CG2	214GLN	CG	1.00
181VA	CG2	214GLN	CD	0.01
181VA	CG2	219MET	CE	0.03
181VA	CG2	229ARG	CZ	0.02
181VA	CG2	230PHE	CD1	0.43
181VA	CG2	230PHE	CE1	0.01
181VA	C	182SER	CA	100.00
181VA	C	182SER	CB	4.11
181VA	C	182SER	C	98.67
182SE	CA	182SER	CB	100.00
182SE	CA	182SER	C	100.00
182SE	CA	234VAL	CG1	0.24
182SE	CB	182SER	C	100.00
182SE	CB	199VAL	CG1	0.02
182SE	CB	199VAL	CG2	0.00
182SE	CB	211LEU	CD1	0.16
182SE	CB	211LEU	CD2	0.03
182SE	CB	213HIS	CD2	0.04
182SE	CB	213HIS	CE1	0.00
182SE	CB	234VAL	CG1	0.06
182SE	C	183VAL	CA	100.00
182SE	C	183VAL	CB	12.05
182SE	C	183VAL	CG1	1.45
182SE	C	183VAL	CG2	5.94
182SE	C	183VAL	C	91.91
183VA	CA	183VAL	CB	100.00
183VA	CA	183VAL	CG1	100.00
183VA	CA	183VAL	CG2	100.00
183VA	CA	183VAL	C	100.00
183VA	CB	183VAL	CG1	100.00
183VA	CB	183VAL	CG2	100.00
183VA	CB	183VAL	C	100.00
183VA	CB	235THR	CB	0.04
183VA	CG1	183VAL	CG2	100.00
183VA	CG1	183VAL	C	92.28
183VA	CG1	214GLN	CB	0.02
183VA	CG1	216VAL	CA	2.06
183VA	CG1	216VAL	CB	7.82
183VA	CG1	216VAL	CG2	26.36
183VA	CG1	219MET	CB	1.56
183VA	CG1	219MET	CG	2.49
183VA	CG1	219MET	CE	0.01
183VA	CG1	233VAL	CG1	0.01
183VA	CG1	235THR	CB	0.05
183VA	CG1	239PHE	CD1	0.02
183VA	CG1	239PHE	CD2	0.08
183VA	CG1	243LEU	CD1	0.72

183VAL	CG1	239PHE	CD2	0.01	104AR(	CG	104ARG	C	0.02	183VA	CG1	243LEU	CD2	0.09
183VAL	CG1	239PHE	CE2	0.01	104AR(	CG	132ARG	CD	0.02	183VA	CG2	183VAL	C	7.89
183VAL	CG1	243LEU	CD1	0.08	104AR(	CG	244SER	CB	0.14	183VA	CG2	216VAL	CA	0.12
183VAL	CG1	243LEU	CD2	0.03	104AR(	CG	259SER	CB	0.32	183VA	CG2	216VAL	CB	0.02
183VAL	CG2	214GLN	CB	0.00	104AR(	CD	104ARG	CZ	100.00	183VA	CG2	216VAL	CG2	0.02
183VAL	CG2	219MET	CG	0.62	104AR(	CD	132ARG	CD	0.01	183VA	CG2	219MET	CB	0.00
183VAL	CG2	219MET	CE	0.22	104AR(	CD	132ARG	CZ	0.01	183VA	CG2	219MET	CG	0.15
183VAL	CG2	233VAL	CG1	18.65	104AR(	CD	244SER	CB	0.84	183VA	CG2	219MET	CE	0.55
183VAL	CG2	233VAL	CG2	0.07	104AR(	CD	259SER	CB	0.92	183VA	CG2	233VAL	CG1	35.35
183VAL	CG2	235THR	CB	0.00	104AR(	CD	272VAL	CG1	0.10	183VA	CG2	233VAL	CG2	0.28
183VAL	CG2	243LEU	CD1	0.00	104AR(	CD	272VAL	CG2	0.01	183VA	CG2	235THR	CB	0.96
183VAL	C	184ASP	CA	100.00	104AR(	CZ	132ARG	CD	0.02	183VA	CG2	239PHE	CD2	0.00
183VAL	C	184ASP	CB	96.88	104AR(	CZ	132ARG	CZ	0.07	183VA	CG2	243LEU	CD1	0.16
183VAL	C	184ASP	C	3.06	104AR(	CZ	244SER	CB	1.08	183VA	CG2	243LEU	CD2	0.80
184ASP	CA	184ASP	CB	100.00	104AR(	CZ	244SER	C	0.00	183VA	C	184ASP	CA	100.00
184ASP	CA	184ASP	CG	100.00	104AR(	CZ	259SER	CB	0.58	183VA	C	184ASP	CB	41.52
184ASP	CA	184ASP	C	100.00	104AR(	CZ	272VAL	CB	0.00	183VA	C	184ASP	C	59.27
184ASP	CB	184ASP	CG	100.00	104AR(	CZ	272VAL	CG1	4.45	184AS	CA	184ASP	CB	100.00
184ASP	CB	184ASP	C	100.00	104AR(	CZ	272VAL	CG2	0.91	184AS	CA	184ASP	CG	100.00
184ASP	CB	195TRP	CD1	0.07	104AR(	C	105PRO	CA	100.00	184AS	CA	184ASP	C	100.00
184ASP	CG	184ASP	C	100.00	104AR(	C	105PRO	CB	0.12	184AS	CB	184ASP	CG	100.00
184ASP	CG	186ALA	CB	4.68	104AR(	C	105PRO	CD	100.00	184AS	CB	184ASP	C	100.00
184ASP	C	185LYS	CA	100.00	104AR(	C	105PRO	C	100.00	184AS	CB	214GLN	C	0.27
184ASP	C	185LYS	CB	99.90	104AR(	C	129LEU	CD1	0.27	184AS	CG	184ASP	C	100.00
184ASP	C	185LYS	C	0.85	104AR(	C	129LEU	CD2	0.02	184AS	CG	186ALA	CB	0.76
184ASP	C	239PHE	CE2	0.06	105PR(	CA	105PRO	CB	100.00	184AS	CG	196ARG	CG	0.01
184ASP	C	239PHE	CZ	0.09	105PR(	CA	105PRO	CG	100.00	184AS	CG	196ARG	CD	0.04
185LYS	CA	185LYS	CB	100.00	105PR(	CA	105PRO	CD	100.00	184AS	CG	196ARG	CZ	0.29
185LYS	CA	185LYS	CG	100.00	105PR(	CA	105PRO	C	100.00	184AS	C	185LYS	CA	100.00
185LYS	CA	185LYS	CD	59.59	105PR(	CA	129LEU	CD1	0.08	184AS	C	185LYS	CB	99.79
185LYS	CA	185LYS	C	100.00	105PR(	CA	129LEU	CD2	0.01	184AS	C	185LYS	C	1.08
185LYS	CA	215TYR	CD1	0.00	105PR(	CB	105PRO	CG	100.00	184AS	C	216VAL	CG1	0.01
185LYS	CB	185LYS	CG	100.00	105PR(	CB	105PRO	CD	100.00	184AS	C	239PHE	CE1	0.03
185LYS	CB	185LYS	CD	100.00	105PR(	CB	105PRO	C	100.00	184AS	C	239PHE	CE2	0.01
185LYS	CB	185LYS	CE	0.00	105PR(	CB	129LEU	CD1	0.08	185LY	CA	185LYS	CB	100.00
185LYS	CB	185LYS	C	100.00	105PR(	CB	129LEU	CD2	0.04	185LY	CA	185LYS	CG	100.00
185LYS	CB	189LEU	CB	0.02	105PR(	CG	105PRO	CD	100.00	185LY	CA	185LYS	CD	84.22
185LYS	CB	189LEU	CD1	0.04	105PR(	CG	105PRO	C	95.67	185LY	CA	185LYS	CE	0.00
185LYS	CB	216VAL	CG1	0.02	105PR(	CG	129LEU	CD1	0.04	185LY	CA	185LYS	C	100.00
185LYS	CB	239PHE	CE1	0.08	105PR(	CG	258PRO	CG	0.72	185LY	CB	185LYS	CG	100.00
185LYS	CB	239PHE	CE2	1.37	105PR(	CG	258PRO	CD	0.01	185LY	CB	185LYS	CD	100.00
185LYS	CB	239PHE	CZ	1.69	105PR(	CG	258PRO	C	0.00	185LY	CB	185LYS	CE	0.02
185LYS	CG	185LYS	CD	100.00	105PR(	CG	296MET	CE	0.52	185LY	CB	185LYS	C	100.00
185LYS	CG	185LYS	CE	100.00	105PR(	CD	105PRO	C	92.13	185LY	CB	189LEU	CD1	0.04
185LYS	CG	185LYS	C	98.54	105PR(	CD	129LEU	CD1	0.07	185LY	CB	216VAL	CG1	0.11
185LYS	CG	188VAL	CG1	0.43	105PR(	CD	296MET	CE	0.63	185LY	CB	239PHE	CE1	0.00
185LYS	CG	188VAL	CG2	7.32	105PR(	C	106ALA	CA	100.00	185LY	CB	239PHE	CE2	0.58
185LYS	CG	216VAL	CG1	0.09	105PR(	C	106ALA	CB	83.75	185LY	CB	239PHE	CZ	1.52
185LYS	CG	239PHE	CZ	0.00	105PR(	C	106ALA	C	21.76	185LY	CG	185LYS	CD	100.00
185LYS	CD	185LYS	CE	100.00	106AL/	CA	106ALA	CB	100.00	185LY	CG	185LYS	CE	100.00
185LYS	CD	185LYS	C	0.32	106AL/	CA	106ALA	C	100.00	185LY	CG	185LYS	C	99.69
185LYS	CD	188VAL	CG2	2.41	106AL/	CA	257LEU	CD1	0.44	185LY	CG	188VAL	CG1	0.05
185LYS	CD	216VAL	CG1	3.72	106AL/	CA	257LEU	CD2	0.04	185LY	CG	188VAL	CG2	9.16
185LYS	CD	239PHE	CZ	0.05	106AL/	CB	106ALA	C	100.00	185LY	CG	189LEU	CD1	0.20
185LYS	CE	188VAL	CG1	0.20	106AL/	CB	128VAL	CG1	0.15	185LY	CG	216VAL	CG1	0.19
185LYS	CE	188VAL	CG2	6.39	106AL/	CB	128VAL	CG2	0.05	185LY	CG	239PHE	CE2	0.09
185LYS	CE	216VAL	CG1	0.02	106AL/	CB	130ILE	CD	0.39	185LY	CG	239PHE	CZ	0.23
185LYS	C	186ALA	CA	100.00	106AL/	CB	247ALA	CB	4.79	185LY	CD	185LYS	CE	100.00
185LYS	C	186ALA	C	100.00	106AL/	CB	247ALA	C	0.08	185LY	CD	188VAL	CG2	0.76
185LYS	C	189LEU	CB	0.01	106AL/	CB	250LEU	CD1	0.24	185LY	CD	216VAL	CG1	14.61
186ALA	CA	186ALA	CB	100.00	106AL/	CB	250LEU	CD2	0.08	185LY	CE	188VAL	CG1	0.01
186ALA	CA	186ALA	C	100.00	106AL/	CB	257LEU	CD1	0.98	185LY	CE	188VAL	CG2	2.67

186ALA	CA	192GLY	C	3.98	106AL	CB	257LEU	CD2	0.21	185LY	CE	216VAL	CG1	0.96
186ALA	CB	186ALA	C	100.00	106AL	C	107LYS	CA	100.00	185LY	CE	239PHE	CZ	0.00
186ALA	CB	192GLY	C	16.42	106AL	C	107LYS	CB	94.30	185LY	C	186ALA	CA	100.00
186ALA	CB	193GLU	CA	1.14	106AL	C	107LYS	CG	0.66	185LY	C	186ALA	CB	0.00
186ALA	CB	196ARG	CD	6.38	106AL	C	107LYS	CD	0.00	185LY	C	186ALA	C	100.00
186ALA	CB	196ARG	CZ	2.73	106AL	C	107LYS	C	7.35	186AL	CA	186ALA	CB	100.00
186ALA	C	187ASN	CA	100.00	106AL	C	250LEU	CD1	0.06	186AL	CA	186ALA	C	100.00
186ALA	C	187ASN	CB	2.67	106AL	C	250LEU	CD2	0.02	186AL	CA	192GLY	CA	0.00
186ALA	C	187ASN	C	97.18	107LY	CA	107LYS	CB	100.00	186AL	CA	192GLY	C	5.45
186ALA	C	215TYR	CE1	0.10	107LY	CA	107LYS	CG	100.00	186AL	CB	186ALA	C	100.00
187ASN	CA	187ASN	CB	100.00	107LY	CA	107LYS	CD	0.14	186AL	CB	192GLY	CA	0.00
187ASN	CA	187ASN	CG	100.00	107LY	CA	107LYS	C	100.00	186AL	CB	192GLY	C	13.82
187ASN	CA	187ASN	C	100.00	107LY	CA	250LEU	CD2	0.00	186AL	CB	193GLU	CA	3.17
187ASN	CB	187ASN	CG	100.00	107LY	CB	107LYS	CG	100.00	186AL	CB	193GLU	CB	0.00
187ASN	CB	187ASN	C	100.00	107LY	CB	107LYS	CD	100.00	186AL	CB	196ARG	CG	0.02
187ASN	CB	215TYR	CE1	1.64	107LY	CB	107LYS	CE	61.99	186AL	CB	196ARG	CD	5.30
187ASN	CB	215TYR	CE2	0.00	107LY	CB	107LYS	C	100.00	186AL	CB	196ARG	CZ	1.17
187ASN	CB	215TYR	CZ	1.07	107LY	CB	109PHE	CE1	0.01	186AL	C	187ASN	CA	100.00
187ASN	CG	187ASN	C	99.90	107LY	CB	109PHE	CE2	0.00	186AL	C	187ASN	CB	0.13
187ASN	CG	188VAL	CG2	0.01	107LY	CB	109PHE	CZ	0.01	186AL	C	187ASN	C	99.83
187ASN	CG	215TYR	CD1	0.36	107LY	CB	127ASP	CA	0.26	187AS	CA	187ASN	CB	100.00
187ASN	CG	215TYR	CE1	2.57	107LY	CB	127ASP	CB	0.08	187AS	CA	187ASN	CG	100.00
187ASN	CG	215TYR	CE2	0.11	107LY	CB	127ASP	CG	0.03	187AS	CA	187ASN	C	100.00
187ASN	CG	215TYR	CZ	0.42	107LY	CB	257LEU	CD2	0.01	187AS	CB	187ASN	CG	100.00
187ASN	C	188VAL	CA	100.00	107LY	CG	107LYS	CD	100.00	187AS	CB	187ASN	C	100.00
187ASN	C	188VAL	CB	98.82	107LY	CG	107LYS	CE	100.00	187AS	CB	215TYR	CE1	0.16
187ASN	C	188VAL	CG1	66.63	107LY	CG	107LYS	C	98.66	187AS	CB	215TYR	CE2	0.01
187ASN	C	188VAL	CG2	1.12	107LY	CG	108VAL	C	0.08	187AS	CB	215TYR	CZ	0.05
187ASN	C	188VAL	C	0.87	107LY	CG	109PHE	CD1	0.04	187AS	CG	187ASN	C	99.91
188VAL	CA	188VAL	CB	100.00	107LY	CG	109PHE	CD2	0.02	187AS	CG	188VAL	CG2	0.01
188VAL	CA	188VAL	CG1	100.00	107LY	CG	109PHE	CE1	0.33	187AS	CG	215TYR	CD1	0.18
188VAL	CA	188VAL	CG2	100.00	107LY	CG	109PHE	CE2	0.26	187AS	CG	215TYR	CD2	0.00
188VAL	CA	188VAL	C	100.00	107LY	CG	109PHE	CZ	0.26	187AS	CG	215TYR	CE1	0.91
188VAL	CB	188VAL	CG1	100.00	107LY	CG	127ASP	CB	0.00	187AS	CG	215TYR	CE2	0.21
188VAL	CB	188VAL	CG2	100.00	107LY	CG	127ASP	CG	0.00	187AS	CG	215TYR	CZ	0.06
188VAL	CB	188VAL	C	100.00	107LY	CG	257LEU	CD1	0.02	187AS	C	188VAL	CA	100.00
188VAL	CG1	188VAL	CG2	100.00	107LY	CG	257LEU	CD2	0.74	187AS	C	188VAL	CB	98.09
188VAL	CG1	188VAL	C	5.07	107LY	CD	107LYS	CE	100.00	187AS	C	188VAL	CG1	55.64
188VAL	CG2	188VAL	C	97.70	107LY	CD	107LYS	C	0.06	187AS	C	188VAL	CG2	1.90
188VAL	C	189LEU	CA	100.00	107LY	CD	109PHE	CD1	0.14	187AS	C	188VAL	C	1.11
188VAL	C	189LEU	CB	20.89	107LY	CD	109PHE	CD2	0.06	188VA	CA	188VAL	CB	100.00
188VAL	C	189LEU	CG	0.32	107LY	CD	109PHE	CE1	1.27	188VA	CA	188VAL	CG1	100.00
188VAL	C	189LEU	C	86.87	107LY	CD	109PHE	CE2	0.64	188VA	CA	188VAL	CG2	100.00
189LEU	CA	189LEU	CB	100.00	107LY	CD	109PHE	CZ	0.32	188VA	CA	188VAL	C	100.00
189LEU	CA	189LEU	CG	100.00	107LY	CD	127ASP	CG	0.18	188VA	CB	188VAL	CG1	100.00
189LEU	CA	189LEU	CD1	76.04	107LY	CD	312GLU	CD	0.09	188VA	CB	188VAL	CG2	100.00
189LEU	CA	189LEU	CD2	23.34	107LY	CE	109PHE	CD1	0.04	188VA	CB	188VAL	C	100.00
189LEU	CA	189LEU	C	100.00	107LY	CE	109PHE	CD2	0.02	188VA	CG1	188VAL	CG2	100.00
189LEU	CB	189LEU	CG	100.00	107LY	CE	109PHE	CE1	0.26	188VA	CG1	188VAL	C	2.76
189LEU	CB	189LEU	CD1	100.00	107LY	CE	109PHE	CE2	0.24	188VA	CG2	188VAL	C	96.34
189LEU	CB	189LEU	CD2	100.00	107LY	CE	109PHE	CZ	0.14	188VA	C	189LEU	CA	100.00
189LEU	CB	189LEU	C	100.00	107LY	CE	127ASP	CB	0.02	188VA	C	189LEU	CB	71.06
189LEU	CG	189LEU	CD1	100.00	107LY	CE	127ASP	CG	0.51	188VA	C	189LEU	CG	18.46
189LEU	CG	189LEU	CD2	100.00	107LY	CE	312GLU	CD	0.50	188VA	C	189LEU	CD1	0.06
189LEU	CG	189LEU	C	76.27	107LY	C	108VAL	CA	100.00	188VA	C	189LEU	CD2	0.01
189LEU	CD1	189LEU	CD2	100.00	107LY	C	108VAL	CB	21.92	188VA	C	189LEU	C	25.57
189LEU	CD1	238ILE	CD	0.01	107LY	C	108VAL	CG1	0.16	189LE	CA	189LEU	CB	100.00
189LEU	CD1	239PHE	CE2	0.02	107LY	C	108VAL	CG2	13.74	189LE	CA	189LEU	CG	100.00
189LEU	CD1	239PHE	CZ	0.05	107LY	C	108VAL	C	91.44	189LE	CA	189LEU	CD1	2.51
189LEU	CD2	191VAL	CB	0.02	107LY	C	126VAL	CG1	0.00	189LE	CA	189LEU	CD2	96.87
189LEU	CD2	191VAL	CG1	0.02	107LY	C	250LEU	CB	0.01	189LE	CA	189LEU	C	100.00
189LEU	CD2	191VAL	C	0.01	107LY	C	250LEU	CD1	0.00	189LE	CB	189LEU	CG	100.00

189LEU	CD2	192GLY	CA	0.04	107LYS	C	250LEU	CD2	0.03	189LEU	CB	189LEU	CD1	100.00
189LEU	CD2	239PHE	CE2	0.07	108VAL	CA	108VAL	CB	100.00	189LEU	CB	189LEU	CD2	100.00
189LEU	CD2	239PHE	CZ	0.03	108VAL	CA	108VAL	CG1	100.00	189LEU	CB	189LEU	C	100.00
189LEU	C	190GLU	CA	100.00	108VAL	CA	108VAL	CG2	100.00	189LEU	CB	191VAL	CG2	0.02
189LEU	C	190GLU	C	100.00	108VAL	CA	108VAL	C	100.00	189LEU	CG	189LEU	CD1	100.00
190GLU	CA	190GLU	CB	100.00	108VAL	CB	108VAL	CG1	100.00	189LEU	CG	189LEU	CD2	100.00
190GLU	CA	190GLU	CG	100.00	108VAL	CB	108VAL	CG2	100.00	189LEU	CG	189LEU	C	1.94
190GLU	CA	190GLU	CD	98.73	108VAL	CB	108VAL	C	100.00	189LEU	CD1	189LEU	CD2	100.00
190GLU	CA	190GLU	C	100.00	108VAL	CG1	108VAL	CG2	100.00	189LEU	CD1	239PHE	CE1	0.02
190GLU	CB	190GLU	CG	100.00	108VAL	CG1	108VAL	C	94.91	189LEU	CD1	239PHE	CE2	0.26
190GLU	CB	190GLU	CD	100.00	108VAL	CG1	109PHE	C	0.00	189LEU	CD1	239PHE	CZ	0.70
190GLU	CB	190GLU	C	100.00	108VAL	CG1	112LEU	CB	0.06	189LEU	CD2	191VAL	CB	0.00
190GLU	CG	190GLU	CD	100.00	108VAL	CG1	112LEU	C	0.39	189LEU	CD2	191VAL	CG1	0.01
190GLU	CG	190GLU	C	25.70	108VAL	CG1	113GLU	CA	2.40	189LEU	CD2	191VAL	CG2	0.00
190GLU	C	191VAL	CA	100.00	108VAL	CG1	113GLU	CB	0.32	189LEU	CD2	239PHE	CZ	0.01
190GLU	C	191VAL	C	100.00	108VAL	CG1	113GLU	CG	1.16	189LEU	C	190GLU	CA	100.00
191VAL	CA	191VAL	CB	100.00	108VAL	CG1	113GLU	CD	0.00	189LEU	C	190GLU	CB	0.00
191VAL	CA	191VAL	CG1	100.00	108VAL	CG1	116SER	CB	0.00	189LEU	C	190GLU	C	100.00
191VAL	CA	191VAL	CG2	100.00	108VAL	CG1	123ALA	C	0.00	190GLU	CA	190GLU	CB	100.00
191VAL	CA	191VAL	C	100.00	108VAL	CG1	250LEU	CD2	0.00	190GLU	CA	190GLU	CG	100.00
191VAL	CB	191VAL	CG1	100.00	108VAL	CG1	250LEU	C	0.00	190GLU	CA	190GLU	CD	97.35
191VAL	CB	191VAL	CG2	100.00	108VAL	CG1	251PRO	CA	0.01	190GLU	CA	190GLU	C	100.00
191VAL	CB	191VAL	C	100.00	108VAL	CG2	108VAL	C	5.24	190GLU	CB	190GLU	CG	100.00
191VAL	CG1	191VAL	CG2	100.00	108VAL	CG2	112LEU	C	0.01	190GLU	CB	190GLU	CD	100.00
191VAL	CG1	191VAL	C	100.00	108VAL	CG2	113GLU	CG	0.17	190GLU	CB	190GLU	C	100.00
191VAL	C	192GLY	CA	100.00	108VAL	CG2	116SER	CB	0.00	190GLU	CG	190GLU	CD	100.00
191VAL	C	192GLY	C	99.61	108VAL	CG2	126VAL	CB	0.98	190GLU	CG	190GLU	C	2.18
192GLY	CA	192GLY	C	100.00	108VAL	CG2	126VAL	CG1	0.90	190GLU	C	191VAL	CA	100.00
192GLY	CA	195TRP	CB	0.02	108VAL	CG2	126VAL	CG2	0.13	190GLU	C	191VAL	CB	0.00
192GLY	C	193GLU	CA	100.00	108VAL	CG2	250LEU	CB	0.24	190GLU	C	191VAL	C	100.00
192GLY	C	193GLU	C	100.00	108VAL	CG2	250LEU	CG	0.10	191VAL	CA	191VAL	CB	100.00
193GLU	CA	193GLU	CB	100.00	108VAL	CG2	250LEU	CD1	0.24	191VAL	CA	191VAL	CG1	100.00
193GLU	CA	193GLU	CG	100.00	108VAL	CG2	250LEU	CD2	0.68	191VAL	CA	191VAL	CG2	100.00
193GLU	CA	193GLU	CD	80.96	108VAL	CG2	250LEU	C	1.42	191VAL	CA	191VAL	C	100.00
193GLU	CA	193GLU	C	100.00	108VAL	CG2	251PRO	CA	0.07	191VAL	CB	191VAL	CG1	100.00
193GLU	CB	193GLU	CG	100.00	108VAL	C	109PHE	CA	100.00	191VAL	CB	191VAL	CG2	100.00
193GLU	CB	193GLU	CD	100.00	108VAL	C	109PHE	CB	3.32	191VAL	CB	191VAL	C	100.00
193GLU	CB	193GLU	C	100.00	108VAL	C	109PHE	CG	0.13	191VAL	CG1	191VAL	CG2	100.00
193GLU	CG	193GLU	CD	100.00	108VAL	C	109PHE	CD1	0.17	191VAL	CG1	191VAL	C	96.98
193GLU	CG	193GLU	C	94.18	108VAL	C	109PHE	CD2	0.03	191VAL	CG2	191VAL	C	15.54
193GLU	CG	197LYS	CE	0.01	108VAL	C	109PHE	C	96.89	191VAL	C	192GLY	CA	100.00
193GLU	CD	193GLU	C	0.83	109PHE	CA	109PHE	CB	100.00	191VAL	C	192GLY	C	98.61
193GLU	CD	197LYS	CE	0.13	109PHE	CA	109PHE	CG	100.00	192GLY	CA	192GLY	C	100.00
193GLU	C	194PHE	CA	100.00	109PHE	CA	109PHE	CD1	76.32	192GLY	CA	195TRP	CB	0.02
193GLU	C	194PHE	C	100.00	109PHE	CA	109PHE	CD2	68.13	192GLY	C	193GLU	CA	100.00
194PHE	CA	194PHE	CB	100.00	109PHE	CA	109PHE	C	100.00	192GLY	C	193GLU	C	100.00
194PHE	CA	194PHE	CG	100.00	109PHE	CA	110PRO	CD	100.00	193GLU	CA	193GLU	CB	100.00
194PHE	CA	194PHE	CD1	94.13	109PHE	CB	109PHE	CG	100.00	193GLU	CA	193GLU	CG	100.00
194PHE	CA	194PHE	CD2	50.23	109PHE	CB	109PHE	CD1	100.00	193GLU	CA	193GLU	CD	92.81
194PHE	CA	194PHE	C	100.00	109PHE	CB	109PHE	CD2	100.00	193GLU	CA	193GLU	C	100.00
194PHE	CA	197LYS	CG	0.00	109PHE	CB	109PHE	C	100.00	193GLU	CB	193GLU	CG	100.00
194PHE	CB	194PHE	CG	100.00	109PHE	CB	110PRO	CD	15.75	193GLU	CB	193GLU	CD	100.00
194PHE	CB	194PHE	CD1	100.00	109PHE	CB	112LEU	CB	0.02	193GLU	CB	193GLU	C	100.00
194PHE	CB	194PHE	CD2	100.00	109PHE	CB	112LEU	CG	0.10	193GLU	CG	193GLU	CD	100.00
194PHE	CB	194PHE	C	100.00	109PHE	CB	112LEU	CD1	2.41	193GLU	CG	193GLU	C	92.44
194PHE	CG	194PHE	CD1	100.00	109PHE	CB	112LEU	CD2	0.99	193GLU	CG	197LYS	CE	0.01
194PHE	CG	194PHE	CD2	100.00	109PHE	CB	316ALA	CB	0.15	193GLU	CD	193GLU	C	0.02
194PHE	CG	194PHE	CE1	100.00	109PHE	CB	319LEU	CD1	0.09	193GLU	C	194PHE	CA	100.00
194PHE	CG	194PHE	CE2	100.00	109PHE	CG	109PHE	CD1	100.00	193GLU	C	194PHE	C	100.00
194PHE	CG	194PHE	CZ	100.00	109PHE	CG	109PHE	CD2	100.00	194PHE	CA	194PHE	CB	100.00
194PHE	CG	194PHE	C	100.00	109PHE	CG	109PHE	CE1	100.00	194PHE	CA	194PHE	CG	100.00
194PHE	CD1	194PHE	CD2	100.00	109PHE	CG	109PHE	CE2	100.00	194PHE	CA	194PHE	CD1	91.65

194PHE CD1	194PHE	CE1	100.00	109PHI CG	109PHE	CZ	100.00	194PH CA	194PHE	CD2	62.47
194PHE CD1	194PHE	CE2	100.00	109PHI CG	109PHE	C	2.80	194PH CA	194PHE	C	100.00
194PHE CD1	194PHE	CZ	100.00	109PHI CG	112LEU	CD1	0.02	194PH CB	194PHE	CG	100.00
194PHE CD1	194PHE	C	0.49	109PHI CG	316ALA	CB	0.10	194PH CB	194PHE	CD1	100.00
194PHE CD1	197LYS	CE	0.04	109PHI CG	319LEU	CD1	0.28	194PH CB	194PHE	CD2	100.00
194PHE CD2	194PHE	CE1	100.00	109PHI CG	319LEU	CD2	0.00	194PH CB	194PHE	C	100.00
194PHE CD2	194PHE	CE2	100.00	109PHI CD1	109PHE	CD2	100.00	194PH CG	194PHE	CD1	100.00
194PHE CD2	194PHE	CZ	100.00	109PHI CD1	109PHE	CE1	100.00	194PH CG	194PHE	CD2	100.00
194PHE CD2	194PHE	C	63.66	109PHI CD1	109PHE	CE2	100.00	194PH CG	194PHE	CE1	100.00
194PHE CE1	194PHE	CE2	100.00	109PHI CD1	109PHE	CZ	100.00	194PH CG	194PHE	CE2	100.00
194PHE CE1	194PHE	CZ	100.00	109PHI CD1	112LEU	CD1	0.10	194PH CG	194PHE	CZ	100.00
194PHE CE1	197LYS	CE	0.00	109PHI CD1	112LEU	CD2	0.02	194PH CG	194PHE	C	100.00
194PHE CE2	194PHE	CZ	100.00	109PHI CD1	251PRO	CB	0.65	194PH CD1	194PHE	CD2	100.00
194PHE C	195TRP	CA	100.00	109PHI CD1	256LEU	CD1	0.00	194PH CD1	194PHE	CE1	100.00
194PHE C	195TRP	C	100.00	109PHI CD1	312GLU	CG	0.10	194PH CD1	194PHE	CE2	100.00
195TRP CA	195TRP	CB	100.00	109PHI CD1	312GLU	CD	0.02	194PH CD1	194PHE	CZ	100.00
195TRP CA	195TRP	CG	100.00	109PHI CD1	315VAL	CG1	0.00	194PH CD1	194PHE	C	0.52
195TRP CA	195TRP	CD1	93.04	109PHI CD1	316ALA	CA	0.02	194PH CD2	194PHE	CE1	100.00
195TRP CA	195TRP	CD2	5.83	109PHI CD1	316ALA	CB	0.65	194PH CD2	194PHE	CE2	100.00
195TRP CA	195TRP	C	100.00	109PHI CD1	319LEU	CD1	1.09	194PH CD2	194PHE	CZ	100.00
195TRP CB	195TRP	CG	100.00	109PHI CD1	319LEU	CD2	0.07	194PH CD2	194PHE	C	67.32
195TRP CB	195TRP	CD1	100.00	109PHI CD2	109PHE	CE1	100.00	194PH CE1	194PHE	CE2	100.00
195TRP CB	195TRP	CD2	100.00	109PHI CD2	109PHE	CE2	100.00	194PH CE1	194PHE	CZ	100.00
195TRP CB	195TRP	CE3	97.91	109PHI CD2	109PHE	CZ	100.00	194PH CE1	197LYS	CE	0.02
195TRP CB	195TRP	C	100.00	109PHI CD2	109PHE	C	1.42	194PH CE2	194PHE	CZ	100.00
195TRP CG	195TRP	CD1	100.00	109PHI CD2	112LEU	CD1	0.20	194PH C	195TRP	CA	100.00
195TRP CG	195TRP	CD2	100.00	109PHI CD2	112LEU	CD2	0.04	194PH C	195TRP	C	100.00
195TRP CG	195TRP	CE2	100.00	109PHI CD2	251PRO	CB	1.81	195TR CA	195TRP	CB	100.00
195TRP CG	195TRP	CE3	100.00	109PHI CD2	256LEU	CD1	0.01	195TR CA	195TRP	CG	100.00
195TRP CG	195TRP	C	100.00	109PHI CD2	256LEU	CD2	0.02	195TR CA	195TRP	CD1	72.88
195TRP CD1	195TRP	CD2	100.00	109PHI CD2	312GLU	CG	0.00	195TR CA	195TRP	CD2	36.06
195TRP CD1	195TRP	CE2	100.00	109PHI CD2	312GLU	CD	0.00	195TR CA	195TRP	CE3	0.24
195TRP CD1	195TRP	C	91.91	109PHI CD2	316ALA	CA	0.01	195TR CA	195TRP	C	100.00
195TRP CD1	199VAL	CG1	0.58	109PHI CD2	316ALA	CB	0.77	195TR CB	195TRP	CG	100.00
195TRP CD1	199VAL	CG2	1.50	109PHI CD2	319LEU	CD1	2.47	195TR CB	195TRP	CD1	100.00
195TRP CD2	195TRP	CE2	100.00	109PHI CD2	319LEU	CD2	0.10	195TR CB	195TRP	CD2	100.00
195TRP CD2	195TRP	CE3	100.00	109PHI CE1	109PHE	CE2	100.00	195TR CB	195TRP	CE3	97.32
195TRP CD2	195TRP	CZ2	100.00	109PHI CE1	109PHE	CZ	100.00	195TR CB	195TRP	C	100.00
195TRP CD2	195TRP	CZ3	100.00	109PHI CE1	112LEU	CD1	0.34	195TR CG	195TRP	CD1	100.00
195TRP CE2	195TRP	CE3	100.00	109PHI CE1	112LEU	CD2	0.04	195TR CG	195TRP	CD2	100.00
195TRP CE2	195TRP	CZ2	100.00	109PHI CE1	251PRO	CB	1.26	195TR CG	195TRP	CE2	100.00
195TRP CE2	195TRP	CZ3	100.00	109PHI CE1	256LEU	CB	0.04	195TR CG	195TRP	CE3	100.00
195TRP CE2	199VAL	CG1	0.45	109PHI CE1	256LEU	CD1	0.21	195TR CG	195TRP	C	100.00
195TRP CE2	199VAL	CG2	0.08	109PHI CE1	256LEU	CD2	0.09	195TR CD1	195TRP	CD2	100.00
195TRP CE3	195TRP	CZ2	100.00	109PHI CE1	257LEU	CD2	0.07	195TR CD1	195TRP	CE2	100.00
195TRP CE3	195TRP	CZ3	100.00	109PHI CE1	288THR	CG2	0.00	195TR CD1	195TRP	C	80.67
195TRP CZ2	195TRP	CZ3	100.00	109PHI CE1	292LEU	CD1	0.34	195TR CD1	199VAL	CG1	0.37
195TRP CZ2	199VAL	CG1	0.01	109PHI CE1	292LEU	CD2	0.01	195TR CD1	199VAL	CG2	0.32
195TRP CZ2	235THR	CA	0.72	109PHI CE1	312GLU	CG	0.92	195TR CD2	195TRP	CE2	100.00
195TRP CZ2	235THR	C	26.49	109PHI CE1	312GLU	CD	0.12	195TR CD2	195TRP	CE3	100.00
195TRP CZ2	236GLY	CA	0.00	109PHI CE1	315VAL	CG1	0.42	195TR CD2	195TRP	CZ2	100.00
195TRP CZ3	236GLY	CA	0.56	109PHI CE1	315VAL	CG2	0.02	195TR CD2	195TRP	CZ3	100.00
195TRP C	196ARG	CA	100.00	109PHI CE1	316ALA	CA	0.01	195TR CD2	195TRP	C	0.10
195TRP C	196ARG	C	100.00	109PHI CE1	316ALA	CB	0.02	195TR CD2	199VAL	CG2	0.24
195TRP C	199VAL	CG2	0.00	109PHI CE1	319LEU	CD1	1.89	195TR CE2	195TRP	CE3	100.00
196ARG CA	196ARG	CB	100.00	109PHI CE1	319LEU	CD2	0.05	195TR CE2	195TRP	CZ2	100.00
196ARG CA	196ARG	CG	100.00	109PHI CE2	109PHE	CZ	100.00	195TR CE2	195TRP	CZ3	100.00
196ARG CA	196ARG	C	100.00	109PHI CE2	112LEU	CG	0.05	195TR CE2	199VAL	CG1	1.36
196ARG CA	199VAL	CG1	0.01	109PHI CE2	112LEU	CD1	0.75	195TR CE2	199VAL	CG2	1.98
196ARG CA	199VAL	CG2	0.02	109PHI CE2	251PRO	CB	2.81	195TR CE3	195TRP	CZ2	100.00
196ARG CB	196ARG	CG	100.00	109PHI CE2	251PRO	CG	0.01	195TR CE3	195TRP	CZ3	100.00
196ARG CB	196ARG	CD	100.00	109PHI CE2	256LEU	CB	0.03	195TR CE3	198THR	CG2	0.00

196ARG	CB	196ARG	C	100.00
196ARG	CG	196ARG	CD	100.00
196ARG	CG	196ARG	CZ	0.28
196ARG	CG	196ARG	C	100.00
196ARG	CG	213HIS	CG	3.12
196ARG	CG	213HIS	CD2	2.92
196ARG	CG	213HIS	CE1	3.20
196ARG	CD	196ARG	CZ	100.00
196ARG	CD	213HIS	CG	0.02
196ARG	CD	213HIS	CD2	0.25
196ARG	CZ	213HIS	CB	50.68
196ARG	CZ	213HIS	CG	0.01
196ARG	CZ	215TYR	CE1	2.65
196ARG	CZ	215TYR	CZ	0.05
196ARG	C	197LYS	CA	100.00
196ARG	C	197LYS	CB	0.10
196ARG	C	197LYS	C	99.90
196ARG	C	199VAL	CG2	0.00
197LYS	CA	197LYS	CB	100.00
197LYS	CA	197LYS	CG	100.00
197LYS	CA	197LYS	CD	4.35
197LYS	CA	197LYS	CE	0.00
197LYS	CA	197LYS	C	100.00
197LYS	CB	197LYS	CG	100.00
197LYS	CB	197LYS	CD	100.00
197LYS	CB	197LYS	CE	6.71
197LYS	CB	197LYS	C	100.00
197LYS	CG	197LYS	CD	100.00
197LYS	CG	197LYS	CE	100.00
197LYS	CG	197LYS	C	90.22
197LYS	CD	197LYS	CE	100.00
197LYS	CD	197LYS	C	4.14
197LYS	CE	201GLU	CD	0.01
197LYS	C	198THR	CA	100.00
197LYS	C	198THR	CB	0.61
197LYS	C	198THR	C	99.77
198THR	CA	198THR	CB	100.00
198THR	CA	198THR	CG2	100.00
198THR	CA	198THR	C	100.00
198THR	CB	198THR	CG2	100.00
198THR	CB	198THR	C	100.00
198THR	CG2	198THR	C	100.00
198THR	CG2	202VAL	CG2	0.00
198THR	C	199VAL	CA	100.00
198THR	C	199VAL	CB	0.01
198THR	C	199VAL	C	100.00
199VAL	CA	199VAL	CB	100.00
199VAL	CA	199VAL	CG1	100.00
199VAL	CA	199VAL	CG2	100.00
199VAL	CA	199VAL	C	100.00
199VAL	CA	202VAL	CG2	0.01
199VAL	CB	199VAL	CG1	100.00
199VAL	CB	199VAL	CG2	100.00
199VAL	CB	199VAL	C	100.00
199VAL	CB	211LEU	CD1	0.00
199VAL	CG1	199VAL	CG2	100.00
199VAL	CG1	199VAL	C	56.83
199VAL	CG1	211LEU	CD1	3.04
199VAL	CG1	211LEU	CD2	0.05
199VAL	CG1	213HIS	CE1	0.45
199VAL	CG2	199VAL	C	47.37
199VAL	CG2	211LEU	CD1	0.02

109PHI	CE2	256LEU	CD1	0.15
109PHI	CE2	256LEU	CD2	0.16
109PHI	CE2	257LEU	CD2	0.12
109PHI	CE2	292LEU	CD1	0.25
109PHI	CE2	292LEU	CD2	0.00
109PHI	CE2	312GLU	CB	0.00
109PHI	CE2	312GLU	CG	0.46
109PHI	CE2	312GLU	CD	0.05
109PHI	CE2	315VAL	CG1	0.25
109PHI	CE2	315VAL	CG2	0.02
109PHI	CE2	316ALA	CA	0.07
109PHI	CE2	316ALA	CB	0.41
109PHI	CE2	319LEU	CG	0.01
109PHI	CE2	319LEU	CD1	3.08
109PHI	CE2	319LEU	CD2	0.06
109PHI	CZ	112LEU	CD1	0.60
109PHI	CZ	112LEU	CD2	0.01
109PHI	CZ	251PRO	CB	0.01
109PHI	CZ	256LEU	CB	0.01
109PHI	CZ	256LEU	CD1	0.01
109PHI	CZ	256LEU	CD2	0.02
109PHI	CZ	257LEU	CD1	0.00
109PHI	CZ	257LEU	CD2	0.34
109PHI	CZ	288THR	CG2	0.02
109PHI	CZ	292LEU	CD1	4.30
109PHI	CZ	292LEU	CD2	0.10
109PHI	CZ	312GLU	CG	0.08
109PHI	CZ	312GLU	CD	0.00
109PHI	CZ	315VAL	CG1	0.56
109PHI	CZ	315VAL	CG2	0.01
109PHI	CZ	316ALA	CB	0.02
109PHI	CZ	319LEU	CD1	0.49
109PHI	CZ	319LEU	CD2	0.00
109PHI	C	110PRO	CA	100.00
109PHI	C	110PRO	CB	0.07
109PHI	C	110PRO	CD	100.00
109PHI	C	110PRO	C	100.00
109PHI	C	112LEU	CD2	0.02
110PR(	CA	110PRO	CB	100.00
110PR(	CA	110PRO	CG	100.00
110PR(	CA	110PRO	CD	100.00
110PR(	CA	110PRO	C	100.00
110PR(	CA	113GLU	CD	0.04
110PR(	CB	110PRO	CG	100.00
110PR(	CB	110PRO	CD	100.00
110PR(	CB	110PRO	C	100.00
110PR(	CB	320LEU	CD1	0.00
110PR(	CG	110PRO	CD	100.00
110PR(	CG	110PRO	C	89.16
110PR(	CG	316ALA	CB	0.02
110PR(	CG	320LEU	CD1	0.00
110PR(	CD	110PRO	C	84.57
110PR(	CD	316ALA	CB	0.02
110PR(	C	111GLY	CA	100.00
110PR(	C	111GLY	C	63.14
110PR(	C	112LEU	CD2	0.01
110PR(	C	320LEU	CD1	0.00
110PR(	C	320LEU	CD2	0.01
111GL\	CA	111GLY	C	100.00
111GL\	CA	113GLU	CD	0.04
111GL\	CA	319LEU	CB	0.03
111GL\	CA	320LEU	CA	0.00

195TR	CE3	199VAL	CG2	0.02
195TR	CZ2	195TRP	CZ3	100.00
195TR	CZ2	199VAL	CG1	1.82
195TR	CZ2	199VAL	CG2	0.20
195TR	CZ2	234VAL	CG2	0.16
195TR	CZ2	234VAL	C	0.02
195TR	CZ2	235THR	CA	2.69
195TR	CZ2	235THR	C	7.52
195TR	CZ2	236GLY	CA	0.43
195TR	CZ3	198THR	CG2	0.05
195TR	CZ3	199VAL	CG1	0.00
195TR	CZ3	199VAL	CG2	0.00
195TR	CZ3	235THR	C	0.01
195TR	CZ3	236GLY	CA	0.05
195TR	C	196ARG	CA	100.00
195TR	C	196ARG	C	100.00
195TR	C	199VAL	CG2	0.00
196AR	CA	196ARG	CB	100.00
196AR	CA	196ARG	CG	100.00
196AR	CA	196ARG	CD	0.22
196AR	CA	196ARG	C	100.00
196AR	CA	199VAL	CG1	0.02
196AR	CA	199VAL	CG2	0.01
196AR	CA	213HIS	CE1	0.00
196AR	CB	196ARG	CG	100.00
196AR	CB	196ARG	CD	100.00
196AR	CB	196ARG	C	100.00
196AR	CB	213HIS	CE1	0.00
196AR	CG	196ARG	CD	100.00
196AR	CG	196ARG	CZ	28.13
196AR	CG	196ARG	C	89.74
196AR	CG	213HIS	CG	0.27
196AR	CG	213HIS	CD2	0.68
196AR	CG	213HIS	CE1	6.44
196AR	CD	196ARG	CZ	100.00
196AR	CD	213HIS	CG	0.02
196AR	CD	213HIS	CD2	0.10
196AR	CD	213HIS	CE1	0.09
196AR	CZ	213HIS	CB	6.64
196AR	CZ	213HIS	CG	1.54
196AR	CZ	213HIS	CD2	4.30
196AR	CZ	213HIS	CE1	0.03
196AR	CZ	215TYR	CE1	0.16
196AR	C	197LYS	CA	100.00
196AR	C	197LYS	CB	0.02
196AR	C	197LYS	C	99.99
196AR	C	199VAL	CG2	0.01
197LY:	CA	197LYS	CB	100.00
197LY:	CA	197LYS	CG	100.00
197LY:	CA	197LYS	CD	20.27
197LY:	CA	197LYS	CE	0.00
197LY:	CA	197LYS	C	100.00
197LY:	CA	200GLU	CB	0.00
197LY:	CB	197LYS	CG	100.00
197LY:	CB	197LYS	CD	100.00
197LY:	CB	197LYS	CE	5.91
197LY:	CB	197LYS	C	100.00
197LY:	CG	197LYS	CD	100.00
197LY:	CG	197LYS	CE	100.00
197LY:	CG	197LYS	C	85.12
197LY:	CD	197LYS	CE	100.00
197LY:	CD	197LYS	C	22.68

199VAL	CG2	211LEU	CD2	0.07	111GLY	CA	320LEU	CG	0.01	197LYS	CD	201GLU	CD	0.02
199VAL	CG2	213HIS	CE1	1.41	111GLY	CA	320LEU	CD1	0.09	197LYS	CE	197LYS	C	0.02
199VAL	C	200GLU	CA	100.00	111GLY	CA	320LEU	CD2	0.30	197LYS	CE	201GLU	CD	0.08
199VAL	C	200GLU	C	100.00	111GLY	C	112LEU	CA	100.00	197LYS	C	198THR	CA	100.00
199VAL	C	211LEU	CD2	0.66	111GLY	C	112LEU	CB	69.98	197LYS	C	198THR	CB	0.10
200GLU	CA	200GLU	CB	100.00	111GLY	C	112LEU	CG	15.76	197LYS	C	198THR	C	99.98
200GLU	CA	200GLU	CG	100.00	111GLY	C	112LEU	CD1	4.42	198TH	CA	198THR	CB	100.00
200GLU	CA	200GLU	CD	89.18	111GLY	C	112LEU	CD2	1.14	198TH	CA	198THR	CG2	100.00
200GLU	CA	200GLU	C	100.00	111GLY	C	112LEU	C	24.45	198TH	CA	198THR	C	100.00
200GLU	CA	211LEU	CD2	1.42	111GLY	C	113GLU	CD	0.01	198TH	CB	198THR	CG2	100.00
200GLU	CB	200GLU	CG	100.00	111GLY	C	115LEU	CD1	0.00	198TH	CB	198THR	C	100.00
200GLU	CB	200GLU	CD	100.00	111GLY	C	319LEU	CD1	0.01	198TH	CG2	198THR	C	100.00
200GLU	CB	200GLU	C	100.00	111GLY	C	319LEU	CD2	0.00	198TH	CG2	202VAL	CG2	0.00
200GLU	CB	204ARG	CZ	0.00	111GLY	C	320LEU	CD1	0.12	198TH	C	199VAL	CA	100.00
200GLU	CB	211LEU	CD2	0.01	111GLY	C	320LEU	CD2	0.10	198TH	C	199VAL	CB	0.01
200GLU	CG	200GLU	CD	100.00	112LEU	CA	112LEU	CB	100.00	198TH	C	199VAL	C	100.00
200GLU	CG	200GLU	C	99.47	112LEU	CA	112LEU	CG	100.00	199VA	CA	199VAL	CB	100.00
200GLU	CG	204ARG	CD	0.00	112LEU	CA	112LEU	CD1	24.06	199VA	CA	199VAL	CG1	100.00
200GLU	CG	204ARG	CZ	5.73	112LEU	CA	112LEU	CD2	53.33	199VA	CA	199VAL	CG2	100.00
200GLU	CD	200GLU	C	5.29	112LEU	CA	112LEU	C	100.00	199VA	CA	199VAL	C	100.00
200GLU	CD	204ARG	CG	0.11	112LEU	CA	115LEU	CD1	0.07	199VA	CB	199VAL	CG1	100.00
200GLU	CD	204ARG	CD	0.00	112LEU	CA	115LEU	CD2	0.00	199VA	CB	199VAL	CG2	100.00
200GLU	CD	204ARG	CZ	0.02	112LEU	CB	112LEU	CG	100.00	199VA	CB	199VAL	C	100.00
200GLU	CD	211LEU	CD2	0.12	112LEU	CB	112LEU	CD1	100.00	199VA	CB	211LEU	CD1	0.00
200GLU	CD	213HIS	CE1	0.00	112LEU	CB	112LEU	CD2	100.00	199VA	CB	211LEU	CD2	0.01
200GLU	C	201GLU	CA	100.00	112LEU	CB	112LEU	C	100.00	199VA	CG1	199VAL	CG2	100.00
200GLU	C	201GLU	CB	0.04	112LEU	CB	115LEU	CD1	0.04	199VA	CG1	199VAL	C	57.52
200GLU	C	201GLU	C	99.99	112LEU	CB	251PRO	CB	0.01	199VA	CG1	211LEU	CG	0.00
200GLU	C	204ARG	CZ	0.00	112LEU	CB	320LEU	CD1	0.01	199VA	CG1	211LEU	CD1	2.18
201GLU	CA	201GLU	CB	100.00	112LEU	CB	320LEU	CD2	0.03	199VA	CG1	211LEU	CD2	0.16
201GLU	CA	201GLU	CG	100.00	112LEU	CG	112LEU	CD1	100.00	199VA	CG1	213HIS	CE1	0.24
201GLU	CA	201GLU	CD	81.44	112LEU	CG	112LEU	CD2	100.00	199VA	CG2	199VAL	C	60.87
201GLU	CA	201GLU	C	100.00	112LEU	CG	112LEU	C	24.45	199VA	CG2	211LEU	CD1	0.10
201GLU	CA	204ARG	CZ	0.26	112LEU	CG	115LEU	CD1	0.02	199VA	CG2	211LEU	CD2	0.22
201GLU	CB	201GLU	CG	100.00	112LEU	CG	319LEU	CD1	0.03	199VA	CG2	213HIS	CE1	0.69
201GLU	CB	201GLU	CD	100.00	112LEU	CG	320LEU	CG	0.00	199VA	CG2	234VAL	CG1	0.00
201GLU	CB	201GLU	C	100.00	112LEU	CG	320LEU	CD1	0.01	199VA	C	200GLU	CA	100.00
201GLU	CB	204ARG	CZ	0.00	112LEU	CG	320LEU	CD2	0.16	199VA	C	200GLU	CB	0.04
201GLU	CG	201GLU	CD	100.00	112LEU	CD1	112LEU	CD2	100.00	199VA	C	200GLU	C	99.99
201GLU	CG	201GLU	C	90.42	112LEU	CD1	112LEU	C	0.20	199VA	C	211LEU	CD1	0.02
201GLU	CG	204ARG	CZ	0.09	112LEU	CD1	115LEU	CB	0.01	199VA	C	211LEU	CD2	0.34
201GLU	CD	201GLU	C	0.72	112LEU	CD1	115LEU	CD1	0.40	200GL	CA	200GLU	CB	100.00
201GLU	CD	204ARG	CD	0.24	112LEU	CD1	115LEU	CD2	0.28	200GL	CA	200GLU	CG	100.00
201GLU	CD	204ARG	CZ	0.11	112LEU	CD1	251PRO	CB	0.13	200GL	CA	200GLU	CD	73.06
201GLU	C	202VAL	CA	100.00	112LEU	CD1	256LEU	CD1	0.19	200GL	CA	200GLU	C	100.00
201GLU	C	202VAL	CB	0.10	112LEU	CD1	256LEU	CD2	0.08	200GL	CA	211LEU	CD1	0.05
201GLU	C	202VAL	CG2	0.06	112LEU	CD1	316ALA	CA	0.02	200GL	CA	211LEU	CD2	1.88
201GLU	C	202VAL	C	99.94	112LEU	CD1	316ALA	CB	0.22	200GL	CB	200GLU	CG	100.00
202VAL	CA	202VAL	CB	100.00	112LEU	CD1	319LEU	CB	6.64	200GL	CB	200GLU	CD	100.00
202VAL	CA	202VAL	CG1	100.00	112LEU	CD1	319LEU	CB	0.00	200GL	CB	200GLU	C	100.00
202VAL	CA	202VAL	CG2	100.00	112LEU	CD1	319LEU	CB	0.78	200GL	CB	211LEU	CD2	0.07
202VAL	CA	202VAL	C	100.00	112LEU	CD1	319LEU	CB	0.04	200GL	CB	213HIS	CE1	0.00
202VAL	CB	202VAL	CG1	100.00	112LEU	CD1	319LEU	C	0.06	200GL	CG	200GLU	CD	100.00
202VAL	CB	202VAL	CG2	100.00	112LEU	CD1	320LEU	CA	0.01	200GL	CG	200GLU	C	98.26
202VAL	CB	202VAL	C	100.00	112LEU	CD1	320LEU	CG	0.04	200GL	CG	211LEU	CD1	0.00
202VAL	CG1	202VAL	CG2	100.00	112LEU	CD1	320LEU	CD1	0.17	200GL	CG	211LEU	CD2	0.08
202VAL	CG1	202VAL	C	99.49	112LEU	CD1	320LEU	CD2	0.36	200GL	CG	213HIS	CE1	0.01
202VAL	CG1	206TYR	CD1	1.38	112LEU	CD1	327LEU	CD1	0.01	200GL	CD	200GLU	C	70.52
202VAL	CG1	206TYR	CD2	0.72	112LEU	CD1	327LEU	CD2	0.00	200GL	CD	204ARG	CG	0.00
202VAL	CG1	206TYR	CE1	0.37	112LEU	CD2	112LEU	C	0.00	200GL	CD	211LEU	CD2	0.01
202VAL	CG1	206TYR	CE2	0.34	112LEU	CD2	114ARG	CZ	0.01	200GL	C	201GLU	CA	100.00
202VAL	CG1	206TYR	CZ	0.00	112LEU	CD2	115LEU	CD1	1.72	200GL	C	201GLU	CB	0.00

202VAL CG2	202VAL C	0.50	112LEU CD2	115LEU CD2	0.38	200GL C	201GLU C	100.00
202VAL C	203GLY CA	100.00	112LEU CD2	251PRO CA	0.00	200GL C	203GLY CA	0.00
202VAL C	203GLY C	98.37	112LEU CD2	251PRO CB	0.16	201GL CA	201GLU CB	100.00
203GLY CA	203GLY C	100.00	112LEU CD2	256LEU CD1	0.38	201GL CA	201GLU CG	100.00
203GLY CA	206TYR CB	0.00	112LEU CD2	256LEU CD2	0.23	201GL CA	201GLU CD	61.39
203GLY CA	211LEU CD2	0.08	112LEU CD2	316ALA CA	0.02	201GL CA	201GLU C	100.00
203GLY C	204ARG CA	100.00	112LEU CD2	316ALA CB	0.15	201GL CA	204ARG CZ	1.52
203GLY C	204ARG CB	1.57	112LEU CD2	319LEU CB	3.33	201GL CB	201GLU CG	100.00
203GLY C	204ARG CG	0.34	112LEU CD2	319LEU CG	0.00	201GL CB	201GLU CD	100.00
203GLY C	204ARG CD	0.01	112LEU CD2	319LEU CD1	0.63	201GL CB	201GLU C	100.00
203GLY C	204ARG CZ	0.03	112LEU CD2	319LEU CD2	0.08	201GL CB	204ARG CZ	0.02
203GLY C	204ARG C	97.66	112LEU CD2	319LEU C	0.25	201GL CG	201GLU CD	100.00
204ARG CA	204ARG CB	100.00	112LEU CD2	320LEU CA	0.02	201GL CG	201GLU C	94.30
204ARG CA	204ARG CG	100.00	112LEU CD2	320LEU CB	0.00	201GL CG	204ARG CZ	0.40
204ARG CA	204ARG CD	5.40	112LEU CD2	320LEU CG	0.08	201GL CD	201GLU C	0.13
204ARG CA	204ARG C	100.00	112LEU CD2	320LEU CD1	0.40	201GL CD	204ARG CZ	0.72
204ARG CB	204ARG CG	100.00	112LEU CD2	320LEU CD2	2.60	201GL C	202VAL CA	100.00
204ARG CB	204ARG CD	100.00	112LEU CD2	324PRO CG	0.00	201GL C	202VAL CB	0.12
204ARG CB	204ARG CZ	1.43	112LEU CD2	324PRO CD	0.00	201GL C	202VAL CG1	0.04
204ARG CB	204ARG C	100.00	112LEU CD2	327LEU CD1	0.04	201GL C	202VAL CG2	0.03
204ARG CG	204ARG CD	100.00	112LEU CD2	327LEU CD2	0.12	201GL C	202VAL C	99.89
204ARG CG	204ARG CZ	20.62	112LEU C	113GLU CA	100.00	202VA CA	202VAL CB	100.00
204ARG CG	204ARG C	5.84	112LEU C	113GLU C	100.00	202VA CA	202VAL CG1	100.00
204ARG CG	211LEU CD2	0.02	112LEU C	115LEU CD1	0.02	202VA CA	202VAL CG2	100.00
204ARG CD	204ARG CZ	100.00	112LEU C	115LEU CD2	0.01	202VA CA	202VAL C	100.00
204ARG CZ	211LEU CB	0.02	113GLU CA	113GLU CB	100.00	202VA CB	202VAL CG1	100.00
204ARG CZ	211LEU CD2	0.08	113GLU CA	113GLU CG	100.00	202VA CB	202VAL CG2	100.00
204ARG C	205GLY CA	100.00	113GLU CA	113GLU CD	63.53	202VA CB	202VAL C	100.00
204ARG C	205GLY C	52.76	113GLU CA	113GLU C	100.00	202VA CG1	202VAL CG2	100.00
205GLY CA	205GLY C	100.00	113GLU CA	123ALA CB	0.00	202VA CG1	202VAL C	99.43
205GLY C	206TYR CA	100.00	113GLU CB	113GLU CG	100.00	202VA CG1	206TYR CD1	1.40
205GLY C	206TYR CB	34.13	113GLU CB	113GLU CD	100.00	202VA CG1	206TYR CE1	0.31
205GLY C	206TYR CG	3.90	113GLU CB	113GLU C	100.00	202VA CG2	202VAL C	0.75
205GLY C	206TYR CD1	4.92	113GLU CB	120GLU CB	0.02	202VA C	203GLY CA	100.00
205GLY C	206TYR CD2	1.66	113GLU CB	120GLU CG	1.62	202VA C	203GLY C	98.68
205GLY C	206TYR C	68.71	113GLU CB	120GLU CD	0.01	203GL CA	203GLY C	100.00
205GLY C	207PRO CD	6.44	113GLU CB	123ALA CB	0.04	203GL CA	211LEU CD2	0.00
206TYR CA	206TYR CB	100.00	113GLU CB	124ARG CG	0.02	203GL C	204ARG CA	100.00
206TYR CA	206TYR CG	100.00	113GLU CG	113GLU CD	100.00	203GL C	204ARG CB	0.79
206TYR CA	206TYR CD1	74.50	113GLU CG	113GLU C	2.23	203GL C	204ARG CG	0.09
206TYR CA	206TYR CD2	68.13	113GLU CG	123ALA CB	0.02	203GL C	204ARG C	99.00
206TYR CA	206TYR C	100.00	113GLU CG	123ALA C	0.04	204AR CA	204ARG CB	100.00
206TYR CA	207PRO CD	100.00	113GLU CG	124ARG CA	0.28	204AR CA	204ARG CG	100.00
206TYR CB	206TYR CG	100.00	113GLU CG	124ARG CB	0.02	204AR CA	204ARG CD	0.05
206TYR CB	206TYR CD1	100.00	113GLU CG	124ARG CG	0.20	204AR CA	204ARG C	100.00
206TYR CB	206TYR CD2	100.00	113GLU CG	124ARG CZ	0.04	204AR CB	204ARG CG	100.00
206TYR CB	206TYR C	100.00	113GLU CD	113GLU C	0.02	204AR CB	204ARG CD	100.00
206TYR CB	207PRO CD	0.05	113GLU CD	124ARG CB	0.00	204AR CB	204ARG CZ	4.48
206TYR CB	209VAL CG2	0.10	113GLU CD	124ARG CG	0.04	204AR CB	204ARG C	100.00
206TYR CG	206TYR CD1	100.00	113GLU CD	124ARG CZ	0.79	204AR CG	204ARG CD	100.00
206TYR CG	206TYR CD2	100.00	113GLU C	114ARG CA	100.00	204AR CG	204ARG CZ	45.88
206TYR CG	206TYR CE1	100.00	113GLU C	114ARG CB	0.15	204AR CG	204ARG C	3.22
206TYR CG	206TYR CE2	100.00	113GLU C	114ARG CG	0.02	204AR CD	204ARG CZ	100.00
206TYR CG	206TYR CZ	100.00	113GLU C	114ARG C	99.78	204AR C	205GLY CA	100.00
206TYR CG	206TYR C	11.99	113GLU C	120GLU CB	0.01	204AR C	205GLY C	72.03
206TYR CG	207PRO CD	0.16	113GLU C	120GLU CG	0.04	205GL CA	205GLY C	100.00
206TYR CD1	206TYR CD2	100.00	114ARG CA	114ARG CB	100.00	205GL C	206TYR CA	100.00
206TYR CD1	206TYR CE1	100.00	114ARG CA	114ARG CG	100.00	205GL C	206TYR CB	79.13
206TYR CD1	206TYR CE2	100.00	114ARG CA	114ARG CD	1.70	205GL C	206TYR CG	17.58
206TYR CD1	206TYR CZ	100.00	114ARG CA	114ARG CZ	0.01	205GL C	206TYR CD1	31.86
206TYR CD1	206TYR C	7.45	114ARG CA	114ARG C	100.00	205GL C	206TYR CD2	0.01
206TYR CD1	207PRO CD	0.26	114ARG CA	120GLU CG	0.08	205GL C	206TYR C	24.85

206TYR	CD1	209VAL	CG2	0.18	114AR(	CA	120GLU	CD	0.02	205GL	C	207PRO	CD	29.95
206TYR	CD2	206TYR	CE1	100.00	114AR(	CB	114ARG	CG	100.00	206TY	CA	206TYR	CB	100.00
206TYR	CD2	206TYR	CE2	100.00	114AR(	CB	114ARG	CD	100.00	206TY	CA	206TYR	CG	100.00
206TYR	CD2	206TYR	CZ	100.00	114AR(	CB	114ARG	CZ	0.96	206TY	CA	206TYR	CD1	81.78
206TYR	CD2	206TYR	C	0.16	114AR(	CB	114ARG	C	100.00	206TY	CA	206TYR	CD2	56.07
206TYR	CD2	207PRO	CD	0.00	114AR(	CB	115LEU	CD2	0.00	206TY	CA	206TYR	C	100.00
206TYR	CD2	209VAL	CB	0.00	114AR(	CB	120GLU	CG	0.01	206TY	CA	207PRO	CD	100.00
206TYR	CD2	209VAL	CG2	0.07	114AR(	CB	120GLU	CD	0.10	206TY	CB	206TYR	CG	100.00
206TYR	CE1	206TYR	CE2	100.00	114AR(	CG	114ARG	CD	100.00	206TY	CB	206TYR	CD1	100.00
206TYR	CE1	206TYR	CZ	100.00	114AR(	CG	114ARG	CZ	19.11	206TY	CB	206TYR	CD2	100.00
206TYR	CE1	209VAL	CB	0.03	114AR(	CG	114ARG	C	51.36	206TY	CB	206TYR	C	100.00
206TYR	CE1	209VAL	CG2	0.39	114AR(	CG	115LEU	CD1	0.02	206TY	CB	209VAL	CG2	0.12
206TYR	CE2	206TYR	CZ	100.00	114AR(	CG	115LEU	CD2	0.03	206TY	CG	206TYR	CD1	100.00
206TYR	CE2	209VAL	CG1	0.00	114AR(	CG	120GLU	CG	0.36	206TY	CG	206TYR	CD2	100.00
206TYR	CE2	209VAL	CG2	0.00	114AR(	CG	120GLU	CD	3.44	206TY	CG	206TYR	CE1	100.00
206TYR	C	207PRO	CA	100.00	114AR(	CD	114ARG	CZ	100.00	206TY	CG	206TYR	CE2	100.00
206TYR	C	207PRO	CB	0.30	114AR(	CD	114ARG	C	0.31	206TY	CG	206TYR	CZ	100.00
206TYR	C	207PRO	CD	100.00	114AR(	CD	115LEU	CD1	0.00	206TY	CD1	206TYR	CD2	100.00
206TYR	C	207PRO	C	100.00	114AR(	CD	120GLU	CG	0.01	206TY	CD1	206TYR	CE1	100.00
207PRO	CA	207PRO	CB	100.00	114AR(	CD	120GLU	CD	0.00	206TY	CD1	206TYR	CE2	100.00
207PRO	CA	207PRO	CG	100.00	114AR(	CZ	115LEU	CD1	0.09	206TY	CD1	206TYR	CZ	100.00
207PRO	CA	207PRO	CD	100.00	114AR(	CZ	115LEU	CD2	0.50	206TY	CD2	206TYR	CE1	100.00
207PRO	CA	207PRO	C	100.00	114AR(	CZ	120GLU	CB	0.00	206TY	CD2	206TYR	CE2	100.00
207PRO	CB	207PRO	CG	100.00	114AR(	CZ	120GLU	CG	0.12	206TY	CD2	206TYR	CZ	100.00
207PRO	CB	207PRO	CD	100.00	114AR(	CZ	120GLU	CD	0.02	206TY	CD2	209VAL	CG2	0.03
207PRO	CB	207PRO	C	100.00	114AR(	CZ	320LEU	CD1	0.00	206TY	CE1	206TYR	CE2	100.00
207PRO	CG	207PRO	CD	100.00	114AR(	CZ	320LEU	CD2	0.08	206TY	CE1	206TYR	CZ	100.00
207PRO	CG	207PRO	C	98.42	114AR(	C	115LEU	CA	100.00	206TY	CE2	206TYR	CZ	100.00
207PRO	CD	207PRO	C	93.65	114AR(	C	115LEU	CB	1.68	206TY	C	207PRO	CA	100.00
207PRO	C	208ASP	CA	100.00	114AR(	C	115LEU	CG	0.16	206TY	C	207PRO	CB	0.19
207PRO	C	208ASP	CB	80.34	114AR(	C	115LEU	CD1	0.16	206TY	C	207PRO	CD	100.00
207PRO	C	208ASP	CG	0.00	114AR(	C	115LEU	CD2	0.04	206TY	C	207PRO	C	100.00
207PRO	C	208ASP	C	94.00	114AR(	C	115LEU	C	97.64	207PR	CA	207PRO	CB	100.00
208ASP	CA	208ASP	CB	100.00	115LE(	CA	115LEU	CB	100.00	207PR	CA	207PRO	CG	100.00
208ASP	CA	208ASP	CG	100.00	115LE(	CA	115LEU	CG	100.00	207PR	CA	207PRO	CD	100.00
208ASP	CA	208ASP	C	100.00	115LE(	CA	115LEU	CD1	44.32	207PR	CA	207PRO	C	100.00
208ASP	CB	208ASP	CG	100.00	115LE(	CA	115LEU	CD2	57.93	207PR	CB	207PRO	CG	100.00
208ASP	CB	208ASP	C	100.00	115LE(	CA	115LEU	C	100.00	207PR	CB	207PRO	CD	100.00
208ASP	CG	208ASP	C	99.73	115LE(	CB	115LEU	CG	100.00	207PR	CB	207PRO	C	100.00
208ASP	C	209VAL	CA	100.00	115LE(	CB	115LEU	CD1	100.00	207PR	CG	207PRO	CD	100.00
208ASP	C	209VAL	CB	10.80	115LE(	CB	115LEU	CD2	100.00	207PR	CG	207PRO	C	98.95
208ASP	C	209VAL	CG1	0.14	115LE(	CB	115LEU	C	100.00	207PR	CD	207PRO	C	90.78
208ASP	C	209VAL	CG2	7.37	115LE(	CB	252GLY	CA	0.05	207PR	C	208ASP	CA	100.00
208ASP	C	209VAL	C	92.82	115LE(	CB	327LEU	CD1	0.08	207PR	C	208ASP	CB	0.52
209VAL	CA	209VAL	CB	100.00	115LE(	CB	327LEU	CD2	0.06	207PR	C	208ASP	C	98.94
209VAL	CA	209VAL	CG1	100.00	115LE(	CG	115LEU	CD1	100.00	208AS	CA	208ASP	CB	100.00
209VAL	CA	209VAL	CG2	100.00	115LE(	CG	115LEU	CD2	100.00	208AS	CA	208ASP	CG	100.00
209VAL	CA	209VAL	C	100.00	115LE(	CG	115LEU	C	24.84	208AS	CA	208ASP	C	100.00
209VAL	CB	209VAL	CG1	100.00	115LE(	CG	327LEU	CD1	0.39	208AS	CB	208ASP	CG	100.00
209VAL	CB	209VAL	CG2	100.00	115LE(	CG	327LEU	CD2	0.10	208AS	CB	208ASP	C	100.00
209VAL	CB	209VAL	C	100.00	115LE(	CD1	115LEU	CD2	100.00	208AS	CG	208ASP	C	99.92
209VAL	CG1	209VAL	CG2	100.00	115LE(	CD1	115LEU	C	0.02	208AS	CG	209VAL	CG2	0.13
209VAL	CG1	209VAL	C	97.61	115LE(	CD1	319LEU	CD1	0.04	208AS	C	209VAL	CA	100.00
209VAL	CG2	209VAL	C	2.36	115LE(	CD1	319LEU	CD2	0.06	208AS	C	209VAL	CB	41.55
209VAL	C	210ALA	CA	100.00	115LE(	CD1	320LEU	CD2	0.06	208AS	C	209VAL	CG1	5.26
209VAL	C	210ALA	CB	7.54	115LE(	CD1	323PRO	CB	0.01	208AS	C	209VAL	CG2	24.05
209VAL	C	210ALA	C	91.78	115LE(	CD1	324PRO	CG	0.12	208AS	C	209VAL	C	69.17
210ALA	CA	210ALA	CB	100.00	115LE(	CD1	324PRO	CD	0.25	209VA	CA	209VAL	CB	100.00
210ALA	CA	210ALA	C	100.00	115LE(	CD1	327LEU	CB	0.00	209VA	CA	209VAL	CG1	100.00
210ALA	CB	210ALA	C	100.00	115LE(	CD1	327LEU	CG	0.03	209VA	CA	209VAL	CG2	100.00
210ALA	C	211LEU	CA	100.00	115LE(	CD1	327LEU	CD1	1.58	209VA	CA	209VAL	C	100.00
210ALA	C	211LEU	CB	86.20	115LE(	CD1	327LEU	CD2	0.52	209VA	CB	209VAL	CG1	100.00

210ALA	C	211LEU	C	18.64	115LEl	CD2	115LEU	C	0.05	209VA	CB	209VAL	CG2	100.00
211LEU	CA	211LEU	CB	100.00	115LEl	CD2	252GLY	CA	0.00	209VA	CB	209VAL	C	100.00
211LEU	CA	211LEU	CG	100.00	115LEl	CD2	252GLY	C	0.04	209VA	CG1	209VAL	CG2	100.00
211LEU	CA	211LEU	CD1	95.68	115LEl	CD2	253SER	CB	0.00	209VA	CG1	209VAL	C	90.25
211LEU	CA	211LEU	CD2	2.81	115LEl	CD2	319LEU	CD1	0.00	209VA	CG2	209VAL	C	9.71
211LEU	CA	211LEU	C	100.00	115LEl	CD2	319LEU	CD2	0.02	209VA	C	210ALA	CA	100.00
211LEU	CB	211LEU	CG	100.00	115LEl	CD2	320LEU	CD2	0.00	209VA	C	210ALA	CB	5.67
211LEU	CB	211LEU	CD1	100.00	115LEl	CD2	323PRO	CB	0.01	209VA	C	210ALA	C	95.03
211LEU	CB	211LEU	CD2	100.00	115LEl	CD2	324PRO	CG	0.09	210AL.	CA	210ALA	CB	100.00
211LEU	CB	211LEU	C	100.00	115LEl	CD2	324PRO	CD	0.21	210AL.	CA	210ALA	C	100.00
211LEU	CG	211LEU	CD1	100.00	115LEl	CD2	327LEU	CB	0.02	210AL.	CB	210ALA	C	100.00
211LEU	CG	211LEU	CD2	100.00	115LEl	CD2	327LEU	CG	0.03	210AL.	C	211LEU	CA	100.00
211LEU	CG	211LEU	C	99.99	115LEl	CD2	327LEU	CD1	0.87	210AL.	C	211LEU	CB	37.43
211LEU	CG	213HIS	CE1	0.20	115LEl	CD2	327LEU	CD2	0.60	210AL.	C	211LEU	C	68.96
211LEU	CD1	211LEU	CD2	100.00	115LEl	CD2	327LEU	C	0.00	211LEI	CA	211LEU	CB	100.00
211LEU	CD1	211LEU	C	1.75	115LEl	C	116SER	CA	100.00	211LEI	CA	211LEU	CG	100.00
211LEU	CD1	213HIS	CE1	0.13	115LEl	C	116SER	CB	0.47	211LEI	CA	211LEU	CD1	94.65
211LEU	CD2	211LEU	C	2.91	115LEl	C	116SER	C	99.62	211LEI	CA	211LEU	CD2	13.99
211LEU	CD2	212GLU	C	0.04	115LEl	C	252GLY	CA	1.78	211LEI	CA	211LEU	C	100.00
211LEU	CD2	213HIS	CE1	10.40	116SEF	CA	116SER	CB	100.00	211LEI	CB	211LEU	CG	100.00
211LEU	C	212GLU	CA	100.00	116SEF	CA	116SER	C	100.00	211LEI	CB	211LEU	CD1	100.00
211LEU	C	212GLU	CB	85.88	116SEF	CA	117PRO	CD	100.00	211LEI	CB	211LEU	CD2	100.00
211LEU	C	212GLU	C	19.11	116SEF	CA	252GLY	CA	0.04	211LEI	CB	211LEU	C	100.00
212GLU	CA	212GLU	CB	100.00	116SEF	CB	116SER	C	100.00	211LEI	CG	211LEU	CD1	100.00
212GLU	CA	212GLU	CG	100.00	116SEF	CB	117PRO	CD	3.34	211LEI	CG	211LEU	CD2	100.00
212GLU	CA	212GLU	CD	0.02	116SEF	CB	123ALA	CB	7.46	211LEI	CG	211LEU	C	98.93
212GLU	CA	212GLU	C	100.00	116SEF	C	117PRO	CA	100.00	211LEI	CG	213HIS	CE1	0.16
212GLU	CB	212GLU	CG	100.00	116SEF	C	117PRO	CB	0.10	211LEI	CD1	211LEU	CD2	100.00
212GLU	CB	212GLU	CD	100.00	116SEF	C	117PRO	CD	100.00	211LEI	CD1	211LEU	C	12.66
212GLU	CB	212GLU	C	100.00	116SEF	C	117PRO	C	100.00	211LEI	CD1	212GLU	C	0.02
212GLU	CG	212GLU	CD	100.00	117PR(	CA	117PRO	CB	100.00	211LEI	CD1	213HIS	CD2	0.07
212GLU	CG	212GLU	C	99.76	117PR(	CA	117PRO	CG	100.00	211LEI	CD1	213HIS	CE1	3.20
212GLU	C	213HIS	CA	100.00	117PR(	CA	117PRO	CD	100.00	211LEI	CD2	211LEU	C	1.60
212GLU	C	213HIS	CB	22.52	117PR(	CA	117PRO	C	100.00	211LEI	CD2	212GLU	C	0.03
212GLU	C	213HIS	CG	5.14	117PR(	CB	117PRO	CG	100.00	211LEI	CD2	213HIS	CD2	0.00
212GLU	C	213HIS	CD2	0.08	117PR(	CB	117PRO	CD	100.00	211LEI	CD2	213HIS	CE1	3.57
212GLU	C	213HIS	C	81.42	117PR(	CB	117PRO	C	100.00	211LEI	C	212GLU	CA	100.00
213HIS	CA	213HIS	CB	100.00	117PR(	CG	117PRO	CD	100.00	211LEI	C	212GLU	CB	78.32
213HIS	CA	213HIS	CG	100.00	117PR(	CG	117PRO	C	95.35	211LEI	C	212GLU	C	30.71
213HIS	CA	213HIS	CD2	5.08	117PR(	CG	249VAL	CG1	1.28	212GL	CA	212GLU	CB	100.00
213HIS	CA	213HIS	C	100.00	117PR(	CG	249VAL	CG2	0.56	212GL	CA	212GLU	CG	100.00
213HIS	CB	213HIS	CG	100.00	117PR(	CG	250LEU	CD2	0.01	212GL	CA	212GLU	CD	0.05
213HIS	CB	213HIS	CD2	100.00	117PR(	CD	117PRO	C	85.12	212GL	CA	212GLU	C	100.00
213HIS	CB	213HIS	CE1	0.12	117PR(	CD	249VAL	CG1	0.04	212GL	CB	212GLU	CG	100.00
213HIS	CB	213HIS	C	100.00	117PR(	CD	250LEU	CA	0.00	212GL	CB	212GLU	CD	100.00
213HIS	CG	213HIS	CD2	100.00	117PR(	CD	252GLY	CA	0.09	212GL	CB	212GLU	C	100.00
213HIS	CG	213HIS	CE1	100.00	117PR(	C	118LEU	CA	100.00	212GL	CG	212GLU	CD	100.00
213HIS	CD2	213HIS	CE1	100.00	117PR(	C	118LEU	CB	0.03	212GL	CG	212GLU	C	99.54
213HIS	C	214GLN	CA	100.00	117PR(	C	118LEU	CG	0.00	212GL	CG	214GLN	CG	0.01
213HIS	C	214GLN	CB	73.41	117PR(	C	118LEU	CD1	0.02	212GL	C	213HIS	CA	100.00
213HIS	C	214GLN	CG	11.11	117PR(	C	118LEU	C	99.92	212GL	C	213HIS	CB	11.93
213HIS	C	214GLN	C	22.62	118LEl	CA	118LEU	CB	100.00	212GL	C	213HIS	CG	2.10
214GLN	CA	214GLN	CB	100.00	118LEl	CA	118LEU	CG	100.00	212GL	C	213HIS	CD2	0.27
214GLN	CA	214GLN	CG	100.00	118LEl	CA	118LEU	CD1	20.67	212GL	C	213HIS	C	91.04
214GLN	CA	214GLN	CD	84.62	118LEl	CA	118LEU	CD2	75.97	213HI!	CA	213HIS	CB	100.00
214GLN	CA	214GLN	C	100.00	118LEl	CA	118LEU	C	100.00	213HI!	CA	213HIS	CG	100.00
214GLN	CB	214GLN	CG	100.00	118LEl	CB	118LEU	CG	100.00	213HI!	CA	213HIS	CD2	13.29
214GLN	CB	214GLN	CD	100.00	118LEl	CB	118LEU	CD1	100.00	213HI!	CA	213HIS	C	100.00
214GLN	CB	214GLN	C	100.00	118LEl	CB	118LEU	CD2	100.00	213HI!	CB	213HIS	CG	100.00
214GLN	CB	219MET	CG	0.02	118LEl	CB	118LEU	C	100.00	213HI!	CB	213HIS	CD2	100.00
214GLN	CG	214GLN	CD	100.00	118LEl	CB	122ILE	CG2	0.00	213HI!	CB	213HIS	CE1	0.12
214GLN	CG	214GLN	C	14.42	118LEl	CB	123ALA	CB	0.04	213HI!	CB	213HIS	C	100.00

214GLN	CG	219MET	CB	0.11	118LEU	CG	118LEU	CD1	100.00	213HIS	CG	213HIS	CD2	100.00
214GLN	CG	219MET	CG	0.01	118LEU	CG	118LEU	CD2	100.00	213HIS	CG	213HIS	CE1	100.00
214GLN	CD	218ALA	CB	0.01	118LEU	CG	118LEU	C	1.44	213HIS	CD2	213HIS	CE1	100.00
214GLN	CD	219MET	CA	0.00	118LEU	CG	250LEU	CD2	0.05	213HIS	C	214GLN	CA	100.00
214GLN	CD	219MET	CB	0.01	118LEU	CD1	118LEU	CD2	100.00	213HIS	C	214GLN	CB	51.76
214GLN	CD	219MET	CG	0.00	118LEU	CD1	122ILE	CG2	0.11	213HIS	C	214GLN	CG	7.12
214GLN	CD	222HIS	CD2	0.04	118LEU	CD1	122ILE	CD	0.00	213HIS	C	214GLN	C	50.68
214GLN	CD	230PHE	CE1	0.00	118LEU	CD1	123ALA	CA	0.03	214GL	CA	214GLN	CB	100.00
214GLN	C	215TYR	CA	100.00	118LEU	CD1	227PRO	CG	8.28	214GL	CA	214GLN	CG	100.00
214GLN	C	215TYR	CB	0.12	118LEU	CD1	227PRO	CD	1.90	214GL	CA	214GLN	CD	84.31
214GLN	C	215TYR	C	99.96	118LEU	CD1	249VAL	CG1	0.00	214GL	CA	214GLN	C	100.00
215TYR	CA	215TYR	CB	100.00	118LEU	CD1	250LEU	CD1	0.38	214GL	CB	214GLN	CG	100.00
215TYR	CA	215TYR	CG	100.00	118LEU	CD1	250LEU	CD2	12.95	214GL	CB	214GLN	CD	100.00
215TYR	CA	215TYR	CD1	99.98	118LEU	CD2	122ILE	CG2	0.74	214GL	CB	214GLN	C	100.00
215TYR	CA	215TYR	CD2	2.10	118LEU	CD2	122ILE	CD	0.05	214GL	CB	219MET	CB	0.00
215TYR	CA	215TYR	C	100.00	118LEU	CD2	122ILE	C	0.01	214GL	CB	219MET	CG	0.25
215TYR	CB	215TYR	CG	100.00	118LEU	CD2	123ALA	CA	0.01	214GL	CB	219MET	CE	0.16
215TYR	CB	215TYR	CD1	100.00	118LEU	CD2	224VAL	CA	0.00	214GL	CG	214GLN	CD	100.00
215TYR	CB	215TYR	CD2	100.00	118LEU	CD2	224VAL	CG1	0.00	214GL	CG	214GLN	C	0.84
215TYR	CB	215TYR	C	100.00	118LEU	CD2	224VAL	CG2	0.01	214GL	CG	219MET	CG	0.00
215TYR	CG	215TYR	CD1	100.00	118LEU	CD2	224VAL	C	0.02	214GL	CG	219MET	CE	0.00
215TYR	CG	215TYR	CD2	100.00	118LEU	CD2	227PRO	CG	1.64	214GL	CD	219MET	CG	0.01
215TYR	CG	215TYR	CE1	100.00	118LEU	CD2	227PRO	CD	1.70	214GL	CD	222HIS	CD2	0.03
215TYR	CG	215TYR	CE2	100.00	118LEU	CD2	250LEU	CD2	0.20	214GL	CD	230PHE	CE1	0.05
215TYR	CG	215TYR	CZ	100.00	118LEU	C	119LYS	CA	100.00	214GL	C	215TYR	CA	100.00
215TYR	CD1	215TYR	CD2	100.00	118LEU	C	119LYS	C	100.00	214GL	C	215TYR	CB	0.05
215TYR	CD1	215TYR	CE1	100.00	119LYS	CA	119LYS	CB	100.00	214GL	C	215TYR	CD1	0.00
215TYR	CD1	215TYR	CE2	100.00	119LYS	CA	119LYS	CG	100.00	214GL	C	215TYR	CD2	0.00
215TYR	CD1	215TYR	CZ	100.00	119LYS	CA	119LYS	CD	0.54	214GL	C	215TYR	C	99.99
215TYR	CD2	215TYR	CE1	100.00	119LYS	CA	119LYS	C	100.00	215TY	CA	215TYR	CB	100.00
215TYR	CD2	215TYR	CE2	100.00	119LYS	CB	119LYS	CG	100.00	215TY	CA	215TYR	CG	100.00
215TYR	CD2	215TYR	CZ	100.00	119LYS	CB	119LYS	CD	100.00	215TY	CA	215TYR	CD1	64.86
215TYR	CE1	215TYR	CE2	100.00	119LYS	CB	119LYS	CE	19.88	215TY	CA	215TYR	CD2	44.71
215TYR	CE1	215TYR	CZ	100.00	119LYS	CB	119LYS	C	100.00	215TY	CA	215TYR	C	100.00
215TYR	CE2	215TYR	CZ	100.00	119LYS	CB	122ILE	CG1	0.07	215TY	CB	215TYR	CG	100.00
215TYR	C	216VAL	CA	100.00	119LYS	CB	122ILE	CD	0.20	215TY	CB	215TYR	CD1	100.00
215TYR	C	216VAL	C	100.00	119LYS	CG	119LYS	CD	100.00	215TY	CB	215TYR	CD2	100.00
216VAL	CA	216VAL	CB	100.00	119LYS	CG	119LYS	CE	100.00	215TY	CB	215TYR	C	100.00
216VAL	CA	216VAL	CG1	100.00	119LYS	CG	119LYS	C	7.52	215TY	CB	217ASP	CG	0.00
216VAL	CA	216VAL	CG2	100.00	119LYS	CG	122ILE	CG1	0.10	215TY	CB	218ALA	CB	0.02
216VAL	CA	216VAL	C	100.00	119LYS	CG	122ILE	CD	0.11	215TY	CG	215TYR	CD1	100.00
216VAL	CB	216VAL	CG1	100.00	119LYS	CD	119LYS	CE	100.00	215TY	CG	215TYR	CD2	100.00
216VAL	CB	216VAL	CG2	100.00	119LYS	CD	122ILE	CD	0.01	215TY	CG	215TYR	CE1	100.00
216VAL	CB	216VAL	C	100.00	119LYS	CE	122ILE	CG1	0.01	215TY	CG	215TYR	CE2	100.00
216VAL	CB	239PHE	CD2	0.09	119LYS	CE	122ILE	CD	0.08	215TY	CG	215TYR	CZ	100.00
216VAL	CB	239PHE	CE2	0.06	119LYS	C	120GLU	CA	100.00	215TY	CD1	215TYR	CD2	100.00
216VAL	CB	239PHE	CZ	0.01	119LYS	C	120GLU	CB	0.00	215TY	CD1	215TYR	CE1	100.00
216VAL	CG1	216VAL	CG2	100.00	119LYS	C	120GLU	C	100.00	215TY	CD1	215TYR	CE2	100.00
216VAL	CG1	216VAL	C	99.92	120GLU	CA	120GLU	CB	100.00	215TY	CD1	215TYR	CZ	100.00
216VAL	CG1	239PHE	CG	0.03	120GLU	CA	120GLU	CG	100.00	215TY	CD2	215TYR	CE1	100.00
216VAL	CG1	239PHE	CD1	0.03	120GLU	CA	120GLU	CD	14.31	215TY	CD2	215TYR	CE2	100.00
216VAL	CG1	239PHE	CD2	0.80	120GLU	CA	120GLU	C	100.00	215TY	CD2	215TYR	CZ	100.00
216VAL	CG1	239PHE	CE1	0.35	120GLU	CA	123ALA	CB	0.01	215TY	CE1	215TYR	CE2	100.00
216VAL	CG1	239PHE	CE2	1.45	120GLU	CB	120GLU	CG	100.00	215TY	CE1	215TYR	CZ	100.00
216VAL	CG1	239PHE	CZ	0.81	120GLU	CB	120GLU	CD	100.00	215TY	CE2	215TYR	CZ	100.00
216VAL	CG2	216VAL	C	100.00	120GLU	CB	120GLU	C	100.00	215TY	C	216VAL	CA	100.00
216VAL	CG2	239PHE	CB	1.50	120GLU	CB	124ARG	CZ	0.00	215TY	C	216VAL	C	100.00
216VAL	CG2	239PHE	CG	2.50	120GLU	CG	120GLU	CD	100.00	216VA	CA	216VAL	CB	100.00
216VAL	CG2	239PHE	CD1	0.25	120GLU	CG	120GLU	C	90.95	216VA	CA	216VAL	CG1	100.00
216VAL	CG2	239PHE	CD2	1.89	120GLU	CG	124ARG	CZ	0.10	216VA	CA	216VAL	CG2	100.00
216VAL	CG2	239PHE	CE2	0.06	120GLU	CD	120GLU	C	9.75	216VA	CA	216VAL	C	100.00
216VAL	CG2	243LEU	CD1	0.39	120GLU	CD	124ARG	CZ	0.08	216VA	CA	219MET	CB	0.00

216VAL	CG2	243LEU CD2	0.11	120GLU	C	121GLU CA	100.00	216VA CB	216VAL	CG1	100.00
216VAL	C	217ASP CA	100.00	120GLU	C	121GLU CB	0.12	216VA CB	216VAL	CG2	100.00
216VAL	C	217ASP CB	0.00	120GLU	C	121GLU C	99.96	216VA CB	216VAL	C	100.00
216VAL	C	217ASP C	100.00	120GLU	C	123ALA CB	0.00	216VA CB	239PHE	CE1	0.07
217ASP	CA	217ASP CB	100.00	121GLU	CA	121GLU CB	100.00	216VA CB	239PHE	CE2	0.04
217ASP	CA	217ASP CG	100.00	121GLU	CA	121GLU CG	100.00	216VA CG1	216VAL	CG2	100.00
217ASP	CA	217ASP C	100.00	121GLU	CA	121GLU CD	95.85	216VA CG1	216VAL	C	99.94
217ASP	CB	217ASP CG	100.00	121GLU	CA	121GLU C	100.00	216VA CG1	239PHE	CD1	0.06
217ASP	CB	217ASP C	100.00	121GLU	CA	124ARG CZ	0.26	216VA CG1	239PHE	CE1	0.92
217ASP	CG	217ASP C	99.57	121GLU	CB	121GLU CG	100.00	216VA CG1	239PHE	CE2	0.28
217ASP	C	218ALA CA	100.00	121GLU	CB	121GLU CD	100.00	216VA CG1	239PHE	CZ	0.84
217ASP	C	218ALA CB	0.12	121GLU	CB	121GLU C	100.00	216VA CG2	216VAL	C	100.00
217ASP	C	218ALA C	99.88	121GLU	CG	121GLU CD	100.00	216VA CG2	239PHE	CB	0.15
217ASP	C	221MET CE	0.00	121GLU	CG	121GLU C	83.18	216VA CG2	239PHE	CG	1.54
218ALA	CA	218ALA CB	100.00	121GLU	CG	124ARG CZ	0.02	216VA CG2	239PHE	CD1	1.69
218ALA	CA	218ALA C	100.00	121GLU	CD	121GLU C	0.28	216VA CG2	239PHE	CD2	0.29
218ALA	CB	218ALA C	100.00	121GLU	CD	124ARG CD	0.03	216VA CG2	239PHE	CE1	0.03
218ALA	C	219MET CA	100.00	121GLU	CD	124ARG CZ	0.21	216VA CG2	239PHE	CE2	0.02
218ALA	C	219MET CB	0.04	121GLU	C	122ILE CA	100.00	216VA CG2	239PHE	CZ	0.00
218ALA	C	219MET C	99.98	121GLU	C	122ILE CB	4.68	216VA CG2	243LEU	CD1	0.11
218ALA	C	221MET CB	0.00	121GLU	C	122ILE CG1	1.19	216VA C	217ASP	CA	100.00
218ALA	C	222HIS CD2	0.76	121GLU	C	122ILE CG2	1.43	216VA C	217ASP	C	100.00
219MET	CA	219MET CB	100.00	121GLU	C	122ILE CD	0.04	217AS CA	217ASP	CB	100.00
219MET	CA	219MET CG	100.00	121GLU	C	122ILE C	95.62	217AS CA	217ASP	CG	100.00
219MET	CA	219MET CE	0.00	122ILE	CA	122ILE CB	100.00	217AS CA	217ASP	C	100.00
219MET	CA	219MET C	100.00	122ILE	CA	122ILE CG1	100.00	217AS CB	217ASP	CG	100.00
219MET	CA	222HIS CD2	0.00	122ILE	CA	122ILE CG2	100.00	217AS CB	217ASP	C	100.00
219MET	CB	219MET CG	100.00	122ILE	CA	122ILE CD	18.42	217AS CG	217ASP	C	98.00
219MET	CB	219MET CE	1.53	122ILE	CA	122ILE C	100.00	217AS C	218ALA	CA	100.00
219MET	CB	219MET C	100.00	122ILE	CB	122ILE CG1	100.00	217AS C	218ALA	CB	0.24
219MET	CG	219MET CE	100.00	122ILE	CB	122ILE CG2	100.00	217AS C	218ALA	C	99.80
219MET	CG	219MET C	99.91	122ILE	CB	122ILE CD	100.00	217AS C	221MET	CE	0.01
219MET	CG	223LEU CD1	0.01	122ILE	CB	122ILE C	100.00	218AL CA	218ALA	CB	100.00
219MET	CG	230PHE CE1	2.43	122ILE	CG1	122ILE CG2	100.00	218AL CA	218ALA	C	100.00
219MET	CG	230PHE CE2	0.04	122ILE	CG1	122ILE CD	100.00	218AL CA	221MET	CB	0.01
219MET	CG	230PHE CZ	3.30	122ILE	CG1	122ILE C	7.83	218AL CA	221MET	CE	0.02
219MET	CG	243LEU CD1	0.01	122ILE	CG2	122ILE CD	97.22	218AL CB	218ALA	C	100.00
219MET	CE	219MET C	0.29	122ILE	CG2	122ILE C	92.12	218AL C	219MET	CA	100.00
219MET	CE	223LEU CG	0.02	122ILE	CG2	225ARG C	0.01	218AL C	219MET	CB	0.02
219MET	CE	223LEU CD1	0.90	122ILE	CG2	226SER CA	0.00	218AL C	219MET	C	99.98
219MET	CE	223LEU CD2	0.08	122ILE	CG2	226SER CB	0.01	218AL C	221MET	CG	0.00
219MET	CE	230PHE CG	0.51	122ILE	CG2	227PRO CG	0.02	218AL C	222HIS	CD2	0.32
219MET	CE	230PHE CD1	4.40	122ILE	CG2	227PRO CD	2.53	219ME CA	219MET	CB	100.00
219MET	CE	230PHE CD2	0.65	122ILE	CD	122ILE C	0.15	219ME CA	219MET	CG	100.00
219MET	CE	230PHE CE1	7.85	122ILE	CD	225ARG C	0.02	219ME CA	219MET	C	100.00
219MET	CE	230PHE CE2	1.55	122ILE	C	123ALA CA	100.00	219ME CA	222HIS	CB	0.00
219MET	CE	230PHE CZ	2.52	122ILE	C	123ALA CB	0.42	219ME CA	222HIS	CD2	0.01
219MET	CE	233VAL CG1	3.18	122ILE	C	123ALA C	99.54	219ME CB	219MET	CG	100.00
219MET	CE	233VAL CG2	5.17	123ALA	CA	123ALA CB	100.00	219ME CB	219MET	CE	9.20
219MET	CE	243LEU CD1	0.06	123ALA	CA	123ALA C	100.00	219ME CB	219MET	C	100.00
219MET	CE	243LEU CD2	0.07	123ALA	CA	126VAL CG2	0.04	219ME CG	219MET	CE	100.00
219MET	C	220ALA CA	100.00	123ALA	CB	123ALA C	100.00	219ME CG	219MET	C	99.34
219MET	C	220ALA C	100.00	123ALA	C	124ARG CA	100.00	219ME CG	223LEU	CD1	0.01
220ALA	CA	220ALA CB	100.00	123ALA	C	124ARG CB	2.45	219ME CG	230PHE	CE1	3.59
220ALA	CA	220ALA C	100.00	123ALA	C	124ARG CG	0.04	219ME CG	230PHE	CE2	0.22
220ALA	CA	223LEU CB	0.00	123ALA	C	124ARG C	98.52	219ME CG	230PHE	CZ	8.33
220ALA	CA	243LEU CD2	0.01	123ALA	C	126VAL CG2	1.96	219ME CG	243LEU	CD1	0.00
220ALA	CA	246LEU CD2	0.00	124ARG	CA	124ARG CB	100.00	219ME CG	243LEU	CD2	0.02
220ALA	CB	220ALA C	100.00	124ARG	CA	124ARG CG	100.00	219ME CE	219MET	C	0.46
220ALA	CB	243LEU CD1	0.22	124ARG	CA	124ARG CD	0.18	219ME CE	223LEU	CG	0.01
220ALA	CB	243LEU CD2	0.07	124ARG	CA	124ARG C	100.00	219ME CE	223LEU	CD1	0.47
220ALA	CB	246LEU CD1	0.01	124ARG	CB	124ARG CG	100.00	219ME CE	223LEU	CD2	0.07

220ALA CB	246LEU CD2	0.22	124AR(CB	124ARG	CD	100.00	219ME CE	230PHE	CG	0.30
220ALA C	221MET CA	100.00	124AR(CB	124ARG	CZ	7.21	219ME CE	230PHE	CD1	2.45
220ALA C	221MET CB	0.03	124AR(CB	124ARG	C	100.00	219ME CE	230PHE	CD2	0.89
220ALA C	221MET C	99.98	124AR(CG	124ARG	CD	100.00	219ME CE	230PHE	CE1	5.12
221MET CA	221MET CB	100.00	124AR(CG	124ARG	CZ	28.05	219ME CE	230PHE	CE2	2.21
221MET CA	221MET CG	100.00	124AR(CG	124ARG	C	80.88	219ME CE	230PHE	CZ	1.86
221MET CA	221MET C	100.00	124AR(CD	124ARG	CZ	100.00	219ME CE	233VAL	CG1	3.01
221MET CB	221MET CG	100.00	124AR(C	125GLY	CA	100.00	219ME CE	233VAL	CG2	3.74
221MET CB	221MET CE	23.50	124AR(C	125GLY	C	48.17	219ME CE	243LEU	CD1	0.23
221MET CB	221MET C	100.00	125GL\ CA	125GLY	C	100.00	219ME CE	243LEU	CD2	0.32
221MET CB	225ARG CZ	0.13	125GL\ CA	228ALA	CB	0.04	219ME C	220ALA	CA	100.00
221MET CG	221MET CE	100.00	125GL\ C	126VAL	CA	100.00	219ME C	220ALA	C	100.00
221MET CG	221MET C	90.15	125GL\ C	126VAL	CB	2.61	220AL. CA	220ALA	CB	100.00
221MET CG	225ARG CZ	0.02	125GL\ C	126VAL	CG2	1.06	220AL. CA	220ALA	C	100.00
221MET CE	221MET C	0.02	125GL\ C	126VAL	C	98.90	220AL. CA	223LEU	CD1	0.01
221MET CE	222HIS CD2	0.01	125GL\ C	228ALA	CB	4.22	220AL. CA	243LEU	CD2	0.00
221MET CE	222HIS CE1	0.13	126VAI CA	126VAL	CB	100.00	220AL. CA	246LEU	CD2	0.03
221MET CE	225ARG CD	0.02	126VAI CA	126VAL	CG1	100.00	220AL. CB	220ALA	C	100.00
221MET CE	225ARG CZ	0.49	126VAI CA	126VAL	CG2	100.00	220AL. CB	243LEU	CG	0.00
221MET C	222HIS CA	100.00	126VAI CA	126VAL	C	100.00	220AL. CB	243LEU	CD1	0.07
221MET C	222HIS CB	1.06	126VAI CA	228ALA	CB	0.01	220AL. CB	243LEU	CD2	0.12
221MET C	222HIS C	98.68	126VAI CB	126VAL	CG1	100.00	220AL. CB	246LEU	CD1	0.26
221MET C	224VAL CG2	0.01	126VAI CB	126VAL	CG2	100.00	220AL. CB	246LEU	CD2	0.75
222HIS CA	222HIS CB	100.00	126VAI CB	126VAL	C	100.00	220AL. C	221MET	CA	100.00
222HIS CA	222HIS CG	100.00	126VAI CG1	126VAL	CG2	100.00	220AL. C	221MET	CB	0.01
222HIS CA	222HIS CD2	31.50	126VAI CG1	126VAL	C	100.00	220AL. C	221MET	C	100.00
222HIS CA	222HIS C	100.00	126VAI CG1	128VAL	CG1	0.02	221ME CA	221MET	CB	100.00
222HIS CA	225ARG CG	0.01	126VAI CG1	128VAL	CG2	0.47	221ME CA	221MET	CG	100.00
222HIS CA	225ARG CZ	0.00	126VAI CG1	227PRO	CB	0.38	221ME CA	221MET	C	100.00
222HIS CB	222HIS CG	100.00	126VAI CG1	227PRO	CG	0.29	221ME CB	221MET	CG	100.00
222HIS CB	222HIS CD2	100.00	126VAI CG1	250LEU	CD1	2.92	221ME CB	221MET	CE	71.12
222HIS CB	222HIS CE1	0.20	126VAI CG1	250LEU	CD2	2.07	221ME CB	221MET	C	100.00
222HIS CB	222HIS C	100.00	126VAI CG2	227PRO	CB	0.41	221ME CG	221MET	CE	100.00
222HIS CB	230PHE CE2	0.07	126VAI CG2	227PRO	CG	1.84	221ME CG	221MET	C	83.55
222HIS CB	230PHE CZ	0.30	126VAI CG2	227PRO	C	0.00	221ME CG	225ARG	CD	0.00
222HIS CG	222HIS CD2	100.00	126VAI CG2	228ALA	CA	0.02	221ME CE	221MET	C	0.02
222HIS CG	222HIS CE1	100.00	126VAI CG2	228ALA	CB	0.31	221ME CE	222HIS	CD2	0.01
222HIS CG	222HIS C	92.41	126VAI C	127ASP	CA	100.00	221ME CE	222HIS	CE1	0.07
222HIS CG	230PHE CE2	0.04	126VAI C	127ASP	CB	99.97	221ME C	222HIS	CA	100.00
222HIS CG	230PHE CZ	0.06	126VAI C	127ASP	C	0.58	221ME C	222HIS	CB	1.12
222HIS CD2	222HIS CE1	100.00	127ASI CA	127ASP	CB	100.00	221ME C	222HIS	C	98.77
222HIS CD2	222HIS C	15.55	127ASI CA	127ASP	CG	100.00	221ME C	224VAL	CG2	0.02
222HIS CD2	225ARG CZ	0.34	127ASI CA	127ASP	C	100.00	222HIS CA	222HIS	CB	100.00
222HIS CD2	226SER CB	0.10	127ASI CB	127ASP	CG	100.00	222HIS CA	222HIS	CG	100.00
222HIS CD2	229ARG CB	0.51	127ASI CB	127ASP	C	100.00	222HIS CA	222HIS	CD2	23.12
222HIS CD2	229ARG CG	0.85	127ASI CG	127ASP	C	99.98	222HIS CA	222HIS	C	100.00
222HIS CD2	229ARG CD	1.26	127ASI CG	176ARG	CZ	0.01	222HIS CA	225ARG	CB	0.00
222HIS CD2	230PHE CE2	1.78	127ASI C	128VAL	CA	100.00	222HIS CA	225ARG	CG	0.01
222HIS CD2	230PHE CZ	1.49	127ASI C	128VAL	CB	38.56	222HIS CA	225ARG	CZ	0.00
222HIS CE1	225ARG CB	0.02	127ASI C	128VAL	CG1	1.18	222HIS CB	222HIS	CG	100.00
222HIS CE1	225ARG CG	0.00	127ASI C	128VAL	CG2	6.41	222HIS CB	222HIS	CD2	100.00
222HIS CE1	225ARG CD	0.01	127ASI C	128VAL	C	66.17	222HIS CB	222HIS	CE1	0.21
222HIS CE1	225ARG CZ	0.02	128VAI CA	128VAL	CB	100.00	222HIS CB	222HIS	C	100.00
222HIS CE1	226SER CB	1.33	128VAI CA	128VAL	CG1	100.00	222HIS CB	230PHE	CE2	0.10
222HIS CE1	229ARG CB	0.10	128VAI CA	128VAL	CG2	100.00	222HIS CB	230PHE	CZ	0.22
222HIS CE1	229ARG CG	0.36	128VAI CA	128VAL	C	100.00	222HIS CG	222HIS	CD2	100.00
222HIS CE1	229ARG CD	1.10	128VAI CB	128VAL	CG1	100.00	222HIS CG	222HIS	CE1	100.00
222HIS CE1	229ARG CZ	0.11	128VAI CB	128VAL	CG2	100.00	222HIS CG	222HIS	C	95.35
222HIS CE1	230PHE CZ	0.00	128VAI CB	128VAL	C	100.00	222HIS CG	230PHE	CE2	0.34
222HIS C	223LEU CA	100.00	128VAI CB	130ILE	CD	0.00	222HIS CG	230PHE	CZ	0.67
222HIS C	223LEU C	100.00	128VAI CG1	128VAL	CG2	100.00	222HIS CD2	222HIS	CE1	100.00
222HIS C	225ARG CB	0.00	128VAI CG1	128VAL	C	95.92	222HIS CD2	222HIS	C	7.56

223LEU	CA	223LEU	CB	100.00	128VAl	CG1	130ILE	CG1	0.44	222HIS	CD2	225ARG	CZ	0.00
223LEU	CA	223LEU	CG	100.00	128VAl	CG1	130ILE	CD	0.89	222HIS	CD2	226SER	CB	0.09
223LEU	CA	223LEU	CD1	77.85	128VAl	CG1	230PHE	CB	0.46	222HIS	CD2	226SER	C	0.02
223LEU	CA	223LEU	CD2	19.06	128VAl	CG1	230PHE	C	0.04	222HIS	CD2	229ARG	CB	0.02
223LEU	CA	223LEU	C	100.00	128VAl	CG1	231ASP	CA	0.00	222HIS	CD2	229ARG	CG	0.05
223LEU	CA	230PHE	CE2	0.17	128VAl	CG1	231ASP	C	0.00	222HIS	CD2	229ARG	CD	0.25
223LEU	CB	223LEU	CG	100.00	128VAl	CG1	233VAL	CG2	2.00	222HIS	CD2	230PHE	CE1	0.00
223LEU	CB	223LEU	CD1	100.00	128VAl	CG1	250LEU	CD1	0.02	222HIS	CD2	230PHE	CE2	3.48
223LEU	CB	223LEU	CD2	100.00	128VAl	CG1	250LEU	CD2	0.03	222HIS	CD2	230PHE	CZ	6.22
223LEU	CB	223LEU	C	100.00	128VAl	CG2	128VAL	C	38.92	222HIS	CE1	225ARG	CB	0.00
223LEU	CB	250LEU	CD1	0.00	128VAl	CG2	130ILE	CG1	0.01	222HIS	CE1	225ARG	CG	0.01
223LEU	CG	223LEU	CD1	100.00	128VAl	CG2	130ILE	CD	0.05	222HIS	CE1	225ARG	CD	0.03
223LEU	CG	223LEU	CD2	100.00	128VAl	CG2	227PRO	CB	0.01	222HIS	CE1	225ARG	CZ	0.18
223LEU	CG	223LEU	C	83.63	128VAl	CG2	230PHE	CB	2.08	222HIS	CE1	226SER	CB	1.75
223LEU	CG	230PHE	CE2	0.02	128VAl	CG2	230PHE	C	0.20	222HIS	CE1	226SER	C	0.00
223LEU	CG	250LEU	CD1	0.07	128VAl	CG2	231ASP	C	0.02	222HIS	CE1	229ARG	CB	0.18
223LEU	CD1	223LEU	CD2	100.00	128VAl	CG2	233VAL	CG2	2.42	222HIS	CE1	229ARG	CG	0.36
223LEU	CD1	223LEU	C	1.44	128VAl	CG2	250LEU	CD2	0.01	222HIS	CE1	229ARG	CD	1.14
223LEU	CD1	227PRO	CB	0.05	128VAl	C	129LEU	CA	100.00	222HIS	CE1	230PHE	CE1	0.01
223LEU	CD1	230PHE	CG	0.00	128VAl	C	129LEU	CB	39.35	222HIS	CE1	230PHE	CE2	0.16
223LEU	CD1	230PHE	CD2	4.02	128VAl	C	129LEU	C	72.47	222HIS	CE1	230PHE	CZ	0.33
223LEU	CD1	230PHE	CE2	0.45	129LEl	CA	129LEU	CB	100.00	222HIS	C	223LEU	CA	100.00
223LEU	CD1	243LEU	CD1	0.23	129LEl	CA	129LEU	CG	100.00	222HIS	C	223LEU	CB	0.00
223LEU	CD1	243LEU	CD2	1.48	129LEl	CA	129LEU	CD1	91.62	222HIS	C	223LEU	C	100.00
223LEU	CD1	246LEU	CD2	0.10	129LEl	CA	129LEU	CD2	8.62	222HIS	C	225ARG	CB	0.00
223LEU	CD1	250LEU	CD1	0.19	129LEl	CA	129LEU	C	100.00	222HIS	C	230PHE	CE2	0.00
223LEU	CD1	250LEU	CD2	0.01	129LEl	CB	129LEU	CG	100.00	223LEl	CA	223LEU	CB	100.00
223LEU	CD2	223LEU	C	3.17	129LEl	CB	129LEU	CD1	100.00	223LEl	CA	223LEU	CG	100.00
223LEU	CD2	224VAL	CG1	0.02	129LEl	CB	129LEU	CD2	100.00	223LEl	CA	223LEU	CD1	57.01
223LEU	CD2	227PRO	CB	0.07	129LEl	CB	129LEU	C	100.00	223LEl	CA	223LEU	CD2	38.29
223LEU	CD2	230PHE	CD2	0.95	129LEl	CB	176ARG	CZ	0.94	223LEl	CA	223LEU	C	100.00
223LEU	CD2	230PHE	CE2	0.68	129LEl	CG	129LEU	CD1	100.00	223LEl	CA	230PHE	CE2	0.11
223LEU	CD2	243LEU	CD1	0.88	129LEl	CG	129LEU	CD2	100.00	223LEl	CB	223LEU	CG	100.00
223LEU	CD2	243LEU	CD2	0.22	129LEl	CG	129LEU	C	96.18	223LEl	CB	223LEU	CD1	100.00
223LEU	CD2	246LEU	CB	0.00	129LEl	CG	131VAL	CG2	0.05	223LEl	CB	223LEU	CD2	100.00
223LEU	CD2	246LEU	CG	0.05	129LEl	CG	176ARG	CZ	0.20	223LEl	CB	223LEU	C	100.00
223LEU	CD2	246LEU	CD1	0.02	129LEl	CD1	129LEU	CD2	100.00	223LEl	CB	230PHE	CE2	0.01
223LEU	CD2	246LEU	CD2	1.02	129LEl	CD1	129LEU	C	5.53	223LEl	CB	246LEU	CD2	0.05
223LEU	CD2	247ALA	CA	0.00	129LEl	CD1	130ILE	C	0.01	223LEl	CG	223LEU	CD1	100.00
223LEU	CD2	247ALA	CB	0.08	129LEl	CD1	131VAL	CG2	0.51	223LEl	CG	223LEU	CD2	100.00
223LEU	CD2	249VAL	CG2	0.01	129LEl	CD1	172ALA	CB	0.12	223LEl	CG	223LEU	C	64.27
223LEU	CD2	250LEU	CD1	0.12	129LEl	CD1	176ARG	CD	0.07	223LEl	CG	230PHE	CD2	0.01
223LEU	CD2	250LEU	CD2	0.01	129LEl	CD1	176ARG	CZ	0.09	223LEl	CG	230PHE	CE2	0.04
223LEU	C	224VAL	CA	100.00	129LEl	CD1	296MET	CE	0.01	223LEl	CG	230PHE	CZ	0.00
223LEU	C	224VAL	CB	14.12	129LEl	CD2	129LEU	C	2.78	223LEl	CD1	223LEU	CD2	100.00
223LEU	C	224VAL	CG1	5.18	129LEl	CD2	130ILE	C	0.01	223LEl	CD1	223LEU	C	0.47
223LEU	C	224VAL	CG2	1.14	129LEl	CD2	131VAL	CG2	1.24	223LEl	CD1	227PRO	CB	0.09
223LEU	C	224VAL	C	89.40	129LEl	CD2	172ALA	CB	8.28	223LEl	CD1	230PHE	CG	0.05
224VAL	CA	224VAL	CB	100.00	129LEl	CD2	176ARG	CD	0.02	223LEl	CD1	230PHE	CD2	3.26
224VAL	CA	224VAL	CG1	100.00	129LEl	CD2	176ARG	CZ	0.04	223LEl	CD1	230PHE	CE2	0.46
224VAL	CA	224VAL	CG2	100.00	129LEl	CD2	232VAL	CG1	0.00	223LEl	CD1	230PHE	CZ	0.01
224VAL	CA	224VAL	C	100.00	129LEl	CD2	232VAL	CG2	0.01	223LEl	CD1	243LEU	CG	0.03
224VAL	CB	224VAL	CG1	100.00	129LEl	C	130ILE	CA	100.00	223LEl	CD1	243LEU	CD1	0.87
224VAL	CB	224VAL	CG2	100.00	129LEl	C	130ILE	CB	10.62	223LEl	CD1	243LEU	CD2	0.58
224VAL	CB	224VAL	C	100.00	129LEl	C	130ILE	CG1	5.97	223LEl	CD1	246LEU	CB	0.01
224VAL	CG1	224VAL	CG2	100.00	129LEl	C	130ILE	CD	0.06	223LEl	CD1	246LEU	CG	0.02
224VAL	CG1	224VAL	C	27.51	129LEl	C	130ILE	C	97.39	223LEl	CD1	246LEU	CD1	0.01
224VAL	CG1	246LEU	CD1	0.09	130ILE	CA	130ILE	CB	100.00	223LEl	CD1	246LEU	CD2	0.39
224VAL	CG1	246LEU	CD2	0.14	130ILE	CA	130ILE	CG1	100.00	223LEl	CD1	250LEU	CD1	0.02
224VAL	CG1	249VAL	CG2	0.00	130ILE	CA	130ILE	CG2	100.00	223LEl	CD2	223LEU	C	3.74
224VAL	CG1	250LEU	CD2	0.05	130ILE	CA	130ILE	CD	19.47	223LEl	CD2	224VAL	CG1	0.02
224VAL	CG2	224VAL	C	72.17	130ILE	CA	130ILE	C	100.00	223LEl	CD2	227PRO	CB	0.10

224VAL	CG2	246LEU	CD1	0.00
224VAL	CG2	246LEU	CD2	0.01
224VAL	C	225ARG	CA	100.00
224VAL	C	225ARG	CB	32.92
224VAL	C	225ARG	CG	6.95
224VAL	C	225ARG	C	64.65
225ARG	CA	225ARG	CB	100.00
225ARG	CA	225ARG	CG	100.00
225ARG	CA	225ARG	CD	5.90
225ARG	CA	225ARG	C	100.00
225ARG	CB	225ARG	CG	100.00
225ARG	CB	225ARG	CD	100.00
225ARG	CB	225ARG	CZ	0.75
225ARG	CB	225ARG	C	100.00
225ARG	CG	225ARG	CD	100.00
225ARG	CG	225ARG	CZ	10.16
225ARG	CG	225ARG	C	42.09
225ARG	CD	225ARG	CZ	100.00
225ARG	CD	225ARG	C	0.06
225ARG	C	226SER	CA	100.00
225ARG	C	226SER	CB	99.89
225ARG	C	226SER	C	0.46
225ARG	C	227PRO	CD	0.90
226SER	CA	226SER	CB	100.00
226SER	CA	226SER	C	100.00
226SER	CA	227PRO	CD	100.00
226SER	CB	226SER	C	100.00
226SER	CB	229ARG	CB	0.00
226SER	C	227PRO	CA	100.00
226SER	C	227PRO	CB	0.02
226SER	C	227PRO	CD	100.00
226SER	C	227PRO	C	100.00
227PRO	CA	227PRO	CB	100.00
227PRO	CA	227PRO	CG	100.00
227PRO	CA	227PRO	CD	100.00
227PRO	CA	227PRO	C	100.00
227PRO	CA	230PHE	CE2	0.00
227PRO	CB	227PRO	CG	100.00
227PRO	CB	227PRO	CD	100.00
227PRO	CB	227PRO	C	100.00
227PRO	CB	250LEU	CD1	1.03
227PRO	CB	250LEU	CD2	5.02
227PRO	CG	227PRO	CD	100.00
227PRO	CG	227PRO	C	85.05
227PRO	CG	250LEU	CD1	0.10
227PRO	CG	250LEU	CD2	3.45
227PRO	CD	227PRO	C	58.38
227PRO	C	228ALA	CA	100.00
227PRO	C	228ALA	CB	0.03
227PRO	C	228ALA	C	99.99
228ALA	CA	228ALA	CB	100.00
228ALA	CA	228ALA	C	100.00
228ALA	CB	228ALA	C	100.00
228ALA	C	229ARG	CA	100.00
228ALA	C	229ARG	CB	1.14
228ALA	C	229ARG	C	98.81
229ARG	CA	229ARG	CB	100.00
229ARG	CA	229ARG	CG	100.00
229ARG	CA	229ARG	CD	44.90
229ARG	CA	229ARG	C	100.00
229ARG	CB	229ARG	CG	100.00
229ARG	CB	229ARG	CD	100.00

130ILE	CA	233VAL	CG2	0.01
130ILE	CB	130ILE	CG1	100.00
130ILE	CB	130ILE	CG2	100.00
130ILE	CB	130ILE	CD	100.00
130ILE	CB	130ILE	C	100.00
130ILE	CG1	130ILE	CG2	100.00
130ILE	CG1	130ILE	CD	100.00
130ILE	CG1	233VAL	CB	0.01
130ILE	CG1	233VAL	CG1	0.01
130ILE	CG1	233VAL	CG2	0.10
130ILE	CG2	130ILE	CD	83.04
130ILE	CG2	130ILE	C	100.00
130ILE	CG2	131VAL	C	0.01
130ILE	CG2	132ARG	CG	0.00
130ILE	CG2	233VAL	CG1	0.01
130ILE	CG2	235THR	CG2	10.22
130ILE	CG2	240GLY	CA	0.01
130ILE	CG2	243LEU	CB	0.00
130ILE	CG2	243LEU	CD2	0.01
130ILE	CG2	244SER	CB	0.00
130ILE	CD	233VAL	CB	0.10
130ILE	CD	233VAL	CG1	0.11
130ILE	CD	233VAL	CG2	0.17
130ILE	CD	243LEU	CB	0.04
130ILE	CD	243LEU	CG	0.00
130ILE	CD	243LEU	CD1	0.01
130ILE	CD	243LEU	CD2	0.05
130ILE	CD	243LEU	C	0.35
130ILE	CD	244SER	CA	0.32
130ILE	CD	244SER	CB	0.01
130ILE	CD	247ALA	CB	5.41
130ILE	C	131VAL	CA	100.00
130ILE	C	131VAL	CB	5.36
130ILE	C	131VAL	CG2	3.25
130ILE	C	131VAL	C	98.33
131VAL	CA	131VAL	CB	100.00
131VAL	CA	131VAL	CG1	100.00
131VAL	CA	131VAL	CG2	100.00
131VAL	CA	131VAL	C	100.00
131VAL	CB	131VAL	CG1	100.00
131VAL	CB	131VAL	CG2	100.00
131VAL	CB	131VAL	C	100.00
131VAL	CB	234VAL	CG2	0.03
131VAL	CG1	131VAL	CG2	100.00
131VAL	CG1	131VAL	C	100.00
131VAL	CG1	165VAL	CG1	0.67
131VAL	CG1	165VAL	CG2	0.40
131VAL	CG1	168VAL	CG1	1.01
131VAL	CG1	168VAL	CG2	0.23
131VAL	CG1	234VAL	CG1	0.00
131VAL	CG1	234VAL	CG2	0.44
131VAL	CG2	168VAL	CG1	0.02
131VAL	CG2	169ALA	CA	0.00
131VAL	CG2	169ALA	CB	0.13
131VAL	CG2	232VAL	CG1	0.10
131VAL	CG2	232VAL	CG2	0.01
131VAL	C	132ARG	CA	100.00
131VAL	C	132ARG	CB	14.68
131VAL	C	132ARG	CG	0.02
131VAL	C	132ARG	C	91.30
132AR	CA	132ARG	CB	100.00
132AR	CA	132ARG	CG	100.00

223LE	CD2	230PHE	CG	0.01
223LE	CD2	230PHE	CD2	2.65
223LE	CD2	230PHE	CE2	0.77
223LE	CD2	243LEU	CG	0.00
223LE	CD2	243LEU	CD1	0.44
223LE	CD2	243LEU	CD2	0.13
223LE	CD2	246LEU	CB	0.05
223LE	CD2	246LEU	CG	0.08
223LE	CD2	246LEU	CD1	0.02
223LE	CD2	246LEU	CD2	0.82
223LE	CD2	246LEU	C	0.02
223LE	CD2	247ALA	CA	0.02
223LE	CD2	247ALA	CB	0.13
223LE	CD2	249VAL	CG1	0.02
223LE	CD2	249VAL	CG2	0.03
223LE	CD2	250LEU	CD1	0.06
223LE	CD2	250LEU	CD2	0.00
223LE	C	224VAL	CA	100.00
223LE	C	224VAL	CB	32.60
223LE	C	224VAL	CG1	15.80
223LE	C	224VAL	CG2	1.34
223LE	C	224VAL	C	73.91
224VA	CA	224VAL	CB	100.00
224VA	CA	224VAL	CG1	100.00
224VA	CA	224VAL	CG2	100.00
224VA	CA	224VAL	C	100.00
224VA	CB	224VAL	CG1	100.00
224VA	CB	224VAL	CG2	100.00
224VA	CB	224VAL	C	100.00
224VA	CG1	224VAL	CG2	100.00
224VA	CG1	224VAL	C	8.16
224VA	CG1	246LEU	CG	0.00
224VA	CG1	246LEU	CD1	0.14
224VA	CG1	246LEU	CD2	0.47
224VA	CG1	249VAL	CG1	0.41
224VA	CG1	249VAL	CG2	0.04
224VA	CG2	224VAL	C	91.57
224VA	CG2	246LEU	CD2	0.02
224VA	CG2	249VAL	CG1	0.00
224VA	C	225ARG	CA	100.00
224VA	C	225ARG	CB	63.59
224VA	C	225ARG	CG	12.49
224VA	C	225ARG	CD	0.00
224VA	C	225ARG	C	33.82
225AR	CA	225ARG	CB	100.00
225AR	CA	225ARG	CG	100.00
225AR	CA	225ARG	CD	0.68
225AR	CA	225ARG	C	100.00
225AR	CB	225ARG	CG	100.00
225AR	CB	225ARG	CD	100.00
225AR	CB	225ARG	CZ	1.25
225AR	CB	225ARG	C	100.00
225AR	CG	225ARG	CD	100.00
225AR	CG	225ARG	CZ	13.93
225AR	CG	225ARG	C	48.22
225AR	CD	225ARG	CZ	100.00
225AR	CD	225ARG	C	0.06
225AR	C	226SER	CA	100.00
225AR	C	226SER	CB	99.93
225AR	C	226SER	C	0.23
225AR	C	227PRO	CD	1.85
226SE	CA	226SER	CB	100.00

229ARG CB	229ARG C	100.00	132AR(CA	132ARG CD	5.81	226SE CA	226SER C	100.00
229ARG CB	230PHE CD2	0.08	132AR(CA	132ARG C	100.00	226SE CA	227PRO CD	100.00
229ARG CB	230PHE CE2	0.06	132AR(CA	235THR CG2	0.01	226SE CB	226SER C	100.00
229ARG CG	229ARG CD	100.00	132AR(CB	132ARG CG	100.00	226SE C	227PRO CA	100.00
229ARG CG	229ARG CZ	20.23	132AR(CB	132ARG CD	100.00	226SE C	227PRO CB	0.04
229ARG CG	229ARG C	99.90	132AR(CB	132ARG CZ	0.02	226SE C	227PRO CD	100.00
229ARG CG	230PHE CD1	0.07	132AR(CB	132ARG C	100.00	226SE C	227PRO C	100.00
229ARG CG	230PHE CD2	0.12	132AR(CG	132ARG CD	100.00	227PR CA	227PRO CB	100.00
229ARG CG	230PHE CE1	0.67	132AR(CG	132ARG CZ	21.18	227PR CA	227PRO CG	100.00
229ARG CG	230PHE CE2	0.54	132AR(CG	132ARG C	96.17	227PR CA	227PRO CD	100.00
229ARG CG	230PHE CZ	0.60	132AR(CG	133GLU C	0.01	227PR CA	227PRO C	100.00
229ARG CD	229ARG CZ	100.00	132AR(CG	235THR CG2	0.00	227PR CA	230PHE CD2	0.16
229ARG CD	229ARG C	49.27	132AR(CG	236GLY C	0.01	227PR CA	230PHE CE2	0.01
229ARG CD	230PHE CG	0.02	132AR(CG	240GLY CA	0.06	227PR CB	227PRO CG	100.00
229ARG CD	230PHE CD1	2.16	132AR(CG	240GLY C	0.79	227PR CB	227PRO CD	100.00
229ARG CD	230PHE CD2	0.03	132AR(CD	132ARG CZ	100.00	227PR CB	227PRO C	100.00
229ARG CD	230PHE CE1	8.19	132AR(CD	132ARG C	12.49	227PR CB	230PHE CD2	0.08
229ARG CD	230PHE CE2	0.29	132AR(CD	133GLU C	0.02	227PR CB	230PHE CE2	0.00
229ARG CD	230PHE CZ	2.35	132AR(CD	134LEU CD1	0.23	227PR CB	250LEU CD1	3.86
229ARG CZ	230PHE CD1	0.76	132AR(CD	134LEU CD2	0.04	227PR CB	250LEU CD2	3.65
229ARG CZ	230PHE CE1	4.22	132AR(CD	240GLY C	0.44	227PR CG	227PRO CD	100.00
229ARG CZ	230PHE CZ	0.01	132AR(CD	241ASP CA	0.02	227PR CG	227PRO C	91.40
229ARG C	230PHE CA	100.00	132AR(CD	241ASP CB	0.00	227PR CG	250LEU CG	0.02
229ARG C	230PHE CB	6.54	132AR(CD	244SER CB	0.00	227PR CG	250LEU CD1	2.19
229ARG C	230PHE CG	1.26	132AR(CZ	134LEU CG	0.02	227PR CG	250LEU CD2	5.89
229ARG C	230PHE CD1	0.03	132AR(CZ	134LEU CD1	8.79	227PR CD	227PRO C	64.14
229ARG C	230PHE CD2	0.04	132AR(CZ	134LEU CD2	0.24	227PR C	228ALA CA	100.00
229ARG C	230PHE C	94.56	132AR(CZ	240GLY C	0.00	227PR C	228ALA CB	0.00
230PHE CA	230PHE CB	100.00	132AR(CZ	241ASP CA	0.09	227PR C	228ALA C	100.00
230PHE CA	230PHE CG	100.00	132AR(CZ	241ASP CB	2.74	228AL CA	228ALA CB	100.00
230PHE CA	230PHE CD1	99.16	132AR(CZ	241ASP CG	0.33	228AL CA	228ALA C	100.00
230PHE CA	230PHE CD2	39.20	132AR(C	133GLU CA	100.00	228AL CB	228ALA C	100.00
230PHE CA	230PHE C	100.00	132AR(C	133GLU CB	0.24	228AL C	229ARG CA	100.00
230PHE CB	230PHE CG	100.00	132AR(C	133GLU C	99.93	228AL C	229ARG CB	0.51
230PHE CB	230PHE CD1	100.00	133GL(CA	133GLU CB	100.00	228AL C	229ARG C	99.54
230PHE CB	230PHE CD2	100.00	133GL(CA	133GLU CG	100.00	229AR CA	229ARG CB	100.00
230PHE CB	230PHE C	100.00	133GL(CA	133GLU CD	48.91	229AR CA	229ARG CG	100.00
230PHE CB	233VAL CG1	0.02	133GL(CA	133GLU C	100.00	229AR CA	229ARG CD	23.52
230PHE CB	233VAL CG2	0.38	133GL(CA	165VAL CG1	0.24	229AR CA	229ARG C	100.00
230PHE CG	230PHE CD1	100.00	133GL(CA	165VAL CG2	0.02	229AR CB	229ARG CG	100.00
230PHE CG	230PHE CD2	100.00	133GL(CB	133GLU CG	100.00	229AR CB	229ARG CD	100.00
230PHE CG	230PHE CE1	100.00	133GL(CB	133GLU CD	100.00	229AR CB	229ARG CZ	0.00
230PHE CG	230PHE CE2	100.00	133GL(CB	133GLU C	100.00	229AR CB	229ARG C	100.00
230PHE CG	230PHE CZ	100.00	133GL(CB	165VAL CG1	0.05	229AR CG	229ARG CD	100.00
230PHE CG	233VAL CG1	0.00	133GL(CB	165VAL CG2	0.03	229AR CG	229ARG CZ	14.63
230PHE CG	233VAL CG2	0.01	133GL(CB	236GLY CA	1.13	229AR CG	229ARG C	99.96
230PHE CD1	230PHE CD2	100.00	133GL(CB	236GLY C	0.91	229AR CG	230PHE CD1	0.05
230PHE CD1	230PHE CE1	100.00	133GL(CG	133GLU CD	100.00	229AR CG	230PHE CD2	0.03
230PHE CD1	230PHE CE2	100.00	133GL(CG	133GLU C	97.84	229AR CG	230PHE CE1	0.03
230PHE CD1	230PHE CZ	100.00	133GL(CG	135THR CG2	0.05	229AR CG	230PHE CE2	0.05
230PHE CD1	233VAL CG1	0.94	133GL(CG	165VAL CB	0.02	229AR CG	230PHE CZ	0.01
230PHE CD1	233VAL CG2	0.58	133GL(CG	165VAL CG1	0.44	229AR CD	229ARG CZ	100.00
230PHE CD2	230PHE CE1	100.00	133GL(CG	165VAL CG2	1.16	229AR CD	229ARG C	38.13
230PHE CD2	230PHE CE2	100.00	133GL(CG	195TRP CZ3	0.01	229AR CD	230PHE CD1	0.30
230PHE CD2	230PHE CZ	100.00	133GL(CD	133GLU C	59.14	229AR CD	230PHE CD2	0.01
230PHE CE1	230PHE CE2	100.00	133GL(CD	135THR CG2	0.10	229AR CD	230PHE CE1	2.24
230PHE CE1	230PHE CZ	100.00	133GL(CD	136GLY CA	0.03	229AR CD	230PHE CE2	0.09
230PHE CE2	230PHE CZ	100.00	133GL(CD	157TYR CE1	0.18	229AR CD	230PHE CZ	0.63
230PHE C	231ASP CA	100.00	133GL(CD	157TYR CE2	0.12	229AR CZ	230PHE CD1	0.74
230PHE C	231ASP CB	85.59	133GL(CD	165VAL CG2	0.01	229AR CZ	230PHE CE1	3.75
230PHE C	231ASP C	19.54	133GL(CD	237ASN CB	0.18	229AR CZ	230PHE CZ	0.01
231ASP CA	231ASP CB	100.00	133GL(C	134LEU CA	100.00	229AR C	230PHE CA	100.00

231ASP CA	231ASP CG	100.00	133GLU C	134LEU CB	93.08	229AR C	230PHE CB	3.22
231ASP CA	231ASP C	100.00	133GLU C	134LEU CG	0.22	229AR C	230PHE CG	0.57
231ASP CB	231ASP CG	100.00	133GLU C	134LEU CD1	0.00	229AR C	230PHE CD1	0.08
231ASP CB	231ASP C	100.00	133GLU C	134LEU C	6.78	229AR C	230PHE CD2	0.02
231ASP CG	231ASP C	99.99	134LEU CA	134LEU CB	100.00	229AR C	230PHE C	97.21
231ASP C	232VAL CA	100.00	134LEU CA	134LEU CG	100.00	230PH CA	230PHE CB	100.00
231ASP C	232VAL CB	49.88	134LEU CA	134LEU CD1	81.98	230PH CA	230PHE CG	100.00
231ASP C	232VAL CG2	38.86	134LEU CA	134LEU CD2	9.57	230PH CA	230PHE CD1	98.86
231ASP C	232VAL C	61.50	134LEU CA	134LEU C	100.00	230PH CA	230PHE CD2	25.48
232VAL CA	232VAL CB	100.00	134LEU CB	134LEU CG	100.00	230PH CA	230PHE C	100.00
232VAL CA	232VAL CG1	100.00	134LEU CB	134LEU CD1	100.00	230PH CB	230PHE CG	100.00
232VAL CA	232VAL CG2	100.00	134LEU CB	134LEU CD2	100.00	230PH CB	230PHE CD1	100.00
232VAL CA	232VAL C	100.00	134LEU CB	134LEU C	100.00	230PH CB	230PHE CD2	100.00
232VAL CB	232VAL CG1	100.00	134LEU CB	135THR CG2	0.00	230PH CB	230PHE C	100.00
232VAL CB	232VAL CG2	100.00	134LEU CG	134LEU CD1	100.00	230PH CB	233VAL CG1	0.00
232VAL CB	232VAL C	100.00	134LEU CG	134LEU CD2	100.00	230PH CB	233VAL CG2	0.02
232VAL CG1	232VAL CG2	100.00	134LEU CG	134LEU C	87.64	230PH CG	230PHE CD1	100.00
232VAL CG1	232VAL C	100.00	134LEU CD1	134LEU CD2	100.00	230PH CG	230PHE CD2	100.00
232VAL C	233VAL CA	100.00	134LEU CD1	134LEU C	0.14	230PH CG	230PHE CE1	100.00
232VAL C	233VAL CB	39.19	134LEU CD1	237ASN CG	0.01	230PH CG	230PHE CE2	100.00
232VAL C	233VAL CG1	3.59	134LEU CD2	134LEU C	0.34	230PH CG	230PHE CZ	100.00
232VAL C	233VAL CG2	21.88	134LEU CD2	261SER CB	0.34	230PH CG	230PHE C	0.05
232VAL C	233VAL C	75.65	134LEU C	135THR CA	100.00	230PH CD1	230PHE CD2	100.00
233VAL CA	233VAL CB	100.00	134LEU C	135THR CB	11.00	230PH CD1	230PHE CE1	100.00
233VAL CA	233VAL CG1	100.00	134LEU C	135THR CG2	5.50	230PH CD1	230PHE CE2	100.00
233VAL CA	233VAL CG2	100.00	134LEU C	135THR C	90.22	230PH CD1	230PHE CZ	100.00
233VAL CA	233VAL C	100.00	134LEU C	140PHE CE2	0.01	230PH CD1	233VAL CG1	0.03
233VAL CB	233VAL CG1	100.00	135THR CA	135THR CB	100.00	230PH CD1	233VAL CG2	0.13
233VAL CB	233VAL CG2	100.00	135THR CA	135THR CG2	100.00	230PH CD2	230PHE CE1	100.00
233VAL CB	233VAL C	100.00	135THR CA	135THR C	100.00	230PH CD2	230PHE CE2	100.00
233VAL CG1	233VAL CG2	100.00	135THR CA	140PHE CG	0.00	230PH CD2	230PHE CZ	100.00
233VAL CG1	233VAL C	92.33	135THR CA	140PHE CD1	0.00	230PH CD2	230PHE C	0.03
233VAL CG1	235THR CG2	0.00	135THR CB	135THR CG2	100.00	230PH CD2	233VAL CG2	0.00
233VAL CG1	243LEU CD2	0.19	135THR CB	135THR C	100.00	230PH CE1	230PHE CE2	100.00
233VAL CG2	233VAL C	7.68	135THR CB	140PHE CD1	0.01	230PH CE1	230PHE CZ	100.00
233VAL C	234VAL CA	100.00	135THR CB	140PHE CE1	0.00	230PH CE2	230PHE CZ	100.00
233VAL C	234VAL CB	86.52	135THR CB	161GLU CG	0.99	230PH C	231ASP CA	100.00
233VAL C	234VAL CG1	0.02	135THR CB	161GLU CD	0.10	230PH C	231ASP CB	88.44
233VAL C	234VAL C	24.36	135THR CG2	135THR C	2.32	230PH C	231ASP C	18.28
234VAL CA	234VAL CB	100.00	135THR CG2	140PHE CE1	0.01	231AS CA	231ASP CB	100.00
234VAL CA	234VAL CG1	100.00	135THR CG2	140PHE CE2	0.00	231AS CA	231ASP CG	100.00
234VAL CA	234VAL CG2	100.00	135THR CG2	140PHE CZ	0.01	231AS CA	231ASP C	100.00
234VAL CA	234VAL C	100.00	135THR CG2	161GLU CB	0.01	231AS CB	231ASP CG	100.00
234VAL CB	234VAL CG1	100.00	135THR CG2	161GLU CG	2.14	231AS CB	231ASP C	100.00
234VAL CB	234VAL CG2	100.00	135THR CG2	161GLU CD	0.16	231AS CG	231ASP C	99.98
234VAL CB	234VAL C	100.00	135THR CG2	161GLU C	0.01	231AS CG	232VAL CG2	0.00
234VAL CG1	234VAL CG2	100.00	135THR CG2	165VAL CG1	0.40	231AS C	232VAL CA	100.00
234VAL CG1	234VAL C	99.56	135THR CG2	165VAL CG2	3.64	231AS C	232VAL CB	42.51
234VAL CG2	234VAL C	99.98	135THR C	136GLY CA	100.00	231AS C	232VAL CG1	0.02
234VAL C	235THR CA	100.00	135THR C	136GLY C	32.85	231AS C	232VAL CG2	25.67
234VAL C	235THR CB	100.00	135THR C	140PHE CB	0.01	231AS C	232VAL C	65.73
234VAL C	235THR CG2	0.43	135THR C	140PHE CD1	0.03	232VA CA	232VAL CB	100.00
234VAL C	235THR C	0.10	135THR C	140PHE CD2	0.06	232VA CA	232VAL CG1	100.00
235THR CA	235THR CB	100.00	135THR C	140PHE CE1	0.02	232VA CA	232VAL CG2	100.00
235THR CA	235THR CG2	100.00	135THR C	140PHE CE2	0.02	232VA CA	232VAL C	100.00
235THR CA	235THR C	100.00	136GLY CA	136GLY C	100.00	232VA CB	232VAL CG1	100.00
235THR CB	235THR CG2	100.00	136GLY CA	139TYR CE1	0.02	232VA CB	232VAL CG2	100.00
235THR CB	235THR C	100.00	136GLY CA	139TYR CZ	0.01	232VA CB	232VAL C	100.00
235THR CB	243LEU CD1	0.00	136GLY CA	140PHE CB	0.48	232VA CG1	232VAL CG2	100.00
235THR CG2	235THR C	99.84	136GLY CA	140PHE CG	1.12	232VA CG1	232VAL C	99.66
235THR CG2	239PHE CB	0.01	136GLY CA	140PHE CD1	1.43	232VA CG2	232VAL C	11.70
235THR CG2	239PHE C	0.46	136GLY CA	140PHE CD2	1.13	232VA C	233VAL CA	100.00

235THR	CG2	240GLY	CA	23.47
235THR	CG2	243LEU	CD1	0.03
235THR	CG2	243LEU	CD2	0.19
235THR	C	236GLY	CA	100.00
235THR	C	236GLY	C	92.90
236GLY	CA	236GLY	C	100.00
236GLY	C	237ASN	CA	100.00
236GLY	C	237ASN	CB	26.41
236GLY	C	237ASN	CG	0.00
236GLY	C	237ASN	C	76.72
237ASN	CA	237ASN	CB	100.00
237ASN	CA	237ASN	CG	100.00
237ASN	CA	237ASN	C	100.00
237ASN	CB	237ASN	CG	100.00
237ASN	CB	237ASN	C	100.00
237ASN	CG	237ASN	C	85.88
237ASN	C	238ILE	CA	100.00
237ASN	C	238ILE	CB	0.00
237ASN	C	238ILE	C	100.00
238ILE	CA	238ILE	CB	100.00
238ILE	CA	238ILE	CG1	100.00
238ILE	CA	238ILE	CG2	100.00
238ILE	CA	238ILE	CD	2.36
238ILE	CA	238ILE	C	100.00
238ILE	CB	238ILE	CG1	100.00
238ILE	CB	238ILE	CG2	100.00
238ILE	CB	238ILE	CD	100.00
238ILE	CB	238ILE	C	100.00
238ILE	CG1	238ILE	CG2	100.00
238ILE	CG1	238ILE	CD	100.00
238ILE	CG1	238ILE	C	100.00
238ILE	CG1	239PHE	CD1	0.01
238ILE	CG1	239PHE	CE1	0.14
238ILE	CG1	239PHE	CZ	0.00
238ILE	CG1	242ILE	CD	0.01
238ILE	CG2	238ILE	CD	97.75
238ILE	CG2	238ILE	C	98.97
238ILE	CG2	239PHE	CG	0.05
238ILE	CG2	239PHE	CD1	0.25
238ILE	CG2	239PHE	CD2	1.94
238ILE	CG2	239PHE	CE1	0.12
238ILE	CG2	239PHE	CE2	0.47
238ILE	CG2	239PHE	CZ	0.02
238ILE	CD	238ILE	C	0.24
238ILE	CD	239PHE	CD1	0.01
238ILE	CD	239PHE	CE1	0.24
238ILE	CD	239PHE	CE2	0.04
238ILE	CD	239PHE	CZ	0.38
238ILE	CD	242ILE	CD	0.01
238ILE	C	239PHE	CA	100.00
238ILE	C	239PHE	CB	3.34
238ILE	C	239PHE	CG	0.08
238ILE	C	239PHE	CD1	0.44
238ILE	C	239PHE	C	96.28
239PHE	CA	239PHE	CB	100.00
239PHE	CA	239PHE	CG	100.00
239PHE	CA	239PHE	CD1	95.74
239PHE	CA	239PHE	CD2	6.94
239PHE	CA	239PHE	C	100.00
239PHE	CA	242ILE	CD	0.00
239PHE	CB	239PHE	CG	100.00
239PHE	CB	239PHE	CD1	100.00
136GL\	CA	140PHE	CE1	0.29
136GL\	CA	140PHE	CE2	0.12
136GL\	CA	140PHE	CZ	0.43
136GL\	CA	157TYR	CE1	0.02
136GL\	C	137GLY	CA	100.00
136GL\	C	137GLY	C	52.44
136GL\	C	139TYR	CE1	0.08
136GL\	C	139TYR	CE2	0.03
136GL\	C	139TYR	CZ	0.04
136GL\	C	140PHE	CB	2.19
136GL\	C	140PHE	CG	0.04
136GL\	C	140PHE	CD1	0.71
136GL\	C	140PHE	CD2	0.28
136GL\	C	140PHE	CE1	0.04
136GL\	C	140PHE	CE2	0.02
136GL\	C	140PHE	CZ	0.01
136GL\	C	157TYR	CE1	0.09
136GL\	C	157TYR	CZ	0.04
136GL\	C	237ASN	CG	0.03
137GL\	CA	137GLY	C	100.00
137GL\	CA	139TYR	CD1	0.02
137GL\	CA	139TYR	CD2	0.02
137GL\	CA	139TYR	CE1	0.42
137GL\	CA	139TYR	CE2	0.67
137GL\	CA	139TYR	CZ	0.24
137GL\	CA	140PHE	C	0.02
137GL\	CA	141GLY	CA	0.02
137GL\	CA	155GLU	CD	0.03
137GL\	CA	157TYR	CE1	0.00
137GL\	CA	157TYR	CE2	0.08
137GL\	CA	157TYR	CZ	0.05
137GL\	CA	237ASN	CB	0.98
137GL\	CA	237ASN	CG	6.40
137GL\	CA	238ILE	CG1	0.28
137GL\	CA	238ILE	CD	0.21
137GL\	C	138ILE	CA	100.00
137GL\	C	138ILE	CB	9.94
137GL\	C	138ILE	CG1	3.39
137GL\	C	138ILE	CD	0.08
137GL\	C	138ILE	C	100.00
137GL\	C	139TYR	CD1	0.13
137GL\	C	139TYR	CD2	0.12
137GL\	C	139TYR	CE1	0.01
137GL\	C	139TYR	CE2	0.02
137GL\	C	140PHE	C	0.05
137GL\	C	141GLY	CA	0.00
137GL\	C	155GLU	CD	0.20
137GL\	C	238ILE	CD	0.00
138ILE	CA	138ILE	CB	100.00
138ILE	CA	138ILE	CG1	100.00
138ILE	CA	138ILE	CG2	100.00
138ILE	CA	138ILE	CD	12.22
138ILE	CA	138ILE	C	100.00
138ILE	CA	155GLU	CD	0.14
138ILE	CB	138ILE	CG1	100.00
138ILE	CB	138ILE	CG2	100.00
138ILE	CB	138ILE	CD	100.00
138ILE	CB	138ILE	C	100.00
138ILE	CB	139TYR	CD1	0.03
138ILE	CB	155GLU	CA	0.01
138ILE	CB	155GLU	CG	0.00
138ILE	CB	155GLU	CD	0.02
232VA	C	233VAL	CB	55.96
232VA	C	233VAL	CG1	2.65
232VA	C	233VAL	CG2	37.77
232VA	C	233VAL	C	62.44
233VA	CA	233VAL	CB	100.00
233VA	CA	233VAL	CG1	100.00
233VA	CA	233VAL	CG2	100.00
233VA	CA	233VAL	C	100.00
233VA	CB	233VAL	CG1	100.00
233VA	CB	233VAL	CG2	100.00
233VA	CB	233VAL	C	100.00
233VA	CG1	233VAL	CG2	100.00
233VA	CG1	233VAL	C	96.46
233VA	CG1	243LEU	CD1	0.00
233VA	CG1	243LEU	CD2	0.08
233VA	CG2	233VAL	C	3.88
233VA	CG2	243LEU	CD2	0.01
233VA	C	234VAL	CA	100.00
233VA	C	234VAL	CB	85.17
233VA	C	234VAL	CG1	0.03
233VA	C	234VAL	C	28.42
234VA	CA	234VAL	CB	100.00
234VA	CA	234VAL	CG1	100.00
234VA	CA	234VAL	CG2	100.00
234VA	CA	234VAL	C	100.00
234VA	CB	234VAL	CG1	100.00
234VA	CB	234VAL	CG2	100.00
234VA	CB	234VAL	C	100.00
234VA	CG1	234VAL	CG2	100.00
234VA	CG1	234VAL	C	99.52
234VA	CG2	234VAL	C	99.98
234VA	C	235THR	CA	100.00
234VA	C	235THR	CB	99.96
234VA	C	235THR	CG2	0.14
234VA	C	235THR	C	0.21
235TH	CA	235THR	CB	100.00
235TH	CA	235THR	CG2	100.00
235TH	CA	235THR	C	100.00
235TH	CB	235THR	CG2	100.00
235TH	CB	235THR	C	100.00
235TH	CB	243LEU	CD1	0.00
235TH	CB	243LEU	CD2	0.00
235TH	CG2	235THR	C	99.88
235TH	CG2	239PHE	CB	0.02
235TH	CG2	239PHE	C	0.16
235TH	CG2	240GLY	CA	11.78
235TH	CG2	243LEU	CD1	0.03
235TH	CG2	243LEU	CD2	0.12
235TH	C	236GLY	CA	100.00
235TH	C	236GLY	C	91.44
236GL	CA	236GLY	C	100.00
236GL	C	237ASN	CA	100.00
236GL	C	237ASN	CB	45.74
236GL	C	237ASN	CG	0.02
236GL	C	237ASN	C	56.75
237AS	CA	237ASN	CB	100.00
237AS	CA	237ASN	CG	100.00
237AS	CA	237ASN	C	100.00
237AS	CB	237ASN	CG	100.00
237AS	CB	237ASN	C	100.00
237AS	CG	237ASN	C	90.95
237AS	C	238ILE	CA	100.00

239PHE CB	239PHE	CD2	100.00	138ILE CB	238ILE	CG2	0.02	237AS C	238ILE	CB	0.00
239PHE CB	239PHE	C	100.00	138ILE CB	238ILE	CD	0.08	237AS C	238ILE	C	100.00
239PHE CB	243LEU	CD1	0.34	138ILE CG1	138ILE	CG2	100.00	237AS C	240GLY	CA	0.00
239PHE CG	239PHE	CD1	100.00	138ILE CG1	138ILE	CD	100.00	238ILE CA	238ILE	CB	100.00
239PHE CG	239PHE	CD2	100.00	138ILE CG1	138ILE	C	50.52	238ILE CA	238ILE	CG1	100.00
239PHE CG	239PHE	CE1	100.00	138ILE CG1	139TYR	CD1	0.07	238ILE CA	238ILE	CG2	100.00
239PHE CG	239PHE	CE2	100.00	138ILE CG1	139TYR	CE1	0.02	238ILE CA	238ILE	CD	1.80
239PHE CG	239PHE	CZ	100.00	138ILE CG1	153ASN	CB	0.50	238ILE CA	238ILE	C	100.00
239PHE CG	239PHE	C	3.50	138ILE CG1	153ASN	CG	0.01	238ILE CB	238ILE	CG1	100.00
239PHE CD1	239PHE	CD2	100.00	138ILE CG1	154THR	C	0.10	238ILE CB	238ILE	CG2	100.00
239PHE CD1	239PHE	CE1	100.00	138ILE CG1	155GLU	CA	0.00	238ILE CB	238ILE	CD	100.00
239PHE CD1	239PHE	CE2	100.00	138ILE CG1	155GLU	CB	0.09	238ILE CB	238ILE	C	100.00
239PHE CD1	239PHE	CZ	100.00	138ILE CG1	155GLU	CG	0.06	238ILE CG1	238ILE	CG2	100.00
239PHE CD2	239PHE	CE1	100.00	138ILE CG1	155GLU	CD	0.92	238ILE CG1	238ILE	CD	100.00
239PHE CD2	239PHE	CE2	100.00	138ILE CG1	237ASN	CG	0.01	238ILE CG1	238ILE	C	100.00
239PHE CD2	239PHE	CZ	100.00	138ILE CG1	238ILE	CG1	0.23	238ILE CG1	239PHE	CD1	0.01
239PHE CD2	239PHE	C	2.17	138ILE CG1	238ILE	CG2	0.00	238ILE CG1	239PHE	CE1	0.02
239PHE CE1	239PHE	CE2	100.00	138ILE CG1	238ILE	CD	0.26	238ILE CG1	239PHE	CE2	0.01
239PHE CE1	239PHE	CZ	100.00	138ILE CG2	138ILE	CD	93.23	238ILE CG1	239PHE	CZ	0.01
239PHE CE2	239PHE	CZ	100.00	138ILE CG2	138ILE	C	83.05	238ILE CG1	242ILE	CD	0.08
239PHE C	240GLY	CA	100.00	138ILE CG2	139TYR	CG	0.02	238ILE CG2	238ILE	CD	98.89
239PHE C	240GLY	C	97.92	138ILE CG2	139TYR	CD1	0.20	238ILE CG2	238ILE	C	98.97
239PHE C	243LEU	CB	0.01	138ILE CG2	139TYR	CD2	1.36	238ILE CG2	239PHE	CG	0.00
239PHE C	243LEU	CG	0.01	138ILE CG2	139TYR	CE1	0.09	238ILE CG2	239PHE	CD1	0.26
239PHE C	243LEU	CD1	0.11	138ILE CG2	139TYR	CE2	1.81	238ILE CG2	239PHE	CD2	1.81
240GLY CA	240GLY	C	100.00	138ILE CG2	139TYR	CZ	0.47	238ILE CG2	239PHE	CE1	0.14
240GLY CA	243LEU	CB	0.01	138ILE CG2	153ASN	CB	0.29	238ILE CG2	239PHE	CE2	0.70
240GLY C	241ASP	CA	100.00	138ILE CG2	153ASN	CG	0.07	238ILE CG2	239PHE	CZ	0.02
240GLY C	241ASP	C	100.00	138ILE CG2	154THR	C	0.08	238ILE CD	238ILE	C	0.96
240GLY C	243LEU	CB	0.00	138ILE CG2	155GLU	CA	0.12	238ILE CD	239PHE	CD1	0.01
241ASP CA	241ASP	CB	100.00	138ILE CG2	155GLU	CB	0.20	238ILE CD	239PHE	CE1	0.02
241ASP CA	241ASP	CG	100.00	138ILE CG2	155GLU	CG	0.08	238ILE CD	239PHE	CE2	0.02
241ASP CA	241ASP	C	100.00	138ILE CG2	155GLU	CD	0.22	238ILE CD	239PHE	CZ	0.04
241ASP CB	241ASP	CG	100.00	138ILE CG2	238ILE	CB	0.05	238ILE CD	242ILE	CD	0.02
241ASP CB	241ASP	C	100.00	138ILE CG2	238ILE	CG1	0.63	238ILE C	239PHE	CA	100.00
241ASP CG	241ASP	C	99.98	138ILE CG2	238ILE	CG2	1.24	238ILE C	239PHE	CB	1.18
241ASP C	242ILE	CA	100.00	138ILE CG2	238ILE	CD	1.06	238ILE C	239PHE	CG	0.09
241ASP C	242ILE	CB	0.06	138ILE CD	138ILE	C	3.53	238ILE C	239PHE	CD1	0.09
241ASP C	242ILE	CG1	0.02	138ILE CD	139TYR	CD1	0.08	238ILE C	239PHE	C	98.22
241ASP C	242ILE	CD	0.01	138ILE CD	139TYR	CD2	0.02	239PH CA	239PHE	CB	100.00
241ASP C	242ILE	C	99.97	138ILE CD	139TYR	CE1	0.01	239PH CA	239PHE	CG	100.00
242ILE CA	242ILE	CB	100.00	138ILE CD	139TYR	CE2	0.00	239PH CA	239PHE	CD1	93.45
242ILE CA	242ILE	CG1	100.00	138ILE CD	153ASN	CB	0.06	239PH CA	239PHE	CD2	18.83
242ILE CA	242ILE	CG2	100.00	138ILE CD	153ASN	CG	0.00	239PH CA	239PHE	C	100.00
242ILE CA	242ILE	CD	7.32	138ILE CD	153ASN	C	0.02	239PH CA	242ILE	CD	0.01
242ILE CA	242ILE	C	100.00	138ILE CD	154THR	C	0.02	239PH CB	239PHE	CG	100.00
242ILE CB	242ILE	CG1	100.00	138ILE CD	155GLU	CB	0.08	239PH CB	239PHE	CD1	100.00
242ILE CB	242ILE	CG2	100.00	138ILE CD	155GLU	CG	0.01	239PH CB	239PHE	CD2	100.00
242ILE CB	242ILE	CD	100.00	138ILE CD	155GLU	CD	0.08	239PH CB	239PHE	C	100.00
242ILE CB	242ILE	C	100.00	138ILE CD	237ASN	CB	0.98	239PH CB	243LEU	CD1	0.58
242ILE CG1	242ILE	CG2	100.00	138ILE CD	237ASN	CG	0.26	239PH CG	239PHE	CD1	100.00
242ILE CG1	242ILE	CD	100.00	138ILE CD	238ILE	CB	0.03	239PH CG	239PHE	CD2	100.00
242ILE CG2	242ILE	CD	92.02	138ILE CD	238ILE	CG1	1.33	239PH CG	239PHE	CE1	100.00
242ILE CG2	242ILE	C	100.00	138ILE CD	238ILE	CG2	0.04	239PH CG	239PHE	CE2	100.00
242ILE CG2	246LEU	CD1	0.07	138ILE CD	238ILE	CD	0.81	239PH CG	239PHE	CZ	100.00
242ILE CG2	246LEU	CD2	0.00	138ILE CD	239PHE	CD1	0.01	239PH CD1	239PHE	CD2	100.00
242ILE C	243LEU	CA	100.00	138ILE CD	239PHE	CD2	0.00	239PH CD1	239PHE	CE1	100.00
242ILE C	243LEU	C	100.00	138ILE CD	239PHE	CE1	0.44	239PH CD1	239PHE	CE2	100.00
243LEU CA	243LEU	CB	100.00	138ILE CD	239PHE	CE2	0.41	239PH CD1	239PHE	CZ	100.00
243LEU CA	243LEU	CG	100.00	138ILE CD	239PHE	CZ	1.44	239PH CD1	242ILE	CD	0.00
243LEU CA	243LEU	CD1	52.23	138ILE C	139TYR	CA	100.00	239PH CD2	239PHE	CE1	100.00
243LEU CA	243LEU	CD2	48.57	138ILE C	139TYR	CB	3.14	239PH CD2	239PHE	CE2	100.00

243LEU	CA	243LEU	C	100.00	138ILE	C	139TYR	CG	0.02	239PH	CD2	239PHE	CZ	100.00
243LEU	CA	246LEU	CB	0.01	138ILE	C	139TYR	CD1	0.09	239PH	CE1	239PHE	CE2	100.00
243LEU	CA	246LEU	CD2	0.00	138ILE	C	139TYR	CD2	0.01	239PH	CE1	239PHE	CZ	100.00
243LEU	CB	243LEU	CG	100.00	138ILE	C	139TYR	C	95.92	239PH	CE2	239PHE	CZ	100.00
243LEU	CB	243LEU	CD1	100.00	139TYF	CA	139TYR	CB	100.00	239PH	C	240GLY	CA	100.00
243LEU	CB	243LEU	CD2	100.00	139TYF	CA	139TYR	CG	100.00	239PH	C	240GLY	C	98.50
243LEU	CB	243LEU	C	100.00	139TYF	CA	139TYR	CD1	70.35	239PH	C	242ILE	CD	0.00
243LEU	CG	243LEU	CD1	100.00	139TYF	CA	139TYR	CD2	61.39	239PH	C	243LEU	CD1	0.12
243LEU	CG	243LEU	CD2	100.00	139TYF	CA	139TYR	C	100.00	240GL	CA	240GLY	C	100.00
243LEU	CG	243LEU	C	48.35	139TYF	CA	144ARG	CD	0.00	240GL	CA	243LEU	CB	0.02
243LEU	CD1	243LEU	CD2	100.00	139TYF	CA	144ARG	CZ	0.01	240GL	C	241ASP	CA	100.00
243LEU	CD1	243LEU	C	1.13	139TYF	CB	139TYR	CG	100.00	240GL	C	241ASP	C	100.00
243LEU	CD1	246LEU	CD1	0.01	139TYF	CB	139TYR	CD1	100.00	241AS	CA	241ASP	CB	100.00
243LEU	CD1	246LEU	CD2	0.01	139TYF	CB	139TYR	CD2	100.00	241AS	CA	241ASP	CG	100.00
243LEU	CD2	243LEU	C	1.70	139TYF	CB	139TYR	C	100.00	241AS	CA	241ASP	C	100.00
243LEU	CD2	246LEU	CD2	0.06	139TYF	CB	140PHE	CD1	0.03	241AS	CB	241ASP	CG	100.00
243LEU	C	244SER	CA	100.00	139TYF	CB	140PHE	CD2	0.31	241AS	CB	241ASP	C	100.00
243LEU	C	244SER	CB	0.01	139TYF	CB	140PHE	CE1	0.02	241AS	CG	241ASP	C	99.99
243LEU	C	244SER	C	100.00	139TYF	CB	140PHE	CE2	0.16	241AS	C	242ILE	CA	100.00
244SER	CA	244SER	CB	100.00	139TYF	CB	140PHE	CZ	0.00	241AS	C	242ILE	CB	0.18
244SER	CA	244SER	C	100.00	139TYF	CB	144ARG	CZ	0.08	241AS	C	242ILE	CG1	0.08
244SER	CB	244SER	C	100.00	139TYF	CG	139TYR	CD1	100.00	241AS	C	242ILE	CD	0.00
244SER	C	245ASP	CA	100.00	139TYF	CG	139TYR	CD2	100.00	241AS	C	242ILE	C	99.88
244SER	C	245ASP	CB	0.57	139TYF	CG	139TYR	CE1	100.00	242ILE	CA	242ILE	CB	100.00
244SER	C	245ASP	CG	0.00	139TYF	CG	139TYR	CE2	100.00	242ILE	CA	242ILE	CG1	100.00
244SER	C	245ASP	C	99.09	139TYF	CG	139TYR	CZ	100.00	242ILE	CA	242ILE	CG2	100.00
245ASP	CA	245ASP	CB	100.00	139TYF	CG	139TYR	C	87.91	242ILE	CA	242ILE	CD	6.42
245ASP	CA	245ASP	CG	100.00	139TYF	CG	140PHE	CD1	0.00	242ILE	CA	242ILE	C	100.00
245ASP	CA	245ASP	C	100.00	139TYF	CG	140PHE	CD2	0.00	242ILE	CB	242ILE	CG1	100.00
245ASP	CA	248SER	CB	0.00	139TYF	CD1	139TYR	CD2	100.00	242ILE	CB	242ILE	CG2	100.00
245ASP	CA	254LEU	CD2	0.06	139TYF	CD1	139TYR	CE1	100.00	242ILE	CB	242ILE	CD	100.00
245ASP	CB	245ASP	CG	100.00	139TYF	CD1	139TYR	CE2	100.00	242ILE	CB	242ILE	C	100.00
245ASP	CB	245ASP	C	100.00	139TYF	CD1	139TYR	CZ	100.00	242ILE	CG1	242ILE	CG2	100.00
245ASP	CB	254LEU	CD2	1.05	139TYF	CD1	139TYR	C	10.08	242ILE	CG1	242ILE	CD	100.00
245ASP	CG	245ASP	C	0.14	139TYF	CD1	140PHE	CB	0.02	242ILE	CG1	242ILE	C	0.00
245ASP	CG	254LEU	CD1	0.04	139TYF	CD1	140PHE	CG	0.22	242ILE	CG2	242ILE	CD	95.96
245ASP	CG	254LEU	CD2	0.34	139TYF	CD1	140PHE	CD1	1.20	242ILE	CG2	242ILE	C	99.99
245ASP	C	246LEU	CA	100.00	139TYF	CD1	140PHE	CD2	2.76	242ILE	CG2	243LEU	CD1	0.00
245ASP	C	246LEU	CB	0.00	139TYF	CD1	140PHE	CE1	0.46	242ILE	CG2	246LEU	CD1	0.02
245ASP	C	246LEU	C	100.00	139TYF	CD1	140PHE	CE2	1.00	242ILE	C	243LEU	CA	100.00
245ASP	C	254LEU	CD2	0.00	139TYF	CD1	140PHE	CZ	0.05	242ILE	C	243LEU	C	100.00
246LEU	CA	246LEU	CB	100.00	139TYF	CD1	238ILE	CD	0.56	243LEI	CA	243LEU	CB	100.00
246LEU	CA	246LEU	CG	100.00	139TYF	CD2	139TYR	CE1	100.00	243LEI	CA	243LEU	CG	100.00
246LEU	CA	246LEU	CD1	72.69	139TYF	CD2	139TYR	CE2	100.00	243LEI	CA	243LEU	CD1	52.24
246LEU	CA	246LEU	CD2	25.41	139TYF	CD2	139TYR	CZ	100.00	243LEI	CA	243LEU	CD2	46.33
246LEU	CA	246LEU	C	100.00	139TYF	CD2	139TYR	C	31.42	243LEI	CA	243LEU	C	100.00
246LEU	CA	249VAL	CG2	0.07	139TYF	CD2	140PHE	CB	0.08	243LEI	CA	246LEU	CB	0.00
246LEU	CB	246LEU	CG	100.00	139TYF	CD2	140PHE	CG	0.18	243LEI	CB	243LEU	CG	100.00
246LEU	CB	246LEU	CD1	100.00	139TYF	CD2	140PHE	CD1	4.51	243LEI	CB	243LEU	CD1	100.00
246LEU	CB	246LEU	CD2	100.00	139TYF	CD2	140PHE	CD2	0.90	243LEI	CB	243LEU	CD2	100.00
246LEU	CB	246LEU	C	100.00	139TYF	CD2	140PHE	CE1	1.98	243LEI	CB	243LEU	C	100.00
246LEU	CB	250LEU	CD1	0.01	139TYF	CD2	140PHE	CE2	0.46	243LEI	CG	243LEU	CD1	100.00
246LEU	CG	246LEU	CD1	100.00	139TYF	CD2	140PHE	CZ	0.11	243LEI	CG	243LEU	CD2	100.00
246LEU	CG	246LEU	CD2	100.00	139TYF	CD2	237ASN	CB	0.01	243LEI	CG	243LEU	C	49.55
246LEU	CG	246LEU	C	87.84	139TYF	CD2	237ASN	CG	0.05	243LEI	CD1	243LEU	CD2	100.00
246LEU	CG	250LEU	CD1	0.01	139TYF	CD2	238ILE	CD	0.03	243LEI	CD1	243LEU	C	0.72
246LEU	CD1	246LEU	CD2	100.00	139TYF	CE1	139TYR	CE2	100.00	243LEI	CD1	246LEU	CD2	0.02
246LEU	CD1	246LEU	C	12.52	139TYF	CE1	139TYR	CZ	100.00	243LEI	CD2	243LEU	C	0.80
246LEU	CD2	246LEU	C	14.16	139TYF	CE1	140PHE	CB	0.00	243LEI	CD2	246LEU	CD1	0.00
246LEU	CD2	250LEU	CD1	0.04	139TYF	CE1	140PHE	CG	0.01	243LEI	CD2	246LEU	CD2	0.05
246LEU	CD2	250LEU	CD2	0.00	139TYF	CE1	140PHE	CD1	0.75	243LEI	C	244SER	CA	100.00
246LEU	C	247ALA	CA	100.00	139TYF	CE1	140PHE	CD2	1.74	243LEI	C	244SER	CB	0.00

246LEU	C	247ALA	CB	0.02	139TYF	CE1	140PHE	CE1	1.43	243LE	C	244SER	C	100.00
246LEU	C	247ALA	C	100.00	139TYF	CE1	140PHE	CE2	2.40	244SE	CA	244SER	CB	100.00
246LEU	C	249VAL	CG1	0.00	139TYF	CE1	140PHE	CZ	0.33	244SE	CA	244SER	C	100.00
246LEU	C	249VAL	CG2	0.01	139TYF	CE1	237ASN	CG	0.00	244SE	CB	244SER	C	100.00
247ALA	CA	247ALA	CB	100.00	139TYF	CE1	238ILE	CG1	0.03	244SE	C	245ASP	CA	100.00
247ALA	CA	247ALA	C	100.00	139TYF	CE1	238ILE	CD	3.35	244SE	C	245ASP	CB	0.27
247ALA	CA	250LEU	CD1	0.07	139TYF	CE2	139TYR	CZ	100.00	244SE	C	245ASP	CG	0.04
247ALA	CB	247ALA	C	100.00	139TYF	CE2	140PHE	CB	0.01	244SE	C	245ASP	C	99.61
247ALA	CB	250LEU	CD1	0.00	139TYF	CE2	140PHE	CG	0.01	245AS	CA	245ASP	CB	100.00
247ALA	C	248SER	CA	100.00	139TYF	CE2	140PHE	CD1	3.39	245AS	CA	245ASP	CG	100.00
247ALA	C	248SER	CB	0.96	139TYF	CE2	140PHE	CD2	0.75	245AS	CA	245ASP	C	100.00
247ALA	C	248SER	C	99.29	139TYF	CE2	140PHE	CE1	7.32	245AS	CA	254LEU	CD1	0.02
247ALA	C	251PRO	CD	0.01	139TYF	CE2	140PHE	CE2	2.26	245AS	CA	254LEU	CD2	0.21
248SER	CA	248SER	CB	100.00	139TYF	CE2	140PHE	CZ	1.64	245AS	CB	245ASP	CG	100.00
248SER	CA	248SER	C	100.00	139TYF	CE2	237ASN	CB	0.01	245AS	CB	245ASP	C	100.00
248SER	CA	251PRO	CD	0.01	139TYF	CE2	237ASN	CG	0.02	245AS	CB	254LEU	CD1	0.01
248SER	CA	257LEU	CD1	0.10	139TYF	CE2	238ILE	CA	0.00	245AS	CB	254LEU	CD2	0.54
248SER	CA	257LEU	CD2	0.01	139TYF	CE2	238ILE	CG2	0.02	245AS	CG	245ASP	C	0.01
248SER	CB	248SER	C	100.00	139TYF	CE2	238ILE	CD	0.31	245AS	CG	254LEU	CD1	0.17
248SER	CB	254LEU	CB	0.02	139TYF	CE2	241ASP	CG	0.04	245AS	CG	254LEU	CD2	1.27
248SER	CB	254LEU	CG	0.13	139TYF	CZ	140PHE	CD1	0.05	245AS	C	246LEU	CA	100.00
248SER	CB	254LEU	CD1	0.64	139TYF	CZ	140PHE	CD2	0.04	245AS	C	246LEU	CB	0.01
248SER	CB	254LEU	CD2	2.70	139TYF	CZ	140PHE	CE1	0.70	245AS	C	246LEU	C	99.98
248SER	CB	257LEU	CD1	1.18	139TYF	CZ	140PHE	CE2	0.28	246LE	CA	246LEU	CB	100.00
248SER	CB	257LEU	CD2	0.14	139TYF	CZ	140PHE	CZ	0.08	246LE	CA	246LEU	CG	100.00
248SER	C	249VAL	CA	100.00	139TYF	CZ	238ILE	CG1	0.00	246LE	CA	246LEU	CD1	66.18
248SER	C	249VAL	CB	1.59	139TYF	CZ	238ILE	CD	0.23	246LE	CA	246LEU	CD2	32.20
248SER	C	249VAL	CG1	0.36	139TYF	C	140PHE	CA	100.00	246LE	CA	246LEU	C	100.00
248SER	C	249VAL	CG2	0.01	139TYF	C	140PHE	CB	64.89	246LE	CB	246LEU	CG	100.00
248SER	C	249VAL	C	98.38	139TYF	C	140PHE	CG	8.06	246LE	CB	246LEU	CD1	100.00
248SER	C	251PRO	CD	0.76	139TYF	C	140PHE	CD1	1.69	246LE	CB	246LEU	CD2	100.00
248SER	C	254LEU	CB	0.00	139TYF	C	140PHE	CD2	1.87	246LE	CB	246LEU	C	100.00
248SER	C	254LEU	CD1	0.05	139TYF	C	140PHE	C	35.99	246LE	CG	246LEU	CD1	100.00
248SER	C	254LEU	CD2	0.30	139TYF	C	144ARG	CD	0.12	246LE	CG	246LEU	CD2	100.00
249VAL	CA	249VAL	CB	100.00	139TYF	C	144ARG	CZ	0.03	246LE	CG	246LEU	C	94.21
249VAL	CA	249VAL	CG1	100.00	140PHI	CA	140PHE	CB	100.00	246LE	CD1	246LEU	CD2	100.00
249VAL	CA	249VAL	CG2	100.00	140PHI	CA	140PHE	CG	100.00	246LE	CD1	246LEU	C	11.19
249VAL	CA	249VAL	C	100.00	140PHI	CA	140PHE	CD1	57.86	246LE	CD2	246LEU	C	26.57
249VAL	CA	254LEU	CD1	0.02	140PHI	CA	140PHE	CD2	62.14	246LE	C	247ALA	CA	100.00
249VAL	CA	254LEU	CD2	0.04	140PHI	CA	140PHE	C	100.00	246LE	C	247ALA	CB	0.02
249VAL	CB	249VAL	CG1	100.00	140PHI	CA	144ARG	CZ	0.07	246LE	C	247ALA	C	99.98
249VAL	CB	249VAL	CG2	100.00	140PHI	CB	140PHE	CG	100.00	246LE	C	249VAL	CG2	0.01
249VAL	CB	249VAL	C	100.00	140PHI	CB	140PHE	CD1	100.00	247AL	CA	247ALA	CB	100.00
249VAL	CB	254LEU	CD2	0.01	140PHI	CB	140PHE	CD2	100.00	247AL	CA	247ALA	C	100.00
249VAL	CG1	249VAL	CG2	100.00	140PHI	CB	140PHE	C	100.00	247AL	CA	250LEU	CD2	0.02
249VAL	CG1	249VAL	C	32.16	140PHI	CG	140PHE	CD1	100.00	247AL	CB	247ALA	C	100.00
249VAL	CG1	250LEU	CD2	0.04	140PHI	CG	140PHE	CD2	100.00	247AL	CB	250LEU	CD1	0.00
249VAL	CG1	254LEU	CB	0.00	140PHI	CG	140PHE	CE1	100.00	247AL	C	248SER	CA	100.00
249VAL	CG1	254LEU	CD1	0.12	140PHI	CG	140PHE	CE2	100.00	247AL	C	248SER	CB	0.26
249VAL	CG1	254LEU	CD2	0.22	140PHI	CG	140PHE	CZ	100.00	247AL	C	248SER	C	99.77
249VAL	CG2	249VAL	C	90.88	140PHI	CG	140PHE	C	41.48	247AL	C	250LEU	CD2	0.00
249VAL	CG2	250LEU	CD1	0.01	140PHI	CD1	140PHE	CD2	100.00	247AL	C	257LEU	CD1	0.01
249VAL	CG2	250LEU	CD2	0.00	140PHI	CD1	140PHE	CE1	100.00	248SE	CA	248SER	CB	100.00
249VAL	CG2	254LEU	CD2	0.02	140PHI	CD1	140PHE	CE2	100.00	248SE	CA	248SER	C	100.00
249VAL	C	250LEU	CA	100.00	140PHI	CD1	140PHE	CZ	100.00	248SE	CA	251PRO	CD	0.01
249VAL	C	250LEU	CB	0.04	140PHI	CD1	140PHE	C	1.52	248SE	CA	257LEU	CD1	0.02
249VAL	C	250LEU	C	99.98	140PHI	CD1	144ARG	CD	0.00	248SE	CB	248SER	C	100.00
249VAL	C	251PRO	CD	4.51	140PHI	CD1	144ARG	CZ	0.05	248SE	CB	254LEU	CA	0.23
250LEU	CA	250LEU	CB	100.00	140PHI	CD2	140PHE	CE1	100.00	248SE	CB	254LEU	CB	0.08
250LEU	CA	250LEU	CG	100.00	140PHI	CD2	140PHE	CE2	100.00	248SE	CB	254LEU	CG	0.23
250LEU	CA	250LEU	CD1	5.81	140PHI	CD2	140PHE	CZ	100.00	248SE	CB	254LEU	CD1	1.64
250LEU	CA	250LEU	CD2	93.46	140PHI	CD2	140PHE	C	1.28	248SE	CB	254LEU	CD2	4.79

250LEU	CA	250LEU	C	100.00	140PHI	CD2	144ARG	CZ	0.04	248SE	CB	257LEU	CD1	1.21
250LEU	CA	251PRO	CD	100.00	140PHI	CE1	140PHE	CE2	100.00	248SE	CB	257LEU	CD2	0.04
250LEU	CB	250LEU	CG	100.00	140PHI	CE1	140PHE	CZ	100.00	248SE	C	249VAL	CA	100.00
250LEU	CB	250LEU	CD1	100.00	140PHI	CE1	144ARG	CD	0.01	248SE	C	249VAL	CB	0.05
250LEU	CB	250LEU	CD2	100.00	140PHI	CE1	144ARG	CZ	0.08	248SE	C	249VAL	CG1	0.02
250LEU	CB	250LEU	C	100.00	140PHI	CE2	140PHE	CZ	100.00	248SE	C	249VAL	CG2	0.00
250LEU	CB	251PRO	CD	82.03	140PHI	CE2	144ARG	CZ	0.00	248SE	C	249VAL	C	99.96
250LEU	CG	250LEU	CD1	100.00	140PHI	CZ	144ARG	CZ	0.02	248SE	C	251PRO	CD	0.91
250LEU	CG	250LEU	CD2	100.00	140PHI	C	141GLY	CA	100.00	248SE	C	254LEU	CD1	0.01
250LEU	CG	250LEU	C	5.68	140PHI	C	141GLY	C	58.48	249VA	CA	249VAL	CB	100.00
250LEU	CG	251PRO	CD	0.01	140PHI	C	144ARG	CZ	0.08	249VA	CA	249VAL	CG1	100.00
250LEU	CD1	250LEU	CD2	100.00	141GL	CA	141GLY	C	100.00	249VA	CA	249VAL	CG2	100.00
250LEU	CD1	250LEU	C	0.48	141GL	CA	156ARG	CD	0.00	249VA	CA	249VAL	C	100.00
250LEU	CD2	250LEU	C	0.75	141GL	C	142GLU	CA	100.00	249VA	CB	249VAL	CG1	100.00
250LEU	C	251PRO	CA	100.00	141GL	C	142GLU	CB	20.06	249VA	CB	249VAL	CG2	100.00
250LEU	C	251PRO	CB	0.04	141GL	C	142GLU	CG	0.59	249VA	CB	249VAL	C	100.00
250LEU	C	251PRO	CD	100.00	141GL	C	142GLU	CD	0.07	249VA	CG1	249VAL	CG2	100.00
250LEU	C	251PRO	C	100.00	141GL	C	142GLU	C	84.75	249VA	CG1	249VAL	C	63.17
251PRO	CA	251PRO	CB	100.00	141GL	C	144ARG	CD	0.01	249VA	CG1	250LEU	CD2	0.05
251PRO	CA	251PRO	CG	100.00	141GL	C	144ARG	CZ	0.02	249VA	CG2	249VAL	C	71.40
251PRO	CA	251PRO	CD	100.00	142GLI	CA	142GLU	CB	100.00	249VA	CG2	250LEU	CD1	0.00
251PRO	CA	251PRO	C	100.00	142GLI	CA	142GLU	CG	100.00	249VA	CG2	250LEU	CD2	0.02
251PRO	CB	251PRO	CG	100.00	142GLI	CA	142GLU	CD	93.08	249VA	C	250LEU	CA	100.00
251PRO	CB	251PRO	CD	100.00	142GLI	CA	142GLU	C	100.00	249VA	C	250LEU	C	100.00
251PRO	CB	251PRO	C	100.00	142GLI	CA	143PRO	CA	100.00	249VA	C	251PRO	CD	0.32
251PRO	CB	257LEU	CD1	0.01	142GLI	CA	143PRO	C	39.81	249VA	C	252GLY	CA	0.04
251PRO	CB	257LEU	CD2	0.27	142GLI	CB	142GLU	CG	100.00	250LEI	CA	250LEU	CB	100.00
251PRO	CG	251PRO	CD	100.00	142GLI	CB	142GLU	CD	100.00	250LEI	CA	250LEU	CG	100.00
251PRO	CG	251PRO	C	80.65	142GLI	CB	142GLU	C	100.00	250LEI	CA	250LEU	CD1	2.20
251PRO	CG	257LEU	CG	0.03	142GLI	CB	143PRO	CA	4.64	250LEI	CA	250LEU	CD2	98.33
251PRO	CG	257LEU	CD1	3.97	142GLI	CB	143PRO	C	0.00	250LEI	CA	250LEU	C	100.00
251PRO	CG	257LEU	CD2	2.39	142GLI	CG	142GLU	CD	100.00	250LEI	CA	251PRO	CD	100.00
251PRO	CD	251PRO	C	63.60	142GLI	CG	142GLU	C	67.19	250LEI	CB	250LEU	CG	100.00
251PRO	C	252GLY	CA	100.00	142GLI	CG	143PRO	CA	1.72	250LEI	CB	250LEU	CD1	100.00
251PRO	C	252GLY	C	47.74	142GLI	CG	144ARG	CG	0.00	250LEI	CB	250LEU	CD2	100.00
251PRO	C	256LEU	CD1	0.01	142GLI	CD	142GLU	C	0.14	250LEI	CB	250LEU	C	100.00
252GLY	CA	252GLY	C	100.00	142GLI	CD	143PRO	CA	0.44	250LEI	CB	251PRO	CD	94.99
252GLY	CA	254LEU	CD1	0.00	142GLI	CD	144ARG	CG	0.18	250LEI	CG	250LEU	CD1	100.00
252GLY	CA	327LEU	CD1	0.02	142GLI	CD	144ARG	CD	0.04	250LEI	CG	250LEU	CD2	100.00
252GLY	CA	327LEU	CD2	0.06	142GLI	C	143PRO	CA	100.00	250LEI	CG	250LEU	C	0.00
252GLY	C	253SER	CA	100.00	142GLI	C	143PRO	CB	0.10	250LEI	CG	251PRO	CD	0.01
252GLY	C	253SER	CB	82.00	142GLI	C	143PRO	CG	0.00	250LEI	CD1	250LEU	CD2	100.00
252GLY	C	253SER	C	19.89	142GLI	C	143PRO	CD	100.00	250LEI	C	251PRO	CA	100.00
252GLY	C	254LEU	CD1	0.10	142GLI	C	143PRO	C	99.94	250LEI	C	251PRO	CB	0.05
252GLY	C	254LEU	CD2	0.03	142GLI	C	154THR	CB	0.09	250LEI	C	251PRO	CD	100.00
252GLY	C	327LEU	CD1	0.14	143PR(	CA	143PRO	CB	100.00	250LEI	C	251PRO	C	100.00
252GLY	C	327LEU	CD2	0.62	143PR(	CA	143PRO	CG	100.00	251PR	CA	251PRO	CB	100.00
253SER	CA	253SER	CB	100.00	143PR(	CA	143PRO	CD	100.00	251PR	CA	251PRO	CG	100.00
253SER	CA	253SER	C	100.00	143PR(	CA	143PRO	C	100.00	251PR	CA	251PRO	CD	100.00
253SER	CB	253SER	C	100.00	143PR(	CB	143PRO	CG	100.00	251PR	CA	251PRO	C	100.00
253SER	CB	256LEU	CB	0.03	143PR(	CB	143PRO	CD	100.00	251PR	CB	251PRO	CG	100.00
253SER	CB	256LEU	CG	0.09	143PR(	CB	143PRO	C	100.00	251PR	CB	251PRO	CD	100.00
253SER	CB	256LEU	CD1	3.92	143PR(	CB	152TRP	CD1	0.12	251PR	CB	251PRO	C	100.00
253SER	CB	256LEU	CD2	0.06	143PR(	CG	143PRO	CD	100.00	251PR	CB	256LEU	CB	0.18
253SER	CB	257LEU	CG	0.02	143PR(	CG	143PRO	C	97.37	251PR	CB	256LEU	CD1	0.10
253SER	CB	257LEU	CD1	0.00	143PR(	CG	152TRP	CD1	0.12	251PR	CB	256LEU	CD2	0.04
253SER	CB	257LEU	CD2	0.06	143PR(	CG	154THR	CB	0.01	251PR	CB	257LEU	CD2	0.04
253SER	CB	327LEU	CD1	0.55	143PR(	CD	143PRO	C	91.88	251PR	CG	251PRO	CD	100.00
253SER	CB	327LEU	CD2	0.44	143PR(	CD	154THR	CB	0.17	251PR	CG	251PRO	C	96.91
253SER	C	254LEU	CA	100.00	143PR(	CD	154THR	CG2	0.00	251PR	CG	256LEU	CD1	0.00
253SER	C	254LEU	CB	0.62	143PR(	C	144ARG	CA	100.00	251PR	CG	257LEU	CG	0.00
253SER	C	254LEU	CG	0.03	143PR(	C	144ARG	CB	81.74	251PR	CG	257LEU	CD1	4.82

253SER	C	254LEU	CD1	0.03	143PR	C	144ARG	CG	2.04	251PR CG	257LEU CD2	1.31
253SER	C	254LEU	C	99.40	143PR	C	144ARG	CD	0.01	251PR CD	251PRO C	85.85
253SER	C	257LEU	CD1	0.04	143PR	C	144ARG	C	23.26	251PR C	252GLY CA	100.00
254LEU	CA	254LEU	CB	100.00	143PR	C	152TRP	CD1	0.05	251PR C	252GLY C	27.52
254LEU	CA	254LEU	CG	100.00	144AR	CA	144ARG	CB	100.00	251PR C	256LEU CD1	0.41
254LEU	CA	254LEU	CD1	20.55	144AR	CA	144ARG	CG	100.00	252GL CA	252GLY C	100.00
254LEU	CA	254LEU	CD2	70.31	144AR	CA	144ARG	CD	2.97	252GL CA	327LEU CD1	0.03
254LEU	CA	254LEU	C	100.00	144AR	CA	144ARG	CZ	0.00	252GL CA	327LEU CD2	0.08
254LEU	CA	257LEU	CD1	0.08	144AR	CA	144ARG	C	100.00	252GL C	253SER CA	100.00
254LEU	CB	254LEU	CG	100.00	144AR	CA	152TRP	CD1	0.74	252GL C	253SER CB	99.22
254LEU	CB	254LEU	CD1	100.00	144AR	CA	152TRP	CE2	0.00	252GL C	253SER C	1.23
254LEU	CB	254LEU	CD2	100.00	144AR	CB	144ARG	CG	100.00	252GL C	327LEU CD1	0.22
254LEU	CB	254LEU	C	100.00	144AR	CB	144ARG	CD	100.00	252GL C	327LEU CD2	0.84
254LEU	CG	254LEU	CD1	100.00	144AR	CB	144ARG	CZ	1.48	253SE CA	253SER CB	100.00
254LEU	CG	254LEU	CD2	100.00	144AR	CB	144ARG	C	100.00	253SE CA	253SER C	100.00
254LEU	CG	254LEU	C	17.74	144AR	CB	153ASN	CG	0.00	253SE CB	253SER C	100.00
254LEU	CD1	254LEU	CD2	100.00	144AR	CG	144ARG	CD	100.00	253SE CB	256LEU CG	0.02
254LEU	CD1	254LEU	C	3.05	144AR	CG	144ARG	CZ	29.24	253SE CB	256LEU CD1	1.15
254LEU	CD1	272VAL	CG1	0.31	144AR	CG	144ARG	C	86.31	253SE CB	256LEU CD2	0.03
254LEU	CD2	254LEU	C	0.94	144AR	CD	144ARG	CZ	100.00	253SE CB	324PRO CG	0.00
254LEU	CD2	272VAL	CG1	0.15	144AR	CD	144ARG	C	0.33	253SE CB	326ASP CB	0.05
254LEU	C	255GLY	CA	100.00	144AR	CZ	144ARG	C	0.00	253SE CB	326ASP CG	0.00
254LEU	C	255GLY	C	48.69	144AR	CZ	145GLY	CA	0.01	253SE CB	326ASP C	0.01
254LEU	C	257LEU	CD1	1.71	144AR	C	145GLY	CA	100.00	253SE CB	327LEU CG	0.05
254LEU	C	257LEU	CD2	0.01	144AR	C	145GLY	C	12.44	253SE CB	327LEU CD1	2.71
254LEU	C	272VAL	CG1	0.02	144AR	C	152TRP	CD1	1.12	253SE CB	327LEU CD2	0.54
255GLY	CA	255GLY	C	100.00	144AR	C	152TRP	CE2	0.13	253SE C	254LEU CA	100.00
255GLY	CA	272VAL	CG1	0.01	144AR	C	152TRP	CZ2	0.02	253SE C	254LEU CB	0.05
255GLY	CA	272VAL	C	0.01	145GL	CA	145GLY	C	100.00	253SE C	254LEU CD1	0.01
255GLY	CA	273HIS	CA	0.06	145GL	CA	152TRP	CG	0.00	253SE C	254LEU C	99.95
255GLY	CA	273HIS	CB	0.90	145GL	CA	152TRP	CD1	0.33	254LE CA	254LEU CB	100.00
255GLY	C	256LEU	CA	100.00	145GL	CA	152TRP	CD2	0.32	254LE CA	254LEU CG	100.00
255GLY	C	256LEU	CB	41.70	145GL	CA	152TRP	CE2	7.29	254LE CA	254LEU CD1	23.42
255GLY	C	256LEU	CG	10.56	145GL	CA	152TRP	CE3	0.04	254LE CA	254LEU CD2	72.19
255GLY	C	256LEU	CD1	0.27	145GL	CA	152TRP	CZ2	1.28	254LE CA	254LEU C	100.00
255GLY	C	256LEU	CD2	0.17	145GL	CA	152TRP	CZ3	0.05	254LE CA	257LEU CD1	0.06
255GLY	C	256LEU	C	49.56	145GL	C	146MET	CA	100.00	254LE CA	257LEU CD2	0.00
255GLY	C	273HIS	CB	0.03	145GL	C	146MET	CB	94.46	254LE CB	254LEU CG	100.00
256LEU	CA	256LEU	CB	100.00	145GL	C	146MET	CG	0.11	254LE CB	254LEU CD1	100.00
256LEU	CA	256LEU	CG	100.00	145GL	C	146MET	C	7.76	254LE CB	254LEU CD2	100.00
256LEU	CA	256LEU	CD1	6.43	145GL	C	152TRP	CD2	0.04	254LE CB	254LEU C	100.00
256LEU	CA	256LEU	CD2	93.04	145GL	C	152TRP	CE2	0.58	254LE CB	272VAL CG1	0.00
256LEU	CA	256LEU	C	100.00	145GL	C	152TRP	CE3	0.22	254LE CB	273HIS C	0.01
256LEU	CB	256LEU	CG	100.00	145GL	C	152TRP	CZ2	0.31	254LE CB	274GLY CA	0.92
256LEU	CB	256LEU	CD1	100.00	145GL	C	152TRP	CZ3	0.22	254LE CG	254LEU CD1	100.00
256LEU	CB	256LEU	CD2	100.00	146ME	CA	146MET	CB	100.00	254LE CG	254LEU CD2	100.00
256LEU	CB	256LEU	C	100.00	146ME	CA	146MET	CG	100.00	254LE CG	254LEU C	21.20
256LEU	CB	288THR	CG2	0.01	146ME	CA	146MET	CE	0.01	254LE CG	272VAL CG1	0.16
256LEU	CG	256LEU	CD1	100.00	146ME	CA	146MET	C	100.00	254LE CG	274GLY CA	0.01
256LEU	CG	256LEU	CD2	100.00	146ME	CA	151ALA	CB	0.20	254LE CD1	254LEU CD2	100.00
256LEU	CG	256LEU	C	4.05	146ME	CA	152TRP	CZ3	0.08	254LE CD1	254LEU C	0.38
256LEU	CG	288THR	CG2	0.02	146ME	CB	146MET	CG	100.00	254LE CD1	272VAL CG1	0.46
256LEU	CD1	256LEU	CD2	100.00	146ME	CB	146MET	CE	32.32	254LE CD1	273HIS CA	0.00
256LEU	CD1	288THR	CB	0.05	146ME	CB	146MET	C	100.00	254LE CD1	273HIS C	0.02
256LEU	CD1	288THR	CG2	0.13	146ME	CB	151ALA	CA	0.06	254LE CD1	274GLY CA	1.22
256LEU	CD1	319LEU	CD1	0.09	146ME	CB	151ALA	CB	1.12	254LE CD1	274GLY C	0.00
256LEU	CD1	319LEU	CD2	0.15	146ME	CG	146MET	CE	100.00	254LE CD2	254LEU C	1.30
256LEU	CD1	324PRO	CG	0.00	146ME	CG	146MET	C	93.99	254LE CD2	257LEU CD1	0.00
256LEU	CD1	327LEU	CD1	0.85	146ME	CG	151ALA	CB	0.46	254LE CD2	272VAL CG1	0.57
256LEU	CD1	327LEU	CD2	0.06	146ME	CE	146MET	C	0.01	254LE CD2	273HIS C	0.08
256LEU	CD2	286ASN	CG	0.17	146ME	CE	147SER	C	0.00	254LE CD2	274GLY CA	1.02
256LEU	CD2	288THR	CB	0.68	146ME	C	147SER	CA	100.00	254LE CD2	274GLY C	0.00

256LEU	CD2	288THR	CG2	0.28	146ME	C	147SER	CB	91.19	254LE	C	255GLY	CA	100.00
256LEU	CD2	319LEU	CD1	0.06	146ME	C	147SER	C	15.84	254LE	C	255GLY	C	77.38
256LEU	CD2	319LEU	CD2	0.22	146ME	C	152TRP	CZ3	0.12	254LE	C	257LEU	CD1	0.57
256LEU	CD2	324PRO	CG	0.02	147SE	CA	147SER	CB	100.00	254LE	C	257LEU	CD2	0.18
256LEU	CD2	327LEU	CD1	0.02	147SE	CA	147SER	C	100.00	254LE	C	272VAL	CG1	0.03
256LEU	CD2	327LEU	CD2	0.01	147SE	CB	147SER	C	100.00	255GL	CA	255GLY	C	100.00
256LEU	C	257LEU	CA	100.00	147SE	CB	152TRP	CZ3	0.02	255GL	CA	272VAL	C	0.05
256LEU	C	257LEU	CB	12.95	147SE	C	148GLU	CA	100.00	255GL	CA	273HIS	CA	0.16
256LEU	C	257LEU	CG	1.29	147SE	C	148GLU	CB	2.36	255GL	CA	273HIS	CD2	0.04
256LEU	C	257LEU	CD1	1.74	147SE	C	148GLU	C	98.42	255GL	CA	273HIS	C	0.33
256LEU	C	257LEU	CD2	0.05	148GL	CA	148GLU	CB	100.00	255GL	CA	289ALA	CB	0.00
256LEU	C	257LEU	C	84.46	148GL	CA	148GLU	CG	100.00	255GL	C	256LEU	CA	100.00
256LEU	C	289ALA	CA	0.14	148GL	CA	148GLU	CD	97.90	255GL	C	256LEU	CB	15.03
256LEU	C	289ALA	CB	2.58	148GL	CA	148GLU	C	100.00	255GL	C	256LEU	CG	1.38
257LEU	CA	257LEU	CB	100.00	148GL	CB	148GLU	CG	100.00	255GL	C	256LEU	CD1	1.53
257LEU	CA	257LEU	CG	100.00	148GL	CB	148GLU	CD	100.00	255GL	C	256LEU	CD2	0.08
257LEU	CA	257LEU	CD1	40.43	148GL	CB	148GLU	C	100.00	255GL	C	256LEU	C	80.78
257LEU	CA	257LEU	CD2	50.09	148GL	CG	148GLU	CD	100.00	255GL	C	327LEU	CD2	0.01
257LEU	CA	257LEU	C	100.00	148GL	CG	148GLU	C	80.26	256LE	CA	256LEU	CB	100.00
257LEU	CA	258PRO	CD	100.00	148GL	CD	148GLU	C	2.81	256LE	CA	256LEU	CG	100.00
257LEU	CB	257LEU	CG	100.00	148GL	C	149ALA	CA	100.00	256LE	CA	256LEU	CD1	16.94
257LEU	CB	257LEU	CD1	100.00	148GL	C	149ALA	CB	34.07	256LE	CA	256LEU	CD2	82.53
257LEU	CB	257LEU	CD2	100.00	148GL	C	149ALA	C	63.61	256LE	CA	256LEU	C	100.00
257LEU	CB	257LEU	C	100.00	149AL	CA	149ALA	CB	100.00	256LE	CA	288THR	CG2	0.01
257LEU	CB	258PRO	CD	2.26	149AL	CA	149ALA	C	100.00	256LE	CA	292LEU	CD1	0.02
257LEU	CB	272VAL	CB	0.05	149AL	CB	149ALA	C	100.00	256LE	CB	256LEU	CG	100.00
257LEU	CB	272VAL	CG1	0.01	149AL	C	150GLU	CA	100.00	256LE	CB	256LEU	CD1	100.00
257LEU	CB	272VAL	CG2	0.23	149AL	C	150GLU	CB	99.70	256LE	CB	256LEU	CD2	100.00
257LEU	CG	257LEU	CD1	100.00	149AL	C	150GLU	CG	2.14	256LE	CB	256LEU	C	100.00
257LEU	CG	257LEU	CD2	100.00	149AL	C	150GLU	C	0.30	256LE	CB	257LEU	CD2	0.01
257LEU	CG	257LEU	C	0.22	150GL	CA	150GLU	CB	100.00	256LE	CB	288THR	CG2	0.05
257LEU	CD1	257LEU	CD2	100.00	150GL	CA	150GLU	CG	100.00	256LE	CG	256LEU	CD1	100.00
257LEU	CD1	257LEU	C	0.04	150GL	CA	150GLU	CD	1.04	256LE	CG	256LEU	CD2	100.00
257LEU	CD1	272VAL	CB	0.00	150GL	CA	150GLU	C	100.00	256LE	CG	256LEU	C	4.00
257LEU	CD1	272VAL	CG1	0.04	150GL	CB	150GLU	CG	100.00	256LE	CG	319LEU	CD1	0.00
257LEU	CD1	272VAL	CG2	0.00	150GL	CB	150GLU	CD	100.00	256LE	CG	319LEU	CD2	0.05
257LEU	C	258PRO	CA	100.00	150GL	CB	150GLU	C	100.00	256LE	CG	327LEU	CD1	0.00
257LEU	C	258PRO	CB	1.12	150GL	CB	152TRP	CZ3	0.04	256LE	CG	327LEU	CD2	0.01
257LEU	C	258PRO	CD	100.00	150GL	CG	150GLU	CD	100.00	256LE	CD1	256LEU	CD2	100.00
257LEU	C	258PRO	C	100.00	150GL	CG	150GLU	C	91.30	256LE	CD1	256LEU	C	0.56
257LEU	C	272VAL	CG2	0.08	150GL	CG	152TRP	CE3	0.01	256LE	CD1	257LEU	CD2	0.00
257LEU	C	289ALA	CB	0.23	150GL	CG	152TRP	CZ3	0.10	256LE	CD1	288THR	CB	0.01
258PRO	CA	258PRO	CB	100.00	150GL	CD	152TRP	CE3	0.02	256LE	CD1	288THR	CG2	0.08
258PRO	CA	258PRO	CG	100.00	150GL	CD	152TRP	CZ3	0.14	256LE	CD1	292LEU	CD1	0.01
258PRO	CA	258PRO	CD	100.00	150GL	C	151ALA	CA	100.00	256LE	CD1	319LEU	CD1	0.06
258PRO	CA	258PRO	C	100.00	150GL	C	151ALA	CB	93.00	256LE	CD1	319LEU	CD2	0.22
258PRO	CB	258PRO	CG	100.00	150GL	C	151ALA	C	6.75	256LE	CD1	324PRO	CB	0.00
258PRO	CB	258PRO	CD	100.00	150GL	C	152TRP	CZ3	0.10	256LE	CD1	324PRO	CG	0.70
258PRO	CB	258PRO	C	100.00	151AL	CA	151ALA	CB	100.00	256LE	CD1	324PRO	CD	0.02
258PRO	CB	292LEU	CB	0.05	151AL	CA	151ALA	C	100.00	256LE	CD1	326ASP	CB	0.01
258PRO	CB	292LEU	C	3.96	151AL	CA	152TRP	CE3	2.46	256LE	CD1	327LEU	CD1	0.36
258PRO	CB	293SER	CA	1.56	151AL	CB	151ALA	C	100.00	256LE	CD1	327LEU	CD2	0.03
258PRO	CB	293SER	CB	0.00	151AL	C	152TRP	CA	100.00	256LE	CD2	256LEU	C	0.07
258PRO	CB	296MET	CE	0.01	151AL	C	152TRP	CB	99.36	256LE	CD2	286ASN	CG	0.00
258PRO	CG	258PRO	CD	100.00	151AL	C	152TRP	CG	0.06	256LE	CD2	288THR	CB	0.20
258PRO	CG	258PRO	C	99.99	151AL	C	152TRP	CD2	0.03	256LE	CD2	288THR	CG2	0.52
258PRO	CG	292LEU	CB	0.19	151AL	C	152TRP	CE3	19.30	256LE	CD2	292LEU	CD1	0.00
258PRO	CG	296MET	CE	0.11	151AL	C	152TRP	C	0.24	256LE	CD2	319LEU	CD1	3.48
258PRO	CD	258PRO	C	99.96	152TR	CA	152TRP	CB	100.00	256LE	CD2	319LEU	CD2	4.33
258PRO	CD	292LEU	CB	0.04	152TR	CA	152TRP	CG	100.00	256LE	CD2	324PRO	CG	0.11
258PRO	CD	292LEU	CD1	0.01	152TR	CA	152TRP	CD1	54.12	256LE	CD2	327LEU	CD1	0.54
258PRO	C	259SER	CA	100.00	152TR	CA	152TRP	CD2	21.28	256LE	CD2	327LEU	CD2	0.03

258PRO	C	259SER	CB	99.99	152TRI	CA	152TRP	C	100.00	256LEI	C	257LEU	CA	100.00
258PRO	C	259SER	C	0.20	152TRI	CB	152TRP	CG	100.00	256LEI	C	257LEU	CB	39.45
258PRO	C	272VAL	CG2	1.72	152TRI	CB	152TRP	CD1	100.00	256LEI	C	257LEU	CG	9.73
259SER	CA	259SER	CB	100.00	152TRI	CB	152TRP	CD2	100.00	256LEI	C	257LEU	CD1	4.00
259SER	CA	259SER	C	100.00	152TRI	CB	152TRP	CE3	97.54	256LEI	C	257LEU	CD2	0.81
259SER	CB	259SER	C	100.00	152TRI	CB	152TRP	C	100.00	256LEI	C	257LEU	C	57.29
259SER	CB	272VAL	CG2	5.52	152TRI	CG	152TRP	CD1	100.00	256LEI	C	258PRO	CD	0.00
259SER	C	260ALA	CA	100.00	152TRI	CG	152TRP	CD2	100.00	256LEI	C	289ALA	CA	0.01
259SER	C	260ALA	CB	62.00	152TRI	CG	152TRP	CE2	100.00	256LEI	C	289ALA	CB	0.51
259SER	C	260ALA	C	44.69	152TRI	CG	152TRP	CE3	100.00	256LEI	C	292LEU	CD1	2.03
259SER	C	296MET	CE	0.04	152TRI	CG	152TRP	C	99.57	257LEI	CA	257LEU	CB	100.00
260ALA	CA	260ALA	CB	100.00	152TRI	CD1	152TRP	CD2	100.00	257LEI	CA	257LEU	CG	100.00
260ALA	CA	260ALA	C	100.00	152TRI	CD1	152TRP	CE2	100.00	257LEI	CA	257LEU	CD1	39.04
260ALA	CA	269PHE	CD2	0.10	152TRI	CD1	152TRP	CE3	0.00	257LEI	CA	257LEU	CD2	60.11
260ALA	CA	269PHE	CE2	0.01	152TRI	CD1	152TRP	C	21.67	257LEI	CA	257LEU	C	100.00
260ALA	CB	260ALA	C	100.00	152TRI	CD2	152TRP	CE2	100.00	257LEI	CA	258PRO	CD	100.00
260ALA	CB	262LEU	CD1	0.56	152TRI	CD2	152TRP	CE3	100.00	257LEI	CB	257LEU	CG	100.00
260ALA	CB	262LEU	CD2	0.02	152TRI	CD2	152TRP	CZ2	100.00	257LEI	CB	257LEU	CD1	100.00
260ALA	CB	269PHE	CG	0.00	152TRI	CD2	152TRP	CZ3	100.00	257LEI	CB	257LEU	CD2	100.00
260ALA	CB	269PHE	CD1	0.00	152TRI	CD2	152TRP	C	0.00	257LEI	CB	257LEU	C	100.00
260ALA	CB	269PHE	CD2	18.46	152TRI	CE2	152TRP	CE3	100.00	257LEI	CB	258PRO	CD	2.34
260ALA	CB	269PHE	CE2	35.53	152TRI	CE2	152TRP	CZ2	100.00	257LEI	CB	272VAL	CB	0.00
260ALA	CB	269PHE	CZ	0.41	152TRI	CE2	152TRP	CZ3	100.00	257LEI	CB	272VAL	CG2	0.05
260ALA	CB	296MET	CE	7.07	152TRI	CE3	152TRP	CZ2	100.00	257LEI	CG	257LEU	CD1	100.00
260ALA	CB	300HIS	CD2	0.07	152TRI	CE3	152TRP	CZ3	100.00	257LEI	CG	257LEU	CD2	100.00
260ALA	CB	300HIS	CE1	0.07	152TRI	CZ2	152TRP	CZ3	100.00	257LEI	CG	257LEU	C	4.02
260ALA	C	261SER	CA	100.00	152TRI	C	153ASN	CA	100.00	257LEI	CD1	257LEU	CD2	100.00
260ALA	C	261SER	CB	27.41	152TRI	C	153ASN	CB	81.20	257LEI	CD1	257LEU	C	0.81
260ALA	C	261SER	C	78.98	152TRI	C	153ASN	CG	3.44	257LEI	CD1	272VAL	CB	0.03
260ALA	C	262LEU	CD1	0.01	152TRI	C	153ASN	C	9.89	257LEI	CD1	272VAL	CG1	0.04
261SER	CA	261SER	CB	100.00	153ASI	CA	153ASN	CB	100.00	257LEI	CD1	272VAL	CG2	0.02
261SER	CA	261SER	C	100.00	153ASI	CA	153ASN	CG	100.00	257LEI	CD2	257LEU	C	0.90
261SER	CB	261SER	C	100.00	153ASI	CA	153ASN	C	100.00	257LEI	CD2	272VAL	CG1	0.00
261SER	C	262LEU	CA	100.00	153ASI	CB	153ASN	CG	100.00	257LEI	C	258PRO	CA	100.00
261SER	C	262LEU	CB	3.07	153ASI	CB	153ASN	C	100.00	257LEI	C	258PRO	CB	1.19
261SER	C	262LEU	CG	0.24	153ASI	CG	153ASN	C	0.03	257LEI	C	258PRO	CD	100.00
261SER	C	262LEU	CD1	1.06	153ASI	C	154THR	CA	100.00	257LEI	C	258PRO	C	100.00
261SER	C	262LEU	CD2	0.08	153ASI	C	154THR	CB	52.79	257LEI	C	289ALA	CB	0.26
261SER	C	262LEU	C	96.95	153ASI	C	154THR	C	56.54	258PR	CA	258PRO	CB	100.00
262LEU	CA	262LEU	CB	100.00	154THI	CA	154THR	CB	100.00	258PR	CA	258PRO	CG	100.00
262LEU	CA	262LEU	CG	100.00	154THI	CA	154THR	CG2	100.00	258PR	CA	258PRO	CD	100.00
262LEU	CA	262LEU	CD1	17.03	154THI	CA	154THR	C	100.00	258PR	CA	258PRO	C	100.00
262LEU	CA	262LEU	CD2	82.59	154THI	CB	154THR	CG2	100.00	258PR	CB	258PRO	CG	100.00
262LEU	CA	262LEU	C	100.00	154THI	CB	154THR	C	100.00	258PR	CB	258PRO	CD	100.00
262LEU	CB	262LEU	CG	100.00	154THI	CG2	154THR	C	100.00	258PR	CB	258PRO	C	100.00
262LEU	CB	262LEU	CD1	100.00	154THI	CG2	155GLU	C	0.02	258PR	CB	292LEU	CB	0.01
262LEU	CB	262LEU	CD2	100.00	154THI	CG2	156ARG	CB	0.01	258PR	CB	292LEU	C	3.00
262LEU	CB	262LEU	C	100.00	154THI	CG2	156ARG	CG	0.10	258PR	CB	293SER	CA	2.84
262LEU	CG	262LEU	CD1	100.00	154THI	C	155GLU	CA	100.00	258PR	CB	293SER	CB	0.16
262LEU	CG	262LEU	CD2	100.00	154THI	C	155GLU	CB	24.98	258PR	CB	296MET	CE	0.03
262LEU	CG	262LEU	C	0.36	154THI	C	155GLU	C	81.87	258PR	CG	258PRO	CD	100.00
262LEU	CG	300HIS	CE1	0.01	155GLI	CA	155GLU	CB	100.00	258PR	CG	258PRO	C	99.96
262LEU	CD1	262LEU	CD2	100.00	155GLI	CA	155GLU	CG	100.00	258PR	CG	292LEU	CB	0.05
262LEU	CD1	267PRO	CB	0.06	155GLI	CA	155GLU	CD	99.53	258PR	CG	292LEU	C	0.00
262LEU	CD1	300HIS	CD2	0.01	155GLI	CA	155GLU	C	100.00	258PR	CG	296MET	CE	0.10
262LEU	CD1	300HIS	CE1	0.33	155GLI	CB	155GLU	CG	100.00	258PR	CD	258PRO	C	99.76
262LEU	CD2	262LEU	C	0.13	155GLI	CB	155GLU	CD	100.00	258PR	CD	292LEU	CB	0.04
262LEU	CD2	267PRO	CB	1.17	155GLI	CB	155GLU	C	100.00	258PR	C	259SER	CA	100.00
262LEU	CD2	300HIS	CD2	0.21	155GLI	CG	155GLU	CD	100.00	258PR	C	259SER	CB	99.98
262LEU	CD2	300HIS	CE1	5.98	155GLI	CG	155GLU	C	98.20	258PR	C	259SER	C	0.35
262LEU	C	263GLY	CA	100.00	155GLI	CG	157TYR	CD1	0.12	258PR	C	272VAL	CG2	1.11
262LEU	C	263GLY	C	11.99	155GLI	CG	157TYR	CD2	0.06	259SE	CA	259SER	CB	100.00

263GLY	CA	263GLY	C	100.00
263GLY	C	264ARG	CA	100.00
263GLY	C	264ARG	CB	0.68
263GLY	C	264ARG	CG	0.09
263GLY	C	264ARG	C	99.54
264ARG	CA	264ARG	CB	100.00
264ARG	CA	264ARG	CG	100.00
264ARG	CA	264ARG	CD	0.67
264ARG	CA	264ARG	CZ	0.00
264ARG	CA	264ARG	C	100.00
264ARG	CB	264ARG	CG	100.00
264ARG	CB	264ARG	CD	100.00
264ARG	CB	264ARG	CZ	2.57
264ARG	CB	264ARG	C	100.00
264ARG	CG	264ARG	CD	100.00
264ARG	CG	264ARG	CZ	42.40
264ARG	CG	264ARG	C	25.98
264ARG	CD	264ARG	CZ	100.00
264ARG	CD	264ARG	C	0.13
264ARG	CZ	264ARG	C	0.20
264ARG	CZ	265GLY	CA	0.14
264ARG	C	265GLY	CA	100.00
264ARG	C	265GLY	C	61.52
265GLY	CA	265GLY	C	100.00
265GLY	C	266THR	CA	100.00
265GLY	C	266THR	CB	3.02
265GLY	C	266THR	C	97.64
266THR	CA	266THR	CB	100.00
266THR	CA	266THR	CG2	100.00
266THR	CA	266THR	C	100.00
266THR	CA	267PRO	CD	100.00
266THR	CB	266THR	CG2	100.00
266THR	CB	266THR	C	100.00
266THR	CB	267PRO	CD	0.01
266THR	CG2	266THR	C	99.98
266THR	CG2	267PRO	CD	13.46
266THR	C	267PRO	CA	100.00
266THR	C	267PRO	CB	0.09
266THR	C	267PRO	CD	100.00
266THR	C	267PRO	C	100.00
267PRO	CA	267PRO	CB	100.00
267PRO	CA	267PRO	CG	100.00
267PRO	CA	267PRO	CD	100.00
267PRO	CA	267PRO	C	100.00
267PRO	CB	267PRO	CG	100.00
267PRO	CB	267PRO	CD	100.00
267PRO	CB	267PRO	C	100.00
267PRO	CB	269PHE	CE1	0.31
267PRO	CB	269PHE	CZ	0.05
267PRO	CB	300HIS	CD2	0.00
267PRO	CG	267PRO	CD	100.00
267PRO	CG	267PRO	C	99.16
267PRO	CG	269PHE	CE1	0.20
267PRO	CG	269PHE	CZ	0.02
267PRO	CG	300HIS	CD2	0.03
267PRO	CG	300HIS	CE1	0.04
267PRO	CG	301ALA	CB	0.02
267PRO	CD	267PRO	C	95.62
267PRO	C	268VAL	CA	100.00
267PRO	C	268VAL	CB	49.70
267PRO	C	268VAL	CG2	31.77
267PRO	C	268VAL	C	67.85

155GL	CG	157TYR	CE1	2.22
155GL	CG	157TYR	CE2	1.50
155GL	CG	157TYR	CZ	0.01
155GL	CG	191VAL	CB	0.00
155GL	CG	191VAL	CG1	0.03
155GL	CG	191VAL	CG2	0.12
155GL	CG	238ILE	CG2	0.02
155GL	CG	238ILE	CD	0.02
155GL	CD	155GLU	C	0.34
155GL	CD	157TYR	CD1	0.09
155GL	CD	157TYR	CD2	0.09
155GL	CD	157TYR	CE1	5.64
155GL	CD	157TYR	CE2	2.65
155GL	CD	157TYR	CZ	0.12
155GL	C	156ARG	CA	100.00
155GL	C	156ARG	CB	95.04
155GL	C	156ARG	CG	9.94
155GL	C	156ARG	C	6.28
156AR	CA	156ARG	CB	100.00
156AR	CA	156ARG	CG	100.00
156AR	CA	156ARG	CD	2.42
156AR	CA	156ARG	C	100.00
156AR	CB	156ARG	CG	100.00
156AR	CB	156ARG	CD	100.00
156AR	CB	156ARG	CZ	0.48
156AR	CB	156ARG	C	100.00
156AR	CG	156ARG	CD	100.00
156AR	CG	156ARG	CZ	39.14
156AR	CG	156ARG	C	82.87
156AR	CD	156ARG	CZ	100.00
156AR	CD	156ARG	C	2.74
156AR	CZ	158SER	CB	0.31
156AR	C	157TYR	CA	100.00
156AR	C	157TYR	CB	50.22
156AR	C	157TYR	CG	4.28
156AR	C	157TYR	CD1	2.66
156AR	C	157TYR	CD2	1.57
156AR	C	157TYR	C	48.58
157TY	CA	157TYR	CB	100.00
157TY	CA	157TYR	CG	100.00
157TY	CA	157TYR	CD1	59.77
157TY	CA	157TYR	CD2	51.24
157TY	CA	157TYR	C	100.00
157TY	CB	157TYR	CG	100.00
157TY	CB	157TYR	CD1	100.00
157TY	CB	157TYR	CD2	100.00
157TY	CB	157TYR	C	100.00
157TY	CB	162VAL	CG1	0.13
157TY	CB	162VAL	CG2	0.52
157TY	CB	194PHE	CD2	1.08
157TY	CB	194PHE	CE2	1.22
157TY	CG	157TYR	CD1	100.00
157TY	CG	157TYR	CD2	100.00
157TY	CG	157TYR	CE1	100.00
157TY	CG	157TYR	CE2	100.00
157TY	CG	157TYR	CZ	100.00
157TY	CG	157TYR	C	0.02
157TY	CG	162VAL	CG1	1.82
157TY	CG	162VAL	CG2	1.99
157TY	CG	191VAL	CB	0.16
157TY	CG	191VAL	CG1	0.04
157TY	CD1	157TYR	CD2	100.00

259SE	CA	259SER	C	100.00
259SE	CA	296MET	CE	0.00
259SE	CB	259SER	C	100.00
259SE	CB	272VAL	CG1	0.00
259SE	CB	272VAL	CG2	3.67
259SE	C	260ALA	CA	100.00
259SE	C	260ALA	CB	91.90
259SE	C	260ALA	C	10.13
259SE	C	296MET	CE	0.12
260AL	CA	260ALA	CB	100.00
260AL	CA	260ALA	C	100.00
260AL	CB	260ALA	C	100.00
260AL	CB	262LEU	CD1	1.11
260AL	CB	262LEU	CD2	0.01
260AL	CB	269PHE	CG	0.01
260AL	CB	269PHE	CD2	24.82
260AL	CB	269PHE	CE2	20.57
260AL	CB	269PHE	CZ	0.01
260AL	CB	296MET	CE	9.28
260AL	C	261SER	CA	100.00
260AL	C	261SER	CB	44.32
260AL	C	261SER	C	64.96
260AL	C	262LEU	CD1	0.02
261SE	CA	261SER	CB	100.00
261SE	CA	261SER	C	100.00
261SE	CB	261SER	C	100.00
261SE	C	262LEU	CA	100.00
261SE	C	262LEU	CB	9.31
261SE	C	262LEU	CG	0.43
261SE	C	262LEU	CD1	2.98
261SE	C	262LEU	CD2	0.16
261SE	C	262LEU	C	89.21
262LE	CA	262LEU	CB	100.00
262LE	CA	262LEU	CG	100.00
262LE	CA	262LEU	CD1	31.05
262LE	CA	262LEU	CD2	66.85
262LE	CA	262LEU	C	100.00
262LE	CB	262LEU	CG	100.00
262LE	CB	262LEU	CD1	100.00
262LE	CB	262LEU	CD2	100.00
262LE	CB	262LEU	C	100.00
262LE	CG	262LEU	CD1	100.00
262LE	CG	262LEU	CD2	100.00
262LE	CG	262LEU	C	9.25
262LE	CG	267PRO	CB	0.00
262LE	CG	300HIS	CE1	0.01
262LE	CD1	262LEU	CD2	100.00
262LE	CD1	262LEU	C	0.92
262LE	CD1	267PRO	CB	0.17
262LE	CD1	300HIS	CD2	0.00
262LE	CD1	300HIS	CE1	1.26
262LE	CD2	262LEU	C	2.98
262LE	CD2	267PRO	CB	2.57
262LE	CD2	300HIS	CD2	0.44
262LE	CD2	300HIS	CE1	4.70
262LE	C	263GLY	CA	100.00
262LE	C	263GLY	C	0.69
263GL	CA	263GLY	C	100.00
263GL	C	264ARG	CA	100.00
263GL	C	264ARG	CB	0.29
263GL	C	264ARG	C	99.78
264AR	CA	264ARG	CB	100.00

267PRO C	269PHE CE1	0.02	157TYF CD1	157TYR	CE1	100.00	264AR CA	264ARG	CG	100.00
268VAL CA	268VAL CB	100.00	157TYF CD1	157TYR	CE2	100.00	264AR CA	264ARG	CD	21.02
268VAL CA	268VAL CG1	100.00	157TYF CD1	157TYR	CZ	100.00	264AR CA	264ARG	C	100.00
268VAL CA	268VAL CG2	100.00	157TYF CD1	161GLU	CB	0.00	264AR CB	264ARG	CG	100.00
268VAL CA	268VAL C	100.00	157TYF CD1	162VAL	CB	0.01	264AR CB	264ARG	CD	100.00
268VAL CB	268VAL CG1	100.00	157TYF CD1	162VAL	CG1	2.46	264AR CB	264ARG	CZ	0.71
268VAL CB	268VAL CG2	100.00	157TYF CD1	162VAL	CG2	4.56	264AR CB	264ARG	C	100.00
268VAL CB	268VAL C	100.00	157TYF CD1	191VAL	CB	1.45	264AR CG	264ARG	CD	100.00
268VAL CG1	268VAL CG2	100.00	157TYF CD1	191VAL	CG1	0.80	264AR CG	264ARG	CZ	18.16
268VAL CG1	268VAL C	100.00	157TYF CD1	191VAL	CG2	0.00	264AR CG	264ARG	C	98.99
268VAL CG1	270GLU CB	0.00	157TYF CD1	191VAL	C	0.00	264AR CD	264ARG	CZ	100.00
268VAL C	269PHE CA	100.00	157TYF CD1	195TRP	CE3	0.01	264AR CD	264ARG	C	37.21
268VAL C	269PHE CB	16.87	157TYF CD1	195TRP	CZ3	0.02	264AR CZ	264ARG	C	0.32
268VAL C	269PHE CG	2.93	157TYF CD2	157TYR	CE1	100.00	264AR CZ	265GLY	CA	0.96
268VAL C	269PHE CD1	8.84	157TYF CD2	157TYR	CE2	100.00	264AR C	265GLY	CA	100.00
268VAL C	269PHE C	89.34	157TYF CD2	157TYR	CZ	100.00	264AR C	265GLY	C	77.47
269PHE CA	269PHE CB	100.00	157TYF CD2	161GLU	CB	0.01	265GL CA	265GLY	C	100.00
269PHE CA	269PHE CG	100.00	157TYF CD2	162VAL	CB	0.02	265GL C	266THR	CA	100.00
269PHE CA	269PHE CD1	96.65	157TYF CD2	162VAL	CG1	2.34	265GL C	266THR	CB	0.13
269PHE CA	269PHE CD2	84.62	157TYF CD2	162VAL	CG2	4.78	265GL C	266THR	C	99.97
269PHE CA	269PHE C	100.00	157TYF CD2	191VAL	CB	1.05	266TH CA	266THR	CB	100.00
269PHE CB	269PHE CG	100.00	157TYF CD2	191VAL	CG1	0.55	266TH CA	266THR	CG2	100.00
269PHE CB	269PHE CD1	100.00	157TYF CD2	191VAL	CG2	0.00	266TH CA	266THR	C	100.00
269PHE CB	269PHE CD2	100.00	157TYF CD2	191VAL	C	0.03	266TH CA	267PRO	CD	100.00
269PHE CB	269PHE C	100.00	157TYF CD2	195TRP	CE3	0.16	266TH CB	266THR	CG2	100.00
269PHE CB	297MET CE	0.38	157TYF CD2	195TRP	CZ3	0.00	266TH CB	266THR	C	100.00
269PHE CG	269PHE CD1	100.00	157TYF CE1	157TYR	CE2	100.00	266TH CG2	266THR	C	100.00
269PHE CG	269PHE CD2	100.00	157TYF CE1	157TYR	CZ	100.00	266TH CG2	267PRO	CD	5.47
269PHE CG	269PHE CE1	100.00	157TYF CE1	161GLU	CB	0.00	266TH C	267PRO	CA	100.00
269PHE CG	269PHE CE2	100.00	157TYF CE1	161GLU	CG	0.00	266TH C	267PRO	CB	0.05
269PHE CG	269PHE CZ	100.00	157TYF CE1	162VAL	CG1	0.26	266TH C	267PRO	CD	100.00
269PHE CG	297MET CA	0.00	157TYF CE1	162VAL	CG2	0.07	266TH C	267PRO	C	100.00
269PHE CG	297MET CB	0.18	157TYF CE1	191VAL	CB	0.20	267PR CA	267PRO	CB	100.00
269PHE CG	297MET CG	0.04	157TYF CE1	191VAL	CG1	0.00	267PR CA	267PRO	CG	100.00
269PHE CG	297MET CE	0.47	157TYF CE1	191VAL	CG2	0.09	267PR CA	267PRO	CD	100.00
269PHE CD1	269PHE CD2	100.00	157TYF CE1	195TRP	CB	0.01	267PR CA	267PRO	C	100.00
269PHE CD1	269PHE CE1	100.00	157TYF CE1	195TRP	CD2	0.00	267PR CB	267PRO	CG	100.00
269PHE CD1	269PHE CE2	100.00	157TYF CE1	195TRP	CE3	3.63	267PR CB	267PRO	CD	100.00
269PHE CD1	269PHE CZ	100.00	157TYF CE1	195TRP	CZ3	1.16	267PR CB	267PRO	C	100.00
269PHE CD1	297MET CA	0.00	157TYF CE2	157TYR	CZ	100.00	267PR CB	269PHE	CE1	0.56
269PHE CD1	297MET CB	0.68	157TYF CE2	162VAL	CG1	0.41	267PR CB	269PHE	CZ	0.06
269PHE CD1	297MET CG	1.78	157TYF CE2	162VAL	CG2	0.22	267PR CB	300HIS	CD2	0.00
269PHE CD1	297MET CE	2.53	157TYF CE2	191VAL	CB	0.19	267PR CB	300HIS	CE1	0.01
269PHE CD2	269PHE CE1	100.00	157TYF CE2	191VAL	CG2	0.09	267PR CG	267PRO	CD	100.00
269PHE CD2	269PHE CE2	100.00	157TYF CE2	191VAL	C	0.06	267PR CG	267PRO	C	99.34
269PHE CD2	269PHE CZ	100.00	157TYF CE2	195TRP	CB	0.01	267PR CG	269PHE	CE1	0.22
269PHE CD2	296MET CB	0.13	157TYF CE2	195TRP	CD2	0.00	267PR CG	269PHE	CZ	0.00
269PHE CD2	296MET C	0.07	157TYF CE2	195TRP	CE3	5.41	267PR CG	300HIS	CD2	0.06
269PHE CD2	297MET CA	0.38	157TYF CE2	195TRP	CZ3	0.62	267PR CG	300HIS	CE1	0.00
269PHE CD2	297MET CB	0.08	157TYF CZ	162VAL	CG1	0.02	267PR CG	301ALA	CB	0.21
269PHE CD2	297MET CG	0.02	157TYF CZ	162VAL	CG2	0.00	267PR CD	267PRO	C	96.39
269PHE CE1	269PHE CE2	100.00	157TYF CZ	191VAL	CB	0.02	267PR C	268VAL	CA	100.00
269PHE CE1	269PHE CZ	100.00	157TYF CZ	191VAL	CG2	0.05	267PR C	268VAL	CB	35.25
269PHE CE1	297MET CA	0.07	157TYF CZ	195TRP	CE3	2.28	267PR C	268VAL	CG2	23.57
269PHE CE1	297MET CB	0.07	157TYF CZ	195TRP	CZ3	2.30	267PR C	268VAL	C	79.66
269PHE CE1	297MET CG	0.73	157TYF C	158SER	CA	100.00	267PR C	269PHE	CE1	0.02
269PHE CE1	300HIS CB	0.00	157TYF C	158SER	CB	46.13	268VA CA	268VAL	CB	100.00
269PHE CE1	300HIS CD2	0.02	157TYF C	158SER	C	61.43	268VA CA	268VAL	CG1	100.00
269PHE CE1	301ALA CB	0.22	157TYF C	194PHE	CE2	0.71	268VA CA	268VAL	CG2	100.00
269PHE CE2	269PHE CZ	100.00	158SEF CA	158SER	CB	100.00	268VA CA	268VAL	C	100.00
269PHE CE2	296MET CB	0.05	158SEF CA	158SER	C	100.00	268VA CB	268VAL	CG1	100.00
269PHE CE2	296MET CG	0.01	158SEF CA	194PHE	CE2	0.11	268VA CB	268VAL	CG2	100.00

269PHE	CE2	296MET	C	7.37	158SEI	CA	194PHE	CZ	0.01	268VA	CB	268VAL	C	100.00
269PHE	CE2	297MET	CA	12.97	158SEI	CB	158SER	C	100.00	268VA	CG1	268VAL	CG2	100.00
269PHE	CE2	297MET	CB	0.04	158SEI	CB	160PRO	CD	0.00	268VA	CG1	268VAL	C	100.00
269PHE	CE2	297MET	CG	0.02	158SEI	CB	161GLU	CB	0.01	268VA	C	269PHE	CA	100.00
269PHE	CE2	300HIS	CB	0.03	158SEI	CB	161GLU	CD	0.22	268VA	C	269PHE	CB	18.46
269PHE	CE2	300HIS	CG	0.00	158SEI	C	159LYS	CA	100.00	268VA	C	269PHE	CG	4.50
269PHE	CE2	300HIS	CD2	0.01	158SEI	C	159LYS	C	100.00	268VA	C	269PHE	CD1	8.20
269PHE	CZ	297MET	CA	2.08	158SEI	C	160PRO	CD	18.79	268VA	C	269PHE	C	86.39
269PHE	CZ	297MET	CB	0.02	158SEI	C	194PHE	CE2	1.08	269PH	CA	269PHE	CB	100.00
269PHE	CZ	297MET	CG	0.04	158SEI	C	194PHE	CZ	0.18	269PH	CA	269PHE	CG	100.00
269PHE	CZ	300HIS	CB	0.69	159LYS	CA	159LYS	CB	100.00	269PH	CA	269PHE	CD1	94.59
269PHE	CZ	300HIS	CG	0.22	159LYS	CA	159LYS	CG	100.00	269PH	CA	269PHE	CD2	89.80
269PHE	CZ	300HIS	CD2	0.92	159LYS	CA	159LYS	CD	19.40	269PH	CA	269PHE	C	100.00
269PHE	CZ	301ALA	CB	0.31	159LYS	CA	159LYS	CE	0.01	269PH	CB	269PHE	CG	100.00
269PHE	C	270GLU	CA	100.00	159LYS	CA	159LYS	C	100.00	269PH	CB	269PHE	CD1	100.00
269PHE	C	270GLU	CB	91.50	159LYS	CA	160PRO	CD	100.00	269PH	CB	269PHE	CD2	100.00
269PHE	C	270GLU	CG	0.03	159LYS	CA	162VAL	CG2	0.00	269PH	CB	269PHE	C	100.00
269PHE	C	270GLU	C	10.78	159LYS	CA	194PHE	CE2	0.06	269PH	CB	297MET	CE	0.19
270GLU	CA	270GLU	CB	100.00	159LYS	CA	194PHE	CZ	1.58	269PH	CG	269PHE	CD1	100.00
270GLU	CA	270GLU	CG	100.00	159LYS	CB	159LYS	CG	100.00	269PH	CG	269PHE	CD2	100.00
270GLU	CA	270GLU	C	100.00	159LYS	CB	159LYS	CD	100.00	269PH	CG	269PHE	CE1	100.00
270GLU	CA	271PRO	CD	100.00	159LYS	CB	159LYS	CE	4.60	269PH	CG	269PHE	CE2	100.00
270GLU	CB	270GLU	CG	100.00	159LYS	CB	159LYS	C	100.00	269PH	CG	269PHE	CZ	100.00
270GLU	CB	270GLU	CD	100.00	159LYS	CB	160PRO	CD	69.71	269PH	CG	297MET	CB	0.10
270GLU	CB	270GLU	C	100.00	159LYS	CB	194PHE	CE2	0.07	269PH	CG	297MET	CG	0.02
270GLU	CB	271PRO	CD	0.48	159LYS	CB	194PHE	CZ	1.45	269PH	CG	297MET	CE	0.30
270GLU	CG	270GLU	CD	100.00	159LYS	CG	159LYS	CD	100.00	269PH	CD1	269PHE	CD2	100.00
270GLU	CG	270GLU	C	99.82	159LYS	CG	159LYS	CE	100.00	269PH	CD1	269PHE	CE1	100.00
270GLU	CG	271PRO	CD	5.43	159LYS	CG	159LYS	C	94.38	269PH	CD1	269PHE	CE2	100.00
270GLU	C	271PRO	CA	100.00	159LYS	CG	160PRO	CD	0.11	269PH	CD1	269PHE	CZ	100.00
270GLU	C	271PRO	CB	0.16	159LYS	CG	194PHE	CE1	0.03	269PH	CD1	297MET	CA	0.01
270GLU	C	271PRO	CD	100.00	159LYS	CG	194PHE	CE2	0.05	269PH	CD1	297MET	CB	0.70
270GLU	C	271PRO	C	100.00	159LYS	CG	194PHE	CZ	0.92	269PH	CD1	297MET	CG	2.36
271PRO	CA	271PRO	CB	100.00	159LYS	CG	198THR	CG2	1.67	269PH	CD1	297MET	CE	2.09
271PRO	CA	271PRO	CG	100.00	159LYS	CD	159LYS	CE	100.00	269PH	CD2	269PHE	CE1	100.00
271PRO	CA	271PRO	CD	100.00	159LYS	CD	159LYS	C	5.30	269PH	CD2	269PHE	CE2	100.00
271PRO	CA	271PRO	C	100.00	159LYS	CD	160PRO	CD	0.00	269PH	CD2	269PHE	CZ	100.00
271PRO	CB	271PRO	CG	100.00	159LYS	CD	163GLU	CD	0.00	269PH	CD2	296MET	CB	0.05
271PRO	CB	271PRO	CD	100.00	159LYS	CD	194PHE	CE1	0.04	269PH	CD2	296MET	CG	0.01
271PRO	CB	271PRO	C	100.00	159LYS	CD	194PHE	CZ	0.33	269PH	CD2	296MET	C	0.07
271PRO	CB	273HIS	CG	0.00	159LYS	CD	198THR	CG2	0.06	269PH	CD2	297MET	CA	0.29
271PRO	CB	273HIS	CD2	9.58	159LYS	CE	159LYS	C	0.01	269PH	CD2	297MET	CB	0.03
271PRO	CB	273HIS	CE1	2.34	159LYS	CE	163GLU	CD	0.01	269PH	CD2	297MET	CG	0.02
271PRO	CB	289ALA	CB	21.42	159LYS	CE	194PHE	CE1	0.04	269PH	CE1	269PHE	CE2	100.00
271PRO	CB	289ALA	C	5.74	159LYS	CE	194PHE	CZ	0.02	269PH	CE1	269PHE	CZ	100.00
271PRO	CB	290ALA	CA	0.02	159LYS	CE	198THR	CG2	0.10	269PH	CE1	297MET	CA	0.10
271PRO	CG	271PRO	CD	100.00	159LYS	CE	201GLU	CD	0.75	269PH	CE1	297MET	CB	0.08
271PRO	CG	271PRO	C	99.48	159LYS	CE	202VAL	CG1	0.00	269PH	CE1	297MET	CG	0.93
271PRO	CG	273HIS	CD2	7.37	159LYS	C	160PRO	CA	100.00	269PH	CE1	301ALA	CB	1.04
271PRO	CG	273HIS	CE1	1.46	159LYS	C	160PRO	CB	0.12	269PH	CE2	269PHE	CZ	100.00
271PRO	CD	271PRO	C	97.66	159LYS	C	160PRO	CD	100.00	269PH	CE2	296MET	CB	0.04
271PRO	C	272VAL	CA	100.00	159LYS	C	160PRO	C	100.00	269PH	CE2	296MET	CG	0.04
271PRO	C	272VAL	CB	4.08	160PR(	CA	160PRO	CB	100.00	269PH	CE2	296MET	C	7.29
271PRO	C	272VAL	CG2	2.47	160PR(	CA	160PRO	CG	100.00	269PH	CE2	297MET	CA	8.20
271PRO	C	272VAL	C	99.04	160PR(	CA	160PRO	CD	100.00	269PH	CE2	297MET	CB	0.03
272VAL	CA	272VAL	CB	100.00	160PR(	CA	160PRO	C	100.00	269PH	CE2	300HIS	CB	0.02
272VAL	CA	272VAL	CG1	100.00	160PR(	CA	163GLU	CB	0.03	269PH	CE2	300HIS	CG	0.00
272VAL	CA	272VAL	CG2	100.00	160PR(	CB	160PRO	CG	100.00	269PH	CZ	297MET	CA	1.84
272VAL	CA	272VAL	C	100.00	160PR(	CB	160PRO	CD	100.00	269PH	CZ	297MET	CB	0.01
272VAL	CB	272VAL	CG1	100.00	160PR(	CB	160PRO	C	100.00	269PH	CZ	297MET	CG	0.02
272VAL	CB	272VAL	CG2	100.00	160PR(	CB	164ARG	CD	0.02	269PH	CZ	300HIS	CB	0.37
272VAL	CB	272VAL	C	100.00	160PR(	CB	164ARG	CZ	0.21	269PH	CZ	300HIS	CG	0.09

272VAL	CG1	272VAL CG2	100.00	160PR(	CG	160PRO CD	100.00	269PH	CZ	300HIS CD2	0.76
272VAL	CG1	272VAL C	100.00	160PR(	CG	160PRO C	85.02	269PH	CZ	301ALA CB	0.80
272VAL	C	273HIS CA	100.00	160PR(	CG	164ARG CZ	0.01	269PH	C	270GLU CA	100.00
272VAL	C	273HIS CB	54.37	160PR(	CD	160PRO C	69.39	269PH	C	270GLU CB	98.63
272VAL	C	273HIS CG	0.56	160PR(	C	161GLU CA	100.00	269PH	C	270GLU CG	0.01
272VAL	C	273HIS CD2	0.15	160PR(	C	161GLU CB	0.38	269PH	C	270GLU C	1.90
272VAL	C	273HIS C	46.01	160PR(	C	161GLU CG	0.01	270GL	CA	270GLU CB	100.00
273HIS	CA	273HIS CB	100.00	160PR(	C	161GLU C	99.80	270GL	CA	270GLU CG	100.00
273HIS	CA	273HIS CG	100.00	160PR(	C	163GLU CB	0.02	270GL	CA	270GLU C	100.00
273HIS	CA	273HIS CD2	92.36	160PR(	C	164ARG CZ	0.01	270GL	CA	271PRO CD	100.00
273HIS	CA	273HIS C	100.00	161GLl	CA	161GLU CB	100.00	270GL	CB	270GLU CG	100.00
273HIS	CB	273HIS CG	100.00	161GLl	CA	161GLU CG	100.00	270GL	CB	270GLU CD	100.00
273HIS	CB	273HIS CD2	100.00	161GLl	CA	161GLU CD	71.13	270GL	CB	270GLU C	100.00
273HIS	CB	273HIS CE1	0.03	161GLl	CA	161GLU C	100.00	270GL	CB	271PRO CD	0.25
273HIS	CB	273HIS C	100.00	161GLl	CA	164ARG CD	0.00	270GL	CG	270GLU CD	100.00
273HIS	CB	289ALA CB	0.01	161GLl	CA	164ARG CZ	0.06	270GL	CG	270GLU C	99.94
273HIS	CG	273HIS CD2	100.00	161GLl	CB	161GLU CG	100.00	270GL	CG	271PRO CD	7.88
273HIS	CG	273HIS CE1	100.00	161GLl	CB	161GLU CD	100.00	270GL	C	271PRO CA	100.00
273HIS	CG	273HIS C	99.11	161GLl	CB	161GLU C	100.00	270GL	C	271PRO CB	0.08
273HIS	CG	289ALA CB	0.21	161GLl	CG	161GLU CD	100.00	270GL	C	271PRO CD	100.00
273HIS	CD2	273HIS CE1	100.00	161GLl	CG	161GLU C	66.11	270GL	C	271PRO C	100.00
273HIS	CD2	273HIS C	71.19	161GLl	CD	164ARG CD	1.04	270GL	C	293SER CB	0.01
273HIS	CD2	289ALA CB	0.19	161GLl	CD	164ARG CZ	0.04	271PR	CA	271PRO CB	100.00
273HIS	CE1	289ALA CB	0.19	161GLl	C	162VAL CA	100.00	271PR	CA	271PRO CG	100.00
273HIS	CE1	290ALA CB	0.02	161GLl	C	162VAL CB	1.64	271PR	CA	271PRO CD	100.00
273HIS	C	274GLY CA	100.00	161GLl	C	162VAL CG1	0.07	271PR	CA	271PRO C	100.00
273HIS	C	274GLY C	62.86	161GLl	C	162VAL CG2	0.12	271PR	CA	289ALA CB	0.15
274GLY	CA	274GLY C	100.00	161GLl	C	162VAL C	98.62	271PR	CB	271PRO CG	100.00
274GLY	C	275SER CA	100.00	161GLl	C	164ARG CB	0.01	271PR	CB	271PRO CD	100.00
274GLY	C	275SER CB	39.48	161GLl	C	165VAL CG2	0.01	271PR	CB	271PRO C	100.00
274GLY	C	275SER C	66.01	162VAl	CA	162VAL CB	100.00	271PR	CB	273HIS CG	0.00
275SER	CA	275SER CB	100.00	162VAl	CA	162VAL CG1	100.00	271PR	CB	273HIS CD2	0.02
275SER	CA	275SER C	100.00	162VAl	CA	162VAL CG2	100.00	271PR	CB	273HIS CE1	3.80
275SER	CA	279ILE CD	0.00	162VAl	CA	162VAL C	100.00	271PR	CB	289ALA CB	16.55
275SER	CB	275SER C	100.00	162VAl	CA	195TRP CZ3	0.01	271PR	CB	289ALA C	1.81
275SER	C	276ALA CA	100.00	162VAl	CB	162VAL CG1	100.00	271PR	CB	290ALA CA	0.04
275SER	C	276ALA CB	87.79	162VAl	CB	162VAL CG2	100.00	271PR	CG	271PRO CD	100.00
275SER	C	276ALA C	19.18	162VAl	CB	162VAL C	100.00	271PR	CG	271PRO C	99.09
275SER	C	277PRO CD	0.02	162VAl	CB	195TRP CE3	0.16	271PR	CG	273HIS CD2	0.06
276ALA	CA	276ALA CB	100.00	162VAl	CB	195TRP CZ3	0.14	271PR	CG	273HIS CE1	5.13
276ALA	CA	276ALA C	100.00	162VAl	CB	198THR CG2	0.22	271PR	CG	290ALA CB	0.01
276ALA	CA	277PRO CD	100.00	162VAl	CG1	162VAL CG2	100.00	271PR	CD	271PRO C	97.42
276ALA	CB	276ALA C	100.00	162VAl	CG1	162VAL C	82.12	271PR	C	272VAL CA	100.00
276ALA	CB	277PRO CD	31.46	162VAl	CG1	194PHE CE2	0.16	271PR	C	272VAL CB	9.53
276ALA	CB	279ILE CB	0.01	162VAl	CG1	195TRP CD2	0.69	271PR	C	272VAL CG1	0.22
276ALA	CB	279ILE CG1	0.24	162VAl	CG1	195TRP CE2	0.00	271PR	C	272VAL CG2	6.44
276ALA	CB	279ILE CD	0.74	162VAl	CG1	195TRP CE3	13.72	271PR	C	272VAL C	95.23
276ALA	C	277PRO CA	100.00	162VAl	CG1	195TRP CZ3	1.68	271PR	C	273HIS CE1	0.00
276ALA	C	277PRO CB	0.02	162VAl	CG1	198THR CB	0.78	271PR	C	289ALA CB	0.00
276ALA	C	277PRO CD	100.00	162VAl	CG1	198THR CG2	5.89	272VA	CA	272VAL CB	100.00
276ALA	C	277PRO C	100.00	162VAl	CG1	199VAL CG1	0.16	272VA	CA	272VAL CG1	100.00
277PRO	CA	277PRO CB	100.00	162VAl	CG1	199VAL CG2	1.07	272VA	CA	272VAL CG2	100.00
277PRO	CA	277PRO CG	100.00	162VAl	CG2	162VAL C	23.88	272VA	CA	272VAL C	100.00
277PRO	CA	277PRO CD	100.00	162VAl	CG2	194PHE CE2	0.16	272VA	CB	272VAL CG1	100.00
277PRO	CA	277PRO C	100.00	162VAl	CG2	195TRP CD2	0.26	272VA	CB	272VAL CG2	100.00
277PRO	CB	277PRO CG	100.00	162VAl	CG2	195TRP CE3	4.47	272VA	CB	272VAL C	100.00
277PRO	CB	277PRO CD	100.00	162VAl	CG2	195TRP CZ3	0.84	272VA	CG1	272VAL CG2	100.00
277PRO	CB	277PRO C	100.00	162VAl	CG2	198THR CB	0.07	272VA	CG1	272VAL C	98.82
277PRO	CG	277PRO CD	100.00	162VAl	CG2	198THR CG2	0.94	272VA	CG2	272VAL C	1.18
277PRO	CG	277PRO C	26.69	162VAl	CG2	199VAL CG2	0.08	272VA	C	273HIS CA	100.00
277PRO	CD	277PRO C	17.28	162VAl	C	163GLU CA	100.00	272VA	C	273HIS CB	61.62
277PRO	C	278ASP CA	100.00	162VAl	C	163GLU CB	0.03	272VA	C	273HIS CG	27.60

277PRO	C	278ASP	CB	0.33	162VAL	C	163GLU	C	100.00	272VA	C	273HIS	CD2	8.67
277PRO	C	278ASP	C	99.72	162VAL	C	165VAL	CG1	0.02	272VA	C	273HIS	C	37.38
278ASP	CA	278ASP	CB	100.00	162VAL	C	165VAL	CG2	0.00	273HIS	CA	273HIS	CB	100.00
278ASP	CA	278ASP	CG	100.00	162VAL	C	198THR	CG2	0.02	273HIS	CA	273HIS	CG	100.00
278ASP	CA	278ASP	C	100.00	163GLU	CA	163GLU	CB	100.00	273HIS	CA	273HIS	CD2	50.20
278ASP	CB	278ASP	CG	100.00	163GLU	CA	163GLU	CG	100.00	273HIS	CA	273HIS	C	100.00
278ASP	CB	278ASP	C	100.00	163GLU	CA	163GLU	CD	21.99	273HIS	CB	273HIS	CG	100.00
278ASP	CG	278ASP	C	98.46	163GLU	CA	163GLU	C	100.00	273HIS	CB	273HIS	CD2	100.00
278ASP	CG	279ILE	CG2	0.01	163GLU	CA	198THR	CG2	0.38	273HIS	CB	273HIS	CE1	0.02
278ASP	CG	282LYS	CE	0.21	163GLU	CB	163GLU	CG	100.00	273HIS	CB	273HIS	C	100.00
278ASP	C	279ILE	CA	100.00	163GLU	CB	163GLU	CD	100.00	273HIS	CB	275SER	C	0.14
278ASP	C	279ILE	CB	5.24	163GLU	CB	163GLU	C	100.00	273HIS	CB	276ALA	CA	0.03
278ASP	C	279ILE	CG1	0.16	163GLU	CB	167ARG	CZ	0.02	273HIS	CB	276ALA	CB	0.04
278ASP	C	279ILE	CG2	2.74	163GLU	CB	198THR	CG2	0.43	273HIS	CB	277PRO	CD	0.01
278ASP	C	279ILE	CD	0.00	163GLU	CB	202VAL	CG2	0.01	273HIS	CG	273HIS	CD2	100.00
278ASP	C	279ILE	C	94.81	163GLU	CG	163GLU	CD	100.00	273HIS	CG	273HIS	CE1	100.00
278ASP	C	282LYS	CE	0.00	163GLU	CG	163GLU	C	95.35	273HIS	CG	273HIS	C	1.60
279ILE	CA	279ILE	CB	100.00	163GLU	CG	167ARG	CG	0.01	273HIS	CG	276ALA	CB	0.03
279ILE	CA	279ILE	CG1	100.00	163GLU	CG	167ARG	CD	0.06	273HIS	CG	277PRO	CD	0.02
279ILE	CA	279ILE	CG2	100.00	163GLU	CG	167ARG	CZ	0.03	273HIS	CD2	273HIS	CE1	100.00
279ILE	CA	279ILE	CD	8.83	163GLU	CG	198THR	CG2	0.33	273HIS	CD2	276ALA	CB	0.07
279ILE	CA	279ILE	C	100.00	163GLU	CG	202VAL	CB	0.04	273HIS	CD2	277PRO	CG	0.01
279ILE	CA	282LYS	CB	0.03	163GLU	CG	202VAL	CG1	1.82	273HIS	CD2	277PRO	CD	0.02
279ILE	CA	284ILE	CD	0.00	163GLU	CG	202VAL	CG2	5.06	273HIS	CD2	289ALA	CB	0.12
279ILE	CB	279ILE	CG1	100.00	163GLU	CD	163GLU	C	22.62	273HIS	CE1	276ALA	CB	0.00
279ILE	CB	279ILE	CG2	100.00	163GLU	CD	167ARG	CG	0.18	273HIS	CE1	289ALA	CB	0.00
279ILE	CB	279ILE	CD	100.00	163GLU	CD	167ARG	CD	0.14	273HIS	C	274GLY	CA	100.00
279ILE	CB	279ILE	C	100.00	163GLU	CD	167ARG	CZ	0.17	273HIS	C	274GLY	C	78.47
279ILE	CB	284ILE	CG2	0.00	163GLU	CD	202VAL	CG1	0.45	274GL	CA	274GLY	C	100.00
279ILE	CB	284ILE	CD	0.05	163GLU	CD	202VAL	CG2	0.18	274GL	C	275SER	CA	100.00
279ILE	CG1	279ILE	CG2	100.00	163GLU	C	164ARG	CA	100.00	274GL	C	275SER	CB	68.33
279ILE	CG1	279ILE	CD	100.00	163GLU	C	164ARG	CB	0.00	274GL	C	275SER	C	36.44
279ILE	CG1	279ILE	C	67.15	163GLU	C	164ARG	C	100.00	275SE	CA	275SER	CB	100.00
279ILE	CG2	279ILE	CD	97.38	164ARG	CA	164ARG	CB	100.00	275SE	CA	275SER	C	100.00
279ILE	CG2	279ILE	C	32.78	164ARG	CA	164ARG	CG	100.00	275SE	CB	275SER	C	100.00
279ILE	CG2	282LYS	CD	0.00	164ARG	CA	164ARG	C	100.00	275SE	C	276ALA	CA	100.00
279ILE	CG2	284ILE	CB	0.00	164ARG	CA	167ARG	CZ	0.34	275SE	C	276ALA	CB	12.56
279ILE	CG2	284ILE	CG1	0.16	164ARG	CB	164ARG	CG	100.00	275SE	C	276ALA	C	89.64
279ILE	CG2	284ILE	CG2	0.10	164ARG	CB	164ARG	CD	100.00	275SE	C	277PRO	CD	0.74
279ILE	CG2	284ILE	CD	0.52	164ARG	CB	164ARG	CZ	2.20	276AL	CA	276ALA	CB	100.00
279ILE	CG2	284ILE	C	0.08	164ARG	CB	164ARG	C	100.00	276AL	CA	276ALA	C	100.00
279ILE	CG2	285ALA	CA	0.05	164ARG	CB	167ARG	CZ	0.03	276AL	CA	277PRO	CD	100.00
279ILE	CG2	285ALA	CB	0.04	164ARG	CG	164ARG	CD	100.00	276AL	CB	276ALA	C	100.00
279ILE	CD	279ILE	C	1.43	164ARG	CG	164ARG	CZ	8.15	276AL	CB	277PRO	CD	2.52
279ILE	CD	282LYS	CD	0.00	164ARG	CG	164ARG	C	29.75	276AL	CB	279ILE	CG1	0.08
279ILE	CD	284ILE	CD	0.05	164ARG	CG	167ARG	CZ	0.04	276AL	CB	279ILE	CD	0.09
279ILE	CD	284ILE	C	0.00	164ARG	CD	164ARG	CZ	100.00	276AL	C	277PRO	CA	100.00
279ILE	CD	285ALA	CA	0.01	164ARG	CD	264ARG	CZ	0.00	276AL	C	277PRO	CB	0.32
279ILE	C	280ALA	CA	100.00	164ARG	CZ	264ARG	CD	0.19	276AL	C	277PRO	CD	100.00
279ILE	C	280ALA	CB	0.64	164ARG	CZ	264ARG	CZ	1.86	276AL	C	277PRO	C	100.00
279ILE	C	280ALA	C	99.57	164ARG	C	165VAL	CA	100.00	277PR	CA	277PRO	CB	100.00
279ILE	C	282LYS	CB	0.03	164ARG	C	165VAL	CB	0.23	277PR	CA	277PRO	CG	100.00
280ALA	CA	280ALA	CB	100.00	164ARG	C	165VAL	CG1	0.02	277PR	CA	277PRO	CD	100.00
280ALA	CA	280ALA	C	100.00	164ARG	C	165VAL	CG2	0.04	277PR	CA	277PRO	C	100.00
280ALA	CB	280ALA	C	100.00	164ARG	C	165VAL	C	99.86	277PR	CB	277PRO	CG	100.00
280ALA	C	281GLY	CA	100.00	165VAL	CA	165VAL	CB	100.00	277PR	CB	277PRO	CD	100.00
280ALA	C	281GLY	C	53.04	165VAL	CA	165VAL	CG1	100.00	277PR	CB	277PRO	C	100.00
281GLY	CA	281GLY	C	100.00	165VAL	CA	165VAL	CG2	100.00	277PR	CB	279ILE	CG1	0.19
281GLY	C	282LYS	CA	100.00	165VAL	CA	165VAL	C	100.00	277PR	CB	279ILE	CD	0.27
281GLY	C	282LYS	CB	5.86	165VAL	CA	168VAL	CG1	0.02	277PR	CB	280ALA	CB	0.00
281GLY	C	282LYS	CG	0.72	165VAL	CA	168VAL	CG2	0.01	277PR	CG	277PRO	CD	100.00
281GLY	C	282LYS	CD	0.00	165VAL	CB	165VAL	CG1	100.00	277PR	CG	277PRO	C	98.70

281GLY	C	282LYS	C	94.18	165VAl	CB	165VAL	CG2	100.00	277PR	CG	279ILE	CG1	0.29
282LYS	CA	282LYS	CB	100.00	165VAl	CB	165VAL	C	100.00	277PR	CG	279ILE	CG2	0.01
282LYS	CA	282LYS	CG	100.00	165VAl	CG1	165VAL	CG2	100.00	277PR	CG	279ILE	CD	3.76
282LYS	CA	282LYS	CD	1.44	165VAl	CG1	165VAL	C	93.03	277PR	CG	280ALA	CB	0.01
282LYS	CA	282LYS	CE	0.01	165VAl	CG1	195TRP	CZ2	0.00	277PR	CD	277PRO	C	95.33
282LYS	CA	282LYS	C	100.00	165VAl	CG1	195TRP	CZ3	0.08	277PR	C	278ASP	CA	100.00
282LYS	CB	282LYS	CG	100.00	165VAl	CG1	234VAL	CG1	0.08	277PR	C	278ASP	CB	10.18
282LYS	CB	282LYS	CD	100.00	165VAl	CG1	234VAL	CG2	1.51	277PR	C	278ASP	C	93.20
282LYS	CB	282LYS	CE	6.40	165VAl	CG2	165VAL	C	16.71	277PR	C	279ILE	CD	0.00
282LYS	CB	282LYS	C	100.00	165VAl	CG2	195TRP	CZ3	0.07	278AS	CA	278ASP	CB	100.00
282LYS	CB	284ILE	CG1	0.33	165VAl	CG2	234VAL	CG2	0.05	278AS	CA	278ASP	CG	100.00
282LYS	CB	284ILE	CG2	0.01	165VAl	C	166ALA	CA	100.00	278AS	CA	278ASP	C	100.00
282LYS	CB	284ILE	CD	0.46	165VAl	C	166ALA	CB	0.02	278AS	CB	278ASP	CG	100.00
282LYS	CG	282LYS	CD	100.00	165VAl	C	166ALA	C	99.99	278AS	CB	278ASP	C	100.00
282LYS	CG	282LYS	CE	100.00	165VAl	C	168VAL	CG1	0.03	278AS	CG	278ASP	C	98.37
282LYS	CG	282LYS	C	36.01	165VAl	C	168VAL	CG2	0.04	278AS	CG	282LYS	CE	0.01
282LYS	CG	284ILE	CG1	0.03	166AL/	CA	166ALA	CB	100.00	278AS	C	279ILE	CA	100.00
282LYS	CG	284ILE	CD	0.04	166AL/	CA	166ALA	C	100.00	278AS	C	279ILE	CB	79.10
282LYS	CD	282LYS	CE	100.00	166AL/	CB	166ALA	C	100.00	278AS	C	279ILE	CG1	0.23
282LYS	CD	284ILE	CG1	0.06	166AL/	CB	170PHE	CE1	0.09	278AS	C	279ILE	CG2	62.36
282LYS	CD	284ILE	CG2	0.01	166AL/	CB	170PHE	CE2	0.14	278AS	C	279ILE	C	20.48
282LYS	CD	284ILE	CD	0.81	166AL/	CB	170PHE	CZ	0.24	279ILE	CA	279ILE	CB	100.00
282LYS	CE	284ILE	CG1	0.01	166AL/	CB	199VAL	CG1	0.06	279ILE	CA	279ILE	CG1	100.00
282LYS	CE	284ILE	CD	0.21	166AL/	CB	199VAL	CG2	0.28	279ILE	CA	279ILE	CG2	100.00
282LYS	C	283GLY	CA	100.00	166AL/	CB	202VAL	CG1	0.42	279ILE	CA	279ILE	CD	3.95
282LYS	C	283GLY	C	69.72	166AL/	CB	202VAL	CG2	0.90	279ILE	CA	279ILE	C	100.00
283GLY	CA	283GLY	C	100.00	166AL/	C	167ARG	CA	100.00	279ILE	CA	282LYS	CG	0.01
283GLY	CA	333THR	CB	0.00	166AL/	C	167ARG	CB	0.03	279ILE	CB	279ILE	CG1	100.00
283GLY	C	284ILE	CA	100.00	166AL/	C	167ARG	CG	0.01	279ILE	CB	279ILE	CG2	100.00
283GLY	C	284ILE	CB	52.41	166AL/	C	167ARG	C	99.97	279ILE	CB	279ILE	CD	100.00
283GLY	C	284ILE	CG1	1.51	166AL/	C	170PHE	CD1	0.15	279ILE	CB	279ILE	C	100.00
283GLY	C	284ILE	CG2	35.06	166AL/	C	170PHE	CD2	0.66	279ILE	CB	282LYS	CD	0.00
283GLY	C	284ILE	CD	0.00	166AL/	C	170PHE	CE1	0.02	279ILE	CB	284ILE	CG1	0.00
283GLY	C	284ILE	C	44.91	166AL/	C	170PHE	CE2	0.15	279ILE	CB	284ILE	CG2	0.00
283GLY	C	332GLY	CA	0.05	167AR(	CA	167ARG	CB	100.00	279ILE	CB	284ILE	CD	0.06
284ILE	CA	284ILE	CB	100.00	167AR(	CA	167ARG	CG	100.00	279ILE	CG1	279ILE	CG2	100.00
284ILE	CA	284ILE	CG1	100.00	167AR(	CA	167ARG	CD	9.68	279ILE	CG1	279ILE	CD	100.00
284ILE	CA	284ILE	CG2	100.00	167AR(	CA	167ARG	C	100.00	279ILE	CG1	279ILE	C	99.44
284ILE	CA	284ILE	CD	16.18	167AR(	CA	170PHE	CD2	0.03	279ILE	CG2	279ILE	CD	95.82
284ILE	CA	284ILE	C	100.00	167AR(	CA	202VAL	CG1	0.02	279ILE	CG2	279ILE	C	0.01
284ILE	CA	332GLY	CA	0.02	167AR(	CB	167ARG	CG	100.00	279ILE	CG2	282LYS	CD	0.14
284ILE	CB	284ILE	CG1	100.00	167AR(	CB	167ARG	CD	100.00	279ILE	CG2	284ILE	CG1	0.02
284ILE	CB	284ILE	CG2	100.00	167AR(	CB	167ARG	CZ	1.62	279ILE	CG2	284ILE	CG2	0.10
284ILE	CB	284ILE	CD	100.00	167AR(	CB	167ARG	C	100.00	279ILE	CG2	284ILE	CD	0.58
284ILE	CB	284ILE	C	100.00	167AR(	CB	202VAL	CG1	0.00	279ILE	CG2	284ILE	C	0.00
284ILE	CB	325PRO	CB	0.00	167AR(	CG	167ARG	CD	100.00	279ILE	CD	279ILE	C	2.49
284ILE	CB	325PRO	CG	0.08	167AR(	CG	167ARG	CZ	44.45	279ILE	CD	284ILE	CB	0.00
284ILE	CG1	284ILE	CG2	100.00	167AR(	CG	167ARG	C	30.39	279ILE	CD	284ILE	CG1	0.16
284ILE	CG1	284ILE	CD	100.00	167AR(	CG	202VAL	CB	0.00	279ILE	CD	284ILE	CG2	0.02
284ILE	CG1	284ILE	C	87.89	167AR(	CG	202VAL	CG1	0.58	279ILE	CD	284ILE	CD	0.21
284ILE	CG1	325PRO	CG	0.01	167AR(	CG	202VAL	CG2	0.12	279ILE	CD	284ILE	C	0.43
284ILE	CG2	284ILE	CD	87.35	167AR(	CG	206TYR	CE1	0.06	279ILE	CD	285ALA	CA	1.63
284ILE	CG2	284ILE	C	11.18	167AR(	CG	206TYR	CE2	0.01	279ILE	CD	285ALA	CB	0.51
284ILE	CG2	325PRO	CB	0.00	167AR(	CG	206TYR	CZ	0.01	279ILE	CD	326ASP	CB	0.04
284ILE	CG2	325PRO	CG	0.51	167AR(	CD	167ARG	CZ	100.00	279ILE	CD	326ASP	CG	0.10
284ILE	CG2	325PRO	CD	0.00	167AR(	CD	167ARG	C	0.48	279ILE	C	280ALA	CA	100.00
284ILE	CG2	330SER	CA	0.01	167AR(	CD	202VAL	CG1	0.02	279ILE	C	280ALA	CB	0.45
284ILE	CG2	330SER	CB	0.00	167AR(	CD	206TYR	CD1	0.00	279ILE	C	280ALA	C	99.62
284ILE	CG2	330SER	C	0.01	167AR(	CD	206TYR	CE1	0.00	279ILE	C	282LYS	CG	0.04
284ILE	CD	284ILE	C	9.69	167AR(	CZ	168VAL	CG1	0.06	279ILE	C	282LYS	CE	0.01
284ILE	CD	285ALA	CA	0.00	167AR(	CZ	202VAL	CB	0.18	279ILE	C	284ILE	CG1	0.00
284ILE	CD	325PRO	CG	0.10	167AR(	CZ	202VAL	CG1	1.90	280AL.	CA	280ALA	CB	100.00

284ILE	C	285ALA	CA	100.00	167AR(	CZ	202VAL	CG2	1.42	280AL.	CA	280ALA	C	100.00
284ILE	C	285ALA	CB	0.57	167AR(	CZ	206TYR	CD1	0.03	280AL.	CB	280ALA	C	100.00
284ILE	C	285ALA	C	99.54	167AR(	CZ	206TYR	CE1	0.06	280AL.	C	281GLY	CA	100.00
284ILE	C	325PRO	CG	0.00	167AR(	C	168VAL	CA	100.00	280AL.	C	281GLY	C	56.53
284ILE	C	332GLY	CA	0.02	167AR(	C	168VAL	CB	0.16	281GL	CA	281GLY	C	100.00
285ALA	CA	285ALA	CB	100.00	167AR(	C	168VAL	CG1	0.05	281GL	C	282LYS	CA	100.00
285ALA	CA	285ALA	C	100.00	167AR(	C	168VAL	CG2	0.01	281GL	C	282LYS	CB	14.60
285ALA	CB	285ALA	C	100.00	167AR(	C	168VAL	C	99.88	281GL	C	282LYS	CG	2.39
285ALA	C	286ASN	CA	100.00	168VAI	CA	168VAL	CB	100.00	281GL	C	282LYS	CD	0.03
285ALA	C	286ASN	CB	6.07	168VAI	CA	168VAL	CG1	100.00	281GL	C	282LYS	CE	0.00
285ALA	C	286ASN	C	95.90	168VAI	CA	168VAL	CG2	100.00	281GL	C	282LYS	C	85.27
286ASN	CA	286ASN	CB	100.00	168VAI	CA	168VAL	C	100.00	282LY:	CA	282LYS	CB	100.00
286ASN	CA	286ASN	CG	100.00	168VAI	CB	168VAL	CG1	100.00	282LY:	CA	282LYS	CG	100.00
286ASN	CA	286ASN	C	100.00	168VAI	CB	168VAL	CG2	100.00	282LY:	CA	282LYS	CD	9.62
286ASN	CA	287PRO	CD	100.00	168VAI	CB	168VAL	C	100.00	282LY:	CA	282LYS	CE	0.01
286ASN	CB	286ASN	CG	100.00	168VAI	CB	262LEU	CD1	0.12	282LY:	CA	282LYS	C	100.00
286ASN	CB	286ASN	C	100.00	168VAI	CB	262LEU	CD2	0.01	282LY:	CB	282LYS	CG	100.00
286ASN	CB	324PRO	CB	0.04	168VAI	CG1	168VAL	CG2	100.00	282LY:	CB	282LYS	CD	100.00
286ASN	CB	324PRO	CG	0.01	168VAI	CG1	168VAL	C	63.68	282LY:	CB	282LYS	CE	5.63
286ASN	CB	331ALA	CB	0.00	168VAI	CG1	262LEU	CD1	1.98	282LY:	CB	282LYS	C	100.00
286ASN	CG	286ASN	C	99.76	168VAI	CG1	262LEU	CD2	0.58	282LY:	CB	284ILE	CG1	0.66
286ASN	CG	287PRO	CD	5.10	168VAI	CG2	168VAL	C	45.71	282LY:	CB	284ILE	CG2	0.00
286ASN	CG	324PRO	CB	0.02	168VAI	CG2	262LEU	CD1	0.63	282LY:	CB	284ILE	CD	0.36
286ASN	CG	324PRO	CG	0.13	168VAI	CG2	262LEU	CD2	0.53	282LY:	CG	282LYS	CD	100.00
286ASN	CG	336PHE	CE1	0.01	168VAI	C	169ALA	CA	100.00	282LY:	CG	282LYS	CE	100.00
286ASN	CG	336PHE	CE2	0.02	168VAI	C	169ALA	CB	0.02	282LY:	CG	282LYS	C	23.65
286ASN	CG	336PHE	CZ	0.00	168VAI	C	169ALA	C	100.00	282LY:	CG	284ILE	CG1	0.04
286ASN	C	287PRO	CA	100.00	169AL/	CA	169ALA	CB	100.00	282LY:	CG	284ILE	CG2	0.00
286ASN	C	287PRO	CB	1.62	169AL/	CA	169ALA	C	100.00	282LY:	CG	284ILE	CD	0.05
286ASN	C	287PRO	CD	100.00	169AL/	CA	232VAL	CG1	0.02	282LY:	CD	282LYS	CE	100.00
286ASN	C	287PRO	C	99.93	169AL/	CB	169ALA	C	100.00	282LY:	CD	282LYS	C	0.00
287PRO	CA	287PRO	CB	100.00	169AL/	CB	180VAL	CG1	0.31	282LY:	CD	284ILE	CG1	0.03
287PRO	CA	287PRO	CG	100.00	169AL/	CB	180VAL	CG2	0.08	282LY:	CD	284ILE	CG2	0.02
287PRO	CA	287PRO	CD	100.00	169AL/	CB	232VAL	CG1	0.46	282LY:	CD	284ILE	CD	0.33
287PRO	CA	287PRO	C	100.00	169AL/	CB	234VAL	CB	1.47	282LY:	CE	284ILE	CG2	0.01
287PRO	CB	287PRO	CG	100.00	169AL/	CB	234VAL	CG1	1.49	282LY:	CE	284ILE	CD	0.10
287PRO	CB	287PRO	CD	100.00	169AL/	CB	234VAL	CG2	7.93	282LY:	C	283GLY	CA	100.00
287PRO	CB	287PRO	C	100.00	169AL/	C	170PHE	CA	100.00	282LY:	C	283GLY	C	66.93
287PRO	CB	291ILE	CD	2.33	169AL/	C	170PHE	CB	0.00	283GL	CA	283GLY	C	100.00
287PRO	CB	333THR	CG2	0.00	169AL/	C	170PHE	CD1	0.00	283GL	CA	333THR	CB	0.02
287PRO	CG	287PRO	CD	100.00	169AL/	C	170PHE	C	100.00	283GL	CA	333THR	CG2	0.01
287PRO	CG	287PRO	C	99.81	169AL/	C	180VAL	CG1	0.03	283GL	C	284ILE	CA	100.00
287PRO	CG	291ILE	CD	4.62	169AL/	C	180VAL	CG2	0.39	283GL	C	284ILE	CB	70.01
287PRO	CG	333THR	CG2	0.05	170PHI	CA	170PHE	CB	100.00	283GL	C	284ILE	CG1	0.20
287PRO	CG	336PHE	CB	0.00	170PHI	CA	170PHE	CG	100.00	283GL	C	284ILE	CG2	49.26
287PRO	CG	336PHE	CG	0.00	170PHI	CA	170PHE	CD1	79.85	283GL	C	284ILE	CD	0.00
287PRO	CG	336PHE	CD2	12.59	170PHI	CA	170PHE	CD2	66.48	283GL	C	284ILE	C	27.57
287PRO	CG	336PHE	CE2	0.04	170PHI	CA	170PHE	C	100.00	283GL	C	332GLY	CA	0.00
287PRO	CD	287PRO	C	92.52	170PHI	CA	173ALA	CB	0.00	283GL	C	333THR	CB	0.10
287PRO	CD	336PHE	CB	0.02	170PHI	CA	180VAL	CG2	0.02	283GL	C	333THR	CG2	0.28
287PRO	CD	336PHE	CG	0.05	170PHI	CA	209VAL	CG2	0.02	284ILE	CA	284ILE	CB	100.00
287PRO	CD	336PHE	CD2	6.98	170PHI	CB	170PHE	CG	100.00	284ILE	CA	284ILE	CG1	100.00
287PRO	CD	336PHE	CE2	0.02	170PHI	CB	170PHE	CD1	100.00	284ILE	CA	284ILE	CG2	100.00
287PRO	C	288THR	CA	100.00	170PHI	CB	170PHE	CD2	100.00	284ILE	CA	284ILE	CD	2.78
287PRO	C	288THR	C	100.00	170PHI	CB	170PHE	C	100.00	284ILE	CA	284ILE	C	100.00
287PRO	C	291ILE	CD	0.08	170PHI	CB	206TYR	CG	0.60	284ILE	CB	284ILE	CG1	100.00
288THR	CA	288THR	CB	100.00	170PHI	CB	206TYR	CD1	2.04	284ILE	CB	284ILE	CG2	100.00
288THR	CA	288THR	CG2	100.00	170PHI	CB	206TYR	CD2	1.38	284ILE	CB	284ILE	CD	100.00
288THR	CA	288THR	C	100.00	170PHI	CB	206TYR	CE1	5.33	284ILE	CB	284ILE	C	100.00
288THR	CB	288THR	CG2	100.00	170PHI	CB	206TYR	CE2	2.88	284ILE	CB	325PRO	CG1	0.02
288THR	CB	288THR	C	100.00	170PHI	CB	206TYR	CZ	5.11	284ILE	CB	325PRO	CD	0.00
288THR	CG2	288THR	C	99.88	170PHI	CB	209VAL	CG2	0.04	284ILE	CG1	284ILE	CG2	100.00

288THR CG2	292LEU CD1	7.37	170PHI CG	170PHE	CD1	100.00	284ILE CG1	284ILE	CD	100.00
288THR CG2	315VAL CB	0.04	170PHI CG	170PHE	CD2	100.00	284ILE CG1	284ILE	C	97.46
288THR CG2	315VAL CG1	0.01	170PHI CG	170PHE	CE1	100.00	284ILE CG2	284ILE	CD	95.28
288THR CG2	315VAL CG2	0.08	170PHI CG	170PHE	CE2	100.00	284ILE CG2	284ILE	C	1.49
288THR CG2	336PHE CE2	0.02	170PHI CG	170PHE	CZ	100.00	284ILE CG2	325PRO	CG	0.44
288THR CG2	336PHE CZ	0.11	170PHI CG	170PHE	C	0.01	284ILE CG2	325PRO	CD	0.01
288THR C	289ALA CA	100.00	170PHI CG	180VAL	CG2	0.03	284ILE CD	284ILE	C	2.26
288THR C	289ALA C	100.00	170PHI CG	202VAL	CG1	0.02	284ILE CD	325PRO	CG	0.07
289ALA CA	289ALA CB	100.00	170PHI CG	203GLY	CA	0.00	284ILE C	285ALA	CA	100.00
289ALA CA	289ALA C	100.00	170PHI CG	206TYR	CB	0.00	284ILE C	285ALA	CB	0.24
289ALA CB	289ALA C	100.00	170PHI CG	206TYR	CG	0.02	284ILE C	285ALA	C	99.85
289ALA C	290ALA CA	100.00	170PHI CG	206TYR	CD1	0.28	284ILE C	331ALA	CB	0.02
289ALA C	290ALA CB	0.03	170PHI CG	206TYR	CD2	0.09	284ILE C	332GLY	CA	0.00
289ALA C	290ALA C	100.00	170PHI CG	206TYR	CE1	0.12	285AL. CA	285ALA	CB	100.00
290ALA CA	290ALA CB	100.00	170PHI CG	206TYR	CE2	0.05	285AL. CA	285ALA	C	100.00
290ALA CA	290ALA C	100.00	170PHI CG	206TYR	CZ	0.01	285AL. CB	285ALA	C	100.00
290ALA CB	290ALA C	100.00	170PHI CG	209VAL	CG1	0.00	285AL. CB	333THR	CG2	0.12
290ALA C	291ILE CA	100.00	170PHI CG	209VAL	CG2	0.04	285AL. C	286ASN	CA	100.00
290ALA C	291ILE C	100.00	170PHI CD1	170PHE	CD2	100.00	285AL. C	286ASN	CB	1.32
291ILE CA	291ILE CB	100.00	170PHI CD1	170PHE	CE1	100.00	285AL. C	286ASN	CG	0.01
291ILE CA	291ILE CG1	100.00	170PHI CD1	170PHE	CE2	100.00	285AL. C	286ASN	C	99.61
291ILE CA	291ILE CG2	100.00	170PHI CD1	170PHE	CZ	100.00	285AL. C	333THR	CG2	0.01
291ILE CA	291ILE CD	0.12	170PHI CD1	180VAL	CG1	0.08	286AS CA	286ASN	CB	100.00
291ILE CA	291ILE C	100.00	170PHI CD1	180VAL	CG2	4.18	286AS CA	286ASN	CG	100.00
291ILE CB	291ILE CG1	100.00	170PHI CD1	202VAL	CG1	0.76	286AS CA	286ASN	C	100.00
291ILE CB	291ILE CG2	100.00	170PHI CD1	202VAL	CG2	0.08	286AS CA	287PRO	CD	100.00
291ILE CB	291ILE CD	100.00	170PHI CD1	203GLY	CA	0.05	286AS CA	324PRO	CG	0.02
291ILE CB	291ILE C	100.00	170PHI CD1	206TYR	CB	1.46	286AS CA	331ALA	CB	0.01
291ILE CG1	291ILE CG2	100.00	170PHI CD1	206TYR	CG	6.66	286AS CB	286ASN	CG	100.00
291ILE CG1	291ILE CD	100.00	170PHI CD1	206TYR	CD1	3.13	286AS CB	286ASN	C	100.00
291ILE CG2	291ILE CD	100.00	170PHI CD1	206TYR	CD2	5.33	286AS CB	288THR	CG2	0.00
291ILE CG2	291ILE C	100.00	170PHI CD1	206TYR	CE1	0.54	286AS CB	324PRO	CB	0.01
291ILE CG2	311VAL CG1	43.55	170PHI CD1	206TYR	CE2	0.39	286AS CB	324PRO	CG	0.51
291ILE CG2	315VAL CG1	17.96	170PHI CD1	206TYR	CZ	0.11	286AS CB	325PRO	CD	0.01
291ILE CD	315VAL CG1	0.03	170PHI CD1	209VAL	CB	0.34	286AS CB	326ASP	CB	0.17
291ILE CD	336PHE CD2	0.00	170PHI CD1	209VAL	CG1	17.21	286AS CB	326ASP	CG	0.02
291ILE CD	336PHE CE2	0.01	170PHI CD1	209VAL	CG2	1.26	286AS CB	331ALA	CB	0.31
291ILE C	292LEU CA	100.00	170PHI CD2	170PHE	CE1	100.00	286AS CG	286ASN	C	72.16
291ILE C	292LEU C	100.00	170PHI CD2	170PHE	CE2	100.00	286AS CG	287PRO	CD	0.04
292LEU CA	292LEU CB	100.00	170PHI CD2	170PHE	CZ	100.00	286AS CG	288THR	CB	0.03
292LEU CA	292LEU CG	100.00	170PHI CD2	180VAL	CG1	0.19	286AS CG	288THR	CG2	0.26
292LEU CA	292LEU CD2	99.94	170PHI CD2	180VAL	CG2	1.73	286AS CG	324PRO	CB	0.52
292LEU CA	292LEU C	100.00	170PHI CD2	202VAL	CG1	0.51	286AS CG	324PRO	CG	2.66
292LEU CA	295ALA CB	0.14	170PHI CD2	202VAL	CG2	0.00	286AS CG	325PRO	CD	0.08
292LEU CB	292LEU CG	100.00	170PHI CD2	203GLY	CA	0.06	286AS CG	326ASP	CB	0.02
292LEU CB	292LEU CD1	100.00	170PHI CD2	206TYR	CB	0.23	286AS CG	326ASP	CG	0.00
292LEU CB	292LEU CD2	100.00	170PHI CD2	206TYR	CG	0.57	286AS CG	331ALA	CB	1.09
292LEU CB	292LEU C	100.00	170PHI CD2	206TYR	CD1	1.22	286AS CG	336PHE	CG	0.01
292LEU CG	292LEU CD1	100.00	170PHI CD2	206TYR	CD2	0.45	286AS CG	336PHE	CD1	0.02
292LEU CG	292LEU CD2	100.00	170PHI CD2	206TYR	CE1	0.54	286AS C	287PRO	CA	100.00
292LEU CD1	292LEU CD2	100.00	170PHI CD2	206TYR	CE2	0.22	286AS C	287PRO	CB	2.86
292LEU CD2	295ALA CB	0.02	170PHI CD2	206TYR	CZ	0.07	286AS C	287PRO	CD	100.00
292LEU CD2	311VAL CG1	0.01	170PHI CD2	209VAL	CB	0.10	286AS C	287PRO	C	99.87
292LEU CD2	312GLU CA	0.02	170PHI CD2	209VAL	CG1	2.55	287PR CA	287PRO	CB	100.00
292LEU CD2	312GLU CB	0.01	170PHI CD2	209VAL	CG2	1.35	287PR CA	287PRO	CG	100.00
292LEU CD2	312GLU CG	1.36	170PHI CE1	170PHE	CE2	100.00	287PR CA	287PRO	CD	100.00
292LEU CD2	312GLU CD	0.01	170PHI CE1	170PHE	CZ	100.00	287PR CA	287PRO	C	100.00
292LEU CD2	315VAL CG2	1.06	170PHI CE1	180VAL	CB	0.21	287PR CB	287PRO	CG	100.00
292LEU C	293SER CA	100.00	170PHI CE1	180VAL	CG1	0.90	287PR CB	287PRO	CD	100.00
292LEU C	293SER C	100.00	170PHI CE1	180VAL	CG2	2.09	287PR CB	287PRO	C	100.00
293SER CA	293SER CB	100.00	170PHI CE1	199VAL	CG1	0.01	287PR CB	291ILE	CD	1.40
293SER CA	293SER C	100.00	170PHI CE1	199VAL	CG2	0.00	287PR CB	333THR	CG2	0.02

293SER CB	293SER C	100.00	170PHI CE1	202VAL	CB	0.00	287PR CB	336PHE	CD2	0.00
293SER C	294ALA CA	100.00	170PHI CE1	202VAL	CG1	0.21	287PR CG	287PRO	CD	100.00
293SER C	294ALA CB	0.06	170PHI CE1	202VAL	CG2	0.16	287PR CG	287PRO	C	99.99
293SER C	294ALA C	99.98	170PHI CE1	202VAL	C	0.03	287PR CG	291ILE	CD	1.70
293SER C	296MET CB	0.00	170PHI CE1	203GLY	CA	8.64	287PR CG	333THR	CG2	0.05
294ALA CA	294ALA CB	100.00	170PHI CE1	203GLY	C	0.24	287PR CG	336PHE	CB	0.99
294ALA CA	294ALA C	100.00	170PHI CE1	206TYR	CB	0.40	287PR CG	336PHE	CG	0.72
294ALA CA	297MET CB	0.00	170PHI CE1	206TYR	CG	0.25	287PR CG	336PHE	CD1	0.03
294ALA CB	294ALA C	100.00	170PHI CE1	206TYR	CD1	0.15	287PR CG	336PHE	CD2	16.49
294ALA C	295ALA CA	100.00	170PHI CE1	206TYR	CD2	0.09	287PR CG	336PHE	CE2	0.10
294ALA C	295ALA CB	0.08	170PHI CE1	206TYR	CE1	0.01	287PR CG	337THR	CG2	0.01
294ALA C	295ALA C	99.85	170PHI CE1	209VAL	CB	0.12	287PR CD	287PRO	C	98.75
294ALA C	297MET CB	0.00	170PHI CE1	209VAL	CG1	7.15	287PR CD	331ALA	CB	0.01
295ALA CA	295ALA CB	100.00	170PHI CE1	209VAL	CG2	0.05	287PR CD	332GLY	C	0.02
295ALA CA	295ALA C	100.00	170PHI CE1	211LEU	CD1	0.03	287PR CD	333THR	CG2	0.00
295ALA CA	298LEU CB	0.00	170PHI CE1	211LEU	CD2	0.03	287PR CD	336PHE	CB	0.08
295ALA CA	298LEU CD1	0.01	170PHI CE2	170PHE	CZ	100.00	287PR CD	336PHE	CG	0.04
295ALA CA	311VAL CG2	0.00	170PHI CE2	180VAL	CB	0.51	287PR CD	336PHE	CD1	0.10
295ALA CB	295ALA C	100.00	170PHI CE2	180VAL	CG1	1.67	287PR CD	336PHE	CD2	0.78
295ALA CB	308ALA CA	0.01	170PHI CE2	180VAL	CG2	2.55	287PR CD	336PHE	CE2	0.01
295ALA CB	311VAL CB	0.19	170PHI CE2	199VAL	CG1	0.00	287PR C	288THR	CA	100.00
295ALA CB	311VAL CG1	1.49	170PHI CE2	202VAL	CB	0.00	287PR C	288THR	C	100.00
295ALA CB	311VAL CG2	0.27	170PHI CE2	202VAL	CG1	0.55	287PR C	291ILE	CD	0.02
295ALA C	296MET CA	100.00	170PHI CE2	202VAL	CG2	0.20	288TH CA	288THR	CB	100.00
295ALA C	296MET C	100.00	170PHI CE2	202VAL	C	0.06	288TH CA	288THR	CG2	100.00
295ALA C	298LEU CB	0.00	170PHI CE2	203GLY	CA	11.86	288TH CA	288THR	C	100.00
295ALA C	308ALA CB	0.01	170PHI CE2	203GLY	C	0.13	288TH CB	288THR	CG2	100.00
296MET CA	296MET CB	100.00	170PHI CE2	206TYR	CB	0.04	288TH CB	288THR	C	100.00
296MET CA	296MET CG	100.00	170PHI CE2	206TYR	CG	0.04	288TH CB	319LEU	CD2	0.01
296MET CA	296MET C	100.00	170PHI CE2	206TYR	CD1	0.06	288TH CB	336PHE	CZ	0.00
296MET CB	296MET CG	100.00	170PHI CE2	206TYR	CD2	0.02	288TH CG2	288THR	C	44.58
296MET CB	296MET CE	1.73	170PHI CE2	206TYR	CE2	0.01	288TH CG2	292LEU	CD1	1.42
296MET CB	296MET C	100.00	170PHI CE2	209VAL	CB	0.01	288TH CG2	315VAL	CG1	2.01
296MET CG	296MET CE	100.00	170PHI CE2	209VAL	CG1	0.98	288TH CG2	315VAL	CG2	0.01
296MET CG	296MET C	93.88	170PHI CE2	209VAL	CG2	0.09	288TH CG2	319LEU	CD1	0.06
296MET CG	300HIS CD2	0.19	170PHI CE2	211LEU	CD1	0.06	288TH CG2	319LEU	CD2	1.54
296MET CG	300HIS CE1	0.01	170PHI CE2	211LEU	CD2	0.02	288TH CG2	324PRO	CG	0.00
296MET CE	300HIS CD2	0.06	170PHI CZ	180VAL	CB	0.18	288TH CG2	331ALA	CB	0.02
296MET CE	300HIS CE1	0.03	170PHI CZ	180VAL	CG1	0.50	288TH CG2	336PHE	CD2	0.01
296MET C	297MET CA	100.00	170PHI CZ	180VAL	CG2	0.82	288TH CG2	336PHE	CE1	1.16
296MET C	297MET CB	0.00	170PHI CZ	199VAL	CG1	0.10	288TH CG2	336PHE	CE2	6.02
296MET C	297MET C	100.00	170PHI CZ	202VAL	CG1	0.01	288TH CG2	336PHE	CZ	18.24
297MET CA	297MET CB	100.00	170PHI CZ	202VAL	CG2	0.04	288TH C	289ALA	CA	100.00
297MET CA	297MET CG	100.00	170PHI CZ	202VAL	C	0.01	288TH C	289ALA	CB	0.54
297MET CA	297MET C	100.00	170PHI CZ	203GLY	CA	51.41	288TH C	289ALA	C	99.35
297MET CB	297MET CG	100.00	170PHI CZ	203GLY	C	1.15	288TH C	292LEU	CD1	0.01
297MET CB	297MET CE	86.43	170PHI CZ	204ARG	CA	0.01	289AL CA	289ALA	CB	100.00
297MET CB	297MET C	100.00	170PHI CZ	206TYR	CB	0.00	289AL CA	289ALA	C	100.00
297MET CG	297MET CE	100.00	170PHI CZ	209VAL	CG1	0.01	289AL CB	289ALA	C	100.00
297MET CG	297MET C	97.31	170PHI CZ	211LEU	CB	0.01	289AL C	290ALA	CA	100.00
297MET CG	301ALA CB	0.15	170PHI CZ	211LEU	CG	0.00	289AL C	290ALA	CB	0.01
297MET CG	302PHE CE1	0.03	170PHI CZ	211LEU	CD1	0.46	289AL C	290ALA	C	100.00
297MET CG	302PHE CE2	0.05	170PHI CZ	211LEU	CD2	0.05	289AL C	292LEU	CB	0.00
297MET CG	302PHE CZ	0.06	170PHI C	171GLU	CA	100.00	290AL CA	290ALA	CB	100.00
297MET CE	297MET C	0.01	170PHI C	171GLU	CB	0.16	290AL CA	290ALA	C	100.00
297MET CE	298LEU CD1	0.01	170PHI C	171GLU	C	99.91	290AL CB	290ALA	C	100.00
297MET CE	298LEU CD2	0.00	170PHI C	206TYR	CD1	0.01	290AL C	291ILE	CA	100.00
297MET CE	301ALA CB	0.00	170PHI C	206TYR	CD2	0.01	290AL C	291ILE	C	100.00
297MET CE	302PHE CD1	0.00	170PHI C	206TYR	CE1	0.15	291ILE CA	291ILE	CB	100.00
297MET CE	302PHE CD2	0.01	170PHI C	206TYR	CE2	0.18	291ILE CA	291ILE	CG1	100.00
297MET CE	302PHE CE1	0.21	170PHI C	206TYR	CZ	0.08	291ILE CA	291ILE	CG2	100.00
297MET CE	302PHE CE2	0.04	171GLU CA	171GLU	CB	100.00	291ILE CA	291ILE	CD	0.45

297MET	CE	302PHE	CZ	0.05	171GL	CA	171GLU	CG	100.00	291ILE	CA	291ILE	C	100.00
297MET	C	298LEU	CA	100.00	171GL	CA	171GLU	CD	98.38	291ILE	CB	291ILE	CG1	100.00
297MET	C	298LEU	C	100.00	171GL	CA	171GLU	C	100.00	291ILE	CB	291ILE	CG2	100.00
297MET	C	302PHE	CD2	0.00	171GL	CA	174ARG	CZ	0.02	291ILE	CB	291ILE	CD	100.00
298LEU	CA	298LEU	CB	100.00	171GL	CB	171GLU	CG	100.00	291ILE	CB	291ILE	C	100.00
298LEU	CA	298LEU	CG	100.00	171GL	CB	171GLU	CD	100.00	291ILE	CB	315VAL	CG2	0.01
298LEU	CA	298LEU	CD1	31.15	171GL	CB	171GLU	C	100.00	291ILE	CG1	291ILE	CG2	100.00
298LEU	CA	298LEU	CD2	68.63	171GL	CG	171GLU	CD	100.00	291ILE	CG1	291ILE	CD	100.00
298LEU	CA	298LEU	C	100.00	171GL	CG	171GLU	C	87.65	291ILE	CG2	291ILE	CD	99.98
298LEU	CA	302PHE	CD1	0.04	171GL	CG	174ARG	CZ	0.01	291ILE	CG2	291ILE	C	100.00
298LEU	CA	302PHE	CD2	0.00	171GL	CG	206TYR	CE1	0.00	291ILE	CG2	311VAL	CG1	9.92
298LEU	CB	298LEU	CG	100.00	171GL	CD	171GLU	C	1.62	291ILE	CG2	315VAL	CG2	25.36
298LEU	CB	298LEU	CD1	100.00	171GL	CD	174ARG	CD	0.21	291ILE	CD	315VAL	CG2	0.01
298LEU	CB	298LEU	CD2	100.00	171GL	CD	174ARG	CZ	0.16	291ILE	CD	336PHE	CD2	2.86
298LEU	CB	298LEU	C	100.00	171GL	CD	175LYS	CE	0.00	291ILE	CD	336PHE	CE2	0.67
298LEU	CB	302PHE	CZ	0.00	171GL	CD	206TYR	CE1	0.00	291ILE	CD	337THR	CG2	0.44
298LEU	CB	308ALA	CB	0.05	171GL	CD	206TYR	CE2	0.03	291ILE	C	292LEU	CA	100.00
298LEU	CG	298LEU	CD1	100.00	171GL	CD	300HIS	CE1	0.00	291ILE	C	292LEU	C	100.00
298LEU	CG	298LEU	CD2	100.00	171GL	C	172ALA	CA	100.00	292LE	CA	292LEU	CB	100.00
298LEU	CG	298LEU	C	26.59	171GL	C	172ALA	CB	0.04	292LE	CA	292LEU	CG	100.00
298LEU	CD1	298LEU	CD2	100.00	171GL	C	172ALA	C	99.96	292LE	CA	292LEU	CD2	99.97
298LEU	CD1	298LEU	C	0.39	172AL	CA	172ALA	CB	100.00	292LE	CA	292LEU	C	100.00
298LEU	CD1	302PHE	CB	0.08	172AL	CA	172ALA	C	100.00	292LE	CA	295ALA	CB	0.09
298LEU	CD1	302PHE	CG	0.21	172AL	CB	172ALA	C	100.00	292LE	CB	292LEU	CG	100.00
298LEU	CD1	302PHE	CD1	0.23	172AL	CB	232VAL	CG1	0.02	292LE	CB	292LEU	CD1	100.00
298LEU	CD1	302PHE	CD2	0.21	172AL	CB	232VAL	CG2	0.02	292LE	CB	292LEU	CD2	100.00
298LEU	CD1	302PHE	CE1	0.03	172AL	CB	300HIS	CE1	0.00	292LE	CB	292LEU	C	100.00
298LEU	CD1	302PHE	CE2	0.01	172AL	C	173ALA	CA	100.00	292LE	CG	292LEU	CD1	100.00
298LEU	CD1	304LEU	CB	0.04	172AL	C	173ALA	CB	0.02	292LE	CG	292LEU	CD2	100.00
298LEU	CD1	304LEU	CD1	0.09	172AL	C	173ALA	C	99.98	292LE	CG	315VAL	CG1	0.01
298LEU	CD1	307LEU	CB	0.16	172AL	C	175LYS	CG	0.00	292LE	CD1	292LEU	CD2	100.00
298LEU	CD1	307LEU	CG	0.01	173AL	CA	173ALA	CB	100.00	292LE	CD1	315VAL	CG1	0.06
298LEU	CD1	307LEU	CD2	0.03	173AL	CA	173ALA	C	100.00	292LE	CD2	295ALA	CB	0.00
298LEU	CD1	307LEU	C	0.07	173AL	CA	232VAL	CG2	0.36	292LE	CD2	312GLU	CA	0.00
298LEU	CD1	308ALA	CA	0.00	173AL	CB	173ALA	C	100.00	292LE	CD2	312GLU	CB	0.01
298LEU	CD1	311VAL	CG2	0.02	173AL	CB	178LYS	C	0.01	292LE	CD2	312GLU	CG	1.73
298LEU	CD2	298LEU	C	0.61	173AL	CB	179HIS	C	0.00	292LE	CD2	315VAL	CB	0.02
298LEU	CD2	302PHE	CG	0.10	173AL	CB	180VAL	CB	0.00	292LE	CD2	315VAL	CG1	0.80
298LEU	CD2	302PHE	CD1	0.98	173AL	CB	180VAL	CG1	0.40	292LE	CD2	315VAL	CG2	0.02
298LEU	CD2	302PHE	CD2	0.12	173AL	CB	180VAL	CG2	19.27	292LE	C	293SER	CA	100.00
298LEU	CD2	302PHE	CE1	0.27	173AL	CB	209VAL	CG1	8.84	292LE	C	293SER	CB	0.00
298LEU	CD2	302PHE	CE2	0.04	173AL	CB	209VAL	CG2	0.51	292LE	C	293SER	C	100.00
298LEU	CD2	302PHE	CZ	0.02	173AL	CB	232VAL	CG1	0.06	293SE	CA	293SER	CB	100.00
298LEU	CD2	304LEU	CB	0.06	173AL	CB	232VAL	CG2	0.24	293SE	CA	293SER	C	100.00
298LEU	CD2	304LEU	CD1	0.30	173AL	C	174ARG	CA	100.00	293SE	CB	293SER	C	100.00
298LEU	CD2	304LEU	CD2	0.00	173AL	C	174ARG	CB	0.02	293SE	C	294ALA	CA	100.00
298LEU	CD2	307LEU	CB	1.31	173AL	C	174ARG	C	99.98	293SE	C	294ALA	CB	0.02
298LEU	CD2	307LEU	CG	0.02	173AL	C	209VAL	CG1	0.06	293SE	C	294ALA	C	100.00
298LEU	CD2	307LEU	CD1	0.01	173AL	C	209VAL	CG2	0.00	294AL	CA	294ALA	CB	100.00
298LEU	CD2	307LEU	CD2	0.15	174AR	CA	174ARG	CB	100.00	294AL	CA	294ALA	C	100.00
298LEU	CD2	307LEU	C	0.20	174AR	CA	174ARG	CG	100.00	294AL	CA	297MET	CB	0.00
298LEU	CD2	308ALA	CA	0.00	174AR	CA	174ARG	CD	0.55	294AL	CB	294ALA	C	100.00
298LEU	CD2	308ALA	CB	0.01	174AR	CA	174ARG	C	100.00	294AL	C	295ALA	CA	100.00
298LEU	C	299GLU	CA	100.00	174AR	CA	209VAL	CG1	0.12	294AL	C	295ALA	CB	0.03
298LEU	C	299GLU	CB	0.08	174AR	CA	209VAL	CG2	0.32	294AL	C	295ALA	C	99.97
298LEU	C	299GLU	C	99.98	174AR	CB	174ARG	CG	100.00	295AL	CA	295ALA	CB	100.00
299GLU	CA	299GLU	CB	100.00	174AR	CB	174ARG	CD	100.00	295AL	CA	295ALA	C	100.00
299GLU	CA	299GLU	CG	100.00	174AR	CB	174ARG	CZ	12.70	295AL	CA	298LEU	CD1	0.01
299GLU	CA	299GLU	CD	49.87	174AR	CB	174ARG	C	100.00	295AL	CB	295ALA	C	100.00
299GLU	CA	299GLU	C	100.00	174AR	CB	209VAL	CG1	0.03	295AL	CB	308ALA	CA	0.01
299GLU	CA	308ALA	CB	0.01	174AR	CB	209VAL	CG2	0.16	295AL	CB	308ALA	CB	0.01
299GLU	CB	299GLU	CG	100.00	174AR	CG	174ARG	CD	100.00	295AL	CB	311VAL	CB	0.06

299GLU	CB	299GLU	CD	100.00	174AR(	CG	174ARG	CZ	36.99	295AL.	CB	311VAL	CG1	3.46
299GLU	CB	299GLU	C	100.00	174AR(	CG	174ARG	C	45.22	295AL.	CB	311VAL	CG2	0.12
299GLU	CB	300HIS	CD2	0.04	174AR(	CG	206TYR	CE1	0.00	295AL.	CB	312GLU	CG	0.02
299GLU	CB	308ALA	CB	0.72	174AR(	CG	206TYR	CE2	0.00	295AL.	C	296MET	CA	100.00
299GLU	CG	299GLU	CD	100.00	174AR(	CG	208ASP	CB	0.03	295AL.	C	296MET	C	100.00
299GLU	CG	299GLU	C	97.79	174AR(	CG	209VAL	CG1	0.14	295AL.	C	308ALA	CB	0.00
299GLU	CG	300HIS	CD2	0.27	174AR(	CG	209VAL	CG2	0.91	296ME	CA	296MET	CB	100.00
299GLU	CG	300HIS	CE1	0.06	174AR(	CD	174ARG	CZ	100.00	296ME	CA	296MET	CG	100.00
299GLU	CG	305VAL	CG1	0.36	174AR(	CD	174ARG	C	0.48	296ME	CA	296MET	C	100.00
299GLU	CG	305VAL	CG2	0.18	174AR(	CD	206TYR	CD1	0.00	296ME	CB	296MET	CG	100.00
299GLU	CG	308ALA	CB	0.16	174AR(	CD	206TYR	CD2	0.00	296ME	CB	296MET	CE	1.84
299GLU	CD	299GLU	C	39.74	174AR(	CD	206TYR	CE1	0.05	296ME	CB	296MET	C	100.00
299GLU	CD	300HIS	CD2	0.19	174AR(	CD	206TYR	CE2	0.06	296ME	CG	296MET	CE	100.00
299GLU	CD	300HIS	CE1	0.17	174AR(	CD	208ASP	CB	0.02	296ME	CG	296MET	C	97.57
299GLU	CD	305VAL	CG1	0.06	174AR(	CD	209VAL	CG1	0.00	296ME	CG	300HIS	CD2	1.64
299GLU	CD	309ARG	CZ	0.21	174AR(	CD	209VAL	CG2	0.02	296ME	CG	300HIS	CE1	0.01
299GLU	C	300HIS	CA	100.00	174AR(	CZ	175LYS	CG	0.01	296ME	CE	300HIS	CD2	0.11
299GLU	C	300HIS	CB	3.95	174AR(	CZ	175LYS	CE	0.03	296ME	CE	300HIS	CE1	0.07
299GLU	C	300HIS	CG	0.70	174AR(	CZ	206TYR	CD1	0.12	296ME	C	297MET	CA	100.00
299GLU	C	300HIS	CD2	0.01	174AR(	CZ	206TYR	CD2	0.03	296ME	C	297MET	C	100.00
299GLU	C	300HIS	C	95.34	174AR(	CZ	206TYR	CE1	0.76	297ME	CA	297MET	CB	100.00
300HIS	CA	300HIS	CB	100.00	174AR(	CZ	206TYR	CE2	1.24	297ME	CA	297MET	CG	100.00
300HIS	CA	300HIS	CG	100.00	174AR(	CZ	206TYR	CZ	0.24	297ME	CA	297MET	C	100.00
300HIS	CA	300HIS	CD2	31.39	174AR(	CZ	207PRO	CB	0.04	297ME	CB	297MET	CG	100.00
300HIS	CA	300HIS	C	100.00	174AR(	CZ	207PRO	CG	0.11	297ME	CB	297MET	CE	86.53
300HIS	CB	300HIS	CG	100.00	174AR(	CZ	208ASP	CB	0.01	297ME	CB	297MET	C	100.00
300HIS	CB	300HIS	CD2	100.00	174AR(	C	175LYS	CA	100.00	297ME	CG	297MET	CE	100.00
300HIS	CB	300HIS	CE1	0.23	174AR(	C	175LYS	CB	40.33	297ME	CG	297MET	C	99.73
300HIS	CB	300HIS	C	100.00	174AR(	C	175LYS	CG	0.35	297ME	CG	301ALA	CB	0.30
300HIS	CG	300HIS	CD2	100.00	174AR(	C	175LYS	C	58.95	297ME	CG	302PHE	CE1	0.03
300HIS	CG	300HIS	CE1	100.00	174AR(	C	178LYS	CE	0.00	297ME	CG	302PHE	CE2	0.04
300HIS	CG	300HIS	C	90.78	175LYS	CA	175LYS	CB	100.00	297ME	CG	302PHE	CZ	0.13
300HIS	CD2	300HIS	CE1	100.00	175LYS	CA	175LYS	CG	100.00	297ME	CE	297MET	C	0.04
300HIS	CD2	300HIS	C	37.57	175LYS	CA	175LYS	CD	0.89	297ME	CE	298LEU	CD2	0.04
300HIS	CD2	301ALA	CA	0.04	175LYS	CA	175LYS	C	100.00	297ME	CE	302PHE	CG	0.00
300HIS	CD2	301ALA	CB	0.00	175LYS	CB	175LYS	CG	100.00	297ME	CE	302PHE	CD1	0.01
300HIS	C	301ALA	CA	100.00	175LYS	CB	175LYS	CD	100.00	297ME	CE	302PHE	CD2	0.06
300HIS	C	301ALA	CB	17.46	175LYS	CB	175LYS	CE	18.05	297ME	CE	302PHE	CE1	0.26
300HIS	C	301ALA	C	82.66	175LYS	CB	175LYS	C	100.00	297ME	CE	302PHE	CE2	0.66
301ALA	CA	301ALA	CB	100.00	175LYS	CB	305VAL	CG1	0.01	297ME	CE	302PHE	CZ	0.13
301ALA	CA	301ALA	C	100.00	175LYS	CG	175LYS	CD	100.00	297ME	C	298LEU	CA	100.00
301ALA	CB	301ALA	C	100.00	175LYS	CG	175LYS	CE	100.00	297ME	C	298LEU	CB	0.01
301ALA	CB	302PHE	CD1	0.09	175LYS	CG	175LYS	C	78.76	297ME	C	298LEU	C	100.00
301ALA	CB	302PHE	CD2	0.08	175LYS	CG	176ARG	CG	0.00	298LE	CA	298LEU	CB	100.00
301ALA	CB	302PHE	CE1	0.00	175LYS	CG	305VAL	CG2	0.01	298LE	CA	298LEU	CG	100.00
301ALA	CB	302PHE	CE2	0.03	175LYS	CD	175LYS	CE	100.00	298LE	CA	298LEU	CD1	10.79
301ALA	C	302PHE	CA	100.00	175LYS	CD	175LYS	C	0.08	298LE	CA	298LEU	CD2	89.01
301ALA	C	302PHE	CB	21.21	175LYS	CD	299GLU	CG	0.02	298LE	CA	298LEU	C	100.00
301ALA	C	302PHE	CG	1.02	175LYS	CD	299GLU	CD	0.01	298LE	CA	302PHE	CB	0.00
301ALA	C	302PHE	CD1	0.47	175LYS	CD	300HIS	CD2	0.00	298LE	CA	302PHE	CD1	0.03
301ALA	C	302PHE	CD2	0.20	175LYS	CD	300HIS	CE1	0.04	298LE	CA	302PHE	CD2	0.00
301ALA	C	302PHE	C	78.95	175LYS	CD	305VAL	CG2	0.02	298LE	CB	298LEU	CG	100.00
302PHE	CA	302PHE	CB	100.00	175LYS	CE	299GLU	CD	0.02	298LE	CB	298LEU	CD1	100.00
302PHE	CA	302PHE	CG	100.00	175LYS	CE	300HIS	CE1	0.02	298LE	CB	298LEU	CD2	100.00
302PHE	CA	302PHE	CD1	81.29	175LYS	CE	305VAL	CG2	0.01	298LE	CB	298LEU	C	100.00
302PHE	CA	302PHE	CD2	48.51	175LYS	C	176ARG	CA	100.00	298LE	CB	308ALA	CB	0.05
302PHE	CA	302PHE	C	100.00	175LYS	C	176ARG	CB	85.30	298LE	CG	298LEU	CD1	100.00
302PHE	CB	302PHE	CG	100.00	175LYS	C	176ARG	CG	44.21	298LE	CG	298LEU	CD2	100.00
302PHE	CB	302PHE	CD1	100.00	175LYS	C	176ARG	C	22.68	298LE	CG	298LEU	C	7.56
302PHE	CB	302PHE	CD2	100.00	176AR(	CA	176ARG	CB	100.00	298LE	CD1	298LEU	CD2	100.00
302PHE	CB	302PHE	C	100.00	176AR(	CA	176ARG	CG	100.00	298LE	CD1	298LEU	C	0.11
302PHE	CB	304LEU	CG	0.04	176AR(	CA	176ARG	CD	0.00	298LE	CD1	302PHE	CG	0.01

302PHE	CB	304LEU	CD1	1.72	176AR(	CA	176ARG	C	100.00	298LE	CD1	302PHE	CD1	0.10
302PHE	CG	302PHE	CD1	100.00	176AR(	CA	177ARG	CZ	0.04	298LE	CD1	302PHE	CE1	0.01
302PHE	CG	302PHE	CD2	100.00	176AR(	CB	176ARG	CG	100.00	298LE	CD1	302PHE	CZ	0.00
302PHE	CG	302PHE	CE1	100.00	176AR(	CB	176ARG	CD	100.00	298LE	CD1	304LEU	CB	0.03
302PHE	CG	302PHE	CE2	100.00	176AR(	CB	176ARG	C	100.00	298LE	CD1	304LEU	CD1	0.06
302PHE	CG	302PHE	CZ	100.00	176AR(	CB	177ARG	CZ	0.02	298LE	CD1	307LEU	CB	0.26
302PHE	CG	302PHE	C	17.18	176AR(	CB	231ASP	CG	0.24	298LE	CD1	307LEU	CG	0.01
302PHE	CG	304LEU	CG	0.00	176AR(	CB	232VAL	CG2	0.01	298LE	CD1	307LEU	CD1	0.00
302PHE	CG	304LEU	CD1	0.68	176AR(	CG	176ARG	CD	100.00	298LE	CD1	307LEU	CD2	0.06
302PHE	CG	304LEU	CD2	0.06	176AR(	CG	176ARG	CZ	67.51	298LE	CD1	307LEU	C	0.13
302PHE	CD1	302PHE	CD2	100.00	176AR(	CG	176ARG	C	0.30	298LE	CD1	308ALA	CA	0.00
302PHE	CD1	302PHE	CE1	100.00	176AR(	CG	177ARG	CZ	0.00	298LE	CD1	311VAL	CG2	0.49
302PHE	CD1	302PHE	CE2	100.00	176AR(	CG	232VAL	CG2	0.00	298LE	CD2	298LEU	C	0.33
302PHE	CD1	302PHE	CZ	100.00	176AR(	CD	176ARG	CZ	100.00	298LE	CD2	302PHE	CG	0.09
302PHE	CD1	302PHE	C	5.03	176AR(	CD	231ASP	CG	0.04	298LE	CD2	302PHE	CD1	1.20
302PHE	CD1	304LEU	CG	0.62	176AR(	CD	232VAL	CG1	0.01	298LE	CD2	302PHE	CD2	0.34
302PHE	CD1	304LEU	CD1	1.60	176AR(	CD	232VAL	CG2	31.88	298LE	CD2	302PHE	CE1	0.24
302PHE	CD1	304LEU	CD2	0.22	176AR(	C	177ARG	CA	100.00	298LE	CD2	302PHE	CE2	0.06
302PHE	CD1	307LEU	CD1	0.02	176AR(	C	177ARG	CB	2.81	298LE	CD2	304LEU	CB	0.02
302PHE	CD2	302PHE	CE1	100.00	176AR(	C	177ARG	CG	0.89	298LE	CD2	304LEU	CD1	0.31
302PHE	CD2	302PHE	CE2	100.00	176AR(	C	177ARG	CD	0.05	298LE	CD2	304LEU	CD2	0.00
302PHE	CD2	302PHE	CZ	100.00	176AR(	C	177ARG	CZ	0.07	298LE	CD2	307LEU	CB	0.19
302PHE	CD2	302PHE	C	3.24	176AR(	C	177ARG	C	96.92	298LE	CD2	307LEU	CG	0.09
302PHE	CD2	304LEU	CG	0.23	177AR(	CA	177ARG	CB	100.00	298LE	CD2	307LEU	CD1	0.01
302PHE	CD2	304LEU	CD1	1.52	177AR(	CA	177ARG	CG	100.00	298LE	CD2	307LEU	CD2	0.18
302PHE	CD2	304LEU	CD2	0.07	177AR(	CA	177ARG	CD	8.54	298LE	CD2	307LEU	C	0.08
302PHE	CE1	302PHE	CE2	100.00	177AR(	CA	177ARG	CZ	0.07	298LE	CD2	308ALA	CA	0.02
302PHE	CE1	302PHE	CZ	100.00	177AR(	CA	177ARG	C	100.00	298LE	CD2	308ALA	CB	0.00
302PHE	CE1	304LEU	CB	0.01	177AR(	CB	177ARG	CG	100.00	298LE	CD2	311VAL	CG2	0.02
302PHE	CE1	304LEU	CG	0.08	177AR(	CB	177ARG	CD	100.00	298LE	C	299GLU	CA	100.00
302PHE	CE1	304LEU	CD1	0.71	177AR(	CB	177ARG	CZ	0.09	298LE	C	299GLU	CB	0.04
302PHE	CE1	304LEU	CD2	0.19	177AR(	CB	177ARG	C	100.00	298LE	C	299GLU	C	99.99
302PHE	CE1	307LEU	CD1	0.07	177AR(	CB	231ASP	CB	0.00	299GL	CA	299GLU	CB	100.00
302PHE	CE2	302PHE	CZ	100.00	177AR(	CB	231ASP	CG	1.32	299GL	CA	299GLU	CG	100.00
302PHE	CE2	304LEU	CB	0.00	177AR(	CG	177ARG	CD	100.00	299GL	CA	299GLU	CD	1.75
302PHE	CE2	304LEU	CG	0.08	177AR(	CG	177ARG	CZ	18.94	299GL	CA	299GLU	C	100.00
302PHE	CE2	304LEU	CD1	0.85	177AR(	CG	177ARG	C	1.32	299GL	CA	308ALA	CB	0.00
302PHE	CE2	304LEU	CD2	0.06	177AR(	CG	231ASP	CB	0.02	299GL	CB	299GLU	CG	100.00
302PHE	CZ	304LEU	CG	0.00	177AR(	CG	231ASP	CG	5.53	299GL	CB	299GLU	CD	100.00
302PHE	CZ	304LEU	CD1	0.36	177AR(	CD	177ARG	CZ	100.00	299GL	CB	299GLU	C	100.00
302PHE	CZ	304LEU	CD2	0.06	177AR(	CD	231ASP	CB	0.84	299GL	CB	300HIS	CD2	0.07
302PHE	C	303GLY	CA	100.00	177AR(	CD	231ASP	CG	6.28	299GL	CB	305VAL	CG1	0.01
302PHE	C	303GLY	C	68.72	177AR(	CZ	231ASP	CA	0.01	299GL	CB	308ALA	CB	1.37
302PHE	C	304LEU	CD1	0.02	177AR(	CZ	231ASP	CB	7.46	299GL	CG	299GLU	CD	100.00
303GLY	CA	303GLY	C	100.00	177AR(	CZ	231ASP	CG	0.30	299GL	CG	299GLU	C	99.29
303GLY	C	304LEU	CA	100.00	177AR(	C	178LYS	CA	100.00	299GL	CG	300HIS	CD2	0.59
303GLY	C	304LEU	CB	14.10	177AR(	C	178LYS	CB	99.99	299GL	CG	300HIS	CE1	0.02
303GLY	C	304LEU	CG	2.50	177AR(	C	178LYS	CG	32.05	299GL	CG	305VAL	CB	0.00
303GLY	C	304LEU	CD1	0.16	177AR(	C	178LYS	CD	0.07	299GL	CG	305VAL	CG1	0.25
303GLY	C	304LEU	CD2	0.19	177AR(	C	178LYS	C	98.08	299GL	CG	305VAL	CG2	0.04
303GLY	C	304LEU	C	90.25	178LYS	CA	178LYS	CB	100.00	299GL	CG	308ALA	CB	0.30
303GLY	C	305VAL	CG2	0.01	178LYS	CA	178LYS	CG	100.00	299GL	CD	299GLU	C	1.15
304LEU	CA	304LEU	CB	100.00	178LYS	CA	178LYS	CD	1.32	299GL	CD	300HIS	CD2	0.10
304LEU	CA	304LEU	CG	100.00	178LYS	CA	178LYS	C	100.00	299GL	CD	300HIS	CE1	0.04
304LEU	CA	304LEU	CD1	4.97	178LYS	CB	178LYS	CG	100.00	299GL	CD	305VAL	CG1	0.02
304LEU	CA	304LEU	CD2	94.60	178LYS	CB	178LYS	CD	100.00	299GL	CD	308ALA	CB	0.03
304LEU	CA	304LEU	C	100.00	178LYS	CB	178LYS	CE	18.44	299GL	CD	309ARG	CZ	0.16
304LEU	CB	304LEU	CG	100.00	178LYS	CB	178LYS	C	100.00	299GL	C	300HIS	CA	100.00
304LEU	CB	304LEU	CD1	100.00	178LYS	CB	208ASP	C	0.00	299GL	C	300HIS	CB	4.88
304LEU	CB	304LEU	CD2	100.00	178LYS	CB	209VAL	CG2	0.01	299GL	C	300HIS	CG	0.71
304LEU	CB	304LEU	C	100.00	178LYS	CG	178LYS	CD	100.00	299GL	C	300HIS	CD2	0.04
304LEU	CB	307LEU	CB	0.01	178LYS	CG	178LYS	CE	100.00	299GL	C	300HIS	C	93.97

304LEU	CB	307LEU	CD1	0.24	178LYS	CG	178LYS	C	2.07	300HIS	CA	300HIS	CB	100.00
304LEU	CB	307LEU	CD2	0.01	178LYS	CG	209VAL	CG2	0.00	300HIS	CA	300HIS	CG	100.00
304LEU	CG	304LEU	CD1	100.00	178LYS	CD	178LYS	CE	100.00	300HIS	CA	300HIS	CD2	34.81
304LEU	CG	304LEU	CD2	100.00	178LYS	CD	208ASP	CB	0.02	300HIS	CA	300HIS	C	100.00
304LEU	CG	304LEU	C	0.06	178LYS	CD	208ASP	CG	0.90	300HIS	CB	300HIS	CG	100.00
304LEU	CD1	304LEU	CD2	100.00	178LYS	CE	208ASP	CG	0.56	300HIS	CB	300HIS	CD2	100.00
304LEU	CD1	307LEU	CD1	0.53	178LYS	CE	209VAL	CG2	0.00	300HIS	CB	300HIS	CE1	0.24
304LEU	CD1	307LEU	CD2	0.22	178LYS	C	179HIS	CA	100.00	300HIS	CB	300HIS	C	100.00
304LEU	CD2	304LEU	C	0.04	178LYS	C	179HIS	CB	92.20	300HIS	CG	300HIS	CD2	100.00
304LEU	CD2	306GLU	CG	0.00	178LYS	C	179HIS	C	10.03	300HIS	CG	300HIS	CE1	100.00
304LEU	CD2	306GLU	CD	0.01	178LYS	C	209VAL	CG1	0.59	300HIS	CG	300HIS	C	85.00
304LEU	CD2	307LEU	CD1	0.30	178LYS	C	209VAL	CG2	0.02	300HIS	CD2	300HIS	CE1	100.00
304LEU	CD2	307LEU	CD2	0.06	179HIS	CA	179HIS	CB	100.00	300HIS	CD2	300HIS	C	41.09
304LEU	C	305VAL	CA	100.00	179HIS	CA	179HIS	CG	100.00	300HIS	CD2	301ALA	CA	0.04
304LEU	C	305VAL	CB	0.10	179HIS	CA	179HIS	CD2	27.31	300HIS	C	301ALA	CA	100.00
304LEU	C	305VAL	CG1	0.02	179HIS	CA	179HIS	C	100.00	300HIS	C	301ALA	CB	13.89
304LEU	C	305VAL	CG2	0.04	179HIS	CB	179HIS	CG	100.00	300HIS	C	301ALA	C	86.60
304LEU	C	305VAL	C	99.94	179HIS	CB	179HIS	CD2	100.00	301AL	CA	301ALA	CB	100.00
304LEU	C	306GLU	CD	0.01	179HIS	CB	179HIS	CE1	0.42	301AL	CA	301ALA	C	100.00
305VAL	CA	305VAL	CB	100.00	179HIS	CB	179HIS	C	100.00	301AL	CB	301ALA	C	100.00
305VAL	CA	305VAL	CG1	100.00	179HIS	CG	179HIS	CD2	100.00	301AL	CB	302PHE	CD1	0.07
305VAL	CA	305VAL	CG2	100.00	179HIS	CG	179HIS	CE1	100.00	301AL	CB	302PHE	CD2	0.01
305VAL	CA	305VAL	C	100.00	179HIS	CG	179HIS	C	99.98	301AL	CB	302PHE	CE1	0.01
305VAL	CA	308ALA	CB	0.00	179HIS	CG	210ALA	CB	0.00	301AL	CB	302PHE	CE2	0.00
305VAL	CB	305VAL	CG1	100.00	179HIS	CD2	179HIS	CE1	100.00	301AL	C	302PHE	CA	100.00
305VAL	CB	305VAL	CG2	100.00	179HIS	CD2	179HIS	C	16.94	301AL	C	302PHE	CB	9.58
305VAL	CB	305VAL	C	100.00	179HIS	CD2	180VAL	C	0.07	301AL	C	302PHE	CG	0.18
305VAL	CB	309ARG	CZ	0.01	179HIS	CD2	181VAL	CG2	3.28	301AL	C	302PHE	CD1	0.18
305VAL	CG1	305VAL	CG2	100.00	179HIS	CD2	210ALA	CB	0.36	301AL	C	302PHE	CD2	0.08
305VAL	CG1	305VAL	C	68.20	179HIS	CD2	210ALA	C	0.01	301AL	C	302PHE	C	90.56
305VAL	CG1	309ARG	CG	0.07	179HIS	CD2	229ARG	CG	0.00	302PH	CA	302PHE	CB	100.00
305VAL	CG1	309ARG	CZ	5.36	179HIS	CD2	229ARG	CZ	0.00	302PH	CA	302PHE	CG	100.00
305VAL	CG2	305VAL	C	31.73	179HIS	CE1	180VAL	C	0.00	302PH	CA	302PHE	CD1	67.62
305VAL	CG2	309ARG	CZ	0.04	179HIS	CE1	181VAL	CG1	0.01	302PH	CA	302PHE	CD2	63.79
305VAL	C	306GLU	CA	100.00	179HIS	CE1	181VAL	CG2	3.93	302PH	CA	302PHE	C	100.00
305VAL	C	306GLU	C	100.00	179HIS	CE1	210ALA	CB	0.03	302PH	CB	302PHE	CG	100.00
305VAL	C	308ALA	CB	0.00	179HIS	CE1	211LEU	C	2.66	302PH	CB	302PHE	CD1	100.00
305VAL	C	309ARG	CZ	0.04	179HIS	CE1	212GLU	CB	14.95	302PH	CB	302PHE	CD2	100.00
306GLU	CA	306GLU	CB	100.00	179HIS	CE1	212GLU	CD	0.00	302PH	CB	302PHE	C	100.00
306GLU	CA	306GLU	CG	100.00	179HIS	CE1	229ARG	CZ	0.69	302PH	CB	304LEU	CG	0.09
306GLU	CA	306GLU	CD	86.44	179HIS	C	180VAL	CA	100.00	302PH	CB	304LEU	CD1	2.45
306GLU	CA	306GLU	C	100.00	179HIS	C	180VAL	CB	30.17	302PH	CB	304LEU	CD2	0.02
306GLU	CA	309ARG	CZ	0.00	179HIS	C	180VAL	CG1	1.89	302PH	CG	302PHE	CD1	100.00
306GLU	CB	306GLU	CG	100.00	179HIS	C	180VAL	CG2	16.01	302PH	CG	302PHE	CD2	100.00
306GLU	CB	306GLU	CD	100.00	179HIS	C	180VAL	C	78.27	302PH	CG	302PHE	CE1	100.00
306GLU	CB	306GLU	C	100.00	180VAL	CA	180VAL	CB	100.00	302PH	CG	302PHE	CE2	100.00
306GLU	CG	306GLU	CD	100.00	180VAL	CA	180VAL	CG1	100.00	302PH	CG	302PHE	CZ	100.00
306GLU	CG	306GLU	C	88.02	180VAL	CA	180VAL	CG2	100.00	302PH	CG	302PHE	C	8.47
306GLU	CG	307LEU	CD1	0.36	180VAL	CA	180VAL	C	100.00	302PH	CG	304LEU	CG	0.01
306GLU	CD	306GLU	C	0.09	180VAL	CB	180VAL	CG1	100.00	302PH	CG	304LEU	CD1	0.44
306GLU	CD	309ARG	CZ	0.04	180VAL	CB	180VAL	CG2	100.00	302PH	CD1	302PHE	CD2	100.00
306GLU	C	307LEU	CA	100.00	180VAL	CB	180VAL	C	100.00	302PH	CD1	302PHE	CE1	100.00
306GLU	C	307LEU	CB	0.49	180VAL	CB	211LEU	CD1	0.02	302PH	CD1	302PHE	CE2	100.00
306GLU	C	307LEU	CG	0.01	180VAL	CB	211LEU	CD2	0.00	302PH	CD1	302PHE	CZ	100.00
306GLU	C	307LEU	CD1	0.04	180VAL	CG1	180VAL	CG2	100.00	302PH	CD1	302PHE	C	1.99
306GLU	C	307LEU	CD2	0.00	180VAL	CG1	180VAL	C	95.76	302PH	CD1	304LEU	CG	0.12
306GLU	C	307LEU	C	99.53	180VAL	CG1	181VAL	C	0.07	302PH	CD1	304LEU	CD1	1.04
307LEU	CA	307LEU	CB	100.00	180VAL	CG1	209VAL	CG1	0.12	302PH	CD1	304LEU	CD2	0.02
307LEU	CA	307LEU	CG	100.00	180VAL	CG1	209VAL	CG2	0.01	302PH	CD2	302PHE	CE1	100.00
307LEU	CA	307LEU	CD1	54.74	180VAL	CG1	211LEU	CD1	0.77	302PH	CD2	302PHE	CE2	100.00
307LEU	CA	307LEU	CD2	44.85	180VAL	CG1	211LEU	CD2	0.06	302PH	CD2	302PHE	CZ	100.00
307LEU	CA	307LEU	C	100.00	180VAL	CG1	232VAL	CG1	0.16	302PH	CD2	302PHE	C	2.66

307LEU	CB	307LEU	CG	100.00	180VAI	CG1	234VAL	CG1	10.19	302PH	CD2	304LEU	CG	0.23
307LEU	CB	307LEU	CD1	100.00	180VAI	CG1	234VAL	CG2	3.15	302PH	CD2	304LEU	CD1	1.30
307LEU	CB	307LEU	CD2	100.00	180VAI	CG2	180VAL	C	7.28	302PH	CD2	304LEU	CD2	0.01
307LEU	CB	307LEU	C	100.00	180VAI	CG2	181VAL	C	0.00	302PH	CE1	302PHE	CE2	100.00
307LEU	CG	307LEU	CD1	100.00	180VAI	CG2	209VAL	CG1	3.77	302PH	CE1	302PHE	CZ	100.00
307LEU	CG	307LEU	CD2	100.00	180VAI	CG2	209VAL	CG2	0.14	302PH	CE1	304LEU	CB	0.00
307LEU	CG	307LEU	C	20.03	180VAI	CG2	211LEU	CA	0.00	302PH	CE1	304LEU	CG	0.01
307LEU	CD1	307LEU	CD2	100.00	180VAI	CG2	211LEU	CB	0.26	302PH	CE1	304LEU	CD1	0.46
307LEU	CD1	307LEU	C	0.06	180VAI	CG2	211LEU	CD1	0.00	302PH	CE1	304LEU	CD2	0.01
307LEU	CD1	310LYS	CD	0.02	180VAI	CG2	232VAL	CG1	0.19	302PH	CE2	302PHE	CZ	100.00
307LEU	CD2	307LEU	C	0.18	180VAI	CG2	234VAL	CG1	0.13	302PH	CE2	304LEU	CG	0.01
307LEU	CD2	310LYS	CD	0.01	180VAI	CG2	234VAL	CG2	0.04	302PH	CE2	304LEU	CD1	0.42
307LEU	CD2	310LYS	CE	0.02	180VAI	C	181VAL	CA	100.00	302PH	CE2	304LEU	CD2	0.01
307LEU	C	308ALA	CA	100.00	180VAI	C	181VAL	CB	15.08	302PH	CZ	304LEU	CD1	0.06
307LEU	C	308ALA	CB	0.00	180VAI	C	181VAL	CG1	0.00	302PH	C	303GLY	CA	100.00
307LEU	C	308ALA	C	100.00	180VAI	C	181VAL	CG2	6.22	302PH	C	303GLY	C	75.32
308ALA	CA	308ALA	CB	100.00	180VAI	C	181VAL	C	95.67	302PH	C	304LEU	CD1	0.01
308ALA	CA	308ALA	C	100.00	181VAI	CA	181VAL	CB	100.00	302PH	C	304LEU	CD2	0.00
308ALA	CB	308ALA	C	100.00	181VAI	CA	181VAL	CG1	100.00	303GL	CA	303GLY	C	100.00
308ALA	C	309ARG	CA	100.00	181VAI	CA	181VAL	CG2	100.00	303GL	C	304LEU	CA	100.00
308ALA	C	309ARG	C	100.00	181VAI	CA	181VAL	C	100.00	303GL	C	304LEU	CB	9.79
309ARG	CA	309ARG	CB	100.00	181VAI	CB	181VAL	CG1	100.00	303GL	C	304LEU	CG	1.40
309ARG	CA	309ARG	CG	100.00	181VAI	CB	181VAL	CG2	100.00	303GL	C	304LEU	CD1	0.09
309ARG	CA	309ARG	CD	50.17	181VAI	CB	181VAL	C	100.00	303GL	C	304LEU	CD2	0.04
309ARG	CA	309ARG	C	100.00	181VAI	CG1	181VAL	CG2	100.00	303GL	C	304LEU	C	93.72
309ARG	CA	312GLU	CB	0.02	181VAI	CG1	181VAL	C	99.94	303GL	C	305VAL	CG2	0.01
309ARG	CB	309ARG	CG	100.00	181VAI	CG1	183VAL	CG2	0.62	304LE	CA	304LEU	CB	100.00
309ARG	CB	309ARG	CD	100.00	181VAI	CG1	212GLU	CD	0.00	304LE	CA	304LEU	CG	100.00
309ARG	CB	309ARG	CZ	2.52	181VAI	CG1	214GLN	CB	0.33	304LE	CA	304LEU	CD1	3.07
309ARG	CB	309ARG	C	100.00	181VAI	CG1	214GLN	CG	13.78	304LE	CA	304LEU	CD2	96.82
309ARG	CG	309ARG	CD	100.00	181VAI	CG1	214GLN	CD	0.57	304LE	CA	304LEU	C	100.00
309ARG	CG	309ARG	CZ	56.63	181VAI	CG1	219MET	CE	0.24	304LE	CB	304LEU	CG	100.00
309ARG	CG	309ARG	C	11.48	181VAI	CG1	230PHE	CD1	0.02	304LE	CB	304LEU	CD1	100.00
309ARG	CD	309ARG	CZ	100.00	181VAI	CG1	230PHE	CE1	0.02	304LE	CB	304LEU	CD2	100.00
309ARG	CD	312GLU	CD	0.05	181VAI	CG1	233VAL	CG1	0.00	304LE	CB	304LEU	C	100.00
309ARG	C	310LYS	CA	100.00	181VAI	CG1	233VAL	CG2	0.02	304LE	CB	307LEU	CB	0.01
309ARG	C	310LYS	CB	0.02	181VAI	CG2	181VAL	C	0.60	304LE	CB	307LEU	CD1	0.13
309ARG	C	310LYS	C	100.00	181VAI	CG2	212GLU	CD	0.20	304LE	CB	307LEU	CD2	0.00
309ARG	C	312GLU	CB	0.00	181VAI	CG2	214GLN	CB	0.01	304LE	CG	304LEU	CD1	100.00
310LYS	CA	310LYS	CB	100.00	181VAI	CG2	214GLN	CG	0.17	304LE	CG	304LEU	CD2	100.00
310LYS	CA	310LYS	CG	100.00	181VAI	CG2	214GLN	CD	0.00	304LE	CD1	304LEU	CD2	100.00
310LYS	CA	310LYS	CD	23.63	181VAI	CG2	229ARG	CZ	0.06	304LE	CD1	307LEU	CD1	0.45
310LYS	CA	310LYS	CE	1.05	181VAI	CG2	230PHE	CD1	0.12	304LE	CD1	307LEU	CD2	0.15
310LYS	CA	310LYS	C	100.00	181VAI	CG2	230PHE	CE1	0.01	304LE	CD2	306GLU	CD	0.01
310LYS	CB	310LYS	CG	100.00	181VAI	CG2	233VAL	CG2	0.00	304LE	CD2	307LEU	CD1	0.13
310LYS	CB	310LYS	CD	100.00	181VAI	C	182SER	CA	100.00	304LE	CD2	307LEU	CD2	0.07
310LYS	CB	310LYS	CE	7.82	181VAI	C	182SER	CB	7.92	304LE	C	305VAL	CA	100.00
310LYS	CB	310LYS	C	100.00	181VAI	C	182SER	C	97.57	304LE	C	305VAL	CB	0.01
310LYS	CB	344LEU	CD1	0.14	181VAI	C	234VAL	CG1	0.00	304LE	C	305VAL	C	99.99
310LYS	CB	344LEU	CD2	0.08	182SEF	CA	182SER	CB	100.00	304LE	C	306GLU	CD	0.00
310LYS	CG	310LYS	CD	100.00	182SEF	CA	182SER	C	100.00	305VA	CA	305VAL	CB	100.00
310LYS	CG	310LYS	CE	100.00	182SEF	CA	234VAL	CG1	1.78	305VA	CA	305VAL	CG1	100.00
310LYS	CG	310LYS	C	83.49	182SEF	CA	234VAL	CG2	0.04	305VA	CA	305VAL	CG2	100.00
310LYS	CG	344LEU	CD1	0.16	182SEF	CB	182SER	C	100.00	305VA	CA	305VAL	C	100.00
310LYS	CG	344LEU	CD2	0.24	182SEF	CB	199VAL	CG1	0.30	305VA	CB	305VAL	CG1	100.00
310LYS	CD	310LYS	CE	100.00	182SEF	CB	211LEU	CD1	0.06	305VA	CB	305VAL	CG2	100.00
310LYS	CD	310LYS	C	0.17	182SEF	CB	211LEU	CD2	0.00	305VA	CB	305VAL	C	100.00
310LYS	CD	344LEU	CD1	0.08	182SEF	CB	234VAL	CG1	0.88	305VA	CG1	305VAL	CG2	100.00
310LYS	CD	344LEU	CD2	0.03	182SEF	CB	234VAL	CG2	0.02	305VA	CG1	305VAL	C	64.79
310LYS	CD	344LEU	C	0.01	182SEF	C	183VAL	CA	100.00	305VA	CG1	309ARG	CG	0.00
310LYS	CD	345ALA	C	0.03	182SEF	C	183VAL	CB	46.97	305VA	CG2	305VAL	C	35.13
310LYS	CE	310LYS	C	0.01	182SEF	C	183VAL	CG1	0.20	305VA	CG2	306GLU	CD	0.02

310LYS	CE	313ASP	CB	0.00	182SEI	C	183VAL	CG2	36.26	305VA	C	306GLU	CA	100.00
310LYS	CE	344LEU	CD1	0.01	182SEI	C	183VAL	C	69.75	305VA	C	306GLU	CB	0.10
310LYS	CE	344LEU	CD2	0.00	183VAI	CA	183VAL	CB	100.00	305VA	C	306GLU	C	99.97
310LYS	CE	344LEU	C	0.78	183VAI	CA	183VAL	CG1	100.00	306GL	CA	306GLU	CB	100.00
310LYS	CE	345ALA	CA	0.00	183VAI	CA	183VAL	CG2	100.00	306GL	CA	306GLU	CG	100.00
310LYS	CE	345ALA	C	1.25	183VAI	CA	183VAL	C	100.00	306GL	CA	306GLU	CD	90.52
310LYS	C	311VAL	CA	100.00	183VAI	CB	183VAL	CG1	100.00	306GL	CA	306GLU	C	100.00
310LYS	C	311VAL	CB	0.01	183VAI	CB	183VAL	CG2	100.00	306GL	CB	306GLU	CG	100.00
310LYS	C	311VAL	C	100.00	183VAI	CB	183VAL	C	100.00	306GL	CB	306GLU	CD	100.00
310LYS	C	344LEU	CD1	1.00	183VAI	CB	235THR	CB	0.05	306GL	CB	306GLU	C	100.00
310LYS	C	344LEU	CD2	0.25	183VAI	CG1	183VAL	CG2	100.00	306GL	CG	306GLU	CD	100.00
311VAL	CA	311VAL	CB	100.00	183VAI	CG1	183VAL	C	99.15	306GL	CG	306GLU	C	95.97
311VAL	CA	311VAL	CG1	100.00	183VAI	CG1	214GLN	CB	0.00	306GL	CG	307LEU	CD1	0.02
311VAL	CA	311VAL	CG2	100.00	183VAI	CG1	215TYR	C	0.10	306GL	CD	306GLU	C	0.11
311VAL	CA	311VAL	C	100.00	183VAI	CG1	216VAL	CA	14.78	306GL	CD	310LYS	CE	0.00
311VAL	CA	344LEU	CD1	0.14	183VAI	CG1	216VAL	CB	2.89	306GL	C	307LEU	CA	100.00
311VAL	CB	311VAL	CG1	100.00	183VAI	CG1	216VAL	CG2	2.37	306GL	C	307LEU	CB	0.13
311VAL	CB	311VAL	CG2	100.00	183VAI	CG1	219MET	CB	0.39	306GL	C	307LEU	CD1	0.01
311VAL	CB	311VAL	C	100.00	183VAI	CG1	219MET	CG	0.33	306GL	C	307LEU	C	99.86
311VAL	CG1	311VAL	CG2	100.00	183VAI	CG1	219MET	CE	0.00	307LEI	CA	307LEU	CB	100.00
311VAL	CG1	311VAL	C	99.78	183VAI	CG1	233VAL	CG1	0.14	307LEI	CA	307LEU	CG	100.00
311VAL	CG1	315VAL	CG1	0.00	183VAI	CG1	235THR	CB	0.14	307LEI	CA	307LEU	CD1	43.61
311VAL	CG1	344LEU	CD1	0.00	183VAI	CG1	235THR	CG2	0.01	307LEI	CA	307LEU	CD2	57.41
311VAL	CG2	311VAL	C	0.22	183VAI	CG1	239PHE	CD2	0.00	307LEI	CA	307LEU	C	100.00
311VAL	CG2	344LEU	CD1	0.62	183VAI	CG1	243LEU	CD1	0.16	307LEI	CB	307LEU	CG	100.00
311VAL	CG2	344LEU	CD2	0.07	183VAI	CG1	243LEU	CD2	0.01	307LEI	CB	307LEU	CD1	100.00
311VAL	C	312GLU	CA	100.00	183VAI	CG2	183VAL	C	2.96	307LEI	CB	307LEU	CD2	100.00
311VAL	C	312GLU	C	100.00	183VAI	CG2	215TYR	C	0.01	307LEI	CB	307LEU	C	100.00
312GLU	CA	312GLU	CB	100.00	183VAI	CG2	216VAL	CA	0.74	307LEI	CG	307LEU	CD1	100.00
312GLU	CA	312GLU	CG	100.00	183VAI	CG2	216VAL	CB	0.03	307LEI	CG	307LEU	CD2	100.00
312GLU	CA	312GLU	CD	0.22	183VAI	CG2	216VAL	CG2	0.01	307LEI	CG	307LEU	C	34.65
312GLU	CA	312GLU	C	100.00	183VAI	CG2	219MET	CB	0.08	307LEI	CD1	307LEU	CD2	100.00
312GLU	CA	315VAL	CG2	0.03	183VAI	CG2	219MET	CG	0.72	307LEI	CD1	307LEU	C	0.19
312GLU	CB	312GLU	CG	100.00	183VAI	CG2	219MET	CE	0.18	307LEI	CD1	310LYS	CD	0.06
312GLU	CB	312GLU	CD	100.00	183VAI	CG2	233VAL	CG1	19.86	307LEI	CD1	310LYS	CE	0.03
312GLU	CB	312GLU	C	100.00	183VAI	CG2	233VAL	CG2	0.04	307LEI	CD1	344LEU	CD2	0.01
312GLU	CG	312GLU	CD	100.00	183VAI	CG2	235THR	CB	0.08	307LEI	CD2	307LEU	C	1.42
312GLU	CG	312GLU	C	83.32	183VAI	CG2	243LEU	CD1	0.00	307LEI	CD2	310LYS	CD	0.04
312GLU	CD	312GLU	C	0.06	183VAI	C	184ASP	CA	100.00	307LEI	CD2	310LYS	CE	0.10
312GLU	C	313ASP	CA	100.00	183VAI	C	184ASP	CB	98.38	307LEI	CD2	311VAL	CG2	0.02
312GLU	C	313ASP	CB	0.08	183VAI	C	184ASP	C	1.62	307LEI	C	308ALA	CA	100.00
312GLU	C	313ASP	C	99.98	184ASI	CA	184ASP	CB	100.00	307LEI	C	308ALA	C	100.00
312GLU	C	315VAL	CG2	0.03	184ASI	CA	184ASP	CG	100.00	308AL	CA	308ALA	CB	100.00
313ASP	CA	313ASP	CB	100.00	184ASI	CA	184ASP	C	100.00	308AL	CA	308ALA	C	100.00
313ASP	CA	313ASP	CG	100.00	184ASI	CA	239PHE	CE2	0.00	308AL	CB	308ALA	C	100.00
313ASP	CA	313ASP	C	100.00	184ASI	CB	184ASP	CG	100.00	308AL	CB	309ARG	CZ	0.08
313ASP	CB	313ASP	CG	100.00	184ASI	CB	184ASP	C	100.00	308AL	C	309ARG	CA	100.00
313ASP	CB	313ASP	C	100.00	184ASI	CB	195TRP	CD1	0.53	308AL	C	309ARG	CB	0.00
313ASP	CG	313ASP	C	99.99	184ASI	CG	184ASP	C	100.00	308AL	C	309ARG	CZ	0.00
313ASP	CG	317LYS	CD	0.03	184ASI	CG	186ALA	CB	8.36	308AL	C	309ARG	C	99.99
313ASP	CG	317LYS	CE	1.38	184ASI	CG	196ARG	CZ	0.00	308AL	C	312GLU	CD	0.00
313ASP	C	314ALA	CA	100.00	184ASI	C	185LYS	CA	100.00	309AR	CA	309ARG	CB	100.00
313ASP	C	314ALA	CB	0.01	184ASI	C	185LYS	CB	99.88	309AR	CA	309ARG	CG	100.00
313ASP	C	314ALA	C	99.99	184ASI	C	185LYS	C	1.74	309AR	CA	309ARG	CD	98.76
314ALA	CA	314ALA	CB	100.00	184ASI	C	239PHE	CE1	0.07	309AR	CA	309ARG	CZ	0.00
314ALA	CA	314ALA	C	100.00	184ASI	C	239PHE	CE2	0.18	309AR	CA	309ARG	C	100.00
314ALA	CA	343HIS	CB	0.02	184ASI	C	239PHE	CZ	0.02	309AR	CA	312GLU	CB	0.00
314ALA	CA	343HIS	CD2	0.02	185LYS	CA	185LYS	CB	100.00	309AR	CB	309ARG	CG	100.00
314ALA	CB	314ALA	C	100.00	185LYS	CA	185LYS	CG	100.00	309AR	CB	309ARG	CD	100.00
314ALA	CB	340VAL	CG1	0.69	185LYS	CA	185LYS	CD	80.78	309AR	CB	309ARG	C	100.00
314ALA	CB	343HIS	CB	1.03	185LYS	CA	185LYS	CE	0.00	309AR	CG	309ARG	CD	100.00
314ALA	CB	343HIS	C	0.50	185LYS	CA	185LYS	C	100.00	309AR	CG	309ARG	CZ	99.46

314ALA	CB	344LEU	CA	0.02	185LYS	CA	215TYR	CD1	0.01	309AR	CD	309ARG	CZ	100.00
314ALA	CB	344LEU	CB	0.02	185LYS	CB	185LYS	CG	100.00	309AR	C	310LYS	CA	100.00
314ALA	CB	344LEU	CG1	0.19	185LYS	CB	185LYS	CD	100.00	309AR	C	310LYS	CB	0.02
314ALA	CB	344LEU	CD1	1.35	185LYS	CB	185LYS	CE	0.15	309AR	C	310LYS	C	99.99
314ALA	CB	344LEU	CD2	0.50	185LYS	CB	185LYS	C	100.00	310LY	CA	310LYS	CB	100.00
314ALA	C	315VAL	CA	100.00	185LYS	CB	189LEU	CD1	0.42	310LY	CA	310LYS	CG	100.00
314ALA	C	315VAL	C	100.00	185LYS	CB	189LEU	CD2	0.00	310LY	CA	310LYS	CD	1.29
315VAL	CA	315VAL	CB	100.00	185LYS	CB	216VAL	CG1	0.01	310LY	CA	310LYS	C	100.00
315VAL	CA	315VAL	CG1	100.00	185LYS	CB	239PHE	CE1	0.10	310LY	CB	310LYS	CG	100.00
315VAL	CA	315VAL	CG2	100.00	185LYS	CB	239PHE	CE2	0.48	310LY	CB	310LYS	CD	100.00
315VAL	CA	315VAL	C	100.00	185LYS	CB	239PHE	CZ	1.28	310LY	CB	310LYS	CE	5.63
315VAL	CB	315VAL	CG1	100.00	185LYS	CG	185LYS	CD	100.00	310LY	CB	310LYS	C	100.00
315VAL	CB	315VAL	CG2	100.00	185LYS	CG	185LYS	CE	100.00	310LY	CB	344LEU	CD1	0.11
315VAL	CB	315VAL	C	100.00	185LYS	CG	185LYS	C	99.33	310LY	CB	344LEU	CD2	0.17
315VAL	CG1	315VAL	CG2	100.00	185LYS	CG	188VAL	CG1	0.50	310LY	CG	310LYS	CD	100.00
315VAL	CG1	340VAL	CG1	0.40	185LYS	CG	188VAL	CG2	10.59	310LY	CG	310LYS	CE	100.00
315VAL	CG1	340VAL	CG2	0.22	185LYS	CG	189LEU	CD1	0.12	310LY	CG	310LYS	C	78.04
315VAL	CG2	315VAL	C	99.98	185LYS	CG	189LEU	CD2	0.00	310LY	CG	344LEU	CD1	0.16
315VAL	C	316ALA	CA	100.00	185LYS	CG	215TYR	CD1	0.00	310LY	CG	344LEU	CD2	0.77
315VAL	C	316ALA	C	100.00	185LYS	CG	216VAL	CG1	0.01	310LY	CD	310LYS	CE	100.00
315VAL	C	319LEU	CD1	0.01	185LYS	CD	185LYS	CE	100.00	310LY	CD	310LYS	C	0.01
316ALA	CA	316ALA	CB	100.00	185LYS	CD	185LYS	C	0.01	310LY	CD	344LEU	CD1	0.04
316ALA	CA	316ALA	C	100.00	185LYS	CD	188VAL	CG1	0.00	310LY	CD	344LEU	CD2	0.18
316ALA	CB	316ALA	C	100.00	185LYS	CD	188VAL	CG2	0.22	310LY	CD	345ALA	C	0.03
316ALA	C	317LYS	CA	100.00	185LYS	CD	215TYR	CB	0.00	310LY	CE	344LEU	CD1	0.01
316ALA	C	317LYS	CB	0.07	185LYS	CD	215TYR	CD1	0.01	310LY	CE	344LEU	CD2	0.04
316ALA	C	317LYS	CG	0.01	185LYS	CD	216VAL	CG1	1.73	310LY	CE	344LEU	C	0.04
316ALA	C	317LYS	C	99.95	185LYS	CE	187ASN	CG	0.00	310LY	CE	345ALA	C	0.61
317LYS	CA	317LYS	CB	100.00	185LYS	CE	188VAL	CG1	0.19	310LY	C	311VAL	CA	100.00
317LYS	CA	317LYS	CG	100.00	185LYS	CE	188VAL	CG2	1.30	310LY	C	311VAL	C	100.00
317LYS	CA	317LYS	CD	0.26	185LYS	CE	216VAL	CG1	0.01	310LY	C	344LEU	CD1	0.32
317LYS	CA	317LYS	C	100.00	185LYS	CE	217ASP	CG	0.00	310LY	C	344LEU	CD2	0.65
317LYS	CA	320LEU	CB	0.01	185LYS	C	186ALA	CA	100.00	311VA	CA	311VAL	CB	100.00
317LYS	CA	320LEU	CD1	0.01	185LYS	C	186ALA	C	100.00	311VA	CA	311VAL	CG1	100.00
317LYS	CB	317LYS	CG	100.00	185LYS	C	189LEU	CB	0.01	311VA	CA	311VAL	CG2	100.00
317LYS	CB	317LYS	CD	100.00	185LYS	C	189LEU	CD1	0.00	311VA	CA	311VAL	C	100.00
317LYS	CB	317LYS	CE	1.17	186ALA	CA	186ALA	CB	100.00	311VA	CA	344LEU	CD1	0.03
317LYS	CB	317LYS	C	100.00	186ALA	CA	186ALA	C	100.00	311VA	CB	311VAL	CG1	100.00
317LYS	CB	343HIS	CB	0.07	186ALA	CA	192GLY	C	2.78	311VA	CB	311VAL	CG2	100.00
317LYS	CB	343HIS	CG	6.29	186ALA	CB	186ALA	C	100.00	311VA	CB	311VAL	C	100.00
317LYS	CB	343HIS	CD2	21.37	186ALA	CB	192GLY	CA	0.00	311VA	CG1	311VAL	CG2	100.00
317LYS	CB	343HIS	CE1	2.00	186ALA	CB	192GLY	C	12.73	311VA	CG1	311VAL	C	99.78
317LYS	CG	317LYS	CD	100.00	186ALA	CB	193GLU	CA	1.75	311VA	CG1	315VAL	CG2	0.03
317LYS	CG	317LYS	CE	100.00	186ALA	CB	196ARG	CB	0.00	311VA	CG2	311VAL	C	0.22
317LYS	CG	317LYS	C	0.32	186ALA	CB	196ARG	CD	3.24	311VA	CG2	344LEU	CD1	0.26
317LYS	CG	343HIS	CE1	0.00	186ALA	CB	196ARG	CZ	1.97	311VA	CG2	344LEU	CD2	0.07
317LYS	CD	317LYS	CE	100.00	186ALA	C	187ASN	CA	100.00	311VA	C	312GLU	CA	100.00
317LYS	CD	343HIS	CB	0.02	186ALA	C	187ASN	CB	2.64	311VA	C	312GLU	C	100.00
317LYS	CD	343HIS	CG	0.10	186ALA	C	187ASN	CG	0.00	312GL	CA	312GLU	CB	100.00
317LYS	CD	343HIS	CD2	0.00	186ALA	C	187ASN	C	97.11	312GL	CA	312GLU	CG	100.00
317LYS	CD	343HIS	CE1	0.55	186ALA	C	215TYR	CE1	0.06	312GL	CA	312GLU	C	100.00
317LYS	CE	343HIS	CE1	0.01	187ASN	CA	187ASN	CB	100.00	312GL	CB	312GLU	CG	100.00
317LYS	C	318ALA	CA	100.00	187ASN	CA	187ASN	CG	100.00	312GL	CB	312GLU	CD	100.00
317LYS	C	318ALA	CB	0.07	187ASN	CA	187ASN	C	100.00	312GL	CB	312GLU	C	100.00
317LYS	C	318ALA	C	99.86	187ASN	CB	187ASN	CG	100.00	312GL	CG	312GLU	CD	100.00
317LYS	C	320LEU	CB	0.01	187ASN	CB	187ASN	C	100.00	312GL	CG	312GLU	C	28.69
317LYS	C	343HIS	CD2	3.38	187ASN	CB	215TYR	CE1	0.52	312GL	C	313ASP	CA	100.00
317LYS	C	343HIS	CE1	0.02	187ASN	CB	215TYR	CE2	0.00	312GL	C	313ASP	CB	0.01
318ALA	CA	318ALA	CB	100.00	187ASN	CB	215TYR	CZ	0.20	312GL	C	313ASP	C	100.00
318ALA	CA	318ALA	C	100.00	187ASN	CG	187ASN	C	99.85	313AS	CA	313ASP	CB	100.00
318ALA	CA	339THR	CG2	0.70	187ASN	CG	188VAL	CG1	0.00	313AS	CA	313ASP	CG	100.00
318ALA	CA	343HIS	CD2	0.08	187ASN	CG	188VAL	CG2	0.04	313AS	CA	313ASP	C	100.00

318ALA	CA	343HIS	CE1	0.00	187ASI	CG	215TYR	CD1	0.26	313AS	CB	313ASP	CG	100.00
318ALA	CB	318ALA	C	100.00	187ASI	CG	215TYR	CD2	0.01	313AS	CB	313ASP	C	100.00
318ALA	CB	336PHE	CD1	0.05	187ASI	CG	215TYR	CE1	2.67	313AS	CG	313ASP	C	99.92
318ALA	CB	336PHE	CD2	0.00	187ASI	CG	215TYR	CE2	0.08	313AS	CG	317LYS	CD	0.02
318ALA	CB	336PHE	CE1	11.10	187ASI	CG	215TYR	CZ	0.51	313AS	CG	317LYS	CE	1.33
318ALA	CB	336PHE	CE2	2.06	187ASI	C	188VAL	CA	100.00	313AS	C	314ALA	CA	100.00
318ALA	CB	336PHE	CZ	25.29	187ASI	C	188VAL	CB	96.24	313AS	C	314ALA	C	100.00
318ALA	CB	339THR	CB	0.00	187ASI	C	188VAL	CG1	49.30	314AL	CA	314ALA	CB	100.00
318ALA	CB	339THR	CG2	2.39	187ASI	C	188VAL	CG2	2.45	314AL	CA	314ALA	C	100.00
318ALA	CB	340VAL	CG2	18.65	187ASI	C	188VAL	C	2.73	314AL	CA	343HIS	CB	0.08
318ALA	C	319LEU	CA	100.00	188VAI	CA	188VAL	CB	100.00	314AL	CA	343HIS	CD2	0.01
318ALA	C	319LEU	C	100.00	188VAI	CA	188VAL	CG1	100.00	314AL	CB	314ALA	C	100.00
318ALA	C	336PHE	CE1	9.72	188VAI	CA	188VAL	CG2	100.00	314AL	CB	340VAL	CG1	0.37
318ALA	C	336PHE	CZ	0.36	188VAI	CA	188VAL	C	100.00	314AL	CB	343HIS	CB	2.92
319LEU	CA	319LEU	CB	100.00	188VAI	CB	188VAL	CG1	100.00	314AL	CB	343HIS	CD2	0.00
319LEU	CA	319LEU	CG	100.00	188VAI	CB	188VAL	CG2	100.00	314AL	CB	343HIS	C	0.29
319LEU	CA	319LEU	CD1	5.64	188VAI	CB	188VAL	C	100.00	314AL	CB	344LEU	CA	0.03
319LEU	CA	319LEU	CD2	93.76	188VAI	CG1	188VAL	CG2	100.00	314AL	CB	344LEU	CB	0.06
319LEU	CA	319LEU	C	100.00	188VAI	CG1	188VAL	C	9.68	314AL	CB	344LEU	CG	0.78
319LEU	CA	336PHE	CZ	0.00	188VAI	CG1	189LEU	CD1	0.01	314AL	CB	344LEU	CD1	0.43
319LEU	CB	319LEU	CG	100.00	188VAI	CG2	188VAL	C	94.77	314AL	CB	344LEU	CD2	1.71
319LEU	CB	319LEU	CD1	100.00	188VAI	CG2	189LEU	CG	0.00	314AL	C	315VAL	CA	100.00
319LEU	CB	319LEU	CD2	100.00	188VAI	CG2	189LEU	CD1	0.34	314AL	C	315VAL	CB	0.01
319LEU	CB	319LEU	C	100.00	188VAI	CG2	189LEU	CD2	0.03	314AL	C	315VAL	C	100.00
319LEU	CG	319LEU	CD1	100.00	188VAI	C	189LEU	CA	100.00	315VA	CA	315VAL	CB	100.00
319LEU	CG	319LEU	CD2	100.00	188VAI	C	189LEU	CB	18.46	315VA	CA	315VAL	CG1	100.00
319LEU	CG	319LEU	C	5.75	188VAI	C	189LEU	CG	2.78	315VA	CA	315VAL	CG2	100.00
319LEU	CG	324PRO	CD	0.00	188VAI	C	189LEU	CD1	0.41	315VA	CA	315VAL	C	100.00
319LEU	CG	327LEU	CD1	0.00	188VAI	C	189LEU	CD2	0.06	315VA	CB	315VAL	CG1	100.00
319LEU	CG	336PHE	CE1	0.02	188VAI	C	189LEU	C	80.79	315VA	CB	315VAL	CG2	100.00
319LEU	CD1	319LEU	CD2	100.00	189LEL	CA	189LEU	CB	100.00	315VA	CB	315VAL	C	100.00
319LEU	CD1	324PRO	CD	0.62	189LEL	CA	189LEU	CG	100.00	315VA	CG1	315VAL	CG2	100.00
319LEU	CD1	327LEU	CD1	0.00	189LEL	CA	189LEU	CD1	31.23	315VA	CG1	315VAL	C	100.00
319LEU	CD1	336PHE	CE1	0.02	189LEL	CA	189LEU	CD2	68.24	315VA	CG1	319LEU	CD1	1.72
319LEU	CD1	336PHE	CZ	0.02	189LEL	CA	189LEU	C	100.00	315VA	CG2	315VAL	C	2.35
319LEU	CD2	319LEU	C	0.12	189LEL	CB	189LEU	CG	100.00	315VA	CG2	336PHE	CE2	0.00
319LEU	CD2	324PRO	CG	0.16	189LEL	CB	189LEU	CD1	100.00	315VA	CG2	340VAL	CG1	0.16
319LEU	CD2	324PRO	CD	9.09	189LEL	CB	189LEU	CD2	100.00	315VA	CG2	340VAL	CG2	0.28
319LEU	CD2	327LEU	CD1	0.12	189LEL	CB	189LEU	C	100.00	315VA	C	316ALA	CA	100.00
319LEU	CD2	327LEU	CD2	0.06	189LEL	CB	191VAL	CG2	0.04	315VA	C	316ALA	CB	0.00
319LEU	CD2	336PHE	CE1	1.31	189LEL	CB	192GLY	CA	0.04	315VA	C	316ALA	C	100.00
319LEU	CD2	336PHE	CZ	1.30	189LEL	CG	189LEU	CD1	100.00	316AL	CA	316ALA	CB	100.00
319LEU	C	320LEU	CA	100.00	189LEL	CG	189LEU	CD2	100.00	316AL	CA	316ALA	C	100.00
319LEU	C	320LEU	CB	0.22	189LEL	CG	189LEU	C	7.92	316AL	CB	316ALA	C	100.00
319LEU	C	320LEU	CG	0.00	189LEL	CG	191VAL	CG2	0.45	316AL	C	317LYS	CA	100.00
319LEU	C	320LEU	CD1	0.00	189LEL	CD1	189LEU	CD2	100.00	316AL	C	317LYS	CB	0.09
319LEU	C	320LEU	C	99.68	189LEL	CD1	189LEU	C	0.09	316AL	C	317LYS	CG	0.00
320LEU	CA	320LEU	CB	100.00	189LEL	CD1	191VAL	CG2	0.33	316AL	C	317LYS	C	99.91
320LEU	CA	320LEU	CG	100.00	189LEL	CD1	192GLY	CA	0.01	316AL	C	320LEU	CD1	0.00
320LEU	CA	320LEU	CD1	35.89	189LEL	CD1	238ILE	CD	0.14	317LY	CA	317LYS	CB	100.00
320LEU	CA	320LEU	CD2	61.09	189LEL	CD1	239PHE	CE1	0.30	317LY	CA	317LYS	CG	100.00
320LEU	CA	320LEU	C	100.00	189LEL	CD1	239PHE	CE2	0.30	317LY	CA	317LYS	CD	0.21
320LEU	CB	320LEU	CG	100.00	189LEL	CD1	239PHE	CZ	1.41	317LY	CA	317LYS	C	100.00
320LEU	CB	320LEU	CD1	100.00	189LEL	CD2	191VAL	CG2	0.84	317LY	CA	320LEU	CD1	0.01
320LEU	CB	320LEU	CD2	100.00	189LEL	CD2	192GLY	CA	0.00	317LY	CB	317LYS	CG	100.00
320LEU	CB	320LEU	C	100.00	189LEL	CD2	238ILE	CD	0.06	317LY	CB	317LYS	CD	100.00
320LEU	CG	320LEU	CD1	100.00	189LEL	CD2	239PHE	CE1	0.17	317LY	CB	317LYS	CE	1.14
320LEU	CG	320LEU	CD2	100.00	189LEL	CD2	239PHE	CE2	0.18	317LY	CB	317LYS	C	100.00
320LEU	CG	320LEU	C	43.53	189LEL	CD2	239PHE	CZ	0.76	317LY	CB	343HIS	CB	0.01
320LEU	CD1	320LEU	CD2	100.00	189LEL	C	190GLU	CA	100.00	317LY	CB	343HIS	CG	2.97
320LEU	CD1	320LEU	C	0.90	189LEL	C	190GLU	CB	0.00	317LY	CB	343HIS	CD2	17.17
320LEU	CD2	320LEU	C	5.38	189LEL	C	190GLU	C	100.00	317LY	CB	343HIS	CE1	2.72

320LEU C	321GLU	CA	100.00	190GL CA	190GLU	CB	100.00	317LY CG	317LYS	CD	100.00
320LEU C	321GLU	CB	4.76	190GL CA	190GLU	CG	100.00	317LY CG	317LYS	CE	100.00
320LEU C	321GLU	CG	0.01	190GL CA	190GLU	CD	97.25	317LY CG	317LYS	C	0.37
320LEU C	321GLU	C	95.90	190GL CA	190GLU	C	100.00	317LY CG	343HIS	CG	0.00
321GLU CA	321GLU	CB	100.00	190GL CB	190GLU	CG	100.00	317LY CG	343HIS	CD2	0.02
321GLU CA	321GLU	CG	100.00	190GL CB	190GLU	CD	100.00	317LY CG	343HIS	CE1	0.03
321GLU CA	321GLU	CD1	55.83	190GL CB	190GLU	C	100.00	317LY CD	317LYS	CE	100.00
321GLU CA	321GLU	C	100.00	190GL CG	190GLU	CD	100.00	317LY CD	343HIS	CG	0.11
321GLU CB	321GLU	CG	100.00	190GL CG	190GLU	C	6.67	317LY CD	343HIS	CD2	0.02
321GLU CB	321GLU	CD1	100.00	190GL C	191VAL	CA	100.00	317LY CD	343HIS	CE1	0.91
321GLU CB	321GLU	C	100.00	190GL C	191VAL	C	100.00	317LY CE	343HIS	CE1	0.02
321GLU CB	339THR	CG2	0.34	191VA CA	191VAL	CB	100.00	317LY C	318ALA	CA	100.00
321GLU CG	321GLU	CD	100.00	191VA CA	191VAL	CG1	100.00	317LY C	318ALA	CB	0.06
321GLU CG	321GLU	C	92.27	191VA CA	191VAL	CG2	100.00	317LY C	318ALA	C	99.95
321GLU CG	339THR	CG2	1.04	191VA CA	191VAL	C	100.00	317LY C	343HIS	CD2	1.60
321GLU CD	321GLU	C	0.12	191VA CA	194PHE	CB	0.00	318AL CA	318ALA	CB	100.00
321GLU CD	339THR	CG2	2.82	191VA CB	191VAL	CG1	100.00	318AL CA	318ALA	C	100.00
321GLU CD	342ARG	CZ	0.00	191VA CB	191VAL	CG2	100.00	318AL CA	339THR	CG2	1.14
321GLU CD	343HIS	CE1	0.02	191VA CB	191VAL	C	100.00	318AL CA	343HIS	CD2	0.05
321GLU C	322THR	CA	100.00	191VA CG1	191VAL	CG2	100.00	318AL CB	318ALA	C	100.00
321GLU C	322THR	CB	97.81	191VA CG1	191VAL	C	0.26	318AL CB	336PHE	CD1	0.03
321GLU C	322THR	CG2	67.35	191VA CG2	191VAL	C	99.74	318AL CB	336PHE	CD2	0.03
321GLU C	322THR	C	4.98	191VA CG2	238ILE	CD	0.02	318AL CB	336PHE	CE1	11.74
321GLU C	323PRO	CD	4.25	191VA C	192GLY	CA	100.00	318AL CB	336PHE	CE2	2.14
322THR CA	322THR	CB	100.00	191VA C	192GLY	C	95.04	318AL CB	336PHE	CZ	21.49
322THR CA	322THR	CG2	100.00	191VA C	194PHE	CB	0.00	318AL CB	339THR	CB	0.01
322THR CA	322THR	C	100.00	192GL CA	192GLY	C	100.00	318AL CB	339THR	CG2	5.97
322THR CA	323PRO	CD	100.00	192GL CA	195TRP	CB	0.01	318AL CB	340VAL	CG2	11.84
322THR CA	331ALA	CB	0.14	192GL C	193GLU	CA	100.00	318AL C	319LEU	CA	100.00
322THR CB	322THR	CG2	100.00	192GL C	193GLU	C	100.00	318AL C	319LEU	CB	0.01
322THR CB	322THR	C	100.00	193GL CA	193GLU	CB	100.00	318AL C	319LEU	C	99.99
322THR CB	331ALA	CB	1.05	193GL CA	193GLU	CG	100.00	318AL C	336PHE	CE1	4.81
322THR CB	336PHE	CD1	0.00	193GL CA	193GLU	CD	70.35	318AL C	336PHE	CZ	0.20
322THR CG2	331ALA	CB	0.07	193GL CA	193GLU	C	100.00	319LE CA	319LEU	CB	100.00
322THR CG2	335ALA	CB	0.00	193GL CB	193GLU	CG	100.00	319LE CA	319LEU	CG	100.00
322THR CG2	339THR	CG2	0.00	193GL CB	193GLU	CD	100.00	319LE CA	319LEU	CD1	9.36
322THR C	323PRO	CA	100.00	193GL CB	193GLU	C	100.00	319LE CA	319LEU	CD2	90.20
322THR C	323PRO	CB	0.09	193GL CG	193GLU	CD	100.00	319LE CA	319LEU	C	100.00
322THR C	323PRO	CD	100.00	193GL CG	193GLU	C	95.05	319LE CA	336PHE	CE1	0.01
322THR C	323PRO	C	100.00	193GL CG	197LYS	CE	0.02	319LE CA	336PHE	CZ	0.01
322THR C	331ALA	CB	2.60	193GL CD	193GLU	C	0.44	319LE CB	319LEU	CG	100.00
323PRO CA	323PRO	CB	100.00	193GL CD	197LYS	CD	0.00	319LE CB	319LEU	CD1	100.00
323PRO CA	323PRO	CG	100.00	193GL CD	197LYS	CE	0.07	319LE CB	319LEU	CD2	100.00
323PRO CA	323PRO	CD	100.00	193GL C	194PHE	CA	100.00	319LE CB	319LEU	C	100.00
323PRO CA	323PRO	C	100.00	193GL C	194PHE	CB	0.01	319LE CG	319LEU	CD1	100.00
323PRO CA	324PRO	CD	100.00	193GL C	194PHE	C	100.00	319LE CG	319LEU	CD2	100.00
323PRO CB	323PRO	CG	100.00	194PH CA	194PHE	CB	100.00	319LE CG	319LEU	C	9.46
323PRO CB	323PRO	CD	100.00	194PH CA	194PHE	CG	100.00	319LE CG	327LEU	CD2	0.01
323PRO CB	323PRO	C	100.00	194PH CA	194PHE	CD1	92.68	319LE CG	336PHE	CE1	0.04
323PRO CB	324PRO	CD	34.80	194PH CA	194PHE	CD2	57.15	319LE CG	336PHE	CZ	0.01
323PRO CB	327LEU	CB	0.29	194PH CA	194PHE	C	100.00	319LE CD1	319LEU	CD2	100.00
323PRO CB	327LEU	CD1	0.07	194PH CB	194PHE	CG	100.00	319LE CD1	319LEU	C	0.04
323PRO CB	327LEU	CD2	0.12	194PH CB	194PHE	CD1	100.00	319LE CD1	324PRO	CD	0.34
323PRO CG	323PRO	CD	100.00	194PH CB	194PHE	CD2	100.00	319LE CD1	327LEU	CD1	0.01
323PRO CG	323PRO	C	94.94	194PH CB	194PHE	C	100.00	319LE CD1	327LEU	CD2	0.09
323PRO CG	329GLY	CA	0.13	194PH CG	194PHE	CD1	100.00	319LE CD1	336PHE	CE1	0.10
323PRO CD	323PRO	C	89.19	194PH CG	194PHE	CD2	100.00	319LE CD1	336PHE	CZ	0.01
323PRO C	324PRO	CA	100.00	194PH CG	194PHE	CE1	100.00	319LE CD2	319LEU	C	0.10
323PRO C	324PRO	CB	0.16	194PH CG	194PHE	CE2	100.00	319LE CD2	323PRO	CB	0.01
323PRO C	324PRO	CD	100.00	194PH CG	194PHE	CZ	100.00	319LE CD2	324PRO	CB	0.00
323PRO C	324PRO	C	100.00	194PH CG	194PHE	C	99.99	319LE CD2	324PRO	CG	0.08
323PRO C	331ALA	CB	0.74	194PH CD1	194PHE	CD2	100.00	319LE CD2	324PRO	CD	4.74

324PRO	CA	324PRO	CB	100.00	194PHI	CD1	194PHE	CE1	100.00	319LEI	CD2	327LEU	CD1	0.54
324PRO	CA	324PRO	CG	100.00	194PHI	CD1	194PHE	CE2	100.00	319LEI	CD2	327LEU	CD2	0.94
324PRO	CA	324PRO	CD	100.00	194PHI	CD1	194PHE	CZ	100.00	319LEI	CD2	336PHE	CE1	1.66
324PRO	CA	324PRO	C	100.00	194PHI	CD1	194PHE	C	0.48	319LEI	CD2	336PHE	CE2	0.00
324PRO	CA	325PRO	CD	100.00	194PHI	CD1	197LYS	CD	0.00	319LEI	CD2	336PHE	CZ	1.02
324PRO	CA	331ALA	CB	0.02	194PHI	CD1	197LYS	CE	0.02	319LEI	C	320LEU	CA	100.00
324PRO	CB	324PRO	CG	100.00	194PHI	CD2	194PHE	CE1	100.00	319LEI	C	320LEU	CB	0.38
324PRO	CB	324PRO	CD	100.00	194PHI	CD2	194PHE	CE2	100.00	319LEI	C	320LEU	CG	0.02
324PRO	CB	324PRO	C	100.00	194PHI	CD2	194PHE	CZ	100.00	319LEI	C	320LEU	CD1	0.02
324PRO	CB	325PRO	CD	64.02	194PHI	CD2	194PHE	C	67.67	319LEI	C	320LEU	C	99.43
324PRO	CG	324PRO	CD	100.00	194PHI	CE1	194PHE	CE2	100.00	320LEI	CA	320LEU	CB	100.00
324PRO	CG	324PRO	C	96.92	194PHI	CE1	194PHE	CZ	100.00	320LEI	CA	320LEU	CG	100.00
324PRO	CG	327LEU	CB	0.00	194PHI	CE1	197LYS	CE	0.01	320LEI	CA	320LEU	CD1	16.49
324PRO	CG	327LEU	CG	0.01	194PHI	CE2	194PHE	CZ	100.00	320LEI	CA	320LEU	CD2	82.69
324PRO	CG	327LEU	CD1	0.56	194PHI	C	195TRP	CA	100.00	320LEI	CA	320LEU	C	100.00
324PRO	CG	327LEU	CD2	0.12	194PHI	C	195TRP	CB	0.00	320LEI	CB	320LEU	CG	100.00
324PRO	CD	324PRO	C	91.22	194PHI	C	195TRP	C	100.00	320LEI	CB	320LEU	CD1	100.00
324PRO	CD	327LEU	CB	0.01	195TRI	CA	195TRP	CB	100.00	320LEI	CB	320LEU	CD2	100.00
324PRO	CD	327LEU	CD1	0.12	195TRI	CA	195TRP	CG	100.00	320LEI	CB	320LEU	C	100.00
324PRO	CD	327LEU	CD2	0.01	195TRI	CA	195TRP	CD1	84.81	320LEI	CB	320LEU	CD1	100.00
324PRO	C	325PRO	CA	100.00	195TRI	CA	195TRP	CD2	9.56	320LEI	CB	320LEU	CD2	100.00
324PRO	C	325PRO	CB	0.00	195TRI	CA	195TRP	CE3	0.06	320LEI	CB	320LEU	C	16.45
324PRO	C	325PRO	CD	100.00	195TRI	CA	195TRP	C	100.00	320LEI	CD1	320LEU	CD2	100.00
324PRO	C	325PRO	C	100.00	195TRI	CB	195TRP	CG	100.00	320LEI	CD2	320LEU	C	0.20
324PRO	C	328GLY	C	0.00	195TRI	CB	195TRP	CD1	100.00	320LEI	CD2	320LEU	C	0.99
324PRO	C	329GLY	C	0.04	195TRI	CB	195TRP	CD2	100.00	320LEI	C	321GLU	CA	100.00
325PRO	CA	325PRO	CB	100.00	195TRI	CB	195TRP	CE3	96.34	320LEI	C	321GLU	CB	6.94
325PRO	CA	325PRO	CG	100.00	195TRI	CB	195TRP	C	100.00	320LEI	C	321GLU	C	93.93
325PRO	CA	325PRO	CD	100.00	195TRI	CG	195TRP	CD1	100.00	321GL	CA	321GLU	CB	100.00
325PRO	CA	325PRO	C	100.00	195TRI	CG	195TRP	CD2	100.00	321GL	CA	321GLU	CG	100.00
325PRO	CA	329GLY	C	0.01	195TRI	CG	195TRP	CE2	100.00	321GL	CA	321GLU	CD	52.00
325PRO	CB	325PRO	CG	100.00	195TRI	CG	195TRP	CE3	100.00	321GL	CA	321GLU	C	100.00
325PRO	CB	325PRO	CD	100.00	195TRI	CG	195TRP	C	100.00	321GL	CB	321GLU	CG	100.00
325PRO	CB	325PRO	C	100.00	195TRI	CD1	195TRP	CD2	100.00	321GL	CB	321GLU	CD	100.00
325PRO	CB	329GLY	C	0.00	195TRI	CD1	195TRP	CE2	100.00	321GL	CB	321GLU	C	100.00
325PRO	CG	325PRO	CD	100.00	195TRI	CD1	195TRP	CE3	0.00	321GL	CB	339THR	CG2	0.12
325PRO	CG	325PRO	C	39.64	195TRI	CD1	195TRP	C	90.88	321GL	CG	321GLU	CD	100.00
325PRO	CD	325PRO	C	23.05	195TRI	CD1	199VAL	CG1	0.02	321GL	CG	321GLU	C	97.98
325PRO	CD	330SER	CA	0.00	195TRI	CD1	199VAL	CG2	0.54	321GL	CG	322THR	CG2	0.01
325PRO	CD	330SER	C	0.00	195TRI	CD2	195TRP	CE2	100.00	321GL	CG	339THR	CG2	0.60
325PRO	C	326ASP	CA	100.00	195TRI	CD2	195TRP	CE3	100.00	321GL	CD	321GLU	C	0.08
325PRO	C	326ASP	CB	0.76	195TRI	CD2	195TRP	CZ2	100.00	321GL	CD	339THR	CG2	2.38
325PRO	C	326ASP	C	99.59	195TRI	CD2	195TRP	CZ3	100.00	321GL	CD	343HIS	CE1	0.00
326ASP	CA	326ASP	CB	100.00	195TRI	CD2	195TRP	C	0.10	321GL	C	322THR	CA	100.00
326ASP	CA	326ASP	CG	100.00	195TRI	CD2	199VAL	CG2	0.06	321GL	C	322THR	CB	97.73
326ASP	CA	326ASP	C	100.00	195TRI	CE2	195TRP	CE3	100.00	321GL	C	322THR	CG2	66.68
326ASP	CB	326ASP	CG	100.00	195TRI	CE2	195TRP	CZ2	100.00	321GL	C	322THR	C	4.82
326ASP	CB	326ASP	C	100.00	195TRI	CE2	195TRP	CZ3	100.00	321GL	C	323PRO	CD	4.79
326ASP	CG	326ASP	C	97.95	195TRI	CE2	199VAL	CG1	0.01	322TH	CA	322THR	CB	100.00
326ASP	CG	327LEU	CD1	0.01	195TRI	CE2	199VAL	CG2	0.96	322TH	CA	322THR	CG2	100.00
326ASP	CG	327LEU	CD2	0.00	195TRI	CE3	195TRP	CZ2	100.00	322TH	CA	322THR	C	100.00
326ASP	C	327LEU	CA	100.00	195TRI	CE3	195TRP	CZ3	100.00	322TH	CA	323PRO	CD	100.00
326ASP	C	327LEU	CB	11.64	195TRI	CE3	199VAL	CG2	0.10	322TH	CA	331ALA	CB	0.11
326ASP	C	327LEU	CG	1.14	195TRI	CZ2	195TRP	CZ3	100.00	322TH	CB	322THR	CG2	100.00
326ASP	C	327LEU	CD1	0.21	195TRI	CZ2	199VAL	CG2	0.38	322TH	CB	322THR	C	100.00
326ASP	C	327LEU	CD2	0.02	195TRI	CZ2	234VAL	CG1	0.56	322TH	CB	323PRO	CD	0.04
326ASP	C	327LEU	C	85.70	195TRI	CZ2	234VAL	CG2	0.16	322TH	CB	331ALA	CB	0.24
327LEU	CA	327LEU	CB	100.00	195TRI	CZ2	234VAL	C	0.04	322TH	CG2	335ALA	CB	0.00
327LEU	CA	327LEU	CG	100.00	195TRI	CZ2	235THR	CA	1.54	322TH	C	323PRO	CA	100.00
327LEU	CA	327LEU	CD1	20.15	195TRI	CZ2	235THR	C	5.90	322TH	C	323PRO	CB	0.11
327LEU	CA	327LEU	CD2	77.92	195TRI	CZ2	236GLY	CA	0.01	322TH	C	323PRO	CD	100.00
327LEU	CA	327LEU	C	100.00	195TRI	CZ3	199VAL	CG2	0.15	322TH	C	323PRO	C	100.00

327LEU	CB	327LEU	CG	100.00	195TRI	CZ3	236GLY	CA	0.37	322TH	C	331ALA	CB	2.05
327LEU	CB	327LEU	CD1	100.00	195TRI	C	196ARG	CA	100.00	323PR	CA	323PRO	CB	100.00
327LEU	CB	327LEU	CD2	100.00	195TRI	C	196ARG	C	100.00	323PR	CA	323PRO	CG	100.00
327LEU	CB	327LEU	C	100.00	195TRI	C	199VAL	CG1	0.00	323PR	CA	323PRO	CD	100.00
327LEU	CG	327LEU	CD1	100.00	195TRI	C	199VAL	CG2	0.00	323PR	CA	323PRO	C	100.00
327LEU	CG	327LEU	CD2	100.00	196AR(	CA	196ARG	CB	100.00	323PR	CA	324PRO	CD	100.00
327LEU	CG	327LEU	C	7.71	196AR(	CA	196ARG	CG	100.00	323PR	CB	323PRO	CG	100.00
327LEU	CD1	327LEU	CD2	100.00	196AR(	CA	196ARG	C	100.00	323PR	CB	323PRO	CD	100.00
327LEU	CD1	327LEU	C	2.15	196AR(	CA	199VAL	CG1	0.03	323PR	CB	323PRO	C	100.00
327LEU	CD2	327LEU	C	0.89	196AR(	CA	199VAL	CG2	0.03	323PR	CB	324PRO	CD	45.79
327LEU	C	328GLY	CA	100.00	196AR(	CB	196ARG	CG	100.00	323PR	CB	327LEU	CB	0.02
327LEU	C	328GLY	C	46.57	196AR(	CB	196ARG	CD	100.00	323PR	CB	327LEU	CD1	0.00
328GLY	CA	328GLY	C	100.00	196AR(	CB	196ARG	C	100.00	323PR	CB	327LEU	CD2	0.80
328GLY	C	329GLY	CA	100.00	196AR(	CG	196ARG	CD	100.00	323PR	CB	329GLY	CA	0.00
328GLY	C	329GLY	C	35.20	196AR(	CG	196ARG	CZ	0.66	323PR	CG	323PRO	CD	100.00
329GLY	CA	329GLY	C	100.00	196AR(	CG	196ARG	C	99.99	323PR	CG	323PRO	C	95.49
329GLY	C	330SER	CA	100.00	196AR(	CG	213HIS	CG	1.78	323PR	CG	329GLY	CA	0.05
329GLY	C	330SER	CB	99.56	196AR(	CG	213HIS	CD2	2.08	323PR	CD	323PRO	C	91.21
329GLY	C	330SER	C	0.82	196AR(	CG	213HIS	CE1	5.09	323PR	C	324PRO	CA	100.00
330SER	CA	330SER	CB	100.00	196AR(	CD	196ARG	CZ	100.00	323PR	C	324PRO	CB	0.22
330SER	CA	330SER	C	100.00	196AR(	CD	213HIS	CG	0.03	323PR	C	324PRO	CD	100.00
330SER	CB	330SER	C	100.00	196AR(	CD	213HIS	CD2	0.21	323PR	C	324PRO	C	100.00
330SER	C	331ALA	CA	100.00	196AR(	CZ	213HIS	CB	28.29	323PR	C	331ALA	CB	0.89
330SER	C	331ALA	CB	46.55	196AR(	CZ	213HIS	CG	0.01	324PR	CA	324PRO	CB	100.00
330SER	C	331ALA	C	53.54	196AR(	CZ	215TYR	CE1	1.22	324PR	CA	324PRO	CG	100.00
331ALA	CA	331ALA	CB	100.00	196AR(	CZ	215TYR	CE2	0.01	324PR	CA	324PRO	CD	100.00
331ALA	CA	331ALA	C	100.00	196AR(	CZ	215TYR	CZ	0.18	324PR	CA	324PRO	C	100.00
331ALA	CB	331ALA	C	100.00	196AR(	C	197LYS	CA	100.00	324PR	CA	325PRO	CD	100.00
331ALA	CB	336PHE	CD1	0.28	196AR(	C	197LYS	CB	0.00	324PR	CA	331ALA	CB	0.13
331ALA	C	332GLY	CA	100.00	196AR(	C	197LYS	C	99.98	324PR	CB	324PRO	CG	100.00
331ALA	C	332GLY	C	56.15	197LYS	CA	197LYS	CB	100.00	324PR	CB	324PRO	CD	100.00
332GLY	CA	332GLY	C	100.00	197LYS	CA	197LYS	CG	100.00	324PR	CB	324PRO	C	100.00
332GLY	C	333THR	CA	100.00	197LYS	CA	197LYS	CD	12.97	324PR	CB	325PRO	CD	53.64
332GLY	C	333THR	CB	0.21	197LYS	CA	197LYS	CE	0.03	324PR	CB	327LEU	CD1	0.00
332GLY	C	333THR	C	99.89	197LYS	CA	197LYS	C	100.00	324PR	CB	331ALA	CB	0.00
333THR	CA	333THR	CB	100.00	197LYS	CB	197LYS	CG	100.00	324PR	CG	324PRO	CD	100.00
333THR	CA	333THR	CG2	100.00	197LYS	CB	197LYS	CD	100.00	324PR	CG	324PRO	C	93.83
333THR	CA	333THR	C	100.00	197LYS	CB	197LYS	CE	4.37	324PR	CG	327LEU	CB	0.01
333THR	CB	333THR	CG2	100.00	197LYS	CB	197LYS	C	100.00	324PR	CG	327LEU	CG	0.00
333THR	CB	333THR	C	100.00	197LYS	CG	197LYS	CD	100.00	324PR	CG	327LEU	CD1	0.54
333THR	CG2	333THR	C	99.64	197LYS	CG	197LYS	CE	100.00	324PR	CG	327LEU	CD2	0.02
333THR	C	334GLU	CA	100.00	197LYS	CG	197LYS	C	87.56	324PR	CD	324PRO	C	88.26
333THR	C	334GLU	CB	0.06	197LYS	CG	201GLU	CD	0.01	324PR	CD	327LEU	CB	0.01
333THR	C	334GLU	C	99.98	197LYS	CD	197LYS	CE	100.00	324PR	CD	327LEU	CD1	0.07
334GLU	CA	334GLU	CB	100.00	197LYS	CD	197LYS	C	9.20	324PR	CD	327LEU	CD2	0.01
334GLU	CA	334GLU	CG	100.00	197LYS	CE	197LYS	C	0.03	324PR	CD	331ALA	CB	0.01
334GLU	CA	334GLU	CD	99.20	197LYS	CE	201GLU	CD	0.02	324PR	CD	336PHE	CD1	0.02
334GLU	CA	334GLU	C	100.00	197LYS	C	198THR	CA	100.00	324PR	CD	336PHE	CE1	0.04
334GLU	CB	334GLU	CG	100.00	197LYS	C	198THR	CB	0.23	324PR	C	325PRO	CA	100.00
334GLU	CB	334GLU	CD	100.00	197LYS	C	198THR	C	99.93	324PR	C	325PRO	CD	100.00
334GLU	CB	334GLU	C	100.00	198THI	CA	198THR	CB	100.00	324PR	C	325PRO	C	100.00
334GLU	CG	334GLU	CD	100.00	198THI	CA	198THR	CG2	100.00	324PR	C	329GLY	C	0.00
334GLU	CG	334GLU	C	97.86	198THI	CA	198THR	C	100.00	325PR	CA	325PRO	CB	100.00
334GLU	CD	334GLU	C	0.36	198THI	CA	201GLU	CB	0.00	325PR	CA	325PRO	CG	100.00
334GLU	C	335ALA	CA	100.00	198THI	CB	198THR	CG2	100.00	325PR	CA	325PRO	CD	100.00
334GLU	C	335ALA	CB	0.01	198THI	CB	198THR	C	100.00	325PR	CA	325PRO	C	100.00
334GLU	C	335ALA	C	100.00	198THI	CG2	198THR	C	100.00	325PR	CA	328GLY	CA	0.00
335ALA	CA	335ALA	CB	100.00	198THI	CG2	202VAL	CG2	0.14	325PR	CA	329GLY	C	0.04
335ALA	CA	335ALA	C	100.00	198THI	C	199VAL	CA	100.00	325PR	CB	325PRO	CG	100.00
335ALA	CA	338ALA	CB	0.00	198THI	C	199VAL	CB	0.02	325PR	CB	325PRO	CD	100.00
335ALA	CB	335ALA	C	100.00	198THI	C	199VAL	C	99.99	325PR	CB	325PRO	C	100.00
335ALA	C	336PHE	CA	100.00	198THI	C	201GLU	CB	0.00	325PR	CG	325PRO	CD	100.00

335ALA C	336PHE	C	100.00	199VAl CA	199VAL	CB	100.00	325PR CG	325PRO	C	50.84
336PHE CA	336PHE	CB	100.00	199VAl CA	199VAL	CG1	100.00	325PR CG	330SER	C	0.01
336PHE CA	336PHE	CG	100.00	199VAl CA	199VAL	CG2	100.00	325PR CG	331ALA	CB	0.20
336PHE CA	336PHE	CD1	87.01	199VAl CA	199VAL	C	100.00	325PR CD	325PRO	C	31.53
336PHE CA	336PHE	CD2	93.45	199VAl CA	202VAL	CG1	0.01	325PR CD	330SER	C	0.04
336PHE CA	336PHE	C	100.00	199VAl CA	202VAL	CG2	0.00	325PR CD	331ALA	CB	0.40
336PHE CB	336PHE	CG	100.00	199VAl CB	199VAL	CG1	100.00	325PR CD	331ALA	C	0.00
336PHE CB	336PHE	CD1	100.00	199VAl CB	199VAL	CG2	100.00	325PR C	326ASP	CA	100.00
336PHE CB	336PHE	CD2	100.00	199VAl CB	199VAL	C	100.00	325PR C	326ASP	CB	0.02
336PHE CB	336PHE	C	100.00	199VAl CG1	199VAL	CG2	100.00	325PR C	326ASP	C	99.99
336PHE CG	336PHE	CD1	100.00	199VAl CG1	199VAL	C	95.13	325PR C	328GLY	CA	0.00
336PHE CG	336PHE	CD2	100.00	199VAl CG1	211LEU	CD1	5.92	326AS CA	326ASP	CB	100.00
336PHE CG	336PHE	CE1	100.00	199VAl CG1	211LEU	CD2	0.24	326AS CA	326ASP	CG	100.00
336PHE CG	336PHE	CE2	100.00	199VAl CG1	213HIS	CE1	0.83	326AS CA	326ASP	C	100.00
336PHE CG	336PHE	CZ	100.00	199VAl CG2	199VAL	C	8.52	326AS CB	326ASP	CG	100.00
336PHE CG	336PHE	C	99.97	199VAl CG2	211LEU	CD1	0.00	326AS CB	326ASP	C	100.00
336PHE CD1	336PHE	CD2	100.00	199VAl CG2	213HIS	CE1	0.20	326AS CG	326ASP	C	95.50
336PHE CD1	336PHE	CE1	100.00	199VAl C	200GLU	CA	100.00	326AS CG	327LEU	CD1	0.00
336PHE CD1	336PHE	CE2	100.00	199VAl C	200GLU	CB	0.02	326AS C	327LEU	CA	100.00
336PHE CD1	336PHE	CZ	100.00	199VAl C	200GLU	C	100.00	326AS C	327LEU	CB	8.48
336PHE CD1	336PHE	C	0.01	199VAl C	211LEU	CD1	0.00	326AS C	327LEU	CG	0.66
336PHE CD2	336PHE	CE1	100.00	199VAl C	211LEU	CD2	0.04	326AS C	327LEU	CD1	0.05
336PHE CD2	336PHE	CE2	100.00	200GLl CA	200GLU	CB	100.00	326AS C	327LEU	CD2	0.01
336PHE CD2	336PHE	CZ	100.00	200GLl CA	200GLU	CG	100.00	326AS C	327LEU	C	88.74
336PHE CD2	336PHE	C	92.45	200GLl CA	200GLU	CD	90.62	327LE CA	327LEU	CB	100.00
336PHE CD2	340VAL	CG2	2.87	200GLl CA	200GLU	C	100.00	327LE CA	327LEU	CG	100.00
336PHE CE1	336PHE	CE2	100.00	200GLl CA	211LEU	CD1	0.04	327LE CA	327LEU	CD1	30.10
336PHE CE1	336PHE	CZ	100.00	200GLl CA	211LEU	CD2	0.20	327LE CA	327LEU	CD2	68.21
336PHE CE2	336PHE	CZ	100.00	200GLl CB	200GLU	CG	100.00	327LE CA	327LEU	C	100.00
336PHE CE2	340VAL	CG2	21.73	200GLl CB	200GLU	CD	100.00	327LE CB	327LEU	C	100.00
336PHE CZ	340VAL	CG2	0.14	200GLl CB	200GLU	C	100.00	327LE CB	327LEU	CD1	100.00
336PHE C	337THR	CA	100.00	200GLl CB	211LEU	CD1	0.00	327LE CB	327LEU	CD2	100.00
336PHE C	337THR	CB	0.00	200GLl CG	200GLU	CD	100.00	327LE CB	327LEU	C	100.00
336PHE C	337THR	C	100.00	200GLl CG	200GLU	C	99.81	327LE CG	327LEU	CD1	100.00
337THR CA	337THR	CB	100.00	200GLl CG	204ARG	CB	0.00	327LE CG	327LEU	CD2	100.00
337THR CA	337THR	CG2	100.00	200GLl CG	204ARG	CG	0.07	327LE CG	327LEU	C	27.71
337THR CA	337THR	C	100.00	200GLl CG	204ARG	CZ	0.22	327LE CD1	327LEU	CD2	100.00
337THR CB	337THR	CG2	100.00	200GLl CG	211LEU	CD1	0.03	327LE CD1	327LEU	C	0.62
337THR CB	337THR	C	100.00	200GLl CD	200GLU	C	0.12	327LE CD2	327LEU	C	0.88
337THR CG2	337THR	C	100.00	200GLl CD	204ARG	CB	0.04	327LE C	328GLY	CA	100.00
337THR CG2	341LEU	CD1	0.09	200GLl CD	204ARG	CG	0.88	327LE C	328GLY	C	42.21
337THR C	338ALA	CA	100.00	200GLl CD	204ARG	CD	0.00	328GL CA	328GLY	C	100.00
337THR C	338ALA	C	100.00	200GLl CD	204ARG	CZ	0.03	328GL C	329GLY	CA	100.00
338ALA CA	338ALA	CB	100.00	200GLl CD	211LEU	CD1	0.04	328GL C	329GLY	C	31.61
338ALA CA	338ALA	C	100.00	200GLl CD	211LEU	CD2	0.00	329GL CA	329GLY	C	100.00
338ALA CB	338ALA	C	100.00	200GLl C	201GLU	CA	100.00	329GL C	330SER	CA	100.00
338ALA CB	342ARG	CZ	0.01	200GLl C	201GLU	CB	0.61	329GL C	330SER	CB	99.03
338ALA C	339THR	CA	100.00	200GLl C	201GLU	C	99.55	329GL C	330SER	C	1.51
338ALA C	339THR	CB	0.11	200GLl C	204ARG	CB	0.02	330SE CA	330SER	CB	100.00
338ALA C	339THR	C	99.98	200GLl C	204ARG	CG	0.00	330SE CA	330SER	C	100.00
339THR CA	339THR	CB	100.00	201GLl CA	201GLU	CB	100.00	330SE CB	330SER	C	100.00
339THR CA	339THR	CG2	100.00	201GLl CA	201GLU	CG	100.00	330SE C	331ALA	CA	100.00
339THR CA	339THR	C	100.00	201GLl CA	201GLU	CD	44.13	330SE C	331ALA	CB	65.16
339THR CA	342ARG	CZ	0.02	201GLl CA	201GLU	C	100.00	330SE C	331ALA	C	35.15
339THR CB	339THR	CG2	100.00	201GLl CB	201GLU	CG	100.00	331AL CA	331ALA	CB	100.00
339THR CB	339THR	C	100.00	201GLl CB	201GLU	CD	100.00	331AL CA	331ALA	C	100.00
339THR CG2	339THR	C	99.99	201GLl CB	201GLU	C	100.00	331AL CB	331ALA	C	100.00
339THR CG2	342ARG	CZ	0.02	201GLl CG	201GLU	CD	100.00	331AL CB	335ALA	CB	0.00
339THR CG2	343HIS	CD2	0.87	201GLl CG	201GLU	C	92.58	331AL CB	336PHE	CD1	0.59
339THR CG2	343HIS	CE1	0.10	201GLl CD	201GLU	C	0.67	331AL CB	336PHE	CE1	0.11
339THR C	340VAL	CA	100.00	201GLl CD	204ARG	CD	0.00	331AL CB	336PHE	CZ	0.02
339THR C	340VAL	C	100.00	201GLl C	202VAL	CA	100.00	331AL C	332GLY	CA	100.00

339THR	C	343HIS	CD2	0.01	201GL	C	202VAL	CB	9.96	331AL	C	332GLY	C	43.69
340VAL	CA	340VAL	CB	100.00	201GL	C	202VAL	CG1	4.85	331AL	C	335ALA	CB	0.00
340VAL	CA	340VAL	CG1	100.00	201GL	C	202VAL	CG2	0.13	332GL	CA	332GLY	C	100.00
340VAL	CA	340VAL	CG2	100.00	201GL	C	202VAL	C	90.36	332GL	CA	335ALA	CB	0.03
340VAL	CA	340VAL	C	100.00	202VA	CA	202VAL	CB	100.00	332GL	C	333THR	CA	100.00
340VAL	CB	340VAL	CG1	100.00	202VA	CA	202VAL	CG1	100.00	332GL	C	333THR	CB	0.02
340VAL	CB	340VAL	CG2	100.00	202VA	CA	202VAL	CG2	100.00	332GL	C	333THR	C	99.98
340VAL	CB	340VAL	C	100.00	202VA	CA	202VAL	C	100.00	332GL	C	336PHE	CB	0.00
340VAL	CG1	340VAL	CG2	100.00	202VA	CB	202VAL	CG1	100.00	333TH	CA	333THR	CB	100.00
340VAL	CG1	340VAL	C	100.00	202VA	CB	202VAL	CG2	100.00	333TH	CA	333THR	CG2	100.00
340VAL	CG1	344LEU	CD1	0.21	202VA	CB	202VAL	C	100.00	333TH	CA	333THR	C	100.00
340VAL	C	341LEU	C	100.00	202VA	CG1	202VAL	CG2	100.00	333TH	CB	333THR	CG2	100.00
340VAL	C	341LEU	C	100.00	202VA	CG1	202VAL	C	45.30	333TH	CB	333THR	C	100.00
340VAL	C	344LEU	CD1	0.00	202VA	CG1	206TYR	CD1	0.08	333TH	CG2	333THR	C	76.66
341LEU	CA	341LEU	C	100.00	202VA	CG1	206TYR	CD2	0.11	333TH	C	334GLU	CA	100.00
341LEU	CA	341LEU	CG	100.00	202VA	CG1	206TYR	CE1	0.04	333TH	C	334GLU	CB	0.67
341LEU	CA	341LEU	CD1	1.85	202VA	CG1	206TYR	CE2	0.10	333TH	C	334GLU	C	99.55
341LEU	CA	341LEU	CD2	98.07	202VA	CG1	206TYR	CZ	0.00	334GL	CA	334GLU	CB	100.00
341LEU	CA	341LEU	C	100.00	202VA	CG2	202VAL	C	61.08	334GL	CA	334GLU	CG	100.00
341LEU	CB	341LEU	CG	100.00	202VA	C	203GLY	CA	100.00	334GL	CA	334GLU	CD	99.06
341LEU	CB	341LEU	CD1	100.00	202VA	C	203GLY	C	60.50	334GL	CA	334GLU	C	100.00
341LEU	CB	341LEU	CD2	100.00	203GL	CA	203GLY	C	100.00	334GL	CB	334GLU	CG	100.00
341LEU	CB	341LEU	C	100.00	203GL	CA	206TYR	CB	0.00	334GL	CB	334GLU	CD	100.00
341LEU	CG	341LEU	CD1	100.00	203GL	CA	206TYR	CD1	0.00	334GL	CB	334GLU	C	100.00
341LEU	CG	341LEU	CD2	100.00	203GL	CA	211LEU	CD1	0.03	334GL	CG	334GLU	CD	100.00
341LEU	CG	341LEU	C	0.68	203GL	CA	211LEU	CD2	0.15	334GL	CG	334GLU	C	85.05
341LEU	CD1	341LEU	CD2	100.00	203GL	C	204ARG	CA	100.00	334GL	CD1	334GLU	C	0.88
341LEU	CD1	341LEU	C	0.01	203GL	C	204ARG	CB	3.02	334GL	C	335ALA	CA	100.00
341LEU	CD2	341LEU	C	0.03	203GL	C	204ARG	CG	0.63	334GL	C	335ALA	CB	0.07
341LEU	CD2	344LEU	CD1	0.01	203GL	C	204ARG	CD	0.05	334GL	C	335ALA	C	99.93
341LEU	CD2	344LEU	CD2	0.10	203GL	C	204ARG	CZ	0.02	335AL	CA	335ALA	CB	100.00
341LEU	C	342ARG	CA	100.00	203GL	C	204ARG	C	97.79	335AL	CA	335ALA	C	100.00
341LEU	C	342ARG	CB	0.52	203GL	C	206TYR	CB	0.01	335AL	CB	335ALA	C	100.00
341LEU	C	342ARG	CG	0.01	203GL	C	211LEU	CD1	0.18	335AL	C	336PHE	CA	100.00
341LEU	C	342ARG	C	99.52	203GL	C	211LEU	CD2	1.02	335AL	C	336PHE	CB	0.03
341LEU	C	344LEU	CB	0.00	204AR	CA	204ARG	CB	100.00	335AL	C	336PHE	C	99.98
342ARG	CA	342ARG	CB	100.00	204AR	CA	204ARG	CG	100.00	336PH	CA	336PHE	CB	100.00
342ARG	CA	342ARG	CG	100.00	204AR	CA	204ARG	CD	86.84	336PH	CA	336PHE	CG	100.00
342ARG	CA	342ARG	CD	2.37	204AR	CA	204ARG	CZ	0.02	336PH	CA	336PHE	CD1	83.56
342ARG	CA	342ARG	CZ	0.09	204AR	CA	204ARG	C	100.00	336PH	CA	336PHE	CD2	89.89
342ARG	CA	342ARG	C	100.00	204AR	CA	211LEU	CB	0.00	336PH	CA	336PHE	C	100.00
342ARG	CB	342ARG	CG	100.00	204AR	CA	211LEU	CD2	0.01	336PH	CB	336PHE	CG	100.00
342ARG	CB	342ARG	CD	100.00	204AR	CB	204ARG	CG	100.00	336PH	CB	336PHE	CD1	100.00
342ARG	CB	342ARG	CZ	1.11	204AR	CB	204ARG	CD	100.00	336PH	CB	336PHE	CD2	100.00
342ARG	CB	342ARG	C	100.00	204AR	CB	204ARG	CZ	0.07	336PH	CB	336PHE	C	100.00
342ARG	CG	342ARG	CD	100.00	204AR	CB	204ARG	C	100.00	336PH	CG	336PHE	CD1	100.00
342ARG	CG	342ARG	CZ	13.05	204AR	CB	211LEU	CD2	0.07	336PH	CG	336PHE	CD2	100.00
342ARG	CG	342ARG	C	96.36	204AR	CG	204ARG	CD	100.00	336PH	CG	336PHE	CE1	100.00
342ARG	CG	343HIS	CE1	0.01	204AR	CG	204ARG	CZ	4.80	336PH	CG	336PHE	CE2	100.00
342ARG	CD	342ARG	CZ	100.00	204AR	CG	204ARG	C	72.97	336PH	CG	336PHE	CZ	100.00
342ARG	CD	342ARG	C	0.65	204AR	CG	211LEU	CB	0.00	336PH	CG	336PHE	C	99.72
342ARG	CD	343HIS	CD2	0.00	204AR	CG	211LEU	CD2	0.02	336PH	CD1	336PHE	CD2	100.00
342ARG	CD	343HIS	CE1	0.06	204AR	CD	204ARG	CZ	100.00	336PH	CD1	336PHE	CE1	100.00
342ARG	CZ	343HIS	CD2	0.00	204AR	CD	204ARG	C	0.65	336PH	CD1	336PHE	CE2	100.00
342ARG	CZ	343HIS	CE1	2.04	204AR	CD	211LEU	CD2	0.01	336PH	CD1	336PHE	CZ	100.00
342ARG	CZ	345ALA	CB	0.01	204AR	CZ	211LEU	CB	0.30	336PH	CD1	336PHE	C	0.04
342ARG	C	343HIS	CA	100.00	204AR	CZ	211LEU	CG	0.13	336PH	CD2	336PHE	CE1	100.00
342ARG	C	343HIS	CB	5.02	204AR	CZ	211LEU	CD1	0.13	336PH	CD2	336PHE	CE2	100.00
342ARG	C	343HIS	CG	0.66	204AR	CZ	211LEU	CD2	2.53	336PH	CD2	336PHE	CZ	100.00
342ARG	C	343HIS	CD2	0.01	204AR	CZ	213HIS	CD2	0.01	336PH	CD2	336PHE	C	87.10
342ARG	C	343HIS	C	93.61	204AR	C	205GLY	CA	100.00	336PH	CD2	340VAL	CG2	2.29
343HIS	CA	343HIS	CB	100.00	204AR	C	205GLY	C	54.51	336PH	CE1	336PHE	CE2	100.00

343HIS	CA	343HIS	CG	100.00
343HIS	CA	343HIS	CD2	2.09
343HIS	CA	343HIS	C	100.00
343HIS	CB	343HIS	CG	100.00
343HIS	CB	343HIS	CD2	100.00
343HIS	CB	343HIS	CE1	0.13
343HIS	CB	343HIS	C	100.00
343HIS	CG	343HIS	CD2	100.00
343HIS	CG	343HIS	CE1	100.00
343HIS	CG	343HIS	C	0.01
343HIS	CD2	343HIS	CE1	100.00
343HIS	C	344LEU	CA	100.00
343HIS	C	344LEU	CB	4.26
343HIS	C	344LEU	CG	0.30
343HIS	C	344LEU	CD1	0.01
343HIS	C	344LEU	CD2	0.00
343HIS	C	344LEU	C	95.56
343HIS	C	345ALA	CB	0.00
344LEU	CA	344LEU	CB	100.00
344LEU	CA	344LEU	CG	100.00
344LEU	CA	344LEU	CD1	68.35
344LEU	CA	344LEU	CD2	28.39
344LEU	CA	344LEU	C	100.00
344LEU	CB	344LEU	CG	100.00
344LEU	CB	344LEU	CD1	100.00
344LEU	CB	344LEU	CD2	100.00
344LEU	CB	344LEU	C	100.00
344LEU	CG	344LEU	CD1	100.00
344LEU	CG	344LEU	CD2	100.00
344LEU	CG	344LEU	C	69.94
344LEU	CD1	344LEU	CD2	100.00
344LEU	CD1	344LEU	C	0.14
344LEU	CD2	344LEU	C	0.61
344LEU	C	345ALA	CA	100.00
344LEU	C	345ALA	CB	45.62
344LEU	C	345ALA	C	55.88
345ALA	CA	345ALA	CB	100.00
345ALA	CA	345ALA	C	100.00
345ALA	CB	345ALA	C	100.00

205GL	CA	205GLY	C	100.00
205GL	C	206TYR	CA	100.00
205GL	C	206TYR	CB	6.85
205GL	C	206TYR	CG	0.12
205GL	C	206TYR	CD1	0.15
205GL	C	206TYR	CD2	0.06
205GL	C	206TYR	C	94.64
205GL	C	207PRO	CD	0.16
206TY	CA	206TYR	CB	100.00
206TY	CA	206TYR	CG	100.00
206TY	CA	206TYR	CD1	74.32
206TY	CA	206TYR	CD2	79.11
206TY	CA	206TYR	C	100.00
206TY	CA	207PRO	CD	100.00
206TY	CB	206TYR	CG	100.00
206TY	CB	206TYR	CD1	100.00
206TY	CB	206TYR	CD2	100.00
206TY	CB	206TYR	C	100.00
206TY	CB	207PRO	CD	0.06
206TY	CB	209VAL	CG1	0.01
206TY	CB	209VAL	CG2	0.37
206TY	CG	206TYR	CD1	100.00
206TY	CG	206TYR	CD2	100.00
206TY	CG	206TYR	CE1	100.00
206TY	CG	206TYR	CE2	100.00
206TY	CG	206TYR	CZ	100.00
206TY	CG	206TYR	C	90.20
206TY	CG	207PRO	CD	5.35
206TY	CG	209VAL	CG1	0.02
206TY	CG	209VAL	CG2	0.00
206TY	CD1	206TYR	CD2	100.00
206TY	CD1	206TYR	CE1	100.00
206TY	CD1	206TYR	CE2	100.00
206TY	CD1	206TYR	CZ	100.00
206TY	CD1	206TYR	C	22.56
206TY	CD1	207PRO	CD	2.96
206TY	CD1	207PRO	C	0.01
206TY	CD1	209VAL	CB	0.18
206TY	CD1	209VAL	CG1	0.50
206TY	CD1	209VAL	CG2	1.54
206TY	CD2	206TYR	CE1	100.00
206TY	CD2	206TYR	CE2	100.00
206TY	CD2	206TYR	CZ	100.00
206TY	CD2	206TYR	C	34.99
206TY	CD2	207PRO	CD	4.19
206TY	CD2	207PRO	C	0.02
206TY	CD2	209VAL	CB	0.20
206TY	CD2	209VAL	CG1	0.35
206TY	CD2	209VAL	CG2	2.11
206TY	CE1	206TYR	CE2	100.00
206TY	CE1	206TYR	CZ	100.00
206TY	CE1	207PRO	CD	0.01
206TY	CE1	209VAL	CB	0.03
206TY	CE1	209VAL	CG1	0.01
206TY	CE1	209VAL	CG2	1.04
206TY	CE2	206TYR	CZ	100.00
206TY	CE2	209VAL	CB	0.04
206TY	CE2	209VAL	CG1	0.04
206TY	CE2	209VAL	CG2	1.54
206TY	C	207PRO	CA	100.00
206TY	C	207PRO	CB	0.23
206TY	C	207PRO	CD	100.00

336PH	CE1	336PHE	CZ	100.00
336PH	CE2	336PHE	CZ	100.00
336PH	CE2	340VAL	CG2	16.84
336PH	CZ	340VAL	CG2	0.12
336PH	C	337THR	CA	100.00
336PH	C	337THR	C	100.00
337TH	CA	337THR	CB	100.00
337TH	CA	337THR	CG2	100.00
337TH	CA	337THR	C	100.00
337TH	CB	337THR	CG2	100.00
337TH	CB	337THR	C	100.00
337TH	CG2	337THR	C	56.11
337TH	CG2	341LEU	CD1	0.02
337TH	C	338ALA	CA	100.00
337TH	C	338ALA	CB	0.00
337TH	C	338ALA	C	100.00
337TH	C	341LEU	CD1	0.02
338AL	CA	338ALA	CB	100.00
338AL	CA	338ALA	C	100.00
338AL	CB	338ALA	C	100.00
338AL	CB	342ARG	CZ	0.01
338AL	C	339THR	CA	100.00
338AL	C	339THR	CB	0.35
338AL	C	339THR	CG2	0.06
338AL	C	339THR	C	99.81
338AL	C	342ARG	CZ	0.02
339TH	CA	339THR	CB	100.00
339TH	CA	339THR	CG2	100.00
339TH	CA	339THR	C	100.00
339TH	CA	342ARG	CZ	0.08
339TH	CB	339THR	CG2	100.00
339TH	CB	339THR	C	100.00
339TH	CG2	339THR	C	95.14
339TH	CG2	342ARG	CZ	0.08
339TH	CG2	343HIS	CD2	0.64
339TH	C	340VAL	CA	100.00
339TH	C	340VAL	C	100.00
339TH	C	343HIS	CD2	0.29
340VA	CA	340VAL	CB	100.00
340VA	CA	340VAL	CG1	100.00
340VA	CA	340VAL	CG2	100.00
340VA	CA	340VAL	C	100.00
340VA	CB	340VAL	CG1	100.00
340VA	CB	340VAL	CG2	100.00
340VA	CB	340VAL	C	100.00
340VA	CG1	340VAL	CG2	100.00
340VA	CG1	340VAL	C	100.00
340VA	CG1	344LEU	CD1	1.41
340VA	C	341LEU	CA	100.00
340VA	C	341LEU	CB	0.00
340VA	C	341LEU	C	99.99
341LE	CA	341LEU	CB	100.00
341LE	CA	341LEU	CG	100.00
341LE	CA	341LEU	CD1	3.30
341LE	CA	341LEU	CD2	96.52
341LE	CA	341LEU	C	100.00
341LE	CB	341LEU	CG	100.00
341LE	CB	341LEU	CD1	100.00
341LE	CB	341LEU	CD2	100.00
341LE	CB	341LEU	C	100.00
341LE	CG	341LEU	CD1	100.00
341LE	CG	341LEU	CD2	100.00

206TYF	C	207PRO	C	100.00	341LEI	CG	341LEU	C	0.24
206TYF	C	209VAL	CG2	0.00	341LEI	CD1	341LEU	CD2	100.00
207PR(	CA	207PRO	CB	100.00	341LEI	CD2	341LEU	C	0.00
207PR(	CA	207PRO	CG	100.00	341LEI	CD2	344LEU	CD1	0.08
207PR(	CA	207PRO	CD	100.00	341LEI	CD2	344LEU	CD2	0.10
207PR(	CA	207PRO	C	100.00	341LEI	C	342ARG	CA	100.00
207PR(	CB	207PRO	CG	100.00	341LEI	C	342ARG	CB	1.91
207PR(	CB	207PRO	CD	100.00	341LEI	C	342ARG	CG	0.00
207PR(	CB	207PRO	C	100.00	341LEI	C	342ARG	C	98.26
207PR(	CG	207PRO	CD	100.00	342AR	CA	342ARG	CB	100.00
207PR(	CG	207PRO	C	95.91	342AR	CA	342ARG	CG	100.00
207PR(	CD	207PRO	C	89.71	342AR	CA	342ARG	CD	1.43
207PR(	C	208ASP	CA	100.00	342AR	CA	342ARG	C	100.00
207PR(	C	208ASP	CB	99.66	342AR	CA	345ALA	CB	0.00
207PR(	C	208ASP	CG	0.77	342AR	CB	342ARG	CG	100.00
207PR(	C	208ASP	C	97.44	342AR	CB	342ARG	CD	100.00
208ASF	CA	208ASP	CB	100.00	342AR	CB	342ARG	CZ	1.18
208ASF	CA	208ASP	CG	100.00	342AR	CB	342ARG	C	100.00
208ASF	CA	208ASP	C	100.00	342AR	CG	342ARG	CD	100.00
208ASF	CB	208ASP	CG	100.00	342AR	CG	342ARG	CZ	16.51
208ASF	CB	208ASP	C	100.00	342AR	CG	342ARG	C	94.68
208ASF	CG	208ASP	C	93.26	342AR	CG	343HIS	CE1	0.00
208ASF	C	209VAL	CA	100.00	342AR	CD	342ARG	CZ	100.00
208ASF	C	209VAL	CB	36.10	342AR	CD	342ARG	C	0.70
208ASF	C	209VAL	CG1	3.94	342AR	CD	343HIS	CD2	0.00
208ASF	C	209VAL	CG2	21.18	342AR	CD	343HIS	CE1	0.04
208ASF	C	209VAL	C	68.73	342AR	CZ	343HIS	CE1	0.94
209VAI	CA	209VAL	CB	100.00	342AR	C	343HIS	CA	100.00
209VAI	CA	209VAL	CG1	100.00	342AR	C	343HIS	CB	2.78
209VAI	CA	209VAL	CG2	100.00	342AR	C	343HIS	CG	0.25
209VAI	CA	209VAL	C	100.00	342AR	C	343HIS	CD2	0.00
209VAI	CB	209VAL	CG1	100.00	342AR	C	343HIS	C	96.10
209VAI	CB	209VAL	CG2	100.00	342AR	C	345ALA	CB	0.00
209VAI	CB	209VAL	C	100.00	343HI:	CA	343HIS	CB	100.00
209VAI	CG1	209VAL	CG2	100.00	343HI:	CA	343HIS	CG	100.00
209VAI	CG1	209VAL	C	84.90	343HI:	CA	343HIS	CD2	1.81
209VAI	CG1	210ALA	C	0.01	343HI:	CA	343HIS	C	100.00
209VAI	CG1	211LEU	CD1	0.00	343HI:	CB	343HIS	CG	100.00
209VAI	CG1	211LEU	CD2	0.05	343HI:	CB	343HIS	CD2	100.00
209VAI	CG2	209VAL	C	17.94	343HI:	CB	343HIS	CE1	0.16
209VAI	C	210ALA	CA	100.00	343HI:	CB	343HIS	C	100.00
209VAI	C	210ALA	CB	20.44	343HI:	CG	343HIS	CD2	100.00
209VAI	C	210ALA	C	77.80	343HI:	CG	343HIS	CE1	100.00
210AL/	CA	210ALA	CB	100.00	343HI:	CD2	343HIS	CE1	100.00
210AL/	CA	210ALA	C	100.00	343HI:	C	344LEU	CA	100.00
210AL/	CB	210ALA	C	100.00	343HI:	C	344LEU	CB	5.49
210AL/	C	211LEU	CA	100.00	343HI:	C	344LEU	CG	0.74
210AL/	C	211LEU	CB	78.38	343HI:	C	344LEU	C	93.68
210AL/	C	211LEU	CG	0.02	344LEI	CA	344LEU	CB	100.00
210AL/	C	211LEU	CD2	0.12	344LEI	CA	344LEU	CG	100.00
210AL/	C	211LEU	C	23.50	344LEI	CA	344LEU	CD1	19.39
211LEI	CA	211LEU	CB	100.00	344LEI	CA	344LEU	CD2	79.18
211LEI	CA	211LEU	CG	100.00	344LEI	CA	344LEU	C	100.00
211LEI	CA	211LEU	CD1	89.10	344LEI	CB	344LEU	CG	100.00
211LEI	CA	211LEU	CD2	8.54	344LEI	CB	344LEU	CD1	100.00
211LEI	CA	211LEU	C	100.00	344LEI	CB	344LEU	CD2	100.00
211LEI	CB	211LEU	CG	100.00	344LEI	CB	344LEU	C	100.00
211LEI	CB	211LEU	CD1	100.00	344LEI	CG	344LEU	CD1	100.00
211LEI	CB	211LEU	CD2	100.00	344LEI	CG	344LEU	CD2	100.00
211LEI	CB	211LEU	C	100.00	344LEI	CG	344LEU	C	22.15
211LEI	CG	211LEU	CD1	100.00	344LEI	CD1	344LEU	CD2	100.00
211LEI	CG	211LEU	CD2	100.00	344LEI	CD1	344LEU	C	0.09

	211LEU	CG	211LEU	C	97.35
	211LEU	CG	213HIS	CE1	0.04
	211LEU	CD1	211LEU	CD2	100.00
	211LEU	CD1	211LEU	C	6.46
	211LEU	CD1	212GLU	C	0.00
	211LEU	CD1	213HIS	CD2	0.01
	211LEU	CD1	213HIS	CE1	1.88
	211LEU	CD2	211LEU	C	0.26
	211LEU	CD2	212GLU	C	0.00
	211LEU	CD2	213HIS	CE1	2.43
	211LEU	C	212GLU	CA	100.00
	211LEU	C	212GLU	CB	92.55
	211LEU	C	212GLU	C	10.70
	212GLU	CA	212GLU	CB	100.00
	212GLU	CA	212GLU	CG	100.00
	212GLU	CA	212GLU	C	100.00
	212GLU	CB	212GLU	CG	100.00
	212GLU	CB	212GLU	CD	100.00
	212GLU	CB	212GLU	C	100.00
	212GLU	CG	212GLU	CD	100.00
	212GLU	CG	212GLU	C	99.70
	212GLU	CG	214GLN	CG	0.02
	212GLU	C	213HIS	CA	100.00
	212GLU	C	213HIS	CB	23.95
	212GLU	C	213HIS	CG	4.24
	212GLU	C	213HIS	CD2	0.07
	212GLU	C	213HIS	C	76.24
	213HIS	CA	213HIS	CB	100.00
	213HIS	CA	213HIS	CG	100.00
	213HIS	CA	213HIS	CD2	3.90
	213HIS	CA	213HIS	C	100.00
	213HIS	CB	213HIS	CG	100.00
	213HIS	CB	213HIS	CD2	100.00
	213HIS	CB	213HIS	CE1	0.14
	213HIS	CB	213HIS	C	100.00
	213HIS	CG	213HIS	CD2	100.00
	213HIS	CG	213HIS	CE1	100.00
	213HIS	CD2	213HIS	CE1	100.00
	213HIS	C	214GLN	CA	100.00
	213HIS	C	214GLN	CB	73.01
	213HIS	C	214GLN	CG	15.40
	213HIS	C	214GLN	C	20.87
	214GLN	CA	214GLN	CB	100.00
	214GLN	CA	214GLN	CG	100.00
	214GLN	CA	214GLN	CD	83.59
	214GLN	CA	214GLN	C	100.00
	214GLN	CB	214GLN	CG	100.00
	214GLN	CB	214GLN	CD	100.00
	214GLN	CB	214GLN	C	100.00
	214GLN	CB	219MET	CG	0.03
	214GLN	CG	214GLN	CD	100.00
	214GLN	CG	214GLN	C	2.32
	214GLN	CG	219MET	CB	0.03
	214GLN	CG	219MET	CG	0.02
	214GLN	CD	219MET	CA	0.00
	214GLN	CD	219MET	CB	0.00
	214GLN	CD	219MET	CG	0.00
	214GLN	CD	222HIS	CD2	0.01
	214GLN	C	215TYR	CA	100.00
	214GLN	C	215TYR	CB	0.58
	214GLN	C	215TYR	CG	0.02
	214GLN	C	215TYR	CD1	0.00

344LEU	CD2	344LEU	C	1.60
344LEU	C	345ALA	CA	100.00
344LEU	C	345ALA	CB	56.31
344LEU	C	345ALA	C	44.93
345ALA	CA	345ALA	CB	100.00
345ALA	CA	345ALA	C	100.00
345ALA	CB	345ALA	C	100.00

214GLI	C	215TYR	CD2	0.01
214GLI	C	215TYR	C	99.58
215TYI	CA	215TYR	CB	100.00
215TYI	CA	215TYR	CG	100.00
215TYI	CA	215TYR	CD1	99.69
215TYI	CA	215TYR	CD2	9.58
215TYI	CA	215TYR	C	100.00
215TYI	CB	215TYR	CG	100.00
215TYI	CB	215TYR	CD1	100.00
215TYI	CB	215TYR	CD2	100.00
215TYI	CB	215TYR	C	100.00
215TYI	CG	215TYR	CD1	100.00
215TYI	CG	215TYR	CD2	100.00
215TYI	CG	215TYR	CE1	100.00
215TYI	CG	215TYR	CE2	100.00
215TYI	CG	215TYR	CZ	100.00
215TYI	CD1	215TYR	CD2	100.00
215TYI	CD1	215TYR	CE1	100.00
215TYI	CD1	215TYR	CE2	100.00
215TYI	CD1	215TYR	CZ	100.00
215TYI	CD2	215TYR	CE1	100.00
215TYI	CD2	215TYR	CE2	100.00
215TYI	CD2	215TYR	CZ	100.00
215TYI	CE1	215TYR	CE2	100.00
215TYI	CE1	215TYR	CZ	100.00
215TYI	CE2	215TYR	CZ	100.00
215TYI	C	216VAL	CA	100.00
215TYI	C	216VAL	C	100.00
216VAI	CA	216VAL	CB	100.00
216VAI	CA	216VAL	CG1	100.00
216VAI	CA	216VAL	CG2	100.00
216VAI	CA	216VAL	C	100.00
216VAI	CB	216VAL	CG1	100.00
216VAI	CB	216VAL	CG2	100.00
216VAI	CB	216VAL	C	100.00
216VAI	CB	239PHE	CD1	0.01
216VAI	CB	239PHE	CD2	0.02
216VAI	CB	239PHE	CE1	0.08
216VAI	CB	239PHE	CE2	0.14
216VAI	CB	239PHE	CZ	0.01
216VAI	CG1	216VAL	CG2	100.00
216VAI	CG1	216VAL	C	99.86
216VAI	CG1	239PHE	CD1	0.11
216VAI	CG1	239PHE	CD2	0.02
216VAI	CG1	239PHE	CE1	1.05
216VAI	CG1	239PHE	CE2	0.73
216VAI	CG1	239PHE	CZ	0.47
216VAI	CG2	216VAL	C	100.00
216VAI	CG2	239PHE	CB	0.06
216VAI	CG2	239PHE	CG	0.36
216VAI	CG2	239PHE	CD1	0.75
216VAI	CG2	239PHE	CD2	0.54
216VAI	CG2	239PHE	CE1	0.06
216VAI	CG2	239PHE	CE2	0.06
216VAI	CG2	239PHE	CZ	0.01
216VAI	CG2	243LEU	CD1	0.22
216VAI	CG2	243LEU	CD2	0.04
216VAI	C	217ASP	CA	100.00
216VAI	C	217ASP	C	99.98
217ASI	CA	217ASP	CB	100.00
217ASI	CA	217ASP	CG	100.00
217ASI	CA	217ASP	C	100.00

217ASI	CB	217ASP	CG	100.00
217ASI	CB	217ASP	C	100.00
217ASI	CG	217ASP	C	99.50
217ASI	C	218ALA	CA	100.00
217ASI	C	218ALA	CB	0.22
217ASI	C	218ALA	C	99.76
218ALA	CA	218ALA	CB	100.00
218ALA	CA	218ALA	C	100.00
218ALA	CA	221MET	CE	0.00
218ALA	CB	218ALA	C	100.00
218ALA	C	219MET	CA	100.00
218ALA	C	219MET	CB	0.10
218ALA	C	219MET	C	99.97
218ALA	C	222HIS	CD2	0.24
219ME	CA	219MET	CB	100.00
219ME	CA	219MET	CG	100.00
219ME	CA	219MET	C	100.00
219ME	CB	219MET	CG	100.00
219ME	CB	219MET	CE	2.37
219ME	CB	219MET	C	100.00
219ME	CG	219MET	CE	100.00
219ME	CG	219MET	C	99.58
219ME	CG	230PHE	CE1	3.36
219ME	CG	230PHE	CE2	0.03
219ME	CG	230PHE	CZ	3.37
219ME	CG	243LEU	CD1	0.04
219ME	CG	243LEU	CD2	0.02
219ME	CE	219MET	C	0.56
219ME	CE	223LEU	CB	0.01
219ME	CE	223LEU	CG	0.04
219ME	CE	223LEU	CD1	1.38
219ME	CE	223LEU	CD2	0.26
219ME	CE	230PHE	CG	0.95
219ME	CE	230PHE	CD1	5.76
219ME	CE	230PHE	CD2	1.20
219ME	CE	230PHE	CE1	8.66
219ME	CE	230PHE	CE2	2.06
219ME	CE	230PHE	CZ	2.75
219ME	CE	233VAL	CG1	3.78
219ME	CE	233VAL	CG2	6.02
219ME	CE	243LEU	CD1	0.08
219ME	CE	243LEU	CD2	0.28
219ME	C	220ALA	CA	100.00
219ME	C	220ALA	C	100.00
220ALA	CA	220ALA	CB	100.00
220ALA	CA	220ALA	C	100.00
220ALA	CA	243LEU	CD1	0.02
220ALA	CA	243LEU	CD2	0.20
220ALA	CA	246LEU	CD2	0.00
220ALA	CB	220ALA	C	100.00
220ALA	CB	243LEU	CD1	0.17
220ALA	CB	243LEU	CD2	1.42
220ALA	CB	246LEU	CD1	0.00
220ALA	CB	246LEU	CD2	0.11
220ALA	C	221MET	CA	100.00
220ALA	C	221MET	CB	0.03
220ALA	C	221MET	C	100.00
221ME	CA	221MET	CB	100.00
221ME	CA	221MET	CG	100.00
221ME	CA	221MET	C	100.00
221ME	CB	221MET	CG	100.00
221ME	CB	221MET	CE	21.41

	221ME CB	221MET	C	100.00		
	221ME CB	225ARG	CZ	0.06		
	221ME CG	221MET	CE	100.00		
	221ME CG	221MET	C	97.78		
	221ME CG	225ARG	CZ	0.01		
	221ME CE	221MET	C	0.06		
	221ME CE	222HIS	CB	0.00		
	221ME CE	222HIS	CD2	0.01		
	221ME CE	222HIS	CE1	0.01		
	221ME CE	225ARG	CD	0.02		
	221ME CE	225ARG	CZ	0.09		
	221ME C	222HIS	CA	100.00		
	221ME C	222HIS	CB	1.10		
	221ME C	222HIS	C	99.10		
	221ME C	224VAL	CG2	0.03		
	222HIS CA	222HIS	CB	100.00		
	222HIS CA	222HIS	CG	100.00		
	222HIS CA	222HIS	CD2	41.50		
	222HIS CA	222HIS	C	100.00		
	222HIS CA	225ARG	CB	0.03		
	222HIS CB	222HIS	CG	100.00		
	222HIS CB	222HIS	CD2	100.00		
	222HIS CB	222HIS	CE1	0.36		
	222HIS CB	222HIS	C	100.00		
	222HIS CB	230PHE	CE2	0.03		
	222HIS CB	230PHE	CZ	0.14		
	222HIS CG	222HIS	CD2	100.00		
	222HIS CG	222HIS	CE1	100.00		
	222HIS CG	222HIS	C	97.52		
	222HIS CG	225ARG	CZ	0.01		
	222HIS CG	230PHE	CE2	0.08		
	222HIS CG	230PHE	CZ	0.16		
	222HIS CD2	222HIS	CE1	100.00		
	222HIS CD2	222HIS	C	17.93		
	222HIS CD2	225ARG	CZ	0.14		
	222HIS CD2	226SER	CB	0.06		
	222HIS CD2	229ARG	CB	0.53		
	222HIS CD2	229ARG	CG	0.63		
	222HIS CD2	229ARG	CD	1.07		
	222HIS CD2	230PHE	CE2	2.52		
	222HIS CD2	230PHE	CZ	1.54		
	222HIS CE1	225ARG	CD	0.04		
	222HIS CE1	225ARG	CZ	0.06		
	222HIS CE1	226SER	CB	0.74		
	222HIS CE1	229ARG	CB	0.14		
	222HIS CE1	229ARG	CG	0.93		
	222HIS CE1	229ARG	CD	2.12		
	222HIS CE1	229ARG	CZ	0.01		
	222HIS CE1	230PHE	CE2	0.01		
	222HIS C	223LEU	CA	100.00		
	222HIS C	223LEU	C	100.00		
	222HIS C	230PHE	CE2	0.01		
	223LEU CA	223LEU	CB	100.00		
	223LEU CA	223LEU	CG	100.00		
	223LEU CA	223LEU	CD1	84.09		
	223LEU CA	223LEU	CD2	14.42		
	223LEU CA	223LEU	C	100.00		
	223LEU CA	230PHE	CE2	0.31		
	223LEU CB	223LEU	CG	100.00		
	223LEU CB	223LEU	CD1	100.00		
	223LEU CB	223LEU	CD2	100.00		
	223LEU CB	223LEU	C	100.00		

	223LEU	CB	230PHE	CE2	0.00	
	223LEU	CB	246LEU	CD2	0.00	
	223LEU	CG	223LEU	CD1	100.00	
	223LEU	CG	223LEU	CD2	100.00	
	223LEU	CG	223LEU	C	93.56	
	223LEU	CG	230PHE	CE2	0.02	
	223LEU	CG	243LEU	CD2	0.01	
	223LEU	CG	246LEU	CD2	0.01	
	223LEU	CD1	223LEU	CD2	100.00	
	223LEU	CD1	223LEU	C	4.58	
	223LEU	CD1	224VAL	CG1	0.02	
	223LEU	CD1	227PRO	CB	0.09	
	223LEU	CD1	230PHE	CG	0.03	
	223LEU	CD1	230PHE	CD2	4.89	
	223LEU	CD1	230PHE	CE2	0.35	
	223LEU	CD1	243LEU	CG	0.01	
	223LEU	CD1	243LEU	CD1	0.09	
	223LEU	CD1	243LEU	CD2	0.37	
	223LEU	CD1	246LEU	CD1	0.00	
	223LEU	CD1	246LEU	CD2	0.11	
	223LEU	CD1	247ALA	CB	0.00	
	223LEU	CD1	250LEU	CD2	0.00	
	223LEU	CD2	223LEU	C	5.10	
	223LEU	CD2	224VAL	CG1	0.01	
	223LEU	CD2	224VAL	CG2	0.00	
	223LEU	CD2	227PRO	CB	0.01	
	223LEU	CD2	230PHE	CD2	0.59	
	223LEU	CD2	230PHE	CE2	0.21	
	223LEU	CD2	243LEU	CG	0.06	
	223LEU	CD2	243LEU	CD1	0.48	
	223LEU	CD2	243LEU	CD2	1.12	
	223LEU	CD2	246LEU	CB	0.15	
	223LEU	CD2	246LEU	CG	0.06	
	223LEU	CD2	246LEU	CD1	0.18	
	223LEU	CD2	246LEU	CD2	1.32	
	223LEU	CD2	246LEU	C	0.01	
	223LEU	CD2	247ALA	CA	0.00	
	223LEU	CD2	247ALA	CB	0.02	
	223LEU	CD2	250LEU	CD1	0.01	
	223LEU	C	224VAL	CA	100.00	
	223LEU	C	224VAL	CB	10.67	
	223LEU	C	224VAL	CG1	3.32	
	223LEU	C	224VAL	CG2	1.30	
	223LEU	C	224VAL	C	91.54	
	224VAL	CA	224VAL	CB	100.00	
	224VAL	CA	224VAL	CG1	100.00	
	224VAL	CA	224VAL	CG2	100.00	
	224VAL	CA	224VAL	C	100.00	
	224VAL	CB	224VAL	CG1	100.00	
	224VAL	CB	224VAL	CG2	100.00	
	224VAL	CB	224VAL	C	100.00	
	224VAL	CG1	224VAL	CG2	100.00	
	224VAL	CG1	224VAL	C	24.11	
	224VAL	CG1	246LEU	CG	0.04	
	224VAL	CG1	246LEU	CD1	0.28	
	224VAL	CG1	246LEU	CD2	0.34	
	224VAL	CG1	250LEU	CD1	0.00	
	224VAL	CG2	224VAL	C	76.14	
	224VAL	CG2	246LEU	CG	0.01	
	224VAL	CG2	246LEU	CD1	0.02	
	224VAL	CG2	246LEU	CD2	0.20	
	224VAL	C	225ARG	CA	100.00	

224VAl	C	225ARG	CB	18.71
224VAl	C	225ARG	CG	0.77
224VAl	C	225ARG	C	81.76
225AR(	CA	225ARG	CB	100.00
225AR(	CA	225ARG	CG	100.00
225AR(	CA	225ARG	CD	0.46
225AR(	CA	225ARG	C	100.00
225AR(	CB	225ARG	CG	100.00
225AR(	CB	225ARG	CD	100.00
225AR(	CB	225ARG	CZ	0.05
225AR(	CB	225ARG	C	100.00
225AR(	CG	225ARG	CD	100.00
225AR(	CG	225ARG	CZ	1.92
225AR(	CG	225ARG	C	61.71
225AR(	CD	225ARG	CZ	100.00
225AR(	CD	225ARG	C	0.08
225AR(	C	226SER	CA	100.00
225AR(	C	226SER	CB	99.96
225AR(	C	226SER	C	0.32
225AR(	C	227PRO	CD	0.48
226SEI	CA	226SER	CB	100.00
226SEI	CA	226SER	C	100.00
226SEI	CA	227PRO	CD	100.00
226SEI	CB	226SER	C	100.00
226SEI	C	227PRO	CA	100.00
226SEI	C	227PRO	CB	0.06
226SEI	C	227PRO	CD	100.00
226SEI	C	227PRO	C	100.00
227PR(	CA	227PRO	CB	100.00
227PR(	CA	227PRO	CG	100.00
227PR(	CA	227PRO	CD	100.00
227PR(	CA	227PRO	C	100.00
227PR(	CB	227PRO	CG	100.00
227PR(	CB	227PRO	CD	100.00
227PR(	CB	227PRO	C	100.00
227PR(	CB	250LEU	CG	0.04
227PR(	CB	250LEU	CD1	0.46
227PR(	CB	250LEU	CD2	0.55
227PR(	CG	227PRO	CD	100.00
227PR(	CG	227PRO	C	74.75
227PR(	CG	250LEU	CD1	0.27
227PR(	CG	250LEU	CD2	0.54
227PR(	CD	227PRO	C	52.60
227PR(	C	228ALA	CA	100.00
227PR(	C	228ALA	CB	0.03
227PR(	C	228ALA	C	99.97
228AL/	CA	228ALA	CB	100.00
228AL/	CA	228ALA	C	100.00
228AL/	CB	228ALA	C	100.00
228AL/	C	229ARG	CA	100.00
228AL/	C	229ARG	CB	2.22
228AL/	C	229ARG	C	97.85
229AR(	CA	229ARG	CB	100.00
229AR(	CA	229ARG	CG	100.00
229AR(	CA	229ARG	CD	39.52
229AR(	CA	229ARG	C	100.00
229AR(	CB	229ARG	CG	100.00
229AR(	CB	229ARG	CD	100.00
229AR(	CB	229ARG	C	100.00
229AR(	CB	230PHE	CD2	0.08
229AR(	CB	230PHE	CE2	0.07
229AR(	CG	229ARG	CD	100.00

229AR(	CG	229ARG	CZ	8.90
229AR(	CG	229ARG	C	99.98
229AR(	CG	230PHE	CD1	0.07
229AR(	CG	230PHE	CD2	0.16
229AR(	CG	230PHE	CE1	0.48
229AR(	CG	230PHE	CE2	0.54
229AR(	CG	230PHE	CZ	0.53
229AR(	CD	229ARG	CZ	100.00
229AR(	CD	229ARG	C	52.09
229AR(	CD	230PHE	CG	0.01
229AR(	CD	230PHE	CD1	1.12
229AR(	CD	230PHE	CD2	0.03
229AR(	CD	230PHE	CE1	5.88
229AR(	CD	230PHE	CE2	0.32
229AR(	CD	230PHE	CZ	2.42
229AR(	CZ	230PHE	CD1	0.45
229AR(	CZ	230PHE	CE1	3.88
229AR(	CZ	230PHE	CZ	0.02
229AR(	C	230PHE	CA	100.00
229AR(	C	230PHE	CB	8.77
229AR(	C	230PHE	CG	1.80
229AR(	C	230PHE	CD1	0.12
229AR(	C	230PHE	CD2	0.08
229AR(	C	230PHE	C	92.57
230PHI	CA	230PHE	CB	100.00
230PHI	CA	230PHE	CG	100.00
230PHI	CA	230PHE	CD1	98.97
230PHI	CA	230PHE	CD2	43.41
230PHI	CA	230PHE	C	100.00
230PHI	CB	230PHE	CG	100.00
230PHI	CB	230PHE	CD1	100.00
230PHI	CB	230PHE	CD2	100.00
230PHI	CB	230PHE	C	100.00
230PHI	CB	233VAL	CG2	0.19
230PHI	CG	230PHE	CD1	100.00
230PHI	CG	230PHE	CD2	100.00
230PHI	CG	230PHE	CE1	100.00
230PHI	CG	230PHE	CE2	100.00
230PHI	CG	230PHE	CZ	100.00
230PHI	CD1	230PHE	CD2	100.00
230PHI	CD1	230PHE	CE1	100.00
230PHI	CD1	230PHE	CE2	100.00
230PHI	CD1	230PHE	CZ	100.00
230PHI	CD1	233VAL	CG1	0.07
230PHI	CD1	233VAL	CG2	0.35
230PHI	CD2	230PHE	CE1	100.00
230PHI	CD2	230PHE	CE2	100.00
230PHI	CD2	230PHE	CZ	100.00
230PHI	CE1	230PHE	CE2	100.00
230PHI	CE1	230PHE	CZ	100.00
230PHI	CE2	230PHE	CZ	100.00
230PHI	C	231ASP	CA	100.00
230PHI	C	231ASP	CB	88.90
230PHI	C	231ASP	CG	0.01
230PHI	C	231ASP	C	15.34
231ASI	CA	231ASP	CB	100.00
231ASI	CA	231ASP	CG	100.00
231ASI	CA	231ASP	C	100.00
231ASI	CB	231ASP	CG	100.00
231ASI	CB	231ASP	C	100.00
231ASI	CG	231ASP	C	99.82
231ASI	C	232VAL	CA	100.00

231ASI	C	232VAL	CB	55.77
231ASI	C	232VAL	CG1	0.03
231ASI	C	232VAL	CG2	41.30
231ASI	C	232VAL	C	54.64
232VAI	CA	232VAL	CB	100.00
232VAI	CA	232VAL	CG1	100.00
232VAI	CA	232VAL	CG2	100.00
232VAI	CA	232VAL	C	100.00
232VAI	CB	232VAL	CG1	100.00
232VAI	CB	232VAL	CG2	100.00
232VAI	CB	232VAL	C	100.00
232VAI	CG1	232VAL	CG2	100.00
232VAI	CG1	232VAL	C	99.79
232VAI	CG2	232VAL	C	3.54
232VAI	C	233VAL	CA	100.00
232VAI	C	233VAL	CB	49.63
232VAI	C	233VAL	CG1	0.91
232VAI	C	233VAL	CG2	33.41
232VAI	C	233VAL	C	66.10
233VAI	CA	233VAL	CB	100.00
233VAI	CA	233VAL	CG1	100.00
233VAI	CA	233VAL	CG2	100.00
233VAI	CA	233VAL	C	100.00
233VAI	CB	233VAL	CG1	100.00
233VAI	CB	233VAL	CG2	100.00
233VAI	CB	233VAL	C	100.00
233VAI	CG1	233VAL	CG2	100.00
233VAI	CG1	233VAL	C	98.62
233VAI	CG1	235THR	CG2	0.02
233VAI	CG1	243LEU	CD1	0.02
233VAI	CG1	243LEU	CD2	0.22
233VAI	CG2	233VAL	C	1.42
233VAI	C	234VAL	CA	100.00
233VAI	C	234VAL	CB	75.26
233VAI	C	234VAL	CG1	0.03
233VAI	C	234VAL	CG2	2.74
233VAI	C	234VAL	C	40.61
234VAI	CA	234VAL	CB	100.00
234VAI	CA	234VAL	CG1	100.00
234VAI	CA	234VAL	CG2	100.00
234VAI	CA	234VAL	C	100.00
234VAI	CB	234VAL	CG1	100.00
234VAI	CB	234VAL	CG2	100.00
234VAI	CB	234VAL	C	100.00
234VAI	CG1	234VAL	CG2	100.00
234VAI	CG1	234VAL	C	99.16
234VAI	CG2	234VAL	C	87.53
234VAI	C	235THR	CA	100.00
234VAI	C	235THR	CB	100.00
234VAI	C	235THR	CG2	2.29
234VAI	C	235THR	C	0.12
235THI	CA	235THR	CB	100.00
235THI	CA	235THR	CG2	100.00
235THI	CA	235THR	C	100.00
235THI	CB	235THR	CG2	100.00
235THI	CB	235THR	C	100.00
235THI	CB	243LEU	CD1	0.01
235THI	CB	243LEU	CD2	0.00
235THI	CG2	235THR	C	99.02
235THI	CG2	239PHE	CB	0.08
235THI	CG2	239PHE	C	0.35
235THI	CG2	240GLY	CA	33.85

235THI	CG2	243LEU	CD1	0.07
235THI	CG2	243LEU	CD2	0.01
235THI	C	236GLY	CA	100.00
235THI	C	236GLY	C	89.14
236GL'	CA	236GLY	C	100.00
236GL'	CA	239PHE	CB	0.00
236GL'	C	237ASN	CA	100.00
236GL'	C	237ASN	CB	0.22
236GL'	C	237ASN	C	99.88
237ASI	CA	237ASN	CB	100.00
237ASI	CA	237ASN	CG	100.00
237ASI	CA	237ASN	C	100.00
237ASI	CB	237ASN	CG	100.00
237ASI	CB	237ASN	C	100.00
237ASI	CG	237ASN	C	96.02
237ASI	CG	238ILE	CD	0.00
237ASI	C	238ILE	CA	100.00
237ASI	C	238ILE	CB	1.92
237ASI	C	238ILE	CG1	0.63
237ASI	C	238ILE	CG2	0.01
237ASI	C	238ILE	CD	0.04
237ASI	C	238ILE	C	99.00
238ILE	CA	238ILE	CB	100.00
238ILE	CA	238ILE	CG1	100.00
238ILE	CA	238ILE	CG2	100.00
238ILE	CA	238ILE	CD	48.35
238ILE	CA	238ILE	C	100.00
238ILE	CA	241ASP	CB	0.01
238ILE	CB	238ILE	CG1	100.00
238ILE	CB	238ILE	CG2	100.00
238ILE	CB	238ILE	CD	100.00
238ILE	CB	238ILE	C	100.00
238ILE	CB	239PHE	CE1	0.00
238ILE	CG1	238ILE	CG2	100.00
238ILE	CG1	238ILE	CD	100.00
238ILE	CG1	238ILE	C	17.08
238ILE	CG1	239PHE	CD1	0.00
238ILE	CG1	239PHE	CD2	0.19
238ILE	CG1	239PHE	CE2	0.16
238ILE	CG1	239PHE	CZ	0.00
238ILE	CG1	242ILE	CD	0.01
238ILE	CG2	238ILE	CD	96.13
238ILE	CG2	238ILE	C	91.72
238ILE	CG2	239PHE	CG	0.00
238ILE	CG2	239PHE	CD1	0.23
238ILE	CG2	239PHE	CD2	0.24
238ILE	CG2	239PHE	CE1	0.23
238ILE	CG2	239PHE	CE2	0.24
238ILE	CG2	239PHE	CZ	0.11
238ILE	CG2	242ILE	CD	0.28
238ILE	CD	238ILE	C	0.35
238ILE	CD	239PHE	CD1	0.01
238ILE	CD	239PHE	CD2	0.04
238ILE	CD	239PHE	CE1	0.06
238ILE	CD	239PHE	CE2	0.39
238ILE	CD	239PHE	CZ	0.25
238ILE	CD	242ILE	CD	0.00
238ILE	C	239PHE	CA	100.00
238ILE	C	239PHE	CB	1.19
238ILE	C	239PHE	CG	0.21
238ILE	C	239PHE	CD1	0.35
238ILE	C	239PHE	CD2	0.01

238ILE	C	239PHE	C	98.34
238ILE	C	242ILE	CD	0.01
239PHI	CA	239PHE	CB	100.00
239PHI	CA	239PHE	CG	100.00
239PHI	CA	239PHE	CD1	62.73
239PHI	CA	239PHE	CD2	46.78
239PHI	CA	239PHE	C	100.00
239PHI	CA	242ILE	CD	0.01
239PHI	CB	239PHE	CG	100.00
239PHI	CB	239PHE	CD1	100.00
239PHI	CB	239PHE	CD2	100.00
239PHI	CB	239PHE	C	100.00
239PHI	CB	242ILE	CD	0.00
239PHI	CB	243LEU	CD1	0.01
239PHI	CG	239PHE	CD1	100.00
239PHI	CG	239PHE	CD2	100.00
239PHI	CG	239PHE	CE1	100.00
239PHI	CG	239PHE	CE2	100.00
239PHI	CG	239PHE	CZ	100.00
239PHI	CG	239PHE	C	0.14
239PHI	CD1	239PHE	CD2	100.00
239PHI	CD1	239PHE	CE1	100.00
239PHI	CD1	239PHE	CE2	100.00
239PHI	CD1	239PHE	CZ	100.00
239PHI	CD2	239PHE	CE1	100.00
239PHI	CD2	239PHE	CE2	100.00
239PHI	CD2	239PHE	CZ	100.00
239PHI	CD2	239PHE	C	0.04
239PHI	CE1	239PHE	CE2	100.00
239PHI	CE1	239PHE	CZ	100.00
239PHI	CE2	239PHE	CZ	100.00
239PHI	C	240GLY	CA	100.00
239PHI	C	240GLY	C	93.04
239PHI	C	243LEU	CD1	0.01
240GLY	CA	240GLY	C	100.00
240GLY	C	241ASP	CA	100.00
240GLY	C	241ASP	CB	0.04
240GLY	C	241ASP	C	99.98
241ASI	CA	241ASP	CB	100.00
241ASI	CA	241ASP	CG	100.00
241ASI	CA	241ASP	C	100.00
241ASI	CB	241ASP	CG	100.00
241ASI	CB	241ASP	C	100.00
241ASI	CG	241ASP	C	99.94
241ASI	C	242ILE	CA	100.00
241ASI	C	242ILE	CB	0.01
241ASI	C	242ILE	C	100.00
242ILE	CA	242ILE	CB	100.00
242ILE	CA	242ILE	CG1	100.00
242ILE	CA	242ILE	CG2	100.00
242ILE	CA	242ILE	CD	0.20
242ILE	CA	242ILE	C	100.00
242ILE	CB	242ILE	CG1	100.00
242ILE	CB	242ILE	CG2	100.00
242ILE	CB	242ILE	CD	100.00
242ILE	CB	242ILE	C	100.00
242ILE	CG1	242ILE	CG2	100.00
242ILE	CG1	242ILE	CD	100.00
242ILE	CG2	242ILE	CD	99.62
242ILE	CG2	242ILE	C	100.00
242ILE	CG2	243LEU	CD1	0.00
242ILE	CG2	246LEU	CD1	0.09

242ILE	CG2	246LEU	CD2	0.04
242ILE	C	243LEU	CA	100.00
242ILE	C	243LEU	CB	0.01
242ILE	C	243LEU	CD2	0.00
242ILE	C	243LEU	C	99.99
242ILE	C	246LEU	CD2	0.00
243LEU	CA	243LEU	CB	100.00
243LEU	CA	243LEU	CG	100.00
243LEU	CA	243LEU	CD1	20.96
243LEU	CA	243LEU	CD2	78.93
243LEU	CA	243LEU	C	100.00
243LEU	CB	243LEU	CG	100.00
243LEU	CB	243LEU	CD1	100.00
243LEU	CB	243LEU	CD2	100.00
243LEU	CB	243LEU	C	100.00
243LEU	CG	243LEU	CD1	100.00
243LEU	CG	243LEU	CD2	100.00
243LEU	CG	243LEU	C	11.09
243LEU	CD1	243LEU	CD2	100.00
243LEU	CD1	243LEU	C	0.11
243LEU	CD2	243LEU	C	0.34
243LEU	CD2	246LEU	CD1	0.00
243LEU	CD2	246LEU	CD2	0.06
243LEU	C	244SER	CA	100.00
243LEU	C	244SER	CB	0.02
243LEU	C	244SER	C	99.98
244SER	CA	244SER	CB	100.00
244SER	CA	244SER	C	100.00
244SER	CB	244SER	C	100.00
244SER	C	245ASP	CA	100.00
244SER	C	245ASP	CB	0.15
244SER	C	245ASP	CG	0.02
244SER	C	245ASP	C	99.86
245ASP	CA	245ASP	CB	100.00
245ASP	CA	245ASP	CG	100.00
245ASP	CA	245ASP	C	100.00
245ASP	CB	245ASP	CG	100.00
245ASP	CB	245ASP	C	100.00
245ASP	CG	245ASP	C	0.02
245ASP	C	246LEU	CA	100.00
245ASP	C	246LEU	CB	0.02
245ASP	C	246LEU	C	99.99
246LEU	CA	246LEU	CB	100.00
246LEU	CA	246LEU	CG	100.00
246LEU	CA	246LEU	CD1	76.25
246LEU	CA	246LEU	CD2	20.25
246LEU	CA	246LEU	C	100.00
246LEU	CB	246LEU	CG	100.00
246LEU	CB	246LEU	CD1	100.00
246LEU	CB	246LEU	CD2	100.00
246LEU	CB	246LEU	C	100.00
246LEU	CG	246LEU	CD1	100.00
246LEU	CG	246LEU	CD2	100.00
246LEU	CG	246LEU	C	86.10
246LEU	CD1	246LEU	CD2	100.00
246LEU	CD1	246LEU	C	6.10
246LEU	CD2	246LEU	C	6.97
246LEU	C	247ALA	CA	100.00
246LEU	C	247ALA	CB	1.24
246LEU	C	247ALA	C	98.60
246LEU	C	249VAL	CG2	0.00
247ALA	CA	247ALA	CB	100.00

247ALA	CA	247ALA	C	100.00
247ALA	CA	250LEU	CD1	0.32
247ALA	CA	250LEU	CD2	0.00
247ALA	CB	247ALA	C	100.00
247ALA	CB	250LEU	CD1	0.06
247ALA	CB	257LEU	CD2	0.00
247ALA	C	248SER	CA	100.00
247ALA	C	248SER	CB	0.30
247ALA	C	248SER	C	99.77
247ALA	C	250LEU	CB	0.00
247ALA	C	250LEU	CG	0.00
247ALA	C	250LEU	CD1	0.01
247ALA	C	257LEU	CD2	0.01
248SER	CA	248SER	CB	100.00
248SER	CA	248SER	C	100.00
248SER	CA	257LEU	CD1	0.93
248SER	CA	257LEU	CD2	0.00
248SER	CB	248SER	C	100.00
248SER	CB	254LEU	CG	0.00
248SER	CB	254LEU	CD1	0.02
248SER	CB	254LEU	CD2	0.19
248SER	CB	257LEU	CG	0.01
248SER	CB	257LEU	CD1	0.33
248SER	CB	257LEU	CD2	0.08
248SER	CB	272VAL	CG1	0.01
248SER	C	249VAL	CA	100.00
248SER	C	249VAL	CB	0.78
248SER	C	249VAL	CG1	0.13
248SER	C	249VAL	CG2	0.10
248SER	C	249VAL	C	99.12
248SER	C	251PRO	CD	0.02
248SER	C	254LEU	CB	0.03
248SER	C	254LEU	CD2	0.31
248SER	C	257LEU	CD1	0.00
249VAL	CA	249VAL	CB	100.00
249VAL	CA	249VAL	CG1	100.00
249VAL	CA	249VAL	CG2	100.00
249VAL	CA	249VAL	C	100.00
249VAL	CA	254LEU	CD1	0.00
249VAL	CA	254LEU	CD2	0.08
249VAL	CB	249VAL	CG1	100.00
249VAL	CB	249VAL	CG2	100.00
249VAL	CB	249VAL	C	100.00
249VAL	CG1	249VAL	CG2	100.00
249VAL	CG1	249VAL	C	37.95
249VAL	CG1	254LEU	CA	0.01
249VAL	CG1	254LEU	CB	0.02
249VAL	CG1	254LEU	CG	0.02
249VAL	CG1	254LEU	CD1	0.09
249VAL	CG1	254LEU	CD2	3.00
249VAL	CG2	249VAL	C	62.10
249VAL	CG2	250LEU	CD1	0.04
249VAL	CG2	250LEU	CD2	0.02
249VAL	CG2	254LEU	CD1	0.01
249VAL	CG2	254LEU	CD2	0.80
249VAL	C	250LEU	CA	100.00
249VAL	C	250LEU	C	100.00
249VAL	C	251PRO	CD	34.26
250LEU	CA	250LEU	CB	100.00
250LEU	CA	250LEU	CG	100.00
250LEU	CA	250LEU	CD1	9.47
250LEU	CA	250LEU	CD2	90.55

250LEU	CA	250LEU	C	100.00
250LEU	CA	251PRO	CD	100.00
250LEU	CB	250LEU	CG	100.00
250LEU	CB	250LEU	CD1	100.00
250LEU	CB	250LEU	CD2	100.00
250LEU	CB	250LEU	C	100.00
250LEU	CB	251PRO	CD	52.05
250LEU	CG	250LEU	CD1	100.00
250LEU	CG	250LEU	CD2	100.00
250LEU	CG	250LEU	C	1.66
250LEU	CD1	250LEU	CD2	100.00
250LEU	CD1	250LEU	C	0.11
250LEU	CD1	251PRO	CD	0.00
250LEU	CD2	250LEU	C	0.04
250LEU	C	251PRO	CA	100.00
250LEU	C	251PRO	CB	0.15
250LEU	C	251PRO	CD	100.00
250LEU	C	251PRO	C	100.00
251PRO	CA	251PRO	CB	100.00
251PRO	CA	251PRO	CG	100.00
251PRO	CA	251PRO	CD	100.00
251PRO	CA	251PRO	C	100.00
251PRO	CB	251PRO	CG	100.00
251PRO	CB	251PRO	CD	100.00
251PRO	CB	251PRO	C	100.00
251PRO	CB	256LEU	CB	0.01
251PRO	CB	256LEU	CD1	0.36
251PRO	CB	256LEU	CD2	0.14
251PRO	CG	251PRO	CD	100.00
251PRO	CG	251PRO	C	91.01
251PRO	CG	256LEU	CB	0.27
251PRO	CG	256LEU	CD1	0.76
251PRO	CG	256LEU	CD2	0.07
251PRO	CG	257LEU	CG	0.02
251PRO	CG	257LEU	CD1	0.62
251PRO	CG	257LEU	CD2	0.75
251PRO	CD	251PRO	C	71.40
251PRO	CD	257LEU	CD1	0.05
251PRO	CD	257LEU	CD2	0.01
251PRO	C	252GLY	CA	100.00
251PRO	C	252GLY	C	30.07
251PRO	C	256LEU	CD1	0.10
251PRO	C	327LEU	CD1	0.00
252GLY	CA	252GLY	C	100.00
252GLY	CA	327LEU	CD1	0.16
252GLY	CA	327LEU	CD2	0.17
252GLY	C	253SER	CA	100.00
252GLY	C	253SER	CB	92.68
252GLY	C	253SER	C	9.14
252GLY	C	327LEU	CD1	0.66
252GLY	C	327LEU	CD2	0.92
253SER	CA	253SER	CB	100.00
253SER	CA	253SER	C	100.00
253SER	CA	326ASP	CG	0.01
253SER	CA	327LEU	CD2	0.03
253SER	CB	253SER	C	100.00
253SER	CB	256LEU	CB	0.00
253SER	CB	256LEU	CG	0.02
253SER	CB	256LEU	CD1	0.76
253SER	CB	256LEU	CD2	0.08
253SER	CB	324PRO	CB	0.94
253SER	CB	324PRO	CG	5.85

253SEI	CB	326ASP	CB	0.38
253SEI	CB	326ASP	CG	0.06
253SEI	CB	327LEU	CB	0.01
253SEI	CB	327LEU	CG	0.02
253SEI	CB	327LEU	CD1	0.51
253SEI	CB	327LEU	CD2	0.31
253SEI	C	254LEU	CA	100.00
253SEI	C	254LEU	CB	1.99
253SEI	C	254LEU	CG	0.14
253SEI	C	254LEU	CD1	0.08
253SEI	C	254LEU	CD2	0.03
253SEI	C	254LEU	C	97.98
253SEI	C	256LEU	CD1	0.14
253SEI	C	256LEU	CD2	0.20
254LEU	CA	254LEU	CB	100.00
254LEU	CA	254LEU	CG	100.00
254LEU	CA	254LEU	CD1	8.26
254LEU	CA	254LEU	CD2	89.03
254LEU	CA	254LEU	C	100.00
254LEU	CB	254LEU	CG	100.00
254LEU	CB	254LEU	CD1	100.00
254LEU	CB	254LEU	CD2	100.00
254LEU	CB	254LEU	C	100.00
254LEU	CB	276ALA	CB	0.00
254LEU	CB	279ILE	CG1	0.18
254LEU	CB	279ILE	CG2	1.95
254LEU	CB	279ILE	CD	0.33
254LEU	CB	326ASP	CG1	0.02
254LEU	CG	254LEU	CD1	100.00
254LEU	CG	254LEU	CD2	100.00
254LEU	CG	254LEU	C	6.86
254LEU	CG	276ALA	CB	0.00
254LEU	CG	279ILE	CD	0.04
254LEU	CG	326ASP	CG1	0.00
254LEU	CD1	254LEU	CD2	100.00
254LEU	CD1	254LEU	C	0.99
254LEU	CD1	272VAL	CG1	0.02
254LEU	CD1	275SER	CB	0.02
254LEU	CD1	276ALA	CB	0.89
254LEU	CD1	278ASP	CB	0.61
254LEU	CD1	278ASP	CG	0.05
254LEU	CD1	279ILE	CG1	0.26
254LEU	CD1	279ILE	CG2	0.45
254LEU	CD1	279ILE	CD	0.14
254LEU	CD1	326ASP	CG1	0.01
254LEU	CD2	254LEU	C	0.07
254LEU	CD2	275SER	CB	0.07
254LEU	CD2	276ALA	CB	0.03
254LEU	CD2	279ILE	CG2	0.04
254LEU	CD2	279ILE	CD	0.04
254LEU	CD2	326ASP	CB	0.00
254LEU	CD2	326ASP	CG1	0.03
254LEU	C	255GLY	CA	100.00
254LEU	C	255GLY	C	50.62
254LEU	C	256LEU	CD1	0.09
254LEU	C	256LEU	CD2	0.04
254LEU	C	257LEU	CD2	0.06
254LEU	C	272VAL	CG1	0.00
254LEU	C	279ILE	CG2	0.28
254LEU	C	279ILE	CD	0.00
255GLY	CA	255GLY	C	100.00
255GLY	CA	272VAL	CG1	0.03

255GL\	CA	272VAL	CG2	0.18
255GL\	CA	272VAL	C	0.05
255GL\	CA	273HIS	CA	0.15
255GL\	CA	273HIS	CB	1.13
255GL\	CA	273HIS	CG	0.30
255GL\	CA	273HIS	CD2	0.72
255GL\	CA	273HIS	CE1	0.45
255GL\	CA	279ILE	CG2	0.35
255GL\	CA	279ILE	CD	0.20
255GL\	C	256LEU	CA	100.00
255GL\	C	256LEU	CB	29.45
255GL\	C	256LEU	CG	2.46
255GL\	C	256LEU	CD1	2.14
255GL\	C	256LEU	CD2	0.54
255GL\	C	256LEU	C	62.74
255GL\	C	272VAL	CG1	0.08
255GL\	C	272VAL	CG2	0.06
255GL\	C	273HIS	CD2	0.00
255GL\	C	273HIS	CE1	0.00
255GL\	C	289ALA	CB	2.07
256LEU	CA	256LEU	CB	100.00
256LEU	CA	256LEU	CG	100.00
256LEU	CA	256LEU	CD1	22.07
256LEU	CA	256LEU	CD2	75.00
256LEU	CA	256LEU	C	100.00
256LEU	CA	288THR	CG2	0.08
256LEU	CA	289ALA	CB	0.01
256LEU	CB	256LEU	CG	100.00
256LEU	CB	256LEU	CD1	100.00
256LEU	CB	256LEU	CD2	100.00
256LEU	CB	256LEU	C	100.00
256LEU	CB	288THR	CG2	0.06
256LEU	CG	256LEU	CD1	100.00
256LEU	CG	256LEU	CD2	100.00
256LEU	CG	256LEU	C	14.17
256LEU	CG	288THR	CG2	0.11
256LEU	CD1	256LEU	CD2	100.00
256LEU	CD1	256LEU	C	4.86
256LEU	CD1	257LEU	CD1	0.02
256LEU	CD1	257LEU	CD2	0.01
256LEU	CD1	286ASN	CG	0.01
256LEU	CD1	288THR	CB	0.04
256LEU	CD1	288THR	CG2	0.34
256LEU	CD1	315VAL	CG1	0.00
256LEU	CD1	319LEU	CD1	0.12
256LEU	CD1	319LEU	CD2	0.10
256LEU	CD1	324PRO	CB	0.00
256LEU	CD1	324PRO	CG	0.02
256LEU	CD1	327LEU	CD1	0.02
256LEU	CD1	327LEU	CD2	0.01
256LEU	CD2	256LEU	C	0.23
256LEU	CD2	286ASN	CB	0.01
256LEU	CD2	286ASN	CG	0.14
256LEU	CD2	288THR	CB	0.43
256LEU	CD2	288THR	CG2	0.46
256LEU	CD2	292LEU	CD1	0.02
256LEU	CD2	315VAL	CG1	0.04
256LEU	CD2	319LEU	CD1	0.44
256LEU	CD2	319LEU	CD2	0.74
256LEU	CD2	324PRO	CB	0.04
256LEU	CD2	324PRO	CG	0.09
256LEU	CD2	327LEU	CD1	0.00

256LEU	C	257LEU	CA	100.00
256LEU	C	257LEU	CB	38.17
256LEU	C	257LEU	CG	12.62
256LEU	C	257LEU	CD1	0.13
256LEU	C	257LEU	CD2	0.08
256LEU	C	257LEU	C	60.66
256LEU	C	258PRO	CD	0.01
256LEU	C	289ALA	CA	0.24
256LEU	C	289ALA	CB	1.30
256LEU	C	292LEU	CD1	1.10
257LEU	CA	257LEU	CB	100.00
257LEU	CA	257LEU	CG	100.00
257LEU	CA	257LEU	CD1	10.14
257LEU	CA	257LEU	CD2	96.18
257LEU	CA	257LEU	C	100.00
257LEU	CA	258PRO	CD	100.00
257LEU	CA	292LEU	CD1	0.00
257LEU	CB	257LEU	CG	100.00
257LEU	CB	257LEU	CD1	100.00
257LEU	CB	257LEU	CD2	100.00
257LEU	CB	257LEU	C	100.00
257LEU	CB	258PRO	CD	8.32
257LEU	CB	272VAL	CG1	0.00
257LEU	CB	272VAL	CG2	0.10
257LEU	CG	257LEU	CD1	100.00
257LEU	CG	257LEU	CD2	100.00
257LEU	CG	257LEU	C	0.02
257LEU	CD1	257LEU	CD2	100.00
257LEU	CD1	258PRO	CD	0.00
257LEU	CD2	258PRO	CD	0.01
257LEU	C	258PRO	CA	100.00
257LEU	C	258PRO	CB	1.86
257LEU	C	258PRO	CD	100.00
257LEU	C	258PRO	C	99.99
257LEU	C	272VAL	CG2	0.08
257LEU	C	289ALA	CB	0.96
258PRO	CA	258PRO	CB	100.00
258PRO	CA	258PRO	CG	100.00
258PRO	CA	258PRO	CD	100.00
258PRO	CA	258PRO	C	100.00
258PRO	CB	258PRO	CG	100.00
258PRO	CB	258PRO	CD	100.00
258PRO	CB	258PRO	C	100.00
258PRO	CB	292LEU	CB	0.02
258PRO	CB	292LEU	C	2.75
258PRO	CB	293SER	CA	1.32
258PRO	CB	293SER	CB	0.09
258PRO	CB	296MET	CE	0.02
258PRO	CG	258PRO	CD	100.00
258PRO	CG	258PRO	C	99.94
258PRO	CG	292LEU	CB	0.25
258PRO	CG	292LEU	C	0.00
258PRO	CG	296MET	CG	0.01
258PRO	CG	296MET	CE	0.10
258PRO	CD	258PRO	C	99.74
258PRO	CD	292LEU	CB	0.18
258PRO	CD	292LEU	CD1	0.01
258PRO	CD	292LEU	CD2	0.01
258PRO	C	259SER	CA	100.00
258PRO	C	259SER	CB	99.97
258PRO	C	259SER	C	0.14
258PRO	C	272VAL	CG1	0.05

258PR(	C	272VAL	CG2	0.84
259SEI	CA	259SER	CB	100.00
259SEI	CA	259SER	C	100.00
259SEI	CA	296MET	CE	0.00
259SEI	CB	259SER	C	100.00
259SEI	CB	272VAL	CG1	1.76
259SEI	CB	272VAL	CG2	2.70
259SEI	C	260ALA	CA	100.00
259SEI	C	260ALA	CB	92.47
259SEI	C	260ALA	C	8.18
259SEI	C	296MET	CE	0.09
260AL/	CA	260ALA	CB	100.00
260AL/	CA	260ALA	C	100.00
260AL/	CA	269PHE	CD2	0.00
260AL/	CB	260ALA	C	100.00
260AL/	CB	262LEU	CD1	0.63
260AL/	CB	262LEU	CD2	0.06
260AL/	CB	269PHE	CA	0.02
260AL/	CB	269PHE	CG	0.03
260AL/	CB	269PHE	CD2	21.63
260AL/	CB	269PHE	CE2	17.30
260AL/	CB	269PHE	CZ	0.02
260AL/	CB	296MET	CE	8.24
260AL/	CB	300HIS	CD2	0.03
260AL/	CB	300HIS	CE1	0.02
260AL/	C	261SER	CA	100.00
260AL/	C	261SER	CB	32.24
260AL/	C	261SER	C	71.78
260AL/	C	262LEU	CD1	0.02
260AL/	C	262LEU	CD2	0.01
261SEI	CA	261SER	CB	100.00
261SEI	CA	261SER	C	100.00
261SEI	CB	261SER	C	100.00
261SEI	C	262LEU	CA	100.00
261SEI	C	262LEU	CB	29.12
261SEI	C	262LEU	CG	1.21
261SEI	C	262LEU	CD1	2.60
261SEI	C	262LEU	CD2	0.61
261SEI	C	262LEU	C	69.73
262LEU	CA	262LEU	CB	100.00
262LEU	CA	262LEU	CG	100.00
262LEU	CA	262LEU	CD1	31.63
262LEU	CA	262LEU	CD2	66.00
262LEU	CA	262LEU	C	100.00
262LEU	CB	262LEU	CG	100.00
262LEU	CB	262LEU	CD1	100.00
262LEU	CB	262LEU	CD2	100.00
262LEU	CB	262LEU	C	100.00
262LEU	CG	262LEU	CD1	100.00
262LEU	CG	262LEU	CD2	100.00
262LEU	CG	262LEU	C	24.58
262LEU	CG	300HIS	CE1	0.04
262LEU	CD1	262LEU	CD2	100.00
262LEU	CD1	262LEU	C	6.44
262LEU	CD1	267PRO	CB	0.08
262LEU	CD1	300HIS	CD2	0.02
262LEU	CD1	300HIS	CE1	1.83
262LEU	CD2	262LEU	C	3.76
262LEU	CD2	267PRO	CB	1.76
262LEU	CD2	300HIS	CD2	0.56
262LEU	CD2	300HIS	CE1	6.48
262LEU	C	263GLY	CA	100.00

262LEU	C	263GLY	C	1.52
263GLY	CA	263GLY	C	100.00
263GLY	C	264ARG	CA	100.00
263GLY	C	264ARG	CB	1.77
263GLY	C	264ARG	CG	0.18
263GLY	C	264ARG	C	98.31
264ARG	CA	264ARG	CB	100.00
264ARG	CA	264ARG	CG	100.00
264ARG	CA	264ARG	CD	3.71
264ARG	CA	264ARG	C	100.00
264ARG	CB	264ARG	CG	100.00
264ARG	CB	264ARG	CD	100.00
264ARG	CB	264ARG	CZ	2.56
264ARG	CB	264ARG	C	100.00
264ARG	CG	264ARG	CD	100.00
264ARG	CG	264ARG	CZ	24.04
264ARG	CG	264ARG	C	70.24
264ARG	CD	264ARG	CZ	100.00
264ARG	CD	264ARG	C	5.64
264ARG	CZ	264ARG	C	0.14
264ARG	CZ	265GLY	CA	0.44
264ARG	C	265GLY	CA	100.00
264ARG	C	265GLY	C	71.11
265GLY	CA	265GLY	C	100.00
265GLY	C	266THR	CA	100.00
265GLY	C	266THR	CB	0.30
265GLY	C	266THR	CG2	0.02
265GLY	C	266THR	C	99.85
266THR	CA	266THR	CB	100.00
266THR	CA	266THR	CG2	100.00
266THR	CA	266THR	C	100.00
266THR	CA	267PRO	CD	100.00
266THR	CB	266THR	CG2	100.00
266THR	CB	266THR	C	100.00
266THR	CB	267PRO	CD	0.30
266THR	CG2	266THR	C	97.23
266THR	CG2	267PRO	CD	4.52
266THR	C	267PRO	CA	100.00
266THR	C	267PRO	CB	0.10
266THR	C	267PRO	CD	100.00
266THR	C	267PRO	C	100.00
267PRO	CA	267PRO	CB	100.00
267PRO	CA	267PRO	CG	100.00
267PRO	CA	267PRO	CD	100.00
267PRO	CA	267PRO	C	100.00
267PRO	CB	267PRO	CG	100.00
267PRO	CB	267PRO	CD	100.00
267PRO	CB	267PRO	C	100.00
267PRO	CB	269PHE	CE1	1.00
267PRO	CB	269PHE	CZ	0.25
267PRO	CB	300HIS	CD2	0.05
267PRO	CB	300HIS	CE1	0.12
267PRO	CG	267PRO	CD	100.00
267PRO	CG	267PRO	C	98.66
267PRO	CG	269PHE	CE1	0.48
267PRO	CG	269PHE	CZ	0.11
267PRO	CG	300HIS	CD2	0.20
267PRO	CG	300HIS	CE1	0.09
267PRO	CG	301ALA	CB	0.07
267PRO	CD	267PRO	C	95.46
267PRO	C	268VAL	CA	100.00
267PRO	C	268VAL	CB	44.25

267PR(	C	268VAL	CG2	32.25
267PR(	C	268VAL	C	71.44
267PR(	C	269PHE	CD1	0.00
267PR(	C	269PHE	CE1	0.02
268VAL	CA	268VAL	CB	100.00
268VAL	CA	268VAL	CG1	100.00
268VAL	CA	268VAL	CG2	100.00
268VAL	CA	268VAL	C	100.00
268VAL	CB	268VAL	CG1	100.00
268VAL	CB	268VAL	CG2	100.00
268VAL	CB	268VAL	C	100.00
268VAL	CG1	268VAL	CG2	100.00
268VAL	CG1	268VAL	C	100.00
268VAL	CG1	270GLU	CB	0.00
268VAL	C	269PHE	CA	100.00
268VAL	C	269PHE	CB	18.96
268VAL	C	269PHE	CG	6.12
268VAL	C	269PHE	CD1	8.82
268VAL	C	269PHE	CD2	0.00
268VAL	C	269PHE	C	85.80
269PHI	CA	269PHE	CB	100.00
269PHI	CA	269PHE	CG	100.00
269PHI	CA	269PHE	CD1	92.02
269PHI	CA	269PHE	CD2	92.54
269PHI	CA	269PHE	C	100.00
269PHI	CB	269PHE	CG	100.00
269PHI	CB	269PHE	CD1	100.00
269PHI	CB	269PHE	CD2	100.00
269PHI	CB	269PHE	C	100.00
269PHI	CB	297MET	CE	0.12
269PHI	CG	269PHE	CD1	100.00
269PHI	CG	269PHE	CD2	100.00
269PHI	CG	269PHE	CE1	100.00
269PHI	CG	269PHE	CE2	100.00
269PHI	CG	269PHE	CZ	100.00
269PHI	CG	297MET	CB	0.11
269PHI	CG	297MET	CG	0.04
269PHI	CG	297MET	CE	0.18
269PHI	CD1	269PHE	CD2	100.00
269PHI	CD1	269PHE	CE1	100.00
269PHI	CD1	269PHE	CE2	100.00
269PHI	CD1	269PHE	CZ	100.00
269PHI	CD1	297MET	CA	0.01
269PHI	CD1	297MET	CB	0.90
269PHI	CD1	297MET	CG	2.12
269PHI	CD1	297MET	CE	2.36
269PHI	CD2	269PHE	CE1	100.00
269PHI	CD2	269PHE	CE2	100.00
269PHI	CD2	269PHE	CZ	100.00
269PHI	CD2	296MET	CB	0.21
269PHI	CD2	296MET	CG	0.03
269PHI	CD2	296MET	C	0.04
269PHI	CD2	297MET	CA	0.16
269PHI	CD2	297MET	CB	0.04
269PHI	CD2	297MET	CG	0.02
269PHI	CE1	269PHE	CE2	100.00
269PHI	CE1	269PHE	CZ	100.00
269PHI	CE1	297MET	CA	0.22
269PHI	CE1	297MET	CB	0.19
269PHI	CE1	297MET	CG	1.51
269PHI	CE1	297MET	CE	0.00
269PHI	CE1	300HIS	CD2	0.00

269PHI	CE1	301ALA	CB	0.34
269PHI	CE2	269PHE	CZ	100.00
269PHI	CE2	296MET	CB	0.18
269PHI	CE2	296MET	CG	0.10
269PHI	CE2	296MET	CE	0.00
269PHI	CE2	296MET	C	8.50
269PHI	CE2	297MET	CA	7.41
269PHI	CE2	297MET	CB	0.04
269PHI	CE2	300HIS	CB	0.06
269PHI	CE2	300HIS	CG	0.01
269PHI	CE2	300HIS	CD2	0.03
269PHI	CZ	296MET	C	0.00
269PHI	CZ	297MET	CA	2.75
269PHI	CZ	297MET	CB	0.04
269PHI	CZ	297MET	CG	0.03
269PHI	CZ	300HIS	CB	0.55
269PHI	CZ	300HIS	CG	0.22
269PHI	CZ	300HIS	CD2	0.84
269PHI	CZ	300HIS	CE1	0.00
269PHI	CZ	301ALA	CB	0.46
269PHI	C	270GLU	CA	100.00
269PHI	C	270GLU	CB	99.22
269PHI	C	270GLU	CG	0.02
269PHI	C	270GLU	C	1.24
270GLI	CA	270GLU	CB	100.00
270GLI	CA	270GLU	CG	100.00
270GLI	CA	270GLU	C	100.00
270GLI	CA	271PRO	CD	100.00
270GLI	CB	270GLU	CG	100.00
270GLI	CB	270GLU	CD	100.00
270GLI	CB	270GLU	C	100.00
270GLI	CB	271PRO	CD	0.25
270GLI	CG	270GLU	CD	100.00
270GLI	CG	270GLU	C	99.87
270GLI	CG	271PRO	CD	3.14
270GLI	C	271PRO	CA	100.00
270GLI	C	271PRO	CB	0.24
270GLI	C	271PRO	CD	100.00
270GLI	C	271PRO	C	100.00
270GLI	C	293SER	CB	0.01
271PR(	CA	271PRO	CB	100.00
271PR(	CA	271PRO	CG	100.00
271PR(	CA	271PRO	CD	100.00
271PR(	CA	271PRO	C	100.00
271PR(	CA	289ALA	CB	0.03
271PR(	CB	271PRO	CG	100.00
271PR(	CB	271PRO	CD	100.00
271PR(	CB	271PRO	C	100.00
271PR(	CB	273HIS	CD2	0.11
271PR(	CB	273HIS	CE1	5.05
271PR(	CB	289ALA	CB	28.12
271PR(	CB	289ALA	C	1.24
271PR(	CB	290ALA	CA	0.02
271PR(	CB	290ALA	CB	0.00
271PR(	CG	271PRO	CD	100.00
271PR(	CG	271PRO	C	99.29
271PR(	CG	273HIS	CD2	0.09
271PR(	CG	273HIS	CE1	3.90
271PR(	CG	290ALA	CB	0.01
271PR(	CD	271PRO	C	98.29
271PR(	C	272VAL	CA	100.00
271PR(	C	272VAL	CB	3.20

271PR(	C	272VAL	CG1	0.59
271PR(	C	272VAL	CG2	1.17
271PR(	C	272VAL	C	98.61
271PR(	C	273HIS	CD2	0.00
272VAI	CA	272VAL	CB	100.00
272VAI	CA	272VAL	CG1	100.00
272VAI	CA	272VAL	CG2	100.00
272VAI	CA	272VAL	C	100.00
272VAI	CB	272VAL	CG1	100.00
272VAI	CB	272VAL	CG2	100.00
272VAI	CB	272VAL	C	100.00
272VAI	CG1	272VAL	CG2	100.00
272VAI	CG1	272VAL	C	51.46
272VAI	CG1	279ILE	CD	0.02
272VAI	CG2	272VAL	C	49.01
272VAI	C	273HIS	CA	100.00
272VAI	C	273HIS	CB	35.90
272VAI	C	273HIS	CG	10.24
272VAI	C	273HIS	CD2	3.25
272VAI	C	273HIS	C	64.94
273HIS	CA	273HIS	CB	100.00
273HIS	CA	273HIS	CG	100.00
273HIS	CA	273HIS	CD2	10.10
273HIS	CA	273HIS	C	100.00
273HIS	CA	279ILE	CD	0.05
273HIS	CB	273HIS	CG	100.00
273HIS	CB	273HIS	CD2	100.00
273HIS	CB	273HIS	CE1	0.02
273HIS	CB	273HIS	C	100.00
273HIS	CB	279ILE	CD	0.00
273HIS	CB	284ILE	CG2	0.11
273HIS	CB	289ALA	CB	0.22
273HIS	CG	273HIS	CD2	100.00
273HIS	CG	273HIS	CE1	100.00
273HIS	CG	273HIS	C	71.78
273HIS	CG	284ILE	CG2	0.00
273HIS	CG	285ALA	CB	0.02
273HIS	CG	286ASN	CB	0.06
273HIS	CD2	273HIS	CE1	100.00
273HIS	CD2	273HIS	C	2.37
273HIS	CD2	274GLY	CA	0.00
273HIS	CD2	279ILE	CB	0.02
273HIS	CD2	279ILE	CG2	0.02
273HIS	CD2	279ILE	CD	0.04
273HIS	CD2	279ILE	C	0.02
273HIS	CD2	284ILE	CG2	0.00
273HIS	CD2	285ALA	CB	0.08
273HIS	CD2	286ASN	CB	0.19
273HIS	CD2	289ALA	CB	0.57
273HIS	CE1	279ILE	CA	0.04
273HIS	CE1	279ILE	CB	0.57
273HIS	CE1	279ILE	CG1	0.02
273HIS	CE1	279ILE	CG2	4.16
273HIS	CE1	279ILE	CD	0.80
273HIS	CE1	279ILE	C	0.01
273HIS	CE1	280ALA	CA	0.00
273HIS	CE1	280ALA	CB	0.07
273HIS	CE1	280ALA	C	0.04
273HIS	CE1	281GLY	CA	0.00
273HIS	CE1	284ILE	CG2	0.28
273HIS	CE1	285ALA	CA	0.16
273HIS	CE1	285ALA	CB	0.90

273HIS	CE1	285ALA	C	0.01
273HIS	CE1	286ASN	CB	5.89
273HIS	CE1	289ALA	CB	0.11
273HIS	C	274GLY	CA	100.00
273HIS	C	274GLY	C	42.07
273HIS	C	279ILE	CD	0.03
274GLY	CA	274GLY	C	100.00
274GLY	CA	279ILE	CD	0.04
274GLY	CA	280ALA	CA	0.00
274GLY	CA	280ALA	CB	0.27
274GLY	C	275SER	CA	100.00
274GLY	C	275SER	CB	31.87
274GLY	C	275SER	C	85.46
274GLY	C	279ILE	CD	0.04
274GLY	C	280ALA	CB	0.08
275SER	CA	275SER	CB	100.00
275SER	CA	275SER	C	100.00
275SER	CA	280ALA	CB	0.05
275SER	CB	275SER	C	100.00
275SER	CB	279ILE	CD	0.01
275SER	CB	280ALA	CB	0.08
275SER	C	276ALA	CA	100.00
275SER	C	276ALA	CB	37.28
275SER	C	276ALA	C	64.00
275SER	C	277PRO	CD	0.02
275SER	C	280ALA	CB	0.34
276ALA	CA	276ALA	CB	100.00
276ALA	CA	276ALA	C	100.00
276ALA	CA	277PRO	CD	100.00
276ALA	CB	276ALA	C	100.00
276ALA	CB	277PRO	CD	22.36
276ALA	CB	279ILE	CG1	0.82
276ALA	CB	279ILE	CD	0.39
276ALA	C	277PRO	CA	100.00
276ALA	C	277PRO	CB	0.08
276ALA	C	277PRO	CD	100.00
276ALA	C	277PRO	C	100.00
276ALA	C	280ALA	CB	0.06
277PRO	CA	277PRO	CB	100.00
277PRO	CA	277PRO	CG	100.00
277PRO	CA	277PRO	CD	100.00
277PRO	CA	277PRO	C	100.00
277PRO	CA	280ALA	CB	0.02
277PRO	CB	277PRO	CG	100.00
277PRO	CB	277PRO	CD	100.00
277PRO	CB	277PRO	C	100.00
277PRO	CB	282LYS	CB	0.08
277PRO	CB	282LYS	CG	0.06
277PRO	CB	282LYS	CD	0.02
277PRO	CB	282LYS	CE	0.01
277PRO	CG	277PRO	CD	100.00
277PRO	CG	277PRO	C	57.65
277PRO	CG	282LYS	CE	0.00
277PRO	CD	277PRO	C	44.02
277PRO	C	278ASP	CA	100.00
277PRO	C	278ASP	CB	14.46
277PRO	C	278ASP	CG	0.00
277PRO	C	278ASP	C	98.46
277PRO	C	280ALA	CB	0.00
277PRO	C	282LYS	CB	0.26
277PRO	C	282LYS	CG	0.02
278ASP	CA	278ASP	CB	100.00

278ASI	CA	278ASP	CG	100.00
278ASI	CA	278ASP	C	100.00
278ASI	CA	282LYS	CD	0.00
278ASI	CB	278ASP	CG	100.00
278ASI	CB	278ASP	C	100.00
278ASI	CG	278ASP	C	75.30
278ASI	CG	279ILE	CD	0.00
278ASI	CG	282LYS	CD	0.10
278ASI	CG	282LYS	CE	0.37
278ASI	C	279ILE	CA	100.00
278ASI	C	279ILE	CB	34.45
278ASI	C	279ILE	CG1	3.79
278ASI	C	279ILE	CG2	17.25
278ASI	C	279ILE	CD	0.12
278ASI	C	279ILE	C	66.13
278ASI	C	282LYS	CB	0.01
278ASI	C	282LYS	CD	0.02
278ASI	C	283GLY	C	0.00
279ILE	CA	279ILE	CB	100.00
279ILE	CA	279ILE	CG1	100.00
279ILE	CA	279ILE	CG2	100.00
279ILE	CA	279ILE	CD	31.94
279ILE	CA	279ILE	C	100.00
279ILE	CA	284ILE	CG1	0.01
279ILE	CA	284ILE	CD	0.00
279ILE	CA	285ALA	CB	0.00
279ILE	CB	279ILE	CG1	100.00
279ILE	CB	279ILE	CG2	100.00
279ILE	CB	279ILE	CD	100.00
279ILE	CB	279ILE	C	100.00
279ILE	CB	284ILE	CG2	0.02
279ILE	CB	284ILE	CD	0.01
279ILE	CG1	279ILE	CG2	100.00
279ILE	CG1	279ILE	CD	100.00
279ILE	CG1	279ILE	C	52.71
279ILE	CG1	284ILE	CG2	0.00
279ILE	CG2	279ILE	CD	89.80
279ILE	CG2	279ILE	C	46.59
279ILE	CG2	282LYS	CD	0.01
279ILE	CG2	284ILE	CB	0.26
279ILE	CG2	284ILE	CG1	0.04
279ILE	CG2	284ILE	CG2	0.85
279ILE	CG2	284ILE	CD	0.17
279ILE	CG2	285ALA	CA	0.01
279ILE	CG2	285ALA	CB	0.02
279ILE	CG2	325PRO	CG	0.02
279ILE	CG2	326ASP	CA	0.01
279ILE	CG2	326ASP	CB	0.06
279ILE	CG2	326ASP	CG	0.07
279ILE	CD	279ILE	C	5.40
279ILE	CD	280ALA	CB	0.02
279ILE	CD	284ILE	CG1	0.00
279ILE	CD	284ILE	CG2	0.04
279ILE	CD	284ILE	CD	0.02
279ILE	CD	285ALA	CA	0.00
279ILE	CD	285ALA	CB	0.01
279ILE	CD	326ASP	CB	0.10
279ILE	CD	326ASP	CG	2.11
279ILE	C	280ALA	CA	100.00
279ILE	C	280ALA	CB	20.38
279ILE	C	280ALA	C	79.16
279ILE	C	282LYS	CB	0.00

279ILE	C	283GLY	C	0.01
279ILE	C	284ILE	CG1	0.01
279ILE	C	285ALA	CB	0.02
280ALA	CA	280ALA	CB	100.00
280ALA	CA	280ALA	C	100.00
280ALA	CA	285ALA	CB	0.00
280ALA	CB	280ALA	C	100.00
280ALA	CB	282LYS	CE	0.00
280ALA	CB	284ILE	C	0.00
280ALA	CB	285ALA	CB	0.03
280ALA	C	281GLY	CA	100.00
280ALA	C	281GLY	C	53.66
280ALA	C	283GLY	CA	0.01
281GLY	CA	281GLY	C	100.00
281GLY	CA	284ILE	CD	0.00
281GLY	C	282LYS	CA	100.00
281GLY	C	282LYS	CB	17.16
281GLY	C	282LYS	CG	2.18
281GLY	C	282LYS	CD	0.02
281GLY	C	282LYS	C	83.36
282LYS	CA	282LYS	CB	100.00
282LYS	CA	282LYS	CG	100.00
282LYS	CA	282LYS	CD	3.87
282LYS	CA	282LYS	C	100.00
282LYS	CB	282LYS	CG	100.00
282LYS	CB	282LYS	CD	100.00
282LYS	CB	282LYS	CE	12.43
282LYS	CB	282LYS	C	100.00
282LYS	CB	284ILE	CG1	0.14
282LYS	CB	284ILE	CD	1.94
282LYS	CG	282LYS	CD	100.00
282LYS	CG	282LYS	CE	100.00
282LYS	CG	282LYS	C	39.31
282LYS	CG	284ILE	CG1	0.02
282LYS	CG	284ILE	CD	0.06
282LYS	CG	326ASP	CG1	0.00
282LYS	CD	282LYS	CE	100.00
282LYS	CD	282LYS	C	0.41
282LYS	CD	284ILE	CG1	0.03
282LYS	CD	284ILE	CG2	0.01
282LYS	CD	284ILE	CD	0.36
282LYS	CE	282LYS	C	0.00
282LYS	CE	284ILE	CG1	0.01
282LYS	CE	284ILE	CD	0.04
282LYS	C	283GLY	CA	100.00
282LYS	C	283GLY	C	58.93
282LYS	C	284ILE	CD	0.00
283GLY	CA	283GLY	C	100.00
283GLY	CA	325PRO	CG	0.04
283GLY	CA	325PRO	CD	0.03
283GLY	CA	332GLY	CA	0.00
283GLY	CA	333THR	CB	0.00
283GLY	CA	333THR	CG2	0.00
283GLY	C	284ILE	CA	100.00
283GLY	C	284ILE	CB	13.04
283GLY	C	284ILE	CG1	2.99
283GLY	C	284ILE	CG2	5.07
283GLY	C	284ILE	CD	0.08
283GLY	C	284ILE	C	89.11
283GLY	C	325PRO	CG	0.03
283GLY	C	325PRO	CD	0.03
283GLY	C	326ASP	CB	0.00

283GLY	C	332GLY	CA	0.02
283GLY	C	333THR	CG2	0.05
284ILE	CA	284ILE	CB	100.00
284ILE	CA	284ILE	CG1	100.00
284ILE	CA	284ILE	CG2	100.00
284ILE	CA	284ILE	CD	13.51
284ILE	CA	284ILE	C	100.00
284ILE	CA	325PRO	CG	0.00
284ILE	CA	332GLY	CA	0.01
284ILE	CB	284ILE	CG1	100.00
284ILE	CB	284ILE	CG2	100.00
284ILE	CB	284ILE	CD	100.00
284ILE	CB	284ILE	C	100.00
284ILE	CB	325PRO	CB	0.04
284ILE	CB	325PRO	CG	0.05
284ILE	CB	325PRO	CD	0.00
284ILE	CB	333THR	CG2	0.01
284ILE	CG1	284ILE	CG2	100.00
284ILE	CG1	284ILE	CD	100.00
284ILE	CG1	284ILE	C	31.79
284ILE	CG1	325PRO	CB	0.34
284ILE	CG1	325PRO	CG	0.43
284ILE	CG1	332GLY	CA	0.00
284ILE	CG1	333THR	CG2	0.04
284ILE	CG2	284ILE	CD	94.63
284ILE	CG2	284ILE	C	68.01
284ILE	CG2	285ALA	C	0.02
284ILE	CG2	286ASN	CB	0.02
284ILE	CG2	325PRO	CB	0.23
284ILE	CG2	325PRO	CG	1.23
284ILE	CG2	325PRO	CD	0.01
284ILE	CG2	326ASP	CA	0.00
284ILE	CG2	326ASP	CB	0.01
284ILE	CG2	326ASP	CG	0.01
284ILE	CG2	330SER	CB	0.00
284ILE	CG2	331ALA	CB	0.02
284ILE	CG2	331ALA	C	0.01
284ILE	CG2	332GLY	CA	0.12
284ILE	CG2	332GLY	C	0.04
284ILE	CG2	333THR	CG2	1.35
284ILE	CD	284ILE	C	1.93
284ILE	CD	285ALA	C	0.00
284ILE	CD	286ASN	CB	0.01
284ILE	CD	325PRO	CB	0.62
284ILE	CD	325PRO	CG	0.48
284ILE	CD	325PRO	C	0.30
284ILE	CD	326ASP	CA	0.58
284ILE	CD	326ASP	CB	0.79
284ILE	CD	326ASP	CG	0.26
284ILE	CD	330SER	CB	0.06
284ILE	CD	330SER	C	0.02
284ILE	CD	331ALA	CA	0.01
284ILE	CD	331ALA	CB	0.03
284ILE	CD	331ALA	C	0.04
284ILE	CD	332GLY	CA	0.19
284ILE	CD	332GLY	C	0.07
284ILE	CD	333THR	CG2	0.31
284ILE	CD	336PHE	CE1	0.00
284ILE	C	285ALA	CA	100.00
284ILE	C	285ALA	CB	39.02
284ILE	C	285ALA	C	64.07
284ILE	C	325PRO	CG	0.00

284ILE	C	332GLY	CA	0.01
284ILE	C	333THR	CG2	0.00
285AL/	CA	285ALA	CB	100.00
285AL/	CA	285ALA	C	100.00
285AL/	CB	285ALA	C	100.00
285AL/	CB	333THR	CG2	0.94
285AL/	C	286ASN	CA	100.00
285AL/	C	286ASN	CB	7.77
285AL/	C	286ASN	CG	0.02
285AL/	C	286ASN	C	94.25
285AL/	C	325PRO	CG	0.02
285AL/	C	333THR	CG2	0.11
286ASI	CA	286ASN	CB	100.00
286ASI	CA	286ASN	CG	100.00
286ASI	CA	286ASN	C	100.00
286ASI	CA	287PRO	CD	100.00
286ASI	CA	325PRO	CG	0.00
286ASI	CA	333THR	CG2	0.00
286ASI	CB	286ASN	CG	100.00
286ASI	CB	286ASN	C	100.00
286ASI	CB	287PRO	CD	0.10
286ASI	CB	324PRO	CB	0.03
286ASI	CB	324PRO	CG	0.01
286ASI	CB	325PRO	CG	0.11
286ASI	CB	325PRO	CD	0.06
286ASI	CB	331ALA	CB	0.01
286ASI	CB	336PHE	CE1	0.00
286ASI	CG	286ASN	C	98.99
286ASI	CG	287PRO	CD	4.94
286ASI	CG	288THR	CG2	0.00
286ASI	CG	324PRO	CB	0.16
286ASI	CG	324PRO	CG	0.09
286ASI	CG	325PRO	CG	0.14
286ASI	CG	325PRO	CD1	1.16
286ASI	CG	331ALA	CB	2.48
286ASI	CG	336PHE	CG	0.00
286ASI	CG	336PHE	CD1	0.22
286ASI	CG	336PHE	CD2	0.00
286ASI	CG	336PHE	CE1	0.05
286ASI	CG	336PHE	CE2	0.01
286ASI	CG	336PHE	CZ	0.02
286ASI	C	287PRO	CA	100.00
286ASI	C	287PRO	CB	2.07
286ASI	C	287PRO	CD	100.00
286ASI	C	287PRO	C	99.88
287PR(	CA	287PRO	CB	100.00
287PR(	CA	287PRO	CG	100.00
287PR(	CA	287PRO	CD	100.00
287PR(	CA	287PRO	C	100.00
287PR(	CB	287PRO	CG	100.00
287PR(	CB	287PRO	CD	100.00
287PR(	CB	287PRO	C	100.00
287PR(	CB	290ALA	CB	0.00
287PR(	CB	291ILE	CD	0.62
287PR(	CB	333THR	CG2	0.00
287PR(	CB	336PHE	CD2	0.01
287PR(	CB	337THR	CG2	0.04
287PR(	CG	287PRO	CD	100.00
287PR(	CG	287PRO	C	98.94
287PR(	CG	291ILE	CD	0.30
287PR(	CG	332GLY	C	0.01
287PR(	CG	333THR	CG2	0.05

287PR(	CG	336PHE	CB	3.91
287PR(	CG	336PHE	CG	0.83
287PR(	CG	336PHE	CD1	0.45
287PR(	CG	336PHE	CD2	3.26
287PR(	CG	336PHE	CE1	0.01
287PR(	CG	336PHE	CE2	0.04
287PR(	CG	337THR	CG2	0.74
287PR(	CD	287PRO	C	91.12
287PR(	CD	324PRO	CB	0.00
287PR(	CD	331ALA	CB	0.00
287PR(	CD	331ALA	C	0.04
287PR(	CD	332GLY	C	0.08
287PR(	CD	333THR	CB	0.02
287PR(	CD	333THR	CG2	0.22
287PR(	CD	336PHE	CB	0.16
287PR(	CD	336PHE	CG	0.04
287PR(	CD	336PHE	CD1	1.92
287PR(	CD	336PHE	CD2	0.34
287PR(	CD	336PHE	CE1	0.11
287PR(	CD	336PHE	CE2	0.01
287PR(	CD	337THR	CG2	0.00
287PR(	C	288THR	CA	100.00
287PR(	C	288THR	CB	0.24
287PR(	C	288THR	C	99.77
287PR(	C	291ILE	CD	0.08
288THI	CA	288THR	CB	100.00
288THI	CA	288THR	CG2	100.00
288THI	CA	288THR	C	100.00
288THI	CA	315VAL	CG2	0.00
288THI	CA	336PHE	CE2	0.01
288THI	CA	336PHE	CZ	0.00
288THI	CB	288THR	CG2	100.00
288THI	CB	288THR	C	100.00
288THI	CB	315VAL	CG1	0.08
288THI	CB	315VAL	CG2	0.06
288THI	CB	336PHE	CZ	0.00
288THI	CG2	288THR	C	68.49
288THI	CG2	292LEU	CD1	1.26
288THI	CG2	315VAL	CB	0.01
288THI	CG2	315VAL	CG1	9.42
288THI	CG2	315VAL	CG2	0.17
288THI	CG2	319LEU	CG	0.00
288THI	CG2	319LEU	CD1	0.02
288THI	CG2	319LEU	CD2	0.03
288THI	CG2	336PHE	CB	0.01
288THI	CG2	336PHE	CG	0.05
288THI	CG2	336PHE	CD2	4.10
288THI	CG2	336PHE	CE1	0.01
288THI	CG2	336PHE	CE2	5.42
288THI	CG2	336PHE	CZ	3.52
288THI	C	289ALA	CA	100.00
288THI	C	289ALA	CB	0.00
288THI	C	289ALA	C	100.00
288THI	C	292LEU	CD1	0.00
289AL/	CA	289ALA	CB	100.00
289AL/	CA	289ALA	C	100.00
289AL/	CB	289ALA	C	100.00
289AL/	C	290ALA	CA	100.00
289AL/	C	290ALA	CB	0.06
289AL/	C	290ALA	C	99.99
290AL/	CA	290ALA	CB	100.00
290AL/	CA	290ALA	C	100.00

290ALA	CA	293SER	CB	0.00
290ALA	CB	290ALA	C	100.00
290ALA	C	291ILE	CA	100.00
290ALA	C	291ILE	C	100.00
291ILE	CA	291ILE	CB	100.00
291ILE	CA	291ILE	CG1	100.00
291ILE	CA	291ILE	CG2	100.00
291ILE	CA	291ILE	CD	1.20
291ILE	CA	291ILE	C	100.00
291ILE	CB	291ILE	CG1	100.00
291ILE	CB	291ILE	CG2	100.00
291ILE	CB	291ILE	CD	100.00
291ILE	CB	291ILE	C	100.00
291ILE	CB	315VAL	CG2	0.01
291ILE	CG1	291ILE	CG2	100.00
291ILE	CG1	291ILE	CD	100.00
291ILE	CG1	291ILE	C	0.02
291ILE	CG1	336PHE	CE2	0.00
291ILE	CG2	291ILE	CD	99.95
291ILE	CG2	291ILE	C	100.00
291ILE	CG2	311VAL	CG1	10.20
291ILE	CG2	315VAL	CG1	1.34
291ILE	CG2	315VAL	CG2	23.06
291ILE	CG2	340VAL	CG1	0.01
291ILE	CD	315VAL	CG1	0.00
291ILE	CD	315VAL	CG2	0.00
291ILE	CD	336PHE	CD2	1.58
291ILE	CD	336PHE	CE2	0.33
291ILE	CD	337THR	CG2	0.62
291ILE	CD	340VAL	CG1	0.00
291ILE	C	292LEU	CA	100.00
291ILE	C	292LEU	CB	0.01
291ILE	C	292LEU	C	99.98
292LEU	CA	292LEU	CB	100.00
292LEU	CA	292LEU	CG	100.00
292LEU	CA	292LEU	CD1	0.59
292LEU	CA	292LEU	CD2	99.60
292LEU	CA	292LEU	C	100.00
292LEU	CA	295ALA	CB	0.13
292LEU	CB	292LEU	CG	100.00
292LEU	CB	292LEU	CD1	100.00
292LEU	CB	292LEU	CD2	100.00
292LEU	CB	292LEU	C	100.00
292LEU	CG	292LEU	CD1	100.00
292LEU	CG	292LEU	CD2	100.00
292LEU	CG	292LEU	C	0.10
292LEU	CG	315VAL	CG1	0.02
292LEU	CD1	292LEU	CD2	100.00
292LEU	CD1	292LEU	C	0.01
292LEU	CD1	312GLU	CG	0.00
292LEU	CD1	315VAL	CG1	0.34
292LEU	CD1	315VAL	CG2	0.02
292LEU	CD2	292LEU	C	0.00
292LEU	CD2	295ALA	CB	0.03
292LEU	CD2	311VAL	CG1	0.00
292LEU	CD2	312GLU	CA	0.17
292LEU	CD2	312GLU	CB	0.07
292LEU	CD2	312GLU	CG	1.66
292LEU	CD2	312GLU	CD	0.03
292LEU	CD2	315VAL	CB	0.02
292LEU	CD2	315VAL	CG1	0.44
292LEU	CD2	315VAL	CG2	0.48

292LEU	C	293SER	CA	100.00
292LEU	C	293SER	C	100.00
293SER	CA	293SER	CB	100.00
293SER	CA	293SER	C	100.00
293SER	CB	293SER	C	100.00
293SER	C	294ALA	CA	100.00
293SER	C	294ALA	CB	0.02
293SER	C	294ALA	C	99.98
294ALA	CA	294ALA	CB	100.00
294ALA	CA	294ALA	C	100.00
294ALA	CA	297MET	CB	0.01
294ALA	CB	294ALA	C	100.00
294ALA	C	295ALA	CA	100.00
294ALA	C	295ALA	CB	0.06
294ALA	C	295ALA	C	99.95
294ALA	C	297MET	CB	0.01
295ALA	CA	295ALA	CB	100.00
295ALA	CA	295ALA	C	100.00
295ALA	CB	295ALA	C	100.00
295ALA	CB	308ALA	CA	0.00
295ALA	CB	311VAL	CB	0.32
295ALA	CB	311VAL	CG1	3.32
295ALA	CB	311VAL	CG2	0.21
295ALA	C	296MET	CA	100.00
295ALA	C	296MET	C	100.00
295ALA	C	308ALA	CB	0.01
296MET	CA	296MET	CB	100.00
296MET	CA	296MET	CG	100.00
296MET	CA	296MET	C	100.00
296MET	CA	299GLU	CB	0.00
296MET	CB	296MET	CG	100.00
296MET	CB	296MET	CE	2.20
296MET	CB	296MET	C	100.00
296MET	CG	296MET	CE	100.00
296MET	CG	296MET	C	94.54
296MET	CG	300HIS	CD2	0.12
296MET	CG	300HIS	CE1	0.00
296MET	CE	300HIS	CD2	0.00
296MET	CE	300HIS	CE1	0.03
296MET	C	297MET	CA	100.00
296MET	C	297MET	CB	0.01
296MET	C	297MET	C	100.00
296MET	C	299GLU	CB	0.00
297MET	CA	297MET	CB	100.00
297MET	CA	297MET	CG	100.00
297MET	CA	297MET	C	100.00
297MET	CB	297MET	CG	100.00
297MET	CB	297MET	CE	84.35
297MET	CB	297MET	C	100.00
297MET	CG	297MET	CE	100.00
297MET	CG	297MET	C	99.12
297MET	CG	301ALA	CB	0.13
297MET	CG	302PHE	CE1	0.16
297MET	CG	302PHE	CE2	0.16
297MET	CG	302PHE	CZ	0.30
297MET	CE	297MET	C	0.02
297MET	CE	298LEU	CD1	0.00
297MET	CE	298LEU	CD2	0.06
297MET	CE	302PHE	CD1	0.01
297MET	CE	302PHE	CD2	0.01
297MET	CE	302PHE	CE1	0.14
297MET	CE	302PHE	CE2	0.12

297ME	CE	302PHE	CZ	0.07
297ME	C	298LEU	CA	100.00
297ME	C	298LEU	CD1	0.00
297ME	C	298LEU	C	100.00
297ME	C	302PHE	CD1	0.00
297ME	C	302PHE	CD2	0.00
298LEU	CA	298LEU	CB	100.00
298LEU	CA	298LEU	CG	100.00
298LEU	CA	298LEU	CD1	11.26
298LEU	CA	298LEU	CD2	88.36
298LEU	CA	298LEU	C	100.00
298LEU	CA	302PHE	CB	0.02
298LEU	CA	302PHE	CD1	0.04
298LEU	CA	302PHE	CD2	0.02
298LEU	CB	298LEU	CG	100.00
298LEU	CB	298LEU	CD1	100.00
298LEU	CB	298LEU	CD2	100.00
298LEU	CB	298LEU	C	100.00
298LEU	CB	308ALA	CA	0.02
298LEU	CB	308ALA	CB	0.42
298LEU	CG	298LEU	CD1	100.00
298LEU	CG	298LEU	CD2	100.00
298LEU	CG	298LEU	C	7.84
298LEU	CG	304LEU	CD1	0.00
298LEU	CD1	298LEU	CD2	100.00
298LEU	CD1	298LEU	C	0.11
298LEU	CD1	302PHE	CB	0.03
298LEU	CD1	302PHE	CG	0.06
298LEU	CD1	302PHE	CD1	0.14
298LEU	CD1	302PHE	CD2	0.17
298LEU	CD1	302PHE	CE1	0.01
298LEU	CD1	302PHE	CE2	0.04
298LEU	CD1	304LEU	CB	0.01
298LEU	CD1	304LEU	CD1	0.04
298LEU	CD1	307LEU	CB	0.12
298LEU	CD1	307LEU	CG	0.01
298LEU	CD1	307LEU	CD2	0.07
298LEU	CD1	307LEU	C	0.12
298LEU	CD1	311VAL	CG2	0.70
298LEU	CD2	298LEU	C	0.42
298LEU	CD2	302PHE	CB	0.01
298LEU	CD2	302PHE	CG	0.11
298LEU	CD2	302PHE	CD1	1.04
298LEU	CD2	302PHE	CD2	0.60
298LEU	CD2	302PHE	CE1	0.17
298LEU	CD2	302PHE	CE2	0.13
298LEU	CD2	302PHE	CZ	0.01
298LEU	CD2	304LEU	CB	0.06
298LEU	CD2	304LEU	CD1	0.42
298LEU	CD2	304LEU	CD2	0.01
298LEU	CD2	307LEU	CB	0.37
298LEU	CD2	307LEU	CG	0.04
298LEU	CD2	307LEU	CD1	0.01
298LEU	CD2	307LEU	CD2	0.19
298LEU	CD2	307LEU	C	0.16
298LEU	CD2	308ALA	CA	0.01
298LEU	CD2	311VAL	CG2	0.01
298LEU	C	299GLU	CA	100.00
298LEU	C	299GLU	CB	0.08
298LEU	C	299GLU	C	99.99
298LEU	C	308ALA	CB	0.02
299GLU	CA	299GLU	CB	100.00

299GLU	CA	299GLU	CG	100.00
299GLU	CA	299GLU	CD	46.63
299GLU	CA	299GLU	C	100.00
299GLU	CA	305VAL	CG1	0.00
299GLU	CA	308ALA	CB	0.14
299GLU	CB	299GLU	CG	100.00
299GLU	CB	299GLU	CD	100.00
299GLU	CB	299GLU	C	100.00
299GLU	CB	308ALA	CB	2.91
299GLU	CG	299GLU	CD	100.00
299GLU	CG	299GLU	C	96.05
299GLU	CG	300HIS	CD2	0.05
299GLU	CG	305VAL	CG1	0.38
299GLU	CG	305VAL	CG2	0.04
299GLU	CG	308ALA	CB	0.68
299GLU	CD	299GLU	C	32.17
299GLU	CD	300HIS	CD2	0.03
299GLU	CD	300HIS	CE1	0.02
299GLU	CD	305VAL	CG1	0.09
299GLU	CD	308ALA	CB	0.00
299GLU	CD	309ARG	CZ	0.04
299GLU	C	300HIS	CA	100.00
299GLU	C	300HIS	CB	3.00
299GLU	C	300HIS	C	97.20
300HIS	CA	300HIS	CB	100.00
300HIS	CA	300HIS	CG	100.00
300HIS	CA	300HIS	CD2	34.95
300HIS	CA	300HIS	C	100.00
300HIS	CB	300HIS	CG	100.00
300HIS	CB	300HIS	CD2	100.00
300HIS	CB	300HIS	CE1	0.34
300HIS	CB	300HIS	C	100.00
300HIS	CG	300HIS	CD2	100.00
300HIS	CG	300HIS	CE1	100.00
300HIS	CG	300HIS	C	98.88
300HIS	CD2	300HIS	CE1	100.00
300HIS	CD2	300HIS	C	43.93
300HIS	CD2	301ALA	CA	0.06
300HIS	CD2	301ALA	CB	0.00
300HIS	C	301ALA	CA	100.00
300HIS	C	301ALA	CB	15.58
300HIS	C	301ALA	C	84.74
301ALA	CA	301ALA	CB	100.00
301ALA	CA	301ALA	C	100.00
301ALA	CB	301ALA	C	100.00
301ALA	CB	302PHE	CD1	0.11
301ALA	CB	302PHE	CD2	0.07
301ALA	CB	302PHE	CE1	0.02
301ALA	CB	302PHE	CE2	0.02
301ALA	C	302PHE	CA	100.00
301ALA	C	302PHE	CB	24.36
301ALA	C	302PHE	CG	1.34
301ALA	C	302PHE	CD1	0.79
301ALA	C	302PHE	CD2	0.34
301ALA	C	302PHE	C	76.31
302PHE	CA	302PHE	CB	100.00
302PHE	CA	302PHE	CG	100.00
302PHE	CA	302PHE	CD1	64.32
302PHE	CA	302PHE	CD2	65.27
302PHE	CA	302PHE	C	100.00
302PHE	CB	302PHE	CG	100.00
302PHE	CB	302PHE	CD1	100.00

302PHI	CB	302PHE	CD2	100.00
302PHI	CB	302PHE	C	100.00
302PHI	CB	304LEU	CG	0.13
302PHI	CB	304LEU	CD1	2.44
302PHI	CG	302PHE	CD1	100.00
302PHI	CG	302PHE	CD2	100.00
302PHI	CG	302PHE	CE1	100.00
302PHI	CG	302PHE	CE2	100.00
302PHI	CG	302PHE	CZ	100.00
302PHI	CG	302PHE	C	17.16
302PHI	CG	304LEU	CG	0.00
302PHI	CG	304LEU	CD1	1.17
302PHI	CG	304LEU	CD2	0.00
302PHI	CD1	302PHE	CD2	100.00
302PHI	CD1	302PHE	CE1	100.00
302PHI	CD1	302PHE	CE2	100.00
302PHI	CD1	302PHE	CZ	100.00
302PHI	CD1	302PHE	C	3.60
302PHI	CD1	304LEU	CB	0.00
302PHI	CD1	304LEU	CG	0.34
302PHI	CD1	304LEU	CD1	1.92
302PHI	CD1	304LEU	CD2	0.02
302PHI	CD1	307LEU	CD1	0.00
302PHI	CD2	302PHE	CE1	100.00
302PHI	CD2	302PHE	CE2	100.00
302PHI	CD2	302PHE	CZ	100.00
302PHI	CD2	302PHE	C	5.30
302PHI	CD2	304LEU	CG	0.52
302PHI	CD2	304LEU	CD1	3.01
302PHI	CD2	304LEU	CD2	0.10
302PHI	CD2	307LEU	CD1	0.00
302PHI	CE1	302PHE	CE2	100.00
302PHI	CE1	302PHE	CZ	100.00
302PHI	CE1	304LEU	CB	0.01
302PHI	CE1	304LEU	CG	0.01
302PHI	CE1	304LEU	CD1	0.68
302PHI	CE1	304LEU	CD2	0.02
302PHI	CE1	307LEU	CD1	0.00
302PHI	CE2	302PHE	CZ	100.00
302PHI	CE2	304LEU	CB	0.06
302PHI	CE2	304LEU	CG	0.04
302PHI	CE2	304LEU	CD1	0.95
302PHI	CE2	304LEU	CD2	0.08
302PHI	CE2	307LEU	CD1	0.02
302PHI	CE2	307LEU	CD2	0.02
302PHI	CZ	304LEU	CB	0.00
302PHI	CZ	304LEU	CD1	0.09
302PHI	CZ	304LEU	CD2	0.01
302PHI	C	303GLY	CA	100.00
302PHI	C	303GLY	C	71.20
302PHI	C	304LEU	CD1	0.01
303GL\	CA	303GLY	C	100.00
303GL\	C	304LEU	CA	100.00
303GL\	C	304LEU	CB	10.16
303GL\	C	304LEU	CG	1.57
303GL\	C	304LEU	CD1	0.22
303GL\	C	304LEU	CD2	0.08
303GL\	C	304LEU	C	93.52
303GL\	C	305VAL	CG1	0.01
303GL\	C	305VAL	CG2	0.01
304LEU	CA	304LEU	CB	100.00
304LEU	CA	304LEU	CG	100.00

304LEU	CA	304LEU	CD1	5.68
304LEU	CA	304LEU	CD2	93.54
304LEU	CA	304LEU	C	100.00
304LEU	CB	304LEU	CG	100.00
304LEU	CB	304LEU	CD1	100.00
304LEU	CB	304LEU	CD2	100.00
304LEU	CB	304LEU	C	100.00
304LEU	CB	307LEU	CB	0.03
304LEU	CB	307LEU	CD1	0.17
304LEU	CB	307LEU	CD2	0.00
304LEU	CG	304LEU	CD1	100.00
304LEU	CG	304LEU	CD2	100.00
304LEU	CG	304LEU	C	0.54
304LEU	CD1	304LEU	CD2	100.00
304LEU	CD1	304LEU	C	0.01
304LEU	CD1	307LEU	CD1	0.54
304LEU	CD1	307LEU	CD2	0.36
304LEU	CD2	304LEU	C	0.05
304LEU	CD2	307LEU	CB	0.03
304LEU	CD2	307LEU	CD1	0.18
304LEU	CD2	307LEU	CD2	0.10
304LEU	C	305VAL	CA	100.00
304LEU	C	305VAL	CB	0.02
304LEU	C	305VAL	CG2	0.00
304LEU	C	305VAL	C	99.99
305VAL	CA	305VAL	CB	100.00
305VAL	CA	305VAL	CG1	100.00
305VAL	CA	305VAL	CG2	100.00
305VAL	CA	305VAL	C	100.00
305VAL	CA	308ALA	CB	0.01
305VAL	CB	305VAL	CG1	100.00
305VAL	CB	305VAL	CG2	100.00
305VAL	CB	305VAL	C	100.00
305VAL	CB	309ARG	CZ	0.18
305VAL	CG1	305VAL	CG2	100.00
305VAL	CG1	305VAL	C	49.95
305VAL	CG1	309ARG	CD	0.02
305VAL	CG1	309ARG	CZ	3.95
305VAL	CG2	305VAL	C	50.04
305VAL	CG2	306GLU	CD	0.00
305VAL	CG2	309ARG	CZ	0.30
305VAL	C	306GLU	CA	100.00
305VAL	C	306GLU	CB	0.16
305VAL	C	306GLU	C	99.96
305VAL	C	309ARG	CZ	0.09
306GLU	CA	306GLU	CB	100.00
306GLU	CA	306GLU	CG	100.00
306GLU	CA	306GLU	CD	96.40
306GLU	CA	306GLU	C	100.00
306GLU	CA	309ARG	CZ	0.02
306GLU	CB	306GLU	CG	100.00
306GLU	CB	306GLU	CD	100.00
306GLU	CB	306GLU	C	100.00
306GLU	CG	306GLU	CD	100.00
306GLU	CG	306GLU	C	86.89
306GLU	CD	306GLU	C	0.42
306GLU	CD	309ARG	CD	0.04
306GLU	CD	309ARG	CZ	0.05
306GLU	CD	310LYS	CE	0.00
306GLU	C	307LEU	CA	100.00
306GLU	C	307LEU	CB	0.21
306GLU	C	307LEU	CG	0.00

306GLU	C	307LEU	CD1	0.02
306GLU	C	307LEU	CD2	0.01
306GLU	C	307LEU	C	99.83
307LEU	CA	307LEU	CB	100.00
307LEU	CA	307LEU	CG	100.00
307LEU	CA	307LEU	CD1	49.49
307LEU	CA	307LEU	CD2	51.57
307LEU	CA	307LEU	C	100.00
307LEU	CA	310LYS	CB	0.00
307LEU	CB	307LEU	CG	100.00
307LEU	CB	307LEU	CD1	100.00
307LEU	CB	307LEU	CD2	100.00
307LEU	CB	307LEU	C	100.00
307LEU	CG	307LEU	CD1	100.00
307LEU	CG	307LEU	CD2	100.00
307LEU	CG	307LEU	C	35.07
307LEU	CD1	307LEU	CD2	100.00
307LEU	CD1	307LEU	C	0.41
307LEU	CD1	310LYS	CD	0.02
307LEU	CD1	311VAL	CG2	0.00
307LEU	CD1	344LEU	CD2	0.03
307LEU	CD2	307LEU	C	1.34
307LEU	CD2	310LYS	CD	0.02
307LEU	CD2	310LYS	CE	0.01
307LEU	CD2	311VAL	CG2	0.01
307LEU	C	308ALA	CA	100.00
307LEU	C	308ALA	CB	0.01
307LEU	C	308ALA	C	99.99
307LEU	C	310LYS	CB	0.00
308ALA	CA	308ALA	CB	100.00
308ALA	CA	308ALA	C	100.00
308ALA	CB	308ALA	C	100.00
308ALA	C	309ARG	CA	100.00
308ALA	C	309ARG	CB	0.02
308ALA	C	309ARG	C	100.00
309ARG	CA	309ARG	CB	100.00
309ARG	CA	309ARG	CG	100.00
309ARG	CA	309ARG	CD	9.28
309ARG	CA	309ARG	C	100.00
309ARG	CA	312GLU	CB	0.01
309ARG	CB	309ARG	CG	100.00
309ARG	CB	309ARG	CD	100.00
309ARG	CB	309ARG	CZ	4.49
309ARG	CB	309ARG	C	100.00
309ARG	CG	309ARG	CD	100.00
309ARG	CG	309ARG	CZ	42.10
309ARG	CG	309ARG	C	39.59
309ARG	CD	309ARG	CZ	100.00
309ARG	CD	309ARG	C	0.18
309ARG	CD	312GLU	CD	0.02
309ARG	CZ	309ARG	C	0.00
309ARG	CZ	310LYS	CG	0.02
309ARG	C	310LYS	CA	100.00
309ARG	C	310LYS	CB	0.04
309ARG	C	310LYS	C	99.97
309ARG	C	312GLU	CB	0.00
310LYS	CA	310LYS	CB	100.00
310LYS	CA	310LYS	CG	100.00
310LYS	CA	310LYS	CD	2.81
310LYS	CA	310LYS	CE	0.01
310LYS	CA	310LYS	C	100.00
310LYS	CB	310LYS	CG	100.00

310LYS	CB	310LYS	CD	100.00
310LYS	CB	310LYS	CE	5.99
310LYS	CB	310LYS	C	100.00
310LYS	CB	343HIS	CE1	0.00
310LYS	CB	344LEU	CD1	0.03
310LYS	CB	344LEU	CD2	0.22
310LYS	CG	310LYS	CD	100.00
310LYS	CG	310LYS	CE	100.00
310LYS	CG	310LYS	C	83.45
310LYS	CG	343HIS	CD2	0.24
310LYS	CG	343HIS	CE1	1.32
310LYS	CG	344LEU	CD1	0.02
310LYS	CG	344LEU	CD2	1.20
310LYS	CD	310LYS	CE	100.00
310LYS	CD	310LYS	C	0.02
310LYS	CD	344LEU	CA	0.00
310LYS	CD	344LEU	CD1	0.01
310LYS	CD	344LEU	CD2	0.42
310LYS	CD	344LEU	C	0.02
310LYS	CE	343HIS	CD2	0.02
310LYS	CE	343HIS	CE1	0.09
310LYS	CE	344LEU	CD2	0.07
310LYS	CE	344LEU	C	0.04
310LYS	CE	345ALA	C	0.04
310LYS	C	311VAL	CA	100.00
310LYS	C	311VAL	CB	0.02
310LYS	C	311VAL	CG2	0.01
310LYS	C	311VAL	C	100.00
310LYS	C	344LEU	CD1	0.04
310LYS	C	344LEU	CD2	0.42
311VAL	CA	311VAL	CB	100.00
311VAL	CA	311VAL	CG1	100.00
311VAL	CA	311VAL	CG2	100.00
311VAL	CA	311VAL	C	100.00
311VAL	CA	314ALA	CB	0.00
311VAL	CA	344LEU	CD1	0.01
311VAL	CA	344LEU	CD2	0.01
311VAL	CB	311VAL	CG1	100.00
311VAL	CB	311VAL	CG2	100.00
311VAL	CB	311VAL	C	100.00
311VAL	CG1	311VAL	CG2	100.00
311VAL	CG1	311VAL	C	99.72
311VAL	CG1	340VAL	CG1	0.00
311VAL	CG1	344LEU	CD2	0.01
311VAL	CG2	311VAL	C	0.27
311VAL	CG2	344LEU	CD1	0.25
311VAL	CG2	344LEU	CD2	0.75
311VAL	C	312GLU	CA	100.00
311VAL	C	312GLU	CB	0.00
311VAL	C	312GLU	C	100.00
312GLU	CA	312GLU	CB	100.00
312GLU	CA	312GLU	CG	100.00
312GLU	CA	312GLU	CD	0.71
312GLU	CA	312GLU	C	100.00
312GLU	CA	315VAL	CG1	0.00
312GLU	CA	315VAL	CG2	0.00
312GLU	CB	312GLU	CG	100.00
312GLU	CB	312GLU	CD	100.00
312GLU	CB	312GLU	C	100.00
312GLU	CG	312GLU	CD	100.00
312GLU	CG	312GLU	C	94.36
312GLU	CD	312GLU	C	0.08

312GLU	C	313ASP	CA	100.00
312GLU	C	313ASP	CB	0.03
312GLU	C	313ASP	C	99.98
313ASP	CA	313ASP	CB	100.00
313ASP	CA	313ASP	CG	100.00
313ASP	CA	313ASP	C	100.00
313ASP	CA	316ALA	CB	0.00
313ASP	CB	313ASP	CG	100.00
313ASP	CB	313ASP	C	100.00
313ASP	CB	343HIS	CE1	0.01
313ASP	CG	313ASP	C	99.98
313ASP	CG	317LYS	CG	0.00
313ASP	CG	317LYS	CD	0.05
313ASP	CG	317LYS	CE	0.60
313ASP	CG	343HIS	CD2	0.00
313ASP	CG	343HIS	CE1	0.04
313ASP	C	314ALA	CA	100.00
313ASP	C	314ALA	C	100.00
313ASP	C	343HIS	CD2	0.03
313ASP	C	343HIS	CE1	0.00
314ALA	CA	314ALA	CB	100.00
314ALA	CA	314ALA	C	100.00
314ALA	CA	343HIS	CB	0.00
314ALA	CA	343HIS	CG	0.64
314ALA	CA	343HIS	CD2	13.85
314ALA	CA	343HIS	CE1	0.21
314ALA	CB	314ALA	C	100.00
314ALA	CB	340VAL	CG1	2.02
314ALA	CB	340VAL	CG2	0.03
314ALA	CB	343HIS	CB	4.34
314ALA	CB	343HIS	CG	5.66
314ALA	CB	343HIS	CD2	5.14
314ALA	CB	343HIS	CE1	0.19
314ALA	CB	343HIS	C	0.00
314ALA	CB	344LEU	CG	0.08
314ALA	CB	344LEU	CD1	0.42
314ALA	CB	344LEU	CD2	0.28
314ALA	C	315VAL	CA	100.00
314ALA	C	315VAL	CB	0.02
314ALA	C	315VAL	C	100.00
314ALA	C	340VAL	CG2	0.04
315VAL	CA	315VAL	CB	100.00
315VAL	CA	315VAL	CG1	100.00
315VAL	CA	315VAL	CG2	100.00
315VAL	CA	315VAL	C	100.00
315VAL	CA	318ALA	CB	0.01
315VAL	CB	315VAL	CG1	100.00
315VAL	CB	315VAL	CG2	100.00
315VAL	CB	315VAL	C	100.00
315VAL	CB	340VAL	CG2	0.00
315VAL	CG1	315VAL	CG2	100.00
315VAL	CG1	315VAL	C	93.36
315VAL	CG1	319LEU	CD1	0.94
315VAL	CG1	336PHE	CZ	0.00
315VAL	CG1	340VAL	CG1	0.02
315VAL	CG1	340VAL	CG2	0.02
315VAL	CG2	315VAL	C	8.66
315VAL	CG2	336PHE	CE2	0.01
315VAL	CG2	336PHE	CZ	0.00
315VAL	CG2	340VAL	CG1	0.06
315VAL	CG2	340VAL	CG2	0.44
315VAL	C	316ALA	CA	100.00

315VAI	C	316ALA	C	100.00
315VAI	C	318ALA	CB	0.00
316ALA	CA	316ALA	CB	100.00
316ALA	CA	316ALA	C	100.00
316ALA	CB	316ALA	C	100.00
316ALA	C	317LYS	CA	100.00
316ALA	C	317LYS	CB	0.42
316ALA	C	317LYS	CG	0.05
316ALA	C	317LYS	C	99.51
316ALA	C	320LEU	CD1	0.01
317LYS	CA	317LYS	CB	100.00
317LYS	CA	317LYS	CG	100.00
317LYS	CA	317LYS	CD	26.95
317LYS	CA	317LYS	CE	0.48
317LYS	CA	317LYS	C	100.00
317LYS	CA	320LEU	CB	0.00
317LYS	CA	320LEU	CD1	0.01
317LYS	CB	317LYS	CG	100.00
317LYS	CB	317LYS	CD	100.00
317LYS	CB	317LYS	CE	2.08
317LYS	CB	317LYS	C	100.00
317LYS	CB	343HIS	CG	0.00
317LYS	CB	343HIS	CD2	0.06
317LYS	CB	343HIS	CE1	0.04
317LYS	CG	317LYS	CD	100.00
317LYS	CG	317LYS	CE	100.00
317LYS	CG	317LYS	C	1.51
317LYS	CG	343HIS	CD2	0.01
317LYS	CG	343HIS	CE1	0.56
317LYS	CD	317LYS	CE	100.00
317LYS	CD	317LYS	C	0.00
317LYS	CD	343HIS	CG	0.06
317LYS	CD	343HIS	CD2	0.83
317LYS	CD	343HIS	CE1	6.00
317LYS	CE	343HIS	CG	0.00
317LYS	CE	343HIS	CD2	0.01
317LYS	CE	343HIS	CE1	2.00
317LYS	C	318ALA	CA	100.00
317LYS	C	318ALA	CB	0.17
317LYS	C	318ALA	C	99.78
317LYS	C	320LEU	CB	0.00
317LYS	C	343HIS	CD2	0.02
318ALA	CA	318ALA	CB	100.00
318ALA	CA	318ALA	C	100.00
318ALA	CA	339THR	CG2	0.70
318ALA	CA	343HIS	CD2	0.00
318ALA	CB	318ALA	C	100.00
318ALA	CB	336PHE	CD1	0.10
318ALA	CB	336PHE	CD2	0.28
318ALA	CB	336PHE	CE1	14.00
318ALA	CB	336PHE	CE2	1.74
318ALA	CB	336PHE	CZ	16.96
318ALA	CB	339THR	CB	0.08
318ALA	CB	339THR	CG2	3.08
318ALA	CB	339THR	C	0.01
318ALA	CB	340VAL	CG2	4.92
318ALA	C	319LEU	CA	100.00
318ALA	C	319LEU	C	100.00
318ALA	C	336PHE	CE1	3.63
318ALA	C	336PHE	CE2	0.13
318ALA	C	336PHE	CZ	0.24
319LEU	CA	319LEU	CB	100.00

319LEU	CA	319LEU	CG	100.00
319LEU	CA	319LEU	CD1	3.91
319LEU	CA	319LEU	CD2	96.59
319LEU	CA	319LEU	C	100.00
319LEU	CA	336PHE	CZ	0.00
319LEU	CB	319LEU	CG	100.00
319LEU	CB	319LEU	CD1	100.00
319LEU	CB	319LEU	CD2	100.00
319LEU	CB	319LEU	C	100.00
319LEU	CG	319LEU	CD1	100.00
319LEU	CG	319LEU	CD2	100.00
319LEU	CG	319LEU	C	2.64
319LEU	CG	336PHE	CE1	0.01
319LEU	CG	336PHE	CE2	0.00
319LEU	CD1	319LEU	CD2	100.00
319LEU	CD1	319LEU	C	0.12
319LEU	CD1	324PRO	CD	0.16
319LEU	CD1	336PHE	CE1	0.02
319LEU	CD1	336PHE	CE2	0.01
319LEU	CD1	336PHE	CZ	0.02
319LEU	CD2	319LEU	C	0.10
319LEU	CD2	324PRO	CB	0.07
319LEU	CD2	324PRO	CG	0.44
319LEU	CD2	324PRO	CD	2.38
319LEU	CD2	327LEU	CD1	0.06
319LEU	CD2	327LEU	CD2	0.07
319LEU	CD2	336PHE	CE1	0.71
319LEU	CD2	336PHE	CE2	0.82
319LEU	CD2	336PHE	CZ	0.91
319LEU	C	320LEU	CA	100.00
319LEU	C	320LEU	CB	1.21
319LEU	C	320LEU	CG	0.04
319LEU	C	320LEU	CD1	0.07
319LEU	C	320LEU	CD2	0.00
319LEU	C	320LEU	C	98.24
320LEU	CA	320LEU	CB	100.00
320LEU	CA	320LEU	CG	100.00
320LEU	CA	320LEU	CD1	23.03
320LEU	CA	320LEU	CD2	75.26
320LEU	CA	320LEU	C	100.00
320LEU	CB	320LEU	CG	100.00
320LEU	CB	320LEU	CD1	100.00
320LEU	CB	320LEU	CD2	100.00
320LEU	CB	320LEU	C	100.00
320LEU	CG	320LEU	CD1	100.00
320LEU	CG	320LEU	CD2	100.00
320LEU	CG	320LEU	C	24.16
320LEU	CD1	320LEU	CD2	100.00
320LEU	CD1	320LEU	C	0.56
320LEU	CD2	320LEU	C	1.44
320LEU	C	321GLU	CA	100.00
320LEU	C	321GLU	CB	4.81
320LEU	C	321GLU	C	95.84
321GLU	CA	321GLU	CB	100.00
321GLU	CA	321GLU	CG	100.00
321GLU	CA	321GLU	CD	74.57
321GLU	CA	321GLU	C	100.00
321GLU	CB	321GLU	CG	100.00
321GLU	CB	321GLU	CD	100.00
321GLU	CB	321GLU	C	100.00
321GLU	CB	339THR	CG2	0.13
321GLU	CG	321GLU	CD	100.00

321GLU	CG	321GLU	C	98.26
321GLU	CG	322THR	CG2	0.00
321GLU	CG	339THR	CG2	1.32
321GLU	CD	321GLU	C	0.13
321GLU	CD	339THR	CG2	0.82
321GLU	CD	342ARG	CZ	0.20
321GLU	C	322THR	CA	100.00
321GLU	C	322THR	CB	95.14
321GLU	C	322THR	CG2	45.66
321GLU	C	322THR	C	9.64
321GLU	C	323PRO	CD	2.41
322THR	CA	322THR	CB	100.00
322THR	CA	322THR	CG2	100.00
322THR	CA	322THR	C	100.00
322THR	CA	323PRO	CD	100.00
322THR	CA	331ALA	CB	0.06
322THR	CB	322THR	CG2	100.00
322THR	CB	322THR	C	100.00
322THR	CB	323PRO	CD	0.72
322THR	CB	331ALA	CB	0.29
322THR	CB	336PHE	CD1	0.00
322THR	CB	336PHE	CE2	0.00
322THR	CB	336PHE	CZ	0.01
322THR	CG2	322THR	C	19.86
322THR	CG2	323PRO	CD	0.00
322THR	CG2	329GLY	C	0.01
322THR	CG2	330SER	CB	0.00
322THR	CG2	331ALA	CB	0.14
322THR	CG2	335ALA	CB	0.20
322THR	CG2	335ALA	C	0.00
322THR	CG2	336PHE	CG	0.08
322THR	CG2	336PHE	CD1	0.18
322THR	CG2	336PHE	CD2	0.39
322THR	CG2	336PHE	CE1	0.63
322THR	CG2	336PHE	CE2	1.17
322THR	CG2	336PHE	CZ	0.94
322THR	CG2	339THR	CG2	0.04
322THR	C	323PRO	CA	100.00
322THR	C	323PRO	CB	0.13
322THR	C	323PRO	CD	100.00
322THR	C	323PRO	C	100.00
322THR	C	331ALA	CB	0.72
323PRO	CA	323PRO	CB	100.00
323PRO	CA	323PRO	CG	100.00
323PRO	CA	323PRO	CD	100.00
323PRO	CA	323PRO	C	100.00
323PRO	CA	324PRO	CD	100.00
323PRO	CB	323PRO	CG	100.00
323PRO	CB	323PRO	CD	100.00
323PRO	CB	323PRO	C	100.00
323PRO	CB	324PRO	CD	57.55
323PRO	CB	327LEU	CB	0.06
323PRO	CB	327LEU	CD1	0.03
323PRO	CB	327LEU	CD2	0.35
323PRO	CB	327LEU	C	0.01
323PRO	CB	329GLY	CA	0.00
323PRO	CB	329GLY	C	0.00
323PRO	CG	323PRO	CD	100.00
323PRO	CG	323PRO	C	85.92
323PRO	CG	327LEU	CD1	0.00
323PRO	CG	327LEU	CD2	0.00
323PRO	CG	328GLY	C	0.01

323PR(	CG	329GLY	CA	0.21
323PR(	CG	329GLY	C	1.78
323PR(	CD	323PRO	C	82.06
323PR(	CD	329GLY	C	0.00
323PR(	C	324PRO	CA	100.00
323PR(	C	324PRO	CB	0.14
323PR(	C	324PRO	CD	100.00
323PR(	C	324PRO	C	100.00
323PR(	C	331ALA	CB	0.10
323PR(	C	336PHE	CZ	0.02
324PR(	CA	324PRO	CB	100.00
324PR(	CA	324PRO	CG	100.00
324PR(	CA	324PRO	CD	100.00
324PR(	CA	324PRO	C	100.00
324PR(	CA	325PRO	CD	100.00
324PR(	CA	331ALA	CB	0.03
324PR(	CA	336PHE	CE1	0.00
324PR(	CA	336PHE	CZ	0.02
324PR(	CB	324PRO	CG	100.00
324PR(	CB	324PRO	CD	100.00
324PR(	CB	324PRO	C	100.00
324PR(	CB	325PRO	CD	38.95
324PR(	CB	327LEU	CD1	0.02
324PR(	CB	327LEU	CD2	0.01
324PR(	CB	331ALA	CB	0.00
324PR(	CB	336PHE	CZ	0.00
324PR(	CG	324PRO	CD	100.00
324PR(	CG	324PRO	C	81.21
324PR(	CG	327LEU	CB	0.01
324PR(	CG	327LEU	CG	0.00
324PR(	CG	327LEU	CD1	0.38
324PR(	CG	327LEU	CD2	0.04
324PR(	CD	324PRO	C	72.98
324PR(	CD	327LEU	CB	0.00
324PR(	CD	327LEU	CD1	0.11
324PR(	CD	327LEU	CD2	0.02
324PR(	CD	336PHE	CZ	0.00
324PR(	C	325PRO	CA	100.00
324PR(	C	325PRO	CB	0.06
324PR(	C	325PRO	CD	100.00
324PR(	C	325PRO	C	100.00
324PR(	C	329GLY	C	0.01
325PR(	CA	325PRO	CB	100.00
325PR(	CA	325PRO	CG	100.00
325PR(	CA	325PRO	CD	100.00
325PR(	CA	325PRO	C	100.00
325PR(	CA	328GLY	CA	0.02
325PR(	CA	328GLY	C	0.01
325PR(	CA	329GLY	C	0.14
325PR(	CA	330SER	CB	0.08
325PR(	CB	325PRO	CG	100.00
325PR(	CB	325PRO	CD	100.00
325PR(	CB	325PRO	C	100.00
325PR(	CB	330SER	CB	0.14
325PR(	CG	325PRO	CD	100.00
325PR(	CG	325PRO	C	66.65
325PR(	CG	330SER	CB	0.12
325PR(	CG	330SER	C	0.01
325PR(	CG	331ALA	CA	0.00
325PR(	CG	331ALA	CB	0.08
325PR(	CG	333THR	CG2	0.10
325PR(	CG	336PHE	CZ	0.01

325PR(	CD	325PRO	C	46.85
325PR(	CD	330SER	CB	0.15
325PR(	CD	330SER	C	0.26
325PR(	CD	331ALA	CB	7.44
325PR(	CD	336PHE	CE1	0.01
325PR(	CD	336PHE	CZ	0.04
325PR(	C	326ASP	CA	100.00
325PR(	C	326ASP	CB	0.34
325PR(	C	326ASP	C	99.73
325PR(	C	328GLY	CA	0.00
326ASI	CA	326ASP	CB	100.00
326ASI	CA	326ASP	CG	100.00
326ASI	CA	326ASP	C	100.00
326ASI	CB	326ASP	CG	100.00
326ASI	CB	326ASP	C	100.00
326ASI	CG	326ASP	C	96.99
326ASI	CG	327LEU	CD1	0.01
326ASI	C	327LEU	CA	100.00
326ASI	C	327LEU	CB	3.80
326ASI	C	327LEU	CG	0.30
326ASI	C	327LEU	CD1	0.15
326ASI	C	327LEU	CD2	0.08
326ASI	C	327LEU	C	94.99
327LEU	CA	327LEU	CB	100.00
327LEU	CA	327LEU	CG	100.00
327LEU	CA	327LEU	CD1	33.50
327LEU	CA	327LEU	CD2	65.96
327LEU	CA	327LEU	C	100.00
327LEU	CB	327LEU	CG	100.00
327LEU	CB	327LEU	CD1	100.00
327LEU	CB	327LEU	CD2	100.00
327LEU	CB	327LEU	C	100.00
327LEU	CG	327LEU	CD1	100.00
327LEU	CG	327LEU	CD2	100.00
327LEU	CG	327LEU	C	28.67
327LEU	CD1	327LEU	CD2	100.00
327LEU	CD1	327LEU	C	0.99
327LEU	CD2	327LEU	C	3.47
327LEU	C	328GLY	CA	100.00
327LEU	C	328GLY	C	44.35
328GL\	CA	328GLY	C	100.00
328GL\	C	329GLY	CA	100.00
328GL\	C	329GLY	C	36.14
329GL\	CA	329GLY	C	100.00
329GL\	C	330SER	CA	100.00
329GL\	C	330SER	CB	67.37
329GL\	C	330SER	C	33.21
330SEI	CA	330SER	CB	100.00
330SEI	CA	330SER	C	100.00
330SEI	CB	330SER	C	100.00
330SEI	CB	336PHE	CE1	0.01
330SEI	CB	336PHE	CE2	0.01
330SEI	CB	336PHE	CZ	0.05
330SEI	C	331ALA	C	100.00
330SEI	C	331ALA	CB	59.32
330SEI	C	331ALA	C	41.08
331AL/	CA	331ALA	CB	100.00
331AL/	CA	331ALA	C	100.00
331AL/	CA	336PHE	CD1	0.00
331AL/	CB	331ALA	C	100.00
331AL/	CB	335ALA	CB	4.65
331AL/	CB	336PHE	CG	0.02

331ALA	CB	336PHE	CD1	0.88
331ALA	CB	336PHE	CE1	0.02
331ALA	C	332GLY	CA	100.00
331ALA	C	332GLY	C	49.88
331ALA	C	335ALA	CB	0.37
332GLY	CA	332GLY	C	100.00
332GLY	CA	335ALA	CB	0.01
332GLY	C	333THR	CA	100.00
332GLY	C	333THR	CB	2.01
332GLY	C	333THR	CG2	1.07
332GLY	C	333THR	C	98.21
332GLY	C	336PHE	CD1	0.04
333THR	CA	333THR	CB	100.00
333THR	CA	333THR	CG2	100.00
333THR	CA	333THR	C	100.00
333THR	CA	336PHE	CD1	0.01
333THR	CB	333THR	CG2	100.00
333THR	CB	333THR	C	100.00
333THR	CG2	333THR	C	9.09
333THR	C	334GLU	CA	100.00
333THR	C	334GLU	CB	11.59
333THR	C	334GLU	C	90.84
334GLU	CA	334GLU	CB	100.00
334GLU	CA	334GLU	CG	100.00
334GLU	CA	334GLU	CD	97.63
334GLU	CA	334GLU	C	100.00
334GLU	CB	334GLU	CG	100.00
334GLU	CB	334GLU	CD	100.00
334GLU	CB	334GLU	C	100.00
334GLU	CG	334GLU	CD	100.00
334GLU	CG	334GLU	C	96.25
334GLU	CD	334GLU	C	0.78
334GLU	CD	338ALA	CB	0.00
334GLU	C	335ALA	CA	100.00
334GLU	C	335ALA	CB	0.89
334GLU	C	335ALA	C	99.10
335ALA	CA	335ALA	CB	100.00
335ALA	CA	335ALA	C	100.00
335ALA	CB	335ALA	C	100.00
335ALA	C	336PHE	CA	100.00
335ALA	C	336PHE	CB	0.41
335ALA	C	336PHE	CG	0.10
335ALA	C	336PHE	CD1	0.01
335ALA	C	336PHE	CD2	0.02
335ALA	C	336PHE	C	99.51
336PHE	CA	336PHE	C	100.00
336PHE	CA	336PHE	CG	100.00
336PHE	CA	336PHE	CD1	74.88
336PHE	CA	336PHE	CD2	83.15
336PHE	CA	336PHE	C	100.00
336PHE	CB	336PHE	CG	100.00
336PHE	CB	336PHE	CD1	100.00
336PHE	CB	336PHE	CD2	100.00
336PHE	CB	336PHE	C	100.00
336PHE	CG	336PHE	CD1	100.00
336PHE	CG	336PHE	CD2	100.00
336PHE	CG	336PHE	CE1	100.00
336PHE	CG	336PHE	CE2	100.00
336PHE	CG	336PHE	CZ	100.00
336PHE	CG	336PHE	C	78.26
336PHE	CD1	336PHE	CD2	100.00
336PHE	CD1	336PHE	CE1	100.00

336PHI	CD1	336PHE	CE2	100.00
336PHI	CD1	336PHE	CZ	100.00
336PHI	CD1	336PHE	C	0.05
336PHI	CD2	336PHE	CE1	100.00
336PHI	CD2	336PHE	CE2	100.00
336PHI	CD2	336PHE	CZ	100.00
336PHI	CD2	336PHE	C	59.77
336PHI	CD2	340VAL	CG2	1.10
336PHI	CE1	336PHE	CE2	100.00
336PHI	CE1	336PHE	CZ	100.00
336PHI	CE1	340VAL	CG2	0.00
336PHI	CE2	336PHE	CZ	100.00
336PHI	CE2	340VAL	CG2	7.40
336PHI	CZ	340VAL	CG2	0.06
336PHI	C	337THR	CA	100.00
336PHI	C	337THR	CB	6.54
336PHI	C	337THR	CG2	2.22
336PHI	C	337THR	C	94.88
336PHI	C	340VAL	CG2	0.11
337THI	CA	337THR	CB	100.00
337THI	CA	337THR	CG2	100.00
337THI	CA	337THR	C	100.00
337THI	CB	337THR	CG2	100.00
337THI	CB	337THR	C	100.00
337THI	CB	341LEU	CD1	0.00
337THI	CG2	337THR	C	61.66
337THI	CG2	341LEU	CD1	0.14
337THI	CG2	341LEU	CD2	0.00
337THI	C	338ALA	CA	100.00
337THI	C	338ALA	CB	0.01
337THI	C	338ALA	C	100.00
337THI	C	341LEU	CD1	0.08
337THI	C	341LEU	CD2	0.00
338ALA	CA	338ALA	CB	100.00
338ALA	CA	338ALA	C	100.00
338ALA	CA	341LEU	CG	0.00
338ALA	CA	341LEU	CD1	0.00
338ALA	CB	338ALA	C	100.00
338ALA	CB	342ARG	CZ	0.10
338ALA	C	339THR	CA	100.00
338ALA	C	339THR	CB	0.71
338ALA	C	339THR	CG2	0.12
338ALA	C	339THR	C	99.53
339THI	CA	339THR	CB	100.00
339THI	CA	339THR	CG2	100.00
339THI	CA	339THR	C	100.00
339THI	CA	342ARG	CZ	0.04
339THI	CB	339THR	CG2	100.00
339THI	CB	339THR	C	100.00
339THI	CB	342ARG	CZ	0.01
339THI	CG2	339THR	C	61.91
339THI	CG2	342ARG	CZ	1.66
339THI	CG2	343HIS	CD2	0.00
339THI	C	340VAL	CA	100.00
339THI	C	340VAL	CB	0.20
339THI	C	340VAL	CG2	0.03
339THI	C	340VAL	C	99.94
339THI	C	342ARG	CB	0.01
340VAI	CA	340VAL	CB	100.00
340VAI	CA	340VAL	CG1	100.00
340VAI	CA	340VAL	CG2	100.00
340VAI	CA	340VAL	C	100.00

340VAI	CB	340VAL	CG1	100.00
340VAI	CB	340VAL	CG2	100.00
340VAI	CB	340VAL	C	100.00
340VAI	CG1	340VAL	CG2	100.00
340VAI	CG1	340VAL	C	100.00
340VAI	CG1	344LEU	CD1	2.33
340VAI	CG1	344LEU	CD2	0.00
340VAI	CG2	340VAL	C	0.01
340VAI	C	341LEU	CA	100.00
340VAI	C	341LEU	CB	0.04
340VAI	C	341LEU	C	99.98
340VAI	C	344LEU	CD2	0.00
341LEU	CA	341LEU	CB	100.00
341LEU	CA	341LEU	CG	100.00
341LEU	CA	341LEU	CD1	9.80
341LEU	CA	341LEU	CD2	88.74
341LEU	CA	341LEU	C	100.00
341LEU	CA	344LEU	CD1	0.01
341LEU	CB	341LEU	CG	100.00
341LEU	CB	341LEU	CD1	100.00
341LEU	CB	341LEU	CD2	100.00
341LEU	CB	341LEU	C	100.00
341LEU	CG	341LEU	CD1	100.00
341LEU	CG	341LEU	CD2	100.00
341LEU	CG	341LEU	C	7.81
341LEU	CD1	341LEU	CD2	100.00
341LEU	CD1	341LEU	C	5.04
341LEU	CD1	345ALA	CB	0.02
341LEU	CD2	341LEU	C	0.20
341LEU	CD2	344LEU	CD1	0.05
341LEU	CD2	344LEU	CD2	0.02
341LEU	CD2	345ALA	CB	0.02
341LEU	C	342ARG	CA	100.00
341LEU	C	342ARG	CB	1.39
341LEU	C	342ARG	CG	0.09
341LEU	C	342ARG	C	98.60
341LEU	C	345ALA	CB	0.00
342ARG	CA	342ARG	CB	100.00
342ARG	CA	342ARG	CG	100.00
342ARG	CA	342ARG	CD	1.50
342ARG	CA	342ARG	CZ	0.01
342ARG	CA	342ARG	C	100.00
342ARG	CB	342ARG	CG	100.00
342ARG	CB	342ARG	CD	100.00
342ARG	CB	342ARG	CZ	0.24
342ARG	CB	342ARG	C	100.00
342ARG	CG	342ARG	CD	100.00
342ARG	CG	342ARG	CZ	3.94
342ARG	CG	342ARG	C	86.81
342ARG	CD	342ARG	CZ	100.00
342ARG	CD	342ARG	C	0.62
342ARG	C	343HIS	CA	100.00
342ARG	C	343HIS	CB	4.09
342ARG	C	343HIS	CG	0.00
342ARG	C	343HIS	C	95.81
342ARG	C	345ALA	CB	0.02
343HIS	CA	343HIS	CB	100.00
343HIS	CA	343HIS	CG	100.00
343HIS	CA	343HIS	CD2	7.08
343HIS	CA	343HIS	C	100.00
343HIS	CB	343HIS	CG	100.00
343HIS	CB	343HIS	CD2	100.00

343HIS	CB	343HIS	CE1	0.32
343HIS	CB	343HIS	C	100.00
343HIS	CG	343HIS	CD2	100.00
343HIS	CG	343HIS	CE1	100.00
343HIS	CG	343HIS	C	99.36
343HIS	CD2	343HIS	CE1	100.00
343HIS	CD2	343HIS	C	6.49
343HIS	CD2	344LEU	CD1	0.01
343HIS	CD2	344LEU	CD2	0.01
343HIS	C	344LEU	CA	100.00
343HIS	C	344LEU	CB	8.61
343HIS	C	344LEU	CG	1.46
343HIS	C	344LEU	CD1	0.06
343HIS	C	344LEU	CD2	0.07
343HIS	C	344LEU	C	90.02
344LEU	CA	344LEU	CB	100.00
344LEU	CA	344LEU	CG	100.00
344LEU	CA	344LEU	CD1	6.91
344LEU	CA	344LEU	CD2	92.21
344LEU	CA	344LEU	C	100.00
344LEU	CB	344LEU	CG	100.00
344LEU	CB	344LEU	CD1	100.00
344LEU	CB	344LEU	CD2	100.00
344LEU	CB	344LEU	C	100.00
344LEU	CG	344LEU	CD1	100.00
344LEU	CG	344LEU	CD2	100.00
344LEU	CG	344LEU	C	4.60
344LEU	CD1	344LEU	CD2	100.00
344LEU	CD1	344LEU	C	1.34
344LEU	CD1	345ALA	CB	0.01
344LEU	CD2	344LEU	C	0.15
344LEU	C	345ALA	CA	100.00
344LEU	C	345ALA	CB	60.88
344LEU	C	345ALA	C	40.30
345ALA	C	345ALA	CB	100.00
345ALA	C	345ALA	C	100.00
345ALA	C	345ALA	C	100.00

4) *Mut* 337 K

Drnona	D	Arnona	A	percen
1MET	CA	1MET	CB	100.00
1MET	CA	1MET	CG	100.00
1MET	CA	1MET	CE	0.00
1MET	CA	1MET	C	100.00
1MET	CA	65GLU	CD	0.18
1MET	CA	302PHE	CE1	0.02
1MET	CA	302PHE	CE2	0.05
1MET	CA	302PHE	CZ	0.01
1MET	CB	1MET	CG	100.00
1MET	CB	1MET	CE	20.99
1MET	CB	1MET	C	100.00
1MET	CB	32LEU	CD1	0.00
1MET	CB	32LEU	CD2	0.02
1MET	CB	34LEU	CB	0.02
1MET	CB	34LEU	CG	0.00
1MET	CB	34LEU	CD1	0.00
1MET	CB	34LEU	CD2	0.08
1MET	CB	35ALA	CB	0.00
1MET	CB	65GLU	CG	0.00
1MET	CB	65GLU	CD	0.02
1MET	CB	302PHE	CG	0.00
1MET	CB	302PHE	CD1	0.16
1MET	CB	302PHE	CD2	0.10
1MET	CB	302PHE	CE1	4.01
1MET	CB	302PHE	CE2	1.92
1MET	CB	302PHE	CZ	2.42
1MET	CG	1MET	CE	100.00
1MET	CG	1MET	C	84.25
1MET	CG	3VAL	CG1	0.00
1MET	CG	3VAL	CG2	0.06
1MET	CG	32LEU	CD2	0.01
1MET	CG	33GLY	C	0.02
1MET	CG	34LEU	CB	0.01
1MET	CG	34LEU	CD1	0.02
1MET	CG	34LEU	CD2	0.17
1MET	CG	65GLU	CB	3.37
1MET	CG	65GLU	CG	0.28
1MET	CG	65GLU	CD	0.10
1MET	CG	302PHE	CG	0.04
1MET	CG	302PHE	CD1	0.42
1MET	CG	302PHE	CD2	0.47
1MET	CG	302PHE	CE1	1.44
1MET	CG	302PHE	CE2	1.20
1MET	CG	302PHE	CZ	1.04
1MET	CE	1MET	C	0.00
1MET	CE	3VAL	CB	0.00
1MET	CE	3VAL	CG1	0.11
1MET	CE	3VAL	CG2	1.63
1MET	CE	23LEU	CD1	0.00
1MET	CE	23LEU	CD2	0.02
1MET	CE	32LEU	CB	0.01
1MET	CE	32LEU	CG	0.00
1MET	CE	32LEU	CD1	0.06
1MET	CE	32LEU	CD2	0.03
1MET	CE	32LEU	C	0.02
1MET	CE	33GLY	CA	0.00
1MET	CE	33GLY	C	0.07
1MET	CE	34LEU	CA	0.00
1MET	CE	34LEU	CB	0.00

1MET	CE	34LEU	CG	0.00
1MET	CE	34LEU	CD1	0.11
1MET	CE	34LEU	CD2	0.22
1MET	CE	65GLU	CB	0.23
1MET	CE	65GLU	CG	0.53
1MET	CE	65GLU	CD	0.03
1MET	CE	65GLU	C	0.04
1MET	CE	66ALA	CB	1.72
1MET	CE	297MET	CB	0.00
1MET	CE	297MET	CG	0.17
1MET	CE	297MET	CE	0.20
1MET	CE	298LEU	CD1	0.03
1MET	CE	298LEU	CD2	0.04
1MET	CE	301ALA	CB	2.48
1MET	CE	301ALA	C	0.46
1MET	CE	302PHE	CA	0.03
1MET	CE	302PHE	CB	0.08
1MET	CE	302PHE	CG	0.62
1MET	CE	302PHE	CD1	1.97
1MET	CE	302PHE	CD2	2.21
1MET	CE	302PHE	CE1	2.51
1MET	CE	302PHE	CE2	3.76
1MET	CE	302PHE	CZ	1.98
1MET	CE	304LEU	CD1	0.15
1MET	C	2LYS	CA	100.00
1MET	C	2LYS	CB	22.28
1MET	C	2LYS	CG	0.56
1MET	C	2LYS	CD	0.00
1MET	C	2LYS	C	80.43
1MET	C	34LEU	CD1	0.00
1MET	C	34LEU	CD2	0.01
1MET	C	302PHE	CE1	0.00
1MET	C	302PHE	CE2	0.01
1MET	C	302PHE	CZ	0.06
2LYS	CA	2LYS	CB	100.00
2LYS	CA	2LYS	CG	100.00
2LYS	CA	2LYS	CD	2.50
2LYS	CA	2LYS	C	100.00
2LYS	CA	35ALA	CB	0.01
2LYS	CB	2LYS	CG	100.00
2LYS	CB	2LYS	CD	100.00
2LYS	CB	2LYS	CE	21.00
2LYS	CB	2LYS	C	100.00
2LYS	CB	35ALA	CB	0.03
2LYS	CB	35ALA	C	0.00
2LYS	CB	63GLU	C	0.00
2LYS	CB	64ALA	CA	0.02
2LYS	CB	65GLU	CD	0.01
2LYS	CG	2LYS	CD	100.00
2LYS	CG	2LYS	CE	100.00
2LYS	CG	2LYS	C	54.26
2LYS	CG	35ALA	CB	0.05
2LYS	CG	37GLU	CB	0.00
2LYS	CG	64ALA	CA	0.00
2LYS	CG	64ALA	CB	0.01
2LYS	CG	65GLU	CD	0.02
2LYS	CD	2LYS	CE	100.00
2LYS	CD	2LYS	C	0.12
2LYS	CD	35ALA	CB	0.02
2LYS	CD	37GLU	CB	0.07
2LYS	CD	37GLU	CD	0.01
2LYS	CD	63GLU	CG	0.04

2LYS	CD	63GLU	CD	0.10
2LYS	CD	64ALA	CB	0.01
2LYS	CE	35ALA	CB	0.05
2LYS	CE	36TYR	C	0.01
2LYS	CE	37GLU	CB	0.54
2LYS	CE	37GLU	CG	0.04
2LYS	CE	37GLU	CD	0.08
2LYS	CE	63GLU	CB	0.00
2LYS	CE	63GLU	CG	0.04
2LYS	CE	63GLU	CD	0.12
2LYS	CE	63GLU	C	0.01
2LYS	CE	64ALA	CB	0.00
2LYS	CE	65GLU	CD	0.02
2LYS	C	3VAL	CA	100.00
2LYS	C	3VAL	CB	33.47
2LYS	C	3VAL	CG1	3.54
2LYS	C	3VAL	CG2	17.96
2LYS	C	3VAL	C	75.38
2LYS	C	34LEU	CD2	0.00
2LYS	C	64ALA	CB	0.06
2LYS	C	302PHE	CZ	0.00
3VAL	CA	3VAL	CB	100.00
3VAL	CA	3VAL	CG1	100.00
3VAL	CA	3VAL	CG2	100.00
3VAL	CA	3VAL	C	100.00
3VAL	CA	64ALA	CB	0.01
3VAL	CB	3VAL	CG1	100.00
3VAL	CB	3VAL	CG2	100.00
3VAL	CB	3VAL	C	100.00
3VAL	CB	34LEU	CD1	0.04
3VAL	CB	36TYR	CB	0.01
3VAL	CB	36TYR	CD2	0.05
3VAL	CB	36TYR	CE2	0.01
3VAL	CB	68LEU	CD1	0.04
3VAL	CB	297MET	CE	0.00
3VAL	CB	302PHE	CZ	0.00
3VAL	CG:	3VAL	CG2	100.00
3VAL	CG:	3VAL	C	91.73
3VAL	CG:	4ALA	C	0.02
3VAL	CG:	5VAL	CG2	0.45
3VAL	CG:	34LEU	CD1	0.12
3VAL	CG:	34LEU	CD2	0.28
3VAL	CG:	35ALA	C	0.00
3VAL	CG:	36TYR	CA	0.02
3VAL	CG:	36TYR	CB	0.20
3VAL	CG:	36TYR	CD2	0.16
3VAL	CG:	36TYR	CE2	0.04
3VAL	CG:	66ALA	CB	0.02
3VAL	CG:	66ALA	C	0.01
3VAL	CG:	68LEU	CB	1.32
3VAL	CG:	68LEU	CG	0.25
3VAL	CG:	68LEU	CD1	1.78
3VAL	CG:	68LEU	CD2	0.01
3VAL	CG:	297MET	CE	0.78
3VAL	CG:	302PHE	CE1	0.44
3VAL	CG:	302PHE	CE2	0.01
3VAL	CG:	302PHE	CZ	0.46
3VAL	CG:	3VAL	C	11.22
3VAL	CG:	5VAL	CG2	0.01
3VAL	CG:	23LEU	CD2	0.00
3VAL	CG:	34LEU	CB	0.01
3VAL	CG:	34LEU	CG	0.01

3VAL	CG2	34LEU	CD1	2.90
3VAL	CG2	34LEU	CD2	0.89
3VAL	CG2	36TYR	CA	0.04
3VAL	CG2	36TYR	CB	0.41
3VAL	CG2	36TYR	CD2	0.65
3VAL	CG2	36TYR	CE1	0.00
3VAL	CG2	36TYR	CE2	0.10
3VAL	CG2	36TYR	CZ	0.01
3VAL	CG2	66ALA	CB	0.00
3VAL	CG2	66ALA	C	0.01
3VAL	CG2	68LEU	CG	0.04
3VAL	CG2	68LEU	CD1	0.25
3VAL	CG2	297MET	CE	0.97
3VAL	CG2	302PHE	CE1	0.40
3VAL	CG2	302PHE	CE2	0.20
3VAL	CG2	302PHE	CZ	1.43
3VAL	C	4ALA	CA	100.00
3VAL	C	4ALA	CB	3.28
3VAL	C	4ALA	C	98.19
3VAL	C	64ALA	CB	0.51
4ALA	CA	4ALA	CB	100.00
4ALA	CA	4ALA	C	100.00
4ALA	CA	39PHE	CD2	0.10
4ALA	CA	39PHE	CE2	0.19
4ALA	CB	4ALA	C	100.00
4ALA	CB	6LEU	CD1	0.22
4ALA	CB	39PHE	CD1	0.03
4ALA	CB	39PHE	CD2	2.41
4ALA	CB	39PHE	CE1	0.20
4ALA	CB	39PHE	CE2	6.09
4ALA	CB	39PHE	CZ	0.04
4ALA	CB	60GLY	C	0.04
4ALA	CB	61VAL	CB	0.00
4ALA	CB	61VAL	CG1	0.09
4ALA	CB	61VAL	CG2	0.01
4ALA	CB	64ALA	CB	13.46
4ALA	CB	67VAL	CG2	0.02
4ALA	C	5VAL	CA	100.00
4ALA	C	5VAL	CB	22.60
4ALA	C	5VAL	CG2	13.67
4ALA	C	5VAL	C	92.21
4ALA	C	6LEU	CD1	0.06
5VAL	CA	5VAL	CB	100.00
5VAL	CA	5VAL	CG1	100.00
5VAL	CA	5VAL	CG2	100.00
5VAL	CA	5VAL	C	100.00
5VAL	CB	5VAL	CG1	100.00
5VAL	CB	5VAL	CG2	100.00
5VAL	CB	5VAL	C	100.00
5VAL	CB	36TYR	CD2	0.00
5VAL	CB	38VAL	CG2	0.01
5VAL	CG1	5VAL	CG2	100.00
5VAL	CG1	5VAL	C	99.98
5VAL	CG1	7PRO	CB	0.00
5VAL	CG1	7PRO	CD	0.00
5VAL	CG1	16THR	CG2	34.56
5VAL	CG1	20LEU	CD1	0.38
5VAL	CG1	20LEU	CD2	0.00
5VAL	CG1	38VAL	CA	0.00
5VAL	CG1	38VAL	CG1	0.02
5VAL	CG1	38VAL	CG2	0.04
5VAL	CG1	68LEU	CB	0.00

5VAL	CG1	68LEU	CD2	0.02
5VAL	CG2	5VAL	C	0.01
5VAL	CG2	16THR	CG2	0.00
5VAL	CG2	20LEU	CD1	0.30
5VAL	CG2	20LEU	CD2	0.14
5VAL	CG2	23LEU	CD1	0.00
5VAL	CG2	36TYR	CB	0.34
5VAL	CG2	36TYR	CG	0.06
5VAL	CG2	36TYR	CD1	1.14
5VAL	CG2	36TYR	CD2	1.75
5VAL	CG2	36TYR	C	0.02
5VAL	CG2	38VAL	CG2	0.01
5VAL	CG2	68LEU	CD1	0.05
5VAL	CG2	68LEU	CD2	0.01
5VAL	C	6LEU	CA	100.00
5VAL	C	6LEU	CB	17.28
5VAL	C	6LEU	CG	0.31
5VAL	C	6LEU	CD1	19.71
5VAL	C	6LEU	CD2	0.10
5VAL	C	6LEU	C	92.64
5VAL	C	7PRO	CD	0.65
6LEU	CA	6LEU	CB	100.00
6LEU	CA	6LEU	CG	100.00
6LEU	CA	6LEU	CD1	87.44
6LEU	CA	6LEU	CD2	12.09
6LEU	CA	6LEU	C	100.00
6LEU	CA	7PRO	CD	100.00
6LEU	CB	6LEU	CG	100.00
6LEU	CB	6LEU	CD1	100.00
6LEU	CB	6LEU	CD2	100.00
6LEU	CB	6LEU	C	100.00
6LEU	CB	41PHE	CB	0.32
6LEU	CB	41PHE	CG	0.01
6LEU	CB	41PHE	CD1	0.00
6LEU	CB	41PHE	CD2	0.22
6LEU	CB	69LEU	CD1	4.01
6LEU	CB	69LEU	CD2	0.08
6LEU	CG	6LEU	CD1	100.00
6LEU	CG	6LEU	CD2	100.00
6LEU	CG	41PHE	CB	0.30
6LEU	CG	41PHE	CG	0.02
6LEU	CG	41PHE	CD1	0.01
6LEU	CG	57THR	CG2	1.89
6LEU	CG	67VAL	CG1	0.02
6LEU	CD1	6LEU	CD2	100.00
6LEU	CD1	39PHE	CB	0.06
6LEU	CD1	39PHE	CG	0.01
6LEU	CD1	39PHE	CD1	0.02
6LEU	CD1	39PHE	CD2	0.08
6LEU	CD1	41PHE	CB	0.00
6LEU	CD1	41PHE	CD1	0.00
6LEU	CD1	53PHE	CD2	0.04
6LEU	CD1	53PHE	CE1	0.06
6LEU	CD1	53PHE	CE2	0.12
6LEU	CD1	53PHE	CZ	0.02
6LEU	CD1	57THR	CG2	0.97
6LEU	CD1	61VAL	CG1	5.43
6LEU	CD1	61VAL	CG2	0.07
6LEU	CD1	67VAL	CG1	4.06
6LEU	CD1	69LEU	CD1	0.35
6LEU	CD1	69LEU	CD2	0.05
6LEU	CD1	93LEU	CD1	0.15

6LEU	CD1	93LEU	CD2	0.54
6LEU	CD2	39PHE	CB	0.33
6LEU	CD2	41PHE	CB	0.62
6LEU	CD2	41PHE	CG	1.27
6LEU	CD2	41PHE	CD1	1.78
6LEU	CD2	41PHE	CD2	0.34
6LEU	CD2	41PHE	CE1	0.12
6LEU	CD2	41PHE	CE2	0.01
6LEU	CD2	53PHE	CB	0.00
6LEU	CD2	53PHE	CD1	0.08
6LEU	CD2	53PHE	CD2	0.16
6LEU	CD2	53PHE	CE1	0.09
6LEU	CD2	53PHE	CE2	0.17
6LEU	CD2	53PHE	CZ	0.01
6LEU	CD2	57THR	CG2	11.26
6LEU	CD2	61VAL	CG1	1.06
6LEU	CD2	67VAL	CG1	0.10
6LEU	CD2	69LEU	CD1	2.03
6LEU	CD2	69LEU	CD2	0.04
6LEU	CD2	93LEU	CD1	6.56
6LEU	CD2	93LEU	CD2	4.30
6LEU	C	7PRO	CA	100.00
6LEU	C	7PRO	CB	0.19
6LEU	C	7PRO	CD	100.00
6LEU	C	7PRO	C	100.00
6LEU	C	41PHE	CD2	0.03
7PRO	CA	7PRO	CB	100.00
7PRO	CA	7PRO	CG	100.00
7PRO	CA	7PRO	CD	100.00
7PRO	CA	7PRO	C	100.00
7PRO	CA	16THR	CG2	0.00
7PRO	CB	7PRO	CG	100.00
7PRO	CB	7PRO	CD	100.00
7PRO	CB	7PRO	C	100.00
7PRO	CB	12GLY	C	0.01
7PRO	CB	13PRO	CA	0.36
7PRO	CB	13PRO	CB	0.12
7PRO	CB	16THR	CB	0.01
7PRO	CB	16THR	CG2	0.02
7PRO	CB	38VAL	CG1	0.01
7PRO	CG	7PRO	CD	100.00
7PRO	CG	7PRO	C	89.73
7PRO	CG	38VAL	CG1	0.92
7PRO	CD	7PRO	C	72.65
7PRO	CD	38VAL	CG1	0.15
7PRO	CD	41PHE	CB	0.01
7PRO	C	8GLY	CA	100.00
7PRO	C	8GLY	C	84.94
7PRO	C	12GLY	CA	0.00
7PRO	C	13PRO	CB	0.00
7PRO	C	13PRO	CD	0.00
8GLY	CA	8GLY	C	100.00
8GLY	CA	41PHE	CD1	0.04
8GLY	CA	41PHE	CD2	1.01
8GLY	CA	41PHE	CE2	0.06
8GLY	CA	41PHE	C	0.02
8GLY	CA	42GLY	CA	0.00
8GLY	CA	70GLY	C	0.56
8GLY	CA	71SER	C	0.01
8GLY	CA	72VAL	CG1	6.44
8GLY	CA	72VAL	CG2	0.34
8GLY	C	9ASP	CA	100.00

8GLY	C	9ASP	CB	98.34
8GLY	C	9ASP	CG	0.03
8GLY	C	9ASP	C	2.54
8GLY	C	12GLY	CA	0.02
8GLY	C	13PRO	CG	0.12
8GLY	C	13PRO	CD	0.30
8GLY	C	41PHE	CD1	0.00
8GLY	C	42GLY	CA	0.21
8GLY	C	72VAL	CG1	0.17
8GLY	C	72VAL	CG2	0.00
9ASP	CA	9ASP	CB	100.00
9ASP	CA	9ASP	CG	100.00
9ASP	CA	9ASP	C	100.00
9ASP	CA	13PRO	CG	0.01
9ASP	CA	13PRO	CD	0.10
9ASP	CB	9ASP	CG	100.00
9ASP	CB	9ASP	C	100.00
9ASP	CB	42GLY	CA	0.21
9ASP	CB	42GLY	C	0.02
9ASP	CB	43GLY	CA	0.04
9ASP	CB	72VAL	CG1	0.06
9ASP	CB	77TRP	CZ3	0.02
9ASP	CG	9ASP	C	98.58
9ASP	CG	43GLY	CA	0.00
9ASP	CG	76LYS	CD	0.01
9ASP	CG	76LYS	CE	0.26
9ASP	CG	77TRP	CZ2	0.01
9ASP	CG	77TRP	CZ3	0.13
9ASP	C	10GLY	CA	100.00
9ASP	C	10GLY	C	69.87
9ASP	C	13PRO	CD	0.10
10GLY	CA	10GLY	C	100.00
10GLY	CA	73GLY	C	0.00
10GLY	CA	275SER	CB	0.00
10GLY	CA	275SER	C	0.04
10GLY	CA	276ALA	C	0.01
10GLY	CA	280ALA	CB	0.07
10GLY	C	11ILE	CA	100.00
10GLY	C	11ILE	CB	5.35
10GLY	C	11ILE	CG1	3.16
10GLY	C	11ILE	CG2	0.14
10GLY	C	11ILE	CD	0.01
10GLY	C	11ILE	C	95.86
10GLY	C	277PRO	CA	0.00
10GLY	C	280ALA	CB	0.61
10GLY	C	280ALA	C	0.00
10GLY	C	283GLY	CA	0.03
11ILE	CA	11ILE	CB	100.00
11ILE	CA	11ILE	CG1	100.00
11ILE	CA	11ILE	CG2	100.00
11ILE	CA	11ILE	CD	3.89
11ILE	CA	11ILE	C	100.00
11ILE	CA	280ALA	CB	0.12
11ILE	CB	11ILE	CG1	100.00
11ILE	CB	11ILE	CG2	100.00
11ILE	CB	11ILE	CD	100.00
11ILE	CB	11ILE	C	100.00
11ILE	CB	71SER	CB	0.00
11ILE	CB	273HIS	CD2	0.01
11ILE	CB	273HIS	CE1	0.12
11ILE	CB	285ALA	CB	0.02
11ILE	CG1	11ILE	CG2	100.00

11ILE	CG:	11ILE	CD	100.00
11ILE	CG:	11ILE	C	15.64
11ILE	CG:	15VAL	CG1	0.03
11ILE	CG:	71SER	CB	0.07
11ILE	CG:	273HIS	CD2	0.00
11ILE	CG:	273HIS	CE1	0.03
11ILE	CG:	274GLY	C	0.01
11ILE	CG:	275SER	C	0.00
11ILE	CG:	279ILE	CD	0.09
11ILE	CG:	280ALA	CA	0.00
11ILE	CG:	280ALA	CB	0.33
11ILE	CG:	283GLY	CA	0.01
11ILE	CG:	283GLY	C	0.39
11ILE	CG:	284ILE	C	0.01
11ILE	CG:	285ALA	CB	0.05
11ILE	CG:	11ILE	CD	95.72
11ILE	CG:	11ILE	C	84.56
11ILE	CG:	15VAL	CG1	9.11
11ILE	CG:	15VAL	CG2	0.34
11ILE	CG:	71SER	CB	0.02
11ILE	CG:	273HIS	CB	0.02
11ILE	CG:	273HIS	CG	0.09
11ILE	CG:	273HIS	CD2	0.76
11ILE	CG:	273HIS	CE1	2.64
11ILE	CG:	276ALA	CB	0.01
11ILE	CG:	279ILE	CB	0.01
11ILE	CG:	279ILE	CG1	0.02
11ILE	CG:	279ILE	CG2	0.08
11ILE	CG:	279ILE	CD	0.02
11ILE	CG:	279ILE	C	0.00
11ILE	CG:	280ALA	CA	0.07
11ILE	CG:	280ALA	CB	0.26
11ILE	CG:	280ALA	C	0.02
11ILE	CG:	281GLY	CA	0.00
11ILE	CG:	283GLY	CA	0.01
11ILE	CG:	283GLY	C	0.07
11ILE	CG:	284ILE	C	0.10
11ILE	CG:	285ALA	CA	0.08
11ILE	CG:	285ALA	CB	5.61
11ILE	CD	11ILE	C	0.56
11ILE	CD	15VAL	CG1	0.05
11ILE	CD	15VAL	CG2	0.04
11ILE	CD	71SER	CB	0.12
11ILE	CD	72VAL	C	0.01
11ILE	CD	73GLY	C	0.03
11ILE	CD	74GLY	CA	0.02
11ILE	CD	273HIS	CB	0.25
11ILE	CD	273HIS	CG	0.54
11ILE	CD	273HIS	CD2	0.42
11ILE	CD	273HIS	CE1	1.13
11ILE	CD	273HIS	C	0.72
11ILE	CD	274GLY	CA	0.18
11ILE	CD	274GLY	C	4.90
11ILE	CD	275SER	CA	0.01
11ILE	CD	275SER	C	0.01
11ILE	CD	276ALA	CB	0.06
11ILE	CD	279ILE	CG1	0.20
11ILE	CD	279ILE	CD	1.55
11ILE	CD	280ALA	CB	0.07
11ILE	CD	283GLY	CA	0.01
11ILE	CD	283GLY	C	0.06
11ILE	CD	284ILE	C	0.01

11ILE	CD	285ALA	CB	0.78
11ILE	C	12GLY	CA	100.00
11ILE	C	12GLY	C	99.95
11ILE	C	13PRO	CD	7.65
11ILE	C	15VAL	CG2	0.02
11ILE	C	280ALA	CB	0.00
12GLY	CA	12GLY	C	100.00
12GLY	CA	13PRO	CD	100.00
12GLY	CA	15VAL	CG2	0.19
12GLY	CA	70GLY	CA	0.06
12GLY	CA	70GLY	C	0.04
12GLY	CA	71SER	CB	0.02
12GLY	CA	273HIS	CD2	0.01
12GLY	CA	273HIS	CE1	0.00
12GLY	C	13PRO	CA	100.00
12GLY	C	13PRO	CB	0.03
12GLY	C	13PRO	CD	100.00
12GLY	C	13PRO	C	100.00
12GLY	C	15VAL	CG2	0.00
13PRO	CA	13PRO	CB	100.00
13PRO	CA	13PRO	CG	100.00
13PRO	CA	13PRO	CD	100.00
13PRO	CA	13PRO	C	100.00
13PRO	CA	16THR	CG2	0.03
13PRO	CB	13PRO	CG	100.00
13PRO	CB	13PRO	CD	100.00
13PRO	CB	13PRO	C	100.00
13PRO	CG	13PRO	CD	100.00
13PRO	CG	13PRO	C	45.25
13PRO	CD	13PRO	C	31.63
13PRO	C	14GLU	CA	100.00
13PRO	C	14GLU	CB	0.06
13PRO	C	14GLU	C	99.97
13PRO	C	16THR	CG2	0.02
14GLU	CA	14GLU	CB	100.00
14GLU	CA	14GLU	CG	100.00
14GLU	CA	14GLU	CD	98.00
14GLU	CA	14GLU	C	100.00
14GLU	CB	14GLU	CG	100.00
14GLU	CB	14GLU	CD	100.00
14GLU	CB	14GLU	C	100.00
14GLU	CB	280ALA	CB	0.43
14GLU	CB	284ILE	CD	0.05
14GLU	CB	285ALA	CB	0.02
14GLU	CB	333THR	CG2	0.00
14GLU	CG	14GLU	CD	100.00
14GLU	CG	14GLU	C	95.30
14GLU	CG	280ALA	CB	0.12
14GLU	CG	280ALA	C	0.00
14GLU	CG	281GLY	CA	0.04
14GLU	CG	284ILE	CG2	0.04
14GLU	CG	284ILE	CD	0.22
14GLU	CG	285ALA	CB	0.32
14GLU	CG	333THR	CB	0.11
14GLU	CG	333THR	CG2	0.48
14GLU	CD	14GLU	C	0.06
14GLU	CD	280ALA	CA	0.00
14GLU	CD	280ALA	CB	0.01
14GLU	CD	281GLY	CA	0.17
14GLU	CD	283GLY	CA	0.02
14GLU	CD	284ILE	CD	0.01
14GLU	CD	333THR	CB	0.00

14GLU	CD	333THR	CG2	0.09
14GLU	C	15VAL	CA	100.00
14GLU	C	15VAL	CB	0.39
14GLU	C	15VAL	CG1	0.13
14GLU	C	15VAL	C	99.72
14GLU	C	333THR	CG2	0.04
15VAL	CA	15VAL	CB	100.00
15VAL	CA	15VAL	CG1	100.00
15VAL	CA	15VAL	CG2	100.00
15VAL	CA	15VAL	C	100.00
15VAL	CA	18ALA	CB	0.01
15VAL	CB	15VAL	CG1	100.00
15VAL	CB	15VAL	CG2	100.00
15VAL	CB	15VAL	C	100.00
15VAL	CB	273HIS	CE1	0.26
15VAL	CB	287PRO	CB	0.02
15VAL	CB	290ALA	CB	0.12
15VAL	CG:	15VAL	CG2	100.00
15VAL	CG:	15VAL	C	4.06
15VAL	CG:	271PRO	CG	0.20
15VAL	CG:	273HIS	CD2	0.00
15VAL	CG:	273HIS	CE1	0.53
15VAL	CG:	284ILE	CD	0.03
15VAL	CG:	285ALA	CB	1.37
15VAL	CG:	285ALA	C	0.03
15VAL	CG:	286ASN	C	0.01
15VAL	CG:	287PRO	CA	0.25
15VAL	CG:	287PRO	CB	0.02
15VAL	CG:	290ALA	CB	0.18
15VAL	CG:	333THR	CG2	0.49
15VAL	CG:	15VAL	C	96.03
15VAL	CG:	70GLY	CA	0.00
15VAL	CG:	70GLY	C	0.00
15VAL	CG:	271PRO	CG	0.30
15VAL	CG:	273HIS	CB	0.01
15VAL	CG:	273HIS	CG	0.09
15VAL	CG:	273HIS	CD2	1.98
15VAL	CG:	273HIS	CE1	1.18
15VAL	CG:	285ALA	CB	0.17
15VAL	CG:	290ALA	CB	0.15
15VAL	C	16THR	CA	100.00
15VAL	C	16THR	CB	0.04
15VAL	C	16THR	C	99.98
15VAL	C	290ALA	CB	0.10
16THR	CA	16THR	CB	100.00
16THR	CA	16THR	CG2	100.00
16THR	CA	16THR	C	100.00
16THR	CB	16THR	CG2	100.00
16THR	CB	16THR	C	100.00
16THR	CG:	16THR	C	100.00
16THR	CG:	20LEU	CD1	0.40
16THR	CG:	68LEU	CG	0.03
16THR	CG:	68LEU	CD1	0.01
16THR	CG:	68LEU	CD2	1.23
16THR	C	17GLU	CA	100.00
16THR	C	17GLU	CB	0.00
16THR	C	17GLU	C	100.00
17GLU	CA	17GLU	CB	100.00
17GLU	CA	17GLU	CG	100.00
17GLU	CA	17GLU	CD	99.60
17GLU	CA	17GLU	C	100.00
17GLU	CB	17GLU	CG	100.00

17GLU	CB	17GLU	CD	100.00
17GLU	CB	17GLU	C	100.00
17GLU	CG	17GLU	CD	100.00
17GLU	CG	17GLU	C	81.66
17GLU	CG	21LYS	CE	0.06
17GLU	CD	17GLU	C	4.94
17GLU	CD	20LEU	CD1	0.01
17GLU	CD	21LYS	CE	0.00
17GLU	C	18ALA	CA	100.00
17GLU	C	18ALA	CB	0.01
17GLU	C	18ALA	C	99.99
17GLU	C	21LYS	CE	0.01
18ALA	CA	18ALA	CB	100.00
18ALA	CA	18ALA	C	100.00
18ALA	CA	337THR	CG2	0.13
18ALA	CB	18ALA	C	100.00
18ALA	CB	287PRO	CB	14.64
18ALA	CB	287PRO	CG	1.53
18ALA	CB	290ALA	CB	0.57
18ALA	CB	291ILE	CG1	0.01
18ALA	CB	333THR	CB	0.02
18ALA	CB	333THR	CG2	0.94
18ALA	CB	337THR	CG2	4.50
18ALA	C	19ALA	CA	100.00
18ALA	C	19ALA	CB	0.08
18ALA	C	19ALA	C	99.94
18ALA	C	21LYS	CB	0.01
18ALA	C	290ALA	CB	0.02
18ALA	C	291ILE	CG1	0.01
18ALA	C	291ILE	CD	0.11
18ALA	C	337THR	CG2	0.01
19ALA	CA	19ALA	CB	100.00
19ALA	CA	19ALA	C	100.00
19ALA	CA	291ILE	CG1	0.02
19ALA	CA	291ILE	CD	0.01
19ALA	CA	294ALA	CB	0.00
19ALA	CB	19ALA	C	100.00
19ALA	CB	68LEU	CD1	0.02
19ALA	CB	68LEU	CD2	0.24
19ALA	CB	290ALA	CB	0.08
19ALA	CB	290ALA	C	1.20
19ALA	CB	291ILE	CA	0.01
19ALA	CB	291ILE	CG1	0.03
19ALA	CB	294ALA	CB	0.08
19ALA	C	20LEU	CA	100.00
19ALA	C	20LEU	CB	0.03
19ALA	C	20LEU	C	99.99
19ALA	C	22VAL	CG1	0.01
19ALA	C	22VAL	CG2	0.00
19ALA	C	23LEU	CD1	0.02
20LEU	CA	20LEU	CB	100.00
20LEU	CA	20LEU	CG	100.00
20LEU	CA	20LEU	CD1	1.79
20LEU	CA	20LEU	CD2	98.68
20LEU	CA	20LEU	C	100.00
20LEU	CB	20LEU	CG	100.00
20LEU	CB	20LEU	CD1	100.00
20LEU	CB	20LEU	CD2	100.00
20LEU	CB	20LEU	C	100.00
20LEU	CB	24ARG	CZ	9.41
20LEU	CG	20LEU	CD1	100.00
20LEU	CG	20LEU	CD2	100.00

20LEU	CG	20LEU	C	0.75
20LEU	CG	24ARG	CZ	0.03
20LEU	CG	68LEU	CD1	0.06
20LEU	CG	68LEU	CD2	0.28
20LEU	CD1	20LEU	CD2	100.00
20LEU	CD1	20LEU	C	0.03
20LEU	CD1	24ARG	CZ	0.01
20LEU	CD1	36TYR	CB	0.00
20LEU	CD1	36TYR	CG	0.01
20LEU	CD1	36TYR	CD1	0.12
20LEU	CD1	36TYR	CD2	0.10
20LEU	CD1	36TYR	CE1	0.40
20LEU	CD1	36TYR	CE2	0.69
20LEU	CD1	36TYR	CZ	0.11
20LEU	CD1	38VAL	CG1	0.05
20LEU	CD1	38VAL	CG2	0.41
20LEU	CD1	68LEU	CD1	0.03
20LEU	CD1	68LEU	CD2	0.08
20LEU	CD2	20LEU	C	0.05
20LEU	CD2	23LEU	CD1	0.06
20LEU	CD2	23LEU	CD2	0.00
20LEU	CD2	24ARG	CZ	0.14
20LEU	CD2	34LEU	CD2	0.01
20LEU	CD2	36TYR	CB	0.10
20LEU	CD2	36TYR	CG	0.96
20LEU	CD2	36TYR	CD1	2.20
20LEU	CD2	36TYR	CD2	4.37
20LEU	CD2	36TYR	CE1	1.54
20LEU	CD2	36TYR	CE2	3.93
20LEU	CD2	36TYR	CZ	0.60
20LEU	CD2	38VAL	CG2	0.04
20LEU	CD2	68LEU	CD1	1.18
20LEU	CD2	68LEU	CD2	1.29
20LEU	C	21LYS	CA	100.00
20LEU	C	21LYS	C	100.00
20LEU	C	24ARG	CD	0.01
20LEU	C	24ARG	CZ	0.04
21LYS	CA	21LYS	CB	100.00
21LYS	CA	21LYS	CG	100.00
21LYS	CA	21LYS	CD	3.22
21LYS	CA	21LYS	C	100.00
21LYS	CA	24ARG	CZ	0.01
21LYS	CB	21LYS	CG	100.00
21LYS	CB	21LYS	CD	100.00
21LYS	CB	21LYS	CE	9.74
21LYS	CB	21LYS	C	100.00
21LYS	CB	337THR	CG2	0.36
21LYS	CB	341LEU	CD1	0.01
21LYS	CG	21LYS	CD	100.00
21LYS	CG	21LYS	CE	100.00
21LYS	CG	21LYS	C	27.28
21LYS	CG	24ARG	CZ	0.00
21LYS	CG	337THR	CB	0.00
21LYS	CG	337THR	CG2	0.02
21LYS	CG	341LEU	CD1	0.04
21LYS	CG	341LEU	CD2	0.11
21LYS	CD	21LYS	CE	100.00
21LYS	CD	337THR	CB	0.36
21LYS	CD	337THR	CG2	0.46
21LYS	CD	341LEU	CD1	0.07
21LYS	CE	334GLU	CD	0.11
21LYS	CE	337THR	CB	0.00

21LYS	CE	337THR	CG2	0.01
21LYS	CE	341LEU	CD1	0.05
21LYS	CE	341LEU	CD2	0.02
21LYS	C	22VAL	CA	100.00
21LYS	C	22VAL	CB	0.20
21LYS	C	22VAL	CG2	0.09
21LYS	C	22VAL	C	99.88
21LYS	C	341LEU	CD1	0.01
21LYS	C	341LEU	CD2	0.11
22VAL	CA	22VAL	CB	100.00
22VAL	CA	22VAL	CG1	100.00
22VAL	CA	22VAL	CG2	100.00
22VAL	CA	22VAL	C	100.00
22VAL	CA	341LEU	CD2	0.06
22VAL	CA	344LEU	CD1	0.04
22VAL	CB	22VAL	CG1	100.00
22VAL	CB	22VAL	CG2	100.00
22VAL	CB	22VAL	C	100.00
22VAL	CB	294ALA	CB	0.04
22VAL	CB	344LEU	CD1	0.21
22VAL	CB	344LEU	CD2	0.00
22VAL	CG1	22VAL	CG2	100.00
22VAL	CG1	22VAL	C	87.93
22VAL	CG1	26LEU	CD1	0.49
22VAL	CG1	291ILE	CG2	0.04
22VAL	CG1	291ILE	CD	0.00
22VAL	CG1	294ALA	CB	0.34
22VAL	CG1	311VAL	CG1	0.98
22VAL	CG1	311VAL	CG2	3.12
22VAL	CG1	337THR	CA	0.01
22VAL	CG1	337THR	CB	0.00
22VAL	CG1	337THR	CG2	0.00
22VAL	CG1	337THR	C	0.00
22VAL	CG1	340VAL	CG1	0.66
22VAL	CG1	341LEU	CD1	0.07
22VAL	CG1	341LEU	CD2	0.04
22VAL	CG1	344LEU	CD1	1.42
22VAL	CG1	344LEU	CD2	0.54
22VAL	CG2	22VAL	C	12.82
22VAL	CG2	291ILE	CG1	0.06
22VAL	CG2	291ILE	CG2	0.42
22VAL	CG2	291ILE	CD	0.21
22VAL	CG2	294ALA	CB	1.49
22VAL	CG2	311VAL	CG1	0.03
22VAL	CG2	311VAL	CG2	0.04
22VAL	CG2	337THR	CB	0.01
22VAL	CG2	337THR	CG2	0.12
22VAL	CG2	340VAL	CG1	0.56
22VAL	CG2	341LEU	CD1	0.23
22VAL	CG2	341LEU	CD2	0.07
22VAL	CG2	344LEU	CD1	0.11
22VAL	CG2	344LEU	CD2	0.00
22VAL	C	23LEU	CA	100.00
22VAL	C	23LEU	CB	0.02
22VAL	C	23LEU	C	99.99
22VAL	C	26LEU	CD1	0.00
23LEU	CA	23LEU	CB	100.00
23LEU	CA	23LEU	CG	100.00
23LEU	CA	23LEU	CD1	2.08
23LEU	CA	23LEU	CD2	97.59
23LEU	CA	23LEU	C	100.00
23LEU	CA	32LEU	CD1	0.01

23LEU	CB	23LEU	CG	100.00
23LEU	CB	23LEU	CD1	100.00
23LEU	CB	23LEU	CD2	100.00
23LEU	CB	23LEU	C	100.00
23LEU	CB	32LEU	CD1	0.02
23LEU	CB	32LEU	CD2	0.02
23LEU	CB	34LEU	CD1	0.10
23LEU	CB	34LEU	CD2	1.24
23LEU	CB	36TYR	CD1	0.01
23LEU	CB	36TYR	CD2	0.04
23LEU	CB	36TYR	CE1	0.01
23LEU	CB	36TYR	CE2	0.08
23LEU	CB	36TYR	CZ	0.08
23LEU	CG	23LEU	CD1	100.00
23LEU	CG	23LEU	CD2	100.00
23LEU	CG	23LEU	C	1.40
23LEU	CG	34LEU	CD1	0.00
23LEU	CG	34LEU	CD2	0.01
23LEU	CG	294ALA	CB	0.04
23LEU	CD1	23LEU	CD2	100.00
23LEU	CD1	23LEU	C	0.01
23LEU	CD1	32LEU	CD1	0.08
23LEU	CD1	32LEU	CD2	0.02
23LEU	CD1	34LEU	CD1	0.07
23LEU	CD1	34LEU	CD2	0.02
23LEU	CD1	36TYR	CB	0.04
23LEU	CD1	36TYR	CG	0.02
23LEU	CD1	36TYR	CD1	0.01
23LEU	CD1	36TYR	CD2	0.02
23LEU	CD1	36TYR	CE1	0.01
23LEU	CD1	36TYR	CE2	0.06
23LEU	CD1	36TYR	CZ	0.01
23LEU	CD1	68LEU	CD1	0.25
23LEU	CD1	68LEU	CD2	0.05
23LEU	CD1	294ALA	CB	0.80
23LEU	CD1	297MET	CE	3.01
23LEU	CD1	298LEU	CD1	0.02
23LEU	CD2	23LEU	C	0.04
23LEU	CD2	26LEU	CD1	0.01
23LEU	CD2	32LEU	CD1	1.03
23LEU	CD2	32LEU	CD2	0.16
23LEU	CD2	34LEU	CD1	0.18
23LEU	CD2	34LEU	CD2	0.05
23LEU	CD2	36TYR	CB	0.02
23LEU	CD2	36TYR	CG	0.02
23LEU	CD2	36TYR	CD1	0.01
23LEU	CD2	36TYR	CD2	0.07
23LEU	CD2	36TYR	CE1	0.01
23LEU	CD2	36TYR	CE2	0.01
23LEU	CD2	68LEU	CD1	0.00
23LEU	CD2	294ALA	CB	0.16
23LEU	CD2	297MET	CE	0.66
23LEU	CD2	298LEU	CG	0.22
23LEU	CD2	298LEU	CD1	1.59
23LEU	CD2	298LEU	CD2	0.88
23LEU	CD2	302PHE	CE1	0.00
23LEU	CD2	302PHE	CE2	0.02
23LEU	CD2	302PHE	CZ	0.01
23LEU	C	24ARG	CA	100.00
23LEU	C	24ARG	C	100.00
23LEU	C	34LEU	CD1	0.04
23LEU	C	34LEU	CD2	1.20

23LEU	C	36TYR	CE1	0.01
23LEU	C	36TYR	CE2	0.01
23LEU	C	36TYR	CZ	0.00
24ARG	CA	24ARG	CB	100.00
24ARG	CA	24ARG	CG	100.00
24ARG	CA	24ARG	C	100.00
24ARG	CA	34LEU	CD2	0.12
24ARG	CB	24ARG	CG	100.00
24ARG	CB	24ARG	CD	100.00
24ARG	CB	24ARG	CZ	0.06
24ARG	CB	24ARG	C	100.00
24ARG	CG	24ARG	CD	100.00
24ARG	CG	24ARG	CZ	8.77
24ARG	CG	24ARG	C	2.11
24ARG	CG	34LEU	CD1	0.00
24ARG	CG	34LEU	CD2	0.70
24ARG	CG	36TYR	CD1	0.00
24ARG	CG	36TYR	CE1	0.55
24ARG	CG	36TYR	CE2	0.03
24ARG	CG	36TYR	CZ	0.07
24ARG	CD	24ARG	CZ	100.00
24ARG	CD	36TYR	CE1	0.12
24ARG	CD	36TYR	CE2	0.00
24ARG	CD	36TYR	CZ	0.08
24ARG	CZ	36TYR	CE1	0.01
24ARG	C	25ALA	CA	100.00
24ARG	C	25ALA	CB	0.05
24ARG	C	25ALA	C	99.95
25ALA	CA	25ALA	CB	100.00
25ALA	CA	25ALA	C	100.00
25ALA	CA	345ALA	CB	0.00
25ALA	CB	25ALA	C	100.00
25ALA	CB	340VAL	CG1	0.01
25ALA	CB	341LEU	CG	0.16
25ALA	CB	341LEU	CD1	1.25
25ALA	CB	341LEU	CD2	20.66
25ALA	CB	344LEU	CB	0.07
25ALA	CB	344LEU	CD1	0.33
25ALA	CB	344LEU	CD2	0.60
25ALA	CB	345ALA	CB	0.04
25ALA	C	26LEU	CA	100.00
25ALA	C	26LEU	CB	0.13
25ALA	C	26LEU	CG	0.01
25ALA	C	26LEU	C	99.85
25ALA	C	344LEU	CD1	0.01
25ALA	C	344LEU	CD2	0.02
26LEU	CA	26LEU	CB	100.00
26LEU	CA	26LEU	CG	100.00
26LEU	CA	26LEU	CD1	14.86
26LEU	CA	26LEU	CD2	84.67
26LEU	CA	26LEU	C	100.00
26LEU	CA	344LEU	CD2	0.00
26LEU	CB	26LEU	CG	100.00
26LEU	CB	26LEU	CD1	100.00
26LEU	CB	26LEU	CD2	100.00
26LEU	CB	26LEU	C	100.00
26LEU	CB	32LEU	CG	0.00
26LEU	CB	32LEU	CD1	1.08
26LEU	CB	32LEU	CD2	1.89
26LEU	CB	34LEU	CD1	0.23
26LEU	CB	34LEU	CD2	0.03
26LEU	CB	307LEU	CD2	0.00

26LEU	CG	26LEU	CD1	100.00
26LEU	CG	26LEU	CD2	100.00
26LEU	CG	26LEU	C	13.37
26LEU	CG	32LEU	CD1	0.01
26LEU	CG	32LEU	CD2	0.03
26LEU	CG	344LEU	CD1	0.00
26LEU	CG	344LEU	CD2	0.01
26LEU	CD1	26LEU	CD2	100.00
26LEU	CD1	26LEU	C	0.18
26LEU	CD1	30GLU	CB	0.10
26LEU	CD1	30GLU	CG	0.24
26LEU	CD1	32LEU	CD1	0.02
26LEU	CD1	32LEU	CD2	0.01
26LEU	CD1	34LEU	CD1	0.02
26LEU	CD1	298LEU	CD1	7.48
26LEU	CD1	298LEU	CD2	0.37
26LEU	CD1	307LEU	CB	0.00
26LEU	CD1	307LEU	CD1	0.03
26LEU	CD1	307LEU	CD2	0.05
26LEU	CD1	310LYS	CG	0.00
26LEU	CD1	310LYS	CD	0.02
26LEU	CD1	310LYS	CE	0.01
26LEU	CD1	311VAL	CG1	0.04
26LEU	CD1	311VAL	CG2	4.50
26LEU	CD1	341LEU	CD2	0.03
26LEU	CD1	344LEU	CD1	0.04
26LEU	CD1	344LEU	CD2	0.08
26LEU	CD2	26LEU	C	0.22
26LEU	CD2	30GLU	CB	0.09
26LEU	CD2	30GLU	CG	0.09
26LEU	CD2	32LEU	CG	0.00
26LEU	CD2	32LEU	CD1	0.04
26LEU	CD2	32LEU	CD2	0.10
26LEU	CD2	298LEU	CD1	1.25
26LEU	CD2	298LEU	CD2	0.14
26LEU	CD2	307LEU	CG	0.02
26LEU	CD2	307LEU	CD1	0.25
26LEU	CD2	307LEU	CD2	0.34
26LEU	CD2	310LYS	CG	0.00
26LEU	CD2	310LYS	CD	0.03
26LEU	CD2	310LYS	CE	0.04
26LEU	CD2	311VAL	CG1	0.04
26LEU	CD2	311VAL	CG2	0.84
26LEU	CD2	341LEU	CD2	0.00
26LEU	CD2	344LEU	CB	0.01
26LEU	CD2	344LEU	CG	0.00
26LEU	CD2	344LEU	CD1	0.12
26LEU	CD2	344LEU	CD2	0.50
26LEU	C	27ASP	CA	100.00
26LEU	C	27ASP	CB	1.20
26LEU	C	27ASP	C	98.87
26LEU	C	32LEU	CB	0.01
26LEU	C	32LEU	CD1	0.09
26LEU	C	32LEU	CD2	0.58
26LEU	C	34LEU	CD1	0.01
27ASP	CA	27ASP	CB	100.00
27ASP	CA	27ASP	CG	100.00
27ASP	CA	27ASP	C	100.00
27ASP	CA	31GLY	CA	0.06
27ASP	CA	32LEU	CB	0.32
27ASP	CA	32LEU	CG	0.00
27ASP	CA	32LEU	CD1	0.04

27ASP	CA	32LEU	CD2	0.21
27ASP	CB	27ASP	CG	100.00
27ASP	CB	27ASP	C	100.00
27ASP	CB	32LEU	CB	0.03
27ASP	CB	32LEU	CG	0.02
27ASP	CB	32LEU	CD1	0.06
27ASP	CB	32LEU	CD2	0.02
27ASP	CB	34LEU	CB	0.03
27ASP	CB	34LEU	CD1	0.07
27ASP	CB	34LEU	CD2	0.03
27ASP	CB	36TYR	CE2	0.02
27ASP	CG	27ASP	C	99.97
27ASP	CG	32LEU	CB	0.01
27ASP	CG	32LEU	CD1	0.00
27ASP	CG	34LEU	CB	3.61
27ASP	CG	34LEU	CG	0.01
27ASP	CG	34LEU	CD1	0.01
27ASP	CG	36TYR	CE2	0.14
27ASP	C	28GLU	CA	100.00
27ASP	C	28GLU	CB	2.68
27ASP	C	28GLU	CD	0.00
27ASP	C	28GLU	C	97.78
28GLU	CA	28GLU	CB	100.00
28GLU	CA	28GLU	CG	100.00
28GLU	CA	28GLU	CD	93.14
28GLU	CA	28GLU	C	100.00
28GLU	CB	28GLU	CG	100.00
28GLU	CB	28GLU	CD	100.00
28GLU	CB	28GLU	C	100.00
28GLU	CG	28GLU	CD	100.00
28GLU	CG	28GLU	C	95.64
28GLU	CD	28GLU	C	1.78
28GLU	C	29ALA	CA	100.00
28GLU	C	29ALA	CB	8.76
28GLU	C	29ALA	C	90.65
29ALA	CA	29ALA	CB	100.00
29ALA	CA	29ALA	C	100.00
29ALA	CB	29ALA	C	100.00
29ALA	CB	344LEU	CB	0.02
29ALA	CB	344LEU	CD1	0.01
29ALA	CB	344LEU	CD2	0.01
29ALA	CB	344LEU	C	0.02
29ALA	CB	345ALA	CA	0.08
29ALA	CB	345ALA	CB	0.47
29ALA	CB	345ALA	C	0.15
29ALA	C	30GLU	CA	100.00
29ALA	C	30GLU	CB	62.00
29ALA	C	30GLU	CG	0.93
29ALA	C	30GLU	CD	0.02
29ALA	C	30GLU	C	43.04
30GLU	CA	30GLU	CB	100.00
30GLU	CA	30GLU	CG	100.00
30GLU	CA	30GLU	CD	97.27
30GLU	CA	30GLU	C	100.00
30GLU	CB	30GLU	CG	100.00
30GLU	CB	30GLU	CD	100.00
30GLU	CB	30GLU	C	100.00
30GLU	CB	32LEU	CG	0.01
30GLU	CB	32LEU	CD1	0.06
30GLU	CB	32LEU	CD2	0.08
30GLU	CB	307LEU	CD1	0.26
30GLU	CB	307LEU	CD2	0.47

30GLU	CG	30GLU	CD	100.00
30GLU	CG	30GLU	C	73.75
30GLU	CG	32LEU	CG	0.06
30GLU	CG	32LEU	CD1	0.07
30GLU	CG	32LEU	CD2	0.33
30GLU	CG	307LEU	CD1	0.33
30GLU	CG	307LEU	CD2	0.85
30GLU	CG	310LYS	CE	0.01
30GLU	CD	30GLU	C	1.72
30GLU	CD	32LEU	CG	0.00
30GLU	CD	32LEU	CD2	0.02
30GLU	CD	307LEU	CD1	0.08
30GLU	CD	307LEU	CD2	0.14
30GLU	CD	310LYS	CD	0.03
30GLU	CD	310LYS	CE	0.04
30GLU	C	31GLY	CA	100.00
30GLU	C	31GLY	C	65.27
31GLY	CA	31GLY	C	100.00
31GLY	C	32LEU	CA	100.00
31GLY	C	32LEU	CB	42.58
31GLY	C	32LEU	CG	3.18
31GLY	C	32LEU	CD1	0.17
31GLY	C	32LEU	CD2	0.08
31GLY	C	32LEU	C	57.79
32LEU	CA	32LEU	CB	100.00
32LEU	CA	32LEU	CG	100.00
32LEU	CA	32LEU	CD1	15.64
32LEU	CA	32LEU	CD2	76.92
32LEU	CA	32LEU	C	100.00
32LEU	CA	302PHE	CE1	0.00
32LEU	CA	302PHE	CZ	0.02
32LEU	CA	304LEU	CD1	0.03
32LEU	CB	32LEU	CG	100.00
32LEU	CB	32LEU	CD1	100.00
32LEU	CB	32LEU	CD2	100.00
32LEU	CB	32LEU	C	100.00
32LEU	CB	34LEU	CG	0.01
32LEU	CB	34LEU	CD1	0.41
32LEU	CB	34LEU	CD2	0.07
32LEU	CB	302PHE	CD1	0.00
32LEU	CB	302PHE	CD2	0.00
32LEU	CB	302PHE	CE1	0.46
32LEU	CB	302PHE	CE2	0.30
32LEU	CB	302PHE	CZ	0.30
32LEU	CB	304LEU	CD1	1.64
32LEU	CB	304LEU	CD2	0.01
32LEU	CB	307LEU	CD1	0.05
32LEU	CB	307LEU	CD2	0.01
32LEU	CG	32LEU	CD1	100.00
32LEU	CG	32LEU	CD2	100.00
32LEU	CG	32LEU	C	25.69
32LEU	CG	34LEU	CD1	0.01
32LEU	CG	298LEU	CD1	0.01
32LEU	CG	298LEU	CD2	0.00
32LEU	CG	302PHE	CE1	0.10
32LEU	CG	302PHE	CE2	0.04
32LEU	CG	302PHE	CZ	0.12
32LEU	CG	304LEU	CD1	0.02
32LEU	CG	307LEU	CD1	0.01
32LEU	CG	307LEU	CD2	0.02
32LEU	CD1	32LEU	CD2	100.00
32LEU	CD1	32LEU	C	3.16

32LEU	CD1	33GLY	C	0.00
32LEU	CD1	34LEU	CB	0.05
32LEU	CD1	34LEU	CG	0.00
32LEU	CD1	34LEU	CD1	0.90
32LEU	CD1	34LEU	CD2	0.03
32LEU	CD1	36TYR	CD2	0.13
32LEU	CD1	36TYR	CE2	0.32
32LEU	CD1	298LEU	CD1	0.10
32LEU	CD1	298LEU	CD2	2.39
32LEU	CD1	302PHE	CG	0.03
32LEU	CD1	302PHE	CD1	0.44
32LEU	CD1	302PHE	CD2	0.22
32LEU	CD1	302PHE	CE1	2.04
32LEU	CD1	302PHE	CE2	0.48
32LEU	CD1	302PHE	CZ	1.73
32LEU	CD1	304LEU	CD1	1.82
32LEU	CD1	304LEU	CD2	0.05
32LEU	CD1	307LEU	CD1	0.35
32LEU	CD1	307LEU	CD2	0.48
32LEU	CD2	32LEU	C	4.54
32LEU	CD2	33GLY	C	0.01
32LEU	CD2	34LEU	CB	0.04
32LEU	CD2	34LEU	CG	0.00
32LEU	CD2	34LEU	CD1	0.09
32LEU	CD2	34LEU	CD2	0.01
32LEU	CD2	36TYR	CG	0.00
32LEU	CD2	36TYR	CD2	0.08
32LEU	CD2	36TYR	CE2	0.07
32LEU	CD2	298LEU	CD1	0.24
32LEU	CD2	298LEU	CD2	5.58
32LEU	CD2	302PHE	CG	0.01
32LEU	CD2	302PHE	CD1	0.09
32LEU	CD2	302PHE	CD2	0.08
32LEU	CD2	302PHE	CE1	0.33
32LEU	CD2	302PHE	CE2	0.39
32LEU	CD2	302PHE	CZ	0.28
32LEU	CD2	304LEU	CD1	6.75
32LEU	CD2	304LEU	CD2	0.10
32LEU	CD2	307LEU	CG	0.06
32LEU	CD2	307LEU	CD1	4.39
32LEU	CD2	307LEU	CD2	3.61
32LEU	C	33GLY	CA	100.00
32LEU	C	33GLY	C	79.10
32LEU	C	34LEU	CD1	0.00
32LEU	C	34LEU	CD2	0.01
32LEU	C	302PHE	CE1	0.10
32LEU	C	302PHE	CE2	0.08
32LEU	C	302PHE	CZ	0.16
33GLY	CA	33GLY	C	100.00
33GLY	C	34LEU	CA	100.00
33GLY	C	34LEU	CB	6.82
33GLY	C	34LEU	CG	0.18
33GLY	C	34LEU	CD1	0.06
33GLY	C	34LEU	CD2	0.02
33GLY	C	34LEU	C	93.72
33GLY	C	302PHE	CE1	0.02
33GLY	C	302PHE	CE2	0.01
33GLY	C	302PHE	CZ	0.02
34LEU	CA	34LEU	CB	100.00
34LEU	CA	34LEU	CG	100.00
34LEU	CA	34LEU	CD1	63.17
34LEU	CA	34LEU	CD2	31.39

34LEU	CA	34LEU	C	100.00
34LEU	CA	302PHE	CZ	0.00
34LEU	CB	34LEU	CG	100.00
34LEU	CB	34LEU	CD1	100.00
34LEU	CB	34LEU	CD2	100.00
34LEU	CB	34LEU	C	100.00
34LEU	CB	36TYR	CE1	0.13
34LEU	CB	36TYR	CE2	0.78
34LEU	CB	36TYR	CZ	0.95
34LEU	CB	302PHE	CD2	0.00
34LEU	CB	302PHE	CE1	0.04
34LEU	CB	302PHE	CE2	0.02
34LEU	CB	302PHE	CZ	0.02
34LEU	CG	34LEU	CD1	100.00
34LEU	CG	34LEU	CD2	100.00
34LEU	CG	34LEU	C	66.98
34LEU	CG	36TYR	CD1	0.02
34LEU	CG	36TYR	CD2	0.02
34LEU	CG	36TYR	CE2	0.00
34LEU	CG	302PHE	CD1	0.00
34LEU	CG	302PHE	CD2	0.00
34LEU	CG	302PHE	CE1	0.02
34LEU	CG	302PHE	CE2	0.02
34LEU	CG	302PHE	CZ	0.03
34LEU	CD1	34LEU	CD2	100.00
34LEU	CD1	34LEU	C	3.62
34LEU	CD1	36TYR	CD1	0.16
34LEU	CD1	36TYR	CD2	0.06
34LEU	CD1	36TYR	CE1	0.00
34LEU	CD1	36TYR	CE2	0.06
34LEU	CD1	36TYR	CZ	0.04
34LEU	CD1	297MET	CG	0.00
34LEU	CD1	297MET	CE	0.15
34LEU	CD1	298LEU	CD1	0.20
34LEU	CD1	298LEU	CD2	0.19
34LEU	CD1	302PHE	CG	0.02
34LEU	CD1	302PHE	CD1	0.22
34LEU	CD1	302PHE	CD2	0.07
34LEU	CD1	302PHE	CE1	0.44
34LEU	CD1	302PHE	CE2	0.23
34LEU	CD1	302PHE	CZ	0.50
34LEU	CD1	304LEU	CD1	0.06
34LEU	CD1	307LEU	CD2	0.00
34LEU	CD2	34LEU	C	1.91
34LEU	CD2	35ALA	C	0.01
34LEU	CD2	36TYR	CG	0.00
34LEU	CD2	36TYR	CD1	0.40
34LEU	CD2	36TYR	CD2	0.56
34LEU	CD2	36TYR	CE1	0.07
34LEU	CD2	36TYR	CE2	0.18
34LEU	CD2	36TYR	CZ	0.02
34LEU	CD2	65GLU	CB	0.01
34LEU	CD2	297MET	CE	0.22
34LEU	CD2	298LEU	CD1	0.08
34LEU	CD2	298LEU	CD2	0.25
34LEU	CD2	302PHE	CB	0.00
34LEU	CD2	302PHE	CG	0.23
34LEU	CD2	302PHE	CD1	0.59
34LEU	CD2	302PHE	CD2	0.52
34LEU	CD2	302PHE	CE1	1.61
34LEU	CD2	302PHE	CE2	1.42
34LEU	CD2	302PHE	CZ	1.68

34LEU	CD2	304LEU	CD1	0.49
34LEU	C	35ALA	CA	100.00
34LEU	C	35ALA	CB	45.79
34LEU	C	35ALA	C	59.93
34LEU	C	36TYR	CE1	0.00
35ALA	CA	35ALA	CB	100.00
35ALA	CA	35ALA	C	100.00
35ALA	CA	36TYR	CD1	0.00
35ALA	CA	36TYR	CD2	0.01
35ALA	CB	35ALA	C	100.00
35ALA	C	36TYR	CA	100.00
35ALA	C	36TYR	CB	81.84
35ALA	C	36TYR	CG	2.84
35ALA	C	36TYR	CD1	0.85
35ALA	C	36TYR	CD2	1.33
35ALA	C	36TYR	C	20.50
36TYR	CA	36TYR	CB	100.00
36TYR	CA	36TYR	CG	100.00
36TYR	CA	36TYR	CD1	68.05
36TYR	CA	36TYR	CD2	76.36
36TYR	CA	36TYR	C	100.00
36TYR	CB	36TYR	CG	100.00
36TYR	CB	36TYR	CD1	100.00
36TYR	CB	36TYR	CD2	100.00
36TYR	CB	36TYR	C	100.00
36TYR	CB	38VAL	CG2	0.00
36TYR	CG	36TYR	CD1	100.00
36TYR	CG	36TYR	CD2	100.00
36TYR	CG	36TYR	CE1	100.00
36TYR	CG	36TYR	CE2	100.00
36TYR	CG	36TYR	CZ	100.00
36TYR	CG	36TYR	C	79.70
36TYR	CD1	36TYR	CD2	100.00
36TYR	CD1	36TYR	CE1	100.00
36TYR	CD1	36TYR	CE2	100.00
36TYR	CD1	36TYR	CZ	100.00
36TYR	CD1	36TYR	C	22.01
36TYR	CD1	38VAL	CG1	0.00
36TYR	CD1	38VAL	CG2	0.20
36TYR	CD2	36TYR	CE1	100.00
36TYR	CD2	36TYR	CE2	100.00
36TYR	CD2	36TYR	CZ	100.00
36TYR	CD2	36TYR	C	20.78
36TYR	CD2	38VAL	CG2	0.28
36TYR	CD2	68LEU	CD1	0.00
36TYR	CE1	36TYR	CE2	100.00
36TYR	CE1	36TYR	CZ	100.00
36TYR	CE1	38VAL	CG1	0.00
36TYR	CE1	38VAL	CG2	0.56
36TYR	CE2	36TYR	CZ	100.00
36TYR	CE2	38VAL	CG2	0.75
36TYR	CE2	68LEU	CD1	0.00
36TYR	C	37GLU	CA	100.00
36TYR	C	37GLU	CB	69.69
36TYR	C	37GLU	C	43.21
37GLU	CA	37GLU	CB	100.00
37GLU	CA	37GLU	CG	100.00
37GLU	CA	37GLU	CD	94.67
37GLU	CA	37GLU	C	100.00
37GLU	CB	37GLU	CG	100.00
37GLU	CB	37GLU	CD	100.00
37GLU	CB	37GLU	C	100.00

37GLU	CB	39PHE	CD1	0.00
37GLU	CB	39PHE	CE1	0.16
37GLU	CB	39PHE	CE2	2.72
37GLU	CB	39PHE	CZ	1.78
37GLU	CG	37GLU	CD	100.00
37GLU	CG	37GLU	C	98.94
37GLU	CG	39PHE	CD1	0.05
37GLU	CG	39PHE	CD2	0.06
37GLU	CG	39PHE	CE1	4.92
37GLU	CG	39PHE	CE2	9.41
37GLU	CG	39PHE	CZ	22.09
37GLU	CD	37GLU	C	0.01
37GLU	CD	39PHE	CE1	0.11
37GLU	CD	39PHE	CE2	0.02
37GLU	CD	39PHE	CZ	0.40
37GLU	C	38VAL	CA	100.00
37GLU	C	38VAL	CB	4.22
37GLU	C	38VAL	CG1	0.08
37GLU	C	38VAL	CG2	2.39
37GLU	C	38VAL	C	98.62
37GLU	C	39PHE	CD2	0.01
37GLU	C	39PHE	CE1	0.00
37GLU	C	39PHE	CE2	0.08
38VAL	CA	38VAL	CB	100.00
38VAL	CA	38VAL	CG1	100.00
38VAL	CA	38VAL	CG2	100.00
38VAL	CA	38VAL	C	100.00
38VAL	CB	38VAL	CG1	100.00
38VAL	CB	38VAL	CG2	100.00
38VAL	CB	38VAL	C	100.00
38VAL	CG:	38VAL	CG2	100.00
38VAL	CG:	38VAL	C	96.76
38VAL	CG:	38VAL	C	3.20
38VAL	C	39PHE	CA	100.00
38VAL	C	39PHE	CB	24.53
38VAL	C	39PHE	CG	5.78
38VAL	C	39PHE	CD1	0.14
38VAL	C	39PHE	CD2	1.49
38VAL	C	39PHE	C	75.93
39PHE	CA	39PHE	CB	100.00
39PHE	CA	39PHE	CG	100.00
39PHE	CA	39PHE	CD1	94.51
39PHE	CA	39PHE	CD2	51.84
39PHE	CA	39PHE	C	100.00
39PHE	CA	40PRO	CD	100.00
39PHE	CB	39PHE	CG	100.00
39PHE	CB	39PHE	CD1	100.00
39PHE	CB	39PHE	CD2	100.00
39PHE	CB	39PHE	C	100.00
39PHE	CB	40PRO	CD	14.53
39PHE	CB	57THR	CA	0.03
39PHE	CB	57THR	CG2	1.03
39PHE	CG	39PHE	CD1	100.00
39PHE	CG	39PHE	CD2	100.00
39PHE	CG	39PHE	CE1	100.00
39PHE	CG	39PHE	CE2	100.00
39PHE	CG	39PHE	CZ	100.00
39PHE	CG	57THR	CG2	0.22
39PHE	CG	60GLY	CA	0.03
39PHE	CG	61VAL	CG1	0.04
39PHE	CD:	39PHE	CD2	100.00
39PHE	CD:	39PHE	CE1	100.00

39PHE	CD1	39PHE	CE2	100.00
39PHE	CD1	39PHE	CZ	100.00
39PHE	CD1	56PRO	CB	0.01
39PHE	CD1	56PRO	C	0.78
39PHE	CD1	57THR	CA	0.67
39PHE	CD1	57THR	CB	0.00
39PHE	CD1	57THR	CG2	0.18
39PHE	CD1	60GLY	CA	0.10
39PHE	CD1	61VAL	CG1	0.04
39PHE	CD2	39PHE	CE1	100.00
39PHE	CD2	39PHE	CE2	100.00
39PHE	CD2	39PHE	CZ	100.00
39PHE	CD2	56PRO	C	0.03
39PHE	CD2	57THR	CA	0.04
39PHE	CD2	57THR	CG2	0.05
39PHE	CD2	60GLY	CA	0.11
39PHE	CD2	61VAL	CG1	0.08
39PHE	CE1	39PHE	CE2	100.00
39PHE	CE1	39PHE	CZ	100.00
39PHE	CE1	56PRO	CB	0.00
39PHE	CE1	56PRO	C	0.32
39PHE	CE1	57THR	CA	0.04
39PHE	CE1	60GLY	CA	10.46
39PHE	CE1	60GLY	C	0.10
39PHE	CE1	61VAL	CG1	0.02
39PHE	CE2	39PHE	CZ	100.00
39PHE	CE2	56PRO	C	0.04
39PHE	CE2	60GLY	CA	6.35
39PHE	CE2	60GLY	C	1.24
39PHE	CE2	61VAL	CG1	0.03
39PHE	CZ	56PRO	C	0.01
39PHE	CZ	57THR	CA	0.00
39PHE	CZ	60GLY	CA	34.97
39PHE	CZ	60GLY	C	1.15
39PHE	CZ	61VAL	CG1	0.02
39PHE	CZ	63GLU	CG	0.00
39PHE	C	40PRO	CA	100.00
39PHE	C	40PRO	CB	0.10
39PHE	C	40PRO	CD	100.00
39PHE	C	40PRO	C	100.00
39PHE	C	57THR	CG2	0.00
40PRO	CA	40PRO	CB	100.00
40PRO	CA	40PRO	CG	100.00
40PRO	CA	40PRO	CD	100.00
40PRO	CA	40PRO	C	100.00
40PRO	CB	40PRO	CG	100.00
40PRO	CB	40PRO	CD	100.00
40PRO	CB	40PRO	C	100.00
40PRO	CB	44ALA	CB	0.01
40PRO	CB	45ALA	CB	0.01
40PRO	CB	49PHE	CD2	0.00
40PRO	CB	49PHE	CE1	0.00
40PRO	CB	49PHE	CE2	0.08
40PRO	CB	49PHE	CZ	0.00
40PRO	CB	54PRO	CG	0.03
40PRO	CG	40PRO	CD	100.00
40PRO	CG	40PRO	C	90.23
40PRO	CG	49PHE	CE1	0.03
40PRO	CG	49PHE	CE2	0.29
40PRO	CG	49PHE	CZ	0.04
40PRO	CG	54PRO	CG	0.30
40PRO	CG	54PRO	CD	0.00

40PRO	CG	56PRO	CB	0.00
40PRO	CG	56PRO	CG	0.45
40PRO	CG	57THR	CG2	0.00
40PRO	CD	40PRO	C	79.70
40PRO	CD	56PRO	CG	0.03
40PRO	CD	57THR	CG2	0.03
40PRO	C	41PHE	CA	100.00
40PRO	C	41PHE	CB	99.41
40PRO	C	41PHE	C	1.50
40PRO	C	54PRO	CG	0.03
41PHE	CA	41PHE	CB	100.00
41PHE	CA	41PHE	CG	100.00
41PHE	CA	41PHE	CD1	87.14
41PHE	CA	41PHE	CD2	37.13
41PHE	CA	41PHE	C	100.00
41PHE	CB	41PHE	CG	100.00
41PHE	CB	41PHE	CD1	100.00
41PHE	CB	41PHE	CD2	100.00
41PHE	CB	41PHE	C	100.00
41PHE	CG	41PHE	CD1	100.00
41PHE	CG	41PHE	CD2	100.00
41PHE	CG	41PHE	CE1	100.00
41PHE	CG	41PHE	CE2	100.00
41PHE	CG	41PHE	CZ	100.00
41PHE	CG	41PHE	C	99.92
41PHE	CG	53PHE	CD1	0.01
41PHE	CG	53PHE	CD2	0.00
41PHE	CG	53PHE	CE1	0.00
41PHE	CG	57THR	CG2	0.00
41PHE	CG	72VAL	CG1	0.00
41PHE	CG	72VAL	CG2	0.00
41PHE	CD1	41PHE	CD2	100.00
41PHE	CD1	41PHE	CE1	100.00
41PHE	CD1	41PHE	CE2	100.00
41PHE	CD1	41PHE	CZ	100.00
41PHE	CD1	41PHE	C	4.74
41PHE	CD1	53PHE	CB	0.53
41PHE	CD1	53PHE	CG	0.01
41PHE	CD1	53PHE	CD1	0.16
41PHE	CD1	53PHE	CD2	0.14
41PHE	CD1	53PHE	CE1	0.06
41PHE	CD1	53PHE	CE2	0.03
41PHE	CD1	57THR	CG2	0.02
41PHE	CD1	69LEU	CD1	0.13
41PHE	CD1	72VAL	CG1	0.35
41PHE	CD1	72VAL	CG2	0.00
41PHE	CD1	93LEU	CD1	0.07
41PHE	CD2	41PHE	CE1	100.00
41PHE	CD2	41PHE	CE2	100.00
41PHE	CD2	41PHE	CZ	100.00
41PHE	CD2	41PHE	C	16.96
41PHE	CD2	53PHE	CB	0.12
41PHE	CD2	53PHE	CD1	0.02
41PHE	CD2	53PHE	CE1	0.00
41PHE	CD2	69LEU	CD1	1.32
41PHE	CD2	69LEU	CD2	0.07
41PHE	CD2	72VAL	CG1	2.95
41PHE	CD2	72VAL	CG2	0.20
41PHE	CD2	93LEU	CD1	0.01
41PHE	CE1	41PHE	CE2	100.00
41PHE	CE1	41PHE	CZ	100.00
41PHE	CE1	46ILE	CD	0.06

41PHE	CE1	52PRO	CA	0.00
41PHE	CE1	52PRO	CB	0.11
41PHE	CE1	52PRO	C	0.01
41PHE	CE1	53PHE	CB	0.64
41PHE	CE1	53PHE	CG	0.08
41PHE	CE1	53PHE	CD1	0.32
41PHE	CE1	53PHE	CD2	0.13
41PHE	CE1	53PHE	CE1	0.10
41PHE	CE1	53PHE	CE2	0.02
41PHE	CE1	69LEU	CD1	1.76
41PHE	CE1	69LEU	CD2	0.01
41PHE	CE1	72VAL	CG1	2.28
41PHE	CE1	72VAL	CG2	0.08
41PHE	CE1	89GLY	C	0.08
41PHE	CE1	90LEU	CD1	0.02
41PHE	CE1	90LEU	CD2	0.38
41PHE	CE1	93LEU	CD1	3.08
41PHE	CE1	93LEU	CD2	0.55
41PHE	CE2	41PHE	CZ	100.00
41PHE	CE2	53PHE	CB	0.05
41PHE	CE2	53PHE	CG	0.00
41PHE	CE2	53PHE	CD1	0.01
41PHE	CE2	53PHE	CE1	0.00
41PHE	CE2	69LEU	CG	0.01
41PHE	CE2	69LEU	CD1	12.22
41PHE	CE2	69LEU	CD2	0.78
41PHE	CE2	72VAL	CG1	12.41
41PHE	CE2	72VAL	CG2	3.82
41PHE	CE2	89GLY	C	0.00
41PHE	CE2	90LEU	CD1	0.22
41PHE	CE2	90LEU	CD2	0.86
41PHE	CE2	93LEU	CD1	0.36
41PHE	CE2	93LEU	CD2	0.15
41PHE	CZ	46ILE	CD	0.00
41PHE	CZ	69LEU	CG	0.00
41PHE	CZ	69LEU	CD1	0.34
41PHE	CZ	69LEU	CD2	0.06
41PHE	CZ	72VAL	CG1	1.04
41PHE	CZ	72VAL	CG2	2.53
41PHE	CZ	89GLY	C	0.42
41PHE	CZ	90LEU	CA	0.01
41PHE	CZ	90LEU	CG	0.01
41PHE	CZ	90LEU	CD1	0.44
41PHE	CZ	90LEU	CD2	1.92
41PHE	CZ	93LEU	CD1	1.94
41PHE	CZ	93LEU	CD2	1.02
41PHE	C	42GLY	CA	100.00
41PHE	C	42GLY	C	22.82
41PHE	C	45ALA	CB	0.02
41PHE	C	72VAL	CG1	0.01
42GLY	CA	42GLY	C	100.00
42GLY	CA	72VAL	CG1	1.57
42GLY	CA	72VAL	CG2	0.02
42GLY	CA	77TRP	CZ3	0.00
42GLY	CA	86PRO	CB	0.00
42GLY	C	43GLY	CA	100.00
42GLY	C	43GLY	C	45.49
42GLY	C	46ILE	CD	0.01
42GLY	C	72VAL	CB	0.00
42GLY	C	72VAL	CG1	5.59
42GLY	C	72VAL	CG2	0.01
42GLY	C	86PRO	CB	0.06

42GLY	C	86PRO	CG	0.02
43GLY	CA	43GLY	C	100.00
43GLY	CA	46ILE	CG1	0.00
43GLY	CA	46ILE	CG2	0.01
43GLY	CA	46ILE	CD	0.32
43GLY	CA	72VAL	CG1	0.55
43GLY	CA	72VAL	CG2	0.00
43GLY	CA	77TRP	CD2	0.01
43GLY	CA	77TRP	CE2	0.03
43GLY	CA	77TRP	CE3	0.00
43GLY	CA	77TRP	CZ2	1.31
43GLY	CA	77TRP	CZ3	0.05
43GLY	CA	86PRO	CB	0.60
43GLY	CA	86PRO	CG	0.26
43GLY	C	44ALA	CA	100.00
43GLY	C	44ALA	CB	0.24
43GLY	C	44ALA	C	99.79
43GLY	C	46ILE	CG1	0.00
43GLY	C	46ILE	CD	0.05
43GLY	C	77TRP	CE2	0.02
43GLY	C	77TRP	CZ2	0.78
43GLY	C	77TRP	CZ3	0.03
44ALA	CA	44ALA	CB	100.00
44ALA	CA	44ALA	C	100.00
44ALA	CA	77TRP	CZ2	0.03
44ALA	CB	44ALA	C	100.00
44ALA	CB	77TRP	CZ2	0.01
44ALA	C	45ALA	CA	100.00
44ALA	C	45ALA	CB	0.02
44ALA	C	45ALA	C	99.98
45ALA	CA	45ALA	CB	100.00
45ALA	CA	45ALA	C	100.00
45ALA	CA	49PHE	CD1	0.04
45ALA	CA	49PHE	CD2	0.02
45ALA	CA	49PHE	CE1	1.07
45ALA	CA	49PHE	CE2	0.80
45ALA	CA	49PHE	CZ	0.71
45ALA	CB	45ALA	C	100.00
45ALA	CB	49PHE	CD1	0.02
45ALA	CB	49PHE	CD2	0.01
45ALA	CB	49PHE	CE1	3.04
45ALA	CB	49PHE	CE2	2.55
45ALA	CB	49PHE	CZ	7.90
45ALA	CB	51GLU	CB	0.00
45ALA	CB	51GLU	CG	0.00
45ALA	CB	52PRO	CA	0.01
45ALA	CB	52PRO	C	0.03
45ALA	CB	53PHE	C	0.01
45ALA	CB	54PRO	CG	0.05
45ALA	CB	54PRO	CD	0.05
45ALA	C	46ILE	CA	100.00
45ALA	C	46ILE	CB	0.01
45ALA	C	46ILE	C	100.00
46ILE	CA	46ILE	CB	100.00
46ILE	CA	46ILE	CG1	100.00
46ILE	CA	46ILE	CG2	100.00
46ILE	CA	46ILE	CD	5.25
46ILE	CA	46ILE	C	100.00
46ILE	CA	50GLY	CA	0.04
46ILE	CB	46ILE	CG1	100.00
46ILE	CB	46ILE	CG2	100.00
46ILE	CB	46ILE	CD	100.00

46ILE	CB	46ILE	C	100.00
46ILE	CB	86PRO	CB	0.00
46ILE	CG1	46ILE	CG2	100.00
46ILE	CG1	46ILE	CD	100.00
46ILE	CG1	46ILE	C	1.08
46ILE	CG1	51GLU	C	0.01
46ILE	CG1	52PRO	CA	0.07
46ILE	CG1	52PRO	CB	0.04
46ILE	CG1	52PRO	CD	0.00
46ILE	CG1	88THR	CG2	0.23
46ILE	CG1	89GLY	CA	0.00
46ILE	CG2	46ILE	CD	93.07
46ILE	CG2	46ILE	C	99.41
46ILE	CG2	51GLU	C	0.00
46ILE	CG2	52PRO	CA	0.02
46ILE	CG2	77TRP	CD1	0.00
46ILE	CG2	77TRP	CE2	0.01
46ILE	CG2	77TRP	CZ2	0.08
46ILE	CG2	84ILE	CA	0.09
46ILE	CG2	84ILE	CB	0.01
46ILE	CG2	84ILE	CG1	0.00
46ILE	CG2	84ILE	CG2	0.02
46ILE	CG2	84ILE	CD	0.00
46ILE	CG2	84ILE	C	0.12
46ILE	CG2	85ARG	C	0.00
46ILE	CG2	86PRO	CA	0.06
46ILE	CG2	86PRO	CB	0.01
46ILE	CG2	88THR	CG2	12.21
46ILE	CG2	89GLY	CA	0.06
46ILE	CD	52PRO	CA	0.01
46ILE	CD	52PRO	CB	0.01
46ILE	CD	72VAL	CG1	0.00
46ILE	CD	72VAL	CG2	0.04
46ILE	CD	77TRP	CZ2	0.06
46ILE	CD	84ILE	C	0.02
46ILE	CD	85ARG	C	0.10
46ILE	CD	86PRO	CA	0.61
46ILE	CD	86PRO	CB	0.12
46ILE	CD	86PRO	CG	0.02
46ILE	CD	86PRO	CD	0.09
46ILE	CD	88THR	CG2	1.43
46ILE	CD	88THR	C	0.04
46ILE	CD	89GLY	CA	0.27
46ILE	CD	90LEU	CD1	0.00
46ILE	C	47ASP	CA	100.00
46ILE	C	47ASP	CB	1.10
46ILE	C	47ASP	C	99.20
46ILE	C	84ILE	CG2	0.00
46ILE	C	84ILE	CD	0.00
47ASP	CA	47ASP	CB	100.00
47ASP	CA	47ASP	CG	100.00
47ASP	CA	47ASP	C	100.00
47ASP	CA	84ILE	CG2	0.07
47ASP	CB	47ASP	CG	100.00
47ASP	CB	47ASP	C	100.00
47ASP	CB	77TRP	CD1	0.00
47ASP	CB	77TRP	CE2	0.01
47ASP	CB	77TRP	CZ2	0.98
47ASP	CB	84ILE	CG2	0.02
47ASP	CB	84ILE	CD	0.00
47ASP	CG	47ASP	C	99.90
47ASP	CG	76LYS	CD	0.00

47ASP	CG	76LYS	CE	0.23
47ASP	CG	77TRP	CD1	0.00
47ASP	CG	77TRP	CE2	0.04
47ASP	CG	77TRP	CZ2	0.58
47ASP	CG	84ILE	CG2	0.00
47ASP	CG	84ILE	CD	0.00
47ASP	C	48ALA	CA	100.00
47ASP	C	48ALA	CB	1.25
47ASP	C	48ALA	C	98.50
48ALA	CA	48ALA	CB	100.00
48ALA	CA	48ALA	C	100.00
48ALA	CB	48ALA	C	100.00
48ALA	CB	49PHE	CG	0.02
48ALA	CB	49PHE	CD1	3.88
48ALA	CB	49PHE	CD2	4.61
48ALA	CB	49PHE	CE1	0.23
48ALA	CB	49PHE	CE2	0.32
48ALA	CB	49PHE	CZ	0.00
48ALA	C	49PHE	CA	100.00
48ALA	C	49PHE	CB	93.78
48ALA	C	49PHE	CG	2.88
48ALA	C	49PHE	CD1	1.60
48ALA	C	49PHE	CD2	2.10
48ALA	C	49PHE	C	5.94
48ALA	C	84ILE	CG2	0.00
49PHE	CA	49PHE	CB	100.00
49PHE	CA	49PHE	CG	100.00
49PHE	CA	49PHE	CD1	65.55
49PHE	CA	49PHE	CD2	68.49
49PHE	CA	49PHE	C	100.00
49PHE	CB	49PHE	CG	100.00
49PHE	CB	49PHE	CD1	100.00
49PHE	CB	49PHE	CD2	100.00
49PHE	CB	49PHE	C	100.00
49PHE	CB	51GLU	CB	0.00
49PHE	CB	51GLU	CG	0.02
49PHE	CB	54PRO	CB	0.36
49PHE	CB	54PRO	CG	0.01
49PHE	CG	49PHE	CD1	100.00
49PHE	CG	49PHE	CD2	100.00
49PHE	CG	49PHE	CE1	100.00
49PHE	CG	49PHE	CE2	100.00
49PHE	CG	49PHE	CZ	100.00
49PHE	CG	49PHE	C	84.93
49PHE	CG	54PRO	CB	0.74
49PHE	CG	54PRO	CG	0.10
49PHE	CD1	49PHE	CD2	100.00
49PHE	CD1	49PHE	CE1	100.00
49PHE	CD1	49PHE	CE2	100.00
49PHE	CD1	49PHE	CZ	100.00
49PHE	CD1	49PHE	C	8.89
49PHE	CD1	51GLU	CB	1.10
49PHE	CD1	51GLU	CG	0.19
49PHE	CD1	54PRO	CB	0.30
49PHE	CD1	54PRO	CG	0.16
49PHE	CD2	49PHE	CE1	100.00
49PHE	CD2	49PHE	CE2	100.00
49PHE	CD2	49PHE	CZ	100.00
49PHE	CD2	49PHE	C	12.48
49PHE	CD2	51GLU	CB	0.88
49PHE	CD2	51GLU	CG	0.02
49PHE	CD2	54PRO	CB	0.47

49PHE	CD2	54PRO	CG	0.34
49PHE	CE1	49PHE	CE2	100.00
49PHE	CE1	49PHE	CZ	100.00
49PHE	CE1	51GLU	CB	13.89
49PHE	CE1	51GLU	CG	0.68
49PHE	CE1	51GLU	CD	0.01
49PHE	CE1	53PHE	C	0.00
49PHE	CE1	54PRO	CA	0.00
49PHE	CE1	54PRO	CB	0.06
49PHE	CE1	54PRO	CG	0.02
49PHE	CE1	56PRO	CG	0.09
49PHE	CE1	56PRO	CD	0.09
49PHE	CE2	49PHE	CZ	100.00
49PHE	CE2	51GLU	CB	13.48
49PHE	CE2	51GLU	CG	0.22
49PHE	CE2	51GLU	CD	0.01
49PHE	CE2	54PRO	CB	0.02
49PHE	CE2	54PRO	CG	0.07
49PHE	CE2	56PRO	CG	0.04
49PHE	CZ	51GLU	CB	5.08
49PHE	CZ	51GLU	CG	0.39
49PHE	CZ	51GLU	CD	0.02
49PHE	CZ	53PHE	C	0.00
49PHE	CZ	54PRO	CA	0.00
49PHE	CZ	54PRO	CB	0.04
49PHE	CZ	56PRO	CG	0.27
49PHE	CZ	56PRO	CD	0.11
49PHE	C	50GLY	CA	100.00
49PHE	C	50GLY	C	63.22
50GLY	CA	50GLY	C	100.00
50GLY	CA	84ILE	CD	0.00
50GLY	CA	88THR	CG2	0.01
50GLY	C	51GLU	CA	100.00
50GLY	C	51GLU	CB	3.70
50GLY	C	51GLU	CG	0.04
50GLY	C	51GLU	CD	0.01
50GLY	C	51GLU	C	97.47
51GLU	CA	51GLU	CB	100.00
51GLU	CA	51GLU	CG	100.00
51GLU	CA	51GLU	CD	95.12
51GLU	CA	51GLU	C	100.00
51GLU	CA	52PRO	CD	100.00
51GLU	CB	51GLU	CG	100.00
51GLU	CB	51GLU	CD	100.00
51GLU	CB	51GLU	C	100.00
51GLU	CB	52PRO	CD	1.27
51GLU	CB	54PRO	CA	0.00
51GLU	CG	51GLU	CD	100.00
51GLU	CG	51GLU	C	91.74
51GLU	CG	52PRO	CD	0.64
51GLU	CG	53PHE	C	0.31
51GLU	CG	54PRO	CA	0.32
51GLU	CG	54PRO	CB	0.00
51GLU	CD	51GLU	C	0.06
51GLU	CD	52PRO	CD	0.34
51GLU	CD	54PRO	CA	0.01
51GLU	CD	58ARG	CZ	0.03
51GLU	C	52PRO	CA	100.00
51GLU	C	52PRO	CB	0.12
51GLU	C	52PRO	CD	100.00
51GLU	C	52PRO	C	100.00
52PRO	CA	52PRO	CB	100.00

52PRO	CA	52PRO	CG	100.00
52PRO	CA	52PRO	CD	100.00
52PRO	CA	52PRO	C	100.00
52PRO	CA	89GLY	CA	0.02
52PRO	CB	52PRO	CG	100.00
52PRO	CB	52PRO	CD	100.00
52PRO	CB	52PRO	C	100.00
52PRO	CB	53PHE	CD1	0.15
52PRO	CB	53PHE	CD2	0.22
52PRO	CB	53PHE	CE1	0.00
52PRO	CB	53PHE	CE2	0.02
52PRO	CB	86PRO	CA	0.04
52PRO	CB	86PRO	CB	0.08
52PRO	CB	88THR	CG2	0.16
52PRO	CB	88THR	C	0.01
52PRO	CB	89GLY	CA	4.28
52PRO	CB	92SER	CB	4.29
52PRO	CB	93LEU	CA	0.10
52PRO	CB	93LEU	CB	0.05
52PRO	CB	93LEU	CD1	0.02
52PRO	CG	52PRO	CD	100.00
52PRO	CG	52PRO	C	90.57
52PRO	CG	53PHE	CD1	0.03
52PRO	CG	53PHE	CD2	0.07
52PRO	CG	53PHE	CE1	0.04
52PRO	CG	53PHE	CE2	0.08
52PRO	CG	53PHE	CZ	0.02
52PRO	CG	58ARG	CZ	0.04
52PRO	CG	86PRO	CA	0.00
52PRO	CG	88THR	CG2	0.51
52PRO	CG	89GLY	CA	0.10
52PRO	CG	89GLY	C	0.00
52PRO	CG	92SER	CB	0.24
52PRO	CG	92SER	C	0.00
52PRO	CG	93LEU	CA	0.00
52PRO	CG	93LEU	CB	0.00
52PRO	CG	93LEU	CG	0.00
52PRO	CG	93LEU	CD1	0.01
52PRO	CD	52PRO	C	78.08
52PRO	CD	58ARG	CZ	0.00
52PRO	CD	92SER	CB	0.01
52PRO	C	53PHE	CA	100.00
52PRO	C	53PHE	CB	96.60
52PRO	C	53PHE	CG	1.51
52PRO	C	53PHE	CD1	2.14
52PRO	C	53PHE	CD2	3.98
52PRO	C	53PHE	C	5.51
52PRO	C	54PRO	CD	0.63
52PRO	C	89GLY	CA	0.03
53PHE	CA	53PHE	CB	100.00
53PHE	CA	53PHE	CG	100.00
53PHE	CA	53PHE	CD1	56.31
53PHE	CA	53PHE	CD2	57.81
53PHE	CA	53PHE	C	100.00
53PHE	CA	54PRO	CD	100.00
53PHE	CB	53PHE	CG	100.00
53PHE	CB	53PHE	CD1	100.00
53PHE	CB	53PHE	CD2	100.00
53PHE	CB	53PHE	C	100.00
53PHE	CB	54PRO	CD	13.95
53PHE	CB	57THR	CB	0.00
53PHE	CB	57THR	CG2	0.29

53PHE	CB	58ARG	CA	0.01
53PHE	CB	58ARG	CG	0.01
53PHE	CB	89GLY	CA	0.16
53PHE	CB	92SER	CB	0.11
53PHE	CB	93LEU	CD1	0.01
53PHE	CB	93LEU	CD2	0.00
53PHE	CG	53PHE	CD1	100.00
53PHE	CG	53PHE	CD2	100.00
53PHE	CG	53PHE	CE1	100.00
53PHE	CG	53PHE	CE2	100.00
53PHE	CG	53PHE	CZ	100.00
53PHE	CG	53PHE	C	95.09
53PHE	CG	54PRO	CD	0.00
53PHE	CG	57THR	CB	0.02
53PHE	CG	57THR	CG2	1.14
53PHE	CG	58ARG	CB	0.00
53PHE	CG	58ARG	CG	0.05
53PHE	CG	92SER	CB	0.03
53PHE	CG	93LEU	CG	0.01
53PHE	CG	93LEU	CD1	0.39
53PHE	CG	93LEU	CD2	0.15
53PHE	CD1	53PHE	CD2	100.00
53PHE	CD1	53PHE	CE1	100.00
53PHE	CD1	53PHE	CE2	100.00
53PHE	CD1	53PHE	CZ	100.00
53PHE	CD1	53PHE	C	7.65
53PHE	CD1	54PRO	CD	0.02
53PHE	CD1	57THR	CB	0.18
53PHE	CD1	57THR	CG2	1.11
53PHE	CD1	57THR	C	0.04
53PHE	CD1	58ARG	CA	0.15
53PHE	CD1	58ARG	CB	0.07
53PHE	CD1	58ARG	CG	2.05
53PHE	CD1	58ARG	CD	0.06
53PHE	CD1	89GLY	C	0.00
53PHE	CD1	92SER	CB	0.14
53PHE	CD1	93LEU	CB	0.00
53PHE	CD1	93LEU	CG	0.07
53PHE	CD1	93LEU	CD1	1.25
53PHE	CD1	93LEU	CD2	0.36
53PHE	CD2	53PHE	CE1	100.00
53PHE	CD2	53PHE	CE2	100.00
53PHE	CD2	53PHE	CZ	100.00
53PHE	CD2	53PHE	C	3.88
53PHE	CD2	57THR	CB	0.10
53PHE	CD2	57THR	CG2	1.92
53PHE	CD2	57THR	C	0.01
53PHE	CD2	58ARG	CA	0.07
53PHE	CD2	58ARG	CB	0.03
53PHE	CD2	58ARG	CG	1.79
53PHE	CD2	58ARG	CD	0.02
53PHE	CD2	58ARG	CZ	0.49
53PHE	CD2	89GLY	CA	0.01
53PHE	CD2	92SER	CB	0.32
53PHE	CD2	93LEU	CA	0.00
53PHE	CD2	93LEU	CB	0.02
53PHE	CD2	93LEU	CG	0.14
53PHE	CD2	93LEU	CD1	1.07
53PHE	CD2	93LEU	CD2	0.42
53PHE	CE1	53PHE	CE2	100.00
53PHE	CE1	53PHE	CZ	100.00
53PHE	CE1	57THR	CB	0.10

53PHE	CE1	57THR	CG2	0.46
53PHE	CE1	57THR	C	0.05
53PHE	CE1	58ARG	CA	0.97
53PHE	CE1	58ARG	CB	0.28
53PHE	CE1	58ARG	CG	4.93
53PHE	CE1	58ARG	CD	0.42
53PHE	CE1	58ARG	CZ	0.21
53PHE	CE1	61VAL	CG1	0.01
53PHE	CE1	61VAL	CG2	0.06
53PHE	CE1	92SER	CB	0.48
53PHE	CE1	92SER	C	0.42
53PHE	CE1	93LEU	CA	1.40
53PHE	CE1	93LEU	CB	0.24
53PHE	CE1	93LEU	CG	0.13
53PHE	CE1	93LEU	CD1	3.70
53PHE	CE1	93LEU	CD2	0.65
53PHE	CE1	96SER	CB	0.10
53PHE	CE1	97GLN	CD	0.00
53PHE	CE2	53PHE	CZ	100.00
53PHE	CE2	57THR	CB	0.00
53PHE	CE2	57THR	CG2	0.08
53PHE	CE2	57THR	C	0.08
53PHE	CE2	58ARG	CA	1.16
53PHE	CE2	58ARG	CB	0.21
53PHE	CE2	58ARG	CG	3.98
53PHE	CE2	58ARG	CD	0.24
53PHE	CE2	58ARG	CZ	0.24
53PHE	CE2	61VAL	CG1	0.02
53PHE	CE2	61VAL	CG2	0.07
53PHE	CE2	92SER	CB	0.22
53PHE	CE2	92SER	C	0.32
53PHE	CE2	93LEU	CA	3.34
53PHE	CE2	93LEU	CB	0.59
53PHE	CE2	93LEU	CG	0.37
53PHE	CE2	93LEU	CD1	3.64
53PHE	CE2	93LEU	CD2	0.63
53PHE	CE2	96SER	CB	0.02
53PHE	CZ	57THR	CB	0.02
53PHE	CZ	57THR	CG2	0.12
53PHE	CZ	57THR	C	0.03
53PHE	CZ	58ARG	CA	0.14
53PHE	CZ	58ARG	CB	0.14
53PHE	CZ	58ARG	CG	2.42
53PHE	CZ	58ARG	CD	0.10
53PHE	CZ	58ARG	CZ	0.14
53PHE	CZ	61VAL	CG1	0.03
53PHE	CZ	61VAL	CG2	0.03
53PHE	CZ	92SER	CB	0.01
53PHE	CZ	92SER	C	0.03
53PHE	CZ	93LEU	CA	0.62
53PHE	CZ	93LEU	CB	0.17
53PHE	CZ	93LEU	CG	0.26
53PHE	CZ	93LEU	CD1	3.44
53PHE	CZ	93LEU	CD2	0.47
53PHE	CZ	96SER	CB	0.52
53PHE	CZ	97GLN	CD	0.00
53PHE	C	54PRO	CA	100.00
53PHE	C	54PRO	CB	1.08
53PHE	C	54PRO	CD	100.00
53PHE	C	54PRO	C	99.99
54PRO	CA	54PRO	CB	100.00
54PRO	CA	54PRO	CG	100.00

54PRO	CA	54PRO	CD	100.00
54PRO	CA	54PRO	C	100.00
54PRO	CB	54PRO	CG	100.00
54PRO	CB	54PRO	CD	100.00
54PRO	CB	54PRO	C	100.00
54PRO	CB	56PRO	CD	0.00
54PRO	CG	54PRO	CD	100.00
54PRO	CG	54PRO	C	95.20
54PRO	CG	57THR	CG2	0.01
54PRO	CD	54PRO	C	88.32
54PRO	CD	57THR	CB	0.04
54PRO	CD	57THR	CG2	0.01
54PRO	C	55GLU	CA	100.00
54PRO	C	55GLU	C	100.00
54PRO	C	56PRO	CD	22.53
54PRO	C	58ARG	CG	0.11
54PRO	C	58ARG	CD	0.00
54PRO	C	58ARG	CZ	0.12
55GLU	CA	55GLU	CB	100.00
55GLU	CA	55GLU	CG	100.00
55GLU	CA	55GLU	CD	89.65
55GLU	CA	55GLU	C	100.00
55GLU	CA	56PRO	CD	100.00
55GLU	CA	58ARG	CB	0.00
55GLU	CA	58ARG	CZ	1.81
55GLU	CB	55GLU	CG	100.00
55GLU	CB	55GLU	CD	100.00
55GLU	CB	55GLU	C	100.00
55GLU	CB	56PRO	CD	73.64
55GLU	CG	55GLU	CD	100.00
55GLU	CG	55GLU	C	83.68
55GLU	CG	56PRO	CD	0.22
55GLU	CG	58ARG	CZ	0.02
55GLU	CG	59LYS	CD	0.01
55GLU	CG	59LYS	CE	0.04
55GLU	CD	55GLU	C	7.03
55GLU	CD	58ARG	CD	0.02
55GLU	CD	58ARG	CZ	0.26
55GLU	CD	59LYS	CG	0.02
55GLU	CD	59LYS	CD	0.08
55GLU	CD	59LYS	CE	0.77
55GLU	C	56PRO	CA	100.00
55GLU	C	56PRO	CB	0.06
55GLU	C	56PRO	CD	100.00
55GLU	C	56PRO	C	100.00
56PRO	CA	56PRO	CB	100.00
56PRO	CA	56PRO	CG	100.00
56PRO	CA	56PRO	CD	100.00
56PRO	CA	56PRO	C	100.00
56PRO	CB	56PRO	CG	100.00
56PRO	CB	56PRO	CD	100.00
56PRO	CB	56PRO	C	100.00
56PRO	CG	56PRO	CD	100.00
56PRO	CG	56PRO	C	87.43
56PRO	CD	56PRO	C	74.09
56PRO	C	57THR	CA	100.00
56PRO	C	57THR	CB	0.06
56PRO	C	57THR	C	99.99
57THR	CA	57THR	CB	100.00
57THR	CA	57THR	CG2	100.00
57THR	CA	57THR	C	100.00
57THR	CB	57THR	CG2	100.00

57THR	CB	57THR	C	100.00
57THR	CG2	57THR	C	97.00
57THR	CG2	61VAL	CG1	0.04
57THR	CG2	93LEU	CD1	0.01
57THR	C	58ARG	CA	100.00
57THR	C	58ARG	CB	0.02
57THR	C	58ARG	C	99.98
58ARG	CA	58ARG	CB	100.00
58ARG	CA	58ARG	CG	100.00
58ARG	CA	58ARG	CD	1.36
58ARG	CA	58ARG	C	100.00
58ARG	CB	58ARG	CG	100.00
58ARG	CB	58ARG	CD	100.00
58ARG	CB	58ARG	CZ	7.72
58ARG	CB	58ARG	C	100.00
58ARG	CG	58ARG	CD	100.00
58ARG	CG	58ARG	CZ	39.85
58ARG	CG	58ARG	C	44.25
58ARG	CG	97GLN	CD	0.00
58ARG	CD	58ARG	CZ	100.00
58ARG	CD	58ARG	C	0.09
58ARG	CD	96SER	CB	0.00
58ARG	CZ	59LYS	CG	0.01
58ARG	CZ	96SER	CB	0.02
58ARG	C	59LYS	CA	100.00
58ARG	C	59LYS	CB	13.50
58ARG	C	59LYS	CG	0.68
58ARG	C	59LYS	CD	0.02
58ARG	C	59LYS	C	86.74
58ARG	C	61VAL	CG2	0.01
59LYS	CA	59LYS	CB	100.00
59LYS	CA	59LYS	CG	100.00
59LYS	CA	59LYS	CD	11.20
59LYS	CA	59LYS	CE	0.06
59LYS	CA	59LYS	C	100.00
59LYS	CB	59LYS	CG	100.00
59LYS	CB	59LYS	CD	100.00
59LYS	CB	59LYS	CE	12.13
59LYS	CB	59LYS	C	100.00
59LYS	CG	59LYS	CD	100.00
59LYS	CG	59LYS	CE	100.00
59LYS	CG	59LYS	C	23.75
59LYS	CD	59LYS	CE	100.00
59LYS	CD	59LYS	C	0.25
59LYS	CE	59LYS	C	0.00
59LYS	CE	62GLU	CD	0.01
59LYS	C	60GLY	CA	100.00
59LYS	C	60GLY	C	71.24
59LYS	C	62GLU	CB	0.03
59LYS	C	63GLU	CB	0.02
60GLY	CA	60GLY	C	100.00
60GLY	C	61VAL	CA	100.00
60GLY	C	61VAL	CB	0.58
60GLY	C	61VAL	CG1	0.27
60GLY	C	61VAL	C	99.50
61VAL	CA	61VAL	CB	100.00
61VAL	CA	61VAL	CG1	100.00
61VAL	CA	61VAL	CG2	100.00
61VAL	CA	61VAL	C	100.00
61VAL	CA	64ALA	CB	0.00
61VAL	CB	61VAL	CG1	100.00
61VAL	CB	61VAL	CG2	100.00

61VAL	CB	61VAL	C	100.00
61VAL	CB	67VAL	CG2	0.36
61VAL	CG:	61VAL	CG2	100.00
61VAL	CG:	61VAL	C	2.89
61VAL	CG:	67VAL	CG1	0.46
61VAL	CG:	67VAL	CG2	2.11
61VAL	CG:	93LEU	CD1	0.27
61VAL	CG:	93LEU	CD2	0.02
61VAL	CG:	97GLN	CG	0.01
61VAL	CG:	97GLN	CD	0.08
61VAL	CG:	61VAL	C	97.04
61VAL	CG:	93LEU	CD1	0.12
61VAL	CG:	93LEU	CD2	0.01
61VAL	CG:	96SER	CB	0.03
61VAL	CG:	97GLN	CG	0.03
61VAL	CG:	97GLN	CD	0.34
61VAL	C	62GLU	CA	100.00
61VAL	C	62GLU	CB	4.31
61VAL	C	62GLU	CG	0.04
61VAL	C	62GLU	C	96.52
61VAL	C	67VAL	CG2	0.00
62GLU	CA	62GLU	CB	100.00
62GLU	CA	62GLU	CG	100.00
62GLU	CA	62GLU	CD	93.04
62GLU	CA	62GLU	C	100.00
62GLU	CB	62GLU	CG	100.00
62GLU	CB	62GLU	CD	100.00
62GLU	CB	62GLU	C	100.00
62GLU	CG	62GLU	CD	100.00
62GLU	CG	62GLU	C	86.30
62GLU	CG	265GLY	CA	0.01
62GLU	CD	62GLU	C	1.00
62GLU	CD	265GLY	CA	0.03
62GLU	CD	266THR	CB	0.02
62GLU	C	63GLU	CA	100.00
62GLU	C	63GLU	CB	30.35
62GLU	C	63GLU	CG	0.24
62GLU	C	63GLU	CD	0.01
62GLU	C	63GLU	C	73.53
62GLU	C	266THR	CG2	0.01
63GLU	CA	63GLU	CB	100.00
63GLU	CA	63GLU	CG	100.00
63GLU	CA	63GLU	CD	81.28
63GLU	CA	63GLU	C	100.00
63GLU	CB	63GLU	CG	100.00
63GLU	CB	63GLU	CD	100.00
63GLU	CB	63GLU	C	100.00
63GLU	CG	63GLU	CD	100.00
63GLU	CG	63GLU	C	85.77
63GLU	CD	63GLU	C	3.44
63GLU	C	64ALA	CA	100.00
63GLU	C	64ALA	CB	40.82
63GLU	C	64ALA	C	59.22
64ALA	CA	64ALA	CB	100.00
64ALA	CA	64ALA	C	100.00
64ALA	CB	64ALA	C	100.00
64ALA	CB	66ALA	C	0.01
64ALA	CB	67VAL	CG2	0.14
64ALA	CB	266THR	CG2	0.00
64ALA	C	65GLU	CA	100.00
64ALA	C	65GLU	CB	7.23
64ALA	C	65GLU	CG	0.07

64ALA	C	65GLU	CD	0.04
64ALA	C	65GLU	C	93.54
64ALA	C	266THR	CG2	0.52
65GLU	CA	65GLU	CB	100.00
65GLU	CA	65GLU	CG	100.00
65GLU	CA	65GLU	CD	91.37
65GLU	CA	65GLU	C	100.00
65GLU	CA	266THR	CG2	0.11
65GLU	CB	65GLU	CG	100.00
65GLU	CB	65GLU	CD	100.00
65GLU	CB	65GLU	C	100.00
65GLU	CB	302PHE	CE1	0.41
65GLU	CB	302PHE	CE2	1.60
65GLU	CB	302PHE	CZ	1.17
65GLU	CG	65GLU	CD	100.00
65GLU	CG	65GLU	C	70.87
65GLU	CG	301ALA	CB	0.01
65GLU	CG	302PHE	CD1	0.02
65GLU	CG	302PHE	CD2	0.17
65GLU	CG	302PHE	CE1	0.07
65GLU	CG	302PHE	CE2	0.57
65GLU	CG	302PHE	CZ	0.04
65GLU	CD	65GLU	C	0.03
65GLU	CD	302PHE	CE1	0.00
65GLU	CD	302PHE	CE2	0.01
65GLU	C	66ALA	CA	100.00
65GLU	C	66ALA	CB	99.26
65GLU	C	66ALA	C	0.86
65GLU	C	266THR	CB	0.03
65GLU	C	266THR	CG2	5.82
66ALA	CA	66ALA	CB	100.00
66ALA	CA	66ALA	C	100.00
66ALA	CA	266THR	CG2	2.24
66ALA	CB	66ALA	C	100.00
66ALA	CB	267PRO	CG	0.06
66ALA	CB	269PHE	CD1	0.06
66ALA	CB	269PHE	CE1	2.38
66ALA	CB	297MET	CG	3.58
66ALA	CB	297MET	CE	0.56
66ALA	CB	301ALA	CB	0.35
66ALA	CB	302PHE	CE1	0.24
66ALA	CB	302PHE	CE2	0.15
66ALA	CB	302PHE	CZ	0.27
66ALA	C	67VAL	CA	100.00
66ALA	C	67VAL	CB	39.87
66ALA	C	67VAL	CG2	28.38
66ALA	C	67VAL	C	75.99
66ALA	C	266THR	CG2	0.70
66ALA	C	297MET	CE	0.02
67VAL	CA	67VAL	CB	100.00
67VAL	CA	67VAL	CG1	100.00
67VAL	CA	67VAL	CG2	100.00
67VAL	CA	67VAL	C	100.00
67VAL	CB	67VAL	CG1	100.00
67VAL	CB	67VAL	CG2	100.00
67VAL	CB	67VAL	C	100.00
67VAL	CB	268VAL	CG1	0.00
67VAL	CB	268VAL	CG2	0.10
67VAL	CG:	67VAL	CG2	100.00
67VAL	CG:	67VAL	C	100.00
67VAL	CG:	68LEU	C	0.08
67VAL	CG:	69LEU	CB	0.00

67VAL	CG1	69LEU	CG	0.00
67VAL	CG1	69LEU	CD1	0.12
67VAL	CG1	69LEU	CD2	0.08
67VAL	CG1	268VAL	CG1	0.14
67VAL	CG1	268VAL	CG2	0.02
67VAL	CG2	97GLN	CD	0.01
67VAL	CG2	266THR	CB	0.05
67VAL	CG2	266THR	CG2	2.17
67VAL	CG2	268VAL	CG2	0.22
67VAL	C	68LEU	CA	100.00
67VAL	C	68LEU	CB	11.52
67VAL	C	68LEU	CG	0.00
67VAL	C	68LEU	CD1	0.04
67VAL	C	68LEU	CD2	0.00
67VAL	C	68LEU	C	95.80
67VAL	C	297MET	CE	0.34
68LEU	CA	68LEU	CB	100.00
68LEU	CA	68LEU	CG	100.00
68LEU	CA	68LEU	CD1	61.58
68LEU	CA	68LEU	CD2	37.56
68LEU	CA	68LEU	C	100.00
68LEU	CA	297MET	CE	0.01
68LEU	CB	68LEU	CG	100.00
68LEU	CB	68LEU	CD1	100.00
68LEU	CB	68LEU	CD2	100.00
68LEU	CB	68LEU	C	100.00
68LEU	CB	297MET	CE	0.04
68LEU	CG	68LEU	CD1	100.00
68LEU	CG	68LEU	CD2	100.00
68LEU	CG	68LEU	C	58.97
68LEU	CG	297MET	CE	0.12
68LEU	CD1	68LEU	CD2	100.00
68LEU	CD1	68LEU	C	1.54
68LEU	CD1	269PHE	CB	0.17
68LEU	CD1	269PHE	C	0.00
68LEU	CD1	293SER	CB	0.06
68LEU	CD1	293SER	C	0.02
68LEU	CD1	294ALA	CA	0.02
68LEU	CD1	294ALA	CB	0.01
68LEU	CD1	297MET	CG	0.00
68LEU	CD1	297MET	CE	1.17
68LEU	CD2	68LEU	C	2.52
68LEU	CD2	269PHE	CB	0.06
68LEU	CD2	294ALA	CA	0.00
68LEU	CD2	294ALA	CB	0.01
68LEU	CD2	297MET	CE	0.36
68LEU	C	69LEU	CA	100.00
68LEU	C	69LEU	CB	45.96
68LEU	C	69LEU	CD1	0.00
68LEU	C	69LEU	C	63.45
69LEU	CA	69LEU	CB	100.00
69LEU	CA	69LEU	CG	100.00
69LEU	CA	69LEU	CD1	90.04
69LEU	CA	69LEU	CD2	7.24
69LEU	CA	69LEU	C	100.00
69LEU	CB	69LEU	CG	100.00
69LEU	CB	69LEU	CD1	100.00
69LEU	CB	69LEU	CD2	100.00
69LEU	CB	69LEU	C	100.00
69LEU	CB	268VAL	CG1	4.33
69LEU	CB	270GLU	CB	0.00
69LEU	CG	69LEU	CD1	100.00

69LEU	CG	69LEU	CD2	100.00
69LEU	CG	69LEU	C	93.98
69LEU	CG	90LEU	CD1	0.00
69LEU	CG	90LEU	CD2	0.02
69LEU	CG	268VAL	CG1	0.08
69LEU	CG	270GLU	CD	0.00
69LEU	CD1	69LEU	CD2	100.00
69LEU	CD1	69LEU	C	0.76
69LEU	CD1	90LEU	CD1	0.16
69LEU	CD1	90LEU	CD2	0.17
69LEU	CD1	93LEU	CD1	0.55
69LEU	CD1	93LEU	CD2	5.58
69LEU	CD1	268VAL	CG1	1.54
69LEU	CD1	270GLU	CG	0.00
69LEU	CD1	270GLU	CD	0.02
69LEU	CD2	69LEU	C	1.66
69LEU	CD2	70GLY	C	0.16
69LEU	CD2	90LEU	CG	0.01
69LEU	CD2	90LEU	CD1	1.16
69LEU	CD2	90LEU	CD2	0.92
69LEU	CD2	93LEU	CD1	0.06
69LEU	CD2	93LEU	CD2	1.71
69LEU	CD2	268VAL	CG1	10.49
69LEU	CD2	270GLU	CB	0.00
69LEU	CD2	270GLU	CG	0.49
69LEU	CD2	270GLU	CD	1.62
69LEU	C	70GLY	CA	100.00
69LEU	C	70GLY	C	45.52
70GLY	CA	70GLY	C	100.00
70GLY	CA	271PRO	CG	0.00
70GLY	CA	271PRO	CD	0.00
70GLY	CA	273HIS	CE1	0.14
70GLY	C	71SER	CA	100.00
70GLY	C	71SER	CB	8.85
70GLY	C	71SER	C	91.72
70GLY	C	273HIS	CE1	0.03
71SER	CA	71SER	CB	100.00
71SER	CA	71SER	C	100.00
71SER	CA	90LEU	CD1	0.01
71SER	CA	90LEU	CD2	0.00
71SER	CA	273HIS	CE1	0.01
71SER	CB	71SER	C	100.00
71SER	CB	90LEU	CD1	0.01
71SER	CB	90LEU	CD2	0.06
71SER	CB	273HIS	CG	0.00
71SER	CB	273HIS	CD2	0.41
71SER	CB	273HIS	CE1	5.54
71SER	CB	274GLY	CA	0.01
71SER	CB	274GLY	C	0.23
71SER	CB	275SER	CA	0.01
71SER	CB	275SER	CB	0.02
71SER	C	72VAL	CA	100.00
71SER	C	72VAL	CB	66.84
71SER	C	72VAL	CG1	31.44
71SER	C	72VAL	CG2	3.62
71SER	C	72VAL	C	35.63
72VAL	CA	72VAL	CB	100.00
72VAL	CA	72VAL	CG1	100.00
72VAL	CA	72VAL	CG2	100.00
72VAL	CA	72VAL	C	100.00
72VAL	CA	77TRP	CZ3	0.00
72VAL	CB	72VAL	CG1	100.00

72VAL	CB	72VAL	CG2	100.00
72VAL	CB	72VAL	C	100.00
72VAL	CB	86PRO	CB	0.75
72VAL	CB	86PRO	CG	0.08
72VAL	CG:	72VAL	CG2	100.00
72VAL	CG:	72VAL	C	9.68
72VAL	CG:	77TRP	CZ2	0.15
72VAL	CG:	77TRP	CZ3	0.34
72VAL	CG:	86PRO	CB	1.40
72VAL	CG:	86PRO	CG	1.15
72VAL	CG:	86PRO	C	0.00
72VAL	CG:	90LEU	CD2	0.00
72VAL	CG:	72VAL	C	89.83
72VAL	CG:	86PRO	CB	5.40
72VAL	CG:	86PRO	CG	0.06
72VAL	CG:	86PRO	C	1.36
72VAL	CG:	87GLU	CA	0.01
72VAL	CG:	90LEU	CB	0.04
72VAL	CG:	90LEU	CG	0.62
72VAL	CG:	90LEU	CD1	2.50
72VAL	CG:	90LEU	CD2	0.17
72VAL	C	73GLY	CA	100.00
72VAL	C	73GLY	C	25.63
72VAL	C	77TRP	CE3	0.01
72VAL	C	77TRP	CZ3	0.94
72VAL	C	86PRO	CB	0.01
72VAL	C	86PRO	CG	0.09
72VAL	C	275SER	CB	1.41
73GLY	CA	73GLY	C	100.00
73GLY	CA	77TRP	CD2	0.00
73GLY	CA	77TRP	CE3	2.95
73GLY	CA	77TRP	CZ3	1.78
73GLY	CA	86PRO	CB	0.15
73GLY	CA	86PRO	CG	0.84
73GLY	CA	275SER	CB	0.08
73GLY	C	74GLY	CA	100.00
73GLY	C	74GLY	C	19.50
73GLY	C	77TRP	CB	0.00
73GLY	C	77TRP	CE3	4.87
73GLY	C	77TRP	CZ3	4.11
73GLY	C	275SER	CB	1.35
74GLY	CA	74GLY	C	100.00
74GLY	CA	75PRO	CD	100.00
74GLY	CA	77TRP	CE3	0.02
74GLY	CA	77TRP	CZ3	0.01
74GLY	CA	275SER	CB	0.14
74GLY	CA	283GLY	CA	0.01
74GLY	C	75PRO	CA	100.00
74GLY	C	75PRO	CB	0.14
74GLY	C	75PRO	CD	100.00
74GLY	C	75PRO	C	100.00
74GLY	C	77TRP	CE3	0.03
74GLY	C	77TRP	CZ3	0.02
75PRO	CA	75PRO	CB	100.00
75PRO	CA	75PRO	CG	100.00
75PRO	CA	75PRO	CD	100.00
75PRO	CA	75PRO	C	100.00
75PRO	CA	78ASP	CB	0.04
75PRO	CB	75PRO	CG	100.00
75PRO	CB	75PRO	CD	100.00
75PRO	CB	75PRO	C	100.00
75PRO	CB	281GLY	C	0.00

75PRO	CG	75PRO	CD	100.00
75PRO	CG	75PRO	C	63.45
75PRO	CG	276ALA	CB	0.02
75PRO	CG	276ALA	C	0.01
75PRO	CG	277PRO	CA	0.00
75PRO	CG	277PRO	CB	0.06
75PRO	CG	277PRO	CG	0.20
75PRO	CG	277PRO	CD	0.98
75PRO	CG	280ALA	CB	0.00
75PRO	CG	281GLY	CA	0.10
75PRO	CG	281GLY	C	0.13
75PRO	CG	282LYS	C	0.06
75PRO	CG	283GLY	CA	0.08
75PRO	CD	75PRO	C	48.10
75PRO	CD	275SER	CB	0.00
75PRO	CD	276ALA	CA	0.02
75PRO	CD	276ALA	CB	0.01
75PRO	CD	277PRO	CB	0.04
75PRO	CD	277PRO	CG	0.31
75PRO	CD	277PRO	CD	3.62
75PRO	CD	281GLY	CA	0.02
75PRO	CD	282LYS	C	0.02
75PRO	CD	283GLY	CA	0.10
75PRO	C	76LYS	CA	100.00
75PRO	C	76LYS	CB	22.96
75PRO	C	76LYS	CG	1.74
75PRO	C	76LYS	C	93.99
75PRO	C	77TRP	CE3	0.00
76LYS	CA	76LYS	CB	100.00
76LYS	CA	76LYS	CG	100.00
76LYS	CA	76LYS	CD	3.25
76LYS	CA	76LYS	CE	0.00
76LYS	CA	76LYS	C	100.00
76LYS	CB	76LYS	CG	100.00
76LYS	CB	76LYS	CD	100.00
76LYS	CB	76LYS	CE	23.26
76LYS	CB	76LYS	C	100.00
76LYS	CB	77TRP	CD2	0.20
76LYS	CB	77TRP	CE2	0.04
76LYS	CB	77TRP	CE3	2.26
76LYS	CB	77TRP	CZ2	0.01
76LYS	CB	77TRP	CZ3	0.36
76LYS	CG	76LYS	CD	100.00
76LYS	CG	76LYS	CE	100.00
76LYS	CG	76LYS	C	84.22
76LYS	CG	77TRP	CD2	0.49
76LYS	CG	77TRP	CE2	0.17
76LYS	CG	77TRP	CE3	4.21
76LYS	CG	77TRP	CZ2	0.02
76LYS	CG	77TRP	CZ3	1.02
76LYS	CD	76LYS	CE	100.00
76LYS	CD	76LYS	C	1.79
76LYS	CD	77TRP	CD1	0.01
76LYS	CD	77TRP	CD2	0.08
76LYS	CD	77TRP	CE2	0.71
76LYS	CD	77TRP	CE3	0.20
76LYS	CD	77TRP	CZ2	0.73
76LYS	CD	77TRP	CZ3	0.93
76LYS	CE	76LYS	C	0.00
76LYS	CE	77TRP	CD1	0.03
76LYS	CE	77TRP	CD2	0.02
76LYS	CE	77TRP	CE2	1.08

76LYS	CE	77TRP	CE3	0.38
76LYS	CE	77TRP	CZ2	3.59
76LYS	CE	77TRP	CZ3	7.04
76LYS	C	77TRP	CA	100.00
76LYS	C	77TRP	CB	41.06
76LYS	C	77TRP	CG	15.19
76LYS	C	77TRP	CD1	0.39
76LYS	C	77TRP	CD2	1.10
76LYS	C	77TRP	CE3	0.22
76LYS	C	77TRP	C	53.08
77TRP	CA	77TRP	CB	100.00
77TRP	CA	77TRP	CG	100.00
77TRP	CA	77TRP	CD1	90.18
77TRP	CA	77TRP	CD2	4.91
77TRP	CA	77TRP	CE3	0.00
77TRP	CA	77TRP	C	100.00
77TRP	CA	80LEU	CD1	0.21
77TRP	CA	80LEU	CD2	0.00
77TRP	CB	77TRP	CG	100.00
77TRP	CB	77TRP	CD1	100.00
77TRP	CB	77TRP	CD2	100.00
77TRP	CB	77TRP	CE3	97.32
77TRP	CB	77TRP	C	100.00
77TRP	CB	85ARG	CB	0.18
77TRP	CB	85ARG	CG	0.01
77TRP	CB	85ARG	CD	0.09
77TRP	CB	86PRO	CG	0.02
77TRP	CB	86PRO	CD	1.20
77TRP	CG	77TRP	CD1	100.00
77TRP	CG	77TRP	CD2	100.00
77TRP	CG	77TRP	CE2	100.00
77TRP	CG	77TRP	CE3	100.00
77TRP	CG	77TRP	C	0.26
77TRP	CG	85ARG	CB	0.13
77TRP	CG	86PRO	CG	0.11
77TRP	CG	86PRO	CD	16.58
77TRP	CD1	77TRP	CD2	100.00
77TRP	CD1	77TRP	CE2	100.00
77TRP	CD1	77TRP	C	0.03
77TRP	CD1	80LEU	CD1	0.75
77TRP	CD1	80LEU	CD2	0.50
77TRP	CD1	84ILE	CG2	0.00
77TRP	CD1	84ILE	CD	0.01
77TRP	CD1	85ARG	CA	0.07
77TRP	CD1	85ARG	CB	0.40
77TRP	CD1	85ARG	CG	0.00
77TRP	CD1	86PRO	CG	0.08
77TRP	CD1	86PRO	CD	7.43
77TRP	CD2	77TRP	CE2	100.00
77TRP	CD2	77TRP	CE3	100.00
77TRP	CD2	77TRP	CZ2	100.00
77TRP	CD2	77TRP	CZ3	100.00
77TRP	CD2	86PRO	CG	6.55
77TRP	CD2	86PRO	CD	15.28
77TRP	CE2	77TRP	CE3	100.00
77TRP	CE2	77TRP	CZ2	100.00
77TRP	CE2	77TRP	CZ3	100.00
77TRP	CE2	80LEU	CD2	0.00
77TRP	CE2	86PRO	CG	6.56
77TRP	CE2	86PRO	CD	6.38
77TRP	CE3	77TRP	CZ2	100.00
77TRP	CE3	77TRP	CZ3	100.00

77TRP	CE3	86PRO	CG	7.38
77TRP	CE3	86PRO	CD	1.11
77TRP	CZ2	77TRP	CZ3	100.00
77TRP	CZ2	80LEU	CD2	0.00
77TRP	CZ2	86PRO	CG	3.73
77TRP	CZ2	86PRO	CD	0.31
77TRP	CZ3	86PRO	CG	2.32
77TRP	CZ3	86PRO	CD	0.02
77TRP	C	78ASP	CA	100.00
77TRP	C	78ASP	CB	1.56
77TRP	C	78ASP	C	98.78
77TRP	C	80LEU	CD1	0.06
77TRP	C	80LEU	CD2	0.06
77TRP	C	85ARG	CD	0.02
78ASP	CA	78ASP	CB	100.00
78ASP	CA	78ASP	CG	100.00
78ASP	CA	78ASP	C	100.00
78ASP	CB	78ASP	CG	100.00
78ASP	CB	78ASP	C	100.00
78ASP	CG	78ASP	C	99.14
78ASP	C	79GLY	CA	100.00
78ASP	C	79GLY	C	46.05
78ASP	C	80LEU	CD1	0.00
78ASP	C	80LEU	CD2	0.01
78ASP	C	85ARG	CD	0.00
79GLY	CA	79GLY	C	100.00
79GLY	C	80LEU	CA	100.00
79GLY	C	80LEU	CB	5.21
79GLY	C	80LEU	CG	0.68
79GLY	C	80LEU	CD1	0.39
79GLY	C	80LEU	CD2	0.07
79GLY	C	80LEU	C	94.34
80LEU	CA	80LEU	CB	100.00
80LEU	CA	80LEU	CG	100.00
80LEU	CA	80LEU	CD1	24.00
80LEU	CA	80LEU	CD2	74.93
80LEU	CA	80LEU	C	100.00
80LEU	CA	81PRO	CD	100.00
80LEU	CB	80LEU	CG	100.00
80LEU	CB	80LEU	CD1	100.00
80LEU	CB	80LEU	CD2	100.00
80LEU	CB	80LEU	C	100.00
80LEU	CB	81PRO	CD	34.61
80LEU	CB	84ILE	CG1	0.02
80LEU	CB	84ILE	CG2	0.03
80LEU	CB	84ILE	CD	0.01
80LEU	CB	85ARG	CG	0.10
80LEU	CG	80LEU	CD1	100.00
80LEU	CG	80LEU	CD2	100.00
80LEU	CG	80LEU	C	0.70
80LEU	CG	81PRO	CD	0.02
80LEU	CG	84ILE	CD	0.02
80LEU	CD1	80LEU	CD2	100.00
80LEU	CD1	80LEU	C	0.04
80LEU	CD1	81PRO	CD	0.00
80LEU	CD1	84ILE	CG1	0.00
80LEU	CD1	84ILE	CG2	0.07
80LEU	CD1	84ILE	CD	0.06
80LEU	CD1	84ILE	C	0.02
80LEU	CD1	85ARG	CG	0.02
80LEU	CD2	80LEU	C	0.01
80LEU	CD2	81PRO	CD	0.03

80LEU	CD2	84ILE	CG1	0.00
80LEU	CD2	84ILE	CG2	0.05
80LEU	CD2	84ILE	CD	0.54
80LEU	CD2	84ILE	C	0.01
80LEU	CD2	85ARG	CA	0.00
80LEU	CD2	85ARG	CB	0.01
80LEU	CD2	85ARG	CG	0.00
80LEU	CD2	85ARG	CD	0.00
80LEU	C	81PRO	CA	100.00
80LEU	C	81PRO	CB	0.08
80LEU	C	81PRO	CD	100.00
80LEU	C	81PRO	C	100.00
81PRO	CA	81PRO	CB	100.00
81PRO	CA	81PRO	CG	100.00
81PRO	CA	81PRO	CD	100.00
81PRO	CA	81PRO	C	100.00
81PRO	CB	81PRO	CG	100.00
81PRO	CB	81PRO	CD	100.00
81PRO	CB	81PRO	C	100.00
81PRO	CB	83LYS	CG	0.03
81PRO	CB	83LYS	CE	0.02
81PRO	CB	84ILE	CD	0.01
81PRO	CG	81PRO	CD	100.00
81PRO	CG	81PRO	C	90.24
81PRO	CG	83LYS	CG	0.03
81PRO	CG	83LYS	CE	1.67
81PRO	CG	84ILE	CG1	0.04
81PRO	CG	84ILE	CG2	0.01
81PRO	CG	84ILE	CD	0.07
81PRO	CD	81PRO	C	84.20
81PRO	CD	84ILE	CG1	0.05
81PRO	CD	84ILE	CG2	0.04
81PRO	CD	84ILE	CD	0.02
81PRO	C	82ARG	CA	100.00
81PRO	C	82ARG	C	100.00
81PRO	C	85ARG	CD	0.00
82ARG	CA	82ARG	CB	100.00
82ARG	CA	82ARG	CG	100.00
82ARG	CA	82ARG	C	100.00
82ARG	CA	85ARG	CG	0.02
82ARG	CA	85ARG	CD	0.01
82ARG	CA	85ARG	CZ	8.10
82ARG	CB	82ARG	CG	100.00
82ARG	CB	82ARG	CD	100.00
82ARG	CB	82ARG	CZ	0.47
82ARG	CB	82ARG	C	100.00
82ARG	CB	85ARG	CZ	0.72
82ARG	CG	82ARG	CD	100.00
82ARG	CG	82ARG	CZ	4.43
82ARG	CG	82ARG	C	99.48
82ARG	CG	85ARG	CZ	0.04
82ARG	CG	87GLU	CB	0.35
82ARG	CG	87GLU	CG	0.28
82ARG	CG	87GLU	CD	0.28
82ARG	CG	91LEU	CD1	0.11
82ARG	CD	82ARG	CZ	100.00
82ARG	CD	87GLU	CD	0.01
82ARG	CD	91LEU	CD1	0.64
82ARG	CD	91LEU	CD2	0.12
82ARG	CD	144ARG	CZ	0.82
82ARG	CD	188VAL	CG1	0.03
82ARG	CD	188VAL	CG2	0.02

82ARG	CZ	87GLU	CB	0.09
82ARG	CZ	87GLU	CG	0.08
82ARG	CZ	87GLU	CD	0.03
82ARG	CZ	91LEU	CD1	2.88
82ARG	CZ	91LEU	CD2	0.94
82ARG	CZ	140PHE	CD1	0.03
82ARG	CZ	140PHE	CE1	0.49
82ARG	CZ	140PHE	CZ	0.10
82ARG	CZ	144ARG	CG	0.00
82ARG	CZ	144ARG	CD	0.22
82ARG	CZ	144ARG	CZ	3.16
82ARG	CZ	188VAL	CB	0.01
82ARG	CZ	188VAL	CG1	21.24
82ARG	CZ	188VAL	CG2	1.42
82ARG	C	83LYS	CA	100.00
82ARG	C	83LYS	CB	0.02
82ARG	C	83LYS	C	99.98
83LYS	CA	83LYS	CB	100.00
83LYS	CA	83LYS	CG	100.00
83LYS	CA	83LYS	CD	0.14
83LYS	CA	83LYS	C	100.00
83LYS	CB	83LYS	CG	100.00
83LYS	CB	83LYS	CD	100.00
83LYS	CB	83LYS	CE	13.76
83LYS	CB	83LYS	C	100.00
83LYS	CG	83LYS	CD	100.00
83LYS	CG	83LYS	CE	100.00
83LYS	CG	83LYS	C	97.29
83LYS	CG	84ILE	CG1	0.06
83LYS	CG	84ILE	CD	0.02
83LYS	CD	83LYS	CE	100.00
83LYS	CD	83LYS	C	0.14
83LYS	CD	84ILE	CG1	0.01
83LYS	CE	84ILE	CG2	0.01
83LYS	C	84ILE	CA	100.00
83LYS	C	84ILE	CB	85.67
83LYS	C	84ILE	CG1	1.26
83LYS	C	84ILE	CG2	52.12
83LYS	C	84ILE	C	13.56
83LYS	C	88THR	CB	0.02
83LYS	C	88THR	CG2	0.05
84ILE	CA	84ILE	CB	100.00
84ILE	CA	84ILE	CG1	100.00
84ILE	CA	84ILE	CG2	100.00
84ILE	CA	84ILE	CD	1.72
84ILE	CA	84ILE	C	100.00
84ILE	CB	84ILE	CG1	100.00
84ILE	CB	84ILE	CG2	100.00
84ILE	CB	84ILE	CD	100.00
84ILE	CB	84ILE	C	100.00
84ILE	CG:	84ILE	CG2	100.00
84ILE	CG:	84ILE	CD	100.00
84ILE	CG:	84ILE	C	92.08
84ILE	CG:	84ILE	CD	97.98
84ILE	CG:	84ILE	C	9.58
84ILE	CD	84ILE	C	1.10
84ILE	C	85ARG	CA	100.00
84ILE	C	85ARG	CB	7.91
84ILE	C	85ARG	CG	1.09
84ILE	C	85ARG	C	92.88
85ARG	CA	85ARG	CB	100.00
85ARG	CA	85ARG	CG	100.00

85ARG	CA	85ARG	C	100.00
85ARG	CA	86PRO	CD	100.00
85ARG	CB	85ARG	CG	100.00
85ARG	CB	85ARG	CD	100.00
85ARG	CB	85ARG	C	100.00
85ARG	CB	86PRO	CD	18.63
85ARG	CB	87GLU	CB	0.01
85ARG	CB	87GLU	CG	0.06
85ARG	CB	87GLU	CD	0.09
85ARG	CG	85ARG	CD	100.00
85ARG	CG	85ARG	CZ	38.79
85ARG	CG	85ARG	C	4.41
85ARG	CG	86PRO	CD	0.13
85ARG	CD	85ARG	CZ	100.00
85ARG	CZ	87GLU	CD	0.04
85ARG	C	86PRO	CA	100.00
85ARG	C	86PRO	CB	0.01
85ARG	C	86PRO	CD	100.00
85ARG	C	86PRO	C	100.00
86PRO	CA	86PRO	CB	100.00
86PRO	CA	86PRO	CG	100.00
86PRO	CA	86PRO	CD	100.00
86PRO	CA	86PRO	C	100.00
86PRO	CB	86PRO	CG	100.00
86PRO	CB	86PRO	CD	100.00
86PRO	CB	86PRO	C	100.00
86PRO	CB	90LEU	CD1	0.10
86PRO	CG	86PRO	CD	100.00
86PRO	CG	86PRO	C	27.41
86PRO	CD	86PRO	C	15.35
86PRO	C	87GLU	CA	100.00
86PRO	C	87GLU	CB	0.00
86PRO	C	87GLU	C	100.00
86PRO	C	88THR	CG2	0.00
86PRO	C	90LEU	CD1	0.02
87GLU	CA	87GLU	CB	100.00
87GLU	CA	87GLU	CG	100.00
87GLU	CA	87GLU	CD	0.54
87GLU	CA	87GLU	C	100.00
87GLU	CA	90LEU	CB	0.00
87GLU	CA	90LEU	CD1	0.01
87GLU	CB	87GLU	CG	100.00
87GLU	CB	87GLU	CD	100.00
87GLU	CB	87GLU	C	100.00
87GLU	CB	90LEU	CD1	0.00
87GLU	CG	87GLU	CD	100.00
87GLU	CG	87GLU	C	82.99
87GLU	CG	91LEU	CD1	0.01
87GLU	C	88THR	CA	100.00
87GLU	C	88THR	CB	0.11
87GLU	C	88THR	C	99.94
87GLU	C	91LEU	CD1	0.10
88THR	CA	88THR	CB	100.00
88THR	CA	88THR	CG2	100.00
88THR	CA	88THR	C	100.00
88THR	CA	91LEU	CD1	0.00
88THR	CB	88THR	CG2	100.00
88THR	CB	88THR	C	100.00
88THR	CG:	88THR	C	100.00
88THR	C	89GLY	CA	100.00
88THR	C	89GLY	C	93.30
88THR	C	91LEU	CB	0.01

89GLY	CA	89GLY	C	100.00
89GLY	CA	92SER	CB	0.00
89GLY	C	90LEU	CA	100.00
89GLY	C	90LEU	CB	0.12
89GLY	C	90LEU	C	99.88
90LEU	CA	90LEU	CB	100.00
90LEU	CA	90LEU	CG	100.00
90LEU	CA	90LEU	CD1	36.91
90LEU	CA	90LEU	CD2	61.51
90LEU	CA	90LEU	C	100.00
90LEU	CB	90LEU	CG	100.00
90LEU	CB	90LEU	CD1	100.00
90LEU	CB	90LEU	CD2	100.00
90LEU	CB	90LEU	C	100.00
90LEU	CB	94ARG	CZ	0.84
90LEU	CG	90LEU	CD1	100.00
90LEU	CG	90LEU	CD2	100.00
90LEU	CG	90LEU	C	37.68
90LEU	CG	94ARG	CZ	1.48
90LEU	CD1	90LEU	CD2	100.00
90LEU	CD1	90LEU	C	1.20
90LEU	CD1	93LEU	CD2	0.01
90LEU	CD1	94ARG	CD	0.01
90LEU	CD1	94ARG	CZ	0.74
90LEU	CD2	90LEU	C	1.74
90LEU	CD2	91LEU	CD1	0.00
90LEU	CD2	94ARG	CD	0.02
90LEU	CD2	94ARG	CZ	3.43
90LEU	CD2	140PHE	CE1	0.00
90LEU	CD2	140PHE	CZ	0.02
90LEU	CD2	270GLU	CD	0.04
90LEU	C	91LEU	CA	100.00
90LEU	C	91LEU	C	100.00
90LEU	C	94ARG	CD	0.02
91LEU	CA	91LEU	CB	100.00
91LEU	CA	91LEU	CG	100.00
91LEU	CA	91LEU	CD1	52.75
91LEU	CA	91LEU	CD2	47.38
91LEU	CA	91LEU	C	100.00
91LEU	CA	140PHE	CZ	0.00
91LEU	CB	91LEU	CG	100.00
91LEU	CB	91LEU	CD1	100.00
91LEU	CB	91LEU	CD2	100.00
91LEU	CB	91LEU	C	100.00
91LEU	CB	140PHE	CD1	0.00
91LEU	CB	140PHE	CE1	0.00
91LEU	CB	140PHE	CZ	0.01
91LEU	CG	91LEU	CD1	100.00
91LEU	CG	91LEU	CD2	100.00
91LEU	CG	91LEU	C	28.52
91LEU	CG	95LYS	CE	0.01
91LEU	CG	140PHE	CD1	0.02
91LEU	CG	140PHE	CE1	0.05
91LEU	CG	140PHE	CE2	0.00
91LEU	CG	140PHE	CZ	0.04
91LEU	CD1	91LEU	CD2	100.00
91LEU	CD1	91LEU	C	3.23
91LEU	CD1	95LYS	CG	0.01
91LEU	CD1	95LYS	CD	0.08
91LEU	CD1	95LYS	CE	0.04
91LEU	CD1	135THR	CA	0.04
91LEU	CD1	135THR	CB	0.05

91LEU	CD1	135THR	CG2	0.12
91LEU	CD1	135THR	C	0.01
91LEU	CD1	140PHE	CB	0.00
91LEU	CD1	140PHE	CG	0.14
91LEU	CD1	140PHE	CD1	0.48
91LEU	CD1	140PHE	CD2	0.07
91LEU	CD1	140PHE	CE1	1.48
91LEU	CD1	140PHE	CE2	0.05
91LEU	CD1	140PHE	CZ	0.50
91LEU	CD2	91LEU	C	2.85
91LEU	CD2	94ARG	CZ	0.06
91LEU	CD2	95LYS	CG	0.00
91LEU	CD2	95LYS	CD	0.02
91LEU	CD2	95LYS	CE	0.02
91LEU	CD2	135THR	CA	0.03
91LEU	CD2	135THR	CB	0.01
91LEU	CD2	135THR	CG2	0.02
91LEU	CD2	135THR	C	0.01
91LEU	CD2	140PHE	CB	0.02
91LEU	CD2	140PHE	CG	0.03
91LEU	CD2	140PHE	CD1	0.48
91LEU	CD2	140PHE	CD2	0.05
91LEU	CD2	140PHE	CE1	2.48
91LEU	CD2	140PHE	CE2	0.04
91LEU	CD2	140PHE	CZ	1.06
91LEU	C	92SER	CA	100.00
91LEU	C	92SER	CB	0.16
91LEU	C	92SER	C	99.89
92SER	CA	92SER	CB	100.00
92SER	CA	92SER	C	100.00
92SER	CB	92SER	C	100.00
92SER	C	93LEU	CA	100.00
92SER	C	93LEU	CB	0.01
92SER	C	93LEU	C	100.00
93LEU	CA	93LEU	CB	100.00
93LEU	CA	93LEU	CG	100.00
93LEU	CA	93LEU	CD1	64.17
93LEU	CA	93LEU	CD2	27.73
93LEU	CA	93LEU	C	100.00
93LEU	CB	93LEU	CG	100.00
93LEU	CB	93LEU	CD1	100.00
93LEU	CB	93LEU	CD2	100.00
93LEU	CB	93LEU	C	100.00
93LEU	CG	93LEU	CD1	100.00
93LEU	CG	93LEU	CD2	100.00
93LEU	CG	93LEU	C	93.80
93LEU	CD1	93LEU	CD2	100.00
93LEU	CD1	93LEU	C	7.34
93LEU	CD1	94ARG	CG	0.00
93LEU	CD1	97GLN	CG	0.08
93LEU	CD1	97GLN	CD	0.01
93LEU	CD1	268VAL	CG2	0.00
93LEU	CD2	93LEU	C	22.46
93LEU	CD2	97GLN	CG	0.06
93LEU	CD2	97GLN	CD	0.01
93LEU	CD2	99LEU	CD1	0.04
93LEU	CD2	268VAL	CG1	0.01
93LEU	CD2	268VAL	CG2	0.18
93LEU	C	94ARG	CA	100.00
93LEU	C	94ARG	C	100.00
94ARG	CA	94ARG	CB	100.00
94ARG	CA	94ARG	CG	100.00

94ARG	CA	94ARG	CD	0.01
94ARG	CA	94ARG	C	100.00
94ARG	CA	99LEU	CD1	0.60
94ARG	CB	94ARG	CG	100.00
94ARG	CB	94ARG	CD	100.00
94ARG	CB	94ARG	C	100.00
94ARG	CB	99LEU	CD1	0.28
94ARG	CB	134LEU	CD2	0.00
94ARG	CB	135THR	CG2	2.70
94ARG	CG	94ARG	CD	100.00
94ARG	CG	94ARG	CZ	0.78
94ARG	CG	99LEU	CD1	0.93
94ARG	CG	134LEU	CD1	0.01
94ARG	CG	134LEU	CD2	0.04
94ARG	CD	94ARG	CZ	100.00
94ARG	CD	99LEU	CD1	0.01
94ARG	CD	134LEU	CG	0.02
94ARG	CD	134LEU	CD1	0.62
94ARG	CD	134LEU	CD2	0.77
94ARG	CD	135THR	CG2	0.00
94ARG	CZ	134LEU	CB	0.01
94ARG	CZ	134LEU	CG	0.06
94ARG	CZ	134LEU	CD1	2.06
94ARG	CZ	134LEU	CD2	1.82
94ARG	C	95LYS	CA	100.00
94ARG	C	95LYS	CB	0.52
94ARG	C	95LYS	CG	0.02
94ARG	C	95LYS	C	99.63
94ARG	C	135THR	CG2	3.42
95LYS	CA	95LYS	CB	100.00
95LYS	CA	95LYS	CG	100.00
95LYS	CA	95LYS	CD	43.29
95LYS	CA	95LYS	CE	0.50
95LYS	CA	95LYS	C	100.00
95LYS	CA	135THR	CG2	0.03
95LYS	CB	95LYS	CG	100.00
95LYS	CB	95LYS	CD	100.00
95LYS	CB	95LYS	CE	13.80
95LYS	CB	95LYS	C	100.00
95LYS	CG	95LYS	CD	100.00
95LYS	CG	95LYS	CE	100.00
95LYS	CG	95LYS	C	62.08
95LYS	CG	135THR	CB	0.00
95LYS	CG	135THR	CG2	0.22
95LYS	CD	95LYS	CE	100.00
95LYS	CD	95LYS	C	1.27
95LYS	CD	135THR	CB	0.03
95LYS	CD	135THR	CG2	0.02
95LYS	CE	135THR	CG2	0.00
95LYS	CE	140PHE	CD1	0.10
95LYS	CE	140PHE	CE1	0.09
95LYS	CE	140PHE	CE2	0.00
95LYS	C	96SER	CA	100.00
95LYS	C	96SER	CB	0.36
95LYS	C	96SER	C	99.71
95LYS	C	98ASP	CA	0.00
96SER	CA	96SER	CB	100.00
96SER	CA	96SER	C	100.00
96SER	CB	96SER	C	100.00
96SER	C	97GLN	CA	100.00
96SER	C	97GLN	CB	2.79
96SER	C	97GLN	CG	0.44

96SER	C	97GLN	CD	0.04
96SER	C	97GLN	C	97.45
97GLN	CA	97GLN	CB	100.00
97GLN	CA	97GLN	CG	100.00
97GLN	CA	97GLN	CD	97.31
97GLN	CA	97GLN	C	100.00
97GLN	CB	97GLN	CG	100.00
97GLN	CB	97GLN	CD	100.00
97GLN	CB	97GLN	C	100.00
97GLN	CB	99LEU	CG	0.16
97GLN	CB	99LEU	CD1	0.15
97GLN	CB	99LEU	CD2	3.09
97GLN	CB	268VAL	CG2	0.01
97GLN	CG	97GLN	CD	100.00
97GLN	CG	97GLN	C	12.37
97GLN	CG	99LEU	CG	0.02
97GLN	CG	99LEU	CD1	0.06
97GLN	CG	99LEU	CD2	0.45
97GLN	CG	265GLY	C	0.01
97GLN	CG	268VAL	CG2	0.13
97GLN	CD	99LEU	CD2	0.00
97GLN	CD	266THR	CB	0.02
97GLN	CD	268VAL	CG2	0.27
97GLN	C	98ASP	CA	100.00
97GLN	C	98ASP	CB	99.22
97GLN	C	98ASP	C	99.72
97GLN	C	264ARG	CG	0.01
97GLN	C	264ARG	CD	0.00
98ASP	CA	98ASP	CB	100.00
98ASP	CA	98ASP	CG	100.00
98ASP	CA	98ASP	C	100.00
98ASP	CB	98ASP	CG	100.00
98ASP	CB	98ASP	C	100.00
98ASP	CB	264ARG	CB	0.12
98ASP	CB	264ARG	CG	0.02
98ASP	CB	264ARG	CD	0.06
98ASP	CB	264ARG	CZ	0.50
98ASP	CG	98ASP	C	99.30
98ASP	CG	164ARG	CZ	0.00
98ASP	CG	264ARG	CB	0.02
98ASP	CG	264ARG	CD	0.09
98ASP	CG	264ARG	CZ	0.01
98ASP	C	99LEU	CA	100.00
98ASP	C	99LEU	CB	0.52
98ASP	C	99LEU	CG	0.06
98ASP	C	99LEU	CD2	0.01
98ASP	C	99LEU	C	99.60
98ASP	C	263GLY	CA	0.14
99LEU	CA	99LEU	CB	100.00
99LEU	CA	99LEU	CG	100.00
99LEU	CA	99LEU	CD1	0.01
99LEU	CA	99LEU	CD2	99.83
99LEU	CA	99LEU	C	100.00
99LEU	CA	262LEU	C	0.00
99LEU	CA	263GLY	CA	1.04
99LEU	CB	99LEU	CG	100.00
99LEU	CB	99LEU	CD1	100.00
99LEU	CB	99LEU	CD2	100.00
99LEU	CB	99LEU	C	100.00
99LEU	CB	134LEU	CD1	0.10
99LEU	CB	134LEU	CD2	2.23
99LEU	CB	135THR	CG2	0.02

99LEU	CB	262LEU	C	0.01
99LEU	CG	99LEU	CD1	100.00
99LEU	CG	99LEU	CD2	100.00
99LEU	CD:	99LEU	CD2	100.00
99LEU	CD:	134LEU	CG	0.00
99LEU	CD:	134LEU	CD1	0.32
99LEU	CD:	134LEU	CD2	2.89
99LEU	CD:	261SER	CB	2.33
99LEU	CD:	268VAL	CB	0.12
99LEU	CD:	268VAL	CG1	0.01
99LEU	CD:	268VAL	CG2	7.36
99LEU	CD:	261SER	CB	0.48
99LEU	CD:	261SER	C	0.18
99LEU	CD:	262LEU	CA	0.02
99LEU	CD:	262LEU	C	2.30
99LEU	CD:	263GLY	CA	1.17
99LEU	CD:	265GLY	CA	0.01
99LEU	CD:	265GLY	C	0.01
99LEU	CD:	268VAL	CG2	0.55
99LEU	C	100PHE	CA	100.00
99LEU	C	100PHE	CB	46.33
99LEU	C	100PHE	CG	0.42
99LEU	C	100PHE	CD1	0.08
99LEU	C	100PHE	CD2	0.89
99LEU	C	100PHE	C	52.34
99LEU	C	134LEU	CD1	0.01
99LEU	C	134LEU	CD2	0.12
100PHE	CA	100PHE	CB	100.00
100PHE	CA	100PHE	CG	100.00
100PHE	CA	100PHE	CD1	83.61
100PHE	CA	100PHE	CD2	36.75
100PHE	CA	100PHE	C	100.00
100PHE	CA	133GLU	CD	0.12
100PHE	CA	134LEU	CD2	0.01
100PHE	CA	164ARG	CZ	0.04
100PHE	CB	100PHE	CG	100.00
100PHE	CB	100PHE	CD1	100.00
100PHE	CB	100PHE	CD2	100.00
100PHE	CB	100PHE	C	100.00
100PHE	CB	133GLU	CD	0.06
100PHE	CB	135THR	CG2	0.01
100PHE	CB	164ARG	CB	0.09
100PHE	CB	164ARG	CG	0.09
100PHE	CB	164ARG	CD	0.36
100PHE	CB	164ARG	CZ	6.42
100PHE	CB	168VAL	CG2	0.68
100PHE	CB	264ARG	CZ	0.01
100PHE	CG	100PHE	CD1	100.00
100PHE	CG	100PHE	CD2	100.00
100PHE	CG	100PHE	CE1	100.00
100PHE	CG	100PHE	CE2	100.00
100PHE	CG	100PHE	CZ	100.00
100PHE	CG	100PHE	C	74.87
100PHE	CG	164ARG	CB	1.94
100PHE	CG	164ARG	CG	0.83
100PHE	CG	164ARG	CD	1.55
100PHE	CG	164ARG	CZ	0.53
100PHE	CG	263GLY	CA	0.04
100PHE	CD:	100PHE	CD2	100.00
100PHE	CD:	100PHE	CE1	100.00
100PHE	CD:	100PHE	CE2	100.00
100PHE	CD:	100PHE	CZ	100.00

100PHE	CD1	100PHE	C	56.67
100PHE	CD1	101ALA	CA	0.00
100PHE	CD1	101ALA	CB	1.66
100PHE	CD1	164ARG	CA	0.00
100PHE	CD1	164ARG	CB	6.60
100PHE	CD1	164ARG	CG	4.86
100PHE	CD1	164ARG	CD	2.47
100PHE	CD1	164ARG	CZ	1.94
100PHE	CD1	164ARG	C	1.95
100PHE	CD1	165VAL	CA	0.00
100PHE	CD1	165VAL	CG1	0.01
100PHE	CD1	168VAL	CG2	0.17
100PHE	CD1	262LEU	CB	0.04
100PHE	CD1	262LEU	CG	0.00
100PHE	CD1	262LEU	CD1	0.00
100PHE	CD1	262LEU	CD2	0.04
100PHE	CD1	262LEU	C	0.01
100PHE	CD1	263GLY	CA	0.06
100PHE	CD1	263GLY	C	0.14
100PHE	CD2	100PHE	CE1	100.00
100PHE	CD2	100PHE	CE2	100.00
100PHE	CD2	100PHE	CZ	100.00
100PHE	CD2	100PHE	C	0.85
100PHE	CD2	101ALA	CB	0.02
100PHE	CD2	164ARG	CB	0.26
100PHE	CD2	164ARG	CG	0.61
100PHE	CD2	164ARG	CD	2.07
100PHE	CD2	164ARG	CZ	28.59
100PHE	CD2	164ARG	C	0.01
100PHE	CD2	168VAL	CG2	0.07
100PHE	CD2	262LEU	CB	0.27
100PHE	CD2	262LEU	CD1	0.02
100PHE	CD2	262LEU	CD2	0.08
100PHE	CD2	262LEU	C	0.04
100PHE	CD2	263GLY	CA	1.26
100PHE	CD2	263GLY	C	0.12
100PHE	CD2	264ARG	CZ	0.19
100PHE	CE1	100PHE	CE2	100.00
100PHE	CE1	100PHE	CZ	100.00
100PHE	CE1	101ALA	CB	0.53
100PHE	CE1	164ARG	CB	1.94
100PHE	CE1	164ARG	CG	2.11
100PHE	CE1	164ARG	CD	0.30
100PHE	CE1	164ARG	CZ	0.43
100PHE	CE1	164ARG	C	2.23
100PHE	CE1	165VAL	CA	0.00
100PHE	CE1	165VAL	CG1	0.01
100PHE	CE1	168VAL	CG1	0.47
100PHE	CE1	168VAL	CG2	1.49
100PHE	CE1	262LEU	CB	0.21
100PHE	CE1	262LEU	CG	0.00
100PHE	CE1	262LEU	CD1	0.20
100PHE	CE1	262LEU	CD2	0.40
100PHE	CE1	262LEU	C	0.04
100PHE	CE1	263GLY	CA	0.61
100PHE	CE1	263GLY	C	2.26
100PHE	CE1	264ARG	CA	0.13
100PHE	CE1	264ARG	CB	0.00
100PHE	CE2	100PHE	CZ	100.00
100PHE	CE2	164ARG	CB	0.02
100PHE	CE2	164ARG	CG	0.06
100PHE	CE2	164ARG	CD	0.36

100PHE	CE2	164ARG	CZ	1.73
100PHE	CE2	164ARG	C	0.01
100PHE	CE2	167ARG	CZ	0.01
100PHE	CE2	168VAL	CG1	0.06
100PHE	CE2	262LEU	CB	0.27
100PHE	CE2	262LEU	CD2	0.06
100PHE	CE2	262LEU	C	0.21
100PHE	CE2	263GLY	CA	9.65
100PHE	CE2	263GLY	C	18.90
100PHE	CE2	264ARG	CA	0.03
100PHE	CE2	264ARG	CB	0.00
100PHE	CE2	264ARG	CG	0.10
100PHE	CE2	264ARG	CD	0.01
100PHE	CE2	264ARG	CZ	1.67
100PHE	CZ	164ARG	CB	0.11
100PHE	CZ	164ARG	CG	0.20
100PHE	CZ	164ARG	CD	0.16
100PHE	CZ	164ARG	CZ	0.01
100PHE	CZ	164ARG	C	0.00
100PHE	CZ	168VAL	CG1	0.01
100PHE	CZ	168VAL	CG2	0.06
100PHE	CZ	262LEU	CB	0.41
100PHE	CZ	262LEU	CG	0.01
100PHE	CZ	262LEU	CD1	0.08
100PHE	CZ	262LEU	CD2	0.64
100PHE	CZ	262LEU	C	0.02
100PHE	CZ	263GLY	CA	3.29
100PHE	CZ	263GLY	C	5.40
100PHE	CZ	264ARG	CA	0.54
100PHE	CZ	264ARG	CB	0.04
100PHE	CZ	264ARG	CG	0.09
100PHE	CZ	264ARG	CZ	0.06
100PHE	C	101ALA	CA	100.00
100PHE	C	101ALA	CB	77.04
100PHE	C	101ALA	C	25.58
100PHE	C	133GLU	CD	0.00
100PHE	C	134LEU	CB	0.10
100PHE	C	134LEU	CD1	0.01
100PHE	C	134LEU	CD2	0.05
100PHE	C	165VAL	CG1	0.02
101ALA	CA	101ALA	CB	100.00
101ALA	CA	101ALA	C	100.00
101ALA	CA	131VAL	CG2	0.00
101ALA	CA	134LEU	CB	0.02
101ALA	CA	134LEU	CD2	0.18
101ALA	CA	165VAL	CG1	0.01
101ALA	CB	101ALA	C	100.00
101ALA	CB	103LEU	CD1	0.32
101ALA	CB	103LEU	CD2	0.08
101ALA	CB	131VAL	CG1	1.82
101ALA	CB	131VAL	CG2	0.33
101ALA	CB	132ARG	C	0.02
101ALA	CB	165VAL	CG1	0.24
101ALA	CB	165VAL	CG2	0.12
101ALA	CB	168VAL	CB	0.02
101ALA	CB	168VAL	CG1	1.21
101ALA	CB	168VAL	CG2	1.34
101ALA	CB	262LEU	CB	0.02
101ALA	CB	262LEU	CG	0.01
101ALA	CB	262LEU	CD1	0.09
101ALA	CB	262LEU	CD2	0.04
101ALA	C	102ASN	CA	100.00

101ALA	C	102ASN	CB	17.42
101ALA	C	102ASN	C	86.78
101ALA	C	103LEU	CD1	0.05
101ALA	C	134LEU	CB	0.08
101ALA	C	134LEU	CG	0.01
101ALA	C	134LEU	CD1	0.05
101ALA	C	134LEU	CD2	2.78
101ALA	C	262LEU	CD2	0.00
102ASN	CA	102ASN	CB	100.00
102ASN	CA	102ASN	CG	100.00
102ASN	CA	102ASN	C	100.00
102ASN	CA	134LEU	CD2	0.02
102ASN	CA	261SER	CB	0.00
102ASN	CB	102ASN	CG	100.00
102ASN	CB	102ASN	C	100.00
102ASN	CB	132ARG	CG	0.05
102ASN	CB	132ARG	CD	0.04
102ASN	CB	132ARG	CZ	0.10
102ASN	CB	134LEU	CB	0.00
102ASN	CB	134LEU	CG	0.03
102ASN	CB	134LEU	CD1	0.28
102ASN	CB	134LEU	CD2	0.19
102ASN	CG	102ASN	C	99.82
102ASN	CG	132ARG	CZ	0.37
102ASN	CG	134LEU	CD1	0.09
102ASN	CG	134LEU	CD2	0.04
102ASN	CG	259SER	CB	0.01
102ASN	C	103LEU	CA	100.00
102ASN	C	103LEU	CB	28.67
102ASN	C	103LEU	CG	1.06
102ASN	C	103LEU	CD1	1.51
102ASN	C	103LEU	CD2	0.54
102ASN	C	103LEU	C	73.74
103LEU	CA	103LEU	CB	100.00
103LEU	CA	103LEU	CG	100.00
103LEU	CA	103LEU	CD1	32.49
103LEU	CA	103LEU	CD2	58.58
103LEU	CA	103LEU	C	100.00
103LEU	CA	129LEU	CD1	0.00
103LEU	CB	103LEU	CG	100.00
103LEU	CB	103LEU	CD1	100.00
103LEU	CB	103LEU	CD2	100.00
103LEU	CB	103LEU	C	100.00
103LEU	CB	129LEU	CD1	0.03
103LEU	CB	129LEU	CD2	0.03
103LEU	CB	131VAL	CG2	0.02
103LEU	CB	172LEU	CD1	0.00
103LEU	CB	260ALA	C	0.00
103LEU	CB	262LEU	CD1	0.01
103LEU	CB	296MET	CE	0.20
103LEU	CG	103LEU	CD1	100.00
103LEU	CG	103LEU	CD2	100.00
103LEU	CG	103LEU	C	22.93
103LEU	CG	129LEU	CD1	0.00
103LEU	CG	131VAL	CG2	0.01
103LEU	CG	172LEU	CD1	0.04
103LEU	CG	262LEU	CD1	0.08
103LEU	CG	262LEU	CD2	0.09
103LEU	CG	296MET	CE	0.01
103LEU	CD1	103LEU	CD2	100.00
103LEU	CD1	103LEU	C	3.54
103LEU	CD1	105PRO	CD	0.00

103LEU	CD1	129LEU	CB	0.00
103LEU	CD1	129LEU	CG	0.04
103LEU	CD1	129LEU	CD1	0.14
103LEU	CD1	129LEU	CD2	0.08
103LEU	CD1	131VAL	CG2	0.20
103LEU	CD1	168VAL	CG1	0.06
103LEU	CD1	172LEU	CD1	1.08
103LEU	CD1	172LEU	CD2	0.18
103LEU	CD1	260ALA	CB	0.06
103LEU	CD1	260ALA	C	0.02
103LEU	CD1	261SER	CA	0.08
103LEU	CD1	261SER	C	0.28
103LEU	CD1	262LEU	CB	0.01
103LEU	CD1	262LEU	CG	0.13
103LEU	CD1	262LEU	CD1	2.12
103LEU	CD1	262LEU	CD2	0.47
103LEU	CD1	296MET	CE	2.19
103LEU	CD1	300HIS	CD2	0.00
103LEU	CD1	300HIS	CE1	3.15
103LEU	CD2	103LEU	C	0.04
103LEU	CD2	129LEU	CG	0.00
103LEU	CD2	129LEU	CD1	0.02
103LEU	CD2	129LEU	CD2	0.07
103LEU	CD2	131VAL	CB	0.11
103LEU	CD2	131VAL	CG1	0.21
103LEU	CD2	131VAL	CG2	2.00
103LEU	CD2	168VAL	CG1	0.58
103LEU	CD2	172LEU	CG	0.00
103LEU	CD2	172LEU	CD1	10.07
103LEU	CD2	172LEU	CD2	0.60
103LEU	CD2	260ALA	CB	0.01
103LEU	CD2	260ALA	C	0.09
103LEU	CD2	261SER	CA	0.02
103LEU	CD2	261SER	C	0.04
103LEU	CD2	262LEU	CB	0.01
103LEU	CD2	262LEU	CG	0.15
103LEU	CD2	262LEU	CD1	1.83
103LEU	CD2	262LEU	CD2	0.36
103LEU	CD2	296MET	CE	0.36
103LEU	CD2	300HIS	CD2	0.00
103LEU	CD2	300HIS	CE1	0.88
103LEU	C	104ARG	CA	100.00
103LEU	C	104ARG	CB	72.68
103LEU	C	104ARG	CG	4.72
103LEU	C	104ARG	C	39.50
103LEU	C	105PRO	CD	0.62
104ARG	CA	104ARG	CB	100.00
104ARG	CA	104ARG	CG	100.00
104ARG	CA	104ARG	CD	1.39
104ARG	CA	104ARG	C	100.00
104ARG	CA	105PRO	CD	100.00
104ARG	CA	259SER	CB	0.00
104ARG	CB	104ARG	CG	100.00
104ARG	CB	104ARG	CD	100.00
104ARG	CB	104ARG	CZ	0.00
104ARG	CB	104ARG	C	100.00
104ARG	CB	105PRO	CD	0.02
104ARG	CB	257LEU	CD1	0.01
104ARG	CG	104ARG	CD	100.00
104ARG	CG	104ARG	CZ	1.40
104ARG	CG	104ARG	C	67.95
104ARG	CG	105PRO	CD	0.05

104AR(	CG	257LEU	CB	0.00
104AR(	CG	257LEU	CD1	0.62
104AR(	CG	257LEU	CD2	0.23
104AR(	CG	259SER	CB	0.22
104AR(	CD	104ARG	CZ	100.00
104AR(	CD	244SER	CB	0.04
104AR(	CD	257LEU	CB	0.00
104AR(	CD	257LEU	CD1	0.27
104AR(	CD	257LEU	CD2	0.39
104AR(	CD	272VAL	CG2	0.30
104AR(	CZ	244SER	CB	2.73
104AR(	CZ	244SER	C	0.03
104AR(	CZ	245ASP	CG	0.00
104AR(	CZ	254LEU	CD1	0.01
104AR(	CZ	254LEU	CD2	0.00
104AR(	CZ	257LEU	CD1	0.00
104AR(	CZ	257LEU	CD2	0.02
104AR(	CZ	259SER	CB	0.02
104AR(	CZ	272VAL	CG1	2.04
104AR(	CZ	272VAL	CG2	8.88
104AR(	C	105PRO	CA	100.00
104AR(	C	105PRO	CB	0.39
104AR(	C	105PRO	CG	0.00
104AR(	C	105PRO	CD	100.00
104AR(	C	105PRO	C	100.00
104AR(	C	129LEU	CD1	0.74
104AR(	C	129LEU	CD2	0.06
105PR(	CA	105PRO	CB	100.00
105PR(	CA	105PRO	CG	100.00
105PR(	CA	105PRO	CD	100.00
105PR(	CA	105PRO	C	100.00
105PR(	CA	129LEU	CD1	0.19
105PR(	CA	129LEU	CD2	0.06
105PR(	CB	105PRO	CG	100.00
105PR(	CB	105PRO	CD	100.00
105PR(	CB	105PRO	C	100.00
105PR(	CB	107LYS	CB	0.00
105PR(	CB	107LYS	CE	0.01
105PR(	CB	129LEU	CD1	0.21
105PR(	CB	129LEU	CD2	0.21
105PR(	CG	105PRO	CD	100.00
105PR(	CG	105PRO	C	92.69
105PR(	CG	129LEU	CD1	0.09
105PR(	CG	129LEU	CD2	0.02
105PR(	CG	258PRO	CG	5.28
105PR(	CG	258PRO	CD	0.11
105PR(	CG	296MET	CE	1.80
105PR(	CD	105PRO	C	92.72
105PR(	CD	129LEU	CD1	0.20
105PR(	CD	129LEU	CD2	0.01
105PR(	CD	258PRO	CG	0.04
105PR(	CD	296MET	CE	2.13
105PR(	C	106ALA	CA	100.00
105PR(	C	106ALA	CB	44.95
105PR(	C	106ALA	C	64.46
105PR(	C	257LEU	CD1	0.06
105PR(	C	257LEU	CD2	0.00
106ALA	CA	106ALA	CB	100.00
106ALA	CA	106ALA	C	100.00
106ALA	CA	250LEU	CD1	0.02
106ALA	CA	257LEU	CD1	0.46
106ALA	CA	257LEU	CD2	1.42

106ALA	CB	106ALA	C	100.00
106ALA	CB	128VAL	CG1	0.08
106ALA	CB	128VAL	CG2	0.03
106ALA	CB	130ILE	CD	1.00
106ALA	CB	247ALA	CB	0.88
106ALA	CB	247ALA	C	0.01
106ALA	CB	250LEU	CD1	0.78
106ALA	CB	250LEU	CD2	0.25
106ALA	CB	257LEU	CG	0.01
106ALA	CB	257LEU	CD1	0.29
106ALA	CB	257LEU	CD2	2.98
106ALA	C	107LYS	CA	100.00
106ALA	C	107LYS	CB	64.47
106ALA	C	107LYS	CG	0.85
106ALA	C	107LYS	C	40.31
106ALA	C	126VAL	CG1	0.01
106ALA	C	250LEU	CD1	0.08
107LYS	CA	107LYS	CB	100.00
107LYS	CA	107LYS	CG	100.00
107LYS	CA	107LYS	CD	0.40
107LYS	CA	107LYS	C	100.00
107LYS	CB	107LYS	CG	100.00
107LYS	CB	107LYS	CD	100.00
107LYS	CB	107LYS	CE	21.87
107LYS	CB	107LYS	C	100.00
107LYS	CB	109PHE	CD2	0.00
107LYS	CB	109PHE	CE1	0.02
107LYS	CB	109PHE	CE2	0.08
107LYS	CB	109PHE	CZ	0.03
107LYS	CB	127ASP	CA	0.06
107LYS	CB	127ASP	CB	0.03
107LYS	CG	107LYS	CD	100.00
107LYS	CG	107LYS	CE	100.00
107LYS	CG	107LYS	C	95.44
107LYS	CG	108VAL	C	0.06
107LYS	CG	109PHE	CD1	0.04
107LYS	CG	109PHE	CD2	0.02
107LYS	CG	109PHE	CE1	0.16
107LYS	CG	109PHE	CE2	0.19
107LYS	CG	109PHE	CZ	0.09
107LYS	CG	127ASP	CB	0.00
107LYS	CG	127ASP	CG	0.02
107LYS	CD	107LYS	CE	100.00
107LYS	CD	107LYS	C	0.07
107LYS	CD	109PHE	CD1	0.07
107LYS	CD	109PHE	CD2	0.04
107LYS	CD	109PHE	CE1	0.55
107LYS	CD	109PHE	CE2	0.47
107LYS	CD	109PHE	CZ	0.23
107LYS	CD	127ASP	CB	0.00
107LYS	CD	127ASP	CG	0.18
107LYS	CD	312GLU	CD	0.16
107LYS	CE	109PHE	CD1	0.03
107LYS	CE	109PHE	CD2	0.03
107LYS	CE	109PHE	CE1	0.40
107LYS	CE	109PHE	CE2	0.35
107LYS	CE	109PHE	CZ	0.20
107LYS	CE	127ASP	CG	0.18
107LYS	CE	312GLU	CD	0.57
107LYS	C	108VAL	CA	100.00
107LYS	C	108VAL	CB	26.69
107LYS	C	108VAL	CG2	21.32

107LYS	C	108VAL	C	86.65
108VAL	CA	108VAL	CB	100.00
108VAL	CA	108VAL	CG1	100.00
108VAL	CA	108VAL	CG2	100.00
108VAL	CA	108VAL	C	100.00
108VAL	CA	112LEU	CD1	0.01
108VAL	CA	112LEU	CD2	0.00
108VAL	CB	108VAL	CG1	100.00
108VAL	CB	108VAL	CG2	100.00
108VAL	CB	108VAL	C	100.00
108VAL	CB	126VAL	CB	0.01
108VAL	CB	126VAL	CG2	0.02
108VAL	CG1	108VAL	CG2	100.00
108VAL	CG1	108VAL	C	99.99
108VAL	CG1	112LEU	CB	0.73
108VAL	CG1	112LEU	CD1	0.00
108VAL	CG1	112LEU	C	0.86
108VAL	CG1	113GLU	CA	1.58
108VAL	CG1	113GLU	CB	0.50
108VAL	CG1	113GLU	CG	0.76
108VAL	CG1	123ALA	CB	0.01
108VAL	CG1	123ALA	C	0.01
108VAL	CG2	116SER	CB	0.00
108VAL	CG2	126VAL	CB	1.88
108VAL	CG2	126VAL	CG1	2.19
108VAL	CG2	126VAL	CG2	0.37
108VAL	CG2	250LEU	CB	0.30
108VAL	CG2	250LEU	CD1	0.02
108VAL	CG2	250LEU	CD2	0.24
108VAL	CG2	250LEU	C	11.72
108VAL	CG2	251PRO	CA	0.49
108VAL	CG2	251PRO	CB	0.01
108VAL	CG2	251PRO	CD	0.05
108VAL	C	109PHE	CA	100.00
108VAL	C	109PHE	CB	12.98
108VAL	C	109PHE	CG	0.32
108VAL	C	109PHE	CD1	0.14
108VAL	C	109PHE	CD2	0.55
108VAL	C	109PHE	C	87.81
109PHE	CA	109PHE	CB	100.00
109PHE	CA	109PHE	CG	100.00
109PHE	CA	109PHE	CD1	62.35
109PHE	CA	109PHE	CD2	60.67
109PHE	CA	109PHE	C	100.00
109PHE	CA	110PRO	CD	100.00
109PHE	CB	109PHE	CG	100.00
109PHE	CB	109PHE	CD1	100.00
109PHE	CB	109PHE	CD2	100.00
109PHE	CB	109PHE	C	100.00
109PHE	CB	110PRO	CD	35.49
109PHE	CB	112LEU	CB	0.02
109PHE	CB	112LEU	CD1	1.54
109PHE	CB	112LEU	CD2	0.01
109PHE	CB	312GLU	CG	0.03
109PHE	CB	312GLU	CD	0.05
109PHE	CB	316ALA	CB	1.06
109PHE	CB	319LEU	CD1	0.08
109PHE	CG	109PHE	CD1	100.00
109PHE	CG	109PHE	CD2	100.00
109PHE	CG	109PHE	CE1	100.00
109PHE	CG	109PHE	CE2	100.00
109PHE	CG	109PHE	CZ	100.00

109PHE	CG	109PHE	C	19.44
109PHE	CG	110PRO	CD	0.00
109PHE	CG	112LEU	CD1	0.08
109PHE	CG	256LEU	CD2	0.00
109PHE	CG	312GLU	CG	0.30
109PHE	CG	312GLU	CD	0.01
109PHE	CG	316ALA	CB	0.77
109PHE	CG	319LEU	CD1	0.10
109PHE	CG	319LEU	CD2	0.01
109PHE	CD1	109PHE	CD2	100.00
109PHE	CD1	109PHE	CE1	100.00
109PHE	CD1	109PHE	CE2	100.00
109PHE	CD1	109PHE	CZ	100.00
109PHE	CD1	109PHE	C	9.36
109PHE	CD1	112LEU	CB	0.02
109PHE	CD1	112LEU	CG	0.02
109PHE	CD1	112LEU	CD1	1.29
109PHE	CD1	112LEU	CD2	0.66
109PHE	CD1	251PRO	CB	0.01
109PHE	CD1	256LEU	CD1	0.01
109PHE	CD1	256LEU	CD2	0.03
109PHE	CD1	312GLU	CG	0.49
109PHE	CD1	312GLU	CD	0.09
109PHE	CD1	315VAL	CG1	0.01
109PHE	CD1	316ALA	CA	0.06
109PHE	CD1	316ALA	CB	2.41
109PHE	CD1	319LEU	CD1	1.60
109PHE	CD1	319LEU	CD2	0.03
109PHE	CD2	109PHE	CE1	100.00
109PHE	CD2	109PHE	CE2	100.00
109PHE	CD2	109PHE	CZ	100.00
109PHE	CD2	109PHE	C	0.43
109PHE	CD2	110PRO	CD	0.01
109PHE	CD2	112LEU	CD1	0.98
109PHE	CD2	112LEU	CD2	0.56
109PHE	CD2	251PRO	CB	0.02
109PHE	CD2	256LEU	CD2	0.01
109PHE	CD2	312GLU	CG	1.32
109PHE	CD2	312GLU	CD	0.25
109PHE	CD2	315VAL	CG1	0.00
109PHE	CD2	316ALA	CA	0.06
109PHE	CD2	316ALA	CB	2.05
109PHE	CD2	319LEU	CD1	1.26
109PHE	CD2	319LEU	CD2	0.01
109PHE	CE1	109PHE	CE2	100.00
109PHE	CE1	109PHE	CZ	100.00
109PHE	CE1	112LEU	CB	0.02
109PHE	CE1	112LEU	CG	0.15
109PHE	CE1	112LEU	CD1	3.49
109PHE	CE1	112LEU	CD2	0.21
109PHE	CE1	251PRO	CB	0.02
109PHE	CE1	256LEU	CD1	0.04
109PHE	CE1	256LEU	CD2	0.09
109PHE	CE1	292LEU	CD1	0.20
109PHE	CE1	292LEU	CD2	0.04
109PHE	CE1	312GLU	CB	0.02
109PHE	CE1	312GLU	CG	2.76
109PHE	CE1	312GLU	CD	0.46
109PHE	CE1	315VAL	CG1	0.44
109PHE	CE1	316ALA	CA	0.09
109PHE	CE1	316ALA	CB	0.69
109PHE	CE1	319LEU	CD1	1.84

109PHE	CE1	319LEU	CD2	0.01
109PHE	CE2	109PHE	CZ	100.00
109PHE	CE2	112LEU	CB	0.00
109PHE	CE2	112LEU	CD1	2.34
109PHE	CE2	112LEU	CD2	0.70
109PHE	CE2	251PRO	CB	0.06
109PHE	CE2	256LEU	CD1	0.00
109PHE	CE2	256LEU	CD2	0.06
109PHE	CE2	257LEU	CD1	0.01
109PHE	CE2	292LEU	CD1	0.35
109PHE	CE2	292LEU	CD2	0.18
109PHE	CE2	312GLU	CB	0.01
109PHE	CE2	312GLU	CG	4.33
109PHE	CE2	312GLU	CD	0.52
109PHE	CE2	312GLU	C	0.00
109PHE	CE2	315VAL	CG1	0.19
109PHE	CE2	315VAL	CG2	0.01
109PHE	CE2	316ALA	CA	0.04
109PHE	CE2	316ALA	CB	0.29
109PHE	CE2	319LEU	CD1	1.39
109PHE	CZ	112LEU	CB	0.00
109PHE	CZ	112LEU	CG	0.00
109PHE	CZ	112LEU	CD1	2.89
109PHE	CZ	112LEU	CD2	0.37
109PHE	CZ	256LEU	CD1	0.00
109PHE	CZ	256LEU	CD2	0.03
109PHE	CZ	288THR	CG2	0.00
109PHE	CZ	292LEU	CD1	1.93
109PHE	CZ	292LEU	CD2	1.78
109PHE	CZ	312GLU	CB	0.01
109PHE	CZ	312GLU	CG	1.62
109PHE	CZ	312GLU	CD	0.03
109PHE	CZ	315VAL	CG1	0.93
109PHE	CZ	315VAL	CG2	0.00
109PHE	CZ	316ALA	CA	0.01
109PHE	CZ	316ALA	CB	0.01
109PHE	CZ	319LEU	CD1	0.20
109PHE	C	110PRO	CA	100.00
109PHE	C	110PRO	CB	0.06
109PHE	C	110PRO	CD	100.00
109PHE	C	110PRO	C	100.00
109PHE	C	112LEU	CD1	0.00
110PRO	CA	110PRO	CB	100.00
110PRO	CA	110PRO	CG	100.00
110PRO	CA	110PRO	CD	100.00
110PRO	CA	110PRO	C	100.00
110PRO	CA	113GLU	CG	0.00
110PRO	CA	113GLU	CD	0.06
110PRO	CA	124ARG	CZ	0.05
110PRO	CB	110PRO	CG	100.00
110PRO	CB	110PRO	CD	100.00
110PRO	CB	110PRO	C	100.00
110PRO	CB	124ARG	CZ	0.30
110PRO	CG	110PRO	CD	100.00
110PRO	CG	110PRO	C	75.69
110PRO	CG	124ARG	CG	0.00
110PRO	CG	124ARG	CZ	0.03
110PRO	CG	316ALA	CA	0.00
110PRO	CG	316ALA	CB	0.01
110PRO	CD	110PRO	C	70.61
110PRO	CD	316ALA	CB	0.02
110PRO	C	111GLY	CA	100.00

110PR(	C	111GLY	C	50.21
110PR(	C	112LEU	CD1	0.00
110PR(	C	320LEU	CD1	0.00
110PR(	C	320LEU	CD2	0.00
111GLY	CA	111GLY	C	100.00
111GLY	CA	113GLU	CD	0.14
111GLY	CA	114ARG	CZ	0.38
111GLY	CA	319LEU	CB	0.00
111GLY	CA	319LEU	CD1	0.00
111GLY	CA	319LEU	CD2	0.02
111GLY	CA	320LEU	CA	0.00
111GLY	CA	320LEU	CG	0.04
111GLY	CA	320LEU	CD1	0.38
111GLY	CA	320LEU	CD2	0.83
111GLY	C	112LEU	CA	100.00
111GLY	C	112LEU	CB	39.29
111GLY	C	112LEU	CG	11.37
111GLY	C	112LEU	CD1	4.20
111GLY	C	112LEU	CD2	0.13
111GLY	C	112LEU	C	56.51
111GLY	C	113GLU	CD	0.09
111GLY	C	114ARG	CD	0.00
111GLY	C	319LEU	CD1	0.08
111GLY	C	319LEU	CD2	0.36
111GLY	C	320LEU	CD1	0.05
111GLY	C	320LEU	CD2	0.50
112LEU	CA	112LEU	CB	100.00
112LEU	CA	112LEU	CG	100.00
112LEU	CA	112LEU	CD1	41.53
112LEU	CA	112LEU	CD2	49.61
112LEU	CA	112LEU	C	100.00
112LEU	CA	115LEU	CD1	0.06
112LEU	CA	319LEU	CD1	0.01
112LEU	CB	112LEU	CG	100.00
112LEU	CB	112LEU	CD1	100.00
112LEU	CB	112LEU	CD2	100.00
112LEU	CB	112LEU	C	100.00
112LEU	CB	115LEU	CD1	0.02
112LEU	CB	319LEU	CD1	0.05
112LEU	CG	112LEU	CD1	100.00
112LEU	CG	112LEU	CD2	100.00
112LEU	CG	112LEU	C	22.50
112LEU	CG	115LEU	CD1	0.05
112LEU	CG	115LEU	CD2	0.00
112LEU	CG	251PRO	CB	0.01
112LEU	CG	319LEU	CD1	0.30
112LEU	CG	319LEU	CD2	0.03
112LEU	CG	320LEU	CG	0.01
112LEU	CG	320LEU	CD1	0.02
112LEU	CG	320LEU	CD2	0.10
112LEU	CD1	112LEU	CD2	100.00
112LEU	CD1	112LEU	C	0.35
112LEU	CD1	115LEU	CB	0.01
112LEU	CD1	115LEU	CD1	0.22
112LEU	CD1	115LEU	CD2	0.02
112LEU	CD1	251PRO	CA	0.02
112LEU	CD1	251PRO	CB	0.23
112LEU	CD1	256LEU	CD1	0.18
112LEU	CD1	256LEU	CD2	0.24
112LEU	CD1	316ALA	CA	0.05
112LEU	CD1	316ALA	CB	0.30
112LEU	CD1	319LEU	CB	3.57

112LEU	CD1	319LEU	CD1	2.06
112LEU	CD1	319LEU	CD2	0.18
112LEU	CD1	319LEU	C	0.01
112LEU	CD1	320LEU	CA	0.01
112LEU	CD1	320LEU	CB	0.00
112LEU	CD1	320LEU	CG	0.04
112LEU	CD1	320LEU	CD1	0.18
112LEU	CD1	320LEU	CD2	0.34
112LEU	CD1	324PRO	CG	0.00
112LEU	CD1	327LEU	CD1	0.04
112LEU	CD1	327LEU	CD2	0.62
112LEU	CD2	112LEU	C	0.49
112LEU	CD2	115LEU	CD1	2.42
112LEU	CD2	115LEU	CD2	0.13
112LEU	CD2	251PRO	CA	0.18
112LEU	CD2	251PRO	CB	2.35
112LEU	CD2	253SER	CB	0.03
112LEU	CD2	256LEU	CD1	0.88
112LEU	CD2	256LEU	CD2	0.51
112LEU	CD2	316ALA	CA	0.00
112LEU	CD2	319LEU	CB	3.31
112LEU	CD2	319LEU	CG	0.02
112LEU	CD2	319LEU	CD1	3.06
112LEU	CD2	319LEU	CD2	0.41
112LEU	CD2	319LEU	C	0.18
112LEU	CD2	320LEU	CA	0.03
112LEU	CD2	320LEU	CB	0.00
112LEU	CD2	320LEU	CG	0.05
112LEU	CD2	320LEU	CD1	0.20
112LEU	CD2	320LEU	CD2	2.02
112LEU	CD2	324PRO	CG	0.01
112LEU	CD2	327LEU	CD1	0.17
112LEU	CD2	327LEU	CD2	0.30
112LEU	C	113GLU	CA	100.00
112LEU	C	113GLU	CB	0.02
112LEU	C	113GLU	C	99.99
112LEU	C	115LEU	CD1	0.02
113GLU	CA	113GLU	CB	100.00
113GLU	CA	113GLU	CG	100.00
113GLU	CA	113GLU	CD	71.21
113GLU	CA	113GLU	C	100.00
113GLU	CA	123ALA	CB	0.02
113GLU	CB	113GLU	CG	100.00
113GLU	CB	113GLU	CD	100.00
113GLU	CB	113GLU	C	100.00
113GLU	CB	120GLU	CB	0.01
113GLU	CB	120GLU	CG	2.77
113GLU	CB	123ALA	CB	1.12
113GLU	CB	123ALA	C	0.01
113GLU	CB	124ARG	CB	0.00
113GLU	CB	124ARG	CG	0.02
113GLU	CG	113GLU	CD	100.00
113GLU	CG	113GLU	C	36.07
113GLU	CG	120GLU	CA	0.01
113GLU	CG	120GLU	CB	0.10
113GLU	CG	120GLU	CG	1.30
113GLU	CG	123ALA	CB	0.07
113GLU	CG	123ALA	C	0.14
113GLU	CG	124ARG	CA	0.34
113GLU	CG	124ARG	CB	0.02
113GLU	CG	124ARG	CG	0.14
113GLU	CG	124ARG	CZ	0.09

113GLU	CD	113GLU	C	0.31
113GLU	CD	114ARG	CG	0.06
113GLU	CD	124ARG	CB	0.00
113GLU	CD	124ARG	CG	0.37
113GLU	CD	124ARG	CZ	0.08
113GLU	C	114ARG	CA	100.00
113GLU	C	114ARG	CB	0.13
113GLU	C	114ARG	CG	0.02
113GLU	C	114ARG	C	99.78
113GLU	C	120GLU	CB	0.01
113GLU	C	120GLU	CG	0.03
114ARG	CA	114ARG	CB	100.00
114ARG	CA	114ARG	CG	100.00
114ARG	CA	114ARG	CD	2.48
114ARG	CA	114ARG	C	100.00
114ARG	CA	120GLU	CB	0.00
114ARG	CA	120GLU	CG	0.06
114ARG	CB	114ARG	CG	100.00
114ARG	CB	114ARG	CD	100.00
114ARG	CB	114ARG	CZ	1.32
114ARG	CB	114ARG	C	100.00
114ARG	CB	115LEU	CD1	0.00
114ARG	CB	115LEU	CD2	0.00
114ARG	CB	120GLU	CG	0.00
114ARG	CB	120GLU	CD	0.02
114ARG	CG	114ARG	CD	100.00
114ARG	CG	114ARG	CZ	11.39
114ARG	CG	114ARG	C	25.74
114ARG	CG	115LEU	CD1	0.01
114ARG	CG	120GLU	CB	0.04
114ARG	CG	120GLU	CG	0.46
114ARG	CG	120GLU	CD	1.95
114ARG	CD	114ARG	CZ	100.00
114ARG	CD	114ARG	C	0.08
114ARG	CD	120GLU	CG	0.01
114ARG	CD	120GLU	CD	0.01
114ARG	CD	320LEU	CD1	0.00
114ARG	CD	320LEU	CD2	0.17
114ARG	CZ	115LEU	CD2	0.02
114ARG	CZ	120GLU	CG	0.04
114ARG	CZ	120GLU	CD	0.02
114ARG	CZ	320LEU	CD1	0.17
114ARG	CZ	320LEU	CD2	1.07
114ARG	C	115LEU	CA	100.00
114ARG	C	115LEU	CB	4.36
114ARG	C	115LEU	CG	0.48
114ARG	C	115LEU	CD1	0.24
114ARG	C	115LEU	CD2	0.12
114ARG	C	115LEU	C	93.71
114ARG	C	119LYS	CE	0.00
115LEU	CA	115LEU	CB	100.00
115LEU	CA	115LEU	CG	100.00
115LEU	CA	115LEU	CD1	17.45
115LEU	CA	115LEU	CD2	81.27
115LEU	CA	115LEU	C	100.00
115LEU	CB	115LEU	CG	100.00
115LEU	CB	115LEU	CD1	100.00
115LEU	CB	115LEU	CD2	100.00
115LEU	CB	115LEU	C	100.00
115LEU	CB	252GLY	CA	0.00
115LEU	CB	327LEU	CG	0.01
115LEU	CB	327LEU	CD1	0.43

115LEU	CB	327LEU	CD2	0.47
115LEU	CG	115LEU	CD1	100.00
115LEU	CG	115LEU	CD2	100.00
115LEU	CG	115LEU	C	1.98
115LEU	CG	327LEU	CD1	0.04
115LEU	CG	327LEU	CD2	0.01
115LEU	CD1	115LEU	CD2	100.00
115LEU	CD1	115LEU	C	0.00
115LEU	CD1	256LEU	CD1	0.02
115LEU	CD1	256LEU	CD2	0.01
115LEU	CD1	319LEU	CB	0.03
115LEU	CD1	319LEU	CG	0.04
115LEU	CD1	319LEU	CD1	0.05
115LEU	CD1	319LEU	CD2	0.68
115LEU	CD1	320LEU	CD1	0.01
115LEU	CD1	320LEU	CD2	0.24
115LEU	CD1	323PRO	CB	0.14
115LEU	CD1	323PRO	CG	0.01
115LEU	CD1	324PRO	CG	0.18
115LEU	CD1	324PRO	CD	0.56
115LEU	CD1	327LEU	CG	0.04
115LEU	CD1	327LEU	CD1	0.22
115LEU	CD1	327LEU	CD2	1.34
115LEU	CD1	327LEU	C	0.00
115LEU	CD2	115LEU	C	0.02
115LEU	CD2	252GLY	C	0.01
115LEU	CD2	253SER	CB	0.01
115LEU	CD2	256LEU	CD1	0.02
115LEU	CD2	319LEU	CD1	0.00
115LEU	CD2	319LEU	CD2	0.02
115LEU	CD2	320LEU	CD2	0.02
115LEU	CD2	323PRO	CB	0.18
115LEU	CD2	323PRO	CG	0.03
115LEU	CD2	324PRO	CG	0.07
115LEU	CD2	324PRO	CD	0.09
115LEU	CD2	327LEU	CB	0.04
115LEU	CD2	327LEU	CG	0.02
115LEU	CD2	327LEU	CD1	0.24
115LEU	CD2	327LEU	CD2	0.40
115LEU	C	116SER	CA	100.00
115LEU	C	116SER	CB	0.52
115LEU	C	116SER	C	99.66
115LEU	C	252GLY	CA	0.06
115LEU	C	327LEU	CD2	0.00
116SER	CA	116SER	CB	100.00
116SER	CA	116SER	C	100.00
116SER	CA	117PRO	CD	100.00
116SER	CB	116SER	C	100.00
116SER	CB	117PRO	CD	2.06
116SER	CB	123ALA	CB	2.31
116SER	C	117PRO	CA	100.00
116SER	C	117PRO	CB	0.13
116SER	C	117PRO	CD	100.00
116SER	C	117PRO	C	100.00
117PRO	CA	117PRO	CB	100.00
117PRO	CA	117PRO	CG	100.00
117PRO	CA	117PRO	CD	100.00
117PRO	CA	117PRO	C	100.00
117PRO	CA	122ILE	CD	1.95
117PRO	CB	117PRO	CG	100.00
117PRO	CB	117PRO	CD	100.00
117PRO	CB	117PRO	C	100.00

117PR(	CB	118LEU	CD1	0.00
117PR(	CB	118LEU	CD2	0.03
117PR(	CB	122ILE	CG2	0.00
117PR(	CB	122ILE	CD	11.22
117PR(	CB	225ARG	CA	0.01
117PR(	CB	225ARG	CB	0.00
117PR(	CG	117PRO	CD	100.00
117PR(	CG	117PRO	C	97.60
117PR(	CG	118LEU	CD1	0.01
117PR(	CG	249VAL	CG1	2.69
117PR(	CG	249VAL	CG2	1.35
117PR(	CG	252GLY	CA	0.00
117PR(	CG	224VAL	CG1	0.10
117PR(	CG	224VAL	CG2	0.00
117PR(	CG	225ARG	CG	0.00
117PR(	CD	117PRO	C	90.19
117PR(	CD	249VAL	CG1	0.04
117PR(	CD	249VAL	CG2	0.04
117PR(	CD	250LEU	CA	0.00
117PR(	CD	251PRO	C	0.00
117PR(	CD	252GLY	CA	2.99
117PR(	C	118LEU	CA	100.00
117PR(	C	118LEU	CB	0.08
117PR(	C	118LEU	CG	0.00
117PR(	C	118LEU	CD1	0.00
117PR(	C	118LEU	C	99.92
117PR(	C	118LEU	CD1	0.02
117PR(	C	118LEU	CD2	0.15
117PR(	C	119LYS	CG	0.01
117PR(	C	119LYS	CD	0.22
117PR(	C	119LYS	CE	0.03
118LEL	CA	118LEU	CB	100.00
118LEL	CA	118LEU	CG	100.00
118LEL	CA	118LEU	CD1	20.98
118LEL	CA	118LEU	CD2	77.34
118LEL	CA	118LEU	C	100.00
118LEL	CB	118LEU	CG	100.00
118LEL	CB	118LEU	CD1	100.00
118LEL	CB	118LEU	CD2	100.00
118LEL	CB	118LEU	C	100.00
118LEL	CB	122ILE	CG2	0.01
118LEL	CB	123ALA	CB	0.03
118LEL	CG	118LEU	CD1	100.00
118LEL	CG	118LEU	CD2	100.00
118LEL	CG	118LEU	C	1.48
118LEL	CG	122ILE	CD	0.01
118LEL	CG	250LEU	CD2	0.00
118LEL	CG	118LEU	CD2	0.01
118LEL	CD:	118LEU	CD2	100.00
118LEL	CD:	118LEU	C	0.04
118LEL	CD:	122ILE	CG2	0.14
118LEL	CD:	122ILE	CD	0.04
118LEL	CD:	122ILE	C	0.00
118LEL	CD:	123ALA	CA	0.02
118LEL	CD:	123ALA	CB	0.06
118LEL	CD:	126VAL	CG2	0.01
118LEL	CD:	224VAL	CA	0.00
118LEL	CD:	224VAL	CB	0.00
118LEL	CD:	224VAL	CG1	0.13
118LEL	CD:	224VAL	C	0.02
118LEL	CD:	227PRO	CG	0.77
118LEL	CD:	227PRO	CD	0.05

118LEU	CD1	249VAL	CG1	0.18
118LEU	CD1	249VAL	CG2	0.06
118LEU	CD1	249VAL	C	0.00
118LEU	CD1	250LEU	CA	0.00
118LEU	CD1	250LEU	CG	0.01
118LEU	CD1	250LEU	CD1	0.09
118LEU	CD1	250LEU	CD2	4.62
118LEU	CD1	118LEU	CD1	0.02
118LEU	CD1	118LEU	CD2	0.20
118LEU	CD1	224VAL	CG1	0.06
118LEU	CD2	122ILE	CG1	0.01
118LEU	CD2	122ILE	CG2	0.29
118LEU	CD2	122ILE	CD	0.13
118LEU	CD2	122ILE	C	0.01
118LEU	CD2	123ALA	CA	0.00
118LEU	CD2	123ALA	CB	0.01
118LEU	CD2	224VAL	CA	0.01
118LEU	CD2	224VAL	CB	0.00
118LEU	CD2	224VAL	CG1	0.83
118LEU	CD2	224VAL	C	0.17
118LEU	CD2	227PRO	CG	0.44
118LEU	CD2	227PRO	CD	0.08
118LEU	CD2	249VAL	CG1	0.01
118LEU	CD2	250LEU	CG	0.00
118LEU	CD2	250LEU	CD2	0.72
118LEU	CD2	117PRO	CB	0.02
118LEU	CD2	117PRO	C	0.09
118LEU	CD2	118LEU	CG	0.00
118LEU	CD2	118LEU	CD1	0.17
118LEU	CD2	118LEU	CD2	1.12
118LEU	CD2	224VAL	CG1	0.22
118LEU	C	119LYS	CA	100.00
118LEU	C	119LYS	CB	0.01
118LEU	C	119LYS	C	100.00
118LEU	C	119LYS	CG	0.03
118LEU	C	119LYS	CD	0.01
118LEU	C	119LYS	CE	0.02
119LYS	CA	119LYS	CB	100.00
119LYS	CA	119LYS	CG	100.00
119LYS	CA	119LYS	CD	2.60
119LYS	CA	119LYS	CE	0.12
119LYS	CA	119LYS	C	100.00
119LYS	CA	119LYS	CG	0.02
119LYS	CB	119LYS	CG	100.00
119LYS	CB	119LYS	CD	100.00
119LYS	CB	119LYS	CE	24.10
119LYS	CB	119LYS	C	100.00
119LYS	CB	122ILE	CG1	0.27
119LYS	CB	122ILE	CD	0.23
119LYS	CB	117PRO	C	0.00
119LYS	CG	119LYS	CD	100.00
119LYS	CG	119LYS	CE	100.00
119LYS	CG	119LYS	C	7.59
119LYS	CG	121GLU	CD	0.02
119LYS	CG	122ILE	CG1	0.02
119LYS	CG	122ILE	CD	0.16
119LYS	CG	117PRO	C	0.00
119LYS	CG	118LEU	C	0.05
119LYS	CG	119LYS	CA	0.06
119LYS	CG	119LYS	CG	0.10
119LYS	CD	119LYS	CE	100.00
119LYS	CD	121GLU	CG	0.00

119LYS	CD	122ILE	CD	0.06
119LYS	CD	117PRO	C	0.05
119LYS	CD	118LEU	C	0.01
119LYS	CD	119LYS	CA	0.00
119LYS	CD	120GLU	CD	0.01
119LYS	CE	122ILE	CD	0.01
119LYS	CE	117PRO	C	0.08
119LYS	CE	118LEU	C	0.01
119LYS	CE	119LYS	CB	0.00
119LYS	CE	120GLU	CD	1.36
119LYS	C	120GLU	CA	100.00
119LYS	C	120GLU	C	100.00
120GLU	CA	120GLU	CB	100.00
120GLU	CA	120GLU	CG	100.00
120GLU	CA	120GLU	CD	42.37
120GLU	CA	120GLU	C	100.00
120GLU	CB	120GLU	CG	100.00
120GLU	CB	120GLU	CD	100.00
120GLU	CB	120GLU	C	100.00
120GLU	CB	124ARG	CZ	0.77
120GLU	CG	120GLU	CD	100.00
120GLU	CG	120GLU	C	53.32
120GLU	CG	124ARG	CZ	0.04
120GLU	CD	120GLU	C	3.22
120GLU	CD	124ARG	CZ	0.08
120GLU	CD	119LYS	CE	1.26
120GLU	C	121GLU	CA	100.00
120GLU	C	121GLU	CB	0.14
120GLU	C	121GLU	C	99.95
120GLU	C	124ARG	CZ	0.07
121GLU	CA	121GLU	CB	100.00
121GLU	CA	121GLU	CG	100.00
121GLU	CA	121GLU	CD	97.22
121GLU	CA	121GLU	C	100.00
121GLU	CA	124ARG	CZ	0.02
121GLU	CB	121GLU	CG	100.00
121GLU	CB	121GLU	CD	100.00
121GLU	CB	121GLU	C	100.00
121GLU	CB	124ARG	CZ	0.00
121GLU	CG	121GLU	CD	100.00
121GLU	CG	121GLU	C	75.95
121GLU	CG	122ILE	CG1	0.00
121GLU	CG	122ILE	CD	0.01
121GLU	CD	121GLU	C	0.35
121GLU	CD	124ARG	CD	0.00
121GLU	CD	124ARG	CZ	0.06
121GLU	C	122ILE	CA	100.00
121GLU	C	122ILE	CB	7.38
121GLU	C	122ILE	CG1	0.58
121GLU	C	122ILE	CG2	3.64
121GLU	C	122ILE	CD	0.03
121GLU	C	122ILE	C	92.37
121GLU	C	124ARG	CB	0.01
122ILE	CA	122ILE	CB	100.00
122ILE	CA	122ILE	CG1	100.00
122ILE	CA	122ILE	CG2	100.00
122ILE	CA	122ILE	CD	17.07
122ILE	CA	122ILE	C	100.00
122ILE	CB	122ILE	CG1	100.00
122ILE	CB	122ILE	CG2	100.00
122ILE	CB	122ILE	CD	100.00
122ILE	CB	122ILE	C	100.00

122ILE	CG:	122ILE	CG2	100.00
122ILE	CG:	122ILE	CD	100.00
122ILE	CG:	122ILE	C	30.73
122ILE	CG:	117PRO	CB	0.02
122ILE	CG:	122ILE	CD	97.58
122ILE	CG:	122ILE	C	69.21
122ILE	CG:	224VAL	C	0.04
122ILE	CG:	225ARG	C	0.09
122ILE	CG:	227PRO	CG	0.16
122ILE	CG:	227PRO	CD	6.60
122ILE	CG:	250LEU	CD2	0.01
122ILE	CD	122ILE	C	0.71
122ILE	CD	224VAL	C	0.00
122ILE	CD	225ARG	CA	0.01
122ILE	CD	225ARG	C	0.07
122ILE	CD	227PRO	CG	0.00
122ILE	CD	227PRO	CD	0.02
122ILE	CD	117PRO	CA	1.08
122ILE	CD	117PRO	CB	12.08
122ILE	C	123ALA	CA	100.00
122ILE	C	123ALA	CB	1.59
122ILE	C	123ALA	C	98.35
122ILE	C	126VAL	CG2	0.00
123ALA	CA	123ALA	CB	100.00
123ALA	CA	123ALA	C	100.00
123ALA	CA	126VAL	CG2	0.09
123ALA	CA	250LEU	CD2	0.00
123ALA	CB	123ALA	C	100.00
123ALA	CB	250LEU	CD2	0.00
123ALA	C	124ARG	CA	100.00
123ALA	C	124ARG	CB	5.34
123ALA	C	124ARG	CG	0.68
123ALA	C	124ARG	C	95.71
123ALA	C	126VAL	CG2	2.02
124ARG	CA	124ARG	CB	100.00
124ARG	CA	124ARG	CG	100.00
124ARG	CA	124ARG	CD	2.76
124ARG	CA	124ARG	C	100.00
124ARG	CB	124ARG	CG	100.00
124ARG	CB	124ARG	CD	100.00
124ARG	CB	124ARG	CZ	14.68
124ARG	CB	124ARG	C	100.00
124ARG	CG	124ARG	CD	100.00
124ARG	CG	124ARG	CZ	43.01
124ARG	CG	124ARG	C	65.77
124ARG	CD	124ARG	CZ	100.00
124ARG	CD	124ARG	C	0.04
124ARG	C	125GLY	CA	100.00
124ARG	C	125GLY	C	52.15
125GLY	CA	125GLY	C	100.00
125GLY	CA	228ALA	CB	0.11
125GLY	C	126VAL	CA	100.00
125GLY	C	126VAL	CB	3.73
125GLY	C	126VAL	CG2	1.98
125GLY	C	126VAL	C	98.30
125GLY	C	228ALA	CA	0.00
125GLY	C	228ALA	CB	3.41
126VAL	CA	126VAL	CB	100.00
126VAL	CA	126VAL	CG1	100.00
126VAL	CA	126VAL	CG2	100.00
126VAL	CA	126VAL	C	100.00
126VAL	CB	126VAL	CG1	100.00

126VAL	CB	126VAL	CG2	100.00
126VAL	CB	126VAL	C	100.00
126VAL	CG:	126VAL	CG2	100.00
126VAL	CG:	126VAL	C	100.00
126VAL	CG:	128VAL	CG1	0.08
126VAL	CG:	128VAL	CG2	1.17
126VAL	CG:	227PRO	CB	0.17
126VAL	CG:	227PRO	CG	0.04
126VAL	CG:	250LEU	CB	0.09
126VAL	CG:	250LEU	CG	0.00
126VAL	CG:	250LEU	CD1	1.78
126VAL	CG:	250LEU	CD2	1.10
126VAL	CG:	227PRO	CB	1.25
126VAL	CG:	227PRO	CG	7.63
126VAL	CG:	227PRO	C	0.01
126VAL	CG:	228ALA	CA	0.04
126VAL	CG:	228ALA	CB	0.10
126VAL	CG:	250LEU	CB	0.01
126VAL	CG:	250LEU	CD1	0.00
126VAL	CG:	250LEU	CD2	0.03
126VAL	C	127ASP	CA	100.00
126VAL	C	127ASP	CB	99.90
126VAL	C	127ASP	C	0.82
127ASF	CA	127ASP	CB	100.00
127ASF	CA	127ASP	CG	100.00
127ASF	CA	127ASP	C	100.00
127ASF	CB	127ASP	CG	100.00
127ASF	CB	127ASP	C	100.00
127ASF	CG	127ASP	C	99.99
127ASF	CG	176ARG	CZ	0.07
127ASF	C	128VAL	CA	100.00
127ASF	C	128VAL	CB	27.09
127ASF	C	128VAL	CG1	1.14
127ASF	C	128VAL	CG2	6.16
127ASF	C	128VAL	C	77.00
128VAL	CA	128VAL	CB	100.00
128VAL	CA	128VAL	CG1	100.00
128VAL	CA	128VAL	CG2	100.00
128VAL	CA	128VAL	C	100.00
128VAL	CB	128VAL	CG1	100.00
128VAL	CB	128VAL	CG2	100.00
128VAL	CB	128VAL	C	100.00
128VAL	CB	130ILE	CD	0.00
128VAL	CB	230PHE	CE2	0.01
128VAL	CB	250LEU	CD1	0.00
128VAL	CB	250LEU	CD2	0.00
128VAL	CG:	128VAL	CG2	100.00
128VAL	CG:	128VAL	C	96.14
128VAL	CG:	130ILE	CG1	0.32
128VAL	CG:	130ILE	CD	2.90
128VAL	CG:	230PHE	CB	0.74
128VAL	CG:	230PHE	CD2	0.07
128VAL	CG:	230PHE	CE2	0.13
128VAL	CG:	230PHE	C	0.01
128VAL	CG:	231ASP	C	0.01
128VAL	CG:	233VAL	CG1	0.03
128VAL	CG:	233VAL	CG2	2.41
128VAL	CG:	250LEU	CD1	0.54
128VAL	CG:	250LEU	CD2	0.06
128VAL	CG:	128VAL	C	23.76
128VAL	CG:	129LEU	C	0.00
128VAL	CG:	130ILE	CG1	0.01

128VAL	CG2	130ILE	CD	0.11
128VAL	CG2	230PHE	CB	2.20
128VAL	CG2	230PHE	CD2	0.18
128VAL	CG2	230PHE	CE2	0.13
128VAL	CG2	230PHE	C	0.05
128VAL	CG2	231ASP	C	0.01
128VAL	CG2	233VAL	CG2	1.21
128VAL	CG2	250LEU	CD1	0.62
128VAL	CG2	250LEU	CD2	0.26
128VAL	C	129LEU	CA	100.00
128VAL	C	129LEU	CB	45.02
128VAL	C	129LEU	CG	0.02
128VAL	C	129LEU	CD1	0.01
128VAL	C	129LEU	CD2	0.01
128VAL	C	129LEU	C	65.78
129LEU	CA	129LEU	CB	100.00
129LEU	CA	129LEU	CG	100.00
129LEU	CA	129LEU	CD1	76.82
129LEU	CA	129LEU	CD2	23.13
129LEU	CA	129LEU	C	100.00
129LEU	CB	129LEU	CG	100.00
129LEU	CB	129LEU	CD1	100.00
129LEU	CB	129LEU	CD2	100.00
129LEU	CB	129LEU	C	100.00
129LEU	CB	176ARG	CZ	2.11
129LEU	CG	129LEU	CD1	100.00
129LEU	CG	129LEU	CD2	100.00
129LEU	CG	129LEU	C	90.07
129LEU	CG	131VAL	CG2	0.05
129LEU	CG	172LEU	CD2	0.01
129LEU	CG	176ARG	CZ	0.18
129LEU	CD1	129LEU	CD2	100.00
129LEU	CD1	129LEU	C	6.48
129LEU	CD1	130ILE	C	0.02
129LEU	CD1	131VAL	CG1	0.03
129LEU	CD1	131VAL	CG2	0.34
129LEU	CD1	172LEU	CB	0.01
129LEU	CD1	172LEU	CG	0.01
129LEU	CD1	172LEU	CD1	0.08
129LEU	CD1	172LEU	CD2	0.26
129LEU	CD1	176ARG	CD	0.09
129LEU	CD1	176ARG	CZ	1.16
129LEU	CD1	296MET	CE	0.01
129LEU	CD2	129LEU	C	10.94
129LEU	CD2	130ILE	C	0.04
129LEU	CD2	131VAL	CG1	0.03
129LEU	CD2	131VAL	CG2	2.82
129LEU	CD2	172LEU	CB	0.34
129LEU	CD2	172LEU	CG	0.02
129LEU	CD2	172LEU	CD1	0.63
129LEU	CD2	172LEU	CD2	0.64
129LEU	CD2	176ARG	CD	0.05
129LEU	CD2	176ARG	CZ	0.53
129LEU	CD2	232VAL	CG1	0.02
129LEU	CD2	232VAL	CG2	0.04
129LEU	CD2	296MET	CE	0.01
129LEU	CD2	300HIS	CE1	0.02
129LEU	C	130ILE	CA	100.00
129LEU	C	130ILE	CB	14.69
129LEU	C	130ILE	CG1	9.58
129LEU	C	130ILE	CD	0.33
129LEU	C	130ILE	C	93.84

130ILE	CA	130ILE	CB	100.00
130ILE	CA	130ILE	CG1	100.00
130ILE	CA	130ILE	CG2	100.00
130ILE	CA	130ILE	CD	51.63
130ILE	CA	130ILE	C	100.00
130ILE	CA	233VAL	CG2	0.01
130ILE	CB	130ILE	CG1	100.00
130ILE	CB	130ILE	CG2	100.00
130ILE	CB	130ILE	CD	100.00
130ILE	CB	130ILE	C	100.00
130ILE	CG:	130ILE	CG2	100.00
130ILE	CG:	130ILE	CD	100.00
130ILE	CG:	233VAL	CB	0.01
130ILE	CG:	233VAL	CG1	0.01
130ILE	CG:	233VAL	CG2	0.07
130ILE	CG:	247ALA	CB	0.54
130ILE	CG:	130ILE	CD	72.91
130ILE	CG:	130ILE	C	100.00
130ILE	CG:	132ARG	CB	0.00
130ILE	CG:	132ARG	CG	0.00
130ILE	CG:	233VAL	CG1	0.00
130ILE	CG:	235THR	CG2	0.89
130ILE	CG:	243LEU	CG	0.00
130ILE	CG:	243LEU	CD1	0.02
130ILE	CG:	243LEU	CD2	0.06
130ILE	CG:	244SER	CA	0.03
130ILE	CG:	244SER	CB	0.81
130ILE	CD	233VAL	CB	0.23
130ILE	CD	233VAL	CG1	0.11
130ILE	CD	233VAL	CG2	0.60
130ILE	CD	243LEU	CG	0.00
130ILE	CD	243LEU	CD2	0.02
130ILE	CD	243LEU	C	0.04
130ILE	CD	244SER	CA	0.05
130ILE	CD	244SER	CB	0.02
130ILE	CD	247ALA	CB	14.26
130ILE	C	131VAL	CA	100.00
130ILE	C	131VAL	CB	28.12
130ILE	C	131VAL	CG1	0.09
130ILE	C	131VAL	CG2	14.28
130ILE	C	131VAL	C	78.35
131VAL	CA	131VAL	CB	100.00
131VAL	CA	131VAL	CG1	100.00
131VAL	CA	131VAL	CG2	100.00
131VAL	CA	131VAL	C	100.00
131VAL	CB	131VAL	CG1	100.00
131VAL	CB	131VAL	CG2	100.00
131VAL	CB	131VAL	C	100.00
131VAL	CB	169ALA	CB	0.02
131VAL	CB	234VAL	CG2	0.02
131VAL	CG:	131VAL	CG2	100.00
131VAL	CG:	131VAL	C	98.59
131VAL	CG:	165VAL	CG1	0.08
131VAL	CG:	165VAL	CG2	0.70
131VAL	CG:	168VAL	CG1	0.52
131VAL	CG:	168VAL	CG2	0.13
131VAL	CG:	169ALA	CB	0.56
131VAL	CG:	172LEU	CD1	0.00
131VAL	CG:	232VAL	CG1	0.02
131VAL	CG:	234VAL	CB	0.01
131VAL	CG:	234VAL	CG2	0.17
131VAL	CG:	131VAL	C	10.27

131VAL	CG:	165VAL	CG2	0.02
131VAL	CG:	168VAL	CG2	0.00
131VAL	CG:	169ALA	CB	0.15
131VAL	CG:	172LEU	CD1	1.16
131VAL	CG:	172LEU	CD2	0.16
131VAL	CG:	232VAL	CG1	0.58
131VAL	C	132ARG	CA	100.00
131VAL	C	132ARG	CB	96.96
131VAL	C	132ARG	CG	0.41
131VAL	C	132ARG	C	4.39
132AR(	CA	132ARG	CB	100.00
132AR(	CA	132ARG	CG	100.00
132AR(	CA	132ARG	CD	29.26
132AR(	CA	132ARG	C	100.00
132AR(	CA	165VAL	CG1	0.04
132AR(	CA	165VAL	CG2	0.02
132AR(	CB	132ARG	CG	100.00
132AR(	CB	132ARG	CD	100.00
132AR(	CB	132ARG	CZ	0.03
132AR(	CB	132ARG	C	100.00
132AR(	CB	235THR	CG2	0.65
132AR(	CB	235THR	C	0.02
132AR(	CB	240GLY	CA	0.03
132AR(	CG	132ARG	CD	100.00
132AR(	CG	132ARG	CZ	16.24
132AR(	CG	132ARG	C	98.89
132AR(	CG	133GLU	C	0.01
132AR(	CG	134LEU	CD1	0.00
132AR(	CG	134LEU	CD2	0.00
132AR(	CG	235THR	CG2	0.01
132AR(	CG	236GLY	C	0.01
132AR(	CG	240GLY	CA	0.02
132AR(	CG	240GLY	C	0.04
132AR(	CG	244SER	CB	0.11
132AR(	CD	132ARG	CZ	100.00
132AR(	CD	132ARG	C	27.75
132AR(	CD	133GLU	C	0.06
132AR(	CD	134LEU	CD1	0.03
132AR(	CD	134LEU	CD2	0.01
132AR(	CD	235THR	CG2	0.00
132AR(	CD	236GLY	C	0.01
132AR(	CD	237ASN	CG	0.01
132AR(	CD	240GLY	CA	0.01
132AR(	CD	240GLY	C	0.64
132AR(	CD	241ASP	CA	0.01
132AR(	CD	244SER	CB	0.06
132AR(	CZ	134LEU	CD1	1.62
132AR(	CZ	134LEU	CD2	0.10
132AR(	CZ	240GLY	C	0.04
132AR(	CZ	241ASP	CA	1.08
132AR(	CZ	241ASP	CB	0.63
132AR(	CZ	241ASP	CG	0.02
132AR(	CZ	244SER	CB	1.99
132AR(	C	133GLU	CA	100.00
132AR(	C	133GLU	CB	0.35
132AR(	C	133GLU	C	99.84
132AR(	C	165VAL	CG1	0.34
132AR(	C	165VAL	CG2	2.51
133GLU	CA	133GLU	CB	100.00
133GLU	CA	133GLU	CG	100.00
133GLU	CA	133GLU	CD	98.49
133GLU	CA	133GLU	C	100.00

133GLU	CA	165VAL	CG1	0.01
133GLU	CA	165VAL	CG2	0.03
133GLU	CB	133GLU	CG	100.00
133GLU	CB	133GLU	CD	100.00
133GLU	CB	133GLU	C	100.00
133GLU	CB	157TYR	CE1	0.00
133GLU	CB	165VAL	CG1	0.01
133GLU	CB	165VAL	CG2	0.10
133GLU	CB	236GLY	CA	0.02
133GLU	CB	236GLY	C	0.22
133GLU	CB	237ASN	CB	0.01
133GLU	CB	237ASN	CG	0.30
133GLU	CG	133GLU	CD	100.00
133GLU	CG	133GLU	C	21.79
133GLU	CG	136GLY	CA	0.12
133GLU	CG	136GLY	C	0.06
133GLU	CG	157TYR	CE1	0.01
133GLU	CG	165VAL	CB	0.01
133GLU	CG	165VAL	CG1	0.26
133GLU	CG	165VAL	CG2	0.78
133GLU	CD	133GLU	C	2.69
133GLU	CD	135THR	C	0.00
133GLU	CD	136GLY	CA	1.26
133GLU	CD	157TYR	CE1	0.04
133GLU	CD	165VAL	CG1	0.01
133GLU	CD	165VAL	CG2	0.46
133GLU	C	134LEU	CA	100.00
133GLU	C	134LEU	CB	90.90
133GLU	C	134LEU	CG	0.26
133GLU	C	134LEU	CD2	0.06
133GLU	C	134LEU	C	8.81
133GLU	C	237ASN	CG	0.02
134LEU	CA	134LEU	CB	100.00
134LEU	CA	134LEU	CG	100.00
134LEU	CA	134LEU	CD1	80.40
134LEU	CA	134LEU	CD2	18.91
134LEU	CA	134LEU	C	100.00
134LEU	CA	140PHE	CD2	0.00
134LEU	CA	140PHE	CE1	0.00
134LEU	CA	140PHE	CE2	0.02
134LEU	CB	134LEU	CG	100.00
134LEU	CB	134LEU	CD1	100.00
134LEU	CB	134LEU	CD2	100.00
134LEU	CB	134LEU	C	100.00
134LEU	CB	135THR	CG2	0.01
134LEU	CG	134LEU	CD1	100.00
134LEU	CG	134LEU	CD2	100.00
134LEU	CG	134LEU	C	94.13
134LEU	CD1	134LEU	CD2	100.00
134LEU	CD1	134LEU	C	4.45
134LEU	CD1	135THR	CG2	0.03
134LEU	CD1	261SER	CB	0.42
134LEU	CD2	134LEU	C	0.55
134LEU	CD2	261SER	CA	0.00
134LEU	CD2	261SER	CB	5.08
134LEU	C	135THR	CA	100.00
134LEU	C	135THR	CB	79.97
134LEU	C	135THR	CG2	54.41
134LEU	C	135THR	C	20.59
134LEU	C	140PHE	CD2	0.05
134LEU	C	140PHE	CE1	0.00
134LEU	C	140PHE	CE2	0.35

134LEU	C	140PHE	CZ	0.30
135THF	CA	135THR	CB	100.00
[truncated: 554,416 more chars]
