# Supplementary material for: Deciphering the Dynamics of Non-Covalent Interactions Affecting Thermal Stability of a Protein: Molecular Dynamics Study on Point Mutant of Thermus thermophilus Isopropylmalate Dehydrogenase
Source: PLoS One. 2015 Dec 11;10(12):e0144294. doi: 10.1371/journal.pone.0144294 (PMC4689552; doi:10.1371/journal.pone.0144294)
Supplement: S9 Table — (PDF) [file pone.0144294.s011.pdf]

S9 Table. Difference in unique NCI of *mut* and *wt* at 337 K and 300K after removing the interactions having percentage of existence less than or equal to 10 %.

	$mut_{337K-300K}$[1]	$wt_{337K-300K}$[1]
**Saltbridges**[a]	41	14
**Hydrogen bonds (HBs)**[b]	121	41
**HBs**[c] $_{mainch-sidech}$	27	-6
**HBs**[d] $_{sidech-sidech}$	63	27
**HBs**[e] $_{mainch-mainch}$	38	20
**HBs**[f] $_{pol-pol}$	0	3
**HBs**[g] $_{char-char}$	63	31
**Hydrophobic contacts**[h]	81	43

[a],[b], [c], [d], [e], [f], [g],[h] The definition is as per Table 2.
